# Hypoxia‐Induced circPRELID2 Promotes Gastric Cancer Metastasis by Facilitating ZEB2 Translation via PCBP1 O‐GlcNAcylation

**DOI:** 10.1002/advs.202505396

**Published:** 2025-10-21

**Authors:** Pengshan Zhang, Zai Luo, Yitian Xu, Yuan Zhang, Renchao Zhang, Nadina Paerhati, Shaopeng Zhang, Qianqian Cai, Zhengjun Qiu, Chen Huang

**Affiliations:** ^1^ Department of General Surgery Shanghai General Hospital Shanghai Jiao Tong University School of Medicine Shanghai 200080 China

**Keywords:** circPRELID2, gastric cancer, O‐GlcNAcylation, PCBP1, ZEB2 translation

## Abstract

Circular RNAs (circRNAs) are involved in the occurrence and development of various carcinomas. However, the biogenesis, function and underlying mechanism of hypoxia‐induced circRNA in gastric cancer (GC) are poorly understood. Here, a novel circRNA, circPRELID2, which is upregulated by HIF1A under hypoxic conditions, is identified. circPRELID2 is highly expressed in GC tissues, and positively correlates with lymph node invasion, vascular invasion in GC patients. Elevated circPRELID2 promotes the epithelial–mesenchymal transition (EMT) and metastasis of GC cells both in vitro and in vivo under hypoxic conditions. Mechanistically, HIF1A directly binds to the PRELID2 promoter to transcriptionally increase the expression of PRELID2 pre‐mRNA in response to hypoxia. Moreover, protein kinase DYRK1A mediates SFPQ phosphorylation to promote the interaction between SFPQ and SAM68, further forming a DYRK1A‐SFPQ‐SAM68 ternary complex. Subsequently, the DYRK1A‐SFPQ‐SAM68 complex binds to Alu‐containing introns flanking the circPRELID2‐forming exons in the *PRELID2* pre‐mRNA to promote circPRELID2 circularization. Furthermore, circPRELID2 interacts with PCBP1 and promotes the cytoplasmic retention of PCBP1. CircPRELID2 enhances OGT‐mediated PCBP1 O‐GlcNAcylation at Threonine 99 site in the cytoplasm, which disrupts the binding of PCBP1 to the 3′‐UTR of *ZEB2*, resulting in the reversal of ZEB2 translation silencing, and ultimately promoting the EMT and metastasis of GC cells. These findings reveal a new regulatory mechanism of circRNA biogenesis, and also uncover that circPRELID2 participates in the translation of ZEB2 during GC metastasis via the modulation of PCBP1 O‐GlcNAcylation, which provides a promising prognostic biomarker and therapeutic target for GC metastasis.

## Graphical abstract

Under hypoxia, HIF1A is directly bound to the *PRELID2* promoter to increase *PRELID2* pre‐mRNA expression, followed by the DYRK1A–SFPQ–SAM68 complex binding to Alu‐containing introns in *PRELID2* pre‐mRNA, leading to circPRELID2 circularization. CircPRELID2 interacts with PCBP1 and promotes its cytoplasmic retention. In addition, circPRELID2 enhances OGT‐mediated PCBP1 O‐GlcNAcylation at Thr99 site in the cytoplasm, disrupting the binding of PCBP1 to the 3′‐UTR of *ZEB2* and reversing ZEB2 translation silencing.

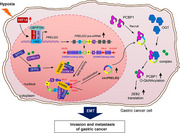

## 1 Introduction

Gastric cancer (GC) is one of the most prevalent solid tumors of the digestive system, ranking fifth globally in both incidence and mortality among all malignancies.^[^ ^1^ ^]^ Despite advances in surgical techniques, immunotherapy, and molecular targeted therapies, the 5‐year survival rate for GC patients remains poor due to a high propensity for metastasis.^[^ ^2^, ^3^, ^4^ ^]^ The aggressive invasion and metastasis of GC cells have become the leading cause of recurrence and death in GC patients.^[^ ^4^, ^5^ ^]^ Therefore, elucidating the molecular mechanisms underlying GC cell invasion and metastasis is critical for improving clinical outcomes.

Intratumoral hypoxia is a hallmark of solid tumors, including gastric cancer. Emerging evidence highlights hypoxia as a key driver of glucose metabolism, angiogenesis, cell survival, and metastasis.^[^ ^6^, ^7^ ^]^ Under hypoxic conditions, hypoxia‐inducible factor 1 (HIF1)–composed of an oxygen‐sensitive HIF‐1α subunit and a constitutively expressed HIF‐1β subunit–transcriptionally activates target genes by binding to hypoxia response elements (HREs) in promoter regions, in cooperation with the coactivator p300/CBP.^[^ ^6^, ^7^, ^8^ ^]^ While the role of HIF1 in regulating mRNA expression during gastric cancer metastasis is well‐established,^[^ ^9^, ^10^, ^11^ ^]^ it is important to note that pre‐mRNA processing generates not only mature mRNA but also substantial amounts of circular RNA (circRNA). However, the mechanisms underlying hypoxia‐mediated circRNA biogenesis remain poorly understood. Circular RNAs (circRNAs) are evolutionarily conserved noncoding RNAs with tissue‐specific expression patterns. They are characterized by covalently closed loop structures that lack a 5′ cap and a 3′ polyA tail, formed through the back‐splicing of pre‐mRNA in eukaryotes, which provides them resistance to RNA exonucleases.^[^ ^12^, ^13^ ^]^ Increasing evidence indicates that circRNAs play roles in various physiological and pathological processes, including tissue development, cardiovascular disease, and tumorigenesis,^[^ ^12^, ^13^, ^14^ ^]^ However, their functions in the hypoxic tumor microenvironment remains unclear. Recently, scattered reports have revealed that hypoxia‐related circRNAs participate in tumor progression in response to hypoxia. For example, circDENND2A is overexpressed in glioma cells under hypoxia and promotes the migration and invasion of glioma cells via sponging miR‐625‐5p.^[^ ^15^ ^]^ And we reported that circTDRD3 is upregulated under hypoxic conditions, promoting the growth and metastasis of colorectal cancer cells through a positive HIF1A/PTBP1/circTDRD3/miR‐1231/HIF1A feedback loop.^[^ ^16^ ^]^ While current research on hypoxia‐related circRNAs largely centers on their miRNA‐sponging activity, alternative functional mechanisms remain unexplored. Although an increasing number of circRNAs act as key player to the progression of GC,^[^ ^17^, ^18^, ^19^, ^20^ ^]^ the regulatory role of hypoxia‐induced circRNAs in GC metastasis is yet to be elucidated.

CircRNAs are primarily generated through back‐splicing of pre‐mRNAs in eukaryotes, a process wherein a downstream 5′ splice donor site covalently links to an upstream 3′ splice acceptor, forming a closed loop of one or multiple exons via spliceosomal machinery.^[^ ^12^, ^13^, ^14^, ^21^ ^]^ This back‐splicing competes with canonical linear splicing of the host gene mRNA despite sharing the same canonical spliceosomal machinery.^[^ ^22^ ^]^ The back‐splicing reaction can mediate the formation of a loop between introns flanking the circularizing exon(s), which can be facilitated by inverted repeat intron sequences, especially inverted repeat Alus (IRAlus) located in the flanking introns.^[^ ^12^, ^21^, ^22^ ^]^ Furthermore, dimerization of RNA‐binding proteins (RBPs) may bring back‐splicing sites into close proximity to promote circRNA biogenesis. Recently, a small number of RBPs mediating circRNA biogenesis, including QKI, NOVA2 and FUS, have been identified.^[^ ^23^, ^24^, ^25^ ^]^ However, whether RBPs and Alus elements cooperatively regulate this process remains unclear.

Here, based on RNA‐seq data from GC tissues and hypoxic GC cells, we identified a novel hypoxia‐induced circRNA termed circPRELID2 (hsa_circ_0074389), derived from exons 2, 3, 4, and 5 of the *PRELID2* gene. Through integrated molecular, cellular, and biochemical analyses, we identified the oncogenic role of circPRELID2, generated through the collaborative induction of DYRK1A‐SFPQ‐SAM68 ternary complex and IRAlus, in the metastasis of GC under hypoxia and further elucidated the mechanism by which circPRELID2/OGT/PCBP1/ZEB2 axis contributes to the translation of ZEB2 protein.

## 2 Results

### 2.1 Identification of circPRELID2 in GC Cells in Response to Hypoxia

**Figure 1:**
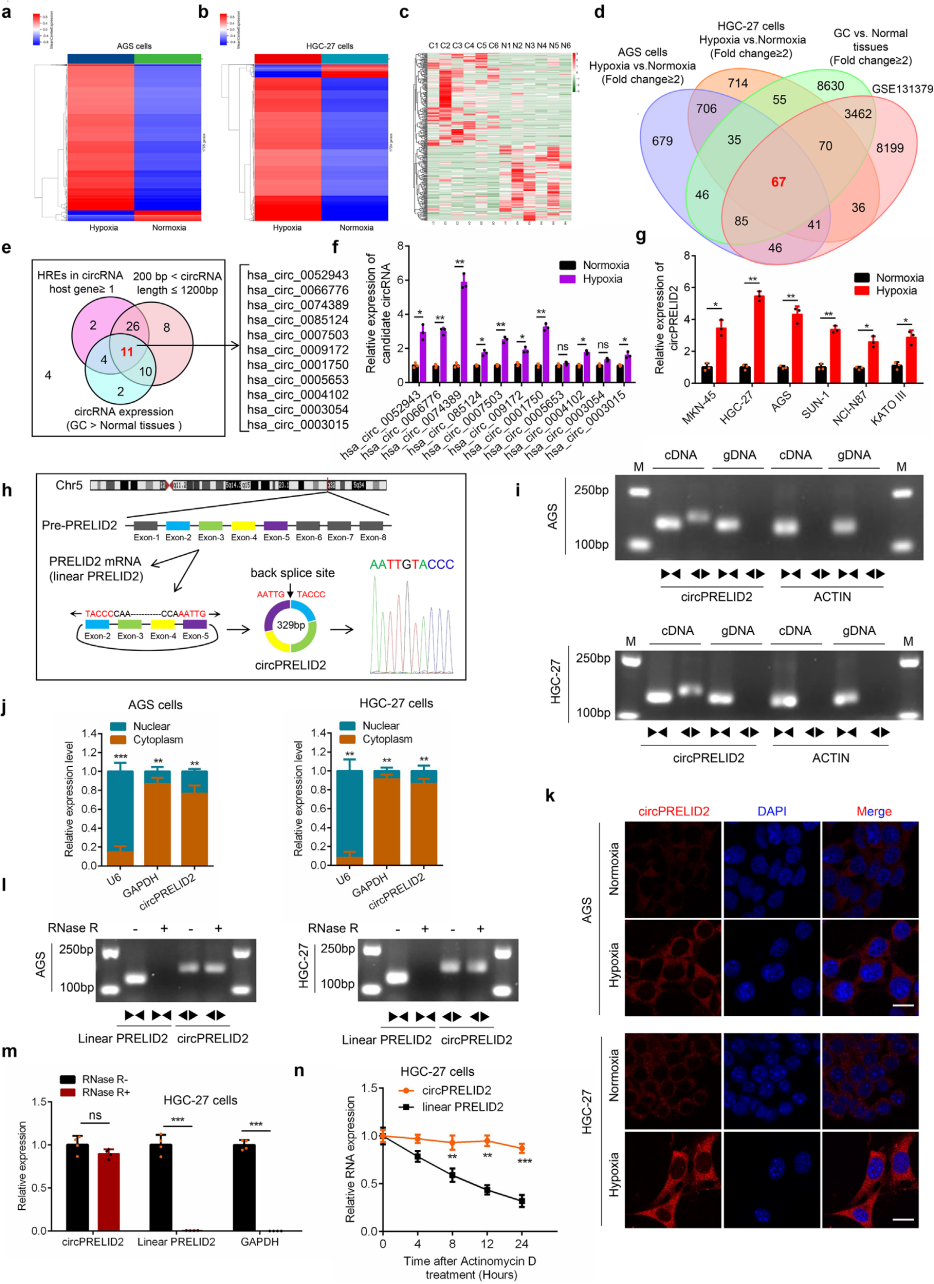
Identification of circPRELID2 in gastric cancer cells in response to hypoxia. a,b) Heatmaps of the differentially expressed circRNAs between two different GC cell lines subjected to normoxia or hypoxia for 24 hr. c) Heatmap showing the significantly dysregulated circRNAs in six pairs of GC tissues and matched adjacent normal tissues. d) Schematic diagram showing the overlap of the 67 candidate circRNAs in (a), (b), (c) and GEO dataset. e) 11 candidate circRNAs were identified from the overlapping 67 circRNAs shown in (d), based on the exclusion criteria presented. f) qRT‐PCR analyses of the relative expression of the 11 candidate circRNAs in HGC‐27 cells under normoxia or hypoxia. g) Relative expression of hsa_circ_0074389 (circPRELID2) was detected via qRT‐PCR in multiple GC cell lines subjected to normoxia or hypoxia. h) Schematic illustration exhibiting the biogenesis of circPRELID2 from its host gene, *PRELID2*, along with the back‐splicing junction site verified by Sanger sequencing. i) CircPRELID2 and ACTIN were amplified from cDNA and gDNA of AGS and HGC‐27 cells, respectively, using divergent and convergent primers. j) Subcellular distribution of circPRELID2 in AGS and HGC‐27 cells detected through nuclear/cytoplasmic fractionation. k) FISH assay showing the subcellular localization of circPRELID2 in AGS and HGC‐27 cells under normoxia or hypoxia. Scale bar = 25 µm. l,m) Relative expression of circPRELID2 and *PRELID2* mRNA in AGS and HGC‐27 cells treated with or without RNase R. n) qRT‐PCR analysis of the relative expression levels of circPRELID2 and *PRELID2* mRNA in HGC‐27 cells treated with actinomycin D for the indicated times. Data were shown as the mean ± SD; *p < 0.05, **p < 0.01, ***p < 0.001.

**Figure 2:**
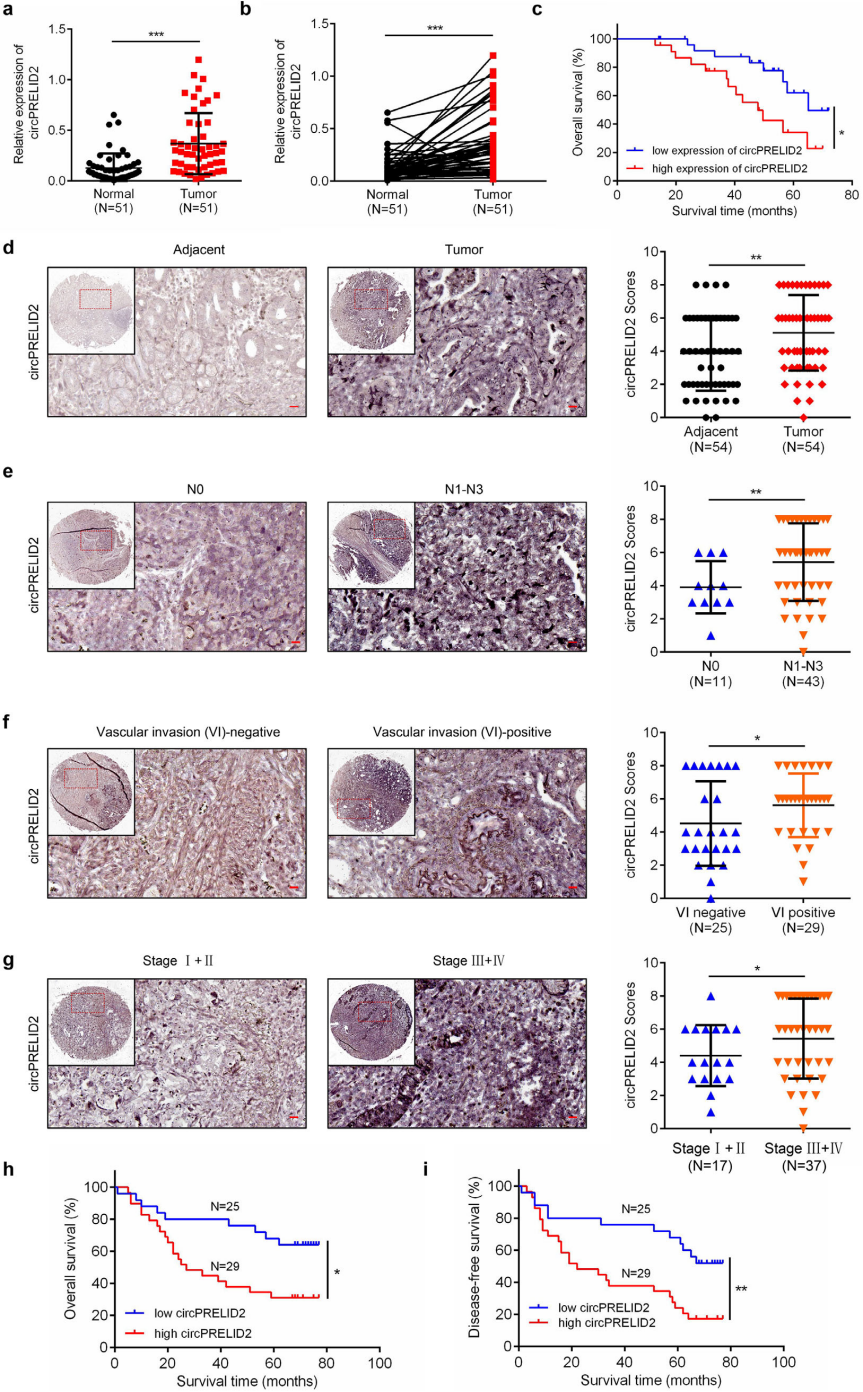
CircPRELID2 is highly expressed in GC tissues and correlates with clinical characteristics. a,b) Relative expression of circPRELID2 in 51 GC tissues and matched adjacent normal tissues was analyzed via qRT‐PCR. c) Kaplan–Meier plots of overall survival of GC patients with low and high circPRELID2 expression. d–g) In situ hybridization (ISH) assays were performed to examine the expression of circPRELID2 on TMA comprising 54 pairs of adjacent normal tissues and GC tissues based on differences in lymph node invasion (N0 versus N1‐3), vascular invasion (VI) and TNM stage. Left, representative ISH staining images; scale bar = 20 µm. Right panel, the quantification of ISH scores of circPRELID2 was shown. h,i) Kaplan–Meier survival analysis showing the overall survival (OS) and disease‐free survival (DFS) of GC patients with low and high circPRELID2 expression based on ISH scores of circPRELID2 obtained from GC TMA. Data were shown as the mean ± SD; *p < 0.05, **p < 0.01, ***p < 0.001.

To identify crucial hypoxia‐responsive circRNAs in gastric cancer, we conducted RNA‐seq analysis to examine the circRNA expression profiles in two different gastric cell lines under normoxia or hypoxia and found that, compared to normoxia, 1705 and 1724 circRNAs were differentially expressed in the two different gastric cell lines under hypoxia, respectively, based on the cut‐off criteria of a fold‐change ≥ 2.0 and *P* < 0.05 (**Figure** **1**a,b). Additionally, RNA‐seq of ribosomal RNA‐depleted total RNA from six pairs of GC tissues and adjacent normal tissues yielded a circRNA profiling database of 12450 differentially expressed circRNAs (Figure 1c). By intersecting these sequencing data with differentially expressed circRNAs from various human tumor cells subjected to hypoxic treatments (GSE131379),^[^ ^26^ ^]^ we identified 67 dysregulated circRNAs in hypoxic GC cells (Figure 1d), including 63 upregulated and 4 downregulated circRNAs. Subsequently, based on the cut‐off criteria of the number of HREs in the promoter region of the circRNA host gene≥ 1, being highly expressed in GC tissues and 200 bp < circRNA length ≤ 1200 bp, 11 candidate circRNAs were identified for further validation (Figure 1e). Consistent with the RNA‐Seq results, 9 out of the 11 candidate circRNAs were significantly upregulated in hypoxia‐treated HGC‐27 cells, and hsa_circ_0074389 (circPRELID2) was the most significantly upregulated circRNA (Figure 1f). Then, we selected the top five candidate circRNAs for continued validation and found that the expression levels of hsa_circ_0052943, hsa_circ_0066776, hsa_circ_0074389, hsa_circ_0007503 and hsa_circ_0001750 were markedly elevated in AGS and HGC‐27 cells under hypoxia, and hsa_circ_0074389 (circPRELID2) remained the one with the highest expression (Figure S1a,b, Supporting Information). In addition, we detected the expression level of these five candidate circRNAs in a cohort of 51 pairs of fresh‐frozen GC tissues and matched adjacent normal tissues and analyzed the correlation of these candidate circRNAs with overall survival of GC patients. We found that hsa_circ_0052943, hsa_circ_0074389, hsa_circ_0007503 and hsa_circ_0001750 were significantly elevated in GC tissues, strikingly, of which hsa_circ_0074389 (circPRELID2) was expressed at the highest level. Notably, only hsa_circ_0074389 (circPRELID2) and hsa_circ_0001750 exhibited a significantly negative correlation with overall survival of GC patients among these circRNAs (**Figures** **2**a,b and S1, Supporting Information). Given its expression abundance in GC tissues and fold‐change under hypoxia, we selected circPRELID2 for further study. To validate these findings, the expression of circPRELID2 was further analyzed in various GC cell lines subjected to normoxia or hypoxia, and the results indicated that circPRELID2 expression was markedly increased in all tested GC cell lines (MKN‐45, HGC‐27, AGS, SNU‐1, NCl‐N87, and KATOIII) exposed to hypoxia (Figure 1g). These results suggested that circPRELID2 is an important hypoxia‐associated circRNA in GC.

### 2.2 Characterization and Clinical Features of circPRELID2

According to circBase database (https://www.circrna.org/),^[^ ^27^ ^]^ circPRELID2 is derived from exons 2–5 of the *PRELID2* gene (chr5) through head‐to‐tail back‐splicing, forming a 329‐bp circular transcript. And its head‐to‐tail back splicing junction was confirmed via Sanger sequencing (Figure 1h). To exclude the possibility that the observed head‐to‐tail splicing resulted from genomic rearrangements or trans‐splicing, convergent primers and divergent primers were designed to amplify *PRELID2* mRNA and circPRELID2, respectively. Agarose gel electrophoresis of the RT‐PCR products indicated that circPRELID2 could be amplified with divergent primers in cDNA, but not in gDNA from AGS and HGC‐27 cells (Figure 1i). Furthermore, nuclear‐cytoplasmic fractionation assay verified that circPRELID2 was mainly localized in the cytoplasm of AGS and HGC‐27 cells, which was validated by FISH assay (Figure 1j,k), suggesting its potential cytoplasmic function. Moreover, RNA extracted from GC cells was treated with RNase R. Interestingly, circPRELID2 was resistant to digestion by RNase R, while *PRELID2* mRNA decreased markedly (Figure 1l,m). To further verify the stability of circPRELID2, we incubated HGC‐27 cells with actinomycin D, a transcription inhibitor, and found that circPRELID2 exhibited greater stability than *PRELID2* mRNA (Figure 1n). Collectively, these results revealed that circPRELID2 is an abundant and stable circRNA located predominantly in the cytoplasm of GC cells.

### 2.3 CircPRELID2 Is Highly Expressed in GC Tissues and Is Correlated with Clinical Characteristics

To further explore the expression pattern and clinical significance of circPRELID2 in GC tissues, we detected the expression level of circPRELID2 in a cohort of 51 pairs of fresh‐frozen GC tissues and matched adjacent normal tissues, and found that circPRELID2 was significantly upregulated in GC samples, in line with the RNA‐seq results (Figure 2a,b). GC patients with higher circPRELID2 levels exhibited significantly worse overall survival (OS) compared to those with lower circPRELID2 expression (Figure 2c). CircPRELID2 expression was significantly positively correlated with lymph node invasion, vascular invasion and proximal location (Table S4, Supporting Information). Furthermore, we performed in situ hybridization (ISH) assay to examine circPRELID2 expression level on gastric cancer tissue microarray (TMA) containing another separate cohort of 54 GC tissues and paired adjacent normal tissues, and found that circPRELID2 was significantly elevated in GC tissues (Figure 2d). Notably, circPRELID2 levels were higher in GC patients with lymph node invasion (N1‐N3), vascular invasion (Positive) or stage III–IV compared to those with no lymph node invasion (N0), no vascular invasion (Negative) or stage I–II, respectively (Figure 2e–g). Meanwhile, Kaplan–Meier survival analysis based on GC TMA also showed that GC patients with higher circPRELID2 expression had worse overall survival (OS) and disease‐free survival (DFS) compared to those with lower expression (Figure 2h,i). These findings suggested that circPRELID2 was significantly upregulated in GC and might serve as a prognostic marker for GC patients.

### 2.4 HIF1A Combines with the *PRELID2* Promoter to Increase the Expression of circPRELID2 under Hypoxia

**Figure 3:**
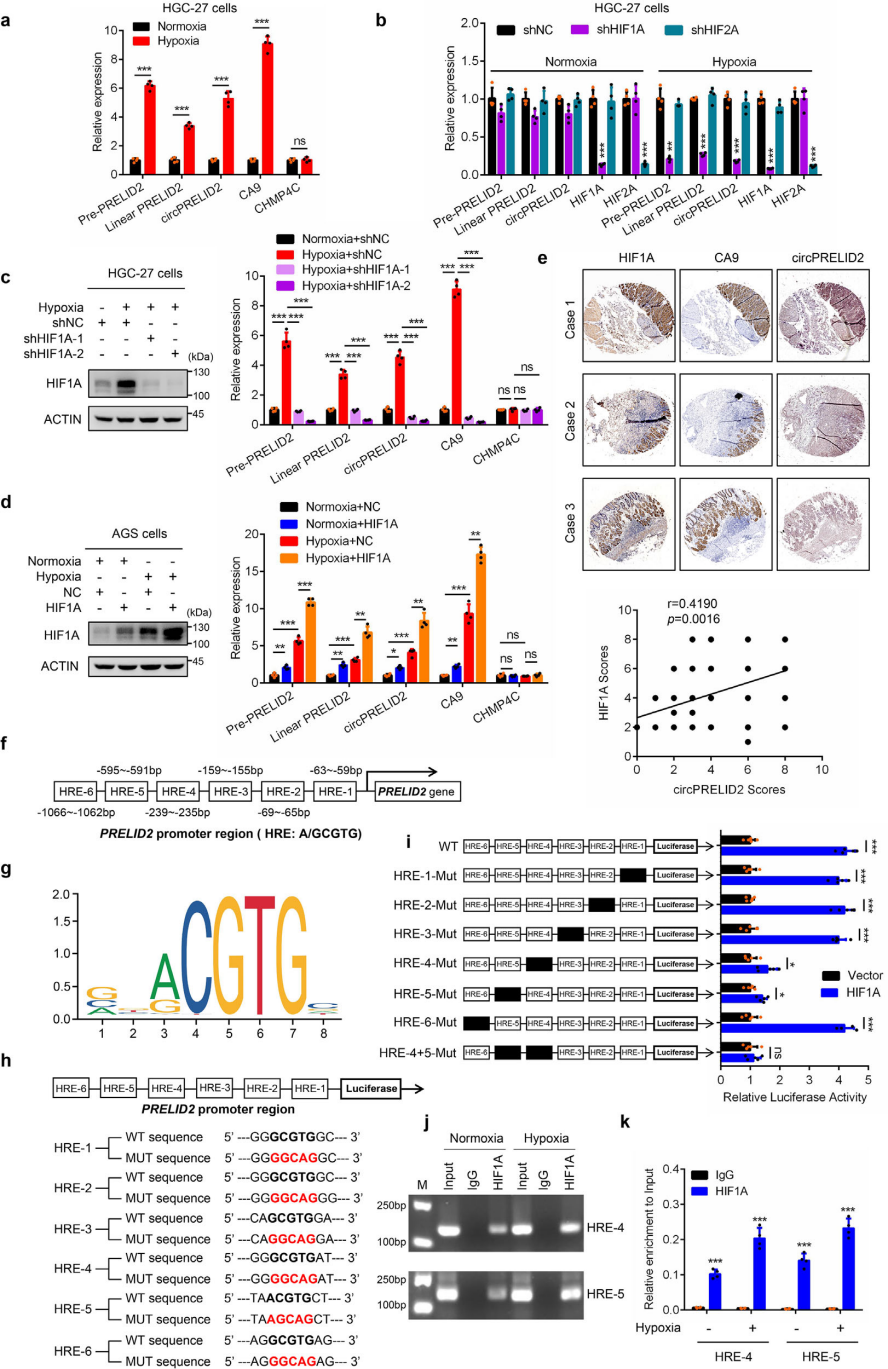
HIF1A combines with the *PRELID2* promoter to increase the expression of circPRELID2 under hypoxia. a) qRT‐PCR analyses of the relative expression of pre‐*PRELID2*, linear *PRELID2*, and circPRELID2 in HGC‐27 cells cultured under normoxia or hypoxia. CA9 and CHMP4C were set as positive and negative control) respectively. b) The relative expression of pre‐*PRELID2*, linear PRELID2, and circPRELID2 in HGC‐27 cells stably transfected shNC, shHIF1A or shHIF2A under normoxia or hypoxia were analyzed by qRT‐PCR. c,d) qRT‐PCR analyses of the relative expression of pre‐*PRELID2*, linear *PRELID2*, and circPRELID2 in hypoxic HGC‐27 or AGS cells following knockdown (c) or overexpression (d) of HIF1A. e) ISH and IHC assays were performed to examine the expression of HIF1A, CA9 and circPRELID2 on GC TMA containing 54 pairs of adjacent normal tissues and GC tissues. Upper panel, representative ISH staining images of HIF1A, CA9 and circPRELID2 were shown. Lower panel, the correlation between HIF1A and circPRELID2 based on ISH scores was analyzed. f) Schematic representation showing putative hypoxia response elements (HREs) in the promoter region (−2000 to −1) of *PRELID2*. g) Schematic diagram showing the conserved HRE specifically bound by HIF1A. h) Dual‐luciferase reporter vectors with the wild‐type (WT) or six putative HRE mutants in the *PRELID2* promoter region were constructed, respectively. i) Dual‐luciferase reporter assay was performed to detect the relative activity of the six putative HREs of *PRELID2* in AGS cells treated under the indicated conditions. j,k) ChIP analysis of the binding capacity of HIF1A to the putative HREs of *PRELID2* in HGC‐27 cells cultured under normoxia or hypoxia. Data were shown as the mean ± SD; *p < 0.05, **p < 0.01, ***p < 0.001.

To further characterize how circPRELID2 is induced by hypoxia, we cultured HGC‐27 and AGS cells under normoxia or hypoxia for 24 h. We observed a significant increase in pre‐*PRELID2*, linear PRELID2 (*PRELID2* mRNA), and circPRELID2 expression in hypoxic conditions compared to normoxia (**Figures** **3**a and S2a, Supporting Information), which was consistent with the most recent studies indicating that PRELID2 is induced by hypoxia.^[^ ^28^ ^]^ Additionally, hypoxia increased pre‐*PRELID2*, *PRELID2* mRNA, and circPRELID2 levels in a time‐dependent manner (Figure S2b, Supporting Information). These findings suggested that hypoxia may induce circPRELID2 expression through upregulating pre‐*PRELID2*.

Furthermore, we found that the depletion of HIF1A, but not HIF2A, resulted in a significant decrease in pre‐*PRELID2*, linear PRELID2, and circPRELID2 under hypoxia (Figure 3b,c), while HIF1A overexpression resulted in the opposite effect (Figure 3d). To further investigate the expression of circPRELID2 in hypoxia regions of GC tissues, we performed ISH and immunohistochemical analysis in the GC TMA, and found that circPRELID2 was significantly increased in hypoxia areas. Expectedly, the expression of circPRELID2 was positively correlated with HIF1A levels in the GC TMA (Figure 3e). Next, the JASPAR database (http://jaspar.genereg.net/) was utilized to examine the binding motif of transcription factor HIF1A on the promoter of *PRELID2*. As shown in Figure 3f,g, six putative HREs were identified within the promoter region of *PRELID2*. Then we mutated these six HREs of the *PRELID2* promoter and subcloned into luciferase reporter vector, respectively (Figure 3h), followed by dual‐luciferase reporter analysis. We found that under hypoxic conditions, HIF1A overexpression significantly elevated the *PRELID2* promoter activity. HRE‐4 or HRE‐5 mutant, rather than HRE‐1, 2, 3, or 6 mutant, markedly decreased the *PRELID2* promoter activity. Interestingly, HRE‐4/HRE‐5 double mutant completely abolished the *PRELID2* promoter activity (Figure 3i). ChIP assay further verified the direct binding of HIF1A to the HRE‐4 and HRE‐5 sites of the *PRELID2* promoter under both normoxia and hypoxia (Figure 3j,k). These data demonstrated that HIF1A directly interacted with *PRELID2* promoter and transcriptionally regulated circPRELID2 expression under hypoxia.

### 2.5 DYRK1A–SFPQ–SAM68 Complex Binds Alu‐Containing Introns in *PRELID2* and Promotes circPRELID2 Circularization

**Figure 4:**
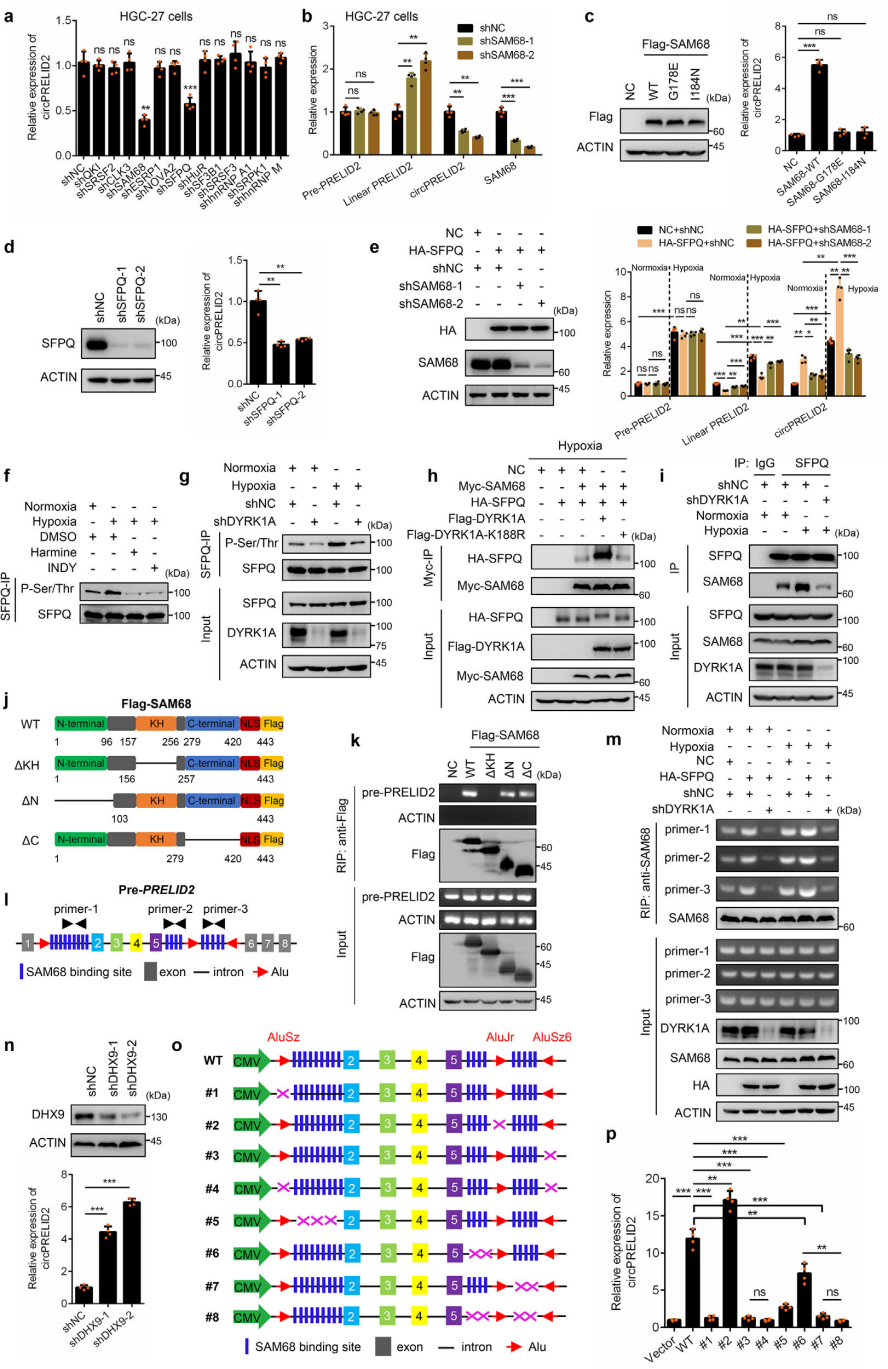
The DYRK1A–SFPQ–SAM68 complex binds Alu‐containing introns in *PRELID2* and promotes circPRELID2 circularization. a) qRT‐PCR analyses were performed to analyze the effect of candidate RNA‐binding proteins on circPRELID2 expression in HGC‐27 cells. b) qRT‐PCR analysis was used to detect the relative expression of pre‐*PRELID2*, linear PRELID2, and circPRELID2 in HGC‐27 cells following the knockdown of SAM68. c) The relative expression of circPRELID2 in AGS cells transfected with SAM68 wild‐type (WT) or its RNA‐binding mutants (G178E, I184N) was detected by qRT‐PCR. d) qRT‐PCR analysis examining the relative expression of circPRELID2 in HGC‐27 shNC or shSFPQ cells constructed using the lentiviral system. e) The stable AGS cells expressing NC or HA‐SFPQ alone or in combination with SAM68 shRNA were cultured under normoxia or hypoxia for 24hr. Then the relative expression of circPRELID2 was analyzed by qRT‐PCR. f) HGC‐27 cells were incubated with 15 µM INDY or 15 µM harmine under normoxia or hypoxia) and then subjected to immunoprecipitation and western blot analyses with the indicated antibodies. g) Lysates from HGC‐27‐shNC and shDYRK1A cells cultured under normoxia or hypoxia were subjected to immunoprecipitation with anti‐SFPQ antibody, followed by western blotting analysis of the phosphorylation of SFPQ. h) AGS cells transfected with NC, HA‐SFPQ, Myc‐SAM68 and/or Flag‐DYRK1A wild‐type (WT) or its catalytically inactive mutant K188R under hypoxia were Myc‐affinity purified, followed by immunoblotting analysis with the indicated antibodies. i) Lysates from HGC‐27‐shNC and shDYRK1A cells subjected to normoxia or hypoxia for 24 h were immunoprecipitated with normal rabbit IgG or with SFPQ antibody, followed by western blot analyses with the indicated antibodies. j) Schematic diagram of SAM68 and its truncations. All constructs were Flag‐tagged at the C‐terminus. k) AGS cells were transfected with Flag‐SAM68 or Flag‐SAM68 deletion constructs, and then RNA immunoprecipitation (RIP) assay was performed using Flag antibody to examine the binding of SAM68 to pre‐*PRELID2*. l) Schematic representation of the pre‐*PRELID2* sequence structure. m) The stable HGC‐27 cells expressing NC or HA‐SFPQ alone or in combination with DYRK1A shRNA were cultured under normoxia or hypoxia for 24hr. Then RIP analysis was performed to examine the SAM68‐binding sites in pre‐*PRELID2*. The locations of primer‐1, primer‐2, and primer‐3 in pre‐*PRELID2* are illustrated in (l). n) qRT‐PCR analysis of circPRELID2 expression in AGS‐shNC or shDHX9 cells. o) Schematic illustration of *PRELID2* minigene expression vectors with wild‐type or deletions of genomic sequences for *PRELID2* circular RNA recapitulation. p) qRT‐PCR analysis was employed to detect the relative expression of circPRELID2 in AGS cells transfected with *PRELID2* minigene expression vectors, as shown in (o). Data were shown as the mean ± SD; ns indicated no significance, *P < 0.05, **P < 0.01, ***P < 0.001.

The biogenesis of circRNAs in mammalian cells is governed by both transcriptional and post‐transcriptional regulation.^[^ ^12^, ^21^, ^22^ ^]^ RNA‐binding proteins (RBPs) are involved post‐transcriptionally in the biogenesis of exon‐derived circRNAs.^[^ ^21^, ^22^, ^23^, ^24^, ^25^ ^]^ We hypothesized that the biogenesis of circPRELID2 could potentially be influenced by certain RNA‐binding proteins. Given the enhanced production of circPRELID2 in hypoxic conditions, we hypothesized that these RNA‐binding proteins might be regulated by hypoxia. Therefore, we conducted a comprehensive literature search regarding the regulation of RBPs or splicing factors by hypoxia,^[^ ^29^, ^30^ ^]^ while also investigating whether these RBPs have potential binding sites on the *PRELID2* gene. Additionally, we searched for studies that report on the regulatory roles of these RBPs in circRNA expression. Considering these aspects, we ultimately focused on a number of hypoxia‐regulated RBPs including QKI, SRSF2, CLK3, SAM68, ESRP1, NOVA2, SFPQ, HuR, etc. Among these RBPs, only knockdown of SAM68 or SFPQ significantly reduced the expression of circPRELID2 in HGC‐27 cells (**Figure** **4**a). Furthermore, we found that SAM68 depletion elevated the expression of linear PRELID2 while decreasing circPRELID2 levels, with no effect on pre‐*PRELID2* expression in HGC‐27 cells (Figure 4b). To investigate whether the RNA‐binding activity of SAM68 was involved in circPRELID2 biogenesis, we constructed two different SAM68 mutants (SAM68‐G178E and SAM68‐I184N) that were previously shown to be defective in RNA binding.^[^ ^31^, ^32^ ^]^ We found that wild‐type SAM68, but not its G178E or I184N mutant, notably enhanced the expression of circPRELID2 in AGS cells (Figure 4c). Similarly, we verified that knockdown of SFPQ led to a reduction in circPRELID2 levels in HGC‐27 cells (Figure 4d). Interestingly, we analyzed the LC‐MS/MS mass spectrometry data which identified the protein components of SAM68 RNP complex^[^ ^33^ ^]^ and the BioGRID interaction database (https://thebiogrid.org/), a database of protein and genetic interactions that primarily relies on literature mining, and found that SFPQ is a potential interaction partner of SAM68 (Figure S3a, Supporting Information). To validate this interaction, we co‐overexpressed Flag‐SAM68 and HA‐SFPQ, and observed that SFPQ was able to pull down SAM68 (Figure S3b, Supporting Information). Furthermore, SFPQ overexpression led to markedly increased expression of circPRELID2 and a significant reduction in *PRELID2* mRNA, which could be rescued by SAM68 knockdown in AGS cells, particularly under hypoxia, indicating that SFPQ was involved in SAM68‐mediated circPRELID2 biogenesis under hypoxia (Figure 4e).

In addition, we found that hypoxia promotes the interaction between SFPQ and SAM68 more effectively than normoxia (Figure 4i). To further investigate how hypoxia boosts their interaction, we treated HGC‐27 cells under normoxic or hypoxic conditions for 24 h, and found hypoxia enhanced the phosphorylation level of SFPQ (Figure 4f). Then, we attempted to identify the protein kinase (s) responsible for SFPQ phosphorylation. Interestingly, in our previous study regarding Dual‐specificity tyrosine phosphorylation‐regulated kinase 1A (DYRK1A),^[^ ^34^ ^]^ an evolutionarily conserved protein kinase belonging to the DYRK family, we constructed stable HEK293T cells transformed with Flag‐DYRK1A, then also affinity‐purified Flag‐DYRK1A from whole cell lysate and analyzed the eluates via SDS‐PAGE and silver staining (Figure S3c, Supporting Information). In addition to a number of known DYRK1A interactors, including DCAF7, CBP and TRAF2,^[^ ^34^, ^35^, ^36^ ^]^ we identified SFPQ and SAM68 as potential DYRK1A‐binding proteins. To verify the interaction between DYRK1A and SFPQ, we performed co‐immunoprecipitation assay in HEK293T cells transfected with Flag‐DYRK1A and/or HA‐SFPQ, and observed that DYRK1A pulled down SFPQ (Figure S3d, Supporting Information). Strikingly, the overexpression of DYRK1A‐WT led to a small upshift in the SFPQ protein based on western blotting (Figure S3d,e, Supporting Information), whereas the catalytically inactive DYRK1A‐K188R did not (Figure S3e, Supporting Information). DYRK1A, a member of the CMGC group of serine/threonine kinases, can phosphorylate some of its substrates at multiple residues,^[^ ^34^, ^35^, ^36^ ^]^ we reasoned that DYRK1A might phosphorylate SFPQ at multiple residues, leading to reduced migration on SDS denaturing gels. In addition, the λ‐phosphatase treatment of lysates generated from cells overexpressing DYRK1A‐WT led to increased migration of SFPQ signals comparable to that of NC or DYRK1A‐K188R samples (Figure S3e, Supporting Information). Furthermore, we found that DYRK1A, but not the catalytically inactive DYRK1A‐K188R, was able to phosphorylate SFPQ (Figure 3f). To examine whether DYRK1A is involved in hypoxia‐mediated SFPQ phosphorylation, we inhibited DYRK1A kinase activity in HGC‐27 cells with two different DYRK1A inhibitors Harmine or INDY under hypoxia, and found that DYRK1A inhibition could significantly suppress hypoxia‐facilitated SFPQ phosphorylation (Figure 4f). Moreover, we observed that hypoxia cannot rescue the reduced SFPQ phosphorylation level caused by DYRK1A knockdown in HGC‐27 cells, indicating that hypoxia‐facilitated SFPQ phosphorylation was mediated through DYRK1A (Figure 4g). To determine whether the phosphorylation of SFPQ is required for the binding of SFPQ to SAM68 under hypoxia, we co‐expressed Myc‐SAM68, HA‐SFPQ and/or Flag‐DYRK1A (WT and K188R) in AGS cells and affinity‐purified Myc‐SAM68, then analyzed the enrichment of SFPQ. We found that DYRK1A‐WT, but not the catalytically inactive DYRK1A‐K188R, promoted the interaction between SFPQ and SAM68 under hypoxia (Figure 4h). As expected, DYRK1A knockdown abolished hypoxia‐enhanced interaction between SFPQ and SAM68 (Figure 4i). Furthermore, we found DYRK1A was markedly upregulated in various GC cell lines compared to normal GES‐1 cells (Figure 3g). DYRK1A inhibition reduced circPRELID2 expression and elevated *PRELID2* mRNA levels without affecting pre‐*PRELID2* expression in HGC‐27 cells under hypoxia, implying DYRK1A is indeed involved in circPRELID2 biogenesis under hypoxia (Figure S3h, Supporting Information). Additionally, we found that the interaction between DYRK1A and either SFPQ or SAM68 does not depend on pre‐*PRELID2*, whereas the mutual binding between SFPQ and SAM68 is diminished in the absence of pre‐*PRELID2* (Figure S3i,j, Supporting Information). Together, these data suggested that under hypoxic conditions, protein kinase DYRK1A‐mediated SFPQ phosphorylation promotes the interaction between SFPQ and SAM68 to form a DYRK1A‐SFPQ‐SAM68 ternary complex, which is engaged in circPRELID2 circularization.

Next, we tried to investigate how this DYRK1A‐SFPQ‐SAM68 ternary complex binds to pre‐*PRELID2* to facilitate circPRELID2 circularization. As Figure 4c suggested, the RNA‐binding activity of SAM68 was required for circPRELID2 biogenesis, implying that SAM68 may directly bind to pre‐*PRELID2* to facilitate circPRELID2 circularization. SAM68 recognizes the bipartite (A/U)AA‐N>15‐(A/U)AA RNA sequence for binding.^[^ ^37^ ^]^ We identified nine candidate SAM68‐binding sites located upstream of the circPRELID2‐generating splice sites, as well as nine potential binding sites downstream (Data S5, Supporting Information, red frame). We thus investigated whether SAM68 indeed binds to pre‐*PRELID2*, and identified the domain(s) of SAM68 responsible for this interaction. We constructed three Flag‐SAM68 truncations as follows: KH domain deletion (ΔKH), N‐terminal deletion (ΔN), and C‐terminal deletion (ΔC) (Figure 4j). RIP assays revealed that wild‐type SAM68 could interact efficiently with pre‐*PRELID2* in AGS cells (Figure 4k). In contrast, SAM68‐ΔN and ΔC mutants showed diminished interactions, and SAM68‐ΔKH exhibited nearly abolished interactions (Figure 4k), indicating that the KH domain is the main region of SAM68 for the interaction with pre‐*PRELID2*. To further verify the interaction between SAM68 and its candidate binding sites in pre‐*PRELID2*, we established stable HGC‐27 cells expressing NC or HA‐SFPQ alone or in combination with DYRK1A shRNA, and cultured them under normoxia or hypoxia for 24 h, showing that SAM68 indeed could bind to these candidate SAM68‐binding sites in pre‐*PRELID2*, and SFPQ promoted this interaction, which could be reversed by DYRK1A knockdown under hypoxia (Figure 4l,m). These results demonstrated that DYRK1A‐SFPQ‐SAM68 complex could interact with pre‐*PRELID2* to facilitate circPRELID2 circularization under hypoxia.

However, we found that knocking down SAM68 or SFPQ did not completely inhibit the formation of circPRELID2 (Figure 4a,b,d), suggesting that other mechanisms may also be involved in its circularization. Interestingly, using UCSC Genome Browser software (http://genome.ucsc.edu/), we found three Alu elements (AluSz, AluJr, and AluSz6) in the introns flanking the upstream and downstream splice sites, with forward Alu Sz in the upstream intron and reverse AluJr and Alu Sz6 in the downstream intron, and their sequences were highly reverse complementary (Figure 4l, Figure 4 and Data S5, green framebox, Supporting Information). Flanking introns containing repeat Alu elements play an important role in circRNA biogenesis.^[^ ^12^, ^21^, ^22^ ^]^ DExH‐box helicase 9 (DHX9), an important RNA helicase, decreases the biogenesis of circRNAs by binding to the inverted repeat Alu (IRAlu) sequences located in the flanking introns to loosen IRAlu pairs.^[^ ^38^ ^]^ We then explored whether circPRELID2 biogenesis was partially regulated by these IRAlu elements. As expected, knockdown of DHX9 with two independent shRNAs markedly increased circPRELID2 expression in AGS cells (Figure 4n). To further verify the synergistic role of the DYRK1A‐SFPQ‐SAM68 complex and inverted‐repeat Alu elements in circPRELID2 biogenesis, wild‐type (spanning from intron 2 to intron 6 of the *PRELID2* gene) and a series of deletion constructs (group #1– #8) of the *PRELID2* minigene were individually cloned into pZW1 expression vector (Figure 4o). After separate transfection with these nine constructs in AGS cells, qRT‐PCR analysis showed that circPRELID2 was produced in clones where flanking IRAlus were formed, including the wild‐type (Figure 4p, group WT), and in which Alu deletions retained a pair of IRAlus formed across flanking introns (Figure 4p, group #2). In contrast, circPRELID2 was not detected in clones where deletions eliminated IRAlu pairing across flanking introns (Figure 4p, groups #1, #3, and #4). Interestingly, deletion of AluJr facilitated circPRELID2 formation, indicating that AluJr plays an inhibitory role in circPRELID2 circularization, potentially through competitive pairing with AluSz6 (Figure 4p, group #2). In addition, deletion of the SAM68‐binding sites in the upstream flanking intron significantly reduced the expression of circPRELID2 (Figure 4p, group #5). Moreover, deletion of the first cluster of downstream SAM68‐binding sites moderately inhibited circPRELID2 production (Figure 4p, group #6), whereas deletion of the second cluster of downstream SAM68‐binding sites completely abolished circPRELID2 formation (Figure 4p, groups #7, and #8) compared to that of the WT construct, indicating that in addition to IRAlu pairing, the binding of DYRK1A‐SFPQ‐SAM68 complex to pre‐*PRELID2* played important roles in circPRELID2 formation, probably through inhibiting competitive pairing between AluJr and AluSz6 or bringing back‐splicing sites into close proximity via dimerization of upstream and downstream SAM68. These results indicated that the DYRK1A‐SFPQ‐SAM68 complex binds to Alu‐containing introns flanking the circPRELID2‐forming exons in *PRELID2* pre‐mRNA to promote circPRELID2 circularization.

### 2.6 CircPRELID2 Promotes the Epithelial–Mesenchymal Transition (EMT) and Metastasis of GC Cells In Vitro and In Vivo Under Hypoxic Conditions

To investigate the role of circPRELID2 in GC progression, we first detected its endogenous expression in normal human gastric epithelial cell line (GES‐1) and six GC cell lines, and found that compared to GES‐1 cells, circPRELID2 expression was significantly upregulated in these GC cell lines, with HGC‐27 showing the highest expression and AGS/MKN‐45 the lowest (Figure S5a, Supporting Information). Therefore, we selected HGC‐27 and AGS cell lines to explore the downstream functions and mechanisms of circPRELID2. Furthermore, two shRNAs specifically targeting the back‐splice junction region of circPRELID2 were designed. shcircPRELID2‐1 and shcircPRELID2‐2 successfully knocked down circPRELID2, but did not alter *PRELID2* mRNA levels in HGC‐27 cells (Figure S5b, Supporting Information). Full‐length circPRELID2 was cloned into the pLC5‐ciR circRNA lentiviral vector, and circPRELID2 overexpression did not alter the expression of *PRELID2* mRNA in AGS cells (Figure S5c, Supporting Information). These results indicated that the knockdown or overexpression of circPRELID2 had no effect on the expression of the host gene *PRELID2*.

In vitro studies showed that circPRELID2 knockdown significantly inhibited the EMT phenotypes induced by hypoxia, while having almost no effect on cell proliferation (Figure S5d,e, Supporting Information). Moreover, we found that circPRELID2 silencing markedly suppressed hypoxia‐facilitated migration and invasion of HGC‐27 cells (Figure S5f,g, Supporting Information). And hypoxia resulted in a significant increase in ZEB2, N‐Cadherin and Vimentin protein levels, but reduced E‐Cadherin level, which could be counteracted by circPRELID2 knockdown (Figure S5h, Supporting Information). Conversely, circPRELID2 overexpression promoted the migration and invasion of AGS cells induced by hypoxia (Figure S5i,j, Supporting Information). And overexpression of circPRELID2 significantly amplified the hypoxia‐induced upregulation of ZEB2, N‐Cadherin and Vimentin protein levels, while promoting a decrease in E‐Cadherin levels (Figure S5k, Supporting Information).

**Figure 5:**
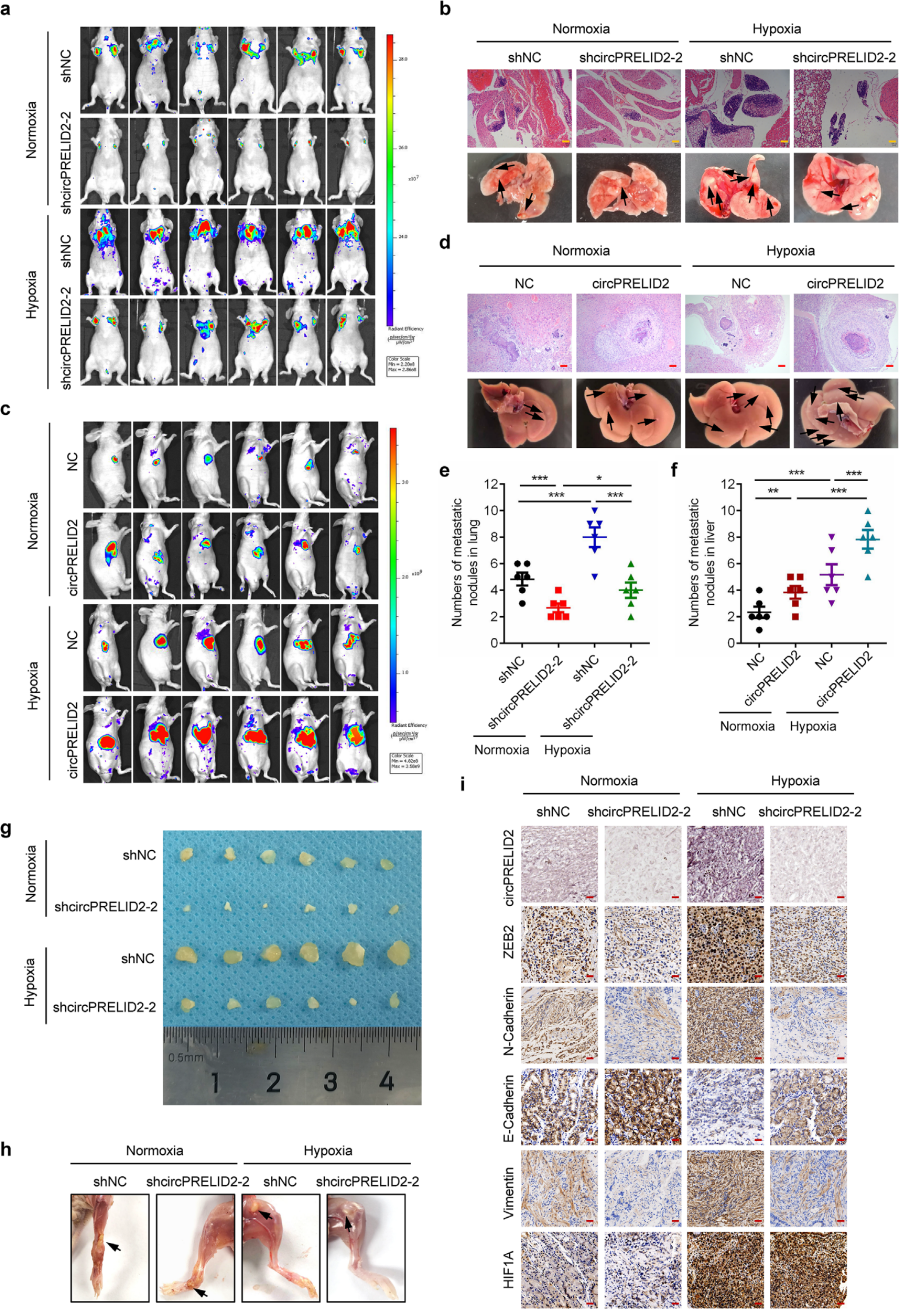
CircPRELID2 promotes metastasis of gastric cancer in vivo under hypoxic conditions. a,b) The lung metastasis capacity of HGC‐27‐shNC and shcircPRELID2 cells was evaluated in lung metastasis mouse model generated under normoxic or hypoxic conditions. Representative bioluminescent images of the lungs in these four groups kept in normoxia or hypoxia were shown (a). Representative photographs of lung metastatic nodules and HE staining were shown (b), scale bar = 100 µm. c,d) A liver metastasis model was generated under normoxia or hypoxia to assess the liver metastasis capacity of AGS cells stably overexpressing NC or circPRELID2, respectively. Representative bioluminescent images of livers in these four groups kept in normoxia or hypoxia were shown (c). Representative pictures of liver metastatic nodules and HE staining were shown (d), scale bar = 100 µm. e,f) The number of metastatic nodules formed in the lungs (a) or livers (c) of nude mice, maintained under normoxic or hypoxic conditions, was shown for these groups following the knockdown or overexpression of circPRELID2. g,h) Lymph node (LN) metastasis capacity of HGC‐27‐shNC or shcircPRELID2 cells was detected after the injection of these cells into the footpad region of the hind limb of BALB/c nude mice maintained under normoxic or hypoxic conditions. Representative images of LN metastatic nodules in these four groups were shown in (g). Representative photographs of the hind limb with LN metastatic nodules were shown (h). i) ISH and IHC staining showing the expression of circPRELID2, ZEB2, N‐Cadherin, Vimentin, E‐Cadherin, and HIF1A in the metastatic nodules generated by HGC‐27‐shNC or shcircPRELID2 cells in the lymph node metastasis mouse model maintained under normoxic or hypoxic conditions. Scale bar = 50 µm. Data were shown as the mean ± SD, *p < 0.05, **p < 0.01, ***p < 0.001.

In addition, we explored the in vivo role of circPRELID2 in GC cells metastasis and found that hypoxia significantly enhanced the lung metastasis capacity of HGC‐27 cells in mouse models (**Figure** **5**a,b,e), an effect abolished by circPRELID2 depletion. Similar results were observed in lymph node metastasis models under hypoxia (Figure 5g,h). Conversely, circPRELID2 overexpression markedly enhanced the metastasis capability of AGS cells in a liver metastasis mouse model generated under normoxic or hypoxic conditions (Figure 5c,d,f). Furthermore, metastatic lesions generated by HGC‐27 cells in the lymph node metastatic mouse model maintained under hypoxic conditions exhibited elevated ZEB2 and reduced E‐Cadherin protein levels compared to those in normoxic conditions, which could be counteracted by circPRELID2 silencing (Figure 5i). Taken together, these data demonstrated that circPRELID2 promotes the EMT and metastasis of GC cells in vitro and in vivo under hypoxic conditions.

### 2.7 CircPRELID2 Interacts with Polyc‐binding Protein 1 (PCBP1) and Promotes its Cytoplasmic Retention

**Figure 6:**
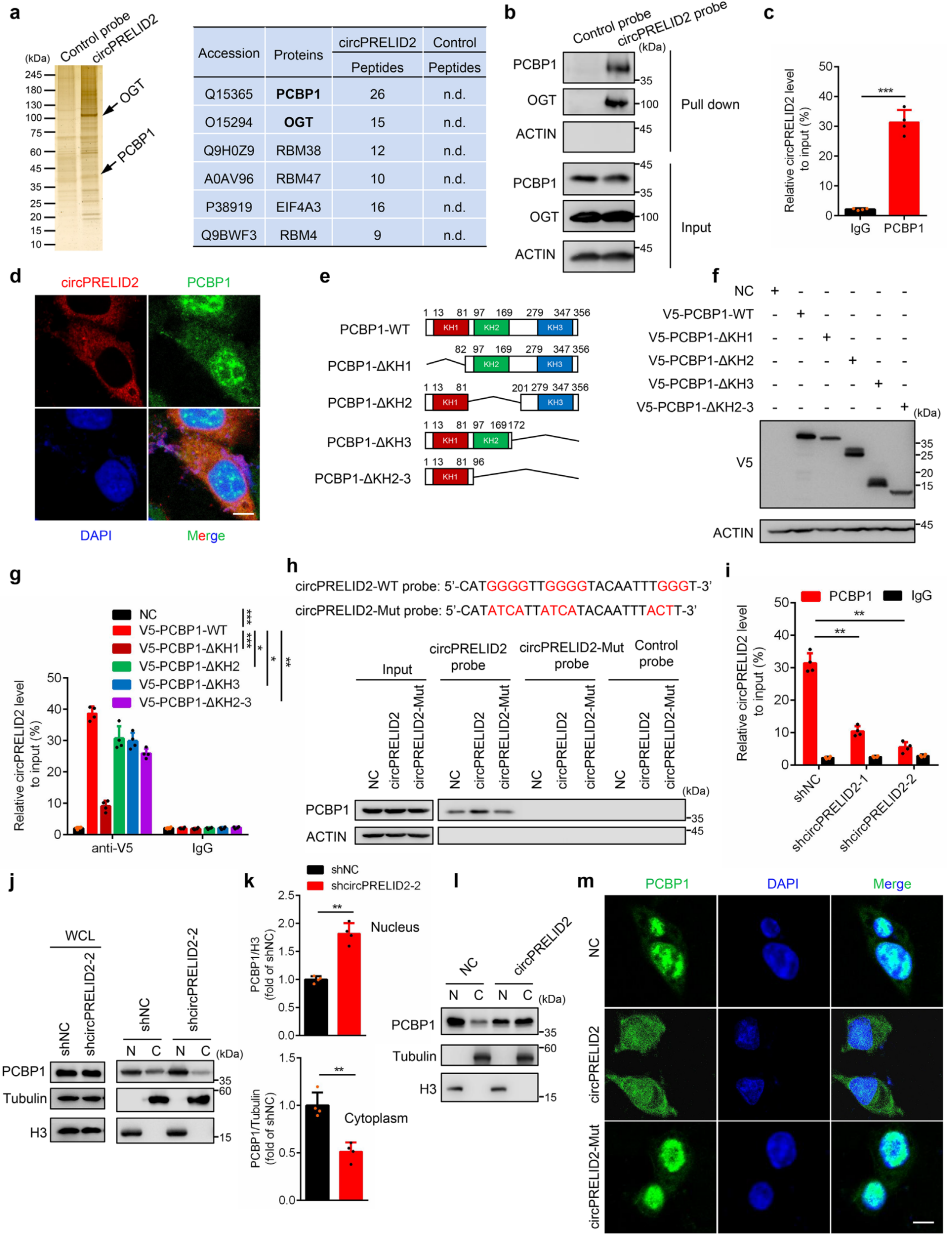
CircPRELID2 interacts with polyC‐binding protein 1 (PCBP1) and promotes its cytoplasmic retention. a) RNA pull‐down assays were performed to examine the functional proteins pulled down by control or circPRELID2 probes in HGC‐27 cells stably overexpressing circPRELID2. The immunoprecipitated proteins were analyzed through silver staining and mass spectrometry. b) The specific binding of PCBP1 or OGT to circPRELID2 in HGC‐27 cells was verified via RNA pull‐down assay. c) RIP/qRT‐PCR assays were performed to detect the interaction between circPRELID2 and PCBP1 in AGS cells. d) RNA FISH‐immunofluorescence analysis of the colocalization of circPRELID2 and PCBP1 in HGC‐27 cells; scale bar = 20 µm. e) Schematic diagram showing PCBP1‐WT and its KH truncations. All constructs were V5‐tagged at the C‐terminus. f,g) HGC‐27 cells were transfected with V5‐PCBP1 or its KH truncations. After 48 h of transfection, RIP assay was performed to examine the binding of PCBP1 to circPRELID2. h) Top, the sequences of circPRELID2‐WT or Mut probe used for RNA pull‐down assays. Bottom, RNA pull‐down assays were performed in AGS cells transfected with NC, circPRELID2‐WT or circPRELID2‐Mut to detect the interaction between circPRELID2 and PCBP1. i) RIP/qRT‐PCR assays were performed to examine the specific interaction between PCBP1 and circPRELID2 in HGC‐27‐shNC and shcircPRELID2 cells. j,k) Nuclear/cytoplasmic fractionation assays were performed to detect the protein level of PCBP1 in the cytoplasm (C) and nucleus (N) of HGC‐27 cells after knockdown of circPRELID2. WCL, whole‐cell lysate. l) The distribution of nuclear (N) and cytoplasmic (C) PCBP1 in AGS cells stably overexpressing circPRELID2 was analyzed. m) Immunofluorescence analysis of PCBP1 localization in AGS cells stably overexpressing NC, circPRELID2‐WT, or circPRELID2‐Mut. Scale bar = 20 µm. Data were shown as the mean ± SD; *p < 0.05, **p < 0.01, ***p < 0.001.

To further explore the potential molecular mechanisms by which circPRELID2 regulates GC metastasis, we initially predicted the potential targets of circPRELID2 using miRNA target prediction tools, including circBank, circInteractome, CSCD and miRDB. After intersecting the results from these prediction tools, no overlapping targets were identified (Figure S6a, Supporting Information). Furthermore, our RIP experiments demonstrated that AGO2 does not interact with circPRELID2, and similar results were obtained in RNA pull‐down assays. These findings indicate that circPRELID2 cannot function as a miRNA sponge (Figure S6b,c, Supporting Information). To investigate whether circPRELID2 exerts its function by interacting with proteins, we performed RNA pull‐down assays and mass spectrometry analysis to identify circPRELID2‐interacting proteins (**Figure** **6**a). We speculated that the most abundant protein, PCBP1, is the putative circPRELID2‐binding protein. We next conducted RNA pull‐down assays and demonstrated that PCBP1 interacted with circPRELID2 in HGC‐27 cells (Figure 6b). RIP assays further confirmed that circPRELID2 was enriched in PCBP1‐immunoprecipitated complexes compared to control IgG samples (Figure 6c). In addition, RNA FISH immunofluorescence analysis showed that circPRELID2 and PCBP1 colocalized in the cytoplasm of GC cells (Figure 6d). Collectively, these results indicated that circPRELID2/PCBP1 forms an RNA–protein complex in the cytoplasm.

The RNA‐binding protein PCBP1, a tumor suppressor that is mainly localized in the nucleus and cytoplasm, primarily contains triple hnRNP K homology (KH) domains which are critical for binding RNA.^[^ ^39^ ^]^ To determine the KH domain/s of PCBP1 responsible for the interaction with circPRELID2, we constructed several PCBP1 KH domain truncations as follows: KH1 domain deletion (ΔKH1), KH2 domain deletion (ΔKH2), KH3 domain deletion (ΔKH3), and KH2/KH3 domain double deletion (ΔKH2‐3) (Figure 6e). RIP assays showed that KH1 was the main domain of PCBP1 that recruited circPRELID2 (Figure 6f,g). PCBP1, also known as polyC binding protein 1, is characterized by its high affinity and sequence‐specific interaction with the poly‐cytosine (poly‐C) region of RNA, ssDNA, and dsDNA.^[^ ^39^, ^40^ ^]^ Known PCBP1‐binding sequences in targets such as P63, Sortilin, Androgen receptor, P21 and BAT^[^ ^41^, ^42^, ^43^, ^44^, ^45^ ^]^ were listed (Figure S6d, Supporting Information). Surprisingly, by browsing the exon 5−exon 2 junction sequence in circPRELID2, we found that the 5′‐A**CCC**AAAUUGUA**CCCC**AA**CCCC**AUG‐3′ sequence located at the back‐splicing junction site is a putative binding motif of PCBP1 (Figure S6d, Supporting Information). Therefore, we constructed a circPRELID2‐Mut plasmid with mutations in the polyC sites, hereafter referred to as circPRELID2‐Mut (Figure S6e, Supporting Information). RNA pull‐down assays demonstrated that wild‐type circPRELID2, but not circPRELID2‐Mut, could interact with PCBP1 (Figure 6h). The specificity of this interaction was further validated by the reduced enrichment ability of PCBP1 in HGC‐27 cells following the knockdown of circPRELID2 (Figure 6i).

Subsequently, we examined the potential effects of circPRELID2 on PCBP1 function and found that circPRELID2 silencing reduced cytoplasmic PCBP1 levels and induced its nuclear translocation, whereas circPRELID2 overexpression significantly enhanced PCBP1 cytoplasmic retention (Figure 6j–l). Immunofluorescence analysis further confirmed that wild‐type circPRELID2, but not the circPRELID2‐Mut, promoted PCBP1 cytoplasmic localization in AGS cells (Figure 6m). Collectively, these data demonstrated that the binding of circPRELID2 to PCBP1 results in PCBP1 retention in the cytoplasm.

### 2.8 circPRELID2 Enhances OGT‐Mediated PCBP1 O‐GlcNAcylation

**Figure 7:**
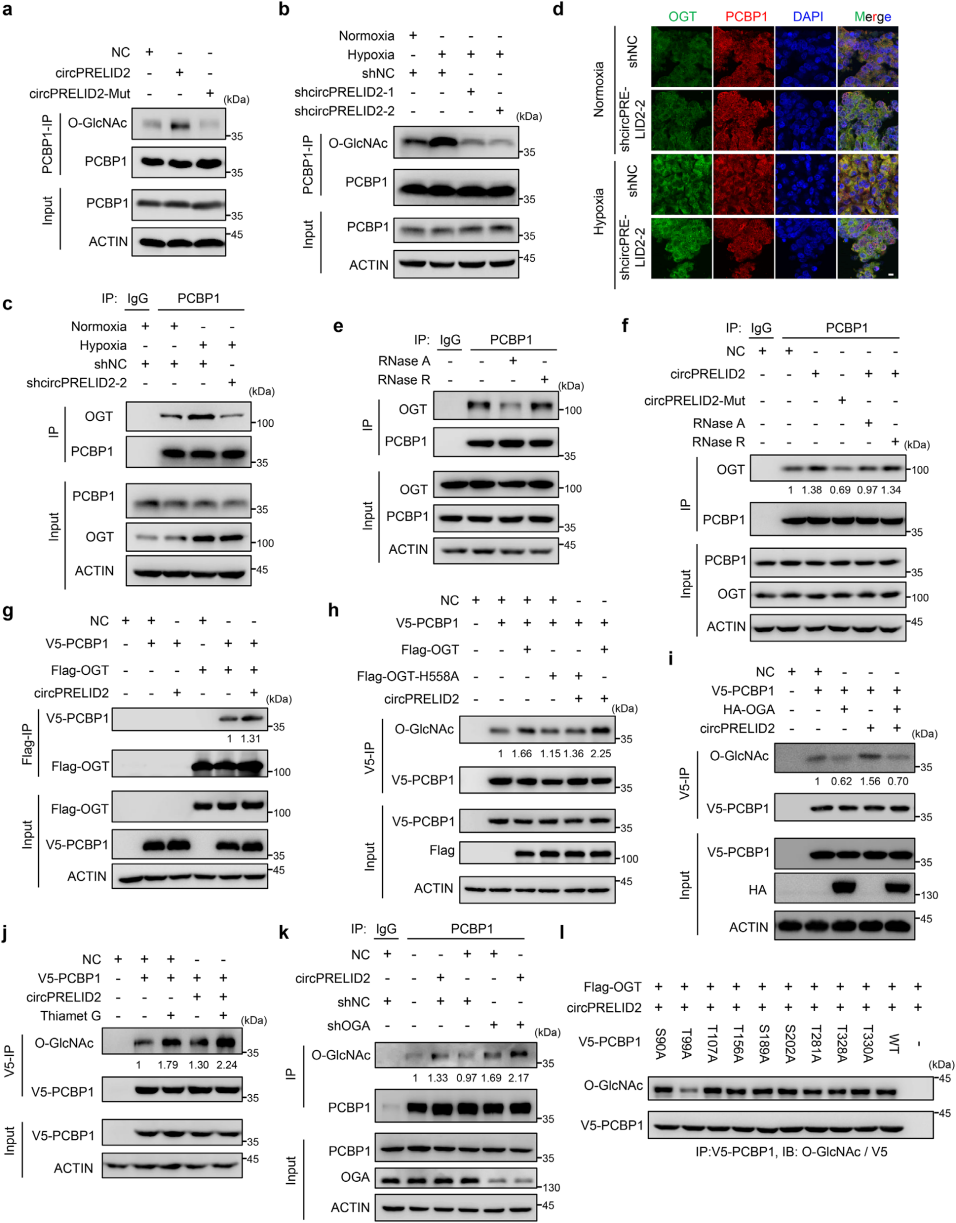
CircPRELID2 enhances OGT‐mediated PCBP1 O‐GlcNAcylation. a) Lysates from AGS cells transfected with NC, circPRELID2‐WT, or circPRELID2‐Mut plasmids were subjected to immunoprecipitation (IP) and immunoblotting analysis. b) Lysates from HGC‐27‐shNC or shcircPRELID2 cells treated with normoxia or hypoxia for 24 h were immunoprecipitated with anti‐PCBP1 antibody followed by immunoblotting with indicated antibody. c) Lysates from HGC‐27‐shNC or shcircPRELID2 cells treated with normoxia or hypoxia were subjected to IP and immunoblotting analysis. d) Immunofluorescence analysis of PCBP1 and OGT localization was performed in metastatic nodules from the four groups constructed in Figure 5g. Scale bar = 20 µm. e) Lysates from HGC‐27 cells were treated with or without RNase A or RNase H and subjected to IP and immunoblotting analyses with the indicated antibodies. f) Lysates from AGS cells transfected with NC, circPRELID2‐WT, or circPRELID2‐Mut were treated with or without RNase A or RNase H and subjected to IP and immunoblotting analyses with the indicated antibodies. g) HEK293T cells were co‐transfected with V5‐PCBP1, Flag‐OGT, and/or circPRELID2 as indicated for 48 h, followed by IP and immunoblotting analysis with the indicated antibodies. h) V5‐PCBP1 was affinity‐purified from AGS cells co‐transfected with V5‐PCBP1 and/or Flag‐OGT, Flag‐OGT‐H558A, and circPRELID2 as indicated for 48 h. Immunoprecipitates were probed with O‐GlcNAc antibody. i) V5‐PCBP1 was co‐transfected with HA‐OGA and/or circPRELID2 into HEK293T cells as indicated for 48 h. Lysates were immunoprecipitated with anti‐V5 antibody, followed by western blotting as indicated. j) AGS cells were transfected with V5‐PCBP1 and/or circPRELID2. After 24 h of transfection, cells were treated with or without 15 µM Thiamet G for 24 h and subjected to IP and western blot analysis. k) AGS‐shNC and shOGA cells transfected with NC or circPRELID2 were lysed and immunoprecipitated with normal IgG or with anti‐PCBP1 antibodies, followed by immunoblotting analysis. l) V5‐tagged wild‐type or different PCBP1 mutants was co‐transfected with Flag‐OGT and circPRELID2 into HEK293T cells for 48 h. Lysates were analyzed as indicated.

We observed that OGT, the only known enzyme that catalyzes the O‐GlcNAcylation of intracellular proteins until now,^[^ ^46^ ^]^ ranked second among circPRELID2‐binding proteins in mass spectrometry (Figure 6a). And RNA pull‐down assays demonstrated that circPRELID2 interacted with OGT (Figure 6b), which is consistent with the observation that OGT can bind to RNA molecules.^[^ ^47^ ^]^ Moreover, bioinformatics analysis of the bioGRID interaction database (https://thebiogrid.org/) and the O‐GlcNAc database (https://oglcnac.org/) showed that OGT is the potential PCBP1‐binding protein. Intriguingly, we found that circPRELID2 significantly increased O‐GlcNAcylation levels in GC cells, an effect that was abrogated by circPRELID2‐Mut (Figure S7a, Supporting Information). To investigate whether OGT mediates PCBP1 O‐GlcNAcylation and whether circPRELID2 is involved in this process, we ectopically expressed circPRELID2‐WT or circPRELID2‐Mut in AGS cells and performed in vivo O‐GlcNAcylation assay. As shown in **Figure** **7**a, PCBP1 could be modified by O‐GlcNAcylation in AGS cells, and circPRELID2‐WT, but not circPRELID2‐Mut, specifically increased the O‐GlcNAcylation level of PCBP1. Likewise, circPRELID2 depletion markedly decreased PCBP1 O‐GlcNAcylation levels in HGC‐27 cells (Figure S7b,c, Supporting Information). OGT overexpression elevated PCBP1 modification, an effect abolished by circPRELID2 depletion (Figure S7c, Supporting Information). OGT silencing could mitigate the increase in PCBP1 O‐GlcNAcylation caused by circPRELID2 overexpression in AGS cells (Figure S7d, Supporting Information). To examine whether hypoxia contributes to the induction of circPRELID2‐enhanced O‐GlcNAcylation of PCBP1, we depleted circPRELID2 in HGC‐27 cells using two specific shRNAs and cultured these cells under normoxia or hypoxia for 24 h, finding that hypoxia promoted PCBP1 O‐GlcNAcylation, which which was attenuated by circPRELID2 silencing (Figure 7b). These results suggested that circPRELID2 was involved in the OGT‐mediated O‐GlcNAcylation of PCBP1.

CircRNAs have been demonstrated to function as scaffolds for target regulation.^[^ ^48^ ^]^ To determine whether circPRELID2 acts as a scaffold to enhance the binding of OGT to PCBP1, co‐immunoprecipitation assays revealed that circPRELID2 knockdown notably reduced the interaction between OGT and PCBP1 at the endogenous protein level in HGC‐27 cells, and circPRELID2 was involved in the hypoxia‐induced binding of OGT to PCBP1 (Figure 7c; Figure S7e, Supporting Information). Moreover, immunofluorescence ofmetastatic nodules generated in Figure 5g showed hypoxia promoted PCBP1 cytoplasmic localization and OGT interaction–an effect reversed by circPRELID2 silencing (Figure 7d). Subsequently, we found that RNase A endonuclease, but not RNase R exonuclease, severely disrupted the interaction between endogenous OGT and PCBP1 because of the resistance of circPRELID2 to RNA exonucleases (Figure 7e). Furthermore, the association between endogenous OGT and PCBP1 was significantly enhanced in AGS cells overexpressing circPRELID2‐WT, rather than circPRELID2‐Mut, which could be reversed by treatment with RNase A, but not with RNase R (Figure 7f). Additionally, overexpressed Flag‐OGT effectively pulled down V5‐PCBP1, which was significantly facilitated by circPRELID2 overexpression (Figure 7g). These results indicated that circPRELID2 promotes the interaction between OGT and PCBP1.

Next, we investigated the role of OGT in PCBP1 O‐GlcNAcylation. Ectopic expression of wild‐type OGT, but not its catalytically inactive mutant (H558A),^[^ ^49^ ^]^ increased the O‐GlcNAcylation of exogenously expressed PCBP1 in AGS cells, an effect potentiated by circPRELID2 (Figure 7h). O‐GlcNAcylation is a dynamic and reversible process, and O‐GlcNAcase (OGA) is able to remove the O‐GlcNAcylation modification of target proteins.^[^ ^46^, ^49^ ^]^ We found that ectopic expression of OGA significantly reduced circPRELID2‐facilitated PCBP1 O‐GlcNAcylation (Figure 7i), whereas the OGA inhibitor Thiamet G^[^ ^50^ ^]^ elevated it (Figure 7j). Consistently, OGA knockdown similarly amplified circPRELID2‐induced PCBP1 O‐GlcNAcylation (Figure 7k). To identify the O‐GlcNAcylation site(s) on PCBP1, nine candidate serine or threonine (Ser/Thr) sites on PCBP1 including Ser90, Thr99, Thr107, Thr156, Ser189, Ser202, Thr281, Thr328, and Thr330 predicted using the YinOYang1.2, NetOGlyc4.0, ISOGlyP and O‐GlcNAc databases, were individually mutated to alanine (A) for O‐GlcNAcylation identification. The O‐GlcNAcylation assays revealed that the mutation of Thr99, but not the other eight residues, resulted in a decrease in PCBP1 O‐GlcNAcylation level, indicating that Thr99 was the primary, albeit not the only, site of PCBP1 O‐GlcNAcylation (Figure 7l). Sequence alignment also revealed that the Thr99 residue on PCBP1 was highly conserved across species (Figure S8a, Supporting Information). These results suggested that circPRELID2 acts as a scaffold to enhance the interaction between OGT and PCBP1, subsequently facilitating OGT‐mediated PCBP1 O‐GlcNAcylation at Thr99.

### 2.9 CircPRELID2‐Enhanced PCBP1 O‐GlcNAcylation Promotes ZEB2 Translation

Numerous studies have indicated that PCBP1, as a tumor suppressor that inhibits tumorigenesis and metastasis, is involved in the transcription, alternative splicing, and translation of many tumor‐associated genes, ferroptosis and autophagy.^[^ ^39^, ^40^ ^]^ Given that in the cytoplasm, circPRELID2 facilitated OGT‐mediated PCBP1 O‐GlcNAcylation, we wondered whether O‐GlcNAcylation of PCBP1 affects its cytoplasmic functions, particularly in translation regulation. To identify the potential target mRNAs which are translationally regulated by PCBP1, we analyzed differentially expressed mRNA transcripts that specifically bind to PCBP1 and exhibited coordinated translational upregulation following PCBP1 depletion based on the GEO database (GSE40466) (Figure S8b, Supporting Information). In addition to a number of reported mRNA transcripts, such as ILEI, EGFR, MOESIN, FAM3C, JAK2 and EIF5A2,^[^ ^44^, ^51^, ^52^ ^]^ we identified ZEB2, an EMT‐related transcription factor that is vital for tumor metastasis,^[^ ^53^ ^]^ as a potential transcript regulated by PCBP1 at the translational level. To identify the genuine downstream target(s), we established stable AGS cells overexpressing circPRELID2‐WT or circPRELID2‐Mut. Among these candidates, only ZEB2 and FAM3C protein levels were significantly elevated by circPRELID2‐WT, not by circPRELID2‐Mut (Figure S8c, Supporting Information). To further examine whether PCBP1 O‐GlcNAcylation affects ZEB2 and FAM3C translation, we knocked down OGT in AGS cells and found that OGT depletion significantly reduced ZEB2 protein levels without impacting FAM3C (Figure S8d, Supporting Information). Furthermore, PCBP1 overexpression notably decreased ZEB2 protein levels, while having no effect on *ZEB2* mRNA levels (Figure S8e, Supporting Information). Therefore, we sought to focus on ZEB2 for further study.

**Figure 8:**
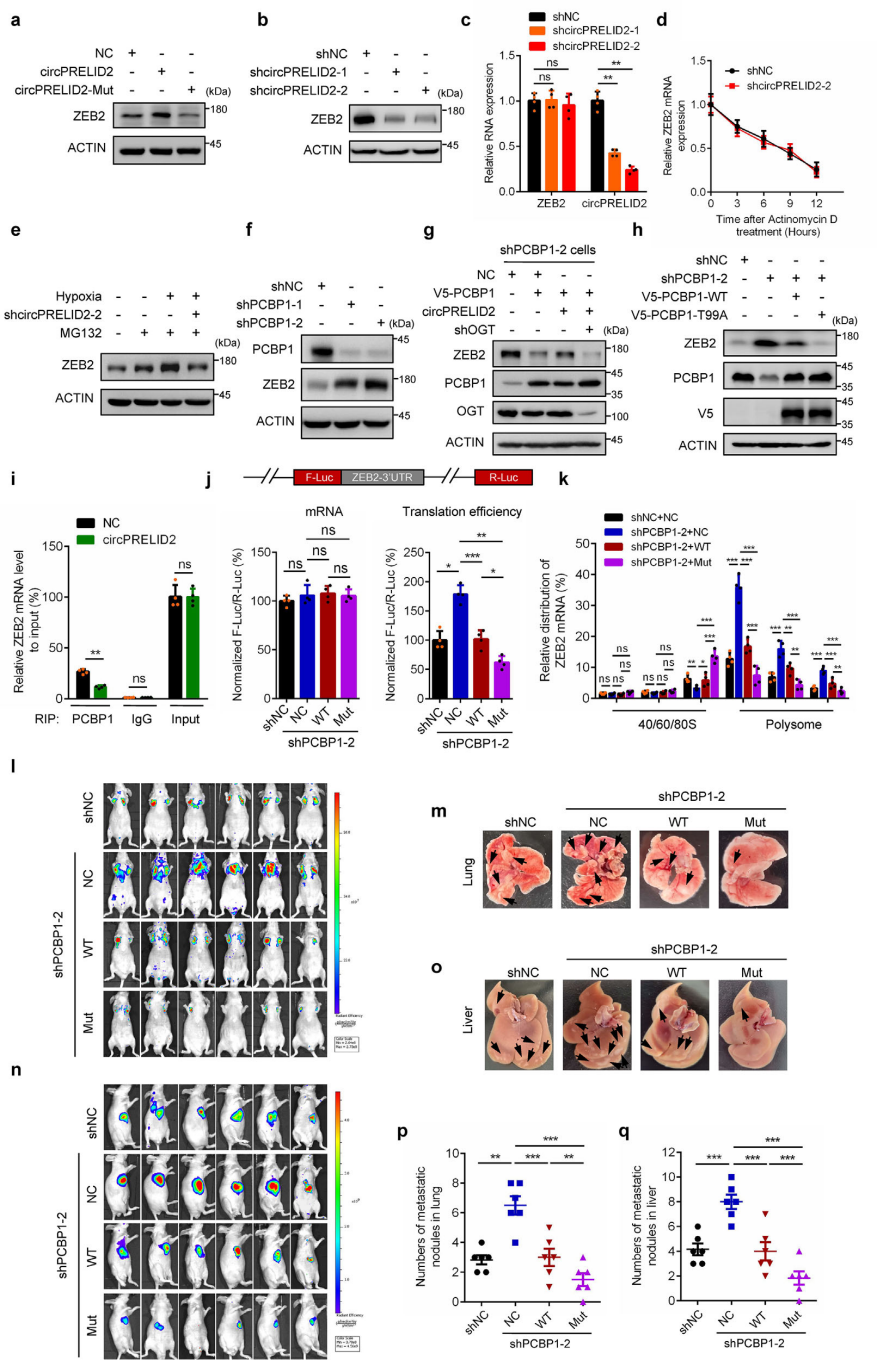
CircPRELID2‐enhanced PCBP1 O‐GlcNAcylation promotes ZEB2 translation. a) Western blotting analysis of ZEB2 protein levels in AGS cells transfected with NC, circPRELID2‐WT, or circPRELID2‐Mut plasmids. b) Immunoblotting analysis showing ZEB2 protein levels in HGC‐27‐shNC or shcircPRELID2 cells. c) qRT‐PCR analysis of the expression of *ZEB2* mRNA upon circPRELID2 knockdown in HGC‐27 cells. d) HGC‐27‐shNC and shcircPRELID2 cells were treated with actinomycin D for the indicated time. And *ZEB2* mRNA level was examined via qRT‐PCR. e) HGC‐27‐shNC and shcircPRELID2 cells cultured under normoxia or hypoxia were treated with or without 15 µM MG132 for 6 h, followed by immunoblotting analysis. f) Immunoblotting analysis of ZEB2 and PCBP1 protein levels in HGC‐27 cells after PCBP1 knockdown. g) V5‐PCBP1 was co‐transfected with circPRELID2 into PCBP1‐silenced AGS cells infected with shNC or shOGT lentivirus as indicated. And ZEB2 protein level was then detected. h) Immunoblotting analysis of ZEB2 protein levels in PCBP1‐silenced HGC‐27 cells after stably re‐expressing V5‐PCBP1‐WT or V5‐PCBP1‐T99A. i) RIP/qRT‐PCR analysis of the binding abilities of PCBP1 and *ZEB2* mRNA in AGS cells stably overexpressing NC or circPRELID2. j) Top, schematic illustration showing *ZEB2*‐3′‐UTR luciferase reporter vector. Bottom‐left, qRT‐PCR analysis showing the mRNA abundance of firefly luciferase (F‐Luc) and Renilla luciferase (R‐Luc). Bottom‐right, translation efficiency was determined as the relative ratio of luciferase intensities divided by the relative mRNA level in PCBP1‐depleted HGC‐27 cells after stably re‐expressing V5‐PCBP1‐WT or V5‐PCBP1‐T99A. k) Polysomes were fractionated through sucrose gradients, and the relative enrichment of *ZEB2* mRNA in ribosome fractions was analyzed via qRT‐PCR. l,m) The lung metastasis capacity of HGC‐27 cells generated in (h) was detected. Representative bioluminescent images of the lungs in these four groups were shown (l). Representative photographs of lung metastatic nodules were shown (m). n,o) The liver metastasis capacity of HGC‐27 cells generated in (h) was assessed. Representative bioluminescent photographs of the livers in the four groups were shown (n). Representative images of metastatic nodules in the livers were shown (o). The black arrows pointed to metastatic nodules. p,q) The number of metastatic nodules formed in the lungs (p) or livers (q) of nude mice in these groups from (l) and (n) was shown, respectively. Data are shown as the mean ± SD, ns, no significance, *p < 0.05, **p < 0.01, ***p < 0.001.

In addition, we validated that ectopically expressed circPRELID2‐WT, but not circPRELID2‐Mut, markedly elevated ZEB2 protein levels in GC cells (**Figure** **8**a; Figure S8f, Supporting Information). Moreover, circPRELID2 knockdown significantly reduced ZEB2 protein levels without affecting *ZEB2* mRNA levels (Figure 8b,c; Figure S8g, Supporting Information). To determine whether this effect was due to the increase in *ZEB2* mRNA stability, HGC‐27‐shNC and shcircPRELID2 cells were treated with actinomycin D for various durations. We found that the half‐life of *ZEB2* mRNA remained unchanged after circPRELID2 knockdown compared to control cells (Figure 8d). Furthermore, ZEB2 protein levels accumulated in HGC‐27 cells treated with the proteasome inhibitor MG132, suggesting that ZEB2 degradation primarily relies on the proteasomal pathway. However, MG132 did not reverse the reduction in ZEB2 protein levels caused by circPRELID2 knockdown under hypoxic conditions, excluding circPRELID2's role in ZEB2 stability regulation (Figure 8e). Additionally, silencing of PCBP1 resulted in a reduction of ZEB2 protein levels in HGC‐27 cells (Figure 8f), which is consistent with the findings in Figure S8e (Supporting Information). Moreover, PCBP1 overexpression led to a significant reduction in ZEB2 protein levels, which could be rescued by circPRELID2 overexpression. Conversely, OGT knockdown reversed the increase in ZEB2 protein levels induced by circPRELID2 (Figure 8g). We also found that, compared to wild‐type PCBP1, PCBP1‐T99A exhibited a significantly greater ability to inhibit ZEB2 translation in PCBP1‐depleted HGC‐27 cells (Figure 8h). These data revealed that PCBP1 suppressed ZEB2 translation, while circPRELID2 and OGT synergistically mediated PCBP1 O‐GlcNAcylation at Thr99 could counteract this inhibition.

Studies have reported that PCBP1, a classical RNA‐binding protein, binds to specific transcripts and has a direct role in translation process.^[^ ^39^, ^40^, ^45^, ^54^ ^]^ To gain mechanistic insights into the regulation of ZEB2 translation by PCBP1 O‐GlcNAcylation, we performed RIP assays and found that PCBP1 interacted with *ZEB2* mRNA. Notably, circPRELID2 overexpression decreased PCBP1's binding affinity to ZEB2 mRNA (Figure 8i). Next, we constructed a pGL3‐*ZEB2* 3′‐UTR luciferase reporter vector to explore whether PCBP1 O‐GlcNAcylation mediates the translation of ZEB2. The dual‐luciferase reporter assay revealed significantly reduced luciferase activity in PCBP1‐depleted GC cells after overexpressing the O‐GlcNAcylation‐deficient PCBP1‐T99A compared to wild‐type PCBP1, with no change in the mRNA levels of firefly (F‐Luc) and Renilla luciferase (R‐Luc) (Figure 8j), demonstrating that the O‐GlcNAcylation site Thr99 in PCBP1 is critical for enhancing ZEB2 translation efficiency. Next, we purified ribosomes, and found that PCBP1 knockdown resulted in the elevated *ZEB2* mRNA levels in translation‐active polysomes (> 80S), based on ribosome profiling. Additionally, we found that ZEB2 mRNA enrichment in polysome fractions significantly decreased with the enforced expression of PCBP1‐T99A in PCBP1‐depleted HGC‐27 cells compared to wild‐type PCBP1 (Figure 8k). We also investigated the impact of PCBP1 O‐GlcNAcylation on GC cell metastasis. PCBP1 knockdown significantly enhanced tumor metastasis compared to control cells, as shown in lung (Figure 8l,m,p) and liver (Figure 8n,o,q) metastasis models. Furthermore, PCBP1‐depleted HGC‐27 cells re‐expressing PCBP1‐T99A exhibited reduced metastatic capacity and fewer metastatic nodules compared to those re‐expressing wild‐type PCBP1 in both lung (Figure 8l,m,p) and liver (Figure 8n,o,q) metastasis models.

To examine whether the O‐GlcNAcylation modification of PCBP1 is also involved in other processes such as transcription, splicing, ferroptosis and autophagy, we sought to compare the effects of reintroducing wild‐type PCBP1 or PCBP1‐T99A in PCBP1‐depleted HGC‐27 cells on these processes. These results showed that there were no significant differences in the regulation of eIF4E transcription, CDK2 exon 5 splicing, autophagy and ferroptosis between wild‐type PCBP1 and PCBP1‐T99A (Figure S9, Supporting Information).

To further investigate the clinical relevance of circPRELID2/OGT/PCBP1/ZEB2 axis, we performed ISH and immunohistochemistry (IHC) staining on gastric cancer tissue microarray (TMA) aforementioned. We found that circPRELID2, OGT and ZEB2 were markedly upregulated, while PCBP1 expression was significantly decreased in GC tissues compared to adjacent normal tissues (Figure S10a,b, Supporting Information). More importantly, the expression of ZEB2 was significantly positively correlated with circPRELID2 or OGT, but negatively correlated with PCBP1 expression (Figure S10c, Supporting Information), supporting that ZEB2 was clinically associated with circPRELID2, OGT and PCBP1 in GC tissues. Taken together, these data suggested that the O‐GlcNAcylation of PCBP1 at Thr99 mediated by OGT disrupts its binding to the 3′‐UTR of *ZEB2*, reversing ZEB2 translation silencing and promoting GC metastasis.

## 3 Discussion

Hypoxia is a prevalent microenvironmental characteristics of solid tumors, which contributes to angiogenesis, cell survival, recurrence, as well as invasion and metastasis by regulating target genes and non‐coding RNAs.^[^ ^6^, ^7^, ^55^ ^]^ So far, numerous lncRNAs and miRNAs modulated by HIF1A have been implicated in cellular adaptation to hypoxia.^[^ ^56^, ^57^ ^]^ However, whether circRNAs participate in hypoxia‐mediated aggressive behaviors in malignancies, particularly in GC, remains poorly understood. Here, we identified a novel hypoxia‐related circRNA, circPRELID2, derived from *PRELID2* gene, which was markedly upregulated in GC tissues and hypoxic GC cells. CircPRELID2 significantly correlated with lymph node invasion, vascular invasion of GC patients, and facilitated the EMT and metastasis of GC cells in vitro and in vivo under hypoxia. Mechanistically, under hypoxia, HIF1A was directly bound to the *PRELID2* promoter to increase *PRELID2* pre‐mRNA expression, followed by the DYRK1A–SFPQ–SAM68 complex binding to Alu‐containing introns in *PRELID2* pre‐mRNA, leading to circPRELID2 circularization. CircPRELID2 interacted with PCBP1 and promoted its cytoplasmic retention. In addition, circPRELID2 enhanced OGT‐mediated PCBP1 O‐GlcNAcylation at Thr99 site in the cytoplasm, disrupting the binding of PCBP1 to the 3′‐UTR of *ZEB2* and reversing ZEB2 translation silencing (Figure S11, Supporting Information). Our findings revealed an oncogenic role of hypoxia‐induced circPRELID2 in GC metastasis, identifying circPRELID2 as a promising prognostic biomarker and therapeutic target for GC metastasis.

DYRK1A is an evolutionarily conserved protein kinase belonging to the DYRK family. Over the past 15 years, more than 30 DYRK1A substrates or binding proteins, located in the nucleus, cytoplasm, vesicles, and cytoskeleton, have been identified.^[^ ^58^, ^59^ ^]^ DYRK1A plays an important role in tumor progression by regulating cellular signaling, chromatin remodeling, as well as alternative splicing.^[^ ^58^, ^59^ ^]^ The consecutive histidine repeat (HIS) domain of DYRK1A mediates its localization to the splicing factor compartment or speckles, which are nuclear structures enriched in pre‐mRNA splicing factors that regulate mRNA splicing.^[^ ^58^, ^60^ ^]^ Several substrate proteins of DYRK1A, including ASF, SC35, SF3b1, SRp55, and 9G8, are involved in alternative splicing and spliceosome assembly.^[^ ^58^, ^59^ ^]^ However, whether DYRK1A participates in circRNA back‐splicing remains unknown. In this study, we demonstrated that protein kinase DYRK1A mediates SFPQ phosphorylation to promote the interaction between SFPQ and SAM68, which facilitates SAM68 binding to the conserved bipartite (A/U)AA‐N>15‐(A/U)AA RNA motifs located upstream and downstream of circPRELID2‐forming splice sites, resulting in the inhibition of competitive pairing between AluJr and AluSz6 and bringing circPRELID2 back‐splicing sites into close proximity, ultimately leading to the back‐splicing of *PRELID2* pre‐mRNA and circPRELID2 circularization (Figure 4; Figure S3, Supporting Information). Moreover, knockdown of SAM68 increased the expression of linear PRELID2 and decreased circPRELID2 levels, but had no effect on pre‐*PRELID2* (Figure 4b), which is consistent with previous finding that circRNA biogenesis competes with linear splicing of the host gene.^[^ ^22^ ^]^ Thus, for the first time, we found that DYRK1A is involved in circRNA back‐splicing.

Furthermore, we identified three Alu elements (AluSz, AluJr, and AluSz6) in the introns flanking upstream and downstream splice sites using the UCSC Genome Browser. Knockdown of DHX9, a known RNA helicase that unhitches IRAlu pairs and suppresses circRNA circularization, significantly facilitated circPRELID2 formation, suggesting that IRAlu elements are also partially involved in circPRELID2 circularization. Additionally, minigene analysis revealed that pairing between AluSz and AluSz6 across flanking introns is indispensable for circPRELID2 formation, while deletion of AluJr significantly promoted circPRELID2 formation, suggesting that AluJr inhibits circPRELID2 circularization potentially through competitive pairing with AluSz6 in downstream flanking introns. Surprisingly, deletion of the upstream SAM68‐binding sites (group #5) or the first cluster of downstream SAM68‐binding sites (group #6) significantly inhibited circPRELID2 generation, whereas deletion of the second cluster of downstream SAM68‐binding sites (groups #7 and #8) that located between AluJr and AluSz6, almost abolished circPRELID2 formation compared to that of the WT construct (Figure 4o,p). Because the RNA‐binding protein SAM68 associates with RNA to form dimers, trimers, and large complexes.^[^ ^37^, ^61^ ^]^ And studies have shown that SAM68 binds close to Alu elements in SMN pre‐mRNAs and promotes SMN circRNAs biogenesis.^[^ ^62^ ^]^ Therefore, we speculate that circPRELID2 biogenesis may occur through the following mechanisms. First, SAM68 binding to the Alu proximal regions, along with its homodimerization, may bring these IRAlus (AluSz and AluSz6) that located in distant introns into close proximity, thereby favoring circPRELID2 biogenesis. Second, the binding of SAM68 to the second cluster of downstream SAM68‐binding sites may block competitive pairing between AluJr and AluSz6, thereby promoting the pairing between AluSz and AluSz6 across the flanking introns, which facilitates circPRELID2 formation. Third, since SAM68 influences the dynamic recruitment of spliceosomal components, including U1snRNP, U2AF65, and U1‐70K, leading to disorders in splicing events.^[^ ^63^, ^64^ ^]^ Therefore, SAM68 may directly affect the back‐splicing of *PRELID2* pre‐mRNA by modulating the spliceosomal machinery. These hypotheses warrant further investigation.

The tumor suppressor PCBP1, which is mainly localized in the nucleus and cytoplasm, represses tumorigenesis and metastasis through regulating transcription, alternative splicing, RNA stability and translation of tumor‐related genes.^[^ ^39^, ^40^ ^]^ However, the regulation of PCBP1 localization and posttranslational modification in the cytoplasm is still vague. Here, we found that circPRELID2 could bind to PCBP1 and mediate the cytoplasmic retention of PCBP1. Furthermore, circPRELID2 enhanced the interaction between OGT and PCBP1 in the cytoplasm, subsequently facilitating OGT‐mediated PCBP1 O‐GlcNAcylation at Thr99, which interferes with PCBP1's binding to the 3′‐UTR element of *ZEB2*. We are the first to uncover PCBP1 O‐GlcNAcylation modification and illustrate a positive regulatory effect of PCBP1 O‐GlcNAcylation on the translation of ZEB2. However, the mechanism by which PCBP1 O‐GlcNAcylation inhibits the binding of PCBP1 to the 3′‐UTR of *ZEB2* needs to be elucidated. Furthermore, we demonstrated that circPRELID2 significantly increased global O‐GlcNAcylation in gastric cancer cells, whereas circPRELID2‐Mut failed to do so (Figure S7a, Supporting Information), suggesting that circPRELID2 may also participate in OGT‐mediated O‐GlcNAcylation of additional substrates. However, whether circPRELID2 regulates OGT enzymatic activity and the precise mechanisms through which it influences O‐GlcNAcylation of other targets remain to be elucidated.

Zinc finger E‐box binding homeobox 2 (ZEB2), an EMT‐related transcription factor, is crucial for EMT and metastasis of multiple tumors.^[^ ^53^ ^]^ Some studies have demonstrated that certain factors, including ncRNAs, are involved in the regulation of ZEB2 expression. For example, members of the miR‐200 family such as miR‐200a, miR‐200b, miR‐200c, miR‐141, and miR‐429, can suppress EMT in multiple cancers by targeting the 3′‐UTR of *ZEB2*.^[^ ^65^ ^]^ In another example, the E3‐ubiquitin ligase SCF^FBXW7^ interacts with ZEB2 and mediates its K48‐linked ubiquitination for degradation in a GSK‐3β phosphorylation‐dependent manner.^[^ ^66^ ^]^ However, there have been few reports on the translational regulation of ZEB2. In this study, we found that circPRELID2 enhances the interaction between OGT and PCBP1, subsequently facilitating OGT‐mediated PCBP1 O‐GlcNAcylation at T99, which disrupts the binding of PCBP1 to the 3′‐UTR element of *ZEB2*, eventually reversing ZEB2 translational silencing and promoting GC EMT and metastasis. We are the first to illustrate a positive regulatory effect of the novel onco‐circRNA circPRELID2 on the translation of *ZEB2* mRNA, facilitated by recruiting and mediating RBP O‐GlcNAcylation, highlighting its potential as a therapeutic target in gastric cancer. Given the intricate pathogenesis of gastric cancer and the limited efficacy of current metastasis‐targeted interventions, innovative therapeutic strategies—such as circRNA‐based modulation, OGT inhibitors, and DYRK1A kinase‐targeted therapy—are urgently needed. These approaches warrant further exploration, particularly as combination therapies to address heterogeneous clinical responses. Furthermore, the mechanism by which PCBP1 O‐GlcNAcylation inhibits the binding of PCBP1 to the 3′‐UTR element of *ZEB2* needs to be elucidated.

In summary, these results reveal that a novel oncogene‐circPRELID2, which is generated by the DYRK1A/SFPQ/SAM68 complex in an Alu elements dependent manner under hypoxia, plays a critical role in facilitating ZEB2 protein translation via circPRELID2/OGT/PCBP1/ZEB2 axis, eventually facilitating the EMT and metastasis of GC cells. More importantly, our findings suggest that circPRELID2 is a potential therapeutic target for GC, and particularly likely for metastatic GC.

## 4 Experimental Section

### Tissue Samples from Gastric Cancer Patients

In this research, two separate groups of human gastric cancer tissues and paired adjacent normal tissues were studied in accordance with the Helsinki Declaration. One group included 51 pairs of fresh‐frozen gastric cancer tissues and matched adjacent normal tissues stored at −80 °C until RNA isolation, which were collected from 51 gastric cancer (GC) patients who received surgical treatment for gastric cancer at Shanghai General Hospital between 2014 and 2019. The other separate group consisted of 54 pairs of gastric cancer tissues and matched adjacent normal tissues which were taken from 54 GC patients who underwent surgical treatment for gastric cancer at Shanghai General Hospital between 2013 and 2016, and these samples were fixed in the formalin, followed by paraffin embedding for tissue microarray (TMA) staining. All gastric cancer pathological diagnoses in this study were confirmed by at least two pathologists according to the 8th edition of the American Joint Commission on Cancer (AJCC) and the Union for International Cancer Control (UICC). Informed consents were signed by all the patients, and this research was approved by the Ethics Committee of Shanghai General Hospital (Research Ethics Approval Code: 2024SQ212).

### Plasmids

The pLC5‐ciR vector (Geneseed, Guangzhou, China) was used to construct circPRELID2‐overexpression plasmid. CircPRELID2 shRNAs targeting the junction region of the circPRELID2 sequence were constructed by RiboBio (Guangzhou, China). Human SAM68 cDNA was subcloned into a pcDNA3.1 vector with a C‐terminal Flag tag to generate Flag‐SAM68. Human SFPQ cDNA was subcloned into pcDNA3 vector and pLVX‐CMV vector with an N‐terminal hemagglutinin (HA) tag to generate HA‐SFPQ. The Flag‐tagged OGT was subcloned into pCMV10 vector. The HA‐tagged OGA was subcloned into pcDNA3 vector. The pLX304‐PCBP1‐V5 expression plasmid was purchased from Thermo Fisher Scientific (Waltham, USA). The HIF1A expression plasmid and PRELID2 luciferase reporter plasmids were purchased from Genechem (Shanghai, China). The *PRELID2* minigene was constructed by subcloning the region from intron 2 to intron 6 of the *PRELID2* gene into the pZW1 expression vector. The pcDNA5‐Flag‐Dyrk1a plasmid has been described previously.^[^ ^34^ ^]^ All the Flag‐SAM68 truncations, circPRELID2 mutants, Flag‐OGT mutants, circPRELID2 minigene deletion constructs, Flag‐Dyrk1a mutants, and V5‐PCBP1 truncations were constructed using site‐directed mutagenesis following the manufacturer's protocol (Toyobo, Japan). All constructs were confirmed through DNA sequencing. The sequences of short hairpin RNA (shRNA) used in this study are listed in Table S1 (Supporting Information).

### Cell Culture, Viral Production, and Infection

HEK293T cells, normal human gastric epithelial cells (GES‐1), and various gastric cancer cell lines (AGS, HGC‐27, MKN‐45, SNU‐1, NCl‐N87, and KATOIII) were obtained from the Culture Collection of Chinese Academy of Sciences (Shanghai, China). The HEK293T cell line was cultured in Dulbecco's modified Eagle's medium, and the other cell lines were cultured in RPMI‐1640 medium supplemented with 10% fetal bovine serum (Gibco, New York, USA), 50 U/mL penicillin, and 50 µg/mL streptomycin (Life‐iLab, Shanghai, China). All these cell lines were maintained at 37 °C with 5% CO_2_ in a humidified chamber. For hypoxia treatments, gastric cancer cell lines were cultured in a hypoxia chamber with 1% O_2_, 94% N_2_, and 5% CO_2_ at 37 °C. Viral production and infection experiments were performed as previously described.^[^ ^34^ ^]^

### Cell Proliferation and Wound Healing Assays

A total of 1 × 10^5^ infected cells were plated into each well of a 6‐well plate. Then, on days 1, 2, and 3 after plating, the cells were digested with trypsin enzyme and resuspended into single‐cell suspension. The cells were counted with hematocytometer by means of trypan blue dye exclusion. Wound healing assays were performed as previously described.^[^ ^67^ ^]^ All these experiments were performed at least in triplicate.

### Transwell and Invasion Assays

For Transwell assay, gastric cancer cells in 150 µL serum‐free RPMI‐1640 medium were seeded into upper chamber, and 500 µL RPMI‐1640 medium supplemented with 10% fetal bovine serum was added to the bottom chamber. After cultured for 24 h, the cells in upper chamber were washed with 1×PBS (Phosphate Buffered Saline), fixed with 4% paraformaldehyde, and stained with 0.1% crystal violet. Then, the cells were photographed and counted in different image fields. For invasion assay, the Matrigel matrix was diluted and added onto the 8 µm pore Transwell filters of the upper chamber, and then placed at 37 °C for 2 h. the other procedures were the same as those for Transwell assay.

### Antibodies

Antibodies used in the study were as follows: anti‐Flag (F3165/F7425, Sigma), anti‐Myc (9B11/71D10, Cell Signaling Technology (CST)), anti‐HIF1A (H1alpha67, Novus), anti‐HA (16B12, Biolegend and H6908, Sigma), anti‐Vimentin (D21H3, CST), anti‐E‐Cadherin (4A2, CST), anti‐N‐Cadherin (D4R1H, CST), anti‐CA9 (GB153899, Servicebio, China), anti‐SAM68 (10222‐1‐AP, Proteintech), anti‐PCBP1 (sc‐393076, Santa Cruz and RN024P, MBL International), anti‐O‐Linked N‐Acetylglucosamine (ab2739, Abcam), anti‐OGT (11576‐2‐AP, Proteintech), anti‐OGA (14711‐1‐AP, Proteintech), anti‐DHX9 (17721‐1‐AP, Proteintech), anti‐p‐Ser/Thr (ab9344, Abcam and 05‐368, Sigma), anti‐SFPQ (15585‐1‐AP, Proteintech), anti‐ZEB2 (14026‐1‐AP, Proteintech and HPA003456, Sigma), anti‐DYRK1A (sc‐100376, Santa Cruz), anti‐FAM3C (14247‐1‐AP, Proteintech), anti‐GNA13 (67188‐1‐Ig, Proteintech), anti‐EGFR (sc‐373746, Santa Cruz), anti‐JAK2 (D2E12, CST), anti‐MOESIN (CY6950, Abways, China), anti‐EIF5A2 (67907‐1‐Ig, Proteintech), anti‐LC3 (D3U4C, CST), anti‐P62 (D1Q5S, CST), anti‐β‐Actin (AC026, ABclonal), anti‐V5 (R960‐25, Thermo Fisher and 30801ES10, Yeasen), anti‐Tubulin (11224‐1‐AP, Proteintech), anti‐H3 (A2348, ABclonal), anti‐mouse/rabbit IgG‐peroxidase secondary antibody (A0545/A9044, Sigma), IPKine HRP conjugated Mouse Anti‐Rabbit IgG light chain specific secondary antibody (A25022, Abbkine), anti‐mouse IgG (light chain specific)‐peroxidase secondary antibody (115‐005‐174, Jackson).

### Immunoblotting

Cells were washed three times with 1×PBS, and lysed in RIPA lysis buffer supplemented with protease inhibitors (Sparkjade, China) and phosphatase inhibitors (MedChemExpress, USA) at 4 °C for 30 min, or lysed in SDS lysis buffer (50 mM Tris‐HCl, pH 6.8, 2% SDS, 6% glycerol) followed by incubation at 98 °C for 10 min. Then after sonicating, the lysates were centrifuged at 13000 rpm at 4 °C for 15 min and quantified with BCA Protein Assay Kit (Thermo Fisher, USA). Protein samples were separated by SDS‐PAGE and transferred onto nitrocellulose membranes (PALL, USA). Then the blots were blocked with 5% non‐fat milk and incubated with primary antibodies at 4 °C overnight, followed by incubation with peroxidase‐labeled secondary antibody at room temperature for 1 h. Finally, the blot signals were detected with an enhanced chemiluminescence (ECL) kit (Vazyme, China) and analyzed with ImageJ software.

### Co‐immunoprecipitation

The treated cells were washed three times with 1×PBS, and lysed in IP lysis buffer (20 mM Tris‐HCl, pH 7.4, 150 mM NaCl, 0.5% Triton X‐100, and 2 mM EDTA) supplemented with protease inhibitors (Sparkjade, Shandong, China) and phosphatase inhibitors (MedChemExpress, USA) at 4 °C for 30 min, then the lysates were centrifuged at 13 000 rpm at 4 °C for 15 min. The supernatant was incubated with anti‐SAM68/anti‐SFPQ/anti‐PCBP1 antibody at 4 °C overnight followed by incubation with protein A/G or protein A beads (Millipore, USA) at 4 °C for 2–4 h, or incubated with anti‐Flag/Myc/HA affinity gel (Sigma, USA) or anti‐V5 agarose affinity gel (Beyotime, China) at 4 °C for 4 h. Then, the beads were washed and eluted with SDS loading buffer, followed by immunoblotting analysis.

### RNA Extraction and qRT‐PCR

Total RNA was extracted by using TRIzol reagent (Life Technologies, USA) according to the manufacturer's protocol and reverse transcribed with Evo M‐MLV RT Kit with gDNA Clean (Accurate biotechnology, Changsha, China). qRT‐PCR was performed with ChamQ SYBR qPCR Master Mix (Vazyme, China) with QuantStudio 6 real time PCR system (Applied Biosystems, USA). The sequences of primers used are listed in Table S2 (Supporting Information).

### RNase R Treatment

Total RNA was equally divided into two groups. One group was incubated with 1.5 µL 20U/µL RNase R (Beyotime, China) at 37 °C for 30 min, and the other group was treated without RNase R. Then, RNA in the two groups was incubated at 70 °C for 10 min to inactivate RNase R, followed by RT‐PCR or qRT‐PCR analysis.

### Actinomycin D Assays

Treated HGC‐27 cells were plated into each well (4 × 10^5^ cells/well) of a 6‐well plate. After cultured for 24 h, these cells were exposed to 2 µg/mL actinomycin D (Sigma, USA) for the indicated times. Then, the cells were collected, and RNA stability was analyzed by qRT‐PCR.

### Fluorescence In Situ Hybridization (FISH)‐Immunofluorescence assay

To detect the colocalization of circPRELID2 with PCBP1 in gastric cancer cells, HGC‐27 and AGS cells were fixed, permeabilized and prehybridized. Then, hybridization with Cy3‐conjugated circPRELID2 probes was performed at 37 °C overnight in a dark chamber. After being washed three times with saline sodium citrate (SCC) buffer, the cells were blocked and incubated with anti‐PCBP1 antibody for 1 h, followed by incubation with 488‐conjugated secondary antibodies (Invitrogen, USA) and DAPI (Invitrogen, USA). Then, the images were acquired via confocal microscopy (Zeiss, Germany). The Cy3‐conjugated circPRELID2 probe sequence is listed in Table S3 (Supporting Information).

### In Situ Hybridization (ISH) and Immunohistochemistry (IHC)

The relative expression of circPRELID2 on gastric cancer tissue microarray (TMA) comprising 54 pairs of gastric cancer tissues and adjacent normal tissues was detected by in situ hybridization with digoxin‐labeled circPRELID2 probe synthesized by Azenta (Suzhou, China) according to the manufacturer's instructions. The total score of circPRELID2 staining intensity on the TMA was calculated by multiplying the positive staining area score (0 = not stained, 1 = 1–25% stained, 2 = 26–50% stained, 3 = 51–75% stained, 4 = 76–100% stained) and intensity staining score (0 = negative, 1 = low, 2 = high). The sequences of digoxin‐labeled circPRELID2 probe are listed in Table S3 (Supporting Information). Immunohistochemistry (IHC) analysis was performed as previously described.^[^ ^67^ ^]^

### RNA Pull‐Down Assays

For biotinylated‐probe RNA pull‐down assay, the biotin‐labeled circPRELID2 probe was synthesized by Genechem (Shanghai, China), and incubated with Dynabeads MyOne Streptavidin C1 beads (Invitrogen, USA) to generate probe‐coated beads. Then, cell lysates were incubated with probe‐coated beads at 4 °C overnight. After washing four times, the enrichment of circPRELID2 in the capture fractions was analyzed by qRT‐PCR. The binding proteins were eluted and detected by immunoblotting or mass spectrometry analysis. The circPRELID2 probe used in this study is listed in Table S3 (Supporting Information).

### RNA‐Binding Protein Immunoprecipitation (RIP) Assay

RNA‐binding protein immunoprecipitation (RIP) assay was performed by using the Magna RIP RNA‐Binding Protein Immunoprecipitation Kit (Millipore, USA) according to the manufacturer's instructions. The immunoprecipitated proteins were subjected to immunoblotting, and the captured RNA was extracted and analyzed by qRT‐PCR.

### Extraction of Nuclear and Cytoplasmic Fractions

Cytoplasmic and nuclear protein extraction assay was performed by using the Nuclear and Cytoplasmic Protein Extraction Kit (Beyotime, China) according to the manufacturer's protocol. Briefly, the cells were washed twice with 1×PBS, and lysed in hypotonically cytoplasmic protein extraction reagent A and B supplemented with protease inhibitors (MedChemExpress, USA) at 4 °C for 15 min. After vortex shaking for 5 s, the lysates were centrifuged at 13 000 rpm at 4 °C for 5 min. The supernatant was collected as the cytoplasmic fraction. Pellets were washed three times with cytoplasmic protein extraction reagent A and resuspended in nuclear protein extraction buffer supplemented with protease inhibitors (MedChemExpress, USA). After sonicating for 40s, the lysates were centrifuged at 13 000 rpm for 15 min and the supernatant was collected as the nuclear fraction.

### Dual‐Luciferase Reporter Assay

Human *PRELID2* promoter containing six candidate HREs and indicated mutants were subcloned into GV238 luciferase reporter vector (GeneChem, China). Cells stably overexpressing NC or HIF1A were co‐transfected with Renilla luciferase plasmid, HRE‐WT or HRE‐Mut luciferase reporter constructs. After 48 h of transfection, the relative luciferase activity was detected by using Dual Luciferase Assay Kit (Promega, USA) according to the manufacturer's instructions and normalized to Renilla luciferase activity.

### Chromatin Immunoprecipitation (ChIP)

HGC‐27 cells were cultured under normoxia or hypoxia for 24 h. Then, The binding capacity of HIF‐1α to the *PRELID2* promoter was analyzed by using SimpleChIP Enzymatic Chromatin IP Kit (CST, USA) with anti‐HIF1A or IgG antibody in accordance with the manufacturer's instructions.

### In Vivo Phosphorylation Assay and In Vivo O‐GlcNAcylation Assay

Cells were washed three times with ice‐cold 1×PBS and then lysed with denaturing lysis buffer (2% SDS, 150 mM NaCl, 20 mM Tris‐HCl, pH 8.0, 2 mM EDTA) supplemented with protease inhibitors and phosphatase inhibitors, followed by heating at 98 °C for 10 min. Then, the lysates were diluted to 0.2% SDS with the same lysis buffer lacking SDS. After sonicating for 40 s, the lysates were centrifuged at 13 000 rpm for 15 min, and the supernatant was incubated with anti‐PCBP1 antibody at 4 °C overnight followed by incubation with protein A/G beads at 4 °C for 4 h, or incubation with anti‐V5/HA affinity gel at 4 °C overnight. Then the captured proteins were eluted and analyzed by immunoblotting. For the in vivo phosphorylation assay, the phosphorylation level of SFPQ was determined by using anti‐p‐Ser/Thr antibody (ab9344, Abcam and 05‐368, Sigma). For in vivo O‐GlcNAcylation assay, anti‐O‐linked N‐acetylglucosamine antibody (ab2739, Abcam) was used to detect PCBP1 O‐GlcNAcylation level.

### Mass Spectrometry

Approximately 2 × 10^8^ cells for each cell line were collected and washed with 1×PBS, then cells were scraped into IP lysis buffer (20 mM Tris‐HCl pH 7.4, 150 mM NaCl, 0.5% Triton X‐100, 2 mM EDTA) supplemented with phosphatase and protease inhibitors (Sigma, USA), and incubated on ice for 30 min. Lysates were centrifuged at 17 000 × g, 4 °C for 15 min, and Flag‐affinity was performed by incubating Flag beads for overlight. Beads were then washed with wash buffer (20 mM Tris‐HCl pH 7.4, 250 mM NaCl, 0.5% Triton X‐100, 2 mM EDTA) three times and analyzed by silver staining and western blotting. Protein mixtures from the purifications were digested with trypsin, and analyzed by an LTQ‐ mass spectrometer (Thermo Fisher Scientific, USA).

### In Vivo Metastasis Assays

All animal studies were performed in line with the experimental animal use guidelines of the National Institutes of Health and were approved by the Animal Care Committee of Shanghai General Hospitall (Research Ethics Approval Code: 2024AWS222). Six‐week‐old male BALB/c nude mice were purchased from Shanghai SLAC Laboratory Animal Co., Ltd. All mice were randomly divided into different groups. For lung metastasis assay, 1 × 10^6^ treated cells were suspended in 150 µL PBS and injected intravenously into the tail vein of mice in these groups. For the liver metastasis model, 2 × 10^6^ treated cells were suspended in 50 µL PBS and injected into the spleen of BALB/c nude mice in different groups. For lymph node metastasis model, 5× 10^5^ treated cells were injected into the footpad region of hind limb of BALB/C nude mice. The cyclic hypoxia treatment was started at day 9 after injection, hypoxic mice were housed in an acrylic chamber with alternating between 10 min of exposure to 7% O_2_, and 10 min of standard laboratory air (21% O_2_), continuing for a total of 4 h each day, whereas normoxic mice were housed in standard laboratory air (21% O_2_) under a 12‐h light and dark cycle.^[^ ^68^ ^]^ Then metastatic nodes were monitored using the IVIS Spectrum in vivo imaging system. After the mice were euthanatized, the lung/liver metastasis capacity of gastric cancer cells was evaluated.

### Statistical Analysis

All statistical analyses were performed using GraphPad Prism 8.0 and SPSS 22.0. Data were shown as the mean ± standard deviation (SD) from at least three independent experiments. Differences between individual groups were analyzed using Student's t‐test and one‐way ANOVA. Kaplan–Meier method and log‐rank test were used to plot overall survival (OS) and disease‐free survival (DFS) curves. Statistical significance was shown as *p < 0.05, ** *p* < 0.01, or *** *p* < 0.001, ns indicated no significance.

## Conflict of Interest

The authors declare no conflict of interest.

## Author Contributions

P.Z., Z.L., Y.X., and Y.Z. contributed equally to this work. P.Z., Z.L., Y.X., and Y.Z. were responsible for the research design. P.Z., Z.L., Y.X., and Y.Z. conducted the experiments. P.Z., N.P., and S.Z. contributed new reagents and analytic tools. P.Z. and Y.X. performed data analysis. R.Z., Q.C., and Z.L. were responsible for collecting and providing clinical samples and associated data. Z.Q. and C.H. offered administrative support and provided suggestions for manuscript revision. P.Z. and C.H. wrote the manuscript.

## Supporting information



Supporting Information

Supporting Information

## Data Availability

The data that support the findings of this study are available from the corresponding author upon reasonable request.
