# Supplementary material for: Hypoxia‐Induced circPRELID2 Promotes Gastric Cancer Metastasis by Facilitating ZEB2 Translation via PCBP1 O‐GlcNAcylation
Source: Adv Sci (Weinh). 2025 Oct 21;12(46):e05396. doi: 10.1002/advs.202505396 (PMC12697806; doi:10.1002/advs.202505396)
Supplement: Supplementary file 1 — Supporting Information [file ADVS-12-e05396-s001.pdf]

Supporting Information for

Hypoxia-Induced circPRELID2 Promotes Gastric Cancer Metastasis by Facilitating ZEB2 Translation via PCBP1 O-GlcNAcylation

Pengshan Zhang# [1], Zai Luo# [1], Yitian Xu# [1], Yuan Zhang# [1], Renchao Zhang[1], Nadina Paerhati[1], Shaopeng Zhang[1], Qianqian Cai[1], Zhengjun Qiu[1], Chen Huang* [1]

1. Department of General Surgery, Shanghai General Hospital, Shanghai Jiao Tong University School of Medicine, Shanghai, 200080, China.

Chen Huang

Email: richard-hc@hotmail.com.

This PDF file includes:

Supplemental Figures 1-11;

Supplemental Table S1-S5;

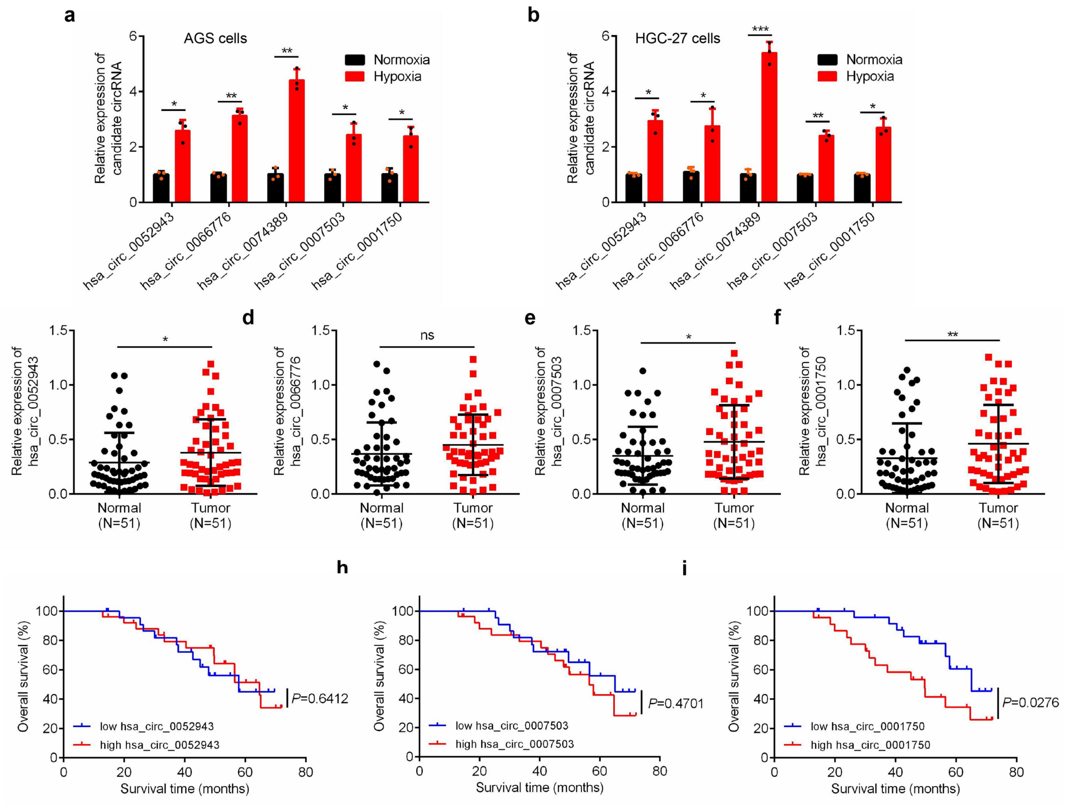

Supplemental Figure 1. Selection of candidate circRNAs in GC tissues. a/b, qRT-PCR analyses of the relative expression of the top five candidate circRNAs in AGS cells (**a**) and HGC-27 cells (**b**) cultured under normoxia or hypoxia. **c-f**, Relative expression of hsa_circ_0052943, hsa_circ_0066776, hsa_circ_0007503 and hsa_circ_0001750 in 51 pairs of GC tissues and matched adjacent normal tissues were detected via qRT-PCR, respectively. **g-i**, Kaplan-Meier overall survival curves of GC patients with low and high expression hsa_circ_0052943, hsa_circ_0007503 or hsa_circ_0001750 were shown, respectively. And ns indicated no significance, *$P < 0.05$, **$P < 0.01$, ***$P < 0.001$.

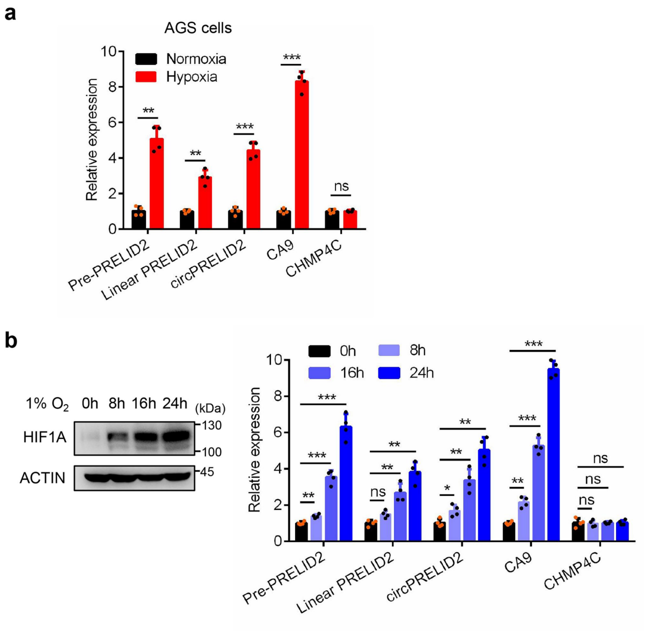

Supplemental Figure 2. Hypoxia facilitates prominently the expression of pre-*PRELID2*, linear PRELID2 (*PRELID2* mRNA), and circPRELID2 in GC cells. a, qRT-PCR analyses of the relative expression of pre-*PRELID2*, linear PRELID2, and circPRELID2 in AGS cells cultured under normoxia or hypoxia for 24h; CA9 was set as positive control, CHMP4C was used as negative control. **b**, qRT-PCR analyses of the relative expression of pre-*PRELID2*, linear PRELID2, and circPRELID2 in HGC-27 cells treated with 1% O_2 for the indicated times. *$P < 0.05$, **$P < 0.01$, ***$P < 0.001$; ns, no significance.

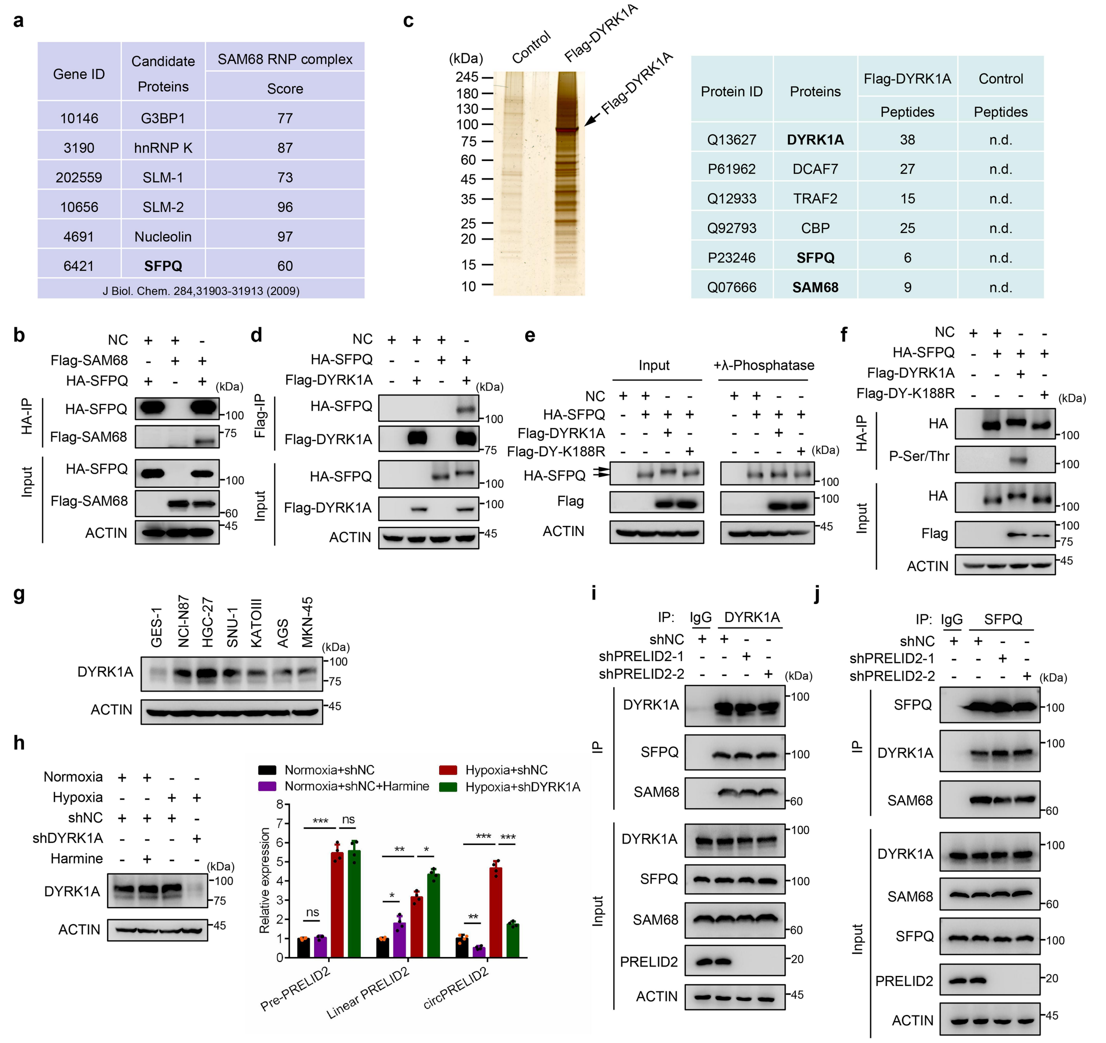

Supplemental Figure 3. Investigating the DYRK1A-SFPQ-SAM68 ternary complex. **a**, Identification of the protein components of SAM68 RNP complex by mass spectrometry. **b**, HEK293T cells were transfected with NC, Flag-SAM68, and/or HA-SFPQ, and IP and western blotting analyses were performed with the indicated antibodies after 48 h of transfection. **c**, Silver staining and relative abundance of the Flag-DYRK1A affinity-purified proteins. HEK293T cells stably expressing NC or Flag-DYRK1A were constructed. Lysates from these cells were Flag-affinity purified, followed by silver staining and mass spectrometry (MS) analysis. **d**, Lysates from HEK293T cells transfected with NC, HA-SFPQ, and/or Flag-DYRK1A were used for IP with anti-Flag antibody, followed by immunoblotting analysis with the indicated antibodies. **e**, Whole cell lysates from HEK293T cells transfected with HA-SFPQ and Flag-DYRK1A or Flag-DYRK1A-K188R were treated with λ-phosphatase and subjected to western blot analyses with the indicated antibodies. The black arrows indicated the slower

migrating SFPQ signal. **f**, HEK293T cells were transfected with HA-SFPQ, Flag-DYRK1A, or Flag-DYRK1A-K188R alone or in combination for 48 h before cells were harvested. Then, HA-SFPQ was affinity-purified for phosphorylation analysis. **g**, The protein level of DYRK1A in multiple GC cell lines and normal human gastric epithelial cells (GES-1) was detected by immunoblotting. **h**, The relative expression of pre-*PRELID2*, linear PRELID2 and circPRELID2 in HGC-27-shNC or shDYRK1A cells treated as indicated were shown. **i/j**, Lysates from HGC-27-shNC or shPRELID2 cells were immunoprecipitated with anti-DYRK1A or SFPQ antibody followed by immunoblotting with indicated antibody.

a

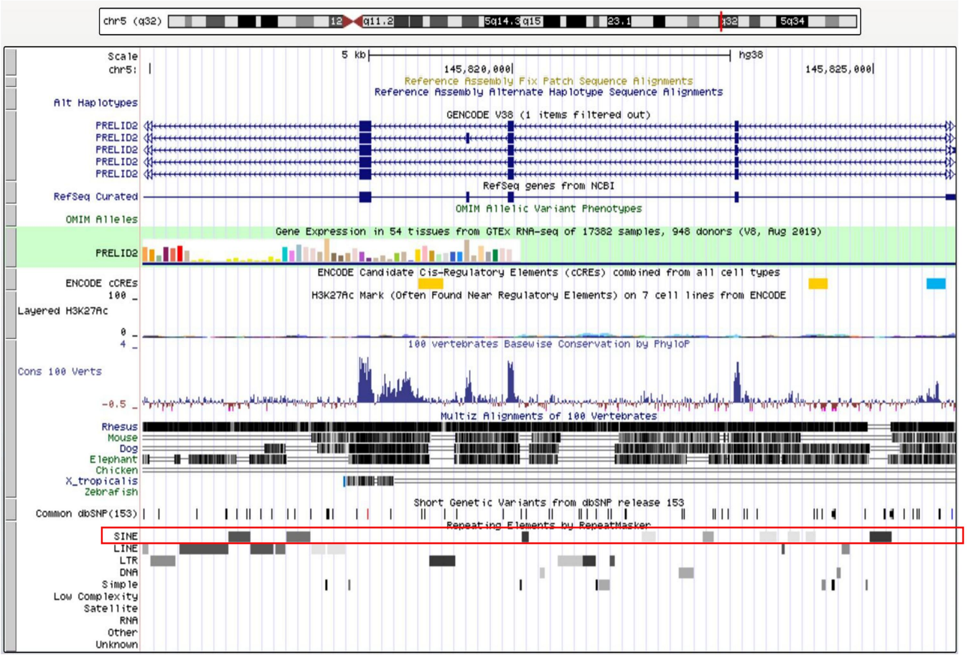

b

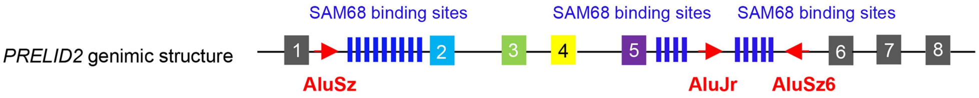

Supplemental Figure 4. Inverted repeat Alu elements in the *PRELID2* gene structure. a, The genomic information of *PRELID2* was shown based on the UCSC Genome Browser. Alu elements were included in short interspersed nuclear elements (SINEs) highlighted in the red box. **b**, Schematic illustration of *PRELID2* gene structure showing the inverted repeat Alu elements (Alu Sz, Alu Jr, and Alu Sz6) in the introns flanking circPRELID2-forming exons.

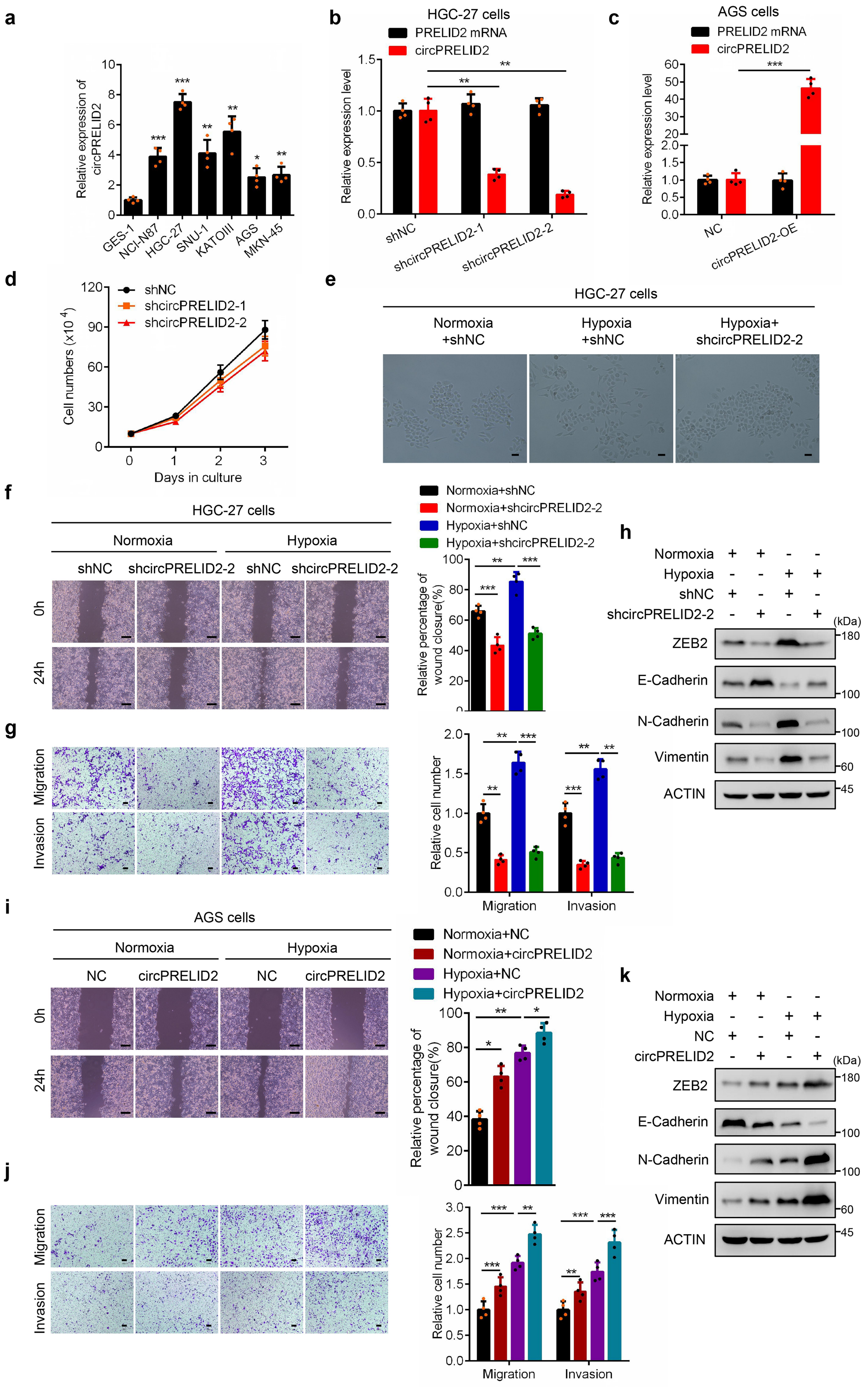

Supplemental Figure 5. CircPRELID2 promotes EMT and metastasis of GC cells *in vitro* under hypoxic conditions. a, Relative expression of circPRELID2 in normal human gastric

epithelial cells (GES-1) and several gastric cell lines was examined via qRT-PCR. **b**, qRT-PCR analyses of the relative expression of *PRELID2* mRNA and circPRELID2 in HGC-27 cells infected with shRNA lentivirus targeting circPRELID2. **c**, Relative expression of *PRELID2* mRNA and circPRELID2 in AGS cells stably overexpressing circPRELID2 were detected by qRT-PCR. **d**, HGC-27 cells were infected with shNC or shcircPRELID2 lentivirus, and then the proliferation of the treated HGC-27 cells was measured. **e**, The EMT morphological changes in HGC-27-shNC or shcircPRELID2 cells subjected to normoxia or hypoxia were shown, scale bar = 25 μm. **f/g**, Wound healing (**f**) and transwell (**g**) assays were performed to detect the migration and invasion ability of HGC-27-shNC and shcircPRELID2 cells subjected to normoxia or hypoxia. Scale bar = 100 μm (**f**) or 50 μm (**g**). **h**, The protein levels of ZEB2, E-Cadherin, N-Cadherin and Vimentin in HGC-27-shNC and shcircPRELID2 cells under normoxic or hypoxic conditions were detected by immunoblotting. **i/j**, The migration (**i**) and invasion (**j**) ability of AGS cells stably transfected with NC or circPRELID2 under normoxic or hypoxic conditions were evaluated. Scale bar = 100 μm (**i**) or 50μm (**j**). **k**, Immunoblotting analyses of the protein levels of ZEB2, E-Cadherin, N-Cadherin and Vimentin in AGS cells stably transfected with NC or circPRELID2 under normoxia or hypoxia.

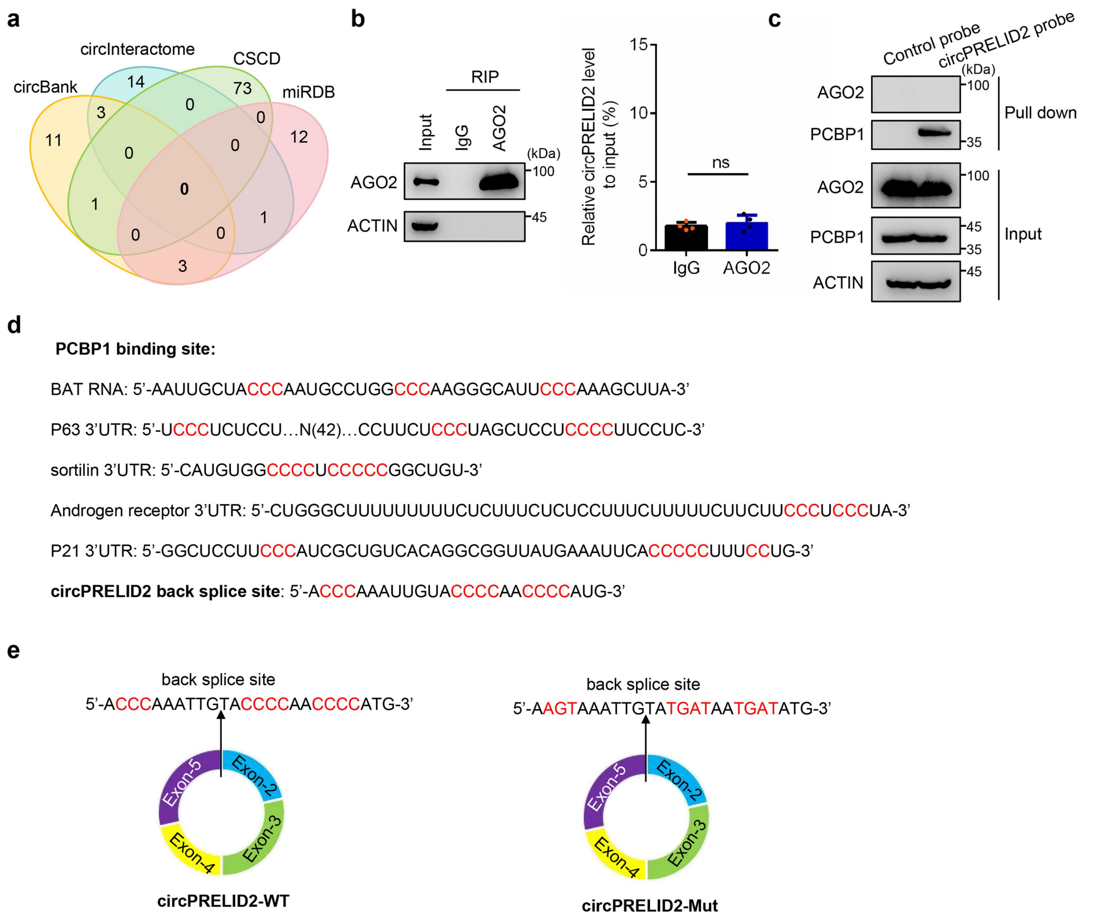

Supplemental Figure 6. The potential binding site of PCBP1 on circPRELID2. a, Schematic illustration showing the overlap of target miRNAs for circPRELID2 predicted by circBank, circInteractome, CSCD and miRDB. **b**, RNA immunoprecipitation (RIP) followed by qRT-PCR analysis was conducted to detect the association of circPRELID2 with AGO2 in HGC-27 cells. **c**, RNA pull-down assays were conducted to detect the specific binding between circPRELID2 and AGO2 in HGC-27 cells. **d**, PCBP1 specifically recognizes and interacts with poly-cytosine (poly-C) regions of target RNA or DNA. A number of known PCBP1 binding sequences in the 3'UTR of target transcripts were listed. And 5′-A**CCC**AAAUUGUA**CCCC**AA**CCCC**AUG-3′ sequence located at the back-splicing junction site of circPRELID2 was a putative binding motif of PCBP1. **e**, Wild-type circPRELID2 and back-splicing junction site-mutated circPRELID2 (circPRELID2-Mut) were shown. The circPRELID2-Mut plasmid was constructed by mutating poly-C sequences in the back-splicing junction site of circPRELID2 to the other three bases.

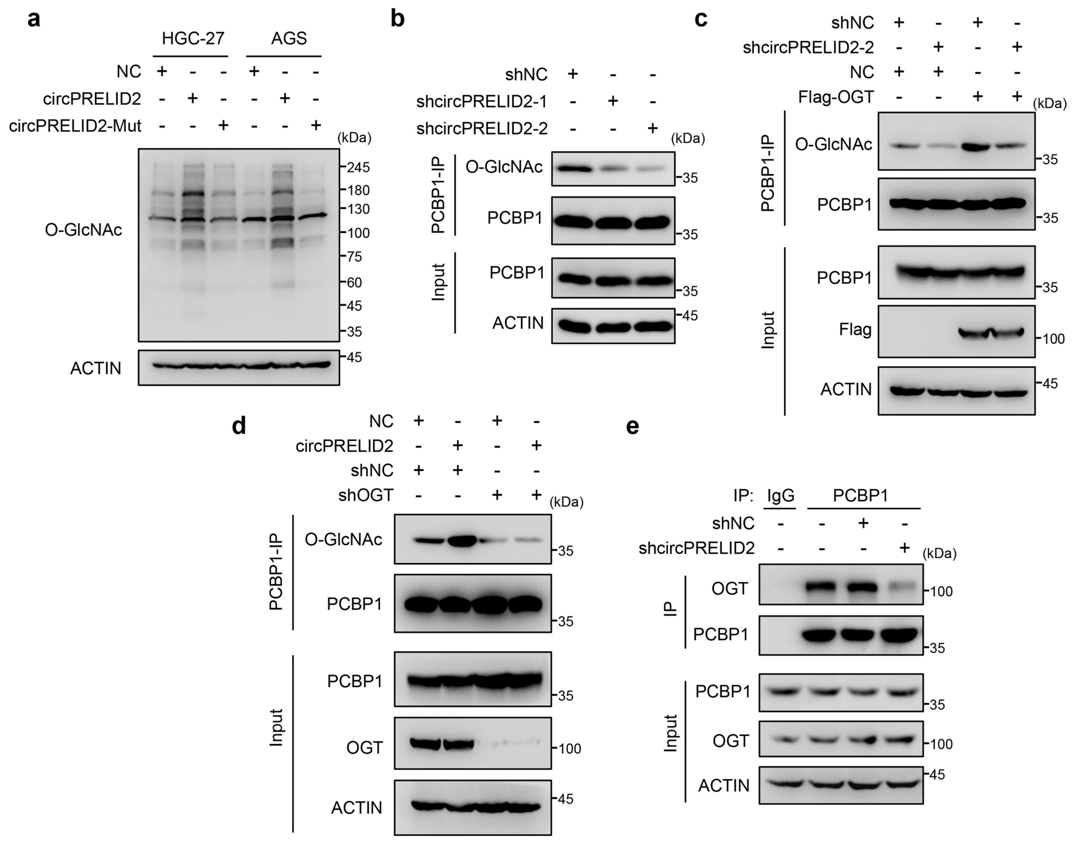

Supplemental Figure 7. CircPRELID2 is required for OGT-mediated PCBP1 O-GlcNAcylation. a, The O-GlcNAc level was detected in AGS and HGC-27 cells transfected with NC, circPRELID2-WT, or circPRELID2-Mut. **b**, Lysates from HGC-27-shNC and shcircPRELID2 cells were subjected to immunoprecipitation with anti-PCBP1 antibody, followed by western blotting with the indicated antibodies to examine the O-GlcNAcylation of PCBP1. **c**, HGC-27-shNC and shcircPRELID2 cells were transfected with NC or Flag-OGT and subjected to IP and immunoblotting analyses with the indicated antibodies. **d**, AGS-shNC and shOGT cells were transfected with NC or circPRELID2. After 48 h of transfection, IP and immunoblotting analyses were performed to detect the O-GlcNAcylation level of PCBP1 with the indicated antibodies. **e**, HGC-27 cells infected with shNC or shcircPRELID2 lentivirus were lysed and immunoprecipitated with normal IgG or with α-PCBP1, followed by western blot analyses with the indicated antibodies.

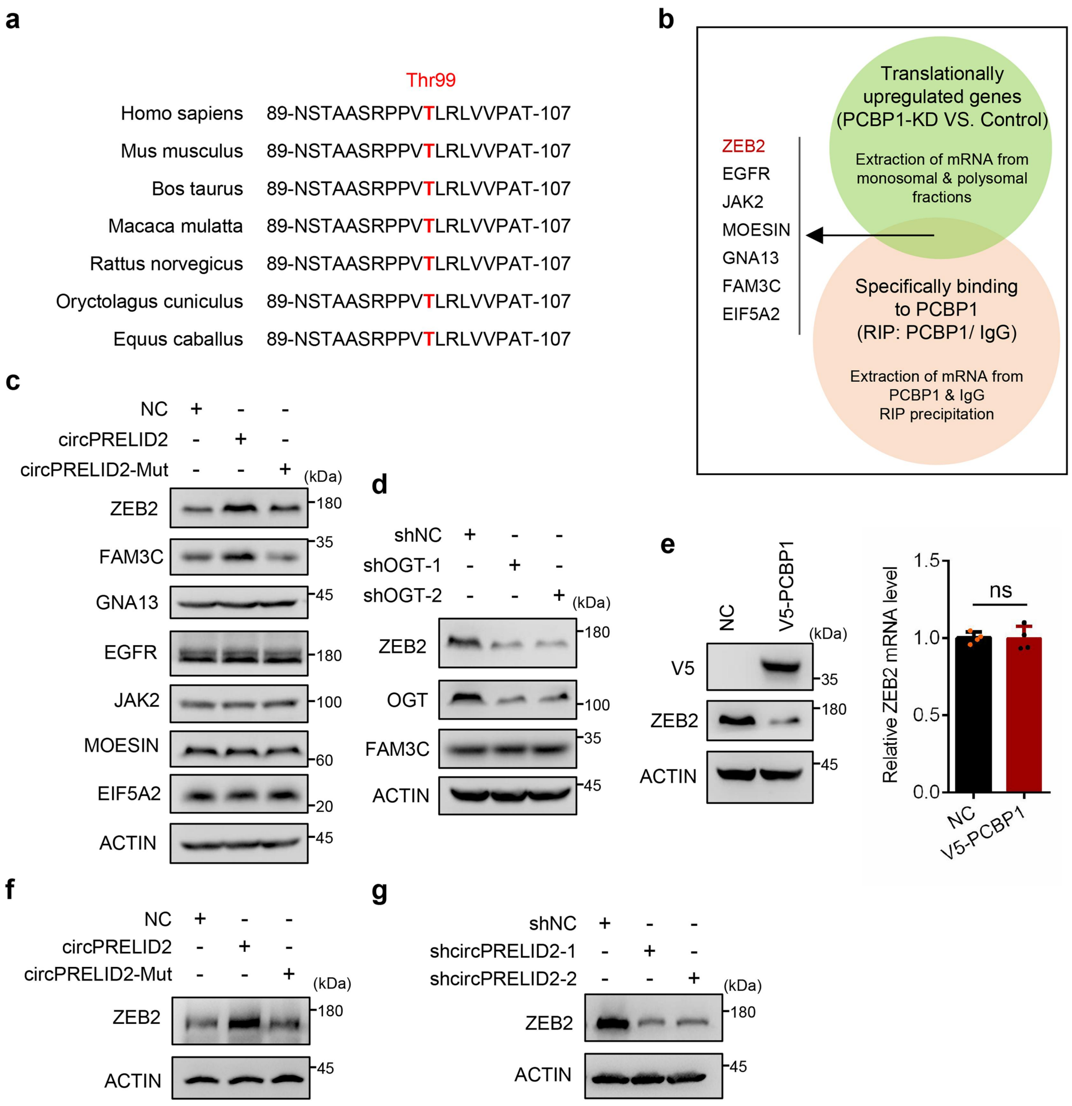

Supplemental Figure 8. Investigation of the downstream targets of PCBP1. a, Sequence alignment of the O-GlcNAcylation site Thr99 of PCBP1 showed that Thr99 residue is highly conserved across species. The Thr99 site was highlighted in bold. **b**, Venn diagram showing the differentially expressed mRNA transcripts that specifically binding to PCBP1 and simultaneously upregulating translationally after depletion of PCBP1 based on GEO database (GSE40466). **c**, Western blotting analysis of candidate protein levels in AGS cells stably transfected with NC, circPRELID2-WT, or circPRELID2-Mut. **d**, Immunoblotting analyses of ZEB2 and FAM3C protein levels in AGS cells infected with shRNA lentiviruses targeting NC or OGT. **e**, The protein and mRNA level of ZEB2 in HGC-27 cells stably overexpressing NC or V5-PCBP1 were analyzed. **e**, Immunoblot analysis of ZEB2 expression in HGC-27 cells stably overexpressing NC, circPRELID2-WT or circPRELID2-Mut. **f**, Western blot analysis of ZEB2

protein levels in AGS cells after knockdown of circPRELID2. g. Western blot analysis of ZEB2 protein levels in HGC-27 cells after overexpression of circPRELID2. $^{*}P < 0.05$, $^{**}P < 0.01$, $^{***}P < 0.001$; ns, no significance.

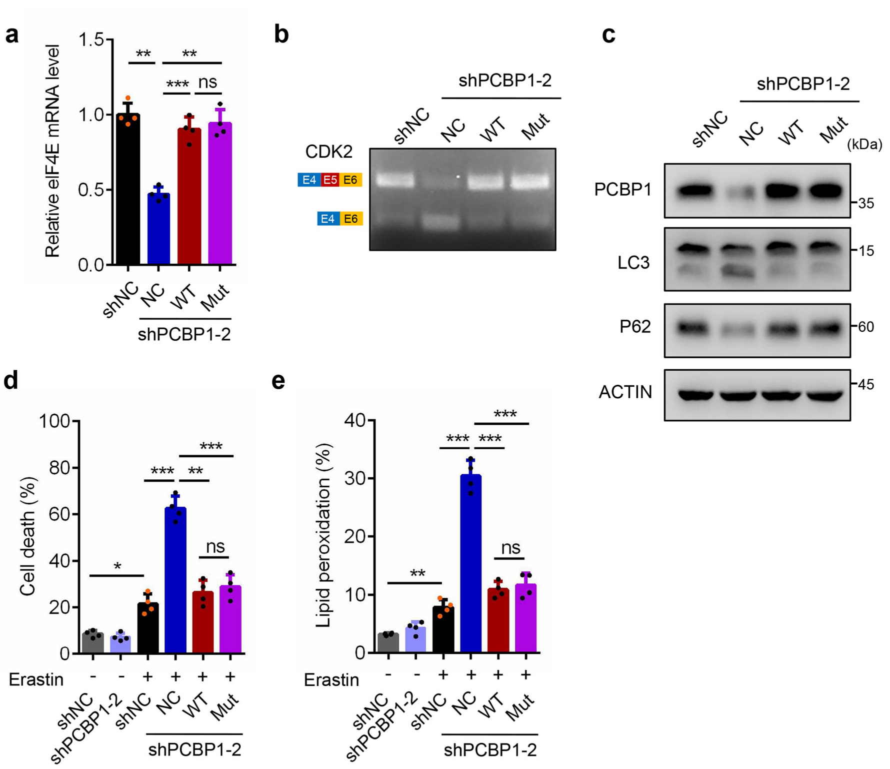

Supplemental Figure 9. Investigating the role of circPRELID2-mediated O-GlcNAcylation of PCBP1 in transcription, splicing, ferroptosis and autophagy. a, qRT-PCR analyses of eIF4E mRNA level in PCBP1-silenced HGC-27 cells after stably re-expressing V5-PCBP1-WT or V5-PCBP1-T99A. **b**, The splicing efficiency of CDK2 exon 5 in PCBP1-silenced HGC-27 cells after stably re-expressing V5-PCBP1-WT or V5-PCBP1-T99A was measured by RT-PCR. **c**, Immunoblotting analyses of LC3 and P62 protein levels in PCBP1-silenced HGC-27 cells after stably re-expressing V5-PCBP1-WT or V5-PCBP1-T99A. **d**, Trypan blue staining assays for stable re-expression of V5-PCBP1-WT or V5-PCBP1-T99A in HGC-27-shPCBP1-2 cells treated with Erastin (10μM), and the cell mortality rate was counted. **e**, The extent of lipid peroxidation in the indicated stable HGC-27 cells treated with Erastin (10μM) was assessed.

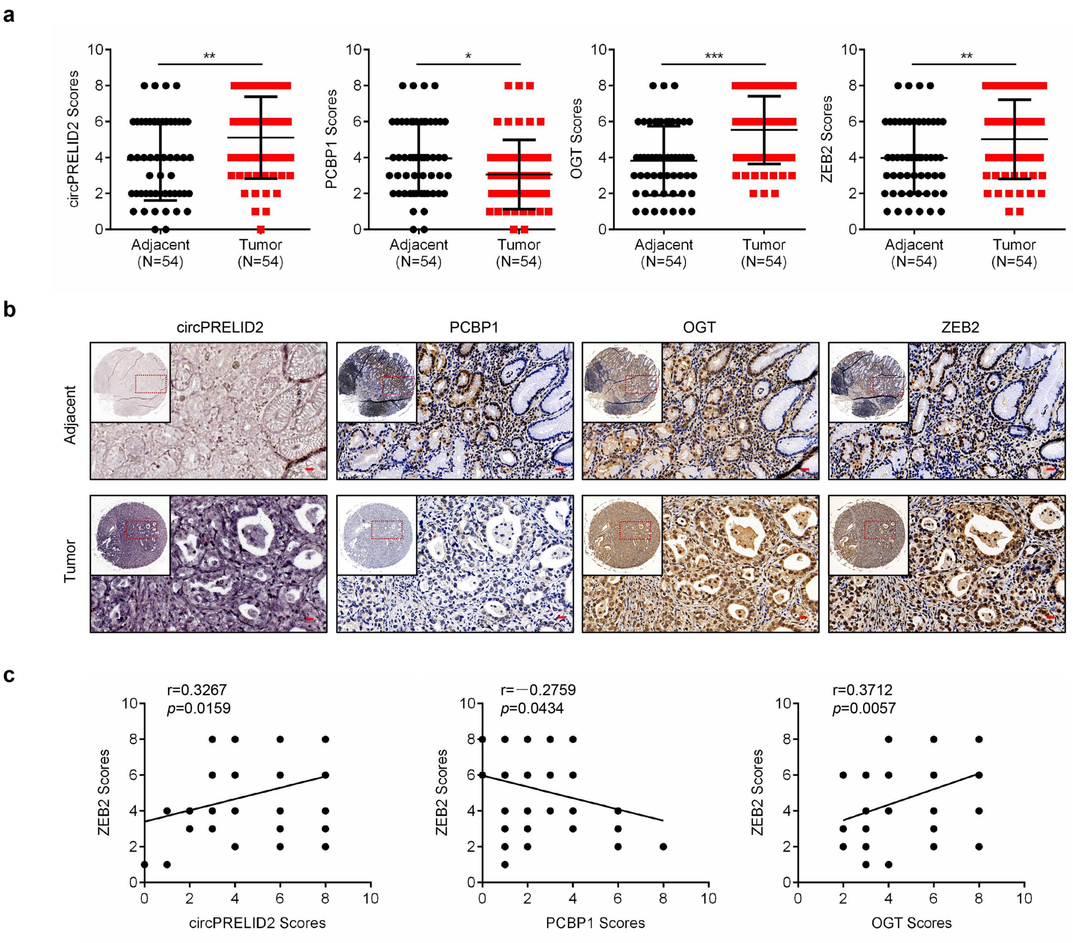

Supplemental Figure 10. The clinical relevance between ZEB2 and circPRELID2, PCBP1 or OGT on gastric cancer tissue microarray. a, ISH and immunohistochemistry (IHC) staining analyses of circPRELID2, PCBP1, OGT and ZEB2 on gastric cancer tissue microarray containing 54 pairs of gastric cancer tissues and adjacent normal tissues. **b**, Representative images of tissue sections which showing the expression of circPRELID2, PCBP1, OGT or ZEB2. Scale bar = 20 μm. **c**, The relevance between ZEB2 and circPRELID2, PCBP1 or OGT on gastric cancer tissue microarray were analyzed.

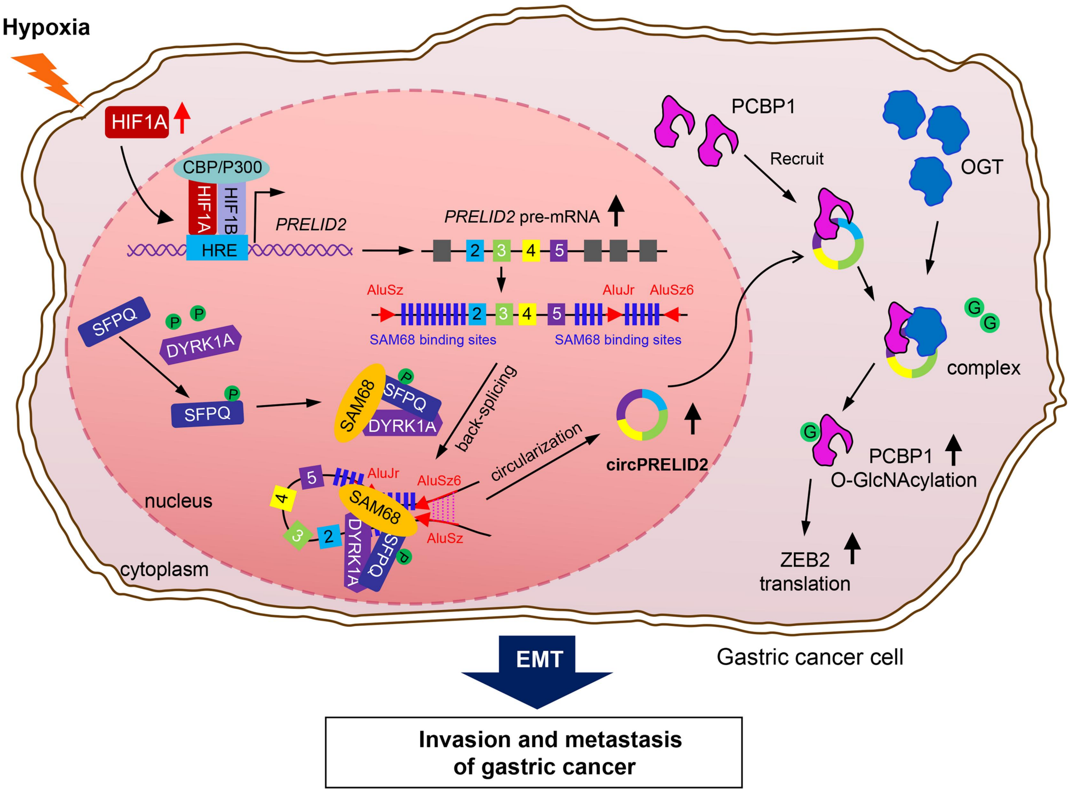

Supplemental Figure 11. Working model of hypoxia-induced circPRELID2 promotes GC metastasis by facilitating ZEB2 translation via PCBP1 O-GlcNAcylation. HIF1A directly combines with the *PRELID2* promoter to transcriptionally increase the expression of *PRELID2* pre-mRNA in response to hypoxia. Moreover, hypoxia-activated protein kinase DYRK1A mediated SFPQ phosphorylation to promote the interaction between SFPQ and SAM68, further forming a DYRK1A-SFPQ-SAM68 ternary complex. And then, the DYRK1A-SFPQ-SAM68 complex binds to Alu-containing introns flanking circPRELID2-forming exons in *PRELID2* pre-mRNA to promote circPRELID2 circularization. Furthermore, circPRELID2 interacts with PCBP1 and promotes its cytoplasmic retention. In addition, circPRELID2 enhances OGT-mediated PCBP1 O-GlcNAcylation at Thr99 site in the cytoplasm, which disrupts the binding of PCBP1 to the 3′-UTR element of *ZEB2*, resulting in the reversal of ZEB2 translation silencing, and ultimately promoting GC EMT and metastasis.

Supplemental Table S1. ShRNAs used in this study.

shRNA	Target sequences（5’-3’）
shHIF1A-1	5’-AATGTGAGTTCGCATCTTGAT-3’
shHIF1A-2	5’-GAGGAAGAACTATGAACATAA-3’
shHIF2A	5’-CGACCTGAAGATTGAAGTGAT-3’
shSAM68-1	5’-TGGACCACAAGGGAATACA-3’
shSAM68-2	5’-TCGGTCAAGATGGAGCCAG-3’
shSFPQ-1	5’-CGCATGGAAGAACTTCACA-3’
shSFPQ-2	5’-TGAAAGATGCAAAAGACAA-3’
shQKI	5’-AGAGCAGAAATCAAATTGA-3’
shSRSF2	5’-CGCACGAAGGTCCAAGTCC-3’
shCLK3	5’-AGCAGAAATATTTCTACAA-3’
shESRP1	5’-TGCACGAAGTGCTAGTTAG-3’
shNOVA2	5’-CGCATCCAGATCTCCAAGA-3’
shHuR	5’-CGCGACTTCAACACCAACA-3’
shSF3B1	5’-CCGACAGTTAGTTGATACT-3’
shSRSF3	5’-AGACTGATAATAAACCTCT-3’
shhnRNP A1	5’-GCCACAACTGTGAAGTTAGAA-3’
shSRPK1	5’-TGACAAAATGTCAAAGAAT-3’
shhnRNP M	5’-AGCTGTGCAAGCTATATCT-3’
shDYRK1A	5’-GGTATTCCACCTGCTCATA-3’
shDHX9-1	5’-AGGAAGAACAAGAAGTGCA-3’
shDHX9-2	5’-ACGAGAACATGGATCAAAT-3’
shcircPRELID2-1	5’-AACCCAAATTGTACCCCAACC-3’
shcircPRELID2-2	5’-GAAAACCCAAATTGTACCCCA-3’
shOGT	5’-TGACCAGTTAGAGAAGAAT-3’
shOGA	5’-CCAAAGTTGTTTCTAAAGA-3’
shPCBP1-1	5’-CGGCTTCTTATGCACGGAA-3’
shPCBP1-2	5’-GGGTGTAAGATCAAAGAGA-3’

Supplemental Table S2. Primers used in this study.

Name	Primers	Sequence (5’-3’)
circPRELID2	Forward primer	5’-GTATGGAAAACCCAAATTGTACCCC-3’
	Reverse primer	5’-AATCACCAGAGTGCTTAAGGACTTCC-3’
PRELID2	Forward primer	5’-CCATACGGAGTCACTGCCTTAC-3’
	Reverse primer	5’-CCTGTGATTGAAATCCTGCCTCT-3’
HIF1A	Forward primer	5’-TATGAGCCAGAAGAACTTTTAGGC-3’
	Reverse primer	5’-CACCTCTTTTGGCAAGCATCCTG-3’
HIF2A	Forward primer	5’-CTGTGTCTGAGAAGAGTAACTTCC-3’
	Reverse primer	5’-TTGCCATAGGCTGAGGACTCCT-3’
CHMP4C	Forward primer	5’-AGACTGAGGAGATGCTGGGCAA-3’
	Reverse primer	5’-TAGTGCCTGTAATGCAGCTCGC-3’
eIF4E	Forward primer	5’-ATGCCTGGCTGTGACTACTCAC-3’
	Reverse primer	5’-GAGGTCACTTCGTCTCTGCTGT-3’
HRE-4	Forward primer	5’-GCTGCCAACCACGGCTTATTCTGTG-3’
	Reverse primer	5’-AGAGCGGCCCTGGTGCCCTC-3’
HRE-5	Forward primer	5’-GAATCCGCAGTGCCCACTACAGC-3’
	Reverse primer	5’-CACCACGGAGCAAGGAACTAGAGGATAC-3’
SAM68	Forward primer	5’-GGCATCCAGAGGATACCTTTGC-3’
	Reverse primer	5’-CCTTGACTCTGGCTGTAATAGCC-3’
SFPQ	Forward primer	5’-ACTCCTCGTCCAGTCATTGTGG-3’
	Reverse primer	5’-CGTACTCAAACGTGCCATGCTG-3’
Primer-1	Forward primer	5’-CAGATACACTATTCATTAGCTGGACAATTTG-3’
	Reverse primer	5’-GTGACAAGGTTGATAATAGCCACTGC-3’
Primer-2	Forward primer	5’-TATACTCATAGAGCCTGTATAGTATGG-3’
	Reverse primer	5’-TTATATGCAATAAAGGAATAAGCTAG-3’
Primer-3	Forward primer	5’-TAGATCCCTACCTCATGTAGAATAC-3’
	Reverse primer	5’-ACAAAAGTTTCTAATTGATGACATC-3’
DHX9	Forward primer	5’-AGCTGTGGCTACAGCGTTCGAT-3’
	Reverse primer	5’-CTGATTCCTCGAATGCCTGCTTC-3’
PCBP1	Forward primer	5’-GGACAACACACCATTTCTCCGC-3’
	Reverse primer	5’-AGCCTTTCACCTCTGGAGAGCT-3’
OGT	Forward primer	5’-CAGGAAGGCTATTGCTGAGAGG-3’
	Reverse primer	5’-CGGAACTCACATATCCTACACGC-3’
OGA	Forward primer	5’-GCAAGAGTTTGGTGTGCCTCATC-3’
	Reverse primer	5’-GTGCTGCAACTAAAGGAGTCCC-3’
ZEB2	Forward primer	5’-AATGCACAGAGTGTGGCAAGGC-3’
	Reverse primer	5’-CTGCTGATGTGCGAACTGTAGG-3’
QKI	Forward primer	5’-TCCGAGGCAAAGGCTCAATGAG-3’
	Reverse primer	5’-GCTCTGTTCTGAGCATCTTCCAC-3’
SRSF2	Forward primer	5’-GCGGTGGCTACGGACGCCG-3’
	Reverse primer	5’-TTCGAGCGGCTGTAGCGAGATC-3’
CLK3	Forward primer	5’-GTTGCCCTGAAGATCATCCGCA-3’

	Reverse primer	5'-CCGTGGAAGTTGAACCAGTCAG-3'
ESRP1	Forward primer	5'-GCACAGAATGCGTTGAGGAAGC-3'
	Reverse primer	5'-GGGTTGGAAGTGGAATGAGAGG-3'
NOVA2	Forward primer	5'-AGCCACCATCAAGCTCTCCAAG-3'
	Reverse primer	5'-CATCGCTTGTGGGATTTCTCGG-3'
HuR	Reverse primer	5'-TGTTCTCTCGGTTTGGGCGGAT-3'
	Forward primer	5'-TCTTCTGCCTCCGACCGTTTGT-3'
SF3B1	Reverse primer	5'-TGGCAAGCGAGACACACTGGTA-3'
	Forward primer	5'-GCCAAAGCACTGATGGTCCGAA-3'
SRSF3	Reverse primer	5'-GATTATCGTAGGAGGAGTCCTCC-3'
	Reverse primer	5'-ACGGCTTGTGATTTCTCTCCCG-3'
hnRNP A1	Forward primer	5'-GCCCTGTCAAAGCAAGAGATGG-3'
	Reverse primer	5'-CGACCACTGAAGTTTCCTCCAC-3'
SRPK1	Forward primer	5'-GGGCATCATCTGCTCAAGTGGA-3'
	Reverse primer	5'-GTCAGTGTGGATGATACGGCAC-3'
hnRNP M	Forward primer	5'-CATGGGTCGATTTGGATCTGGG-3'
	Reverse primer	5'-TCAATGCCAGGACCCATCCTCT-3'
ACTB	Forward primer	5'-CACCATTGGCAATGAGCGGTTC-3'
	Reverse primer	5'-AGGTCTTTGCGGATGTCCACGT-3'

Supplemental Table S3. Probes used in this study.

**FISH/ISH Probes**	**Sequence (5'-3')**
Cy3-labeled circPRELID2 probe	CATGGGGTTGGGGTACAATTTGGGT
Digoxin-labeled circPRELID2 probe	CATGGGGTTGGGGTACAATTTGGGT
**RNA Pull-down assay probes**	**Sequence (5'-3')**
Biotinylated circPRELID2-WT probe	CAT**GGGG**TT**GGGG**TACAATTT**GGG**T
Biotinylated circPRELID2-Mut probe	CAT**ATCA**TT**ATCA**TACAATTT**ACT**T

Supplemental Table S4. Correction between expression of circPRELID2 and clinicopathological features in GC tissues.

Characteristics	Case	circPRELID2 expresson		X^2	*P* value
		Low	High		
**All cases**	51	28	23		
**Gender**					
Male		17	17	0.99	0.32
Female		11	6		
**Age (years)**					
≤60		10	9	0.063	0.802
>60		18	14		
**Tumor size (cm)**					
≤5		22	14	1.906	0.167
>5		6	9		
**T grade**					
T1+T2		13	8	0.707	0.4
T3+T4		15	15		
**Lymph node invasion**					
Negative (N0)		19	6	8.816	**0.003**
Positive (N1-N3)		9	17		
**TNM stage**					
I-II		14	9	0.603	0.438
III-IV		14	14		
**Histological grade**					
Low		8	11	2.003	0.157
Middle-High		20	12		
**Tumor location**					
Proximal		5	10	3.993	**0.046**
Non-proximal		23	13		
**Nerve invasion**					
Yes		12	15	2.534	0.111
No		16	8		
**Vascular invasion**					
Yes		15	19	4.791	**0.029**
No		13	4		

Supplemental Data S5. *PRELID2* gene sequences (Pre-*PRELID2* sequences) and circPRELID2 sequences.

***PRELID2* gene sequences (Pre-*PRELID2* sequences):**

The highlights represent:

Exon

Alu

(A/U)AASAM68 binding sites(A/U)AA

>chromosome:GRCh38:5:145471199:145835969:-1

GATTAACGTGCTGGTTCTCCTAACATCTGTTGGTTGCAGTAAATAATAATACTCGCAACTC
TTTTGTATCCTCTAGTTCCTTGCTCCGTGGTGTTTAACCTTAGCGAGCAAAGGAACTTAC
CTGCAGGTTTTGGTGCACTAAAACGCGGTTCTTATTCCCGAGGCAATCTTTACCTTACAC
GAGGCCCAAACCCAAGCCTCAGAGGCAGCTGCCTGGTGGCATGAGTAATGCTGGCCTT
TGTCACACCAGGCCTTCCTTATTAATACTTTTTTCCCGTGCTGCCAACCACGGCTTATTCT
GTGACTCCAGGCGGTGATACCATAAACGCATAAGCCCAAGGTCCAGGTTTCCTGGGGC
GGGGCGTGATGGCTCCCAGGCAGAGAAGCTTTTCTCGGCAGGCGCGGCCGCGGGAGG
GCACCAGGGCCGCTCTGGCGCCGCCAGCGTGGAGACCGCGGGGCCCCGGGTGCTCT
GGGAGCGCTCCAGCGCCTCCCGCGCTCTGTAACCCCAGGCCCCGGACTCTGGCAGTA
GGGGCGTGGGCGTGGCCGACTGCGCGTTTCTCCGAGTGGCCAATAAAGTTTCGAAAGT
TTGAAAAGCGGAGGAAAAAGGCCACGCCCATGTGGCTGTCC**GAGTGTGGGCGCCTCCG**
GGCCTCTGCGGAGCCCTGGGCAGCTCTGAGCTCGCGGAGGCGTGGCCGGTGCGCGG
GGCCCGCGGCGCGCGGGGATGGGGGTCTCGGTGGATGTGCACCAGGTGTACAAGTA
CCCCTTCGAGCAGGTGGTCGCCAGCTTTCTCCGAAAGGTACCGCCCCGCTTCCACCTT
CCGTATCCCGCGCTGCCTCCGCTGCTTGCCCCTCTCTCCTCTCCTTCATCCACGCGGAA
GCTGGCACCGCCAGTGTCTCTTTCCAGCTGCGGGCTTTGCTTCAGGGATGTGTGGGTC
GAGAGGGATTTCCTCGAAGACTCCCTTTACAGGGAGCGGGAAAAGGGGCCGTGCTTGG
CATCACGTTCACACCTGCCCCAGCTGAAAGAAGAGGCTTTCCTTTTAG**GAAGAGCCTCC**
GGGAATCCCAGCTCGTGAGCTCCGTGGAGTTGCAGATGACCGACGTGTGGAGCTTTC
CGGTTCATCACTGACCCCTTAAAAGCGAGGGGAGGCCATTGGGCAGTCATCCTGGTTT
ACTCAGCATGATGGTTCTGCTGCCCCATCCGCTGCTCCTTATTAAACAGCTGTGGGAC
ATCGTGCAGGTTGCTTACTCTCTTTGTGCCTTAGTTTGCTAATCTGTAAAATGGGGAAA
ATAATAGTACCGACTCATAGAGTTGTTGTGAGGATCCAAATGAGGTAACTTGTGTAAAG
TGCTTAGCACAGTTGTGACCTAAATGAAGAAACTGAGGCAAAATCAATATAAAAAGAGAG
TTCATTCGAGCCAAGTTTGAGGGCTGCATCCTGGGAGTGAAGGTGCCCTGAGTATACAT
TCTGATGAGCAGAAGTCGAAAGTGGGTTTTTAAAGGAGAATAAGAAGCACTTCCTAAGTT
GTTTACCAAGAATTTATATTAAAATAACATAAACTATTGATTGTCTGTACATTGTTCTTTGT
ATCACAAATTCCAGGAACTTGAAGATAATGGGTAAGGCAGCTGGTCAGGAACAAAATGC
CTTTAACCAGTTGCTCCCAGGAATGGGTTGGGTGAGGAGGGGACCACTAAAGTCCTATA
CTCTTGTCTCTCTGGGCTTGATTGATTTTGCATATTTCACTGTAACTCACACAATCTGCTC
TGAGCTATTTTTCTTTTTCTCACTGCTACTGATGTTTAGTTAGGGCTCTAAAATGTTATTGT
TGTTTGTATTACTACAACCGATTTCAATACAGATAATGAACTCTTCCTCAATACTGAGGAG

CCCTGAATCCCAAAGACTGTTTCTAGGATTGTGGGTCCTGATAATAGGAAAATGGGAAAT
GTCTTTGAAAAATCGAAAGCGCCCAGGCCTGGGAGGCAGGACTCCTGGACTCTGATCTG
GTTCTATGCGTGGTTAGTTCTCTGGGTGACCTTGGCTGAGGGTCTTTTCTCTAGGCCTTA
GTTTCCACATCTATAAGATGAAGGGGTTGTACCAGATGATCTTTGAAGGTCCCCTTCGGT
TTTCATAAGCAAAATTTGCAACAGTTGCAATTTGCCTTGGTTAGCAGCCACGGGACATTG
AACTCCATATGCAACCCGGAAGCCAAACTAAGGGAGATGCTGGGAAGATGAAGGTCATT
TGTTAAACGATAAGGTGTAGCCTCCGTTAGAGGAGGTGACACGGTGGCCCTGGTCACAG
AGGAGTGAGGAGGACACTGGTTAACACTTTGTTGCTATAGGGGGCCTCTTTAGAAGGAA
TGTCAGCTTGCTGATATGTTCGTTGATTTTCAGTATCTAGACTAGTGCCTAGCACCTACTA
GGTATTTAATAAAAACCTGAGCAATGAATAAATGAATGAAAGAATGATTTACAGACCTGTT
GTAGACTTTGCCTTAGGCCAGGCTTCTGGGCCCAAAAAGTAACAGCCAACACCCAATTTT
TTTGTTAGAACTGGCACTTTGGAGTCTGGGTACCCTTAAGCCACTGCTCCTTTGTAAAGT
CTTTTTGGATTGTCATCATCAAGAGTCAGTTGATCTCCACCTTCTCAGAACTCACAGGGC
ACTCTGTCTAGGCGTTGCTGACCGTCTGCAGTGTCAGATGGTGACTTCTGTATGTGTTGT
GTTTCCCGTTAGACTCTAAGGTTTTTAAAGGTGAGACTCACTCCTGCAGAAGCACATAAC
ACAATGCCAAACTCTTATTTACGGAGGTCCTGGCGCATTGTCAGCTTTTGGTAAATGCTT
TTCTTTTGTTGAATACTTATCTTCTGTGTGCCAAGCTTTGTGTTAAGTGCTAGAAAAATGT
GGGAGGTCACCGCAGACCCTGTTCTCATGGAACTGATGGTGTGTAGTGGGGAGATTAAC
ATAAATAAATGATGCGCAAATGAACACAAAATTCAAATTGATGATGTGTACTTATGAAAGG
AAAATACATGGTGCTTGAATATAGCAGGGAGGCCTGCTGAAGTCAGAGAGGGTGATCTT
TAGGAAAGGCTTTCCTGAAGAAGTGGCACTGGTGAAATGATGGCTGAAGCATGAATAGA
AGTTTCCTAGGAAGGCTGGGTGTGTGGTGTGTGGTGTGGTGTGTGTGGTGTGGTGTGG
TGTGTGTATGTGCGCTCATGTGTGCTTTCCATACTTTATCATGTTTACTGCTGCCAGCAC
CCTCAGGAAGTAGTTATGATTAATTTCCATTTTACAATATAGAGAAACTGAGGACCAGAG
AAGTCCAAGTGCCTTCCTCAAGTTCCCACAGCTAGCAGGTAGGACAGCCAGGATTTTAA
CACATGGATCCGAAACTCACAGCCTTAGCCACCCGGCTGTGCTGCATTGCATATAATAG
GCACTTCAACGATTATTGAGGTAATACAGGGTTTCAGAATCAACTTTGCCGGCTGGGCAC
GGCAGCTCACTCCTGTAATCCCAGCACTTTGGGAGGCCGAGGCGGGCGGATCACTTGA
GGTCAGGAGTTCAAGACCAGCCTGGCTAACATGGTGAAATACCATCTCTACCAAAAATAC
AAAAAATTAGCCAGGTGTGGTGCCCTATGCCTGTAATCCCAGCTACTCGGGAGGCTAAG
ATGAGAATCACTTGAACCCAAGAGATGGAGGTTGCAGTGAGCTGAGATCATGCCATTGC
ACTCCAGCCTGGGTGACAGAGCAAGACTTCATCTCAAAAACAAAAACAAACAAAAAAACC
CAAAAAACTTGGCTGTGCTCAATGTGTTTTCTTTTGTTCACAGTCTTCTCTCAGGGTTTGC
TCAGCTAAGTGGACCTGTTGACCCTGATATAAAGATATAACTTTGATGTGTGTTAGGGTG
AGAACACAGAATGTTTAAGTTATCTATTGGTATGTAACAAACCACTCCAAAACGTAGCGA
CTTAAACCAATGACAGGCTTTTAATTTTCACAATTCTGTGAGTTGACTAAACTCATGTGGT
TGATTTTTGTGTGCTGTGTGGTATAGCTGAGGTCACTTATGTAGCTGTATTCAGTTAGAA
CCTGTTGTAATTCTTATAAAATATTTAGCAAAGTACCTGGTACATAGTGAATAAGTACTAT
GAAAAGGCAGAACTGGGAAGGGCAGAACTGGGAAACAGAACTGGGGATGTCAGAACTG
GGAAATGGAAGGCAGAACTGGGGAAATGAGAGACAAACATTTGGAGACTAAAGGTCTAG
TGATGGGACAGTGTTAACATCATGCATGTCACTGTACTTCCTTTCTTTCTATTCATTCATA
AAGGAATCCTTCAGAGATTCCTTGGGAGGAACTATGTGACTATTTCTAAGATGTCAGTAA
ACCCATTTGCTGGGTGAATTTTCCCCTTATGTTATTCAAAGCCATGAACTGGTAGTCAGT
AGACCTGGATCTTGATCATTATGCTTTCACTGTTAGGTTGGGTGGCCATGGGAAACAATT

TTCACCATCCTATACTGTGTCCTTGTGCAAATTAGAAAAGGCATCTTTTAGGGGTCCACT
CCATAAGCTCACAGTTCATAGGGCTAGGGGAAAGCAAATGAGACTATTTATGCAAATTAG
AAAAAGTTACTCCTTCCTTTAGGTGGATGCAGTCCTGTGCCCACAGGCCCACTTGACCA
GGAGCCCTTGTGCAGTCAGCAGCATCACAACTGTGTAAGCCCTAGTTTCTCCTCTGTGA
AAATGGTAATGCCACCTACCCCACCTACCTTCCAGGGTTGTTGTGAGGATGTCACATGT
GTTAACAGTTTGAAAAGCATAAGCTTTTTTATAAGCCTAAGGTAGTAAGAATTATCTTTTT
GGCAAAATACACTTTTATTTTTCTTTCAGTCTTTATGATTTTTTTTTCAAAGGAAAGCACTT
GGCAGAAGCGACAATCATATGAGGATCACTGGGAAGCATTTAATCTGATTTTGCACAATG
GTATGTTTGAAGCTGCTTTAGCACTGAGGAAGTTCTATTTTAAATCAGATCTACTGATCTA
TGGCTTTTGGTTTGTTAGGAGTTGCCTTCCAAGTTATAATATACTACGGTTTTTAAAACCT
GAAGAATGTTTGCTCTTCTTACATCTGCCTGTTGAATACCACAGGCAAAATATTAGCTGC
ATGCTTTCAAAGCTAAGTGACACTGTGAAAACAAACAATATGATACTCTTTGTTCCTAAAA
TACAATATTTCTCAATAAACTCTTCATGGAGGAATGTTATGAAACCCAAAATAGTTTATTCA
TTGGATGCTGAACTGTATGTACTTTCCAAGAACTATAGTGTCAATGCACTTGACAGTGTAT
TTTGGTTTCTTTCTTTGTGTGATTCTTAGCTTTCTAGCTCTTATCACTTACCACTGTTTCCT
TCCTTTTACTTAAAATCATCTACAAAGAGAACACAAAGTTGCATAGATGAATGTAGCAGTG
TAGAAAGTGGGTCTTCTCTGGAATCTCACTTAGACCTTTTTAATGGGCTTGCCTTTCATGA
GCTGGATTCAGAGAACTTCTTAAATTTCATCTGCCTGAAATTTAAACTCATTAAAAAGGAT
CCAGAGAGCTATTATATTGACTGGATTTTCTTGTTTATATCTAAATGAGGCCAGAGAAGTA
AAGATTTTTCTTACCTTCAGAAGGTTGACTTTCTTTTTATGCTGTGGTCAAAGGAGTCATA
GTGAATAGAATATCACCTTCATAGGTGGTGATTTTTGTCATTTGTTTCTGATGTATCCCAA
TTACCCAAAGAGATCCTGGAACATAGTAGGTACTCAGCAAATCCTTGCTGAATGAATGAA
TGAACTGACTCTTTTGGGGATTAGCCCTGTGCAATTTTAGATTGAAGTCAGTATGGGATT
CAGAAACATGTCCTTACTTTGGATACTGTAATAAAATGGACAGATTAAAATATTGATAATT
TCTTTGCTTTGACAGAATGACCCTGGGTGTTATTTATTTTGGCTGGGAGCCTGGTGTGCT
GAACAAAATGGTTTGAATGTTTACTCTTAAGCTCATAAAGGGGATTTATGAAGGGCTATTT
CCATTTAGAGTAGAAAGTTGATTGGGTAATTAATAATGTTTCCATTTCTGATCCTATCTTTT
CTCCCTTGCAAATCTGACACTTACATGTTATCTTTTGTCACATGCTGTACTAGTCTTTCTT
CACTTTTGTCTTCCTGTCCTCAGTTCCACTAGAAGATTAGGCATGATGTAAAGGAGAATG
GTGCAGATGTGCAGAATGTGGGCTCTGACATTGGATGGGGTGGGGAAGACAGGGATGG
AGCGCTGTTAGCCCTGTGAGCTTGGACAGGTCAGTTCATTTTTCTGTGCCTCGATTTCCT
CATCTGCAAAATGAGAATAATAATCACTGTCTTCTGGAGTTGTTGGAGAAATTAAATTGGA
TAATGCTTACAGTTATATGTAATATACCTAGCTTAATTTCAGCACACAGCAGGCTCCTTTA
AAGTACTTGTTGAATAATGATAACAACAACAATAATTTGTTGCATTTATTTCATGAACCTGA
AAATAAAAAGCTGAGAGAAGTGGCCTCAGAAATCATTACTAACTTAGAGTAATAAAGGAG
AGAGTGAGCATGAATAAAGGTACACATTTGAATTAGGAATATTTTCTAAATGCCATTTGAA
CAATTGTAAAGTATACTTAGAAACTTATCTAATGGAGATTGTGGCTTAATCAGTGTATAAA
AGACAGAAGATATTGGCCAGGCATGGTGGCTCATGCCTATAATCCAAGCACTTTGCAAG
GCCTAGTTGGGTTGATCACTTGAGCCTAGGAGTTCAAGACCAGCCTGGGCAACAGGGC
AAAAACCCCATCTCTACCACCACCACCACCACCACCACCAACAACAACAAAACCCCAAAC
CACACAGACACAAAAACAAAAAACCCCCAAAATTAGCCGGGGCATGGTGGCACACACCT
GTAGTCCCAGGTACTCGGGAGGCTGAGTTAGGAGGATCGCTTGAGCCGGGAGGCAGAG
GTTGCAGTGAGCTGTAATTGCATCACTGCACTCCAGCGTGGATGACAAAGTGAGACCCA
GTCTCAAAAAAAAAAAAAAAAAAAAAAAAAGATTTTTTTCACTATCTAAGTACATGGACCTG

TCTTCAAGGAGTTCACTGTCAAAAGGGACAGAGGAGGATATATTAATTTTCATGATAAAG
GCTTTCTTGGGGGCATATGGCAGACATTACATTGTAGTATGGGGCAAGATTTATATTTTA
ATTATAAATCCATGAAAGCATTTAAACTATTTAGAAACAGTTGTCTTTTTAAAAACAAGTAA
ATATTCTCTCCAGCACTCTTACTTCTCTGGTGCAGCCCTTTCCCTAATGCTAGTGTGGAG
TGAAGTGTCAGGGAGGCCTTGTATCTTACACAAATTCCGAACTGGTAAAGCACGAGCTT
CTGAGTTTTTTATTAGTCTAGGAAAATCAGATATATTATCTTCTAATCATTTTAAAATCATT
GTGTAAGTATTTTTGGATTGCAGATAGATGCCAGCAATGTATTTAATGCCACACCACAGT
AAATTGCAAGATAAAAACCAAAATCTTGAAACTTTTTAAGTCTTCAAAAGTGTTGATTCATT
TGTTAGTAGCAAAACCAGCTACATTTATTTTCTACTTGCACAGTGCTAGGTATTATACTGT
AATTAAATTGTTTCCTTTAATTCTTATAACAACTCTTTGAGGTAAGTATTTATTCTCATTTTA
CAGATGAAGAAACTAGGGCAGATTAAAGTGATTTGTCCAAGCTTCTCACAGATAGGATTC
CAATTCAGATCTCTTGGGCTCTAAAATTCACTGTTTTCTAACATCGTGTCTTTAATTTTGTT
TTTTATTTCTTTTCATTTATAAATAATTTGACTTATAAAACATTTCAAAAACAATATTGCAGA
AACAAAGAATTCCCACATACTTTTTTCCAGCTTCCCCAAACCACAGTATACTTTTTAAAAC
CAGGAAATTAACACTGATACAATGCTATTAACTAACCTATAGACCCTATTTAGATTTTGTC
AATTGTCCACTAGTATCGTTTTTCCAGTCTAGCATCCAATTCAGGACCCCACATTGCATTT
AGCTGCCAGTGTCCTGCAGTGTAGGACGGTTCCTCAGTCTTTCCTTGCATTTCCGGACC
ATGGTGATTTTGAAGAGTGCTGGGCAGTTATTTGTAGTGTATCTTTAGCTGCACTGGGTT
TATGCATACATTTGATGTTGTGATAAGAGGATTTTCCTCTGTGTTACAATGAAAAACTGTC
TTTTCAAAACCTTCACTATGCCGTTGCTGATTATTGGGTCATTTTTGAAAGAAAGAGGCTG
ATGTGCTGTTGGTTGGCCTTTGTTTGGAACCATAGCTTTTCCTGGCCAGAAGGATCTTGG
ATGATCACCTGGTGGTTCATCACCCCGACCTCACAGATGGCAGCGTTGTAGTCTGCATG
TCTTGGCTCGTTGCCCTTTGTTCTTTCTATTTCATCATCCTATTGAAAGACCAATGAACAG
CCATTCCAAATACTTTTCAACTTAAGTACGTTTTGTCCTTGATCATTGAAATTTTGATTAAT
TTATAACTCGTTTCGTCTTGGTATAAAATCAACTTTGCTTTTCTTCTGAGATAGACTATGGT
GTGGAGCTCATAAAAGGAATTTGGCATAGCTCATGGTGATTTCTTTCTGCTGCTAAACAC
CAAATCTCTTTTCAGCAGCCCCATCTCTTTGCATGTACATGTTTGGCAGCAGCCTAGATA
ACAGATTCCCTTAATGCTTATAACACGGGTATGTTAATATACCAGACTTACTGTAGGTCTT
CTTTGCCTTCTTTGAAGGACTCTTCAGACAGATCTGTCAGTCACCTGCTTCCTTGTACTCT
CTCTGGTAACCTCATTTGTAAATTGGTAGACAGTGACTCCTTTGGATGTCACTGACTGTTT
CTGAGAGGGGAAGCAAATGTTATTAAAAAAAGGCATTGGCTTTTGAGCTTTTTGGAAAGT
TCATATTTTAATAACTCTTTTAAGAGTAGGCTTTCTGAAAGCTTATGGGTTTTTTTTTCCCT
CGGCAAATATGAATGCAAAATGTTATATCTTCATGAAGCTTCTCCCAGAAACATATTGAAT
ATTCTGTAATTAGATGGATTCAGTTTCTTGCCTCTAACTTTTTATATATATGTGTTTGCTTT
AATGCATATTCCCCCCCAATATTATGGAGTTCTTGGCTTATCTTTTAATATGTAAAAACTAA
AGCAAGCAGATACAACAGCTTTAGGCACGTCTACATTTTACCCTTGTTAGACTGTTACAT
ACATATGATATATACAGTGGTATTTGTAGTAGTTTCATGTCTTCGGGAAAGGAAAAGGAG
TTAGTTTACACGTTCTGTGAAGAAATTAAAAATTATAAGGAAATGCAAAATGAAGTCAAAT
ATACCTTTAAAAATCCCTTAAATCATCCATAAAGTAGTATAGCATACATGGCATATGTATG
CAGCCTGTGGGATCGTTCTCTTGCACACTGCAGTTTGTGATCAGCTAAGCCAGGATCAA
GAAAGACAAGGGCTTTTGGCAGATATCTGGGTCTTCAGATCACATTGCTTTCATGAGAGT
CTCTAGTATGAAATGGGGATTGAAGAATCCCATGTGAACCTGCTTGTCTGCGGGCTTTAC
ACTATTATATTTATAGCAGAAATACTCTGTATATTAATACTTGATAG**GTTTCCACGCTTTGA**
GAAGGACTGAATGAGATCATGCGCATGGCAGCTTCTCTAGGATAGCACCCTGCGTGAA

CACAGTAGCTGCTCAGTTAAGTCTTTGATGGGTGTGATCCTGATTCAGCTTGATCAATT
CTGCAGTCATACTGGATTGTGTTTGAAAAGGTGTCCAGAAAAACCTGGCCATCTGCCT
CCTGTCTTCCCAGGTAAGTGTTCCTGGTATTGACCTCTGAGAAGCCTTCCCTCTTTCAAC
TTTTTCTCATGGGAGTTTTGGTAAATTTTCATGAATTAAAACATGAATATTCAGGTTACCAC
AAGATGGCGTCAGATCATTTCAAGTAATGCCAGGTAGGGGGAGCCTCAGTTCAATAAAT
CTTTAAAGAGGTTGAAATATGTGCCTTTCATAGTATGGTAGTTAGCTATTGCAAATAAACC
CCTACTTTCTTCTACTGTTCTTCTATGGAAACTTGAGGCTCTTGGCTGTTCAACTCCAAAA
ATTATGTAAGTGTGAAGTATTTGAGTTTATAATGTTTAAGAATTTCTCTGATAGCTTAGTGA
AGTAATCAGAACTTGAAAAATTTGATCATCATGTCTCCCTGGTGTTTACATTTTCAACGTC
TTTTATAGTTTAGTTATCTTTCTACAACTTATGTTGAATTTGTAATATTCAGTCAATATATAT
TTATGGGTATGTAATGAACAAACTAGAACTGTAGTAAACGAGATAAGCACATTTTACTTTA
GTGTACAAGTTATAATTTATACACATATAATCACAATTCAGAGGACCTGTGTGAGTCTCAA
ATATCTCCCTTGCTTTAGGGGTACTGTGTAAGCGAAAGTCTCCTTTCCTGAGGAGCAGAG
TATCTAAACCTGACCCTCTGTCATGGGCATGTAACTCGGAAGGGCTGAACTTCAAGAAA
GCAATCACTGTAGTTCCAAGTTTTTTTTTTTTTTTTTTTTTTTTTTTTTTTTTTTGAGACAGA
GTCTCACTCTATCACCGAGGCTGGAGTGCAGTGGCATGATCTCGGCTCACTGCAACCTC
CGCCTCCCAGGTTCAAGCAATTCTCCTGCCTCAGCATCCTGAGTAGCTGGGACTACAGG
CATGCACCACCACATCTGGCTAATTTTTGTATTTTTAGTAGAGACAGGGTTTCACTATTTT
GGGCAGGCTGGTCTCAAACTCCTAACCTCAAGTGATCCACCCGCCTCAGCCTCCCAAAG
TGCTGGGATTACAGGCATGAACCTCCATGCCCAGCCAAGCATTTTTTTTTCTAGGGCTAA
AATGAGTGAATGAATGAATATATAAATAAATAGATTTTTAAAAACCAAGTTTTTCATCAGTT
ATCAAGATTTCAGTTTCCAGGATGGAAGTGTGAATGACAGAGTTTACCAACATGAAACAT
GAAGAGATATTGAGGTTGCTGAAAGCTGAGCCACTGCTCTCTTCATAGATTGGTTTCTGA
CTGAAGGCAGGCTCATTTTCCAAATTGATCCCTGAGGTCATTGAGGTTTATATTCCCAAT
TTTTTATTTATCCAGAAAATATTGACTGAGTGCTTTTTATGTGCCAGGCATTACGCTGGCT
GTTGGAGATACAGGAGTAACTGAAATAGACAGTATTCCTGCCCTCAGAGAGCCTGTGTT
CTAATCTAGTGTCTTTTTGGGTCACAGATATCTTTAAGAATGTGTTAAAATGATGAACCCC
TTTCCCTGAAAAATCCATTAATAAATCAATATCACACACACACACACACACACACACAC
ACACACACACGCCTGCTGTGGGTGGAGACCCACAGTCAGAGTTTTGTATTTCACTTCCTA
GGGTTCGGAAAACTTAAAGCTCAAGTTTGCAAAGTGAGGGGCCTTTCTTCTTTCTCCCCT
CAGCCTGCCTGCTGCTTTTGCCTTCTGCTCAGCACTTTGTGATGTCATGGATAAGACTTC
CTGCCAATTTTCTTTTCAGATTTGATCACATGATACGTCGGTAAACCTCAGGGAAGAGAG
TCCAAGGGGGCTCAGTAGGCTTTGGTCAGCAGCTACCTTGTCCTGTGGCTTCTAGGAGG
TCACTTCTGCTCCCAGTGTCTCTCTTTTCACATCTGAGTAGTGGAAAGGATTGTGTTAATT
TAACTCTTAGGTGATTTGGTGGTAGGTTAGGTGGGCAGGCTTTGCAGGCTGGCAGAAAG
TATCGGGGGTAAATTATGTTACCCCCCAGATACACTATTCATTAGCTGGACAATTTGAGC
AAATTATCTAATTTGTCTAAGCCTTATTTTTCATATATGAAGTGGAAAAAATAACACATACC
TTATAGAATGGTTTTGTGAATTAACTGAGCTAATGTATCTAAAGTATTTAAATTGCCTACG
ACAAAAGCAGTGGCTATTATCAACCTTGTCACAGCCAAATTGCTTTTCAGGGGAATTAAA
TTAACATGTTAAATATTTATTGAGTATTTACTATGTGCCTAGCAAAGCATACTTCTATGACT
ATGCTACTGTGATGCTAACCCAGCCACCAGACAATGATATTAGACCACAGTGAAGAGGT
CCACTCTGTATTAAACAGGCCTGGTTTAATTCCCTGGCTCCACCAGGAGCCAGCTGGGT
GACCCTAAGCAATCCAGTCTTCATTCAGTTTCCGTATCTGAAAGATGGGGATGGTGGTAT
CACATAAATCAGTTATAACAGAGAGGCTGTTGGATTTAGACAAGATTGTGCAAGGAAAGC

ATTTAGTACAGTGCCTAGCACAAAGAACACTCTTGATTTATGTTAGGCTGGTTCAATATTA
ATAGTATTATTATTTACTGATTAGCTTAACCTCATCCCCCTCAGGGCTAGTTGGATCTAAA
GAAGGTTTTCCCCAGATTTCATGTAAGTCAAGATGATGATAGCATCTCACTTATCTAGGC
CCAAGTTATAAAATTAAGATCCATGATTCAAAACTGGGCTTTGTTTAGAAGTTTCTTGGAA
AATATTTACTAGTCCTGCAGACAGCTGTGGTTACTTCTAAATAGTTCACCTTGGTAGAAGA
TTAATGGTAATTCTTTTTTATATATTTTTGTTTAG**TACCCCAACCCCATGGATAAAAATGT**
CATCTCAGTAAAAATCATGGAGGAAAAAAGAGGTAAGTTCTCTGTACAGTTTTTTCAGT
AATGATCATTAAATGATAGTGATTTTTGTAAAGATGTGTGTACGTGTGTGTGTGGCCTATT
AGGTTTTTTTTAAAAAATTGTTCACCTGCAGTTTCTTCCATTATTAGTTTTTAAACAGGTAT
ATTTCTGTACTTGAGGTGACTTAGAAAGGTGTGGCAGGTAGAGCACCAGGATTTGTGATA
GTAAAAGGCCTGCTGATGCTTTTTGTCACTTTCTAACTAATAATTGATGACAACACTGAGC
TATATATAATTGTAACTGCTAACATTTTTGAGCACTTAATATGTGGCAGGTACTCTTATTAA
TTCATTTAATATTCAGAACAATGTTTGGAATAGGTGTTAGACTACTCATTTAATGAATGGA
GAAACTGGGATACAGAGAAGTTAAGTGACTTGCTCAAGGTCACATGGCTGTAGCCTATTA
TATCATTTTGTAGATGGCAAAATGCATATGATTAAATACGAGATATGACTATAAAGTGATG
GCTTATGGAATCACAACTACTAGGCAGTGTGGTTTAGCATTGACTAAATCAGGAGTTGAC
AAGCTATGGCTTGTGCTGCCTGTTTTAGTAAATAAAGTTTGACTGGAACATAGTCACATTT
GTTTACATGTCATCTACGACTGCTTCCTCACTACAACAGCAGAGTTGACCAGTTGCCACA
GAGACTTTATTGCCCACAAAACTTAAATTATTTAGCACCTGATTCTTTGCAGAAAATGTTT
ACTGATTCCTGGTCTAGAGCATAGGAATCTCTTCTGTTCTGTGTTATCCCCATCTCCAGC
AAAAACCATTATTAGTGTCAGTGTTTAAACTTGAGAACTGGGCAATGCAGGAAGCTGTGA
CAATGCACATAGTCAATGTACAAAGCTTTGGGGACACTGGGGCCTAGGTCCAGCATGCT
CTTTACCCTTCTTAGGAATCATGAGATGCTACTGTTCATTGTGTGTGACTCTGAAGCCCT
CTCTGTGCTTGGTAAACATAACTGCCTTTTGCTTGTATATGCATTTTCTGTAATTCTATAAC
CATATAGATATATTAAAAATATTTTCTCATGTACCTGCAGTGTGAGAGAAACTTATTATTTG
TCATCAGACAAACTGGATTTCAAATCTTGATTTCATTACTACCTTGGTGACCCAACTTTTT
GGAGCCTCAGTTTCTTCATCTGTCAAACGGGAAAATAATCTTGTTTTTAGGTTGTTAGGAA
GATCAAATGAGAGAACACTGTGCATATTCTAAATTGAAATGTTAATTATAATTTCTGTCAT
CGTCAGGGGCATCTTTGCCTTTGGGAGTGGCTAATCCATTTCGAGTATGTGTGCAGCAA
ACACTGGATGAGATGGCCAAAGGGAGGGCCAATGGGCAGAGGGGTTTGCACCCTATCA
ACAGCTTCTGGGCAGTCAGGGTTTTTGCATCAAGGGTTGGGACTACTATAGCCAGAGCT
TTGAGAAAACCTGTAACAGCATGGCTAAGTCACAGTGATTCACAAAGCAGAAGACTGCA
CTTCAGAATTTATTCTTTTCTCTGCCTCTGCCTTTCTTTTTCTTAACTAACATTGTTTTTTAC
ATCCCTGGCTTAATTCTGGAAACTTCACCGCAGAACAAAATGGTTGCAGTCCTAGTGTTA
CAGTAGGTAGCTAGTCAGGTATGAGCAGGACAGAAGAGGGCTTACACACACACACACAA
ACACGAGGGCTTACACACACATACACAGAAGAGGGCTTACACACATACACACACACAGA
AGAGAGCTTAAACACACAGAAGAGGGCTTACACACACATACACACAGAAGAGGGCTTAC
ACACACATACACACACACAGAAGAGGACTTACACACACACACACAGAAGAGGACTTACA
CACACACACACACACACACACACACACCCAGGAGAGTTGGATGACCATCAGGTGACGGT
CAGGCGGTTGTTAACTGTCTCTCTAAAGTAATAATTGGTCACAGCTGGCACCAGGCAAA
GGCACGCTTCTAATAGATAGAAAATACCTGAAACTGATGATCAGCAGCTTCCTGATAAGA
TCTCAGGAGTTGGGAGAAGTGATGTAAGACCCCGGATATATGCCAACGTGCGTAAAACC
CCAAGTCGAAAGGTCAGACCACACGCCTGCCTTTCAGGTTGCCCGCTTGGCTGTCTTCT
AACTGGTACTTTCCTTCCTTTCATTCCCATTCTAAAGGAAGCGGAATAGCTTTTTCCTGTT

TAGGAAAGTTTATAAAAAGAACAGGAATAGCCATTTCCTATTCTTTTTAAATAAACTTTCAC
TCCTGCTCTAAAACTTGTCTTGGTCCCTCCTTCTGCCTTATGCCCCTCAGTCGAATTCCTT
CTTCTTCTGAGGAGACCATAATTGAGGTTGCTGCAGAACCGTACAGGTTTGCTGCCGGT
AACAATAGGAAGTCTACTCCCTTATGAGAGAGAAATGTGGCTAATATAATTAATTATGGGT
TTAAATTTTGCAGCCAGAAATCTGTGCTTGAAAGTTTGGTACAAAAGGTAGCAGCATCAG
GTTCTCCCTCTTTTTGTTGAAACATTCTCTTTGGAGTTGAGCAGGAGTCCCACTGGTCAT
CGTCATTGGAGTACTGACACGGATAGTCCAGAGCAGCGAACGGTTGTGATTCTTTCATC
CTTGACATTGGAGTGTGCATTCTGAGTTACTGTTGTACCCTTATTCCTTCATCCCAGATAA
GGAATACCTGATTCGGCCCTGAAAAGTGTCAGGGAAACTGAATTTTGAGTTAAACATTTT
CTGTAGGAATCTTGCAAATAGAAAATTGATACTGACTTTAAAAGATTCATTGCGGGAGGC
TGAGGCAGGAGAATCCCTTGAACCCAGGAGGTAGAAGTTGCAGTGAGCCGAGACCGTG
CCATTGCACTCCAGCCTGGGTGTCTCAAAAAAAAAAAACAAAAAACAAAAAACAAAAAAA
CCCCACCCCCGCAAGATTTATTGACACTAAGAAAAGAACACATTTCTTTAATTTTTTTTTG
TGTAAAAAAATTTCTTTTTTAG**ATGAATCAACAGGGGTCATCTACAGAAAGAGGATTGC**
AATCTGTCAGAACGTGGTTCCAGAAATTTTAAGGAAGGTAAGTTCATTTTAAAATGTAA
TCACAGTTGTAACATTGAGTAATTTTTAATAGTCATGAAAAGGAACAGCATTTTCTGAT
GTAAAGGAGCGTTAGAAACTTCCTACTCCTTTTACCTCAGAACCTTCTGAGGAAGAAA
GCTCTTTCTTATCCAACCTCACTTTTAGCAGATGCCACTGGAGATACTTTGTCTCTCTGT
CTTGTGAAGAACTCTTAATGGACATAGAGATATTGTGCAGTTACTTTAAAGATACTTTT
GGCATTTTGCATGAAGTGACTATCTAATCTGTTCATGATTAGAAATTACTCTTAGGTGAGA
ATGATCAGAAATGGGAAGAATGTAAATATTTAGTCTCATCTTTAATAAGAAAGAGAAAAAC
TTCAGTTTCTAATTCACTATCAGAGATAACAGTTATTTCCCAGAGTATAAGGTTCTCCACA
CCCTCTGTAAGTCTTTATGTGTATTTCTCTAAAGTAATATCATTGATGATGAAATCTTCATG
ATAGCAAATTGTTTTCTCTCTTTGAAG**TCCTTAAGCACTCTGGTGATTCTTTGTTGGAAAA**
AGGTGAGTGGAAATTTTTAATCACATTGCTTGACCCATAGAGGGATTTTCAGTGGGAGTC
CTTTTAGAGCTATGAGGAAGTCAGAGGAAAGCATCTGGATATGCCCTTTAAACAGGTCTC
AGAGGGTAGTTTCAACCCAAGAGAGATTCTGTGTGTGATGTTGTGTTACTCCATTCTCAG
GCTACTGATAAAGACATACCTGAGACTGAGTAATTTATAAATAAAAAGAGTTTTAATGGAC
TCACAGTTCCACATGGCTGGGGAGGCCTCATAATCATGGTGGAAGGTGAAGGAGGAGC
CCAGATACATGTTACATGGCAGCAGGCAAGAGAGCGTGTGCAGGGGAACTGCCCTTTAT
AAAACCATCAGATCTGGTGAGACTTATTCACTATCATGGGAACAGCACAGGAAAACCCG
CCCCCGTGATTCAGTTACCTCCCACTGAGTCTCTCCCATGACATGTGGGGATTATGGGA
GCTACAATTCAAGATGAGATTTGGGAGGGGACACAGCCAAACCATATCAGATGTGGAAC
CCAGCTTGGCTCCTGCCGATTAGCTTGCAAATTAAATTGCTGTGCTGTATGTGGAACAAA
TGGCAGCTTCCGTCACTTCCTTATCATGAATTAAAAGACTGTCACATTCTTCAGTGTAATA
AAGCTGATATGACAATGAAAATGAACTTTCTTTTATTTTCCAGATGGTAAAACTTGTTGCA
GTGTAGCAGCAAATTATTACTGGCTTTTTCCAGAGTGTGTACCCTGCCTTTGCCCTATAT
ACTCACTCTGAATCTATTATTTAAAAAGGCATTCAACTTTGTCTTGTGTCTCAATTCTTAAA
TAATTTAAGGACACCTCTGACTTGTACAATTCTGCCTTTGTAGTTTTCCATGTCTTCACCT
AGGAAACCATACATTTCTAGGTATTGGACAGAGTTTAAAATGAAGAAAATATTTTACCTGA
TATTGTTCTGCATGTGAGCTTGTAAGTGGGCTCGATTAGAATCCATCAATTTAAGAAGGC
CATAACCAGCCTTAGAGGAGACGCGAAGGAGGCTGATAATTTGCTTAGTGTTTAGCAGT
TAGGTAAATTAGGCCAGAGACTGTATAGCCTACATAAATCCTGGGTCATTTGCATGTCCT
CACCAGCACTTAGTAAGGTCATAGTATGTTTGATTATCAAAAACGTACAGCCACTGGTGA

GCCATCATCCATGTATCAGGATTACTTGTCCTCTTGGTTATCCAAGACTGAGAGTAACTC
TGGATGCAGTCATTATGTTGCCTGCTCTTCCTAAATTGAAAAAGCCATTTTCTTTCTGTTG
GACAG**GTGAGCATTTTGAAAGTACCTAATATCCAATTAGAAGAGGAGTCATGGCTCAA**
TCCTCGGGAAAGAAACATGGCCATACGGAGTCACTGCCTTACGTGGACACAGTATGCA
TCCATGAAGGAAGAGTCTGTCTTCCGGGAAAGTATGGAAAACCCAAATTGGTAAGACT
CGTGTGTATATGTGTGTGTGTTTTGGTTGCAGGGATGGACACATGAGTACATACATTAAA
AATCTATGGTTCTATACTTATGACCACTGTTTATAACTCATACAAATTCCACATTATTAATT
TTTTAATCACAGTAATTAATGAATTTTTTGCAAGGGAGGTCAGGAAATGATAGAGTTCACC
TTCTGTCTCACCATCTCCCCCAATCCTGTTGTCTTTTTCACCCTCTTGAGAAGTAGTGAGT
GTTATCAGTTTGCTGAATATTCTTCCAGACCTTAAAGAGATACCTTTAGATCCTTATATATA
TACTCATAGAGCCTGTATAGTATGGTTTTGTATATATTTTAAAACATGTATTGTAAGTTATA
AATTAGTTTGTAAATTGTGCACTTCTTTTTTTTTTACTTAATGGAATGTTTCTGAGCTTAAA
CCATGTGATATATATATATATATATATATATATATAAAACTAGCTTATTCCTTTATTGCATATAA
TTCAATCCTGTGACTATATGACATTTTATGTAGCTACTACTTTTTTGATAGACAGATGGTAT
CTAAGTTCTTTTTTACAAACAATATTACAACATTTGTGAAGCATATCTTCCTTGTATATGAT
CCTTTATGTTATATGAGTTGTTTATCTAAAGTATATATATATATATATTTTTTTTTATTTATTT
ATTTATTTTTTTTTGAAATGCAGTCTTGCTCTGTCACCTAGGCTGGAGTGCAGTGGTGTAA
TCATGGCTCGGTGCAGCCTCAACCTCCTGGGCTCAAGTGATCCTCCCACCTCAGCCTCC
TGAGAAGCAGGGACTACAGGCATGTGCCTCAGGAGATGAAGGTTTCAGGCATGTGTCAC
CATGCCTTGTTAATTAAAAAATTTGTTTCGTAGATACGGGGTCTCACTATGTTGCCCAGG
CTGGTCTCAAACTCCTGGCTCAAGTGATTCTCCTACATCAGCCTCCCAAAGTGCTAGGAT
TCCAGGCGTGATCCACTGTGCCTGGCCTAGAGTAGAATTTTTTAATATCAGGTTTATTGA
GATATTATTTGCATACAATAAAACTTACTTTTCTTAGTGTATAGTTCTTTGAGTTTTGACAA
ATATATAGTTGTGCCACCACTGCCACAATTAAACTATAGAACATTTTAGATAGATTTAAAA
CAGTTAATATTACAAGACCAATCAATGGGGAAAGAATATTTCTTTCAACAAATGGTGCTGA
GATAACTGGATATCCACATACGAAAGAATGAATTTAGATCCCTACCTCATGTAGAATACAA
AATTAACTCAAAATAGATCAAATACCTAAATGAAAGAGTTAAAACTATAAGACACTTAGAA
ATAAACATGGGTATAAATCTTTGTCATCTTGGGTTAGGTAGTAATTTCTTAGATATGATAG
CAAAAATTTAAGCAGCAAAATTAAAAATAGATAAAATTGATGTCATCAATTAGAAACTTTTG
TGTTTCAAGGGACACTGTCAAGAAGGTGAAAAACAGGCTGGGCGTGGTGACTCATGCCT
GTAATCCTAGCACTTTGGGAGGCCAAGGCGGGGTGATCACTTGAGTCCAGGAGTTTGAG
ACCACCCTAGTCACATGGCAAAATCCCGTCTCTACTAAAAATACAACAATTAGCCGGGTG
TGGTGGTGCATGCCTATGGTTTCAACTATATGGGTGGCTGAGGCACGAGAATAGCTTGA
ACCCAGGAGACGAAGGTTTCAGTGAGCCGAGATCGCACCATGGCACTCTAGCCTGGGC
AACAGAGCGAGACTGTCTCAAAAAAAAAAAAAAAAAAAAAAAAAAAGACACACAATAACAA
GGATTGGTTGTGGATATGTGGATAAATTAGAACCCTTACATGTTTCTTATGGGAATATAAA
ATGGTGCAGCTGCTTTGGAGAACAGTTTTTCAGATCCTCAAAAAGTTAAACGTAGAATTA
CCACTTGACCCAGCAATTTCACTCCTAGATACTACAAGAAAAATGAGAAAATATATGTCCA
CACAAAAACTTGTACAAAACTTTCATAGCATTATAATAGCCAGAATGTAGAAACAAGCCAA
TGTCCATCAACTGATAAATATGTAGCCCAATGTAATGTATCCACAAAATGGAATATTATTT
AGCTACAAAAAGGAATGGAGTGCTGATTCATGGTTAAACATGGACAAATTTTGAAAATAT
GCTAAATGAAAGAAGGCAGTTACAAAAGGCCACATATTGTATGGGTCCATTTATTTGAAA
AGTCCAGAATGTGTAAATCCATAGTGACAGAATGTAGATTATTGGTTTCCATGGGTTACG
GGGAGAGGGGAGTGATGAGTGACTGCCAGTTGGTACGGAGTTTCTTTTTTGGGGTGATG

AAAATGTCCTGGAATTAGTGTTGATGGTTGCAGAACTCTGCGAATATACTAAACCTCACT
GAATTGTGTACCTTAAAAGGGTGAATTTTATGGTAAGTGAATTATATCTCAATACAGAAAA
CGATATAGCTGATTCTCAGGACATGCATACTTTTATTAGTTTATATAGTGTTTTAGTTTGC
CTGCTATAACAAGAATACCATAGACTGTGTGGTTTACACAGTAAACATTAATTTCTCACAG
ACCTGGAGGCTGGGAAATCCACCATCTAGGCACTGGCCTTTCCAGTGTCTGGTGAAGGC
CATATTTTTGGTTTGCAGATGCTGCCTCCTCATCATTGCCTCACATGGTGGATAGAGTGA
GTTCTAGTCTTGGTTTCTTTTTATAAGGACACCAATCCCAAAATGCAGTCTCTAACTCATG
ACCTCATGAAAACCTAATTACCTCCCAAAGTCTCAGTCTCCTAGTACCATCCCATTGTAG
GTTAAAGTTTCAGCACATGAATTGGGGGTGGCAGGGGAGGGCACCGACATGCAGTCGT
AACATACAGATTCTGTCACTTTGCCTTCCAATGTGTTAATATGCACATTTTACACTGATCA
ACAGCGCATGAGCGTGTTCATTCCCTTACAACAGCGTCAGCACTTATTCACATGCTTTTT
AACTTTTGCCAGTATAATGGGTGAAAAATGATTAATTTTTTTGAGATACATTTAAAAACAAG
TAAAATATAAGCACTAAATGTTAATAGTGATTATTGCTGTGAACTGTGGTTGCTGTGAACT
GGATAATTATCTGTTTTTTTTTGTTATTATTCTGTGTGGTTTGAATTTTTTATTATATACTTA
TCATTTTATGGAAATAGTAAAGCCCTCAAGCCCAAACAACTATAAACCGAAAATTTAAAAT
CCATTCTTAAGCCCAAACCCTTGTCCAGATCATTACTGCTCTCCATAACTTTGGTCCTGTA
GTGATAATTTTAGGATGCTGCAGCAGCCCGCTGAGTGGGTGTGAAGCCATGTCTTATTG
GCATCAGAGGTGGGCTTGCTCTGAACCACCCGCTAGTTTTTCTTTCTTAGTCTCTCTCAT
GATAAGAGTTTCACATAGGGCATAGATGTTTGGGATTAAGTGTAAAGGCTGTGGCCAGA
GAATAGAGCTCCTTTCTTTGTATGTAGAAATAGCAAAAGGATGGAATTGGCTGATTTTGT
GCAAAAAAATCATTTCATCTACTTCGTGTTAGCCTTAATTTTTGCACATATTTGAGCTATTA
GCTAGTCTTAAAAGGTGAACTAAACTTTCATCTGTGATATATAAAATGACAATTAGCTCTG
AAAATCCAGGGAGGACATCCTTTAGGAACAATTTTCCTTTGAGTGTTTTTCAAAAATTATT
TTAAAATAGTCATTCTGATTCTAGAAACAATAATGCATGTGTCTTGTAAAAAAACCAGTTC
TGAGAAAAAAAGGAAAATTAATCATACCTGGAGATATTGTTTGTTTACATGATTTTCTTGG
TCTGTTCCTCATTGAAACTTAGGTTGTTTGTTTTTGCTCTTACAAACAGTGTTGTGGTGAA
AGTCTCTATGATTACATATTTTCCCACTTATTCTGTTACCATTTAAGGTAGATTGAGCAAA
GTGAGATTTCATGGTCAAAGGTTGTATGCATTTTATATTTTGGTACATATGACCAGACTTC
TGTATAAGAAGGTTTGACCAGTTAAATTTTTTTTTTTCTTCCTTTGGTTTGTTATGTTCCAG
TCCTCTCCAAAACACTGCCTTTTCTATCATTGACACAGCAGGCTAACATTTATTGATGCCT
ACTATGTCAAGGTCAAAGCTATATCCTTCATGTGTATTCTTTGGAGGCTTAGGTTAAATAA
CTTGCCCAAGGTGATACAGTCAGTAGATTGCTAAGCCAAGATACGAATGCAGATATTGTA
GACTCCACCTTGAGCACAGTGGTGAGGCAGGAATATGAACATTTACAAAGGATGGGTTT
ATCTTGTTCTGTAGAGTGCTTTGGATCCCAGGTATGAAGAATGGGTTTTGTCCCTTCAGC
AACAAGCAGCCACAAAAAGGTTTCTGATTAGGGGTTGATATGCTGAAAGCTGGGATCCA
CATTCCAGAGAACTTATGTTTCCTTAGCTCTTGACATGGATTTAGAAAGGCATTTAGGCA
GACATTCTAAATGAATGGGTAATGGGATGGGGTGGGGGCATCCAAGAAAGATTCATTAT
TACTTTAATTTCTTCCCTTTGCTAAGGAAATCTGAGAGTAATGGCTAGCCACTGCACCCA
GAATAGGGACAAGTTTTTTTAAAGGGAATAAAAAAGCTGTCAGGGCTACTAAGTGTAAAA
TTAGAC

CircPRELID2 (hsa_circ_0074389):

TACCCCAACCCCATGGATAAAAATGTCATCTCAGTAAAAATCATGGAGGAAAAAAGAGAT
GAATCAACAGGGGTCATCTACAGAAAGAGGATTGCAATCTGTCAGAACGTGGTTCCAGA

AATTTTAAGGAAGTCCTTAAGCACTCTGGTGATTCTTTGTTGGAAAAAGGTGAGCATTTTG
AAAGTACCTAATATCCAATTAGAAGAGGAGTCATGGCTCAATCCTCGGGAAAGAAACATG
GCCATACGGAGTCACTGCCTTACGTGGACACAGTATGCATCCATGAAGGAAGAGTCTGT
CTTCCGGGAAAGTATGGAAAACCCAAATTG
