# Supplementary material for: Hypoxia‐Induced circPRELID2 Promotes Gastric Cancer Metastasis by Facilitating ZEB2 Translation via PCBP1 O‐GlcNAcylation
Source: Adv Sci (Weinh). 2025 Oct 21;12(46):e05396. doi: 10.1002/advs.202505396 (PMC12697806; doi:10.1002/advs.202505396)
Supplement: Supplementary file 2 — Supporting Information [file ADVS-12-e05396-s002.zip › circPRELID2-pull down-Mass Spectrometry-proteins.pdf]

Supplementary Data 2. Mass spectrometry identification of proteins pulled down by circPRELID2 probe					
Accession	Description	Gene name	Peptide count	Unique peptides	Confidence Score
sp|Q15365|PCBP1_HUMAN	Poly(rC)-binding protein 1	PCBP1	26	26	1368.24
sp|O15294|OGT1_HUMAN	UDP-N-acetylglucosamine--peptide N-acetylglucosaminyltrans	OGT	15	15	1131.39
sp|Q9HOZ9|RBM38_HUMAN	RNA-binding motif protein 38	RBM38	12	12	927.1
sp|A0AV96|RBM47_HUMAN	RNA-binding motif protein 47	RBM47	10	10	902.83
sp|P38919|EIF4A3_HUMAN	eukaryotic translation initiation factor 4A, isoform 3	EIF4A3	16	16	1229.37
sp|Q9BWF3|RBM4_HUMAN	RNA-binding motif protein 4	RBM4	9	9	833.22
tr|MOQZM1|MOQZM1_HUMAN	Heterogeneous nuclear ribonucleoprotein M (Fragment)	HNRNPM	11	9	725.46
sp|Q15427|SF3B4_HUMAN	Splicing factor 3B subunit 4	SF3B4	8	8	510.52
sp|P60228|EIF3E_HUMAN	Eukaryotic translation initiation factor 3 subunit E	EIF3E	7	6	424.47
sp|P62249|RS16_HUMAN	40S ribosomal protein S16	RPS16	3	3	203.39
sp|O15042-2|SR140_HUMAN	Isoform 2 of U2 snRNP-associated SURP motif-containing pro	U2SURP	1	1	26.1
sp|Q92841-1|DDX17_HUMAN	Isoform 2 of Probable ATP-dependent RNA helicase DDX17	DDX17	7	5	263.45
sp|Q9UI30|TR112_HUMAN	tRNA methyltransferase 112 homolog	TRMT112	4	4	236.11
sp|P62273|RS29_HUMAN	ribosomal protein S29 isoform 1	RPS29	2	2	72.53
sp|O60828|PQBP1_HUMAN	polyglutamine binding protein 1	PQBP1	1	1	28.77
tr|C9J4Z3|C9J4Z3_HUMAN	60S ribosomal protein L37a	RPL37A	1	1	39.49
sp|Q96MT7-2|WDR52_HUMAN	Isoform 2 of WD repeat-containing protein 52	WDR52	3	3	222.13
sp|P62269|RS18_HUMAN	40S ribosomal protein S18	RPS18	9	9	417.69
tr|D6RIF6|D6RIF6_HUMAN	SLAIN motif-containing protein 2	SLAIN2	4	4	219.27
sp|Q08211|DHX9_HUMAN	ATP-dependent RNA helicase A	DHX9	11	10	524.47
tr|E9PNW8|E9PNW8_HUMAN	Fatty acyl-CoA reductase 1 (Fragment)	FAR1	1	1	28.93
sp|P62241|RS8_HUMAN	40S ribosomal protein S8	RPS8	2	2	53.2
sp|Q9UBM7|DHCR7_HUMAN	7-dehydrocholesterol reductase	DHCR7	5	4	283.66
sp|P07910|HNRPC_HUMAN	heterogeneous nuclear ribonucleoprotein C isoform a	HNRNPC	3	3	210.73
sp|Q13247|SRSF6_HUMAN	arginine/serine-rich splicing factor 6	SRSF6	3	3	129.03
sp|Q9BWC9|CC106_HUMAN	coiled-coil domain containing 106	CCDC106	1	1	33.16
tr|H0Y2W2|H0Y2W2_HUMAN	ATPase family AAA domain-containing protein 3A (Fragment)	ATAD3A	8	8	428.62
sp|P05023-3|AT1A1_HUMAN	Isoform 3 of Sodium/potassium-transporting ATPase subunit	ATP1A1	2	2	49.27
tr|J3QLE5|J3QLE5_HUMAN	Small nuclear ribonucleoprotein-associated protein N (Frag	SNRPN	1	1	32.35
sp|P46087-2|NOP2_HUMAN	Isoform 2 of Probable 28S rRNA (cytosine(4447)-C(5))-methy	NOP2	5	5	347.94
tr|E9PCY7|E9PCY7_HUMAN	Heterogeneous nuclear ribonucleoprotein H	HNRNPH1	4	4	302.33
sp|Q9NXV2|KCTD5_HUMAN	BTB/POZ domain-containing protein KCTD5	KCTD5	9	9	419.01

sp|Q9BRD0|BUD13 HUMAN	BUD13 homolog isoform 1	BUD13	4	4	288.42
sp|O00425|IF2B3 HUMAN	Insulin-like growth factor 2 mRNA-binding protein 3	IGF2BP3	11	9	676.46
tr|B7ZLZ9|B7ZLZ9 HUMAN	N-myc downstream-regulated gene 3 protein	NDRG3	6	6	195.22
sp|Q9BRL6|SRSF8 HUMAN	Serine/arginine-rich splicing factor 8	SRSF8	2	2	52.75
sp|Q9NZI8|IF2B1 HUMAN	Insulin-like growth factor 2 mRNA-binding protein 1	IGF2BP1	3	3	116.49
sp|P31942|HNRH3_HUMAN	heterogeneous nuclear ribonucleoprotein H3 isoform a	HNRNPH3	7	7	385.62
sp|P34932|HSP74 HUMAN	Heat shock 70 kDa protein 4	HSPA4	1	1	24.17
sp|P29692|EF1D HUMAN	Elongation factor 1-delta	EEF1D	4	4	273.32
sp|P55795|HNRH2 HUMAN	Heterogeneous nuclear ribonucleoprotein H2	HNRNPH2	8	8	456.78
sp|P40763|STAT3 HUMAN	Signal transducer and activator of transcription 3	STAT3	2	2	63.36
tr|Q9UQ84|Q9UQ84 HUMAN	Exonuclease 1	EXO1	2	2	38.6
sp|P17980|PRS6A HUMAN	proteasome 26S ATPase subunit 3	PSMC3	6	6	337.82
sp|Q14137|BOP1 HUMAN	block of proliferation 1	BOP1	8	8	351.61
sp|Q13148|TADBP HUMAN	TAR DNA-binding protein 43	TARDBP	1	1	36.25
sp|P51991|ROA3 HUMAN	heterogeneous nuclear ribonucleoprotein A3	HNRNPA3	3	3	87.2
sp|Q07666|KHDR1 HUMAN	KH domain-containing, RNA-binding, signal transduction-ass	KHDRBS1	3	3	116.25
tr|E7EPB3|E7EPB3 HUMAN	60S ribosomal protein L14	RPL14	5	5	277.39
sp|Q8NC51-4|PAIRB HUMAN	Plasminogen activator inhibitor 1 RNA-binding protein	PAIRBP1	6	6	245.27
sp|Q01813|PFKAP HUMAN	ATP-dependent 6-phosphofructokinase, platelet type	PFKP	2	2	52.44
sp|P19013|K2C4 HUMAN	Keratin, type II cytoskeletal 4	KRT4	7	7	443.95
sp|P51532|SMCA4 HUMAN	Transcription activator BRG1	SMARCA4	1	1	24
sp|P26368|U2AF2 HUMAN	Splicing factor U2AF 65 kDa subunit	U2AF2	4	4	235.32
sp|Q14258|TRI25 HUMAN	Tripartite motif-containing protein 25	TRIM25	7	7	429.75
tr|P62244|P62244_HUMAN	40S ribosomal protein S15a	RPS15A	9	9	473.53
sp|Q7Z4H7|HAUS6 HUMAN	HAUS augmin-like complex subunit 6	HAUS6	3	3	137.92
sp|Q02790|FKBP4 HUMAN	Peptidyl-prolyl cis-trans isomerase FKBP4	FKBP4	3	3	102.22
sp|Q96I25|SPF45 HUMAN	RNA-binding motif protein 17	RBM17	5	5	395.53
sp|Q9UEW8|STK39 HUMAN	STE20/SPS1-related proline-alanine-rich protein kinase	STK39	7	7	466.47
sp|Q9BZD4|NUF2 HUMAN	Cell division cycle-associated protein 1	CDCA1	1	1	29.07
tr|C9J4Z3|C9J4Z3 HUMAN	60S ribosomal protein L37a	RPL37A	4	4	315.55
sp|Q00839|HNRPU HUMAN	Heterogeneous nuclear ribonucleoprotein U	HNRNPU	7	6	533.04
sp|O95684|CEP43 HUMAN	FGFR1 oncogene partner	FGFR1OP	1	1	22.19
sp|Q9UHX1|PUF60 HUMAN	poly-U binding splicing factor 60KDa isoform b	PUF60	1	1	25.08
tr|Q0QEN7|Q0QEN7 HUMAN	ATP synthase subunit beta (Fragment)	ATP5B	4	4	189.24

tr|Q3ZTT7|Q3ZTT7_HUMAN	SH3 domain-binding protein 1	SH3BP1	4	4	247.56
sp|O76094|SRP72_HUMAN	Signal recognition particle subunit SRP72	SRP72	2	2	73.11
sp|P61081|UBC12_HUMAN	ubiquitin-conjugating enzyme E2M	UBE2M	1	1	35.32
sp|Q15717|ELAV1_HUMAN	ELAV-like protein 1	ELAVL1	3	3	128.57
sp|P49354|FNTA_HUMAN	Farnesyltransferase, CAAX box, alpha	FNTA	1	1	51.54
sp|Q93096|TP4A1_HUMAN	Protein tyrosine phosphatase type IVA, member 1	PTP4A1	1	1	33.03
sp|Q8IY67|RAVR1_HUMAN	Ribonucleoprotein PTB-binding 1	RAVER1	8	8	524.72
sp|P62195|PRS8_HUMAN	proteasome 26S ATPase subunit 5	PSMC5	6	6	371.91
tr|P49711|P49711_HUMAN	CCCTC-binding factor	CTCF	2	2	84.27
sp|P26640|SYVC_HUMAN	Valine--tRNA ligase	VARS	2	2	65.55
sp|O75165|DJC13_HUMAN	DnaJ homolog subfamily C member 13	DNAJC13	1	1	39.04
sp|P78406|RAE1L_HUMAN	mRNA export factor RAE1	RAE1	3	2	95.54
sp|Q9Y2Z0-2|SUGT1_HUMAN	Isoform 2 of Suppressor of G2 allele of SKP1 homolog	SUGT1	5	5	227.61
sp|Q99496|RING2_HUMAN	E3 ubiquitin-protein ligase RING2	RNF2	1	1	42.12
sp|O95478|NSA2_HUMAN	Ribosome biogenesis protein NSA2 homolog	NSA2	4	4	293.54
sp|Q9UKK9|NUDT5_HUMAN	nudix-type motif 5	NUDT5	1	1	46.85
sp|O14497|ARI1A_HUMAN	AT-rich interactive domain-containing protein 1A	ARID1A	6	6	410.06
sp|O15372|EIF3H_HUMAN	Eukaryotic translation initiation factor 3 subunit H	EIF3H	3	3	245.87
sp|P13861|KAP2_HUMAN	cAMP-dependent protein kinase, regulatory subunit alpha 2	PRKAR2A	3	3	187.52
sp|P42766|RL35_HUMAN	60S ribosomal protein L35	RPL35	1	1	47.74
sp|O76094|SRP72_HUMAN	Signal recognition particle subunit SRP72	SRP72	1	1	22.71
sp|P04264|K2C1_HUMAN	Keratin, type II cytoskeletal 1	KRT1	2	2	87.05
sp|Q96JB3|HIC2_HUMAN	Hypermethylated in cancer 2 protein	HIC2	9	9	589.46
sp|P23284|PPIB_HUMAN	Peptidyl-prolyl cis-trans isomerase B	PPIB	1	1	47.33
sp|P07814|SYEP_HUMAN	glutamyl-prolyl tRNA synthetase	EPRS	3	3	167.36
sp|O14979|HNRDL_HUMAN	Heterogeneous nuclear ribonucleoprotein D-like	HNRPDL	2	2	111.21
tr|F8W6P5|F8W6P5_HUMAN	LVV-hemorphin-7 (Fragment)	HBB	1	1	37.75
sp|Q6P5Z2|PKN3_HUMAN	Protein kinase N3	PKN3	1	1	25.93
sp|P35813|PPM1A_HUMAN	Protein phosphatase 1A	PPM1A	1	1	21.52
tr|A8MXL6|A8MXL6_HUMAN	SEC13 protein isoform 2	SEC13	1	1	55.67
sp|Q9NUQ6|SPS2L_HUMAN	SPATS2-like protein	SPATS2L	2	2	95.02
sp|O95391|SLU7_HUMAN	step II splicing factor SLU7	SLU7	5	5	335
sp|Q8IWE4|DCNL3_HUMAN	DCN1-like protein 3	DCUN1D3	2	2	75.48
sp|Q13310-2|PABP4_HUMAN	Isoform 2 of Polyadenylate-binding protein 4	PABPC4	2	2	97.34

sp|P78371|TCPB_HUMAN	chaperonin containing TCP1, subunit 2	CCT2	1	1	35.11
sp|P24534|EF1B_HUMAN	eukaryotic translation elongation factor 1 beta 2	EEF1B2	5	5	246.85
sp|P46782|RS5_HUMAN	40S ribosomal protein S5	RPS5	2	2	77.53
sp|P40429|RL13A_HUMAN	60S ribosomal protein L13a	RPL13A	4	3	186.24
sp|Q9P2J5|SYLC_HUMAN	leucyl-tRNA synthetase	LARS	1	1	26.92
sp|Q92804|RBP56_HUMAN	TBP-associated factor 15 isoform 1	TAF15	2	2	111.04
tr|H0Y2W2|H0Y2W2_HUMAN	ATPase family AAA domain-containing protein 3A (Fragment)	ATAD3A	1	1	31.42
sp|P07900|HS90A_HUMAN	heat shock 90kDa protein 1, alpha isoform 1	HSP90AA1	2	2	47.57
sp|P24278|ZBT25_HUMAN	Zinc finger and BTB domain-containing protein 25	ZBTB25	3	3	115.72
sp|Q9H0A0|NAT10_HUMAN	N-acetyltransferase 10	NAT10	1	1	29.03
tr|D6RCE2|D6RCE2_HUMAN	SKI3 subunit of superkiller complex protein	SKIC3	6	5	264.56
sp|Q9UKD2|MRT4_HUMAN	mRNA turnover 4 homolog	MRTO4	2	2	124.62
sp|Q8IXT5|RB12B_HUMAN	RNA binding motif protein 12B	RBM12B	8	8	424.48
sp|Q9H7N4|SFR19_HUMAN	SR-related CTD-associated factor 1	SCAF1	2	2	83.61
sp|Q86Y37|CACL1_HUMAN	CDK2-associated and cullin domain-containing protein 1	CACUL1	3	3	173.03
sp|P49792|RBP2_HUMAN	E3 SUMO-protein ligase RanBP2	RANBP2	2	2	131.37
sp|Q07955|SRSF1_HUMAN	splicing factor, arginine/serine-rich 1 isoform 1	SRSF1	6	4	247.24
sp|P42695|CNDD3_HUMAN	Condensin-2 complex subunit D3	NCAPD3	1	1	23.21
sp|P62913|RL11_HUMAN	ribosomal protein L11	RPL11	1	1	44.06
sp|O95433|AHSA1_HUMAN	Activator of 90 kDa heat shock protein ATPase homolog 1	AHSA1	1	1	60.88
sp|Q8NAF0|ZN579_HUMAN	Zinc finger protein 579	ZNF579	1	1	26.73
tr|H0YJX5|H0YJX5_HUMAN	Coiled-coil domain-containing protein 88C	CCDC88C	1	1	32.05
sp|P62280|RS11_HUMAN	40S ribosomal protein S11	RPS11	1	1	53.42
sp|A6NKD9|CC85C_HUMAN	Coiled-coil domain-containing protein 85C	CCDC85C	1	1	22.22
sp|P56537|IF6_HUMAN	eukaryotic translation initiation factor 6 isoform a	EIF6	1	1	48.37
sp|Q8IVW6|ARI3B_HUMAN	AT-rich interactive domain-containing protein 3B	ARID3B	1	1	25.83
