# Supplementary material for: Hypoxia‐Induced circPRELID2 Promotes Gastric Cancer Metastasis by Facilitating ZEB2 Translation via PCBP1 O‐GlcNAcylation
Source: Adv Sci (Weinh). 2025 Oct 21;12(46):e05396. doi: 10.1002/advs.202505396 (PMC12697806; doi:10.1002/advs.202505396)
Supplement: Supplementary file 2 — Supporting Information [file ADVS-12-e05396-s002.zip › HGC-27 cells-comp2_H2_vs._N2_FC2_circRNA.pdf]

ProbeID	FC(H2_vs._N2)	log2FC(H2_vs._	Regula	Case_mean(H2_v	Control_mean(H2	Sequence	TargetID	Chromosome	Start	End	Strand	Length	circRNA_Annotatio	circRNA_BestTran	circRNA_GeneSy	circRNA_Study	circRNA_Ge	Type	823_H.flag	823_N.flag	823_H.normali	823_N.normalized
hsa_gci149762	2.344409817	1.229224784	up	5.370938398	4.141713615	ACCCCACCAAACTACCG	hsa_circ_0000005	chr1	1586822	1650894	-	49639	ALT_DONOR, CDS, c	NM_024011	CDK11A	Memczak2013	728642	circRNA	Detected	Detected	5.370938398	4.141713615
hsa_gci149794	2.33992604	1.22646293	up	6.097518665	4.871055735	AACACAACTAGAGGTAT	hsa_circ_0000095	chr1	95609446	95616975	+	410	ANNOTATED, CDS, c	NM_001199691	TMEM56-RWDD3	Memczak2013, Salz	100527978	circRNA	Detected	Detected	6.097518665	4.871055735
hsa_gci149862	3.197358664	1.676880588	up	5.283945059	3.607064471	CTGTTTCAGTTATGCAA	hsa_circ_0000258	chr10	105197771	105198565	+	180	ANNOTATED, CDS, c	NM_014976	PDCD11	Jeck2013, Memczak	22984	circRNA	Detected	Detected	5.283945059	3.607064471
hsa_gci149872	2.335888027	1.223971119	up	7.23152142	6.007550301	TTTTGTCCTGAAATGTG	hsa_circ_0000285	chr11	33362513	33363232	+	284	ANNOTATED, CDS, c	NM_005734	HIPK3	Memczak2013, Salz	10114	circRNA	Detected	Detected	7.23152142	6.007550301
hsa_gci149936	14.54905755	3.862853797	up	5.07725939	1.214405592	AGTTCAAACGACTAATG	hsa_circ_0000502	chr13	108895879	108903664	+	7785	INTERGENIC	None		Memczak2013		circRNA	Detected	Not Detected	5.07725939	1.214405592
hsa_gci149940	2.243543243	1.165778992	up	5.38030574	4.214526748	AAACTACGAGCCATACA	hsa_circ_0000522	chr14	21825355	21829372	-	867	ANNOTATED, CDS, c	NM_007192	SUPT16H	Jeck2013, Memczak	11198	circRNA	Detected	Detected	5.38030574	4.214526748
hsa_gci149963	2.043191095	1.030824142	up	6.150883656	5.120059514	CCACTCCGATGTGCCCT	hsa_circ_0000593	chr15	41988272	42005694	+	2366	ANNOTATED, CDS, c	NM_001164273	MGA	Memczak2013, Salz	23269	circRNA	Detected	Detected	6.150883656	5.120059514
hsa_gci149987	2.346828347	1.230712323	up	6.974307957	5.743595634	AGACTCCCCGGTCCGGA	hsa_circ_0000651	chr15	90982563	90986710	+	446	ANNOTATED, CDS, c	NM_003870	IQGAP1	Jeck2013, Memczak	8826	circRNA	Detected	Detected	6.974307957	5.743595634
hsa_gci149997	2.008652161	1.006227754	up	8.378160546	7.371932792	TCTACATTTACCTACGT	hsa_circ_0000711	chr16	68155889	68160513	+	1298	ANNOTATED, CDS, c	NM_173165	NFATC3	Jeck2013, Memczak	4775	circRNA	Detected	Detected	8.378160546	7.371932792
hsa_gci149999	2.317886169	1.212809718	up	7.202865955	5.990056237	CTCTTAGGCAGAGTTGT	hsa_circ_0000714	chr16	70294946	70302282	-	823	ANNOTATED, CDS, c	NM_001605	AARS	Memczak2013, Salz	16	circRNA	Detected	Detected	7.202865955	5.990056237
hsa_gci150002	2.657340119	1.409982894	up	8.906958349	7.496975456	TGTCCCCACCTAAATAC	hsa_circ_0000739	chr17	5314022	5320002	-	383	ANNOTATED, CDS, c	NM_002532	NUP88	Memczak2013, Salz	4927	circRNA	Detected	Detected	8.906958349	7.496975456
hsa_gci150021	2.103916489	1.07307744	up	6.217933594	5.144856153	TTCCTCTCCTGCATTGT	hsa_circ_0000809	chr17	76688498	76695050	-	341	ANNOTATED, CDS, c	NM_004762	CYTH1	Memczak2013, Salz	9267	circRNA	Detected	Detected	6.217933594	5.144856153
hsa_gci150047	2.172195861	1.119154193	up	5.138866818	4.019712625	ATTAGGACCCCGATCCA	hsa_circ_0000889	chr19	10283765	10288043	-	275	ANNOTATED, CDS, c	NM_001379	DNMT1	Jeck2013, Memczak	1786	circRNA	Detected	Detected	5.138866818	4.019712625
hsa_gci150072	-6.756222467	-2.756216834	down	1.21888238	3.975099213	ACCTCAGTAAGGTTAAT	hsa_circ_0001002	chr2	48573339	48573890	+	551	ANNOTATED, CDS, c	NM_002158	FOXN2	Jeck2013, Memczak	3344	circRNA	Not Detected	Detected	1.21888238	3.975099213
hsa_gci150096	2.246244036	1.167514673	up	5.808474792	4.640960119	AGGGATACCAGAAGAAC	hsa_circ_0001072	chr2	144966169	144969146	-	267	ANNOTATED, CDS, c	NM_024659	GTDC1	Jeck2013, Memczak	79712	circRNA	Detected	Detected	5.808474792	4.640960119
hsa_gci150105	3.077636371	1.621822784	up	6.438389896	4.816567112	TCTGTAGTGTACCAACC	hsa_circ_0001090	chr2	201719338	201719809	-	163	ANNOTATED, CDS, c	NM_004071	CLK1	Memczak2013, Salz	1195	circRNA	Detected	Detected	6.438389896	4.816567112
hsa_gci150166	2.096121666	1.067722458	up	8.7386945	7.670972042	ACAGGTGTTTCCGTGAA	hsa_circ_0001280	chr3	33725850	33738425	-	449	ANNOTATED, CDS, c	NM_015097	CLASP2	Jeck2013, Memczak	23122	circRNA	Detected	Detected	8.7386945	7.670972042
hsa_gci150180	2.221474778	1.151517761	up	4.033696053	2.882178292	CACAGTAGTATCGGTGA	hsa_circ_0001320	chr3	71064699	71102924	-	692	ANNOTATED, CDS, c	NM_032682	FOXP1	Jeck2013, Memczak	27086	circRNA	Detected	Not Detected	4.033696053	2.882178292
hsa_gci150202	2.122655189	1.085870034	up	6.493116397	5.407246363	TAACAACTCTTTACTGA	hsa_circ_0001369	chr3	183368083	183369064	+	981	ANNOTATED, CDS, c	NM_017644	KLHL24	Memczak2013, Salz	54800	circRNA	Detected	Detected	6.493116397	5.407246363
hsa_gci150217	3.008120781	1.588862495	up	4.630260506	3.041398011	AGTAGAAGCCAGAGTCA	hsa_circ_0001411	chr4	54249939	54256040	+	169	ANNOTATED, CDS, c	NM_001134937	FIP1L1	Jeck2013, Memczak	81608	circRNA	Detected	Not Detected	4.630260506	3.041398011
hsa_gci150233	2.016105372	1.011571044	up	4.640706626	3.629135582	AAATTAGAAGTGGTAGA	hsa_circ_0001447	chr4	146744573	146770713	-	402	ANNOTATED, CDS, c	NM_178835	ZNF827	Jeck2013, Memczak	152485	circRNA	Detected	Detected	4.640706626	3.629135582
hsa_gci150248	2.3103303	1.208099123	up	4.320695642	3.112596519	GAGGTACCGGCAGCCCG	hsa_circ_0001479	chr5	43122140	43162033	+	967	ANNOTATED, CDS, c	NM_003432	ZNF131	Memczak2013, Salz	7690	circRNA	Detected	Not Detected	4.320695642	3.112596519
hsa_gci150274	2.303037586	1.203537956	up	4.500853197	3.29731524	TGTTCGAAGACGATCTG	hsa_circ_0001540	chr5	139819703	139825560	+	530	ANNOTATED, CDS, c	NM_017747	ANKHD1	Jeck2013, Memczak	54882	circRNA	Detected	Detected	4.500853197	3.29731524
hsa_gci150285	-4.901055828	-2.293092581	down	3.304629693	5.597722273	CAACGGTAATGTAATTT	hsa_circ_0001580	chr6	18160113	18166609	+	430	ANNOTATED, CDS, c	NM_153042	KDM1B	Jeck2013, Memczak	221656	circRNA	Not Detected	Detected	3.304629693	5.597722273
hsa_gci150313	2.103269591	1.072633782	up	7.945803774	6.873169992	TGTGATTTCGCAATGTT	hsa_circ_0001655	chr6	156468903	156489628	-	20725	INTERGENIC	None		Memczak2013		circRNA	Detected	Detected	7.945803774	6.873169992
hsa_gci150344	2.811236703	1.491204932	up	4.416131393	2.924926461	GTTCTCTGACGGACGTG	hsa_circ_0001750	chr7	135262543	135277940	+	1182	ANNOTATED, CDS, c	NM_015135	NUP205	Jeck2013, Memczak	23165	circRNA	Detected	Not Detected	4.416131393	2.924926461
hsa_gci150364	2.33188668	1.221497682	up	6.04696274	4.825465058	AGTAAATCATCGTATAA	hsa_circ_0001813	chr8	95839490	95840021	+	213	ANNOTATED, CDS, c	NM_017864	INTS8	Memczak2013, Salz	55656	circRNA	Detected	Detected	6.04696274	4.825465058
hsa_gci150388	2.709010206	1.437765829	up	4.118400209	2.68063438	AGGGTGGTAGGAAGGAG	hsa_circ_0001913	chrX	19701940	19713859	-	336	ANNOTATED, CDS, c	NM_001024666	SH3KBP1	Memczak2013, Salz	30011	circRNA	Detected	Not Detected	4.118400209	2.68063438
hsa_gci150405	2.2853594	1.192421064	up	5.239990688	4.047569624	TGTAGGAAGTTACAATA	hsa_circ_0001962	chr9	114296018	114296633	+	220	ANNOTATED, CDS, c	NM_133464	ZNF483	Jeck2013	158399	circRNA	Detected	Detected	5.239990688	4.047569624
hsa_gci150418	-2.27296713	-1.184576821	down	2.664572391	3.849149212	GTGTCCCTTTATTTTCC	hsa_circ_0001980	chr8	124031434	124042956	-	464	ANNOTATED, CDS, c	NM_024295	DERL1	Jeck2013	79139	circRNA	Not Detected	Detected	2.664572391	3.849149212
hsa_gci150436	-2.352770331	-1.234360496	down	5.09481499	6.329175486	TTAAGAAGGTCCTTCCC	hsa_circ_0002006	chr3	72881519	72893574	-	456	ANNOTATED, CDS, c	NM_018130	SHQ1	Jeck2013	55164	circRNA	Detected	Detected	5.09481499	6.329175486
hsa_gci150442	-2.405362551	-1.266254363	down	1.944900046	3.211154409	TTCAAGTGCTTCTAAAT	hsa_circ_0002015	chr17	53478829	53481229	-	172	ANNOTATED, CDS, c	NM_012329	MMD	Jeck2013	23531	circRNA	Not Detected	Detected	1.944900046	3.211154409
hsa_gci150477	2.350829976	1.2331702	up	9.940971689	8.70780149	ACTTGTAGACCCCCGAG	hsa_circ_0002064	chr9	109734285	109765707	+	762	ANNOTATED, CDS, c	NM_021224	ZNF462	Jeck2013	58499	circRNA	Detected	Detected	9.940971689	8.70780149
hsa_gci150478	2.210331945	1.144263048	up	4.15395515	3.009692102	GGTGTCCTTCGATTTAT	hsa_circ_0002065	chr2	198324655	198334895	+	445	ANNOTATED, CDS, c	NM_025147	COQ10B	Jeck2013	80219	circRNA	Detected	Not Detected	4.15395515	3.009692102
hsa_gci150495	2.992650204	1.581423659	up	5.314309796	3.732886137	TGCATGGTACCGTTATC	hsa_circ_0002091	chr10	17632344	17646046	-	628	ALT_DONOR, CDS, c	NM_014241	HACD1	Jeck2013	9200	circRNA	Detected	Detected	5.314309796	3.732886137
hsa_gci150505	2.509307782	1.327289437	up	8.266354399	6.939064962	TCTCTCTCTAGGACACG	hsa_circ_0002108	chr9	16727794	16749695	-	11539	ALT_ACCEPTOR, CDS	NM_017637	BNC2	Jeck2013	54796	circRNA	Detected	Detected	8.266354399	6.939064962
hsa_gci150524	2.880124722	1.526131288	up	5.410076065	3.883944777	CGTCTCGTCCCAAAGTG	hsa_circ_0002136	chr2	161186190	161188421	-	2231	ALT_ACCEPTOR, ALT	NM_016836	RBMS1	Jeck2013	5937	circRNA	Detected	Detected	5.410076065	3.883944777
hsa_gci150550	-5.686534917	-2.507549816	down	1.411042013	3.918591829	CGTGTTGACAAAGAACA	hsa_circ_0002174	chr3	195686053	195686957	-	904	ALT_DONOR, downst	NR_003264	SDHAP1	Jeck2013	255812	circRNA	Not Detected	Detected	1.411042013	3.918591829
hsa_gci150593	2.534172317	1.341514627	up	5.881193101	4.539678473	TTGTCTCGTCATCCTGG	hsa_circ_0002239	chr1	8037711	8045195	+	329	ALT_DONOR, CDS, c	NM_007262	PARK7	Jeck2013	11315	circRNA	Detected	Detected	5.881193101	4.539678473
hsa_gci150624	-16.70356238	-4.062083915	down	1.375755617	5.437839532	ATCTCGGAAAGTTACGT	hsa_circ_0002292	chr5	36211945	36227667	-	560	ANNOTATED, CDS, c	NM_001085411	NADK2	Jeck2013, Salzman	133686	circRNA	Not Detected	Detected	1.375755617	5.437839532
hsa_gci150641	2.829584295	1.500590117	up	4.902708234	3.402118117	GCTATCATGTACCCTCG	hsa_circ_0002326	chr18	9814016	9816167	+	1127	ALT_DONOR, CDS, c	NM_006868	RAB31	Jeck2013	11031	circRNA	Detected	Detected	4.902708234	3.402118117
hsa_gci150661	2.388131537	1.255882302	up	6.21001675	4.954134449	AAGACAGTCAGTCGAAC	hsa_circ_0002361	chr1	53322669	53329829	+	1070	ANNOTATED, CDS, c	NM_001004339	ZYG11A	Jeck2013	440590	circRNA	Detected	Detected	6.21001675	4.954134449
hsa_gci150667	2.896156482	1.534139555	up	5.071065229	3.536925674	ACAGGTCTTAAATAGTA	hsa_circ_0002370	chr2	63660878	63798760	-	79436	ALT_ACCEPTOR, CDS	NM_015910	WDPCP	Jeck2013	51057	circRNA	Detected	Detected	5.071065229	3.536925674
hsa_gci150668	3.353397383	1.745623455	up	4.142600613	2.396977159	GCAAACCAGTAAAGAAG	hsa_circ_0002371	chr10	70545892	70546449	+	230	ANNOTATED, CDS, c	NM_018237	CCAR1	Jeck2013, Salzman	55749	circRNA	Detected	Not Detected	4.142600613	2.396977159
hsa_gci150675	2.474987029	1.307420965	up	8.778550252	7.471129287	AAATGTGAAGGGAGAGA	hsa_circ_0002379	chr11	47521004	47528449	-	6189	ALT_ACCEPTOR, CDS	NM_001172639	CELF1	Jeck2013	10658	circRNA	Detected	Detected	8.778550252	7.471129287
hsa_gci150678	2.32127713	1.214918772	up	5.59371876	4.378799988	AAACGCTGGTCGGCCCG	hsa_circ_0002385	chr9	20582455	20586148	-	3693	ALT_ACCEPTOR, ALT	NM_004529	MLLT3	Jeck2013	4300	circRNA	Detected	Detected	5.59371876	4.378799988
hsa_gci150708	3.796129789	1.924529319	up	8.531970103	6.607440785	GTAAAAATCCTTGACCC	hsa_circ_0002430	chr20	37179713	37182719	+	363	ANNOTATED, CDS, c	NM_020336	RALGAPB	Jeck2013	57148	circRNA	Detected	Detected	8.531970103	6.607440785
hsa_gci150722	2.433375282	1.282958834	up	7.79266083	6.509701996	TATGTCTCTCTCGTCAG	hsa_circ_0002453	chr3	8977554	8990254	-	756	ANNOTATED, CDS, c	NM_020165	RAD18	Jeck2013	56852	circRNA	Detected	Detected	7.79266083	6.509701996
hsa_gci150726	2.334964055	1.223400341	up	7.822682942	6.599282601	AGACGTAGAGGTAGTTC	hsa_circ_0002459	chr10	13175501	13178897	+	233	ALT_DONOR, CDS, c	NM_001008211	OPTN	Jeck2013	10133	circRNA	Detected	Detected	7.822682942	6.599282601
hsa_gci150754	2.443468593	1.288930561	up	6.493592327	5.204661766	AGACGTTGTAGAAAGTG	hsa_circ_0002497	chr7	91924202	91963034	+	6697	ALT_DONOR, CDS, c	NM_019004	ANKIB1	Jeck2013	54467	circRNA	Detected	Detected	6.493592327	5.204661766

hsa_gci150768	5.100819386	2.350729018	up	5.087264479	2.736535462	AAGTTCCACTTGAAATG	hsa_circ_0002516	chr3	141259374	141278837	+	413	ANNOTATED, CDS, c	NM_006506	RASA2	Jeck2013	5922	circRNA	Detected	Not Detected	5.087264479	2.736535462
hsa_gci150779	2.06210814	1.044119992	up	9.482641752	8.43852176	AAAGGTTAAGGTAGTAC	hsa_circ_0002529	chr5	64587155	64595958	-	289	ANNOTATED, CDS, c	NM_197941	ADAMTS6	Jeck2013	11174	circRNA	Detected	Detected	9.482641752	8.43852176
hsa_gci150817	2.393845268	1.259329903	up	4.382684728	3.123354825	TGCAAGACGATTAAGGC	hsa_circ_0002584	chr12	112757028	112757546	-	518	ANNOTATED, CDS, c	NM_001109662	HECTD4	Jeck2013	283450	circRNA	Detected	Not Detected	4.382684728	3.123354825
hsa_gci150871	2.594851821	1.375652156	up	5.488609357	4.112957201	CCGTCCCTATCGTTGAC	hsa_circ_0002668	chr4	47877143	47888016	-	703	ANNOTATED, CDS, c	NM_152995	NFXL1	Jeck2013, Salzman	152518	circRNA	Detected	Detected	5.488609357	4.112957201
hsa_gci150887	2.698213588	1.432004555	up	5.903018541	4.471013986	CAACACCTACATGTCCC	hsa_circ_0002685	chr7	5256193	5256818	+	195	ANNOTATED, CDS, c	NM_015610	WIPI2	Jeck2013, Salzman	26100	circRNA	Detected	Detected	5.903018541	4.471013986
hsa_gci150904	2.596683213	1.376670021	up	4.60158324	3.224913219	AGGTAGAAACTAAGATA	hsa_circ_0002712	chr12	110922882	110924522	+	436	ANNOTATED, CDS, c	NM_013300	FAM216A	Jeck2013, Salzman	29902	circRNA	Detected	Not Detected	4.60158324	3.224913219
hsa_gci150906	2.586680175	1.371101686	up	4.676911397	3.305809712	ATGTAAGGTCTCTTTTC	hsa_circ_0002714	chr7	80418621	80435074	-	816	ANNOTATED, CDS, c	NM_006379	SEMA3C	Jeck2013, Salzman	10512	circRNA	Detected	Detected	4.676911397	3.305809712
hsa_gci150946	2.736653054	1.452412545	up	4.236059001	2.783646457	CCGACCGAAAGGGGTTT	hsa_circ_0002777	chrX	14814281	14815420	-	1139	INTERGENIC	None		Jeck2013		circRNA	Detected	Not Detected	4.236059001	2.783646457
hsa_gci151013	2.096027332	1.06765753	up	5.409393675	4.341736145	TCAAAGACCCTCTTCCA	hsa_circ_0002886	chr12	83250788	83251359	+	571	ANNOTATED, CDS, c	NM_152588	TMTC2	Jeck2013, Salzman	160335	circRNA	Detected	Detected	5.409393675	4.341736145
hsa_gci151029	9.719449664	3.280874628	up	5.255524854	1.974650226	AGTAGTGACCTCCTACT	hsa_circ_0002914	chr2	11943032	11945282	+	363	ANNOTATED, CDS, c	NM_145693	LPIN1	Jeck2013, Salzman	23175	circRNA	Detected	Not Detected	5.255524854	1.974650226
hsa_gci151040	-3.820180261	-1.933640716	down	1.77276793	3.706408645	CTAGTTGACACTACTAC	hsa_circ_0002938	chr2	36726361	36749456	+	1056	ANNOTATED, CDS, c	NM_016441	CRIM1	Jeck2013, Salzman	51232	circRNA	Not Detected	Detected	1.77276793	3.706408645
hsa_gci151042	4.505489355	2.17168381	up	4.323754634	2.152070824	CTCTTAGACATGCTTCC	hsa_circ_0002941	chr19	6742977	6743847	+	445	ANNOTATED, CDS, c	NM_004240	TRIP10	Jeck2013, Salzman	9322	circRNA	Detected	Not Detected	4.323754634	2.152070824
hsa_gci151055	2.092400271	1.065158862	up	9.670305433	8.605146571	CCTACCTCCTCCAAACT	hsa_circ_0002963	chr9	112918598	112918777	+	179	ANNOTATED, CDS, c	NM_007203	PALM2-AKAP2	Jeck2013	445815	circRNA	Detected	Detected	9.670305433	8.605146571
hsa_gci151075	2.202065424	1.138857333	up	5.513800522	4.374943189	AATGACCGACCCACTAG	hsa_circ_0003007	chr15	101104896	101105470	-	574	INTERGENIC	None		Jeck2013		circRNA	Detected	Detected	5.513800522	4.374943189
hsa_gci151079	2.067252861	1.047714866	up	5.990559959	4.942845092	AATAGGAAACGTCAGAC	hsa_circ_0003015	chr11	12901254	12923660	+	543	ANNOTATED, CDS, c	NM_021961	TEAD1	Jeck2013	7003	circRNA	Detected	Detected	5.990559959	4.942845092
hsa_gci151103	2.600801583	1.378956339	up	5.550860816	4.171904477	AGGACCCACGACAAAGA	hsa_circ_0003054	chr22	46085591	46114373	+	612	ANNOTATED, CDS, c	NM_013236	ATXN10	Jeck2013, Salzman	25814	circRNA	Detected	Detected	5.550860816	4.171904477
hsa_gci151149	2.873476048	1.522797023	up	7.848826086	6.326029062	TCTTACACACAAACATT	hsa_circ_0003123	chr7	72193789	72209578	-	229	ANNOTATED, CDS, c	NM_001145440	TYW1B	Jeck2013, Salzman	441250	circRNA	Detected	Detected	7.848826086	6.326029062
hsa_gci151180	2.581634699	1.368284874	up	4.149636735	2.781351862	AAAGAGATCAAAACATT	hsa_circ_0003170	chr13	99157168	99171727	-	14559	ALT_DONOR, CDS, c	NM_001032296	STK24	Jeck2013	8428	circRNA	Detected	Not Detected	4.149636735	2.781351862
hsa_gci151203	2.997555509	1.58378647	up	4.266165399	2.682378929	AGGGCATTCTACAACAT	hsa_circ_0003200	chr14	74358729	74364965	+	780	ANNOTATED, CDS, c	NM_001242924	ZNF410	Jeck2013, Salzman	57862	circRNA	Detected	Not Detected	4.266165399	2.682378929
hsa_gci151209	-3.826716038	-1.93610685	down	1.310468051	3.246574901	CCAGTGTCTTCTAAACC	hsa_circ_0003209	chr20	30370051	30385318	+	891	ANNOTATED, CDS, c	NM_012112	TPX2	Jeck2013, Salzman	22974	circRNA	Not Detected	Detected	1.310468051	3.246574901
hsa_gci151231	2.93653493	1.554114799	up	5.001449646	3.447334847	CTTTCGGTCCACCCAAG	hsa_circ_0003244	chr8	131130406	131138335	-	499	ANNOTATED, CDS, c	NM_001247996	ASAP1	Jeck2013, Salzman	50807	circRNA	Detected	Detected	5.001449646	3.447334847
hsa_gci151232	2.436368297	1.284732237	up	6.169278343	4.884546106	TAATTCGACAGACTTTA	hsa_circ_0003245	chr13	30127897	30128810	-	913	ALT_ACCEPTOR, cod	NM_003045	SLC7A1	Jeck2013	6541	circRNA	Detected	Detected	6.169278343	4.884546106
hsa_gci151235	2.558854724	1.355498242	up	8.530563157	7.175064915	TTGATCAACGATCCTGT	hsa_circ_0003251	chr12	1003727	1006847	+	939	ANNOTATED, CDS, c	NM_001184985	WNK1	Jeck2013	65125	circRNA	Detected	Detected	8.530563157	7.175064915
hsa_gci151288	2.398659667	1.262228476	up	5.712210291	4.449981816	TAAATGTCATACCGTAC	hsa_circ_0003324	chr7	107593974	107601094	-	970	ANNOTATED, CDS, c	NM_002291	LAMB1	Jeck2013, Salzman	3912	circRNA	Detected	Detected	5.712210291	4.449981816
hsa_gci151322	3.391384934	1.761874544	up	4.454295889	2.692421345	GCGACCCGGAAACCCCT	hsa_circ_0003387	chr12	111947352	111951343	-	560	ANNOTATED, CDS, c	NM_002973	ATXN2	Jeck2013	6311	circRNA	Detected	Not Detected	4.454295889	2.692421345
hsa_gci151324	-4.19986113	-2.070341625	down	1.598416341	3.668757966	TATTACTATTTCTTAAA	hsa_circ_0003390	chr4	128590190	128609022	+	477	ANNOTATED, CDS, c	NM_015693	INTU	Jeck2013, Salzman	27152	circRNA	Not Detected	Detected	1.598416341	3.668757966
hsa_gci151330	2.480276181	1.310500775	up	9.568149749	8.257648974	GGTATAGTCGTGGATCC	hsa_circ_0003400	chr3	3197902	3215945	-	576	ANNOTATED, CDS, c	NM_016302	CRBN	Jeck2013, Salzman	51185	circRNA	Detected	Detected	9.568149749	8.257648974
hsa_gci151336	2.961113974	1.566140021	up	6.130771857	4.564631836	TCAGTCTAGAGGTTAGA	hsa_circ_0003408	chr13	23927923	23945304	-	2014	ANNOTATED, CDS, c	NM_014363	SACS	Jeck2013	26278	circRNA	Detected	Detected	6.130771857	4.564631836
hsa_gci151354	2.18420281	1.127106821	up	5.378399052	4.25129223	GTGACACAAGAGTTTAT	hsa_circ_0003438	chr16	70294946	70296427	-	293	ANNOTATED, CDS, c	NM_001605	AARS	Jeck2013, Salzman	16	circRNA	Detected	Detected	5.378399052	4.25129223
hsa_gci151367	-2.484677749	-1.313058753	down	2.475807473	3.788866226	CTAAAAAGGCCCAATAT	hsa_circ_0003454	chr15	72208710	72231268	-	383	ANNOTATED, CDS, c	NM_006901	MYO9A	Jeck2013, Salzman	4649	circRNA	Not Detected	Detected	2.475807473	3.788866226
hsa_gci151372	2.14015687	1.097716548	up	4.195861639	3.098145091	GGGGGAGGTAACAAAAG	hsa_circ_0003461	chr4	184129105	184130178	+	281	ANNOTATED, CDS, c	NM_024949	WWC2	Jeck2013, Salzman	80014	circRNA	Detected	Not Detected	4.195861639	3.098145091
hsa_gci151378	3.412929744	1.771010717	up	4.905425836	3.134415119	TTGGTGAGGTCTTGGGA	hsa_circ_0003471	chr13	111546455	111546740	-	285	ALT_ACCEPTOR, ALT	NM_017664	ANKRD10	Jeck2013	55608	circRNA	Detected	Not Detected	4.905425836	3.134415119
hsa_gci151419	2.656741339	1.409657773	up	7.098118086	5.688460313	TCCCTACCCAAGATTAA	hsa_circ_0003542	chr5	177053436	177054615	-	303	ANNOTATED, INTERN	NR_026921	LOC202181	Jeck2013, Salzman	202181	circRNA	Detected	Detected	7.098118086	5.688460313
hsa_gci151446	2.669859214	1.416763668	up	5.108338544	3.691574876	CGTAAAGTGGTAGTGAA	hsa_circ_0003587	chr13	30801548	30815223	-	655	ANNOTATED, CDS, c	NM_032116	KATNAL1	Jeck2013, Salzman	84056	circRNA	Detected	Detected	5.108338544	3.691574876
hsa_gci151448	2.128473659	1.089819236	up	6.127828971	5.038009734	GGACTATGACATTATTT	hsa_circ_0003590	chr15	78837210	78838106	+	220	ANNOTATED, CDS, c	NM_002789	PSMA4	Jeck2013, Salzman	5685	circRNA	Detected	Detected	6.127828971	5.038009734
hsa_gci151467	2.332716621	1.222011059	up	4.793633247	3.571622188	AAAGTAAAAGACTTCGA	hsa_circ_0003618	chr22	28692185	28693840	-	404	ANNOTATED, CDS, c	NM_001145418	TTC28	Jeck2013	23331	circRNA	Detected	Detected	4.793633247	3.571622188
hsa_gci151470	-2.687075588	-1.426036905	down	3.458797365	4.884834271	TCCACCACTTATCCCCA	hsa_circ_0003624	chr9	33886878	33900269	+	185	ANNOTATED, CDS, c	NM_017811	UBE2R2	Jeck2013, Salzman	54926	circRNA	Not Detected	Detected	3.458797365	4.884834271
hsa_gci151502	2.255021304	1.173141063	up	6.132781692	4.959640629	AGTAATAGAAATCGTAC	hsa_circ_0003669	chr3	108388527	108396445	+	460	ANNOTATED, CDS, c	NM_014648	DZIP3	Jeck2013	9666	circRNA	Detected	Detected	6.132781692	4.959640629
hsa_gci151504	118.3838713	6.88732873	up	8.228645259	1.341316529	TAAGGGGTGACGACTTA	hsa_circ_0003671	chr13	108175911	108176384	-	473	ALT_ACCEPTOR, ALT	NM_001080396	FAM155A	Jeck2013	728215	circRNA	Detected	Not Detected	8.228645259	1.341316529
hsa_gci151514	2.323007979	1.21599411	up	6.919064221	5.703070112	ACGAGGGTAACGCAAAG	hsa_circ_0003681	chr16	71736500	71748704	-	424	ANNOTATED, CDS, c	NM_015020	PHLPP2	Jeck2013, Salzman	23035	circRNA	Detected	Detected	6.919064221	5.703070112
hsa_gci151552	3.010565482	1.590034498	up	6.515423151	4.925388653	TCACTCCTCCTAAAGAT	hsa_circ_0003740	chr1	226016425	226019660	+	369	ANNOTATED, CDS, c	NM_000120	EPHX1	Jeck2013, Salzman	2052	circRNA	Detected	Detected	6.515423151	4.925388653
hsa_gci151577	2.446457999	1.290694514	up	8.059935133	6.769240619	TAAACTTGTAGTTTTGA	hsa_circ_0003782	chr9	20819794	20874806	+	862	ANNOTATED, CDS, c	NM_017794	FOCAD	Jeck2013	54914	circRNA	Detected	Detected	8.059935133	6.769240619
hsa_gci151598	2.097405353	1.06860571	up	4.524415998	3.455810289	CTAGTCCACGATTCACA	hsa_circ_0003810	chr6	79770194	79770535	-	250	ANNOTATED, CDS, c	NM_017934	PHIP	Jeck2013, Salzman	55023	circRNA	Detected	Detected	4.524415998	3.455810289
hsa_gci151623	-4.243624017	-2.08529684	down	1.426758296	3.512055136	AAATGTGACCTGACTTT	hsa_circ_0003855	chr12	12672795	12674397	-	732	ANNOTATED, CDS, c	NM_030640	DUSP16	Jeck2013, Salzman	80824	circRNA	Not Detected	Detected	1.426758296	3.512055136
hsa_gci151628	2.457800504	1.297367819	up	6.583762656	5.286394837	GGACACCGAAAACCGAA	hsa_circ_0003861	chr9	74309424	74313120	-	319	ANNOTATED, CDS, c	NM_013390	CEMIP2	Jeck2013, Salzman	23670	circRNA	Detected	Detected	6.583762656	5.286394837
hsa_gci151665	2.304598653	1.204515527	up	9.184590942	7.980075415	TTATTCCAGTGTAAGTG	hsa_circ_0003913	chr10	79253555	79254861	-	1306	ALT_ACCEPTOR, ALT	NM_001014797	KCNMA1	Jeck2013	3778	circRNA	Detected	Detected	9.184590942	7.980075415
hsa_gci151679	2.733027875	1.450500174	up	5.335475214	3.884975039	TCAATAATACTCGATTT	hsa_circ_0003931	chr7	23381682	23385681	-	385	ANNOTATED, CDS, c	NM_006547	IGF2BP3	Jeck2013, Salzman	10643	circRNA	Detected	Detected	5.335475214	3.884975039
hsa_gci151687	2.13921337	1.097080387	up	7.820211693	6.723131306	AGTCCATGTGACACCGT	hsa_circ_0003948	chr7	6505701	6509385	-	412	ANNOTATED, CDS, c	NM_006854	KDELR2	Jeck2013, Salzman	11014	circRNA	Detected	Detected	7.820211693	6.723131306
hsa_gci151694	2.025796167	1.018489019	up	4.740896354	3.722407334	GTGGGGGCTCACCGAGG	hsa_circ_0003962	chr7	66520821	66548526	+	282	ANNOTATED, CDS, c	NM_018264	TYW1	Jeck2013	55253	circRNA	Detected	Detected	4.740896354	3.722407334
hsa_gci151705	2.112513366	1.078960469	up	5.82408004	4.74511957	TTGAGGTTCATGAAGTA	hsa_circ_0003977	chr4	100829867	100844342	-	332	ANNOTATED, CDS, c	NM_001031723	DNAJB14	Jeck2013, Salzman	79982	circRNA	Detected	Detected	5.82408004	4.74511957
hsa_gci151716	2.252460524	1.171501822	up	4.452456251	3.280954429	CAGTATAAGGTAAGAAA	hsa_circ_0003994	chr2	201281101	201284219	+	297	ANNOTATED, CDS, c	NM_001100422	SPATS2L	Jeck2013, Salzman	26010	circRNA	Detected	Detected	4.452456251	3.280954429

hsa_gci151751	2.322881512	1.215915566	up	6.679703171	5.463787605	CGACTTATCCCAGGGTG	hsa_circ_0004044	chr2	224743245	224760347	-	777	ALT_DONOR, CDS, c	NM_020830	WDFY1	Jeck2013	57590	circRNA	Detected	Detected	6.679703171	5.463787605
hsa_gci151779	2.177149955	1.122440779	up	8.726933092	7.604492313	CCGTCTCGACCCTTTCC	hsa_circ_0004094	chr7	18694385	18696610	+	2225	ALT_ACCEPTOR, ALT	NM_178423	HDAC9	Jeck2013	9734	circRNA	Detected	Detected	8.726933092	7.604492313
hsa_gci151783	2.177611723	1.122746738	up	8.972138443	7.849391705	AAGTAGGCGAGGAGACC	hsa_circ_0004102	chr10	128768965	128807068	+	1012	ANNOTATED, CDS, c	NM_001380	DOCK1	Jeck2013	1793	circRNA	Detected	Detected	8.972138443	7.849391705
hsa_gci151818	-10.55555808	-3.399930953	down	1.216953861	4.616884814	GGACAAAGGCTCCTTAA	hsa_circ_0004149	chr12	27135705	27152609	-	709	ANNOTATED, CDS, c	NM_016551	TM7SF3	Jeck2013	51768	circRNA	Not Detected	Detected	1.216953861	4.616884814
hsa_gci151875	2.554463543	1.353020346	up	5.062005426	3.70898508	AGACGTCCTTCTAATAG	hsa_circ_0004234	chr20	31518203	31519142	+	939	INTERGENIC	None		Jeck2013		circRNA	Detected	Detected	5.062005426	3.70898508
hsa_gci151900	-3.314402946	-1.728749008	down	2.765569308	4.494318316	GACAGAGGTACGTTCTT	hsa_circ_0004273	chr17	60111147	60112969	-	344	ANNOTATED, CDS, c	NM_005121	MED13	Jeck2013, Salzman	9969	circRNA	Not Detected	Detected	2.765569308	4.494318316
hsa_gci151946	-2.122923826	-1.086052606	down	4.540929498	5.626982104	AAGATGTTAAGAAGGTC	hsa_circ_0004340	chr3	72890195	72893574	-	343	ANNOTATED, CDS, c	NM_018130	SHQ1	Jeck2013	55164	circRNA	Detected	Detected	4.540929498	5.626982104
hsa_gci151960	2.577980874	1.366241561	up	7.133903911	5.767662351	GGAGTTCTCAACCGTCC	hsa_circ_0004366	chr8	100286425	100287482	+	309	ANNOTATED, CDS, c	NM_017890	VPS13B	Jeck2013, Salzman	157680	circRNA	Detected	Detected	7.133903911	5.767662351
hsa_gci151961	2.969581905	1.570259825	up	5.071331557	3.501071732	GCTCCGGGGAACAGAAG	hsa_circ_0004367	chr15	66015185	66048810	-	1610	ANNOTATED, CDS, c	NM_001144823	DENND4A	Jeck2013, Salzman	10260	circRNA	Detected	Detected	5.071331557	3.501071732
hsa_gci151968	2.433568318	1.283073276	up	5.06004214	3.776968864	GTAACCACCACTCCCGT	hsa_circ_0004380	chr8	141407718	141415797	-	248	ANNOTATED, CDS, c	NM_031466	TRAPPC9	Jeck2013, Salzman	83696	circRNA	Detected	Detected	5.06004214	3.776968864
hsa_gci151970	2.565820032	1.359419982	up	7.875902062	6.51648208	TCCTGAGGCCTCTCAAG	hsa_circ_0004384	chr2	172182350	172188377	-	377	ANNOTATED, CDS, c	NM_024770	METTL8	Jeck2013	79828	circRNA	Detected	Detected	7.875902062	6.51648208
hsa_gci151975	2.350052115	1.23269275	up	6.408805709	5.176112959	AACACCTCTTCACTTGT	hsa_circ_0004390	chr1	85331067	85331821	-	754	ANNOTATED, CDS, c	NM_012152	LPAR3	Jeck2013	23566	circRNA	Detected	Detected	6.408805709	5.176112959
hsa_gci151976	5.754075226	2.524584082	up	6.020834823	3.496250741	GCTCCGGGGAACAGAAG	hsa_circ_0004393	chr15	66048477	66048810	-	333	ANNOTATED, CDS, c	NM_001144823	DENND4A	Jeck2013, Salzman	10260	circRNA	Detected	Detected	6.020834823	3.496250741
hsa_gci152044	3.350554859	1.744400029	up	7.385856237	5.641456208	TCTAGAAACGAACGTAT	hsa_circ_0004496	chr4	177632652	177650900	-	557	ANNOTATED, CDS, c	NM_005429	VEGFC	Jeck2013	7424	circRNA	Detected	Detected	7.385856237	5.641456208
hsa_gci152046	-2.067343065	-1.047777817	down	3.876466203	4.92424402	AGACTTTGCACCCGTCA	hsa_circ_0004499	chr20	17933230	17936119	-	357	ANNOTATED, CDS, c	NM_152227	SNX5	Jeck2013, Salzman	27131	circRNA	Not Detected	Detected	3.876466203	4.92424402
hsa_gci152060	2.280274177	1.189207302	up	5.285546795	4.096339493	CCCCAAATCCCCGATGG	hsa_circ_0004524	chr3	138289159	138291774	-	470	ANNOTATED, CDS, c	NM_024491	CEP70	Jeck2013, Salzman	80321	circRNA	Detected	Detected	5.285546795	4.096339493
hsa_gci152077	2.415346569	1.272230211	up	4.615423433	3.343193222	AGGAAGAAACTGAGACT	hsa_circ_0004554	chr2	230666967	230668943	-	556	ANNOTATED, CDS, c	NM_004238	TRIP12	Jeck2013, Salzman	9320	circRNA	Detected	Detected	4.615423433	3.343193222
hsa_gci152098	5.455325765	2.44766535	up	6.678154227	4.230488877	CGGACTAAAACGATCCA	hsa_circ_0004590	chr12	78334098	78392256	+	637	ANNOTATED, CDS, c	NM_014903	NAV3	Jeck2013, Salzman	89795	circRNA	Detected	Detected	6.678154227	4.230488877
hsa_gci152104	2.433850691	1.283240666	up	7.373464843	6.090224177	ATTCTAACTTATTCTTG	hsa_circ_0004598	chr11	122665409	122669742	+	470	ANNOTATED, CDS, c	NM_032873	UBASH3B	Jeck2013	84959	circRNA	Detected	Detected	7.373464843	6.090224177
hsa_gci152131	2.632678579	1.396531395	up	5.909724748	4.513193352	ACAACGTTCTGCTACAA	hsa_circ_0004643	chr2	72786581	72802797	-	246	ANNOTATED, CDS, c	NM_015189	EXOC6B	Jeck2013, Salzman	23233	circRNA	Detected	Detected	5.909724748	4.513193352
hsa_gci152138	2.333644222	1.222584631	up	6.713179045	5.490594414	AGAAAGAAACATCACAG	hsa_circ_0004651	chr4	83279811	83280792	-	331	ANNOTATED, CDS, c	NM_031370	HNRNPD	Jeck2013, Salzman	3184	circRNA	Detected	Detected	6.713179045	5.490594414
hsa_gci152168	2.153905804	1.106955158	up	6.832470264	5.725515105	TCGATATTTGGAACAGG	hsa_circ_0004703	chr7	5941287	5949747	+	390	ANNOTATED, CDS, c	NM_015622	CCZ1	Jeck2013, Salzman	51622	circRNA	Detected	Detected	6.832470264	5.725515105
hsa_gci152183	2.106890899	1.075115609	up	5.27355763	4.198442021	ACACCGACCATCCACGA	hsa_circ_0004729	chr2	159854989	159857701	+	2712	ALT_ACCEPTOR, cod	NM_033394	TANC1	Jeck2013	85461	circRNA	Detected	Detected	5.27355763	4.198442021
hsa_gci152188	2.007210635	1.00519202	up	4.352031431	3.346839411	GAATAAGAAGAGTAGTT	hsa_circ_0004735	chr9	22046749	22112394	+	1795	ANNOTATED, INTERN	NR_003529	CDKN2B-AS1	Jeck2013	100048912	circRNA	Detected	Detected	4.352031431	3.346839411
hsa_gci152191	2.025913386	1.018572496	up	7.692115994	6.673543498	TTTAGACTAGAAAACTC	hsa_circ_0004738	chr5	170667931	170669824	+	354	ANNOTATED, CDS, c	NM_022897	RANBP17	Jeck2013, Salzman	64901	circRNA	Detected	Detected	7.692115994	6.673543498
hsa_gci152200	2.032220751	1.023057124	up	5.355042413	4.331985288	GGGAAGGAGTAGTCTAT	hsa_circ_0004751	chr17	35310185	35311207	+	549	ANNOTATED, CDS, c	NM_012138	AATF	Jeck2013	26574	circRNA	Detected	Detected	5.355042413	4.331985288
hsa_gci152204	9.576650246	3.259521113	up	6.732649262	3.473128149	TGTATCTCGACTCACTG	hsa_circ_0004755	chr7	157013382	157024021	+	567	ANNOTATED, CDS, c	NM_014671	UBE3C	Jeck2013	9690	circRNA	Detected	Detected	6.732649262	3.473128149
hsa_gci152227	2.274391951	1.185480898	up	7.579539991	6.394059092	AGTAGTTACAGTGAAAA	hsa_circ_0004796	chr17	45741524	45755779	+	1567	ANNOTATED, CDS, c	NM_002265	KPNB1	Jeck2013, Salzman	3837	circRNA	Detected	Detected	7.579539991	6.394059092
hsa_gci152297	2.125158136	1.087570198	up	5.824578751	4.737008553	GACGTAGGACCCCTACC	hsa_circ_0004899	chr11	57563048	57569668	+	1153	ANNOTATED, CDS, c	NM_001085458	CTNND1	Jeck2013	1500	circRNA	Detected	Detected	5.824578751	4.737008553
hsa_gci152345	4.001188919	2.000428748	up	4.297688599	2.297259851	AGGGGAAGAATAAGAAG	hsa_circ_0004972	chr12	53410256	53413810	+	464	ANNOTATED, CDS, c	NM_001417	EIF4B	Jeck2013, Salzman	1975	circRNA	Detected	Not Detected	4.297688599	2.297259851
hsa_gci152357	2.303717173	1.203963608	up	5.85179736	4.647833752	ACCGAGTAGACCCCGGT	hsa_circ_0004994	chr20	33954359	33971936	-	335	ANNOTATED, CDS, c	NM_018244	UQCC1	Jeck2013, Salzman	55245	circRNA	Detected	Detected	5.85179736	4.647833752
hsa_gci152358	6.137201872	2.617581039	up	4.433459929	1.81587889	TTTAGGTAGGAGGTGTG	hsa_circ_0004996	chr1	179100445	179102509	-	234	ANNOTATED, CDS, c	NM_007314	ABL2	Jeck2013, Salzman	27	circRNA	Detected	Not Detected	4.433459929	1.81587889
hsa_gci152365	16.2599799	4.023253569	up	5.230389554	1.207135985	TGGAGTCCTCGATTACT	hsa_circ_0005009	chr3	179131199	179144030	-	741	ANNOTATED, CDS, c	NM_021629	GNB4	Jeck2013	59345	circRNA	Detected	Not Detected	5.230389554	1.207135985
hsa_gci152381	2.255348407	1.173350319	up	5.953315311	4.779964993	CCCGTCGTAACATTATT	hsa_circ_0005033	chr7	27668989	27672045	-	213	ALT_ACCEPTOR, CDS	NM_152740	HIBADH	Jeck2013	11112	circRNA	Detected	Detected	5.953315311	4.779964993
hsa_gci152384	28.14443384	4.814777722	up	6.040044476	1.225266754	CCAGGTGTCAAAAGTTT	hsa_circ_0005040	chr14	25443870	25446449	-	2579	ALT_ACCEPTOR, CDS	NM_014178	STXBP6	Jeck2013	29091	circRNA	Detected	Not Detected	6.040044476	1.225266754
hsa_gci152406	2.040455537	1.028891273	up	5.705246637	4.676355363	CCACCCGTCTTACTTAC	hsa_circ_0005070	chr8	103341317	103357773	-	590	ANNOTATED, CDS, c	NM_015902	UBR5	Jeck2013, Salzman	51366	circRNA	Detected	Detected	5.705246637	4.676355363
hsa_gci152416	2.541497961	1.345679073	up	4.575647319	3.229968246	AAAGAAGTAAAACATTT	hsa_circ_0005085	chr2	9419445	9437574	+	219	ANNOTATED, CDS, c	NM_003887	ASAP2	Jeck2013, Salzman	8853	circRNA	Detected	Detected	4.575647319	3.229968246
hsa_gci152430	2.3225913	1.215735309	up	6.882375363	5.666640054	TCTGATTTGAAGACTTC	hsa_circ_0005108	chr14	39870379	39871715	-	797	ANNOTATED, CDS, c	NM_203301	FBXO33	Jeck2013	254170	circRNA	Detected	Detected	6.882375363	5.666640054
hsa_gci152436	6.052561735	2.59754589	up	3.820535215	1.222989325	CGGGAGACGTAGACACC	hsa_circ_0005117	chr2	48555699	48556261	+	562	ALT_DONOR, coding	NM_002158	FOXN2	Jeck2013	3344	circRNA	Detected	Not Detected	3.820535215	1.222989325
hsa_gci152438	2.148896082	1.103595717	up	5.84752131	4.743925593	TCCCAAGGATTTTATTT	hsa_circ_0005119	chr9	113734352	113771481	-	35869	ALT_ACCEPTOR, CDS	NM_057159	LPAR1	Jeck2013	1902	circRNA	Detected	Detected	5.84752131	4.743925593
hsa_gci152463	2.157325488	1.10924386	up	4.631342299	3.522098439	CTCCTCGTGCCACGACA	hsa_circ_0005161	chr13	114174930	114188555	+	314	ANNOTATED, CDS, c	NM_017905	TMCO3	Jeck2013, Salzman	55002	circRNA	Detected	Detected	4.631342299	3.522098439
hsa_gci152466	-4.707825207	-2.235060757	down	1.251113598	3.486174355	TTTTACCTCTAATAGGT	hsa_circ_0005164	chr10	76598440	76603236	+	949	ANNOTATED, CDS, c	NM_012330	KAT6B	Jeck2013, Salzman	23522	circRNA	Not Detected	Detected	1.251113598	3.486174355
hsa_gci152468	2.896765621	1.53444296	up	5.725898139	4.191455178	CCTGTATGAAAACCGCC	hsa_circ_0005166	chr12	63359304	63359768	+	464	INTERGENIC	None		Jeck2013		circRNA	Detected	Detected	5.725898139	4.191455178
hsa_gci152488	2.87998392	1.526060757	up	5.115375168	3.589314412	TATGTTAAACGTTTCTA	hsa_circ_0005198	chr13	25072253	25077915	-	592	ANNOTATED, CDS, c	NM_006437	PARP4	Jeck2013	143	circRNA	Detected	Detected	5.115375168	3.589314412
hsa_gci152497	3.067448795	1.617039261	up	8.331127542	6.714088281	CTTACAAGAGAAAGTCT	hsa_circ_0005211	chr15	59470591	59480415	-	244	ANNOTATED, CDS, c	NM_004998	MYO1E	Jeck2013	4643	circRNA	Detected	Detected	8.331127542	6.714088281
hsa_gci152513	3.132492704	1.647311149	up	6.796565698	5.149254549	GGGCTCCCGAAGAACCA	hsa_circ_0005230	chr1	172109619	172113577	-	3958	ALT_ACCEPTOR, ALT	NR_038397	DNM3OS	Jeck2013	100628315	circRNA	Detected	Detected	6.796565698	5.149254549
hsa_gci152518	3.564830245	1.833833378	up	4.693373691	2.859540313	TGTACCACTCCGACTCC	hsa_circ_0005237	chr12	112130500	112147488	+	703	ANNOTATED, CDS, c	NM_001136538	ACAD10	Jeck2013, Salzman	80724	circRNA	Detected	Not Detected	4.693373691	2.859540313
hsa_gci152543	2.318165991	1.212983874	up	4.050994058	2.838010184	GAACTACTGTCTCTGTC	hsa_circ_0005279	chr12	110764194	110765822	+	551	ANNOTATED, CDS, c	NM_170665	ATP2A2	Jeck2013, Salzman	488	circRNA	Detected	Not Detected	4.050994058	2.838010184
hsa_gci152577	4.032408098	2.011641653	up	5.244873275	3.233231622	AAGTCTCAAAGGTGTCT	hsa_circ_0005326	chr12	58142321	58143287	-	266	ALT_DONOR, CDS, c	NM_000075	CDK4	Jeck2013	1019	circRNA	Detected	Detected	5.244873275	3.233231622
hsa_gci152587	2.152317198	1.105890711	up	7.672090464	6.566199753	AGCCTACTCAGGGTAGT	hsa_circ_0005352	chr8	25340914	25341726	+	333	ANNOTATED, CDS, c	NM_152562	CDCA2	Jeck2013	157313	circRNA	Detected	Detected	7.672090464	6.566199753
hsa_gci152593	2.160470058	1.111345237	up	4.762633749	3.651288512	TATAGACCGACGGTCTG	hsa_circ_0005363	chr12	78388582	78415642	+	1352	ANNOTATED, CDS, c	NM_014903	NAV3	Jeck2013, Salzman	89795	circRNA	Detected	Detected	4.762633749	3.651288512

hsa_gci152615	2.017366355	1.012473102	up	6.643775825	5.631302723	AACTTATAGGAGAGTTA	hsa_circ_0005390	chr8	131414130	131440068	-	25938	ALT_ACCEPTOR, cod	NM_001247996	ASAP1	Jeck2013	50807	circRNA	Detected	Detected	6.643775825	5.631302723
hsa_gci152620	2.420632879	1.275384292	up	9.085148607	7.809764314	CTTACCACTAAAGAAGA	hsa_circ_0005397	chr17	30500849	30503232	+	233	ANNOTATED, CDS, c	NM_001033568	RHOT1	Jeck2013, Salzman	55288	circRNA	Detected	Detected	9.085148607	7.809764314
hsa_gci152630	2.193883615	1.133486993	up	5.934778049	4.801291056	GAAGATCGAGGAGAGCA	hsa_circ_0005414	chr3	32479446	32483505	+	4059	ALT_ACCEPTOR, CDS	NM_138410	CMTM7	Jeck2013	112616	circRNA	Detected	Detected	5.934778049	4.801291056
hsa_gci152631	-4.032161751	-2.011553514	down	1.265419727	3.276973241	GGACGGTGTCGAGCTCC	hsa_circ_0005417	chr15	43102811	43109295	-	285	ANNOTATED, CDS, c	NM_173500	TTBK2	Jeck2013, Salzman	146057	circRNA	Not Detected	Detected	1.265419727	3.276973241
hsa_gci152654	4.024060729	2.008652078	up	6.804851376	4.796199298	GACTAAAACGATCCATC	hsa_circ_0005450	chr12	78334098	78401225	+	1664	ANNOTATED, CDS, c	NM_014903	NAV3	Jeck2013, Salzman	89795	circRNA	Detected	Detected	6.804851376	4.796199298
hsa_gci152656	2.143907635	1.100242752	up	7.440164769	6.339922017	CTCTCTCGACTCGACAA	hsa_circ_0005452	chr3	86996159	87018273	-	592	ALT_DONOR, CDS, c	NM_016206	VGLL3	Jeck2013	389136	circRNA	Detected	Detected	7.440164769	6.339922017
hsa_gci152663	5.114428735	2.354573105	up	4.811768368	2.457195263	TGGACTTCACTGAGCCC	hsa_circ_0005468	chr11	125513686	125514538	+	419	ANNOTATED, CDS, c	NM_001114122	CHEK1	Jeck2013	1111	circRNA	Detected	Not Detected	4.811768368	2.457195263
hsa_gci152667	3.094419992	1.629669021	up	8.49659114	6.866922119	CGAAGGTCGTTGAGGGA	hsa_circ_0005472	chr6	160103505	160106065	-	345	ANNOTATED, CDS, c	NM_001024465	SOD2	Jeck2013, Salzman	6648	circRNA	Detected	Detected	8.49659114	6.866922119
hsa_gci152668	2.304868322	1.204684331	up	6.003555217	4.798870886	TAGGAGAGAAATCCTAG	hsa_circ_0005474	chr10	93711159	93717050	+	500	ANNOTATED, CDS, c	NM_003972	BTAF1	Jeck2013	9044	circRNA	Detected	Detected	6.003555217	4.798870886
hsa_gci152696	2.298241176	1.200530201	up	9.173105414	7.972575212	GAGTCTTATCTAAGGTG	hsa_circ_0005518	chrX	53622146	53634660	-	1061	ANNOTATED, CDS, c	NM_031407	HUWE1	Jeck2013	10075	circRNA	Detected	Detected	9.173105414	7.972575212
hsa_gci152726	6.902166071	2.787049186	up	4.007370852	1.220321665	GGAAGAAGTTACAGTAG	hsa_circ_0005558	chr15	81262802	81274523	-	9249	ALT_DONOR, CDS, c	NM_015154	MESD	Jeck2013	23184	circRNA	Detected	Not Detected	4.007370852	1.220321665
hsa_gci152746	3.017934735	1.593561607	up	7.014315941	5.420754335	TTAGTGTAATCGGTCCC	hsa_circ_0005585	chr5	43675612	43704502	+	623	ALT_DONOR, CDS, c	NM_012343	NNT	Jeck2013	23530	circRNA	Detected	Detected	7.014315941	5.420754335
hsa_gci152756	3.767353378	1.913551365	up	9.877253642	7.963702277	TGTTACTATTACCTTTG	hsa_circ_0005601	chr7	6465618	6474651	-	637	ANNOTATED, CDS, c	NM_139179	DAGLB	Jeck2013, Salzman	221955	circRNA	Detected	Detected	9.877253642	7.963702277
hsa_gci152771	3.238966502	1.695533548	up	4.087456177	2.391922629	AGTTTCTACTTTCTGTA	hsa_circ_0005624	chr10	71912018	71921687	-	826	ALT_DONOR, CDS, c	NM_001142648	SAR1A	Jeck2013	56681	circRNA	Detected	Not Detected	4.087456177	2.391922629
hsa_gci152794	2.282263132	1.190465136	up	7.326991565	6.136526429	TGACCTTCTTCAAAATA	hsa_circ_0005653	chr18	196636	204692	+	701	ANNOTATED, CDS, c	NM_005151	USP14	Jeck2013, Salzman	9097	circRNA	Detected	Detected	7.326991565	6.136526429
hsa_gci152811	2.263539272	1.178580337	up	7.605961543	6.427381206	GAGGTGAGTCGATGGTA	hsa_circ_0005677	chr20	33077070	33092208	+	396	ANNOTATED, CDS, c	NM_031483	ITCH	Jeck2013	83737	circRNA	Detected	Detected	7.605961543	6.427381206
hsa_gci152866	6.408223577	2.679924483	up	3.937390923	1.25746644	AAAGGAGGTCTTTTGTC	hsa_circ_0005757	chrY	15024874	15026561	+	322	ANNOTATED, CDS, c	NM_001122665	DDX3Y	Jeck2013, Salzman	8653	circRNA	Detected	Not Detected	3.937390923	1.25746644
hsa_gci152903	2.326910283	1.218415587	up	5.243753285	4.025337699	ACTTACTTTGTCAGTTG	hsa_circ_0005821	chr9	111843118	111870850	-	870	ANNOTATED, CDS, c	NM_032012	TMEM245	Jeck2013, Salzman	23731	circRNA	Detected	Detected	5.243753285	4.025337699
hsa_gci152909	2.677641562	1.42096285	up	5.597991931	4.177029082	TAATATATGTCGAAAAC	hsa_circ_0005828	chr10	88231964	88233730	-	386	ANNOTATED, CDS, c	NM_015045	WAPL	Jeck2013, Salzman	23063	circRNA	Detected	Detected	5.597991931	4.177029082
hsa_gci152932	-3.69793989	-1.886721774	down	1.221179634	3.107901408	AAACGAAACTTCAGGGC	hsa_circ_0005868	chr20	33057852	33069011	+	669	ANNOTATED, CDS, c	NM_031483	ITCH	Jeck2013, Salzman	83737	circRNA	Not Detected	Detected	1.221179634	3.107901408
hsa_gci152965	2.551135399	1.351139471	up	5.244523221	3.89338375	AAAGTCCGTTCAATAAC	hsa_circ_0005917	chr6	10702601	10702872	+	196	ANNOTATED, CDS, c	NM_017906	PAK1IP1	Jeck2013	55003	circRNA	Detected	Detected	5.244523221	3.89338375
hsa_gci152969	2.0610895	1.043407154	up	7.603733748	6.560326594	TCCTCCGGAAGTTAAAG	hsa_circ_0005927	chr8	42259305	42260979	+	379	ANNOTATED, CDS, c	NM_001135694	VDAC3	Jeck2013, Salzman	7419	circRNA	Detected	Detected	7.603733748	6.560326594
hsa_gci153007	2.442013637	1.288071257	up	7.91092923	6.622857973	ATATAAGTTGTACCGAA	hsa_circ_0005984	chr8	100443764	100494030	+	788	ANNOTATED, CDS, c	NM_017890	VPS13B	Jeck2013, Salzman	157680	circRNA	Detected	Detected	7.91092923	6.622857973
hsa_gci153008	2.961161876	1.566163359	up	6.225883529	4.659720169	CCACCGGCGGTGTCCGA	hsa_circ_0005986	chr1	14057494	14068652	+	375	ANNOTATED, CDS, c	NM_012231	PRDM2	Jeck2013, Salzman	7799	circRNA	Detected	Detected	6.225883529	4.659720169
hsa_gci153017	-4.388330938	-2.133672328	down	1.22956834	3.363240668	GCCGGCATATCTGTAGA	hsa_circ_0005996	chr20	45874751	45875261	-	510	ANNOTATED, CDS, c	NM_183047	ZMYND8	Jeck2013	23613	circRNA	Not Detected	Detected	1.22956834	3.363240668
hsa_gci153029	3.027349928	1.598055444	up	6.963185927	5.365130482	AACACGGTTAAAGTTTT	hsa_circ_0006020	chrX	16836696	16838378	+	396	ANNOTATED, CDS, c	NM_018360	TXLNG	Jeck2013	55787	circRNA	Detected	Detected	6.963185927	5.365130482
hsa_gci153033	2.074142779	1.052515209	up	7.110668283	6.058153074	TCAATGGAATAAAAAAT	hsa_circ_0006024	chr5	93111864	93120205	-	202	ANNOTATED, CDS, c	NM_032042	FAM172A	Jeck2013, Salzman	83989	circRNA	Detected	Detected	7.110668283	6.058153074
hsa_gci153048	2.316929202	1.212213961	up	3.992277113	2.780063152	ATTATTAAGACAGTCGA	hsa_circ_0006042	chr7	27668989	27672060	-	228	ALT_ACCEPTOR, CDS	NM_152740	HIBADH	Jeck2013	11112	circRNA	Detected	Not Detected	3.992277113	2.780063152
hsa_gci153069	2.320403798	1.214375886	up	7.782238996	6.56786311	AGAAACAACTGACAAAG	hsa_circ_0006077	chr12	133324417	133324917	-	383	ANNOTATED, CDS, c	NM_015114	ANKLE2	Jeck2013, Salzman	23141	circRNA	Detected	Detected	7.782238996	6.56786311
hsa_gci153083	2.308264256	1.206808397	up	3.821525249	2.614716853	GTGAAAACAGTAACAAG	hsa_circ_0006101	chr2	201784944	201802689	-	1013	ANNOTATED, INTERN	NR_033915	ORC2	Jeck2013	4999	circRNA	Detected	Not Detected	3.821525249	2.614716853
hsa_gci153086	2.74769055	1.458219534	up	8.267608119	6.809388585	TATAGTAGGAGTCTTTC	hsa_circ_0006106	chr9	112898406	112918777	+	2592	ANNOTATED, CDS, c	NM_007203	PALM2-AKAP2	Jeck2013	445815	circRNA	Detected	Detected	8.267608119	6.809388585
hsa_gci153141	3.585807509	1.842298045	up	4.676044221	2.833746176	TGGTACCACCTATGGAC	hsa_circ_0006184	chr12	51398570	51404549	-	467	ANNOTATED, CDS, c	NM_001174129	SLC11A2	Jeck2013	4891	circRNA	Detected	Not Detected	4.676044221	2.833746176
hsa_gci153142	2.775852457	1.472930887	up	6.648794264	5.175863376	AGGAATTTTGAATCGAA	hsa_circ_0006187	chr6	73843143	73879577	+	330	ANNOTATED, CDS, c	NM_001160133	KCNQ5	Jeck2013	56479	circRNA	Detected	Detected	6.648794264	5.175863376
hsa_gci153162	-3.888614567	-1.959256244	down	1.31267603	3.271932275	AATGATTTGCCCTGCAC	hsa_circ_0006215	chr3	27478878	27490288	-	286	ANNOTATED, CDS, c	NM_003615	SLC4A7	Jeck2013, Salzman	9497	circRNA	Not Detected	Detected	1.31267603	3.271932275
hsa_gci153165	2.754948611	1.462025408	up	8.57291651	7.110891102	GAAAGTCAATTTAGAGG	hsa_circ_0006218	chr14	74398205	74398815	+	610	ALT_DONOR, coding	NM_001242924	ZNF410	Jeck2013	57862	circRNA	Detected	Detected	8.57291651	7.110891102
hsa_gci153174	9.628895065	3.267370255	up	6.840272064	3.572901809	GGTGTGTACAGACCTAC	hsa_circ_0006230	chr7	157013382	157047004	+	1036	ANNOTATED, CDS, c	NM_014671	UBE3C	Jeck2013	9690	circRNA	Detected	Detected	6.840272064	3.572901809
hsa_gci153206	-8.417327756	-3.073362294	down	1.407088153	4.480450447	GGACCAGACAAGGGGTC	hsa_circ_0006283	chr18	44681169	44683908	-	1536	ALT_DONOR, CDS, c	NM_016097	IER3IP1	Jeck2013	51124	circRNA	Not Detected	Detected	1.407088153	4.480450447
hsa_gci153232	-65.59661094	-6.035549375	down	1.384036638	7.419586012	TGTCTCCCAGGTTCAGG	hsa_circ_0006322	chrY	7209155	7239930	+	441	ANNOTATED, INTERN	NR_028062	PRKY	Jeck2013, Salzman	5616	circRNA	Not Detected	Detected	1.384036638	7.419586012
hsa_gci153233	2.569544477	1.361512624	up	4.95877935	3.597266726	TATCCCGAGTTCCGAAG	hsa_circ_0006323	chr1	97981281	98015300	-	401	ANNOTATED, CDS, c	NM_000110	DPYD	Jeck2013	1806	circRNA	Detected	Detected	4.95877935	3.597266726
hsa_gci153234	3.32114944	1.731682641	up	6.975079388	5.243396747	AAGTTTGGTCCCTTCCA	hsa_circ_0006324	chr1	197611840	197627499	-	376	ANNOTATED, CDS, c	NM_001195215	DENND1B	Jeck2013, Salzman	163486	circRNA	Detected	Detected	6.975079388	5.243396747
hsa_gci153241	2.362198651	1.240130294	up	9.082994926	7.842864632	GAGTAAAAAGTGAACAG	hsa_circ_0006334	chr5	137527162	137537137	-	1009	ANNOTATED, CDS, c	NM_004661	CDC23	Jeck2013	8697	circRNA	Detected	Detected	9.082994926	7.842864632
hsa_gci153246	2.835265176	1.503483673	up	7.266530312	5.763046639	TCTGTCCAGTGTTCCAA	hsa_circ_0006343	chr15	42556287	42560230	-	200	ANNOTATED, CDS, c	NM_015497	TMEM87A	Jeck2013	25963	circRNA	Detected	Detected	7.266530312	5.763046639
hsa_gci153258	2.561385498	1.3569244	up	7.105437583	5.748513183	CCGTTCCATTTTCTCTA	hsa_circ_0006360	chr1	150390071	150418877	+	665	ANNOTATED, CDS, c	NM_015203	RPRD2	Jeck2013	23248	circRNA	Detected	Detected	7.105437583	5.748513183
hsa_gci153260	2.57735259	1.365889916	up	6.551602413	5.185712496	TCATTGTGAGGAGATCC	hsa_circ_0006362	chr4	123856868	123900541	+	722	ANNOTATED, CDS, c	NM_145207	SPATA5	Jeck2013, Salzman	166378	circRNA	Detected	Detected	6.551602413	5.185712496
hsa_gci153269	-2.195650823	-1.134648639	down	2.538664246	3.673312885	TCCAGCAAGAGGAAGTA	hsa_circ_0006382	chr19	8520288	8528570	+	325	ANNOTATED, CDS, c	NM_005968	HNRNPM	Jeck2013, Salzman	4670	circRNA	Not Detected	Detected	2.538664246	3.673312885
hsa_gci153291	-3.720582144	-1.895528372	down	1.346987109	3.242515481	AAATGCAAGACTTGTAT	hsa_circ_0006422	chr2	36737125	36749456	+	927	ANNOTATED, CDS, c	NM_016441	CRIM1	Jeck2013, Salzman	51232	circRNA	Not Detected	Detected	1.346987109	3.242515481
hsa_gci153308	2.001461184	1.001053637	up	8.426687009	7.425633372	TATGTGGAAATCATAGT	hsa_circ_0006445	chr2	159851413	159857701	+	6288	ALT_ACCEPTOR, cod	NM_033394	TANC1	Jeck2013	85461	circRNA	Detected	Detected	8.426687009	7.425633372
hsa_gci153329	-3.803341971	-1.927267662	down	1.240171468	3.16743913	AGGGGGAAGTGTTTTAG	hsa_circ_0006484	chr20	43132455	43141635	-	854	ANNOTATED, CDS, c	NM_006811	SERINC3	Jeck2013	10955	circRNA	Not Detected	Detected	1.240171468	3.16743913
hsa_gci153338	2.072405008	1.051305975	up	3.999734484	2.948428509	GTTTTCACGTAGACGTC	hsa_circ_0006496	chr3	142462326	142467302	+	305	ANNOTATED, CDS, c	NM_001251845	TRPC1	Jeck2013	7220	circRNA	Detected	Not Detected	3.999734484	2.948428509
hsa_gci153350	2.368699934	1.24409545	up	5.857597008	4.613501558	CGGCCAAGAGTATCTTG	hsa_circ_0006520	chr10	99236439	99238146	-	422	ANNOTATED, CDS, c	NM_022362	MMS19	Jeck2013, Salzman	64210	circRNA	Detected	Detected	5.857597008	4.613501558
hsa_gci153362	2.964732512	1.567901946	up	4.931365728	3.363463782	ATAATACTATTGAGGAA	hsa_circ_0006534	chr10	27406504	27410377	-	484	ANNOTATED, CDS, c	NM_139312	YME1L1	Jeck2013, Salzman	10730	circRNA	Detected	Detected	4.931365728	3.363463782

hsa_gci153466	2.920728098	1.546328058	up	7.805165839	6.258837781	TTTCAGCCCGAAGCATC	hsa_circ_0006704	chr20	17933230	17934761	-	246	ANNOTATED, CDS, c	NM_152227	SNX5	Jeck2013, Salzman	27131	circRNA	Detected	Detected	7.805165839	6.258837781
hsa_gci153485	2.049392975	1.035196651	up	11.77740408	10.74220743	TTCTCTATAGTTGGTCC	hsa_circ_0006728	chr9	33351557	33354890	+	449	ANNOTATED, CDS, c	NM_002504	NFX1	Jeck2013, Salzman	4799	circRNA	Detected	Detected	11.77740408	10.74220743
hsa_gci153505	2.4959042	1.31956256	up	6.243996447	4.924433886	GTGGGTGGAGGTGTGAC	hsa_circ_0006757	chr6	34574331	34574681	-	350	ANNOTATED, CDS, c	NM_024294	C6orf106	Jeck2013, Salzman	64771	circRNA	Detected	Detected	6.243996447	4.924433886
hsa_gci153527	-6.883430372	-2.783127714	down	1.222875502	4.006003216	TGTACTACTCTTCCGGG	hsa_circ_0006790	chr2	32142994	32145979	-	225	ANNOTATED, CDS, c	NM_015955	MEMO1	Jeck2013, Salzman	51072	circRNA	Not Detected	Detected	1.222875502	4.006003216
hsa_gci153533	2.34557451	1.229941331	up	5.605430953	4.375489622	TGTTAACCTCTTCACTG	hsa_circ_0006800	chr2	32713654	32728303	+	1531	ANNOTATED, CDS, c	NM_016252	BIRC6	Jeck2013	57448	circRNA	Detected	Detected	5.605430953	4.375489622
hsa_gci153544	2.343642749	1.228752671	up	8.388925756	7.160173086	TAGTGTCCGAAGTAAAG	hsa_circ_0006817	chr1	150990287	150991145	+	296	ANNOTATED, CDS, c	NM_021222	PRUNE1	Jeck2013	58497	circRNA	Detected	Detected	8.388925756	7.160173086
hsa_gci153574	2.795395063	1.483052188	up	4.262264884	2.779212696	ACGTGGATCATGAGGTC	hsa_circ_0006865	chr4	39843567	39846421	-	255	ANNOTATED, CDS, c	NM_001100399	PDS5A	Jeck2013	23244	circRNA	Detected	Not Detected	4.262264884	2.779212696
hsa_gci153588	2.522207377	1.3346869	up	4.475241186	3.140554286	GGAGGTAGGTATTACTT	hsa_circ_0006886	chr16	57197912	57207781	-	562	ANNOTATED, CDS, c	NM_024946	FAM192A	Jeck2013	80011	circRNA	Detected	Not Detected	4.475241186	3.140554286
hsa_gci153595	2.723479909	1.445451224	up	4.670025206	3.224573981	GTAGGCCATCGAAGAAC	hsa_circ_0006898	chr21	37775055	37783902	+	398	ANNOTATED, CDS, c	NM_005441	CHAF1B	Jeck2013	8208	circRNA	Detected	Detected	4.670025206	3.224573981
hsa_gci153610	2.078305884	1.055408005	up	5.542368983	4.486960978	ATTTAAGAAGAAATTTG	hsa_circ_0006921	chr11	103182606	103194718	+	546	ANNOTATED, CDS, c	NM_001080463	DYNC2H1	Jeck2013, Salzman	79659	circRNA	Detected	Detected	5.542368983	4.486960978
hsa_gci153613	2.064708757	1.045938293	up	6.437591974	5.391653681	TTTGCTCTTCGTTTCTT	hsa_circ_0006925	chr12	65702308	65722383	+	314	ANNOTATED, CDS, c	NM_001193460	MSRB3	Jeck2013	253827	circRNA	Detected	Detected	6.437591974	5.391653681
hsa_gci153649	7.052115905	2.818056186	up	4.793963768	1.975907582	GTGTTGGTATCCCTGCT	hsa_circ_0006980	chr1	179100445	179100616	-	171	ANNOTATED, CDS, c	NM_007314	ABL2	Jeck2013, Salzman	27	circRNA	Detected	Not Detected	4.793963768	1.975907582
hsa_gci153659	2.152238991	1.105838288	up	9.785597325	8.679759037	TTAAGGTTTAAGACTGT	hsa_circ_0006992	chr19	34954930	34957919	+	243	ANNOTATED, CDS, c	NM_005499	UBA2	Jeck2013, Salzman	10054	circRNA	Detected	Detected	9.785597325	8.679759037
hsa_gci153668	2.091487095	1.064529096	up	9.009694741	7.945165645	TGACCTGTAGAACCACT	hsa_circ_0007011	chr2	181846744	181848820	+	270	ANNOTATED, CDS, c	NM_006357	UBE2E3	Jeck2013, Salzman	10477	circRNA	Detected	Detected	9.009694741	7.945165645
hsa_gci153677	2.165012007	1.114375026	up	6.077429323	4.963054297	TCTGTACTCCATCGTTA	hsa_circ_0007026	chr20	45891031	45905539	-	623	ANNOTATED, CDS, c	NM_183047	ZMYND8	Jeck2013, Salzman	23613	circRNA	Detected	Detected	6.077429323	4.963054297
hsa_gci153694	4.822513375	2.26978524	up	4.792747299	2.52296206	AAAGACACTCCTGACGT	hsa_circ_0007055	chr11	10019812	10024236	-	356	ANNOTATED, CDS, c	NM_030962	SBF2	Jeck2013, Salzman	81846	circRNA	Detected	Not Detected	4.792747299	2.52296206
hsa_gci153734	2.496486483	1.319899095	up	7.399885354	6.079986259	TGTGACAGTGTAGCTTT	hsa_circ_0007124	chr3	104536316	104548557	-	12241	ALT_ACCEPTOR, ALT	TCONS_00006568		Jeck2013		circRNA	Detected	Detected	7.399885354	6.079986259
hsa_gci153743	3.958979105	1.985128453	up	4.839627606	2.854499153	AGAGGTCCCAGCACTAG	hsa_circ_0007138	chr6	128625812	128643455	-	17643	ALT_DONOR, CDS, c	NM_001135648	PTPRK	Jeck2013	5796	circRNA	Detected	Not Detected	4.839627606	2.854499153
hsa_gci153757	-6.531008041	-2.707305684	down	1.418887037	4.126192721	GACGTAATAGGACACTT	hsa_circ_0007156	chr11	9829546	9830549	-	288	ANNOTATED, CDS, c	NM_030962	SBF2	Jeck2013	81846	circRNA	Not Detected	Detected	1.418887037	4.126192721
hsa_gci153764	-2.248228259	-1.168788518	down	2.619842834	3.788631352	CTTTTGTGACTGTTTTA	hsa_circ_0007165	chr6	73713630	73830300	+	822	ANNOTATED, CDS, c	NM_001160133	KCNQ5	Jeck2013, Salzman	56479	circRNA	Not Detected	Detected	2.619842834	3.788631352
hsa_gci153803	2.622189958	1.390772202	up	7.217054148	5.826281946	CAAAGGAGTAGTTATGA	hsa_circ_0007227	chr1	21329205	21437876	-	362	ANNOTATED, CDS, c	NM_001198803	EIF4G3	Jeck2013, Salzman	8672	circRNA	Detected	Detected	7.217054148	5.826281946
hsa_gci153812	2.514633314	1.33034804	up	8.300187835	6.969839795	ACGGGACAGGTGAAAAA	hsa_circ_0007242	chr10	13169744	13178897	+	523	ALT_DONOR, CDS, c	NM_001008211	OPTN	Jeck2013	10133	circRNA	Detected	Detected	8.300187835	6.969839795
hsa_gci153819	3.948114462	1.981163816	up	7.923868901	5.942705085	TCTTACACACAAACATT	hsa_circ_0007252	chr7	66532271	66548526	+	229	ANNOTATED, CDS, c	NM_018264	TYW1	Jeck2013, Salzman	55253	circRNA	Detected	Detected	7.923868901	5.942705085
hsa_gci153820	7.570788912	2.920443644	up	4.125489002	1.205045358	CCCACGGCAAGTAAAAG	hsa_circ_0007254	chr11	47508301	47510576	-	320	ANNOTATED, CDS, c	NM_001172640	CELF1	Jeck2013, Salzman	10658	circRNA	Detected	Not Detected	4.125489002	1.205045358
hsa_gci153831	2.585693745	1.370551409	up	6.810777594	5.440226185	AAGAGTTCTAGTTACTA	hsa_circ_0007272	chr17	58346810	58348842	-	240	ANNOTATED, CDS, c	NM_032582	USP32	Jeck2013, Salzman	84669	circRNA	Detected	Detected	6.810777594	5.440226185
hsa_gci153835	3.434285689	1.780010058	up	4.024123654	2.244113596	TGCCTCGGTCAGTGTAC	hsa_circ_0007280	chr13	33716446	33741759	-	218	ANNOTATED, CDS, c	NM_001243476	STARD13	Jeck2013, Salzman	90627	circRNA	Detected	Not Detected	4.024123654	2.244113596
hsa_gci153852	6.547378535	2.71091739	up	3.979100128	1.268182738	AGACAAGAGGTGGTCTG	hsa_circ_0007301	chr1	59787207	59797704	+	10497	ALT_DONOR, CDS, c	NM_001113411	FGGY	Jeck2013	55277	circRNA	Detected	Not Detected	3.979100128	1.268182738
hsa_gci153880	3.306047427	1.725107421	up	10.15230308	8.427195658	GCTCCTCGTCCTGGGTT	hsa_circ_0007344	chr2	10740978	10747437	-	396	ANNOTATED, CDS, c	NM_024894	NOL10	Jeck2013	79954	circRNA	Detected	Detected	10.15230308	8.427195658
hsa_gci153899	2.359575323	1.238527226	up	6.866290606	5.62776338	GACTCGTCCCTCCTAAA	hsa_circ_0007379	chr14	35020919	35024118	-	3199	INTERGENIC	None		Jeck2013		circRNA	Detected	Detected	6.866290606	5.62776338
hsa_gci153901	2.670944006	1.417349732	up	4.472886311	3.055536579	TCCGAGCCGTTATAAGT	hsa_circ_0007383	chr11	118453893	118455359	+	551	ANNOTATED, CDS, c	NM_001655	ARCN1	Jeck2013, Salzman	372	circRNA	Detected	Not Detected	4.472886311	3.055536579
hsa_gci153917	2.549478583	1.350202219	up	4.278850115	2.928647896	CAAACTATAGGTCTCCT	hsa_circ_0007409	chr8	30332294	30336893	+	180	ANNOTATED, CDS, c	NM_006867	RBPMS	Jeck2013, Salzman	11030	circRNA	Detected	Not Detected	4.278850115	2.928647896
hsa_gci153958	2.205528598	1.141124467	up	7.623324892	6.482200425	AAACTGAAAGACGGTTT	hsa_circ_0007467	chr15	83858112	83861060	-	2948	ALT_ACCEPTOR, ALT	NM_016073	HDGFL3	Jeck2013	50810	circRNA	Detected	Detected	7.623324892	6.482200425
hsa_gci153982	2.193634412	1.133323108	up	7.548565624	6.415242515	CGTATGAGTAGAAGAGG	hsa_circ_0007497	chr14	21840032	21841589	-	264	ANNOTATED, CDS, c	NM_007192	SUPT16H	Jeck2013	11198	circRNA	Detected	Detected	7.548565624	6.415242515
hsa_gci153985	2.306898882	1.205954768	up	6.703765985	5.497811217	AAAGAACACCTCTCATT	hsa_circ_0007503	chr10	28872327	28884970	+	645	ANNOTATED, INTERN	NR_024557	WAC	Jeck2013, Salzman	51322	circRNA	Detected	Detected	6.703765985	5.497811217
hsa_gci153994	2.469935935	1.304473622	up	5.289543486	3.985069864	GCACATTGACCTGTAGA	hsa_circ_0007516	chr1	33099245	33100393	-	230	ANNOTATED, CDS, c	NM_178547	ZBTB8OS	Jeck2013, Salzman	339487	circRNA	Detected	Detected	5.289543486	3.985069864
hsa_gci154001	2.250066332	1.169967533	up	7.194685402	6.024717869	TTTCTCTTGACCTCTTG	hsa_circ_0007528	chr18	59739905	59763183	-	595	ANNOTATED, CDS, c	NM_176787	PIGN	Jeck2013, Salzman	23556	circRNA	Detected	Detected	7.194685402	6.024717869
hsa_gci154005	-3.034128235	-1.601282061	down	3.722874975	5.324157037	AGGTTAGGAGTACAGGT	hsa_circ_0007535	chr18	33722243	33739978	+	1100	ANNOTATED, CDS, c	NM_001242875	ELP2	Jeck2013, Salzman	55250	circRNA	Not Detected	Detected	3.722874975	5.324157037
hsa_gci154014	2.157416507	1.109304728	up	4.355483001	3.246178274	ACTGCATTCAAAAACGT	hsa_circ_0007548	chr16	74666420	74670475	-	383	ANNOTATED, CDS, c	NM_018124	RFWD3	Jeck2013, Salzman	55159	circRNA	Detected	Detected	4.355483001	3.246178274
hsa_gci154030	-3.988158046	-1.995722583	down	1.33960476	3.335327343	TAAGAACAAGTTCATCT	hsa_circ_0007576	chr1	41512078	41514562	-	393	ANNOTATED, CDS, c	NM_001172218	SCMH1	Jeck2013, Salzman	22955	circRNA	Not Detected	Detected	1.33960476	3.335327343
hsa_gci154042	2.765476964	1.467528325	up	5.44167839	3.974150066	TTGTATTAGACCTTTTT	hsa_circ_0007592	chr9	4860124	4860901	+	777	ALT_DONOR, CDS, c	NM_005772	RCL1	Jeck2013	10171	circRNA	Detected	Detected	5.44167839	3.974150066
hsa_gci154047	2.082686012	1.058445354	up	7.945165645	6.886720291	AAGACTCCCAACGAAAA	hsa_circ_0007600	chr1	21377358	21386287	-	8929	ALT_ACCEPTOR, cod	NM_001198801	EIF4G3	Jeck2013	8672	circRNA	Detected	Detected	7.945165645	6.886720291
hsa_gci154053	2.02799863	1.020056678	up	8.397296176	7.377239498	CCCGTTCACAACCCCCT	hsa_circ_0007608	chr1	155340294	155340774	-	354	ANNOTATED, CDS, c	NM_018489	ASH1L	Jeck2013, Salzman	55870	circRNA	Detected	Detected	8.397296176	7.377239498
hsa_gci154064	2.194377842	1.13381196	up	8.349576123	7.215764163	TCACTATTCGACTATCC	hsa_circ_0007629	chr3	124692574	124696790	-	263	ANNOTATED, CDS, c	NM_020733	HEG1	Jeck2013, Salzman	57493	circRNA	Detected	Detected	8.349576123	7.215764163
hsa_gci154075	2.333385514	1.222424684	up	8.454178443	7.231753759	GTTGTCTAATTGAAATC	hsa_circ_0007643	chr17	1264385	1265302	-	314	ANNOTATED, INTERN	NR_024058	YWHAE	Jeck2013, Salzman	7531	circRNA	Detected	Detected	8.454178443	7.231753759
hsa_gci154096	2.189513221	1.130610161	up	8.133017071	7.00240691	GGTACCTGTCAGATGAA	hsa_circ_0007672	chr7	65705311	65706257	+	946	ANNOTATED, CDS, c	NM_003596	TPST1	Jeck2013	8460	circRNA	Detected	Detected	8.133017071	7.00240691
hsa_gci154100	10.73030101	3.423618642	up	6.732220895	3.308602252	CTCCGAAGAATCGTTAG	hsa_circ_0007676	chr4	83867410	83900159	-	484	ANNOTATED, CDS, c	NM_194282	LIN54	Jeck2013, Salzman	132660	circRNA	Detected	Detected	6.732220895	3.308602252
hsa_gci154106	2.409931624	1.268992214	up	4.73727458	3.468282366	CAGAAAAACATATTGAG	hsa_circ_0007687	chr10	70411602	70427013	+	397	ANNOTATED, CDS, c	NM_030625	TET1	Jeck2013	80312	circRNA	Detected	Detected	4.73727458	3.468282366
hsa_gci154109	2.255319041	1.173331534	up	4.221893138	3.048561604	ACCCCCGACGAAGTCAA	hsa_circ_0007691	chr5	153413350	153414506	-	396	ALT_ACCEPTOR, CDS	NM_018691	FAM114A2	Jeck2013	10827	circRNA	Detected	Not Detected	4.221893138	3.048561604
hsa_gci154133	2.719162014	1.443162113	up	3.841997002	2.398834889	GGTCATGTTAGAGTCCT	hsa_circ_0007723	chr12	28458581	28460682	+	467	ANNOTATED, CDS, c	NM_018318	CCDC91	Jeck2013, Salzman	55297	circRNA	Detected	Not Detected	3.841997002	2.398834889
hsa_gci154135	2.223541938	1.152859615	up	4.613356473	3.460496858	CCCGCGGTAATTCTAGT	hsa_circ_0007726	chr5	126140467	126147590	+	580	ANNOTATED, CDS, c	NM_005573	LMNB1	Jeck2013, Salzman	4001	circRNA	Detected	Detected	4.613356473	3.460496858
hsa_gci154138	2.124538924	1.087149776	up	5.549086625	4.461936849	TAAGAGAACCAGTTCAG	hsa_circ_0007733	chrX	117788564	117788924	+	274	ANNOTATED, CDS, c	NM_144658	DOCK11	Jeck2013, Salzman	139818	circRNA	Detected	Detected	5.549086625	4.461936849

hsa_gci154141	16.6584129	4.058179052	up	5.343586247	1.285407195	CGAAGTTCGGTTGGAGT	hsa_circ_0007739	chr1	220179447	220180680	-	345	ANNOTATED, CDS, c	NM_004446	EPRS	Jeck2013, Salzman	2058	circRNA	Detected	Not Detected	5.343586247	1.285407195
hsa_gci154147	2.224874887	1.15372421	up	9.354975593	8.201251383	GAACTCCTCCTCAGAAA	hsa_circ_0007749	chr18	20572710	20576425	+	1108	ANNOTATED, CDS, c	NM_002894	RBBP8	Jeck2013, Salzman	5932	circRNA	Detected	Detected	9.354975593	8.201251383
hsa_gci154158	2.507368115	1.326173819	up	8.334695304	7.008521485	TTACGTAAATGTCGTGG	hsa_circ_0007767	chr11	77824931	77832220	-	409	ANNOTATED, CDS, c	NM_001007027	ALG8	Jeck2013, Salzman	79053	circRNA	Detected	Detected	8.334695304	7.008521485
hsa_gci154165	2.323929416	1.216566251	up	5.548946405	4.332380154	CAAACTGTCATGGCCGT	hsa_circ_0007776	chr12	112096539	112098479	-	325	ANNOTATED, CDS, c	NM_006768	BRAP	Jeck2013, Salzman	8315	circRNA	Detected	Detected	5.548946405	4.332380154
hsa_gci154169	7.287965754	2.86551618	up	5.170763362	2.305247182	ACTATGTTACTGTGTTT	hsa_circ_0007782	chr14	78036726	78045452	-	429	ANNOTATED, CDS, c	NM_004863	SPTLC2	Jeck2013	9517	circRNA	Detected	Not Detected	5.170763362	2.305247182
hsa_gci154202	4.343645375	2.118906323	up	4.120379736	2.001473413	ATCGTCGACAGACATTT	hsa_circ_0007838	chr12	48465449	48468576	-	443	ANNOTATED, CDS, c	NM_014554	SENP1	Jeck2013, Salzman	29843	circRNA	Detected	Not Detected	4.120379736	2.001473413
hsa_gci154210	-4.635973159	-2.212872214	down	1.548828042	3.761700255	TACCTGCGGACACACCC	hsa_circ_0007847	chr14	25325141	25326363	-	297	ANNOTATED, CDS, c	NM_014178	STXBP6	Jeck2013, Salzman	29091	circRNA	Not Detected	Detected	1.548828042	3.761700255
hsa_gci154229	7.979801958	2.996352942	up	4.302827759	1.306474817	AGGGTACTTACACGCTA	hsa_circ_0007869	chr7	102874246	102898171	-	699	ANNOTATED, INTERN	NR_027768	DPY19L2P2	Jeck2013	349152	circRNA	Detected	Not Detected	4.302827759	1.306474817
hsa_gci154233	2.275080822	1.185917798	up	7.033583617	5.84766582	AACATATCTCAACTGGT	hsa_circ_0007876	chr10	104122231	104125356	+	623	ANNOTATED, CDS, c	NM_004193	GBF1	Jeck2013, Salzman	8729	circRNA	Detected	Detected	7.033583617	5.84766582
hsa_gci154247	2.513945603	1.329953433	up	4.82511589	3.495162457	ACTTCTTCCTTGTGCTG	hsa_circ_0007898	chr11	17121406	17134213	-	797	ANNOTATED, CDS, c	NM_002645	PIK3C2A	Jeck2013, Salzman	5286	circRNA	Detected	Detected	4.82511589	3.495162457
hsa_gci154259	2.172917872	1.119633647	up	7.149155361	6.029521714	ATGTCGTAAAAAGTACA	hsa_circ_0007912	chr3	137940767	137942575	+	313	ANNOTATED, CDS, c	NM_015396	ARMC8	Jeck2013, Salzman	25852	circRNA	Detected	Detected	7.149155361	6.029521714
hsa_gci154262	3.613717144	1.853483587	up	5.884553858	4.031070272	CCTCCAGTAGTTTCTCG	hsa_circ_0007916	chr2	239093821	239103511	-	477	ANNOTATED, CDS, c	NM_030768	ILKAP	Jeck2013, Salzman	80895	circRNA	Detected	Detected	5.884553858	4.031070272
hsa_gci154288	2.350692747	1.23308598	up	6.549425312	5.316339332	TCGGTGTGCCCGGAGTG	hsa_circ_0007956	chr18	47017995	47018203	-	208	ALT_DONOR, coding	NM_000985	RPL17	Jeck2013	6139	circRNA	Detected	Detected	6.549425312	5.316339332
hsa_gci154292	2.36945087	1.244552747	up	5.705793268	4.461240521	TAATTCTTGGTGCCTCC	hsa_circ_0007966	chrX	107083899	107148856	+	1069	ANNOTATED, CDS, c	NM_012216	MID2	Jeck2013	11043	circRNA	Detected	Detected	5.705793268	4.461240521
hsa_gci154343	2.200990867	1.138153159	up	7.695221278	6.55706812	GAGTCCATAAGCCAATA	hsa_circ_0008048	chr3	159530546	159584070	+	27576	ALT_ACCEPTOR, CDS	NM_001197109	SCHIP1	Jeck2013	29970	circRNA	Detected	Detected	7.695221278	6.55706812
hsa_gci154344	2.887096762	1.529619461	up	8.37638626	6.8467668	AAACGTCGGAGGAAACC	hsa_circ_0008050	chr9	123215733	123220900	-	591	ANNOTATED, CDS, c	NM_018249	CDK5RAP2	Jeck2013	55755	circRNA	Detected	Detected	8.37638626	6.8467668
hsa_gci154366	2.15097518	1.104990878	up	5.818675432	4.713684554	TAATTCCAGTAGTATCG	hsa_circ_0008083	chr2	15601324	15618413	-	1256	ANNOTATED, CDS, c	NM_015909	NBAS	Jeck2013, Salzman	51594	circRNA	Detected	Detected	5.818675432	4.713684554
hsa_gci154384	2.575876367	1.365063351	up	4.487738502	3.122675151	CTAATTATATCCCATAG	hsa_circ_0008115	chr10	26993605	26998697	+	305	ANNOTATED, CDS, c	NM_014317	PDSS1	Jeck2013, Salzman	23590	circRNA	Detected	Not Detected	4.487738502	3.122675151
hsa_gci154387	3.646779844	1.866623107	up	7.217258335	5.350635229	TGTAGAGATTTACAGTT	hsa_circ_0008122	chr19	23316881	23318845	+	223	ANNOTATED, INTERN	TCONS_12_00012420		Jeck2013		circRNA	Detected	Detected	7.217258335	5.350635229
hsa_gci154416	3.631598461	1.860604695	up	4.241884091	2.381279396	GTTCTAAGTCCTTATTT	hsa_circ_0008160	chr21	38439561	38441924	-	192	ANNOTATED, INTERN	NR_028352	PIGP	Jeck2013, Salzman	51227	circRNA	Detected	Not Detected	4.241884091	2.381279396
hsa_gci154419	-2.405683993	-1.266447145	down	2.150205998	3.416653143	GTGAAAGAAGGACTTCG	hsa_circ_0008165	chr4	85781564	85853516	-	405	ANNOTATED, CDS, c	NM_014991	WDFY3	Jeck2013	23001	circRNA	Not Detected	Detected	2.150205998	3.416653143
hsa_gci154458	2.521639792	1.334362206	up	6.334124095	4.999761889	GTGAGGTCTTGAGGTCG	hsa_circ_0008217	chr8	103335538	103359320	-	1398	ANNOTATED, CDS, c	NM_015902	UBR5	Jeck2013	51366	circRNA	Detected	Detected	6.334124095	4.999761889
hsa_gci154461	2.952951248	1.562157538	up	5.241441031	3.679283493	TCTCGGTTTACGACCTC	hsa_circ_0008221	chr12	11199618	11273779	-	74161	ALT_ACCEPTOR, INT	NR_037918	PRH1-PRR4	Jeck2013	100533464	circRNA	Detected	Detected	5.241441031	3.679283493
hsa_gci154466	-2.215039583	-1.14733248	down	2.297259851	3.444592331	TTAGTAAGGACCTATAG	hsa_circ_0008233	chr8	131352654	131374017	-	17837	ALT_DONOR, CDS, c	NM_001247996	ASAP1	Jeck2013	50807	circRNA	Not Detected	Detected	2.297259851	3.444592331
hsa_gci154471	2.234624624	1.160032505	up	8.328506806	7.168474301	CTCTCCCACCGAACGAT	hsa_circ_0008246	chr4	6995910	7016284	+	914	ANNOTATED, CDS, c	NM_020773	TBC1D14	Jeck2013, Salzman	57533	circRNA	Detected	Detected	8.328506806	7.168474301
hsa_gci154509	2.835515821	1.503611206	up	6.918763614	5.415152408	TGAGAATCCTTTTTTAG	hsa_circ_0008305	chr8	141799572	141840625	-	584	ANNOTATED, CDS, c	NM_001199649	PTK2	Jeck2013, Salzman	5747	circRNA	Detected	Detected	6.918763614	5.415152408
hsa_gci154516	2.542894887	1.346471828	up	8.21755496	6.871083132	CCCTTCCCACAAAGGTG	hsa_circ_0008313	chr6	2397454	2399271	+	1730	ALT_ACCEPTOR, INT	TCONS_00011149		Jeck2013		circRNA	Detected	Detected	8.21755496	6.871083132
hsa_gci154518	2.469290229	1.304096414	up	7.425633372	6.121536958	GATATACTCTTGAAACG	hsa_circ_0008316	chr8	95547066	95556170	-	421	ANNOTATED, CDS, c	NM_015496	VIRMA	Jeck2013, Salzman	25962	circRNA	Detected	Detected	7.425633372	6.121536958
hsa_gci154533	4.554454769	2.187278354	up	4.018554603	1.831276249	AGACAAGTTTCGACCTC	hsa_circ_0008341	chr4	83748521	83750211	-	323	ANNOTATED, CDS, c	NM_014933	SEC31A	Jeck2013	22872	circRNA	Detected	Not Detected	4.018554603	1.831276249
hsa_gci154534	3.132036933	1.647101225	up	4.638764625	2.9916634	GGTCACTCTTAGGATTA	hsa_circ_0008342	chr11	77330650	77340944	-	611	ANNOTATED, CDS, c	NM_001293	CLNS1A	Jeck2013, Salzman	1207	circRNA	Detected	Not Detected	4.638764625	2.9916634
hsa_gci154571	2.813661491	1.49244877	up	4.408919198	2.916470428	GACTCTGGGTGTCTTGA	hsa_circ_0008406	chr14	73749066	73763993	-	862	ANNOTATED, CDS, c	NM_001005743	NUMB	Jeck2013	8650	circRNA	Detected	Not Detected	4.408919198	2.916470428
hsa_gci154578	2.231210135	1.157826393	up	6.539926094	5.382099701	TCGTAGAAAGTGCTCCC	hsa_circ_0008419	chr7	133002037	133059756	+	526	ANNOTATED, CDS, c	NM_021807	EXOC4	Jeck2013, Salzman	60412	circRNA	Detected	Detected	6.539926094	5.382099701
hsa_gci154596	2.45183519	1.293862006	up	4.239394315	2.945532309	TTCTACCTGAGGTCTAT	hsa_circ_0008448	chr12	28458581	28515448	+	545	ANNOTATED, CDS, c	NM_018318	CCDC91	Jeck2013	55297	circRNA	Detected	Not Detected	4.239394315	2.945532309
hsa_gci154632	3.640731396	1.864228306	up	4.031692451	2.167464144	ACGTTGTCCGGACCCTC	hsa_circ_0008501	chr1	8601272	8674745	-	434	ANNOTATED, CDS, c	NM_012102	RERE	Jeck2013, Salzman	473	circRNA	Detected	Not Detected	4.031692451	2.167464144
hsa_gci154690	2.571237662	1.362462966	up	6.609786894	5.247323927	GTTCCTTCCAAAGTCTT	hsa_circ_0008598	chr13	30104674	30127997	-	818	ANNOTATED, CDS, c	NM_003045	SLC7A1	Jeck2013	6541	circRNA	Detected	Detected	6.609786894	5.247323927
hsa_gci154711	2.244774943	1.166570811	up	4.663092012	3.496521201	GGGTACCAGGTACGAAA	hsa_circ_0008625	chr17	57987922	58003943	+	388	ANNOTATED, CDS, c	NM_003161	RPS6KB1	Jeck2013	6198	circRNA	Detected	Detected	4.663092012	3.496521201
hsa_gci154712	-2.532802911	-1.340734818	down	2.172584201	3.51331902	TTCAACGAGAATAAAGG	hsa_circ_0008626	chr12	109212024	109212334	-	310	ALT_ACCEPTOR, CDS	NM_018984	SSH1	Jeck2013	54434	circRNA	Not Detected	Detected	2.172584201	3.51331902
hsa_gci154737	2.006984297	1.005029329	up	4.576227748	3.571198419	TATTTCTTTTACGGTTC	hsa_circ_0008659	chr1	214564330	214571344	-	318	ANNOTATED, CDS, c	NM_005401	PTPN14	Jeck2013, Salzman	5784	circRNA	Detected	Detected	4.576227748	3.571198419
hsa_gci154790	2.216018381	1.147969848	up	5.140915806	3.992945958	TAGAGTCGTTATCGTAG	hsa_circ_0008750	chr3	136191254	136196254	-	303	ANNOTATED, CDS, c	NM_005862	STAG1	Jeck2013, Salzman	10274	circRNA	Detected	Detected	5.140915806	3.992945958
hsa_gci154791	2.236881785	1.161489015	up	11.58260705	10.42111803	CTCATACACACCGATCC	hsa_circ_0008751	chr7	90585011	90613556	+	215	ANNOTATED, CDS, c	NM_012395	CDK14	Jeck2013, Salzman	5218	circRNA	Detected	Detected	11.58260705	10.42111803
hsa_gci154806	2.312163493	1.209243414	up	4.22577911	3.016535696	TTCTACGTTCCCTCGGG	hsa_circ_0008778	chr8	141874410	141935848	-	571	ANNOTATED, CDS, c	NM_001199649	PTK2	Jeck2013	5747	circRNA	Detected	Not Detected	4.22577911	3.016535696
hsa_gci154808	3.944383958	1.979799995	up	4.755687764	2.77588777	GATGAGTCTGGACGTTC	hsa_circ_0008782	chr9	86294689	86294952	-	263	ANNOTATED, CDS, c	NM_013438	UBQLN1	Jeck2013	29979	circRNA	Detected	Not Detected	4.755687764	2.77588777
hsa_gci154823	8.166770197	3.029765633	up	4.236262091	1.206496459	TACCACCTATGGACTCA	hsa_circ_0008802	chr12	51402258	51404549	-	221	ANNOTATED, CDS, c	NM_001174129	SLC11A2	Jeck2013, Salzman	4891	circRNA	Detected	Not Detected	4.236262091	1.206496459
hsa_gci154835	2.604894675	1.381225041	up	6.086320569	4.705095528	CGGGTCGGCCTGGCTCC	hsa_circ_0008819	chr5	109103235	109110666	+	539	ANNOTATED, CDS, c	NM_002372	MAN2A1	Jeck2013	4124	circRNA	Detected	Detected	6.086320569	4.705095528
hsa_gci154839	2.502722115	1.323498113	up	5.92092076	4.597422648	ACAAAACAAGCGAGATT	hsa_circ_0008828	chr4	47905209	47907363	-	241	ANNOTATED, CDS, c	NM_152995	NFXL1	Jeck2013, Salzman	152518	circRNA	Detected	Detected	5.92092076	4.597422648
hsa_gci154845	2.439333367	1.286486935	up	10.12422503	8.837738092	GACAGACCAACTCTGAG	hsa_circ_0008837	chr10	882342	890995	-	320	ANNOTATED, CDS, c	NM_015155	LARP4B	Jeck2013	23185	circRNA	Detected	Detected	10.12422503	8.837738092
hsa_gci154852	2.004127233	1.002974102	up	5.023355114	4.020381012	AAGGAGGAAAGGACAAA	hsa_circ_0008851	chr10	35426709	35441308	+	4111	ALT_DONOR, CDS, c	NM_183013	CREM	Jeck2013	1390	circRNA	Detected	Detected	5.023355114	4.020381012
hsa_gci154860	3.173921074	1.666266253	up	7.621790447	5.955524194	TATCCGTCCCCAATGTG	hsa_circ_0008864	chr4	20568884	20591360	+	457	ANNOTATED, CDS, c	NM_004787	SLIT2	Jeck2013	9353	circRNA	Detected	Detected	7.621790447	5.955524194
hsa_gci154869	4.479196581	2.163239984	up	7.061998224	4.89875824	TGATTCCGTCTGTAGTT	hsa_circ_0008877	chr12	78360008	78415642	+	1609	ANNOTATED, CDS, c	NM_014903	NAV3	Jeck2013, Salzman	89795	circRNA	Detected	Detected	7.061998224	4.89875824
hsa_gci154874	10.55423244	3.399749757	up	4.604559497	1.204809739	TCCATACCAAAACTCAT	hsa_circ_0008882	chrM	13418	13499	+	81	INTERGENIC	None		Jeck2013		circRNA	Detected	Not Detected	4.604559497	1.204809739
hsa_gci154887	2.362074849	1.240054681	up	9.513116066	8.273061385	TATTTTCAGTTAATAAA	hsa_circ_0008903	chr5	6737622	6746540	+	743	ANNOTATED, CDS, c	NM_006999	PAPD7	Jeck2013, Salzman	11044	circRNA	Detected	Detected	9.513116066	8.273061385

hsa_gci154914	2.019604285	1.014072643	up	6.640294066	5.626221423	TTAGCCGTTTCCAGTTA	hsa_circ_0008943	chrX	10031484	10084547	+	1158	ANNOTATED, CDS, c	NM_015691	WWC3	Jeck2013, Salzman	55841	circRNA	Detected	Detected	6.640294066	5.626221423
hsa_gci154939	2.696430047	1.431050607	up	9.091014095	7.659963488	AAGGGTTACCCGAGATT	hsa_circ_0008980	chr20	45021731	45023142	-	264	ANNOTATED, CDS, c	NM_182764	ELMO2	Jeck2013, Salzman	63916	circRNA	Detected	Detected	9.091014095	7.659963488
hsa_gci154952	2.319344652	1.213717219	up	6.499264013	5.285546795	CCACTCCCACTAGAACT	hsa_circ_0009001	chr3	104536316	104574295	-	37979	ALT_ACCEPTOR, ALT	TCONS_00006568		Jeck2013		circRNA	Detected	Detected	6.499264013	5.285546795
hsa_gci154965	2.039829724	1.028448727	up	4.127060333	3.098611606	GCAGTTGTAAAGTAGAA	hsa_circ_0009022	chr18	9583114	9595100	-	815	ANNOTATED, CDS, c	NM_005134	PPP4R1	Jeck2013, Salzman	9989	circRNA	Detected	Not Detected	4.127060333	3.098611606
hsa_gci154987	2.973209791	1.572021264	up	6.014714753	4.442693489	TTATAAGACAGGACTTA	hsa_circ_0009056	chr8	118830673	118834836	-	348	ANNOTATED, CDS, c	NM_000127	EXT1	Jeck2013	2131	circRNA	Detected	Detected	6.014714753	4.442693489
hsa_gci154999	2.610636794	1.384401756	up	5.785944721	4.401542965	CGACGGTAATAAAACGA	hsa_circ_0009071	chr8	141799572	141900868	-	1209	ANNOTATED, CDS, c	NM_001199649	PTK2	Jeck2013, Salzman	5747	circRNA	Detected	Detected	5.785944721	4.401542965
hsa_gci155019	2.801881393	1.486395886	up	6.871083132	5.384687246	GAAAACCGTTTATTGCT	hsa_circ_0009102	chr8	141799572	141874498	-	815	ANNOTATED, CDS, c	NM_001199649	PTK2	Jeck2013, Salzman	5747	circRNA	Detected	Detected	6.871083132	5.384687246
hsa_gci155064	2.037551684	1.026836655	up	7.503991062	6.477154407	CGATGGTGAAAGAAGAC	hsa_circ_0009172	chr10	70218860	70229920	-	645	ANNOTATED, CDS, c	NM_001080449	DNA2	Jeck2013, Salzman	1763	circRNA	Detected	Detected	7.503991062	6.477154407
hsa_gci155080	2.17855408	1.123370927	up	7.218164561	6.094793634	ACTCGTCGAAAAAACTT	hsa_circ_0009303	chr1	1599765	1601590	-	273	ANNOTATED, CDS, c	NM_001110781	SLC35E2B	Salzman2013	728661	circRNA	Detected	Detected	7.218164561	6.094793634
hsa_gci155082	99.61136753	6.638238485	up	8.05205403	1.413815545	CTTACCAGAAGGTCAGA	hsa_circ_0009325	chr1	1716724	1718492	-	1768	ANNOTATED, coding	NM_002074	GNB1	Salzman2013	2782	circRNA	Detected	Not Detected	8.05205403	1.413815545
hsa_gci155091	2.027342746	1.019590014	up	8.755420978	7.735830964	TTATGTACATTTAAACA	hsa_circ_0009361	chr1	1749275	1770677	-	191	ANNOTATED, CDS, c	NM_002074	GNB1	Salzman2013	2782	circRNA	Detected	Detected	8.755420978	7.735830964
hsa_gci155093	6.232909353	2.639905731	up	3.898657348	1.258751617	CAGGGGAAGTCTGTAAG	hsa_circ_0009376	chr1	2327222	2330953	+	293	ANNOTATED, CDS, c	NM_007033	RER1	Salzman2013	11079	circRNA	Detected	Not Detected	3.898657348	1.258751617
hsa_gci155099	2.098699467	1.069495588	up	6.457608656	5.388113068	AAAGGCGGGGTTCCCCT	hsa_circ_0009422	chr1	3696783	3700675	-	1426	ANNOTATED, CDS, c	NM_020710	LRRC47	Salzman2013	57470	circRNA	Detected	Detected	6.457608656	5.388113068
hsa_gci155101	2.243024964	1.165445677	up	7.288652904	6.123207227	GACGACCGTCCGTGGGT	hsa_circ_0009424	chr1	3696783	3713068	-	2648	ANNOTATED, CDS, c	NM_020710	LRRC47	Salzman2013	57470	circRNA	Detected	Detected	7.288652904	6.123207227
hsa_gci155103	2.948571	1.560015934	up	5.271780432	3.711764498	GAAACGTTGGACAGAAA	hsa_circ_0009435	chr1	3761470	3764177	-	279	ANNOTATED, CDS, c	NM_014704	CEP104	Salzman2013	9731	circRNA	Detected	Detected	5.271780432	3.711764498
hsa_gci155107	2.254196363	1.172613194	up	4.502545214	3.32993202	GTAGTCTCACCCATTAG	hsa_circ_0009515	chr1	6581406	6589231	-	4969	ANNOTATED, CDS, c	NM_024654	NOL9	Salzman2013	79707	circRNA	Detected	Detected	4.502545214	3.32993202
hsa_gci155111	2.870172196	1.521137294	up	6.630314245	5.109176951	TCTAGTGTAAGGTTTAT	hsa_circ_0009528	chr1	6592027	6601987	-	670	ANNOTATED, CDS, c	NM_024654	NOL9	Salzman2013	79707	circRNA	Detected	Detected	6.630314245	5.109176951
hsa_gci155112	2.13406478	1.09360397	up	5.480884126	4.387280156	CTAGTGTAAGGTTTATC	hsa_circ_0009530	chr1	6593339	6601987	-	260	ANNOTATED, CDS, c	NM_024654	NOL9	Salzman2013	79707	circRNA	Detected	Detected	5.480884126	4.387280156
hsa_gci155122	2.781604047	1.475917071	up	3.864328851	2.38841178	TCTAATGGAGGACCATC	hsa_circ_0009555	chr1	8029404	8045342	+	606	ANNOTATED, CDS, c	NM_007262	PARK7	Salzman2013	11315	circRNA	Detected	Not Detected	3.864328851	2.38841178
hsa_gci155124	2.273047698	1.184627958	up	7.119149006	5.934521047	CCACTTGCCCCGGACGA	hsa_circ_0009559	chr1	8412463	8416306	-	3045	ANNOTATED, CDS, c	NM_012102	RERE	Salzman2013	473	circRNA	Detected	Detected	7.119149006	5.934521047
hsa_gci155126	2.508297406	1.326708417	up	9.665013001	8.338304584	GTTTCGCGTCCCGCCTC	hsa_circ_0009563	chr1	8412463	8483747	-	6308	ANNOTATED, CDS, c	NM_001042682	RERE	Salzman2013	473	circRNA	Detected	Detected	9.665013001	8.338304584
hsa_gci155127	2.2294963	1.156717806	up	10.24957021	9.092852408	CGTTTATACAGTCTTTT	hsa_circ_0009571	chr1	8425871	8601377	-	722	ANNOTATED, CDS, c	NM_012102	RERE	Salzman2013	473	circRNA	Detected	Detected	10.24957021	9.092852408
hsa_gci155148	2.063944355	1.045404075	up	6.350158191	5.304754116	TTATCAAAATAACTACA	hsa_circ_0009627	chr1	8927175	8930569	-	263	ANNOTATED, CDS, c	NM_001428	ENO1	Salzman2013	2023	circRNA	Detected	Detected	6.350158191	5.304754116
hsa_gci155152	2.490597272	1.316491758	up	4.409127561	3.092635803	GAGGGTCGCTTCCAACA	hsa_circ_0009642	chr1	9323567	9331394	+	7827	ANNOTATED, CDS, c	NM_004285	H6PD	Salzman2013	9563	circRNA	Detected	Not Detected	4.409127561	3.092635803
hsa_gci155158	2.75535686	1.462239182	up	7.512096463	6.049857281	CGAAAGTAATAAAAAGT	hsa_circ_0009699	chr1	10204997	10207148	+	228	ANNOTATED, CDS, c	NM_001105562	UBE4B	Salzman2013	10277	circRNA	Detected	Detected	7.512096463	6.049857281
hsa_gci155159	2.424303665	1.27757042	up	6.48050327	5.20293285	ATTTCCGTCACAATATG	hsa_circ_0009700	chr1	10209241	10211619	+	335	ANNOTATED, CDS, c	NM_001105562	UBE4B	Salzman2013	10277	circRNA	Detected	Detected	6.48050327	5.20293285
hsa_gci155160	2.927808684	1.549821285	up	4.769268573	3.219447289	CTTAAATTTCCGTCACA	hsa_circ_0009701	chr1	10209241	10221344	+	607	ANNOTATED, CDS, c	NM_001105562	UBE4B	Salzman2013	10277	circRNA	Detected	Not Detected	4.769268573	3.219447289
hsa_gci155162	2.615090459	1.386860852	up	6.090501682	4.70364083	CAGGTTAACCTCTCTAT	hsa_circ_0009721	chr1	10431198	10435431	+	584	ANNOTATED, CDS, c	NM_015074	KIF1B	Salzman2013	23095	circRNA	Detected	Detected	6.090501682	4.70364083
hsa_gci155167	3.068356398	1.617466066	up	3.858077012	2.240610947	AAACGTCGTTACGGAAC	hsa_circ_0009731	chr1	10468127	10480201	+	1398	ANNOTATED, CDS, c	NM_002631	PGD	Salzman2013	5226	circRNA	Detected	Not Detected	3.858077012	2.240610947
hsa_gci155187	2.273895573	1.185166001	up	6.443309848	5.258143847	TAAGGGTCCCTACCGGT	hsa_circ_0009945	chr1	12252919	12269277	+	3035	ANNOTATED, CDS, c	NM_001066	TNFRSF1B	Salzman2013	7133	circRNA	Detected	Detected	6.443309848	5.258143847
hsa_gci155191	5.528105904	2.466785254	up	5.640158203	3.173372949	AAGCTCCTTCTCTGAGA	hsa_circ_0009962	chr1	12326937	12395884	+	6957	ANNOTATED, CDS, c	NM_015378	VPS13D	Salzman2013	55187	circRNA	Detected	Not Detected	5.640158203	3.173372949
hsa_gci155195	2.079530444	1.056257806	up	5.224029041	4.167771235	TATCCTCTCAGACTATA	hsa_circ_0009995	chr1	12382592	12383837	+	286	ANNOTATED, CDS, c	NM_015378	VPS13D	Salzman2013	55187	circRNA	Detected	Detected	5.224029041	4.167771235
hsa_gci155197	2.005868917	1.004227329	up	7.836181521	6.831954192	CGCGAACCCCCAAAAGT	hsa_circ_0009999	chr1	12401836	12445432	+	2858	ANNOTATED, CDS, c	NM_015378	VPS13D	Salzman2013	55187	circRNA	Detected	Detected	7.836181521	6.831954192
hsa_gci155215	3.395814007	1.763757443	up	4.747738759	2.983981317	ACCGTCCCTAGAGTAGG	hsa_circ_0010063	chr1	16042731	16061264	+	3835	ANNOTATED, CDS, c	NM_015164	PLEKHM2	Salzman2013	23207	circRNA	Detected	Not Detected	4.747738759	2.983981317
hsa_gci155224	2.788470384	1.479473948	up	7.347111821	5.867637873	CAGGGAGGCGACCCCGC	hsa_circ_0010099	chr1	16095022	16113084	+	2612	ANNOTATED, CDS, c	NM_017556	FBLIM1	Salzman2013	54751	circRNA	Detected	Detected	7.347111821	5.867637873
hsa_gci155240	2.331088425	1.221003731	up	9.294482195	8.073478464	TGTCATCTTAGACCCCA	hsa_circ_0010353	chr1	19495928	19501527	-	968	ANNOTATED, CDS, c	NM_020765	UBR4	Salzman2013	23352	circRNA	Detected	Detected	9.294482195	8.073478464
hsa_gci155255	2.459970035	1.298640742	up	4.511539328	3.212898586	TAGTCCGAAGGTGGTTC	hsa_circ_0010440	chr1	21076215	21076375	-	160	ANNOTATED, CDS, c	NM_016287	HP1BP3	Salzman2013	50809	circRNA	Detected	Detected	4.511539328	3.212898586
hsa_gci155264	2.238254049	1.162373796	up	7.371182032	6.208808236	GAAGTCAAAGGAGTAGT	hsa_circ_0010475	chr1	21267983	21437876	-	1860	ANNOTATED, CDS, c	NM_001198803	EIF4G3	Salzman2013	8672	circRNA	Detected	Detected	7.371182032	6.208808236
hsa_gci155291	4.724461882	2.240150015	up	5.003881667	2.763731651	AGTCGTGCGGTTGCTCT	hsa_circ_0010844	chr1	23408712	23410184	+	660	ANNOTATED, CDS, c	NM_001009999	KDM1A	Salzman2013	23028	circRNA	Detected	Not Detected	5.003881667	2.763731651
hsa_gci155295	2.989387119	1.579849735	up	5.029368277	3.449518542	AACCGTAGGTCCACTGG	hsa_circ_0010857	chr1	23755055	23760862	-	2170	ANNOTATED, CDS, c	NM_017707	ASAP3	Salzman2013	55616	circRNA	Detected	Detected	5.029368277	3.449518542
hsa_gci155298	2.882971316	1.527556483	up	5.378995547	3.851439064	GAAGGCCGGCTCCCGGA	hsa_circ_0010876	chr1	24018268	24022915	+	634	ANNOTATED, CDS, c	NM_000975	RPL11	Salzman2013	6135	circRNA	Detected	Detected	5.378995547	3.851439064
hsa_gci155319	2.168777064	1.116881761	up	6.412160884	5.295279123	AAACTACCCGACCTTTC	hsa_circ_0010978	chr1	26581661	26605298	+	3595	ALT_DONOR, CDS, c	NM_022778	CEP85	Salzman2013	64793	circRNA	Detected	Detected	6.412160884	5.295279123
hsa_gci155321	2.577550824	1.366000875	up	4.142522435	2.77652156	AACAAGGTGTCGGAGGT	hsa_circ_0010985	chr1	26607548	26608013	+	465	ANNOTATED, CDS, c	NM_031286	SH3BGRL3	Salzman2013	83442	circRNA	Detected	Not Detected	4.142522435	2.77652156
hsa_gci155322	2.94487551	1.558206648	up	6.514354095	4.956147448	AGAAAACCAAATCGACG	hsa_circ_0010988	chr1	26644410	26647014	+	505	ANNOTATED, CDS, c	NM_001803	CD52	Salzman2013	1043	circRNA	Detected	Detected	6.514354095	4.956147448
hsa_gci155324	2.322741323	1.215828494	up	4.952187527	3.736359033	GGAACCCACCTCTTGAC	hsa_circ_0011020	chr1	27100292	27100389	+	97	ANNOTATED, CDS, c	NM_006015	ARID1A	Salzman2013	8289	circRNA	Detected	Detected	4.952187527	3.736359033
hsa_gci155330	10.39045953	3.377187555	up	4.879846437	1.502658882	CGACCCGGAGTACTAAG	hsa_circ_0011038	chr1	27268243	27272887	+	848	ANNOTATED, CDS, c	NM_006600	NUDC	Salzman2013	10726	circRNA	Detected	Not Detected	4.879846437	1.502658882
hsa_gci155342	2.498082396	1.320821063	up	4.794415923	3.47359486	GGCCGCTGACGCTCAGT	hsa_circ_0011081	chr1	27992571	27994886	-	565	ANNOTATED, CDS, c	NM_022873	IFI6	Salzman2013	2537	circRNA	Detected	Detected	4.794415923	3.47359486
hsa_gci155345	2.106886701	1.075112735	up	7.615507822	6.540395087	AAGAGGCCGACTTCCGA	hsa_circ_0011084	chr1	27992571	27998724	-	860	ANNOTATED, CDS, c	NM_022873	IFI6	Salzman2013	2537	circRNA	Detected	Detected	7.615507822	6.540395087
hsa_gci155361	2.810946735	1.491056116	up	4.778394954	3.287338838	CTCTAGGACAAAGAAAA	hsa_circ_0011159	chr1	28931883	28948677	-	534	ANNOTATED, CDS, c	NM_001135218	TAF12	Salzman2013	6883	circRNA	Detected	Detected	4.778394954	3.287338838
hsa_gci155386	-2.449009046	-1.292198103	down	4.076496012	5.368694115	GTGGGCTGTCCTACTGT	hsa_circ_0011271	chr1	31810021	31837780	+	1342	ANNOTATED, CDS, c	NM_016505	ZCCHC17	Salzman2013	51538	circRNA	Detected	Detected	4.076496012	5.368694115
hsa_gci155396	2.409607041	1.268797891	up	11.05676912	9.787971233	GGGCTCTTCCCCATCGT	hsa_circ_0011299	chr1	32050751	32053287	+	1254	ANNOTATED, CDS, c	NM_022164	TINAGL1	Salzman2013	64129	circRNA	Detected	Detected	11.05676912	9.787971233

hsa_gci155400	2.115326692	1.080880491	up	5.386216465	4.305335974	AGTTAGTTTCGTTCACG	hsa_circ_0011331	chr1	32372021	32375713	-	2615	ANNOTATED, CDS, c	NM_080391	PTP4A2	Salzman2013	8073	circRNA	Detected	Detected	5.386216465	4.305335974
hsa_gci155432	-2.340459179	-1.226791603	down	2.737042116	3.963833718	AGGGGTCCTGTTCAGCT	hsa_circ_0011429	chr1	33123027	33135164	+	802	ANNOTATED, CDS, c	NM_005610	RBBP4	Salzman2013	5928	circRNA	Not Detected	Detected	2.737042116	3.963833718
hsa_gci155438	2.265669061	1.179937147	up	7.733006032	6.553068885	CCTGGGCAAAACTTATC	hsa_circ_0011439	chr1	33240839	33256855	-	1738	ANNOTATED, CDS, c	NM_003680	YARS	Salzman2013	8565	circRNA	Detected	Detected	7.733006032	6.553068885
hsa_gci155443	5.023247678	2.328620411	up	7.449662023	5.121041612	AAGTTTGAAGTCAAAGA	hsa_circ_0011461	chr1	33476825	33497262	-	2701	ANNOTATED, ncRNA,	NR_037592	AK2	Salzman2013	204	circRNA	Detected	Detected	7.449662023	5.121041612
hsa_gci155444	2.214360046	1.146889818	up	5.601530005	4.454640187	CAGCGGACACACCTAAG	hsa_circ_0011474	chr1	33760537	33766320	+	2028	ANNOTATED, CDS, c	NM_152493	ZNF362	Salzman2013	149076	circRNA	Detected	Detected	5.601530005	4.454640187
hsa_gci155459	2.325554039	1.217574464	up	6.006195327	4.788620863	ACGGTTCAGTACAAATA	hsa_circ_0011555	chr1	35854510	35855699	+	324	ANNOTATED, CDS, c	NM_005095	ZMYM4	Salzman2013	9202	circRNA	Detected	Detected	6.006195327	4.788620863
hsa_gci155465	2.472253847	1.305826885	up	5.969115581	4.663288696	AGAAAAGTGAGTAGAAT	hsa_circ_0011581	chr1	36096874	36102033	-	194	ANNOTATED, CDS, c	NM_002794	PSMB2	Salzman2013	5690	circRNA	Detected	Detected	5.969115581	4.663288696
hsa_gci155485	2.929578125	1.550692924	up	8.747607961	7.196915038	CTCATGCGTCCGGTCCC	hsa_circ_0011715	chr1	38171112	38175391	+	1612	ANNOTATED, CDS, c	NM_018101	CDCA8	Salzman2013	55143	circRNA	Detected	Detected	8.747607961	7.196915038
hsa_gci155502	11.00840846	3.460534001	up	4.683367298	1.222833296	AAGTCGTATCGACCATA	hsa_circ_0011794	chr1	39758437	39793025	+	2700	ANNOTATED, CDS, c	NM_012090	MACF1	Salzman2013	23499	circRNA	Detected	Not Detected	4.683367298	1.222833296
hsa_gci155508	6.29159371	2.653425509	up	3.869047212	1.215621702	TAGAGGGTCTTGAAGGA	hsa_circ_0011812	chr1	39784124	39793025	+	832	ANNOTATED, CDS, c	NM_012090	MACF1	Salzman2013	23499	circRNA	Detected	Not Detected	3.869047212	1.215621702
hsa_gci155520	2.076357007	1.054054521	up	6.503693641	5.44963912	CGTAGTAAGTAAAGAAA	hsa_circ_0011876	chr1	40530145	40538321	+	2106	ANNOTATED, CDS, c	NM_006367	CAP1	Salzman2013	10487	circRNA	Detected	Detected	6.503693641	5.44963912
hsa_gci155531	2.706519214	1.43643863	up	4.295185897	2.858747267	CAAAAGTGTAGGTAATC	hsa_circ_0011947	chr1	41578954	41625605	-	991	ANNOTATED, CDS, c	NM_001031694	SCMH1	Salzman2013	22955	circRNA	Detected	Not Detected	4.295185897	2.858747267
hsa_gci155540	2.524715061	1.336120575	up	5.359572488	4.023451913	TCTACCGGTGTCGGAGT	hsa_circ_0012124	chr1	44442261	44443967	+	628	ALT_DONOR, CDS, c	NM_004047	ATP6V0B	Salzman2013	533	circRNA	Detected	Detected	5.359572488	4.023451913
hsa_gci155543	-2.229887697	-1.156971054	down	2.568678219	3.725649273	GACACTCTCGACCTTTC	hsa_circ_0012142	chr1	44750506	44818597	-	696	ANNOTATED, CDS, c	NM_024066	ERI3	Salzman2013	79033	circRNA	Not Detected	Detected	2.568678219	3.725649273
hsa_gci155573	3.3288635	1.735029714	up	4.743925593	3.008895879	TAAACTTTCCGGTGTGC	hsa_circ_0012271	chr1	46120861	46126897	-	1287	ANNOTATED, CDS, c	NM_021639	GPBP1L1	Salzman2013	60313	circRNA	Detected	Not Detected	4.743925593	3.008895879
hsa_gci155578	2.25144439	1.170850844	up	3.987023255	2.816172412	TGGAGGTGATGGAGTCC	hsa_circ_0012339	chr1	47023089	47028515	-	1787	ANNOTATED, CDS, c	NM_003684	MKNK1	Salzman2013	8569	circRNA	Detected	Not Detected	3.987023255	2.816172412
hsa_gci155579	3.269715674	1.709165188	up	5.388991491	3.679826303	GTAGGGTGACGGGTTCA	hsa_circ_0012340	chr1	47023089	47030808	-	1883	ANNOTATED, CDS, c	NM_003684	MKNK1	Salzman2013	8569	circRNA	Detected	Detected	5.388991491	3.679826303
hsa_gci155668	2.934955377	1.553338569	up	4.183116953	2.629778385	GCTGAAGAAGTCCATTA	hsa_circ_0012715	chr1	56989494	56990226	-	336	ANNOTATED, CDS, c	NM_003713	PLPP3	Salzman2013	8613	circRNA	Detected	Not Detected	4.183116953	2.629778385
hsa_gci155684	2.179155971	1.12376946	up	6.424248751	5.300479292	AAGAAAAAGTAATACCT	hsa_circ_0012793	chr1	62713143	62737261	-	983	ANNOTATED, CDS, c	NM_181712	KANK4	Salzman2013	163782	circRNA	Detected	Detected	6.424248751	5.300479292
hsa_gci155698	2.00412537	1.00297276	up	5.507209298	4.504236538	GAAAAGTTGTCTTTGTT	hsa_circ_0012869	chr1	65323338	65330655	-	468	ANNOTATED, CDS, c	NM_002227	JAK1	Salzman2013	3716	circRNA	Detected	Detected	5.507209298	4.504236538
hsa_gci155699	2.482851978	1.311998254	up	5.620321144	4.308322891	TTAACCACTTTATCTTG	hsa_circ_0012872	chr1	65323338	65339206	-	1129	ANNOTATED, CDS, c	NM_002227	JAK1	Salzman2013	3716	circRNA	Detected	Detected	5.620321144	4.308322891
hsa_gci155708	2.510965747	1.328242348	up	4.648575561	3.320333213	TTGAATTGTTTCGTCTG	hsa_circ_0012934	chr1	70694104	70703235	+	515	ANNOTATED, CDS, c	NM_004768	SRSF11	Salzman2013	9295	circRNA	Detected	Detected	4.648575561	3.320333213
hsa_gci155725	2.157524066	1.109376652	up	4.783171093	3.673794441	TGTCAATCTATTACGAA	hsa_circ_0012998	chr1	78191306	78205102	-	1078	ANNOTATED, CDS, c	NM_015017	USP33	Salzman2013	23032	circRNA	Detected	Detected	4.783171093	3.673794441
hsa_gci155736	-3.30606936	-1.725116992	down	2.38939186	4.114508852	TCCATGGAGGAACAGGT	hsa_circ_0013044	chr1	78478734	78482995	+	1867	ANNOTATED, CDS, c	NM_007034	DNAJB4	Salzman2013	11080	circRNA	Not Detected	Detected	2.38939186	4.114508852
hsa_gci155737	2.643973794	1.402707878	up	10.52619588	9.123488	AGTAACTAGAAGTATAG	hsa_circ_0013048	chr1	82302569	82372915	+	387	ANNOTATED, CDS, c	NM_012302	ADGRL2	Salzman2013	23266	circRNA	Detected	Detected	10.52619588	9.123488
hsa_gci155740	5.223227883	2.384941648	up	5.296781605	2.911839957	TTCTTTAAATTTCTTCG	hsa_circ_0013058	chr1	85331067	85331842	-	775	ALT_ACCEPTOR, CDS	NM_012152	LPAR3	Salzman2013	23566	circRNA	Detected	Not Detected	5.296781605	2.911839957
hsa_gci155742	2.843128377	1.507479243	up	6.408599764	4.901120521	GTACGAGTGCCCCTAGT	hsa_circ_0013066	chr1	85784167	85787251	-	3084	ANNOTATED, CDS, c	NM_001134445	DDAH1	Salzman2013	23576	circRNA	Detected	Detected	6.408599764	4.901120521
hsa_gci155782	2.507740314	1.326387959	up	6.177475435	4.851087475	GAGGTCTACGGTCCCTT	hsa_circ_0013205	chr1	93811477	93828148	+	3208	ANNOTATED, CDS, c	NM_001938	DR1	Salzman2013	1810	circRNA	Detected	Detected	6.177475435	4.851087475
hsa_gci155785	2.016558764	1.011895448	up	6.90238783	5.890492382	GTGTGTCGTCCTCCGTT	hsa_circ_0013220	chr1	94360169	94360284	-	115	ANNOTATED, CDS, c	NM_002061	GCLM	Salzman2013	2730	circRNA	Detected	Detected	6.90238783	5.890492382
hsa_gci155793	2.138130191	1.096349702	up	6.087523948	4.991174247	AGGTTATGTTTTAATAT	hsa_circ_0013247	chr1	95616880	95616975	+	95	ANNOTATED, CDS, c	NM_001199679	TMEM56	Salzman2013	148534	circRNA	Detected	Detected	6.087523948	4.991174247
hsa_gci155797	3.043564134	1.605761767	up	7.974342836	6.36858107	GACCCCCTTTGTAAACA	hsa_circ_0013257	chr1	97564044	97915779	-	1026	ANNOTATED, CDS, c	NM_000110	DPYD	Salzman2013	1806	circRNA	Detected	Detected	7.974342836	6.36858107
hsa_gci155827	2.526421333	1.337095258	up	6.259786863	4.922691604	GACCTCCCCGATTACGA	hsa_circ_0013350	chr1	101377667	101383683	+	322	ANNOTATED, CDS, c	NM_133496	SLC30A7	Salzman2013	148867	circRNA	Detected	Detected	6.259786863	4.922691604
hsa_gci155843	2.496981948	1.320185391	up	6.583507086	5.263321695	ATTTGTAACGTGTCGAA	hsa_circ_0013408	chr1	109553537	109554340	-	803	ANNOTATED, CDS, c	NM_001142550	WDR47	Salzman2013	22911	circRNA	Detected	Detected	6.583507086	5.263321695
hsa_gci155851	5.20588148	2.380142467	up	4.375407254	1.995264787	GTAGACTGTCCCAGGGC	hsa_circ_0013477	chr1	110943876	110946658	-	447	ANNOTATED, CDS, c	NM_006402	LAMTOR5	Salzman2013	10542	circRNA	Detected	Not Detected	4.375407254	1.995264787
hsa_gci155874	2.229579377	1.156771563	up	6.98233846	5.825566897	AAGTCTTTTCTACCGTA	hsa_circ_0013584	chr1	113655100	113662145	+	1173	ANNOTATED, CDS, c	NM_014813	LRIG2	Salzman2013	9860	circRNA	Detected	Detected	6.98233846	5.825566897
hsa_gci155883	2.875503354	1.52381452	up	5.859621413	4.335806893	CCGTGGCATAATGTGGT	hsa_circ_0013634	chr1	114969798	115007010	-	894	ANNOTATED, CDS, c	NM_015906	TRIM33	Salzman2013	51592	circRNA	Detected	Detected	5.859621413	4.335806893
hsa_gci155891	4.411818531	2.14137345	up	4.123757601	1.98238415	AAATGCTACTTCAAGTA	hsa_circ_0013678	chr1	116927404	116939356	+	1850	ANNOTATED, CDS, c	NM_001160233	ATP1A1	Salzman2013	476	circRNA	Detected	Not Detected	4.123757601	1.98238415
hsa_gci155895	3.378476155	1.756372673	up	7.30625339	5.549880718	CCCCTGAGAGTGGTGTG	hsa_circ_0013702	chr1	117484336	117532972	+	5974	ANNOTATED, CDS, c	NM_020440	PTGFRN	Salzman2013	5738	circRNA	Detected	Detected	7.30625339	5.549880718
hsa_gci155896	2.07468738	1.052893963	up	6.723131306	5.670237343	CGGGTGCACGTCCCTCA	hsa_circ_0013703	chr1	117487300	117532972	+	5605	ANNOTATED, CDS, c	NM_020440	PTGFRN	Salzman2013	5738	circRNA	Detected	Detected	6.723131306	5.670237343
hsa_gci155898	6.618021009	2.726399871	up	3.94514335	1.218743479	ACTCGCGTTAGTAACAG	hsa_circ_0013723	chr1	117624449	117634572	+	1022	ANNOTATED, CDS, c	NM_003594	TTF2	Salzman2013	8458	circRNA	Detected	Not Detected	3.94514335	1.218743479
hsa_gci155921	2.624553735	1.392072136	up	6.976106321	5.584034186	TAGGTCCTTGCGATTGT	hsa_circ_0013884	chr1	145440631	145441053	+	309	ANNOTATED, CDS, c	NM_006472	TXNIP	Salzman2013	10628	circRNA	Detected	Detected	6.976106321	5.584034186
hsa_gci155927	2.577868431	1.366178634	up	4.495251102	3.129072469	ATTTTAGAACTTTTTTG	hsa_circ_0013919	chr1	145663158	145682094	+	281	ANNOTATED, CDS, c	NM_014455	RNF115	Salzman2013	27246	circRNA	Detected	Not Detected	4.495251102	3.129072469
hsa_gci155928	6.727979915	2.750173399	up	4.921277847	2.171104448	AGATTTTAGAACTTTTT	hsa_circ_0013920	chr1	145663158	145687091	+	564	ANNOTATED, CDS, c	NM_014455	RNF115	Salzman2013	27246	circRNA	Detected	Not Detected	4.921277847	2.171104448
hsa_gci155937	2.602699765	1.380008899	up	7.577577967	6.197569069	GTAGTAACCGTTCCATT	hsa_circ_0014012	chr1	150390071	150390201	+	130	ANNOTATED, CDS, c	NM_015203	RPRD2	Salzman2013	23248	circRNA	Detected	Detected	7.577577967	6.197569069
hsa_gci155944	2.996665254	1.583357935	up	4.555226806	2.971868871	CCGGGCCACTCAATTTC	hsa_circ_0014038	chr1	150618700	150667326	-	2966	ANNOTATED, CDS, c	NM_018178	GOLPH3L	Salzman2013	55204	circRNA	Detected	Not Detected	4.555226806	2.971868871
hsa_gci155965	2.190391141	1.131188517	up	5.421212588	4.290024071	TAAAGACATGTAGAACG	hsa_circ_0014152	chr1	151317203	151317664	-	203	ANNOTATED, CDS, c	NM_000449	RFX5	Salzman2013	5993	circRNA	Detected	Detected	5.421212588	4.290024071
hsa_gci155980	2.244554364	1.166429039	up	5.090642458	3.924213419	GGACGGTACATTTGTAA	hsa_circ_0014189	chr1	151630710	151630903	+	193	ANNOTATED, CDS, c	NM_030918	SNX27	Salzman2013	81609	circRNA	Detected	Detected	5.090642458	3.924213419
hsa_gci155984	2.66776152	1.415629705	up	4.832167096	3.416537391	GTCCAGGAAGTAATAAA	hsa_circ_0014201	chr1	151955385	151955800	-	415	ANNOTATED, CDS, c	NM_002966	S100A10	Salzman2013	6281	circRNA	Detected	Detected	4.832167096	3.416537391
hsa_gci155989	3.469602111	1.794770226	up	4.542922825	2.748152599	AAGGGCCGGGTCGGAAC	hsa_circ_0014206	chr1	152004981	152009511	-	594	ANNOTATED, CDS, c	NM_005620	S100A11	Salzman2013	6282	circRNA	Detected	Not Detected	4.542922825	2.748152599
hsa_gci155998	2.490192734	1.316257407	up	7.126119156	5.809861748	GACCGAACTGGGCCCCT	hsa_circ_0014254	chr1	153634513	153641033	-	1423	ANNOTATED, CDS, c	NM_004515	ILF2	Salzman2013	3608	circRNA	Detected	Detected	7.126119156	5.809861748
hsa_gci156015	3.682615124	1.880730627	up	6.589988177	4.70925755	GTTCAGATTAAACAAGA	hsa_circ_0014372	chr1	154199757	154201201	+	189	ANNOTATED, CDS, c	NM_014847	UBAP2L	Salzman2013	9898	circRNA	Detected	Detected	6.589988177	4.70925755

hsa_gci156032	2.028902219	1.020699338	up	6.605837097	5.585137759	GGAAGCCCTGTGAGGTC	hsa_circ_0014455	chr1	154934774	154940273	-	2403	ALT_DONOR, CDS, c	NM_001130040	SHC1	Salzman2013	6464	circRNA	Detected	Detected	6.605837097	5.585137759
hsa_gci156044	2.715821635	1.441388732	up	4.418358409	2.976969677	ACGGGGGTGCACTATGG	hsa_circ_0014517	chr1	155225769	155232176	-	1572	ANNOTATED, CDS, c	NM_005698	SCAMP3	Salzman2013	10067	circRNA	Detected	Not Detected	4.418358409	2.976969677
hsa_gci156047	3.053086668	1.610268544	up	7.65031302	6.040044476	TGTGAGTCGTTTCTAGT	hsa_circ_0014552	chr1	155313104	155340774	-	1961	ANNOTATED, CDS, c	NM_018489	ASH1L	Salzman2013	55870	circRNA	Detected	Detected	7.65031302	6.040044476
hsa_gci156051	3.253139932	1.701832881	up	8.266138232	6.564305352	ACAACCCCCTTGTGAGT	hsa_circ_0014570	chr1	155324263	155340774	-	881	ANNOTATED, CDS, c	NM_018489	ASH1L	Salzman2013	55870	circRNA	Detected	Detected	8.266138232	6.564305352
hsa_gci156055	2.164614768	1.114110294	up	4.405986218	3.291875924	CCCCAAAGTCTTCCTGA	hsa_circ_0014578	chr1	155340294	155385714	-	858	ANNOTATED, CDS, c	NM_018489	ASH1L	Salzman2013	55870	circRNA	Detected	Detected	4.405986218	3.291875924
hsa_gci156060	2.226074222	1.154501696	up	5.191545797	4.037044101	AAATAGTCTCCAACCGA	hsa_circ_0014586	chr1	155385534	155429689	-	1024	ANNOTATED, CDS, c	NM_018489	ASH1L	Salzman2013	55870	circRNA	Detected	Detected	5.191545797	4.037044101
hsa_gci156071	3.570528857	1.836137778	up	4.167771235	2.331633456	CCGTTCGAAGTGACTTA	hsa_circ_0014615	chr1	155695172	155697529	+	333	ANNOTATED, CDS, c	NM_001199849	DAP3	Salzman2013	7818	circRNA	Detected	Not Detected	4.167771235	2.331633456
hsa_gci156085	2.155512592	1.108030991	up	8.438777879	7.330746888	CTCAATCAAGACCGTTG	hsa_circ_0014700	chr1	156252703	156262234	+	2226	ANNOTATED, ncRNA,	NR_026678	TMEM79	Salzman2013	84283	circRNA	Detected	Detected	8.438777879	7.330746888
hsa_gci156093	2.352320343	1.234084542	up	7.340745874	6.106661332	TGGCGATGATTCCCTAC	hsa_circ_0014721	chr1	156294762	156307580	-	504	ANNOTATED, INTERN	NR_036565	CCT3	Salzman2013	7203	circRNA	Detected	Detected	7.340745874	6.106661332
hsa_gci156098	2.619363176	1.389216104	up	7.605205938	6.215989834	TTCCCTACCCGGTTCTT	hsa_circ_0014726	chr1	156303337	156307580	-	386	ANNOTATED, INTERN	NR_036565	CCT3	Salzman2013	7203	circRNA	Detected	Detected	7.605205938	6.215989834
hsa_gci156101	10.14244965	3.342334235	up	4.560795446	1.21846121	CACAGAAACCCCGGTAC	hsa_circ_0014778	chr1	156707093	156707326	-	233	ANNOTATED, CDS, c	NM_145729	MRPL24	Salzman2013	79590	circRNA	Detected	Not Detected	4.560795446	1.21846121
hsa_gci156107	2.546876006	1.348728724	up	4.104041873	2.755313148	CTTCTCCAGAAACCCCG	hsa_circ_0014787	chr1	156711898	156714938	-	1901	ANNOTATED, CDS, c	NM_004494	HDGF	Salzman2013	3068	circRNA	Detected	Not Detected	4.104041873	2.755313148
hsa_gci156116	2.604697304	1.381115724	up	4.935496857	3.554381133	CGGGGAGCCCCATGTCA	hsa_circ_0014855	chr1	159887902	159889625	-	1107	ANNOTATED, CDS, c	NM_003564	TAGLN2	Salzman2013	8407	circRNA	Detected	Detected	4.935496857	3.554381133
hsa_gci156122	3.981601237	1.99334874	up	8.912112354	6.918763614	CAATCCTTCTTTGAACC	hsa_circ_0014868	chr1	160185504	160208537	-	2948	ANNOTATED, ncRNA,	NR_028103	DCAF8	Salzman2013	50717	circRNA	Detected	Detected	8.912112354	6.918763614
hsa_gci156123	2.38868526	1.256216772	up	7.029863163	5.77364639	TTACTACAAACGACATC	hsa_circ_0014869	chr1	160185504	160213824	-	3697	ANNOTATED, ncRNA,	NR_028103	DCAF8	Salzman2013	50717	circRNA	Detected	Detected	7.029863163	5.77364639
hsa_gci156131	4.54802878	2.185241384	up	4.949210375	2.763968991	CCCACTTTCGACTCGGA	hsa_circ_0014920	chr1	160279981	160310084	-	1103	ANNOTATED, CDS, c	NM_001098398	COPA	Salzman2013	1314	circRNA	Detected	Not Detected	4.949210375	2.763968991
hsa_gci156137	2.137877091	1.096178913	up	6.949892702	5.853713789	GGACGGCTTCCGCCAAC	hsa_circ_0014963	chr1	161070345	161072165	-	517	ANNOTATED, CDS, c	NM_012394	PFDN2	Salzman2013	5202	circRNA	Detected	Detected	6.949892702	5.853713789
hsa_gci156156	2.787490045	1.478966653	up	7.622877341	6.143910688	AAGCAAAAGAGATGGTG	hsa_circ_0015050	chr1	162557264	162560301	+	524	ANNOTATED, CDS, c	NM_003115	UAP1	Salzman2013	6675	circRNA	Detected	Detected	7.622877341	6.143910688
hsa_gci156158	2.641820345	1.40153236	up	6.082806711	4.681274351	CCCTTTTGAGTAGAAGA	hsa_circ_0015052	chr1	162560112	162560301	+	189	ANNOTATED, CDS, c	NM_003115	UAP1	Salzman2013	6675	circRNA	Detected	Detected	6.082806711	4.681274351
hsa_gci156178	2.475136298	1.307507972	up	6.82715849	5.519650518	ACTGTGATTAGAAATCC	hsa_circ_0015150	chr1	168260397	168274434	+	713	ANNOTATED, CDS, c	NM_005149	TBX19	Salzman2013	9095	circRNA	Detected	Detected	6.82715849	5.519650518
hsa_gci156188	2.237892564	1.162140778	up	8.417187293	7.255046516	AACATCAACAGTAACGG	hsa_circ_0015187	chr1	169951112	169961396	-	615	ANNOTATED, CDS, c	NM_001204517	KIFAP3	Salzman2013	22920	circRNA	Detected	Detected	8.417187293	7.255046516
hsa_gci156202	3.739539505	1.902860624	up	6.587822682	4.684962058	ATATCAGACAATGTCTG	hsa_circ_0015235	chr1	171537385	171553665	+	1191	ANNOTATED, CDS, c	NM_015172	PRRC2C	Salzman2013	23215	circRNA	Detected	Detected	6.587822682	4.684962058
hsa_gci156208	2.2513202	1.170771262	up	4.396311084	3.225539822	CATTTCCATGACATTGG	hsa_circ_0015251	chr1	171556141	171557644	+	450	ANNOTATED, CDS, c	NM_015172	PRRC2C	Salzman2013	23215	circRNA	Detected	Detected	4.396311084	3.225539822
hsa_gci156214	2.602489055	1.379892096	up	4.542998039	3.163105943	AAATGTTGTATGTGCGG	hsa_circ_0015260	chr1	172520651	172548407	+	1436	ANNOTATED, CDS, c	NM_014283	SUCO	Salzman2013	51430	circRNA	Detected	Not Detected	4.542998039	3.163105943
hsa_gci156231	3.581082295	1.840395673	up	4.372737686	2.532342013	CCATCAGGTGGACCTCT	hsa_circ_0015329	chr1	174200282	174210795	+	386	ANNOTATED, CDS, c	NM_014857	RABGAP1L	Salzman2013	9910	circRNA	Detected	Not Detected	4.372737686	2.532342013
hsa_gci156240	2.216304596	1.148156171	up	3.959624004	2.811467833	GGACCTCCAACCGACAT	hsa_circ_0015365	chr1	176050287	176085817	-	309	ANNOTATED, CDS, c	NM_022457	COP1	Salzman2013	64326	circRNA	Detected	Not Detected	3.959624004	2.811467833
hsa_gci156249	2.156815443	1.108902731	up	5.324918632	4.2160159	GTATAGTTTCGTCTACC	hsa_circ_0015379	chr1	176145045	176153828	-	158	ANNOTATED, CDS, c	NM_022457	COP1	Salzman2013	64326	circRNA	Detected	Detected	5.324918632	4.2160159
hsa_gci156262	4.875032144	2.285411732	up	5.372438538	3.087026806	GGTAGAAATTTGGTGGC	hsa_circ_0015435	chr1	179100445	179198819	-	678	ANNOTATED, CDS, c	NM_007314	ABL2	Salzman2013	27	circRNA	Detected	Not Detected	5.372438538	3.087026806
hsa_gci156271	2.712858345	1.439813718	up	6.394059092	4.954245374	TTCCTTCTCTTAGTCGT	hsa_circ_0015455	chr1	179316734	179320597	+	479	ANNOTATED, CDS, c	NM_003101	SOAT1	Salzman2013	6646	circRNA	Detected	Detected	6.394059092	4.954245374
hsa_gci156276	2.860418228	1.516226102	up	3.935874701	2.419648599	GAAACAGTCTAAGAGGT	hsa_circ_0015480	chr1	179984983	179991983	+	1334	ANNOTATED, CDS, c	NM_014810	CEP350	Salzman2013	9857	circRNA	Detected	Not Detected	3.935874701	2.419648599
hsa_gci156299	2.332883528	1.222114281	up	6.511657767	5.289543486	ACGTCCCCATCTTAGAC	hsa_circ_0015573	chr1	182845581	182846038	+	169	ANNOTATED, INTERN	NR_033302	DHX9	Salzman2013	1660	circRNA	Detected	Detected	6.511657767	5.289543486
hsa_gci156338	-3.486921774	-1.801953999	down	1.722470618	3.524424617	TGTAGTCCCCGTTACTT	hsa_circ_0015791	chr1	197611840	197614873	-	165	ANNOTATED, CDS, c	NM_001195215	DENND1B	Salzman2013	163486	circRNA	Not Detected	Detected	1.722470618	3.524424617
hsa_gci156340	2.290801828	1.19585266	up	5.982823568	4.786970908	GAAGTTTGGTCCCTTCC	hsa_circ_0015796	chr1	197621364	197627499	-	151	ANNOTATED, CDS, c	NM_001195215	DENND1B	Salzman2013	163486	circRNA	Detected	Detected	5.982823568	4.786970908
hsa_gci156358	-2.493287528	-1.318049265	down	2.059439288	3.377488553	CAAGTACACCATCACTT	hsa_circ_0015857	chr1	200906307	200926954	+	12475	ALT_ACCEPTOR, ALT	TCONS_12_00000747		Salzman2013		circRNA	Not Detected	Detected	2.059439288	3.377488553
hsa_gci156359	2.607690396	1.382772592	up	12.01350689	10.63073429	TTGTCCCGTGGGTGGTG	hsa_circ_0015869	chr1	201103899	201113079	-	1176	ANNOTATED, CDS, c	NM_016456	TMEM9	Salzman2013	252839	circRNA	Detected	Detected	12.01350689	10.63073429
hsa_gci156390	2.984518346	1.577498122	up	4.591057377	3.013559255	TTTAGGTGGAAGAAGAC	hsa_circ_0016035	chr1	202722032	202742416	-	1296	ANNOTATED, CDS, c	NM_006618	KDM5B	Salzman2013	10765	circRNA	Detected	Not Detected	4.591057377	3.013559255
hsa_gci156395	2.392101944	1.258278874	up	4.612368795	3.354089921	GTTCTTTAAGTTAAAAT	hsa_circ_0016049	chr1	202742245	202743863	-	294	ANNOTATED, CDS, c	NM_006618	KDM5B	Salzman2013	10765	circRNA	Detected	Detected	4.612368795	3.354089921
hsa_gci156398	2.273143896	1.184689013	up	4.452745162	3.268056149	CCTTCACGCCCAGGTGT	hsa_circ_0016068	chr1	203274663	203278729	+	2711	ANNOTATED, CDS, c	NM_006763	BTG2	Salzman2013	7832	circRNA	Detected	Detected	4.452745162	3.268056149
hsa_gci156401	2.251724106	1.171030071	up	5.778322486	4.607292415	TAGACGTCCGTCGTCTA	hsa_circ_0016076	chr1	203669333	203677232	+	908	ANNOTATED, CDS, c	NM_001001396	ATP2B4	Salzman2013	493	circRNA	Detected	Detected	5.778322486	4.607292415
hsa_gci156412	2.215683902	1.147752075	up	5.058032007	3.910279932	TGAAGAGAAGGAATCCA	hsa_circ_0016197	chr1	205132850	205156934	-	1292	ANNOTATED, CDS, c	NM_015375	DSTYK	Salzman2013	25778	circRNA	Detected	Detected	5.058032007	3.910279932
hsa_gci156416	2.627444816	1.393660464	up	6.432441587	5.038781124	TTAAAAGAATCACCCTC	hsa_circ_0016215	chr1	205689628	205696933	-	315	ANNOTATED, CDS, c	NM_022731	NUCKS1	Salzman2013	64710	circRNA	Detected	Detected	6.432441587	5.038781124
hsa_gci156417	2.735465292	1.451786251	up	4.667799229	3.216012979	GAGGTTACCGATGTCCC	hsa_circ_0016250	chr1	206769066	206778860	-	1087	ANNOTATED, CDS, c	NM_006893	EIF2D	Salzman2013	1939	circRNA	Detected	Detected	4.667799229	3.216012979
hsa_gci156425	2.431466368	1.281826636	up	5.606872416	4.32504578	AGACCCACCCGAGACAA	hsa_circ_0016270	chr1	206904984	206907630	+	2415	ANNOTATED, CDS, c	NM_004759	MAPKAPK2	Salzman2013	9261	circRNA	Detected	Detected	5.606872416	4.32504578
hsa_gci156443	2.413279384	1.270994945	up	7.521193219	6.250198274	GTTTAGACCCCCTCTTT	hsa_circ_0016404	chr1	212977661	212977993	+	166	ANNOTATED, CDS, c	NM_001146171	TATDN3	Salzman2013	128387	circRNA	Detected	Detected	7.521193219	6.250198274
hsa_gci156453	2.774983027	1.472478947	up	9.588220065	8.115741117	TCGTTTAAGTTTTGGGT	hsa_circ_0016417	chr1	213061830	213062599	+	218	ANNOTATED, CDS, c	NM_014053	FLVCR1	Salzman2013	28982	circRNA	Detected	Detected	9.588220065	8.115741117
hsa_gci156496	-2.502900587	-1.323600989	down	2.223550326	3.547151315	AGGAAGAAAATTTGACT	hsa_circ_0016708	chr1	226453913	226488906	-	905	ANNOTATED, CDS, c	NM_173083	LIN9	Salzman2013	286826	circRNA	Not Detected	Detected	2.223550326	3.547151315
hsa_gci156499	2.091618582	1.064619792	up	8.36691735	7.302297558	CTAGTCCACGAGCCCCT	hsa_circ_0016729	chr1	227171461	227175246	+	1590	ANNOTATED, CDS, c	NM_020247	COQ8A	Salzman2013	56997	circRNA	Detected	Detected	8.36691735	7.302297558
hsa_gci156520	2.191442504	1.131880828	up	7.601148789	6.469267962	CCGTTAAGAGAGGTTTT	hsa_circ_0016869	chr1	230804421	230805281	+	289	ANNOTATED, CDS, c	NM_007357	COG2	Salzman2013	22796	circRNA	Detected	Detected	7.601148789	6.469267962
hsa_gci156525	2.54137621	1.345609959	up	5.468905998	4.123296039	GTCTTCTTACGACCGGC	hsa_circ_0016893	chr1	231406503	231408137	+	323	ANNOTATED, CDS, c	NM_014236	GNPAT	Salzman2013	8443	circRNA	Detected	Detected	5.468905998	4.123296039
hsa_gci156531	2.124027499	1.086802445	up	7.182859013	6.096056569	TCATGGTTTGACGAATA	hsa_circ_0016904	chr1	231700273	231702269	+	1996	ANNOTATED, CDS, c	NM_005999	TSNAX	Salzman2013	7257	circRNA	Detected	Detected	7.182859013	6.096056569
hsa_gci156569	2.964964817	1.568014986	up	4.998385116	3.430370131	AAATATTTATGTGTATA	hsa_circ_0017027	chr1	234565832	234603396	-	1510	ANNOTATED, CDS, c	NM_005646	TARBP1	Salzman2013	6894	circRNA	Detected	Detected	4.998385116	3.430370131

hsa_gci156577	-3.67289177	-1.876916384	down	1.405822852	3.282739236	AGATGGAAAGACTATAG	hsa_circ_0017053	chr1	235590454	235597595	+	277	ANNOTATED, CDS, c	NM_001079515	TBCE	Salzman2013	6905	circRNA	Not Detected	Detected	1.405822852	3.282739236
hsa_gci156582	2.023806197	1.017071142	up	7.683499183	6.666428041	ATCTAAAGGTGACTCCT	hsa_circ_0017069	chr1	235634163	235658138	-	650	ANNOTATED, CDS, c	NM_152490	B3GALNT2	Salzman2013	148789	circRNA	Detected	Detected	7.683499183	6.666428041
hsa_gci156599	2.444933866	1.289795442	up	7.348974901	6.059179459	CTTCCACCCCTTATTAG	hsa_circ_0017214	chr1	243242289	243260601	-	1562	ANNOTATED, INTERN	TCONS_12_00002816		Salzman2013		circRNA	Detected	Detected	7.348974901	6.059179459
hsa_gci156625	-12.00649521	-3.585743173	down	1.246423923	4.832167096	GAGATGAACCCTTGTAA	hsa_circ_0017308	chr1	246711846	246720023	-	302	ANNOTATED, CDS, c	NM_022366	TFB2M	Salzman2013	64216	circRNA	Not Detected	Detected	1.246423923	4.832167096
hsa_gci156643	3.358130326	1.747658221	up	3.859402313	2.111744092	GGTGACCCTCTTCACAT	hsa_circ_0017410	chr10	888871	931702	-	687	ANNOTATED, CDS, c	NM_015155	LARP4B	Salzman2013	23185	circRNA	Detected	Not Detected	3.859402313	2.111744092
hsa_gci156644	2.682373163	1.423509954	up	4.554885059	3.131375105	AGACTTTGGAATCAACG	hsa_circ_0017412	chr10	909682	910210	-	289	ANNOTATED, CDS, c	NM_015155	LARP4B	Salzman2013	23185	circRNA	Detected	Not Detected	4.554885059	3.131375105
hsa_gci156677	-2.752318724	-1.460647547	down	3.467070002	4.927717549	GGAGAGGCTTCCCTTGT	hsa_circ_0017551	chr10	5468617	5471192	+	127	ANNOTATED, CDS, c	NM_001047160	NET1	Salzman2013	10276	circRNA	Not Detected	Detected	3.467070002	4.927717549
hsa_gci156690	6.299554721	2.655249857	up	3.873182538	1.217932682	CCTACGTAGAAACTGA	hsa_circ_0017598	chr10	5947999	5969504	+	2672	ANNOTATED, CDS, c	NM_032807	FBH1	Salzman2013	84893	circRNA	Detected	Not Detected	3.873182538	1.217932682
hsa_gci156697	2.146535546	1.102010064	up	6.232076666	5.130066602	TCTTTAAGACAGGTCAG	hsa_circ_0017632	chr10	7269816	7327916	-	767	ANNOTATED, CDS, c	NM_001029880	SFMBT2	Salzman2013	57713	circRNA	Detected	Detected	6.232076666	5.130066602
hsa_gci156700	2.120672774	1.084522026	up	7.995639094	6.911117067	TACTCTTTAAGACAGGT	hsa_circ_0017636	chr10	7285519	7327916	-	684	ANNOTATED, CDS, c	NM_001029880	SFMBT2	Salzman2013	57713	circRNA	Detected	Detected	7.995639094	6.911117067
hsa_gci156708	2.057841728	1.041132026	up	8.240057589	7.198925563	TTAAGTAGGACGTCTTT	hsa_circ_0017649	chr10	7412242	7423911	-	246	ANNOTATED, CDS, c	NM_001029880	SFMBT2	Salzman2013	57713	circRNA	Detected	Detected	8.240057589	7.198925563
hsa_gci156760	2.813425828	1.492327929	up	5.345034272	3.852706342	CAGTAACAAGGCCAACC	hsa_circ_0017878	chr10	17276691	17279592	+	841	ANNOTATED, CDS, c	NM_003380	VIM	Salzman2013	7431	circRNA	Detected	Detected	5.345034272	3.852706342
hsa_gci156763	2.019906099	1.014288227	up	7.753793462	6.739505235	GGAGTCCAAGTCCCTCC	hsa_circ_0017882	chr10	17277844	17279592	+	494	ANNOTATED, CDS, c	NM_003380	VIM	Salzman2013	7431	circRNA	Detected	Detected	7.753793462	6.739505235
hsa_gci156796	2.293459369	1.197525349	up	6.26745663	5.069931281	CACCCGACTCCTCGTCA	hsa_circ_0018042	chr10	27493368	27494177	-	161	ANNOTATED, CDS, c	NM_145698	ACBD5	Salzman2013	91452	circRNA	Detected	Detected	6.26745663	5.069931281
hsa_gci156823	2.20801602	1.14275064	up	6.936891029	5.794140389	GCACTACACCTAGGACG	hsa_circ_0018167	chr10	34398487	34806087	-	5458	ANNOTATED, CDS, c	NM_019619	PARD3	Salzman2013	56288	circRNA	Detected	Detected	6.936891029	5.794140389
hsa_gci156833	8.980516785	3.166798468	up	7.062960108	3.896161641	ACGACCGGGCTCCTACT	hsa_circ_0018200	chr10	35772331	35805551	+	211	ANNOTATED, CDS, c	NM_145012	CCNY	Salzman2013	219771	circRNA	Detected	Detected	7.062960108	3.896161641
hsa_gci156845	8.988607605	3.16809765	up	4.488643649	1.320545999	TAAAAGAATATGGTCGC	hsa_circ_0018243	chr10	43315955	43316136	+	181	ANNOTATED, CDS, c	NM_014753	BMS1	Salzman2013	9790	circRNA	Detected	Not Detected	4.488643649	1.320545999
hsa_gci156864	3.787272712	1.92115931	up	7.174503346	5.253344036	AGTCCGAATTCCCAGTT	hsa_circ_0018369	chr10	51581269	51590734	+	3067	ANNOTATED, CDS, c	NM_001145260	NCOA4	Salzman2013	8031	circRNA	Detected	Detected	7.174503346	5.253344036
hsa_gci156871	3.116954639	1.64013716	up	4.291736002	2.651598842	CGGGTAAGTCCCTAGCA	hsa_circ_0018397	chr10	52071021	52350007	-	1578	ANNOTATED, CDS, c	NM_147156	SGMS1	Salzman2013	259230	circRNA	Detected	Not Detected	4.291736002	2.651598842
hsa_gci156872	2.638876784	1.399923989	up	5.610961066	4.211037077	CGGGTAAGTCCCTAGCA	hsa_circ_0018401	chr10	52193235	52350007	-	452	ANNOTATED, coding	NM_147156	SGMS1	Salzman2013	259230	circRNA	Detected	Detected	5.610961066	4.211037077
hsa_gci156878	2.544983197	1.347656131	up	7.868883702	6.521227571	AGAAAGGTCCGCGGCTC	hsa_circ_0018417	chr10	58117198	58121034	-	1851	ANNOTATED, CDS, c	NM_032997	ZWINT	Salzman2013	11130	circRNA	Detected	Detected	7.868883702	6.521227571
hsa_gci156931	2.951151465	1.561277968	up	8.556833241	6.995555273	AGAGGGTCCGTGAGTCG	hsa_circ_0018616	chr10	71124538	71161637	+	3125	ANNOTATED, CDS, c	NM_033500	HK1	Salzman2013	3098	circRNA	Detected	Detected	8.556833241	6.995555273
hsa_gci156936	3.702588367	1.888534168	up	5.315923366	3.427389198	ATAATTACGAATGATGT	hsa_circ_0018635	chr10	71917519	71921687	-	364	ANNOTATED, CDS, c	NM_001142648	SAR1A	Salzman2013	56681	circRNA	Detected	Detected	5.315923366	3.427389198
hsa_gci156942	2.393161301	1.258917639	up	11.21116084	9.952243199	CCTCGGTCTCCCTCAAC	hsa_circ_0018655	chr10	72163860	72188374	+	7531	ANNOTATED, CDS, c	NM_004096	EIF4EBP2	Salzman2013	1979	circRNA	Detected	Detected	11.21116084	9.952243199
hsa_gci156945	-5.120358558	-2.35624484	down	1.788044775	4.144289615	CCGTGTGAGTACCCACG	hsa_circ_0018677	chr10	72643266	72643805	-	539	ANNOTATED, CDS, c	NM_000281	PCBD1	Salzman2013	5092	circRNA	Not Detected	Detected	1.788044775	4.144289615
hsa_gci156946	3.191193928	1.674096284	up	4.024815899	2.350719615	CGGACGTCCCGGTGAGA	hsa_circ_0018678	chr10	72643266	72644989	-	620	ANNOTATED, CDS, c	NM_000281	PCBD1	Salzman2013	5092	circRNA	Detected	Not Detected	4.024815899	2.350719615
hsa_gci156955	2.014165587	1.010182294	up	9.930027077	8.919844783	GCCACTGTTGCAGAAAC	hsa_circ_0018697	chr10	73576054	73591677	-	2553	ANNOTATED, CDS, c	NM_001042465	PSAP	Salzman2013	5660	circRNA	Detected	Detected	9.930027077	8.919844783
hsa_gci156962	2.021805859	1.015644471	up	10.52610651	9.510462039	GTTGCAGAAACGTATAC	hsa_circ_0018721	chr10	73585593	73591677	-	603	ANNOTATED, CDS, c	NM_001042465	PSAP	Salzman2013	5660	circRNA	Detected	Detected	10.52610651	9.510462039
hsa_gci156982	4.782840539	2.257867692	up	6.624565164	4.366697472	AAAGTAGTCGTTACACC	hsa_circ_0018824	chr10	75302560	75302889	-	197	ANNOTATED, CDS, c	NM_152586	USP54	Salzman2013	159195	circRNA	Detected	Detected	6.624565164	4.366697472
hsa_gci156990	2.462853028	1.300330537	up	8.517384685	7.217054148	TAAGGTCTTTCCGTCTT	hsa_circ_0018841	chr10	75458908	75481262	-	1216	ANNOTATED, ncRNA,	NR_026592	BMS1P4	Salzman2013	729096	circRNA	Detected	Detected	8.517384685	7.217054148
hsa_gci156991	2.654223033	1.408289605	up	7.612071167	6.203781562	TTGTTCATTAAGATTAC	hsa_circ_0018843	chr10	75458908	75488345	-	1884	ANNOTATED, ncRNA,	NR_026592	BMS1P4	Salzman2013	729096	circRNA	Detected	Detected	7.612071167	6.203781562
hsa_gci157001	2.640630141	1.400882245	up	5.347346884	3.946464639	ACGTAGAAGACTCTTCG	hsa_circ_0018984	chr10	82248972	82269227	+	467	ANNOTATED, CDS, c	NM_030927	TSPAN14	Salzman2013	81619	circRNA	Detected	Detected	5.347346884	3.946464639
hsa_gci157009	12.31016512	3.621778209	up	6.27196589	2.650187681	ACTGTGGTCAAAGTATA	hsa_circ_0019014	chr10	88231964	88277848	-	2052	ANNOTATED, CDS, c	NM_015045	WAPL	Salzman2013	23063	circRNA	Detected	Not Detected	6.27196589	2.650187681
hsa_gci157027	2.437886055	1.285630697	up	4.818789194	3.533158496	CAAGGCGAAAGGGTACA	hsa_circ_0019091	chr10	92982445	93008317	+	448	ANNOTATED, CDS, c	NM_032373	PCGF5	Salzman2013	84333	circRNA	Detected	Detected	4.818789194	3.533158496
hsa_gci157038	2.285131862	1.192277418	up	6.146979444	4.954702027	AATAGGTCCTCCCTAAA	hsa_circ_0019133	chr10	93754291	93757508	+	461	ANNOTATED, CDS, c	NM_003972	BTAF1	Salzman2013	9044	circRNA	Detected	Detected	6.146979444	4.954702027
hsa_gci157059	2.461975246	1.299816256	up	5.133950649	3.834134392	AACAGCTACTGTTCCGA	hsa_circ_0019249	chr10	96997329	97007123	-	809	ANNOTATED, CDS, c	NM_020992	PDLIM1	Salzman2013	9124	circRNA	Detected	Detected	5.133950649	3.834134392
hsa_gci157069	-8.307151801	-3.054353918	down	2.462531716	5.516885634	AATTATTACGTCCAGGT	hsa_circ_0019316	chr10	98667021	98711953	+	661	ANNOTATED, CDS, c	NM_032440	LCOR	Salzman2013	84458	circRNA	Not Detected	Detected	2.462531716	5.516885634
hsa_gci157070	2.07406636	1.052462054	up	11.33482592	10.28236387	GTGTCCCTGACCTCCGT	hsa_circ_0019327	chr10	99116457	99139527	-	2754	ANNOTATED, CDS, c	NM_015179	RRP12	Salzman2013	23223	circRNA	Detected	Detected	11.33482592	10.28236387
hsa_gci157078	2.012424396	1.008934584	up	6.721351698	5.712417114	TATAACATCTGACACAC	hsa_circ_0019368	chr10	99236439	99237161	-	249	ALT_ACCEPTOR, CDS	NM_022362	MMS19	Salzman2013	64210	circRNA	Detected	Detected	6.721351698	5.712417114
hsa_gci157110	9.666727502	3.273027574	up	4.466809266	1.193781693	CCTCGTCGGGTCGGTGT	hsa_circ_0019565	chr10	103190101	103317070	+	5960	ANNOTATED, CDS, c	NM_033637	BTRC	Salzman2013	8945	circRNA	Detected	Not Detected	4.466809266	1.193781693
hsa_gci157114	2.266972223	1.180766714	up	4.714920434	3.53415372	TCGACCACAGATAGATC	hsa_circ_0019580	chr10	103368591	103369410	+	390	ANNOTATED, CDS, c	NM_015448	DPCD	Salzman2013	25911	circRNA	Detected	Detected	4.714920434	3.53415372
hsa_gci157130	2.319955239	1.21409697	up	5.115889556	3.901792585	GTAGTTTTTCGTTTCGT	hsa_circ_0019618	chr10	103567486	103567658	-	172	ANNOTATED, CDS, c	NM_012215	MGEA5	Salzman2013	10724	circRNA	Detected	Detected	5.115889556	3.901792585
hsa_gci157141	3.418447801	1.773341396	up	4.905993121	3.132651725	CGGGTAGTCCCACCCGA	hsa_circ_0019660	chr10	103867324	103868080	-	756	ANNOTATED, CDS, c	NM_003893	LDB1	Salzman2013	8861	circRNA	Detected	Not Detected	4.905993121	3.132651725
hsa_gci157153	2.144858814	1.100882685	up	5.901440884	4.800558199	GTGAGGTCCTCCAACTG	hsa_circ_0019787	chr10	104899162	104934739	-	199	ANNOTATED, CDS, c	NM_012229	NT5C2	Salzman2013	22978	circRNA	Detected	Detected	5.901440884	4.800558199
hsa_gci157174	2.006905449	1.004972649	up	6.861934417	5.856961769	CGGGGCCCCCGCGCGAA	hsa_circ_0019973	chr10	106014920	106027222	+	751	ANNOTATED, CDS, c	NM_001191003	GSTO1	Salzman2013	9446	circRNA	Detected	Detected	6.861934417	5.856961769
hsa_gci157177	3.025672964	1.597256059	up	5.457170237	3.859914177	CCAGTCCACCATTAACA	hsa_circ_0019987	chr10	111624523	111625070	-	547	ANNOTATED, CDS, c	NM_020383	XPNPEP1	Salzman2013	7511	circRNA	Detected	Detected	5.457170237	3.859914177
hsa_gci157191	2.486657868	1.314208025	up	6.325308552	5.011100528	CCCACCTTTGCCCGCGA	hsa_circ_0020064	chr10	116190868	116331165	-	7008	ANNOTATED, CDS, c	NM_001003408	ABLIM1	Salzman2013	3983	circRNA	Detected	Detected	6.325308552	5.011100528
hsa_gci157211	2.068690156	1.048717577	up	8.525338305	7.476620727	CCTGAGATCATCTGTAA	hsa_circ_0020148	chr10	120824910	120825082	-	172	ANNOTATED, CDS, c	NM_003750	EIF3A	Salzman2013	8661	circRNA	Detected	Detected	8.525338305	7.476620727
hsa_gci157212	2.270942515	1.183291187	up	8.357794533	7.174503346	ACGGTTGGAGTTCTTTA	hsa_circ_0020150	chr10	120824910	120833449	-	1073	ANNOTATED, CDS, c	NM_003750	EIF3A	Salzman2013	8661	circRNA	Detected	Detected	8.357794533	7.174503346
hsa_gci157218	2.774043871	1.471990604	up	6.358665587	4.886674984	GGGTTCCTTACCGTGAG	hsa_circ_0020169	chr10	120927214	120936665	-	1512	ANNOTATED, CDS, c	NM_006793	PRDX3	Salzman2013	10935	circRNA	Detected	Detected	6.358665587	4.886674984
hsa_gci157228	2.273934254	1.185190542	up	8.096472841	6.911282298	AAAGTGGTGTGTTTATT	hsa_circ_0020209	chr10	122273422	122278408	+	155	ANNOTATED, CDS, c	NM_001030059	PLPP4	Salzman2013	196051	circRNA	Detected	Detected	8.096472841	6.911282298

hsa_gci157269	2.534889223	1.341922701	up	8.95978552	7.617862819	CTACCCCTGCCATGTGT	hsa_circ_0020367	chr10	127483448	127505201	-	793	ALT_ACCEPTOR, CDS	NM_000375	UROS	Salzman2013	7390	circRNA	Detected	Detected	8.95978552	7.617862819
hsa_gci157295	5.41064802	2.435801392	up	6.074024867	3.638223475	AGACCGTAGTATCAATA	hsa_circ_0020414	chr10	128768965	129224275	+	4805	ANNOTATED, CDS, c	NM_001380	DOCK1	Salzman2013	1793	circRNA	Detected	Detected	6.074024867	3.638223475
hsa_gci157342	2.557074257	1.354494057	up	6.457956287	5.10346223	GTGGACTTCACAGTCGA	hsa_circ_0020613	chr11	238997	252980	+	1416	ALT_DONOR, CDS, c	NM_002817	PSMD13	Salzman2013	5719	circRNA	Detected	Detected	6.457956287	5.10346223
hsa_gci157351	2.84104986	1.506424151	up	3.999401954	2.492977804	GGCTCTCCGACGTTATG	hsa_circ_0020656	chr11	494511	499185	-	1122	ANNOTATED, CDS, c	NM_002939	RNH1	Salzman2013	6050	circRNA	Detected	Not Detected	3.999401954	2.492977804
hsa_gci157354	2.05835172	1.041489523	up	7.522668333	6.48117881	CGACTCTATTTTGTCGT	hsa_circ_0020683	chr11	700141	704129	+	1372	ANNOTATED, CDS, c	NM_001042463	TMEM80	Salzman2013	283232	circRNA	Detected	Detected	7.522668333	6.48117881
hsa_gci157368	2.976718944	1.573723012	up	8.333958472	6.76023546	AAATGACGAAATCCATT	hsa_circ_0020822	chr11	2991032	2993473	-	361	ANNOTATED, CDS, c	NM_005969	NAP1L4	Salzman2013	4676	circRNA	Detected	Detected	8.333958472	6.76023546
hsa_gci157374	4.396430328	2.136332606	up	6.231853949	4.095521343	CCTCGTCCGTATAGACA	hsa_circ_0020859	chr11	3712576	3712719	-	143	ANNOTATED, CDS, c	NM_016320	NUP98	Salzman2013	4928	circRNA	Detected	Detected	6.231853949	4.095521343
hsa_gci157379	2.821111526	1.496263701	up	4.196955783	2.700692082	AGTCATTCGTTACTACG	hsa_circ_0020874	chr11	3726429	3726586	-	157	ANNOTATED, CDS, c	NM_016320	NUP98	Salzman2013	4928	circRNA	Detected	Not Detected	4.196955783	2.700692082
hsa_gci157381	2.778520543	1.474316907	up	7.366547746	5.892230839	ATCATTCTCGGAAGACC	hsa_circ_0020879	chr11	3726429	3752808	-	1540	ANNOTATED, CDS, c	NM_016320	NUP98	Salzman2013	4928	circRNA	Detected	Detected	7.366547746	5.892230839
hsa_gci157386	3.294222343	1.719937933	up	5.611342055	3.891404123	CAATGAGGTTTATTAGG	hsa_circ_0020897	chr11	3752620	3784269	-	833	ANNOTATED, CDS, c	NM_005387	NUP98	Salzman2013	4928	circRNA	Detected	Detected	5.611342055	3.891404123
hsa_gci157388	4.198169562	2.069760438	up	4.866963803	2.797203366	ATTGAGGTCGAAACTGG	hsa_circ_0020899	chr11	3752620	3794969	-	1286	ANNOTATED, CDS, c	NM_005387	NUP98	Salzman2013	4928	circRNA	Detected	Not Detected	4.866963803	2.797203366
hsa_gci157398	-2.335549296	-1.223761896	down	2.849178904	4.0729408	ACATGGTGGAGTAGGTG	hsa_circ_0020936	chr11	4076755	4095909	+	584	ANNOTATED, CDS, c	NM_003156	STIM1	Salzman2013	6786	circRNA	Not Detected	Detected	2.849178904	4.0729408
hsa_gci157411	2.175672975	1.121461722	up	8.059082242	6.93762052	TTCGACAGTTTTTTAGA	hsa_circ_0020967	chr11	5289579	5290906	-	471	ANNOTATED, CDS, c	NM_005330	HBE1	Salzman2013	3046	circRNA	Detected	Detected	8.059082242	6.93762052
hsa_gci157418	3.217628703	1.685997856	up	7.477822251	5.791824394	GGCGGGGCCTTCATTGG	hsa_circ_0021002	chr11	6502676	6505911	+	2842	ANNOTATED, CDS, c	NM_012192	TIMM10B	Salzman2013	26515	circRNA	Detected	Detected	7.477822251	5.791824394
hsa_gci157420	3.691634997	1.884259917	up	4.551549382	2.667289465	GACTTGCCTACAGGCTC	hsa_circ_0021043	chr11	6625206	6632099	+	1911	ANNOTATED, CDS, c	NM_001014795	ILK	Salzman2013	3611	circRNA	Detected	Not Detected	4.551549382	2.667289465
hsa_gci157429	2.001451012	1.001046305	up	7.77213665	6.771090345	GTCTGTACGACGTCCGA	hsa_circ_0021116	chr11	9002122	9005107	-	2985	ANNOTATED, CDS, c	NM_020645	NRIP3	Salzman2013	56675	circRNA	Detected	Detected	7.77213665	6.771090345
hsa_gci157432	2.112051139	1.078644767	up	7.853046831	6.774402064	GAAAATATCGGCTCGCA	hsa_circ_0021120	chr11	9002122	9025596	-	3809	ANNOTATED, CDS, c	NM_020645	NRIP3	Salzman2013	56675	circRNA	Detected	Detected	7.853046831	6.774402064
hsa_gci157446	2.79737778	1.4840751	up	8.887784649	7.403709549	TTCCTTTCTCTTCGCGG	hsa_circ_0021159	chr11	9406168	9431633	+	621	ANNOTATED, CDS, c	NM_006391	IPO7	Salzman2013	10527	circRNA	Detected	Detected	8.887784649	7.403709549
hsa_gci157451	2.663874789	1.413526272	up	8.091502841	6.677976568	AGACCCGTCGTCACCAT	hsa_circ_0021181	chr11	9446460	9446809	+	194	ANNOTATED, CDS, c	NM_006391	IPO7	Salzman2013	10527	circRNA	Detected	Detected	8.091502841	6.677976568
hsa_gci157458	6.170477946	2.62538224	up	4.492211259	1.866829018	AGACACTCCTGACGTGT	hsa_circ_0021225	chr11	9983503	10024236	-	1241	ANNOTATED, CDS, c	NM_030962	SBF2	Salzman2013	81846	circRNA	Detected	Not Detected	4.492211259	1.866829018
hsa_gci157479	2.837956882	1.50485267	up	8.030948789	6.526096119	AGGAGTCGGAGAATGGC	hsa_circ_0021318	chr11	13435076	13484838	-	1065	ANNOTATED, CDS, c	NM_032320	BTBD10	Salzman2013	84280	circRNA	Detected	Detected	8.030948789	6.526096119
hsa_gci157483	3.089780842	1.627504512	up	5.89002944	4.262524928	AGTCGTCCAACTCGTCC	hsa_circ_0021330	chr11	14526421	14526794	-	373	ANNOTATED, CDS, c	NM_148976	PSMA1	Salzman2013	5682	circRNA	Detected	Detected	5.89002944	4.262524928
hsa_gci157491	2.220010543	1.150566528	up	8.009924878	6.85935835	TCCCCCTGTCGTAATCT	hsa_circ_0021386	chr11	17126715	17134213	-	360	ANNOTATED, CDS, c	NM_002645	PIK3C2A	Salzman2013	5286	circRNA	Detected	Detected	8.009924878	6.85935835
hsa_gci157504	5.245437904	2.391063218	up	3.943343912	1.552280694	TACTGGTCGAACCTCAA	hsa_circ_0021450	chr11	18422383	18424560	+	348	ANNOTATED, CDS, c	NM_001165414	LDHA	Salzman2013	3939	circRNA	Detected	Not Detected	3.943343912	1.552280694
hsa_gci157519	-4.122176404	-2.043406245	down	1.240436621	3.283842865	TACGAATAACTGAAAAA	hsa_circ_0021506	chr11	22225349	22249132	+	608	ANNOTATED, CDS, c	NM_213599	ANO5	Salzman2013	203859	circRNA	Not Detected	Detected	1.240436621	3.283842865
hsa_gci157534	2.499101792	1.321409666	up	7.70702202	6.385612355	GTTCTGAACACTCCGGT	hsa_circ_0021598	chr11	33307958	33363232	+	1899	ANNOTATED, CDS, c	NM_005734	HIPK3	Salzman2013	10114	circRNA	Detected	Detected	7.70702202	6.385612355
hsa_gci157538	2.677397339	1.420831258	up	7.707378992	6.286547734	ACGGAGTACGAGAAGGT	hsa_circ_0021604	chr11	33360302	33363232	+	556	ANNOTATED, CDS, c	NM_005734	HIPK3	Salzman2013	10114	circRNA	Detected	Detected	7.707378992	6.286547734
hsa_gci157551	2.67928782	1.421849569	up	5.947098563	4.525248993	ACTACCCTGACCAGACC	hsa_circ_0021659	chr11	34118025	34120607	+	1659	ANNOTATED, CDS, c	NM_203364	CAPRIN1	Salzman2013	4076	circRNA	Detected	Detected	5.947098563	4.525248993
hsa_gci157557	2.118636134	1.083135833	up	7.228824906	6.145689073	TTTCAGACCTAGAACCT	hsa_circ_0021688	chr11	34156058	34158573	+	375	ANNOTATED, CDS, c	NM_024662	NAT10	Salzman2013	55226	circRNA	Detected	Detected	7.228824906	6.145689073
hsa_gci157563	2.296362969	1.199350696	up	8.92256277	7.723212075	TTATTGAGAAGCTTATA	hsa_circ_0021713	chr11	34991685	34999729	+	207	ANNOTATED, CDS, c	NM_003477	PDHX	Salzman2013	8050	circRNA	Detected	Detected	8.92256277	7.723212075
hsa_gci157586	3.151844821	1.656196506	up	7.977526065	6.321329558	ATAATACCAAGTGTACC	hsa_circ_0021859	chr11	46529740	46568846	-	1875	ANNOTATED, CDS, c	NM_017749	AMBRA1	Salzman2013	55626	circRNA	Detected	Detected	7.977526065	6.321329558
hsa_gci157589	2.470824468	1.304992523	up	7.170585447	5.865592924	GTCCACACACTGTTCAA	hsa_circ_0021880	chr11	46698631	46717305	-	3142	ANNOTATED, CDS, c	NM_004308	ARHGAP1	Salzman2013	392	circRNA	Detected	Detected	7.170585447	5.865592924
hsa_gci157603	2.497197438	1.320309891	up	9.320963776	8.000653885	GTTCTGCATATATAACC	hsa_circ_0022018	chr11	47644252	47644328	-	76	ANNOTATED, CDS, c	NM_014342	MTCH2	Salzman2013	23788	circRNA	Detected	Detected	9.320963776	8.000653885
hsa_gci157613	2.78451463	1.477425873	up	7.834442972	6.357017099	TATAAAGCAGGATAACA	hsa_circ_0022036	chr11	47653205	47660603	-	340	ANNOTATED, CDS, c	NM_014342	MTCH2	Salzman2013	23788	circRNA	Detected	Detected	7.834442972	6.357017099
hsa_gci157614	2.36514456	1.241928365	up	7.24492751	6.002999144	GATAACAACCTCCTTCT	hsa_circ_0022039	chr11	47660250	47660603	-	192	ANNOTATED, CDS, c	NM_014342	MTCH2	Salzman2013	23788	circRNA	Detected	Detected	7.24492751	6.002999144
hsa_gci157636	2.677130582	1.420687511	up	5.825053557	4.404366046	TTTTTGGTAGTCGCCCA	hsa_circ_0022129	chr11	57176647	57193825	-	1551	ANNOTATED, CDS, c	NM_014096	SLC43A3	Salzman2013	29015	circRNA	Detected	Detected	5.825053557	4.404366046
hsa_gci157644	2.061060642	1.043386953	up	12.37938137	11.33599441	GCTAACCGACGCGACCC	hsa_circ_0022192	chr11	58910555	58922511	+	3659	ANNOTATED, CDS, c	NM_001142520	FAM111A	Salzman2013	63901	circRNA	Detected	Detected	12.37938137	11.33599441
hsa_gci157650	-4.894781227	-2.29124438	down	2.448299552	4.739543931	TGTCTTTACCGACTACT	hsa_circ_0022225	chr11	60688357	60690915	+	1773	ANNOTATED, CDS, c	NM_024092	TMEM109	Salzman2013	79073	circRNA	Not Detected	Detected	2.448299552	4.739543931
hsa_gci157665	2.641883946	1.401567092	up	8.363078416	6.961511323	GGCCGACATCAACACAG	hsa_circ_0022399	chr11	61640997	61643448	-	466	ANNOTATED, CDS, c	NM_021727	FADS3	Salzman2013	3995	circRNA	Detected	Detected	8.363078416	6.961511323
hsa_gci157668	2.146198065	1.101783223	up	11.22073454	10.11895131	GGTCCCACTCGTCGCCG	hsa_circ_0022402	chr11	61640997	61645059	-	818	ANNOTATED, CDS, c	NM_021727	FADS3	Salzman2013	3995	circRNA	Detected	Detected	11.22073454	10.11895131
hsa_gci157670	2.960925679	1.566048278	up	5.303840414	3.737792136	ACCGGGTCCAGTACGAC	hsa_circ_0022404	chr11	61640997	61646319	-	1104	ANNOTATED, CDS, c	NM_021727	FADS3	Salzman2013	3995	circRNA	Detected	Detected	5.303840414	3.737792136
hsa_gci157671	2.417529277	1.273533361	up	5.846179811	4.572646449	AAAGAAGGACCGGCGAA	hsa_circ_0022423	chr11	62201015	62201363	-	348	ANNOTATED, CDS, c	NM_024060	AHNAK	Salzman2013	79026	circRNA	Detected	Detected	5.846179811	4.572646449
hsa_gci157683	-3.825537702	-1.935662542	down	1.337281742	3.272944284	CTTTCAAGATGAAGAAG	hsa_circ_0022510	chr11	62462039	62489852	-	1827	ANNOTATED, INTERN	NR_037946	HNRNPUL2-BSCL	Salzman2013	100534595	circRNA	Not Detected	Detected	1.337281742	3.272944284
hsa_gci157689	2.005526567	1.003981078	up	6.205501821	5.201520744	CAACCTCAATCCTAGTT	hsa_circ_0022519	chr11	62532811	62534187	+	394	ANNOTATED, CDS, c	NM_002696	POLR2G	Salzman2013	5436	circRNA	Detected	Detected	6.205501821	5.201520744
hsa_gci157699	2.148377005	1.103247185	up	5.261516278	4.158269094	GTTGCACGCGCATTTAG	hsa_circ_0022567	chr11	62621990	62622938	-	211	ANNOTATED, INTERN	NR_003098	SNHG1	Salzman2013	23642	circRNA	Detected	Detected	5.261516278	4.158269094
hsa_gci157747	2.585612205	1.370505913	up	10.0101426	8.639636689	CCGTATTTAGTAAAACG	hsa_circ_0022899	chr11	65622284	65623201	-	712	ANNOTATED, CDS, c	NM_005507	CFL1	Salzman2013	1072	circRNA	Detected	Detected	10.0101426	8.639636689
hsa_gci157748	2.440193838	1.286995754	up	5.851293035	4.564297281	AACTTGTGGAACTACTG	hsa_circ_0022900	chr11	65622284	65623713	-	1020	ANNOTATED, CDS, c	NM_005507	CFL1	Salzman2013	1072	circRNA	Detected	Detected	5.851293035	4.564297281
hsa_gci157764	-2.086297799	-1.060945104	down	2.232172592	3.293117696	TAGAGAACTTCGTACAT	hsa_circ_0022974	chr11	66104803	66106256	-	662	ANNOTATED, CDS, c	NM_015399	BRMS1	Salzman2013	25855	circRNA	Not Detected	Detected	2.232172592	3.293117696
hsa_gci157773	2.111376262	1.0781837	up	4.526811769	3.448628069	GTCGGGTGTGTGTAGGG	hsa_circ_0023016	chr11	66413497	66413944	+	447	ANNOTATED, coding	NM_002896	RBM4	Salzman2013	5936	circRNA	Detected	Detected	4.526811769	3.448628069
hsa_gci157787	2.275415423	1.186129963	up	7.943414484	6.757284522	TGGTCGCCCATGTCCTG	hsa_circ_0023100	chr11	67256737	67258579	+	829	ANNOTATED, CDS, c	NM_003977	AIP	Salzman2013	9049	circRNA	Detected	Detected	7.943414484	6.757284522
hsa_gci157806	2.476027395	1.308027277	up	6.080500306	4.77247303	TCCTCCCGCGACGGAGC	hsa_circ_0023240	chr11	68452391	68458643	+	560	ANNOTATED, CDS, c	NM_015973	GAL	Salzman2013	51083	circRNA	Detected	Detected	6.080500306	4.77247303

hsa_gci157809	2.058225513	1.041401062	up	7.061227932	6.01982687	GCCAGCTCCCGTGGCCG	hsa_circ_0023245	chr11	68458384	68458643	+	259	ANNOTATED, CDS, c	NM_015973	GAL	Salzman2013	51083	circRNA	Detected	Detected	7.061227932	6.01982687
hsa_gci157818	2.415917662	1.272571286	up	5.895618366	4.62304708	GCGTGAAGACAAGGAGC	hsa_circ_0023305	chr11	69457798	69469242	+	3882	ANNOTATED, CDS, c	NM_053056	CCND1	Salzman2013	595	circRNA	Detected	Detected	5.895618366	4.62304708
hsa_gci157820	2.478264071	1.309329922	up	9.347188327	8.037858405	TGACGAAAAAACGTGAA	hsa_circ_0023323	chr11	70183470	70194526	+	735	ANNOTATED, CDS, c	NM_003626	PPFIA1	Salzman2013	8500	circRNA	Detected	Detected	9.347188327	8.037858405
hsa_gci157826	2.248596669	1.169024908	up	5.563156197	4.394131289	ACAACCCAACGCTAAAA	hsa_circ_0023367	chr11	71145456	71156004	-	2398	ANNOTATED, CDS, c	NM_001360	DHCR7	Salzman2013	1717	circRNA	Detected	Detected	5.563156197	4.394131289
hsa_gci157833	2.314212486	1.210521336	up	6.996993515	5.786472179	GTGTCGGTTCGCCCACA	hsa_circ_0023421	chr11	71808337	71809461	-	750	ANNOTATED, CDS, c	NM_017907	LAMTOR1	Salzman2013	55004	circRNA	Detected	Detected	6.996993515	5.786472179
hsa_gci157838	2.320813896	1.214630839	up	7.161595455	5.946964616	CACGCACGCACGCACGG	hsa_circ_0023506	chr11	73418464	73472201	-	1016	ANNOTATED, CDS, c	NM_002869	RAB6A	Salzman2013	5870	circRNA	Detected	Detected	7.161595455	5.946964616
hsa_gci157850	2.807892782	1.489487848	up	4.658544044	3.169056196	TTCAAAGACTGTCTCCA	hsa_circ_0023552	chr11	73820078	73850873	-	1479	ANNOTATED, CDS, c	NM_015531	C2CD3	Salzman2013	26005	circRNA	Detected	Detected	4.658544044	3.169056196
hsa_gci157863	2.601312081	1.379239491	up	6.558543059	5.179303568	CCCACCTGTCGTCCAGT	hsa_circ_0023572	chr11	73964536	73965748	+	1212	ANNOTATED, CDS, c	NM_016147	PPME1	Salzman2013	51400	circRNA	Detected	Detected	6.558543059	5.179303568
hsa_gci157872	3.273561111	1.710860911	up	6.2392983	4.528437389	CATTCTACTTTTGTGTT	hsa_circ_0023596	chr11	74563033	74574066	-	261	ANNOTATED, CDS, c	NM_182969	XRRA1	Salzman2013	143570	circRNA	Detected	Detected	6.2392983	4.528437389
hsa_gci157879	3.016625847	1.59293577	up	6.477645049	4.88470928	TCTCGTTGTAGGTCTGA	hsa_circ_0023617	chr11	75116657	75116733	+	76	ANNOTATED, coding	NM_001005	RPS3	Salzman2013	6188	circRNA	Detected	Detected	6.477645049	4.88470928
hsa_gci157889	2.300948302	1.202228569	up	6.163808544	4.961579974	AAAGAGAAAGAAAAATA	hsa_circ_0023683	chr11	77069942	77091039	-	407	ANNOTATED, CDS, c	NM_001128620	PAK1	Salzman2013	5058	circRNA	Detected	Detected	6.163808544	4.961579974
hsa_gci157899	-3.993694958	-1.997724143	down	1.27613375	3.273857893	ACCATAGGTGATAGCGT	hsa_circ_0023700	chr11	77383086	77404656	-	1036	ANNOTATED, CDS, c	NM_016578	RSF1	Salzman2013	51773	circRNA	Not Detected	Detected	1.27613375	3.273857893
hsa_gci157926	2.281909431	1.190241532	up	4.629319432	3.4390779	ACATGACTTTTACTTAT	hsa_circ_0023780	chr11	77820487	77830295	-	560	ANNOTATED, CDS, c	NM_001007027	ALG8	Salzman2013	79053	circRNA	Detected	Detected	4.629319432	3.4390779
hsa_gci157937	2.270341196	1.182909128	up	4.3904743	3.207565172	TTTGTGAACTCATTTTC	hsa_circ_0023812	chr11	78180292	78204241	-	337	ANNOTATED, CDS, c	NM_024678	NARS2	Salzman2013	79731	circRNA	Detected	Detected	4.3904743	3.207565172
hsa_gci157949	2.331662262	1.221358831	up	10.75798866	9.536629832	GTCCTTCCTTTAGTGGA	hsa_circ_0023874	chr11	85374567	85374645	-	78	ANNOTATED, INTERN	NR_028025	CREBZF	Salzman2013	58487	circRNA	Detected	Detected	10.75798866	9.536629832
hsa_gci157967	2.940142123	1.555885895	up	6.1904543	4.634568405	CCCACACCTTATCCTGT	hsa_circ_0023911	chr11	85692787	85780923	-	1561	ANNOTATED, CDS, c	NM_001206947	PICALM	Salzman2013	8301	circRNA	Detected	Detected	6.1904543	4.634568405
hsa_gci157972	2.0803637	1.05683577	up	4.289124077	3.232288307	ATCCTCAAAAAGACACA	hsa_circ_0023921	chr11	85707868	85726006	-	806	ANNOTATED, CDS, c	NM_007166	PICALM	Salzman2013	8301	circRNA	Detected	Detected	4.289124077	3.232288307
hsa_gci157983	2.847144592	1.509515762	up	7.961701598	6.452185836	CGGCCATAAGTAAGAGA	hsa_circ_0023943	chr11	85737333	85780923	-	381	ANNOTATED, CDS, c	NM_001206947	PICALM	Salzman2013	8301	circRNA	Detected	Detected	7.961701598	6.452185836
hsa_gci157985	2.544339692	1.347291296	up	7.929361743	6.582070447	CGGCCATAAGTAAGAGA	hsa_circ_0023946	chr11	85742510	85780923	-	305	ANNOTATED, CDS, c	NM_001206947	PICALM	Salzman2013	8301	circRNA	Detected	Detected	7.929361743	6.582070447
hsa_gci157992	2.748890147	1.458849254	up	7.157339411	5.698490157	CCGGAGCCCCTTCGGCG	hsa_circ_0023967	chr11	86511490	86522273	+	3804	ANNOTATED, CDS, c	NM_007173	PRSS23	Salzman2013	11098	circRNA	Detected	Detected	7.157339411	5.698490157
hsa_gci158044	2.493745775	1.318314397	up	7.460360198	6.142045801	TGTCTGATACATCAAGA	hsa_circ_0024247	chr11	108559662	108594189	+	1117	ANNOTATED, CDS, c	NM_004398	DDX10	Salzman2013	1662	circRNA	Detected	Detected	7.460360198	6.142045801
hsa_gci158049	2.176663393	1.122118322	up	4.437376322	3.315258001	TTATGACTGTGTTCGAA	hsa_circ_0024273	chr11	111625222	111626174	-	342	ANNOTATED, CDS, c	NM_002716	PPP2R1B	Salzman2013	5519	circRNA	Detected	Detected	4.437376322	3.315258001
hsa_gci158090	2.877854468	1.524993637	up	5.482034816	3.957041178	GCAGGTGTCCAATTACA	hsa_circ_0024476	chr11	118461055	118461221	+	166	ANNOTATED, CDS, c	NM_001655	ARCN1	Salzman2013	372	circRNA	Detected	Detected	5.482034816	3.957041178
hsa_gci158110	2.064625474	1.045880098	up	8.768451698	7.722571599	GAAGGTACGGAATATCC	hsa_circ_0024604	chr11	120276826	120278532	+	110	ANNOTATED, CDS, c	NM_015313	ARHGEF12	Salzman2013	23365	circRNA	Detected	Detected	8.768451698	7.722571599
hsa_gci158144	2.661563566	1.412274023	up	7.483162392	6.070888369	GCAGGTCCCTCTATAAA	hsa_circ_0024783	chr11	125479328	125479484	+	156	ANNOTATED, CDS, c	NM_152713	STT3A	Salzman2013	3703	circRNA	Detected	Detected	7.483162392	6.070888369
hsa_gci158185	2.073561995	1.052111181	up	5.139451663	4.087340481	CAACACCGCCTACCTCA	hsa_circ_0025032	chr12	2966846	2975687	-	2517	ANNOTATED, CDS, c	NM_202002	FOXM1	Salzman2013	2305	circRNA	Detected	Detected	5.139451663	4.087340481
hsa_gci158191	2.408414719	1.268083839	up	6.54298428	5.27490044	TGACTGAACCGACTTGA	hsa_circ_0025061	chr12	4609303	4628046	-	712	ANNOTATED, CDS, c	NM_020374	C12orf4	Salzman2013	57102	circRNA	Detected	Detected	6.54298428	5.27490044
hsa_gci158210	3.27079474	1.709641225	up	8.875155277	7.165514052	AGTCGAAAGTACAGTGA	hsa_circ_0025214	chr12	6679247	6680198	-	776	ANNOTATED, CDS, c	NM_001273	CHD4	Salzman2013	1108	circRNA	Detected	Detected	8.875155277	7.165514052
hsa_gci158250	2.27079751	1.183199065	up	5.481877643	4.298678578	GGTTTACGACCTCTTGG	hsa_circ_0025463	chr12	11199618	11199787	-	169	ANNOTATED, INTERN	NR_037918	PRH1-PRR4	Salzman2013	100533464	circRNA	Detected	Detected	5.481877643	4.298678578
hsa_gci158251	2.802972857	1.486957774	up	7.65837253	6.171414756	CGACCAAGAAGTCAGCC	hsa_circ_0025472	chr12	12277498	12279855	-	466	ANNOTATED, CDS, c	NM_002336	LRP6	Salzman2013	4040	circRNA	Detected	Detected	7.65837253	6.171414756
hsa_gci158260	2.13770402	1.096062116	up	3.998785895	2.902723779	CTCTCCACCATTATTAG	hsa_circ_0025499	chr12	12653452	12715448	-	1294	ANNOTATED, CDS, c	NM_030640	DUSP16	Salzman2013	80824	circRNA	Detected	Not Detected	3.998785895	2.902723779
hsa_gci158291	-2.189395835	-1.130532813	down	2.825229049	3.955761861	ATCACCTCCTCAACGGT	hsa_circ_0025625	chr12	24982756	24989530	-	302	ANNOTATED, CDS, c	NM_005504	BCAT1	Salzman2013	586	circRNA	Not Detected	Detected	2.825229049	3.955761861
hsa_gci158326	2.579149039	1.366895143	up	6.615672654	5.248777511	CAGTCTGTCTTTCCTTC	hsa_circ_0025803	chr12	31555431	31600703	-	1320	ANNOTATED, CDS, c	NM_144973	DENND5B	Salzman2013	160518	circRNA	Detected	Detected	6.615672654	5.248777511
hsa_gci158331	2.568021132	1.360657074	up	4.747678793	3.387021719	CGGAAGACGGTGTCAGT	hsa_circ_0025826	chr12	31600471	31613315	-	957	ANNOTATED, CDS, c	NM_144973	DENND5B	Salzman2013	160518	circRNA	Detected	Detected	4.747678793	3.387021719
hsa_gci158332	3.159620534	1.659751303	up	5.22953797	3.569786667	TTTCACACCACTACGGA	hsa_circ_0025829	chr12	31604873	31613315	-	725	ANNOTATED, CDS, c	NM_144973	DENND5B	Salzman2013	160518	circRNA	Detected	Detected	5.22953797	3.569786667
hsa_gci158350	2.151642117	1.105438134	up	10.59572612	9.490287987	GGGCGCCGGCGGGGCGG	hsa_circ_0025928	chr12	44187525	44200178	-	3066	ANNOTATED, CDS, c	NM_001242397	TWF1	Salzman2013	5756	circRNA	Detected	Detected	10.59572612	9.490287987
hsa_gci158362	4.106684907	2.037974258	up	7.714119953	5.676145695	TTTTACTTTAGAACCTG	hsa_circ_0025986	chr12	46751970	46757609	-	3491	ANNOTATED, CDS, c	NM_018976	SLC38A2	Salzman2013	54407	circRNA	Detected	Detected	7.714119953	5.676145695
hsa_gci158366	2.342480079	1.228036779	up	10.91302131	9.68498453	AGAACCTGTGTAAGTAG	hsa_circ_0025993	chr12	46757508	46757609	-	101	ANNOTATED, CDS, c	NM_018976	SLC38A2	Salzman2013	54407	circRNA	Detected	Detected	10.91302131	9.68498453
hsa_gci158372	7.041301129	2.815842042	up	4.099083334	1.283241292	GGTCTAGAACTTGAACA	hsa_circ_0026043	chr12	49082240	49094856	-	6384	ANNOTATED, CDS, c	NM_001240	CCNT1	Salzman2013	904	circRNA	Detected	Not Detected	4.099083334	1.283241292
hsa_gci158379	2.257072401	1.174452697	up	3.997361843	2.822909146	ACCGGACCAGGGACCAT	hsa_circ_0026073	chr12	49329991	49334971	-	3377	ANNOTATED, CDS, c	NM_001659	ARF3	Salzman2013	377	circRNA	Detected	Not Detected	3.997361843	2.822909146
hsa_gci158380	2.24191751	1.164733196	up	8.027003305	6.862270109	TACTTCCTTCATATGTG	hsa_circ_0026080	chr12	49399525	49399635	-	110	ANNOTATED, CDS, c	NM_001206710	PRKAG1	Salzman2013	5571	circRNA	Detected	Detected	8.027003305	6.862270109
hsa_gci158381	2.087855618	1.062021948	up	6.043180957	4.981159009	CGATAGACTTCTTTACT	hsa_circ_0026081	chr12	49399525	49406893	-	159	ANNOTATED, CDS, c	NM_001206710	PRKAG1	Salzman2013	5571	circRNA	Detected	Detected	6.043180957	4.981159009
hsa_gci158434	6.617892775	2.726371917	up	5.092534159	2.366162242	CGTGGTACCACCTATGG	hsa_circ_0026294	chr12	51390599	51404549	-	869	ANNOTATED, CDS, c	NM_001174129	SLC11A2	Salzman2013	4891	circRNA	Detected	Not Detected	5.092534159	2.366162242
hsa_gci158440	2.534182339	1.341520333	up	11.09181673	9.750296397	GCGCCCCAACCGCGTCT	hsa_circ_0026317	chr12	51639132	51664202	-	1087	ANNOTATED, CDS, c	NM_001031628	SMAGP	Salzman2013	57228	circRNA	Detected	Detected	11.09181673	9.750296397
hsa_gci158442	2.648273488	1.405052118	up	8.852801165	7.447749048	GAAAGAAGAATTAAAGG	hsa_circ_0026334	chr12	52139686	52188425	+	2797	ANNOTATED, CDS, c	NM_014191	SCN8A	Salzman2013	6334	circRNA	Detected	Detected	8.852801165	7.447749048
hsa_gci158443	3.405525218	1.767877315	up	4.606332733	2.838455418	ATTATACCGTCGACTAT	hsa_circ_0026349	chr12	52385646	52385777	+	131	ANNOTATED, CDS, c	NM_020328	ACVR1B	Salzman2013	91	circRNA	Detected	Not Detected	4.606332733	2.838455418
hsa_gci158445	3.978893432	1.992367259	up	6.625477453	4.633110193	GGCCCTCGACGACTGTG	hsa_circ_0026371	chr12	52679696	52680277	-	581	ANNOTATED, CDS, c	NM_002281	KRT81	Salzman2013	3887	circRNA	Detected	Detected	6.625477453	4.633110193
hsa_gci158449	2.145475858	1.101297667	up	5.25578927	4.154491603	GGTATCGGTGACCTCTG	hsa_circ_0026391	chr12	52840434	52841209	-	775	ANNOTATED, CDS, c	NM_005555	KRT6B	Salzman2013	3854	circRNA	Detected	Detected	5.25578927	4.154491603
hsa_gci158463	-2.055711192	-1.039637593	down	3.647582065	4.687219658	ACGAGGTCCTTGGCATG	hsa_circ_0026472	chr12	53290970	53295856	-	1369	ANNOTATED, CDS, c	NM_002273	KRT8	Salzman2013	3856	circRNA	Not Detected	Detected	3.647582065	4.687219658
hsa_gci158466	3.42971061	1.778086851	up	5.183935558	3.405848708	CTCCTTCACCTGTCGTA	hsa_circ_0026476	chr12	53290970	53343626	-	46554	ALT_ACCEPTOR, CDS	NM_002273	KRT8	Salzman2013	3856	circRNA	Detected	Detected	5.183935558	3.405848708
hsa_gci158489	2.027543347	1.019732758	up	6.039620555	5.019887796	CACCCGGGGGCCTGCTG	hsa_circ_0026577	chr12	53646601	53648190	+	1589	ANNOTATED, CDS, c	NM_001170790	MFSD5	Salzman2013	84975	circRNA	Detected	Detected	6.039620555	5.019887796

hsa_gci158492	3.466457055	1.793461887	up	4.258186728	2.46472484	CTGGTACCTCACTCGGA	hsa_circ_0026605	chr12	53701239	53702133	-	493	ANNOTATED, CDS, c	NM_015665	AAAS	Salzman2013	8086	circRNA	Detected	Not Detected	4.258186728	2.46472484
hsa_gci158493	4.734792988	2.24330135	up	5.414268861	3.170967511	CCCCTGTGTCCAGAGAC	hsa_circ_0026609	chr12	53701239	53703065	-	864	ANNOTATED, CDS, c	NM_015665	AAAS	Salzman2013	8086	circRNA	Detected	Not Detected	5.414268861	3.170967511
hsa_gci158515	2.110742474	1.07775057	up	5.150461122	4.072710551	TGGTGGTGCCGACGGTG	hsa_circ_0026713	chr12	54676583	54679030	+	1145	ANNOTATED, CDS, c	NM_031157	HNRNPA1	Salzman2013	3178	circRNA	Detected	Detected	5.150461122	4.072710551
hsa_gci158516	2.294903154	1.198433272	up	11.69594692	10.49751365	TATAGGTGGTGGTGGTG	hsa_circ_0026714	chr12	54676583	54680872	+	2987	ALT_DONOR, CDS, c	NM_031157	HNRNPA1	Salzman2013	3178	circRNA	Detected	Detected	11.69594692	10.49751365
hsa_gci158518	-2.563098712	-1.357889042	down	2.609724139	3.967613181	CGATGACGACGACGACC	hsa_circ_0026717	chr12	54678041	54679030	+	758	ANNOTATED, CDS, c	NM_031157	HNRNPA1	Salzman2013	3178	circRNA	Not Detected	Detected	2.609724139	3.967613181
hsa_gci158522	2.4430298	1.288671461	up	6.155006729	4.866335268	CGAAACTGTCATATGTC	hsa_circ_0026724	chr12	54734330	54736071	+	151	ANNOTATED, CDS, c	NM_016057	COPZ1	Salzman2013	22818	circRNA	Detected	Detected	6.155006729	4.866335268
hsa_gci158534	2.142512507	1.099303626	up	10.49126928	9.391965657	CCCTGCACCCCGTCGTG	hsa_circ_0026807	chr12	56437902	56438007	+	105	ANNOTATED, CDS, c	NM_001029	RPS26	Salzman2013	6231	circRNA	Detected	Detected	10.49126928	9.391965657
hsa_gci158537	2.268002409	1.181422173	up	8.145521481	6.964099308	CTTCAAGGCAGATGCCG	hsa_circ_0026827	chr12	56510373	56511616	+	584	ANNOTATED, CDS, c	NM_001035267	RPL41	Salzman2013	6171	circRNA	Detected	Detected	8.145521481	6.964099308
hsa_gci158548	2.074402514	1.052695859	up	5.612792615	4.560096756	TCGGTCGTCCACTTAAG	hsa_circ_0026851	chr12	56526204	56532623	+	1551	ANNOTATED, CDS, c	NM_001184796	ESYT1	Salzman2013	23344	circRNA	Detected	Detected	5.612792615	4.560096756
hsa_gci158557	2.407857744	1.267750161	up	4.335070678	3.067320517	TCACAGGAAGGTCGCGC	hsa_circ_0026881	chr12	56546203	56551771	+	1064	ANNOTATED, CDS, c	NM_001199629	MYL6B	Salzman2013	140465	circRNA	Detected	Not Detected	4.335070678	3.067320517
hsa_gci158568	2.215270278	1.147482728	up	8.979898655	7.832415927	GGTAGACGACTCGGTTA	hsa_circ_0027028	chr12	56956200	56975047	+	556	ANNOTATED, CDS, c	NM_002898	RBMS2	Salzman2013	5939	circRNA	Detected	Detected	8.979898655	7.832415927
hsa_gci158570	2.354380055	1.235347226	up	9.238181128	8.002833902	TCATACCGGAATCACCT	hsa_circ_0027034	chr12	56962758	56975047	+	389	ANNOTATED, CDS, c	NM_002898	RBMS2	Salzman2013	5939	circRNA	Detected	Detected	9.238181128	8.002833902
hsa_gci158576	2.437800561	1.285580102	up	6.059520895	4.773940793	GTCTTCCCTAAGCCGGG	hsa_circ_0027070	chr12	57031958	57036364	-	787	ANNOTATED, CDS, c	NM_001686	ATP5F1B	Salzman2013	506	circRNA	Detected	Detected	6.059520895	4.773940793
hsa_gci158578	3.146722673	1.653850037	up	4.232032522	2.578182486	GGTCGTGGTGGTTTTTC	hsa_circ_0027072	chr12	57031958	57037371	-	1131	ANNOTATED, CDS, c	NM_001686	ATP5F1B	Salzman2013	506	circRNA	Detected	Not Detected	4.232032522	2.578182486
hsa_gci158591	2.694973091	1.430270868	up	4.156413942	2.726143074	GACGTGGTGTCCCGTAC	hsa_circ_0027272	chr12	57898007	57910438	+	1484	ANNOTATED, CDS, c	NM_004990	MARS	Salzman2013	4141	circRNA	Detected	Not Detected	4.156413942	2.726143074
hsa_gci158605	2.395592354	1.260382433	up	10.5472882	9.286905772	CTGTCAGCCCTCGAAAC	hsa_circ_0027333	chr12	58213709	58240747	-	5020	ANNOTATED, CDS, c	NM_005730	CTDSP2	Salzman2013	10106	circRNA	Detected	Detected	10.5472882	9.286905772
hsa_gci158623	-3.241902327	-1.696840626	down	2.841196483	4.538037108	AGACCAGACCCAGACGT	hsa_circ_0027425	chr12	65107221	65113962	-	3539	ANNOTATED, CDS, c	NM_002076	GNS	Salzman2013	2799	circRNA	Not Detected	Detected	2.841196483	4.538037108
hsa_gci158639	3.418141287	1.773212031	up	8.377190548	6.603978517	TGACCTCAAAAAGACAA	hsa_circ_0027465	chr12	69050880	69051493	+	613	ALT_DONOR, CDS, c	NM_015646	RAP1B	Salzman2013	5908	circRNA	Detected	Detected	8.377190548	6.603978517
hsa_gci158651	2.346624116	1.230586768	up	7.025117754	5.794530986	CGTGCCCTTCAGGTACA	hsa_circ_0027498	chr12	69326457	69326620	-	163	ANNOTATED, CDS, c	NM_001874	CPM	Salzman2013	1368	circRNA	Detected	Detected	7.025117754	5.794530986
hsa_gci158671	2.849407232	1.510661824	up	4.598943249	3.088281426	TCCAAAAACTCTCGTAA	hsa_circ_0027605	chr12	76443346	76454059	-	1072	ALT_DONOR, CDS, c	NM_004537	NAP1L1	Salzman2013	4673	circRNA	Detected	Not Detected	4.598943249	3.088281426
hsa_gci158679	2.560310869	1.35631899	up	5.040712833	3.684393843	TATTAAAAGTCCTTCTC	hsa_circ_0027628	chr12	78225068	78415642	+	2196	ANNOTATED, CDS, c	NM_014903	NAV3	Salzman2013	89795	circRNA	Detected	Detected	5.040712833	3.684393843
hsa_gci158680	3.04181122	1.60493062	up	5.879124425	4.274193805	CCAACCGGGTCAGTCAC	hsa_circ_0027635	chr12	78334098	78569207	+	4860	ANNOTATED, CDS, c	NM_014903	NAV3	Salzman2013	89795	circRNA	Detected	Detected	5.879124425	4.274193805
hsa_gci158681	4.548962437	2.185537522	up	6.607440785	4.421903263	AAACGATCCATCACCAA	hsa_circ_0027638	chr12	78334098	78592456	+	6209	ANNOTATED, CDS, c	NM_014903	NAV3	Salzman2013	89795	circRNA	Detected	Detected	6.607440785	4.421903263
hsa_gci158692	2.048255304	1.034395551	up	4.76744379	3.733048239	ACGAGACGAAGCCCCTC	hsa_circ_0027684	chr12	83379703	83424658	+	204	ANNOTATED, CDS, c	NM_152588	TMTC2	Salzman2013	160335	circRNA	Detected	Detected	4.76744379	3.733048239
hsa_gci158708	2.510985784	1.328253861	up	4.560850928	3.232597068	CGGTGACCACCGAAGAA	hsa_circ_0027755	chr12	95500729	95605043	-	3401	ANNOTATED, CDS, c	NM_018351	FGD6	Salzman2013	55785	circRNA	Detected	Detected	4.560850928	3.232597068
hsa_gci158727	3.142589056	1.651953629	up	4.566471831	2.914518202	TCCAGAAACACCCTTGA	hsa_circ_0027803	chr12	96672038	96683065	-	3084	ANNOTATED, CDS, c	NM_001170464	CDK17	Salzman2013	5128	circRNA	Detected	Not Detected	4.566471831	2.914518202
hsa_gci158728	4.305551815	2.106198151	up	4.349104216	2.242906066	ACACCGAACTTTTTAGA	hsa_circ_0027810	chr12	96704829	96728643	-	572	ANNOTATED, CDS, c	NM_001170464	CDK17	Salzman2013	5128	circRNA	Detected	Not Detected	4.349104216	2.242906066
hsa_gci158734	2.681116551	1.422833936	up	6.176461821	4.753627885	TTGACAGACACGTCAAG	hsa_circ_0027838	chr12	99128568	100219167	-	4382	ANNOTATED, CDS, c	NM_152788	ANKS1B	Salzman2013	56899	circRNA	Detected	Detected	6.176461821	4.753627885
hsa_gci158738	2.340030245	1.226527177	up	5.749476452	4.522949276	CCCTGTTTGTCTACCCT	hsa_circ_0027852	chr12	100491142	100497729	-	462	ANNOTATED, CDS, c	NM_015054	UHRF1BP1L	Salzman2013	23074	circRNA	Detected	Detected	5.749476452	4.522949276
hsa_gci158739	2.343588831	1.22871948	up	6.044157653	4.815438174	TTTGTCTACCCTACACA	hsa_circ_0027853	chr12	100492129	100497729	-	321	ANNOTATED, CDS, c	NM_015054	UHRF1BP1L	Salzman2013	23074	circRNA	Detected	Detected	6.044157653	4.815438174
hsa_gci158740	4.074933097	2.026776373	up	5.703962171	3.677185798	TGTTTGTCTACCCTACA	hsa_circ_0027854	chr12	100496543	100497729	-	131	ANNOTATED, CDS, c	NM_015054	UHRF1BP1L	Salzman2013	23074	circRNA	Detected	Detected	5.703962171	3.677185798
hsa_gci158756	2.470778018	1.3049654	up	5.95915339	4.654187989	ACTTTTAATTTATCGGA	hsa_circ_0027921	chr12	102492883	102512361	-	514	ANNOTATED, CDS, c	NM_024057	NUP37	Salzman2013	79023	circRNA	Detected	Detected	5.95915339	4.654187989
hsa_gci158759	2.184344553	1.127200441	up	8.907426674	7.780226233	TCAGAGCGCCCTTTGTA	hsa_circ_0027939	chr12	104335609	104341703	+	1367	ANNOTATED, CDS, c	NM_003299	HSP90B1	Salzman2013	7184	circRNA	Detected	Detected	8.907426674	7.780226233
hsa_gci158760	2.200646186	1.137927211	up	9.960886021	8.82295881	TGCATATTTTTCGAACT	hsa_circ_0027944	chr12	104370695	104374740	+	455	ANNOTATED, CDS, c	NM_003211	TDG	Salzman2013	6996	circRNA	Detected	Detected	9.960886021	8.82295881
hsa_gci158783	3.276127471	1.711991492	up	5.428544202	3.71655271	CCTTAACGGTGACCCTC	hsa_circ_0028052	chr12	108145191	108147886	-	981	ANNOTATED, CDS, c	NM_012406	PRDM4	Salzman2013	11108	circRNA	Detected	Detected	5.428544202	3.71655271
hsa_gci158790	2.004213214	1.003035995	up	5.557553574	4.554517579	GAAACCATCTCCTCTAC	hsa_circ_0028073	chr12	109048081	109096774	-	2535	ALT_ACCEPTOR, CDS	NM_014325	CORO1C	Salzman2013	23603	circRNA	Detected	Detected	5.557553574	4.554517579
hsa_gci158797	2.87415331	1.523137019	up	4.868437805	3.345300786	TGTATTTTTGCAAGGGG	hsa_circ_0028156	chr12	110405262	110429565	-	712	ANNOTATED, CDS, c	NM_057169	GIT2	Salzman2013	9815	circRNA	Detected	Detected	4.868437805	3.345300786
hsa_gci158833	2.256780566	1.174266147	up	6.433502121	5.259235974	AGTATATATGGAGGTAA	hsa_circ_0028268	chr12	111951160	111990781	-	1210	ANNOTATED, CDS, c	NM_002973	ATXN2	Salzman2013	6311	circRNA	Detected	Detected	6.433502121	5.259235974
hsa_gci158838	2.10561803	1.074243748	up	8.074340105	7.000096357	AGTTGTCTCTCTTTTTA	hsa_circ_0028287	chr12	112143541	112153766	+	749	ANNOTATED, CDS, c	NM_001136538	ACAD10	Salzman2013	80724	circRNA	Detected	Detected	8.074340105	7.000096357
hsa_gci158839	2.000917903	1.000661975	up	5.517362835	4.51670086	GAAGATTAAGGAAATGA	hsa_circ_0028289	chr12	112150301	112171872	+	850	ANNOTATED, CDS, c	NM_001136538	ACAD10	Salzman2013	80724	circRNA	Detected	Detected	5.517362835	4.51670086
hsa_gci158840	2.39163457	1.25799697	up	5.951530257	4.693533288	TTAGAACCGTTTAGGTT	hsa_circ_0028310	chr12	112308888	112308984	+	96	ANNOTATED, CDS, c	NM_139078	MAPKAPK5	Salzman2013	8550	circRNA	Detected	Detected	5.951530257	4.693533288
hsa_gci158862	2.473723308	1.306684141	up	7.045738038	5.739053898	GAGGTCCCACCGCACCT	hsa_circ_0028480	chr12	113705647	113728048	+	1498	ANNOTATED, CDS, c	NM_001143819	TPCN1	Salzman2013	53373	circRNA	Detected	Detected	7.045738038	5.739053898
hsa_gci158863	2.806183513	1.488609358	up	7.2369116	5.748302242	AATCCCCTCTTGTTCAT	hsa_circ_0028487	chr12	113710462	113711473	+	194	ANNOTATED, CDS, c	NM_001143819	TPCN1	Salzman2013	53373	circRNA	Detected	Detected	7.2369116	5.748302242
hsa_gci158868	3.089852876	1.627538146	up	8.141437855	6.513899709	CCTAACCCGACCCGGCG	hsa_circ_0028560	chr12	116413319	116413543	-	224	ANNOTATED, CDS, c	NM_015335	MED13L	Salzman2013	23389	circRNA	Detected	Detected	8.141437855	6.513899709
hsa_gci158883	2.117838295	1.082592439	up	4.637159821	3.554567382	TAAGTCCCAGACACCGG	hsa_circ_0028627	chr12	118456876	118458790	+	282	ANNOTATED, CDS, c	NM_181578	RFC5	Salzman2013	5985	circRNA	Detected	Detected	4.637159821	3.554567382
hsa_gci158886	3.226723289	1.690069863	up	4.609317985	2.919248121	GTAAAAACGATGAAGCC	hsa_circ_0028635	chr12	118480645	118481182	-	377	ANNOTATED, CDS, c	NM_018639	WSB2	Salzman2013	55884	circRNA	Detected	Not Detected	4.609317985	2.919248121
hsa_gci158905	-5.581130275	-2.480557322	down	1.375963542	3.856520864	CTGACTCTGTCGCACAT	hsa_circ_0028811	chr12	120599683	120611630	-	1150	ANNOTATED, CDS, c	NM_006836	GCN1	Salzman2013	10985	circRNA	Not Detected	Detected	1.375963542	3.856520864
hsa_gci158906	2.829093293	1.500339752	up	4.200260172	2.69992042	CGGTAAAAACTAGACCT	hsa_circ_0028876	chr12	120875903	120878529	+	545	ANNOTATED, CDS, c	NM_004373	COX6A1	Salzman2013	1337	circRNA	Detected	Not Detected	4.200260172	2.69992042
hsa_gci158907	2.334576965	1.223161151	up	4.265123523	3.041962372	TCGCGCTGCTTCTTCCA	hsa_circ_0028877	chr12	120876181	120878529	+	416	ANNOTATED, CDS, c	NM_004373	COX6A1	Salzman2013	1337	circRNA	Detected	Not Detected	4.265123523	3.041962372
hsa_gci158908	3.750332783	1.907018618	up	4.269800719	2.362782101	ATACCAATGGTAGAGGG	hsa_circ_0028878	chr12	120878256	120878529	+	273	ANNOTATED, CDS, c	NM_004373	COX6A1	Salzman2013	1337	circRNA	Detected	Not Detected	4.269800719	2.362782101
hsa_gci158910	2.420055406	1.275040078	up	7.322242703	6.047202625	GGCCGGGGTCCGAGAGG	hsa_circ_0028882	chr12	120907659	120936298	+	809	ANNOTATED, CDS, c	NM_001037494	DYNLL1	Salzman2013	8655	circRNA	Detected	Detected	7.322242703	6.047202625

hsa_gci158922	-3.662950556	-1.873006227	down	1.22109668	3.094102907	ATCTCACGTGTCTGGAA	hsa_circ_0028922	chr12	121132897	121139667	+	5607	ANNOTATED, CDS, c	NM_014730	MLEC	Salzman2013	9761	circRNA	Not Detected	Detected	1.22109668	3.094102907
hsa_gci158935	2.411906606	1.270174044	up	7.124604996	5.854430951	TCAGCCACTTTGTTGGA	hsa_circ_0028989	chr12	121970710	121972495	-	248	ANNOTATED, CDS, c	NM_032590	KDM2B	Salzman2013	84678	circRNA	Detected	Detected	7.124604996	5.854430951
hsa_gci158959	2.20676641	1.141933926	up	5.9600682	4.818134274	ACAAACTCTTACATAGA	hsa_circ_0029130	chr12	123061441	123109235	+	4020	ANNOTATED, CDS, c	NM_014708	KNTC1	Salzman2013	9735	circRNA	Detected	Detected	5.9600682	4.818134274
hsa_gci158962	2.366959798	1.243035203	up	5.877526029	4.634490827	AAAGGAATCTATTCCTT	hsa_circ_0029142	chr12	123100016	123103092	+	176	ANNOTATED, CDS, c	NM_014708	KNTC1	Salzman2013	9735	circRNA	Detected	Detected	5.877526029	4.634490827
hsa_gci158966	-3.652002734	-1.868687845	down	1.357128619	3.225816464	AACCGTAACAAAAAGGT	hsa_circ_0029179	chr12	123645682	123707674	-	4265	ALT_ACCEPTOR, CDS	NM_022782	MPHOSPH9	Salzman2013	10198	circRNA	Not Detected	Detected	1.357128619	3.225816464
hsa_gci158976	-2.836707985	-1.504217645	down	4.207234751	5.711452395	GGTTTAACTTATCGACA	hsa_circ_0029197	chr12	123694602	123707674	-	2078	ALT_ACCEPTOR, CDS	NM_022782	MPHOSPH9	Salzman2013	10198	circRNA	Detected	Detected	4.207234751	5.711452395
hsa_gci158982	2.466309126	1.302353638	up	6.143341336	4.840987698	AGTCCTCGTCATTGAAC	hsa_circ_0029211	chr12	123799919	123800207	-	185	ANNOTATED, CDS, c	NM_001167856	SBNO1	Salzman2013	55206	circRNA	Detected	Detected	6.143341336	4.840987698
hsa_gci158998	2.135500463	1.094574211	up	7.176773085	6.082198874	CCTTAACCGTTATAAGT	hsa_circ_0029263	chr12	124156602	124171582	+	574	ANNOTATED, CDS, c	NM_024809	TCTN2	Salzman2013	79867	circRNA	Detected	Detected	7.176773085	6.082198874
hsa_gci159001	2.353661605	1.234906913	up	7.281869653	6.04696274	CTTCGTCCTGGCAGACT	hsa_circ_0029269	chr12	124171382	124171582	+	200	ANNOTATED, CDS, c	NM_024809	TCTN2	Salzman2013	79867	circRNA	Detected	Detected	7.281869653	6.04696274
hsa_gci159010	2.095927208	1.067588613	up	7.823994273	6.756405661	TAGTTTGTACCAGTCGG	hsa_circ_0029387	chr12	129277738	129293501	-	1647	ANNOTATED, CDS, c	NM_145648	SLC15A4	Salzman2013	121260	circRNA	Detected	Detected	7.823994273	6.756405661
hsa_gci159021	2.005136332	1.003700331	up	6.218742156	5.215041825	AGTGTCCAGTAGTAGGA	hsa_circ_0029430	chr12	131360426	131360826	+	400	ANNOTATED, CDS, c	NM_006325	RAN	Salzman2013	5901	circRNA	Detected	Detected	6.218742156	5.215041825
hsa_gci159022	2.235927415	1.160873355	up	8.070578468	6.909705113	GGCCGGTACGCGCCGCT	hsa_circ_0029450	chr12	132414451	132428406	+	1485	ANNOTATED, CDS, c	NM_025215	PUS1	Salzman2013	80324	circRNA	Detected	Detected	8.070578468	6.909705113
hsa_gci159024	2.380981753	1.251556564	up	6.516963445	5.26540688	GAATAAGTGGACCAAAC	hsa_circ_0029486	chr12	133200347	133202903	-	1466	ANNOTATED, CDS, c	NM_006231	POLE	Salzman2013	5426	circRNA	Detected	Detected	6.516963445	5.26540688
hsa_gci159028	2.031000926	1.022190898	up	6.649717534	5.627526636	GTGACCTCCTTTGTAGA	hsa_circ_0029491	chr12	133200347	133235260	-	5225	ALT_ACCEPTOR, CDS	NM_006231	POLE	Salzman2013	5426	circRNA	Detected	Detected	6.649717534	5.627526636
hsa_gci159029	3.345790149	1.742346961	up	9.25634378	7.513996819	CGGTCGGTAGAGGTACC	hsa_circ_0029492	chr12	133200347	133241048	-	5328	ANNOTATED, CDS, c	NM_006231	POLE	Salzman2013	5426	circRNA	Detected	Detected	9.25634378	7.513996819
hsa_gci159040	3.179674247	1.668878971	up	5.914486509	4.245607538	TGGTCGATTATAGTACT	hsa_circ_0029579	chr12	133393125	133398897	-	589	ANNOTATED, CDS, c	NM_005895	GOLGA3	Salzman2013	2802	circRNA	Detected	Detected	5.914486509	4.245607538
hsa_gci159047	2.811372099	1.491274414	up	5.59421411	4.102939696	CTGTCCCGACTTCCGCT	hsa_circ_0029619	chr13	20534097	20544211	+	116	ANNOTATED, coding	NM_003453	ZMYM2	Salzman2013	7750	circRNA	Detected	Detected	5.59421411	4.102939696
hsa_gci159083	2.767993826	1.468840725	up	3.96467913	2.495838405	CTTCATGAAGGTTCTAA	hsa_circ_0029784	chr13	25881943	25912869	+	1523	ANNOTATED, CDS, c	NM_014089	NUP58	Salzman2013	9818	circRNA	Detected	Not Detected	3.96467913	2.495838405
hsa_gci159096	4.142922217	2.050648734	up	5.188088177	3.137439443	ATTTCGTGGAGGTAGGT	hsa_circ_0029837	chr13	28748408	28846450	+	1861	ALT_DONOR, CDS, c	NM_175854	PAN3	Salzman2013	255967	circRNA	Detected	Not Detected	5.188088177	3.137439443
hsa_gci159129	3.01916222	1.594148275	up	5.837572306	4.243424031	AATACAGATGATAACCC	hsa_circ_0029930	chr13	32899212	32900750	+	315	ANNOTATED, CDS, c	NM_000059	BRCA2	Salzman2013	675	circRNA	Detected	Detected	5.837572306	4.243424031
hsa_gci159155	-2.382244517	-1.252321501	down	2.138970216	3.391291717	TGAAACGTTATCCTCCG	hsa_circ_0029984	chr13	35622699	35644989	+	848	ANNOTATED, CDS, c	NM_015678	NBEA	Salzman2013	26960	circRNA	Not Detected	Detected	2.138970216	3.391291717
hsa_gci159158	2.302493023	1.203196785	up	5.031597836	3.828401051	TCGTGTCCTCCGTCATG	hsa_circ_0030003	chr13	37427555	37447052	-	848	ANNOTATED, CDS, c	NM_001127217	SMAD9	Salzman2013	4093	circRNA	Detected	Detected	5.031597836	3.828401051
hsa_gci159162	2.388196561	1.255921583	up	6.35108652	5.095164937	AGCCGGGTCCTTTTTAT	hsa_circ_0030026	chr13	39596462	39605768	-	619	ANNOTATED, CDS, c	NM_025138	PROSER1	Salzman2013	80209	circRNA	Detected	Detected	6.35108652	5.095164937
hsa_gci159167	2.126650926	1.088583245	up	6.526876077	5.438292832	AATCACGTCTTGAACCA	hsa_circ_0030038	chr13	40293372	40365802	+	3873	ANNOTATED, CDS, c	NM_001145079	COG6	Salzman2013	57511	circRNA	Detected	Detected	6.526876077	5.438292832
hsa_gci159173	2.026476799	1.018973659	up	5.250680486	4.231706827	CGACCCCTTAGACCAGC	hsa_circ_0030051	chr13	41515056	41518061	-	727	ANNOTATED, CDS, c	NM_172373	ELF1	Salzman2013	1997	circRNA	Detected	Detected	5.250680486	4.231706827
hsa_gci159175	3.044975247	1.6064305	up	7.486083478	5.879652978	GACGTTGGTCGCACCCG	hsa_circ_0030064	chr13	41834628	41836467	-	1839	ALT_ACCEPTOR, CDS	NM_004294	MTRF1	Salzman2013	9617	circRNA	Detected	Detected	7.486083478	5.879652978
hsa_gci159194	2.095138341	1.067045507	up	9.558227949	8.491182442	CGCATTGTAGCGCCCCC	hsa_circ_0030172	chr13	45978461	45980112	-	181	ANNOTATED, CDS, c	NM_001010875	SLC25A30	Salzman2013	253512	circRNA	Detected	Detected	9.558227949	8.491182442
hsa_gci159213	2.411316503	1.269821028	up	4.937055301	3.667234273	TTATATGACTTATTGCG	hsa_circ_0030240	chr13	49580287	49688867	+	291	ANNOTATED, CDS, c	NM_001079673	FNDC3A	Salzman2013	22862	circRNA	Detected	Detected	4.937055301	3.667234273
hsa_gci159240	2.581128957	1.368002223	up	6.73674174	5.368739517	ATCGCGGCAATCACGGT	hsa_circ_0030356	chr13	53196123	53203917	+	981	ALT_DONOR, coding	NM_001011724	HNRNPA1L2	Salzman2013	144983	circRNA	Detected	Detected	6.73674174	5.368739517
hsa_gci159246	2.658431767	1.410575438	up	8.402968931	6.992393493	CTACAGTGACCCCTCCT	hsa_circ_0030380	chr13	61018816	61041513	+	369	ANNOTATED, CDS, c	NM_001146070	TDRD3	Salzman2013	81550	circRNA	Detected	Detected	8.402968931	6.992393493
hsa_gci159248	2.852427292	1.512190113	up	5.038450672	3.526260559	AGTATCGCATCTCTCAA	hsa_circ_0030387	chr13	64560503	64608670	-	1108	ANNOTATED, ncRNA,	TCONS_12_00007568		Salzman2013		circRNA	Detected	Detected	5.038450672	3.526260559
hsa_gci159272	2.020472529	1.014692736	up	6.010094101	4.995401365	GACTGTTGTTTCGGTAG	hsa_circ_0030491	chr13	77713330	77837924	-	6228	ANNOTATED, CDS, c	NM_015057	MYCBP2	Salzman2013	23077	circRNA	Detected	Detected	6.010094101	4.995401365
hsa_gci159278	2.225416463	1.154075346	up	8.063089229	6.909013883	TGGTCGTTTATGTTCCC	hsa_circ_0030511	chr13	77791974	77807398	-	428	ANNOTATED, CDS, c	NM_015057	MYCBP2	Salzman2013	23077	circRNA	Detected	Detected	8.063089229	6.909013883
hsa_gci159279	2.016249074	1.011673871	up	7.662108908	6.650435038	CTACCAAGTATTTTTAT	hsa_circ_0030514	chr13	77798585	77807398	-	310	ANNOTATED, CDS, c	NM_015057	MYCBP2	Salzman2013	23077	circRNA	Detected	Detected	7.662108908	6.650435038
hsa_gci159286	2.075330767	1.053341292	up	6.285985543	5.232644252	CCCCTCGACAGATCCAA	hsa_circ_0030545	chr13	80055258	80122529	+	987	ANNOTATED, CDS, c	NM_019080	NDFIP2	Salzman2013	54602	circRNA	Detected	Detected	6.285985543	5.232644252
hsa_gci159292	2.163213765	1.113176237	up	8.16971363	7.056537393	TCCACCGTGCCCTATTT	hsa_circ_0030552	chr13	80107457	80117817	+	353	ANNOTATED, CDS, c	NM_019080	NDFIP2	Salzman2013	54602	circRNA	Detected	Detected	8.16971363	7.056537393
hsa_gci159293	6.773492488	2.759899895	up	4.437923838	1.678023942	ACCTGTGAGAAGGAGGA	hsa_circ_0030553	chr13	80113816	80113910	+	94	ANNOTATED, CDS, c	NM_019080	NDFIP2	Salzman2013	54602	circRNA	Detected	Not Detected	4.437923838	1.678023942
hsa_gci159306	3.560673159	1.832150014	up	8.303638643	6.471488629	CCGGTTAAGGGGCTCGT	hsa_circ_0030593	chr13	95822785	95839146	-	471	ANNOTATED, CDS, c	NM_005845	ABCC4	Salzman2013	10257	circRNA	Detected	Detected	8.303638643	6.471488629
hsa_gci159307	-4.853712704	-2.279088716	down	1.224831271	3.503919987	TCCGGAACATCTCAACC	hsa_circ_0030594	chr13	95822785	95840796	-	561	ANNOTATED, CDS, c	NM_005845	ABCC4	Salzman2013	10257	circRNA	Not Detected	Detected	1.224831271	3.503919987
hsa_gci159319	2.022152565	1.015891848	up	10.29867596	9.282784112	AGAGAAACCATTTCCTA	hsa_circ_0030654	chr13	98009735	98046374	+	3084	ANNOTATED, CDS, c	NM_144778	MBNL2	Salzman2013	10150	circRNA	Detected	Detected	10.29867596	9.282784112
hsa_gci159321	4.382362243	2.131708741	up	6.671528279	4.539819537	CCGGACCCTATAAGAGT	hsa_circ_0030662	chr13	98634756	98670958	+	2746	ANNOTATED, CDS, c	NM_002271	IPO5	Salzman2013	3843	circRNA	Detected	Detected	6.671528279	4.539819537
hsa_gci159353	2.899648173	1.535877862	up	7.327203845	5.791325982	CCAGTGTAAATCAAAGC	hsa_circ_0030777	chr13	101077886	101101559	+	153	ANNOTATED, CDS, c	NM_000282	PCCA	Salzman2013	5095	circRNA	Detected	Detected	7.327203845	5.791325982
hsa_gci159362	2.207836489	1.142633331	up	5.353010991	4.21037766	AGGGCCGACGGGATGCA	hsa_circ_0030869	chr13	111088615	111165373	+	5248	ANNOTATED, CDS, c	NM_001846	COL4A2	Salzman2013	1284	circRNA	Detected	Detected	5.353010991	4.21037766
hsa_gci159370	2.181235413	1.125145483	up	6.820243348	5.695097864	AAGCACTCACCGTGCCG	hsa_circ_0030900	chr13	111530886	111536140	-	1669	ANNOTATED, CDS, c	NM_017664	ANKRD10	Salzman2013	55608	circRNA	Detected	Detected	6.820243348	5.695097864
hsa_gci159385	2.348986278	1.232038286	up	4.435436134	3.203397848	CAGTTAACGAGATTAAT	hsa_circ_0030981	chr13	113832487	113838801	-	681	ANNOTATED, CDS, c	NM_001127203	PCID2	Salzman2013	55795	circRNA	Detected	Detected	4.435436134	3.203397848
hsa_gci159392	3.442205848	1.783333375	up	6.409654173	4.626320798	CCCTCGGCAACCGTCGA	hsa_circ_0031005	chr13	113975871	113977741	+	1316	ANNOTATED, CDS, c	NM_005561	LAMP1	Salzman2013	3916	circRNA	Detected	Detected	6.409654173	4.626320798
hsa_gci159400	2.230831928	1.157581824	up	9.436534713	8.278952889	GCCGTCTTACTCTCTGT	hsa_circ_0031034	chr13	114202617	114204544	+	955	ANNOTATED, CDS, c	NM_017905	TMCO3	Salzman2013	55002	circRNA	Detected	Detected	9.436534713	8.278952889
hsa_gci159416	2.000158962	1.000114662	up	8.094569674	7.094455012	AATCCTAAAAAAGAAGT	hsa_circ_0031189	chr14	21868437	21871822	-	1292	ALT_DONOR, CDS, c	NM_001170629	CHD8	Salzman2013	57680	circRNA	Detected	Detected	8.094569674	7.094455012
hsa_gci159422	3.033245827	1.600862425	up	8.385357774	6.784495349	AGAGGTCTTGAGAATAG	hsa_circ_0031209	chr14	23033806	23034037	-	231	ANNOTATED, coding	NM_001344	DAD1	Salzman2013	1603	circRNA	Detected	Detected	8.385357774	6.784495349
hsa_gci159446	2.081673989	1.057744146	up	5.937845882	4.880101737	GACAGAGGACACGTCCG	hsa_circ_0031294	chr14	24027903	24028787	+	884	ANNOTATED, CDS, c	NM_024328	THTPA	Salzman2013	79178	circRNA	Detected	Detected	5.937845882	4.880101737
hsa_gci159447	2.661385167	1.412177318	up	9.71080273	8.298625412	AAGTAACGTCAAGTGAC	hsa_circ_0031323	chr14	24606908	24608176	+	784	ANNOTATED, CDS, c	NM_176783	PSME1	Salzman2013	5720	circRNA	Detected	Detected	9.71080273	8.298625412

hsa_gci159467	2.159226252	1.110514422	up	9.285329115	8.174814693	TTCAATACTGTTCCCTA	hsa_circ_0031448	chr14	31535331	31535540	+	209	ANNOTATED, CDS, c	NM_007077	AP4S1	Salzman2013	11154	circRNA	Detected	Detected	9.285329115	8.174814693
hsa_gci159489	2.769615391	1.469685647	up	5.773305713	4.303620066	GAGAAGAGTCATTGTTC	hsa_circ_0031540	chr14	31787429	31806820	-	810	ANNOTATED, CDS, c	NM_015473	HEATR5A	Salzman2013	25938	circRNA	Detected	Detected	5.773305713	4.303620066
hsa_gci159492	-3.106236622	-1.635167733	down	2.127809474	3.762977207	TGTTGTCGTCTTGAGCT	hsa_circ_0031544	chr14	31803517	31844174	-	4823	ALT_DONOR, CDS, c	NM_015473	HEATR5A	Salzman2013	25938	circRNA	Not Detected	Detected	2.127809474	3.762977207
hsa_gci159512	2.544262192	1.347247351	up	4.160446615	2.813199264	GAAGGAGCAAACCCATC	hsa_circ_0031615	chr14	35227909	35231429	-	610	ANNOTATED, CDS, c	NM_013448	BAZ1A	Salzman2013	11177	circRNA	Detected	Not Detected	4.160446615	2.813199264
hsa_gci159516	4.222177406	2.077987199	up	4.287213298	2.2092261	CCGACAACGTGTCTTAC	hsa_circ_0031632	chr14	35261980	35331528	-	1397	ANNOTATED, CDS, c	NM_013448	BAZ1A	Salzman2013	11177	circRNA	Detected	Not Detected	4.287213298	2.2092261
hsa_gci159529	2.102265853	1.071945125	up	8.826754197	7.754809072	GGTCTCCACTCACCTGA	hsa_circ_0031665	chr14	36017713	36147308	-	3181	ANNOTATED, CDS, c	NM_014990	RALGAPA1	Salzman2013	253959	circRNA	Detected	Detected	8.826754197	7.754809072
hsa_gci159534	2.190281314	1.131116177	up	8.779676733	7.648560555	ACTCGCCCCTTCATAAA	hsa_circ_0031675	chr14	36133847	36159209	-	1544	ANNOTATED, CDS, c	NM_014990	RALGAPA1	Salzman2013	253959	circRNA	Detected	Detected	8.779676733	7.648560555
hsa_gci159538	2.504603841	1.324582427	up	4.182283221	2.857700793	AACGTAGGTGGACTGAA	hsa_circ_0031693	chr14	36302183	36304129	+	208	ANNOTATED, CDS, c	NM_032352	BRMS1L	Salzman2013	84312	circRNA	Detected	Not Detected	4.182283221	2.857700793
hsa_gci159558	3.891207636	1.960217965	up	4.402676734	2.442458769	GCCTAGTCTGGCACGGC	hsa_circ_0031775	chr14	50050289	50052767	-	204	ANNOTATED, CDS, c	NM_001032	RPS29	Salzman2013	6235	circRNA	Detected	Not Detected	4.402676734	2.442458769
hsa_gci159566	2.353073704	1.23454651	up	6.086461612	4.851915102	CGTACCCGTCGTGCTCC	hsa_circ_0031797	chr14	50246312	50249856	+	773	ANNOTATED, CDS, c	NM_014315	KLHDC2	Salzman2013	23588	circRNA	Detected	Detected	6.086461612	4.851915102
hsa_gci159591	2.365950082	1.242419635	up	4.063618368	2.821198732	TATACCGTCTCGGTCCT	hsa_circ_0031884	chr14	51374988	51387785	-	1719	ANNOTATED, CDS, c	NM_002863	PYGL	Salzman2013	5836	circRNA	Detected	Not Detected	4.063618368	2.821198732
hsa_gci159618	3.334803749	1.737601862	up	6.767413368	5.029811506	TATAACGGGTGTCCCTC	hsa_circ_0032014	chr14	55467220	55467710	-	249	ANNOTATED, CDS, c	NM_007086	WDHD1	Salzman2013	11169	circRNA	Detected	Detected	6.767413368	5.029811506
hsa_gci159655	2.192553435	1.132612003	up	5.184568048	4.051956045	CCGCCGAATCTCGCCAG	hsa_circ_0032156	chr14	64063756	64108591	-	3111	ANNOTATED, CDS, c	NM_080666	WDR89	Salzman2013	112840	circRNA	Detected	Detected	5.184568048	4.051956045
hsa_gci159673	2.0204252	1.014658941	up	6.539624146	5.524965205	AGGAAATCAAAGAGATC	hsa_circ_0032276	chr14	68143518	68157138	-	2022	ANNOTATED, CDS, c	NM_016026	RDH11	Salzman2013	51109	circRNA	Detected	Detected	6.539624146	5.524965205
hsa_gci159688	2.850630036	1.511280814	up	5.52656757	4.015286756	CGCACCACCACTAAAAC	hsa_circ_0032348	chr14	69919957	69921614	+	155	ANNOTATED, CDS, c	NM_018375	SLC39A9	Salzman2013	55334	circRNA	Detected	Detected	5.52656757	4.015286756
hsa_gci159762	2.128598142	1.089903609	up	4.522949276	3.433045667	GACGACTTTGTTAGACT	hsa_circ_0032674	chr14	75576387	75587283	-	729	ANNOTATED, CDS, c	NM_033116	NEK9	Salzman2013	91754	circRNA	Detected	Detected	4.522949276	3.433045667
hsa_gci159783	2.87227689	1.522194833	up	4.084598337	2.562403504	GGTTGTCCAGACCGGAC	hsa_circ_0032780	chr14	77931881	77935815	+	679	ANNOTATED, CDS, c	NM_012111	AHSA1	Salzman2013	10598	circRNA	Detected	Not Detected	4.084598337	2.562403504
hsa_gci159796	2.295921697	1.199073439	up	5.941386377	4.742312938	ATGATCTTCAGCCCTCC	hsa_circ_0032849	chr14	88450737	88454867	-	387	ANNOTATED, CDS, c	NM_000153	GALC	Salzman2013	2581	circRNA	Detected	Detected	5.941386377	4.742312938
hsa_gci159831	2.666094643	1.414727995	up	5.5921558	4.177427805	CCGGACAACTAGTGTTA	hsa_circ_0033007	chr14	93180167	93183806	-	307	ANNOTATED, CDS, c	NM_001008530	LGMN	Salzman2013	5641	circRNA	Detected	Detected	5.5921558	4.177427805
hsa_gci159835	-2.122747161	-1.085932543	down	2.127875778	3.213808321	ACGAGAGTCGTTACTAT	hsa_circ_0033033	chr14	93703895	93730339	-	6960	ANNOTATED, CDS, c	NM_001002860	BTBD7	Salzman2013	55727	circRNA	Not Detected	Detected	2.127875778	3.213808321
hsa_gci159836	2.516841519	1.331614376	up	7.099880225	5.768265849	GGCGGTAAAACTGAGGA	hsa_circ_0033034	chr14	93703895	93799385	-	8448	ANNOTATED, CDS, c	NM_001002860	BTBD7	Salzman2013	55727	circRNA	Detected	Detected	7.099880225	5.768265849
hsa_gci159838	4.156533429	2.055380815	up	4.446134394	2.39075358	TAGGTCGTCGGAGAAAA	hsa_circ_0033041	chr14	93730130	93762503	-	1477	ANNOTATED, CDS, c	NM_001002860	BTBD7	Salzman2013	55727	circRNA	Detected	Not Detected	4.446134394	2.39075358
hsa_gci159839	3.522559777	1.816624189	up	5.522331543	3.705707354	GGTGAAGCCACCTGTAT	hsa_circ_0033059	chr14	94517267	94521530	-	860	ANNOTATED, CDS, c	NM_020414	DDX24	Salzman2013	57062	circRNA	Detected	Detected	5.522331543	3.705707354
hsa_gci159859	2.229663085	1.156825727	up	4.216337461	3.059511734	GTCTTTTGCGTACGAAT	hsa_circ_0033174	chr14	100800124	100803539	-	1390	ANNOTATED, CDS, c	NM_004184	WARS	Salzman2013	7453	circRNA	Detected	Not Detected	4.216337461	3.059511734
hsa_gci159861	2.418267187	1.273973652	up	5.872191116	4.598217463	CCTAAACAAGTCGTCTG	hsa_circ_0033178	chr14	100800124	100835595	-	2576	ANNOTATED, CDS, c	NM_004184	WARS	Salzman2013	7453	circRNA	Detected	Detected	5.872191116	4.598217463
hsa_gci159862	2.308970253	1.207249587	up	5.792688429	4.585438842	GGTTACTTTGGGAGAAC	hsa_circ_0033179	chr14	100800124	100841927	-	2884	ANNOTATED, CDS, c	NM_004184	WARS	Salzman2013	7453	circRNA	Detected	Detected	5.792688429	4.585438842
hsa_gci159867	2.129562609	1.090557146	up	7.933635888	6.843078742	TGTGGTTGTAGAAATGA	hsa_circ_0033188	chr14	100820529	100828258	-	697	ALT_DONOR, CDS, c	NM_004184	WARS	Salzman2013	7453	circRNA	Detected	Detected	7.933635888	6.843078742
hsa_gci159908	2.157893681	1.109623785	up	10.03073298	8.921109198	CAAATAAACAGTAGATC	hsa_circ_0033406	chr14	102842986	102843277	+	291	ANNOTATED, CDS, c	NM_014844	TECPR2	Salzman2013	9895	circRNA	Detected	Detected	10.03073298	8.921109198
hsa_gci159909	2.29649611	1.19943434	up	10.96152372	9.762089383	TCCTTACAAATAAACAG	hsa_circ_0033408	chr14	102842986	102931626	+	3861	ANNOTATED, CDS, c	NM_014844	TECPR2	Salzman2013	9895	circRNA	Detected	Detected	10.96152372	9.762089383
hsa_gci159922	3.014892062	1.592106352	up	5.619804584	4.027698232	GCAAAGACTACCGTCAT	hsa_circ_0033481	chr14	103928741	103941547	+	999	ANNOTATED, CDS, c	NM_001128918	MARK3	Salzman2013	4140	circRNA	Detected	Detected	5.619804584	4.027698232
hsa_gci159930	3.213624359	1.684201302	up	4.688504899	3.004303597	GTCACAGAAACAGGAGG	hsa_circ_0033506	chr14	104128452	104152568	+	1803	ANNOTATED, CDS, c	NM_005552	KLC1	Salzman2013	3831	circRNA	Detected	Not Detected	4.688504899	3.004303597
hsa_gci159944	3.049055848	1.608362576	up	8.982338236	7.373975661	CCGGAGCCCTCGCCGAA	hsa_circ_0033590	chr14	105715118	105717430	+	1968	ANNOTATED, CDS, c	NM_033271	BTBD6	Salzman2013	90135	circRNA	Detected	Detected	8.982338236	7.373975661
hsa_gci159952	2.615347829	1.387002831	up	8.167768162	6.780765332	AGACAGAAGTTTCAATC	hsa_circ_0034067	chr15	22925975	22926065	+	90	ANNOTATED, CDS, c	NM_014608	CYFIP1	Salzman2013	23191	circRNA	Detected	Detected	8.167768162	6.780765332
hsa_gci159971	2.597018807	1.376856461	up	8.658602399	7.281745937	CAAGTCCTCGTGCCCTT	hsa_circ_0034189	chr15	28090104	28096621	-	188	ANNOTATED, CDS, c	NM_000275	OCA2	Salzman2013	4948	circRNA	Detected	Detected	8.658602399	7.281745937
hsa_gci159977	3.834189498	1.938921641	up	4.856206336	2.917284695	CTGCGTCGGAGGGTTCG	hsa_circ_0034269	chr15	28834637	28836269	+	1023	ANNOTATED, ncRNA,	TCONS_12_00009409		Salzman2013		circRNA	Detected	Not Detected	4.856206336	2.917284695
hsa_gci159978	2.272610602	1.184350508	up	4.282068074	3.097717566	AGAGCAGGTGGTCTATA	hsa_circ_0034284	chr15	30010224	30065560	-	3890	ANNOTATED, CDS, c	NM_003257	TJP1	Salzman2013	7082	circRNA	Detected	Not Detected	4.282068074	3.097717566
hsa_gci159980	2.424674274	1.277790952	up	4.528070519	3.250279567	AGGTGGTCTATAACGTT	hsa_circ_0034291	chr15	30034845	30065560	-	1066	ANNOTATED, CDS, c	NM_003257	TJP1	Salzman2013	7082	circRNA	Detected	Detected	4.528070519	3.250279567
hsa_gci159990	4.027348426	2.009830292	up	7.060915939	5.051085646	CCTACCCGACTCGTCCA	hsa_circ_0034350	chr15	34633916	34634309	-	393	ANNOTATED, CDS, c	NM_018648	NOP10	Salzman2013	55505	circRNA	Detected	Detected	7.060915939	5.051085646
hsa_gci159994	2.107169401	1.075306301	up	5.999079685	4.923773384	TATTATCTGAACCTCCG	hsa_circ_0034402	chr15	36983885	36989591	+	198	ANNOTATED, CDS, c	NM_032499	C15orf41	Salzman2013	84529	circRNA	Detected	Detected	5.999079685	4.923773384
hsa_gci159999	2.350021957	1.232674236	up	6.865666192	5.632991956	CACTCCTCCTGTGACCA	hsa_circ_0034460	chr15	39876188	39886641	+	2802	ANNOTATED, CDS, c	NM_003246	THBS1	Salzman2013	7057	circRNA	Detected	Detected	6.865666192	5.632991956
hsa_gci160006	2.496002496	1.319619377	up	6.38638219	5.066762813	CTACTGGTGCCCCTGGT	hsa_circ_0034484	chr15	39880242	39889668	+	4317	ANNOTATED, CDS, c	NM_003246	THBS1	Salzman2013	7057	circRNA	Detected	Detected	6.38638219	5.066762813
hsa_gci160012	2.254853656	1.173033803	up	8.241211566	7.068177763	CAGGGTAGGCACGTCCC	hsa_circ_0034515	chr15	39882005	39889668	+	3685	ANNOTATED, CDS, c	NM_003246	THBS1	Salzman2013	7057	circRNA	Detected	Detected	8.241211566	7.068177763
hsa_gci160054	-3.620092502	-1.856026562	down	1.972746514	3.828773076	ACACCTCTCGTCCGGAC	hsa_circ_0034673	chr15	41361767	41379880	-	1245	ANNOTATED, CDS, c	NM_017553	INO80	Salzman2013	54617	circRNA	Not Detected	Detected	1.972746514	3.828773076
hsa_gci160056	2.821058602	1.496236635	up	5.113178929	3.616942293	GCTTATGCGTCCGCGGT	hsa_circ_0034690	chr15	41624891	41673248	+	2454	ANNOTATED, CDS, c	NM_016359	NUSAP1	Salzman2013	51203	circRNA	Detected	Detected	5.113178929	3.616942293
hsa_gci160060	2.936405708	1.554051311	up	7.775681297	6.221629986	GTCGTCCGACCATCGGT	hsa_circ_0034708	chr15	41809374	41819499	-	2936	ANNOTATED, CDS, c	NM_015540	RPAP1	Salzman2013	26015	circRNA	Detected	Detected	7.775681297	6.221629986
hsa_gci160062	2.128549867	1.089870889	up	8.593263714	7.503392825	ACTCCCCAGTGCCCGTT	hsa_circ_0034710	chr15	41809374	41822177	-	3604	ANNOTATED, CDS, c	NM_015540	RPAP1	Salzman2013	26015	circRNA	Detected	Detected	8.593263714	7.503392825
hsa_gci160065	-2.864430764	-1.518248467	down	2.411876557	3.930125024	ATGTGCCCTCAGTAGAA	hsa_circ_0034735	chr15	41988272	42000406	+	1361	ANNOTATED, CDS, c	NM_001164273	MGA	Salzman2013	23269	circRNA	Not Detected	Detected	2.411876557	3.930125024
hsa_gci160070	11.42328901	3.513906188	up	4.728702776	1.214796589	CGGTGACCTTAAACTCG	hsa_circ_0034756	chr15	42040834	42050037	+	1979	ANNOTATED, CDS, c	NM_001164273	MGA	Salzman2013	23269	circRNA	Detected	Not Detected	4.728702776	1.214796589
hsa_gci160073	2.158935321	1.110320023	up	8.230776521	7.120456498	ATGGTCCAATATAAGTT	hsa_circ_0034799	chr15	42525410	42560230	-	857	ANNOTATED, CDS, c	NM_015497	TMEM87A	Salzman2013	25963	circRNA	Detected	Detected	8.230776521	7.120456498
hsa_gci160074	2.811301017	1.491237937	up	8.691734991	7.200497054	TTTCTGTCCAGTGTTCC	hsa_circ_0034802	chr15	42531867	42560230	-	479	ANNOTATED, CDS, c	NM_015497	TMEM87A	Salzman2013	25963	circRNA	Detected	Detected	8.691734991	7.200497054
hsa_gci160075	2.866930788	1.519507076	up	7.124517363	5.605010286	TCTGTCCAGTGTTCCAA	hsa_circ_0034803	chr15	42553155	42560230	-	299	ANNOTATED, CDS, c	NM_015497	TMEM87A	Salzman2013	25963	circRNA	Detected	Detected	7.124517363	5.605010286

hsa_gci160076	2.30322224	1.203653625	up	8.607645916	7.403992291	TTTAAAGTCGGTCCTCT	hsa_circ_0034805	chr15	42579888	42603044	+	856	ANNOTATED, CDS, c	NM_198141	GANC	Salzman2013	2595	circRNA	Detected	Detected	8.607645916	7.403992291
hsa_gci160079	3.092606233	1.628823154	up	5.036633161	3.407810007	TTGACCTGTTGACAGTG	hsa_circ_0034815	chr15	42727581	42732011	-	1118	ANNOTATED, CDS, c	NM_022473	ZNF106	Salzman2013	64397	circRNA	Detected	Detected	5.036633161	3.407810007
hsa_gci160084	2.337158829	1.22475578	up	5.925994814	4.701239034	TTATTTAGATGTTTGTA	hsa_circ_0034867	chr15	43086841	43132631	-	763	ANNOTATED, CDS, c	NM_173500	TTBK2	Salzman2013	146057	circRNA	Detected	Detected	5.925994814	4.701239034
hsa_gci160092	2.047652497	1.033970899	up	5.642357985	4.608387086	TACTTCCTAGAATGCCC	hsa_circ_0034890	chr15	43250210	43350627	-	3742	ANNOTATED, CDS, c	NM_174916	UBR1	Salzman2013	197131	circRNA	Detected	Detected	5.642357985	4.608387086
hsa_gci160103	-3.987101474	-1.995340323	down	1.382507607	3.37784793	AAACAAGATGATGTTGG	hsa_circ_0034960	chr15	43670044	43675702	+	339	ANNOTATED, CDS, c	NM_014444	TUBGCP4	Salzman2013	27229	circRNA	Not Detected	Detected	1.382507607	3.37784793
hsa_gci160120	2.21038286	1.14429628	up	7.578052388	6.433756108	CTTCGAGTCGTTCAACG	hsa_circ_0035051	chr15	44620882	44630515	+	420	ANNOTATED, CDS, c	NM_138423	CASC4	Salzman2013	113201	circRNA	Detected	Detected	7.578052388	6.433756108
hsa_gci160123	2.714679915	1.440782102	up	6.231391235	4.790609134	GTGCAAGTTAAGATGAG	hsa_circ_0035057	chr15	44776421	44783197	+	505	ANNOTATED, CDS, c	NM_016396	CTDSPL2	Salzman2013	51496	circRNA	Detected	Detected	6.231391235	4.790609134
hsa_gci160124	-3.975369649	-1.991089015	down	1.207110189	3.198199204	TGAGGCAAAACTTCCTA	hsa_circ_0035058	chr15	44776421	44792011	+	783	ANNOTATED, CDS, c	NM_016396	CTDSPL2	Salzman2013	51496	circRNA	Not Detected	Detected	1.207110189	3.198199204
hsa_gci160126	2.622287765	1.390826013	up	4.62860957	3.237783557	ACAACCCAACTTGTAGA	hsa_circ_0035074	chr15	44859621	44865018	-	549	ANNOTATED, CDS, c	NM_025137	SPG11	Salzman2013	80208	circRNA	Detected	Detected	4.62860957	3.237783557
hsa_gci160127	2.268951361	1.182025683	up	6.481077473	5.29905179	ACTCCATAGAGGTCTCC	hsa_circ_0035085	chr15	44881449	44912601	-	2286	ANNOTATED, CDS, c	NM_025137	SPG11	Salzman2013	80208	circRNA	Detected	Detected	6.481077473	5.29905179
hsa_gci160155	2.087693715	1.06191007	up	3.871248993	2.809338922	ACAACTCATAACAACAA	hsa_circ_0035212	chr15	50724534	50763992	+	1076	ANNOTATED, CDS, c	NM_001128611	USP8	Salzman2013	9101	circRNA	Detected	Not Detected	3.871248993	2.809338922
hsa_gci160162	2.20993871	1.144006359	up	5.803903757	4.659897398	GATAGGTCCTAAAGACC	hsa_circ_0035239	chr15	50905903	50955243	-	1767	ANNOTATED, CDS, c	NM_017672	TRPM7	Salzman2013	54822	circRNA	Detected	Detected	5.803903757	4.659897398
hsa_gci160167	-2.140491869	-1.097942355	down	3.955119834	5.053062189	AGTAGTTTGATTACCTT	hsa_circ_0035261	chr15	51018517	51041067	-	969	ANNOTATED, CDS, c	NM_032802	SPPL2A	Salzman2013	84888	circRNA	Detected	Detected	3.955119834	5.053062189
hsa_gci160236	2.873664219	1.522891496	up	6.306987276	4.78409578	ATATCCGAAACTGTCTG	hsa_circ_0035560	chr15	60653139	60674640	-	309	ANNOTATED, CDS, c	NM_001136015	ANXA2	Salzman2013	302	circRNA	Detected	Detected	6.306987276	4.78409578
hsa_gci160244	2.729921444	1.448859437	up	5.672811288	4.223951851	ACGAACTATATAAGAAA	hsa_circ_0035602	chr15	62966025	63012079	+	2203	ANNOTATED, CDS, c	NM_015059	TLN2	Salzman2013	83660	circRNA	Detected	Detected	5.672811288	4.223951851
hsa_gci160250	3.166495313	1.662886944	up	6.772969417	5.110082473	AAAGAAAAGGAGAAGGT	hsa_circ_0035626	chr15	63447819	63448720	-	220	ANNOTATED, CDS, c	NM_015920	RPS27L	Salzman2013	51065	circRNA	Detected	Detected	6.772969417	5.110082473
hsa_gci160253	2.968417121	1.569693833	up	7.479635117	5.909941284	GTACACACACGTCATGT	hsa_circ_0035636	chr15	63615729	63631144	-	3072	ANNOTATED, CDS, c	NM_001218	CA12	Salzman2013	771	circRNA	Detected	Detected	7.479635117	5.909941284
hsa_gci160261	2.022840993	1.01638292	up	5.405203096	4.388820176	GAAAACAACCTGGCCGT	hsa_circ_0035650	chr15	63824845	63866602	+	1005	ANNOTATED, CDS, c	NM_006537	USP3	Salzman2013	9960	circRNA	Detected	Detected	5.405203096	4.388820176
hsa_gci160285	3.652652633	1.86894456	up	6.34804365	4.47909909	CCGATGCTCGATGAAGT	hsa_circ_0035775	chr15	63970043	64005864	-	2920	ANNOTATED, CDS, c	NM_003922	HERC1	Salzman2013	8925	circRNA	Detected	Detected	6.34804365	4.47909909
hsa_gci160289	5.619931571	2.490552564	up	5.747834953	3.257282388	ACTTCTTGGTGTTGGAT	hsa_circ_0035783	chr15	63970043	64041990	-	5168	ANNOTATED, CDS, c	NM_003922	HERC1	Salzman2013	8925	circRNA	Detected	Detected	5.747834953	3.257282388
hsa_gci160294	2.489146538	1.315651165	up	5.972350977	4.656699812	GAAGTGCCCGACTCTCC	hsa_circ_0035800	chr15	63998978	64005864	-	486	ANNOTATED, CDS, c	NM_003922	HERC1	Salzman2013	8925	circRNA	Detected	Detected	5.972350977	4.656699812
hsa_gci160320	3.992585595	1.997323337	up	5.587813097	3.59048976	GCTCCGGGGAACAGAAG	hsa_circ_0035938	chr15	66007815	66048810	-	1975	ANNOTATED, CDS, c	NM_001144823	DENND4A	Salzman2013	10260	circRNA	Detected	Detected	5.587813097	3.59048976
hsa_gci160328	2.625210679	1.392433207	up	5.521434531	4.129001324	GCTCCGGGGAACAGAAG	hsa_circ_0035952	chr15	66030044	66048810	-	1062	ANNOTATED, CDS, c	NM_001144823	DENND4A	Salzman2013	10260	circRNA	Detected	Detected	5.521434531	4.129001324
hsa_gci160329	2.639929235	1.400499258	up	5.500361732	4.099862474	GCTCCGGGGAACAGAAG	hsa_circ_0035956	chr15	66044716	66048810	-	583	ANNOTATED, CDS, c	NM_001144823	DENND4A	Salzman2013	10260	circRNA	Detected	Detected	5.500361732	4.099862474
hsa_gci160356	2.133566539	1.093267104	up	6.242945	5.149677896	TCAGTTTAGGAACCATT	hsa_circ_0036069	chr15	69629679	69652470	+	327	ANNOTATED, CDS, c	NM_001104554	PAQR5	Salzman2013	54852	circRNA	Detected	Detected	6.242945	5.149677896
hsa_gci160408	2.146884135	1.102244332	up	5.729786736	4.627542404	CCCTGTGTGCTGATTAG	hsa_circ_0036348	chr15	75699395	75705386	-	934	ANNOTATED, CDS, c	NM_001145358	SIN3A	Salzman2013	25942	circRNA	Detected	Detected	5.729786736	4.627542404
hsa_gci160409	2.562512441	1.357559009	up	4.570943871	3.213384862	GATTAGTGAGGACCCCA	hsa_circ_0036353	chr15	75703832	75705386	-	535	ANNOTATED, CDS, c	NM_001145358	SIN3A	Salzman2013	25942	circRNA	Detected	Detected	4.570943871	3.213384862
hsa_gci160414	2.59996314	1.37849117	up	7.696993007	6.318501837	CCCTAACCGGAAGGTGA	hsa_circ_0036367	chr15	75942096	75950968	+	2484	ALT_ACCEPTOR, CDS	NM_153271	SNX33	Salzman2013	257364	circRNA	Detected	Detected	7.696993007	6.318501837
hsa_gci160450	2.192581588	1.132630528	up	5.40649171	4.273861182	GAGTATCTTAGCGCTTA	hsa_circ_0036443	chr15	78396990	78403618	-	1140	ANNOTATED, CDS, c	NM_006383	CIB2	Salzman2013	10518	circRNA	Detected	Detected	5.40649171	4.273861182
hsa_gci160455	2.134323853	1.093779101	up	8.494731534	7.400952433	GTCCATTTACAAGAACG	hsa_circ_0036471	chr15	78780988	78789653	+	830	ANNOTATED, CDS, c	NM_004136	IREB2	Salzman2013	3658	circRNA	Detected	Detected	8.494731534	7.400952433
hsa_gci160456	3.380779895	1.757356093	up	8.465895626	6.708539533	GGCGGTGTCCGGAGCCT	hsa_circ_0036477	chr15	79051544	79056187	-	686	ANNOTATED, CDS, c	NM_014272	ADAMTS7	Salzman2013	11173	circRNA	Detected	Detected	8.465895626	6.708539533
hsa_gci160462	2.218601516	1.149650568	up	8.505400476	7.355749908	ATGACCAGAACATCCGA	hsa_circ_0036538	chr15	81229014	81235443	+	848	ANNOTATED, CDS, c	NM_018689	CEMIP	Salzman2013	57214	circRNA	Detected	Detected	8.505400476	7.355749908
hsa_gci160481	2.655544608	1.409007764	up	4.325389267	2.916381502	GATGAAAGGACCCGTCG	hsa_circ_0036628	chr15	85657103	85659350	+	350	ANNOTATED, CDS, c	NM_002605	PDE8A	Salzman2013	5151	circRNA	Detected	Not Detected	4.325389267	2.916381502
hsa_gci160492	-8.550458797	-3.096001834	down	3.638356152	6.734357986	CCCTCATTAGAAGGTTA	hsa_circ_0036670	chr15	89169376	89175512	+	3029	ANNOTATED, CDS, c	NM_022767	AEN	Salzman2013	64782	circRNA	Not Detected	Detected	3.638356152	6.734357986
hsa_gci160495	2.288007038	1.19409149	up	10.63882196	9.444730467	TCATAGTAATAATAAAG	hsa_circ_0036680	chr15	89633513	89698765	+	1042	ANNOTATED, CDS, c	NM_007011	ABHD2	Salzman2013	11057	circRNA	Detected	Detected	10.63882196	9.444730467
hsa_gci160497	2.14281181	1.099505153	up	9.499734764	8.400229611	CCAGTCTAGAGAATAAG	hsa_circ_0036683	chr15	89656955	89698765	+	644	ANNOTATED, CDS, c	NM_007011	ABHD2	Salzman2013	11057	circRNA	Detected	Detected	9.499734764	8.400229611
hsa_gci160499	2.015511559	1.011146057	up	10.5997326	9.588586541	CGTCTACTGGTACCACT	hsa_circ_0036688	chr15	89698597	89698765	+	168	ANNOTATED, CDS, c	NM_007011	ABHD2	Salzman2013	11057	circRNA	Detected	Detected	10.5997326	9.588586541
hsa_gci160515	2.511788872	1.328715204	up	7.801287147	6.472571943	GGTTCCCTCGGTTTCGA	hsa_circ_0036757	chr15	90627211	90628619	-	678	ANNOTATED, CDS, c	NM_002168	IDH2	Salzman2013	3418	circRNA	Detected	Detected	7.801287147	6.472571943
hsa_gci160543	2.692646982	1.429025099	up	3.818416959	2.38939186	AGGCCTCGTTACCGTAG	hsa_circ_0036840	chr15	91029261	91045475	+	3188	ANNOTATED, CDS, c	NM_003870	IQGAP1	Salzman2013	8826	circRNA	Detected	Not Detected	3.818416959	2.38939186
hsa_gci160550	2.167944484	1.116327813	up	6.899128574	5.782800761	GAGAAGAAAGTTACCCG	hsa_circ_0036863	chr15	91161114	91188577	+	4452	ANNOTATED, CDS, c	NM_022769	CRTC3	Salzman2013	64784	circRNA	Detected	Detected	6.899128574	5.782800761
hsa_gci160594	-3.820320522	-1.933693684	down	1.219732471	3.153426156	CCCGTGTGTTCTGTGCT	hsa_circ_0037293	chr16	1469752	1470745	-	891	ALT_ACCEPTOR, ALT	NM_001010878	C16orf91	Salzman2013	283951	circRNA	Not Detected	Detected	1.219732471	3.153426156
hsa_gci160612	2.129636415	1.090607146	up	7.489811082	6.399203936	CCTAGAGAAATGACTAT	hsa_circ_0037533	chr16	2498855	2499916	+	393	ANNOTATED, CDS, c	NM_001761	CCNF	Salzman2013	899	circRNA	Detected	Detected	7.489811082	6.399203936
hsa_gci160613	-5.213139873	-2.382152569	down	1.25742416	3.639576729	AGGGCCTACCAGTCCTA	hsa_circ_0037534	chr16	2498855	2508859	+	3105	ANNOTATED, CDS, c	NM_001761	CCNF	Salzman2013	899	circRNA	Not Detected	Detected	1.25742416	3.639576729
hsa_gci160616	2.324606459	1.216986498	up	4.102939696	2.885953197	GTTCGACCATATAATAC	hsa_circ_0037556	chr16	2631295	2647276	+	769	ANNOTATED, CDS, c	NM_002613	PDPK1	Salzman2013	5170	circRNA	Detected	Not Detected	4.102939696	2.885953197
hsa_gci160623	6.879727925	2.782351511	up	10.14889926	7.366547746	CACCCACAGGTCAGCCT	hsa_circ_0037618	chr16	3708037	3712133	-	413	ANNOTATED, CDS, c	NM_016292	TRAP1	Salzman2013	10131	circRNA	Detected	Detected	10.14889926	7.366547746
hsa_gci160625	2.557299535	1.354621153	up	4.136685212	2.782064059	TCCCCTTACAGGAGTGA	hsa_circ_0037621	chr16	3708037	3721792	-	1042	ANNOTATED, CDS, c	NM_016292	TRAP1	Salzman2013	10131	circRNA	Detected	Not Detected	4.136685212	2.782064059
hsa_gci160644	2.438962797	1.286267752	up	8.014918265	6.728650513	AGAACCGAGGTATCTTC	hsa_circ_0037785	chr16	8722586	8722967	+	381	ANNOTATED, CDS, c	NM_024109	METTL22	Salzman2013	79091	circRNA	Detected	Detected	8.014918265	6.728650513
hsa_gci160663	3.328354215	1.734808978	up	7.317490089	5.582681111	ACCATAATAGTGTAGAA	hsa_circ_0037870	chr16	11791980	11830089	-	853	ANNOTATED, CDS, c	NM_015914	TXNDC11	Salzman2013	51061	circRNA	Detected	Detected	7.317490089	5.582681111
hsa_gci160687	2.101463054	1.071394093	up	5.746728089	4.675333997	TGAAACGACTATTGATA	hsa_circ_0037958	chr16	14645877	14698083	-	778	ANNOTATED, CDS, c	NM_002582	PARN	Salzman2013	5073	circRNA	Detected	Detected	5.746728089	4.675333997
hsa_gci160691	2.30394586	1.204106816	up	5.023869302	3.819762486	CCCAAAGCCCTTTTGTA	hsa_circ_0037968	chr16	14680174	14721044	-	717	ANNOTATED, CDS, c	NM_002582	PARN	Salzman2013	5073	circRNA	Detected	Detected	5.023869302	3.819762486
hsa_gci160703	5.08144967	2.345240137	up	5.084569141	2.739329004	GTCAAAGAAGTAGAAGT	hsa_circ_0038020	chr16	15162025	15168718	-	695	ANNOTATED, CDS, c	NM_018427	RRN3	Salzman2013	54700	circRNA	Detected	Not Detected	5.084569141	2.739329004

hsa_gci160716	2.925205357	1.548537909	up	7.861752453	6.313214543	TTGAGGTGGGATCCCCT	hsa_circ_0038167	chr16	16333707	16388663	+	3921	ALT_DONOR, CDS, c	NM_001004067	NOMO3	Salzman2013	408050	circRNA	Detected	Detected	7.861752453	6.313214543
hsa_gci160719	2.961394538	1.56627671	up	8.662957818	7.096681108	GCCCCTTCGCTTACTAA	hsa_circ_0038178	chr16	16355358	16388663	+	2956	ALT_DONOR, CDS, c	NM_001004067	NOMO3	Salzman2013	408050	circRNA	Detected	Detected	8.662957818	7.096681108
hsa_gci160741	2.854875037	1.513427598	up	6.755360689	5.241933091	AGTGTAGAAGTTGCTCT	hsa_circ_0038293	chr16	18882122	18882210	-	88	ANNOTATED, CDS, c	NM_015092	SMG1	Salzman2013	23049	circRNA	Detected	Detected	6.755360689	5.241933091
hsa_gci160761	2.926406479	1.549130174	up	7.238578322	5.689448148	TTGTATTTTTGAGTCGA	hsa_circ_0038367	chr16	19659105	19663412	+	292	ANNOTATED, CDS, c	NM_020314	VPS35L	Salzman2013	57020	circRNA	Detected	Detected	7.238578322	5.689448148
hsa_gci160768	2.778330157	1.474218049	up	5.912248519	4.43803047	GTCCGGCCTGGTTTGGT	hsa_circ_0038399	chr16	20912074	20927036	+	528	ANNOTATED, CDS, c	NM_001128301	LYRM1	Salzman2013	57149	circRNA	Detected	Detected	5.912248519	4.43803047
hsa_gci160778	2.098041834	1.069043445	up	5.314140175	4.24509673	TTTAATGGACCCAGCCG	hsa_circ_0038425	chr16	21475023	21495856	-	1815	ANNOTATED, INTERN	NR_027155		Salzman2013		circRNA	Detected	Detected	5.314140175	4.24509673
hsa_gci160781	2.609506582	1.383777041	up	3.967020376	2.583243335	CTACAACACGTAGTTCA	hsa_circ_0038432	chr16	21483993	21498937	-	4007	ALT_ACCEPTOR, INT	NR_027155		Salzman2013		circRNA	Detected	Not Detected	3.967020376	2.583243335
hsa_gci160790	2.323673125	1.216407137	up	5.315777296	4.09937016	TCGACACCGAAATTGAA	hsa_circ_0038457	chr16	21968555	21994668	+	1505	ANNOTATED, CDS, c	NM_003366	UQCRC2	Salzman2013	7385	circRNA	Detected	Detected	5.315777296	4.09937016
hsa_gci160805	2.103461211	1.072765214	up	5.143047594	4.070282379	AATTTAATGGACCCAGC	hsa_circ_0038524	chr16	22466022	22466926	+	320	ANNOTATED, INTERN	NR_027154		Salzman2013		circRNA	Detected	Detected	5.143047594	4.070282379
hsa_gci160822	2.155808326	1.108228913	up	4.270653619	3.162424705	CATTCTCCGAAGGCACC	hsa_circ_0038616	chr16	23581800	23585710	+	3910	ANNOTATED, CDS, c	NM_019116	UBFD1	Salzman2013	56061	circRNA	Detected	Detected	4.270653619	3.162424705
hsa_gci160828	2.061307886	1.043560008	up	11.5657859	10.52222589	CCGTCCCCCAAGAGGTT	hsa_circ_0038635	chr16	23698789	23701688	+	1080	ANNOTATED, CDS, c	NM_005030	PLK1	Salzman2013	5347	circRNA	Detected	Detected	11.5657859	10.52222589
hsa_gci160836	2.242508836	1.16511367	up	5.248321958	4.083208288	ACATTCGACCCGGTCTC	hsa_circ_0038667	chr16	24564833	24570998	+	544	ANNOTATED, CDS, c	NM_006910	RBBP6	Salzman2013	5930	circRNA	Detected	Detected	5.248321958	4.083208288
hsa_gci160853	3.575929985	1.83831849	up	5.125939408	3.287620918	GCCCGTCGCGTGGTGCG	hsa_circ_0038732	chr16	27471933	27501064	-	3916	ANNOTATED, CDS, c	NM_001520	GTF3C1	Salzman2013	2975	circRNA	Detected	Detected	5.125939408	3.287620918
hsa_gci160854	3.060776745	1.613897818	up	5.918668166	4.304770348	GGCCCCGCGTACGCGTT	hsa_circ_0038736	chr16	27471933	27561251	-	7107	ANNOTATED, CDS, c	NM_001520	GTF3C1	Salzman2013	2975	circRNA	Detected	Detected	5.918668166	4.304770348
hsa_gci160861	2.176501423	1.122010964	up	7.097275966	5.975265003	ACAGTGTCCCCTGACCT	hsa_circ_0038790	chr16	28117703	28181230	-	2207	ANNOTATED, CDS, c	NM_015171	XPO6	Salzman2013	23214	circRNA	Detected	Detected	7.097275966	5.975265003
hsa_gci160864	2.40485867	1.265952111	up	5.657051558	4.391099447	ATAGACTCTAGGCATGT	hsa_circ_0038799	chr16	28128638	28133083	-	238	ANNOTATED, CDS, c	NM_015171	XPO6	Salzman2013	23214	circRNA	Detected	Detected	5.657051558	4.391099447
hsa_gci160870	2.939178386	1.555412923	up	7.199251644	5.643838721	GTCCCCTGACCTAGTTG	hsa_circ_0038817	chr16	28157414	28181230	-	929	ANNOTATED, CDS, c	NM_015171	XPO6	Salzman2013	23214	circRNA	Detected	Detected	7.199251644	5.643838721
hsa_gci160872	-4.462972225	-2.158004828	down	1.290964108	3.448968936	CTCGCACTGAGTCCCTT	hsa_circ_0038833	chr16	28488599	28493703	-	648	ANNOTATED, CDS, c	NM_001042432	CLN3	Salzman2013	1201	circRNA	Not Detected	Detected	1.290964108	3.448968936
hsa_gci160882	2.441218448	1.287601398	up	4.272017329	2.984415931	CGTACCGGGGTTTGGCC	hsa_circ_0038952	chr16	29869677	29872580	-	1296	ANNOTATED, CDS, c	NM_006319	CDIPT	Salzman2013	10423	circRNA	Detected	Not Detected	4.272017329	2.984415931
hsa_gci160886	2.803191286	1.487070195	up	9.948156548	8.461086353	CGCCGACCGGTCCGCCC	hsa_circ_0039060	chr16	30663116	30667734	+	1837	ANNOTATED, CDS, c	NM_024031	PRR14	Salzman2013	78994	circRNA	Detected	Detected	9.948156548	8.461086353
hsa_gci160889	2.01355024	1.009741469	up	8.367979824	7.358238355	GAGTTTCTGTCCCATTC	hsa_circ_0039118	chr16	30899115	30899311	-	196	ANNOTATED, CDS, c	NM_004765	BCL7C	Salzman2013	9274	circRNA	Detected	Detected	8.367979824	7.358238355
hsa_gci160894	2.006940839	1.00499809	up	5.123074068	4.118075978	CATTCCCTGCACGCCTT	hsa_circ_0039144	chr16	31102174	31106276	-	998	ANNOTATED, CDS, c	NM_024006	VKORC1	Salzman2013	79001	circRNA	Detected	Detected	5.123074068	4.118075978
hsa_gci160895	6.401758352	2.67846822	up	3.936487095	1.258018875	GAAACCCAACGAACAAC	hsa_circ_0039151	chr16	31193718	31193985	+	177	ANNOTATED, CDS, c	NM_004960	FUS	Salzman2013	2521	circRNA	Detected	Not Detected	3.936487095	1.258018875
hsa_gci160914	2.769168184	1.469452678	up	6.266794909	4.797342231	GCCCAGTTGCTTTGGGC	hsa_circ_0039213	chr16	46723557	46732306	+	1687	ANNOTATED, ncRNA,	NR_037620	ORC6	Salzman2013	23594	circRNA	Detected	Detected	6.266794909	4.797342231
hsa_gci160927	3.749483725	1.906691961	up	4.38464772	2.477955758	CTAATTACGAAAATTCT	hsa_circ_0039260	chr16	47531309	47533805	+	229	ANNOTATED, CDS, c	NM_001031835	PHKB	Salzman2013	5257	circRNA	Detected	Not Detected	4.38464772	2.477955758
hsa_gci160958	2.191948	1.132213573	up	4.690923102	3.558709528	CCGCCTGTCGACGATTT	hsa_circ_0039387	chr16	53504657	53514681	+	558	ANNOTATED, CDS, c	NM_005611	RBL2	Salzman2013	5934	circRNA	Detected	Detected	4.690923102	3.558709528
hsa_gci160964	2.316230013	1.211778527	up	6.219125253	5.007346726	GGTCCTTGCTTCTGTCC	hsa_circ_0039430	chr16	56403104	56423287	-	430	ANNOTATED, CDS, c	NM_001144	AMFR	Salzman2013	267	circRNA	Detected	Detected	6.219125253	5.007346726
hsa_gci160971	2.050672296	1.036096963	up	7.412362099	6.376265136	GCACCACCTCACGTCGC	hsa_circ_0039459	chr16	56642477	56643409	+	419	ANNOTATED, CDS, c	NM_005953	MT2A	Salzman2013	4502	circRNA	Detected	Detected	7.412362099	6.376265136
hsa_gci160989	8.634176355	3.11005856	up	5.104648171	1.994589611	CCCCGTCTTGAGGAGTG	hsa_circ_0039579	chr16	57503076	57505921	+	1460	ANNOTATED, CDS, c	NM_032940	POLR2C	Salzman2013	5432	circRNA	Detected	Not Detected	5.104648171	1.994589611
hsa_gci160992	2.389977321	1.256996928	up	6.974723319	5.71772639	GCGTCGTCGTGGAGTCG	hsa_circ_0039606	chr16	58054044	58055527	+	1483	ANNOTATED, CDS, c	NM_024598	USB1	Salzman2013	79650	circRNA	Detected	Detected	6.974723319	5.71772639
hsa_gci160993	3.491116165	1.803688363	up	4.459591378	2.655903015	ACTCCTATCAAAGACTA	hsa_circ_0039615	chr16	58555086	58594266	-	5073	ANNOTATED, CDS, c	NM_016284	CNOT1	Salzman2013	23019	circRNA	Detected	Not Detected	4.459591378	2.655903015
hsa_gci161028	2.638653998	1.399802185	up	4.284228087	2.884425901	CAAGAAGAAATGGTCCC	hsa_circ_0039690	chr16	58594115	58633415	-	2304	ANNOTATED, CDS, c	NM_016284	CNOT1	Salzman2013	23019	circRNA	Detected	Not Detected	4.284228087	2.884425901
hsa_gci161035	20.24932555	4.339801951	up	6.73628788	2.396485929	GGAACAGGTTTAAAAAA	hsa_circ_0039710	chr16	58741034	58756182	-	2087	ANNOTATED, CDS, c	NM_002080	GOT2	Salzman2013	2806	circRNA	Detected	Not Detected	6.73628788	2.396485929
hsa_gci161037	2.147984394	1.102983512	up	5.926420248	4.823436737	ATTCCTGTCCCTGAAGG	hsa_circ_0039712	chr16	58741034	58768246	-	2447	ANNOTATED, CDS, c	NM_002080	GOT2	Salzman2013	2806	circRNA	Detected	Detected	5.926420248	4.823436737
hsa_gci161049	2.327322166	1.218670933	up	9.339717818	8.121046885	ACGCGAAGGCGAGGTTG	hsa_circ_0039769	chr16	66965957	66968320	-	849	ANNOTATED, ncRNA,	NR_024525	FAM96B	Salzman2013	51647	circRNA	Detected	Detected	9.339717818	8.121046885
hsa_gci161056	2.049206958	1.035065696	up	8.993185119	7.958119423	CCTGTGGTCTAACCGGG	hsa_circ_0039806	chr16	67263291	67271472	-	2988	ANNOTATED, CDS, c	NM_013241	FHOD1	Salzman2013	29109	circRNA	Detected	Detected	8.993185119	7.958119423
hsa_gci161057	3.035065157	1.601727489	up	5.767226624	4.165499135	AGGTGTTTCTCGTAGTC	hsa_circ_0039808	chr16	67263291	67272031	-	3195	ANNOTATED, CDS, c	NM_013241	FHOD1	Salzman2013	29109	circRNA	Detected	Detected	5.767226624	4.165499135
hsa_gci161073	3.380331255	1.75716463	up	7.356054702	5.598890072	ACATGAAGAGTTATACT	hsa_circ_0039933	chr16	68208276	68217269	+	324	ANNOTATED, CDS, c	NM_004555	NFATC3	Salzman2013	4775	circRNA	Detected	Detected	7.356054702	5.598890072
hsa_gci161106	3.486803391	1.801905018	up	7.781368228	5.979463211	GCTGAAGGAGGCCGTTC	hsa_circ_0040054	chr16	69354953	69358946	+	1197	ANNOTATED, CDS, c	NM_013245	VPS4A	Salzman2013	27183	circRNA	Detected	Detected	7.781368228	5.979463211
hsa_gci161115	2.090378414	1.063764132	up	5.062275077	3.998510945	TTACATCGACCAGGTCT	hsa_circ_0040080	chr16	69729038	69729591	+	553	ANNOTATED, CDS, c	NM_173215	NFAT5	Salzman2013	10725	circRNA	Detected	Detected	5.062275077	3.998510945
hsa_gci161116	2.437496843	1.28540035	up	8.307561122	7.022160772	GAGGTGACCTCGGTACA	hsa_circ_0040088	chr16	69775773	69788829	-	1716	ANNOTATED, CDS, c	NM_014062	NOB1	Salzman2013	28987	circRNA	Detected	Detected	8.307561122	7.022160772
hsa_gci161142	2.519415869	1.333089281	up	4.966115312	3.633026031	TTAAACTATATTCATTG	hsa_circ_0040264	chr16	70575571	70578436	+	262	ANNOTATED, CDS, c	NM_012426	SF3B3	Salzman2013	23450	circRNA	Detected	Detected	4.966115312	3.633026031
hsa_gci161161	2.110698149	1.077720273	up	7.542449694	6.464729421	TAAGAATGACATACGAC	hsa_circ_0040369	chr16	71772844	71773005	-	161	ANNOTATED, CDS, c	NM_001030007	AP1G1	Salzman2013	164	circRNA	Detected	Detected	7.542449694	6.464729421
hsa_gci161172	4.251391171	2.087935008	up	9.287186652	7.199251644	CCCTATCTAGACGTCTC	hsa_circ_0040420	chr16	72139903	72146811	+	1612	ANNOTATED, CDS, c	NM_014003	DHX38	Salzman2013	9785	circRNA	Detected	Detected	9.287186652	7.199251644
hsa_gci161176	3.150653288	1.655651002	up	9.905389727	8.249738725	CCCGTCGCACCCGTTGT	hsa_circ_0040439	chr16	74333473	74340186	+	1995	ALT_ACCEPTOR, CDS	NM_002811	PSMD7	Salzman2013	5713	circRNA	Detected	Detected	9.905389727	8.249738725
hsa_gci161221	4.401172155	2.137887805	up	4.616884814	2.478997009	GAAATGACAACTTCAGA	hsa_circ_0040667	chr16	84100093	84103643	-	571	ANNOTATED, CDS, c	NM_003791	MBTPS1	Salzman2013	8720	circRNA	Detected	Not Detected	4.616884814	2.478997009
hsa_gci161227	2.044879592	1.032015896	up	9.832929251	8.800913356	GAGCGGCCCCTGCTACC	hsa_circ_0040694	chr16	84599203	84651189	-	1599	ANNOTATED, CDS, c	NM_021149	COTL1	Salzman2013	23406	circRNA	Detected	Detected	9.832929251	8.800913356
hsa_gci161240	2.055866941	1.039746894	up	5.350909353	4.311162459	TAATGACTGTGACGACT	hsa_circ_0040778	chr16	87457415	87493737	-	270	ANNOTATED, CDS, c	NM_015144	ZCCHC14	Salzman2013	23174	circRNA	Detected	Detected	5.350909353	4.311162459
hsa_gci161246	2.023129269	1.016588505	up	5.426210124	4.409621619	GCCCTGGGGTGCTTCTT	hsa_circ_0040796	chr16	87863628	87871547	-	3428	ANNOTATED, CDS, c	NM_003486	SLC7A5	Salzman2013	8140	circRNA	Detected	Detected	5.426210124	4.409621619
hsa_gci161248	2.505865268	1.325308848	up	10.16599657	8.840687725	ACTACCGGTCCCCGTCC	hsa_circ_0040798	chr16	87863628	87873431	-	3656	ANNOTATED, CDS, c	NM_003486	SLC7A5	Salzman2013	8140	circRNA	Detected	Detected	10.16599657	8.840687725
hsa_gci161258	2.368242826	1.243817014	up	9.71793708	8.474120066	GCCCGTCGCGGCTCCGA	hsa_circ_0040946	chr16	89598870	89624174	+	1911	ANNOTATED, CDS, c	NM_003119	SPG7	Salzman2013	6687	circRNA	Detected	Detected	9.71793708	8.474120066

hsa_gci161259	2.657164038	1.409887294	up	7.155941374	5.74605408	GCCGCAAACAGGCTCCC	hsa_circ_0040953	chr16	89619386	89624174	+	1282	ANNOTATED, CDS, c	NM_003119	SPG7	Salzman2013	6687	circRNA	Detected	Detected	7.155941374	5.74605408
hsa_gci161260	2.028454444	1.020380902	up	4.065872082	3.045491179	TCAAATAGTGAAGGAAA	hsa_circ_0040954	chr16	89623294	89624174	+	880	ANNOTATED, CDS, c	NM_003119	SPG7	Salzman2013	6687	circRNA	Detected	Not Detected	4.065872082	3.045491179
hsa_gci161276	2.352374978	1.23411805	up	7.230045574	5.995927524	CCCGTGCCAGGACTCTC	hsa_circ_0041123	chr16	89986997	90002505	+	3245	ALT_ACCEPTOR, CDS	NM_001197181	TUBB3	Salzman2013	10381	circRNA	Detected	Detected	7.230045574	5.995927524
hsa_gci161289	2.008390106	1.006039523	up	9.559229711	8.553190189	CGATGAAAGAAGTAACT	hsa_circ_0041193	chr17	1264385	1268352	-	514	ANNOTATED, INTERN	NR_024058	YWHAE	Salzman2013	7531	circRNA	Detected	Detected	9.559229711	8.553190189
hsa_gci161290	2.203000622	1.139469903	up	9.00905198	7.869582078	TGTTGACGAGTCGAGAT	hsa_circ_0041194	chr17	1264385	1303556	-	763	ANNOTATED, ncRNA,	NR_024058	YWHAE	Salzman2013	7531	circRNA	Detected	Detected	9.00905198	7.869582078
hsa_gci161292	2.163750187	1.113533944	up	7.295147953	6.181614008	TTAAGTCGGCACCCCGC	hsa_circ_0041197	chr17	1367479	1369046	-	1567	ANNOTATED, CDS, c	NM_001080779	MYO1C	Salzman2013	4641	circRNA	Detected	Detected	7.295147953	6.181614008
hsa_gci161296	2.236280518	1.161101171	up	4.453687884	3.292586713	GAACTTGACCCACCGCT	hsa_circ_0041204	chr17	1367479	1375522	-	2935	ANNOTATED, CDS, c	NM_001080779	MYO1C	Salzman2013	4641	circRNA	Detected	Detected	4.453687884	3.292586713
hsa_gci161306	2.174988916	1.121008049	up	3.944606343	2.823598294	GGAGTGAGAGACGGGTC	hsa_circ_0041326	chr17	1646129	1658559	+	2284	ANNOTATED, CDS, c	NM_000934	SERPINF2	Salzman2013	5345	circRNA	Detected	Not Detected	3.944606343	2.823598294
hsa_gci161307	2.164143802	1.113796366	up	8.006435812	6.892639446	CGCGAGTGTCCCCTCTT	hsa_circ_0041332	chr17	1648468	1658559	+	2144	ANNOTATED, CDS, c	NM_000934	SERPINF2	Salzman2013	5345	circRNA	Detected	Detected	8.006435812	6.892639446
hsa_gci161321	2.183688141	1.126766836	up	4.381031058	3.254264223	AACCGTATAGTACGTAG	hsa_circ_0041377	chr17	1963132	2196271	-	3762	ANNOTATED, CDS, c	NM_001170957	SMG6	Salzman2013	23293	circRNA	Detected	Detected	4.381031058	3.254264223
hsa_gci161326	2.238514738	1.162541817	up	8.499421627	7.336879811	CCGAGGCCCCCAAGGTC	hsa_circ_0041405	chr17	2278785	2284348	+	2590	ANNOTATED, CDS, c	NM_014853	SGSM2	Salzman2013	9905	circRNA	Detected	Detected	8.499421627	7.336879811
hsa_gci161342	2.954692151	1.563007824	up	4.257297944	2.69429012	GTCCAGTGGTCACGAAG	hsa_circ_0041570	chr17	4574678	4576440	-	1603	ANNOTATED, CDS, c	NM_014389	PELP1	Salzman2013	27043	circRNA	Detected	Not Detected	4.257297944	2.69429012
hsa_gci161343	2.070920105	1.050271897	up	3.912368372	2.862096475	TACTCCTACCAGGCCGA	hsa_circ_0041571	chr17	4574678	4576969	-	1866	ANNOTATED, CDS, c	NM_014389	PELP1	Salzman2013	27043	circRNA	Detected	Not Detected	3.912368372	2.862096475
hsa_gci161346	3.362848474	1.749683774	up	8.73477845	6.985094675	GTCGGCCCTTATTGTGA	hsa_circ_0041576	chr17	4574678	4579802	-	2746	ANNOTATED, CDS, c	NM_014389	PELP1	Salzman2013	27043	circRNA	Detected	Detected	8.73477845	6.985094675
hsa_gci161349	3.212345919	1.683627257	up	4.540096801	2.856469544	GTGGGCGCCTGCGTGGA	hsa_circ_0041592	chr17	4636827	4643223	-	2324	ANNOTATED, CDS, c	NM_022059	CXCL16	Salzman2013	58191	circRNA	Detected	Not Detected	4.540096801	2.856469544
hsa_gci161354	2.460042303	1.298683124	up	4.252977011	2.954293887	GAGACCGGAGGCGGCGA	hsa_circ_0041620	chr17	4843629	4848517	+	1708	ANNOTATED, CDS, c	NM_015528	RNF167	Salzman2013	26001	circRNA	Detected	Not Detected	4.252977011	2.954293887
hsa_gci161363	2.736444896	1.452302805	up	8.044126562	6.591823757	ATTTAAACAGTTGAAAA	hsa_circ_0041641	chr17	4856566	4859157	+	883	ALT_DONOR, CDS, c	NM_001976	ENO3	Salzman2013	2027	circRNA	Detected	Detected	8.044126562	6.591823757
hsa_gci161370	2.574093361	1.36406438	up	8.866285527	7.502221147	TGTCCCCACCTAAATAC	hsa_circ_0041700	chr17	5307418	5320002	-	895	ANNOTATED, CDS, c	NM_002532	NUP88	Salzman2013	4927	circRNA	Detected	Detected	8.866285527	7.502221147
hsa_gci161371	2.839878792	1.505829356	up	8.332514239	6.826684883	CTAAAGTTTGTCCCCAC	hsa_circ_0041702	chr17	5308376	5320002	-	747	ANNOTATED, CDS, c	NM_002532	NUP88	Salzman2013	4927	circRNA	Detected	Detected	8.332514239	6.826684883
hsa_gci161380	2.519709888	1.333257635	up	7.522222918	6.188965283	GTACTCTTCCGGTCTTA	hsa_circ_0041758	chr17	6920579	6920843	+	264	ANNOTATED, ncRNA,	NR_037717		Salzman2013		circRNA	Detected	Detected	7.522222918	6.188965283
hsa_gci161397	3.105676526	1.634907573	up	10.47231682	8.837409245	CAACCGCTCATCGACCC	hsa_circ_0041891	chr17	7416339	7417935	+	1596	ANNOTATED, CDS, c	NM_000937	POLR2A	Salzman2013	5430	circRNA	Detected	Detected	10.47231682	8.837409245
hsa_gci161403	3.590782813	1.844298395	up	7.750378006	5.906079611	CCTACCCCTAATCGTCA	hsa_circ_0041908	chr17	7473673	7482324	+	2679	ANNOTATED, ncRNA,	NR_037926	SENP3-EIF4A1	Salzman2013	100533955	circRNA	Detected	Detected	7.750378006	5.906079611
hsa_gci161408	2.061346134	1.043586777	up	4.899666069	3.856079292	GTCATGTTTGTAGAACT	hsa_circ_0041926	chr17	7480373	7481562	+	562	ANNOTATED, INTERN	NR_037926	SENP3-EIF4A1	Salzman2013	100533955	circRNA	Detected	Detected	4.899666069	3.856079292
hsa_gci161414	-2.925202555	-1.548536527	down	2.871742583	4.420279111	GTTTGGCAGGTGGAGCT	hsa_circ_0041936	chr17	7481659	7482324	+	665	ANNOTATED, ncRNA,	NR_037926	SENP3-EIF4A1	Salzman2013	100533955	circRNA	Not Detected	Detected	2.871742583	4.420279111
hsa_gci161421	-2.291374998	-1.196213584	down	3.348717411	4.544930995	TAAGAAGTGGTGGTCGA	hsa_circ_0041982	chr17	7833662	7834052	-	390	ANNOTATED, CDS, c	NM_021210	TRAPPC1	Salzman2013	58485	circRNA	Not Detected	Detected	3.348717411	4.544930995
hsa_gci161429	2.417627638	1.273592058	up	6.620506455	5.346914396	CGATCCCGGAGTCTTGA	hsa_circ_0042065	chr17	9153787	9479275	-	968	ANNOTATED, CDS, c	NM_004853	STX8	Salzman2013	9482	circRNA	Detected	Detected	6.620506455	5.346914396
hsa_gci161430	6.148967685	2.620344225	up	10.55164189	7.931297667	TGAGTAGTCCGACCTGG	hsa_circ_0042066	chr17	9395145	9408479	-	218	ANNOTATED, CDS, c	NM_004853	STX8	Salzman2013	9482	circRNA	Detected	Detected	10.55164189	7.931297667
hsa_gci161436	3.407538114	1.768729794	up	4.20646967	2.437739876	AAGTCAAAAGGACTACT	hsa_circ_0042097	chr17	11984672	12013743	+	467	ANNOTATED, CDS, c	NM_003010	MAP2K4	Salzman2013	6416	circRNA	Detected	Not Detected	4.20646967	2.437739876
hsa_gci161451	2.713401826	1.440102711	up	8.30944551	6.869342799	GTTATTTGTTAGTACAA	hsa_circ_0042183	chr17	16097775	16097953	-	178	ANNOTATED, CDS, c	NM_006311	NCOR1	Salzman2013	9611	circRNA	Detected	Detected	8.30944551	6.869342799
hsa_gci161463	2.074555116	1.052801986	up	6.085927927	5.033125941	TACCTTCCCGTCCGAAT	hsa_circ_0042253	chr17	17165279	17168295	-	321	ANNOTATED, CDS, c	NM_003653	COPS3	Salzman2013	8533	circRNA	Detected	Detected	6.085927927	5.033125941
hsa_gci161469	4.158233765	2.055970865	up	6.594822621	4.538851756	CCGTCGAGGTCCATCGG	hsa_circ_0042302	chr17	18148152	18151347	-	1919	ANNOTATED, CDS, c	NM_002018	FLII	Salzman2013	2314	circRNA	Detected	Detected	6.594822621	4.538851756
hsa_gci161478	3.104061453	1.63415712	up	5.310436749	3.676279629	CTGCATTTCGGGACTTT	hsa_circ_0042349	chr17	18607931	18639431	+	19452	ALT_ACCEPTOR, CDS	NM_001037330	TRIM16L	Salzman2013	147166	circRNA	Detected	Detected	5.310436749	3.676279629
hsa_gci161505	-2.835975324	-1.50384498	down	2.663109757	4.166954736	GCGACGACGGGTTCACG	hsa_circ_0042448	chr17	20902905	20911309	-	3913	ANNOTATED, CDS, c	NM_015276	USP22	Salzman2013	23326	circRNA	Not Detected	Detected	2.663109757	4.166954736
hsa_gci161507	2.020699771	1.014854986	up	4.107993454	3.093138467	TCACGAGACCAGACCAC	hsa_circ_0042461	chr17	20911182	20911309	-	127	ANNOTATED, CDS, c	NM_015276	USP22	Salzman2013	23326	circRNA	Detected	Not Detected	4.107993454	3.093138467
hsa_gci161520	-2.186221546	-1.128439607	down	4.487326718	5.615766326	TGTCCCATATCTCATGT	hsa_circ_0042521	chr17	26512204	26512291	+	87	ANNOTATED, CDS, c	NM_016231	NLK	Salzman2013	51701	circRNA	Detected	Detected	4.487326718	5.615766326
hsa_gci161529	2.123474	1.086426444	up	6.638469782	5.552043338	ACAAGTCTTGATCCCTC	hsa_circ_0042599	chr17	26946877	26966478	-	4437	ANNOTATED, CDS, c	NM_014680	KIAA0100	Salzman2013	9703	circRNA	Detected	Detected	6.638469782	5.552043338
hsa_gci161533	2.687608911	1.426323219	up	6.125353405	4.699030186	GGAGGTCCGCCAGTAGT	hsa_circ_0042657	chr17	27001554	27029249	+	5519	ANNOTATED, CDS, c	NM_003170	SUPT6H	Salzman2013	6830	circRNA	Detected	Detected	6.125353405	4.699030186
hsa_gci161542	2.846156423	1.509014954	up	4.35981017	2.850795216	GGGTCGCCATAGGGTTT	hsa_circ_0042686	chr17	27041298	27042723	-	828	ANNOTATED, CDS, c	NM_031934	RAB34	Salzman2013	83871	circRNA	Detected	Not Detected	4.35981017	2.850795216
hsa_gci161549	2.111854838	1.078510672	up	7.295284473	6.216773801	GTCGCCCCGACGTTGCA	hsa_circ_0042721	chr17	27206356	27211333	-	2378	ANNOTATED, CDS, c	NM_004475	FLOT2	Salzman2013	2319	circRNA	Detected	Detected	7.295284473	6.216773801
hsa_gci161558	3.623045036	1.857202739	up	6.059993174	4.202790435	CACAGCGAGGTCTCTCC	hsa_circ_0042793	chr17	27952964	28004759	-	8768	ANNOTATED, CDS, c	NM_033389	SSH2	Salzman2013	85464	circRNA	Detected	Detected	6.059993174	4.202790435
hsa_gci161563	2.297465817	1.200043396	up	5.614850619	4.414807223	GCGAGGTCTCTCCGTTT	hsa_circ_0042810	chr17	27994147	28004759	-	424	ANNOTATED, CDS, c	NM_033389	SSH2	Salzman2013	85464	circRNA	Detected	Detected	5.614850619	4.414807223
hsa_gci161564	3.345570888	1.742252413	up	5.888203754	4.145951341	CACAGCGAGGTCTCTCC	hsa_circ_0042812	chr17	28000929	28004759	-	3043	ALT_DONOR, CDS, c	NM_033389	SSH2	Salzman2013	85464	circRNA	Detected	Detected	5.888203754	4.145951341
hsa_gci161626	2.161818807	1.112245609	up	9.106831808	7.994586199	AAGAAGACGGGCCCCTC	hsa_circ_0043001	chr17	30500849	30502381	+	126	ANNOTATED, CDS, c	NM_001033568	RHOT1	Salzman2013	55288	circRNA	Detected	Detected	9.106831808	7.994586199
hsa_gci161627	2.416090821	1.272674687	up	9.46247449	8.189799803	ACCTTACCACTAAAGAA	hsa_circ_0043002	chr17	30500849	30503034	+	180	ANNOTATED, CDS, c	NM_001033568	RHOT1	Salzman2013	55288	circRNA	Detected	Detected	9.46247449	8.189799803
hsa_gci161643	-3.772216933	-1.915412645	down	1.319809671	3.235222316	CCAGAGGTACCGTGGTT	hsa_circ_0043077	chr17	31260191	31268667	+	1206	ANNOTATED, CDS, c	NM_015544	TMEM98	Salzman2013	26022	circRNA	Not Detected	Detected	1.319809671	3.235222316
hsa_gci161649	3.500108512	1.80739965	up	9.147936768	7.340537118	CAACGTCCGTGGCTACT	hsa_circ_0043132	chr17	33998772	34001340	+	293	ANNOTATED, CDS, c	NM_001030006	AP2B1	Salzman2013	163	circRNA	Detected	Detected	9.147936768	7.340537118
hsa_gci161672	2.452947181	1.294516169	up	4.933728928	3.639212759	GGAGGTCCGTGACCGTG	hsa_circ_0043261	chr17	35620587	35634898	-	719	ANNOTATED, CDS, c	NM_198839	ACACA	Salzman2013	31	circRNA	Detected	Detected	4.933728928	3.639212759
hsa_gci161674	2.027796299	1.019912734	up	8.850451866	7.830539131	CGCGAGGTCCCGACTCG	hsa_circ_0043271	chr17	35767311	35787108	+	561	ANNOTATED, CDS, c	NM_001488	TADA2A	Salzman2013	6871	circRNA	Detected	Detected	8.850451866	7.830539131
hsa_gci161680	2.572465929	1.36315197	up	7.107258532	5.744106562	AGAACTCGTCAGGTCGA	hsa_circ_0043279	chr17	35797838	35802753	+	339	ANNOTATED, CDS, c	NM_001488	TADA2A	Salzman2013	6871	circRNA	Detected	Detected	7.107258532	5.744106562
hsa_gci161681	2.372490573	1.246402355	up	8.111360232	6.864957877	AAGAACTCGTCAGGTCG	hsa_circ_0043280	chr17	35797838	35804870	+	412	ANNOTATED, CDS, c	NM_001488	TADA2A	Salzman2013	6871	circRNA	Detected	Detected	8.111360232	6.864957877
hsa_gci161695	2.362942152	1.24058431	up	6.775864113	5.535279803	CTACCCTCGTTAGGCCC	hsa_circ_0043332	chr17	36522169	36533668	+	429	ANNOTATED, CDS, c	NM_014598	SOCS7	Salzman2013	30837	circRNA	Detected	Detected	6.775864113	5.535279803

hsa_gci161719	-2.416308754	-1.272804813	down	2.767252576	4.040057389	ATACATGAACTCTCGAA	hsa_circ_0043405	chr17	37420427	37437716	-	582	ANNOTATED, CDS, c	NM_032875	FBXL20	Salzman2013	84961	circRNA	Not Detected	Detected	2.767252576	4.040057389
hsa_gci161721	2.947426882	1.559456024	up	8.983699289	7.424243265	TGTCGCCGTGTCCCATT	hsa_circ_0043410	chr17	37437641	37459892	-	592	ANNOTATED, CDS, c	NM_032875	FBXL20	Salzman2013	84961	circRNA	Detected	Detected	8.983699289	7.424243265
hsa_gci161740	12.37270958	3.629089575	up	5.854622004	2.225532429	AAAGAGGTATTGCTCCT	hsa_circ_0043450	chr17	37649003	37667883	+	660	ANNOTATED, CDS, c	NM_016507	CDK12	Salzman2013	51755	circRNA	Detected	Not Detected	5.854622004	2.225532429
hsa_gci161741	2.360393672	1.239027496	up	5.815946178	4.576918682	TTCCCGATGGTGTTTCA	hsa_circ_0043451	chr17	37673692	37673809	+	117	ANNOTATED, CDS, c	NM_016507	CDK12	Salzman2013	51755	circRNA	Detected	Detected	5.815946178	4.576918682
hsa_gci161745	2.577628949	1.366044602	up	5.962337158	4.596292556	GAGGGGTGTAGAGGGGC	hsa_circ_0043476	chr17	37895023	37903538	+	2191	ANNOTATED, CDS, c	NM_001242442	GRB7	Salzman2013	2886	circRNA	Detected	Detected	5.962337158	4.596292556
hsa_gci161766	2.076267795	1.053992533	up	5.347129722	4.29313719	GAGGGCCAAGTTAAGAA	hsa_circ_0043596	chr17	39679868	39680520	-	507	ANNOTATED, CDS, c	NM_002276	KRT19	Salzman2013	3880	circRNA	Detected	Detected	5.347129722	4.29313719
hsa_gci161775	6.439410333	2.686928585	up	4.133222904	1.446294319	GCCAAGAAAGAACATGA	hsa_circ_0043633	chr17	39775691	39776810	-	272	ANNOTATED, CDS, c	NM_000422	KRT17	Salzman2013	3872	circRNA	Detected	Not Detected	4.133222904	1.446294319
hsa_gci161780	2.202833676	1.139360569	up	5.160187151	4.020826582	CAAGAGGTGGCTGCTCA	hsa_circ_0043656	chr17	39910858	39914036	-	1602	ANNOTATED, CDS, c	NM_002230	JUP	Salzman2013	3728	circRNA	Detected	Detected	5.160187151	4.020826582
hsa_gci161783	2.481101018	1.310980476	up	9.413966329	8.102985853	TATCTTGTCCTGGCTGA	hsa_circ_0043660	chr17	39910858	39923832	-	2668	ANNOTATED, CDS, c	NM_002230	JUP	Salzman2013	3728	circRNA	Detected	Detected	9.413966329	8.102985853
hsa_gci161796	2.543859473	1.347018976	up	5.099519654	3.752500678	CAGAGACACGGTTTCTG	hsa_circ_0043704	chr17	40023178	40055038	-	3007	ANNOTATED, CDS, c	NM_001096	ACLY	Salzman2013	47	circRNA	Detected	Detected	5.099519654	3.752500678
hsa_gci161804	3.631374066	1.860515549	up	7.414517696	5.554002146	CCCCGTGCACCGTCATT	hsa_circ_0043763	chr17	40265128	40271454	-	2171	ANNOTATED, CDS, c	NM_021078	KAT2A	Salzman2013	2648	circRNA	Detected	Detected	7.414517696	5.554002146
hsa_gci161811	2.38627064	1.254757676	up	4.614070659	3.359312982	AAGTGTAGACCTAAATT	hsa_circ_0043825	chr17	40618446	40653322	+	1908	ANNOTATED, CDS, c	NM_001130020	ATP6V0A1	Salzman2013	535	circRNA	Detected	Detected	4.614070659	3.359312982
hsa_gci161824	3.097601093	1.631151367	up	5.480417345	3.849265978	GGTCCTTTGGGCCTTCG	hsa_circ_0043902	chr17	40925453	40931617	+	1101	ANNOTATED, CDS, c	NM_032353	VPS25	Salzman2013	84313	circRNA	Detected	Detected	5.480417345	3.849265978
hsa_gci161826	5.212966022	2.382104457	up	4.033960825	1.651856369	AACAAGACCGGTGAGGA	hsa_circ_0043908	chr17	40928262	40931617	+	719	ANNOTATED, CDS, c	NM_032353	VPS25	Salzman2013	84313	circRNA	Detected	Not Detected	4.033960825	1.651856369
hsa_gci161834	3.33920561	1.739504929	up	6.41350179	4.67399686	GTCTCGTACCGACATCC	hsa_circ_0043930	chr17	41151949	41154971	+	364	ANNOTATED, CDS, c	NM_000988	RPL27	Salzman2013	6155	circRNA	Detected	Detected	6.41350179	4.67399686
hsa_gci161840	-6.33714694	-2.663833468	down	1.309892811	3.973726279	TTATAAAGTCACAGGCA	hsa_circ_0043947	chr17	41199659	41215968	-	393	ANNOTATED, CDS, c	NM_007300	BRCA1	Salzman2013	672	circRNA	Not Detected	Detected	1.309892811	3.973726279
hsa_gci161852	2.834970918	1.503333935	up	4.716784545	3.21345061	AGACGTTCCCGAACAAA	hsa_circ_0044000	chr17	41598736	41601680	+	1062	ANNOTATED, CDS, c	NM_004941	DHX8	Salzman2013	1659	circRNA	Detected	Detected	4.716784545	3.21345061
hsa_gci161860	3.834931371	1.93920076	up	8.025891602	6.086690842	CGAGAAGTGGTCGAGTA	hsa_circ_0044053	chr17	42396992	42398099	-	765	ANNOTATED, CDS, c	NM_001143780	SLC25A39	Salzman2013	51629	circRNA	Detected	Detected	8.025891602	6.086690842
hsa_gci161863	2.229180259	1.156513283	up	6.894240445	5.737727162	GGAAGTGGTGCAGGTCC	hsa_circ_0044056	chr17	42396992	42400711	-	1371	ANNOTATED, CDS, c	NM_001143780	SLC25A39	Salzman2013	51629	circRNA	Detected	Detected	6.894240445	5.737727162
hsa_gci161866	2.122907337	1.0860414	up	7.201859746	6.115818346	ATAGTCCCGTGACCTAC	hsa_circ_0044066	chr17	42427595	42430470	+	1737	ANNOTATED, CDS, c	NM_002087	GRN	Salzman2013	2896	circRNA	Detected	Detected	7.201859746	6.115818346
hsa_gci161878	-12.23934909	-3.61345493	down	1.388351224	5.001806154	TAAGACTGGACGCAGAG	hsa_circ_0044188	chr17	44144914	44172067	-	27153	INTERGENIC	None		Salzman2013		circRNA	Not Detected	Detected	1.388351224	5.001806154
hsa_gci161907	4.115144579	2.040943118	up	6.714366792	4.673423674	ATTGTCCAGGATTCAGG	hsa_circ_0044331	chr17	46018888	46026674	+	3465	ANNOTATED, CDS, c	NM_018129	PNPO	Salzman2013	55163	circRNA	Detected	Detected	6.714366792	4.673423674
hsa_gci161909	3.246291078	1.698792364	up	7.056383077	5.357590712	GTTGTACGCTCGTCTCC	hsa_circ_0044333	chr17	46021981	46026674	+	3049	ANNOTATED, CDS, c	NM_018129	PNPO	Salzman2013	55163	circRNA	Detected	Detected	7.056383077	5.357590712
hsa_gci161910	2.749856141	1.459356146	up	8.581538079	7.122181933	TGTTCCCTTCGTTTCCC	hsa_circ_0044334	chr17	46022924	46026674	+	2949	ANNOTATED, CDS, c	NM_018129	PNPO	Salzman2013	55163	circRNA	Detected	Detected	8.581538079	7.122181933
hsa_gci161916	2.288534848	1.194424261	up	4.819363021	3.62493876	CCTCTTTACTTATTACG	hsa_circ_0044367	chr17	46970770	46973232	+	521	ANNOTATED, CDS, c	NM_005175	ATP5MC1	Salzman2013	516	circRNA	Detected	Detected	4.819363021	3.62493876
hsa_gci161919	3.004581823	1.587164211	up	8.432139046	6.844974835	GACGACGAACTCTCTGC	hsa_circ_0044371	chr17	46973016	46973232	+	216	ANNOTATED, CDS, c	NM_005175	ATP5MC1	Salzman2013	516	circRNA	Detected	Detected	8.432139046	6.844974835
hsa_gci161920	2.675570742	1.419846674	up	6.844160823	5.424314148	AAGGACCTCCTCCGAGG	hsa_circ_0044375	chr17	46988169	47006422	+	2674	ANNOTATED, CDS, c	NM_023079	UBE2Z	Salzman2013	65264	circRNA	Detected	Detected	6.844160823	5.424314148
hsa_gci161934	2.632379632	1.396367564	up	6.821467937	5.425100373	GTCGTAGTTTCGCTCGC	hsa_circ_0044420	chr17	47481419	47486520	-	1360	ANNOTATED, CDS, c	NM_002634	PHB	Salzman2013	5245	circRNA	Detected	Detected	6.821467937	5.425100373
hsa_gci161942	-5.894267283	-2.559312483	down	2.960205555	5.519518038	GTAAATGGGCCTGAGCC	hsa_circ_0044493	chr17	48158673	48167849	+	1743	ANNOTATED, CDS, c	NM_002204	ITGA3	Salzman2013	3675	circRNA	Not Detected	Detected	2.960205555	5.519518038
hsa_gci161952	2.322303853	1.215556748	up	4.203749445	2.988192697	AACTAAAAAATTGAGGA	hsa_circ_0044635	chr17	49052131	49071296	-	1513	ANNOTATED, CDS, c	NM_001251971	SPAG9	Salzman2013	9043	circRNA	Detected	Not Detected	4.203749445	2.988192697
hsa_gci161960	2.017659841	1.01268297	up	5.175572044	4.162889075	CATGGATCGGTGTCCCG	hsa_circ_0044656	chr17	49231585	49249105	+	1168	ANNOTATED, ncRNA,	NR_037149	NME1-NME2	Salzman2013	654364	circRNA	Detected	Detected	5.175572044	4.162889075
hsa_gci161988	2.163283074	1.11322246	up	4.224211343	3.110988883	GTCGCGGCTCGCGCCGG	hsa_circ_0044753	chr17	56048909	56065615	-	4633	ANNOTATED, CDS, c	NM_007146	VEZF1	Salzman2013	7716	circRNA	Detected	Not Detected	4.224211343	3.110988883
hsa_gci161998	2.750283152	1.459580157	up	5.709798439	4.250218282	CACGGACGAGGGTCTGA	hsa_circ_0044801	chr17	57075560	57093160	-	1686	ANNOTATED, CDS, c	NM_015294	TRIM37	Salzman2013	4591	circRNA	Detected	Detected	5.709798439	4.250218282
hsa_gci162011	2.961540018	1.566347581	up	4.627427113	3.061079532	GGGAGTGCGTCTCCAGA	hsa_circ_0044838	chr17	57430575	57479095	+	5099	ANNOTATED, CDS, c	NM_001005404	YPEL2	Salzman2013	388403	circRNA	Detected	Not Detected	4.627427113	3.061079532
hsa_gci162018	2.267418146	1.18105047	up	4.320162491	3.139112021	GGACCCACTGTTCAAGG	hsa_circ_0044867	chr17	57774666	57784856	-	798	ANNOTATED, CDS, c	NM_016077	PTRH2	Salzman2013	51651	circRNA	Detected	Detected	4.320162491	3.139112021
hsa_gci162025	2.089293158	1.063014938	up	7.279532907	6.216517969	CGAAGACGTCTCTACCT	hsa_circ_0044889	chr17	57915655	57917952	+	927	ANNOTATED, CDS, c	NM_030938	VMP1	Salzman2013	81671	circRNA	Detected	Detected	7.279532907	6.216517969
hsa_gci162035	2.277305969	1.187328139	up	5.750864499	4.56353636	GATTATGGGCGAAATTT	hsa_circ_0044927	chr17	58275620	58292135	-	1567	ANNOTATED, CDS, c	NM_032582	USP32	Salzman2013	84669	circRNA	Detected	Detected	5.750864499	4.56353636
hsa_gci162052	5.308385792	2.408273224	up	6.815475653	4.407202429	ACCCAGAAGAACCCCTT	hsa_circ_0044976	chr17	58756813	58952099	+	666	ANNOTATED, CDS, c	NM_001099432	BCAS3	Salzman2013	54828	circRNA	Detected	Detected	6.815475653	4.407202429
hsa_gci162056	2.538671524	1.344073738	up	6.947484745	5.603411007	TGTGTTGTCATGCCACC	hsa_circ_0044989	chr17	58945936	58952099	+	185	ANNOTATED, CDS, c	NM_001099432	BCAS3	Salzman2013	54828	circRNA	Detected	Detected	6.947484745	5.603411007
hsa_gci162057	2.819993298	1.495691734	up	7.359290351	5.863598617	TTCACTTATGTCTAGGT	hsa_circ_0044990	chr17	58945936	58967132	+	262	ANNOTATED, CDS, c	NM_001099432	BCAS3	Salzman2013	54828	circRNA	Detected	Detected	7.359290351	5.863598617
hsa_gci162059	2.120519705	1.08441789	up	9.346927451	8.262509561	ACTTTAAACACCTCCTC	hsa_circ_0045000	chr17	59112026	59118253	+	392	ANNOTATED, CDS, c	NM_001099432	BCAS3	Salzman2013	54828	circRNA	Detected	Detected	9.346927451	8.262509561
hsa_gci162061	2.016841484	1.012097698	up	12.31806121	11.30596351	GATCTTGTCACCGGTCC	hsa_circ_0045013	chr17	59820373	59886118	-	1752	ANNOTATED, CDS, c	NM_032043	BRIP1	Salzman2013	83990	circRNA	Detected	Detected	12.31806121	11.30596351
hsa_gci162082	2.450978345	1.293357738	up	5.73514954	4.441791802	GCCCGTACTGATCATCC	hsa_circ_0045087	chr17	60064367	60064489	-	122	ANNOTATED, CDS, c	NM_005121	MED13	Salzman2013	9969	circRNA	Detected	Detected	5.73514954	4.441791802
hsa_gci162083	2.135866834	1.094821701	up	4.731043034	3.636221333	CCTCGTCCTGTTCACAA	hsa_circ_0045096	chr17	60106901	60112969	-	813	ANNOTATED, CDS, c	NM_005121	MED13	Salzman2013	9969	circRNA	Detected	Detected	4.731043034	3.636221333
hsa_gci162102	2.002038832	1.001469957	up	6.69196191	5.690491953	GTTCACATCCGTAATAG	hsa_circ_0045194	chr17	61417461	61432751	+	1007	ANNOTATED, CDS, c	NM_025185	TANC2	Salzman2013	26115	circRNA	Detected	Detected	6.69196191	5.690491953
hsa_gci162115	2.016542026	1.011883473	up	6.680784736	5.668901264	GTTTACCACCTATCAGT	hsa_circ_0045234	chr17	61882430	61884014	+	225	ANNOTATED, CDS, c	NM_007372	DDX42	Salzman2013	11325	circRNA	Detected	Detected	6.680784736	5.668901264
hsa_gci162117	2.11094495	1.077888956	up	6.544879626	5.466990671	TCGGTAGTCACAGTTTA	hsa_circ_0045241	chr17	61896792	61902796	-	2513	ANNOTATED, CDS, c	NM_017647	FTSJ3	Salzman2013	117246	circRNA	Detected	Detected	6.544879626	5.466990671
hsa_gci162135	9.14460232	3.192920432	up	5.233627047	2.040706615	TAGACCGAACCCTGTCC	hsa_circ_0045360	chr17	64222145	64224314	-	274	ANNOTATED, CDS, c	NM_000042	APOH	Salzman2013	350	circRNA	Detected	Not Detected	5.233627047	2.040706615
hsa_gci162138	2.000277238	1.000199971	up	9.790833529	8.790633558	GGCATTCACACCTAAAA	hsa_circ_0045373	chr17	64637472	64806862	+	8438	ANNOTATED, CDS, c	NM_002737	PRKCA	Salzman2013	5578	circRNA	Detected	Detected	9.790833529	8.790633558
hsa_gci162139	2.571347483	1.362524584	up	8.768903224	7.406378639	ACCCCTGTAACTACTGC	hsa_circ_0045374	chr17	64641500	64806862	+	8326	ANNOTATED, CDS, c	NM_002737	PRKCA	Salzman2013	5578	circRNA	Detected	Detected	8.768903224	7.406378639
hsa_gci162173	2.421638609	1.275983582	up	5.826905637	4.550922055	CCCTTTTCCTGCTTTTT	hsa_circ_0045539	chr17	72199794	72206019	+	392	ANNOTATED, CDS, c	NM_000999	RPL38	Salzman2013	6169	circRNA	Detected	Detected	5.826905637	4.550922055

hsa_gci162174	2.316197518	1.211758287	up	6.953991145	5.742232858	GCGACCCGAGACCGCGC	hsa_circ_0045541	chr17	72200056	72206019	+	302	ANNOTATED, CDS, c	NM_000999	RPL38	Salzman2013	6169	circRNA	Detected	Detected	6.953991145	5.742232858
hsa_gci162175	2.173398216	1.119952533	up	4.495151007	3.375198474	GCCCGACACTCGTCCTT	hsa_circ_0045543	chr17	72200268	72206019	+	261	ANNOTATED, CDS, c	NM_000999	RPL38	Salzman2013	6169	circRNA	Detected	Detected	4.495151007	3.375198474
hsa_gci162178	2.121285757	1.084938979	up	5.513099371	4.428160392	GGGGGGTCCTGGTGATG	hsa_circ_0045581	chr17	73015794	73017356	+	654	ANNOTATED, CDS, c	NM_001545	MRPL58	Salzman2013	3396	circRNA	Detected	Detected	5.513099371	4.428160392
hsa_gci162186	-3.950288977	-1.981958195	down	2.298397598	4.280355793	AGCCATCAAAAATGAGT	hsa_circ_0045608	chr17	73208086	73221924	+	697	ANNOTATED, CDS, c	NM_024844	NUP85	Salzman2013	79902	circRNA	Not Detected	Detected	2.298397598	4.280355793
hsa_gci162190	4.824873595	2.270491146	up	8.198039423	5.927548277	ACAACCCGGTCCCGAGG	hsa_circ_0045682	chr17	73725345	73753899	+	5166	ANNOTATED, CDS, c	NM_000213	ITGB4	Salzman2013	3691	circRNA	Detected	Detected	8.198039423	5.927548277
hsa_gci162199	2.52287685	1.335069785	up	7.854122534	6.519052748	CGTAGCGACCCTCGATT	hsa_circ_0045822	chr17	74559200	74561430	+	2230	ANNOTATED, ncRNA,	NR_038108		Salzman2013		circRNA	Detected	Detected	7.854122534	6.519052748
hsa_gci162205	2.292341136	1.196821756	up	9.773514646	8.57669289	GGTTAGCAGCCCCCTAT	hsa_circ_0045849	chr17	75085234	75089429	+	223	ANNOTATED, coding	NM_001204410	SEC14L1	Salzman2013	6397	circRNA	Detected	Detected	9.773514646	8.57669289
hsa_gci162207	3.502616594	1.808433075	up	4.8643285	3.055895425	GCCTCCTTGGCTTCTTA	hsa_circ_0045854	chr17	75196565	75201428	+	350	ANNOTATED, CDS, c	NM_001204410	SEC14L1	Salzman2013	6397	circRNA	Detected	Not Detected	4.8643285	3.055895425
hsa_gci162210	2.971644908	1.571261733	up	5.401167731	3.829905998	GAGTCCGGGAAACTTGT	hsa_circ_0045942	chr17	76849058	76867088	-	3122	ANNOTATED, CDS, c	NM_003255	TIMP2	Salzman2013	7077	circRNA	Detected	Detected	5.401167731	3.829905998
hsa_gci162218	3.474197254	1.796679668	up	4.028537673	2.231858005	GAAGGAAGCGAAGGAGG	hsa_circ_0045957	chr17	76987801	76988222	-	421	ALT_ACCEPTOR, ALT	NM_138793	CANT1	Salzman2013	124583	circRNA	Detected	Not Detected	4.028537673	2.231858005
hsa_gci162228	2.167178526	1.115818003	up	9.387257642	8.271439639	ATCGGCTTTTTGGGGTG	hsa_circ_0046110	chr17	79224670	79249951	-	1958	ANNOTATED, CDS, c	NM_138570	SLC38A10	Salzman2013	124565	circRNA	Detected	Detected	9.387257642	8.271439639
hsa_gci162230	2.037317308	1.026670694	up	6.361351867	5.334681172	ACGGCGTTCTAAGGTAT	hsa_circ_0046144	chr17	79477504	79478134	-	537	ANNOTATED, INTERN	NR_037688	ACTG1	Salzman2013	71	circRNA	Detected	Detected	6.361351867	5.334681172
hsa_gci162231	2.257330333	1.174617555	up	9.60987927	8.435261715	TACCGGCCCCACAACTT	hsa_circ_0046145	chr17	79477504	79478652	-	976	ANNOTATED, INTERN	NR_037688	ACTG1	Salzman2013	71	circRNA	Detected	Detected	9.60987927	8.435261715
hsa_gci162241	2.158951203	1.110330636	up	8.382956106	7.272625471	AAGTGCCCTCCGAGCTC	hsa_circ_0046188	chr17	79555969	79575848	-	846	ANNOTATED, CDS, c	NM_017921	NPLOC4	Salzman2013	55666	circRNA	Detected	Detected	8.382956106	7.272625471
hsa_gci162260	2.134543229	1.093927381	up	7.01442967	5.92050229	ACTTTATTGCTAAGTCT	hsa_circ_0046435	chr17	80540616	80545148	+	877	ANNOTATED, CDS, c	NM_004514	FOXK2	Salzman2013	3607	circRNA	Detected	Detected	7.01442967	5.92050229
hsa_gci162297	2.613482717	1.385973619	up	4.849174963	3.463201344	ACTTCTAGACCTGAGTC	hsa_circ_0046592	chr17	80863811	80901062	+	2498	ANNOTATED, CDS, c	NM_005993	TBCD	Salzman2013	6904	circRNA	Detected	Detected	4.849174963	3.463201344
hsa_gci162320	2.392656174	1.258613095	up	4.429374089	3.170760994	CAGAAGGAGGGGGGATA	hsa_circ_0046752	chr18	3247527	3256234	+	1229	ANNOTATED, CDS, c	NM_006471	MYL12A	Salzman2013	10627	circRNA	Detected	Detected	4.429374089	3.170760994
hsa_gci162368	2.610239052	1.384181938	up	4.77479577	3.390613832	GTTATAATTTGGTTTGC	hsa_circ_0046964	chr18	12356692	12360050	-	537	ANNOTATED, CDS, c	NM_006796	AFG3L2	Salzman2013	10939	circRNA	Detected	Detected	4.77479577	3.390613832
hsa_gci162369	2.694708441	1.430129186	up	5.37545584	3.945326654	ATTGTTTAGCCATTTCA	hsa_circ_0046965	chr18	12356692	12371690	-	1050	ANNOTATED, CDS, c	NM_006796	AFG3L2	Salzman2013	10939	circRNA	Detected	Detected	5.37545584	3.945326654
hsa_gci162421	2.210111704	1.144119289	up	8.155682008	7.011562719	AGTGGTTCCCCCGAGTA	hsa_circ_0047135	chr18	20570899	20576425	+	1221	ANNOTATED, CDS, c	NM_002894	RBBP8	Salzman2013	5932	circRNA	Detected	Detected	8.155682008	7.011562719
hsa_gci162425	5.543913734	2.470904809	up	5.095691641	2.624786833	GTTTTCTACAGATCGGC	hsa_circ_0047167	chr18	21115432	21121172	-	1104	ANNOTATED, CDS, c	NM_000271	NPC1	Salzman2013	4864	circRNA	Detected	Not Detected	5.095691641	2.624786833
hsa_gci162490	2.16006384	1.111073952	up	8.074259878	6.963185927	GCAAGACCTCGGCCCTG	hsa_circ_0047467	chr18	34376033	34409158	-	2065	ANNOTATED, CDS, c	NM_015476	TPGS2	Salzman2013	25941	circRNA	Detected	Detected	8.074259878	6.963185927
hsa_gci162529	2.142903918	1.099567165	up	6.599760516	5.500193351	TGACTATATAGGTCCTC	hsa_circ_0047610	chr18	45391429	45391504	-	75	ANNOTATED, CDS, c	NM_001003652	SMAD2	Salzman2013	4087	circRNA	Detected	Detected	6.599760516	5.500193351
hsa_gci162572	2.388400289	1.256044648	up	11.01875502	9.762710368	AACAGGAATCATTCTTC	hsa_circ_0047786	chr18	55398645	55401328	+	2683	ANTISENSE, CDS, c	NM_005603	ATP8B1	Salzman2013	5205	circRNA	Detected	Detected	11.01875502	9.762710368
hsa_gci162591	2.082287222	1.058169082	up	5.97218592	4.914016838	CCGTGTTGGTTCCTTCT	hsa_circ_0047850	chr18	59757708	59763183	-	206	ANNOTATED, CDS, c	NM_176787	PIGN	Salzman2013	23556	circRNA	Detected	Detected	5.97218592	4.914016838
hsa_gci162598	3.111726972	1.637715482	up	4.530345845	2.892630363	GAAAGGCATCAAGTTTG	hsa_circ_0047870	chr18	59888269	59899660	+	1004	ANNOTATED, CDS, c	NM_020854	KIAA1468	Salzman2013	57614	circRNA	Detected	Not Detected	4.530345845	2.892630363
hsa_gci162599	4.109288906	2.038888763	up	5.190498654	3.151609891	TACCTCCTCGCTAGACG	hsa_circ_0047880	chr18	60015400	60017170	+	208	ANNOTATED, CDS, c	NM_003839	TNFRSF11A	Salzman2013	8792	circRNA	Detected	Detected	5.190498654	3.151609891
hsa_gci162612	2.512718434	1.329249017	up	5.691646899	4.362397882	TGAAGATAGTGTATTCA	hsa_circ_0047964	chr18	72589152	72601994	+	372	ANNOTATED, CDS, c	NM_017757	ZNF407	Salzman2013	55628	circRNA	Detected	Detected	5.691646899	4.362397882
hsa_gci162619	2.698882941	1.432362404	up	4.199737559	2.767375155	ATCACAAGAGGCGTAAG	hsa_circ_0047976	chr18	74561481	74620529	+	2496	ANNOTATED, CDS, c	NM_007345	ZNF236	Salzman2013	7776	circRNA	Detected	Not Detected	4.199737559	2.767375155
hsa_gci162634	2.674895564	1.419482566	up	7.000939037	5.581456472	GTTCGTGTCTCGGGTAC	hsa_circ_0048035	chr19	305574	311939	-	1864	ANNOTATED, CDS, c	NM_017550	MIER2	Salzman2013	54531	circRNA	Detected	Detected	7.000939037	5.581456472
hsa_gci162664	2.294293427	1.198049915	up	11.09651209	9.89846217	TATACGGCTTGACAAGG	hsa_circ_0048392	chr19	2233154	2233904	-	750	ANNOTATED, CDS, c	NM_018049	PLEKHJ1	Salzman2013	55111	circRNA	Detected	Detected	11.09651209	9.89846217
hsa_gci162667	2.437042501	1.285131411	up	8.750082152	7.464950741	GCGGGGCGCGGGAAACT	hsa_circ_0048406	chr19	2321519	2328614	-	520	ANNOTATED, CDS, c	NM_016199	LSM7	Salzman2013	51690	circRNA	Detected	Detected	8.750082152	7.464950741
hsa_gci162669	2.240992377	1.164137742	up	4.564297281	3.40015954	GGGGCGTGGTCGACGGG	hsa_circ_0048410	chr19	2428163	2431900	-	3018	ANNOTATED, CDS, c	NM_032737	LMNB2	Salzman2013	84823	circRNA	Detected	Detected	4.564297281	3.40015954
hsa_gci162671	4.72797603	2.241222721	up	6.181101627	3.939878906	GGAACCCGGTCGGCGCA	hsa_circ_0048496	chr19	3574380	3579081	+	1369	ANNOTATED, CDS, c	NM_006339	HMG20B	Salzman2013	10362	circRNA	Detected	Detected	6.181101627	3.939878906
hsa_gci162676	2.383628265	1.25315926	up	6.01511985	4.76196059	GAGCGCCATGCTCTGCT	hsa_circ_0048534	chr19	3976053	3978170	-	1362	ANNOTATED, CDS, c	NM_001961	EEF2	Salzman2013	1938	circRNA	Detected	Detected	6.01511985	4.76196059
hsa_gci162683	2.108384553	1.076138028	up	5.623291004	4.547152977	GGTGCCATGGCTCCTGT	hsa_circ_0048541	chr19	3976053	3982422	-	2463	ANNOTATED, CDS, c	NM_001961	EEF2	Salzman2013	1938	circRNA	Detected	Detected	5.623291004	4.547152977
hsa_gci162687	2.037171446	1.026567401	up	7.020780022	5.994212621	CTGCTGCCGCCTTCTCA	hsa_circ_0048545	chr19	3976053	3985461	-	3158	ANNOTATED, CDS, c	NM_001961	EEF2	Salzman2013	1938	circRNA	Detected	Detected	7.020780022	5.994212621
hsa_gci162696	2.108221919	1.076026738	up	8.840467158	7.76444042	CCCCACGTCCACCTACA	hsa_circ_0048617	chr19	4445002	4447622	-	1279	ANNOTATED, CDS, c	NM_025241	UBXN6	Salzman2013	80700	circRNA	Detected	Detected	8.840467158	7.76444042
hsa_gci162762	3.342137926	1.740771273	up	5.913677907	4.172906635	CGTCAGCCCTCATGAGG	hsa_circ_0049298	chr19	10745431	10755235	+	2472	ANNOTATED, CDS, c	NM_020428	SLC44A2	Salzman2013	57153	circRNA	Detected	Detected	5.913677907	4.172906635
hsa_gci162771	3.154074597	1.657216782	up	9.106302353	7.449085571	ATACAGCCCCTTCCGAA	hsa_circ_0049449	chr19	11453451	11456091	-	509	ANNOTATED, CDS, c	NM_198536	TMEM205	Salzman2013	374882	circRNA	Detected	Detected	9.106302353	7.449085571
hsa_gci162779	2.203959894	1.140097971	up	5.723658773	4.583560802	CCGTCTCTACCTGTCGC	hsa_circ_0049535	chr19	12778880	12779239	-	359	ANNOTATED, CDS, c	NM_016145	WDR83OS	Salzman2013	51398	circRNA	Detected	Detected	5.723658773	4.583560802
hsa_gci162789	4.026773063	2.009624168	up	6.799601926	4.789977758	AATGGCCTCCGGTACTG	hsa_circ_0049605	chr19	12907633	12912084	-	867	ANNOTATED, CDS, c	NM_005809	PRDX2	Salzman2013	7001	circRNA	Detected	Detected	6.799601926	4.789977758
hsa_gci162792	2.330062287	1.220368521	up	4.298136348	3.077767827	CACAAACCTAAGCTAGG	hsa_circ_0049639	chr19	13049947	13055304	+	1740	ANNOTATED, CDS, c	NM_004343	CALR	Salzman2013	811	circRNA	Detected	Not Detected	4.298136348	3.077767827
hsa_gci162796	3.929677349	1.974410863	up	7.898624872	5.924214009	CCCAGTCCCTATACGAG	hsa_circ_0049648	chr19	13051354	13055304	+	1129	ANNOTATED, CDS, c	NM_004343	CALR	Salzman2013	811	circRNA	Detected	Detected	7.898624872	5.924214009
hsa_gci162800	2.04802586	1.034233932	up	7.949601162	6.91536723	TGCCGACAACCCGACTG	hsa_circ_0049672	chr19	13255223	13259905	-	450	ANNOTATED, CDS, c	NM_003765	STX10	Salzman2013	8677	circRNA	Detected	Detected	7.949601162	6.91536723
hsa_gci162804	3.107845029	1.635914566	up	6.291902557	4.65598799	CCCCAAGCTCCGGTTCT	hsa_circ_0049720	chr19	14230320	14231477	-	1157	ANNOTATED, CDS, c	NM_018154	ASF1B	Salzman2013	55723	circRNA	Detected	Detected	6.291902557	4.65598799
hsa_gci162808	3.090034136	1.627622776	up	5.154014617	3.526391841	GTTTGAGGTCTGGCCCG	hsa_circ_0049758	chr19	14588570	14606944	-	1929	ANNOTATED, CDS, c	NM_005716	GIPC1	Salzman2013	10755	circRNA	Detected	Detected	5.154014617	3.526391841
hsa_gci162829	2.624751356	1.392180762	up	7.364366681	5.97218592	CGGGGGCCAGGTGGTGC	hsa_circ_0050019	chr19	17690290	17693965	+	2315	ANNOTATED, CDS, c	NM_024656	COLGALT1	Salzman2013	79709	circRNA	Detected	Detected	7.364366681	5.97218592
hsa_gci162837	2.326539543	1.218185708	up	5.724445823	4.506260115	CCGGGGGCGTCCATATC	hsa_circ_0050095	chr19	18285849	18288927	+	827	ANNOTATED, CDS, c	NM_006332	IFI30	Salzman2013	10437	circRNA	Detected	Detected	5.724445823	4.506260115
hsa_gci162842	2.131511628	1.091876925	up	8.122210703	7.030333778	GAGAAGTCCCAGACTTA	hsa_circ_0050178	chr19	19040009	19042220	-	521	ANNOTATED, ncRNA,	NR_027297	HOMER3	Salzman2013	9454	circRNA	Detected	Detected	8.122210703	7.030333778
hsa_gci162843	2.475308919	1.307608585	up	8.14874892	6.841140334	GACCTTGAAGAGCCGTT	hsa_circ_0050181	chr19	19040009	19045219	-	1025	ANNOTATED, ncRNA,	NR_027297	HOMER3	Salzman2013	9454	circRNA	Detected	Detected	8.14874892	6.841140334

hsa_gci162849	2.488553406	1.315307349	up	7.547161297	6.231853949	GAGGTCTCTCTTAAGAT	hsa_circ_0050274	chr19	20295164	20296891	+	223	ANNOTATED, CDS, c	NM_052852	ZNF486	Salzman2013	90649	circRNA	Detected	Detected	7.547161297	6.231853949
hsa_gci162851	2.004428288	1.003190803	up	7.32602613	6.322835327	GAATCTTTGTCGTTATG	hsa_circ_0050282	chr19	21216891	21216990	+	99	ANNOTATED, CDS, c	NM_025189	ZNF430	Salzman2013	80264	circRNA	Detected	Detected	7.32602613	6.322835327
hsa_gci162852	2.028477762	1.020397486	up	8.321693529	7.301296043	GGACCTCGAGTCCGACG	hsa_circ_0050284	chr19	21324839	21326446	+	243	ANNOTATED, CDS, c	NM_133473	ZNF431	Salzman2013	170959	circRNA	Detected	Detected	8.321693529	7.301296043
hsa_gci162855	2.866795785	1.519439138	up	6.981899587	5.462460449	CTTAAGATACCGGTGTA	hsa_circ_0050299	chr19	22000689	22002023	-	226	ANNOTATED, CDS, c	NM_003423	ZNF43	Salzman2013	7594	circRNA	Detected	Detected	6.981899587	5.462460449
hsa_gci162859	2.529235012	1.338701096	up	8.189799803	6.851098708	CGGGCCCCCAGGTTACC	hsa_circ_0050320	chr19	28283907	28284140	-	233	ANNOTATED, INTERN	TCONS_00027260		Salzman2013		circRNA	Detected	Detected	8.189799803	6.851098708
hsa_gci162869	2.077643513	1.054948135	up	7.717790272	6.662842138	CCTCATACCCATTTACA	hsa_circ_0050430	chr19	33482728	33493906	-	884	ANNOTATED, CDS, c	NM_033103	RHPN2	Salzman2013	85415	circRNA	Detected	Detected	7.717790272	6.662842138
hsa_gci162872	-5.395050581	-2.431636486	down	1.802375653	4.234012139	GTGAGTTGCTAGAGACC	hsa_circ_0050447	chr19	33877854	33968996	-	1374	ANNOTATED, CDS, c	NM_000285	PEPD	Salzman2013	5184	circRNA	Not Detected	Detected	1.802375653	4.234012139
hsa_gci162873	2.100506378	1.070737167	up	4.48339413	3.412656963	GAGGCCAGAGCAGGACC	hsa_circ_0050454	chr19	34287750	34306666	+	4932	ANNOTATED, CDS, c	NM_024076	KCTD15	Salzman2013	79047	circRNA	Detected	Detected	4.48339413	3.412656963
hsa_gci162887	2.782339139	1.476298281	up	5.20404955	3.727751269	TCATCGCAGGAGGGTGG	hsa_circ_0050497	chr19	34884152	34893318	+	3250	ANNOTATED, CDS, c	NM_001184722	GPI	Salzman2013	2821	circRNA	Detected	Detected	5.20404955	3.727751269
hsa_gci162890	-58.60160769	-5.872868339	down	1.382450709	7.255319048	ACTTGTGTTATCTCAAC	hsa_circ_0050503	chr19	34890460	34893318	+	2656	ANNOTATED, CDS, c	NM_001184722	GPI	Salzman2013	2821	circRNA	Not Detected	Detected	1.382450709	7.255319048
hsa_gci162903	2.653889144	1.408108109	up	7.932951932	6.524843823	TATTGGTCATATTTGAC	hsa_circ_0050523	chr19	34924252	34957919	+	1448	ANNOTATED, CDS, c	NM_005499	UBA2	Salzman2013	10054	circRNA	Detected	Detected	7.932951932	6.524843823
hsa_gci162906	2.505322178	1.324996142	up	6.952240266	5.627244123	CGGTCCGTGTAAGATAA	hsa_circ_0050529	chr19	34925772	34960798	+	2237	ANNOTATED, CDS, c	NM_005499	UBA2	Salzman2013	10054	circRNA	Detected	Detected	6.952240266	5.627244123
hsa_gci162914	2.519855139	1.333340799	up	5.749689325	4.416348527	CAAACAGGTAACAGTTG	hsa_circ_0050547	chr19	34941169	34957919	+	970	ANNOTATED, CDS, c	NM_005499	UBA2	Salzman2013	10054	circRNA	Detected	Detected	5.749689325	4.416348527
hsa_gci162916	2.360456024	1.239065606	up	10.0926345	8.853568894	ATGGTATCGTTTAAAAA	hsa_circ_0050558	chr19	34951374	34957919	+	340	ANNOTATED, CDS, c	NM_005499	UBA2	Salzman2013	10054	circRNA	Detected	Detected	10.0926345	8.853568894
hsa_gci162918	2.151764166	1.105519967	up	6.701059287	5.595539321	GGAGCCACCACCAACGT	hsa_circ_0050600	chr19	35988118	35988440	-	322	ANNOTATED, coding	NM_033317	DMKN	Salzman2013	93099	circRNA	Detected	Detected	6.701059287	5.595539321
hsa_gci162943	2.425860882	1.278496817	up	6.907767294	5.629270476	TCGTTTCATTATGAACT	hsa_circ_0050820	chr19	38798067	38799968	-	492	ANNOTATED, CDS, c	NM_033557	YIF1B	Salzman2013	90522	circRNA	Detected	Detected	6.907767294	5.629270476
hsa_gci162947	2.452434962	1.294214877	up	7.340087934	6.045873057	GTAACTTGTTGAAATGC	hsa_circ_0050868	chr19	39090564	39092165	-	223	ANNOTATED, CDS, c	NM_007181	MAP4K1	Salzman2013	11184	circRNA	Detected	Detected	7.340087934	6.045873057
hsa_gci162948	3.221521912	1.687742408	up	6.771729491	5.083987084	AAAGGCCGACGGTGCAC	hsa_circ_0050880	chr19	39109721	39127595	+	879	ANNOTATED, CDS, c	NM_013234	EIF3K	Salzman2013	27335	circRNA	Detected	Detected	6.771729491	5.083987084
hsa_gci162950	-4.245411326	-2.085904339	down	1.224339118	3.310243457	GACTTTCTTCCGACCCA	hsa_circ_0050887	chr19	39114716	39123136	+	263	ANNOTATED, CDS, c	NM_013234	EIF3K	Salzman2013	27335	circRNA	Not Detected	Detected	1.224339118	3.310243457
hsa_gci162951	3.803442822	1.927305917	up	4.345550853	2.418244936	TTTCTTCCGACCCAACT	hsa_circ_0050889	chr19	39114716	39127595	+	534	ANNOTATED, CDS, c	NM_013234	EIF3K	Salzman2013	27335	circRNA	Detected	Not Detected	4.345550853	2.418244936
hsa_gci162953	2.276014564	1.186509789	up	3.851221159	2.664711369	GGTTCTCCAGGTACAAA	hsa_circ_0050892	chr19	39123069	39127595	+	338	ANNOTATED, CDS, c	NM_013234	EIF3K	Salzman2013	27335	circRNA	Detected	Not Detected	3.851221159	2.664711369
hsa_gci162959	2.082070437	1.058018877	up	5.46167615	4.403657273	TCGTTAGTGCCCGAACC	hsa_circ_0050928	chr19	39306061	39308004	-	704	ANNOTATED, CDS, c	NM_001398	ECH1	Salzman2013	1891	circRNA	Detected	Detected	5.46167615	4.403657273
hsa_gci162964	2.428604598	1.280127624	up	6.634345148	5.354217525	ATGTTACACATCTACAG	hsa_circ_0050989	chr19	39907190	39911492	+	853	ANNOTATED, CDS, c	NM_022835	PLEKHG2	Salzman2013	64857	circRNA	Detected	Detected	6.634345148	5.354217525
hsa_gci162971	3.538666127	1.82320565	up	8.069906216	6.246700566	CTTCAACCAGTGTAGTG	hsa_circ_0051017	chr19	39963048	39967308	+	1583	ANNOTATED, CDS, c	NM_003169	SUPT5H	Salzman2013	6829	circRNA	Detected	Detected	8.069906216	6.246700566
hsa_gci162990	2.25340525	1.17210679	up	5.994212621	4.822105831	GTTTGACCCTCTTCTAC	hsa_circ_0051141	chr19	41263236	41271297	+	1001	ANNOTATED, CDS, c	NM_004596	SNRPA	Salzman2013	6626	circRNA	Detected	Detected	5.994212621	4.822105831
hsa_gci162996	2.349097657	1.23210669	up	7.231186375	5.999079685	CTTTCGACGTCCCTCCA	hsa_circ_0051164	chr19	41727050	41767671	+	4239	ANNOTATED, CDS, c	NM_021913	AXL	Salzman2013	558	circRNA	Detected	Detected	7.231186375	5.999079685
hsa_gci163008	5.080678721	2.345021237	up	4.231456645	1.886435408	GTGTGCCTGTACAAGAG	hsa_circ_0051203	chr19	41762356	41767671	+	2511	ANNOTATED, CDS, c	NM_021913	AXL	Salzman2013	558	circRNA	Detected	Not Detected	4.231456645	1.886435408
hsa_gci163025	2.449694827	1.292602036	up	5.841296281	4.548694246	GACATGAACCCCCACAA	hsa_circ_0051340	chr19	44047463	44055839	-	900	ANNOTATED, CDS, c	NM_006297	XRCC1	Salzman2013	7515	circRNA	Detected	Detected	5.841296281	4.548694246
hsa_gci163026	-6.537681547	-2.708779105	down	1.368962919	4.077742024	CTGTCTCGCTCTGAGGT	hsa_circ_0051370	chr19	44337656	44341331	+	337	ANNOTATED, CDS, c	NM_181845	ZNF283	Salzman2013	284349	circRNA	Not Detected	Detected	1.368962919	4.077742024
hsa_gci163031	3.310584188	1.727085819	up	9.205659399	7.47857358	TGCACGTCCCGCCACTC	hsa_circ_0051394	chr19	45260583	45263301	+	1070	ANNOTATED, CDS, c	NM_005178	BCL3	Salzman2013	602	circRNA	Detected	Detected	9.205659399	7.47857358
hsa_gci163040	2.222222674	1.152003386	up	8.340405431	7.188402044	TGTCAGACAAGAGGTGT	hsa_circ_0051545	chr19	46190711	46192605	-	569	ANNOTATED, CDS, c	NM_177542	SNRPD2	Salzman2013	6633	circRNA	Detected	Detected	8.340405431	7.188402044
hsa_gci163041	-3.664994691	-1.873811108	down	2.929219618	4.803030727	TTTCCTTTCCGTCAGAC	hsa_circ_0051546	chr19	46190711	46195443	-	868	ANNOTATED, CDS, c	NM_177542	SNRPD2	Salzman2013	6633	circRNA	Not Detected	Detected	2.929219618	4.803030727
hsa_gci163045	2.280825309	1.189555953	up	6.753314518	5.563758565	GGCGCGCGCGGGGCGGA	hsa_circ_0051592	chr19	47104511	47114039	+	2259	ANNOTATED, CDS, c	NM_005184	CALM3	Salzman2013	808	circRNA	Detected	Detected	6.753314518	5.563758565
hsa_gci163047	2.32608734	1.217905268	up	8.678265466	7.460360198	CGTGAAGTAGTAGTCGC	hsa_circ_0051620	chr19	47278139	47280662	-	1195	ANNOTATED, CDS, c	NM_005628	SLC1A5	Salzman2013	6510	circRNA	Detected	Detected	8.678265466	7.460360198
hsa_gci163048	2.210784868	1.144558643	up	5.405505619	4.260946976	GGAGGTGGAGGACGGGG	hsa_circ_0051621	chr19	47278139	47285806	-	1596	ANNOTATED, CDS, c	NM_005628	SLC1A5	Salzman2013	6510	circRNA	Detected	Detected	5.405505619	4.260946976
hsa_gci163052	3.524904193	1.817584045	up	7.712558469	5.894974424	CCCAGGTGGCCCAGGTC	hsa_circ_0051648	chr19	47567446	47575378	-	4276	ANNOTATED, CDS, c	NM_015168	ZC3H4	Salzman2013	23211	circRNA	Detected	Detected	7.712558469	5.894974424
hsa_gci163062	2.103557884	1.072831518	up	9.827912364	8.755080847	CGCCGGCCCCTTGAGTA	hsa_circ_0051706	chr19	48284120	48287943	+	625	ANNOTATED, CDS, c	NM_003009	SELENOW	Salzman2013	6415	circRNA	Detected	Detected	9.827912364	8.755080847
hsa_gci163067	2.15404019	1.107045168	up	6.967134772	5.860089605	GGGAACCTCGCCTGCAT	hsa_circ_0051757	chr19	49118587	49118704	-	117	ANNOTATED, CDS, c	NM_000979	RPL18	Salzman2013	6141	circRNA	Detected	Detected	6.967134772	5.860089605
hsa_gci163074	2.915351954	1.543670063	up	7.997051226	6.453381163	GGACAACGTGATTGTGC	hsa_circ_0051777	chr19	49298318	49300590	-	848	ANNOTATED, ncRNA,	NR_028450	BCAT2	Salzman2013	587	circRNA	Detected	Detected	7.997051226	6.453381163
hsa_gci163086	2.102221044	1.071914373	up	6.020159154	4.948244781	ACTACTTTTGGAAGGGA	hsa_circ_0051875	chr19	49952348	49952911	-	563	ALT_DONOR, CDS, c	NM_017916	PIH1D1	Salzman2013	55011	circRNA	Detected	Detected	6.020159154	4.948244781
hsa_gci163088	2.039465343	1.028190991	up	4.554517579	3.526326588	GGGTCCTCTACCGGAGC	hsa_circ_0051891	chr19	49993106	49995564	+	1089	ANNOTATED, CDS, c	NM_012423	RPL13A	Salzman2013	23521	circRNA	Detected	Detected	4.554517579	3.526326588
hsa_gci163093	2.055961679	1.039813375	up	3.905583489	2.865770114	TTGGAGCACTCGGTCCG	hsa_circ_0051904	chr19	49994681	49995564	+	702	ANNOTATED, CDS, c	NM_012423	RPL13A	Salzman2013	23521	circRNA	Detected	Not Detected	3.905583489	2.865770114
hsa_gci163095	2.501674572	1.32289413	up	11.13647475	9.81358062	CCAAAGGAATAGCTAAT	hsa_circ_0051907	chr19	49999633	50002944	+	609	ANNOTATED, CDS, c	NM_001015	RPS11	Salzman2013	6205	circRNA	Detected	Detected	11.13647475	9.81358062
hsa_gci163098	2.833575969	1.502623882	up	4.460862227	2.958238345	TAGAAGTAGAACCAGTG	hsa_circ_0051911	chr19	50001173	50002944	+	306	ANNOTATED, CDS, c	NM_001015	RPS11	Salzman2013	6205	circRNA	Detected	Not Detected	4.460862227	2.958238345
hsa_gci163104	2.018183001	1.013056998	up	6.764265615	5.751208616	CGAGTCCTTCAGACAAC	hsa_circ_0051932	chr19	50170278	50177173	+	811	ANNOTATED, CDS, c	NM_001040668	BCL2L12	Salzman2013	83596	circRNA	Detected	Detected	6.764265615	5.751208616
hsa_gci163108	2.077402532	1.05478079	up	5.765115578	4.710334788	ATCCGTCCTTTGTCGTC	hsa_circ_0052029	chr19	51300960	51302215	-	1255	ANNOTATED, CDS, c	NM_199249	C19orf48	Salzman2013	84798	circRNA	Detected	Detected	5.765115578	4.710334788
hsa_gci163117	3.378918546	1.756561572	up	7.135683151	5.379121579	TCCCGGTCCTCCTGGAG	hsa_circ_0052078	chr19	52709215	52729678	+	2045	ANNOTATED, CDS, c	NM_014225	PPP2R1A	Salzman2013	5518	circRNA	Detected	Detected	7.135683151	5.379121579
hsa_gci163124	-4.081025734	-2.028931808	down	1.253465121	3.282396929	TCCGAAGAAGGAATCCT	hsa_circ_0052104	chr19	52960120	53020131	+	6709	ANNOTATED, CDS, c	NM_001099694	ZNF578	Salzman2013	147660	circRNA	Not Detected	Detected	1.253465121	3.282396929
hsa_gci163136	2.075876837	1.053720851	up	9.529567507	8.475846656	CCCCTCTTCTCGTCACC	hsa_circ_0052264	chr19	56172403	56186082	+	1742	ANNOTATED, CDS, c	NM_007279	U2AF2	Salzman2013	11338	circRNA	Detected	Detected	9.529567507	8.475846656
hsa_gci163140	2.329829941	1.220224653	up	10.13851547	8.91829082	CCGTCTCAGCCCCTGGT	hsa_circ_0052270	chr19	56179872	56186082	+	1334	ANNOTATED, CDS, c	NM_007279	U2AF2	Salzman2013	11338	circRNA	Detected	Detected	10.13851547	8.91829082
hsa_gci163142	2.031470324	1.02252429	up	7.391228907	6.368704617	GGTTTTACTGGCGGTGC	hsa_circ_0052300	chr19	58193356	58269527	+	72059	ALT_DONOR, CDS, c	NM_138347	ZNF551	Salzman2013	90233	circRNA	Detected	Detected	7.391228907	6.368704617

hsa_gci163143	2.723874989	1.445660493	up	6.983729894	5.538069401	AGTTATAACGACACACA	hsa_circ_0052303	chr19	58231118	58235831	-	3320	ALT_ACCEPTOR, CDS	NM_024833	ZNF671	Salzman2013	79891	circRNA	Detected	Detected	6.983729894	5.538069401
hsa_gci163151	2.09043477	1.063803026	up	5.612142889	4.548339862	GCGAGCGGGCGCGCGGT	hsa_circ_0052368	chr19	59055835	59062082	+	2959	ANNOTATED, CDS, c	NM_005762	TRIM28	Salzman2013	10155	circRNA	Detected	Detected	5.612142889	4.548339862
hsa_gci163152	2.880351798	1.526245029	up	9.535700306	8.009455277	CGAACGTGTGCCCTGTC	hsa_circ_0052370	chr19	59056791	59062082	+	2330	ANNOTATED, CDS, c	NM_005762	TRIM28	Salzman2013	10155	circRNA	Detected	Detected	9.535700306	8.009455277
hsa_gci163162	2.077638469	1.054944632	up	7.954435769	6.899491137	CATCTTCGAGTGACCGT	hsa_circ_0052400	chr19	59062932	59065603	-	776	ANNOTATED, CDS, c	NM_014453	CHMP2A	Salzman2013	27243	circRNA	Detected	Detected	7.954435769	6.899491137
hsa_gci163168	2.226595805	1.154839689	up	7.532699289	6.3778596	GTTCCGGTCGTTTAGTA	hsa_circ_0052436	chr2	1635658	1657557	-	4811	ANNOTATED, CDS, c	NM_012293	PXDN	Salzman2013	7837	circRNA	Detected	Detected	7.532699289	6.3778596
hsa_gci163201	2.081096646	1.057343965	up	7.799581893	6.742237928	CCCTTCAGCCCCAAGAC	hsa_circ_0052598	chr2	10580507	10580994	-	487	ANNOTATED, CDS, c	NM_002539	ODC1	Salzman2013	4953	circRNA	Detected	Detected	7.799581893	6.742237928
hsa_gci163228	2.38899984	1.256406757	up	5.538728929	4.282322171	TGTAGGAATTATTAGGG	hsa_circ_0052766	chr2	15601324	15644337	-	1454	ANNOTATED, CDS, c	NM_015909	NBAS	Salzman2013	51594	circRNA	Detected	Detected	5.538728929	4.282322171
hsa_gci163229	2.454910081	1.295670182	up	5.659072419	4.363402237	AGGTGGTTGTTCATTTT	hsa_circ_0052767	chr2	15601324	15651474	-	1593	ANNOTATED, CDS, c	NM_015909	NBAS	Salzman2013	51594	circRNA	Detected	Detected	5.659072419	4.363402237
hsa_gci163230	2.00585343	1.004216191	up	4.936429199	3.932213009	TACAAAAACCAACGGAG	hsa_circ_0052771	chr2	15601324	15698758	-	2222	ANNOTATED, CDS, c	NM_015909	NBAS	Salzman2013	51594	circRNA	Detected	Detected	4.936429199	3.932213009
hsa_gci163231	3.188773615	1.673001678	up	5.118376319	3.445374641	GAACCATCGAGACTAAA	hsa_circ_0052778	chr2	15615810	15674765	-	694	ANNOTATED, CDS, c	NM_015909	NBAS	Salzman2013	51594	circRNA	Detected	Detected	5.118376319	3.445374641
hsa_gci163232	2.119706711	1.083864663	up	7.830943234	6.747078571	TTTCCTACGTGGTACAA	hsa_circ_0052780	chr2	15615810	15698758	-	1224	ANNOTATED, CDS, c	NM_015909	NBAS	Salzman2013	51594	circRNA	Detected	Detected	7.830943234	6.747078571
hsa_gci163235	11.16119108	3.48041909	up	4.6799719	1.19955281	TGTCGAACACGTTAGAG	hsa_circ_0052793	chr2	15735268	15735677	+	116	ANNOTATED, CDS, c	NM_004939	DDX1	Salzman2013	1653	circRNA	Detected	Not Detected	4.6799719	1.19955281
hsa_gci163238	2.479885088	1.310273271	up	5.98825908	4.677985809	TAGCCCGTCTCACTGTA	hsa_circ_0052805	chr2	15768761	15768963	+	116	ANNOTATED, CDS, c	NM_004939	DDX1	Salzman2013	1653	circRNA	Detected	Detected	5.98825908	4.677985809
hsa_gci163240	2.741780416	1.455113033	up	6.229202647	4.774089613	GACCTCAGGTTCCGGTC	hsa_circ_0052819	chr2	20232410	20234823	-	825	ANNOTATED, CDS, c	NM_014713	LAPTM4A	Salzman2013	9741	circRNA	Detected	Detected	6.229202647	4.774089613
hsa_gci163246	2.346531462	1.230529804	up	6.657823848	5.427294044	GGTAGAACTAGAAGTCC	hsa_circ_0052831	chr2	20400557	20405185	-	2836	ANNOTATED, CDS, c	NM_001006946	SDC1	Salzman2013	6382	circRNA	Detected	Detected	6.657823848	5.427294044
hsa_gci163273	-4.841737506	-2.275524866	down	1.227690743	3.503215609	TTATTCGCACCGGACAT	hsa_circ_0052934	chr2	23977048	24033372	-	2068	ANNOTATED, CDS, c	NM_017552	ATAD2B	Salzman2013	54454	circRNA	Not Detected	Detected	1.227690743	3.503215609
hsa_gci163274	3.989697452	1.996279348	up	8.118005212	6.121725864	TTCTCGTTCCTCCCAAC	hsa_circ_0052943	chr2	24021007	24056948	-	1072	ANNOTATED, CDS, c	NM_017552	ATAD2B	Salzman2013	54454	circRNA	Detected	Detected	8.118005212	6.121725864
hsa_gci163276	4.070752144	2.025295383	up	7.539595519	5.514300137	AGGTAGGTATTCTCGTT	hsa_circ_0052948	chr2	24046127	24056948	-	563	ANNOTATED, CDS, c	NM_017552	ATAD2B	Salzman2013	54454	circRNA	Detected	Detected	7.539595519	5.514300137
hsa_gci163278	2.022392891	1.016063298	up	5.097503275	4.081439977	CCTACCGAAGCTTCCCG	hsa_circ_0052951	chr2	24046127	24110855	-	1713	ANNOTATED, CDS, c	NM_017552	ATAD2B	Salzman2013	54454	circRNA	Detected	Detected	5.097503275	4.081439977
hsa_gci163289	2.791753157	1.481171386	up	4.433098381	2.951926995	GCCGGTCGAGGAAATCC	hsa_circ_0052996	chr2	25013135	25013450	-	315	ANNOTATED, CDS, c	NM_001013663	PTRHD1	Salzman2013	391356	circRNA	Detected	Not Detected	4.433098381	2.951926995
hsa_gci163303	-2.788728897	-1.479607691	down	2.782286482	4.261894174	ACGTACTGTATGTCTCT	hsa_circ_0053087	chr2	26587169	26609404	+	1038	ANNOTATED, CDS, c	NM_033505	SELENOI	Salzman2013	85465	circRNA	Not Detected	Detected	2.782286482	4.261894174
hsa_gci163306	2.345391301	1.22982864	up	5.143505868	3.913677228	GAGTTGAGGGTGTTGAC	hsa_circ_0053090	chr2	26597907	26606215	+	158	ANNOTATED, CDS, c	NM_033505	SELENOI	Salzman2013	85465	circRNA	Detected	Detected	5.143505868	3.913677228
hsa_gci163311	3.613715538	1.853482945	up	7.494676666	5.641193721	CTACTAGCTTCGTTGGC	hsa_circ_0053121	chr2	27262608	27264565	+	1405	ANNOTATED, CDS, c	NM_017727	TMEM214	Salzman2013	54867	circRNA	Detected	Detected	7.494676666	5.641193721
hsa_gci163312	3.099667287	1.632113368	up	8.166886421	6.534773054	GACTAAGTGCCTTAGCC	hsa_circ_0053123	chr2	27263212	27264565	+	1139	ANNOTATED, CDS, c	NM_017727	TMEM214	Salzman2013	54867	circRNA	Detected	Detected	8.166886421	6.534773054
hsa_gci163316	2.452337932	1.294157796	up	4.216891039	2.922733243	GTGTGTGAGCGTGTCAA	hsa_circ_0053153	chr2	27422454	27429810	-	2449	ANNOTATED, ncRNA,	NR_028323	SLC5A6	Salzman2013	8884	circRNA	Detected	Not Detected	4.216891039	2.922733243
hsa_gci163317	3.80118821	1.92645046	up	3.972249134	2.045798674	TCAAGGTGTGTGAGCGT	hsa_circ_0053164	chr2	27428217	27429810	-	341	ANNOTATED, INTERN	NR_028323	SLC5A6	Salzman2013	8884	circRNA	Detected	Not Detected	3.972249134	2.045798674
hsa_gci163322	2.061398185	1.043623206	up	6.677591862	5.633968656	GAGGTTCCATACCCCTC	hsa_circ_0053212	chr2	27461909	27466654	+	1884	ANNOTATED, CDS, c	NM_004341	CAD	Salzman2013	790	circRNA	Detected	Detected	6.677591862	5.633968656
hsa_gci163323	2.888894135	1.530517337	up	4.518262299	2.987744963	CTCCTACTTGACTGGTT	hsa_circ_0053221	chr2	27466065	27466654	+	466	ANNOTATED, CDS, c	NM_004341	CAD	Salzman2013	790	circRNA	Detected	Not Detected	4.518262299	2.987744963
hsa_gci163325	4.998547484	2.321508927	up	3.9121293	1.590620373	GGTCGGAGGATGGTGTC	hsa_circ_0053224	chr2	27532359	27535639	-	765	ANNOTATED, CDS, c	NM_002437	MPV17	Salzman2013	4358	circRNA	Detected	Not Detected	3.9121293	1.590620373
hsa_gci163360	2.748686957	1.45874261	up	7.502221147	6.043478537	ACCGGCTAGAGTCTATC	hsa_circ_0053374	chr2	30457270	30482899	+	2704	ANNOTATED, CDS, c	NM_030915	LBH	Salzman2013	81606	circRNA	Detected	Detected	7.502221147	6.043478537
hsa_gci163367	2.549809336	1.350389372	up	4.554759598	3.204370225	AACAGACACTCTGGCAT	hsa_circ_0053400	chr2	32248971	32264395	-	545	ANNOTATED, CDS, c	NM_032574	DPY30	Salzman2013	84661	circRNA	Detected	Detected	4.554759598	3.204370225
hsa_gci163380	2.827638517	1.499597699	up	4.882384342	3.382786643	TGGTTATTGTACCCCAC	hsa_circ_0053431	chr2	32431954	32434630	+	117	ANNOTATED, CDS, c	NM_001193513	SLC30A6	Salzman2013	55676	circRNA	Detected	Detected	4.882384342	3.382786643
hsa_gci163385	-2.800488873	-1.485678696	down	2.786132257	4.271810953	AAGTGGACAGGTGGACC	hsa_circ_0053445	chr2	32602655	32658872	+	3084	ANNOTATED, CDS, c	NM_016252	BIRC6	Salzman2013	57448	circRNA	Not Detected	Detected	2.786132257	4.271810953
hsa_gci163396	2.423922759	1.277343726	up	7.776362052	6.499018326	TCTCTATATGACTGTAA	hsa_circ_0053462	chr2	32602655	32728303	+	9174	ANNOTATED, CDS, c	NM_016252	BIRC6	Salzman2013	57448	circRNA	Detected	Detected	7.776362052	6.499018326
hsa_gci163494	5.352560837	2.420229288	up	3.947032645	1.526803356	GGAGCACCGTAAACGTT	hsa_circ_0053757	chr2	32703702	32754891	+	5026	ANNOTATED, CDS, c	NM_016252	BIRC6	Salzman2013	57448	circRNA	Detected	Not Detected	3.947032645	1.526803356
hsa_gci163515	2.607658827	1.382755127	up	6.894310235	5.511555107	ACGTCCCTTCTCTGTAC	hsa_circ_0053836	chr2	32740056	32744013	+	1055	ANNOTATED, CDS, c	NM_016252	BIRC6	Salzman2013	57448	circRNA	Detected	Detected	6.894310235	5.511555107
hsa_gci163516	2.419691045	1.274822851	up	6.989280952	5.714458101	AACGTCCCTTCTCTGTA	hsa_circ_0053839	chr2	32740056	32800433	+	2787	ANNOTATED, CDS, c	NM_016252	BIRC6	Salzman2013	57448	circRNA	Detected	Detected	6.989280952	5.714458101
hsa_gci163529	2.431806068	1.282028181	up	7.121457606	5.839429425	TGAAGTTGTCCGTGTCC	hsa_circ_0053921	chr2	33488360	33614373	+	2316	ANNOTATED, CDS, c	NM_206943	LTBP1	Salzman2013	4052	circRNA	Detected	Detected	7.121457606	5.839429425
hsa_gci163535	2.13496849	1.094214777	up	9.603128792	8.508914015	AGAAGAAACCCAACTAG	hsa_circ_0053940	chr2	33585663	33590570	+	711	ANNOTATED, CDS, c	NM_206943	LTBP1	Salzman2013	4052	circRNA	Detected	Detected	9.603128792	8.508914015
hsa_gci163546	2.187162219	1.129060227	up	4.355252944	3.226192717	TGGTGTATTCGGTCTCT	hsa_circ_0053988	chr2	36691676	36771641	+	1877	ANNOTATED, CDS, c	NM_016441	CRIM1	Salzman2013	51232	circRNA	Detected	Not Detected	4.355252944	3.226192717
hsa_gci163550	2.233181763	1.15910068	up	4.735662128	3.576561448	ATTTATGCGTCCGACCG	hsa_circ_0053994	chr2	36704031	36771641	+	1755	ANNOTATED, CDS, c	NM_016441	CRIM1	Salzman2013	51232	circRNA	Detected	Detected	4.735662128	3.576561448
hsa_gci163583	2.968198082	1.569587373	up	8.14087842	6.571291047	CCAGCACTTTATCTGGA	hsa_circ_0054147	chr2	38970740	38973889	-	1679	ANNOTATED, CDS, c	NM_001031684	SRSF7	Salzman2013	6432	circRNA	Detected	Detected	8.14087842	6.571291047
hsa_gci163626	2.29443003	1.198135812	up	8.062423349	6.864287538	ATAGACGAAAGAGGTCG	hsa_circ_0054322	chr2	43805651	43818093	-	645	ANNOTATED, CDS, c	NM_001083953	THADA	Salzman2013	63892	circRNA	Detected	Detected	8.062423349	6.864287538
hsa_gci163634	4.641027345	2.214444198	up	4.319495051	2.105050853	ACTTAGATTCGCCAGTA	hsa_circ_0054365	chr2	44170825	44172556	-	294	ANNOTATED, CDS, c	NM_133259	LRPPRC	Salzman2013	10128	circRNA	Detected	Not Detected	4.319495051	2.105050853
hsa_gci163679	2.474393421	1.307074902	up	4.444033246	3.136958344	AAAGGCCCCTAGAGACG	hsa_circ_0054539	chr2	54093221	54115949	-	1240	ANNOTATED, CDS, c	NM_014614	PSME4	Salzman2013	23198	circRNA	Detected	Not Detected	4.444033246	3.136958344
hsa_gci163695	-2.283361803	-1.191159476	down	3.940113652	5.131273128	GGGAGTCTCTGGTCTCC	hsa_circ_0054654	chr2	56097854	56151298	-	1821	ANNOTATED, CDS, c	NM_001039348	EFEMP1	Salzman2013	2202	circRNA	Not Detected	Detected	3.940113652	5.131273128
hsa_gci163796	3.022112878	1.595557547	up	7.515082121	5.919524574	ACCGACGACGAATAACG	hsa_circ_0055025	chr2	68740679	68794519	+	844	ANNOTATED, CDS, c	NM_173545	APLF	Salzman2013	200558	circRNA	Detected	Detected	7.515082121	5.919524574
hsa_gci163797	2.756219176	1.462690617	up	6.397446241	4.934755625	TCATAGAAGGTGTCTTT	hsa_circ_0055034	chr2	69350148	69351775	+	149	ANNOTATED, CDS, c	NM_032208	ANTXR1	Salzman2013	84168	circRNA	Detected	Detected	6.397446241	4.934755625
hsa_gci163827	3.034330878	1.601378412	up	3.848910247	2.247531835	CCCGGTTGACGACGTAC	hsa_circ_0055192	chr2	73118475	73119289	+	814	ANNOTATED, CDS, c	NM_003124	SPR	Salzman2013	6697	circRNA	Detected	Not Detected	3.848910247	2.247531835
hsa_gci163832	2.082959269	1.058634629	up	6.758589457	5.699954829	TCTCTCGCCGCCTAGTA	hsa_circ_0055215	chr2	73455133	73455702	-	569	ANNOTATED, CDS, c	NM_032319	PRADC1	Salzman2013	84279	circRNA	Detected	Detected	6.758589457	5.699954829
hsa_gci163857	2.450701702	1.293194891	up	4.244656527	2.951461636	ACAGACCAAACAGACGG	hsa_circ_0055388	chr2	85133126	85133799	+	402	ANNOTATED, CDS, c	NM_021103	TMSB10	Salzman2013	9168	circRNA	Detected	Not Detected	4.244656527	2.951461636

hsa_gci163878	2.725592241	1.446569746	up	5.668731514	4.222161769	CTAGTTTGTCCGCTTAT	hsa_circ_0055500	chr2	86355063	86362110	+	682	ANNOTATED, CDS, c	NM_017952	PTCD3	Salzman2013	55037	circRNA	Detected	Detected	5.668731514	4.222161769
hsa_gci163896	2.376206492	1.248660212	up	8.940120481	7.69146027	AAGGTTGGCGAAGTAGA	hsa_circ_0055628	chr2	96858106	96861287	-	553	ANNOTATED, CDS, c	NM_020151	STARD7	Salzman2013	56910	circRNA	Detected	Detected	8.940120481	7.69146027
hsa_gci163900	2.872077013	1.522094435	up	6.163260949	4.641166514	CCGTGTCCATCGTCCGA	hsa_circ_0055703	chr2	97541618	97544230	-	658	ANNOTATED, CDS, c	NM_001122646	FAM178B	Salzman2013	51252	circRNA	Detected	Detected	6.163260949	4.641166514
hsa_gci163901	2.729638172	1.448709727	up	11.30596351	9.857253785	CCGACTCGCTCCTGTCA	hsa_circ_0055704	chr2	97541618	97559788	-	784	ANNOTATED, CDS, c	NM_001122646	FAM178B	Salzman2013	51252	circRNA	Detected	Detected	11.30596351	9.857253785
hsa_gci163909	2.33949544	1.226197417	up	5.901816109	4.675618692	ACATATCAAAACTTTAT	hsa_circ_0055732	chr2	97817615	97833504	+	532	ANNOTATED, CDS, c	NM_001164315	ANKRD36	Salzman2013	375248	circRNA	Detected	Detected	5.901816109	4.675618692
hsa_gci163932	3.014707175	1.592017877	up	6.581057885	4.989040008	CCTGTCGGCGTGTGCCT	hsa_circ_0055879	chr2	101622420	101622885	+	465	ANNOTATED, CDS, c	NM_001099693	RPL31	Salzman2013	6160	circRNA	Detected	Detected	6.581057885	4.989040008
hsa_gci163935	2.288101928	1.194151322	up	4.159725628	2.965574307	CGACGAGGGTCATTGTC	hsa_circ_0055883	chr2	101638142	101638983	-	307	ANNOTATED, CDS, c	NM_001102426	TBC1D8	Salzman2013	11138	circRNA	Detected	Not Detected	4.159725628	2.965574307
hsa_gci163939	2.339293547	1.22607291	up	9.817883824	8.591810914	CCTCCGCCACCTGCTTA	hsa_circ_0055897	chr2	101881306	101886778	+	1604	ANNOTATED, CDS, c	NM_017546	CNOT11	Salzman2013	55571	circRNA	Detected	Detected	9.817883824	8.591810914
hsa_gci163973	11.20663548	3.486281304	up	4.832899733	1.346618429	TCCGAGGAAGAGGTTCT	hsa_circ_0056026	chr2	111395408	111399811	-	1043	ANNOTATED, CDS, c	NM_004336	BUB1	Salzman2013	699	circRNA	Detected	Not Detected	4.832899733	1.346618429
hsa_gci163985	2.21441567	1.146926057	up	10.44888219	9.301956129	ACTACTTGGTTTTTCTG	hsa_circ_0056079	chr2	112582707	112622528	-	2056	ANNOTATED, CDS, c	NM_022662	ANAPC1	Salzman2013	64682	circRNA	Detected	Detected	10.44888219	9.301956129
hsa_gci163995	-2.152456493	-1.105984077	down	3.509697982	4.615682059	CACTGTCTCGCTCCGAG	hsa_circ_0056165	chr2	113996665	113998402	+	1737	ALT_ACCEPTOR, ALT	NR_015377		Salzman2013		circRNA	Not Detected	Detected	3.509697982	4.615682059
hsa_gci164008	2.225540083	1.154155485	up	5.533254841	4.379099356	CGATTTTCCCGTCGTCA	hsa_circ_0056239	chr2	120639672	120640199	+	174	ANNOTATED, CDS, c	NM_002830	PTPN4	Salzman2013	5775	circRNA	Detected	Detected	5.533254841	4.379099356
hsa_gci164013	3.017177774	1.593199704	up	5.947271714	4.354072011	TTAGGTAATTAAAGGCA	hsa_circ_0056254	chr2	120689999	120725548	+	1624	ANNOTATED, CDS, c	NM_002830	PTPN4	Salzman2013	5775	circRNA	Detected	Detected	5.947271714	4.354072011
hsa_gci164020	2.041918665	1.029925401	up	6.240496515	5.210571114	GATAAGTCGTTTAGTTA	hsa_circ_0056282	chr2	120885263	120925083	+	667	ANNOTATED, CDS, c	NM_020909	EPB41L5	Salzman2013	57669	circRNA	Detected	Detected	6.240496515	5.210571114
hsa_gci164021	2.721061398	1.44416951	up	6.591029801	5.146860291	TAATAAGAGTAAATTGT	hsa_circ_0056283	chr2	120918456	120925083	+	211	ANNOTATED, CDS, c	NM_020909	EPB41L5	Salzman2013	57669	circRNA	Detected	Detected	6.591029801	5.146860291
hsa_gci164029	-2.30131056	-1.202455687	down	2.358335546	3.560791233	TAAACGTAGGAAAGGGG	hsa_circ_0056330	chr2	128028911	128030537	-	215	ANNOTATED, CDS, c	NM_000122	ERCC3	Salzman2013	2071	circRNA	Not Detected	Detected	2.358335546	3.560791233
hsa_gci164050	-4.381816609	-2.131529105	down	2.097835259	4.229364364	GCGACGGAAACAAAGAG	hsa_circ_0056455	chr2	130948041	130948300	+	259	ANNOTATED, CDS, c	NM_025029	MZT2B	Salzman2013	80097	circRNA	Not Detected	Detected	2.097835259	4.229364364
hsa_gci164097	4.317734581	2.110274561	up	7.98882978	5.878555219	CCTGTAATTTGACGACG	hsa_circ_0056612	chr2	136690352	136719068	-	347	ANNOTATED, CDS, c	NM_001349	DARS	Salzman2013	1615	circRNA	Detected	Detected	7.98882978	5.878555219
hsa_gci164098	5.247546494	2.391643044	up	7.807171334	5.41552829	TGTAATTTGACGACGAC	hsa_circ_0056613	chr2	136700947	136719068	-	206	ANNOTATED, CDS, c	NM_001349	DARS	Salzman2013	1615	circRNA	Detected	Detected	7.807171334	5.41552829
hsa_gci164131	2.493319146	1.31806756	up	4.799180141	3.481112581	CCTGTTCGCGTGTGACC	hsa_circ_0056818	chr2	160053118	160089170	+	4259	ANNOTATED, CDS, c	NM_001145909	TANC1	Salzman2013	85461	circRNA	Detected	Detected	4.799180141	3.481112581
hsa_gci164132	2.503410611	1.323894942	up	7.446116345	6.122221403	GGAGAAGGACGCCGCCG	hsa_circ_0056819	chr2	160074007	160089170	+	3994	ANNOTATED, CDS, c	NM_001145909	TANC1	Salzman2013	85461	circRNA	Detected	Detected	7.446116345	6.122221403
hsa_gci164151	2.423347668	1.277001397	up	8.659491417	7.382490019	CGTTACGGAGCATAAGG	hsa_circ_0056942	chr2	169547543	169551561	+	144	ANNOTATED, CDS, c	NM_203463	CERS6	Salzman2013	253782	circRNA	Detected	Detected	8.659491417	7.382490019
hsa_gci164172	2.262463763	1.177894685	up	6.110049729	4.932155044	TTATAGAAAGTAAATTA	hsa_circ_0057023	chr2	171862656	171867964	-	570	ANNOTATED, CDS, c	NM_012290	TLK1	Salzman2013	9874	circRNA	Detected	Detected	6.110049729	4.932155044
hsa_gci164187	2.950052343	1.560740552	up	4.023451913	2.46271136	GGCCTTCACTGGCGTGT	hsa_circ_0057071	chr2	172778934	172809519	+	385	ANNOTATED, CDS, c	NM_003642	HAT1	Salzman2013	8520	circRNA	Detected	Not Detected	4.023451913	2.46271136
hsa_gci164211	3.180837136	1.669406505	up	4.09521306	2.425806554	AAATTAAGGTAGTGGAG	hsa_circ_0057132	chr2	175094035	175111543	-	245	ANNOTATED, CDS, c	NM_013341	OLA1	Salzman2013	29789	circRNA	Detected	Not Detected	4.09521306	2.425806554
hsa_gci164221	2.319612587	1.213883872	up	4.731992023	3.518108151	TTGGACTCCAAATTCTC	hsa_circ_0057182	chr2	178362416	178408564	+	6333	ANNOTATED, CDS, c	NM_003659	AGPS	Salzman2013	8540	circRNA	Detected	Detected	4.731992023	3.518108151
hsa_gci164222	3.459575522	1.790595035	up	7.715657772	5.925062737	ATGTATCATAAGGTTTC	hsa_circ_0057185	chr2	178372697	178408564	+	6073	ANNOTATED, CDS, c	NM_003659	AGPS	Salzman2013	8540	circRNA	Detected	Detected	7.715657772	5.925062737
hsa_gci164239	3.342682437	1.741006302	up	5.813280483	4.072274181	GTGTTAGTAGACCGGTT	hsa_circ_0057287	chr2	183853757	183866771	-	435	ANNOTATED, CDS, c	NM_205842	NCKAP1	Salzman2013	10787	circRNA	Detected	Detected	5.813280483	4.072274181
hsa_gci164282	2.00082589	1.000595631	up	7.556763854	6.556168223	TTCGTCCGATTACTGTC	hsa_circ_0057556	chr2	196571339	196571509	+	170	ANNOTATED, CDS, c	NM_001127257	SLC39A10	Salzman2013	57181	circRNA	Detected	Detected	7.556763854	6.556168223
hsa_gci164300	2.508455254	1.326799203	up	6.574957294	5.24815809	CGCCACGCGCAGCCCCG	hsa_circ_0057667	chr2	198351307	198364998	-	2301	ANNOTATED, CDS, c	NM_199440	HSPD1	Salzman2013	3329	circRNA	Detected	Detected	6.574957294	5.24815809
hsa_gci164304	2.192395241	1.132507908	up	7.789768289	6.657260381	CCATTGGCTTCGTAAAG	hsa_circ_0057679	chr2	198360017	198363574	-	512	ANNOTATED, CDS, c	NM_002156	HSPD1	Salzman2013	3329	circRNA	Detected	Detected	7.789768289	6.657260381
hsa_gci164308	2.062497677	1.044392495	up	6.24577809	5.201385596	GCGCGCGTCGCACGCAT	hsa_circ_0057694	chr2	200775978	200792996	+	3728	ANNOTATED, CDS, c	NM_153689	C2orf69	Salzman2013	205327	circRNA	Detected	Detected	6.24577809	5.201385596
hsa_gci164354	2.460905297	1.299189139	up	4.511304515	3.212115375	CGTCCTGGTATAAACGG	hsa_circ_0057928	chr2	207026069	207027653	+	521	ANNOTATED, CDS, c	NM_001959	EEF1B2	Salzman2013	1933	circRNA	Detected	Not Detected	4.511304515	3.212115375
hsa_gci164363	4.114878317	2.040849768	up	4.462378239	2.42152847	CCCTTACAGACGAACAC	hsa_circ_0057964	chr2	208725828	208842310	-	1498	ANNOTATED, CDS, c	NM_001080475	PLEKHM3	Salzman2013	389072	circRNA	Detected	Not Detected	4.462378239	2.42152847
hsa_gci164368	12.83305911	3.681793212	up	4.881176848	1.199383636	TCTTGGTCTAGATAGGT	hsa_circ_0057980	chr2	209163364	209167077	+	409	ANNOTATED, CDS, c	NM_015040	PIKFYVE	Salzman2013	200576	circRNA	Detected	Not Detected	4.881176848	1.199383636
hsa_gci164417	2.528766954	1.338434087	up	7.281353662	5.942919575	GTAACCACACTGCTCCC	hsa_circ_0058218	chr2	219114086	219114644	+	202	ANNOTATED, CDS, c	NM_152862	ARPC2	Salzman2013	10109	circRNA	Detected	Detected	7.281353662	5.942919575
hsa_gci164425	2.386324485	1.254790229	up	4.202459701	2.947669471	TATTTTTCAACTAATGA	hsa_circ_0058280	chr2	219452308	219458999	+	470	ANNOTATED, CDS, c	NM_005444	CNOT9	Salzman2013	9125	circRNA	Detected	Not Detected	4.202459701	2.947669471
hsa_gci164426	4.850151763	2.278029891	up	8.576032922	6.298003032	TTCTAGAAGACCTCCTA	hsa_circ_0058281	chr2	219457026	219457430	+	191	ANNOTATED, CDS, c	NM_005444	CNOT9	Salzman2013	9125	circRNA	Detected	Detected	8.576032922	6.298003032
hsa_gci164455	2.300431185	1.2019043	up	4.094621386	2.892717085	GTACCTCTATCTAAAGT	hsa_circ_0058517	chr2	228356262	228399741	+	1110	ANNOTATED, CDS, c	NM_001135187	AGFG1	Salzman2013	3267	circRNA	Detected	Not Detected	4.094621386	2.892717085
hsa_gci164472	2.183632592	1.126730136	up	4.1601944	3.033464264	GAGAGAGAACTTCCCTG	hsa_circ_0058559	chr2	230662405	230744844	-	3448	ANNOTATED, CDS, c	NM_004238	TRIP12	Salzman2013	9320	circRNA	Detected	Not Detected	4.1601944	3.033464264
hsa_gci164480	5.549819535	2.47244086	up	5.674596652	3.202155793	CCGCGCCGTCATTGAAG	hsa_circ_0058580	chr2	230679988	230786655	-	1600	ANNOTATED, CDS, c	NM_004238	TRIP12	Salzman2013	9320	circRNA	Detected	Detected	5.674596652	3.202155793
hsa_gci164495	2.34358095	1.228714628	up	9.028427478	7.79971285	TCTACCGAAAAGTCTCG	hsa_circ_0058679	chr2	232573234	232578250	+	1207	ANNOTATED, CDS, c	NM_001099285	PTMA	Salzman2013	5757	circRNA	Detected	Detected	9.028427478	7.79971285
hsa_gci164515	3.158507962	1.659243209	up	5.376029468	3.716786259	CCGGTCACTCCCTGTGT	hsa_circ_0058813	chr2	236945204	236949485	+	246	ANNOTATED, CDS, c	NM_001037131	AGAP1	Salzman2013	116987	circRNA	Detected	Detected	5.376029468	3.716786259
hsa_gci164519	2.038746221	1.027682203	up	6.578291664	5.550609461	CACCCTCGGGTCGGTCA	hsa_circ_0058856	chr2	238939196	238940895	+	91	ANNOTATED, INTERN	NR_037904	UBE2F-SCLY	Salzman2013	100533179	circRNA	Detected	Detected	6.578291664	5.550609461
hsa_gci164532	2.495503533	1.319330946	up	9.298058283	7.978727337	TACTGGAGCCCCTCCAT	hsa_circ_0058938	chr2	241404889	241407495	+	2197	ANNOTATED, CDS, c	NM_002081	GPC1	Salzman2013	2817	circRNA	Detected	Detected	9.298058283	7.978727337
hsa_gci164534	2.001260651	1.000909081	up	4.673423674	3.672514593	GTGCGTCGGAGTTTAAC	hsa_circ_0058963	chr2	241666242	241666357	-	115	ANNOTATED, CDS, c	NM_001244008	KIF1A	Salzman2013	547	circRNA	Detected	Detected	4.673423674	3.672514593
hsa_gci164557	2.298952247	1.200976499	up	4.543886403	3.342909904	TTGACTAACCTAGGTTG	hsa_circ_0059063	chr2	242283166	242293441	+	2479	ANNOTATED, CDS, c	NM_001008491	SEPT2	Salzman2013	4735	circRNA	Detected	Detected	4.543886403	3.342909904
hsa_gci164568	2.949678584	1.560557758	up	9.951753704	8.391195946	GTCCCGAAGGACCTGCC	hsa_circ_0059147	chr20	371930	378203	+	1655	ANNOTATED, CDS, c	NM_021158	TRIB3	Salzman2013	57761	circRNA	Detected	Detected	9.951753704	8.391195946
hsa_gci164573	2.340368493	1.226735701	up	8.161642196	6.934906495	GGGGAGGATGGTCCTCC	hsa_circ_0059160	chr20	463337	524482	-	2836	ANNOTATED, CDS, c	NM_177559	CSNK2A1	Salzman2013	1457	circRNA	Detected	Detected	8.161642196	6.934906495
hsa_gci164576	2.824824288	1.498161131	up	7.116235546	5.618074415	TCGACCCCCATTCTGGA	hsa_circ_0059168	chr20	472897	489304	-	730	ANNOTATED, CDS, c	NM_177559	CSNK2A1	Salzman2013	1457	circRNA	Detected	Detected	7.116235546	5.618074415
hsa_gci164577	2.772200219	1.471031458	up	7.588363972	6.117332514	CGACCCCCATTCTGGAA	hsa_circ_0059169	chr20	476362	489304	-	619	ANNOTATED, CDS, c	NM_177559	CSNK2A1	Salzman2013	1457	circRNA	Detected	Detected	7.588363972	6.117332514

hsa_gci164578	23.99458183	4.584636765	up	5.80890026	1.224263495	AAAGTCTGGACAAAAGA	hsa_circ_0059171	chr20	489094	489304	-	210	ANNOTATED, CDS, c	NM_177559	CSNK2A1	Salzman2013	1457	circRNA	Detected	Not Detected	5.80890026	1.224263495
hsa_gci164585	2.582049841	1.368516849	up	6.914199299	5.54568245	GGAATTAATATTTTTCG	hsa_circ_0059187	chr20	2473344	2474690	-	453	ANNOTATED, CDS, c	NM_024325	ZNF343	Salzman2013	79175	circRNA	Detected	Detected	6.914199299	5.54568245
hsa_gci164604	2.486649391	1.314203107	up	5.783097199	4.468894092	CCGGTCCTCGACCACCT	hsa_circ_0059264	chr20	3199162	3204506	+	688	ANNOTATED, CDS, c	NM_033453	ITPA	Salzman2013	3704	circRNA	Detected	Detected	5.783097199	4.468894092
hsa_gci164608	2.698259454	1.432029079	up	7.849639332	6.417610253	CCCTCCGGCCAATGTTA	hsa_circ_0059274	chr20	3259557	3270863	-	412	ANNOTATED, CDS, c	NM_001009984	C20orf194	Salzman2013	25943	circRNA	Detected	Detected	7.849639332	6.417610253
hsa_gci164624	2.791960563	1.481278563	up	8.693659358	7.212380795	CGACTGACGCGGGCCGC	hsa_circ_0059343	chr20	3925823	3996216	-	437	ANNOTATED, CDS, c	NM_001134338	RNF24	Salzman2013	11237	circRNA	Detected	Detected	8.693659358	7.212380795
hsa_gci164644	2.462179666	1.29993604	up	9.15505115	7.855115111	CGACTCCGAGGGCTGGT	hsa_circ_0059413	chr20	6055491	6104191	-	5151	ANNOTATED, CDS, c	NM_017671	FERMT1	Salzman2013	55612	circRNA	Detected	Detected	9.15505115	7.855115111
hsa_gci164655	2.041046203	1.029308841	up	4.993079366	3.963770525	AATTTCGCTTTGTATAA	hsa_circ_0059486	chr20	16385457	16387101	-	172	ANNOTATED, CDS, c	NM_024704	KIF16B	Salzman2013	55614	circRNA	Detected	Detected	4.993079366	3.963770525
hsa_gci164657	4.758586137	2.250532986	up	5.355248987	3.104716001	GACTTCGCTCCCTAGTT	hsa_circ_0059497	chr20	17922243	17937681	-	1921	ANNOTATED, CDS, c	NM_152227	SNX5	Salzman2013	27131	circRNA	Detected	Not Detected	5.355248987	3.104716001
hsa_gci164658	2.988745291	1.579539952	up	4.977783873	3.398243921	TCGCTCCCTAGTTGTAA	hsa_circ_0059500	chr20	17927811	17937681	-	1345	ALT_DONOR, CDS, c	NM_152227	SNX5	Salzman2013	27131	circRNA	Detected	Detected	4.977783873	3.398243921
hsa_gci164663	3.120258676	1.641665637	up	5.04288891	3.401223273	ACTTCGCTCCCTAGTTG	hsa_circ_0059512	chr20	17933230	17937681	-	462	ANNOTATED, CDS, c	NM_152227	SNX5	Salzman2013	27131	circRNA	Detected	Detected	5.04288891	3.401223273
hsa_gci164673	2.213933703	1.14661202	up	6.197879835	5.051267815	GTCCCGCTCTTCGGGTC	hsa_circ_0059559	chr20	19998438	20014273	+	1173	ANNOTATED, CDS, c	NM_181527	NAA20	Salzman2013	51126	circRNA	Detected	Detected	6.197879835	5.051267815
hsa_gci164674	2.414643679	1.271810311	up	7.836863158	6.565052847	GAGACCGGTCACCCGCT	hsa_circ_0059561	chr20	20006320	20014273	+	901	ANNOTATED, CDS, c	NM_181527	NAA20	Salzman2013	51126	circRNA	Detected	Detected	7.836863158	6.565052847
hsa_gci164676	2.945994602	1.558754787	up	5.554495813	3.995741026	ATTACATGTGTCCGAAA	hsa_circ_0059570	chr20	20553518	20616261	-	2096	ANNOTATED, CDS, c	NM_020343	RALGAPA2	Salzman2013	57186	circRNA	Detected	Detected	5.554495813	3.995741026
hsa_gci164691	2.104993115	1.073815514	up	4.18390213	3.110086616	AGGTTCAGATTGGGGTT	hsa_circ_0059639	chr20	25249764	25278648	+	3677	ANNOTATED, CDS, c	NM_002862	PYGB	Salzman2013	5834	circRNA	Detected	Not Detected	4.18390213	3.110086616
hsa_gci164698	2.022512833	1.016148857	up	5.147833286	4.131684429	GGTCCCTTCATAGCTAT	hsa_circ_0059659	chr20	25282854	25287551	-	290	ANNOTATED, CDS, c	NM_001042472	ABHD12	Salzman2013	26090	circRNA	Detected	Detected	5.147833286	4.131684429
hsa_gci164714	-4.243080774	-2.085112143	down	1.279895631	3.365007774	CGGAGAAACATCATTCA	hsa_circ_0059716	chr20	30354358	30389603	+	2735	ANNOTATED, CDS, c	NM_012112	TPX2	Salzman2013	22974	circRNA	Not Detected	Detected	1.279895631	3.365007774
hsa_gci164716	2.560709393	1.356543535	up	5.245313723	3.888770188	GAGAAGAGGTCGAGAAC	hsa_circ_0059719	chr20	30363669	30389603	+	2356	ANNOTATED, CDS, c	NM_012112	TPX2	Salzman2013	22974	circRNA	Detected	Detected	5.245313723	3.888770188
hsa_gci164727	-4.92666027	-2.30060999	down	1.814686492	4.115296483	TTCCAAGGACTTGATCT	hsa_circ_0059736	chr20	30386167	30389603	+	1019	ANNOTATED, CDS, c	NM_012112	TPX2	Salzman2013	22974	circRNA	Not Detected	Detected	1.814686492	4.115296483
hsa_gci164751	3.63039059	1.860124775	up	7.497188602	5.637063827	GCGAAGTCACGGACCTC	hsa_circ_0059889	chr20	32436272	32442173	+	1292	ANNOTATED, CDS, c	NM_176812	CHMP4B	Salzman2013	128866	circRNA	Detected	Detected	7.497188602	5.637063827
hsa_gci164759	2.497694714	1.320597151	up	5.645107287	4.324510136	TGAGTGTCCCCATTGCT	hsa_circ_0059935	chr20	33000320	33001685	+	263	ANNOTATED, CDS, c	NM_031483	ITCH	Salzman2013	83737	circRNA	Detected	Detected	5.645107287	4.324510136
hsa_gci164789	2.219896135	1.150492177	up	6.295017244	5.144525067	CGTCCCGGCGTTAGAAT	hsa_circ_0060063	chr20	33954359	33962059	-	131	ANNOTATED, CDS, c	NM_018244	UQCC1	Salzman2013	55245	circRNA	Detected	Detected	6.295017244	5.144525067
hsa_gci164798	5.277095482	2.399744087	up	6.605087943	4.205343856	CCTGTCCTTGTCATCGT	hsa_circ_0060088	chr20	34130071	34145405	+	1209	ANNOTATED, CDS, c	NM_198398	ERGIC3	Salzman2013	51614	circRNA	Detected	Detected	6.605087943	4.205343856
hsa_gci164808	4.191275513	2.06738936	up	7.030333778	4.962944418	AGCACGACTCACCATCT	hsa_circ_0060106	chr20	34145195	34145405	+	210	ANNOTATED, CDS, c	NM_198398	ERGIC3	Salzman2013	51614	circRNA	Detected	Detected	7.030333778	4.962944418
hsa_gci164820	-2.38436999	-1.25360812	down	3.213263753	4.466871874	AAAGTCAGTAGTTGCCC	hsa_circ_0060147	chr20	34389412	34505584	+	2036	ANNOTATED, CDS, c	NM_016436	PHF20	Salzman2013	51230	circRNA	Not Detected	Detected	3.213263753	4.466871874
hsa_gci164851	2.714670976	1.440777351	up	6.248196163	4.807418812	CGGGACGGAAGGGCCCC	hsa_circ_0060303	chr20	36145818	36156083	-	2042	ANNOTATED, CDS, c	NM_001167822	BLCAP	Salzman2013	10904	circRNA	Detected	Detected	6.248196163	4.807418812
hsa_gci164854	2.338129481	1.225354825	up	7.964593639	6.739238813	TTATGTCAAACAACCGT	hsa_circ_0060309	chr20	36405746	36488438	+	780	ANNOTATED, CDS, c	NM_030877	CTNNBL1	Salzman2013	56259	circRNA	Detected	Detected	7.964593639	6.739238813
hsa_gci164864	2.005045048	1.003634651	up	8.780524253	7.776889602	CGCCCATCCCTACGGGT	hsa_circ_0060326	chr20	36756863	36770601	-	2962	ANNOTATED, CDS, c	NM_004613	TGM2	Salzman2013	7052	circRNA	Detected	Detected	8.780524253	7.776889602
hsa_gci164880	2.338629748	1.225663473	up	7.098000155	5.872336682	AACTTCGTGAAAAGATA	hsa_circ_0060448	chr20	42295708	42345122	+	2713	ANNOTATED, CDS, c	NM_002466	MYBL2	Salzman2013	4605	circRNA	Detected	Detected	7.098000155	5.872336682
hsa_gci164893	2.185692359	1.128090353	up	6.064970916	4.936880563	CCGGAAACTGTCACAGA	hsa_circ_0060532	chr20	43804501	43805185	+	451	ANNOTATED, CDS, c	NM_002638	PI3	Salzman2013	5266	circRNA	Detected	Detected	6.064970916	4.936880563
hsa_gci164898	2.434202248	1.283449041	up	8.154274775	6.870825734	CCGCGCGTCCGACGCCG	hsa_circ_0060542	chr20	43953928	43977064	-	2612	ANNOTATED, CDS, c	NM_002999	SDC4	Salzman2013	6385	circRNA	Detected	Detected	8.154274775	6.870825734
hsa_gci164933	3.405912377	1.76804132	up	4.914651123	3.146609803	TAAATCAAGATGTCATC	hsa_circ_0060767	chr20	47733662	47770608	-	1445	ANNOTATED, CDS, c	NM_001037328	STAU1	Salzman2013	6780	circRNA	Detected	Detected	4.914651123	3.146609803
hsa_gci164934	-3.138564741	-1.650104968	down	1.73067581	3.380780778	ACGTGTCACGTAAATCA	hsa_circ_0060772	chr20	47752369	47770608	-	404	ANNOTATED, CDS, c	NM_017453	STAU1	Salzman2013	6780	circRNA	Not Detected	Detected	1.73067581	3.380780778
hsa_gci164940	2.201944394	1.138778037	up	6.322242068	5.183464032	GTTTTCCGCCCGTTAAA	hsa_circ_0060787	chr20	47862438	47868159	-	3906	ANNOTATED, CDS, c	NM_021035	ZNFX1	Salzman2013	57169	circRNA	Detected	Detected	6.322242068	5.183464032
hsa_gci164977	2.037394753	1.026725535	up	5.015335568	3.988610033	GGCGTGAGACCGGACCC	hsa_circ_0060948	chr20	54944444	54965721	-	2194	ANNOTATED, CDS, c	NM_198433	AURKA	Salzman2013	6790	circRNA	Detected	Detected	5.015335568	3.988610033
hsa_gci164992	2.575559606	1.364885929	up	5.518443799	4.153557871	AAATGGTCTAAGAGGTC	hsa_circ_0060995	chr20	57470666	57485136	+	834	ANNOTATED, coding	NM_016592	GNAS	Salzman2013	2778	circRNA	Detected	Detected	5.518443799	4.153557871
hsa_gci164998	2.665756274	1.414544883	up	5.802154607	4.387609725	GTCTTCTCAATAGTCTT	hsa_circ_0061046	chr20	60835036	60871269	+	3732	ANNOTATED, CDS, c	NM_144498	OSBPL2	Salzman2013	9885	circRNA	Detected	Detected	5.802154607	4.387609725
hsa_gci164999	2.758072614	1.46366044	up	5.80475704	4.3410966	AGGTCAGTCCCGGGGGT	hsa_circ_0061062	chr20	60882426	60883918	+	784	ANNOTATED, CDS, c	NM_007002	ADRM1	Salzman2013	11047	circRNA	Detected	Detected	5.80475704	4.3410966
hsa_gci165001	3.019434009	1.594278142	up	6.407193064	4.812914921	TCGTGTGACCGGTCCAG	hsa_circ_0061065	chr20	60883076	60883918	+	469	ANNOTATED, CDS, c	NM_007002	ADRM1	Salzman2013	11047	circRNA	Detected	Detected	6.407193064	4.812914921
hsa_gci165003	2.399359238	1.262649178	up	8.320735085	7.058085907	CGATCAGTGGGCCGTCT	hsa_circ_0061107	chr20	60962120	60963576	+	396	ANNOTATED, CDS, c	NM_001024	RPS21	Salzman2013	6227	circRNA	Detected	Detected	8.320735085	7.058085907
hsa_gci165004	2.600973533	1.379051719	up	8.836593898	7.457542179	GCAGCAAGACGTAAAGC	hsa_circ_0061110	chr20	60962376	60963576	+	324	ANNOTATED, CDS, c	NM_001024	RPS21	Salzman2013	6227	circRNA	Detected	Detected	8.836593898	7.457542179
hsa_gci165007	5.024320809	2.328928585	up	5.054628852	2.725700267	GGTAGCCGGAACCGGTT	hsa_circ_0061113	chr20	60963364	60963576	+	120	ANNOTATED, CDS, c	NM_001024	RPS21	Salzman2013	6227	circRNA	Detected	Not Detected	5.054628852	2.725700267
hsa_gci165023	2.665800208	1.41456866	up	5.075053797	3.660485137	AACTGTCTGTAGGGCTG	hsa_circ_0061223	chr20	62698267	62703700	+	932	ANNOTATED, CDS, c	NM_003195	TCEA2	Salzman2013	6919	circRNA	Detected	Detected	5.075053797	3.660485137
hsa_gci165025	3.200374166	1.678240585	up	7.251493898	5.573253313	CGTGTAGATGTGCTCCC	hsa_circ_0061229	chr20	62703524	62703700	+	176	ANNOTATED, CDS, c	NM_003195	TCEA2	Salzman2013	6919	circRNA	Detected	Detected	7.251493898	5.573253313
hsa_gci165077	2.200569027	1.137876627	up	8.109439897	6.97156327	AAGATGAACGTAAGGTC	hsa_circ_0061505	chr21	34652056	34669520	+	1505	ANNOTATED, CDS, c	NM_000628	IL10RB	Salzman2013	3588	circRNA	Detected	Detected	8.109439897	6.97156327
hsa_gci165099	2.25857413	1.175412266	up	5.817589033	4.642176767	ACCTATTAGACCTCTTT	hsa_circ_0061607	chr21	37619814	37626207	+	562	ANNOTATED, CDS, c	NM_005128	DOPEY2	Salzman2013	9980	circRNA	Detected	Detected	5.817589033	4.642176767
hsa_gci165135	2.274407563	1.185490801	up	10.90936004	9.723869239	AAGTCACTTGAAGTCCC	hsa_circ_0061750	chr21	40648098	40650739	-	213	ANNOTATED, CDS, c	NM_033656	BRWD1	Salzman2013	54014	circRNA	Detected	Detected	10.90936004	9.723869239
hsa_gci165163	2.051797321	1.036888227	up	5.385592146	4.348703919	CAGTAAAGGTCCTCAGC	hsa_circ_0061889	chr21	45104433	45115960	+	4082	ANNOTATED, CDS, c	NM_015056	RRP1B	Salzman2013	23076	circRNA	Detected	Detected	5.385592146	4.348703919
hsa_gci165177	2.166769868	1.115545934	up	8.333040204	7.217494271	GCCCCCAGTGCACCGAC	hsa_circ_0061977	chr21	46225531	46238044	-	1807	ANNOTATED, CDS, c	NM_006936	SUMO3	Salzman2013	6612	circRNA	Detected	Detected	8.333040204	7.217494271
hsa_gci165216	2.184166614	1.127082913	up	8.088900565	6.961817652	TTTTCTCCAGCCCTCCA	hsa_circ_0062349	chr22	21072980	21159453	-	4078	ANNOTATED, CDS, c	NM_058004	PI4KA	Salzman2013	5297	circRNA	Detected	Detected	8.088900565	6.961817652
hsa_gci165222	2.176515691	1.122020421	up	6.761986058	5.639965637	CGAGCGCGAGGGTCCCC	hsa_circ_0062421	chr22	21982377	21984340	-	1338	ANNOTATED, CDS, c	NM_001017964	YDJC	Salzman2013	150223	circRNA	Detected	Detected	6.761986058	5.639965637
hsa_gci165235	2.318564243	1.213231701	up	7.36582616	6.152594459	GTGGTACCCGGAACAAG	hsa_circ_0062449	chr22	22311402	22324778	-	2288	ANNOTATED, CDS, c	NM_003935	TOP3B	Salzman2013	8940	circRNA	Detected	Detected	7.36582616	6.152594459

hsa_gci165241	2.210761555	1.14454343	up	9.168703963	8.024160534	GCTGGAGAAGTCCCGTC	hsa_circ_0062555	chr22	24108020	24108213	-	193	ANNOTATED, CDS, c	NM_213720	CHCHD10	Salzman2013	400916	circRNA	Detected	Detected	9.168703963	8.024160534
hsa_gci165242	2.250910956	1.170508986	up	4.85943804	3.688929054	GGCGCGTCCTTCATCCG	hsa_circ_0062558	chr22	24125597	24126503	+	906	ANNOTATED, CDS, c	NM_005940	MMP11	Salzman2013	4320	circRNA	Detected	Detected	4.85943804	3.688929054
hsa_gci165247	2.651237662	1.406666002	up	6.412989543	5.006323541	GTGGTATGGTCGTCCTG	hsa_circ_0062585	chr22	24459421	24574596	+	5399	ANNOTATED, CDS, c	NM_001199281	CABIN1	Salzman2013	23523	circRNA	Detected	Detected	6.412989543	5.006323541
hsa_gci165255	2.760726926	1.465048193	up	4.908182936	3.443134743	CGGGGTGGGAGAGCGGG	hsa_circ_0062614	chr22	24936405	24951275	-	3607	ANNOTATED, CDS, c	NM_031444	GUCD1	Salzman2013	83606	circRNA	Detected	Detected	4.908182936	3.443134743
hsa_gci165271	2.771609448	1.470723979	up	4.048465352	2.577741373	CGTCGGCGGGTCGCGGA	hsa_circ_0062722	chr22	29190547	29196560	-	1820	ANNOTATED, CDS, c	NM_005080	XBP1	Salzman2013	7494	circRNA	Detected	Not Detected	4.048465352	2.577741373
hsa_gci165290	3.922867613	1.971908649	up	4.032212872	2.060304224	AAGAGTATCATGGTCAA	hsa_circ_0062885	chr22	31654276	31656063	+	435	ANNOTATED, CDS, c	NM_016733	LIMK2	Salzman2013	3985	circRNA	Detected	Not Detected	4.032212872	2.060304224
hsa_gci165296	-2.08099199	-1.057271412	down	2.316638438	3.37390985	TGACGTGGTAGGAATTT	hsa_circ_0062916	chr22	31924675	31927115	+	246	ANNOTATED, CDS, c	NM_001007467	SFI1	Salzman2013	9814	circRNA	Not Detected	Detected	2.316638438	3.37390985
hsa_gci165301	4.06827087	2.024415738	up	4.810971711	2.786555973	CTCCTCTGCGCCGGCGA	hsa_circ_0062966	chr22	32340478	32353590	+	1793	ANNOTATED, CDS, c	NM_003405	YWHAH	Salzman2013	7533	circRNA	Detected	Not Detected	4.810971711	2.786555973
hsa_gci165307	2.331278367	1.22112128	up	5.504998828	4.283877548	AAGGGGTGGTGGAACCG	hsa_circ_0062991	chr22	33245438	33259028	+	4179	ANNOTATED, CDS, c	NM_000362	TIMP3	Salzman2013	7078	circRNA	Detected	Detected	5.504998828	4.283877548
hsa_gci165314	2.998671222	1.584323352	up	5.436545915	3.852222563	AGAAATGTCTTGTGGAC	hsa_circ_0063022	chr22	35679954	35684400	+	423	ANNOTATED, INTERN	NR_027780	HMGXB4	Salzman2013	10042	circRNA	Detected	Detected	5.436545915	3.852222563
hsa_gci165322	13.29431116	3.732737121	up	6.401704174	2.668967053	CCACTGAATATCGGTCC	hsa_circ_0063085	chr22	36677322	36745300	-	7293	ANNOTATED, CDS, c	NM_002473	MYH9	Salzman2013	4627	circRNA	Detected	Not Detected	6.401704174	2.668967053
hsa_gci165328	-3.987388886	-1.995444317	down	1.784497141	3.779941457	ACCTAGTGCCCACAGTA	hsa_circ_0063174	chr22	36914809	36922178	-	869	ANNOTATED, CDS, c	NM_003753	EIF3D	Salzman2013	8664	circRNA	Not Detected	Detected	1.784497141	3.779941457
hsa_gci165372	-2.706480749	-1.436418126	down	2.037240441	3.473658568	AACCGTTTTCGTCCAGT	hsa_circ_0063420	chr22	40749076	40759075	+	744	ANNOTATED, CDS, c	NM_000026	ADSL	Salzman2013	158	circRNA	Not Detected	Detected	2.037240441	3.473658568
hsa_gci165376	-2.039090909	-1.027926097	down	4.962408218	5.990334314	CATACAGCCACGTTTAA	hsa_circ_0063434	chr22	40757276	40759075	+	309	ANNOTATED, CDS, c	NM_000026	ADSL	Salzman2013	158	circRNA	Detected	Detected	4.962408218	5.990334314
hsa_gci165387	3.944887623	1.979984203	up	5.130255575	3.150271371	CTTCACGGACAGGTTTC	hsa_circ_0063581	chr22	41994031	41997194	-	3163	ANNOTATED, CDS, c	NM_015704	DESI1	Salzman2013	27351	circRNA	Detected	Detected	5.130255575	3.150271371
hsa_gci165390	2.842830258	1.50732796	up	7.442683314	5.935355354	CGTCGTGCCCCAAGAGT	hsa_circ_0063594	chr22	42054255	42060052	+	642	ANNOTATED, CDS, c	NM_001469	XRCC6	Salzman2013	2547	circRNA	Detected	Detected	7.442683314	5.935355354
hsa_gci165400	3.683831314	1.881207001	up	5.30713472	3.425927719	AAGTGTCAGTAGAACCA	hsa_circ_0063649	chr22	42979726	42991620	-	2523	ANNOTATED, CDS, c	NM_032311	POLDIP3	Salzman2013	84271	circRNA	Detected	Detected	5.30713472	3.425927719
hsa_gci165405	2.414476691	1.271710536	up	7.860738569	6.589028033	ATCTCTCTCTAGTGCAT	hsa_circ_0063683	chr22	43459818	43460330	-	244	ANNOTATED, INTERN	NR_027779	TTLL1	Salzman2013	25809	circRNA	Detected	Detected	7.860738569	6.589028033
hsa_gci165426	2.463063072	1.300453572	up	9.355522502	8.05506893	ATGTAACCGCCCTTCCA	hsa_circ_0063853	chr22	47287161	47308084	+	307	ANNOTATED, CDS, c	NM_014346	TBC1D22A	Salzman2013	25771	circRNA	Detected	Detected	9.355522502	8.05506893
hsa_gci165428	6.890544469	2.784617985	up	7.168845343	4.384227359	TGTGTCCCGAATCGTGC	hsa_circ_0063876	chr22	50356294	50357720	+	1352	ALT_ACCEPTOR, CDS	NM_001001852	PIM3	Salzman2013	415116	circRNA	Detected	Detected	7.168845343	4.384227359
hsa_gci165434	2.501316965	1.322687886	up	6.713463854	5.390775968	CCTTGTGCATCAGACAT	hsa_circ_0063920	chr22	50713407	50720510	-	3125	ANNOTATED, CDS, c	NM_012401	PLXNB2	Salzman2013	23654	circRNA	Detected	Detected	6.713463854	5.390775968
hsa_gci165440	2.186938492	1.128912645	up	6.754678239	5.625765594	TGTTGACTACAAGCTGA	hsa_circ_0064027	chr3	4355330	4358949	+	1169	ANNOTATED, CDS, c	NM_001243723	SETMAR	Salzman2013	6419	circRNA	Detected	Detected	6.754678239	5.625765594
hsa_gci165447	5.646716752	2.497412265	up	4.919057951	2.421645686	ACTACGTCGCACCGCAT	hsa_circ_0064091	chr3	5252804	5252901	+	97	ANNOTATED, CDS, c	NM_014674	EDEM1	Salzman2013	9695	circRNA	Detected	Not Detected	4.919057951	2.421645686
hsa_gci165463	2.024636391	1.017662834	up	6.772201371	5.754538537	CGCCCCGGAGCTCTCTG	hsa_circ_0064201	chr3	10068182	10143614	+	7183	ALT_ACCEPTOR, CDS	NM_001018115	FANCD2	Salzman2013	2177	circRNA	Detected	Detected	6.772201371	5.754538537
hsa_gci165474	2.268008656	1.181426147	up	7.60549924	6.424073093	GAGTCCCCCGGGTTCAA	hsa_circ_0064254	chr3	10119764	10143614	+	2165	ANNOTATED, CDS, c	NM_001018115	FANCD2	Salzman2013	2177	circRNA	Detected	Detected	7.60549924	6.424073093
hsa_gci165480	2.250408966	1.170187205	up	8.492583117	7.322395911	GGAGGCCCGTCGTTGCG	hsa_circ_0064278	chr3	10342614	10362858	-	1599	ANNOTATED, ncRNA,	NR_024272	SEC13	Salzman2013	6396	circRNA	Detected	Detected	8.492583117	7.322395911
hsa_gci165485	2.449892535	1.292718466	up	6.415585486	5.122867019	CAATGTCCCTAGTATAT	hsa_circ_0064307	chr3	11372813	11389509	+	606	ANNOTATED, CDS, c	NM_006395	ATG7	Salzman2013	10533	circRNA	Detected	Detected	6.415585486	5.122867019
hsa_gci165516	2.278682002	1.188199605	up	6.823300733	5.635101128	GTACGTAATTTTAAGAG	hsa_circ_0064524	chr3	16310632	16313229	+	341	ANNOTATED, CDS, c	NM_138381	OXNAD1	Salzman2013	92106	circRNA	Detected	Detected	6.823300733	5.635101128
hsa_gci165546	2.957490682	1.564373623	up	7.826728855	6.262355231	ACACAACTAGATATCAA	hsa_circ_0064659	chr3	31617887	31665353	+	1413	ANNOTATED, CDS, c	NM_178862	STT3B	Salzman2013	201595	circRNA	Detected	Detected	7.826728855	6.262355231
hsa_gci165555	3.171307105	1.665077593	up	7.887281327	6.222203734	GCGTGTGTCTACTTCCG	hsa_circ_0064680	chr3	32483331	32496333	+	956	ANNOTATED, CDS, c	NM_138410	CMTM7	Salzman2013	112616	circRNA	Detected	Detected	7.887281327	6.222203734
hsa_gci165580	2.564238014	1.35853018	up	5.789069953	4.430539773	AAAGGTAGGTGACTAAG	hsa_circ_0064770	chr3	33623312	33686395	-	1635	ANNOTATED, CDS, c	NM_015097	CLASP2	Salzman2013	23122	circRNA	Detected	Detected	5.789069953	4.430539773
hsa_gci165595	2.248050975	1.168674749	up	4.145501306	2.976826556	CAAAATCAAAGACCTCG	hsa_circ_0064807	chr3	36484855	36485132	+	277	ANNOTATED, CDS, c	NM_003149	STAC	Salzman2013	6769	circRNA	Detected	Not Detected	4.145501306	2.976826556
hsa_gci165605	2.930646391	1.551218904	up	13.08288079	11.53166188	GGTCCTGACGTGTCCGT	hsa_circ_0064851	chr3	38022232	38025960	+	3728	ANNOTATED, CDS, c	NM_001008392	CTDSPL	Salzman2013	10217	circRNA	Detected	Detected	13.08288079	11.53166188
hsa_gci165606	2.747841817	1.458298956	up	9.597436301	8.139137345	CATTAGTAGTCTCTGTT	hsa_circ_0064864	chr3	38182247	38184512	+	1982	ANNOTATED, CDS, c	NM_001172567	MYD88	Salzman2013	4615	circRNA	Detected	Detected	9.597436301	8.139137345
hsa_gci165611	2.3457049	1.230021527	up	7.12082396	5.890802433	TTTTAAAGGGAAATTCA	hsa_circ_0064903	chr3	39449111	39453552	+	826	ANNOTATED, CDS, c	NM_002295	RPSA	Salzman2013	3921	circRNA	Detected	Detected	7.12082396	5.890802433
hsa_gci165617	2.168287108	1.1165558	up	4.244119871	3.127564071	AGACGGAAGACGGTCCG	hsa_circ_0064923	chr3	40502897	40503859	+	584	ANNOTATED, CDS, c	NM_001034996	RPL14	Salzman2013	9045	circRNA	Detected	Not Detected	4.244119871	3.127564071
hsa_gci165619	2.568741308	1.361061607	up	5.816494752	4.455433145	GTTTTGTCAACATACCA	hsa_circ_0064931	chr3	41265511	41279567	+	2185	ANNOTATED, CDS, c	NM_001904	CTNNB1	Salzman2013	1499	circRNA	Detected	Detected	5.816494752	4.455433145
hsa_gci165627	2.399740499	1.262878405	up	4.426766515	3.163888109	GCCATGTTGGAAGTTGA	hsa_circ_0064945	chr3	41277214	41281939	+	1769	ANNOTATED, CDS, c	NM_001904	CTNNB1	Salzman2013	1499	circRNA	Detected	Not Detected	4.426766515	3.163888109
hsa_gci165647	2.277749227	1.18760892	up	8.076873454	6.889264534	AGTGACACTAGGGGTAG	hsa_circ_0065024	chr3	44790235	44795860	-	4954	ANNOTATED, CDS, c	NM_020696	KIAA1143	Salzman2013	57456	circRNA	Detected	Detected	8.076873454	6.889264534
hsa_gci165652	2.242222701	1.164929576	up	5.145770956	3.98084138	GAAACCCGTGACGGGGA	hsa_circ_0065042	chr3	44956752	44986784	-	12106	ANNOTATED, CDS, c	NM_016598	ZDHHC3	Salzman2013	51304	circRNA	Detected	Detected	5.145770956	3.98084138
hsa_gci165656	2.090927871	1.064143296	up	6.99013811	5.925994814	CGACCCATTCGTACAAG	hsa_circ_0065082	chr3	45527183	45542071	+	742	ANNOTATED, CDS, c	NM_015340	LARS2	Salzman2013	23395	circRNA	Detected	Detected	6.99013811	5.925994814
hsa_gci165679	2.063966387	1.045419476	up	7.51041517	6.464995694	TGCTATGCACAGTTAGT	hsa_circ_0065244	chr3	47651555	47719801	-	1586	ANNOTATED, CDS, c	NM_003074	SMARCC1	Salzman2013	6599	circRNA	Detected	Detected	7.51041517	6.464995694
hsa_gci165702	2.45874443	1.297921785	up	7.429453696	6.131531911	GTAAAGATAGAGGAACC	hsa_circ_0065334	chr3	47898920	47958664	-	2496	ANNOTATED, CDS, c	NM_002375	MAP4	Salzman2013	4134	circRNA	Detected	Detected	7.429453696	6.131531911
hsa_gci165710	3.915610949	1.969237427	up	8.94360056	6.974363132	CCGTGGAGCATCGCCCT	hsa_circ_0065496	chr3	48725435	48727146	-	947	ANNOTATED, CDS, c	NM_016291	IP6K2	Salzman2013	51447	circRNA	Detected	Detected	8.94360056	6.974363132
hsa_gci165713	2.17210079	1.119091049	up	6.462677296	5.343586247	CACCTATCTGATCGACG	hsa_circ_0065500	chr3	48725435	48754920	-	2012	ALT_ACCEPTOR, CDS	NM_016291	IP6K2	Salzman2013	51447	circRNA	Detected	Detected	6.462677296	5.343586247
hsa_gci165719	3.236678275	1.694513969	up	5.983864281	4.289350312	TGACGACAACGAACCCC	hsa_circ_0065534	chr3	49061761	49062702	-	593	ANNOTATED, CDS, c	NM_000884	IMPDH2	Salzman2013	3615	circRNA	Detected	Detected	5.983864281	4.289350312
hsa_gci165720	2.451861695	1.293877601	up	4.316400378	3.022522776	CTACGGACGTCCCCGAT	hsa_circ_0065537	chr3	49061761	49064480	-	1068	ANNOTATED, CDS, c	NM_000884	IMPDH2	Salzman2013	3615	circRNA	Detected	Not Detected	4.316400378	3.022522776
hsa_gci165724	2.569862882	1.361691385	up	7.570812971	6.209121586	CCGGAAAGTGTGAGACC	hsa_circ_0065550	chr3	49067141	49068077	-	936	ANNOTATED, CDS, c	NM_017730	QRICH1	Salzman2013	54870	circRNA	Detected	Detected	7.570812971	6.209121586
hsa_gci165751	3.303249337	1.723885872	up	6.416340674	4.692454802	GGTCCCTCAGTAGGCTC	hsa_circ_0065692	chr3	49714335	49720934	+	1481	ANNOTATED, CDS, c	NM_001640	APEH	Salzman2013	327	circRNA	Detected	Detected	6.416340674	4.692454802
hsa_gci165759	3.312917292	1.728102186	up	4.637343834	2.909241648	TGGAGACTATTACGCAC	hsa_circ_0065708	chr3	49720669	49720934	+	265	ANNOTATED, CDS, c	NM_001640	APEH	Salzman2013	327	circRNA	Detected	Not Detected	4.637343834	2.909241648
hsa_gci165761	2.039966255	1.028545288	up	9.487608044	8.459062756	TTTTCTTCTGCTGCCTC	hsa_circ_0065727	chr3	49738923	49758962	+	2892	ANNOTATED, CDS, c	NM_022064	RNF123	Salzman2013	63891	circRNA	Detected	Detected	9.487608044	8.459062756

hsa_gci165767	2.980324449	1.575469397	up	5.653788493	4.078319097	AGACTCCGACTCCCATC	hsa_circ_0065776	chr3	50095160	50114685	+		1798	ANNOTATED, CDS, c	NM_005777	RBM6	Salzman2013	10180	circRNA	Detected	Detected	5.653788493	4.078319097
hsa_gci165775	2.90194964	1.537022483	up	8.931833229	7.394810746	GAAAAGGACTTTAACAA	hsa_circ_0065800	chr3	50141680	50147121	+		711	ANNOTATED, CDS, c	NM_005778	RBM5	Salzman2013	10181	circRNA	Detected	Detected	8.931833229	7.394810746
hsa_gci165782	2.623846635	1.391683396	up	8.414083894	7.022400498	TTGACGACTATTAGGAC	hsa_circ_0065817	chr3	50145664	50147121	+		159	ANNOTATED, CDS, c	NM_005778	RBM5	Salzman2013	10181	circRNA	Detected	Detected	8.414083894	7.022400498
hsa_gci165784	2.341255492	1.227282379	up	4.609805165	3.382522786	CACCAGTAAGGGGTTCC	hsa_circ_0065851	chr3	50330258	50333050	-		1689	ANNOTATED, CDS, c	NM_003549	HYAL3	Salzman2013	8372	circRNA	Detected	Detected	4.609805165	3.382522786
hsa_gci165785	2.366164684	1.242550488	up	5.024003197	3.781452709	TCCGAGCCGACAGAGGT	hsa_circ_0065853	chr3	50362340	50365668	-		1682	ANNOTATED, CDS, c	NM_007275	TUSC2	Salzman2013	11334	circRNA	Detected	Detected	5.024003197	3.781452709
hsa_gci165796	2.02221312	1.01593505	up	7.11396239	6.098027339	GTGTAGCCGTGAATAGT	hsa_circ_0065937	chr3	51718428	51738339	+		991	ANNOTATED, CDS, c	NM_001243725	TEX264	Salzman2013	51368	circRNA	Detected	Detected	7.11396239	6.098027339
hsa_gci165797	2.092365213	1.06513469	up	5.832449576	4.767314886	TAACTAATCCACCTACG	hsa_circ_0065941	chr3	51967445	51969275	-		533	ANNOTATED, CDS, c	NM_004704	RRP9	Salzman2013	9136	circRNA	Detected	Detected	5.832449576	4.767314886
hsa_gci165801	2.322043799	1.215395185	up	6.290363459	5.074968274	CCGGCGCGCCCGGGGTC	hsa_circ_0065975	chr3	52082936	52090461	-		3233	ANNOTATED, CDS, c	NM_001947	DUSP7	Salzman2013	1849	circRNA	Detected	Detected	6.290363459	5.074968274
hsa_gci165807	-4.136483212	-2.048404727	down	1.237005295	3.285410022	TCTGTCGGTCACAAGGA	hsa_circ_0066000	chr3	52282971	52284611	+		1183	ALT_DONOR, CDS, c	NM_144641	PPM1M	Salzman2013	132160	circRNA	Not Detected	Detected	1.237005295	3.285410022
hsa_gci165814	2.560479949	1.356414261	up	8.404425411	7.04801115	TGTCCCTGTAGAAGAAA	hsa_circ_0066078	chr3	52610556	52613215	-		304	ANNOTATED, CDS, c	NM_018313	PBRM1	Salzman2013	55193	circRNA	Detected	Detected	8.404425411	7.04801115
hsa_gci165854	2.395248334	1.260175239	up	6.296659056	5.036483817	AACATGTTTAACTAACA	hsa_circ_0066385	chr3	58260384	58271180	+		314	ANNOTATED, CDS, c	NM_020676	ABHD6	Salzman2013	57406	circRNA	Detected	Detected	6.296659056	5.036483817
hsa_gci165859	6.257541317	2.645595912	up	8.708828364	6.063232452	GGTCAGCTCCCTAAACG	hsa_circ_0066438	chr3	64004274	64008199	-		681	ANNOTATED, CDS, c	NM_014814	PSMD6	Salzman2013	9861	circRNA	Detected	Detected	8.708828364	6.063232452
hsa_gci165867	2.173659932	1.120126249	up	4.366018065	3.245891816	TAGAAGACCTCCAATTA	hsa_circ_0066491	chr3	67568670	67660020	-		576	ANNOTATED, CDS, c	NM_003848	SUCLG2	Salzman2013	8801	circRNA	Detected	Detected	4.366018065	3.245891816
hsa_gci165891	-2.066634808	-1.047283475	down	2.551749566	3.599033041	TCCAACTCATTCAAAAC	hsa_circ_0066618	chr3	98530037	98530398	-		213	ANNOTATED, CDS, c	NM_080927	DCBLD2	Salzman2013	131566	circRNA	Not Detected	Detected	2.551749566	3.599033041
hsa_gci165918	2.193139743	1.132997741	up	6.173199234	5.040201494	ACCTTAAACTGACGAGC	hsa_circ_0066694	chr3	101399933	101401751	-		327	ANNOTATED, CDS, c	NM_000986	RPL24	Salzman2013	6152	circRNA	Detected	Detected	6.173199234	5.040201494
hsa_gci165941	3.407448952	1.768692044	up	7.872887545	6.104195501	CACCGAGGTGTTATTAA	hsa_circ_0066776	chr3	110830876	110845182	+		909	ANNOTATED, CDS, c	NM_015480	NECTIN3	Salzman2013	25945	circRNA	Detected	Detected	7.872887545	6.104195501
hsa_gci165955	2.001076233	1.000776129	up	7.736970285	6.736194156	TCCCCGTCCAGTGTCTG	hsa_circ_0066847	chr3	114107851	114219238	-		141	ANNOTATED, coding	NM_001164343	ZBTB20	Salzman2013	26137	circRNA	Detected	Detected	7.736970285	6.736194156
hsa_gci165968	2.038093641	1.027220338	up	6.610987067	5.583766728	TAGTCTGTCAAGTTACT	hsa_circ_0066911	chr3	120122088	120122201	-		113	ANNOTATED, CDS, c	NM_007085	FSTL1	Salzman2013	11167	circRNA	Detected	Detected	6.610987067	5.583766728
hsa_gci165969	2.211088337	1.144756665	up	9.082061081	7.937304416	GAATGCCTAAAGAAGAA	hsa_circ_0066912	chr3	120122088	120129831	-		396	ANNOTATED, CDS, c	NM_007085	FSTL1	Salzman2013	11167	circRNA	Detected	Detected	9.082061081	7.937304416
hsa_gci165993	2.585266641	1.370313086	up	7.761058806	6.39074572	TTGAAAAGTCCGCTTTC	hsa_circ_0066993	chr3	122180070	122215417	-		437	ANNOTATED, INTERN	NR_026698	KPNA1	Salzman2013	3836	circRNA	Detected	Detected	7.761058806	6.39074572
hsa_gci165994	2.613366114	1.38590925	up	7.584376887	6.198467637	GCTTTCAAGAGAAAAGG	hsa_circ_0066994	chr3	122215283	122215417	-		134	ANNOTATED, INTERN	NR_026698	KPNA1	Salzman2013	3836	circRNA	Detected	Detected	7.584376887	6.198467637
hsa_gci166028	2.900894524	1.53649784	up	7.873592175	6.337094334	GACCCCGTGCCGACTAA	hsa_circ_0067215	chr3	128338812	128344855	-		1129	ANNOTATED, CDS, c	NM_002950	RPN1	Salzman2013	6184	circRNA	Detected	Detected	7.873592175	6.337094334
hsa_gci166034	2.279581622	1.188769067	up	4.684962058	3.496192991	AGGAGTAGAAGAGGGAA	hsa_circ_0067229	chr3	128350790	128363826	-		582	ANNOTATED, CDS, c	NM_002950	RPN1	Salzman2013	6184	circRNA	Detected	Detected	4.684962058	3.496192991
hsa_gci166036	3.728611614	1.898638528	up	7.365021588	5.46638306	GCGGGGCTCGCCTTCAT	hsa_circ_0067232	chr3	128444978	128533641	+		2232	ANNOTATED, CDS, c	NM_004637	RAB7A	Salzman2013	7879	circRNA	Detected	Detected	7.365021588	5.46638306
hsa_gci166043	7.681352457	2.941360349	up	4.622296338	1.680935989	AAGTAATTTATGTGCCC	hsa_circ_0067252	chr3	128971123	128971798	+		233	ANNOTATED, CDS, c	NM_016128	COPG1	Salzman2013	22820	circRNA	Detected	Not Detected	4.622296338	1.680935989
hsa_gci166069	6.454425123	2.690288605	up	3.896861304	1.2065727	TCGTAGTAACAGTGGAC	hsa_circ_0067434	chr3	133894452	133901915	-		473	ANNOTATED, CDS, c	NM_001005861	RYK	Salzman2013	6259	circRNA	Detected	Not Detected	3.896861304	1.2065727
hsa_gci166097	2.204884163	1.140702863	up	7.532974739	6.392271876	TCAGTTTTTGGTAGCAT	hsa_circ_0067535	chr3	138426000	138431146	-		228	ANNOTATED, CDS, c	NM_006219	PIK3CB	Salzman2013	5291	circRNA	Detected	Detected	7.532974739	6.392271876
hsa_gci166098	2.173365714	1.119930958	up	8.393151615	7.273220657	CCCGTCCCGAGACAAGT	hsa_circ_0067536	chr3	138452202	138453646	-		249	ANNOTATED, CDS, c	NM_006219	PIK3CB	Salzman2013	5291	circRNA	Detected	Detected	8.393151615	7.273220657
hsa_gci166120	2.253640382	1.17225732	up	4.738627386	3.566370067	ACTCCCTCACCAATGTA	hsa_circ_0067619	chr3	142116170	142123918	-		626	ANNOTATED, CDS, c	NM_019001	XRN1	Salzman2013	54464	circRNA	Detected	Detected	4.738627386	3.566370067
hsa_gci166144	3.29913328	1.722087062	up	8.008172191	6.286085129	GATACCTCTCGTCGTAG	hsa_circ_0067718	chr3	149563797	149677923	+		797	ANNOTATED, CDS, c	NM_007282	RNF13	Salzman2013	11342	circRNA	Detected	Detected	8.008172191	6.286085129
hsa_gci166145	2.222693864	1.152309257	up	7.268922198	6.116612942	ACGTCCCTCCAGTAGTT	hsa_circ_0067720	chr3	149570302	149629870	+		492	ANNOTATED, CDS, c	NM_007282	RNF13	Salzman2013	11342	circRNA	Detected	Detected	7.268922198	6.116612942
hsa_gci166146	2.488316373	1.315169926	up	7.027478825	5.712308898	CGTCCCTCCAGTAGTTT	hsa_circ_0067721	chr3	149570302	149639014	+		586	ANNOTATED, CDS, c	NM_007282	RNF13	Salzman2013	11342	circRNA	Detected	Detected	7.027478825	5.712308898
hsa_gci166162	3.244160816	1.697845337	up	7.180392158	5.482546821	AAGAAGTCGTGTATAAT	hsa_circ_0067790	chr3	155639975	155643155	+		242	ANNOTATED, CDS, c	NM_003875	GMPS	Salzman2013	8833	circRNA	Detected	Detected	7.180392158	5.482546821
hsa_gci166163	5.69230842	2.509013832	up	7.664310558	5.155296726	GGGACGCCGGCGGCATA	hsa_circ_0067793	chr3	156257928	156272973	-		3716	ANNOTATED, CDS, c	NM_007107	SSR3	Salzman2013	6747	circRNA	Detected	Detected	7.664310558	5.155296726
hsa_gci166164	3.017730823	1.593464126	up	8.638005832	7.044541707	ACGGTATAAGCGGTCAT	hsa_circ_0067794	chr3	156262096	156271570	-		358	ANNOTATED, CDS, c	NM_007107	SSR3	Salzman2013	6747	circRNA	Detected	Detected	8.638005832	7.044541707
hsa_gci166178	3.944029146	1.979670213	up	7.068177763	5.08850755	AAATCCCGAGCCACTTT	hsa_circ_0067883	chr3	169700494	169706147	+		479	ANNOTATED, CDS, c	NM_003262	SEC62	Salzman2013	7095	circRNA	Detected	Detected	7.068177763	5.08850755
hsa_gci166197	2.233609107	1.159376729	up	6.240268109	5.08089138	ACAATTGAAACCTGTCT	hsa_circ_0067962	chr3	171451233	171455872	-		637	ANNOTATED, CDS, c	NM_002662	PLD1	Salzman2013	5337	circRNA	Detected	Detected	6.240268109	5.08089138
hsa_gci166214	2.248179423	1.168757179	up	4.111974843	2.943217664	ACCTCACCTCGAAAATC	hsa_circ_0068021	chr3	172486796	172491812	+		257	ANNOTATED, CDS, c	NM_018098	ECT2	Salzman2013	1894	circRNA	Detected	Not Detected	4.111974843	2.943217664
hsa_gci166220	2.754074878	1.461567784	up	6.768702504	5.30713472	CGTTCCGGGCCGACGGC	hsa_circ_0068042	chr3	176738541	176914266	-		104347	ALT_ACCEPTOR, CDS	NM_024665	TBL1XR1	Salzman2013	79718	circRNA	Detected	Detected	6.768702504	5.30713472
hsa_gci166230	2.367853059	1.243579555	up	10.36470098	9.121121424	GGCTCTCGCCCCTAACT	hsa_circ_0068067	chr3	178735010	178743013	-		8003	ANNOTATED, CDS, c	NM_152240	ZMAT3	Salzman2013	64393	circRNA	Detected	Detected	10.36470098	9.121121424
hsa_gci166234	3.102227715	1.633304589	up	5.309941943	3.676637354	CCGTTTCCCGCCAGTGA	hsa_circ_0068079	chr3	179065479	179066751	+		238	ANNOTATED, CDS, c	NM_033540	MFN1	Salzman2013	55669	circRNA	Detected	Detected	5.309941943	3.676637354
hsa_gci166261	2.919810254	1.545874618	up	4.578327908	3.032453291	AAACTATTATCAGAGTT	hsa_circ_0068176	chr3	183435438	183480067	+		1847	ANNOTATED, CDS, c	NM_018023	YEATS2	Salzman2013	55689	circRNA	Detected	Not Detected	4.578327908	3.032453291
hsa_gci166262	3.37091128	1.753138657	up	4.851485341	3.098346684	CAAGAAACTATTATCAG	hsa_circ_0068177	chr3	183435438	183493911	+		2477	ANNOTATED, CDS, c	NM_018023	YEATS2	Salzman2013	55689	circRNA	Detected	Not Detected	4.851485341	3.098346684
hsa_gci166278	5.292146726	2.403853061	up	5.584474059	3.180620998	GTCGACGCTCCTGACAG	hsa_circ_0068240	chr3	183547172	183551613	-		525	ANNOTATED, CDS, c	NM_018622	PARL	Salzman2013	55486	circRNA	Detected	Detected	5.584474059	3.180620998
hsa_gci166290	2.328507209	1.219405349	up	6.230999911	5.011594562	AACACAAGTCACCCCAA	hsa_circ_0068290	chr3	183899938	183901879	+		820	ANNOTATED, CDS, c	NM_004068	AP2M1	Salzman2013	1173	circRNA	Detected	Detected	6.230999911	5.011594562
hsa_gci166309	2.050711174	1.036124314	up	5.334681172	4.298556858	AAATGTTCCGAAGTCCC	hsa_circ_0068376	chr3	184654033	184689540	+		522	ANNOTATED, CDS, c	NM_001009921	VPS8	Salzman2013	23355	circRNA	Detected	Detected	5.334681172	4.298556858
hsa_gci166324	7.859812885	2.974494967	up	5.886207991	2.911713023	TCCACCCCGTACTTCGT	hsa_circ_0068428	chr3	185764105	185798964	-		3623	ANNOTATED, CDS, c	NM_004454	ETV5	Salzman2013	2119	circRNA	Detected	Not Detected	5.886207991	2.911713023
hsa_gci166376	2.547795537	1.349249505	up	8.568672281	7.219422776	ACTATATCTTTAACGGG	hsa_circ_0068609	chr3	195785154	195785503	-		141	ANNOTATED, CDS, c	NM_003234	TFRC	Salzman2013	7037	circRNA	Detected	Detected	8.568672281	7.219422776
hsa_gci166378	2.605689536	1.381665199	up	6.228183992	4.846518792	ACAAAAGGTCAGTCTCC	hsa_circ_0068611	chr3	195785154	195792471	-		637	ANNOTATED, CDS, c	NM_003234	TFRC	Salzman2013	7037	circRNA	Detected	Detected	6.228183992	4.846518792
hsa_gci166379	10.96068683	3.454266299	up	4.684685371	1.230419072	GGTCCGACTTGGCCCAT	hsa_circ_0068624	chr3	195794902	195802231	-		864	ANNOTATED, CDS, c	NM_003234	TFRC	Salzman2013	7037	circRNA	Detected	Not Detected	4.684685371	1.230419072
hsa_gci166384	-2.125514284	-1.087811954	down	3.10038734	4.188199295	TCGAAGATCGACCGACA	hsa_circ_0068643	chr3	196089164	196089558	-		394	ANNOTATED, CDS, c	NM_015562	UBXN7	Salzman2013	26043	circRNA	Not Detected	Detected	3.10038734	4.188199295

hsa_gci166389	2.835254001	1.503477987	up	3.897176261	2.393698273	CCCCGTGCTTGAAGAAG	hsa_circ_0068651	chr3	196098766	196129890	-	394	ANNOTATED, CDS, c	NM_015562	UBXN7	Salzman2013	26043	circRNA	Detected	Not Detected	3.897176261	2.393698273
hsa_gci166418	7.678603294	2.940843915	up	4.142973496	1.202129581	CACGTCGACAGATACAT	hsa_circ_0068743	chr3	197562544	197592342	+	663	ANNOTATED, CDS, c	NM_032773	LRCH3	Salzman2013	84859	circRNA	Detected	Not Detected	4.142973496	1.202129581
hsa_gci166430	2.920829446	1.546378119	up	7.241930417	5.695552299	AGGGACTTACAATTCTC	hsa_circ_0068784	chr4	337570	338219	+	223	ANNOTATED, CDS, c	NM_003441	ZNF141	Salzman2013	7700	circRNA	Detected	Detected	7.241930417	5.695552299
hsa_gci166438	2.159786242	1.110888533	up	8.761512155	7.650623622	CGCCTTCTCCAGTCGCG	hsa_circ_0068881	chr4	1813205	1838299	-	4571	ANNOTATED, CDS, c	NM_012318	LETM1	Salzman2013	3954	circRNA	Detected	Detected	8.761512155	7.650623622
hsa_gci166447	3.112601884	1.638121061	up	10.86541873	9.227297669	TCGGTGGTTCGCTCCTC	hsa_circ_0068920	chr4	2626988	2702271	+	4020	ALT_ACCEPTOR, CDS	NM_003704	FAM193A	Salzman2013	8603	circRNA	Detected	Detected	10.86541873	9.227297669
hsa_gci166452	3.952869406	1.982900292	up	8.986864185	7.003963892	TAGAACCTGGGCAGGGC	hsa_circ_0068961	chr4	3076407	3109150	+	892	ANNOTATED, CDS, c	NM_002111	HTT	Salzman2013	3064	circRNA	Detected	Detected	8.986864185	7.003963892
hsa_gci166453	2.110104266	1.077314288	up	6.738516788	5.6612025	CGACTTTCAAGAAAGAA	hsa_circ_0068963	chr4	3088665	3124663	+	1058	ANNOTATED, CDS, c	NM_002111	HTT	Salzman2013	3064	circRNA	Detected	Detected	6.738516788	5.6612025
hsa_gci166457	2.504939979	1.324776036	up	3.917096291	2.592320256	CACTGCAGACAATACGA	hsa_circ_0069006	chr4	3144492	3201666	+	2631	ANNOTATED, CDS, c	NM_002111	HTT	Salzman2013	3064	circRNA	Detected	Not Detected	3.917096291	2.592320256
hsa_gci166468	2.857589237	1.514798552	up	4.31472381	2.799925259	GCACCACGCCGTCTTGG	hsa_circ_0069053	chr4	5627475	5667376	-	1176	ANNOTATED, CDS, c	NM_001166136	EVC2	Salzman2013	132884	circRNA	Detected	Not Detected	4.31472381	2.799925259
hsa_gci166481	3.23486392	1.693705024	up	7.887074689	6.193369665	GTCTACAGTTTCTCGAT	hsa_circ_0069116	chr4	7006570	7012508	+	377	ANNOTATED, CDS, c	NM_020773	TBC1D14	Salzman2013	57533	circRNA	Detected	Detected	7.887074689	6.193369665
hsa_gci166482	2.390385853	1.257243515	up	4.723825483	3.466581968	TGGAGAGGAACCGAGCC	hsa_circ_0069117	chr4	7006570	7016284	+	487	ANNOTATED, CDS, c	NM_020773	TBC1D14	Salzman2013	57533	circRNA	Detected	Detected	4.723825483	3.466581968
hsa_gci166487	2.536601198	1.342896718	up	5.130424074	3.787527356	CCTCAAGTTCTTTCTCT	hsa_circ_0069140	chr4	7811340	7873807	-	1056	ANNOTATED, CDS, c	NM_001134647	AFAP1	Salzman2013	60312	circRNA	Detected	Detected	5.130424074	3.787527356
hsa_gci166495	-2.60281162	-1.3800709	down	2.760076617	4.140147517	GGGTGTCTTCTCGGTGA	hsa_circ_0069197	chr4	10075962	10099515	-	2483	ANNOTATED, CDS, c	NM_017491	WDR1	Salzman2013	9948	circRNA	Not Detected	Detected	2.760076617	4.140147517
hsa_gci166496	2.120472956	1.084386084	up	5.17735473	4.092968647	AGTCGTTCCGCGACGTG	hsa_circ_0069212	chr4	10441503	10456612	-	6844	ANNOTATED, CDS, c	NM_053042	ZNF518B	Salzman2013	85460	circRNA	Detected	Detected	5.17735473	4.092968647
hsa_gci166497	-2.843597005	-1.507717021	down	1.824979525	3.332696546	AGGGTTCCTAAAGTCGT	hsa_circ_0069217	chr4	13369346	13378246	-	1101	ANNOTATED, CDS, c	NM_004249	RAB28	Salzman2013	9364	circRNA	Not Detected	Detected	1.824979525	3.332696546
hsa_gci166502	-4.234463945	-2.082179346	down	1.24131438	3.323493726	GTAGGTCCGTGTCTTTC	hsa_circ_0069239	chr4	16204084	16204203	-	119	ANNOTATED, CDS, c	NM_153365	TAPT1	Salzman2013	202018	circRNA	Not Detected	Detected	1.24131438	3.323493726
hsa_gci166514	2.189600869	1.130667912	up	5.475962937	4.345295024	TTTTGTGATGTTCTTCC	hsa_circ_0069296	chr4	20493383	20521076	+	355	ANNOTATED, CDS, c	NM_004787	SLIT2	Salzman2013	9353	circRNA	Detected	Detected	5.475962937	4.345295024
hsa_gci166530	2.629319317	1.39468936	up	5.484235249	4.089545889	CCACGTCGTCCTACTGA	hsa_circ_0069383	chr4	26622234	26721747	+	866	ANNOTATED, CDS, c	NM_018317	TBC1D19	Salzman2013	55296	circRNA	Detected	Detected	5.484235249	4.089545889
hsa_gci166531	2.113966147	1.079952274	up	7.113591869	6.033639595	CCTCCACGTCGTCCTAC	hsa_circ_0069384	chr4	26622234	26737109	+	899	ANNOTATED, CDS, c	NM_018317	TBC1D19	Salzman2013	55296	circRNA	Detected	Detected	7.113591869	6.033639595
hsa_gci166539	3.128681196	1.64555466	up	6.581382016	4.935827357	TTTCGTTTTCTGTTTCA	hsa_circ_0069411	chr4	37847253	37857362	+	827	ANNOTATED, CDS, c	NM_018290	PGM2	Salzman2013	55276	circRNA	Detected	Detected	6.581382016	4.935827357
hsa_gci166540	2.746879828	1.457793796	up	5.827741483	4.369947688	GAGTAAAGAGCCAGAGT	hsa_circ_0069421	chr4	38037208	38104778	+	1255	ANNOTATED, CDS, c	NM_015173	TBC1D1	Salzman2013	23216	circRNA	Detected	Detected	5.827741483	4.369947688
hsa_gci166546	2.29713946	1.199838446	up	7.681582958	6.481744512	CCTACTATTACAGACTG	hsa_circ_0069444	chr4	39077584	39098498	+	917	ANNOTATED, CDS, c	NM_015990	KLHL5	Salzman2013	51088	circRNA	Detected	Detected	7.681582958	6.481744512
hsa_gci166556	3.786016076	1.920680537	up	5.247747686	3.32706715	GCATCCCTGACTAGTTT	hsa_circ_0069481	chr4	39471638	39471784	+	146	ANNOTATED, CDS, c	NM_006859	LIAS	Salzman2013	11019	circRNA	Detected	Detected	5.247747686	3.32706715
hsa_gci166564	-4.071731124	-2.025642297	down	1.3672629	3.392905197	TACTAGAAGGGTCGTGG	hsa_circ_0069531	chr4	39915230	39918808	-	257	ANNOTATED, CDS, c	NM_001100399	PDS5A	Salzman2013	23244	circRNA	Not Detected	Detected	1.3672629	3.392905197
hsa_gci166569	2.585677602	1.370542402	up	5.971891103	4.601348701	AAAGTCTCTATTATAGA	hsa_circ_0069546	chr4	40121551	40144481	+	3154	ANNOTATED, CDS, c	NM_018177	N4BP2	Salzman2013	55728	circRNA	Detected	Detected	5.971891103	4.601348701
hsa_gci166574	2.024522079	1.017581377	up	9.530023539	8.512442162	GGGGTCGGCCCTGTCGT	hsa_circ_0069573	chr4	41259131	41270446	+	995	ANNOTATED, CDS, c	NM_004181	UCHL1	Salzman2013	7345	circRNA	Detected	Detected	9.530023539	8.512442162
hsa_gci166575	2.090091764	1.063566284	up	9.962417933	8.898851649	GGGTCGGCCCTGTCGTG	hsa_circ_0069574	chr4	41259625	41270446	+	983	ANNOTATED, CDS, c	NM_004181	UCHL1	Salzman2013	7345	circRNA	Detected	Detected	9.962417933	8.898851649
hsa_gci166587	-3.887574743	-1.958870413	down	1.408627286	3.367497699	GTCGGTCACTCCAGAAG	hsa_circ_0069621	chr4	42626552	42629126	-	314	ANNOTATED, CDS, c	NM_006095	ATP8A1	Salzman2013	10396	circRNA	Not Detected	Detected	1.408627286	3.367497699
hsa_gci166600	11.72294336	3.551262938	up	4.96469631	1.413433372	GACACGACGCTCCAGAA	hsa_circ_0069707	chr4	48712535	48782316	-	402	ANNOTATED, coding	NM_015030	FRYL	Salzman2013	285527	circRNA	Detected	Not Detected	4.96469631	1.413433372
hsa_gci166603	2.09293688	1.065528803	up	4.767314886	3.701786084	ATAAACGGTCACAACTC	hsa_circ_0069714	chr4	52729602	52729673	+	71	ANNOTATED, CDS, c	NM_001040402	DCUN1D4	Salzman2013	23142	circRNA	Detected	Detected	4.767314886	3.701786084
hsa_gci166605	2.714181056	1.440516963	up	4.826113217	3.385596254	ACGGTCACAACTCTCTA	hsa_circ_0069716	chr4	52729602	52740551	+	226	ANNOTATED, CDS, c	NM_001040402	DCUN1D4	Salzman2013	23142	circRNA	Detected	Detected	4.826113217	3.385596254
hsa_gci166617	2.35561211	1.236101996	up	5.612326297	4.376224301	CTCCTTTTGGACCTCCA	hsa_circ_0069751	chr4	54306748	54310270	+	138	ANNOTATED, CDS, c	NM_001134937	FIP1L1	Salzman2013	81608	circRNA	Detected	Detected	5.612326297	4.376224301
hsa_gci166627	2.884730077	1.528436333	up	5.153826957	3.625390624	TTGTGAAGGATATAGGC	hsa_circ_0069804	chr4	56719815	56738124	+	1388	ANNOTATED, CDS, c	NM_001024924	EXOC1	Salzman2013	55763	circRNA	Detected	Detected	5.153826957	3.625390624
hsa_gci166647	2.219952531	1.150528828	up	5.742909052	4.592380224	AACCGTAAAAAGACATA	hsa_circ_0069900	chr4	68539414	68544251	-	288	ANNOTATED, CDS, c	NM_018227	UBA6	Salzman2013	55236	circRNA	Detected	Detected	5.742909052	4.592380224
hsa_gci166648	2.209470484	1.143700658	up	6.406587712	5.262887054	ACGACAACTGTATTACC	hsa_circ_0069901	chr4	68539414	68562425	-	475	ANNOTATED, CDS, c	NM_018227	UBA6	Salzman2013	55236	circRNA	Detected	Detected	6.406587712	5.262887054
hsa_gci166673	2.195411743	1.134491538	up	5.620944001	4.486452463	ACTAATATGACGACATC	hsa_circ_0070025	chr4	76888429	76903190	-	955	ANNOTATED, CDS, c	NM_018115	SDAD1	Salzman2013	55153	circRNA	Detected	Detected	5.620944001	4.486452463
hsa_gci166684	2.376597646	1.248897678	up	5.514854428	4.26595675	TAGACGAGGGGGGTGCG	hsa_circ_0070055	chr4	77870894	77959768	+	5555	ANNOTATED, CDS, c	NM_018243	SEPT11	Salzman2013	55752	circRNA	Detected	Detected	5.514854428	4.26595675
hsa_gci166699	2.604924685	1.381241661	up	4.781812273	3.400570612	CTCCTTTTGACACCTCT	hsa_circ_0070194	chr4	79747190	79793952	+	1615	ANNOTATED, CDS, c	NM_198892	BMP2K	Salzman2013	55589	circRNA	Detected	Detected	4.781812273	3.400570612
hsa_gci166712	2.556767551	1.354321004	up	3.865579411	2.511258407	TATAGCTTCGTGGTGAC	hsa_circ_0070245	chr4	83787960	83788469	-	315	ANNOTATED, CDS, c	NM_014933	SEC31A	Salzman2013	22872	circRNA	Detected	Not Detected	3.865579411	2.511258407
hsa_gci166718	7.664773124	2.938243089	up	6.889123423	3.950880335	TGACACTTTCTTGGGAT	hsa_circ_0070257	chr4	83867410	83891622	-	360	ANNOTATED, CDS, c	NM_194282	LIN54	Salzman2013	132660	circRNA	Detected	Detected	6.889123423	3.950880335
hsa_gci166736	2.28646641	1.193119725	up	6.895504845	5.70238512	TTAAGAGCTTTTTAAGG	hsa_circ_0070343	chr4	85738544	85738738	-	194	ANNOTATED, CDS, c	NM_014991	WDFY3	Salzman2013	23001	circRNA	Detected	Detected	6.895504845	5.70238512
hsa_gci166740	2.261573125	1.177326644	up	5.888799578	4.711472934	GTCCCGGAGTCGATCAC	hsa_circ_0070351	chr4	87556404	87593696	+	299	ANNOTATED, CDS, c	NM_080685	PTPN13	Salzman2013	5783	circRNA	Detected	Detected	5.888799578	4.711472934
hsa_gci166757	-2.380784651	-1.25143713	down	2.79442129	4.045858421	TTCAGAAAACTCGTCTA	hsa_circ_0070420	chr4	89396991	89397181	+	190	ANNOTATED, CDS, c	NM_016323	HERC5	Salzman2013	51191	circRNA	Not Detected	Detected	2.79442129	4.045858421
hsa_gci166778	2.153408557	1.106622062	up	7.465287649	6.358665587	AAGACGGTAAGACTTCA	hsa_circ_0070527	chr4	103446668	103504114	+	934	ANNOTATED, CDS, c	NM_003998	NFKB1	Salzman2013	4790	circRNA	Detected	Detected	7.465287649	6.358665587
hsa_gci166794	-2.099240502	-1.06986746	down	2.63170003	3.70156749	ACGTTAGTAGACTCTCT	hsa_circ_0070590	chr4	107114765	107133992	-	285	ANNOTATED, CDS, c	NM_001163435	TBCK	Salzman2013	93627	circRNA	Not Detected	Detected	2.63170003	3.70156749
hsa_gci166797	2.760781708	1.46507682	up	4.62304708	3.15797026	AACCATCCACTGTAACC	hsa_circ_0070613	chr4	108575850	108622441	-	1041	ANNOTATED, CDS, c	NM_005443	PAPSS1	Salzman2013	9061	circRNA	Detected	Not Detected	4.62304708	3.15797026
hsa_gci166802	-6.123910499	-2.614453198	down	1.392917654	4.007370852	TGTAAAACGTATTGTCC	hsa_circ_0070631	chr4	110394159	110402937	+	288	ANNOTATED, CDS, c	NM_006323	SEC24B	Salzman2013	10427	circRNA	Not Detected	Detected	1.392917654	4.007370852
hsa_gci166842	-2.367926053	-1.243624028	down	2.097956683	3.341580711	AACCTGTTGTCTCCCTC	hsa_circ_0070743	chr4	119549190	119549381	+	191	ANNOTATED, INTERN	TCONS_12_00020705		Salzman2013		circRNA	Not Detected	Detected	2.097956683	3.341580711
hsa_gci166848	2.194303079	1.133762806	up	5.584034186	4.45027138	AGTTACACTGGAGTTTC	hsa_circ_0070771	chr4	120160372	120194863	+	2793	ANNOTATED, CDS, c	NM_019050	USP53	Salzman2013	54532	circRNA	Detected	Detected	5.584034186	4.45027138
hsa_gci166871	4.116412135	2.041387432	up	4.835551455	2.794164023	GACATCTCCCGAACCCT	hsa_circ_0070865	chr4	123200929	123246475	+	2404	ANNOTATED, CDS, c	NM_015312	KIAA1109	Salzman2013	84162	circRNA	Detected	Not Detected	4.835551455	2.794164023
hsa_gci166881	2.514263097	1.330135624	up	8.39962375	7.069488126	AAGATAGTACAAGGAAT	hsa_circ_0070907	chr4	128605563	128609022	+	268	ANNOTATED, CDS, c	NM_015693	INTU	Salzman2013	27152	circRNA	Detected	Detected	8.39962375	7.069488126

hsa_gci166909	2.500299372	1.322100845	up	4.597422648	3.275321802	AACTGTCTTGACGAGAA	hsa_circ_0071037	chr4	143324090	143383879	-	562	ANNOTATED, CDS, c	NM_003866	INPP4B	Salzman2013	8821	circRNA	Detected	Detected	4.597422648	3.275321802
hsa_gci166924	4.023910618	2.008598259	up	7.467569148	5.458970888	AAGGCGACCCGGTGACT	hsa_circ_0071099	chr4	148743877	148744108	+	158	ANNOTATED, CDS, c	NM_024605	ARHGAP10	Salzman2013	79658	circRNA	Detected	Detected	7.467569148	5.458970888
hsa_gci166973	2.253782312	1.172348176	up	9.799072063	8.626723888	TTCTAGAGAACTAATGG	hsa_circ_0071261	chr4	152095821	152108615	-	1403	ANNOTATED, CDS, c	NM_001009555	SH3D19	Salzman2013	152503	circRNA	Detected	Detected	9.799072063	8.626723888
hsa_gci166979	2.426458503	1.278852187	up	7.72310279	6.444250603	CCGCCACGACCCCTTGA	hsa_circ_0071282	chr4	153791904	153833063	+	2612	ANNOTATED, CDS, c	NM_001025595	ARFIP1	Salzman2013	27236	circRNA	Detected	Detected	7.72310279	6.444250603
hsa_gci166985	2.082873795	1.058575426	up	10.45327993	9.394704507	TCTATTTCTACGTAGGG	hsa_circ_0071327	chr4	159076762	159076977	-	215	ANNOTATED, CDS, c	NM_016613	FAM198B	Salzman2013	51313	circRNA	Detected	Detected	10.45327993	9.394704507
hsa_gci166991	2.66311808	1.413116398	up	7.616745518	6.203629121	CAATCTTCAAGAAATTT	hsa_circ_0071351	chr4	160253602	160264556	+	1366	ANNOTATED, CDS, c	NM_014247	RAPGEF2	Salzman2013	9693	circRNA	Detected	Detected	7.616745518	6.203629121
hsa_gci166994	5.11164125	2.353786587	up	8.876990224	6.523203637	GTCTTCACCCAGCAGAA	hsa_circ_0071368	chr4	165997229	166024248	-	4369	ANNOTATED, CDS, c	NM_001100389	TMEM192	Salzman2013	201931	circRNA	Detected	Detected	8.876990224	6.523203637
hsa_gci166995	5.238129683	2.389051778	up	8.442566022	6.053514244	GCAGAAGTTATGAGACC	hsa_circ_0071369	chr4	166006737	166024248	-	650	ANNOTATED, CDS, c	NM_001100389	TMEM192	Salzman2013	201931	circRNA	Detected	Detected	8.442566022	6.053514244
hsa_gci166996	4.875912162	2.285672136	up	9.007572807	6.72190067	TGAGACCCACTATAGGT	hsa_circ_0071370	chr4	166009619	166024248	-	547	ANNOTATED, CDS, c	NM_001100389	TMEM192	Salzman2013	201931	circRNA	Detected	Detected	9.007572807	6.72190067
hsa_gci166997	5.1284366	2.358519088	up	8.980530703	6.622011615	TCTTCACCCAGCAGAAG	hsa_circ_0071371	chr4	166024101	166024248	-	147	ANNOTATED, CDS, c	NM_001100389	TMEM192	Salzman2013	201931	circRNA	Detected	Detected	8.980530703	6.622011615
hsa_gci167013	2.541061648	1.345431376	up	3.997705828	2.652274452	ACCCGCTACAAGTCTCG	hsa_circ_0071454	chr4	174253971	174254146	-	175	ANNOTATED, CDS, c	NM_002129	HMGB2	Salzman2013	3148	circRNA	Detected	Not Detected	3.997705828	2.652274452
hsa_gci167026	-2.683556652	-1.424146345	down	2.393032899	3.817179244	TATAAGAGGTAACGACT	hsa_circ_0071516	chr4	184626131	184626223	+	92	ANNOTATED, CDS, c	NM_021942	TRAPPC11	Salzman2013	60684	circRNA	Not Detected	Detected	2.393032899	3.817179244
hsa_gci167029	2.085054503	1.060085096	up	4.357323558	3.297238462	ACAGAGTTACGGTGTCA	hsa_circ_0071529	chr4	185548849	185552311	-	1927	ANNOTATED, CDS, c	NM_004346	CASP3	Salzman2013	836	circRNA	Detected	Detected	4.357323558	3.297238462
hsa_gci167060	2.027100858	1.019417871	up	6.444588249	5.425170378	TTAGAGAAAACGCACCG	hsa_circ_0071762	chr5	6622128	6632090	-	368	ANNOTATED, CDS, c	NM_017755	NSUN2	Salzman2013	54888	circRNA	Detected	Detected	6.444588249	5.425170378
hsa_gci167061	3.069597039	1.618049278	up	4.250476784	2.632427506	GATTTGTCTTCGAGAAG	hsa_circ_0071820	chr5	9337824	9380135	-	301	ANNOTATED, CDS, c	NM_003966	SEMA5A	Salzman2013	9037	circRNA	Detected	Not Detected	4.250476784	2.632427506
hsa_gci167068	2.826976986	1.499260138	up	4.403055318	2.90379518	TCGGCACTTCCGTATAG	hsa_circ_0071840	chr5	10381911	10387178	+	217	ANNOTATED, CDS, c	NM_005885	MARCH6	Salzman2013	10299	circRNA	Detected	Not Detected	4.403055318	2.90379518
hsa_gci167081	2.278771663	1.188256371	up	8.705872141	7.51761577	GGCTGCACCGCGTCGGC	hsa_circ_0071871	chr5	10679341	10761387	-	2342	ANNOTATED, CDS, c	NM_004394	DAP	Salzman2013	1611	circRNA	Detected	Detected	8.705872141	7.51761577
hsa_gci167093	2.143279287	1.099819857	up	5.41552829	4.315708433	GTTTTACCGATGTTTTT	hsa_circ_0071973	chr5	14741935	14751348	-	495	ANNOTATED, CDS, c	NM_054027	ANKH	Salzman2013	56172	circRNA	Detected	Detected	5.41552829	4.315708433
hsa_gci167103	2.13557731	1.094626126	up	5.474443358	4.379817232	AGGGACGACAGATGACG	hsa_circ_0072004	chr5	16754936	16764505	-	750	ANNOTATED, CDS, c	NM_012334	MYO10	Salzman2013	4651	circRNA	Detected	Detected	5.474443358	4.379817232
hsa_gci167104	3.303087253	1.72381508	up	6.059045235	4.335230155	CGTCTAGACGGGTTTCG	hsa_circ_0072005	chr5	16754936	16766307	-	869	ANNOTATED, CDS, c	NM_012334	MYO10	Salzman2013	4651	circRNA	Detected	Detected	6.059045235	4.335230155
hsa_gci167155	-4.015391132	-2.005540525	down	1.381037088	3.386577613	GTAGTTGTGGTCGACAG	hsa_circ_0072262	chr5	37329291	37333735	-	466	ANNOTATED, CDS, c	NM_153485	NUP155	Salzman2013	9631	circRNA	Not Detected	Detected	1.381037088	3.386577613
hsa_gci167156	3.977513314	1.99186676	up	5.972791481	3.980924721	AGCGTCTTTCTCGGGTC	hsa_circ_0072263	chr5	37331786	37333735	-	282	ANNOTATED, CDS, c	NM_153485	NUP155	Salzman2013	9631	circRNA	Detected	Detected	5.972791481	3.980924721
hsa_gci167177	2.407175289	1.267341202	up	4.18126091	2.913919708	TCCTAAGTAATTTATTT	hsa_circ_0072311	chr5	38903983	38924697	+	1053	ANNOTATED, CDS, c	NM_003999	OSMR	Salzman2013	9180	circRNA	Detected	Not Detected	4.18126091	2.913919708
hsa_gci167188	9.855069637	3.300866066	up	4.558709795	1.257843729	TACTTCTCTGTAATCGT	hsa_circ_0072349	chr5	40728446	40728578	-	132	ANNOTATED, CDS, c	NM_012382	TTC33	Salzman2013	23548	circRNA	Detected	Not Detected	4.558709795	1.257843729
hsa_gci167195	-5.208594256	-2.380894057	down	2.124249039	4.505143096	TAAGTTTCCTTCACTAG	hsa_circ_0072389	chr5	43294157	43299077	-	1193	ANNOTATED, CDS, c	NM_001098272	HMGCS1	Salzman2013	3157	circRNA	Not Detected	Detected	2.124249039	4.505143096
hsa_gci167216	3.364347098	1.750326555	up	10.94657651	9.19624995	ACGACGTCCGTTTGCGG	hsa_circ_0072457	chr5	52856464	52899360	+	205	ANNOTATED, CDS, c	NM_002495	NDUFS4	Salzman2013	4724	circRNA	Detected	Detected	10.94657651	9.19624995
hsa_gci167289	2.431872674	1.282067695	up	9.778658836	8.49659114	TCACGTCTATAGTATAG	hsa_circ_0072683	chr5	64587155	64629912	-	439	ANNOTATED, CDS, c	NM_197941	ADAMTS6	Salzman2013	11174	circRNA	Detected	Detected	9.778658836	8.49659114
hsa_gci167290	2.427156466	1.279267114	up	9.872530829	8.593263714	ATTTCATACCGGTAAAA	hsa_circ_0072684	chr5	64587155	64769779	-	1791	ANNOTATED, CDS, c	NM_197941	ADAMTS6	Salzman2013	11174	circRNA	Detected	Detected	9.872530829	8.593263714
hsa_gci167296	2.186034363	1.128316079	up	7.303157845	6.174841766	ACGTGACCCCTCTAATA	hsa_circ_0072697	chr5	64863339	64868113	+	773	ANNOTATED, CDS, c	NM_015342	PPWD1	Salzman2013	23398	circRNA	Detected	Detected	7.303157845	6.174841766
hsa_gci167315	2.382979424	1.252766495	up	4.572563369	3.319796874	TTTTCCAGCGAAAAGGG	hsa_circ_0072766	chr5	68485374	68506184	+	1389	ANNOTATED, CDS, c	NM_022909	CENPH	Salzman2013	64946	circRNA	Detected	Detected	4.572563369	3.319796874
hsa_gci167341	3.351822103	1.74494558	up	4.798724739	3.053779158	GAGAAGGGCTCTCCTTT	hsa_circ_0072909	chr5	71521883	71591416	-	615	ANNOTATED, CDS, c	NM_015084	MRPS27	Salzman2013	23107	circRNA	Detected	Not Detected	4.798724739	3.053779158
hsa_gci167364	2.394939641	1.259989297	up	8.450653921	7.190664624	CCGACTGTCCCGACCCT	hsa_circ_0072973	chr5	72370568	72374139	+	401	ANNOTATED, CDS, c	NM_138782	FCHO2	Salzman2013	115548	circRNA	Detected	Detected	8.450653921	7.190664624
hsa_gci167366	2.202193956	1.138941538	up	9.769248108	8.63030657	CTAAAGGTTCCGACTGT	hsa_circ_0072977	chr5	72370568	72386349	+	3286	ANNOTATED, CDS, c	NM_138782	FCHO2	Salzman2013	115548	circRNA	Detected	Detected	9.769248108	8.63030657
hsa_gci167382	2.590161371	1.373041983	up	6.255714473	4.88267249	TTATGCAGAGCGACTCT	hsa_circ_0073044	chr5	74676896	74677902	-	259	ANNOTATED, CDS, c	NM_001130105	COL4A3BP	Salzman2013	10087	circRNA	Detected	Detected	6.255714473	4.88267249
hsa_gci167402	2.173444615	1.119983332	up	9.995725614	8.875742282	TTATCCGTCGCCCCTCG	hsa_circ_0073135	chr5	78908242	78964851	+	1901	ANNOTATED, CDS, c	NM_001114394	PAPD4	Salzman2013	167153	circRNA	Detected	Detected	9.995725614	8.875742282
hsa_gci167439	2.338591646	1.225639967	up	8.535349786	7.309709819	GCAGAGTCACCAGAGGA	hsa_circ_0073358	chr5	90664540	90679149	-	4161	ANNOTATED, CDS, c	NM_020801	ARRDC3	Salzman2013	57561	circRNA	Detected	Detected	8.535349786	7.309709819
hsa_gci167522	2.892652392	1.532392966	up	5.596202004	4.063809038	CCGCGGTAATTCTAGTC	hsa_circ_0073746	chr5	126140467	126156827	+	1027	ANNOTATED, CDS, c	NM_005573	LMNB1	Salzman2013	4001	circRNA	Detected	Detected	5.596202004	4.063809038
hsa_gci167523	2.875921619	1.524024357	up	4.17606331	2.652038953	TCGAACTAGACCCGCGG	hsa_circ_0073747	chr5	126140467	126161799	+	1252	ANNOTATED, CDS, c	NM_005573	LMNB1	Salzman2013	4001	circRNA	Detected	Not Detected	4.17606331	2.652038953
hsa_gci167524	-3.739375644	-1.902797406	down	1.342392495	3.245189902	CTACAGTTTGACCGAAC	hsa_circ_0073755	chr5	126862309	126874835	+	532	ANNOTATED, CDS, c	NM_130809	PRRC1	Salzman2013	133619	circRNA	Not Detected	Detected	1.342392495	3.245189902
hsa_gci167528	2.545879757	1.348164282	up	6.244715143	4.896550861	ACTCACTTTGTGCCCGG	hsa_circ_0073804	chr5	127782173	127800614	-	324	ANNOTATED, CDS, c	NM_001999	FBN2	Salzman2013	2201	circRNA	Detected	Detected	6.244715143	4.896550861
hsa_gci167533	-5.632231195	-2.493706556	down	1.243028425	3.736734981	TCCAAAGTGAGTTTTTA	hsa_circ_0073823	chr5	130651668	130651837	+	169	ANNOTATED, coding	NM_020240	CDC42SE2	Salzman2013	56990	circRNA	Not Detected	Detected	1.243028425	3.736734981
hsa_gci167549	4.767074733	2.253104242	up	4.290953692	2.03784945	GTTCCGAAGGTAAAACA	hsa_circ_0073871	chr5	131528303	131553041	-	1828	ANNOTATED, CDS, c	NM_004199	P4HA2	Salzman2013	8974	circRNA	Detected	Not Detected	4.290953692	2.03784945
hsa_gci167565	2.064313856	1.045662333	up	6.773701811	5.728039478	CGTCTCCACCTAAGTGA	hsa_circ_0073960	chr5	134002512	134015418	+	816	ANNOTATED, CDS, c	NM_021982	SEC24A	Salzman2013	10802	circRNA	Detected	Detected	6.773701811	5.728039478
hsa_gci167566	2.634858989	1.397725755	up	7.243113425	5.845387671	TTCAACATCCGTCTCCA	hsa_circ_0073961	chr5	134002512	134023989	+	1158	ANNOTATED, CDS, c	NM_021982	SEC24A	Salzman2013	10802	circRNA	Detected	Detected	7.243113425	5.845387671
hsa_gci167574	2.427235122	1.279313867	up	6.039177743	4.759863877	TGTAGTTTATGTTTCTC	hsa_circ_0073981	chr5	134022479	134033747	+	775	ANNOTATED, CDS, c	NM_021982	SEC24A	Salzman2013	10802	circRNA	Detected	Detected	6.039177743	4.759863877
hsa_gci167593	2.332182324	1.221680579	up	6.085547103	4.863866524	CTGAGGGTCCCAGAGCA	hsa_circ_0074040	chr5	135382023	135399507	+	2346	ANNOTATED, CDS, c	NM_000358	TGFBI	Salzman2013	7045	circRNA	Detected	Detected	6.085547103	4.863866524
hsa_gci167594	2.321704727	1.215184503	up	3.978278357	2.763093855	ACGGTTGATACACCCTT	hsa_circ_0074043	chr5	135383941	135399507	+	3059	ALT_ACCEPTOR, CDS	NM_000358	TGFBI	Salzman2013	7045	circRNA	Detected	Not Detected	3.978278357	2.763093855
hsa_gci167650	3.441792513	1.783160128	up	5.12062514	3.337465012	TGTCGTAATTGTAGGGG	hsa_circ_0074256	chr5	139819703	139919441	+	7450	ANNOTATED, CDS, c	NM_017747	ANKHD1	Salzman2013	54882	circRNA	Detected	Detected	5.12062514	3.337465012
hsa_gci167661	2.476729281	1.308436184	up	7.1305497	5.822113517	GGACCCTCTCCAGGAGT	hsa_circ_0074306	chr5	140894587	140951607	-	3291	ANNOTATED, CDS, c	NM_005219	DIAPH1	Salzman2013	1729	circRNA	Detected	Detected	7.1305497	5.822113517
hsa_gci167676	5.385987941	2.429211	up	4.239489161	1.810278162	TCGTTTGGGAATGTATT	hsa_circ_0074380	chr5	142779220	142815077	-	2178	ANNOTATED, CDS, c	NM_001018077	NR3C1	Salzman2013	2908	circRNA	Detected	Not Detected	4.239489161	1.810278162
hsa_gci167678	2.267499798	1.181102422	up	12.43423442	11.253132	CCCGTGGCCACCCGCCC	hsa_circ_0074383	chr5	143537728	143550278	-	3447	ANNOTATED, CDS, c	NM_001024947	YIPF5	Salzman2013	81555	circRNA	Detected	Detected	12.43423442	11.253132

hsa_gci167679	2.401337976	1.26383847	up	6.313214543	5.049376074	ACGGAGAAACTTACTTG	hsa_circ_0074385	chr5	145144493	145176110	-	176	ANNOTATED, CDS, c	NM_138492	PRELID2	Salzman2013	153768	circRNA	Detected	Detected	6.313214543	5.049376074
hsa_gci167680	5.39131192	2.430636381	up	10.10094489	7.670308511	TCTACTGTAAAAATAGG	hsa_circ_0074389	chr5	145197456	145202697	-	329	ANNOTATED, CDS, c	NM_182960	PRELID2	Salzman2013	153768	circRNA	Detected	Detected	10.10094489	7.670308511
hsa_gci167681	4.468358159	2.159744829	up	7.347288006	5.187543177	CCTCCTCTACAGGTAAA	hsa_circ_0074401	chr5	145515163	145519824	-	122	ANNOTATED, CDS, c	NM_020117	LARS	Salzman2013	51520	circRNA	Detected	Detected	7.347288006	5.187543177
hsa_gci167685	2.612085321	1.385202022	up	4.769062714	3.383860692	ATTCCGACTTCCTAGTC	hsa_circ_0074417	chr5	145598547	145616995	+	1220	ANNOTATED, CDS, c	NM_018989	RBM27	Salzman2013	54439	circRNA	Detected	Detected	4.769062714	3.383860692
hsa_gci167699	2.35192539	1.233842294	up	4.400759851	3.166917556	TATCTGGTTTCCGACCG	hsa_circ_0074483	chr5	147889018	147902909	-	723	ANNOTATED, CDS, c	NM_001040173	HTR4	Salzman2013	3360	circRNA	Detected	Not Detected	4.400759851	3.166917556
hsa_gci167709	2.414190037	1.271539245	up	5.43570826	4.164169015	TCGAGAAACCCTCCGTG	hsa_circ_0074559	chr5	150088308	150095224	-	2714	ANNOTATED, CDS, c	NM_001135644	DCTN4	Salzman2013	51164	circRNA	Detected	Detected	5.43570826	4.164169015
hsa_gci167719	2.253802329	1.172360989	up	6.446625748	5.274264759	ACCCTTGAAGGCTCCTA	hsa_circ_0074629	chr5	150856150	150871940	+	4734	ANNOTATED, CDS, c	NM_078483	SLC36A1	Salzman2013	206358	circRNA	Detected	Detected	6.446625748	5.274264759
hsa_gci167720	-2.47333564	-1.306458031	down	3.07805572	4.384513752	AGGGCTACCTCATGTCG	hsa_circ_0074630	chr5	150858880	150871940	+	4567	ANNOTATED, CDS, c	NM_078483	SLC36A1	Salzman2013	206358	circRNA	Not Detected	Detected	3.07805572	4.384513752
hsa_gci167721	-2.663395144	-1.413266484	down	2.253414003	3.666680487	ACCTTCCTCACCTAAAT	hsa_circ_0074636	chr5	151041008	151043160	-	2152	ANNOTATED, CDS, c	NM_003118	SPARC	Salzman2013	6678	circRNA	Not Detected	Detected	2.253414003	3.666680487
hsa_gci167726	2.997351209	1.583688139	up	7.480639476	5.896951337	CTCAGTTTATACAGGTT	hsa_circ_0074668	chr5	151166132	151179561	+	1004	ANNOTATED, CDS, c	NM_005754	G3BP1	Salzman2013	10146	circRNA	Detected	Detected	7.480639476	5.896951337
hsa_gci167742	2.059513004	1.042303236	up	5.473257672	4.430954436	ATGTTAATCTCCGTAAC	hsa_circ_0074789	chr5	156919980	156926670	-	600	ANNOTATED, CDS, c	NM_033274	ADAM19	Salzman2013	8728	circRNA	Detected	Detected	5.473257672	4.430954436
hsa_gci167761	2.373964435	1.247298322	up	6.669080208	5.421781886	TGTAGACCAGAAGAGAG	hsa_circ_0074854	chr5	162940560	162944680	+	576	ANNOTATED, CDS, c	NM_182796	MAT2B	Salzman2013	27430	circRNA	Detected	Detected	6.669080208	5.421781886
hsa_gci167779	2.404496184	1.265734637	up	5.392670519	4.126935882	GGAATTCTGGTGACCAC	hsa_circ_0075010	chr5	170818709	170833731	+	844	ANNOTATED, CDS, c	NM_001037738	NPM1	Salzman2013	4869	circRNA	Detected	Detected	5.392670519	4.126935882
hsa_gci167784	2.92642111	1.549137387	up	4.208929753	2.659792366	CACGGGTCCTGACAAGT	hsa_circ_0075021	chr5	170833400	170833731	+	331	ANNOTATED, CDS, c	NM_001037738	NPM1	Salzman2013	4869	circRNA	Detected	Not Detected	4.208929753	2.659792366
hsa_gci167818	2.176987903	1.122333391	up	4.494318316	3.371984925	TAGGTAGGACCCGGCCG	hsa_circ_0075150	chr5	176562087	176563031	+	944	ANNOTATED, CDS, c	NM_022455	NSD1	Salzman2013	64324	circRNA	Detected	Detected	4.494318316	3.371984925
hsa_gci167831	2.550946028	1.351032375	up	6.806132571	5.455100196	TGTATTGCCCCACCTCA	hsa_circ_0075225	chr5	176939496	176942822	-	1115	ANNOTATED, CDS, c	NM_016222	DDX41	Salzman2013	51428	circRNA	Detected	Detected	6.806132571	5.455100196
hsa_gci167833	2.195879715	1.134799029	up	7.680566147	6.545767118	CGTGCGGGTCGAGGTGT	hsa_circ_0075238	chr5	177019212	177023099	+	1393	ANNOTATED, CDS, c	NM_017510	TMED9	Salzman2013	54732	circRNA	Detected	Detected	7.680566147	6.545767118
hsa_gci167847	2.975519619	1.57314163	up	4.424830204	2.851688574	CCACCCGTTCAAAGTCT	hsa_circ_0075343	chr5	179665331	179707608	-	1179	ANNOTATED, CDS, c	NM_139069	MAPK9	Salzman2013	5601	circRNA	Detected	Not Detected	4.424830204	2.851688574
hsa_gci167848	2.137921099	1.096208611	up	7.204065261	6.10785665	TTATAGGGCTTCCCACC	hsa_circ_0075344	chr5	179666923	179707608	-	1107	ANNOTATED, CDS, c	NM_139069	MAPK9	Salzman2013	5601	circRNA	Detected	Detected	7.204065261	6.10785665
hsa_gci167859	3.554601473	1.82968782	up	6.054280931	4.224593111	AGGTCGGACCCGCTGTC	hsa_circ_0075399	chr5	180666872	180667345	-	473	ALT_ACCEPTOR, ALT	NM_006098	RACK1	Salzman2013	10399	circRNA	Detected	Detected	6.054280931	4.224593111
hsa_gci167875	2.436701272	1.284929395	up	6.681870207	5.396940812	ACTTGGTCATGTCGCCC	hsa_circ_0075493	chr6	3015763	3019994	+	498	ANNOTATED, CDS, c	NM_000904	NQO2	Salzman2013	4835	circRNA	Detected	Detected	6.681870207	5.396940812
hsa_gci167881	2.154410749	1.107293333	up	5.027042652	3.919749319	TACAACGTCGCGTGCTC	hsa_circ_0075523	chr6	4954131	4955778	+	1647	ANNOTATED, CDS, c	NM_004824	CDYL	Salzman2013	9425	circRNA	Detected	Detected	5.027042652	3.919749319
hsa_gci167898	2.433178598	1.28284222	up	7.176323444	5.893481224	ACAATCTTTTGTAAAGG	hsa_circ_0075648	chr6	13579682	13601181	+	1051	ANNOTATED, CDS, c	NM_012241	SIRT5	Salzman2013	23408	circRNA	Detected	Detected	7.176323444	5.893481224
hsa_gci167924	-5.32283979	-2.412196144	down	1.261060134	3.673256278	AGAGGTTCGACTGACCA	hsa_circ_0075737	chr6	17669523	17675264	-	291	ANNOTATED, CDS, c	NM_005124	NUP153	Salzman2013	9972	circRNA	Not Detected	Detected	1.261060134	3.673256278
hsa_gci167948	2.202285793	1.139001701	up	5.901113003	4.762111302	TTTAATCTACACCAACT	hsa_circ_0075831	chr6	22020567	22191885	+	678	ANNOTATED, INTERN	NR_015410	CASC15	Salzman2013	401237	circRNA	Detected	Detected	5.901113003	4.762111302
hsa_gci167958	2.067816798	1.048108373	up	5.090219837	4.042111464	GTGTCTGGGATTAGAGG	hsa_circ_0075932	chr6	31473919	31478901	+	4982	INTERGENIC	None		Salzman2013		circRNA	Detected	Detected	5.090219837	4.042111464
hsa_gci167989	3.209492241	1.682345073	up	4.58018473	2.897839657	GACTCGCCGACTCTTGA	hsa_circ_0076198	chr6	36862279	36870210	+	5344	ALT_ACCEPTOR, CDS	NM_152734	C6orf89	Salzman2013	221477	circRNA	Detected	Not Detected	4.58018473	2.897839657
hsa_gci167990	2.461547249	1.299565432	up	5.297154086	3.997588654	GGTACAGAAGGTTATAT	hsa_circ_0076200	chr6	36867201	36882469	+	714	ANNOTATED, CDS, c	NM_152734	C6orf89	Salzman2013	221477	circRNA	Detected	Detected	5.297154086	3.997588654
hsa_gci167998	2.667321429	1.415391689	up	4.652574498	3.237182809	AGGACGTCCAAGAGGAC	hsa_circ_0076234	chr6	37429324	37449284	+	2776	ANNOTATED, CDS, c	NM_015050	CMTR1	Salzman2013	23070	circRNA	Detected	Detected	4.652574498	3.237182809
hsa_gci168002	2.466694696	1.302579164	up	8.971856325	7.669277161	CCGCGACCCCAAGTGTG	hsa_circ_0076277	chr6	39156746	39159531	-	2785	ANNOTATED, CDS, c	NM_003740	KCNK5	Salzman2013	8645	circRNA	Detected	Detected	8.971856325	7.669277161
hsa_gci168010	2.313917318	1.210337314	up	10.22741457	9.017077258	AGAGGGTTAAAGACTTT	hsa_circ_0076324	chr6	42023268	42023752	+	162	ANNOTATED, CDS, c	NM_138572	TAF8	Salzman2013	129685	circRNA	Detected	Detected	10.22741457	9.017077258
hsa_gci168046	2.836652287	1.504189318	up	6.516012055	5.011822737	ACTGTCTAGTAGTTTCG	hsa_circ_0076442	chr6	42993774	42997337	+	806	ANNOTATED, CDS, c	NM_033112	RRP36	Salzman2013	88745	circRNA	Detected	Detected	6.516012055	5.011822737
hsa_gci168053	2.172473007	1.119338252	up	6.920250594	5.800912343	GTCCTTGTGGACCCCCT	hsa_circ_0076490	chr6	43146543	43149244	+	2489	ANNOTATED, CDS, c	NM_003131	SRF	Salzman2013	6722	circRNA	Detected	Detected	6.920250594	5.800912343
hsa_gci168061	2.064722132	1.045947638	up	6.652077003	5.606129365	CTCAACGAAGTGTACCA	hsa_circ_0076546	chr6	43492844	43516197	-	1314	ANNOTATED, CDS, c	NM_020750	XPO5	Salzman2013	57510	circRNA	Detected	Detected	6.652077003	5.606129365
hsa_gci168084	2.960800692	1.565987378	up	8.47912613	6.913138752	ACCCCGAGTAGCCCTCT	hsa_circ_0076698	chr6	47199267	47202634	-	1692	ANNOTATED, CDS, c	NM_014452	TNFRSF21	Salzman2013	27242	circRNA	Detected	Detected	8.47912613	6.913138752
hsa_gci168086	2.124414996	1.087065619	up	9.131094224	8.044028606	CCCGCGTCCCTGTGTGC	hsa_circ_0076701	chr6	47199267	47277680	-	3634	ANNOTATED, CDS, c	NM_014452	TNFRSF21	Salzman2013	27242	circRNA	Detected	Detected	9.131094224	8.044028606
hsa_gci168094	3.128184736	1.645325714	up	7.968854759	6.323529045	CGTTGCCTACCCAGAGA	hsa_circ_0076751	chr6	52138027	52141996	-	643	ANNOTATED, CDS, c	NM_002388	MCM3	Salzman2013	4172	circRNA	Detected	Detected	7.968854759	6.323529045
hsa_gci168095	6.511142119	2.702910629	up	3.905330839	1.20242021	GACATCGAGTCGCAGAC	hsa_circ_0076760	chr6	52288743	52319085	+	2110	ANNOTATED, INTERN	NR_033327	EFHC1	Salzman2013	114327	circRNA	Detected	Not Detected	3.905330839	1.20242021
hsa_gci168102	3.181161603	1.669553662	up	4.897343421	3.227789758	CAGAAAGGAAGCCCTCC	hsa_circ_0076784	chr6	53132195	53134068	-	1873	ANNOTATED, CDS, c	NM_001242828	ELOVL5	Salzman2013	60481	circRNA	Detected	Detected	4.897343421	3.227789758
hsa_gci168123	2.500355153	1.322133031	up	5.748513183	4.426380151	CCGGTCGACCTCCCTGT	hsa_circ_0076878	chr6	56955608	56963939	+	165	ANNOTATED, CDS, c	NM_001031623	ZNF451	Salzman2013	26036	circRNA	Detected	Detected	5.748513183	4.426380151
hsa_gci168125	3.566295382	1.8344262	up	5.101165124	3.266738923	CACGGGTTATTTATGGC	hsa_circ_0076889	chr6	57038233	57050012	+	10143	ALT_ACCEPTOR, CDS	NM_004282	BAG2	Salzman2013	9532	circRNA	Detected	Detected	5.101165124	3.266738923
hsa_gci168132	4.026519835	2.00953344	up	4.94682146	2.93728802	AGAAAGTAGTCTGACGT	hsa_circ_0076905	chr6	57372287	57499035	+	606	ANNOTATED, CDS, c	NM_000947	PRIM2	Salzman2013	5558	circRNA	Detected	Not Detected	4.94682146	2.93728802
hsa_gci168171	2.035425222	1.02533022	up	3.90696877	2.88163855	AAAGAAGTAATATGCAA	hsa_circ_0077089	chr6	76368977	76380436	+	842	ANNOTATED, CDS, c	NM_015571	SENP6	Salzman2013	26054	circRNA	Detected	Not Detected	3.90696877	2.88163855
hsa_gci168175	3.244208119	1.697866373	up	8.455900771	6.758034398	GTTTTTGGTCACACGTA	hsa_circ_0077097	chr6	76412360	76423297	+	690	ANNOTATED, CDS, c	NM_015571	SENP6	Salzman2013	26054	circRNA	Detected	Detected	8.455900771	6.758034398
hsa_gci168180	8.163898436	3.029258234	up	4.243844657	1.214586423	GTCCACGATTCACAGAC	hsa_circ_0077117	chr6	79664948	79770535	-	3714	ANNOTATED, CDS, c	NM_017934	PHIP	Salzman2013	55023	circRNA	Detected	Not Detected	4.243844657	1.214586423
hsa_gci168195	2.501426869	1.322751275	up	7.168474301	5.845723026	TCGGGTCGCGGCGGGCC	hsa_circ_0077155	chr6	80340999	80413369	+	4646	ANNOTATED, CDS, c	NM_031469	SH3BGRL2	Salzman2013	83699	circRNA	Detected	Detected	7.168474301	5.845723026
hsa_gci168202	2.030163787	1.021596124	up	8.262509561	7.240913437	ATTTGTAACTTTAGTCA	hsa_circ_0077186	chr6	83667030	83763957	-	875	ANNOTATED, CDS, c	NM_198920	UBE3D	Salzman2013	90025	circRNA	Detected	Detected	8.262509561	7.240913437
hsa_gci168204	4.838466867	2.274549983	up	3.919659223	1.645109241	GTCGTTGACCCCGTTCA	hsa_circ_0077200	chr6	83835218	83857010	+	4247	ANNOTATED, CDS, c	NM_001199942	DOPEY1	Salzman2013	23033	circRNA	Detected	Not Detected	3.919659223	1.645109241
hsa_gci168214	5.080453223	2.344957204	up	5.470317314	3.125360109	CGGCACACCAAGAGGTG	hsa_circ_0077259	chr6	87795215	87795551	-	336	ANNOTATED, CDS, c	NM_001252383	CGA	Salzman2013	1081	circRNA	Detected	Not Detected	5.470317314	3.125360109
hsa_gci168215	-2.099163546	-1.069814571	down	2.30059863	3.370413201	AGACTATTGAGTTGTCC	hsa_circ_0077264	chr6	87926016	87953329	+	555	ANNOTATED, CDS, c	NM_015021	ZNF292	Salzman2013	23036	circRNA	Not Detected	Detected	2.30059863	3.370413201
hsa_gci168252	2.344394359	1.229215271	up	4.449117059	3.219901788	ACGACTGGGAACCTCCA	hsa_circ_0077434	chr6	99990262	100016690	-	2307	ANNOTATED, CDS, c	NM_005190	CCNC	Salzman2013	892	circRNA	Detected	Detected	4.449117059	3.219901788

hsa_gci168280	2.751254146	1.460089414	up	7.655458829	6.195369414	TTTTAGACGTCAATAGT	hsa_circ_0077526	chr6	105563560	105564743	-	310	ANNOTATED, CDS, c	NM_001199563	BVES	Salzman2013	11149	circRNA	Detected	Detected	7.655458829	6.195369414
hsa_gci168281	3.63175947	1.860668657	up	6.611671414	4.751002757	TTTGTTAGCGGCCATGT	hsa_circ_0077527	chr6	105563560	105573453	-	607	ANNOTATED, CDS, c	NM_001199563	BVES	Salzman2013	11149	circRNA	Detected	Detected	6.611671414	4.751002757
hsa_gci168287	2.702834747	1.434473307	up	7.271349922	5.836876615	CCTCCTGCGGTATAACC	hsa_circ_0077547	chr6	107585389	107780779	-	10763	ALT_DONOR, CDS, c	NM_020381	PDSS2	Salzman2013	57107	circRNA	Detected	Detected	7.271349922	5.836876615
hsa_gci168294	5.164157955	2.368533129	up	5.464881703	3.096348574	GCAAGCAAGACACGGTC	hsa_circ_0077564	chr6	108532716	108533458	-	742	ANNOTATED, CDS, c	NM_003795	SNX3	Salzman2013	8724	circRNA	Detected	Not Detected	5.464881703	3.096348574
hsa_gci168317	2.728714785	1.448221607	up	6.685246548	5.237024941	TAGTAAAAACTAACGTT	hsa_circ_0077671	chr6	112460953	112462705	-	443	ANNOTATED, CDS, c	NM_001105206	LAMA4	Salzman2013	3910	circRNA	Detected	Detected	6.685246548	5.237024941
hsa_gci168320	3.651198869	1.86837025	up	5.539543898	3.671173648	TCGTTCGCCCTGGAGTA	hsa_circ_0077698	chr6	116747736	116759442	+	3395	ANNOTATED, CDS, c	NM_013352	DSE	Salzman2013	29940	circRNA	Detected	Detected	5.539543898	3.671173648
hsa_gci168352	2.340772566	1.226984766	up	3.954244405	2.727259639	GGTCCCAGCACTAGACT	hsa_circ_0077815	chr6	128540066	128643455	-	645	ANNOTATED, CDS, c	NM_001135648	PTPRK	Salzman2013	5796	circRNA	Detected	Not Detected	3.954244405	2.727259639
hsa_gci168361	2.314986937	1.211004053	up	6.496966136	5.285962083	GCCACCCAATGCCCACT	hsa_circ_0077878	chr6	133135870	133138703	+	458	ANNOTATED, CDS, c	NM_001016	RPS12	Salzman2013	6206	circRNA	Detected	Detected	6.496966136	5.285962083
hsa_gci168367	2.269139112	1.182145058	up	4.301178562	3.119033504	ACCCCACCAAAATTTTA	hsa_circ_0077892	chr6	134301219	134305809	+	525	ANNOTATED, CDS, c	NM_001253676	TBPL1	Salzman2013	9519	circRNA	Detected	Not Detected	4.301178562	3.119033504
hsa_gci168387	2.775826728	1.472917515	up	4.751371592	3.278454077	ACCACACCCTTTCTCTA	hsa_circ_0078032	chr6	142231034	142231148	-	114	INTERGENIC	None		Salzman2013		circRNA	Detected	Detected	4.751371592	3.278454077
hsa_gci168403	3.295585093	1.720534622	up	5.037717035	3.317182413	AACTATCCCTTTAGGTA	hsa_circ_0078149	chr6	147527106	147556467	+	180	ANNOTATED, CDS, c	NM_001127715	STXBP5	Salzman2013	134957	circRNA	Detected	Detected	5.037717035	3.317182413
hsa_gci168406	2.111380763	1.078186776	up	7.358728834	6.280542059	CGGTCCCGACTCCCAAC	hsa_circ_0078156	chr6	147527106	147660374	+	2056	ANNOTATED, CDS, c	NM_001127715	STXBP5	Salzman2013	134957	circRNA	Detected	Detected	7.358728834	6.280542059
hsa_gci168447	3.144630663	1.652890582	up	4.932506086	3.279615504	CTTTAGCCAGTAAACAT	hsa_circ_0078292	chr6	151277130	151281551	+	218	ANNOTATED, CDS, c	NM_001242767	MTHFD1L	Salzman2013	25902	circRNA	Detected	Detected	4.932506086	3.279615504
hsa_gci168461	2.067616875	1.047968882	up	4.322164519	3.274195637	TATCAGTAAGTCCCTTA	hsa_circ_0078364	chr6	155095122	155114110	+	445	ANNOTATED, CDS, c	NM_014892	SCAF8	Salzman2013	22828	circRNA	Detected	Not Detected	4.322164519	3.274195637
hsa_gci168470	2.148023472	1.103009758	up	8.941747558	7.8387378	AGGAGGCTCCGGTTCTG	hsa_circ_0078407	chr6	157357968	157473538	+	52122	ALT_ACCEPTOR, ALT	NM_020732	ARID1B	Salzman2013	57492	circRNA	Detected	Detected	8.941747558	7.8387378
hsa_gci168471	3.477515824	1.79805708	up	9.423022048	7.624964968	GATGGGTCCCTGTCACC	hsa_circ_0078431	chr6	158049380	158094977	+	1725	ANNOTATED, CDS, c	NM_024630	ZDHHC14	Salzman2013	79683	circRNA	Detected	Detected	9.423022048	7.624964968
hsa_gci168512	2.98397904	1.577237402	up	4.200522978	2.623285576	GGTTCCTTTCGGTTAGA	hsa_circ_0078529	chr6	159186772	159197536	-	2236	ANNOTATED, CDS, c	NM_003379	EZR	Salzman2013	7430	circRNA	Detected	Not Detected	4.200522978	2.623285576
hsa_gci168516	2.584378402	1.369817324	up	7.748479544	6.37866222	CCCGGGGCCTCGAGTCG	hsa_circ_0078543	chr6	160148151	160177351	+	2121	ANNOTATED, CDS, c	NM_004906	WTAP	Salzman2013	9589	circRNA	Detected	Detected	7.748479544	6.37866222
hsa_gci168564	15.26920246	3.932552804	up	5.725515105	1.792962301	GAAACCGGGTCAACAAT	hsa_circ_0078905	chr6_mann	2039210	2041289	+	2079	INTERGENIC	None		Salzman2013		circRNA	Detected	Not Detected	5.725515105	1.792962301
hsa_gci168566	2.641005682	1.401087405	up	7.993825634	6.592738228	CTACACTCACTGTCCCT	hsa_circ_0079040	chr7	618892	647089	-	451	ANNOTATED, CDS, c	NM_001164761	PRKAR1B	Salzman2013	5575	circRNA	Detected	Detected	7.993825634	6.592738228
hsa_gci168570	2.045113838	1.03218115	up	5.477599281	4.44541813	GCGATGGTCCAAGAAAC	hsa_circ_0079130	chr7	1606969	1607486	-	517	ANNOTATED, CDS, c	NM_032302	PSMG3	Salzman2013	84262	circRNA	Detected	Detected	5.477599281	4.44541813
hsa_gci168572	3.0337976	1.601124839	up	6.919748405	5.318623566	GCGGCGCGGGCCTTCAA	hsa_circ_0079133	chr7	1606969	1609668	-	778	ANNOTATED, CDS, c	NM_001134340	PSMG3	Salzman2013	84262	circRNA	Detected	Detected	6.919748405	5.318623566
hsa_gci168582	2.733606135	1.450805391	up	5.349303137	3.898497747	TACAACACCTACATGTC	hsa_circ_0079242	chr7	5256193	5273486	+	3892	ANNOTATED, CDS, c	NM_015610	WIPI2	Salzman2013	26100	circRNA	Detected	Detected	5.349303137	3.898497747
hsa_gci168600	12.07081581	3.593451279	up	5.66441416	2.070962881	CTTTTTATAAAGAACCG	hsa_circ_0079321	chr7	6068245	6068663	-	198	ANNOTATED, CDS, c	NM_014413	EIF2AK1	Salzman2013	27102	circRNA	Detected	Not Detected	5.66441416	2.070962881
hsa_gci168609	2.229702782	1.156851413	up	6.418856133	5.26200472	AAACGGGGCCCTCCAAT	hsa_circ_0079349	chr7	6438292	6443598	+	1932	ANNOTATED, CDS, c	NM_018890	RAC1	Salzman2013	5879	circRNA	Detected	Detected	6.418856133	5.26200472
hsa_gci168644	-8.210014179	-3.037384714	down	1.154012572	4.191397286	TGTACTCCTAAGAGGTT	hsa_circ_0079450	chr7	12370508	12419192	-	4284	ANNOTATED, CDS, c	NM_001135924	VWDE	Salzman2013	221806	circRNA	Not Detected	Detected	1.154012572	4.191397286
hsa_gci168652	2.389201856	1.256528747	up	4.128241577	2.87171283	AGGGACTTCTGACACCC	hsa_circ_0079491	chr7	16714036	16721029	+	281	ANNOTATED, CDS, c	NM_001159767	BZW2	Salzman2013	28969	circRNA	Detected	Not Detected	4.128241577	2.87171283
hsa_gci168688	3.303313215	1.72391377	up	7.262802271	5.5388885	AAGTCAAACAGATCACG	hsa_circ_0079591	chr7	23358756	23391205	-	919	ANNOTATED, CDS, c	NM_006547	IGF2BP3	Salzman2013	10643	circRNA	Detected	Detected	7.262802271	5.5388885
hsa_gci168693	2.282249152	1.190456299	up	6.548130466	5.357674168	GGCACGTCAAGATAAAA	hsa_circ_0079603	chr7	23458394	23508161	-	110	ANNOTATED, CDS, c	NM_006547	IGF2BP3	Salzman2013	10643	circRNA	Detected	Detected	6.548130466	5.357674168
hsa_gci168718	2.668541606	1.416051505	up	4.075525628	2.659474124	ATGAACCCCACGAAATA	hsa_circ_0079713	chr7	30638411	30649346	+	659	ANNOTATED, CDS, c	NM_002047	GARS	Salzman2013	2617	circRNA	Detected	Not Detected	4.075525628	2.659474124
hsa_gci168729	8.557316421	3.097158438	up	4.551342726	1.454184288	GGGTCAGTCCTCGTCTA	hsa_circ_0079747	chr7	30915109	30915271	+	162	ANNOTATED, INTERN	NR_037598	INMT-MINDY4	Salzman2013	100526825	circRNA	Detected	Not Detected	4.551342726	1.454184288
hsa_gci168741	2.307227061	1.206159991	up	6.606480252	5.400320261	TTTTTGAAAGTGCCTCT	hsa_circ_0079786	chr7	32672154	32756421	-	773	ANNOTATED, INTERN	NR_036680	DPY19L1P1	Salzman2013	100129460	circRNA	Detected	Detected	6.606480252	5.400320261
hsa_gci168742	2.44242655	1.288315177	up	7.254452609	5.966137432	TTTTTGAAAGTGCCTCT	hsa_circ_0079787	chr7	32674863	32756421	-	695	ANNOTATED, INTERN	NR_036680	DPY19L1P1	Salzman2013	100129460	circRNA	Detected	Detected	7.254452609	5.966137432
hsa_gci168754	2.516821368	1.331602824	up	5.401661787	4.070058963	TTGGTTTTTGTAGGAAC	hsa_circ_0079832	chr7	34985278	34994390	-	305	ANNOTATED, CDS, c	NM_015283	DPY19L1	Salzman2013	23333	circRNA	Detected	Detected	5.401661787	4.070058963
hsa_gci168755	2.429360043	1.28057632	up	7.648727211	6.368150891	TGTAATGTGCCCATGTG	hsa_circ_0079833	chr7	34985278	35013217	-	722	ANNOTATED, CDS, c	NM_015283	DPY19L1	Salzman2013	23333	circRNA	Detected	Detected	7.648727211	6.368150891
hsa_gci168756	2.893598784	1.532864897	up	7.549530324	6.016665427	TGTGACGACGTTTTTGT	hsa_circ_0079835	chr7	34985278	35058208	-	1246	ANNOTATED, CDS, c	NM_015283	DPY19L1	Salzman2013	23333	circRNA	Detected	Detected	7.549530324	6.016665427
hsa_gci168759	2.181484662	1.12531033	up	7.018290843	5.892980513	CGGGCCTCGCCGGTGAA	hsa_circ_0079847	chr7	35050079	35077653	-	690	ANNOTATED, CDS, c	NM_015283	DPY19L1	Salzman2013	23333	circRNA	Detected	Detected	7.018290843	5.892980513
hsa_gci168778	3.000744883	1.585320669	up	6.382564461	4.797243792	GCGGTGTCTAGTGAAGG	hsa_circ_0079912	chr7	39663151	39747723	+	2810	ANNOTATED, CDS, c	NM_005402	RALA	Salzman2013	5898	circRNA	Detected	Detected	6.382564461	4.797243792
hsa_gci168795	2.137520957	1.095938565	up	6.285549452	5.189610888	CATCTCTTCCGGGAGAC	hsa_circ_0079962	chr7	43678858	43680248	-	499	ANNOTATED, CDS, c	NM_018224	COA1	Salzman2013	55744	circRNA	Detected	Detected	6.285549452	5.189610888
hsa_gci168798	4.151897059	2.053770674	up	5.076904902	3.023134228	AGAGCCGAGGTCCTGTC	hsa_circ_0079972	chr7	43906156	43908975	-	606	ANNOTATED, CDS, c	NM_001204871	URGCP-MRPS24	Salzman2013	100534592	circRNA	Detected	Not Detected	5.076904902	3.023134228
hsa_gci168807	2.141062249	1.098326741	up	8.422581088	7.324254347	CCCTGAGGACATTGAAT	hsa_circ_0080044	chr7	44605402	44611335	-	1197	ANNOTATED, CDS, c	NM_019082	DDX56	Salzman2013	54606	circRNA	Detected	Detected	8.422581088	7.324254347
hsa_gci168827	2.616952547	1.387887764	up	6.735162918	5.347275154	TGGCGTCCCTTAGTGGT	hsa_circ_0080185	chr7	47476860	47520739	-	315	ANNOTATED, CDS, c	NM_022748	TNS3	Salzman2013	64759	circRNA	Detected	Detected	6.735162918	5.347275154
hsa_gci168843	8.068987454	3.012387647	up	4.270258226	1.25787058	ACTAAAAGGTAGAGAGG	hsa_circ_0080286	chr7	56169265	56169554	-	289	ANNOTATED, CDS, c	NM_016139	CHCHD2	Salzman2013	51142	circRNA	Detected	Not Detected	4.270258226	1.25787058
hsa_gci168844	2.561990755	1.357265269	up	6.00215973	4.64489446	GACGACACGACCGACCC	hsa_circ_0080287	chr7	56169265	56170704	-	434	ANNOTATED, CDS, c	NM_016139	CHCHD2	Salzman2013	51142	circRNA	Detected	Detected	6.00215973	4.64489446
hsa_gci168867	2.211043215	1.144727223	up	7.924877217	6.780149994	TGTGTTCAGGTTTAGTA	hsa_circ_0080373	chr7	66409962	66413681	+	437	ANNOTATED, CDS, c	NM_017994	TMEM248	Salzman2013	55069	circRNA	Detected	Detected	7.924877217	6.780149994
hsa_gci168873	3.632427137	1.860933859	up	5.437839532	3.576905673	TATGCACCGTCCATCTT	hsa_circ_0080465	chr7	72483620	72519759	+	36139	INTERGENIC	None		Salzman2013		circRNA	Detected	Detected	5.437839532	3.576905673
hsa_gci168883	-2.578725257	-1.366658074	down	2.402875677	3.769533751	TAGTTAGTAGGCACTCC	hsa_circ_0080503	chr7	73100965	73105342	+	373	ANNOTATED, INTERN	NR_037776	BUD23	Salzman2013	114049	circRNA	Not Detected	Detected	2.402875677	3.769533751
hsa_gci168885	2.369206477	1.244403935	up	5.10320392	3.858799984	TACCGGTGGTCGTGACG	hsa_circ_0080535	chr7	73601940	73611429	+	2479	ANNOTATED, CDS, c	NM_022170	EIF4H	Salzman2013	7458	circRNA	Detected	Detected	5.10320392	3.858799984
hsa_gci168886	4.408359172	2.140241773	up	5.055369355	2.915127582	TGAACAAGGGACACTTT	hsa_circ_0080594	chr7	73949442	73954298	+	344	ANNOTATED, CDS, c	NM_001199207	GTF2IRD1	Salzman2013	9569	circRNA	Detected	Not Detected	5.055369355	2.915127582
hsa_gci168894	2.743702165	1.456123882	up	9.805443264	8.349319382	GGCAGAGTTTGTAAAGC	hsa_circ_0080655	chr7	74601105	74610433	-	1737	ALT_DONOR, INTERN	NR_002206	GTF2IP1	Salzman2013	2970	circRNA	Detected	Detected	9.805443264	8.349319382
hsa_gci168895	2.067102493	1.047609923	up	4.913412757	3.865802834	CAGGCACTAAAACCGTA	hsa_circ_0080656	chr7	74601105	74612761	-	1987	ALT_DONOR, INTERN	NR_002206	GTF2IP1	Salzman2013	2970	circRNA	Detected	Detected	4.913412757	3.865802834

hsa_gci168913	2.643174708	1.402271787	up	6.952389072	5.550117284	GTGTGACGGGTCGCGGA	hsa_circ_0080773	chr7	75677392	75695930	+	1305	ANNOTATED, CDS, c	NM_005918	MDH2	Salzman2013	4191	circRNA	Detected	Detected	6.952389072	5.550117284
hsa_gci168916	2.519703475	1.333253963	up	7.080977901	5.747723937	GGTGGAACCCCCACGTA	hsa_circ_0080783	chr7	75693656	75695930	+	586	ANNOTATED, CDS, c	NM_005918	MDH2	Salzman2013	4191	circRNA	Detected	Detected	7.080977901	5.747723937
hsa_gci168928	-2.557261243	-1.35459955	down	2.428955326	3.783554876	AGAGCCGTCCGGTAATG	hsa_circ_0080840	chr7	77214859	77221573	+	111	ANNOTATED, CDS, c	NM_002835	PTPN12	Salzman2013	5782	circRNA	Not Detected	Detected	2.428955326	3.783554876
hsa_gci168948	2.161964821	1.112343048	up	7.60262103	6.490277982	TATATGTAAGGTCTCTT	hsa_circ_0080903	chr7	80427407	80435074	-	593	ANNOTATED, CDS, c	NM_006379	SEMA3C	Salzman2013	10512	circRNA	Detected	Detected	7.60262103	6.490277982
hsa_gci168949	2.217063859	1.148650325	up	6.516342517	5.367692192	GTTAGTACTTTTGAACC	hsa_circ_0080904	chr7	80427407	80440017	-	684	ANNOTATED, CDS, c	NM_006379	SEMA3C	Salzman2013	10512	circRNA	Detected	Detected	6.516342517	5.367692192
hsa_gci168950	2.76656156	1.468094026	up	8.202900119	6.734806093	ACTTCATAAGTCTCCAG	hsa_circ_0080905	chr7	80427407	80458061	-	1028	ANNOTATED, CDS, c	NM_006379	SEMA3C	Salzman2013	10512	circRNA	Detected	Detected	8.202900119	6.734806093
hsa_gci168967	2.335304513	1.223610683	up	6.326814603	5.10320392	AGTGTCGGACCCCTGAG	hsa_circ_0080970	chr7	87837821	87838767	-	173	ANNOTATED, CDS, c	NM_003130	SRI	Salzman2013	6717	circRNA	Detected	Detected	6.326814603	5.10320392
hsa_gci169040	2.272956687	1.184570193	up	4.032995833	2.84842564	CCACTACACGACCCGGT	hsa_circ_0081393	chr7	99690403	99693093	-	716	ANNOTATED, CDS, c	NM_182776	MCM7	Salzman2013	4176	circRNA	Detected	Not Detected	4.032995833	2.84842564
hsa_gci169054	2.932509705	1.552135883	up	6.40226798	4.850132097	CCCTGTGTCAGACCGGG	hsa_circ_0081485	chr7	100201581	100205798	+	1218	ANNOTATED, CDS, c	NM_002593	PCOLCE	Salzman2013	5118	circRNA	Detected	Detected	6.40226798	4.850132097
hsa_gci169055	2.971394987	1.571140395	up	9.411381902	7.840241507	TTGACCTGCTCCTCAAG	hsa_circ_0081487	chr7	100204038	100205798	+	697	ANNOTATED, CDS, c	NM_002593	PCOLCE	Salzman2013	5118	circRNA	Detected	Detected	9.411381902	7.840241507
hsa_gci169061	4.186591419	2.065776128	up	6.309452634	4.243676506	GCATCGGCAACCCCTTC	hsa_circ_0081497	chr7	100276020	100276792	+	692	ANNOTATED, CDS, c	NM_005273	GNB2	Salzman2013	2783	circRNA	Detected	Detected	6.309452634	4.243676506
hsa_gci169064	2.169152499	1.117131483	up	5.16204159	4.044910107	GGGGACCCCGGACCATT	hsa_circ_0081542	chr7	100465532	100471076	+	1423	ALT_ACCEPTOR, CDS	NM_003302	TRIP6	Salzman2013	7205	circRNA	Detected	Detected	5.16204159	4.044910107
hsa_gci169067	2.900119167	1.536112182	up	6.827695335	5.291583152	CCGACCGAGTCCCCGTG	hsa_circ_0081545	chr7	100467991	100471076	+	847	ANNOTATED, CDS, c	NM_003302	TRIP6	Salzman2013	7205	circRNA	Detected	Detected	6.827695335	5.291583152
hsa_gci169076	2.224362429	1.153391874	up	5.867546886	4.714155012	GGAACAATTACCACTAG	hsa_circ_0081681	chr7	102178365	102210371	-	1530	ANNOTATED, CDS, c	NM_001097615	POLR2J3	Salzman2013	548644	circRNA	Detected	Detected	5.867546886	4.714155012
hsa_gci169112	3.199003888	1.677622746	up	8.114356582	6.436733836	GGTAGAAAAGGATAAAG	hsa_circ_0081852	chr7	105112578	105146720	-	839	ANNOTATED, CDS, c	NM_019042	PUS7	Salzman2013	54517	circRNA	Detected	Detected	8.114356582	6.436733836
hsa_gci169129	2.056094637	1.03990667	up	4.435830372	3.395923702	TTCCTAGAACCCTTAAC	hsa_circ_0081942	chr7	107615430	107638937	-	1269	ANNOTATED, CDS, c	NM_002291	LAMB1	Salzman2013	3912	circRNA	Detected	Detected	4.435830372	3.395923702
hsa_gci169144	2.391204405	1.25773746	up	9.40375235	8.14601489	GTTCGTGTCGCCCCCGG	hsa_circ_0082002	chr7	116339124	116340338	+	1214	ANNOTATED, CDS, c	NM_001127500	MET	Salzman2013	4233	circRNA	Detected	Detected	9.40375235	8.14601489
hsa_gci169145	2.569749673	1.361627829	up	9.102028705	7.740400876	TCCACGTTCGTGTCGCC	hsa_circ_0082003	chr7	116339124	116397828	+	2116	ANNOTATED, CDS, c	NM_001127500	MET	Salzman2013	4233	circRNA	Detected	Detected	9.102028705	7.740400876
hsa_gci169157	3.909044802	1.96681612	up	6.501540589	4.53472447	GAATCTTCTGTCCGTTT	hsa_circ_0082036	chr7	120686940	120687047	+	107	ANNOTATED, CDS, c	NM_024913	CPED1	Salzman2013	79974	circRNA	Detected	Detected	6.501540589	4.53472447
hsa_gci169172	2.147990485	1.102987603	up	5.217371739	4.114384136	TTCCTTACAGTATGTCC	hsa_circ_0082139	chr7	127447537	127484477	+	191	ANNOTATED, CDS, c	NM_014390	SND1	Salzman2013	27044	circRNA	Detected	Detected	5.217371739	4.114384136
hsa_gci169175	2.102914546	1.072390226	up	5.669416775	4.597026549	GTACATTCCTTACAGTA	hsa_circ_0082142	chr7	127447537	127569382	+	517	ANNOTATED, CDS, c	NM_014390	SND1	Salzman2013	27044	circRNA	Detected	Detected	5.669416775	4.597026549
hsa_gci169176	2.355707281	1.236160281	up	5.798946036	4.562785755	TCCTTACAGTATGTCCC	hsa_circ_0082147	chr7	127447537	127732659	+	2130	ANNOTATED, CDS, c	NM_014390	SND1	Salzman2013	27044	circRNA	Detected	Detected	5.798946036	4.562785755
hsa_gci169208	3.31140757	1.727444589	up	4.789125296	3.061680706	GGAGGGTCCTAGCATTG	hsa_circ_0082317	chr7	129519407	129520811	-	152	ANNOTATED, CDS, c	NM_003344	UBE2H	Salzman2013	7328	circRNA	Detected	Not Detected	4.789125296	3.061680706
hsa_gci169218	-2.046427182	-1.033107333	down	2.695942343	3.729049675	TATTCCGTAATTCCGAC	hsa_circ_0082366	chr7	129841689	129842503	-	374	ANNOTATED, CDS, c	NM_032842	TMEM209	Salzman2013	84928	circRNA	Not Detected	Detected	2.695942343	3.729049675
hsa_gci169223	2.631305665	1.395778848	up	7.645640379	6.24986153	CGATGGCCTCCTAGTCC	hsa_circ_0082390	chr7	130794854	131084192	+	839	ANNOTATED, CDS, c	NM_001145354	MKLN1	Salzman2013	4289	circRNA	Detected	Detected	7.645640379	6.24986153
hsa_gci169224	2.221206328	1.151343411	up	8.69187833	7.540534919	AGTAAACCAAACAGGTG	hsa_circ_0082396	chr7	131060182	131060252	+	70	ANNOTATED, CDS, c	NM_001145354	MKLN1	Salzman2013	4289	circRNA	Detected	Detected	8.69187833	7.540534919
hsa_gci169229	2.268713699	1.181874559	up	4.059046367	2.877171808	CTACGATTTCAATTTCG	hsa_circ_0082408	chr7	131082025	131084192	+	303	ANNOTATED, CDS, c	NM_001145354	MKLN1	Salzman2013	4289	circRNA	Detected	Not Detected	4.059046367	2.877171808
hsa_gci169241	2.213068512	1.146048114	up	7.809182956	6.663134842	AAACATTGTAGTTAGTC	hsa_circ_0082444	chr7	133002037	133041327	+	351	ANNOTATED, CDS, c	NM_021807	EXOC4	Salzman2013	60412	circRNA	Detected	Detected	7.809182956	6.663134842
hsa_gci169242	2.574775075	1.364446408	up	8.006875182	6.642428774	AGTTAGTCTCCTTGTCT	hsa_circ_0082446	chr7	133002037	133314894	+	858	ANNOTATED, CDS, c	NM_021807	EXOC4	Salzman2013	60412	circRNA	Detected	Detected	8.006875182	6.642428774
hsa_gci169274	3.04668463	1.607240171	up	4.000884669	2.393644498	TGCTTCCTCCACGGCAT	hsa_circ_0082595	chr7	138235807	138268744	+	1800	ANNOTATED, CDS, c	NM_015905	TRIM24	Salzman2013	8805	circRNA	Detected	Not Detected	4.000884669	2.393644498
hsa_gci169275	2.163870782	1.113614349	up	10.70649729	9.592882941	TAAGACTTTCAAATAGT	hsa_circ_0082597	chr7	138252225	138252399	+	174	ANNOTATED, CDS, c	NM_015905	TRIM24	Salzman2013	8805	circRNA	Detected	Detected	10.70649729	9.592882941
hsa_gci169280	2.696250143	1.430954348	up	7.750808811	6.319854463	TCTCTTGGAGTACTCTT	hsa_circ_0082633	chr7	138768525	138774505	-	389	ANNOTATED, CDS, c	NM_020119	ZC3HAV1	Salzman2013	56829	circRNA	Detected	Detected	7.750808811	6.319854463
hsa_gci169302	3.225596203	1.689565846	up	8.148087842	6.458521997	CACGTGTGGTGGTGGCC	hsa_circ_0082734	chr7	140402665	140406446	+	333	ANNOTATED, CDS, c	NM_004546	NDUFB2	Salzman2013	4708	circRNA	Detected	Detected	8.148087842	6.458521997
hsa_gci169304	2.366068466	1.242491821	up	7.023725928	5.781234107	CCTCTGCCTGACCACTC	hsa_circ_0082737	chr7	140433812	140494267	-	1905	ANNOTATED, CDS, c	NM_004333	BRAF	Salzman2013	673	circRNA	Detected	Detected	7.023725928	5.781234107
hsa_gci169325	2.169929668	1.117648283	up	11.2343859	10.11673762	GGTGAGCGGGTTCATTC	hsa_circ_0082792	chr7	141443360	141450257	+	465	ANNOTATED, CDS, c	NM_003143	SSBP1	Salzman2013	6742	circRNA	Detected	Detected	11.2343859	10.11673762
hsa_gci169336	2.392858007	1.25873479	up	5.389647521	4.130912732	CAGGAACTACCGGCTCA	hsa_circ_0082849	chr7	148700153	148703145	-	1539	ANNOTATED, CDS, c	NM_004911	PDIA4	Salzman2013	9601	circRNA	Detected	Detected	5.389647521	4.130912732
hsa_gci169357	2.532149978	1.340362857	up	3.993658004	2.653295147	CCCGGAAGTTGAAGGTA	hsa_circ_0082986	chr7	151855947	151948051	-	10049	ANNOTATED, CDS, c	NM_170606	KMT2C	Salzman2013	58508	circRNA	Detected	Not Detected	3.993658004	2.653295147
hsa_gci169374	2.214808972	1.147182271	up	3.79700845	2.649826179	TTTAAGGAACTGATATA	hsa_circ_0083068	chr7	152520426	152549336	+	393	ANNOTATED, CDS, c	NM_020445	ACTR3B	Salzman2013	57180	circRNA	Detected	Not Detected	3.79700845	2.649826179
hsa_gci169378	-3.651649409	-1.868548261	down	1.449742544	3.318290804	ACTGACAGCTATGTCCC	hsa_circ_0083082	chr7	155093275	155101945	+	2387	ANNOTATED, CDS, c	NM_005542	INSIG1	Salzman2013	3638	circRNA	Not Detected	Detected	1.449742544	3.318290804
hsa_gci169379	2.387887091	1.255734622	up	4.209342378	2.953607756	GGCGCGGTCCGAATAGT	hsa_circ_0083085	chr7	155457868	155465609	+	128	ANNOTATED, CDS, c	NM_053043	RBM33	Salzman2013	155435	circRNA	Detected	Not Detected	4.209342378	2.953607756
hsa_gci169418	2.145610937	1.101388496	up	4.417934667	3.316546171	TCGAAGTTATTTTACAT	hsa_circ_0083202	chr7	158536219	158557544	-	807	ANNOTATED, CDS, c	NM_020728	ESYT2	Salzman2013	57488	circRNA	Detected	Detected	4.417934667	3.316546171
hsa_gci169460	2.176466577	1.121987865	up	6.963957071	5.841969205	GGAGCAGACTTAGCAGT	hsa_circ_0083383	chr8	12955907	12958279	-	1601	ANNOTATED, CDS, c	NM_182643	DLC1	Salzman2013	10395	circRNA	Detected	Detected	6.963957071	5.841969205
hsa_gci169461	3.032435365	1.600476895	up	6.18089975	4.580422854	CTTGTACCTCCCGTGCT	hsa_circ_0083385	chr8	12955907	12973753	-	1918	ANNOTATED, CDS, c	NM_001164271	DLC1	Salzman2013	10395	circRNA	Detected	Detected	6.18089975	4.580422854
hsa_gci169486	2.541070752	1.345436545	up	9.758021056	8.412584511	TCTACGAGACTTCTAAT	hsa_circ_0083438	chr8	17512069	17613470	-	3368	ANNOTATED, CDS, c	NM_001001924	MTUS1	Salzman2013	57509	circRNA	Detected	Detected	9.758021056	8.412584511
hsa_gci169487	2.072775309	1.051563735	up	5.942507217	4.890943482	CTAAGAAAACGGACGAG	hsa_circ_0083439	chr8	17532694	17542051	-	282	ANNOTATED, CDS, c	NM_001001924	MTUS1	Salzman2013	57509	circRNA	Detected	Detected	5.942507217	4.890943482
hsa_gci169504	2.1698207	1.117575833	up	4.169762599	3.052186767	AGAAGTTATCAAAGTCG	hsa_circ_0083495	chr8	19679949	19694671	+	1198	ANNOTATED, CDS, c	NM_018142	INTS10	Salzman2013	55174	circRNA	Detected	Not Detected	4.169762599	3.052186767
hsa_gci169539	2.48001137	1.310346735	up	4.623589099	3.313242364	TTTAGAGCTTCGAACAT	hsa_circ_0083726	chr8	25232079	25237910	+	301	ANNOTATED, CDS, c	NM_024940	DOCK5	Salzman2013	80005	circRNA	Detected	Detected	4.623589099	3.313242364
hsa_gci169560	2.7473867	1.458059987	up	8.642965395	7.184905408	ACTAAAGATATACCCTG	hsa_circ_0083861	chr8	30557597	30560757	-	203	ANNOTATED, CDS, c	NM_000637	GSR	Salzman2013	2936	circRNA	Detected	Detected	8.642965395	7.184905408
hsa_gci169565	2.37753143	1.249464413	up	9.991316078	8.741851665	CCACTGACATGGTACTA	hsa_circ_0083894	chr8	31004568	31015046	+	599	ANNOTATED, CDS, c	NM_000553	WRN	Salzman2013	7486	circRNA	Detected	Detected	9.991316078	8.741851665
hsa_gci169571	2.324089335	1.216665525	up	10.18323407	8.966568541	CGGTGATCGGCCCCGAC	hsa_circ_0083933	chr8	37623043	37637286	+	2393	ANNOTATED, CDS, c	NM_007198	PLPBP	Salzman2013	11212	circRNA	Detected	Detected	10.18323407	8.966568541
hsa_gci169585	2.68709199	1.426045712	up	8.075238204	6.649192492	TAAAGAAAAGGTAGAAA	hsa_circ_0084003	chr8	38271435	38285611	-	1844	ANNOTATED, CDS, c	NM_023110	FGFR1	Salzman2013	2260	circRNA	Detected	Detected	8.075238204	6.649192492

hsa_gci169600	2.79101528	1.480790023	up	7.012372032	5.531582009	TGAGGCCTCCGACGTCC	hsa_circ_0084061	chr8	41119475	41166990	-	4467	ANNOTATED, CDS, c	NM_003012	SFRP1	Salzman2013	6422	circRNA	Detected	Detected	7.012372032	5.531582009
hsa_gci169604	3.072928765	1.619614323	up	4.779599446	3.159985123	GGGGGACCTAGAGTCGT	hsa_circ_0084092	chr8	42022588	42028701	+	2796	ANNOTATED, CDS, c	NM_001134296	AP3M2	Salzman2013	10947	circRNA	Detected	Not Detected	4.779599446	3.159985123
hsa_gci169623	2.36203151	1.240028211	up	6.193369665	4.953341454	TACAAAACTACATGTAA	hsa_circ_0084140	chr8	42725146	42729149	-	185	ANNOTATED, CDS, c	NM_001160223	RNF170	Salzman2013	81790	circRNA	Detected	Detected	6.193369665	4.953341454
hsa_gci169642	2.458208578	1.297607333	up	9.293541451	7.995934118	GACCGCTCAATCGGCTT	hsa_circ_0084207	chr8	48685668	48690435	-	1599	ANNOTATED, CDS, c	NM_006904	PRKDC	Salzman2013	5591	circRNA	Detected	Detected	9.293541451	7.995934118
hsa_gci169667	3.181188362	1.669565798	up	9.178678333	7.509112535	GTCGGGCTCTTTCTCGA	hsa_circ_0084333	chr8	48752576	48830943	-	5034	ANNOTATED, CDS, c	NM_006904	PRKDC	Salzman2013	5591	circRNA	Detected	Detected	9.178678333	7.509112535
hsa_gci169677	2.237064346	1.161606754	up	7.109916481	5.948309727	AACGGAAATCATGTTTT	hsa_circ_0084401	chr8	48805819	48856589	-	3005	ALT_DONOR, CDS, c	NM_006904	PRKDC	Salzman2013	5591	circRNA	Detected	Detected	7.109916481	5.948309727
hsa_gci169679	2.081317125	1.057496802	up	7.333785838	6.276289036	TAAATCGTCGAGATAGA	hsa_circ_0084403	chr8	48805819	48868508	-	3402	ALT_DONOR, CDS, c	NM_006904	PRKDC	Salzman2013	5591	circRNA	Detected	Detected	7.333785838	6.276289036
hsa_gci169776	2.483681352	1.312480093	up	6.942899583	5.63041949	GAGTAAAGTAGGTCGGA	hsa_circ_0084790	chr8	77616277	77690675	+	3371	ANNOTATED, CDS, c	NM_024721	ZFHX4	Salzman2013	79776	circRNA	Detected	Detected	6.942899583	5.63041949
hsa_gci169777	2.611583277	1.384924708	up	4.818272376	3.433347667	AACGATGTACCCCTAGA	hsa_circ_0084791	chr8	77616277	77779521	+	13617	ANNOTATED, CDS, c	NM_024721	ZFHX4	Salzman2013	79776	circRNA	Detected	Detected	4.818272376	3.433347667
hsa_gci169779	2.850041154	1.510982752	up	7.324632387	5.813649635	ATCCTTTGGGTTCGTAC	hsa_circ_0084795	chr8	77775329	77779521	+	4192	ANNOTATED, CDS, c	NM_024721	ZFHX4	Salzman2013	79776	circRNA	Detected	Detected	7.324632387	5.813649635
hsa_gci169783	2.635724508	1.398199584	up	4.144097106	2.745897521	TTTTAAGTCGATTTTGG	hsa_circ_0084819	chr8	87386258	87437547	+	1178	ANNOTATED, CDS, c	NM_007013	WWP1	Salzman2013	11059	circRNA	Detected	Not Detected	4.144097106	2.745897521
hsa_gci169824	2.123058236	1.086143945	up	7.25596591	6.169821965	CGCTCGCGCGCCCGTGC	hsa_circ_0084963	chr8	96145948	96168913	+	2965	ANNOTATED, CDS, c	NM_024613	PLEKHF2	Salzman2013	79666	circRNA	Detected	Detected	7.25596591	6.169821965
hsa_gci169842	2.314706539	1.210829299	up	4.673838219	3.46300892	AAGCACATTGAGGTAAG	hsa_circ_0085010	chr8	98787808	98864830	+	2238	ANNOTATED, CDS, c	NM_018407	LAPTM4B	Salzman2013	55353	circRNA	Detected	Detected	4.673838219	3.46300892
hsa_gci169847	3.353250084	1.745560083	up	6.085811977	4.340251894	TAGGTCCGACGCGCAAC	hsa_circ_0085016	chr8	98828285	98864830	+	1524	ANNOTATED, CDS, c	NM_018407	LAPTM4B	Salzman2013	55353	circRNA	Detected	Detected	6.085811977	4.340251894
hsa_gci169852	2.063670086	1.045212349	up	7.400726413	6.355514065	CGGAATCCTCGCCCTGC	hsa_circ_0085023	chr8	99053937	99057627	-	471	ANNOTATED, CDS, c	NM_000989	RPL30	Salzman2013	6156	circRNA	Detected	Detected	7.400726413	6.355514065
hsa_gci169856	2.219521513	1.150248692	up	4.651652239	3.501403547	GAAAGGGCATTGAGGCG	hsa_circ_0085040	chr8	99466860	99837909	-	2826	ANNOTATED, CDS, c	NM_006281	STK3	Salzman2013	6788	circRNA	Detected	Detected	4.651652239	3.501403547
hsa_gci169863	2.825203422	1.498354749	up	7.072193643	5.573838893	TAAATGTTGAGCAGATT	hsa_circ_0085057	chr8	100115180	100454863	+	3033	ANNOTATED, CDS, c	NM_017890	VPS13B	Salzman2013	157680	circRNA	Detected	Detected	7.072193643	5.573838893
hsa_gci169867	2.320262052	1.214287753	up	8.90823642	7.693948667	TCAGGACGGACCTTTGG	hsa_circ_0085070	chr8	100182266	100454863	+	1237	ANNOTATED, CDS, c	NM_017890	VPS13B	Salzman2013	157680	circRNA	Detected	Detected	8.90823642	7.693948667
hsa_gci169870	2.318365485	1.213108022	up	7.782449735	6.569341713	TATAAGTTGTACCGAAA	hsa_circ_0085081	chr8	100443764	100454863	+	363	ANNOTATED, CDS, c	NM_017890	VPS13B	Salzman2013	157680	circRNA	Detected	Detected	7.782449735	6.569341713
hsa_gci169871	2.429544009	1.280685566	up	8.437595114	7.156909548	ATTGTCCTTGACTGTTA	hsa_circ_0085082	chr8	100443764	100479862	+	584	ANNOTATED, CDS, c	NM_017890	VPS13B	Salzman2013	157680	circRNA	Detected	Detected	8.437595114	7.156909548
hsa_gci169873	2.043247606	1.030864044	up	7.222397265	6.191533221	CCGAAATTGTCCTTGAC	hsa_circ_0085087	chr8	100443764	100673719	+	3039	ANNOTATED, CDS, c	NM_017890	VPS13B	Salzman2013	157680	circRNA	Detected	Detected	7.222397265	6.191533221
hsa_gci169884	2.04351659	1.031053956	up	5.603969898	4.572915943	CGTGTAGGTTTACGATA	hsa_circ_0085124	chr8	101281012	101287389	-	517	ANNOTATED, CDS, c	NM_183419	RNF19A	Salzman2013	25897	circRNA	Detected	Detected	5.603969898	4.572915943
hsa_gci169885	2.866421779	1.51925091	up	6.245906258	4.726655347	CTAAATGTCTTCTGACG	hsa_circ_0085125	chr8	101281012	101300495	-	1284	ANNOTATED, CDS, c	NM_183419	RNF19A	Salzman2013	25897	circRNA	Detected	Detected	6.245906258	4.726655347
hsa_gci169907	3.341017795	1.740287667	up	5.311202447	3.57091478	GTGAGGTCTTGAGGTCG	hsa_circ_0085213	chr8	103297740	103359320	-	5098	ANNOTATED, CDS, c	NM_015902	UBR5	Salzman2013	51366	circRNA	Detected	Detected	5.311202447	3.57091478
hsa_gci169909	4.478134482	2.162897854	up	4.985087317	2.822189463	TTATTACTGTGAAGACC	hsa_circ_0085243	chr8	103312227	103358623	-	2530	ANNOTATED, CDS, c	NM_015902	UBR5	Salzman2013	51366	circRNA	Detected	Not Detected	4.985087317	2.822189463
hsa_gci169910	-3.81478188	-1.93160057	down	1.534036871	3.46563744	ACAGCCGTAGTTCCTCT	hsa_circ_0085248	chr8	103323526	103357773	-	1880	ANNOTATED, CDS, c	NM_015902	UBR5	Salzman2013	51366	circRNA	Not Detected	Detected	1.534036871	3.46563744
hsa_gci169911	2.571215086	1.362450299	up	6.697278318	5.334828019	ACTTAAAACAAGGTAGG	hsa_circ_0085254	chr8	103339969	103341627	-	383	ANNOTATED, CDS, c	NM_015902	UBR5	Salzman2013	51366	circRNA	Detected	Detected	6.697278318	5.334828019
hsa_gci169918	2.249981196	1.169912944	up	6.065723856	4.895810912	ATAAACAAGTCGAAGAG	hsa_circ_0085269	chr8	103358463	103373854	-	674	ANNOTATED, CDS, c	NM_015902	UBR5	Salzman2013	51366	circRNA	Detected	Detected	6.065723856	4.895810912
hsa_gci169934	2.150047071	1.104368245	up	11.46717847	10.36281023	GTAAAACTCTCGACTAG	hsa_circ_0085362	chr8	116599227	116635985	-	2821	ANNOTATED, CDS, c	NM_014112	TRPS1	Salzman2013	7227	circRNA	Detected	Detected	11.46717847	10.36281023
hsa_gci169957	3.11276466	1.638196506	up	4.717016063	3.078819557	CTTTCCAGGTAACCAAC	hsa_circ_0085458	chr8	124346117	124348772	-	427	ANNOTATED, CDS, c	NM_014109	ATAD2	Salzman2013	29028	circRNA	Detected	Not Detected	4.717016063	3.078819557
hsa_gci169961	2.163741124	1.113527901	up	9.256834738	8.143306837	GCTCAGTGATGACACCT	hsa_circ_0085473	chr8	124371460	124373886	-	333	ANNOTATED, CDS, c	NM_014109	ATAD2	Salzman2013	29028	circRNA	Detected	Detected	9.256834738	8.143306837
hsa_gci169986	2.433741429	1.283175898	up	5.957014117	4.673838219	AACCACCCCGAACACTT	hsa_circ_0085558	chr8	129108763	129113499	+	411	ANNOTATED, ncRNA,	NR_003367	PVT1	Salzman2013	5820	circRNA	Detected	Detected	5.957014117	4.673838219
hsa_gci170013	2.377772239	1.249610529	up	7.055995904	5.806385375	TCGTTAGTTTTGTATGT	hsa_circ_0085633	chr8	132958729	132958880	+	151	ANNOTATED, CDS, c	NM_015137	EFR3A	Salzman2013	23167	circRNA	Detected	Detected	7.055995904	5.806385375
hsa_gci170026	2.329242136	1.219860623	up	4.586226844	3.366366222	ACATTGACCTTCTACGT	hsa_circ_0085724	chr8	141668480	141935848	-	4403	ANNOTATED, CDS, c	NM_001199649	PTK2	Salzman2013	5747	circRNA	Detected	Detected	4.586226844	3.366366222
hsa_gci170029	2.862308978	1.517179415	up	7.761175862	6.243996447	AAGTAGATATTAGAGTC	hsa_circ_0085744	chr8	141727696	141774389	-	965	ANNOTATED, CDS, c	NM_001199649	PTK2	Salzman2013	5747	circRNA	Detected	Detected	7.761175862	6.243996447
hsa_gci170043	2.882468467	1.527304826	up	6.6738348	5.146529974	AAGTCCGTCATGTCTAA	hsa_circ_0085828	chr8	144102765	144103827	+	930	ALT_ACCEPTOR, CDS	NM_002346	LY6E	Salzman2013	4061	circRNA	Detected	Detected	6.6738348	5.146529974
hsa_gci170045	2.045911639	1.032743838	up	4.073443044	3.040699206	TCCGACGCCACCTAGAA	hsa_circ_0085863	chr8	144806102	144810893	-	4791	ANNOTATED, CDS, c	NM_198488	FAM83H	Salzman2013	286077	circRNA	Detected	Not Detected	4.073443044	3.040699206
hsa_gci170046	2.445989076	1.290417961	up	9.399439447	8.109021487	TACAACCCCGGTCCCCG	hsa_circ_0085866	chr8	144806102	144812767	-	5543	ANNOTATED, CDS, c	NM_198488	FAM83H	Salzman2013	286077	circRNA	Detected	Detected	9.399439447	8.109021487
hsa_gci170048	-2.038980308	-1.027847842	down	2.901267403	3.929115245	CCAGTCGTGATCCTTGT	hsa_circ_0085935	chr8	145066045	145067583	+	1198	ANNOTATED, CDS, c	NM_000837	GRINA	Salzman2013	2907	circRNA	Not Detected	Detected	2.901267403	3.929115245
hsa_gci170052	2.218587712	1.149641592	up	6.148309258	4.998667667	GGGGACCCCGCGGTGCT	hsa_circ_0085952	chr8	145149941	145152428	+	1228	ALT_ACCEPTOR, CDS	NM_001916	CYC1	Salzman2013	1537	circRNA	Detected	Detected	6.148309258	4.998667667
hsa_gci170056	2.221338603	1.151429323	up	6.618293816	5.466864493	CGTGTGTAGGAACCGAT	hsa_circ_0085960	chr8	145151936	145152428	+	395	ANNOTATED, CDS, c	NM_001916	CYC1	Salzman2013	1537	circRNA	Detected	Detected	6.618293816	5.466864493
hsa_gci170066	2.774863087	1.47241659	up	4.845485593	3.373069003	CTTGAGTCAGGCCGGCC	hsa_circ_0086065	chr8	145618445	145634733	-	4494	ANNOTATED, CDS, c	NM_013291	CPSF1	Salzman2013	29894	circRNA	Detected	Detected	4.845485593	3.373069003
hsa_gci170072	3.942273414	1.979027837	up	8.125889102	6.146861265	TTTCTCCTTTGTGCCGC	hsa_circ_0086161	chr8	146015153	146017805	-	875	ANNOTATED, CDS, c	NM_000973	RPL8	Salzman2013	6132	circRNA	Detected	Detected	8.125889102	6.146861265
hsa_gci170076	-2.048849636	-1.03481411	down	2.654538735	3.689352845	TTTAAGAGTTATCGAGA	hsa_circ_0086185	chr9	121960	172172	-	743	ANNOTATED, CDS, c	NM_001145355	CBWD1	Salzman2013	55871	circRNA	Not Detected	Detected	2.654538735	3.689352845
hsa_gci170112	2.83149787	1.501565445	up	4.71516825	3.213602805	CGAACTCTACACGGACT	hsa_circ_0086368	chr9	14081841	14113080	-	6567	ANNOTATED, CDS, c	NM_001190737	NFIB	Salzman2013	4781	circRNA	Detected	Detected	4.71516825	3.213602805
hsa_gci170117	2.015659789	1.011252156	up	4.239247763	3.227995608	GACAACTCGTCGGGAGT	hsa_circ_0086391	chr9	14804953	14806844	-	383	ANNOTATED, CDS, c	NM_144966	FREM1	Salzman2013	158326	circRNA	Detected	Detected	4.239247763	3.227995608
hsa_gci170121	2.002642555	1.001904942	up	4.096408056	3.094503113	GAACGGCGTCTGGTACT	hsa_circ_0086411	chr9	15874529	15920420	+	285	ANNOTATED, CDS, c	NM_173550	CCDC171	Salzman2013	203238	circRNA	Detected	Not Detected	4.096408056	3.094503113
hsa_gci170136	2.279725279	1.188859981	up	6.048668135	4.859808154	CGTCGGTCACCGACCCT	hsa_circ_0086475	chr9	19376253	19379616	-	781	ANNOTATED, CDS, c	NM_001010	RPS6	Salzman2013	6194	circRNA	Detected	Detected	6.048668135	4.859808154
hsa_gci170154	2.127287513	1.089015034	up	5.933162008	4.844146974	ACAAGTTCGTGTCAATA	hsa_circ_0086512	chr9	20758088	20789607	+	1063	ANNOTATED, CDS, c	NM_017794	FOCAD	Salzman2013	54914	circRNA	Detected	Detected	5.933162008	4.844146974
hsa_gci170166	2.619231311	1.389143473	up	5.367692192	3.978548718	ACCGGTCCCTAGATATA	hsa_circ_0086561	chr9	20866927	20933102	+	1301	ANNOTATED, CDS, c	NM_017794	FOCAD	Salzman2013	54914	circRNA	Detected	Detected	5.367692192	3.978548718
hsa_gci170175	-4.154771959	-2.054769295	down	2.280630054	4.335399348	AGAGTAGTTTTCCCTGT	hsa_circ_0086589	chr9	22046749	22065756	+	1350	ANNOTATED, INTERN	NR_003529	CDKN2B-AS1	Salzman2013	100048912	circRNA	Not Detected	Detected	2.280630054	4.335399348

hsa_gci170184	2.478199744	1.309292474	up	5.894262685	4.584970211	TATTGTGTAGAGCCCTC	hsa_circ_0086651	chr9	33318728	33352717	+	1141	ANNOTATED, CDS, c	NM_002504	NFX1	Salzman2013	4799	circRNA	Detected	Detected	5.894262685	4.584970211
hsa_gci170188	-3.373952755	-1.754439772	down	2.61878963	4.373229402	ACCACTTATCCCCATCA	hsa_circ_0086681	chr9	33886878	33886965	+	87	ANNOTATED, CDS, c	NM_017811	UBE2R2	Salzman2013	54926	circRNA	Not Detected	Detected	2.61878963	4.373229402
hsa_gci170196	2.329288202	1.219889155	up	6.324625151	5.104735996	ATCTTAGACTTATCAGA	hsa_circ_0086717	chr9	33953282	33971752	-	481	ANNOTATED, CDS, c	NM_018449	UBAP2	Salzman2013	55833	circRNA	Detected	Detected	6.324625151	5.104735996
hsa_gci170205	2.532837815	1.3407547	up	6.304243135	4.963488434	TTATTACATACATGTTT	hsa_circ_0086735	chr9	33986757	34017187	-	561	ANNOTATED, CDS, c	NM_018449	UBAP2	Salzman2013	55833	circRNA	Detected	Detected	6.304243135	4.963488434
hsa_gci170212	2.349869477	1.232580625	up	7.872280249	6.639699624	CCGACGCTACCGGAGCC	hsa_circ_0086755	chr9	34368906	34372872	-	3966	ALT_ACCEPTOR, CDS	NM_020702	MYORG	Salzman2013	57462	circRNA	Detected	Detected	7.872280249	6.639699624
hsa_gci170214	3.279497332	1.713474701	up	7.647638982	5.934164281	GCCCGTCGTACGAGTCC	hsa_circ_0086760	chr9	34613547	34614765	-	537	ANNOTATED, CDS, c	NM_024348	DCTN3	Salzman2013	11258	circRNA	Detected	Detected	7.647638982	5.934164281
hsa_gci170224	2.833575451	1.502623618	up	8.899833323	7.397209704	CAGATTGTGTCCCTACT	hsa_circ_0086794	chr9	35099888	35102191	-	1031	ANNOTATED, CDS, c	NM_013442	STOML2	Salzman2013	30968	circRNA	Detected	Detected	8.899833323	7.397209704
hsa_gci170289	4.084302762	2.030089814	up	5.261760383	3.231670569	CGTTGGCGCTTCGCCGC	hsa_circ_0087217	chr9	77561498	77567802	-	2325	ANNOTATED, CDS, c	NM_017998	C9orf40	Salzman2013	55071	circRNA	Detected	Not Detected	5.261760383	3.231670569
hsa_gci170305	2.745382071	1.457006941	up	5.254625806	3.797618866	GAACGTCTTTTTAGTGG	hsa_circ_0087273	chr9	80919650	80923499	+	549	ANNOTATED, CDS, c	NM_058179	PSAT1	Salzman2013	29968	circRNA	Detected	Detected	5.254625806	3.797618866
hsa_gci170313	5.880404033	2.555915284	up	4.149804392	1.593889108	CCTCTGGTACAGGTACA	hsa_circ_0087301	chr9	82267507	82341656	+	3529	ANNOTATED, CDS, c	NM_007005	TLE4	Salzman2013	7091	circRNA	Detected	Not Detected	4.149804392	1.593889108
hsa_gci170321	2.424676398	1.277792215	up	4.337094016	3.059301801	GACAGAAAAGTGAACGG	hsa_circ_0087331	chr9	84267098	84300811	-	277	ALT_DONOR, CDS, c	NM_005077	TLE1	Salzman2013	7088	circRNA	Detected	Detected	4.337094016	3.059301801
hsa_gci170350	2.213122561	1.146083349	up	5.993588414	4.847505065	GTAGGTGTCGAAAACTA	hsa_circ_0087452	chr9	94056664	94118252	-	1963	ALT_DONOR, CDS, c	NM_001698	AUH	Salzman2013	549	circRNA	Detected	Detected	5.993588414	4.847505065
hsa_gci170376	2.131267619	1.091711761	up	10.97618948	9.884477718	AAGGTTCCTTTTAACCA	hsa_circ_0087619	chr9	99107580	99130587	-	433	ANNOTATED, CDS, c	NM_007001	SLC35D2	Salzman2013	11046	circRNA	Detected	Detected	10.97618948	9.884477718
hsa_gci170407	2.384819379	1.253880004	up	4.307361832	3.053481828	CCGATATAACCCGAACC	hsa_circ_0087742	chr9	102590322	102596341	+	1861	ANNOTATED, CDS, c	NM_173199	NR4A3	Salzman2013	8013	circRNA	Detected	Not Detected	4.307361832	3.053481828
hsa_gci170408	2.647393328	1.404572554	up	5.856006593	4.451434039	ACTCCCAACCCATAAAC	hsa_circ_0087749	chr9	102768787	102784508	-	730	ANNOTATED, CDS, c	NM_015051	ERP44	Salzman2013	23071	circRNA	Detected	Detected	5.856006593	4.451434039
hsa_gci170409	2.876500612	1.524314777	up	6.236520063	4.712205285	ACTCCCAACCCATAAAC	hsa_circ_0087750	chr9	102778603	102784508	-	476	ANNOTATED, CDS, c	NM_015051	ERP44	Salzman2013	23071	circRNA	Detected	Detected	6.236520063	4.712205285
hsa_gci170415	2.450181052	1.292888359	up	9.493630037	8.200741678	GCCCGTCCTTATTGGTT	hsa_circ_0087765	chr9	103070781	103072661	-	263	ANNOTATED, CDS, c	NM_017746	TEX10	Salzman2013	54881	circRNA	Detected	Detected	9.493630037	8.200741678
hsa_gci170452	2.519291712	1.333018183	up	4.781372913	3.44835473	GTATCGGTTGGTGGTTG	hsa_circ_0087912	chr9	111812562	111870850	-	1544	ANNOTATED, CDS, c	NM_032012	TMEM245	Salzman2013	23731	circRNA	Detected	Detected	4.781372913	3.44835473
hsa_gci170456	2.806535032	1.488790068	up	8.423784885	6.934994818	TATTGGTCCTTATGACC	hsa_circ_0087920	chr9	111812849	111868889	-	1280	ANNOTATED, CDS, c	NM_032012	TMEM245	Salzman2013	23731	circRNA	Detected	Detected	8.423784885	6.934994818
hsa_gci170458	2.769427343	1.469587689	up	5.999478158	4.529890469	ACTTTGTCAGTTGTGTA	hsa_circ_0087927	chr9	111819470	111870850	-	1275	ANNOTATED, CDS, c	NM_032012	TMEM245	Salzman2013	23731	circRNA	Detected	Detected	5.999478158	4.529890469
hsa_gci170462	2.198300487	1.136388603	up	5.63153961	4.495151007	CTTTGTCAGTTGTGTAT	hsa_circ_0087938	chr9	111868787	111870850	-	220	ANNOTATED, CDS, c	NM_032012	TMEM245	Salzman2013	23731	circRNA	Detected	Detected	5.63153961	4.495151007
hsa_gci170473	2.929329941	1.550570698	up	6.882658722	5.332088023	GGGAACTTTATGGTGAC	hsa_circ_0087991	chr9	114145490	114154104	-	711	ANNOTATED, CDS, c	NM_001080398	ECPAS	Salzman2013	23392	circRNA	Detected	Detected	6.882658722	5.332088023
hsa_gci170488	2.319466301	1.213792885	up	11.98718933	10.77339644	GATGTAAAAATGAAAGG	hsa_circ_0088030	chr9	114337013	114348445	-	551	ANNOTATED, CDS, c	NM_001146108	PTGR1	Salzman2013	22949	circRNA	Detected	Detected	11.98718933	10.77339644
hsa_gci170494	2.332356315	1.221788206	up	8.60872554	7.386937334	AATTAGAAACAGTCTTA	hsa_circ_0088046	chr9	114860749	114875148	-	588	ANNOTATED, CDS, c	NM_022486	SUSD1	Salzman2013	64420	circRNA	Detected	Detected	8.60872554	7.386937334
hsa_gci170502	2.369807381	1.244769801	up	8.834764515	7.589994714	AAGTTCTGACACGGTTT	hsa_circ_0088072	chr9	115013208	115015068	-	286	ANNOTATED, CDS, c	NM_001244898	PTBP3	Salzman2013	9991	circRNA	Detected	Detected	8.834764515	7.589994714
hsa_gci170503	2.149146492	1.103763824	up	5.711815865	4.608052041	TAGAGCAAATTTAAAGA	hsa_circ_0088075	chr9	115013208	115038293	-	768	ANNOTATED, CDS, c	NM_001244898	PTBP3	Salzman2013	9991	circRNA	Detected	Detected	5.711815865	4.608052041
hsa_gci170505	2.233988951	1.15962205	up	8.870718185	7.711096135	ACGACAGTAAGGGTAAT	hsa_circ_0088080	chr9	115024714	115038293	-	482	ANNOTATED, CDS, c	NM_001244898	PTBP3	Salzman2013	9991	circRNA	Detected	Detected	8.870718185	7.711096135
hsa_gci170509	-3.142321639	-1.651830858	down	2.599611971	4.251442829	GCAATTACTACGGTCTT	hsa_circ_0088085	chr9	115060120	115060196	-	76	ANNOTATED, CDS, c	NM_001244898	PTBP3	Salzman2013	9991	circRNA	Not Detected	Detected	2.599611971	4.251442829
hsa_gci170521	2.759500437	1.464407114	up	4.285308904	2.82090179	GTTAGACTGACGTTTTT	hsa_circ_0088135	chr9	116046574	116050544	+	368	ANNOTATED, CDS, c	NM_004697	PRPF4	Salzman2013	9128	circRNA	Detected	Not Detected	4.285308904	2.82090179
hsa_gci170534	3.635340216	1.862090386	up	5.062578031	3.200487644	TCTACTGTACGACTAGG	hsa_circ_0088225	chr9	118989709	119115195	+	2064	ANNOTATED, CDS, c	NM_002581	PAPPA	Salzman2013	5069	circRNA	Detected	Detected	5.062578031	3.200487644
hsa_gci170541	2.692404829	1.428895349	up	10.21789123	8.788995877	CATCCGACGAGGGTGTC	hsa_circ_0088277	chr9	123151146	123206020	-	3024	ANNOTATED, CDS, c	NM_018249	CDK5RAP2	Salzman2013	55755	circRNA	Detected	Detected	10.21789123	8.788995877
hsa_gci170595	2.381881573	1.252101685	up	7.925267211	6.673165526	ACAATTCGTCGAATATT	hsa_circ_0088471	chr9	126429304	126439068	-	205	ANNOTATED, CDS, c	NM_020946	DENND1A	Salzman2013	57706	circRNA	Detected	Detected	7.925267211	6.673165526
hsa_gci170615	2.053546617	1.038117698	up	4.001842781	2.963725083	TTCTCCTCCAGTGTTTG	hsa_circ_0088553	chr9	127923119	127933459	-	162	ANNOTATED, CDS, c	NM_001123355	PPP6C	Salzman2013	5537	circRNA	Detected	Not Detected	4.001842781	2.963725083
hsa_gci170618	2.312873071	1.209686094	up	3.914411643	2.704725549	GGGGGTTAGTGTTGTAG	hsa_circ_0088576	chr9	128069691	128070159	+	325	ANNOTATED, CDS, c	NM_015635	GAPVD1	Salzman2013	26130	circRNA	Detected	Not Detected	3.914411643	2.704725549
hsa_gci170633	2.387910032	1.255748482	up	8.288993419	7.033244937	TGCGGTCGGCCCTGTCG	hsa_circ_0088667	chr9	130267622	130271410	-	2581	ALT_DONOR, CDS, c	NM_022833	FAM129B	Salzman2013	64855	circRNA	Detected	Detected	8.288993419	7.033244937
hsa_gci170634	2.180385168	1.124583012	up	8.257648974	7.133065963	TGCGTGTGGAGACGGAA	hsa_circ_0088668	chr9	130267622	130272612	-	2769	ALT_DONOR, CDS, c	NM_022833	FAM129B	Salzman2013	64855	circRNA	Detected	Detected	8.257648974	7.133065963
hsa_gci170651	2.091662254	1.064649915	up	8.450918195	7.38626828	GTAGCAATTAGTCGTGG	hsa_circ_0088795	chr9	131151539	131153015	+	1476	ANNOTATED, CDS, c	NM_001135947	URM1	Salzman2013	81605	circRNA	Detected	Detected	8.450918195	7.38626828
hsa_gci170652	2.412514367	1.270537534	up	3.842473147	2.571935613	CGAACCCTTCTCTGTAT	hsa_circ_0088807	chr9	131271154	131271376	+	222	ANNOTATED, CDS, c	NM_001003722	GLE1	Salzman2013	2733	circRNA	Detected	Not Detected	3.842473147	2.571935613
hsa_gci170660	2.299011719	1.20101382	up	6.215707921	5.014694101	GAAGGTCGGCGACTGGG	hsa_circ_0088875	chr9	131381149	131395944	+	2165	ANNOTATED, CDS, c	NM_001130438	SPTAN1	Salzman2013	6709	circRNA	Detected	Detected	6.215707921	5.014694101
hsa_gci170663	2.616391614	1.387578496	up	6.760017034	5.372438538	GTTTTCGGAGGCCGCCC	hsa_circ_0088911	chr9	131549509	131572711	+	3879	ANNOTATED, CDS, c	NM_018201	TBC1D13	Salzman2013	54662	circRNA	Detected	Detected	6.760017034	5.372438538
hsa_gci170668	2.686088779	1.425506989	up	4.885588074	3.460081085	TGGTCCTACTCCCGTGA	hsa_circ_0088942	chr9	131731678	131744980	+	872	ANNOTATED, CDS, c	NM_015354	NUP188	Salzman2013	23511	circRNA	Detected	Detected	4.885588074	3.460081085
hsa_gci170680	2.097057615	1.068366499	up	7.636386847	6.568020348	GCCGGTTCGCCGTCCTT	hsa_circ_0089050	chr9	132565431	132573560	+	2769	ANNOTATED, CDS, c	NM_014506	TOR1B	Salzman2013	27348	circRNA	Detected	Detected	7.636386847	6.568020348
hsa_gci170697	2.813380104	1.492304482	up	9.253363288	7.761058806	GAGGACCTCCAGCCCCT	hsa_circ_0089187	chr9	134133464	134136737	-	3273	ANNOTATED, CDS, c	NM_033387	FAM78A	Salzman2013	286336	circRNA	Detected	Detected	9.253363288	7.761058806
hsa_gci170717	2.437648604	1.285490171	up	7.692355043	6.406864872	CTTAAAGAGGAACGTGA	hsa_circ_0089302	chr9	135553363	135565470	+	3250	ANNOTATED, CDS, c	NM_012204	GTF3C4	Salzman2013	9329	circRNA	Detected	Detected	7.692355043	6.406864872
hsa_gci170718	2.023297112	1.016708189	up	7.456598601	6.439890412	TGTCCTCTTCTCCTTAT	hsa_circ_0089304	chr9	135759297	135765418	+	607	ANNOTATED, CDS, c	NM_018956	SPACA9	Salzman2013	11092	circRNA	Detected	Detected	7.456598601	6.439890412
hsa_gci170732	3.185755875	1.671635717	up	5.255905749	3.584270031	GAAGGCAGCGGTAGGCG	hsa_circ_0089470	chr9	138392482	138396519	+	1505	ANNOTATED, CDS, c	NM_016034	MRPS2	Salzman2013	51116	circRNA	Detected	Detected	5.255905749	3.584270031
hsa_gci170741	3.023059463	1.596009357	up	6.347460125	4.751450769	AGCTTGGGGTAGAGGTA	hsa_circ_0089501	chr9	138824814	138831605	-	773	ANNOTATED, CDS, c	NM_016172	UBAC1	Salzman2013	10422	circRNA	Detected	Detected	6.347460125	4.751450769
hsa_gci170752	2.946351233	1.558929424	up	5.173691141	3.614761718	GAACTTCGAAGCGTGCT	hsa_circ_0089563	chr9	139567594	139572008	-	1264	ANNOTATED, CDS, c	NM_006412	AGPAT2	Salzman2013	10555	circRNA	Detected	Detected	5.173691141	3.614761718
hsa_gci170779	2.125156883	1.087569348	up	6.387968315	5.300398967	CGGAGGTGGGGTAACCC	hsa_circ_0089844	chrX	6966960	6968513	-	1553	ANNOTATED, CDS, c	NM_001135565	PUDP	Salzman2013	8226	circRNA	Detected	Detected	6.387968315	5.300398967
hsa_gci170795	2.590098935	1.373007206	up	5.904037317	4.531030111	CAAAGGGGTAGTTTCCT	hsa_circ_0089977	chrX	18264696	18275150	-	549	ANNOTATED, CDS, c	NM_006089	SCML2	Salzman2013	10389	circRNA	Detected	Detected	5.904037317	4.531030111
hsa_gci170822	2.635117428	1.397867254	up	5.031484491	3.633617237	GACAGTCGGGCCGTGTT	hsa_circ_0090123	chrX	24089674	24096927	+	2432	ANNOTATED, CDS, c	NM_001415	EIF2S3	Salzman2013	1968	circRNA	Detected	Detected	5.031484491	3.633617237

hsa_gci170824	3.197762824	1.677062939	up	5.212893754	3.535830815	CGCCTCAGCCAACGACG	hsa_circ_0090134	chrX	24483343	24568583	+	12779	ANNOTATED, CDS, c	NM_001142386	PDK3	Salzman2013	5165	circRNA	Detected	Detected	5.212893754	3.535830815
hsa_gci170843	2.443829272	1.289143501	up	6.464789516	5.175646015	CAACGGCCCCTTAAAAG	hsa_circ_0090228	chrX	40988252	40988398	+	146	ANNOTATED, CDS, c	NM_001039590	USP9X	Salzman2013	8239	circRNA	Detected	Detected	6.464789516	5.175646015
hsa_gci170844	2.547609645	1.349144239	up	7.975217376	6.626073137	CCCTCAACGGCCCCTTA	hsa_circ_0090229	chrX	40988252	40990789	+	226	ANNOTATED, CDS, c	NM_001039590	USP9X	Salzman2013	8239	circRNA	Detected	Detected	7.975217376	6.626073137
hsa_gci170862	5.264082185	2.396182013	up	5.498829489	3.102647476	TCAAAAGAAAACTCCGT	hsa_circ_0090306	chrX	41495830	41530783	-	486	ANNOTATED, CDS, c	NM_003688	CASK	Salzman2013	8573	circRNA	Detected	Not Detected	5.498829489	3.102647476
hsa_gci170864	2.194939371	1.13418109	up	9.116460931	7.982279841	GGCTTTACCTATACAAA	hsa_circ_0090314	chrX	43571118	43606068	+	3586	ANNOTATED, CDS, c	NM_000240	MAOA	Salzman2013	4128	circRNA	Detected	Detected	9.116460931	7.982279841
hsa_gci170866	2.001641702	1.001183752	up	9.724913894	8.723730142	GCTCTGGTAAATTCTGA	hsa_circ_0090320	chrX	43603355	43606068	+	2518	ANNOTATED, CDS, c	NM_000240	MAOA	Salzman2013	4128	circRNA	Detected	Detected	9.724913894	8.723730142
hsa_gci170869	2.090549278	1.063882051	up	3.998210242	2.934328191	GTTGGTTTCGATTGACT	hsa_circ_0090328	chrX	44894175	44913200	+	311	ANNOTATED, CDS, c	NM_021140	KDM6A	Salzman2013	7403	circRNA	Detected	Not Detected	3.998210242	2.934328191
hsa_gci170887	2.553787642	1.352638564	up	9.957271883	8.604633319	CAAACGTCCCCTACCTA	hsa_circ_0090452	chrX	47445919	47446190	+	271	ANNOTATED, CDS, c	NM_003254	TIMP1	Salzman2013	7076	circRNA	Detected	Detected	9.957271883	8.604633319
hsa_gci170903	2.06840325	1.048517477	up	8.508029765	7.459512289	TCCACTCTTCAAAGGAA	hsa_circ_0090595	chrX	51641676	51645450	+	773	ANNOTATED, CDS, c	NM_001005333	MAGED1	Salzman2013	9500	circRNA	Detected	Detected	8.508029765	7.459512289
hsa_gci170915	2.163424633	1.113316863	up	4.691066231	3.577749368	AGGAGTAGGAGTCCCAG	hsa_circ_0090739	chrX	53588716	53589205	-	303	ANNOTATED, CDS, c	NM_031407	HUWE1	Salzman2013	10075	circRNA	Detected	Detected	4.691066231	3.577749368
hsa_gci170922	10.31157336	3.366192573	up	9.053213906	5.687021333	TCGGTACCCGGTCAGCA	hsa_circ_0090796	chrX	53654360	53657986	-	727	ANNOTATED, CDS, c	NM_031407	HUWE1	Salzman2013	10075	circRNA	Detected	Detected	9.053213906	5.687021333
hsa_gci170952	2.117894018	1.082630397	up	6.185257912	5.102627515	TTGACCAGGTACCCGAG	hsa_circ_0091002	chrX	70516414	70521018	+	1858	ANNOTATED, CDS, c	NM_001145408	NONO	Salzman2013	4841	circRNA	Detected	Detected	6.185257912	5.102627515
hsa_gci170953	2.550934811	1.351026031	up	6.162794399	4.811768368	TCCCCCGTACCGTGGAG	hsa_circ_0091007	chrX	70518556	70521018	+	1337	ANNOTATED, CDS, c	NM_001145408	NONO	Salzman2013	4841	circRNA	Detected	Detected	6.162794399	4.811768368
hsa_gci170958	2.526929226	1.337385258	up	8.150258778	6.81287352	GTTGTACCCGGCCTTGA	hsa_circ_0091027	chrX	70752911	70765701	+	1985	ALT_DONOR, CDS, c	NM_181672	OGT	Salzman2013	8473	circRNA	Detected	Detected	8.150258778	6.81287352
hsa_gci170961	2.063975931	1.045426147	up	6.359735943	5.314309796	CAAGACAAATTAACGGT	hsa_circ_0091030	chrX	70757678	70765701	+	1529	ALT_DONOR, CDS, c	NM_181672	OGT	Salzman2013	8473	circRNA	Detected	Detected	6.359735943	5.314309796
hsa_gci170963	2.071235761	1.050491779	up	6.626073137	5.575581358	TTAACTATATTCGTAGG	hsa_circ_0091035	chrX	70774362	70775235	+	276	ANNOTATED, CDS, c	NM_181672	OGT	Salzman2013	8473	circRNA	Detected	Detected	6.626073137	5.575581358
hsa_gci170967	4.018624657	2.006701835	up	7.528136366	5.521434531	GGAAAGGAGCCCCTTCT	hsa_circ_0091048	chrX	71492452	71492622	-	170	ANNOTATED, CDS, c	NM_001007	RPS4X	Salzman2013	6191	circRNA	Detected	Detected	7.528136366	5.521434531
hsa_gci170987	2.535643577	1.342351967	up	7.721900803	6.379548836	GCCGCCGTCGTTGTTGG	hsa_circ_0091119	chrX	77166193	77275895	+	2941	ANNOTATED, CDS, c	NM_000052	ATP7A	Salzman2013	538	circRNA	Detected	Detected	7.721900803	6.379548836
hsa_gci170992	2.747101392	1.457910159	up	6.969000562	5.511090403	AACGGTCGGTAGTCCCG	hsa_circ_0091130	chrX	77258569	77275895	+	1238	ANNOTATED, CDS, c	NM_000052	ATP7A	Salzman2013	538	circRNA	Detected	Detected	6.969000562	5.511090403
hsa_gci171012	2.566436435	1.359766528	up	7.664815137	6.305048608	TGTAGAAGTACGTTTAG	hsa_circ_0091200	chrX	85211157	85219057	-	852	ANNOTATED, CDS, c	NM_000390	CHM	Salzman2013	1121	circRNA	Detected	Detected	7.664815137	6.305048608
hsa_gci171025	2.514862997	1.330479808	up	6.863597254	5.533117447	GCGGCGCCTCTGTGAAT	hsa_circ_0091272	chrX	102631955	102633001	+	690	ANNOTATED, CDS, c	NM_206915	BEX3	Salzman2013	27018	circRNA	Detected	Detected	6.863597254	5.533117447
hsa_gci171030	-3.203357181	-1.679584671	down	1.660265126	3.339849796	GGACAGACGTTACCTCC	hsa_circ_0091293	chrX	106888406	106894256	+	1421	ANNOTATED, CDS, c	NM_002764	PRPS1	Salzman2013	5631	circRNA	Not Detected	Detected	1.660265126	3.339849796
hsa_gci171062	-2.599054872	-1.377987093	down	2.240530847	3.61851794	AGGTCTCCACCTTACAA	hsa_circ_0091432	chrX	119386523	119392251	+	5085	ANNOTATED, CDS, c	NM_001184742	ZBTB33	Salzman2013	10009	circRNA	Not Detected	Detected	2.240530847	3.61851794
hsa_gci171079	5.733846396	2.519503257	up	3.915126601	1.395623344	GCACGCCCGGCGTTTCG	hsa_circ_0091505	chrX	129040096	129053465	+	842	ANNOTATED, CDS, c	NM_006649	UTP14A	Salzman2013	10813	circRNA	Detected	Not Detected	3.915126601	1.395623344
hsa_gci171086	2.018909005	1.013575888	up	6.187103307	5.173527419	CGGGTCCTCGTCTCGTC	hsa_circ_0091533	chrX	130843525	130928494	-	974	ANNOTATED, INTERN	NR_026975		Salzman2013		circRNA	Detected	Detected	6.187103307	5.173527419
hsa_gci171089	2.046248467	1.032981336	up	5.548432619	4.515451283	TCGGACCCGCTGTCTTA	hsa_circ_0091539	chrX	130883333	130928143	-	926	ANNOTATED, INTERN	TCONS_12_00030860		Salzman2013		circRNA	Detected	Detected	5.548432619	4.515451283
hsa_gci171090	-3.619880303	-1.855941993	down	1.517675464	3.373617457	TGGCTGGAGTCCCTAGG	hsa_circ_0091546	chrX	130902576	130918067	-	301	ANNOTATED, INTERN	NR_026975		Salzman2013		circRNA	Not Detected	Detected	1.517675464	3.373617457
hsa_gci171094	2.190824867	1.131474161	up	6.808051871	5.67657771	GGTCCTCGTCTCGTCGA	hsa_circ_0091558	chrX	130928324	130928494	-	170	ANNOTATED, INTERN	TCONS_12_00030860		Salzman2013		circRNA	Detected	Detected	6.808051871	5.67657771
hsa_gci171112	2.80473487	1.4878644	up	9.617930073	8.130065673	CAGTATTTGCTTCTCCG	hsa_circ_0091625	chrX	135044230	135056134	-	3648	ANNOTATED, CDS, c	NM_173470	MMGT1	Salzman2013	93380	circRNA	Detected	Detected	9.617930073	8.130065673
hsa_gci171130	2.788947949	1.479721009	up	5.800657216	4.320936207	GCAGCATGGTCCAGACC	hsa_circ_0091710	chrX	151130894	151131115	-	221	ANNOTATED, CDS, c	NM_004961	GABRE	Salzman2013	2564	circRNA	Detected	Detected	5.800657216	4.320936207
hsa_gci171132	2.671372383	1.417581099	up	7.580842047	6.163260949	CGTCCTCGACCCGTTAC	hsa_circ_0091721	chrX	151934651	151936231	-	1580	ANNOTATED, CDS, c	NM_005362	MAGEA3	Salzman2013	4102	circRNA	Detected	Detected	7.580842047	6.163260949
hsa_gci171139	2.072812205	1.051589415	up	6.372589741	5.321000326	GGTCTTTCCCTACCCGA	hsa_circ_0091733	chrX	152037327	152037907	+	580	ANNOTATED, CDS, c	NM_001129765	NSDHL	Salzman2013	50814	circRNA	Detected	Detected	6.372589741	5.321000326
hsa_gci171144	3.746997123	1.90573487	up	6.056902171	4.151167301	TAGTAGTATGAAGGCTT	hsa_circ_0091763	chrX	152965946	152981144	-	993	ANNOTATED, CDS, c	NM_001139457	BCAP31	Salzman2013	10134	circRNA	Detected	Detected	6.056902171	4.151167301
hsa_gci171153	2.084586791	1.059761439	up	7.877432431	6.817670992	TGTCTCTTCCCCGACGT	hsa_circ_0091833	chrX	153576899	153581043	-	1882	ANNOTATED, CDS, c	NM_001110556	FLNA	Salzman2013	2316	circRNA	Detected	Detected	7.877432431	6.817670992
hsa_gci171158	2.087913667	1.062062059	up	6.667266247	5.605204188	TAGTACTCATCCGTCGT	hsa_circ_0091843	chrX	153576899	153586723	-	3663	ANNOTATED, CDS, c	NM_001110556	FLNA	Salzman2013	2316	circRNA	Detected	Detected	6.667266247	5.605204188
hsa_gci171165	-3.758117298	-1.910010097	down	1.287421723	3.19743182	GTCAGTCCTGTGTCCGT	hsa_circ_0091856	chrX	153576899	153596106	-	7639	ANNOTATED, CDS, c	NM_001110556	FLNA	Salzman2013	2316	circRNA	Not Detected	Detected	1.287421723	3.19743182
hsa_gci171178	3.262395743	1.705931798	up	6.922190566	5.216258768	TCCGGTGGGTGGTCGTG	hsa_circ_0092090	chrX	153696109	153701985	+	2988	ANNOTATED, CDS, c	NM_017514	PLXNA3	Salzman2013	55558	circRNA	Detected	Detected	6.922190566	5.216258768
hsa_gci171179	2.671605825	1.417707165	up	6.134056928	4.716349763	TCTTCGCGTCGCCGTCC	hsa_circ_0092094	chrX	153699447	153701985	+	1417	ANNOTATED, CDS, c	NM_017514	PLXNA3	Salzman2013	55558	circRNA	Detected	Detected	6.134056928	4.716349763
hsa_gci171184	2.555555997	1.353637204	up	6.290951653	4.937314449	GAGGTAGTGAAGGTCGG	hsa_circ_0092166	chrX	154528097	154528458	-	236	ANNOTATED, CDS, c	NM_001289	CLIC2	Salzman2013	1193	circRNA	Detected	Detected	6.290951653	4.937314449
hsa_gci171187	2.788479131	1.479478474	up	4.011639097	2.532160623	GGTCGGTTAAGTTTAGT	hsa_circ_0092169	chrX	154741333	154743926	-	400	ANNOTATED, CDS, c	NM_018196	TMLHE	Salzman2013	55217	circRNA	Detected	Not Detected	4.011639097	2.532160623
hsa_gci171192	2.176981279	1.122329001	up	5.293645279	4.171316278	GAGAAATTTGAACCTCC	hsa_circ_0092203	chrY	1472031	1511210	-	1903	ANNOTATED, CDS, c	NM_004192	ASMTL	Salzman2013	8623	circRNA	Detected	Detected	5.293645279	4.171316278
hsa_gci171200	5.64154528	2.496090386	up	4.331633565	1.835543178	AGAAATACATCAATAGG	hsa_circ_0092232	chrY	14821320	14821476	+	156	ANNOTATED, CDS, c	NM_004654	USP9Y	Salzman2013	8287	circRNA	Detected	Not Detected	4.331633565	1.835543178
