# Supplementary material for: Hypoxia‐Induced circPRELID2 Promotes Gastric Cancer Metastasis by Facilitating ZEB2 Translation via PCBP1 O‐GlcNAcylation
Source: Adv Sci (Weinh). 2025 Oct 21;12(46):e05396. doi: 10.1002/advs.202505396 (PMC12697806; doi:10.1002/advs.202505396)

Figure 1

Fig.1i

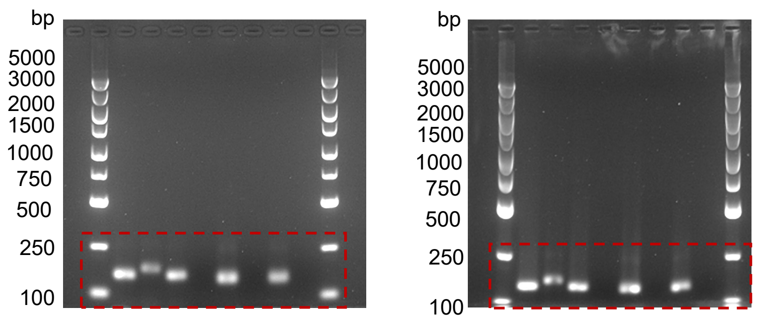

Fig.1l

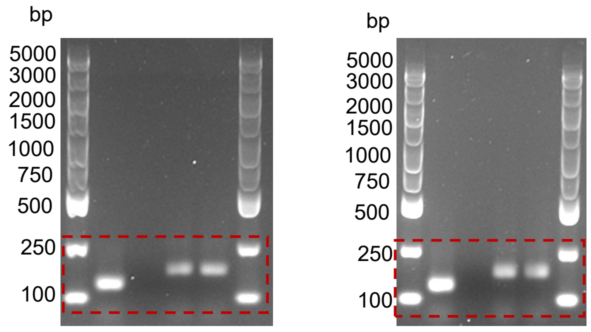

Figure 3

Fig.3c

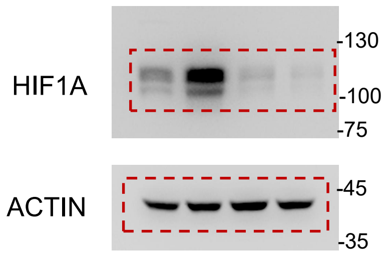

Fig.3d

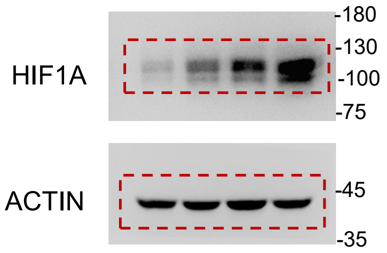

Fig.3j

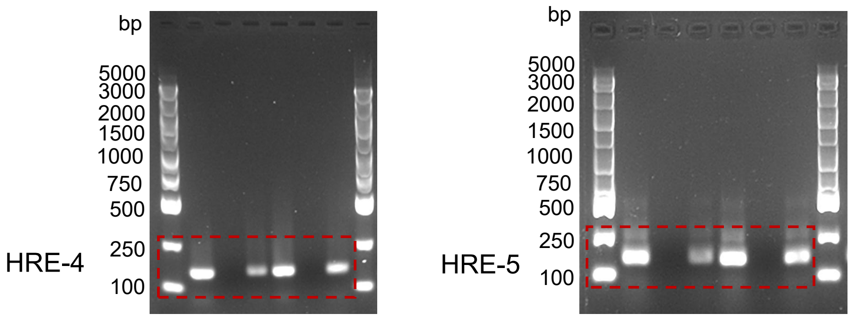

Figure 4

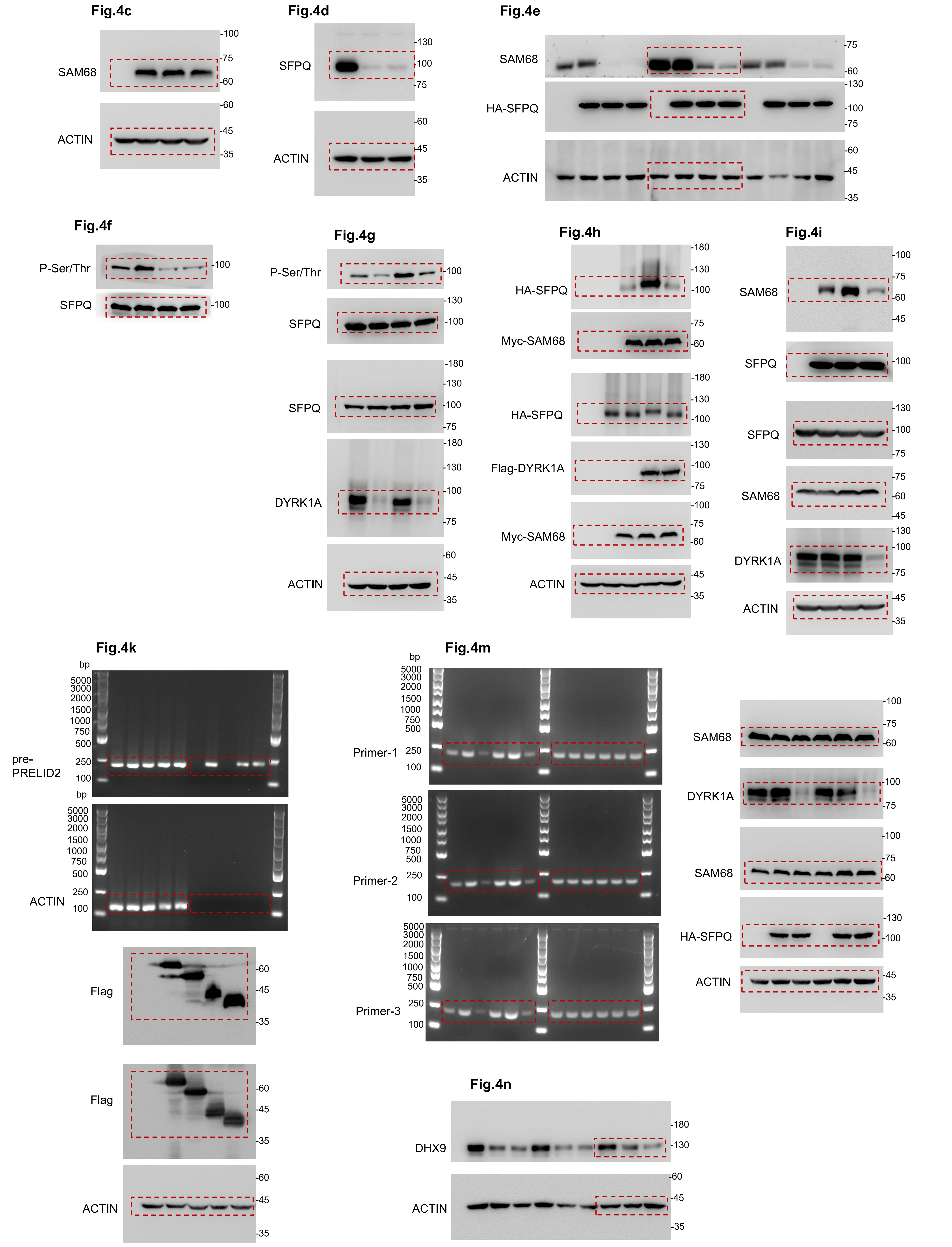

Fig.4c
SAM68
ACTIN
Fig.4d
SFPQ
ACTIN
Fig.4e
SAM68
HA-SFPQ
ACTIN
Fig.4f
P-Ser/Thr
SFPQ
Fig.4g
P-Ser/Thr
SFPQ
SFPQ
DYRK1A
ACTIN
Fig.4h
HA-SFPQ
Myc-SAM68
HA-SFPQ
Flag-DYRK1A
Myc-SAM68
ACTIN
Fig.4i
SAM68
SFPQ
SFPQ
SAM68
DYRK1A
ACTIN
Fig.4k
pre-PRELID2
ACTIN
Flag
Flag
ACTIN
Fig.4m
Primer-1
Primer-2
Primer-3
SAM68
DYRK1A
SAM68
HA-SFPQ
ACTIN
Fig.4n
DHX9
ACTIN

Figure 6

Fig.6b

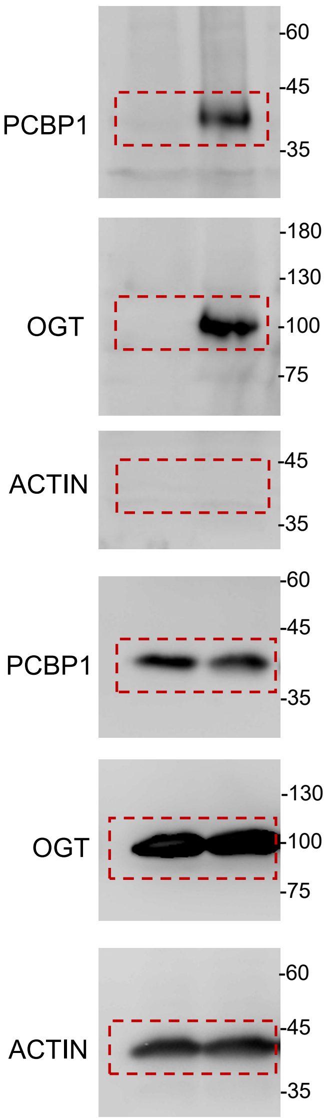

Fig.6f

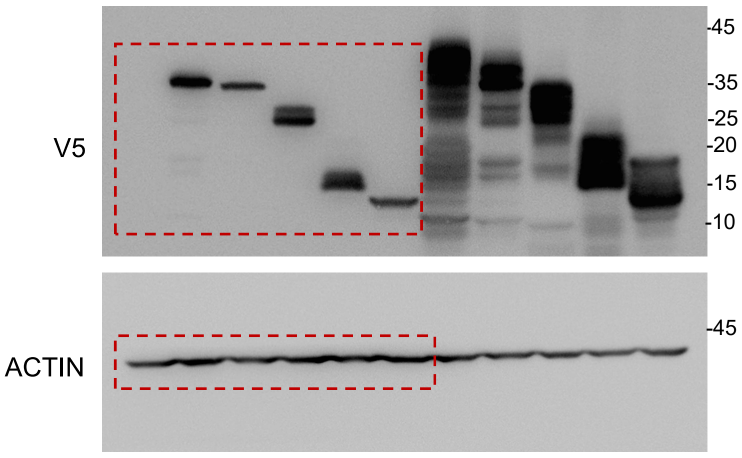

Fig.6h

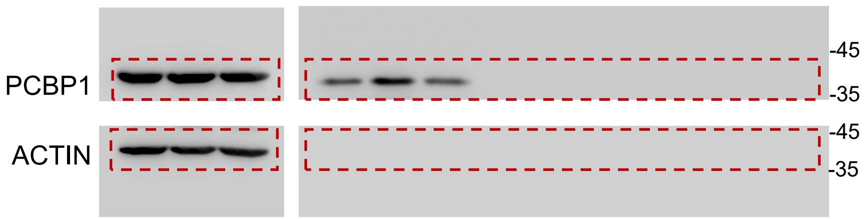

Fig.6j

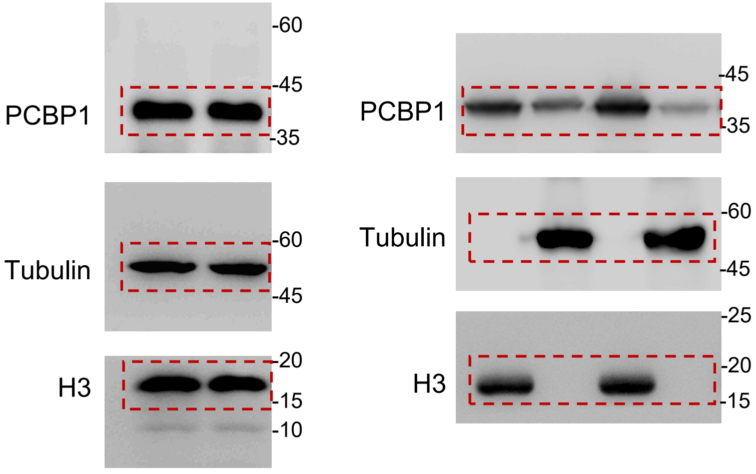

Fig.6l

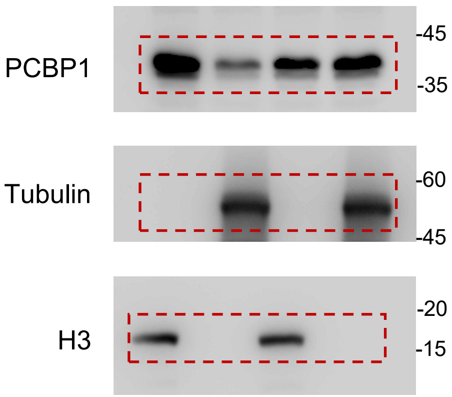

Figure 7

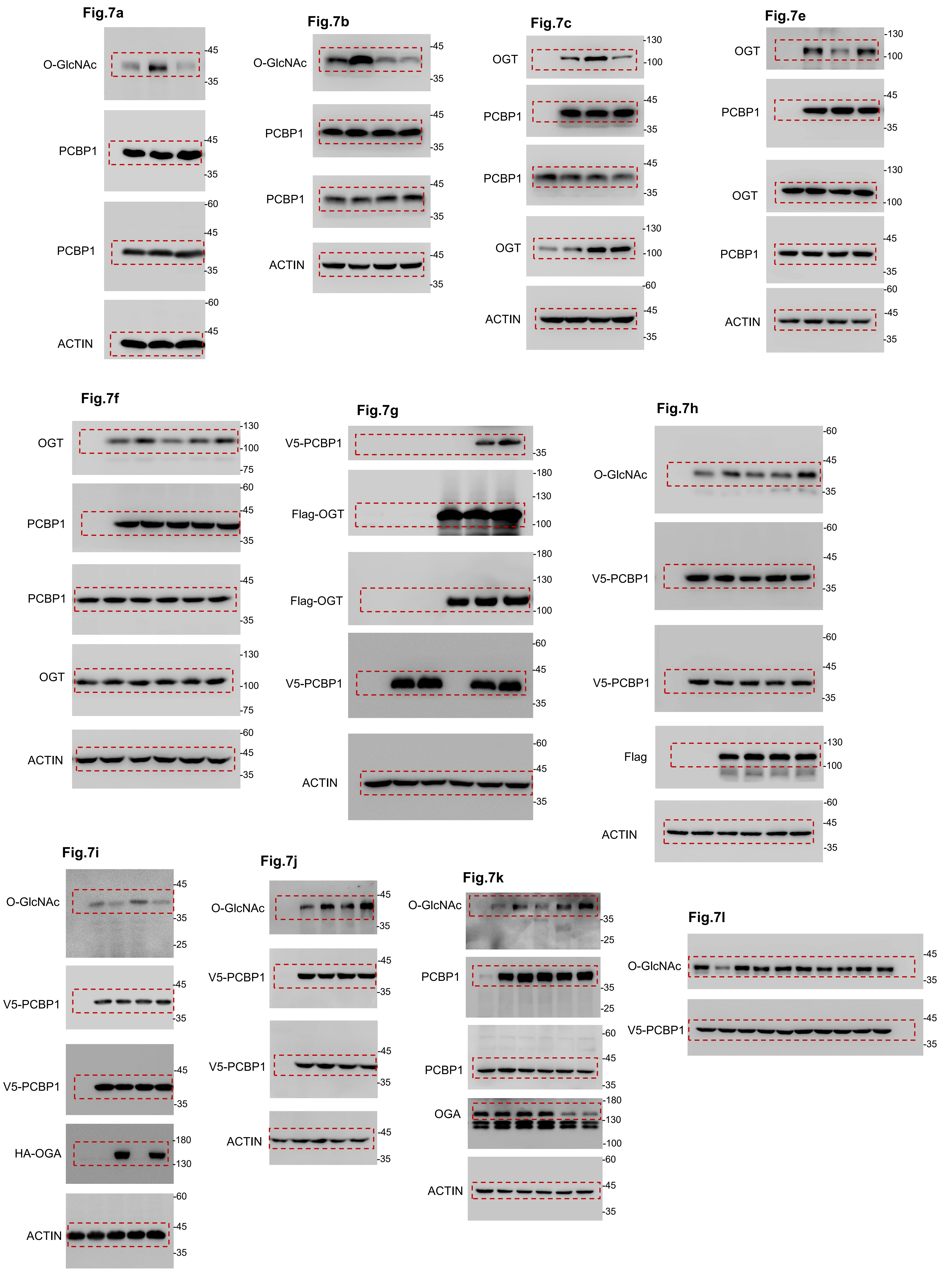
Fig.7a
O-GlcNAc
PCBP1
PCBP1
ACTIN
-45
-35
-60
Fig.7b
O-GlcNAc
PCBP1
PCBP1
ACTIN
Fig.7c
OGT
PCBP1
PCBP1
OGT
ACTIN
-130
-100
Fig.7e
OGT
PCBP1
OGT
PCBP1
ACTIN
Fig.7f
OGT
PCBP1
PCBP1
OGT
ACTIN
-75
Fig.7g
V5-PCBP1
Flag-OGT
Flag-OGT
V5-PCBP1
ACTIN
-180
Fig.7h
O-GlcNAc
V5-PCBP1
V5-PCBP1
Flag
ACTIN
Fig.7i
O-GlcNAc
V5-PCBP1
V5-PCBP1
HA-OGA
ACTIN
-25
Fig.7j
O-GlcNAc
V5-PCBP1
V5-PCBP1
ACTIN
Fig.7k
O-GlcNAc
PCBP1
PCBP1
OGA
ACTIN
Fig.7l
O-GlcNAc
V5-PCBP1

Figure 8

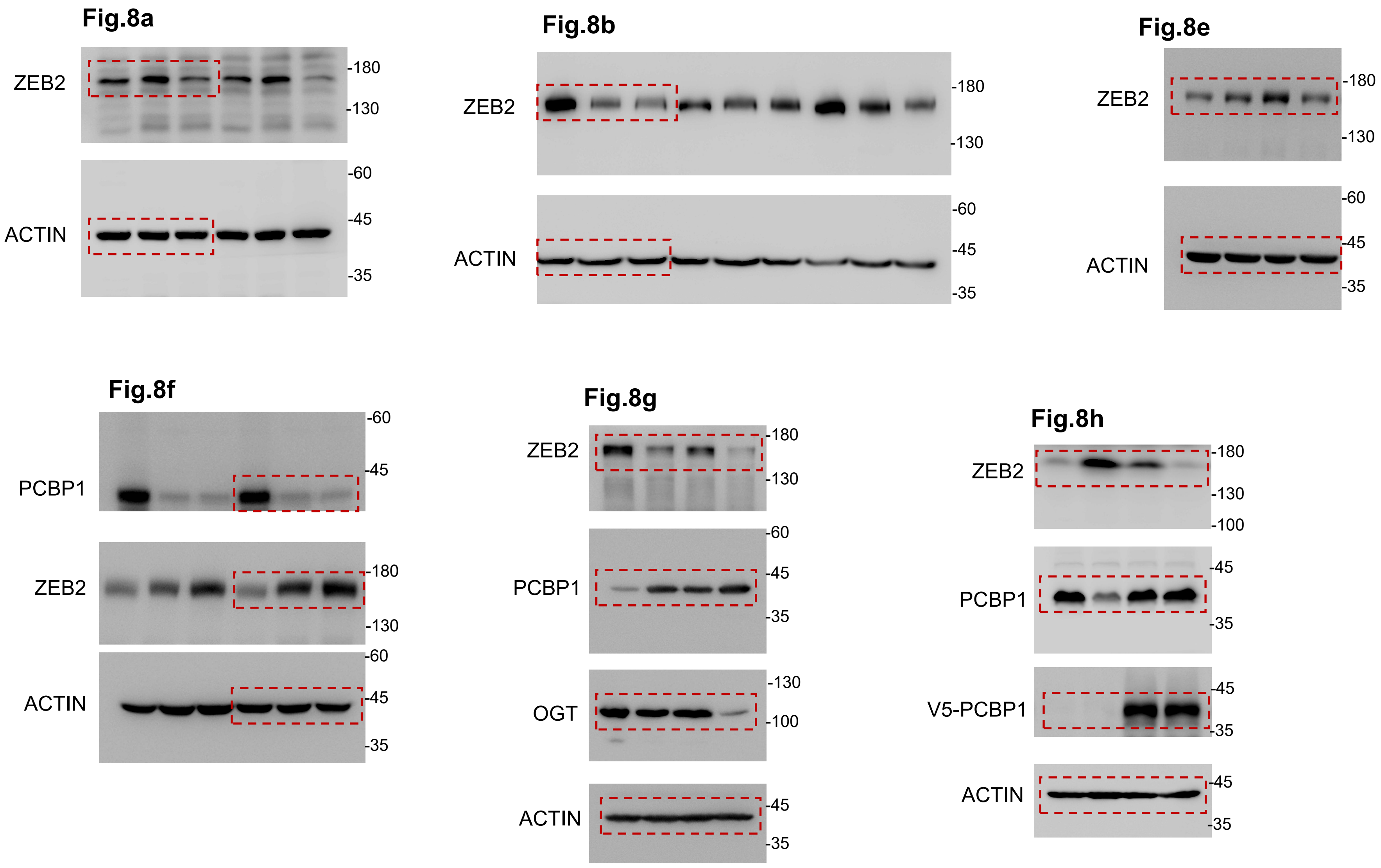

Fig.8a
ZEB2
ACTIN
-180
-130
-60
-45
-35
Fig.8b
ZEB2
ACTIN
-180
-130
-60
-45
-35
Fig.8e
ZEB2
ACTIN
-180
-130
-60
-45
-35
Fig.8f
PCBP1
ZEB2
ACTIN
-60
-45
-180
-130
-60
-45
-35
Fig.8g
ZEB2
PCBP1
OGT
ACTIN
-180
-130
-60
-45
-35
-130
-100
-45
-35
Fig.8h
ZEB2
PCBP1
V5-PCBP1
ACTIN
-180
-130
-100
-45
-35
-45
-35
-45
-35

Supplementary Figure 2

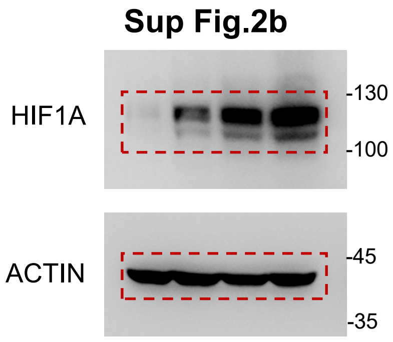

Supplementary Figure 3

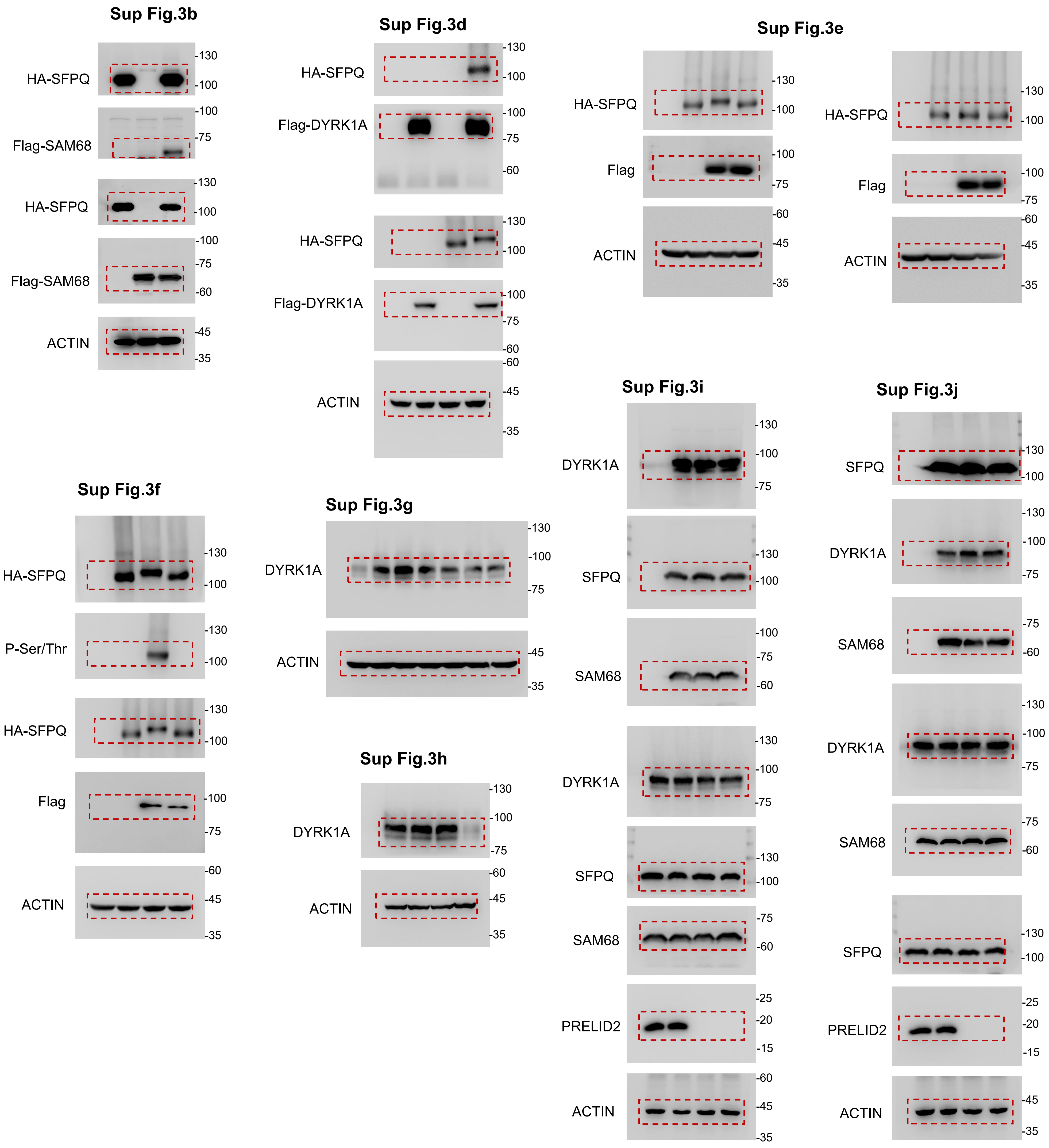

Supplementary Figure 5

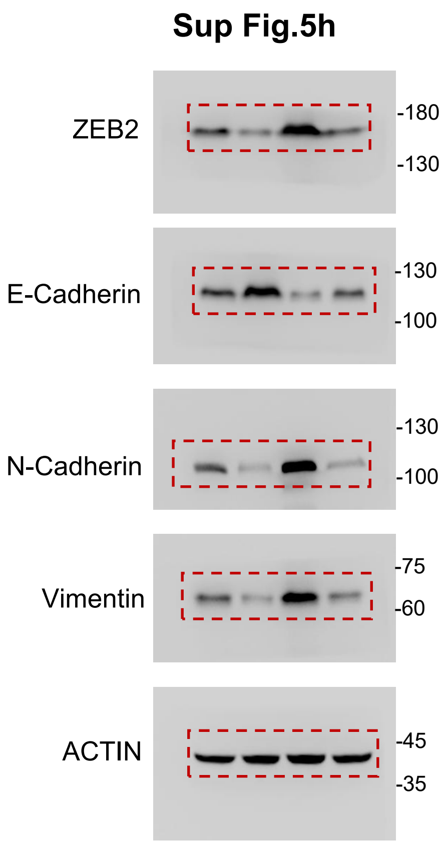

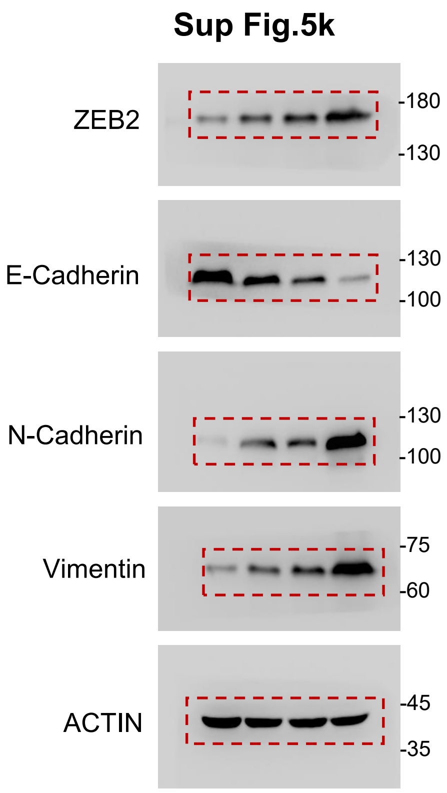

Supplementary Figure 6

Sup Fig. 6b

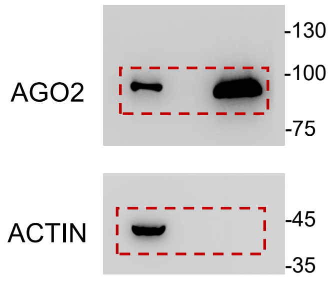

Sup Fig. 6c

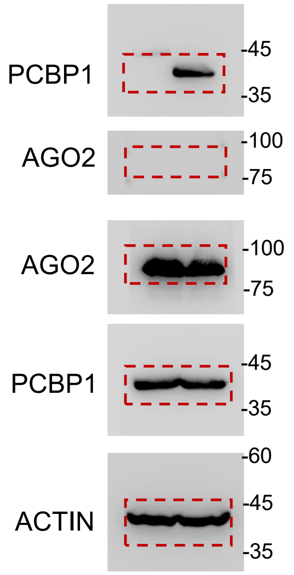

Supplementary Figure 7

Sup Fig. 7a

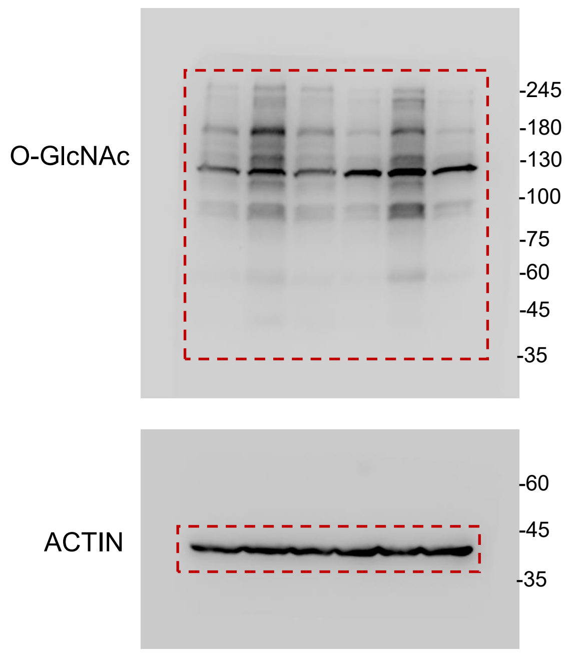

Sup Fig. 7b

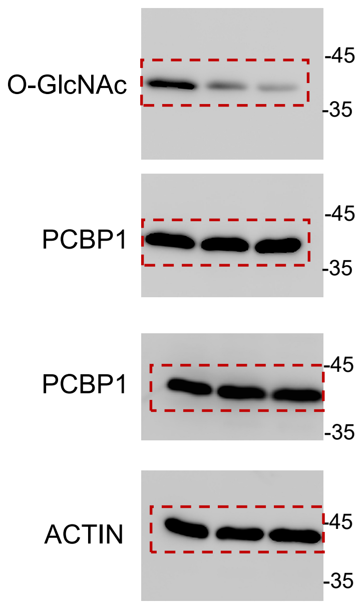

Sup Fig. 7c

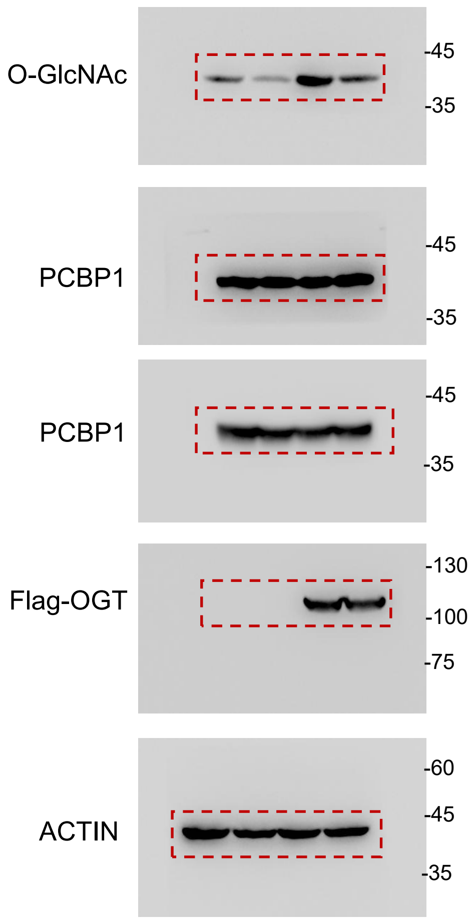

Sup Fig. 7d

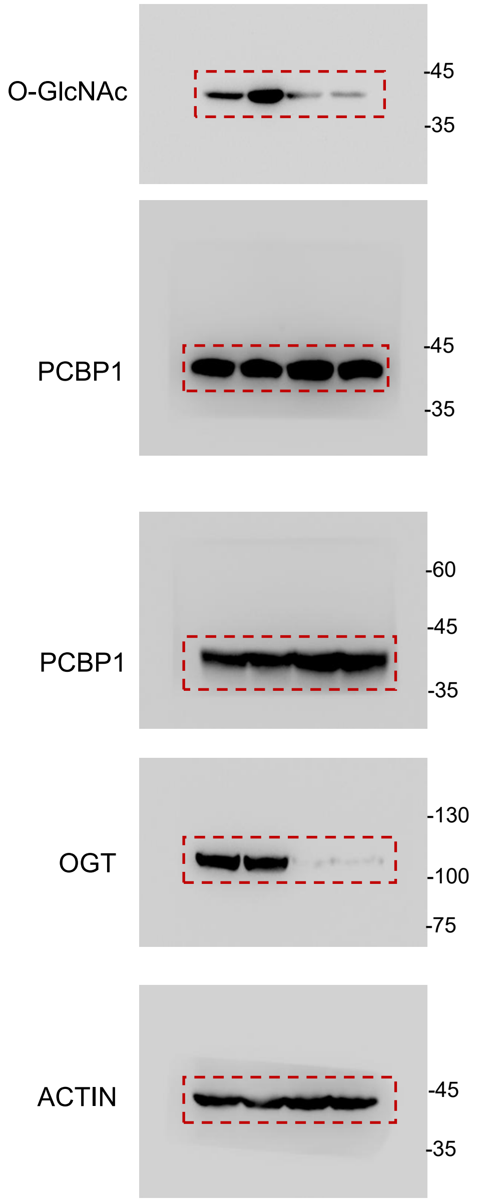

Sup Fig. 7e

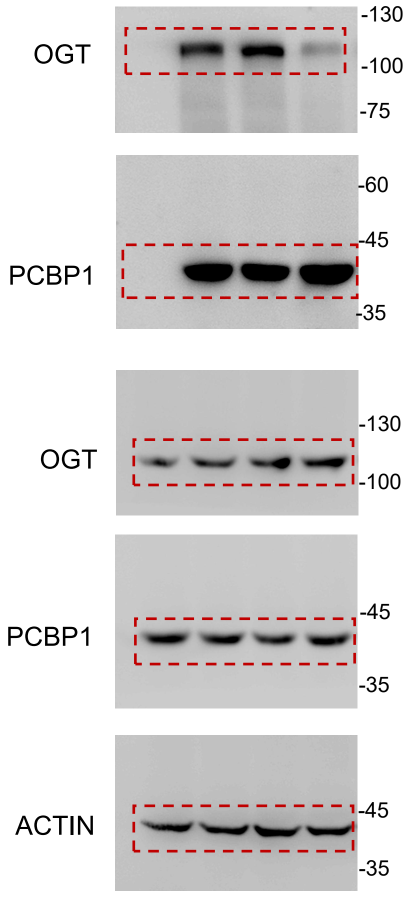

Supplementary Figure 8

Sup Fig. 8c

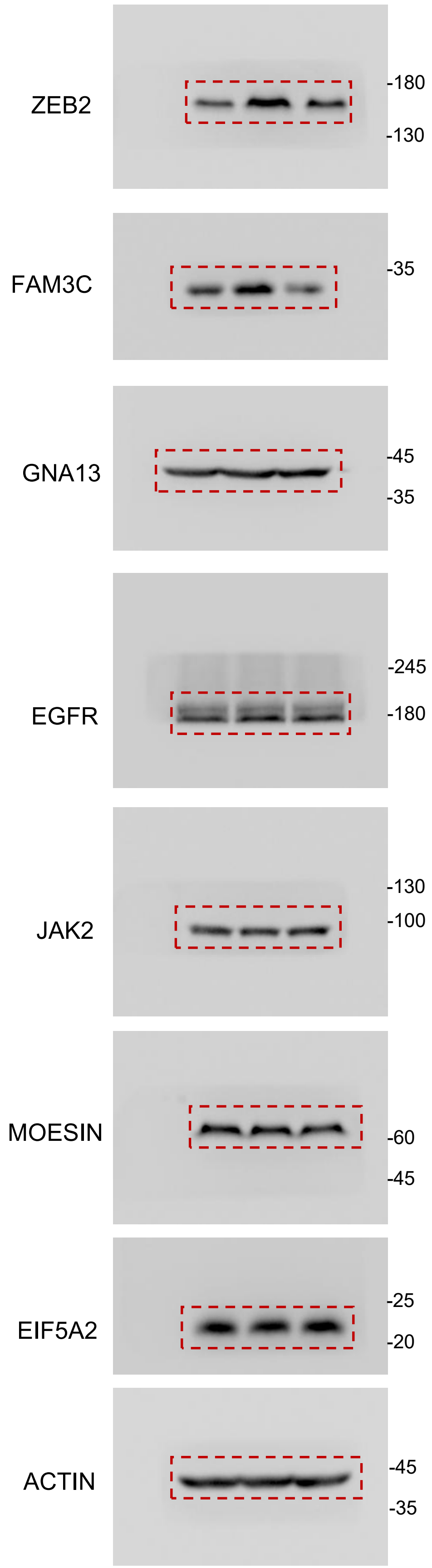

Sup Fig. 8d

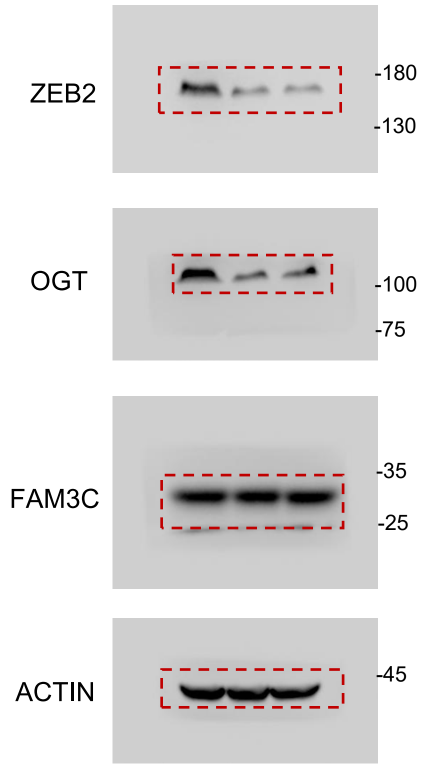

Sup Fig. 8e

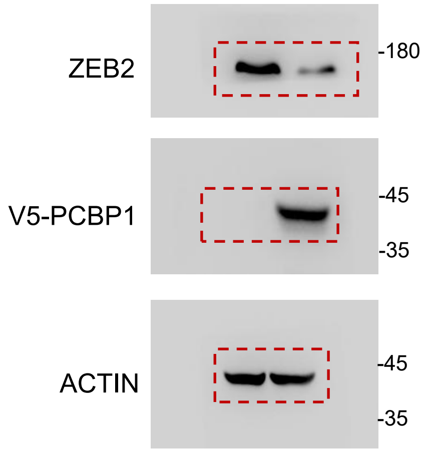

Sup Fig. 8f

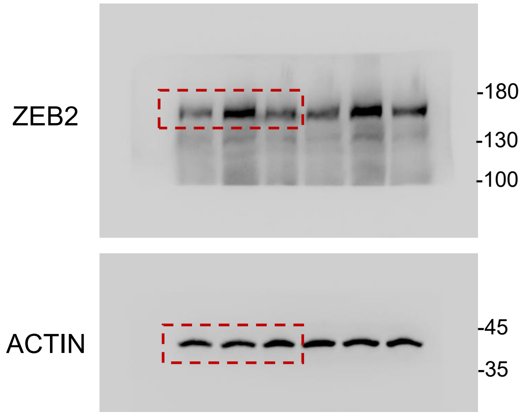

Sup Fig. 8g

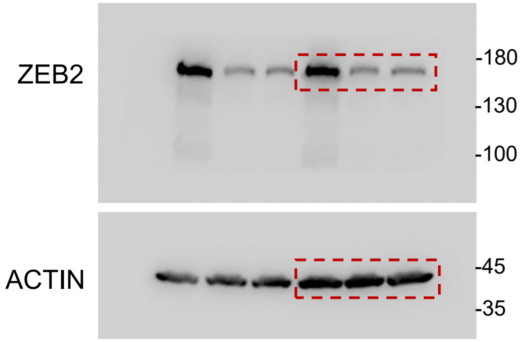

Supplementary Figure 9

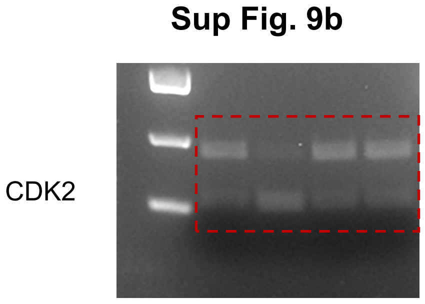

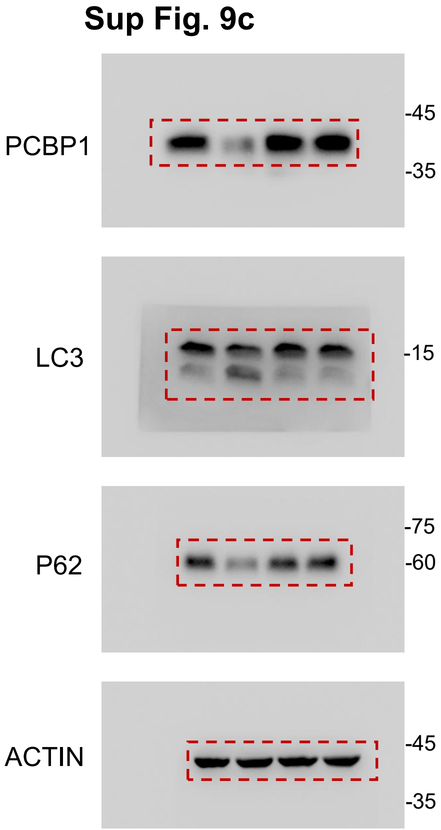

Supplement: Supplementary file 2 — Supporting Information [file ADVS-12-e05396-s002.zip › Original Images of Unaltered Blots.pdf]
