# Supplementary material for: Hypoxia‐Induced circPRELID2 Promotes Gastric Cancer Metastasis by Facilitating ZEB2 Translation via PCBP1 O‐GlcNAcylation
Source: Adv Sci (Weinh). 2025 Oct 21;12(46):e05396. doi: 10.1002/advs.202505396 (PMC12697806; doi:10.1002/advs.202505396)
Supplement: Supplementary file 2 — Supporting Information [file ADVS-12-e05396-s002.zip › AGS cells-comp1_H1_vs._N1_FC2_circRNA.pdf]

| ProbeID | FC(H1_vs._N1) | log2FC(H1_vs._N1) | Regulation(H1_vs. | Case_mean(H1_vs._N1) | Control_mean(H1 | Sequence | TargetID | Chromosome | Start | End | Strand | Length | circRNA_Annotatio | circRNA_BestT | circRNA_GeneSy | circRNA_Study | circRNA_GeneID | Type | 7901_H.flag | 7901_N.flag | 7901_H.normalized | 7901_N.normalized |
|---|---|---|---|---|---|---|---|---|---|---|---|---|---|---|---|---|---|---|---|---|---|---|
| hsa_gci149763 | 2.496991587 | 1.320190961 | up | 5.367437739 | 4.047246778 | CTACCAGGGAGA | hsa_circ_0000012 | chr1 | 8616533 | 8617582 | - | 203 | ANNOTATED, CDS, c | NM_012102 | RERE | Memczak2013, Salz | 473 | circRNA | Detected | Detected | 5.367437739 | 4.047246778 |
| hsa_gci149794 | 2.906628948 | 1.539346913 | up | 6.173634929 | 4.634288016 | AACACAACTAGA | hsa_circ_0000095 | chr1 | 95609446 | 95616975 | + | 410 | ANNOTATED, CDS, c | NM_001199691 | TMEM56-RWDD3 | Memczak2013, Salz | 100527978 | circRNA | Detected | Detected | 6.173634929 | 4.634288016 |
| hsa_gci149862 | 2.008712317 | 1.00627096 | up | 5.154572505 | 4.148301545 | CTGTTTCAGTTA | hsa_circ_0000258 | chr10 | 105197771 | 105198565 | + | 180 | ANNOTATED, CDS, c | NM_014976 | PDCD11 | Jeck2013, Memczak | 22984 | circRNA | Detected | Detected | 5.154572505 | 4.148301545 |
| hsa_gci149869 | 2.215273501 | 1.147484827 | up | 4.307021362 | 3.159536535 | TGGACAGAAGAA | hsa_circ_0000280 | chr11 | 18312988 | 18314523 | - | 656 | ANNOTATED, CDS, c | NM_181508 | HPS5 | Jeck2013, Memczak | 11234 | circRNA | Detected | Not Detected | 4.307021362 | 3.159536535 |
| hsa_gci149870 | 9.691089301 | 3.276658837 | up | 4.495364508 | 1.218705671 | ACGAACTCGGTA | hsa_circ_0000283 | chr11 | 33127112 | 33127610 | - | 229 | ANNOTATED, CDS, c | NM_001326 | CSTF3 | Jeck2013, Memczak | 1479 | circRNA | Detected | Not Detected | 4.495364508 | 1.218705671 |
| hsa_gci149872 | 2.086089622 | 1.06080114 | up | 7.196371143 | 6.135570003 | TTTTGTCCTGAA | hsa_circ_0000285 | chr11 | 33362513 | 33363232 | + | 284 | ANNOTATED, CDS, c | NM_005734 | HIPK3 | Memczak2013, Salz | 10114 | circRNA | Detected | Detected | 7.196371143 | 6.135570003 |
| hsa_gci149884 | 2.188743673 | 1.130103008 | up | 11.39980601 | 10.269703 | CGTCCGTCATAC | hsa_circ_0000344 | chr11 | 77394754 | 77404656 | - | 350 | ANNOTATED, CDS, c | NM_016578 | RSF1 | Jeck2013, Memczak | 51773 | circRNA | Detected | Detected | 11.39980601 | 10.269703 |
| hsa_gci149940 | 2.1837786 | 1.126826597 | up | 6.187103307 | 5.06027671 | AAACTACGAGCC | hsa_circ_0000522 | chr14 | 21825355 | 21829372 | - | 867 | ANNOTATED, CDS, c | NM_007192 | SUPT16H | Jeck2013, Memczak | 11198 | circRNA | Detected | Detected | 6.187103307 | 5.06027671 |
| hsa_gci149963 | 2.135872374 | 1.094825444 | up | 6.033504659 | 4.938679215 | CCACTCCGATGT | hsa_circ_0000593 | chr15 | 41988272 | 42005694 | + | 2366 | ANNOTATED, CDS, c | NM_001164273 | MGA | Memczak2013, Salz | 23269 | circRNA | Detected | Detected | 6.033504659 | 4.938679215 |
| hsa_gci149964 | 2.030657587 | 1.02194699 | up | 9.727416265 | 8.705469275 | ACAGACGGTAGA | hsa_circ_0000595 | chr15 | 44615162 | 44630515 | + | 475 | ANNOTATED, CDS, c | NM_138423 | CASC4 | Memczak2013, Salz | 113201 | circRNA | Detected | Detected | 9.727416265 | 8.705469275 |
| hsa_gci149965 | 2.019949281 | 1.014319069 | up | 8.05506893 | 7.040749862 | CACGAGAGTTAA | hsa_circ_0000596 | chr15 | 44624185 | 44630515 | + | 317 | ANNOTATED, CDS, c | NM_138423 | CASC4 | Memczak2013, Salz | 113201 | circRNA | Detected | Detected | 8.05506893 | 7.040749862 |
| hsa_gci149987 | 3.072672734 | 1.619494116 | up | 7.116437968 | 5.496943852 | AGACTCCCCGGT | hsa_circ_0000651 | chr15 | 90982563 | 90986710 | + | 446 | ANNOTATED, CDS, c | NM_003870 | IQGAP1 | Jeck2013, Memczak | 8826 | circRNA | Detected | Detected | 7.116437968 | 5.496943852 |
| hsa_gci149997 | 2.087940219 | 1.062080406 | up | 8.313333597 | 7.251253191 | TCTACATTTACC | hsa_circ_0000711 | chr16 | 68155889 | 68160513 | + | 1298 | ANNOTATED, CDS, c | NM_173165 | NFATC3 | Jeck2013, Memczak | 4775 | circRNA | Detected | Detected | 8.313333597 | 7.251253191 |
| hsa_gci149999 | 2.357745442 | 1.237407964 | up | 7.12082396 | 5.883415997 | CTCTTAGGCAGA | hsa_circ_0000714 | chr16 | 70294946 | 70302282 | - | 823 | ANNOTATED, CDS, c | NM_001605 | AARS | Memczak2013, Salz | 16 | circRNA | Detected | Detected | 7.12082396 | 5.883415997 |
| hsa_gci150002 | 2.157803334 | 1.109563381 | up | 8.692595883 | 7.583032502 | TGTCCCCACCTA | hsa_circ_0000739 | chr17 | 5314022 | 5320002 | - | 383 | ANNOTATED, CDS, c | NM_002532 | NUP88 | Memczak2013, Salz | 4927 | circRNA | Detected | Detected | 8.692595883 | 7.583032502 |
| hsa_gci150004 | 3.282788019 | 1.714921593 | up | 6.807983728 | 5.093062135 | AAATCAACAACA | hsa_circ_0000745 | chr17 | 20107645 | 20109225 | + | 1580 | ANNOTATED, CDS, c | NM_001033554 | SPECC1 | Memczak2013, Salz | 92521 | circRNA | Detected | Detected | 6.807983728 | 5.093062135 |
| hsa_gci150044 | 2.808222227 | 1.489657107 | up | 6.091005808 | 4.601348701 | AAGCAATGGTGA | hsa_circ_0000880 | chr19 | 5604593 | 5604947 | - | 263 | ANNOTATED, CDS, c | NM_014649 | SAFB2 | Memczak2013 | 9667 | circRNA | Detected | Detected | 6.091005808 | 4.601348701 |
| hsa_gci150072 | -6.567453442 | -2.715334068 | down | 1.182780075 | 3.898114142 | ACCTCAGTAAGG | hsa_circ_0001002 | chr2 | 48573339 | 48573890 | + | 551 | ANNOTATED, CDS, c | NM_002158 | FOXN2 | Jeck2013, Memczak | 3344 | circRNA | Not Detected | Detected | 1.182780075 | 3.898114142 |
| hsa_gci150081 | 2.039158771 | 1.027974109 | up | 6.437704595 | 5.409730486 | TAACGAAACGAT | hsa_circ_0001020 | chr2 | 64083439 | 64085070 | + | 236 | ANNOTATED, CDS, c | NM_006759 | UGP2 | Jeck2013, Memczak | 7360 | circRNA | Detected | Detected | 6.437704595 | 5.409730486 |
| hsa_gci150096 | 3.783950107 | 1.919893066 | up | 6.192675158 | 4.272782092 | AGGGATACCAGA | hsa_circ_0001072 | chr2 | 144966169 | 144969146 | - | 267 | ANNOTATED, CDS, c | NM_024659 | GTDC1 | Jeck2013, Memczak | 79712 | circRNA | Detected | Detected | 6.192675158 | 4.272782092 |
| hsa_gci150105 | 2.569525932 | 1.361502212 | up | 6.368359542 | 5.00685733 | TCTGTAGTGTAC | hsa_circ_0001090 | chr2 | 201719338 | 201719809 | - | 163 | ANNOTATED, CDS, c | NM_004071 | CLK1 | Memczak2013, Salz | 1195 | circRNA | Detected | Detected | 6.368359542 | 5.00685733 |
| hsa_gci150117 | 2.826289549 | 1.498909275 | up | 4.689237846 | 3.190328571 | CCGTAAGACGAG | hsa_circ_0001115 | chr2 | 234358627 | 234360706 | + | 376 | ANNOTATED, CDS, c | NM_152879 | DGKD | Jeck2013, Memczak | 8527 | circRNA | Detected | Not Detected | 4.689237846 | 3.190328571 |
| hsa_gci150166 | 2.276493265 | 1.186813191 | up | 8.173253727 | 6.986440536 | ACAGGTGTTTCC | hsa_circ_0001280 | chr3 | 33725850 | 33738425 | - | 449 | ANNOTATED, CDS, c | NM_015097 | CLASP2 | Jeck2013, Memczak | 23122 | circRNA | Detected | Detected | 8.173253727 | 6.986440536 |
| hsa_gci150178 | 2.431566503 | 1.28188605 | up | 7.871212705 | 6.589326655 | TTACCGACCTTT | hsa_circ_0001316 | chr3 | 56703727 | 56707753 | - | 404 | ANNOTATED, CDS, c | NM_001112736 | FAM208A | Jeck2013, Memczak | 23272 | circRNA | Detected | Detected | 7.871212705 | 6.589326655 |
| hsa_gci150192 | 2.220823957 | 1.151095036 | up | 4.046177407 | 2.895082371 | GTCTCTGCAGTC | hsa_circ_0001348 | chr3 | 152017193 | 152018156 | + | 963 | ANNOTATED, CDS, c | NM_207296 | MBNL1 | Memczak2013, Salz | 4154 | circRNA | Detected | Not Detected | 4.046177407 | 2.895082371 |
| hsa_gci150202 | 3.266421663 | 1.707711041 | up | 6.83719102 | 5.12947998 | TAACAACTCTTT | hsa_circ_0001369 | chr3 | 183368083 | 183369064 | + | 981 | ANNOTATED, CDS, c | NM_017644 | KLHL24 | Memczak2013, Salz | 54800 | circRNA | Detected | Detected | 6.83719102 | 5.12947998 |
| hsa_gci150209 | 2.319786461 | 1.21399201 | up | 4.42381484 | 3.209822831 | AAAGGTAGTCAG | hsa_circ_0001394 | chr4 | 6925099 | 6925838 | + | 739 | ANNOTATED, CDS, c | NM_001113361 | TBC1D14 | Jeck2013, Memczak | 57533 | circRNA | Detected | Detected | 4.42381484 | 3.209822831 |
| hsa_gci150217 | 3.523861298 | 1.81715714 | up | 4.925058548 | 3.107901408 | AGTAGAAGCCAG | hsa_circ_0001411 | chr4 | 54249939 | 54256040 | + | 169 | ANNOTATED, CDS, c | NM_001134937 | FIP1L1 | Jeck2013, Memczak | 81608 | circRNA | Detected | Not Detected | 4.925058548 | 3.107901408 |
| hsa_gci150260 | 2.259800409 | 1.176195356 | up | 7.010635036 | 5.83443968 | AAACAGTTTTAA | hsa_circ_0001501 | chr5 | 77423853 | 77461496 | - | 801 | ANNOTATED, CDS, c | NM_003664 | AP3B1 | Jeck2013, Memczak | 8546 | circRNA | Detected | Detected | 7.010635036 | 5.83443968 |
| hsa_gci150272 | 2.035283097 | 1.025229479 | up | 4.16114325 | 3.13591377 | TTTAGGTCTTCT | hsa_circ_0001537 | chr5 | 137726701 | 137735701 | + | 1819 | ANNOTATED, CDS, c | NM_016604 | KDM3B | Memczak2013, Salz | 51780 | circRNA | Detected | Not Detected | 4.16114325 | 3.13591377 |
| hsa_gci150274 | 2.241482384 | 1.164453161 | up | 5.176430157 | 4.011976996 | TGTTCGAAGACG | hsa_circ_0001540 | chr5 | 139819703 | 139825560 | + | 530 | ANNOTATED, CDS, c | NM_017747 | ANKHD1 | Jeck2013, Memczak | 54882 | circRNA | Detected | Detected | 5.176430157 | 4.011976996 |
| hsa_gci150283 | 2.637960537 | 1.399422982 | up | 8.658602399 | 7.259179416 | GGGCTTCCCACC | hsa_circ_0001566 | chr5 | 179688683 | 179707608 | - | 497 | ANNOTATED, CDS, c | NM_139069 | MAPK9 | Jeck2013, Memczak | 5601 | circRNA | Detected | Detected | 8.658602399 | 7.259179416 |
| hsa_gci150313 | 2.491423009 | 1.316969992 | up | 8.256242794 | 6.939272802 | TGTGATTTCGCA | hsa_circ_0001655 | chr6 | 156468903 | 156489628 | - | 20725 | INTERGENIC | None |  | Memczak2013 |  | circRNA | Detected | Detected | 8.256242794 | 6.939272802 |
| hsa_gci150344 | 2.775002419 | 1.472489029 | up | 4.516347202 | 3.043858172 | GTTCTCTGACGG | hsa_circ_0001750 | chr7 | 135262543 | 135277940 | + | 1182 | ANNOTATED, CDS, c | NM_015135 | NUP205 | Jeck2013, Memczak | 23165 | circRNA | Detected | Not Detected | 4.516347202 | 3.043858172 |
| hsa_gci150364 | 2.581121118 | 1.367997841 | up | 6.270347067 | 4.902349226 | AGTAAATCATCG | hsa_circ_0001813 | chr8 | 95839490 | 95840021 | + | 213 | ANNOTATED, CDS, c | NM_017864 | INTS8 | Memczak2013, Salz | 55656 | circRNA | Detected | Detected | 6.270347067 | 4.902349226 |
| hsa_gci150369 | 3.672586153 | 1.876796334 | up | 4.23776912 | 2.360972786 | AAGTTCAGCAAA | hsa_circ_0001839 | chr9 | 6880011 | 6893232 | + | 292 | ANNOTATED, CDS, c | NM_001146694 | KDM4C | Jeck2013, Memczak | 23081 | circRNA | Detected | Not Detected | 4.23776912 | 2.360972786 |
| hsa_gci150454 | 2.165230015 | 1.114520293 | up | 8.407772445 | 7.293252152 | TACCTCGTCTCT | hsa_circ_0002034 | chr3 | 155706088 | 155706162 | + | 74 | INTERGENIC | None |  | Jeck2013 |  | circRNA | Detected | Detected | 8.407772445 | 7.293252152 |
| hsa_gci150477 | 2.086851193 | 1.06132773 | up | 9.712343979 | 8.651016248 | ACTTGTAGACCC | hsa_circ_0002064 | chr9 | 109734285 | 109765707 | + | 762 | ANNOTATED, CDS, c | NM_021224 | ZNF462 | Jeck2013 | 58499 | circRNA | Detected | Detected | 9.712343979 | 8.651016248 |
| hsa_gci150505 | 3.380774296 | 1.757353704 | up | 7.38662418 | 5.629270476 | TCTCTCTCTAGG | hsa_circ_0002108 | chr9 | 16727794 | 16749695 | - | 11539 | ALT_ACCEPTOR, CDS | NM_017637 | BNC2 | Jeck2013 | 54796 | circRNA | Detected | Detected | 7.38662418 | 5.629270476 |
| hsa_gci150522 | 2.389003607 | 1.256409032 | up | 5.284107264 | 4.027698232 | GTTTTACCCACC | hsa_circ_0002134 | chr3 | 61608136 | 61609229 | + | 1093 | ALT_ACCEPTOR, ALT | NM_002841 | PTPRG | Jeck2013 | 5793 | circRNA | Detected | Detected | 5.284107264 | 4.027698232 |
| hsa_gci150524 | 3.183131959 | 1.670446966 | up | 5.654415647 | 3.983968681 | CGTCTCGTCCCA | hsa_circ_0002136 | chr2 | 161186190 | 161188421 | - | 2231 | ALT_ACCEPTOR, ALT | NM_016836 | RBMS1 | Jeck2013 | 5937 | circRNA | Detected | Detected | 5.654415647 | 3.983968681 |
| hsa_gci150641 | 2.298718901 | 1.200830057 | up | 5.118787901 | 3.917957844 | GCTATCATGTAC | hsa_circ_0002326 | chr18 | 9814016 | 9816167 | + | 1127 | ALT_DONOR, CDS, c | NM_006868 | RAB31 | Jeck2013 | 11031 | circRNA | Detected | Detected | 5.118787901 | 3.917957844 |
| hsa_gci150662 | 2.037244442 | 1.026619095 | up | 5.74398582 | 4.717366725 | GTTTGACAGGTT | hsa_circ_0002362 | chr2 | 9458652 | 9468040 | + | 341 | ANNOTATED, CDS, c | NM_003887 | ASAP2 | Jeck2013 | 8853 | circRNA | Detected | Detected | 5.74398582 | 4.717366725 |
| hsa_gci150667 | 3.625812917 | 1.858304487 | up | 5.31507235 | 3.456767863 | ACAGGTCTTAAA | hsa_circ_0002370 | chr2 | 63660878 | 63798760 | - | 79436 | ALT_ACCEPTOR, CDS | NM_015910 | WDPCP | Jeck2013 | 51057 | circRNA | Detected | Detected | 5.31507235 | 3.456767863 |
| hsa_gci150675 | 2.813314177 | 1.492270675 | up | 8.639255333 | 7.146984658 | AAATGTGAAGGG | hsa_circ_0002379 | chr11 | 47521004 | 47528449 | - | 6189 | ALT_ACCEPTOR, CDS | NM_001172639 | CELF1 | Jeck2013 | 10658 | circRNA | Detected | Detected | 8.639255333 | 7.146984658 |
| hsa_gci150686 | 2.539302562 | 1.344432305 | up | 6.235375787 | 4.890943482 | AAGTGACCTCTT | hsa_circ_0002399 | chr7 | 32598170 | 32599076 | + | 606 | ANNOTATED, CDS, c | NM_015060 | AVL9 | Jeck2013 | 23080 | circRNA | Detected | Detected | 6.235375787 | 4.890943482 |
| hsa_gci150708 | 2.295656303 | 1.198906664 | up | 8.064572856 | 6.865666192 | GTAAAAATCCTT | hsa_circ_0002430 | chr20 | 37179713 | 37182719 | + | 363 | ANNOTATED, CDS, c | NM_020336 | RALGAPB | Jeck2013 | 57148 | circRNA | Detected | Detected | 8.064572856 | 6.865666192 |
| hsa_gci150710 | 2.029259181 | 1.020953141 | up | 7.770507558 | 6.749554417 | AGTTCGTTAGAG | hsa_circ_0002432 | chr5 | 37213660 | 37224694 | - | 481 | ALT_ACCEPTOR, CDS | NM_023073 | CPLANE1 | Jeck2013 | 65250 | circRNA | Detected | Detected | 7.770507558 | 6.749554417 |
| hsa_gci150722 | 3.917624421 | 1.969979094 | up | 6.263116284 | 4.29313719 | TATGTCTCTCTC | hsa_circ_0002453 | chr3 | 8977554 | 8990254 | - | 756 | ANNOTATED, CDS, c | NM_020165 | RAD18 | Jeck2013 | 56852 | circRNA | Detected | Detected | 6.263116284 | 4.29313719 |
| hsa_gci150726 | 2.426739448 | 1.279019219 | up | 7.472549414 | 6.193530195 | AGACGTAGAGGT | hsa_circ_0002459 | chr10 | 13175501 | 13178897 | + | 233 | ALT_DONOR, CDS, c | NM_001008211 | OPTN | Jeck2013 | 10133 | circRNA | Detected | Detected | 7.472549414 | 6.193530195 |

| | | | | | | | | | | | | | | | | | | | | | | |
|---|---|---|---|---|---|---|---|---|---|---|---|---|---|---|---|---|---|---|---|---|---|---|
| hsa_gci150733 | 2.054081594 | 1.03849349 | up | 5.652564149 | 4.614070659 | GGTACCTCATCT | hsa_circ_0002471 | chr1 | 92539907 | 92539981 | - | 74 | INTERGENIC | None | | Jeck2013 | | circRNA | Detected | Detected | 5.652564149 | 4.614070659 |
| hsa_gci150820 | -3.271200324 | -1.709820111 | down | 1.789152049 | 3.49897216 | GTATACCGTGGG | hsa_circ_0002592 | chr13 | 60435540 | 60490383 | - | 567 | ANNOTATED, CDS, c | NM_001042517 | DIAPH3 | Jeck2013 | 81624 | circRNA | Not Detected | Detected | 1.789152049 | 3.49897216 |
| hsa_gci150887 | 2.5148101 | 1.330449462 | up | 6.339523397 | 5.009073934 | CAACACCTACAT | hsa_circ_0002685 | chr7 | 5256193 | 5256818 | + | 195 | ANNOTATED, CDS, c | NM_015610 | WIPI2 | Jeck2013, Salzman | 26100 | circRNA | Detected | Detected | 6.339523397 | 5.009073934 |
| hsa_gci150906 | 2.24455373 | 1.166428632 | up | 4.982684246 | 3.816255614 | ATGTAAGGTCTC | hsa_circ_0002714 | chr7 | 80418621 | 80435074 | - | 816 | ANNOTATED, CDS, c | NM_006379 | SEMA3C | Jeck2013, Salzman | 10512 | circRNA | Detected | Detected | 4.982684246 | 3.816255614 |
| hsa_gci150916 | 2.702193683 | 1.434131085 | up | 4.858089593 | 3.423958507 | GGTTACAGACCG | hsa_circ_0002727 | chr10 | 88230748 | 88233730 | - | 498 | ANNOTATED, CDS, c | NM_015045 | WAPL | Jeck2013, Salzman | 23063 | circRNA | Detected | Detected | 4.858089593 | 3.423958507 |
| hsa_gci150967 | 2.482292432 | 1.311673085 | up | 8.429419589 | 7.117746504 | GACCTAGCTCGA | hsa_circ_0002809 | chr17 | 57076741 | 57094785 | - | 634 | ANNOTATED, CDS, c | NM_001005207 | TRIM37 | Jeck2013 | 4591 | circRNA | Detected | Detected | 8.429419589 | 7.117746504 |
| hsa_gci150986 | 2.016093126 | 1.011562281 | up | 11.58853886 | 10.57697658 | TCCGTCTGTCCT | hsa_circ_0002842 | chrX | 64295353 | 64333693 | + | 22960 | ALT_ACCEPTOR, ALT | TCONS_00017187 | | Jeck2013 | | circRNA | Detected | Detected | 11.58853886 | 10.57697658 |
| hsa_gci151005 | -3.427378938 | -1.777105708 | down | 1.538152293 | 3.315258001 | AAGAAATGAAAC | hsa_circ_0002873 | chr12 | 12788710 | 12788908 | + | 198 | ANNOTATED, CDS, c | NM_001310 | CREBL2 | Jeck2013 | 1389 | circRNA | Not Detected | Detected | 1.538152293 | 3.315258001 |
| hsa_gci151040 | -4.104372801 | -2.037161777 | down | 1.240013749 | 3.277175526 | CTAGTTGACACT | hsa_circ_0002938 | chr2 | 36726361 | 36749456 | + | 1056 | ANNOTATED, CDS, c | NM_016441 | CRIM1 | Jeck2013, Salzman | 51232 | circRNA | Not Detected | Detected | 1.240013749 | 3.277175526 |
| hsa_gci151055 | 3.31867859 | 1.730608913 | up | 9.666294956 | 7.935686044 | CCTACCTCCTCC | hsa_circ_0002963 | chr9 | 112918598 | 112918777 | + | 179 | ANNOTATED, CDS, c | NM_007203 | PALM2-AKAP2 | Jeck2013 | 445815 | circRNA | Detected | Detected | 9.666294956 | 7.935686044 |
| hsa_gci151079 | 2.195579071 | 1.134601492 | up | 5.759044578 | 4.624443086 | AATAGGAAACGT | hsa_circ_0003015 | chr11 | 12901254 | 12923660 | + | 543 | ANNOTATED, CDS, c | NM_021961 | TEAD1 | Jeck2013 | 7003 | circRNA | Detected | Detected | 5.759044578 | 4.624443086 |
| hsa_gci151090 | 4.01633773 | 2.005880589 | up | 5.416485083 | 3.410604494 | AATCCTATAGCT | hsa_circ_0003035 | chr5 | 73128162 | 73154044 | + | 1023 | ANNOTATED, CDS, c | NM_001080479 | ARHGEF28 | Jeck2013 | 64283 | circRNA | Detected | Detected | 5.416485083 | 3.410604494 |
| hsa_gci151103 | 2.196490104 | 1.135199999 | up | 5.644948774 | 4.509748774 | AGGACCCACGAC | hsa_circ_0003054 | chr22 | 46085591 | 46114373 | + | 612 | ANNOTATED, CDS, c | NM_013236 | ATXN10 | Jeck2013, Salzman | 25814 | circRNA | Detected | Detected | 5.644948774 | 4.509748774 |
| hsa_gci151151 | 3.361388277 | 1.749057199 | up | 4.097324531 | 2.348267331 | AATTTACCTGGT | hsa_circ_0003125 | chrX | 11056722 | 11057325 | - | 603 | ALT_ACCEPTOR, ALT | TCONS_00016946 | | Jeck2013 | | circRNA | Detected | Not Detected | 4.097324531 | 2.348267331 |
| hsa_gci151202 | 2.243002832 | 1.165431442 | up | 4.313513252 | 3.14808181 | GACGTCCAGTAT | hsa_circ_0003199 | chr17 | 78704359 | 78705925 | + | 1566 | ALT_DONOR, CDS, c | NM_020761 | RPTOR | Jeck2013 | 57521 | circRNA | Detected | Not Detected | 4.313513252 | 3.14808181 |
| hsa_gci151209 | -4.514959553 | -2.174713064 | down | 1.31980117 | 3.494514234 | CCAGTGTCTTCT | hsa_circ_0003209 | chr20 | 30370051 | 30385318 | + | 891 | ANNOTATED, CDS, c | NM_012112 | TPX2 | Jeck2013, Salzman | 22974 | circRNA | Not Detected | Detected | 1.31980117 | 3.494514234 |
| hsa_gci151235 | 2.046332561 | 1.033040625 | up | 8.321693529 | 7.288652904 | TTGATCAACGAT | hsa_circ_0003251 | chr12 | 1003727 | 1006847 | + | 939 | ANNOTATED, CDS, c | NM_001184985 | WNK1 | Jeck2013 | 65125 | circRNA | Detected | Detected | 8.321693529 | 7.288652904 |
| hsa_gci151271 | 2.635387603 | 1.398015164 | up | 5.240807618 | 3.842792454 | TCCGGTGTCGCT | hsa_circ_0003303 | chr22 | 46096161 | 46114373 | + | 337 | ANNOTATED, CDS, c | NM_013236 | ATXN10 | Jeck2013, Salzman | 25814 | circRNA | Detected | Detected | 5.240807618 | 3.842792454 |
| hsa_gci151286 | 2.615285548 | 1.386968475 | up | 6.068326868 | 4.681358393 | AAGTGTCACATT | hsa_circ_0003322 | chr20 | 34446223 | 34459751 | + | 942 | ANNOTATED, CDS, c | NM_016436 | PHF20 | Jeck2013, Salzman | 51230 | circRNA | Detected | Detected | 6.068326868 | 4.681358393 |
| hsa_gci151330 | 2.286361481 | 1.193053517 | up | 9.254692506 | 8.06163899 | GGTATAGTCGTG | hsa_circ_0003400 | chr3 | 3197902 | 3215945 | - | 576 | ANNOTATED, CDS, c | NM_016302 | CRBN | Jeck2013, Salzman | 51185 | circRNA | Detected | Detected | 9.254692506 | 8.06163899 |
| hsa_gci151343 | 3.057984345 | 1.612581021 | up | 8.538945122 | 6.926364101 | TGTCTTTTCGGA | hsa_circ_0003420 | chr16 | 14947882 | 16345820 | + | 1211004 | ALT_ACCEPTOR, ALT | NM_004996 | ABCC1 | Jeck2013 | 4363 | circRNA | Detected | Detected | 8.538945122 | 6.926364101 |
| hsa_gci151344 | 3.25204506 | 1.701347247 | up | 7.114884347 | 5.4135371 | GAAATTTAAGTG | hsa_circ_0003422 | chr17 | 28937959 | 29348067 | + | 353077 | ALT_ACCEPTOR, ALT | NM_024857 | ATAD5 | Jeck2013 | 79915 | circRNA | Detected | Detected | 7.114884347 | 5.4135371 |
| hsa_gci151347 | 3.221542118 | 1.687751456 | up | 4.091811882 | 2.404060425 | TTTACTTACCCG | hsa_circ_0003427 | chr16 | 14989970 | 16387509 | + | 1210605 | ALT_ACCEPTOR, ALT | NM_004996 | ABCC1 | Jeck2013 | 4363 | circRNA | Detected | Not Detected | 4.091811882 | 2.404060425 |
| hsa_gci151352 | 2.716363973 | 1.441676803 | up | 6.540754665 | 5.099077861 | GTAAGTATTTAC | hsa_circ_0003436 | chr3 | 23541098 | 23574192 | + | 281 | ANNOTATED, CDS, c | NM_152653 | UBE2E2 | Jeck2013 | 7325 | circRNA | Detected | Detected | 6.540754665 | 5.099077861 |
| hsa_gci151367 | 2.443746417 | 1.289094587 | up | 4.273510518 | 2.984415931 | CTAAAAAGGCCC | hsa_circ_0003454 | chr15 | 72208710 | 72231268 | - | 383 | ANNOTATED, CDS, c | NM_006901 | MYO9A | Jeck2013, Salzman | 4649 | circRNA | Detected | Not Detected | 4.273510518 | 2.984415931 |
| hsa_gci151378 | 2.868887387 | 1.520491339 | up | 5.400080362 | 3.879589023 | TTGGTGAGGTCT | hsa_circ_0003471 | chr13 | 111546455 | 111546740 | - | 285 | ALT_ACCEPTOR, ALT | NM_017664 | ANKRD10 | Jeck2013 | 55608 | circRNA | Detected | Detected | 5.400080362 | 3.879589023 |
| hsa_gci151431 | 4.176632052 | 2.062340052 | up | 4.065001617 | 2.002661565 | ATACAGCTCCCA | hsa_circ_0003564 | chr16 | 3554719 | 3558468 | + | 377 | ANNOTATED, CDS, c | NM_015041 | CLUAP1 | Jeck2013 | 23059 | circRNA | Detected | Not Detected | 4.065001617 | 2.002661565 |
| hsa_gci151448 | 2.273805948 | 1.185109137 | up | 6.224261456 | 5.039152319 | GGACTATGACAT | hsa_circ_0003590 | chr15 | 78837210 | 78838106 | + | 220 | ANNOTATED, CDS, c | NM_002789 | PSMA4 | Jeck2013, Salzman | 5685 | circRNA | Detected | Detected | 6.224261456 | 5.039152319 |
| hsa_gci151459 | 2.957029739 | 1.564148753 | up | 4.671632546 | 3.107483793 | ATTGTCCATTCT | hsa_circ_0003604 | chr15 | 40678562 | 40679407 | + | 181 | ANNOTATED, CDS, c | NM_001142761 | KNSTRN | Jeck2013 | 90417 | circRNA | Detected | Not Detected | 4.671632546 | 3.107483793 |
| hsa_gci151463 | 2.685460459 | 1.425169479 | up | 6.264797085 | 4.839627606 | AAGTTTGATGGG | hsa_circ_0003613 | chr1 | 52283737 | 52299842 | - | 649 | ANNOTATED, CDS, c | NM_002525 | NRDC | Jeck2013 | 4898 | circRNA | Detected | Detected | 6.264797085 | 4.839627606 |
| hsa_gci151467 | 2.190603311 | 1.131328255 | up | 4.589414213 | 3.458085957 | AAAGTAAAAGAC | hsa_circ_0003618 | chr22 | 28692185 | 28693840 | - | 404 | ANNOTATED, CDS, c | NM_001145418 | TTC28 | Jeck2013 | 23331 | circRNA | Detected | Detected | 4.589414213 | 3.458085957 |
| hsa_gci151470 | -2.118371673 | -1.082955736 | down | 3.983968681 | 5.066924416 | TCCACCACTTAT | hsa_circ_0003624 | chr9 | 33886878 | 33900269 | + | 185 | ANNOTATED, CDS, c | NM_017811 | UBE2R2 | Jeck2013, Salzman | 54926 | circRNA | Detected | Detected | 3.983968681 | 5.066924416 |
| hsa_gci151479 | 2.929266732 | 1.550539567 | up | 4.350740443 | 2.800200876 | TCATAAGTCTCC | hsa_circ_0003634 | chr7 | 80418621 | 80458061 | - | 1251 | ANNOTATED, CDS, c | NM_006379 | SEMA3C | Jeck2013, Salzman | 10512 | circRNA | Detected | Not Detected | 4.350740443 | 2.800200876 |
| hsa_gci151496 | 8.580954634 | 3.101138157 | up | 4.337842578 | 1.236704421 | GGGTAGCGGAAC | hsa_circ_0003656 | chr7 | 104925455 | 104937980 | - | 12525 | ALT_ACCEPTOR, ALT | NM_182692 | SRPK2 | Jeck2013 | 6733 | circRNA | Detected | Not Detected | 4.337842578 | 1.236704421 |
| hsa_gci151502 | 2.412860437 | 1.27074447 | up | 6.075959541 | 4.805215071 | AGTAATAGAAAT | hsa_circ_0003669 | chr3 | 108388527 | 108396445 | + | 460 | ANNOTATED, CDS, c | NM_014648 | DZIP3 | Jeck2013 | 9666 | circRNA | Detected | Detected | 6.075959541 | 4.805215071 |
| hsa_gci151514 | 3.247023129 | 1.699117661 | up | 6.941415979 | 5.242298318 | ACGAGGGTAACG | hsa_circ_0003681 | chr16 | 71736500 | 71748704 | - | 424 | ANNOTATED, CDS, c | NM_015020 | PHLPP2 | Jeck2013, Salzman | 23035 | circRNA | Detected | Detected | 6.941415979 | 5.242298318 |
| hsa_gci151543 | 3.301015024 | 1.722909705 | up | 4.9442805 | 3.221370795 | CGTTACATTATC | hsa_circ_0003727 | chr1 | 51246528 | 51323669 | - | 7465 | ALT_DONOR, CDS, c | NM_007051 | FAF1 | Jeck2013 | 11124 | circRNA | Detected | Detected | 4.9442805 | 3.221370795 |
| hsa_gci151552 | 3.894749131 | 1.961530404 | up | 6.308601663 | 4.347071258 | TCACTCCTCCTA | hsa_circ_0003740 | chr1 | 226016425 | 226019660 | + | 369 | ANNOTATED, CDS, c | NM_000120 | EPHX1 | Jeck2013, Salzman | 2052 | circRNA | Detected | Detected | 6.308601663 | 4.347071258 |
| hsa_gci151558 | 2.673264892 | 1.4186028 | up | 5.012054861 | 3.593452061 | TATAAACACAAA | hsa_circ_0003751 | chr1 | 41578954 | 41627104 | - | 1084 | ANNOTATED, CDS, c | NM_001031694 | SCMH1 | Jeck2013, Salzman | 22955 | circRNA | Detected | Detected | 5.012054861 | 3.593452061 |
| hsa_gci151565 | 2.618344955 | 1.388655178 | up | 4.219888418 | 2.83123324 | CTTAAGACAGTA | hsa_circ_0003761 | chr5 | 80021271 | 80040434 | + | 423 | ANNOTATED, CDS, c | NM_002439 | MSH3 | Jeck2013 | 4437 | circRNA | Detected | Not Detected | 4.219888418 | 2.83123324 |
| hsa_gci151577 | 2.09801059 | 1.06902196 | up | 7.799035575 | 6.730013615 | TAAACTTGTAGT | hsa_circ_0003782 | chr9 | 20819794 | 20874806 | + | 862 | ANNOTATED, CDS, c | NM_017794 | FOCAD | Jeck2013 | 54914 | circRNA | Detected | Detected | 7.799035575 | 6.730013615 |
| hsa_gci151579 | 2.268076017 | 1.181468995 | up | 6.358665587 | 5.177196593 | ACACTCCATAGA | hsa_circ_0003784 | chr15 | 44907560 | 44912601 | - | 418 | ANNOTATED, CDS, c | NM_025137 | SPG11 | Jeck2013, Salzman | 80208 | circRNA | Detected | Detected | 6.358665587 | 5.177196593 |
| hsa_gci151607 | 4.11677849 | 2.041515824 | up | 5.632810787 | 3.591294963 | TTCAGAAGCTCT | hsa_circ_0003822 | chr8 | 59477577 | 59484873 | + | 255 | ANNOTATED, CDS, c | NM_005625 | SDCBP | Jeck2013 | 6386 | circRNA | Detected | Detected | 5.632810787 | 3.591294963 |
| hsa_gci151628 | 2.429895824 | 1.280894463 | up | 6.086690842 | 4.805796379 | GGACACCGAAAA | hsa_circ_0003861 | chr9 | 74309424 | 74313120 | - | 319 | ANNOTATED, CDS, c | NM_013390 | CEMIP2 | Jeck2013, Salzman | 23670 | circRNA | Detected | Detected | 6.086690842 | 4.805796379 |
| hsa_gci151651 | 2.148884387 | 1.103587866 | up | 8.836593898 | 7.733006032 | TACCGAAGCAAC | hsa_circ_0003892 | chr19 | 11230767 | 11238761 | + | 544 | ANNOTATED, CDS, c | NM_000527 | LDLR | Jeck2013, Salzman | 3949 | circRNA | Detected | Detected | 8.836593898 | 7.733006032 |
| hsa_gci151665 | 2.224294992 | 1.153348135 | up | 8.880009274 | 7.72666114 | TTATTCCAGTGT | hsa_circ_0003913 | chr10 | 79253555 | 79254861 | - | 1306 | ALT_ACCEPTOR, ALT | NM_001014797 | KCNMA1 | Jeck2013 | 3778 | circRNA | Detected | Detected | 8.880009274 | 7.72666114 |
| hsa_gci151694 | 2.206158757 | 1.141536612 | up | 4.666391772 | 3.52485516 | GTGGGGGCTCAC | hsa_circ_0003962 | chr7 | 66520821 | 66548526 | + | 282 | ANNOTATED, CDS, c | NM_018264 | TYW1 | Jeck2013 | 55253 | circRNA | Detected | Detected | 4.666391772 | 3.52485516 |
| hsa_gci151705 | 2.630899893 | 1.395556355 | up | 6.228945248 | 4.833388893 | TTGAGGTTCATG | hsa_circ_0003977 | chr4 | 100829867 | 100844342 | - | 332 | ANNOTATED, CDS, c | NM_001031723 | DNAJB14 | Jeck2013, Salzman | 79982 | circRNA | Detected | Detected | 6.228945248 | 4.833388893 |
| hsa_gci151733 | 2.323856126 | 1.216520752 | up | 7.638027353 | 6.421506602 | AGTTGTAGTAGT | hsa_circ_0004016 | chr8 | 1824736 | 1846691 | + | 971 | ANNOTATED, CDS, c | NM_014629 | ARHGEF10 | Jeck2013 | 9639 | circRNA | Detected | Detected | 7.638027353 | 6.421506602 |
| hsa_gci151748 | 3.961668307 | 1.986108095 | up | 4.231427986 | 2.245319891 | TAAACAAAGTTA | hsa_circ_0004039 | chr1 | 236969443 | 236979843 | + | 515 | ANNOTATED, CDS, c | NM_000254 | MTR | Jeck2013, Salzman | 4548 | circRNA | Detected | Not Detected | 4.231427986 | 2.245319891 |
| hsa_gci151751 | 2.137364561 | 1.095833003 | up | 6.85037154 | 5.754538537 | CGACTTATCCCA | hsa_circ_0004044 | chr2 | 224743245 | 224760347 | - | 777 | ALT_DONOR, CDS, c | NM_020830 | WDFY1 | Jeck2013 | 57590 | circRNA | Detected | Detected | 6.85037154 | 5.754538537 |

| | | | | | | | | | | | | | | | | | | | | | | |
|---|---|---|---|---|---|---|---|---|---|---|---|---|---|---|---|---|---|---|---|---|---|---|
| hsa_gci151779 | 2.335427392 | 1.223686593 | up | 8.383341352 | 7.15965476 | CCGTCTCGACCC | hsa_circ_0004094 | chr7 | 18694385 | 18696610 | + | 2225 | ALT_ACCEPTOR, ALT | NM_178423 | HDAC9 | Jeck2013 | 9734 | circRNA | Detected | Detected | 8.383341352 | 7.15965476 |
| hsa_gci151783 | 2.442918581 | 1.288605782 | up | 8.88585484 | 7.597249059 | AAGTAGGCGAGG | hsa_circ_0004102 | chr10 | 128768965 | 128807068 | + | 1012 | ANNOTATED, CDS, c | NM_001380 | DOCK1 | Jeck2013 | 1793 | circRNA | Detected | Detected | 8.88585484 | 7.597249059 |
| hsa_gci151804 | 2.353817059 | 1.235002197 | up | 5.395448812 | 4.160446615 | ACCGCCTCACCT | hsa_circ_0004130 | chrX | 23731251 | 23740870 | - | 262 | ANNOTATED, CDS, c | NM_001037171 | ACOT9 | Jeck2013 | 23597 | circRNA | Detected | Detected | 5.395448812 | 4.160446615 |
| hsa_gci151809 | -2.77840989 | -1.474259451 | down | 1.916772091 | 3.391031543 | CCTATTAGGAAA | hsa_circ_0004137 | chr14 | 23375403 | 23378804 | - | 335 | ANNOTATED, CDS, c | NM_001077351 | RBM23 | Jeck2013, Salzman | 55147 | circRNA | Not Detected | Detected | 1.916772091 | 3.391031543 |
| hsa_gci151830 | 7.236330516 | 2.855258304 | up | 4.259316276 | 1.404057972 | TAACAAGCGTAT | hsa_circ_0004173 | chr4 | 13338516 | 13383218 | - | 32035 | ALT_DONOR, CDS, c | NM_004249 | RAB28 | Jeck2013 | 9364 | circRNA | Detected | Not Detected | 4.259316276 | 1.404057972 |
| hsa_gci151837 | 7.838560718 | 2.970588777 | up | 4.18213064 | 1.211541862 | AGACGGAGGTGT | hsa_circ_0004186 | chr12 | 112884079 | 112894702 | + | 1577 | ALT_DONOR, CDS, c | NM_002834 | PTPN11 | Jeck2013 | 5781 | circRNA | Detected | Not Detected | 4.18213064 | 1.211541862 |
| hsa_gci151847 | 3.785266661 | 1.920394937 | up | 4.688938077 | 2.76854314 | CGGTAGAAAGAA | hsa_circ_0004196 | chr13 | 24164288 | 24190184 | + | 393 | ANNOTATED, CDS, c | NM_148957 | TNFRSF19 | Jeck2013, Salzman | 55504 | circRNA | Detected | Not Detected | 4.688938077 | 2.76854314 |
| hsa_gci151875 | 2.201817349 | 1.138694795 | up | 5.492032728 | 4.353337932 | AGACGTCCTTCT | hsa_circ_0004234 | chr20 | 31518203 | 31519142 | + | 939 | INTERGENIC | None | | Jeck2013 | | circRNA | Detected | Detected | 5.492032728 | 4.353337932 |
| hsa_gci151930 | 7.461880721 | 2.899539299 | up | 4.279174772 | 1.379635474 | AAGTTTCGGTTT | hsa_circ_0004310 | chr14 | 100847246 | 100848083 | + | 837 | ANNOTATED, CDS, c | NM_024515 | WDR25 | Jeck2013 | 79446 | circRNA | Detected | Not Detected | 4.279174772 | 1.379635474 |
| hsa_gci151961 | 3.257966757 | 1.703971883 | up | 5.673541619 | 3.969569736 | GCTCCGGGGAAC | hsa_circ_0004367 | chr15 | 66015185 | 66048810 | - | 1610 | ANNOTATED, CDS, c | NM_001144823 | DENND4A | Jeck2013, Salzman | 10260 | circRNA | Detected | Detected | 5.673541619 | 3.969569736 |
| hsa_gci151968 | 2.172582296 | 1.119410826 | up | 4.856145807 | 3.736734981 | GTAACCACCACT | hsa_circ_0004380 | chr8 | 141407718 | 141415797 | - | 248 | ANNOTATED, CDS, c | NM_031466 | TRAPPC9 | Jeck2013, Salzman | 83696 | circRNA | Detected | Detected | 4.856145807 | 3.736734981 |
| hsa_gci151970 | 3.615787517 | 1.8543099 | up | 7.942926085 | 6.088616185 | TCCTGAGGCCTC | hsa_circ_0004384 | chr2 | 172182350 | 172188377 | - | 377 | ANNOTATED, CDS, c | NM_024770 | METTL8 | Jeck2013 | 79828 | circRNA | Detected | Detected | 7.942926085 | 6.088616185 |
| hsa_gci151973 | 2.401134379 | 1.263716146 | up | 6.582339712 | 5.318623566 | TCCTAGAATCAT | hsa_circ_0004387 | chr10 | 75506387 | 75506762 | + | 287 | ANNOTATED, CDS, c | NM_004922 | SEC24C | Jeck2013 | 9632 | circRNA | Detected | Detected | 6.582339712 | 5.318623566 |
| hsa_gci151975 | 3.307774785 | 1.72586101 | up | 5.827591589 | 4.10173058 | AACACCTCTTCA | hsa_circ_0004390 | chr1 | 85331067 | 85331821 | - | 754 | ANNOTATED, CDS, c | NM_012152 | LPAR3 | Jeck2013 | 23566 | circRNA | Detected | Detected | 5.827591589 | 4.10173058 |
| hsa_gci152005 | 2.428993818 | 1.280358818 | up | 5.052944977 | 3.772586159 | TAGTTTCGTCTA | hsa_circ_0004436 | chr1 | 176118141 | 176153828 | - | 424 | ANNOTATED, CDS, c | NM_022457 | COP1 | Jeck2013 | 64326 | circRNA | Detected | Detected | 5.052944977 | 3.772586159 |
| hsa_gci152044 | 2.496382807 | 1.319839181 | up | 7.568777007 | 6.248937826 | TCTAGAAACGAA | hsa_circ_0004496 | chr4 | 177632652 | 177650900 | - | 557 | ANNOTATED, CDS, c | NM_005429 | VEGFC | Jeck2013 | 7424 | circRNA | Detected | Detected | 7.568777007 | 6.248937826 |
| hsa_gci152077 | 2.323835369 | 1.216507865 | up | 4.957200134 | 3.740692269 | AGGAAGAAACTG | hsa_circ_0004554 | chr2 | 230666967 | 230668943 | - | 556 | ANNOTATED, CDS, c | NM_004238 | TRIP12 | Jeck2013, Salzman | 9320 | circRNA | Detected | Detected | 4.957200134 | 3.740692269 |
| hsa_gci152098 | 2.083039369 | 1.058690106 | up | 5.564168176 | 4.50547807 | CGGACTAAAACG | hsa_circ_0004590 | chr12 | 78334098 | 78392256 | + | 637 | ANNOTATED, CDS, c | NM_014903 | NAV3 | Jeck2013, Salzman | 89795 | circRNA | Detected | Detected | 5.564168176 | 4.50547807 |
| hsa_gci152104 | 2.637864259 | 1.399370327 | up | 7.563951524 | 6.164581197 | ATTCTAACTTAT | hsa_circ_0004598 | chr11 | 122665409 | 122669742 | + | 470 | ANNOTATED, CDS, c | NM_032873 | UBASH3B | Jeck2013 | 84959 | circRNA | Detected | Detected | 7.563951524 | 6.164581197 |
| hsa_gci152112 | 2.367271058 | 1.243224908 | up | 4.499709004 | 3.256484096 | GGTCTTCCCACC | hsa_circ_0004613 | chr4 | 114430793 | 114438813 | - | 383 | ANNOTATED, CDS, c | NM_001221 | CAMK2D | Jeck2013 | 817 | circRNA | Detected | Detected | 4.499709004 | 3.256484096 |
| hsa_gci152147 | 2.214159104 | 1.146758894 | up | 7.205195467 | 6.058436572 | CTTGTAACACGA | hsa_circ_0004668 | chr4 | 103720563 | 103731012 | - | 374 | ANNOTATED, CDS, c | NM_181892 | UBE2D3 | Jeck2013, Salzman | 7323 | circRNA | Detected | Detected | 7.205195467 | 6.058436572 |
| hsa_gci152166 | 2.193606126 | 1.133304505 | up | 9.269521017 | 8.136216512 | GGATTTCTCTTC | hsa_circ_0004698 | chr7 | 38247047 | 38259261 | + | 707 | ANNOTATED, CDS, c | NM_032016 | STARD3NL | Jeck2013 | 83930 | circRNA | Detected | Detected | 9.269521017 | 8.136216512 |
| hsa_gci152168 | 2.507062484 | 1.325997953 | up | 7.061764114 | 5.735766161 | TCGATATTTGGA | hsa_circ_0004703 | chr7 | 5941287 | 5949747 | + | 390 | ANNOTATED, CDS, c | NM_015622 | CCZ1 | Jeck2013, Salzman | 51622 | circRNA | Detected | Detected | 7.061764114 | 5.735766161 |
| hsa_gci152188 | 6.432239793 | 2.685321191 | up | 5.0993463 | 2.414025109 | GAATAAGAAGAG | hsa_circ_0004735 | chr9 | 22046749 | 22112394 | + | 1795 | ANNOTATED, INTERN | NR_003529 | CDKN2B-AS1 | Jeck2013 | 100048912 | circRNA | Detected | Not Detected | 5.0993463 | 2.414025109 |
| hsa_gci152191 | 3.51219831 | 1.812374307 | up | 8.077918464 | 6.265544158 | TTTAGACTAGAA | hsa_circ_0004738 | chr5 | 170667931 | 170669824 | + | 354 | ANNOTATED, CDS, c | NM_022897 | RANBP17 | Jeck2013, Salzman | 64901 | circRNA | Detected | Detected | 8.077918464 | 6.265544158 |
| hsa_gci152198 | 5.073771778 | 2.343058627 | up | 4.530345845 | 2.187287218 | TGTATGGACCTT | hsa_circ_0004749 | chr1 | 89401781 | 89420908 | - | 662 | ALT_DONOR, CDS, c | NM_001008661 | KYAT3 | Jeck2013 | 56267 | circRNA | Detected | Not Detected | 4.530345845 | 2.187287218 |
| hsa_gci152200 | 2.252807477 | 1.171724028 | up | 5.188176573 | 4.016452545 | GGGAAGGAGTAG | hsa_circ_0004751 | chr17 | 35310185 | 35311207 | + | 549 | ANNOTATED, CDS, c | NM_012138 | AATF | Jeck2013 | 26574 | circRNA | Detected | Detected | 5.188176573 | 4.016452545 |
| hsa_gci152204 | 2.299109316 | 1.201075063 | up | 6.54699835 | 5.345923286 | TGTATCTCGACT | hsa_circ_0004755 | chr7 | 157013382 | 157024021 | + | 567 | ANNOTATED, CDS, c | NM_014671 | UBE3C | Jeck2013 | 9690 | circRNA | Detected | Detected | 6.54699835 | 5.345923286 |
| hsa_gci152227 | 2.127926096 | 1.089448046 | up | 7.562784513 | 6.473336467 | AGTAGTTACAGT | hsa_circ_0004796 | chr17 | 45741524 | 45755779 | + | 1567 | ANNOTATED, CDS, c | NM_002265 | KPNB1 | Jeck2013, Salzman | 3837 | circRNA | Detected | Detected | 7.562784513 | 6.473336467 |
| hsa_gci152291 | -5.240508753 | -2.389706876 | down | 1.262525406 | 3.652232283 | GTCTTGAGTCGT | hsa_circ_0004892 | chr4 | 155122899 | 155128205 | + | 5306 | INTERGENIC | None | | Jeck2013 | | circRNA | Not Detected | Detected | 1.262525406 | 3.652232283 |
| hsa_gci152297 | 2.572640567 | 1.363249907 | up | 6.306987276 | 4.943737369 | GACGTAGGACCC | hsa_circ_0004899 | chr11 | 57563048 | 57569668 | + | 1153 | ANNOTATED, CDS, c | NM_001085458 | CTNND1 | Jeck2013 | 1500 | circRNA | Detected | Detected | 6.306987276 | 4.943737369 |
| hsa_gci152343 | 2.704549075 | 1.435388076 | up | 4.882245014 | 3.446856939 | TTACATCAGTCG | hsa_circ_0004970 | chr14 | 53339516 | 53340944 | - | 175 | ANNOTATED, CDS, c | NM_001134999 | FERMT2 | Jeck2013, Salzman | 10979 | circRNA | Detected | Detected | 4.882245014 | 3.446856939 |
| hsa_gci152368 | 2.191893862 | 1.132177941 | up | 8.280825187 | 7.148647246 | AATTGTTAAAAT | hsa_circ_0005013 | chr8 | 26439484 | 26441499 | + | 274 | ANNOTATED, CDS, c | NM_001197293 | DPYSL2 | Jeck2013, Salzman | 1808 | circRNA | Detected | Detected | 8.280825187 | 7.148647246 |
| hsa_gci152377 | 7.755205272 | 2.955164968 | up | 4.29313719 | 1.337972221 | TGTATGTCTCTT | hsa_circ_0005029 | chr2 | 26587169 | 26596497 | + | 516 | ANNOTATED, CDS, c | NM_033505 | SELENOI | Jeck2013, Salzman | 85465 | circRNA | Detected | Not Detected | 4.29313719 | 1.337972221 |
| hsa_gci152406 | 2.281802545 | 1.190173954 | up | 5.816494752 | 4.626320798 | CCACCCGTCTTA | hsa_circ_0005070 | chr8 | 103341317 | 103357773 | - | 590 | ANNOTATED, CDS, c | NM_015902 | UBR5 | Jeck2013, Salzman | 51366 | circRNA | Detected | Detected | 5.816494752 | 4.626320798 |
| hsa_gci152416 | 2.182170187 | 1.125763622 | up | 4.628003465 | 3.502239844 | AAAGAAGTAAAA | hsa_circ_0005085 | chr2 | 9419445 | 9437574 | + | 219 | ANNOTATED, CDS, c | NM_003887 | ASAP2 | Jeck2013, Salzman | 8853 | circRNA | Detected | Detected | 4.628003465 | 3.502239844 |
| hsa_gci152422 | -2.087557317 | -1.061815809 | down | 5.373601483 | 6.435417292 | TCTGGTTTGGTT | hsa_circ_0005095 | chrX | 44870205 | 44879975 | + | 180 | ANNOTATED, CDS, c | NM_021140 | KDM6A | Jeck2013, Salzman | 7403 | circRNA | Detected | Detected | 5.373601483 | 6.435417292 |
| hsa_gci152430 | 4.268757673 | 2.093816266 | up | 5.133058448 | 3.039242182 | TCTGATTTGAAG | hsa_circ_0005108 | chr14 | 39870379 | 39871715 | - | 797 | ANNOTATED, CDS, c | NM_203301 | FBXO33 | Jeck2013 | 254170 | circRNA | Detected | Not Detected | 5.133058448 | 3.039242182 |
| hsa_gci152438 | 2.252059598 | 1.171245007 | up | 5.720974475 | 4.549729468 | TCCCAAGGATTT | hsa_circ_0005119 | chr9 | 113734352 | 113771481 | - | 35869 | ALT_ACCEPTOR, CDS | NM_057159 | LPAR1 | Jeck2013 | 1902 | circRNA | Detected | Detected | 5.720974475 | 4.549729468 |
| hsa_gci152463 | -3.447356761 | -1.785490609 | down | 2.177108758 | 3.962599366 | CTCCTCGTGCCA | hsa_circ_0005161 | chr13 | 114174930 | 114188555 | + | 314 | ANNOTATED, CDS, c | NM_017905 | TMCO3 | Jeck2013, Salzman | 55002 | circRNA | Not Detected | Detected | 2.177108758 | 3.962599366 |
| hsa_gci152468 | 2.137974705 | 1.096244784 | up | 5.650762363 | 4.554517579 | CCTGTATGAAAA | hsa_circ_0005166 | chr12 | 63359304 | 63359768 | + | 464 | INTERGENIC | None | | Jeck2013 | | circRNA | Detected | Detected | 5.650762363 | 4.554517579 |
| hsa_gci152497 | 2.035815976 | 1.025607157 | up | 7.871681789 | 6.846074632 | CTTACAAGAGAA | hsa_circ_0005211 | chr15 | 59470591 | 59480415 | - | 244 | ANNOTATED, CDS, c | NM_004998 | MYO1E | Jeck2013 | 4643 | circRNA | Detected | Detected | 7.871681789 | 6.846074632 |
| hsa_gci152504 | 2.047019689 | 1.033524979 | up | 5.554306281 | 4.520781303 | TGAGTGTCACCC | hsa_circ_0005220 | chr16 | 88008653 | 88014733 | + | 248 | ANNOTATED, CDS, c | NM_001173540 | BANP | Jeck2013, Salzman | 54971 | circRNA | Detected | Detected | 5.554306281 | 4.520781303 |
| hsa_gci152513 | 2.018611449 | 1.013363241 | up | 6.482432449 | 5.469069208 | GGGCTCCCGAAG | hsa_circ_0005230 | chr1 | 172109619 | 172113577 | - | 3958 | ALT_ACCEPTOR, ALT | NR_038397 | DNM3OS | Jeck2013 | 100628315 | circRNA | Detected | Detected | 6.482432449 | 5.469069208 |
| hsa_gci152522 | 2.5020904 | 1.323133915 | up | 4.983305177 | 3.660171262 | ACGATCGTAACT | hsa_circ_0005246 | chr1 | 44785299 | 44804994 | - | 395 | ANNOTATED, CDS, c | NM_024066 | ERI3 | Jeck2013, Salzman | 79033 | circRNA | Detected | Detected | 4.983305177 | 3.660171262 |
| hsa_gci152534 | 2.02386667 | 1.01711425 | up | 6.73628788 | 5.719173629 | AATTAATACGAG | hsa_circ_0005265 | chr20 | 2928627 | 2945848 | + | 554 | ANNOTATED, CDS, c | NM_080841 | PTPRA | Jeck2013, Salzman | 5786 | circRNA | Detected | Detected | 6.73628788 | 5.719173629 |
| hsa_gci152560 | 2.112407992 | 1.078888505 | up | 4.262052758 | 3.183164253 | CGTTTGCCCCAA | hsa_circ_0005301 | chr15 | 64668941 | 64669104 | - | 163 | ANNOTATED, CDS, c | NM_014736 | PCLAF | Jeck2013 | 9768 | circRNA | Detected | Not Detected | 4.262052758 | 3.183164253 |
| hsa_gci152569 | 2.507891681 | 1.326475038 | up | 8.043506176 | 6.717031138 | TACATCAGAAAA | hsa_circ_0005316 | chrX | 46712910 | 46719537 | + | 781 | ANNOTATED, CDS, c | NM_006915 | RP2 | Jeck2013 | 6102 | circRNA | Detected | Detected | 8.043506176 | 6.717031138 |
| hsa_gci152587 | 2.32053944 | 1.214460218 | up | 7.605205938 | 6.39074572 | AGCCTACTCAGG | hsa_circ_0005352 | chr8 | 25340914 | 25341726 | + | 333 | ANNOTATED, CDS, c | NM_152562 | CDCA2 | Jeck2013 | 157313 | circRNA | Detected | Detected | 7.605205938 | 6.39074572 |
| hsa_gci152593 | 3.371580285 | 1.753424952 | up | 4.860643698 | 3.107218746 | TATAGACCGACG | hsa_circ_0005363 | chr12 | 78388582 | 78415642 | + | 1352 | ANNOTATED, CDS, c | NM_014903 | NAV3 | Jeck2013, Salzman | 89795 | circRNA | Detected | Not Detected | 4.860643698 | 3.107218746 |
| hsa_gci152620 | 2.154634877 | 1.107443412 | up | 8.631974933 | 7.524531522 | CTTACCACTAAA | hsa_circ_0005397 | chr17 | 30500849 | 30503232 | + | 233 | ANNOTATED, CDS, c | NM_001033568 | RHOT1 | Jeck2013, Salzman | 55288 | circRNA | Detected | Detected | 8.631974933 | 7.524531522 |

| | | | | | | | | | | | | | | | | | | | | | | |
|---|---|---|---|---|---|---|---|---|---|---|---|---|---|---|---|---|---|---|---|---|---|---|
| hsa_gci152656 | 4.454656028 | 2.155314037 | up | 6.826247298 | 4.670933262 | CTCTCTCGACTC | hsa_circ_0005452 | chr3 | 86996159 | 87018273 | - | 592 | ALT_DONOR, CDS, c | NM_016206 | VGLL3 | Jeck2013 | 389136 | circRNA | Detected | Detected | 6.826247298 | 4.670933262 |
| hsa_gci152667 | 2.049147006 | 1.035023487 | up | 8.23652047 | 7.201496983 | CGAAGGTCGTTG | hsa_circ_0005472 | chr6 | 160103505 | 160106065 | - | 345 | ANNOTATED, CDS, c | NM_001024465 | SOD2 | Jeck2013, Salzman | 6648 | circRNA | Detected | Detected | 8.23652047 | 7.201496983 |
| hsa_gci152668 | 4.622711354 | 2.208739282 | up | 6.671117521 | 4.462378239 | TAGGAGAGAAAT | hsa_circ_0005474 | chr10 | 93711159 | 93717050 | + | 500 | ANNOTATED, CDS, c | NM_003972 | BTAF1 | Jeck2013 | 9044 | circRNA | Detected | Detected | 6.671117521 | 4.462378239 |
| hsa_gci152723 | 3.14942011 | 1.655086216 | up | 4.498406111 | 2.843319896 | CTCCTTACAAAG | hsa_circ_0005554 | chr14 | 101446913 | 101455127 | + | 8214 | ALT_ACCEPTOR, ALT | NR_003219 | SNORD114-26 | Jeck2013 | 767606 | circRNA | Detected | Not Detected | 4.498406111 | 2.843319896 |
| hsa_gci152746 | 2.913184412 | 1.54259703 | up | 6.983729894 | 5.441132864 | TTAGTGTAATCG | hsa_circ_0005585 | chr5 | 43675612 | 43704502 | + | 623 | ALT_DONOR, CDS, c | NM_012343 | NNT | Jeck2013 | 23530 | circRNA | Detected | Detected | 6.983729894 | 5.441132864 |
| hsa_gci152748 | 2.238043843 | 1.162238299 | up | 5.628863477 | 4.466625178 | GTTAGGACACCC | hsa_circ_0005587 | chr20 | 34243123 | 34246936 | - | 228 | ALT_ACCEPTOR, INT | NR_037188 | CPNE1 | Jeck2013, Salzman | 8904 | circRNA | Detected | Detected | 5.628863477 | 4.466625178 |
| hsa_gci152755 | 3.940681508 | 1.978445153 | up | 6.220789804 | 4.24234465 | TCACCCCAGGTC | hsa_circ_0005596 | chr9 | 72879219 | 72882891 | + | 195 | ANNOTATED, CDS, c | NM_015110 | SMC5 | Jeck2013 | 23137 | circRNA | Detected | Detected | 6.220789804 | 4.24234465 |
| hsa_gci152756 | 2.262183156 | 1.177715741 | up | 9.189832432 | 8.012116691 | TGTTACTATTAC | hsa_circ_0005601 | chr7 | 6465618 | 6474651 | - | 637 | ANNOTATED, CDS, c | NM_139179 | DAGLB | Jeck2013, Salzman | 221955 | circRNA | Detected | Detected | 9.189832432 | 8.012116691 |
| hsa_gci152771 | 2.003590918 | 1.002587977 | up | 4.134222651 | 3.131634674 | AGTTTCTACTTT | hsa_circ_0005624 | chr10 | 71912018 | 71921687 | - | 826 | ALT_DONOR, CDS, c | NM_001142648 | SAR1A | Jeck2013 | 56681 | circRNA | Detected | Not Detected | 4.134222651 | 3.131634674 |
| hsa_gci152774 | 2.367049781 | 1.243090048 | up | 9.233811707 | 7.990721659 | AAGTAGATGAAA | hsa_circ_0005627 | chr16 | 14687157 | 14698083 | - | 216 | ANNOTATED, CDS, c | NM_002582 | PARN | Jeck2013, Salzman | 5073 | circRNA | Detected | Detected | 9.233811707 | 7.990721659 |
| hsa_gci152794 | 3.609960981 | 1.851983244 | up | 7.313319169 | 5.461335925 | TGACCTTCTTCA | hsa_circ_0005653 | chr18 | 196636 | 204692 | + | 701 | ANNOTATED, CDS, c | NM_005151 | USP14 | Jeck2013, Salzman | 9097 | circRNA | Detected | Detected | 7.313319169 | 5.461335925 |
| hsa_gci152831 | 2.02887325 | 1.020678738 | up | 4.986723047 | 3.966044309 | AACACCGTTCTA | hsa_circ_0005704 | chr20 | 10536878 | 10541468 | + | 211 | ANNOTATED, CDS, c | NM_001009608 | SLX4IP | Jeck2013 | 128710 | circRNA | Detected | Detected | 4.986723047 | 3.966044309 |
| hsa_gci152863 | 5.361493038 | 2.42263481 | up | 4.7546828 | 2.33204799 | AAAGGGGGATCC | hsa_circ_0005754 | chr15 | 33076591 | 33076875 | + | 284 | ANTISENSE, coding | NM_001103184 | FMN1 | Jeck2013 | 342184 | circRNA | Detected | Not Detected | 4.7546828 | 2.33204799 |
| hsa_gci152883 | 2.347154793 | 1.23091299 | up | 8.184553388 | 6.953640399 | TGTGGTTTTCGA | hsa_circ_0005785 | chr12 | 110819556 | 110834257 | - | 1031 | ANNOTATED, CDS, c | NM_016238 | ANAPC7 | Jeck2013, Salzman | 51434 | circRNA | Detected | Detected | 8.184553388 | 6.953640399 |
| hsa_gci152903 | 2.234568001 | 1.159995948 | up | 5.283945059 | 4.123949111 | ACTTACTTTGTC | hsa_circ_0005821 | chr9 | 111843118 | 111870850 | - | 870 | ANNOTATED, CDS, c | NM_032012 | TMEM245 | Jeck2013, Salzman | 23731 | circRNA | Detected | Detected | 5.283945059 | 4.123949111 |
| hsa_gci152909 | 2.549281508 | 1.350090694 | up | 5.728785756 | 4.378695062 | TAATATATGTCG | hsa_circ_0005828 | chr10 | 88231964 | 88233730 | - | 386 | ANNOTATED, CDS, c | NM_015045 | WAPL | Jeck2013, Salzman | 23063 | circRNA | Detected | Detected | 5.728785756 | 4.378695062 |
| hsa_gci152936 | 2.103946389 | 1.073097944 | up | 9.124780319 | 8.051682376 | CCTTCCCCACTT | hsa_circ_0005873 | chr3 | 197597030 | 197602646 | + | 4570 | ALT_DONOR, CDS, c | NM_032773 | LRCH3 | Jeck2013, Salzman | 84859 | circRNA | Detected | Detected | 9.124780319 | 8.051682376 |
| hsa_gci152969 | 3.40073844 | 1.765848049 | up | 6.447678661 | 4.681830612 | TCCTCCGGAAGT | hsa_circ_0005927 | chr8 | 42259305 | 42260979 | + | 379 | ANNOTATED, CDS, c | NM_001135694 | VDAC3 | Jeck2013, Salzman | 7419 | circRNA | Detected | Detected | 6.447678661 | 4.681830612 |
| hsa_gci152995 | 2.035839743 | 1.025624 | up | 5.622758182 | 4.597134182 | ATGCCCCTTTAC | hsa_circ_0005966 | chr13 | 20576989 | 20580726 | + | 665 | ANNOTATED, CDS, c | NM_003453 | ZMYM2 | Jeck2013 | 7750 | circRNA | Detected | Detected | 5.622758182 | 4.597134182 |
| hsa_gci153007 | 2.330737791 | 1.22078671 | up | 7.850086898 | 6.629300189 | ATATAAGTTGTA | hsa_circ_0005984 | chr8 | 100443764 | 100494030 | + | 788 | ANNOTATED, CDS, c | NM_017890 | VPS13B | Jeck2013, Salzman | 157680 | circRNA | Detected | Detected | 7.850086898 | 6.629300189 |
| hsa_gci153008 | 3.052087863 | 1.609796495 | up | 6.511281363 | 4.901484868 | CCACCGGCGGTG | hsa_circ_0005986 | chr1 | 14057494 | 14068652 | + | 375 | ANNOTATED, CDS, c | NM_012231 | PRDM2 | Jeck2013, Salzman | 7799 | circRNA | Detected | Detected | 6.511281363 | 4.901484868 |
| hsa_gci153011 | 2.54692516 | 1.348756567 | up | 5.084714127 | 3.73595756 | TAGTTTTACCCA | hsa_circ_0005989 | chr3 | 61608136 | 61608814 | + | 678 | ALT_ACCEPTOR, ALT | NM_002841 | PTPRG | Jeck2013 | 5793 | circRNA | Detected | Detected | 5.084714127 | 3.73595756 |
| hsa_gci153018 | -3.890967327 | -1.960128866 | down | 2.219574569 | 4.179703435 | GTGTAATGTGCC | hsa_circ_0005997 | chr7 | 35009044 | 35013217 | - | 192 | ANNOTATED, CDS, c | NM_015283 | DPY19L1 | Jeck2013, Salzman | 23333 | circRNA | Not Detected | Detected | 2.219574569 | 4.179703435 |
| hsa_gci153029 | 2.13747616 | 1.095908329 | up | 5.303840414 | 4.207932085 | AACACGGTTAAA | hsa_circ_0006020 | chrX | 16836696 | 16838378 | + | 396 | ANNOTATED, CDS, c | NM_018360 | TXLNG | Jeck2013 | 55787 | circRNA | Detected | Detected | 5.303840414 | 4.207932085 |
| hsa_gci153033 | 2.666323482 | 1.414851821 | up | 7.516035997 | 6.101184176 | TCAATGGAATAA | hsa_circ_0006024 | chr5 | 93111864 | 93120205 | - | 202 | ANNOTATED, CDS, c | NM_032042 | FAM172A | Jeck2013, Salzman | 83989 | circRNA | Detected | Detected | 7.516035997 | 6.101184176 |
| hsa_gci153059 | -12.25786101 | -3.615635347 | down | 1.297777411 | 4.913412757 | TCTCGTAGATTT | hsa_circ_0006063 | chr10 | 26789747 | 26792203 | + | 371 | ANNOTATED, CDS, c | NM_019043 | APBB1IP | Jeck2013, Salzman | 54518 | circRNA | Not Detected | Detected | 1.297777411 | 4.913412757 |
| hsa_gci153069 | 2.15109079 | 1.105068417 | up | 7.538314567 | 6.43324615 | AGAAACAACTGA | hsa_circ_0006077 | chr12 | 133324417 | 133324917 | - | 383 | ANNOTATED, CDS, c | NM_015114 | ANKLE2 | Jeck2013, Salzman | 23141 | circRNA | Detected | Detected | 7.538314567 | 6.43324615 |
| hsa_gci153086 | 2.845045855 | 1.508451905 | up | 7.965923617 | 6.457471711 | TATAGTAGGAGT | hsa_circ_0006106 | chr9 | 112898406 | 112918777 | + | 2592 | ANNOTATED, CDS, c | NM_007203 | PALM2-AKAP2 | Jeck2013 | 445815 | circRNA | Detected | Detected | 7.965923617 | 6.457471711 |
| hsa_gci153141 | 2.204696091 | 1.140579799 | up | 4.875864968 | 3.735285169 | TGGTACCACCTA | hsa_circ_0006184 | chr12 | 51398570 | 51404549 | - | 467 | ANNOTATED, CDS, c | NM_001174129 | SLC11A2 | Jeck2013 | 4891 | circRNA | Detected | Detected | 4.875864968 | 3.735285169 |
| hsa_gci153165 | 2.502527812 | 1.323386103 | up | 8.018439518 | 6.695053415 | GAAAGTCAATTT | hsa_circ_0006218 | chr14 | 74398205 | 74398815 | + | 610 | ALT_DONOR, coding | NM_001242924 | ZNF410 | Jeck2013 | 57862 | circRNA | Detected | Detected | 8.018439518 | 6.695053415 |
| hsa_gci153172 | 2.007965069 | 1.005734172 | up | 4.701700433 | 3.695966261 | TCTTTATGAAAG | hsa_circ_0006226 | chr3 | 61607643 | 61609229 | + | 1586 | ALT_ACCEPTOR, ALT | NM_002841 | PTPRG | Jeck2013 | 5793 | circRNA | Detected | Detected | 4.701700433 | 3.695966261 |
| hsa_gci153177 | 6.784492679 | 2.76224094 | up | 4.012246466 | 1.250005526 | CCGTCCAAAGCC | hsa_circ_0006238 | chr12 | 49888523 | 49893988 | + | 575 | ANNOTATED, CDS, c | NM_023071 | SPATS2 | Jeck2013, Salzman | 65244 | circRNA | Detected | Not Detected | 4.012246466 | 1.250005526 |
| hsa_gci153201 | 2.419684698 | 1.274819067 | up | 5.950863287 | 4.676044221 | AGAGATCGTCGT | hsa_circ_0006278 | chr14 | 68151731 | 68157138 | - | 400 | ANNOTATED, CDS, c | NM_016026 | RDH11 | Jeck2013, Salzman | 51109 | circRNA | Detected | Detected | 5.950863287 | 4.676044221 |
| hsa_gci153232 | -5.04444249 | -2.334694832 | down | 1.643413002 | 3.978107834 | TGTCTCCCAGGT | hsa_circ_0006322 | chrY | 7209155 | 7239930 | + | 441 | ANNOTATED, INTERN | NR_028062 | PRKY | Jeck2013, Salzman | 5616 | circRNA | Not Detected | Detected | 1.643413002 | 3.978107834 |
| hsa_gci153234 | 2.051285024 | 1.036527967 | up | 7.781123048 | 6.744595082 | AAGTTTGGTCCC | hsa_circ_0006324 | chr1 | 197611840 | 197627499 | - | 376 | ANNOTATED, CDS, c | NM_001195215 | DENND1B | Jeck2013, Salzman | 163486 | circRNA | Detected | Detected | 7.781123048 | 6.744595082 |
| hsa_gci153241 | 2.622387681 | 1.390880982 | up | 9.084829649 | 7.693948667 | GAGTAAAAAGTG | hsa_circ_0006334 | chr5 | 137527162 | 137537137 | - | 1009 | ANNOTATED, CDS, c | NM_004661 | CDC23 | Jeck2013 | 8697 | circRNA | Detected | Detected | 9.084829649 | 7.693948667 |
| hsa_gci153246 | 2.781433402 | 1.475828563 | up | 7.074122754 | 5.598294191 | TCTGTCCAGTGT | hsa_circ_0006343 | chr15 | 42556287 | 42560230 | - | 200 | ANNOTATED, CDS, c | NM_015497 | TMEM87A | Jeck2013 | 25963 | circRNA | Detected | Detected | 7.074122754 | 5.598294191 |
| hsa_gci153254 | 7.055586673 | 2.818766048 | up | 4.147202083 | 1.328436035 | AGAGGACGGTGT | hsa_circ_0006355 | chrX | 154736558 | 154766779 | - | 13300 | ALT_ACCEPTOR, CDS | NM_018196 | TMLHE | Jeck2013 | 55217 | circRNA | Detected | Not Detected | 4.147202083 | 1.328436035 |
| hsa_gci153258 | 2.128086161 | 1.089556563 | up | 6.833902932 | 5.744346369 | CCGTTCCATTTT | hsa_circ_0006360 | chr1 | 150390071 | 150418877 | + | 665 | ANNOTATED, CDS, c | NM_015203 | RPRD2 | Jeck2013 | 23248 | circRNA | Detected | Detected | 6.833902932 | 5.744346369 |
| hsa_gci153277 | 3.943004362 | 1.979295307 | up | 4.190563711 | 2.211268404 | GATCACAGCAGA | hsa_circ_0006396 | chr12 | 112116954 | 112121111 | - | 551 | ANNOTATED, CDS, c | NM_006768 | BRAP | Jeck2013, Salzman | 8315 | circRNA | Detected | Not Detected | 4.190563711 | 2.211268404 |
| hsa_gci153313 | 7.934293812 | 2.988101822 | up | 4.407943654 | 1.419841832 | TGGGTGGTTCTA | hsa_circ_0006451 | chr10 | 71119652 | 71124658 | + | 269 | ANNOTATED, CDS, c | NM_033500 | HK1 | Jeck2013, Salzman | 3098 | circRNA | Detected | Not Detected | 4.407943654 | 1.419841832 |
| hsa_gci153329 | 2.443275043 | 1.288816279 | up | 4.317310324 | 3.028494045 | AGGGGGAAGTGT | hsa_circ_0006484 | chr20 | 43132455 | 43141635 | - | 854 | ANNOTATED, CDS, c | NM_006811 | SERINC3 | Jeck2013 | 10955 | circRNA | Detected | Not Detected | 4.317310324 | 3.028494045 |
| hsa_gci153344 | 8.642945074 | 3.111522993 | up | 4.311026226 | 1.199503233 | ACAAGTTTCAGC | hsa_circ_0006509 | chr15 | 72338064 | 72338975 | - | 911 | ANNOTATED, CDS, c | NM_006901 | MYO9A | Jeck2013, Salzman | 4649 | circRNA | Detected | Not Detected | 4.311026226 | 1.199503233 |
| hsa_gci153390 | 2.06048362 | 1.042982995 | up | 6.887818908 | 5.844835913 | TAAAAAGAAGTA | hsa_circ_0006578 | chr1 | 36823839 | 36828257 | - | 1666 | ALT_ACCEPTOR, CDS | NM_032017 | STK40 | Jeck2013 | 83931 | circRNA | Detected | Detected | 6.887818908 | 5.844835913 |
| hsa_gci153452 | 3.39418378 | 1.763064683 | up | 8.849300446 | 7.086235763 | AGGGAACAGTTC | hsa_circ_0006682 | chr17 | 45041062 | 45042405 | + | 1343 | INTERGENIC | None | | Jeck2013 | | circRNA | Detected | Detected | 8.849300446 | 7.086235763 |
| hsa_gci153466 | 2.69594592 | 1.430791557 | up | 7.799800134 | 6.369008577 | TTTCAGCCCGAA | hsa_circ_0006704 | chr20 | 17933230 | 17934761 | - | 246 | ANNOTATED, CDS, c | NM_152227 | SNX5 | Jeck2013, Salzman | 27131 | circRNA | Detected | Detected | 7.799800134 | 6.369008577 |
| hsa_gci153505 | 2.427158344 | 1.279268231 | up | 6.704438608 | 5.425170378 | GTGGGTGGAGGT | hsa_circ_0006757 | chr6 | 34574331 | 34574681 | - | 350 | ANNOTATED, CDS, c | NM_024294 | C6orf106 | Jeck2013, Salzman | 64771 | circRNA | Detected | Detected | 6.704438608 | 5.425170378 |
| hsa_gci153506 | 6.626115357 | 2.72816332 | up | 4.169155579 | 1.440992259 | TTAAATAGTACC | hsa_circ_0006759 | chr2 | 63711737 | 63798760 | - | 78995 | ALT_ACCEPTOR, CDS | NM_015910 | WDPCP | Jeck2013 | 51057 | circRNA | Detected | Not Detected | 4.169155579 | 1.440992259 |
| hsa_gci153544 | 2.001427061 | 1.00102904 | up | 8.027458796 | 7.026429756 | TAGTGTCCGAAG | hsa_circ_0006817 | chr1 | 150990287 | 150991145 | + | 296 | ANNOTATED, CDS, c | NM_021222 | PRUNE1 | Jeck2013 | 58497 | circRNA | Detected | Detected | 8.027458796 | 7.026429756 |
| hsa_gci153605 | -4.16581929 | -2.058600258 | down | 1.165649773 | 3.224250031 | TCACACTTCTGT | hsa_circ_0006914 | chr18 | 46798547 | 46812986 | - | 488 | ANNOTATED, CDS, c | NM_017653 | DYM | Jeck2013, Salzman | 54808 | circRNA | Not Detected | Detected | 1.165649773 | 3.224250031 |
| hsa_gci153610 | 2.109254264 | 1.076733018 | up | 5.589262102 | 4.512529084 | ATTTAAGAAGAA | hsa_circ_0006921 | chr11 | 103182606 | 103194718 | + | 546 | ANNOTATED, CDS, c | NM_001080463 | DYNC2H1 | Jeck2013, Salzman | 79659 | circRNA | Detected | Detected | 5.589262102 | 4.512529084 |

| | | | | | | | | | | | | | | | | | | | | | | |
|---|---|---|---|---|---|---|---|---|---|---|---|---|---|---|---|---|---|---|---|---|---|---|
| hsa_gci153622 | 2.243710326 | 1.165886429 | up | 9.264404743 | 8.098518314 | GGTAATCTAAAA | hsa_circ_0006946 | chr2 | 101521194 | 101554304 | + | 385 | ANNOTATED, CDS, c | NM_002518 | NPAS2 | Jeck2013, Salzman | 4862 | circRNA | Detected | Detected | 9.264404743 | 8.098518314 |
| hsa_gci153668 | 2.738048549 | 1.453148028 | up | 8.621749774 | 7.168601746 | TGACCTGTAGAA | hsa_circ_0007011 | chr2 | 181846744 | 181848820 | + | 270 | ANNOTATED, CDS, c | NM_006357 | UBE2E3 | Jeck2013, Salzman | 10477 | circRNA | Detected | Detected | 8.621749774 | 7.168601746 |
| hsa_gci153677 | 2.445228882 | 1.289969513 | up | 6.38638219 | 5.096412677 | TCTGTACTCCAT | hsa_circ_0007026 | chr20 | 45891031 | 45905539 | - | 623 | ANNOTATED, CDS, c | NM_183047 | ZMYND8 | Jeck2013, Salzman | 23613 | circRNA | Detected | Detected | 6.38638219 | 5.096412677 |
| hsa_gci153682 | 2.602549369 | 1.379925531 | up | 7.34153243 | 5.961606899 | ATCACAAGAGAC | hsa_circ_0007033 | chr8 | 72964773 | 72974600 | + | 9827 | ALT_DONOR, downst | NR_033652 | | Jeck2013 | | circRNA | Detected | Detected | 7.34153243 | 5.961606899 |
| hsa_gci153694 | 2.900105505 | 1.536105386 | up | 5.303381568 | 3.767276182 | AAAGACACTCCT | hsa_circ_0007055 | chr11 | 10019812 | 10024236 | - | 356 | ANNOTATED, CDS, c | NM_030962 | SBF2 | Jeck2013, Salzman | 81846 | circRNA | Detected | Detected | 5.303381568 | 3.767276182 |
| hsa_gci153700 | 3.307277482 | 1.725644093 | up | 8.798334458 | 7.072690365 | TAGACCCAAACG | hsa_circ_0007074 | chr1 | 54348615 | 54348949 | - | 334 | ALT_DONOR, INTERN | NR_036639 | YIPF1 | Jeck2013 | 54432 | circRNA | Detected | Detected | 8.798334458 | 7.072690365 |
| hsa_gci153711 | 2.385766463 | 1.254452828 | up | 7.076566345 | 5.822113517 | ACCTCCTCTTTA | hsa_circ_0007087 | chr3 | 196528797 | 196530035 | + | 249 | ANNOTATED, CDS, c | NM_002577 | PAK2 | Jeck2013 | 5062 | circRNA | Detected | Detected | 7.076566345 | 5.822113517 |
| hsa_gci153715 | 2.022923145 | 1.01644151 | up | 7.463383902 | 6.446942392 | GGAGTACCACCT | hsa_circ_0007092 | chr2 | 100065797 | 100081447 | - | 360 | ANNOTATED, CDS, c | NM_016316 | REV1 | Jeck2013, Salzman | 51455 | circRNA | Detected | Detected | 7.463383902 | 6.446942392 |
| hsa_gci153734 | 2.822988179 | 1.497223087 | up | 7.391000956 | 5.893777869 | TGTGACAGTGTA | hsa_circ_0007124 | chr3 | 104536316 | 104548557 | - | 12241 | ALT_ACCEPTOR, ALT | TCONS_00006568 | | Jeck2013 | | circRNA | Detected | Detected | 7.391000956 | 5.893777869 |
| hsa_gci153743 | 3.92036176 | 1.970986788 | up | 5.277383265 | 3.306396476 | AGAGGTCCCAGC | hsa_circ_0007138 | chr6 | 128625812 | 128643455 | - | 17643 | ALT_DONOR, CDS, c | NM_001135648 | PTPRK | Jeck2013 | 5796 | circRNA | Detected | Detected | 5.277383265 | 3.306396476 |
| hsa_gci153749 | -3.034321989 | -1.601374186 | down | 1.89150905 | 3.492883236 | CCTAAGTCACCC | hsa_circ_0007144 | chr18 | 8076452 | 8143777 | + | 859 | ANNOTATED, CDS, c | NM_001105244 | PTPRM | Jeck2013, Salzman | 5797 | circRNA | Not Detected | Detected | 1.89150905 | 3.492883236 |
| hsa_gci153812 | 2.04755657 | 1.033903311 | up | 7.515082121 | 6.48117881 | ACGGGACAGGTG | hsa_circ_0007242 | chr10 | 13169744 | 13178897 | + | 523 | ALT_DONOR, CDS, c | NM_001008211 | OPTN | Jeck2013 | 10133 | circRNA | Detected | Detected | 7.515082121 | 6.48117881 |
| hsa_gci153817 | -2.179924394 | -1.124278099 | down | 2.177612056 | 3.301890155 | CGAATAGGTGGG | hsa_circ_0007249 | chr1 | 31810021 | 31821821 | + | 440 | ANNOTATED, CDS, c | NM_016505 | ZCCHC17 | Jeck2013 | 51538 | circRNA | Not Detected | Detected | 2.177612056 | 3.301890155 |
| hsa_gci153819 | 2.495530859 | 1.319346744 | up | 7.724773451 | 6.405426707 | TCTTACACACAA | hsa_circ_0007252 | chr7 | 66532271 | 66548526 | + | 229 | ANNOTATED, CDS, c | NM_018264 | TYW1 | Jeck2013, Salzman | 55253 | circRNA | Detected | Detected | 7.724773451 | 6.405426707 |
| hsa_gci153831 | 2.234197759 | 1.159756891 | up | 6.773437439 | 5.613680548 | AAGAGTTCTAGT | hsa_circ_0007272 | chr17 | 58346810 | 58348842 | - | 240 | ANNOTATED, CDS, c | NM_032582 | USP32 | Jeck2013, Salzman | 84669 | circRNA | Detected | Detected | 6.773437439 | 5.613680548 |
| hsa_gci153850 | 3.197750799 | 1.677057514 | up | 5.993588414 | 4.3165309 | GACAGGTCGTGA | hsa_circ_0007298 | chr9 | 98002930 | 98011651 | - | 423 | ANNOTATED, CDS, c | NM_000136 | FANCC | Jeck2013 | 2176 | circRNA | Detected | Detected | 5.993588414 | 4.3165309 |
| hsa_gci153866 | 2.269012684 | 1.182064674 | up | 6.79302574 | 5.610961066 | GACCCTACTCGT | hsa_circ_0007319 | chr7 | 5097409 | 6771528 | + | 1660819 | ALT_ACCEPTOR, ALT | TCONS_12_00025633 | | Jeck2013 | | circRNA | Detected | Detected | 6.79302574 | 5.610961066 |
| hsa_gci153880 | 2.250538619 | 1.170270322 | up | 9.776377461 | 8.60610714 | GCTCCTCGTCCT | hsa_circ_0007344 | chr2 | 10740978 | 10747437 | - | 396 | ANNOTATED, CDS, c | NM_024894 | NOL10 | Jeck2013 | 79954 | circRNA | Detected | Detected | 9.776377461 | 8.60610714 |
| hsa_gci153899 | 2.552340593 | 1.35182086 | up | 6.706961009 | 5.355140149 | GACTCGTCCCTC | hsa_circ_0007379 | chr14 | 35020919 | 35024118 | - | 3199 | INTERGENIC | None | | Jeck2013 | | circRNA | Detected | Detected | 6.706961009 | 5.355140149 |
| hsa_gci153907 | -8.781420193 | -3.134454281 | down | 1.29192587 | 4.426380151 | ACACCGTATCAA | hsa_circ_0007391 | chr1 | 31742004 | 31764853 | - | 587 | ANNOTATED, CDS, c | NM_004814 | SNRNP40 | Jeck2013, Salzman | 9410 | circRNA | Not Detected | Detected | 1.29192587 | 4.426380151 |
| hsa_gci153923 | -2.150876036 | -1.104924378 | down | 3.284500302 | 4.38942468 | AAACCCTCCTGT | hsa_circ_0007418 | chr1 | 22816372 | 22818026 | + | 900 | ANNOTATED, CDS, c | NM_001083621 | ZBTB40 | Jeck2013, Salzman | 9923 | circRNA | Not Detected | Detected | 3.284500302 | 4.38942468 |
| hsa_gci153925 | 15.92283557 | 3.993025371 | up | 5.174091132 | 1.181065761 | CGTTGTTGGTAA | hsa_circ_0007420 | chr5 | 140788834 | 140790187 | + | 1353 | ALT_ACCEPTOR, CDS | NM_018926 | PCDHGB6 | Jeck2013 | 56100 | circRNA | Detected | Not Detected | 5.174091132 | 1.181065761 |
| hsa_gci153958 | 2.126847021 | 1.088716267 | up | 7.413341418 | 6.324625151 | AAACTGAAAGAC | hsa_circ_0007467 | chr15 | 83858112 | 83861060 | - | 2948 | ALT_ACCEPTOR, ALT | NM_016073 | HDGFL3 | Jeck2013 | 50810 | circRNA | Detected | Detected | 7.413341418 | 6.324625151 |
| hsa_gci153982 | 2.312530475 | 1.209472378 | up | 7.559457563 | 6.349985185 | CGTATGAGTAGA | hsa_circ_0007497 | chr14 | 21840032 | 21841589 | - | 264 | ANNOTATED, CDS, c | NM_007192 | SUPT16H | Jeck2013 | 11198 | circRNA | Detected | Detected | 7.559457563 | 6.349985185 |
| hsa_gci153985 | 2.802694267 | 1.486814376 | up | 6.934504039 | 5.447689663 | AAAGAACACCTC | hsa_circ_0007503 | chr10 | 28872327 | 28884970 | + | 645 | ANNOTATED, INTERN | NR_024557 | WAC | Jeck2013, Salzman | 51322 | circRNA | Detected | Detected | 6.934504039 | 5.447689663 |
| hsa_gci154001 | 2.540018198 | 1.344838833 | up | 7.215764163 | 5.87092533 | TTTCTCTTGACC | hsa_circ_0007528 | chr18 | 59739905 | 59763183 | - | 595 | ANNOTATED, CDS, c | NM_176787 | PIGN | Jeck2013, Salzman | 23556 | circRNA | Detected | Detected | 7.215764163 | 5.87092533 |
| hsa_gci154005 | -3.332261688 | -1.736501702 | down | 3.025894059 | 4.762395762 | AGGTTAGGAGTA | hsa_circ_0007535 | chr18 | 33722243 | 33739978 | + | 1100 | ANNOTATED, CDS, c | NM_001242875 | ELP2 | Jeck2013, Salzman | 55250 | circRNA | Not Detected | Detected | 3.025894059 | 4.762395762 |
| hsa_gci154014 | 2.624433107 | 1.392005826 | up | 4.799815832 | 3.407810007 | ACTGCATTCAAA | hsa_circ_0007548 | chr16 | 74666420 | 74670475 | - | 383 | ANNOTATED, CDS, c | NM_018124 | RFWD3 | Jeck2013, Salzman | 55159 | circRNA | Detected | Detected | 4.799815832 | 3.407810007 |
| hsa_gci154037 | 2.085599176 | 1.060461918 | up | 9.958247416 | 8.897785498 | ACCCTGATCGTT | hsa_circ_0007586 | chr16 | 4029116 | 4033441 | - | 369 | ANNOTATED, CDS, c | NM_001116 | ADCY9 | Jeck2013 | 115 | circRNA | Detected | Detected | 9.958247416 | 8.897785498 |
| hsa_gci154047 | 2.573745239 | 1.363869256 | up | 7.80670035 | 6.442831094 | AAGACTCCCAAC | hsa_circ_0007600 | chr1 | 21377358 | 21386287 | - | 8929 | ALT_ACCEPTOR, cod | NM_001198801 | EIF4G3 | Jeck2013 | 8672 | circRNA | Detected | Detected | 7.80670035 | 6.442831094 |
| hsa_gci154053 | 2.053016285 | 1.037745071 | up | 8.294371159 | 7.256626088 | CCCGTTCACAAC | hsa_circ_0007608 | chr1 | 155340294 | 155340774 | - | 354 | ANNOTATED, CDS, c | NM_018489 | ASH1L | Jeck2013, Salzman | 55870 | circRNA | Detected | Detected | 8.294371159 | 7.256626088 |
| hsa_gci154064 | 2.621956077 | 1.390643518 | up | 8.135664149 | 6.745020631 | TCACTATTCGAC | hsa_circ_0007629 | chr3 | 124692574 | 124696790 | - | 263 | ANNOTATED, CDS, c | NM_020733 | HEG1 | Jeck2013, Salzman | 57493 | circRNA | Detected | Detected | 8.135664149 | 6.745020631 |
| hsa_gci154075 | 2.167985862 | 1.116355349 | up | 7.790411756 | 6.674056407 | GTTGTCTAATTG | hsa_circ_0007643 | chr17 | 1264385 | 1265302 | - | 314 | ANNOTATED, INTERN | NR_024058 | YWHAE | Jeck2013, Salzman | 7531 | circRNA | Detected | Detected | 7.790411756 | 6.674056407 |
| hsa_gci154084 | 3.039424162 | 1.603798022 | up | 4.626320798 | 3.022522776 | GAAAAGAAAGCG | hsa_circ_0007656 | chr14 | 31424825 | 31425448 | - | 178 | ANNOTATED, CDS, c | NM_001083893 | STRN3 | Jeck2013, Salzman | 29966 | circRNA | Detected | Not Detected | 4.626320798 | 3.022522776 |
| hsa_gci154096 | 2.228006255 | 1.155753283 | up | 7.940805659 | 6.785052377 | GGTACCTGTCAG | hsa_circ_0007672 | chr7 | 65705311 | 65706257 | + | 946 | ANNOTATED, CDS, c | NM_003596 | TPST1 | Jeck2013 | 8460 | circRNA | Detected | Detected | 7.940805659 | 6.785052377 |
| hsa_gci154100 | 2.464640478 | 1.301377213 | up | 6.649768131 | 5.348390918 | CTCCGAAGAATC | hsa_circ_0007676 | chr4 | 83867410 | 83900159 | - | 484 | ANNOTATED, CDS, c | NM_194282 | LIN54 | Jeck2013, Salzman | 132660 | circRNA | Detected | Detected | 6.649768131 | 5.348390918 |
| hsa_gci154106 | 2.096652535 | 1.068087792 | up | 4.825465058 | 3.757377266 | CAGAAAAACATA | hsa_circ_0007687 | chr10 | 70411602 | 70427013 | + | 397 | ANNOTATED, CDS, c | NM_030625 | TET1 | Jeck2013 | 80312 | circRNA | Detected | Detected | 4.825465058 | 3.757377266 |
| hsa_gci154123 | 2.136002472 | 1.094913317 | up | 7.166370415 | 6.071457098 | TTAGAGTTGAAG | hsa_circ_0007708 | chr12 | 123262037 | 123265877 | + | 360 | ANNOTATED, CDS, c | NM_201435 | CCDC62 | Jeck2013, Salzman | 84660 | circRNA | Detected | Detected | 7.166370415 | 6.071457098 |
| hsa_gci154147 | 2.794956233 | 1.482825692 | up | 8.976346631 | 7.49352094 | GAACTCCTCCTC | hsa_circ_0007749 | chr18 | 20572710 | 20576425 | + | 1108 | ANNOTATED, CDS, c | NM_002894 | RBBP8 | Jeck2013, Salzman | 5932 | circRNA | Detected | Detected | 8.976346631 | 7.49352094 |
| hsa_gci154151 | 2.350803519 | 1.233153963 | up | 5.845387671 | 4.612233708 | CCACAAAACCCT | hsa_circ_0007755 | chr1 | 145592632 | 145595220 | - | 441 | ALT_DONOR, CDS, c | NM_006468 | POLR3C | Jeck2013 | 10623 | circRNA | Detected | Detected | 5.845387671 | 4.612233708 |
| hsa_gci154158 | 2.700356068 | 1.433149653 | up | 8.238405683 | 6.80525603 | TTACGTAAATGT | hsa_circ_0007767 | chr11 | 77824931 | 77832220 | - | 409 | ANNOTATED, CDS, c | NM_001007027 | ALG8 | Jeck2013, Salzman | 79053 | circRNA | Detected | Detected | 8.238405683 | 6.80525603 |
| hsa_gci154160 | 4.135613866 | 2.04810149 | up | 5.694304546 | 3.646203056 | ATGTCTACACAC | hsa_circ_0007770 | chr16 | 71799391 | 71803602 | - | 182 | ANNOTATED, CDS, c | NM_001030007 | AP1G1 | Jeck2013, Salzman | 164 | circRNA | Detected | Detected | 5.694304546 | 3.646203056 |
| hsa_gci154163 | 2.864434949 | 1.518250575 | up | 5.649759024 | 4.131508449 | GATAGCTTTCAT | hsa_circ_0007774 | chr2 | 109116173 | 110604786 | - | 1397925 | ALT_ACCEPTOR, ALT | NM_022336 | EDAR | Jeck2013 | 10913 | circRNA | Detected | Detected | 5.649759024 | 4.131508449 |
| hsa_gci154172 | 2.082821078 | 1.058538912 | up | 7.213212015 | 6.154673103 | ATCGGAAGAAGT | hsa_circ_0007791 | chr5 | 72347175 | 72348334 | + | 234 | ANNOTATED, CDS, c | NM_138782 | FCHO2 | Jeck2013 | 115548 | circRNA | Detected | Detected | 7.213212015 | 6.154673103 |
| hsa_gci154196 | 2.094403184 | 1.066539196 | up | 5.26932963 | 4.202790435 | GAGAGGTCCTCC | hsa_circ_0007831 | chr17 | 27244302 | 27254081 | - | 886 | ANNOTATED, CDS, c | NM_001033561 | PHF12 | Jeck2013 | 57649 | circRNA | Detected | Detected | 5.26932963 | 4.202790435 |
| hsa_gci154247 | 2.587466458 | 1.371540161 | up | 4.554078141 | 3.18253798 | ACTTCTTCCTTG | hsa_circ_0007898 | chr11 | 17121406 | 17134213 | - | 797 | ANNOTATED, CDS, c | NM_002645 | PIK3C2A | Jeck2013, Salzman | 5286 | circRNA | Detected | Detected | 4.554078141 | 3.18253798 |
| hsa_gci154254 | -8.431644131 | -3.075813977 | down | 1.292356202 | 4.368170179 | CGATTACGGACG | hsa_circ_0007905 | chr1 | 180953812 | 180962561 | - | 391 | ANNOTATED, CDS, c | NM_005819 | STX6 | Jeck2013, Salzman | 10228 | circRNA | Not Detected | Detected | 1.292356202 | 4.368170179 |
| hsa_gci154259 | 2.588044851 | 1.37186262 | up | 6.662842138 | 5.290979518 | ATGTCGTAAAAA | hsa_circ_0007912 | chr3 | 137940767 | 137942575 | + | 313 | ANNOTATED, CDS, c | NM_015396 | ARMC8 | Jeck2013, Salzman | 25852 | circRNA | Detected | Detected | 6.662842138 | 5.290979518 |
| hsa_gci154286 | 2.065498764 | 1.046490197 | up | 8.255808335 | 7.209318138 | GCCTTCTCCCAC | hsa_circ_0007954 | chr8 | 25149539 | 25154164 | + | 285 | ANNOTATED, CDS, c | NM_024940 | DOCK5 | Jeck2013 | 80005 | circRNA | Detected | Detected | 8.255808335 | 7.209318138 |
| hsa_gci154295 | 2.558428542 | 1.355257939 | up | 9.670305433 | 8.315047494 | ACAAATTTTTTC | hsa_circ_0007970 | chr4 | 146435589 | 146436165 | + | 576 | ANNOTATED, CDS, c | NM_005900 | SMAD1 | Jeck2013, Salzman | 4086 | circRNA | Detected | Detected | 9.670305433 | 8.315047494 |
| hsa_gci154343 | 2.302218525 | 1.20302478 | up | 7.5998431 | 6.39681832 | GAGTCCATAAGC | hsa_circ_0008048 | chr3 | 159530546 | 159584070 | + | 27576 | ALT_ACCEPTOR, CDS | NM_001197109 | SCHIP1 | Jeck2013 | 29970 | circRNA | Detected | Detected | 7.5998431 | 6.39681832 |

| | | | | | | | | | | | | | | | | | | | | | | |
|---|---|---|---|---|---|---|---|---|---|---|---|---|---|---|---|---|---|---|---|---|---|---|
| hsa_gci154344 | 2.447104486 | 1.291075702 | up | 8.187389282 | 6.89631358 | AAACGTCGGAGG | hsa_circ_0008050 | chr9 | 123215733 | 123220900 | - | 591 | ANNOTATED, CDS, c | NM_018249 | CDK5RAP2 | Jeck2013 | 55755 | circRNA | Detected | Detected | 8.187389282 | 6.89631358 |
| hsa_gci154349 | 2.53986119 | 1.344749652 | up | 4.891273886 | 3.546524233 | GTTTACCAGATA | hsa_circ_0008058 | chr5 | 109049220 | 109051965 | + | 400 | ANNOTATED, CDS, c | NM_002372 | MAN2A1 | Jeck2013 | 4124 | circRNA | Detected | Detected | 4.891273886 | 3.546524233 |
| hsa_gci154366 | 2.333854477 | 1.222714607 | up | 5.816555038 | 4.593840431 | TAATTCCAGTAG | hsa_circ_0008083 | chr2 | 15601324 | 15618413 | - | 1256 | ANNOTATED, CDS, c | NM_015909 | NBAS | Jeck2013, Salzman | 51594 | circRNA | Detected | Detected | 5.816555038 | 4.593840431 |
| hsa_gci154387 | 2.298800695 | 1.200881391 | up | 6.352459085 | 5.151577694 | TGTAGAGATTTA | hsa_circ_0008122 | chr19 | 23316881 | 23318845 | + | 223 | ANNOTATED, INTERN | TCONS_12_00012420 | | Jeck2013 | | circRNA | Detected | Detected | 6.352459085 | 5.151577694 |
| hsa_gci154390 | 2.665918852 | 1.414632867 | up | 4.728398284 | 3.313765418 | GTTAGCTCTTCG | hsa_circ_0008127 | chr4 | 20359791 | 20365907 | + | 6116 | ALT_ACCEPTOR, ALT | NM_004787 | SLIT2 | Jeck2013 | 9353 | circRNA | Detected | Detected | 4.728398284 | 3.313765418 |
| hsa_gci154406 | 2.69087826 | 1.428077123 | up | 6.200427413 | 4.77235029 | AACTCGTCCCGA | hsa_circ_0008148 | chr10 | 93754291 | 93768032 | + | 614 | ANNOTATED, CDS, c | NM_003972 | BTAF1 | Jeck2013 | 9044 | circRNA | Detected | Detected | 6.200427413 | 4.77235029 |
| hsa_gci154413 | -4.640207876 | -2.214189438 | down | 1.176492778 | 3.390682216 | CGTCTTGCTCCT | hsa_circ_0008157 | chr11 | 118425173 | 118430579 | - | 671 | ANNOTATED, CDS, c | NM_020153 | IFT46 | Jeck2013, Salzman | 56912 | circRNA | Not Detected | Detected | 1.176492778 | 3.390682216 |
| hsa_gci154438 | 2.105954446 | 1.07447423 | up | 5.364852882 | 4.290378652 | CAACTTGGACAA | hsa_circ_0008189 | chr11 | 76224429 | 76239510 | + | 831 | ANNOTATED, CDS, c | NM_020193 | EMSY | Jeck2013 | 56946 | circRNA | Detected | Detected | 5.364852882 | 4.290378652 |
| hsa_gci154451 | 4.520896448 | 2.176608873 | up | 4.234012139 | 2.057403266 | GTGACATAATAC | hsa_circ_0008206 | chr7 | 151927007 | 151935911 | - | 444 | ANNOTATED, CDS, c | NM_170606 | KMT2C | Jeck2013, Salzman | 58508 | circRNA | Detected | Not Detected | 4.234012139 | 2.057403266 |
| hsa_gci154458 | 2.625291839 | 1.392477808 | up | 6.507678676 | 5.115200868 | GTGAGGTCTTGA | hsa_circ_0008217 | chr8 | 103335538 | 103359320 | - | 1398 | ANNOTATED, CDS, c | NM_015902 | UBR5 | Jeck2013 | 51366 | circRNA | Detected | Detected | 6.507678676 | 5.115200868 |
| hsa_gci154468 | -6.937242702 | -2.794362359 | down | 1.291557919 | 4.085920277 | GCCAAGACGAGA | hsa_circ_0008243 | chr4 | 148785996 | 148803083 | + | 548 | ANNOTATED, CDS, c | NM_024605 | ARHGAP10 | Jeck2013 | 79658 | circRNA | Not Detected | Detected | 1.291557919 | 4.085920277 |
| hsa_gci154471 | 3.11726511 | 1.640280855 | up | 7.81010282 | 6.169821965 | CTCTCCCACCGA | hsa_circ_0008246 | chr4 | 6995910 | 7016284 | + | 914 | ANNOTATED, CDS, c | NM_020773 | TBC1D14 | Jeck2013, Salzman | 57533 | circRNA | Detected | Detected | 7.81010282 | 6.169821965 |
| hsa_gci154504 | 2.14565672 | 1.10141928 | up | 4.740996009 | 3.639576729 | AGGAATTCACTC | hsa_circ_0008297 | chrY | 15021270 | 15024974 | + | 434 | ANNOTATED, CDS, c | NM_001122665 | DDX3Y | Jeck2013, Salzman | 8653 | circRNA | Detected | Detected | 4.740996009 | 3.639576729 |
| hsa_gci154509 | 2.105072476 | 1.073869905 | up | 6.115029712 | 5.041159807 | TGAGAATCCTTT | hsa_circ_0008305 | chr8 | 141799572 | 141840625 | - | 584 | ANNOTATED, CDS, c | NM_001199649 | PTK2 | Jeck2013, Salzman | 5747 | circRNA | Detected | Detected | 6.115029712 | 5.041159807 |
| hsa_gci154518 | 2.071451494 | 1.050642038 | up | 7.065668371 | 6.015026334 | GATATACTCTTG | hsa_circ_0008316 | chr8 | 95547066 | 95556170 | - | 421 | ANNOTATED, CDS, c | NM_015496 | VIRMA | Jeck2013, Salzman | 25962 | circRNA | Detected | Detected | 7.065668371 | 6.015026334 |
| hsa_gci154524 | 2.204199154 | 1.14025458 | up | 7.774599728 | 6.634345148 | AAGAGTATGAAA | hsa_circ_0008323 | chr22 | 31947382 | 31971378 | + | 6085 | ALT_ACCEPTOR, CDS | NM_001007467 | SFI1 | Jeck2013 | 9814 | circRNA | Detected | Detected | 7.774599728 | 6.634345148 |
| hsa_gci154533 | 2.111771388 | 1.078453663 | up | 4.040306489 | 2.961852826 | AGACAAGTTTCG | hsa_circ_0008341 | chr4 | 83748521 | 83750211 | - | 323 | ANNOTATED, CDS, c | NM_014933 | SEC31A | Jeck2013 | 22872 | circRNA | Detected | Not Detected | 4.040306489 | 2.961852826 |
| hsa_gci154534 | 2.151195937 | 1.105138935 | up | 5.244523221 | 4.139384285 | GGTCACTCTTAG | hsa_circ_0008342 | chr11 | 77330650 | 77340944 | - | 611 | ANNOTATED, CDS, c | NM_001293 | CLNS1A | Jeck2013, Salzman | 1207 | circRNA | Detected | Detected | 5.244523221 | 4.139384285 |
| hsa_gci154559 | 7.3391115 | 2.875605416 | up | 4.130912732 | 1.255307316 | GACCTTCTTGAA | hsa_circ_0008384 | chr1 | 71530651 | 71535045 | - | 490 | ALT_DONOR, CDS, c | NM_005455 | ZRANB2 | Jeck2013 | 9406 | circRNA | Detected | Not Detected | 4.130912732 | 1.255307316 |
| hsa_gci154560 | 2.177938708 | 1.122963354 | up | 4.649953992 | 3.526990638 | TATTTACCATAT | hsa_circ_0008385 | chr8 | 37971709 | 37993284 | + | 1134 | ANNOTATED, CDS, c | NM_001105214 | ASH2L | Jeck2013 | 9070 | circRNA | Detected | Detected | 4.649953992 | 3.526990638 |
| hsa_gci154563 | 2.553532007 | 1.352494142 | up | 7.900624609 | 6.548130466 | GTCGGAGGTACA | hsa_circ_0008393 | chr10 | 101728871 | 101731891 | - | 278 | ANNOTATED, CDS, c | NM_015221 | DNMBP | Jeck2013 | 23268 | circRNA | Detected | Detected | 7.900624609 | 6.548130466 |
| hsa_gci154578 | 2.383831105 | 1.253282024 | up | 6.94735803 | 5.694076006 | TCGTAGAAAGTG | hsa_circ_0008419 | chr7 | 133002037 | 133059756 | + | 526 | ANNOTATED, CDS, c | NM_021807 | EXOC4 | Jeck2013, Salzman | 60412 | circRNA | Detected | Detected | 6.94735803 | 5.694076006 |
| hsa_gci154604 | 2.246745918 | 1.167836981 | up | 5.473119886 | 4.305282904 | TCTTCAATACAA | hsa_circ_0008457 | chr11 | 47521004 | 47522484 | - | 224 | ANNOTATED, CDS, c | NM_001172639 | CELF1 | Jeck2013, Salzman | 10658 | circRNA | Detected | Detected | 5.473119886 | 4.305282904 |
| hsa_gci154628 | 2.287791871 | 1.193955811 | up | 7.65031302 | 6.456357209 | TAAAAGGTGAAA | hsa_circ_0008496 | chr8 | 101721360 | 101725409 | - | 693 | ANNOTATED, CDS, c | NM_002568 | PABPC1 | Jeck2013, Salzman | 26986 | circRNA | Detected | Detected | 7.65031302 | 6.456357209 |
| hsa_gci154632 | -4.939561032 | -2.304382838 | down | 1.284714689 | 3.589097527 | ACGTTGTCCGGA | hsa_circ_0008501 | chr1 | 8601272 | 8674745 | - | 434 | ANNOTATED, CDS, c | NM_012102 | RERE | Jeck2013, Salzman | 473 | circRNA | Not Detected | Detected | 1.284714689 | 3.589097527 |
| hsa_gci154639 | 2.749435681 | 1.459135537 | up | 5.637063827 | 4.17792829 | ACCGGGTCAGTC | hsa_circ_0008509 | chr12 | 78334098 | 78415642 | + | 1780 | ANNOTATED, CDS, c | NM_014903 | NAV3 | Jeck2013, Salzman | 89795 | circRNA | Detected | Detected | 5.637063827 | 4.17792829 |
| hsa_gci154656 | 4.850035479 | 2.277995301 | up | 4.486960978 | 2.208965677 | CATAATATAGTC | hsa_circ_0008540 | chr7 | 102944288 | 102944937 | + | 279 | ANNOTATED, CDS, c | NM_004279 | PMPCB | Jeck2013, Salzman | 9512 | circRNA | Detected | Not Detected | 4.486960978 | 2.208965677 |
| hsa_gci154674 | 3.39410483 | 1.763031124 | up | 7.7650062 | 6.001975076 | CCGTCCGGTAAG | hsa_circ_0008571 | chr9 | 5021962 | 5055788 | + | 1081 | ANNOTATED, CDS, c | NM_004972 | JAK2 | Jeck2013 | 3717 | circRNA | Detected | Detected | 7.7650062 | 6.001975076 |
| hsa_gci154728 | 5.398400688 | 2.432532063 | up | 4.465096658 | 2.032564595 | TTTCGTAAGATT | hsa_circ_0008650 | chr1 | 246350044 | 246357265 | - | 7221 | ALT_ACCEPTOR, ALT | NM_001167740 | SMYD3 | Jeck2013 | 64754 | circRNA | Detected | Not Detected | 4.465096658 | 2.032564595 |
| hsa_gci154748 | 2.607169471 | 1.382484364 | up | 4.607397583 | 3.224913219 | TTCTACTTAGCC | hsa_circ_0008676 | chr4 | 72205086 | 72222904 | + | 477 | ANNOTATED, CDS, c | NM_001098484 | SLC4A4 | Jeck2013 | 8671 | circRNA | Detected | Not Detected | 4.607397583 | 3.224913219 |
| hsa_gci154762 | 2.484233087 | 1.312800543 | up | 6.003555217 | 4.690754674 | AGTAAAAGATAG | hsa_circ_0008698 | chr9 | 36352738 | 36353328 | - | 269 | ANNOTATED, CDS, c | NM_194328 | RNF38 | Jeck2013, Salzman | 152006 | circRNA | Detected | Detected | 6.003555217 | 4.690754674 |
| hsa_gci154766 | -4.219831038 | -2.077185235 | down | 1.886901122 | 3.964086356 | AATTATTTCTGA | hsa_circ_0008707 | chr20 | 47708582 | 47711500 | + | 461 | ANNOTATED, CDS, c | NM_001316 | CSE1L | Jeck2013, Salzman | 1434 | circRNA | Not Detected | Detected | 1.886901122 | 3.964086356 |
| hsa_gci154772 | 3.036773456 | 1.602539287 | up | 5.715043172 | 4.112503885 | GAGTTTTGTTTG | hsa_circ_0008717 | chr1 | 229665945 | 229678118 | - | 724 | ANNOTATED, CDS, c | NM_012089 | ABCB10 | Jeck2013 | 23456 | circRNA | Detected | Detected | 5.715043172 | 4.112503885 |
| hsa_gci154806 | 4.92350562 | 2.299685905 | up | 5.088154332 | 2.788468428 | TTCTACGTTCCC | hsa_circ_0008778 | chr8 | 141874410 | 141935848 | - | 571 | ANNOTATED, CDS, c | NM_001199649 | PTK2 | Jeck2013 | 5747 | circRNA | Detected | Not Detected | 5.088154332 | 2.788468428 |
| hsa_gci154808 | 2.240916589 | 1.16408895 | up | 4.992158526 | 3.828069576 | GATGAGTCTGGA | hsa_circ_0008782 | chr9 | 86294689 | 86294952 | - | 263 | ANNOTATED, CDS, c | NM_013438 | UBQLN1 | Jeck2013 | 29979 | circRNA | Detected | Detected | 4.992158526 | 3.828069576 |
| hsa_gci154823 | 2.969708445 | 1.570321299 | up | 4.845182554 | 3.274861255 | TACCACCTATGG | hsa_circ_0008802 | chr12 | 51402258 | 51404549 | - | 221 | ANNOTATED, CDS, c | NM_001174129 | SLC11A2 | Jeck2013, Salzman | 4891 | circRNA | Detected | Detected | 4.845182554 | 3.274861255 |
| hsa_gci154839 | 2.121459858 | 1.08505738 | up | 5.658282766 | 4.573225385 | ACAAAACAAGCG | hsa_circ_0008828 | chr4 | 47905209 | 47907363 | - | 241 | ANNOTATED, CDS, c | NM_152995 | NFXL1 | Jeck2013, Salzman | 152518 | circRNA | Detected | Detected | 5.658282766 | 4.573225385 |
| hsa_gci154845 | 2.2186742 | 1.149697831 | up | 9.801960432 | 8.652262601 | GACAGACCAACT | hsa_circ_0008837 | chr10 | 882342 | 890995 | - | 320 | ANNOTATED, CDS, c | NM_015155 | LARP4B | Jeck2013 | 23185 | circRNA | Detected | Detected | 9.801960432 | 8.652262601 |
| hsa_gci154860 | 2.296601949 | 1.199500828 | up | 6.996993515 | 5.797492686 | TATCCGTCCCCA | hsa_circ_0008864 | chr4 | 20568884 | 20591360 | + | 457 | ANNOTATED, CDS, c | NM_004787 | SLIT2 | Jeck2013 | 9353 | circRNA | Detected | Detected | 6.996993515 | 5.797492686 |
| hsa_gci154869 | 2.657358079 | 1.409992644 | up | 6.377558606 | 4.967565962 | TGATTCCGTCTG | hsa_circ_0008877 | chr12 | 78360008 | 78415642 | + | 1609 | ANNOTATED, CDS, c | NM_014903 | NAV3 | Jeck2013, Salzman | 89795 | circRNA | Detected | Detected | 6.377558606 | 4.967565962 |
| hsa_gci154894 | 2.2179163 | 1.149204922 | up | 11.10037645 | 9.951171526 | CGTGAAGAACTT | hsa_circ_0008915 | chr10 | 126631025 | 126655350 | + | 1039 | ALT_ACCEPTOR, CDS | NM_017580 | ZRANB1 | Jeck2013, Salzman | 54764 | circRNA | Detected | Detected | 11.10037645 | 9.951171526 |
| hsa_gci154907 | 3.136841133 | 1.649312465 | up | 6.357616148 | 4.708303683 | AAACATTGCCCT | hsa_circ_0008933 | chr8 | 37330595 | 37333530 | - | 304 | ANNOTATED, INTERN | TCONS_00014973 | | Jeck2013 | | circRNA | Detected | Detected | 6.357616148 | 4.708303683 |
| hsa_gci154925 | 2.332839309 | 1.222086935 | up | 4.046927165 | 2.824840231 | AGTCGAATTTAC | hsa_circ_0008961 | chr2 | 191520702 | 191537878 | + | 1201 | ANNOTATED, CDS, c | NM_005966 | NAB1 | Jeck2013 | 4664 | circRNA | Detected | Not Detected | 4.046927165 | 2.824840231 |
| hsa_gci154939 | 2.752403344 | 1.460691902 | up | 8.954943299 | 7.494251397 | AAGGGTTACCCG | hsa_circ_0008980 | chr20 | 45021731 | 45023142 | - | 264 | ANNOTATED, CDS, c | NM_182764 | ELMO2 | Jeck2013, Salzman | 63916 | circRNA | Detected | Detected | 8.954943299 | 7.494251397 |
| hsa_gci154948 | 2.635733009 | 1.398204238 | up | 4.062136623 | 2.663932385 | AGTCAAAAGGAC | hsa_circ_0008996 | chr17 | 11984672 | 11999011 | + | 295 | ANNOTATED, CDS, c | NM_003010 | MAP2K4 | Jeck2013, Salzman | 6416 | circRNA | Detected | Not Detected | 4.062136623 | 2.663932385 |
| hsa_gci154952 | 2.048669608 | 1.034687338 | up | 6.284916633 | 5.250229295 | CCACTCCCACTA | hsa_circ_0009001 | chr3 | 104536316 | 104574295 | - | 37979 | ALT_ACCEPTOR, ALT | TCONS_00006568 | | Jeck2013 | | circRNA | Detected | Detected | 6.284916633 | 5.250229295 |
| hsa_gci154965 | 2.350870202 | 1.233194886 | up | 4.291392362 | 3.058197476 | GCAGTTGTAAAG | hsa_circ_0009022 | chr18 | 9583114 | 9595100 | - | 815 | ANNOTATED, CDS, c | NM_005134 | PPP4R1 | Jeck2013, Salzman | 9989 | circRNA | Detected | Not Detected | 4.291392362 | 3.058197476 |
| hsa_gci154984 | 2.156991417 | 1.109020436 | up | 7.051527653 | 5.942507217 | ACGGGTGTCCCT | hsa_circ_0009051 | chr14 | 55462320 | 55467710 | - | 460 | ANNOTATED, CDS, c | NM_007086 | WDHD1 | Jeck2013, Salzman | 11169 | circRNA | Detected | Detected | 7.051527653 | 5.942507217 |
| hsa_gci155019 | 2.177617808 | 1.12275077 | up | 6.310927343 | 5.188176573 | GAAAACCGTTTA | hsa_circ_0009102 | chr8 | 141799572 | 141874498 | - | 815 | ANNOTATED, CDS, c | NM_001199649 | PTK2 | Jeck2013, Salzman | 5747 | circRNA | Detected | Detected | 6.310927343 | 5.188176573 |
| hsa_gci155026 | 3.049846324 | 1.60873655 | up | 6.338974735 | 4.730238186 | GAAGTTCATTAA | hsa_circ_0009112 | chr7 | 91972337 | 91981956 | + | 610 | ANNOTATED, CDS, c | NM_019004 | ANKIB1 | Jeck2013 | 54467 | circRNA | Detected | Detected | 6.338974735 | 4.730238186 |
| hsa_gci155062 | 2.59396582 | 1.37515947 | up | 6.804766967 | 5.429607497 | TCTCGTATTATC | hsa_circ_0009169 | chr3 | 186988982 | 187003844 | - | 14862 | ALT_DONOR, CDS, c | NM_001879 | MASP1 | Jeck2013 | 5648 | circRNA | Detected | Detected | 6.804766967 | 5.429607497 |

| | | | | | | | | | | | | | | | | | | | | | | | |
|---|---|---|---|---|---|---|---|---|---|---|---|---|---|---|---|---|---|---|---|---|---|---|---|
| hsa_gci155064 | 3.70018341 | 1.887596784 | up | 6.258513579 | 4.370916795 | CGATGGTGAAAG | hsa_circ_0009172 | chr10 | 70218860 | 70229920 | - | 645 | ANNOTATED, CDS, c | NM_001080449 | DNA2 | Jeck2013, Salzman | 1763 | circRNA | Detected | Detected | 6.258513579 | 4.370916795 |
| hsa_gci155080 | 2.58305106 | 1.369076162 | up | 7.220212364 | 5.851136202 | ACTCGTCGAAAA | hsa_circ_0009303 | chr1 | 1599765 | 1601590 | - | 273 | ANNOTATED, CDS, c | NM_001110781 | SLC35E2B | Salzman2013 | 728661 | circRNA | Detected | Detected | 7.220212364 | 5.851136202 |
| hsa_gci155088 | -2.570152318 | -1.361853862 | down | 2.079333213 | 3.441187075 | AAGTCTTCCCGA | hsa_circ_0009337 | chr1 | 1718760 | 1770677 | - | 1127 | ANNOTATED, CDS, c | NM_002074 | GNB1 | Salzman2013 | 2782 | circRNA | Not Detected | Detected | 2.079333213 | 3.441187075 |
| hsa_gci155091 | 4.619915457 | 2.207866451 | up | 7.625139759 | 5.417273308 | TTATGTACATTT | hsa_circ_0009361 | chr1 | 1749275 | 1770677 | - | 191 | ANNOTATED, CDS, c | NM_002074 | GNB1 | Salzman2013 | 2782 | circRNA | Detected | Detected | 7.625139759 | 5.417273308 |
| hsa_gci155092 | -3.708973833 | -1.89102009 | down | 2.852183213 | 4.743203303 | AGACCAGACCAG | hsa_circ_0009362 | chr1 | 1756835 | 1770677 | - | 152 | ANNOTATED, CDS, c | NM_002074 | GNB1 | Salzman2013 | 2782 | circRNA | Not Detected | Detected | 2.852183213 | 4.743203303 |
| hsa_gci155100 | 2.577927015 | 1.366211419 | up | 4.01312826 | 2.646916841 | CGAGTCGACCAA | hsa_circ_0009423 | chr1 | 3696783 | 3703874 | - | 2005 | ANNOTATED, CDS, c | NM_020710 | LRRC47 | Salzman2013 | 57470 | circRNA | Detected | Not Detected | 4.01312826 | 2.646916841 |
| hsa_gci155101 | 4.413163615 | 2.141813235 | up | 7.795917478 | 5.654104243 | GACGACCGTCCG | hsa_circ_0009424 | chr1 | 3696783 | 3713068 | - | 2648 | ANNOTATED, CDS, c | NM_020710 | LRRC47 | Salzman2013 | 57470 | circRNA | Detected | Detected | 7.795917478 | 5.654104243 |
| hsa_gci155107 | 2.679922492 | 1.422191276 | up | 4.885200196 | 3.46300892 | GTAGTCTCACCC | hsa_circ_0009515 | chr1 | 6581406 | 6589231 | - | 4969 | ANNOTATED, CDS, c | NM_024654 | NOL9 | Salzman2013 | 79707 | circRNA | Detected | Detected | 4.885200196 | 3.46300892 |
| hsa_gci155111 | 2.342350174 | 1.22795677 | up | 6.530414742 | 5.302457972 | TCTAGTGTAAGG | hsa_circ_0009528 | chr1 | 6592027 | 6601987 | - | 670 | ANNOTATED, CDS, c | NM_024654 | NOL9 | Salzman2013 | 79707 | circRNA | Detected | Detected | 6.530414742 | 5.302457972 |
| hsa_gci155124 | 2.377640103 | 1.249530355 | up | 7.434788266 | 6.185257912 | CCACTTGCCCCG | hsa_circ_0009559 | chr1 | 8412463 | 8416306 | - | 3045 | ANNOTATED, CDS, c | NM_012102 | RERE | Salzman2013 | 473 | circRNA | Detected | Detected | 7.434788266 | 6.185257912 |
| hsa_gci155127 | 2.142437121 | 1.099252863 | up | 9.895939154 | 8.796686291 | CGTTTATACAGT | hsa_circ_0009571 | chr1 | 8425871 | 8601377 | - | 722 | ANNOTATED, CDS, c | NM_012102 | RERE | Salzman2013 | 473 | circRNA | Detected | Detected | 9.895939154 | 8.796686291 |
| hsa_gci155148 | 3.274163796 | 1.711126497 | up | 7.062508106 | 5.351381609 | TTATCAAAATAA | hsa_circ_0009627 | chr1 | 8927175 | 8930569 | - | 263 | ANNOTATED, CDS, c | NM_001428 | ENO1 | Salzman2013 | 2023 | circRNA | Detected | Detected | 7.062508106 | 5.351381609 |
| hsa_gci155157 | 2.100574298 | 1.070783815 | up | 5.957458799 | 4.886674984 | AGAAACCGTCGT | hsa_circ_0009693 | chr1 | 10186851 | 10186935 | + | 84 | ANNOTATED, CDS, c | NM_001105562 | UBE4B | Salzman2013 | 10277 | circRNA | Detected | Detected | 5.957458799 | 4.886674984 |
| hsa_gci155159 | 2.028473529 | 1.020394476 | up | 6.169821965 | 5.149427489 | ATTTCCGTCACA | hsa_circ_0009700 | chr1 | 10209241 | 10211619 | + | 335 | ANNOTATED, CDS, c | NM_001105562 | UBE4B | Salzman2013 | 10277 | circRNA | Detected | Detected | 6.169821965 | 5.149427489 |
| hsa_gci155160 | 2.800057494 | 1.48545645 | up | 4.626916906 | 3.141460456 | CTTAAATTTCCG | hsa_circ_0009701 | chr1 | 10209241 | 10221344 | + | 607 | ANNOTATED, CDS, c | NM_001105562 | UBE4B | Salzman2013 | 10277 | circRNA | Detected | Not Detected | 4.626916906 | 3.141460456 |
| hsa_gci155162 | 2.027876236 | 1.019969605 | up | 5.447193084 | 4.427223479 | CAGGTTAACCTC | hsa_circ_0009721 | chr1 | 10431198 | 10435431 | + | 584 | ANNOTATED, CDS, c | NM_015074 | KIF1B | Salzman2013 | 23095 | circRNA | Detected | Detected | 5.447193084 | 4.427223479 |
| hsa_gci155188 | 2.007108725 | 1.00511877 | up | 11.33941088 | 10.33429211 | GGAATAGCCGTC | hsa_circ_0009946 | chr1 | 12262023 | 12269277 | + | 2686 | ANNOTATED, CDS, c | NM_001066 | TNFRSF1B | Salzman2013 | 7133 | circRNA | Detected | Detected | 11.33941088 | 10.33429211 |
| hsa_gci155191 | 3.693916173 | 1.885151127 | up | 5.729058835 | 3.843907707 | AAGCTCCTTCTC | hsa_circ_0009962 | chr1 | 12326937 | 12395884 | + | 6957 | ANNOTATED, CDS, c | NM_015378 | VPS13D | Salzman2013 | 55187 | circRNA | Detected | Detected | 5.729058835 | 3.843907707 |
| hsa_gci155195 | 2.583309754 | 1.369220641 | up | 5.739326864 | 4.370106222 | TATCCTCTCAGA | hsa_circ_0009995 | chr1 | 12382592 | 12383837 | + | 286 | ANNOTATED, CDS, c | NM_015378 | VPS13D | Salzman2013 | 55187 | circRNA | Detected | Detected | 5.739326864 | 4.370106222 |
| hsa_gci155197 | 2.153690945 | 1.106811238 | up | 7.619450513 | 6.512639275 | CGCGAACCCCCA | hsa_circ_0009999 | chr1 | 12401836 | 12445432 | + | 2858 | ANNOTATED, CDS, c | NM_015378 | VPS13D | Salzman2013 | 55187 | circRNA | Detected | Detected | 7.619450513 | 6.512639275 |
| hsa_gci155216 | 2.848670452 | 1.510288733 | up | 6.841634253 | 5.33134552 | AACTCCCGGTCC | hsa_circ_0010071 | chr1 | 16044387 | 16061264 | + | 3618 | ANNOTATED, CDS, c | NM_015164 | PLEKHM2 | Salzman2013 | 23207 | circRNA | Detected | Detected | 6.841634253 | 5.33134552 |
| hsa_gci155224 | 2.034578401 | 1.024729874 | up | 7.259736943 | 6.235007069 | CAGGGAGGCGAC | hsa_circ_0010099 | chr1 | 16095022 | 16113084 | + | 2612 | ANNOTATED, CDS, c | NM_017556 | FBLIM1 | Salzman2013 | 54751 | circRNA | Detected | Detected | 7.259736943 | 6.235007069 |
| hsa_gci155226 | 2.684222244 | 1.424504126 | up | 4.71516825 | 3.290664124 | CCGAGCTGTCTG | hsa_circ_0010103 | chr1 | 16111042 | 16113084 | + | 2042 | ANNOTATED, CDS, c | NM_017556 | FBLIM1 | Salzman2013 | 54751 | circRNA | Detected | Detected | 4.71516825 | 3.290664124 |
| hsa_gci155240 | 2.302405517 | 1.203141954 | up | 8.896345334 | 7.69320338 | TGTCATCTTAGA | hsa_circ_0010353 | chr1 | 19495928 | 19501527 | - | 968 | ANNOTATED, CDS, c | NM_020765 | UBR4 | Salzman2013 | 23352 | circRNA | Detected | Detected | 8.896345334 | 7.69320338 |
| hsa_gci155255 | 2.779219833 | 1.474679955 | up | 5.579615972 | 4.104936017 | TAGTCCGAAGGT | hsa_circ_0010440 | chr1 | 21076215 | 21076375 | - | 160 | ANNOTATED, CDS, c | NM_016287 | HP1BP3 | Salzman2013 | 50809 | circRNA | Detected | Detected | 5.579615972 | 4.104936017 |
| hsa_gci155264 | 2.113224021 | 1.079445714 | up | 7.195075212 | 6.115629498 | GAAGTCAAAGGA | hsa_circ_0010475 | chr1 | 21267983 | 21437876 | - | 1860 | ANNOTATED, CDS, c | NM_001198803 | EIF4G3 | Salzman2013 | 8672 | circRNA | Detected | Detected | 7.195075212 | 6.115629498 |
| hsa_gci155297 | 2.089039305 | 1.062839637 | up | 4.990917662 | 3.928078025 | CGGAAGGTCGGT | hsa_circ_0010861 | chr1 | 23755055 | 23768223 | - | 3334 | ANNOTATED, CDS, c | NM_017707 | ASAP3 | Salzman2013 | 55616 | circRNA | Detected | Detected | 4.990917662 | 3.928078025 |
| hsa_gci155298 | 2.281791967 | 1.190167266 | up | 5.388991491 | 4.198824225 | GAAGGCCGGCTC | hsa_circ_0010876 | chr1 | 24018268 | 24022915 | + | 634 | ANNOTATED, CDS, c | NM_000975 | RPL11 | Salzman2013 | 6135 | circRNA | Detected | Detected | 5.388991491 | 4.198824225 |
| hsa_gci155322 | 6.392062121 | 2.676281429 | up | 7.149878325 | 4.473596896 | AGAAAACCAAAT | hsa_circ_0010988 | chr1 | 26644410 | 26647014 | + | 505 | ANNOTATED, CDS, c | NM_001803 | CD52 | Salzman2013 | 1043 | circRNA | Detected | Detected | 7.149878325 | 4.473596896 |
| hsa_gci155325 | 2.063436913 | 1.045049331 | up | 6.542580137 | 5.497530806 | TCGAACGCTCGG | hsa_circ_0011023 | chr1 | 27158937 | 27182211 | + | 2709 | ANNOTATED, CDS, c | NM_032283 | ZDHHC18 | Salzman2013 | 84243 | circRNA | Detected | Detected | 6.542580137 | 5.497530806 |
| hsa_gci155328 | 2.165767028 | 1.11487806 | up | 6.696080149 | 5.581202089 | CTTCCTTCGACT | hsa_circ_0011035 | chr1 | 27250579 | 27272887 | + | 1130 | ANNOTATED, CDS, c | NM_006600 | NUDC | Salzman2013 | 10726 | circRNA | Detected | Detected | 6.696080149 | 5.581202089 |
| hsa_gci155340 | 2.133150109 | 1.092985491 | up | 9.336864732 | 8.243879241 | TTACAAGACTTA | hsa_circ_0011074 | chr1 | 27739065 | 27755443 | - | 867 | ANNOTATED, CDS, c | NM_006990 | WASF2 | Salzman2013 | 10163 | circRNA | Detected | Detected | 9.336864732 | 8.243879241 |
| hsa_gci155342 | 2.283992153 | 1.191557694 | up | 5.374674647 | 4.183116953 | GGCCGCTGACGC | hsa_circ_0011081 | chr1 | 27992571 | 27994886 | - | 565 | ANNOTATED, CDS, c | NM_022873 | IFI6 | Salzman2013 | 2537 | circRNA | Detected | Detected | 5.374674647 | 4.183116953 |
| hsa_gci155355 | 2.330808258 | 1.220830327 | up | 5.296613336 | 4.075783008 | GCGGGGCCAGCT | hsa_circ_0011126 | chr1 | 28562871 | 28564616 | + | 1745 | ANNOTATED, CDS, c | NM_178191 | ATP5IF1 | Salzman2013 | 93974 | circRNA | Detected | Detected | 5.296613336 | 4.075783008 |
| hsa_gci155392 | 2.069927019 | 1.049579903 | up | 6.065723856 | 5.016143954 | CCTCTCACTTCT | hsa_circ_0011286 | chr1 | 31899500 | 31907527 | + | 1239 | ANNOTATED, CDS, c | NM_001199039 | SERINC2 | Salzman2013 | 347735 | circRNA | Detected | Detected | 6.065723856 | 5.016143954 |
| hsa_gci155399 | 2.071675748 | 1.050798215 | up | 5.374344462 | 4.323546247 | CGTGCCGAGGAA | hsa_circ_0011325 | chr1 | 32256024 | 32262316 | - | 1685 | ANNOTATED, CDS, c | NM_144569 | SPOCD1 | Salzman2013 | 90853 | circRNA | Detected | Detected | 5.374344462 | 4.323546247 |
| hsa_gci155428 | 8.384663562 | 3.067752896 | up | 4.455433145 | 1.387680249 | GAGGCGCGGTCT | hsa_circ_0011413 | chr1 | 32799439 | 32801834 | - | 1546 | ANNOTATED, CDS, c | NM_023009 | MARCKSL1 | Salzman2013 | 65108 | circRNA | Detected | Not Detected | 4.455433145 | 1.387680249 |
| hsa_gci155432 | -3.176881332 | -1.667611201 | down | 2.090558237 | 3.758169438 | AGGGGTCCTGTT | hsa_circ_0011429 | chr1 | 33123027 | 33135164 | + | 802 | ANNOTATED, CDS, c | NM_005610 | RBBP4 | Salzman2013 | 5928 | circRNA | Not Detected | Detected | 2.090558237 | 3.758169438 |
| hsa_gci155438 | 3.315827988 | 1.729369167 | up | 7.881467129 | 6.152097962 | CCTGGGCAAAAC | hsa_circ_0011439 | chr1 | 33240839 | 33256855 | - | 1738 | ANNOTATED, CDS, c | NM_003680 | YARS | Salzman2013 | 8565 | circRNA | Detected | Detected | 7.881467129 | 6.152097962 |
| hsa_gci155439 | 2.136104854 | 1.094982465 | up | 6.429266294 | 5.334283829 | GCTCAGTCGGCC | hsa_circ_0011449 | chr1 | 33283175 | 33324476 | + | 4298 | ANNOTATED, CDS, c | NM_022753 | S100PBP | Salzman2013 | 64766 | circRNA | Detected | Detected | 6.429266294 | 5.334283829 |
| hsa_gci155443 | 2.496482905 | 1.319897028 | up | 6.529205559 | 5.209308531 | AAGTTTGAAGTC | hsa_circ_0011461 | chr1 | 33476825 | 33497262 | - | 2701 | ANNOTATED, ncRNA, | NR_037592 | AK2 | Salzman2013 | 204 | circRNA | Detected | Detected | 6.529205559 | 5.209308531 |
| hsa_gci155445 | 2.807389228 | 1.489229099 | up | 8.01461312 | 6.525384021 | GACCCACTGTCT | hsa_circ_0011519 | chr1 | 35558920 | 35563167 | + | 1087 | ALT_ACCEPTOR, CDS | NM_024772 | ZMYM1 | Salzman2013 | 79830 | circRNA | Detected | Detected | 8.01461312 | 6.525384021 |
| hsa_gci155465 | 2.340219272 | 1.226643713 | up | 6.168079927 | 4.941436214 | AGAAAAGTGAGT | hsa_circ_0011581 | chr1 | 36096874 | 36102033 | - | 194 | ANNOTATED, CDS, c | NM_002794 | PSMB2 | Salzman2013 | 5690 | circRNA | Detected | Detected | 6.168079927 | 4.941436214 |
| hsa_gci155485 | 2.414152702 | 1.271516933 | up | 8.393151615 | 7.121634681 | CTCATGCGTCCG | hsa_circ_0011715 | chr1 | 38171112 | 38175391 | + | 1612 | ANNOTATED, CDS, c | NM_018101 | CDCA8 | Salzman2013 | 55143 | circRNA | Detected | Detected | 8.393151615 | 7.121634681 |
| hsa_gci155514 | 7.979164691 | 2.996237724 | up | 6.121938425 | 3.125700701 | ACTCCACCGTGC | hsa_circ_0011821 | chr1 | 39816456 | 39824936 | + | 1279 | ANNOTATED, CDS, c | NM_012090 | MACF1 | Salzman2013 | 23499 | circRNA | Detected | Not Detected | 6.121938425 | 3.125700701 |
| hsa_gci155518 | 2.601066237 | 1.379103139 | up | 9.116255608 | 7.737152469 | GGAGAAGCGGTG | hsa_circ_0011873 | chr1 | 40506254 | 40538321 | + | 2773 | ANNOTATED, CDS, c | NM_006367 | CAP1 | Salzman2013 | 10487 | circRNA | Detected | Detected | 9.116255608 | 7.737152469 |
| hsa_gci155532 | 2.129643234 | 1.090611765 | up | 4.159936558 | 3.069324793 | AGATACGATATA | hsa_circ_0011955 | chr1 | 42664772 | 42671534 | - | 231 | ANNOTATED, CDS, c | NM_001198851 | FOXJ3 | Salzman2013 | 22887 | circRNA | Detected | Not Detected | 4.159936558 | 3.069324793 |
| hsa_gci155536 | 2.195892308 | 1.134807303 | up | 6.531967315 | 5.397160012 | AATAAGAAGAAA | hsa_circ_0011982 | chr1 | 43161869 | 43162615 | + | 393 | ANNOTATED, CDS, c | NM_004559 | YBX1 | Salzman2013 | 4904 | circRNA | Detected | Detected | 6.531967315 | 5.397160012 |
| hsa_gci155579 | 2.507606651 | 1.326311061 | up | 5.828037118 | 4.501726057 | GTAGGGTGACGG | hsa_circ_0012340 | chr1 | 47023089 | 47030808 | - | 1883 | ANNOTATED, CDS, c | NM_003684 | MKNK1 | Salzman2013 | 8569 | circRNA | Detected | Detected | 5.828037118 | 4.501726057 |
| hsa_gci155611 | 2.383779268 | 1.253250652 | up | 5.345923286 | 4.092672635 | TTTACTAAGTAA | hsa_circ_0012415 | chr1 | 51826842 | 51831701 | - | 425 | ANNOTATED, CDS, c | NM_001981 | EPS15 | Salzman2013 | 2060 | circRNA | Detected | Detected | 5.345923286 | 4.092672635 |
| hsa_gci155638 | -2.129161035 | -1.090285069 | down | 2.785538346 | 3.875823415 | TCTAGACCTACT | hsa_circ_0012548 | chr1 | 52954580 | 52959334 | - | 249 | ANNOTATED, CDS, c | NM_001009881 | ZCCHC11 | Salzman2013 | 23318 | circRNA | Not Detected | Detected | 2.785538346 | 3.875823415 |

| | | | | | | | | | | | | | | | | | | | | | |
|---|---|---|---|---|---|---|---|---|---|---|---|---|---|---|---|---|---|---|---|---|---|
| hsa_gci155653 | 2.014205125 | 1.010210613 | up | 4.450726718 | 3.440516104 | GACTTTTTTGTC | hsa_circ_0012599 | chr1 | 54252830 | 54254869 | - | 165 | ANNOTATED, CDS, c | NM_018087 | NDC1 | Salzman2013 | 55706 | circRNA | Detected | Detected | 4.450726718 | 3.440516104 |
| hsa_gci155663 | 3.2563196 | 1.703242303 | up | 9.229229177 | 7.525986873 | ACCGTAGTCCCT | hsa_circ_0012634 | chr1 | 54506428 | 54509198 | - | 317 | ANNOTATED, CDS, c | NM_004872 | TMEM59 | Salzman2013 | 9528 | circRNA | Detected | Detected | 9.229229177 | 7.525986873 |
| hsa_gci155676 | 2.593259929 | 1.374766818 | up | 8.475166149 | 7.10039933 | CAGTGAACCGAC | hsa_circ_0012757 | chr1 | 61743191 | 61892228 | + | 953 | ANNOTATED, CDS, c | NM_001134673 | NFIA | Salzman2013 | 4774 | circRNA | Detected | Detected | 8.475166149 | 7.10039933 |
| hsa_gci155684 | 2.269018276 | 1.182068229 | up | 6.401126532 | 5.219058303 | AAGAAAAAGTAA | hsa_circ_0012793 | chr1 | 62713143 | 62737261 | - | 983 | ANNOTATED, CDS, c | NM_181712 | KANK4 | Salzman2013 | 163782 | circRNA | Detected | Detected | 6.401126532 | 5.219058303 |
| hsa_gci155703 | 2.908773087 | 1.540410757 | up | 6.205501821 | 4.665091065 | GCCCCTCATTGT | hsa_circ_0012907 | chr1 | 67356836 | 67359096 | - | 298 | ANNOTATED, CDS, c | NM_024763 | WDR78 | Salzman2013 | 79819 | circRNA | Detected | Detected | 6.205501821 | 4.665091065 |
| hsa_gci155707 | 7.803453582 | 2.96411276 | up | 4.305335974 | 1.341223214 | CTACTCTGACCG | hsa_circ_0012933 | chr1 | 70694104 | 70701233 | + | 387 | ANNOTATED, CDS, c | NM_004768 | SRSF11 | Salzman2013 | 9295 | circRNA | Detected | Not Detected | 4.305335974 | 1.341223214 |
| hsa_gci155708 | 2.98588232 | 1.578157307 | up | 5.095164937 | 3.51700763 | TTGAATTGTTTC | hsa_circ_0012934 | chr1 | 70694104 | 70703235 | + | 515 | ANNOTATED, CDS, c | NM_004768 | SRSF11 | Salzman2013 | 9295 | circRNA | Detected | Detected | 5.095164937 | 3.51700763 |
| hsa_gci155721 | 2.464218425 | 1.30113014 | up | 6.534127064 | 5.232996924 | GTCCTCCCATAA | hsa_circ_0012981 | chr1 | 78041752 | 78045313 | - | 351 | ANNOTATED, CDS, c | NM_015534 | ZZZ3 | Salzman2013 | 26009 | circRNA | Detected | Detected | 6.534127064 | 5.232996924 |
| hsa_gci155740 | 2.123669437 | 1.086559219 | up | 4.155419147 | 3.068859928 | TTCTTTAAATTT | hsa_circ_0013058 | chr1 | 85331067 | 85331842 | - | 775 | ALT_ACCEPTOR, CDS | NM_012152 | LPAR3 | Salzman2013 | 23566 | circRNA | Detected | Not Detected | 4.155419147 | 3.068859928 |
| hsa_gci155742 | 2.112291339 | 1.078808833 | up | 6.412030921 | 5.333222088 | GTACGAGTGCCC | hsa_circ_0013066 | chr1 | 85784167 | 85787251 | - | 3084 | ANNOTATED, CDS, c | NM_001134445 | DDAH1 | Salzman2013 | 23576 | circRNA | Detected | Detected | 6.412030921 | 5.333222088 |
| hsa_gci155775 | 2.462080532 | 1.299877951 | up | 4.626658755 | 3.326780803 | GGAAGAAGACTC | hsa_circ_0013189 | chr1 | 93307322 | 93307481 | + | 159 | ANNOTATED, CDS, c | NM_000969 | RPL5 | Salzman2013 | 6125 | circRNA | Detected | Detected | 4.626658755 | 3.326780803 |
| hsa_gci155793 | 2.181860848 | 1.125559094 | up | 6.052843676 | 4.927284582 | AGGTTATGTTTT | hsa_circ_0013247 | chr1 | 95616880 | 95616975 | + | 95 | ANNOTATED, CDS, c | NM_001199679 | TMEM56 | Salzman2013 | 148534 | circRNA | Detected | Detected | 6.052843676 | 4.927284582 |
| hsa_gci155808 | 2.191097278 | 1.131653537 | up | 4.297051905 | 3.165398368 | AACTATAACTTG | hsa_circ_0013307 | chr1 | 100568510 | 100576039 | - | 1005 | ANNOTATED, CDS, c | NM_194292 | SASS6 | Salzman2013 | 163786 | circRNA | Detected | Not Detected | 4.297051905 | 3.165398368 |
| hsa_gci155828 | 2.657309345 | 1.409966186 | up | 6.52022523 | 5.110259044 | TCAGAGAGTACC | hsa_circ_0013351 | chr1 | 101377667 | 101387397 | + | 458 | ANNOTATED, CDS, c | NM_133496 | SLC30A7 | Salzman2013 | 148867 | circRNA | Detected | Detected | 6.52022523 | 5.110259044 |
| hsa_gci155841 | 2.174740997 | 1.120843592 | up | 10.91302131 | 9.792177716 | ACGTATAGCGGA | hsa_circ_0013398 | chr1 | 109482257 | 109486679 | - | 702 | ANNOTATED, CDS, c | NM_001048210 | CLCC1 | Salzman2013 | 23155 | circRNA | Detected | Detected | 10.91302131 | 9.792177716 |
| hsa_gci155843 | 2.953649301 | 1.562498539 | up | 6.581468771 | 5.018970232 | ATTTGTAACGTG | hsa_circ_0013408 | chr1 | 109553537 | 109554340 | - | 803 | ANNOTATED, CDS, c | NM_001142550 | WDR47 | Salzman2013 | 22911 | circRNA | Detected | Detected | 6.581468771 | 5.018970232 |
| hsa_gci155874 | 2.638230084 | 1.399570389 | up | 7.067200838 | 5.667630448 | AAGTCTTTTCTA | hsa_circ_0013584 | chr1 | 113655100 | 113662145 | + | 1173 | ANNOTATED, CDS, c | NM_014813 | LRIG2 | Salzman2013 | 9860 | circRNA | Detected | Detected | 7.067200838 | 5.667630448 |
| hsa_gci155895 | 2.432901606 | 1.282677974 | up | 7.132129054 | 5.84945108 | CCCCTGAGAGTG | hsa_circ_0013702 | chr1 | 117484336 | 117532972 | + | 5974 | ANNOTATED, CDS, c | NM_020440 | PTGFRN | Salzman2013 | 5738 | circRNA | Detected | Detected | 7.132129054 | 5.84945108 |
| hsa_gci155896 | 2.046471777 | 1.03313877 | up | 6.913666467 | 5.880527696 | CGGGTGCACGTC | hsa_circ_0013703 | chr1 | 117487300 | 117532972 | + | 5605 | ANNOTATED, CDS, c | NM_020440 | PTGFRN | Salzman2013 | 5738 | circRNA | Detected | Detected | 6.913666467 | 5.880527696 |
| hsa_gci155927 | 2.49266675 | 1.317690019 | up | 4.166603612 | 2.848913593 | ATTTTAGAACTT | hsa_circ_0013919 | chr1 | 145663158 | 145682094 | + | 281 | ANNOTATED, CDS, c | NM_014455 | RNF115 | Salzman2013 | 27246 | circRNA | Detected | Not Detected | 4.166603612 | 2.848913593 |
| hsa_gci155949 | 2.197910164 | 1.13613242 | up | 9.315259855 | 8.179127435 | CTCCCCTCGTCA | hsa_circ_0014094 | chr1 | 151032150 | 151040973 | + | 2158 | ANNOTATED, CDS, c | NM_006818 | MLLT11 | Salzman2013 | 10962 | circRNA | Detected | Detected | 9.315259855 | 8.179127435 |
| hsa_gci155968 | 2.159378779 | 1.11061633 | up | 4.671125524 | 3.560509194 | GCTCCTGACTCC | hsa_circ_0014159 | chr1 | 151372456 | 151374412 | + | 762 | ANNOTATED, CDS, c | NM_002796 | PSMB4 | Salzman2013 | 5692 | circRNA | Detected | Detected | 4.671125524 | 3.560509194 |
| hsa_gci155998 | 2.476171314 | 1.308111131 | up | 7.113751644 | 5.805640513 | GACCGAACTGGG | hsa_circ_0014254 | chr1 | 153634513 | 153641033 | - | 1423 | ANNOTATED, CDS, c | NM_004515 | ILF2 | Salzman2013 | 3608 | circRNA | Detected | Detected | 7.113751644 | 5.805640513 |
| hsa_gci155999 | 2.122089069 | 1.085485211 | up | 7.549287466 | 6.463802255 | CTGGGCCCCTTT | hsa_circ_0014255 | chr1 | 153634513 | 153641052 | - | 1442 | ALT_ACCEPTOR, CDS | NM_004515 | ILF2 | Salzman2013 | 3608 | circRNA | Detected | Detected | 7.549287466 | 6.463802255 |
| hsa_gci156002 | 2.29927195 | 1.201177113 | up | 4.680933094 | 3.479755981 | AGTGTAACAAAT | hsa_circ_0014260 | chr1 | 153635180 | 153640553 | - | 799 | ANNOTATED, CDS, c | NM_004515 | ILF2 | Salzman2013 | 3608 | circRNA | Detected | Detected | 4.680933094 | 3.479755981 |
| hsa_gci156032 | 2.271012337 | 1.183335544 | up | 7.080977901 | 5.897642357 | GGAAGCCCTGTG | hsa_circ_0014455 | chr1 | 154934774 | 154940273 | - | 2403 | ALT_DONOR, CDS, c | NM_001130040 | SHC1 | Salzman2013 | 6464 | circRNA | Detected | Detected | 7.080977901 | 5.897642357 |
| hsa_gci156043 | 2.98855446 | 1.579447833 | up | 8.318953068 | 6.739505235 | CATCTCCCCCGG | hsa_circ_0014515 | chr1 | 155225769 | 155228745 | - | 950 | ANNOTATED, CDS, c | NM_005698 | SCAMP3 | Salzman2013 | 10067 | circRNA | Detected | Detected | 8.318953068 | 6.739505235 |
| hsa_gci156051 | 2.00407181 | 1.002934204 | up | 8.017250146 | 7.014315941 | ACAACCCCCTTG | hsa_circ_0014570 | chr1 | 155324263 | 155340774 | - | 881 | ANNOTATED, CDS, c | NM_018489 | ASH1L | Salzman2013 | 55870 | circRNA | Detected | Detected | 8.017250146 | 7.014315941 |
| hsa_gci156055 | 2.122999641 | 1.086104128 | up | 4.520781303 | 3.434677175 | CCCCAAAGTCTT | hsa_circ_0014578 | chr1 | 155340294 | 155385714 | - | 858 | ANNOTATED, CDS, c | NM_018489 | ASH1L | Salzman2013 | 55870 | circRNA | Detected | Detected | 4.520781303 | 3.434677175 |
| hsa_gci156071 | 3.168697333 | 1.663889863 | up | 4.309855473 | 2.645965609 | CCGTTCGAAGTG | hsa_circ_0014615 | chr1 | 155695172 | 155697529 | + | 333 | ANNOTATED, CDS, c | NM_001199849 | DAP3 | Salzman2013 | 7818 | circRNA | Detected | Not Detected | 4.309855473 | 2.645965609 |
| hsa_gci156085 | 2.348983847 | 1.232036792 | up | 8.276976262 | 7.044939469 | CTCAATCAAGAC | hsa_circ_0014700 | chr1 | 156252703 | 156262234 | + | 2226 | ANNOTATED, ncRNA, | NR_026678 | TMEM79 | Salzman2013 | 84283 | circRNA | Detected | Detected | 8.276976262 | 7.044939469 |
| hsa_gci156091 | -3.397370793 | -1.764418684 | down | 1.681712769 | 3.446131453 | ACCTGAACCGGC | hsa_circ_0014718 | chr1 | 156294762 | 156303434 | - | 215 | ANNOTATED, INTERN | NR_036564 | CCT3 | Salzman2013 | 7203 | circRNA | Not Detected | Detected | 1.681712769 | 3.446131453 |
| hsa_gci156093 | 2.134011144 | 1.09356771 | up | 6.89181195 | 5.79824424 | TGGCGATGATTC | hsa_circ_0014721 | chr1 | 156294762 | 156307580 | - | 504 | ANNOTATED, INTERN | NR_036565 | CCT3 | Salzman2013 | 7203 | circRNA | Detected | Detected | 6.89181195 | 5.79824424 |
| hsa_gci156098 | 2.52401449 | 1.335720192 | up | 7.397209704 | 6.061489512 | TTCCCTACCCGG | hsa_circ_0014726 | chr1 | 156303337 | 156307580 | - | 386 | ANNOTATED, INTERN | NR_036565 | CCT3 | Salzman2013 | 7203 | circRNA | Detected | Detected | 7.397209704 | 6.061489512 |
| hsa_gci156116 | 2.610267814 | 1.384197835 | up | 5.358783743 | 3.974585908 | CGGGGAGCCCCA | hsa_circ_0014855 | chr1 | 159887902 | 159889625 | - | 1107 | ANNOTATED, CDS, c | NM_003564 | TAGLN2 | Salzman2013 | 8407 | circRNA | Detected | Detected | 5.358783743 | 3.974585908 |
| hsa_gci156122 | 2.004472134 | 1.003222361 | up | 8.03035234 | 7.027129978 | CAATCCTTCTTT | hsa_circ_0014868 | chr1 | 160185504 | 160208537 | - | 2948 | ANNOTATED, ncRNA, | NR_028103 | DCAF8 | Salzman2013 | 50717 | circRNA | Detected | Detected | 8.03035234 | 7.027129978 |
| hsa_gci156137 | 2.544607008 | 1.347442862 | up | 7.03348804 | 5.686045178 | GGACGGCTTCCG | hsa_circ_0014963 | chr1 | 161070345 | 161072165 | - | 517 | ANNOTATED, CDS, c | NM_012394 | PFDN2 | Salzman2013 | 5202 | circRNA | Detected | Detected | 7.03348804 | 5.686045178 |
| hsa_gci156139 | -4.90092057 | -2.293052765 | down | 1.457170077 | 3.750222842 | TATTCCGTGTTT | hsa_circ_0014971 | chr1 | 161123533 | 161128646 | + | 1122 | ANNOTATED, CDS, c | NM_016406 | UFC1 | Salzman2013 | 51506 | circRNA | Not Detected | Detected | 1.457170077 | 3.750222842 |
| hsa_gci156156 | 2.127693438 | 1.0892903 | up | 7.545330277 | 6.456039978 | AAGCAAAAGAGA | hsa_circ_0015050 | chr1 | 162557264 | 162560301 | + | 524 | ANNOTATED, CDS, c | NM_003115 | UAP1 | Salzman2013 | 6675 | circRNA | Detected | Detected | 7.545330277 | 6.456039978 |
| hsa_gci156163 | 2.444831063 | 1.289734779 | up | 5.300020065 | 4.010285286 | TTTCTAGGTCGT | hsa_circ_0015089 | chr1 | 166827355 | 166833158 | - | 623 | ANNOTATED, CDS, c | NM_053053 | TADA1 | Salzman2013 | 117143 | circRNA | Detected | Detected | 5.300020065 | 4.010285286 |
| hsa_gci156173 | 2.244493616 | 1.166389993 | up | 6.664833641 | 5.498443648 | GGGACGGCCGGC | hsa_circ_0015138 | chr1 | 168148170 | 168171351 | + | 3031 | ANNOTATED, CDS, c | NM_152902 | TIPRL | Salzman2013 | 261726 | circRNA | Detected | Detected | 6.664833641 | 5.498443648 |
| hsa_gci156178 | 2.391611398 | 1.257982992 | up | 6.755360689 | 5.497377697 | ACTGTGATTAGA | hsa_circ_0015150 | chr1 | 168260397 | 168274434 | + | 713 | ANNOTATED, CDS, c | NM_005149 | TBX19 | Salzman2013 | 9095 | circRNA | Detected | Detected | 6.755360689 | 5.497377697 |
| hsa_gci156183 | 2.478336811 | 1.309372266 | up | 4.845848524 | 3.536476258 | TATGACCTATTT | hsa_circ_0015164 | chr1 | 169446392 | 169446995 | - | 603 | ANNOTATED, CDS, c | NM_006996 | SLC19A2 | Salzman2013 | 10560 | circRNA | Detected | Detected | 4.845848524 | 3.536476258 |
| hsa_gci156188 | 4.067658743 | 2.024198649 | up | 8.224478878 | 6.200280229 | AACATCAACAGT | hsa_circ_0015187 | chr1 | 169951112 | 169961396 | - | 615 | ANNOTATED, CDS, c | NM_001204517 | KIFAP3 | Salzman2013 | 22920 | circRNA | Detected | Detected | 8.224478878 | 6.200280229 |
| hsa_gci156202 | 2.471336648 | 1.30529155 | up | 6.315458998 | 5.010167449 | ATATCAGACAAT | hsa_circ_0015235 | chr1 | 171537385 | 171553665 | + | 1191 | ANNOTATED, CDS, c | NM_015172 | PRRC2C | Salzman2013 | 23215 | circRNA | Detected | Detected | 6.315458998 | 5.010167449 |
| hsa_gci156207 | 2.036686525 | 1.026223946 | up | 5.964141588 | 4.937917642 | TATTTTCGACCG | hsa_circ_0015247 | chr1 | 171553094 | 171557644 | + | 790 | ANNOTATED, CDS, c | NM_015172 | PRRC2C | Salzman2013 | 23215 | circRNA | Detected | Detected | 5.964141588 | 4.937917642 |
| hsa_gci156244 | 2.242932212 | 1.165386018 | up | 4.971992717 | 3.806606698 | ACAGTACATACG | hsa_circ_0015369 | chr1 | 176050287 | 176153828 | - | 870 | ANNOTATED, CDS, c | NM_022457 | COP1 | Salzman2013 | 64326 | circRNA | Detected | Detected | 4.971992717 | 3.806606698 |
| hsa_gci156253 | 2.501778114 | 1.322953841 | up | 6.374762083 | 5.051808242 | TAGACTGTTTAT | hsa_circ_0015404 | chr1 | 178846632 | 178852668 | + | 297 | ANNOTATED, CDS, c | NM_152663 | RALGPS2 | Salzman2013 | 55103 | circRNA | Detected | Detected | 6.374762083 | 5.051808242 |
| hsa_gci156268 | 2.329946727 | 1.220296969 | up | 4.265208822 | 3.044911853 | CTCACCCGGAAG | hsa_circ_0015449 | chr1 | 179311248 | 179312770 | + | 207 | ANNOTATED, CDS, c | NM_003101 | SOAT1 | Salzman2013 | 6646 | circRNA | Detected | Not Detected | 4.265208822 | 3.044911853 |
| hsa_gci156273 | 2.095928328 | 1.067589384 | up | 7.693948667 | 6.626359283 | ACACAGAACTCC | hsa_circ_0015458 | chr1 | 179851419 | 179889211 | + | 3807 | ANNOTATED, CDS, c | NM_015602 | TOR1AIP1 | Salzman2013 | 26092 | circRNA | Detected | Detected | 7.693948667 | 6.626359283 |
| hsa_gci156278 | 3.719941638 | 1.895279987 | up | 6.548020429 | 4.652740442 | TACTCTCATGTC | hsa_circ_0015491 | chr1 | 180047597 | 180049796 | + | 357 | ANNOTATED, CDS, c | NM_014810 | CEP350 | Salzman2013 | 9857 | circRNA | Detected | Detected | 6.548020429 | 4.652740442 |

| | | | | | | | | | | | | | | | | | | | | | | |
|---|---|---|---|---|---|---|---|---|---|---|---|---|---|---|---|---|---|---|---|---|---|---|
| hsa_gci156299 | 2.404727169 | 1.265873221 | up | 6.81430584 | 5.548432619 | ACGTCCCCATCT | hsa_circ_0015573 | chr1 | 182845581 | 182846038 | + | 169 | ANNOTATED, INTERN | NR_033302 | DHX9 | Salzman2013 | 1660 | circRNA | Detected | Detected | 6.81430584 | 5.548432619 |
| hsa_gci156340 | 2.432700832 | 1.282558912 | up | 7.354318025 | 6.071759114 | GAAGTTTGGTCC | hsa_circ_0015796 | chr1 | 197621364 | 197627499 | - | 151 | ANNOTATED, CDS, c | NM_001195215 | DENND1B | Salzman2013 | 163486 | circRNA | Detected | Detected | 7.354318025 | 6.071759114 |
| hsa_gci156358 | -7.521585998 | -2.9110369 | down | 1.312118456 | 4.223155356 | CAAGTACACCAT | hsa_circ_0015857 | chr1 | 200906307 | 200926954 | + | 12475 | ALT_ACCEPTOR, ALT | TCONS_12_00000747 | | Salzman2013 | | circRNA | Not Detected | Detected | 1.312118456 | 4.223155356 |
| hsa_gci156366 | 3.14813621 | 1.654497963 | up | 4.177767052 | 2.523269089 | GACCCTGACTTC | hsa_circ_0015899 | chr1 | 201755556 | 201796102 | + | 9830 | ANNOTATED, CDS, c | NM_020443 | NAV1 | Salzman2013 | 89796 | circRNA | Detected | Not Detected | 4.177767052 | 2.523269089 |
| hsa_gci156368 | 6.860835956 | 2.778384372 | up | 4.17606331 | 1.397678938 | GAAAGACAGGTA | hsa_circ_0015911 | chr1 | 201786215 | 201796102 | + | 7336 | ANNOTATED, CDS, c | NM_020443 | NAV1 | Salzman2013 | 89796 | circRNA | Detected | Not Detected | 4.17606331 | 1.397678938 |
| hsa_gci156395 | 2.971400987 | 1.571143308 | up | 5.155642425 | 3.584499117 | GTTCTTTAAGTT | hsa_circ_0016049 | chr1 | 202742245 | 202743863 | - | 294 | ANNOTATED, CDS, c | NM_006618 | KDM5B | Salzman2013 | 10765 | circRNA | Detected | Detected | 5.155642425 | 3.584499117 |
| hsa_gci156398 | 2.025095872 | 1.01799021 | up | 4.83009223 | 3.812102021 | CCTTCACGCCCA | hsa_circ_0016068 | chr1 | 203274663 | 203278729 | + | 2711 | ANNOTATED, CDS, c | NM_006763 | BTG2 | Salzman2013 | 7832 | circRNA | Detected | Detected | 4.83009223 | 3.812102021 |
| hsa_gci156410 | 8.969903569 | 3.165092476 | up | 4.401845524 | 1.236753049 | AAACCATCACTT | hsa_circ_0016158 | chr1 | 204494611 | 204501374 | + | 378 | ANNOTATED, CDS, c | NM_002393 | MDM4 | Salzman2013 | 4194 | circRNA | Detected | Not Detected | 4.401845524 | 1.236753049 |
| hsa_gci156416 | 2.4527043 | 1.294373312 | up | 6.322242068 | 5.027868757 | TTAAAAGAATCA | hsa_circ_0016215 | chr1 | 205689628 | 205696933 | - | 315 | ANNOTATED, CDS, c | NM_022731 | NUCKS1 | Salzman2013 | 64710 | circRNA | Detected | Detected | 6.322242068 | 5.027868757 |
| hsa_gci156419 | -3.047590332 | -1.607668984 | down | 3.121033793 | 4.728702776 | GTGGTCAGGTCC | hsa_circ_0016256 | chr1 | 206773616 | 206781679 | - | 617 | ANNOTATED, CDS, c | NM_006893 | EIF2D | Salzman2013 | 1939 | circRNA | Not Detected | Detected | 3.121033793 | 4.728702776 |
| hsa_gci156435 | 3.815708806 | 1.931951077 | up | 6.456976992 | 4.525025915 | GTCGCTCAAGAA | hsa_circ_0016363 | chr1 | 211836114 | 211843749 | - | 1353 | ALT_DONOR, CDS, c | NM_002497 | NEK2 | Salzman2013 | 4751 | circRNA | Detected | Detected | 6.456976992 | 4.525025915 |
| hsa_gci156453 | 2.170183195 | 1.117816832 | up | 9.155675237 | 8.037858405 | TCGTTTAAGTTT | hsa_circ_0016417 | chr1 | 213061830 | 213062599 | + | 218 | ANNOTATED, CDS, c | NM_014053 | FLVCR1 | Salzman2013 | 28982 | circRNA | Detected | Detected | 9.155675237 | 8.037858405 |
| hsa_gci156460 | 2.329146669 | 1.219801491 | up | 5.519518038 | 4.299716548 | ACCCACGTTTAC | hsa_circ_0016461 | chr1 | 215749243 | 215760028 | + | 634 | ANNOTATED, CDS, c | NM_016121 | KCTD3 | Salzman2013 | 51133 | circRNA | Detected | Detected | 5.519518038 | 4.299716548 |
| hsa_gci156488 | 2.230656047 | 1.157468076 | up | 7.986499179 | 6.829031102 | TTTAAAGGATGA | hsa_circ_0016652 | chr1 | 225600155 | 225611791 | - | 1098 | ANNOTATED, CDS, c | NM_002296 | LBR | Salzman2013 | 3930 | circRNA | Detected | Detected | 7.986499179 | 6.829031102 |
| hsa_gci156520 | 2.065114942 | 1.046222083 | up | 7.491108784 | 6.444886701 | CCGTTAAGAGAG | hsa_circ_0016869 | chr1 | 230804421 | 230805281 | + | 289 | ANNOTATED, CDS, c | NM_007357 | COG2 | Salzman2013 | 22796 | circRNA | Detected | Detected | 7.491108784 | 6.444886701 |
| hsa_gci156531 | 2.551828842 | 1.351531567 | up | 7.340537118 | 5.989005551 | TCATGGTTTGAC | hsa_circ_0016904 | chr1 | 231700273 | 231702269 | + | 1996 | ANNOTATED, CDS, c | NM_005999 | TSNAX | Salzman2013 | 7257 | circRNA | Detected | Detected | 7.340537118 | 5.989005551 |
| hsa_gci156578 | 2.581917229 | 1.368442751 | up | 5.34754288 | 3.979100128 | TCTGAACCATTG | hsa_circ_0017059 | chr1 | 235600636 | 235602237 | + | 307 | ANNOTATED, CDS, c | NM_001079515 | TBCE | Salzman2013 | 6905 | circRNA | Detected | Detected | 5.34754288 | 3.979100128 |
| hsa_gci156580 | 4.605913896 | 2.203487441 | up | 5.186503353 | 2.983015912 | GCCTGTCTGACT | hsa_circ_0017067 | chr1 | 235634163 | 235647831 | - | 401 | ANNOTATED, CDS, c | NM_152490 | B3GALNT2 | Salzman2013 | 148789 | circRNA | Detected | Not Detected | 5.186503353 | 2.983015912 |
| hsa_gci156582 | 2.222443162 | 1.152146523 | up | 7.642869781 | 6.490723258 | ATCTAAAGGTGA | hsa_circ_0017069 | chr1 | 235634163 | 235658138 | - | 650 | ANNOTATED, CDS, c | NM_152490 | B3GALNT2 | Salzman2013 | 148789 | circRNA | Detected | Detected | 7.642869781 | 6.490723258 |
| hsa_gci156597 | 2.952461691 | 1.561918341 | up | 4.394070822 | 2.832152482 | TATGCTAATAAG | hsa_circ_0017210 | chr1 | 243232882 | 243254898 | - | 1073 | ANNOTATED, INTERN | TCONS_12_00002816 | | Salzman2013 | | circRNA | Detected | Not Detected | 4.394070822 | 2.832152482 |
| hsa_gci156674 | 2.105556887 | 1.074201854 | up | 10.22213862 | 9.147936768 | AGTAGTCTTTTA | hsa_circ_0017538 | chr10 | 5019891 | 5020158 | + | 267 | ANNOTATED, CDS, c | NM_001353 | AKR1C1 | Salzman2013 | 1645 | circRNA | Detected | Detected | 10.22213862 | 9.147936768 |
| hsa_gci156677 | -2.751734004 | -1.460341019 | down | 3.71867745 | 5.179018469 | GGAGAGGCTTCC | hsa_circ_0017551 | chr10 | 5468617 | 5471192 | + | 127 | ANNOTATED, CDS, c | NM_001047160 | NET1 | Salzman2013 | 10276 | circRNA | Not Detected | Detected | 3.71867745 | 5.179018469 |
| hsa_gci156698 | 2.593676717 | 1.374998669 | up | 8.646156184 | 7.271157515 | TTTCACTAACAA | hsa_circ_0017633 | chr10 | 7285519 | 7285667 | - | 148 | ANNOTATED, CDS, c | NM_001029880 | SFMBT2 | Salzman2013 | 57713 | circRNA | Detected | Detected | 8.646156184 | 7.271157515 |
| hsa_gci156699 | 2.186491061 | 1.12861745 | up | 8.611223022 | 7.482605572 | TCCGGTAGAAGT | hsa_circ_0017635 | chr10 | 7285519 | 7318951 | - | 348 | ANNOTATED, CDS, c | NM_001029880 | SFMBT2 | Salzman2013 | 57713 | circRNA | Detected | Detected | 8.611223022 | 7.482605572 |
| hsa_gci156700 | 2.328827935 | 1.21960405 | up | 7.931497706 | 6.711893655 | TACTCTTTAAGA | hsa_circ_0017636 | chr10 | 7285519 | 7327916 | - | 684 | ANNOTATED, CDS, c | NM_001029880 | SFMBT2 | Salzman2013 | 57713 | circRNA | Detected | Detected | 7.931497706 | 6.711893655 |
| hsa_gci156708 | 2.10021312 | 1.070535733 | up | 8.054832608 | 6.984296874 | TTAAGTAGGACG | hsa_circ_0017649 | chr10 | 7412242 | 7423911 | - | 246 | ANNOTATED, CDS, c | NM_001029880 | SFMBT2 | Salzman2013 | 57713 | circRNA | Detected | Detected | 8.054832608 | 6.984296874 |
| hsa_gci156719 | -3.84700803 | -1.943736841 | down | 1.388599097 | 3.332335938 | CTAGACCAGGAA | hsa_circ_0017702 | chr10 | 11971863 | 12009453 | - | 1856 | ANNOTATED, CDS, c | NM_080599 | UPF2 | Salzman2013 | 26019 | circRNA | Not Detected | Detected | 1.388599097 | 3.332335938 |
| hsa_gci156721 | 2.050987077 | 1.036318402 | up | 5.361473042 | 4.32515464 | GAGGGGAAATCT | hsa_circ_0017713 | chr10 | 12021055 | 12056183 | - | 808 | ANNOTATED, CDS, c | NM_080599 | UPF2 | Salzman2013 | 26019 | circRNA | Detected | Detected | 5.361473042 | 4.32515464 |
| hsa_gci156760 | 2.691433532 | 1.428374797 | up | 5.351573281 | 3.923198484 | CAGTAACAAGGC | hsa_circ_0017878 | chr10 | 17276691 | 17279592 | + | 841 | ANNOTATED, CDS, c | NM_003380 | VIM | Salzman2013 | 7431 | circRNA | Detected | Detected | 5.351573281 | 3.923198484 |
| hsa_gci156796 | 2.090690303 | 1.063979369 | up | 5.764798155 | 4.700818785 | CACCCGACTCCT | hsa_circ_0018042 | chr10 | 27493368 | 27494177 | - | 161 | ANNOTATED, CDS, c | NM_145698 | ACBD5 | Salzman2013 | 91452 | circRNA | Detected | Detected | 5.764798155 | 4.700818785 |
| hsa_gci156823 | 2.680278016 | 1.422382654 | up | 6.957335323 | 5.534952669 | GCACTACACCTA | hsa_circ_0018167 | chr10 | 34398487 | 34806087 | - | 5458 | ANNOTATED, CDS, c | NM_019619 | PARD3 | Salzman2013 | 56288 | circRNA | Detected | Detected | 6.957335323 | 5.534952669 |
| hsa_gci156833 | 3.204904589 | 1.680281408 | up | 5.967124609 | 4.286843201 | ACGACCGGGCTC | hsa_circ_0018200 | chr10 | 35772331 | 35805551 | + | 211 | ANNOTATED, CDS, c | NM_145012 | CCNY | Salzman2013 | 219771 | circRNA | Detected | Detected | 5.967124609 | 4.286843201 |
| hsa_gci156835 | 2.661223461 | 1.412089657 | up | 5.234624431 | 3.822534773 | TACTACCAATTA | hsa_circ_0018202 | chr10 | 35790428 | 35819171 | + | 350 | ANNOTATED, CDS, c | NM_145012 | CCNY | Salzman2013 | 219771 | circRNA | Detected | Detected | 5.234624431 | 3.822534773 |
| hsa_gci156840 | 2.020225121 | 1.014516066 | up | 6.211168326 | 5.19665226 | CAAAAACTACAC | hsa_circ_0018207 | chr10 | 35814891 | 35819171 | + | 214 | ANNOTATED, CDS, c | NM_145012 | CCNY | Salzman2013 | 219771 | circRNA | Detected | Detected | 6.211168326 | 5.19665226 |
| hsa_gci156872 | 2.143102658 | 1.099700959 | up | 5.520113392 | 4.420412433 | CGGGTAAGTCCC | hsa_circ_0018401 | chr10 | 52193235 | 52350007 | - | 452 | ANNOTATED, coding | NM_147156 | SGMS1 | Salzman2013 | 259230 | circRNA | Detected | Detected | 5.520113392 | 4.420412433 |
| hsa_gci156878 | 3.704753382 | 1.889377509 | up | 7.909536663 | 6.020159154 | AGAAAGGTCCGC | hsa_circ_0018417 | chr10 | 58117198 | 58121034 | - | 1851 | ANNOTATED, CDS, c | NM_032997 | ZWINT | Salzman2013 | 11130 | circRNA | Detected | Detected | 7.909536663 | 6.020159154 |
| hsa_gci156880 | 2.351236055 | 1.233419387 | up | 8.286918984 | 7.053499597 | TACACGAAGAAC | hsa_circ_0018445 | chr10 | 62645869 | 62745703 | - | 1737 | ANNOTATED, INTERN | NR_024554 | RHOBTB1 | Salzman2013 | 9886 | circRNA | Detected | Detected | 8.286918984 | 7.053499597 |
| hsa_gci156885 | 2.726522108 | 1.447061854 | up | 6.926600342 | 5.479538489 | GCGGCTCCGGTG | hsa_circ_0018458 | chr10 | 64893006 | 64914786 | + | 1896 | ANNOTATED, CDS, c | NM_030759 | NRBF2 | Salzman2013 | 29982 | circRNA | Detected | Detected | 6.926600342 | 5.479538489 |
| hsa_gci156886 | 3.111262104 | 1.637499938 | up | 4.891930862 | 3.254430924 | AAGTGTGTGAAG | hsa_circ_0018463 | chr10 | 64944367 | 64954135 | - | 1317 | ANNOTATED, CDS, c | NM_032776 | JMJD1C | Salzman2013 | 221037 | circRNA | Detected | Detected | 4.891930862 | 3.254430924 |
| hsa_gci156915 | -3.811835163 | -1.930485733 | down | 1.35017478 | 3.280660513 | CCGACACAATTC | hsa_circ_0018550 | chr10 | 70500385 | 70502326 | + | 227 | ANNOTATED, CDS, c | NM_018237 | CCAR1 | Salzman2013 | 55749 | circRNA | Not Detected | Detected | 1.35017478 | 3.280660513 |
| hsa_gci156928 | 2.160039007 | 1.111057366 | up | 4.291024233 | 3.179966868 | ACTTAAAGTAGT | hsa_circ_0018606 | chr10 | 70954943 | 70956839 | + | 170 | ANNOTATED, CDS, c | NM_003171 | SUPV3L1 | Salzman2013 | 6832 | circRNA | Detected | Detected | 4.291024233 | 3.179966868 |
| hsa_gci156941 | -3.890072624 | -1.959797089 | down | 1.231349985 | 3.191147075 | CACCATGAAGTT | hsa_circ_0018652 | chr10 | 71977567 | 71978573 | - | 174 | ANNOTATED, CDS, c | NM_021129 | PPA1 | Salzman2013 | 5464 | circRNA | Not Detected | Detected | 1.231349985 | 3.191147075 |
| hsa_gci156942 | 2.014124789 | 1.010153071 | up | 10.54300834 | 9.532855273 | CCTCGGTCTCCC | hsa_circ_0018655 | chr10 | 72163860 | 72188374 | + | 7531 | ANNOTATED, CDS, c | NM_004096 | EIF4EBP2 | Salzman2013 | 1979 | circRNA | Detected | Detected | 10.54300834 | 9.532855273 |
| hsa_gci156956 | 2.526539981 | 1.33716301 | up | 8.699083329 | 7.36192032 | CCTGGCCCGGCC | hsa_circ_0018698 | chr10 | 73576054 | 73594262 | - | 2687 | ANNOTATED, CDS, c | NM_001042465 | PSAP | Salzman2013 | 5660 | circRNA | Detected | Detected | 8.699083329 | 7.36192032 |
| hsa_gci156990 | 2.650332713 | 1.406173482 | up | 8.231498201 | 6.825324719 | TAAGGTCTTTCC | hsa_circ_0018841 | chr10 | 75458908 | 75481262 | - | 1216 | ANNOTATED, ncRNA, | NR_026592 | BMS1P4 | Salzman2013 | 729096 | circRNA | Detected | Detected | 8.231498201 | 6.825324719 |
| hsa_gci156991 | 2.809167923 | 1.490142867 | up | 7.360513362 | 5.870370495 | TTGTTCATTAAG | hsa_circ_0018843 | chr10 | 75458908 | 75488345 | - | 1884 | ANNOTATED, ncRNA, | NR_026592 | BMS1P4 | Salzman2013 | 729096 | circRNA | Detected | Detected | 7.360513362 | 5.870370495 |
| hsa_gci157009 | 2.539229484 | 1.344390785 | up | 5.991202998 | 4.646812212 | ACTGTGGTCAAA | hsa_circ_0019014 | chr10 | 88231964 | 88277848 | - | 2052 | ANNOTATED, CDS, c | NM_015045 | WAPL | Salzman2013 | 23063 | circRNA | Detected | Detected | 5.991202998 | 4.646812212 |
| hsa_gci157027 | 3.174084909 | 1.666340722 | up | 5.50669072 | 3.840349998 | CAAGGCGAAAGG | hsa_circ_0019091 | chr10 | 92982445 | 93008317 | + | 448 | ANNOTATED, CDS, c | NM_032373 | PCGF5 | Salzman2013 | 84333 | circRNA | Detected | Detected | 5.50669072 | 3.840349998 |
| hsa_gci157028 | 2.23669701 | 1.161369838 | up | 5.296189992 | 4.134820154 | AAGTTTCACGAC | hsa_circ_0019093 | chr10 | 93000240 | 93008317 | + | 153 | ANNOTATED, CDS, c | NM_032373 | PCGF5 | Salzman2013 | 84333 | circRNA | Detected | Detected | 5.296189992 | 4.134820154 |
| hsa_gci157074 | 2.004013218 | 1.002892024 | up | 5.712210291 | 4.709318267 | CCGGTTCTGTTA | hsa_circ_0019342 | chr10 | 99195751 | 99197507 | - | 693 | ALT_DONOR, CDS, c | NM_016046 | EXOSC1 | Salzman2013 | 51013 | circRNA | Detected | Detected | 5.712210291 | 4.709318267 |
| hsa_gci157078 | 2.176941009 | 1.122302314 | up | 6.758137681 | 5.635835367 | TATAACATCTGA | hsa_circ_0019368 | chr10 | 99236439 | 99237161 | - | 249 | ALT_ACCEPTOR, CDS | NM_022362 | MMS19 | Salzman2013 | 64210 | circRNA | Detected | Detected | 6.758137681 | 5.635835367 |

| | | | | | | | | | | | | | | | | | | | | | | |
|---|---|---|---|---|---|---|---|---|---|---|---|---|---|---|---|---|---|---|---|---|---|---|
| hsa_gci157081 | 3.359411362 | 1.748208465 | up | 5.442095594 | 3.693887129 | GCTCCGACCCCA | hsa_circ_0019379 | chr10 | 99437180 | 99439670 | - | 897 | ALT_ACCEPTOR, CDS | NM_021732 | AVPI1 | Salzman2013 | 60370 | circRNA | Detected | Detected | 5.442095594 | 3.693887129 |
| hsa_gci157088 | 2.362562453 | 1.240352467 | up | 6.512039841 | 5.271687374 | CCGTCCTCATCC | hsa_circ_0019440 | chr10 | 101553648 | 101569975 | + | 1432 | ANNOTATED, CDS, c | NM_000392 | ABCC2 | Salzman2013 | 1244 | circRNA | Detected | Detected | 6.512039841 | 5.271687374 |
| hsa_gci157130 | 6.769992617 | 2.759154261 | up | 5.488916677 | 2.729762416 | GTAGTTTTTCGT | hsa_circ_0019618 | chr10 | 103567486 | 103567658 | - | 172 | ANNOTATED, CDS, c | NM_012215 | MGEA5 | Salzman2013 | 10724 | circRNA | Detected | Not Detected | 5.488916677 | 2.729762416 |
| hsa_gci157153 | 2.686025192 | 1.425472836 | up | 5.762343127 | 4.336870291 | GTGAGGTCCTCC | hsa_circ_0019787 | chr10 | 104899162 | 104934739 | - | 199 | ANNOTATED, CDS, c | NM_012229 | NT5C2 | Salzman2013 | 22978 | circRNA | Detected | Detected | 5.762343127 | 4.336870291 |
| hsa_gci157155 | -8.112027741 | -3.020062586 | down | 1.346492732 | 4.366555318 | TCAAGCTCTGGT | hsa_circ_0019789 | chr10 | 105062552 | 105099378 | - | 7049 | ALT_ACCEPTOR, CDS | NM_001011663 | PCGF6 | Salzman2013 | 84108 | circRNA | Not Detected | Detected | 1.346492732 | 4.366555318 |
| hsa_gci157173 | 2.670803808 | 1.417274002 | up | 4.651939572 | 3.234665569 | TCCCCGACACAG | hsa_circ_0019972 | chr10 | 106014467 | 106027222 | + | 1004 | ANNOTATED, CDS, c | NM_004832 | GSTO1 | Salzman2013 | 9446 | circRNA | Detected | Not Detected | 4.651939572 | 3.234665569 |
| hsa_gci157174 | 2.885488774 | 1.528815718 | up | 6.917807209 | 5.388991491 | CGGGGCCCCCGC | hsa_circ_0019973 | chr10 | 106014920 | 106027222 | + | 751 | ANNOTATED, CDS, c | NM_001191003 | GSTO1 | Salzman2013 | 9446 | circRNA | Detected | Detected | 6.917807209 | 5.388991491 |
| hsa_gci157177 | 3.875324948 | 1.954317286 | up | 5.427758126 | 3.47344084 | CCAGTCCACCAT | hsa_circ_0019987 | chr10 | 111624523 | 111625070 | - | 547 | ANNOTATED, CDS, c | NM_020383 | XPNPEP1 | Salzman2013 | 7511 | circRNA | Detected | Detected | 5.427758126 | 3.47344084 |
| hsa_gci157197 | 2.097143049 | 1.068425273 | up | 6.154673103 | 5.08624783 | TATCTTTTGGTT | hsa_circ_0020082 | chr10 | 116595295 | 116620648 | + | 1794 | ANNOTATED, CDS, c | NM_020940 | FAM160B1 | Salzman2013 | 57700 | circRNA | Detected | Detected | 6.154673103 | 5.08624783 |
| hsa_gci157202 | 3.222663295 | 1.688253463 | up | 4.223938016 | 2.535684553 | TCTTCCAAGACA | hsa_circ_0020094 | chr10 | 116879948 | 116925405 | + | 799 | ANNOTATED, CDS, c | NM_207303 | ATRNL1 | Salzman2013 | 26033 | circRNA | Detected | Not Detected | 4.223938016 | 2.535684553 |
| hsa_gci157211 | 2.121706981 | 1.085225426 | up | 8.281311989 | 7.196086562 | CCTGAGATCATC | hsa_circ_0020148 | chr10 | 120824910 | 120825082 | - | 172 | ANNOTATED, CDS, c | NM_003750 | EIF3A | Salzman2013 | 8661 | circRNA | Detected | Detected | 8.281311989 | 7.196086562 |
| hsa_gci157218 | 2.386077516 | 1.254640913 | up | 6.192356101 | 4.937715188 | GGGTTCCTTACC | hsa_circ_0020169 | chr10 | 120927214 | 120936665 | - | 1512 | ANNOTATED, CDS, c | NM_006793 | PRDX3 | Salzman2013 | 10935 | circRNA | Detected | Detected | 6.192356101 | 4.937715188 |
| hsa_gci157223 | 2.055638504 | 1.03958658 | up | 5.493456067 | 4.453869487 | GCGACCCTGCAC | hsa_circ_0020180 | chr10 | 121336591 | 121356541 | - | 1476 | ANNOTATED, CDS, c | NM_001033925 | TIAL1 | Salzman2013 | 7073 | circRNA | Detected | Detected | 5.493456067 | 4.453869487 |
| hsa_gci157227 | 2.219584117 | 1.150289384 | up | 4.200522978 | 3.050233594 | CGGCGGTGACGG | hsa_circ_0020203 | chr10 | 121685547 | 121693299 | + | 902 | ANNOTATED, CDS, c | NM_007190 | SEC23IP | Salzman2013 | 11196 | circRNA | Detected | Not Detected | 4.200522978 | 3.050233594 |
| hsa_gci157228 | 2.485889238 | 1.313762017 | up | 8.143038081 | 6.829276065 | AAAGTGGTGTGT | hsa_circ_0020209 | chr10 | 122273422 | 122278408 | + | 155 | ANNOTATED, CDS, c | NM_001030059 | PLPP4 | Salzman2013 | 196051 | circRNA | Detected | Detected | 8.143038081 | 6.829276065 |
| hsa_gci157256 | 2.575803518 | 1.365022549 | up | 6.64697592 | 5.281953371 | ACGGGGCGCCTC | hsa_circ_0020332 | chr10 | 126490353 | 126525239 | + | 2992 | ANNOTATED, CDS, c | NM_032182 | ABRAXAS2 | Salzman2013 | 23172 | circRNA | Detected | Detected | 6.64697592 | 5.281953371 |
| hsa_gci157259 | 2.775868788 | 1.472939375 | up | 6.959486427 | 5.486547052 | AGAACCTGACAA | hsa_circ_0020340 | chr10 | 126662213 | 126662967 | + | 271 | ANNOTATED, CDS, c | NM_017580 | ZRANB1 | Salzman2013 | 54764 | circRNA | Detected | Detected | 6.959486427 | 5.486547052 |
| hsa_gci157269 | 2.13476396 | 1.094076561 | up | 8.582078241 | 7.48800168 | CTACCCCTGCCA | hsa_circ_0020367 | chr10 | 127483448 | 127505201 | - | 793 | ALT_ACCEPTOR, CDS | NM_000375 | UROS | Salzman2013 | 7390 | circRNA | Detected | Detected | 8.582078241 | 7.48800168 |
| hsa_gci157335 | 2.464193435 | 1.301115509 | up | 10.82705637 | 9.525940863 | TGGACCGTCCGT | hsa_circ_0020573 | chr10 | 135123279 | 135126666 | + | 828 | ANNOTATED, CDS, c | NM_145806 | ZNF511 | Salzman2013 | 118472 | circRNA | Detected | Detected | 10.82705637 | 9.525940863 |
| hsa_gci157341 | 2.08148636 | 1.057614105 | up | 6.010516321 | 4.952902216 | AACAGAGAATCT | hsa_circ_0020600 | chr11 | 164244 | 169052 | - | 458 | ANNOTATED, INTERN | TCONS_12_00005353 | | Salzman2013 | | circRNA | Detected | Detected | 6.010516321 | 4.952902216 |
| hsa_gci157353 | 2.150638811 | 1.104765252 | up | 6.267841974 | 5.163076722 | CCACCTCCACTT | hsa_circ_0020658 | chr11 | 494511 | 502181 | - | 1584 | ANNOTATED, CDS, c | NM_203383 | RNH1 | Salzman2013 | 6050 | circRNA | Detected | Detected | 6.267841974 | 5.163076722 |
| hsa_gci157356 | 2.770833523 | 1.470320034 | up | 6.34924626 | 4.878926226 | AAAGGTAAGTCG | hsa_circ_0020707 | chr11 | 811596 | 812876 | + | 265 | ANNOTATED, CDS, c | NM_001004 | RPLP2 | Salzman2013 | 6181 | circRNA | Detected | Detected | 6.34924626 | 4.878926226 |
| hsa_gci157357 | 2.051661719 | 1.036792877 | up | 9.178230732 | 8.141437855 | CGTTCGAACGGT | hsa_circ_0020708 | chr11 | 812534 | 812876 | + | 216 | ANNOTATED, CDS, c | NM_001004 | RPLP2 | Salzman2013 | 6181 | circRNA | Detected | Detected | 9.178230732 | 8.141437855 |
| hsa_gci157374 | 2.452692361 | 1.294366289 | up | 5.623291004 | 4.328924715 | CCTCGTCCGTAT | hsa_circ_0020859 | chr11 | 3712576 | 3712719 | - | 143 | ANNOTATED, CDS, c | NM_016320 | NUP98 | Salzman2013 | 4928 | circRNA | Detected | Detected | 5.623291004 | 4.328924715 |
| hsa_gci157381 | 2.58814728 | 1.371919717 | up | 7.2369116 | 5.864991883 | ATCATTCTCGGA | hsa_circ_0020879 | chr11 | 3726429 | 3752808 | - | 1540 | ANNOTATED, CDS, c | NM_016320 | NUP98 | Salzman2013 | 4928 | circRNA | Detected | Detected | 7.2369116 | 5.864991883 |
| hsa_gci157384 | 2.051480915 | 1.036665733 | up | 5.039294193 | 4.002628461 | TCGAAACTGGTA | hsa_circ_0020892 | chr11 | 3744386 | 3794969 | - | 1702 | ANNOTATED, CDS, c | NM_005387 | NUP98 | Salzman2013 | 4928 | circRNA | Detected | Detected | 5.039294193 | 4.002628461 |
| hsa_gci157406 | 2.077180821 | 1.05462681 | up | 5.751597311 | 4.696970501 | CGGTTTTGCCAG | hsa_circ_0020959 | chr11 | 5269501 | 5269717 | - | 216 | ANNOTATED, CDS, c | NM_000559 | HBG1 | Salzman2013 | 3047 | circRNA | Detected | Detected | 5.751597311 | 4.696970501 |
| hsa_gci157411 | 3.189061765 | 1.67313204 | up | 8.244549682 | 6.571417643 | TTCGACAGTTTT | hsa_circ_0020967 | chr11 | 5289579 | 5290906 | - | 471 | ANNOTATED, CDS, c | NM_005330 | HBE1 | Salzman2013 | 3046 | circRNA | Detected | Detected | 8.244549682 | 6.571417643 |
| hsa_gci157415 | 2.516402049 | 1.331362442 | up | 7.951238452 | 6.619876009 | CCGTAGGTCTGT | hsa_circ_0020994 | chr11 | 6416354 | 6416931 | - | 577 | ANNOTATED, CDS, c | NM_001164 | APBB1 | Salzman2013 | 322 | circRNA | Detected | Detected | 7.951238452 | 6.619876009 |
| hsa_gci157418 | 2.403620405 | 1.265209074 | up | 7.510099863 | 6.244890788 | GGCGGGGCCTTC | hsa_circ_0021002 | chr11 | 6502676 | 6505911 | + | 2842 | ANNOTATED, CDS, c | NM_012192 | TIMM10B | Salzman2013 | 26515 | circRNA | Detected | Detected | 7.510099863 | 6.244890788 |
| hsa_gci157429 | 2.282341888 | 1.19051492 | up | 7.600095221 | 6.409580301 | GTCTGTACGACG | hsa_circ_0021116 | chr11 | 9002122 | 9005107 | - | 2985 | ANNOTATED, CDS, c | NM_020645 | NRIP3 | Salzman2013 | 56675 | circRNA | Detected | Detected | 7.600095221 | 6.409580301 |
| hsa_gci157432 | 2.092728432 | 1.065385109 | up | 7.537609308 | 6.472224199 | GAAAATATCGGC | hsa_circ_0021120 | chr11 | 9002122 | 9025596 | - | 3809 | ANNOTATED, CDS, c | NM_020645 | NRIP3 | Salzman2013 | 56675 | circRNA | Detected | Detected | 7.537609308 | 6.472224199 |
| hsa_gci157442 | -3.035705172 | -1.602031683 | down | 1.913052904 | 3.515084587 | AAGGACGGAATT | hsa_circ_0021151 | chr11 | 9302200 | 9305140 | - | 2940 | ANNOTATED, CDS, c | NM_015012 | TMEM41B | Salzman2013 | 440026 | circRNA | Not Detected | Detected | 1.913052904 | 3.515084587 |
| hsa_gci157446 | 2.800190556 | 1.485525007 | up | 8.922804416 | 7.437279409 | TTCCTTTCTCTT | hsa_circ_0021159 | chr11 | 9406168 | 9431633 | + | 621 | ANNOTATED, CDS, c | NM_006391 | IPO7 | Salzman2013 | 10527 | circRNA | Detected | Detected | 8.922804416 | 7.437279409 |
| hsa_gci157451 | 2.536125985 | 1.342626415 | up | 7.831599904 | 6.48897349 | AGACCCGTCGTC | hsa_circ_0021181 | chr11 | 9446460 | 9446809 | + | 194 | ANNOTATED, CDS, c | NM_006391 | IPO7 | Salzman2013 | 10527 | circRNA | Detected | Detected | 7.831599904 | 6.48897349 |
| hsa_gci157468 | 2.143806715 | 1.100174839 | up | 4.096806084 | 2.996631245 | GCCGGGCCAACG | hsa_circ_0021252 | chr11 | 10818592 | 10830582 | - | 3911 | ANNOTATED, CDS, c | NM_001418 | EIF4G2 | Salzman2013 | 1982 | circRNA | Detected | Not Detected | 4.096806084 | 2.996631245 |
| hsa_gci157479 | 2.798169841 | 1.484483533 | up | 8.56498088 | 7.080497347 | AGGAGTCGGAGA | hsa_circ_0021318 | chr11 | 13435076 | 13484838 | - | 1065 | ANNOTATED, CDS, c | NM_032320 | BTBD10 | Salzman2013 | 84280 | circRNA | Detected | Detected | 8.56498088 | 7.080497347 |
| hsa_gci157515 | 2.010173012 | 1.007319677 | up | 9.971464302 | 8.964144625 | AGAAAACGTAGA | hsa_circ_0021492 | chr11 | 20075625 | 20083905 | + | 502 | ANNOTATED, CDS, c | NM_001244963 | NAV2 | Salzman2013 | 89797 | circRNA | Detected | Detected | 9.971464302 | 8.964144625 |
| hsa_gci157528 | 2.331775588 | 1.221428949 | up | 4.591057377 | 3.369628427 | TCTAGACAAAGT | hsa_circ_0021570 | chr11 | 32948702 | 32956981 | + | 3968 | ANNOTATED, CDS, c | NM_001076786 | QSER1 | Salzman2013 | 79832 | circRNA | Detected | Detected | 4.591057377 | 3.369628427 |
| hsa_gci157534 | 2.103800781 | 1.072998095 | up | 7.631012147 | 6.558014052 | GTTCTGAACACT | hsa_circ_0021598 | chr11 | 33307958 | 33363232 | + | 1899 | ANNOTATED, CDS, c | NM_005734 | HIPK3 | Salzman2013 | 10114 | circRNA | Detected | Detected | 7.631012147 | 6.558014052 |
| hsa_gci157538 | 2.054295466 | 1.038643697 | up | 7.264292442 | 6.225648744 | ACGGAGTACGAG | hsa_circ_0021604 | chr11 | 33360302 | 33363232 | + | 556 | ANNOTATED, CDS, c | NM_005734 | HIPK3 | Salzman2013 | 10114 | circRNA | Detected | Detected | 7.264292442 | 6.225648744 |
| hsa_gci157546 | 2.092405511 | 1.065162475 | up | 4.368170179 | 3.303007705 | TACCGTCCTAGT | hsa_circ_0021650 | chr11 | 34107851 | 34107960 | + | 109 | ANNOTATED, CDS, c | NM_005898 | CAPRIN1 | Salzman2013 | 4076 | circRNA | Detected | Detected | 4.368170179 | 3.303007705 |
| hsa_gci157550 | 2.048227245 | 1.034375787 | up | 6.910389656 | 5.876013868 | AAGACCAAGTAA | hsa_circ_0021656 | chr11 | 34113452 | 34120607 | + | 1810 | ANNOTATED, CDS, c | NM_203364 | CAPRIN1 | Salzman2013 | 4076 | circRNA | Detected | Detected | 6.910389656 | 5.876013868 |
| hsa_gci157563 | 2.082333873 | 1.058201403 | up | 8.71277965 | 7.654578247 | TTATTGAGAAGC | hsa_circ_0021713 | chr11 | 34991685 | 34999729 | + | 207 | ANNOTATED, CDS, c | NM_003477 | PDHX | Salzman2013 | 8050 | circRNA | Detected | Detected | 8.71277965 | 7.654578247 |
| hsa_gci157578 | 3.346584602 | 1.742689486 | up | 6.493396225 | 4.750706738 | CGACCGACGACA | hsa_circ_0021782 | chr11 | 43471631 | 43472815 | + | 244 | ANNOTATED, CDS, c | NM_018259 | TTC17 | Salzman2013 | 55761 | circRNA | Detected | Detected | 6.493396225 | 4.750706738 |
| hsa_gci157584 | 2.437632632 | 1.285480718 | up | 7.781368228 | 6.49588751 | ATTATATCTATA | hsa_circ_0021855 | chr11 | 46515157 | 46568846 | - | 2057 | ANNOTATED, CDS, c | NM_017749 | AMBRA1 | Salzman2013 | 55626 | circRNA | Detected | Detected | 7.781368228 | 6.49588751 |
| hsa_gci157585 | -4.397866461 | -2.136803798 | down | 1.274146424 | 3.410950222 | TACGTCGTCCTC | hsa_circ_0021858 | chr11 | 46529740 | 46529920 | - | 180 | ANNOTATED, CDS, c | NM_017749 | AMBRA1 | Salzman2013 | 55626 | circRNA | Not Detected | Detected | 1.274146424 | 3.410950222 |
| hsa_gci157586 | 2.101745501 | 1.071587985 | up | 7.782788781 | 6.711200796 | ATAATACCAAGT | hsa_circ_0021859 | chr11 | 46529740 | 46568846 | - | 1875 | ANNOTATED, CDS, c | NM_017749 | AMBRA1 | Salzman2013 | 55626 | circRNA | Detected | Detected | 7.782788781 | 6.711200796 |
| hsa_gci157613 | 3.142412279 | 1.651872472 | up | 7.347111821 | 5.695239349 | TATAAAGCAGGA | hsa_circ_0022036 | chr11 | 47653205 | 47660603 | - | 340 | ANNOTATED, CDS, c | NM_014342 | MTCH2 | Salzman2013 | 23788 | circRNA | Detected | Detected | 7.347111821 | 5.695239349 |
| hsa_gci157614 | 3.174134069 | 1.666363066 | up | 7.303157845 | 5.636794779 | GATAACAACCTC | hsa_circ_0022039 | chr11 | 47660250 | 47660603 | - | 192 | ANNOTATED, CDS, c | NM_014342 | MTCH2 | Salzman2013 | 23788 | circRNA | Detected | Detected | 7.303157845 | 5.636794779 |
| hsa_gci157630 | 2.008827321 | 1.006353555 | up | 4.056398758 | 3.050045203 | GACCGTCTCCGC | hsa_circ_0022109 | chr11 | 57093458 | 57095900 | - | 1071 | ANNOTATED, CDS, c | NM_003146 | SSRP1 | Salzman2013 | 6749 | circRNA | Detected | Not Detected | 4.056398758 | 3.050045203 |

| | | | | | | | | | | | | | | | | | | | | | | |
|---|---|---|---|---|---|---|---|---|---|---|---|---|---|---|---|---|---|---|---|---|---|---|
| hsa_gci157635 | 2.0149257 | 1.010726641 | up | 6.454399199 | 5.443672558 | GAACGGTTTCAC | hsa_circ_0022128 | chr11 | 57176647 | 57176771 | - | 124 | ANNOTATED, CDS, c | NM_014096 | SLC43A3 | Salzman2013 | 29015 | circRNA | Detected | Detected | 6.454399199 | 5.443672558 |
| hsa_gci157636 | 2.368902562 | 1.244218859 | up | 5.730671322 | 4.486452463 | TTTTTGGTAGTC | hsa_circ_0022129 | chr11 | 57176647 | 57193825 | - | 1551 | ANNOTATED, CDS, c | NM_014096 | SLC43A3 | Salzman2013 | 29015 | circRNA | Detected | Detected | 5.730671322 | 4.486452463 |
| hsa_gci157637 | 3.568187042 | 1.835191242 | up | 5.351829564 | 3.516638322 | AACGCATTCCCC | hsa_circ_0022138 | chr11 | 57252003 | 57259335 | - | 1374 | ANNOTATED, CDS, c | NM_003627 | SLC43A1 | Salzman2013 | 8501 | circRNA | Detected | Detected | 5.351829564 | 3.516638322 |
| hsa_gci157641 | 2.240913349 | 1.164086864 | up | 5.264355871 | 4.100269006 | CTCAACGAACCA | hsa_circ_0022176 | chr11 | 57506602 | 57508445 | + | 1005 | ANNOTATED, ncRNA, | NR_026593 | TMX2 | Salzman2013 | 51075 | circRNA | Detected | Detected | 5.264355871 | 4.100269006 |
| hsa_gci157650 | -2.321036346 | -1.214769115 | down | 3.95183012 | 5.166599235 | TGTCTTTACCGA | hsa_circ_0022225 | chr11 | 60688357 | 60690915 | + | 1773 | ANNOTATED, CDS, c | NM_024092 | TMEM109 | Salzman2013 | 79073 | circRNA | Not Detected | Detected | 3.95183012 | 5.166599235 |
| hsa_gci157658 | 5.123957386 | 2.357258477 | up | 4.229608945 | 1.872350467 | ACTTCGGTAGAC | hsa_circ_0022355 | chr11 | 61556601 | 61556707 | - | 106 | ANNOTATED, coding | NM_014206 | TMEM258 | Salzman2013 | 746 | circRNA | Detected | Not Detected | 4.229608945 | 1.872350467 |
| hsa_gci157665 | 2.356457545 | 1.23661969 | up | 8.030506558 | 6.793886869 | GGCCGACATCAA | hsa_circ_0022399 | chr11 | 61640997 | 61643448 | - | 466 | ANNOTATED, CDS, c | NM_021727 | FADS3 | Salzman2013 | 3995 | circRNA | Detected | Detected | 8.030506558 | 6.793886869 |
| hsa_gci157671 | 2.125206221 | 1.087602841 | up | 5.474443358 | 4.386840517 | AAAGAAGGACCG | hsa_circ_0022423 | chr11 | 62201015 | 62201363 | - | 348 | ANNOTATED, CDS, c | NM_024060 | AHNAK | Salzman2013 | 79026 | circRNA | Detected | Detected | 5.474443358 | 4.386840517 |
| hsa_gci157677 | 2.220573443 | 1.150932287 | up | 8.132831875 | 6.981899587 | AGGTAGTAGTGG | hsa_circ_0022437 | chr11 | 62327072 | 62339373 | - | 1221 | ANNOTATED, CDS, c | NM_001404 | EEF1G | Salzman2013 | 1937 | circRNA | Detected | Detected | 8.132831875 | 6.981899587 |
| hsa_gci157689 | 3.393295099 | 1.7626869 | up | 6.358865399 | 4.596178499 | CAACCTCAATCC | hsa_circ_0022519 | chr11 | 62532811 | 62534187 | + | 394 | ANNOTATED, CDS, c | NM_002696 | POLR2G | Salzman2013 | 5436 | circRNA | Detected | Detected | 6.358865399 | 4.596178499 |
| hsa_gci157692 | 4.950491754 | 2.307571842 | up | 4.050316778 | 1.742744937 | CTAAGTCCTGTT | hsa_circ_0022527 | chr11 | 62568800 | 62568889 | - | 89 | ANNOTATED, CDS, c | NM_001081491 | NXF1 | Salzman2013 | 10482 | circRNA | Detected | Not Detected | 4.050316778 | 1.742744937 |
| hsa_gci157699 | 2.471087223 | 1.305145935 | up | 6.03203602 | 4.726890085 | GTTGCACGCGCA | hsa_circ_0022567 | chr11 | 62621990 | 62622938 | - | 211 | ANNOTATED, INTERN | NR_003098 | SNHG1 | Salzman2013 | 23642 | circRNA | Detected | Detected | 6.03203602 | 4.726890085 |
| hsa_gci157707 | 2.1473567 | 1.102561859 | up | 5.84945108 | 4.746889221 | TTCGGTTTTACC | hsa_circ_0022624 | chr11 | 63742078 | 63744015 | + | 507 | ANNOTATED, CDS, c | NM_004074 | COX8A | Salzman2013 | 1351 | circRNA | Detected | Detected | 5.84945108 | 4.746889221 |
| hsa_gci157714 | 2.183511709 | 1.126650267 | up | 4.424830204 | 3.298179937 | GTACTAGGCCCC | hsa_circ_0022639 | chr11 | 63963116 | 63972020 | + | 1551 | ANNOTATED, CDS, c | NM_006819 | STIP1 | Salzman2013 | 10963 | circRNA | Detected | Detected | 4.424830204 | 3.298179937 |
| hsa_gci157732 | 2.276704375 | 1.186946973 | up | 8.10330959 | 6.916362617 | CCCGGTCCCTTG | hsa_circ_0022774 | chr11 | 64888098 | 64888508 | - | 236 | ANNOTATED, CDS, c | NM_001997 | FAU | Salzman2013 | 2197 | circRNA | Detected | Detected | 8.10330959 | 6.916362617 |
| hsa_gci157735 | -2.778809272 | -1.474466816 | down | 2.16189125 | 3.636358066 | GAGTACGTTTGT | hsa_circ_0022796 | chr11 | 64978730 | 64979477 | + | 747 | ANNOTATED, CDS, c | NM_001198868 | CAPN1 | Salzman2013 | 823 | circRNA | Not Detected | Detected | 2.16189125 | 3.636358066 |
| hsa_gci157740 | 2.410025671 | 1.269048514 | up | 6.093416928 | 4.824368414 | CGTCCCATCCTC | hsa_circ_0022820 | chr11 | 65108436 | 65120451 | + | 2204 | ANNOTATED, CDS, c | NM_006268 | DPF2 | Salzman2013 | 5977 | circRNA | Detected | Detected | 6.093416928 | 4.824368414 |
| hsa_gci157773 | 5.745600178 | 2.522457603 | up | 4.153557871 | 1.631100268 | GTCGGGTGTGTG | hsa_circ_0023016 | chr11 | 66413497 | 66413944 | + | 447 | ANNOTATED, coding | NM_002896 | RBM4 | Salzman2013 | 5936 | circRNA | Detected | Not Detected | 4.153557871 | 1.631100268 |
| hsa_gci157780 | 2.400316105 | 1.263224411 | up | 7.968079385 | 6.704854974 | CTTAGCGGTCGG | hsa_circ_0023045 | chr11 | 66624875 | 66627946 | + | 2528 | ANNOTATED, CDS, c | NM_024036 | LRFN4 | Salzman2013 | 78999 | circRNA | Detected | Detected | 7.968079385 | 6.704854974 |
| hsa_gci157787 | 2.392856976 | 1.258734168 | up | 7.511845945 | 6.253111777 | TGGTCGCCCATG | hsa_circ_0023100 | chr11 | 67256737 | 67258579 | + | 829 | ANNOTATED, CDS, c | NM_003977 | AIP | Salzman2013 | 9049 | circRNA | Detected | Detected | 7.511845945 | 6.253111777 |
| hsa_gci157812 | 2.669486147 | 1.416562062 | up | 4.526811769 | 3.110249707 | TTGTTGAAGTCG | hsa_circ_0023255 | chr11 | 68529002 | 68529155 | - | 153 | ANNOTATED, CDS, c | NM_001876 | CPT1A | Salzman2013 | 1374 | circRNA | Detected | Not Detected | 4.526811769 | 3.110249707 |
| hsa_gci157820 | 2.461454482 | 1.299511061 | up | 8.600807104 | 7.301296043 | TGACGAAAAAAC | hsa_circ_0023323 | chr11 | 70183470 | 70194526 | + | 735 | ANNOTATED, CDS, c | NM_003626 | PPFIA1 | Salzman2013 | 8500 | circRNA | Detected | Detected | 8.600807104 | 7.301296043 |
| hsa_gci157831 | 2.689491521 | 1.427333441 | up | 7.227990657 | 5.800657216 | CGGAGGGACTCG | hsa_circ_0023413 | chr11 | 71713910 | 71720433 | - | 2291 | ANNOTATED, CDS, c | NM_006185 | NUMA1 | Salzman2013 | 4926 | circRNA | Detected | Detected | 7.227990657 | 5.800657216 |
| hsa_gci157833 | 2.526331075 | 1.337043716 | up | 6.919470559 | 5.582426843 | GTGTCGGTTCGC | hsa_circ_0023421 | chr11 | 71808337 | 71809461 | - | 750 | ANNOTATED, CDS, c | NM_017907 | LAMTOR1 | Salzman2013 | 55004 | circRNA | Detected | Detected | 6.919470559 | 5.582426843 |
| hsa_gci157838 | 2.227049256 | 1.155133467 | up | 6.863306516 | 5.708173049 | CACGCACGCACG | hsa_circ_0023506 | chr11 | 73418464 | 73472201 | - | 1016 | ANNOTATED, CDS, c | NM_002869 | RAB6A | Salzman2013 | 5870 | circRNA | Detected | Detected | 6.863306516 | 5.708173049 |
| hsa_gci157856 | 2.983589753 | 1.577049177 | up | 6.58529873 | 5.008249553 | TGGTCTCCACCG | hsa_circ_0023564 | chr11 | 73879388 | 73879658 | - | 270 | ANNOTATED, CDS, c | NM_015531 | C2CD3 | Salzman2013 | 26005 | circRNA | Detected | Detected | 6.58529873 | 5.008249553 |
| hsa_gci157872 | 2.421886122 | 1.276131031 | up | 5.746573905 | 4.470442874 | CATTCTACTTTT | hsa_circ_0023596 | chr11 | 74563033 | 74574066 | - | 261 | ANNOTATED, CDS, c | NM_182969 | XRRA1 | Salzman2013 | 143570 | circRNA | Detected | Detected | 5.746573905 | 4.470442874 |
| hsa_gci157879 | 2.641732596 | 1.40148444 | up | 5.829644832 | 4.428160392 | TCTCGTTGTAGG | hsa_circ_0023617 | chr11 | 75116657 | 75116733 | + | 76 | ANNOTATED, coding | NM_001005 | RPS3 | Salzman2013 | 6188 | circRNA | Detected | Detected | 5.829644832 | 4.428160392 |
| hsa_gci157885 | 2.287884695 | 1.194014345 | up | 6.075191193 | 4.881176848 | TGGACAACACAC | hsa_circ_0023655 | chr11 | 76224429 | 76227356 | + | 321 | ANNOTATED, CDS, c | NM_020193 | EMSY | Salzman2013 | 56946 | circRNA | Detected | Detected | 6.075191193 | 4.881176848 |
| hsa_gci157889 | 9.612813894 | 3.264958803 | up | 8.459062756 | 5.194103953 | AAAGAGAAAGAA | hsa_circ_0023683 | chr11 | 77069942 | 77091039 | - | 407 | ANNOTATED, CDS, c | NM_001128620 | PAK1 | Salzman2013 | 5058 | circRNA | Detected | Detected | 8.459062756 | 5.194103953 |
| hsa_gci157900 | 2.277278505 | 1.18731074 | up | 11.39040216 | 10.20309142 | CGTCCGTCATAC | hsa_circ_0023701 | chr11 | 77386080 | 77404656 | - | 847 | ANNOTATED, CDS, c | NM_016578 | RSF1 | Salzman2013 | 51773 | circRNA | Detected | Detected | 11.39040216 | 10.20309142 |
| hsa_gci157908 | 2.393660672 | 1.259218648 | up | 5.639104838 | 4.379886189 | AAGTCAGAGTGT | hsa_circ_0023737 | chr11 | 77632385 | 77692622 | - | 1518 | ANNOTATED, CDS, c | NM_033547 | INTS4 | Salzman2013 | 92105 | circRNA | Detected | Detected | 5.639104838 | 4.379886189 |
| hsa_gci157926 | 2.97878825 | 1.574725572 | up | 4.643205158 | 3.068479586 | ACATGACTTTTA | hsa_circ_0023780 | chr11 | 77820487 | 77830295 | - | 560 | ANNOTATED, CDS, c | NM_001007027 | ALG8 | Salzman2013 | 79053 | circRNA | Detected | Not Detected | 4.643205158 | 3.068479586 |
| hsa_gci157932 | -4.732631652 | -2.242642639 | down | 1.176736605 | 3.419379244 | ACCCTACGAACG | hsa_circ_0023800 | chr11 | 78147006 | 78180359 | - | 1184 | ANNOTATED, CDS, c | NM_024678 | NARS2 | Salzman2013 | 79731 | circRNA | Not Detected | Detected | 1.176736605 | 3.419379244 |
| hsa_gci157937 | 2.410466158 | 1.269312175 | up | 4.353337932 | 3.084025758 | TTTGTGAACTCA | hsa_circ_0023812 | chr11 | 78180292 | 78204241 | - | 337 | ANNOTATED, CDS, c | NM_024678 | NARS2 | Salzman2013 | 79731 | circRNA | Detected | Not Detected | 4.353337932 | 3.084025758 |
| hsa_gci157946 | 45.78497984 | 5.516802482 | up | 6.756405661 | 1.239603178 | TTTCGAGCCAGT | hsa_circ_0023866 | chr11 | 83173044 | 85309832 | - | 2913 | ANNOTATED, CDS, c | NM_001142699 | DLG2 | Salzman2013 | 1740 | circRNA | Detected | Not Detected | 6.756405661 | 1.239603178 |
| hsa_gci157954 | -4.692423727 | -2.230333295 | down | 1.73343723 | 3.963770525 | CGACACGTTGAC | hsa_circ_0023894 | chr11 | 85687665 | 85695016 | - | 455 | ANNOTATED, CDS, c | NM_001008660 | PICALM | Salzman2013 | 8301 | circRNA | Not Detected | Detected | 1.73343723 | 3.963770525 |
| hsa_gci157983 | 2.273653588 | 1.185012463 | up | 7.808884345 | 6.623871882 | CGGCCATAAGTA | hsa_circ_0023943 | chr11 | 85737333 | 85780923 | - | 381 | ANNOTATED, CDS, c | NM_001206947 | PICALM | Salzman2013 | 8301 | circRNA | Detected | Detected | 7.808884345 | 6.623871882 |
| hsa_gci157985 | 2.251698776 | 1.171013842 | up | 7.73606669 | 6.565052847 | CGGCCATAAGTA | hsa_circ_0023946 | chr11 | 85742510 | 85780923 | - | 305 | ANNOTATED, CDS, c | NM_001206947 | PICALM | Salzman2013 | 8301 | circRNA | Detected | Detected | 7.73606669 | 6.565052847 |
| hsa_gci157992 | 2.403412331 | 1.265084179 | up | 7.334963871 | 6.069879692 | CCGGAGCCCCTT | hsa_circ_0023967 | chr11 | 86511490 | 86522273 | + | 3804 | ANNOTATED, CDS, c | NM_007173 | PRSS23 | Salzman2013 | 11098 | circRNA | Detected | Detected | 7.334963871 | 6.069879692 |
| hsa_gci158020 | 2.094178544 | 1.066384448 | up | 5.707198837 | 4.64081439 | TATTTGCAAACT | hsa_circ_0024143 | chr11 | 103173821 | 103194718 | + | 944 | ANNOTATED, CDS, c | NM_001080463 | DYNC2H1 | Salzman2013 | 79659 | circRNA | Detected | Detected | 5.707198837 | 4.64081439 |
| hsa_gci158044 | 2.314735085 | 1.210847091 | up | 7.255781391 | 6.0449343 | TGTCTGATACAT | hsa_circ_0024247 | chr11 | 108559662 | 108594189 | + | 1117 | ANNOTATED, CDS, c | NM_004398 | DDX10 | Salzman2013 | 1662 | circRNA | Detected | Detected | 7.255781391 | 6.0449343 |
| hsa_gci158050 | 2.017890494 | 1.012847885 | up | 5.231354353 | 4.218506468 | TTTGCCACAGGC | hsa_circ_0024277 | chr11 | 111652918 | 111742305 | - | 6159 | ANNOTATED, CDS, c | NM_024740 | ALG9 | Salzman2013 | 79796 | circRNA | Detected | Detected | 5.231354353 | 4.218506468 |
| hsa_gci158081 | 2.065871549 | 1.046750554 | up | 6.091544157 | 5.044793603 | GAATAACTGGCC | hsa_circ_0024443 | chr11 | 118354897 | 118363945 | + | 1092 | ANNOTATED, CDS, c | NM_001197104 | KMT2A | Salzman2013 | 4297 | circRNA | Detected | Detected | 6.091544157 | 5.044793603 |
| hsa_gci158090 | 2.61707943 | 1.387957712 | up | 5.754749627 | 4.366791915 | GCAGGTGTCCAA | hsa_circ_0024476 | chr11 | 118461055 | 118461221 | + | 166 | ANNOTATED, CDS, c | NM_001655 | ARCN1 | Salzman2013 | 372 | circRNA | Detected | Detected | 5.754749627 | 4.366791915 |
| hsa_gci158144 | 2.35789199 | 1.237497633 | up | 7.462623054 | 6.225125421 | GCAGGTCCCTCT | hsa_circ_0024783 | chr11 | 125479328 | 125479484 | + | 156 | ANNOTATED, CDS, c | NM_152713 | STT3A | Salzman2013 | 3703 | circRNA | Detected | Detected | 7.462623054 | 6.225125421 |
| hsa_gci158154 | 2.134340922 | 1.093790639 | up | 7.454538908 | 6.36074827 | TTTATGTGTAGT | hsa_circ_0024871 | chr11 | 129996594 | 130000061 | + | 494 | ANNOTATED, CDS, c | NM_001642 | APLP2 | Salzman2013 | 334 | circRNA | Detected | Detected | 7.454538908 | 6.36074827 |
| hsa_gci158155 | 2.086436114 | 1.061040746 | up | 6.67946217 | 5.618421424 | CTTTATGTGTAG | hsa_circ_0024872 | chr11 | 129996594 | 130005610 | + | 747 | ANNOTATED, CDS, c | NM_001642 | APLP2 | Salzman2013 | 334 | circRNA | Detected | Detected | 6.67946217 | 5.618421424 |
| hsa_gci158189 | 2.009862178 | 1.007096575 | up | 5.524064426 | 4.516967851 | CACTGCGTTCGA | hsa_circ_0025055 | chr12 | 4382901 | 4414522 | + | 6522 | ANNOTATED, CDS, c | NM_001759 | CCND2 | Salzman2013 | 894 | circRNA | Detected | Detected | 5.524064426 | 4.516967851 |
| hsa_gci158191 | 2.945190131 | 1.558360772 | up | 6.493116397 | 4.934755625 | TGACTGAACCGA | hsa_circ_0025061 | chr12 | 4609303 | 4628046 | - | 712 | ANNOTATED, CDS, c | NM_020374 | C12orf4 | Salzman2013 | 57102 | circRNA | Detected | Detected | 6.493116397 | 4.934755625 |
| hsa_gci158210 | 2.176238341 | 1.121836569 | up | 8.684333596 | 7.562497027 | AGTCGAAAGTAC | hsa_circ_0025214 | chr12 | 6679247 | 6680198 | - | 776 | ANNOTATED, CDS, c | NM_001273 | CHD4 | Salzman2013 | 1108 | circRNA | Detected | Detected | 8.684333596 | 7.562497027 |

| | | | | | | | | | | | | | | | | | | | | | | |
|---|---|---|---|---|---|---|---|---|---|---|---|---|---|---|---|---|---|---|---|---|---|---|
| hsa_gci158225 | 2.168069794 | 1.1164112 | up | 6.141485521 | 5.025074321 | GGAGAGACAGTC | hsa_circ_0025304 | chr12 | 7074514 | 7077184 | - | 756 | ANNOTATED, CDS, c | NM_001144831 | PHB2 | Salzman2013 | 11331 | circRNA | Detected | Detected | 6.141485521 | 5.025074321 |
| hsa_gci158237 | 2.026738617 | 1.01916004 | up | 4.425432433 | 3.406272392 | CCCACGGGGTGT | hsa_circ_0025354 | chr12 | 8071823 | 8082467 | - | 3003 | ANNOTATED, CDS, c | NM_006931 | SLC2A3 | Salzman2013 | 6515 | circRNA | Detected | Detected | 4.425432433 | 3.406272392 |
| hsa_gci158251 | 2.637751012 | 1.399308389 | up | 7.374886585 | 5.975578196 | CGACCAAGAAGT | hsa_circ_0025472 | chr12 | 12277498 | 12279855 | - | 466 | ANNOTATED, CDS, c | NM_002336 | LRP6 | Salzman2013 | 4040 | circRNA | Detected | Detected | 7.374886585 | 5.975578196 |
| hsa_gci158264 | 2.366994014 | 1.243056058 | up | 7.793142992 | 6.550086934 | TAGTAAATGTCA | hsa_circ_0025516 | chr12 | 14609494 | 14635024 | + | 2190 | ALT_DONOR, CDS, c | NM_018179 | ATF7IP | Salzman2013 | 55729 | circRNA | Detected | Detected | 7.793142992 | 6.550086934 |
| hsa_gci158285 | 2.86025889 | 1.516145735 | up | 4.569390007 | 3.053244271 | TTCCTTAGGTCC | hsa_circ_0025608 | chr12 | 22622642 | 22659753 | - | 1495 | ANNOTATED, CDS, c | NM_014802 | C2CD5 | Salzman2013 | 9847 | circRNA | Detected | Not Detected | 4.569390007 | 3.053244271 |
| hsa_gci158287 | 2.114992754 | 1.080652721 | up | 5.525651764 | 4.444999043 | AGGTCCTCCTTT | hsa_circ_0025612 | chr12 | 22637630 | 22659753 | - | 512 | ANNOTATED, CDS, c | NM_014802 | C2CD5 | Salzman2013 | 9847 | circRNA | Detected | Detected | 5.525651764 | 4.444999043 |
| hsa_gci158300 | -3.909753466 | -1.96707764 | down | 1.328379201 | 3.295456841 | CCTCTTCCTTTG | hsa_circ_0025705 | chr12 | 27132716 | 27143560 | - | 499 | ANNOTATED, CDS, c | NM_016551 | TM7SF3 | Salzman2013 | 51768 | circRNA | Not Detected | Detected | 1.328379201 | 3.295456841 |
| hsa_gci158326 | 2.765112929 | 1.467338402 | up | 6.59196716 | 5.124628758 | CAGTCTGTCTTT | hsa_circ_0025803 | chr12 | 31555431 | 31600703 | - | 1320 | ANNOTATED, CDS, c | NM_144973 | DENND5B | Salzman2013 | 160518 | circRNA | Detected | Detected | 6.59196716 | 5.124628758 |
| hsa_gci158362 | 3.022303552 | 1.595648568 | up | 7.698841589 | 6.103193021 | TTTTACTTTAGA | hsa_circ_0025986 | chr12 | 46751970 | 46757609 | - | 3491 | ANNOTATED, CDS, c | NM_018976 | SLC38A2 | Salzman2013 | 54407 | circRNA | Detected | Detected | 7.698841589 | 6.103193021 |
| hsa_gci158380 | 2.871344987 | 1.521726678 | up | 7.669520806 | 6.147794128 | TACTTCCTTCAT | hsa_circ_0026080 | chr12 | 49399525 | 49399635 | - | 110 | ANNOTATED, CDS, c | NM_001206710 | PRKAG1 | Salzman2013 | 5571 | circRNA | Detected | Detected | 7.669520806 | 6.147794128 |
| hsa_gci158385 | 2.144594442 | 1.10070485 | up | 4.273059471 | 3.172354622 | CTCGACCGCCAT | hsa_circ_0026124 | chr12 | 49521566 | 49523173 | - | 1304 | ANNOTATED, CDS, c | NM_006082 | TUBA1B | Salzman2013 | 10376 | circRNA | Detected | Not Detected | 4.273059471 | 3.172354622 |
| hsa_gci158418 | 2.790153053 | 1.480344263 | up | 4.74308153 | 3.262737267 | CCGGTTAAGGTA | hsa_circ_0026213 | chr12 | 50523580 | 50531603 | - | 1383 | ANNOTATED, CDS, c | NM_147190 | CERS5 | Salzman2013 | 91012 | circRNA | Detected | Detected | 4.74308153 | 3.262737267 |
| hsa_gci158419 | 2.13209421 | 1.092271187 | up | 4.341306644 | 3.249035457 | GGTAGAATGCGG | hsa_circ_0026216 | chr12 | 50523580 | 50561097 | - | 1943 | ANNOTATED, CDS, c | NM_147190 | CERS5 | Salzman2013 | 91012 | circRNA | Detected | Detected | 4.341306644 | 3.249035457 |
| hsa_gci158420 | 2.654033844 | 1.408186768 | up | 6.974307957 | 5.566121189 | AGGAGCTACGGT | hsa_circ_0026218 | chr12 | 50529514 | 50537840 | - | 675 | ANNOTATED, CDS, c | NM_147190 | CERS5 | Salzman2013 | 91012 | circRNA | Detected | Detected | 6.974307957 | 5.566121189 |
| hsa_gci158421 | 2.435309694 | 1.284105249 | up | 6.821081206 | 5.536975957 | ACAGGAGCTACG | hsa_circ_0026219 | chr12 | 50531510 | 50537840 | - | 439 | ANNOTATED, CDS, c | NM_147190 | CERS5 | Salzman2013 | 91012 | circRNA | Detected | Detected | 6.821081206 | 5.536975957 |
| hsa_gci158438 | 2.097821035 | 1.068891607 | up | 4.078988326 | 3.010096719 | CGAGACCGACCA | hsa_circ_0026308 | chr12 | 51495717 | 51497986 | - | 234 | ANNOTATED, CDS, c | NM_005653 | TFCP2 | Salzman2013 | 7024 | circRNA | Detected | Not Detected | 4.078988326 | 3.010096719 |
| hsa_gci158440 | 2.696985043 | 1.431347521 | up | 10.85829479 | 9.426947264 | GCGCCCCAACCG | hsa_circ_0026317 | chr12 | 51639132 | 51664202 | - | 1087 | ANNOTATED, CDS, c | NM_001031628 | SMAGP | Salzman2013 | 57228 | circRNA | Detected | Detected | 10.85829479 | 9.426947264 |
| hsa_gci158442 | 2.429810218 | 1.280843635 | up | 8.501971905 | 7.22112827 | GAAAGAAGAATT | hsa_circ_0026334 | chr12 | 52139686 | 52188425 | + | 2797 | ANNOTATED, CDS, c | NM_014191 | SCN8A | Salzman2013 | 6334 | circRNA | Detected | Detected | 8.501971905 | 7.22112827 |
| hsa_gci158445 | 3.107765144 | 1.635877482 | up | 6.667160987 | 5.031283504 | GGCCCTCGACGA | hsa_circ_0026371 | chr12 | 52679696 | 52680277 | - | 581 | ANNOTATED, CDS, c | NM_002281 | KRT81 | Salzman2013 | 3887 | circRNA | Detected | Detected | 6.667160987 | 5.031283504 |
| hsa_gci158469 | 2.944147854 | 1.557850125 | up | 5.428860888 | 3.871010763 | AAACGTACCTCA | hsa_circ_0026493 | chr12 | 53346505 | 53346685 | + | 180 | ANNOTATED, CDS, c | NM_000224 | KRT18 | Salzman2013 | 3875 | circRNA | Detected | Detected | 5.428860888 | 3.871010763 |
| hsa_gci158474 | 142.9410356 | 7.159276335 | up | 8.680409145 | 1.52113281 | AGAATAAGAAGA | hsa_circ_0026502 | chr12 | 53410256 | 53432195 | + | 1563 | ANNOTATED, CDS, c | NM_001417 | EIF4B | Salzman2013 | 1975 | circRNA | Detected | Not Detected | 8.680409145 | 1.52113281 |
| hsa_gci158489 | 2.056002241 | 1.039841837 | up | 5.928954847 | 4.88911301 | CACCCGGGGGCC | hsa_circ_0026577 | chr12 | 53646601 | 53648190 | + | 1589 | ANNOTATED, CDS, c | NM_001170790 | MFSD5 | Salzman2013 | 84975 | circRNA | Detected | Detected | 5.928954847 | 4.88911301 |
| hsa_gci158493 | 6.232845447 | 2.639890939 | up | 6.179186081 | 3.539295142 | CCCCTGTGTCCA | hsa_circ_0026609 | chr12 | 53701239 | 53703065 | - | 864 | ANNOTATED, CDS, c | NM_015665 | AAAS | Salzman2013 | 8086 | circRNA | Detected | Detected | 6.179186081 | 3.539295142 |
| hsa_gci158495 | 2.011148334 | 1.008019493 | up | 10.99933557 | 9.991316078 | TCCTTCTCCTCC | hsa_circ_0026636 | chr12 | 53839798 | 53840427 | + | 629 | ANNOTATED, CDS, c | NM_018457 | PRR13 | Salzman2013 | 54458 | circRNA | Detected | Detected | 10.99933557 | 9.991316078 |
| hsa_gci158504 | 2.128162991 | 1.089608647 | up | 4.158750999 | 3.069142351 | TCCATTCCCCTG | hsa_circ_0026680 | chr12 | 54058943 | 54063125 | - | 463 | ANNOTATED, CDS, c | NM_001002031 | ATP5MC2 | Salzman2013 | 517 | circRNA | Detected | Not Detected | 4.158750999 | 3.069142351 |
| hsa_gci158515 | 2.470693436 | 1.304916012 | up | 5.855838067 | 4.550922055 | TGGTGGTGCCGA | hsa_circ_0026713 | chr12 | 54676583 | 54679030 | + | 1145 | ANNOTATED, CDS, c | NM_031157 | HNRNPA1 | Salzman2013 | 3178 | circRNA | Detected | Detected | 5.855838067 | 4.550922055 |
| hsa_gci158522 | 2.040676334 | 1.029047379 | up | 5.846715627 | 4.817668249 | CGAAACTGTCAT | hsa_circ_0026724 | chr12 | 54734330 | 54736071 | + | 151 | ANNOTATED, CDS, c | NM_016057 | COPZ1 | Salzman2013 | 22818 | circRNA | Detected | Detected | 5.846715627 | 4.817668249 |
| hsa_gci158526 | 2.287646582 | 1.193864188 | up | 8.949607226 | 7.755743038 | GACTATGAGAAG | hsa_circ_0026784 | chr12 | 56119229 | 56119410 | - | 181 | ANNOTATED, CDS, c | NM_001780 | CD63 | Salzman2013 | 967 | circRNA | Detected | Detected | 8.949607226 | 7.755743038 |
| hsa_gci158534 | 2.15120429 | 1.105144538 | up | 10.2584032 | 9.153258663 | CCCTGCACCCCG | hsa_circ_0026807 | chr12 | 56437902 | 56438007 | + | 105 | ANNOTATED, CDS, c | NM_001029 | RPS26 | Salzman2013 | 6231 | circRNA | Detected | Detected | 10.2584032 | 9.153258663 |
| hsa_gci158537 | 3.040022823 | 1.604082155 | up | 7.554184977 | 5.950102823 | CTTCAAGGCAGA | hsa_circ_0026827 | chr12 | 56510373 | 56511616 | + | 584 | ANNOTATED, CDS, c | NM_001035267 | RPL41 | Salzman2013 | 6171 | circRNA | Detected | Detected | 7.554184977 | 5.950102823 |
| hsa_gci158548 | 3.524714527 | 1.817506416 | up | 6.255050796 | 4.43754438 | TCGGTCGTCCAC | hsa_circ_0026851 | chr12 | 56526204 | 56532623 | + | 1551 | ANNOTATED, CDS, c | NM_001184796 | ESYT1 | Salzman2013 | 23344 | circRNA | Detected | Detected | 6.255050796 | 4.43754438 |
| hsa_gci158558 | 2.640736253 | 1.400940218 | up | 5.425394102 | 4.024453885 | GAGTCTGCGAAT | hsa_circ_0026884 | chr12 | 56551481 | 56551771 | + | 144 | ANNOTATED, CDS, c | NM_001199629 | MYL6B | Salzman2013 | 140465 | circRNA | Detected | Detected | 5.425394102 | 4.024453885 |
| hsa_gci158568 | 2.965182196 | 1.568120754 | up | 9.110996842 | 7.542876088 | GGTAGACGACTC | hsa_circ_0027028 | chr12 | 56956200 | 56975047 | + | 556 | ANNOTATED, CDS, c | NM_002898 | RBMS2 | Salzman2013 | 5939 | circRNA | Detected | Detected | 9.110996842 | 7.542876088 |
| hsa_gci158570 | 2.570176418 | 1.36186739 | up | 9.021384122 | 7.659516732 | TCATACCGGAAT | hsa_circ_0027034 | chr12 | 56962758 | 56975047 | + | 389 | ANNOTATED, CDS, c | NM_002898 | RBMS2 | Salzman2013 | 5939 | circRNA | Detected | Detected | 9.021384122 | 7.659516732 |
| hsa_gci158577 | 2.255638282 | 1.173535733 | up | 4.542922825 | 3.369387092 | GGTCCACCAAGT | hsa_circ_0027071 | chr12 | 57031958 | 57036615 | - | 946 | ANNOTATED, CDS, c | NM_001686 | ATP5F1B | Salzman2013 | 506 | circRNA | Detected | Detected | 4.542922825 | 3.369387092 |
| hsa_gci158639 | 3.456227452 | 1.789198163 | up | 7.778506705 | 5.989308542 | TGACCTCAAAAA | hsa_circ_0027465 | chr12 | 69050880 | 69051493 | + | 613 | ALT_DONOR, CDS, c | NM_015646 | RAP1B | Salzman2013 | 5908 | circRNA | Detected | Detected | 7.778506705 | 5.989308542 |
| hsa_gci158650 | 2.123650071 | 1.086546063 | up | 6.613024394 | 5.526478331 | CGTCGTCGAGGG | hsa_circ_0027495 | chr12 | 69252702 | 69265736 | - | 831 | ANNOTATED, CDS, c | NM_001874 | CPM | Salzman2013 | 1368 | circRNA | Detected | Detected | 6.613024394 | 5.526478331 |
| hsa_gci158651 | 2.334150726 | 1.222897725 | up | 7.242471168 | 6.019573444 | CGTGCCCTTCAG | hsa_circ_0027498 | chr12 | 69326457 | 69326620 | - | 163 | ANNOTATED, CDS, c | NM_001874 | CPM | Salzman2013 | 1368 | circRNA | Detected | Detected | 7.242471168 | 6.019573444 |
| hsa_gci158671 | 4.674465426 | 2.224801387 | up | 4.958427481 | 2.733626093 | TCCAAAAACTCT | hsa_circ_0027605 | chr12 | 76443346 | 76454059 | - | 1072 | ALT_DONOR, CDS, c | NM_004537 | NAP1L1 | Salzman2013 | 4673 | circRNA | Detected | Not Detected | 4.958427481 | 2.733626093 |
| hsa_gci158679 | 2.007433482 | 1.005352184 | up | 4.852340221 | 3.846988038 | TATTAAAAGTCC | hsa_circ_0027628 | chr12 | 78225068 | 78415642 | + | 2196 | ANNOTATED, CDS, c | NM_014903 | NAV3 | Salzman2013 | 89795 | circRNA | Detected | Detected | 4.852340221 | 3.846988038 |
| hsa_gci158680 | 2.605216493 | 1.381403265 | up | 4.852959779 | 3.471556513 | CCAACCGGGTCA | hsa_circ_0027635 | chr12 | 78334098 | 78569207 | + | 4860 | ANNOTATED, CDS, c | NM_014903 | NAV3 | Salzman2013 | 89795 | circRNA | Detected | Detected | 4.852959779 | 3.471556513 |
| hsa_gci158681 | 2.276705205 | 1.186947499 | up | 5.448727803 | 4.261780304 | AAACGATCCATC | hsa_circ_0027638 | chr12 | 78334098 | 78592456 | + | 6209 | ANNOTATED, CDS, c | NM_014903 | NAV3 | Salzman2013 | 89795 | circRNA | Detected | Detected | 5.448727803 | 4.261780304 |
| hsa_gci158708 | 3.809444673 | 1.929580702 | up | 4.703206134 | 2.773625432 | CGGTGACCACCG | hsa_circ_0027755 | chr12 | 95500729 | 95605043 | - | 3401 | ANNOTATED, CDS, c | NM_018351 | FGD6 | Salzman2013 | 55785 | circRNA | Detected | Not Detected | 4.703206134 | 2.773625432 |
| hsa_gci158714 | 2.193465017 | 1.133211697 | up | 11.87029254 | 10.73708084 | GGTCTAGGACAT | hsa_circ_0027767 | chr12 | 95645715 | 95668665 | + | 1033 | ANNOTATED, INTERN | NR_038241 | VEZT | Salzman2013 | 55591 | circRNA | Detected | Detected | 11.87029254 | 10.73708084 |
| hsa_gci158727 | 2.686294085 | 1.425617254 | up | 4.224211343 | 2.798594089 | TCCAGAAACACC | hsa_circ_0027803 | chr12 | 96672038 | 96683065 | - | 3084 | ANNOTATED, CDS, c | NM_001170464 | CDK17 | Salzman2013 | 5128 | circRNA | Detected | Not Detected | 4.224211343 | 2.798594089 |
| hsa_gci158734 | 2.379627461 | 1.250735733 | up | 6.135570003 | 4.884834271 | TTGACAGACACG | hsa_circ_0027838 | chr12 | 99128568 | 100219167 | - | 4382 | ANNOTATED, CDS, c | NM_152788 | ANKS1B | Salzman2013 | 56899 | circRNA | Detected | Detected | 6.135570003 | 4.884834271 |
| hsa_gci158739 | 2.37594771 | 1.248503086 | up | 6.196998279 | 4.948495193 | TTTGTCTACCCT | hsa_circ_0027853 | chr12 | 100492129 | 100497729 | - | 321 | ANNOTATED, CDS, c | NM_015054 | UHRF1BP1L | Salzman2013 | 23074 | circRNA | Detected | Detected | 6.196998279 | 4.948495193 |
| hsa_gci158740 | 3.427006195 | 1.7769488 | up | 6.392964938 | 4.616016138 | TGTTTGTCTACC | hsa_circ_0027854 | chr12 | 100496543 | 100497729 | - | 131 | ANNOTATED, CDS, c | NM_015054 | UHRF1BP1L | Salzman2013 | 23074 | circRNA | Detected | Detected | 6.392964938 | 4.616016138 |
| hsa_gci158759 | 2.113298634 | 1.079496651 | up | 8.647848355 | 7.568351704 | TCAGAGCGCCCT | hsa_circ_0027939 | chr12 | 104335609 | 104341703 | + | 1367 | ANNOTATED, CDS, c | NM_003299 | HSP90B1 | Salzman2013 | 7184 | circRNA | Detected | Detected | 8.647848355 | 7.568351704 |
| hsa_gci158760 | 2.542507744 | 1.346252168 | up | 9.767093501 | 8.420841333 | TGCATATTTTTC | hsa_circ_0027944 | chr12 | 104370695 | 104374740 | + | 455 | ANNOTATED, CDS, c | NM_003211 | TDG | Salzman2013 | 6996 | circRNA | Detected | Detected | 9.767093501 | 8.420841333 |
| hsa_gci158762 | 3.666984975 | 1.874594355 | up | 4.663288696 | 2.788694342 | TGAACTCTACCA | hsa_circ_0027950 | chr12 | 104480624 | 104480792 | + | 168 | ANNOTATED, CDS, c | NM_013320 | HCFC2 | Salzman2013 | 29915 | circRNA | Detected | Not Detected | 4.663288696 | 2.788694342 |

| | | | | | | | | | | | | | | | | | | | | | |
|---|---|---|---|---|---|---|---|---|---|---|---|---|---|---|---|---|---|---|---|---|---|
| hsa_gci158765 | 3.186488524 | 1.671967465 | up | 4.828544929 | 3.156577464 | TAAAAATCATCG | hsa_circ_0027958 | chr12 | 104705067 | 104733051 | + | 1467 | ANNOTATED, CDS, c | NM_003330 | TXNRD1 | Salzman2013 | 7296 | circRNA | Detected | Not Detected | 4.828544929 | 3.156577464 |
| hsa_gci158771 | 2.299151241 | 1.201101372 | up | 7.112960531 | 5.911859159 | CTGGGCGGCCTG | hsa_circ_0028002 | chr12 | 105724413 | 105765296 | + | 1366 | ANNOTATED, CDS, c | NM_001145199 | C12orf75 | Salzman2013 | 387882 | circRNA | Detected | Detected | 7.112960531 | 5.911859159 |
| hsa_gci158779 | 2.387215636 | 1.25532889 | up | 10.23032025 | 8.974991363 | GTCGTAGGTCCT | hsa_circ_0028034 | chr12 | 108010865 | 108053419 | + | 3238 | ANNOTATED, CDS, c | NM_001018072 | BTBD11 | Salzman2013 | 121551 | circRNA | Detected | Detected | 10.23032025 | 8.974991363 |
| hsa_gci158797 | 2.167262628 | 1.115873989 | up | 5.057923888 | 3.942049899 | TGTATTTTTGCA | hsa_circ_0028156 | chr12 | 110405262 | 110429565 | - | 712 | ANNOTATED, CDS, c | NM_057169 | GIT2 | Salzman2013 | 9815 | circRNA | Detected | Detected | 5.057923888 | 3.942049899 |
| hsa_gci158800 | 2.806808692 | 1.488930735 | up | 4.580772937 | 3.091842202 | GGATAAATGACT | hsa_circ_0028160 | chr12 | 110426767 | 110429565 | - | 353 | ANNOTATED, CDS, c | NM_057169 | GIT2 | Salzman2013 | 9815 | circRNA | Detected | Not Detected | 4.580772937 | 3.091842202 |
| hsa_gci158807 | 2.278240236 | 1.187919885 | up | 8.072241757 | 6.884321872 | ATGAAGACTCCA | hsa_circ_0028182 | chr12 | 110813870 | 110834257 | - | 1407 | ANNOTATED, CDS, c | NM_016238 | ANAPC7 | Salzman2013 | 51434 | circRNA | Detected | Detected | 8.072241757 | 6.884321872 |
| hsa_gci158816 | 2.045245708 | 1.032274173 | up | 11.50007384 | 10.46779967 | TGTCCGTCCACT | hsa_circ_0028202 | chr12 | 110893390 | 110895439 | - | 338 | ANNOTATED, CDS, c | NM_001164372 | GPN3 | Salzman2013 | 51184 | circRNA | Detected | Detected | 11.50007384 | 10.46779967 |
| hsa_gci158833 | 2.328683416 | 1.219514519 | up | 6.560757207 | 5.341242688 | AGTATATATGGA | hsa_circ_0028268 | chr12 | 111951160 | 111990781 | - | 1210 | ANNOTATED, CDS, c | NM_002973 | ATXN2 | Salzman2013 | 6311 | circRNA | Detected | Detected | 6.560757207 | 5.341242688 |
| hsa_gci158838 | 2.059781778 | 1.042491501 | up | 8.074259878 | 7.031768378 | AGTTGTCTCTCT | hsa_circ_0028287 | chr12 | 112143541 | 112153766 | + | 749 | ANNOTATED, CDS, c | NM_001136538 | ACAD10 | Salzman2013 | 80724 | circRNA | Detected | Detected | 8.074259878 | 7.031768378 |
| hsa_gci158839 | 2.498966219 | 1.321331399 | up | 5.775453513 | 4.454122114 | GAAGATTAAGGA | hsa_circ_0028289 | chr12 | 112150301 | 112171872 | + | 850 | ANNOTATED, CDS, c | NM_001136538 | ACAD10 | Salzman2013 | 80724 | circRNA | Detected | Detected | 5.775453513 | 4.454122114 |
| hsa_gci158850 | -4.058667233 | -2.021006059 | down | 1.22891535 | 3.24992141 | AATAAAAGGGCT | hsa_circ_0028336 | chr12 | 112512478 | 112513593 | - | 202 | ANNOTATED, CDS, c | NM_024953 | NAA25 | Salzman2013 | 80018 | circRNA | Not Detected | Detected | 1.22891535 | 3.24992141 |
| hsa_gci158857 | 2.078547806 | 1.05557593 | up | 5.233627047 | 4.178051117 | AGGCTGCCCAAG | hsa_circ_0028411 | chr12 | 112743867 | 112757546 | - | 1158 | ANNOTATED, CDS, c | NM_001109662 | HECTD4 | Salzman2013 | 283450 | circRNA | Detected | Detected | 5.233627047 | 4.178051117 |
| hsa_gci158863 | 2.130831271 | 1.091416359 | up | 7.076084433 | 5.984668074 | AATCCCCTCTTG | hsa_circ_0028487 | chr12 | 113710462 | 113711473 | + | 194 | ANNOTATED, CDS, c | NM_001143819 | TPCN1 | Salzman2013 | 53373 | circRNA | Detected | Detected | 7.076084433 | 5.984668074 |
| hsa_gci158868 | 2.157059562 | 1.109066013 | up | 8.107745439 | 6.998679425 | CCTAACCCGACC | hsa_circ_0028560 | chr12 | 116413319 | 116413543 | - | 224 | ANNOTATED, CDS, c | NM_015335 | MED13L | Salzman2013 | 23389 | circRNA | Detected | Detected | 8.107745439 | 6.998679425 |
| hsa_gci158905 | -3.948446194 | -1.981285031 | down | 1.22275845 | 3.204043481 | CTGACTCTGTCG | hsa_circ_0028811 | chr12 | 120599683 | 120611630 | - | 1150 | ANNOTATED, CDS, c | NM_006836 | GCN1 | Salzman2013 | 10985 | circRNA | Not Detected | Detected | 1.22275845 | 3.204043481 |
| hsa_gci158925 | 2.578518132 | 1.36654219 | up | 5.740450966 | 4.373908776 | TCGACAGTCTCG | hsa_circ_0028925 | chr12 | 121157722 | 121161443 | + | 3721 | ANNOTATED, CDS, c | NM_001080533 | UNC119B | Salzman2013 | 84747 | circRNA | Detected | Detected | 5.740450966 | 4.373908776 |
| hsa_gci158933 | 2.118581592 | 1.083098692 | up | 5.490594414 | 4.407495723 | TGTAGAACCTGT | hsa_circ_0028972 | chr12 | 121768385 | 121773526 | - | 363 | ANNOTATED, CDS, c | NM_016237 | ANAPC5 | Salzman2013 | 51433 | circRNA | Detected | Detected | 5.490594414 | 4.407495723 |
| hsa_gci158935 | 2.335325435 | 1.223623608 | up | 6.61327113 | 5.389647521 | TCAGCCACTTTG | hsa_circ_0028989 | chr12 | 121970710 | 121972495 | - | 248 | ANNOTATED, CDS, c | NM_032590 | KDM2B | Salzman2013 | 84678 | circRNA | Detected | Detected | 6.61327113 | 5.389647521 |
| hsa_gci158959 | 2.468031049 | 1.303360545 | up | 5.946964616 | 4.643604071 | ACAAACTCTTAC | hsa_circ_0029130 | chr12 | 123061441 | 123109235 | + | 4020 | ANNOTATED, CDS, c | NM_014708 | KNTC1 | Salzman2013 | 9735 | circRNA | Detected | Detected | 5.946964616 | 4.643604071 |
| hsa_gci158976 | -2.175229967 | -1.121167932 | down | 3.624668816 | 4.745836748 | GGTTTAACTTAT | hsa_circ_0029197 | chr12 | 123694602 | 123707674 | - | 2078 | ALT_ACCEPTOR, CDS | NM_022782 | MPHOSPH9 | Salzman2013 | 10198 | circRNA | Not Detected | Detected | 3.624668816 | 4.745836748 |
| hsa_gci158982 | 3.137697306 | 1.649706182 | up | 6.060564634 | 4.410858452 | AGTCCTCGTCAT | hsa_circ_0029211 | chr12 | 123799919 | 123800207 | - | 185 | ANNOTATED, CDS, c | NM_001167856 | SBNO1 | Salzman2013 | 55206 | circRNA | Detected | Detected | 6.060564634 | 4.410858452 |
| hsa_gci158998 | 2.785824604 | 1.478104429 | up | 7.304682081 | 5.826577652 | CCTTAACCGTTA | hsa_circ_0029263 | chr12 | 124156602 | 124171582 | + | 574 | ANNOTATED, CDS, c | NM_024809 | TCTN2 | Salzman2013 | 79867 | circRNA | Detected | Detected | 7.304682081 | 5.826577652 |
| hsa_gci159001 | 2.522414602 | 1.334805427 | up | 7.460360198 | 6.125554771 | CTTCGTCCTGGC | hsa_circ_0029269 | chr12 | 124171382 | 124171582 | + | 200 | ANNOTATED, CDS, c | NM_024809 | TCTN2 | Salzman2013 | 79867 | circRNA | Detected | Detected | 7.460360198 | 6.125554771 |
| hsa_gci159003 | 2.73512514 | 1.451606842 | up | 10.6402362 | 9.188629354 | GAAAGACCTTTT | hsa_circ_0029284 | chr12 | 124420954 | 124428901 | - | 1695 | ALT_ACCEPTOR, CDS | NM_025140 | CCDC92 | Salzman2013 | 80212 | circRNA | Detected | Detected | 10.6402362 | 9.188629354 |
| hsa_gci159010 | 2.61061597 | 1.384390248 | up | 7.797588165 | 6.413197916 | TAGTTTGTACCA | hsa_circ_0029387 | chr12 | 129277738 | 129293501 | - | 1647 | ANNOTATED, CDS, c | NM_145648 | SLC15A4 | Salzman2013 | 121260 | circRNA | Detected | Detected | 7.797588165 | 6.413197916 |
| hsa_gci159017 | 2.104106818 | 1.073207947 | up | 4.943737369 | 3.870529423 | GGCAGGCTGACT | hsa_circ_0029425 | chr12 | 131356616 | 131360826 | + | 1071 | ANNOTATED, CDS, c | NM_006325 | RAN | Salzman2013 | 5901 | circRNA | Detected | Detected | 4.943737369 | 3.870529423 |
| hsa_gci159019 | 2.706137581 | 1.436235188 | up | 4.382812516 | 2.946577327 | TGTAATACTACC | hsa_circ_0029427 | chr12 | 131359090 | 131359278 | + | 188 | ANNOTATED, CDS, c | NM_006325 | RAN | Salzman2013 | 5901 | circRNA | Detected | Not Detected | 4.382812516 | 2.946577327 |
| hsa_gci159021 | 2.153623104 | 1.106765792 | up | 6.249182897 | 5.142417105 | AGTGTCCAGTAG | hsa_circ_0029430 | chr12 | 131360426 | 131360826 | + | 400 | ANNOTATED, CDS, c | NM_006325 | RAN | Salzman2013 | 5901 | circRNA | Detected | Detected | 6.249182897 | 5.142417105 |
| hsa_gci159022 | 2.139538657 | 1.097299746 | up | 7.864548652 | 6.767248907 | GGCCGGTACGCG | hsa_circ_0029450 | chr12 | 132414451 | 132428406 | + | 1485 | ANNOTATED, CDS, c | NM_025215 | PUS1 | Salzman2013 | 80324 | circRNA | Detected | Detected | 7.864548652 | 6.767248907 |
| hsa_gci159024 | 2.45565033 | 1.296105144 | up | 6.778983995 | 5.482878851 | GAATAAGTGGAC | hsa_circ_0029486 | chr12 | 133200347 | 133202903 | - | 1466 | ANNOTATED, CDS, c | NM_006231 | POLE | Salzman2013 | 5426 | circRNA | Detected | Detected | 6.778983995 | 5.482878851 |
| hsa_gci159025 | 2.329438221 | 1.219982069 | up | 4.289590954 | 3.069608885 | CTACTGGGGTCC | hsa_circ_0029488 | chr12 | 133200347 | 133214725 | - | 2244 | ANNOTATED, CDS, c | NM_006231 | POLE | Salzman2013 | 5426 | circRNA | Detected | Not Detected | 4.289590954 | 3.069608885 |
| hsa_gci159026 | -2.260294793 | -1.176510944 | down | 2.697500808 | 3.874011753 | GTTCCGACCCGT | hsa_circ_0029489 | chr12 | 133200347 | 133219582 | - | 3245 | ANNOTATED, CDS, c | NM_006231 | POLE | Salzman2013 | 5426 | circRNA | Not Detected | Detected | 2.697500808 | 3.874011753 |
| hsa_gci159027 | 3.28461165 | 1.715722806 | up | 6.466843983 | 4.751121177 | GTGGTCCCGGGC | hsa_circ_0029490 | chr12 | 133200347 | 133220146 | - | 3506 | ANNOTATED, CDS, c | NM_006231 | POLE | Salzman2013 | 5426 | circRNA | Detected | Detected | 6.466843983 | 4.751121177 |
| hsa_gci159028 | 2.251788461 | 1.171071303 | up | 6.602622038 | 5.431550735 | GTGACCTCCTTT | hsa_circ_0029491 | chr12 | 133200347 | 133235260 | - | 5225 | ALT_ACCEPTOR, CDS | NM_006231 | POLE | Salzman2013 | 5426 | circRNA | Detected | Detected | 6.602622038 | 5.431550735 |
| hsa_gci159029 | 2.383366993 | 1.253001116 | up | 8.425471181 | 7.172470065 | CGGTCGGTAGAG | hsa_circ_0029492 | chr12 | 133200347 | 133241048 | - | 5328 | ANNOTATED, CDS, c | NM_006231 | POLE | Salzman2013 | 5426 | circRNA | Detected | Detected | 8.425471181 | 7.172470065 |
| hsa_gci159040 | 2.013071133 | 1.009398152 | up | 6.0919003 | 5.082502148 | TGGTCGATTATA | hsa_circ_0029579 | chr12 | 133393125 | 133398897 | - | 589 | ANNOTATED, CDS, c | NM_005895 | GOLGA3 | Salzman2013 | 2802 | circRNA | Detected | Detected | 6.0919003 | 5.082502148 |
| hsa_gci159044 | 2.264425279 | 1.179144934 | up | 5.606129365 | 4.426984431 | TTGTAGTAGAAT | hsa_circ_0029607 | chr13 | 20277008 | 20356931 | - | 1912 | ANNOTATED, CDS, c | NM_001042414 | PSPC1 | Salzman2013 | 55269 | circRNA | Detected | Detected | 5.606129365 | 4.426984431 |
| hsa_gci159068 | 3.014791913 | 1.592058428 | up | 6.347746192 | 4.755687764 | AGAGTAACCTAT | hsa_circ_0029696 | chr13 | 21735928 | 21746820 | - | 726 | ANNOTATED, CDS, c | NM_145061 | SKA3 | Salzman2013 | 221150 | circRNA | Detected | Detected | 6.347746192 | 4.755687764 |
| hsa_gci159101 | -9.299595182 | -3.217167916 | down | 1.173931531 | 4.391099447 | CCTTCTGGGTCG | hsa_circ_0029851 | chr13 | 28830428 | 28845003 | + | 958 | ANNOTATED, CDS, c | NM_175854 | PAN3 | Salzman2013 | 255967 | circRNA | Not Detected | Detected | 1.173931531 | 4.391099447 |
| hsa_gci159117 | 2.437388058 | 1.285335962 | up | 8.683767863 | 7.398431902 | TCGTGACCGTCC | hsa_circ_0029876 | chr13 | 30083550 | 30090383 | - | 5279 | ANNOTATED, CDS, c | NM_003045 | SLC7A1 | Salzman2013 | 6541 | circRNA | Detected | Detected | 8.683767863 | 7.398431902 |
| hsa_gci159158 | 3.337611824 | 1.738816174 | up | 6.053703159 | 4.314886985 | TCGTGTCCTCCG | hsa_circ_0030003 | chr13 | 37427555 | 37447052 | - | 848 | ANNOTATED, CDS, c | NM_001127217 | SMAD9 | Salzman2013 | 4093 | circRNA | Detected | Detected | 6.053703159 | 4.314886985 |
| hsa_gci159160 | 7.117090355 | 2.831287552 | up | 6.221901383 | 3.390613832 | TTGACCCCGAGC | hsa_circ_0030024 | chr13 | 39584001 | 39605768 | - | 4224 | ANNOTATED, CDS, c | NM_025138 | PROSER1 | Salzman2013 | 80209 | circRNA | Detected | Detected | 6.221901383 | 3.390613832 |
| hsa_gci159162 | 3.34272402 | 1.741024249 | up | 6.247616673 | 4.506592424 | AGCCGGGTCCTT | hsa_circ_0030026 | chr13 | 39596462 | 39605768 | - | 619 | ANNOTATED, CDS, c | NM_025138 | PROSER1 | Salzman2013 | 80209 | circRNA | Detected | Detected | 6.247616673 | 4.506592424 |
| hsa_gci159164 | 4.150484993 | 2.053279928 | up | 4.335631635 | 2.282351707 | GGGGTCCAAGTC | hsa_circ_0030034 | chr13 | 40268770 | 40301685 | + | 752 | ANNOTATED, CDS, c | NM_001145079 | COG6 | Salzman2013 | 57511 | circRNA | Detected | Not Detected | 4.335631635 | 2.282351707 |
| hsa_gci159167 | 2.194809519 | 1.134095738 | up | 6.543031567 | 5.40893583 | AATCACGTCTTG | hsa_circ_0030038 | chr13 | 40293372 | 40365802 | + | 3873 | ANNOTATED, CDS, c | NM_001145079 | COG6 | Salzman2013 | 57511 | circRNA | Detected | Detected | 6.543031567 | 5.40893583 |
| hsa_gci159175 | 2.239416909 | 1.163123138 | up | 7.142024008 | 5.978900871 | GACGTTGGTCGC | hsa_circ_0030064 | chr13 | 41834628 | 41836467 | - | 1839 | ALT_ACCEPTOR, CDS | NM_004294 | MTRF1 | Salzman2013 | 9617 | circRNA | Detected | Detected | 7.142024008 | 5.978900871 |
| hsa_gci159188 | 2.647528946 | 1.404646457 | up | 4.161810236 | 2.757163778 | TAACCTACCATA | hsa_circ_0030131 | chr13 | 42739466 | 42742954 | + | 512 | ANNOTATED, CDS, c | NM_178009 | DGKH | Salzman2013 | 160851 | circRNA | Detected | Not Detected | 4.161810236 | 2.757163778 |
| hsa_gci159194 | 2.059727066 | 1.042453179 | up | 9.249239509 | 8.206786331 | CGCATTGTAGCG | hsa_circ_0030172 | chr13 | 45978461 | 45980112 | - | 181 | ANNOTATED, CDS, c | NM_001010875 | SLC25A30 | Salzman2013 | 253512 | circRNA | Detected | Detected | 9.249239509 | 8.206786331 |
| hsa_gci159204 | 2.682648642 | 1.423658111 | up | 4.863782532 | 3.440124421 | TCTAAGGTTTAA | hsa_circ_0030214 | chr13 | 47297355 | 47303119 | + | 248 | ANNOTATED, CDS, c | NM_001164211 | LRCH1 | Salzman2013 | 23143 | circRNA | Detected | Detected | 4.863782532 | 3.440124421 |
| hsa_gci159235 | 36.10440675 | 5.174103032 | up | 7.373091168 | 2.198988136 | AGTAATCAAACG | hsa_circ_0030342 | chr13 | 52992126 | 53001354 | - | 377 | ANNOTATED, CDS, c | NM_016075 | VPS36 | Salzman2013 | 51028 | circRNA | Detected | Not Detected | 7.373091168 | 2.198988136 |
| hsa_gci159241 | 3.523771781 | 1.817120491 | up | 7.010004715 | 5.192884224 | AGTCCTGAGTCC | hsa_circ_0030358 | chr13 | 53201572 | 53203917 | + | 705 | ALT_DONOR, coding | NM_001011724 | HNRNPA1L2 | Salzman2013 | 144983 | circRNA | Detected | Detected | 7.010004715 | 5.192884224 |

| | | | | | | | | | | | | | | | | | | | | | | | |
|---|---|---|---|---|---|---|---|---|---|---|---|---|---|---|---|---|---|---|---|---|---|---|---|
| hsa_gci159248 | 2.677393682 | 1.420829287 | up | 5.405309554 | 3.984480266 | AGTATCGCATCT | hsa_circ_0030387 | chr13 | 64560503 | 64608670 | - | 1108 | ANNOTATED, ncRNA, | TCONS_12_00007568 | | Salzman2013 | | circRNA | Detected | Detected | 5.405309554 | 3.984480266 |
| hsa_gci159278 | 2.406802081 | 1.267117509 | up | 8.179453689 | 6.91233618 | TGGTCGTTTATG | hsa_circ_0030511 | chr13 | 77791974 | 77807398 | - | 428 | ANNOTATED, CDS, c | NM_015057 | MYCBP2 | Salzman2013 | 23077 | circRNA | Detected | Detected | 8.179453689 | 6.91233618 |
| hsa_gci159279 | 2.805261806 | 1.488135419 | up | 7.645024679 | 6.15688926 | CTACCAAGTATT | hsa_circ_0030514 | chr13 | 77798585 | 77807398 | - | 310 | ANNOTATED, CDS, c | NM_015057 | MYCBP2 | Salzman2013 | 23077 | circRNA | Detected | Detected | 7.645024679 | 6.15688926 |
| hsa_gci159285 | 2.07592577 | 1.053754857 | up | 4.462665167 | 3.40891031 | GTACTAGTTTTC | hsa_circ_0030538 | chr13 | 79940315 | 79941007 | - | 255 | ALT_DONOR, CDS, c | NM_022118 | RBM26 | Salzman2013 | 64062 | circRNA | Detected | Detected | 4.462665167 | 3.40891031 |
| hsa_gci159292 | 2.95651989 | 1.563899983 | up | 8.383705236 | 6.819805253 | TCCACCGTGCCC | hsa_circ_0030552 | chr13 | 80107457 | 80117817 | + | 353 | ANNOTATED, CDS, c | NM_019080 | NDFIP2 | Salzman2013 | 54602 | circRNA | Detected | Detected | 8.383705236 | 6.819805253 |
| hsa_gci159306 | 2.717631411 | 1.442349799 | up | 8.178236041 | 6.735886243 | CCGGTTAAGGGG | hsa_circ_0030593 | chr13 | 95822785 | 95839146 | - | 471 | ANNOTATED, CDS, c | NM_005845 | ABCC4 | Salzman2013 | 10257 | circRNA | Detected | Detected | 8.178236041 | 6.735886243 |
| hsa_gci159319 | 2.055299799 | 1.03934885 | up | 10.22545849 | 9.186109642 | AGAGAAACCATT | hsa_circ_0030654 | chr13 | 98009735 | 98046374 | + | 3084 | ANNOTATED, CDS, c | NM_144778 | MBNL2 | Salzman2013 | 10150 | circRNA | Detected | Detected | 10.22545849 | 9.186109642 |
| hsa_gci159321 | 2.215631961 | 1.147718255 | up | 6.3641188 | 5.216400545 | CCGGACCCTATA | hsa_circ_0030662 | chr13 | 98634756 | 98670958 | + | 2746 | ANNOTATED, CDS, c | NM_002271 | IPO5 | Salzman2013 | 3843 | circRNA | Detected | Detected | 6.3641188 | 5.216400545 |
| hsa_gci159353 | 2.232212142 | 1.158474143 | up | 7.157088827 | 5.998614684 | CCAGTGTAAATC | hsa_circ_0030777 | chr13 | 101077886 | 101101559 | + | 153 | ANNOTATED, CDS, c | NM_000282 | PCCA | Salzman2013 | 5095 | circRNA | Detected | Detected | 7.157088827 | 5.998614684 |
| hsa_gci159360 | 2.028416703 | 1.020354059 | up | 5.201855646 | 4.181501587 | GGGCCCGAGCAG | hsa_circ_0030834 | chr13 | 110406183 | 110438914 | - | 6998 | ANNOTATED, CDS, c | NM_003749 | IRS2 | Salzman2013 | 8660 | circRNA | Detected | Detected | 5.201855646 | 4.181501587 |
| hsa_gci159364 | 2.311441863 | 1.208793077 | up | 10.6428253 | 9.434032225 | GGCCCTTCCTCG | hsa_circ_0030880 | chr13 | 111175412 | 111214071 | - | 1509 | ANNOTATED, CDS, c | NM_017817 | RAB20 | Salzman2013 | 55647 | circRNA | Detected | Detected | 10.6428253 | 9.434032225 |
| hsa_gci159370 | 2.113989359 | 1.079968115 | up | 6.792178407 | 5.712210291 | AAGCACTCACCG | hsa_circ_0030900 | chr13 | 111530886 | 111536140 | - | 1669 | ANNOTATED, CDS, c | NM_017664 | ANKRD10 | Salzman2013 | 55608 | circRNA | Detected | Detected | 6.792178407 | 5.712210291 |
| hsa_gci159389 | 119.2548545 | 6.897904185 | up | 8.748396017 | 1.850491833 | CGACGACTGCGT | hsa_circ_0030997 | chr13 | 113960799 | 113977741 | + | 2198 | ANNOTATED, CDS, c | NM_005561 | LAMP1 | Salzman2013 | 3916 | circRNA | Detected | Not Detected | 8.748396017 | 1.850491833 |
| hsa_gci159392 | 2.836766836 | 1.504247575 | up | 6.360194209 | 4.855946634 | CCCTCGGCAACC | hsa_circ_0031005 | chr13 | 113975871 | 113977741 | + | 1316 | ANNOTATED, CDS, c | NM_005561 | LAMP1 | Salzman2013 | 3916 | circRNA | Detected | Detected | 6.360194209 | 4.855946634 |
| hsa_gci159405 | 2.33603703 | 1.224063144 | up | 5.277677347 | 4.053614204 | TTCGTAACATTG | hsa_circ_0031088 | chr13 | 115030624 | 115038150 | + | 583 | ANNOTATED, CDS, c | NM_001078645 | CDC16 | Salzman2013 | 8881 | circRNA | Detected | Detected | 5.277677347 | 4.053614204 |
| hsa_gci159411 | 2.966749759 | 1.568883243 | up | 4.235672035 | 2.666788793 | AAGAGGCCCACG | hsa_circ_0031158 | chr14 | 21821861 | 21829372 | - | 1127 | ANNOTATED, CDS, c | NM_007192 | SUPT16H | Salzman2013 | 11198 | circRNA | Detected | Not Detected | 4.235672035 | 2.666788793 |
| hsa_gci159412 | 2.345051275 | 1.229619468 | up | 6.160985195 | 4.931365728 | CCTCTGAGAGAA | hsa_circ_0031171 | chr14 | 21853352 | 21861413 | - | 1846 | ANNOTATED, CDS, c | NM_001170629 | CHD8 | Salzman2013 | 57680 | circRNA | Detected | Detected | 6.160985195 | 4.931365728 |
| hsa_gci159415 | 2.020310033 | 1.014576703 | up | 6.690498745 | 5.675922041 | AACTGTTTCTTA | hsa_circ_0031182 | chr14 | 21853352 | 21883152 | - | 6197 | ANNOTATED, CDS, c | NM_001170629 | CHD8 | Salzman2013 | 57680 | circRNA | Detected | Detected | 6.690498745 | 5.675922041 |
| hsa_gci159416 | 2.435287115 | 1.284091873 | up | 7.921169851 | 6.637077978 | AATCCTAAAAAA | hsa_circ_0031189 | chr14 | 21868437 | 21871822 | - | 1292 | ALT_DONOR, CDS, c | NM_001170629 | CHD8 | Salzman2013 | 57680 | circRNA | Detected | Detected | 7.921169851 | 6.637077978 |
| hsa_gci159422 | 2.554607423 | 1.353101603 | up | 7.976293194 | 6.62319159 | AGAGGTCTTGAG | hsa_circ_0031209 | chr14 | 23033806 | 23034037 | - | 231 | ANNOTATED, coding | NM_001344 | DAD1 | Salzman2013 | 1603 | circRNA | Detected | Detected | 7.976293194 | 6.62319159 |
| hsa_gci159423 | 2.191033767 | 1.131611719 | up | 6.456740877 | 5.325129158 | AGACATAAGAGT | hsa_circ_0031210 | chr14 | 23033806 | 23044133 | - | 406 | ANNOTATED, CDS, c | NM_001344 | DAD1 | Salzman2013 | 1603 | circRNA | Detected | Detected | 6.456740877 | 5.325129158 |
| hsa_gci159424 | 2.405406278 | 1.266280589 | up | 7.196629169 | 5.93034858 | TATAAACTTCAC | hsa_circ_0031212 | chr14 | 23235730 | 23237831 | + | 1070 | ALT_DONOR, CDS, c | NM_005015 | OXA1L | Salzman2013 | 5018 | circRNA | Detected | Detected | 7.196629169 | 5.93034858 |
| hsa_gci159446 | 3.633511594 | 1.86136451 | up | 6.351561658 | 4.490197148 | GACAGAGGACAC | hsa_circ_0031294 | chr14 | 24027903 | 24028787 | + | 884 | ANNOTATED, CDS, c | NM_024328 | THTPA | Salzman2013 | 79178 | circRNA | Detected | Detected | 6.351561658 | 4.490197148 |
| hsa_gci159447 | 2.67215684 | 1.418004688 | up | 9.050183894 | 7.632179206 | AAGTAACGTCAA | hsa_circ_0031323 | chr14 | 24606908 | 24608176 | + | 784 | ANNOTATED, CDS, c | NM_176783 | PSME1 | Salzman2013 | 5720 | circRNA | Detected | Detected | 9.050183894 | 7.632179206 |
| hsa_gci159453 | 2.248075386 | 1.168690415 | up | 8.940607174 | 7.771916759 | TTATGAAGAAGT | hsa_circ_0031353 | chr14 | 24652455 | 24652719 | - | 164 | ANNOTATED, CDS, c | NM_024658 | IPO4 | Salzman2013 | 79711 | circRNA | Detected | Detected | 8.940607174 | 7.771916759 |
| hsa_gci159455 | 2.195305064 | 1.134421434 | up | 11.69281586 | 10.55839443 | CTGTTTATCAGA | hsa_circ_0031388 | chr14 | 24759803 | 24760201 | - | 398 | ANNOTATED, CDS, c | NM_001136050 | DHRS1 | Salzman2013 | 115817 | circRNA | Detected | Detected | 11.69281586 | 10.55839443 |
| hsa_gci159465 | 2.288969639 | 1.194698327 | up | 4.289350312 | 3.094651985 | ATCTTTACGTTA | hsa_circ_0031441 | chr14 | 31380982 | 31425448 | - | 1316 | ANNOTATED, CDS, c | NM_001083893 | STRN3 | Salzman2013 | 29966 | circRNA | Detected | Not Detected | 4.289350312 | 3.094651985 |
| hsa_gci159467 | 2.850061312 | 1.510992956 | up | 8.904992751 | 7.393999795 | TTCAATACTGTT | hsa_circ_0031448 | chr14 | 31535331 | 31535540 | + | 209 | ANNOTATED, CDS, c | NM_007077 | AP4S1 | Salzman2013 | 11154 | circRNA | Detected | Detected | 8.904992751 | 7.393999795 |
| hsa_gci159468 | 3.423782556 | 1.775591079 | up | 8.94406538 | 7.168474301 | ATGTCAAGTTTT | hsa_circ_0031449 | chr14 | 31535331 | 31542179 | + | 365 | ANNOTATED, CDS, c | NM_007077 | AP4S1 | Salzman2013 | 11154 | circRNA | Detected | Detected | 8.94406538 | 7.168474301 |
| hsa_gci159489 | 2.531703034 | 1.340108188 | up | 5.600243394 | 4.260135206 | GAGAAGAGTCAT | hsa_circ_0031540 | chr14 | 31787429 | 31806820 | - | 810 | ANNOTATED, CDS, c | NM_015473 | HEATR5A | Salzman2013 | 25938 | circRNA | Detected | Detected | 5.600243394 | 4.260135206 |
| hsa_gci159508 | -2.668404953 | -1.415977624 | down | 2.648696376 | 4.064674001 | CAGAACCACCAC | hsa_circ_0031600 | chr14 | 35030617 | 35074923 | - | 2661 | ANNOTATED, CDS, c | NM_021249 | SNX6 | Salzman2013 | 58533 | circRNA | Not Detected | Detected | 2.648696376 | 4.064674001 |
| hsa_gci159512 | 2.432012756 | 1.282150796 | up | 4.174781159 | 2.892630363 | GAAGGAGCAAAC | hsa_circ_0031615 | chr14 | 35227909 | 35231429 | - | 610 | ANNOTATED, CDS, c | NM_013448 | BAZ1A | Salzman2013 | 11177 | circRNA | Detected | Not Detected | 4.174781159 | 2.892630363 |
| hsa_gci159514 | -13.88666895 | -3.795628671 | down | 1.316492373 | 5.112121043 | AAGCAAGTTTTT | hsa_circ_0031622 | chr14 | 35234226 | 35331528 | - | 3436 | ANNOTATED, CDS, c | NM_013448 | BAZ1A | Salzman2013 | 11177 | circRNA | Not Detected | Detected | 1.316492373 | 5.112121043 |
| hsa_gci159527 | 4.373305447 | 2.128724115 | up | 5.234797191 | 3.106073077 | ACCAGTCACGGA | hsa_circ_0031663 | chr14 | 35870715 | 35873004 | - | 1230 | ANNOTATED, CDS, c | NM_020529 | NFKBIA | Salzman2013 | 4792 | circRNA | Detected | Not Detected | 5.234797191 | 3.106073077 |
| hsa_gci159534 | 2.573330124 | 1.363636547 | up | 8.601030991 | 7.237394444 | ACTCGCCCCTTC | hsa_circ_0031675 | chr14 | 36133847 | 36159209 | - | 1544 | ANNOTATED, CDS, c | NM_014990 | RALGAPA1 | Salzman2013 | 253959 | circRNA | Detected | Detected | 8.601030991 | 7.237394444 |
| hsa_gci159550 | 2.20144969 | 1.138453875 | up | 5.944159359 | 4.805705484 | AAGTCAGACTTC | hsa_circ_0031748 | chr14 | 39777652 | 39796226 | + | 777 | ANNOTATED, CDS, c | NM_001247989 | MIA2 | Salzman2013 | 4253 | circRNA | Detected | Detected | 5.944159359 | 4.805705484 |
| hsa_gci159551 | 2.03804085 | 1.027182969 | up | 5.855203374 | 4.828020406 | ATTGGCTATTAA | hsa_circ_0031749 | chr14 | 39782543 | 39796226 | + | 638 | ANNOTATED, CDS, c | NM_001247989 | MIA2 | Salzman2013 | 4253 | circRNA | Detected | Detected | 5.855203374 | 4.828020406 |
| hsa_gci159558 | 2.059304724 | 1.042157327 | up | 4.057134879 | 3.014977552 | GCCTAGTCTGGC | hsa_circ_0031775 | chr14 | 50050289 | 50052767 | - | 204 | ANNOTATED, CDS, c | NM_001032 | RPS29 | Salzman2013 | 6235 | circRNA | Detected | Not Detected | 4.057134879 | 3.014977552 |
| hsa_gci159564 | 2.428829904 | 1.280261459 | up | 6.496520227 | 5.216258768 | GGCGGGCGGCTC | hsa_circ_0031792 | chr14 | 50234786 | 50249856 | + | 1806 | ANNOTATED, CDS, c | NM_014315 | KLHDC2 | Salzman2013 | 23588 | circRNA | Detected | Detected | 6.496520227 | 5.216258768 |
| hsa_gci159577 | 2.743302998 | 1.455913977 | up | 5.052092828 | 3.596178851 | GGTAAATAGTTA | hsa_circ_0031830 | chr14 | 50647289 | 50667802 | - | 756 | ANNOTATED, CDS, c | NM_006939 | SOS2 | Salzman2013 | 6655 | circRNA | Detected | Detected | 5.052092828 | 3.596178851 |
| hsa_gci159579 | 2.144747802 | 1.100808013 | up | 5.485772684 | 4.384964671 | AAGTAATCGACT | hsa_circ_0031836 | chr14 | 50667670 | 50671127 | - | 258 | ANNOTATED, CDS, c | NM_006939 | SOS2 | Salzman2013 | 6655 | circRNA | Detected | Detected | 5.485772684 | 4.384964671 |
| hsa_gci159585 | 2.784155995 | 1.477240047 | up | 6.627181627 | 5.14994158 | ACGTATGTATCC | hsa_circ_0031856 | chr14 | 50912796 | 50949134 | - | 863 | ANNOTATED, CDS, c | NM_198794 | MAP4K5 | Salzman2013 | 11183 | circRNA | Detected | Detected | 6.627181627 | 5.14994158 |
| hsa_gci159611 | 2.534992545 | 1.341981504 | up | 6.99013811 | 5.648156605 | TCGAGTAGTTTA | hsa_circ_0031983 | chr14 | 54898825 | 54903155 | - | 182 | ANNOTATED, CDS, c | NM_005776 | CNIH1 | Salzman2013 | 10175 | circRNA | Detected | Detected | 6.99013811 | 5.648156605 |
| hsa_gci159620 | 3.995020528 | 1.998202916 | up | 8.66363826 | 6.665435343 | ACCGTCCGTACA | hsa_circ_0032017 | chr14 | 55475033 | 55493521 | - | 520 | ANNOTATED, CDS, c | NM_007086 | WDHD1 | Salzman2013 | 11169 | circRNA | Detected | Detected | 8.66363826 | 6.665435343 |
| hsa_gci159625 | 2.665291753 | 1.414293465 | up | 4.23845057 | 2.824157105 | GAGACCGAGCAA | hsa_circ_0032052 | chr14 | 57740961 | 57747140 | + | 874 | ANNOTATED, CDS, c | NM_018229 | AP5M1 | Salzman2013 | 55745 | circRNA | Detected | Not Detected | 4.23845057 | 2.824157105 |
| hsa_gci159655 | 3.408579739 | 1.769170733 | up | 5.558640753 | 3.78947002 | CCGCCGAATCTC | hsa_circ_0032156 | chr14 | 64063756 | 64108591 | - | 3111 | ANNOTATED, CDS, c | NM_080666 | WDR89 | Salzman2013 | 112840 | circRNA | Detected | Detected | 5.558640753 | 3.78947002 |
| hsa_gci159668 | 4.996397585 | 2.320888283 | up | 5.15852941 | 2.837641127 | TGTGGTGCTCTT | hsa_circ_0032254 | chr14 | 67431907 | 67525503 | + | 316 | ANNOTATED, CDS, c | NM_020806 | GPHN | Salzman2013 | 10243 | circRNA | Detected | Not Detected | 5.15852941 | 2.837641127 |
| hsa_gci159670 | 2.351818272 | 1.233776586 | up | 8.392414994 | 7.158638408 | CAAAAAGTGTTT | hsa_circ_0032261 | chr14 | 67736417 | 67770316 | + | 1277 | ANNOTATED, CDS, c | NM_022474 | MPP5 | Salzman2013 | 64398 | circRNA | Detected | Detected | 8.392414994 | 7.158638408 |
| hsa_gci159674 | 2.095914433 | 1.067579819 | up | 4.611933538 | 3.544353719 | GGGCCGTTCGAT | hsa_circ_0032277 | chr14 | 68151731 | 68159310 | - | 661 | ANNOTATED, CDS, c | NM_016026 | RDH11 | Salzman2013 | 51109 | circRNA | Detected | Detected | 4.611933538 | 3.544353719 |
| hsa_gci159675 | -4.673725351 | -2.224572958 | down | 2.293900431 | 4.518473388 | ATGTGTGGGGTG | hsa_circ_0032278 | chr14 | 68151731 | 68159769 | - | 780 | ANNOTATED, CDS, c | NM_016026 | RDH11 | Salzman2013 | 51109 | circRNA | Not Detected | Detected | 2.293900431 | 4.518473388 |
| hsa_gci159762 | 2.604033174 | 1.380747828 | up | 4.375275248 | 2.99452742 | GACGACTTTGTT | hsa_circ_0032674 | chr14 | 75576387 | 75587283 | - | 729 | ANNOTATED, CDS, c | NM_033116 | NEK9 | Salzman2013 | 91754 | circRNA | Detected | Not Detected | 4.375275248 | 2.99452742 |

| | | | | | | | | | | | | | | | | | | | | | | |
|---|---|---|---|---|---|---|---|---|---|---|---|---|---|---|---|---|---|---|---|---|---|---|
| hsa_gci159806 | 2.588165935 | 1.371930116 | up | 6.353535206 | 4.98160509 | GGTAGTCGTTAA | hsa_circ_0032901 | chr14 | 90450859 | 90485762 | + | 760 | ANNOTATED, CDS, c | NM_018319 | TDP1 | Salzman2013 | 55775 | circRNA | Detected | Detected | 6.353535206 | 4.98160509 |
| hsa_gci159836 | 2.504568121 | 1.324561851 | up | 7.100570321 | 5.776008469 | GGCGGTAAAACT | hsa_circ_0033034 | chr14 | 93703895 | 93799385 | - | 8448 | ANNOTATED, CDS, c | NM_001002860 | BTBD7 | Salzman2013 | 55727 | circRNA | Detected | Detected | 7.100570321 | 5.776008469 |
| hsa_gci159839 | 5.104186544 | 2.351681057 | up | 5.607408235 | 3.255727179 | GGTGAAGCCACC | hsa_circ_0033059 | chr14 | 94517267 | 94521530 | - | 860 | ANNOTATED, CDS, c | NM_020414 | DDX24 | Salzman2013 | 57062 | circRNA | Detected | Not Detected | 5.607408235 | 3.255727179 |
| hsa_gci159842 | 2.312814143 | 1.209649336 | up | 5.615130923 | 4.405481587 | CAGCATTCAAGA | hsa_circ_0033075 | chr14 | 95552564 | 95557000 | - | 4436 | ANNOTATED, CDS, c | NM_177438 | DICER1 | Salzman2013 | 23405 | circRNA | Detected | Detected | 5.615130923 | 4.405481587 |
| hsa_gci159860 | 2.1781777 | 1.123121657 | up | 5.778322486 | 4.65520083 | CAATTTATGTAG | hsa_circ_0033176 | chr14 | 100800124 | 100820206 | - | 1961 | ANNOTATED, CDS, c | NM_004184 | WARS | Salzman2013 | 7453 | circRNA | Detected | Detected | 5.778322486 | 4.65520083 |
| hsa_gci159861 | 3.011906167 | 1.590676825 | up | 6.085927927 | 4.495251102 | CCTAAACAAGTC | hsa_circ_0033178 | chr14 | 100800124 | 100835595 | - | 2576 | ANNOTATED, CDS, c | NM_004184 | WARS | Salzman2013 | 7453 | circRNA | Detected | Detected | 6.085927927 | 4.495251102 |
| hsa_gci159862 | 2.171384585 | 1.118615272 | up | 5.912248519 | 4.793633247 | GGTTACTTTGGG | hsa_circ_0033179 | chr14 | 100800124 | 100841927 | - | 2884 | ANNOTATED, CDS, c | NM_004184 | WARS | Salzman2013 | 7453 | circRNA | Detected | Detected | 5.912248519 | 4.793633247 |
| hsa_gci159865 | 2.547367827 | 1.349007293 | up | 8.351414203 | 7.00240691 | AAGTATCCGTAG | hsa_circ_0033186 | chr14 | 100820529 | 100820903 | - | 374 | ALT_DONOR, CDS, c | NM_004184 | WARS | Salzman2013 | 7453 | circRNA | Detected | Detected | 8.351414203 | 7.00240691 |
| hsa_gci159866 | 2.614651494 | 1.386618663 | up | 6.096953451 | 4.710334788 | GCCAAATAATCG | hsa_circ_0033187 | chr14 | 100820529 | 100826999 | - | 483 | ALT_DONOR, CDS, c | NM_004184 | WARS | Salzman2013 | 7453 | circRNA | Detected | Detected | 6.096953451 | 4.710334788 |
| hsa_gci159867 | 2.20105741 | 1.138196775 | up | 7.982512017 | 6.844315242 | TGTGGTTGTAGA | hsa_circ_0033188 | chr14 | 100820529 | 100828258 | - | 697 | ALT_DONOR, CDS, c | NM_004184 | WARS | Salzman2013 | 7453 | circRNA | Detected | Detected | 7.982512017 | 6.844315242 |
| hsa_gci159909 | 2.11979001 | 1.083921356 | up | 10.47231682 | 9.388395462 | TCCTTACAAATA | hsa_circ_0033408 | chr14 | 102842986 | 102931626 | + | 3861 | ANNOTATED, CDS, c | NM_014844 | TECPR2 | Salzman2013 | 9895 | circRNA | Detected | Detected | 10.47231682 | 9.388395462 |
| hsa_gci159913 | 4.184312791 | 2.064990702 | up | 4.250081546 | 2.185090844 | GGCACCCGAAAC | hsa_circ_0033433 | chr14 | 103398715 | 103416241 | - | 3160 | ANNOTATED, CDS, c | NM_006035 | CDC42BPB | Salzman2013 | 9578 | circRNA | Detected | Not Detected | 4.250081546 | 2.185090844 |
| hsa_gci159944 | 2.246523927 | 1.167694427 | up | 9.147338835 | 7.979644407 | CCGGAGCCCTCG | hsa_circ_0033590 | chr14 | 105715118 | 105717430 | + | 1968 | ANNOTATED, CDS, c | NM_033271 | BTBD6 | Salzman2013 | 90135 | circRNA | Detected | Detected | 9.147338835 | 7.979644407 |
| hsa_gci159952 | 2.206076148 | 1.14148259 | up | 8.317121056 | 7.175638467 | AGACAGAAGTTT | hsa_circ_0034067 | chr15 | 22925975 | 22926065 | + | 90 | ANNOTATED, CDS, c | NM_014608 | CYFIP1 | Salzman2013 | 23191 | circRNA | Detected | Detected | 8.317121056 | 7.175638467 |
| hsa_gci159965 | 2.042379121 | 1.030250695 | up | 5.481815175 | 4.451564481 | AGCATACTTTTA | hsa_circ_0034144 | chr15 | 25342467 | 25351441 | + | 8974 | ALT_ACCEPTOR, ALT | NR_003340 | SNORD116-26 | Salzman2013 | 100033438 | circRNA | Detected | Detected | 5.481815175 | 4.451564481 |
| hsa_gci159990 | 2.154688441 | 1.107479276 | up | 6.513806258 | 5.406326982 | CCTACCCGACTC | hsa_circ_0034350 | chr15 | 34633916 | 34634309 | - | 393 | ANNOTATED, CDS, c | NM_018648 | NOP10 | Salzman2013 | 55505 | circRNA | Detected | Detected | 6.513806258 | 5.406326982 |
| hsa_gci159994 | 2.915002542 | 1.543497141 | up | 6.197226783 | 4.653729641 | TATTATCTGAAC | hsa_circ_0034402 | chr15 | 36983885 | 36989591 | + | 198 | ANNOTATED, CDS, c | NM_032499 | C15orf41 | Salzman2013 | 84529 | circRNA | Detected | Detected | 6.197226783 | 4.653729641 |
| hsa_gci159999 | 3.871629999 | 1.952941085 | up | 6.889264534 | 4.936323449 | CACTCCTCCTGT | hsa_circ_0034460 | chr15 | 39876188 | 39886641 | + | 2802 | ANNOTATED, CDS, c | NM_003246 | THBS1 | Salzman2013 | 7057 | circRNA | Detected | Detected | 6.889264534 | 4.936323449 |
| hsa_gci160000 | 2.220539551 | 1.150910268 | up | 8.1632823 | 7.012372032 | GGTAAGTGGTGC | hsa_circ_0034461 | chr15 | 39876188 | 39889668 | + | 4908 | ANNOTATED, CDS, c | NM_003246 | THBS1 | Salzman2013 | 7057 | circRNA | Detected | Detected | 8.1632823 | 7.012372032 |
| hsa_gci160006 | 2.006540444 | 1.004710235 | up | 6.177139563 | 5.172429328 | CTACTGGTGCCC | hsa_circ_0034484 | chr15 | 39880242 | 39889668 | + | 4317 | ANNOTATED, CDS, c | NM_003246 | THBS1 | Salzman2013 | 7057 | circRNA | Detected | Detected | 6.177139563 | 5.172429328 |
| hsa_gci160009 | 2.19466187 | 1.133998682 | up | 4.547610008 | 3.413611326 | GTTTCGTCCCTA | hsa_circ_0034502 | chr15 | 39881159 | 39886641 | + | 1860 | ANNOTATED, CDS, c | NM_003246 | THBS1 | Salzman2013 | 7057 | circRNA | Detected | Detected | 4.547610008 | 3.413611326 |
| hsa_gci160054 | 3.754663034 | 1.908683438 | up | 4.138569947 | 2.22988651 | ACACCTCTCGTC | hsa_circ_0034673 | chr15 | 41361767 | 41379880 | - | 1245 | ANNOTATED, CDS, c | NM_017553 | INO80 | Salzman2013 | 54617 | circRNA | Detected | Not Detected | 4.138569947 | 2.22988651 |
| hsa_gci160056 | 6.180035966 | 2.627615234 | up | 5.488609357 | 2.860994123 | GCTTATGCGTCC | hsa_circ_0034690 | chr15 | 41624891 | 41673248 | + | 2454 | ANNOTATED, CDS, c | NM_016359 | NUSAP1 | Salzman2013 | 51203 | circRNA | Detected | Not Detected | 5.488609357 | 2.860994123 |
| hsa_gci160059 | 2.701009973 | 1.433498966 | up | 6.703577894 | 5.270078927 | ATCACGTCCTCC | hsa_circ_0034705 | chr15 | 41809374 | 41809889 | - | 515 | ANNOTATED, CDS, c | NM_015540 | RPAP1 | Salzman2013 | 26015 | circRNA | Detected | Detected | 6.703577894 | 5.270078927 |
| hsa_gci160060 | 2.404120133 | 1.265508989 | up | 8.233796012 | 6.968287023 | GTCGTCCGACCA | hsa_circ_0034708 | chr15 | 41809374 | 41819499 | - | 2936 | ANNOTATED, CDS, c | NM_015540 | RPAP1 | Salzman2013 | 26015 | circRNA | Detected | Detected | 8.233796012 | 6.968287023 |
| hsa_gci160062 | 2.763449805 | 1.466470408 | up | 8.595980732 | 7.129510324 | ACTCCCCAGTGC | hsa_circ_0034710 | chr15 | 41809374 | 41822177 | - | 3604 | ANNOTATED, CDS, c | NM_015540 | RPAP1 | Salzman2013 | 26015 | circRNA | Detected | Detected | 8.595980732 | 7.129510324 |
| hsa_gci160073 | 2.38160891 | 1.251936524 | up | 7.794003432 | 6.542066908 | ATGGTCCAATAT | hsa_circ_0034799 | chr15 | 42525410 | 42560230 | - | 857 | ANNOTATED, CDS, c | NM_015497 | TMEM87A | Salzman2013 | 25963 | circRNA | Detected | Detected | 7.794003432 | 6.542066908 |
| hsa_gci160074 | 2.739059353 | 1.453680528 | up | 8.479817403 | 7.026136875 | TTTCTGTCCAGT | hsa_circ_0034802 | chr15 | 42531867 | 42560230 | - | 479 | ANNOTATED, CDS, c | NM_015497 | TMEM87A | Salzman2013 | 25963 | circRNA | Detected | Detected | 8.479817403 | 7.026136875 |
| hsa_gci160075 | 2.872951146 | 1.52253346 | up | 6.905455301 | 5.38292184 | TCTGTCCAGTGT | hsa_circ_0034803 | chr15 | 42553155 | 42560230 | - | 299 | ANNOTATED, CDS, c | NM_015497 | TMEM87A | Salzman2013 | 25963 | circRNA | Detected | Detected | 6.905455301 | 5.38292184 |
| hsa_gci160079 | 2.600157069 | 1.378598776 | up | 5.214067346 | 3.835468571 | TTGACCTGTTGA | hsa_circ_0034815 | chr15 | 42727581 | 42732011 | - | 1118 | ANNOTATED, CDS, c | NM_022473 | ZNF106 | Salzman2013 | 64397 | circRNA | Detected | Detected | 5.214067346 | 3.835468571 |
| hsa_gci160092 | 2.488327138 | 1.315176167 | up | 6.010094101 | 4.694917934 | TACTTCCTAGAA | hsa_circ_0034890 | chr15 | 43250210 | 43350627 | - | 3742 | ANNOTATED, CDS, c | NM_174916 | UBR1 | Salzman2013 | 197131 | circRNA | Detected | Detected | 6.010094101 | 4.694917934 |
| hsa_gci160108 | 2.606332526 | 1.38202116 | up | 6.565956718 | 5.183935558 | CGTGGGCCGCGT | hsa_circ_0034972 | chr15 | 43692241 | 43694048 | + | 313 | ANNOTATED, CDS, c | NM_014444 | TUBGCP4 | Salzman2013 | 27229 | circRNA | Detected | Detected | 6.565956718 | 5.183935558 |
| hsa_gci160120 | 2.472643117 | 1.306054027 | up | 8.027221243 | 6.721167217 | CTTCGAGTCGTT | hsa_circ_0035051 | chr15 | 44620882 | 44630515 | + | 420 | ANNOTATED, CDS, c | NM_138423 | CASC4 | Salzman2013 | 113201 | circRNA | Detected | Detected | 8.027221243 | 6.721167217 |
| hsa_gci160123 | 2.05546398 | 1.039464091 | up | 6.462677296 | 5.423213205 | GTGCAAGTTAAG | hsa_circ_0035057 | chr15 | 44776421 | 44783197 | + | 505 | ANNOTATED, CDS, c | NM_016396 | CTDSPL2 | Salzman2013 | 51496 | circRNA | Detected | Detected | 6.462677296 | 5.423213205 |
| hsa_gci160127 | 2.111215991 | 1.078074183 | up | 6.172889173 | 5.09481499 | ACTCCATAGAGG | hsa_circ_0035085 | chr15 | 44881449 | 44912601 | - | 2286 | ANNOTATED, CDS, c | NM_025137 | SPG11 | Salzman2013 | 80208 | circRNA | Detected | Detected | 6.172889173 | 5.09481499 |
| hsa_gci160138 | 2.755144447 | 1.462127958 | up | 5.954546718 | 4.492418759 | TTATAGGTTTAT | hsa_circ_0035142 | chr15 | 48733917 | 48760299 | - | 1581 | ANNOTATED, CDS, c | NM_000138 | FBN1 | Salzman2013 | 2200 | circRNA | Detected | Detected | 5.954546718 | 4.492418759 |
| hsa_gci160146 | 2.647042697 | 1.404381466 | up | 4.431214574 | 3.026833108 | ATAGTTAGACCT | hsa_circ_0035190 | chr15 | 49528047 | 49611313 | + | 27280 | ALT_DONOR, CDS, c | NM_001001556 | GALK2 | Salzman2013 | 2585 | circRNA | Detected | Not Detected | 4.431214574 | 3.026833108 |
| hsa_gci160148 | 2.776531136 | 1.473283574 | up | 4.167049474 | 2.6937659 | TACAATTTTTCC | hsa_circ_0035196 | chr15 | 50168474 | 50171656 | - | 330 | ANNOTATED, CDS, c | NM_024837 | ATP8B4 | Salzman2013 | 79895 | circRNA | Detected | Not Detected | 4.167049474 | 2.6937659 |
| hsa_gci160155 | 2.397759387 | 1.261686893 | up | 4.543343591 | 3.281656697 | ACAACTCATAAC | hsa_circ_0035212 | chr15 | 50724534 | 50763992 | + | 1076 | ANNOTATED, CDS, c | NM_001128611 | USP8 | Salzman2013 | 9101 | circRNA | Detected | Detected | 4.543343591 | 3.281656697 |
| hsa_gci160179 | 2.076531149 | 1.054175513 | up | 7.466550242 | 6.412374729 | GTCCGTCTTCCC | hsa_circ_0035291 | chr15 | 52161413 | 52192495 | + | 753 | ANNOTATED, CDS, c | NM_014547 | TMOD3 | Salzman2013 | 29766 | circRNA | Detected | Detected | 7.466550242 | 6.412374729 |
| hsa_gci160180 | 2.193191484 | 1.133031777 | up | 6.005109545 | 4.872077768 | GGGACGTCCGTC | hsa_circ_0035292 | chr15 | 52161413 | 52194233 | + | 898 | ANNOTATED, CDS, c | NM_014547 | TMOD3 | Salzman2013 | 29766 | circRNA | Detected | Detected | 6.005109545 | 4.872077768 |
| hsa_gci160181 | 2.584485508 | 1.369877113 | up | 5.623558664 | 4.253681552 | ACTACAGACCAA | hsa_circ_0035293 | chr15 | 52161413 | 52204331 | + | 4257 | ANNOTATED, CDS, c | NM_014547 | TMOD3 | Salzman2013 | 29766 | circRNA | Detected | Detected | 5.623558664 | 4.253681552 |
| hsa_gci160209 | -4.753037766 | -2.248849865 | down | 1.3513227 | 3.600172565 | TTTTGATGACGA | hsa_circ_0035426 | chr15 | 57535669 | 57535748 | + | 79 | ANNOTATED, CDS, c | NM_207037 | TCF12 | Salzman2013 | 6938 | circRNA | Not Detected | Detected | 1.3513227 | 3.600172565 |
| hsa_gci160214 | 2.0442405 | 1.031564936 | up | 11.48484487 | 10.45327993 | GACGATCCGGAC | hsa_circ_0035457 | chr15 | 58974394 | 59042177 | - | 769 | ANNOTATED, CDS, c | NM_001110 | ADAM10 | Salzman2013 | 102 | circRNA | Detected | Detected | 11.48484487 | 10.45327993 |
| hsa_gci160229 | 2.536648286 | 1.342923499 | up | 4.620661996 | 3.277738497 | TCCCGTATCTAT | hsa_circ_0035529 | chr15 | 59510089 | 59548577 | - | 870 | ANNOTATED, CDS, c | NM_004998 | MYO1E | Salzman2013 | 4643 | circRNA | Detected | Detected | 4.620661996 | 3.277738497 |
| hsa_gci160232 | 2.187053728 | 1.128988663 | up | 6.452439099 | 5.323450436 | TATCCGAAACTG | hsa_circ_0035543 | chr15 | 60641273 | 60674640 | - | 912 | ANNOTATED, CDS, c | NM_001136015 | ANXA2 | Salzman2013 | 302 | circRNA | Detected | Detected | 6.452439099 | 5.323450436 |
| hsa_gci160236 | 2.194030024 | 1.133583269 | up | 6.271515183 | 5.137931915 | ATATCCGAAACT | hsa_circ_0035560 | chr15 | 60653139 | 60674640 | - | 309 | ANNOTATED, CDS, c | NM_001136015 | ANXA2 | Salzman2013 | 302 | circRNA | Detected | Detected | 6.271515183 | 5.137931915 |
| hsa_gci160253 | 2.072853154 | 1.051617916 | up | 7.227142828 | 6.175524912 | GTACACACACGT | hsa_circ_0035636 | chr15 | 63615729 | 63631144 | - | 3072 | ANNOTATED, CDS, c | NM_001218 | CA12 | Salzman2013 | 771 | circRNA | Detected | Detected | 7.227142828 | 6.175524912 |
| hsa_gci160261 | 4.307210878 | 2.106753959 | up | 6.027709664 | 3.920955705 | GAAAACAACCTG | hsa_circ_0035650 | chr15 | 63824845 | 63866602 | + | 1005 | ANNOTATED, CDS, c | NM_006537 | USP3 | Salzman2013 | 9960 | circRNA | Detected | Detected | 6.027709664 | 3.920955705 |
| hsa_gci160280 | 2.08567024 | 1.060511075 | up | 6.864957877 | 5.804446802 | TCTCTCTTCCCT | hsa_circ_0035761 | chr15 | 63966540 | 63981898 | - | 1909 | ANNOTATED, CDS, c | NM_003922 | HERC1 | Salzman2013 | 8925 | circRNA | Detected | Detected | 6.864957877 | 5.804446802 |
| hsa_gci160311 | -2.884690612 | -1.528416596 | down | 1.762489429 | 3.290906025 | TCATCCTACCTG | hsa_circ_0035897 | chr15 | 65756098 | 65772737 | - | 853 | ANNOTATED, CDS, c | NM_130434 | DPP8 | Salzman2013 | 54878 | circRNA | Not Detected | Detected | 1.762489429 | 3.290906025 |

| | | | | | | | | | | | | | | | | | | | | | | |
|---|---|---|---|---|---|---|---|---|---|---|---|---|---|---|---|---|---|---|---|---|---|---|
| hsa_gci160320 | 2.473187807 | 1.306371798 | up | 5.829321074 | 4.522949276 | GCTCCGGGGAAC | hsa_circ_0035938 | chr15 | 66007815 | 66048810 | - | 1975 | ANNOTATED, CDS, c | NM_001144823 | DENND4A | Salzman2013 | 10260 | circRNA | Detected | Detected | 5.829321074 | 4.522949276 |
| hsa_gci160328 | 3.900085682 | 1.963505819 | up | 5.963616213 | 4.000110394 | GCTCCGGGGAAC | hsa_circ_0035952 | chr15 | 66030044 | 66048810 | - | 1062 | ANNOTATED, CDS, c | NM_001144823 | DENND4A | Salzman2013 | 10260 | circRNA | Detected | Detected | 5.963616213 | 4.000110394 |
| hsa_gci160356 | 3.139801237 | 1.650673233 | up | 6.109026453 | 4.458353219 | TCAGTTTAGGAA | hsa_circ_0036069 | chr15 | 69629679 | 69652470 | + | 327 | ANNOTATED, CDS, c | NM_001104554 | PAQR5 | Salzman2013 | 54852 | circRNA | Detected | Detected | 6.109026453 | 4.458353219 |
| hsa_gci160358 | 2.120947059 | 1.08470861 | up | 6.698183368 | 5.613474757 | CTCGTGGAACCG | hsa_circ_0036088 | chr15 | 69745158 | 69747884 | + | 512 | ANNOTATED, CDS, c | NM_001003 | RPLP1 | Salzman2013 | 6176 | circRNA | Detected | Detected | 6.698183368 | 5.613474757 |
| hsa_gci160367 | 2.466742241 | 1.302606971 | up | 5.897449529 | 4.594842558 | CGACTCTTTCTC | hsa_circ_0036113 | chr15 | 71229099 | 71256271 | + | 210 | ANNOTATED, CDS, c | NM_001199017 | LRRC49 | Salzman2013 | 54839 | circRNA | Detected | Detected | 5.897449529 | 4.594842558 |
| hsa_gci160393 | 2.289501556 | 1.195033546 | up | 6.764265615 | 5.569232069 | CGTTAGACCCCG | hsa_circ_0036230 | chr15 | 72855734 | 72858946 | + | 150 | ANNOTATED, CDS, c | NM_005744 | ARIH1 | Salzman2013 | 25820 | circRNA | Detected | Detected | 6.764265615 | 5.569232069 |
| hsa_gci160397 | 2.722274783 | 1.444812698 | up | 6.307513294 | 4.862700596 | ACGGCCCCAAAA | hsa_circ_0036240 | chr15 | 72987517 | 73009191 | + | 439 | ANNOTATED, INTERN | NR_045565 | BBS4 | Salzman2013 | 585 | circRNA | Detected | Detected | 6.307513294 | 4.862700596 |
| hsa_gci160407 | 2.340315479 | 1.226703021 | up | 5.715854169 | 4.489151148 | GGCCTAACAACG | hsa_circ_0036338 | chr15 | 75672969 | 75688837 | - | 1529 | ANNOTATED, CDS, c | NM_001145358 | SIN3A | Salzman2013 | 25942 | circRNA | Detected | Detected | 5.715854169 | 4.489151148 |
| hsa_gci160414 | 2.686766928 | 1.425871176 | up | 7.852018894 | 6.426147718 | CCCTAACCGGAA | hsa_circ_0036367 | chr15 | 75942096 | 75950968 | + | 2484 | ALT_ACCEPTOR, CDS | NM_153271 | SNX33 | Salzman2013 | 257364 | circRNA | Detected | Detected | 7.852018894 | 6.426147718 |
| hsa_gci160415 | 2.348499717 | 1.231739419 | up | 6.299669871 | 5.067930451 | ACCTACTACAGT | hsa_circ_0036368 | chr15 | 75942871 | 75950968 | + | 1709 | ALT_ACCEPTOR, CDS | NM_153271 | SNX33 | Salzman2013 | 257364 | circRNA | Detected | Detected | 6.299669871 | 5.067930451 |
| hsa_gci160446 | 4.324275334 | 2.112458385 | up | 4.704990051 | 2.592531666 | CCACACCCATTT | hsa_circ_0036438 | chr15 | 78308887 | 78337410 | - | 1261 | ANNOTATED, CDS, c | NM_144572 | TBC1D2B | Salzman2013 | 23102 | circRNA | Detected | Not Detected | 4.704990051 | 2.592531666 |
| hsa_gci160455 | 2.410644041 | 1.269418636 | up | 8.389122875 | 7.119704239 | GTCCATTTACAA | hsa_circ_0036471 | chr15 | 78780988 | 78789653 | + | 830 | ANNOTATED, CDS, c | NM_004136 | IREB2 | Salzman2013 | 3658 | circRNA | Detected | Detected | 8.389122875 | 7.119704239 |
| hsa_gci160456 | 2.428799041 | 1.280243126 | up | 8.104160631 | 6.823917505 | GGCGGTGTCCGG | hsa_circ_0036477 | chr15 | 79051544 | 79056187 | - | 686 | ANNOTATED, CDS, c | NM_014272 | ADAMTS7 | Salzman2013 | 11173 | circRNA | Detected | Detected | 8.104160631 | 6.823917505 |
| hsa_gci160492 | -3.927513111 | -1.973616092 | down | 4.051898086 | 6.025514177 | CCCTCATTAGAA | hsa_circ_0036670 | chr15 | 89169376 | 89175512 | + | 3029 | ANNOTATED, CDS, c | NM_022767 | AEN | Salzman2013 | 64782 | circRNA | Not Detected | Detected | 4.051898086 | 6.025514177 |
| hsa_gci160506 | 2.007743358 | 1.005574867 | up | 4.673225128 | 3.667650261 | ATAGGTTCCACC | hsa_circ_0036726 | chr15 | 90125916 | 90126196 | + | 280 | ANNOTATED, CDS, c | NM_152259 | TICRR | Salzman2013 | 90381 | circRNA | Detected | Detected | 4.673225128 | 3.667650261 |
| hsa_gci160512 | 70.74177442 | 6.144490502 | up | 7.361776665 | 1.217286164 | AGGTCTCAAAGG | hsa_circ_0036747 | chr15 | 90373830 | 90414778 | - | 5225 | ANNOTATED, CDS, c | NM_001199058 | C15orf38-AP3S | Salzman2013 | 100526783 | circRNA | Detected | Not Detected | 7.361776665 | 1.217286164 |
| hsa_gci160515 | 2.772009359 | 1.470932128 | up | 8.091840202 | 6.620908074 | GGTTCCCTCGGT | hsa_circ_0036757 | chr15 | 90627211 | 90628619 | - | 678 | ANNOTATED, CDS, c | NM_002168 | IDH2 | Salzman2013 | 3418 | circRNA | Detected | Detected | 8.091840202 | 6.620908074 |
| hsa_gci160529 | 2.024234217 | 1.017376229 | up | 7.087027977 | 6.069651748 | AGACTCCCCGGT | hsa_circ_0036803 | chr15 | 90982563 | 91045475 | + | 6642 | ANNOTATED, CDS, c | NM_003870 | IQGAP1 | Salzman2013 | 8826 | circRNA | Detected | Detected | 7.087027977 | 6.069651748 |
| hsa_gci160542 | 2.421936134 | 1.276160822 | up | 6.927878873 | 5.651718052 | GCGGACGAGTCC | hsa_circ_0036837 | chr15 | 91025191 | 91045475 | + | 3780 | ANNOTATED, CDS, c | NM_003870 | IQGAP1 | Salzman2013 | 8826 | circRNA | Detected | Detected | 6.927878873 | 5.651718052 |
| hsa_gci160550 | 2.055685181 | 1.039619339 | up | 6.40693319 | 5.367313851 | GAGAAGAAAGTT | hsa_circ_0036863 | chr15 | 91161114 | 91188577 | + | 4452 | ANNOTATED, CDS, c | NM_022769 | CRTC3 | Salzman2013 | 64784 | circRNA | Detected | Detected | 6.40693319 | 5.367313851 |
| hsa_gci160554 | 4.040523376 | 2.01454218 | up | 4.986860362 | 2.972318182 | TTCGGAAGGAGC | hsa_circ_0036881 | chr15 | 91424515 | 91426687 | + | 2172 | ANNOTATED, CDS, c | NM_002569 | FURIN | Salzman2013 | 5045 | circRNA | Detected | Not Detected | 4.986860362 | 2.972318182 |
| hsa_gci160555 | 2.547592079 | 1.349134292 | up | 8.346013795 | 6.996879503 | GGTTTTAGAAAA | hsa_circ_0036903 | chr15 | 91488121 | 91497323 | + | 2185 | ANNOTATED, CDS, c | NM_001039675 | UNC45A | Salzman2013 | 55898 | circRNA | Detected | Detected | 8.346013795 | 6.996879503 |
| hsa_gci160582 | 10.31707978 | 3.366962772 | up | 4.616851095 | 1.249888323 | CGACAGGGTGTA | hsa_circ_0037087 | chr15 | 101812389 | 101814886 | - | 838 | ANNOTATED, CDS, c | NM_018445 | SELENOS | Salzman2013 | 55829 | circRNA | Detected | Not Detected | 4.616851095 | 1.249888323 |
| hsa_gci160605 | 2.798802313 | 1.484809589 | up | 5.771844091 | 4.287034502 | GCCGGGTTGAAC | hsa_circ_0037373 | chr16 | 2012061 | 2014366 | - | 742 | ANNOTATED, CDS, c | NM_002952 | RPS2 | Salzman2013 | 6187 | circRNA | Detected | Detected | 5.771844091 | 4.287034502 |
| hsa_gci160612 | 2.439840347 | 1.286786747 | up | 7.553188473 | 6.266401726 | CCTAGAGAAATG | hsa_circ_0037533 | chr16 | 2498855 | 2499916 | + | 393 | ANNOTATED, CDS, c | NM_001761 | CCNF | Salzman2013 | 899 | circRNA | Detected | Detected | 7.553188473 | 6.266401726 |
| hsa_gci160623 | 2.386737742 | 1.25504005 | up | 8.246298593 | 6.991258543 | CACCCACAGGTC | hsa_circ_0037618 | chr16 | 3708037 | 3712133 | - | 413 | ANNOTATED, CDS, c | NM_016292 | TRAP1 | Salzman2013 | 10131 | circRNA | Detected | Detected | 8.246298593 | 6.991258543 |
| hsa_gci160631 | 2.921145387 | 1.546534164 | up | 4.62860957 | 3.082075406 | TGGTTAACACAA | hsa_circ_0037676 | chr16 | 3830732 | 3832927 | - | 493 | ANNOTATED, CDS, c | NM_004380 | CREBBP | Salzman2013 | 1387 | circRNA | Detected | Not Detected | 4.62860957 | 3.082075406 |
| hsa_gci160644 | 2.120124706 | 1.084149127 | up | 7.85117988 | 6.767030754 | AGAACCGAGGTA | hsa_circ_0037785 | chr16 | 8722586 | 8722967 | + | 381 | ANNOTATED, CDS, c | NM_024109 | METTL22 | Salzman2013 | 79091 | circRNA | Detected | Detected | 7.85117988 | 6.767030754 |
| hsa_gci160663 | 3.107254851 | 1.635640574 | up | 7.343129428 | 5.707488854 | ACCATAATAGTG | hsa_circ_0037870 | chr16 | 11791980 | 11830089 | - | 853 | ANNOTATED, CDS, c | NM_015914 | TXNDC11 | Salzman2013 | 51061 | circRNA | Detected | Detected | 7.343129428 | 5.707488854 |
| hsa_gci160687 | 2.67552763 | 1.419823427 | up | 5.940119441 | 4.520296013 | TGAAACGACTAT | hsa_circ_0037958 | chr16 | 14645877 | 14698083 | - | 778 | ANNOTATED, CDS, c | NM_002582 | PARN | Salzman2013 | 5073 | circRNA | Detected | Detected | 5.940119441 | 4.520296013 |
| hsa_gci160702 | 2.425551464 | 1.27831279 | up | 5.801525489 | 4.523212698 | GGCAATAGTTGG | hsa_circ_0038019 | chr16 | 15162025 | 15166937 | - | 556 | ANNOTATED, CDS, c | NM_018427 | RRN3 | Salzman2013 | 54700 | circRNA | Detected | Detected | 5.801525489 | 4.523212698 |
| hsa_gci160716 | 3.37561016 | 1.755148301 | up | 8.130065673 | 6.374917373 | TTGAGGTGGGAT | hsa_circ_0038167 | chr16 | 16333707 | 16388663 | + | 3921 | ALT_DONOR, CDS, c | NM_001004067 | NOMO3 | Salzman2013 | 408050 | circRNA | Detected | Detected | 8.130065673 | 6.374917373 |
| hsa_gci160719 | 2.574973975 | 1.364557851 | up | 8.337022828 | 6.972464976 | GCCCCTTCGCTT | hsa_circ_0038178 | chr16 | 16355358 | 16388663 | + | 2956 | ALT_DONOR, CDS, c | NM_001004067 | NOMO3 | Salzman2013 | 408050 | circRNA | Detected | Detected | 8.337022828 | 6.972464976 |
| hsa_gci160749 | 2.015826981 | 1.011371817 | up | 7.546015784 | 6.534643967 | GCACCGCTGTCG | hsa_circ_0038318 | chr16 | 19125962 | 19132952 | + | 6990 | ANNOTATED, ncRNA, | NR_028028 | ITPRIPL2 | Salzman2013 | 162073 | circRNA | Detected | Detected | 7.546015784 | 6.534643967 |
| hsa_gci160778 | 2.240122442 | 1.16357759 | up | 5.289871735 | 4.126294145 | TTTAATGGACCG | hsa_circ_0038425 | chr16 | 21475023 | 21495856 | - | 1815 | ANNOTATED, INTERN | NR_027155 | | Salzman2013 | | circRNA | Detected | Detected | 5.289871735 | 4.126294145 |
| hsa_gci160803 | 3.128326771 | 1.645391218 | up | 6.802049061 | 5.156657843 | CCCCGACCAGAT | hsa_circ_0038486 | chr16 | 22217591 | 22300066 | + | 7412 | ANNOTATED, CDS, c | NM_013302 | EEF2K | Salzman2013 | 29904 | circRNA | Detected | Detected | 6.802049061 | 5.156657843 |
| hsa_gci160805 | 2.612682247 | 1.385531676 | up | 5.507209298 | 4.121677623 | AATTTAATGGAC | hsa_circ_0038524 | chr16 | 22466022 | 22466926 | + | 320 | ANNOTATED, INTERN | NR_027154 | | Salzman2013 | | circRNA | Detected | Detected | 5.507209298 | 4.121677623 |
| hsa_gci160815 | 2.042720837 | 1.030492055 | up | 7.02957174 | 5.999079685 | AACGAGACAAAG | hsa_circ_0038557 | chr16 | 23117533 | 23117626 | - | 93 | ANNOTATED, CDS, c | NM_020718 | USP31 | Salzman2013 | 57478 | circRNA | Detected | Detected | 7.02957174 | 5.999079685 |
| hsa_gci160816 | 2.333145765 | 1.222276443 | up | 7.588363972 | 6.366087529 | GACGTCACGTAA | hsa_circ_0038559 | chr16 | 23117533 | 23119504 | - | 320 | ANNOTATED, CDS, c | NM_020718 | USP31 | Salzman2013 | 57478 | circRNA | Detected | Detected | 7.588363972 | 6.366087529 |
| hsa_gci160836 | 2.292194105 | 1.196729218 | up | 5.79743878 | 4.600709562 | ACATTCGACCCG | hsa_circ_0038667 | chr16 | 24564833 | 24570998 | + | 544 | ANNOTATED, CDS, c | NM_006910 | RBBP6 | Salzman2013 | 5930 | circRNA | Detected | Detected | 5.79743878 | 4.600709562 |
| hsa_gci160852 | 2.218013809 | 1.149268348 | up | 5.817589033 | 4.668320686 | GCCTACGTCGGC | hsa_circ_0038730 | chr16 | 27471933 | 27472883 | - | 950 | ANNOTATED, CDS, c | NM_001520 | GTF3C1 | Salzman2013 | 2975 | circRNA | Detected | Detected | 5.817589033 | 4.668320686 |
| hsa_gci160854 | 3.209617893 | 1.682401554 | up | 5.918125231 | 4.235723677 | GGCCCCGCGTAC | hsa_circ_0038736 | chr16 | 27471933 | 27561251 | - | 7107 | ANNOTATED, CDS, c | NM_001520 | GTF3C1 | Salzman2013 | 2975 | circRNA | Detected | Detected | 5.918125231 | 4.235723677 |
| hsa_gci160864 | 2.13823402 | 1.096419758 | up | 5.5636363 | 4.467216541 | ATAGACTCTAGG | hsa_circ_0038799 | chr16 | 28128638 | 28133083 | - | 238 | ANNOTATED, CDS, c | NM_015171 | XPO6 | Salzman2013 | 23214 | circRNA | Detected | Detected | 5.5636363 | 4.467216541 |
| hsa_gci160870 | 3.319480452 | 1.730957456 | up | 7.115644702 | 5.384687246 | GTCCCCTGACCT | hsa_circ_0038817 | chr16 | 28157414 | 28181230 | - | 929 | ANNOTATED, CDS, c | NM_015171 | XPO6 | Salzman2013 | 23214 | circRNA | Detected | Detected | 7.115644702 | 5.384687246 |
| hsa_gci160886 | 2.244396572 | 1.166327614 | up | 9.431530698 | 8.265203084 | CGCCGACCGGTC | hsa_circ_0039060 | chr16 | 30663116 | 30667734 | + | 1837 | ANNOTATED, CDS, c | NM_024031 | PRR14 | Salzman2013 | 78994 | circRNA | Detected | Detected | 9.431530698 | 8.265203084 |
| hsa_gci160914 | 3.019871655 | 1.594487236 | up | 6.258108938 | 4.663621701 | GCCCAGTTGCTT | hsa_circ_0039213 | chr16 | 46723557 | 46732306 | + | 1687 | ANNOTATED, ncRNA, | NR_037620 | ORC6 | Salzman2013 | 23594 | circRNA | Detected | Detected | 6.258108938 | 4.663621701 |
| hsa_gci160971 | 3.225812255 | 1.689662475 | up | 7.17169729 | 5.482034816 | GCACCACCTCAC | hsa_circ_0039459 | chr16 | 56642477 | 56643409 | + | 419 | ANNOTATED, CDS, c | NM_005953 | MT2A | Salzman2013 | 4502 | circRNA | Detected | Detected | 7.17169729 | 5.482034816 |
| hsa_gci160991 | 10.70457507 | 3.420155622 | up | 4.713276195 | 1.293120573 | CTTGACCCGTAC | hsa_circ_0039605 | chr16 | 58051237 | 58055527 | + | 1673 | ANNOTATED, CDS, c | NM_024598 | USB1 | Salzman2013 | 79650 | circRNA | Detected | Not Detected | 4.713276195 | 1.293120573 |
| hsa_gci160996 | 3.224959378 | 1.689280988 | up | 5.829061508 | 4.13978052 | CTATCAAAGACT | hsa_circ_0039626 | chr16 | 58562378 | 58594266 | - | 4474 | ANNOTATED, CDS, c | NM_016284 | CNOT1 | Salzman2013 | 23019 | circRNA | Detected | Detected | 5.829061508 | 4.13978052 |
| hsa_gci161022 | 3.753579698 | 1.908267116 | up | 6.743254334 | 4.834987219 | AAATATTCCCTT | hsa_circ_0039681 | chr16 | 58594115 | 58614664 | - | 915 | ANNOTATED, CDS, c | NM_016284 | CNOT1 | Salzman2013 | 23019 | circRNA | Detected | Detected | 6.743254334 | 4.834987219 |
| hsa_gci161025 | 3.482211199 | 1.800003706 | up | 4.041201699 | 2.241197993 | TAGACCTTCGAA | hsa_circ_0039686 | chr16 | 58594115 | 58620652 | - | 1697 | ANNOTATED, CDS, c | NM_016284 | CNOT1 | Salzman2013 | 23019 | circRNA | Detected | Not Detected | 4.041201699 | 2.241197993 |

| | | | | | | | | | | | | | | | | | | | | | | |
|---|---|---|---|---|---|---|---|---|---|---|---|---|---|---|---|---|---|---|---|---|---|---|
| hsa_gci161037 | 2.783483985 | 1.476891782 | up | 6.074522749 | 4.597630966 | ATTCCTGTCCCT | hsa_circ_0039712 | chr16 | 58741034 | 58768246 | - | 2447 | ANNOTATED, CDS, c | NM_002080 | GOT2 | Salzman2013 | 2806 | circRNA | Detected | Detected | 6.074522749 | 4.597630966 |
| hsa_gci161048 | 2.648081035 | 1.404947271 | up | 4.600226696 | 3.195279425 | TGTCGGTGACAT | hsa_circ_0039767 | chr16 | 66965957 | 66967642 | - | 418 | ANNOTATED, ncRNA, | NR_024525 | FAM96B | Salzman2013 | 51647 | circRNA | Detected | Detected | 4.600226696 | 3.195279425 |
| hsa_gci161049 | 2.065483101 | 1.046479257 | up | 8.936902042 | 7.890422785 | ACGCGAAGGCGA | hsa_circ_0039769 | chr16 | 66965957 | 66968320 | - | 849 | ANNOTATED, ncRNA, | NR_024525 | FAM96B | Salzman2013 | 51647 | circRNA | Detected | Detected | 8.936902042 | 7.890422785 |
| hsa_gci161056 | 2.310838854 | 1.208416657 | up | 8.91557026 | 7.707153603 | CCTGTGGTCTAA | hsa_circ_0039806 | chr16 | 67263291 | 67271472 | - | 2988 | ANNOTATED, CDS, c | NM_013241 | FHOD1 | Salzman2013 | 29109 | circRNA | Detected | Detected | 8.91557026 | 7.707153603 |
| hsa_gci161057 | 3.364373856 | 1.75033803 | up | 5.942507217 | 4.192169188 | AGGTGTTTCTCG | hsa_circ_0039808 | chr16 | 67263291 | 67272031 | - | 3195 | ANNOTATED, CDS, c | NM_013241 | FHOD1 | Salzman2013 | 29109 | circRNA | Detected | Detected | 5.942507217 | 4.192169188 |
| hsa_gci161094 | 2.464565007 | 1.301333035 | up | 7.640307771 | 6.338974735 | GCTTTGACAGAA | hsa_circ_0040021 | chr16 | 69184439 | 69184807 | + | 264 | ANNOTATED, CDS, c | NM_032830 | UTP4 | Salzman2013 | 84916 | circRNA | Detected | Detected | 7.640307771 | 6.338974735 |
| hsa_gci161106 | 2.678623172 | 1.421491638 | up | 7.265231775 | 5.843740138 | GTCGAAGGAGGC | hsa_circ_0040054 | chr16 | 69354953 | 69358946 | + | 1197 | ANNOTATED, CDS, c | NM_013245 | VPS4A | Salzman2013 | 27183 | circRNA | Detected | Detected | 7.265231775 | 5.843740138 |
| hsa_gci161109 | 2.659757597 | 1.411294769 | up | 8.271702274 | 6.860407505 | TATGTTTATTAC | hsa_circ_0040064 | chr16 | 69404111 | 69406258 | - | 508 | ALT_DONOR, CDS, c | NM_005652 | TERF2 | Salzman2013 | 7014 | circRNA | Detected | Detected | 8.271702274 | 6.860407505 |
| hsa_gci161115 | 2.152496423 | 1.10601084 | up | 5.249832198 | 4.143821358 | TTACATCGACCA | hsa_circ_0040080 | chr16 | 69729038 | 69729591 | + | 553 | ANNOTATED, CDS, c | NM_173215 | NFAT5 | Salzman2013 | 10725 | circRNA | Detected | Detected | 5.249832198 | 4.143821358 |
| hsa_gci161116 | 2.051840254 | 1.036918414 | up | 8.09768332 | 7.060764906 | GAGGTGACCTCG | hsa_circ_0040088 | chr16 | 69775773 | 69788829 | - | 1716 | ANNOTATED, CDS, c | NM_014062 | NOB1 | Salzman2013 | 28987 | circRNA | Detected | Detected | 8.09768332 | 7.060764906 |
| hsa_gci161139 | 2.104617579 | 1.073558111 | up | 6.405646135 | 5.332088023 | AGGGTGCCCTTT | hsa_circ_0040251 | chr16 | 70562775 | 70573110 | + | 997 | ANNOTATED, CDS, c | NM_012426 | SF3B3 | Salzman2013 | 23450 | circRNA | Detected | Detected | 6.405646135 | 5.332088023 |
| hsa_gci161142 | 2.628535181 | 1.394259045 | up | 4.094496486 | 2.700237442 | TTAAACTATATT | hsa_circ_0040264 | chr16 | 70575571 | 70578436 | + | 262 | ANNOTATED, CDS, c | NM_012426 | SF3B3 | Salzman2013 | 23450 | circRNA | Detected | Not Detected | 4.094496486 | 2.700237442 |
| hsa_gci161161 | 2.016780658 | 1.012054187 | up | 7.311086592 | 6.299032405 | TAAGAATGACAT | hsa_circ_0040369 | chr16 | 71772844 | 71773005 | - | 161 | ANNOTATED, CDS, c | NM_001030007 | AP1G1 | Salzman2013 | 164 | circRNA | Detected | Detected | 7.311086592 | 6.299032405 |
| hsa_gci161162 | 2.497969854 | 1.320756066 | up | 7.225981786 | 5.90522572 | TAAAGACACTCC | hsa_circ_0040372 | chr16 | 71773136 | 71803602 | - | 1551 | ANNOTATED, CDS, c | NM_001030007 | AP1G1 | Salzman2013 | 164 | circRNA | Detected | Detected | 7.225981786 | 5.90522572 |
| hsa_gci161166 | 2.210620373 | 1.144451295 | up | 6.306335031 | 5.161883737 | CCTCCTGATGTC | hsa_circ_0040379 | chr16 | 71787735 | 71803602 | - | 728 | ANNOTATED, CDS, c | NM_001030007 | AP1G1 | Salzman2013 | 164 | circRNA | Detected | Detected | 6.306335031 | 5.161883737 |
| hsa_gci161190 | 2.146984278 | 1.102311627 | up | 5.337808267 | 4.23549664 | TAGACAAGGAAG | hsa_circ_0040487 | chr16 | 74666420 | 74671868 | - | 498 | ANNOTATED, CDS, c | NM_018124 | RFWD3 | Salzman2013 | 55159 | circRNA | Detected | Detected | 5.337808267 | 4.23549664 |
| hsa_gci161211 | 2.285770251 | 1.192680402 | up | 4.477180703 | 3.284500302 | ACCTCAAGAAGG | hsa_circ_0040609 | chr16 | 81888048 | 81892768 | + | 286 | ANNOTATED, CDS, c | NM_002661 | PLCG2 | Salzman2013 | 5336 | circRNA | Detected | Detected | 4.477180703 | 3.284500302 |
| hsa_gci161240 | 2.817361923 | 1.494344906 | up | 5.891417332 | 4.397072425 | TAATGACTGTGA | hsa_circ_0040778 | chr16 | 87457415 | 87493737 | - | 270 | ANNOTATED, CDS, c | NM_015144 | ZCCHC14 | Salzman2013 | 23174 | circRNA | Detected | Detected | 5.891417332 | 4.397072425 |
| hsa_gci161246 | 2.09004587 | 1.063534605 | up | 5.926708612 | 4.863174007 | GCCCTGGGGTGC | hsa_circ_0040796 | chr16 | 87863628 | 87871547 | - | 3428 | ANNOTATED, CDS, c | NM_003486 | SLC7A5 | Salzman2013 | 8140 | circRNA | Detected | Detected | 5.926708612 | 4.863174007 |
| hsa_gci161248 | 2.254676091 | 1.172920189 | up | 9.857253785 | 8.684333596 | ACTACCGGTCCC | hsa_circ_0040798 | chr16 | 87863628 | 87873431 | - | 3656 | ANNOTATED, CDS, c | NM_003486 | SLC7A5 | Salzman2013 | 8140 | circRNA | Detected | Detected | 9.857253785 | 8.684333596 |
| hsa_gci161258 | 2.15995546 | 1.111001563 | up | 9.367679285 | 8.256677722 | GCCCGTCGCGGC | hsa_circ_0040946 | chr16 | 89598870 | 89624174 | + | 1911 | ANNOTATED, CDS, c | NM_003119 | SPG7 | Salzman2013 | 6687 | circRNA | Detected | Detected | 9.367679285 | 8.256677722 |
| hsa_gci161259 | 2.344423366 | 1.229233121 | up | 7.070529403 | 5.841296281 | GCCGCAAACAGG | hsa_circ_0040953 | chr16 | 89619386 | 89624174 | + | 1282 | ANNOTATED, CDS, c | NM_003119 | SPG7 | Salzman2013 | 6687 | circRNA | Detected | Detected | 7.070529403 | 5.841296281 |
| hsa_gci161262 | 3.201023777 | 1.678533393 | up | 4.808706651 | 3.130173258 | GCCCGTGTGAGA | hsa_circ_0040969 | chr16 | 89710843 | 89715905 | - | 2116 | ANNOTATED, CDS, c | NM_002768 | CHMP1A | Salzman2013 | 5119 | circRNA | Detected | Not Detected | 4.808706651 | 3.130173258 |
| hsa_gci161269 | 19.54077596 | 4.288415853 | up | 5.528991545 | 1.240575692 | CGTAGACTTGTC | hsa_circ_0041027 | chr16 | 89824984 | 89858955 | - | 1975 | ANNOTATED, CDS, c | NM_000135 | FANCA | Salzman2013 | 2175 | circRNA | Detected | Not Detected | 5.528991545 | 1.240575692 |
| hsa_gci161273 | 5.181525151 | 2.373376809 | up | 4.937715188 | 2.564338379 | CTCAAACGGTGA | hsa_circ_0041081 | chr16 | 89950989 | 89977792 | + | 1823 | ANNOTATED, CDS, c | NM_014972 | TCF25 | Salzman2013 | 22980 | circRNA | Detected | Not Detected | 4.937715188 | 2.564338379 |
| hsa_gci161276 | 2.28342059 | 1.191196618 | up | 7.181530933 | 5.990334314 | CCCGTGCCAGGA | hsa_circ_0041123 | chr16 | 89986997 | 90002505 | + | 3245 | ALT_ACCEPTOR, CDS | NM_001197181 | TUBB3 | Salzman2013 | 10381 | circRNA | Detected | Detected | 7.181530933 | 5.990334314 |
| hsa_gci161283 | 2.368113013 | 1.243737932 | up | 4.213568641 | 2.969830708 | TCGCGTACGACG | hsa_circ_0041134 | chr16 | 90028419 | 90034468 | + | 2646 | ANNOTATED, CDS, c | NM_001242820 | DEF8 | Salzman2013 | 54849 | circRNA | Detected | Not Detected | 4.213568641 | 2.969830708 |
| hsa_gci161289 | 2.623544804 | 1.391517428 | up | 8.634431246 | 7.242913818 | CGATGAAAGAAG | hsa_circ_0041193 | chr17 | 1264385 | 1268352 | - | 514 | ANNOTATED, INTERN | NR_024058 | YWHAE | Salzman2013 | 7531 | circRNA | Detected | Detected | 8.634431246 | 7.242913818 |
| hsa_gci161290 | 2.19684235 | 1.135431343 | up | 8.388340722 | 7.252909379 | TGTTGACGAGTC | hsa_circ_0041194 | chr17 | 1264385 | 1303556 | - | 763 | ANNOTATED, ncRNA, | NR_024058 | YWHAE | Salzman2013 | 7531 | circRNA | Detected | Detected | 8.388340722 | 7.252909379 |
| hsa_gci161292 | 2.194278168 | 1.133746427 | up | 7.44307253 | 6.309326103 | TTAAGTCGGCAC | hsa_circ_0041197 | chr17 | 1367479 | 1369046 | - | 1567 | ANNOTATED, CDS, c | NM_001080779 | MYO1C | Salzman2013 | 4641 | circRNA | Detected | Detected | 7.44307253 | 6.309326103 |
| hsa_gci161301 | 2.2992905 | 1.201188753 | up | 9.46247449 | 8.261285737 | AACTATGAGTAG | hsa_circ_0041252 | chr17 | 1437392 | 1461853 | - | 748 | ANNOTATED, CDS, c | NM_006224 | PITPNA | Salzman2013 | 5306 | circRNA | Detected | Detected | 9.46247449 | 8.261285737 |
| hsa_gci161307 | 2.138722078 | 1.09674902 | up | 8.118387111 | 7.021638091 | CGCGAGTGTCCC | hsa_circ_0041332 | chr17 | 1648468 | 1658559 | + | 2144 | ANNOTATED, CDS, c | NM_000934 | SERPINF2 | Salzman2013 | 5345 | circRNA | Detected | Detected | 8.118387111 | 7.021638091 |
| hsa_gci161320 | 3.243052302 | 1.697352291 | up | 4.618712648 | 2.921360357 | GAACCAGTAGGA | hsa_circ_0041375 | chr17 | 1963132 | 2090125 | - | 2927 | ANNOTATED, CDS, c | NM_001170957 | SMG6 | Salzman2013 | 23293 | circRNA | Detected | Not Detected | 4.618712648 | 2.921360357 |
| hsa_gci161321 | 2.464606595 | 1.301357379 | up | 4.39950247 | 3.098145091 | AACCGTATAGTA | hsa_circ_0041377 | chr17 | 1963132 | 2196271 | - | 3762 | ANNOTATED, CDS, c | NM_001170957 | SMG6 | Salzman2013 | 23293 | circRNA | Detected | Not Detected | 4.39950247 | 3.098145091 |
| hsa_gci161326 | 2.111687877 | 1.07839661 | up | 8.579422063 | 7.501025453 | CCGAGGCCCCCA | hsa_circ_0041405 | chr17 | 2278785 | 2284348 | + | 2590 | ANNOTATED, CDS, c | NM_014853 | SGSM2 | Salzman2013 | 9905 | circRNA | Detected | Detected | 8.579422063 | 7.501025453 |
| hsa_gci161335 | 2.404467161 | 1.265717223 | up | 6.008678947 | 4.742961724 | AATCATTATCTT | hsa_circ_0041501 | chr17 | 3967654 | 3968123 | - | 469 | ANNOTATED, CDS, c | NM_015113 | ZZEF1 | Salzman2013 | 23140 | circRNA | Detected | Detected | 6.008678947 | 4.742961724 |
| hsa_gci161346 | 2.014152653 | 1.01017303 | up | 8.113793319 | 7.103620289 | GTCGGCCCTTAT | hsa_circ_0041576 | chr17 | 4574678 | 4579802 | - | 2746 | ANNOTATED, CDS, c | NM_014389 | PELP1 | Salzman2013 | 27043 | circRNA | Detected | Detected | 8.113793319 | 7.103620289 |
| hsa_gci161363 | 2.438304856 | 1.285878514 | up | 7.804333708 | 6.518455194 | ATTTAAACAGTT | hsa_circ_0041641 | chr17 | 4856566 | 4859157 | + | 883 | ALT_DONOR, CDS, c | NM_001976 | ENO3 | Salzman2013 | 2027 | circRNA | Detected | Detected | 7.804333708 | 6.518455194 |
| hsa_gci161364 | 5.118295471 | 2.355663434 | up | 4.644126345 | 2.288462911 | CAGGGAGAATAA | hsa_circ_0041646 | chr17 | 4862520 | 4862938 | - | 418 | ANNOTATED, CDS, c | NM_004890 | SPAG7 | Salzman2013 | 9552 | circRNA | Detected | Not Detected | 4.644126345 | 2.288462911 |
| hsa_gci161370 | 2.305703682 | 1.205207117 | up | 8.705469275 | 7.500262158 | TGTCCCCACCTA | hsa_circ_0041700 | chr17 | 5307418 | 5320002 | - | 895 | ANNOTATED, CDS, c | NM_002532 | NUP88 | Salzman2013 | 4927 | circRNA | Detected | Detected | 8.705469275 | 7.500262158 |
| hsa_gci161380 | 2.708641139 | 1.437569267 | up | 7.523745834 | 6.086176567 | GTACTCTTCCGG | hsa_circ_0041758 | chr17 | 6920579 | 6920843 | + | 264 | ANNOTATED, ncRNA, | NR_037717 | | Salzman2013 | | circRNA | Detected | Detected | 7.523745834 | 6.086176567 |
| hsa_gci161397 | 2.258261016 | 1.175212246 | up | 9.859378629 | 8.684166383 | CAACCGCTCATC | hsa_circ_0041891 | chr17 | 7416339 | 7417935 | + | 1596 | ANNOTATED, CDS, c | NM_000937 | POLR2A | Salzman2013 | 5430 | circRNA | Detected | Detected | 9.859378629 | 8.684166383 |
| hsa_gci161399 | 3.455024174 | 1.788695805 | up | 6.371630248 | 4.582934443 | TTTTGGCAACCC | hsa_circ_0041900 | chr17 | 7467941 | 7480010 | + | 2079 | ANNOTATED, INTERN | NR_037926 | SENP3-EIF4A1 | Salzman2013 | 100533955 | circRNA | Detected | Detected | 6.371630248 | 4.582934443 |
| hsa_gci161400 | 2.119275129 | 1.083570894 | up | 6.869946152 | 5.786375258 | ACCCGTCTCATG | hsa_circ_0041901 | chr17 | 7467941 | 7482324 | + | 3306 | ANNOTATED, ncRNA, | NR_037926 | SENP3-EIF4A1 | Salzman2013 | 100533955 | circRNA | Detected | Detected | 6.869946152 | 5.786375258 |
| hsa_gci161403 | 2.390105126 | 1.257074075 | up | 7.274547408 | 6.017473333 | CCTACCCCTAAT | hsa_circ_0041908 | chr17 | 7473673 | 7482324 | + | 2679 | ANNOTATED, ncRNA, | NR_037926 | SENP3-EIF4A1 | Salzman2013 | 100533955 | circRNA | Detected | Detected | 7.274547408 | 6.017473333 |
| hsa_gci161428 | 3.525581395 | 1.817861189 | up | 4.812290468 | 2.99442928 | GTTAAAGAACCC | hsa_circ_0042064 | chr17 | 9153787 | 9471787 | - | 801 | ANNOTATED, CDS, c | NM_004853 | STX8 | Salzman2013 | 9482 | circRNA | Detected | Not Detected | 4.812290468 | 2.99442928 |
| hsa_gci161429 | 2.002840301 | 1.002047391 | up | 6.469404623 | 5.467357233 | CGATCCCGGAGT | hsa_circ_0042065 | chr17 | 9153787 | 9479275 | - | 968 | ANNOTATED, CDS, c | NM_004853 | STX8 | Salzman2013 | 9482 | circRNA | Detected | Detected | 6.469404623 | 5.467357233 |
| hsa_gci161448 | -3.619234764 | -1.855684692 | down | 1.824979525 | 3.680664218 | TTTACTCAAAAA | hsa_circ_0042172 | chr17 | 16001680 | 16052831 | - | 2026 | ANNOTATED, CDS, c | NM_001190440 | NCOR1 | Salzman2013 | 9611 | circRNA | Not Detected | Detected | 1.824979525 | 3.680664218 |
| hsa_gci161451 | 2.785627889 | 1.478002552 | up | 7.970353024 | 6.492350472 | GTTATTTGTTAG | hsa_circ_0042183 | chr17 | 16097775 | 16097953 | - | 178 | ANNOTATED, CDS, c | NM_006311 | NCOR1 | Salzman2013 | 9611 | circRNA | Detected | Detected | 7.970353024 | 6.492350472 |
| hsa_gci161467 | 2.356614547 | 1.236715808 | up | 6.488380373 | 5.251664565 | ACGAGTCCCTGA | hsa_circ_0042279 | chr17 | 18110128 | 18113267 | + | 1891 | ANNOTATED, CDS, c | NM_017758 | ALKBH5 | Salzman2013 | 54890 | circRNA | Detected | Detected | 6.488380373 | 5.251664565 |
| hsa_gci161469 | 2.259698484 | 1.176130284 | up | 6.044356391 | 4.868226107 | CCGTCGAGGTCC | hsa_circ_0042302 | chr17 | 18148152 | 18151347 | - | 1919 | ANNOTATED, CDS, c | NM_002018 | FLII | Salzman2013 | 2314 | circRNA | Detected | Detected | 6.044356391 | 4.868226107 |

| | | | | | | | | | | | | | | | | | | | | | | |
|---|---|---|---|---|---|---|---|---|---|---|---|---|---|---|---|---|---|---|---|---|---|---|
| hsa_gci161478 | 2.826163313 | 1.498844836 | up | 5.988604774 | 4.489759938 | CTGCATTTCGGG | hsa_circ_0042349 | chr17 | 18607931 | 18639431 | + | 19452 | ALT_ACCEPTOR, CDS | NM_001037330 | TRIM16L | Salzman2013 | 147166 | circRNA | Detected | Detected | 5.988604774 | 4.489759938 |
| hsa_gci161490 | 2.226601988 | 1.154843695 | up | 4.335895138 | 3.181051443 | GTTCTTTTCCTA | hsa_circ_0042398 | chr17 | 19720061 | 19729499 | - | 309 | ANNOTATED, CDS, c | NM_014683 | ULK2 | Salzman2013 | 9706 | circRNA | Detected | Not Detected | 4.335895138 | 3.181051443 |
| hsa_gci161520 | -2.008415913 | -1.006058061 | down | 4.210771756 | 5.216829817 | TGTCCCATATCT | hsa_circ_0042521 | chr17 | 26512204 | 26512291 | + | 87 | ANNOTATED, CDS, c | NM_016231 | NLK | Salzman2013 | 51701 | circRNA | Detected | Detected | 4.210771756 | 5.216829817 |
| hsa_gci161525 | 2.282868093 | 1.190847502 | up | 6.713179045 | 5.522331543 | TGTCCGTGGTAC | hsa_circ_0042547 | chr17 | 26880405 | 26897981 | - | 2326 | ANNOTATED, CDS, c | NM_033198 | PIGS | Salzman2013 | 94005 | circRNA | Detected | Detected | 6.713179045 | 5.522331543 |
| hsa_gci161527 | 2.086288382 | 1.060938592 | up | 5.419389571 | 4.358450979 | GACTGGGGGTCT | hsa_circ_0042552 | chr17 | 26900132 | 26900952 | - | 707 | ANNOTATED, CDS, c | NM_005165 | ALDOC | Salzman2013 | 230 | circRNA | Detected | Detected | 5.419389571 | 4.358450979 |
| hsa_gci161529 | 2.673737582 | 1.418857877 | up | 6.940885467 | 5.52202759 | ACAAGTCTTGAT | hsa_circ_0042599 | chr17 | 26946877 | 26966478 | - | 4437 | ANNOTATED, CDS, c | NM_014680 | KIAA0100 | Salzman2013 | 9703 | circRNA | Detected | Detected | 6.940885467 | 5.52202759 |
| hsa_gci161533 | 2.146620144 | 1.102066921 | up | 6.298466556 | 5.196399635 | GGAGGTCCGCCA | hsa_circ_0042657 | chr17 | 27001554 | 27029249 | + | 5519 | ANNOTATED, CDS, c | NM_003170 | SUPT6H | Salzman2013 | 6830 | circRNA | Detected | Detected | 6.298466556 | 5.196399635 |
| hsa_gci161534 | 2.581337395 | 1.368118722 | up | 10.26015434 | 8.892035614 | TCCGTTATTTTC | hsa_circ_0042663 | chr17 | 27005562 | 27029249 | + | 4671 | ANNOTATED, CDS, c | NM_003170 | SUPT6H | Salzman2013 | 6830 | circRNA | Detected | Detected | 10.26015434 | 8.892035614 |
| hsa_gci161558 | 5.098028418 | 2.349939416 | up | 6.767030754 | 4.417091338 | CACAGCGAGGTC | hsa_circ_0042793 | chr17 | 27952964 | 28004759 | - | 8768 | ANNOTATED, CDS, c | NM_033389 | SSH2 | Salzman2013 | 85464 | circRNA | Detected | Detected | 6.767030754 | 4.417091338 |
| hsa_gci161563 | 2.008359228 | 1.006017342 | up | 6.005037073 | 4.999019731 | GCGAGGTCTCTC | hsa_circ_0042810 | chr17 | 27994147 | 28004759 | - | 424 | ANNOTATED, CDS, c | NM_033389 | SSH2 | Salzman2013 | 85464 | circRNA | Detected | Detected | 6.005037073 | 4.999019731 |
| hsa_gci161564 | 2.539353642 | 1.344461326 | up | 6.238892023 | 4.894430698 | CACAGCGAGGTC | hsa_circ_0042812 | chr17 | 28000929 | 28004759 | - | 3043 | ALT_DONOR, CDS, c | NM_033389 | SSH2 | Salzman2013 | 85464 | circRNA | Detected | Detected | 6.238892023 | 4.894430698 |
| hsa_gci161565 | 2.014986511 | 1.010770181 | up | 12.07541075 | 11.06464057 | ACTTGTAACGAA | hsa_circ_0042815 | chr17 | 28002847 | 28022541 | - | 1312 | ALT_DONOR, CDS, c | NM_033389 | SSH2 | Salzman2013 | 85464 | circRNA | Detected | Detected | 12.07541075 | 11.06464057 |
| hsa_gci161568 | 7.395907901 | 2.886727259 | up | 4.152423868 | 1.265696609 | CTCATTTTACTT | hsa_circ_0042819 | chr17 | 28003837 | 28022541 | - | 322 | ANNOTATED, CDS, c | NM_033389 | SSH2 | Salzman2013 | 85464 | circRNA | Detected | Not Detected | 4.152423868 | 1.265696609 |
| hsa_gci161569 | 8.25042377 | 3.044468223 | up | 4.266165399 | 1.221697176 | ACTTGTAACGAA | hsa_circ_0042823 | chr17 | 28011580 | 28022541 | - | 187 | ANNOTATED, CDS, c | NM_033389 | SSH2 | Salzman2013 | 85464 | circRNA | Detected | Not Detected | 4.266165399 | 1.221697176 |
| hsa_gci161584 | 2.032232122 | 1.023065197 | up | 7.776089357 | 6.753024161 | TTCCTCGCTGAA | hsa_circ_0042869 | chr17 | 29171852 | 29206505 | + | 1709 | ANNOTATED, CDS, c | NM_024857 | ATAD5 | Salzman2013 | 79915 | circRNA | Detected | Detected | 7.776089357 | 6.753024161 |
| hsa_gci161589 | 2.767753608 | 1.468715517 | up | 6.004508153 | 4.535792636 | CTTTCCGAGAAG | hsa_circ_0042877 | chr17 | 29325679 | 29326929 | + | 1250 | ANNOTATED, CDS, c | NM_001184992 | RNF135 | Salzman2013 | 84282 | circRNA | Detected | Detected | 6.004508153 | 4.535792636 |
| hsa_gci161626 | 2.03788831 | 1.027074984 | up | 8.876466689 | 7.849391705 | AAGAAGACGGGC | hsa_circ_0043001 | chr17 | 30500849 | 30502381 | + | 126 | ANNOTATED, CDS, c | NM_001033568 | RHOT1 | Salzman2013 | 55288 | circRNA | Detected | Detected | 8.876466689 | 7.849391705 |
| hsa_gci161627 | 2.290532285 | 1.195682898 | up | 9.058937667 | 7.863254769 | ACCTTACCACTA | hsa_circ_0043002 | chr17 | 30500849 | 30503034 | + | 180 | ANNOTATED, CDS, c | NM_001033568 | RHOT1 | Salzman2013 | 55288 | circRNA | Detected | Detected | 9.058937667 | 7.863254769 |
| hsa_gci161645 | 2.430541854 | 1.281277978 | up | 4.80102253 | 3.519744553 | AACTTTCGGTTT | hsa_circ_0043082 | chr17 | 33310020 | 33313150 | + | 695 | ANNOTATED, CDS, c | NM_013975 | LIG3 | Salzman2013 | 3980 | circRNA | Detected | Detected | 4.80102253 | 3.519744553 |
| hsa_gci161649 | 2.103429649 | 1.072743567 | up | 8.257648974 | 7.184905408 | CAACGTCCGTGG | hsa_circ_0043132 | chr17 | 33998772 | 34001340 | + | 293 | ANNOTATED, CDS, c | NM_001030006 | AP2B1 | Salzman2013 | 163 | circRNA | Detected | Detected | 8.257648974 | 7.184905408 |
| hsa_gci161651 | 2.456500618 | 1.296604602 | up | 5.063101372 | 3.76649677 | CGGACCTAAAGG | hsa_circ_0043138 | chr17 | 34147027 | 34149837 | + | 437 | ANNOTATED, CDS, c | NM_139215 | TAF15 | Salzman2013 | 8148 | circRNA | Detected | Detected | 5.063101372 | 3.76649677 |
| hsa_gci161672 | 2.249297428 | 1.169474444 | up | 5.720276169 | 4.550801726 | GGAGGTCCGTGA | hsa_circ_0043261 | chr17 | 35620587 | 35634898 | - | 719 | ANNOTATED, CDS, c | NM_198839 | ACACA | Salzman2013 | 31 | circRNA | Detected | Detected | 5.720276169 | 4.550801726 |
| hsa_gci161681 | 2.35975485 | 1.238636989 | up | 8.031089905 | 6.792452917 | AAGAACTCGTCA | hsa_circ_0043280 | chr17 | 35797838 | 35804870 | + | 412 | ANNOTATED, CDS, c | NM_001488 | TADA2A | Salzman2013 | 6871 | circRNA | Detected | Detected | 8.031089905 | 6.792452917 |
| hsa_gci161689 | 2.316219102 | 1.211771731 | up | 4.062566947 | 2.850795216 | ACGACGACAAGT | hsa_circ_0043297 | chr17 | 35936438 | 35945538 | - | 530 | ANNOTATED, CDS, c | NM_007247 | SYNRG | Salzman2013 | 11276 | circRNA | Detected | Not Detected | 4.062566947 | 2.850795216 |
| hsa_gci161700 | 2.56930237 | 1.361376685 | up | 5.939302186 | 4.577925501 | GTCCAAGTCGGC | hsa_circ_0043368 | chr17 | 36912135 | 36920478 | + | 488 | ANNOTATED, CDS, c | NM_002795 | PSMB3 | Salzman2013 | 5691 | circRNA | Detected | Detected | 5.939302186 | 4.577925501 |
| hsa_gci161716 | 2.091162644 | 1.064305274 | up | 5.17829603 | 4.113990756 | GCCCGGAGTCGG | hsa_circ_0043401 | chr17 | 37360777 | 37360980 | + | 203 | ANNOTATED, CDS, c | NM_000981 | RPL19 | Salzman2013 | 6143 | circRNA | Detected | Detected | 5.17829603 | 4.113990756 |
| hsa_gci161721 | 2.225034281 | 1.153827564 | up | 8.852329499 | 7.698501935 | TGTCGCCGTGTC | hsa_circ_0043410 | chr17 | 37437641 | 37459892 | - | 592 | ANNOTATED, CDS, c | NM_032875 | FBXL20 | Salzman2013 | 84961 | circRNA | Detected | Detected | 8.852329499 | 7.698501935 |
| hsa_gci161731 | 3.451781359 | 1.787341085 | up | 4.212705759 | 2.425364674 | TCCAAGTAATTT | hsa_circ_0043428 | chr17 | 37579579 | 37584043 | - | 446 | ANNOTATED, CDS, c | NM_004774 | MED1 | Salzman2013 | 5469 | circRNA | Detected | Not Detected | 4.212705759 | 2.425364674 |
| hsa_gci161745 | 2.26835546 | 1.181646734 | up | 6.170943639 | 4.989296905 | GAGGGGTGTAGA | hsa_circ_0043476 | chr17 | 37895023 | 37903538 | + | 2191 | ANNOTATED, CDS, c | NM_001242442 | GRB7 | Salzman2013 | 2886 | circRNA | Detected | Detected | 6.170943639 | 4.989296905 |
| hsa_gci161765 | 2.152700169 | 1.106147393 | up | 6.37635028 | 5.270202887 | CAGAAGGTTCCG | hsa_circ_0043595 | chr17 | 39679868 | 39680249 | - | 381 | ANNOTATED, CDS, c | NM_002276 | KRT19 | Salzman2013 | 3880 | circRNA | Detected | Detected | 6.37635028 | 5.270202887 |
| hsa_gci161767 | 2.46456165 | 1.30133107 | up | 6.827695335 | 5.526364265 | GAGTCGCATGAC | hsa_circ_0043597 | chr17 | 39679868 | 39680792 | - | 669 | ANNOTATED, CDS, c | NM_002276 | KRT19 | Salzman2013 | 3880 | circRNA | Detected | Detected | 6.827695335 | 5.526364265 |
| hsa_gci161769 | 2.554564821 | 1.353077544 | up | 4.405130351 | 3.052052807 | ACCTCAAGAGTT | hsa_circ_0043599 | chr17 | 39679868 | 39681525 | - | 909 | ANNOTATED, CDS, c | NM_002276 | KRT19 | Salzman2013 | 3880 | circRNA | Detected | Not Detected | 4.405130351 | 3.052052807 |
| hsa_gci161770 | 3.183397392 | 1.670567263 | up | 6.899065579 | 5.228498315 | AGACCATGAGGT | hsa_circ_0043603 | chr17 | 39679868 | 39925929 | - | 242759 | ALT_ACCEPTOR, CDS | NM_002276 | KRT19 | Salzman2013 | 3880 | circRNA | Detected | Detected | 6.899065579 | 5.228498315 |
| hsa_gci161780 | 2.241502841 | 1.164466328 | up | 5.290199504 | 4.125733177 | CAAGAGGTGGCT | hsa_circ_0043656 | chr17 | 39910858 | 39914036 | - | 1602 | ANNOTATED, CDS, c | NM_002230 | JUP | Salzman2013 | 3728 | circRNA | Detected | Detected | 5.290199504 | 4.125733177 |
| hsa_gci161785 | 2.284787477 | 1.192059977 | up | 6.358521247 | 5.16646127 | GACAACATCTCG | hsa_circ_0043678 | chr17 | 39958204 | 39967286 | - | 1703 | ANNOTATED, CDS, c | NM_006455 | P3H4 | Salzman2013 | 10609 | circRNA | Detected | Detected | 6.358521247 | 5.16646127 |
| hsa_gci161794 | 4.235702632 | 2.082601308 | up | 4.570444266 | 2.487842957 | CCTAGAGAAACA | hsa_circ_0043702 | chr17 | 40023178 | 40048700 | - | 2589 | ANNOTATED, CDS, c | NM_001096 | ACLY | Salzman2013 | 47 | circRNA | Detected | Not Detected | 4.570444266 | 2.487842957 |
| hsa_gci161816 | -2.766750232 | -1.468192411 | down | 2.64912414 | 4.117316551 | GTCCGGGTTCCG | hsa_circ_0043841 | chr17 | 40690356 | 40696466 | + | 1911 | ANNOTATED, CDS, c | NM_000263 | NAGLU | Salzman2013 | 4669 | circRNA | Not Detected | Detected | 2.64912414 | 4.117316551 |
| hsa_gci161819 | 3.568028587 | 1.835127174 | up | 5.251664565 | 3.416537391 | GGGGGGTCGTTA | hsa_circ_0043861 | chr17 | 40764444 | 40767256 | + | 1154 | ANNOTATED, CDS, c | NM_001070 | TUBG1 | Salzman2013 | 7283 | circRNA | Detected | Not Detected | 5.251664565 | 3.416537391 |
| hsa_gci161825 | 2.204048855 | 1.140156203 | up | 6.315011028 | 5.174854825 | ACAGGTGTAAGC | hsa_circ_0043903 | chr17 | 40925750 | 40931617 | + | 1008 | ANNOTATED, CDS, c | NM_032353 | VPS25 | Salzman2013 | 84313 | circRNA | Detected | Detected | 6.315011028 | 5.174854825 |
| hsa_gci161828 | 2.264241227 | 1.179027668 | up | 7.034394223 | 5.855366556 | TGCGAGGACCCT | hsa_circ_0043911 | chr17 | 40949651 | 40950185 | - | 534 | ANNOTATED, CDS, c | NM_001040431 | COA3 | Salzman2013 | 28958 | circRNA | Detected | Detected | 7.034394223 | 5.855366556 |
| hsa_gci161835 | 2.243777638 | 1.165929709 | up | 5.231273964 | 4.065344255 | AGGAATAACTGC | hsa_circ_0043931 | chr17 | 41154689 | 41154971 | + | 194 | ANNOTATED, CDS, c | NM_000988 | RPL27 | Salzman2013 | 6155 | circRNA | Detected | Detected | 5.231273964 | 4.065344255 |
| hsa_gci161837 | 2.586099868 | 1.370777989 | up | 4.234929554 | 2.864151564 | TAAGAAGAAGAG | hsa_circ_0043938 | chr17 | 41166621 | 41168161 | - | 1540 | ANNOTATED, CDS, c | NM_006373 | VAT1 | Salzman2013 | 10493 | circRNA | Detected | Not Detected | 4.234929554 | 2.864151564 |
| hsa_gci161841 | 2.070854937 | 1.050226497 | up | 5.064577732 | 4.014351235 | ACCAAAGAAGGT | hsa_circ_0043949 | chr17 | 41201137 | 41209152 | - | 213 | ANNOTATED, CDS, c | NM_007300 | BRCA1 | Salzman2013 | 672 | circRNA | Detected | Detected | 5.064577732 | 4.014351235 |
| hsa_gci161843 | 2.752617629 | 1.460804216 | up | 4.240016912 | 2.779212696 | ACTACCCGTAAA | hsa_circ_0043954 | chr17 | 41219624 | 41226538 | - | 590 | ANNOTATED, CDS, c | NM_007300 | BRCA1 | Salzman2013 | 672 | circRNA | Detected | Not Detected | 4.240016912 | 2.779212696 |
| hsa_gci161850 | 2.116539063 | 1.081707116 | up | 11.56050371 | 10.47879659 | GTAGTAACGACG | hsa_circ_0043987 | chr17 | 41577323 | 41601680 | + | 2730 | ANNOTATED, CDS, c | NM_004941 | DHX8 | Salzman2013 | 1659 | circRNA | Detected | Detected | 11.56050371 | 10.47879659 |
| hsa_gci161851 | 41.47900312 | 5.374309317 | up | 6.60701394 | 1.232704623 | CCGACTCCACTG | hsa_circ_0043993 | chr17 | 41585176 | 41601680 | + | 2019 | ANNOTATED, CDS, c | NM_004941 | DHX8 | Salzman2013 | 1659 | circRNA | Detected | Not Detected | 6.60701394 | 1.232704623 |
| hsa_gci161857 | 4.181930163 | 2.064168969 | up | 4.451696244 | 2.387527274 | AGAGATCAAACA | hsa_circ_0044037 | chr17 | 42266302 | 42269099 | + | 1885 | ANNOTATED, CDS, c | NM_177441 | TMUB2 | Salzman2013 | 79089 | circRNA | Detected | Not Detected | 4.451696244 | 2.387527274 |
| hsa_gci161860 | 2.476963087 | 1.308572369 | up | 7.085091628 | 5.776519259 | CGAGAAGTGGTC | hsa_circ_0044053 | chr17 | 42396992 | 42398099 | - | 765 | ANNOTATED, CDS, c | NM_001143780 | SLC25A39 | Salzman2013 | 51629 | circRNA | Detected | Detected | 7.085091628 | 5.776519259 |
| hsa_gci161866 | 3.843496803 | 1.942419469 | up | 8.042578375 | 6.100158906 | ATAGTCCCGTGA | hsa_circ_0044066 | chr17 | 42427595 | 42430470 | + | 1737 | ANNOTATED, CDS, c | NM_002087 | GRN | Salzman2013 | 2896 | circRNA | Detected | Detected | 8.042578375 | 6.100158906 |
| hsa_gci161872 | 2.532272997 | 1.340432946 | up | 8.072653841 | 6.732220895 | AAGGGTTTGAGT | hsa_circ_0044105 | chr17 | 42937272 | 42971893 | - | 1864 | ANNOTATED, CDS, c | NM_004247 | EFTUD2 | Salzman2013 | 9343 | circRNA | Detected | Detected | 8.072653841 | 6.732220895 |
| hsa_gci161907 | 2.74863731 | 1.458716552 | up | 6.477009569 | 5.018293018 | ATTGTCCAGGAT | hsa_circ_0044331 | chr17 | 46018888 | 46026674 | + | 3465 | ANNOTATED, CDS, c | NM_018129 | PNPO | Salzman2013 | 55163 | circRNA | Detected | Detected | 6.477009569 | 5.018293018 |

| | | | | | | | | | | | | | | | | | | | | | | |
|---|---|---|---|---|---|---|---|---|---|---|---|---|---|---|---|---|---|---|---|---|---|---|
| hsa_gci161910 | 2.842266324 | 1.507041743 | up | 8.625665053 | 7.118623309 | TGTTCCCTTCGT | hsa_circ_0044334 | chr17 | 46022924 | 46026674 | + | 2949 | ANNOTATED, CDS, c | NM_018129 | PNPO | Salzman2013 | 55163 | circRNA | Detected | Detected | 8.625665053 | 7.118623309 |
| hsa_gci161915 | 3.024052515 | 1.596483193 | up | 4.389716168 | 2.793232974 | GAAGGGGGTGGG | hsa_circ_0044366 | chr17 | 46970147 | 46973232 | + | 646 | ANNOTATED, CDS, c | NM_005175 | ATP5MC1 | Salzman2013 | 516 | circRNA | Detected | Not Detected | 4.389716168 | 2.793232974 |
| hsa_gci161916 | 2.827480726 | 1.49951719 | up | 5.427033639 | 3.927516449 | CCTCTTTACTTA | hsa_circ_0044367 | chr17 | 46970770 | 46973232 | + | 521 | ANNOTATED, CDS, c | NM_005175 | ATP5MC1 | Salzman2013 | 516 | circRNA | Detected | Detected | 5.427033639 | 3.927516449 |
| hsa_gci161919 | 2.123001806 | 1.086105598 | up | 8.051204349 | 6.965098751 | GACGACGAACTC | hsa_circ_0044371 | chr17 | 46973016 | 46973232 | + | 216 | ANNOTATED, CDS, c | NM_005175 | ATP5MC1 | Salzman2013 | 516 | circRNA | Detected | Detected | 8.051204349 | 6.965098751 |
| hsa_gci161920 | 2.754402437 | 1.461739363 | up | 6.975432827 | 5.513693465 | AAGGACCTCCTC | hsa_circ_0044375 | chr17 | 46988169 | 47006422 | + | 2674 | ANNOTATED, CDS, c | NM_023079 | UBE2Z | Salzman2013 | 65264 | circRNA | Detected | Detected | 6.975432827 | 5.513693465 |
| hsa_gci161939 | 2.214719315 | 1.147123869 | up | 4.300657497 | 3.153533628 | TGAGGTTCGGTG | hsa_circ_0044445 | chr17 | 48141891 | 48148294 | + | 417 | ANNOTATED, CDS, c | NM_002204 | ITGA3 | Salzman2013 | 3675 | circRNA | Detected | Not Detected | 4.300657497 | 3.153533628 |
| hsa_gci161942 | -4.281364798 | -2.098070767 | down | 3.499921164 | 5.597991931 | GTAAATGGGCCT | hsa_circ_0044493 | chr17 | 48158673 | 48167849 | + | 1743 | ANNOTATED, CDS, c | NM_002204 | ITGA3 | Salzman2013 | 3675 | circRNA | Not Detected | Detected | 3.499921164 | 5.597991931 |
| hsa_gci161960 | 3.081905151 | 1.623822462 | up | 5.987681523 | 4.363859061 | CATGGATCGGTG | hsa_circ_0044656 | chr17 | 49231585 | 49249105 | + | 1168 | ANNOTATED, ncRNA, | NR_037149 | NME1-NME2 | Salzman2013 | 654364 | circRNA | Detected | Detected | 5.987681523 | 4.363859061 |
| hsa_gci161987 | -2.595136345 | -1.375810338 | down | 1.887838499 | 3.263648837 | CGGACGACAACC | hsa_circ_0044752 | chr17 | 56048909 | 56060754 | - | 4457 | ANNOTATED, CDS, c | NM_007146 | VEZF1 | Salzman2013 | 7716 | circRNA | Not Detected | Detected | 1.887838499 | 3.263648837 |
| hsa_gci161993 | 2.215323224 | 1.147517209 | up | 7.659174976 | 6.511657767 | TAGTGTTAACAG | hsa_circ_0044773 | chr17 | 56424548 | 56428869 | - | 217 | ANNOTATED, CDS, c | NM_003168 | SUPT4H1 | Salzman2013 | 6827 | circRNA | Detected | Detected | 7.659174976 | 6.511657767 |
| hsa_gci161995 | 4.665540583 | 2.222044252 | up | 11.39108164 | 9.169037386 | GGTGAGAACCGT | hsa_circ_0044781 | chr17 | 56566892 | 56586202 | - | 5426 | ANNOTATED, CDS, c | NM_004687 | MTMR4 | Salzman2013 | 9110 | circRNA | Detected | Detected | 11.39108164 | 9.169037386 |
| hsa_gci162005 | -3.862989993 | -1.94971794 | down | 2.464300373 | 4.414018313 | TGCTGTCTCGCT | hsa_circ_0044828 | chr17 | 57165651 | 57167453 | - | 1802 | ALT_ACCEPTOR, CDS | NM_015294 | TRIM37 | Salzman2013 | 4591 | circRNA | Not Detected | Detected | 2.464300373 | 4.414018313 |
| hsa_gci162011 | 2.586945764 | 1.371249808 | up | 4.853890311 | 3.482640503 | GGGAGTGCGTCT | hsa_circ_0044838 | chr17 | 57430575 | 57479095 | + | 5099 | ANNOTATED, CDS, c | NM_001005404 | YPEL2 | Salzman2013 | 388403 | circRNA | Detected | Detected | 4.853890311 | 3.482640503 |
| hsa_gci162025 | 2.562265455 | 1.357419949 | up | 7.118436214 | 5.761016265 | CGAAGACGTCTC | hsa_circ_0044889 | chr17 | 57915655 | 57917952 | + | 927 | ANNOTATED, CDS, c | NM_030938 | VMP1 | Salzman2013 | 81671 | circRNA | Detected | Detected | 7.118436214 | 5.761016265 |
| hsa_gci162035 | 2.228611609 | 1.156145213 | up | 5.758158442 | 4.602013229 | GATTATGGGCGA | hsa_circ_0044927 | chr17 | 58275620 | 58292135 | - | 1567 | ANNOTATED, CDS, c | NM_032582 | USP32 | Salzman2013 | 84669 | circRNA | Detected | Detected | 5.758158442 | 4.602013229 |
| hsa_gci162045 | 3.005418729 | 1.587566008 | up | 6.464462022 | 4.876896015 | TATCAAATGGAA | hsa_circ_0044954 | chr17 | 58348710 | 58366043 | - | 292 | ANNOTATED, CDS, c | NM_032582 | USP32 | Salzman2013 | 84669 | circRNA | Detected | Detected | 6.464462022 | 4.876896015 |
| hsa_gci162056 | 2.391839625 | 1.258120659 | up | 7.159015297 | 5.900894638 | TGTGTTGTCATG | hsa_circ_0044989 | chr17 | 58945936 | 58952099 | + | 185 | ANNOTATED, CDS, c | NM_001099432 | BCAS3 | Salzman2013 | 54828 | circRNA | Detected | Detected | 7.159015297 | 5.900894638 |
| hsa_gci162057 | 2.379893773 | 1.25089718 | up | 7.095495217 | 5.844598037 | TTCACTTATGTC | hsa_circ_0044990 | chr17 | 58945936 | 58967132 | + | 262 | ANNOTATED, CDS, c | NM_001099432 | BCAS3 | Salzman2013 | 54828 | circRNA | Detected | Detected | 7.095495217 | 5.844598037 |
| hsa_gci162059 | 2.6070534 | 1.382420134 | up | 8.884276965 | 7.501856832 | ACTTTAAACACC | hsa_circ_0045000 | chr17 | 59112026 | 59118253 | + | 392 | ANNOTATED, CDS, c | NM_001099432 | BCAS3 | Salzman2013 | 54828 | circRNA | Detected | Detected | 8.884276965 | 7.501856832 |
| hsa_gci162061 | 2.197611748 | 1.135936528 | up | 11.99864852 | 10.86271199 | GATCTTGTCACC | hsa_circ_0045013 | chr17 | 59820373 | 59886118 | - | 1752 | ANNOTATED, CDS, c | NM_032043 | BRIP1 | Salzman2013 | 83990 | circRNA | Detected | Detected | 11.99864852 | 10.86271199 |
| hsa_gci162072 | -3.957968694 | -1.984760201 | down | 1.350567142 | 3.335327343 | CGGAGGAGGTTC | hsa_circ_0045049 | chr17 | 59967146 | 60002460 | - | 1691 | ANNOTATED, INTERN | NR_026641 | INTS2 | Salzman2013 | 57508 | circRNA | Not Detected | Detected | 1.350567142 | 3.335327343 |
| hsa_gci162081 | 2.227437649 | 1.155385048 | up | 5.976067161 | 4.820682113 | CACTTCCTCGTC | hsa_circ_0045084 | chr17 | 60050087 | 60112969 | - | 3497 | ANNOTATED, CDS, c | NM_005121 | MED13 | Salzman2013 | 9969 | circRNA | Detected | Detected | 5.976067161 | 4.820682113 |
| hsa_gci162102 | 2.667316698 | 1.41538913 | up | 6.723131306 | 5.307742175 | GTTCACATCCGT | hsa_circ_0045194 | chr17 | 61417461 | 61432751 | + | 1007 | ANNOTATED, CDS, c | NM_025185 | TANC2 | Salzman2013 | 26115 | circRNA | Detected | Detected | 6.723131306 | 5.307742175 |
| hsa_gci162115 | 2.141897837 | 1.098889669 | up | 6.618293816 | 5.519404147 | GTTTACCACCTA | hsa_circ_0045234 | chr17 | 61882430 | 61884014 | + | 225 | ANNOTATED, CDS, c | NM_007372 | DDX42 | Salzman2013 | 11325 | circRNA | Detected | Detected | 6.618293816 | 5.519404147 |
| hsa_gci162117 | 2.203307898 | 1.139671116 | up | 6.314199548 | 5.174528432 | TCGGTAGTCACA | hsa_circ_0045241 | chr17 | 61896792 | 61902796 | - | 2513 | ANNOTATED, CDS, c | NM_017647 | FTSJ3 | Salzman2013 | 117246 | circRNA | Detected | Detected | 6.314199548 | 5.174528432 |
| hsa_gci162126 | 3.268366967 | 1.708569976 | up | 4.506704016 | 2.79813404 | GAGTCTTCTGTT | hsa_circ_0045308 | chr17 | 62515438 | 62522318 | + | 679 | ANNOTATED, CDS, c | NM_138363 | CEP95 | Salzman2013 | 90799 | circRNA | Detected | Not Detected | 4.506704016 | 2.79813404 |
| hsa_gci162127 | 3.405455624 | 1.767847833 | up | 4.640481456 | 2.872633623 | CCAAGAGGAAAG | hsa_circ_0045318 | chr17 | 62567915 | 62579662 | - | 531 | ANNOTATED, CDS, c | NM_022739 | SMURF2 | Salzman2013 | 64750 | circRNA | Detected | Not Detected | 4.640481456 | 2.872633623 |
| hsa_gci162138 | 2.482046227 | 1.311529985 | up | 9.67203949 | 8.360509505 | GGCATTCACACC | hsa_circ_0045373 | chr17 | 64637472 | 64806862 | + | 8438 | ANNOTATED, CDS, c | NM_002737 | PRKCA | Salzman2013 | 5578 | circRNA | Detected | Detected | 9.67203949 | 8.360509505 |
| hsa_gci162139 | 2.231538006 | 1.158038378 | up | 8.413084894 | 7.255046516 | ACCCCTGTAACT | hsa_circ_0045374 | chr17 | 64641500 | 64806862 | + | 8326 | ANNOTATED, CDS, c | NM_002737 | PRKCA | Salzman2013 | 5578 | circRNA | Detected | Detected | 8.413084894 | 7.255046516 |
| hsa_gci162145 | 2.473718338 | 1.306681242 | up | 6.079154272 | 4.77247303 | TAACGTGAAATT | hsa_circ_0045409 | chr17 | 65162593 | 65163912 | - | 465 | ANNOTATED, CDS, c | NM_014877 | HELZ | Salzman2013 | 9931 | circRNA | Detected | Detected | 6.079154272 | 4.77247303 |
| hsa_gci162160 | 2.924909429 | 1.548391952 | up | 7.045146324 | 5.496754372 | ATCGTAAGAGCA | hsa_circ_0045476 | chr17 | 66033225 | 66039115 | + | 689 | ANNOTATED, CDS, c | NM_002266 | KPNA2 | Salzman2013 | 3838 | circRNA | Detected | Detected | 7.045146324 | 5.496754372 |
| hsa_gci162167 | 3.244770665 | 1.698116515 | up | 6.002615865 | 4.304499351 | TCCGGTCCATTC | hsa_circ_0045505 | chr17 | 66431760 | 66432620 | - | 191 | ANNOTATED, CDS, c | NM_017983 | WIPI1 | Salzman2013 | 55062 | circRNA | Detected | Detected | 6.002615865 | 4.304499351 |
| hsa_gci162173 | 2.609729529 | 1.383900295 | up | 6.062928242 | 4.679027947 | CCCTTTTCCTGC | hsa_circ_0045539 | chr17 | 72199794 | 72206019 | + | 392 | ANNOTATED, CDS, c | NM_000999 | RPL38 | Salzman2013 | 6169 | circRNA | Detected | Detected | 6.062928242 | 4.679027947 |
| hsa_gci162174 | 2.321923628 | 1.21532052 | up | 6.921033102 | 5.705712581 | GCGACCCGAGAC | hsa_circ_0045541 | chr17 | 72200056 | 72206019 | + | 302 | ANNOTATED, CDS, c | NM_000999 | RPL38 | Salzman2013 | 6169 | circRNA | Detected | Detected | 6.921033102 | 5.705712581 |
| hsa_gci162178 | 2.318232968 | 1.213025555 | up | 5.434148135 | 4.22112258 | GGGGGGTCCTGG | hsa_circ_0045581 | chr17 | 73015794 | 73017356 | + | 654 | ANNOTATED, CDS, c | NM_001545 | MRPL58 | Salzman2013 | 3396 | circRNA | Detected | Detected | 5.434148135 | 4.22112258 |
| hsa_gci162190 | 2.167104768 | 1.115768901 | up | 7.339916018 | 6.224147116 | ACAACCCGGTCC | hsa_circ_0045682 | chr17 | 73725345 | 73753899 | + | 5166 | ANNOTATED, CDS, c | NM_000213 | ITGB4 | Salzman2013 | 3691 | circRNA | Detected | Detected | 7.339916018 | 6.224147116 |
| hsa_gci162192 | 2.202369367 | 1.139056449 | up | 10.01404718 | 8.874990732 | AGGTGCTATCCC | hsa_circ_0045703 | chr17 | 73748526 | 73753899 | + | 1756 | ANNOTATED, CDS, c | NM_000213 | ITGB4 | Salzman2013 | 3691 | circRNA | Detected | Detected | 10.01404718 | 8.874990732 |
| hsa_gci162194 | 2.459445457 | 1.298333061 | up | 4.175057386 | 2.876724326 | AACTCACAAAAA | hsa_circ_0045768 | chr17 | 74060056 | 74063411 | - | 310 | ANNOTATED, CDS, c | NM_014230 | SRP68 | Salzman2013 | 6730 | circRNA | Detected | Not Detected | 4.175057386 | 2.876724326 |
| hsa_gci162199 | 2.051463952 | 1.036653803 | up | 7.785176404 | 6.7485226 | CGTAGCGACCCT | hsa_circ_0045822 | chr17 | 74559200 | 74561430 | + | 2230 | ANNOTATED, ncRNA, | NR_038108 | | Salzman2013 | | circRNA | Detected | Detected | 7.785176404 | 6.7485226 |
| hsa_gci162205 | 2.125875484 | 1.088057098 | up | 9.404745964 | 8.316688866 | GGTTAGCAGCCC | hsa_circ_0045849 | chr17 | 75085234 | 75089429 | + | 223 | ANNOTATED, coding | NM_001204410 | SEC14L1 | Salzman2013 | 6397 | circRNA | Detected | Detected | 9.404745964 | 8.316688866 |
| hsa_gci162212 | 2.465801811 | 1.302056848 | up | 4.808847651 | 3.506790803 | CCGACTTAATGC | hsa_circ_0045945 | chr17 | 76849058 | 76918483 | - | 51705 | ALT_ACCEPTOR, CDS | NM_003255 | TIMP2 | Salzman2013 | 7077 | circRNA | Detected | Detected | 4.808847651 | 3.506790803 |
| hsa_gci162228 | 2.771321334 | 1.470574001 | up | 9.002192474 | 7.531618473 | ATCGGCTTTTTG | hsa_circ_0046110 | chr17 | 79224670 | 79249951 | - | 1958 | ANNOTATED, CDS, c | NM_138570 | SLC38A10 | Salzman2013 | 124565 | circRNA | Detected | Detected | 9.002192474 | 7.531618473 |
| hsa_gci162230 | 2.54694987 | 1.348770564 | up | 6.397760155 | 5.048989591 | ACGGCGTTCTAA | hsa_circ_0046144 | chr17 | 79477504 | 79478134 | - | 537 | ANNOTATED, INTERN | NR_037688 | ACTG1 | Salzman2013 | 71 | circRNA | Detected | Detected | 6.397760155 | 5.048989591 |
| hsa_gci162231 | 2.574094704 | 1.364065133 | up | 9.490287987 | 8.126222854 | TACCGGCCCCAC | hsa_circ_0046145 | chr17 | 79477504 | 79478652 | - | 976 | ANNOTATED, INTERN | NR_037688 | ACTG1 | Salzman2013 | 71 | circRNA | Detected | Detected | 9.490287987 | 8.126222854 |
| hsa_gci162241 | 2.039997108 | 1.028567107 | up | 8.486826669 | 7.458259563 | AAGTGCCCTCCG | hsa_circ_0046188 | chr17 | 79555969 | 79575848 | - | 846 | ANNOTATED, CDS, c | NM_017921 | NPLOC4 | Salzman2013 | 55666 | circRNA | Detected | Detected | 8.486826669 | 7.458259563 |
| hsa_gci162251 | 2.17275539 | 1.119525764 | up | 4.892494351 | 3.772968586 | CGAGTCCAGCCG | hsa_circ_0046277 | chr17 | 79869814 | 79873388 | - | 1273 | ANNOTATED, CDS, c | NM_016538 | SIRT7 | Salzman2013 | 51547 | circRNA | Detected | Detected | 4.892494351 | 3.772968586 |
| hsa_gci162259 | 2.381033331 | 1.251587816 | up | 10.93616769 | 9.684579875 | GAGAACCAGTAG | hsa_circ_0046419 | chr17 | 80417867 | 80430574 | + | 493 | ANNOTATED, CDS, c | NM_001038618 | NARF | Salzman2013 | 26502 | circRNA | Detected | Detected | 10.93616769 | 9.684579875 |
| hsa_gci162260 | 2.742981712 | 1.455745003 | up | 5.672082464 | 4.216337461 | ACTTTATTGCTA | hsa_circ_0046435 | chr17 | 80540616 | 80545148 | + | 877 | ANNOTATED, CDS, c | NM_004514 | FOXK2 | Salzman2013 | 3607 | circRNA | Detected | Detected | 5.672082464 | 4.216337461 |
| hsa_gci162321 | 2.043806052 | 1.031258297 | up | 5.486547052 | 4.455288754 | GTCTCAACGTCC | hsa_circ_0046753 | chr18 | 3252272 | 3256234 | + | 1807 | ALT_ACCEPTOR, CDS | NM_006471 | MYL12A | Salzman2013 | 10627 | circRNA | Detected | Detected | 5.486547052 | 4.455288754 |
| hsa_gci162359 | 2.586115406 | 1.370786657 | up | 4.162329504 | 2.791542846 | CCTAAGTCGCAT | hsa_circ_0046904 | chr18 | 9845578 | 9862553 | + | 3439 | ANNOTATED, CDS, c | NM_006868 | RAB31 | Salzman2013 | 11031 | circRNA | Detected | Not Detected | 4.162329504 | 2.791542846 |
| hsa_gci162368 | 3.882724166 | 1.95706922 | up | 5.042358597 | 3.085289377 | GTTATAATTTGG | hsa_circ_0046964 | chr18 | 12356692 | 12360050 | - | 537 | ANNOTATED, CDS, c | NM_006796 | AFG3L2 | Salzman2013 | 10939 | circRNA | Detected | Not Detected | 5.042358597 | 3.085289377 |

| | | | | | | | | | | | | | | | | | | | | | | |
|---|---|---|---|---|---|---|---|---|---|---|---|---|---|---|---|---|---|---|---|---|---|---|
| hsa_gci162369 | 2.943770245 | 1.557665076 | up | 5.97178475 | 4.414119674 | ATTGTTTAGCCA | hsa_circ_0046965 | chr18 | 12356692 | 12371690 | - | 1050 | ANNOTATED, CDS, c | NM_006796 | AFG3L2 | Salzman2013 | 10939 | circRNA | Detected | Detected | 5.97178475 | 4.414119674 |
| hsa_gci162407 | 2.715411042 | 1.441170601 | up | 5.839429425 | 4.398258824 | ACAGAGGTTCAG | hsa_circ_0047090 | chr18 | 19243638 | 19244191 | - | 553 | ALT_DONOR, CDS, c | NM_138340 | ABHD3 | Salzman2013 | 171586 | circRNA | Detected | Detected | 5.839429425 | 4.398258824 |
| hsa_gci162421 | 2.494582631 | 1.318798458 | up | 8.737080794 | 7.418282336 | AGTGGTTCCCCC | hsa_circ_0047135 | chr18 | 20570899 | 20576425 | + | 1221 | ANNOTATED, CDS, c | NM_002894 | RBBP8 | Salzman2013 | 5932 | circRNA | Detected | Detected | 8.737080794 | 7.418282336 |
| hsa_gci162428 | -4.158755779 | -2.056151966 | down | 1.436565939 | 3.492717905 | TATAACGATAGC | hsa_circ_0047179 | chr18 | 21131590 | 21137154 | - | 773 | ANNOTATED, CDS, c | NM_000271 | NPC1 | Salzman2013 | 4864 | circRNA | Not Detected | Detected | 1.436565939 | 3.492717905 |
| hsa_gci162442 | 2.018166688 | 1.013045337 | up | 5.166057217 | 4.15301188 | CCCATCAATCCT | hsa_circ_0047304 | chr18 | 23596216 | 23612496 | - | 2262 | ANNOTATED, CDS, c | NM_001007559 | SS18 | Salzman2013 | 6760 | circRNA | Detected | Detected | 5.166057217 | 4.15301188 |
| hsa_gci162490 | 2.387569012 | 1.255542434 | up | 8.023993705 | 6.76845127 | GCAAGACCTCGG | hsa_circ_0047467 | chr18 | 34376033 | 34409158 | - | 2065 | ANNOTATED, CDS, c | NM_015476 | TPGS2 | Salzman2013 | 25941 | circRNA | Detected | Detected | 8.023993705 | 6.76845127 |
| hsa_gci162502 | 2.285866934 | 1.192741423 | up | 5.398395617 | 4.205654194 | TGAACCTACTAA | hsa_circ_0047502 | chr18 | 40847856 | 40854305 | - | 3639 | ALT_ACCEPTOR, CDS | NM_020783 | SYT4 | Salzman2013 | 6860 | circRNA | Detected | Detected | 5.398395617 | 4.205654194 |
| hsa_gci162529 | 2.113157578 | 1.079400353 | up | 6.635128945 | 5.555728592 | TGACTATATAGG | hsa_circ_0047610 | chr18 | 45391429 | 45391504 | - | 75 | ANNOTATED, CDS, c | NM_001003652 | SMAD2 | Salzman2013 | 4087 | circRNA | Detected | Detected | 6.635128945 | 5.555728592 |
| hsa_gci162533 | 2.010321351 | 1.007426136 | up | 5.020675224 | 4.013249088 | AAGTATACATTA | hsa_circ_0047643 | chr18 | 46783379 | 46860223 | - | 966 | ANNOTATED, CDS, c | NM_017653 | DYM | Salzman2013 | 54808 | circRNA | Detected | Detected | 5.020675224 | 4.013249088 |
| hsa_gci162536 | 2.391051241 | 1.257645048 | up | 7.851363579 | 6.593718531 | AAGTATACATTA | hsa_circ_0047650 | chr18 | 46858233 | 46860223 | - | 269 | ANNOTATED, CDS, c | NM_017653 | DYM | Salzman2013 | 54808 | circRNA | Detected | Detected | 7.851363579 | 6.593718531 |
| hsa_gci162572 | 2.083604709 | 1.059081602 | up | 9.935324341 | 8.876242738 | AACAGGAATCAT | hsa_circ_0047786 | chr18 | 55398645 | 55401328 | + | 2683 | ANTISENSE, CDS, c | NM_005603 | ATP8B1 | Salzman2013 | 5205 | circRNA | Detected | Detected | 9.935324341 | 8.876242738 |
| hsa_gci162573 | -2.739366327 | -1.453842206 | down | 1.971377811 | 3.425220017 | AAAGTCTCTTTT | hsa_circ_0047809 | chr18 | 56530060 | 56606853 | + | 3252 | ANNOTATED, CDS, c | NM_018181 | ZNF532 | Salzman2013 | 55205 | circRNA | Not Detected | Detected | 1.971377811 | 3.425220017 |
| hsa_gci162587 | 130.9987843 | 7.033409613 | up | 8.270160422 | 1.236750809 | CGGCATGGGCGG | hsa_circ_0047834 | chr18 | 57567659 | 57571538 | + | 3879 | ALT_ACCEPTOR, CDS | NM_021127 | PMAIP1 | Salzman2013 | 5366 | circRNA | Detected | Not Detected | 8.270160422 | 1.236750809 |
| hsa_gci162590 | 3.942908647 | 1.979260285 | up | 7.366598874 | 5.387338589 | TCTCTTGACCTC | hsa_circ_0047848 | chr18 | 59752441 | 59763183 | - | 349 | ANNOTATED, CDS, c | NM_176787 | PIGN | Salzman2013 | 23556 | circRNA | Detected | Detected | 7.366598874 | 5.387338589 |
| hsa_gci162591 | 2.152337238 | 1.105904144 | up | 6.00916317 | 4.903259027 | CCGTGTTGGTTC | hsa_circ_0047850 | chr18 | 59757708 | 59763183 | - | 206 | ANNOTATED, CDS, c | NM_176787 | PIGN | Salzman2013 | 23556 | circRNA | Detected | Detected | 6.00916317 | 4.903259027 |
| hsa_gci162598 | 2.88382526 | 1.52798375 | up | 4.812738712 | 3.284754962 | GAAAGGCATCAA | hsa_circ_0047870 | chr18 | 59888269 | 59899660 | + | 1004 | ANNOTATED, CDS, c | NM_020854 | KIAA1468 | Salzman2013 | 57614 | circRNA | Detected | Detected | 4.812738712 | 3.284754962 |
| hsa_gci162608 | -14.24381526 | -3.832263724 | down | 1.230925849 | 5.063189573 | ACTTCTGTGCCC | hsa_circ_0047940 | chr18 | 67806834 | 67817969 | - | 859 | ANNOTATED, CDS, c | NM_173630 | RTTN | Salzman2013 | 25914 | circRNA | Not Detected | Detected | 1.230925849 | 5.063189573 |
| hsa_gci162610 | -8.703005223 | -3.121513662 | down | 1.291075486 | 4.412589148 | TGCCTATAGGAC | hsa_circ_0047946 | chr18 | 67807335 | 67857955 | - | 1663 | ANNOTATED, CDS, c | NM_173630 | RTTN | Salzman2013 | 25914 | circRNA | Not Detected | Detected | 1.291075486 | 4.412589148 |
| hsa_gci162612 | 2.779990803 | 1.47508011 | up | 5.78818271 | 4.3131026 | TGAAGATAGTGT | hsa_circ_0047964 | chr18 | 72589152 | 72601994 | + | 372 | ANNOTATED, CDS, c | NM_017757 | ZNF407 | Salzman2013 | 55628 | circRNA | Detected | Detected | 5.78818271 | 4.3131026 |
| hsa_gci162619 | 2.248146505 | 1.168736054 | up | 4.205751504 | 3.03701545 | ATCACAAGAGGC | hsa_circ_0047976 | chr18 | 74561481 | 74620529 | + | 2496 | ANNOTATED, CDS, c | NM_007345 | ZNF236 | Salzman2013 | 7776 | circRNA | Detected | Not Detected | 4.205751504 | 3.03701545 |
| hsa_gci162634 | 2.172407463 | 1.119294725 | up | 7.170585447 | 6.051290722 | GTTCGTGTCTCG | hsa_circ_0048035 | chr19 | 305574 | 311939 | - | 1864 | ANNOTATED, CDS, c | NM_017550 | MIER2 | Salzman2013 | 54531 | circRNA | Detected | Detected | 7.170585447 | 6.051290722 |
| hsa_gci162642 | 2.408720189 | 1.268266812 | up | 11.13647475 | 9.868207938 | CAAGTTCGGCGG | hsa_circ_0048161 | chr19 | 1074797 | 1086627 | + | 2929 | ANNOTATED, CDS, c | NM_012292 | ARHGAP45 | Salzman2013 | 23526 | circRNA | Detected | Detected | 11.13647475 | 9.868207938 |
| hsa_gci162651 | 2.133939398 | 1.093519206 | up | 4.494892565 | 3.40137336 | ACTAGAGTAGTT | hsa_circ_0048312 | chr19 | 2110134 | 2138713 | - | 2982 | ANNOTATED, CDS, c | NM_003938 | AP3D1 | Salzman2013 | 8943 | circRNA | Detected | Detected | 4.494892565 | 3.40137336 |
| hsa_gci162652 | 2.184936422 | 1.127591301 | up | 6.232892678 | 5.105301377 | GAACTAGAGTAG | hsa_circ_0048319 | chr19 | 2115536 | 2138713 | - | 2053 | ANNOTATED, CDS, c | NM_003938 | AP3D1 | Salzman2013 | 8943 | circRNA | Detected | Detected | 6.232892678 | 5.105301377 |
| hsa_gci162667 | 2.422012187 | 1.276206125 | up | 8.7471149 | 7.470908776 | GCGGGGCGCGGG | hsa_circ_0048406 | chr19 | 2321519 | 2328614 | - | 520 | ANNOTATED, CDS, c | NM_016199 | LSM7 | Salzman2013 | 51690 | circRNA | Detected | Detected | 8.7471149 | 7.470908776 |
| hsa_gci162678 | -2.514140128 | -1.330065062 | down | 3.492398715 | 4.822463777 | CTACCCGAGGTG | hsa_circ_0048536 | chr19 | 3976053 | 3980064 | - | 1729 | ANNOTATED, CDS, c | NM_001961 | EEF2 | Salzman2013 | 1938 | circRNA | Not Detected | Detected | 3.492398715 | 4.822463777 |
| hsa_gci162683 | 2.578290207 | 1.366414659 | up | 5.619965222 | 4.253550562 | GGTGCCATGGCT | hsa_circ_0048541 | chr19 | 3976053 | 3982422 | - | 2463 | ANNOTATED, CDS, c | NM_001961 | EEF2 | Salzman2013 | 1938 | circRNA | Detected | Detected | 5.619965222 | 4.253550562 |
| hsa_gci162687 | 2.325386019 | 1.217470226 | up | 7.552424658 | 6.334954432 | CTGCTGCCGCCT | hsa_circ_0048545 | chr19 | 3976053 | 3985461 | - | 3158 | ANNOTATED, CDS, c | NM_001961 | EEF2 | Salzman2013 | 1938 | circRNA | Detected | Detected | 7.552424658 | 6.334954432 |
| hsa_gci162690 | 3.583518476 | 1.841376793 | up | 5.516223766 | 3.674846973 | TGTACCACTTTG | hsa_circ_0048593 | chr19 | 4324039 | 4324664 | - | 368 | ANNOTATED, CDS, c | NM_017720 | STAP2 | Salzman2013 | 55620 | circRNA | Detected | Detected | 5.516223766 | 3.674846973 |
| hsa_gci162696 | 2.588094372 | 1.371890225 | up | 8.613429346 | 7.241539121 | CCCCACGTCCAC | hsa_circ_0048617 | chr19 | 4445002 | 4447622 | - | 1279 | ANNOTATED, CDS, c | NM_025241 | UBXN6 | Salzman2013 | 80700 | circRNA | Detected | Detected | 8.613429346 | 7.241539121 |
| hsa_gci162700 | 2.129203456 | 1.090313813 | up | 8.573402828 | 7.483089015 | CCGCTCCCTGTG | hsa_circ_0048654 | chr19 | 4657556 | 4670415 | - | 1051 | ANNOTATED, CDS, c | NM_019107 | MYDGF | Salzman2013 | 56005 | circRNA | Detected | Detected | 8.573402828 | 7.483089015 |
| hsa_gci162719 | 2.063538998 | 1.045120703 | up | 7.839203818 | 6.794083115 | TCGACGCGTTTG | hsa_circ_0048850 | chr19 | 6532796 | 6535939 | + | 1360 | ANNOTATED, CDS, c | NM_003811 | TNFSF9 | Salzman2013 | 8744 | circRNA | Detected | Detected | 7.839203818 | 6.794083115 |
| hsa_gci162736 | 3.343541801 | 1.741377154 | up | 6.112724856 | 4.371347702 | GAAGGAGGTGGA | hsa_circ_0049077 | chr19 | 8550486 | 8554002 | + | 1283 | ANNOTATED, CDS, c | NM_005968 | HNRNPM | Salzman2013 | 4670 | circRNA | Detected | Detected | 6.112724856 | 4.371347702 |
| hsa_gci162737 | 2.679754935 | 1.422101071 | up | 6.532553732 | 5.110452661 | ACCTTTTCAAAC | hsa_circ_0049086 | chr19 | 8661425 | 8662201 | - | 274 | ANNOTATED, CDS, c | NM_030957 | ADAMTS10 | Salzman2013 | 81794 | circRNA | Detected | Detected | 6.532553732 | 5.110452661 |
| hsa_gci162746 | 2.005411186 | 1.003898075 | up | 5.753729017 | 4.749830942 | TATGGGACACGT | hsa_circ_0049188 | chr19 | 10244021 | 10305755 | - | 5400 | ANNOTATED, CDS, c | NM_001130823 | DNMT1 | Salzman2013 | 1786 | circRNA | Detected | Detected | 5.753729017 | 4.749830942 |
| hsa_gci162752 | -2.252817583 | -1.171730499 | down | 2.130609701 | 3.3023402 | AGTCTTAGTGCA | hsa_circ_0049239 | chr19 | 10395078 | 10397291 | + | 2002 | ANNOTATED, CDS, c | NM_000201 | ICAM1 | Salzman2013 | 3383 | circRNA | Not Detected | Detected | 2.130609701 | 3.3023402 |
| hsa_gci162753 | -4.314123906 | -2.109067613 | down | 1.16454544 | 3.273613052 | TACGTCGCGACG | hsa_circ_0049250 | chr19 | 10501808 | 10502382 | - | 574 | ANNOTATED, CDS, c | NM_007065 | CDC37 | Salzman2013 | 11140 | circRNA | Not Detected | Detected | 1.16454544 | 3.273613052 |
| hsa_gci162762 | 2.569116159 | 1.361272121 | up | 6.02642447 | 4.665152348 | CGTCAGCCCTCA | hsa_circ_0049298 | chr19 | 10745431 | 10755235 | + | 2472 | ANNOTATED, CDS, c | NM_020428 | SLC44A2 | Salzman2013 | 57153 | circRNA | Detected | Detected | 6.02642447 | 4.665152348 |
| hsa_gci162775 | -9.372002033 | -3.228357267 | down | 1.855438145 | 5.083795412 | CTCTGGTCGGAC | hsa_circ_0049502 | chr19 | 12037388 | 12061578 | + | 23283 | ALT_ACCEPTOR, CDS | NM_144566 | ZNF700 | Salzman2013 | 90592 | circRNA | Not Detected | Detected | 1.855438145 | 5.083795412 |
| hsa_gci162779 | 2.963414404 | 1.567260387 | up | 5.852940912 | 4.285680525 | CCGTCTCTACCT | hsa_circ_0049535 | chr19 | 12778880 | 12779239 | - | 359 | ANNOTATED, CDS, c | NM_016145 | WDR83OS | Salzman2013 | 51398 | circRNA | Detected | Detected | 5.852940912 | 4.285680525 |
| hsa_gci162789 | 3.498430965 | 1.806708023 | up | 7.069595077 | 5.262887054 | AATGGCCTCCGG | hsa_circ_0049605 | chr19 | 12907633 | 12912084 | - | 867 | ANNOTATED, CDS, c | NM_005809 | PRDX2 | Salzman2013 | 7001 | circRNA | Detected | Detected | 7.069595077 | 5.262887054 |
| hsa_gci162796 | 2.032081161 | 1.022958024 | up | 7.025117754 | 6.00215973 | CCCAGTCCCTAT | hsa_circ_0049648 | chr19 | 13051354 | 13055304 | + | 1129 | ANNOTATED, CDS, c | NM_004343 | CALR | Salzman2013 | 811 | circRNA | Detected | Detected | 7.025117754 | 6.00215973 |
| hsa_gci162800 | 2.233437729 | 1.159266031 | up | 6.522771182 | 5.363505151 | TGCCGACAACCC | hsa_circ_0049672 | chr19 | 13255223 | 13259905 | - | 450 | ANNOTATED, CDS, c | NM_003765 | STX10 | Salzman2013 | 8677 | circRNA | Detected | Detected | 6.522771182 | 5.363505151 |
| hsa_gci162801 | 2.743491511 | 1.456013112 | up | 6.816276147 | 5.360263035 | ACTTGAACGGAC | hsa_circ_0049673 | chr19 | 13255223 | 13260407 | - | 545 | ANNOTATED, CDS, c | NM_003765 | STX10 | Salzman2013 | 8677 | circRNA | Detected | Detected | 6.816276147 | 5.360263035 |
| hsa_gci162804 | 3.128401153 | 1.64542552 | up | 5.955280993 | 4.309855473 | CCCCAAGCTCCG | hsa_circ_0049720 | chr19 | 14230320 | 14231477 | - | 1157 | ANNOTATED, CDS, c | NM_018154 | ASF1B | Salzman2013 | 55723 | circRNA | Detected | Detected | 5.955280993 | 4.309855473 |
| hsa_gci162808 | 2.714127643 | 1.440488571 | up | 5.051967923 | 3.611479352 | GTTTGAGGTCTG | hsa_circ_0049758 | chr19 | 14588570 | 14606944 | - | 1929 | ANNOTATED, CDS, c | NM_005716 | GIPC1 | Salzman2013 | 10755 | circRNA | Detected | Detected | 5.051967923 | 3.611479352 |
| hsa_gci162828 | 2.945886455 | 1.558701825 | up | 9.203302681 | 7.644600857 | GGCGGCGGAGTC | hsa_circ_0050017 | chr19 | 17688169 | 17693965 | + | 2555 | ANNOTATED, CDS, c | NM_024656 | COLGALT1 | Salzman2013 | 79709 | circRNA | Detected | Detected | 9.203302681 | 7.644600857 |
| hsa_gci162836 | -4.328977152 | -2.114026186 | down | 1.296755959 | 3.410782145 | TCAATGGTCCGT | hsa_circ_0050088 | chr19 | 18283166 | 18288927 | + | 2444 | ALT_ACCEPTOR, CDS | NM_006332 | IFI30 | Salzman2013 | 10437 | circRNA | Not Detected | Detected | 1.296755959 | 3.410782145 |
| hsa_gci162837 | 2.283268339 | 1.191100421 | up | 5.580218972 | 4.389118551 | CCGGGGGCGTCC | hsa_circ_0050095 | chr19 | 18285849 | 18288927 | + | 827 | ANNOTATED, CDS, c | NM_006332 | IFI30 | Salzman2013 | 10437 | circRNA | Detected | Detected | 5.580218972 | 4.389118551 |
| hsa_gci162842 | 2.255347516 | 1.173349748 | up | 8.287312138 | 7.11396239 | GAGAAGTCCCAG | hsa_circ_0050178 | chr19 | 19040009 | 19042220 | - | 521 | ANNOTATED, ncRNA, | NR_027297 | HOMER3 | Salzman2013 | 9454 | circRNA | Detected | Detected | 8.287312138 | 7.11396239 |
| hsa_gci162843 | 2.28253722 | 1.190638386 | up | 8.106291661 | 6.915653276 | GACCTTGAAGAG | hsa_circ_0050181 | chr19 | 19040009 | 19045219 | - | 1025 | ANNOTATED, ncRNA, | NR_027297 | HOMER3 | Salzman2013 | 9454 | circRNA | Detected | Detected | 8.106291661 | 6.915653276 |

| | | | | | | | | | | | | | | | | | | | | | | |
|---|---|---|---|---|---|---|---|---|---|---|---|---|---|---|---|---|---|---|---|---|---|---|
| hsa_gci162849 | 2.282328511 | 1.190506464 | up | 7.298773163 | 6.1082667 | GAGGTCTCTCTT | hsa_circ_0050274 | chr19 | 20295164 | 20296891 | + | 223 | ANNOTATED, CDS, c | NM_052852 | ZNF486 | Salzman2013 | 90649 | circRNA | Detected | Detected | 7.298773163 | 6.1082667 |
| hsa_gci162851 | 2.515949711 | 1.331103086 | up | 7.830121411 | 6.499018326 | GAATCTTTGTCG | hsa_circ_0050282 | chr19 | 21216891 | 21216990 | + | 99 | ANNOTATED, CDS, c | NM_025189 | ZNF430 | Salzman2013 | 80264 | circRNA | Detected | Detected | 7.830121411 | 6.499018326 |
| hsa_gci162856 | 2.687197179 | 1.426102186 | up | 5.538339857 | 4.112237671 | CGGTGTAGAGAT | hsa_circ_0050306 | chr19 | 23316881 | 23317008 | + | 127 | ANNOTATED, INTERN | TCONS_12_00012420 | | Salzman2013 | | circRNA | Detected | Detected | 5.538339857 | 4.112237671 |
| hsa_gci162859 | 2.03682928 | 1.026325064 | up | 7.990282135 | 6.963957071 | CGGGCCCCCAGG | hsa_circ_0050320 | chr19 | 28283907 | 28284140 | - | 233 | ANNOTATED, INTERN | TCONS_00027260 | | Salzman2013 | | circRNA | Detected | Detected | 7.990282135 | 6.963957071 |
| hsa_gci162869 | 2.428085889 | 1.279819455 | up | 7.350302698 | 6.070483243 | CCTCATACCCAT | hsa_circ_0050430 | chr19 | 33482728 | 33493906 | - | 884 | ANNOTATED, CDS, c | NM_033103 | RHPN2 | Salzman2013 | 85415 | circRNA | Detected | Detected | 7.350302698 | 6.070483243 |
| hsa_gci162872 | -2.060778585 | -1.043189506 | down | 3.285631624 | 4.32882113 | GTGAGTTGCTAG | hsa_circ_0050447 | chr19 | 33877854 | 33968996 | - | 1374 | ANNOTATED, CDS, c | NM_000285 | PEPD | Salzman2013 | 5184 | circRNA | Not Detected | Detected | 3.285631624 | 4.32882113 |
| hsa_gci162882 | 2.379515979 | 1.250668143 | up | 6.111311841 | 4.860643698 | TATACGGGTACC | hsa_circ_0050480 | chr19 | 34857249 | 34893318 | + | 3932 | ANNOTATED, CDS, c | NM_000175 | GPI | Salzman2013 | 2821 | circRNA | Detected | Detected | 6.111311841 | 4.860643698 |
| hsa_gci162884 | 2.334253496 | 1.222961244 | up | 5.173527419 | 3.950566176 | CAGTGGCGAGGC | hsa_circ_0050491 | chr19 | 34868407 | 34893318 | + | 3652 | ANNOTATED, CDS, c | NM_000175 | GPI | Salzman2013 | 2821 | circRNA | Detected | Detected | 5.173527419 | 3.950566176 |
| hsa_gci162903 | 2.46060389 | 1.29901243 | up | 7.164199508 | 5.865187078 | TATTGGTCATAT | hsa_circ_0050523 | chr19 | 34924252 | 34957919 | + | 1448 | ANNOTATED, CDS, c | NM_005499 | UBA2 | Salzman2013 | 10054 | circRNA | Detected | Detected | 7.164199508 | 5.865187078 |
| hsa_gci162906 | 2.760383074 | 1.464868492 | up | 7.592157368 | 6.127288877 | CGGTCCGTGTAA | hsa_circ_0050529 | chr19 | 34925772 | 34960798 | + | 2237 | ANNOTATED, CDS, c | NM_005499 | UBA2 | Salzman2013 | 10054 | circRNA | Detected | Detected | 7.592157368 | 6.127288877 |
| hsa_gci162909 | 2.789683713 | 1.480101562 | up | 7.112013546 | 5.631911984 | AGAGACCCAGCC | hsa_circ_0050535 | chr19 | 34929549 | 34945258 | + | 673 | ANNOTATED, CDS, c | NM_005499 | UBA2 | Salzman2013 | 10054 | circRNA | Detected | Detected | 7.112013546 | 5.631911984 |
| hsa_gci162910 | 4.04215194 | 2.015123552 | up | 4.951515245 | 2.936391693 | CAGCCGAATCCT | hsa_circ_0050537 | chr19 | 34929549 | 34955036 | + | 1145 | ANNOTATED, CDS, c | NM_005499 | UBA2 | Salzman2013 | 10054 | circRNA | Detected | Not Detected | 4.951515245 | 2.936391693 |
| hsa_gci162914 | 2.035096131 | 1.025096944 | up | 5.60455987 | 4.579462926 | CAAACAGGTAAC | hsa_circ_0050547 | chr19 | 34941169 | 34957919 | + | 970 | ANNOTATED, CDS, c | NM_005499 | UBA2 | Salzman2013 | 10054 | circRNA | Detected | Detected | 5.60455987 | 4.579462926 |
| hsa_gci162920 | 2.323693846 | 1.216420002 | up | 6.513033337 | 5.296613336 | GGTCCACCCTTT | hsa_circ_0050609 | chr19 | 36037427 | 36038429 | + | 608 | ANNOTATED, CDS, c | NM_001242597 | TMEM147 | Salzman2013 | 10430 | circRNA | Detected | Detected | 6.513033337 | 5.296613336 |
| hsa_gci162935 | -4.078328197 | -2.027977879 | down | 1.20407953 | 3.232057409 | GACTAGCCAGTC | hsa_circ_0050756 | chr19 | 36636877 | 36641251 | + | 792 | ALT_DONOR, CDS, c | NM_001749 | CAPNS1 | Salzman2013 | 826 | circRNA | Not Detected | Detected | 1.20407953 | 3.232057409 |
| hsa_gci162943 | 2.136614185 | 1.09532642 | up | 6.757504075 | 5.662177655 | TCGTTTCATTAT | hsa_circ_0050820 | chr19 | 38798067 | 38799968 | - | 492 | ANNOTATED, CDS, c | NM_033557 | YIF1B | Salzman2013 | 90522 | circRNA | Detected | Detected | 6.757504075 | 5.662177655 |
| hsa_gci162947 | 2.40179112 | 1.264110687 | up | 6.231853949 | 4.967743262 | GTAACTTGTTGA | hsa_circ_0050868 | chr19 | 39090564 | 39092165 | - | 223 | ANNOTATED, CDS, c | NM_007181 | MAP4K1 | Salzman2013 | 11184 | circRNA | Detected | Detected | 6.231853949 | 4.967743262 |
| hsa_gci162964 | 2.116124106 | 1.081424241 | up | 6.07568763 | 4.994263389 | ATGTTACACATC | hsa_circ_0050989 | chr19 | 39907190 | 39911492 | + | 853 | ANNOTATED, CDS, c | NM_022835 | PLEKHG2 | Salzman2013 | 64857 | circRNA | Detected | Detected | 6.07568763 | 4.994263389 |
| hsa_gci162966 | 2.698049799 | 1.431916977 | up | 8.562814673 | 7.130897696 | CCATTATCCGGT | hsa_circ_0050998 | chr19 | 39923846 | 39924209 | - | 260 | ANNOTATED, CDS, c | NM_001020 | RPS16 | Salzman2013 | 6217 | circRNA | Detected | Detected | 8.562814673 | 7.130897696 |
| hsa_gci162967 | 2.730009178 | 1.448905801 | up | 4.92203395 | 3.473128149 | GGCTCTTCGTCT | hsa_circ_0050999 | chr19 | 39923846 | 39924401 | - | 357 | ANNOTATED, CDS, c | NM_001020 | RPS16 | Salzman2013 | 6217 | circRNA | Detected | Detected | 4.92203395 | 3.473128149 |
| hsa_gci162971 | 3.545239731 | 1.825883187 | up | 7.889666485 | 6.063783298 | CTTCAACCAGTG | hsa_circ_0051017 | chr19 | 39963048 | 39967308 | + | 1583 | ANNOTATED, CDS, c | NM_003169 | SUPT5H | Salzman2013 | 6829 | circRNA | Detected | Detected | 7.889666485 | 6.063783298 |
| hsa_gci162976 | 2.448362297 | 1.291817057 | up | 7.001863861 | 5.710046805 | AAGGTCCGAGCC | hsa_circ_0051033 | chr19 | 40325092 | 40329845 | - | 673 | ANNOTATED, CDS, c | NM_001436 | FBL | Salzman2013 | 2091 | circRNA | Detected | Detected | 7.001863861 | 5.710046805 |
| hsa_gci163009 | 2.252370372 | 1.171444079 | up | 4.933036976 | 3.761592897 | AACACCGTTAGA | hsa_circ_0051204 | chr19 | 41763397 | 41767671 | + | 2351 | ANNOTATED, CDS, c | NM_021913 | AXL | Salzman2013 | 558 | circRNA | Detected | Detected | 4.933036976 | 3.761592897 |
| hsa_gci163026 | -2.096466373 | -1.06795969 | down | 3.100921752 | 4.168881442 | CTGTCTCGCTCT | hsa_circ_0051370 | chr19 | 44337656 | 44341331 | + | 337 | ANNOTATED, CDS, c | NM_181845 | ZNF283 | Salzman2013 | 284349 | circRNA | Not Detected | Detected | 3.100921752 | 4.168881442 |
| hsa_gci163028 | 2.131047652 | 1.091562854 | up | 11.43852206 | 10.3469592 | CGTTTCCCACCG | hsa_circ_0051380 | chr19 | 45161029 | 45162362 | + | 502 | ANNOTATED, CDS, c | NM_001135770 | PVR | Salzman2013 | 5817 | circRNA | Detected | Detected | 11.43852206 | 10.3469592 |
| hsa_gci163031 | 2.293101067 | 1.197299942 | up | 8.749291718 | 7.551991776 | TGCACGTCCCGC | hsa_circ_0051394 | chr19 | 45260583 | 45263301 | + | 1070 | ANNOTATED, CDS, c | NM_005178 | BCL3 | Salzman2013 | 602 | circRNA | Detected | Detected | 8.749291718 | 7.551991776 |
| hsa_gci163034 | 3.378742202 | 1.756486277 | up | 6.865123375 | 5.108637098 | AAGAGTGGCACC | hsa_circ_0051444 | chr19 | 45682002 | 45685058 | + | 2600 | ANNOTATED, CDS, c | NM_212550 | BLOC1S3 | Salzman2013 | 388552 | circRNA | Detected | Detected | 6.865123375 | 5.108637098 |
| hsa_gci163040 | 2.454637581 | 1.295510031 | up | 8.147567721 | 6.85205769 | TGTCAGACAAGA | hsa_circ_0051545 | chr19 | 46190711 | 46192605 | - | 569 | ANNOTATED, CDS, c | NM_177542 | SNRPD2 | Salzman2013 | 6633 | circRNA | Detected | Detected | 8.147567721 | 6.85205769 |
| hsa_gci163045 | 2.389549564 | 1.256738692 | up | 7.124604996 | 5.867866303 | GGCGCGCGCGGG | hsa_circ_0051592 | chr19 | 47104511 | 47114039 | + | 2259 | ANNOTATED, CDS, c | NM_005184 | CALM3 | Salzman2013 | 808 | circRNA | Detected | Detected | 7.124604996 | 5.867866303 |
| hsa_gci163048 | 2.652505098 | 1.407355524 | up | 5.619804584 | 4.21244906 | GGAGGTGGAGGA | hsa_circ_0051621 | chr19 | 47278139 | 47285806 | - | 1596 | ANNOTATED, CDS, c | NM_005628 | SLC1A5 | Salzman2013 | 6510 | circRNA | Detected | Detected | 5.619804584 | 4.21244906 |
| hsa_gci163051 | 2.382782992 | 1.252647567 | up | 11.28338055 | 10.03073298 | CAAGTGTCTGTA | hsa_circ_0051640 | chr19 | 47341422 | 47342056 | - | 443 | ANNOTATED, CDS, c | NM_004069 | AP2S1 | Salzman2013 | 1175 | circRNA | Detected | Detected | 11.28338055 | 10.03073298 |
| hsa_gci163060 | 2.791822745 | 1.481207347 | up | 10.20309142 | 8.721884072 | CCGCGGTTCCGC | hsa_circ_0051704 | chr19 | 48281841 | 48287943 | + | 880 | ANNOTATED, CDS, c | NM_003009 | SELENOW | Salzman2013 | 6415 | circRNA | Detected | Detected | 10.20309142 | 8.721884072 |
| hsa_gci163062 | 2.294490613 | 1.198173905 | up | 9.615164273 | 8.416990368 | CGCCGGCCCCTT | hsa_circ_0051706 | chr19 | 48284120 | 48287943 | + | 625 | ANNOTATED, CDS, c | NM_003009 | SELENOW | Salzman2013 | 6415 | circRNA | Detected | Detected | 9.615164273 | 8.416990368 |
| hsa_gci163074 | 5.530548604 | 2.467422596 | up | 8.185054956 | 5.71763236 | GGACAACGTGAT | hsa_circ_0051777 | chr19 | 49298318 | 49300590 | - | 848 | ANNOTATED, ncRNA, | NR_028450 | BCAT2 | Salzman2013 | 587 | circRNA | Detected | Detected | 8.185054956 | 5.71763236 |
| hsa_gci163086 | 4.122436517 | 2.043497277 | up | 6.582169374 | 4.538672097 | ACTACTTTTGGA | hsa_circ_0051875 | chr19 | 49952348 | 49952911 | - | 563 | ALT_DONOR, CDS, c | NM_017916 | PIH1D1 | Salzman2013 | 55011 | circRNA | Detected | Detected | 6.582169374 | 4.538672097 |
| hsa_gci163089 | 2.478706634 | 1.309587532 | up | 5.8128036 | 4.503216067 | AAGTGTCGCATG | hsa_circ_0051893 | chr19 | 49993488 | 49995564 | + | 1016 | ANNOTATED, CDS, c | NM_012423 | RPL13A | Salzman2013 | 23521 | circRNA | Detected | Detected | 5.8128036 | 4.503216067 |
| hsa_gci163114 | -2.17285557 | -1.119592282 | down | 2.148969192 | 3.268561473 | GGATCGCTGGTT | hsa_circ_0052067 | chr19 | 52693054 | 52729678 | + | 2509 | ANNOTATED, CDS, c | NM_014225 | PPP2R1A | Salzman2013 | 5518 | circRNA | Not Detected | Detected | 2.148969192 | 3.268561473 |
| hsa_gci163117 | 3.495940945 | 1.805680814 | up | 6.817275377 | 5.011594562 | TCCCGGTCCTCC | hsa_circ_0052078 | chr19 | 52709215 | 52729678 | + | 2045 | ANNOTATED, CDS, c | NM_014225 | PPP2R1A | Salzman2013 | 5518 | circRNA | Detected | Detected | 6.817275377 | 5.011594562 |
| hsa_gci163126 | 2.871655498 | 1.521882685 | up | 5.488096585 | 3.966213901 | TATATACGTAAC | hsa_circ_0052114 | chr19 | 53231982 | 53233258 | - | 202 | ANNOTATED, coding | NM_001161499 | ZNF611 | Salzman2013 | 81856 | circRNA | Detected | Detected | 5.488096585 | 3.966213901 |
| hsa_gci163142 | 2.273284063 | 1.18477797 | up | 7.782977702 | 6.598199732 | GGTTTTACTGGC | hsa_circ_0052300 | chr19 | 58193356 | 58269527 | + | 72059 | ALT_DONOR, CDS, c | NM_138347 | ZNF551 | Salzman2013 | 90233 | circRNA | Detected | Detected | 7.782977702 | 6.598199732 |
| hsa_gci163152 | 2.473573416 | 1.30659672 | up | 9.252228116 | 7.945631396 | CGAACGTGTGCC | hsa_circ_0052370 | chr19 | 59056791 | 59062082 | + | 2330 | ANNOTATED, CDS, c | NM_005762 | TRIM28 | Salzman2013 | 10155 | circRNA | Detected | Detected | 9.252228116 | 7.945631396 |
| hsa_gci163159 | 5.485236012 | 2.455553697 | up | 4.834408182 | 2.378854485 | GGGAGTGGACTC | hsa_circ_0052391 | chr19 | 59060354 | 59062082 | + | 1261 | ANNOTATED, CDS, c | NM_005762 | TRIM28 | Salzman2013 | 10155 | circRNA | Detected | Not Detected | 4.834408182 | 2.378854485 |
| hsa_gci163162 | 3.553168093 | 1.829105941 | up | 8.192893633 | 6.363787691 | CATCTTCGAGTG | hsa_circ_0052400 | chr19 | 59062932 | 59065603 | - | 776 | ANNOTATED, CDS, c | NM_014453 | CHMP2A | Salzman2013 | 27243 | circRNA | Detected | Detected | 8.192893633 | 6.363787691 |
| hsa_gci163186 | 3.001022508 | 1.585454139 | up | 5.984668074 | 4.399213934 | GTAAGGAAGATC | hsa_circ_0052544 | chr2 | 9570051 | 9574089 | + | 495 | ANNOTATED, CDS, c | NM_016207 | CPSF3 | Salzman2013 | 51692 | circRNA | Detected | Detected | 5.984668074 | 4.399213934 |
| hsa_gci163201 | 2.035551138 | 1.025419466 | up | 7.622411852 | 6.596992386 | CCCTTCAGCCCC | hsa_circ_0052598 | chr2 | 10580507 | 10580994 | - | 487 | ANNOTATED, CDS, c | NM_002539 | ODC1 | Salzman2013 | 4953 | circRNA | Detected | Detected | 7.622411852 | 6.596992386 |
| hsa_gci163228 | 2.125856481 | 1.088044202 | up | 5.121740256 | 4.033696053 | TGTAGGAATTAT | hsa_circ_0052766 | chr2 | 15601324 | 15644337 | - | 1454 | ANNOTATED, CDS, c | NM_015909 | NBAS | Salzman2013 | 51594 | circRNA | Detected | Detected | 5.121740256 | 4.033696053 |
| hsa_gci163231 | 2.222314218 | 1.152062817 | up | 4.345878802 | 3.193815985 | GAACCATCGAGA | hsa_circ_0052778 | chr2 | 15615810 | 15674765 | - | 694 | ANNOTATED, CDS, c | NM_015909 | NBAS | Salzman2013 | 51594 | circRNA | Detected | Detected | 4.345878802 | 3.193815985 |
| hsa_gci163238 | 2.533381244 | 1.341064202 | up | 6.463394735 | 5.122330534 | TAGCCCGTCTCA | hsa_circ_0052805 | chr2 | 15768761 | 15768963 | + | 116 | ANNOTATED, CDS, c | NM_004939 | DDX1 | Salzman2013 | 1653 | circRNA | Detected | Detected | 6.463394735 | 5.122330534 |
| hsa_gci163240 | 2.063224136 | 1.044900556 | up | 5.975265003 | 4.930364447 | GACCTCAGGTTC | hsa_circ_0052819 | chr2 | 20232410 | 20234823 | - | 825 | ANNOTATED, CDS, c | NM_014713 | LAPTM4A | Salzman2013 | 9741 | circRNA | Detected | Detected | 5.975265003 | 4.930364447 |
| hsa_gci163246 | 2.874857831 | 1.523490613 | up | 7.153494672 | 5.630004059 | GGTAGAACTAGA | hsa_circ_0052831 | chr2 | 20400557 | 20405185 | - | 2836 | ANNOTATED, CDS, c | NM_001006946 | SDC1 | Salzman2013 | 6382 | circRNA | Detected | Detected | 7.153494672 | 5.630004059 |
| hsa_gci163270 | 2.468241875 | 1.303483778 | up | 6.188965283 | 4.885481505 | TCTGTAAGCAAG | hsa_circ_0052877 | chr2 | 20939730 | 20939965 | - | 235 | ANNOTATED, CDS, c | NM_021925 | LDAH | Salzman2013 | 60526 | circRNA | Detected | Detected | 6.188965283 | 4.885481505 |

| | | | | | | | | | | | | | | | | | | | | | | |
|---|---|---|---|---|---|---|---|---|---|---|---|---|---|---|---|---|---|---|---|---|---|---|
| hsa_gci163272 | 2.648139118 | 1.404978915 | up | 5.265878102 | 3.860899187 | TCAACATACACA | hsa_circ_0052907 | chr2 | 21237944 | 21265348 | - | 3575 | ANNOTATED, CDS, c | NM_000384 | APOB | Salzman2013 | 338 | circRNA | Detected | Detected | 5.265878102 | 3.860899187 |
| hsa_gci163274 | 3.569012886 | 1.83552511 | up | 7.759739119 | 5.924214009 | TTCTCGTTCCTC | hsa_circ_0052943 | chr2 | 24021007 | 24056948 | - | 1072 | ANNOTATED, CDS, c | NM_017552 | ATAD2B | Salzman2013 | 54454 | circRNA | Detected | Detected | 7.759739119 | 5.924214009 |
| hsa_gci163276 | 2.311513465 | 1.208837767 | up | 6.558933133 | 5.350095366 | AGGTAGGTATTC | hsa_circ_0052948 | chr2 | 24046127 | 24056948 | - | 563 | ANNOTATED, CDS, c | NM_017552 | ATAD2B | Salzman2013 | 54454 | circRNA | Detected | Detected | 6.558933133 | 5.350095366 |
| hsa_gci163278 | 2.215139705 | 1.14739769 | up | 5.413738035 | 4.266340345 | CCTACCGAAGCT | hsa_circ_0052951 | chr2 | 24046127 | 24110855 | - | 1713 | ANNOTATED, CDS, c | NM_017552 | ATAD2B | Salzman2013 | 54454 | circRNA | Detected | Detected | 5.413738035 | 4.266340345 |
| hsa_gci163303 | 2.78441484 | 1.477374169 | up | 4.468894092 | 2.991519922 | ACGTACTGTATG | hsa_circ_0053087 | chr2 | 26587169 | 26609404 | + | 1038 | ANNOTATED, CDS, c | NM_033505 | SELENOI | Salzman2013 | 85465 | circRNA | Detected | Not Detected | 4.468894092 | 2.991519922 |
| hsa_gci163306 | 3.722066408 | 1.896103796 | up | 5.52461843 | 3.628514634 | GAGTTGAGGGTG | hsa_circ_0053090 | chr2 | 26597907 | 26606215 | + | 158 | ANNOTATED, CDS, c | NM_033505 | SELENOI | Salzman2013 | 85465 | circRNA | Detected | Detected | 5.52461843 | 3.628514634 |
| hsa_gci163311 | 2.404492311 | 1.265732313 | up | 6.757064345 | 5.491332032 | CTACTAGCTTCG | hsa_circ_0053121 | chr2 | 27262608 | 27264565 | + | 1405 | ANNOTATED, CDS, c | NM_017727 | TMEM214 | Salzman2013 | 54867 | circRNA | Detected | Detected | 6.757064345 | 5.491332032 |
| hsa_gci163312 | 2.354602925 | 1.235483787 | up | 7.759268058 | 6.523784271 | GACTAAGTGCCT | hsa_circ_0053123 | chr2 | 27263212 | 27264565 | + | 1139 | ANNOTATED, CDS, c | NM_017727 | TMEM214 | Salzman2013 | 54867 | circRNA | Detected | Detected | 7.759268058 | 6.523784271 |
| hsa_gci163313 | 2.547832352 | 1.349270351 | up | 8.839950833 | 7.490680482 | GTTTCCTCGAGG | hsa_circ_0053127 | chr2 | 27277497 | 27282403 | + | 1669 | ANNOTATED, CDS, c | NM_001035507 | AGBL5 | Salzman2013 | 60509 | circRNA | Detected | Detected | 8.839950833 | 7.490680482 |
| hsa_gci163320 | 4.585640316 | 2.197123199 | up | 4.261780304 | 2.064657105 | AGACCCACTTTG | hsa_circ_0053192 | chr2 | 27446784 | 27466654 | + | 5951 | ANNOTATED, CDS, c | NM_004341 | CAD | Salzman2013 | 790 | circRNA | Detected | Not Detected | 4.261780304 | 2.064657105 |
| hsa_gci163322 | 3.161382414 | 1.66055556 | up | 7.146806184 | 5.486250624 | GAGGTTCCATAC | hsa_circ_0053212 | chr2 | 27461909 | 27466654 | + | 1884 | ANNOTATED, CDS, c | NM_004341 | CAD | Salzman2013 | 790 | circRNA | Detected | Detected | 7.146806184 | 5.486250624 |
| hsa_gci163325 | 2.764404679 | 1.466968826 | up | 4.282662671 | 2.815693845 | GGTCGGAGGATG | hsa_circ_0053224 | chr2 | 27532359 | 27535639 | - | 765 | ANNOTATED, CDS, c | NM_002437 | MPV17 | Salzman2013 | 4358 | circRNA | Detected | Not Detected | 4.282662671 | 2.815693845 |
| hsa_gci163328 | 2.515256747 | 1.330705672 | up | 8.245814925 | 6.915109253 | TTATCGTGGAAC | hsa_circ_0053238 | chr2 | 27587218 | 27587765 | - | 429 | ANNOTATED, CDS, c | NM_001034116 | EIF2B4 | Salzman2013 | 8890 | circRNA | Detected | Detected | 8.245814925 | 6.915109253 |
| hsa_gci163329 | 2.553876937 | 1.352689008 | up | 8.347717527 | 6.995028519 | AGCTTCAAGACT | hsa_circ_0053239 | chr2 | 27587218 | 27590068 | - | 735 | ANNOTATED, CDS, c | NM_001034116 | EIF2B4 | Salzman2013 | 8890 | circRNA | Detected | Detected | 8.347717527 | 6.995028519 |
| hsa_gci163357 | 2.391446697 | 1.257883635 | up | 8.583764509 | 7.325880874 | AAGTGTAACGTT | hsa_circ_0053365 | chr2 | 29355017 | 29366811 | + | 612 | ANNOTATED, CDS, c | NM_024692 | CLIP4 | Salzman2013 | 79745 | circRNA | Detected | Detected | 8.583764509 | 7.325880874 |
| hsa_gci163360 | 3.037993818 | 1.603118934 | up | 7.174503346 | 5.571384412 | ACCGGCTAGAGT | hsa_circ_0053374 | chr2 | 30457270 | 30482899 | + | 2704 | ANNOTATED, CDS, c | NM_030915 | LBH | Salzman2013 | 81606 | circRNA | Detected | Detected | 7.174503346 | 5.571384412 |
| hsa_gci163368 | -2.764240729 | -1.466883261 | down | 2.222568128 | 3.689451389 | CTCGGGACGTGG | hsa_circ_0053401 | chr2 | 32248971 | 32264844 | - | 800 | ANNOTATED, CDS, c | NM_032574 | DPY30 | Salzman2013 | 84661 | circRNA | Not Detected | Detected | 2.222568128 | 3.689451389 |
| hsa_gci163415 | 2.304314514 | 1.204337643 | up | 11.54252854 | 10.3381909 | GTTCATTTTGTG | hsa_circ_0053513 | chr2 | 32620578 | 32620661 | + | 83 | ANNOTATED, CDS, c | NM_016252 | BIRC6 | Salzman2013 | 57448 | circRNA | Detected | Detected | 11.54252854 | 10.3381909 |
| hsa_gci163416 | 2.653384592 | 1.4078338 | up | 9.374254925 | 7.966421124 | TTCATTTTGTGT | hsa_circ_0053514 | chr2 | 32620578 | 32693851 | + | 5176 | ANNOTATED, CDS, c | NM_016252 | BIRC6 | Salzman2013 | 57448 | circRNA | Detected | Detected | 9.374254925 | 7.966421124 |
| hsa_gci163515 | 2.12588307 | 1.088062247 | up | 6.880114961 | 5.792052714 | ACGTCCCTTCTC | hsa_circ_0053836 | chr2 | 32740056 | 32744013 | + | 1055 | ANNOTATED, CDS, c | NM_016252 | BIRC6 | Salzman2013 | 57448 | circRNA | Detected | Detected | 6.880114961 | 5.792052714 |
| hsa_gci163516 | 2.013959477 | 1.010034655 | up | 6.973250447 | 5.963215791 | AACGTCCCTTCT | hsa_circ_0053839 | chr2 | 32740056 | 32800433 | + | 2787 | ANNOTATED, CDS, c | NM_016252 | BIRC6 | Salzman2013 | 57448 | circRNA | Detected | Detected | 6.973250447 | 5.963215791 |
| hsa_gci163535 | 2.265693508 | 1.179952713 | up | 9.301956129 | 8.122003415 | AGAAGAAACCCA | hsa_circ_0053940 | chr2 | 33585663 | 33590570 | + | 711 | ANNOTATED, CDS, c | NM_206943 | LTBP1 | Salzman2013 | 4052 | circRNA | Detected | Detected | 9.301956129 | 8.122003415 |
| hsa_gci163574 | 2.707046472 | 1.436719654 | up | 7.055681033 | 5.618961379 | TGCTTAGACCTT | hsa_circ_0054126 | chr2 | 37516498 | 37518142 | - | 290 | ANNOTATED, CDS, c | NM_005813 | PRKD3 | Salzman2013 | 23683 | circRNA | Detected | Detected | 7.055681033 | 5.618961379 |
| hsa_gci163575 | 2.272753256 | 1.184441065 | up | 4.090423834 | 2.905982769 | TCTTAAAACAGT | hsa_circ_0054127 | chr2 | 37516498 | 37520414 | - | 429 | ANNOTATED, CDS, c | NM_005813 | PRKD3 | Salzman2013 | 23683 | circRNA | Detected | Not Detected | 4.090423834 | 2.905982769 |
| hsa_gci163583 | 2.065281484 | 1.046338425 | up | 7.435243418 | 6.388904993 | CCAGCACTTTAT | hsa_circ_0054147 | chr2 | 38970740 | 38973889 | - | 1679 | ANNOTATED, CDS, c | NM_001031684 | SRSF7 | Salzman2013 | 6432 | circRNA | Detected | Detected | 7.435243418 | 6.388904993 |
| hsa_gci163595 | 2.219322931 | 1.150119607 | up | 7.418282336 | 6.268162728 | CCCTTCCCTCGG | hsa_circ_0054220 | chr2 | 39743554 | 39745851 | + | 256 | ANNOTATED, INTERN | NR_037875 | LOC728730 | Salzman2013 | 728730 | circRNA | Detected | Detected | 7.418282336 | 6.268162728 |
| hsa_gci163626 | 2.397109406 | 1.261295756 | up | 7.492295667 | 6.230999911 | ATAGACGAAAGA | hsa_circ_0054322 | chr2 | 43805651 | 43818093 | - | 645 | ANNOTATED, CDS, c | NM_001083953 | THADA | Salzman2013 | 63892 | circRNA | Detected | Detected | 7.492295667 | 6.230999911 |
| hsa_gci163634 | -4.180636898 | -2.063722746 | down | 1.181824744 | 3.24554749 | ACTTAGATTCGC | hsa_circ_0054365 | chr2 | 44170825 | 44172556 | - | 294 | ANNOTATED, CDS, c | NM_133259 | LRPPRC | Salzman2013 | 10128 | circRNA | Not Detected | Detected | 1.181824744 | 3.24554749 |
| hsa_gci163662 | 3.006484106 | 1.588077332 | up | 4.823763621 | 3.235686289 | AGGAAACTAAAA | hsa_circ_0054476 | chr2 | 48023032 | 48023202 | + | 170 | ANNOTATED, CDS, c | NM_000179 | MSH6 | Salzman2013 | 2956 | circRNA | Detected | Not Detected | 4.823763621 | 3.235686289 |
| hsa_gci163768 | -2.32541557 | -1.21748856 | down | 2.168107694 | 3.385596254 | ACCATCTAGGGT | hsa_circ_0054926 | chr2 | 63609040 | 63714628 | - | 1464 | ANNOTATED, CDS, c | NM_015910 | WDPCP | Salzman2013 | 51057 | circRNA | Not Detected | Detected | 2.168107694 | 3.385596254 |
| hsa_gci163789 | 2.981584089 | 1.576079026 | up | 4.102157146 | 2.52607812 | TATATGGCCACC | hsa_circ_0054999 | chr2 | 66670033 | 66739426 | + | 405 | ANNOTATED, CDS, c | NM_002398 | MEIS1 | Salzman2013 | 4211 | circRNA | Detected | Not Detected | 4.102157146 | 2.52607812 |
| hsa_gci163796 | 3.257505251 | 1.703767504 | up | 7.536855421 | 5.833087917 | ACCGACGACGAA | hsa_circ_0055025 | chr2 | 68740679 | 68794519 | + | 844 | ANNOTATED, CDS, c | NM_173545 | APLF | Salzman2013 | 200558 | circRNA | Detected | Detected | 7.536855421 | 5.833087917 |
| hsa_gci163832 | 2.029266391 | 1.020958267 | up | 6.799789873 | 5.778831606 | TCTCTCGCCGCC | hsa_circ_0055215 | chr2 | 73455133 | 73455702 | - | 569 | ANNOTATED, CDS, c | NM_032319 | PRADC1 | Salzman2013 | 84279 | circRNA | Detected | Detected | 6.799789873 | 5.778831606 |
| hsa_gci163838 | -3.275563657 | -1.711743186 | down | 1.519573634 | 3.23131682 | TAAGGTTTTTCC | hsa_circ_0055251 | chr2 | 74056531 | 74087278 | + | 1336 | ANNOTATED, CDS, c | NM_006463 | STAMBP | Salzman2013 | 10617 | circRNA | Not Detected | Detected | 1.519573634 | 3.23131682 |
| hsa_gci163863 | 2.322449764 | 1.215647391 | up | 4.094909459 | 2.879262069 | ATGTTCAGAACA | hsa_circ_0055418 | chr2 | 85768755 | 85769095 | + | 257 | ANNOTATED, CDS, c | NM_005911 | MAT2A | Salzman2013 | 4144 | circRNA | Detected | Not Detected | 4.094909459 | 2.879262069 |
| hsa_gci163878 | 3.193770544 | 1.675260666 | up | 5.951269663 | 4.276008997 | CTAGTTTGTCCG | hsa_circ_0055500 | chr2 | 86355063 | 86362110 | + | 682 | ANNOTATED, CDS, c | NM_017952 | PTCD3 | Salzman2013 | 55037 | circRNA | Detected | Detected | 5.951269663 | 4.276008997 |
| hsa_gci163895 | 2.165729219 | 1.114852874 | up | 7.016401818 | 5.901548944 | GGGTCCTTGTCC | hsa_circ_0055625 | chr2 | 96808907 | 96810112 | - | 1089 | ANNOTATED, CDS, c | NM_004418 | DUSP2 | Salzman2013 | 1844 | circRNA | Detected | Detected | 7.016401818 | 5.901548944 |
| hsa_gci163900 | 3.598010033 | 1.847199209 | up | 7.092072484 | 5.244873275 | CCGTGTCCATCG | hsa_circ_0055703 | chr2 | 97541618 | 97544230 | - | 658 | ANNOTATED, CDS, c | NM_001122646 | FAM178B | Salzman2013 | 51252 | circRNA | Detected | Detected | 7.092072484 | 5.244873275 |
| hsa_gci163912 | 2.534301174 | 1.341587983 | up | 6.873169992 | 5.531582009 | ATCGTCGTTTTG | hsa_circ_0055754 | chr2 | 98262520 | 98264657 | + | 505 | ANNOTATED, CDS, c | NM_001862 | COX5B | Salzman2013 | 1329 | circRNA | Detected | Detected | 6.873169992 | 5.531582009 |
| hsa_gci163913 | 2.154978011 | 1.107673148 | up | 6.461492129 | 5.353818981 | TCATGTAACATA | hsa_circ_0055756 | chr2 | 98263806 | 98264657 | + | 299 | ANNOTATED, CDS, c | NM_001862 | COX5B | Salzman2013 | 1329 | circRNA | Detected | Detected | 6.461492129 | 5.353818981 |
| hsa_gci163914 | 2.23245188 | 1.158629079 | up | 6.164581197 | 5.005952118 | CCATAACAGGAG | hsa_circ_0055757 | chr2 | 98264458 | 98264657 | + | 199 | ANNOTATED, CDS, c | NM_001862 | COX5B | Salzman2013 | 1329 | circRNA | Detected | Detected | 6.164581197 | 5.005952118 |
| hsa_gci163929 | 6.193922143 | 2.630853249 | up | 4.941523433 | 2.310670184 | GGCGTCAACGTT | hsa_circ_0055876 | chr2 | 101618690 | 101622885 | + | 785 | ANNOTATED, CDS, c | NM_001099693 | RPL31 | Salzman2013 | 6160 | circRNA | Detected | Not Detected | 4.941523433 | 2.310670184 |
| hsa_gci163985 | 2.018478718 | 1.013268376 | up | 9.921177064 | 8.907908688 | ACTACTTGGTTT | hsa_circ_0056079 | chr2 | 112582707 | 112622528 | - | 2056 | ANNOTATED, CDS, c | NM_022662 | ANAPC1 | Salzman2013 | 64682 | circRNA | Detected | Detected | 9.921177064 | 8.907908688 |
| hsa_gci164013 | 2.559182677 | 1.355683132 | up | 5.623079306 | 4.267396174 | TTAGGTAATTAA | hsa_circ_0056254 | chr2 | 120689999 | 120725548 | + | 1624 | ANNOTATED, CDS, c | NM_002830 | PTPN4 | Salzman2013 | 5775 | circRNA | Detected | Detected | 5.623079306 | 4.267396174 |
| hsa_gci164020 | 3.032295672 | 1.600410434 | up | 6.744935501 | 5.144525067 | GATAAGTCGTTT | hsa_circ_0056282 | chr2 | 120885263 | 120925083 | + | 667 | ANNOTATED, CDS, c | NM_020909 | EPB41L5 | Salzman2013 | 57669 | circRNA | Detected | Detected | 6.744935501 | 5.144525067 |
| hsa_gci164021 | 2.543955039 | 1.347073173 | up | 6.765602484 | 5.418529311 | TAATAAGAGTAA | hsa_circ_0056283 | chr2 | 120918456 | 120925083 | + | 211 | ANNOTATED, CDS, c | NM_020909 | EPB41L5 | Salzman2013 | 57669 | circRNA | Detected | Detected | 6.765602484 | 5.418529311 |
| hsa_gci164045 | -3.639529449 | -1.863751938 | down | 1.318580177 | 3.182332115 | GTAGGTGGAAGT | hsa_circ_0056432 | chr2 | 128922303 | 128953249 | + | 7776 | ANNOTATED, ncRNA, | NR_027671 | UGGT1 | Salzman2013 | 56886 | circRNA | Not Detected | Detected | 1.318580177 | 3.182332115 |
| hsa_gci164055 | 2.262728797 | 1.178063679 | up | 7.017900817 | 5.839837138 | TGTCCTAAGTAC | hsa_circ_0056481 | chr2 | 131810463 | 131840230 | - | 611 | ANNOTATED, CDS, c | NM_001009993 | FAM168B | Salzman2013 | 130074 | circRNA | Detected | Detected | 7.017900817 | 5.839837138 |
| hsa_gci164070 | 2.881024199 | 1.526581778 | up | 4.645245284 | 3.118663506 | GCCCAAGTCATG | hsa_circ_0056529 | chr2 | 135107375 | 135212192 | + | 6888 | ANNOTATED, CDS, c | NM_002410 | MGAT5 | Salzman2013 | 4249 | circRNA | Detected | Not Detected | 4.645245284 | 3.118663506 |
| hsa_gci164084 | 14.69581617 | 3.87733358 | up | 5.124536564 | 1.247202984 | AAGTGTGTGACG | hsa_circ_0056565 | chr2 | 136374237 | 136393568 | + | 594 | ANNOTATED, CDS, c | NM_015361 | R3HDM1 | Salzman2013 | 23518 | circRNA | Detected | Not Detected | 5.124536564 | 1.247202984 |
| hsa_gci164095 | 2.250797881 | 1.17043651 | up | 4.455952225 | 3.285515715 | ACACATCTGACA | hsa_circ_0056591 | chr2 | 136505836 | 136537955 | + | 1306 | ANNOTATED, CDS, c | NM_014607 | UBXN4 | Salzman2013 | 23190 | circRNA | Detected | Detected | 4.455952225 | 3.285515715 |

| | | | | | | | | | | | | | | | | | | | | | | | | |
|---|---|---|---|---|---|---|---|---|---|---|---|---|---|---|---|---|---|---|---|---|---|---|---|---|
| hsa_gci164100 | 3.216682517 | 1.685573551 | up | 6.442534074 | 4.756960523 | AGGTTAGACCGA | hsa_circ_0056664 | chr2 | 144966169 | 144993304 | - | 347 | ANNOTATED, CDS, c | NM_001164629 | GTDC1 | Salzman2013 | 79712 | circRNA | Detected | Detected | 6.442534074 | 4.756960523 |
| hsa_gci164109 | 2.207002039 | 1.142087962 | up | 4.441090734 | 3.299002771 | GGTTTCCCAGGG | hsa_circ_0056697 | chr2 | 150426146 | 150436162 | - | 1078 | ANNOTATED, CDS, c | NM_015702 | MMADHC | Salzman2013 | 27249 | circRNA | Detected | Detected | 4.441090734 | 3.299002771 |
| hsa_gci164119 | 2.338619964 | 1.225657436 | up | 8.816900303 | 7.591242867 | TATTCATCTCAG | hsa_circ_0056754 | chr2 | 153591507 | 153591640 | + | 133 | ANNOTATED, CDS, c | NM_152522 | ARL6IP6 | Salzman2013 | 151188 | circRNA | Detected | Detected | 8.816900303 | 7.591242867 |
| hsa_gci164123 | 4.367982753 | 2.12696716 | up | 4.492044086 | 2.365076926 | GCCTGCCGGCGG | hsa_circ_0056785 | chr2 | 159522924 | 159537940 | + | 1753 | ANNOTATED, CDS, c | NM_003628 | PKP4 | Salzman2013 | 8502 | circRNA | Detected | Not Detected | 4.492044086 | 2.365076926 |
| hsa_gci164131 | 2.507125868 | 1.326034427 | up | 4.547768698 | 3.22173427 | CCTGTTCGCGTG | hsa_circ_0056818 | chr2 | 160053118 | 160089170 | + | 4259 | ANNOTATED, CDS, c | NM_001145909 | TANC1 | Salzman2013 | 85461 | circRNA | Detected | Detected | 4.547768698 | 3.22173427 |
| hsa_gci164145 | 2.381765123 | 1.252031149 | up | 5.705246637 | 4.453215488 | GATGTTAACAGA | hsa_circ_0056923 | chr2 | 167151108 | 167159812 | - | 277 | ANNOTATED, CDS, c | NM_002977 | SCN9A | Salzman2013 | 6335 | circRNA | Detected | Detected | 5.705246637 | 4.453215488 |
| hsa_gci164151 | 2.555928967 | 1.353847742 | up | 8.855291655 | 7.501443913 | CGTTACGGAGCA | hsa_circ_0056942 | chr2 | 169547543 | 169551561 | + | 144 | ANNOTATED, CDS, c | NM_203463 | CERS6 | Salzman2013 | 253782 | circRNA | Detected | Detected | 8.855291655 | 7.501443913 |
| hsa_gci164172 | 2.217311691 | 1.148811586 | up | 6.29185918 | 5.143047594 | TTATAGAAAGTA | hsa_circ_0057023 | chr2 | 171862656 | 171867964 | - | 570 | ANNOTATED, CDS, c | NM_012290 | TLK1 | Salzman2013 | 9874 | circRNA | Detected | Detected | 6.29185918 | 5.143047594 |
| hsa_gci164217 | 3.50756166 | 1.810468465 | up | 4.703053795 | 2.892585329 | ACAGTTAGTTTA | hsa_circ_0057168 | chr2 | 178097119 | 178098999 | - | 549 | ANNOTATED, CDS, c | NM_006164 | NFE2L2 | Salzman2013 | 4780 | circRNA | Detected | Not Detected | 4.703053795 | 2.892585329 |
| hsa_gci164222 | 2.797915035 | 1.484352153 | up | 7.356054702 | 5.871702549 | ATGTATCATAAG | hsa_circ_0057185 | chr2 | 178372697 | 178408564 | + | 6073 | ANNOTATED, CDS, c | NM_003659 | AGPS | Salzman2013 | 8540 | circRNA | Detected | Detected | 7.356054702 | 5.871702549 |
| hsa_gci164239 | 3.01757532 | 1.593389782 | up | 5.921531374 | 4.328141593 | GTGTTAGTAGAC | hsa_circ_0057287 | chr2 | 183853757 | 183866771 | - | 435 | ANNOTATED, CDS, c | NM_205842 | NCKAP1 | Salzman2013 | 10787 | circRNA | Detected | Detected | 5.921531374 | 4.328141593 |
| hsa_gci164242 | 2.636347894 | 1.398540762 | up | 6.080652575 | 4.682111814 | GGTTCCGCACCC | hsa_circ_0057291 | chr2 | 183866680 | 183866771 | - | 91 | ANNOTATED, CDS, c | NM_205842 | NCKAP1 | Salzman2013 | 10787 | circRNA | Detected | Detected | 6.080652575 | 4.682111814 |
| hsa_gci164245 | 2.575221704 | 1.364696641 | up | 8.150258778 | 6.785562136 | TCGTAGTAACCA | hsa_circ_0057297 | chr2 | 183993014 | 183995845 | + | 871 | ALT_DONOR, CDS, c | NM_138285 | NUP35 | Salzman2013 | 129401 | circRNA | Detected | Detected | 8.150258778 | 6.785562136 |
| hsa_gci164269 | 6.063871454 | 2.600239171 | up | 5.513693465 | 2.913454294 | TTCTTGTTTTTG | hsa_circ_0057509 | chr2 | 191523784 | 191557492 | + | 4015 | ALT_ACCEPTOR, CDS | NM_005966 | NAB1 | Salzman2013 | 4664 | circRNA | Detected | Not Detected | 5.513693465 | 2.913454294 |
| hsa_gci164282 | 2.19156391 | 1.131960751 | up | 7.261713232 | 6.12975248 | TTCGTCCGATTA | hsa_circ_0057556 | chr2 | 196571339 | 196571509 | + | 170 | ANNOTATED, CDS, c | NM_001127257 | SLC39A10 | Salzman2013 | 57181 | circRNA | Detected | Detected | 7.261713232 | 6.12975248 |
| hsa_gci164300 | 2.161963746 | 1.11234233 | up | 6.106366299 | 4.994023969 | CGCCACGCGCAG | hsa_circ_0057667 | chr2 | 198351307 | 198364998 | - | 2301 | ANNOTATED, CDS, c | NM_199440 | HSPD1 | Salzman2013 | 3329 | circRNA | Detected | Detected | 6.106366299 | 4.994023969 |
| hsa_gci164330 | 2.290533213 | 1.195683482 | up | 6.619656962 | 5.423973481 | GGTTACACTCTG | hsa_circ_0057802 | chr2 | 203332241 | 203332412 | + | 171 | ANNOTATED, CDS, c | NM_001204 | BMPR2 | Salzman2013 | 659 | circRNA | Detected | Detected | 6.619656962 | 5.423973481 |
| hsa_gci164371 | 3.687161117 | 1.882510459 | up | 5.517944238 | 3.635433779 | TCGACCCCAGTA | hsa_circ_0058010 | chr2 | 210962809 | 211019335 | - | 1579 | ANNOTATED, CDS, c | NM_152519 | KANSL1L | Salzman2013 | 151050 | circRNA | Detected | Detected | 5.517944238 | 3.635433779 |
| hsa_gci164414 | 2.409464956 | 1.268712818 | up | 8.407177977 | 7.138465158 | TTCGGAGGTACA | hsa_circ_0058197 | chr2 | 217536827 | 217542954 | - | 4898 | ANNOTATED, CDS, c | NM_000599 | IGFBP5 | Salzman2013 | 3488 | circRNA | Detected | Detected | 8.407177977 | 7.138465158 |
| hsa_gci164417 | 2.171663409 | 1.118800514 | up | 7.254624734 | 6.13582422 | GTAACCACACTG | hsa_circ_0058218 | chr2 | 219114086 | 219114644 | + | 202 | ANNOTATED, CDS, c | NM_152862 | ARPC2 | Salzman2013 | 10109 | circRNA | Detected | Detected | 7.254624734 | 6.13582422 |
| hsa_gci164423 | 3.454224808 | 1.78836198 | up | 5.044793603 | 3.256431624 | TTTATAAATGTT | hsa_circ_0058275 | chr2 | 219447693 | 219449444 | + | 226 | ANNOTATED, CDS, c | NM_005444 | CNOT9 | Salzman2013 | 9125 | circRNA | Detected | Not Detected | 5.044793603 | 3.256431624 |
| hsa_gci164426 | 4.359020493 | 2.124003986 | up | 8.682937119 | 6.558933133 | TTCTAGAAGACC | hsa_circ_0058281 | chr2 | 219457026 | 219457430 | + | 191 | ANNOTATED, CDS, c | NM_005444 | CNOT9 | Salzman2013 | 9125 | circRNA | Detected | Detected | 8.682937119 | 6.558933133 |
| hsa_gci164449 | 2.465561813 | 1.301916423 | up | 5.512294082 | 4.21037766 | CAGTCAAAGTCC | hsa_circ_0058493 | chr2 | 227729319 | 227732034 | + | 656 | ANNOTATED, CDS, c | NM_001167608 | RHBDD1 | Salzman2013 | 84236 | circRNA | Detected | Detected | 5.512294082 | 4.21037766 |
| hsa_gci164455 | 2.903783261 | 1.537933774 | up | 4.082571115 | 2.54463734 | GTACCTCTATCT | hsa_circ_0058517 | chr2 | 228356262 | 228399741 | + | 1110 | ANNOTATED, CDS, c | NM_001135187 | AGFG1 | Salzman2013 | 3267 | circRNA | Detected | Not Detected | 4.082571115 | 2.54463734 |
| hsa_gci164457 | 2.454509176 | 1.29543456 | up | 4.442477824 | 3.147043264 | CTCTATCTAAAG | hsa_circ_0058519 | chr2 | 228356262 | 228416833 | + | 1442 | ANNOTATED, CDS, c | NM_001135187 | AGFG1 | Salzman2013 | 3267 | circRNA | Detected | Not Detected | 4.442477824 | 3.147043264 |
| hsa_gci164487 | 2.440080947 | 1.286929008 | up | 6.651280898 | 5.36435189 | GTGCCCCAGCAA | hsa_circ_0058639 | chr2 | 232010953 | 232011070 | + | 117 | ANNOTATED, CDS, c | NM_002807 | PSMD1 | Salzman2013 | 5707 | circRNA | Detected | Detected | 6.651280898 | 5.36435189 |
| hsa_gci164489 | 2.813579479 | 1.492406717 | up | 5.653459472 | 4.161052754 | CAAGTGCCCCAG | hsa_circ_0058641 | chr2 | 232010953 | 232026223 | + | 390 | ANNOTATED, CDS, c | NM_002807 | PSMD1 | Salzman2013 | 5707 | circRNA | Detected | Detected | 5.653459472 | 4.161052754 |
| hsa_gci164495 | 2.441314775 | 1.287658323 | up | 8.739901105 | 7.452242782 | TCTACCGAAAAG | hsa_circ_0058679 | chr2 | 232573234 | 232578250 | + | 1207 | ANNOTATED, CDS, c | NM_001099285 | PTMA | Salzman2013 | 5757 | circRNA | Detected | Detected | 8.739901105 | 7.452242782 |
| hsa_gci164510 | 2.462685864 | 1.300232612 | up | 5.090642458 | 3.790409846 | GAAAAGTGTGCC | hsa_circ_0058773 | chr2 | 234359567 | 234360706 | + | 226 | ANNOTATED, CDS, c | NM_152879 | DGKD | Salzman2013 | 8527 | circRNA | Detected | Detected | 5.090642458 | 3.790409846 |
| hsa_gci164519 | 2.23484701 | 1.160176072 | up | 6.573914108 | 5.413738035 | CACCCTCGGGTC | hsa_circ_0058856 | chr2 | 238939196 | 238940895 | + | 91 | ANNOTATED, INTERN | NR_037904 | UBE2F-SCLY | Salzman2013 | 100533179 | circRNA | Detected | Detected | 6.573914108 | 5.413738035 |
| hsa_gci164532 | 2.313893407 | 1.210322406 | up | 8.714720776 | 7.50439837 | TACTGGAGCCCC | hsa_circ_0058938 | chr2 | 241404889 | 241407495 | + | 2197 | ANNOTATED, CDS, c | NM_002081 | GPC1 | Salzman2013 | 2817 | circRNA | Detected | Detected | 8.714720776 | 7.50439837 |
| hsa_gci164534 | 2.313295825 | 1.20994977 | up | 4.399889567 | 3.189939797 | GTGCGTCGGAGT | hsa_circ_0058963 | chr2 | 241666242 | 241666357 | - | 115 | ANNOTATED, CDS, c | NM_001244008 | KIF1A | Salzman2013 | 547 | circRNA | Detected | Detected | 4.399889567 | 3.189939797 |
| hsa_gci164567 | 2.200699003 | 1.137961836 | up | 8.440059647 | 7.30209781 | GTCGGTCTCCCC | hsa_circ_0059145 | chr20 | 368654 | 378203 | + | 1946 | ANNOTATED, CDS, c | NM_021158 | TRIB3 | Salzman2013 | 57761 | circRNA | Detected | Detected | 8.440059647 | 7.30209781 |
| hsa_gci164573 | 3.533393686 | 1.821054502 | up | 8.127389533 | 6.306335031 | GGGGAGGATGGT | hsa_circ_0059160 | chr20 | 463337 | 524482 | - | 2836 | ANNOTATED, CDS, c | NM_177559 | CSNK2A1 | Salzman2013 | 1457 | circRNA | Detected | Detected | 8.127389533 | 6.306335031 |
| hsa_gci164576 | 2.501573454 | 1.322835815 | up | 7.224060377 | 5.901224562 | TCGACCCCCATT | hsa_circ_0059168 | chr20 | 472897 | 489304 | - | 730 | ANNOTATED, CDS, c | NM_177559 | CSNK2A1 | Salzman2013 | 1457 | circRNA | Detected | Detected | 7.224060377 | 5.901224562 |
| hsa_gci164577 | 2.218103671 | 1.149326796 | up | 7.487627323 | 6.338300527 | CGACCCCCATTC | hsa_circ_0059169 | chr20 | 476362 | 489304 | - | 619 | ANNOTATED, CDS, c | NM_177559 | CSNK2A1 | Salzman2013 | 1457 | circRNA | Detected | Detected | 7.487627323 | 6.338300527 |
| hsa_gci164582 | 2.286819361 | 1.19334241 | up | 8.176212863 | 6.982870453 | GAACCCCTTCCA | hsa_circ_0059179 | chr20 | 1349620 | 1373525 | - | 1422 | ANNOTATED, CDS, c | NM_000801 | FKBP1A | Salzman2013 | 2280 | circRNA | Detected | Detected | 8.176212863 | 6.982870453 |
| hsa_gci164585 | 2.709136374 | 1.437833018 | up | 7.319026119 | 5.881193101 | GGAATTAATATT | hsa_circ_0059187 | chr20 | 2473344 | 2474690 | - | 453 | ANNOTATED, CDS, c | NM_024325 | ZNF343 | Salzman2013 | 79175 | circRNA | Detected | Detected | 7.319026119 | 5.881193101 |
| hsa_gci164608 | 2.51362917 | 1.329771828 | up | 7.939558722 | 6.609786894 | CCCTCCGGCCAA | hsa_circ_0059274 | chr20 | 3259557 | 3270863 | - | 412 | ANNOTATED, CDS, c | NM_001009984 | C20orf194 | Salzman2013 | 25943 | circRNA | Detected | Detected | 7.939558722 | 6.609786894 |
| hsa_gci164618 | 2.174538734 | 1.120709407 | up | 8.470135352 | 7.349425945 | TGAGAGGAGCCG | hsa_circ_0059300 | chr20 | 3527930 | 3619582 | + | 3313 | ANNOTATED, CDS, c | NM_139321 | ATRN | Salzman2013 | 8455 | circRNA | Detected | Detected | 8.470135352 | 7.349425945 |
| hsa_gci164624 | 2.230255985 | 1.15720931 | up | 8.987330721 | 7.830121411 | CGACTGACGCGG | hsa_circ_0059343 | chr20 | 3925823 | 3996216 | - | 437 | ANNOTATED, CDS, c | NM_001134338 | RNF24 | Salzman2013 | 11237 | circRNA | Detected | Detected | 8.987330721 | 7.830121411 |
| hsa_gci164629 | 2.150002372 | 1.104338252 | up | 4.080917021 | 2.976578769 | TCGAAAATTCAT | hsa_circ_0059354 | chr20 | 4760668 | 4766974 | - | 4418 | ANNOTATED, CDS, c | NM_170774 | RASSF2 | Salzman2013 | 9770 | circRNA | Detected | Not Detected | 4.080917021 | 2.976578769 |
| hsa_gci164644 | 2.90490249 | 1.538489737 | up | 9.388395462 | 7.849905725 | CGACTCCGAGGG | hsa_circ_0059413 | chr20 | 6055491 | 6104191 | - | 5151 | ANNOTATED, CDS, c | NM_017671 | FERMT1 | Salzman2013 | 55612 | circRNA | Detected | Detected | 9.388395462 | 7.849905725 |
| hsa_gci164655 | 2.079078224 | 1.055944039 | up | 5.193088441 | 4.137144402 | AATTTCGCTTTG | hsa_circ_0059486 | chr20 | 16385457 | 16387101 | - | 172 | ANNOTATED, CDS, c | NM_024704 | KIF16B | Salzman2013 | 55614 | circRNA | Detected | Detected | 5.193088441 | 4.137144402 |
| hsa_gci164657 | 2.142257612 | 1.099131978 | up | 4.666112081 | 3.566980102 | GACTTCGCTCCC | hsa_circ_0059497 | chr20 | 17922243 | 17937681 | - | 1921 | ANNOTATED, CDS, c | NM_152227 | SNX5 | Salzman2013 | 27131 | circRNA | Detected | Detected | 4.666112081 | 3.566980102 |
| hsa_gci164661 | 2.277433125 | 1.187408691 | up | 6.381214824 | 5.193806133 | ATCCTCGTCCAC | hsa_circ_0059507 | chr20 | 17930775 | 17934761 | - | 524 | ANNOTATED, CDS, c | NM_152227 | SNX5 | Salzman2013 | 27131 | circRNA | Detected | Detected | 6.381214824 | 5.193806133 |
| hsa_gci164663 | 3.096196583 | 1.630497074 | up | 4.707714412 | 3.077217338 | ACTTCGCTCCCT | hsa_circ_0059512 | chr20 | 17933230 | 17937681 | - | 462 | ANNOTATED, CDS, c | NM_152227 | SNX5 | Salzman2013 | 27131 | circRNA | Detected | Not Detected | 4.707714412 | 3.077217338 |
| hsa_gci164673 | 3.141381387 | 1.651399107 | up | 6.199825337 | 4.548426229 | GTCCCGCTCTTC | hsa_circ_0059559 | chr20 | 19998438 | 20014273 | + | 1173 | ANNOTATED, CDS, c | NM_181527 | NAA20 | Salzman2013 | 51126 | circRNA | Detected | Detected | 6.199825337 | 4.548426229 |
| hsa_gci164674 | 2.185310112 | 1.127838024 | up | 7.573015412 | 6.445177388 | GAGACCGGTCAC | hsa_circ_0059561 | chr20 | 20006320 | 20014273 | + | 901 | ANNOTATED, CDS, c | NM_181527 | NAA20 | Salzman2013 | 51126 | circRNA | Detected | Detected | 7.573015412 | 6.445177388 |
| hsa_gci164675 | 2.166010352 | 1.115040138 | up | 7.916307915 | 6.801267777 | TCGCCCCACCTG | hsa_circ_0059567 | chr20 | 20512330 | 20527476 | - | 426 | ANNOTATED, CDS, c | NM_020343 | RALGAPA2 | Salzman2013 | 57186 | circRNA | Detected | Detected | 7.916307915 | 6.801267777 |
| hsa_gci164677 | 2.455041311 | 1.295747301 | up | 6.036232365 | 4.740485064 | ACCGACCATCCT | hsa_circ_0059571 | chr20 | 20553518 | 20620544 | - | 2351 | ANNOTATED, CDS, c | NM_020343 | RALGAPA2 | Salzman2013 | 57186 | circRNA | Detected | Detected | 6.036232365 | 4.740485064 |

| | | | | | | | | | | | | | | | | | | | | | | |
|---|---|---|---|---|---|---|---|---|---|---|---|---|---|---|---|---|---|---|---|---|---|---|
| hsa_gci164687 | 2.109793674 | 1.077101918 | up | 9.790833529 | 8.713731611 | ATAAACCTACGT | hsa_circ_0059622 | chr20 | 24943579 | 24959518 | - | 1908 | ANNOTATED, CDS, c | NM_020531 | APMAP | Salzman2013 | 57136 | circRNA | Detected | Detected | 9.790833529 | 8.713731611 |
| hsa_gci164694 | 2.131503537 | 1.091871449 | up | 5.592823567 | 4.500952117 | AAGTCGACCTAC | hsa_circ_0059647 | chr20 | 25259692 | 25278648 | + | 3023 | ANNOTATED, CDS, c | NM_002862 | PYGB | Salzman2013 | 5834 | circRNA | Detected | Detected | 5.592823567 | 4.500952117 |
| hsa_gci164704 | 4.040730169 | 2.014616015 | up | 4.702529586 | 2.687913572 | GGTCCGTGTGGT | hsa_circ_0059700 | chr20 | 30136831 | 30149539 | + | 494 | ANNOTATED, CDS, c | NM_178580 | HM13 | Salzman2013 | 81502 | circRNA | Detected | Not Detected | 4.702529586 | 2.687913572 |
| hsa_gci164716 | 2.313461143 | 1.210052868 | up | 4.80294736 | 3.592894492 | GAGAAGAGGTCG | hsa_circ_0059719 | chr20 | 30363669 | 30389603 | + | 2356 | ANNOTATED, CDS, c | NM_012112 | TPX2 | Salzman2013 | 22974 | circRNA | Detected | Detected | 4.80294736 | 3.592894492 |
| hsa_gci164717 | 2.479444422 | 1.310016887 | up | 5.385492664 | 4.075475777 | TAAAGAAGTGTC | hsa_circ_0059721 | chr20 | 30365289 | 30389603 | + | 2234 | ANNOTATED, CDS, c | NM_012112 | TPX2 | Salzman2013 | 22974 | circRNA | Detected | Detected | 5.385492664 | 4.075475777 |
| hsa_gci164719 | 2.27760222 | 1.187515805 | up | 9.15505115 | 7.967535346 | CGAATTGTTACC | hsa_circ_0059723 | chr20 | 30366615 | 30389603 | + | 2082 | ANNOTATED, CDS, c | NM_012112 | TPX2 | Salzman2013 | 22974 | circRNA | Detected | Detected | 9.15505115 | 7.967535346 |
| hsa_gci164720 | 3.049470552 | 1.608558784 | up | 4.024901721 | 2.416342937 | CTCGTTCTGGGA | hsa_circ_0059724 | chr20 | 30368741 | 30389603 | + | 3220 | ALT_ACCEPTOR, CDS | NM_012112 | TPX2 | Salzman2013 | 22974 | circRNA | Detected | Not Detected | 4.024901721 | 2.416342937 |
| hsa_gci164721 | 2.842522999 | 1.507172022 | up | 8.456291165 | 6.949119143 | ACGTTTAGAACC | hsa_circ_0059727 | chr20 | 30370051 | 30389603 | + | 1910 | ANNOTATED, CDS, c | NM_012112 | TPX2 | Salzman2013 | 22974 | circRNA | Detected | Detected | 8.456291165 | 6.949119143 |
| hsa_gci164726 | 2.276623825 | 1.186895929 | up | 7.390677491 | 6.203781562 | GTTCGACGAAGA | hsa_circ_0059735 | chr20 | 30385206 | 30389603 | + | 1131 | ANNOTATED, CDS, c | NM_012112 | TPX2 | Salzman2013 | 22974 | circRNA | Detected | Detected | 7.390677491 | 6.203781562 |
| hsa_gci164734 | 2.617455083 | 1.38816478 | up | 5.476414959 | 4.088250179 | TACAGGAAGGCC | hsa_circ_0059760 | chr20 | 30734587 | 30746317 | + | 686 | ANNOTATED, CDS, c | NM_014742 | TM9SF4 | Salzman2013 | 9777 | circRNA | Detected | Detected | 5.476414959 | 4.088250179 |
| hsa_gci164750 | 2.942318521 | 1.556953434 | up | 7.197768258 | 5.640814824 | CGGGCCGGCGCG | hsa_circ_0059888 | chr20 | 32399109 | 32442173 | + | 1647 | ANNOTATED, CDS, c | NM_176812 | CHMP4B | Salzman2013 | 128866 | circRNA | Detected | Detected | 7.197768258 | 5.640814824 |
| hsa_gci164751 | 2.447184058 | 1.291122614 | up | 7.295147953 | 6.004025339 | GCGAAGTCACGG | hsa_circ_0059889 | chr20 | 32436272 | 32442173 | + | 1292 | ANNOTATED, CDS, c | NM_176812 | CHMP4B | Salzman2013 | 128866 | circRNA | Detected | Detected | 7.295147953 | 6.004025339 |
| hsa_gci164757 | 2.937295955 | 1.554488635 | up | 5.204939519 | 3.650450884 | GTGTTCCGGTGG | hsa_circ_0059931 | chr20 | 32957199 | 33059320 | + | 1667 | ANNOTATED, CDS, c | NM_031483 | ITCH | Salzman2013 | 83737 | circRNA | Detected | Detected | 5.204939519 | 3.650450884 |
| hsa_gci164759 | 2.20817959 | 1.142857511 | up | 6.182151704 | 5.039294193 | TGAGTGTCCCCA | hsa_circ_0059935 | chr20 | 33000320 | 33001685 | + | 263 | ANNOTATED, CDS, c | NM_031483 | ITCH | Salzman2013 | 83737 | circRNA | Detected | Detected | 6.182151704 | 5.039294193 |
| hsa_gci164772 | 2.06521539 | 1.046292254 | up | 4.166954736 | 3.120662482 | ACCCCGTATGAC | hsa_circ_0059974 | chr20 | 33148345 | 33225789 | - | 1314 | ANNOTATED, CDS, c | NM_080476 | PIGU | Salzman2013 | 128869 | circRNA | Detected | Not Detected | 4.166954736 | 3.120662482 |
| hsa_gci164777 | 2.273568103 | 1.18495822 | up | 6.059993174 | 4.875034954 | CGTTCTTTTTAT | hsa_circ_0059981 | chr20 | 33176256 | 33203943 | - | 253 | ANNOTATED, CDS, c | NM_080476 | PIGU | Salzman2013 | 128869 | circRNA | Detected | Detected | 6.059993174 | 4.875034954 |
| hsa_gci164779 | 2.562514999 | 1.357560449 | up | 8.348260454 | 6.990700005 | TCAAAGGAAACT | hsa_circ_0059991 | chr20 | 33320362 | 33324562 | - | 106 | ANNOTATED, CDS, c | NM_014071 | NCOA6 | Salzman2013 | 23054 | circRNA | Detected | Detected | 8.348260454 | 6.990700005 |
| hsa_gci164803 | 2.171443055 | 1.118654119 | up | 6.382564461 | 5.263910342 | GAAGGGTCCCCG | hsa_circ_0060096 | chr20 | 34136560 | 34145405 | + | 670 | ANNOTATED, CDS, c | NM_198398 | ERGIC3 | Salzman2013 | 51614 | circRNA | Detected | Detected | 6.382564461 | 5.263910342 |
| hsa_gci164809 | 2.409982385 | 1.269022602 | up | 5.552325758 | 4.283303157 | TTAAAGGAATTG | hsa_circ_0060122 | chr20 | 34243123 | 34246904 | - | 196 | ANNOTATED, INTERN | NR_037188 | CPNE1 | Salzman2013 | 8904 | circRNA | Detected | Detected | 5.552325758 | 4.283303157 |
| hsa_gci164820 | -2.844288342 | -1.508067727 | down | 2.805445525 | 4.313513252 | AAAGTCAGTAGT | hsa_circ_0060147 | chr20 | 34389412 | 34505584 | + | 2036 | ANNOTATED, CDS, c | NM_016436 | PHF20 | Salzman2013 | 51230 | circRNA | Not Detected | Detected | 2.805445525 | 4.313513252 |
| hsa_gci164832 | 2.548825468 | 1.349832588 | up | 10.08461104 | 8.73477845 | CGGCTGAGAAGC | hsa_circ_0060190 | chr20 | 35202956 | 35240960 | + | 1090 | ANNOTATED, CDS, c | NM_001199535 | | Salzman2013 | | circRNA | Detected | Detected | 10.08461104 | 8.73477845 |
| hsa_gci164834 | -4.457097977 | -2.156104675 | down | 1.343721202 | 3.499825877 | AGACAGCCTTGG | hsa_circ_0060193 | chr20 | 35236117 | 35238133 | + | 345 | ANNOTATED, INTERN | NR_026562 | RAB5IF | Salzman2013 | 55969 | circRNA | Not Detected | Detected | 1.343721202 | 3.499825877 |
| hsa_gci164851 | 2.278741822 | 1.188237479 | up | 6.409654173 | 5.221416694 | CGGGACGGAAGG | hsa_circ_0060303 | chr20 | 36145818 | 36156083 | - | 2042 | ANNOTATED, CDS, c | NM_001167822 | BLCAP | Salzman2013 | 10904 | circRNA | Detected | Detected | 6.409654173 | 5.221416694 |
| hsa_gci164854 | 2.161742921 | 1.112194965 | up | 7.632520615 | 6.52032565 | TTATGTCAAACA | hsa_circ_0060309 | chr20 | 36405746 | 36488438 | + | 780 | ANNOTATED, CDS, c | NM_030877 | CTNNBL1 | Salzman2013 | 56259 | circRNA | Detected | Detected | 7.632520615 | 6.52032565 |
| hsa_gci164864 | 2.408431224 | 1.268093726 | up | 8.58749393 | 7.319400204 | CGCCCATCCCTA | hsa_circ_0060326 | chr20 | 36756863 | 36770601 | - | 2962 | ANNOTATED, CDS, c | NM_004613 | TGM2 | Salzman2013 | 7052 | circRNA | Detected | Detected | 8.58749393 | 7.319400204 |
| hsa_gci164885 | 2.134401648 | 1.093831686 | up | 7.973946646 | 6.880114961 | GACCTTCTCAAA | hsa_circ_0060469 | chr20 | 42343773 | 42345122 | + | 674 | ANNOTATED, CDS, c | NM_002466 | MYBL2 | Salzman2013 | 4605 | circRNA | Detected | Detected | 7.973946646 | 6.880114961 |
| hsa_gci164888 | 2.08701315 | 1.06143969 | up | 4.813147324 | 3.751707634 | GTGTCTGCGTGC | hsa_circ_0060488 | chr20 | 43135467 | 43150726 | - | 917 | ANNOTATED, CDS, c | NM_006811 | SERINC3 | Salzman2013 | 10955 | circRNA | Detected | Detected | 4.813147324 | 3.751707634 |
| hsa_gci164934 | -4.750660025 | -2.248127966 | down | 1.284506695 | 3.53263466 | ACGTGTCACGTA | hsa_circ_0060772 | chr20 | 47752369 | 47770608 | - | 404 | ANNOTATED, CDS, c | NM_017453 | STAU1 | Salzman2013 | 6780 | circRNA | Not Detected | Detected | 1.284506695 | 3.53263466 |
| hsa_gci164942 | -2.225547499 | -1.154160292 | down | 2.203247672 | 3.357407964 | GTGGGTGGGCGT | hsa_circ_0060794 | chr20 | 47862438 | 47881401 | - | 5120 | ANNOTATED, CDS, c | NM_021035 | ZNFX1 | Salzman2013 | 57169 | circRNA | Not Detected | Detected | 2.203247672 | 3.357407964 |
| hsa_gci164992 | 2.221040653 | 1.1512358 | up | 5.015633754 | 3.864397955 | AAATGGTCTAAG | hsa_circ_0060995 | chr20 | 57470666 | 57485136 | + | 834 | ANNOTATED, coding | NM_016592 | GNAS | Salzman2013 | 2778 | circRNA | Detected | Detected | 5.015633754 | 3.864397955 |
| hsa_gci164998 | 2.314800306 | 1.21088774 | up | 6.04776771 | 4.83687997 | GTCTTCTCAATA | hsa_circ_0061046 | chr20 | 60835036 | 60871269 | + | 3732 | ANNOTATED, CDS, c | NM_144498 | OSBPL2 | Salzman2013 | 9885 | circRNA | Detected | Detected | 6.04776771 | 4.83687997 |
| hsa_gci164999 | 2.116779856 | 1.081871238 | up | 5.965868245 | 4.883997007 | AGGTCAGTCCCG | hsa_circ_0061062 | chr20 | 60882426 | 60883918 | + | 784 | ANNOTATED, CDS, c | NM_007002 | ADRM1 | Salzman2013 | 11047 | circRNA | Detected | Detected | 5.965868245 | 4.883997007 |
| hsa_gci165000 | 2.073683961 | 1.052196038 | up | 6.61843614 | 5.566240103 | ACGGCTGACCGA | hsa_circ_0061064 | chr20 | 60882651 | 60883918 | + | 702 | ANNOTATED, CDS, c | NM_007002 | ADRM1 | Salzman2013 | 11047 | circRNA | Detected | Detected | 6.61843614 | 5.566240103 |
| hsa_gci165001 | 4.782385933 | 2.257730559 | up | 6.459797178 | 4.20206662 | TCGTGTGACCGG | hsa_circ_0061065 | chr20 | 60883076 | 60883918 | + | 469 | ANNOTATED, CDS, c | NM_007002 | ADRM1 | Salzman2013 | 11047 | circRNA | Detected | Detected | 6.459797178 | 4.20206662 |
| hsa_gci165003 | 2.067373274 | 1.047798897 | up | 8.14087842 | 7.093079523 | CGATCAGTGGGC | hsa_circ_0061107 | chr20 | 60962120 | 60963576 | + | 396 | ANNOTATED, CDS, c | NM_001024 | RPS21 | Salzman2013 | 6227 | circRNA | Detected | Detected | 8.14087842 | 7.093079523 |
| hsa_gci165004 | 2.637993175 | 1.399440832 | up | 8.558456129 | 7.159015297 | GCAGCAAGACGT | hsa_circ_0061110 | chr20 | 60962376 | 60963576 | + | 324 | ANNOTATED, CDS, c | NM_001024 | RPS21 | Salzman2013 | 6227 | circRNA | Detected | Detected | 8.558456129 | 7.159015297 |
| hsa_gci165008 | 2.502248124 | 1.323224855 | up | 6.378369276 | 5.055144422 | CTCCGCTGGGGG | hsa_circ_0061130 | chr20 | 61482845 | 61493115 | - | 3995 | ALT_DONOR, CDS, c | NM_006602 | TCFL5 | Salzman2013 | 10732 | circRNA | Detected | Detected | 6.378369276 | 5.055144422 |
| hsa_gci165025 | 2.067158185 | 1.047648792 | up | 6.961511323 | 5.913862531 | CGTGTAGATGTG | hsa_circ_0061229 | chr20 | 62703524 | 62703700 | + | 176 | ANNOTATED, CDS, c | NM_003195 | TCEA2 | Salzman2013 | 6919 | circRNA | Detected | Detected | 6.961511323 | 5.913862531 |
| hsa_gci165035 | 2.771566213 | 1.470701474 | up | 6.067728024 | 4.597026549 | TGGCGGGGGTGA | hsa_circ_0061267 | chr21 | 15743436 | 15755509 | - | 3986 | ANNOTATED, CDS, c | NM_006948 | HSPA13 | Salzman2013 | 6782 | circRNA | Detected | Detected | 6.067728024 | 4.597026549 |
| hsa_gci165043 | 2.995033104 | 1.582571949 | up | 6.602230866 | 5.019658917 | TCAAAGGTAATG | hsa_circ_0061284 | chr21 | 17138315 | 17198684 | + | 1343 | ANNOTATED, CDS, c | NM_013396 | USP25 | Salzman2013 | 29761 | circRNA | Detected | Detected | 6.602230866 | 5.019658917 |
| hsa_gci165065 | 2.675131864 | 1.419610007 | up | 6.436333251 | 5.016723243 | GTATCAGTTTAG | hsa_circ_0061421 | chr21 | 33057361 | 33069063 | - | 1711 | ANNOTATED, CDS, c | NM_020706 | SCAF4 | Salzman2013 | 57466 | circRNA | Detected | Detected | 6.436333251 | 5.016723243 |
| hsa_gci165077 | 2.527005877 | 1.337429019 | up | 8.389442019 | 7.052013 | AAGATGAACGTA | hsa_circ_0061505 | chr21 | 34652056 | 34669520 | + | 1505 | ANNOTATED, CDS, c | NM_000628 | IL10RB | Salzman2013 | 3588 | circRNA | Detected | Detected | 8.389442019 | 7.052013 |
| hsa_gci165182 | 3.241348072 | 1.696593953 | up | 5.679074389 | 3.982480436 | TTTTATGAAAAC | hsa_circ_0062003 | chr21 | 46591524 | 46605006 | + | 1732 | ANNOTATED, CDS, c | NM_015833 | ADARB1 | Salzman2013 | 104 | circRNA | Detected | Detected | 5.679074389 | 3.982480436 |
| hsa_gci165183 | 3.152581501 | 1.656533668 | up | 5.646947377 | 3.99041371 | TTTTATGAAAAC | hsa_circ_0062004 | chr21 | 46591524 | 46624651 | + | 1914 | ANNOTATED, CDS, c | NM_015833 | ADARB1 | Salzman2013 | 104 | circRNA | Detected | Detected | 5.646947377 | 3.99041371 |
| hsa_gci165190 | -2.574431483 | -1.364253874 | down | 2.879590783 | 4.243844657 | TGACGGGACCGA | hsa_circ_0062045 | chr21 | 47421874 | 47424963 | + | 2167 | ANNOTATED, CDS, c | NM_001848 | COL6A1 | Salzman2013 | 1291 | circRNA | Not Detected | Detected | 2.879590783 | 4.243844657 |
| hsa_gci165209 | 2.373554925 | 1.247049435 | up | 5.03515031 | 3.788100875 | CACTTATAGTTG | hsa_circ_0062252 | chr22 | 19393308 | 19398301 | - | 360 | ANNOTATED, CDS, c | NM_003325 | HIRA | Salzman2013 | 7290 | circRNA | Detected | Detected | 5.03515031 | 3.788100875 |
| hsa_gci165216 | 2.040002465 | 1.028570896 | up | 7.935686044 | 6.907115148 | TTTTCTCCAGCC | hsa_circ_0062349 | chr22 | 21072980 | 21159453 | - | 4078 | ANNOTATED, CDS, c | NM_058004 | PI4KA | Salzman2013 | 5297 | circRNA | Detected | Detected | 7.935686044 | 6.907115148 |
| hsa_gci165222 | 2.345384798 | 1.22982464 | up | 6.845417129 | 5.615592489 | CGAGCGCGAGGG | hsa_circ_0062421 | chr22 | 21982377 | 21984340 | - | 1338 | ANNOTATED, CDS, c | NM_001017964 | YDJC | Salzman2013 | 150223 | circRNA | Detected | Detected | 6.845417129 | 5.615592489 |
| hsa_gci165235 | 2.251408761 | 1.170828013 | up | 7.36606824 | 6.195240227 | GTGGTACCCGGA | hsa_circ_0062449 | chr22 | 22311402 | 22324778 | - | 2288 | ANNOTATED, CDS, c | NM_003935 | TOP3B | Salzman2013 | 8940 | circRNA | Detected | Detected | 7.36606824 | 6.195240227 |
| hsa_gci165241 | 2.420395371 | 1.27524273 | up | 9.135425116 | 7.860182386 | GCTGGAGAAGTC | hsa_circ_0062555 | chr22 | 24108020 | 24108213 | - | 193 | ANNOTATED, CDS, c | NM_213720 | CHCHD10 | Salzman2013 | 400916 | circRNA | Detected | Detected | 9.135425116 | 7.860182386 |
| hsa_gci165246 | 2.341201637 | 1.227249192 | up | 4.449981816 | 3.222732623 | CGTTCGAGAGAG | hsa_circ_0062583 | chr22 | 24451335 | 24574596 | + | 6289 | ANNOTATED, CDS, c | NM_001199281 | CABIN1 | Salzman2013 | 23523 | circRNA | Detected | Not Detected | 4.449981816 | 3.222732623 |

| | | | | | | | | | | | | | | | | | | | | | | | |
|---|---|---|---|---|---|---|---|---|---|---|---|---|---|---|---|---|---|---|---|---|---|---|---|
| hsa_gci165284 | -3.351065324 | -1.74461981 | down | 2.302949814 | 4.047569624 | AGTTGGTCAAGT | hsa_circ_0062847 | chr22 | 31322599 | 31354680 | - | 3221 | ANNOTATED, CDS, c | NM_014941 | MORC2 | Salzman2013 | 22880 | circRNA | Not Detected | Detected | 2.302949814 | 4.047569624 |
| hsa_gci165289 | 2.271969927 | 1.183943738 | up | 7.987260324 | 6.803316585 | ACTATCTTACCT | hsa_circ_0062876 | chr22 | 31500762 | 31501678 | - | 466 | ANNOTATED, CDS, c | NM_080430 | SELENOM | Salzman2013 | 140606 | circRNA | Detected | Detected | 7.987260324 | 6.803316585 |
| hsa_gci165322 | 2.626434769 | 1.393105754 | up | 4.829613305 | 3.436507551 | CCACTGAATATC | hsa_circ_0063085 | chr22 | 36677322 | 36745300 | - | 7293 | ANNOTATED, CDS, c | NM_002473 | MYH9 | Salzman2013 | 4627 | circRNA | Detected | Detected | 4.829613305 | 3.436507551 |
| hsa_gci165325 | 3.575386868 | 1.838099355 | up | 6.653832279 | 4.815732925 | TACCACTGAATA | hsa_circ_0063149 | chr22 | 36723505 | 36745300 | - | 537 | ANNOTATED, CDS, c | NM_002473 | MYH9 | Salzman2013 | 4627 | circRNA | Detected | Detected | 6.653832279 | 4.815732925 |
| hsa_gci165390 | 2.141699347 | 1.098755968 | up | 7.172326636 | 6.073570669 | CGTCGTGCCCCA | hsa_circ_0063594 | chr22 | 42054255 | 42060052 | + | 642 | ANNOTATED, CDS, c | NM_001469 | XRCC6 | Salzman2013 | 2547 | circRNA | Detected | Detected | 7.172326636 | 6.073570669 |
| hsa_gci165405 | 3.797486102 | 1.925044685 | up | 6.711650976 | 4.786606291 | ATCTCTCTCTAG | hsa_circ_0063683 | chr22 | 43459818 | 43460330 | - | 244 | ANNOTATED, INTERN | NR_027779 | TTLL1 | Salzman2013 | 25809 | circRNA | Detected | Detected | 6.711650976 | 4.786606291 |
| hsa_gci165408 | 2.930267042 | 1.551032147 | up | 5.575297316 | 4.02426517 | GGTGACTGGTCG | hsa_circ_0063686 | chr22 | 43558808 | 43559248 | + | 440 | ANNOTATED, CDS, c | NM_000714 | TSPO | Salzman2013 | 706 | circRNA | Detected | Detected | 5.575297316 | 4.02426517 |
| hsa_gci165415 | 2.624469672 | 1.392025926 | up | 5.4470619 | 4.055035974 | CGAGTGTCGACC | hsa_circ_0063765 | chr22 | 46067916 | 46136418 | + | 1200 | ALT_ACCEPTOR, CDS | NM_013236 | ATXN10 | Salzman2013 | 25814 | circRNA | Detected | Detected | 5.4470619 | 4.055035974 |
| hsa_gci165424 | 2.29920266 | 1.201133636 | up | 6.938396103 | 5.737262467 | TATGTCCGTGTC | hsa_circ_0063841 | chr22 | 47106982 | 47116912 | - | 427 | ANNOTATED, CDS, c | NM_022766 | CERK | Salzman2013 | 64781 | circRNA | Detected | Detected | 6.938396103 | 5.737262467 |
| hsa_gci165428 | 3.559153251 | 1.831534054 | up | 7.204200934 | 5.37266688 | TGTGTCCCGAAT | hsa_circ_0063876 | chr22 | 50356294 | 50357720 | + | 1352 | ALT_ACCEPTOR, CDS | NM_001001852 | PIM3 | Salzman2013 | 415116 | circRNA | Detected | Detected | 7.204200934 | 5.37266688 |
| hsa_gci165434 | 3.293592318 | 1.719661989 | up | 6.793568773 | 5.073906784 | CCTTGTGCATCA | hsa_circ_0063920 | chr22 | 50713407 | 50720510 | - | 3125 | ANNOTATED, CDS, c | NM_012401 | PLXNB2 | Salzman2013 | 23654 | circRNA | Detected | Detected | 6.793568773 | 5.073906784 |
| hsa_gci165440 | 2.519680544 | 1.333240834 | up | 6.59428381 | 5.261042975 | TGTTGACTACAA | hsa_circ_0064027 | chr3 | 4355330 | 4358949 | + | 1169 | ANNOTATED, CDS, c | NM_001243723 | SETMAR | Salzman2013 | 6419 | circRNA | Detected | Detected | 6.59428381 | 5.261042975 |
| hsa_gci165448 | 2.495193857 | 1.319151906 | up | 8.545133692 | 7.225981786 | ACCCGTCGCCGA | hsa_circ_0064097 | chr3 | 8918879 | 8977739 | - | 4945 | ANNOTATED, CDS, c | NM_020165 | RAD18 | Salzman2013 | 56852 | circRNA | Detected | Detected | 8.545133692 | 7.225981786 |
| hsa_gci165463 | 2.312714779 | 1.209587353 | up | 7.113278744 | 5.903691391 | CGCCCCGGAGCT | hsa_circ_0064201 | chr3 | 10068182 | 10143614 | + | 7183 | ALT_ACCEPTOR, CDS | NM_001018115 | FANCD2 | Salzman2013 | 2177 | circRNA | Detected | Detected | 7.113278744 | 5.903691391 |
| hsa_gci165474 | 2.115831163 | 1.081224509 | up | 7.672254311 | 6.591029801 | GAGTCCCCCGGG | hsa_circ_0064254 | chr3 | 10119764 | 10143614 | + | 2165 | ANNOTATED, CDS, c | NM_001018115 | FANCD2 | Salzman2013 | 2177 | circRNA | Detected | Detected | 7.672254311 | 6.591029801 |
| hsa_gci165516 | 2.869396491 | 1.520747332 | up | 7.302297558 | 5.781550225 | GTACGTAATTTT | hsa_circ_0064524 | chr3 | 16310632 | 16313229 | + | 341 | ANNOTATED, CDS, c | NM_138381 | OXNAD1 | Salzman2013 | 92106 | circRNA | Detected | Detected | 7.302297558 | 5.781550225 |
| hsa_gci165533 | 2.222960097 | 1.152482052 | up | 8.935856748 | 7.783374696 | GACTCCGGCGTC | hsa_circ_0064592 | chr3 | 25639474 | 25705831 | - | 5232 | ALT_ACCEPTOR, CDS | NM_001068 | TOP2B | Salzman2013 | 7155 | circRNA | Detected | Detected | 8.935856748 | 7.783374696 |
| hsa_gci165546 | 2.816768528 | 1.494041013 | up | 7.615261974 | 6.121220961 | ACACAACTAGAT | hsa_circ_0064659 | chr3 | 31617887 | 31665353 | + | 1413 | ANNOTATED, CDS, c | NM_178862 | STT3B | Salzman2013 | 201595 | circRNA | Detected | Detected | 7.615261974 | 6.121220961 |
| hsa_gci165555 | 2.297511316 | 1.200071967 | up | 7.352938295 | 6.152866327 | GCGTGTGTCTAC | hsa_circ_0064680 | chr3 | 32483331 | 32496333 | + | 956 | ANNOTATED, CDS, c | NM_138410 | CMTM7 | Salzman2013 | 112616 | circRNA | Detected | Detected | 7.352938295 | 6.152866327 |
| hsa_gci165560 | 2.507577189 | 1.326294111 | up | 4.785290252 | 3.458996141 | ACATCTGTCCGT | hsa_circ_0064694 | chr3 | 32746302 | 32758729 | + | 627 | ANNOTATED, CDS, c | NM_015442 | CNOT10 | Salzman2013 | 25904 | circRNA | Detected | Detected | 4.785290252 | 3.458996141 |
| hsa_gci165569 | 2.986120792 | 1.578272525 | up | 9.32689235 | 7.748619824 | GACGTGTCCCCC | hsa_circ_0064721 | chr3 | 33174046 | 33175757 | + | 230 | ANNOTATED, CDS, c | NM_006371 | CRTAP | Salzman2013 | 10491 | circRNA | Detected | Detected | 9.32689235 | 7.748619824 |
| hsa_gci165589 | 2.471566673 | 1.305425825 | up | 8.73305869 | 7.427632865 | ACAGAAACCGTA | hsa_circ_0064789 | chr3 | 33725850 | 33731424 | - | 370 | ANNOTATED, CDS, c | NM_015097 | CLASP2 | Salzman2013 | 23122 | circRNA | Detected | Detected | 8.73305869 | 7.427632865 |
| hsa_gci165606 | 2.207184633 | 1.142207317 | up | 9.397873987 | 8.25566667 | CATTAGTAGTCT | hsa_circ_0064864 | chr3 | 38182247 | 38184512 | + | 1982 | ANNOTATED, CDS, c | NM_001172567 | MYD88 | Salzman2013 | 4615 | circRNA | Detected | Detected | 9.397873987 | 8.25566667 |
| hsa_gci165611 | 2.0383428 | 1.027396698 | up | 7.060614204 | 6.033217505 | TTTTAAAGGGAA | hsa_circ_0064903 | chr3 | 39449111 | 39453552 | + | 826 | ANNOTATED, CDS, c | NM_002295 | RPSA | Salzman2013 | 3921 | circRNA | Detected | Detected | 7.060614204 | 6.033217505 |
| hsa_gci165617 | 2.488599293 | 1.315333951 | up | 4.268337152 | 2.953003202 | AGACGGAAGACG | hsa_circ_0064923 | chr3 | 40502897 | 40503859 | + | 584 | ANNOTATED, CDS, c | NM_001034996 | RPL14 | Salzman2013 | 9045 | circRNA | Detected | Not Detected | 4.268337152 | 2.953003202 |
| hsa_gci165619 | 2.887243504 | 1.529692786 | up | 6.477154407 | 4.947461621 | GTTTTGTCAACA | hsa_circ_0064931 | chr3 | 41265511 | 41279567 | + | 2185 | ANNOTATED, CDS, c | NM_001904 | CTNNB1 | Salzman2013 | 1499 | circRNA | Detected | Detected | 6.477154407 | 4.947461621 |
| hsa_gci165626 | 2.076581512 | 1.054210503 | up | 5.998614684 | 4.944404181 | AAACGCCCTGTT | hsa_circ_0064944 | chr3 | 41275629 | 41281939 | + | 1928 | ANNOTATED, CDS, c | NM_001904 | CTNNB1 | Salzman2013 | 1499 | circRNA | Detected | Detected | 5.998614684 | 4.944404181 |
| hsa_gci165627 | 2.677056056 | 1.420647348 | up | 4.694842985 | 3.274195637 | GCCATGTTGGAA | hsa_circ_0064945 | chr3 | 41277214 | 41281939 | + | 1769 | ANNOTATED, CDS, c | NM_001904 | CTNNB1 | Salzman2013 | 1499 | circRNA | Detected | Not Detected | 4.694842985 | 3.274195637 |
| hsa_gci165647 | 2.171817948 | 1.118903175 | up | 7.975759431 | 6.856856256 | AGTGACACTAGG | hsa_circ_0065024 | chr3 | 44790235 | 44795860 | - | 4954 | ANNOTATED, CDS, c | NM_020696 | KIAA1143 | Salzman2013 | 57456 | circRNA | Detected | Detected | 7.975759431 | 6.856856256 |
| hsa_gci165656 | 2.555748565 | 1.353745911 | up | 7.096188654 | 5.742442743 | CGACCCATTCGT | hsa_circ_0065082 | chr3 | 45527183 | 45542071 | + | 742 | ANNOTATED, CDS, c | NM_015340 | LARS2 | Salzman2013 | 23395 | circRNA | Detected | Detected | 7.096188654 | 5.742442743 |
| hsa_gci165679 | 2.496999129 | 1.320195318 | up | 7.619865189 | 6.299669871 | TGCTATGCACAG | hsa_circ_0065244 | chr3 | 47651555 | 47719801 | - | 1586 | ANNOTATED, CDS, c | NM_003074 | SMARCC1 | Salzman2013 | 6599 | circRNA | Detected | Detected | 7.619865189 | 6.299669871 |
| hsa_gci165702 | 2.74971714 | 1.459283218 | up | 7.18257635 | 5.723293132 | GTAAAGATAGAG | hsa_circ_0065334 | chr3 | 47898920 | 47958664 | - | 2496 | ANNOTATED, CDS, c | NM_002375 | MAP4 | Salzman2013 | 4134 | circRNA | Detected | Detected | 7.18257635 | 5.723293132 |
| hsa_gci165704 | 2.563533875 | 1.358133962 | up | 7.430933523 | 6.072799561 | AAACGTAGACGT | hsa_circ_0065336 | chr3 | 47908735 | 47913590 | - | 744 | ANNOTATED, CDS, c | NM_002375 | MAP4 | Salzman2013 | 4134 | circRNA | Detected | Detected | 7.430933523 | 6.072799561 |
| hsa_gci165710 | 3.602141158 | 1.848854718 | up | 8.609357711 | 6.760502993 | CCGTGGAGCATC | hsa_circ_0065496 | chr3 | 48725435 | 48727146 | - | 947 | ANNOTATED, CDS, c | NM_016291 | IP6K2 | Salzman2013 | 51447 | circRNA | Detected | Detected | 8.609357711 | 6.760502993 |
| hsa_gci165713 | 2.937577292 | 1.554626812 | up | 6.914199299 | 5.359572488 | CACCTATCTGAT | hsa_circ_0065500 | chr3 | 48725435 | 48754920 | - | 2012 | ALT_ACCEPTOR, CDS | NM_016291 | IP6K2 | Salzman2013 | 51447 | circRNA | Detected | Detected | 6.914199299 | 5.359572488 |
| hsa_gci165719 | 3.084909728 | 1.625228273 | up | 4.432304666 | 2.807076392 | TGACGACAACGA | hsa_circ_0065534 | chr3 | 49061761 | 49062702 | - | 593 | ANNOTATED, CDS, c | NM_000884 | IMPDH2 | Salzman2013 | 3615 | circRNA | Detected | Not Detected | 4.432304666 | 2.807076392 |
| hsa_gci165724 | 2.44543647 | 1.290091985 | up | 7.582112998 | 6.292021012 | CCGGAAAGTGTG | hsa_circ_0065550 | chr3 | 49067141 | 49068077 | - | 936 | ANNOTATED, CDS, c | NM_017730 | QRICH1 | Salzman2013 | 54870 | circRNA | Detected | Detected | 7.582112998 | 6.292021012 |
| hsa_gci165747 | 2.362761464 | 1.240473987 | up | 8.432139046 | 7.191665059 | TCCTGTCCGTCG | hsa_circ_0065677 | chr3 | 49713488 | 49720934 | + | 1916 | ANNOTATED, CDS, c | NM_001640 | APEH | Salzman2013 | 327 | circRNA | Detected | Detected | 8.432139046 | 7.191665059 |
| hsa_gci165761 | 2.841248097 | 1.506524813 | up | 9.21692951 | 7.710404697 | TTTTCTTCTGCT | hsa_circ_0065727 | chr3 | 49738923 | 49758962 | + | 2892 | ANNOTATED, CDS, c | NM_022064 | RNF123 | Salzman2013 | 63891 | circRNA | Detected | Detected | 9.21692951 | 7.710404697 |
| hsa_gci165764 | 2.277172342 | 1.187243482 | up | 8.061121117 | 6.873877635 | AAGTCGAATAAT | hsa_circ_0065744 | chr3 | 49785250 | 49785601 | - | 351 | ANNOTATED, CDS, c | NM_001242829 | IP6K1 | Salzman2013 | 9807 | circRNA | Detected | Detected | 8.061121117 | 6.873877635 |
| hsa_gci165767 | 2.085172464 | 1.060166713 | up | 5.849111778 | 4.788945065 | AGACTCCGACTC | hsa_circ_0065776 | chr3 | 50095160 | 50114685 | + | 1798 | ANNOTATED, CDS, c | NM_005777 | RBM6 | Salzman2013 | 10180 | circRNA | Detected | Detected | 5.849111778 | 4.788945065 |
| hsa_gci165775 | 2.54303771 | 1.346552856 | up | 8.612265975 | 7.26571312 | GAAAAGGACTTT | hsa_circ_0065800 | chr3 | 50141680 | 50147121 | + | 711 | ANNOTATED, CDS, c | NM_005778 | RBM5 | Salzman2013 | 10181 | circRNA | Detected | Detected | 8.612265975 | 7.26571312 |
| hsa_gci165782 | 2.822497933 | 1.496972524 | up | 8.51629816 | 7.019325636 | TTGACGACTATT | hsa_circ_0065817 | chr3 | 50145664 | 50147121 | + | 159 | ANNOTATED, CDS, c | NM_005778 | RBM5 | Salzman2013 | 10181 | circRNA | Detected | Detected | 8.51629816 | 7.019325636 |
| hsa_gci165793 | -4.295158137 | -2.102711249 | down | 1.161264837 | 3.263976085 | GTACATGGTGAT | hsa_circ_0065912 | chr3 | 51474985 | 51517852 | - | 1136 | ANNOTATED, CDS, c | NM_014703 | DCAF1 | Salzman2013 | 9730 | circRNA | Not Detected | Detected | 1.161264837 | 3.263976085 |
| hsa_gci165796 | 2.25053323 | 1.170266867 | up | 7.207402004 | 6.037135137 | GTGTAGCCGTGA | hsa_circ_0065937 | chr3 | 51718428 | 51738339 | + | 991 | ANNOTATED, CDS, c | NM_001243725 | TEX264 | Salzman2013 | 51368 | circRNA | Detected | Detected | 7.207402004 | 6.037135137 |
| hsa_gci165801 | 2.145530483 | 1.101334399 | up | 6.377368254 | 5.276033855 | CCGGCGCGCCCG | hsa_circ_0065975 | chr3 | 52082936 | 52090461 | - | 3233 | ANNOTATED, CDS, c | NM_001947 | DUSP7 | Salzman2013 | 1849 | circRNA | Detected | Detected | 6.377368254 | 5.276033855 |
| hsa_gci165802 | 2.496688827 | 1.320016023 | up | 6.343056985 | 5.023040962 | GTCGCCCTTCTT | hsa_circ_0065983 | chr3 | 52232687 | 52240767 | + | 1374 | ANNOTATED, CDS, c | NM_000688 | ALAS1 | Salzman2013 | 211 | circRNA | Detected | Detected | 6.343056985 | 5.023040962 |
| hsa_gci165826 | 2.750373921 | 1.459627771 | up | 8.139844418 | 6.680216647 | TCTATGATATAT | hsa_circ_0066184 | chr3 | 53338206 | 53353500 | - | 320 | ANNOTATED, CDS, c | NM_018403 | DCP1A | Salzman2013 | 55802 | circRNA | Detected | Detected | 8.139844418 | 6.680216647 |
| hsa_gci165854 | 2.25370514 | 1.172298775 | up | 6.199596654 | 5.027297879 | AACATGTTTAAC | hsa_circ_0066385 | chr3 | 58260384 | 58271180 | + | 314 | ANNOTATED, CDS, c | NM_020676 | ABHD6 | Salzman2013 | 57406 | circRNA | Detected | Detected | 6.199596654 | 5.027297879 |
| hsa_gci165859 | 2.42358637 | 1.277143497 | up | 7.11736412 | 5.840220623 | GGTCAGCTCCCT | hsa_circ_0066438 | chr3 | 64004274 | 64008199 | - | 681 | ANNOTATED, CDS, c | NM_014814 | PSMD6 | Salzman2013 | 9861 | circRNA | Detected | Detected | 7.11736412 | 5.840220623 |
| hsa_gci165864 | 2.561092487 | 1.356759353 | up | 8.867174523 | 7.51041517 | GGTCCCTACCAT | hsa_circ_0066480 | chr3 | 66501982 | 66502057 | - | 75 | ANNOTATED, CDS, c | NM_015541 | LRIG1 | Salzman2013 | 26018 | circRNA | Detected | Detected | 8.867174523 | 7.51041517 |

| | | | | | | | | | | | | | | | | | | | | | | |
|---|---|---|---|---|---|---|---|---|---|---|---|---|---|---|---|---|---|---|---|---|---|---|
| hsa_gci165867 | 2.977054432 | 1.5738856 | up | 4.56353636 | 2.989650761 | TAGAAGACCTCC | hsa_circ_0066491 | chr3 | 67568670 | 67660020 | - | 576 | ANNOTATED, CDS, c | NM_003848 | SUCLG2 | Salzman2013 | 8801 | circRNA | Detected | Not Detected | 4.56353636 | 2.989650761 |
| hsa_gci165868 | 2.013277551 | 1.009546077 | up | 10.06129381 | 9.051747729 | TGTATACAGTTT | hsa_circ_0066494 | chr3 | 67721305 | 67721428 | + | 123 | INTERGENIC | None | | Salzman2013 | | circRNA | Detected | Detected | 10.06129381 | 9.051747729 |
| hsa_gci165892 | 2.233975295 | 1.159613231 | up | 5.881193101 | 4.721579869 | CACAGGTGTAGG | hsa_circ_0066621 | chr3 | 98530311 | 98600611 | - | 1245 | ANNOTATED, CDS, c | NM_080927 | DCBLD2 | Salzman2013 | 131566 | circRNA | Detected | Detected | 5.881193101 | 4.721579869 |
| hsa_gci165906 | 2.488180375 | 1.315091074 | up | 9.331976897 | 8.016885823 | TTCCAAGGTCCC | hsa_circ_0066658 | chr3 | 100274052 | 100287811 | + | 737 | ANNOTATED, CDS, c | NM_018004 | TMEM45A | Salzman2013 | 55076 | circRNA | Detected | Detected | 9.331976897 | 8.016885823 |
| hsa_gci165918 | 2.135694756 | 1.094705465 | up | 5.902247323 | 4.807541858 | ACCTTAAACTGA | hsa_circ_0066694 | chr3 | 101399933 | 101401751 | - | 327 | ANNOTATED, CDS, c | NM_000986 | RPL24 | Salzman2013 | 6152 | circRNA | Detected | Detected | 5.902247323 | 4.807541858 |
| hsa_gci165939 | 2.509688565 | 1.327508347 | up | 5.215198114 | 3.887689767 | TGTCCCTGTTCT | hsa_circ_0066766 | chr3 | 108330020 | 108343371 | + | 354 | ANNOTATED, CDS, c | NM_014648 | DZIP3 | Salzman2013 | 9666 | circRNA | Detected | Detected | 5.215198114 | 3.887689767 |
| hsa_gci165941 | 3.353632454 | 1.745724583 | up | 7.652079051 | 5.906354468 | CACCGAGGTGTT | hsa_circ_0066776 | chr3 | 110830876 | 110845182 | + | 909 | ANNOTATED, CDS, c | NM_015480 | NECTIN3 | Salzman2013 | 25945 | circRNA | Detected | Detected | 7.652079051 | 5.906354468 |
| hsa_gci165955 | 2.533662378 | 1.341224292 | up | 7.694759497 | 6.353535206 | TCCCCGTCCAGT | hsa_circ_0066847 | chr3 | 114107851 | 114219238 | - | 141 | ANNOTATED, coding | NM_001164343 | ZBTB20 | Salzman2013 | 26137 | circRNA | Detected | Detected | 7.694759497 | 6.353535206 |
| hsa_gci165968 | 3.125654122 | 1.644158142 | up | 7.028969163 | 5.384811021 | TAGTCTGTCAAG | hsa_circ_0066911 | chr3 | 120122088 | 120122201 | - | 113 | ANNOTATED, CDS, c | NM_007085 | FSTL1 | Salzman2013 | 11167 | circRNA | Detected | Detected | 7.028969163 | 5.384811021 |
| hsa_gci165969 | 2.272312918 | 1.18416152 | up | 8.79436113 | 7.610199609 | GAATGCCTAAAG | hsa_circ_0066912 | chr3 | 120122088 | 120129831 | - | 396 | ANNOTATED, CDS, c | NM_007085 | FSTL1 | Salzman2013 | 11167 | circRNA | Detected | Detected | 8.79436113 | 7.610199609 |
| hsa_gci165971 | 2.385106658 | 1.254053783 | up | 6.815767798 | 5.561714015 | TGTTGTAACTTG | hsa_circ_0066917 | chr3 | 120357301 | 120360540 | - | 232 | ANNOTATED, CDS, c | NM_000187 | HGD | Salzman2013 | 3081 | circRNA | Detected | Detected | 6.815767798 | 5.561714015 |
| hsa_gci165993 | 3.010417114 | 1.589963396 | up | 8.120141746 | 6.53017835 | TTGAAAAGTCCG | hsa_circ_0066993 | chr3 | 122180070 | 122215417 | - | 437 | ANNOTATED, INTERN | NR_026698 | KPNA1 | Salzman2013 | 3836 | circRNA | Detected | Detected | 8.120141746 | 6.53017835 |
| hsa_gci165994 | 2.403998037 | 1.265435718 | up | 7.768916899 | 6.50348118 | GCTTTCAAGAGA | hsa_circ_0066994 | chr3 | 122215283 | 122215417 | - | 134 | ANNOTATED, INTERN | NR_026698 | KPNA1 | Salzman2013 | 3836 | circRNA | Detected | Detected | 7.768916899 | 6.50348118 |
| hsa_gci166003 | 7.183781465 | 2.844743463 | up | 4.155608327 | 1.310864864 | CCCCAAAAGTTT | hsa_circ_0067089 | chr3 | 124810282 | 124998091 | - | 68514 | ALT_ACCEPTOR, CDS | NM_024628 | SLC12A8 | Salzman2013 | 84561 | circRNA | Detected | Not Detected | 4.155608327 | 1.310864864 |
| hsa_gci166021 | 2.550481174 | 1.350769452 | up | 6.374124566 | 5.023355114 | GCACTCCCCTAC | hsa_circ_0067194 | chr3 | 127783627 | 127806651 | - | 16820 | ALT_DONOR, CDS, c | NM_003707 | RUVBL1 | Salzman2013 | 8607 | circRNA | Detected | Detected | 6.374124566 | 5.023355114 |
| hsa_gci166028 | 2.093576597 | 1.065969703 | up | 7.554350076 | 6.488380373 | GACCCCGTGCCG | hsa_circ_0067215 | chr3 | 128338812 | 128344855 | - | 1129 | ANNOTATED, CDS, c | NM_002950 | RPN1 | Salzman2013 | 6184 | circRNA | Detected | Detected | 7.554350076 | 6.488380373 |
| hsa_gci166036 | 2.233923819 | 1.159579988 | up | 7.04041108 | 5.880831092 | GCGGGGCTCGCC | hsa_circ_0067232 | chr3 | 128444978 | 128533641 | + | 2232 | ANNOTATED, CDS, c | NM_004637 | RAB7A | Salzman2013 | 7879 | circRNA | Detected | Detected | 7.04041108 | 5.880831092 |
| hsa_gci166097 | 2.031614214 | 1.022626473 | up | 7.362941517 | 6.340315044 | TCAGTTTTTGGT | hsa_circ_0067535 | chr3 | 138426000 | 138431146 | - | 228 | ANNOTATED, CDS, c | NM_006219 | PIK3CB | Salzman2013 | 5291 | circRNA | Detected | Detected | 7.362941517 | 6.340315044 |
| hsa_gci166098 | 2.221325972 | 1.151421119 | up | 8.19961089 | 7.048189771 | CCCGTCCCGAGA | hsa_circ_0067536 | chr3 | 138452202 | 138453646 | - | 249 | ANNOTATED, CDS, c | NM_006219 | PIK3CB | Salzman2013 | 5291 | circRNA | Detected | Detected | 8.19961089 | 7.048189771 |
| hsa_gci166144 | 2.180366641 | 1.124570753 | up | 7.750048206 | 6.625477453 | GATACCTCTCGT | hsa_circ_0067718 | chr3 | 149563797 | 149677923 | + | 797 | ANNOTATED, CDS, c | NM_007282 | RNF13 | Salzman2013 | 11342 | circRNA | Detected | Detected | 7.750048206 | 6.625477453 |
| hsa_gci166145 | 2.215674025 | 1.147745645 | up | 7.387316201 | 6.239570557 | ACGTCCCTCCAG | hsa_circ_0067720 | chr3 | 149570302 | 149629870 | + | 492 | ANNOTATED, CDS, c | NM_007282 | RNF13 | Salzman2013 | 11342 | circRNA | Detected | Detected | 7.387316201 | 6.239570557 |
| hsa_gci166146 | 2.326286164 | 1.218028578 | up | 6.986912758 | 5.76888418 | CGTCCCTCCAGT | hsa_circ_0067721 | chr3 | 149570302 | 149639014 | + | 586 | ANNOTATED, CDS, c | NM_007282 | RNF13 | Salzman2013 | 11342 | circRNA | Detected | Detected | 6.986912758 | 5.76888418 |
| hsa_gci166162 | 2.252484389 | 1.171517107 | up | 6.909545869 | 5.738028761 | AAGAAGTCGTGT | hsa_circ_0067790 | chr3 | 155639975 | 155643155 | + | 242 | ANNOTATED, CDS, c | NM_003875 | GMPS | Salzman2013 | 8833 | circRNA | Detected | Detected | 6.909545869 | 5.738028761 |
| hsa_gci166163 | 2.330820297 | 1.220837779 | up | 6.154371962 | 4.933534182 | GGGACGCCGGCG | hsa_circ_0067793 | chr3 | 156257928 | 156272973 | - | 3716 | ANNOTATED, CDS, c | NM_007107 | SSR3 | Salzman2013 | 6747 | circRNA | Detected | Detected | 6.154371962 | 4.933534182 |
| hsa_gci166164 | 2.884471853 | 1.528307186 | up | 8.790633558 | 7.262326372 | ACGGTATAAGCG | hsa_circ_0067794 | chr3 | 156262096 | 156271570 | - | 358 | ANNOTATED, CDS, c | NM_007107 | SSR3 | Salzman2013 | 6747 | circRNA | Detected | Detected | 8.790633558 | 7.262326372 |
| hsa_gci166172 | 2.367252286 | 1.243213467 | up | 4.019633411 | 2.776419944 | TATATCGGTGCA | hsa_circ_0067842 | chr3 | 160138527 | 160146730 | + | 938 | ANNOTATED, CDS, c | NM_005496 | SMC4 | Salzman2013 | 10051 | circRNA | Detected | Not Detected | 4.019633411 | 2.776419944 |
| hsa_gci166178 | 2.289713296 | 1.195166964 | up | 6.858431094 | 5.663264131 | AAATCCCGAGCC | hsa_circ_0067883 | chr3 | 169700494 | 169706147 | + | 479 | ANNOTATED, CDS, c | NM_003262 | SEC62 | Salzman2013 | 7095 | circRNA | Detected | Detected | 6.858431094 | 5.663264131 |
| hsa_gci166179 | 2.684748891 | 1.424787157 | up | 4.876235615 | 3.451448458 | TCTTCACCTCTC | hsa_circ_0067884 | chr3 | 169805367 | 169867032 | - | 12224 | ANNOTATED, CDS, c | NM_024947 | PHC3 | Salzman2013 | 80012 | circRNA | Detected | Detected | 4.876235615 | 3.451448458 |
| hsa_gci166206 | 2.337868559 | 1.22519382 | up | 6.806132571 | 5.58093875 | CTGGTGGTGGGC | hsa_circ_0067988 | chr3 | 171965322 | 172118492 | + | 6578 | ANNOTATED, CDS, c | NM_022763 | FNDC3B | Salzman2013 | 64778 | circRNA | Detected | Detected | 6.806132571 | 5.58093875 |
| hsa_gci166221 | 2.010536293 | 1.007580379 | up | 10.36821912 | 9.360638743 | GGGGTTTTATTG | hsa_circ_0068043 | chr3 | 176738541 | 176915048 | - | 6550 | ANNOTATED, CDS, c | NM_024665 | TBL1XR1 | Salzman2013 | 79718 | circRNA | Detected | Detected | 10.36821912 | 9.360638743 |
| hsa_gci166230 | 2.145636765 | 1.101405863 | up | 10.12194987 | 9.020544011 | GGCTCTCGCCCC | hsa_circ_0068067 | chr3 | 178735010 | 178743013 | - | 8003 | ANNOTATED, CDS, c | NM_152240 | ZMAT3 | Salzman2013 | 64393 | circRNA | Detected | Detected | 10.12194987 | 9.020544011 |
| hsa_gci166248 | 2.38353698 | 1.253104008 | up | 6.815112915 | 5.562008907 | TGTACTCATACC | hsa_circ_0068145 | chr3 | 182733005 | 182746977 | - | 4102 | ALT_ACCEPTOR, CDS | NM_020166 | MCCC1 | Salzman2013 | 56922 | circRNA | Detected | Detected | 6.815112915 | 5.562008907 |
| hsa_gci166251 | 2.08476451 | 1.05988443 | up | 10.269703 | 9.20981857 | AATTTTCCCAAA | hsa_circ_0068153 | chr3 | 183005267 | 183015456 | + | 10189 | ANTISENSE, CDS, c | NM_015078 | MCF2L2 | Salzman2013 | 23101 | circRNA | Detected | Detected | 10.269703 | 9.20981857 |
| hsa_gci166261 | 2.433324835 | 1.282928925 | up | 4.301278073 | 3.018349149 | AAACTATTATCA | hsa_circ_0068176 | chr3 | 183435438 | 183480067 | + | 1847 | ANNOTATED, CDS, c | NM_018023 | YEATS2 | Salzman2013 | 55689 | circRNA | Detected | Not Detected | 4.301278073 | 3.018349149 |
| hsa_gci166262 | 2.184498198 | 1.127301916 | up | 4.511162608 | 3.383860692 | CAAGAAACTATT | hsa_circ_0068177 | chr3 | 183435438 | 183493911 | + | 2477 | ANNOTATED, CDS, c | NM_018023 | YEATS2 | Salzman2013 | 55689 | circRNA | Detected | Detected | 4.511162608 | 3.383860692 |
| hsa_gci166282 | 2.118999436 | 1.083383204 | up | 6.421506602 | 5.338123398 | TTATCGTCGACT | hsa_circ_0068249 | chr3 | 183551279 | 183584553 | - | 707 | ANNOTATED, CDS, c | NM_018622 | PARL | Salzman2013 | 55486 | circRNA | Detected | Detected | 6.421506602 | 5.338123398 |
| hsa_gci166307 | 2.217043685 | 1.148637198 | up | 6.97936074 | 5.830723543 | TGATACTGTCCG | hsa_circ_0068367 | chr3 | 184603897 | 184618760 | + | 412 | ANNOTATED, CDS, c | NM_001009921 | VPS8 | Salzman2013 | 23355 | circRNA | Detected | Detected | 6.97936074 | 5.830723543 |
| hsa_gci166309 | 2.550124837 | 1.350567873 | up | 5.574519724 | 4.223951851 | AAATGTTCCGAA | hsa_circ_0068376 | chr3 | 184654033 | 184689540 | + | 522 | ANNOTATED, CDS, c | NM_001009921 | VPS8 | Salzman2013 | 23355 | circRNA | Detected | Detected | 5.574519724 | 4.223951851 |
| hsa_gci166310 | 2.079390863 | 1.056160967 | up | 5.62249606 | 4.566335093 | GAAATGTTCCGA | hsa_circ_0068377 | chr3 | 184654033 | 184700882 | + | 687 | ANNOTATED, CDS, c | NM_001009921 | VPS8 | Salzman2013 | 23355 | circRNA | Detected | Detected | 5.62249606 | 4.566335093 |
| hsa_gci166324 | 2.831946132 | 1.501793823 | up | 4.949210375 | 3.447416552 | TCCACCCCGTAC | hsa_circ_0068428 | chr3 | 185764105 | 185798964 | - | 3623 | ANNOTATED, CDS, c | NM_004454 | ETV5 | Salzman2013 | 2119 | circRNA | Detected | Detected | 4.949210375 | 3.447416552 |
| hsa_gci166340 | 2.080882045 | 1.057195188 | up | 4.268890699 | 3.211695511 | AGAGAGAGGAAG | hsa_circ_0068467 | chr3 | 186505283 | 186505671 | + | 170 | ANNOTATED, CDS, c | NM_001967 | EIF4A2 | Salzman2013 | 1974 | circRNA | Detected | Detected | 4.268890699 | 3.211695511 |
| hsa_gci166364 | 2.195141424 | 1.134313889 | up | 4.347916695 | 3.213602805 | CCGCCGGTACTT | hsa_circ_0068561 | chr3 | 194789012 | 194991895 | - | 2721 | ANNOTATED, CDS, c | NM_152531 | XXYLT1 | Salzman2013 | 152002 | circRNA | Detected | Detected | 4.347916695 | 3.213602805 |
| hsa_gci166376 | 2.287606731 | 1.193839056 | up | 8.359692147 | 7.165853091 | ACTATATCTTTA | hsa_circ_0068609 | chr3 | 195785154 | 195785503 | - | 141 | ANNOTATED, CDS, c | NM_003234 | TFRC | Salzman2013 | 7037 | circRNA | Detected | Detected | 8.359692147 | 7.165853091 |
| hsa_gci166438 | 2.458429338 | 1.297736889 | up | 8.946297444 | 7.648560555 | CGCCTTCTCCAG | hsa_circ_0068881 | chr4 | 1813205 | 1838299 | - | 4571 | ANNOTATED, CDS, c | NM_012318 | LETM1 | Salzman2013 | 3954 | circRNA | Detected | Detected | 8.946297444 | 7.648560555 |
| hsa_gci166439 | -4.392552929 | -2.13505967 | down | 1.380615807 | 3.515675477 | GTCCTCAAGTTC | hsa_circ_0068887 | chr4 | 1894508 | 1906105 | + | 1254 | ANNOTATED, CDS, c | NM_133330 | NSD2 | Salzman2013 | 7468 | circRNA | Not Detected | Detected | 1.380615807 | 3.515675477 |
| hsa_gci166452 | 3.192166108 | 1.674535726 | up | 8.473230443 | 6.798694717 | TAGAACCTGGGC | hsa_circ_0068961 | chr4 | 3076407 | 3109150 | + | 892 | ANNOTATED, CDS, c | NM_002111 | HTT | Salzman2013 | 3064 | circRNA | Detected | Detected | 8.473230443 | 6.798694717 |
| hsa_gci166453 | 2.33015314 | 1.220424774 | up | 7.022160772 | 5.801735998 | CGACTTTCAAGA | hsa_circ_0068963 | chr4 | 3088665 | 3124663 | + | 1058 | ANNOTATED, CDS, c | NM_002111 | HTT | Salzman2013 | 3064 | circRNA | Detected | Detected | 7.022160772 | 5.801735998 |
| hsa_gci166464 | -3.678304823 | -1.879041042 | down | 1.764358124 | 3.643399165 | TACTAAATCTTT | hsa_circ_0069040 | chr4 | 4650703 | 4650836 | + | 133 | ANNOTATED, INTERN | NR_037888 | | Salzman2013 | | circRNA | Not Detected | Detected | 1.764358124 | 3.643399165 |
| hsa_gci166476 | 2.273974429 | 1.185216031 | up | 4.713815027 | 3.528598996 | TCCACCTCTCAA | hsa_circ_0069103 | chr4 | 6925099 | 7000929 | + | 1180 | ANNOTATED, CDS, c | NM_020773 | TBC1D14 | Salzman2013 | 57533 | circRNA | Detected | Detected | 4.713815027 | 3.528598996 |
| hsa_gci166477 | 2.182616576 | 1.126058712 | up | 6.524843823 | 5.398785111 | ATCTCCACCTCT | hsa_circ_0069104 | chr4 | 6925099 | 7002978 | + | 1287 | ANNOTATED, CDS, c | NM_020773 | TBC1D14 | Salzman2013 | 57533 | circRNA | Detected | Detected | 6.524843823 | 5.398785111 |
| hsa_gci166487 | 2.375475352 | 1.248216237 | up | 5.523755715 | 4.275539477 | CCTCAAGTTCTT | hsa_circ_0069140 | chr4 | 7811340 | 7873807 | - | 1056 | ANNOTATED, CDS, c | NM_001134647 | AFAP1 | Salzman2013 | 60312 | circRNA | Detected | Detected | 5.523755715 | 4.275539477 |

| | | | | | | | | | | | | | | | | | | | | | | |
|---|---|---|---|---|---|---|---|---|---|---|---|---|---|---|---|---|---|---|---|---|---|---|
| hsa_gci166492 | 2.863547639 | 1.517803605 | up | 4.568568326 | 3.050764721 | AAGTTCTTTCTC | hsa_circ_0069152 | chr4 | 7870348 | 7873807 | - | 227 | ANNOTATED, CDS, c | NM_001134647 | AFAP1 | Salzman2013 | 60312 | circRNA | Detected | Not Detected | 4.568568326 | 3.050764721 |
| hsa_gci166496 | 2.566551475 | 1.359831196 | up | 5.90423175 | 4.544400555 | AGTCGTTCCGCG | hsa_circ_0069212 | chr4 | 10441503 | 10456612 | - | 6844 | ANNOTATED, CDS, c | NM_053042 | ZNF518B | Salzman2013 | 85460 | circRNA | Detected | Detected | 5.90423175 | 4.544400555 |
| hsa_gci166498 | 2.441851529 | 1.287975483 | up | 5.985611972 | 4.697636489 | GGGGCGGGGGAT | hsa_circ_0069220 | chr4 | 13369346 | 13485989 | - | 1811 | ANNOTATED, CDS, c | NM_004249 | RAB28 | Salzman2013 | 9364 | circRNA | Detected | Detected | 5.985611972 | 4.697636489 |
| hsa_gci166502 | 6.488541669 | 2.697894262 | up | 4.314886985 | 1.616992723 | GTAGGTCCGTGT | hsa_circ_0069239 | chr4 | 16204084 | 16204203 | - | 119 | ANNOTATED, CDS, c | NM_153365 | TAPT1 | Salzman2013 | 202018 | circRNA | Detected | Not Detected | 4.314886985 | 1.616992723 |
| hsa_gci166514 | 2.638172088 | 1.399538674 | up | 5.522918942 | 4.123380267 | TTTTGTGATGTT | hsa_circ_0069296 | chr4 | 20493383 | 20521076 | + | 355 | ANNOTATED, CDS, c | NM_004787 | SLIT2 | Salzman2013 | 9353 | circRNA | Detected | Detected | 5.522918942 | 4.123380267 |
| hsa_gci166520 | 2.158866214 | 1.110273841 | up | 5.534386335 | 4.424112494 | CGGCGGACCCAC | hsa_circ_0069317 | chr4 | 22388998 | 22404422 | - | 2063 | ANNOTATED, CDS, c | NM_145290 | ADGRA3 | Salzman2013 | 166647 | circRNA | Detected | Detected | 5.534386335 | 4.424112494 |
| hsa_gci166531 | 2.005086109 | 1.003664195 | up | 6.920250594 | 5.916586399 | CCTCCACGTCGT | hsa_circ_0069384 | chr4 | 26622234 | 26737109 | + | 899 | ANNOTATED, CDS, c | NM_018317 | TBC1D19 | Salzman2013 | 55296 | circRNA | Detected | Detected | 6.920250594 | 5.916586399 |
| hsa_gci166539 | 2.54418001 | 1.34720075 | up | 6.563343412 | 5.216142662 | TTTCGTTTTCTG | hsa_circ_0069411 | chr4 | 37847253 | 37857362 | + | 827 | ANNOTATED, CDS, c | NM_018290 | PGM2 | Salzman2013 | 55276 | circRNA | Detected | Detected | 6.563343412 | 5.216142662 |
| hsa_gci166540 | 2.122712846 | 1.085909221 | up | 5.600446804 | 4.514537583 | GAGTAAAGAGCC | hsa_circ_0069421 | chr4 | 38037208 | 38104778 | + | 1255 | ANNOTATED, CDS, c | NM_015173 | TBC1D1 | Salzman2013 | 23216 | circRNA | Detected | Detected | 5.600446804 | 4.514537583 |
| hsa_gci166550 | 3.595319874 | 1.846120132 | up | 5.005000254 | 3.158880122 | TTAACGGTCAGA | hsa_circ_0069454 | chr4 | 39226503 | 39233892 | + | 774 | ANNOTATED, CDS, c | NM_025132 | WDR19 | Salzman2013 | 57728 | circRNA | Detected | Not Detected | 5.005000254 | 3.158880122 |
| hsa_gci166565 | 20.90963424 | 4.386095921 | up | 5.625482812 | 1.239386891 | ACGTTCCAGTTT | hsa_circ_0069532 | chr4 | 39915230 | 39922019 | - | 338 | ANNOTATED, CDS, c | NM_001100399 | PDS5A | Salzman2013 | 23244 | circRNA | Detected | Not Detected | 5.625482812 | 1.239386891 |
| hsa_gci166569 | 2.309841586 | 1.207793912 | up | 6.035612955 | 4.827819043 | AAAGTCTCTATT | hsa_circ_0069546 | chr4 | 40121551 | 40144481 | + | 3154 | ANNOTATED, CDS, c | NM_018177 | N4BP2 | Salzman2013 | 55728 | circRNA | Detected | Detected | 6.035612955 | 4.827819043 |
| hsa_gci166613 | 2.119043227 | 1.083413018 | up | 5.841969205 | 4.758556187 | TTGGGTATTAAT | hsa_circ_0069745 | chr4 | 54257175 | 54294350 | + | 669 | ANNOTATED, CDS, c | NM_030917 | FIP1L1 | Salzman2013 | 81608 | circRNA | Detected | Detected | 5.841969205 | 4.758556187 |
| hsa_gci166615 | 2.361568933 | 1.239745648 | up | 6.358285931 | 5.118540283 | TATCAATATCAG | hsa_circ_0069748 | chr4 | 54292038 | 54294350 | + | 251 | ANNOTATED, CDS, c | NM_030917 | FIP1L1 | Salzman2013 | 81608 | circRNA | Detected | Detected | 6.358285931 | 5.118540283 |
| hsa_gci166647 | 3.269032316 | 1.708863639 | up | 5.782800761 | 4.073937122 | AACCGTAAAAAG | hsa_circ_0069900 | chr4 | 68539414 | 68544251 | - | 288 | ANNOTATED, CDS, c | NM_018227 | UBA6 | Salzman2013 | 55236 | circRNA | Detected | Detected | 5.782800761 | 4.073937122 |
| hsa_gci166673 | 2.06347892 | 1.0450787 | up | 5.272204332 | 4.227125631 | ACTAATATGACG | hsa_circ_0070025 | chr4 | 76888429 | 76903190 | - | 955 | ANNOTATED, CDS, c | NM_018115 | SDAD1 | Salzman2013 | 55153 | circRNA | Detected | Detected | 5.272204332 | 4.227125631 |
| hsa_gci166716 | -4.064203962 | -2.022972805 | down | 1.39736491 | 3.420337716 | AATTATACATAG | hsa_circ_0070251 | chr4 | 83793096 | 83820890 | - | 9433 | ALT_ACCEPTOR, CDS | NM_014933 | SEC31A | Salzman2013 | 22872 | circRNA | Not Detected | Detected | 1.39736491 | 3.420337716 |
| hsa_gci166718 | 2.349770195 | 1.23251967 | up | 6.743610072 | 5.511090403 | TGACACTTTCTT | hsa_circ_0070257 | chr4 | 83867410 | 83891622 | - | 360 | ANNOTATED, CDS, c | NM_194282 | LIN54 | Salzman2013 | 132660 | circRNA | Detected | Detected | 6.743610072 | 5.511090403 |
| hsa_gci166727 | 2.194224738 | 1.133711298 | up | 9.113451939 | 7.979740641 | TTGTCCCCCTCA | hsa_circ_0070292 | chr4 | 85617122 | 85724620 | - | 6472 | ANNOTATED, CDS, c | NM_014991 | WDFY3 | Salzman2013 | 23001 | circRNA | Detected | Detected | 9.113451939 | 7.979740641 |
| hsa_gci166733 | 2.254513418 | 1.172816096 | up | 10.97188816 | 9.799072063 | TCCCATTTGTCC | hsa_circ_0070327 | chr4 | 85662913 | 85724620 | - | 3805 | ANNOTATED, CDS, c | NM_014991 | WDFY3 | Salzman2013 | 23001 | circRNA | Detected | Detected | 10.97188816 | 9.799072063 |
| hsa_gci166734 | 2.147297304 | 1.102521954 | up | 10.57616151 | 9.473639552 | CCCATTTGTCCC | hsa_circ_0070332 | chr4 | 85693987 | 85724620 | - | 2420 | ANNOTATED, CDS, c | NM_014991 | WDFY3 | Salzman2013 | 23001 | circRNA | Detected | Detected | 10.57616151 | 9.473639552 |
| hsa_gci166740 | 3.570048602 | 1.835943715 | up | 5.473908532 | 3.637964817 | GTCCCGGAGTCG | hsa_circ_0070351 | chr4 | 87556404 | 87593696 | + | 299 | ANNOTATED, CDS, c | NM_080685 | PTPN13 | Salzman2013 | 5783 | circRNA | Detected | Detected | 5.473908532 | 3.637964817 |
| hsa_gci166757 | -2.647482152 | -1.404620958 | down | 2.447104159 | 3.851725117 | TTCAGAAAACTC | hsa_circ_0070420 | chr4 | 89396991 | 89397181 | + | 190 | ANNOTATED, CDS, c | NM_016323 | HERC5 | Salzman2013 | 51191 | circRNA | Not Detected | Detected | 2.447104159 | 3.851725117 |
| hsa_gci166764 | -4.353931428 | -2.122318686 | down | 1.280328342 | 3.402647028 | CCGAACCCACGT | hsa_circ_0070467 | chr4 | 95494501 | 95507595 | + | 672 | ANNOTATED, CDS, c | NM_006457 | PDLIM5 | Salzman2013 | 10611 | circRNA | Not Detected | Detected | 1.280328342 | 3.402647028 |
| hsa_gci166788 | 2.007433626 | 1.005352287 | up | 8.046966149 | 7.041613861 | ATCCTTACGGTA | hsa_circ_0070568 | chr4 | 106367539 | 106374799 | - | 219 | ANNOTATED, CDS, c | NM_176869 | PPA2 | Salzman2013 | 27068 | circRNA | Detected | Detected | 8.046966149 | 7.041613861 |
| hsa_gci166820 | -8.319737424 | -3.056537997 | down | 1.293706403 | 4.3502444 | AACTTTGGTGGG | hsa_circ_0070679 | chr4 | 113483526 | 113484430 | - | 259 | ANNOTATED, CDS, c | NM_018392 | ZGRF1 | Salzman2013 | 55345 | circRNA | Not Detected | Detected | 1.293706403 | 4.3502444 |
| hsa_gci166835 | 2.920655742 | 1.546292318 | up | 5.977857056 | 4.431564738 | GCATTACACGTC | hsa_circ_0070707 | chr4 | 114161640 | 114209642 | + | 1584 | ANNOTATED, CDS, c | NM_001148 | ANK2 | Salzman2013 | 287 | circRNA | Detected | Detected | 5.977857056 | 4.431564738 |
| hsa_gci166839 | 3.112469741 | 1.638059812 | up | 7.10039933 | 5.462339519 | AGTTTGAGTAAA | hsa_circ_0070733 | chr4 | 119237336 | 119259469 | - | 790 | ANNOTATED, CDS, c | NM_003619 | PRSS12 | Salzman2013 | 8492 | circRNA | Detected | Detected | 7.10039933 | 5.462339519 |
| hsa_gci166841 | 2.983027182 | 1.576777124 | up | 4.583861385 | 3.007084261 | GTCCCGTTCCAG | hsa_circ_0070741 | chr4 | 119539150 | 119549381 | + | 9488 | ALT_ACCEPTOR, ncR | TCONS_12_00020705 | | Salzman2013 | | circRNA | Detected | Not Detected | 4.583861385 | 3.007084261 |
| hsa_gci166842 | -2.5102569 | -1.327835017 | down | 2.049653536 | 3.377488553 | AACCTGTTGTCT | hsa_circ_0070743 | chr4 | 119549190 | 119549381 | + | 191 | ANNOTATED, INTERN | TCONS_12_00020705 | | Salzman2013 | | circRNA | Not Detected | Detected | 2.049653536 | 3.377488553 |
| hsa_gci166843 | -2.012386304 | -1.008907276 | down | 4.245607538 | 5.254514814 | ACTATCGTGATG | hsa_circ_0070744 | chr4 | 119549190 | 119551501 | + | 249 | ANNOTATED, INTERN | TCONS_12_00020705 | | Salzman2013 | | circRNA | Detected | Detected | 4.245607538 | 5.254514814 |
| hsa_gci166870 | 3.479910405 | 1.799050163 | up | 5.892599053 | 4.093548891 | ATCTCCCGAACC | hsa_circ_0070864 | chr4 | 123200929 | 123236843 | + | 1948 | ANNOTATED, CDS, c | NM_015312 | KIAA1109 | Salzman2013 | 84162 | circRNA | Detected | Detected | 5.892599053 | 4.093548891 |
| hsa_gci166881 | 2.423430098 | 1.277050469 | up | 8.357794533 | 7.080744064 | AAGATAGTACAA | hsa_circ_0070907 | chr4 | 128605563 | 128609022 | + | 268 | ANNOTATED, CDS, c | NM_015693 | INTU | Salzman2013 | 27152 | circRNA | Detected | Detected | 8.357794533 | 7.080744064 |
| hsa_gci166883 | 2.126403931 | 1.088415677 | up | 7.026685349 | 5.938269672 | TTCTATGTTGTA | hsa_circ_0070916 | chr4 | 128806862 | 128815036 | + | 2225 | ANNOTATED, CDS, c | NM_014264 | PLK4 | Salzman2013 | 10733 | circRNA | Detected | Detected | 7.026685349 | 5.938269672 |
| hsa_gci166912 | 2.500915665 | 1.322456408 | up | 6.804030447 | 5.481574039 | TTTCAGACACAG | hsa_circ_0071062 | chr4 | 146054801 | 146062791 | - | 5303 | ANNOTATED, CDS, c | NM_001102653 | OTUD4 | Salzman2013 | 54726 | circRNA | Detected | Detected | 6.804030447 | 5.481574039 |
| hsa_gci166920 | 2.62007026 | 1.3896055 | up | 4.655706211 | 3.266100711 | AGAAAGAGAGTT | hsa_circ_0071090 | chr4 | 147177977 | 147215247 | - | 2304 | ALT_DONOR, CDS, c | NM_001029998 | SLC10A7 | Salzman2013 | 84068 | circRNA | Detected | Detected | 4.655706211 | 3.266100711 |
| hsa_gci166936 | 4.496292948 | 2.168736035 | up | 4.779964993 | 2.611228958 | ATTTTCGTTCCG | hsa_circ_0071168 | chr4 | 151509199 | 151682999 | - | 783 | ANNOTATED, CDS, c | NM_006726 | LRBA | Salzman2013 | 987 | circRNA | Detected | Not Detected | 4.779964993 | 2.611228958 |
| hsa_gci166973 | 2.438672699 | 1.286096143 | up | 9.219968509 | 7.933872366 | TTCTAGAGAACT | hsa_circ_0071261 | chr4 | 152095821 | 152108615 | - | 1403 | ANNOTATED, CDS, c | NM_001009555 | SH3D19 | Salzman2013 | 152503 | circRNA | Detected | Detected | 9.219968509 | 7.933872366 |
| hsa_gci166991 | 2.550702689 | 1.350894748 | up | 7.417911043 | 6.067016295 | CAATCTTCAAGA | hsa_circ_0071351 | chr4 | 160253602 | 160264556 | + | 1366 | ANNOTATED, CDS, c | NM_014247 | RAPGEF2 | Salzman2013 | 9693 | circRNA | Detected | Detected | 7.417911043 | 6.067016295 |
| hsa_gci166995 | 2.189184875 | 1.130393794 | up | 7.974342836 | 6.843949042 | GCAGAAGTTATG | hsa_circ_0071369 | chr4 | 166006737 | 166024248 | - | 650 | ANNOTATED, CDS, c | NM_001100389 | TMEM192 | Salzman2013 | 201931 | circRNA | Detected | Detected | 7.974342836 | 6.843949042 |
| hsa_gci166997 | 2.1498699 | 1.104249358 | up | 8.43852176 | 7.334272403 | TCTTCACCCAGC | hsa_circ_0071371 | chr4 | 166024101 | 166024248 | - | 147 | ANNOTATED, CDS, c | NM_001100389 | TMEM192 | Salzman2013 | 201931 | circRNA | Detected | Detected | 8.43852176 | 7.334272403 |
| hsa_gci167048 | 2.365025232 | 1.241855576 | up | 4.100602843 | 2.858747267 | AGAAGAGAACCA | hsa_circ_0071654 | chr5 | 619104 | 624694 | + | 430 | ANNOTATED, CDS, c | NM_018140 | CEP72 | Salzman2013 | 55722 | circRNA | Detected | Not Detected | 4.100602843 | 2.858747267 |
| hsa_gci167081 | 2.533518919 | 1.341142602 | up | 8.432625345 | 7.091482743 | GGCTGCACCGCG | hsa_circ_0071871 | chr5 | 10679341 | 10761387 | - | 2342 | ANNOTATED, CDS, c | NM_004394 | DAP | Salzman2013 | 1611 | circRNA | Detected | Detected | 8.432625345 | 7.091482743 |
| hsa_gci167103 | 2.284087162 | 1.191617706 | up | 5.40649171 | 4.214874004 | AGGGACGACAGA | hsa_circ_0072004 | chr5 | 16754936 | 16764505 | - | 750 | ANNOTATED, CDS, c | NM_012334 | MYO10 | Salzman2013 | 4651 | circRNA | Detected | Detected | 5.40649171 | 4.214874004 |
| hsa_gci167123 | 2.277514507 | 1.187460244 | up | 6.430856991 | 5.243396747 | ATTGGACGAAAC | hsa_circ_0072098 | chr5 | 32415073 | 32415292 | - | 219 | ANNOTATED, CDS, c | NM_016107 | ZFR | Salzman2013 | 51663 | circRNA | Detected | Detected | 6.430856991 | 5.243396747 |
| hsa_gci167134 | 2.184887752 | 1.127559163 | up | 5.84266796 | 4.715108796 | AGGGTAAAGGGT | hsa_circ_0072160 | chr5 | 36953719 | 36961685 | + | 537 | ANNOTATED, CDS, c | NM_015384 | NIPBL | Salzman2013 | 25836 | circRNA | Detected | Detected | 5.84266796 | 4.715108796 |
| hsa_gci167150 | 2.603277056 | 1.38032886 | up | 4.251946793 | 2.871617933 | GTCAACCGTCCC | hsa_circ_0072251 | chr5 | 37318089 | 37324209 | - | 214 | ANNOTATED, CDS, c | NM_153485 | NUP155 | Salzman2013 | 9631 | circRNA | Detected | Not Detected | 4.251946793 | 2.871617933 |
| hsa_gci167194 | 2.412412951 | 1.270476886 | up | 4.628956644 | 3.358479758 | AACATTTTTAGT | hsa_circ_0072387 | chr5 | 43294157 | 43297268 | - | 609 | ANNOTATED, CDS, c | NM_001098272 | HMGCS1 | Salzman2013 | 3157 | circRNA | Detected | Detected | 4.628956644 | 3.358479758 |
| hsa_gci167195 | -2.883695226 | -1.527918696 | down | 3.337803789 | 4.865722485 | TAAGTTTCCTTC | hsa_circ_0072389 | chr5 | 43294157 | 43299077 | - | 1193 | ANNOTATED, CDS, c | NM_001098272 | HMGCS1 | Salzman2013 | 3157 | circRNA | Not Detected | Detected | 3.337803789 | 4.865722485 |
| hsa_gci167246 | 3.492321581 | 1.804186412 | up | 5.871702549 | 4.067516137 | CCTGAGGCAGGG | hsa_circ_0072564 | chr5 | 58289198 | 58511794 | - | 560 | ANNOTATED, CDS, c | NM_001197220 | PDE4D | Salzman2013 | 5144 | circRNA | Detected | Detected | 5.871702549 | 4.067516137 |
| hsa_gci167282 | 2.197631051 | 1.1359492 | up | 4.047789157 | 2.911839957 | AAGGTTAAGGTA | hsa_circ_0072658 | chr5 | 64466443 | 64595958 | - | 2021 | ANNOTATED, CDS, c | NM_197941 | ADAMTS6 | Salzman2013 | 11174 | circRNA | Detected | Not Detected | 4.047789157 | 2.911839957 |

| | | | | | | | | | | | | | | | | | | | | | | |
|---|---|---|---|---|---|---|---|---|---|---|---|---|---|---|---|---|---|---|---|---|---|---|
| hsa_gci167290 | 2.067425034 | 1.047835018 | up | 9.458629636 | 8.410794618 | ATTTCATACCGG | hsa_circ_0072684 | chr5 | 64587155 | 64769779 | - | 1791 | ANNOTATED, CDS, c | NM_197941 | ADAMTS6 | Salzman2013 | 11174 | circRNA | Detected | Detected | 9.458629636 | 8.410794618 |
| hsa_gci167293 | 2.082789628 | 1.058517127 | up | 5.060665703 | 4.002148576 | GTTCTATTCAGT | hsa_circ_0072687 | chr5 | 64747301 | 64766969 | - | 976 | ANNOTATED, CDS, c | NM_197941 | ADAMTS6 | Salzman2013 | 11174 | circRNA | Detected | Detected | 5.060665703 | 4.002148576 |
| hsa_gci167325 | 2.065987705 | 1.046831669 | up | 6.295688278 | 5.248856609 | GTGTTATTAGTA | hsa_circ_0072793 | chr5 | 68809774 | 68830666 | + | 308 | ANNOTATED, CDS, c | NM_002538 | OCLN | Salzman2013 | 100506658 | circRNA | Detected | Detected | 6.295688278 | 5.248856609 |
| hsa_gci167341 | 2.166851409 | 1.115600224 | up | 4.952187527 | 3.836587303 | GAGAAGGGCTCT | hsa_circ_0072909 | chr5 | 71521883 | 71591416 | - | 615 | ANNOTATED, CDS, c | NM_015084 | MRPS27 | Salzman2013 | 23107 | circRNA | Detected | Detected | 4.952187527 | 3.836587303 |
| hsa_gci167349 | -4.69361761 | -2.23070031 | down | 1.33350095 | 3.564201259 | CGTCGTCGTCTT | hsa_circ_0072933 | chr5 | 72182896 | 72189073 | + | 746 | ANNOTATED, CDS, c | NM_002270 | TNPO1 | Salzman2013 | 3842 | circRNA | Not Detected | Detected | 1.33350095 | 3.564201259 |
| hsa_gci167364 | 2.527088191 | 1.337476013 | up | 8.26202251 | 6.924546498 | CCGACTGTCCCG | hsa_circ_0072973 | chr5 | 72370568 | 72374139 | + | 401 | ANNOTATED, CDS, c | NM_138782 | FCHO2 | Salzman2013 | 115548 | circRNA | Detected | Detected | 8.26202251 | 6.924546498 |
| hsa_gci167366 | 2.721600861 | 1.444455503 | up | 9.498830879 | 8.054375377 | CTAAAGGTTCCG | hsa_circ_0072977 | chr5 | 72370568 | 72386349 | + | 3286 | ANNOTATED, CDS, c | NM_138782 | FCHO2 | Salzman2013 | 115548 | circRNA | Detected | Detected | 9.498830879 | 8.054375377 |
| hsa_gci167427 | 3.365401238 | 1.75077852 | up | 5.840734118 | 4.089955598 | CCTAAGTCCTAC | hsa_circ_0073255 | chr5 | 86679529 | 86682720 | + | 235 | ANNOTATED, CDS, c | NM_002890 | RASA1 | Salzman2013 | 5921 | circRNA | Detected | Detected | 5.840734118 | 4.089955598 |
| hsa_gci167428 | 3.115080435 | 1.639269416 | up | 4.993739729 | 3.354470314 | GGTTATACCCTA | hsa_circ_0073259 | chr5 | 86700660 | 86700824 | - | 164 | ANNOTATED, CDS, c | NM_001199189 | CCNH | Salzman2013 | 902 | circRNA | Detected | Detected | 4.993739729 | 3.354470314 |
| hsa_gci167429 | 3.138385463 | 1.650022558 | up | 5.883447575 | 4.233425016 | TCCCGCGGGTGA | hsa_circ_0073261 | chr5 | 86705106 | 86708850 | - | 553 | ANNOTATED, CDS, c | NM_001239 | CCNH | Salzman2013 | 902 | circRNA | Detected | Detected | 5.883447575 | 4.233425016 |
| hsa_gci167439 | 2.318362973 | 1.213106458 | up | 8.504397826 | 7.291291367 | GCAGAGTCACCA | hsa_circ_0073358 | chr5 | 90664540 | 90679149 | - | 4161 | ANNOTATED, CDS, c | NM_020801 | ARRDC3 | Salzman2013 | 57561 | circRNA | Detected | Detected | 8.504397826 | 7.291291367 |
| hsa_gci167523 | 2.17985937 | 1.124235065 | up | 4.292660761 | 3.168425696 | TCGAACTAGACC | hsa_circ_0073747 | chr5 | 126140467 | 126161799 | + | 1252 | ANNOTATED, CDS, c | NM_005573 | LMNB1 | Salzman2013 | 4001 | circRNA | Detected | Not Detected | 4.292660761 | 3.168425696 |
| hsa_gci167528 | 2.063311652 | 1.044961749 | up | 6.206148268 | 5.161186519 | ACTCACTTTGTG | hsa_circ_0073804 | chr5 | 127782173 | 127800614 | - | 324 | ANNOTATED, CDS, c | NM_001999 | FBN2 | Salzman2013 | 2201 | circRNA | Detected | Detected | 6.206148268 | 5.161186519 |
| hsa_gci167539 | 3.026099367 | 1.597459362 | up | 7.60549924 | 6.008039878 | GCGGACCCCGAC | hsa_circ_0073845 | chr5 | 130977406 | 131046354 | - | 5850 | ANNOTATED, CDS, c | NM_133372 | FNIP1 | Salzman2013 | 96459 | circRNA | Detected | Detected | 7.60549924 | 6.008039878 |
| hsa_gci167540 | 2.036361534 | 1.025993719 | up | 7.998960003 | 6.972966284 | TGTGAAACGACT | hsa_circ_0073847 | chr5 | 130977406 | 131054753 | - | 6017 | ANNOTATED, CDS, c | NM_133372 | FNIP1 | Salzman2013 | 96459 | circRNA | Detected | Detected | 7.998960003 | 6.972966284 |
| hsa_gci167551 | 3.263968106 | 1.70662696 | up | 4.342852042 | 2.636225082 | ACGACGGGTGGT | hsa_circ_0073884 | chr5 | 132091696 | 132097588 | - | 3410 | ANNOTATED, CDS, c | NM_015146 | SEPT8 | Salzman2013 | 23176 | circRNA | Detected | Not Detected | 4.342852042 | 2.636225082 |
| hsa_gci167651 | 2.73041873 | 1.449122216 | up | 6.443309848 | 4.994187632 | TCTACCTTATCC | hsa_circ_0074275 | chr5 | 139916922 | 139917819 | + | 401 | ALT_ACCEPTOR, ALT | NM_020690 | ANKHD1-EIF4EB | Salzman2013 | 404734 | circRNA | Detected | Detected | 6.443309848 | 4.994187632 |
| hsa_gci167676 | 2.233673007 | 1.159418002 | up | 4.429788983 | 3.270370982 | TCGTTTGGGAAT | hsa_circ_0074380 | chr5 | 142779220 | 142815077 | - | 2178 | ANNOTATED, CDS, c | NM_001018077 | NR3C1 | Salzman2013 | 2908 | circRNA | Detected | Not Detected | 4.429788983 | 3.270370982 |
| hsa_gci167680 | 3.160138238 | 1.659987669 | up | 9.442437404 | 7.782449735 | TCTACTGTAAAA | hsa_circ_0074389 | chr5 | 145197456 | 145202697 | - | 329 | ANNOTATED, CDS, c | NM_182960 | PRELID2 | Salzman2013 | 153768 | circRNA | Detected | Detected | 9.442437404 | 7.782449735 |
| hsa_gci167681 | 3.47152046 | 1.795567674 | up | 7.300336434 | 5.50476876 | CCTCCTCTACAG | hsa_circ_0074401 | chr5 | 145515163 | 145519824 | - | 122 | ANNOTATED, CDS, c | NM_020117 | LARS | Salzman2013 | 51520 | circRNA | Detected | Detected | 7.300336434 | 5.50476876 |
| hsa_gci167709 | 2.664054284 | 1.41362348 | up | 5.662586966 | 4.248963486 | TCGAGAAACCCT | hsa_circ_0074559 | chr5 | 150088308 | 150095224 | - | 2714 | ANNOTATED, CDS, c | NM_001135644 | DCTN4 | Salzman2013 | 51164 | circRNA | Detected | Detected | 5.662586966 | 4.248963486 |
| hsa_gci167751 | 2.070637113 | 1.050074738 | up | 8.028129918 | 6.97805518 | TCACTCCCTCCA | hsa_circ_0074834 | chr5 | 159437506 | 159492550 | + | 1386 | ANNOTATED, CDS, c | NM_003314 | TTC1 | Salzman2013 | 7265 | circRNA | Detected | Detected | 8.028129918 | 6.97805518 |
| hsa_gci167755 | 2.389264754 | 1.256566727 | up | 10.36143255 | 9.10486582 | CGCGTTAGACCA | hsa_circ_0074845 | chr5 | 159854721 | 159855746 | + | 297 | ANNOTATED, CDS, c | NM_004219 | PTTG1 | Salzman2013 | 9232 | circRNA | Detected | Detected | 10.36143255 | 9.10486582 |
| hsa_gci167803 | -4.178919783 | -2.063130065 | down | 1.247990483 | 3.311120549 | AGGAACCAGACC | hsa_circ_0075092 | chr5 | 175716656 | 175717958 | + | 1302 | ALT_ACCEPTOR, ALT | NM_198567 | SIMC1 | Salzman2013 | 375484 | circRNA | Not Detected | Detected | 1.247990483 | 3.311120549 |
| hsa_gci167826 | 2.05244572 | 1.037344068 | up | 7.04016827 | 6.002824202 | TAACAAGTGGTA | hsa_circ_0075177 | chr5 | 176758562 | 176764525 | - | 1082 | ANNOTATED, CDS, c | NM_006816 | LMAN2 | Salzman2013 | 10960 | circRNA | Detected | Detected | 7.04016827 | 6.002824202 |
| hsa_gci167831 | 2.006892589 | 1.004963404 | up | 6.787435134 | 5.78247173 | TGTATTGCCCCA | hsa_circ_0075225 | chr5 | 176939496 | 176942822 | - | 1115 | ANNOTATED, CDS, c | NM_016222 | DDX41 | Salzman2013 | 51428 | circRNA | Detected | Detected | 6.787435134 | 5.78247173 |
| hsa_gci167833 | 2.299542368 | 1.201346779 | up | 7.545075306 | 6.343728527 | CGTGCGGGTCGA | hsa_circ_0075238 | chr5 | 177019212 | 177023099 | + | 1393 | ANNOTATED, CDS, c | NM_017510 | TMED9 | Salzman2013 | 54732 | circRNA | Detected | Detected | 7.545075306 | 6.343728527 |
| hsa_gci167847 | 7.202232355 | 2.848444144 | up | 4.050068263 | 1.20162412 | CCACCCGTTCAA | hsa_circ_0075343 | chr5 | 179665331 | 179707608 | - | 1179 | ANNOTATED, CDS, c | NM_139069 | MAPK9 | Salzman2013 | 5601 | circRNA | Detected | Not Detected | 4.050068263 | 1.20162412 |
| hsa_gci167859 | 2.484501119 | 1.312956192 | up | 6.034935475 | 4.721979284 | AGGTCGGACCCG | hsa_circ_0075399 | chr5 | 180666872 | 180667345 | - | 473 | ALT_ACCEPTOR, ALT | NM_006098 | RACK1 | Salzman2013 | 10399 | circRNA | Detected | Detected | 6.034935475 | 4.721979284 |
| hsa_gci167869 | 2.985743252 | 1.578090112 | up | 6.136360215 | 4.558270104 | CCACTAGGACAA | hsa_circ_0075445 | chr6 | 629834 | 637861 | - | 465 | ANNOTATED, CDS, c | NM_018303 | EXOC2 | Salzman2013 | 55770 | circRNA | Detected | Detected | 6.136360215 | 4.558270104 |
| hsa_gci167871 | 2.589815586 | 1.372849371 | up | 5.885494187 | 4.512644816 | ATGACGAAATAG | hsa_circ_0075457 | chr6 | 2779496 | 2784637 | + | 466 | ANNOTATED, CDS, c | NM_020135 | WRNIP1 | Salzman2013 | 56897 | circRNA | Detected | Detected | 5.885494187 | 4.512644816 |
| hsa_gci167875 | 2.528178909 | 1.338098561 | up | 6.91233618 | 5.574237619 | ACTTGGTCATGT | hsa_circ_0075493 | chr6 | 3015763 | 3019994 | + | 498 | ANNOTATED, CDS, c | NM_000904 | NQO2 | Salzman2013 | 4835 | circRNA | Detected | Detected | 6.91233618 | 5.574237619 |
| hsa_gci167948 | 2.912042254 | 1.542031289 | up | 6.063338584 | 4.521307295 | TTTAATCTACAC | hsa_circ_0075831 | chr6 | 22020567 | 22191885 | + | 678 | ANNOTATED, INTERN | NR_015410 | CASC15 | Salzman2013 | 401237 | circRNA | Detected | Detected | 6.063338584 | 4.521307295 |
| hsa_gci167951 | 2.162923332 | 1.112982528 | up | 7.860906366 | 6.747923838 | GTACTTAGAGGT | hsa_circ_0075859 | chr6 | 25426728 | 25435832 | + | 182 | ANNOTATED, CDS, c | NM_017640 | CARMIL1 | Salzman2013 | 55604 | circRNA | Detected | Detected | 7.860906366 | 6.747923838 |
| hsa_gci167958 | 3.34162882 | 1.740551491 | up | 5.094486118 | 3.353934627 | GTGTCTGGGATT | hsa_circ_0075932 | chr6 | 31473919 | 31478901 | + | 4982 | INTERGENIC | None | | Salzman2013 | | circRNA | Detected | Detected | 5.094486118 | 3.353934627 |
| hsa_gci167964 | -2.430797837 | -1.281429913 | down | 2.55790215 | 3.839332063 | AACAGACCCCTT | hsa_circ_0076054 | chr6 | 34789429 | 34789732 | + | 202 | ANNOTATED, CDS, c | NM_017754 | UHRF1BP1 | Salzman2013 | 54887 | circRNA | Not Detected | Detected | 2.55790215 | 3.839332063 |
| hsa_gci167972 | 2.066825262 | 1.047416422 | up | 4.80708855 | 3.759672127 | TCCAGACTCTCG | hsa_circ_0076134 | chr6 | 35426094 | 35434881 | + | 1380 | ALT_ACCEPTOR, CDS | NM_021922 | FANCE | Salzman2013 | 2178 | circRNA | Detected | Detected | 4.80708855 | 3.759672127 |
| hsa_gci167987 | 4.898655126 | 2.292385727 | up | 5.716866139 | 3.424480412 | TGTAGGGGTCGG | hsa_circ_0076194 | chr6 | 36651873 | 36655116 | + | 2039 | ANNOTATED, CDS, c | NM_001220778 | CDKN1A | Salzman2013 | 1026 | circRNA | Detected | Detected | 5.716866139 | 3.424480412 |
| hsa_gci167989 | -2.048752438 | -1.034745666 | down | 2.856054997 | 3.890800663 | GACTCGCCGACT | hsa_circ_0076198 | chr6 | 36862279 | 36870210 | + | 5344 | ALT_ACCEPTOR, CDS | NM_152734 | C6orf89 | Salzman2013 | 221477 | circRNA | Not Detected | Detected | 2.856054997 | 3.890800663 |
| hsa_gci167990 | 2.006121508 | 1.00440899 | up | 4.404979602 | 3.400570612 | GGTACAGAAGGT | hsa_circ_0076200 | chr6 | 36867201 | 36882469 | + | 714 | ANNOTATED, CDS, c | NM_152734 | C6orf89 | Salzman2013 | 221477 | circRNA | Detected | Detected | 4.404979602 | 3.400570612 |
| hsa_gci167992 | 2.103963562 | 1.073109719 | up | 5.371849057 | 4.298739338 | TAGCCGAGTACT | hsa_circ_0076208 | chr6 | 36935916 | 36949448 | + | 1560 | ANNOTATED, CDS, c | NM_014341 | MTCH1 | Salzman2013 | 23787 | circRNA | Detected | Detected | 5.371849057 | 4.298739338 |
| hsa_gci168000 | 2.37795333 | 1.249720401 | up | 5.558915728 | 4.309195327 | ACCTTATCTGCT | hsa_circ_0076254 | chr6 | 38545375 | 38565897 | - | 1181 | ANNOTATED, CDS, c | NM_052893 | BTBD9 | Salzman2013 | 114781 | circRNA | Detected | Detected | 5.558915728 | 4.309195327 |
| hsa_gci168002 | 2.184775148 | 1.127484808 | up | 8.91330137 | 7.785816561 | CCGCGACCCCAA | hsa_circ_0076277 | chr6 | 39156746 | 39159531 | - | 2785 | ANNOTATED, CDS, c | NM_003740 | KCNK5 | Salzman2013 | 8645 | circRNA | Detected | Detected | 8.91330137 | 7.785816561 |
| hsa_gci168010 | 2.155719397 | 1.1081694 | up | 9.754185522 | 8.646016122 | AGAGGGTTAAAG | hsa_circ_0076324 | chr6 | 42023268 | 42023752 | + | 162 | ANNOTATED, CDS, c | NM_138572 | TAF8 | Salzman2013 | 129685 | circRNA | Detected | Detected | 9.754185522 | 8.646016122 |
| hsa_gci168046 | 2.675876652 | 1.420011614 | up | 6.888635489 | 5.468623874 | ACTGTCTAGTAG | hsa_circ_0076442 | chr6 | 42993774 | 42997337 | + | 806 | ANNOTATED, CDS, c | NM_033112 | RRP36 | Salzman2013 | 88745 | circRNA | Detected | Detected | 6.888635489 | 5.468623874 |
| hsa_gci168053 | 2.436730848 | 1.284946905 | up | 6.866922119 | 5.581975214 | GTCCTTGTGGAC | hsa_circ_0076490 | chr6 | 43146543 | 43149244 | + | 2489 | ANNOTATED, CDS, c | NM_003131 | SRF | Salzman2013 | 6722 | circRNA | Detected | Detected | 6.866922119 | 5.581975214 |
| hsa_gci168063 | 2.544303039 | 1.347270513 | up | 5.463432371 | 4.116161859 | CCTCTCCTCAAC | hsa_circ_0076560 | chr6 | 43514307 | 43516197 | - | 482 | ANNOTATED, CDS, c | NM_020750 | XPO5 | Salzman2013 | 57510 | circRNA | Detected | Detected | 5.463432371 | 4.116161859 |
| hsa_gci168066 | 2.675334298 | 1.419719175 | up | 6.878481767 | 5.458762591 | TTTTCACTGTCT | hsa_circ_0076569 | chr6 | 43517225 | 43517671 | - | 132 | ANNOTATED, CDS, c | NM_020750 | XPO5 | Salzman2013 | 57510 | circRNA | Detected | Detected | 6.878481767 | 5.458762591 |
| hsa_gci168085 | 2.381639549 | 1.251955084 | up | 4.345806667 | 3.093851583 | ACAAGACCGACT | hsa_circ_0076700 | chr6 | 47199267 | 47254331 | - | 3105 | ANNOTATED, CDS, c | NM_014452 | TNFRSF21 | Salzman2013 | 27242 | circRNA | Detected | Not Detected | 4.345806667 | 3.093851583 |
| hsa_gci168094 | 2.727063068 | 1.447348066 | up | 7.759097143 | 6.311749077 | CGTTGCCTACCC | hsa_circ_0076751 | chr6 | 52138027 | 52141996 | - | 643 | ANNOTATED, CDS, c | NM_002388 | MCM3 | Salzman2013 | 4172 | circRNA | Detected | Detected | 7.759097143 | 6.311749077 |
| hsa_gci168123 | 2.195357344 | 1.13445579 | up | 5.401253123 | 4.266797333 | CCGGTCGACCTC | hsa_circ_0076878 | chr6 | 56955608 | 56963939 | + | 165 | ANNOTATED, CDS, c | NM_001031623 | ZNF451 | Salzman2013 | 26036 | circRNA | Detected | Detected | 5.401253123 | 4.266797333 |

| | | | | | | | | | | | | | | | | | | | | | | |
|---|---|---|---|---|---|---|---|---|---|---|---|---|---|---|---|---|---|---|---|---|---|---|
| hsa_gci168132 | 2.701886234 | 1.43396693 | up | 5.23987948 | 3.805912551 | AGAAAGTAGTCT | hsa_circ_0076905 | chr6 | 57372287 | 57499035 | + | 606 | ANNOTATED, CDS, c | NM_000947 | PRIM2 | Salzman2013 | 5558 | circRNA | Detected | Detected | 5.23987948 | 3.805912551 |
| hsa_gci168175 | 3.542789914 | 1.824885917 | up | 8.735275572 | 6.910389656 | GTTTTTGGTCAC | hsa_circ_0077097 | chr6 | 76412360 | 76423297 | + | 690 | ANNOTATED, CDS, c | NM_015571 | SENP6 | Salzman2013 | 26054 | circRNA | Detected | Detected | 8.735275572 | 6.910389656 |
| hsa_gci168195 | 2.435001564 | 1.283922699 | up | 7.179099161 | 5.895176462 | TCGGGTCGCGGC | hsa_circ_0077155 | chr6 | 80340999 | 80413369 | + | 4646 | ANNOTATED, CDS, c | NM_031469 | SH3BGRL2 | Salzman2013 | 83699 | circRNA | Detected | Detected | 7.179099161 | 5.895176462 |
| hsa_gci168196 | 2.637901268 | 1.399390568 | up | 4.484608993 | 3.085218425 | AGGGGTCTCGCT | hsa_circ_0077167 | chr6 | 80878591 | 81053529 | + | 710 | ANNOTATED, CDS, c | NM_000056 | BCKDHB | Salzman2013 | 594 | circRNA | Detected | Not Detected | 4.484608993 | 3.085218425 |
| hsa_gci168202 | 2.308758825 | 1.207117476 | up | 8.096595131 | 6.889477655 | ATTTGTAACTTT | hsa_circ_0077186 | chr6 | 83667030 | 83763957 | - | 875 | ANNOTATED, CDS, c | NM_198920 | UBE3D | Salzman2013 | 90025 | circRNA | Detected | Detected | 8.096595131 | 6.889477655 |
| hsa_gci168214 | 2.243750735 | 1.165912412 | up | 4.113581883 | 2.947669471 | CGGCACACCAAG | hsa_circ_0077259 | chr6 | 87795215 | 87795551 | - | 336 | ANNOTATED, CDS, c | NM_001252383 | CGA | Salzman2013 | 1081 | circRNA | Detected | Not Detected | 4.113581883 | 2.947669471 |
| hsa_gci168219 | 103.2699993 | 6.690277391 | up | 7.907717142 | 1.217439751 | TACCAGTAGTCA | hsa_circ_0077286 | chr6 | 88362833 | 88367736 | + | 312 | ANNOTATED, CDS, c | NM_181837 | ORC3 | Salzman2013 | 23595 | circRNA | Detected | Not Detected | 7.907717142 | 1.217439751 |
| hsa_gci168250 | 2.480205312 | 1.310459552 | up | 10.63390425 | 9.323444698 | TTTTTAACACCG | hsa_circ_0077431 | chr6 | 99979238 | 99981059 | + | 1726 | ANNOTATED, CDS, c | NM_001195131 | | Salzman2013 | | circRNA | Detected | Detected | 10.63390425 | 9.323444698 |
| hsa_gci168251 | 2.577649615 | 1.366056169 | up | 9.278308521 | 7.912252352 | AGAACCTAAGTA | hsa_circ_0077433 | chr6 | 99979507 | 99981059 | + | 1552 | ANNOTATED, CDS, c | NM_001195131 | | Salzman2013 | | circRNA | Detected | Detected | 9.278308521 | 7.912252352 |
| hsa_gci168252 | 3.042109479 | 1.605072074 | up | 4.379817232 | 2.774745158 | ACGACTGGGAAC | hsa_circ_0077434 | chr6 | 99990262 | 100016690 | - | 2307 | ANNOTATED, CDS, c | NM_005190 | CCNC | Salzman2013 | 892 | circRNA | Detected | Not Detected | 4.379817232 | 2.774745158 |
| hsa_gci168275 | 2.682392495 | 1.423520352 | up | 5.078129473 | 3.654609121 | AAAGAAATTGTC | hsa_circ_0077515 | chr6 | 105259185 | 105297121 | - | 396 | ANNOTATED, CDS, c | NM_020771 | HACE1 | Salzman2013 | 57531 | circRNA | Detected | Detected | 5.078129473 | 3.654609121 |
| hsa_gci168277 | 2.966344873 | 1.568686338 | up | 8.451925485 | 6.883239147 | TTATTAAATGAG | hsa_circ_0077519 | chr6 | 105280916 | 105291173 | - | 208 | ANNOTATED, CDS, c | NM_020771 | HACE1 | Salzman2013 | 57531 | circRNA | Detected | Detected | 8.451925485 | 6.883239147 |
| hsa_gci168283 | 2.403236746 | 1.264978777 | up | 5.477293936 | 4.212315159 | TCGTTTGGTTAA | hsa_circ_0077537 | chr6 | 106740902 | 106740981 | - | 79 | ANNOTATED, CDS, c | NM_004849 | ATG5 | Salzman2013 | 9474 | circRNA | Detected | Detected | 5.477293936 | 4.212315159 |
| hsa_gci168287 | 2.108975489 | 1.076542328 | up | 7.056383077 | 5.979840749 | CCTCCTGCGGTA | hsa_circ_0077547 | chr6 | 107585389 | 107780779 | - | 10763 | ALT_DONOR, CDS, c | NM_020381 | PDSS2 | Salzman2013 | 57107 | circRNA | Detected | Detected | 7.056383077 | 5.979840749 |
| hsa_gci168317 | 2.719400294 | 1.443288531 | up | 6.774712387 | 5.331423856 | TAGTAAAAACTA | hsa_circ_0077671 | chr6 | 112460953 | 112462705 | - | 443 | ANNOTATED, CDS, c | NM_001105206 | LAMA4 | Salzman2013 | 3910 | circRNA | Detected | Detected | 6.774712387 | 5.331423856 |
| hsa_gci168318 | 3.06957227 | 1.618037637 | up | 4.467216541 | 2.849178904 | CCAACGATTATT | hsa_circ_0077687 | chr6 | 114178526 | 114184652 | + | 4291 | ANNOTATED, CDS, c | NM_002356 | MARCKS | Salzman2013 | 4082 | circRNA | Detected | Not Detected | 4.467216541 | 2.849178904 |
| hsa_gci168320 | 4.460640949 | 2.157251026 | up | 7.179223125 | 5.021972099 | TCGTTCGCCCTG | hsa_circ_0077698 | chr6 | 116747736 | 116759442 | + | 3395 | ANNOTATED, CDS, c | NM_013352 | DSE | Salzman2013 | 29940 | circRNA | Detected | Detected | 7.179223125 | 5.021972099 |
| hsa_gci168348 | 2.050044906 | 1.035655512 | up | 4.19678255 | 3.161127038 | CGGTTGCCTTGC | hsa_circ_0077799 | chr6 | 126661252 | 126669754 | + | 550 | ANNOTATED, CDS, c | NM_001012507 | CENPW | Salzman2013 | 387103 | circRNA | Detected | Not Detected | 4.19678255 | 3.161127038 |
| hsa_gci168353 | 2.723573864 | 1.445500994 | up | 4.876443406 | 3.430942412 | AGGTCCCAGCAC | hsa_circ_0077817 | chr6 | 128643183 | 128643455 | - | 272 | ANNOTATED, CDS, c | NM_001135648 | PTPRK | Salzman2013 | 5796 | circRNA | Detected | Detected | 4.876443406 | 3.430942412 |
| hsa_gci168389 | 4.685842915 | 2.228308591 | up | 6.264985773 | 4.036677182 | CTCACCCGTCTC | hsa_circ_0078037 | chr6 | 142519575 | 142525202 | + | 258 | ANNOTATED, CDS, c | NM_016485 | VTA1 | Salzman2013 | 51534 | circRNA | Detected | Detected | 6.264985773 | 4.036677182 |
| hsa_gci168400 | 2.136064019 | 1.094954886 | up | 5.144525067 | 4.049570181 | AAATCGGTGGTT | hsa_circ_0078142 | chr6 | 146215285 | 146244921 | - | 1293 | ANNOTATED, CDS, c | NM_001042683 | SHPRH | Salzman2013 | 257218 | circRNA | Detected | Detected | 5.144525067 | 4.049570181 |
| hsa_gci168406 | 2.215424701 | 1.147583292 | up | 7.531171542 | 6.38358825 | CGGTCCCGACTC | hsa_circ_0078156 | chr6 | 147527106 | 147660374 | + | 2056 | ANNOTATED, CDS, c | NM_001127715 | STXBP5 | Salzman2013 | 134957 | circRNA | Detected | Detected | 7.531171542 | 6.38358825 |
| hsa_gci168409 | 7.278624364 | 2.863665812 | up | 4.18021303 | 1.316547218 | GAATATACCTGG | hsa_circ_0078168 | chr6 | 147583425 | 147635167 | + | 727 | ANNOTATED, CDS, c | NM_001127715 | STXBP5 | Salzman2013 | 134957 | circRNA | Detected | Not Detected | 4.18021303 | 1.316547218 |
| hsa_gci168415 | 2.443216724 | 1.288781842 | up | 5.987182755 | 4.698400913 | ACTCTCTCTTGT | hsa_circ_0078201 | chr6 | 149699153 | 149700654 | + | 1501 | ANNOTATED, CDS, c | NM_015093 | TAB2 | Salzman2013 | 23118 | circRNA | Detected | Detected | 5.987182755 | 4.698400913 |
| hsa_gci168419 | 2.556428027 | 1.354129409 | up | 4.966581792 | 3.612452383 | GGAAAGGTCGAG | hsa_circ_0078221 | chr6 | 149982050 | 150005728 | - | 3711 | ANNOTATED, CDS, c | NM_004690 | LATS1 | Salzman2013 | 9113 | circRNA | Detected | Detected | 4.966581792 | 3.612452383 |
| hsa_gci168433 | 2.09408296 | 1.066318597 | up | 5.717970836 | 4.651652239 | TGAAATAGACAG | hsa_circ_0078247 | chr6 | 150092297 | 150123552 | + | 666 | ANNOTATED, CDS, c | NM_005389 | PCMT1 | Salzman2013 | 5110 | circRNA | Detected | Detected | 5.717970836 | 4.651652239 |
| hsa_gci168436 | 2.726922705 | 1.447273807 | up | 4.542998039 | 3.095724231 | AGTATTCCCCGA | hsa_circ_0078253 | chr6 | 150117550 | 150123667 | + | 418 | ANNOTATED, CDS, c | NM_001252053 | PCMT1 | Salzman2013 | 5110 | circRNA | Detected | Not Detected | 4.542998039 | 3.095724231 |
| hsa_gci168470 | 2.935002735 | 1.553361848 | up | 8.61097232 | 7.057610472 | AGGAGGCTCCGG | hsa_circ_0078407 | chr6 | 157357968 | 157473538 | + | 52122 | ALT_ACCEPTOR, ALT | NM_020732 | ARID1B | Salzman2013 | 57492 | circRNA | Detected | Detected | 8.61097232 | 7.057610472 |
| hsa_gci168471 | 2.094270262 | 1.066447632 | up | 8.635115794 | 7.568668163 | GATGGGTCCCTG | hsa_circ_0078431 | chr6 | 158049380 | 158094977 | + | 1725 | ANNOTATED, CDS, c | NM_024630 | ZDHHC14 | Salzman2013 | 79683 | circRNA | Detected | Detected | 8.635115794 | 7.568668163 |
| hsa_gci168475 | 3.805798982 | 1.928199362 | up | 4.629492889 | 2.701293527 | AATAAAGGTCCA | hsa_circ_0078437 | chr6 | 158288578 | 158331057 | + | 937 | ANNOTATED, CDS, c | NM_016224 | SNX9 | Salzman2013 | 51429 | circRNA | Detected | Not Detected | 4.629492889 | 2.701293527 |
| hsa_gci168481 | 2.323080893 | 1.216039392 | up | 6.430106738 | 5.214067346 | GGGGTCGTCTAC | hsa_circ_0078475 | chr6 | 158900782 | 158932856 | + | 8736 | ANNOTATED, CDS, c | NM_020245 | TULP4 | Salzman2013 | 56995 | circRNA | Detected | Detected | 6.430106738 | 5.214067346 |
| hsa_gci168505 | 2.018197733 | 1.01306753 | up | 6.693001108 | 5.679933578 | CTTTTAAAGGCC | hsa_circ_0078512 | chr6 | 159028275 | 159046786 | + | 536 | ANNOTATED, CDS, c | NM_020823 | TMEM181 | Salzman2013 | 57583 | circRNA | Detected | Detected | 6.693001108 | 5.679933578 |
| hsa_gci168512 | 2.559291102 | 1.355744253 | up | 4.414807223 | 3.05906297 | GGTTCCTTTCGG | hsa_circ_0078529 | chr6 | 159186772 | 159197536 | - | 2236 | ANNOTATED, CDS, c | NM_003379 | EZR | Salzman2013 | 7430 | circRNA | Detected | Not Detected | 4.414807223 | 3.05906297 |
| hsa_gci168516 | 2.311643411 | 1.208918868 | up | 8.110043687 | 6.901124819 | CCCGGGGCCTCG | hsa_circ_0078543 | chr6 | 160148151 | 160177351 | + | 2121 | ANNOTATED, CDS, c | NM_004906 | WTAP | Salzman2013 | 9589 | circRNA | Detected | Detected | 8.110043687 | 6.901124819 |
| hsa_gci168547 | 2.08567519 | 1.060514499 | up | 6.132781692 | 5.072267193 | GTATTGACTTGA | hsa_circ_0078770 | chr6 | 170626457 | 170667406 | + | 2304 | ANNOTATED, CDS, c | NM_032448 | FAM120B | Salzman2013 | 84498 | circRNA | Detected | Detected | 6.132781692 | 5.072267193 |
| hsa_gci168566 | 2.762206155 | 1.465820998 | up | 7.722777182 | 6.256956184 | CTACACTCACTG | hsa_circ_0079040 | chr7 | 618892 | 647089 | - | 451 | ANNOTATED, CDS, c | NM_001164761 | PRKAR1B | Salzman2013 | 5575 | circRNA | Detected | Detected | 7.722777182 | 6.256956184 |
| hsa_gci168572 | 2.352241433 | 1.234036146 | up | 6.823917505 | 5.58988136 | GCGGCGCGGGCC | hsa_circ_0079133 | chr7 | 1606969 | 1609668 | - | 778 | ANNOTATED, CDS, c | NM_001134340 | PSMG3 | Salzman2013 | 84262 | circRNA | Detected | Detected | 6.823917505 | 5.58988136 |
| hsa_gci168582 | 2.269775428 | 1.182549564 | up | 5.499620611 | 4.317071047 | TACAACACCTAC | hsa_circ_0079242 | chr7 | 5256193 | 5273486 | + | 3892 | ANNOTATED, CDS, c | NM_015610 | WIPI2 | Salzman2013 | 26100 | circRNA | Detected | Detected | 5.499620611 | 4.317071047 |
| hsa_gci168607 | 2.42312921 | 1.276871337 | up | 5.248321958 | 3.971450622 | GGTAGATGGTAT | hsa_circ_0079346 | chr7 | 6431554 | 6431672 | + | 118 | ANNOTATED, CDS, c | NM_018890 | RAC1 | Salzman2013 | 5879 | circRNA | Detected | Detected | 5.248321958 | 3.971450622 |
| hsa_gci168609 | 3.343758545 | 1.741470673 | up | 6.823300733 | 5.08183006 | AAACGGGGCCCT | hsa_circ_0079349 | chr7 | 6438292 | 6443598 | + | 1932 | ANNOTATED, CDS, c | NM_018890 | RAC1 | Salzman2013 | 5879 | circRNA | Detected | Detected | 6.823300733 | 5.08183006 |
| hsa_gci168616 | 2.840737161 | 1.506265352 | up | 7.914096741 | 6.407831389 | GTCCATGTGACA | hsa_circ_0079371 | chr7 | 6500711 | 6509385 | - | 2507 | ANNOTATED, CDS, c | NM_006854 | KDELR2 | Salzman2013 | 11014 | circRNA | Detected | Detected | 7.914096741 | 6.407831389 |
| hsa_gci168637 | 2.504712789 | 1.324645182 | up | 7.548085383 | 6.223440201 | TTCCCCGTCCTT | hsa_circ_0079422 | chr7 | 8257934 | 8275635 | - | 658 | ANNOTATED, CDS, c | NM_004968 | ICA1 | Salzman2013 | 3382 | circRNA | Detected | Detected | 7.548085383 | 6.223440201 |
| hsa_gci168639 | 2.754634505 | 1.461860909 | up | 4.964532014 | 3.502671105 | GATCGTTTCGCT | hsa_circ_0079433 | chr7 | 11068307 | 11091407 | + | 1164 | ANNOTATED, CDS, c | NM_014660 | PHF14 | Salzman2013 | 9678 | circRNA | Detected | Detected | 4.964532014 | 3.502671105 |
| hsa_gci168645 | 2.371343469 | 1.24570464 | up | 4.177835559 | 2.932130919 | AAGTGGTTCTAG | hsa_circ_0079456 | chr7 | 12379947 | 12410490 | - | 2779 | ANNOTATED, CDS, c | NM_001135924 | VWDE | Salzman2013 | 221806 | circRNA | Detected | Not Detected | 4.177835559 | 2.932130919 |
| hsa_gci168688 | 2.16555715 | 1.114738246 | up | 6.589988177 | 5.47524993 | AAGTCAAACAGA | hsa_circ_0079591 | chr7 | 23358756 | 23391205 | - | 919 | ANNOTATED, CDS, c | NM_006547 | IGF2BP3 | Salzman2013 | 10643 | circRNA | Detected | Detected | 6.589988177 | 5.47524993 |
| hsa_gci168693 | 2.001214657 | 1.000875924 | up | 7.009383081 | 6.008507157 | GGCACGTCAAGA | hsa_circ_0079603 | chr7 | 23458394 | 23508161 | - | 110 | ANNOTATED, CDS, c | NM_006547 | IGF2BP3 | Salzman2013 | 10643 | circRNA | Detected | Detected | 7.009383081 | 6.008507157 |
| hsa_gci168721 | 3.417800587 | 1.773068225 | up | 4.104849071 | 2.331780846 | TAGTCGTGTAGG | hsa_circ_0079724 | chr7 | 30661008 | 30661116 | + | 108 | ANNOTATED, CDS, c | NM_002047 | GARS | Salzman2013 | 2617 | circRNA | Detected | Not Detected | 4.104849071 | 2.331780846 |
| hsa_gci168740 | 2.383916883 | 1.253333936 | up | 6.828915293 | 5.575581358 | GCCTCTCCACCA | hsa_circ_0079785 | chr7 | 32662972 | 32678977 | - | 349 | ANNOTATED, INTERN | NR_036680 | DPY19L1P1 | Salzman2013 | 100129460 | circRNA | Detected | Detected | 6.828915293 | 5.575581358 |
| hsa_gci168741 | 2.661980249 | 1.412499867 | up | 7.117746504 | 5.705246637 | TTTTTGAAAGTG | hsa_circ_0079786 | chr7 | 32672154 | 32756421 | - | 773 | ANNOTATED, INTERN | NR_036680 | DPY19L1P1 | Salzman2013 | 100129460 | circRNA | Detected | Detected | 7.117746504 | 5.705246637 |
| hsa_gci168751 | -5.269154066 | -2.397571364 | down | 1.297170879 | 3.694742243 | CACCACAGGTGT | hsa_circ_0079824 | chr7 | 34977605 | 35013217 | - | 1268 | ANNOTATED, CDS, c | NM_015283 | DPY19L1 | Salzman2013 | 23333 | circRNA | Not Detected | Detected | 1.297170879 | 3.694742243 |
| hsa_gci168754 | 2.316006878 | 1.211639538 | up | 5.457961004 | 4.246321466 | TTGGTTTTTGTA | hsa_circ_0079832 | chr7 | 34985278 | 34994390 | - | 305 | ANNOTATED, CDS, c | NM_015283 | DPY19L1 | Salzman2013 | 23333 | circRNA | Detected | Detected | 5.457961004 | 4.246321466 |

| | | | | | | | | | | | | | | | | | | | | | | |
|---|---|---|---|---|---|---|---|---|---|---|---|---|---|---|---|---|---|---|---|---|---|---|
| hsa_gci168755 | 2.708257174 | 1.437364743 | up | 7.592658161 | 6.155293418 | TGTAATGTGCCC | hsa_circ_0079833 | chr7 | 34985278 | 35013217 | - | 722 | ANNOTATED, CDS, c | NM_015283 | DPY19L1 | Salzman2013 | 23333 | circRNA | Detected | Detected | 7.592658161 | 6.155293418 |
| hsa_gci168756 | 2.317605298 | 1.212634887 | up | 7.285826028 | 6.073191141 | TGTGACGACGTT | hsa_circ_0079835 | chr7 | 34985278 | 35058208 | - | 1246 | ANNOTATED, CDS, c | NM_015283 | DPY19L1 | Salzman2013 | 23333 | circRNA | Detected | Detected | 7.285826028 | 6.073191141 |
| hsa_gci168778 | 2.67389626 | 1.418943494 | up | 6.278294051 | 4.859350557 | GCGGTGTCTAGT | hsa_circ_0079912 | chr7 | 39663151 | 39747723 | + | 2810 | ANNOTATED, CDS, c | NM_005402 | RALA | Salzman2013 | 5898 | circRNA | Detected | Detected | 6.278294051 | 4.859350557 |
| hsa_gci168798 | 2.501398418 | 1.322734866 | up | 4.485338308 | 3.162603442 | AGAGCCGAGGTC | hsa_circ_0079972 | chr7 | 43906156 | 43908975 | - | 606 | ANNOTATED, CDS, c | NM_001204871 | URGCP-MRPS24 | Salzman2013 | 100534592 | circRNA | Detected | Not Detected | 4.485338308 | 3.162603442 |
| hsa_gci168802 | 2.886624199 | 1.5293833 | up | 4.967234142 | 3.437850843 | GAAAGAAGGGAA | hsa_circ_0080007 | chr7 | 44146144 | 44154159 | + | 3523 | ANNOTATED, CDS, c | NM_001129 | AEBP1 | Salzman2013 | 165 | circRNA | Detected | Detected | 4.967234142 | 3.437850843 |
| hsa_gci168807 | 2.841169097 | 1.506484699 | up | 8.624377978 | 7.117893279 | CCCTGAGGACAT | hsa_circ_0080044 | chr7 | 44605402 | 44611335 | - | 1197 | ANNOTATED, CDS, c | NM_019082 | DDX56 | Salzman2013 | 54606 | circRNA | Detected | Detected | 8.624377978 | 7.117893279 |
| hsa_gci168827 | 2.615464298 | 1.387067077 | up | 6.811229547 | 5.42416247 | TGGCGTCCCTTA | hsa_circ_0080185 | chr7 | 47476860 | 47520739 | - | 315 | ANNOTATED, CDS, c | NM_022748 | TNS3 | Salzman2013 | 64759 | circRNA | Detected | Detected | 6.811229547 | 5.42416247 |
| hsa_gci168844 | 2.66218914 | 1.412613074 | up | 5.979463211 | 4.566850137 | GACGACACGACC | hsa_circ_0080287 | chr7 | 56169265 | 56170704 | - | 434 | ANNOTATED, CDS, c | NM_016139 | CHCHD2 | Salzman2013 | 51142 | circRNA | Detected | Detected | 5.979463211 | 4.566850137 |
| hsa_gci168845 | 2.798348683 | 1.484575738 | up | 6.929625881 | 5.445050143 | CACGTCGAGAGT | hsa_circ_0080288 | chr7 | 56169265 | 56172168 | - | 684 | ANNOTATED, CDS, c | NM_016139 | CHCHD2 | Salzman2013 | 51142 | circRNA | Detected | Detected | 6.929625881 | 5.445050143 |
| hsa_gci168867 | 2.113662927 | 1.079745324 | up | 7.741018443 | 6.66127312 | TGTGTTCAGGTT | hsa_circ_0080373 | chr7 | 66409962 | 66413681 | + | 437 | ANNOTATED, CDS, c | NM_017994 | TMEM248 | Salzman2013 | 55069 | circRNA | Detected | Detected | 7.741018443 | 6.66127312 |
| hsa_gci168873 | 2.075162999 | 1.053224661 | up | 5.433990997 | 4.380766336 | TATGCACCGTCC | hsa_circ_0080465 | chr7 | 72483620 | 72519759 | + | 36139 | INTERGENIC | None | | Salzman2013 | | circRNA | Detected | Detected | 5.433990997 | 4.380766336 |
| hsa_gci168877 | 2.225384135 | 1.154054389 | up | 7.465872747 | 6.311818359 | AAGAGTATGGTC | hsa_circ_0080479 | chr7 | 72861593 | 72922801 | - | 3620 | ANNOTATED, CDS, c | NM_032408 | BAZ1B | Salzman2013 | 9031 | circRNA | Detected | Detected | 7.465872747 | 6.311818359 |
| hsa_gci168879 | 2.403428927 | 1.265094141 | up | 4.173051034 | 2.907956893 | CATAAGCAAGAA | hsa_circ_0080483 | chr7 | 72877251 | 72884813 | - | 656 | ANNOTATED, CDS, c | NM_032408 | BAZ1B | Salzman2013 | 9031 | circRNA | Detected | Not Detected | 4.173051034 | 2.907956893 |
| hsa_gci168881 | 2.521976776 | 1.33455499 | up | 8.402637451 | 7.068082461 | AGGTCTTGTTCG | hsa_circ_0080493 | chr7 | 72912826 | 72922801 | - | 347 | ANNOTATED, CDS, c | NM_032408 | BAZ1B | Salzman2013 | 9031 | circRNA | Detected | Detected | 8.402637451 | 7.068082461 |
| hsa_gci168894 | 2.015571031 | 1.011188627 | up | 9.850311779 | 8.839123153 | GGCAGAGTTTGT | hsa_circ_0080655 | chr7 | 74601105 | 74610433 | - | 1737 | ALT_DONOR, INTERN | NR_002206 | GTF2IP1 | Salzman2013 | 2970 | circRNA | Detected | Detected | 9.850311779 | 8.839123153 |
| hsa_gci168913 | 2.076455192 | 1.05412274 | up | 6.733549445 | 5.679426704 | GTGTGACGGGTC | hsa_circ_0080773 | chr7 | 75677392 | 75695930 | + | 1305 | ANNOTATED, CDS, c | NM_005918 | MDH2 | Salzman2013 | 4191 | circRNA | Detected | Detected | 6.733549445 | 5.679426704 |
| hsa_gci168914 | 2.148438984 | 1.103288805 | up | 6.503369167 | 5.400080362 | AGTCCAGGCTCC | hsa_circ_0080778 | chr7 | 75686727 | 75695930 | + | 984 | ANNOTATED, CDS, c | NM_005918 | MDH2 | Salzman2013 | 4191 | circRNA | Detected | Detected | 6.503369167 | 5.400080362 |
| hsa_gci168939 | 2.195856088 | 1.134783506 | up | 4.66085813 | 3.526074623 | ATAACCCACATT | hsa_circ_0080883 | chr7 | 80371853 | 80418844 | - | 3481 | ANNOTATED, CDS, c | NM_006379 | SEMA3C | Salzman2013 | 10512 | circRNA | Detected | Detected | 4.66085813 | 3.526074623 |
| hsa_gci168950 | 2.245696939 | 1.167163246 | up | 7.901117449 | 6.733954202 | ACTTCATAAGTC | hsa_circ_0080905 | chr7 | 80427407 | 80458061 | - | 1028 | ANNOTATED, CDS, c | NM_006379 | SEMA3C | Salzman2013 | 10512 | circRNA | Detected | Detected | 7.901117449 | 6.733954202 |
| hsa_gci168963 | 2.077452013 | 1.054815152 | up | 8.288993419 | 7.234178267 | GGATAGAGGCGT | hsa_circ_0080950 | chr7 | 86792810 | 86815296 | + | 1543 | ANNOTATED, INTERN | NR_024549 | DMTF1 | Salzman2013 | 9988 | circRNA | Detected | Detected | 8.288993419 | 7.234178267 |
| hsa_gci168969 | 2.345247705 | 1.229740308 | up | 10.15230308 | 8.92256277 | GTCGGGTACCGT | hsa_circ_0080977 | chr7 | 90041957 | 90045268 | + | 3311 | ANNOTATED, CDS, c | NM_001185072 | CLDN12 | Salzman2013 | 9069 | circRNA | Detected | Detected | 10.15230308 | 8.92256277 |
| hsa_gci168970 | 2.460163738 | 1.298754338 | up | 4.615423433 | 3.316669095 | ACTTAATTAGGG | hsa_circ_0080978 | chr7 | 90193048 | 90356126 | + | 146122 | ALT_ACCEPTOR, CDS | NM_012395 | CDK14 | Salzman2013 | 5218 | circRNA | Detected | Detected | 4.615423433 | 3.316669095 |
| hsa_gci168972 | 3.012942362 | 1.591173074 | up | 5.902199301 | 4.311026226 | TCGAAAAGGTTT | hsa_circ_0080983 | chr7 | 90376995 | 90585132 | + | 578 | ANNOTATED, CDS, c | NM_012395 | CDK14 | Salzman2013 | 5218 | circRNA | Detected | Detected | 5.902199301 | 4.311026226 |
| hsa_gci168973 | 2.371724337 | 1.245936337 | up | 5.292312419 | 4.046376083 | AGCGTCGTTGAC | hsa_circ_0080985 | chr7 | 90546915 | 90585132 | + | 245 | ANNOTATED, CDS, c | NM_012395 | CDK14 | Salzman2013 | 5218 | circRNA | Detected | Detected | 5.292312419 | 4.046376083 |
| hsa_gci169009 | 2.772215001 | 1.471039151 | up | 4.090783012 | 2.619743861 | CCACTTCGTCAG | hsa_circ_0081215 | chr7 | 97920422 | 97920839 | + | 417 | ANNOTATED, CDS, c | NM_015379 | BRI3 | Salzman2013 | 25798 | circRNA | Detected | Not Detected | 4.090783012 | 2.619743861 |
| hsa_gci169010 | 2.066682786 | 1.047316968 | up | 5.974486189 | 4.927169221 | CATAAGATACTT | hsa_circ_0081236 | chr7 | 98490046 | 98560050 | + | 6547 | ANNOTATED, CDS, c | NM_001244580 | TRRAP | Salzman2013 | 8295 | circRNA | Detected | Detected | 5.974486189 | 4.927169221 |
| hsa_gci169024 | 2.757529064 | 1.463376092 | up | 8.178012756 | 6.714636664 | GCAAAAAGTGAT | hsa_circ_0081292 | chr7 | 98547282 | 98547464 | + | 182 | ANNOTATED, CDS, c | NM_001244580 | TRRAP | Salzman2013 | 8295 | circRNA | Detected | Detected | 8.178012756 | 6.714636664 |
| hsa_gci169049 | 3.826142595 | 1.935890642 | up | 4.528926851 | 2.593036209 | TAGTAGGACCAT | hsa_circ_0081407 | chr7 | 99691795 | 99697716 | - | 1892 | ANNOTATED, CDS, c | NM_182776 | MCM7 | Salzman2013 | 4176 | circRNA | Detected | Not Detected | 4.528926851 | 2.593036209 |
| hsa_gci169067 | 2.946633023 | 1.559067397 | up | 6.436018829 | 4.876951432 | CCGACCGAGTCC | hsa_circ_0081545 | chr7 | 100467991 | 100471076 | + | 847 | ANNOTATED, CDS, c | NM_003302 | TRIP6 | Salzman2013 | 7205 | circRNA | Detected | Detected | 6.436018829 | 4.876951432 |
| hsa_gci169071 | 2.129953915 | 1.090822216 | up | 7.466066846 | 6.37524463 | CGCGCTGCACCT | hsa_circ_0081576 | chr7 | 100778995 | 100782547 | + | 2033 | ANNOTATED, CDS, c | NM_000602 | SERPINE1 | Salzman2013 | 5054 | circRNA | Detected | Detected | 7.466066846 | 6.37524463 |
| hsa_gci169076 | 2.492329015 | 1.317494533 | up | 5.620133492 | 4.30263896 | GGAACAATTACC | hsa_circ_0081681 | chr7 | 102178365 | 102210371 | - | 1530 | ANNOTATED, CDS, c | NM_001097615 | POLR2J3 | Salzman2013 | 548644 | circRNA | Detected | Detected | 5.620133492 | 4.30263896 |
| hsa_gci169082 | 2.443155095 | 1.288745451 | up | 4.411802671 | 3.12305722 | TCAGGAATGCTG | hsa_circ_0081744 | chr7 | 102944857 | 102944937 | + | 80 | ANNOTATED, CDS, c | NM_004279 | PMPCB | Salzman2013 | 9512 | circRNA | Detected | Not Detected | 4.411802671 | 3.12305722 |
| hsa_gci169085 | 2.915107259 | 1.543548967 | up | 4.156336406 | 2.612787439 | GTATTCAAAAGT | hsa_circ_0081756 | chr7 | 102994367 | 103004754 | + | 686 | ANNOTATED, CDS, c | NM_002803 | PSMC2 | Salzman2013 | 5701 | circRNA | Detected | Not Detected | 4.156336406 | 2.612787439 |
| hsa_gci169095 | 3.014583988 | 1.591958924 | up | 4.098455793 | 2.506496868 | TTTACAGAAGTG | hsa_circ_0081808 | chr7 | 104678572 | 104707254 | + | 685 | ANNOTATED, CDS, c | NM_182931 | KMT2E | Salzman2013 | 55904 | circRNA | Detected | Not Detected | 4.098455793 | 2.506496868 |
| hsa_gci169112 | 2.533637596 | 1.34121018 | up | 7.677042845 | 6.335832664 | GGTAGAAAAGGA | hsa_circ_0081852 | chr7 | 105112578 | 105146720 | - | 839 | ANNOTATED, CDS, c | NM_019042 | PUS7 | Salzman2013 | 54517 | circRNA | Detected | Detected | 7.677042845 | 6.335832664 |
| hsa_gci169129 | 2.269531141 | 1.182394284 | up | 5.187543177 | 4.005148893 | TTCCTAGAACCC | hsa_circ_0081942 | chr7 | 107615430 | 107638937 | - | 1269 | ANNOTATED, CDS, c | NM_002291 | LAMB1 | Salzman2013 | 3912 | circRNA | Detected | Detected | 5.187543177 | 4.005148893 |
| hsa_gci169144 | 2.112407878 | 1.078888427 | up | 9.252891491 | 8.174003064 | GTTCGTGTCGCC | hsa_circ_0082002 | chr7 | 116339124 | 116340338 | + | 1214 | ANNOTATED, CDS, c | NM_001127500 | MET | Salzman2013 | 4233 | circRNA | Detected | Detected | 9.252891491 | 8.174003064 |
| hsa_gci169145 | 2.527019356 | 1.337436715 | up | 8.858629934 | 7.521193219 | TCCACGTTCGTG | hsa_circ_0082003 | chr7 | 116339124 | 116397828 | + | 2116 | ANNOTATED, CDS, c | NM_001127500 | MET | Salzman2013 | 4233 | circRNA | Detected | Detected | 8.858629934 | 7.521193219 |
| hsa_gci169159 | -3.110160387 | -1.63698898 | down | 2.300139064 | 3.937128045 | AGTACGTAGTCA | hsa_circ_0082049 | chr7 | 120770178 | 120937498 | + | 3466 | ANNOTATED, CDS, c | NM_024913 | CPED1 | Salzman2013 | 79974 | circRNA | Not Detected | Detected | 2.300139064 | 3.937128045 |
| hsa_gci169200 | 2.638678674 | 1.399815677 | up | 7.895467712 | 6.495652036 | TGACAACTTGAA | hsa_circ_0082284 | chr7 | 128845043 | 128851611 | + | 1399 | ANNOTATED, CDS, c | NM_005631 | SMO | Salzman2013 | 6608 | circRNA | Detected | Detected | 7.895467712 | 6.495652036 |
| hsa_gci169202 | 4.055680605 | 2.019944041 | up | 4.183301612 | 2.163357572 | GAAAAGACACAT | hsa_circ_0082302 | chr7 | 129269918 | 129330386 | + | 706 | ANNOTATED, CDS, c | NM_001040110 | NRF1 | Salzman2013 | 4899 | circRNA | Detected | Not Detected | 4.183301612 | 2.163357572 |
| hsa_gci169207 | 9.637818234 | 3.268706593 | up | 4.621342568 | 1.352635975 | GAGGGTCCTAGC | hsa_circ_0082316 | chr7 | 129497350 | 129520811 | - | 245 | ANNOTATED, CDS, c | NM_003344 | UBE2H | Salzman2013 | 7328 | circRNA | Detected | Not Detected | 4.621342568 | 1.352635975 |
| hsa_gci169208 | 4.265279316 | 2.092640221 | up | 5.921413297 | 3.828773076 | GGAGGGTCCTAG | hsa_circ_0082317 | chr7 | 129519407 | 129520811 | - | 152 | ANNOTATED, CDS, c | NM_003344 | UBE2H | Salzman2013 | 7328 | circRNA | Detected | Detected | 5.921413297 | 3.828773076 |
| hsa_gci169218 | 2.077876891 | 1.05511018 | up | 4.533631577 | 3.478521397 | TATTCCGTAATT | hsa_circ_0082366 | chr7 | 129841689 | 129842503 | - | 374 | ANNOTATED, CDS, c | NM_032842 | TMEM209 | Salzman2013 | 84928 | circRNA | Detected | Detected | 4.533631577 | 3.478521397 |
| hsa_gci169223 | 2.413314616 | 1.271016007 | up | 7.669967621 | 6.398951614 | CGATGGCCTCCT | hsa_circ_0082390 | chr7 | 130794854 | 131084192 | + | 839 | ANNOTATED, CDS, c | NM_001145354 | MKLN1 | Salzman2013 | 4289 | circRNA | Detected | Detected | 7.669967621 | 6.398951614 |
| hsa_gci169224 | 2.235415191 | 1.160542813 | up | 8.450918195 | 7.290375382 | AGTAAACCAAAC | hsa_circ_0082396 | chr7 | 131060182 | 131060252 | + | 70 | ANNOTATED, CDS, c | NM_001145354 | MKLN1 | Salzman2013 | 4289 | circRNA | Detected | Detected | 8.450918195 | 7.290375382 |
| hsa_gci169240 | 2.304989467 | 1.204760158 | up | 7.722571599 | 6.517811442 | AAACATTGTAGT | hsa_circ_0082443 | chr7 | 133002037 | 133002144 | + | 107 | ANNOTATED, CDS, c | NM_021807 | EXOC4 | Salzman2013 | 60412 | circRNA | Detected | Detected | 7.722571599 | 6.517811442 |
| hsa_gci169242 | 2.155328202 | 1.107907572 | up | 7.790914832 | 6.68300726 | AGTTAGTCTCCT | hsa_circ_0082446 | chr7 | 133002037 | 133314894 | + | 858 | ANNOTATED, CDS, c | NM_021807 | EXOC4 | Salzman2013 | 60412 | circRNA | Detected | Detected | 7.790914832 | 6.68300726 |
| hsa_gci169274 | 2.408936554 | 1.268396397 | up | 4.133370155 | 2.864973759 | TGCTTCCTCCAC | hsa_circ_0082595 | chr7 | 138235807 | 138268744 | + | 1800 | ANNOTATED, CDS, c | NM_015905 | TRIM24 | Salzman2013 | 8805 | circRNA | Detected | Not Detected | 4.133370155 | 2.864973759 |
| hsa_gci169275 | 2.167536103 | 1.116056024 | up | 10.41002694 | 9.293970917 | TAAGACTTTCAA | hsa_circ_0082597 | chr7 | 138252225 | 138252399 | + | 174 | ANNOTATED, CDS, c | NM_015905 | TRIM24 | Salzman2013 | 8805 | circRNA | Detected | Detected | 10.41002694 | 9.293970917 |
| hsa_gci169280 | 2.217471764 | 1.148915734 | up | 7.409943158 | 6.261027424 | TCTCTTGGAGTA | hsa_circ_0082633 | chr7 | 138768525 | 138774505 | - | 389 | ANNOTATED, CDS, c | NM_020119 | ZC3HAV1 | Salzman2013 | 56829 | circRNA | Detected | Detected | 7.409943158 | 6.261027424 |

| | | | | | | | | | | | | | | | | | | | | | | |
|---|---|---|---|---|---|---|---|---|---|---|---|---|---|---|---|---|---|---|---|---|---|---|
| hsa_gci169287 | 2.613008123 | 1.385711609 | up | 9.101074898 | 7.715363289 | ATCCGTCCGTAT | hsa_circ_0082660 | chr7 | 139059146 | 139102475 | + | 2601 | ALT_ACCEPTOR, CDS | NM_016019 | LUC7L2 | Salzman2013 | 51631 | circRNA | Detected | Detected | 9.101074898 | 7.715363289 |
| hsa_gci169328 | 3.265110578 | 1.707131852 | up | 5.541502284 | 3.834370432 | AAGGTGAGGAGG | hsa_circ_0082816 | chr7 | 143554192 | 143557540 | - | 829 | ANNOTATED, CDS, c | NM_014719 | TCAF1 | Salzman2013 | 9747 | circRNA | Detected | Detected | 5.541502284 | 3.834370432 |
| hsa_gci169334 | 3.214438273 | 1.684566647 | up | 6.246700566 | 4.562133919 | TTCTCGTCCACT | hsa_circ_0082845 | chr7 | 148529725 | 148534092 | - | 4367 | ALT_ACCEPTOR, CDS | NM_004456 | EZH2 | Salzman2013 | 2146 | circRNA | Detected | Detected | 6.246700566 | 4.562133919 |
| hsa_gci169344 | 2.127046973 | 1.088851894 | up | 4.05347402 | 2.964622126 | CGCCATGGTGTC | hsa_circ_0082905 | chr7 | 150750898 | 150752460 | - | 593 | ANNOTATED, CDS, c | NM_004935 | CDK5 | Salzman2013 | 1020 | circRNA | Detected | Not Detected | 4.05347402 | 2.964622126 |
| hsa_gci169390 | 2.051062763 | 1.03637164 | up | 5.214774079 | 4.178402439 | TCCAACCTTTTT | hsa_circ_0083123 | chr7 | 156555801 | 156589186 | - | 300 | ANNOTATED, CDS, c | NM_022458 | LMBR1 | Salzman2013 | 64327 | circRNA | Detected | Detected | 5.214774079 | 4.178402439 |
| hsa_gci169403 | 7.487433857 | 2.904471353 | up | 4.135637748 | 1.231166395 | TCCTCTTGGGGA | hsa_circ_0083161 | chr7 | 157000091 | 157000629 | + | 391 | ANNOTATED, CDS, c | NM_014671 | UBE3C | Salzman2013 | 9690 | circRNA | Detected | Not Detected | 4.135637748 | 1.231166395 |
| hsa_gci169418 | 2.616030952 | 1.387379611 | up | 4.188306966 | 2.800927356 | TCGAAGTTATTT | hsa_circ_0083202 | chr7 | 158536219 | 158557544 | - | 807 | ANNOTATED, CDS, c | NM_020728 | ESYT2 | Salzman2013 | 57488 | circRNA | Detected | Not Detected | 4.188306966 | 2.800927356 |
| hsa_gci169461 | 2.004769761 | 1.003436559 | up | 6.124993013 | 5.121556454 | CTTGTACCTCCC | hsa_circ_0083385 | chr8 | 12955907 | 12973753 | - | 1918 | ANNOTATED, CDS, c | NM_001164271 | DLC1 | Salzman2013 | 10395 | circRNA | Detected | Detected | 6.124993013 | 5.121556454 |
| hsa_gci169486 | 2.243566128 | 1.165793707 | up | 9.240133813 | 8.074340105 | TCTACGAGACTT | hsa_circ_0083438 | chr8 | 17512069 | 17613470 | - | 3368 | ANNOTATED, CDS, c | NM_001001924 | MTUS1 | Salzman2013 | 57509 | circRNA | Detected | Detected | 9.240133813 | 8.074340105 |
| hsa_gci169490 | 3.352607207 | 1.745283466 | up | 4.249682696 | 2.50439923 | GAGACTTCTAAT | hsa_circ_0083444 | chr8 | 17601112 | 17613470 | - | 2441 | ANNOTATED, CDS, c | NM_001001924 | MTUS1 | Salzman2013 | 57509 | circRNA | Detected | Not Detected | 4.249682696 | 2.50439923 |
| hsa_gci169531 | 2.148583503 | 1.103385847 | up | 4.919904011 | 3.816518163 | TGTTTTAATTAC | hsa_circ_0083673 | chr8 | 23291605 | 23299579 | - | 1179 | ALT_DONOR, CDS, c | NM_004901 | ENTPD4 | Salzman2013 | 9583 | circRNA | Detected | Detected | 4.919904011 | 3.816518163 |
| hsa_gci169546 | 3.682938428 | 1.880857278 | up | 8.085793498 | 6.20493622 | GTTCCTCTACAG | hsa_circ_0083775 | chr8 | 27514298 | 27517056 | + | 1143 | ANNOTATED, CDS, c | NM_016240 | SCARA3 | Salzman2013 | 51435 | circRNA | Detected | Detected | 8.085793498 | 6.20493622 |
| hsa_gci169547 | 3.612214053 | 1.852883387 | up | 8.040985651 | 6.188102264 | CCAGTTCCTCTA | hsa_circ_0083776 | chr8 | 27514298 | 27530537 | + | 3264 | ANNOTATED, CDS, c | NM_016240 | SCARA3 | Salzman2013 | 51435 | circRNA | Detected | Detected | 8.040985651 | 6.188102264 |
| hsa_gci169553 | 3.430171779 | 1.778280827 | up | 4.298739338 | 2.520458512 | TCTGTCTAGATA | hsa_circ_0083818 | chr8 | 28692808 | 28695293 | - | 227 | ANNOTATED, CDS, c | NM_018250 | INTS9 | Salzman2013 | 55756 | circRNA | Detected | Not Detected | 4.298739338 | 2.520458512 |
| hsa_gci169558 | 2.935588656 | 1.553649827 | up | 4.028805774 | 2.475155947 | TTATCTTGTTTC | hsa_circ_0083841 | chr8 | 29961815 | 29962002 | + | 187 | ANNOTATED, CDS, c | NM_015344 | LEPROTL1 | Salzman2013 | 23484 | circRNA | Detected | Not Detected | 4.028805774 | 2.475155947 |
| hsa_gci169565 | 2.268303773 | 1.18161386 | up | 9.638756215 | 8.457142355 | CCACTGACATGG | hsa_circ_0083894 | chr8 | 31004568 | 31015046 | + | 599 | ANNOTATED, CDS, c | NM_000553 | WRN | Salzman2013 | 7486 | circRNA | Detected | Detected | 9.638756215 | 8.457142355 |
| hsa_gci169568 | 2.992889415 | 1.581538973 | up | 6.772464549 | 5.190925576 | TCAGGGTCCGGT | hsa_circ_0083910 | chr8 | 33408561 | 33416289 | - | 245 | ANNOTATED, CDS, c | NM_024787 | RNF122 | Salzman2013 | 79845 | circRNA | Detected | Detected | 6.772464549 | 5.190925576 |
| hsa_gci169571 | 2.317733592 | 1.212714748 | up | 9.510462039 | 8.297747292 | CGGTGATCGGCC | hsa_circ_0083933 | chr8 | 37623043 | 37637286 | + | 2393 | ANNOTATED, CDS, c | NM_007198 | PLPBP | Salzman2013 | 11212 | circRNA | Detected | Detected | 9.510462039 | 8.297747292 |
| hsa_gci169575 | 41.07876145 | 5.360320779 | up | 6.593255511 | 1.232934732 | GACTCGTCTTTT | hsa_circ_0083962 | chr8 | 37978449 | 37978667 | + | 218 | ANNOTATED, CDS, c | NM_001105214 | ASH2L | Salzman2013 | 9070 | circRNA | Detected | Not Detected | 6.593255511 | 1.232934732 |
| hsa_gci169585 | 2.418316346 | 1.27400298 | up | 7.975952142 | 6.701949162 | TAAAGAAAAGGT | hsa_circ_0084003 | chr8 | 38271435 | 38285611 | - | 1844 | ANNOTATED, CDS, c | NM_023110 | FGFR1 | Salzman2013 | 2260 | circRNA | Detected | Detected | 7.975952142 | 6.701949162 |
| hsa_gci169595 | 2.260372431 | 1.176560498 | up | 5.613474757 | 4.436914259 | AGGGAGTTACGT | hsa_circ_0084051 | chr8 | 38947565 | 38948865 | + | 230 | ANNOTATED, CDS, c | NM_003816 | ADAM9 | Salzman2013 | 8754 | circRNA | Detected | Detected | 5.613474757 | 4.436914259 |
| hsa_gci169600 | 2.506952628 | 1.325934735 | up | 6.710247594 | 5.384312859 | TGAGGCCTCCGA | hsa_circ_0084061 | chr8 | 41119475 | 41166990 | - | 4467 | ANNOTATED, CDS, c | NM_003012 | SFRP1 | Salzman2013 | 6422 | circRNA | Detected | Detected | 6.710247594 | 5.384312859 |
| hsa_gci169604 | 2.481002596 | 1.310923245 | up | 4.324694742 | 3.013771497 | GGGGGACCTAGA | hsa_circ_0084092 | chr8 | 42022588 | 42028701 | + | 2796 | ANNOTATED, CDS, c | NM_001134296 | AP3M2 | Salzman2013 | 10947 | circRNA | Detected | Not Detected | 4.324694742 | 3.013771497 |
| hsa_gci169607 | 2.530910247 | 1.339656346 | up | 7.232636859 | 5.892980513 | AGTAAATCATGT | hsa_circ_0084108 | chr8 | 42214837 | 42218883 | + | 144 | ANNOTATED, CDS, c | NM_002690 | POLB | Salzman2013 | 5423 | circRNA | Detected | Detected | 7.232636859 | 5.892980513 |
| hsa_gci169613 | 3.795536953 | 1.924303997 | up | 6.754678239 | 4.830374241 | ACTGGTCTTCAT | hsa_circ_0084119 | chr8 | 42256229 | 42260979 | + | 585 | ANNOTATED, CDS, c | NM_001135694 | VDAC3 | Salzman2013 | 7419 | circRNA | Detected | Detected | 6.754678239 | 4.830374241 |
| hsa_gci169623 | 2.429576902 | 1.280705098 | up | 5.555898893 | 4.275193795 | TACAAAACTACA | hsa_circ_0084140 | chr8 | 42725146 | 42729149 | - | 185 | ANNOTATED, CDS, c | NM_001160223 | RNF170 | Salzman2013 | 81790 | circRNA | Detected | Detected | 5.555898893 | 4.275193795 |
| hsa_gci169642 | 2.171799342 | 1.118890815 | up | 9.028427478 | 7.909536663 | GACCGCTCAATC | hsa_circ_0084207 | chr8 | 48685668 | 48690435 | - | 1599 | ANNOTATED, CDS, c | NM_006904 | PRKDC | Salzman2013 | 5591 | circRNA | Detected | Detected | 9.028427478 | 7.909536663 |
| hsa_gci169663 | 5.033676863 | 2.331612606 | up | 4.345295024 | 2.013682419 | TTTGGCTTTAGA | hsa_circ_0084301 | chr8 | 48719697 | 48869991 | - | 9592 | ANNOTATED, CDS, c | NM_006904 | PRKDC | Salzman2013 | 5591 | circRNA | Detected | Not Detected | 4.345295024 | 2.013682419 |
| hsa_gci169667 | 2.036210863 | 1.02588697 | up | 8.807560577 | 7.781673607 | GTCGGGCTCTTT | hsa_circ_0084333 | chr8 | 48752576 | 48830943 | - | 5034 | ANNOTATED, CDS, c | NM_006904 | PRKDC | Salzman2013 | 5591 | circRNA | Detected | Detected | 8.807560577 | 7.781673607 |
| hsa_gci169677 | 2.36877675 | 1.244142236 | up | 6.701949162 | 5.457806927 | AACGGAAATCAT | hsa_circ_0084401 | chr8 | 48805819 | 48856589 | - | 3005 | ALT_DONOR, CDS, c | NM_006904 | PRKDC | Salzman2013 | 5591 | circRNA | Detected | Detected | 6.701949162 | 5.457806927 |
| hsa_gci169678 | 2.030161929 | 1.021594804 | up | 7.725617802 | 6.704022998 | TTAAGTAGGTAC | hsa_circ_0084402 | chr8 | 48805819 | 48867006 | - | 3327 | ALT_DONOR, CDS, c | NM_006904 | PRKDC | Salzman2013 | 5591 | circRNA | Detected | Detected | 7.725617802 | 6.704022998 |
| hsa_gci169679 | 2.964825074 | 1.567946988 | up | 7.326991565 | 5.759044578 | TAAATCGTCGAG | hsa_circ_0084403 | chr8 | 48805819 | 48868508 | - | 3402 | ALT_DONOR, CDS, c | NM_006904 | PRKDC | Salzman2013 | 5591 | circRNA | Detected | Detected | 7.326991565 | 5.759044578 |
| hsa_gci169680 | 2.581750508 | 1.36834959 | up | 8.564216828 | 7.195867238 | ATGTATTTTTGA | hsa_circ_0084404 | chr8 | 48805819 | 48869823 | - | 3495 | ALT_DONOR, CDS, c | NM_006904 | PRKDC | Salzman2013 | 5591 | circRNA | Detected | Detected | 8.564216828 | 7.195867238 |
| hsa_gci169720 | 3.274137457 | 1.711114891 | up | 4.007171419 | 2.296056528 | TGAACGGGAGGT | hsa_circ_0084545 | chr8 | 56737139 | 56738005 | + | 866 | ANNOTATED, CDS, c | NM_024831 | TGS1 | Salzman2013 | 96764 | circRNA | Detected | Not Detected | 4.007171419 | 2.296056528 |
| hsa_gci169724 | 2.15136832 | 1.105254539 | up | 9.009694741 | 7.904440202 | GGCCATAGGAAT | hsa_circ_0084553 | chr8 | 56982261 | 56986718 | - | 461 | ANNOTATED, CDS, c | NM_001146227 | RPS20 | Salzman2013 | 6224 | circRNA | Detected | Detected | 9.009694741 | 7.904440202 |
| hsa_gci169835 | 2.001321959 | 1.000953277 | up | 6.49588751 | 5.494934233 | GGACGTACGAAC | hsa_circ_0084991 | chr8 | 97345684 | 97346774 | + | 1090 | ANNOTATED, CDS, c | NM_014754 | PTDSS1 | Salzman2013 | 9791 | circRNA | Detected | Detected | 6.49588751 | 5.494934233 |
| hsa_gci169856 | -2.434719031 | -1.283755294 | down | 3.978863861 | 5.262619154 | GAAAGGGCATTG | hsa_circ_0085040 | chr8 | 99466860 | 99837909 | - | 2826 | ANNOTATED, CDS, c | NM_006281 | STK3 | Salzman2013 | 6788 | circRNA | Detected | Detected | 3.978863861 | 5.262619154 |
| hsa_gci169865 | 3.083802388 | 1.624710319 | up | 5.514707231 | 3.889996912 | ACCTGACATTAT | hsa_circ_0085061 | chr8 | 100146859 | 100182391 | + | 1127 | ANNOTATED, CDS, c | NM_017890 | VPS13B | Salzman2013 | 157680 | circRNA | Detected | Detected | 5.514707231 | 3.889996912 |
| hsa_gci169867 | 2.622551036 | 1.390970849 | up | 8.606735012 | 7.215764163 | TCAGGACGGACC | hsa_circ_0085070 | chr8 | 100182266 | 100454863 | + | 1237 | ANNOTATED, CDS, c | NM_017890 | VPS13B | Salzman2013 | 157680 | circRNA | Detected | Detected | 8.606735012 | 7.215764163 |
| hsa_gci169870 | 2.318787972 | 1.213370907 | up | 7.94867471 | 6.735303802 | TATAAGTTGTAC | hsa_circ_0085081 | chr8 | 100443764 | 100454863 | + | 363 | ANNOTATED, CDS, c | NM_017890 | VPS13B | Salzman2013 | 157680 | circRNA | Detected | Detected | 7.94867471 | 6.735303802 |
| hsa_gci169871 | 2.038827034 | 1.027739388 | up | 8.081943292 | 7.054203904 | ATTGTCCTTGAC | hsa_circ_0085082 | chr8 | 100443764 | 100479862 | + | 584 | ANNOTATED, CDS, c | NM_017890 | VPS13B | Salzman2013 | 157680 | circRNA | Detected | Detected | 8.081943292 | 7.054203904 |
| hsa_gci169872 | 2.289412466 | 1.194977406 | up | 7.358238355 | 6.163260949 | TGTACCGAAATT | hsa_circ_0085086 | chr8 | 100443764 | 100533238 | + | 1738 | ANNOTATED, CDS, c | NM_017890 | VPS13B | Salzman2013 | 157680 | circRNA | Detected | Detected | 7.358238355 | 6.163260949 |
| hsa_gci169873 | 2.076555593 | 1.054192495 | up | 6.988713543 | 5.934521047 | CCGAAATTGTCC | hsa_circ_0085087 | chr8 | 100443764 | 100673719 | + | 3039 | ANNOTATED, CDS, c | NM_017890 | VPS13B | Salzman2013 | 157680 | circRNA | Detected | Detected | 6.988713543 | 5.934521047 |
| hsa_gci169884 | 3.191357916 | 1.674170418 | up | 6.252226243 | 4.578055824 | CGTGTAGGTTTA | hsa_circ_0085124 | chr8 | 101281012 | 101287389 | - | 517 | ANNOTATED, CDS, c | NM_183419 | RNF19A | Salzman2013 | 25897 | circRNA | Detected | Detected | 6.252226243 | 4.578055824 |
| hsa_gci169885 | 2.904126397 | 1.538104246 | up | 6.416177245 | 4.878073 | CTAAATGTCTTC | hsa_circ_0085125 | chr8 | 101281012 | 101300495 | - | 1284 | ANNOTATED, CDS, c | NM_183419 | RNF19A | Salzman2013 | 25897 | circRNA | Detected | Detected | 6.416177245 | 4.878073 |
| hsa_gci169889 | 2.127841826 | 1.089390911 | up | 5.401661787 | 4.312270876 | CGACGTCCTGCA | hsa_circ_0085131 | chr8 | 101715143 | 101719033 | - | 909 | ANNOTATED, CDS, c | NM_002568 | PABPC1 | Salzman2013 | 26986 | circRNA | Detected | Detected | 5.401661787 | 4.312270876 |
| hsa_gci169905 | 2.194489325 | 1.133885252 | up | 10.82091969 | 9.687034442 | GGGTGTCGCCCC | hsa_circ_0085208 | chr8 | 103292621 | 103311775 | - | 2895 | ANNOTATED, CDS, c | NM_015902 | UBR5 | Salzman2013 | 51366 | circRNA | Detected | Detected | 10.82091969 | 9.687034442 |
| hsa_gci169907 | 2.119307831 | 1.083593155 | up | 5.920093988 | 4.836500833 | GTGAGGTCTTGA | hsa_circ_0085213 | chr8 | 103297740 | 103359320 | - | 5098 | ANNOTATED, CDS, c | NM_015902 | UBR5 | Salzman2013 | 51366 | circRNA | Detected | Detected | 5.920093988 | 4.836500833 |
| hsa_gci169918 | 3.11695039 | 1.640135193 | up | 6.236038651 | 4.595903458 | ATAAACAAGTCG | hsa_circ_0085269 | chr8 | 103358463 | 103373854 | - | 674 | ANNOTATED, CDS, c | NM_015902 | UBR5 | Salzman2013 | 51366 | circRNA | Detected | Detected | 6.236038651 | 4.595903458 |
| hsa_gci169941 | 3.067923243 | 1.617262388 | up | 4.577925501 | 2.960663113 | GTTGTGTTCCCT | hsa_circ_0085406 | chr8 | 120814033 | 120814265 | - | 232 | ANNOTATED, CDS, c | NM_003184 | TAF2 | Salzman2013 | 6873 | circRNA | Detected | Not Detected | 4.577925501 | 2.960663113 |
| hsa_gci169946 | 2.024325102 | 1.017441002 | up | 5.894825406 | 4.877384405 | AATAAGAGTCCT | hsa_circ_0085429 | chr8 | 122629344 | 122641580 | - | 729 | ANNOTATED, CDS, c | NM_005328 | HAS2 | Salzman2013 | 3037 | circRNA | Detected | Detected | 5.894825406 | 4.877384405 |

| | | | | | | | | | | | | | | | | | | | | | |
|---|---|---|---|---|---|---|---|---|---|---|---|---|---|---|---|---|---|---|---|---|---|
| hsa_gci169961 | 2.208183962 | 1.142860367 | up | 9.046814163 | 7.903953797 | GCTCAGTGATGA | hsa_circ_0085473 | chr8 | 124371460 | 124373886 | - | 333 | ANNOTATED, CDS, c | NM_014109 | ATAD2 | Salzman2013 | 29028 | circRNA | Detected | Detected | 9.046814163 | 7.903953797 |
| hsa_gci169977 | 2.26067992 | 1.176756741 | up | 8.41432832 | 7.237571578 | CCCGTAGCAGCG | hsa_circ_0085535 | chr8 | 128750493 | 128753680 | + | 1811 | ANNOTATED, CDS, c | NM_002467 | MYC | Salzman2013 | 4609 | circRNA | Detected | Detected | 8.41432832 | 7.237571578 |
| hsa_gci169994 | 2.781572846 | 1.475900888 | up | 4.961081874 | 3.485180986 | GATATGTCCAGT | hsa_circ_0085584 | chr8 | 131128923 | 131138335 | - | 683 | ANNOTATED, CDS, c | NM_001247996 | ASAP1 | Salzman2013 | 50807 | circRNA | Detected | Detected | 4.961081874 | 3.485180986 |
| hsa_gci170009 | -3.855537651 | -1.946932057 | down | 1.604245905 | 3.551177962 | GTCGTCGTCGTA | hsa_circ_0085627 | chr8 | 132952745 | 132999949 | + | 2055 | ANNOTATED, CDS, c | NM_015137 | EFR3A | Salzman2013 | 23167 | circRNA | Not Detected | Detected | 1.604245905 | 3.551177962 |
| hsa_gci170012 | 2.253344026 | 1.172067592 | up | 7.848062686 | 6.675995094 | TCAAAAGTGTTC | hsa_circ_0085632 | chr8 | 132956991 | 132958880 | + | 279 | ANNOTATED, CDS, c | NM_015137 | EFR3A | Salzman2013 | 23167 | circRNA | Detected | Detected | 7.848062686 | 6.675995094 |
| hsa_gci170042 | 2.502417871 | 1.323322721 | up | 7.805165839 | 6.481843118 | AAGAAGAGGTCC | hsa_circ_0085810 | chr8 | 143692409 | 143693613 | - | 954 | ANNOTATED, coding | NM_015193 | ARC | Salzman2013 | 23237 | circRNA | Detected | Detected | 7.805165839 | 6.481843118 |
| hsa_gci170043 | 2.129750017 | 1.090684102 | up | 6.53535979 | 5.444675688 | AAGTCCGTCATG | hsa_circ_0085828 | chr8 | 144102765 | 144103827 | + | 930 | ALT_ACCEPTOR, CDS | NM_002346 | LY6E | Salzman2013 | 4061 | circRNA | Detected | Detected | 6.53535979 | 5.444675688 |
| hsa_gci170045 | 2.559244334 | 1.35571789 | up | 4.333463154 | 2.977745264 | TCCGACGCCACC | hsa_circ_0085863 | chr8 | 144806102 | 144810893 | - | 4791 | ANNOTATED, CDS, c | NM_198488 | FAM83H | Salzman2013 | 286077 | circRNA | Detected | Not Detected | 4.333463154 | 2.977745264 |
| hsa_gci170046 | 2.253297152 | 1.17203758 | up | 8.86228483 | 7.690247249 | TACAACCCCGGT | hsa_circ_0085866 | chr8 | 144806102 | 144812767 | - | 5543 | ANNOTATED, CDS, c | NM_198488 | FAM83H | Salzman2013 | 286077 | circRNA | Detected | Detected | 8.86228483 | 7.690247249 |
| hsa_gci170049 | 2.38558649 | 1.254343992 | up | 4.850876961 | 3.596532968 | ACCGCCAGTCCT | hsa_circ_0085937 | chr8 | 145066412 | 145067583 | + | 997 | ANNOTATED, CDS, c | NM_000837 | GRINA | Salzman2013 | 2907 | circRNA | Detected | Detected | 4.850876961 | 3.596532968 |
| hsa_gci170051 | 2.728395687 | 1.448052887 | up | 5.788304782 | 4.340251894 | CACAGGTGACGC | hsa_circ_0085939 | chr8 | 145066859 | 145067583 | + | 724 | ANNOTATED, CDS, c | NM_000837 | GRINA | Salzman2013 | 2907 | circRNA | Detected | Detected | 5.788304782 | 4.340251894 |
| hsa_gci170052 | 2.850244807 | 1.511085837 | up | 6.595456975 | 5.084371137 | GGGGACCCCGCG | hsa_circ_0085952 | chr8 | 145149941 | 145152428 | + | 1228 | ALT_ACCEPTOR, CDS | NM_001916 | CYC1 | Salzman2013 | 1537 | circRNA | Detected | Detected | 6.595456975 | 5.084371137 |
| hsa_gci170076 | 4.234801065 | 2.082294199 | up | 4.919719015 | 2.837424816 | TTTAAGAGTTAT | hsa_circ_0086185 | chr9 | 121960 | 172172 | - | 743 | ANNOTATED, CDS, c | NM_001145355 | CBWD1 | Salzman2013 | 55871 | circRNA | Detected | Not Detected | 4.919719015 | 2.837424816 |
| hsa_gci170089 | 2.599568045 | 1.378271919 | up | 6.444250603 | 5.065978684 | GCAATAAAACAG | hsa_circ_0086258 | chr9 | 4823547 | 4849550 | + | 835 | ANNOTATED, CDS, c | NM_005772 | RCL1 | Salzman2013 | 10171 | circRNA | Detected | Detected | 6.444250603 | 5.065978684 |
| hsa_gci170097 | 2.433935544 | 1.283290963 | up | 5.023355114 | 3.740064151 | ATCAGGACCCCG | hsa_circ_0086293 | chr9 | 5825091 | 5831028 | - | 430 | ANNOTATED, CDS, c | NM_024896 | ERMP1 | Salzman2013 | 79956 | circRNA | Detected | Detected | 5.023355114 | 3.740064151 |
| hsa_gci170098 | 3.696470478 | 1.886148391 | up | 7.823994273 | 5.937845882 | CCATATAGGTAA | hsa_circ_0086296 | chr9 | 6420911 | 6421142 | + | 231 | ANNOTATED, CDS, c | NM_152896 | UHRF2 | Salzman2013 | 115426 | circRNA | Detected | Detected | 7.823994273 | 5.937845882 |
| hsa_gci170136 | 2.55793727 | 1.354980884 | up | 5.992140705 | 4.637159821 | CGTCGGTCACCG | hsa_circ_0086475 | chr9 | 19376253 | 19379616 | - | 781 | ANNOTATED, CDS, c | NM_001010 | RPS6 | Salzman2013 | 6194 | circRNA | Detected | Detected | 5.992140705 | 4.637159821 |
| hsa_gci170137 | 2.050615463 | 1.036056979 | up | 5.552043338 | 4.515986359 | GGAGGCTCCGCG | hsa_circ_0086476 | chr9 | 19376253 | 19380235 | - | 829 | ANNOTATED, CDS, c | NM_001010 | RPS6 | Salzman2013 | 6194 | circRNA | Detected | Detected | 5.552043338 | 4.515986359 |
| hsa_gci170147 | 2.092233211 | 1.065043671 | up | 6.128638851 | 5.06359518 | ACATACACACGT | hsa_circ_0086499 | chr9 | 20715320 | 20981685 | + | 4670 | ANNOTATED, CDS, c | NM_017794 | FOCAD | Salzman2013 | 54914 | circRNA | Detected | Detected | 6.128638851 | 5.06359518 |
| hsa_gci170159 | 2.086002219 | 1.060740693 | up | 8.902438658 | 7.841697965 | TCGCTTACGTTT | hsa_circ_0086550 | chr9 | 20862576 | 20874806 | + | 397 | ANNOTATED, CDS, c | NM_017794 | FOCAD | Salzman2013 | 54914 | circRNA | Detected | Detected | 8.902438658 | 7.841697965 |
| hsa_gci170162 | 4.232047953 | 2.081355974 | up | 8.814629567 | 6.733273593 | CGAAACCTCTCT | hsa_circ_0086554 | chr9 | 20862576 | 20948392 | + | 1878 | ANNOTATED, CDS, c | NM_017794 | FOCAD | Salzman2013 | 54914 | circRNA | Detected | Detected | 8.814629567 | 6.733273593 |
| hsa_gci170163 | 4.119194404 | 2.042362215 | up | 9.384838418 | 7.342476202 | AAACCTCTCTCT | hsa_circ_0086555 | chr9 | 20862576 | 20949674 | + | 2028 | ANNOTATED, CDS, c | NM_017794 | FOCAD | Salzman2013 | 54914 | circRNA | Detected | Detected | 9.384838418 | 7.342476202 |
| hsa_gci170181 | 2.921411414 | 1.546665543 | up | 6.542066908 | 4.995401365 | CTGTCAAATAGT | hsa_circ_0086623 | chr9 | 33038682 | 33039062 | + | 380 | ANNOTATED, CDS, c | NM_001539 | DNAJA1 | Salzman2013 | 3301 | circRNA | Detected | Detected | 6.542066908 | 4.995401365 |
| hsa_gci170184 | 3.471498393 | 1.795558504 | up | 6.206026109 | 4.410467605 | TATTGTGTAGAG | hsa_circ_0086651 | chr9 | 33318728 | 33352717 | + | 1141 | ANNOTATED, CDS, c | NM_002504 | NFX1 | Salzman2013 | 4799 | circRNA | Detected | Detected | 6.206026109 | 4.410467605 |
| hsa_gci170186 | 2.002941238 | 1.002120096 | up | 11.30455098 | 10.30243089 | CACGTTCCCCCT | hsa_circ_0086657 | chr9 | 33347035 | 33352717 | + | 385 | ANNOTATED, CDS, c | NM_002504 | NFX1 | Salzman2013 | 4799 | circRNA | Detected | Detected | 11.30455098 | 10.30243089 |
| hsa_gci170188 | -7.546157188 | -2.915742152 | down | 1.253413427 | 4.169155579 | ACCACTTATCCC | hsa_circ_0086681 | chr9 | 33886878 | 33886965 | + | 87 | ANNOTATED, CDS, c | NM_017811 | UBE2R2 | Salzman2013 | 54926 | circRNA | Not Detected | Detected | 1.253413427 | 4.169155579 |
| hsa_gci170205 | 2.070695655 | 1.050115526 | up | 6.387127905 | 5.33701238 | TTATTACATACA | hsa_circ_0086735 | chr9 | 33986757 | 34017187 | - | 561 | ANNOTATED, CDS, c | NM_018449 | UBAP2 | Salzman2013 | 55833 | circRNA | Detected | Detected | 6.387127905 | 5.33701238 |
| hsa_gci170212 | 2.232688315 | 1.158781864 | up | 7.836758432 | 6.677976568 | CCGACGCTACCG | hsa_circ_0086755 | chr9 | 34368906 | 34372872 | - | 3966 | ALT_ACCEPTOR, CDS | NM_020702 | MYORG | Salzman2013 | 57462 | circRNA | Detected | Detected | 7.836758432 | 6.677976568 |
| hsa_gci170214 | 2.252661522 | 1.171630555 | up | 7.201859746 | 6.030229191 | GCCCGTCGTACG | hsa_circ_0086760 | chr9 | 34613547 | 34614765 | - | 537 | ANNOTATED, CDS, c | NM_024348 | DCTN3 | Salzman2013 | 11258 | circRNA | Detected | Detected | 7.201859746 | 6.030229191 |
| hsa_gci170224 | 2.295669692 | 1.198915077 | up | 8.654241529 | 7.455326452 | CAGATTGTGTCC | hsa_circ_0086794 | chr9 | 35099888 | 35102191 | - | 1031 | ANNOTATED, CDS, c | NM_013442 | STOML2 | Salzman2013 | 30968 | circRNA | Detected | Detected | 8.654241529 | 7.455326452 |
| hsa_gci170247 | 2.278680059 | 1.188198375 | up | 5.183464032 | 3.995265656 | ATCTCTACGACT | hsa_circ_0087024 | chr9 | 36369715 | 36390613 | - | 558 | ANNOTATED, CDS, c | NM_194328 | RNF38 | Salzman2013 | 152006 | circRNA | Detected | Detected | 5.183464032 | 3.995265656 |
| hsa_gci170289 | 3.081829477 | 1.623787037 | up | 4.947280763 | 3.323493726 | CGTTGGCGCTTC | hsa_circ_0087217 | chr9 | 77561498 | 77567802 | - | 2325 | ANNOTATED, CDS, c | NM_017998 | C9orf40 | Salzman2013 | 55071 | circRNA | Detected | Not Detected | 4.947280763 | 3.323493726 |
| hsa_gci170321 | 2.083427592 | 1.058958961 | up | 4.265850708 | 3.206891747 | GACAGAAAAGTG | hsa_circ_0087331 | chr9 | 84267098 | 84300811 | - | 277 | ALT_DONOR, CDS, c | NM_005077 | TLE1 | Salzman2013 | 7088 | circRNA | Detected | Detected | 4.265850708 | 3.206891747 |
| hsa_gci170324 | -3.046314308 | -1.607064802 | down | 2.154635454 | 3.761700255 | AGTGAACGGTCA | hsa_circ_0087335 | chr9 | 84267128 | 84300811 | - | 247 | ANNOTATED, CDS, c | NM_005077 | TLE1 | Salzman2013 | 7088 | circRNA | Not Detected | Detected | 2.154635454 | 3.761700255 |
| hsa_gci170350 | 2.644578429 | 1.403037761 | up | 5.712629515 | 4.309591753 | GTAGGTGTCGAA | hsa_circ_0087452 | chr9 | 94056664 | 94118252 | - | 1963 | ALT_DONOR, CDS, c | NM_001698 | AUH | Salzman2013 | 549 | circRNA | Detected | Detected | 5.712629515 | 4.309591753 |
| hsa_gci170389 | 13.41954447 | 3.746263795 | up | 4.962944418 | 1.216680623 | ACGAGAGGTTTC | hsa_circ_0087673 | chr9 | 100403843 | 100410505 | + | 774 | ANNOTATED, CDS, c | NM_002486 | NCBP1 | Salzman2013 | 4686 | circRNA | Detected | Not Detected | 4.962944418 | 1.216680623 |
| hsa_gci170402 | 2.227991732 | 1.155743879 | up | 10.59756967 | 9.441825789 | ACGGTGACCCCC | hsa_circ_0087735 | chr9 | 101984827 | 101992901 | + | 483 | ANNOTATED, CDS, c | NM_006808 | SEC61B | Salzman2013 | 10952 | circRNA | Detected | Detected | 10.59756967 | 9.441825789 |
| hsa_gci170455 | 2.53734864 | 1.343321764 | up | 6.856254783 | 5.512933019 | AGGATCTTAAAA | hsa_circ_0087915 | chr9 | 111812849 | 111822732 | - | 354 | ANNOTATED, CDS, c | NM_032012 | TMEM245 | Salzman2013 | 23731 | circRNA | Detected | Detected | 6.856254783 | 5.512933019 |
| hsa_gci170456 | 2.223590025 | 1.152890816 | up | 7.396398726 | 6.24350791 | TATTGGTCCTTA | hsa_circ_0087920 | chr9 | 111812849 | 111868889 | - | 1280 | ANNOTATED, CDS, c | NM_032012 | TMEM245 | Salzman2013 | 23731 | circRNA | Detected | Detected | 7.396398726 | 6.24350791 |
| hsa_gci170458 | 2.501704001 | 1.322911101 | up | 6.295207867 | 4.972296766 | ACTTTGTCAGTT | hsa_circ_0087927 | chr9 | 111819470 | 111870850 | - | 1275 | ANNOTATED, CDS, c | NM_032012 | TMEM245 | Salzman2013 | 23731 | circRNA | Detected | Detected | 6.295207867 | 4.972296766 |
| hsa_gci170462 | 2.752912316 | 1.460958659 | up | 5.703438914 | 4.242480255 | CTTTGTCAGTTG | hsa_circ_0087938 | chr9 | 111868787 | 111870850 | - | 220 | ANNOTATED, CDS, c | NM_032012 | TMEM245 | Salzman2013 | 23731 | circRNA | Detected | Detected | 5.703438914 | 4.242480255 |
| hsa_gci170473 | 2.665657333 | 1.414491336 | up | 6.834420211 | 5.419928876 | GGGAACTTTATG | hsa_circ_0087991 | chr9 | 114145490 | 114154104 | - | 711 | ANNOTATED, CDS, c | NM_001080398 | ECPAS | Salzman2013 | 23392 | circRNA | Detected | Detected | 6.834420211 | 5.419928876 |
| hsa_gci170495 | 2.032128379 | 1.022991547 | up | 8.528060512 | 7.505068965 | CGGTCTTTGAAG | hsa_circ_0088048 | chr9 | 114860749 | 114905903 | - | 1101 | ANNOTATED, CDS, c | NM_022486 | SUSD1 | Salzman2013 | 64420 | circRNA | Detected | Detected | 8.528060512 | 7.505068965 |
| hsa_gci170499 | 3.084517286 | 1.625044731 | up | 4.109906596 | 2.484861865 | ACGGTCTTCTTT | hsa_circ_0088063 | chr9 | 114986412 | 115060196 | - | 1493 | ANNOTATED, CDS, c | NM_001163790 | PTBP3 | Salzman2013 | 9991 | circRNA | Detected | Not Detected | 4.109906596 | 2.484861865 |
| hsa_gci170502 | 2.467101845 | 1.302817273 | up | 8.533556659 | 7.230739386 | AAGTTCTGACAC | hsa_circ_0088072 | chr9 | 115013208 | 115015068 | - | 286 | ANNOTATED, CDS, c | NM_001244898 | PTBP3 | Salzman2013 | 9991 | circRNA | Detected | Detected | 8.533556659 | 7.230739386 |
| hsa_gci170504 | 4.122201359 | 2.043414979 | up | 7.987740114 | 5.944325136 | GTCTTCGGTAAA | hsa_circ_0088079 | chr9 | 115024714 | 115030475 | - | 312 | ANNOTATED, CDS, c | NM_001244898 | PTBP3 | Salzman2013 | 9991 | circRNA | Detected | Detected | 7.987740114 | 5.944325136 |
| hsa_gci170505 | 3.113482844 | 1.63852933 | up | 8.6100926 | 6.97156327 | ACGACAGTAAGG | hsa_circ_0088080 | chr9 | 115024714 | 115038293 | - | 482 | ANNOTATED, CDS, c | NM_001244898 | PTBP3 | Salzman2013 | 9991 | circRNA | Detected | Detected | 8.6100926 | 6.97156327 |
| hsa_gci170509 | 9.356764974 | 3.226009816 | up | 4.639062466 | 1.41305265 | GCAATTACTACG | hsa_circ_0088085 | chr9 | 115060120 | 115060196 | - | 76 | ANNOTATED, CDS, c | NM_001244898 | PTBP3 | Salzman2013 | 9991 | circRNA | Detected | Not Detected | 4.639062466 | 1.41305265 |
| hsa_gci170516 | 2.083004721 | 1.058666109 | up | 4.219251919 | 3.160585809 | TTTAGTTTGTCT | hsa_circ_0088110 | chr9 | 115920006 | 115923978 | + | 257 | ANNOTATED, CDS, c | NM_001860 | SLC31A2 | Salzman2013 | 1318 | circRNA | Detected | Not Detected | 4.219251919 | 3.160585809 |
| hsa_gci170517 | 2.038740897 | 1.027678436 | up | 7.375229252 | 6.347550816 | AACGTCTCACAA | hsa_circ_0088114 | chr9 | 115948715 | 115950771 | - | 1567 | ALT_DONOR, CDS, c | NM_015258 | FKBP15 | Salzman2013 | 23307 | circRNA | Detected | Detected | 7.375229252 | 6.347550816 |
| hsa_gci170558 | -3.64599449 | -1.866312381 | down | 1.539730736 | 3.406043117 | CGTCCGCATAAC | hsa_circ_0088357 | chr9 | 124906337 | 124922098 | - | 843 | ANNOTATED, CDS, c | NM_014222 | NDUFA8 | Salzman2013 | 4702 | circRNA | Not Detected | Detected | 1.539730736 | 3.406043117 |

| | | | | | | | | | | | | | | | | | | | | | | |
|---|---|---|---|---|---|---|---|---|---|---|---|---|---|---|---|---|---|---|---|---|---|---|
| hsa_gci170591 | 2.331881745 | 1.221494628 | up | 8.709121951 | 7.487627323 | TACAATTCGTCG | hsa_circ_0088462 | chr9 | 126392654 | 126439068 | - | 417 | ANNOTATED, CDS, c | NM_020946 | DENND1A | Salzman2013 | 57706 | circRNA | Detected | Detected | 8.709121951 | 7.487627323 |
| hsa_gci170595 | 2.317073998 | 1.212304119 | up | 7.927474624 | 6.715170505 | ACAATTCGTCGA | hsa_circ_0088471 | chr9 | 126429304 | 126439068 | - | 205 | ANNOTATED, CDS, c | NM_020946 | DENND1A | Salzman2013 | 57706 | circRNA | Detected | Detected | 7.927474624 | 6.715170505 |
| hsa_gci170633 | 2.420361121 | 1.275222315 | up | 7.884494391 | 6.609272076 | TGCGGTCGGCCC | hsa_circ_0088667 | chr9 | 130267622 | 130271410 | - | 2581 | ALT_DONOR, CDS, c | NM_022833 | FAM129B | Salzman2013 | 64855 | circRNA | Detected | Detected | 7.884494391 | 6.609272076 |
| hsa_gci170646 | 2.002390915 | 1.00172365 | up | 8.592259415 | 7.590535765 | CCTCGCGGTCCC | hsa_circ_0088771 | chr9 | 131038865 | 131051268 | + | 724 | ANNOTATED, CDS, c | NM_001040011 | SWI5 | Salzman2013 | 375757 | circRNA | Detected | Detected | 8.592259415 | 7.590535765 |
| hsa_gci170651 | 2.5328143 | 1.340741306 | up | 8.463013462 | 7.122272156 | GTAGCAATTAGT | hsa_circ_0088795 | chr9 | 131151539 | 131153015 | + | 1476 | ANNOTATED, CDS, c | NM_001135947 | URM1 | Salzman2013 | 81605 | circRNA | Detected | Detected | 8.463013462 | 7.122272156 |
| hsa_gci170660 | 2.587998544 | 1.371836806 | up | 6.151545813 | 4.779709007 | GAAGGTCGGCGA | hsa_circ_0088875 | chr9 | 131381149 | 131395944 | + | 2165 | ANNOTATED, CDS, c | NM_001130438 | SPTAN1 | Salzman2013 | 6709 | circRNA | Detected | Detected | 6.151545813 | 4.779709007 |
| hsa_gci170663 | 2.720159427 | 1.443691209 | up | 7.228221444 | 5.784530235 | GTTTTCGGAGGC | hsa_circ_0088911 | chr9 | 131549509 | 131572711 | + | 3879 | ANNOTATED, CDS, c | NM_018201 | TBC1D13 | Salzman2013 | 54662 | circRNA | Detected | Detected | 7.228221444 | 5.784530235 |
| hsa_gci170680 | 2.086981863 | 1.061418063 | up | 7.538060788 | 6.476642726 | GCCGGTTCGCCG | hsa_circ_0089050 | chr9 | 132565431 | 132573560 | + | 2769 | ANNOTATED, CDS, c | NM_014506 | TOR1B | Salzman2013 | 27348 | circRNA | Detected | Detected | 7.538060788 | 6.476642726 |
| hsa_gci170693 | 2.231692141 | 1.158138023 | up | 7.111820835 | 5.953682812 | GCCCCCGAGGGC | hsa_circ_0089136 | chr9 | 133748246 | 133763062 | + | 4478 | ANNOTATED, CDS, c | NM_007313 | ABL1 | Salzman2013 | 25 | circRNA | Detected | Detected | 7.111820835 | 5.953682812 |
| hsa_gci170718 | 2.0383031 | 1.0273686 | up | 7.515522795 | 6.488154196 | TGTCCTCTTCTC | hsa_circ_0089304 | chr9 | 135759297 | 135765418 | + | 607 | ANNOTATED, CDS, c | NM_018956 | SPACA9 | Salzman2013 | 11092 | circRNA | Detected | Detected | 7.515522795 | 6.488154196 |
| hsa_gci170724 | 2.523913561 | 1.335662502 | up | 5.760608587 | 4.424946085 | CCTCGGTGGAAG | hsa_circ_0089340 | chr9 | 136215776 | 136218280 | + | 857 | ANNOTATED, CDS, c | NM_000972 | RPL7A | Salzman2013 | 6130 | circRNA | Detected | Detected | 5.760608587 | 4.424946085 |
| hsa_gci170779 | 2.196307004 | 1.135079731 | up | 6.373733326 | 5.238653595 | CGGAGGTGGGGT | hsa_circ_0089844 | chrX | 6966960 | 6968513 | - | 1553 | ANNOTATED, CDS, c | NM_001135565 | PUDP | Salzman2013 | 8226 | circRNA | Detected | Detected | 6.373733326 | 5.238653595 |
| hsa_gci170782 | 2.176292486 | 1.121872463 | up | 9.030727693 | 7.90885523 | GGTCCTTGACCT | hsa_circ_0089861 | chrX | 9862397 | 9917481 | + | 6906 | ANNOTATED, CDS, c | NM_001649 | SHROOM2 | Salzman2013 | 357 | circRNA | Detected | Detected | 9.030727693 | 7.90885523 |
| hsa_gci170795 | 2.249519149 | 1.169616648 | up | 6.114334925 | 4.944718278 | CAAAGGGGTAGT | hsa_circ_0089977 | chrX | 18264696 | 18275150 | - | 549 | ANNOTATED, CDS, c | NM_006089 | SCML2 | Salzman2013 | 10389 | circRNA | Detected | Detected | 6.114334925 | 4.944718278 |
| hsa_gci170804 | 3.265338022 | 1.707232345 | up | 6.255572207 | 4.548339862 | TGGGTTCTCACG | hsa_circ_0090051 | chrX | 21886584 | 21887796 | + | 300 | ANNOTATED, CDS, c | NM_015884 | MBTPS2 | Salzman2013 | 51360 | circRNA | Detected | Detected | 6.255572207 | 4.548339862 |
| hsa_gci170806 | 2.190944336 | 1.131552831 | up | 6.849279222 | 5.71772639 | CGGTCCCTCTCA | hsa_circ_0090069 | chrX | 22108546 | 22117269 | + | 416 | ANNOTATED, CDS, c | NM_000444 | PHEX | Salzman2013 | 5251 | circRNA | Detected | Detected | 6.849279222 | 5.71772639 |
| hsa_gci170810 | 2.06246224 | 1.044367706 | up | 4.95877935 | 3.914411643 | AGAACCGCCTCA | hsa_circ_0090091 | chrX | 23722810 | 23740870 | - | 858 | ANNOTATED, CDS, c | NM_001037171 | ACOT9 | Salzman2013 | 23597 | circRNA | Detected | Detected | 4.95877935 | 3.914411643 |
| hsa_gci170818 | 2.266436166 | 1.180425528 | up | 5.420442441 | 4.240016912 | GGTCCACAAGGC | hsa_circ_0090115 | chrX | 24080532 | 24096927 | + | 2966 | ANNOTATED, CDS, c | NM_001415 | EIF2S3 | Salzman2013 | 1968 | circRNA | Detected | Detected | 5.420442441 | 4.240016912 |
| hsa_gci170822 | 3.442476929 | 1.783446986 | up | 5.175863376 | 3.392416391 | GACAGTCGGGCC | hsa_circ_0090123 | chrX | 24089674 | 24096927 | + | 2432 | ANNOTATED, CDS, c | NM_001415 | EIF2S3 | Salzman2013 | 1968 | circRNA | Detected | Detected | 5.175863376 | 3.392416391 |
| hsa_gci170839 | 2.001276348 | 1.000920397 | up | 4.927169221 | 3.926248825 | TTAAGTTCTTAT | hsa_circ_0090220 | chrX | 40982723 | 40982977 | + | 254 | ANNOTATED, CDS, c | NM_001039590 | USP9X | Salzman2013 | 8239 | circRNA | Detected | Detected | 4.927169221 | 3.926248825 |
| hsa_gci170843 | 2.673434571 | 1.418694369 | up | 6.822548967 | 5.403854598 | CAACGGCCCCTT | hsa_circ_0090228 | chrX | 40988252 | 40988398 | + | 146 | ANNOTATED, CDS, c | NM_001039590 | USP9X | Salzman2013 | 8239 | circRNA | Detected | Detected | 6.822548967 | 5.403854598 |
| hsa_gci170844 | 2.553453561 | 1.352449821 | up | 8.101514931 | 6.74906511 | CCCTCAACGGCC | hsa_circ_0090229 | chrX | 40988252 | 40990789 | + | 226 | ANNOTATED, CDS, c | NM_001039590 | USP9X | Salzman2013 | 8239 | circRNA | Detected | Detected | 8.101514931 | 6.74906511 |
| hsa_gci170848 | -2.096385726 | -1.067904191 | down | 2.547711826 | 3.615616017 | TATTTTGTGTAC | hsa_circ_0090252 | chrX | 41012200 | 41031211 | + | 1385 | ANNOTATED, CDS, c | NM_001039590 | USP9X | Salzman2013 | 8239 | circRNA | Not Detected | Detected | 2.547711826 | 3.615616017 |
| hsa_gci170863 | 2.141099708 | 1.098351982 | up | 8.687048207 | 7.588696225 | CTCAAACCGTCG | hsa_circ_0090312 | chrX | 43542760 | 43606068 | + | 3819 | ANNOTATED, CDS, c | NM_000240 | MAOA | Salzman2013 | 4128 | circRNA | Detected | Detected | 8.687048207 | 7.588696225 |
| hsa_gci170865 | 2.123839669 | 1.08667486 | up | 8.679897866 | 7.593223006 | TCCCTAGCGTAA | hsa_circ_0090317 | chrX | 43590940 | 43606068 | + | 3097 | ANNOTATED, CDS, c | NM_000240 | MAOA | Salzman2013 | 4128 | circRNA | Detected | Detected | 8.679897866 | 7.593223006 |
| hsa_gci170866 | 2.194289372 | 1.133753794 | up | 9.346393016 | 8.212639222 | GCTCTGGTAAAT | hsa_circ_0090320 | chrX | 43603355 | 43606068 | + | 2518 | ANNOTATED, CDS, c | NM_000240 | MAOA | Salzman2013 | 4128 | circRNA | Detected | Detected | 9.346393016 | 8.212639222 |
| hsa_gci170867 | -3.037648471 | -1.602954925 | down | 3.276451843 | 4.879406768 | TTCATCTGGTTT | hsa_circ_0090324 | chrX | 44870205 | 44896934 | + | 270 | ANNOTATED, CDS, c | NM_021140 | KDM6A | Salzman2013 | 7403 | circRNA | Not Detected | Detected | 3.276451843 | 4.879406768 |
| hsa_gci170880 | 2.531734725 | 1.340126247 | up | 8.25001732 | 6.909891073 | GACGTACTACCC | hsa_circ_0090405 | chrX | 47062341 | 47074527 | + | 2143 | ANNOTATED, CDS, c | NM_003334 | UBA1 | Salzman2013 | 7317 | circRNA | Detected | Detected | 8.25001732 | 6.909891073 |
| hsa_gci170883 | 2.075722855 | 1.053613832 | up | 5.91272485 | 4.859111018 | GAATATGGTCAC | hsa_circ_0090426 | chrX | 47098468 | 47107727 | + | 2985 | ANNOTATED, CDS, c | NM_004651 | USP11 | Salzman2013 | 8237 | circRNA | Detected | Detected | 5.91272485 | 4.859111018 |
| hsa_gci170887 | 2.794242555 | 1.48245726 | up | 9.43899015 | 7.95653289 | CAAACGTCCCCT | hsa_circ_0090452 | chrX | 47445919 | 47446190 | + | 271 | ANNOTATED, CDS, c | NM_003254 | TIMP1 | Salzman2013 | 7076 | circRNA | Detected | Detected | 9.43899015 | 7.95653289 |
| hsa_gci170903 | 2.520722311 | 1.333837196 | up | 8.229544212 | 6.895707016 | TCCACTCTTCAA | hsa_circ_0090595 | chrX | 51641676 | 51645450 | + | 773 | ANNOTATED, CDS, c | NM_001005333 | MAGED1 | Salzman2013 | 9500 | circRNA | Detected | Detected | 8.229544212 | 6.895707016 |
| hsa_gci170907 | 2.49697139 | 1.320179291 | up | 9.463217372 | 8.143038081 | AGGGGTCGGGAA | hsa_circ_0090621 | chrX | 53295505 | 53310796 | - | 956 | ANNOTATED, CDS, c | NM_001243197 | IQSEC2 | Salzman2013 | 23096 | circRNA | Detected | Detected | 9.463217372 | 8.143038081 |
| hsa_gci170915 | 2.129363099 | 1.090421979 | up | 4.972296766 | 3.881874787 | AGGAGTAGGAGT | hsa_circ_0090739 | chrX | 53588716 | 53589205 | - | 303 | ANNOTATED, CDS, c | NM_031407 | HUWE1 | Salzman2013 | 10075 | circRNA | Detected | Detected | 4.972296766 | 3.881874787 |
| hsa_gci170922 | 2.209572305 | 1.143767142 | up | 7.688813861 | 6.545046719 | TCGGTACCCGGT | hsa_circ_0090796 | chrX | 53654360 | 53657986 | - | 727 | ANNOTATED, CDS, c | NM_031407 | HUWE1 | Salzman2013 | 10075 | circRNA | Detected | Detected | 7.688813861 | 6.545046719 |
| hsa_gci170928 | 3.148114438 | 1.654487985 | up | 5.833549359 | 4.179061374 | CGTCGAGATGCC | hsa_circ_0090839 | chrX | 54837478 | 54842445 | + | 1199 | ALT_ACCEPTOR, CDS | NM_201222 | MAGED2 | Salzman2013 | 10916 | circRNA | Detected | Detected | 5.833549359 | 4.179061374 |
| hsa_gci170931 | 2.472164697 | 1.305774859 | up | 4.540997175 | 3.235222316 | CTAACGATAACC | hsa_circ_0090845 | chrX | 54841093 | 54842440 | + | 685 | ALT_DONOR, CDS, c | NM_201222 | MAGED2 | Salzman2013 | 10916 | circRNA | Detected | Detected | 4.540997175 | 3.235222316 |
| hsa_gci170933 | 2.420539625 | 1.275328712 | up | 6.36074827 | 5.085419558 | GGCTGGACTCCT | hsa_circ_0090854 | chrX | 55511048 | 55515421 | - | 4373 | ANNOTATED, CDS, c | NM_201286 | USP51 | Salzman2013 | 158880 | circRNA | Detected | Detected | 6.36074827 | 5.085419558 |
| hsa_gci170943 | 2.076682664 | 1.054280776 | up | 4.866904699 | 3.812623924 | AGTGTGCGTGTG | hsa_circ_0090885 | chrX | 64936679 | 64947771 | + | 180 | ANNOTATED, CDS, c | NM_002444 | MSN | Salzman2013 | 4478 | circRNA | Detected | Detected | 4.866904699 | 3.812623924 |
| hsa_gci170949 | -2.014585637 | -1.010483133 | down | 3.448298627 | 4.45878176 | AAGAAGAGGTAG | hsa_circ_0090953 | chrX | 69561648 | 69624672 | + | 1765 | ANNOTATED, CDS, c | NM_012310 | KIF4A | Salzman2013 | 24137 | circRNA | Not Detected | Detected | 3.448298627 | 4.45878176 |
| hsa_gci170958 | 2.165858593 | 1.114939053 | up | 7.88820991 | 6.773270856 | GTTGTACCCGGC | hsa_circ_0091027 | chrX | 70752911 | 70765701 | + | 1985 | ALT_DONOR, CDS, c | NM_181672 | OGT | Salzman2013 | 8473 | circRNA | Detected | Detected | 7.88820991 | 6.773270856 |
| hsa_gci170960 | 2.698171704 | 1.43198216 | up | 6.934994818 | 5.503012657 | CTTTCGTATGCA | hsa_circ_0091029 | chrX | 70756027 | 70765701 | + | 1710 | ALT_DONOR, CDS, c | NM_181672 | OGT | Salzman2013 | 8473 | circRNA | Detected | Detected | 6.934994818 | 5.503012657 |
| hsa_gci170961 | 2.267091562 | 1.180842659 | up | 6.349761913 | 5.168919254 | CAAGACAAATTA | hsa_circ_0091030 | chrX | 70757678 | 70765701 | + | 1529 | ALT_DONOR, CDS, c | NM_181672 | OGT | Salzman2013 | 8473 | circRNA | Detected | Detected | 6.349761913 | 5.168919254 |
| hsa_gci170963 | 3.267274645 | 1.708087732 | up | 6.524654843 | 4.816567112 | TTAACTATATTC | hsa_circ_0091035 | chrX | 70774362 | 70775235 | + | 276 | ANNOTATED, CDS, c | NM_181672 | OGT | Salzman2013 | 8473 | circRNA | Detected | Detected | 6.524654843 | 4.816567112 |
| hsa_gci170967 | 5.693303325 | 2.509265965 | up | 7.77790968 | 5.268643715 | GGAAAGGAGCCC | hsa_circ_0091048 | chrX | 71492452 | 71492622 | - | 170 | ANNOTATED, CDS, c | NM_001007 | RPS4X | Salzman2013 | 6191 | circRNA | Detected | Detected | 7.77790968 | 5.268643715 |
| hsa_gci170992 | 2.606849749 | 1.382307433 | up | 6.969839795 | 5.587532362 | AACGGTCGGTAG | hsa_circ_0091130 | chrX | 77258569 | 77275895 | + | 1238 | ANNOTATED, CDS, c | NM_000052 | ATP7A | Salzman2013 | 538 | circRNA | Detected | Detected | 6.969839795 | 5.587532362 |
| hsa_gci171000 | 2.414327819 | 1.27162158 | up | 5.887045013 | 4.615423433 | ATAATAACTACT | hsa_circ_0091156 | chrX | 77378331 | 77378871 | + | 295 | ANNOTATED, CDS, c | NM_000291 | PGK1 | Salzman2013 | 5230 | circRNA | Detected | Detected | 5.887045013 | 4.615423433 |
| hsa_gci171012 | 2.158171018 | 1.109809192 | up | 7.439627544 | 6.329818352 | TGTAGAAGTACG | hsa_circ_0091200 | chrX | 85211157 | 85219057 | - | 852 | ANNOTATED, CDS, c | NM_000390 | CHM | Salzman2013 | 1121 | circRNA | Detected | Detected | 7.439627544 | 6.329818352 |
| hsa_gci171025 | 2.261865629 | 1.177513226 | up | 6.578291664 | 5.400778438 | GCGGCGCCTCTG | hsa_circ_0091272 | chrX | 102631955 | 102633001 | + | 690 | ANNOTATED, CDS, c | NM_206915 | BEX3 | Salzman2013 | 27018 | circRNA | Detected | Detected | 6.578291664 | 5.400778438 |
| hsa_gci171041 | -4.675357299 | -2.225076622 | down | 1.454005803 | 3.679082426 | ACTCCCCACTAT | hsa_circ_0091351 | chrX | 114357089 | 114384633 | - | 649 | ANNOTATED, CDS, c | NM_020871 | LRCH2 | Salzman2013 | 57631 | circRNA | Not Detected | Detected | 1.454005803 | 3.679082426 |
| hsa_gci171049 | 2.014998362 | 1.010778666 | up | 4.14642559 | 3.135646924 | CCGGTTTCTGAT | hsa_circ_0091404 | chrX | 118370210 | 118378429 | + | 1916 | ANNOTATED, CDS, c | NM_006667 | PGRMC1 | Salzman2013 | 10857 | circRNA | Detected | Not Detected | 4.14642559 | 3.135646924 |
| hsa_gci171060 | 8.139814355 | 3.024995891 | up | 4.310225387 | 1.285229496 | CGAGGAACCAGT | hsa_circ_0091424 | chrX | 118967988 | 118985836 | - | 2148 | ANNOTATED, CDS, c | NM_080632 | UPF3B | Salzman2013 | 65109 | circRNA | Detected | Not Detected | 4.310225387 | 1.285229496 |

| | | | | | | | | | | | | | | | | | | | | | | |
|---|---|---|---|---|---|---|---|---|---|---|---|---|---|---|---|---|---|---|---|---|---|---|
| hsa_gci171062 | -2.972614651 | -1.571732454 | down | 2.445700216 | 4.017432671 | AGGTCTCCACCT | hsa_circ_0091432 | chrX | 119386523 | 119392251 | + | 5085 | ANNOTATED, CDS, c | NM_001184742 | ZBTB33 | Salzman2013 | 10009 | circRNA | Not Detected | Detected | 2.445700216 | 4.017432671 |
| hsa_gci171073 | 2.077966144 | 1.055172149 | up | 4.625537227 | 3.570365078 | TCCATTGGTTCG | hsa_circ_0091477 | chrX | 128630726 | 128645960 | - | 996 | ANNOTATED, CDS, c | NM_003069 | SMARCA1 | Salzman2013 | 6594 | circRNA | Detected | Detected | 4.625537227 | 3.570365078 |
| hsa_gci171086 | 2.259801861 | 1.176196283 | up | 5.955795729 | 4.779599446 | CGGGTCCTCGTC | hsa_circ_0091533 | chrX | 130843525 | 130928494 | - | 974 | ANNOTATED, INTERN | NR_026975 | | Salzman2013 | | circRNA | Detected | Detected | 5.955795729 | 4.779599446 |
| hsa_gci171088 | 3.010051216 | 1.589788035 | up | 7.251253191 | 5.661465156 | ATAACCCCTGGC | hsa_circ_0091537 | chrX | 130883333 | 130918067 | - | 702 | ANNOTATED, INTERN | NR_026975 | | Salzman2013 | | circRNA | Detected | Detected | 7.251253191 | 5.661465156 |
| hsa_gci171093 | 3.174023449 | 1.666312787 | up | 4.659074674 | 2.992761887 | AACTTCTTGTAT | hsa_circ_0091554 | chrX | 130919184 | 130928494 | - | 394 | ANNOTATED, INTERN | TCONS_12_00030860 | | Salzman2013 | | circRNA | Detected | Not Detected | 4.659074674 | 2.992761887 |
| hsa_gci171094 | 2.00523978 | 1.00377476 | up | 5.223729884 | 4.219955124 | GGTCCTCGTCTC | hsa_circ_0091558 | chrX | 130928324 | 130928494 | - | 170 | ANNOTATED, INTERN | TCONS_12_00030860 | | Salzman2013 | | circRNA | Detected | Detected | 5.223729884 | 4.219955124 |
| hsa_gci171095 | 3.403352315 | 1.766956507 | up | 6.412499054 | 4.645542547 | GTCCCTGGAGTG | hsa_circ_0091560 | chrX | 130928351 | 130939065 | - | 732 | ANNOTATED, INTERN | NR_026975 | | Salzman2013 | | circRNA | Detected | Detected | 6.412499054 | 4.645542547 |
| hsa_gci171101 | -3.901207387 | -1.963920694 | down | 1.402656664 | 3.366577358 | ATCAAGAACCGT | hsa_circ_0091580 | chrX | 132826396 | 132888203 | - | 1024 | ANNOTATED, CDS, c | NM_001164617 | GPC3 | Salzman2013 | 2719 | circRNA | Not Detected | Detected | 1.402656664 | 3.366577358 |
| hsa_gci171105 | 2.109224772 | 1.076712846 | up | 4.221715874 | 3.145003029 | GACCGAATATAG | hsa_circ_0091589 | chrX | 133632419 | 133634698 | + | 763 | ANNOTATED, CDS, c | NM_000194 | HPRT1 | Salzman2013 | 3251 | circRNA | Detected | Not Detected | 4.221715874 | 3.145003029 |
| hsa_gci171112 | 2.142141016 | 1.099053455 | up | 9.120805881 | 8.021752427 | CAGTATTTGCTT | hsa_circ_0091625 | chrX | 135044230 | 135056134 | - | 3648 | ANNOTATED, CDS, c | NM_173470 | MMGT1 | Salzman2013 | 93380 | circRNA | Detected | Detected | 9.120805881 | 8.021752427 |
| hsa_gci171126 | 3.559923094 | 1.831846074 | up | 4.474756906 | 2.642910831 | GAGAACCAGCAC | hsa_circ_0091694 | chrX | 149962163 | 149963959 | - | 219 | ANNOTATED, CDS, c | NM_001242614 | CD99L2 | Salzman2013 | 83692 | circRNA | Detected | Not Detected | 4.474756906 | 2.642910831 |
| hsa_gci171128 | 2.214257825 | 1.146823218 | up | 4.265656755 | 3.118833538 | GCGATCGACGGA | hsa_circ_0091698 | chrX | 150151762 | 150159248 | + | 3556 | ANNOTATED, CDS, c | NM_005342 | HMGB3 | Salzman2013 | 3149 | circRNA | Detected | Not Detected | 4.265656755 | 3.118833538 |
| hsa_gci171132 | 2.270084997 | 1.182746316 | up | 7.522668333 | 6.339922017 | CGTCCTCGACCC | hsa_circ_0091721 | chrX | 151934651 | 151936231 | - | 1580 | ANNOTATED, CDS, c | NM_005362 | MAGEA3 | Salzman2013 | 4102 | circRNA | Detected | Detected | 7.522668333 | 6.339922017 |
| hsa_gci171144 | 2.418155402 | 1.273906962 | up | 5.773470286 | 4.499563324 | TAGTAGTATGAA | hsa_circ_0091763 | chrX | 152965946 | 152981144 | - | 993 | ANNOTATED, CDS, c | NM_001139457 | BCAP31 | Salzman2013 | 10134 | circRNA | Detected | Detected | 5.773470286 | 4.499563324 |
| hsa_gci171153 | 2.69840805 | 1.432108528 | up | 8.006435812 | 6.574327284 | TGTCTCTTCCCC | hsa_circ_0091833 | chrX | 153576899 | 153581043 | - | 1882 | ANNOTATED, CDS, c | NM_001110556 | FLNA | Salzman2013 | 2316 | circRNA | Detected | Detected | 8.006435812 | 6.574327284 |
| hsa_gci171158 | 2.087539309 | 1.061803364 | up | 6.40836641 | 5.346563045 | TAGTACTCATCC | hsa_circ_0091843 | chrX | 153576899 | 153586723 | - | 3663 | ANNOTATED, CDS, c | NM_001110556 | FLNA | Salzman2013 | 2316 | circRNA | Detected | Detected | 6.40836641 | 5.346563045 |
| hsa_gci171160 | 2.290674237 | 1.195772304 | up | 5.773305713 | 4.577533409 | CGGAGTGCCTTC | hsa_circ_0091845 | chrX | 153576899 | 153588273 | - | 4456 | ANNOTATED, CDS, c | NM_001110556 | FLNA | Salzman2013 | 2316 | circRNA | Detected | Detected | 5.773305713 | 4.577533409 |
| hsa_gci171164 | 2.660941335 | 1.411936704 | up | 7.920256419 | 6.508319715 | CGTGAGCCCAAT | hsa_circ_0091853 | chrX | 153576899 | 153593854 | - | 6832 | ANNOTATED, CDS, c | NM_001110556 | FLNA | Salzman2013 | 2316 | circRNA | Detected | Detected | 7.920256419 | 6.508319715 |
| hsa_gci171178 | 2.162164093 | 1.112476018 | up | 6.581382016 | 5.468905998 | TCCGGTGGGTGG | hsa_circ_0092090 | chrX | 153696109 | 153701985 | + | 2988 | ANNOTATED, CDS, c | NM_017514 | PLXNA3 | Salzman2013 | 55558 | circRNA | Detected | Detected | 6.581382016 | 5.468905998 |
| hsa_gci171179 | 2.294403052 | 1.198118848 | up | 6.144940308 | 4.94682146 | TCTTCGCGTCGC | hsa_circ_0092094 | chrX | 153699447 | 153701985 | + | 1417 | ANNOTATED, CDS, c | NM_017514 | PLXNA3 | Salzman2013 | 55558 | circRNA | Detected | Detected | 6.144940308 | 4.94682146 |
| hsa_gci171180 | 2.078909914 | 1.055827243 | up | 4.520208253 | 3.46438101 | CGCTTTCCGTAC | hsa_circ_0092133 | chrX | 153845864 | 153846529 | - | 426 | ANNOTATED, CDS, c | NM_001327 | CTAG1B | Salzman2013 | 1485 | circRNA | Detected | Detected | 4.520208253 | 3.46438101 |
| hsa_gci171184 | 2.683147602 | 1.423926421 | up | 6.324625151 | 4.90069873 | GAGGTAGTGAAG | hsa_circ_0092166 | chrX | 154528097 | 154528458 | - | 236 | ANNOTATED, CDS, c | NM_001289 | CLIC2 | Salzman2013 | 1193 | circRNA | Detected | Detected | 6.324625151 | 4.90069873 |
| hsa_gci171195 | 2.84511397 | 1.508486445 | up | 7.496095309 | 5.987608864 | GAAGAACCCCGG | hsa_circ_0092220 | chrY | 2710205 | 2734997 | + | 851 | ANNOTATED, CDS, c | NM_001008 | RPS4Y1 | Salzman2013 | 6192 | circRNA | Detected | Detected | 7.496095309 | 5.987608864 |
