# Supplementary material for: Hypoxia‐Induced circPRELID2 Promotes Gastric Cancer Metastasis by Facilitating ZEB2 Translation via PCBP1 O‐GlcNAcylation
Source: Adv Sci (Weinh). 2025 Oct 21;12(46):e05396. doi: 10.1002/advs.202505396 (PMC12697806; doi:10.1002/advs.202505396)
Supplement: Supplementary file 2 — Supporting Information [file ADVS-12-e05396-s002.zip › Flag-DYRK1A-Mass Spectrometry-proteins.pdf]

Supplementary Data 3. Mass spectrometry identification of potential binding proteins of DYRK1A					
Accession	Description	Gene name	Peptide count	Unique peptides	Confidence Score
Q13627	dual specificity tyrosine phosphorylation regulated kinase 1A	DYRK1A	38	38	2449.42
P21333	filamin A	FLNA	29	26	1842.59
P61962	DDB1 and CUL4 associated factor 7	DCAF7	27	27	1578.53
P24941	cyclin-dependent kinase 2 isoform 2	CDK2	13	13	1032.6
P06400	retinoblastoma 1	RB1	19	16	1319.69
P23246	splicing factor proline and glutamine rich	SFPQ	6	6	474.41
Q9GZS3	WD repeat-containing protein 61	WDR61	7	5	733.03
Q12933	TNF receptor-associated factor 2	TRAF2	15	15	1273.28
P20248	Cyclin-A2	CCNA2	17	16	1240.54
Q9BRK4	leucine zipper, putative tumor suppressor 2	LZTS2	8	8	749.38
Q8NCN4	ring finger protein 169	RNF169	12	12	937.32
Q9Y4B4	Helicase ARIP4	RAD54L2	3	3	224.5
Q92793	CREB binding protein isoform a	CBP	25	25	1420.82
P30281	G1/S-specific cyclin-D3	CCND3	9	9	831.44
Q12815	Trophinin-associated protein	TROAP	7	7	503.58
O00716	E2F transcription factor 3	E2F3	2	2	104.35
P31689	DnaJ heat shock protein family (Hsp40) member A1	DNAJA1	5	5	395.84
Q9BYW2	SET domain containing 2	SETD2	12	11	739.80
P52272	heterogeneous nuclear ribonucleoprotein M	HNRNPM	7	7	471.55
tr|E9PQN2|E9PQN2_HUMAN	Bcl-2-associated transcription factor 1	BCLAF1	4	4	320.27
P48382	regulatory factor X, 5	RFX5	4	4	394.42
O60341	amine oxidase (flavin containing) domain 2 isoform b	KDM1A	8	6	438.18
Q2KHR2	regulatory factor X domain containing 2 [Homo sapiens]	RFX7	3	3	263.21
Q86VQ1	glucocorticoid induced transcript 1 [Homo sapiens]	GLCCI1	5	4	394.5
Q15776	zinc finger protein 192	ZNF192	3	3	238.21
Q07666	KH RNA binding domain containing, signal transduction associated	SAM68	9	9	943.53
P04637	tumor protein p53 isoform a	TP53	22	20	1343.49
O15042	U2 snRNP associated SURP domain containing	U2SURP	2	2	104.27
Q9NWA0	mediator complex subunit 9	MED9	7	7	590.22
Q01081	U2 small nuclear RNA auxillary factor 1 isoform a	U2AF1	3	3	136.55
Q15293	reticulocalbin 1	RCN1	5	5	348.76
P62847	ribosomal protein S24	RPS24	7	7	402.32

tr|A0A024RD18|A0A024RD18	Serine/threonine kinase 38	STK38	5	5	374.85
P07203	glutathione peroxidase 1 isoform 1	GPX1	3	3	224.87
P01106	myc proto-oncogene protein	MYC	14	11	632.33
Q92804	TATA-box binding protein associated factor 15	TAF15	5	5	394.28
Q5PRF9	sterile alpha motif domain containing 4B	SAMD4B	3	3	204.93
Q8IXK0	Polyhomeotic-like protein 2	PHC2	3	3	173.43
tr|A0A024R534|A0A024R534	Metastasis associated 1 family, member 2	MTA2	7	7	482.88
Q96T58	spen homolog, transcriptional regulator	SPEN	6	5	395.32
Q9UN86	G3BP stress granule assembly factor 2	G3BP2	2	2	82.82
Q15424	scaffold attachment factor B	SAFB	10	8	639.21
Q92628	hypothetical protein LOC9778	KIAA0232	2	2	104.2
Q71SY5	mediator complex subunit 25	MED25	5	5	319.49
tr|A0A024RAN7|A0A024RAN7	Ankyrin repeat, family A (RFXANK-like), 2,	ANKRA2	4	4	247.52
P11908	phosphoribosyl pyrophosphate synthetase 2 isoform 2	PRPS2	7	5	347.37
tr|E7EMB3|E7EMB3_HUMAN	Calmodulin	CALM2	3	3	194.26
P25787	proteasome subunit alpha type 2	PSMA2	3	3	134.94
P00338	lactate dehydrogenase A	LDHA	8	8	630.57
Q96T17	MAP7 domain containing 2	MAP7D2	4	4	249.04
Q96FJ2	dynein light chain LC8-type 2	DYNLL2	7	7	427.23
tr|H3BSS5|H3BSS5_HUMAN	BTB/POZ domain-containing protein KCTD5	KCTD5	3	3	134.58
Q9HAH7	fibrosin	FBRS	6	6	374.20
P41743	Protein kinase C iota type	PRKCI	8	8	549.22
Q9NYF3	family with sequence similarity 53 member C	FAM53C	13	13	963.45
tr|Q5VWV2|Q5VWV2_HUMAN	Partitioning defective 3 homolog	PARD3	10	9	832.03
Q00610	Clathrin heavy chain	CLTC	4	4	214.94
Q9Y4A5	Transformation/transcription domain-associated protein	TRRAP	1	1	35.2
P22061	protein-L-isoaspartate(D-aspartate) O-methyltransferase	PCMT1	1	1	29.04
Q9H4W6	Transcription factor COE3	EBF3	3	3	147.53
Q14980	nuclear mitotic apparatus protein 1	NUMA1	5	4	273.2
P17844	DEAD-box helicase 5	DDX5	2	2	43.43
Q9C0C7	activating molecule in beclin-1-regulated autophagy	AMBRA1	6	6	380.05
Q6W2J9	BCL6 corepressor	BCOR	4	4	284.91
P19105	myosin light chain 12A	MYL12A	6	6	302.4
tr|Q2NKY5|Q2NKY5_HUMAN	TUBB6 protein	TUBB6	4	4	285.83

P29590	promyelocytic leukemia	PML	7	7	384.61
Q96HC4	PDZ and LIM domain 5	PDLIM5	8	8	664.7
Q01804	OTU deubiquitinase 4	OTUD4	3	3	240.88
Q04637	eukaryotic translation initiation factor 4 gamma 1	EIF4G1	5	5	333.53
P13647	Keratin, type II cytoskeletal 5	KRT5	3	3	204.49
Q14244	microtubule associated protein 7	MAP7	9	9	822.01
tr|A4D2J0|A4D2J0_HUMAN	SNARE protein Ykt6	YKT6	3	3	202.3
Q13885	tubulin beta 2A class IIa	TUBB2A	7	7	418.34
tr|E7EUN2|E7EUN2_HUMAN	Arf-GAP with GTPase, ANK repeat and PH domain-containing protein	AGAP1	2	2	83.46
O95684	FGFR1 oncogene partner	FGFR1OP	6	6	384.07
Q16527	cysteine and glycine rich protein 2	CSRP2	1	1	20.92
Q03701	CCAAT/enhancer-binding protein zeta	CEBPZ	3	3	201.4
P23527	Histone H2B type 1-O	HIST1H2BO	3	3	149.68
P40424	PBX homeobox 1	PBX1	6	6	403.47
P35579	myosin heavy chain 9	MYH9	12	9	728.94
Q13227	G protein pathway suppressor 2	GPS2	4	4	259.44
P26641	Elongation factor 1-gamma	EEF1G	4	3	284.06
P29558	RNA binding motif single stranded interacting protein 1	RBMS1	2	2	63.43
P27348	tyrosine 3-monooxygenase/tryptophan 5-monooxygenase activation p	YWHAQ	5	5	337.39
P08670	vimentin	VIM	3	3	148.58
Q8N7X1	RNA-binding motif protein, X-linked-like-3	RBMXL3	1	1	42.11
Q01469	fatty acid binding protein 5	FABP5	1	1	28.74
P62937	peptidylprolyl isomerase A	PPIA	2	2	120.92
Q9BQG0	Myb-binding protein 1A	MYBBP1A	4	3	231.4
Q7Z6Z7	HECT, UBA and WWE domain containing 1, E3 ubiquitin protein liga	HUWE1	2	2	131.38
Q14566	minichromosome maintenance complex component 6	MCM6	5	4	236.43
Q96HS1	PGAM family member 5, mitochondrial serine/threonine protein pho	PGAM5	2	2	73.2
Q92945	KH-type splicing regulatory protein	KHSRP	6	5	302.47
Q96AV8	Transcription factor E2F7	E2F7	3	3	200.3
P46940	IQ motif containing GTPase activating protein 1	IQGAP1	7	6	520.89
Q13363	C-terminal-binding protein 1	CTBP1	3	3	284.22
O15234	cancer susceptibility 3	CASC3	2	2	142.43
P47756	capping actin protein of muscle Z-line beta subunit	CAPZB	1	1	39.04
P19474	E3 ubiquitin-protein ligase TRIM21	TRIM21	4	4	284.81

Q8NEJ9	neuroguidin	NGDN	1	1	42.05
Q8NAF0	Zinc finger protein 579	ZNF579	5	5	381.32
O00571	DEAD-box helicase 3, X-linked	DDX3X	1	1	22.42
P18621	ribosomal protein L17	RPL17	6	6	444.93
Q5T7B8	kinesin family member 24	KIF24	3	3	184.72
P11413	glucose-6-phosphate 1-dehydrogenase	G6PD	3	3	153.2
Q92499	DEAD-box helicase 1	DDX1	5	5	393.84
Q15007	Wilms tumor 1 associated protein	WTAP	1	1	30.31
tr|E7EQS8|E7EQS8_HUMAN	Methylcytosine dioxygenase TET2	TET2	4	4	219.34
P52597	heterogeneous nuclear ribonucleoprotein F	HNRNPF	11	9	571.7
tr|H3BUW8|H3BUW8_HUMAN	Lon protease homolog 2, peroxisomal	LONP2	2	2	88.27
P16989	Y-box binding protein 3	YBX3	5	5	379.02
P62424	ribosomal protein L7a	RPL7A	3	3	149.43
tr|E9PQ57|E9PQ57_HUMAN	mRNA export factor	RAE1	6	6	432.77
Q08945	structure specific recognition protein 1	SSRP1	6	5	302.41
Q13310	poly(A) binding protein cytoplasmic 4	PABPC4	3	3	139.43
Q6ZW49	PAX interacting protein 1	PAXIP1	1	1	32.7
Q07020	ribosomal protein L18	RPL18	8	8	702.29
Q12888	tumor protein p53 binding protein 1	TP53BP1	2	2	161.45
P16401	histone cluster 1 H1 family member b	HIST1H1B	1	1	34.03
Q9H8S9	MOB kinase activator 1A	MOB1A	3	3	183.99
tr|B7WPL0|B7WPL0_HUMAN	Synembryn-B	RIC8B	5	5	348.46
P38646	heat shock protein family A (Hsp70) member 9	HSPA9	1	1	27.72
Q9UGU0	transcription factor 20	TCF20	6	6	492.25
O00287	Regulatory factor X-associated protein	RFXAP	2	2	103.64
tr|A0A024R1Q8|A0A024R1Q8	Ribosomal protein L23, isoform CRA_b	RPL23	2	2	83.07
Q9Y262	eukaryotic translation initiation factor 3 subunit L	EIF3L	4	4	246.75
Q9NR12	PDZ and LIM domain 7	PDLIM7	1	1	30.53
Q9H0H5	Rac GTPase activating protein 1	RACGAP1	3	2	117.79
Q9Y224	RNA transcription, translation and transport factor	RTRAF	3	3	140.56
Q16555	dihydropyrimidinase like 2	DPYSL2	4	4	240.43
Q6PJG2	ELM2 and Myb/SANT domain containing 1	ELMSAN1	1	1	40.88
P60866	ribosomal protein S20	RPS20	5	5	310.22
Q8TAQ2	SWI/SNF related, matrix associated, actin dependent regulator o	SMARCC2	8	8	638.43

P62244	ribosomal protein S15a	RPS15A	1	1	24.37
P68371	tubulin beta 4B class IVb	TUBB4B	3	3	147.56
Q9BTC0	death inducer-obliterator 1	DIDO1	6	6	493.07
tr|A0A068F7M9|A0A068F7M9	FH1/FH2 domain-containing protein 1 variant	FHOD1	2	2	93.74
P02751	fibronectin 1	FN1	7	7	584.39
P33176	kinesin family member 5B	KIF5B	3	3	175.95
tr|B3KNL2|B3KNL2_HUMAN	RuvB-like 2 (E. coli), isoform CRA_d	RUVBL2	1	1	36.23
P22626	heterogeneous nuclear ribonucleoprotein A2/B1	HNRNPA2B1	1	1	23.42
P21980	transglutaminase 2	TGM2	4	3	258.77
tr|F8VZX2|F8VZX2_HUMAN	Poly(rC)-binding protein 2	PCBP2	3	3	157.39
Q63ZY3	KN motif and ankyrin repeat domains 2	KANK2	3	3	160.44
P61981	tyrosine 3-monooxygenase/tryptophan 5-monooxygenase activation	YWHAG	9	7	742.97
Q8IY81	pre-rRNA processing protein FTSJ3	FTSJ3	2	2	90.81
Q5TGY3	AT hook, DNA binding motif, containing 1	AHDC1	1	1	34.59
Q9UQ35	serine/arginine repetitive matrix 2	SRRM2	3	3	168.65
tr|B2R806|B2R806_HUMAN	Eukaryotic translation initiation factor 3 subunit E	EIF3E	5	5	402.41
P35637	FUS RNA binding protein	FUS	3	3	157.39
Q16891	inner membrane mitochondrial protein	IMMT	1	1	40.45
P07996	thrombospondin 1	THBS1	1	1	23.43
A6H8Y1	B double prime 1, subunit of RNA polymerase III transcription i	BDP1	3	3	147.97
P09493	tropomyosin 1 (alpha)	TPM1	5	5	368.88
P62906	ribosomal protein L10a	RPL10A	2	1	63.2
P57678	Gem-associated protein 4	GEMIN4	2	2	94.45
P62241	ribosomal protein S8	RPS8	2	2	101.37
P05141	solute carrier family 25 member 5	SLC25A5	4	4	301.66
Q9UEG4	zinc finger protein 629	ZNF629	1	1	29.93
P63261	actin gamma 1	ACTG1	3	3	195.32
P60660	myosin light chain 6	MYL6	3	3	164.96
P11142	heat shock protein family A (Hsp70) member 8	HSPA8	2	2	88.05
Q8NEY8	periphilin 1	PPHLN1	2	2	63.48
tr|H3BU53|H3BU53_HUMAN	MAX gene-associated protein (Fragment)	MGA	4	4	294.02
Q9UGN5	poly(ADP-ribose) polymerase 2	PARP2	5	4	381.47
P61247	ribosomal protein S3A	RPS3A	1	1	24.57
P26373	ribosomal protein L13	RPL13	1	1	21.33
tr|E9PAU2|E9PAU2_HUMAN	Ribonucleoprotein PTB-binding 1	RAVER1	7	5	501.8

P15170	G1 to S phase transition 1	GSPT1	4	4	329.05
P14618	pyruvate kinase, muscle	PKM	5	5	439.24
P22695	ubiquinol-cytochrome c reductase core protein 2	UQCRC2	2	2	72.46
Q9UNX3	ribosomal protein L26 like 1	RPL26L1	1	1	24.55
Q9BVS5	tRNA methyltransferase 61B	TRMT61B	6	5	461.04
P62899	ribosomal protein L31	RPL31	3	3	155.53
Q12849	G-rich RNA sequence binding factor 1	GRSF1	3	3	172.43
P16403	histone cluster 1 H1 family member c	HIST1H1C	1	1	24.58
Q9C0J8	pre-mRNA 3' end processing protein WDR33	WDR33	2	2	69.3
O75821	eukaryotic translation initiation factor 3 subunit G	EIF3G	2	2	72.23
A6ND36	family with sequence similarity 83 member G	FAM83G	1	1	34.87
O60506	synaptotagmin binding cytoplasmic RNA interacting protein	SYNCRIP	4	4	372.03
Q9ULX6	A-kinase anchor protein 8-like	AKAP8L	9	8	730.66
O15027	SEC16 homolog A, endoplasmic reticulum export factor	SEC16A	2	2	123.22
tr|A0A024RBJ3|A0A024RBJ3	Anaphase promoting complex subunit 7	ANAPC7	3	2	130.83
P15924	desmoplakin	DSP	1	1	35.71
Q15645	thyroid hormone receptor interactor 13	TRIP13	2	2	69.52
P49790	Nucleoporin 153kDa	NUP153	2	2	83.94
tr|C9JFV4|C9JFV4_HUMAN	Proline-, glutamic acid- and leucine-rich protein 1	PELP1	1	1	29.57
Q13263	tripartite motif containing 28	TRIM28	3	3	184.42
Q86Y91	kinesin family member 18B	KIF18B	2	2	84.09
O14979	heterogeneous nuclear ribonucleoprotein D like	HNRNPDL	6	5	438.76
O75362	zinc finger protein 217	ZNF217	1	1	24.6
tr|D6REX3|D6REX3_HUMAN	Protein transport protein Sec31A	SEC31A	2	2	77.88
P12273	Prolactin-inducible protein	PIP	1	1	24.59
Q9BVP2	G protein nucleolar 3	GNL3	1	1	36.63
P40429	ribosomal protein L13a	RPL13A	1	1	29.32
O14545	TRAF-type zinc finger domain containing 1	TRAFD1	1	1	26.11
Q9UKM9	RALY heterogeneous nuclear ribonucleoprotein	RALY	1	1	34.96
P11387	DNA topoisomerase I	TOP1	1	1	38.31
Q9Y520	proline rich coiled-coil 2C	PRRC2C	1	1	22.87
Q2TAM9	tumor suppressor candidate 1	TUSC1	1	1	29.45
tr|A0A024R667|A0A024R667	Chromosome 14 open reading frame 150	C14orf150	1	1	36.91
P0DMV8	heat shock protein family A (Hsp70) member 1A	HSPA1A	1	1	20.04
Q9BQE3	tubulin alpha 1c	TUBA1C	1	1	27.67

O43390	heterogeneous nuclear ribonucleoprotein R	HNRNPR	1	1	25.43
P10809	heat shock protein family D (Hsp60) member 1	HSPD1	1	1	35.28
P53621	coatomer protein complex subunit alpha	COPA	1	1	31.77
P62753	ribosomal protein S6	RPS6	1	1	35.83
Q71U36	tubulin alpha 1a	TUBA1A	1	1	33.65
tr|A0A0C4DG89|A0A0C4DG89	Probable ATP-dependent RNA helicase DDX46	DDX46	1	1	27.49
