# Supplementary material for: Hypoxia‐Induced circPRELID2 Promotes Gastric Cancer Metastasis by Facilitating ZEB2 Translation via PCBP1 O‐GlcNAcylation
Source: Adv Sci (Weinh). 2025 Oct 21;12(46):e05396. doi: 10.1002/advs.202505396 (PMC12697806; doi:10.1002/advs.202505396)
Supplement: Supplementary file 2 — Supporting Information [file ADVS-12-e05396-s002.zip › RPM_anno-Gastric cancer-6N_vs._6C.pdf]

id	25C	25N	42C	42N	50C	50N	53C	53N	55C	55N	63C	63N	circbase_id	circRNA_chr	circRNA_strand	circRNA_start	circRNA_end	genomic_length	circRNA_length	Type	best.transcript	gene
circRNA_00001	0.144025172	0.153524756	0.196277805	0.1754076	0.18056803	0.144990365	0.090444084	0.122917773	0.172163486	0.242069794	0.141786611	0.0412728	hsa_circ_0002333	NC_000001.11	-	805799	810170	4372	4372	intergenic		
circRNA_00002	0.072012586	0.112584821	0.468885868	0.0825448	0.20063115	0.196772639	0.060296056	0.204862955	0.13165443	0.191638587	0.10127615	0.113500201	hsa_circ_0000002	NC_000001.11	-	1223244	1223968	725	251	sense-overlappin	NM_016176.3	SDF4
circRNA_00003	0.041150049	0	0	0	0	0	0	0	0	0	0	0	hsa_circ_0008063	NC_000001.11	-	1256992	1257310	319	242	sense-overlappin	NM_058167.2	UBE2J2
circRNA_00004	0	0	0	0	0	0	0	0	0	0.030258724	0	0	hsa_circ_0000004	NC_000001.11	+	1495485	1529331	33847	18398	sense-overlappin	NM_001170535.1	ATAD3A
circRNA_00005	0.020575025	0	0	0	0	0	0	0	0	0.030258724	0	0	-	NC_000001.11	-	1561332	1564916	3585	3585	sense-overlappin	NM_014188.2	SSU72
circRNA_00006	0	0	0	0	0.03009467	0	0	0	0	0	0	0	-	NC_000001.11	-	1648995	1716478	67484	55900	sense-overlappin	NM_024011.2	CDK11A
circRNA_00007	0.041150049	0	0	0	0	0	0	0	0	0	0	0	-	NC_000001.11	-	1655369	1657498	2130	287	sense-overlappin	NM_033489.2	CDK11B
circRNA_00008	0	0	0	0	0.09028402	0.082851637	0	0	0	0	0	0.175409401	hsa_circ_0000006	NC_000001.11	-	1669664	1734835	65172	43786	sense-overlappin	NM_001290264.1	SLC35E2B
circRNA_00009	0	0	0	0	0.02006311	0	0	0	0	0	0	0	-	NC_000001.11	-	1692449	1739704	47256	33853	sense-overlappin	NM_001199787.1	SLC35E2
circRNA_00010	0	0	0	0	0	0	0	0	0	0	0	0.051591	-	NC_000001.11	-	1716151	1716478	328	328	sense-overlappin	NM_024011.2	CDK11A
circRNA_00011	0	0	0	0	0	0	0	0	0	0	0.030382845	0	-	NC_000001.11	-	1761952	1779971	18020	370	sense-overlappin	NM_001198994.1	NADK
circRNA_00012	0	0	0.065425935	0	0	0	0	0	0	0	0.030382845	0	hsa_circ_0000007	NC_000001.11	-	1804419	1806538	2120	227	sense-overlappin	NM_001282538.1	GNB1
circRNA_00013	0.041150049	0	0.04361729	0.0412724	0.02006311	0	0.020098685	0.020486295	0	0	0	0	hsa_circ_0009353	NC_000001.11	-	1804419	1817875	13457	373	sense-overlappin	NM_001282539.1	GNB1
circRNA_00014	0	0	0.076330258	0.0412724	0.03009467	0.041425819	0.020098685	0	0	0.030258724	0.050638075	0	hsa_circ_0000008	NC_000001.11	-	1815756	1825499	9744	249	sense-overlappin	NM_001282539.1	GNB1
circRNA_00015	0	0.030704951	0.065425935	0	0.06018934	0.051782273	0.110542769	0.020486295	0.091145375	0.040344966	0.030382845	0	hsa_circ_0008702	NC_000001.11	-	1815756	1839238	23483	298	sense-overlappin	NM_002074.4	GNB1
circRNA_00016	0	0	0	0	0.03009467	0	0	0	0	0	0	0	hsa_circ_0009361	NC_000001.11	-	1817837	1839238	21402	191	sense-overlappin	NM_002074.4	GNB1
circRNA_00017	0	0	0.021808645	0	0	0	0	0	0	0	0	0	-	NC_000001.11	-	1839190	1853297	14108	14108	sense-overlappin	NM_001282538.1	GNB1
circRNA_00018	0	0	0	0	0	0	0	0.030729443	0	0	0	0	-	NC_000001.11	-	1867190	1868285	1096	1096	intronic	NM_001282538.1	GNB1
circRNA_00019	0	0	0.054521613	0	0	0	0	0	0	0	0	0	hsa_circ_0007120	NC_000001.11	+	2302978	2304585	1608	798	sense-overlappin	NM_003036.3	SKI
circRNA_00020	0	0.020469968	0	0	0	0	0	0	0	0	0	0	-	NC_000001.11	+	3186125	3186474	350	350	exonic	XM_005244774.3	PRDM16
circRNA_00021	0	0	0.021808645	0	0	0	0	0	0	0	0	0	hsa_circ_0009461	NC_000001.11	-	5947104	5961949	14846	602	sense-overlappin	NM_001291594.1	NPHP4
circRNA_00022	0	0	0.054521613	0	0	0	0	0	0	0	0	0	hsa_circ_0009463	NC_000001.11	-	5947104	5986327	39224	1157	sense-overlappin	NM_015102.4	NPHP4
circRNA_00023	0	0	0.021808645	0	0	0	0	0	0	0	0	0	hsa_circ_0009135	NC_000001.11	-	5961794	5969259	7466	394	sense-overlappin	NM_001291593.1	NPHP4
circRNA_00024	0	0	0.021808645	0	0	0	0	0	0	0	0	0	-	NC_000001.11	-	5961794	5986232	24439	616	sense-overlappin	NM_001291593.1	NPHP4
circRNA_00025	0	0	0.098138903	0	0	0	0	0	0	0	0	0	hsa_circ_0005719	NC_000001.11	-	5961794	5986327	24534	711	sense-overlappin	NM_001291593.1	NPHP4
circRNA_00026	0	0	0	0.0412724	0	0	0	0	0	0	0	0	hsa_circ_0007427	NC_000001.11	-	6231902	6233643	1742	388	sense-overlappin	NM_012405.3	ICMT
circRNA_00027	0	0	0.021808645	0	0	0	0	0	0	0	0	0	-	NC_000001.11	-	6533280	6549698	16419	621	sense-overlappin	NM_024654.4	NOL9
circRNA_00028	0.082300098	0	0.098138903	0.0206362	0	0	0	0	0	0	0	0	hsa_circ_0008737	NC_000001.11	+	6806983	6825210	18228	295	sense-overlappin	XM_011541085.1	CAMTA1
circRNA_00029	0	0	0.021808645	0	0	0	0	0	0	0	0	0	-	NC_000001.11	+	6819320	6888899	69580	64739	sense-overlappin	NM_015215.3	CAMTA1
circRNA_00030	0.164600197	0.16375974	0.250799418	0.2476343	0.29091517	0.289980731	0.110542769	0.174133512	0.091145375	0.292501001	0.06076569	0.103182001	hsa_circ_0006354	NC_000001.11	+	7777160	7778169	1010	211	sense-overlappin	NM_004781.3	VAMP3
circRNA_00031	0	0	0.032712968	0	0	0	0	0	0	0	0	0	-	NC_000001.11	+	7835762	7837149	1388	335	sense-overlappin	XM_011542384.1	PER3
circRNA_00032	0	0	0	0.0412724	0.03009467	0.041425819	0	0	0	0.07060369	0	0	hsa_circ_0009581	NC_000001.11	-	8495063	8541318	46256	379	sense-overlappin	XM_005263464.1	RERE
circRNA_00033	0.020575025	0	0.021808645	0.0722267	0	0.020712909	0.020098685	0	0	0.080689931	0.04051046	0	hsa_circ_0006837	NC_000001.11	-	8495063	8557523	62461	582	sense-overlappin	XM_005263464.1	RERE
circRNA_00034	0	0.030704951	0	0.0206362	0	0	0.030148028	0	0	0	0	0	hsa_circ_0005829	NC_000001.11	-	8508627	8557523	48897	357	sense-overlappin	XM_005263464.1	RERE
circRNA_00035	0.102875123	0.040939935	0.08723458	0.1650895	0.04012623	0.062138728	0.040197371	0.061458886	0.070890847	0.121034897	0.050638075	0.0206364	hsa_circ_0002158	NC_000001.11	-	8541214	8557523	16310	308	sense-overlappin	XM_005263464.1	RERE
circRNA_00036	0	0.040939935	0.119947548	0.0619086	0.02006311	0	0.050246713	0.040972591	0.060763583	0.060517448	0.02025523	0	hsa_circ_0008501	NC_000001.11	-	8541214	8614686	73473	434	sense-overlappin	XM_005263464.1	RERE
circRNA_00037	0	0	0.065425935	0	0	0	0	0	0	0	0	0	hsa_circ_0009594	NC_000001.11	-	8614561	8624380	9820	197	sense-overlappin	XM_005263464.1	RERE
circRNA_00038	0.020575025	0	0.054521613	0	0	0	0	0	0.040509055	0	0.030382845	0	hsa_circ_0005039	NC_000001.11	-	8614561	8656441	41881	666	sense-overlappin	XM_005263464.1	RERE
circRNA_00039	0.051437562	0	0.185373483	0.0722267	0.09028402	0.051782273	0.030148028	0	0.030381792	0.040344966	0.06076569	0.0206364	-	NC_000001.11	-	8655973	8656441	469	469	exonic	XM_005263464.1	RERE
circRNA_00040	0	0	0	0	0.02006311	0	0	0	0	0	0	0	hsa_circ_0009654	NC_000001.11	+	9710105	9710596	492	349	sense-overlappin	XM_006710688.1	PIK3CD
circRNA_00041	0	0	0	0	0	0.020712909	0	0	0	0	0	0	hsa_circ_0009040	NC_000001.11	-	9740658	9741256	599	599	sense-overlappin	NM_001009566.2	CLSTN1
circRNA_00042	0.030862537	0	0.032712968	0.0515905	0	0	0	0	0	0	0.02025523	0	hsa_circ_0009674	NC_000001.11	-	9871187	9877989	6803	296	sense-overlappin	NM_001012329.1	CTNNBIP1
circRNA_00043	0	0	0	0	0	0	0	0	0	0.030258724	0	0	hsa_circ_0009677	NC_000001.11	-	9931891	9932898	1008	178	sense-overlappin	XM_005263506.1	LZIC
circRNA_00044	0.082300098	0.204699675	0.26170374	0.1754076	0.11034713	0.103564547	0.180888168	0.122917773	0.162036222	0.211811069	0.182297071	0.123818401	hsa_circ_0000014	NC_000001.11	-	9931891	9934860	2970	277	sense-overlappin	XM_005263506.1	LZIC
circRNA_00045	0	0	0.04361729	0.0309543	0	0.051782273	0	0.030729443	0.030381792	0.050431207	0.04051046	0	hsa_circ_0007803	NC_000001.11	-	9931891	9935627	3737	413	sense-overlappin	XM_005263506.1	LZIC
circRNA_00046	0	0	0	0	0	0.020712909	0	0.030729443	0	0	0	0	-	NC_000001.11	-	9931891	9936627	4737	522	sense-overlappin	XM_005263506.1	LZIC
circRNA_00047	0	0	0.032712968	0.0206362	0	0	0.030148028	0.040972591	0	0.030258724	0	0	-	NC_000001.11	+	9972018	9981170	9153	495	sense-overlappin	NM_001297778.1	NMNAT1
circRNA_00048	0	0.071644886	0.196277805	0.0309543	0.02006311	0.020712909	0	0.030729443	0.050636319	0.040344966	0	0	hsa_circ_0005199	NC_000001.11	+	10095461	10105744	10284	598	sense-overlappin	NM_001105562.2	UBE4B
circRNA_00049	0	0	0	0	0	0	0	0	0	0	0.02025523	0	hsa_circ_0007962	NC_000001.11	+	10117459	10135186	17728	1028	sense-overlappin	NM_001105562.2	UBE4B
circRNA_00050	0	0	0	0.0206362	0	0	0	0	0	0	0	0	hsa_circ_0006955	NC_000001.11	+	10134988	10137205	2218	338	sense-overlappin	NM_001105562.2	UBE4B
circRNA_00051	0	0	0	0	0.02006311	0	0	0	0	0	0	0	-	NC_000001.11	+	10275428	10282533	7106	432	sense-overlappin	NM_183416.3	KIF1B
circRNA_00052	0	0	0	0	0.02006311	0	0	0	0	0	0	0	hsa_circ_0000015	NC_000001.11	+	10433842	10442441	8600	402	sense-overlappin	NM_199294.2	APITD1
circRNA_00053	0	0	0.021808645	0	0.02006311	0	0	0	0	0	0.030382845	0	hsa_circ_0006905	NC_000001.11	+	10440347	10442441	2095	244	sense-overlappin	NM_199294.2	APITD1

circRNA_00054	0.020575025	0	0	0	0	0	0	0	0	0	0	0	-	NC_000001.11	-	10665083	10665347	265	265	exonic	NM_001079843.2	CASZ1
circRNA_00055	0	0	0	0	0	0	0	0	0	0.040344966	0	0	hsa_circ_0002448	NC_000001.11	-	11073934	11076948	3015	278	sense-overlappin	XM_005263476.3	EXOSC10
circRNA_00056	0	0	0.021808645	0	0	0	0	0	0	0	0	0	hsa_circ_0009792	NC_000001.11	-	11121246	11122126	881	271	sense-overlappin	NM_004958.3	MTOR
circRNA_00057	0	0	0	0	0.02006311	0	0	0	0	0	0	0	hsa_circ_0006576	NC_000001.11	-	11124498	11133197	8700	1416	sense-overlappin	NM_004958.3	MTOR
circRNA_00058	0	0	0.032712968	0	0	0	0	0	0	0	0	0	-	NC_000001.11	-	11204561	11209458	4898	290	sense-overlappin	NM_004958.3	MTOR
circRNA_00059	0	0	0	0.0206362	0	0	0	0	0	0	0	0	-	NC_000001.11	+	11911510	11911879	370	370	intergenic		
circRNA_00060	0	0	0	0.0206362	0	0	0	0	0	0	0	0	hsa_circ_0009910	NC_000001.11	+	11989165	11992690	3526	315	sense-overlappin	NM_001127660.1	MFN2
circRNA_00061	0.072012586	0.071644886	0	0.0722267	0.02006311	0.113921001	0	0	0.060763583	0.030258724	0.030382845	0.0412728	hsa_circ_0006470	NC_000001.11	+	12001401	12002103	703	344	sense-overlappin	NM_001127660.1	MFN2
circRNA_00062	0.164600197	0.051174919	0.08723458	0.2063619	0.02006311	0.031069364	0.050246713	0.061458886	0.070890847	0.121034897	0.04051046	0.051591	hsa_circ_0009964	NC_000001.11	+	12275825	12278038	2214	2214	exonic	NM_015378.3	VPS13D
circRNA_00063	0	0	0	0	0	0	0.020098685	0	0	0	0	0	hsa_circ_0007677	NC_000001.11	+	12299202	12304728	5527	406	sense-overlappin	NM_015378.3	VPS13D
circRNA_00064	0	0	0	0.0412724	0	0	0	0	0	0	0	0	hsa_circ_0008275	NC_000001.11	+	12299202	12308641	9440	617	sense-overlappin	NM_015378.3	VPS13D
circRNA_00065	0	0	0	0	0	0	0	0	0	0.020172483	0	0	hsa_circ_0010020	NC_000001.11	-	12572728	12579412	6685	485	sense-overlappin	XM_005263533.3	DHRS3
circRNA_00066	0.030862537	0.020469968	0	0.0309543	0	0.020712909	0.020098685	0	0	0	0	0	hsa_circ_0010023	NC_000001.11	-	12578718	12579412	695	359	sense-overlappin	XM_005263533.3	DHRS3
circRNA_00067	0	0	0	0.0206362	0	0	0	0	0	0	0	0	-	NC_000001.11	+	12587286	12587610	325	325	antisense	XM_005263533.3	DHRS3
circRNA_00068	0	0	0.04361729	0.0309543	0	0	0	0	0	0.030258724	0	0.0206364	hsa_circ_0010028	NC_000001.11	+	13715541	13742157	26617	449	sense-overlappin	NM_001135610.1	PRDM2
circRNA_00069	0	0	0.109043225	0.0309543	0	0.020712909	0	0.020486295	0	0.050431207	0	0	hsa_circ_0005986	NC_000001.11	+	13731000	13742157	11158	375	sense-overlappin	NM_001135610.1	PRDM2
circRNA_00070	0	0	0	0	0	0.031069364	0.020098685	0	0	0.030258724	0	0	hsa_circ_0004861	NC_000001.11	+	13731000	13749487	18488	502	sense-overlappin	NM_001135610.1	PRDM2
circRNA_00071	0	0.020469968	0	0	0	0	0	0	0	0	0	0	hsa_circ_0005888	NC_000001.11	+	13769118	13773188	4071	167	sense-overlappin	XM_005245998.2	PRDM2
circRNA_00072	0	0.030704951	0	0.0206362	0	0	0	0	0	0.030258724	0	0	-	NC_000001.11	+	13769118	13782831	13714	4581	sense-overlappin	XM_005245998.2	PRDM2
circRNA_00073	0	0	0.098138903	0.0825448	0	0.020712909	0.020098685	0.030729443	0	0.020172483	0.02025523	0	-	NC_000001.11	+	13773078	13782831	9754	4525	sense-overlappin	NM_015866.4	PRDM2
circRNA_00074	0	0	0.054521613	0.0619086	0.06018934	0	0	0	0.050636319	0.020172483	0	0.0206364	hsa_circ_0010029	NC_000001.11	+	13778418	13782831	4414	4414	exonic	NM_015866.4	PRDM2
circRNA_00075	0	0	0.04361729	0.0206362	0.02006311	0.020712909	0	0	0	0.07060369	0	0	hsa_circ_0000018	NC_000001.11	+	15534237	15536814	2578	407	sense-overlappin	NM_001287811.1	DNAJC16
circRNA_00076	0	0	0	0	0	0	0	0	0	0.030258724	0	0	hsa_circ_0002898	NC_000001.11	+	15536475	15536814	340	340	exonic	NM_001287811.1	DNAJC16
circRNA_00077	0	0	0	0	0	0	0	0.020486295	0	0	0	0	hsa_circ_0010048	NC_000001.11	+	15626669	15643650	16982	751	sense-overlappin	NM_032341.4	DDI2
circRNA_00078	0	0	0.021808645	0	0	0	0	0	0	0	0.02025523	0	hsa_circ_0010052	NC_000001.11	+	15630325	15651895	21571	915	sense-overlappin	NM_032341.4	DDI2
circRNA_00079	0	0.020469968	0.032712968	0.0412724	0.03009467	0.062138728	0	0.020486295	0	0.060517448	0	0	hsa_circ_0000019	NC_000001.11	+	15638307	15643650	5344	257	sense-overlappin	NM_032341.4	DDI2
circRNA_00080	0	0	0	0.0206362	0.02006311	0	0	0	0	0.040344966	0.04051046	0.0309546	hsa_circ_0000020	NC_000001.11	+	15638307	15651895	13589	551	sense-overlappin	NM_032341.4	DDI2
circRNA_00081	0	0	0	0	0	0	0.020098685	0	0	0	0	0	hsa_circ_0010059	NC_000001.11	+	15643522	15651895	8374	423	sense-overlappin	NM_032341.4	DDI2
circRNA_00082	0.102875123	0.051174919	0.054521613	0.0412724	0	0.051782273	0.020098685	0	0	0.080689931	0.030382845	0.0309546	hsa_circ_0000021	NC_000001.11	+	15717893	15721388	3496	435	sense-overlappin	XR_946590.1	
circRNA_00083	0	0	0.04361729	0.0206362	0	0	0	0	0	0	0	0	hsa_circ_0002237	NC_000001.11	+	15872816	15876678	3863	798	sense-overlappin	NM_015001.2	SPEN
circRNA_00084	0	0	0.021808645	0	0	0	0	0	0	0	0	0	hsa_circ_0010111	NC_000001.11	+	15909321	15920983	11663	868	sense-overlappin	NM_015001.2	SPEN
circRNA_00085	0	0	0	0	0	0	0	0	0	0.040344966	0	0	hsa_circ_0010113	NC_000001.11	+	15911101	15916279	5179	353	sense-overlappin	NM_015001.2	SPEN
circRNA_00086	0	0	0.04361729	0.0309543	0	0	0.040197371	0	0	0	0	0	hsa_circ_0003567	NC_000001.11	-	16201782	16202574	793	239	sense-overlappin	XM_011540706.1	ARHGEF19
circRNA_00087	0	0	0	0.0206362	0	0	0	0	0	0	0	0	hsa_circ_0010144	NC_000001.11	-	16294783	16315435	20653	519	sense-overlappin	XM_006710698.2	FBXO42
circRNA_00088	0	0	0	0.0206362	0	0	0	0	0	0	0	0	-	NC_000001.11	-	16294783	16316571	21789	654	sense-overlappin	XM_006710698.2	FBXO42
circRNA_00089	0	0	0	0	0	0	0	0	0	0	0.02025523	0	-	NC_000001.11	+	16380248	16380397	150	150	intronic	NM_001114600.2	SZRD1
circRNA_00090	0.030862537	0.020469968	0.021808645	0	0	0	0	0	0	0	0	0	hsa_circ_0005562	NC_000001.11	+	17580553	17588479	7927	300	sense-overlappin	XM_006710728.1	ARHGEF10L
circRNA_00091	0.020575025	0	0	0	0	0	0	0	0	0	0	0	-	NC_000001.11	-	17633641	17641692	8052	2989	antisense	XR_946688.1	
circRNA_00092	0	0	0.065425935	0	0.02006311	0	0	0	0	0	0	0	-	NC_000001.11	-	18918362	18919773	1412	237	sense-overlappin	NM_001136265.1	IFFO2
circRNA_00093	0	0	0	0	0	0	0	0	0	0	0.02025523	0	-	NC_000001.11	-	19174319	19187301	12983	1488	sense-overlappin	NM_020765.2	UBR4
circRNA_00094	0	0	0.021808645	0.0412724	0	0	0	0	0	0	0	0	hsa_circ_0003777	NC_000001.11	-	19183811	19185286	1476	434	sense-overlappin	NM_020765.2	UBR4
circRNA_00095	0	0	0	0	0	0	0.030148028	0	0	0	0	0	-	NC_000001.11	-	19473646	19474239	594	594	intronic	NM_001282162.1	CAPZB
circRNA_00096	0	0	0.032712968	0	0	0	0	0	0	0	0	0	-	NC_000001.11	-	19770542	19787117	16576	556	sense-overlappin	XM_011541180.1	TMCO4
circRNA_00097	0	0	0	0	0	0.020712909	0	0	0	0	0	0	hsa_circ_0007296	NC_000001.11	-	20744744	20749882	5139	734	sense-overlappin	XM_005245878.3	HP1BP3
circRNA_00098	0	0	0	0	0	0	0	0	0.020254528	0	0	0	-	NC_000001.11	-	20744744	20776750	32007	1519	sense-overlappin	XM_005245878.3	HP1BP3
circRNA_00099	0	0	0.04361729	0	0.05015779	0	0	0	0	0	0	0	-	NC_000001.11	-	20744744	20780540	35797	1815	sense-overlappin	XM_005245878.3	HP1BP3
circRNA_00100	0	0	0	0	0	0	0.030148028	0	0	0	0	0	hsa_circ_0008765	NC_000001.11	-	20749723	20749975	253	253	sense-overlappin	XM_005245878.3	HP1BP3
circRNA_00101	0	0.020469968	0.032712968	0.0515905	0.03009467	0.051782273	0.030148028	0	0.040509055	0.020172483	0.050638075	0	hsa_circ_0000023	NC_000001.11	-	20749723	20773610	23888	791	sense-overlappin	XM_005245878.3	HP1BP3
circRNA_00102	0.092587611	0.040939935	0.119947548	0.1547714	0.12037869	0.196772639	0.090444084	0.081945182	0.111399902	0.110948655	0.030382845	0.0619092	hsa_circ_0000024	NC_000001.11	-	20757166	20773610	16445	631	sense-overlappin	XM_005245878.3	HP1BP3
circRNA_00103	0	0	0	0	0	0	0.020098685	0	0	0.020172483	0	0	hsa_circ_0004493	NC_000001.11	-	20757166	20776750	19585	785	sense-overlappin	XM_005245878.3	HP1BP3
circRNA_00104	0	0	0	0	0	0	0.020098685	0	0	0	0	0	hsa_circ_0002437	NC_000001.11	-	20757166	20780540	23375	1081	sense-overlappin	XM_005245878.3	HP1BP3
circRNA_00105	0	0	0.032712968	0.0515905	0	0	0	0	0.030381792	0.040344966	0.04051046	0	hsa_circ_0002238	NC_000001.11	-	20765377	20773610	8234	540	sense-overlappin	XM_005245878.3	HP1BP3
circRNA_00106	0	0	0	0	0	0	0.050246713	0	0	0	0	0	-	NC_000001.11	-	20765377	20776750	11374	694	sense-overlappin	XM_005245878.3	HP1BP3
circRNA_00107	0.216037758	0.112584821	0.04361729	0.0928628	0.13041025	0.165703275	0.120592112	0.051215739	0.060763583	0.201724828	0.050638075	0.082545601	hsa_circ_0005782	NC_000001.11	-	20770930	20773610	2681	304	sense-overlappin	XM_005245878.3	HP1BP3
circRNA_00108	0	0	0.021808645	0	0	0	0	0	0	0	0	0	hsa_circ_0010458	NC_000001.11	-	20840856	20865262	24407	1439	sense-overlappin	NM_003760.4	EIF4G3

circRNA_00109	0	0	0	0	0.03009467	0	0	0	0	0	0	0	hsa_circ_0010466	NC_000001.11	-	20893517	20895501	1985	254	sense-overlappin	NM_003760.4	EIF4G3
circRNA_00110	0	0	0	0	0	0	0.020098685	0	0	0	0	0	hsa_circ_0007214	NC_000001.11	-	20893517	20904971	11455	590	sense-overlappin	NM_003760.4	EIF4G3
circRNA_00111	0	0	0.021808645	0	0.02006311	0	0	0	0	0	0	0.0206364	hsa_circ_0010467	NC_000001.11	-	20893517	20942330	48814	1430	sense-overlappin	NM_003760.4	EIF4G3
circRNA_00112	0	0	0.021808645	0	0.02006311	0.041425819	0	0	0	0	0.02025523	0	hsa_circ_0000025	NC_000001.11	-	20969474	20981227	11754	516	sense-overlappin	NM_003760.4	EIF4G3
circRNA_00113	0	0	0	0	0	0	0	0	0.020254528	0	0.02025523	0	hsa_circ_0008071	NC_000001.11	-	21001199	21002808	1610	210	sense-overlappin	XM_011542373.1	EIF4G3
circRNA_00114	0.030862537	0	0	0	0	0	0.020098685	0	0	0	0	0.0206364	hsa_circ_0005075	NC_000001.11	-	21050866	21089213	38348	205	sense-overlappin	NM_001198801.1	EIF4G3
circRNA_00115	0	0	0.04361729	0	0.09028402	0	0.060296056	0	0	0.040344966	0	0	hsa_circ_0000026	NC_000001.11	-	21050866	21111383	60518	266	sense-overlappin	XM_011542373.1	EIF4G3
circRNA_00116	0	0.040939935	0	0	0	0	0	0.061458886	0	0	0	0.0412728	hsa_circ_0005735	NC_000001.11	-	21089138	21111383	22246	137	sense-overlappin	XM_011542373.1	EIF4G3
circRNA_00117	0	0	0.032712968	0	0	0	0	0.040972591	0	0	0	0.0309546	hsa_circ_0000027	NC_000001.11	-	21221747	21228041	6295	466	sense-overlappin	NM_001113347.1	ECE1
circRNA_00118	0	0	0.021808645	0	0	0	0	0	0	0	0	0	hsa_circ_0007877	NC_000001.11	-	21247221	21258839	11619	548	sense-overlappin	NM_001113347.1	ECE1
circRNA_00119	0.051437562	0	0.098138903	0	0	0	0.030148028	0	0.030381792	0	0	0	hsa_circ_0002402	NC_000001.11	-	21272699	21290156	17458	442	sense-overlappin	NM_001113349.1	ECE1
circRNA_00120	0.051437562	0	0	0	0.02006311	0	0	0	0	0	0	0	hsa_circ_0010523	NC_000001.11	-	21680808	21695221	14414	358	sense-overlappin	NM_032236.5	USP48
circRNA_00121	0	0	0.021808645	0	0	0	0	0	0.020254528	0	0	0	-	NC_000001.11	-	21701498	21703618	2121	212	sense-overlappin	NM_032236.5	USP48
circRNA_00122	0	0.061409903	0.065425935	0.0206362	0.05015779	0.041425819	0.020098685	0.081945182	0.030381792	0.080689931	0	0.0619092	hsa_circ_0000028	NC_000001.11	-	21715389	21721764	6376	315	sense-overlappin	NM_032236.5	USP48
circRNA_00123	0	0.040939935	0.032712968	0.0515905	0	0.031069364	0	0.020486295	0	0	0	0.0412728	-	NC_000001.11	-	21715389	21724095	8707	513	sense-overlappin	NM_032236.5	USP48
circRNA_00124	0	0	0.021808645	0	0	0	0	0	0	0	0	0	-	NC_000001.11	-	21715389	21757783	42395	1829	sense-overlappin	NM_032236.5	USP48
circRNA_00125	0	0	0	0	0.04012623	0	0	0	0	0	0.030382845	0.051591	hsa_circ_0004808	NC_000001.11	-	21721036	21721764	729	246	sense-overlappin	NM_032236.5	USP48
circRNA_00126	0.020575025	0	0	0	0	0.020712909	0	0	0	0	0	0	hsa_circ_0002166	NC_000001.11	-	21748138	21751615	3478	243	sense-overlappin	NM_032236.5	USP48
circRNA_00127	0	0	0.021808645	0	0	0	0	0	0	0	0	0	-	NC_000001.11	-	21751507	21757783	6277	640	sense-overlappin	NM_032236.5	USP48
circRNA_00128	0	0	0.054521613	0	0	0	0	0	0	0	0	0	hsa_circ_0010735	NC_000001.11	-	21861757	21862115	359	215	sense-overlappin	NM_001291860.1	HSPG2
circRNA_00129	0	0	0.021808645	0	0	0	0	0	0	0	0	0	-	NC_000001.11	+	21978369	22012767	34399	15417	sense-overlappin	XM_011541132.1	CELA3B
circRNA_00130	0.030862537	0.020469968	0.17446916	0.0722267	0.02006311	0	0.020098685	0	0.020254528	0.020172483	0.02025523	0	hsa_circ_0005576	NC_000001.11	+	22078429	22086866	8438	536	sense-overlappin	NM_044472.2	CDC42
circRNA_00131	0	0	0	0	0	0	0	0	0	0	0.030382845	0	hsa_circ_0007604	NC_000001.11	+	22078429	22091501	13073	610	sense-overlappin	NM_001039802.1	CDC42
circRNA_00132	0	0	0	0	0.02006311	0	0	0	0	0	0	0	-	NC_000001.11	+	22180655	22181150	496	496	intergenic		
circRNA_00133	0	0	0.08723458	0.0309543	0	0	0	0	0.020254528	0	0	0	hsa_circ_0007418	NC_000001.11	+	22489880	22491533	1654	900	sense-overlappin	XM_011542499.1	ZBTB40
circRNA_00134	0	0	0	0	0.02006311	0	0	0	0	0	0.04051046	0	hsa_circ_0004085	NC_000001.11	+	22781421	22785076	3656	750	sense-overlappin	XM_006710442.2	EPHB2
circRNA_00135	0.030862537	0	0	0	0	0	0.020098685	0	0	0.040344966	0	0	-	NC_000001.11	+	23030469	23044486	14018	226	sense-overlappin	XM_006710474.2	KDM1A
circRNA_00136	0.072012586	0.194464692	0.196277805	0.2063619	0.1404418	0.113921001	0.070345399	0.081945182	0.13165443	0.181552345	0.06076569	0.092863801	hsa_circ_0009061	NC_000001.11	+	23030469	23050520	20052	360	sense-overlappin	XM_006710474.2	KDM1A
circRNA_00137	0	0.030704951	0.054521613	0.0309543	0.02006311	0.020712909	0	0.051215739	0.030381792	0	0.04051046	0.0412728	hsa_circ_0007822	NC_000001.11	+	23030469	23059167	28699	816	sense-overlappin	XM_006710474.2	KDM1A
circRNA_00138	0	0	0.032712968	0	0	0	0	0	0	0	0	0	hsa_circ_0003889	NC_000001.11	+	23050387	23059167	8781	590	sense-overlappin	XM_006710474.2	KDM1A
circRNA_00139	0	0	0	0.0412724	0	0	0	0	0	0.030258724	0	0	hsa_circ_0003685	NC_000001.11	+	23055069	23069151	14083	623	sense-overlappin	NM_001009999.2	KDM1A
circRNA_00140	0	0	0	0.0309543	0	0.051782273	0	0.030729443	0	0.020172483	0	0	hsa_circ_0010835	NC_000001.11	+	23071225	23072197	973	209	sense-overlappin	XM_006710474.2	KDM1A
circRNA_00141	0	0	0	0.0206362	0	0	0	0	0	0	0	0	-	NC_000001.11	+	23750845	23752518	1674	1298	sense-overlappin	NM_003198.2	TCEB3
circRNA_00142	0	0	0.021808645	0	0	0	0	0	0	0	0	0	hsa_circ_0010900	NC_000001.11	-	23810736	23820593	9858	501	sense-overlappin	NM_000191.2	HMGCL
circRNA_00143	0.041150049	0	0.021808645	0.0206362	0	0	0	0	0	0.050431207	0	0	hsa_circ_0003757	NC_000001.11	-	23814190	23820593	6404	437	sense-overlappin	NM_000191.2	HMGCL
circRNA_00144	0.020575025	0	0.021808645	0	0	0	0	0	0	0	0	0	hsa_circ_0010906	NC_000001.11	-	24169417	24180854	11438	309	sense-overlappin	NM_170743.3	IFNLR1
circRNA_00145	0.123450148	0.08187987	0.196277805	0.0619086	0.0702209	0.103564547	0.050246713	0.030729443	0.020254528	0.110948655	0	0.051591	hsa_circ_0003553	NC_000001.11	+	24514314	24514567	254	254	exonic	NM_001251979.1	RCAN3
circRNA_00146	0	0	0.054521613	0.0928628	0	0.020712909	0	0	0	0.020172483	0	0	hsa_circ_0004802	NC_000001.11	+	24531218	24533254	2037	346	sense-overlappin	NM_001251979.1	RCAN3
circRNA_00147	0	0	0	0	0	0	0.020098685	0	0	0	0	0	hsa_circ_0010919	NC_000001.11	+	24660719	24663224	2506	355	sense-overlappin	XM_011540479.1	SRRM1
circRNA_00148	0	0	0	0	0	0.020712909	0	0	0	0	0	0	-	NC_000001.11	-	25225002	25228235	3234	308	sense-overlappin	NM_015484.4	SYF2
circRNA_00149	0	0	0	0	0	0.020712909	0	0	0	0.040344966	0	0	-	NC_000001.11	-	25227442	25228235	794	209	sense-overlappin	NM_015484.4	SYF2
circRNA_00150	0	0	0	0	0	0.041425819	0	0	0	0	0	0	-	NC_000001.11	-	25242374	25243633	1260	416	sense-overlappin	NM_020317.3	RSRP1
circRNA_00151	0	0.020469968	0	0	0	0.041425819	0	0	0	0	0	0	hsa_circ_0007054	NC_000001.11	+	25340474	25351693	11220	287	sense-overlappin	NM_014313.3	TMEM50A
circRNA_00152	0	0	0	0.0309543	0	0	0	0	0	0	0	0	hsa_circ_0010935	NC_000001.11	+	25446762	25458892	12131	1074	sense-overlappin	NM_018202.5	TMEM57
circRNA_00153	0	0	0.021808645	0	0	0	0	0	0	0	0	0	-	NC_000001.11	+	25553922	25557267	3346	371	sense-overlappin	XM_006710559.2	LDLRAP1
circRNA_00154	0	0	0.021808645	0	0	0	0	0	0	0	0	0	hsa_circ_0002868	NC_000001.11	+	26255171	26259802	4632	1133	sense-overlappin	XM_005245969.2	CEP85
circRNA_00155	0.041150049	0	0	0	0.03009467	0	0	0.030729443	0	0	0.030382845	0	hsa_circ_0000033	NC_000001.11	+	26257597	26259802	2206	438	sense-overlappin	XM_005245969.2	CEP85
circRNA_00156	0	0	0	0	0	0	0	0	0	0.020172483	0	0	-	NC_000001.11	+	26268483	26269614	1132	308	sense-overlappin	XM_005245969.2	CEP85
circRNA_00157	0	0	0	0	0	0	0	0	0	0.030258724	0	0	-	NC_000001.11	+	26440048	26441019	972	972	intronic	NM_001243564.1	DHDDS
circRNA_00158	0.051437562	0.092114854	0.021808645	0.0412724	0.04012623	0.051782273	0	0	0	0.050431207	0.04051046	0.082545601	hsa_circ_0000034	NC_000001.11	+	26446316	26447660	1345	219	sense-overlappin	NM_024887.3	DHDDS
circRNA_00159	0	0.020469968	0	0	0.03009467	0.062138728	0	0	0	0	0.02025523	0	-	NC_000001.11	-	26453609	26463433	9825	7694	antisense	NM_001243564.1	DHDDS
circRNA_00160	0.020575025	0.030704951	0.098138903	0.0412724	0.04012623	0	0	0.061458886	0	0	0	0.0412728	hsa_circ_0008494	NC_000001.11	+	26729651	26732792	3142	783	sense-overlappin	NM_006015.4	ARID1A
circRNA_00161	0	0	0	0	0.02006311	0	0	0	0	0	0	0	hsa_circ_0006913	NC_000001.11	+	26760856	26763285	2430	812	sense-overlappin	NM_006015.4	ARID1A
circRNA_00162	0.041150049	0	0	0.0309543	0.0702209	0	0	0	0.050636319	0.020172483	0	0	hsa_circ_0003940	NC_000001.11	+	26941457	26941818	362	270	sense-overlappin	XM_011540529.1	NUDC
circRNA_00163	0.257187808	0.153524756	0.152660515	0.2269981	0.11034713	0.248554912	0.110542769	0.133160921	0.121527166	0.201724828	0.050638075	0.123818401	hsa_circ_0005087	NC_000001.11	+	26942660	26943065	406	312	sense-overlappin	XM_011540529.1	NUDC
circRNA_00164	0	0	0	0	0	0.041425819	0	0	0	0	0	0	hsa_circ_0007283	NC_000001.11	-	27105885	27109777	3893	672	sense-overlappin	XM_011542021.1	SLC9A1
circRNA_00165	0	0	0	0	0	0	0	0	0	0	0	0.0412728	-	NC_000001.11	+	27303621	27306272	2652	455	sense-overlappin	NM_001276252.1	WDTC1

circRNA_00166	0	0	0	0	0	0	0	0	0.040509055	0.060517448	0	0	-	NC_000001.11	-	27370596	27374451	3856	567	sense-overlappin	NM_003665.2	FCN3
circRNA_00167	0	0	0.04361729	0	0.03009467	0	0	0	0	0	0.02025523	0	hsa_circ_0002470	NC_000001.11	-	27668226	27669346	1121	330	sense-overlappin	NM_002038.3	IFI6
circRNA_00168	0.051437562	0.040939935	0.021808645	0.1031809	0.02006311	0.031069364	0.030148028	0.040972591	0.060763583	0.040344966	0.030382845	0	hsa_circ_0008126	NC_000001.11	+	27789562	27793632	4071	170	sense-overlappin	NM_177424.2	STX12
circRNA_00169	0	0	0	0	0	0	0	0	0.020254528	0	0	0	hsa_circ_0005866	NC_000001.11	-	27906928	27907282	355	216	sense-overlappin	XM_011541897.1	RPA2
circRNA_00170	0	0	0	0	0	0.020712909	0	0	0	0	0	0	hsa_circ_0008057	NC_000001.11	-	28035544	28048426	12883	328	sense-overlappin	NM_001282561.1	EYA3
circRNA_00171	0.041150049	0	0.08723458	0	0	0.020712909	0	0.020486295	0.040509055	0.020172483	0	0	hsa_circ_0007895	NC_000001.11	-	28035544	28058094	22551	435	sense-overlappin	XM_011541004.1	EYA3
circRNA_00172	0	0	0	0	0	0	0.020098685	0	0	0	0	0	-	NC_000001.11	-	28202013	28202389	377	377	intronic	NM_014280.2	DNAJC8
circRNA_00173	0.185175222	0.071644886	0	0.0412724	0.05015779	0.093208092	0	0.030729443	0.030381792	0.060517448	0.02025523	0.0309546	hsa_circ_0005406	NC_000001.11	+	28459085	28466535	7451	574	sense-overlappin	NM_001048183.1	PHACTR4
circRNA_00174	0	0	0.021808645	0.0309543	0	0	0.060296056	0	0	0	0	0	-	NC_000001.11	+	28459085	28466768	7684	807	sense-overlappin	NM_001048183.1	PHACTR4
circRNA_00175	0	0	0.021808645	0	0.03009467	0	0	0	0	0	0	0	hsa_circ_0002909	NC_000001.11	+	28473554	28474151	598	598	exonic	NM_001048183.1	PHACTR4
circRNA_00176	0	0	0	0	0.02006311	0.031069364	0	0.020486295	0.020254528	0	0	0	hsa_circ_0000038	NC_000001.11	+	28473554	28476291	2738	783	sense-overlappin	NM_001048183.1	PHACTR4
circRNA_00177	0	0	0	0.0412724	0	0	0	0	0	0	0	0.0206364	hsa_circ_0011137	NC_000001.11	+	28473554	28480604	7051	937	sense-overlappin	NM_001048183.1	PHACTR4
circRNA_00178	0	0.020469968	0	0	0	0	0	0	0	0	0	0	-	NC_000001.11	+	28476107	28480604	4498	339	sense-overlappin	NM_001048183.1	PHACTR4
circRNA_00179	0	0	0	0	0	0	0	0	0.030381792	0	0	0	hsa_circ_0011153	NC_000001.11	+	28560633	28561398	766	301	sense-overlappin	NR_003109.1	
circRNA_00180	0.020575025	0	0	0	0	0	0	0	0	0	0	0	hsa_circ_0011159	NC_000001.11	-	28605372	28622165	16794	534	sense-overlappin	NM_001135218.1	TAF12
circRNA_00181	0	0	0	0	0	0	0	0	0.030381792	0	0	0	hsa_circ_0011162	NC_000001.11	-	28617953	28622165	4213	330	sense-overlappin	NM_001135218.1	TAF12
circRNA_00182	0	0	0.032712968	0	0.02006311	0	0	0	0	0	0	0	hsa_circ_0011163	NC_000001.11	+	28683583	28697084	13502	628	sense-overlappin	XM_011540520.1	GMEB1
circRNA_00183	0	0	0.021808645	0	0	0	0	0	0	0	0	0	-	NC_000001.11	+	28683583	28710642	27060	1021	sense-overlappin	XM_011540520.1	GMEB1
circRNA_00184	0	0	0	0	0	0	0	0	0	0.020172483	0	0	-	NC_000001.11	+	28704192	28710642	6451	261	sense-overlappin	XM_011540520.1	GMEB1
circRNA_00185	0.061725074	0.040939935	0.032712968	0.0206362	0	0.051782273	0	0	0	0	0	0	hsa_circ_0000039	NC_000001.11	+	28742403	28743986	1584	1584	exonic	NM_001173128.1	YTHDF2
circRNA_00186	0	0.020469968	0	0.0206362	0	0	0	0	0	0.040344966	0.02025523	0	hsa_circ_0011167	NC_000001.11	+	28987431	28987905	475	475	exonic	NM_001166005.1	EPB41
circRNA_00187	0	0	0.032712968	0	0	0	0	0	0	0	0	0	hsa_circ_0011168	NC_000001.11	+	28987431	28997319	9889	873	sense-overlappin	NM_004437.3	EPB41
circRNA_00188	0	0	0	0	0.03009467	0	0	0	0	0	0	0	hsa_circ_0000041	NC_000001.11	+	28993330	29018442	25113	656	sense-overlappin	NM_001166005.1	EPB41
circRNA_00189	0	0	0.021808645	0	0	0	0	0	0	0	0	0	-	NC_000001.11	+	28993330	29035923	42594	995	sense-overlappin	NM_001166005.1	EPB41
circRNA_00190	0	0.030704951	0.032712968	0	0	0	0.020098685	0.040972591	0	0.030258724	0.02025523	0.0412728	hsa_circ_0000042	NC_000001.11	+	29035826	29039426	3601	271	sense-overlappin	NM_001166005.1	EPB41
circRNA_00191	0	0.020469968	0.054521613	0	0.11034713	0.041425819	0.020098685	0	0	0.020172483	0	0	-	NC_000001.11	-	29148080	29154910	6831	1452	sense-overlappin	NM_005626.4	SRSF4
circRNA_00192	0.072012586	0.061409903	0.021808645	0.0928628	0.03009467	0.113921001	0.030148028	0.061458886	0.030381792	0.040344966	0.04051046	0	hsa_circ_0006602	NC_000001.11	-	29154696	29154910	215	215	exonic	NM_005626.4	SRSF4
circRNA_00193	0	0	0.021808645	0	0	0	0	0	0	0	0	0	-	NC_000001.11	-	30950133	30952363	2231	259	sense-overlappin	NM_001020658.1	PUM1
circRNA_00194	0	0	0.021808645	0	0	0	0	0	0	0	0	0	hsa_circ_0011240	NC_000001.11	-	30964674	30981405	16732	1165	sense-overlappin	NM_001020658.1	PUM1
circRNA_00195	0	0	0.032712968	0	0	0	0	0	0	0	0	0	-	NC_000001.11	-	30988309	30988788	480	480	intronic	NM_001020658.1	PUM1
circRNA_00196	0.113162635	0.040939935	0.141756193	0.0825448	0.03009467	0.051782273	0.030148028	0.071702034	0.040509055	0.060517448	0.070893305	0.0206364	hsa_circ_0000043	NC_000001.11	-	30992390	30995220	2831	438	sense-overlappin	NM_001020658.1	PUM1
circRNA_00197	0.030862537	0	0	0	0	0	0	0	0	0	0.02025523	0	hsa_circ_0006636	NC_000001.11	-	30992390	31007099	14710	723	sense-overlappin	NM_001020658.1	PUM1
circRNA_00198	0.082300098	0	0.054521613	0.0515905	0	0.051782273	0.030148028	0.020486295	0.050636319	0.020172483	0	0.0412728	hsa_circ_0000044	NC_000001.11	-	30992390	31007102	14713	726	sense-overlappin	NM_001020658.1	PUM1
circRNA_00199	0.051437562	0	0.032712968	0.0206362	0	0	0.020098685	0	0	0	0	0	hsa_circ_0004949	NC_000001.11	-	31005853	31007102	1250	288	sense-overlappin	NM_001020658.1	PUM1
circRNA_00200	0	0	0.021808645	0	0	0	0	0	0	0	0	0	-	NC_000001.11	-	31028796	31059577	30782	443	sense-overlappin	NM_001020658.1	PUM1
circRNA_00201	0	0	0	0	0.02006311	0	0	0	0	0	0	0	hsa_circ_0000045	NC_000001.11	-	31059204	31059577	374	374	exonic	NM_001020658.1	PUM1
circRNA_00202	0.072012586	0	0.076330258	0	0	0	0	0	0	0	0	0.0206364	hsa_circ_0007249	NC_000001.11	+	31337175	31348974	11800	440	sense-overlappin	NM_001282566.1	ZCCHC17
circRNA_00203	0	0	0	0	0	0	0	0	0.020254528	0	0	0	hsa_circ_0011279	NC_000001.11	+	31423693	31426823	3131	741	sense-overlappin	NM_001199038.1	SERINC2
circRNA_00204	0	0	0	0	0	0	0	0	0.040509055	0	0	0	-	NC_000001.11	-	31907661	31919658	11998	2202	sense-overlappin	XR_946766.1	
circRNA_00205	0	0	0	0	0	0	0.020098685	0	0	0	0	0	hsa_circ_0004893	NC_000001.11	-	31911696	31919658	7963	913	sense-overlappin	NM_001195100.1	PTP4A2
circRNA_00206	0.051437562	0.040939935	0	0	0	0.041425819	0	0	0	0	0	0	hsa_circ_0005067	NC_000001.11	-	31915891	31919658	3768	786	sense-overlappin	NM_001195100.1	PTP4A2
circRNA_00207	0.195462734	0.122819805	0.17446916	0.2579524	0.15047336	0.15534682	0.060296056	0.030729443	0.050636319	0.171466104	0.10127615	0.072227401	hsa_circ_0007364	NC_000001.11	-	31915895	31919658	3764	782	sense-overlappin	NM_001195100.1	PTP4A2
circRNA_00208	0	0	0.021808645	0	0	0.031069364	0	0.020486295	0.050636319	0.030258724	0	0.0309546	hsa_circ_0007777	NC_000001.11	+	32030298	32033334	3037	389	sense-overlappin	NM_006559.2	KHDRBS1
circRNA_00209	0	0	0	0	0	0	0.020098685	0	0	0	0	0	hsa_circ_0003446	NC_000001.11	+	32030298	32038619	8322	793	sense-overlappin	NM_006559.2	KHDRBS1
circRNA_00210	0	0	0	0.0309543	0	0.020712909	0	0	0	0	0	0	hsa_circ_0004289	NC_000001.11	+	32030298	32045390	15093	1105	sense-overlappin	NR_073499.1	
circRNA_00211	0	0	0.076330258	0	0	0	0	0	0	0.020172483	0	0	hsa_circ_0007231	NC_000001.11	+	32036910	32038619	1710	404	sense-overlappin	NM_001271878.1	KHDRBS1
circRNA_00212	0	0	0	0	0	0.020712909	0	0	0	0	0	0	hsa_circ_0002467	NC_000001.11	+	32036910	32039573	2664	463	sense-overlappin	NM_001271878.1	KHDRBS1
circRNA_00213	0	0	0.032712968	0	0	0	0	0	0	0	0	0	-	NC_000001.11	+	32109450	32152557	43108	43108	sense-overlappin	XM_011541160.1	KPNA6
circRNA_00214	0	0	0	0	0	0.031069364	0	0	0	0	0	0	-	NC_000001.11	+	32154588	32159531	4944	554	sense-overlappin	NM_012316.4	KPNA6
circRNA_00215	0.041150049	0	0	0	0	0	0	0	0	0	0	0	hsa_circ_0003238	NC_000001.11	+	32187954	32190249	2296	366	sense-overlappin	XM_011540932.1	TXLNA
circRNA_00216	0	0.030704951	0	0	0	0	0	0	0	0	0	0	hsa_circ_0011385	NC_000001.11	+	32226171	32226530	360	278	sense-overlappin	XM_011542354.1	EIF3I
circRNA_00217	0	0	0	0	0	0	0	0	0.030381792	0	0	0	-	NC_000001.11	-	32234080	32235567	1488	1488	intergenic		
circRNA_00218	0	0	0	0	0.03009467	0.041425819	0.050246713	0	0.030381792	0	0	0	hsa_circ_0011399	NC_000001.11	+	32279671	32280210	540	363	sense-overlappin	NM_005356.4	LCK
circRNA_00219	0	0.040939935	0	0	0	0	0	0	0	0	0	0	hsa_circ_0005339	NC_000001.11	+	32326939	32327677	739	281	sense-overlappin	NM_004964.2	HDAC1
circRNA_00220	0.020575025	0	0	0	0	0	0	0	0	0	0	0	hsa_circ_0011414	NC_000001.11	-	32383830	32386895	3066	336	sense-overlappin	NM_001143888.2	BSDC1

circRNA_00221	0	0	0.032712968	0	0	0	0	0	0	0.020172483	0	0	hsa_circ_0011422	NC_000001.11	+		32592931	32593754	824	824	exonic	NM_001040441.2	ZBTB8A
circRNA_00222	0	0	0	0	0	0	0.020098685	0	0	0.030258724	0	0	-	NC_000001.11	-		32631827	32634110	2284	296	sense-overlappin	XM_011541330.1	ZBTB8OS
circRNA_00223	0	0	0	0	0	0.020712909	0	0	0	0	0	0	-	NC_000001.11	-		32633645	32634110	466	243	sense-overlappin	XM_011541330.1	ZBTB8OS
circRNA_00224	0	0	0	0	0.02006311	0	0	0	0	0	0	0	hsa_circ_0008672	NC_000001.11	+		32668225	32672901	4677	902	sense-overlappin	NM_001135255.1	RBBP4
circRNA_00225	0	0	0	0.0309543	0.02006311	0	0	0	0	0	0	0	-	NC_000001.11	-		32779382	32781145	1764	434	sense-overlappin	XM_011542347.1	YARS
circRNA_00226	0	0	0.021808645	0	0	0	0	0	0	0	0	0	hsa_circ_0011451	NC_000001.11	+		32825313	32853166	27854	1305	sense-overlappin	XM_011541963.1	S100PBP
circRNA_00227	0	0	0	0	0	0	0	0	0	0	0	0.0309546	-	NC_000001.11	+		32894844	32895657	814	274	antisense	NM_033504.2	TMEM54
circRNA_00228	0	0	0.021808645	0	0	0	0	0	0	0	0	0.0206364	hsa_circ_0004013	NC_000001.11	-		32942252	32949774	7523	978	sense-overlappin	XM_006710356.2	RNF19B
circRNA_00229	0	0	0	0	0	0	0	0.020486295	0	0	0	0	hsa_circ_0011458	NC_000001.11	-		32944019	32945628	1610	256	sense-overlappin	XM_006710356.2	RNF19B
circRNA_00230	0.092587611	0.030704951	0.141756193	0.0206362	0.08025246	0.020712909	0.15074014	0.051215739	0.060763583	0.050431207	0.04051046	0.0309546	hsa_circ_0000048	NC_000001.11	-		32948222	32949774	1553	351	sense-overlappin	XM_006710356.2	RNF19B
circRNA_00231	0	0	0	0.0206362	0	0	0	0	0	0	0	0	hsa_circ_0011462	NC_000001.11	-		33013207	33014594	1388	269	sense-overlappin	NM_001199199.1	AK2
circRNA_00232	0.082300098	0.030704951	0.054521613	0	0.02006311	0.020712909	0.020098685	0.020486295	0.081018111	0.050431207	0.04051046	0	hsa_circ_0009027	NC_000001.11	+		33294937	33295305	369	238	sense-overlappin	XM_005270507.1	ZNF362
circRNA_00233	0	0	0	0.0309543	0	0	0	0	0	0	0	0	-	NC_000001.11	-		35003968	35015162	11195	1564	sense-overlappin	NM_007167.3	ZMYM6
circRNA_00234	0	0	0	0.0206362	0	0	0	0	0	0	0	0	-	NC_000001.11	-		35014697	35020467	5771	702	sense-overlappin	NM_007167.3	ZMYM6
circRNA_00235	0	0	0	0.0619086	0	0	0	0	0	0	0	0	-	NC_000001.11	+		35104295	35104769	475	388	sense-overlappin	NM_001289088.1	ZMYM1
circRNA_00236	0	0	0	0	0.03009467	0	0	0	0.020254528	0	0	0	hsa_circ_0003730	NC_000001.11	+		35104295	35110447	6153	542	sense-overlappin	NM_001289088.1	ZMYM1
circRNA_00237	0	0	0.04361729	0	0	0	0	0	0	0	0	0	-	NC_000001.11	-		35177989	35188090	10102	3796	sense-overlappin	XM_005271112.3	SFPQ
circRNA_00238	0	0	0	0	0	0	0	0	0	0.020172483	0	0	-	NC_000001.11	+		35293026	35295988	2963	2963	sense-overlappin	XM_011542423.1	ZMYM4
circRNA_00239	0.072012586	0.112584821	0.119947548	0.1960438	0.04012623	0.103564547	0.050246713	0.071702034	0.162036222	0.171466104	0.06076569	0.092863801	hsa_circ_0011536	NC_000001.11	+		35358925	35361789	2865	755	sense-overlappin	XR_246305.3	
circRNA_00240	0	0	0	0	0	0	0.020098685	0	0	0	0	0	hsa_circ_0011537	NC_000001.11	+		35358925	35370627	11703	1096	sense-overlappin	XR_246305.3	
circRNA_00241	0	0	0	0	0.02006311	0	0	0	0	0	0	0	hsa_circ_0002043	NC_000001.11	+		35381259	35381758	500	388	sense-overlappin	XR_246305.3	
circRNA_00242	0.030862537	0.020469968	0.054521613	0.0619086	0.06018934	0.062138728	0.030148028	0.051215739	0.020254528	0.030258724	0	0.051591	hsa_circ_0000051	NC_000001.11	+		35381259	35389082	7824	1255	sense-overlappin	XR_246305.3	
circRNA_00243	0	0	0.021808645	0.0206362	0	0	0	0	0	0	0	0	hsa_circ_0011542	NC_000001.11	+		35381259	35390098	8840	1406	sense-overlappin	XR_246305.3	
circRNA_00244	0.020575025	0	0	0	0	0	0	0	0	0	0	0	-	NC_000001.11	+		35392212	35399576	7365	941	sense-overlappin	XR_946801.1	
circRNA_00245	0	0	0	0	0	0.041425819	0	0	0	0	0	0	-	NC_000001.11	+		35396552	35399576	3025	617	sense-overlappin	XR_946801.1	
circRNA_00246	0	0	0	0.0309543	0	0.020712909	0.020098685	0.020486295	0	0.040344966	0.02025523	0.051591	hsa_circ_0004709	NC_000001.11	+		35413972	35415714	1743	361	sense-overlappin	XR_946801.1	
circRNA_00247	0.041150049	0	0	0	0.04012623	0	0	0	0.020254528	0.030258724	0	0.0309546	hsa_circ_0011571	NC_000001.11	-		35470863	35479212	8350	447	sense-overlappin	XM_006710907.1	KIAA0319L
circRNA_00248	0	0.030704951	0.054521613	0.0309543	0	0	0	0.030729443	0.030381792	0	0	0	hsa_circ_0011572	NC_000001.11	-		35474805	35479212	4408	349	sense-overlappin	XM_006710907.1	KIAA0319L
circRNA_00249	0	0	0	0	0.03009467	0	0	0.020486295	0.020254528	0	0	0	hsa_circ_0004660	NC_000001.11	-		35478966	35479212	247	247	exonic	XM_006710907.1	KIAA0319L
circRNA_00250	0	0	0.032712968	0	0	0	0	0	0	0	0	0	hsa_circ_0011597	NC_000001.11	+		35816882	35835993	19112	1705	sense-overlappin	XM_005270578.2	AGO4
circRNA_00251	0	0	0.032712968	0.0206362	0	0	0	0	0	0	0	0	-	NC_000001.11	+		35831427	35835993	4567	876	sense-overlappin	XM_005270578.2	AGO4
circRNA_00252	0	0	0	0	0	0	0	0.020486295	0	0	0	0	hsa_circ_0011625	NC_000001.11	+		35901474	35902337	864	386	sense-overlappin	XM_011541237.1	AGO1
circRNA_00253	0	0	0	0	0	0	0	0	0	0.040344966	0	0	hsa_circ_0011635	NC_000001.11	+		36004341	36009594	5254	491	sense-overlappin	XM_005270575.2	AGO3
circRNA_00254	0	0	0	0	0	0.041425819	0	0	0	0	0	0	hsa_circ_0011636	NC_000001.11	+		36004341	36014048	9708	748	sense-overlappin	XM_005270575.2	AGO3
circRNA_00255	0	0	0.032712968	0	0.04012623	0	0	0	0	0	0	0	hsa_circ_0011637	NC_000001.11	+		36004341	36027298	22958	933	sense-overlappin	XM_005270575.2	AGO3
circRNA_00256	0	0	0	0.0309543	0	0	0	0	0	0.020172483	0	0.0206364	hsa_circ_0006578	NC_000001.11	-		36358239	36362656	4418	1681	sense-overlappin	NM_001282546.1	STK40
circRNA_00257	0.051437562	0.051174919	0	0.0515905	0	0.020712909	0.040197371	0.030729443	0.020254528	0.060517448	0	0.0206364	hsa_circ_0000053	NC_000001.11	-		36361221	36362656	1436	1436	sense-overlappin	NM_001282546.1	STK40
circRNA_00258	0	0	0	0	0	0	0	0	0	0.020172483	0	0	-	NC_000001.11	+		36407380	36413357	5978	5978	intergenic		
circRNA_00259	0	0.020469968	0	0	0	0	0	0.020486295	0	0	0	0	-	NC_000001.11	-		37587311	37589082	1772	1772	sense-overlappin	NM_013285.2	GNL2
circRNA_00260	0.020575025	0	0.032712968	0.0412724	0	0	0	0	0	0.050431207	0	0	hsa_circ_0005807	NC_000001.11	-		37866459	37874088	7630	628	sense-overlappin	XM_011541389.1	INPP5B
circRNA_00261	0	0.020469968	0	0	0	0	0	0	0	0	0	0	-	NC_000001.11	-		37990696	37994676	3981	3981	intergenic		
circRNA_00262	0.020575025	0	0	0	0	0	0	0	0	0	0	0	hsa_circ_0003529	NC_000001.11	+		38998171	39001106	2936	276	sense-overlappin	NM_024595.2	AKIRIN1
circRNA_00263	0	0	0	0	0	0	0	0	0	0.020172483	0	0	-	NC_000001.11	+		39331203	39340953	9751	5967	sense-overlappin	XM_006710527.2	MACF1
circRNA_00264	0.020575025	0	0	0	0	0	0	0	0	0	0	0	-	NC_000001.11	+		39331203	39347210	16008	6201	sense-overlappin	XM_006710527.2	MACF1
circRNA_00265	0.030862537	0	0	0	0	0	0	0	0	0	0	0	-	NC_000001.11	+		39340502	39340953	452	366	sense-overlappin	XM_006710527.2	MACF1
circRNA_00266	0	0.040939935	0	0	0	0	0	0	0	0	0	0	hsa_circ_0011821	NC_000001.11	+		39350785	39359264	8480	1279	sense-overlappin	XM_006710527.2	MACF1
circRNA_00267	0.020575025	0	0	0	0	0	0	0	0	0	0	0	-	NC_000001.11	+		39370030	39372596	2567	275	sense-overlappin	XM_006710527.2	MACF1
circRNA_00268	0	0	0	0.0206362	0	0	0	0	0	0	0	0	hsa_circ_0011837	NC_000001.11	+		39385434	39388658	3225	1968	sense-overlappin	XM_006710527.2	MACF1
circRNA_00269	0	0	0	0	0.04012623	0	0	0	0	0	0	0	hsa_circ_0011843	NC_000001.11	+		39439274	39439500	227	227	exonic	XM_006710527.2	MACF1
circRNA_00270	0.020575025	0	0	0	0	0.020712909	0	0	0	0	0	0	-	NC_000001.11	+		39804878	39808198	3321	352	sense-overlappin	XM_011542545.1	LOC105378951
circRNA_00271	0	0	0	0	0	0	0	0	0	0.020172483	0	0	hsa_circ_0006164	NC_000001.11	-		39841135	39857417	16283	1839	sense-overlappin	NM_017646.4	TRIT1
circRNA_00272	0	0	0	0	0	0	0	0	0.030381792	0	0	0	hsa_circ_0011865	NC_000001.11	-		39850119	39857417	7299	529	sense-overlappin	NM_017646.4	TRIT1
circRNA_00273	0.020575025	0	0.032712968	0	0.02006311	0.020712909	0	0.020486295	0	0	0	0	hsa_circ_0000057	NC_000001.11	-		39853970	39857417	3448	240	sense-overlappin	NM_017646.4	TRIT1
circRNA_00274	0.020575025	0	0	0	0	0.041425819	0	0.020486295	0.020254528	0.060517448	0	0	hsa_circ_0009142	NC_000001.11	+		40064227	40064559	333	230	sense-overlappin	XM_011540509.1	CAP1
circRNA_00275	0.030862537	0	0.08723458	0.0206362	0.0702209	0.020712909	0.040197371	0	0.030381792	0.050431207	0	0	hsa_circ_0006373	NC_000001.11	+		40189055	40202614	13560	573	sense-overlappin	NM_012421.3	RLF
circRNA_00276	0.030862537	0	0.021808645	0	0	0	0	0	0	0	0	0	hsa_circ_0007302	NC_000001.11	+		40190772	40202614	11843	418	sense-overlappin	NM_012421.3	RLF

circRNA_00277	0	0.020469968	0	0.0206362	0	0	0	0	0.020254528	0	0	0	hsa_circ_0008985	NC_000001.11	+	40267786	40272035	4250	499	sense-overlappin	NM_005857.4	ZMPSTE24
circRNA_00278	0	0	0	0	0	0	0	0	0	0.020172483	0	0	-	NC_000001.11	+	40267786	40281527	13742	684	sense-overlappin	NM_005857.4	ZMPSTE24
circRNA_00279	0	0	0	0	0	0	0	0	0	0.020172483	0	0	hsa_circ_0003276	NC_000001.11	+	40406736	40409835	3100	299	sense-overlappin	XM_011541959.1	SMAP2
circRNA_00280	0	0	0.250799418	0.0206362	0	0	0	0.030729443	0	0	0.02025523	0	-	NC_000001.11	+	40514515	40515918	1404	1404	exonic	XM_005271125.2	EXO5
circRNA_00281	0	0	0.054521613	0	0	0	0	0	0	0	0	0	-	NC_000001.11	+	40514515	40517186	2672	2672	exonic	XM_005271125.2	EXO5
circRNA_00282	0	0	0.032712968	0	0	0	0	0	0	0	0	0	-	NC_000001.11	-	40641709	40647842	6134	423	sense-overlappin	XM_011542480.1	RIMS3
circRNA_00283	0	0	0.032712968	0	0	0	0	0.020486295	0	0	0	0	-	NC_000001.11	+	40738836	40753246	14411	395	sense-overlappin	NM_001142587.1	NFYC
circRNA_00284	0	0	0.109043225	0	0	0	0.030148028	0	0	0	0	0	hsa_circ_0005157	NC_000001.11	+	41008794	41009589	796	242	sense-overlappin	NM_001905.3	CTPS1
circRNA_00285	0.051437562	0	0.032712968	0	0	0	0	0	0	0	0	0	hsa_circ_0008296	NC_000001.11	+	41008794	41010254	1461	336	sense-overlappin	NM_001905.3	CTPS1
circRNA_00286	0.421788005	0.225169643	0.796015543	0.2579524	0.11034713	0.227842003	0.130641455	0.174133512	0.293690652	0.282414759	0.192424686	0.185727601	hsa_circ_0000061	NC_000001.11	-	41070595	41075451	4857	360	sense-overlappin	NM_001172222.1	SCMH1
circRNA_00287	0	0	0.021808645	0	0	0	0	0	0	0	0	0	hsa_circ_0011943	NC_000001.11	-	41070595	41161432	90838	1444	sense-overlappin	NM_001031694.2	SCMH1
circRNA_00288	0.061725074	0	0.065425935	0	0	0	0	0	0	0	0	0	hsa_circ_0004628	NC_000001.11	-	41113283	41117010	3728	333	sense-overlappin	NM_001031694.2	SCMH1
circRNA_00289	0.123450148	0	0.054521613	0.0309543	0	0	0	0	0.020254528	0	0	0	hsa_circ_0000063	NC_000001.11	-	41113283	41143112	29830	568	sense-overlappin	NM_001031694.2	SCMH1
circRNA_00290	0	0	0.032712968	0	0	0	0	0	0	0	0	0	hsa_circ_0003751	NC_000001.11	-	41113283	41161432	48150	1084	sense-overlappin	NM_001031694.2	SCMH1
circRNA_00291	0.020575025	0	0	0.0309543	0	0	0	0	0	0	0	0	hsa_circ_0011950	NC_000001.11	-	41575544	41585318	9775	5728	sense-overlappin	XM_006710808.2	HIVEP3
circRNA_00292	0	0	0.032712968	0	0	0	0	0	0	0	0	0	-	NC_000001.11	-	42188737	42227966	39230	1201	sense-overlappin	NM_001198851.1	FOXJ3
circRNA_00293	0	0	0.054521613	0	0	0	0	0	0	0	0	0	hsa_circ_0011957	NC_000001.11	-	42227883	42278672	50790	484	sense-overlappin	NM_001198851.1	FOXJ3
circRNA_00294	0	0	0.021808645	0	0.02006311	0	0	0	0	0	0	0	-	NC_000001.11	-	42227883	42278784	50902	596	sense-overlappin	NM_001198851.1	FOXJ3
circRNA_00295	0	0	0.065425935	0.0515905	0	0.020712909	0	0	0	0	0	0	hsa_circ_0011958	NC_000001.11	-	42227883	42311110	83228	545	sense-overlappin	NM_001198851.1	FOXJ3
circRNA_00296	0	0	0.04361729	0.0515905	0	0	0	0	0	0	0	0	hsa_circ_0011959	NC_000001.11	-	42227883	42323827	95945	799	sense-overlappin	NM_001198851.1	FOXJ3
circRNA_00297	0	0	0.032712968	0	0	0	0	0	0	0	0	0	-	NC_000001.11	-	42257456	42311110	53655	8120	sense-overlappin	NM_001198851.1	FOXJ3
circRNA_00298	0.061725074	0.122819805	0.730589608	0.1238171	0.03009467	0.020712909	0.040197371	0.040972591	0.030381792	0.030258724	0	0	hsa_circ_0006848	NC_000001.11	-	42265115	42278672	13558	400	sense-overlappin	NM_001198851.1	FOXJ3
circRNA_00299	0.061725074	0	0.545216125	0.0825448	0.02006311	0	0	0	0.030381792	0.040344966	0.030382845	0	-	NC_000001.11	-	42265115	42278784	13670	512	sense-overlappin	NM_001198851.1	FOXJ3
circRNA_00300	0	0.030704951	0.52340748	0.2889066	0.02006311	0.082851637	0	0.040972591	0.050636319	0.050431207	0	0.0206364	hsa_circ_0004416	NC_000001.11	-	42265115	42311110	45996	461	sense-overlappin	NM_001198851.1	FOXJ3
circRNA_00301	0	0	0.141756193	0.0928628	0	0	0	0	0	0	0	0	-	NC_000001.11	-	42265115	42323827	58713	715	sense-overlappin	NM_001198851.1	FOXJ3
circRNA_00302	0	0	0.065425935	0	0	0	0	0	0	0	0	0	-	NC_000001.11	-	42278348	42278672	325	325	exonic	NM_001198851.1	FOXJ3
circRNA_00303	0	0	0.032712968	0	0	0	0	0	0	0	0	0	-	NC_000001.11	-	42278348	42278784	437	437	exonic	NM_001198851.1	FOXJ3
circRNA_00304	0	0	0.04361729	0	0.03009467	0	0	0	0	0	0	0	hsa_circ_0011960	NC_000001.11	-	42278348	42311110	32763	386	sense-overlappin	NM_001198851.1	FOXJ3
circRNA_00305	0	0	0.054521613	0.0206362	0	0	0	0	0	0	0	0	-	NC_000001.11	-	42278348	42323827	45480	640	sense-overlappin	NM_001198851.1	FOXJ3
circRNA_00306	0	0	0.065425935	0	0	0	0	0	0	0	0	0	-	NC_000001.11	-	42311050	42323827	12778	315	sense-overlappin	NM_001198851.1	FOXJ3
circRNA_00307	0	0	0.032712968	0	0	0	0	0	0	0	0	0	-	NC_000001.11	+	42399404	42399634	231	231	exonic	NM_173642.3	RIMKLA
circRNA_00308	0.020575025	0	0.021808645	0	0	0	0	0	0	0	0	0	-	NC_000001.11	+	42480461	42498916	18456	547	sense-overlappin	XM_011542066.1	CCDC30
circRNA_00309	0	0.020469968	0.08723458	0.0206362	0	0	0.040197371	0	0	0.060517448	0	0	hsa_circ_0004752	NC_000001.11	+	42773563	42775479	1917	490	sense-overlappin	NM_024097.3	C1orf50
circRNA_00310	0	0	0	0	0	0	0	0	0	0.030258724	0	0	-	NC_000001.11	+	42773563	42775522	1960	533	sense-overlappin	NM_024097.3	C1orf50
circRNA_00311	0.020575025	0	0	0	0	0	0	0	0	0	0	0	-	NC_000001.11	+	43409354	43409449	96	96	intronic	XM_011541104.1	SZT2
circRNA_00312	0	0	0.032712968	0.0309543	0	0	0.020098685	0	0	0	0	0	hsa_circ_0006595	NC_000001.11	+	43920404	43920928	525	294	sense-overlappin	XM_011541982.1	ST3GAL3
circRNA_00313	0	0	0	0	0	0	0	0	0	0	0	0.0206364	hsa_circ_0012133	NC_000001.11	-	44002313	44002985	673	372	sense-overlappin	NM_001261380.1	SLC6A9
circRNA_00314	0	0	0.021808645	0	0	0	0	0	0	0	0	0	-	NC_000001.11	+	44091350	44105514	14165	14165	intronic	XM_011540700.1	KLF17
circRNA_00315	0	0.040939935	0	0	0	0	0	0	0	0.020172483	0	0	hsa_circ_0007437	NC_000001.11	-	44284835	44339322	54488	620	sense-overlappin	NM_001301698.1	ERI3
circRNA_00316	0	0	0.032712968	0	0	0	0	0.030729443	0	0	0	0	hsa_circ_0012144	NC_000001.11	-	44308310	44319744	11435	269	sense-overlappin	NM_001301698.1	ERI3
circRNA_00317	0.051437562	0.020469968	0.119947548	0.0515905	0	0	0	0	0	0	0.02025523	0.0309546	hsa_circ_0007693	NC_000001.11	-	44308310	44339322	31013	547	sense-overlappin	NM_001301698.1	ERI3
circRNA_00318	0	0	0.109043225	0	0	0	0.020098685	0	0	0	0	0	hsa_circ_0002563	NC_000001.11	+	44757556	44758140	585	247	sense-overlappin	XM_011540540.1	KIF2C
circRNA_00319	0	0	0	0.0206362	0	0	0	0	0	0.040344966	0	0.0412728	-	NC_000001.11	-	45087881	45088237	357	357	exonic	XM_011541860.1	ZSWIM5
circRNA_00320	0	0.020469968	0.032712968	0	0	0	0	0	0	0	0	0	-	NC_000001.11	-	45330516	45334511	3996	1482	sense-overlappin	XM_011541497.1	MUTYH
circRNA_00321	0	0	0	0.0206362	0	0	0	0	0	0	0	0	-	NC_000001.11	-	45421725	45457871	36147	430	sense-overlappin	XM_005270355.2	TESK2
circRNA_00322	0	0	0	0	0	0	0	0	0	0.040344966	0	0	-	NC_000001.11	-	45457564	45457871	308	308	exonic	XM_005270355.2	TESK2
circRNA_00323	0	0	0	0	0	0	0	0.020486295	0	0	0	0	-	NC_000001.11	+	45509625	45509866	242	242	exonic	NM_015506.2	MMACHC
circRNA_00324	0	0	0	0	0	0	0	0	0	0.020172483	0	0	-	NC_000001.11	-	45509645	45509884	240	240	antisense	NM_015506.2	MMACHC
circRNA_00325	0	0	0.065425935	0	0.02006311	0	0.020098685	0.020486295	0	0.020172483	0	0	hsa_circ_0000067	NC_000001.11	+	45566569	45567020	452	272	sense-overlappin	NM_001202413.1	AKR1A1
circRNA_00326	0	0	0	0	0	0	0.030148028	0	0	0	0	0	hsa_circ_0002053	NC_000001.11	+	45614096	45616393	2298	573	sense-overlappin	NM_001195193.1	NASP
circRNA_00327	0	0.020469968	0	0	0	0	0	0	0	0	0	0	hsa_circ_0005361	NC_000001.11	-	45633489	45642499	9011	567	sense-overlappin	NM_021639.4	GPBP1L1
circRNA_00328	0.082300098	0.061409903	0.08723458	0.1031809	0.08025246	0.165703275	0.070345399	0.071702034	0.020254528	0.07060369	0.06076569	0.0206364	hsa_circ_0008774	NC_000001.11	-	45640210	45642499	2290	267	sense-overlappin	NM_021639.4	GPBP1L1
circRNA_00329	0	0	0.021808645	0.0515905	0.03009467	0	0	0	0	0	0	0	hsa_circ_0000068	NC_000001.11	-	45659028	45661225	2198	1157	sense-overlappin	NM_021639.4	GPBP1L1
circRNA_00330	0	0	0	0	0.02006311	0	0	0	0	0	0	0	hsa_circ_0012277	NC_000001.11	+	45824433	45829581	5149	291	sense-overlappin	XM_011541065.1	MAST2
circRNA_00331	0	0	0.032712968	0	0	0	0	0	0	0	0	0	-	NC_000001.11	-	46043654	46080750	37097	1299	sense-overlappin	NM_003629.3	PIK3R3

circRNA_00332	0	0	0	0.0515905	0	0	0.040197371	0.020486295	0.050636319	0.020172483	0.030382845	0	hsa_circ_0012300	NC_000001.11	-	46055795	46080750	24956	835	sense-overlappin	NM_003629.3	PIK3R3
circRNA_00333	0	0	0	0	0	0	0	0	0	0.020172483	0	0	-	NC_000001.11	+	46309897	46310316	420	310	sense-overlappin	NM_001297565.1	UQCRH
circRNA_00334	0	0	0.021808645	0	0	0	0	0	0	0	0	0	-	NC_000001.11	-	46632759	46635970	3212	3212	sense-overlappin	NM_001243728.1	ATPAF1
circRNA_00335	0	0	0	0	0	0	0	0	0	0.040344966	0	0	-	NC_000001.11	-	46678469	46691937	13469	901	sense-overlappin	NM_014774.2	EFCAB14
circRNA_00336	0	0	0	0	0.02006311	0	0	0	0	0	0	0	-	NC_000001.11	+	47055404	47068763	13360	12406	sense-overlappin	XM_011540913.1	CYP4Z1
circRNA_00337	0	0	0	0	0.03009467	0	0	0	0	0	0	0	-	NC_000001.11	+	47055404	47084978	29575	12859	sense-overlappin	XM_011540913.1	CYP4Z1
circRNA_00338	0	0	0.021808645	0	0.02006311	0	0	0	0	0	0	0	-	NC_000001.11	-	47260289	47272241	11953	863	sense-overlappin	XM_011541999.1	STIL
circRNA_00339	0	0	0.098138903	0	0.03009467	0	0.020098685	0.020486295	0	0	0	0	hsa_circ_0000069	NC_000001.11	-	47280241	47282459	2219	1084	sense-overlappin	XM_011541999.1	STIL
circRNA_00340	0	0	0.08723458	0	0	0	0	0	0	0	0	0	hsa_circ_0002632	NC_000001.11	-	47295765	47301748	5984	379	sense-overlappin	NM_001282938.1	STIL
circRNA_00341	0	0	0.021808645	0	0	0	0	0	0	0	0	0	hsa_circ_0007322	NC_000001.11	-	47301561	47310362	8802	496	sense-overlappin	NM_001048166.1	STIL
circRNA_00342	0	0.020469968	0.04361729	0.0309543	0.02006311	0.041425819	0.020098685	0.030729443	0	0.07060369	0	0	hsa_circ_0008201	NC_000001.11	+	47368469	47375293	6825	474	sense-overlappin	NM_016308.2	CMPK1
circRNA_00343	0	0.040939935	0	0	0	0	0	0	0	0	0	0	hsa_circ_0004741	NC_000001.11	+	47374909	47375293	385	174	sense-overlappin	NM_001136140.1	CMPK1
circRNA_00344	0	0	0	0	0	0	0	0	0	0.020172483	0	0	-	NC_000001.11	-	48305787	48359770	53984	377	sense-overlappin	XM_006710699.1	SPATA6
circRNA_00345	0.072012586	0.051174919	0.109043225	0.1857257	0.17053648	0	0.100493427	0.061458886	0.192418013	0.131121138	0.08102092	0.103182001	hsa_circ_0008202	NC_000001.11	-	48355670	48359770	4101	285	sense-overlappin	XM_006710699.1	SPATA6
circRNA_00346	0	0.040939935	0.032712968	0.0412724	0	0.020712909	0	0	0.040509055	0.020172483	0	0.0412728	hsa_circ_0012386	NC_000001.11	-	48355670	48403882	48213	789	sense-overlappin	XM_006710699.1	SPATA6
circRNA_00347	0	0	0.054521613	0	0	0	0	0	0	0.020172483	0.030382845	0	-	NC_000001.11	-	48385309	48403882	18574	504	sense-overlappin	XM_006710699.1	SPATA6
circRNA_00348	0	0	0.032712968	0	0	0	0	0	0	0	0	0	-	NC_000001.11	-	48395267	48403882	8616	463	sense-overlappin	XM_006710699.1	SPATA6
circRNA_00349	0	0	0.032712968	0	0	0	0	0.030729443	0	0	0	0	hsa_circ_0012389	NC_000001.11	-	48451552	48453131	1580	187	sense-overlappin	XM_006710699.1	SPATA6
circRNA_00350	0	0	0.04361729	0	0.02006311	0.051782273	0	0	0	0	0	0	hsa_circ_0008981	NC_000001.11	-	50490588	50535457	44870	248	sense-overlappin	NM_007051.2	FAF1
circRNA_00351	0	0	0	0	0	0	0	0	0	0	0	0.0309546	hsa_circ_0012396	NC_000001.11	-	50535369	50584811	49443	654	sense-overlappin	NM_007051.2	FAF1
circRNA_00352	0	0	0.021808645	0	0	0	0	0	0	0.020172483	0	0	hsa_circ_0012397	NC_000001.11	-	50535369	50596216	60848	750	sense-overlappin	NM_007051.2	FAF1
circRNA_00353	0	0	0.04361729	0.0206362	0	0	0	0	0	0	0	0	hsa_circ_0012400	NC_000001.11	-	50539592	50596216	56625	661	sense-overlappin	NM_007051.2	FAF1
circRNA_00354	0.020575025	0	0.076330258	0	0.03009467	0	0	0	0	0	0	0	hsa_circ_0008197	NC_000001.11	-	50567077	50596216	29140	524	sense-overlappin	NM_007051.2	FAF1
circRNA_00355	0	0	0	0.0206362	0.02006311	0	0	0.020486295	0	0	0.02025523	0	hsa_circ_0004619	NC_000001.11	-	50655442	50744775	89334	377	sense-overlappin	NM_007051.2	FAF1
circRNA_00356	0	0	0.021808645	0	0	0	0	0	0.020254528	0.020172483	0	0	hsa_circ_0005191	NC_000001.11	-	50788000	50801677	13678	253	sense-overlappin	NM_007051.2	FAF1
circRNA_00357	0	0	0	0	0	0	0	0	0	0.020172483	0	0	-	NC_000001.11	-	51365953	51402525	36573	405	sense-overlappin	NM_001159969.1	EPS15
circRNA_00358	0	0	0	0	0	0	0	0	0.030381792	0	0	0	hsa_circ_0012421	NC_000001.11	-	51394381	51400953	6573	237	sense-overlappin	NM_001159969.1	EPS15
circRNA_00359	0	0.030704951	0.021808645	0.0309543	0	0	0	0	0	0	0	0.0309546	hsa_circ_0012423	NC_000001.11	-	51394381	51421858	27478	1079	sense-overlappin	NM_001159969.1	EPS15
circRNA_00360	0	0.030704951	0.032712968	0	0	0	0.060296056	0.030729443	0.040509055	0	0	0.0412728	hsa_circ_0012425	NC_000001.11	-	51394381	51448135	53755	1558	sense-overlappin	XM_011540991.1	EPS15
circRNA_00361	0.051437562	0.051174919	0.076330258	0.0825448	0.04012623	0.020712909	0.090444084	0.020486295	0.030381792	0.040344966	0.04051046	0	hsa_circ_0005567	NC_000001.11	-	51402435	51408332	5898	607	sense-overlappin	NM_001159969.1	EPS15
circRNA_00362	0	0	0	0	0	0	0	0.020486295	0	0	0	0	-	NC_000001.11	-	51402435	51429567	27133	8551	sense-overlappin	XM_006710446.1	EPS15
circRNA_00363	0	0	0.032712968	0	0.03009467	0.041425819	0	0	0	0	0	0.0412728	hsa_circ_0004877	NC_000001.11	-	51405905	51408332	2428	402	sense-overlappin	NM_001159969.1	EPS15
circRNA_00364	0	0	0	0	0	0	0	0	0.050636319	0	0	0	hsa_circ_0006125	NC_000001.11	-	51440347	51448135	7789	479	sense-overlappin	XM_011540991.1	EPS15
circRNA_00365	0.030862537	0	0.021808645	0	0	0	0.020098685	0.030729443	0.040509055	0.080689931	0	0	hsa_circ_0007330	NC_000001.11	-	51461091	51465326	4236	252	sense-overlappin	NM_001981.2	EPS15
circRNA_00366	0	0	0.021808645	0	0	0	0	0	0	0	0	0	hsa_circ_0006350	NC_000001.11	-	51461091	51481314	20224	528	sense-overlappin	NM_001981.2	EPS15
circRNA_00367	0	0	0.021808645	0	0	0	0	0	0	0	0	0	-	NC_000001.11	+	51669434	51673571	4138	4138	sense-overlappin	NM_024586.5	OSBPL9
circRNA_00368	0.020575025	0	0	0	0	0	0	0	0	0	0	0	-	NC_000001.11	+	51745536	51750195	4660	225	sense-overlappin	NM_024586.5	OSBPL9
circRNA_00369	0	0	0	0	0	0.031069364	0	0	0	0	0	0	hsa_circ_0012460	NC_000001.11	+	51750145	51761971	11827	286	sense-overlappin	NM_024586.5	OSBPL9
circRNA_00370	0	0	0.021808645	0	0.02006311	0	0	0	0	0	0	0	hsa_circ_0008022	NC_000001.11	+	51750145	51765981	15837	446	sense-overlappin	NM_024586.5	OSBPL9
circRNA_00371	0	0	0.04361729	0.0309543	0	0	0	0	0.020254528	0	0.02025523	0	hsa_circ_0002039	NC_000001.11	+	51772070	51772723	654	232	sense-overlappin	NM_024586.5	OSBPL9
circRNA_00372	0	0.030704951	0	0	0	0	0	0	0	0	0	0	-	NC_000001.11	-	51803814	51834170	30357	1601	sense-overlappin	XM_005270903.1	NRD1
circRNA_00373	0	0	0	0	0	0.062138728	0	0	0	0	0	0	hsa_circ_0007430	NC_000001.11	-	51823664	51834170	10507	447	sense-overlappin	XM_005270903.1	NRD1
circRNA_00374	0.020575025	0.020469968	0.04361729	0.0515905	0.03009467	0.051782273	0.100493427	0.020486295	0	0.151293621	0	0.0619092	hsa_circ_0009076	NC_000001.11	-	51827796	51834170	6375	228	sense-overlappin	XM_005270903.1	NRD1
circRNA_00375	0	0	0	0.0206362	0	0	0	0	0	0	0	0	-	NC_000001.11	-	51836131	51840514	4384	606	sense-overlappin	XM_011541525.1	NRD1
circRNA_00376	0	0	0	0	0	0.031069364	0	0	0	0	0	0	-	NC_000001.11	+	52073130	52074458	1329	1329	intronic	NM_001136497.2	BTF3L4
circRNA_00377	0	0	0.021808645	0	0	0	0	0	0	0	0	0	hsa_circ_0012492	NC_000001.11	+	52233171	52239595	6425	2214	sense-overlappin	NM_004799.3	ZFYVE9
circRNA_00378	0	0	0	0	0	0.020712909	0	0	0	0	0	0	-	NC_000001.11	-	52445787	52461769	15983	753	sense-overlappin	NM_001009881.2	ZCCHC11
circRNA_00379	0	0	0	0	0	0	0	0	0	0.030258724	0	0	-	NC_000001.11	-	52458336	52461769	3434	366	sense-overlappin	NM_001009881.2	ZCCHC11
circRNA_00380	0	0	0.021808645	0	0	0	0	0	0	0	0	0	-	NC_000001.11	-	52471952	52475535	3584	855	sense-overlappin	NM_001009881.2	ZCCHC11
circRNA_00381	0	0.020469968	0.021808645	0.0309543	0	0.031069364	0.020098685	0	0.040509055	0	0	0	hsa_circ_0012545	NC_000001.11	-	52477708	52481923	4216	508	sense-overlappin	NM_001009881.2	ZCCHC11
circRNA_00382	0	0	0	0	0	0	0	0	0	0	0.02025523	0	hsa_circ_0008815	NC_000001.11	-	52477708	52489035	11328	635	sense-overlappin	NM_001009881.2	ZCCHC11
circRNA_00383	0	0	0.032712968	0	0	0	0	0	0	0	0	0	-	NC_000001.11	-	52481423	52509712	28290	966	sense-overlappin	NM_001009881.2	ZCCHC11
circRNA_00384	0	0	0	0.0309543	0	0	0	0	0	0	0	0	-	NC_000001.11	-	52488909	52509712	20804	633	sense-overlappin	NM_001009881.2	ZCCHC11
circRNA_00385	0	0	0	0	0	0	0	0	0	0	0	0.0412728	-	NC_000001.11	-	52490732	52509712	18981	506	sense-overlappin	NM_001009881.2	ZCCHC11
circRNA_00386	0.154312685	0.092114854	0.098138903	0.0722267	0.02006311	0.103564547	0.030148028	0.081945182	0	0.121034897	0.070893305	0	hsa_circ_0003632	NC_000001.11	-	52493611	52509712	16102	436	sense-overlappin	NM_001009881.2	ZCCHC11
circRNA_00387	0	0	0.08723458	0.0309543	0.04012623	0	0	0	0	0.030258724	0	0.0206364	-	NC_000001.11	-	52509596	52526373	16778	1092	sense-overlappin	NM_001009881.2	ZCCHC11

circRNA_00388	0	0	0.021808645	0	0	0	0	0	0	0	0	0	-	NC_000001.11	-	52509596	52535122	25527	9841	sense-overlappin	NM_001009881.2	ZCCHC11
circRNA_00389	0	0.020469968	0.163564838	0	0.03009467	0.051782273	0.040197371	0.09218833	0.020254528	0.040344966	0.030382845	0.051591	hsa_circ_0012553	NC_000001.11	-	52515891	52526373	10483	975	sense-overlappin	NM_001009881.2	ZCCHC11
circRNA_00390	0	0	0	0	0	0.020712909	0	0	0	0	0	0	hsa_circ_0003996	NC_000001.11	+	52801819	52802139	321	210	sense-overlappin	NM_024646.2	ZYG11B
circRNA_00391	0	0	0.076330258	0	0	0	0	0	0	0	0	0	hsa_circ_0006906	NC_000001.11	-	52993403	52993702	300	300	antisense	NM_001007098.2	SCP2
circRNA_00392	0	0	0	0.0206362	0	0	0	0	0	0	0	0	-	NC_000001.11	-	53233542	53235635	2094	170	sense-overlappin	NM_002370.3	MAGOH
circRNA_00393	0	0	0	0	0.02006311	0	0	0	0	0	0	0	-	NC_000001.11	-	53302012	53302440	429	429	intronic	XM_005271174.2	LRP8
circRNA_00394	0	0	0	0	0	0	0	0	0.030381792	0	0	0	hsa_circ_0002079	NC_000001.11	-	53594108	53600278	6171	1061	sense-overlappin	NM_147193.2	GLIS1
circRNA_00395	0	0	0	0	0	0	0	0	0	0.040344966	0	0	-	NC_000001.11	-	53923904	53940097	16194	333	sense-overlappin	NM_016126.2	HSPB11
circRNA_00396	0.030862537	0	0	0	0.02006311	0.072495183	0	0	0	0	0	0	hsa_circ_0012634	NC_000001.11	-	54040756	54043525	2770	320	sense-overlappin	XM_005271351.1	TMEM59
circRNA_00397	0	0	0	0	0	0	0	0	0	0.030258724	0	0	hsa_circ_0004839	NC_000001.11	-	54040756	54047372	6617	521	sense-overlappin	XM_005271350.1	TMEM59
circRNA_00398	0.020575025	0	0.021808645	0	0	0	0	0	0	0	0	0	-	NC_000001.11	+	54651949	54673186	21238	1804	sense-overlappin	NM_001039464.3	MROH7
circRNA_00399	0	0	0	0	0.02006311	0	0	0	0	0	0	0	-	NC_000001.11	-	55134070	55138718	4649	564	sense-overlappin	XM_005270690.2	USP24
circRNA_00400	0	0	0	0	0	0	0	0.030729443	0	0	0	0	-	NC_000001.11	-	58253807	58263863	10057	10057	intergenic		
circRNA_00401	0	0	0.119947548	0	0	0	0	0	0	0	0	0	-	NC_000001.11	-	58462045	58539306	77262	20507	sense-overlappin	NM_145243.4	OMA1
circRNA_00402	0.041150049	0	0	0.0206362	0	0	0.020098685	0	0	0.030258724	0.030382845	0	hsa_circ_0006281	NC_000001.11	-	58506060	58534331	28272	636	sense-overlappin	NM_145243.4	OMA1
circRNA_00403	0.061725074	0.08187987	0.228990773	0.1754076	0.05015779	0.041425819	0.080394741	0.09218833	0.030381792	0.211811069	0.091148535	0.0412728	hsa_circ_0000072	NC_000001.11	-	58506060	58536741	30682	865	sense-overlappin	NM_145243.4	OMA1
circRNA_00404	0	0	0.272608063	0	0	0	0	0	0	0	0	0	hsa_circ_0002316	NC_000001.11	-	58506060	58539310	33251	1381	sense-overlappin	NM_145243.4	OMA1
circRNA_00405	0	0	0.032712968	0.0206362	0	0.041425819	0	0	0	0	0.04051046	0	hsa_circ_0000073	NC_000001.11	-	58527261	58536741	9481	715	sense-overlappin	NM_145243.4	OMA1
circRNA_00406	0.020575025	0	0	0	0.05015779	0.020712909	0	0.020486295	0	0	0	0	-	NC_000001.11	-	58533949	58539306	5358	1027	sense-overlappin	NM_145243.4	OMA1
circRNA_00407	0	0	0	0	0	0	0	0	0	0	0.02025523	0	-	NC_000001.11	-	58536509	58539306	2798	745	sense-overlappin	NM_145243.4	OMA1
circRNA_00408	0	0	0	0	0	0	0	0	0	0.020172483	0	0	hsa_circ_0012732	NC_000001.11	-	58671870	58685251	13382	1262	sense-overlappin	XM_011540574.1	MYSM1
circRNA_00409	0	0	0	0	0	0	0	0	0	0	0.030382845	0	hsa_circ_0012742	NC_000001.11	-	58681785	58685251	3467	860	sense-overlappin	XM_011540574.1	MYSM1
circRNA_00410	0	0	0	0	0	0	0.020098685	0	0	0	0	0	hsa_circ_0002222	NC_000001.11	-	58685153	58690249	5097	453	sense-overlappin	XM_011540574.1	MYSM1
circRNA_00411	0	0	0.032712968	0	0	0	0	0	0	0	0	0	-	NC_000001.11	+	58714832	58718748	3917	3917	intergenic		
circRNA_00412	0	0	0	0	0	0	0.030148028	0	0	0	0	0	-	NC_000001.11	+	59316146	59346398	30253	5869	sense-overlappin	XM_011541731.1	FGGY
circRNA_00413	0	0	0	0	0	0	0	0	0	0	0.02025523	0	hsa_circ_0012746	NC_000001.11	+	59321536	59340069	18534	327	sense-overlappin	XM_011541731.1	FGGY
circRNA_00414	0.113162635	0.071644886	0.065425935	0.0619086	0.05015779	0.020712909	0.040197371	0.040972591	0.060763583	0.050431207	0.02025523	0.0619092	hsa_circ_0000075	NC_000001.11	+	59321536	59346398	24863	479	sense-overlappin	XM_011541731.1	FGGY
circRNA_00415	0.092587611	0.040939935	0.076330258	0.1031809	0.04012623	0.062138728	0	0.051215739	0	0.090776173	0.050638075	0.0206364	hsa_circ_0005354	NC_000001.11	+	59321536	59378837	57302	568	sense-overlappin	XM_011541731.1	FGGY
circRNA_00416	0.030862537	0.08187987	0	0.0515905	0.03009467	0.041425819	0.050246713	0	0	0.030258724	0	0	hsa_circ_0000076	NC_000001.11	+	59339958	59346398	6441	264	sense-overlappin	XM_011541731.1	FGGY
circRNA_00417	0.123450148	0.122819805	0.196277805	0.3611333	0.11034713	0.196772639	0.100493427	0.102431477	0.030381792	0.14120738	0.091148535	0.0412728	hsa_circ_0006633	NC_000001.11	+	59339958	59378837	38880	353	sense-overlappin	XM_011541731.1	FGGY
circRNA_00418	0	0	0	0	0	0	0.020098685	0	0	0	0	0	-	NC_000001.11	+	59346247	59378837	32591	241	sense-overlappin	XM_011541731.1	FGGY
circRNA_00419	0	0	0	0.0412724	0	0	0	0	0	0	0	0	-	NC_000001.11	+	59828780	59843598	14819	639	sense-overlappin	NM_015888.4	HOOK1
circRNA_00420	0	0	0.021808645	0	0	0	0	0	0	0	0	0	-	NC_000001.11	-	59900965	59916100	15136	1271	sense-overlappin	XR_946558.1	
circRNA_00421	0	0	0.032712968	0	0	0	0	0	0	0	0	0	-	NC_000001.11	-	60512662	60600250	87589	87589	intergenic		
circRNA_00422	0	0	0.032712968	0	0	0	0	0	0	0	0	0	-	NC_000001.11	-	60528919	60600250	71332	71332	intergenic		
circRNA_00423	0	0	0	0	0	0.020712909	0	0	0	0	0	0	-	NC_000001.11	-	61694697	61696534	1838	1838	sense-overlappin	XR_246299.2	
circRNA_00424	0	0	0	0	0	0	0	0.020486295	0	0	0	0	-	NC_000001.11	-	61694697	61709437	14741	275	sense-overlappin	XR_246299.2	
circRNA_00425	0	0	0	0	0	0	0.020098685	0	0	0	0	0	hsa_circ_0012779	NC_000001.11	+	61856030	61884408	28379	1019	sense-overlappin	NM_176877.2	INADL
circRNA_00426	0	0	0.021808645	0	0	0	0	0	0	0	0	0	-	NC_000001.11	+	61856030	61901459	45430	1269	sense-overlappin	NM_176877.2	INADL
circRNA_00427	0.020575025	0	0	0	0	0	0.030148028	0	0	0	0	0	hsa_circ_0012782	NC_000001.11	+	61899583	61901459	1877	250	sense-overlappin	NM_176877.2	INADL
circRNA_00428	0	0	0.021808645	0	0	0	0	0	0	0	0	0	-	NC_000001.11	+	61990168	62051058	60891	455	sense-overlappin	NM_176877.2	INADL
circRNA_00429	0	0.020469968	0	0	0	0	0	0	0	0	0	0	-	NC_000001.11	+	62056685	62061276	4592	4592	intronic	NM_176877.2	INADL
circRNA_00430	0	0	0	0	0.04012623	0	0	0	0	0	0.02025523	0	-	NC_000001.11	+	62441488	62442299	812	226	sense-overlappin	NM_001017415.1	USP1
circRNA_00431	0	0	0	0	0	0	0	0.020486295	0	0	0	0.051591	-	NC_000001.11	+	62444738	62447511	2774	863	sense-overlappin	NM_001017415.1	USP1
circRNA_00432	0	0	0	0	0	0	0	0.020486295	0	0	0	0	hsa_circ_0000080	NC_000001.11	+	62444738	62448666	3929	1065	sense-overlappin	NM_001017415.1	USP1
circRNA_00433	0	0	0.021808645	0	0	0	0	0	0	0	0	0	-	NC_000001.11	+	62447341	62448666	1326	373	sense-overlappin	NM_001017415.1	USP1
circRNA_00434	0	0	0	0	0	0	0	0	0	0.030258724	0	0	hsa_circ_0012799	NC_000001.11	-	62473982	62477834	3853	713	sense-overlappin	NM_001271999.1	DOCK7
circRNA_00435	0	0	0	0	0	0	0	0	0	0.030258724	0	0	hsa_circ_0002032	NC_000001.11	-	62577262	62586624	9363	430	sense-overlappin	NM_001271999.1	DOCK7
circRNA_00436	0	0	0.021808645	0	0	0	0	0	0	0	0	0	hsa_circ_0012814	NC_000001.11	-	62577262	62625401	48140	830	sense-overlappin	NM_001271999.1	DOCK7
circRNA_00437	0	0	0.021808645	0	0	0	0	0	0	0	0	0	hsa_circ_0012818	NC_000001.11	-	62618706	62654159	35454	1538	sense-overlappin	NM_001271999.1	DOCK7
circRNA_00438	0	0.020469968	0	0	0	0	0	0	0	0	0	0	hsa_circ_0012819	NC_000001.11	-	62618706	62663130	44425	1644	sense-overlappin	NM_001271999.1	DOCK7
circRNA_00439	0.030862537	0	0	0	0	0	0	0	0	0	0	0	-	NC_000001.11	+	62803719	62834852	31134	1157	sense-overlappin	NM_032852.3	ATG4C
circRNA_00440	0.041150049	0	0.021808645	0	0	0	0	0	0	0	0	0	hsa_circ_0012824	NC_000001.11	+	62803719	62841547	37829	1277	sense-overlappin	NM_032852.3	ATG4C
circRNA_00441	0	0	0.021808645	0	0	0	0	0	0	0	0	0	hsa_circ_0012828	NC_000001.11	+	62829040	62841547	12508	413	sense-overlappin	NM_032852.3	ATG4C
circRNA_00442	0	0	0.032712968	0.0412724	0	0	0	0	0	0.020172483	0	0	hsa_circ_0004586	NC_000001.11	-	63478764	63508570	29807	249	sense-overlappin	XM_006710514.2	ITGB3BP

circRNA_00443	0.030862537	0.040939935	0	0.0825448	0	0.020712909	0.020098685	0	0.020254528	0.030258724	0.02025523	0.0309546	hsa_circ_0000081	NC_000001.11	+		64009305	64050716	41412	391	sense-overlappin	NM_001083592.1	ROR1
circRNA_00444	0	0	0	0	0	0	0.020098685	0	0	0	0	0	hsa_circ_0000082	NC_000001.11	+		64049691	64050716	1026	319	sense-overlappin	NM_001083592.1	ROR1
circRNA_00445	0	0	0	0.0309543	0	0	0	0	0	0	0	0	-	NC_000001.11	+		64137369	64159192	21824	904	sense-overlappin	NM_005012.3	ROR1
circRNA_00446	0	0	0	0.0206362	0	0	0	0	0	0	0	0	hsa_circ_0012844	NC_000001.11	+		64550594	64602912	52319	319	sense-overlappin	NM_020925.2	CACHD1
circRNA_00447	0	0	0	0.0412724	0	0	0	0	0	0	0	0	-	NC_000001.11	+		64582156	64602912	20757	256	sense-overlappin	NM_020925.2	CACHD1
circRNA_00448	0	0	0	0.0206362	0	0	0	0	0	0	0	0	-	NC_000001.11	+		64602806	64641969	39164	746	sense-overlappin	NM_020925.2	CACHD1
circRNA_00449	0	0	0	0.0825448	0	0.031069364	0	0	0	0	0	0.0206364	-	NC_000001.11	+		64629355	64641969	12615	639	sense-overlappin	NM_020925.2	CACHD1
circRNA_00450	0	0.020469968	0	0	0	0	0	0	0	0	0.030382845	0	hsa_circ_0007009	NC_000001.11	+		64666057	64675983	9927	699	sense-overlappin	NM_020925.2	CACHD1
circRNA_00451	0	0	0	0	0	0	0.020098685	0	0	0	0	0	-	NC_000001.11	+		64768656	64789514	20859	856	sense-overlappin	XM_006710738.2	RAVER2
circRNA_00452	0	0	0	0	0	0	0	0.030729443	0	0	0	0	hsa_circ_0012855	NC_000001.11	+		64777623	64781571	3949	662	sense-overlappin	XM_006710738.2	RAVER2
circRNA_00453	0	0	0.163564838	0	0	0.031069364	0	0	0.030381792	0	0	0	hsa_circ_0012856	NC_000001.11	+		64777623	64789514	11892	789	sense-overlappin	XM_006710738.2	RAVER2
circRNA_00454	0	0	0.04361729	0	0	0	0	0	0	0	0	0	hsa_circ_0012859	NC_000001.11	+		64802976	64814840	11865	824	sense-overlappin	XM_006710738.2	RAVER2
circRNA_00455	0	0	0	0	0	0	0	0	0	0	0.030382845	0	hsa_circ_0012860	NC_000001.11	+		64812738	64814840	2103	249	sense-overlappin	XM_006710738.2	RAVER2
circRNA_00456	0	0.020469968	0	0	0	0	0	0	0	0	0	0	-	NC_000001.11	-		64989435	64995088	5654	5654	intergenic		
circRNA_00457	0	0.030704951	0.021808645	0.0722267	0.06018934	0.031069364	0	0.030729443	0	0.030258724	0.10127615	0	hsa_circ_0002454	NC_000001.11	+		65364635	65366196	1562	350	sense-overlappin	XM_011542485.1	DNAJC6
circRNA_00458	0.092587611	0	0	0.0206362	0.03009467	0	0	0	0	0	0	0	hsa_circ_0008433	NC_000001.11	+		65913245	65918835	5591	351	sense-overlappin	NM_001037341.1	PDE4B
circRNA_00459	0.092587611	0.020469968	0.08723458	0.0825448	0.03009467	0.113921001	0.170838825	0.122917773	0.121527166	0.201724828	0	0.103182001	hsa_circ_0006677	NC_000001.11	-		66891154	66905375	14222	473	sense-overlappin	NM_024763.4	WDR78
circRNA_00460	0	0	0.054521613	0.0309543	0	0	0	0	0	0	0	0	-	NC_000001.11	+		66940028	66963160	23133	641	sense-overlappin	XM_011541867.1	MIER1
circRNA_00461	0	0	0.04361729	0.0412724	0	0	0.030148028	0	0.020254528	0.030258724	0	0.0206364	-	NC_000001.11	+		66946150	66959743	13594	506	sense-overlappin	NM_001077700.2	MIER1
circRNA_00462	0	0	0	0	0	0.020712909	0	0	0	0	0	0	-	NC_000001.11	+		66946150	66976722	30573	1036	sense-overlappin	NM_001077700.2	MIER1
circRNA_00463	0	0	0	0	0	0.020712909	0	0	0	0	0	0	-	NC_000001.11	+		66958059	66959743	1685	360	sense-overlappin	NM_001077700.2	MIER1
circRNA_00464	0.082300098	0.051174919	0.338033998	0.1238171	0.1404418	0.082851637	0.060296056	0.071702034	0.121527166	0.191638587	0.02025523	0	-	NC_000001.11	+		66958059	66963160	5102	433	sense-overlappin	NM_001077700.2	MIER1
circRNA_00465	0.020575025	0	0	0	0	0	0	0	0	0	0	0	-	NC_000001.11	-		68126000	68159247	33248	1473	sense-overlappin	NM_001193334.1	WLS
circRNA_00466	0	0.020469968	0	0	0	0	0	0	0	0	0	0	-	NC_000001.11	-		68137780	68155260	17481	1012	sense-overlappin	NM_001002292.3	WLS
circRNA_00467	0	0	0	0	0.02006311	0	0	0	0	0	0	0	hsa_circ_0012937	NC_000001.11	-		70270856	70306290	35435	786	sense-overlappin	NM_030816.4	ANKRD13C
circRNA_00468	0	0	0.021808645	0.0206362	0	0	0	0	0	0	0	0	hsa_circ_0012938	NC_000001.11	-		70270856	70315566	44711	918	sense-overlappin	NM_030816.4	ANKRD13C
circRNA_00469	0	0	0.04361729	0	0	0	0	0	0	0	0	0	hsa_circ_0002231	NC_000001.11	-		70292388	70306290	13903	506	sense-overlappin	NM_030816.4	ANKRD13C
circRNA_00470	0.082300098	0.040939935	0.054521613	0.1857257	0.08025246	0.062138728	0.040197371	0.020486295	0.081018111	0.060517448	0.04051046	0.0206364	hsa_circ_0000085	NC_000001.11	-		70292388	70315566	23179	638	sense-overlappin	NM_030816.4	ANKRD13C
circRNA_00471	0	0	0.021808645	0.0206362	0	0	0	0	0	0	0	0	hsa_circ_0012942	NC_000001.11	-		70296130	70315566	19437	476	sense-overlappin	NM_030816.4	ANKRD13C
circRNA_00472	0	0	0	0	0	0	0.050246713	0	0	0.020172483	0	0	hsa_circ_0003566	NC_000001.11	-		70300764	70315566	14803	344	sense-overlappin	NM_030816.4	ANKRD13C
circRNA_00473	0	0	0	0	0	0	0.030148028	0	0	0	0	0	hsa_circ_0012947	NC_000001.11	+		70417937	70424416	6480	338	sense-overlappin	NM_001902.5	CTH
circRNA_00474	0	0	0	0	0	0	0	0	0	0	0.02025523	0	-	NC_000001.11	+		74236102	74271708	35607	404	sense-overlappin	NM_001199327.1	FPGT-TNNI3K
circRNA_00475	0	0	0	0	0	0.051782273	0	0	0	0	0	0	-	NC_000001.11	+		75728401	75750546	22146	1014	sense-overlappin	NM_001286043.1	ACADM
circRNA_00476	0	0	0.032712968	0.0309543	0	0	0	0	0	0.020172483	0	0	hsa_circ_0012963	NC_000001.11	+		75732644	75734871	2228	449	sense-overlappin	NM_001286043.1	ACADM
circRNA_00477	0	0	0	0	0	0.041425819	0	0	0	0	0	0	-	NC_000001.11	+		75732644	75750546	17903	926	sense-overlappin	NM_001286043.1	ACADM
circRNA_00478	0	0.020469968	0	0.0309543	0	0.031069364	0	0	0	0.020172483	0	0	hsa_circ_0008129	NC_000001.11	+		75739980	75750546	10567	477	sense-overlappin	NM_000016.5	ACADM
circRNA_00479	0	0	0	0	0	0	0	0	0.020254528	0.030258724	0	0	hsa_circ_0012964	NC_000001.11	+		75745806	75750546	4741	346	sense-overlappin	NM_000016.5	ACADM
circRNA_00480	0	0	0	0	0	0.020712909	0	0	0	0	0	0	-	NC_000001.11	+		75749419	75750546	1128	237	sense-overlappin	NM_000016.5	ACADM
circRNA_00481	0.020575025	0	0	0	0	0	0	0	0	0	0	0	-	NC_000001.11	+		77044204	77068843	24640	6383	sense-overlappin	XR_946772.1	
circRNA_00482	0	0	0.021808645	0	0	0	0	0	0	0	0	0	hsa_circ_0012975	NC_000001.11	-		77154449	77210489	56041	893	sense-overlappin	NM_005482.2	PIGK
circRNA_00483	0	0	0	0.0309543	0	0	0	0	0	0	0	0	-	NC_000001.11	-		77616741	77641655	24915	17016	sense-overlappin	XM_005270725.2	ZZZ3
circRNA_00484	0	0	0.021808645	0.0309543	0	0	0	0	0	0	0	0	hsa_circ_0012986	NC_000001.11	-		77631835	77641655	9821	1922	sense-overlappin	XM_005270725.2	ZZZ3
circRNA_00485	0.020575025	0	0.065425935	0.1238171	0	0.041425819	0.060296056	0.030729443	0.040509055	0.060517448	0	0.0412728	-	NC_000001.11	-		77631850	77641655	9806	1907	sense-overlappin	XM_005270725.2	ZZZ3
circRNA_00486	0.030862537	0	0	0	0.05015779	0	0	0	0	0	0.02025523	0	hsa_circ_0006395	NC_000001.11	-		77633115	77641655	8541	642	sense-overlappin	XM_005270725.2	ZZZ3
circRNA_00487	0	0	0	0	0	0	0	0	0.030381792	0.030258724	0	0	-	NC_000001.11	-		77701369	77713281	11913	294	sense-overlappin	XM_005270648.1	USP33
circRNA_00488	0	0	0	0.0412724	0	0.020712909	0	0	0.020254528	0.030258724	0	0.0309546	-	NC_000001.11	-		77711747	77714783	3037	361	sense-overlappin	XM_005270648.1	USP33
circRNA_00489	0	0.051174919	0.021808645	0.0928628	0.02006311	0.051782273	0	0	0	0.040344966	0	0	hsa_circ_0005374	NC_000001.11	-		77711747	77715868	4122	488	sense-overlappin	XM_005270648.1	USP33
circRNA_00490	0	0	0	0	0	0	0	0	0.020254528	0	0	0	-	NC_000001.11	-		77713200	77714783	1584	252	sense-overlappin	XM_005270648.1	USP33
circRNA_00491	0.051437562	0	0.054521613	0.0722267	0.02006311	0.020712909	0.020098685	0	0.020254528	0.040344966	0.030382845	0	hsa_circ_0000087	NC_000001.11	-		77717867	77725762	7896	759	sense-overlappin	XM_005270648.1	USP33
circRNA_00492	0	0	0	0	0	0.020712909	0	0	0	0	0	0	-	NC_000001.11	-		77729860	77736158	6299	366	sense-overlappin	XM_005270648.1	USP33
circRNA_00493	0	0	0	0	0	0	0.030148028	0	0	0	0	0	hsa_circ_0013000	NC_000001.11	-		77740877	77741748	872	249	sense-overlappin	XM_005270648.1	USP33
circRNA_00494	0	0	0	0	0	0	0	0.020486295	0	0	0	0	-	NC_000001.11	+		77783238	77807101	23864	556	sense-overlappin	NM_001270384.1	FAM73A
circRNA_00495	0.030862537	0	0	0	0	0	0	0	0	0.020172483	0	0	-	NC_000001.11	+		77801331	77807101	5771	442	sense-overlappin	XM_011541423.1	FAM73A
circRNA_00496	0.051437562	0.020469968	0.04361729	0.0619086	0.02006311	0.041425819	0.030148028	0.051215739	0.081018111	0.040344966	0.02025523	0	hsa_circ_0002570	NC_000001.11	+		77801331	77815231	13901	700	sense-overlappin	XM_011541423.1	FAM73A
circRNA_00497	0	0	0.021808645	0	0	0	0	0	0	0	0	0	-	NC_000001.11	+		77803270	77813867	10598	398	sense-overlappin	XM_011541423.1	FAM73A

circRNA_00498	0	0	0	0	0	0.031069364	0	0	0	0.050431207	0	0	hsa_circ_0008606	NC_000001.11	+	77858938	77864028	5091	513	sense-overlappin	XM_011541423.1	FAM73A
circRNA_00499	0	0	0	0.0619086	0	0	0	0	0	0.040344966	0	0	hsa_circ_0013012	NC_000001.11	+	77858938	77866391	7454	567	sense-overlappin	XM_011541423.1	FAM73A
circRNA_00500	0	0.020469968	0	0	0	0	0.020098685	0	0	0.030258724	0	0	-	NC_000001.11	+	77873004	77875111	2108	383	sense-overlappin	XM_011541423.1	FAM73A
circRNA_00501	0	0	0	0	0	0	0.020098685	0	0	0	0	0	-	NC_000001.11	+	77916055	77918273	2219	499	sense-overlappin	NM_144573.3	NEXN
circRNA_00502	0	0	0	0.0412724	0	0	0	0	0	0.030258724	0	0	hsa_circ_0013048	NC_000001.11	+	81836885	81907230	70346	387	sense-overlappin	XM_011541093.1	ADGRL2
circRNA_00503	0	0	0	0	0	0	0	0	0	0.020172483	0	0	-	NC_000001.11	+	81936728	81956060	19333	1733	sense-overlappin	XM_011541093.1	ADGRL2
circRNA_00504	0.020575025	0.020469968	0	0.0412724	0	0.020712909	0	0.051215739	0.020254528	0.060517448	0	0.0206364	-	NC_000001.11	-	83911165	83947282	36118	1439	sense-overlappin	NM_024686.4	TTLL7
circRNA_00505	0	0.030704951	0.032712968	0.0309543	0	0	0	0	0	0	0	0	-	NC_000001.11	+	84179177	84197824	18648	596	sense-overlappin	NM_207578.2	PRKACB
circRNA_00506	0.020575025	0	0.032712968	0.0309543	0	0	0	0	0	0	0	0	hsa_circ_0004083	NC_000001.11	+	84179177	84214317	35141	884	sense-overlappin	NM_002731.3	PRKACB
circRNA_00507	0	0	0	0	0	0	0	0	0.020254528	0	0	0	-	NC_000001.11	+	84179177	84235364	56188	1069	sense-overlappin	NM_002731.3	PRKACB
circRNA_00508	0	0	0	0	0	0	0	0	0	0.060517448	0	0	-	NC_000001.11	-	84655832	84671308	15477	1478	sense-overlappin	NM_001166417.1	SSX2IP
circRNA_00509	0.041150049	0.030704951	0.032712968	0.0309543	0	0.041425819	0	0.030729443	0.040509055	0.030258724	0	0.0309546	hsa_circ_0004390	NC_000001.11	-	84865385	84866138	754	754	exonic	XM_011541149.1	LPAR3
circRNA_00510	0	0	0	0	0	0	0	0	0	0	0	0.0309546	-	NC_000001.11	-	85029106	85045362	16257	993	sense-overlappin	XM_011541742.1	MCOLN3
circRNA_00511	0.030862537	0	0.04361729	0	0	0	0	0	0	0	0	0	-	NC_000001.11	-	85182098	85190752	8655	1799	sense-overlappin	NM_032184.1	SYDE2
circRNA_00512	0	0	0.065425935	0.0206362	0	0	0.040197371	0	0.020254528	0	0	0	hsa_circ_0004607	NC_000001.11	-	85350415	85358847	8433	294	sense-overlappin	XM_006710544.1	DDAH1
circRNA_00513	0	0	0.021808645	0	0	0.020712909	0	0	0	0.020172483	0	0	hsa_circ_0005110	NC_000001.11	-	85775674	85786461	10788	423	sense-overlappin	NM_152890.5	COL24A1
circRNA_00514	0	0	0	0	0	0	0	0	0	0	0	0.0206364	-	NC_000001.11	-	86352859	86354692	1834	289	sense-overlappin	NM_001184765.1	ODF2L
circRNA_00515	0	0	0	0	0	0.020712909	0.020098685	0	0	0	0	0	hsa_circ_0013081	NC_000001.11	+	86715724	86719635	3912	271	sense-overlappin	NM_001206651.1	SH3GLB1
circRNA_00516	0	0	0.021808645	0	0	0	0	0	0	0.020172483	0.02025523	0	-	NC_000001.11	+	86715724	86724405	8682	498	sense-overlappin	NM_001206651.1	SH3GLB1
circRNA_00517	0	0	0.021808645	0	0	0	0	0	0	0	0	0	-	NC_000001.11	+	86715724	86743235	27512	1089	sense-overlappin	NM_001206652.1	SH3GLB1
circRNA_00518	0	0	0	0	0	0	0	0	0.030381792	0	0	0.0206364	hsa_circ_0013084	NC_000001.11	+	86719507	86724405	4899	356	sense-overlappin	NM_001206651.1	SH3GLB1
circRNA_00519	0.030862537	0	0.021808645	0	0.02006311	0	0	0	0	0	0	0	hsa_circ_0000089	NC_000001.11	+	88740988	88741288	301	301	exonic	XM_005271031.1	PKN2
circRNA_00520	0	0	0	0	0	0	0	0.030729443	0	0	0	0	hsa_circ_0013092	NC_000001.11	+	88740988	88760376	19389	456	sense-overlappin	XM_005271031.1	PKN2
circRNA_00521	0	0	0	0.0206362	0	0.031069364	0	0	0.020254528	0	0	0	-	NC_000001.11	+	88740988	88771879	30892	937	sense-overlappin	XM_005271031.1	PKN2
circRNA_00522	0	0.092114854	0	0.1031809	0	0	0.050246713	0.020486295	0.070890847	0	0	0.0412728	hsa_circ_0013093	NC_000001.11	+	88740988	88786213	45226	1185	sense-overlappin	XM_005271031.1	PKN2
circRNA_00523	0	0.040939935	0.032712968	0.0309543	0	0	0	0.020486295	0	0	0.02025523	0.0309546	hsa_circ_0002008	NC_000001.11	+	88770352	88771879	1528	481	sense-overlappin	XM_005271031.1	PKN2
circRNA_00524	0	0	0.054521613	0.0412724	0.05015779	0	0	0	0	0	0	0	hsa_circ_0005900	NC_000001.11	+	88770352	88786213	15862	729	sense-overlappin	XM_005271031.1	PKN2
circRNA_00525	0.020575025	0	0	0	0	0	0	0	0	0	0	0	hsa_circ_0013107	NC_000001.11	+	88807313	88822003	14691	539	sense-overlappin	XM_005271031.1	PKN2
circRNA_00526	0	0	0	0.0309543	0	0	0	0	0	0	0	0	-	NC_000001.11	-	88853107	88857487	4381	522	sense-overlappin	NM_001514.5	GTF2B
circRNA_00527	0	0	0	0.0206362	0.02006311	0	0	0.040972591	0	0	0	0	-	NC_000001.11	-	88936099	88969467	33369	1350	sense-overlappin	NM_001008661.2	CCBL2
circRNA_00528	0	0	0	0	0	0	0.020098685	0	0	0	0	0	-	NC_000001.11	-	88949091	88969467	20377	1042	sense-overlappin	NM_001008661.2	CCBL2
circRNA_00529	0	0	0.021808645	0	0	0	0.030148028	0	0	0	0	0	-	NC_000001.11	+	89568247	89584789	16543	2379	sense-overlappin	XM_011541146.1	LRRC8B
circRNA_00530	0	0.020469968	0	0	0	0	0	0	0.020254528	0	0	0	-	NC_000001.11	+	89686470	89714851	28382	2195	sense-overlappin	XM_011542283.1	LRRC8C
circRNA_00531	0	0	0.021808645	0	0	0	0	0	0	0	0	0	-	NC_000001.11	-	90916640	90918154	1515	454	sense-overlappin	NM_016620.3	ZNF644
circRNA_00532	0	0	0.032712968	0.0412724	0.02006311	0	0	0	0	0	0	0	-	NC_000001.11	-	90916640	90941309	24670	4221	sense-overlappin	XM_011542261.1	ZNF644
circRNA_00533	0	0	0	0	0.02006311	0	0	0	0	0.030258724	0	0	-	NC_000001.11	-	90916640	90982370	65731	4282	sense-overlappin	XM_011542261.1	ZNF644
circRNA_00534	0	0	0	0	0	0	0	0	0	0.030258724	0	0	-	NC_000001.11	-	90935408	90941309	5902	3767	sense-overlappin	XM_011542261.1	ZNF644
circRNA_00535	0	0	0	0.0309543	0	0	0	0	0	0	0	0	-	NC_000001.11	-	90935408	90982370	46963	3828	sense-overlappin	XM_011542261.1	ZNF644
circRNA_00536	0	0	0.141756193	0	0	0	0.020098685	0	0	0	0	0	-	NC_000001.11	-	90937485	90941309	3825	3644	sense-overlappin	NM_201269.2	ZNF644
circRNA_00537	0	0	0.065425935	0.0309543	0.04012623	0	0.050246713	0	0.050636319	0.040344966	0	0.0206364	-	NC_000001.11	-	90937485	90982370	44886	3705	sense-overlappin	NM_201269.2	ZNF644
circRNA_00538	0.030862537	0	0	0	0	0	0	0.020486295	0	0	0	0	-	NC_000001.11	-	91343430	91353299	9870	650	sense-overlappin	XM_011540859.1	HFM1
circRNA_00539	0	0	0	0	0	0	0	0.020486295	0	0	0	0	-	NC_000001.11	-	91375358	91385834	10477	1191	sense-overlappin	XM_011540855.1	HFM1
circRNA_00540	0	0	0.032712968	0	0	0	0	0	0	0.020172483	0	0	-	NC_000001.11	-	91861471	91864279	2809	2809	sense-overlappin	XM_006710868.1	TGFBR3
circRNA_00541	0	0	0	0	0	0	0	0	0	0	0	0.0412728	-	NC_000001.11	+	92088698	92129776	41079	603	sense-overlappin	NM_183242.3	BTBD8
circRNA_00542	0	0	0.065425935	0	0	0.041425819	0	0	0	0.040344966	0.030382845	0	hsa_circ_0013148	NC_000001.11	-	92262863	92271652	8790	738	sense-overlappin	NM_053274.2	GLMN
circRNA_00543	0	0.040939935	0	0	0	0	0	0	0	0	0	0	hsa_circ_0013153	NC_000001.11	+	92320599	92345914	25316	1200	sense-overlappin	NM_024813.2	RPAP2
circRNA_00544	0	0.020469968	0	0	0	0	0	0	0	0	0	0	-	NC_000001.11	+	92323445	92345914	22470	1164	sense-overlappin	NM_024813.2	RPAP2
circRNA_00545	0	0.020469968	0	0.0206362	0	0	0	0	0	0	0.030382845	0.0309546	hsa_circ_0004463	NC_000001.11	+	92333391	92345914	12524	233	sense-overlappin	NM_024813.2	RPAP2
circRNA_00546	0.061725074	0.061409903	0.327129675	0.113499	0.03009467	0.124277456	0.040197371	0.081945182	0.111399902	0.100862414	0.06076569	0	hsa_circ_0000091	NC_000001.11	+	92333391	92380873	47483	445	sense-overlappin	XR_246295.1	
circRNA_00547	0	0	0	0	0.04012623	0.020712909	0.030148028	0.020486295	0.040509055	0	0	0.0206364	hsa_circ_0013162	NC_000001.11	-	92563642	92607727	44086	339	sense-overlappin	XM_011542100.1	EVI5
circRNA_00548	0.030862537	0	0.054521613	0	0.02006311	0.041425819	0	0.061458886	0.060763583	0.030258724	0	0.0206364	hsa_circ_0013163	NC_000001.11	-	92563642	92636336	72695	774	sense-overlappin	XM_011542100.1	EVI5
circRNA_00549	0	0	0	0	0	0	0	0	0	0	0	0.0309546	-	NC_000001.11	-	92563642	92639237	75596	3675	sense-overlappin	XM_011542100.1	EVI5
circRNA_00550	0	0	0	0	0	0	0	0.020486295	0	0	0.02025523	0	hsa_circ_0005371	NC_000001.11	-	92605307	92607727	2421	243	sense-overlappin	XM_011542100.1	EVI5
circRNA_00551	0	0.030704951	0	0	0.02006311	0	0	0	0	0	0	0	hsa_circ_0013166	NC_000001.11	-	92605307	92625934	20628	543	sense-overlappin	XM_011542100.1	EVI5
circRNA_00552	0.051437562	0.040939935	0.021808645	0.0412724	0	0.031069364	0	0	0	0	0	0.0309546	hsa_circ_0013167	NC_000001.11	-	92605307	92636336	31030	678	sense-overlappin	XM_011542100.1	EVI5

circRNA_00553	0	0	0	0	0	0	0	0	0	0	0	0.0206364	-	NC_000001.11	-	92605307	92639237	33931	3579	sense-overlappin	XM_011542100.1	EVI5
circRNA_00554	0	0.020469968	0.021808645	0.0206362	0	0	0	0.020486295	0	0	0	0	hsa_circ_0013168	NC_000001.11	-	92605307	92704744	99438	2002	sense-overlappin	XM_011542100.1	EVI5
circRNA_00555	0	0	0	0	0.02006311	0	0	0	0.020254528	0	0	0	-	NC_000001.11	-	92608666	92609439	774	774	intronic	XM_011542100.1	EVI5
circRNA_00556	0	0	0	0	0	0	0	0	0.020254528	0	0	0	hsa_circ_0005886	NC_000001.11	-	92624176	92636336	12161	435	sense-overlappin	XM_011542100.1	EVI5
circRNA_00557	0	0	0	0	0.04012623	0	0	0	0	0	0	0	-	NC_000001.11	-	92624176	92704744	80569	1759	sense-overlappin	XM_011542100.1	EVI5
circRNA_00558	0.020575025	0	0	0	0.02006311	0	0	0	0	0	0	0	-	NC_000001.11	-	92662719	92704744	42026	1324	sense-overlappin	XM_011542100.1	EVI5
circRNA_00559	0	0.051174919	0	0.0206362	0	0	0	0.040972591	0	0	0.02025523	0	-	NC_000001.11	-	92663420	92704744	41325	1177	sense-overlappin	XM_011542100.1	EVI5
circRNA_00560	0	0	0.021808645	0.0412724	0	0	0	0	0	0.020172483	0	0	-	NC_000001.11	-	92665939	92704744	38806	1144	sense-overlappin	XM_011542100.1	EVI5
circRNA_00561	0	0	0.021808645	0	0	0	0	0	0	0	0	0	-	NC_000001.11	-	92677158	92704744	27587	1090	sense-overlappin	XM_011542100.1	EVI5
circRNA_00562	0	0	0.065425935	0	0.02006311	0	0	0	0	0.040344966	0	0	hsa_circ_0013171	NC_000001.11	-	92693802	92704744	10943	1029	sense-overlappin	XM_011542100.1	EVI5
circRNA_00563	0	0	0	0	0	0	0.020098685	0	0	0	0	0	hsa_circ_0013172	NC_000001.11	-	92693802	92736627	42826	1259	sense-overlappin	XM_011542100.1	EVI5
circRNA_00564	0	0	0	0	0	0	0	0	0	0	0.030382845	0	hsa_circ_0013174	NC_000001.11	-	92736398	92736627	230	230	exonic	XM_011542100.1	EVI5
circRNA_00565	0	0	0.021808645	0	0	0	0	0	0	0	0	0	-	NC_000001.11	-	93154656	93160226	5571	564	sense-overlappin	NM_001167830.1	TMED5
circRNA_00566	0	0	0	0	0	0	0	0.020486295	0	0	0	0	-	NC_000001.11	+	93217738	93226449	8712	462	sense-overlappin	XM_011541365.1	CCDC18
circRNA_00567	0	0	0	0	0.02006311	0	0	0	0	0	0	0	-	NC_000001.11	+	93221609	93264901	43293	1923	sense-overlappin	XM_011541365.1	CCDC18
circRNA_00568	0	0	0.021808645	0	0.03009467	0	0.020098685	0	0	0	0	0.0309546	hsa_circ_0004535	NC_000001.11	-	93305388	93325895	20508	20508	intergenic		
circRNA_00569	0	0	0	0	0	0	0	0	0	0	0	0.0206364	hsa_circ_0013204	NC_000001.11	-	93324635	93325895	1261	1261	intergenic		
circRNA_00570	0	0	0.032712968	0	0	0	0	0.020486295	0	0.020172483	0	0	hsa_circ_0006427	NC_000001.11	-	93567279	93567851	573	325	sense-overlappin	XM_011542252.1	BCAR3
circRNA_00571	0.020575025	0.020469968	0	0	0	0.031069364	0	0	0.030381792	0	0.04051046	0.0206364	hsa_circ_0013218	NC_000001.11	-	93876268	93877862	1595	1595	exonic	NM_014597.4	DNTTIP2
circRNA_00572	0	0.030704951	0	0	0	0	0	0	0	0	0	0	hsa_circ_0013222	NC_000001.11	-	93894614	93897898	3285	378	sense-overlappin	XM_011541261.1	GCLM
circRNA_00573	0	0.020469968	0.021808645	0	0	0	0.030148028	0	0	0.020172483	0	0	hsa_circ_0003513	NC_000001.11	-	93894614	93901669	7056	463	sense-overlappin	XM_011541261.1	GCLM
circRNA_00574	0	0	0	0.0206362	0	0.020712909	0	0	0	0	0	0	hsa_circ_0013225	NC_000001.11	-	94201720	94231643	29924	1313	sense-overlappin	NM_004815.3	ARHGAP29
circRNA_00575	0	0	0	0	0	0.020712909	0	0	0	0	0	0	-	NC_000001.11	-	94203930	94205683	1754	252	sense-overlappin	XM_006711048.2	ARHGAP29
circRNA_00576	0	0	0	0	0.02006311	0	0	0	0	0	0	0	hsa_circ_0003310	NC_000001.11	-	94220258	94231643	11386	372	sense-overlappin	NM_004815.3	ARHGAP29
circRNA_00577	0	0	0	0	0.02006311	0	0	0	0	0	0	0	-	NC_000001.11	+	94455733	94468007	12275	297	sense-overlappin	XM_006710802.2	ABCD3
circRNA_00578	0.041150049	0	0	0	0	0	0	0	0	0	0	0	-	NC_000001.11	+	94455733	94480606	24874	789	sense-overlappin	XM_006710802.2	ABCD3
circRNA_00579	0	0	0	0.0309543	0	0	0	0	0	0	0	0	hsa_circ_0013232	NC_000001.11	+	94458607	94480606	22000	717	sense-overlappin	XM_006710802.2	ABCD3
circRNA_00580	0	0	0	0.0309543	0	0.051782273	0	0	0	0	0.06076569	0	-	NC_000001.11	+	94458607	94483239	24633	787	sense-overlappin	XM_006710802.2	ABCD3
circRNA_00581	0	0	0.021808645	0.0412724	0	0	0	0	0	0	0	0	-	NC_000001.11	+	94458607	94491247	32641	1276	sense-overlappin	XM_006710802.2	ABCD3
circRNA_00582	0	0	0	0.0825448	0	0	0	0	0	0	0	0	hsa_circ_0013235	NC_000001.11	+	94487542	94491247	3706	489	sense-overlappin	XM_006710802.2	ABCD3
circRNA_00583	0	0.030704951	0	0.0206362	0.02006311	0	0	0	0	0	0	0	-	NC_000001.11	+	94498602	94499614	1013	354	sense-overlappin	XM_006710802.2	ABCD3
circRNA_00584	0	0	0	0.0206362	0	0	0	0	0	0	0	0	hsa_circ_0006524	NC_000001.11	+	94498602	94506642	8041	459	sense-overlappin	XM_006710802.2	ABCD3
circRNA_00585	0	0	0	0	0	0.031069364	0	0	0	0	0	0	-	NC_000001.11	+	95138275	95151419	13145	6026	sense-overlappin	NM_001199679.1	TMEM56
circRNA_00586	0	0	0	0	0	0.020712909	0	0	0	0	0	0	-	NC_000001.11	+	95138275	95173889	35615	6100	sense-overlappin	NM_001199679.1	TMEM56
circRNA_00587	0.030862537	0.040939935	0.185373483	0.0928628	0.12037869	0.020712909	0.050246713	0.020486295	0.050636319	0.030258724	0	0.0309546	hsa_circ_0000095	NC_000001.11	+	95143891	95151419	7529	410	sense-overlappin	NM_001199679.1	TMEM56
circRNA_00588	0	0	0.065425935	0.0619086	0	0.062138728	0	0	0	0.030258724	0	0.0412728	hsa_circ_0005720	NC_000001.11	+	95143891	95173889	29999	484	sense-overlappin	NM_001199679.1	TMEM56
circRNA_00589	0	0	0	0	0.02006311	0	0	0	0	0	0.030382845	0	hsa_circ_0003580	NC_000001.11	+	96769703	96770851	1149	317	sense-overlappin	NM_001300985.1	PTBP2
circRNA_00590	0.030862537	0	0	0	0	0	0	0	0.020254528	0.050431207	0	0	hsa_circ_0013252	NC_000001.11	+	96769703	96785254	15552	789	sense-overlappin	NM_001300985.1	PTBP2
circRNA_00591	0	0	0	0	0	0	0	0.020486295	0	0	0	0	-	NC_000001.11	-	97364961	97365110	150	150	intronic	NM_000110.3	DPYD
circRNA_00592	0	0	0	0	0.02006311	0	0	0	0	0	0	0	hsa_circ_0006323	NC_000001.11	-	97515726	97549744	34019	401	sense-overlappin	NM_000110.3	DPYD
circRNA_00593	0	0	0	0	0	0	0	0	0.020254528	0.050431207	0	0	-	NC_000001.11	-	97549560	97573970	24411	396	sense-overlappin	NM_000110.3	DPYD
circRNA_00594	0	0	0	0	0	0	0	0	0	0	0.030382845	0	hsa_circ_0003268	NC_000001.11	-	97549560	97595166	45607	674	sense-overlappin	NM_000110.3	DPYD
circRNA_00595	0	0	0	0	0	0	0	0	0	0	0	0.0206364	-	NC_000001.11	-	97573760	97595166	21407	489	sense-overlappin	NM_000110.3	DPYD
circRNA_00596	0	0	0	0	0	0	0.020098685	0	0	0	0	0	hsa_circ_0004161	NC_000001.11	-	97679095	97699547	20453	367	sense-overlappin	NM_000110.3	DPYD
circRNA_00597	0	0	0.021808645	0	0	0	0	0	0	0	0	0	hsa_circ_0013269	NC_000001.11	+	98684885	98698905	14021	858	sense-overlappin	XM_006710677.1	SNX7
circRNA_00598	0	0	0.065425935	0	0	0	0	0	0	0	0	0	-	NC_000001.11	+	98691075	98701903	10829	762	sense-overlappin	XM_006710677.1	SNX7
circRNA_00599	0	0	0	0	0	0	0	0	0	0	0.02025523	0	-	NC_000001.11	+	99861503	99881691	20189	2226	sense-overlappin	XM_005270557.1	AGL
circRNA_00600	0	0	0	0	0	0	0	0	0	0	0.02025523	0	hsa_circ_0013290	NC_000001.11	+	99870400	99877828	7429	947	sense-overlappin	XM_005270557.1	AGL
circRNA_00601	0	0	0	0.0206362	0	0	0	0	0	0	0	0	-	NC_000001.11	+	99993537	100017815	24279	905	sense-overlappin	XM_005270691.3	SLC35A3
circRNA_00602	0	0	0	0.0412724	0	0	0	0.020486295	0	0	0	0	hsa_circ_0013298	NC_000001.11	+	99993537	99999415	5879	360	sense-overlappin	XM_005270691.3	SLC35A3
circRNA_00603	0	0	0	0.0206362	0	0	0	0	0	0	0	0	-	NC_000001.11	+	100049909	100061949	12041	402	sense-overlappin	XM_011541957.1	HIAT1
circRNA_00604	0	0	0.065425935	0	0	0	0.050246713	0	0	0	0	0	hsa_circ_0000096	NC_000001.11	+	100049909	100069685	19777	807	sense-overlappin	XM_005271118.1	HIAT1
circRNA_00605	0	0	0	0	0	0	0	0	0.020254528	0	0	0	-	NC_000001.11	+	100390732	100442996	52265	303	sense-overlappin	XM_011542344.1	CDC14A
circRNA_00606	0	0	0	0.0206362	0	0.031069364	0	0	0.020254528	0	0	0	hsa_circ_0000097	NC_000001.11	+	100424222	100442996	18775	210	sense-overlappin	XM_011542344.1	CDC14A
circRNA_00607	0.051437562	0	0.08723458	0.0619086	0.06018934	0.020712909	0	0.040972591	0.040509055	0.030258724	0	0	hsa_circ_0000098	NC_000001.11	+	100906852	100921841	14990	660	sense-overlappin	XM_011540779.1	SLC30A7

circRNA_00608	0	0	0	0	0	0	0.020098685	0	0	0	0	0	hsa_circ_0013353	NC_000001.11	+	100913663	100921841	8179	331	sense-overlappin	XM_011540779.1	SLC30A7
circRNA_00609	0	0	0.021808645	0	0	0	0	0	0	0	0	0	-	NC_000001.11	+	102997816	103004196	6381	1674	antisense	NM_001190709.1	COL11A1
circRNA_00610	0.164600197	0.040939935	0.021808645	0.0309543	0	0.041425819	0.040197371	0.051215739	0.060763583	0.100862414	0.030382845	0.0412728	hsa_circ_0000099	NC_000001.11	+	103565435	103575540	10106	1307	sense-overlappin	NM_020978.4	AMY2B
circRNA_00611	0.030862537	0	0	0	0	0	0	0	0	0	0	0	hsa_circ_0002286	NC_000001.11	+	107324282	107324922	641	641	exonic	XM_011541023.1	NTNG1
circRNA_00612	0	0	0	0.0309543	0.02006311	0	0	0.030729443	0	0	0	0	-	NC_000001.11	-	107683488	107705061	21574	275	sense-overlappin	NM_006113.4	VAV3
circRNA_00613	0	0	0	0	0	0	0	0.020486295	0	0	0	0	-	NC_000001.11	-	107760784	107770728	9945	462	sense-overlappin	NM_006113.4	VAV3
circRNA_00614	0.092587611	0	0.08723458	0.0412724	0.04012623	0.093208092	0.020098685	0	0.050636319	0.080689931	0.04051046	0	hsa_circ_0004270	NC_000001.11	-	108148279	108161293	13015	532	sense-overlappin	NM_213651.2	SLC25A24
circRNA_00615	0	0	0.032712968	0.0206362	0	0	0	0	0	0	0	0	-	NC_000001.11	-	108154983	108185954	30972	639	sense-overlappin	NM_213651.2	SLC25A24
circRNA_00616	0	0	0.08723458	0	0	0	0	0	0	0	0	0	-	NC_000001.11	+	108331171	108338690	7520	7520	intergenic		
circRNA_00617	0	0	0	0	0	0	0	0	0	0.030258724	0	0	-	NC_000001.11	+	108624645	108629657	5013	588	sense-overlappin	NM_001010883.2	FAM102B
circRNA_00618	0	0	0	0	0	0	0	0	0.020254528	0	0	0	hsa_circ_0013382	NC_000001.11	+	108752257	108760085	7829	389	sense-overlappin	NM_007269.2	STXBP3
circRNA_00619	0	0	0.032712968	0	0	0	0	0	0	0	0	0	-	NC_000001.11	+	108782422	108794907	12486	301	sense-overlappin	NM_007269.2	STXBP3
circRNA_00620	0	0	0	0	0.02006311	0	0	0	0	0	0	0	-	NC_000001.11	+	108793582	108800305	6724	572	sense-overlappin	NM_007269.2	STXBP3
circRNA_00621	0	0.020469968	0	0	0	0	0	0	0	0	0	0	-	NC_000001.11	+	108882580	108929975	47396	5033	sense-overlappin	XM_011541301.1	GPSM2
circRNA_00622	0	0	0.021808645	0	0	0	0	0	0	0	0	0	hsa_circ_0013386	NC_000001.11	+	108885275	108904254	18980	1440	sense-overlappin	XM_011541301.1	GPSM2
circRNA_00623	0	0	0	0	0	0	0	0.020486295	0	0	0	0	-	NC_000001.11	+	108931063	108932922	1860	1860	antisense	NM_001278202.1	CLCC1
circRNA_00624	0	0	0	0.0206362	0	0	0	0	0	0	0	0	-	NC_000001.11	+	109161894	109173837	11944	799	sense-overlappin	NM_020775.4	KIAA1324
circRNA_00625	0	0	0	0.0412724	0	0	0	0	0	0	0.02025523	0	-	NC_000001.11	+	109171866	109173837	1972	485	sense-overlappin	NM_001284352.1	KIAA1324
circRNA_00626	0	0	0	0	0	0	0	0.020486295	0	0	0	0	hsa_circ_0007868	NC_000001.11	+	109230878	109231786	909	300	sense-overlappin	XM_006710813.2	SARS
circRNA_00627	0	0	0	0	0.02006311	0	0	0	0	0	0	0	hsa_circ_0013431	NC_000001.11	-	109316850	109317969	1120	226	sense-overlappin	NM_001205228.1	SORT1
circRNA_00628	0	0	0	0	0.02006311	0	0	0	0	0	0	0	-	NC_000001.11	-	109342014	109345878	3865	273	sense-overlappin	NM_001205228.1	SORT1
circRNA_00629	0	0	0	0	0.02006311	0	0	0	0	0	0	0	hsa_circ_0004571	NC_000001.11	+	109573737	109574037	301	185	sense-overlappin	NM_006496.3	GNAI3
circRNA_00630	0	0	0	0	0	0	0	0	0	0	0.02025523	0	-	NC_000001.11	+	109659790	109690564	30775	28651	sense-overlappin	NM_000561.3	GSTM1
circRNA_00631	0	0	0	0	0	0	0	0	0	0.040344966	0	0	-	NC_000001.11	+	109661165	109671583	10419	7518	sense-overlappin	NM_000848.3	GSTM2
circRNA_00632	0	0	0	0	0	0	0	0	0.020254528	0	0	0.072227401	-	NC_000001.11	+	109661165	109690564	29400	27276	sense-overlappin	NM_000561.3	GSTM1
circRNA_00633	0.041150049	0.051174919	0	0	0	0	0.020098685	0.020486295	0	0.060517448	0.151914226	0	-	NC_000001.11	+	109665011	109690564	25554	23430	sense-overlappin	NM_000561.3	GSTM1
circRNA_00634	0	0	0	0	0	0.020712909	0.030148028	0.020486295	0	0.080689931	0	0	hsa_circ_0000105	NC_000001.11	+	110891392	110892533	1142	269	sense-overlappin	NM_001040033.1	CD53
circRNA_00635	0	0.020469968	0	0	0.04012623	0.020712909	0	0	0.040509055	0.07060369	0.02025523	0	hsa_circ_0000106	NC_000001.11	+	111147642	111161296	13655	702	sense-overlappin	NM_001007794.1	CEPT1
circRNA_00636	0	0	0	0.6706761	0	0.020712909	0	0	0	0.020172483	0	0	-	NC_000001.11	+	111282981	111315435	32455	8518	sense-overlappin	NM_201653.3	CHIA
circRNA_00637	0	0	0	0.0206362	0	0	0	0	0.030381792	0	0	0	hsa_circ_0000107	NC_000001.11	-	112555868	112616895	61028	1191	sense-overlappin	XM_011541628.1	ST7L
circRNA_00638	0	0	0.054521613	0	0	0	0	0	0	0	0	0	-	NC_000001.11	-	112576986	112591603	14618	623	sense-overlappin	XM_011541628.1	ST7L
circRNA_00639	0	0	0	0	0	0.020712909	0	0	0	0	0	0	-	NC_000001.11	-	112581992	112598086	16095	563	sense-overlappin	XM_011541628.1	ST7L
circRNA_00640	0.030862537	0	0	0	0	0	0	0	0	0	0	0	-	NC_000001.11	-	112591525	112616895	25371	496	sense-overlappin	XM_011541628.1	ST7L
circRNA_00641	0	0	0.04361729	0	0	0	0	0	0	0	0	0	hsa_circ_0013524	NC_000001.11	-	112597971	112616895	18925	417	sense-overlappin	XM_011541628.1	ST7L
circRNA_00642	0.041150049	0	0.04361729	0.0515905	0	0.041425819	0.050246713	0	0	0.030258724	0	0	hsa_circ_0000109	NC_000001.11	+	112653598	112659779	6182	430	sense-overlappin	NM_006135.2	CAPZA1
circRNA_00643	0	0.020469968	0	0	0	0	0.030148028	0	0	0	0	0	hsa_circ_0005664	NC_000001.11	+	112653598	112667145	13548	549	sense-overlappin	XM_011542225.1	CAPZA1
circRNA_00644	0.041150049	0	0	0	0	0	0	0	0	0	0	0	-	NC_000001.11	+	113549515	113622994	73480	1044	sense-overlappin	XR_946601.1	
circRNA_00645	0.030862537	0	0	0	0	0	0	0	0	0	0	0	-	NC_000001.11	+	113585387	113590658	5272	385	sense-overlappin	XR_946601.1	
circRNA_00646	0	0	0	0	0	0	0	0	0	0	0	0.0206364	-	NC_000001.11	+	113614601	113622994	8394	342	sense-overlappin	XR_946601.1	
circRNA_00647	0	0	0.032712968	0.0309543	0	0	0	0	0	0	0	0	hsa_circ_0006583	NC_000001.11	+	113671734	113673465	1732	374	sense-overlappin	XM_005270737.2	MAGI3
circRNA_00648	0	0	0	0	0	0	0	0	0	0.020172483	0	0	-	NC_000001.11	-	113705890	113712113	6224	888	sense-overlappin	NM_006608.2	PHTF1
circRNA_00649	0	0	0.04361729	0	0	0.020712909	0	0	0	0	0	0.0412728	-	NC_000001.11	-	113825142	113834439	9298	387	sense-overlappin	XM_011541224.1	PTPN22
circRNA_00650	0.102875123	0.102349838	0.04361729	0.1238171	0.06018934	0.113921001	0.080394741	0.061458886	0.101272639	0.171466104	0.04051046	0.072227401	hsa_circ_0000110	NC_000001.11	-	113829592	113834439	4848	356	sense-overlappin	XM_011541224.1	PTPN22
circRNA_00651	0	0	0	0.0206362	0	0	0	0.020486295	0	0	0	0	hsa_circ_0013608	NC_000001.11	-	113829592	113855049	25458	1710	sense-overlappin	XM_011541224.1	PTPN22
circRNA_00652	0	0	0	0	0	0	0	0	0.020254528	0	0	0	-	NC_000001.11	-	113833111	113855049	21939	1513	sense-overlappin	XM_011541224.1	PTPN22
circRNA_00653	0	0.020469968	0	0.0206362	0.02006311	0.041425819	0	0.020486295	0	0.171466104	0.02025523	0	-	NC_000001.11	-	113834910	113855049	20140	1354	sense-overlappin	XM_011541224.1	PTPN22
circRNA_00654	0	0.020469968	0	0	0	0.020712909	0	0	0	0	0	0	-	NC_000001.11	-	113837590	113838620	1031	895	sense-overlappin	XM_011541224.1	PTPN22
circRNA_00655	0	0	0	0.0309543	0	0.020712909	0	0.030729443	0.040509055	0.07060369	0	0.0412728	hsa_circ_0000111	NC_000001.11	-	113848540	113855049	6510	375	sense-overlappin	XM_011541224.1	PTPN22
circRNA_00656	0	0	0	0	0	0.020712909	0	0	0	0	0	0	hsa_circ_0013609	NC_000001.11	-	113854471	113855049	579	210	sense-overlappin	XM_011541224.1	PTPN22
circRNA_00657	0	0	0	0	0	0	0	0.020486295	0	0	0	0	-	NC_000001.11	-	113856382	113858573	2192	474	sense-overlappin	XM_011541224.1	PTPN22
circRNA_00658	0.072012586	0.030704951	0.152660515	0.0928628	0.04012623	0	0.060296056	0.030729443	0.050636319	0.121034897	0	0.0412728	hsa_circ_0004127	NC_000001.11	-	114463104	114464388	1285	397	sense-overlappin	NM_015906.3	TRIM33
circRNA_00659	0	0	0.021808645	0.0309543	0	0	0	0	0	0.020172483	0	0	hsa_circ_0013644	NC_000001.11	-	114719579	114720717	1139	343	sense-overlappin	NM_001007553.2	CSDE1
circRNA_00660	0	0	0	0.0206362	0	0	0.020098685	0	0	0.030258724	0	0	hsa_circ_0000115	NC_000001.11	-	114733732	114738072	4341	638	sense-overlappin	NM_001007553.2	CSDE1
circRNA_00661	0	0	0	0	0	0	0	0	0	0.020172483	0	0	-	NC_000001.11	+	114739339	114747588	8250	6614	antisense	NM_001130523.2	CSDE1
circRNA_00662	0.041150049	0	0	0	0	0	0	0	0	0	0	0	hsa_circ_0002623	NC_000001.11	+	115659641	115664268	4628	735	sense-overlappin	NM_001172411.1	VANGL1

circRNA_00663	0	0	0.032712968	0	0.02006311	0	0	0	0	0	0	0	-	NC_000001.11	+	117396952	117420649	23698	5787	sense-overlappin	XR_946528.1	
circRNA_00664	0.061725074	0.102349838	0.501598835	0.1857257	0.0702209	0.072495183	0.15074014	0.051215739	0.050636319	0.090776173	0.070893305	0.072227401	hsa_circ_0000116	NC_000001.11	+	117402186	117405645	3460	353	sense-overlappin	XR_946528.1	
circRNA_00665	0.164600197	0.419634334	1.286710056	0.7429028	0.43135697	0.497109824	0.623059246	0.389239614	0.334199707	0.675778174	0.293700836	0.165091201	hsa_circ_0000117	NC_000001.11	+	117402186	117414831	12646	472	sense-overlappin	XR_946528.1	
circRNA_00666	0.689263325	1.565952516	3.467574558	1.9810741	1.75552256	1.625963383	1.547598772	0.962855888	0.779799317	1.573453658	0.658294978	0.660364805	hsa_circ_0000118	NC_000001.11	+	117402186	117420649	18464	553	sense-overlappin	XR_946528.1	
circRNA_00667	0.257187808	0.24563961	1.766500246	0.660358	0.97306107	0.48675337	0.351726994	0.174133512	0.425345082	0.43370838	0.12153138	0.103182001	hsa_circ_0000119	NC_000001.11	+	117402186	117442325	40140	648	sense-overlappin	XR_946528.1	
circRNA_00668	0	0	0.032712968	0	0	0	0	0	0	0	0	0	-	NC_000001.11	+	117402186	117442335	40150	658	sense-overlappin	XR_946528.1	
circRNA_00669	0	0.051174919	0.163564838	0.0515905	0.06018934	0.020712909	0.040197371	0.020486295	0	0.050431207	0	0	hsa_circ_0000120	NC_000001.11	+	117402186	117466427	64242	866	sense-overlappin	XM_011540536.1	MAN1A2
circRNA_00670	0	0	0	0	0.03009467	0	0	0	0	0	0	0	hsa_circ_0013734	NC_000001.11	+	117405549	117420649	15101	297	sense-overlappin	XR_946528.1	
circRNA_00671	0	0	0.032712968	0	0	0	0	0	0	0	0	0	hsa_circ_0013738	NC_000001.11	+	117414713	117442325	27613	295	sense-overlappin	XR_946528.1	
circRNA_00672	0	0	0.021808645	0.0619086	0	0.062138728	0	0.020486295	0	0.040344966	0	0	hsa_circ_0002059	NC_000001.11	+	117460489	117502970	42482	843	sense-overlappin	XM_011540536.1	MAN1A2
circRNA_00673	0	0	0	0	0	0.041425819	0.030148028	0	0	0	0	0	-	NC_000001.11	+	117460489	117522937	62449	956	sense-overlappin	XM_006710302.1	MAN1A2
circRNA_00674	0	0	0	0	0.02006311	0	0	0	0	0	0	0	-	NC_000001.11	+	117493147	117502970	9824	625	sense-overlappin	XM_011540536.1	MAN1A2
circRNA_00675	0	0.051174919	0	0	0	0	0	0	0	0	0.030382845	0.0206364	hsa_circ_0013751	NC_000001.11	-	117881823	117920424	38602	1449	sense-overlappin	XM_011541624.1	GDAP2
circRNA_00676	0	0	0	0	0.03009467	0	0	0	0.020254528	0.040344966	0	0	hsa_circ_0006188	NC_000001.11	-	117911994	117918736	6743	463	sense-overlappin	XM_011541624.1	GDAP2
circRNA_00677	0	0	0	0	0	0	0	0	0	0	0.04051046	0	hsa_circ_0013756	NC_000001.11	+	117933288	117938558	5271	611	sense-overlappin	NM_006784.2	WDR3
circRNA_00678	0	0	0	0	0	0	0	0	0	0	0.02025523	0	-	NC_000001.11	-	119755427	119759989	4563	628	sense-overlappin	NM_005518.3	HMGCS2
circRNA_00679	0.020575025	0	0.032712968	0.0619086	0	0.041425819	0	0	0.020254528	0.020172483	0	0	-	NC_000001.11	-	145771711	145788966	17256	378	sense-overlappin	XM_005272952.3	RNF115
circRNA_00680	0	0	0.032712968	0	0	0	0	0	0	0	0	0	-	NC_000001.11	-	145771711	145794196	22486	5608	sense-overlappin	XM_005272952.3	RNF115
circRNA_00681	0	0	0	0	0	0	0	0	0	0.020172483	0	0	hsa_circ_0013912	NC_000001.11	+	145833260	145833582	323	198	sense-overlappin	NM_006468.7	POLR3C
circRNA_00682	0	0	0.021808645	0.0412724	0.04012623	0.062138728	0.040197371	0	0	0.060517448	0	0	hsa_circ_0003693	NC_000001.11	+	145839890	145841071	1182	302	sense-overlappin	NM_006468.7	POLR3C
circRNA_00683	0	0.020469968	0	0.0412724	0.03009467	0.020712909	0.050246713	0.030729443	0.050636319	0.040344966	0.030382845	0.0309546	hsa_circ_0007755	NC_000001.11	+	145839890	145842477	2588	441	sense-overlappin	NM_006468.7	POLR3C
circRNA_00684	0	0.020469968	0	0	0	0	0	0	0	0	0	0	-	NC_000001.11	+	145840922	145842477	1556	289	sense-overlappin	NM_006468.7	POLR3C
circRNA_00685	0	0	0	0.0206362	0	0	0	0	0	0	0	0	-	NC_000001.11	+	145974825	145975436	612	297	sense-overlappin	XM_005277431.3	POLR3GL
circRNA_00686	0	0	0.021808645	0	0.04012623	0.020712909	0	0	0.020254528	0	0	0	-	NC_000001.11	+	146968448	146972960	4513	813	sense-overlappin	NM_001278141.1	NBPF12
circRNA_00687	0	0.020469968	0.119947548	0.0722267	0	0.020712909	0	0	0	0	0	0	hsa_circ_0004220	NC_000001.11	+	147252623	147259918	7296	449	sense-overlappin	XR_922001.1	
circRNA_00688	0	0	0	0	0	0	0	0	0	0.030258724	0	0	-	NC_000001.11	+	147252623	147268878	16256	958	sense-overlappin	XR_922001.1	
circRNA_00689	0	0	0.032712968	0	0	0	0	0	0	0	0	0	-	NC_000001.11	+	147252623	147276257	23635	1412	sense-overlappin	XR_922001.1	
circRNA_00690	0	0	0.021808645	0	0	0	0	0	0	0	0	0	-	NC_000001.11	+	147252623	147280191	27569	1578	sense-overlappin	NM_004284.4	CHD1L
circRNA_00691	0	0	0	0	0	0	0	0	0	0	0	0.0206364	-	NC_000001.11	+	147272382	147294517	22136	4317	sense-overlappin	NM_001256336.1	CHD1L
circRNA_00692	0	0	0	0.0619086	0.0702209	0.051782273	0.020098685	0.051215739	0.030381792	0.020172483	0	0	hsa_circ_0013953	NC_000001.11	+	147275354	147285487	10134	748	sense-overlappin	NM_001256336.1	CHD1L
circRNA_00693	0	0	0	0	0.02006311	0	0	0	0.020254528	0	0	0	hsa_circ_0006296	NC_000001.11	+	147286298	147287733	1436	302	sense-overlappin	XM_006711638.1	CHD1L
circRNA_00694	0	0	0	0.0515905	0	0	0	0	0.020254528	0	0	0	-	NC_000001.11	-	147648246	147659775	11530	924	sense-overlappin	XM_011509601.1	ACP6
circRNA_00695	0	0	0.021808645	0	0	0	0	0	0	0	0	0	hsa_circ_0013958	NC_000001.11	-	147658960	147659775	816	340	sense-overlappin	XM_011509601.1	ACP6
circRNA_00696	0	0	0.032712968	0	0	0.062138728	0	0	0	0.080689931	0	0	-	NC_000001.11	+	149655699	149669310	13612	13612	intergenic		
circRNA_00697	0.061725074	0	0	0	0.04012623	0	0	0	0	0	0	0	-	NC_000001.11	-	149959684	149964349	4666	241	sense-overlappin	NM_020205.3	OTUD7B
circRNA_00698	0	0	0	0	0.02006311	0	0	0	0	0	0	0	hsa_circ_0003619	NC_000001.11	-	149964222	149965878	1657	230	sense-overlappin	NM_020205.3	OTUD7B
circRNA_00699	0	0	0	0	0	0	0	0	0	0	0.02025523	0	-	NC_000001.11	+	150077669	150110627	32959	1280	sense-overlappin	NR_103998.1	
circRNA_00700	0	0	0	0	0.02006311	0	0	0	0	0	0	0	-	NC_000001.11	-	150226608	150226721	114	114	exonic	NM_001136479.2	ANP32E
circRNA_00701	0	0	0	0	0.03009467	0	0	0	0	0	0	0	-	NC_000001.11	-	150226608	150229237	2630	354	sense-overlappin	NM_001136479.2	ANP32E
circRNA_00702	0.020575025	0	0.04361729	0	0	0	0.020098685	0	0	0	0.030382845	0	hsa_circ_0004300	NC_000001.11	-	150230571	150231926	1356	273	sense-overlappin	NM_001136479.2	ANP32E
circRNA_00703	0	0	0	0	0	0.051782273	0	0	0	0	0	0	-	NC_000001.11	+	150334935	150335241	307	307	exonic	NM_004698.2	PRPF3
circRNA_00704	0	0	0	0	0	0	0	0	0	0	0	0.051591	hsa_circ_0014008	NC_000001.11	+	150334935	150338326	3392	474	sense-overlappin	NM_004698.2	PRPF3
circRNA_00705	0	0	0.04361729	0	0	0	0	0.030729443	0.020254528	0	0	0	-	NC_000001.11	+	150440923	150446401	5479	535	sense-overlappin	NM_015203.4	RPRD2
circRNA_00706	0	0	0	0.0515905	0.02006311	0	0	0	0	0	0	0	-	NC_000001.11	+	150444251	150446401	2151	303	sense-overlappin	NM_001297673.1	RPRD2
circRNA_00707	0	0	0	0	0.02006311	0	0	0	0	0	0	0	-	NC_000001.11	+	150460060	150464727	4668	459	sense-overlappin	NM_001297673.1	RPRD2
circRNA_00708	0	0.040939935	0.065425935	0.0206362	0	0	0	0.020486295	0	0.030258724	0	0	hsa_circ_0003952	NC_000001.11	+	150492411	150496908	4498	325	sense-overlappin	NM_025150.4	TARS2
circRNA_00709	0	0	0	0	0.02006311	0	0	0	0	0	0	0	hsa_circ_0014022	NC_000001.11	+	150492411	150497747	5337	543	sense-overlappin	NM_025150.4	TARS2
circRNA_00710	0	0	0	0	0	0	0	0	0	0.020172483	0	0	-	NC_000001.11	-	150661814	150663763	1950	247	sense-overlappin	NM_018178.5	GOLPH3L
circRNA_00711	0	0	0	0	0	0	0	0	0	0.050431207	0	0	hsa_circ_0006494	NC_000001.11	-	150817920	150826617	8698	338	sense-overlappin	NM_001197325.1	ARNT
circRNA_00712	0	0	0	0	0	0	0	0	0	0.020172483	0	0	hsa_circ_0003439	NC_000001.11	+	150927704	150941428	13725	558	sense-overlappin	NM_001243491.1	SETDB1
circRNA_00713	0	0	0.021808645	0	0	0	0	0	0	0	0	0	hsa_circ_0006352	NC_000001.11	+	150951365	150962719	11355	1078	sense-overlappin	NM_001145415.1	SETDB1
circRNA_00714	0	0	0.021808645	0	0	0	0	0	0	0	0	0	hsa_circ_0014085	NC_000001.11	+	151017812	151028944	11133	894	sense-overlappin	NM_021222.2	PRUNE
circRNA_00715	0	0	0	0.0309543	0	0.031069364	0.030148028	0	0.020254528	0.040344966	0	0	hsa_circ_0006760	NC_000001.11	+	151024611	151025673	1063	344	sense-overlappin	NM_001303229.1	PRUNE
circRNA_00716	0	0	0	0	0	0	0	0	0	0.040344966	0	0	hsa_circ_0000126	NC_000001.11	+	151024611	151027327	2717	439	sense-overlappin	NM_001303229.1	PRUNE
circRNA_00717	0	0	0	0	0	0	0	0	0	0.020172483	0	0	hsa_circ_0014086	NC_000001.11	+	151024611	151028944	4334	598	sense-overlappin	NM_001303229.1	PRUNE

circRNA_00718	0	0	0	0	0	0	0	0	0	0	0.030382845	0	hsa_circ_0014088	NC_000001.11	+	151027233	151028944	1712	254	sense-overlappin	NM_001303229.1	PRUNE
circRNA_00719	0	0	0.021808645	0	0	0	0	0	0	0	0	0	-	NC_000001.11	+	151088158	151107222	19065	1002	sense-overlappin	XM_005244880.3	GABPB2
circRNA_00720	0	0	0.021808645	0	0	0	0	0	0	0	0	0	hsa_circ_0000127	NC_000001.11	+	151088190	151098002	9813	670	sense-overlappin	XM_005244880.3	GABPB2
circRNA_00721	0	0	0.065425935	0	0	0	0	0	0	0.030258724	0	0	-	NC_000001.11	+	151088190	151107222	19033	970	sense-overlappin	XM_005244880.3	GABPB2
circRNA_00722	0	0	0	0.0309543	0	0	0	0	0	0.040344966	0.030382845	0	-	NC_000001.11	+	151088190	151118139	29950	1278	sense-overlappin	XM_005244880.3	GABPB2
circRNA_00723	0	0	0	0	0	0.031069364	0	0	0	0	0	0	-	NC_000001.11	+	151117392	151118139	748	308	sense-overlappin	NM_144618.2	GABPB2
circRNA_00724	0.092587611	0	0.032712968	0.0515905	0.08025246	0.103564547	0.090444084	0.040972591	0.030381792	0.060517448	0.030382845	0.051591	hsa_circ_0000128	NC_000001.11	+	151166934	151167414	481	276	sense-overlappin	NM_001204848.1	TNFAIP8L2-SCNM1
circRNA_00725	0	0	0	0	0	0.020712909	0	0	0.030381792	0	0	0	-	NC_000001.11	-	151202700	151203275	576	576	antisense	XM_011510044.1	PIP5K1A
circRNA_00726	0	0	0	0	0	0	0	0	0	0.020172483	0	0	-	NC_000001.11	+	151223259	151225564	2306	2215	sense-overlappin	XM_006711568.2	PIP5K1A
circRNA_00727	0	0	0.032712968	0	0	0	0	0	0.030381792	0	0	0	hsa_circ_0014118	NC_000001.11	+	151224245	151232703	8459	554	sense-overlappin	XM_006711568.2	PIP5K1A
circRNA_00728	0	0	0.021808645	0	0	0	0	0	0	0	0	0	hsa_circ_0007228	NC_000001.11	+	151224248	151232703	8456	551	sense-overlappin	XM_006711568.2	PIP5K1A
circRNA_00729	0	0	0	0.0206362	0	0	0	0	0	0	0	0	hsa_circ_0014123	NC_000001.11	+	151224248	151234496	10249	851	sense-overlappin	XM_006711568.2	PIP5K1A
circRNA_00730	0	0	0.032712968	0	0	0.020712909	0	0	0	0	0	0	hsa_circ_0014132	NC_000001.11	+	151242123	151242567	445	277	sense-overlappin	XM_011510044.1	PIP5K1A
circRNA_00731	0.030862537	0	0	0	0	0	0	0	0	0	0	0	hsa_circ_0014144	NC_000001.11	+	151288528	151289271	744	356	sense-overlappin	NM_001304764.1	ZNF687
circRNA_00732	0	0	0.021808645	0	0	0.082851637	0	0	0.050636319	0.050431207	0	0	hsa_circ_0006892	NC_000001.11	-	151315573	151316509	937	937	exonic	NM_001198773.1	PI4KB
circRNA_00733	0	0	0	0.0206362	0	0	0	0	0	0	0	0	hsa_circ_0008701	NC_000001.11	-	151423397	151430841	7445	1395	sense-overlappin	XM_011509331.1	POGZ
circRNA_00734	0	0	0.032712968	0	0	0	0	0	0	0.020172483	0	0	hsa_circ_0014174	NC_000001.11	-	151423397	151442205	18809	1679	sense-overlappin	XM_005244999.1	POGZ
circRNA_00735	0.030862537	0	0.021808645	0	0.02006311	0	0	0	0	0	0	0	hsa_circ_0002520	NC_000001.11	-	151427823	151442205	14383	1079	sense-overlappin	XM_005244999.1	POGZ
circRNA_00736	0	0	0	0.0206362	0	0	0	0	0	0	0	0	hsa_circ_0000130	NC_000001.11	+	151638888	151639119	232	232	exonic	XM_005245511.3	SNX27
circRNA_00737	0	0	0.119947548	0	0	0	0.030148028	0	0	0.030258724	0	0	hsa_circ_0000131	NC_000001.11	+	151638888	151668635	29748	838	sense-overlappin	XM_005245511.3	SNX27
circRNA_00738	0	0	0	0.0309543	0	0	0	0.020486295	0	0	0	0	hsa_circ_0014188	NC_000001.11	+	151638888	151693483	54596	1267	sense-overlappin	XM_005245511.3	SNX27
circRNA_00739	0	0	0.021808645	0	0	0	0	0	0	0	0	0	-	NC_000001.11	+	151658235	151662270	4036	363	sense-overlappin	XM_005245511.3	SNX27
circRNA_00740	0	0	0.021808645	0	0.02006311	0	0	0	0	0.020172483	0	0	hsa_circ_0002394	NC_000001.11	+	151658235	151668635	10401	606	sense-overlappin	XM_005245511.3	SNX27
circRNA_00741	0.061725074	0	0	0	0	0	0	0	0	0	0	0	-	NC_000001.11	+	152248895	152251758	2864	2864	intergenic		
circRNA_00742	0	0	0	0	0	0.041425819	0	0	0	0	0	0	-	NC_000001.11	+	152248895	152253510	4616	4616	intergenic		
circRNA_00743	0	0	0	0	0	0	0	0	0	0.040344966	0	0	-	NC_000001.11	+	153642243	153642429	187	187	exonic	NM_001206612.1	CHTOP
circRNA_00744	0	0.020469968	0.065425935	0.0722267	0.02006311	0.062138728	0.050246713	0	0.020254528	0	0	0	hsa_circ_0000133	NC_000001.11	-	153665220	153668557	3338	469	sense-overlappin	NM_001267809.1	ILF2
circRNA_00745	0	0	0	0	0	0	0	0	0	0.020172483	0	0	-	NC_000001.11	-	153667555	153668557	1003	286	sense-overlappin	NM_001267809.1	ILF2
circRNA_00746	0	0	0	0	0.06018934	0.031069364	0	0	0	0	0	0	hsa_circ_0008368	NC_000001.11	-	154172908	154173201	294	189	sense-overlappin	NM_001278191.1	TPM3
circRNA_00747	0	0	0	0	0	0.041425819	0	0	0	0	0	0	-	NC_000001.11	-	154172908	154176248	3341	323	sense-overlappin	NM_001278191.1	TPM3
circRNA_00748	0.020575025	0	0	0	0	0	0	0	0	0	0	0	-	NC_000001.11	-	154206989	154207103	115	115	exonic	NM_001098616.2	C1orf43
circRNA_00749	0	0	0	0	0.02006311	0	0	0	0	0	0	0	-	NC_000001.11	+	154225084	154237136	12053	776	sense-overlappin	XM_011510214.1	UBAP2L
circRNA_00750	0	0	0	0.0515905	0.03009467	0.051782273	0	0	0.050636319	0.030258724	0.04051046	0.0309546	-	NC_000001.11	+	154234591	154235291	701	298	sense-overlappin	XM_011510214.1	UBAP2L
circRNA_00751	0	0	0	0	0	0	0	0.020486295	0	0	0	0	hsa_circ_0014375	NC_000001.11	+	154234591	154246375	11785	768	sense-overlappin	XM_011510214.1	UBAP2L
circRNA_00752	0	0	0.021808645	0	0	0	0	0	0	0	0	0	hsa_circ_0006433	NC_000001.11	+	154249239	154251653	2415	650	sense-overlappin	XM_011510214.1	UBAP2L
circRNA_00753	0.020575025	0	0	0.0309543	0	0	0	0	0	0	0	0	hsa_circ_0004717	NC_000001.11	+	154251041	154251653	613	451	sense-overlappin	XM_011510214.1	UBAP2L
circRNA_00754	0	0	0.054521613	0	0	0	0	0	0	0	0	0	hsa_circ_0005423	NC_000001.11	-	154588551	154597267	8717	951	sense-overlappin	NM_001193495.1	ADAR
circRNA_00755	0	0	0	0	0	0	0.020098685	0	0	0	0	0	-	NC_000001.11	-	155337589	155339368	1780	506	sense-overlappin	XM_011509770.1	ASH1L
circRNA_00756	0	0	0.021808645	0	0	0	0	0	0	0	0	0	-	NC_000001.11	-	155352706	155354630	1925	311	sense-overlappin	XM_011509770.1	ASH1L
circRNA_00757	0.041150049	0	0	0	0	0	0	0	0	0.020172483	0	0	hsa_circ_0014566	NC_000001.11	-	155352706	155357749	5044	571	sense-overlappin	XM_011509770.1	ASH1L
circRNA_00758	0	0	0	0.0309543	0	0	0	0	0.020254528	0	0	0	hsa_circ_0007608	NC_000001.11	-	155370504	155370983	480	354	sense-overlappin	XM_011509770.1	ASH1L
circRNA_00759	0	0	0.04361729	0	0	0	0.020098685	0	0	0	0	0	hsa_circ_0014577	NC_000001.11	-	155370504	155395553	25050	678	sense-overlappin	XM_011509770.1	ASH1L
circRNA_00760	0	0	0	0	0	0	0	0.020486295	0	0	0	0	hsa_circ_0002897	NC_000001.11	-	155378281	155415923	37643	504	sense-overlappin	XM_011509770.1	ASH1L
circRNA_00761	0	0	0	0.0309543	0	0	0	0	0	0	0	0	-	NC_000001.11	-	155395459	155439068	43610	1017	sense-overlappin	XM_011509770.1	ASH1L
circRNA_00762	0.061725074	0	0	0.0928628	0	0	0.040197371	0.040972591	0	0.050431207	0.050638075	0	hsa_circ_0014584	NC_000001.11	-	155395459	155459898	64440	1119	sense-overlappin	NM_018489.2	ASH1L
circRNA_00763	0	0.040939935	0	0	0	0	0	0	0	0	0	0	-	NC_000001.11	-	155395459	155462599	67141	3820	sense-overlappin	NM_018489.2	ASH1L
circRNA_00764	0	0	0	0.0515905	0	0	0	0	0	0	0	0	hsa_circ_0014585	NC_000001.11	-	155415744	155439068	23325	922	sense-overlappin	XM_011509770.1	ASH1L
circRNA_00765	0	0	0	0.1444533	0	0.020712909	0	0.020486295	0.030381792	0.020172483	0	0	hsa_circ_0014586	NC_000001.11	-	155415744	155459898	44155	1024	sense-overlappin	NM_018489.2	ASH1L
circRNA_00766	0.092587611	0.133054789	0.141756193	0.1857257	0.05015779	0.113921001	0.050246713	0.09218833	0.101272639	0.171466104	0.030382845	0.0412728	hsa_circ_0000137	NC_000001.11	-	155438327	155439068	742	742	exonic	XM_011509770.1	ASH1L
circRNA_00767	0	0	0	0	0	0	0	0	0	0.030258724	0	0	-	NC_000001.11	-	155438327	155440593	2267	2267	sense-overlappin	XM_006711452.2	ASH1L
circRNA_00768	0.072012586	0.143289773	0.098138903	0.2889066	0.12037869	0.124277456	0.100493427	0.040972591	0.101272639	0.131121138	0.091148535	0.165091201	hsa_circ_0003247	NC_000001.11	-	155438327	155459898	21572	844	sense-overlappin	XM_006711452.2	ASH1L
circRNA_00769	0	0	0.054521613	0	0	0	0	0	0	0	0	0	-	NC_000001.11	-	155438327	155477958	39632	917	sense-overlappin	XM_006711452.2	ASH1L
circRNA_00770	0	0	0	0.0309543	0	0	0	0	0	0	0	0	-	NC_000001.11	-	155438327	155482449	44123	5408	sense-overlappin	XM_006711452.2	ASH1L
circRNA_00771	0	0	0.021808645	0	0	0	0	0	0	0	0	0	hsa_circ_0014590	NC_000001.11	-	155477886	155482449	4564	4564	exonic	XM_006711452.2	ASH1L
circRNA_00772	0	0	0	0.0515905	0	0	0.020098685	0	0	0	0	0	hsa_circ_0014591	NC_000001.11	-	155477886	155521618	43733	5083	sense-overlappin	XM_006711452.2	ASH1L
circRNA_00773	0	0	0.032712968	0.0412724	0	0.051782273	0	0	0.020254528	0	0	0	hsa_circ_0014592	NC_000001.11	-	155521100	155521618	519	519	exonic	XM_006711452.2	ASH1L

circRNA_00774	0	0	0	0	0	0.020712909	0	0	0	0	0	0	hsa_circ_0014601	NC_000001.11	-	155668627	155675096	6470	555	sense-overlappin	NM_001198899.1	YY1AP1
circRNA_00775	0	0	0.054521613	0	0	0	0	0	0.020254528	0	0	0	-	NC_000001.11	-	155668627	155680456	11830	980	sense-overlappin	NM_018253.3	YY1AP1
circRNA_00776	0.072012586	0.040939935	0	0.0928628	0.02006311	0.072495183	0	0.040972591	0.030381792	0.050431207	0	0	-	NC_000001.11	-	155670320	155679512	9193	863	sense-overlappin	NM_139121.2	YY1AP1
circRNA_00777	0	0	0.021808645	0	0	0	0	0.020486295	0	0	0	0	hsa_circ_0014604	NC_000001.11	-	155672560	155679512	6953	718	sense-overlappin	NM_139121.2	YY1AP1
circRNA_00778	0.030862537	0	0	0	0	0	0	0	0.020254528	0	0	0	-	NC_000001.11	-	155674685	155679512	4828	871	sense-overlappin	NM_139121.2	YY1AP1
circRNA_00779	0.154312685	0.133054789	0.152660515	0.1960438	0.1404418	0.238198457	0.060296056	0.071702034	0.172163486	0.131121138	0.091148535	0.0619092	hsa_circ_0003608	NC_000001.11	-	155675010	155679512	4503	546	sense-overlappin	NM_139121.2	YY1AP1
circRNA_00780	0	0	0.04361729	0	0.03009467	0	0	0.020486295	0	0.020172483	0	0	-	NC_000001.11	-	155675010	155680456	5447	512	sense-overlappin	NM_018253.3	YY1AP1
circRNA_00781	0.504088103	0.378694399	0.359842643	0.4230419	0.28088361	0.787090555	0.241184224	0.348267023	0.344326971	0.726209381	0.162041841	0.433364403	hsa_circ_0014606	NC_000001.11	-	155676548	155679512	2965	459	sense-overlappin	NM_139121.2	YY1AP1
circRNA_00782	0	0	0.032712968	0	0	0	0	0	0	0	0	0	-	NC_000001.11	-	155676548	155680456	3909	425	sense-overlappin	NM_018253.3	YY1AP1
circRNA_00783	0	0	0	0	0.03009467	0	0.020098685	0	0	0	0	0	-	NC_000001.11	-	155682606	155682867	262	262	intronic	NM_001198903.1	YY1AP1
circRNA_00784	0	0	0	0	0	0.020712909	0	0	0	0.030258724	0	0	hsa_circ_0014611	NC_000001.11	+	155717006	155726019	9014	427	sense-overlappin	XM_011509972.1	DAP3
circRNA_00785	0.061725074	0	0.04361729	0	0.05015779	0.020712909	0	0	0.040509055	0	0	0	hsa_circ_0014613	NC_000001.11	+	155721517	155726019	4503	304	sense-overlappin	XM_011509972.1	DAP3
circRNA_00786	0.030862537	0	0.021808645	0	0	0.020712909	0	0	0.040509055	0.040344966	0.02025523	0	hsa_circ_0014614	NC_000001.11	+	155725382	155726019	638	202	sense-overlappin	XM_011509972.1	DAP3
circRNA_00787	0.092587611	0.020469968	0.054521613	0.0928628	0.02006311	0.113921001	0.070345399	0	0.050636319	0.080689931	0.02025523	0.0206364	hsa_circ_0014624	NC_000001.11	-	155765000	155766709	1710	1710	exonic	NM_001282858.1	GON4L
circRNA_00788	0	0	0	0.0309543	0	0	0	0	0	0	0	0	-	NC_000001.11	-	155767425	155771217	3793	268	sense-overlappin	NM_001282858.1	GON4L
circRNA_00789	0	0	0.021808645	0	0	0	0	0	0	0	0	0	-	NC_000001.11	-	155771067	155773210	2144	296	sense-overlappin	NM_001282858.1	GON4L
circRNA_00790	0	0	0	0.0206362	0	0	0	0	0	0	0	0	-	NC_000001.11	-	155773066	155777820	4755	603	sense-overlappin	NM_001282858.1	GON4L
circRNA_00791	0	0	0	0	0	0.031069364	0	0	0	0.020172483	0	0	-	NC_000001.11	-	155777622	155784089	6468	303	sense-overlappin	NM_001282858.1	GON4L
circRNA_00792	0	0	0.021808645	0	0	0	0	0	0	0	0	0	hsa_circ_0014632	NC_000001.11	-	155795050	155814449	19400	586	sense-overlappin	NM_001282858.1	GON4L
circRNA_00793	0	0	0	0	0.06018934	0	0	0	0	0	0	0	-	NC_000001.11	-	155813634	155814449	816	291	sense-overlappin	NM_001282858.1	GON4L
circRNA_00794	0	0	0	0	0.04012623	0	0	0	0	0.020172483	0	0	-	NC_000001.11	-	155820606	155827028	6423	509	sense-overlappin	NM_001282858.1	GON4L
circRNA_00795	0.185175222	0.184229708	0.185373483	0.2889066	0.36113607	0.362475913	0.200986853	0.143404068	0.202545277	0.211811069	0.111403765	0.082545601	hsa_circ_0000139	NC_000001.11	-	155853276	155853806	531	531	exonic	NM_001282858.1	GON4L
circRNA_00796	0	0	0	0	0.02006311	0	0	0	0	0	0	0	-	NC_000001.11	+	155866635	155868791	2157	2157	sense-overlappin	NM_152280.4	SYT11
circRNA_00797	0.030862537	0	0	0	0.03009467	0	0.040197371	0	0	0.030258724	0	0	hsa_circ_0006575	NC_000001.11	-	155904311	155904804	494	266	sense-overlappin	NM_001256821.1	RIT1
circRNA_00798	0	0	0.032712968	0	0	0.041425819	0	0.020486295	0	0	0	0	hsa_circ_0008998	NC_000001.11	-	155921375	155921918	544	312	sense-overlappin	NM_014949.3	KIAA0907
circRNA_00799	0	0	0	0	0	0.031069364	0	0	0	0	0	0	hsa_circ_0005758	NC_000001.11	-	155921375	155923687	2313	373	sense-overlappin	NM_014949.3	KIAA0907
circRNA_00800	0	0	0	0	0	0	0.020098685	0	0	0	0	0	hsa_circ_0014642	NC_000001.11	-	155925632	155927156	1525	429	sense-overlappin	NM_014949.3	KIAA0907
circRNA_00801	0.020575025	0	0	0.0515905	0.05015779	0.031069364	0.040197371	0	0	0.020172483	0	0	hsa_circ_0008339	NC_000001.11	-	155926676	155927156	481	217	sense-overlappin	NM_014949.3	KIAA0907
circRNA_00802	0	0	0.021808645	0	0	0	0	0	0	0	0	0	hsa_circ_0007393	NC_000001.11	+	156199835	156200472	638	638	exonic	NM_001286184.1	SLC25A44
circRNA_00803	0	0	0.021808645	0	0.05015779	0.031069364	0.030148028	0	0	0	0	0	hsa_circ_0014717	NC_000001.11	-	156320839	156334918	14080	516	sense-overlappin	NM_005998.4	CCT3
circRNA_00804	0.020575025	0	0.04361729	0.0206362	0	0.041425819	0	0	0	0.030258724	0.02025523	0.0309546	hsa_circ_0004680	NC_000001.11	-	156333547	156334918	1372	211	sense-overlappin	NM_005998.4	CCT3
circRNA_00805	0	0	0	0	0	0	0	0	0.020254528	0	0	0	-	NC_000001.11	+	159010003	159032691	22689	1508	sense-overlappin	XM_005245127.2	IFI16
circRNA_00806	0	0	0.032712968	0	0	0	0	0	0	0	0	0	hsa_circ_0004617	NC_000001.11	-	159918763	159920537	1775	665	sense-overlappin	NM_001277224.1	TAGLN2
circRNA_00807	0	0	0	0	0.02006311	0	0	0	0	0	0	0	-	NC_000001.11	-	160017611	160021071	3461	3461	intergenic		
circRNA_00808	0	0	0.021808645	0	0	0	0	0	0	0	0	0	hsa_circ_0014877	NC_000001.11	-	160237135	160240370	3236	910	sense-overlappin	NR_028103.1	
circRNA_00809	0.144025172	0.092114854	0.065425935	0.0619086	0.16050492	0	0.020098685	0	0.020254528	0.030258724	0.02025523	0.0412728	hsa_circ_0008661	NC_000001.11	-	160323431	160332557	9127	320	sense-overlappin	NM_001098398.1	COPA
circRNA_00810	0	0	0	0	0	0	0	0	0.020254528	0	0	0	-	NC_000001.11	-	160619776	160624185	4410	164	sense-overlappin	XM_011509905.1	SLAMF1
circRNA_00811	0	0	0	0	0.02006311	0	0	0	0	0	0	0	hsa_circ_0000145	NC_000001.11	-	161102048	161102375	328	213	sense-overlappin	XM_011509624.1	PFDN2
circRNA_00812	0	0	0	0.0206362	0	0.020712909	0	0	0	0	0	0	hsa_circ_0014982	NC_000001.11	+	161168993	161170024	1032	371	sense-overlappin	XM_011509669.1	PPOX
circRNA_00813	0	0	0.109043225	0.0206362	0.03009467	0.031069364	0	0	0	0.050431207	0.02025523	0	hsa_circ_0003039	NC_000001.11	+	161778244	161784096	5853	272	sense-overlappin	NM_007348.3	ATF6
circRNA_00814	0	0	0	0	0.02006311	0.082851637	0	0	0.020254528	0	0	0	hsa_circ_0007360	NC_000001.11	+	161778244	161802272	24029	827	sense-overlappin	NM_007348.3	ATF6
circRNA_00815	0	0	0	0	0	0	0	0	0	0.030258724	0	0	hsa_circ_0015022	NC_000001.11	+	161791408	161792327	920	334	sense-overlappin	NM_007348.3	ATF6
circRNA_00816	0	0.030704951	0	0.0412724	0.02006311	0	0.020098685	0	0.030381792	0.040344966	0	0	hsa_circ_0008857	NC_000001.11	+	161846449	161863312	16864	532	sense-overlappin	NM_007348.3	ATF6
circRNA_00817	0	0	0	0	0	0	0	0	0.020254528	0	0	0	-	NC_000001.11	-	161997825	162020182	22358	1303	sense-overlappin	NM_001297713.1	OLFML2B
circRNA_00818	0	0	0	0.0206362	0	0	0	0	0	0.020172483	0	0	hsa_circ_0007398	NC_000001.11	+	162576777	162600903	24127	2829	sense-overlappin	XM_005245462.2	UAP1
circRNA_00819	0	0	0	0	0	0	0	0.030729443	0	0	0	0	-	NC_000001.11	+	162576777	162601514	24738	3440	sense-overlappin	XM_005245462.2	UAP1
circRNA_00820	0	0	0.04361729	0	0.03009467	0	0	0	0	0	0	0	hsa_circ_0006758	NC_000001.11	+	165890204	165891322	1119	257	sense-overlappin	NM_012474.4	UCK2
circRNA_00821	0	0	0	0	0	0	0.020098685	0	0	0	0.02025523	0	-	NC_000001.11	-	166862203	166863921	1719	308	sense-overlappin	NM_053053.3	TADA1
circRNA_00822	0	0	0.032712968	0	0	0	0	0	0	0	0	0	-	NC_000001.11	+	167332470	167338258	5789	5789	sense-overlappin	NM_001198786.1	POU2F1
circRNA_00823	0	0	0	0	0	0	0	0.020486295	0	0	0	0	hsa_circ_0015107	NC_000001.11	+	167765583	167773368	7786	514	sense-overlappin	NM_003953.5	MPZL1
circRNA_00824	0.020575025	0	0.109043225	0.0515905	0	0	0	0	0	0	0.030382845	0	hsa_circ_0000153	NC_000001.11	+	167765583	167776166	10584	617	sense-overlappin	NM_003953.5	MPZL1
circRNA_00825	0	0	0	0.0206362	0	0	0	0	0	0	0	0	hsa_circ_0005827	NC_000001.11	+	167765583	167788137	22555	935	sense-overlappin	NM_003953.5	MPZL1
circRNA_00826	0	0.020469968	0	0	0	0	0	0	0	0	0	0	hsa_circ_0007921	NC_000001.11	+	167772275	167776166	3892	450	sense-overlappin	NM_003953.5	MPZL1
circRNA_00827	0	0	0.054521613	0	0	0	0.020098685	0	0	0	0	0	hsa_circ_0000154	NC_000001.11	+	167951800	167975015	23216	341	sense-overlappin	NM_001017977.2	DCAF6
circRNA_00828	0	0	0.141756193	0.113499	0.04012623	0	0.030148028	0	0	0.030258724	0.030382845	0	hsa_circ_0009109	NC_000001.11	+	167966629	167975015	8387	279	sense-overlappin	NM_001017977.2	DCAF6
circRNA_00829	0	0	0.032712968	0	0	0	0	0	0	0	0	0	hsa_circ_0015124	NC_000001.11	+	168002482	168004793	2312	475	sense-overlappin	NM_001017977.2	DCAF6
circRNA_00830	0	0	0	0	0	0	0	0	0	0	0.04051046	0	-	NC_000001.11	+	168011394	168011701	308	308	intronic	NM_001017977.2	DCAF6

circRNA_00831	0	0	0	0.0206362	0	0	0	0	0	0.020172483	0.02025523	0.0309546	hsa_circ_0000155	NC_000001.11	+	168038371	168045227	6857	649	sense-overlappin	NM_001017977.2	DCAF6
circRNA_00832	0	0	0.021808645	0	0	0	0	0	0	0	0	0	hsa_circ_0007277	NC_000001.11	+	168063621	168068463	4843	491	sense-overlappin	NM_001017977.2	DCAF6
circRNA_00833	0	0.020469968	0	0	0	0	0	0	0	0	0	0	hsa_circ_0015136	NC_000001.11	-	168104477	168104894	418	418	exonic	XM_006711253.1	GPR161
circRNA_00834	0	0	0.021808645	0	0	0	0	0	0	0	0	0	-	NC_000001.11	+	168231514	168242987	11474	867	sense-overlappin	NM_199344.2	SFT2D2
circRNA_00835	0	0	0.04361729	0	0	0	0	0	0	0	0	0	hsa_circ_0008944	NC_000001.11	+	168231514	168243069	11556	949	sense-overlappin	NM_199344.2	SFT2D2
circRNA_00836	0	0	0.021808645	0	0	0	0	0	0	0	0	0	-	NC_000001.11	+	168235101	168243069	7969	776	sense-overlappin	NM_199344.2	SFT2D2
circRNA_00837	0	0	0.021808645	0	0	0	0	0	0	0	0	0	-	NC_000001.11	+	168236589	168243069	6481	694	sense-overlappin	NM_199344.2	SFT2D2
circRNA_00838	0	0	0.021808645	0	0	0	0	0	0	0	0	0	-	NC_000001.11	+	169124884	169131481	6598	612	sense-overlappin	NM_001677.3	ATP1B1
circRNA_00839	0	0	0	0	0	0	0	0	0	0	0.04051046	0	hsa_circ_0015154	NC_000001.11	-	169230718	169287408	56691	342	sense-overlappin	XM_005245106.3	NME7
circRNA_00840	0	0	0.021808645	0	0.02006311	0	0	0	0	0	0	0	hsa_circ_0015157	NC_000001.11	-	169323117	169324500	1384	275	sense-overlappin	NM_013330.4	NME7
circRNA_00841	0	0	0.032712968	0	0	0	0	0	0	0	0	0	-	NC_000001.11	+	169836927	169850327	13401	687	sense-overlappin	XM_005245317.3	C1orf112
circRNA_00842	0	0	0	0.0206362	0	0	0	0	0	0	0	0	hsa_circ_0015181	NC_000001.11	-	169875978	169878819	2842	300	sense-overlappin	NM_020423.6	SCYL3
circRNA_00843	0.072012586	0.071644886	0.08723458	0.0412724	0.03009467	0.113921001	0.040197371	0	0.111399902	0.110948655	0.030382845	0.0309546	-	NC_000001.11	-	169978085	170031975	53891	1146	sense-overlappin	XM_011509307.1	KIFAP3
circRNA_00844	0	0	0.021808645	0	0	0	0	0	0	0	0	0	hsa_circ_0015189	NC_000001.11	-	170016462	170024596	8135	342	sense-overlappin	XM_011509307.1	KIFAP3
circRNA_00845	0	0	0.021808645	0	0	0	0	0	0	0	0	0	-	NC_000001.11	+	171512032	171550240	38209	5184	sense-overlappin	XM_005245030.3	PRRC2C
circRNA_00846	0	0	0	0	0	0	0	0	0	0.050431207	0	0	hsa_circ_0015207	NC_000001.11	+	171522177	171523522	1346	305	sense-overlappin	XM_005245030.3	PRRC2C
circRNA_00847	0	0	0.021808645	0.0309543	0	0	0	0	0	0	0	0	hsa_circ_0015210	NC_000001.11	+	171523221	171524965	1745	367	sense-overlappin	XM_005245030.3	PRRC2C
circRNA_00848	0	0	0.032712968	0	0.0702209	0.031069364	0.020098685	0.051215739	0.020254528	0.020172483	0.02025523	0	hsa_circ_0003644	NC_000001.11	+	171523221	171527844	4624	421	sense-overlappin	XM_005245030.3	PRRC2C
circRNA_00849	0.041150049	0	0	0.0309543	0.03009467	0.051782273	0	0	0	0.040344966	0	0	hsa_circ_0015211	NC_000001.11	+	171523221	171532961	9741	1040	sense-overlappin	XM_005245030.3	PRRC2C
circRNA_00850	0	0	0.032712968	0	0	0	0	0	0	0	0	0	-	NC_000001.11	+	171523221	171537473	14253	1671	sense-overlappin	XM_005245030.3	PRRC2C
circRNA_00851	0	0.020469968	0	0	0.04012623	0	0	0	0	0	0	0	-	NC_000001.11	+	171523221	171587751	64531	7254	sense-overlappin	XM_005245015.1	PRRC2C
circRNA_00852	0	0	0.021808645	0	0	0	0	0	0	0	0	0	-	NC_000001.11	+	171524821	171537473	12653	1449	sense-overlappin	XM_005245030.3	PRRC2C
circRNA_00853	0.041150049	0	0.054521613	0	0	0.020712909	0	0	0	0.040344966	0	0	hsa_circ_0004412	NC_000001.11	+	171568247	171575128	6882	397	sense-overlappin	XM_005245030.3	PRRC2C
circRNA_00854	0	0	0	0	0	0	0	0	0	0.030258724	0	0	hsa_circ_0015243	NC_000001.11	+	171579354	171587751	8398	928	sense-overlappin	XM_005245015.1	PRRC2C
circRNA_00855	0.020575025	0.030704951	0	0.0206362	0	0	0	0	0	0.050431207	0.02025523	0	hsa_circ_0005230	NC_000001.11	-	172140480	172144437	3958	3958	antisense	NM_001136127.2	DNM3
circRNA_00856	0.061725074	0.051174919	0.08723458	0.0722267	0.12037869	0.041425819	0.070345399	0.051215739	0.030381792	0.060517448	0	0.082545601	hsa_circ_0000160	NC_000001.11	+	172551512	172557794	6283	670	sense-overlappin	XR_921826.1	
circRNA_00857	0	0	0	0	0.05015779	0	0	0	0	0	0	0	hsa_circ_0015259	NC_000001.11	+	172551512	172570730	19219	987	sense-overlappin	XR_921826.1	
circRNA_00858	0.020575025	0	0.032712968	0	0	0	0	0	0	0	0	0	hsa_circ_0015260	NC_000001.11	+	172551512	172579267	27756	1436	sense-overlappin	XR_921826.1	
circRNA_00859	0.020575025	0	0.04361729	0	0.02006311	0	0	0	0.020254528	0	0	0	hsa_circ_0015261	NC_000001.11	+	172551512	172591071	39560	2851	sense-overlappin	XR_921826.1	
circRNA_00860	0	0	0.021808645	0	0.02006311	0	0	0	0	0	0.030382845	0	hsa_circ_0015262	NC_000001.11	+	172555869	172557794	1926	444	sense-overlappin	NM_016227.3	SUCO
circRNA_00861	0	0	0.021808645	0.0206362	0.02006311	0.020712909	0	0.020486295	0.020254528	0.020172483	0	0	-	NC_000001.11	+	172555869	172570730	14862	761	sense-overlappin	NM_016227.3	SUCO
circRNA_00862	0	0.030704951	0	0	0	0	0	0	0	0	0	0	hsa_circ_0015263	NC_000001.11	+	172555869	172579267	23399	1210	sense-overlappin	XR_921826.1	
circRNA_00863	0	0	0.021808645	0	0.02006311	0.041425819	0	0	0	0	0	0	-	NC_000001.11	+	172555869	172591071	35203	2625	sense-overlappin	XR_921826.1	
circRNA_00864	0	0	0	0	0.03009467	0	0	0	0	0	0	0	hsa_circ_0015264	NC_000001.11	+	172600064	172602787	2724	352	sense-overlappin	NM_016227.3	SUCO
circRNA_00865	0	0	0.109043225	0	0	0	0	0	0	0	0	0	-	NC_000001.11	-	173624787	173627159	2373	234	sense-overlappin	NM_198493.2	ANKRD45
circRNA_00866	0	0	0.021808645	0	0	0	0	0	0	0	0	0	-	NC_000001.11	-	173624787	173677623	52837	2095	sense-overlappin	XM_011509464.1	ANKRD45
circRNA_00867	0	0	0.021808645	0	0	0	0	0	0	0	0	0	-	NC_000001.11	-	173646846	173676391	29546	629	sense-overlappin	XM_011509464.1	ANKRD45
circRNA_00868	0	0	0.021808645	0	0	0	0	0	0	0	0	0	-	NC_000001.11	-	173646846	173678241	31396	2479	sense-overlappin	XM_011509464.1	ANKRD45
circRNA_00869	0	0	0.021808645	0	0	0	0	0	0	0	0	0	-	NC_000001.11	+	173774305	173787962	13658	3250	sense-overlappin	NM_014458.3	KLHL20
circRNA_00870	0	0	0.054521613	0	0	0.041425819	0	0	0	0.020172483	0	0.0309546	hsa_circ_0004927	NC_000001.11	-	173992755	173993135	381	381	sense-overlappin	NM_001300850.1	RC3H1
circRNA_00871	0.020575025	0	0	0	0	0	0	0	0.020254528	0	0	0.0309546	hsa_circ_0008141	NC_000001.11	+	174219125	174221164	2040	364	sense-overlappin	NM_014857.4	RABGAP1L
circRNA_00872	0	0	0.04361729	0	0	0	0	0	0	0	0	0	hsa_circ_0015326	NC_000001.11	+	174219125	174231355	12231	575	sense-overlappin	NM_014857.4	RABGAP1L
circRNA_00873	0	0	0.021808645	0	0	0	0	0	0	0	0	0	-	NC_000001.11	+	174250475	174305127	54653	748	sense-overlappin	NM_014857.4	RABGAP1L
circRNA_00874	0	0.030704951	0	0	0	0	0	0	0	0	0	0	hsa_circ_0000161	NC_000001.11	+	174272414	174278779	6366	337	sense-overlappin	NM_014857.4	RABGAP1L
circRNA_00875	0	0	0	0	0	0.031069364	0	0	0	0.07060369	0	0	hsa_circ_0005089	NC_000001.11	+	174272414	174305127	32714	479	sense-overlappin	NM_014857.4	RABGAP1L
circRNA_00876	0	0	0	0	0	0	0	0	0	0.040344966	0	0	-	NC_000001.11	+	174275833	174371072	95240	506	sense-overlappin	NM_014857.4	RABGAP1L
circRNA_00877	0	0	0.032712968	0	0	0	0	0	0	0	0	0	-	NC_000001.11	+	174969277	174982905	13629	372	sense-overlappin	XM_005245681.1	RABGAP1L
circRNA_00878	0	0	0.021808645	0.0206362	0	0.031069364	0	0	0	0	0.030382845	0.0206364	hsa_circ_0004722	NC_000001.11	-	175986943	175989479	2537	404	sense-overlappin	NM_001001740.3	RFWD2
circRNA_00879	0	0	0	0	0	0	0	0	0.020254528	0	0	0	-	NC_000001.11	-	175986943	176046324	59382	856	sense-overlappin	NM_001001740.3	RFWD2
circRNA_00880	0	0	0	0	0	0	0.020098685	0	0	0	0	0	hsa_circ_0015359	NC_000001.11	-	176027572	176046324	18753	452	sense-overlappin	NM_001001740.3	RFWD2
circRNA_00881	0	0	0.021808645	0	0	0	0.030148028	0	0	0	0	0	hsa_circ_0005357	NC_000001.11	-	176043186	176046324	3139	335	sense-overlappin	NM_001001740.3	RFWD2
circRNA_00882	0	0	0	0	0	0.031069364	0	0	0	0	0	0	hsa_circ_0015365	NC_000001.11	-	176081152	176116681	35530	309	sense-overlappin	NM_001001740.3	RFWD2
circRNA_00883	0	0	0.076330258	0.0928628	0	0	0	0	0	0	0	0.0412728	hsa_circ_0003572	NC_000001.11	-	176081152	176136547	55396	500	sense-overlappin	XM_011509886.1	RFWD2
circRNA_00884	0	0	0	0	0.02006311	0	0	0	0	0	0	0	hsa_circ_0015373	NC_000001.11	-	176116624	176136547	19924	249	sense-overlappin	XM_011509886.1	RFWD2
circRNA_00885	0	0	0	0	0	0.051782273	0	0	0	0	0	0	hsa_circ_0008566	NC_000001.11	-	176135010	176163891	28882	403	sense-overlappin	NM_022457.6	RFWD2
circRNA_00886	0	0	0.032712968	0	0	0	0	0	0	0	0	0	hsa_circ_0005288	NC_000001.11	-	176149006	176176007	27002	352	sense-overlappin	NM_001001740.3	RFWD2
circRNA_00887	0	0	0.08723458	0	0.03009467	0	0	0	0	0	0	0	hsa_circ_0004436	NC_000001.11	-	176149006	176184692	35687	412	sense-overlappin	NM_001001740.3	RFWD2

circRNA_00888	0	0	0	0	0	0	0	0	0	0.030258724	0	0	hsa_circ_0015377	NC_000001.11	-	176162869	176163891	1023	185	sense-overlappin	NM_001001740.3	RFWD2
circRNA_00889	0	0	0.04361729	0	0	0	0.030148028	0	0	0	0	0	hsa_circ_0009064	NC_000001.11	-	176162869	176184692	21824	343	sense-overlappin	NM_001001740.3	RFWD2
circRNA_00890	0	0.020469968	0.021808645	0	0	0	0	0	0	0	0	0	hsa_circ_0002061	NC_000001.11	+	178123056	178125501	2446	2446	intronic	XM_011510166.1	RASAL2
circRNA_00891	0	0	0.021808645	0	0	0	0	0	0	0	0	0	-	NC_000001.11	+	178212513	178300118	87606	71306	sense-overlappin	XM_011510166.1	RASAL2
circRNA_00892	0	0	0.021808645	0	0	0	0.020098685	0	0	0	0	0	hsa_circ_0002846	NC_000001.11	+	178283564	178300118	16555	255	sense-overlappin	XM_011510166.1	RASAL2
circRNA_00893	0	0	0.021808645	0	0	0	0	0	0	0	0	0	-	NC_000001.11	+	178737108	178741609	4502	4502	intronic	NM_001286247.1	RALGPS2
circRNA_00894	0	0	0	0	0	0	0	0.020486295	0	0	0	0	-	NC_000001.11	+	178741488	178785607	44120	35490	sense-overlappin	NM_001286247.1	RALGPS2
circRNA_00895	0	0	0.021808645	0	0	0	0	0	0	0	0	0	-	NC_000001.11	+	178808045	178811404	3360	174	sense-overlappin	NM_001286247.1	RALGPS2
circRNA_00896	0	0	0.021808645	0	0	0	0	0	0	0	0	0	hsa_circ_0015400	NC_000001.11	+	178808045	178833550	25506	394	sense-overlappin	NM_001286247.1	RALGPS2
circRNA_00897	0.051437562	0	0.054521613	0.0412724	0	0	0.020098685	0	0	0	0	0	hsa_circ_0005977	NC_000001.11	+	179043715	179054638	10924	707	sense-overlappin	XM_011510225.1	FAM20B
circRNA_00898	0	0	0.032712968	0	0	0	0	0	0	0	0	0	hsa_circ_0008653	NC_000001.11	+	179063927	179064496	570	364	sense-overlappin	XM_011510225.1	FAM20B
circRNA_00899	0.030862537	0	0	0.0206362	0	0	0	0.020486295	0	0	0	0	-	NC_000001.11	-	179118587	179121867	3281	536	sense-overlappin	NM_001136000.2	ABL2
circRNA_00900	0.030862537	0.030704951	0	0	0	0	0	0	0	0	0	0	hsa_circ_0015430	NC_000001.11	-	179120190	179121867	1678	358	sense-overlappin	NM_001136000.2	ABL2
circRNA_00901	0.030862537	0.030704951	0.098138903	0.0412724	0.02006311	0.020712909	0.050246713	0.061458886	0.050636319	0.060517448	0.04051046	0	hsa_circ_0004996	NC_000001.11	-	179131311	179133374	2064	234	sense-overlappin	NM_001136000.2	ABL2
circRNA_00902	0	0	0.021808645	0	0	0	0	0	0	0	0	0	-	NC_000001.11	+	179335506	179343635	8130	810	sense-overlappin	NM_001252511.1	SOAT1
circRNA_00903	0	0	0	0	0	0	0	0	0	0.020172483	0	0	hsa_circ_0015438	NC_000001.11	+	179337837	179341310	3474	451	sense-overlappin	NM_001252511.1	SOAT1
circRNA_00904	0	0	0	0	0	0	0	0	0.020254528	0	0	0	hsa_circ_0007700	NC_000001.11	+	179337837	179342943	5107	612	sense-overlappin	NM_001252511.1	SOAT1
circRNA_00905	0	0	0	0	0	0	0	0	0	0	0	0.0309546	hsa_circ_0015446	NC_000001.11	+	179341028	179343635	2608	490	sense-overlappin	NM_001252511.1	SOAT1
circRNA_00906	0	0	0	0	0	0	0	0.030729443	0	0.040344966	0.04051046	0	hsa_circ_0015454	NC_000001.11	+	179347600	179350431	2832	333	sense-overlappin	NM_001252511.1	SOAT1
circRNA_00907	0	0	0.021808645	0	0	0	0	0	0	0	0	0	-	NC_000001.11	+	179618599	179669404	50806	2029	sense-overlappin	NM_001199085.1	TDRD5
circRNA_00908	0	0	0.119947548	0	0	0	0	0	0	0	0	0	-	NC_000001.11	+	179662104	179669404	7301	538	sense-overlappin	NM_001199091.1	TDRD5
circRNA_00909	0	0	0	0	0	0	0.040197371	0	0	0	0	0	-	NC_000001.11	+	179893173	179893496	324	324	intronic	XM_011509403.1	TOR1AIP1
circRNA_00910	0	0	0	0	0	0.041425819	0.030148028	0	0	0	0	0	-	NC_000001.11	+	179900126	179908673	8548	297	sense-overlappin	XM_011509403.1	TOR1AIP1
circRNA_00911	0	0	0	0.0309543	0	0	0	0	0	0	0	0	-	NC_000001.11	+	179901302	179908673	7372	255	sense-overlappin	XM_011509403.1	TOR1AIP1
circRNA_00912	0	0	0	0	0	0	0	0	0	0.020172483	0	0	-	NC_000001.11	+	179986169	179992221	6053	405	sense-overlappin	XM_011510193.1	CEP350
circRNA_00913	0	0	0.076330258	0.0412724	0	0	0.020098685	0	0	0.030258724	0	0	-	NC_000001.11	+	179986169	179997175	11007	1028	sense-overlappin	XM_011510193.1	CEP350
circRNA_00914	0	0	0.098138903	0.0309543	0	0.062138728	0	0	0.030381792	0.020172483	0	0.0206364	-	NC_000001.11	+	179986169	180006567	20399	1256	sense-overlappin	XM_011510193.1	CEP350
circRNA_00915	0	0	0.021808645	0	0	0	0	0	0	0	0	0	-	NC_000001.11	+	179986169	180024582	38414	3560	sense-overlappin	XM_011510193.1	CEP350
circRNA_00916	0	0	0.021808645	0	0	0	0	0	0	0	0	0	-	NC_000001.11	+	179990507	179997175	6669	898	sense-overlappin	XM_011510193.1	CEP350
circRNA_00917	0	0	0.021808645	0	0	0	0	0	0	0	0	0	-	NC_000001.11	+	179992062	179997175	5114	783	sense-overlappin	XM_011510193.1	CEP350
circRNA_00918	0	0.061409903	0.04361729	0.0722267	0.04012623	0.072495183	0.050246713	0.030729443	0.040509055	0.040344966	0	0	hsa_circ_0003942	NC_000001.11	+	180003174	180006567	3394	228	sense-overlappin	XM_011510193.1	CEP350
circRNA_00919	0	0	0	0.0722267	0.02006311	0.062138728	0	0.020486295	0	0	0	0	hsa_circ_0015475	NC_000001.11	+	180003174	180024582	21409	2532	sense-overlappin	XM_011510193.1	CEP350
circRNA_00920	0	0	0.021808645	0	0	0	0	0	0	0	0	0	-	NC_000001.11	+	180019949	180021009	1061	1061	exonic	XM_011510193.1	CEP350
circRNA_00921	0	0	0	0	0	0.031069364	0	0	0	0	0	0	-	NC_000001.11	+	180019949	180024582	4634	1376	sense-overlappin	XM_011510193.1	CEP350
circRNA_00922	0	0	0.021808645	0.0309543	0	0	0	0	0	0	0	0	hsa_circ_0006924	NC_000001.11	+	180041138	180048705	7568	682	sense-overlappin	XM_011510193.1	CEP350
circRNA_00923	0	0	0.032712968	0.0309543	0	0	0	0	0	0	0	0	hsa_circ_0015489	NC_000001.11	+	180062220	180065272	3053	305	sense-overlappin	XM_011510193.1	CEP350
circRNA_00924	0	0	0.021808645	0	0	0	0	0	0	0	0	0	hsa_circ_0007652	NC_000001.11	+	180075022	180084178	9157	718	sense-overlappin	XM_011510193.1	CEP350
circRNA_00925	0	0	0	0.0412724	0	0	0	0	0	0	0	0	hsa_circ_0015491	NC_000001.11	+	180078463	180080661	2199	357	sense-overlappin	XM_011510193.1	CEP350
circRNA_00926	0	0	0	0	0	0.031069364	0	0	0	0	0	0	-	NC_000001.11	+	180087578	180090796	3219	223	sense-overlappin	XM_011510193.1	CEP350
circRNA_00927	0.030862537	0	0.021808645	0	0	0	0	0	0	0	0.02025523	0	hsa_circ_0009175	NC_000001.11	-	180397516	180413471	15956	196	sense-overlappin	XR_921977.1	
circRNA_00928	0	0	0.065425935	0	0.03009467	0	0	0	0	0	0	0	hsa_circ_0000164	NC_000001.11	-	180397516	180430262	32747	279	sense-overlappin	XR_921977.1	
circRNA_00929	0.020575025	0.040939935	0.021808645	0	0	0	0	0	0	0	0	0	hsa_circ_0005909	NC_000001.11	+	180824753	180825344	592	371	sense-overlappin	NM_001135669.1	XPR1
circRNA_00930	0.205750246	0.08187987	0.196277805	0.0928628	0.06018934	0.031069364	0.040197371	0.040972591	0.020254528	0.050431207	0.050638075	0.0309546	hsa_circ_0007905	NC_000001.11	-	180984677	180993425	8749	391	sense-overlappin	XM_011509059.1	STX6
circRNA_00931	0.030862537	0	0.098138903	0.0309543	0	0	0.020098685	0	0.030381792	0	0	0	hsa_circ_0006796	NC_000001.11	-	180984677	181005463	20787	656	sense-overlappin	XM_011509058.1	STX6
circRNA_00932	0	0	0.021808645	0	0	0	0	0	0	0	0	0	hsa_circ_0015529	NC_000001.11	-	181002606	181005463	2858	265	sense-overlappin	XM_011509058.1	STX6
circRNA_00933	0	0	0	0	0	0	0.030148028	0	0	0	0	0	hsa_circ_0004573	NC_000001.11	+	183114531	183115637	1107	307	sense-overlappin	NM_002293.3	LAMC1
circRNA_00934	0	0	0	0	0	0	0	0	0	0.020172483	0	0	-	NC_000001.11	+	183478877	183489403	10527	10527	intronic	NM_001174061.1	SMG7
circRNA_00935	0	0	0.021808645	0.0206362	0	0	0	0	0	0	0	0	-	NC_000001.11	-	184694044	184749592	55549	2660	sense-overlappin	NM_025191.3	EDEM3
circRNA_00936	0	0	0.021808645	0	0	0	0	0	0	0	0	0	hsa_circ_0006507	NC_000001.11	-	184706643	184711877	5235	667	sense-overlappin	NM_025191.3	EDEM3
circRNA_00937	0	0	0.021808645	0	0	0	0	0	0	0	0	0	-	NC_000001.11	-	184716888	184749592	32705	1212	sense-overlappin	NM_025191.3	EDEM3
circRNA_00938	0	0	0	0.0309543	0	0.020712909	0	0.020486295	0	0	0	0	-	NC_000001.11	-	184732837	184749592	16756	454	sense-overlappin	NM_025191.3	EDEM3
circRNA_00939	0	0	0	0	0	0	0	0	0	0.050431207	0	0	-	NC_000001.11	-	184734531	184749592	15062	300	sense-overlappin	NM_025191.3	EDEM3
circRNA_00940	0	0	0	0	0	0	0	0	0	0	0	0.0309546	-	NC_000001.11	-	184812115	184812193	79	79	intronic	NM_052966.3	FAM129A
circRNA_00941	0	0	0.021808645	0	0	0	0	0	0	0	0	0	hsa_circ_0002829	NC_000001.11	+	185087552	185093276	5725	466	sense-overlappin	NM_007212.3	RNF2
circRNA_00942	0	0	0	0	0.03009467	0	0	0	0	0	0.030382845	0	-	NC_000001.11	+	185174372	185175113	742	742	exonic	XR_921847.1	
circRNA_00943	0	0	0	0.0309543	0	0	0	0	0	0	0	0	-	NC_000001.11	+	185184243	185222036	37794	1171	sense-overlappin	XR_921847.1	
circRNA_00944	0.020575025	0	0.054521613	0.1031809	0.02006311	0.082851637	0.030148028	0.102431477	0.040509055	0.272328518	0	0.0309546	hsa_circ_0004689	NC_000001.11	+	185214507	185231708	17202	469	sense-overlappin	XR_921847.1	

circRNA_00945	0.020575025	0	0	0	0	0	0	0	0	0	0	0	-	NC_000001.11	-	186318447	186318828	382	253	sense-overlappin	NM_003292.2	TPR
circRNA_00946	0	0	0	0	0.03009467	0	0	0	0	0.020172483	0	0	-	NC_000001.11	-	186341028	186344090	3063	603	sense-overlappin	NM_003292.2	TPR
circRNA_00947	0	0	0	0.0206362	0	0	0.030148028	0	0	0	0	0	hsa_circ_0007538	NC_000001.11	+	186390711	186391791	1081	237	sense-overlappin	NM_001164245.1	C1orf27
circRNA_00948	0	0	0	0	0.02006311	0	0	0	0	0	0	0	-	NC_000001.11	+	186390711	186425297	34587	6102	sense-overlappin	XM_011509660.1	C1orf27
circRNA_00949	0	0	0	0	0	0.041425819	0	0	0	0	0	0	-	NC_000001.11	+	186406083	186411893	5811	269	sense-overlappin	XM_011509661.1	C1orf27
circRNA_00950	0	0.051174919	0.032712968	0.0825448	0.04012623	0.041425819	0.030148028	0.020486295	0.040509055	0.060517448	0	0	hsa_circ_0000166	NC_000001.11	+	186406083	186419077	12995	449	sense-overlappin	XM_011509661.1	C1orf27
circRNA_00951	0	0	0	0	0	0	0	0.030729443	0	0	0	0	-	NC_000001.11	+	186425123	186451274	26152	21412	antisense	NM_002597.4	PDC
circRNA_00952	0	0	0	0	0.02006311	0	0	0	0	0	0	0	-	NC_000001.11	+	186893011	186894211	1201	263	sense-overlappin	NM_024420.2	PLA2G4A
circRNA_00953	0	0	0.098138903	0	0.10031557	0	0	0	0	0	0	0	-	NC_000001.11	+	186893011	186911389	18379	443	sense-overlappin	NM_024420.2	PLA2G4A
circRNA_00954	0	0	0.021808645	0	0	0	0	0	0	0	0	0	-	NC_000001.11	+	189380806	189425475	44670	44670	intergenic		
circRNA_00955	0	0	0.021808645	0	0	0	0	0	0	0	0	0	-	NC_000001.11	+	189745513	189777337	31825	31825	intergenic		
circRNA_00956	0	0	0.021808645	0	0	0	0	0	0	0	0	0	-	NC_000001.11	-	192935829	192957422	21594	21594	intergenic		
circRNA_00957	0	0	0.021808645	0	0	0	0	0	0	0	0	0	-	NC_000001.11	-	193021097	193029657	8561	780	sense-overlappin	XM_011509608.1	UCHL5
circRNA_00958	0	0	0.021808645	0	0	0	0	0	0	0	0	0	hsa_circ_0015733	NC_000001.11	-	193028085	193029657	1573	383	sense-overlappin	NM_001199261.1	UCHL5
circRNA_00959	0	0	0	0	0	0	0	0	0.020254528	0.030258724	0	0	-	NC_000001.11	+	193069034	193077050	8017	1173	sense-overlappin	XM_006711495.2	TROVE2
circRNA_00960	0	0	0.032712968	0	0	0	0	0	0	0	0	0	-	NC_000001.11	+	193069034	193082708	13675	1551	sense-overlappin	XM_006711495.2	TROVE2
circRNA_00961	0	0	0.021808645	0	0.05015779	0.031069364	0	0	0	0	0	0	-	NC_000001.11	+	193075820	193077050	1231	506	sense-overlappin	NM_001173524.1	TROVE2
circRNA_00962	0	0	0	0	0	0	0	0	0.040509055	0	0	0	hsa_circ_0007910	NC_000001.11	-	193096648	193101204	4557	353	sense-overlappin	NM_197962.2	GLRX2
circRNA_00963	0	0	0	0	0	0.020712909	0	0	0	0	0	0	-	NC_000001.11	+	193135391	193152444	17054	665	sense-overlappin	NM_024529.4	CDC73
circRNA_00964	0	0	0	0.0206362	0	0.020712909	0	0	0	0.020172483	0	0	-	NC_000001.11	+	193138085	193142066	3982	306	sense-overlappin	NM_024529.4	CDC73
circRNA_00965	0	0	0.021808645	0	0.02006311	0	0	0.030729443	0	0	0	0	-	NC_000001.11	+	193141850	193152444	10595	460	sense-overlappin	NM_024529.4	CDC73
circRNA_00966	0	0	0.021808645	0	0	0	0	0	0	0	0	0	hsa_circ_0015749	NC_000001.11	+	193203795	193236356	32562	445	sense-overlappin	NM_024529.4	CDC73
circRNA_00967	0	0	0.021808645	0	0	0	0.020098685	0	0	0	0	0	hsa_circ_0003820	NC_000001.11	+	193232993	193236356	3364	263	sense-overlappin	NM_024529.4	CDC73
circRNA_00968	0	0	0	0	0	0	0	0.020486295	0.030381792	0	0	0.0206364	-	NC_000001.11	-	194159280	194167715	8436	8436	intergenic		
circRNA_00969	0	0	0	0	0	0.020712909	0	0	0	0	0	0	-	NC_000001.11	+	196725121	196728522	3402	717	sense-overlappin	NM_000186.3	CFH
circRNA_00970	0	0	0.032712968	0	0	0	0	0	0	0	0	0	-	NC_000001.11	-	197117789	197125191	7403	1129	sense-overlappin	NM_001206846.1	ASPM
circRNA_00971	0	0	0.054521613	0	0	0	0	0	0	0	0	0	-	NC_000001.11	-	197117789	197135242	17454	2039	sense-overlappin	NM_001206846.1	ASPM
circRNA_00972	0	0	0	0	0	0.041425819	0	0	0	0.080689931	0	0	hsa_circ_0015778	NC_000001.11	-	197583152	197611976	28825	376	sense-overlappin	XM_011509247.1	DENND1B
circRNA_00973	0	0	0	0.0309543	0	0	0	0	0	0	0	0	hsa_circ_0015779	NC_000001.11	-	197583152	197617759	34608	477	sense-overlappin	XM_011509247.1	DENND1B
circRNA_00974	0	0	0.021808645	0	0	0	0	0	0	0	0	0	hsa_circ_0000168	NC_000001.11	-	197583152	197645743	62592	642	sense-overlappin	XM_011509247.1	DENND1B
circRNA_00975	0	0	0.021808645	0	0	0	0	0	0	0	0	0	hsa_circ_0015782	NC_000001.11	-	197595208	197617759	22552	375	sense-overlappin	XM_011509247.1	DENND1B
circRNA_00976	0.020575025	0	0.021808645	0	0	0	0	0	0	0	0	0	-	NC_000001.11	-	197607073	197658369	51297	625	sense-overlappin	XM_011509246.1	DENND1B
circRNA_00977	0	0	0.08723458	0	0	0.051782273	0	0	0.040509055	0.090776173	0.02025523	0	hsa_circ_0006324	NC_000001.11	-	197642711	197658369	15659	376	sense-overlappin	XM_011509246.1	DENND1B
circRNA_00978	0.030862537	0	0.021808645	0	0	0	0	0	0	0	0	0	hsa_circ_0006709	NC_000001.11	-	197642711	197715074	72364	590	sense-overlappin	XM_011509246.1	DENND1B
circRNA_00979	0	0	0.076330258	0	0	0	0	0	0	0	0	0	hsa_circ_0015797	NC_000001.11	-	197652235	197674169	21935	321	sense-overlappin	XM_011509247.1	DENND1B
circRNA_00980	0	0	0.054521613	0	0	0	0	0	0	0.020172483	0	0	hsa_circ_0015798	NC_000001.11	-	197652235	197715074	62840	365	sense-overlappin	XM_011509247.1	DENND1B
circRNA_00981	0	0	0	0.0206362	0	0.041425819	0	0	0	0	0	0	hsa_circ_0000169	NC_000001.11	+	198220999	198264235	43237	11954	sense-overlappin	NM_133494.2	NEK7
circRNA_00982	0	0	0.021808645	0	0	0	0.020098685	0.020486295	0	0.030258724	0	0	hsa_circ_0002528	NC_000001.11	+	198232553	198264235	31683	400	sense-overlappin	NM_133494.2	NEK7
circRNA_00983	0	0	0	0	0	0	0	0	0	0.020172483	0	0	hsa_circ_0006267	NC_000001.11	+	198277961	198297240	19280	426	sense-overlappin	NM_133494.2	NEK7
circRNA_00984	0	0	0.032712968	0	0	0	0	0	0	0	0.030382845	0	-	NC_000001.11	+	200043774	200048818	5045	908	sense-overlappin	NM_003822.4	NR5A2
circRNA_00985	0.041150049	0	0.065425935	0	0	0	0	0	0	0	0.04051046	0	hsa_circ_0015814	NC_000001.11	+	200043774	200120955	77182	1176	sense-overlappin	NM_003822.4	NR5A2
circRNA_00986	0	0	0	0	0.02006311	0	0	0	0	0	0	0	-	NC_000001.11	-	200665937	200666751	815	815	exonic	XM_005245520.1	DDX59
circRNA_00987	0.041150049	0	0	0.0309543	0	0.051782273	0	0.051215739	0	0.040344966	0	0	hsa_circ_0015839	NC_000001.11	+	200760839	200815644	54806	506	sense-overlappin	NM_001297707.1	CAMSAP2
circRNA_00988	0.020575025	0	0	0	0	0	0	0	0	0	0	0	hsa_circ_0003736	NC_000001.11	-	201485283	201490344	5062	393	sense-overlappin	NM_001193572.1	CSRP1
circRNA_00989	0	0	0.032712968	0	0	0	0	0	0	0	0	0	hsa_circ_0003115	NC_000001.11	+	201847279	201848594	1316	351	sense-overlappin	NM_018085.4	IPO9
circRNA_00990	0	0	0.032712968	0	0	0	0	0	0	0	0	0	-	NC_000001.11	+	201847279	201853097	5819	527	sense-overlappin	NM_018085.4	IPO9
circRNA_00991	0	0	0.021808645	0	0	0	0	0	0.030381792	0	0	0	-	NC_000001.11	+	201852104	201858994	6891	954	sense-overlappin	NM_018085.4	IPO9
circRNA_00992	0	0	0	0	0.02006311	0	0	0	0	0	0	0	hsa_circ_0015928	NC_000001.11	+	201854595	201858994	4400	778	sense-overlappin	NM_018085.4	IPO9
circRNA_00993	0.051437562	0	0	0.0206362	0	0	0	0	0	0	0	0	-	NC_000001.11	+	201857096	201858994	1899	346	sense-overlappin	NM_018085.4	IPO9
circRNA_00994	0	0	0	0	0	0	0	0	0	0	0	0.0206364	-	NC_000001.11	+	201863448	201871327	7880	1108	sense-overlappin	NM_018085.4	IPO9
circRNA_00995	0	0	0	0	0	0.031069364	0	0	0	0	0	0	hsa_circ_0005356	NC_000001.11	+	202001376	202003461	2086	447	sense-overlappin	NM_020216.3	RNPEP
circRNA_00996	0	0	0.021808645	0	0	0	0	0	0	0	0	0	-	NC_000001.11	-	202154491	202155134	644	644	intronic	XM_011509821.1	PTPN7
circRNA_00997	0	0	0.021808645	0.0206362	0	0	0	0	0	0	0	0	hsa_circ_0015980	NC_000001.11	-	202416787	202428929	12143	630	sense-overlappin	NM_001167857.1	PPP1R12B
circRNA_00998	0	0	0.04361729	0	0	0	0	0	0	0	0	0	hsa_circ_0015985	NC_000001.11	+	202422620	202449171	26552	1611	sense-overlappin	XM_011509576.1	PPP1R12B
circRNA_00999	0	0	0.021808645	0	0	0	0.020098685	0	0	0	0	0	-	NC_000001.11	+	202425566	202431619	6054	600	sense-overlappin	NM_001167857.1	PPP1R12B

circRNA_01000	0.020575025	0	0	0	0	0	0	0	0	0	0	0	-	NC_000001.11	+	202558877	202569197	10321	372	sense-overlappin	NM_002481.3	PPP1R12B
circRNA_01001	0.030862537	0	0	0	0	0	0	0	0	0	0	0	-	NC_000001.11	-	202750659	202764145	13487	1110	sense-overlappin	XM_011509090.1	KDM5B
circRNA_01002	0	0	0	0	0	0.020712909	0	0	0	0	0	0	hsa_circ_0016040	NC_000001.11	-	202755271	202760573	5303	620	sense-overlappin	XM_011509090.1	KDM5B
circRNA_01003	0	0	0	0.0206362	0	0	0	0	0	0.020172483	0	0	hsa_circ_0004981	NC_000001.11	-	202755271	202764145	8875	827	sense-overlappin	XM_011509090.1	KDM5B
circRNA_01004	0	0	0	0.0309543	0	0	0	0	0	0	0	0	hsa_circ_0003126	NC_000001.11	-	202773118	202777094	3977	372	sense-overlappin	XM_011509090.1	KDM5B
circRNA_01005	0.020575025	0	0	0	0	0	0	0	0	0	0	0	hsa_circ_0002815	NC_000001.11	+	203703652	203722689	19038	2087	sense-overlappin	NM_001001396.2	ATP2B4
circRNA_01006	0.123450148	0	0.032712968	0	0	0.020712909	0	0.061458886	0.030381792	0.040344966	0.050638075	0	hsa_circ_0007167	NC_000001.11	+	203707009	203708104	1096	458	sense-overlappin	NM_001001396.2	ATP2B4
circRNA_01007	0	0.040939935	0	0	0	0	0	0	0	0	0	0	hsa_circ_0016105	NC_000001.11	+	203770828	203772147	1320	301	sense-overlappin	XM_006711397.2	LAX1
circRNA_01008	0.020575025	0	0	0	0	0	0	0	0	0	0	0	-	NC_000001.11	+	203818977	203819316	340	340	intronic	XM_005245643.2	ZC3H11A
circRNA_01009	0	0	0	0	0	0	0	0	0.030381792	0	0	0	hsa_circ_0016113	NC_000001.11	+	203833791	203840374	6584	231	sense-overlappin	XM_005245643.2	ZC3H11A
circRNA_01010	0	0.030704951	0.098138903	0.1238171	0	0	0	0	0	0.030258724	0	0	hsa_circ_0004777	NC_000001.11	+	204112915	204114605	1691	419	sense-overlappin	XM_005245623.1	SOX13
circRNA_01011	0	0.030704951	0	0	0	0	0	0	0	0	0	0	hsa_circ_0009157	NC_000001.11	+	204112915	204123804	10890	1373	sense-overlappin	XM_005245623.1	SOX13
circRNA_01012	0	0	0.032712968	0.0309543	0	0	0	0	0.020254528	0	0	0	hsa_circ_0016123	NC_000001.11	+	204116507	204117707	1201	354	sense-overlappin	XM_005245623.1	SOX13
circRNA_01013	0	0	0	0	0	0	0.020098685	0	0	0	0	0	-	NC_000001.11	-	204427648	204430038	2391	307	sense-overlappin	XM_011509632.1	PIK3C2B
circRNA_01014	0	0	0.021808645	0	0	0	0	0.020486295	0	0.060517448	0	0	hsa_circ_0000174	NC_000001.11	-	204468870	204469886	1017	1017	exonic	XM_011509632.1	PIK3C2B
circRNA_01015	0	0	0.021808645	0.0206362	0	0	0	0	0	0	0	0	-	NC_000001.11	-	204489816	204490750	935	799	sense-overlappin	XM_011509630.1	PIK3C2B
circRNA_01016	0	0	0	0	0	0	0	0	0	0.040344966	0	0	-	NC_000001.11	+	204952011	204991306	39296	3045	sense-overlappin	XM_011509318.1	NFASC
circRNA_01017	0	0	0.021808645	0	0	0	0	0	0	0	0	0	hsa_circ_0003527	NC_000001.11	-	205103857	205105168	1312	304	sense-overlappin	NM_001193272.1	RBBP5
circRNA_01018	0.030862537	0	0	0	0	0	0	0	0	0	0	0	-	NC_000001.11	-	205161258	205163955	2698	624	sense-overlappin	XM_011509394.1	DSTYK
circRNA_01019	0	0	0	0	0	0	0	0	0	0	0	0.0412728	-	NC_000001.11	-	205598455	205620838	22384	1352	sense-overlappin	XR_921752.1	
circRNA_01020	0.236612783	0.296814529	0.763302576	0.1960438	0.25078894	0.321050095	0.361776336	0.163890364	0.232927069	0.221897311	0.151914226	0.196045802	hsa_circ_0000175	NC_000001.11	-	205616478	205623891	7414	1373	sense-overlappin	XR_921752.1	
circRNA_01021	0	0	0	0	0.02006311	0	0	0	0	0	0	0	-	NC_000001.11	-	205616478	205627874	11397	1506	sense-overlappin	XM_005244950.3	ELK4
circRNA_01022	0	0	0	0	0	0.020712909	0	0	0	0	0	0	-	NC_000001.11	+	206443613	206443949	337	337	intronic	XM_011509360.1	SRGAP2
circRNA_01023	0.030862537	0	0.021808645	0.0206362	0	0	0	0	0	0.030258724	0	0	-	NC_000001.11	-	207027494	207027679	186	186	exonic	NM_023938.5	C1orf116
circRNA_01024	0.030862537	0	0	0	0	0	0	0	0	0	0.02025523	0.0619092	-	NC_000001.11	-	207130171	207155058	24888	12621	antisense	XM_005273252.3	C4BPA
circRNA_01025	0	0	0	0	0	0	0.020098685	0	0	0	0.030382845	0	-	NC_000001.11	+	207757014	207759724	2711	378	sense-overlappin	XM_011509563.1	CD46
circRNA_01026	0	0	0.021808645	0.0206362	0	0	0	0	0	0	0	0	-	NC_000001.11	+	207757014	207767195	10182	759	sense-overlappin	XM_011509563.1	CD46
circRNA_01027	0	0	0.076330258	0	0	0	0	0	0	0	0	0	hsa_circ_0006827	NC_000001.11	-	207815929	207817414	1486	1486	intergenic		
circRNA_01028	0	0	0.032712968	0	0	0	0	0	0	0	0	0	-	NC_000001.11	-	208092786	208096883	4098	366	sense-overlappin	NM_025179.3	PLXNA2
circRNA_01029	0	0	0.04361729	0	0	0	0	0.030729443	0.040509055	0	0	0	hsa_circ_0002472	NC_000001.11	-	208210280	208218002	7723	1451	sense-overlappin	NM_025179.3	PLXNA2
circRNA_01030	0.051437562	0	0	0.0619086	0.05015779	0.062138728	0.020098685	0.051215739	0.020254528	0.040344966	0.050638075	0.0309546	hsa_circ_0007234	NC_000001.11	+	211353239	211354467	1229	277	sense-overlappin	XM_011509958.1	TRAF5
circRNA_01031	0	0.020469968	0	0	0	0	0	0	0	0	0.02025523	0	-	NC_000001.11	-	211778918	211787727	8810	497	sense-overlappin	NM_014873.2	LPGAT1
circRNA_01032	0	0.08187987	0.250799418	0.0928628	0	0.041425819	0	0	0.091145375	0.121034897	0	0	hsa_circ_0002274	NC_000001.11	-	211778918	211793190	14273	616	sense-overlappin	NM_014873.2	LPGAT1
circRNA_01033	0	0	0	0	0	0	0	0	0	0.030258724	0	0	hsa_circ_0016374	NC_000001.11	-	212006639	212021212	14574	819	sense-overlappin	NR_037667.1	
circRNA_01034	0	0	0.032712968	0	0	0	0	0	0	0	0	0	hsa_circ_0000179	NC_000001.11	+	212044660	212047417	2758	282	sense-overlappin	NM_001286229.1	DTL
circRNA_01035	0	0.040939935	0.076330258	0	0.04012623	0.031069364	0.050246713	0.051215739	0.030381792	0.020172483	0	0.0309546	hsa_circ_0002321	NC_000001.11	+	212329135	212333598	4464	299	sense-overlappin	NM_006243.3	PPP2R5A
circRNA_01036	0	0	0	0	0.02006311	0	0	0	0	0	0	0	hsa_circ_0016394	NC_000001.11	+	212342188	212349242	7055	447	sense-overlappin	NM_006243.3	PPP2R5A
circRNA_01037	0	0	0	0	0	0	0	0	0	0	0	0.0619092	-	NC_000001.11	+	212863708	212872818	9111	430	sense-overlappin	XR_426771.1	
circRNA_01038	0.020575025	0	0.021808645	0	0	0.020712909	0	0	0	0	0	0	-	NC_000001.11	+	212863725	212872818	9094	413	sense-overlappin	XR_426771.1	
circRNA_01039	0	0.020469968	0.032712968	0	0.02006311	0.031069364	0	0	0	0	0	0	hsa_circ_0000182	NC_000001.11	+	212863725	212885396	21672	458	sense-overlappin	NM_014053.3	FLVCR1
circRNA_01040	0	0	0	0	0	0.020712909	0	0	0	0	0	0	-	NC_000001.11	+	212863725	212895354	31630	994	sense-overlappin	NM_014053.3	FLVCR1
circRNA_01041	0	0	0	0	0	0	0	0	0	0.020172483	0	0	-	NC_000001.11	+	212870054	212872818	2765	2765	sense-overlappin	NM_014053.3	FLVCR1
circRNA_01042	0	0	0	0	0	0	0	0	0	0.080689931	0	0	hsa_circ_0016414	NC_000001.11	+	212872678	212889257	16580	642	sense-overlappin	NM_014053.3	FLVCR1
circRNA_01043	0	0	0	0	0	0	0	0	0	0.040344966	0	0	-	NC_000001.11	+	213071006	213117410	46405	553	sense-overlappin	XM_011509411.1	RPS6KC1
circRNA_01044	0	0	0.032712968	0	0	0	0	0	0	0	0	0	-	NC_000001.11	+	213072870	213078079	5210	501	sense-overlappin	NM_001287219.1	RPS6KC1
circRNA_01045	0	0	0.021808645	0.0206362	0	0	0	0	0	0	0	0	-	NC_000001.11	+	213077696	213104569	26874	306	sense-overlappin	XM_011509411.1	RPS6KC1
circRNA_01046	0.051437562	0	0.13085187	0.0412724	0.05015779	0.062138728	0.030148028	0	0.060763583	0.07060369	0	0	hsa_circ_0004849	NC_000001.11	+	213077696	213117410	39715	400	sense-overlappin	XM_011509411.1	RPS6KC1
circRNA_01047	0	0	0	0	0	0	0.020098685	0	0	0	0	0	-	NC_000001.11	+	213077696	213142801	65106	13684	sense-overlappin	NM_001287218.1	RPS6KC1
circRNA_01048	0	0	0.021808645	0	0	0	0	0	0	0	0	0	hsa_circ_0005817	NC_000001.11	+	213104454	213117410	12957	210	sense-overlappin	NM_001136138.2	RPS6KC1
circRNA_01049	0	0	0	0.0206362	0	0	0	0	0	0	0	0	-	NC_000001.11	+	213104454	213176492	72039	782	sense-overlappin	NM_001136138.2	RPS6KC1
circRNA_01050	0	0	0.065425935	0	0.02006311	0.031069364	0	0	0	0	0	0	hsa_circ_0005522	NC_000001.11	+	213117317	213129889	12573	535	sense-overlappin	NM_001287218.1	RPS6KC1
circRNA_01051	0	0	0	0.0206362	0	0	0	0	0.020254528	0.020172483	0	0	hsa_circ_0007772	NC_000001.11	+	213129527	213176492	46966	572	sense-overlappin	NM_001136138.2	RPS6KC1
circRNA_01052	0	0	0	0.0206362	0	0	0	0	0	0	0	0	hsa_circ_0016430	NC_000001.11	+	213230497	213232255	1759	181	sense-overlappin	NM_001136138.2	RPS6KC1
circRNA_01053	0	0	0.021808645	0	0	0	0	0	0	0	0	0	-	NC_000001.11	+	214305187	214336940	31754	1285	sense-overlappin	NM_020197.2	SMYD2

circRNA_01054	0	0	0	0	0	0	0	0.020486295	0	0	0	0	hsa_circ_0016438	NC_000001.11	+	214314762	214318983	4222	297	sense-overlappin	NM_020197.2	SMYD2
circRNA_01055	0	0	0.021808645	0	0	0	0	0	0	0	0	0	-	NC_000001.11	+	214314762	214336940	22179	1221	sense-overlappin	NM_020197.2	SMYD2
circRNA_01056	0	0	0	0	0.02006311	0	0	0	0	0	0	0	hsa_circ_0008659	NC_000001.11	-	214390988	214398001	7014	318	sense-overlappin	XR_247032.3	
circRNA_01057	0	0	0	0.1031809	0	0	0	0	0	0	0	0	hsa_circ_0008788	NC_000001.11	+	215169199	215172183	2985	348	sense-overlappin	XM_011509521.1	KCNK2
circRNA_01058	0	0	0.021808645	0	0	0	0	0	0	0	0	0	-	NC_000001.11	+	215573786	215595471	21686	850	sense-overlappin	XM_005273156.1	KCTD3
circRNA_01059	0	0	0	0	0.02006311	0	0	0	0	0	0	0	-	NC_000001.11	+	215573786	215604302	30517	1226	sense-overlappin	XM_005273156.1	KCTD3
circRNA_01060	0	0	0.054521613	0.0722267	0	0.020712909	0	0	0.020254528	0	0	0	hsa_circ_0000184	NC_000001.11	+	215575901	215579137	3237	352	sense-overlappin	XM_005273156.1	KCTD3
circRNA_01061	0	0	0.08723458	0.0206362	0	0	0	0	0.030381792	0	0	0	hsa_circ_0005521	NC_000001.11	+	215586495	215595471	8977	307	sense-overlappin	XM_005273156.1	KCTD3
circRNA_01062	0	0	0	0.0206362	0	0	0	0	0	0	0	0	-	NC_000001.11	-	216650973	216688916	37944	7554	sense-overlappin	XM_011509272.1	ESRRG
circRNA_01063	0	0	0	0.0206362	0	0	0	0	0	0	0	0	-	NC_000001.11	-	216677076	216682026	4951	547	sense-overlappin	XM_011509272.1	ESRRG
circRNA_01064	0	0.020469968	0	0	0	0.031069364	0	0	0	0	0	0	hsa_circ_0016470	NC_000001.11	-	217491680	217498395	6716	640	sense-overlappin	XR_921857.1	
circRNA_01065	0	0	0.032712968	0.0206362	0	0	0	0	0	0	0	0	hsa_circ_0002843	NC_000001.11	-	217610321	217614202	3882	325	sense-overlappin	NM_018040.3	GPATCH2
circRNA_01066	0.041150049	0	0.054521613	0.0515905	0.04012623	0.041425819	0	0.020486295	0.020254528	0.040344966	0.06076569	0	hsa_circ_0004314	NC_000001.11	+	219193082	219218712	25631	7507	sense-overlappin	XM_006711162.2	LYPLAL1
circRNA_01067	0.051437562	0.071644886	0.109043225	0.0309543	0.06018934	0.020712909	0	0.020486295	0.030381792	0.030258724	0	0	hsa_circ_0004417	NC_000001.11	+	219193082	219241308	48227	30103	sense-overlappin	XM_006711162.2	LYPLAL1
circRNA_01068	0	0	0.021808645	0	0	0	0	0	0	0	0	0	-	NC_000001.11	+	219193130	219218712	25583	7459	sense-overlappin	XM_006711162.2	LYPLAL1
circRNA_01069	0	0	0	0	0	0	0	0	0	0.030258724	0	0	hsa_circ_0006963	NC_000001.11	+	219193130	219241308	48179	30055	sense-overlappin	XM_006711162.2	LYPLAL1
circRNA_01070	0	0	0.021808645	0	0	0.031069364	0	0	0	0	0	0	hsa_circ_0016485	NC_000001.11	-	220005248	220007338	2091	458	sense-overlappin	NM_004446.2	EPRS
circRNA_01071	0	0.030704951	0.04361729	0.0206362	0.02006311	0.041425819	0.030148028	0	0	0.080689931	0	0	hsa_circ_0007739	NC_000001.11	-	220006106	220007338	1233	345	sense-overlappin	NM_004446.2	EPRS
circRNA_01072	0	0	0	0	0	0	0	0	0.030381792	0	0	0	-	NC_000001.11	+	220102129	220102777	649	400	sense-overlappin	NM_018060.3	IARS2
circRNA_01073	0	0	0	0	0.02006311	0	0	0	0	0	0	0	-	NC_000001.11	+	220136809	220139139	2331	361	sense-overlappin	NM_018060.3	IARS2
circRNA_01074	0	0	0	0	0	0.041425819	0	0	0	0	0.02025523	0	hsa_circ_0016508	NC_000001.11	-	220182257	220182931	675	312	sense-overlappin	NM_012414.3	RAB3GAP2
circRNA_01075	0	0	0	0.0206362	0	0	0	0	0	0	0	0	hsa_circ_0002924	NC_000001.11	-	220212887	220213979	1093	206	sense-overlappin	NM_012414.3	RAB3GAP2
circRNA_01076	0	0	0	0.0309543	0	0	0	0.020486295	0	0	0	0	-	NC_000001.11	+	220761658	220770556	8899	480	sense-overlappin	XR_247029.3	
circRNA_01077	0	0	0	0	0.03009467	0	0	0	0	0	0	0	-	NC_000001.11	-	222558131	222564125	5995	634	sense-overlappin	NM_001201536.1	TAF1A
circRNA_01078	0	0	0.021808645	0	0	0	0.020098685	0	0	0	0	0	-	NC_000001.11	+	222724092	222730177	6086	588	sense-overlappin	NM_001288579.1	BROX
circRNA_01079	0	0	0	0.0412724	0	0	0	0	0	0	0	0	-	NC_000001.11	+	222827428	222835215	7788	385	sense-overlappin	XR_921983.1	
circRNA_01080	0	0	0	0	0	0	0	0.020486295	0	0	0	0	-	NC_000001.11	+	222943792	222943927	136	136	intronic	XM_011510073.1	DISP1
circRNA_01081	0	0	0	0	0	0	0.020098685	0	0	0.030258724	0	0	-	NC_000001.11	-	223132475	223137263	4789	528	sense-overlappin	XM_005273241.3	TLR5
circRNA_01082	0	0	0	0.0206362	0.03009467	0	0	0	0	0.050431207	0.02025523	0	hsa_circ_0003914	NC_000001.11	-	223804174	223806947	2774	277	sense-overlappin	NM_001031685.2	TP53BP2
circRNA_01083	0	0.061409903	0	0	0	0	0	0	0	0	0	0	-	NC_000001.11	-	223810431	223818337	7907	329	sense-overlappin	NM_005426.2	TP53BP2
circRNA_01084	0.030862537	0	0	0	0	0	0	0.040972591	0	0.040344966	0	0	-	NC_000001.11	+	224189577	224190319	743	743	exonic	NM_003676.3	DEGS1
circRNA_01085	0	0	0	0	0	0.020712909	0	0	0	0	0	0	hsa_circ_0000188	NC_000001.11	-	224250212	224268133	17922	207	sense-overlappin	NM_001243146.1	NVL
circRNA_01086	0.030862537	0	0	0	0	0	0	0	0	0	0	0	-	NC_000001.11	-	224317694	224317930	237	153	sense-overlappin	NM_001243146.1	NVL
circRNA_01087	0	0	0.021808645	0	0	0	0	0	0	0.020172483	0	0	hsa_circ_0000190	NC_000001.11	+	224365879	224371423	5545	207	sense-overlappin	NM_001277198.1	CNIH4
circRNA_01088	0	0	0	0	0.02006311	0	0	0	0	0	0	0	hsa_circ_0002137	NC_000001.11	+	224730462	224734706	4245	257	sense-overlappin	XM_005273075.2	CNIH3
circRNA_01089	0	0	0.032712968	0	0	0	0	0	0	0	0	0	-	NC_000001.11	+	224952670	224967583	14914	684	sense-overlappin	XM_011544083.1	DNAH14
circRNA_01090	0	0	1.253997088	0	0	0	0	0	0	0	0	0	hsa_circ_0016599	NC_000001.11	+	224952670	224968874	16205	800	sense-overlappin	XM_011544083.1	DNAH14
circRNA_01091	0.020575025	0	3.08592327	0.2682705	0	0.062138728	0	0.040972591	0.030381792	0	0	0.0309546	hsa_circ_0016600	NC_000001.11	+	224952670	224974153	21484	863	sense-overlappin	XM_011544083.1	DNAH14
circRNA_01092	0	0	0.04361729	0	0	0	0	0	0	0	0	0	-	NC_000001.11	+	224952670	224975372	22703	2082	sense-overlappin	XM_011544083.1	DNAH14
circRNA_01093	0.020575025	0.051174919	1.897352116	0.1238171	0	0.041425819	0	0	0	0.030258724	0	0	hsa_circ_0016601	NC_000001.11	+	224952670	225007544	54875	1140	sense-overlappin	XM_011544083.1	DNAH14
circRNA_01094	0	0	0.098138903	0.0309543	0	0	0	0	0	0	0	0	-	NC_000001.11	+	224952670	225023865	71196	1391	sense-overlappin	XM_011544083.1	DNAH14
circRNA_01095	0	0	0.032712968	0	0	0	0	0	0	0	0	0	-	NC_000001.11	+	224952670	225051795	99126	2457	sense-overlappin	XM_011544083.1	DNAH14
circRNA_01096	0	0	0.021808645	0	0	0	0	0	0	0	0	0	-	NC_000001.11	+	224952674	224968874	16201	796	sense-overlappin	XM_011544083.1	DNAH14
circRNA_01097	0	0	0.065425935	0	0	0	0	0	0	0	0	0	-	NC_000001.11	+	224952674	225007544	54871	1136	sense-overlappin	XM_011544083.1	DNAH14
circRNA_01098	0	0	0.065425935	0	0	0	0	0	0	0	0	0	-	NC_000001.11	+	224952685	224968874	16190	785	sense-overlappin	XM_011544083.1	DNAH14
circRNA_01099	0	0	0.163564838	0	0	0	0	0	0	0	0	0	-	NC_000001.11	+	224952685	224974153	21469	848	sense-overlappin	XM_011544083.1	DNAH14
circRNA_01100	0	0	0.065425935	0	0	0	0	0	0	0	0	0	-	NC_000001.11	+	224952685	225007544	54860	1125	sense-overlappin	XM_011544083.1	DNAH14
circRNA_01101	0	0	0.054521613	0	0	0	0	0	0	0	0	0	-	NC_000001.11	+	225002783	225007544	4762	277	sense-overlappin	XM_011544083.1	DNAH14
circRNA_01102	0	0	0.021808645	0	0	0	0	0	0	0	0	0	-	NC_000001.11	+	225002783	225043983	41201	1082	sense-overlappin	XM_011544083.1	DNAH14
circRNA_01103	0	0	0.021808645	0	0	0	0	0	0	0	0	0	-	NC_000001.11	+	225002783	225051795	49013	1594	sense-overlappin	XM_011544083.1	DNAH14
circRNA_01104	0	0	0.04361729	0	0	0	0	0	0	0	0	0	-	NC_000001.11	+	225038694	225043983	5290	554	sense-overlappin	XM_011544083.1	DNAH14
circRNA_01105	0	0	0.04361729	0	0	0	0	0	0	0	0	0	hsa_circ_0016616	NC_000001.11	+	225038694	225097239	58546	2337	sense-overlappin	XM_011544083.1	DNAH14
circRNA_01106	0	0	0.250799418	0	0	0	0.020098685	0	0	0	0	0	-	NC_000001.11	+	225079207	225097239	18033	1271	sense-overlappin	XM_011544083.1	DNAH14
circRNA_01107	0	0	0.054521613	0	0	0	0	0	0	0	0	0	-	NC_000001.11	+	225079207	225123614	44408	1830	sense-overlappin	XM_011544083.1	DNAH14
circRNA_01108	0	0	0.021808645	0	0	0	0	0	0	0	0	0	-	NC_000001.11	+	225079207	225159485	80279	2919	sense-overlappin	XM_011544060.1	DNAH14

circRNA_01109	0	0	0.032712968	0	0	0	0	0	0	0	0	0	-	NC_000001.11	+	225080379	225097239	16861	929	sense-overlappin	XM_011544083.1	DNAH14
circRNA_01110	0	0	0.021808645	0	0	0	0	0	0	0	0	0	-	NC_000001.11	+	225080379	225123614	43236	1488	sense-overlappin	XM_011544083.1	DNAH14
circRNA_01111	0	0	0.054521613	0	0	0	0	0	0	0	0	0	-	NC_000001.11	+	225082549	225123614	41066	1118	sense-overlappin	XM_011544083.1	DNAH14
circRNA_01112	0	0	0.04361729	0	0	0	0	0	0	0	0	0	-	NC_000001.11	+	225082549	225144628	62080	1604	sense-overlappin	XM_011544083.1	DNAH14
circRNA_01113	0	0	0.08723458	0	0	0	0	0	0	0	0	0	-	NC_000001.11	+	225085710	225097239	11530	202	sense-overlappin	XM_011544083.1	DNAH14
circRNA_01114	0	0	0.032712968	0	0	0	0	0	0	0	0	0	-	NC_000001.11	+	225152005	225185425	33421	730	sense-overlappin	XM_011544083.1	DNAH14
circRNA_01115	0	0	0.021808645	0	0	0	0	0	0	0	0	0	-	NC_000001.11	+	225152005	225192911	40907	946	sense-overlappin	XM_011544083.1	DNAH14
circRNA_01116	0	0	0.04361729	0	0	0	0	0	0	0	0	0	-	NC_000001.11	+	225167939	225185425	17487	225	sense-overlappin	XM_011544083.1	DNAH14
circRNA_01117	0	0	0.032712968	0	0	0	0	0	0	0	0	0	-	NC_000001.11	+	225167939	225207220	39282	994	sense-overlappin	XM_011544083.1	DNAH14
circRNA_01118	0	0	0.04361729	0	0	0	0	0	0	0	0	0	-	NC_000001.11	+	225167939	225231151	63213	1073	sense-overlappin	XM_011544083.1	DNAH14
circRNA_01119	0	0	0.13085187	0	0	0	0	0	0	0	0	0	-	NC_000001.11	+	225185291	225207220	21930	904	sense-overlappin	XM_011544083.1	DNAH14
circRNA_01120	0	0	0.021808645	0	0	0	0	0	0	0	0	0	-	NC_000001.11	+	225185291	225266769	81479	2052	sense-overlappin	NM_001373.1	DNAH14
circRNA_01121	0	0	0.065425935	0	0	0	0	0	0	0	0	0	-	NC_000001.11	+	225264197	225290082	25886	1312	sense-overlappin	XM_011544080.1	DNAH14
circRNA_01122	0	0	0.032712968	0	0	0	0	0	0	0	0	0	-	NC_000001.11	+	225264197	225308410	44214	2083	sense-overlappin	XM_011544078.1	DNAH14
circRNA_01123	0.020575025	0	0	0	0	0.020712909	0.020098685	0	0.040509055	0	0	0	hsa_circ_0016650	NC_000001.11	-	225412454	225415332	2879	247	sense-overlappin	XM_011544185.1	LBR
circRNA_01124	0	0	0.032712968	0	0	0	0	0	0	0	0	0	-	NC_000001.11	-	225412454	225419798	7345	718	sense-overlappin	XM_011544185.1	LBR
circRNA_01125	0.051437562	0	0	0	0	0	0	0	0	0	0	0	-	NC_000001.11	-	225507951	225513016	5066	320	sense-overlappin	NM_001008493.1	ENAH
circRNA_01126	0.030862537	0	0.032712968	0	0	0	0	0	0	0	0	0	hsa_circ_0016662	NC_000001.11	-	225554906	225567414	12509	344	sense-overlappin	NM_001008493.1	ENAH
circRNA_01127	0	0	0	0	0	0	0	0.030729443	0	0	0.030382845	0	-	NC_000001.11	-	225987896	225988388	493	493	exonic	NM_152608.3	SDE2
circRNA_01128	0	0	0.04361729	0	0	0	0	0	0	0	0	0	-	NC_000001.11	-	226265533	226301205	35673	1007	sense-overlappin	NM_173083.3	LIN9
circRNA_01129	0	0	0.032712968	0.0206362	0	0	0	0.020486295	0	0	0	0	hsa_circ_0016733	NC_000001.11	-	227004994	227017050	12057	546	sense-overlappin	XM_005273321.2	CDC42BPA
circRNA_01130	0	0	0.021808645	0	0	0	0	0	0	0	0	0	-	NC_000001.11	-	227016080	227035607	19528	1658	sense-overlappin	XM_005273321.2	CDC42BPA
circRNA_01131	0	0	0	0	0	0	0	0	0	0.040344966	0	0	-	NC_000001.11	-	227016080	227048010	31931	1848	sense-overlappin	XM_005273321.2	CDC42BPA
circRNA_01132	0	0	0	0	0.02006311	0	0	0	0	0.020172483	0	0	hsa_circ_0009044	NC_000001.11	-	227026055	227035607	9553	1331	sense-overlappin	XM_005273321.2	CDC42BPA
circRNA_01133	0	0	0	0	0.02006311	0	0	0	0	0	0	0	-	NC_000001.11	-	227069777	227147559	77783	2211	sense-overlappin	XM_005273321.2	CDC42BPA
circRNA_01134	0	0	0.021808645	0	0	0	0	0	0	0	0	0	-	NC_000001.11	-	227112312	227112913	602	354	sense-overlappin	XM_005273321.2	CDC42BPA
circRNA_01135	0	0	0.185373483	0.0412724	0.02006311	0	0	0	0.020254528	0.030258724	0	0	hsa_circ_0003659	NC_000001.11	-	227139576	227147559	7984	697	sense-overlappin	XM_005273321.2	CDC42BPA
circRNA_01136	0	0	0.08723458	0.0206362	0	0	0	0	0	0.050431207	0	0	hsa_circ_0005322	NC_000001.11	-	227193786	227213219	19434	329	sense-overlappin	XM_005273321.2	CDC42BPA
circRNA_01137	0	0	0	0.0309543	0	0	0.030148028	0	0	0	0	0	hsa_circ_0016754	NC_000001.11	-	227193786	227254155	60370	421	sense-overlappin	XM_005273321.2	CDC42BPA
circRNA_01138	0	0	0	0	0	0	0.030148028	0	0	0	0	0	hsa_circ_0016761	NC_000001.11	+	227747692	227767083	19392	1158	sense-overlappin	NM_053052.3	SNAP47
circRNA_01139	0	0	0.032712968	0	0	0	0	0	0	0	0	0	-	NC_000001.11	+	228224459	228264391	39933	2484	sense-overlappin	XM_006711828.2	OBSCN
circRNA_01140	0	0	0.054521613	0	0	0	0	0	0	0	0	0	-	NC_000001.11	+	228264116	228264391	276	276	exonic	XM_011544298.1	OBSCN
circRNA_01141	0	0	0	0	0	0	0.070345399	0	0.020254528	0	0	0	-	NC_000001.11	-	228394737	228395252	516	516	exonic	XM_011544285.1	TRIM11
circRNA_01142	0	0	0.032712968	0	0.03009467	0	0	0	0	0	0	0	-	NC_000001.11	+	229286486	229304000	17515	833	sense-overlappin	NM_004578.3	RAB4A
circRNA_01143	0	0	0.032712968	0	0.02006311	0	0	0	0	0	0	0	hsa_circ_0003896	NC_000001.11	-	229450525	229465519	14995	981	sense-overlappin	NM_018230.2	NUP133
circRNA_01144	0	0	0	0	0.02006311	0	0	0	0	0	0	0	-	NC_000001.11	-	229466634	229487613	20980	1005	sense-overlappin	NM_018230.2	NUP133
circRNA_01145	0	0.030704951	0.032712968	0	0	0.031069364	0	0	0	0.020172483	0	0	hsa_circ_0016867	NC_000001.11	+	230663141	230671640	8500	599	sense-overlappin	NM_001145036.1	COG2
circRNA_01146	0	0.020469968	0.076330258	0.0722267	0	0	0	0.102431477	0	0.040344966	0.02025523	0	hsa_circ_0016868	NC_000001.11	+	230663141	230675124	11984	726	sense-overlappin	NM_001145036.1	COG2
circRNA_01147	0	0	0.065425935	0	0	0.020712909	0	0	0.030381792	0	0	0	hsa_circ_0004531	NC_000001.11	-	230943806	230961303	17498	401	sense-overlappin	NM_024525.4	TTC13
circRNA_01148	0	0	0	0	0	0	0	0	0	0.030258724	0	0	hsa_circ_0000194	NC_000001.11	-	230945389	230961303	15915	308	sense-overlappin	NM_024525.4	TTC13
circRNA_01149	0	0	0	0	0.04012623	0	0	0	0	0	0	0	-	NC_000001.11	-	230948899	230954403	5505	5505	sense-overlappin	NM_001122835.2	TTC13
circRNA_01150	0.041150049	0.061409903	0.239895095	0.1341352	0.29091517	0.103564547	0.070345399	0.051215739	0.121527166	0.131121138	0.050638075	0.051591	hsa_circ_0000195	NC_000001.11	-	230954333	230961303	6971	242	sense-overlappin	NM_001122835.2	TTC13
circRNA_01151	0	0	0	0	0	0	0	0	0	0.030258724	0	0	-	NC_000001.11	-	231364327	231367636	3310	2217	sense-overlappin	XM_005273167.3	EGLN1
circRNA_01152	0	0	0	0	0.02006311	0	0	0	0	0	0	0	-	NC_000001.11	-	231364327	231374099	9773	2474	sense-overlappin	NM_022051.2	EGLN1
circRNA_01153	0.020575025	0.020469968	0	0.0309543	0.06018934	0.041425819	0	0	0	0.020172483	0	0	hsa_circ_0000196	NC_000001.11	-	231370562	231374099	3538	257	sense-overlappin	XM_005273166.3	EGLN1
circRNA_01154	0.020575025	0	0.032712968	0	0.13041025	0.051782273	0.020098685	0.040972591	0	0.030258724	0.030382845	0	hsa_circ_0004834	NC_000001.11	+	231537213	231542611	5399	246	sense-overlappin	NM_005999.2	TSNAX
circRNA_01155	0	0.030704951	0	0	0	0	0	0	0.020254528	0	0	0	-	NC_000001.11	+	231749926	231771070	21145	517	sense-overlappin	NM_001164549.1	DISC1
circRNA_01156	0.020575025	0.030704951	0	0.0825448	0	0.041425819	0.060296056	0.030729443	0	0.020172483	0.02025523	0.072227401	hsa_circ_0007848	NC_000001.11	+	231795242	231818517	23276	347	sense-overlappin	NM_001164544.1	DISC1
circRNA_01157	0	0	0	0	0	0.020712909	0	0	0	0	0	0	-	NC_000001.11	+	231818329	231819150	822	822	exonic	NM_001164544.1	DISC1
circRNA_01158	0	0	0.021808645	0	0	0	0	0	0.020254528	0.020172483	0	0	-	NC_000001.11	-	232460887	232471528	10642	1010	sense-overlappin	NM_020808.3	SIPA1L2
circRNA_01159	0.051437562	0	0.119947548	0	0.02006311	0	0	0	0	0	0	0	-	NC_000001.11	-	232513857	232515608	1752	1752	exonic	XM_005273214.1	SIPA1L2
circRNA_01160	0	0.051174919	0.04361729	0	0	0	0	0	0	0	0	0	-	NC_000001.11	-	232513857	232533583	19727	1846	sense-overlappin	XM_006711798.2	SIPA1L2
circRNA_01161	0	0	0	0	0.05015779	0	0	0	0	0	0	0	hsa_circ_0007041	NC_000001.11	-	233054268	233057290	3023	275	sense-overlappin	XM_011544279.1	PCNXL2
circRNA_01162	0	0	0.032712968	0	0	0	0	0	0	0	0.030382845	0	hsa_circ_0005994	NC_000001.11	-	233090061	233095863	5803	239	sense-overlappin	XM_011544279.1	PCNXL2

circRNA_01163	0	0	0	0	0	0	0	0	0	0.020172483	0	0	hsa_circ_0016952	NC_000001.11	-	233112799	233161363	48565	732	sense-overlappin	XR_949160.1	
circRNA_01164	0.030862537	0	0.021808645	0	0	0	0	0	0	0	0	0	hsa_circ_0006635	NC_000001.11	-	233160283	233208689	48407	826	sense-overlappin	XM_011544278.1	PCNXL2
circRNA_01165	0	0	0.032712968	0	0	0	0	0	0	0	0	0	hsa_circ_0016962	NC_000001.11	-	233160283	233236980	76698	1295	sense-overlappin	XM_011544278.1	PCNXL2
circRNA_01166	0	0	0.04361729	0	0	0	0	0	0	0.040344966	0	0	hsa_circ_0001958	NC_000001.11	-	233177802	233208689	30888	582	sense-overlappin	XM_011544278.1	PCNXL2
circRNA_01167	0	0	0	0.0206362	0	0	0	0	0	0	0	0	hsa_circ_0016965	NC_000001.11	-	233177802	233236980	59179	1051	sense-overlappin	XM_011544278.1	PCNXL2
circRNA_01168	0	0	0	0.0206362	0	0	0	0	0	0.050431207	0	0	hsa_circ_0005223	NC_000001.11	-	233198939	233208689	9751	375	sense-overlappin	XM_011544278.1	PCNXL2
circRNA_01169	0	0	0	0	0.03009467	0	0	0	0	0	0	0.0206364	hsa_circ_0016969	NC_000001.11	-	233198939	233218184	19246	562	sense-overlappin	XM_011544278.1	PCNXL2
circRNA_01170	0	0.020469968	0	0	0	0	0	0	0	0	0	0	hsa_circ_0000198	NC_000001.11	-	233198939	233236980	38042	844	sense-overlappin	XM_011544278.1	PCNXL2
circRNA_01171	0	0	0.032712968	0	0	0	0	0	0	0	0	0	hsa_circ_0006415	NC_000001.11	-	233208518	233236980	28463	641	sense-overlappin	XM_011544278.1	PCNXL2
circRNA_01172	0	0	0.04361729	0.0206362	0.04012623	0.031069364	0.020098685	0.040972591	0.040509055	0.060517448	0	0	hsa_circ_0003314	NC_000001.11	-	233217899	233227371	9473	333	sense-overlappin	XM_011544278.1	PCNXL2
circRNA_01173	0	0	0.032712968	0.0206362	0	0	0	0	0	0	0	0	hsa_circ_0016979	NC_000001.11	-	233217899	233236980	19082	469	sense-overlappin	XM_011544278.1	PCNXL2
circRNA_01174	0	0	0.021808645	0	0	0	0	0	0	0	0	0	hsa_circ_0016989	NC_000001.11	-	233227226	233236980	9755	282	sense-overlappin	XM_011544278.1	PCNXL2
circRNA_01175	0	0	0.032712968	0.0722267	0	0.020712909	0	0	0.020254528	0.030258724	0	0	-	NC_000001.11	+	233346442	233355011	8570	506	sense-overlappin	XM_011544305.1	KIAA1804
circRNA_01176	0	0	0.021808645	0.0928628	0	0	0	0	0	0	0	0	-	NC_000001.11	+	233346442	233362293	15852	747	sense-overlappin	XM_011544305.1	KIAA1804
circRNA_01177	0	0	0.04361729	0.0206362	0	0	0	0	0	0	0	0	-	NC_000001.11	+	233346442	233376527	30086	1119	sense-overlappin	XM_011544305.1	KIAA1804
circRNA_01178	0	0	0.04361729	0.0206362	0	0	0	0	0	0.030258724	0	0	-	NC_000001.11	-	234446803	234472811	26009	1203	sense-overlappin	NM_005646.3	TARBP1
circRNA_01179	0	0	0	0	0	0	0.020098685	0	0	0	0	0	-	NC_000001.11	-	234450428	234472811	22384	930	sense-overlappin	NM_005646.3	TARBP1
circRNA_01180	0	0	0	0	0.02006311	0	0	0	0	0	0	0	-	NC_000001.11	-	235137877	235155112	17236	484	sense-overlappin	NM_015014.2	RBM34
circRNA_01181	0	0	0	0	0	0	0	0	0	0	0	0.0206364	-	NC_000001.11	-	235148404	235155112	6709	336	sense-overlappin	NM_015014.2	RBM34
circRNA_01182	0	0	0	0	0	0	0.020098685	0	0	0.020172483	0	0	hsa_circ_0017041	NC_000001.11	-	235152706	235155112	2407	292	sense-overlappin	NM_015014.2	RBM34
circRNA_01183	0	0	0	0	0.03009467	0	0	0	0.020254528	0.030258724	0	0	-	NC_000001.11	-	235194013	235214026	20014	542	sense-overlappin	XM_011544213.1	ARID4B
circRNA_01184	0	0	0	0	0	0	0	0.030729443	0	0	0	0	-	NC_000001.11	-	235196031	235214026	17996	343	sense-overlappin	XM_011544213.1	ARID4B
circRNA_01185	0	0.020469968	0	0	0	0	0	0	0	0	0	0	-	NC_000001.11	-	235213769	235221662	7894	776	sense-overlappin	XM_011544213.1	ARID4B
circRNA_01186	0	0	0.021808645	0	0	0	0	0	0	0	0	0	-	NC_000001.11	-	235219793	235240451	20659	1137	sense-overlappin	NM_001206794.1	ARID4B
circRNA_01187	0	0	0	0	0.02006311	0	0	0	0	0	0	0	-	NC_000001.11	-	235219793	235260752	40960	1577	sense-overlappin	NM_001206794.1	ARID4B
circRNA_01188	0	0	0	0	0	0	0	0	0	0	0	0.0309546	-	NC_000001.11	-	235267827	235268638	812	812	intronic	NM_001206794.1	ARID4B
circRNA_01189	0	0	0	0	0.02006311	0	0	0	0	0	0	0	-	NC_000001.11	+	235335242	235342883	7642	1037	sense-overlappin	NM_001037277.1	GGPS1
circRNA_01190	0.020575025	0	0	0.0309543	0.0702209	0	0	0.030729443	0	0.020172483	0	0	-	NC_000001.11	+	235341708	235342883	1176	944	sense-overlappin	NM_001037277.1	GGPS1
circRNA_01191	0	0	0	0	0	0	0	0	0	0.020172483	0	0	-	NC_000001.11	-	235762720	235782087	19368	1391	sense-overlappin	NM_000081.3	LYST
circRNA_01192	0	0	0	0.0206362	0	0	0	0	0.020254528	0	0	0	-	NC_000001.11	-	235780865	235782087	1223	352	sense-overlappin	NM_000081.3	LYST
circRNA_01193	0	0	0	0	0	0	0.020098685	0	0	0	0	0	hsa_circ_0017092	NC_000001.11	-	235800320	235801097	778	294	sense-overlappin	NM_000081.3	LYST
circRNA_01194	0	0	0	0.0206362	0	0	0	0	0	0	0	0	-	NC_000001.11	-	235800320	235810534	10215	3723	sense-overlappin	NM_000081.3	LYST
circRNA_01195	0	0.061409903	0.032712968	0	0.02006311	0	0	0	0	0	0	0	hsa_circ_0005899	NC_000001.11	-	235830226	235833667	3442	289	sense-overlappin	NM_000081.3	LYST
circRNA_01196	0	0	0	0	0	0	0	0	0.030381792	0	0	0	hsa_circ_0017105	NC_000001.11	-	236024070	236038253	14184	993	sense-overlappin	XM_011544195.1	NID1
circRNA_01197	0	0	0.032712968	0	0	0	0	0	0	0	0	0	-	NC_000001.11	+	236168706	236180028	11323	423	sense-overlappin	XR_247039.2	
circRNA_01198	0	0	0	0	0.04012623	0	0	0	0	0	0	0	-	NC_000001.11	+	236168706	236183906	15201	552	sense-overlappin	XR_247039.2	
circRNA_01199	0	0	0	0	0	0.031069364	0	0	0	0	0	0	-	NC_000001.11	-	236243422	236253505	10084	283	sense-overlappin	NM_019891.3	ERO1LB
circRNA_01200	0	0	0	0	0.02006311	0	0	0	0	0	0	0	hsa_circ_0003219	NC_000001.11	+	236803428	236806233	2806	305	sense-overlappin	XM_006711769.2	MTR
circRNA_01201	0	0	0	0	0	0	0	0	0	0	0	0.0309546	-	NC_000001.11	+	236803428	236838599	35172	1481	sense-overlappin	XM_006711769.2	MTR
circRNA_01202	0	0	0	0.0206362	0	0	0	0	0	0	0	0	-	NC_000001.11	+	236803428	236853088	49661	1919	sense-overlappin	XM_006711769.2	MTR
circRNA_01203	0.092587611	0	0.098138903	0.0412724	0.04012623	0.093208092	0.020098685	0.030729443	0.020254528	0.100862414	0	0.0619092	hsa_circ_0004039	NC_000001.11	+	236806144	236816543	10400	515	sense-overlappin	XM_006711769.2	MTR
circRNA_01204	0	0	0	0.0206362	0	0	0	0	0	0	0	0	-	NC_000001.11	+	236806144	236863554	57411	2156	sense-overlappin	XM_006711769.2	MTR
circRNA_01205	0	0	0	0	0	0	0	0	0	0	0.02025523	0	hsa_circ_0017155	NC_000001.11	+	236835547	236853088	17542	765	sense-overlappin	XM_006711769.2	MTR
circRNA_01206	0	0	0	0.0206362	0	0.031069364	0	0	0	0	0	0	hsa_circ_0017160	NC_000001.11	+	236850344	236853088	2745	438	sense-overlappin	XM_006711769.2	MTR
circRNA_01207	0	0.020469968	0	0	0	0.041425819	0	0	0	0	0	0.0206364	-	NC_000001.11	+	237566567	237569319	2753	384	sense-overlappin	XM_006711809.2	RYR2
circRNA_01208	0.020575025	0	0	0	0	0	0	0	0	0	0	0	-	NC_000001.11	+	237591739	237610988	19250	780	sense-overlappin	XM_006711809.2	RYR2
circRNA_01209	0	0	0	0	0.02006311	0	0	0	0	0	0	0	-	NC_000001.11	+	239406379	239492807	86429	86429	sense-overlappin	XM_011544041.1	CHRM3
circRNA_01210	0	0	0	0	0.03009467	0	0	0	0	0	0	0	-	NC_000001.11	+	239484175	239492807	8633	8633	sense-overlappin	XM_011544041.1	CHRM3
circRNA_01211	0	0	0	0	0	0	0	0	0.030381792	0	0	0	-	NC_000001.11	+	239492709	239545749	53041	208	sense-overlappin	XM_011544041.1	CHRM3
circRNA_01212	0.061725074	0	0	0	0	0	0	0	0	0	0	0	hsa_circ_0017184	NC_000001.11	+	240206799	240294883	88085	2229	sense-overlappin	NM_020066.4	FMN2
circRNA_01213	0	0.061409903	0	0	0	0	0	0	0	0	0	0	-	NC_000001.11	-	244408712	244417752	9041	1300	sense-overlappin	NM_001126.3	ADSS
circRNA_01214	0	0	0	0	0	0	0.030148028	0	0	0	0	0	-	NC_000001.11	+	244552322	244552814	493	493	exonic	XM_011544142.1	C1orf101
circRNA_01215	0.030862537	0	0.021808645	0	0.03009467	0.041425819	0	0	0.040509055	0.030258724	0	0	hsa_circ_0006080	NC_000001.11	+	244552322	244561145	8824	971	sense-overlappin	XM_011544142.1	C1orf101
circRNA_01216	0	0.030704951	0	0	0	0	0	0	0	0	0	0	-	NC_000001.11	-	244845247	244848856	3610	3610	intergenic		
circRNA_01217	0	0	0	0	0.0702209	0	0	0	0	0	0.030382845	0	hsa_circ_0008294	NC_000001.11	+	245002121	245017326	15206	197	sense-overlappin	XM_011544301.1	EFCAB2

circRNA_01218	0	0	0	0	0	0	0	0	0	0.050431207	0	0	hsa_circ_0006983	NC_000001.11	+	245002121	245022296	20176	5167	sense-overlappin	XM_011544301.1	EFCAB2
circRNA_01219	0.041150049	0	0.021808645	0	0	0	0	0	0	0	0	0	hsa_circ_0002426	NC_000001.11	+	245002121	245059458	57338	270	sense-overlappin	XM_011544301.1	EFCAB2
circRNA_01220	0	0	0	0.0412724	0	0	0	0	0	0.040344966	0	0	-	NC_000001.11	+	245017242	245083688	66447	348	sense-overlappin	XM_011544301.1	EFCAB2
circRNA_01221	0	0	0.098138903	0.0309543	0	0.031069364	0	0	0	0	0	0	hsa_circ_0005090	NC_000001.11	-	245915530	245929937	14408	282	sense-overlappin	NM_022743.2	SMYD3
circRNA_01222	0	0	0.098138903	0	0	0	0	0	0.020254528	0	0	0	hsa_circ_0017306	NC_000001.11	-	246327201	246335474	8274	303	sense-overlappin	NM_022743.2	SMYD3
circRNA_01223	0	0.051174919	0	0	0	0	0	0	0	0	0	0	hsa_circ_0005988	NC_000001.11	-	246437148	246440124	2977	2977	intronic	NM_001167740.1	SMYD3
circRNA_01224	0	0	0	0	0	0	0	0	0	0	0	0.0206364	-	NC_000001.11	-	246540690	246551302	10613	827	sense-overlappin	NM_022366.2	TFB2M
circRNA_01225	0	0	0	0	0.02006311	0	0.030148028	0	0	0.020172483	0	0	hsa_circ_0004005	NC_000001.11	-	246556573	246557534	962	303	sense-overlappin	NM_022366.2	TFB2M
circRNA_01226	0.020575025	0	0.021808645	0.0515905	0	0	0	0	0	0	0	0	hsa_circ_0017310	NC_000001.11	+	246591512	246591941	430	430	exonic	NM_001139459.1	CNST
circRNA_01227	0	0	0.04361729	0	0	0	0	0	0	0	0	0	hsa_circ_0017311	NC_000001.11	+	246621429	246634587	13159	439	sense-overlappin	NM_001139459.1	CNST
circRNA_01228	0	0	0	0	0	0	0	0	0	0	0	0.0619092	-	NC_000001.11	+	246627356	246631924	4569	4569	sense-overlappin	NM_001139459.1	CNST
circRNA_01229	0	0	0.04361729	0	0	0	0	0	0	0	0	0	-	NC_000001.11	+	246631359	246631924	566	566	sense-overlappin	NM_001139459.1	CNST
circRNA_01230	0	0.020469968	0.032712968	0.0515905	0	0	0	0	0	0	0	0	-	NC_000001.11	+	246726892	246740301	13410	324	sense-overlappin	NM_016002.2	SCCPDH
circRNA_01231	0	0	0.04361729	0	0	0	0	0	0	0.050431207	0	0	-	NC_000001.11	+	246726892	246744125	17234	374	sense-overlappin	NM_016002.2	SCCPDH
circRNA_01232	0	0.020469968	0.08723458	0	0.09028402	0.031069364	0.060296056	0.020486295	0.030381792	0.040344966	0.04051046	0	hsa_circ_0002802	NC_000001.11	-	247138716	247159813	21098	230	sense-overlappin	NM_001297567.1	ZNF124
circRNA_01233	0.380637955	0.388929383	1.537509474	0.4230419	0.4714832	0.248554912	0.391924364	0.327780728	0.222799805	0.423622139	0.243062761	0.247636802	hsa_circ_0002922	NC_000001.11	-	247155566	247159813	4248	2026	sense-overlappin	NM_001297568.1	ZNF124
circRNA_01234	0	0	0.04361729	0	0.03009467	0	0	0	0	0	0	0	-	NC_000001.11	-	247155566	247159867	4302	2080	sense-overlappin	NM_001297568.1	ZNF124
circRNA_01235	0.360062931	0.470809253	2.813315207	0.6500399	1.00315575	0.455684006	0.502467134	0.31753758	0.405090554	0.524484553	0.334211296	0.319864203	-	NC_000001.11	-	247156406	247159813	3408	1186	sense-overlappin	NM_001297568.1	ZNF124
circRNA_01236	0	0	0.054521613	0	0	0	0.030148028	0	0	0	0	0	-	NC_000001.11	-	247156406	247159867	3462	1240	sense-overlappin	NM_001297568.1	ZNF124
circRNA_01237	0	0	0.021808645	0	0	0	0	0	0	0	0	0	-	NC_000001.11	-	247156832	247159813	2982	760	sense-overlappin	NM_001297568.1	ZNF124
circRNA_01238	0	0	0.13085187	0.0309543	0.08025246	0.041425819	0.040197371	0.051215739	0	0.030258724	0.050638075	0	hsa_circ_0005667	NC_000001.11	-	247157000	247159813	2814	592	sense-overlappin	NM_001297568.1	ZNF124
circRNA_01239	0	0	0.076330258	0	0.04012623	0	0.020098685	0	0	0	0	0	hsa_circ_0017348	NC_000001.11	-	247159006	247159813	808	188	sense-overlappin	NM_001243740.2	ZNF124
circRNA_01240	0	0	0.021808645	0	0	0	0	0	0	0	0	0	-	NC_000001.11	-	247159006	247159867	862	242	sense-overlappin	NM_001243740.2	ZNF124
circRNA_01241	0	0	0.032712968	0.0206362	0	0	0	0	0	0	0	0	-	NC_000002.12	-	229966	234272	4307	490	sense-overlappin	NM_001282687.1	SH3YL1
circRNA_01242	0.020575025	0	0	0	0	0	0	0	0	0	0	0	-	NC_000002.12	-	1687632	1708796	21165	15878	sense-overlappin	NM_012293.2	PXDN
circRNA_01243	0	0	0	0	0	0	0	0	0	0	0	0.0412728	-	NC_000002.12	+	6951318	6955997	4680	4680	intronic	NM_014746.4	RNF144A
circRNA_01244	0.030862537	0	0	0	0	0	0	0	0	0	0	0	-	NC_000002.12	+	6996916	7030215	33300	758	sense-overlappin	NM_014746.4	RNF144A
circRNA_01245	0.030862537	0	0	0.0206362	0.04012623	0.020712909	0	0	0.101272639	0.040344966	0	0.0309546	hsa_circ_0005315	NC_000002.12	-	8770670	8776892	6223	308	sense-overlappin	NM_020738.2	KIDINS220
circRNA_01246	0	0	0	0.0412724	0	0	0	0	0	0	0	0	-	NC_000002.12	-	8788647	8803127	14481	1184	sense-overlappin	NM_020738.2	KIDINS220
circRNA_01247	0	0	0	0	0	0	0	0.020486295	0	0	0	0	-	NC_000002.12	-	8800400	8803127	2728	297	sense-overlappin	NM_020738.2	KIDINS220
circRNA_01248	0	0	0.032712968	0	0	0	0	0	0	0	0	0	hsa_circ_0052508	NC_000002.12	-	8877030	8958642	81613	615	sense-overlappin	NM_138799.2	MBOAT2
circRNA_01249	0.041150049	0	0.021808645	0	0	0	0	0	0	0.020172483	0	0	hsa_circ_0005079	NC_000002.12	-	8882511	8958642	76132	431	sense-overlappin	NM_138799.2	MBOAT2
circRNA_01250	0	0	0.054521613	0	0	0	0	0	0	0	0	0	hsa_circ_0008847	NC_000002.12	-	8888018	8958642	70625	376	sense-overlappin	NM_138799.2	MBOAT2
circRNA_01251	0.041150049	0	0.370746965	0.0619086	0.06018934	0.113921001	0.070345399	0.040972591	0.060763583	0.14120738	0.06076569	0.072227401	hsa_circ_0000972	NC_000002.12	-	8908621	8958642	50022	320	sense-overlappin	NM_138799.2	MBOAT2
circRNA_01252	0	0	0.021808645	0	0	0	0	0	0	0	0	0	-	NC_000002.12	-	8908621	8974435	65815	16113	sense-overlappin	NM_138799.2	MBOAT2
circRNA_01253	0	0	0.119947548	0	0	0.051782273	0.050246713	0	0	0.020172483	0	0	hsa_circ_0000973	NC_000002.12	-	8939821	8958642	18822	3590	sense-overlappin	NM_138799.2	MBOAT2
circRNA_01254	0	0	0.327129675	0.0515905	0.03009467	0.051782273	0.030148028	0.051215739	0	0	0.050638075	0	hsa_circ_0007334	NC_000002.12	-	8943187	8958642	15456	224	sense-overlappin	NM_138799.2	MBOAT2
circRNA_01255	0	0	0.021808645	0	0	0	0	0	0	0	0	0	-	NC_000002.12	-	8943187	8962618	19432	4200	sense-overlappin	NM_138799.2	MBOAT2
circRNA_01256	0.020575025	0	0.098138903	0	0	0.031069364	0	0	0	0.030258724	0.030382845	0	-	NC_000002.12	-	8943187	8974435	31249	16017	sense-overlappin	NM_138799.2	MBOAT2
circRNA_01257	0	0	0	0	0	0.020712909	0	0	0	0	0	0	-	NC_000002.12	-	8943187	8982144	38958	23726	sense-overlappin	NM_138799.2	MBOAT2
circRNA_01258	0	0	0	0	0	0	0	0.020486295	0	0	0	0	-	NC_000002.12	-	8943187	8987761	44575	29343	sense-overlappin	NM_138799.2	MBOAT2
circRNA_01259	0	0	0.021808645	0	0	0	0	0	0	0	0	0	hsa_circ_0005085	NC_000002.12	+	9279317	9297445	18129	328	sense-overlappin	XM_011510409.1	ASAP2
circRNA_01260	0	0	0	0	0	0	0	0	0	0	0	0.0412728	-	NC_000002.12	+	9289671	9289757	87	87	intronic	NM_001135191.1	ASAP2
circRNA_01261	0	0	0.04361729	0	0	0	0	0	0	0	0	0	-	NC_000002.12	+	9344532	9374944	30413	897	sense-overlappin	NM_001135191.1	ASAP2
circRNA_01262	0	0	0	0	0	0	0	0	0	0.020172483	0	0	hsa_circ_0006089	NC_000002.12	+	9350808	9358889	8082	438	sense-overlappin	NM_001135191.1	ASAP2
circRNA_01263	0	0	0.021808645	0	0	0	0	0	0	0	0	0	hsa_circ_0008768	NC_000002.12	+	9350808	9368519	17712	533	sense-overlappin	NM_001135191.1	ASAP2
circRNA_01264	0	0	0.021808645	0	0	0	0	0	0	0	0	0	hsa_circ_0009090	NC_000002.12	+	9356047	9358889	2843	350	sense-overlappin	NM_001135191.1	ASAP2
circRNA_01265	0.020575025	0	0	0	0	0	0	0	0	0	0	0	hsa_circ_0052529	NC_000002.12	+	9356047	9380808	24762	905	sense-overlappin	NM_001135191.1	ASAP2
circRNA_01266	0	0	0	0	0	0	0	0	0	0.030258724	0	0	-	NC_000002.12	-	9408113	9418732	10620	416	sense-overlappin	NM_004763.3	ITGB1BP1
circRNA_01267	0	0.030704951	0	0	0.02006311	0	0	0	0	0	0	0.0309546	-	NC_000002.12	-	9535834	9536828	995	220	sense-overlappin	NM_003183.4	ADAM17
circRNA_01268	0	0	0	0	0.03009467	0	0	0	0	0	0	0	-	NC_000002.12	+	9995879	9999029	3151	243	sense-overlappin	XM_006711882.1	GRHL1
circRNA_01269	0.030862537	0	0	0	0	0	0	0	0	0	0	0	hsa_circ_0052613	NC_000002.12	-	10644320	10663044	18725	435	sense-overlappin	NM_001261392.1	NOL10
circRNA_01270	0.030862537	0.020469968	0.032712968	0.0309543	0	0	0	0	0	0.030258724	0.04051046	0.0412728	hsa_circ_0000977	NC_000002.12	-	10644320	10668723	24404	562	sense-overlappin	NM_001261392.1	NOL10
circRNA_01271	0	0	0.021808645	0	0	0	0	0	0	0	0	0	-	NC_000002.12	-	10644320	10684612	40293	960	sense-overlappin	NM_024894.3	NOL10

circRNA_01272	0	0	0	0	0	0	0	0	0	0.020172483	0.02025523	0	hsa_circ_0008511	NC_000002.12	-	10659172	10668723	9552	292	sense-overlappin	NM_001261392.1	NOL10
circRNA_01273	0	0	0.021808645	0	0	0	0	0	0	0	0	0	hsa_circ_0052620	NC_000002.12	-	10788697	10789889	1193	299	sense-overlappin	XM_011510308.1	PDIA6
circRNA_01274	0	0	0	0.0206362	0	0.020712909	0	0	0	0	0	0	hsa_circ_0052621	NC_000002.12	-	10788697	10790833	2137	414	sense-overlappin	XM_011510308.1	PDIA6
circRNA_01275	0	0	0	0	0	0	0	0	0.020254528	0	0	0	-	NC_000002.12	-	10788697	10793202	4506	652	sense-overlappin	XM_011510308.1	PDIA6
circRNA_01276	0	0	0	0	0	0	0	0	0.020254528	0	0	0	hsa_circ_0007250	NC_000002.12	-	10791795	10793202	1408	238	sense-overlappin	XM_011510308.1	PDIA6
circRNA_01277	0	0	0.021808645	0	0	0	0	0	0	0	0	0	-	NC_000002.12	-	11200957	11202121	1165	361	sense-overlappin	XM_005246190.3	ROCK2
circRNA_01278	0	0	0	0	0	0	0	0	0	0	0	0.0206364	hsa_circ_0005444	NC_000002.12	-	11235702	11235962	261	261	exonic	XM_005246190.3	ROCK2
circRNA_01279	0.041150049	0.040939935	0.04361729	0	0	0.020712909	0.020098685	0	0	0.020172483	0	0	hsa_circ_0007494	NC_000002.12	-	11286539	11288449	1911	896	sense-overlappin	XM_005246190.3	ROCK2
circRNA_01280	0.030862537	0	0	0.0412724	0	0	0	0	0.030381792	0.040344966	0	0	hsa_circ_0002229	NC_000002.12	+	11765533	11767858	2326	297	sense-overlappin	XM_011510338.1	LPIN1
circRNA_01281	0	0	0	0	0	0.031069364	0	0	0	0	0	0	hsa_circ_0002914	NC_000002.12	+	11802907	11805156	2250	363	sense-overlappin	XM_006711869.1	LPIN1
circRNA_01282	0	0	0.021808645	0	0	0	0	0	0	0	0	0	-	NC_000002.12	-	14986664	14998224	11561	11561	intergenic		
circRNA_01283	0	0	0.021808645	0	0	0	0	0	0	0	0	0	hsa_circ_0052713	NC_000002.12	-	15275484	15309247	33764	1142	sense-overlappin	NM_015909.3	NBAS
circRNA_01284	0	0	0	0.0309543	0	0	0	0	0	0	0	0	hsa_circ_0052736	NC_000002.12	-	15351992	15427794	75803	1840	sense-overlappin	NM_015909.3	NBAS
circRNA_01285	0	0	0.04361729	0	0	0	0	0	0	0	0	0	hsa_circ_0009014	NC_000002.12	-	15374608	15417712	43105	1126	sense-overlappin	NM_015909.3	NBAS
circRNA_01286	0	0	0.032712968	0	0	0	0	0	0	0.030258724	0	0	hsa_circ_0052750	NC_000002.12	-	15394227	15417712	23486	680	sense-overlappin	NM_015909.3	NBAS
circRNA_01287	0.020575025	0	0	0.0309543	0	0.020712909	0	0	0	0	0	0	hsa_circ_0008522	NC_000002.12	-	15394227	15427794	33568	918	sense-overlappin	NM_015909.3	NBAS
circRNA_01288	0	0	0	0.0206362	0	0	0	0	0	0	0	0	-	NC_000002.12	-	15415546	15478289	62744	1854	sense-overlappin	NM_015909.3	NBAS
circRNA_01289	0	0	0	0	0	0	0	0	0	0	0	0.0206364	-	NC_000002.12	-	15415546	15511350	95805	2191	sense-overlappin	NM_015909.3	NBAS
circRNA_01290	0	0.051174919	0.098138903	0.1341352	0.02006311	0.031069364	0.040197371	0	0.020254528	0.080689931	0.030382845	0	hsa_circ_0008083	NC_000002.12	-	15461201	15478289	17089	1256	sense-overlappin	NM_015909.3	NBAS
circRNA_01291	0	0	0.032712968	0	0.05015779	0.062138728	0	0	0.040509055	0	0	0	hsa_circ_0052767	NC_000002.12	-	15461201	15511350	50150	1593	sense-overlappin	NM_015909.3	NBAS
circRNA_01292	0.020575025	0.030704951	0.032712968	0.0515905	0.02006311	0	0.050246713	0.09218833	0	0.050431207	0	0.0309546	hsa_circ_0000978	NC_000002.12	-	15488894	15511350	22457	337	sense-overlappin	NM_015909.3	NBAS
circRNA_01293	0	0	0.021808645	0	0	0	0.040197371	0	0	0	0	0.0206364	hsa_circ_0007128	NC_000002.12	-	15488894	15534641	45748	436	sense-overlappin	NM_015909.3	NBAS
circRNA_01294	0	0	0.021808645	0	0	0	0	0	0	0.040344966	0	0	hsa_circ_0008061	NC_000002.12	-	15488894	15539356	50463	704	sense-overlappin	NM_015909.3	NBAS
circRNA_01295	0.030862537	0	0	0.0309543	0	0	0	0	0	0.040344966	0	0	hsa_circ_0004665	NC_000002.12	-	15504145	15539356	35212	575	sense-overlappin	NM_015909.3	NBAS
circRNA_01296	0	0	0.04361729	0.0309543	0	0	0	0	0	0	0	0	hsa_circ_0052787	NC_000002.12	-	15534543	15539356	4814	367	sense-overlappin	NM_015909.3	NBAS
circRNA_01297	0	0	0.076330258	0.0619086	0.03009467	0	0	0.020486295	0.020254528	0.060517448	0.04051046	0	hsa_circ_0003287	NC_000002.12	-	15551493	15558634	7142	262	sense-overlappin	NM_015909.3	NBAS
circRNA_01298	0	0	0	0	0	0	0.020098685	0	0	0.050431207	0	0	hsa_circ_0005556	NC_000002.12	-	15553426	15558634	5209	218	sense-overlappin	NM_015909.3	NBAS
circRNA_01299	0	0	0.04361729	0	0	0	0	0	0	0	0	0	-	NC_000002.12	-	17665503	17745951	80449	3334	sense-overlappin	XM_011533107.1	SMC6
circRNA_01300	0	0	0.021808645	0	0	0	0	0	0	0	0	0	-	NC_000002.12	+	17761396	17768811	7416	549	sense-overlappin	XM_011532822.1	GEN1
circRNA_01301	0	0	0.021808645	0.0412724	0	0	0	0	0	0	0	0	-	NC_000002.12	+	18644170	18688328	44159	44159	intergenic		
circRNA_01302	0.020575025	0	0	0.0206362	0	0	0.020098685	0	0	0.020172483	0	0	hsa_circ_0005952	NC_000002.12	-	20254863	20256170	1308	386	sense-overlappin	NM_001282790.1	PUM2
circRNA_01303	0	0	0	0	0	0	0	0	0	0.020172483	0	0	-	NC_000002.12	+	20267297	20268380	1084	1084	antisense	NM_001282790.1	PUM2
circRNA_01304	0	0	0.109043225	0.0619086	0.02006311	0	0	0.030729443	0.020254528	0	0	0.051591	hsa_circ_0052852	NC_000002.12	-	20278583	20327378	48796	2100	sense-overlappin	XM_006711975.2	PUM2
circRNA_01305	0	0	0.021808645	0	0	0	0	0	0	0	0	0	-	NC_000002.12	-	20278583	20331937	53355	2196	sense-overlappin	XM_011532719.1	PUM2
circRNA_01306	0	0.020469968	0.032712968	0	0	0	0	0	0	0	0	0	-	NC_000002.12	-	20278583	20333063	54481	2293	sense-overlappin	XM_011532719.1	PUM2
circRNA_01307	0	0	0.076330258	0	0.02006311	0	0	0	0	0	0	0	-	NC_000002.12	-	20282947	20327378	44432	1863	sense-overlappin	NM_001282791.1	PUM2
circRNA_01308	0	0	0.04361729	0	0	0	0	0	0	0	0	0	hsa_circ_0003733	NC_000002.12	-	20297553	20327378	29826	1152	sense-overlappin	NM_001282791.1	PUM2
circRNA_01309	0	0	0.021808645	0	0	0	0	0	0	0	0	0	hsa_circ_0001976	NC_000002.12	-	20307978	20318645	10668	832	sense-overlappin	NM_001282790.1	PUM2
circRNA_01310	0	0.020469968	0.13085187	0	0	0	0	0.040972591	0.040509055	0	0	0.0206364	hsa_circ_0052867	NC_000002.12	-	20307978	20327378	19401	1026	sense-overlappin	NM_001282791.1	PUM2
circRNA_01311	0	0	0.032712968	0	0	0	0	0	0	0	0	0	hsa_circ_0004836	NC_000002.12	-	20307978	20333063	25086	1219	sense-overlappin	XM_011532719.1	PUM2
circRNA_01312	0	0	0	0	0	0	0	0	0.020254528	0	0	0	hsa_circ_0004011	NC_000002.12	-	20326261	20327378	1118	194	sense-overlappin	NM_001282791.1	PUM2
circRNA_01313	0	0	0	0	0	0.020712909	0	0	0	0	0	0	hsa_circ_0052877	NC_000002.12	-	20739971	20740205	235	235	exonic	NM_001282719.1	C2orf43
circRNA_01314	0.020575025	0	0	0	0	0	0	0	0	0	0	0	hsa_circ_0002271	NC_000002.12	-	20774810	20790398	15589	314	sense-overlappin	NM_001282721.1	C2orf43
circRNA_01315	0	0	0	0	0	0	0	0	0	0	0.02025523	0	-	NC_000002.12	+	23555427	23555936	510	510	intronic	NM_052920.1	KLHL29
circRNA_01316	0	0	0	0	0	0	0	0	0	0.020172483	0	0	-	NC_000002.12	-	23792350	23792716	367	367	intronic	XM_006712030.2	ATAD2B
circRNA_01317	0	0	0	0	0	0	0.020098685	0	0.020254528	0	0	0	hsa_circ_0052943	NC_000002.12	-	23798138	23834078	35941	1156	sense-overlappin	NR_125717.1	
circRNA_01318	0	0.030704951	0	0.0309543	0	0	0.060296056	0	0.040509055	0.040344966	0	0	hsa_circ_0003828	NC_000002.12	-	23823258	23823569	312	312	exonic	XM_006712030.2	ATAD2B
circRNA_01319	0.041150049	0	0	0	0	0	0	0	0	0.030258724	0	0	hsa_circ_0052955	NC_000002.12	-	23857415	23888399	30985	1242	sense-overlappin	XM_006712030.2	ATAD2B
circRNA_01320	0	0	0	0.0206362	0.02006311	0	0	0	0	0.020172483	0	0	hsa_circ_0052962	NC_000002.12	-	23875829	23885829	10001	447	sense-overlappin	XM_006712030.2	ATAD2B
circRNA_01321	0	0.030704951	0.032712968	0	0	0	0	0	0.020254528	0.020172483	0.02025523	0	hsa_circ_0052963	NC_000002.12	-	23880639	23885829	5191	371	sense-overlappin	XM_006712030.2	ATAD2B
circRNA_01322	0	0	0	0	0	0	0	0	0	0	0.02025523	0	hsa_circ_0052964	NC_000002.12	-	23880639	23888399	7761	575	sense-overlappin	XM_006712030.2	ATAD2B
circRNA_01323	0	0	0	0	0	0	0	0.020486295	0	0	0	0	hsa_circ_0052965	NC_000002.12	-	23884765	23885829	1065	212	sense-overlappin	XM_006712030.2	ATAD2B
circRNA_01324	0.030862537	0	0.021808645	0.0206362	0.02006311	0	0.020098685	0.061458886	0	0.090776173	0.02025523	0	hsa_circ_0005970	NC_000002.12	+	23958301	23977075	18775	301	sense-overlappin	XM_011532632.1	UBXN2A
circRNA_01325	0.051437562	0	0.119947548	0.1031809	0.03009467	0.041425819	0.060296056	0	0.040509055	0.060517448	0.030382845	0.0412728	hsa_circ_0052967	NC_000002.12	+	23958301	23984831	26531	598	sense-overlappin	XM_011532632.1	UBXN2A

circRNA_01326	0.020575025	0	0.065425935	0.0309543	0.02006311	0	0	0	0	0.050431207	0.02025523	0	hsa_circ_0003947	NC_000002.12	+	23982896	23984831	1936	297	sense-overlappin	XM_011532632.1	UBXN2A
circRNA_01327	0	0	0	0	0	0	0	0	0.030381792	0	0	0	-	NC_000002.12	+	24053902	24063782	9881	240	sense-overlappin	XM_011532699.1	FKBP1B
circRNA_01328	0	0	0.021808645	0	0	0	0	0	0	0	0	0	hsa_circ_0005938	NC_000002.12	+	24060814	24063782	2969	192	sense-overlappin	XM_011532699.1	FKBP1B
circRNA_01329	0.020575025	0	0.021808645	0.0309543	0	0.031069364	0	0	0.040509055	0	0	0	hsa_circ_0007270	NC_000002.12	+	24135119	24139450	4332	342	sense-overlappin	NM_001145710.1	FAM228B
circRNA_01330	0.226325271	0.102349838	0.196277805	0.3508152	0.02006311	0.082851637	0.060296056	0.061458886	0.081018111	0.302587242	0.10127615	0.175409401	hsa_circ_0000982	NC_000002.12	+	24135119	24147086	11968	587	sense-overlappin	NM_001145710.1	FAM228B
circRNA_01331	0	0	0	0.0309543	0	0.031069364	0	0	0	0	0	0	-	NC_000002.12	+	24135119	24161613	26495	695	sense-overlappin	NM_001145710.1	FAM228B
circRNA_01332	0	0	0	0	0	0	0	0	0	0	0.02025523	0.0206364	-	NC_000002.12	+	24137909	24147086	9178	518	sense-overlappin	NM_001145710.1	FAM228B
circRNA_01333	0.030862537	0	0	0	0	0	0	0	0	0	0	0	hsa_circ_0008073	NC_000002.12	+	24139370	24147086	7717	326	sense-overlappin	NM_001291328.1	FAM228B
circRNA_01334	0	0	0.021808645	0	0	0	0	0	0	0.020172483	0	0	hsa_circ_0052978	NC_000002.12	-	24246129	24254431	8303	689	sense-overlappin	NM_006277.2	ITSN2
circRNA_01335	0	0	0	0	0	0	0	0	0	0	0.02025523	0	-	NC_000002.12	-	24257888	24312375	54488	2700	sense-overlappin	NM_006277.2	ITSN2
circRNA_01336	0	0	0	0.0206362	0	0	0	0	0	0	0	0.051591	-	NC_000002.12	-	24270671	24271941	1271	274	sense-overlappin	NM_006277.2	ITSN2
circRNA_01337	0	0.051174919	0	0	0.02006311	0	0	0	0	0.030258724	0	0	hsa_circ_0052983	NC_000002.12	-	24270671	24275849	5179	411	sense-overlappin	NM_006277.2	ITSN2
circRNA_01338	0.030862537	0	0.021808645	0	0	0	0.020098685	0	0	0.030258724	0	0.0412728	hsa_circ_0006934	NC_000002.12	+	24510969	24584560	73592	53547	sense-overlappin	XM_005264625.1	NCOA1
circRNA_01339	0	0	0.032712968	0	0	0	0	0	0	0	0	0	-	NC_000002.12	+	24536715	24584560	47846	27801	sense-overlappin	XM_005264625.1	NCOA1
circRNA_01340	0	0	0	0.0206362	0	0	0	0.040972591	0	0	0	0	-	NC_000002.12	+	24554389	24556055	1667	1667	intronic	XM_005264625.1	NCOA1
circRNA_01341	0	0	0.04361729	0	0	0	0	0.020486295	0	0	0	0	-	NC_000002.12	+	24554389	24564430	10042	10042	sense-overlappin	XM_005264625.1	NCOA1
circRNA_01342	0.030862537	0.051174919	0	0.0206362	0	0	0.030148028	0.020486295	0.020254528	0.020172483	0	0	hsa_circ_0004938	NC_000002.12	+	24554389	24584560	30172	10127	sense-overlappin	XM_005264625.1	NCOA1
circRNA_01343	0	0	0	0	0	0	0	0.020486295	0	0.020172483	0	0	-	NC_000002.12	+	24554389	24593721	39333	19288	sense-overlappin	XM_005264625.1	NCOA1
circRNA_01344	0	0	0	0	0	0	0	0	0.040509055	0	0	0	-	NC_000002.12	+	24556015	24564430	8416	8416	sense-overlappin	XM_005264625.1	NCOA1
circRNA_01345	0	0	0.032712968	0.0619086	0	0.020712909	0.020098685	0	0	0	0.02025523	0	-	NC_000002.12	+	24556015	24584560	28546	8501	sense-overlappin	XM_005264625.1	NCOA1
circRNA_01346	0	0.020469968	0	0	0	0	0.020098685	0	0.020254528	0.020172483	0	0	hsa_circ_0000983	NC_000002.12	+	24563904	24584560	20657	612	sense-overlappin	XM_005264625.1	NCOA1
circRNA_01347	0.030862537	0	0.032712968	0.0206362	0	0.020712909	0.020098685	0	0.030381792	0	0.02025523	0	hsa_circ_0005968	NC_000002.12	+	24564295	24584560	20266	221	sense-overlappin	XM_005264625.1	NCOA1
circRNA_01348	0	0.040939935	0	0.0206362	0.05015779	0.020712909	0.030148028	0	0	0.020172483	0.050638075	0	-	NC_000002.12	+	24564295	24593721	29427	9382	sense-overlappin	XM_005264625.1	NCOA1
circRNA_01349	0	0	0	0	0	0	0	0	0	0.020172483	0	0	-	NC_000002.12	+	24584476	24673463	88988	613	sense-overlappin	XM_005264625.1	NCOA1
circRNA_01350	0	0	0	0	0	0	0.040197371	0	0	0	0	0	-	NC_000002.12	+	24643966	24673463	29498	528	sense-overlappin	XM_005264625.1	NCOA1
circRNA_01351	0	0	0.032712968	0	0	0	0	0	0	0	0	0	-	NC_000002.12	+	24643966	24683128	39163	706	sense-overlappin	XM_005264625.1	NCOA1
circRNA_01352	0	0	0.021808645	0	0	0	0	0	0	0	0	0	hsa_circ_0053040	NC_000002.12	-	25607236	25628384	21149	300	sense-overlappin	NM_001256308.1	DTNB
circRNA_01353	0	0	0.054521613	0	0.03009467	0	0	0	0.020254528	0.020172483	0	0	hsa_circ_0004476	NC_000002.12	-	25799385	25806337	6953	260	sense-overlappin	XM_011532951.1	ASXL2
circRNA_01354	0	0	0.04361729	0.0206362	0	0	0	0	0	0	0	0	-	NC_000002.12	-	25799385	25845563	46179	346	sense-overlappin	NM_018263.4	ASXL2
circRNA_01355	0	0	0	0	0	0	0.040197371	0.030729443	0.020254528	0.040344966	0.030382845	0	-	NC_000002.12	+	26069574	26069800	227	227	intronic	NM_016131.4	RAB10
circRNA_01356	0.051437562	0	0	0	0	0	0	0	0	0	0	0	hsa_circ_0053063	NC_000002.12	-	26230192	26234355	4164	362	sense-overlappin	NM_000182.4	HADHA
circRNA_01357	0	0.020469968	0.032712968	0.0515905	0.05015779	0	0.020098685	0.040972591	0.050636319	0.040344966	0	0	hsa_circ_0008257	NC_000002.12	+	26263380	26277160	13781	333	sense-overlappin	NM_000183.2	HADHB
circRNA_01358	0	0	0	0	0.02006311	0	0	0	0	0	0	0	-	NC_000002.12	+	26279994	26284957	4964	413	sense-overlappin	NM_000183.2	HADHB
circRNA_01359	0	0	0.021808645	0	0	0	0	0	0	0	0	0	hsa_circ_0053083	NC_000002.12	+	26279994	26285571	5578	578	sense-overlappin	NM_000183.2	HADHB
circRNA_01360	0.030862537	0	0	0.0515905	0	0.041425819	0	0.020486295	0.030381792	0.030258724	0	0	hsa_circ_0005029	NC_000002.12	+	26364302	26373629	9328	516	sense-overlappin	NM_033505.2	EPT1
circRNA_01361	0	0	0	0	0	0	0	0	0	0.020172483	0	0	-	NC_000002.12	+	26364302	26375089	10788	566	sense-overlappin	NM_033505.2	EPT1
circRNA_01362	0	0	0.032712968	0	0	0	0	0	0.040509055	0	0	0.051591	hsa_circ_0004269	NC_000002.12	+	26364302	26375148	10847	625	sense-overlappin	NM_033505.2	EPT1
circRNA_01363	0	0	0.032712968	0	0.02006311	0	0	0	0	0	0	0	hsa_circ_0007325	NC_000002.12	+	26373367	26375148	1782	372	sense-overlappin	NM_033505.2	EPT1
circRNA_01364	0	0	0.021808645	0	0	0	0	0	0	0	0	0	-	NC_000002.12	+	27058400	27059404	1005	418	sense-overlappin	XM_011533011.1	AGBL5
circRNA_01365	0	0	0.076330258	0	0	0	0	0	0	0	0	0	-	NC_000002.12	-	27207639	27212146	4508	346	sense-overlappin	NM_021095.2	SLC5A6
circRNA_01366	0	0	0	0	0.02006311	0	0	0	0	0	0	0	-	NC_000002.12	-	27207659	27212146	4488	326	sense-overlappin	NM_021095.2	SLC5A6
circRNA_01367	0.020575025	0.020469968	0.032712968	0	0	0	0	0.040972591	0	0.030258724	0	0	-	NC_000002.12	-	27350314	27350632	319	319	exonic	NM_001521.3	GTF3C2
circRNA_01368	0	0	0	0	0.02006311	0	0	0	0	0	0	0	-	NC_000002.12	+	27433249	27437360	4112	928	sense-overlappin	NM_013392.2	NRBP1
circRNA_01369	0	0	0	0	0	0	0	0	0	0.020172483	0	0	-	NC_000002.12	-	27477217	27484266	7050	1029	sense-overlappin	XM_005264254.1	IFT172
circRNA_01370	0.041150049	0	0.04361729	0.0206362	0	0.031069364	0	0	0	0.060517448	0	0.0412728	hsa_circ_0000985	NC_000002.12	+	27599583	27601442	1860	392	sense-overlappin	NM_001271287.1	ZNF512
circRNA_01371	0	0	0	0	0	0	0	0	0	0.020172483	0.02025523	0	-	NC_000002.12	+	27599583	27603307	3725	659	sense-overlappin	NM_001271287.1	ZNF512
circRNA_01372	0	0	0.032712968	0	0	0	0	0	0	0	0	0	-	NC_000002.12	+	27668843	27677924	9082	619	sense-overlappin	NM_018158.2	SLC4A1AP
circRNA_01373	0	0	0	0	0.02006311	0	0	0	0	0	0	0	-	NC_000002.12	+	27677295	27682359	5065	369	sense-overlappin	NM_018158.2	SLC4A1AP
circRNA_01374	0	0	0	0	0	0.020712909	0	0	0	0	0	0	-	NC_000002.12	+	27677295	27685277	7983	610	sense-overlappin	NM_018158.2	SLC4A1AP
circRNA_01375	0	0	0.032712968	0	0	0	0	0	0	0	0	0	-	NC_000002.12	+	28752038	28799299	47262	1067	sense-overlappin	NM_206876.1	PPP1CB
circRNA_01376	0	0	0.032712968	0	0	0	0	0	0	0	0	0	hsa_circ_0003905	NC_000002.12	+	28776851	28783978	7128	540	sense-overlappin	NM_206876.1	PPP1CB
circRNA_01377	0	0	0.032712968	0	0.02006311	0	0	0	0	0	0	0	hsa_circ_0053325	NC_000002.12	+	28776851	28793997	17147	827	sense-overlappin	NM_206876.1	PPP1CB
circRNA_01378	0	0	0.021808645	0	0	0	0	0	0	0	0	0.0412728	-	NC_000002.12	+	28776851	28799433	22583	1062	sense-overlappin	NM_206876.1	PPP1CB
circRNA_01379	0	0	0	0	0	0	0	0	0	0	0.030382845	0	-	NC_000002.12	+	28781804	28799433	17630	633	sense-overlappin	NM_206876.1	PPP1CB
circRNA_01380	0	0	0.021808645	0	0	0	0	0	0.050636319	0	0	0.0206364	hsa_circ_0007439	NC_000002.12	+	28783907	28788809	4903	224	sense-overlappin	NM_206876.1	PPP1CB

circRNA_01381	0.020575025	0	0	0	0	0	0	0	0	0	0	0	-	NC_000002.12	+	28783907	28799433	15527	594	sense-overlappin	NM_206876.1	PPP1CB
circRNA_01382	0	0	0.021808645	0	0	0	0	0	0	0	0	0	hsa_circ_0002583	NC_000002.12	-	28850328	28865119	14792	691	sense-overlappin	NM_017910.3	TRMT61B
circRNA_01383	0	0	0	0	0.02006311	0	0	0	0	0	0	0	hsa_circ_0002025	NC_000002.12	+	28906460	28914208	7749	383	sense-overlappin	NM_015131.2	WDR43
circRNA_01384	0	0	0.021808645	0	0	0	0	0	0	0	0	0	-	NC_000002.12	+	28906460	28929710	23251	1074	sense-overlappin	NM_015131.2	WDR43
circRNA_01385	0	0	0.021808645	0	0	0	0	0	0	0	0	0.0206364	-	NC_000002.12	+	28941461	28947161	5701	796	sense-overlappin	NM_015131.2	WDR43
circRNA_01386	0	0	0.065425935	0	0	0	0	0	0	0.020172483	0	0	-	NC_000002.12	+	29121374	29135666	14293	663	sense-overlappin	NM_001287528.1	CLIP4
circRNA_01387	0	0	0	0	0	0.041425819	0	0.020486295	0	0	0	0	-	NC_000002.12	+	29121374	29181697	60324	1937	sense-overlappin	XM_005264562.2	CLIP4
circRNA_01388	0	0.020469968	0.021808645	0	0	0	0.020098685	0	0	0.020172483	0	0	hsa_circ_0006348	NC_000002.12	+	29131258	29135666	4409	515	sense-overlappin	NM_001287528.1	CLIP4
circRNA_01389	0	0	0	0.0206362	0	0	0	0	0	0	0	0	hsa_circ_0006908	NC_000002.12	+	29133655	29145367	11713	654	sense-overlappin	NM_001287528.1	CLIP4
circRNA_01390	0	0	0.021808645	0	0	0	0	0	0	0	0	0	hsa_circ_0004337	NC_000002.12	+	30459552	30533314	73763	773	sense-overlappin	NM_001304445.1	LCLAT1
circRNA_01391	0	0	0	0	0.02006311	0	0	0	0	0	0	0	-	NC_000002.12	+	30459552	30552649	93098	20108	sense-overlappin	NM_001304445.1	LCLAT1
circRNA_01392	0	0	0.152660515	0.0206362	0.06018934	0	0	0.030729443	0.030381792	0.030258724	0	0	hsa_circ_0000987	NC_000002.12	+	30525587	30533314	7728	368	sense-overlappin	NM_001002257.2	LCLAT1
circRNA_01393	0	0	0	0	0	0.020712909	0	0	0	0	0	0	hsa_circ_0053375	NC_000002.12	+	30525587	30568176	42590	789	sense-overlappin	XM_011532744.1	LCLAT1
circRNA_01394	0	0	0.021808645	0	0	0	0	0	0	0	0	0	hsa_circ_0003001	NC_000002.12	-	31891992	31932135	40144	437	sense-overlappin	XR_244937.2	
circRNA_01395	0	0	0	0	0	0	0	0	0	0.040344966	0	0	hsa_circ_0053394	NC_000002.12	-	31917926	31943383	25458	376	sense-overlappin	XR_244937.2	
circRNA_01396	0	0	0.021808645	0	0	0	0	0	0	0	0	0	-	NC_000002.12	-	31917926	31950717	32792	7710	sense-overlappin	XR_244937.2	
circRNA_01397	0	0	0	0	0	0.020712909	0	0	0	0	0	0	hsa_circ_0053408	NC_000002.12	+	32087492	32116212	28721	683	sense-overlappin	XM_011533067.1	SPAST
circRNA_01398	0	0	0.021808645	0	0	0	0	0	0	0.030258724	0	0	-	NC_000002.12	+	32098796	32116212	17417	512	sense-overlappin	XM_011533067.1	SPAST
circRNA_01399	0	0	0	0.0206362	0	0	0	0	0	0	0	0	hsa_circ_0053410	NC_000002.12	+	32098796	32128479	29684	659	sense-overlappin	XM_011533067.1	SPAST
circRNA_01400	0	0	0.032712968	0	0	0.020712909	0	0	0	0.020172483	0	0	hsa_circ_0002795	NC_000002.12	+	32114638	32116212	1575	416	sense-overlappin	XM_011533067.1	SPAST
circRNA_01401	0	0	0	0	0	0.020712909	0	0	0	0	0	0	hsa_circ_0053413	NC_000002.12	+	32114638	32128479	13842	563	sense-overlappin	XM_011533067.1	SPAST
circRNA_01402	0	0	0	0	0	0	0	0	0.020254528	0	0	0	hsa_circ_0053419	NC_000002.12	+	32136563	32147258	10696	483	sense-overlappin	XM_005264516.3	SPAST
circRNA_01403	0	0	0.021808645	0	0.03009467	0.020712909	0.040197371	0	0	0	0	0	hsa_circ_0005695	NC_000002.12	+	32174063	32184338	10276	314	sense-overlappin	NM_001193513.1	SLC30A6
circRNA_01404	0.020575025	0.030704951	0.065425935	0.1341352	0	0	0.050246713	0.020486295	0	0	0.030382845	0	hsa_circ_0053441	NC_000002.12	+	32377588	32395593	18006	709	sense-overlappin	NM_016252.3	BIRC6
circRNA_01405	0	0	0	0	0.02006311	0	0	0	0	0	0	0	hsa_circ_0007337	NC_000002.12	+	32377588	32401623	24036	1093	sense-overlappin	NM_016252.3	BIRC6
circRNA_01406	0.041150049	0.020469968	0.032712968	0.0309543	0	0	0.030148028	0.030729443	0.030381792	0.020172483	0	0	hsa_circ_0000989	NC_000002.12	+	32377588	32406557	28970	1152	sense-overlappin	NM_016252.3	BIRC6
circRNA_01407	0	0	0.054521613	0.0206362	0	0.093208092	0.030148028	0	0	0	0	0	hsa_circ_0053442	NC_000002.12	+	32377588	32416163	38576	2547	sense-overlappin	NM_016252.3	BIRC6
circRNA_01408	0	0	0.054521613	0	0	0	0	0	0	0	0	0	hsa_circ_0053450	NC_000002.12	+	32377588	32453942	76355	4428	sense-overlappin	NM_016252.3	BIRC6
circRNA_01409	0	0	0	0	0	0.051782273	0	0	0	0	0	0.0309546	hsa_circ_0053517	NC_000002.12	+	32401163	32433804	32642	2375	sense-overlappin	NM_016252.3	BIRC6
circRNA_01410	0	0	0	0	0.03009467	0	0	0	0	0	0	0	hsa_circ_0000990	NC_000002.12	+	32406499	32416163	9665	1454	sense-overlappin	NM_016252.3	BIRC6
circRNA_01411	0.020575025	0	0.076330258	0.0412724	0	0.031069364	0.020098685	0.020486295	0	0.050431207	0.06076569	0	hsa_circ_0053535	NC_000002.12	+	32414769	32416163	1395	1395	exonic	NM_016252.3	BIRC6
circRNA_01412	0	0.030704951	0	0	0	0	0	0	0	0	0	0	-	NC_000002.12	+	32439508	32441462	1955	313	sense-overlappin	NM_016252.3	BIRC6
circRNA_01413	0	0	0	0.0206362	0	0	0	0	0	0	0	0	hsa_circ_0005207	NC_000002.12	+	32439508	32443588	4081	705	sense-overlappin	NM_016252.3	BIRC6
circRNA_01414	0	0.030704951	0.021808645	0.0515905	0.03009467	0	0.020098685	0	0	0.020172483	0	0	hsa_circ_0053593	NC_000002.12	+	32439508	32453942	14435	1122	sense-overlappin	NM_016252.3	BIRC6
circRNA_01415	0	0	0.021808645	0	0.02006311	0	0	0	0	0	0	0	-	NC_000002.12	+	32439508	32453954	14447	1134	sense-overlappin	NM_016252.3	BIRC6
circRNA_01416	0	0.020469968	0	0	0	0	0	0	0	0	0	0	-	NC_000002.12	+	32439508	32473239	33732	3089	sense-overlappin	NM_016252.3	BIRC6
circRNA_01417	0	0	0	0	0	0	0.020098685	0.030729443	0	0	0	0	-	NC_000002.12	+	32473112	32477583	4472	476	sense-overlappin	NM_016252.3	BIRC6
circRNA_01418	0	0	0	0	0.03009467	0	0	0	0.030381792	0	0	0	-	NC_000002.12	+	32473112	32487801	14690	1376	sense-overlappin	NM_016252.3	BIRC6
circRNA_01419	0	0.051174919	0	0.0309543	0	0	0	0	0	0.020172483	0	0	hsa_circ_0002408	NC_000002.12	+	32478635	32479617	983	340	sense-overlappin	NM_016252.3	BIRC6
circRNA_01420	0.020575025	0	0.04361729	0	0	0.020712909	0	0	0	0	0	0	hsa_circ_0007917	NC_000002.12	+	32478635	32482582	3948	628	sense-overlappin	NM_016252.3	BIRC6
circRNA_01421	0.051437562	0	0	0	0.03009467	0	0	0	0	0.030258724	0	0	hsa_circ_0003288	NC_000002.12	+	32478635	32493667	15033	1400	sense-overlappin	NM_016252.3	BIRC6
circRNA_01422	0	0	0	0	0	0.020712909	0	0	0	0	0	0	-	NC_000002.12	+	32478635	32500109	21475	1963	sense-overlappin	NM_016252.3	BIRC6
circRNA_01423	0	0	0	0.0206362	0	0	0	0	0	0	0	0	-	NC_000002.12	+	32478635	32505205	26571	2632	sense-overlappin	NM_016252.3	BIRC6
circRNA_01424	0	0	0	0	0	0	0	0	0.040509055	0	0	0	-	NC_000002.12	+	32488588	32493667	5080	500	sense-overlappin	NM_016252.3	BIRC6
circRNA_01425	0	0.030704951	0	0.0309543	0.02006311	0.020712909	0	0	0	0.030258724	0	0	hsa_circ_0008585	NC_000002.12	+	32607455	32611582	4128	324	sense-overlappin	NM_016252.3	BIRC6
circRNA_01426	0.020575025	0	0	0	0	0	0	0	0	0	0	0	hsa_circ_0005910	NC_000002.12	+	33186856	33222151	35296	675	sense-overlappin	XM_011532862.1	LTBP1
circRNA_01427	0	0	0	0	0	0	0.020098685	0	0	0	0	0	-	NC_000002.12	+	33239812	33240745	934	934	intronic	XM_011532862.1	LTBP1
circRNA_01428	0.020575025	0	0	0	0	0	0	0	0	0	0	0	-	NC_000002.12	+	33257284	33347510	90227	1833	sense-overlappin	XM_005264317.2	LTBP1
circRNA_01429	0	0	0	0	0	0	0	0	0	0.040344966	0	0	-	NC_000002.12	+	33280039	33315269	35231	738	sense-overlappin	XM_011532862.1	LTBP1
circRNA_01430	0.020575025	0	0	0	0	0	0	0	0	0	0	0	hsa_circ_0053932	NC_000002.12	+	33300451	33300573	123	123	exonic	XM_011532862.1	LTBP1
circRNA_01431	0.020575025	0	0	0	0	0	0	0	0	0	0	0	hsa_circ_0002247	NC_000002.12	+	33300451	33301644	1194	246	sense-overlappin	XM_011532862.1	LTBP1
circRNA_01432	0	0	0	0	0	0.031069364	0	0	0	0	0	0	-	NC_000002.12	+	33342838	33365503	22666	981	sense-overlappin	XM_005264317.2	LTBP1
circRNA_01433	0	0	0	0	0	0	0.020098685	0	0	0	0	0	-	NC_000002.12	+	33523879	33534400	10522	645	sense-overlappin	NM_170672.2	RASGRP3
circRNA_01434	0	0	0.021808645	0	0	0	0	0	0	0	0	0	hsa_circ_0007972	NC_000002.12	+	36378357	36442735	64379	18795	sense-overlappin	XR_939687.1	
circRNA_01435	0	0	0	0	0.02006311	0	0	0	0	0	0	0	-	NC_000002.12	+	36387972	36396787	8816	8816	sense-overlappin	XR_939687.1	

circRNA_01436	0	0	0	0	0.04012623	0	0	0	0	0	0	0	hsa_circ_0002017	NC_000002.12	+	36396614	36396787	174	174	exonic	XR_939687.1	
circRNA_01437	0	0.030704951	0.098138903	0.0206362	0.09028402	0	0.110542769	0.030729443	0.070890847	0.090776173	0.02025523	0.072227401	hsa_circ_0002346	NC_000002.12	+	36396614	36442735	46122	538	sense-overlappin	XR_939687.1	
circRNA_01438	0	0.020469968	0.032712968	0	0	0	0	0	0	0	0	0	hsa_circ_0005579	NC_000002.12	+	36396614	36464655	68042	660	sense-overlappin	XR_939687.1	
circRNA_01439	0	0	0	0	0.03009467	0	0	0	0	0	0	0.0309546	hsa_circ_0002348	NC_000002.12	+	36396614	36479694	83081	1041	sense-overlappin	XR_939687.1	
circRNA_01440	0.082300098	0.020469968	0.065425935	0.0309543	0.0702209	0	0.050246713	0.020486295	0.020254528	0.040344966	0	0	hsa_circ_0007386	NC_000002.12	+	36441258	36442735	1478	364	sense-overlappin	XR_939687.1	
circRNA_01441	0	0	0.032712968	0.0206362	0.03009467	0	0	0	0	0	0	0	hsa_circ_0054034	NC_000002.12	-	36877891	36894033	16143	615	sense-overlappin	XM_011533073.1	STRN
circRNA_01442	0	0	0.021808645	0	0	0	0	0	0	0	0	0	-	NC_000002.12	-	37000586	37002572	1987	495	sense-overlappin	NM_019024.2	HEATR5B
circRNA_01443	0	0	0	0	0	0.020712909	0	0	0	0	0	0.0309546	hsa_circ_0054055	NC_000002.12	-	37003542	37007304	3763	528	sense-overlappin	NM_019024.2	HEATR5B
circRNA_01444	0	0	0	0.0206362	0	0	0	0	0	0	0	0	-	NC_000002.12	-	37019809	37020836	1028	251	sense-overlappin	NM_019024.2	HEATR5B
circRNA_01445	0	0	0.13085187	0	0.04012623	0.020712909	0	0	0.020254528	0	0	0	-	NC_000002.12	-	37101923	37104132	2210	2210	intergenic		
circRNA_01446	0	0	0	0	0.02006311	0	0	0	0	0	0	0	-	NC_000002.12	-	37106068	37106148	81	81	exonic	XM_011532987.1	EIF2AK2
circRNA_01447	0	0	0.021808645	0	0	0	0	0	0	0	0	0	-	NC_000002.12	-	37114731	37122664	7934	469	sense-overlappin	XM_011532987.1	EIF2AK2
circRNA_01448	0	0	0	0	0	0	0	0	0	0.020172483	0	0	hsa_circ_0007047	NC_000002.12	+	37199704	37201726	2023	2023	antisense	NM_005760.2	CEBPZ
circRNA_01449	0	0	0	0	0	0	0.030148028	0	0	0	0	0	-	NC_000002.12	-	37287814	37290999	3186	1832	sense-overlappin	NM_005813.4	PRKD3
circRNA_01450	0.020575025	0.092114854	0.098138903	0.0619086	0.02006311	0.124277456	0.110542769	0.061458886	0.060763583	0.151293621	0	0.0412728	hsa_circ_0000992	NC_000002.12	-	37316237	37317179	943	943	exonic	XM_005264237.2	PRKD3
circRNA_01451	0	0	0.021808645	0	0	0	0	0	0	0	0	0	-	NC_000002.12	+	37929262	38017235	87974	1245	sense-overlappin	NM_001170791.2	RMDN2
circRNA_01452	0	0.040939935	0	0	0	0	0	0	0	0	0	0.0206364	-	NC_000002.12	+	38121985	38131614	9630	9630	intergenic		
circRNA_01453	0	0	0.08723458	0	0	0	0	0	0.020254528	0.020172483	0	0.0206364	hsa_circ_0002918	NC_000002.12	-	38309379	38319019	9641	708	sense-overlappin	XM_011533020.1	ATL2
circRNA_01454	0	0	0	0	0	0.020712909	0	0	0	0	0	0	hsa_circ_0054144	NC_000002.12	-	38585644	38591648	6005	357	sense-overlappin	XM_005264639.2	HNRNPLL
circRNA_01455	0.041150049	0	0	0	0	0	0	0.020486295	0	0	0.02025523	0	-	NC_000002.12	+	38880179	38881578	1400	295	sense-overlappin	NM_001145450.1	MORN2
circRNA_01456	0	0	0	0	0.02006311	0	0	0	0	0	0	0	hsa_circ_0054171	NC_000002.12	-	39006412	39010703	4292	401	sense-overlappin	NM_005633.3	SOS1
circRNA_01457	0	0	0	0.0412724	0	0	0	0	0	0	0	0	-	NC_000002.12	-	39179187	39229588	50402	983	sense-overlappin	XM_011532816.1	CDKL4
circRNA_01458	0	0	0	0	0	0	0	0	0.030381792	0	0	0	-	NC_000002.12	-	39196952	39229588	32637	619	sense-overlappin	XM_011532819.1	CDKL4
circRNA_01459	0	0	0.032712968	0	0	0	0	0	0	0	0	0	-	NC_000002.12	-	39204527	39229588	25062	510	sense-overlappin	XM_011532816.1	CDKL4
circRNA_01460	0	0	0.021808645	0.0309543	0	0.031069364	0	0	0	0.020172483	0	0	-	NC_000002.12	-	39213400	39229588	16189	419	sense-overlappin	XM_011532816.1	CDKL4
circRNA_01461	0	0	0	0	0	0.062138728	0	0	0	0	0	0	hsa_circ_0054190	NC_000002.12	-	39272283	39280356	8074	344	sense-overlappin	NM_001270425.1	MAP4K3
circRNA_01462	0	0	0.04361729	0	0	0	0	0	0	0.020172483	0	0	-	NC_000002.12	-	39272283	39288280	15998	659	sense-overlappin	NM_001270425.1	MAP4K3
circRNA_01463	0.020575025	0	0	0	0	0	0	0	0	0	0	0	hsa_circ_0054196	NC_000002.12	-	39286852	39288280	1429	273	sense-overlappin	NM_001270425.1	MAP4K3
circRNA_01464	0	0	0	0	0	0.031069364	0	0	0	0	0.030382845	0	hsa_circ_0054197	NC_000002.12	-	39286852	39290334	3483	316	sense-overlappin	NM_001270425.1	MAP4K3
circRNA_01465	0	0	0	0	0	0	0.020098685	0	0	0.020172483	0	0	hsa_circ_0054203	NC_000002.12	-	39292773	39309519	16747	274	sense-overlappin	NM_003618.3	MAP4K3
circRNA_01466	0.020575025	0	0.032712968	0	0.02006311	0	0	0	0	0	0	0	-	NC_000002.12	-	39325518	39326277	760	388	sense-overlappin	NM_001270425.1	MAP4K3
circRNA_01467	0.061725074	0	0.109043225	0	0.06018934	0	0.050246713	0	0.030381792	0.040344966	0	0.0309546	hsa_circ_0054211	NC_000002.12	-	39331917	39337581	5665	220	sense-overlappin	NM_001270425.1	MAP4K3
circRNA_01468	0	0	0.04361729	0	0	0	0	0	0	0	0	0	hsa_circ_0054212	NC_000002.12	-	39331917	39356339	24423	376	sense-overlappin	NM_001270425.1	MAP4K3
circRNA_01469	0	0	0.021808645	0	0	0	0	0	0	0	0	0	hsa_circ_0054213	NC_000002.12	-	39331917	39378123	46207	434	sense-overlappin	NM_001270425.1	MAP4K3
circRNA_01470	0.185175222	0.051174919	0.076330258	0.0928628	0.03009467	0.051782273	0	0	0.081018111	0.100862414	0	0	hsa_circ_0054214	NC_000002.12	-	39336920	39337581	662	104	sense-overlappin	NM_001270425.1	MAP4K3
circRNA_01471	0	0	0	0	0	0	0	0	0	0	0	0.0309546	-	NC_000002.12	+	39369291	39374303	5013	5013	antisense	NM_001270425.1	MAP4K3
circRNA_01472	0	0	0	0	0	0	0	0.020486295	0	0	0	0	hsa_circ_0054218	NC_000002.12	+	39453839	39454365	527	527	intergenic		
circRNA_01473	0	0	0	0	0	0.031069364	0	0	0	0	0	0	hsa_circ_0054221	NC_000002.12	+	39704081	39707186	3106	252	sense-overlappin	NM_001167959.1	TMEM178A
circRNA_01474	0.041150049	0	0	0	0	0	0	0	0.030381792	0	0	0	-	NC_000002.12	-	40139401	40164984	25584	507	sense-overlappin	XM_011533059.1	SLC8A1
circRNA_01475	0.020575025	0	0	0	0	0	0	0	0.020254528	0	0	0.0309546	hsa_circ_0054226	NC_000002.12	-	40139401	40177855	38455	734	sense-overlappin	XM_011533048.1	SLC8A1
circRNA_01476	0.030862537	0	0	0	0	0	0	0	0	0.020172483	0	0	-	NC_000002.12	-	40160765	40177855	17091	458	sense-overlappin	XM_011533048.1	SLC8A1
circRNA_01477	0.174887709	0.061409903	0	0.1031809	0.05015779	0.020712909	0.15074014	0.030729443	0.101272639	0.030258724	0	0.185727601	hsa_circ_0005232	NC_000002.12	-	40428473	40430301	1829	1829	exonic	XM_011533048.1	SLC8A1
circRNA_01478	0.833288497	0.358224432	0.152660515	0.2373162	0.16050492	0.46604046	0.633108588	0.389239614	0.941835539	0.474053346	0.273445606	0.619092005	hsa_circ_0000994	NC_000002.12	-	40428473	40430304	1832	1832	exonic	NM_001112800.1	SLC8A1
circRNA_01479	0	0	0.032712968	0	0	0	0	0	0	0	0	0	-	NC_000002.12	+	42256501	42264731	8231	459	sense-overlappin	NM_019063.4	EML4
circRNA_01480	0	0	0	0.0309543	0	0	0	0	0	0	0	0	hsa_circ_0054236	NC_000002.12	+	42280850	42284703	3854	344	sense-overlappin	NM_001145076.2	EML4
circRNA_01481	0	0	0	0	0.03009467	0	0	0	0	0	0	0	hsa_circ_0054242	NC_000002.12	+	42286269	42303446	17178	888	sense-overlappin	NM_001145076.2	EML4
circRNA_01482	0	0	0	0.0206362	0	0.041425819	0	0.020486295	0.020254528	0.020172483	0	0	hsa_circ_0054243	NC_000002.12	+	42286269	42304551	18283	956	sense-overlappin	NM_001145076.2	EML4
circRNA_01483	0	0	0	0.0309543	0	0	0	0	0	0	0	0	hsa_circ_0054248	NC_000002.12	+	42295125	42304551	9427	749	sense-overlappin	NM_001145076.2	EML4
circRNA_01484	0	0	0.032712968	0	0	0	0	0	0	0	0	0	-	NC_000002.12	+	42609458	42659862	50405	512	sense-overlappin	NM_001282755.1	MTA3
circRNA_01485	0	0	0.032712968	0	0	0	0	0	0.020254528	0	0	0	hsa_circ_0054263	NC_000002.12	+	42640173	42659862	19690	385	sense-overlappin	NM_001282755.1	MTA3
circRNA_01486	0	0	0.021808645	0	0	0	0	0	0	0	0	0	hsa_circ_0003990	NC_000002.12	+	42718988	42723035	4048	234	sense-overlappin	NM_001282755.1	MTA3
circRNA_01487	0	0	0.021808645	0	0	0	0	0	0	0	0	0	-	NC_000002.12	-	42788529	42791036	2508	2108	sense-overlappin	XM_011532730.1	HAAO
circRNA_01488	0	0	0	0	0	0.020712909	0	0	0	0	0	0	-	NC_000002.12	-	43397971	43446995	49025	17084	sense-overlappin	NM_001083953.1	THADA
circRNA_01489	0	0	0	0	0	0	0	0	0	0	0.030382845	0	hsa_circ_0054309	NC_000002.12	-	43428100	43430302	2203	222	sense-overlappin	NM_001083953.1	THADA
circRNA_01490	0	0	0.04361729	0	0	0	0	0	0	0	0	0	-	NC_000002.12	-	43485234	43508780	23547	462	sense-overlappin	NM_001083953.1	THADA

circRNA_01491	0	0	0.065425935	0	0	0	0	0	0	0	0	0	hsa_circ_0054313	NC_000002.12	-	43485234	43527988	42755	572	sense-overlappin	NM_001083953.1	THADA
circRNA_01492	0	0	0.032712968	0	0	0	0	0	0	0	0	0	-	NC_000002.12	-	43498833	43508780	9948	370	sense-overlappin	NM_001083953.1	THADA
circRNA_01493	0	0	0.032712968	0	0	0	0	0	0	0	0	0	hsa_circ_0002392	NC_000002.12	-	43498833	43527988	29156	480	sense-overlappin	NM_001083953.1	THADA
circRNA_01494	0	0	0.021808645	0	0	0	0	0	0	0	0	0	-	NC_000002.12	-	43549210	43560385	11176	795	sense-overlappin	NM_001083953.1	THADA
circRNA_01495	0	0.092114854	0	0.0412724	0.02006311	0	0	0.020486295	0.020254528	0	0	0	hsa_circ_0054322	NC_000002.12	-	43578513	43590954	12442	645	sense-overlappin	NM_001083953.1	THADA
circRNA_01496	0	0	0	0	0	0	0	0	0	0.030258724	0	0	-	NC_000002.12	+	43692514	43706416	13903	1635	sense-overlappin	XR_939657.1	
circRNA_01497	0	0	0	0	0	0	0	0	0.030381792	0	0	0	hsa_circ_0007879	NC_000002.12	+	43712225	43738520	26296	822	sense-overlappin	XR_244924.1	
circRNA_01498	0	0.040939935	0	0	0	0	0	0	0	0.040344966	0	0	-	NC_000002.12	+	43712225	43741043	28819	1050	sense-overlappin	XR_244924.1	
circRNA_01499	0	0	0	0	0	0	0	0	0	0.030258724	0	0	-	NC_000002.12	+	43726272	43738520	12249	582	sense-overlappin	XR_244924.1	
circRNA_01500	0	0.040939935	0	0	0	0.020712909	0	0.030729443	0.050636319	0.020172483	0	0	-	NC_000002.12	+	43742741	43745963	3223	432	sense-overlappin	XR_939656.1	
circRNA_01501	0	0	0	0	0	0.020712909	0	0.030729443	0	0	0	0	-	NC_000002.12	+	43745866	43765726	19861	1055	sense-overlappin	NM_172069.3	PLEKHH2
circRNA_01502	0	0	0.021808645	0.0515905	0	0	0	0	0	0	0	0	hsa_circ_0054326	NC_000002.12	+	43794457	43801709	7253	485	sense-overlappin	NM_001193464.1	DYNC2LI1
circRNA_01503	0.020575025	0	0	0.0206362	0	0	0	0	0	0.040344966	0	0	-	NC_000002.12	+	43794457	43805246	10790	676	sense-overlappin	NM_001193464.1	DYNC2LI1
circRNA_01504	0	0	0	0.0515905	0	0	0	0.030729443	0	0	0.02025523	0	hsa_circ_0054345	NC_000002.12	-	43912432	43918398	5967	379	sense-overlappin	NM_133259.3	LRPPRC
circRNA_01505	0	0	0.04361729	0	0	0	0	0	0	0	0	0	hsa_circ_0005539	NC_000002.12	-	43912432	43925961	13530	539	sense-overlappin	NM_133259.3	LRPPRC
circRNA_01506	0	0	0.076330258	0	0	0	0.020098685	0.020486295	0	0	0.02025523	0	-	NC_000002.12	-	43973607	43982434	8828	1220	sense-overlappin	NM_133259.3	LRPPRC
circRNA_01507	0	0	0.032712968	0	0	0	0	0	0	0	0	0	-	NC_000002.12	+	44174762	44180397	5636	5636	intronic	NM_001033557.2	PPM1B
circRNA_01508	0	0.020469968	0.04361729	0	0	0	0.020098685	0	0	0.030258724	0	0	hsa_circ_0004916	NC_000002.12	+	44201186	44209327	8142	978	sense-overlappin	NM_001033557.2	PPM1B
circRNA_01509	0	0	0.032712968	0	0	0	0.020098685	0	0	0	0	0	hsa_circ_0006882	NC_000002.12	+	44201186	44218537	17352	1148	sense-overlappin	NM_001033557.2	PPM1B
circRNA_01510	0.020575025	0	0.076330258	0.0309543	0.02006311	0.041425819	0	0.020486295	0	0.040344966	0	0.051591	hsa_circ_0009062	NC_000002.12	+	44209210	44218537	9328	288	sense-overlappin	NM_001033557.2	PPM1B
circRNA_01511	0	0	0	0	0	0	0	0	0	0.020172483	0	0	-	NC_000002.12	+	44549555	44558013	8459	8459	sense-overlappin	NM_024766.4	CAMKMT
circRNA_01512	0	0.020469968	0	0	0	0	0	0	0	0	0	0	-	NC_000002.12	+	44564776	44651425	86650	86650	intronic	NM_024766.4	CAMKMT
circRNA_01513	0.020575025	0	0.054521613	0	0.02006311	0	0.050246713	0	0	0	0.030382845	0.0206364	hsa_circ_0006530	NC_000002.12	+	44704283	44715353	11071	247	sense-overlappin	NM_024766.4	CAMKMT
circRNA_01514	0	0	0	0	0	0	0	0	0	0	0.02025523	0	hsa_circ_0054420	NC_000002.12	-	45476993	45562756	85764	744	sense-overlappin	NM_018079.4	SRBD1
circRNA_01515	0	0	0.021808645	0	0	0	0	0	0	0.040344966	0	0	hsa_circ_0002200	NC_000002.12	-	45546317	45553730	7414	509	sense-overlappin	XM_011532946.1	SRBD1
circRNA_01516	0	0	0.032712968	0.0309543	0	0	0.040197371	0.030729443	0	0	0	0.0309546	-	NC_000002.12	-	45546317	45562756	16440	613	sense-overlappin	XM_011532946.1	SRBD1
circRNA_01517	0.041150049	0.040939935	0.13085187	0.113499	0.06018934	0.041425819	0	0.051215739	0.040509055	0.020172483	0.030382845	0	hsa_circ_0005542	NC_000002.12	-	45546732	45553730	6999	465	sense-overlappin	NM_018079.4	SRBD1
circRNA_01518	0	0.040939935	0.17446916	0.1341352	0.09028402	0.051782273	0	0.030729443	0.050636319	0.080689931	0.030382845	0	hsa_circ_0000997	NC_000002.12	-	45546732	45562756	16025	569	sense-overlappin	NM_018079.4	SRBD1
circRNA_01519	0	0	0	0	0	0.020712909	0	0	0	0	0	0	-	NC_000002.12	-	45546732	45585774	39043	1226	sense-overlappin	NM_018079.4	SRBD1
circRNA_01520	0	0	0	0	0	0	0	0	0	0	0.02025523	0.051591	hsa_circ_0005377	NC_000002.12	+	45697941	45701491	3551	285	sense-overlappin	XM_011532977.1	PRKCE
circRNA_01521	0	0	0	0	0.03009467	0	0	0	0	0	0	0	hsa_circ_0000998	NC_000002.12	+	45697941	45701524	3584	318	sense-overlappin	XM_011532977.1	PRKCE
circRNA_01522	0	0	0.021808645	0	0	0	0	0	0	0	0	0	-	NC_000002.12	+	46001404	46086362	84959	769	sense-overlappin	XM_005264429.1	PRKCE
circRNA_01523	0	0	0.021808645	0	0	0	0	0	0	0	0	0	hsa_circ_0008979	NC_000002.12	-	46907810	46909177	1368	315	sense-overlappin	NM_001171506.2	MCFD2
circRNA_01524	0	0	0	0	0	0.020712909	0	0	0	0	0	0	hsa_circ_0003478	NC_000002.12	+	46974973	46978907	3935	247	sense-overlappin	NM_001288953.1	TTC7A
circRNA_01525	0	0	0	0.0309543	0	0	0	0	0	0	0	0	-	NC_000002.12	-	47024960	47025264	305	305	antisense	NM_001288953.1	TTC7A
circRNA_01526	0	0	0	0.0206362	0	0	0	0	0	0	0	0	-	NC_000002.12	-	47336409	47336846	438	438	intergenic		
circRNA_01527	0	0.020469968	0.032712968	0	0.03009467	0	0.030148028	0.020486295	0.040509055	0	0.02025523	0.0206364	-	NC_000002.12	+	47373463	47374048	586	349	sense-overlappin	NM_002354.2	EPCAM
circRNA_01528	0	0.020469968	0.021808645	0	0.03009467	0	0	0	0	0	0	0	-	NC_000002.12	+	47373463	47375299	1837	415	sense-overlappin	NM_002354.2	EPCAM
circRNA_01529	0	0	0.021808645	0	0.04012623	0.020712909	0.020098685	0.020486295	0	0	0.030382845	0	-	NC_000002.12	+	47377014	47379969	2956	367	sense-overlappin	NM_002354.2	EPCAM
circRNA_01530	0	0	0.054521613	0	0.04012623	0	0.040197371	0.020486295	0	0	0.050638075	0	-	NC_000002.12	+	47378953	47379969	1017	303	sense-overlappin	NM_002354.2	EPCAM
circRNA_01531	0.041150049	0	0.054521613	0.0309543	0	0.041425819	0.020098685	0.051215739	0	0.030258724	0	0.051591	hsa_circ_0006794	NC_000002.12	-	47822218	47823360	1143	304	sense-overlappin	NM_025133.4	FBXO11
circRNA_01532	0	0.030704951	0	0	0	0	0	0	0	0	0	0	hsa_circ_0054495	NC_000002.12	-	47832769	47839769	7001	921	sense-overlappin	NM_025133.4	FBXO11
circRNA_01533	0.030862537	0	0.076330258	0.0206362	0.04012623	0	0.070345399	0	0	0.060517448	0.06076569	0.0206364	hsa_circ_0007641	NC_000002.12	+	48328561	48346751	18191	878	sense-overlappin	XM_005264283.1	FOXN2
circRNA_01534	0	0	0	0.0309543	0	0	0	0	0	0	0	0	hsa_circ_0006684	NC_000002.12	+	48328561	48359147	30587	979	sense-overlappin	XM_005264283.1	FOXN2
circRNA_01535	0	0	0.054521613	0	0	0	0	0	0	0	0.02025523	0	hsa_circ_0007732	NC_000002.12	+	48328561	48362707	34147	1044	sense-overlappin	XM_005264283.1	FOXN2
circRNA_01536	0	0	0.021808645	0	0	0	0	0	0	0	0	0	hsa_circ_0001002	NC_000002.12	+	48346201	48346751	551	551	exonic	XM_005264282.1	FOXN2
circRNA_01537	0	0	0.032712968	0	0	0	0	0	0	0	0	0	hsa_circ_0008506	NC_000002.12	+	48346201	48362707	16507	717	sense-overlappin	XM_005264282.1	FOXN2
circRNA_01538	0	0	0.032712968	0	0	0	0	0	0.040509055	0	0	0	-	NC_000002.12	+	48451008	48465642	14635	840	sense-overlappin	NM_001135629.2	PPP1R21
circRNA_01539	0.020575025	0	0	0.0206362	0.04012623	0.031069364	0	0	0	0.020172483	0	0	hsa_circ_0008358	NC_000002.12	+	48474683	48491170	16488	584	sense-overlappin	XR_939655.1	
circRNA_01540	0.020575025	0	0.04361729	0	0	0	0	0.020486295	0.020254528	0	0	0	hsa_circ_0006911	NC_000002.12	+	48498493	48511468	12976	621	sense-overlappin	NM_001135629.2	PPP1R21
circRNA_01541	0.082300098	0	0	0	0	0	0	0	0	0	0	0	-	NC_000002.12	+	48580587	48582563	1977	1977	exonic	NM_001198593.1	STON1-GTF2A1L
circRNA_01542	0.041150049	0	0	0	0	0.051782273	0	0	0	0	0	0	hsa_circ_0054522	NC_000002.12	+	48580587	48591855	11269	2180	sense-overlappin	NM_001198593.1	STON1-GTF2A1L
circRNA_01543	0	0	0	0	0	0	0	0.030729443	0	0	0	0	-	NC_000002.12	+	48620851	48671680	50830	1308	sense-overlappin	NM_001198593.1	STON1-GTF2A1L
circRNA_01544	0.041150049	0	0	0	0	0.082851637	0	0	0	0	0.04051046	0	-	NC_000002.12	+	48642402	48671680	29279	1082	sense-overlappin	NM_001198593.1	STON1-GTF2A1L
circRNA_01545	0	0	0	0	0.02006311	0	0	0	0	0	0	0	-	NC_000002.12	-	48708948	48714054	5107	144	sense-overlappin	XM_011532831.1	LHCGR

circRNA_01546	0.020575025	0	0	0	0	0	0	0	0	0	0	0	-	NC_000002.12	+	48767112	48809641	42530	42530	sense-overlappin	NM_001198593.1	STON1-GTF2A1L
circRNA_01547	0.051437562	0	0	0	0	0.062138728	0	0	0	0	0.02025523	0	-	NC_000002.12	+	48799306	48809641	10336	10336	intergenic		
circRNA_01548	0	0	0	0	0	0.041425819	0	0	0	0	0	0	-	NC_000002.12	+	48807660	48809641	1982	1982	intergenic		
circRNA_01549	0.051437562	0	0	0	0	0	0	0	0	0	0	0	hsa_circ_0001004	NC_000002.12	-	50921869	50925955	4087	60	sense-overlappin	NM_001135659.1	NRXN1
circRNA_01550	0	0	0.021808645	0	0	0	0	0	0	0	0	0	-	NC_000002.12	-	53603385	53606080	2696	2696	intergenic		
circRNA_01551	0.020575025	0.030704951	0.032712968	0	0	0	0	0	0.040509055	0.030258724	0.02025523	0	hsa_circ_0003959	NC_000002.12	-	53714384	53716743	2360	376	sense-overlappin	NM_001201965.1	ASB3
circRNA_01552	0	0.030704951	0	0	0	0	0	0	0	0	0	0	hsa_circ_0002416	NC_000002.12	-	53750783	53765585	14803	368	sense-overlappin	NM_016115.4	ASB3
circRNA_01553	0	0	0	0	0	0	0	0.040972591	0	0	0	0	hsa_circ_0054547	NC_000002.12	-	53892808	53893799	992	279	sense-overlappin	NM_014614.2	PSME4
circRNA_01554	0	0	0	0	0	0	0	0	0	0	0.030382845	0	hsa_circ_0004772	NC_000002.12	-	53936087	53937540	1454	289	sense-overlappin	NM_014614.2	PSME4
circRNA_01555	0	0	0	0	0	0.020712909	0	0	0	0	0	0	-	NC_000002.12	+	54038212	54051050	12839	12839	intergenic		
circRNA_01556	0	0.030704951	0	0.0206362	0	0.082851637	0	0	0.030381792	0.030258724	0	0	hsa_circ_0004462	NC_000002.12	+	54050958	54057360	6403	6403	intergenic		
circRNA_01557	0	0.020469968	0.054521613	0	0	0	0	0	0	0	0	0	-	NC_000002.12	+	54928313	54928751	439	329	sense-overlappin	XM_011532836.1	EML6
circRNA_01558	0	0	0.021808645	0	0	0	0	0	0	0	0	0	-	NC_000002.12	+	54959104	54960334	1231	273	sense-overlappin	XM_011532836.1	EML6
circRNA_01559	0.13373766	0.092114854	0.4361729	0.0412724	0.06018934	0.062138728	0.040197371	0	0.060763583	0.07060369	0.172169456	0	hsa_circ_0001006	NC_000002.12	-	54982515	54987698	5184	347	sense-overlappin	NM_007008.2	RTN4
circRNA_01560	0	0	0.065425935	0	0	0	0	0	0	0	0	0	hsa_circ_0054598	NC_000002.12	-	55025086	55028220	3135	2457	sense-overlappin	XM_011532991.1	RTN4
circRNA_01561	0	0	0	0	0	0.020712909	0	0	0	0	0	0	-	NC_000002.12	-	55177602	55205728	28127	24542	sense-overlappin	XM_006711939.2	CLHC1
circRNA_01562	0	0.030704951	0	0	0	0	0	0	0	0	0	0	-	NC_000002.12	-	55206270	55209831	3562	507	sense-overlappin	XM_011532531.1	CLHC1
circRNA_01563	0	0	0	0	0.02006311	0	0	0	0	0	0	0	-	NC_000002.12	-	55206270	55218600	12331	1431	sense-overlappin	XM_011532525.1	CLHC1
circRNA_01564	0	0	0	0	0	0	0.020098685	0	0	0	0	0	-	NC_000002.12	-	55206270	55222493	16224	1088	sense-overlappin	XM_011532525.1	CLHC1
circRNA_01565	0	0	0.021808645	0.0309543	0	0	0.060296056	0.020486295	0.040509055	0	0	0	hsa_circ_0054618	NC_000002.12	-	55332566	55335164	2599	1199	sense-overlappin	XM_011532966.1	CCDC88A
circRNA_01566	0	0	0	0	0	0	0	0	0	0	0	0.0309546	hsa_circ_0054621	NC_000002.12	-	55355579	55364033	8455	398	sense-overlappin	XM_011532966.1	CCDC88A
circRNA_01567	0	0	0	0	0	0	0.020098685	0	0	0	0	0	-	NC_000002.12	-	55363950	55388886	24937	322	sense-overlappin	XM_011532966.1	CCDC88A
circRNA_01568	0	0	0.021808645	0.0309543	0	0	0	0	0	0	0	0	hsa_circ_0003263	NC_000002.12	-	55558775	55573777	15003	848	sense-overlappin	XM_005264448.3	SMEK2
circRNA_01569	0	0	0.021808645	0	0	0	0	0	0	0	0	0	hsa_circ_0054628	NC_000002.12	-	55558775	55604076	45302	2256	sense-overlappin	NM_001122964.2	SMEK2
circRNA_01570	0	0.030704951	0	0	0	0	0	0	0	0	0.02025523	0	-	NC_000002.12	-	55577315	55579781	2467	241	sense-overlappin	XM_005264448.3	SMEK2
circRNA_01571	0	0	0	0	0.02006311	0	0	0	0	0	0	0	-	NC_000002.12	-	55577315	55588956	11642	685	sense-overlappin	XM_005264448.3	SMEK2
circRNA_01572	0	0	0	0	0.02006311	0	0	0	0	0	0	0	-	NC_000002.12	-	55577315	55591575	14261	756	sense-overlappin	XM_005264448.3	SMEK2
circRNA_01573	0	0	0	0	0	0	0	0	0	0.040344966	0	0	hsa_circ_0007650	NC_000002.12	-	55643158	55644720	1563	247	sense-overlappin	XM_005264629.1	PNPT1
circRNA_01574	0	0	0	0	0.02006311	0	0	0	0	0	0	0	-	NC_000002.12	-	55656131	55685048	28918	1144	sense-overlappin	XM_005264629.1	PNPT1
circRNA_01575	0	0	0.032712968	0	0	0	0	0	0	0	0	0	-	NC_000002.12	-	55661956	55673079	11124	568	sense-overlappin	XM_005264629.1	PNPT1
circRNA_01576	0	0	0.119947548	0	0.03009467	0	0	0	0	0	0	0	-	NC_000002.12	+	55759196	55772912	13717	13717	intergenic		
circRNA_01577	0	0	0.032712968	0	0	0	0	0	0	0	0	0	-	NC_000002.12	+	58048827	58089723	40897	685	sense-overlappin	XM_006712092.2	VRK2
circRNA_01578	0.020575025	0.092114854	0.283512385	0	0.11034713	0	0.030148028	0	0.030381792	0	0.030382845	0	-	NC_000002.12	+	58084089	58089723	5635	407	sense-overlappin	NM_001288837.1	VRK2
circRNA_01579	0	0.020469968	0.163564838	0	0	0.020712909	0.020098685	0.020486295	0.020254528	0	0.030382845	0	hsa_circ_0003165	NC_000002.12	-	58198579	58232112	33534	459	sense-overlappin	NM_001114636.1	FANCL
circRNA_01580	0.061725074	0.102349838	0.948676058	0.1031809	0.35110451	0.041425819	0.120592112	0.09218833	0.050636319	0.07060369	0	0.051591	hsa_circ_0004435	NC_000002.12	-	58198594	58232112	33519	444	sense-overlappin	NM_001114636.1	FANCL
circRNA_01581	0	0	0	0	0	0	0.020098685	0	0	0	0	0	hsa_circ_0054677	NC_000002.12	-	58221942	58229874	7933	219	sense-overlappin	NM_001114636.1	FANCL
circRNA_01582	0.164600197	0.092114854	0.730589608	0.1444533	0.52164099	0.258911367	0.241184224	0.133160921	0.162036222	0.221897311	0.232935146	0.092863801	hsa_circ_0001009	NC_000002.12	-	58221942	58232112	10171	278	sense-overlappin	NM_001114636.1	FANCL
circRNA_01583	0	0	0	0	0	0.031069364	0	0	0	0	0	0	-	NC_000002.12	+	60786947	60787620	674	230	sense-overlappin	XM_005264500.2	PAPOLG
circRNA_01584	0	0	0.021808645	0	0	0	0	0	0	0	0	0	-	NC_000002.12	+	60786947	60793715	6769	602	sense-overlappin	XM_005264500.2	PAPOLG
circRNA_01585	0	0.020469968	0	0	0	0	0	0	0.020254528	0.020172483	0	0	hsa_circ_0001011	NC_000002.12	+	60891683	60901083	9401	384	sense-overlappin	NM_001291746.1	REL
circRNA_01586	0	0	0	0	0	0.041425819	0	0	0	0	0	0	-	NC_000002.12	+	60891683	60921999	30317	1314	sense-overlappin	NM_002908.3	REL
circRNA_01587	0	0	0	0.0206362	0	0	0.020098685	0	0.020254528	0	0	0	hsa_circ_0008534	NC_000002.12	+	61031419	61032113	695	695	exonic	NM_002618.3	PEX13
circRNA_01588	0	0	0	0	0	0	0	0	0	0.030258724	0	0	-	NC_000002.12	+	61112518	61118116	5599	3745	sense-overlappin	XM_011533129.1	KIAA1841
circRNA_01589	0.020575025	0	0	0.0619086	0.03009467	0.020712909	0	0.020486295	0.081018111	0.020172483	0.02025523	0	hsa_circ_0001013	NC_000002.12	+	61112522	61118116	5595	3741	sense-overlappin	XM_011533129.1	KIAA1841
circRNA_01590	0.041150049	0	0.141756193	0	0.06018934	0	0	0	0.030381792	0.040344966	0.02025523	0.0412728	hsa_circ_0001014	NC_000002.12	+	61113770	61118116	4347	2493	sense-overlappin	XM_011533129.1	KIAA1841
circRNA_01591	0.041150049	0	0.076330258	0.0722267	0.03009467	0.062138728	0.070345399	0.061458886	0.121527166	0.07060369	0	0.0619092	hsa_circ_0007793	NC_000002.12	+	61115979	61118116	2138	284	sense-overlappin	XM_011533129.1	KIAA1841
circRNA_01592	0	0	0	0	0.03009467	0	0	0	0	0	0	0	-	NC_000002.12	+	61178981	61186497	7517	753	sense-overlappin	XM_011532542.1	AHSA2
circRNA_01593	0	0	0	0	0	0	0	0	0	0.020172483	0	0	-	NC_000002.12	-	61220310	61223296	2987	452	sense-overlappin	NM_014709.3	USP34
circRNA_01594	0	0	0	0	0	0	0.020098685	0	0	0	0	0	-	NC_000002.12	-	61256384	61278443	22060	965	sense-overlappin	NM_014709.3	USP34
circRNA_01595	0	0	0.032712968	0	0.02006311	0	0	0.040972591	0.030381792	0.040344966	0	0	hsa_circ_0007493	NC_000002.12	-	61278165	61281242	3078	435	sense-overlappin	NM_014709.3	USP34
circRNA_01596	0	0	0.021808645	0	0	0	0	0	0	0	0	0	hsa_circ_0006587	NC_000002.12	-	61278165	61284957	6793	684	sense-overlappin	NM_014709.3	USP34
circRNA_01597	0.041150049	0	0	0.0309543	0	0	0	0	0	0	0	0	-	NC_000002.12	-	61284875	61295290	10416	578	sense-overlappin	NM_014709.3	USP34
circRNA_01598	0	0	0.021808645	0	0	0	0	0	0	0.030258724	0	0	-	NC_000002.12	-	61284875	61296925	12051	704	sense-overlappin	NM_014709.3	USP34
circRNA_01599	0	0	0.021808645	0	0	0	0	0	0	0	0	0	-	NC_000002.12	-	61293464	61350693	57230	3297	sense-overlappin	NM_014709.3	USP34
circRNA_01600	0	0	0	0	0	0.020712909	0	0	0	0	0	0	-	NC_000002.12	-	61300951	61301454	504	311	sense-overlappin	NM_014709.3	USP34

circRNA_01601	0	0	0.032712968	0.0206362	0	0	0	0	0	0	0	0	-	NC_000002.12	-	61311540	61350693	39154	2566	sense-overlappin	NM_014709.3	USP34
circRNA_01602	0	0	0	0	0	0	0.020098685	0	0	0	0	0	-	NC_000002.12	-	61325375	61333971	8597	269	sense-overlappin	NM_014709.3	USP34
circRNA_01603	0	0	0	0	0	0.031069364	0	0	0	0	0	0	-	NC_000002.12	-	61325375	61339681	14307	513	sense-overlappin	NM_014709.3	USP34
circRNA_01604	0	0.030704951	0	0	0	0	0	0	0	0	0	0	-	NC_000002.12	-	61331276	61350693	19418	1679	sense-overlappin	NM_014709.3	USP34
circRNA_01605	0	0	0	0	0.02006311	0	0	0	0	0	0	0	-	NC_000002.12	-	61347870	61349285	1416	778	sense-overlappin	NM_014709.3	USP34
circRNA_01606	0	0	0	0	0	0	0.020098685	0	0	0.080689931	0	0	-	NC_000002.12	-	61347870	61350693	2824	1034	sense-overlappin	NM_014709.3	USP34
circRNA_01607	0	0	0	0.0309543	0	0	0	0	0	0	0	0	hsa_circ_0005738	NC_000002.12	-	61370321	61395233	24913	699	sense-overlappin	NM_014709.3	USP34
circRNA_01608	0	0	0.032712968	0	0	0	0.020098685	0	0	0	0	0	-	NC_000002.12	-	61479375	61479675	301	301	intronic	XM_005264546.1	XPO1
circRNA_01609	0	0	0	0	0	0	0.060296056	0	0	0	0	0	hsa_circ_0054853	NC_000002.12	-	61482380	61490776	8397	1085	sense-overlappin	XM_005264546.1	XPO1
circRNA_01610	0	0	0	0	0.04012623	0	0	0.020486295	0.020254528	0	0.02025523	0.0309546	hsa_circ_0002607	NC_000002.12	-	61482957	61490776	7820	925	sense-overlappin	XM_005264546.1	XPO1
circRNA_01611	0	0	0.021808645	0.0515905	0	0	0	0	0	0	0.02025523	0	-	NC_000002.12	-	61482957	61493053	10097	1567	sense-overlappin	XM_005264546.1	XPO1
circRNA_01612	0	0	0.021808645	0	0	0	0	0	0	0	0	0	hsa_circ_0054858	NC_000002.12	-	61482957	61533903	50947	2818	sense-overlappin	NM_003400.3	XPO1
circRNA_01613	0	0	0	0	0.05015779	0	0	0	0	0.020172483	0.030382845	0	hsa_circ_0005050	NC_000002.12	-	61485768	61490776	5009	621	sense-overlappin	XM_005264546.1	XPO1
circRNA_01614	0	0	0	0	0	0.020712909	0	0	0	0	0	0	hsa_circ_0054861	NC_000002.12	-	61485768	61493053	7286	1263	sense-overlappin	XM_005264546.1	XPO1
circRNA_01615	0	0	0	0	0	0.051782273	0	0	0	0.020172483	0	0	hsa_circ_0054876	NC_000002.12	-	61492035	61495613	3579	999	sense-overlappin	XM_005264546.1	XPO1
circRNA_01616	0	0	0.021808645	0	0.03009467	0	0	0	0	0	0	0	hsa_circ_0054877	NC_000002.12	-	61492035	61497007	4973	1128	sense-overlappin	XM_005264546.1	XPO1
circRNA_01617	0	0	0	0	0.02006311	0	0	0	0	0.030258724	0	0	hsa_circ_0006033	NC_000002.12	-	61493894	61495613	1720	357	sense-overlappin	XM_005264546.1	XPO1
circRNA_01618	0	0	0	0	0.04012623	0.031069364	0	0	0	0	0	0	hsa_circ_0006493	NC_000002.12	-	61493894	61497007	3114	486	sense-overlappin	XM_005264546.1	XPO1
circRNA_01619	0	0.020469968	0.065425935	0	0.04012623	0.020712909	0.020098685	0	0	0	0.02025523	0	hsa_circ_0054886	NC_000002.12	-	61495455	61498913	3459	457	sense-overlappin	XM_005264546.1	XPO1
circRNA_01620	0	0	0	0	0	0.020712909	0	0	0	0.020172483	0	0	-	NC_000002.12	-	61495455	61499894	4440	639	sense-overlappin	XM_005264546.1	XPO1
circRNA_01621	0	0	0.021808645	0	0.03009467	0	0	0	0	0	0	0	-	NC_000002.12	-	61498673	61499894	1222	351	sense-overlappin	XM_005264546.1	XPO1
circRNA_01622	0	0	0.021808645	0	0	0	0	0	0	0	0	0	-	NC_000002.12	-	61501996	61533903	31908	414	sense-overlappin	XM_011533098.1	XPO1
circRNA_01623	0	0	0	0	0	0	0	0	0	0	0.02025523	0	-	NC_000002.12	-	61507801	61509110	1310	1310	intronic	XM_005264546.1	XPO1
circRNA_01624	0.102875123	0.071644886	0.512503158	0.1857257	0.38119918	0.289980731	0.211036196	0.194619807	0.253181596	0.201724828	0.141786611	0.134136601	hsa_circ_0001016	NC_000002.12	-	61522611	61526521	3911	175	sense-overlappin	XM_011533098.1	XPO1
circRNA_01625	0.349775418	0.276344562	0.763302576	0.3920876	0.39123074	0.227842003	0.361776336	0.235592398	0.31394518	0.474053346	0.212679916	0.144454801	hsa_circ_0001017	NC_000002.12	-	61522611	61533903	11293	307	sense-overlappin	XM_011533098.1	XPO1
circRNA_01626	0	0	0	0.0206362	0	0	0	0	0.020254528	0	0	0	hsa_circ_0054903	NC_000002.12	-	61872082	61883548	11467	1311	sense-overlappin	NM_006430.3	CCT4
circRNA_01627	0	0	0.021808645	0.0206362	0	0	0	0	0.020254528	0	0	0	hsa_circ_0007473	NC_000002.12	-	61873002	61883548	10547	945	sense-overlappin	NM_006430.3	CCT4
circRNA_01628	0	0	0.021808645	0	0	0	0	0	0	0	0	0	hsa_circ_0005625	NC_000002.12	-	61876095	61880394	4300	647	sense-overlappin	NM_001256721.1	CCT4
circRNA_01629	0	0	0	0.0309543	0.03009467	0	0	0	0	0	0	0	-	NC_000002.12	-	61876095	61883548	7454	737	sense-overlappin	NM_006430.3	CCT4
circRNA_01630	0	0	0.054521613	0.0412724	0.02006311	0	0	0.020486295	0	0.050431207	0	0	-	NC_000002.12	+	62000701	62000982	282	282	exonic	XM_011532560.1	COMMD1
circRNA_01631	0	0	0	0	0	0	0	0	0	0	0	0.0206364	-	NC_000002.12	+	62135831	62141661	5831	5831	sense-overlappin	XM_005264157.3	COMMD1
circRNA_01632	0	0	0.021808645	0	0	0	0	0	0	0	0	0	hsa_circ_0006335	NC_000002.12	+	62747395	62771392	23998	208	sense-overlappin	NM_001142615.2	EHBP1
circRNA_01633	0.020575025	0	0	0	0	0	0	0	0	0.030258724	0	0	-	NC_000002.12	+	62943802	62949162	5361	952	sense-overlappin	NM_001142615.2	EHBP1
circRNA_01634	0.020575025	0	0.054521613	0.0722267	0	0.072495183	0.040197371	0.030729443	0.060763583	0.07060369	0	0	hsa_circ_0005552	NC_000002.12	+	62979188	62996766	17579	751	sense-overlappin	XM_011532713.1	EHBP1
circRNA_01635	0	0	0.04361729	0	0	0	0	0	0	0	0	0	-	NC_000002.12	+	63194600	63201291	6692	6692	antisense	XM_011532881.1	WDPCP
circRNA_01636	0	0	1.210379798	0.0515905	0	0	0	0	0	0	0	0	-	NC_000002.12	+	63196122	63201291	5170	5170	antisense	XM_011532881.1	WDPCP
circRNA_01637	0	0	0.04361729	0	0	0	0	0	0	0	0	0	hsa_circ_0054923	NC_000002.12	-	63313248	63382094	68847	377	sense-overlappin	XM_011532881.1	WDPCP
circRNA_01638	0	0	0.04361729	0	0	0	0	0	0	0	0	0	hsa_circ_0007387	NC_000002.12	-	63404048	63439871	35824	1051	sense-overlappin	XM_011532881.1	WDPCP
circRNA_01639	0	0	0.04361729	0	0.02006311	0.020712909	0	0	0.020254528	0.020172483	0	0	hsa_circ_0003497	NC_000002.12	-	63433745	63439871	6127	441	sense-overlappin	XM_011532881.1	WDPCP
circRNA_01640	0.041150049	0	0.152660515	0.0515905	0.02006311	0.020712909	0	0.020486295	0	0.020172483	0	0	hsa_circ_0005762	NC_000002.12	-	63484604	63492940	8337	309	sense-overlappin	XM_011532881.1	WDPCP
circRNA_01641	0.020575025	0	0.065425935	0.0206362	0	0	0	0	0	0	0	0	hsa_circ_0006759	NC_000002.12	-	63484604	63571626	87023	561	sense-overlappin	XM_011532881.1	WDPCP
circRNA_01642	0	0	0	0	0.04012623	0	0	0	0	0	0	0	-	NC_000002.12	-	63571375	63571626	252	252	exonic	XM_011532881.1	WDPCP
circRNA_01643	0.051437562	0.040939935	0.294416708	0.0619086	0.12037869	0.082851637	0.030148028	0.081945182	0.040509055	0.151293621	0.04051046	0.0412728	hsa_circ_0001020	NC_000002.12	+	63856306	63857936	1631	405	sense-overlappin	XM_006712088.1	UGP2
circRNA_01644	0.041150049	0.020469968	0.021808645	0	0	0	0	0.030729443	0	0	0	0.0206364	hsa_circ_0004227	NC_000002.12	+	63856306	63862928	6623	5397	sense-overlappin	XM_006712088.1	UGP2
circRNA_01645	0	0	0	0	0	0.020712909	0	0	0.020254528	0	0	0	-	NC_000002.12	+	63856306	63884093	27788	725	sense-overlappin	XM_006712088.1	UGP2
circRNA_01646	0	0	0	0.0515905	0	0	0	0	0	0	0	0	-	NC_000002.12	-	63949037	63965966	16930	645	sense-overlappin	XM_006712029.2	VPS54
circRNA_01647	0	0	0.04361729	0	0	0	0	0	0	0	0	0	hsa_circ_0054958	NC_000002.12	-	63962058	63984019	21962	1030	sense-overlappin	XM_006712029.2	VPS54
circRNA_01648	0	0	0	0	0	0	0	0	0	0.020172483	0	0	-	NC_000002.12	-	63972166	63984019	11854	477	sense-overlappin	XM_006712029.2	VPS54
circRNA_01649	0.020575025	0	0.17446916	0.1238171	0.02006311	0.041425819	0.020098685	0	0.030381792	0.020172483	0.030382845	0	hsa_circ_0001021	NC_000002.12	+	64551443	64553409	1967	1967	exonic	XR_244940.2	
circRNA_01650	0	0	0	0	0	0.041425819	0	0	0	0	0	0	hsa_circ_0054961	NC_000002.12	+	64551443	64573068	21626	2426	sense-overlappin	XR_244940.2	
circRNA_01651	0	0	0	0	0	0	0	0.030729443	0	0	0	0	hsa_circ_0054963	NC_000002.12	+	64569092	64573068	3977	307	sense-overlappin	XR_244940.2	
circRNA_01652	0.030862537	0	0.065425935	0.0206362	0.02006311	0.103564547	0.060296056	0	0	0	0.030382845	0	hsa_circ_0006037	NC_000002.12	+	64572946	64585545	12600	392	sense-overlappin	NM_203437.3	AFTPH
circRNA_01653	0	0.051174919	0	0.0309543	0.03009467	0.020712909	0.070345399	0.051215739	0.020254528	0	0	0.072227401	hsa_circ_0054971	NC_000002.12	+	65001448	65010763	9316	273	sense-overlappin	NM_001193493.1	SLC1A4
circRNA_01654	0	0.020469968	0	0	0	0	0	0	0	0	0	0	-	NC_000002.12	+	65003953	65010763	6811	230	sense-overlappin	NM_001193493.1	SLC1A4

circRNA_01655	0	0.020469968	0.021808645	0	0	0	0	0	0.030381792	0	0	0	-	NC_000002.12	-		65090983	65098066	7084	192	sense-overlappin	XM_011533009.1	RAB1A
circRNA_01656	0	0	0	0	0	0.031069364	0	0	0	0	0	0	hsa_circ_0054980	NC_000002.12	+		65239852	65265175	25324	981	sense-overlappin	NM_001005386.2	ACTR2
circRNA_01657	0	0	0.032712968	0	0.03009467	0	0	0	0	0	0	0	hsa_circ_0007961	NC_000002.12	-		65331987	65344896	12910	412	sense-overlappin	XM_005264200.3	SPRED2
circRNA_01658	0	0	0	0	0	0.031069364	0	0	0	0	0	0	hsa_circ_0055002	NC_000002.12	+		66464109	66512294	48186	258	sense-overlappin	XM_005264321.1	MEIS1
circRNA_01659	0	0.020469968	0	0	0	0	0	0	0	0	0	0	hsa_circ_0008158	NC_000002.12	+		66464109	66548019	83911	335	sense-overlappin	XM_005264321.1	MEIS1
circRNA_01660	0	0	0	0	0	0.031069364	0	0	0	0	0	0	-	NC_000002.12	-		68131277	68134799	3523	267	sense-overlappin	NM_138458.3	WDR92
circRNA_01661	0	0	0	0	0.02006311	0	0	0	0	0	0	0	-	NC_000002.12	-		68137299	68141795	4497	353	sense-overlappin	NM_138458.3	WDR92
circRNA_01662	0	0	0	0	0	0	0	0.020486295	0	0	0	0	-	NC_000002.12	-		68141694	68147567	5874	334	sense-overlappin	NM_138458.3	WDR92
circRNA_01663	0	0	0	0	0.02006311	0	0	0	0	0	0	0	-	NC_000002.12	+		68158380	68162663	4284	413	sense-overlappin	NM_020143.2	PNO1
circRNA_01664	0	0	0	0	0.02006311	0	0	0	0	0	0	0	-	NC_000002.12	-		68186468	68188690	2223	422	sense-overlappin	NM_000945.3	PPP3R1
circRNA_01665	0	0.020469968	0.021808645	0.0309543	0.03009467	0.020712909	0	0	0	0.020172483	0	0	hsa_circ_0003874	NC_000002.12	+		68490190	68513680	23491	526	sense-overlappin	NM_173545.2	APLF
circRNA_01666	0	0.040939935	0	0.0309543	0	0	0	0	0.030381792	0.030258724	0	0.0412728	hsa_circ_0055019	NC_000002.12	+		68490190	68526242	36053	708	sense-overlappin	NM_173545.2	APLF
circRNA_01667	0	0	0	0	0	0	0	0	0.020254528	0	0	0	hsa_circ_0055020	NC_000002.12	+		68490190	68538227	48038	1064	sense-overlappin	NM_173545.2	APLF
circRNA_01668	0.144025172	0.040939935	0.076330258	0.0619086	0.03009467	0.124277456	0.020098685	0.030729443	0.141781694	0.100862414	0	0.092863801	hsa_circ_0001023	NC_000002.12	+		68490190	68545312	55123	1190	sense-overlappin	NM_173545.2	APLF
circRNA_01669	0	0	0	0	0	0.020712909	0	0	0	0	0	0	hsa_circ_0055021	NC_000002.12	+		68490190	68567387	77198	1237	sense-overlappin	NM_173545.2	APLF
circRNA_01670	0	0	0	0	0	0	0	0	0.030381792	0.030258724	0	0	-	NC_000002.12	+		68490190	68578003	87814	1421	sense-overlappin	NM_173545.2	APLF
circRNA_01671	0	0	0	0	0	0	0	0	0	0.040344966	0	0	-	NC_000002.12	+		68502731	68526242	23512	636	sense-overlappin	NM_173545.2	APLF
circRNA_01672	0.072012586	0	0	0	0	0.020712909	0	0	0	0	0	0	-	NC_000002.12	+		68567341	68578003	10663	231	sense-overlappin	NM_173545.2	APLF
circRNA_01673	0	0	0	0.0206362	0	0	0	0	0	0	0	0	-	NC_000002.12	-		69326171	69345999	19829	1109	sense-overlappin	NM_001244710.1	GFPT1
circRNA_01674	0	0	0	0.0309543	0	0	0	0	0	0	0	0	hsa_circ_0005551	NC_000002.12	-		69328271	69338565	10295	690	sense-overlappin	NM_001244710.1	GFPT1
circRNA_01675	0.020575025	0	0	0	0	0.031069364	0	0	0	0	0	0	hsa_circ_0055038	NC_000002.12	-		69328271	69345999	17729	884	sense-overlappin	NM_001244710.1	GFPT1
circRNA_01676	0	0.020469968	0.021808645	0.0412724	0.02006311	0	0.030148028	0	0	0.020172483	0	0	hsa_circ_0001025	NC_000002.12	-		69337898	69338565	668	279	sense-overlappin	NM_001244710.1	GFPT1
circRNA_01677	0	0.020469968	0.065425935	0.0619086	0.02006311	0	0	0	0.020254528	0	0	0	hsa_circ_0005504	NC_000002.12	-		69337898	69345999	8102	473	sense-overlappin	NM_001244710.1	GFPT1
circRNA_01678	0	0	0	0.0206362	0	0	0	0	0	0	0	0	hsa_circ_0055048	NC_000002.12	-		69337898	69363670	25773	1259	sense-overlappin	NM_001244710.1	GFPT1
circRNA_01679	0	0	0.021808645	0.0309543	0	0	0	0	0	0.030258724	0	0	hsa_circ_0055049	NC_000002.12	-		69342152	69363670	21519	980	sense-overlappin	NM_001244710.1	GFPT1
circRNA_01680	0	0	0	0.0412724	0	0.051782273	0	0	0	0	0	0	hsa_circ_0055055	NC_000002.12	-		69348171	69363670	15500	786	sense-overlappin	NM_001244710.1	GFPT1
circRNA_01681	0	0	0	0	0	0	0	0.061458886	0	0.020172483	0	0	hsa_circ_0055061	NC_000002.12	-		69356496	69363670	7175	382	sense-overlappin	NM_001244710.1	GFPT1
circRNA_01682	0	0	0.08723458	0.0309543	0.11034713	0	0	0.030729443	0	0.040344966	0	0.0309546	hsa_circ_0002863	NC_000002.12	-		69358329	69363670	5342	320	sense-overlappin	NM_001244710.1	GFPT1
circRNA_01683	0	0	0.032712968	0	0	0	0	0	0	0	0	0	-	NC_000002.12	-		69358329	69370108	11780	428	sense-overlappin	NM_001244710.1	GFPT1
circRNA_01684	0	0.020469968	0.054521613	0	0.03009467	0	0	0	0	0	0	0	hsa_circ_0007046	NC_000002.12	-		69363545	69374113	10569	342	sense-overlappin	NM_001244710.1	GFPT1
circRNA_01685	0	0	0.021808645	0	0	0	0	0	0	0	0	0	hsa_circ_0055095	NC_000002.12	+		69806385	69812709	6325	342	sense-overlappin	XM_011532805.1	ANXA4
circRNA_01686	0	0	0.032712968	0	0	0	0	0	0	0	0	0	-	NC_000002.12	+		69812653	69819338	6686	306	sense-overlappin	XM_011532805.1	ANXA4
circRNA_01687	0	0	0.04361729	0	0.02006311	0	0	0	0	0	0	0	-	NC_000002.12	+		69812653	69820821	8169	429	sense-overlappin	XM_011532805.1	ANXA4
circRNA_01688	0	0	0.021808645	0	0.05015779	0	0	0	0	0	0.02025523	0	-	NC_000002.12	+		69816101	69819338	3238	249	sense-overlappin	XM_011532805.1	ANXA4
circRNA_01689	0	0.020469968	0	0	0	0	0	0	0	0	0	0	hsa_circ_0055102	NC_000002.12	-		69988742	69996888	8147	8147	intergenic		
circRNA_01690	0	0	0.04361729	0	0	0	0	0	0	0	0	0	hsa_circ_0004975	NC_000002.12	-		70160625	70181997	21373	1528	sense-overlappin	XM_005264391.1	C2orf42
circRNA_01691	0	0	0.021808645	0	0	0	0	0	0	0	0	0	hsa_circ_0008220	NC_000002.12	-		70175673	70181997	6325	1051	sense-overlappin	XM_005264391.1	C2orf42
circRNA_01692	0.051437562	0.071644886	0.076330258	0.0825448	0.05015779	0.031069364	0.030148028	0	0.040509055	0.030258724	0	0.0309546	hsa_circ_0055113	NC_000002.12	-		70179532	70181997	2466	946	sense-overlappin	XM_005264391.1	C2orf42
circRNA_01693	0.051437562	0.020469968	0	0	0	0	0	0	0	0.020172483	0	0.0309546	hsa_circ_0002199	NC_000002.12	-		70224554	70230854	6301	470	sense-overlappin	XM_005264531.1	TIA1
circRNA_01694	0	0	0.021808645	0	0	0	0	0	0	0	0	0	hsa_circ_0055145	NC_000002.12	+		71075555	71076701	1147	186	sense-overlappin	XM_005264412.3	NAGK
circRNA_01695	0	0	0	0	0	0	0	0	0.020254528	0	0	0	-	NC_000002.12	+		71355719	71370005	14287	948	sense-overlappin	NM_001014972.2	ZNF638
circRNA_01696	0	0	0.04361729	0.0309543	0	0	0	0	0	0.020172483	0	0	hsa_circ_0055149	NC_000002.12	+		71355719	71380565	24847	1060	sense-overlappin	NM_001014972.2	ZNF638
circRNA_01697	0.020575025	0	0	0	0	0	0	0	0	0	0	0	hsa_circ_0055150	NC_000002.12	+		71355719	71418639	62921	1982	sense-overlappin	NM_001014972.2	ZNF638
circRNA_01698	0.051437562	0	0.021808645	0	0	0	0.020098685	0.020486295	0	0	0	0	hsa_circ_0001028	NC_000002.12	+		71363153	71370005	6853	886	sense-overlappin	NM_001014972.2	ZNF638
circRNA_01699	0	0	0.032712968	0	0	0	0	0	0	0	0	0	-	NC_000002.12	+		71363153	71380565	17413	998	sense-overlappin	NM_001014972.2	ZNF638
circRNA_01700	0	0	0	0.0206362	0	0	0	0	0	0	0	0	-	NC_000002.12	+		71365429	71406262	40834	1418	sense-overlappin	NM_001014972.2	ZNF638
circRNA_01701	0.051437562	0.030704951	0.196277805	0.0928628	0	0.124277456	0.040197371	0.112674625	0.081018111	0.030258724	0.02025523	0.0412728	hsa_circ_0055151	NC_000002.12	+		71396141	71418639	22499	922	sense-overlappin	NM_001014972.2	ZNF638
circRNA_01702	0	0	0	0	0	0	0	0	0	0	0	0.0206364	-	NC_000002.12	+		71418602	71431428	12827	2491	sense-overlappin	NM_001014972.2	ZNF638
circRNA_01703	0.061725074	0	0.021808645	0	0.02006311	0	0	0	0.020254528	0.050431207	0	0	-	NC_000002.12	+		71426460	71427414	955	955	exonic	NM_001014972.2	ZNF638
circRNA_01704	0	0	0	0.0309543	0	0	0.030148028	0	0	0	0	0	-	NC_000002.12	+		71426460	71431428	4969	1162	sense-overlappin	NM_001014972.2	ZNF638
circRNA_01705	0	0	0	0	0	0	0.030148028	0	0	0	0	0	-	NC_000002.12	+		71526220	71539239	13020	427	sense-overlappin	XM_005264585.3	DYSF
circRNA_01706	0	0.092114854	0	0.0309543	0	0.020712909	0.020098685	0	0.020254528	0.060517448	0	0.0309546	-	NC_000002.12	-		72465160	72480750	15591	315	sense-overlappin	XM_005264224.1	EXOC6B
circRNA_01707	0	0	0	0.0206362	0	0	0	0	0	0	0	0	hsa_circ_0004643	NC_000002.12	-		72559453	72575668	16216	246	sense-overlappin	XM_005264223.1	EXOC6B
circRNA_01708	0	0.030704951	0	0	0	0	0	0	0	0	0	0	-	NC_000002.12	-		72578473	72578554	82	82	intronic	XM_005264223.1	EXOC6B
circRNA_01709	0.020575025	0	0	0	0	0	0	0	0	0	0	0	-	NC_000002.12	-		72674532	72733118	58587	43961	sense-overlappin	XM_005264223.1	EXOC6B

circRNA_01710	0	0	0	0	0	0.031069364	0	0	0	0	0	0	-	NC_000002.12	-	72680614	72733118	52505	37879	sense-overlappin	XM_005264223.1	EXOC6B
circRNA_01711	0	0	0	0	0	0.031069364	0	0	0	0.020172483	0	0	-	NC_000002.12	-	72718103	72731245	13143	342	sense-overlappin	XM_005264223.1	EXOC6B
circRNA_01712	0.390925468	0.286579545	0.30532103	0.3817695	0.23072582	0.341763004	0.070345399	0.22534925	0.293690652	0.413535897	0.141786611	0.268273202	hsa_circ_0009043	NC_000002.12	-	72718103	72733118	15016	390	sense-overlappin	XM_005264223.1	EXOC6B
circRNA_01713	0.092587611	0.020469968	0.04361729	0	0.04012623	0	0.040197371	0.030729443	0.060763583	0.040344966	0.02025523	0	hsa_circ_0001030	NC_000002.12	-	72731007	72733118	2112	185	sense-overlappin	XM_005264223.1	EXOC6B
circRNA_01714	0	0.030704951	0	0	0.03009467	0	0	0	0	0	0	0	hsa_circ_0007273	NC_000002.12	-	73088050	73089315	1266	1137	sense-overlappin	XM_011532753.1	RAB11FIP5
circRNA_01715	0.020575025	0	0.021808645	0	0	0	0	0	0	0	0	0	hsa_circ_0055229	NC_000002.12	+	73419123	73432291	13169	982	sense-overlappin	NM_015120.4	ALMS1
circRNA_01716	0.020575025	0	0.021808645	0	0	0	0	0	0	0	0	0	-	NC_000002.12	+	73432129	73455295	23167	6405	sense-overlappin	NM_015120.4	ALMS1
circRNA_01717	0	0.040939935	0	0	0	0	0	0	0.040509055	0	0	0	-	NC_000002.12	+	73432129	73491498	59370	8270	sense-overlappin	NM_015120.4	ALMS1
circRNA_01718	0.072012586	0	0	0	0	0	0	0	0	0	0	0	-	NC_000002.12	+	73432129	73520016	87888	8512	sense-overlappin	NM_015120.4	ALMS1
circRNA_01719	0	0	0	0.0412724	0	0	0	0	0	0.040344966	0	0	hsa_circ_0055235	NC_000002.12	+	73432198	73455295	23098	6336	sense-overlappin	NM_015120.4	ALMS1
circRNA_01720	0	0.030704951	0	0	0	0.031069364	0	0	0	0.020172483	0	0	hsa_circ_0055236	NC_000002.12	+	73432198	73491498	59301	8201	sense-overlappin	NM_015120.4	ALMS1
circRNA_01721	0.020575025	0.020469968	0	0.0412724	0	0	0	0	0	0	0	0	hsa_circ_0055239	NC_000002.12	+	73447960	73455295	7336	6242	sense-overlappin	NM_015120.4	ALMS1
circRNA_01722	0	0	0.021808645	0	0	0	0	0	0	0	0	0	hsa_circ_0055240	NC_000002.12	+	73489634	73491498	1865	1865	exonic	NM_015120.4	ALMS1
circRNA_01723	0	0	0.021808645	0	0	0	0	0	0	0	0	0	hsa_circ_0055241	NC_000002.12	+	73519775	73573424	53650	2008	sense-overlappin	NM_015120.4	ALMS1
circRNA_01724	0	0	0	0	0.05015779	0	0	0	0	0	0	0	hsa_circ_0055243	NC_000002.12	+	73534824	73573424	38601	1766	sense-overlappin	NM_015120.4	ALMS1
circRNA_01725	0	0	0.032712968	0	0	0	0.020098685	0	0	0.020172483	0	0	-	NC_000002.12	+	73557220	73559142	1923	306	sense-overlappin	NM_015120.4	ALMS1
circRNA_01726	0.020575025	0	0	0	0	0	0	0	0	0	0	0	-	NC_000002.12	+	73913485	73914871	1387	354	sense-overlappin	NM_001199893.1	ACTG2
circRNA_01727	0	0	0	0	0	0.020712909	0	0	0	0	0	0	-	NC_000002.12	+	73945258	73945414	157	157	intronic	NM_080916.2	DGUOK
circRNA_01728	0	0	0	0	0	0.020712909	0	0	0	0	0	0	-	NC_000002.12	-	74029690	74029770	81	81	antisense	XM_011532683.1	TET3
circRNA_01729	0	0	0	0	0	0.020712909	0	0.020486295	0	0	0.02025523	0	hsa_circ_0006251	NC_000002.12	+	74046278	74048411	2134	2134	exonic	XM_011532683.1	TET3
circRNA_01730	0	0	0	0	0	0	0	0	0	0.020172483	0	0	hsa_circ_0001032	NC_000002.12	+	74073549	74080591	7043	185	sense-overlappin	XM_011532683.1	TET3
circRNA_01731	0.020575025	0	0.021808645	0	0	0	0	0	0	0	0.02025523	0	hsa_circ_0007911	NC_000002.12	-	74165218	74172752	7535	395	sense-overlappin	XM_011532947.1	MOB1A
circRNA_01732	0	0	0.021808645	0	0	0	0.020098685	0	0	0	0	0	hsa_circ_0008330	NC_000002.12	-	74167014	74172752	5739	261	sense-overlappin	XM_011532947.1	MOB1A
circRNA_01733	0	0	0.021808645	0	0	0	0	0	0	0	0	0	hsa_circ_0008440	NC_000002.12	-	75518056	75522487	4432	276	sense-overlappin	NM_001135032.1	EVA1A
circRNA_01734	0	0	0.021808645	0	0	0	0	0	0	0	0	0	hsa_circ_0055371	NC_000002.12	-	75670138	75672016	1879	214	sense-overlappin	NM_001201334.1	GCFC2
circRNA_01735	0	0.020469968	0	0	0	0	0	0	0	0	0	0.0206364	-	NC_000002.12	-	75694241	75696315	2075	303	sense-overlappin	NM_001201334.1	GCFC2
circRNA_01736	0	0	0.076330258	0	0	0	0	0	0	0	0	0	-	NC_000002.12	+	81548618	81570413	21796	21796	intergenic		
circRNA_01737	0	0	0.065425935	0	0	0	0	0	0	0	0	0	-	NC_000002.12	+	81797818	81798518	701	701	intergenic		
circRNA_01738	0	0	0	0	0	0	0	0	0	0.020172483	0	0	-	NC_000002.12	-	84431508	84449752	18245	728	sense-overlappin	NM_003849.3	SUCLG1
circRNA_01739	0	0	0	0	0	0.041425819	0	0	0	0	0	0	-	NC_000002.12	+	84686484	84701339	14856	998	sense-overlappin	XM_011532649.1	DNAH6
circRNA_01740	0	0	0	0	0.02006311	0	0	0	0	0	0	0	-	NC_000002.12	+	84688439	84701339	12901	924	sense-overlappin	XM_011532649.1	DNAH6
circRNA_01741	0	0	0.032712968	0	0	0	0	0	0	0	0	0	-	NC_000002.12	-	84961674	84963103	1430	1430	intergenic		
circRNA_01742	0	0	0	0	0.03009467	0.031069364	0	0	0	0	0	0	hsa_circ_0004964	NC_000002.12	+	85043564	85049648	6085	560	sense-overlappin	NM_020122.4	KCMF1
circRNA_01743	0	0	0	0	0	0	0.040197371	0	0	0	0	0	hsa_circ_0055397	NC_000002.12	-	85349384	85351021	1638	642	sense-overlappin	NM_017750.3	RETSAT
circRNA_01744	0	0	0	0.1031809	0	0.103564547	0.020098685	0	0	0.020172483	0	0.0412728	hsa_circ_0008059	NC_000002.12	+	85368686	85369830	1145	161	sense-overlappin	XM_005264596.2	ELMOD3
circRNA_01745	0.020575025	0	0.054521613	0.0722267	0.03009467	0.062138728	0	0.051215739	0	0.040344966	0.030382845	0.0309546	hsa_circ_0004726	NC_000002.12	+	85368686	85371562	2877	408	sense-overlappin	XM_005264596.2	ELMOD3
circRNA_01746	0	0	0	0	0.03009467	0	0	0	0	0.040344966	0	0	hsa_circ_0055426	NC_000002.12	-	85555484	85556260	777	186	sense-overlappin	NM_000821.5	GGCX
circRNA_01747	0	0	0	0	0	0	0	0	0	0	0.02025523	0	-	NC_000002.12	+	85596519	85596931	413	241	sense-overlappin	NM_016494.3	RNF181
circRNA_01748	0	0	0.021808645	0	0	0	0	0	0	0	0	0	hsa_circ_0055440	NC_000002.12	+	85619220	85621579	2360	165	sense-overlappin	XM_006711922.1	USP39
circRNA_01749	0	0	0	0	0	0	0.020098685	0	0	0	0	0	-	NC_000002.12	+	85849403	85875085	25683	14369	antisense	XR_939735.1	
circRNA_01750	0	0.030704951	0.032712968	0	0	0	0	0	0	0.030258724	0	0	hsa_circ_0055451	NC_000002.12	-	85861181	85863485	2305	236	sense-overlappin	XM_011533143.1	ST3GAL5
circRNA_01751	0	0	0.021808645	0	0	0	0	0	0	0	0	0	-	NC_000002.12	-	86065274	86081706	16433	1241	sense-overlappin	XM_006711983.2	POLR1A
circRNA_01752	0	0	0	0	0	0	0.020098685	0	0	0	0	0	-	NC_000002.12	-	86098611	86100172	1562	355	sense-overlappin	XM_006711983.2	POLR1A
circRNA_01753	0.030862537	0	0	0.0619086	0.02006311	0	0	0	0	0	0	0	hsa_circ_0055499	NC_000002.12	+	86127161	86127991	831	196	sense-overlappin	NM_017952.5	PTCD3
circRNA_01754	0	0.030704951	0	0	0	0	0	0	0	0	0	0	hsa_circ_0055521	NC_000002.12	+	86206106	86207327	1222	335	sense-overlappin	XM_005264361.2	MRPL35
circRNA_01755	0	0	0	0	0.02006311	0	0	0	0	0.020172483	0	0	hsa_circ_0008804	NC_000002.12	+	86474776	86480362	5587	833	sense-overlappin	XM_011532985.1	KDM3A
circRNA_01756	0	0	0.032712968	0	0	0	0	0	0	0	0	0	-	NC_000002.12	-	86529218	86542312	13095	241	sense-overlappin	NM_001193517.1	CHMP3
circRNA_01757	0	0.020469968	0	0	0	0	0	0	0	0	0	0	-	NC_000002.12	+	86740927	86753558	12632	379	sense-overlappin	NM_022780.3	RMND5A
circRNA_01758	0	0	0	0	0.03009467	0	0	0	0	0.030258724	0	0	-	NC_000002.12	+	86765027	86766024	998	333	sense-overlappin	NM_022780.3	RMND5A
circRNA_01759	0	0	0	0.0206362	0	0	0	0	0	0	0	0	-	NC_000002.12	+	86770023	86771712	1690	258	sense-overlappin	NM_022780.3	RMND5A
circRNA_01760	0	0	0.021808645	0	0	0	0	0	0	0	0	0	hsa_circ_0055553	NC_000002.12	-	88034134	88037051	2918	207	sense-overlappin	NM_001304526.1	KRCC1
circRNA_01761	0	0	0	0	0	0.020712909	0.040197371	0	0	0	0	0	-	NC_000002.12	+	88776501	88792494	15994	15994	intergenic		
circRNA_01762	0	0	0	0	0	0	0.020098685	0	0	0	0	0	-	NC_000002.12	+	88778202	88779319	1118	1118	intergenic		
circRNA_01763	0	0	0	0	0	0	0	0	0	0.040344966	0	0	-	NC_000002.12	+	88780843	88805408	24566	24566	intergenic		
circRNA_01764	0	0.122819805	0	0.0619086	0	0.165703275	0	0.071702034	0	0	0	0.072227401	-	NC_000002.12	+	88782734	88805408	22675	22675	intergenic		
circRNA_01765	0	0.040939935	0	0	0	0.020712909	0.050246713	0.071702034	0	0.07060369	0	0.0412728	-	NC_000002.12	+	88786470	88805408	18939	18939	intergenic		

circRNA_01766	0	0.061409903	0	0.0515905	0	0.15534682	0.15074014	0.133160921	0.030381792	0.201724828	0	0.154773001	-	NC_000002.12	+	88801099	88804880	3782	3782	intergenic		
circRNA_01767	0	0	0	0	0	0	0	0	0.293690652	0.968279174	0.091148535	0	-	NC_000002.12	+	90082996	90159914	76919	76919	intergenic	IGKV1D-17	
circRNA_01768	0.082300098	0	0.032712968	0	0.03009467	0.072495183	0	0	0.020254528	0.030258724	0	0.0309546	-	NC_000002.12	-	94196055	94203287	7233	7233	intergenic		
circRNA_01769	0	0	0.054521613	0	0	0	0	0	0	0	0	0	-	NC_000002.12	+	94209037	94211974	2938	2938	intergenic		
circRNA_01770	0.030862537	0.071644886	0.021808645	0.0722267	0.05015779	0.041425819	0	0.061458886	0.070890847	0.030258724	0.08102092	0.0206364	-	NC_000002.12	-	95148885	95153259	4375	1606	sense-overlappin	XR_923048.1	
circRNA_01771	0	0	0	0	0	0.031069364	0	0	0	0	0	0	-	NC_000002.12	-	95834669	95835127	459	459	intronic	XR_923080.1	
circRNA_01772	0	0	0.141756193	0	0	0.041425819	0	0	0	0.040344966	0.050638075	0	-	NC_000002.12	-	95851166	95860074	8909	1661	sense-overlappin	XR_923080.1	
circRNA_01773	0	0.051174919	0.032712968	0.0206362	0	0.072495183	0	0.040972591	0.060763583	0.040344966	0.02025523	0.0619092	-	NC_000002.12	-	95851694	95860074	8381	1631	sense-overlappin	XR_923080.1	
circRNA_01774	0	0	0	0	0	0	0	0.020486295	0	0	0	0	-	NC_000002.12	-	95855266	95860074	4809	1313	sense-overlappin	XR_923080.1	
circRNA_01775	0	0	0	0	0	0	0	0	0	0	0.04051046	0	-	NC_000002.12	-	95884173	95918042	33870	1938	sense-overlappin	XR_923080.1	
circRNA_01776	0	0.020469968	0	0	0	0	0	0	0	0.050431207	0	0.103182001	-	NC_000002.12	-	95895562	95910445	14884	816	sense-overlappin	XR_923080.1	
circRNA_01777	0	0	0	0	0	0.062138728	0	0	0	0	0	0	-	NC_000002.12	-	95897437	95902958	5522	306	sense-overlappin	XR_923080.1	
circRNA_01778	0	0	0	0	0	0	0	0	0.030381792	0	0.04051046	0.0412728	-	NC_000002.12	-	95897437	95910445	13009	714	sense-overlappin	XR_923080.1	
circRNA_01779	0	0	0	0	0	0	0	0.020486295	0	0	0	0	-	NC_000002.12	-	95899308	95914180	14873	816	sense-overlappin	XR_923080.1	
circRNA_01780	0.061725074	0	0	0	0	0	0	0	0	0	0	0	-	NC_000002.12	-	95914108	95916171	2064	204	sense-overlappin	XR_923080.1	
circRNA_01781	0	0	0.054521613	0	0	0	0	0	0	0	0	0.072227401	-	NC_000002.12	-	95919733	95919920	188	102	sense-overlappin	XR_923080.1	
circRNA_01782	0	0	0	0	0	0	0	0.051215739	0	0	0	0	-	NC_000002.12	-	95958757	95962424	3668	204	sense-overlappin	XR_923080.1	
circRNA_01783	0	0	0.021808645	0	0	0	0	0	0	0	0	0	hsa_circ_0055629	NC_000002.12	-	96194958	96195549	592	259	sense-overlappin	NM_020151.3	STARD7
circRNA_01784	0	0	0.04361729	0	0	0	0	0	0	0	0	0	-	NC_000002.12	+	96236684	96240688	4005	4005	intergenic		
circRNA_01785	0	0	0	0	0	0	0.030148028	0	0	0	0	0	hsa_circ_0007532	NC_000002.12	+	96359045	96360710	1666	379	sense-overlappin	NM_001281710.1	NCAPH
circRNA_01786	0	0	0	0	0	0	0	0.061458886	0	0	0	0	-	NC_000002.12	+	97142640	97211741	69102	2670	sense-overlappin	XR_922919.1	
circRNA_01787	0	0	0	0	0	0	0	0	0	0	0	0.0206364	-	NC_000002.12	+	97189218	97194754	5537	306	sense-overlappin	XM_011511137.1	ANKRD36
circRNA_01788	0	0	0.021808645	0	0	0	0	0	0	0	0	0	-	NC_000002.12	+	97194726	97206135	11410	714	sense-overlappin	XM_011511137.1	ANKRD36
circRNA_01789	0	0	0	0	0.02006311	0	0	0	0	0	0	0	-	NC_000002.12	+	97194845	97196621	1777	102	sense-overlappin	XM_011511137.1	ANKRD36
circRNA_01790	0	0	0.032712968	0	0	0	0	0	0	0	0	0	-	NC_000002.12	+	97194845	97202230	7386	408	sense-overlappin	XM_011511137.1	ANKRD36
circRNA_01791	0	0	0	0	0.02006311	0	0	0	0	0	0	0.0309546	-	NC_000002.12	+	97194845	97204096	9252	510	sense-overlappin	XM_011511137.1	ANKRD36
circRNA_01792	0	0	0	0	0	0	0	0	0	0	0.04051046	0	-	NC_000002.12	+	97194845	97205968	11124	612	sense-overlappin	XM_011511137.1	ANKRD36
circRNA_01793	0	0	0	0	0	0	0	0	0	0	0.02025523	0	-	NC_000002.12	+	97198586	97204096	5511	306	sense-overlappin	XM_011511137.1	ANKRD36
circRNA_01794	0.092587611	0	0.08723458	0	0.06018934	0.062138728	0	0	0	0.050431207	0	0	-	NC_000002.12	+	97200334	97208006	7673	510	sense-overlappin	XM_011511137.1	ANKRD36
circRNA_01795	0	0	0	0	0	0	0.080394741	0	0	0	0	0	-	NC_000002.12	+	97202202	97204263	2062	204	sense-overlappin	XM_011511137.1	ANKRD36
circRNA_01796	0	0	0	0	0	0	0	0	0	0	0.02025523	0	-	NC_000002.12	+	97204068	97206135	2068	204	sense-overlappin	XM_011511137.1	ANKRD36
circRNA_01797	0	0	0	0	0	0	0	0	0	0	0	0.0206364	-	NC_000002.12	+	97206063	97207839	1777	102	sense-overlappin	XM_011511137.1	ANKRD36
circRNA_01798	0	0	0	0	0	0	0	0	0	0	0	0.0412728	-	NC_000002.12	-	97545833	97547608	1776	102	sense-overlappin	XM_011511550.1	ANKRD36B
circRNA_01799	0	0	0	0	0	0	0	0	0	0.030258724	0	0	-	NC_000002.12	-	97545833	97549491	3659	204	sense-overlappin	XM_011511550.1	ANKRD36B
circRNA_01800	0	0.030704951	0	0	0	0	0	0	0	0	0	0	-	NC_000002.12	-	97547703	97549491	1789	102	sense-overlappin	XM_011511550.1	ANKRD36B
circRNA_01801	0.020575025	0	0	0.0412724	0.03009467	0	0	0	0	0	0	0	-	NC_000002.12	-	97813971	97818721	4751	543	sense-overlappin	NM_015348.1	TMEM131
circRNA_01802	0.030862537	0	0	0	0	0	0.020098685	0	0	0	0	0.0309546	-	NC_000002.12	-	97813971	97833426	19456	605	sense-overlappin	NM_015348.1	TMEM131
circRNA_01803	0	0	0.04361729	0	0.03009467	0	0	0	0	0	0	0	-	NC_000002.12	-	97859304	97927487	68184	347	sense-overlappin	XM_005263909.1	TMEM131
circRNA_01804	0	0	0.021808645	0	0	0	0	0	0	0	0	0	-	NC_000002.12	-	97888052	97927487	39436	223	sense-overlappin	XM_005263909.1	TMEM131
circRNA_01805	0	0	0	0	0.03009467	0	0	0	0	0	0	0	-	NC_000002.12	-	98635222	98678471	43250	1374	sense-overlappin	NM_012214.2	MGAT4A
circRNA_01806	0	0	0	0	0	0.031069364	0	0	0	0	0	0	-	NC_000002.12	-	99027120	99117759	90640	10252	sense-overlappin	XM_011511939.1	TSGA10
circRNA_01807	0	0	0	0	0	0	0	0	0	0.020172483	0.02025523	0	-	NC_000002.12	-	99105359	99117759	12401	987	sense-overlappin	XM_011511939.1	TSGA10
circRNA_01808	0.072012586	0.071644886	0.207182128	0.0619086	0.31097828	0.082851637	0.15074014	0.081945182	0.081018111	0.030258724	0.131658996	0.0206364	hsa_circ_0001050	NC_000002.12	-	99169550	99171429	1880	264	sense-overlappin	XM_011510583.1	MITD1
circRNA_01809	0	0.020469968	0.032712968	0	0.03009467	0	0	0	0	0	0	0.0206364	hsa_circ_0001051	NC_000002.12	+	99186177	99195756	9580	564	sense-overlappin	NM_145212.3	MRPL30
circRNA_01810	0	0	0	0.0309543	0.03009467	0	0	0	0	0	0	0	hsa_circ_0008609	NC_000002.12	+	99194751	99195756	1006	405	sense-overlappin	NM_145212.3	MRPL30
circRNA_01811	0	0	0	0.0309543	0	0	0	0	0	0	0	0	hsa_circ_0004147	NC_000002.12	+	99194751	99216114	21364	19366	sense-overlappin	NR_028356.1	
circRNA_01812	0.020575025	0	0	0	0	0	0	0	0	0	0	0	-	NC_000002.12	-	99302690	99304745	2056	2056	sense-overlappin	XM_011510580.1	LYG1
circRNA_01813	0	0	0.021808645	0	0	0	0	0	0	0	0	0	-	NC_000002.12	+	99360236	99371730	11495	1517	sense-overlappin	NM_015904.3	EIF5B
circRNA_01814	0	0	0	0	0	0	0.030148028	0	0	0	0	0	hsa_circ_0055836	NC_000002.12	-	99412731	99418947	6217	341	sense-overlappin	XM_011511340.1	REV1
circRNA_01815	0	0	0	0	0.05015779	0	0.030148028	0	0	0	0	0	hsa_circ_0007900	NC_000002.12	-	99412731	99435941	23211	1083	sense-overlappin	XM_011511339.1	REV1
circRNA_01816	0	0	0	0.0309543	0.03009467	0	0	0	0	0	0	0	hsa_circ_0055837	NC_000002.12	-	99412731	99464985	52255	2287	sense-overlappin	XM_011511336.1	REV1
circRNA_01817	0	0	0.021808645	0	0.03009467	0	0	0	0	0	0	0	hsa_circ_0001052	NC_000002.12	-	99418828	99435941	17114	862	sense-overlappin	XM_011511339.1	REV1
circRNA_01818	0	0.020469968	0	0	0	0	0	0	0	0	0	0	hsa_circ_0003100	NC_000002.12	-	99429840	99435941	6102	331	sense-overlappin	XM_011511339.1	REV1
circRNA_01819	0	0	0	0	0.02006311	0	0	0	0	0	0	0	hsa_circ_0055846	NC_000002.12	-	99434332	99464985	30654	1605	sense-overlappin	XR_427092.1	
circRNA_01820	0	0	0	0	0.02006311	0	0	0	0	0	0	0	hsa_circ_0055850	NC_000002.12	-	99442317	99464985	22669	670	sense-overlappin	XR_427092.1	

circRNA_01821	0.041150049	0	0.04361729	0.0309543	0.11034713	0.020712909	0.030148028	0	0	0.050431207	0	0	hsa_circ_0007092	NC_000002.12	-	99449336	99464985	15650	517	sense-overlappin	XR_427092.1	
circRNA_01822	0.020575025	0.020469968	0	0	0.06018934	0	0.020098685	0	0	0	0	0.0309546	hsa_circ_0001053	NC_000002.12	-	99462496	99464985	2490	191	sense-overlappin	XM_011511336.1	REV1
circRNA_01823	0	0.020469968	0.032712968	0	0	0	0	0	0	0	0.050638075	0	hsa_circ_0006946	NC_000002.12	+	100904733	100937842	33110	385	sense-overlappin	NM_002518.3	NPAS2
circRNA_01824	0	0	0	0	0.04012623	0.020712909	0	0	0	0	0	0	hsa_circ_0055862	NC_000002.12	+	100932910	100949480	16571	417	sense-overlappin	NM_002518.3	NPAS2
circRNA_01825	0.020575025	0	0.04361729	0.0515905	0.04012623	0	0	0	0	0	0	0	hsa_circ_0002401	NC_000002.12	+	101257791	101262691	4901	318	sense-overlappin	NM_017546.4	CNOT11
circRNA_01826	0	0	0	0	0	0	0	0	0	0.020172483	0	0	hsa_circ_0001057	NC_000002.12	+	101257791	101265047	7257	521	sense-overlappin	NM_017546.4	CNOT11
circRNA_01827	0	0	0	0	0	0	0.020098685	0	0	0	0	0	-	NC_000002.12	+	101257791	101266879	9089	724	sense-overlappin	NM_017546.4	CNOT11
circRNA_01828	0	0	0.032712968	0	0	0	0	0	0	0	0	0	-	NC_000002.12	-	101281859	101286177	4319	296	sense-overlappin	XR_922908.1	
circRNA_01829	0	0	0	0.0515905	0	0	0	0	0	0	0	0	hsa_circ_0055903	NC_000002.12	-	101281859	101289055	7197	379	sense-overlappin	XR_922908.1	
circRNA_01830	0.051437562	0	0.065425935	0.0619086	0	0.072495183	0.020098685	0	0.030381792	0.040344966	0.030382845	0	hsa_circ_0055904	NC_000002.12	-	101281859	101295181	13323	699	sense-overlappin	XR_922908.1	
circRNA_01831	0	0	0.032712968	0.0206362	0	0.020712909	0	0	0	0	0	0	hsa_circ_0055906	NC_000002.12	-	101288973	101295181	6209	403	sense-overlappin	XR_922908.1	
circRNA_01832	0	0	0	0	0	0	0.040197371	0.030729443	0	0	0	0	hsa_circ_0001058	NC_000002.12	-	101294014	101295181	1168	320	sense-overlappin	XR_922908.1	
circRNA_01833	0	0.051174919	0	0.1031809	0	0	0	0	0	0	0	0	-	NC_000002.12	+	102508079	102526346	18268	840	sense-overlappin	XM_011511158.1	SLC9A4
circRNA_01834	0	0	0	0.0206362	0	0	0	0	0	0	0	0	-	NC_000002.12	+	102508224	102526346	18123	695	sense-overlappin	XM_011511158.1	SLC9A4
circRNA_01835	0	0	0.032712968	0	0	0	0	0	0	0	0	0	-	NC_000002.12	+	102657564	102665350	7787	715	sense-overlappin	NM_003048.4	SLC9A2
circRNA_01836	0	0.030704951	0	0	0	0	0	0	0	0	0	0	-	NC_000002.12	-	105296356	105298705	2350	350	sense-overlappin	XM_011512193.1	TGFBRAP1
circRNA_01837	0	0	0.021808645	0	0	0	0	0	0	0	0	0	-	NC_000002.12	+	105855048	105855289	242	242	exonic	XM_011511991.1	NCK2
circRNA_01838	0	0	0.032712968	0	0	0	0	0	0	0	0	0	hsa_circ_0055937	NC_000002.12	-	106112646	106166083	53438	785	sense-overlappin	XM_011511902.1	UXS1
circRNA_01839	0	0	0.032712968	0	0	0	0	0	0	0	0	0	hsa_circ_0055940	NC_000002.12	-	106122970	106129778	6809	287	sense-overlappin	NM_001253876.1	UXS1
circRNA_01840	0	0	0.239895095	0.0206362	0.03009467	0.051782273	0.050246713	0.020486295	0.020254528	0.040344966	0	0	hsa_circ_0055945	NC_000002.12	-	106122970	106166083	43114	665	sense-overlappin	XM_011511902.1	UXS1
circRNA_01841	0	0	0.021808645	0	0	0	0	0	0	0	0	0	hsa_circ_0055947	NC_000002.12	-	106129674	106166083	36410	483	sense-overlappin	XM_011511902.1	UXS1
circRNA_01842	0.030862537	0	0.30532103	0.1031809	0.12037869	0.082851637	0.050246713	0.040972591	0.030381792	0.121034897	0.030382845	0.072227401	hsa_circ_0004029	NC_000002.12	-	106145190	106166083	20894	378	sense-overlappin	XM_011511902.1	UXS1
circRNA_01843	0.185175222	0.071644886	0.163564838	0.1547714	0.12037869	0.051782273	0.040197371	0.051215739	0.081018111	0.110948655	0.04051046	0.0309546	hsa_circ_0001060	NC_000002.12	-	106158058	106166083	8026	197	sense-overlappin	XM_011511902.1	UXS1
circRNA_01844	0	0	0	0	0	0	0	0	0.020254528	0	0	0	-	NC_000002.12	+	108451028	108452466	1439	153	sense-overlappin	NM_181453.3	GCC2
circRNA_01845	0.020575025	0	0	0	0.04012623	0	0	0	0	0	0	0	-	NC_000002.12	+	108451028	108475850	24823	2997	sense-overlappin	NM_181453.3	GCC2
circRNA_01846	0	0	0.021808645	0	0	0	0	0	0	0	0	0	-	NC_000002.12	+	108468980	108475850	6871	2844	sense-overlappin	NM_181453.3	GCC2
circRNA_01847	0	0	0.021808645	0	0	0	0	0	0	0	0	0	-	NC_000002.12	+	108729132	108740681	11550	903	sense-overlappin	XM_005264002.1	RANBP2
circRNA_01848	0.113162635	0	0.098138903	0.113499	0.15047336	0.051782273	0.040197371	0.040972591	0.050636319	0.050431207	0	0.0412728	hsa_circ_0006965	NC_000002.12	+	108771701	108773046	1346	443	sense-overlappin	XM_005264002.1	RANBP2
circRNA_01849	0	0	0	0	0.03009467	0	0	0	0	0	0	0	hsa_circ_0055999	NC_000002.12	+	108788852	108794721	5870	425	sense-overlappin	NM_001303105.1	CCDC138
circRNA_01850	0.020575025	0	0	0	0	0	0	0	0	0	0	0	hsa_circ_0056000	NC_000002.12	+	108791675	108816105	24431	940	sense-overlappin	NM_001303105.1	CCDC138
circRNA_01851	0	0	0.054521613	0	0	0	0	0	0	0	0	0	hsa_circ_0056001	NC_000002.12	+	108791675	108856970	65296	1427	sense-overlappin	NM_001303105.1	CCDC138
circRNA_01852	0	0	0	0.0309543	0.03009467	0	0	0	0	0	0	0	hsa_circ_0001061	NC_000002.12	+	108839185	108856970	17786	487	sense-overlappin	NM_001303105.1	CCDC138
circRNA_01853	0	0	0.032712968	0	0	0	0	0	0	0.020172483	0	0	-	NC_000002.12	+	108846738	108856970	10233	370	sense-overlappin	NM_001303105.1	CCDC138
circRNA_01854	0	0	0	0	0.04012623	0	0	0	0	0	0	0	-	NC_000002.12	+	108873451	108876442	2992	494	sense-overlappin	NM_001303106.1	CCDC138
circRNA_01855	0	0.020469968	0	0	0	0	0	0	0	0	0	0	hsa_circ_0003523	NC_000002.12	+	109329224	109347949	18726	18726	sense-overlappin	XM_011511111.1	SH3RF3
circRNA_01856	0	0	0	0	0.02006311	0	0	0	0	0	0	0	hsa_circ_0003065	NC_000002.12	-	109564366	109585838	21473	929	sense-overlappin	XM_011510703.1	10-Sep
circRNA_01857	0	0.020469968	0.119947548	0.0309543	0.04012623	0	0	0	0.030381792	0.07060369	0	0	hsa_circ_0002076	NC_000002.12	-	109564366	109593119	28754	998	sense-overlappin	XM_011510703.1	10-Sep
circRNA_01858	0.020575025	0	0	0	0.03009467	0	0	0	0	0	0	0	hsa_circ_0007654	NC_000002.12	-	109574581	109593119	18539	570	sense-overlappin	XM_011510703.1	10-Sep
circRNA_01859	0.041150049	0	0.098138903	0.0206362	0.09028402	0	0	0	0.030381792	0.040344966	0	0	hsa_circ_0056012	NC_000002.12	-	109585126	109585838	713	314	sense-overlappin	XM_011510703.1	10-Sep
circRNA_01860	0	0	0.076330258	0.113499	0.12037869	0.020712909	0.030148028	0	0	0.040344966	0	0	hsa_circ_0008953	NC_000002.12	-	109585126	109593119	7994	383	sense-overlappin	XM_011510703.1	10-Sep
circRNA_01861	0	0.020469968	0.021808645	0	0	0	0	0	0	0	0.02025523	0	hsa_circ_0056018	NC_000002.12	-	110161603	110163132	1530	180	sense-overlappin	NM_000272.3	NPHP1
circRNA_01862	0	0	0.021808645	0.0619086	0.03009467	0	0	0.071702034	0	0	0	0	hsa_circ_0056019	NC_000002.12	-	110161603	110163135	1533	183	sense-overlappin	NM_000272.3	NPHP1
circRNA_01863	0	0	0	0	0	0	0	0	0	0	0.030382845	0	-	NC_000002.12	+	110637236	110637326	91	91	intergenic		
circRNA_01864	0	0	0.021808645	0	0	0	0	0	0	0	0	0	hsa_circ_0056040	NC_000002.12	-	110666263	110674365	8103	931	sense-overlappin	XR_923001.1	
circRNA_01865	0	0	0	0	0	0	0	0.020486295	0	0	0	0	-	NC_000002.12	+	110987108	111031714	44607	310	sense-overlappin	XM_011511425.1	ACOXL
circRNA_01866	0	0	0	0	0	0	0.020098685	0	0	0	0	0	-	NC_000002.12	+	110987108	111049288	62181	381	sense-overlappin	XM_011511416.1	ACOXL
circRNA_01867	0	0.030704951	0	0	0	0	0	0	0	0	0	0	-	NC_000002.12	+	111929120	111929540	421	421	exonic	XM_005263568.3	MERTK
circRNA_01868	0	0	0	0	0	0	0	0	0	0	0.02025523	0	hsa_circ_0056121	NC_000002.12	+	111944960	111947567	2608	275	sense-overlappin	XM_005263568.3	MERTK
circRNA_01869	0	0	0	0	0.03009467	0	0	0	0	0	0	0	-	NC_000002.12	+	112059977	112067067	7091	285	sense-overlappin	NM_032824.2	TMEM87B
circRNA_01870	0	0	0.076330258	0	0	0	0	0	0	0	0	0	-	NC_000002.12	+	112074912	112107840	32929	1127	sense-overlappin	NM_032824.2	TMEM87B
circRNA_01871	0	0	0	0	0	0.031069364	0	0	0	0.040344966	0	0	-	NC_000002.12	+	112116084	112117940	1857	1857	exonic	NM_032824.2	TMEM87B
circRNA_01872	0.051437562	0.08187987	0.065425935	0	0.09028402	0.072495183	0.030148028	0.051215739	0.091145375	0.090776173	0.070893305	0	hsa_circ_0001062	NC_000002.12	+	112299849	112300029	181	181	exonic	XM_011511156.1	ZC3H6
circRNA_01873	0	0	0.032712968	0	0	0	0	0	0	0	0	0	-	NC_000002.12	+	112299849	112311937	12089	715	sense-overlappin	XM_011511156.1	ZC3H6
circRNA_01874	0	0	0.032712968	0	0	0.031069364	0	0.040972591	0	0	0	0	-	NC_000002.12	+	112299849	112325197	25349	2054	sense-overlappin	XM_011511156.1	ZC3H6

circRNA_01875	0	0.071644886	0.04361729	0.113499	0.04012623	0.093208092	0	0.081945182	0	0.050431207	0	0	-	NC_000002.12	+	112299849	112332282	32434	3332	sense-overlappin	XM_011511156.1	ZC3H6
circRNA_01876	0	0	0	0	0	0	0	0	0.020254528	0.030258724	0	0	-	NC_000002.12	+	112501206	112503181	1976	406	sense-overlappin	NM_153712.4	TTL
circRNA_01877	0	0	0	0	0	0	0	0.020486295	0	0	0	0	-	NC_000002.12	+	113444964	113457204	12241	330	sense-overlappin	XM_011510669.1	CBWD2
circRNA_01878	0	0	0	0	0	0	0	0	0	0	0.02025523	0	-	NC_000002.12	+	113444964	113465173	20210	514	sense-overlappin	XM_011510669.1	CBWD2
circRNA_01879	0	0	0	0	0	0	0	0	0	0	0	0.0206364	-	NC_000002.12	-	113719154	113729505	10352	511	sense-overlappin	NR_104470.1	
circRNA_01880	0	0	0	0	0	0	0	0	0.020254528	0.040344966	0	0	hsa_circ_0008827	NC_000002.12	-	113729401	113735858	6458	379	sense-overlappin	XM_011511922.1	SLC35F5
circRNA_01881	0	0	0	0	0	0	0	0	0.040509055	0	0	0	hsa_circ_0056180	NC_000002.12	-	113729401	113743794	14394	649	sense-overlappin	XM_011511922.1	SLC35F5
circRNA_01882	0.020575025	0	0	0	0	0	0	0	0.030381792	0.020172483	0	0	-	NC_000002.12	-	113830852	113831585	734	734	intergenic		
circRNA_01883	0	0.020469968	0	0	0	0	0	0	0	0	0	0	-	NC_000002.12	-	113835087	113836791	1705	1705	intergenic		
circRNA_01884	0	0	0	0	0.02006311	0	0	0	0	0	0	0	hsa_circ_0056189	NC_000002.12	+	113913172	113917008	3837	181	sense-overlappin	NM_005721.4	ACTR3
circRNA_01885	0	0	0	0	0.02006311	0	0	0	0	0	0	0	-	NC_000002.12	+	113934279	113942359	8081	426	sense-overlappin	NM_005721.4	ACTR3
circRNA_01886	0.030862537	0.020469968	0.054521613	0	0.02006311	0.062138728	0	0	0.050636319	0.020172483	0	0.0412728	hsa_circ_0008712	NC_000002.12	+	113939959	113942359	2401	318	sense-overlappin	NM_005721.4	ACTR3
circRNA_01887	0	0	0	0	0	0	0	0	0	0.020172483	0	0	hsa_circ_0056199	NC_000002.12	+	113939959	113951845	11887	537	sense-overlappin	NM_005721.4	ACTR3
circRNA_01888	0	0	0	0	0.04012623	0	0	0	0	0	0	0	-	NC_000002.12	-	117490977	117521329	30353	30353	intergenic		
circRNA_01889	0	0	0.032712968	0	0	0	0	0	0	0	0	0	-	NC_000002.12	-	117931037	117958481	27445	954	sense-overlappin	NM_019044.4	CCDC93
circRNA_01890	0	0	0	0	0	0.020712909	0	0	0	0	0	0	-	NC_000002.12	+	118103197	118109900	6704	2012	sense-overlappin	NM_016133.2	INSIG2
circRNA_01891	0.020575025	0	0	0	0	0	0	0	0	0	0	0	-	NC_000002.12	-	119336268	119339971	3704	3704	sense-overlappin	XM_011510603.1	C2orf76
circRNA_01892	0	0	0.04361729	0.0515905	0	0	0	0	0	0	0	0	-	NC_000002.12	+	119809837	119885882	76046	692	sense-overlappin	NM_002830.3	PTPN4
circRNA_01893	0	0.040939935	0	0	0.03009467	0	0	0	0	0.040344966	0	0	-	NC_000002.12	+	119877323	119885882	8560	429	sense-overlappin	NM_002830.3	PTPN4
circRNA_01894	0	0	0	0	0.04012623	0	0	0	0	0.020172483	0	0	hsa_circ_0007517	NC_000002.12	+	119881786	119882623	838	219	sense-overlappin	NM_002830.3	PTPN4
circRNA_01895	0	0	0	0.0206362	0	0.020712909	0	0	0	0	0	0	hsa_circ_0056245	NC_000002.12	+	119920069	119934958	14890	527	sense-overlappin	NM_002830.3	PTPN4
circRNA_01896	0	0	0.021808645	0	0.03009467	0	0.030148028	0	0	0.020172483	0.02025523	0	hsa_circ_0056247	NC_000002.12	+	119926598	119934958	8361	354	sense-overlappin	NM_002830.3	PTPN4
circRNA_01897	0	0	0	0.0825448	0.04012623	0	0	0	0	0	0	0	hsa_circ_0056248	NC_000002.12	+	119926598	119945240	18643	514	sense-overlappin	NM_002830.3	PTPN4
circRNA_01898	0	0	0	0.0206362	0	0	0	0	0	0.020172483	0	0	-	NC_000002.12	+	119932424	119946574	14151	586	sense-overlappin	NM_002830.3	PTPN4
circRNA_01899	0	0	0	0	0	0	0	0.030729443	0	0	0	0	hsa_circ_0056264	NC_000002.12	+	120019077	120042110	23034	293	sense-overlappin	NM_001184939.1	EPB41L5
circRNA_01900	0.020575025	0.061409903	0.207182128	0.1341352	0.0702209	0	0.060296056	0.040972591	0.030381792	0	0	0	hsa_circ_0001064	NC_000002.12	+	120073178	120078581	5404	518	sense-overlappin	NM_001184939.1	EPB41L5
circRNA_01901	0	0	0.021808645	0	0	0	0	0	0	0	0	0	-	NC_000002.12	+	120073178	120087240	14063	588	sense-overlappin	NM_001184939.1	EPB41L5
circRNA_01902	0	0	0	0	0.02006311	0	0	0	0	0	0	0	hsa_circ_0056275	NC_000002.12	+	120087171	120093276	6106	375	sense-overlappin	NM_001184939.1	EPB41L5
circRNA_01903	0.051437562	0	0	0.0722267	0.04012623	0	0	0.020486295	0	0.020172483	0.02025523	0	hsa_circ_0056280	NC_000002.12	+	120127688	120146289	18602	456	sense-overlappin	NM_001184937.1	EPB41L5
circRNA_01904	0.051437562	0.030704951	0.032712968	0.2476343	0.08025246	0.041425819	0.040197371	0.061458886	0	0.040344966	0	0	hsa_circ_0006834	NC_000002.12	+	120127688	120175000	47313	958	sense-overlappin	NM_020909.3	EPB41L5
circRNA_01905	0.174887709	0.092114854	0.239895095	0.2992247	0.21066271	0.062138728	0.050246713	0.030729443	0.030381792	0.060517448	0.04051046	0.0412728	hsa_circ_0008278	NC_000002.12	+	120127688	120175004	47317	962	sense-overlappin	NM_020909.3	EPB41L5
circRNA_01906	0	0	0.054521613	0	0	0	0	0	0	0	0	0	hsa_circ_0056285	NC_000002.12	+	120278618	120289757	11140	621	sense-overlappin	XM_011511574.1	RALB
circRNA_01907	0	0	0	0.0206362	0	0	0	0	0	0	0	0	hsa_circ_0007186	NC_000002.12	+	120285874	120289757	3884	387	sense-overlappin	XM_005263724.1	RALB
circRNA_01908	0	0	0.032712968	0	0	0	0	0	0	0	0	0	-	NC_000002.12	+	120891396	120927466	36071	36071	sense-overlappin	XM_006712422.1	GLI2
circRNA_01909	0	0	0	0	0	0	0	0	0	0.040344966	0	0	hsa_circ_0056310	NC_000002.12	-	121457687	121458975	1289	207	sense-overlappin	XM_011510852.1	CLASP1
circRNA_01910	0	0	0.065425935	0	0	0.041425819	0.020098685	0	0	0	0	0	hsa_circ_0002374	NC_000002.12	-	121503167	121530325	27159	517	sense-overlappin	XM_006712373.1	CLASP1
circRNA_01911	0.082300098	0	0.08723458	0.0206362	0.03009467	0	0	0	0	0.090776173	0.050638075	0.0309546	hsa_circ_0007052	NC_000002.12	-	121605701	121606180	480	480	exonic	XM_006712373.1	CLASP1
circRNA_01912	0.072012586	0.020469968	0.065425935	0.0412724	0.05015779	0.072495183	0.050246713	0.040972591	0.030381792	0.121034897	0.030382845	0	hsa_circ_0002847	NC_000002.12	+	121757240	121761524	4285	307	sense-overlappin	NM_001261401.1	TSN
circRNA_01913	0	0	0.065425935	0	0	0	0	0	0	0	0	0	-	NC_000002.12	+	122040191	122043150	2960	2960	intergenic		
circRNA_01914	0	0	0.021808645	0	0	0	0	0	0	0	0	0	-	NC_000002.12	+	122040194	122043150	2957	2957	intergenic		
circRNA_01915	0	0	0.021808645	0	0	0	0	0	0	0	0	0	-	NC_000002.12	+	122233705	122236310	2606	2606	intergenic		
circRNA_01916	0	0	0.032712968	0	0.03009467	0	0	0	0	0	0	0	-	NC_000002.12	-	127329921	127343194	13274	531	sense-overlappin	NM_006609.4	MAP3K2
circRNA_01917	0	0	0.04361729	0	0.02006311	0	0	0.020486295	0	0	0	0	-	NC_000002.12	-	127335870	127343194	7325	329	sense-overlappin	NM_006609.4	MAP3K2
circRNA_01918	0	0	0	0	0.02006311	0	0.030148028	0	0	0	0	0	hsa_circ_0003192	NC_000002.12	-	127763062	127771004	7943	747	sense-overlappin	NM_018383.4	WDR33
circRNA_01919	0	0	0	0	0.02006311	0	0	0.020486295	0	0	0	0	hsa_circ_0056362	NC_000002.12	-	127768189	127771004	2816	401	sense-overlappin	NM_018383.4	WDR33
circRNA_01920	0	0	0.021808645	0	0	0	0.020098685	0	0	0	0	0	-	NC_000002.12	-	127869454	127871359	1906	317	sense-overlappin	XM_011511955.1	AMMECR1L
circRNA_01921	0	0.051174919	0	0.0412724	0	0	0	0	0.020254528	0	0	0	hsa_circ_0056390	NC_000002.12	-	127993187	127996491	3305	264	sense-overlappin	NM_001145928.1	SAP130
circRNA_01922	0	0	0	0.0206362	0	0	0	0	0	0	0	0	hsa_circ_0006319	NC_000002.12	+	128097429	128104014	6586	219	sense-overlappin	NM_020120.3	UGGT1
circRNA_01923	0	0	0	0	0	0	0.060296056	0	0	0	0	0	hsa_circ_0056401	NC_000002.12	+	128097429	128109746	12318	463	sense-overlappin	NM_020120.3	UGGT1
circRNA_01924	0	0	0	0	0	0	0	0.020486295	0	0	0	0	hsa_circ_0056413	NC_000002.12	+	128113084	128145967	32884	1495	sense-overlappin	NM_020120.3	UGGT1
circRNA_01925	0.020575025	0.020469968	0.04361729	0	0.0702209	0	0.040197371	0	0.030381792	0.040344966	0	0.0309546	hsa_circ_0008298	NC_000002.12	+	128164730	128171284	6555	279	sense-overlappin	NM_020120.3	UGGT1
circRNA_01926	0	0	0	0	0	0	0.020098685	0	0	0	0	0	hsa_circ_0005289	NC_000002.12	+	128172573	128174858	2286	435	sense-overlappin	NM_020120.3	UGGT1
circRNA_01927	0	0	0	0	0	0	0.030148028	0	0	0	0	0	-	NC_000002.12	+	128191786	128194312	2527	2527	exonic	NM_020120.3	UGGT1
circRNA_01928	0	0	0	0	0	0.031069364	0	0	0	0	0	0	-	NC_000002.12	+	130358867	130359646	780	321	sense-overlappin	NM_014369.3	PTPN18
circRNA_01929	0	0	0.032712968	0	0.04012623	0	0	0	0	0	0	0	hsa_circ_0003524	NC_000002.12	-	131071855	131082657	10803	165	sense-overlappin	XM_006712265.2	FAM168B

circRNA_01930	0	0	0	0	0	0	0.020098685	0	0	0	0	0	hsa_circ_0056488	NC_000002.12	+	131125753	131130760	5008	296	sense-overlappin	XM_011511387.1	PLEKHB2
circRNA_01931	0	0	0.08723458	0	0	0	0	0	0	0	0	0	-	NC_000002.12	+	131553013	131582129	29117	29117	intergenic		
circRNA_01932	0	0	0.04361729	0	0	0	0	0	0	0	0	0	-	NC_000002.12	+	131564861	131580264	15404	15404	intergenic		
circRNA_01933	0	0	0.065425935	0	0	0	0	0	0	0	0	0	-	NC_000002.12	+	131575035	131582129	7095	7095	intergenic		
circRNA_01934	0	0	0.08723458	0	0	0	0	0	0	0	0	0	-	NC_000002.12	+	131575035	131613103	38069	38069	intergenic		
circRNA_01935	0	0	0	0	0	0	0	0	0	0.030258724	0	0	-	NC_000002.12	+	132645101	132686465	41365	17776	antisense	XM_005263583.3	LYPD1
circRNA_01936	0	0	0	0	0	0	0	0	0	0	0	0.0309546	hsa_circ_0056507	NC_000002.12	-	132725627	132732051	6425	585	sense-overlappin	NM_207363.2	NCKAP5
circRNA_01937	0	0.040939935	0.08723458	0.0412724	0	0.031069364	0.030148028	0.030729443	0.060763583	0.050431207	0	0.0412728	hsa_circ_0001068	NC_000002.12	+	134253095	134254644	1550	507	sense-overlappin	XM_011511204.1	MGAT5
circRNA_01938	0.020575025	0	0	0	0	0.020712909	0	0	0.030381792	0.020172483	0	0	hsa_circ_0002480	NC_000002.12	+	134253095	134270550	17456	672	sense-overlappin	XM_011511204.1	MGAT5
circRNA_01939	0	0	0	0	0	0	0	0	0	0.030258724	0	0	hsa_circ_0008155	NC_000002.12	+	134317529	134318739	1211	167	sense-overlappin	XM_005263669.3	MGAT5
circRNA_01940	0	0	0.032712968	0	0	0	0	0	0	0	0	0	hsa_circ_0004198	NC_000002.12	+	134317529	134362408	44880	974	sense-overlappin	XM_005263669.3	MGAT5
circRNA_01941	0	0	0	0.0206362	0	0	0	0	0	0	0	0	-	NC_000002.12	+	134336217	134362408	26192	807	sense-overlappin	XM_005263669.3	MGAT5
circRNA_01942	0	0	0.032712968	0	0	0	0	0	0	0	0	0	-	NC_000002.12	-	134866542	134869032	2491	2491	antisense	XM_005263586.3	ACMSD
circRNA_01943	0.020575025	0	0.021808645	0	0	0	0	0	0	0	0	0	-	NC_000002.12	-	134866542	134869041	2500	2500	antisense	XM_005263586.3	ACMSD
circRNA_01944	0	0	0.032712968	0	0	0	0	0	0	0	0	0	hsa_circ_0002291	NC_000002.12	+	135090998	135093693	2696	212	sense-overlappin	XM_011510824.1	RAB3GAP1
circRNA_01945	0	0	0	0	0	0	0.020098685	0	0	0	0	0	-	NC_000002.12	+	135129995	135130721	727	263	sense-overlappin	XM_011510824.1	RAB3GAP1
circRNA_01946	0	0	0	0.0206362	0	0	0	0	0	0	0.02025523	0	hsa_circ_0008036	NC_000002.12	+	135129995	135135932	5938	950	sense-overlappin	XM_011510824.1	RAB3GAP1
circRNA_01947	0	0	0	0	0.02006311	0	0	0	0	0	0	0	-	NC_000002.12	-	135335950	135345635	9686	9686	sense-overlappin	NM_001286568.1	ZRANB3
circRNA_01948	0	0.051174919	0.04361729	0.0412724	0.02006311	0	0.030148028	0.020486295	0.040509055	0	0.02025523	0.0309546	hsa_circ_0056558	NC_000002.12	+	135602500	135605016	2517	420	sense-overlappin	NM_001282799.1	R3HDM1
circRNA_01949	0	0	0	0	0	0	0	0.020486295	0	0	0	0	-	NC_000002.12	+	135621494	135680324	58831	2156	sense-overlappin	XR_922894.1	
circRNA_01950	0	0	0	0	0	0	0.020098685	0	0	0	0	0	-	NC_000002.12	+	135631718	135639074	7357	674	sense-overlappin	NM_001282799.1	R3HDM1
circRNA_01951	0	0.020469968	0	0	0	0	0	0	0	0.050431207	0	0	hsa_circ_0001069	NC_000002.12	+	135631718	135639122	7405	722	sense-overlappin	NM_001282799.1	R3HDM1
circRNA_01952	0	0	0.065425935	0.0309543	0.02006311	0.072495183	0.040197371	0.030729443	0.020254528	0.07060369	0.050638075	0	hsa_circ_0001070	NC_000002.12	+	135675332	135680324	4993	307	sense-overlappin	NM_001282799.1	R3HDM1
circRNA_01953	0	0	0	0	0	0.020712909	0	0.020486295	0	0	0	0	hsa_circ_0056586	NC_000002.12	+	135709433	135710231	799	277	sense-overlappin	NM_001282799.1	R3HDM1
circRNA_01954	0	0.020469968	0.04361729	0	0	0	0	0.020486295	0	0.020172483	0.02025523	0.0619092	hsa_circ_0002981	NC_000002.12	+	135748267	135761911	13645	520	sense-overlappin	NM_014607.3	UBXN4
circRNA_01955	0	0	0.032712968	0	0.02006311	0	0.030148028	0	0	0.080689931	0	0	-	NC_000002.12	+	135748267	135772547	24281	868	sense-overlappin	NM_014607.3	UBXN4
circRNA_01956	0.020575025	0	0.021808645	0	0	0	0	0.030729443	0	0	0	0	-	NC_000002.12	+	135769769	135770735	967	220	sense-overlappin	NM_014607.3	UBXN4
circRNA_01957	0.041150049	0	0	0	0	0	0	0.040972591	0	0	0	0	-	NC_000002.12	+	135769769	135772547	2779	348	sense-overlappin	NM_014607.3	UBXN4
circRNA_01958	0	0	0.032712968	0	0	0	0	0	0	0	0	0	hsa_circ_0056594	NC_000002.12	+	135769769	135776351	6583	451	sense-overlappin	NM_014607.3	UBXN4
circRNA_01959	0	0.030704951	0	0	0	0	0	0	0	0	0	0	-	NC_000002.12	+	135769769	135780385	10617	786	sense-overlappin	NM_014607.3	UBXN4
circRNA_01960	0	0	0.065425935	0	0	0.031069364	0	0.020486295	0	0.020172483	0.02025523	0	-	NC_000002.12	-	135862607	135865163	2557	293	sense-overlappin	NM_005915.5	MCM6
circRNA_01961	0	0	0	0	0	0	0	0	0.030381792	0	0	0	-	NC_000002.12	-	135930482	135933990	3509	2442	sense-overlappin	NM_001293312.1	DARS
circRNA_01962	0	0	0.021808645	0	0	0	0	0	0	0	0	0	hsa_circ_0056613	NC_000002.12	-	135943378	135961498	18121	206	sense-overlappin	NM_001293312.1	DARS
circRNA_01963	0	0	0.032712968	0	0	0	0	0	0	0	0	0	-	NC_000002.12	-	139384768	139394689	9922	9922	intergenic		
circRNA_01964	0	0	0	0	0	0	0	0.020486295	0	0	0	0	-	NC_000002.12	+	142954810	142986021	31212	529	sense-overlappin	XM_011512101.1	KYNU
circRNA_01965	0	0	0	0	0	0.041425819	0	0	0	0	0	0	-	NC_000002.12	-	143951982	143970762	18781	418	sense-overlappin	XM_011511863.1	GTDC1
circRNA_01966	0	0.030704951	0	0	0	0	0	0	0	0	0	0	hsa_circ_0008208	NC_000002.12	-	144141882	144235737	93856	1042	sense-overlappin	XM_011511852.1	GTDC1
circRNA_01967	0.020575025	0.030704951	0.098138903	0	0.03009467	0.062138728	0.090444084	0	0	0.07060369	0.02025523	0	hsa_circ_0001072	NC_000002.12	-	144208603	144211579	2977	491	sense-overlappin	XM_011511847.1	GTDC1
circRNA_01968	0.205750246	0.184229708	0.4361729	0.1960438	0.11034713	0.103564547	0.080394741	0.061458886	0.050636319	0.161379862	0.04051046	0.0412728	hsa_circ_0001073	NC_000002.12	+	147896301	147899898	3598	473	sense-overlappin	NM_001278579.1	ACVR2A
circRNA_01969	0	0	0.021808645	0	0	0	0	0	0	0	0	0	-	NC_000002.12	-	147955347	147975975	20629	453	sense-overlappin	NM_001190879.2	ORC4
circRNA_01970	0.020575025	0	0.588833415	0.0309543	0.05015779	0	0	0.051215739	0.040509055	0.040344966	0.02025523	0.0206364	hsa_circ_0001074	NC_000002.12	-	147972739	147975975	3237	242	sense-overlappin	NM_001190879.2	ORC4
circRNA_01971	0.072012586	0.122819805	0.065425935	0.0515905	0.0702209	0.113921001	0.030148028	0.030729443	0.020254528	0.080689931	0.10127615	0.051591	hsa_circ_0004491	NC_000002.12	-	147972739	147982081	9343	396	sense-overlappin	NM_001190879.2	ORC4
circRNA_01972	0	0	0	0	0	0	0	0	0	0	0	0.0206364	hsa_circ_0002616	NC_000002.12	+	148051910	148054449	2540	2540	sense-overlappin	XR_922967.1	
circRNA_01973	0	0	0	0	0.03009467	0	0	0	0	0	0	0	hsa_circ_0001075	NC_000002.12	+	148761782	148765146	3365	474	sense-overlappin	XM_011510941.1	EPC2
circRNA_01974	0	0	0	0	0	0.020712909	0	0	0	0	0	0	-	NC_000002.12	+	148761782	148771387	9606	1054	sense-overlappin	XM_011510941.1	EPC2
circRNA_01975	0	0	0	0	0.02006311	0	0	0	0	0	0	0	-	NC_000002.12	+	149449049	149486345	37297	15893	sense-overlappin	NM_001195685.1	LYPD6
circRNA_01976	0	0	0.04361729	0	0	0	0	0	0	0	0	0	-	NC_000002.12	+	149468645	149486345	17701	15794	sense-overlappin	NM_001195685.1	LYPD6
circRNA_01977	0	0	0	0	0	0	0.030148028	0	0	0	0	0	-	NC_000002.12	-	151275484	151282954	7471	640	sense-overlappin	XM_005246941.2	NMI
circRNA_01978	0	0	0.021808645	0	0	0	0	0	0	0	0	0	-	NC_000002.12	-	151281948	151282896	949	125	sense-overlappin	XM_005246941.2	NMI
circRNA_01979	0	0	0	0	0	0.031069364	0	0	0	0	0	0	-	NC_000002.12	+	151422950	151438746	15797	853	sense-overlappin	NM_001177663.1	RIF1
circRNA_01980	0	0	0	0	0	0.031069364	0	0	0	0	0	0	-	NC_000002.12	+	151438684	151443709	5026	503	sense-overlappin	NM_001177663.1	RIF1
circRNA_01981	0	0	0.032712968	0	0	0	0	0	0	0	0	0	-	NC_000002.12	-	152132114	152150229	18116	985	sense-overlappin	XR_922831.1	
circRNA_01982	0	0	0	0	0.03009467	0	0	0	0	0	0	0	-	NC_000002.12	-	152143827	152150229	6403	664	sense-overlappin	XR_922831.1	
circRNA_01983	0	0	0	0.0412724	0	0	0	0	0	0.020172483	0	0	-	NC_000002.12	-	152144888	152148300	3413	392	sense-overlappin	XR_922831.1	

circRNA_01984	0	0	0.021808645	0	0	0.020712909	0	0	0	0	0	0	-	NC_000002.12	-	152147162	152148300	1139	322	sense-overlappin	XR_922831.1	
circRNA_01985	0	0.020469968	0	0.0515905	0.10031557	0	0	0	0	0.040344966	0	0	-	NC_000002.12	+	152575136	152581049	5914	280	sense-overlappin	XR_241279.2	
circRNA_01986	0	0	0.021808645	0	0	0	0	0	0	0	0	0	-	NC_000002.12	-	155759631	155814067	54437	54437	intergenic		
circRNA_01987	0	0	0	0	0	0	0.020098685	0	0.020254528	0	0	0	hsa_circ_0004955	NC_000002.12	-	157799427	157818559	19133	249	sense-overlappin	XM_005246940.2	ACVR1
circRNA_01988	0	0	0	0	0	0	0	0	0	0.030258724	0	0	hsa_circ_0056768	NC_000002.12	-	158309433	158345318	35886	963	sense-overlappin	XM_005246319.2	CCDC148
circRNA_01989	0	0	0	0	0	0	0.030148028	0	0	0	0	0	-	NC_000002.12	-	158309433	158358570	49138	1085	sense-overlappin	XM_005246319.2	CCDC148
circRNA_01990	0	0	0	0.0309543	0	0	0	0	0	0	0	0	hsa_circ_0006870	NC_000002.12	+	158533180	158603104	69925	285	sense-overlappin	NM_001304971.1	PKP4
circRNA_01991	0.030862537	0	0	0	0	0	0	0	0	0	0.02025523	0	hsa_circ_0056784	NC_000002.12	+	158631753	158640759	9007	542	sense-overlappin	NM_001304971.1	PKP4
circRNA_01992	0	0	0	0	0	0	0.020098685	0	0	0	0	0	-	NC_000002.12	+	158994902	159011440	16539	6423	sense-overlappin	XM_011512048.1	TANC1
circRNA_01993	0	0	0	0	0.02006311	0	0	0	0	0	0	0	hsa_circ_0005102	NC_000002.12	+	159001080	159011440	10361	245	sense-overlappin	XM_011512048.1	TANC1
circRNA_01994	0	0	0	0	0	0.020712909	0	0	0	0	0	0	-	NC_000002.12	+	159001080	159065971	64892	321	sense-overlappin	XM_011512048.1	TANC1
circRNA_01995	0	0	0.021808645	0.0309543	0	0	0.020098685	0.040972591	0	0.030258724	0	0	hsa_circ_0008010	NC_000002.12	+	159136194	159150556	14363	420	sense-overlappin	NM_001145909.1	TANC1
circRNA_01996	0.030862537	0.020469968	0	0	0	0	0.020098685	0	0	0.030258724	0	0	hsa_circ_0004213	NC_000002.12	+	159149142	159150556	1415	318	sense-overlappin	NM_001145909.1	TANC1
circRNA_01997	0.030862537	0.020469968	0.021808645	0.0206362	0.06018934	0	0	0	0	0.020172483	0	0	hsa_circ_0056810	NC_000002.12	+	159169250	159170805	1556	384	sense-overlappin	NM_001145909.1	TANC1
circRNA_01998	0.020575025	0	0	0	0	0	0	0	0	0	0	0	-	NC_000002.12	-	159245766	159248266	2501	2501	intronic	NM_152528.2	WDSUB1
circRNA_01999	0	0	0	0	0.02006311	0	0	0	0	0	0	0	hsa_circ_0056821	NC_000002.12	-	159248372	159259843	11472	503	sense-overlappin	NM_152528.2	WDSUB1
circRNA_02000	0	0	0	0	0	0.051782273	0	0	0	0.100862414	0	0.0309546	-	NC_000002.12	-	159337462	159395834	58373	2756	sense-overlappin	XM_011511057.1	BAZ2B
circRNA_02001	0	0.020469968	0.021808645	0	0	0.020712909	0	0.020486295	0	0	0	0.051591	hsa_circ_0056829	NC_000002.12	-	159337567	159395834	58268	2651	sense-overlappin	XM_011511057.1	BAZ2B
circRNA_02002	0	0	0	0	0	0	0	0	0	0	0	0.0309546	hsa_circ_0002463	NC_000002.12	-	159373045	159374753	1709	208	sense-overlappin	XM_011511057.1	BAZ2B
circRNA_02003	0	0	0.021808645	0	0	0	0	0	0	0	0	0	-	NC_000002.12	-	159446782	159453801	7020	551	sense-overlappin	XM_011511038.1	BAZ2B
circRNA_02004	0	0.030704951	0	0	0	0	0	0	0	0	0	0	-	NC_000002.12	-	159669018	159670052	1035	1035	intronic	XM_011511036.1	BAZ2B
circRNA_02005	0	0	0.054521613	0	0	0	0	0	0	0	0	0	hsa_circ_0056838	NC_000002.12	+	159729009	159752571	23563	1797	sense-overlappin	NM_001282805.1	7-Mar
circRNA_02006	0	0	0.021808645	0	0	0.020712909	0	0	0	0	0	0	hsa_circ_0056841	NC_000002.12	+	159743061	159748903	5843	1460	sense-overlappin	NM_001282805.1	7-Mar
circRNA_02007	0	0	0	0.0206362	0.03009467	0	0	0	0	0	0	0	-	NC_000002.12	+	159745770	159748903	3134	1267	sense-overlappin	NM_001282805.1	7-Mar
circRNA_02008	0	0	0.021808645	0	0.02006311	0	0	0	0	0	0	0	-	NC_000002.12	-	159777938	159783469	5532	429	sense-overlappin	NM_014880.4	CD302
circRNA_02009	0	0	0.076330258	0	0	0	0	0	0.020254528	0	0	0	hsa_circ_0056856	NC_000002.12	-	160123791	160126601	2811	321	sense-overlappin	NM_000888.4	ITGB6
circRNA_02010	0.051437562	0.030704951	0.13085187	0.0309543	0.02006311	0.051782273	0.020098685	0	0.020254528	0	0.02025523	0	hsa_circ_0005227	NC_000002.12	+	161179614	161204793	25180	379	sense-overlappin	XM_005246211.1	TANK
circRNA_02011	0	0	0.021808645	0	0.02006311	0	0	0	0	0	0	0	hsa_circ_0007303	NC_000002.12	+	161367478	161395203	27726	723	sense-overlappin	NM_005805.5	PSMD14
circRNA_02012	0	0	0.04361729	0	0	0	0	0	0	0	0	0	-	NC_000002.12	-	164521980	164527135	5156	335	sense-overlappin	NM_001303422.1	GRB14
circRNA_02013	0	0	0	0.0206362	0	0	0	0	0	0	0	0	-	NC_000002.12	-	164539766	164547816	8051	8051	sense-overlappin	NM_001303422.1	GRB14
circRNA_02014	0.020575025	0	0	0	0	0	0	0	0	0	0	0	-	NC_000002.12	+	164687198	164688381	1184	1184	antisense	NM_001278461.1	COBLL1
circRNA_02015	0	0	0	0.0412724	0	0	0	0	0	0	0.030382845	0.0309546	-	NC_000002.12	-	164692221	164695833	3613	1742	sense-overlappin	NM_001278461.1	COBLL1
circRNA_02016	0.030862537	0.061409903	0	0.1031809	0.05015779	0.031069364	0.050246713	0.030729443	0	0.060517448	0.02025523	0.0619092	-	NC_000002.12	-	164692221	164695836	3616	1745	sense-overlappin	NM_001278461.1	COBLL1
circRNA_02017	0	0	0	0.0206362	0	0	0	0	0	0	0	0	-	NC_000002.12	-	164692221	164700756	8536	2075	sense-overlappin	NM_001278461.1	COBLL1
circRNA_02018	0	0	0	0.0619086	0	0.020712909	0	0	0	0.040344966	0	0.0619092	hsa_circ_0056891	NC_000002.12	-	164692221	164705105	12885	2418	sense-overlappin	NM_014900.4	COBLL1
circRNA_02019	0	0	0	0.0825448	0	0.041425819	0	0.030729443	0.020254528	0.020172483	0	0	hsa_circ_0056892	NC_000002.12	-	164692221	164730115	37895	3226	sense-overlappin	NM_001278458.1	COBLL1
circRNA_02020	0	0	0.032712968	0	0	0	0	0	0	0	0	0	hsa_circ_0056893	NC_000002.12	-	164722075	164730115	8041	805	sense-overlappin	NM_001278458.1	COBLL1
circRNA_02021	0	0	0	0.0206362	0	0	0	0	0	0	0	0	hsa_circ_0056894	NC_000002.12	-	164722075	164743875	21801	994	sense-overlappin	NM_001278458.1	COBLL1
circRNA_02022	0	0	0	0	0.02006311	0	0	0	0	0	0	0	-	NC_000002.12	-	164743687	164779785	36099	36099	sense-overlappin	NM_001278461.1	COBLL1
circRNA_02023	0	0	0	0.0206362	0	0	0	0	0	0	0	0	-	NC_000002.12	-	165842865	165871020	28156	28156	intergenic		
circRNA_02024	0	0	0	0	0	0.031069364	0	0	0	0.020172483	0	0	-	NC_000002.12	-	166447612	166472445	24834	944	sense-overlappin	XM_006712680.1	SCN7A
circRNA_02025	0	0	0	0.0412724	0.03009467	0	0.020098685	0	0.020254528	0.020172483	0	0	-	NC_000002.12	-	168012634	168017095	4462	122	sense-overlappin	XM_005246465.2	STK39
circRNA_02026	0	0	0.054521613	0	0	0	0	0	0	0	0	0	-	NC_000002.12	-	168012634	168065381	52748	256	sense-overlappin	XM_011510966.1	STK39
circRNA_02027	0	0	0.021808645	0	0	0	0	0	0	0	0	0	hsa_circ_0005280	NC_000002.12	-	168012634	168075231	62598	409	sense-overlappin	XM_011510966.1	STK39
circRNA_02028	0.051437562	0.08187987	0.054521613	0.0309543	0.03009467	0	0.040197371	0.020486295	0.020254528	0	0.030382845	0	hsa_circ_0003279	NC_000002.12	-	168063500	168075231	11732	287	sense-overlappin	XM_011510966.1	STK39
circRNA_02029	0.030862537	0.143289773	0.207182128	0	0.03009467	0	0.080394741	0.102431477	0.162036222	0.161379862	0.050638075	0.051591	hsa_circ_0005882	NC_000002.12	-	168063500	168129758	66259	462	sense-overlappin	XM_011510966.1	STK39
circRNA_02030	0	0	0	0.0206362	0	0	0	0	0	0.030258724	0	0	-	NC_000002.12	-	168074982	168129758	54777	328	sense-overlappin	XM_011510966.1	STK39
circRNA_02031	0.041150049	0.092114854	0.054521613	0.0515905	0.02006311	0.041425819	0.050246713	0.020486295	0.081018111	0.060517448	0	0.0619092	hsa_circ_0003296	NC_000002.12	-	168129541	168140758	11218	461	sense-overlappin	XM_005246465.2	STK39
circRNA_02032	0	0.030704951	0	0	0.02006311	0	0	0	0	0	0	0	hsa_circ_0002249	NC_000002.12	-	168129541	168167407	37867	768	sense-overlappin	XM_005246465.2	STK39
circRNA_02033	0	0	0.098138903	0	0	0.031069364	0	0	0	0.040344966	0	0.0206364	hsa_circ_0002193	NC_000002.12	-	168138088	168140758	2671	346	sense-overlappin	XM_005246465.2	STK39
circRNA_02034	0	0	0	0	0	0	0.030148028	0	0.020254528	0	0	0	hsa_circ_0003120	NC_000002.12	-	168138088	168167407	29320	653	sense-overlappin	XM_005246465.2	STK39
circRNA_02035	0.051437562	0	0	0	0	0	0	0.020486295	0.030381792	0	0	0	hsa_circ_0002029	NC_000002.12	-	168138088	168182090	44003	766	sense-overlappin	XM_005246465.2	STK39
circRNA_02036	0	0	0.021808645	0	0	0	0	0	0	0	0	0	-	NC_000002.12	-	168138088	168183602	45515	2278	sense-overlappin	XM_005246465.2	STK39
circRNA_02037	0	0.030704951	0.032712968	0	0.02006311	0	0.050246713	0	0.060763583	0	0.02025523	0	hsa_circ_0007082	NC_000002.12	-	168161787	168167407	5621	307	sense-overlappin	XM_005246465.2	STK39
circRNA_02038	0.030862537	0.040939935	0.032712968	0.1031809	0.04012623	0.062138728	0.110542769	0	0.030381792	0.060517448	0	0.072227401	hsa_circ_0001079	NC_000002.12	-	168161787	168182090	20304	420	sense-overlappin	XM_005246465.2	STK39
circRNA_02039	0	0	0.021808645	0	0	0	0	0	0	0	0	0	-	NC_000002.12	-	168161787	168183602	21816	1932	sense-overlappin	XM_005246465.2	STK39
circRNA_02040	0	0	0	0	0	0	0	0	0	0	0.04051046	0	hsa_circ_0056939	NC_000002.12	+	168547596	168561322	13727	237	sense-overlappin	NM_001256126.1	CERS6
circRNA_02041	0.051437562	0.030704951	0.04361729	0.0309543	0.02006311	0.051782273	0.020098685	0.051215739	0	0.040344966	0.02025523	0	hsa_circ_0007024	NC_000002.12	+	168691034	168717978	26945	380	sense-overlappin	NM_001256126.1	CERS6

circRNA_02042	0	0	0	0	0	0.020712909	0	0	0	0	0	0	-	NC_000002.12	+	169509767	169510888	1122	1122	exonic	NM_006063.2	KLHL41
circRNA_02043	0	0.020469968	0	0	0	0	0	0	0	0	0	0	-	NC_000002.12	-	169540051	169544835	4785	244	sense-overlappin	XM_006712751.2	FASTKD1
circRNA_02044	0	0	0.032712968	0.0206362	0	0	0	0	0	0	0	0	hsa_circ_0001080	NC_000002.12	-	169544721	169557297	12577	845	sense-overlappin	XM_006712751.2	FASTKD1
circRNA_02045	0	0	0	0	0	0	0	0	0.020254528	0	0.02025523	0	hsa_circ_0002377	NC_000002.12	-	169555124	169557297	2174	243	sense-overlappin	XM_006712751.2	FASTKD1
circRNA_02046	0	0.040939935	0.04361729	0	0	0	0	0	0	0	0	0	-	NC_000002.12	+	169603642	169608758	5117	446	sense-overlappin	NM_004792.2	PPIG
circRNA_02047	0.020575025	0	0.283512385	0	0.02006311	0	0	0	0.020254528	0	0.02025523	0	hsa_circ_0004888	NC_000002.12	+	169603642	169614724	11083	616	sense-overlappin	NM_004792.2	PPIG
circRNA_02048	0	0	0.021808645	0	0	0	0	0	0	0	0	0	-	NC_000002.12	+	169606103	169614681	8579	304	sense-overlappin	NM_004792.2	PPIG
circRNA_02049	0	0	0	0	0	0	0.030148028	0	0	0	0	0	-	NC_000002.12	+	169630774	169631933	1160	382	sense-overlappin	NM_004792.2	PPIG
circRNA_02050	0	0	0.021808645	0	0	0	0	0	0	0	0	0	-	NC_000002.12	-	169762940	169763378	439	439	intergenic		
circRNA_02051	0	0	0.021808645	0	0	0	0	0	0	0.040344966	0	0	hsa_circ_0056971	NC_000002.12	+	169872236	169875949	3714	299	sense-overlappin	XR_922842.1	
circRNA_02052	0.030862537	0.040939935	0	0.0206362	0	0.031069364	0	0	0	0	0	0	hsa_circ_0002603	NC_000002.12	+	169872236	169878574	6339	493	sense-overlappin	XR_922842.1	
circRNA_02053	0	0	0	0	0	0.051782273	0	0	0	0	0	0	-	NC_000002.12	+	169905114	169914146	9033	401	sense-overlappin	XR_922842.1	
circRNA_02054	0	0.061409903	0	0	0	0	0	0	0	0	0	0	-	NC_000002.12	+	169905114	169942634	37521	1340	sense-overlappin	XR_922842.1	
circRNA_02055	0	0	0	0	0	0	0	0	0	0	0	0.0309546	hsa_circ_0056999	NC_000002.12	+	170015281	170040985	25705	293	sense-overlappin	XR_922841.1	
circRNA_02056	0	0	0.021808645	0.0309543	0	0	0.020098685	0	0	0	0	0	hsa_circ_0057002	NC_000002.12	+	170055460	170061443	5984	359	sense-overlappin	XR_922841.1	
circRNA_02057	0.020575025	0.020469968	0	0	0	0.031069364	0.020098685	0.020486295	0	0	0.02025523	0.0412728	-	NC_000002.12	-	171016001	171016251	251	251	intronic	NM_001136555.1	TLK1
circRNA_02058	0	0	0	0	0.04012623	0	0	0	0	0	0	0	-	NC_000002.12	-	171022872	171028405	5534	325	sense-overlappin	XR_923070.1	
circRNA_02059	0.113162635	0.061409903	0.021808645	0.0515905	0	0.062138728	0	0.040972591	0.091145375	0.050431207	0	0	hsa_circ_0004442	NC_000002.12	-	171028339	171046362	18024	256	sense-overlappin	NM_001136555.1	TLK1
circRNA_02060	0	0	0.021808645	0	0	0	0	0	0	0	0	0	-	NC_000002.12	-	171028339	171082852	54514	1047	sense-overlappin	XM_005246981.1	TLK1
circRNA_02061	0.041150049	0.030704951	0.021808645	0	0	0	0.020098685	0	0	0	0	0	hsa_circ_0004813	NC_000002.12	-	171046174	171050174	4001	437	sense-overlappin	NM_001136555.1	TLK1
circRNA_02062	0	0	0.021808645	0	0	0	0	0	0	0	0	0	-	NC_000002.12	-	171046174	171082852	36679	980	sense-overlappin	XM_005246981.1	TLK1
circRNA_02063	0	0	0.021808645	0	0	0	0	0	0	0	0	0	-	NC_000002.12	-	171081640	171117857	36218	1332	sense-overlappin	NM_012290.4	TLK1
circRNA_02064	0	0	0	0	0	0	0.020098685	0	0	0	0	0	hsa_circ_0004827	NC_000002.12	-	171082781	171117857	35077	191	sense-overlappin	NM_012290.4	TLK1
circRNA_02065	0	0	0	0	0	0	0	0	0.030381792	0	0	0	hsa_circ_0004384	NC_000002.12	-	171325841	171331867	6027	377	sense-overlappin	NM_024770.3	METTL8
circRNA_02066	0.030862537	0	0.032712968	0	0	0	0.030148028	0	0.040509055	0.060517448	0	0	hsa_circ_0057041	NC_000002.12	-	171337453	171339554	2102	421	sense-overlappin	NM_024770.3	METTL8
circRNA_02067	0	0	0	0	0.02006311	0.020712909	0	0	0	0	0	0	hsa_circ_0005904	NC_000002.12	+	171448681	171458075	9395	411	sense-overlappin	XM_011511883.1	DCAF17
circRNA_02068	0	0	0.021808645	0	0	0	0	0	0	0	0	0	hsa_circ_0057050	NC_000002.12	+	171448681	171469030	20350	660	sense-overlappin	XM_011511883.1	DCAF17
circRNA_02069	0	0	0	0	0	0.031069364	0	0	0	0	0	0	-	NC_000002.12	+	171448681	171473975	25295	770	sense-overlappin	XM_006712767.1	DCAF17
circRNA_02070	0	0	0.032712968	0	0	0	0	0	0.030381792	0	0	0	hsa_circ_0057051	NC_000002.12	+	171448681	171476950	28270	861	sense-overlappin	XM_006712767.1	DCAF17
circRNA_02071	0	0	0.065425935	0.0206362	0.04012623	0.072495183	0	0	0	0.080689931	0.04051046	0.0309546	hsa_circ_0008032	NC_000002.12	+	171925537	171953001	27465	302	sense-overlappin	XM_006712808.2	HAT1
circRNA_02072	0	0.020469968	0	0	0	0	0.030148028	0	0	0	0	0	hsa_circ_0057089	NC_000002.12	+	172558708	172565073	6366	555	sense-overlappin	XM_011511343.1	PDK1
circRNA_02073	0.020575025	0.020469968	0.032712968	0	0.02006311	0	0.030148028	0.051215739	0.030381792	0.060517448	0	0.0206364	-	NC_000002.12	+	172558708	172566933	8226	633	sense-overlappin	XM_011511343.1	PDK1
circRNA_02074	0	0.030704951	0	0	0	0	0.020098685	0	0	0	0	0.082545601	hsa_circ_0057090	NC_000002.12	+	172558708	172570824	12117	809	sense-overlappin	XM_011511343.1	PDK1
circRNA_02075	0	0	0	0	0	0.041425819	0.070345399	0.030729443	0	0	0	0	-	NC_000002.12	+	172558708	172596023	37316	1229	sense-overlappin	XM_011511343.1	PDK1
circRNA_02076	0	0	0	0	0.03009467	0	0	0	0	0	0	0	-	NC_000002.12	+	172562220	172566933	4714	491	sense-overlappin	XM_011511343.1	PDK1
circRNA_02077	0	0	0	0	0.03009467	0	0	0	0	0	0	0	hsa_circ_0057098	NC_000002.12	+	172564503	172596023	31521	955	sense-overlappin	NR_103731.1	
circRNA_02078	0	0	0	0	0	0.031069364	0	0	0	0	0	0	hsa_circ_0057104	NC_000002.12	+	172568741	172593048	24308	401	sense-overlappin	NR_103731.1	
circRNA_02079	0.020575025	0.061409903	0	0.0515905	0.02006311	0.113921001	0.090444084	0.030729443	0.040509055	0.090776173	0	0.092863801	hsa_circ_0057105	NC_000002.12	+	172568741	172596023	27283	596	sense-overlappin	NR_103731.1	
circRNA_02080	0	0	0.032712968	0	0	0.020712909	0	0.020486295	0	0.050431207	0	0.0309546	hsa_circ_0006006	NC_000002.12	+	172570726	172596023	25298	519	sense-overlappin	NR_103731.1	
circRNA_02081	0	0	0	0	0	0.041425819	0	0	0.030381792	0.040344966	0	0	hsa_circ_0057109	NC_000002.12	+	172586278	172596023	9746	420	sense-overlappin	NR_103731.1	
circRNA_02082	0	0.020469968	0.065425935	0	0	0	0.030148028	0.071702034	0.050636319	0	0	0	hsa_circ_0057123	NC_000002.12	+	173358712	173359491	780	363	sense-overlappin	XM_011511957.1	CDCA7
circRNA_02083	0	0.040939935	0	0	0	0	0	0	0	0	0	0	-	NC_000002.12	+	173358712	173367831	9120	1499	sense-overlappin	XM_011511957.1	CDCA7
circRNA_02084	0	0	0	0	0	0	0	0	0	0	0	0.0309546	-	NC_000002.12	+	173365452	173367831	2380	626	sense-overlappin	XM_011511957.1	CDCA7
circRNA_02085	0	0	0	0	0	0	0.020098685	0	0	0	0	0	-	NC_000002.12	-	173940950	173941068	119	119	intronic	NM_001017371.4	SP3
circRNA_02086	0	0	0.141756193	0	0.02006311	0	0.020098685	0.030729443	0	0.040344966	0.030382845	0.0206364	hsa_circ_0002642	NC_000002.12	-	173954873	173956232	1360	1360	exonic	NM_001017371.4	SP3
circRNA_02087	0.041150049	0	0.021808645	0	0.03009467	0	0.040197371	0	0	0	0	0	hsa_circ_0057129	NC_000002.12	-	174123180	174142000	18821	355	sense-overlappin	NM_001011708.1	OLA1
circRNA_02088	0	0	0	0.0309543	0	0	0	0	0	0.050431207	0	0	-	NC_000002.12	-	174439898	174461492	21595	843	sense-overlappin	NM_001033045.3	GPR155
circRNA_02089	0	0	0	0.0515905	0	0.031069364	0	0	0	0	0	0	-	NC_000002.12	-	174459878	174481987	22110	1802	sense-overlappin	NM_001033045.3	GPR155
circRNA_02090	0	0	0	0.0206362	0	0	0	0	0	0	0	0	-	NC_000002.12	-	174472965	174481987	9023	891	sense-overlappin	NM_001033045.3	GPR155
circRNA_02091	0.020575025	0	0	0	0	0	0	0	0	0	0	0	hsa_circ_0004062	NC_000002.12	-	174875788	174878128	2341	2341	sense-overlappin	NM_001822.5	CHN1
circRNA_02092	0.030862537	0	0	0	0	0	0	0	0	0	0	0	hsa_circ_0007941	NC_000002.12	-	174915058	174952202	37145	241	sense-overlappin	NM_001822.5	CHN1
circRNA_02093	0	0	0	0	0.02006311	0	0	0	0	0	0	0	-	NC_000002.12	-	175080660	175097593	16934	537	sense-overlappin	NR_045768.1	
circRNA_02094	0	0	0.032712968	0.0309543	0	0	0	0	0	0	0	0.0619092	hsa_circ_0007967	NC_000002.12	-	175111568	175121540	9973	726	sense-overlappin	NM_001256090.1	ATF2
circRNA_02095	0.020575025	0.020469968	0.054521613	0.0206362	0	0.020712909	0	0	0.020254528	0.020172483	0	0	hsa_circ_0005775	NC_000002.12	-	175970764	175995646	24883	555	sense-overlappin	XM_005246879.3	KIAA1715
circRNA_02096	0.020575025	0	0.021808645	0	0	0	0	0	0	0	0	0	hsa_circ_0008624	NC_000002.12	-	175979810	175995646	15837	514	sense-overlappin	XM_005246879.3	KIAA1715
circRNA_02097	0.020575025	0.040939935	0.239895095	0	0.03009467	0	0.020098685	0	0.030381792	0	0	0	hsa_circ_0057163	NC_000002.12	+	176296860	176328912	32053	502	sense-overlappin	XM_011510497.1	MTX2
circRNA_02098	0.020575025	0.030704951	0.185373483	0.0619086	0.18056803	0.031069364	0	0.020486295	0	0.060517448	0	0	hsa_circ_0006449	NC_000002.12	+	176296860	176337577	40718	790	sense-overlappin	XM_011510497.1	MTX2

circRNA_02099	0	0	0	0	0.02006311	0	0	0	0	0	0	0	-	NC_000002.12	+	176296860	176344711	47852	7924	sense-overlappin	XM_011510497.1	MTX2
circRNA_02100	0	0	0	0.0206362	0	0	0	0	0	0	0	0	-	NC_000002.12	-	177222523	177234271	11749	10035	sense-overlappin	NM_001145412.2	NFE2L2
circRNA_02101	0	0	0	0	0	0	0.020098685	0	0	0.020172483	0.04051046	0	-	NC_000002.12	-	177231889	177234271	2383	669	sense-overlappin	NM_001145412.2	NFE2L2
circRNA_02102	0.041150049	0	0	0	0	0	0	0.020486295	0.020254528	0	0	0	hsa_circ_0057173	NC_000002.12	+	177440965	177462018	21054	359	sense-overlappin	NM_003659.3	AGPS
circRNA_02103	0.072012586	0.040939935	0.054521613	0.0515905	0.05015779	0.051782273	0	0	0.070890847	0.040344966	0.04051046	0.0412728	hsa_circ_0004305	NC_000002.12	+	178535732	178542361	6630	3489	antisense	NM_001256850.1	TTN
circRNA_02104	0	0	0	0.0206362	0	0	0	0	0	0.030258724	0	0	-	NC_000002.12	+	178591139	178600667	9529	5353	antisense	NM_001256850.1	TTN
circRNA_02105	0	0	0.021808645	0	0.03009467	0	0	0	0	0	0	0	hsa_circ_0002087	NC_000002.12	-	179172120	179191866	19747	394	sense-overlappin	NM_178123.4	SESTD1
circRNA_02106	0	0	0	0	0	0	0	0	0	0	0	0.0206364	-	NC_000002.12	-	179945568	179945683	116	116	exonic	XM_005246726.1	CWC22
circRNA_02107	0	0	0	0	0	0	0	0	0	0	0	0.0412728	hsa_circ_0057234	NC_000002.12	-	179973193	179973802	610	223	sense-overlappin	XM_005246726.1	CWC22
circRNA_02108	0	0	0	0	0	0	0	0	0.020254528	0	0	0	-	NC_000002.12	+	181354208	181362918	8711	8711	intergenic		
circRNA_02109	0	0	0	0	0	0	0	0	0.020254528	0	0	0	-	NC_000002.12	+	181474960	181485992	11033	834	sense-overlappin	NM_000885.4	ITGA4
circRNA_02110	0	0	0	0.0309543	0	0	0	0	0.020254528	0.030258724	0	0	hsa_circ_0002844	NC_000002.12	+	181921948	181928278	6331	583	sense-overlappin	NM_001130445.2	SSFA2
circRNA_02111	0	0	0	0	0	0.020712909	0	0	0	0	0	0	hsa_circ_0057256	NC_000002.12	+	182736249	182743712	7464	457	sense-overlappin	NM_018981.2	DNAJC10
circRNA_02112	0	0	0	0	0	0	0	0.020486295	0	0	0	0	-	NC_000002.12	-	182942070	182957596	15527	814	sense-overlappin	NM_013436.4	NCKAP1
circRNA_02113	0.030862537	0	0.021808645	0.0619086	0	0.031069364	0	0	0	0	0	0	hsa_circ_0002092	NC_000002.12	-	182952405	182953331	927	448	sense-overlappin	NM_013436.4	NCKAP1
circRNA_02114	0	0	0.032712968	0	0	0	0	0	0	0.020172483	0	0	-	NC_000002.12	-	182952405	182957596	5192	720	sense-overlappin	NM_013436.4	NCKAP1
circRNA_02115	0	0	0	0.0309543	0	0	0	0	0	0	0	0	hsa_circ_0057276	NC_000002.12	-	182962159	182967361	5203	399	sense-overlappin	NM_013436.4	NCKAP1
circRNA_02116	0	0	0	0	0	0.020712909	0	0	0	0	0	0	-	NC_000002.12	-	182967216	182989186	21971	838	sense-overlappin	NM_013436.4	NCKAP1
circRNA_02117	0	0	0	0	0.02006311	0	0	0	0	0	0	0	hsa_circ_0057292	NC_000002.12	-	183001953	183003325	1373	384	sense-overlappin	NM_013436.4	NCKAP1
circRNA_02118	0	0	0.021808645	0.0515905	0	0	0	0	0	0.040344966	0	0	hsa_circ_0057293	NC_000002.12	-	183001953	183023916	21964	495	sense-overlappin	NM_013436.4	NCKAP1
circRNA_02119	0.030862537	0	0.032712968	0	0	0	0.020098685	0	0	0.020172483	0	0	hsa_circ_0004820	NC_000002.12	+	183128287	183130545	2259	299	sense-overlappin	NM_001287584.1	NUP35
circRNA_02120	0	0	0	0	0	0	0	0	0.040509055	0.030258724	0.02025523	0	hsa_circ_0057298	NC_000002.12	+	183128287	183133623	5337	480	sense-overlappin	NM_001287585.1	NUP35
circRNA_02121	0	0.030704951	0	0	0	0	0	0	0	0	0	0	hsa_circ_0057301	NC_000002.12	+	183133566	183159652	26087	564	sense-overlappin	NM_001287584.1	NUP35
circRNA_02122	0	0	0.021808645	0.0309543	0	0	0	0	0	0	0	0	-	NC_000002.12	+	183151508	183161195	9688	648	sense-overlappin	NM_001287584.1	NUP35
circRNA_02123	0	0	0.109043225	0	0	0	0	0.030729443	0	0	0	0	hsa_circ_0004328	NC_000002.12	+	183151508	183182680	31173	22133	sense-overlappin	XM_011510576.1	NUP35
circRNA_02124	0	0	0.30532103	0	0.02006311	0	0	0	0	0	0	0	-	NC_000002.12	+	183178807	183182680	3874	3874	intergenic		
circRNA_02125	0	0	0.032712968	0	0	0	0	0	0	0	0	0	-	NC_000002.12	+	185737013	185764565	27553	3293	sense-overlappin	NM_173651.2	FSIP2
circRNA_02126	0.030862537	0	0	0	0	0	0	0	0.030381792	0	0	0	-	NC_000002.12	-	186038331	186086370	48040	48040	intergenic		
circRNA_02127	0	0	0	0	0.02006311	0	0	0	0	0	0	0	hsa_circ_0005843	NC_000002.12	-	186081330	186086370	5041	5041	intergenic		
circRNA_02128	0	0	0	0	0	0	0	0	0	0.020172483	0	0	-	NC_000002.12	-	186081330	186178691	97362	97362	intergenic		
circRNA_02129	0	0	0	0	0	0	0	0	0	0.030258724	0	0	hsa_circ_0057319	NC_000002.12	+	186633329	186641588	8260	574	sense-overlappin	NM_002210.4	ITGAV
circRNA_02130	0	0	0	0	0	0	0	0.030729443	0	0	0	0	hsa_circ_0057328	NC_000002.12	+	186740228	186754049	13822	774	sense-overlappin	NM_177454.3	FAM171B
circRNA_02131	0	0	0	0.0206362	0	0	0	0	0	0	0	0	hsa_circ_0003353	NC_000002.12	-	187484124	187503770	19647	630	sense-overlappin	XM_005246819.1	TFPI
circRNA_02132	0	0	0	0.0206362	0	0	0	0	0	0	0	0	-	NC_000002.12	+	188318488	188383889	65402	16107	sense-overlappin	XM_011511327.1	GULP1
circRNA_02133	0	0	0	0.0309543	0	0	0	0	0	0	0	0	hsa_circ_0057340	NC_000002.12	+	188383763	188483492	99730	261	sense-overlappin	NM_001252668.1	GULP1
circRNA_02134	0	0	0	0.0412724	0	0	0	0	0	0	0	0	-	NC_000002.12	+	188517525	188570120	52596	5750	sense-overlappin	NM_001252668.1	GULP1
circRNA_02135	0	0	0	0	0.03009467	0	0	0	0	0	0	0	-	NC_000002.12	+	188529097	188570120	41024	447	sense-overlappin	NM_001252668.1	GULP1
circRNA_02136	0	0	0	0	0	0	0	0	0.020254528	0	0	0	-	NC_000002.12	+	188998266	189005457	7192	1116	sense-overlappin	NM_000090.3	COL3A1
circRNA_02137	0	0	0	0	0	0	0	0	0.020254528	0	0	0	-	NC_000002.12	+	188999285	188999577	293	207	sense-overlappin	NM_000090.3	COL3A1
circRNA_02138	0	0	0	0	0	0	0	0	0.030381792	0	0	0	-	NC_000002.12	+	189006937	189007607	671	162	sense-overlappin	NM_000090.3	COL3A1
circRNA_02139	0	0	0	0.1547714	0	0	0	0	0	0.020172483	0	0.051591	-	NC_000002.12	+	189719572	189720787	1216	411	sense-overlappin	XM_011510695.1	ANKAR
circRNA_02140	0.082300098	0.08187987	0.04361729	0.1547714	0	0.020712909	0.050246713	0.051215739	0.050636319	0.110948655	0.02025523	0.072227401	hsa_circ_0005644	NC_000002.12	+	189728660	189744788	16129	1026	sense-overlappin	XM_011510673.1	ANKAR
circRNA_02141	0	0.020469968	0	0.0206362	0	0	0	0	0	0	0	0	hsa_circ_0005503	NC_000002.12	+	189728660	189755320	26661	11558	sense-overlappin	XM_011510677.1	ANKAR
circRNA_02142	0	0	0	0	0	0.020712909	0	0	0	0	0	0	-	NC_000002.12	-	189753916	189754345	430	354	sense-overlappin	XR_922985.1	
circRNA_02143	0	0	0.021808645	0.0206362	0	0	0.020098685	0	0	0.020172483	0	0.0206364	hsa_circ_0004776	NC_000002.12	+	189791790	189805754	13965	438	sense-overlappin	NM_000534.4	PMS1
circRNA_02144	0.113162635	0.133054789	0.185373483	0.1960438	0.06018934	0.041425819	0.060296056	0.040972591	0.091145375	0.131121138	0.030382845	0.144454801	hsa_circ_0001083	NC_000002.12	+	189791790	189818180	26391	602	sense-overlappin	NM_000534.4	PMS1
circRNA_02145	0	0.020469968	0.032712968	0	0	0	0.040197371	0	0	0	0.02025523	0	-	NC_000002.12	+	189805652	189818180	12529	267	sense-overlappin	NM_000534.4	PMS1
circRNA_02146	0	0	0.04361729	0	0	0	0	0	0	0	0	0	-	NC_000002.12	+	190435977	190437561	1585	1585	exonic	XM_005246656.2	MFSD6
circRNA_02147	0	0	0.021808645	0	0	0	0	0	0	0	0	0	-	NC_000002.12	+	190568073	190573419	5347	5347	intergenic		
circRNA_02148	0	0	0	0	0	0	0.020098685	0	0	0	0	0	-	NC_000002.12	+	190653727	190673152	19426	3451	sense-overlappin	NM_005966.3	NAB1
circRNA_02149	0	0	0.34893832	0.0619086	0.02006311	0.020712909	0.030148028	0.071702034	0	0.040344966	0	0.072227401	hsa_circ_0008961	NC_000002.12	+	190655977	190673152	17176	1201	sense-overlappin	NM_005966.3	NAB1
circRNA_02150	0.277762832	0.307049513	1.799213214	0.2785885	0.11034713	0.113921001	0.100493427	0.184376659	0.26330886	0.242069794	0.12153138	0.092863801	hsa_circ_0002024	NC_000002.12	+	190659158	190673152	13995	1024	sense-overlappin	XM_005246585.1	NAB1
circRNA_02151	0.020575025	0	0	0	0	0.020712909	0	0	0	0	0	0	hsa_circ_0057521	NC_000002.12	+	190900564	190910321	9758	433	sense-overlappin	XR_241306.1	
circRNA_02152	0.072012586	0.16375974	0	0	0.06018934	0.15534682	0.050246713	0.020486295	0.020254528	0.161379862	0.050638075	0.0412728	hsa_circ_0001085	NC_000002.12	+	190900564	190924593	24030	643	sense-overlappin	XM_005246467.2	GLS
circRNA_02153	0	0	0.032712968	0	0.02006311	0	0	0	0	0	0	0	hsa_circ_0057523	NC_000002.12	+	190900564	190931637	31074	1045	sense-overlappin	XM_005246467.2	GLS

circRNA_02154	0	0	0	0	0	0.031069364	0	0	0	0	0	0	hsa_circ_0002428	NC_000002.12	+	190910263	190913364	3102	231	sense-overlappin	XR_241306.1	
circRNA_02155	0	0	0	0.0309543	0	0	0	0	0	0	0	0	-	NC_000002.12	-	190986854	190995219	8366	436	sense-overlappin	NM_007315.3	STAT1
circRNA_02156	0	0	0	0	0	0	0	0	0	0	0	0.0206364	hsa_circ_0057550	NC_000002.12	+	195680032	195681050	1019	1019	exonic	XM_011511505.1	SLC39A10
circRNA_02157	0	0	0.032712968	0	0	0.020712909	0	0.020486295	0	0	0	0	hsa_circ_0057551	NC_000002.12	+	195680032	195683906	3875	1227	sense-overlappin	XM_011511505.1	SLC39A10
circRNA_02158	0	0	0	0	0	0.031069364	0	0	0.030381792	0	0	0.0619092	-	NC_000002.12	-	196139620	196146055	6436	501	sense-overlappin	NM_004226.3	STK17B
circRNA_02159	0.020575025	0	0	0	0.02006311	0	0.030148028	0	0	0	0	0	-	NC_000002.12	-	196220039	196220941	903	262	sense-overlappin	XM_006712648.2	HECW2
circRNA_02160	0	0	0.032712968	0	0	0	0.020098685	0	0	0.030258724	0	0	hsa_circ_0057582	NC_000002.12	-	196271193	196308085	36893	901	sense-overlappin	XM_006712648.2	HECW2
circRNA_02161	0	0	0.021808645	0	0	0	0	0	0	0	0	0	hsa_circ_0001086	NC_000002.12	+	196656633	196677361	20729	1333	sense-overlappin	XM_006712438.2	CCDC150
circRNA_02162	0	0	0.032712968	0	0	0	0	0	0	0	0	0	-	NC_000002.12	-	196875746	196898369	22624	619	sense-overlappin	XM_011511879.1	PGAP1
circRNA_02163	0	0	0.04361729	0.0206362	0	0	0	0	0	0	0	0	hsa_circ_0057607	NC_000002.12	-	196890828	196898369	7542	366	sense-overlappin	XM_011511879.1	PGAP1
circRNA_02164	0	0	0.021808645	0	0.02006311	0	0	0	0	0	0	0	hsa_circ_0057608	NC_000002.12	-	196912882	196922186	9305	586	sense-overlappin	XM_011511879.1	PGAP1
circRNA_02165	0	0.051174919	0.032712968	0.0928628	0	0	0	0.040972591	0	0.020172483	0.030382845	0.0206364	hsa_circ_0004191	NC_000002.12	-	196912882	196923125	10244	1525	sense-overlappin	XM_011511879.1	PGAP1
circRNA_02166	0	0	0	0	0	0	0	0	0	0.020172483	0	0	hsa_circ_0001087	NC_000002.12	-	197007806	197009031	1226	206	sense-overlappin	XM_011512149.1	ANKRD44
circRNA_02167	0	0.030704951	0	0	0	0	0	0	0	0	0	0	-	NC_000002.12	-	197025196	197090032	64837	812	sense-overlappin	XR_923062.1	
circRNA_02168	0	0	0	0	0	0.020712909	0	0	0	0	0	0	-	NC_000002.12	-	197125381	197126037	657	289	sense-overlappin	XM_011512149.1	ANKRD44
circRNA_02169	0	0	0	0	0.02006311	0	0	0	0	0	0	0	hsa_circ_0002065	NC_000002.12	+	197459932	197470171	10240	445	sense-overlappin	NM_025147.3	COQ10B
circRNA_02170	0	0	0	0	0	0.041425819	0	0	0	0	0	0	hsa_circ_0005445	NC_000002.12	+	197503039	197503330	292	212	sense-overlappin	NM_002157.2	HSPE1
circRNA_02171	0	0	0.021808645	0	0	0	0	0	0	0	0	0	hsa_circ_0057681	NC_000002.12	+	197523624	197540437	16814	294	sense-overlappin	NM_001202485.1	HSPE1-MOB4
circRNA_02172	0	0.051174919	0	0	0.05015779	0.020712909	0.030148028	0	0.020254528	0	0	0	hsa_circ_0003915	NC_000002.12	-	199368605	199433514	64910	531	sense-overlappin	NM_001172509.1	SATB2
circRNA_02173	0	0	0	0	0	0	0	0	0.040509055	0	0	0	hsa_circ_0002541	NC_000002.12	-	199940089	199948472	8384	270	sense-overlappin	NM_001039693.2	TYW5
circRNA_02174	0.020575025	0	0.021808645	0	0	0	0	0	0	0	0	0	-	NC_000002.12	+	200389223	200419496	30274	467	sense-overlappin	NM_001282735.1	SPATS2L
circRNA_02175	0	0	0.065425935	0	0	0	0	0	0	0	0	0	hsa_circ_0057701	NC_000002.12	+	200412311	200419496	7186	406	sense-overlappin	NM_001282735.1	SPATS2L
circRNA_02176	0	0	0	0	0	0	0.040197371	0	0	0	0	0	hsa_circ_0003994	NC_000002.12	+	200416379	200419496	3118	297	sense-overlappin	NM_001282735.1	SPATS2L
circRNA_02177	0.041150049	0	0.054521613	0	0.02006311	0	0.020098685	0	0	0	0	0	hsa_circ_0003071	NC_000002.12	+	200419250	200419496	247	247	exonic	NM_001282735.1	SPATS2L
circRNA_02178	0	0	0.021808645	0	0.02006311	0	0	0	0	0	0	0	hsa_circ_0057703	NC_000002.12	+	200439122	200440784	1663	343	sense-overlappin	NM_001282735.1	SPATS2L
circRNA_02179	0.072012586	0.030704951	0.032712968	0.0309543	0.08025246	0.041425819	0.020098685	0.030729443	0.040509055	0.040344966	0	0.0309546	hsa_circ_0004001	NC_000002.12	-	200856682	200856985	304	225	sense-overlappin	NM_001162407.1	CLK1
circRNA_02180	0	0	0	0	0.03009467	0.020712909	0	0	0	0	0	0	-	NC_000002.12	-	200856682	200861862	5181	1057	sense-overlappin	NM_001162407.1	CLK1
circRNA_02181	0	0.030704951	0	0	0	0	0	0	0	0	0	0	-	NC_000002.12	-	200913295	200937935	24641	1163	sense-overlappin	XM_011511254.1	ORC2
circRNA_02182	0	0	0	0.0309543	0	0	0	0	0	0	0	0	hsa_circ_0006101	NC_000002.12	-	200920222	200937966	17745	1013	sense-overlappin	XM_011511254.1	ORC2
circRNA_02183	0	0	0.021808645	0	0	0	0	0	0	0	0	0	-	NC_000002.12	-	200925836	200937966	12131	694	sense-overlappin	XM_011511254.1	ORC2
circRNA_02184	0	0	0	0	0	0	0.020098685	0	0	0	0	0	-	NC_000002.12	-	200933876	200937966	4091	354	sense-overlappin	XM_011511254.1	ORC2
circRNA_02185	0	0.040939935	0	0	0	0	0	0	0	0	0	0	-	NC_000002.12	-	200942685	200958133	15449	491	sense-overlappin	XM_011511254.1	ORC2
circRNA_02186	0	0	0	0	0	0.031069364	0	0	0	0	0	0	hsa_circ_0003858	NC_000002.12	-	201016991	201024039	7049	419	sense-overlappin	XM_005246475.1	FAM126B
circRNA_02187	0	0	0	0.0309543	0	0	0	0	0	0.040344966	0	0	hsa_circ_0057745	NC_000002.12	+	201129729	201133134	3406	524	sense-overlappin	NM_001127184.2	CFLAR
circRNA_02188	0.082300098	0	0	0	0	0	0	0	0	0	0	0.0619092	-	NC_000002.12	+	201129729	201136107	6379	660	sense-overlappin	NM_001127184.2	CFLAR
circRNA_02189	0	0	0.032712968	0	0	0	0	0	0	0.040344966	0	0	hsa_circ_0002860	NC_000002.12	+	201129729	201149835	20107	930	sense-overlappin	NM_003879.5	CFLAR
circRNA_02190	0	0.051174919	0	0	0	0	0	0.020486295	0	0	0	0	hsa_circ_0007530	NC_000002.12	+	201133029	201149835	16807	512	sense-overlappin	NM_001202517.1	CFLAR
circRNA_02191	0	0	0	0	0	0.020712909	0	0	0	0	0	0	-	NC_000002.12	+	201143331	201149835	6505	2234	sense-overlappin	NM_001202517.1	CFLAR
circRNA_02192	0	0.071644886	0.054521613	0.0515905	0.03009467	0.093208092	0.030148028	0.112674625	0.081018111	0.080689931	0.070893305	0.0619092	hsa_circ_0001092	NC_000002.12	+	201145378	201149835	4458	187	sense-overlappin	NM_001202517.1	CFLAR
circRNA_02193	0	0	0	0.0206362	0.04012623	0	0	0.040972591	0	0	0	0	-	NC_000002.12	+	201145378	201154407	9030	4759	sense-overlappin	NM_001202517.1	CFLAR
circRNA_02194	0	0	0	0	0	0	0	0	0.020254528	0	0	0	-	NC_000002.12	+	201160432	201164084	3653	761	sense-overlappin	NM_001202516.1	CFLAR
circRNA_02195	0	0	0	0.0206362	0	0	0.020098685	0.030729443	0	0	0	0	-	NC_000002.12	+	201208075	201208467	393	287	sense-overlappin	XR_923043.1	
circRNA_02196	0	0	0.054521613	0	0	0	0	0	0	0	0	0	-	NC_000002.12	-	201545543	201582217	36675	646	sense-overlappin	NM_001168216.1	ALS2CR11
circRNA_02197	0	0	0.032712968	0	0	0	0	0	0	0	0	0	hsa_circ_0057769	NC_000002.12	-	201565729	201582217	16489	461	sense-overlappin	NM_001168216.1	ALS2CR11
circRNA_02198	0	0	0	0	0	0	0	0	0.020254528	0	0	0	hsa_circ_0057781	NC_000002.12	-	201738670	201744429	5760	419	sense-overlappin	NM_020919.3	ALS2
circRNA_02199	0.020575025	0	0	0	0	0.020712909	0	0	0	0.020172483	0.04051046	0	hsa_circ_0001093	NC_000002.12	-	201760881	201761818	938	938	exonic	NM_020919.3	ALS2
circRNA_02200	0	0	0	0	0	0	0.030148028	0	0	0.020172483	0	0	hsa_circ_0008327	NC_000002.12	+	202290323	202295837	5515	572	sense-overlappin	NM_015934.3	NOP58
circRNA_02201	0	0	0	0	0.03009467	0	0	0	0	0	0	0	-	NC_000002.12	-	202292225	202292404	180	180	antisense	NM_015934.3	NOP58
circRNA_02202	0.102875123	0.122819805	0.185373483	0.1547714	0.10031557	0.082851637	0.200986853	0.081945182	0.13165443	0.121034897	0.172169456	0.144454801	hsa_circ_0003218	NC_000002.12	+	202464809	202467689	2881	342	sense-overlappin	XM_011511688.1	BMPR2
circRNA_02203	0.030862537	0	0.021808645	0	0	0	0.020098685	0	0	0	0	0	hsa_circ_0005078	NC_000002.12	+	202555252	202556531	1280	1280	exonic	XM_011511687.1	BMPR2
circRNA_02204	0	0	0.04361729	0	0	0.020712909	0	0	0	0	0	0	-	NC_000002.12	+	202755538	202759353	3816	491	sense-overlappin	NM_173511.3	FAM117B
circRNA_02205	0	0	0	0	0	0	0	0.020486295	0	0	0	0	-	NC_000002.12	-	202779390	202819899	40510	1233	sense-overlappin	NM_001288623.1	ICA1L
circRNA_02206	0	0.020469968	0	0	0	0	0	0	0	0	0	0	-	NC_000002.12	-	202896065	202897415	1351	271	sense-overlappin	NM_018256.3	WDR12
circRNA_02207	0	0	0	0	0	0	0	0	0	0.040344966	0	0	-	NC_000002.12	+	202917877	202955758	37882	971	sense-overlappin	NM_024744.15	CARF
circRNA_02208	0.020575025	0.030704951	0	0	0	0	0	0	0	0	0	0.0206364	hsa_circ_0057829	NC_000002.12	+	202952559	202954134	1576	251	sense-overlappin	NM_001104586.2	CARF
circRNA_02209	0.020575025	0.020469968	0.098138903	0.0412724	0	0.082851637	0.060296056	0.040972591	0.050636319	0.060517448	0.030382845	0.0412728	hsa_circ_0004919	NC_000002.12	+	202952559	202955758	3200	336	sense-overlappin	NM_001104586.2	CARF

circRNA_02210	0	0.020469968	0.021808645	0.0412724	0	0	0	0.030729443	0	0.07060369	0	0.0309546	hsa_circ_0003747	NC_000002.12	+	202952559	202961426	8868	526	sense-overlappin	NM_001104586.2	CARF
circRNA_02211	0	0	0	0	0.02006311	0	0	0	0	0.020172483	0	0	hsa_circ_0003493	NC_000002.12	+	202954005	202955758	1754	215	sense-overlappin	NM_001104586.2	CARF
circRNA_02212	0	0	0	0	0	0	0	0	0	0.030258724	0	0	-	NC_000002.12	+	202974334	202982441	8108	728	sense-overlappin	NM_001104586.2	CARF
circRNA_02213	0	0	0	0	0	0	0	0	0	0.050431207	0	0	-	NC_000002.12	+	202977269	202982441	5173	565	sense-overlappin	NM_001104586.2	CARF
circRNA_02214	0	0	0	0.1031809	0.02006311	0.020712909	0	0.030729443	0	0.020172483	0.04051046	0	hsa_circ_0005395	NC_000002.12	+	203056427	203057453	1027	210	sense-overlappin	XM_011511658.1	NBEAL1
circRNA_02215	0	0	0	0.0206362	0	0	0	0	0	0	0	0	-	NC_000002.12	+	203056427	203077837	21411	379	sense-overlappin	XM_011511658.1	NBEAL1
circRNA_02216	0	0	0	0	0	0	0.020098685	0	0	0	0	0	hsa_circ_0004584	NC_000002.12	+	203068393	203084569	16177	583	sense-overlappin	XM_011511658.1	NBEAL1
circRNA_02217	0	0	0	0.0206362	0	0	0	0	0	0	0	0	-	NC_000002.12	+	203111979	203113318	1340	424	sense-overlappin	XM_011511658.1	NBEAL1
circRNA_02218	0	0	0	0	0	0	0	0	0	0.020172483	0	0	-	NC_000002.12	+	203111979	203126926	14948	1166	sense-overlappin	XM_011511658.1	NBEAL1
circRNA_02219	0	0	0	0.0206362	0	0	0	0	0	0	0	0	-	NC_000002.12	+	203157699	203175287	17589	877	sense-overlappin	XM_011511658.1	NBEAL1
circRNA_02220	0	0	0	0	0.02006311	0	0	0	0	0	0	0	-	NC_000002.12	+	203193795	203199447	5653	317	sense-overlappin	XM_011511658.1	NBEAL1
circRNA_02221	0	0	0	0	0	0.020712909	0	0.020486295	0	0	0	0	hsa_circ_0057867	NC_000002.12	+	203208637	203209322	686	279	sense-overlappin	XM_011511658.1	NBEAL1
circRNA_02222	0	0.030704951	0	0	0	0	0	0	0	0	0	0	-	NC_000002.12	+	203210958	203213653	2696	285	sense-overlappin	XM_011511658.1	NBEAL1
circRNA_02223	0	0	0.021808645	0	0	0	0	0	0	0	0	0	hsa_circ_0057870	NC_000002.12	+	203245846	203266681	20836	528	sense-overlappin	NM_177538.2	CYP20A1
circRNA_02224	0	0.030704951	0	0	0	0	0	0	0	0	0	0	-	NC_000002.12	+	203297893	203303095	5203	5203	exonic	NM_177538.2	CYP20A1
circRNA_02225	0.030862537	0	0.032712968	0.0206362	0	0	0	0	0	0	0	0	-	NC_000002.12	+	203380208	203402734	22527	907	sense-overlappin	XM_006712183.2	ABI2
circRNA_02226	0	0	0.021808645	0	0	0	0	0	0	0	0	0	hsa_circ_0007951	NC_000002.12	+	203402576	203427269	24694	513	sense-overlappin	XM_005246217.1	ABI2
circRNA_02227	0	0	0.021808645	0	0	0	0	0	0	0	0	0	hsa_circ_0005613	NC_000002.12	-	203489584	203495353	5770	732	sense-overlappin	NM_213589.2	RAPH1
circRNA_02228	0	0	0.021808645	0	0	0	0	0	0	0	0	0	-	NC_000002.12	-	203489584	203497395	7812	813	sense-overlappin	XM_006712695.2	RAPH1
circRNA_02229	0.041150049	0	0.032712968	0	0	0.041425819	0	0	0	0	0	0	-	NC_000002.12	+	204558024	204559867	1844	1844	intronic	NM_001302769.1	PARD3B
circRNA_02230	0	0	0	0.0206362	0	0	0	0	0	0	0	0	-	NC_000002.12	+	204657142	204686282	29141	29141	sense-overlappin	NM_001302769.1	PARD3B
circRNA_02231	0	0	0.032712968	0	0	0	0	0	0	0	0	0	hsa_circ_0006219	NC_000002.12	+	204661809	204686282	24474	24474	sense-overlappin	NM_001302769.1	PARD3B
circRNA_02232	0	0	0.021808645	0	0	0	0	0	0	0	0	0	hsa_circ_0057890	NC_000002.12	+	205158722	205185863	27142	590	sense-overlappin	NM_001302769.1	PARD3B
circRNA_02233	0.020575025	0.040939935	0	0	0	0	0.050246713	0.020486295	0	0.020172483	0	0	hsa_circ_0008172	NC_000002.12	+	205158722	205193320	34599	706	sense-overlappin	NM_001302769.1	PARD3B
circRNA_02234	0	0	0.021808645	0	0	0	0	0	0	0.040344966	0	0	-	NC_000002.12	+	205300530	205301701	1172	445	sense-overlappin	NM_001302769.1	PARD3B
circRNA_02235	0	0	0.032712968	0	0	0	0	0	0	0	0	0	-	NC_000002.12	+	205401013	205440672	39660	414	sense-overlappin	NM_001302769.1	PARD3B
circRNA_02236	0.051437562	0	0.065425935	0.0722267	0.03009467	0.041425819	0.030148028	0.040972591	0	0.020172483	0	0	hsa_circ_0004575	NC_000002.12	+	205440370	205440672	303	303	exonic	NM_001302769.1	PARD3B
circRNA_02237	0	0	0	0	0.05015779	0	0	0.030729443	0	0	0	0	hsa_circ_0006005	NC_000002.12	+	206841107	206881891	40785	38456	sense-overlappin	XM_011510627.1	CPO
circRNA_02238	0	0	0	0	0	0	0	0	0	0	0	0.0206364	-	NC_000002.12	+	206866697	206879440	12744	12744	sense-overlappin	XM_011510627.1	CPO
circRNA_02239	0	0.040939935	0	0	0	0	0	0	0	0	0	0	hsa_circ_0008544	NC_000002.12	+	206866697	206881891	15195	12866	sense-overlappin	XM_011510627.1	CPO
circRNA_02240	0	0	0	0	0	0	0	0.030729443	0	0	0	0.0206364	hsa_circ_0057953	NC_000002.12	+	207567463	207577655	10193	881	sense-overlappin	XR_922862.1	
circRNA_02241	0	0.030704951	0.04361729	0.0515905	0	0	0.020098685	0	0.030381792	0.050431207	0	0	hsa_circ_0002556	NC_000002.12	+	207724800	207742342	17543	419	sense-overlappin	NM_152523.2	CCNYL1
circRNA_02242	0	0	0	0	0.03009467	0	0.040197371	0	0	0	0	0	hsa_circ_0057959	NC_000002.12	+	207726842	207742342	15501	344	sense-overlappin	NM_152523.2	CCNYL1
circRNA_02243	0.174887709	0.204699675	0.207182128	0.1341352	0.03009467	0.134633911	0.070345399	0.133160921	0.050636319	0.14120738	0.091148535	0	hsa_circ_0001095	NC_000002.12	-	207976651	207977586	936	936	exonic	NM_001080475.2	PLEKHM3
circRNA_02244	0	0.020469968	0	0	0	0	0	0	0	0	0	0	-	NC_000002.12	+	208273584	208298779	25196	914	sense-overlappin	XM_011510779.1	PIKFYVE
circRNA_02245	0.041150049	0.030704951	0.098138903	0	0.03009467	0	0	0	0.030381792	0.030258724	0	0	hsa_circ_0004920	NC_000002.12	+	208285726	208288818	3093	298	sense-overlappin	XM_011510779.1	PIKFYVE
circRNA_02246	0	0	0	0	0	0.020712909	0	0	0	0	0	0	-	NC_000002.12	+	208312236	208346147	33912	3573	sense-overlappin	XM_011510779.1	PIKFYVE
circRNA_02247	0.113162635	0	0.032712968	0.0412724	0	0	0	0.040972591	0.020254528	0.07060369	0	0.0619092	hsa_circ_0001097	NC_000002.12	+	208345111	208348023	2913	347	sense-overlappin	XM_011510779.1	PIKFYVE
circRNA_02248	0	0	0.032712968	0	0.02006311	0	0.020098685	0	0	0.030258724	0	0	hsa_circ_0007225	NC_000002.12	+	210015973	210021064	5092	1812	sense-overlappin	NM_001278282.1	RPE
circRNA_02249	0.020575025	0	0	0	0	0	0	0	0	0	0	0	-	NC_000002.12	-	210040420	210075756	35337	479	sense-overlappin	NM_152519.2	KANSL1L
circRNA_02250	0	0	0.032712968	0	0	0	0	0	0	0	0	0	-	NC_000002.12	-	210040420	210098207	57788	601	sense-overlappin	NM_152519.2	KANSL1L
circRNA_02251	0	0	0.021808645	0	0	0	0	0	0	0	0	0	-	NC_000002.12	-	210098086	210104301	6216	320	sense-overlappin	NM_152519.2	KANSL1L
circRNA_02252	0	0	0	0	0	0	0	0	0	0.020172483	0	0	-	NC_000002.12	-	210098086	210154590	56505	1558	sense-overlappin	NM_152519.2	KANSL1L
circRNA_02253	0	0.030704951	0.065425935	0	0.02006311	0.062138728	0.020098685	0.040972591	0	0	0	0	hsa_circ_0058010	NC_000002.12	-	210098086	210154611	56526	1579	sense-overlappin	NM_152519.2	KANSL1L
circRNA_02254	0.072012586	0	0.381651288	0.0722267	0.02006311	0	0.080394741	0	0	0.040344966	0	0	hsa_circ_0002617	NC_000002.12	-	210104104	210154611	50508	1457	sense-overlappin	NM_152519.2	KANSL1L
circRNA_02255	0.030862537	0	0.17446916	0	0	0	0.020098685	0	0	0	0	0	-	NC_000002.12	-	210104104	210155404	51301	2250	sense-overlappin	NM_152519.2	KANSL1L
circRNA_02256	0	0	0.032712968	0	0	0	0	0	0	0	0	0	-	NC_000002.12	-	210104104	210155408	51305	2254	sense-overlappin	NM_152519.2	KANSL1L
circRNA_02257	0	0	0.141756193	0.0309543	0	0	0.020098685	0	0	0	0	0	hsa_circ_0002822	NC_000002.12	-	210129031	210154611	25581	1259	sense-overlappin	NM_152519.2	KANSL1L
circRNA_02258	0	0	0.065425935	0	0	0	0	0	0	0	0	0	-	NC_000002.12	-	210129031	210155404	26374	2052	sense-overlappin	NM_152519.2	KANSL1L
circRNA_02259	0.144025172	0.092114854	0.21808645	0.0722267	0.06018934	0.051782273	0.050246713	0.030729443	0.091145375	0.07060369	0.04051046	0.0619092	hsa_circ_0008459	NC_000002.12	-	210153495	210154611	1117	1117	exonic	NM_152519.2	KANSL1L
circRNA_02260	0	0	0	0	0	0	0.030148028	0	0	0	0	0	-	NC_000002.12	-	210153495	210155404	1910	1910	sense-overlappin	NM_152519.2	KANSL1L
circRNA_02261	0	0	0	0	0	0	0	0	0	0.030258724	0	0	-	NC_000002.12	-	210192804	210220802	27999	1122	sense-overlappin	XM_005246517.3	ACADL
circRNA_02262	0	0	0.032712968	0	0	0	0	0	0.020254528	0	0	0	-	NC_000002.12	-	213310059	213317356	7298	257	sense-overlappin	NM_001025436.2	SPAG16
circRNA_02263	0	0	0.098138903	0	0	0	0	0	0	0	0	0	hsa_circ_0005760	NC_000002.12	+	213310059	213340270	30212	365	sense-overlappin	NM_024532.4	SPAG16
circRNA_02264	0	0	0.021808645	0	0	0	0	0	0	0	0	0	-	NC_000002.12	+	213310059	213343897	33839	3992	sense-overlappin	NM_024532.4	SPAG16

circRNA_02265	0	0	0.076330258	0.0206362	0.04012623	0	0	0.040972591	0.020254528	0.030258724	0	0	hsa_circ_0058039	NC_000002.12	+	213310059	213350645	40587	483	sense-overlappin	NM_024532.4	SPAG16
circRNA_02266	0	0	0.39255561	0.0722267	0.02006311	0.020712909	0	0.040972591	0	0	0	0.0206364	hsa_circ_0004664	NC_000002.12	+	213310059	213364145	54087	553	sense-overlappin	NM_024532.4	SPAG16
circRNA_02267	0.020575025	0.051174919	0.13085187	0.0206362	0.06018934	0.062138728	0	0	0.040509055	0.060517448	0	0	hsa_circ_0058040	NC_000002.12	+	213310059	213375119	65061	663	sense-overlappin	NM_024532.4	SPAG16
circRNA_02268	0	0	0.021808645	0	0	0	0	0	0	0	0	0	-	NC_000002.12	-	214728918	214781509	52592	1728	sense-overlappin	XM_011511567.1	BARD1
circRNA_02269	0	0	0.032712968	0	0	0	0	0	0	0	0	0	hsa_circ_0007935	NC_000002.12	-	214745067	214769312	24246	589	sense-overlappin	XM_011511567.1	BARD1
circRNA_02270	0	0	0.04361729	0	0	0	0	0	0.020254528	0	0	0	hsa_circ_0058042	NC_000002.12	-	214745067	214781509	36443	1539	sense-overlappin	XM_011511567.1	BARD1
circRNA_02271	0.041150049	0	0.185373483	0	0.02006311	0	0	0.030729443	0	0	0	0	hsa_circ_0003963	NC_000002.12	-	214745067	214797117	52051	1745	sense-overlappin	XM_011511567.1	BARD1
circRNA_02272	0	0	0.032712968	0	0	0	0	0	0	0	0	0	hsa_circ_0058047	NC_000002.12	-	214752447	214769312	16866	363	sense-overlappin	XM_011511567.1	BARD1
circRNA_02273	0.020575025	0	0.021808645	0	0	0	0	0	0	0	0	0	-	NC_000002.12	-	214752447	214781509	29063	1313	sense-overlappin	XM_011511567.1	BARD1
circRNA_02274	0.020575025	0	0.032712968	0	0	0	0	0	0	0	0	0	hsa_circ_0058048	NC_000002.12	-	214752447	214792445	39999	1462	sense-overlappin	XM_011511567.1	BARD1
circRNA_02275	0.041150049	0	0.119947548	0.0309543	0.02006311	0	0	0	0.030381792	0.030258724	0	0	hsa_circ_0002999	NC_000002.12	-	214752447	214797117	44671	1519	sense-overlappin	XM_011511567.1	BARD1
circRNA_02276	0.020575025	0.020469968	0.239895095	0.0206362	0.10031557	0.031069364	0.020098685	0.020486295	0.101272639	0.040344966	0	0.0206364	hsa_circ_0001098	NC_000002.12	-	214767482	214781509	14028	1204	sense-overlappin	XM_011511567.1	BARD1
circRNA_02277	0	0	0.065425935	0	0.02006311	0	0	0	0	0	0	0	hsa_circ_0058050	NC_000002.12	-	214767482	214792445	24964	1353	sense-overlappin	XM_011511567.1	BARD1
circRNA_02278	0.020575025	0.071644886	0.26170374	0.0412724	0.06018934	0.051782273	0.040197371	0	0.050636319	0.121034897	0	0	hsa_circ_0058051	NC_000002.12	-	214767482	214797117	29636	1410	sense-overlappin	XM_011511567.1	BARD1
circRNA_02279	0	0	0.065425935	0	0.02006311	0	0	0	0	0	0	0	hsa_circ_0058055	NC_000002.12	-	214780560	214797117	16558	1156	sense-overlappin	XM_011511567.1	BARD1
circRNA_02280	0	0	0.032712968	0	0	0	0	0	0	0	0	0	hsa_circ_0003484	NC_000002.12	-	214792297	214797117	4821	206	sense-overlappin	XM_011511567.1	BARD1
circRNA_02281	0	0	0	0	0	0	0.020098685	0	0	0	0	0	hsa_circ_0058092	NC_000002.12	-	215373322	215391814	18493	2178	sense-overlappin	NM_002026.2	FN1
circRNA_02282	0	0.040939935	0.04361729	0	0	0.031069364	0	0	0	0	0	0	hsa_circ_0058174	NC_000002.12	+	216141186	216148276	7091	328	sense-overlappin	NM_021141.3	XRCC5
circRNA_02283	0	0	0	0	0	0	0.030148028	0	0	0	0	0	hsa_circ_0058182	NC_000002.12	+	216190225	216194986	4762	275	sense-overlappin	NM_021141.3	XRCC5
circRNA_02284	0	0.020469968	0	0	0	0	0	0	0	0	0	0	-	NC_000002.12	-	217425302	217478558	53257	53257	intergenic		
circRNA_02285	0.030862537	0	0	0	0	0.020712909	0	0	0	0	0	0	hsa_circ_0004690	NC_000002.12	-	217880898	217900512	19615	1108	sense-overlappin	XM_011511711.1	TNS1
circRNA_02286	0.051437562	0	0	0	0	0.031069364	0	0.020486295	0	0	0	0	hsa_circ_0008475	NC_000002.12	+	218238664	218239484	821	281	sense-overlappin	NM_152862.2	ARPC2
circRNA_02287	0	0	0	0	0	0	0	0.020486295	0	0	0	0	hsa_circ_0058213	NC_000002.12	+	218238664	218245546	6883	408	sense-overlappin	NM_152862.2	ARPC2
circRNA_02288	0	0	0.021808645	0	0	0	0	0	0	0	0	0	hsa_circ_0058244	NC_000002.12	+	218434526	218435434	909	326	sense-overlappin	NM_007127.2	VIL1
circRNA_02289	0	0	0	0	0	0	0.030148028	0	0	0	0	0	hsa_circ_0058263	NC_000002.12	-	218509979	218562813	52835	1294	sense-overlappin	XM_005246720.1	USP37
circRNA_02290	0	0	0.021808645	0	0	0	0	0	0	0	0	0	hsa_circ_0058275	NC_000002.12	+	218582971	218584721	1751	226	sense-overlappin	XM_011512138.1	RQCD1
circRNA_02291	0	0	0	0.0206362	0	0	0.020098685	0	0	0	0	0	hsa_circ_0007648	NC_000002.12	-	218656150	218656463	314	314	exonic	NM_001105537.2	ZNF142
circRNA_02292	0	0	0	0.0515905	0	0	0	0	0	0	0	0	-	NC_000002.12	-	219047745	219051580	3836	3836	intergenic		
circRNA_02293	0	0	0.021808645	0	0	0	0	0	0	0	0	0	hsa_circ_0058430	NC_000002.12	-	222599928	222648795	48868	1756	sense-overlappin	NR_130154.1	
circRNA_02294	0	0	0.021808645	0	0	0	0	0	0	0	0	0	-	NC_000002.12	-	222613811	222624479	10669	500	sense-overlappin	NM_005687.4	FARSB
circRNA_02295	0	0	0	0	0	0	0	0	0	0.030258724	0	0	-	NC_000002.12	-	222630113	222647325	17213	1259	sense-overlappin	NR_130154.1	
circRNA_02296	0	0	0	0	0.02006311	0	0	0	0	0	0	0	hsa_circ_0058443	NC_000002.12	+	222887830	222909150	21321	584	sense-overlappin	XR_922889.1	
circRNA_02297	0.051437562	0	0.04361729	0.0619086	0.12037869	0.031069364	0	0.040972591	0	0.040344966	0	0	hsa_circ_0004821	NC_000002.12	+	222900674	222909150	8477	525	sense-overlappin	XR_922889.1	
circRNA_02298	0	0	0.021808645	0	0	0	0	0	0	0	0	0	-	NC_000002.12	+	222900674	222924595	23922	1439	sense-overlappin	XR_922889.1	
circRNA_02299	0	0	0.032712968	0.0206362	0	0	0	0	0	0	0	0	-	NC_000002.12	+	222900674	222934687	34014	2152	sense-overlappin	NM_004457.3	ACSL3
circRNA_02300	0	0	0	0	0.02006311	0	0	0	0	0	0	0	hsa_circ_0058451	NC_000002.12	+	222918046	222924595	6550	736	sense-overlappin	XR_922889.1	
circRNA_02301	0	0	0	0	0.03009467	0	0	0	0	0	0	0	hsa_circ_0058454	NC_000002.12	+	222927017	222934687	7671	713	sense-overlappin	NM_004457.3	ACSL3
circRNA_02302	0	0	0.04361729	0	0	0	0	0	0.020254528	0	0	0	hsa_circ_0058456	NC_000002.12	-	223775901	223777869	1969	288	sense-overlappin	NM_001039569.1	AP1S3
circRNA_02303	0	0.020469968	0	0	0	0	0	0.020486295	0	0.040344966	0	0.0206364	hsa_circ_0001102	NC_000002.12	-	223894234	223895630	1397	233	sense-overlappin	NM_020830.4	WDFY1
circRNA_02304	0	0	0	0	0	0	0	0.020486295	0	0	0	0	hsa_circ_0008365	NC_000002.12	-	223991803	224001922	10120	707	sense-overlappin	NR_073116.1	
circRNA_02305	0.020575025	0	0	0	0	0	0	0	0	0	0	0	hsa_circ_0001103	NC_000002.12	-	223998115	224001922	3808	509	sense-overlappin	NR_073116.1	
circRNA_02306	0	0	0.021808645	0	0	0	0	0	0	0	0	0	-	NC_000002.12	-	224495832	224535641	39810	1578	sense-overlappin	NM_001257198.1	CUL3
circRNA_02307	0	0	0.032712968	0	0	0	0	0	0	0	0	0	-	NC_000002.12	-	224528947	224535641	6695	6695	sense-overlappin	NM_001257198.1	CUL3
circRNA_02308	0.082300098	0.061409903	0.17446916	0.0412724	0.11034713	0.020712909	0.120592112	0.030729443	0.030381792	0.020172483	0	0	hsa_circ_0008309	NC_000002.12	-	224535528	224557856	22329	438	sense-overlappin	XM_011511996.1	CUL3
circRNA_02309	0	0	0	0	0	0	0	0	0.020254528	0	0	0	-	NC_000002.12	-	224903060	224906566	3507	3507	intronic	NM_001290263.1	DOCK10
circRNA_02310	0	0.102349838	0.13085187	0.0928628	0.13041025	0.103564547	0.050246713	0.071702034	0.070890847	0.100862414	0.02025523	0.082545601	hsa_circ_0058493	NC_000002.12	+	226864604	226867318	2715	656	sense-overlappin	XR_923039.1	
circRNA_02311	0	0	0	0.0619086	0	0	0.020098685	0	0	0	0.02025523	0	-	NC_000002.12	+	226864604	226867688	3085	1026	sense-overlappin	XR_923039.1	
circRNA_02312	0.072012586	0.225169643	0.141756193	0.1754076	0.03009467	0.207129093	0.100493427	0.133160921	0.101272639	0.151293621	0.04051046	0.051591	hsa_circ_0058495	NC_000002.12	+	226864604	226914351	49748	946	sense-overlappin	XR_923039.1	
circRNA_02313	0.092587611	0.040939935	0.141756193	0.0928628	0.09028402	0.113921001	0.080394741	0.061458886	0.020254528	0.121034897	0.030382845	0.0412728	hsa_circ_0058497	NC_000002.12	+	226906793	226914351	7559	290	sense-overlappin	XR_923039.1	
circRNA_02314	0	0	0.076330258	0	0	0	0	0	0	0	0	0	hsa_circ_0058518	NC_000002.12	+	227491547	227536993	45447	1283	sense-overlappin	NM_001135187.1	AGFG1
circRNA_02315	0	0	0	0	0	0	0	0	0	0.020172483	0.030382845	0	hsa_circ_0058520	NC_000002.12	+	227519948	227524915	4968	433	sense-overlappin	NM_001135187.1	AGFG1
circRNA_02316	0	0	0	0.0206362	0	0	0	0	0	0	0	0	hsa_circ_0058539	NC_000002.12	-	229768616	229771632	3017	313	sense-overlappin	NM_001284215.1	TRIP12
circRNA_02317	0	0.040939935	0	0	0	0	0	0	0.030381792	0.030258724	0	0	-	NC_000002.12	-	229774097	229788940	14844	999	sense-overlappin	NM_001284215.1	TRIP12
circRNA_02318	0	0.040939935	0	0	0	0	0	0	0	0	0	0	-	NC_000002.12	-	229818364	229830839	12476	329	sense-overlappin	NM_001284215.1	TRIP12
circRNA_02319	0	0	0.021808645	0	0	0	0	0	0	0	0	0	-	NC_000002.12	-	229836848	229840927	4080	243	sense-overlappin	NM_001284215.1	TRIP12
circRNA_02320	0.030862537	0	0	0	0	0	0	0	0	0	0	0	-	NC_000002.12	-	229836848	229860531	23684	1085	sense-overlappin	XM_005246960.3	TRIP12
circRNA_02321	0	0	0.08723458	0.0515905	0	0.031069364	0	0	0	0.020172483	0	0	hsa_circ_0007381	NC_000002.12	-	229836848	229880128	43281	1232	sense-overlappin	XM_005246960.3	TRIP12

circRNA_02322	0	0	0	0	0	0	0	0	0	0.030258724	0	0	hsa_circ_0004079	NC_000002.12	-	229840822	229860531	19710	948	sense-overlappin	XM_005246960.3	TRIP12
circRNA_02323	0	0	0.054521613	0	0	0.020712909	0.060296056	0	0	0	0.050638075	0	hsa_circ_0005689	NC_000002.12	-	229858772	229860531	1760	842	sense-overlappin	XM_005246960.3	TRIP12
circRNA_02324	0	0.051174919	0.076330258	0.0825448	0.10031557	0.072495183	0.020098685	0	0.030381792	0.040344966	0.04051046	0.0619092	hsa_circ_0003273	NC_000002.12	-	229858772	229880128	21357	989	sense-overlappin	XM_005246960.3	TRIP12
circRNA_02325	0	0	0	0	0	0	0	0	0	0.020172483	0	0	-	NC_000002.12	-	230045745	230046722	978	978	exonic	NM_152527.4	SLC16A14
circRNA_02326	0	0	0	0	0	0	0	0	0	0.020172483	0	0	-	NC_000002.12	+	230255452	230287966	32515	561	sense-overlappin	XM_011510517.1	SP140
circRNA_02327	0	0.071644886	0.08723458	0.0515905	0.10031557	0.062138728	0.040197371	0.071702034	0	0.040344966	0.030382845	0	hsa_circ_0008382	NC_000002.12	+	230357805	230361697	3893	416	sense-overlappin	XM_006712854.2	SP140L
circRNA_02328	0	0	0	0	0	0	0	0	0	0	0.030382845	0	-	NC_000002.12	+	230357805	230371651	13847	530	sense-overlappin	XM_006712854.2	SP140L
circRNA_02329	0	0	0	0	0	0	0	0	0	0.020172483	0.02025523	0	hsa_circ_0005116	NC_000002.12	+	230442937	230449150	6214	479	sense-overlappin	NM_001206702.1	SP100
circRNA_02330	0.082300098	0.071644886	0.937771736	0.2269981	0.12037869	0.062138728	0.070345399	0.081945182	0.030381792	0.171466104	0.111403765	0.123818401	hsa_circ_0003922	NC_000002.12	+	230442937	230450255	7319	713	sense-overlappin	NM_001206702.1	SP100
circRNA_02331	0.072012586	0	0.032712968	0.0309543	0	0	0	0	0	0.040344966	0	0	-	NC_000002.12	+	230461262	230474447	13186	780	sense-overlappin	NM_001206701.1	SP100
circRNA_02332	0	0	0.032712968	0	0	0	0	0	0	0	0	0	hsa_circ_0003898	NC_000002.12	+	230759959	230793331	33373	441	sense-overlappin	XM_011511350.1	CAB39
circRNA_02333	0	0	0.065425935	0.0619086	0.03009467	0.041425819	0.020098685	0.030729443	0.030381792	0	0.030382845	0.0206364	hsa_circ_0001110	NC_000002.12	+	231075511	231087181	11671	1002	sense-overlappin	NM_001191037.1	PSMD1
circRNA_02334	0	0	0	0	0	0	0	0	0	0.020172483	0	0	-	NC_000002.12	+	231090495	231129904	39410	39410	intronic	NM_001191037.1	PSMD1
circRNA_02335	0.030862537	0	0	0	0	0	0	0	0	0	0	0	hsa_circ_0005845	NC_000002.12	+	231214831	231216793	1963	327	sense-overlappin	NM_001291656.1	ARMC9
circRNA_02336	0.030862537	0	0	0	0	0	0	0	0	0	0	0	hsa_circ_0058686	NC_000002.12	+	231788555	231791808	3254	254	sense-overlappin	XM_006712693.1	COPS7B
circRNA_02337	0	0	0.076330258	0	0	0	0	0	0	0	0	0	hsa_circ_0004862	NC_000002.12	+	232014835	232015671	837	303	sense-overlappin	NM_001257282.1	DIS3L2
circRNA_02338	0	0.040939935	0.13085187	0.0309543	0.03009467	0	0	0	0	0	0	0	hsa_circ_0007321	NC_000002.12	+	232014835	232030080	15246	459	sense-overlappin	NM_001257282.1	DIS3L2
circRNA_02339	0	0	0.021808645	0	0	0	0	0	0	0	0	0	hsa_circ_0058691	NC_000002.12	+	232087487	232087721	235	235	exonic	NM_001257282.1	DIS3L2
circRNA_02340	0	0	0	0	0.02006311	0	0	0	0	0	0	0	-	NC_000002.12	+	232087487	232088040	554	554	sense-overlappin	NM_001257282.1	DIS3L2
circRNA_02341	0	0	0.032712968	0	0	0	0	0	0	0	0	0	-	NC_000002.12	+	232087487	232089270	1784	1784	sense-overlappin	NM_001257282.1	DIS3L2
circRNA_02342	0	0	0	0	0	0	0	0	0.030381792	0	0	0	hsa_circ_0058698	NC_000002.12	+	232130619	232136719	6101	349	sense-overlappin	NM_152383.4	DIS3L2
circRNA_02343	0	0	0.021808645	0	0	0	0	0	0	0	0	0	-	NC_000002.12	+	232703424	232761436	58013	711	sense-overlappin	NM_015575.3	GIGYF2
circRNA_02344	0	0	0	0	0	0	0	0	0	0	0.02025523	0	hsa_circ_0058728	NC_000002.12	+	232747615	232749082	1468	226	sense-overlappin	NM_001103146.1	GIGYF2
circRNA_02345	0	0	0	0	0	0	0	0	0	0.020172483	0	0	-	NC_000002.12	+	232747615	232756334	8720	338	sense-overlappin	NM_001103146.1	GIGYF2
circRNA_02346	0	0	0.021808645	0.0206362	0	0	0	0	0	0.030258724	0	0	hsa_circ_0003341	NC_000002.12	+	232747615	232761436	13822	491	sense-overlappin	NM_001103146.1	GIGYF2
circRNA_02347	0	0	0.021808645	0	0	0	0	0	0	0	0	0	-	NC_000002.12	+	232756223	232819985	63763	2325	sense-overlappin	NM_001103147.1	GIGYF2
circRNA_02348	0	0	0	0	0	0	0	0	0.030381792	0	0	0	hsa_circ_0003937	NC_000002.12	+	232811244	232812491	1248	209	sense-overlappin	NM_001103146.1	GIGYF2
circRNA_02349	0	0	0	0	0	0	0	0.030729443	0	0	0	0	-	NC_000002.12	+	232811244	232839971	28728	991	sense-overlappin	NM_001103146.1	GIGYF2
circRNA_02350	0	0	0.054521613	0.0722267	0.02006311	0	0	0	0	0	0	0	hsa_circ_0001115	NC_000002.12	+	233449982	233452060	2079	376	sense-overlappin	NM_152879.2	DGKD
circRNA_02351	0.030862537	0	0	0	0	0	0	0	0	0	0	0	hsa_circ_0058773	NC_000002.12	+	233450922	233452060	1139	226	sense-overlappin	NM_152879.2	DGKD
circRNA_02352	0	0	0	0.0309543	0	0	0	0	0	0	0	0	hsa_circ_0058779	NC_000002.12	-	233549101	233551519	2419	273	sense-overlappin	NM_018218.2	USP40
circRNA_02353	0	0	0.021808645	0	0	0	0	0	0	0	0	0	hsa_circ_0058799	NC_000002.12	+	235750354	235799522	49169	419	sense-overlappin	NM_001244888.1	AGAP1
circRNA_02354	0	0	0.054521613	0	0	0	0	0	0	0	0	0	hsa_circ_0058806	NC_000002.12	+	235797759	235807331	9573	377	sense-overlappin	NM_001244888.1	AGAP1
circRNA_02355	0	0.030704951	0	0.0412724	0.05015779	0	0	0	0	0	0	0	hsa_circ_0003272	NC_000002.12	+	235930765	235968623	37859	321	sense-overlappin	NM_001037131.2	AGAP1
circRNA_02356	0	0	0	0	0.02006311	0	0	0	0	0	0	0	-	NC_000002.12	-	236324532	236377185	52654	1209	sense-overlappin	NR_073043.1	
circRNA_02357	0	0	0	0.0206362	0	0	0	0	0.020254528	0	0	0	-	NC_000002.12	+	237973092	237973747	656	233	sense-overlappin	NR_103500.1	
circRNA_02358	0	0	0.021808645	0	0	0	0	0	0	0	0	0	-	NC_000002.12	+	238093861	238094522	662	187	sense-overlappin	NM_016510.5	SCLY
circRNA_02359	0	0	0	0.0206362	0	0	0	0.040972591	0	0.020172483	0	0	hsa_circ_0006014	NC_000002.12	-	238173534	238185287	11754	531	sense-overlappin	XM_011511946.1	ILKAP
circRNA_02360	0	0	0	0	0	0.020712909	0	0	0	0	0	0	hsa_circ_0058874	NC_000002.12	-	238182065	238184113	2049	304	sense-overlappin	XM_011511946.1	ILKAP
circRNA_02361	0.041150049	0.030704951	0.141756193	0.0928628	0	0	0.040197371	0.040972591	0.070890847	0.161379862	0.06076569	0	hsa_circ_0001116	NC_000002.12	-	238182065	238185287	3223	411	sense-overlappin	XM_011511946.1	ILKAP
circRNA_02362	0.030862537	0	0.021808645	0	0	0	0	0	0	0	0	0	hsa_circ_0058876	NC_000002.12	-	238182065	238194870	12806	794	sense-overlappin	XM_005246106.1	ILKAP
circRNA_02363	0	0	0	0	0	0.020712909	0	0	0	0	0	0	hsa_circ_0007916	NC_000002.12	-	238185181	238194870	9690	477	sense-overlappin	XR_923033.1	
circRNA_02364	0	0	0	0	0	0	0	0.020486295	0	0	0	0	hsa_circ_0003854	NC_000002.12	-	238275743	238277955	2213	467	sense-overlappin	NM_022817.2	PER2
circRNA_02365	0	0	0	0	0.02006311	0	0	0	0	0	0	0	hsa_circ_0058905	NC_000002.12	-	239156652	239163923	7272	243	sense-overlappin	XM_011512227.1	HDAC4
circRNA_02366	0.041150049	0	0	0	0	0	0	0	0	0	0	0	-	NC_000002.12	-	239156652	239176563	19912	394	sense-overlappin	XM_011512227.1	HDAC4
circRNA_02367	0.020575025	0	0	0	0	0	0	0	0	0	0	0	hsa_circ_0003445	NC_000002.12	-	239176413	239236664	60252	468	sense-overlappin	XM_011512229.1	HDAC4
circRNA_02368	0.061725074	0.112584821	0.272608063	0.1341352	0.04012623	0.144990365	0.040197371	0.051215739	0.070890847	0.14120738	0.06076569	0	hsa_circ_0001118	NC_000002.12	-	239990074	240007370	17297	250	sense-overlappin	NM_004544.3	NDUFA10
circRNA_02369	0	0	0.054521613	0	0	0	0	0	0	0.030258724	0	0.0309546	hsa_circ_0001119	NC_000002.12	-	239990074	240014860	24787	452	sense-overlappin	NM_004544.3	NDUFA10
circRNA_02370	0	0.020469968	0.032712968	0	0	0.020712909	0	0	0	0	0	0	hsa_circ_0058919	NC_000002.12	-	239990074	240022340	32267	924	sense-overlappin	NM_004544.3	NDUFA10
circRNA_02371	0	0	0.08723458	0	0	0	0	0	0	0	0	0	hsa_circ_0058920	NC_000002.12	-	240005210	240007370	2161	141	sense-overlappin	NM_004544.3	NDUFA10
circRNA_02372	0.020575025	0	0	0	0	0	0	0	0	0	0	0	hsa_circ_0058923	NC_000002.12	-	240005210	240022340	17131	815	sense-overlappin	NM_004544.3	NDUFA10
circRNA_02373	0	0	0.054521613	0	0	0.031069364	0	0	0	0	0.02025523	0	hsa_circ_0005059	NC_000002.12	-	240552914	240555055	2142	334	sense-overlappin	NM_001282771.1	ANKMY1
circRNA_02374	0	0.020469968	0	0	0	0	0	0	0	0	0	0	hsa_circ_0006391	NC_000002.12	-	241140521	241140753	233	233	exonic	XM_011510828.1	PASK
circRNA_02375	0	0	0	0.0309543	0	0.041425819	0	0	0	0	0	0	-	NC_000002.12	+	241160332	241163401	3070	280	sense-overlappin	NM_001282414.1	PPP1R7

circRNA_02376	0	0	0.032712968	0	0	0	0	0.020486295	0	0	0	0	hsa_circ_0059027	NC_000002.12	-	241238649	241249980	11332	1377	sense-overlappin	NM_001243900.1	HDLBP
circRNA_02377	0	0	0	0	0	0	0	0	0	0	0	0.0412728	hsa_circ_0059060	NC_000002.12	+	241342992	241343897	906	248	sense-overlappin	NM_001008491.2	2-Sep
circRNA_02378	0	0	0	0	0	0	0	0	0	0.030258724	0	0	hsa_circ_0059061	NC_000002.12	+	241342992	241346249	3258	332	sense-overlappin	NM_001008491.2	2-Sep
circRNA_02379	0	0.020469968	0	0	0	0	0	0	0	0	0	0	hsa_circ_0059062	NC_000002.12	+	241342992	241348191	5200	390	sense-overlappin	NM_001008491.2	2-Sep
circRNA_02380	0.061725074	0	0	0	0	0.062138728	0	0	0	0	0	0	-	NC_000002.12	+	241343752	241348191	4440	288	sense-overlappin	NM_001008491.2	2-Sep
circRNA_02381	0	0	0.021808645	0	0	0	0	0	0	0	0	0	hsa_circ_0059067	NC_000002.12	+	241373084	241373290	207	207	exonic	NM_001282983.1	FARP2
circRNA_02382	0	0.040939935	0.021808645	0	0	0	0	0	0	0	0	0	-	NC_000002.12	+	241403828	241407615	3788	227	sense-overlappin	NM_001282983.1	FARP2
circRNA_02383	0	0	0	0.0206362	0	0	0.020098685	0	0	0.020172483	0	0	hsa_circ_0059071	NC_000002.12	+	241403828	241413421	9594	440	sense-overlappin	NM_001282983.1	FARP2
circRNA_02384	0.051437562	0	0.109043225	0.0309543	0	0.051782273	0	0	0	0	0.04051046	0	hsa_circ_0004924	NC_000002.12	+	241403828	241418109	14282	588	sense-overlappin	NM_001282983.1	FARP2
circRNA_02385	0	0	0.021808645	0	0	0	0	0	0	0	0.02025523	0	hsa_circ_0006511	NC_000002.12	+	241403828	241436538	32711	975	sense-overlappin	NM_001282983.1	FARP2
circRNA_02386	0.030862537	0.020469968	0.021808645	0	0	0	0.040197371	0	0.020254528	0	0.02025523	0	hsa_circ_0059074	NC_000002.12	+	241407537	241413421	5885	292	sense-overlappin	NM_001282983.1	FARP2
circRNA_02387	0	0	0	0.0619086	0	0	0	0	0	0	0	0	-	NC_000002.12	+	241407537	241434321	26785	700	sense-overlappin	NM_001282983.1	FARP2
circRNA_02388	0	0	0.021808645	0	0	0	0	0	0	0	0	0	hsa_circ_0001121	NC_000002.12	+	241407537	241436538	29002	827	sense-overlappin	NM_001282983.1	FARP2
circRNA_02389	0	0	0	0	0.02006311	0	0	0	0	0	0	0	hsa_circ_0059082	NC_000002.12	+	241413307	241431774	18468	359	sense-overlappin	NM_001282983.1	FARP2
circRNA_02390	0	0	0	0	0	0	0	0.020486295	0	0	0	0	-	NC_000002.12	+	241413307	241435030	21724	592	sense-overlappin	NM_001282983.1	FARP2
circRNA_02391	0	0	0.04361729	0	0	0	0	0	0	0	0	0	hsa_circ_0004946	NC_000002.12	-	241601896	241603079	1184	214	sense-overlappin	NM_001164356.1	THAP4
circRNA_02392	0	0.040939935	0	0.0206362	0.02006311	0	0.050246713	0	0	0	0	0	hsa_circ_0007647	NC_000002.12	-	241632917	241634079	1163	1163	exonic	XM_005247016.3	THAP4
circRNA_02393	0	0	0.021808645	0	0	0	0	0	0	0	0	0	hsa_circ_0003662	NC_000002.12	+	241654546	241655343	798	175	sense-overlappin	NM_013325.4	ATG4B
circRNA_02394	0	0	0.021808645	0	0	0	0	0	0	0.020172483	0	0	hsa_circ_0001124	NC_000002.12	+	241704653	241712071	7419	445	sense-overlappin	XR_923041.1	
circRNA_02395	0	0	0	0	0	0	0	0	0	0	0.02025523	0	-	NC_000002.12	+	241754631	241755995	1365	1365	sense-overlappin	NM_001287249.1	D2HGDH
circRNA_02396	0	0	0	0	0	0	0	0	0	0.020172483	0	0	hsa_circ_0059132	NC_000002.12	+	241755045	241756014	970	970	sense-overlappin	NM_001287249.1	D2HGDH
circRNA_02397	0	0	0.054521613	0	0	0	0	0	0	0	0	0	hsa_circ_0004631	NC_000003.12	+	3137260	3140648	3389	333	sense-overlappin	NM_001302946.1	TRNT1
circRNA_02398	0	0.020469968	0.054521613	0.0515905	0.02006311	0.072495183	0	0.040972591	0	0.050431207	0	0.0206364	-	NC_000003.12	+	3137260	3144710	7451	460	sense-overlappin	NM_001302946.1	TRNT1
circRNA_02399	0	0	0	0	0.03009467	0	0.020098685	0	0	0	0	0	-	NC_000003.12	+	3140510	3144710	4201	266	sense-overlappin	NM_001302946.1	TRNT1
circRNA_02400	0	0.071644886	0.054521613	0.1031809	0.03009467	0	0.030148028	0.030729443	0.020254528	0.030258724	0.02025523	0	hsa_circ_0003400	NC_000003.12	-	3156219	3174261	18043	717	sense-overlappin	XM_011533794.1	CRBN
circRNA_02401	0.030862537	0.051174919	0	0	0	0	0	0	0.050636319	0.020172483	0	0	-	NC_000003.12	-	3167634	3175269	7636	761	sense-overlappin	XM_011533793.1	CRBN
circRNA_02402	0	0	0	0	0.02006311	0	0	0	0	0	0	0	-	NC_000003.12	-	3777725	3782648	4924	4924	intergenic		
circRNA_02403	0.051437562	0.051174919	0	0.0412724	0.10031557	0.082851637	0.070345399	0.09218833	0	0	0.050638075	0.0619092	hsa_circ_0003910	NC_000003.12	-	3777725	3789074	11350	11350	intergenic		
circRNA_02404	0	0	0.021808645	0	0	0	0	0	0	0	0	0	-	NC_000003.12	-	3777725	3791453	13729	13729	intergenic		
circRNA_02405	0	0	0	0	0	0	0	0	0	0.020172483	0	0	-	NC_000003.12	-	4362039	4376389	14351	276	sense-overlappin	NM_001164674.1	SUMF1
circRNA_02406	0	0.030704951	0.021808645	0.0619086	0	0	0.020098685	0	0.030381792	0.030258724	0	0	hsa_circ_0001260	NC_000003.12	-	4410865	4420146	9282	435	sense-overlappin	XM_011533623.1	SUMF1
circRNA_02407	0	0	0	0	0	0	0	0	0	0	0	0.0206364	hsa_circ_0064039	NC_000003.12	-	4449266	4453049	3784	249	sense-overlappin	XM_011533623.1	SUMF1
circRNA_02408	0	0	0.032712968	0	0	0	0	0	0	0	0	0	hsa_circ_0005046	NC_000003.12	+	5170503	5174414	3912	388	sense-overlappin	NM_018184.2	ARL8B
circRNA_02409	0.082300098	0.102349838	0.098138903	0.1238171	0.05015779	0.124277456	0.060296056	0.040972591	0.172163486	0.050431207	0	0.051591	hsa_circ_0001263	NC_000003.12	+	5174017	5174414	398	139	sense-overlappin	NM_018184.2	ARL8B
circRNA_02410	0	0	0	0.0206362	0	0	0	0	0	0	0	0	-	NC_000003.12	+	5199592	5208263	8672	927	sense-overlappin	NM_014674.2	EDEM1
circRNA_02411	0.020575025	0.030704951	0.141756193	0	0.06018934	0	0	0.020486295	0.020254528	0.030258724	0.02025523	0	hsa_circ_0001264	NC_000003.12	-	8935871	8941804	5934	623	sense-overlappin	NM_020165.3	RAD18
circRNA_02412	0	0	0.076330258	0	0	0	0	0	0	0	0	0	hsa_circ_0002453	NC_000003.12	-	8935871	8948570	12700	756	sense-overlappin	NM_020165.3	RAD18
circRNA_02413	0	0.030704951	0	0.0309543	0.03009467	0.031069364	0	0	0	0.040344966	0	0	-	NC_000003.12	-	9388087	9390497	2411	2411	intergenic		
circRNA_02414	0	0	0	0.0309543	0	0	0	0	0	0	0	0	hsa_circ_0004211	NC_000003.12	-	9390290	9390497	208	208	intergenic		
circRNA_02415	0	0.020469968	0	0	0	0	0	0	0	0	0	0	-	NC_000003.12	+	9418733	9418882	150	150	intronic	XM_011533936.1	SETD5
circRNA_02416	0	0	0	0	0.02006311	0	0	0	0	0	0	0	hsa_circ_0064139	NC_000003.12	+	9445048	9445740	693	337	sense-overlappin	XM_011533936.1	SETD5
circRNA_02417	0	0	0	0	0	0	0	0	0	0.020172483	0.02025523	0.0206364	hsa_circ_0001265	NC_000003.12	+	9669432	9671170	1739	184	sense-overlappin	NM_001077525.2	MTMR14
circRNA_02418	0	0.040939935	0.021808645	0.0309543	0	0.031069364	0	0.020486295	0.020254528	0.030258724	0	0	hsa_circ_0008027	NC_000003.12	+	9750945	9751949	1005	428	sense-overlappin	NM_002542.5	OGG1
circRNA_02419	0	0	0	0	0	0	0	0	0.050636319	0	0.02025523	0	-	NC_000003.12	-	9762914	9763213	300	214	sense-overlappin	NM_003656.4	CAMK1
circRNA_02420	0	0	0	0	0	0	0	0	0	0	0.02025523	0	hsa_circ_0004355	NC_000003.12	+	9797659	9801756	4098	327	sense-overlappin	NM_001024959.2	ARPC4
circRNA_02421	0	0	0	0	0	0	0.020098685	0	0	0	0	0	hsa_circ_0064272	NC_000003.12	+	10270131	10271015	885	885	exonic	NM_014760.3	TATDN2
circRNA_02422	0	0	0	0.0619086	0	0	0	0	0	0	0	0	hsa_circ_0064307	NC_000003.12	+	11331340	11348035	16696	606	sense-overlappin	NM_001136031.2	ATG7
circRNA_02423	0	0	0	0	0	0.020712909	0	0	0	0	0	0	hsa_circ_0004626	NC_000003.12	+	11331340	11380052	48713	1278	sense-overlappin	NM_001144912.1	ATG7
circRNA_02424	0	0	0.032712968	0	0	0	0	0	0	0	0	0	hsa_circ_0003025	NC_000003.12	+	11331340	11426926	95587	1401	sense-overlappin	NM_006395.2	ATG7
circRNA_02425	0	0	0	0	0	0.020712909	0	0	0	0	0	0	hsa_circ_0064310	NC_000003.12	+	11340645	11342279	1635	236	sense-overlappin	NM_001136031.2	ATG7
circRNA_02426	0	0	0	0.0206362	0	0	0.030148028	0	0	0	0	0	hsa_circ_0007975	NC_000003.12	+	11358418	11364734	6317	591	sense-overlappin	NM_001136031.2	ATG7
circRNA_02427	0	0	0.021808645	0	0	0	0.020098685	0.030729443	0	0	0.02025523	0.0309546	hsa_circ_0008927	NC_000003.12	+	11358418	11380052	21635	672	sense-overlappin	NM_001144912.1	ATG7
circRNA_02428	0	0	0.032712968	0	0	0	0.060296056	0	0	0	0	0	hsa_circ_0008210	NC_000003.12	+	11358418	11426926	68509	795	sense-overlappin	NM_006395.2	ATG7
circRNA_02429	0	0	0	0.0309543	0	0	0	0	0	0	0	0	hsa_circ_0007287	NC_000003.12	-	11807833	11809682	1850	1320	sense-overlappin	NR_104314.1	
circRNA_02430	0	0	0	0	0	0	0	0	0.030381792	0	0	0	hsa_circ_0064335	NC_000003.12	+	12312380	12381491	69112	472	sense-overlappin	NM_138712.3	PPARG

circRNA_02431	0	0.040939935	0	0	0	0	0	0.030729443	0	0	0.04051046	0	hsa_circ_0064336	NC_000003.12	+	12312380	12392752	80373	611	sense-overlappin	NM_138712.3	PPARG
circRNA_02432	0	0.020469968	0	0	0	0	0	0.020486295	0	0	0	0	hsa_circ_0064337	NC_000003.12	+	12312380	12406081	93702	811	sense-overlappin	NM_138712.3	PPARG
circRNA_02433	0	0	0	0	0	0.031069364	0	0	0	0.050431207	0	0	-	NC_000003.12	+	12379704	12392752	13049	537	sense-overlappin	NM_005037.5	PPARG
circRNA_02434	0	0	0	0	0	0	0	0	0	0.030258724	0	0	-	NC_000003.12	+	12405882	12417154	11273	651	sense-overlappin	NM_005037.5	PPARG
circRNA_02435	0	0	0	0	0	0	0	0.020486295	0	0	0	0	hsa_circ_0005028	NC_000003.12	+	12516611	12519197	2587	190	sense-overlappin	XM_005265499.2	TSEN2
circRNA_02436	0	0	0	0.0206362	0	0	0	0	0	0	0	0	-	NC_000003.12	+	12516611	12532773	16163	541	sense-overlappin	NM_001145393.1	TSEN2
circRNA_02437	0	0	0	0	0	0	0	0	0	0.020172483	0	0	-	NC_000003.12	+	12528888	12532773	3886	351	sense-overlappin	NM_001145393.1	TSEN2
circRNA_02438	0	0	0	0	0	0	0	0	0.020254528	0	0	0	hsa_circ_0006619	NC_000003.12	+	12574792	12576741	1950	326	sense-overlappin	NM_001271707.1	MKRN2
circRNA_02439	0	0	0	0	0	0	0	0	0	0.020172483	0	0	-	NC_000003.12	-	13001461	13002069	609	609	intronic	NM_001134382.2	IQSEC1
circRNA_02440	0	0	0.021808645	0	0.03009467	0	0.040197371	0	0.020254528	0.030258724	0	0	-	NC_000003.12	-	13129305	13129525	221	221	intronic	XM_011534309.1	IQSEC1
circRNA_02441	0.030862537	0	0	0	0	0	0	0	0	0.020172483	0	0	hsa_circ_0064416	NC_000003.12	+	13500713	13502980	2268	237	sense-overlappin	XM_005265493.1	HDAC11
circRNA_02442	0.020575025	0	0	0	0	0	0	0	0	0	0	0	-	NC_000003.12	+	14818956	14821596	2641	2641	sense-overlappin	XM_011533422.1	FGD5
circRNA_02443	0	0	0	0	0	0	0	0	0	0	0.02025523	0	hsa_circ_0064455	NC_000003.12	+	15016152	15034809	18658	1099	sense-overlappin	NM_001291694.1	NR2C2
circRNA_02444	0	0	0	0	0.04012623	0.031069364	0	0	0	0	0	0	hsa_circ_0004046	NC_000003.12	+	15028586	15030452	1867	312	sense-overlappin	NM_001291694.1	NR2C2
circRNA_02445	0	0.020469968	0	0	0	0	0	0	0	0	0	0	-	NC_000003.12	+	15230442	15240853	10412	762	sense-overlappin	XR_940400.1	
circRNA_02446	0	0.020469968	0.021808645	0.0206362	0	0.031069364	0	0.020486295	0	0.050431207	0	0	-	NC_000003.12	-	15411245	15415942	4698	506	sense-overlappin	XM_005264867.3	METTL6
circRNA_02447	0	0	0.021808645	0	0	0	0	0	0	0	0	0	hsa_circ_0064491	NC_000003.12	-	15563358	15573242	9885	795	sense-overlappin	NM_001284413.1	HACL1
circRNA_02448	0	0	0.065425935	0	0	0	0	0	0	0	0	0	hsa_circ_0064516	NC_000003.12	-	15735410	15751820	16411	360	sense-overlappin	XM_011533551.1	ANKRD28
circRNA_02449	0	0	0.021808645	0.0206362	0	0	0	0	0	0	0	0	-	NC_000003.12	-	15751750	15814309	62560	19237	sense-overlappin	NM_015199.3	ANKRD28
circRNA_02450	0	0	0.021808645	0	0	0	0	0	0	0	0	0	-	NC_000003.12	-	15795223	15814309	19087	19087	sense-overlappin	NM_015199.3	ANKRD28
circRNA_02451	0	0	0.021808645	0	0	0	0	0	0	0	0	0	-	NC_000003.12	+	15939510	15965796	26287	26287	intergenic		
circRNA_02452	0	0	0.054521613	0.0412724	0.03009467	0.041425819	0.020098685	0.051215739	0	0	0.02025523	0.051591	hsa_circ_0001273	NC_000003.12	+	16286342	16303592	17251	786	sense-overlappin	NM_138381.3	OXNAD1
circRNA_02453	0	0	0	0.0206362	0.03009467	0	0	0	0	0	0	0	-	NC_000003.12	+	16294856	16303592	8737	679	sense-overlappin	NM_138381.3	OXNAD1
circRNA_02454	0	0	0	0.0309543	0	0.041425819	0	0.040972591	0	0.050431207	0.02025523	0	-	NC_000003.12	+	16301626	16302748	1123	352	sense-overlappin	NM_138381.3	OXNAD1
circRNA_02455	0	0	0.021808645	0	0	0	0	0	0	0	0	0	-	NC_000003.12	+	16301626	16303592	1967	537	sense-overlappin	NM_138381.3	OXNAD1
circRNA_02456	0	0	0	0	0	0.051782273	0	0.030729443	0	0.030258724	0	0	-	NC_000003.12	-	16647574	16648612	1039	1039	intergenic		
circRNA_02457	0	0	0.021808645	0.0515905	0	0	0.030148028	0.040972591	0.020254528	0	0	0	hsa_circ_0001274	NC_000003.12	+	17009674	17014911	5238	2691	sense-overlappin	NM_001144382.1	PLCL2
circRNA_02458	0	0.020469968	0	0	0	0	0	0	0	0	0	0	-	NC_000003.12	+	17009674	17068065	58392	2877	sense-overlappin	NM_001144382.1	PLCL2
circRNA_02459	0.020575025	0	0	0	0	0	0	0	0	0	0	0	-	NC_000003.12	-	17185109	17214370	29262	264	sense-overlappin	NM_001134381.1	TBC1D5
circRNA_02460	0	0	0	0	0	0	0	0	0.020254528	0	0	0	hsa_circ_0008286	NC_000003.12	-	17372075	17384015	11941	486	sense-overlappin	NM_001134381.1	TBC1D5
circRNA_02461	0.030862537	0	0	0	0	0	0	0	0	0	0	0	-	NC_000003.12	-	17403181	17406526	3346	342	sense-overlappin	NM_001134381.1	TBC1D5
circRNA_02462	0	0	0	0	0	0	0	0	0.020254528	0	0	0	-	NC_000003.12	-	17470845	17472248	1404	1404	intronic	NM_001134381.1	TBC1D5
circRNA_02463	0	0	0	0.0206362	0	0	0.030148028	0	0	0	0	0	hsa_circ_0002726	NC_000003.12	-	17508474	17562142	53669	350	sense-overlappin	XM_011534285.1	TBC1D5
circRNA_02464	0	0	0.032712968	0.0206362	0	0.062138728	0.020098685	0	0.020254528	0	0	0.0206364	-	NC_000003.12	-	17508474	17586557	78084	691	sense-overlappin	XM_011534285.1	TBC1D5
circRNA_02465	0	0	0	0	0	0	0	0	0	0.030258724	0	0	-	NC_000003.12	-	17622648	17647056	24409	359	sense-overlappin	XM_011534285.1	TBC1D5
circRNA_02466	0	0	0.04361729	0	0	0	0	0	0	0	0	0	-	NC_000003.12	+	18041449	18064062	22614	22614	sense-overlappin	NM_001257177.1	LOC339862
circRNA_02467	0.072012586	0.040939935	0.032712968	0.1031809	0	0.082851637	0	0.040972591	0	0.060517448	0	0	hsa_circ_0064555	NC_000003.12	-	18378170	18420991	42822	1599	sense-overlappin	XM_011533988.1	SATB1
circRNA_02468	0	0.030704951	0	0.0412724	0	0	0	0	0.030381792	0.030258724	0	0	hsa_circ_0064557	NC_000003.12	-	18415111	18420991	5881	663	sense-overlappin	XM_011533988.1	SATB1
circRNA_02469	0	0	0.021808645	0	0	0	0	0	0	0	0	0	-	NC_000003.12	+	19125733	19143147	17415	17415	intergenic		
circRNA_02470	0	0	0.021808645	0	0.04012623	0	0	0	0	0	0.02025523	0	-	NC_000003.12	+	19950806	19951061	256	256	exonic	NM_001292048.1	RAB5A
circRNA_02471	0	0	0	0	0	0	0.020098685	0	0	0	0	0	-	NC_000003.12	+	20071364	20072459	1096	331	sense-overlappin	XM_011534206.1	KAT2B
circRNA_02472	0	0	0	0	0	0	0	0	0	0	0.02025523	0	-	NC_000003.12	+	23289682	23295449	5768	5768	intronic	XM_011534076.1	UBE2E2
circRNA_02473	0	0	0.021808645	0	0	0	0	0	0	0	0	0	hsa_circ_0064577	NC_000003.12	+	23954537	23965162	10626	1423	sense-overlappin	NR_110524.1	
circRNA_02474	0	0	0.021808645	0.0515905	0	0	0	0	0	0	0	0	hsa_circ_0001276	NC_000003.12	+	23954537	23968023	13487	1634	sense-overlappin	NR_110524.1	
circRNA_02475	0	0	0	0	0	0	0	0	0	0	0	0.0206364	-	NC_000003.12	-	25628847	25645455	16609	1822	sense-overlappin	NM_001068.3	TOP2B
circRNA_02476	0	0.020469968	0	0	0	0	0	0	0	0	0	0	hsa_circ_0005018	NC_000003.12	-	27322177	27356904	34728	618	sense-overlappin	XM_011533406.1	NEK10
circRNA_02477	0	0.020469968	0	0	0	0	0	0	0	0	0	0	hsa_circ_0003925	NC_000003.12	-	27344272	27352919	8648	399	sense-overlappin	XR_940384.1	
circRNA_02478	0	0	0.021808645	0	0	0	0	0	0	0	0	0	hsa_circ_0064618	NC_000003.12	-	27383153	27424152	41000	2440	sense-overlappin	XM_005265598.3	SLC4A7
circRNA_02479	0	0.071644886	0.032712968	0	0	0.020712909	0	0	0	0	0	0	hsa_circ_0002901	NC_000003.12	-	27411642	27424152	12511	616	sense-overlappin	XM_005265598.3	SLC4A7
circRNA_02480	0	0	0	0	0	0	0.020098685	0	0.030381792	0.040344966	0	0.0206364	hsa_circ_0001277	NC_000003.12	-	27448651	27452498	3848	229	sense-overlappin	XM_005265598.3	SLC4A7
circRNA_02481	0	0	0.076330258	0	0	0	0	0	0	0	0	0	-	NC_000003.12	-	27916774	27930955	14182	14182	intergenic		
circRNA_02482	0	0	0.032712968	0	0	0	0	0	0	0	0	0	-	NC_000003.12	-	27916774	27987409	70636	70636	intergenic		
circRNA_02483	0	0	0.34893832	0	0.04012623	0	0	0	0	0	0	0	-	NC_000003.12	-	28010470	28028603	18134	18134	intergenic		
circRNA_02484	0	0	0	0	0	0	0	0	0	0.020172483	0	0	-	NC_000003.12	+	28263291	28263380	90	90	exonic	XM_011533402.1	CMC1
circRNA_02485	0	0	0	0	0.02006311	0.020712909	0	0	0	0	0	0	-	NC_000003.12	+	28435110	28492173	57064	325	sense-overlappin	NM_001040432.2	ZCWPW2

circRNA_02486	0	0	0.04361729	0	0	0	0	0	0.020254528	0	0	0	-	NC_000003.12	+	30623199	30650460	27262	435	sense-overlappin	NM_001024847.2	TGFBR2
circRNA_02487	0	0	0.032712968	0	0	0	0	0.020486295	0	0	0	0	hsa_circ_0005224	NC_000003.12	+	30644747	30650460	5714	360	sense-overlappin	XM_011534045.1	TGFBR2
circRNA_02488	0	0	0.054521613	0	0	0	0	0	0	0	0	0	hsa_circ_0064654	NC_000003.12	+	30644747	30674246	29500	1302	sense-overlappin	XM_011534045.1	TGFBR2
circRNA_02489	0	0	0.04361729	0	0	0	0	0	0	0	0	0	hsa_circ_0064656	NC_000003.12	+	30671638	30674246	2609	942	sense-overlappin	XM_011534045.1	TGFBR2
circRNA_02490	0.082300098	0.030704951	0.185373483	0.113499	0.12037869	0.103564547	0.26128291	0.051215739	0.050636319	0.161379862	0.04051046	0	hsa_circ_0001278	NC_000003.12	+	31576396	31580096	3701	397	sense-overlappin	NM_178862.2	STT3B
circRNA_02491	0	0	0	0	0.03009467	0	0.050246713	0.020486295	0.050636319	0	0	0	hsa_circ_0003338	NC_000003.12	+	31576396	31600459	24064	563	sense-overlappin	NM_178862.2	STT3B
circRNA_02492	0	0	0.021808645	0	0	0	0	0	0	0	0	0	-	NC_000003.12	+	31639988	31647091	7104	7104	intergenic		
circRNA_02493	0	0	0.032712968	0	0	0	0	0	0	0	0	0	-	NC_000003.12	-	31830040	31879830	49791	448	sense-overlappin	XM_011533324.1	OSBPL10
circRNA_02494	0	0	0.141756193	0.0309543	0	0	0.040197371	0.030729443	0.020254528	0	0	0	hsa_circ_0008549	NC_000003.12	-	31876433	31879830	3398	256	sense-overlappin	XM_011533324.1	OSBPL10
circRNA_02495	0	0	0	0	0	0	0	0	0	0	0.02025523	0	-	NC_000003.12	+	32282591	32292155	9565	9565	sense-overlappin	XM_011533416.1	CMTM8
circRNA_02496	0	0	0.032712968	0	0	0	0	0	0	0	0	0	-	NC_000003.12	+	32428806	32442013	13208	13208	sense-overlappin	NM_138410.2	CMTM7
circRNA_02497	0	0	0.021808645	0	0	0	0	0	0	0	0	0	hsa_circ_0007783	NC_000003.12	+	32441840	32454542	12703	657	sense-overlappin	NM_138410.2	CMTM7
circRNA_02498	0.020575025	0	0	0	0	0	0	0	0	0	0	0	-	NC_000003.12	-	32532986	32541206	8221	512	sense-overlappin	NM_016141.3	DYNC1LI1
circRNA_02499	0	0	0	0	0.02006311	0	0	0	0	0	0	0	hsa_circ_0064687	NC_000003.12	-	32532986	32545965	12980	860	sense-overlappin	NM_016141.3	DYNC1LI1
circRNA_02500	0	0	0.021808645	0	0	0	0.050246713	0	0	0	0	0	-	NC_000003.12	-	32534511	32545965	11455	748	sense-overlappin	NM_016141.3	DYNC1LI1
circRNA_02501	0.020575025	0	0.032712968	0	0.03009467	0.020712909	0.030148028	0	0	0	0	0	-	NC_000003.12	-	32541037	32545965	4929	518	sense-overlappin	NM_016141.3	DYNC1LI1
circRNA_02502	0	0	0.021808645	0	0	0	0	0	0	0	0	0	-	NC_000003.12	-	32544876	32545965	1090	348	sense-overlappin	NM_016141.3	DYNC1LI1
circRNA_02503	0	0	0	0	0	0	0	0	0	0	0.02025523	0	-	NC_000003.12	+	32685308	32764809	79502	2157	sense-overlappin	NM_015442.2	CNOT10
circRNA_02504	0	0	0	0	0.03009467	0	0	0	0	0	0	0	hsa_circ_0064690	NC_000003.12	+	32703868	32708820	4953	408	sense-overlappin	NM_001256741.1	CNOT10
circRNA_02505	0	0	0	0	0	0	0	0	0	0	0.04051046	0	-	NC_000003.12	+	32703868	32764809	60942	1982	sense-overlappin	NM_015442.2	CNOT10
circRNA_02506	0	0	0	0.0309543	0	0	0	0	0	0	0	0	-	NC_000003.12	+	32708670	32717237	8568	465	sense-overlappin	NM_001256741.1	CNOT10
circRNA_02507	0	0.020469968	0	0	0	0	0	0	0	0	0	0	hsa_circ_0064697	NC_000003.12	+	32716225	32717237	1013	171	sense-overlappin	NM_001256741.1	CNOT10
circRNA_02508	0	0	0.021808645	0	0	0	0	0	0	0	0	0	-	NC_000003.12	+	32720114	32737490	17377	851	sense-overlappin	NM_015442.2	CNOT10
circRNA_02509	0	0	0.032712968	0.0412724	0.03009467	0	0.020098685	0.020486295	0	0.020172483	0	0	-	NC_000003.12	+	32733423	32737490	4068	380	sense-overlappin	NM_015442.2	CNOT10
circRNA_02510	0	0	0	0	0	0	0.030148028	0	0	0	0	0	-	NC_000003.12	+	32757014	32757274	261	261	intronic	NM_001256741.1	CNOT10
circRNA_02511	0	0	0	0	0	0	0	0.030729443	0	0	0	0	-	NC_000003.12	+	32764455	32778860	14406	9279	sense-overlappin	XR_940404.1	
circRNA_02512	0	0	0.021808645	0	0	0	0.020098685	0	0	0	0	0	hsa_circ_0001279	NC_000003.12	-	33068230	33068970	741	212	sense-overlappin	NM_001079811.1	GLB1
circRNA_02513	0.020575025	0	0	0.0206362	0.03009467	0	0	0	0	0	0	0.0619092	-	NC_000003.12	+	33358967	33364719	5753	225	sense-overlappin	NM_012157.3	FBXL2
circRNA_02514	0	0	0	0.0206362	0	0	0	0	0	0	0	0	hsa_circ_0064729	NC_000003.12	-	33396162	33402904	6743	463	sense-overlappin	NM_001128160.1	UBP1
circRNA_02515	0	0	0	0.0206362	0.03009467	0	0	0	0	0	0	0	hsa_circ_0001985	NC_000003.12	-	33409236	33425741	16506	706	sense-overlappin	NM_001128160.1	UBP1
circRNA_02516	0	0	0	0.0206362	0	0	0	0	0	0	0	0	-	NC_000003.12	-	33412722	33425741	13020	335	sense-overlappin	NM_001128160.1	UBP1
circRNA_02517	0	0	0	0.0412724	0	0	0	0	0	0	0	0	-	NC_000003.12	-	33516023	33538942	22920	706	sense-overlappin	XM_006713056.1	CLASP2
circRNA_02518	0	0	0	0	0	0	0	0	0	0.050431207	0	0	-	NC_000003.12	-	33570727	33663515	92789	2263	sense-overlappin	XM_011533505.1	CLASP2
circRNA_02519	0	0.040939935	0	0	0	0	0	0	0	0	0	0	hsa_circ_0064772	NC_000003.12	-	33592395	33644903	52509	1353	sense-overlappin	XM_006713056.1	CLASP2
circRNA_02520	0	0	0	0	0	0	0	0	0.020254528	0	0	0	hsa_circ_0005324	NC_000003.12	-	33606591	33644903	38313	979	sense-overlappin	XM_006713056.1	CLASP2
circRNA_02521	0	0	0.021808645	0	0	0	0	0	0	0	0	0	-	NC_000003.12	-	33619603	33632374	12772	458	sense-overlappin	XM_006713056.1	CLASP2
circRNA_02522	0	0	0.021808645	0	0	0	0	0	0	0	0	0	hsa_circ_0064788	NC_000003.12	-	33644757	33696933	52177	667	sense-overlappin	NM_015097.2	CLASP2
circRNA_02523	0.051437562	0	0.021808645	0	0	0	0	0	0	0	0	0	-	NC_000003.12	-	33663445	33696933	33489	520	sense-overlappin	NM_015097.2	CLASP2
circRNA_02524	0.061725074	0.040939935	0.141756193	0.0515905	0.09028402	0.020712909	0.030148028	0.051215739	0	0.040344966	0.030382845	0.0309546	hsa_circ_0001280	NC_000003.12	-	33684359	33696933	12575	449	sense-overlappin	NM_015097.2	CLASP2
circRNA_02525	0	0	0.032712968	0	0	0	0	0	0	0	0	0	-	NC_000003.12	-	33687060	33696933	9874	351	sense-overlappin	NM_015097.2	CLASP2
circRNA_02526	0	0	0	0	0.02006311	0	0	0	0	0	0	0	-	NC_000003.12	-	33696855	33701200	4346	4346	sense-overlappin	NM_015097.2	CLASP2
circRNA_02527	0	0	0.021808645	0	0	0	0	0	0	0.020172483	0	0	hsa_circ_0064802	NC_000003.12	+	33836044	33838327	2284	347	sense-overlappin	NM_001162429.2	PDCD6IP
circRNA_02528	0.041150049	0	0	0	0	0	0	0	0	0	0	0	-	NC_000003.12	+	37020310	37028932	8623	674	sense-overlappin	NM_000249.3	MLH1
circRNA_02529	0	0	0	0	0	0.020712909	0	0	0	0	0	0	-	NC_000003.12	-	37083636	37096660	13025	405	sense-overlappin	NM_006309.3	LRRFIP2
circRNA_02530	0	0	0.032712968	0	0	0.031069364	0	0	0	0	0	0	hsa_circ_0003264	NC_000003.12	-	37129063	37149038	19976	232	sense-overlappin	NM_006309.3	LRRFIP2
circRNA_02531	0	0	0	0.0825448	0	0	0	0.020486295	0	0	0	0.0206364	hsa_circ_0001282	NC_000003.12	+	37249698	37273601	23904	1952	sense-overlappin	XM_005265069.3	GOLGA4
circRNA_02532	0	0	0	0	0	0	0	0.020486295	0	0	0	0	hsa_circ_0008654	NC_000003.12	+	37273536	37282272	8737	381	sense-overlappin	NM_001172713.1	GOLGA4
circRNA_02533	0	0	0	0.0309543	0	0.031069364	0	0.020486295	0	0	0	0	-	NC_000003.12	+	37273536	37302332	28797	1138	sense-overlappin	NM_001172713.1	GOLGA4
circRNA_02534	0	0	0.08723458	0	0	0	0	0	0	0	0	0	-	NC_000003.12	+	37275819	37276517	699	699	intronic	NM_001172713.1	GOLGA4
circRNA_02535	0	0	0	0.0515905	0	0	0	0	0	0.060517448	0	0	-	NC_000003.12	+	37281958	37302332	20375	1072	sense-overlappin	NM_001172713.1	GOLGA4
circRNA_02536	0	0	0	0.0309543	0	0	0	0	0	0	0	0	-	NC_000003.12	+	37286014	37329093	43080	5715	sense-overlappin	NM_001172713.1	GOLGA4
circRNA_02537	0	0	0.021808645	0.0309543	0	0	0	0	0	0	0	0	-	NC_000003.12	+	37294979	37302332	7354	652	sense-overlappin	NM_001172713.1	GOLGA4
circRNA_02538	0	0	0.032712968	0	0	0	0	0	0	0	0	0	-	NC_000003.12	+	37294979	37319194	24216	963	sense-overlappin	NM_001172713.1	GOLGA4
circRNA_02539	0	0	0	0.0206362	0	0	0	0	0	0	0	0	-	NC_000003.12	+	37323588	37329093	5506	4491	sense-overlappin	NM_001172713.1	GOLGA4
circRNA_02540	0	0	0.021808645	0	0	0	0.030148028	0	0	0	0	0	-	NC_000003.12	+	37379042	37386370	7329	7329	sense-overlappin	NM_178344.2	C3orf35
circRNA_02541	0	0	0.032712968	0	0	0	0	0	0	0	0	0	-	NC_000003.12	+	37383226	37386370	3145	3145	sense-overlappin	NM_178344.2	C3orf35

circRNA_02542	0	0	0	0	0	0	0	0.020486295	0	0	0.02025523	0	-	NC_000003.12	+	37383226	37399659	16434	16434	sense-overlappin	NM_178344.2	C3orf35
circRNA_02543	0.030862537	0	0	0	0	0	0	0	0	0	0	0	-	NC_000003.12	+	37411138	37416724	5587	462	sense-overlappin	NM_001302831.1	C3orf35
circRNA_02544	0	0	0.032712968	0	0	0	0	0	0	0	0	0	hsa_circ_0064869	NC_000003.12	+	38216096	38236961	20866	826	sense-overlappin	XM_011534332.1	OXSR1
circRNA_02545	0	0	0	0.0206362	0	0	0	0	0	0	0	0	-	NC_000003.12	+	40189826	40190463	638	638	exonic	NM_001284423.1	MYRIP
circRNA_02546	0	0.030704951	0	0	0	0	0	0	0	0	0	0	-	NC_000003.12	+	40311572	40311969	398	398	intronic	NM_005875.2	EIF1B
circRNA_02547	0	0	0.021808645	0	0	0	0.020098685	0	0	0	0	0	hsa_circ_0002102	NC_000003.12	+	40461407	40462248	842	464	sense-overlappin	NM_003973.4	RPL14
circRNA_02548	0	0	0.032712968	0	0.03009467	0.041425819	0.030148028	0	0	0	0	0	hsa_circ_0004194	NC_000003.12	+	41224021	41227352	3332	1261	sense-overlappin	XM_006712983.1	CTNNB1
circRNA_02549	0	0	0.021808645	0	0	0	0	0	0	0	0	0	-	NC_000003.12	-	41931844	41938197	6354	313	sense-overlappin	XM_006713215.2	ULK4
circRNA_02550	0	0	0.021808645	0	0	0	0	0	0	0	0	0	-	NC_000003.12	+	42619020	42632823	13804	696	sense-overlappin	XM_011533750.1	NKTR
circRNA_02551	0	0	0	0.0206362	0	0	0	0	0	0	0	0	-	NC_000003.12	-	42735707	42752025	16319	858	sense-overlappin	XR_940385.1	
circRNA_02552	0.072012586	0	0.054521613	0.0825448	0.05015779	0.041425819	0.050246713	0	0.050636319	0.050431207	0.111403765	0.0206364	hsa_circ_0004089	NC_000003.12	+	43299754	43303792	4039	757	sense-overlappin	XM_005265245.2	SNRK
circRNA_02553	0	0	0	0	0	0	0	0.020486295	0	0.030258724	0	0	-	NC_000003.12	+	43299754	43309942	10189	6907	sense-overlappin	XM_005265245.2	SNRK
circRNA_02554	0	0	0	0.0206362	0	0	0	0	0	0	0	0	-	NC_000003.12	+	43340287	43343478	3192	348	sense-overlappin	XM_005265245.2	SNRK
circRNA_02555	0	0	0	0	0	0	0	0.020486295	0	0	0	0	-	NC_000003.12	-	43499991	43544667	44677	44677	sense-overlappin	NM_001204831.1	ANO10
circRNA_02556	0	0	0.032712968	0	0	0	0	0	0	0	0	0	hsa_circ_0009084	NC_000003.12	-	43549720	43565727	16008	696	sense-overlappin	XM_011533887.1	ANO10
circRNA_02557	0	0	0	0	0	0	0	0	0	0	0	0.0412728	hsa_circ_0004022	NC_000003.12	-	43565653	43580472	14820	821	sense-overlappin	NM_001204831.1	ANO10
circRNA_02558	0	0	0.032712968	0	0	0	0	0	0	0	0	0	hsa_circ_0009026	NC_000003.12	-	43598532	43600581	2050	333	sense-overlappin	NM_001204831.1	ANO10
circRNA_02559	0	0.020469968	0.098138903	0.0515905	0	0	0	0	0	0	0	0	hsa_circ_0003533	NC_000003.12	-	43598532	43605863	7332	483	sense-overlappin	NM_001204831.1	ANO10
circRNA_02560	0.020575025	0	0	0	0	0	0	0	0	0	0	0	hsa_circ_0002206	NC_000003.12	+	43711709	43715058	3350	267	sense-overlappin	NM_016006.4	ABHD5
circRNA_02561	0	0	0	0	0	0	0.020098685	0	0	0	0	0	-	NC_000003.12	-	45089054	45091538	2485	454	sense-overlappin	NM_022842.4	CDCP1
circRNA_02562	0	0	0	0.0309543	0	0	0	0	0	0	0	0	-	NC_000003.12	-	45110473	45112445	1973	732	sense-overlappin	NM_022842.4	CDCP1
circRNA_02563	0	0	0.021808645	0.0206362	0	0	0	0	0	0.040344966	0	0	hsa_circ_0005870	NC_000003.12	-	47037666	47046621	8956	511	sense-overlappin	XR_940418.1	
circRNA_02564	0	0	0.054521613	0	0	0.020712909	0	0	0	0	0	0	hsa_circ_0006544	NC_000003.12	-	47037666	47057490	19825	1181	sense-overlappin	XR_940418.1	
circRNA_02565	0	0	0	0	0	0	0	0	0.030381792	0.030258724	0.02025523	0	hsa_circ_0065140	NC_000003.12	-	47037666	47062346	24681	1365	sense-overlappin	XR_940418.1	
circRNA_02566	0.030862537	0.08187987	0.021808645	0.0722267	0.02006311	0.062138728	0	0.040972591	0.040509055	0.131121138	0.030382845	0.0206364	hsa_circ_0004692	NC_000003.12	-	47037666	47067118	29453	1414	sense-overlappin	XR_940418.1	
circRNA_02567	0	0.020469968	0.04361729	0	0.04012623	0	0.030148028	0.030729443	0.050636319	0.030258724	0	0	hsa_circ_0002569	NC_000003.12	-	47056821	47067118	10298	903	sense-overlappin	NM_014159.6	SETD2
circRNA_02568	0.020575025	0	0	0	0	0	0	0	0	0	0	0	-	NC_000003.12	-	47062163	47098081	35919	1278	sense-overlappin	NM_014159.6	SETD2
circRNA_02569	0	0	0	0.0515905	0	0	0	0	0	0	0	0	-	NC_000003.12	-	47067070	47084382	17313	995	sense-overlappin	XR_940419.1	
circRNA_02570	0	0.030704951	0	0	0.04012623	0	0	0	0.020254528	0.050431207	0	0	-	NC_000003.12	-	47067070	47098081	31012	1377	sense-overlappin	XR_940419.1	
circRNA_02571	0	0	0	0	0	0	0	0	0	0	0	0.0206364	-	NC_000003.12	-	47079512	47103423	23912	2793	sense-overlappin	XR_940419.1	
circRNA_02572	0.020575025	0	0	0	0	0	0	0	0	0	0	0	-	NC_000003.12	-	47083720	47088247	4528	918	sense-overlappin	NM_014159.6	SETD2
circRNA_02573	0	0	0	0	0	0	0.020098685	0	0	0	0	0	-	NC_000003.12	-	47086195	47098081	11887	382	sense-overlappin	NM_014159.6	SETD2
circRNA_02574	0	0	0	0	0	0.082851637	0.050246713	0	0.020254528	0.040344966	0	0	hsa_circ_0001289	NC_000003.12	-	47097955	47103423	5469	303	sense-overlappin	NM_014159.6	SETD2
circRNA_02575	0.030862537	0.020469968	0.021808645	0.0619086	0.08025246	0.041425819	0.050246713	0.020486295	0.020254528	0.060517448	0.02025523	0	hsa_circ_0001290	NC_000003.12	-	47097955	47106120	8166	427	sense-overlappin	NM_014159.6	SETD2
circRNA_02576	0.030862537	0	0.032712968	0	0	0	0	0	0.030381792	0	0	0	-	NC_000003.12	-	47105997	47116754	10758	385	sense-overlappin	NM_014159.6	SETD2
circRNA_02577	0	0	0.021808645	0	0	0	0	0	0	0	0	0	hsa_circ_0065172	NC_000003.12	-	47113876	47124548	10673	4628	sense-overlappin	NM_014159.6	SETD2
circRNA_02578	0	0	0	0	0	0.020712909	0	0	0	0	0	0	hsa_circ_0008377	NC_000003.12	+	47169169	47169396	228	228	intergenic		
circRNA_02579	0.020575025	0	0	0	0	0.020712909	0	0	0	0	0	0	-	NC_000003.12	+	47169169	47174263	5095	5095	intergenic		
circRNA_02580	0.061725074	0	0.04361729	0.0515905	0.03009467	0.041425819	0.030148028	0.020486295	0.020254528	0.020172483	0	0	hsa_circ_0007291	NC_000003.12	-	47425485	47428670	3186	785	sense-overlappin	NM_012235.2	SCAP
circRNA_02581	0	0	0	0.0309543	0	0	0	0	0	0	0	0	hsa_circ_0065214	NC_000003.12	-	47425485	47435137	9653	915	sense-overlappin	NM_012235.2	SCAP
circRNA_02582	0	0.020469968	0.032712968	0.0206362	0	0	0	0	0	0	0	0	hsa_circ_0001292	NC_000003.12	-	47435008	47443091	8084	350	sense-overlappin	NM_012235.2	SCAP
circRNA_02583	0	0	0	0	0	0.031069364	0	0	0	0	0	0	hsa_circ_0008997	NC_000003.12	-	47610066	47622341	12276	397	sense-overlappin	NM_003074.3	SMARCC1
circRNA_02584	0	0.051174919	0	0.0309543	0	0	0	0.051215739	0	0	0	0.0412728	hsa_circ_0065243	NC_000003.12	-	47610066	47638780	28715	723	sense-overlappin	NM_003074.3	SMARCC1
circRNA_02585	0	0.040939935	0	0.0825448	0	0.031069364	0	0	0.030381792	0.040344966	0	0	hsa_circ_0065244	NC_000003.12	-	47610066	47678311	68246	1586	sense-overlappin	NM_003074.3	SMARCC1
circRNA_02586	0	0	0.021808645	0	0.03009467	0.051782273	0	0.020486295	0	0	0	0	-	NC_000003.12	-	47622207	47638780	16574	461	sense-overlappin	NM_003074.3	SMARCC1
circRNA_02587	0	0	0.032712968	0	0	0	0	0.030729443	0	0	0	0	hsa_circ_0065249	NC_000003.12	-	47622207	47678311	56105	1324	sense-overlappin	NM_003074.3	SMARCC1
circRNA_02588	0.020575025	0.030704951	0	0	0	0	0	0	0	0.030258724	0	0	hsa_circ_0065251	NC_000003.12	-	47635190	47636136	947	270	sense-overlappin	NM_003074.3	SMARCC1
circRNA_02589	0	0.030704951	0	0	0	0	0	0	0	0	0	0	-	NC_000003.12	-	47635190	47638780	3591	326	sense-overlappin	NM_003074.3	SMARCC1
circRNA_02590	0	0	0	0	0	0	0	0	0	0.030258724	0	0	hsa_circ_0065252	NC_000003.12	-	47635190	47678311	43122	1189	sense-overlappin	NM_003074.3	SMARCC1
circRNA_02591	0	0	0	0.0206362	0	0	0	0	0	0	0	0	-	NC_000003.12	-	47658558	47678311	19754	3599	sense-overlappin	NM_003074.3	SMARCC1
circRNA_02592	0	0	0	0	0.02006311	0	0	0	0	0	0	0	hsa_circ_0003602	NC_000003.12	-	47661294	47678311	17018	863	sense-overlappin	NM_003074.3	SMARCC1
circRNA_02593	0	0	0	0	0.02006311	0	0	0	0.020254528	0.020172483	0	0	hsa_circ_0001294	NC_000003.12	-	47670658	47676782	6125	328	sense-overlappin	NM_003074.3	SMARCC1
circRNA_02594	0	0	0	0	0	0.031069364	0	0	0	0	0	0	hsa_circ_0008639	NC_000003.12	-	47675475	47678311	2837	382	sense-overlappin	NM_003074.3	SMARCC1
circRNA_02595	0	0	0	0.0206362	0	0	0	0	0	0.020172483	0	0	hsa_circ_0001296	NC_000003.12	-	47678198	47686170	7973	308	sense-overlappin	NM_003074.3	SMARCC1
circRNA_02596	0	0	0	0	0	0	0	0	0.020254528	0	0	0	hsa_circ_0006985	NC_000003.12	-	47678198	47693300	15103	406	sense-overlappin	NM_003074.3	SMARCC1
circRNA_02597	0	0	0.021808645	0	0	0	0	0	0	0	0	0	hsa_circ_0065284	NC_000003.12	-	47686049	47693300	7252	220	sense-overlappin	NM_003074.3	SMARCC1

circRNA_02598	0	0	0.032712968	0	0	0.020712909	0	0	0	0	0	0	hsa_circ_0065292	NC_000003.12	-		47706409	47714490	8082	324	sense-overlappin	NM_003074.3	SMARCC1
circRNA_02599	0	0	0	0	0	0	0	0	0	0.030258724	0	0	hsa_circ_0065299	NC_000003.12	-		47720666	47736126	15461	233	sense-overlappin	NM_003074.3	SMARCC1
circRNA_02600	0	0	0	0.0412724	0.02006311	0.020712909	0.020098685	0	0	0	0	0	hsa_circ_0065301	NC_000003.12	-		47720666	47745993	25328	401	sense-overlappin	NM_003074.3	SMARCC1
circRNA_02601	0	0	0.021808645	0.0206362	0.02006311	0.031069364	0	0.051215739	0	0.050431207	0	0	hsa_circ_0005435	NC_000003.12	-		47729025	47772936	43912	495	sense-overlappin	XM_011534034.1	SMARCC1
circRNA_02602	0	0	0	0	0	0	0	0	0	0	0.030382845	0.0619092	hsa_circ_0065307	NC_000003.12	-		47736034	47745993	9960	261	sense-overlappin	NM_003074.3	SMARCC1
circRNA_02603	0	0	0.021808645	0	0	0	0	0	0	0	0	0	hsa_circ_0005367	NC_000003.12	-		47736034	47772936	36903	425	sense-overlappin	XM_011534034.1	SMARCC1
circRNA_02604	0	0	0.021808645	0	0	0	0	0	0	0	0	0	hsa_circ_0007878	NC_000003.12	-		47914817	47918841	4025	1470	sense-overlappin	NM_001134364.1	MAP4
circRNA_02605	0.020575025	0	0	0	0	0	0	0	0	0	0	0	-	NC_000003.12	-		48469361	48469843	483	329	sense-overlappin	NM_001272082.1	SHISA5
circRNA_02606	0.020575025	0	0	0	0	0	0	0	0	0	0	0	-	NC_000003.12	-		48548532	48550234	1703	342	sense-overlappin	XM_005265231.3	PFKFB4
circRNA_02607	0.020575025	0	0	0	0	0	0	0	0	0	0	0	-	NC_000003.12	-		48765004	48765349	346	177	sense-overlappin	XR_940469.1	
circRNA_02608	0	0	0	0	0	0	0	0	0	0	0	0.0206364	hsa_circ_0065505	NC_000003.12	-		48782986	48807684	24699	280	sense-overlappin	XR_940469.1	
circRNA_02609	0	0	0.032712968	0	0	0	0.020098685	0	0	0.050431207	0	0	hsa_circ_0006838	NC_000003.12	+		48922748	48927813	5066	538	sense-overlappin	XM_005264798.1	ARIH2
circRNA_02610	0	0.051174919	0.032712968	0.0206362	0	0	0	0	0	0.020172483	0	0	hsa_circ_0008343	NC_000003.12	+		48979482	48980496	1015	296	sense-overlappin	XM_011533268.1	ARIH2
circRNA_02611	0	0	0.021808645	0	0	0	0	0	0	0	0	0	hsa_circ_0065554	NC_000003.12	-		49044390	49057890	13501	1477	sense-overlappin	XM_006713212.2	QRICH1
circRNA_02612	0.020575025	0.030704951	0	0.0515905	0.03009467	0.113921001	0.040197371	0.040972591	0.030381792	0	0	0	hsa_circ_0001301	NC_000003.12	-		49335020	49335596	577	577	sense-overlappin	NM_003363.3	USP4
circRNA_02613	0	0	0	0	0	0	0.020098685	0	0	0	0	0	-	NC_000003.12	+		49962576	49975392	12817	1549	sense-overlappin	XR_940360.1	
circRNA_02614	0.030862537	0	0.076330258	0.0619086	0	0.020712909	0	0.020486295	0.020254528	0.040344966	0.02025523	0.051591	hsa_circ_0065769	NC_000003.12	+		49967470	49975392	7923	1439	sense-overlappin	XR_940360.1	
circRNA_02615	0	0	0	0	0	0	0.030148028	0.030729443	0.030381792	0	0	0.0309546	hsa_circ_0006213	NC_000003.12	+		50060956	50062108	1153	358	sense-overlappin	XR_940360.1	
circRNA_02616	0.041150049	0.030704951	0.054521613	0.0412724	0.03009467	0.062138728	0.020098685	0.081945182	0	0.110948655	0	0.072227401	hsa_circ_0006531	NC_000003.12	+		50065031	50066502	1472	357	sense-overlappin	XR_940360.1	
circRNA_02617	0.020575025	0	0.032712968	0.0206362	0.02006311	0	0	0.020486295	0	0.030258724	0	0	hsa_circ_0065795	NC_000003.12	+		50093720	50105709	11990	672	sense-overlappin	NM_005778.3	RBM5
circRNA_02618	0	0	0	0.0309543	0	0	0	0	0.020254528	0	0	0	hsa_circ_0001304	NC_000003.12	+		50099982	50105709	5728	516	sense-overlappin	NM_005778.3	RBM5
circRNA_02619	0	0	0	0	0	0	0	0.020486295	0	0	0	0	hsa_circ_0003795	NC_000003.12	+		50105077	50105709	633	227	sense-overlappin	NM_005778.3	RBM5
circRNA_02620	0	0.020469968	0.021808645	0	0	0	0	0.020486295	0.060763583	0.040344966	0	0	hsa_circ_0002479	NC_000003.12	+		50113383	50114251	869	384	sense-overlappin	NM_005778.3	RBM5
circRNA_02621	0.061725074	0	0.04361729	0	0	0	0	0	0	0	0	0	hsa_circ_0007083	NC_000003.12	+		50252100	50253184	1085	346	sense-overlappin	NM_001282619.1	GNAI2
circRNA_02622	0	0	0	0	0.02006311	0	0	0	0	0	0	0	hsa_circ_0007264	NC_000003.12	+		50252100	50257714	5615	974	sense-overlappin	NM_001282619.1	GNAI2
circRNA_02623	0	0	0.065425935	0	0.04012623	0	0.020098685	0	0	0	0	0	hsa_circ_0007485	NC_000003.12	-		51440970	51443903	2934	753	sense-overlappin	NM_001171904.1	VPRBP
circRNA_02624	0.030862537	0.204699675	0.021808645	0.2476343	0	0.124277456	0.120592112	0.122917773	0.040509055	0.191638587	0.070893305	0.268273202	hsa_circ_0001306	NC_000003.12	+		51541498	51552063	10566	10566	sense-overlappin	XM_011533524.1	RAD54L2
circRNA_02625	0	0.051174919	0.04361729	0.0309543	0.03009467	0.062138728	0	0.040972591	0	0.050431207	0	0.0619092	hsa_circ_0001307	NC_000003.12	+		51541498	51590559	49062	346	sense-overlappin	XM_011533524.1	RAD54L2
circRNA_02626	0	0	0	0.0206362	0	0	0	0	0	0	0	0	hsa_circ_0006909	NC_000003.12	+		51541589	51590559	48971	255	sense-overlappin	XM_011533524.1	RAD54L2
circRNA_02627	0	0	0	0	0	0	0	0.020486295	0	0	0	0	hsa_circ_0065926	NC_000003.12	+		51630272	51630931	660	344	sense-overlappin	XM_011533524.1	RAD54L2
circRNA_02628	0	0	0	0.0412724	0.02006311	0	0	0	0.020254528	0.040344966	0	0	hsa_circ_0002338	NC_000003.12	+		52204693	52206751	2059	588	sense-overlappin	NM_000688.5	ALAS1
circRNA_02629	0	0	0	0	0	0	0	0	0	0	0.02025523	0	hsa_circ_0065987	NC_000003.12	+		52204693	52212420	7728	1185	sense-overlappin	NM_000688.5	ALAS1
circRNA_02630	0.030862537	0.040939935	0.04361729	0.0309543	0	0.041425819	0	0	0	0	0	0	hsa_circ_0004912	NC_000003.12	+		52412811	52414587	1777	255	sense-overlappin	XM_011533826.1	PHF7
circRNA_02631	0	0	0	0	0	0	0	0	0.020254528	0	0	0	-	NC_000003.12	-		52641954	52648442	6489	373	sense-overlappin	NM_018313.4	PBRM1
circRNA_02632	0	0	0.021808645	0	0	0	0	0	0	0	0	0	-	NC_000003.12	-		52651742	52679726	27985	729	sense-overlappin	NM_018313.4	PBRM1
circRNA_02633	0	0	0	0	0.03009467	0.031069364	0	0	0	0	0.04051046	0.0309546	-	NC_000003.12	+		52687502	52690704	3203	444	sense-overlappin	NM_014366.4	GNL3
circRNA_02634	0	0	0	0.0515905	0	0.031069364	0	0.040972591	0.020254528	0.050431207	0	0	hsa_circ_0001308	NC_000003.12	-		52737586	52739634	2049	340	sense-overlappin	XM_006713310.1	NEK4
circRNA_02635	0.041150049	0	0	0.1238171	0.02006311	0.031069364	0.030148028	0.061458886	0.081018111	0.171466104	0.04051046	0.0206364	hsa_circ_0001309	NC_000003.12	-		52737586	52741499	3914	429	sense-overlappin	XM_006713310.1	NEK4
circRNA_02636	0	0	0	0	0	0	0	0	0	0.020172483	0	0	hsa_circ_0066108	NC_000003.12	-		52737586	52766375	28790	2073	sense-overlappin	NM_001193533.1	NEK4
circRNA_02637	0	0	0	0	0	0.020712909	0	0	0	0	0	0	-	NC_000003.12	-		52743352	52743448	97	97	exonic	XM_006713310.1	NEK4
circRNA_02638	0.020575025	0	0	0.0309543	0	0	0	0	0	0.020172483	0	0	hsa_circ_0066119	NC_000003.12	-		52760795	52763624	2830	297	sense-overlappin	XM_006713310.1	NEK4
circRNA_02639	0	0	0	0	0	0.020712909	0	0	0	0	0	0	-	NC_000003.12	-		52760795	52766375	5581	603	sense-overlappin	XM_006713310.1	NEK4
circRNA_02640	0	0	0	0.0412724	0	0	0	0	0	0	0	0	hsa_circ_0066143	NC_000003.12	-		52912538	52913617	1080	250	sense-overlappin	XM_005265221.2	SFMBT1
circRNA_02641	0	0	0	0	0	0	0	0	0	0	0.02025523	0	hsa_circ_0001310	NC_000003.12	-		52912538	52916214	3677	315	sense-overlappin	XM_005265221.2	SFMBT1
circRNA_02642	0	0	0.032712968	0	0	0	0	0	0	0	0	0	-	NC_000003.12	-		52918484	52928341	9858	518	sense-overlappin	XM_005265221.2	SFMBT1
circRNA_02643	0	0	0.032712968	0	0	0	0	0	0	0	0	0	-	NC_000003.12	-		52930941	52969258	38318	925	sense-overlappin	XM_005265221.2	SFMBT1
circRNA_02644	0	0	0	0	0	0	0	0	0	0	0	0.0206364	hsa_circ_0066151	NC_000003.12	-		52943353	52969258	25906	494	sense-overlappin	XM_005265221.2	SFMBT1
circRNA_02645	0	0	0.021808645	0	0	0	0	0	0	0	0	0	hsa_circ_0066157	NC_000003.12	+		53178404	53179776	1373	334	sense-overlappin	NM_006254.3	PRKCD
circRNA_02646	0.051437562	0	0.054521613	0.0206362	0.02006311	0	0	0	0	0	0	0	-	NC_000003.12	-		53290791	53292827	2037	825	sense-overlappin	NM_001290204.1	DCP1A
circRNA_02647	0	0	0	0	0	0	0.030148028	0	0	0	0.030382845	0	hsa_circ_0002554	NC_000003.12	-		53874211	53876080	1870	287	sense-overlappin	XM_005265587.3	ACTR8
circRNA_02648	0.030862537	0	0	0.0619086	0	0	0	0	0	0	0	0	-	NC_000003.12	+		56563684	56567053	3370	783	sense-overlappin	XM_011533619.1	CCDC66
circRNA_02649	0	0	0	0.0412724	0	0	0	0	0	0	0	0	-	NC_000003.12	+		56563684	56571302	7619	905	sense-overlappin	XM_011533619.1	CCDC66
circRNA_02650	0	0	0	0.0206362	0	0	0	0	0	0	0	0	-	NC_000003.12	+		56565638	56567053	1416	1226	sense-overlappin	XM_011533612.1	CCDC66
circRNA_02651	0.020575025	0.040939935	0.032712968	0.1238171	0.05015779	0.103564547	0.090444084	0.040972591	0.101272639	0.080689931	0	0.0412728	hsa_circ_0001312	NC_000003.12	+		56566594	56567053	460	270	sense-overlappin	XM_011533612.1	CCDC66
circRNA_02652	0.174887709	0.061409903	0.196277805	0.21668	0.11034713	0.258911367	0.180888168	0.09218833	0.141781694	0.282414759	0.050638075	0.123818401	hsa_circ_0001313	NC_000003.12	+		56592970	56594028	1059	468	sense-overlappin	XM_011533612.1	CCDC66
circRNA_02653	0	0	0.032712968	0	0	0.031069364	0	0	0	0	0	0	-	NC_000003.12	+		56592970	56598300	5331	4740	sense-overlappin	XM_011533612.1	CCDC66

circRNA_02654	0	0	0	0.0206362	0	0	0	0	0	0	0	0	-	NC_000003.12	-	56624479	56673725	49247	4152	sense-overlappin	NM_001112736.1	FAM208A
circRNA_02655	0.072012586	0.051174919	0	0.0722267	0.02006311	0.020712909	0.060296056	0.051215739	0.040509055	0.030258724	0	0.0412728	hsa_circ_0001314	NC_000003.12	-	56627037	56628614	1578	392	sense-overlappin	NM_015224.3	FAM208A
circRNA_02656	0	0	0	0	0	0	0	0.020486295	0	0	0	0	-	NC_000003.12	-	56648822	56660834	12013	249	sense-overlappin	NM_015224.3	FAM208A
circRNA_02657	0	0	0	0	0	0	0	0	0	0	0	0.0206364	hsa_circ_0066230	NC_000003.12	-	56648822	56661017	12196	353	sense-overlappin	NM_015224.3	FAM208A
circRNA_02658	0	0	0	0	0	0.031069364	0	0	0	0	0	0	-	NC_000003.12	-	56660731	56662490	1760	314	sense-overlappin	NM_015224.3	FAM208A
circRNA_02659	0.041150049	0.122819805	0.239895095	0.1341352	0.05015779	0.020712909	0.110542769	0.061458886	0.050636319	0.060517448	0.050638075	0.0619092	hsa_circ_0001315	NC_000003.12	-	56660731	56673725	12995	1037	sense-overlappin	NM_001112736.1	FAM208A
circRNA_02660	0	0	0	0	0.05015779	0	0	0	0	0	0	0	hsa_circ_0066239	NC_000003.12	-	56663541	56673725	10185	723	sense-overlappin	NM_001112736.1	FAM208A
circRNA_02661	0	0	0.021808645	0	0.02006311	0.031069364	0	0	0	0	0	0	hsa_circ_0066241	NC_000003.12	-	56666260	56673725	7466	691	sense-overlappin	NM_001112736.1	FAM208A
circRNA_02662	0	0	0	0	0	0.020712909	0	0	0	0	0	0	-	NC_000003.12	-	56669700	56671692	1993	258	sense-overlappin	XM_011533553.1	FAM208A
circRNA_02663	0	0	0.032712968	0.0309543	0.08025246	0	0.030148028	0.030729443	0.040509055	0.060517448	0	0	hsa_circ_0001316	NC_000003.12	-	56669700	56673725	4026	404	sense-overlappin	XM_011533553.1	FAM208A
circRNA_02664	0	0	0	0	0	0.020712909	0	0	0	0	0	0	-	NC_000003.12	-	56670073	56673725	3653	312	sense-overlappin	XM_011533553.1	FAM208A
circRNA_02665	0	0	0	0.0309543	0	0	0	0	0.020254528	0	0	0	-	NC_000003.12	-	56737185	56745462	8278	429	sense-overlappin	NM_001128616.1	ARHGEF3
circRNA_02666	0	0	0	0	0	0	0	0	0	0	0.02025523	0	hsa_circ_0066243	NC_000003.12	-	56745205	56745462	258	258	exonic	NM_001128616.1	ARHGEF3
circRNA_02667	0.041150049	0.040939935	0.04361729	0.0309543	0	0.062138728	0.020098685	0	0.020254528	0.090776173	0	0.0412728	hsa_circ_0001317	NC_000003.12	+	57242856	57248351	5496	448	sense-overlappin	NM_012096.2	APPL1
circRNA_02668	0.030862537	0	0.032712968	0.0722267	0	0.031069364	0	0	0	0.050431207	0	0	hsa_circ_0066251	NC_000003.12	+	57242856	57267792	24937	1478	sense-overlappin	NM_012096.2	APPL1
circRNA_02669	0	0	0	0	0	0	0.020098685	0	0	0.020172483	0	0	hsa_circ_0005129	NC_000003.12	-	57583898	57584464	567	191	sense-overlappin	NM_001660.3	ARF4
circRNA_02670	0.020575025	0	0	0	0	0	0	0	0	0	0	0	-	NC_000003.12	-	57625517	57625689	173	173	exonic	NM_152678.2	DENND6A
circRNA_02671	0.051437562	0.040939935	0	0.0412724	0.02006311	0	0	0	0	0	0	0	-	NC_000003.12	-	57633265	57641747	8483	316	sense-overlappin	NM_152678.2	DENND6A
circRNA_02672	0	0	0.021808645	0	0	0	0	0	0.020254528	0	0	0	-	NC_000003.12	-	57641653	57661551	19899	619	sense-overlappin	NM_152678.2	DENND6A
circRNA_02673	0	0	0	0	0	0	0	0	0	0.020172483	0	0	hsa_circ_0005702	NC_000003.12	+	57841299	57849816	8518	173	sense-overlappin	XR_427291.2	
circRNA_02674	0	0	0	0	0	0	0	0	0	0	0.02025523	0	hsa_circ_0001975	NC_000003.12	+	57841299	57858159	16861	341	sense-overlappin	XR_427291.2	
circRNA_02675	0	0	0	0.0206362	0	0	0	0	0	0	0	0	hsa_circ_0066273	NC_000003.12	+	57841299	57864716	23418	789	sense-overlappin	XR_427291.2	
circRNA_02676	0	0.020469968	0	0	0	0	0	0	0	0	0	0	-	NC_000003.12	+	57841299	57871698	30400	954	sense-overlappin	XR_427291.2	
circRNA_02677	0.051437562	0	0	0	0	0	0	0.020486295	0	0	0	0	hsa_circ_0066277	NC_000003.12	+	57847197	57858159	10963	268	sense-overlappin	XR_427291.2	
circRNA_02678	0	0	0	0	0	0	0	0.030729443	0	0	0	0	hsa_circ_0066279	NC_000003.12	+	57847197	57864716	17520	716	sense-overlappin	XR_427291.2	
circRNA_02679	0	0.040939935	0	0	0	0	0	0	0	0	0	0	hsa_circ_0066282	NC_000003.12	+	57847197	57896932	49736	1207	sense-overlappin	XR_427291.2	
circRNA_02680	0	0	0	0	0	0.020712909	0	0	0	0	0	0	-	NC_000003.12	+	57860699	57864857	4159	499	sense-overlappin	XR_427291.2	
circRNA_02681	0.020575025	0.030704951	0	0.0412724	0.06018934	0.072495183	0.030148028	0.030729443	0.050636319	0.020172483	0.030382845	0.0619092	hsa_circ_0002693	NC_000003.12	+	57890041	57896932	6892	326	sense-overlappin	XR_427291.2	
circRNA_02682	0.030862537	0	0	0	0	0	0	0	0	0	0	0	-	NC_000003.12	+	57896247	57896932	686	266	sense-overlappin	XR_427291.2	
circRNA_02683	0	0	0	0	0.03009467	0	0	0	0	0	0	0	-	NC_000003.12	+	57907884	57917077	9194	809	sense-overlappin	XM_005265456.3	SLMAP
circRNA_02684	0	0	0.021808645	0	0	0	0	0	0	0	0	0	-	NC_000003.12	+	58008564	58008856	293	293	exonic	NM_001164317.1	FLNB
circRNA_02685	0	0	0.032712968	0	0	0	0	0.030729443	0	0	0	0	hsa_circ_0066290	NC_000003.12	+	58077046	58081776	4731	495	sense-overlappin	NM_001164317.1	FLNB
circRNA_02686	0	0	0	0	0	0.020712909	0	0	0	0	0	0	-	NC_000003.12	+	58089476	58089842	367	367	intronic	NM_001164317.1	FLNB
circRNA_02687	0	0	0	0	0	0	0	0	0	0	0.04051046	0	-	NC_000003.12	-	58429708	58430942	1235	435	sense-overlappin	NM_001173468.1	PDHB
circRNA_02688	0	0	0	0	0	0	0	0	0	0.020172483	0	0	-	NC_000003.12	+	58448063	58450201	2139	2139	intergenic		
circRNA_02689	0	0	0.021808645	0	0	0	0	0	0	0	0	0.0619092	-	NC_000003.12	-	60011371	60014264	2894	288	sense-overlappin	XM_011533486.1	FHIT
circRNA_02690	0	0	0.021808645	0	0	0	0	0	0	0	0	0	-	NC_000003.12	-	60178441	60187471	9031	9031	intronic	XM_011533486.1	FHIT
circRNA_02691	0	0	0.021808645	0	0	0.020712909	0	0	0	0	0	0	-	NC_000003.12	-	60765524	60767098	1575	54	sense-overlappin	XM_011533482.1	FHIT
circRNA_02692	0	0.051174919	0.065425935	0	0	0	0	0.030729443	0	0	0.04051046	0	hsa_circ_0002134	NC_000003.12	+	61622463	61623555	1093	1093	intronic	NM_002841.3	PTPRG
circRNA_02693	0.030862537	0	0	0	0	0	0	0	0.030381792	0	0	0	hsa_circ_0001988	NC_000003.12	+	63898399	63913225	14827	504	sense-overlappin	NM_000333.3	ATXN7
circRNA_02694	0	0	0	0	0	0	0	0	0.020254528	0.020172483	0	0	hsa_circ_0002634	NC_000003.12	+	63898399	63952483	54085	609	sense-overlappin	NM_000333.3	ATXN7
circRNA_02695	0.020575025	0	0.152660515	0.0206362	0.03009467	0.072495183	0.020098685	0.051215739	0.050636319	0	0.050638075	0	hsa_circ_0007761	NC_000003.12	+	63912588	63913225	638	405	sense-overlappin	NM_000333.3	ATXN7
circRNA_02696	0.041150049	0	0	0	0	0	0	0	0	0	0	0	hsa_circ_0066444	NC_000003.12	-	64594258	64596991	2734	339	sense-overlappin	NM_182920.1	ADAMTS9
circRNA_02697	0	0	0	0	0	0	0	0	0	0	0.02025523	0	-	NC_000003.12	+	65018441	65019651	1211	1211	intergenic		
circRNA_02698	0	0	0	0	0	0	0	0	0	0	0.04051046	0	-	NC_000003.12	+	65018441	65050822	32382	32382	intergenic		
circRNA_02699	0	0	0.032712968	0	0	0	0	0	0	0	0	0	-	NC_000003.12	-	65361199	65364946	3748	438	sense-overlappin	XM_011534239.1	MAGI1
circRNA_02700	0	0	0	0	0.03009467	0	0	0	0	0	0	0	-	NC_000003.12	-	65391142	65442849	51708	1338	sense-overlappin	XM_011534239.1	MAGI1
circRNA_02701	0	0	0	0	0	0.020712909	0	0	0	0.020172483	0	0	-	NC_000003.12	-	65391142	65453340	62199	1460	sense-overlappin	XM_011534239.1	MAGI1
circRNA_02702	0	0	0	0.0722267	0	0	0	0	0	0	0	0	hsa_circ_0066452	NC_000003.12	-	65429520	65430881	1362	804	sense-overlappin	XM_011534239.1	MAGI1
circRNA_02703	0	0.020469968	0.021808645	0.0825448	0	0.020712909	0	0	0	0	0	0	hsa_circ_0003540	NC_000003.12	-	65429520	65442849	13330	1089	sense-overlappin	XM_011534239.1	MAGI1
circRNA_02704	0	0	0	0	0.02006311	0	0	0	0	0	0	0	-	NC_000003.12	-	65609744	65622088	12345	12345	sense-overlappin	XM_011534239.1	MAGI1
circRNA_02705	0	0	0	0	0.03009467	0	0	0	0	0	0	0	-	NC_000003.12	-	65717302	65725751	8450	8450	intronic	XM_011534239.1	MAGI1
circRNA_02706	0.020575025	0	0.021808645	0	0	0	0	0	0.030381792	0	0.02025523	0	-	NC_000003.12	+	65870852	65871199	348	348	antisense	XM_011534239.1	MAGI1
circRNA_02707	0	0	0.021808645	0	0	0	0	0	0	0	0	0	-	NC_000003.12	-	65975956	66006013	30058	30058	intronic	XM_011534239.1	MAGI1

circRNA_02708	0.030862537	0	0	0	0	0	0	0	0	0	0	0	-	NC_000003.12	-	66398970	66413014	14045	585	sense-overlappin	NM_015541.2	LRIG1
circRNA_02709	0	0.020469968	0	0	0	0	0.070345399	0	0	0	0	0	hsa_circ_0066474	NC_000003.12	-	66405198	66417266	12069	795	sense-overlappin	NM_015541.2	LRIG1
circRNA_02710	0	0.030704951	0	0	0	0	0	0	0	0	0	0	-	NC_000003.12	-	66462438	66477394	14957	14957	sense-overlappin	NM_015541.2	LRIG1
circRNA_02711	0	0	0	0.0206362	0	0	0	0	0	0	0	0.0309546	hsa_circ_0005688	NC_000003.12	-	67400731	67409034	8304	8304	sense-overlappin	NM_001177599.1	SUCLG2
circRNA_02712	0.020575025	0.061409903	0.021808645	0.0412724	0.02006311	0.020712909	0.020098685	0.020486295	0	0	0	0.0309546	hsa_circ_0003060	NC_000003.12	-	67495798	67498295	2498	305	sense-overlappin	NM_001177599.1	SUCLG2
circRNA_02713	0	0	0.054521613	0	0	0.113921001	0.050246713	0.040972591	0.070890847	0.110948655	0.030382845	0	hsa_circ_0004276	NC_000003.12	-	67495798	67508903	13106	402	sense-overlappin	NM_001177599.1	SUCLG2
circRNA_02714	0	0	0	0.0206362	0	0	0	0.040972591	0	0.050431207	0	0	hsa_circ_0005657	NC_000003.12	-	67495798	67529186	33389	836	sense-overlappin	NM_001177599.1	SUCLG2
circRNA_02715	0	0.020469968	0	0	0	0	0	0	0	0	0	0	-	NC_000003.12	+	67668213	67750660	82448	82448	intergenic		
circRNA_02716	0.020575025	0.020469968	0	0.0206362	0	0.020712909	0	0	0	0	0	0.0206364	-	NC_000003.12	+	67746771	67750660	3890	3890	intergenic		
circRNA_02717	0	0	0.021808645	0	0	0	0	0	0	0	0	0	-	NC_000003.12	-	68988295	69004482	16188	622	sense-overlappin	XM_011533599.1	EOGT
circRNA_02718	0	0	0	0	0	0	0	0.020486295	0	0	0	0	hsa_circ_0066499	NC_000003.12	-	68998011	69004482	6472	316	sense-overlappin	NM_001278689.1	EOGT
circRNA_02719	0	0	0	0	0	0	0	0	0.020254528	0	0	0	hsa_circ_0066502	NC_000003.12	-	69001608	69004482	2875	212	sense-overlappin	NM_001278689.1	EOGT
circRNA_02720	0	0	0	0	0.02006311	0	0	0	0	0	0	0	-	NC_000003.12	-	69023145	69026097	2953	557	sense-overlappin	XM_011534056.1	TMF1
circRNA_02721	0	0	0	0	0	0.041425819	0	0	0	0	0	0	-	NC_000003.12	-	69039551	69044595	5045	480	sense-overlappin	XM_011534056.1	TMF1
circRNA_02722	0	0	0.032712968	0	0	0	0	0	0	0	0	0	-	NC_000003.12	-	69043750	69048562	4813	1445	sense-overlappin	XM_011534056.1	TMF1
circRNA_02723	0	0	0.032712968	0	0	0	0	0	0	0	0	0	hsa_circ_0066526	NC_000003.12	-	70987994	71015653	27660	277	sense-overlappin	NM_001244813.1	FOXP1
circRNA_02724	0	0.030704951	0.021808645	0	0	0	0	0	0.030381792	0	0	0	-	NC_000003.12	-	70987994	71053773	65780	864	sense-overlappin	NM_001244813.1	FOXP1
circRNA_02725	0	0	0	0	0	0	0	0	0.020254528	0	0	0	hsa_circ_0066529	NC_000003.12	-	71015549	71047095	31547	464	sense-overlappin	NM_001244813.1	FOXP1
circRNA_02726	0.051437562	0.071644886	0.119947548	0.0825448	0.02006311	0	0.030148028	0.020486295	0.050636319	0	0	0.0412728	hsa_circ_0001320	NC_000003.12	-	71015549	71053773	38225	692	sense-overlappin	NM_001244813.1	FOXP1
circRNA_02727	0	0	0	0.0412724	0	0	0	0	0	0	0.02025523	0	-	NC_000003.12	-	71041328	71047095	5768	359	sense-overlappin	NM_001244813.1	FOXP1
circRNA_02728	0.072012586	0.102349838	0.250799418	0.1857257	0.10031557	0.144990365	0	0.081945182	0.070890847	0.090776173	0.111403765	0.0412728	hsa_circ_0008234	NC_000003.12	-	71041328	71053773	12446	587	sense-overlappin	NM_001244813.1	FOXP1
circRNA_02729	0	0.020469968	0	0	0	0	0	0	0	0	0	0	hsa_circ_0066532	NC_000003.12	-	71046942	71053773	6832	382	sense-overlappin	NM_001244813.1	FOXP1
circRNA_02730	0	0	0.054521613	0	0	0	0	0	0	0	0.02025523	0	-	NC_000003.12	-	71493426	71501988	8563	8563	sense-overlappin	NM_001244816.1	FOXP1
circRNA_02731	0.030862537	0	0	0	0	0.020712909	0.030148028	0	0	0.030258724	0	0	-	NC_000003.12	-	71684572	71699708	15137	536	sense-overlappin	NM_001134651.1	EIF4E3
circRNA_02732	0	0.030704951	0	0.0206362	0	0	0	0	0	0.020172483	0.04051046	0	hsa_circ_0001322	NC_000003.12	-	71684572	71710484	25913	609	sense-overlappin	NM_001134651.1	EIF4E3
circRNA_02733	0.072012586	0	0	0	0	0.020712909	0.020098685	0	0.020254528	0.030258724	0	0.0206364	hsa_circ_0066535	NC_000003.12	-	71690010	71699708	9699	379	sense-overlappin	NM_001134651.1	EIF4E3
circRNA_02734	0.020575025	0	0	0	0	0	0	0	0	0	0	0	hsa_circ_0066536	NC_000003.12	-	71690010	71710484	20475	452	sense-overlappin	NM_001134651.1	EIF4E3
circRNA_02735	0	0	0.021808645	0	0	0	0	0	0	0	0	0	-	NC_000003.12	-	71710412	71728623	18212	221	sense-overlappin	NM_001134650.1	EIF4E3
circRNA_02736	0	0	0	0	0	0.020712909	0	0	0	0	0	0	-	NC_000003.12	-	71718077	71728623	10547	10547	sense-overlappin	NM_001134650.1	EIF4E3
circRNA_02737	0	0.020469968	0	0	0	0	0	0	0	0	0	0.0206364	hsa_circ_0004340	NC_000003.12	-	72841045	72844423	3379	343	sense-overlappin	NM_018130.2	SHQ1
circRNA_02738	0	0	0	0	0	0	0	0	0	0.030258724	0	0	hsa_circ_0006973	NC_000003.12	-	73602354	73608684	6331	195	sense-overlappin	NM_015009.2	PDZRN3
circRNA_02739	0	0	0.04361729	0	0	0	0	0	0	0	0	0	hsa_circ_0002609	NC_000003.12	-	75741276	75741736	461	220	sense-overlappin	XM_011533247.1	ZNF717
circRNA_02740	0	0	0.021808645	0	0	0	0	0	0	0	0	0	hsa_circ_0003424	NC_000003.12	-	75741276	75741865	590	349	sense-overlappin	XM_011533247.1	ZNF717
circRNA_02741	0	0	0	0	0	0	0	0	0	0.020172483	0	0	hsa_circ_0066556	NC_000003.12	+	77098014	77098340	327	327	exonic	NM_001128929.3	ROBO2
circRNA_02742	0	0	0	0	0	0	0	0	0.050636319	0	0	0	hsa_circ_0066568	NC_000003.12	-	78714397	78717883	3487	388	sense-overlappin	NM_001145845.1	ROBO1
circRNA_02743	0	0	0	0	0	0.031069364	0	0	0	0	0	0	hsa_circ_0004788	NC_000003.12	-	78938601	78938927	327	327	exonic	NM_001145845.1	ROBO1
circRNA_02744	0	0	0.021808645	0	0	0	0	0	0	0	0	0	-	NC_000003.12	-	87643695	87644502	808	808	intergenic		
circRNA_02745	0	0	0.621546383	0	0	0	0	0	0	0	0	0	-	NC_000003.12	-	87890110	87892031	1922	1922	antisense	XM_005264749.2	HTR1F
circRNA_02746	0	0	0.381651288	0	0	0	0	0	0	0	0	0	-	NC_000003.12	-	87891607	87892031	425	425	antisense	XM_005264749.2	HTR1F
circRNA_02747	0.020575025	0	0	0	0	0	0	0	0	0	0	0	-	NC_000003.12	+	89407269	89431359	24091	752	sense-overlappin	NM_005233.5	EPHA3
circRNA_02748	0.030862537	0.040939935	0.196277805	0	0.0702209	0.031069364	0.070345399	0	0	0.030258724	0	0	hsa_circ_0006135	NC_000003.12	+	93995874	94003908	8035	443	sense-overlappin	XM_006713531.2	ARL13B
circRNA_02749	0	0	0	0	0	0	0	0	0	0.020172483	0	0	-	NC_000003.12	+	93995878	94003908	8031	439	sense-overlappin	XM_006713531.2	ARL13B
circRNA_02750	0	0	0	0	0	0	0	0	0	0	0	0.0206364	-	NC_000003.12	+	94003659	94003908	250	250	exonic	XM_006713531.2	ARL13B
circRNA_02751	0	0	0	0	0	0	0	0	0	0.030258724	0	0	-	NC_000003.12	+	97895959	97900485	4527	430	sense-overlappin	NM_153605.3	CRYBG3
circRNA_02752	0	0	0	0	0.03009467	0	0	0.020486295	0	0.040344966	0	0	hsa_circ_0066608	NC_000003.12	+	98768430	98773983	5554	492	sense-overlappin	XM_011512336.1	ST3GAL6
circRNA_02753	0	0.020469968	0	0.0309543	0	0	0	0	0	0	0	0	hsa_circ_0066631	NC_000003.12	-	98849461	98881767	32307	366	sense-overlappin	NM_080927.3	DCBLD2
circRNA_02754	0.030862537	0	0	0	0	0	0	0	0	0	0	0	-	NC_000003.12	-	99797161	99797338	178	178	intergenic		
circRNA_02755	0	0	0	0	0	0	0	0	0	0	0	0.0206364	-	NC_000003.12	-	99848295	99851070	2776	2271	exonic	NM_001282793.1	FILIP1L
circRNA_02756	0.020575025	0	0	0	0	0	0	0	0	0	0	0	hsa_circ_0007601	NC_000003.12	+	100281742	100290701	8960	435	sense-overlappin	NM_001199198.2	TBC1D23
circRNA_02757	0	0	0	0	0.02006311	0	0	0	0	0	0	0	hsa_circ_0066640	NC_000003.12	+	100283607	100299331	15725	821	sense-overlappin	NM_001199198.2	TBC1D23
circRNA_02758	0	0	0	0.0309543	0	0.031069364	0	0	0	0.040344966	0	0	hsa_circ_0005605	NC_000003.12	+	100295087	100298045	2959	399	sense-overlappin	NM_001199198.2	TBC1D23
circRNA_02759	0	0	0	0	0	0	0.030148028	0	0	0	0	0	hsa_circ_0066658	NC_000003.12	+	100555209	100568967	13759	737	sense-overlappin	NM_018004.1	TMEM45A
circRNA_02760	0	0	0	0	0.04012623	0	0.030148028	0	0	0.020172483	0	0	hsa_circ_0008839	NC_000003.12	+	100732508	100736716	4209	306	sense-overlappin	XM_005247066.1	TFG
circRNA_02761	0	0	0	0	0	0	0.020098685	0	0	0.020172483	0	0	-	NC_000003.12	-	100846372	100862385	16014	513	sense-overlappin	XM_005247309.1	ABI3BP
circRNA_02762	0	0	0	0.0206362	0	0	0	0	0	0	0	0	hsa_circ_0066672	NC_000003.12	-	101340095	101341779	1685	251	sense-overlappin	NM_001077203.2	SENP7

circRNA_02763	0	0	0	0	0.03009467	0	0	0	0	0	0	0	-	NC_000003.12	-	101656104	101672213	16110	1881	sense-overlappin	XM_011512689.1	ZBTB11
circRNA_02764	0	0	0	0.0309543	0	0	0	0	0	0.040344966	0	0	-	NC_000003.12	-	101664538	101672213	7676	1490	sense-overlappin	XM_011512689.1	ZBTB11
circRNA_02765	0	0.112584821	0	0.0825448	0.08025246	0.062138728	0.070345399	0.051215739	0.030381792	0.090776173	0.04051046	0.092863801	hsa_circ_0003179	NC_000003.12	-	101671130	101672213	1084	468	sense-overlappin	XM_011512689.1	ZBTB11
circRNA_02766	0.020575025	0	0	0	0	0	0	0	0	0	0	0	hsa_circ_0066701	NC_000003.12	+	101731840	101732654	815	281	sense-overlappin	XM_006713743.2	CEP97
circRNA_02767	0	0	0	0	0	0	0	0	0.020254528	0	0	0	hsa_circ_0004385	NC_000003.12	+	101816795	101821750	4956	554	sense-overlappin	NM_145037.2	NXPE3
circRNA_02768	0	0	0	0.0206362	0	0	0	0	0	0	0	0	-	NC_000003.12	+	105532002	105552585	20584	1270	sense-overlappin	NM_001243281.1	ALCAM
circRNA_02769	0	0	0.021808645	0	0	0	0	0	0	0	0	0	hsa_circ_0066714	NC_000003.12	-	105670233	105681818	11586	514	sense-overlappin	XR_924204.1	
circRNA_02770	0	0	0	0	0	0	0	0	0.020254528	0	0	0	-	NC_000003.12	-	105722398	105722492	95	95	intronic	XM_011513258.1	CBLB
circRNA_02771	0	0	0.054521613	0	0	0	0	0	0	0	0	0	hsa_circ_0066725	NC_000003.12	-	105734009	105776542	42534	784	sense-overlappin	XM_011513258.1	CBLB
circRNA_02772	0.051437562	0.051174919	0.076330258	0.1341352	0	0.062138728	0.090444084	0.102431477	0.020254528	0.110948655	0.050638075	0	hsa_circ_0001324	NC_000003.12	+	107710452	107716849	6398	414	sense-overlappin	NM_001142568.2	BBX
circRNA_02773	0	0.030704951	0.054521613	0.0515905	0	0.041425819	0.060296056	0.061458886	0.050636319	0.030258724	0	0	hsa_circ_0001325	NC_000003.12	+	107710452	107733023	22572	678	sense-overlappin	NM_001142568.2	BBX
circRNA_02774	0	0	0.065425935	0	0	0	0	0	0	0	0	0	hsa_circ_0066761	NC_000003.12	+	108605335	108616657	11323	447	sense-overlappin	XM_005247917.2	DZIP3
circRNA_02775	0	0	0.04361729	0	0	0	0	0	0	0	0	0	-	NC_000003.12	+	108632953	108675873	42921	2085	sense-overlappin	XM_005247917.2	DZIP3
circRNA_02776	0	0	0	0	0	0.020712909	0	0	0	0	0	0	-	NC_000003.12	+	108647943	108662257	14315	631	sense-overlappin	XM_005247917.2	DZIP3
circRNA_02777	0	0	0	0	0	0	0	0	0	0	0.02025523	0	hsa_circ_0003669	NC_000003.12	+	108669681	108677598	7918	460	sense-overlappin	XM_005247917.2	DZIP3
circRNA_02778	0	0.020469968	0	0	0	0	0	0	0	0	0	0	-	NC_000003.12	+	110014689	110014766	78	78	intergenic		
circRNA_02779	0	0	0	0.0412724	0.05015779	0	0.040197371	0	0	0.050431207	0	0	hsa_circ_0004639	NC_000003.12	+	111112030	111122238	10209	757	sense-overlappin	NM_001243286.1	PVRL3
circRNA_02780	0.072012586	0.061409903	0.04361729	0.0412724	0.09028402	0.072495183	0.221085539	0.061458886	0.020254528	0.060517448	0.091148535	0.0206364	hsa_circ_0066776	NC_000003.12	+	111112030	111126335	14306	909	sense-overlappin	NM_001243286.1	PVRL3
circRNA_02781	0	0	0.032712968	0	0	0	0	0	0	0	0	0	-	NC_000003.12	+	111112030	111127882	15853	2456	sense-overlappin	NM_001243286.1	PVRL3
circRNA_02782	0.041150049	0.061409903	0	0.113499	0	0.031069364	0.080394741	0.071702034	0	0.040344966	0.04051046	0	-	NC_000003.12	+	111112030	111147484	35455	1130	sense-overlappin	XM_005247322.3	PVRL3
circRNA_02783	0.020575025	0	0	0	0.02006311	0	0	0	0	0.040344966	0.030382845	0	hsa_circ_0007315	NC_000003.12	+	111144899	111147484	2586	221	sense-overlappin	XM_005247322.3	PVRL3
circRNA_02784	0	0	0.032712968	0	0	0	0	0	0	0	0	0	hsa_circ_0003067	NC_000003.12	+	111903966	111920419	16454	10019	sense-overlappin	NM_001134437.1	PHLDB2
circRNA_02785	0.072012586	0.040939935	0.04361729	0.0722267	0.04012623	0.165703275	0.070345399	0.040972591	0.050636319	0.121034897	0	0.0206364	hsa_circ_0001326	NC_000003.12	+	111913319	111920419	7101	666	sense-overlappin	NM_001134437.1	PHLDB2
circRNA_02786	0	0	0	0	0	0.041425819	0	0	0	0	0	0	hsa_circ_0004125	NC_000003.12	+	111939475	111962312	22838	947	sense-overlappin	NM_001134437.1	PHLDB2
circRNA_02787	0	0	0	0.0206362	0	0	0	0	0	0	0	0	hsa_circ_0005128	NC_000003.12	+	111945268	111962312	17045	680	sense-overlappin	NM_001134437.1	PHLDB2
circRNA_02788	0	0	0.04361729	0.0206362	0	0	0	0	0	0	0	0	-	NC_000003.12	+	112109543	112113145	3603	253	sense-overlappin	NM_024616.2	C3orf52
circRNA_02789	0	0	0.021808645	0	0	0	0	0	0	0	0	0	-	NC_000003.12	-	112266238	112280783	14546	1010	sense-overlappin	XM_011512721.1	SLC9C1
circRNA_02790	0	0	0.04361729	0	0	0	0	0	0	0	0	0	-	NC_000003.12	+	112573889	112665187	91299	83366	sense-overlappin	XM_011512948.1	SLC35A5
circRNA_02791	0	0	0.032712968	0	0	0	0	0	0	0	0	0	-	NC_000003.12	+	112580546	112665187	84642	83298	sense-overlappin	XM_011512948.1	SLC35A5
circRNA_02792	0.061725074	0	0	0	0	0	0.020098685	0	0	0	0	0.0309546	hsa_circ_0007897	NC_000003.12	+	113249722	113250833	1112	457	sense-overlappin	XM_011513301.1	BOC
circRNA_02793	0	0	0	0	0	0	0	0.020486295	0	0	0	0	-	NC_000003.12	-	113326445	113366309	39865	2072	sense-overlappin	NM_001164496.1	CFAP44
circRNA_02794	0	0	0	0.0206362	0	0	0	0	0	0	0	0	-	NC_000003.12	-	113341744	113407041	65298	2547	sense-overlappin	NM_001164496.1	CFAP44
circRNA_02795	0	0	0	0	0	0	0	0	0	0.040344966	0	0	-	NC_000003.12	-	113358745	113366309	7565	621	sense-overlappin	NM_001164496.1	CFAP44
circRNA_02796	0	0	0.021808645	0	0	0	0	0	0	0	0	0	-	NC_000003.12	-	113395750	113407041	11292	1000	sense-overlappin	NM_001164496.1	CFAP44
circRNA_02797	0	0	0	0.0206362	0	0	0	0	0	0	0	0.0206364	-	NC_000003.12	-	113395750	113416627	20878	1320	sense-overlappin	NM_001164496.1	CFAP44
circRNA_02798	0	0	0.054521613	0	0	0	0	0	0	0	0	0	hsa_circ_0066814	NC_000003.12	-	113488945	113499582	10638	464	sense-overlappin	NM_144718.3	SPICE1
circRNA_02799	0	0.051174919	0.981389026	0.0309543	0.08025246	0.031069364	0.020098685	0.040972591	0	0.020172483	0.02025523	0	hsa_circ_0006163	NC_000003.12	-	113488945	113506605	17661	611	sense-overlappin	NM_144718.3	SPICE1
circRNA_02800	0	0	0.054521613	0	0	0	0	0	0	0	0	0	hsa_circ_0066817	NC_000003.12	+	113566420	113567710	1291	293	sense-overlappin	NM_017699.2	SIDT1
circRNA_02801	0	0	0.021808645	0	0	0	0	0	0	0	0	0	-	NC_000003.12	+	113593005	113623733	30729	1321	sense-overlappin	XM_005247558.2	SIDT1
circRNA_02802	0	0	0	0.0309543	0	0	0	0	0	0	0	0.0412728	-	NC_000003.12	-	114018678	114046732	28055	1034	sense-overlappin	XM_011513051.1	KIAA1407
circRNA_02803	0	0	0.021808645	0	0	0	0	0	0	0	0	0	hsa_circ_0066842	NC_000003.12	+	114065237	114080057	14821	740	sense-overlappin	XM_005247769.2	QTRTD1
circRNA_02804	0.030862537	0	0.021808645	0.0722267	0	0	0.020098685	0.020486295	0.040509055	0	0.030382845	0	-	NC_000003.12	-	114693528	114717527	24000	7308	sense-overlappin	NM_001164344.2	ZBTB20
circRNA_02805	0	0	0	0.0206362	0	0	0	0	0	0	0	0	-	NC_000003.12	-	114693528	114747205	53678	3582	sense-overlappin	NM_001164343.2	ZBTB20
circRNA_02806	0.082300098	0.020469968	0.098138903	0.0928628	0.13041025	0.113921001	0.040197371	0.030729443	0.081018111	0.090776173	0	0.082545601	hsa_circ_0001329	NC_000003.12	+	119500695	119504021	3327	323	sense-overlappin	NM_016589.3	TIMMDC1
circRNA_02807	0	0	0	0	0.02006311	0.082851637	0.030148028	0.040972591	0.050636319	0.060517448	0	0.0309546	hsa_circ_0006884	NC_000003.12	+	119500695	119513719	13025	402	sense-overlappin	NM_016589.3	TIMMDC1
circRNA_02808	0.041150049	0.061409903	0.032712968	0.0515905	0.05015779	0.031069364	0.020098685	0.051215739	0.020254528	0.040344966	0	0	hsa_circ_0066874	NC_000003.12	+	119500695	119517315	16621	513	sense-overlappin	NM_016589.3	TIMMDC1
circRNA_02809	0	0	0	0	0.02006311	0	0	0	0	0	0	0	hsa_circ_0008394	NC_000003.12	+	119503532	119504021	490	157	sense-overlappin	NM_016589.3	TIMMDC1
circRNA_02810	0.13373766	0	0	0	0	0	0	0.040972591	0.030381792	0.020172483	0.030382845	0.0206364	hsa_circ_0066875	NC_000003.12	+	119503532	119513719	10188	236	sense-overlappin	NM_016589.3	TIMMDC1
circRNA_02811	0.041150049	0	0.054521613	0.0619086	0	0.082851637	0	0.051215739	0.040509055	0.060517448	0	0	hsa_circ_0001330	NC_000003.12	+	119503532	119517315	13784	347	sense-overlappin	NM_016589.3	TIMMDC1
circRNA_02812	0	0	0	0	0	0	0.030148028	0	0	0	0	0	hsa_circ_0003763	NC_000003.12	-	119843255	119863605	20351	286	sense-overlappin	NM_001146156.1	GSK3B
circRNA_02813	0	0	0	0.0206362	0	0	0	0	0	0	0	0	hsa_circ_0066888	NC_000003.12	-	119843255	119876508	33254	421	sense-overlappin	NM_002093.3	GSK3B
circRNA_02814	0	0	0	0	0	0	0	0.020486295	0	0.030258724	0.030382845	0	hsa_circ_0007986	NC_000003.12	-	119863419	119876508	13090	322	sense-overlappin	XM_006713610.1	GSK3B
circRNA_02815	0	0.051174919	0.08723458	0.0206362	0	0	0	0.030729443	0.040509055	0.030258724	0	0	hsa_circ_0008797	NC_000003.12	-	119863419	119905852	42434	420	sense-overlappin	XM_006713610.1	GSK3B
circRNA_02816	0	0	0.021808645	0	0	0	0	0	0	0	0	0	hsa_circ_0006497	NC_000003.12	-	120002046	120002239	194	194	exonic	XM_006713610.1	GSK3B

circRNA_02817	0.020575025	0	0	0	0	0	0	0	0	0	0	0	hsa_circ_0007524	NC_000003.12	-	120002046	120093593	91548	441	sense-overlappin	XM_006713610.1	GSK3B
circRNA_02818	0	0	0.283512385	0	0	0	0	0	0	0	0	0	hsa_circ_0006498	NC_000003.12	+	120750523	120751000	478	478	exonic	NM_005513.2	GTF2E1
circRNA_02819	0	0	0.054521613	0	0	0	0	0	0	0	0	0	-	NC_000003.12	-	121449315	121487509	38195	1843	sense-overlappin	XM_011512354.1	POLQ
circRNA_02820	0	0	0.032712968	0	0	0	0	0	0	0	0	0	hsa_circ_0066950	NC_000003.12	-	121493478	121498670	5193	563	sense-overlappin	XM_011512351.1	POLQ
circRNA_02821	0	0	0	0	0	0.031069364	0	0	0	0	0	0	-	NC_000003.12	-	121496808	121498670	1863	319	sense-overlappin	XM_011512351.1	POLQ
circRNA_02822	0	0.020469968	0	0	0	0	0	0	0	0	0	0	-	NC_000003.12	-	121496808	121522149	25342	1170	sense-overlappin	XM_011512351.1	POLQ
circRNA_02823	0	0	0	0	0.02006311	0	0	0	0	0	0	0	-	NC_000003.12	-	121522003	121544906	22904	1092	sense-overlappin	XM_011512343.1	POLQ
circRNA_02824	0	0	0	0	0	0	0	0	0	0.060517448	0	0	-	NC_000003.12	-	121635735	121637256	1522	237	sense-overlappin	NM_005335.5	HCLS1
circRNA_02825	0.041150049	0	0	0.0722267	0.02006311	0	0	0	0	0	0.02025523	0	hsa_circ_0066965	NC_000003.12	-	121714861	121730973	16113	1406	sense-overlappin	NM_001256486.1	GOLGB1
circRNA_02826	0	0	0	0	0.03009467	0	0	0	0	0	0	0	-	NC_000003.12	-	121726913	121727032	120	120	exonic	NM_001256486.1	GOLGB1
circRNA_02827	0	0	0.021808645	0	0	0.020712909	0	0.020486295	0	0.030258724	0	0	hsa_circ_0066970	NC_000003.12	+	121844453	121857156	12704	378	sense-overlappin	NM_018456.4	EAF2
circRNA_02828	0	0	0	0	0	0.082851637	0.050246713	0.061458886	0.050636319	0.090776173	0	0.0619092	hsa_circ_0066971	NC_000003.12	+	121844453	121872788	28336	630	sense-overlappin	NM_018456.4	EAF2
circRNA_02829	0	0.030704951	0	0	0	0	0	0	0	0	0	0	-	NC_000003.12	+	121911574	121915693	4120	362	sense-overlappin	NM_021082.3	SLC15A2
circRNA_02830	0	0	0.032712968	0	0.02006311	0	0	0	0	0	0	0	hsa_circ_0066991	NC_000003.12	-	122449574	122467429	17856	911	sense-overlappin	NR_026698.1	
circRNA_02831	0	0	0.021808645	0	0	0	0	0	0	0	0	0	hsa_circ_0066995	NC_000003.12	-	122536168	122540852	4685	696	sense-overlappin	NM_001146102.1	PARP9
circRNA_02832	0	0	0.021808645	0	0	0	0	0	0	0	0	0	-	NC_000003.12	-	122536168	122545489	9322	754	sense-overlappin	NM_001146102.1	PARP9
circRNA_02833	0	0	0	0	0.02006311	0	0	0	0	0	0	0	-	NC_000003.12	+	122685185	122695662	10478	648	sense-overlappin	XM_011512929.1	PARP14
circRNA_02834	0	0	0.04361729	0	0	0	0	0	0	0	0	0	-	NC_000003.12	+	122692301	122695662	3362	480	sense-overlappin	XM_011512929.1	PARP14
circRNA_02835	0	0	0.021808645	0	0	0	0	0	0	0	0	0	hsa_circ_0006132	NC_000003.12	+	122703742	122708268	4527	538	sense-overlappin	XM_011512929.1	PARP14
circRNA_02836	0	0	0.032712968	0	0	0	0	0	0	0	0	0	-	NC_000003.12	-	122740258	122777906	37649	1490	sense-overlappin	NM_024610.5	HSPBAP1
circRNA_02837	0	0	0.032712968	0	0	0	0	0	0	0	0	0	hsa_circ_0067013	NC_000003.12	+	123089168	123092442	3275	215	sense-overlappin	NM_006810.3	PDIA5
circRNA_02838	0	0	0	0	0	0.041425819	0	0	0	0	0	0	hsa_circ_0067017	NC_000003.12	+	123089168	123124343	35176	731	sense-overlappin	NM_006810.3	PDIA5
circRNA_02839	0	0	0	0	0.02006311	0	0	0	0	0	0	0.0309546	-	NC_000003.12	+	123124066	123130616	6551	301	sense-overlappin	NM_006810.3	PDIA5
circRNA_02840	0	0	0.021808645	0	0.03009467	0	0	0	0	0	0	0	-	NC_000003.12	+	123209199	123259589	50391	742	sense-overlappin	NM_012430.4	SEC22A
circRNA_02841	0.051437562	0.020469968	0.04361729	0	0.05015779	0.031069364	0.020098685	0	0	0	0	0	hsa_circ_0002236	NC_000003.12	+	123223559	123259589	36031	541	sense-overlappin	NM_012430.4	SEC22A
circRNA_02842	0.020575025	0	0	0	0	0	0	0	0	0	0	0	-	NC_000003.12	-	123325322	123332675	7354	682	sense-overlappin	XM_011512360.1	ADCY5
circRNA_02843	0.051437562	0	0	0	0	0	0	0	0	0	0	0	hsa_circ_0067047	NC_000003.12	-	123647224	123649194	1971	331	sense-overlappin	NM_053025.3	MYLK
circRNA_02844	0	0.030704951	0.04361729	0.0206362	0.03009467	0.051782273	0	0.040972591	0	0	0.02025523	0	-	NC_000003.12	-	123931102	123949132	18031	1426	sense-overlappin	XM_011513081.1	CCDC14
circRNA_02845	0	0	0.021808645	0	0	0	0	0	0	0	0	0	-	NC_000003.12	-	123933673	123949132	15460	1074	sense-overlappin	XM_011513081.1	CCDC14
circRNA_02846	0.030862537	0	0	0	0	0	0	0	0	0	0	0	-	NC_000003.12	+	124474663	124496413	21751	904	sense-overlappin	XM_006713815.2	KALRN
circRNA_02847	0	0	0	0	0	0.020712909	0	0	0	0	0	0	-	NC_000003.12	+	124735093	124744203	9111	1406	sense-overlappin	NM_000373.3	UMPS
circRNA_02848	0	0	0	0.0412724	0	0	0	0	0	0	0.030382845	0	hsa_circ_0006118	NC_000003.12	+	124737568	124744203	6636	1252	sense-overlappin	NM_000373.3	UMPS
circRNA_02849	0	0	0.021808645	0	0	0	0	0	0	0	0	0	-	NC_000003.12	-	124796388	124848558	52171	1332	sense-overlappin	NM_002213.4	ITGB5
circRNA_02850	0	0	0.021808645	0	0	0	0	0	0	0	0	0	-	NC_000003.12	-	124809022	124848558	39537	902	sense-overlappin	NM_002213.4	ITGB5
circRNA_02851	0	0	0.032712968	0	0	0	0	0	0	0	0	0	-	NC_000003.12	-	124817621	124848558	30938	767	sense-overlappin	NM_002213.4	ITGB5
circRNA_02852	0	0.020469968	0	0	0	0	0	0	0	0	0	0	-	NC_000003.12	+	124913223	124916284	3062	2741	antisense	NM_033049.3	MUC13
circRNA_02853	0	0	0	0	0	0	0	0	0.020254528	0	0	0	-	NC_000003.12	-	125118769	125120686	1918	176	sense-overlappin	NM_001195483.1	SLC12A8
circRNA_02854	0.020575025	0	0	0	0	0	0	0	0	0	0	0	-	NC_000003.12	-	125277726	125277809	84	84	exonic	NM_021964.2	ZNF148
circRNA_02855	0	0	0	0	0	0	0.020098685	0.030729443	0	0	0	0	hsa_circ_0006095	NC_000003.12	-	125277726	125279247	1522	208	sense-overlappin	NM_021964.2	ZNF148
circRNA_02856	0	0	0.032712968	0	0	0	0.030148028	0	0	0	0	0.0412728	hsa_circ_0067096	NC_000003.12	-	125277726	125313656	35931	683	sense-overlappin	NM_021964.2	ZNF148
circRNA_02857	0	0.030704951	0.152660515	0.0206362	0	0	0.020098685	0	0	0	0	0	hsa_circ_0067098	NC_000003.12	-	125277726	125331238	53513	900	sense-overlappin	NM_021964.2	ZNF148
circRNA_02858	0	0	0.021808645	0	0	0	0	0	0	0	0	0	-	NC_000003.12	-	125288103	125331238	43136	692	sense-overlappin	NM_021964.2	ZNF148
circRNA_02859	0	0	0.076330258	0	0	0	0	0	0.020254528	0	0	0	hsa_circ_0007557	NC_000003.12	-	125313308	125323444	10137	485	sense-overlappin	NM_021964.2	ZNF148
circRNA_02860	0	0	0.021808645	0	0	0	0	0	0	0	0	0	-	NC_000003.12	-	125313308	125331233	17926	561	sense-overlappin	NM_021964.2	ZNF148
circRNA_02861	0.061725074	0.102349838	0.556120448	0.113499	0.10031557	0.072495183	0.090444084	0.081945182	0.050636319	0.050431207	0.050638075	0.0309546	hsa_circ_0001333	NC_000003.12	-	125313308	125331238	17931	566	sense-overlappin	NM_021964.2	ZNF148
circRNA_02862	0	0	0	0.0309543	0	0	0	0	0	0	0	0	-	NC_000003.12	-	125451305	125457365	6061	361	sense-overlappin	NM_003794.3	SNX4
circRNA_02863	0	0	0	0	0	0	0	0.030729443	0	0	0	0	-	NC_000003.12	-	125505215	125509533	4319	4319	intronic	NM_003794.3	SNX4
circRNA_02864	0	0	0.021808645	0	0	0	0	0	0	0	0	0	-	NC_000003.12	-	125560379	125582978	22600	991	sense-overlappin	NM_022776.4	OSBPL11
circRNA_02865	0	0	0	0	0.03009467	0	0	0	0	0	0	0	-	NC_000003.12	-	125576189	125580040	3852	433	sense-overlappin	NM_022776.4	OSBPL11
circRNA_02866	0	0	0.021808645	0.0309543	0	0	0	0	0	0	0	0	-	NC_000003.12	-	126607651	126647296	39646	1943	sense-overlappin	NM_052883.1	TXNRD3
circRNA_02867	0.072012586	0	0.47979019	0.0412724	0	0	0	0	0	0.020172483	0.050638075	0	hsa_circ_0008181	NC_000003.12	-	126621742	126647296	25555	1281	sense-overlappin	NM_001173513.1	TXNRD3
circRNA_02868	0	0	0.076330258	0	0	0	0	0	0	0	0	0	hsa_circ_0007858	NC_000003.12	-	126629379	126647296	17918	1047	sense-overlappin	NM_001173513.1	TXNRD3
circRNA_02869	0	0.020469968	0	0	0	0	0	0	0	0	0	0	hsa_circ_0002409	NC_000003.12	-	126642032	126647296	5265	469	sense-overlappin	NM_001173513.1	TXNRD3
circRNA_02870	0	0	0.119947548	0	0	0	0	0	0	0	0	0	hsa_circ_0003181	NC_000003.12	+	126727078	126733222	6145	324	sense-overlappin	NM_032343.2	CHCHD6
circRNA_02871	0	0	0	0.0309543	0	0	0	0	0	0	0	0	hsa_circ_0067163	NC_000003.12	-	127579740	127580163	424	275	sense-overlappin	NM_001136053.2	TPRA1

circRNA_02872	0	0	0.054521613	0	0	0	0	0	0	0	0	0	hsa_circ_0007358	NC_000003.12	-	127710576	127722566	11991	416	sense-overlappin	XM_011512380.1	MGLL
circRNA_02873	0	0	0	0	0.02006311	0	0	0	0	0	0	0	-	NC_000003.12	+	127742485	127742957	473	473	antisense	NM_001256585.1	MGLL
circRNA_02874	0	0	0	0	0	0.020712909	0	0	0	0	0	0.0309546	hsa_circ_0007841	NC_000003.12	+	128060102	128060661	560	264	sense-overlappin	NM_013336.3	SEC61A1
circRNA_02875	0.102875123	0.040939935	0.141756193	0.0619086	0.04012623	0	0.020098685	0	0	0.090776173	0	0	hsa_circ_0008759	NC_000003.12	+	128262128	128264781	2654	262	sense-overlappin	NM_021937.4	EEFSEC
circRNA_02876	0	0	0	0	0	0	0.040197371	0	0	0	0	0	hsa_circ_0067231	NC_000003.12	-	128637799	128644983	7185	372	sense-overlappin	NM_002950.3	RPN1
circRNA_02877	0	0	0.054521613	0.0309543	0.02006311	0	0.020098685	0	0	0	0	0	hsa_circ_0006346	NC_000003.12	+	128795360	128807671	12312	536	sense-overlappin	NM_004637.5	RAB7A
circRNA_02878	0.020575025	0	0	0	0.04012623	0	0	0	0	0	0	0	hsa_circ_0001337	NC_000003.12	+	128795360	128813471	18112	681	sense-overlappin	NM_004637.5	RAB7A
circRNA_02879	0.020575025	0.051174919	0.032712968	0.0412724	0	0	0	0.040972591	0	0	0	0	hsa_circ_0008923	NC_000003.12	+	128806372	128807671	1300	348	sense-overlappin	NM_004637.5	RAB7A
circRNA_02880	0.030862537	0.020469968	0.021808645	0.0412724	0	0.020712909	0	0	0.020254528	0	0	0	hsa_circ_0004271	NC_000003.12	+	129265549	129267099	1551	320	sense-overlappin	XM_011512549.1	COPG1
circRNA_02881	0	0.020469968	0	0	0	0	0	0	0	0	0	0	hsa_circ_0067286	NC_000003.12	+	129288854	129290804	1951	270	sense-overlappin	NM_020187.2	HMCES
circRNA_02882	0.030862537	0.020469968	0.021808645	0	0	0	0	0	0	0.030258724	0	0	hsa_circ_0067291	NC_000003.12	+	129463560	129469417	5858	467	sense-overlappin	NM_001280545.1	IFT122
circRNA_02883	0	0	0	0	0	0.020712909	0	0	0	0	0	0	-	NC_000003.12	-	129585952	129586719	768	363	sense-overlappin	NM_015103.2	PLXND1
circRNA_02884	0	0	0	0	0	0	0	0	0	0	0.030382845	0	-	NC_000003.12	-	129585952	129596062	10111	7075	sense-overlappin	NM_015103.2	PLXND1
circRNA_02885	0	0	0.021808645	0	0	0	0	0	0	0	0	0	hsa_circ_0003665	NC_000003.12	-	129670330	129671264	935	935	exonic	XM_006713552.2	TMCC1
circRNA_02886	0	0	0	0	0	0	0	0.020486295	0	0	0	0	-	NC_000003.12	-	129827803	129828504	702	702	exonic	NM_001017395.3	TMCC1
circRNA_02887	0.020575025	0	0.021808645	0	0	0	0	0.020486295	0	0	0	0	hsa_circ_0067322	NC_000003.12	-	129827803	129828508	706	706	exonic	NM_001017395.3	TMCC1
circRNA_02888	0.072012586	0.020469968	0.04361729	0.0515905	0	0	0.030148028	0	0	0	0.04051046	0	hsa_circ_0067323	NC_000003.12	-	129827803	129832826	5024	759	sense-overlappin	NM_001017395.3	TMCC1
circRNA_02889	0	0	0.021808645	0	0	0	0	0	0	0	0	0	-	NC_000003.12	-	129827803	129880562	52760	1494	sense-overlappin	XM_011512570.1	TMCC1
circRNA_02890	0	0	0.021808645	0	0	0	0	0	0	0	0	0	-	NC_000003.12	-	129838898	129880559	41662	732	sense-overlappin	XM_011512570.1	TMCC1
circRNA_02891	0	0.020469968	0	0	0	0	0	0	0	0	0	0	-	NC_000003.12	-	129838898	129880562	41665	735	sense-overlappin	XM_011512570.1	TMCC1
circRNA_02892	0	0	0.021808645	0.0206362	0	0	0	0	0	0	0	0	-	NC_000003.12	-	129860940	129880559	19620	413	sense-overlappin	XM_011512570.1	TMCC1
circRNA_02893	0	0	0.021808645	0	0	0	0	0	0.020254528	0	0	0	-	NC_000003.12	-	129867010	129880562	13553	343	sense-overlappin	XM_006713545.2	TMCC1
circRNA_02894	0.061725074	0.092114854	0.152660515	0.0722267	0.05015779	0.113921001	0.040197371	0.040972591	0.060763583	0.100862414	0.091148535	0	hsa_circ_0001340	NC_000003.12	-	129880309	129880559	251	251	exonic	NM_001017395.3	TMCC1
circRNA_02895	0	0	0.065425935	0	0.04012623	0.041425819	0	0.020486295	0.020254528	0.020172483	0.04051046	0	-	NC_000003.12	-	129880309	129880562	254	254	exonic	XM_011512574.1	TMCC1
circRNA_02896	0	0	0.04361729	0	0	0	0	0	0	0	0	0	-	NC_000003.12	-	129880309	129884971	4663	4663	sense-overlappin	XM_011512574.1	TMCC1
circRNA_02897	0	0	0.021808645	0	0	0	0	0	0	0	0	0	-	NC_000003.12	-	129965701	129972108	6408	6408	intergenic		
circRNA_02898	0.030862537	0	0.119947548	0	0	0	0	0.020486295	0.030381792	0	0	0	hsa_circ_0005797	NC_000003.12	+	130930416	130941699	11284	525	sense-overlappin	XM_011512687.1	ATP2C1
circRNA_02899	0	0	0.021808645	0	0	0	0	0	0	0	0	0	-	NC_000003.12	+	130930416	130980679	50264	1833	sense-overlappin	XM_005247354.1	ATP2C1
circRNA_02900	0.041150049	0	0	0.0206362	0	0	0	0	0	0	0	0	-	NC_000003.12	+	130967145	130969396	2252	291	sense-overlappin	XM_011512687.1	ATP2C1
circRNA_02901	0	0	0.04361729	0	0	0	0	0	0.020254528	0	0.02025523	0.0206364	hsa_circ_0067347	NC_000003.12	+	131162408	131170887	8480	437	sense-overlappin	NM_145910.3	NEK11
circRNA_02902	0	0	0	0	0	0	0	0	0	0.020172483	0	0	-	NC_000003.12	+	131162408	131243496	81089	659	sense-overlappin	NM_001146003.1	NEK11
circRNA_02903	0	0	0	0	0	0	0	0	0	0	0.030382845	0	-	NC_000003.12	-	131462749	131471279	8531	392	sense-overlappin	NM_007208.3	MRPL3
circRNA_02904	0	0	0	0	0	0	0	0	0	0.030258724	0	0	hsa_circ_0004559	NC_000003.12	-	131468091	131471279	3189	265	sense-overlappin	NM_007208.3	MRPL3
circRNA_02905	0	0	0	0	0	0	0.020098685	0	0	0	0	0	-	NC_000003.12	+	132328267	132356855	28589	1018	sense-overlappin	NM_001099.4	ACPP
circRNA_02906	0	0	0.021808645	0	0	0	0	0	0	0	0	0	hsa_circ_0007818	NC_000003.12	-	132603229	132631479	28251	919	sense-overlappin	NM_032169.4	ACAD11
circRNA_02907	0	0	0	0	0	0	0.020098685	0	0	0	0	0	hsa_circ_0005875	NC_000003.12	-	132618634	132631479	12846	712	sense-overlappin	NM_032169.4	ACAD11
circRNA_02908	0	0	0.032712968	0	0	0	0	0	0	0	0	0	-	NC_000003.12	-	132639492	132644896	5405	553	sense-overlappin	NM_032169.4	ACAD11
circRNA_02909	0	0	0	0	0	0.020712909	0.030148028	0	0	0	0	0	-	NC_000003.12	-	132686260	132716909	30650	2659	sense-overlappin	NM_153240.4	NPHP3
circRNA_02910	0.020575025	0	0	0.0309543	0	0	0	0	0	0.020172483	0	0	-	NC_000003.12	-	132713126	132716909	3784	448	sense-overlappin	NM_153240.4	NPHP3
circRNA_02911	0	0	0	0	0.03009467	0	0	0	0	0	0	0	hsa_circ_0008069	NC_000003.12	-	133623091	133624175	1085	374	sense-overlappin	NM_007027.3	TOPBP1
circRNA_02912	0	0	0	0	0	0	0	0	0	0.040344966	0	0	-	NC_000003.12	-	133947256	133948971	1716	434	sense-overlappin	NM_005630.2	SLCO2A1
circRNA_02913	0.020575025	0	0.163564838	0	0	0	0	0.020486295	0.030381792	0.020172483	0	0	hsa_circ_0003113	NC_000003.12	-	134158121	134195182	37062	1077	sense-overlappin	NM_001005861.2	RYK
circRNA_02914	0.030862537	0.030704951	0.228990773	0.0825448	0.02006311	0.020712909	0.030148028	0.030729443	0.040509055	0.050431207	0.02025523	0	hsa_circ_0003633	NC_000003.12	-	134175609	134195182	19574	796	sense-overlappin	NM_001005861.2	RYK
circRNA_02915	0.092587611	0.030704951	0.065425935	0.1444533	0	0	0.040197371	0	0.081018111	0.060517448	0.04051046	0.0309546	hsa_circ_0005768	NC_000003.12	-	134188837	134195182	6346	323	sense-overlappin	XR_924160.1	
circRNA_02916	0	0	0.032712968	0	0	0	0	0	0	0	0	0	-	NC_000003.12	-	134207472	134211607	4136	289	sense-overlappin	XR_924160.1	
circRNA_02917	0	0	0.021808645	0	0	0	0	0	0	0	0	0	-	NC_000003.12	+	134495296	134507286	11991	247	sense-overlappin	NM_001042383.1	CEP63
circRNA_02918	0	0	0	0.0309543	0	0	0	0	0	0	0.02025523	0	-	NC_000003.12	+	134545586	134552012	6427	912	sense-overlappin	XM_005247795.3	CEP63
circRNA_02919	0	0	0	0.0309543	0	0	0	0	0	0	0	0	-	NC_000003.12	+	136001059	136003493	2435	2435	exonic	XM_011512957.1	PPP2R3A
circRNA_02920	0	0	0	0.0309543	0	0	0	0	0	0	0	0	-	NC_000003.12	+	136026832	136090667	63836	932	sense-overlappin	NM_001190447.1	PPP2R3A
circRNA_02921	0.030862537	0	0	0	0	0.031069364	0	0	0	0	0	0	-	NC_000003.12	+	136102007	136106322	4316	402	sense-overlappin	NM_001190447.1	PPP2R3A
circRNA_02922	0	0	0	0	0.02006311	0	0	0	0	0	0	0	-	NC_000003.12	+	136331486	136331875	390	390	intergenic		
circRNA_02923	0.030862537	0	0.021808645	0	0	0	0	0	0	0	0	0	hsa_circ_0005023	NC_000003.12	-	136377660	136423044	45385	720	sense-overlappin	XM_011512332.1	STAG1
circRNA_02924	0	0	0	0.0206362	0.02006311	0.020712909	0	0	0	0.040344966	0	0	hsa_circ_0067474	NC_000003.12	-	136398749	136423044	24296	627	sense-overlappin	XM_011512332.1	STAG1
circRNA_02925	0	0	0.021808645	0	0.02006311	0	0	0	0	0.030258724	0	0	-	NC_000003.12	-	136417885	136423044	5160	546	sense-overlappin	XM_011512332.1	STAG1
circRNA_02926	0	0	0	0.0206362	0.05015779	0	0	0	0	0	0	0	hsa_circ_0067478	NC_000003.12	-	136417885	136502779	84895	1520	sense-overlappin	XM_011512332.1	STAG1
circRNA_02927	0	0	0.021808645	0	0	0	0	0	0	0	0	0	-	NC_000003.12	-	136433556	136464988	31433	445	sense-overlappin	XM_011512332.1	STAG1

circRNA_02928	0	0.020469968	0.021808645	0	0	0	0	0	0	0	0	0	-	NC_000003.12	-	136443287	136464988	21702	341	sense-overlappin	XM_011512332.1	STAG1
circRNA_02929	0	0	0	0	0	0.020712909	0	0	0	0	0	0	-	NC_000003.12	-	136443287	136502779	59493	870	sense-overlappin	XM_011512332.1	STAG1
circRNA_02930	0	0	0	0	0.02006311	0	0	0	0.030381792	0.020172483	0	0	hsa_circ_0067480	NC_000003.12	-	136464881	136502779	37899	637	sense-overlappin	XM_011512332.1	STAG1
circRNA_02931	0	0	0	0	0	0	0	0	0	0	0.02025523	0	hsa_circ_0008750	NC_000003.12	-	136472413	136477412	5000	303	sense-overlappin	XM_011512332.1	STAG1
circRNA_02932	0	0	0.021808645	0	0	0	0	0	0	0	0	0	-	NC_000003.12	+	136945583	136946295	713	713	exonic	NM_001291999.1	NCK1
circRNA_02933	0	0	0	0	0.0702209	0	0.020098685	0	0	0	0	0	-	NC_000003.12	-	138120419	138131282	10864	4894	sense-overlappin	NM_016161.2	A4GNT
circRNA_02934	0.051437562	0	0	0	0	0	0	0.051215739	0	0	0	0	hsa_circ_0007348	NC_000003.12	+	138209817	138223733	13917	390	sense-overlappin	NM_014154.4	ARMC8
circRNA_02935	0	0	0.021808645	0	0	0	0	0	0	0	0	0	-	NC_000003.12	+	138209817	138235114	25298	564	sense-overlappin	NM_014154.4	ARMC8
circRNA_02936	0	0	0.021808645	0	0.03009467	0	0	0	0	0	0	0	-	NC_000003.12	-	138500110	138532570	32461	1017	sense-overlappin	XM_005247806.2	CEP70
circRNA_02937	0	0	0	0	0	0	0	0	0	0	0.02025523	0	-	NC_000003.12	-	138525490	138529462	3973	252	sense-overlappin	XM_005247806.2	CEP70
circRNA_02938	0	0	0.032712968	0.0206362	0.0702209	0	0	0	0	0.020172483	0	0	-	NC_000003.12	-	138525490	138537347	11858	479	sense-overlappin	XM_005247806.2	CEP70
circRNA_02939	0	0	0.04361729	0	0.02006311	0	0	0	0	0	0	0	-	NC_000003.12	-	138525490	138572932	47443	949	sense-overlappin	XM_005247806.2	CEP70
circRNA_02940	0	0	0.054521613	0	0	0	0	0	0	0	0	0	-	NC_000003.12	-	138525490	138572984	47495	1001	sense-overlappin	NM_001288964.1	CEP70
circRNA_02941	0	0	0	0.0309543	0	0	0	0	0	0	0	0.0206364	-	NC_000003.12	-	138542691	138572932	30242	28097	sense-overlappin	XM_005247806.2	CEP70
circRNA_02942	0	0	0.17446916	0.1444533	0	0	0.100493427	0.122917773	0.050636319	0.151293621	0	0	hsa_circ_0002468	NC_000003.12	-	138570318	138571356	1039	396	sense-overlappin	XM_005247806.2	CEP70
circRNA_02943	0	0	0.021808645	0	0	0	0	0	0.020254528	0	0	0	-	NC_000003.12	-	138570318	138572914	2597	452	sense-overlappin	XM_005247806.2	CEP70
circRNA_02944	0	0	0.21808645	0.2785885	0	0	0.080394741	0.071702034	0.060763583	0.201724828	0	0	hsa_circ_0004524	NC_000003.12	-	138570318	138572932	2615	470	sense-overlappin	XM_005247806.2	CEP70
circRNA_02945	0	0	0.207182128	0.0825448	0	0	0.040197371	0.051215739	0.060763583	0.080689931	0	0	hsa_circ_0003823	NC_000003.12	-	138570318	138572984	2667	522	sense-overlappin	NM_001288964.1	CEP70
circRNA_02946	0	0	0	0	0	0	0	0	0	0.020172483	0	0.0309546	hsa_circ_0007080	NC_000003.12	+	138621407	138622416	1010	362	sense-overlappin	XM_011512950.1	FAIM
circRNA_02947	0	0	0.021808645	0	0	0	0	0	0.040509055	0	0.02025523	0	-	NC_000003.12	-	138681967	138699095	17129	923	sense-overlappin	NM_001256045.1	PIK3CB
circRNA_02948	0	0	0	0	0	0	0.020098685	0	0	0	0	0	-	NC_000003.12	-	138691000	138699095	8096	455	sense-overlappin	NM_001256045.1	PIK3CB
circRNA_02949	0	0	0.021808645	0	0	0	0	0	0	0	0	0	-	NC_000003.12	-	138733361	138737886	4526	429	sense-overlappin	NM_006219.2	PIK3CB
circRNA_02950	0.020575025	0	0	0	0	0	0	0	0	0	0	0	-	NC_000003.12	+	141278342	141279504	1163	286	sense-overlappin	NM_001037172.2	PXYLP1
circRNA_02951	0	0.030704951	0	0	0	0	0	0	0	0	0	0	hsa_circ_0007714	NC_000003.12	+	141368621	141404031	35411	738	sense-overlappin	NM_001080412.2	ZBTB38
circRNA_02952	0	0	0	0	0	0	0	0	0	0.020172483	0	0	hsa_circ_0067581	NC_000003.12	+	141403927	141404031	105	105	exonic	NM_001080412.2	ZBTB38
circRNA_02953	0	0.020469968	0	0	0	0	0	0	0	0	0	0	hsa_circ_0003365	NC_000003.12	+	141501949	141529802	27854	10531	sense-overlappin	NM_001303245.1	RASA2
circRNA_02954	0	0	0	0	0.02006311	0	0	0	0	0	0	0	-	NC_000003.12	+	141501949	141540609	38661	10608	sense-overlappin	NM_001303245.1	RASA2
circRNA_02955	0	0.030704951	0	0	0	0	0	0	0	0.030258724	0.04051046	0	hsa_circ_0001344	NC_000003.12	+	141509568	141529802	20235	423	sense-overlappin	XM_011513059.1	RASA2
circRNA_02956	0	0.020469968	0	0	0	0	0.020098685	0	0.020254528	0	0	0	hsa_circ_0006347	NC_000003.12	+	141509568	141540609	31042	500	sense-overlappin	XM_011513059.1	RASA2
circRNA_02957	0.020575025	0	0.076330258	0.0515905	0	0.051782273	0.020098685	0	0	0	0.04051046	0	hsa_circ_0067582	NC_000003.12	+	141512163	141540609	28447	394	sense-overlappin	NM_001303245.1	RASA2
circRNA_02958	0	0.061409903	0	0	0	0	0.030148028	0.051215739	0.030381792	0.040344966	0.02025523	0	hsa_circ_0005372	NC_000003.12	+	141553857	141559995	6139	336	sense-overlappin	NM_001303245.1	RASA2
circRNA_02959	0	0	0	0	0.03009467	0	0.020098685	0	0	0	0	0	hsa_circ_0005742	NC_000003.12	+	141903620	141907274	3655	237	sense-overlappin	NM_001679.2	ATP1B3
circRNA_02960	0.030862537	0	0	0	0	0.031069364	0	0	0	0	0	0	-	NC_000003.12	-	142054115	142101841	47727	209	sense-overlappin	NM_001178138.1	TFDP2
circRNA_02961	0.030862537	0	0.08723458	0	0	0.020712909	0	0.030729443	0.030381792	0	0	0	hsa_circ_0008304	NC_000003.12	-	142093061	142101841	8781	174	sense-overlappin	XM_011513106.1	TFDP2
circRNA_02962	0	0	0	0	0	0	0	0	0.020254528	0	0	0	-	NC_000003.12	-	142347234	142359931	12698	483	sense-overlappin	XM_011512921.1	XRN1
circRNA_02963	0	0	0.021808645	0	0	0	0	0	0	0	0	0	hsa_circ_0067616	NC_000003.12	-	142355401	142376594	21194	1053	sense-overlappin	XM_011512921.1	XRN1
circRNA_02964	0	0	0.065425935	0.0619086	0	0.031069364	0.050246713	0	0	0.020172483	0	0	hsa_circ_0067619	NC_000003.12	-	142397329	142405076	7748	626	sense-overlappin	XM_011512921.1	XRN1
circRNA_02965	0.020575025	0.030704951	0.021808645	0.0825448	0.02006311	0	0	0.040972591	0.020254528	0.050431207	0.050638075	0	hsa_circ_0067620	NC_000003.12	-	142425222	142426841	1620	319	sense-overlappin	XM_011512921.1	XRN1
circRNA_02966	0	0	0	0	0	0.031069364	0	0	0	0	0	0	hsa_circ_0067627	NC_000003.12	-	142465097	142468068	2972	489	sense-overlappin	XM_011512924.1	ATR
circRNA_02967	0	0	0	0.0515905	0	0	0.020098685	0	0	0	0	0	-	NC_000003.12	-	142512260	142560454	48195	3509	sense-overlappin	XM_011512924.1	ATR
circRNA_02968	0.020575025	0	0	0	0	0	0	0	0	0	0	0	-	NC_000003.12	-	142566121	142572766	6646	4845	sense-overlappin	XM_011512924.1	ATR
circRNA_02969	0	0	0.098138903	0	0	0	0	0	0	0	0	0	-	NC_000003.12	+	142664202	142678113	13912	615	sense-overlappin	NM_001145319.1	PLS1
circRNA_02970	0.082300098	0.051174919	0.141756193	0	0	0	0	0	0.020254528	0.030258724	0	0	hsa_circ_0001345	NC_000003.12	+	142736379	142748460	12082	460	sense-overlappin	XR_924165.1	
circRNA_02971	0	0	0.032712968	0	0	0	0	0	0	0	0	0	-	NC_000003.12	+	142736379	142762313	25935	14313	sense-overlappin	XR_924165.1	
circRNA_02972	0.051437562	0	0	0	0	0	0	0	0	0	0	0	-	NC_000003.12	+	142736379	142792967	56589	1409	sense-overlappin	XR_924165.1	
circRNA_02973	0	0.020469968	0.076330258	0.0412724	0.0702209	0.031069364	0.020098685	0.030729443	0	0.040344966	0.04051046	0	hsa_circ_0006731	NC_000003.12	+	143985543	143989837	4295	632	sense-overlappin	NM_173552.3	C3orf58
circRNA_02974	0	0	0	0	0	0	0	0.030729443	0	0	0	0	-	NC_000003.12	+	143985543	144048812	63270	59607	sense-overlappin	NM_173552.3	C3orf58
circRNA_02975	0	0	0.04361729	0	0	0	0	0	0	0	0	0	-	NC_000003.12	-	146102755	146110448	7694	439	sense-overlappin	XM_005247535.3	PLOD2
circRNA_02976	0.082300098	0	0.109043225	0.1031809	0.02006311	0.062138728	0	0	0.030381792	0.07060369	0	0	hsa_circ_0067682	NC_000003.12	-	146102755	146124229	21475	729	sense-overlappin	XM_005247535.3	PLOD2
circRNA_02977	0.26747532	0.061409903	0.17446916	0.1547714	0.06018934	0.103564547	0.060296056	0.051215739	0.020254528	0.181552345	0.131658996	0.051591	-	NC_000003.12	-	146121112	146124229	3118	290	sense-overlappin	XM_005247535.3	PLOD2
circRNA_02978	0	0	0.021808645	0.0309543	0	0	0	0	0	0	0	0	-	NC_000003.12	-	146189799	146222092	32294	5623	sense-overlappin	XM_011513031.1	PLSCR4
circRNA_02979	0	0	0.032712968	0	0	0	0	0	0	0	0	0	-	NC_000003.12	-	149059718	149060858	1141	215	sense-overlappin	NM_003071.3	HLTF
circRNA_02980	0	0	0	0	0	0.031069364	0	0	0	0	0	0	-	NC_000003.12	-	149522853	149523478	626	626	intronic	NM_001168280.1	WWTR1
circRNA_02981	0	0	0	0	0	0.020712909	0	0	0	0	0	0	-	NC_000003.12	-	149542335	149573000	30666	340	sense-overlappin	NM_001168280.1	WWTR1
circRNA_02982	0.030862537	0	0	0	0	0	0	0	0	0	0	0	-	NC_000003.12	-	149697722	149703328	5607	5607	sense-overlappin	NM_001168278.1	WWTR1

circRNA_02983	0	0.020469968	0.17446916	0.0309543	0.20063115	0.051782273	0	0.020486295	0	0.030258724	0	0.0412728	hsa_circ_0067716	NC_000003.12	+		149846011	149872154	26144	337	sense-overlappin	XM_011512373.1	RNF13
circRNA_02984	0	0	0.021808645	0	0.05015779	0	0	0	0	0	0	0	hsa_circ_0067717	NC_000003.12	+		149846011	149895560	49550	425	sense-overlappin	XM_011512373.1	RNF13
circRNA_02985	0	0	0.032712968	0	0.02006311	0	0.020098685	0	0	0	0	0	hsa_circ_0004565	NC_000003.12	+		149846011	149902162	56152	516	sense-overlappin	XM_011512373.1	RNF13
circRNA_02986	0	0	0.163564838	0	0.11034713	0	0.050246713	0	0	0	0.06076569	0	hsa_circ_0003502	NC_000003.12	+		149846011	149912083	66073	622	sense-overlappin	XM_011512373.1	RNF13
circRNA_02987	0.072012586	0.16375974	0.56702477	0.2992247	0.44138853	0.15534682	0.331628308	0.071702034	0.182290749	0.110948655	0.091148535	0.113500201	hsa_circ_0001346	NC_000003.12	+		149846011	149921227	75217	716	sense-overlappin	XM_011512373.1	RNF13
circRNA_02988	0	0	0.065425935	0	0	0	0	0	0	0	0	0	hsa_circ_0067721	NC_000003.12	+		149852516	149921227	68712	586	sense-overlappin	XM_011512373.1	RNF13
circRNA_02989	0	0	0.021808645	0	0	0	0	0	0	0	0	0	hsa_circ_0067722	NC_000003.12	+		149872029	149902162	30134	305	sense-overlappin	XM_011512373.1	RNF13
circRNA_02990	0	0	0.021808645	0	0	0	0	0	0	0	0	0	hsa_circ_0067724	NC_000003.12	+		149872029	149921227	49199	505	sense-overlappin	XM_011512373.1	RNF13
circRNA_02991	0	0	0.098138903	0.0722267	0.02006311	0	0	0	0.060763583	0.110948655	0	0	hsa_circ_0006801	NC_000003.12	+		149895473	149921227	25755	379	sense-overlappin	XM_011512373.1	RNF13
circRNA_02992	0	0	0	0	0	0	0	0.020486295	0.030381792	0	0	0	hsa_circ_0005342	NC_000003.12	+		149902072	149921227	19156	291	sense-overlappin	XM_011512373.1	RNF13
circRNA_02993	0	0.040939935	0	0	0	0	0	0	0	0	0	0	hsa_circ_0008138	NC_000003.12	+		150562542	150563614	1073	219	sense-overlappin	XM_011513223.1	EIF2A
circRNA_02994	0	0	0	0	0	0.020712909	0	0	0	0	0	0	-	NC_000003.12	+		150571958	150581746	9789	815	sense-overlappin	XM_011513223.1	EIF2A
circRNA_02995	0	0	0	0	0.03009467	0	0	0	0	0	0.030382845	0	-	NC_000003.12	+		150575649	150581746	6098	243	sense-overlappin	XM_011513223.1	EIF2A
circRNA_02996	0	0	0	0	0	0.031069364	0	0	0	0	0	0	-	NC_000003.12	+		150622385	150627914	5530	736	sense-overlappin	NM_016275.3	SELT
circRNA_02997	0	0	0.021808645	0	0	0	0	0	0	0	0	0	-	NC_000003.12	-		151539153	151600951	61799	431	sense-overlappin	XM_011512708.1	IGSF10
circRNA_02998	0	0	0.239895095	0	0	0	0	0	0	0	0	0	-	NC_000003.12	-		151581539	151600951	19413	321	sense-overlappin	XM_011512708.1	IGSF10
circRNA_02999	0	0	0.032712968	0	0	0	0	0	0	0	0	0	-	NC_000003.12	+		151795739	151803925	8187	8187	intergenic		
circRNA_03000	0	0	0	0.0206362	0	0	0	0	0	0	0	0	hsa_circ_0001348	NC_000003.12	+		152299405	152300367	963	963	exonic	XM_006713639.1	MBNL1
circRNA_03001	0	0	0	0	0.03009467	0	0	0	0	0	0	0	-	NC_000003.12	-		152335573	152343068	7496	7496	antisense	XM_011512848.1	MBNL1
circRNA_03002	0.030862537	0.040939935	0.04361729	0	0.02006311	0.020712909	0	0.061458886	0.030381792	0.090776173	0.02025523	0	hsa_circ_0001349	NC_000003.12	+		152414941	152432920	17980	375	sense-overlappin	XM_005247475.2	MBNL1
circRNA_03003	0.041150049	0	0.032712968	0	0	0	0	0	0.020254528	0	0.04051046	0	hsa_circ_0007630	NC_000003.12	+		152445282	152447773	2492	466	sense-overlappin	XM_005247475.2	MBNL1
circRNA_03004	0.020575025	0	0	0	0	0	0	0	0	0	0	0	-	NC_000003.12	+		153172836	153209886	37051	37051	intergenic		
circRNA_03005	0	0	0	0.0206362	0	0	0	0	0.020254528	0	0	0.0206364	hsa_circ_0067755	NC_000003.12	-		154295284	154306295	11012	750	sense-overlappin	NM_001114397.1	DHX36
circRNA_03006	0	0	0.021808645	0	0	0	0	0	0	0	0	0	-	NC_000003.12	-		154309653	154316163	6511	570	sense-overlappin	NM_001114397.1	DHX36
circRNA_03007	0	0	0	0.0206362	0	0	0.020098685	0	0	0	0	0	hsa_circ_0067763	NC_000003.12	-		155549787	155568324	18538	591	sense-overlappin	XM_011512566.1	PLCH1
circRNA_03008	0	0	0	0	0	0	0	0	0	0.020172483	0	0	-	NC_000003.12	-		155828182	155834041	5860	715	sense-overlappin	NM_001190992.1	SLC33A1
circRNA_03009	0	0	0.032712968	0.0206362	0.03009467	0	0	0	0	0.040344966	0	0	-	NC_000003.12	-		155828182	155842619	14438	903	sense-overlappin	NM_001190992.1	SLC33A1
circRNA_03010	0.061725074	0.020469968	0.021808645	0.0309543	0.05015779	0.031069364	0.020098685	0	0	0.030258724	0.050638075	0.0309546	hsa_circ_0001350	NC_000003.12	-		155829688	155834041	4354	519	sense-overlappin	NM_001190992.1	SLC33A1
circRNA_03011	0	0	0	0.0825448	0.04012623	0.031069364	0	0.040972591	0	0	0	0.051591	hsa_circ_0067772	NC_000003.12	-		155829688	155842619	12932	707	sense-overlappin	NM_001190992.1	SLC33A1
circRNA_03012	0	0.061409903	0.04361729	0.0825448	0.05015779	0.072495183	0	0.071702034	0.030381792	0.040344966	0	0.0206364	hsa_circ_0067774	NC_000003.12	-		155833468	155842619	9152	491	sense-overlappin	NM_001190992.1	SLC33A1
circRNA_03013	0	0	0.04361729	0	0	0	0	0	0	0	0	0	-	NC_000003.12	+		155893518	155898041	4524	297	sense-overlappin	XM_011513263.1	GMPS
circRNA_03014	0.020575025	0	0.185373483	0.0206362	0	0.041425819	0	0.030729443	0	0	0	0.0206364	-	NC_000003.12	+		155893518	155911279	17762	859	sense-overlappin	XM_011513263.1	GMPS
circRNA_03015	0	0	0.021808645	0	0	0	0	0	0	0	0	0	-	NC_000003.12	+		155893518	155916192	22675	1185	sense-overlappin	XM_011513263.1	GMPS
circRNA_03016	0	0	0	0	0.02006311	0	0	0	0	0	0	0	-	NC_000003.12	+		155893518	155937881	44364	2244	sense-overlappin	XM_011513263.1	GMPS
circRNA_03017	0	0	0	0	0.03009467	0	0	0	0	0	0	0	-	NC_000003.12	+		155897927	155925366	27440	1351	sense-overlappin	XM_011513263.1	GMPS
circRNA_03018	0	0	0.032712968	0	0	0	0	0	0	0	0	0	hsa_circ_0002832	NC_000003.12	+		155910692	155914570	3879	512	sense-overlappin	XM_011513263.1	GMPS
circRNA_03019	0.020575025	0	0.021808645	0	0	0	0	0	0	0	0	0	hsa_circ_0008184	NC_000003.12	+		155910692	155925366	14675	1034	sense-overlappin	XM_011513263.1	GMPS
circRNA_03020	0	0	0.032712968	0	0	0	0	0	0	0	0	0	hsa_circ_0003017	NC_000003.12	+		155919233	155925366	6134	348	sense-overlappin	XM_011513263.1	GMPS
circRNA_03021	0	0	0	0	0	0.020712909	0	0	0	0.030258724	0	0	hsa_circ_0067790	NC_000003.12	+		155922187	155925366	3180	242	sense-overlappin	XM_011513263.1	GMPS
circRNA_03022	0	0	0.08723458	0.0206362	0	0	0.030148028	0	0	0.020172483	0	0	hsa_circ_0006999	NC_000003.12	+		156694020	156696025	2006	330	sense-overlappin	NM_015508.4	TIPARP
circRNA_03023	0.020575025	0	0	0	0	0	0	0	0	0	0	0	-	NC_000003.12	-		157209793	157216772	6980	6980	intergenic		
circRNA_03024	0.195462734	0.092114854	2.300812049	0.1238171	0.23072582	0.176059729	0.100493427	0.051215739	0.060763583	0.131121138	0.08102092	0.103182001	hsa_circ_0001355	NC_000003.12	+		158122103	158123991	1889	322	sense-overlappin	XM_011512875.1	RSRC1
circRNA_03025	0	0	0.163564838	0	0	0	0	0	0	0	0	0	hsa_circ_0067808	NC_000003.12	+		158122103	158203245	81143	496	sense-overlappin	XM_011512875.1	RSRC1
circRNA_03026	0.030862537	0	0	0	0	0	0	0	0	0	0	0	-	NC_000003.12	+		158742102	158742256	155	155	intergenic		
circRNA_03027	0	0	0.021808645	0	0	0	0	0	0	0	0	0	-	NC_000003.12	-		160366043	160384646	18604	815	sense-overlappin	NM_001190241.1	IFT80
circRNA_03028	0	0.040939935	0.04361729	0	0.03009467	0	0.020098685	0	0	0	0	0.0206364	hsa_circ_0001356	NC_000003.12	+		160413473	160414517	1045	292	sense-overlappin	NM_001002800.2	SMC4
circRNA_03029	0	0	0	0	0	0	0	0.020486295	0	0	0	0	-	NC_000003.12	+		160938713	160961910	23198	23198	sense-overlappin	XM_006713507.2	PPM1L
circRNA_03030	0	0	0.054521613	0	0	0	0	0	0	0	0	0	hsa_circ_0004264	NC_000003.12	+		161224930	161235212	10283	533	sense-overlappin	XM_005247511.1	NMD3
circRNA_03031	0	0.020469968	0	0	0	0	0	0	0	0	0	0	-	NC_000003.12	+		161234727	161238826	4100	396	sense-overlappin	XM_005247511.1	NMD3
circRNA_03032	0	0	0	0	0	0.031069364	0	0	0	0	0	0	-	NC_000003.12	+		164608992	164619440	10449	10449	intergenic		
circRNA_03033	0	0	0.021808645	0	0	0	0	0	0	0	0	0	-	NC_000003.12	-		165502147	165528873	26727	26727	intergenic		
circRNA_03034	0	0	0.021808645	0	0	0	0	0	0	0	0	0	-	NC_000003.12	-		167522315	167537000	14686	951	sense-overlappin	NM_178824.3	WDR49
circRNA_03035	0	0	0.021808645	0	0	0	0	0	0	0	0	0	-	NC_000003.12	-		167522315	167621643	99329	18958	sense-overlappin	NM_178824.3	WDR49
circRNA_03036	0	0	0.021808645	0	0	0	0	0	0	0	0	0	-	NC_000003.12	-		167602127	167604468	2342	317	sense-overlappin	NM_178824.3	WDR49
circRNA_03037	0	0	0.021808645	0	0	0	0	0	0	0	0	0	-	NC_000003.12	-		167695596	167725473	29878	584	sense-overlappin	XM_006713485.2	PDCD10
circRNA_03038	0	0	0.032712968	0.0309543	0	0	0	0.030729443	0.030381792	0	0	0	hsa_circ_0067869	NC_000003.12	-		168024526	168048365	23840	1676	sense-overlappin	XM_005247364.1	GOLIM4

circRNA_03039	0.051437562	0	0.032712968	0.0206362	0	0	0	0.051215739	0.030381792	0.07060369	0.030382845	0	hsa_circ_0004086	NC_000003.12	-	168036836	168041474	4639	326	sense-overlappin	XM_005247364.1	GOLIM4
circRNA_03040	0	0	0	0	0	0	0.040197371	0	0.020254528	0.020172483	0	0	hsa_circ_0067871	NC_000003.12	-	168036836	168048365	11530	656	sense-overlappin	XM_005247364.1	GOLIM4
circRNA_03041	0	0	0	0	0	0.020712909	0	0	0	0	0	0	-	NC_000003.12	-	169381187	169381524	338	338	exonic	NM_004991.3	MECOM
circRNA_03042	0.030862537	0	0.054521613	0.0206362	0.05015779	0.031069364	0	0	0	0	0	0	hsa_circ_0067880	NC_000003.12	+	169975608	169988359	12752	694	sense-overlappin	NM_003262.3	SEC62
circRNA_03043	0	0	0	0.0412724	0	0	0	0	0	0.030258724	0.02025523	0	-	NC_000003.12	+	169976946	169985865	8920	465	sense-overlappin	NM_003262.3	SEC62
circRNA_03044	0.051437562	0.051174919	0.021808645	0.1960438	0.21066271	0.062138728	0.060296056	0.061458886	0.040509055	0.07060369	0.02025523	0.123818401	hsa_circ_0001358	NC_000003.12	+	169976946	169988359	11414	585	sense-overlappin	NM_003262.3	SEC62
circRNA_03045	0	0	0	0.0206362	0	0	0	0	0	0	0	0	-	NC_000003.12	-	170106832	170122744	15913	680	sense-overlappin	XR_924180.1	
circRNA_03046	0	0	0	0	0	0	0	0.020486295	0	0	0	0	hsa_circ_0067894	NC_000003.12	-	170106832	170129552	22721	1597	sense-overlappin	XR_924180.1	
circRNA_03047	0.051437562	0.092114854	0	0.0412724	0	0.072495183	0	0.020486295	0.030381792	0.080689931	0	0.0412728	hsa_circ_0006893	NC_000003.12	-	170113360	170122744	9385	565	sense-overlappin	XR_924180.1	
circRNA_03048	0	0	0	0	0	0.020712909	0	0	0	0	0	0	hsa_circ_0067896	NC_000003.12	-	170113360	170136665	23306	1729	sense-overlappin	XR_924180.1	
circRNA_03049	0	0.020469968	0	0.0206362	0	0.020712909	0.030148028	0	0	0	0	0	hsa_circ_0004616	NC_000003.12	-	170117226	170122744	5519	405	sense-overlappin	XR_924180.1	
circRNA_03050	0.030862537	0	0	0	0.03009467	0.031069364	0	0	0.020254528	0.040344966	0.050638075	0	hsa_circ_0007854	NC_000003.12	-	170122591	170126007	3417	3417	sense-overlappin	XR_924180.1	
circRNA_03051	0.072012586	0	0	0.0206362	0	0.031069364	0	0.020486295	0.030381792	0.040344966	0	0	hsa_circ_0067900	NC_000003.12	-	170122591	170129552	6962	1071	sense-overlappin	XR_924180.1	
circRNA_03052	0	0	0	0	0	0.031069364	0	0	0	0	0	0	hsa_circ_0067901	NC_000003.12	-	170122591	170149244	26654	1576	sense-overlappin	XR_924180.1	
circRNA_03053	0	0	0.04361729	0	0	0	0	0	0	0	0	0	hsa_circ_0067906	NC_000003.12	-	170128684	170178938	50255	1774	sense-overlappin	XR_924180.1	
circRNA_03054	0	0	0	0	0	0	0	0	0	0	0.02025523	0	hsa_circ_0001965	NC_000003.12	-	170136419	170145521	9103	346	sense-overlappin	XR_924180.1	
circRNA_03055	0.102875123	0.153524756	0.370746965	0.1960438	0.27085205	0.186416184	0.140690797	0.122917773	0.081018111	0.282414759	0.12153138	0.051591	hsa_circ_0001359	NC_000003.12	-	170136419	170149244	12826	505	sense-overlappin	XR_924180.1	
circRNA_03056	0	0.020469968	0	0	0.04012623	0.041425819	0	0	0	0.060517448	0	0	hsa_circ_0002622	NC_000003.12	-	170136419	170178938	42520	905	sense-overlappin	XR_924180.1	
circRNA_03057	0.318912882	0.112584821	0.109043225	0.1444533	0.34107295	0.207129093	0.180888168	0.133160921	0.141781694	0.221897311	0.08102092	0.092863801	hsa_circ_0001360	NC_000003.12	-	170145423	170149244	3822	258	sense-overlappin	XR_924180.1	
circRNA_03058	0	0	0.021808645	0	0.02006311	0	0	0	0.020254528	0.030258724	0	0	hsa_circ_0005228	NC_000003.12	-	170145423	170178938	33516	658	sense-overlappin	XR_924180.1	
circRNA_03059	0.020575025	0	0	0	0	0	0	0	0	0	0	0	-	NC_000003.12	-	170146342	170146614	273	273	intronic	XR_924180.1	
circRNA_03060	0	0	0	0	0	0	0	0	0	0.030258724	0	0	hsa_circ_0067918	NC_000003.12	-	170149086	170178938	29853	559	sense-overlappin	XR_924180.1	
circRNA_03061	0	0	0	0	0	0	0	0.020486295	0	0	0	0	hsa_circ_0005362	NC_000003.12	-	170171373	170178938	7566	400	sense-overlappin	XR_924180.1	
circRNA_03062	0	0	0	0	0	0	0	0.030729443	0	0	0	0	hsa_circ_0067934	NC_000003.12	+	170295911	170297393	1483	170	sense-overlappin	NM_002740.5	PRKCI
circRNA_03063	0	0	0	0	0	0	0	0.020486295	0	0	0	0	-	NC_000003.12	+	170359699	170381341	21643	1338	sense-overlappin	NM_001145098.2	SKIL
circRNA_03064	0.030862537	0	0	0	0	0	0.030148028	0	0	0.020172483	0	0.0412728	-	NC_000003.12	+	170359699	170390464	30766	1813	sense-overlappin	NM_001145098.2	SKIL
circRNA_03065	0	0.020469968	0.08723458	0.0206362	0.04012623	0	0.040197371	0.061458886	0.030381792	0	0.030382845	0	-	NC_000003.12	+	170359699	170391260	31562	2038	sense-overlappin	NM_001145098.2	SKIL
circRNA_03066	0	0	0	0.0206362	0	0	0	0	0	0	0	0	-	NC_000003.12	+	170360321	170381341	21021	1207	sense-overlappin	XM_006713735.1	SKIL
circRNA_03067	0	0	0	0	0.02006311	0	0	0	0	0	0	0	-	NC_000003.12	+	170384533	170390464	5932	475	sense-overlappin	XM_006713735.1	SKIL
circRNA_03068	0	0	0.021808645	0	0	0	0	0	0	0	0	0	hsa_circ_0067943	NC_000003.12	-	171123596	171128878	5283	512	sense-overlappin	NM_001161560.1	TNIK
circRNA_03069	0.072012586	0.092114854	0.04361729	0.0619086	0.05015779	0.165703275	0.050246713	0.020486295	0.091145375	0.121034897	0.030382845	0.051591	hsa_circ_0002387	NC_000003.12	-	171188702	171194635	5934	333	sense-overlappin	NM_001161560.1	TNIK
circRNA_03070	0	0	0	0	0.0702209	0	0	0	0	0	0	0	-	NC_000003.12	-	171642840	171677694	34855	726	sense-overlappin	NM_001130081.2	PLD1
circRNA_03071	0	0	0.021808645	0	0	0	0	0	0	0	0	0	-	NC_000003.12	-	171665160	171674613	9454	282	sense-overlappin	XR_924146.1	
circRNA_03072	0	0.020469968	0	0	0	0	0	0	0	0	0	0	hsa_circ_0067956	NC_000003.12	-	171674500	171677694	3195	362	sense-overlappin	NM_001130081.2	PLD1
circRNA_03073	0	0	0	0	0.02006311	0	0	0	0	0	0	0	-	NC_000003.12	-	171674500	171709709	35210	1318	sense-overlappin	NM_002662.4	PLD1
circRNA_03074	0	0	0.021808645	0	0	0	0	0	0	0	0	0	hsa_circ_0067960	NC_000003.12	-	171708755	171726076	17322	539	sense-overlappin	NM_001130081.2	PLD1
circRNA_03075	0	0	0.04361729	0	0.02006311	0	0	0	0	0	0	0	hsa_circ_0067961	NC_000003.12	-	171708755	171738082	29328	1176	sense-overlappin	NM_001130081.2	PLD1
circRNA_03076	0	0	0	0	0.03009467	0	0	0	0	0	0	0	-	NC_000003.12	-	171724696	171738082	13387	789	sense-overlappin	NM_001130081.2	PLD1
circRNA_03077	0	0	0.032712968	0	0.04012623	0	0	0	0	0	0	0	hsa_circ_0067962	NC_000003.12	-	171733444	171738082	4639	637	sense-overlappin	NM_001130081.2	PLD1
circRNA_03078	0	0	0	0.0309543	0.03009467	0	0	0	0	0	0	0.0309546	-	NC_000003.12	+	172060710	172133546	72837	51957	sense-overlappin	NM_022763.3	FNDC3B
circRNA_03079	0	0	0	0	0	0.031069364	0	0	0	0	0	0	-	NC_000003.12	+	172079354	172112590	33237	33237	sense-overlappin	NM_022763.3	FNDC3B
circRNA_03080	0	0	0	0.0206362	0	0	0	0	0	0	0	0	-	NC_000003.12	+	172079354	172133546	54193	33313	sense-overlappin	NM_022763.3	FNDC3B
circRNA_03081	0	0	0	0	0.03009467	0	0	0	0	0	0	0	-	NC_000003.12	+	172081363	172133546	52184	31304	sense-overlappin	NM_022763.3	FNDC3B
circRNA_03082	0.020575025	0.040939935	0.08723458	0.1857257	0.17053648	0.041425819	0	0	0.101272639	0.060517448	0.02025523	0.0412728	hsa_circ_0001361	NC_000003.12	+	172112452	172133546	21095	215	sense-overlappin	NM_022763.3	FNDC3B
circRNA_03083	0	0	0	0.0206362	0	0	0	0	0	0	0	0	-	NC_000003.12	+	172112452	172158792	46341	25461	sense-overlappin	NM_022763.3	FNDC3B
circRNA_03084	0	0	0	0	0.02006311	0	0	0	0	0	0	0	hsa_circ_0067981	NC_000003.12	+	172133471	172226947	93477	153	sense-overlappin	NM_022763.3	FNDC3B
circRNA_03085	0	0	0.021808645	0	0	0	0	0	0	0	0	0	-	NC_000003.12	+	172217808	172218614	807	807	intronic	NM_022763.3	FNDC3B
circRNA_03086	0	0	0	0	0.0702209	0	0.020098685	0	0	0	0	0	hsa_circ_0067985	NC_000003.12	+	172226871	172251541	24671	603	sense-overlappin	NM_022763.3	FNDC3B
circRNA_03087	0	0	0	0	0.03009467	0	0	0	0	0	0	0	hsa_circ_0067986	NC_000003.12	+	172226871	172298787	71917	874	sense-overlappin	NM_022763.3	FNDC3B
circRNA_03088	0.318912882	0.317284497	0.283512385	0.9492647	0.90284017	0.279624276	0.180888168	0.184376659	0.192418013	0.484139587	0.12153138	0.340500603	hsa_circ_0006156	NC_000003.12	+	172247533	172251541	4009	526	sense-overlappin	NM_022763.3	FNDC3B
circRNA_03089	0	0	0	0.0206362	0	0	0	0	0	0	0	0	-	NC_000003.12	+	172247533	172262083	14551	11068	sense-overlappin	NM_022763.3	FNDC3B
circRNA_03090	0	0	0	0	0.02006311	0	0	0	0	0	0	0	-	NC_000003.12	+	172247533	172262838	15306	11823	sense-overlappin	NM_022763.3	FNDC3B
circRNA_03091	0.030862537	0.08187987	0.065425935	0.0928628	0.44138853	0.062138728	0	0.09218833	0	0.080689931	0.02025523	0	hsa_circ_0001362	NC_000003.12	+	172247533	172285984	38452	585	sense-overlappin	NM_022763.3	FNDC3B
circRNA_03092	0	0	0	0	0.08025246	0	0	0	0	0	0	0	-	NC_000003.12	+	172247533	172298787	51255	797	sense-overlappin	NM_022763.3	FNDC3B
circRNA_03093	0	0	0	0	0.03009467	0	0	0	0	0	0	0	hsa_circ_0005700	NC_000003.12	+	172247533	172310881	63349	990	sense-overlappin	NM_022763.3	FNDC3B
circRNA_03094	0.020575025	0.030704951	0.032712968	0.0309543	0.10031557	0.020712909	0.030148028	0	0	0.040344966	0.030382845	0	-	NC_000003.12	+	172251260	172262083	10824	10824	sense-overlappin	NM_022763.3	FNDC3B

circRNA_03095	0.020575025	0	0	0.0825448	0.10031557	0.031069364	0.020098685	0	0	0.030258724	0	0	hsa_circ_0067990	NC_000003.12	+	172251260	172298787	47528	553	sense-overlappin	NM_022763.3	FNDC3B
circRNA_03096	0	0	0.021808645	0.0619086	0.08025246	0	0	0.030729443	0.030381792	0	0	0	hsa_circ_0067991	NC_000003.12	+	172251260	172307501	56242	692	sense-overlappin	NM_022763.3	FNDC3B
circRNA_03097	0.041150049	0.061409903	0.076330258	0.330179	0.39123074	0.072495183	0	0.040972591	0.030381792	0.07060369	0.02025523	0.154773001	hsa_circ_0003692	NC_000003.12	+	172251260	172310881	59622	746	sense-overlappin	NM_022763.3	FNDC3B
circRNA_03098	0	0	0	0	0.02006311	0	0	0	0	0	0	0	-	NC_000003.12	-	172284652	172284807	156	156	antisense	NM_022763.3	FNDC3B
circRNA_03099	0	0	0	0	0.05015779	0	0	0	0.040509055	0	0	0	hsa_circ_0002026	NC_000003.12	+	172285926	172298787	12862	271	sense-overlappin	NM_022763.3	FNDC3B
circRNA_03100	0	0.051174919	0.04361729	0.0309543	0.08025246	0.051782273	0	0.030729443	0.050636319	0.040344966	0	0	hsa_circ_0002422	NC_000003.12	+	172285926	172310881	24956	464	sense-overlappin	NM_022763.3	FNDC3B
circRNA_03101	0	0	0	0	0.0702209	0	0.060296056	0	0	0	0.04051046	0.0206364	hsa_circ_0067997	NC_000003.12	+	172295363	172310881	15519	405	sense-overlappin	NM_022763.3	FNDC3B
circRNA_03102	0	0	0	0	0.03009467	0	0	0	0	0	0	0	hsa_circ_0067998	NC_000003.12	+	172298728	172310881	12154	253	sense-overlappin	NM_022763.3	FNDC3B
circRNA_03103	0	0	0	0	0	0	0	0	0	0.020172483	0	0	-	NC_000003.12	+	172336839	172340321	3483	3483	sense-overlappin	NM_022763.3	FNDC3B
circRNA_03104	0	0	0	0	0.0702209	0	0	0	0	0	0	0	hsa_circ_0068013	NC_000003.12	+	172362633	172381093	18461	508	sense-overlappin	NM_022763.3	FNDC3B
circRNA_03105	0	0.020469968	0	0	0.04012623	0	0.030148028	0	0	0	0	0.0412728	-	NC_000003.12	-	172505974	172506919	946	946	exonic	NM_001190942.1	TNFSF10
circRNA_03106	0	0	0	0.0206362	0.02006311	0	0.020098685	0	0	0	0	0	-	NC_000003.12	-	172505974	172509321	3348	1051	sense-overlappin	NM_003810.3	TNFSF10
circRNA_03107	0	0	0.021808645	0	0	0	0	0	0	0	0	0	-	NC_000003.12	-	172635916	172648114	12199	495	sense-overlappin	NM_001146276.1	NCEH1
circRNA_03108	0.020575025	0	0.08723458	0	0.04012623	0	0	0	0.020254528	0	0	0	hsa_circ_0007042	NC_000003.12	-	172645623	172648114	2492	323	sense-overlappin	NM_001146276.1	NCEH1
circRNA_03109	0	0	0.021808645	0	0.02006311	0	0	0	0	0	0	0	-	NC_000003.12	+	172760156	172784803	24648	1249	sense-overlappin	XM_005247176.1	ECT2
circRNA_03110	0	0	0.04361729	0	0.03009467	0	0	0	0	0.020172483	0	0	-	NC_000003.12	+	172760161	172783909	23749	1147	sense-overlappin	XM_005247176.1	ECT2
circRNA_03111	0	0	0	0	0.02006311	0	0	0	0	0	0	0	-	NC_000003.12	+	172760161	172784803	24643	1244	sense-overlappin	XM_005247176.1	ECT2
circRNA_03112	0	0	0	0	0.02006311	0	0	0	0	0	0	0	-	NC_000003.12	+	172761610	172774022	12413	864	sense-overlappin	XM_005247176.1	ECT2
circRNA_03113	0	0	0.04361729	0	0.03009467	0	0	0	0	0	0	0	-	NC_000003.12	+	172761610	172783909	22300	1044	sense-overlappin	XM_005247176.1	ECT2
circRNA_03114	0	0	0.04361729	0	0.06018934	0	0	0	0	0	0	0	hsa_circ_0003975	NC_000003.12	+	172769007	172786574	17568	616	sense-overlappin	XM_005247176.1	ECT2
circRNA_03115	0	0	0	0	0.06018934	0	0	0	0	0	0	0	-	NC_000003.12	+	172773903	172783909	10007	300	sense-overlappin	XM_005247176.1	ECT2
circRNA_03116	0	0	0	0	0.04012623	0	0	0	0	0	0	0	hsa_circ_0006829	NC_000003.12	+	172782163	172786574	4412	359	sense-overlappin	XM_005247176.1	ECT2
circRNA_03117	0.030862537	0	0	0.0515905	0.02006311	0	0	0.020486295	0	0.07060369	0.02025523	0	-	NC_000003.12	+	174523413	174553757	30345	30345	sense-overlappin	XM_006713560.2	NAALADL2
circRNA_03118	0	0	0.04361729	0.0206362	0	0	0	0	0	0	0	0	-	NC_000003.12	+	175096790	175097291	502	502	exonic	XM_006713560.2	NAALADL2
circRNA_03119	0	0	0	0	0	0.020712909	0	0	0	0	0	0	-	NC_000003.12	-	177079827	177131449	51623	18802	sense-overlappin	XM_005247771.2	TBL1XR1
circRNA_03120	0	0	0	0	0	0	0	0	0	0	0.02025523	0	-	NC_000003.12	+	177492602	177497259	4658	4658	intergenic		
circRNA_03121	0	0	0	0	0	0	0.030148028	0	0	0	0	0	-	NC_000003.12	-	179178607	179250934	72328	39996	antisense	NM_006218.2	PIK3CA
circRNA_03122	0.030862537	0	0.021808645	0.0206362	0.05015779	0.020712909	0	0	0.020254528	0.080689931	0.030382845	0	-	NC_000003.12	+	179203544	179204588	1045	332	sense-overlappin	XM_006713658.2	PIK3CA
circRNA_03123	0	0.040939935	0.04361729	0.0206362	0	0	0.020098685	0	0	0.030258724	0	0	-	NC_000003.12	+	179378341	179392104	13764	942	sense-overlappin	NM_033540.2	MFN1
circRNA_03124	0.051437562	0	0	0	0	0	0	0	0	0	0	0	-	NC_000003.12	-	179405190	179426242	21053	958	sense-overlappin	NM_021629.3	GNB4
circRNA_03125	0.092587611	0	0	0	0.04012623	0.020712909	0.030148028	0.020486295	0.040509055	0.050431207	0.050638075	0	hsa_circ_0001364	NC_000003.12	-	179413412	179419505	6094	603	sense-overlappin	NM_021629.3	GNB4
circRNA_03126	0	0	0	0	0	0	0	0	0.030381792	0	0.02025523	0	hsa_circ_0005009	NC_000003.12	-	179413412	179426242	12831	741	sense-overlappin	NM_021629.3	GNB4
circRNA_03127	0.041150049	0	0.032712968	0.0309543	0	0	0	0	0	0	0	0	hsa_circ_0005292	NC_000003.12	-	179592644	179601790	9147	385	sense-overlappin	NM_020409.2	MRPL47
circRNA_03128	0	0	0.032712968	0	0	0	0	0	0	0	0	0	-	NC_000003.12	+	179614971	179618521	3551	325	sense-overlappin	NM_002492.3	NDUFB5
circRNA_03129	0.041150049	0	0	0.0206362	0	0	0	0.020486295	0.030381792	0	0	0	hsa_circ_0007541	NC_000003.12	+	179764002	179765848	1847	321	sense-overlappin	NM_003940.2	USP13
circRNA_03130	0	0.020469968	0	0	0.04012623	0.041425819	0	0	0	0	0	0	hsa_circ_0068129	NC_000003.12	+	180933334	180935231	1898	147	sense-overlappin	NM_001013438.2	FXR1
circRNA_03131	0	0	0.032712968	0	0.04012623	0.031069364	0.030148028	0	0	0.020172483	0	0	hsa_circ_0002004	NC_000003.12	+	180947865	180949343	1479	432	sense-overlappin	NM_001013438.2	FXR1
circRNA_03132	0	0	0	0.0309543	0	0.103564547	0	0	0.020254528	0.050431207	0	0	hsa_circ_0068142	NC_000003.12	+	182884753	182887713	2961	334	sense-overlappin	NM_014616.2	ATP11B
circRNA_03133	0.020575025	0	0	0	0	0	0	0	0	0	0	0	-	NC_000003.12	+	182884753	182913994	29242	1029	sense-overlappin	XM_005247241.1	ATP11B
circRNA_03134	0	0	0.021808645	0	0	0	0	0	0	0	0	0	-	NC_000003.12	-	182947238	182965753	18516	697	sense-overlappin	XM_011512912.1	DCUN1D1
circRNA_03135	0	0.040939935	0.04361729	0.113499	0	0	0	0	0	0.050431207	0	0	hsa_circ_0008550	NC_000003.12	-	183070999	183071357	359	270	sense-overlappin	NR_120640.1	
circRNA_03136	0	0	0	0.0206362	0	0	0	0	0	0	0	0	-	NC_000003.12	-	183070999	183092545	21547	625	sense-overlappin	NR_120640.1	
circRNA_03137	0	0	0.032712968	0.0309543	0.03009467	0.020712909	0	0	0	0	0	0	hsa_circ_0068147	NC_000003.12	-	183086693	183092545	5853	233	sense-overlappin	NR_120640.1	
circRNA_03138	0.041150049	0.08187987	0.065425935	0.0825448	0.0702209	0.113921001	0.030148028	0.061458886	0.050636319	0.100862414	0.02025523	0.082545601	hsa_circ_0001367	NC_000003.12	+	183643480	183651276	7797	1044	sense-overlappin	XM_011512937.1	KLHL24
circRNA_03139	0	0	0	0.0206362	0	0	0	0	0	0	0	0	hsa_circ_0068165	NC_000003.12	+	183643480	183663642	20163	1229	sense-overlappin	XM_011512937.1	KLHL24
circRNA_03140	0	0	0	0	0.02006311	0.031069364	0	0	0.030381792	0.020172483	0	0	-	NC_000003.12	+	183643480	183665039	21560	1348	sense-overlappin	XM_011512937.1	KLHL24
circRNA_03141	0	0.020469968	0.109043225	0.1857257	0.03009467	0.15534682	0	0.081945182	0.040509055	0.050431207	0.04051046	0.0206364	hsa_circ_0001368	NC_000003.12	+	183643480	183672484	29005	1726	sense-overlappin	XM_011512937.1	KLHL24
circRNA_03142	0	0.030704951	0.032712968	0.0206362	0.03009467	0.082851637	0	0.020486295	0.030381792	0	0	0.051591	hsa_circ_0001369	NC_000003.12	+	183650296	183651276	981	981	exonic	XM_011512937.1	KLHL24
circRNA_03143	0	0	0	0	0	0.020712909	0	0	0	0	0	0	-	NC_000003.12	+	183650296	183654089	3794	3794	sense-overlappin	XM_011512937.1	KLHL24
circRNA_03144	0	0.020469968	0	0	0	0	0	0.020486295	0	0	0	0	hsa_circ_0006667	NC_000003.12	+	183650296	183672484	22189	1663	sense-overlappin	XM_011512937.1	KLHL24
circRNA_03145	0	0	0.032712968	0	0	0	0	0	0	0	0	0	-	NC_000003.12	+	183715144	183724531	9388	669	sense-overlappin	XM_011512966.1	YEATS2
circRNA_03146	0	0	0.032712968	0	0	0	0	0	0	0	0	0	hsa_circ_0068173	NC_000003.12	+	183715144	183728851	13708	831	sense-overlappin	XM_011512966.1	YEATS2
circRNA_03147	0	0	0	0	0	0	0.020098685	0	0	0	0	0	hsa_circ_0006846	NC_000003.12	+	183717651	183722136	4486	437	sense-overlappin	XM_011512966.1	YEATS2
circRNA_03148	0	0	0	0	0	0.020712909	0	0	0	0	0	0	hsa_circ_0068175	NC_000003.12	+	183717651	183752253	34603	1050	sense-overlappin	XM_011512966.1	YEATS2
circRNA_03149	0	0	0.032712968	0	0	0	0	0	0	0	0	0	hsa_circ_0009131	NC_000003.12	+	183736718	183762279	25562	1138	sense-overlappin	XM_011512966.1	YEATS2
circRNA_03150	0	0	0.021808645	0.0206362	0	0	0	0	0	0.030258724	0	0	-	NC_000003.12	+	183747672	183762279	14608	1026	sense-overlappin	XM_011512966.1	YEATS2
circRNA_03151	0	0	0.021808645	0	0	0	0	0	0	0	0	0	hsa_circ_0068191	NC_000003.12	+	183747672	183786301	38630	1992	sense-overlappin	XM_011512966.1	YEATS2

circRNA_03152	0	0	0	0	0.02006311	0	0	0	0	0	0	0	hsa_circ_0006614	NC_000003.12	+	183775915	183790980	15066	729	sense-overlappin	XM_011512966.1	YEATS2
circRNA_03153	0	0	0	0	0	0	0.030148028	0	0	0	0	0	-	NC_000003.12	+	183797923	183798989	1067	228	sense-overlappin	XM_005247597.2	YEATS2
circRNA_03154	0	0	0.021808645	0	0	0	0	0	0	0	0	0	hsa_circ_0068252	NC_000003.12	-	183840570	183844326	3757	317	sense-overlappin	XM_005247582.3	PARL
circRNA_03155	0	0	0.021808645	0	0	0	0	0	0	0	0	0	-	NC_000003.12	-	183840570	183856551	15982	12542	sense-overlappin	XM_005247582.3	PARL
circRNA_03156	0	0	0	0	0	0	0	0.020486295	0	0	0	0	-	NC_000003.12	+	183908028	183909794	1767	1767	intergenic		
circRNA_03157	0	0	0	0	0	0	0	0	0	0.050431207	0	0	-	NC_000003.12	-	183959733	183978651	18919	1450	sense-overlappin	XM_011512316.1	ABCC5
circRNA_03158	0	0	0	0	0	0	0	0.020486295	0	0	0	0	hsa_circ_0068258	NC_000003.12	-	183959733	183983007	23275	2006	sense-overlappin	XM_011512316.1	ABCC5
circRNA_03159	0	0	0	0.0206362	0	0	0	0	0	0	0	0	-	NC_000003.12	-	183992712	183995917	3206	3206	intronic	XM_011512316.1	ABCC5
circRNA_03160	0	0	0	0.0206362	0	0	0	0	0	0	0	0	hsa_circ_0068355	NC_000003.12	+	184824545	184834742	10198	535	sense-overlappin	XR_924117.1	
circRNA_03161	0	0	0	0	0	0	0	0	0.030381792	0	0	0	hsa_circ_0068360	NC_000003.12	+	184849071	184870805	21735	1193	sense-overlappin	XR_924117.1	
circRNA_03162	0	0	0.021808645	0	0	0	0	0	0	0.020172483	0	0	hsa_circ_0068367	NC_000003.12	+	184886110	184900972	14863	591	sense-overlappin	XM_011512601.1	VPS8
circRNA_03163	0	0	0.021808645	0	0	0	0	0	0	0	0	0	-	NC_000003.12	+	184886110	184971752	85643	1865	sense-overlappin	XM_011512601.1	VPS8
circRNA_03164	0	0	0.04361729	0	0	0	0	0	0	0	0	0	-	NC_000003.12	+	184913519	184971752	58234	1274	sense-overlappin	NM_001009921.2	VPS8
circRNA_03165	0	0	0	0	0.02006311	0	0	0	0	0	0	0	hsa_circ_0068373	NC_000003.12	+	184924862	184971752	46891	966	sense-overlappin	NM_001009921.2	VPS8
circRNA_03166	0	0	0.021808645	0	0	0	0	0	0	0	0	0	-	NC_000003.12	+	184924862	184983094	58233	1131	sense-overlappin	NM_001009921.2	VPS8
circRNA_03167	0	0	0	0	0.05015779	0	0	0	0	0	0	0	hsa_circ_0068375	NC_000003.12	+	184936246	184957521	21276	285	sense-overlappin	NM_001009921.2	VPS8
circRNA_03168	0	0	0.04361729	0	0	0	0	0	0	0	0	0	hsa_circ_0068379	NC_000003.12	+	184957374	184971752	14379	385	sense-overlappin	NM_001009921.2	VPS8
circRNA_03169	0	0	0.032712968	0.0206362	0	0.041425819	0.020098685	0	0	0	0	0	-	NC_000003.12	+	185437447	185443636	6190	376	sense-overlappin	NM_001242314.1	MAP3K13
circRNA_03170	0	0	0	0	0	0	0	0.020486295	0	0	0	0	hsa_circ_0068389	NC_000003.12	+	185437447	185451395	13949	803	sense-overlappin	NM_001242314.1	MAP3K13
circRNA_03171	0	0	0	0.0206362	0	0	0	0	0	0	0	0	-	NC_000003.12	+	185437447	185466963	29517	1168	sense-overlappin	NM_001242314.1	MAP3K13
circRNA_03172	0	0	0.04361729	0	0	0	0	0	0	0	0	0	-	NC_000003.12	-	185527484	185535132	7649	579	sense-overlappin	XM_011512531.1	LIPH
circRNA_03173	0.020575025	0	0.04361729	0	0	0	0	0	0	0	0	0.0206364	-	NC_000003.12	-	185533571	185535132	1562	477	sense-overlappin	XM_011512531.1	LIPH
circRNA_03174	0	0	0	0	0.03009467	0	0	0	0	0.020172483	0	0	-	NC_000003.12	+	185590114	185613408	23295	832	sense-overlappin	XM_005247690.2	SENP2
circRNA_03175	0	0.020469968	0	0	0	0	0	0.040972591	0.040509055	0.020172483	0	0	-	NC_000003.12	+	185598412	185613408	14997	776	sense-overlappin	XM_005247690.2	SENP2
circRNA_03176	0	0	0	0	0.02006311	0	0	0	0	0.030258724	0	0	-	NC_000003.12	+	185600765	185613408	12644	575	sense-overlappin	XM_005247690.2	SENP2
circRNA_03177	0	0	0.032712968	0	0	0	0	0	0	0	0	0	-	NC_000003.12	+	185614564	185624082	9519	678	sense-overlappin	NM_021627.2	SENP2
circRNA_03178	0	0	0	0	0	0	0	0	0	0.030258724	0.02025523	0	-	NC_000003.12	+	185617480	185624082	6603	501	sense-overlappin	NM_021627.2	SENP2
circRNA_03179	0	0	0	0	0	0	0	0	0	0.020172483	0	0	hsa_circ_0006840	NC_000003.12	-	185921104	185922126	1023	200	sense-overlappin	NM_001243879.1	TRA2B
circRNA_03180	0	0	0.032712968	0.0619086	0.02006311	0.072495183	0	0	0.020254528	0	0	0	hsa_circ_0006248	NC_000003.12	-	185921104	185923984	2881	389	sense-overlappin	NM_001243879.1	TRA2B
circRNA_03181	0	0	0	0	0.02006311	0	0	0	0	0	0	0	hsa_circ_0005286	NC_000003.12	-	186079817	186081175	1359	418	sense-overlappin	NM_004454.2	ETV5
circRNA_03182	0	0	0	0	0	0	0	0	0	0	0.02025523	0	-	NC_000003.12	+	186581995	186582773	779	141	sense-overlappin	NM_016306.5	DNAJB11
circRNA_03183	0	0	0.032712968	0	0	0	0	0	0	0	0	0	hsa_circ_0068462	NC_000003.12	+	186786502	186787264	763	282	sense-overlappin	NM_001967.3	EIF4A2
circRNA_03184	0	0	0	0	0.03009467	0	0	0	0	0	0	0	hsa_circ_0003878	NC_000003.12	-	186791725	186804724	13000	812	sense-overlappin	NM_002916.3	RFC4
circRNA_03185	0	0	0	0	0.02006311	0	0	0	0	0	0	0	hsa_circ_0068482	NC_000003.12	+	187038742	187051346	12605	887	sense-overlappin	XM_011513085.1	ST6GAL1
circRNA_03186	0	0	0.054521613	0	0	0	0	0	0	0	0	0	-	NC_000003.12	-	187833424	187836179	2756	2756	intergenic		
circRNA_03187	0.030862537	0	0	0	0	0	0	0	0	0	0	0	hsa_circ_0006517	NC_000003.12	+	188484592	188524787	40196	236	sense-overlappin	XM_005247450.3	LPP
circRNA_03188	0.072012586	0.040939935	0	0.0309543	0	0.051782273	0	0	0	0.020172483	0	0.0206364	hsa_circ_0003759	NC_000003.12	+	188609161	188609844	684	684	exonic	XM_005247450.3	LPP
circRNA_03189	0.030862537	0	0	0	0	0	0	0.030729443	0	0	0	0	-	NC_000003.12	+	188708267	188760282	52016	297	sense-overlappin	XM_005247450.3	LPP
circRNA_03190	0.041150049	0	0	0	0	0	0	0	0	0	0	0	hsa_circ_0005842	NC_000003.12	+	188758632	188760282	1651	1651	sense-overlappin	XM_005247450.3	LPP
circRNA_03191	0	0	0	0	0.02006311	0	0	0	0	0	0	0	-	NC_000003.12	+	189127057	189132697	5641	453	sense-overlappin	XM_011512729.1	TPRG1
circRNA_03192	0	0	0.021808645	0	0	0	0	0	0	0	0	0	hsa_circ_0068514	NC_000003.12	+	189737740	189738774	1035	262	sense-overlappin	XM_011513253.1	TP63
circRNA_03193	0	0	0	0	0.02006311	0	0	0	0	0	0	0	-	NC_000003.12	+	189922793	189922864	72	72	intergenic		
circRNA_03194	0	0	0	0	0	0	0.030148028	0	0	0	0	0	-	NC_000003.12	-	194433772	194454392	20621	1615	sense-overlappin	NM_024524.3	ATP13A3
circRNA_03195	0	0	0.021808645	0	0.02006311	0	0	0	0	0.050431207	0	0	hsa_circ_0001376	NC_000003.12	-	194448457	194454392	5936	520	sense-overlappin	NM_024524.3	ATP13A3
circRNA_03196	0	0	0.032712968	0	0.10031557	0.020712909	0.030148028	0	0.020254528	0	0	0.0309546	hsa_circ_0005494	NC_000003.12	-	194459471	194462236	2766	525	sense-overlappin	NM_024524.3	ATP13A3
circRNA_03197	0	0	0	0	0.02006311	0	0	0	0	0	0	0	hsa_circ_0068550	NC_000003.12	-	194604287	194611020	6734	264	sense-overlappin	NM_001011655.2	TMEM44
circRNA_03198	0	0	0.021808645	0	0	0	0	0	0	0	0	0	-	NC_000003.12	-	195278859	195345317	66459	2647	sense-overlappin	XM_011512605.1	ACAP2
circRNA_03199	0	0	0	0	0	0	0.020098685	0	0	0	0	0	-	NC_000003.12	-	195291706	195308837	17132	1311	sense-overlappin	XM_011512605.1	ACAP2
circRNA_03200	0	0	0	0	0.03009467	0	0	0	0	0	0	0	hsa_circ_0002382	NC_000003.12	-	195295708	195302174	6467	556	sense-overlappin	NM_012287.5	ACAP2
circRNA_03201	0	0	0.098138903	0	0	0	0	0	0	0	0	0	-	NC_000003.12	-	195295708	195345317	49610	1408	sense-overlappin	XM_011512605.1	ACAP2
circRNA_03202	0	0	0.032712968	0	0	0	0.020098685	0	0	0	0	0	-	NC_000003.12	-	195301575	195302174	600	279	sense-overlappin	NM_012287.5	ACAP2
circRNA_03203	0	0	0.032712968	0	0	0	0	0	0	0	0	0	-	NC_000003.12	-	195306511	195345317	38807	852	sense-overlappin	XM_011512605.1	ACAP2
circRNA_03204	0	0	0.065425935	0	0	0	0	0	0	0.050431207	0	0	hsa_circ_0007347	NC_000003.12	-	195326885	195345317	18433	459	sense-overlappin	NM_012287.5	ACAP2
circRNA_03205	0	0	0	0	0	0	0	0	0	0.020172483	0	0	-	NC_000003.12	-	195342958	195344608	1651	1651	intronic	NM_012287.5	ACAP2

circRNA_03206	0	0	0.04361729	0	0.02006311	0	0	0	0	0.020172483	0	0	hsa_circ_0001996	NC_000003.12	-	195365482	195392147	26666	15759	sense-overlappin	NM_012287.5	ACAP2
circRNA_03207	0.030862537	0	0.294416708	0.0825448	0.09028402	0.031069364	0.070345399	0.061458886	0.060763583	0.020172483	0.04051046	0.051591	hsa_circ_0007331	NC_000003.12	-	195381009	195392147	11139	232	sense-overlappin	NM_012287.5	ACAP2
circRNA_03208	0	0	0.04361729	0	0.02006311	0	0	0	0	0	0	0	hsa_circ_0009013	NC_000003.12	-	195381009	195412916	31908	303	sense-overlappin	XM_011512604.1	ACAP2
circRNA_03209	0	0	0	0.0206362	0.02006311	0	0	0	0	0	0	0	-	NC_000003.12	-	195516842	195524896	8055	445	sense-overlappin	NM_001291504.1	PPP1R2
circRNA_03210	0.020575025	0	0.04361729	0	0	0	0	0	0	0	0	0	hsa_circ_0001377	NC_000003.12	-	195878253	195888606	10354	1274	sense-overlappin	XM_011512318.1	TNK2
circRNA_03211	0	0	0	0	0.05015779	0.031069364	0	0	0	0	0	0	hsa_circ_0008756	NC_000003.12	-	195959012	195960086	1075	1075	intergenic		
circRNA_03212	0	0	0	0	0	0	0	0	0.020254528	0	0	0	hsa_circ_0068610	NC_000003.12	-	196058284	196060247	1964	209	sense-overlappin	NM_003234.2	TFRC
circRNA_03213	0	0	0.021808645	0	0	0	0	0	0	0	0	0	hsa_circ_0068643	NC_000003.12	-	196362294	196362687	394	394	exonic	NM_015562.1	UBXN7
circRNA_03214	0	0	0	0	0	0	0	0	0	0.020172483	0	0	hsa_circ_0068651	NC_000003.12	-	196371896	196403019	31124	394	sense-overlappin	NM_015562.1	UBXN7
circRNA_03215	0	0	0.021808645	0	0	0	0	0.020486295	0	0	0	0	hsa_circ_0005051	NC_000003.12	-	196391813	196393619	1807	179	sense-overlappin	NM_015562.1	UBXN7
circRNA_03216	0.298337857	0.266109578	0.34893832	0.2373162	0.48151476	0.445327551	0.371825679	0.440455353	0.303817916	0.726209381	0.243062761	0.402409803	hsa_circ_0001380	NC_000003.12	-	196391813	196403019	11207	247	sense-overlappin	NM_015562.1	UBXN7
circRNA_03217	0	0	0	0	0	0.020712909	0	0	0	0	0	0	-	NC_000003.12	-	196391813	196407393	15581	395	sense-overlappin	NM_015562.1	UBXN7
circRNA_03218	0	0	0	0	0.03009467	0	0	0	0	0	0	0	-	NC_000003.12	-	196471640	196488683	17044	1594	sense-overlappin	NM_152617.3	RNF168
circRNA_03219	0	0	0	0	0.02006311	0	0.020098685	0	0	0	0	0	-	NC_000003.12	-	196483770	196488683	4914	379	sense-overlappin	NM_152617.3	RNF168
circRNA_03220	0	0.030704951	0.119947548	0.0412724	0.09028402	0.051782273	0	0.051215739	0	0.030258724	0.02025523	0	-	NC_000003.12	-	196487399	196488683	1285	257	sense-overlappin	NM_152617.3	RNF168
circRNA_03221	0.030862537	0	0	0	0	0	0	0	0	0	0	0	-	NC_000003.12	+	196716858	196731092	14235	575	sense-overlappin	NM_001166304.1	PIGX
circRNA_03222	0	0	0	0	0.03009467	0	0	0	0	0	0	0	-	NC_000003.12	+	196722415	196733815	11401	568	sense-overlappin	NM_001166304.1	PIGX
circRNA_03223	0	0	0.021808645	0	0	0	0	0	0	0	0	0	-	NC_000003.12	+	196779420	196782833	3414	3414	sense-overlappin	NM_002577.4	PAK2
circRNA_03224	0	0	0.032712968	0	0.02006311	0.041425819	0.040197371	0	0.040509055	0.020172483	0	0	hsa_circ_0003036	NC_000003.12	+	196806579	196807914	1336	241	sense-overlappin	NM_002577.4	PAK2
circRNA_03225	0	0	0.04361729	0	0.04012623	0.031069364	0.030148028	0.030729443	0.040509055	0	0.030382845	0.0206364	hsa_circ_0004950	NC_000003.12	+	196806579	196812851	6273	467	sense-overlappin	NM_002577.4	PAK2
circRNA_03226	0	0	0.08723458	0	0	0	0	0	0	0	0	0	hsa_circ_0068669	NC_000003.12	+	196885151	196886694	1544	1544	exonic	NM_152699.4	SENP5
circRNA_03227	0	0	0	0	0	0	0.020098685	0	0	0	0	0	-	NC_000003.12	+	196885151	196903610	18460	1915	sense-overlappin	NM_152699.4	SENP5
circRNA_03228	0	0	0.021808645	0	0	0	0	0	0	0	0	0	-	NC_000003.12	-	197051577	197066754	15178	528	sense-overlappin	NM_001204387.1	DLG1
circRNA_03229	0	0	0	0	0	0	0	0	0	0	0	0.0206364	hsa_circ_0068680	NC_000003.12	-	197051577	197069260	17684	606	sense-overlappin	NM_001204387.1	DLG1
circRNA_03230	0	0	0	0	0.02006311	0	0	0	0	0	0	0	-	NC_000003.12	-	197075837	197119530	43694	874	sense-overlappin	XR_246039.2	
circRNA_03231	0.061725074	0	0.021808645	0.0309543	0.03009467	0	0	0	0	0	0	0	hsa_circ_0007203	NC_000003.12	-	197081051	197119530	38480	740	sense-overlappin	NM_001204387.1	DLG1
circRNA_03232	0	0	0.04361729	0	0	0.020712909	0	0	0	0	0	0	hsa_circ_0007345	NC_000003.12	-	197085580	197119530	33951	673	sense-overlappin	NM_001204387.1	DLG1
circRNA_03233	0	0.020469968	0.065425935	0	0.02006311	0	0.040197371	0	0.020254528	0	0	0	hsa_circ_0008583	NC_000003.12	-	197090912	197119530	28619	496	sense-overlappin	NM_001204387.1	DLG1
circRNA_03234	0	0	0.163564838	0	0.03009467	0.031069364	0.030148028	0	0	0.07060369	0.04051046	0	hsa_circ_0008500	NC_000003.12	-	197104903	197119530	14628	381	sense-overlappin	NM_001204387.1	DLG1
circRNA_03235	0	0	0	0	0.03009467	0	0	0	0	0	0	0	hsa_circ_0068691	NC_000003.12	-	197104903	197142768	37866	1009	sense-overlappin	NM_001204387.1	DLG1
circRNA_03236	0.041150049	0	0.076330258	0.0309543	0	0.020712909	0	0	0.050636319	0	0	0.051591	hsa_circ_0001383	NC_000003.12	-	197115927	197119530	3604	278	sense-overlappin	NM_001204387.1	DLG1
circRNA_03237	0	0	0.054521613	0	0	0	0	0.020486295	0	0	0	0	hsa_circ_0068697	NC_000003.12	-	197119410	197142768	23359	749	sense-overlappin	NM_001204387.1	DLG1
circRNA_03238	0	0	0.04361729	0	0.05015779	0	0.020098685	0	0	0	0	0	-	NC_000003.12	-	197130527	197142768	12242	628	sense-overlappin	NM_001204387.1	DLG1
circRNA_03239	0	0	0.054521613	0.0206362	0.02006311	0	0.050246713	0.040972591	0	0	0	0	-	NC_000003.12	-	197130527	197149796	19270	682	sense-overlappin	NM_001204387.1	DLG1
circRNA_03240	0	0	0	0	0.03009467	0	0	0	0	0	0	0	hsa_circ_0068699	NC_000003.12	-	197136542	197142768	6227	483	sense-overlappin	NM_001204387.1	DLG1
circRNA_03241	0	0	0	0	0	0	0	0	0	0.020172483	0	0	-	NC_000003.12	-	197270700	197282845	12146	12146	sense-overlappin	NM_001098424.1	DLG1
circRNA_03242	0	0	0	0	0.02006311	0	0	0	0	0	0	0	-	NC_000003.12	-	197282679	197297784	15106	461	sense-overlappin	XM_005269289.2	DLG1
circRNA_03243	0	0	0	0	0	0	0	0	0	0.020172483	0	0	-	NC_000003.12	+	197756426	197763638	7213	7213	sense-overlappin	NM_001011537.2	FYTTD1
circRNA_03244	0	0.061409903	0	0	0	0	0	0	0	0	0	0	hsa_circ_0068723	NC_000003.12	+	197814908	197817302	2395	272	sense-overlappin	NM_032773.3	LRCH3
circRNA_03245	0.113162635	0.040939935	0.065425935	0.1031809	0.0702209	0.093208092	0.060296056	0.040972591	0.060763583	0.080689931	0	0.0206364	hsa_circ_0002266	NC_000003.12	+	197814908	197820430	5523	378	sense-overlappin	NM_032773.3	LRCH3
circRNA_03246	0	0	0	0	0.02006311	0.041425819	0	0	0	0	0	0	-	NC_000003.12	+	197814908	197832317	17410	840	sense-overlappin	NM_032773.3	LRCH3
circRNA_03247	0	0	0	0	0.02006311	0	0	0	0.020254528	0	0	0	hsa_circ_0068727	NC_000003.12	+	197817176	197820430	3255	233	sense-overlappin	NM_032773.3	LRCH3
circRNA_03248	0.030862537	0	0.032712968	0.0309543	0.03009467	0	0.020098685	0.020486295	0.070890847	0	0	0	hsa_circ_0006040	NC_000003.12	+	197830770	197839397	8628	441	sense-overlappin	NM_032773.3	LRCH3
circRNA_03249	0.051437562	0.030704951	0	0.0412724	0.04012623	0.020712909	0	0	0	0.030258724	0	0	hsa_circ_0068738	NC_000003.12	+	197830770	197866219	35450	986	sense-overlappin	NM_032773.3	LRCH3
circRNA_03250	0	0	0	0	0	0	0	0	0	0.020172483	0	0	-	NC_000003.12	+	197832197	197839397	7201	347	sense-overlappin	NM_032773.3	LRCH3
circRNA_03251	0.030862537	0	0	0	0	0	0	0	0	0	0	0.0309546	hsa_circ_0008106	NC_000003.12	+	197865423	197871462	6040	414	sense-overlappin	NM_032773.3	LRCH3
circRNA_03252	0.072012586	0.051174919	0.163564838	0.113499	0.09028402	0.093208092	0.070345399	0.081945182	0.050636319	0.181552345	0.050638075	0.113500201	hsa_circ_0008351	NC_000003.12	+	197865423	197875775	10353	492	sense-overlappin	XM_005269362.1	LRCH3
circRNA_03253	0.051437562	0	0	0.0412724	0	0	0	0	0	0	0	0	hsa_circ_0002319	NC_000003.12	+	197866112	197871462	5351	365	sense-overlappin	NM_032773.3	LRCH3
circRNA_03254	0.030862537	0	0	0	0	0	0	0	0	0	0	0	-	NC_000003.12	+	197980326	197985890	5565	380	sense-overlappin	NM_001136049.2	LMLN
circRNA_03255	0	0	0	0.0309543	0	0	0	0	0	0	0	0	hsa_circ_0068772	NC_000003.12	+	197980326	197999342	19017	683	sense-overlappin	NM_001136049.2	LMLN
circRNA_03256	0	0	0	0	0	0	0	0	0	0.030258724	0	0	-	NC_000003.12	+	197983943	198024788	40846	1039	sense-overlappin	NM_001136049.2	LMLN
circRNA_03257	0	0	0	0.0206362	0	0	0	0	0	0	0	0	-	NC_000003.12	+	198003015	198024788	21774	535	sense-overlappin	NM_001136049.2	LMLN
circRNA_03258	0	0	0	0	0	0	0	0.020486295	0	0	0	0	-	NC_000004.12	+	130788	131505	718	223	sense-overlappin	NM_001039127.5	ZNF718
circRNA_03259	0.051437562	0	0.04361729	0.0309543	0	0	0	0.020486295	0	0.020172483	0	0.0309546	-	NC_000004.12	+	253560	254837	1278	1278	intergenic		
circRNA_03260	0.020575025	0	0	0	0	0	0	0	0	0	0	0	-	NC_000004.12	+	505718	506866	1149	1149	sense-overlappin	XM_011513494.1	PIGG

circRNA_03261	0	0	0	0	0	0	0	0	0	0	0	0.0206364	hsa_circ_0068789	NC_000004.12	+	505718	507593	1876	399	sense-overlappin	XM_011513494.1	PIGG
circRNA_03262	0	0.020469968	0	0	0	0	0	0	0	0	0	0	hsa_circ_0068808	NC_000004.12	-	870711	884086	13376	1043	sense-overlappin	XM_011513429.1	GAK
circRNA_03263	0	0.020469968	0	0	0	0	0	0	0	0	0	0	hsa_circ_0068815	NC_000004.12	-	888847	898158	9312	680	sense-overlappin	XM_011513429.1	GAK
circRNA_03264	0.020575025	0	0	0	0	0	0	0	0	0	0	0	-	NC_000004.12	-	898033	904774	6742	264	sense-overlappin	XM_011513429.1	GAK
circRNA_03265	0.020575025	0	0	0	0	0	0	0	0	0.030258724	0	0	hsa_circ_0007179	NC_000004.12	-	898033	904779	6747	269	sense-overlappin	XM_011513429.1	GAK
circRNA_03266	0	0	0	0	0	0	0	0	0.040509055	0	0	0	hsa_circ_0005830	NC_000004.12	-	911673	913668	1996	237	sense-overlappin	XM_011513432.1	GAK
circRNA_03267	0	0	0.032712968	0	0	0	0	0	0	0	0	0	-	NC_000004.12	-	1212368	1241519	29152	1350	sense-overlappin	NM_001012614.1	CTBP1
circRNA_03268	0	0	0.021808645	0	0	0	0	0	0	0	0	0	hsa_circ_0068837	NC_000004.12	-	1225360	1238337	12978	507	sense-overlappin	NM_001012614.1	CTBP1
circRNA_03269	0	0.030704951	0.250799418	0.0206362	0.04012623	0	0.060296056	0	0.040509055	0.060517448	0.02025523	0	hsa_circ_0001386	NC_000004.12	-	1225360	1241519	16160	702	sense-overlappin	NM_001012614.1	CTBP1
circRNA_03270	0	0	0	0.0206362	0	0	0	0	0	0	0	0	-	NC_000004.12	+	1892166	1939778	47613	10370	sense-overlappin	NM_001042424.2	WHSC1
circRNA_03271	0.020575025	0	0.196277805	0.0722267	0	0.051782273	0.020098685	0	0.040509055	0	0	0.0206364	hsa_circ_0008460	NC_000004.12	+	1900626	1904378	3753	789	sense-overlappin	NM_001042424.2	WHSC1
circRNA_03272	0	0	0.04361729	0	0.03009467	0	0	0	0	0	0	0	hsa_circ_0004156	NC_000004.12	+	1900626	1918623	17998	1439	sense-overlappin	NM_001042424.2	WHSC1
circRNA_03273	0	0	0.109043225	0.0206362	0	0	0.030148028	0	0	0	0	0	hsa_circ_0002688	NC_000004.12	+	1900626	1930770	30145	1584	sense-overlappin	NM_001042424.2	WHSC1
circRNA_03274	0.030862537	0.030704951	0.163564838	0.0412724	0	0	0.020098685	0	0.020254528	0.020172483	0.04051046	0.0412728	hsa_circ_0001387	NC_000004.12	+	1900626	1935262	34637	1703	sense-overlappin	NM_001042424.2	WHSC1
circRNA_03275	0	0	0.021808645	0	0	0.020712909	0.040197371	0	0	0	0.06076569	0	hsa_circ_0005881	NC_000004.12	+	1900626	1939778	39153	1910	sense-overlappin	NM_001042424.2	WHSC1
circRNA_03276	0	0	0	0	0	0	0	0	0.020254528	0	0	0	hsa_circ_0003112	NC_000004.12	+	1904216	1918623	14408	813	sense-overlappin	NM_001042424.2	WHSC1
circRNA_03277	0	0	0.032712968	0	0	0	0	0	0	0	0	0	-	NC_000004.12	+	1904216	1935262	31047	1077	sense-overlappin	NM_001042424.2	WHSC1
circRNA_03278	0	0	0.032712968	0.0206362	0	0	0	0	0	0	0	0	hsa_circ_0068896	NC_000004.12	+	1918141	1918623	483	483	exonic	NM_001042424.2	WHSC1
circRNA_03279	0	0.020469968	0	0	0.03009467	0	0	0	0	0.030258724	0	0	hsa_circ_0008872	NC_000004.12	+	2490337	2497121	6785	281	sense-overlappin	NM_001185010.2	RNF4
circRNA_03280	0	0	0	0.0412724	0	0.031069364	0	0	0	0	0	0	hsa_circ_0004803	NC_000004.12	+	2490337	2500738	10402	361	sense-overlappin	NM_001185010.2	RNF4
circRNA_03281	0	0	0.054521613	0	0	0	0	0	0	0	0	0	hsa_circ_0008424	NC_000004.12	+	2625262	2626577	1316	302	sense-overlappin	XM_011513592.1	FAM193A
circRNA_03282	0	0	0.032712968	0.0206362	0	0	0	0	0	0	0	0	-	NC_000004.12	+	2657803	2663288	5486	768	sense-overlappin	XM_011513592.1	FAM193A
circRNA_03283	0	0	0.021808645	0	0.02006311	0	0	0	0	0	0	0	hsa_circ_0006291	NC_000004.12	+	2657803	2672372	14570	1020	sense-overlappin	XM_011513592.1	FAM193A
circRNA_03284	0	0	0	0.0206362	0	0	0	0	0.020254528	0	0	0	hsa_circ_0068926	NC_000004.12	+	2657803	2690970	33168	1492	sense-overlappin	XM_011513592.1	FAM193A
circRNA_03285	0	0	0	0	0	0	0	0	0	0	0	0.0206364	-	NC_000004.12	+	2657803	2700544	42742	3061	sense-overlappin	XM_006713930.2	FAM193A
circRNA_03286	0	0.030704951	0.021808645	0	0.03009467	0	0.050246713	0	0.030381792	0	0	0	hsa_circ_0003758	NC_000004.12	+	2699680	2700544	865	865	exonic	XM_006713930.2	FAM193A
circRNA_03287	0	0	0	0	0	0	0	0	0	0.020172483	0	0.0206364	hsa_circ_0068943	NC_000004.12	+	2875896	2884666	8771	530	sense-overlappin	XM_005247937.1	ADD1
circRNA_03288	0	0.051174919	0	0.0309543	0	0	0.040197371	0	0	0	0	0	hsa_circ_0006737	NC_000004.12	-	2949934	2951245	1312	412	sense-overlappin	NM_001291978.1	NOP14
circRNA_03289	0	0	0	0	0	0	0	0	0	0	0.02025523	0	hsa_circ_0068956	NC_000004.12	-	2949934	2953645	3712	670	sense-overlappin	NM_001291978.1	NOP14
circRNA_03290	0	0	0	0	0	0.031069364	0	0	0	0	0	0	-	NC_000004.12	+	2992215	3013828	21614	597	sense-overlappin	XM_011513457.1	GRK4
circRNA_03291	0.154312685	0.040939935	0.283512385	0.1650895	0.03009467	0.207129093	0.090444084	0.163890364	0.101272639	0.191638587	0.030382845	0.134136601	hsa_circ_0001392	NC_000004.12	+	3086939	3107423	20485	484	sense-overlappin	NM_002111.7	HTT
circRNA_03292	0	0	0	0.0206362	0	0	0	0	0.020254528	0	0	0	-	NC_000004.12	+	3186597	3189093	2497	502	sense-overlappin	NM_002111.7	HTT
circRNA_03293	0	0	0	0.0309543	0	0	0	0	0	0	0	0	hsa_circ_0069018	NC_000004.12	+	3204007	3209949	5943	838	sense-overlappin	NM_002111.7	HTT
circRNA_03294	0	0	0.065425935	0.0412724	0	0	0	0	0	0	0	0	hsa_circ_0002429	NC_000004.12	+	3316070	3318051	1982	1982	exonic	XR_427479.1	
circRNA_03295	0	0	0	0	0	0.031069364	0	0	0	0	0	0	hsa_circ_0005963	NC_000004.12	-	4237827	4246343	8517	410	sense-overlappin	NM_001297551.1	TMEM128
circRNA_03296	0	0	0.021808645	0	0	0	0	0	0	0	0	0	hsa_circ_0069031	NC_000004.12	-	4240321	4246343	6023	301	sense-overlappin	NM_001297551.1	TMEM128
circRNA_03297	0	0	0	0.0206362	0	0	0	0	0	0	0	0	-	NC_000004.12	-	4434770	4457257	22488	404	sense-overlappin	XM_011513484.1	STX18
circRNA_03298	0	0	0	0.0206362	0	0	0	0	0	0	0	0	-	NC_000004.12	-	4457191	4471706	14516	329	sense-overlappin	NM_016930.2	STX18
circRNA_03299	0	0	0	0	0.02006311	0.020712909	0	0	0	0.020172483	0	0	hsa_circ_0009031	NC_000004.12	+	4648977	4649542	566	566	intergenic		
circRNA_03300	0	0	0.04361729	0	0	0	0	0	0	0	0	0	hsa_circ_0004228	NC_000004.12	-	5663107	5665649	2543	275	sense-overlappin	XM_011513393.1	EVC2
circRNA_03301	0	0	0.021808645	0	0	0	0	0	0	0	0	0	hsa_circ_0069064	NC_000004.12	+	5729307	5756362	27056	1263	sense-overlappin	XR_924928.1	
circRNA_03302	0	0	0	0	0	0	0	0	0	0.020172483	0	0	hsa_circ_0069086	NC_000004.12	+	6598198	6600756	2559	291	sense-overlappin	NM_001292038.1	MAN2B2
circRNA_03303	0	0	0.08723458	0.0206362	0	0.031069364	0	0	0	0	0.04051046	0	hsa_circ_0001394	NC_000004.12	+	6923373	6924111	739	739	exonic	XM_011513508.1	TBC1D14
circRNA_03304	0	0	0.021808645	0	0	0	0	0	0	0	0	0	hsa_circ_0069101	NC_000004.12	+	6923373	6967424	44052	860	sense-overlappin	XM_011513508.1	TBC1D14
circRNA_03305	0	0.040939935	0.08723458	0.0515905	0	0	0.020098685	0.040972591	0.020254528	0.030258724	0	0	hsa_circ_0006681	NC_000004.12	+	6994184	7001251	7068	427	sense-overlappin	XM_011513508.1	TBC1D14
circRNA_03306	0.030862537	0	0	0	0	0	0	0	0	0	0	0	hsa_circ_0003201	NC_000004.12	+	6996325	7004924	8600	389	sense-overlappin	XM_011513508.1	TBC1D14
circRNA_03307	0	0	0	0	0	0.031069364	0	0	0	0.030258724	0	0	-	NC_000004.12	+	6999085	7004924	5840	306	sense-overlappin	XM_011513508.1	TBC1D14
circRNA_03308	0	0	0	0.0206362	0	0.020712909	0	0	0	0	0	0	-	NC_000004.12	+	7006632	7014557	7926	508	sense-overlappin	XM_011513508.1	TBC1D14
circRNA_03309	0	0	0	0	0.03009467	0	0	0	0	0	0.02025523	0	hsa_circ_0002515	NC_000004.12	-	7855466	7872080	16615	336	sense-overlappin	NM_001134647.1	AFAP1
circRNA_03310	0	0.020469968	0.08723458	0.0309543	0.16050492	0.041425819	0.050246713	0.030729443	0	0	0.02025523	0	hsa_circ_0069152	NC_000004.12	-	7868622	7872080	3459	227	sense-overlappin	NM_001134647.1	AFAP1
circRNA_03311	0	0	0	0	0	0	0	0	0	0	0.02025523	0	-	NC_000004.12	-	9610284	9610926	643	643	antisense	XM_011513918.1	LOC101928948
circRNA_03312	0	0	0.207182128	0	0	0	0	0.030729443	0	0	0	0	-	NC_000004.12	+	9641071	9641431	361	361	intronic	XM_011513918.1	LOC101928948
circRNA_03313	0	0	1.046814961	0.0515905	0.02006311	0	0.241184224	0.081945182	0.081018111	0.050431207	0.12153138	0.0412728	-	NC_000004.12	+	9641071	9641450	380	380	intronic	XM_011513918.1	LOC101928948
circRNA_03314	0.072012586	0.061409903	0.08723458	0.0309543	0.03009467	0.103564547	0.030148028	0.030729443	0.040509055	0.060517448	0	0.0206364	hsa_circ_0003550	NC_000004.12	-	10097711	10103986	6276	420	sense-overlappin	NM_017491.3	WDR1
circRNA_03315	0	0	0.032712968	0	0	0	0	0	0	0	0	0	-	NC_000004.12	+	11119737	11161821	42085	42085	intergenic		

circRNA_03316	0	0	0	0	0	0.020712909	0	0	0	0	0	0	hsa_circ_0004173	NC_000004.12	-	13336893	13381594	44702	39834	sense-overlappin	XM_011513911.1	RAB28
circRNA_03317	0	0	0	0	0.02006311	0	0	0	0	0	0	0	-	NC_000004.12	-	13371795	13381594	9800	255	sense-overlappin	NM_001159601.1	RAB28
circRNA_03318	0	0	0.021808645	0	0	0	0	0	0	0	0	0	-	NC_000004.12	-	13423412	13428237	4826	4826	sense-overlappin	XR_925363.1	
circRNA_03319	0	0	0	0	0.03009467	0	0	0	0	0	0	0	-	NC_000004.12	-	13576838	13587771	10934	803	sense-overlappin	XM_011513830.1	BOD1L1
circRNA_03320	0	0	0	0	0	0	0	0	0	0.030258724	0	0	hsa_circ_0069226	NC_000004.12	-	15612266	15644708	32443	2019	sense-overlappin	NR_036464.1	
circRNA_03321	0	0	0	0	0	0.020712909	0	0	0	0	0	0	-	NC_000004.12	-	15625252	15630791	5540	1084	sense-overlappin	NM_001193534.1	FBXL5
circRNA_03322	0.072012586	0.020469968	0.141756193	0.0412724	0.03009467	0	0	0	0.040509055	0	0	0.0309546	hsa_circ_0069227	NC_000004.12	-	15625252	15644708	19457	1870	sense-overlappin	NR_036464.1	
circRNA_03323	0	0	0	0	0	0	0	0	0	0.020172483	0	0	-	NC_000004.12	-	15630666	15644657	13992	861	sense-overlappin	NR_036464.1	
circRNA_03324	0	0.020469968	0	0	0	0	0	0	0	0	0	0	-	NC_000004.12	-	15630666	15644708	14043	912	sense-overlappin	NR_036464.1	
circRNA_03325	0	0	0	0	0	0	0	0	0.020254528	0.020172483	0	0	-	NC_000004.12	-	15636494	15644708	8215	786	sense-overlappin	NR_036464.1	
circRNA_03326	0	0	0	0	0	0	0	0	0	0	0	0.0309546	-	NC_000004.12	-	15984263	15987716	3454	297	sense-overlappin	NM_001145849.1	PROM1
circRNA_03327	0	0	0.032712968	0	0	0	0	0	0	0	0	0	-	NC_000004.12	+	16238771	16323036	84266	84266	intergenic		
circRNA_03328	0	0	0	0	0.04012623	0	0	0	0	0	0	0	hsa_circ_0009006	NC_000004.12	-	16585922	16595875	9954	380	sense-overlappin	NM_001130834.2	LDB2
circRNA_03329	0	0	0	0	0	0.031069364	0	0	0	0	0	0	-	NC_000004.12	+	17617815	17618611	797	797	intronic	NM_025205.4	MED28
circRNA_03330	0	0	0	0	0	0.020712909	0	0	0	0	0	0	hsa_circ_0001395	NC_000004.12	+	17814853	17815358	506	231	sense-overlappin	NM_022346.4	NCAPG
circRNA_03331	0	0	0	0.0206362	0	0	0	0	0	0	0	0	-	NC_000004.12	-	17909094	17972885	63792	528	sense-overlappin	XM_011513824.1	LCORL
circRNA_03332	0	0.040939935	0	0.0206362	0	0	0	0	0	0.030258724	0	0	hsa_circ_0003190	NC_000004.12	-	17960265	17972885	12621	1914	sense-overlappin	XM_011513824.1	LCORL
circRNA_03333	0	0.020469968	0.04361729	0.0206362	0.02006311	0	0	0	0	0.050431207	0	0.0206364	hsa_circ_0069285	NC_000004.12	-	17961903	17972885	10983	276	sense-overlappin	XM_011513824.1	LCORL
circRNA_03334	0	0	0	0	0	0	0	0	0	0	0	0.0206364	-	NC_000004.12	-	17961903	18009978	48076	37369	sense-overlappin	XM_011513824.1	LCORL
circRNA_03335	0	0	0	0.0206362	0	0	0	0	0	0	0	0	-	NC_000004.12	+	20707803	20727397	19595	738	sense-overlappin	XM_011513799.1	PACRGL
circRNA_03336	0	0	0.054521613	0	0	0	0	0	0	0	0	0	hsa_circ_0069320	NC_000004.12	-	22389088	22402799	13712	491	sense-overlappin	XM_011513811.1	ADGRA3
circRNA_03337	0	0.020469968	0	0	0	0	0	0.030729443	0	0	0	0	hsa_circ_0002785	NC_000004.12	-	22401431	22402799	1369	249	sense-overlappin	XM_011513811.1	ADGRA3
circRNA_03338	0	0.020469968	0	0	0	0	0	0	0	0	0	0	-	NC_000004.12	+	22735981	22748960	12980	12980	intergenic		
circRNA_03339	0	0	0	0	0	0	0	0.020486295	0	0	0	0	-	NC_000004.12	+	22747296	22748960	1665	1665	intergenic		
circRNA_03340	0	0	0	0	0	0	0.020098685	0	0	0	0	0	hsa_circ_0002518	NC_000004.12	-	24570654	24576678	6025	630	sense-overlappin	NM_001358.2	DHX15
circRNA_03341	0	0	0	0	0.02006311	0	0	0	0	0	0	0	-	NC_000004.12	-	24592994	24597408	4415	4415	intergenic		
circRNA_03342	0	0	0	0.0309543	0	0	0	0	0	0	0	0	-	NC_000004.12	-	24853072	24876697	23626	309	sense-overlappin	XM_011513907.1	CCDC149
circRNA_03343	0.041150049	0.040939935	0	0.0412724	0.02006311	0.062138728	0	0	0.020254528	0.040344966	0	0	hsa_circ_0069335	NC_000004.12	-	25127264	25159107	31844	1006	sense-overlappin	NM_016955.3	SEPSECS
circRNA_03344	0	0	0.04361729	0.0206362	0	0	0.030148028	0	0.020254528	0	0	0	hsa_circ_0006259	NC_000004.12	-	25144774	25159107	14334	912	sense-overlappin	NM_016955.3	SEPSECS
circRNA_03345	0	0	0.021808645	0	0	0	0.030148028	0.020486295	0.020254528	0	0	0	hsa_circ_0069338	NC_000004.12	-	25151960	25159107	7148	690	sense-overlappin	NM_016955.3	SEPSECS
circRNA_03346	0	0	0.021808645	0	0	0	0	0	0	0	0	0	hsa_circ_0069340	NC_000004.12	-	25154998	25159107	4110	587	sense-overlappin	NM_016955.3	SEPSECS
circRNA_03347	0	0	0	0	0	0	0	0	0	0	0.050638075	0	-	NC_000004.12	+	25256543	25263849	7307	454	sense-overlappin	NM_018323.3	PI4K2B
circRNA_03348	0	0	0	0	0	0	0	0	0	0	0	0.0412728	hsa_circ_0001397	NC_000004.12	+	25314046	25315400	1355	202	sense-overlappin	NM_024936.2	ZCCHC4
circRNA_03349	0	0.030704951	0.098138903	0.0412724	0	0.020712909	0.020098685	0.071702034	0.040509055	0.040344966	0	0	hsa_circ_0001398	NC_000004.12	+	25333183	25333988	806	357	sense-overlappin	NM_024936.2	ZCCHC4
circRNA_03350	0	0	0	0.0309543	0	0	0	0	0	0	0	0	hsa_circ_0007107	NC_000004.12	+	25349492	25364505	15014	547	sense-overlappin	XM_011513836.1	ZCCHC4
circRNA_03351	0	0	0	0	0	0	0.020098685	0	0	0	0	0	-	NC_000004.12	-	25757534	25847408	89875	2641	sense-overlappin	XM_011513820.1	SEL1L3
circRNA_03352	0	0	0.021808645	0	0	0	0	0	0	0	0	0	-	NC_000004.12	-	25765326	25790574	25249	999	sense-overlappin	XM_011513820.1	SEL1L3
circRNA_03353	0	0	0	0	0	0	0	0	0	0.030258724	0	0	-	NC_000004.12	-	25782242	25790574	8333	501	sense-overlappin	XM_011513820.1	SEL1L3
circRNA_03354	0	0	0	0	0	0	0	0	0	0.020172483	0	0	-	NC_000004.12	-	25802283	25822128	19846	799	sense-overlappin	XM_011513820.1	SEL1L3
circRNA_03355	0	0	0	0	0	0	0	0	0.030381792	0	0	0	hsa_circ_0069366	NC_000004.12	-	25802283	25830156	27874	858	sense-overlappin	XM_011513820.1	SEL1L3
circRNA_03356	0	0	0	0	0	0.020712909	0	0	0	0	0	0	-	NC_000004.12	-	25802283	25833569	31287	1096	sense-overlappin	XM_011513820.1	SEL1L3
circRNA_03357	0	0.020469968	0	0	0	0	0	0	0	0	0	0	hsa_circ_0069370	NC_000004.12	-	25818138	25822128	3991	407	sense-overlappin	XM_011513820.1	SEL1L3
circRNA_03358	0	0	0	0	0	0	0	0	0.030381792	0	0	0	-	NC_000004.12	-	25818138	25835323	17186	831	sense-overlappin	XM_011513820.1	SEL1L3
circRNA_03359	0	0	0	0	0	0	0.020098685	0	0	0	0	0	-	NC_000004.12	-	25832995	25835323	2329	365	sense-overlappin	XM_011513820.1	SEL1L3
circRNA_03360	0.020575025	0.030704951	0.021808645	0.0206362	0	0.020712909	0.050246713	0.030729443	0.030381792	0.040344966	0.02025523	0.082545601	-	NC_000004.12	-	25847294	25847864	571	571	exonic	NM_015187.4	SEL1L3
circRNA_03361	0	0	0	0	0	0	0	0	0	0.020172483	0	0	-	NC_000004.12	+	26386353	26420725	34373	476	sense-overlappin	XM_005248161.2	RBPJ
circRNA_03362	0	0	0	0	0	0	0.020098685	0	0	0	0	0	hsa_circ_0069377	NC_000004.12	+	26386353	26424743	38391	727	sense-overlappin	XM_005248161.2	RBPJ
circRNA_03363	0	0	0	0	0	0	0.020098685	0	0	0	0	0	-	NC_000004.12	+	26424342	26424743	402	251	sense-overlappin	XM_005248161.2	RBPJ
circRNA_03364	0.020575025	0	0	0	0	0	0	0	0.020254528	0	0	0	-	NC_000004.12	+	26919504	26957726	38223	246	sense-overlappin	NM_001169117.1	STIM2
circRNA_03365	0.020575025	0	0	0	0	0	0	0	0	0	0	0	-	NC_000004.12	+	30920153	30922583	2431	247	sense-overlappin	XM_011513842.1	PCDH7
circRNA_03366	0.061725074	0.08187987	0	0.0825448	0	0.041425819	0.070345399	0.061458886	0.020254528	0.060517448	0	0.0619092	-	NC_000004.12	+	30920153	30950215	30063	15151	sense-overlappin	XM_011513842.1	PCDH7
circRNA_03367	0	0	0	0	0	0	0	0	0.020254528	0	0	0	-	NC_000004.12	-	36177827	36193647	15821	370	sense-overlappin	NM_015230.3	ARAP2
circRNA_03368	0	0	0	0	0.04012623	0	0	0	0	0	0.02025523	0	hsa_circ_0069396	NC_000004.12	-	36210390	36214480	4091	582	sense-overlappin	NM_015230.3	ARAP2
circRNA_03369	0	0.020469968	0.065425935	0	0	0.020712909	0	0	0	0.060517448	0	0	hsa_circ_0069397	NC_000004.12	-	36210390	36229645	19256	1646	sense-overlappin	NM_015230.3	ARAP2
circRNA_03370	0.041150049	0.051174919	0.08723458	0.0515905	0.04012623	0.176059729	0.060296056	0.020486295	0.050636319	0.090776173	0.02025523	0.051591	hsa_circ_0069399	NC_000004.12	-	36228582	36229645	1064	1064	exonic	NM_015230.3	ARAP2
circRNA_03371	0.041150049	0	0.076330258	0.0309543	0	0.072495183	0	0	0	0.040344966	0	0.051591	hsa_circ_0002194	NC_000004.12	-	37631385	37635123	3739	376	sense-overlappin	XR_427509.1	

circRNA_03372	0.164600197	0.276344562	0.39255561	0.5881314	0.15047336	0.828516374	0.190937511	0.430212205	0.344326971	0.726209381	0.12153138	0.361137003	hsa_circ_0001400	NC_000004.12	-	37631385	37638504	7120	434	sense-overlappin	XR_427509.1	
circRNA_03373	0	0	0	0	0	0	0	0.030729443	0	0	0	0	-	NC_000004.12	+	37985897	37985989	93	93	intronic	XM_011513668.1	TBC1D1
circRNA_03374	0	0.020469968	0	0	0	0	0	0	0	0	0	0	-	NC_000004.12	+	38027788	38054338	26551	1122	sense-overlappin	XM_011513668.1	TBC1D1
circRNA_03375	0.020575025	0	0	0	0	0	0	0	0	0	0	0	-	NC_000004.12	+	38030364	38049898	19535	5832	sense-overlappin	XM_011513668.1	TBC1D1
circRNA_03376	0	0	0	0	0	0	0.030148028	0	0	0	0	0	-	NC_000004.12	+	38035588	38049898	14311	608	sense-overlappin	XM_011513668.1	TBC1D1
circRNA_03377	0.030862537	0.030704951	0.032712968	0	0	0	0	0	0.020254528	0	0	0	hsa_circ_0069420	NC_000004.12	+	38035588	38054338	18751	1030	sense-overlappin	XM_011513668.1	TBC1D1
circRNA_03378	0	0	0.021808645	0	0.03009467	0	0.070345399	0.030729443	0	0	0	0.0309546	hsa_circ_0007820	NC_000004.12	+	38054199	38103157	48959	686	sense-overlappin	XM_005262646.1	TBC1D1
circRNA_03379	0	0	0.119947548	0.0412724	0.04012623	0.093208092	0.110542769	0.051215739	0.060763583	0.100862414	0.04051046	0.0412728	hsa_circ_0001402	NC_000004.12	+	38089932	38103157	13226	507	sense-overlappin	XM_005262646.1	TBC1D1
circRNA_03380	0.030862537	0	0.021808645	0	0	0	0	0	0	0	0	0	hsa_circ_0007486	NC_000004.12	+	38089932	38118192	28261	912	sense-overlappin	XM_005262646.1	TBC1D1
circRNA_03381	0	0	0.021808645	0	0	0	0.020098685	0	0	0	0	0	-	NC_000004.12	+	38089932	38125131	35200	1082	sense-overlappin	XM_005262646.1	TBC1D1
circRNA_03382	0	0.020469968	0	0	0	0	0	0	0	0.07060369	0	0.0309546	-	NC_000004.12	-	38790070	38791231	1162	1162	exonic	XR_925165.1	
circRNA_03383	0	0	0	0.0206362	0	0.062138728	0	0.040972591	0	0	0	0.0309546	-	NC_000004.12	-	38826685	38829537	2853	2853	exonic	NM_006068.4	TLR6
circRNA_03384	0	0	0	0	0.03009467	0	0	0	0	0	0	0	-	NC_000004.12	+	38878071	38943515	65445	1658	sense-overlappin	NM_138389.2	FAM114A1
circRNA_03385	0.092587611	0.020469968	0	0	0	0	0	0	0	0.020172483	0	0	-	NC_000004.12	+	38931451	38935790	4340	375	sense-overlappin	NM_138389.2	FAM114A1
circRNA_03386	0	0	0	0	0	0	0	0	0.020254528	0	0	0	hsa_circ_0069438	NC_000004.12	-	38986336	38993998	7663	465	sense-overlappin	XM_011513749.1	TMEM156
circRNA_03387	0	0	0	0	0.03009467	0	0	0	0	0	0	0	hsa_circ_0069442	NC_000004.12	+	39075965	39081239	5275	320	sense-overlappin	XM_011513699.1	KLHL5
circRNA_03388	0.030862537	0.030704951	0	0.0515905	0.06018934	0.020712909	0.050246713	0.030729443	0	0.020172483	0	0	hsa_circ_0069443	NC_000004.12	+	39075965	39086727	10763	730	sense-overlappin	XM_011513699.1	KLHL5
circRNA_03389	0	0	0	0	0	0	0	0	0	0	0	0.0206364	-	NC_000004.12	+	39224884	39228690	3807	503	sense-overlappin	XM_011513725.1	WDR19
circRNA_03390	0	0	0	0	0	0.020712909	0	0	0	0	0	0	hsa_circ_0008596	NC_000004.12	-	39300021	39303151	3131	698	sense-overlappin	NM_001204747.1	RFC1
circRNA_03391	0	0	0	0	0	0	0.020098685	0	0	0	0	0	-	NC_000004.12	-	39304814	39323417	18604	1471	sense-overlappin	NM_001204747.1	RFC1
circRNA_03392	0.051437562	0	0.054521613	0.0412724	0.05015779	0	0	0	0	0.060517448	0	0.0412728	hsa_circ_0001403	NC_000004.12	-	39326563	39327756	1194	311	sense-overlappin	NM_001204747.1	RFC1
circRNA_03393	0	0	0	0	0.03009467	0	0	0	0	0	0	0	-	NC_000004.12	+	39733355	39745810	12456	4218	sense-overlappin	NM_001111112.1	UBE2K
circRNA_03394	0	0.040939935	0	0	0	0	0	0	0	0.020172483	0	0.0619092	-	NC_000004.12	+	39733359	39745810	12452	4214	sense-overlappin	NM_001111112.1	UBE2K
circRNA_03395	0.020575025	0	0	0	0	0	0.020098685	0	0	0	0	0	-	NC_000004.12	+	39737420	39745810	8391	153	sense-overlappin	NM_001111112.1	UBE2K
circRNA_03396	0	0.040939935	0.032712968	0.0515905	0	0.020712909	0	0	0	0.060517448	0	0	hsa_circ_0009154	NC_000004.12	+	39737420	39755739	18320	236	sense-overlappin	NM_001111112.1	UBE2K
circRNA_03397	0	0	0.109043225	0.0825448	0.05015779	0.072495183	0	0.030729443	0.020254528	0.060517448	0	0.0206364	hsa_circ_0002590	NC_000004.12	+	39737420	39774933	37514	336	sense-overlappin	NM_001111112.1	UBE2K
circRNA_03398	0	0	0	0	0	0.020712909	0	0	0.020254528	0	0	0	hsa_circ_0069492	NC_000004.12	+	39737420	39777810	40391	465	sense-overlappin	NM_005339.4	UBE2K
circRNA_03399	0	0	0.04361729	0	0	0	0	0	0	0	0	0	-	NC_000004.12	-	39837478	39842056	4579	840	sense-overlappin	XM_011513674.1	PDS5A
circRNA_03400	0	0	0	0	0	0	0	0	0.030381792	0	0	0	-	NC_000004.12	-	39837856	39842056	4201	462	sense-overlappin	XM_011513674.1	PDS5A
circRNA_03401	0	0	0	0	0	0	0	0	0	0	0.04051046	0	-	NC_000004.12	-	39837856	39845880	8025	671	sense-overlappin	XM_011513674.1	PDS5A
circRNA_03402	0	0	0	0	0	0.020712909	0	0.020486295	0	0	0	0.0309546	hsa_circ_0005668	NC_000004.12	-	39841948	39845880	3933	318	sense-overlappin	XM_011513674.1	PDS5A
circRNA_03403	0	0	0	0	0	0	0	0	0.030381792	0	0	0	-	NC_000004.12	-	39848851	39849652	802	253	sense-overlappin	XM_011513674.1	PDS5A
circRNA_03404	0	0	0	0	0.02006311	0	0	0	0	0	0.02025523	0	-	NC_000004.12	-	39862219	39890364	28146	1316	sense-overlappin	XM_011513674.1	PDS5A
circRNA_03405	0	0	0	0	0.03009467	0	0	0	0	0	0	0	-	NC_000004.12	-	39872986	39874412	1427	283	sense-overlappin	XM_011513674.1	PDS5A
circRNA_03406	0	0	0	0	0	0	0.040197371	0.030729443	0	0	0	0.0309546	-	NC_000004.12	-	39872986	39890364	17379	666	sense-overlappin	XM_011513674.1	PDS5A
circRNA_03407	0	0	0	0.0412724	0	0	0	0	0	0	0	0	-	NC_000004.12	-	39872986	39902460	29475	1051	sense-overlappin	XM_011513674.1	PDS5A
circRNA_03408	0	0	0	0	0	0	0.060296056	0.020486295	0	0	0	0	hsa_circ_0001405	NC_000004.12	-	39898389	39902460	4072	385	sense-overlappin	XM_011513674.1	PDS5A
circRNA_03409	0	0	0.054521613	0	0	0	0	0	0	0	0	0	-	NC_000004.12	-	39904040	39928164	24125	1247	sense-overlappin	XM_011513674.1	PDS5A
circRNA_03410	0.072012586	0.051174919	0.054521613	0.0206362	0.03009467	0.124277456	0.070345399	0.051215739	0.070890847	0	0.091148535	0.092863801	hsa_circ_0007308	NC_000004.12	-	39913611	39925933	12323	563	sense-overlappin	XM_011513674.1	PDS5A
circRNA_03411	0	0	0	0	0	0.020712909	0	0	0	0	0	0	-	NC_000004.12	+	40197306	40197416	111	111	intronic	NM_001278368.1	RHOH
circRNA_03412	0	0	0	0	0.02006311	0	0	0	0	0	0	0	-	NC_000004.12	-	40589870	40596800	6931	6931	sense-overlappin	XM_011513703.1	RBM47
circRNA_03413	0	0.020469968	0	0.0412724	0.03009467	0.031069364	0.030148028	0	0	0.060517448	0.02025523	0	-	NC_000004.12	-	40594258	40596800	2543	2543	sense-overlappin	XM_011513703.1	RBM47
circRNA_03414	0	0	0	0	0	0	0	0	0.030381792	0	0	0	-	NC_000004.12	-	40594258	40599087	4830	4830	sense-overlappin	XM_011513703.1	RBM47
circRNA_03415	0.030862537	0	0	0	0	0	0	0	0	0	0	0	hsa_circ_0069558	NC_000004.12	-	40827132	40830577	3446	203	sense-overlappin	NM_001166051.1	APBB2
circRNA_03416	0	0	0.021808645	0	0	0	0	0	0	0	0	0	hsa_circ_0069561	NC_000004.12	-	40890364	40945070	54707	691	sense-overlappin	NM_001166050.1	APBB2
circRNA_03417	0	0	0	0.0412724	0	0	0	0	0	0	0	0	hsa_circ_0069562	NC_000004.12	-	40890364	40945073	54710	694	sense-overlappin	NM_001166050.1	APBB2
circRNA_03418	0	0	0	0	0	0	0	0.020486295	0	0	0	0	-	NC_000004.12	-	40901819	40903200	1382	1382	intronic	NM_001166050.1	APBB2
circRNA_03419	0.072012586	0	0	0.0412724	0.03009467	0	0.020098685	0	0	0.020172483	0.02025523	0	hsa_circ_0008453	NC_000004.12	-	40934456	40945070	10615	416	sense-overlappin	NM_001166050.1	APBB2
circRNA_03420	0	0	0	0	0	0	0	0	0.020254528	0.020172483	0	0	hsa_circ_0002686	NC_000004.12	-	40934456	40945073	10618	419	sense-overlappin	NM_001166050.1	APBB2
circRNA_03421	0.020575025	0	0.032712968	0	0	0	0	0	0.020254528	0.020172483	0	0	hsa_circ_0005991	NC_000004.12	-	41013583	41014398	816	816	exonic	NM_001166050.1	APBB2
circRNA_03422	0	0	0	0.0206362	0	0	0	0	0	0	0	0	hsa_circ_0069565	NC_000004.12	-	41013583	41033304	19722	885	sense-overlappin	NM_001166050.1	APBB2
circRNA_03423	0	0	0	0.0309543	0	0	0	0	0	0	0	0	hsa_circ_0004124	NC_000004.12	+	41661420	41671594	10175	477	sense-overlappin	NM_001289124.1	LIMCH1
circRNA_03424	0	0	0	0.0412724	0	0	0	0	0.030381792	0	0	0	-	NC_000004.12	+	42001616	42023384	21769	501	sense-overlappin	XM_011513620.1	SLC30A9
circRNA_03425	0	0.020469968	0	0	0	0	0	0	0	0	0	0	hsa_circ_0069590	NC_000004.12	+	42001616	42049479	47864	731	sense-overlappin	XM_011513620.1	SLC30A9
circRNA_03426	0	0	0.032712968	0	0	0	0	0	0	0	0	0	hsa_circ_0069594	NC_000004.12	+	42018111	42023384	5274	336	sense-overlappin	XM_011513620.1	SLC30A9

circRNA_03427	0	0	0	0	0	0.031069364	0	0	0	0	0	0	-	NC_000004.12	+	42075657	42086570	10914	733	sense-overlappin	NM_006345.3	SLC30A9
circRNA_03428	0	0	0	0.0206362	0	0	0	0	0	0	0	0	hsa_circ_0069607	NC_000004.12	-	42485496	42507154	21659	398	sense-overlappin	XM_011513619.1	ATP8A1
circRNA_03429	0	0	0.021808645	0	0	0	0	0	0	0	0.02025523	0	hsa_circ_0069612	NC_000004.12	-	42503450	42524847	21398	429	sense-overlappin	XM_011513619.1	ATP8A1
circRNA_03430	0	0	0	0.0619086	0	0	0	0	0	0	0	0	hsa_circ_0069613	NC_000004.12	-	42503450	42543986	40537	499	sense-overlappin	XM_011513619.1	ATP8A1
circRNA_03431	0	0	0	0	0	0	0	0	0	0	0.02025523	0	hsa_circ_0069615	NC_000004.12	-	42543917	42556040	12124	382	sense-overlappin	XM_011513619.1	ATP8A1
circRNA_03432	0	0	0.021808645	0	0	0	0	0	0	0	0	0	hsa_circ_0069621	NC_000004.12	-	42624536	42627109	2574	314	sense-overlappin	XM_011513619.1	ATP8A1
circRNA_03433	0	0	0	0.0412724	0	0	0	0	0	0	0	0	-	NC_000004.12	-	44293404	44311727	18324	18013	sense-overlappin	XM_011513689.1	KCTD8
circRNA_03434	0	0	0	0.0309543	0	0	0	0	0	0	0	0	-	NC_000004.12	-	44293404	44323645	30242	29931	sense-overlappin	XM_011513689.1	KCTD8
circRNA_03435	0	0	0	0	0	0	0	0.020486295	0	0	0	0	-	NC_000004.12	-	47884346	47890703	6358	464	sense-overlappin	NM_001278624.1	NFXL1
circRNA_03436	0	0.061409903	0.163564838	0.0928628	0.06018934	0.176059729	0.120592112	0.040972591	0.060763583	0.090776173	0	0.082545601	-	NC_000004.12	+	48369849	48383784	13936	971	sense-overlappin	XM_005248121.1	SLAIN2
circRNA_03437	0.072012586	0.040939935	0.08723458	0.0309543	0	0	0.020098685	0.030729443	0	0	0	0	-	NC_000004.12	+	48369849	48394653	24805	1049	sense-overlappin	XM_005248121.1	SLAIN2
circRNA_03438	0	0	0	0.0206362	0	0	0	0	0	0	0	0	-	NC_000004.12	-	48534545	48542121	7577	1113	sense-overlappin	NM_015030.1	FRYL
circRNA_03439	0	0	0	0	0	0	0	0	0	0.030258724	0	0	-	NC_000004.12	-	48590659	48605833	15175	811	sense-overlappin	XM_011513681.1	FRYL
circRNA_03440	0	0	0	0	0.03009467	0	0	0	0	0	0	0	hsa_circ_0004669	NC_000004.12	-	48684673	48687160	2488	2488	sense-overlappin	NM_015030.1	FRYL
circRNA_03441	0.041150049	0.040939935	0.065425935	0.0825448	0.05015779	0	0.040197371	0.030729443	0.050636319	0.050431207	0.04051046	0	hsa_circ_0002021	NC_000004.12	-	48684673	48710698	26026	303	sense-overlappin	NM_015030.1	FRYL
circRNA_03442	0	0	0	0	0.02006311	0	0	0	0	0	0	0	-	NC_000004.12	+	48842636	48857365	14730	561	sense-overlappin	NM_017830.3	OCIAD1
circRNA_03443	0	0	0	0	0.02006311	0	0	0	0	0	0	0	-	NC_000004.12	+	51848205	51891851	43647	644	sense-overlappin	NM_001287757.1	DCUN1D4
circRNA_03444	0	0	0	0	0.04012623	0	0	0	0	0	0	0	-	NC_000004.12	+	51860543	51877854	17312	439	sense-overlappin	XM_011534379.1	DCUN1D4
circRNA_03445	0	0	0	0	0.04012623	0	0	0	0	0	0	0	-	NC_000004.12	+	51860543	51891851	31309	602	sense-overlappin	XM_011534379.1	DCUN1D4
circRNA_03446	0.020575025	0.143289773	0.207182128	0.1960438	0.1404418	0.041425819	0.110542769	0.051215739	0.050636319	0.060517448	0.02025523	0.072227401	hsa_circ_0001410	NC_000004.12	+	51863437	51877854	14418	318	sense-overlappin	NM_001040402.2	DCUN1D4
circRNA_03447	0	0	0.076330258	0.0412724	0.04012623	0.020712909	0	0.030729443	0.020254528	0.020172483	0	0	hsa_circ_0007928	NC_000004.12	+	51863437	51886638	23202	389	sense-overlappin	NM_001040402.2	DCUN1D4
circRNA_03448	0.041150049	0.051174919	0.283512385	0.2063619	0.1404418	0.093208092	0.040197371	0.061458886	0.101272639	0.100862414	0.070893305	0.092863801	hsa_circ_0007646	NC_000004.12	+	51863437	51891851	28415	481	sense-overlappin	NM_001040402.2	DCUN1D4
circRNA_03449	0	0.030704951	0.196277805	0.0619086	0.08025246	0.072495183	0.030148028	0	0.070890847	0.080689931	0	0.0309546	hsa_circ_0069718	NC_000004.12	+	51863437	51899378	35942	590	sense-overlappin	NM_001040402.2	DCUN1D4
circRNA_03450	0	0	0.021808645	0	0	0.020712909	0	0	0	0	0	0	-	NC_000004.12	+	51874271	51899378	25108	479	sense-overlappin	NM_001040402.2	DCUN1D4
circRNA_03451	0	0	0	0	0	0	0.020098685	0	0	0	0	0	hsa_circ_0069725	NC_000004.12	-	52626018	52631144	5127	525	sense-overlappin	NM_001134223.1	USP46
circRNA_03452	0.020575025	0	0	0	0	0	0	0	0	0	0	0	-	NC_000004.12	+	53379073	53399839	20767	730	sense-overlappin	NM_030917.3	FIP1L1
circRNA_03453	0	0	0	0	0	0	0.030148028	0.030729443	0.020254528	0	0	0	hsa_circ_0001411	NC_000004.12	+	53383773	53389873	6101	169	sense-overlappin	XM_005265771.3	FIP1L1
circRNA_03454	0	0	0	0	0	0.020712909	0	0	0	0	0	0	-	NC_000004.12	+	53383773	53396061	12289	5040	sense-overlappin	XM_005265771.3	FIP1L1
circRNA_03455	0	0	0.021808645	0	0	0	0	0	0	0	0	0	hsa_circ_0006805	NC_000004.12	+	53383773	53399839	16067	587	sense-overlappin	XM_005265771.3	FIP1L1
circRNA_03456	0	0.020469968	0	0	0.02006311	0	0	0	0	0	0	0	hsa_circ_0069740	NC_000004.12	+	53383773	53428183	44411	946	sense-overlappin	XM_005265771.3	FIP1L1
circRNA_03457	0	0.061409903	0.032712968	0	0	0	0	0.030729443	0.040509055	0.090776173	0	0.0619092	hsa_circ_0005912	NC_000004.12	+	53399730	53428183	28454	469	sense-overlappin	XM_005265771.3	FIP1L1
circRNA_03458	0	0	0	0	0	0	0	0	0	0	0	0.0206364	hsa_circ_0069747	NC_000004.12	+	53399730	53444103	44374	607	sense-overlappin	XM_005265771.3	FIP1L1
circRNA_03459	0.041150049	0.061409903	0.08723458	0.0412724	0.06018934	0.113921001	0.070345399	0.102431477	0.050636319	0.080689931	0.04051046	0.0309546	hsa_circ_0007476	NC_000004.12	+	53414615	53428183	13569	359	sense-overlappin	XM_005265771.3	FIP1L1
circRNA_03460	0	0	0.021808645	0.0309543	0	0	0	0	0	0	0	0	hsa_circ_0001412	NC_000004.12	+	53414615	53444103	29489	497	sense-overlappin	XM_005265771.3	FIP1L1
circRNA_03461	0.030862537	0	0.065425935	0	0.03009467	0	0.030148028	0	0	0.090776173	0	0	hsa_circ_0069748	NC_000004.12	+	53425872	53428183	2312	251	sense-overlappin	XM_005265771.3	FIP1L1
circRNA_03462	0	0	0.04361729	0	0.03009467	0.020712909	0	0	0	0.030258724	0.030382845	0.0206364	hsa_circ_0001413	NC_000004.12	+	53425872	53444103	18232	389	sense-overlappin	XM_005265771.3	FIP1L1
circRNA_03463	0	0	0	0	0.03009467	0.062138728	0.020098685	0	0	0	0	0.0412728	-	NC_000004.12	+	55403236	55417985	14750	688	sense-overlappin	NR_073070.1	
circRNA_03464	0.308625369	0.173994724	0.239895095	0.1238171	0.15047336	0.093208092	0.140690797	0.112674625	0.070890847	0.262242276	0.141786611	0.154773001	hsa_circ_0001414	NC_000004.12	+	55411614	55417985	6372	585	sense-overlappin	NM_018475.4	TMEM165
circRNA_03465	0.020575025	0	0.021808645	0	0	0	0	0	0	0	0	0	hsa_circ_0069789	NC_000004.12	-	55453054	55456300	3247	414	sense-overlappin	NM_001267843.1	CLOCK
circRNA_03466	0	0	0	0	0	0	0	0.020486295	0	0	0	0	hsa_circ_0069791	NC_000004.12	-	55475963	55489465	13503	640	sense-overlappin	NM_001267843.1	CLOCK
circRNA_03467	0	0	0.021808645	0	0	0	0	0	0	0.040344966	0.02025523	0	hsa_circ_0003936	NC_000004.12	+	55877917	55890386	12470	465	sense-overlappin	XM_011534393.1	EXOC1
circRNA_03468	0	0	0	0.0206362	0	0	0	0	0	0	0	0	-	NC_000004.12	+	55891315	55893780	2466	414	sense-overlappin	XM_005265747.1	EXOC1
circRNA_03469	0	0	0	0	0	0	0	0.020486295	0	0	0	0	-	NC_000004.12	+	55893552	55899884	6333	613	sense-overlappin	XM_005265747.1	EXOC1
circRNA_03470	0	0	0	0	0.02006311	0.020712909	0	0	0	0	0	0	-	NC_000004.12	-	56371451	56378464	7014	510	sense-overlappin	XM_005265724.3	AASDH
circRNA_03471	0	0.020469968	0	0.0206362	0	0	0.030148028	0	0.020254528	0	0	0	-	NC_000004.12	+	56478379	56483270	4892	315	sense-overlappin	NM_006947.3	SRP72
circRNA_03472	0	0	0	0	0	0.020712909	0	0	0	0	0	0	-	NC_000004.12	+	56478379	56484864	6486	444	sense-overlappin	NM_006947.3	SRP72
circRNA_03473	0.030862537	0	0	0	0	0	0	0	0	0	0	0	-	NC_000004.12	+	61934840	61936045	1206	307	sense-overlappin	XM_011531793.1	ADGRL3
circRNA_03474	0	0.040939935	0.04361729	0.0619086	0	0.020712909	0	0.020486295	0.020254528	0.080689931	0	0	hsa_circ_0069863	NC_000004.12	+	61934840	61948276	13437	693	sense-overlappin	XM_011531793.1	ADGRL3
circRNA_03475	0	0	0	0.0309543	0	0.020712909	0	0	0.030381792	0	0	0	hsa_circ_0069864	NC_000004.12	+	61934840	61983603	48764	1124	sense-overlappin	XM_011531793.1	ADGRL3
circRNA_03476	0.041150049	0.071644886	0	0.113499	0	0.072495183	0	0.051215739	0.121527166	0.080689931	0	0.0206364	-	NC_000004.12	+	61934840	61998265	63426	1283	sense-overlappin	XM_011531793.1	ADGRL3
circRNA_03477	0	0	0.021808645	0	0	0	0	0	0	0	0	0	-	NC_000004.12	-	65004403	65051105	46703	46703	intergenic		
circRNA_03478	0	0	0	0	0	0	0	0	0	0.020172483	0	0	hsa_circ_0069868	NC_000004.12	-	67518156	67519502	1347	499	sense-overlappin	NM_001812.2	CENPC
circRNA_03479	0	0	0.021808645	0	0	0	0.020098685	0	0	0	0	0	-	NC_000004.12	+	67720422	67730170	9749	9749	intergenic		
circRNA_03480	0	0	0	0	0.03009467	0	0	0	0	0	0	0	-	NC_000004.12	+	67728540	67732345	3806	3806	intergenic		
circRNA_03481	0	0	0	0	0	0.020712909	0	0	0	0	0	0	-	NC_000004.12	+	67790448	67802411	11964	11964	intergenic		

circRNA_03482	0	0	0	0	0.04012623	0	0	0.020486295	0	0	0	0	hsa_circ_0002048	NC_000004.12	+	70762519	70768661	6143	518	sense-overlappin	XM_011531755.1	RUFY3
circRNA_03483	0	0	0	0	0	0	0.020098685	0	0	0	0	0	-	NC_000004.12	+	70788806	70794894	6089	486	sense-overlappin	XR_938709.1	
circRNA_03484	0	0	0	0	0.02006311	0	0	0	0	0	0	0	-	NC_000004.12	-	70821398	70821757	360	360	intronic	NM_001098477.1	GRSF1
circRNA_03485	0	0	0	0	0	0	0	0	0	0.030258724	0	0	-	NC_000004.12	+	70942823	70975286	32464	567	sense-overlappin	XM_005265709.2	MOB1B
circRNA_03486	0	0	0.021808645	0	0	0	0	0	0	0	0	0	-	NC_000004.12	+	70958874	70970024	11151	261	sense-overlappin	XM_011532412.1	MOB1B
circRNA_03487	0	0.071644886	0	0.0206362	0.02006311	0.062138728	0	0	0.050636319	0.030258724	0	0	hsa_circ_0069922	NC_000004.12	+	71236576	71255399	18824	254	sense-overlappin	NM_001098484.2	SLC4A4
circRNA_03488	0	0	0	0	0.02006311	0	0	0	0	0	0	0	hsa_circ_0008676	NC_000004.12	+	71339370	71357187	17818	477	sense-overlappin	NM_001098484.2	SLC4A4
circRNA_03489	0	0	0	0.0206362	0	0	0	0	0	0	0	0	hsa_circ_0069924	NC_000004.12	+	71339370	71397653	58284	554	sense-overlappin	NM_001098484.2	SLC4A4
circRNA_03490	0	0	0	0	0.02006311	0	0	0	0	0	0	0	-	NC_000004.12	+	71453495	71487018	33524	652	sense-overlappin	NM_001098484.2	SLC4A4
circRNA_03491	0	0	0	0	0.02006311	0	0	0	0	0	0	0	-	NC_000004.12	+	71466444	71472970	6527	406	sense-overlappin	NM_001098484.2	SLC4A4
circRNA_03492	0	0	0	0.0206362	0	0	0	0	0	0	0	0	-	NC_000004.12	+	71486948	71497692	10745	263	sense-overlappin	NM_001098484.2	SLC4A4
circRNA_03493	0	0	0	0.0309543	0.03009467	0	0	0	0.020254528	0.050431207	0	0	hsa_circ_0006693	NC_000004.12	-	73078642	73092300	13659	2081	sense-overlappin	NM_001286771.1	ANKRD17
circRNA_03494	0.051437562	0.092114854	0.13085187	0.0722267	0.11034713	0.134633911	0.070345399	0.112674625	0.070890847	0.14120738	0.06076569	0.0309546	hsa_circ_0001417	NC_000004.12	-	73085249	73092300	7052	1832	sense-overlappin	NM_001286771.1	ANKRD17
circRNA_03495	0	0	0	0	0	0	0	0	0	0	0.04051046	0	hsa_circ_0007410	NC_000004.12	-	73085249	73097272	12024	2138	sense-overlappin	NM_001286771.1	ANKRD17
circRNA_03496	0.102875123	0.143289773	0.13085187	0.0825448	0.12037869	0.144990365	0.140690797	0.143404068	0.081018111	0.211811069	0.091148535	0.134136601	hsa_circ_0007883	NC_000004.12	-	73090667	73092300	1634	1634	exonic	NM_001286771.1	ANKRD17
circRNA_03497	0	0.030704951	0	0.0309543	0	0.020712909	0	0	0.030381792	0.030258724	0	0	hsa_circ_0004148	NC_000004.12	-	73118688	73125312	6625	954	sense-overlappin	NM_001286771.1	ANKRD17
circRNA_03498	0	0	0	0.0515905	0	0	0	0	0	0	0.04051046	0	hsa_circ_0069981	NC_000004.12	+	74174506	74201370	26865	569	sense-overlappin	XR_938736.1	
circRNA_03499	0	0	0	0	0.03009467	0.031069364	0.020098685	0	0	0	0.030382845	0	hsa_circ_0069982	NC_000004.12	+	74174506	74225394	50889	662	sense-overlappin	XR_938736.1	
circRNA_03500	0	0	0	0.0206362	0	0	0	0	0	0	0	0	-	NC_000004.12	-	75080300	75084703	4404	4404	intergenic		
circRNA_03501	0	0	0.032712968	0	0	0	0	0	0	0	0	0	-	NC_000004.12	-	75080324	75084703	4380	4380	intergenic		
circRNA_03502	0	0	0.021808645	0	0	0	0	0	0	0	0	0	hsa_circ_0069998	NC_000004.12	-	75646338	75662049	15712	1200	sense-overlappin	NM_012297.4	G3BP2
circRNA_03503	0	0	0	0	0	0.020712909	0	0	0	0	0	0	hsa_circ_0006977	NC_000004.12	-	75648639	75655870	7232	486	sense-overlappin	NM_012297.4	G3BP2
circRNA_03504	0	0.051174919	0	0.0515905	0.03009467	0.031069364	0	0	0	0.100862414	0	0	hsa_circ_0007096	NC_000004.12	+	75790150	75790797	648	244	sense-overlappin	NM_001290049.1	USO1
circRNA_03505	0	0	0	0.0206362	0	0	0	0	0	0	0	0	hsa_circ_0008757	NC_000004.12	+	75804134	75805303	1170	303	sense-overlappin	NM_001290049.1	USO1
circRNA_03506	0	0	0	0	0	0	0.020098685	0	0	0	0	0	hsa_circ_0070012	NC_000004.12	+	75806486	75812375	5890	510	sense-overlappin	NM_001290049.1	USO1
circRNA_03507	0	0	0	0	0.02006311	0	0	0	0	0	0	0	-	NC_000004.12	+	75811392	75812879	1488	1488	sense-overlappin	NM_001290049.1	USO1
circRNA_03508	0	0	0.032712968	0	0	0	0	0	0	0	0	0	hsa_circ_0070022	NC_000004.12	-	75964135	75982037	17903	1166	sense-overlappin	NM_001288984.1	SDAD1
circRNA_03509	0	0.020469968	0.065425935	0.0619086	0.03009467	0.041425819	0	0.020486295	0	0.040344966	0	0	hsa_circ_0070033	NC_000004.12	-	76124649	76144473	19825	1097	sense-overlappin	NM_017426.3	NUP54
circRNA_03510	0	0	0	0	0	0	0.020098685	0	0	0	0	0	hsa_circ_0070036	NC_000004.12	-	76130656	76144473	13818	989	sense-overlappin	NM_017426.3	NUP54
circRNA_03511	0.123450148	0.143289773	0.152660515	0.0722267	0.0702209	0.041425819	0.110542769	0.102431477	0.081018111	0.07060369	0.04051046	0.051591	hsa_circ_0070039	NC_000004.12	-	76134175	76144473	10299	643	sense-overlappin	NM_017426.3	NUP54
circRNA_03512	0	0.040939935	0.021808645	0.0206362	0.05015779	0	0	0.040972591	0	0.040344966	0	0.0412728	hsa_circ_0070040	NC_000004.12	-	76144149	76144473	325	228	sense-overlappin	NM_017426.3	NUP54
circRNA_03513	0	0	0.032712968	0	0	0	0	0	0	0	0	0	-	NC_000004.12	-	76396269	76403280	7012	361	sense-overlappin	NM_001042784.1	CCDC158
circRNA_03514	0	0	0.032712968	0.0928628	0.04012623	0.020712909	0.020098685	0.030729443	0	0	0	0	hsa_circ_0070049	NC_000004.12	+	76738761	76741926	3166	3166	exonic	NM_020859.3	SHROOM3
circRNA_03515	0	0	0	0	0	0	0.020098685	0	0	0	0	0	hsa_circ_0006989	NC_000004.12	+	76996425	77020670	24246	926	sense-overlappin	NM_018243.2	11-Sep
circRNA_03516	0	0	0	0	0	0.020712909	0	0	0	0	0	0	hsa_circ_0070057	NC_000004.12	+	76996425	77030970	34546	1247	sense-overlappin	NM_018243.2	11-Sep
circRNA_03517	0.020575025	0	0.021808645	0	0	0	0.020098685	0	0	0	0	0	hsa_circ_0008524	NC_000004.12	-	77055146	77066401	11256	733	sense-overlappin	NM_006835.2	CCNI
circRNA_03518	0.020575025	0	0	0	0	0	0	0	0.020254528	0	0	0	-	NC_000004.12	-	77058507	77066405	7899	286	sense-overlappin	XM_011531555.1	CCNI
circRNA_03519	0	0	0.076330258	0.0309543	0.02006311	0.062138728	0.070345399	0.020486295	0.020254528	0.080689931	0	0.0412728	hsa_circ_0006168	NC_000004.12	-	77773081	77776392	3312	395	sense-overlappin	XM_011531808.1	CNOT6L
circRNA_03520	0	0	0	0.0309543	0	0	0	0	0	0	0	0	-	NC_000004.12	+	78595381	78604399	9019	429	sense-overlappin	NM_005139.2	ANXA3
circRNA_03521	0	0	0	0	0	0	0	0.020486295	0	0	0	0	-	NC_000004.12	-	78597863	78598077	215	215	antisense	NM_005139.2	ANXA3
circRNA_03522	0	0.040939935	0.04361729	0.0722267	0	0	0.050246713	0	0.091145375	0.040344966	0.02025523	0	hsa_circ_0070190	NC_000004.12	+	78826037	78851056	25020	705	sense-overlappin	NM_017593.3	BMP2K
circRNA_03523	0	0	0	0	0	0	0.030148028	0	0	0	0	0	hsa_circ_0070196	NC_000004.12	+	78833582	78851056	17475	586	sense-overlappin	NM_017593.3	BMP2K
circRNA_03524	0	0	0	0	0	0	0	0	0	0	0.02025523	0	hsa_circ_0070197	NC_000004.12	+	78842385	78851056	8672	480	sense-overlappin	NM_017593.3	BMP2K
circRNA_03525	0	0	0	0	0	0	0	0	0	0	0.030382845	0	-	NC_000004.12	+	79044975	79086262	41288	41288	intergenic		
circRNA_03526	0	0	0	0	0	0	0.040197371	0	0	0	0	0	-	NC_000004.12	-	81456651	81459514	2864	444	sense-overlappin	NM_001300735.1	RASGEF1B
circRNA_03527	0	0	0	0	0.03009467	0.041425819	0	0	0	0.020172483	0	0	hsa_circ_0003451	NC_000004.12	+	82454722	82457038	2317	257	sense-overlappin	NM_001292017.1	ENOPH1
circRNA_03528	0	0	0	0	0	0.041425819	0	0	0	0	0	0	-	NC_000004.12	-	82824555	82827632	3078	384	sense-overlappin	XM_011531749.1	SEC31A
circRNA_03529	0	0	0	0.0206362	0	0	0	0	0	0	0	0	-	NC_000004.12	-	82844386	82848977	4592	391	sense-overlappin	XM_011531749.1	SEC31A
circRNA_03530	0	0	0	0	0	0	0	0	0	0.020172483	0	0	-	NC_000004.12	-	82844386	82857764	13379	1093	sense-overlappin	XM_011531749.1	SEC31A
circRNA_03531	0	0	0.032712968	0.0412724	0.02006311	0	0	0	0	0	0	0.0206364	hsa_circ_0070245	NC_000004.12	-	82866808	82867316	509	315	sense-overlappin	XM_011531749.1	SEC31A
circRNA_03532	0.072012586	0	0.054521613	0.1031809	0.09028402	0.041425819	0	0	0.101272639	0.020172483	0.04051046	0.0412728	hsa_circ_0003549	NC_000004.12	-	82871944	82875822	3879	380	sense-overlappin	XM_011531749.1	SEC31A
circRNA_03533	0	0	0	0	0	0	0	0	0	0.040344966	0	0.0206364	hsa_circ_0002671	NC_000004.12	-	82874611	82875822	1212	237	sense-overlappin	XM_011531749.1	SEC31A
circRNA_03534	0	0	0	0.0309543	0	0	0	0	0	0.030258724	0	0	hsa_circ_0003716	NC_000004.12	-	82874611	82881940	7330	643	sense-overlappin	XM_011531749.1	SEC31A
circRNA_03535	0	0	0	0	0.03009467	0	0	0	0.020254528	0	0	0	hsa_circ_0070253	NC_000004.12	-	82878730	82880922	2193	323	sense-overlappin	XM_011531749.1	SEC31A
circRNA_03536	0.020575025	0	0	0.0412724	0.02006311	0.041425819	0.020098685	0.020486295	0	0.050431207	0	0	hsa_circ_0001421	NC_000004.12	-	82878730	82881937	3208	403	sense-overlappin	XM_011531749.1	SEC31A
circRNA_03537	0	0	0.021808645	0.113499	0.09028402	0.051782273	0.080394741	0.040972591	0.121527166	0	0.02025523	0.0619092	hsa_circ_0006618	NC_000004.12	-	82878730	82881940	3211	406	sense-overlappin	XM_011531749.1	SEC31A

circRNA_03538	0	0	0	0	0.03009467	0	0	0	0	0	0	0	-	NC_000004.12	-	82930943	82946474	15532	1097	sense-overlappin	XM_006714081.2	LIN54
circRNA_03539	0	0.020469968	0	0	0	0	0.020098685	0	0	0	0	0	hsa_circ_0007676	NC_000004.12	-	82946258	82979006	32749	484	sense-overlappin	XM_006714081.2	LIN54
circRNA_03540	0	0	0	0.0309543	0	0	0	0	0	0	0	0	hsa_circ_0003148	NC_000004.12	-	82970327	82979006	8680	267	sense-overlappin	XM_006714081.2	LIN54
circRNA_03541	0	0	0.021808645	0	0	0	0	0	0	0	0	0	-	NC_000004.12	+	83056926	83068522	11597	677	sense-overlappin	NM_016129.2	COPS4
circRNA_03542	0	0	0	0	0	0	0.020098685	0	0	0	0	0	-	NC_000004.12	-	83463494	83469151	5658	320	sense-overlappin	NM_139076.2	FAM175A
circRNA_03543	0	0	0	0	0	0	0	0.020486295	0	0	0	0	hsa_circ_0009036	NC_000004.12	-	83470203	83472288	2086	261	sense-overlappin	NM_139076.2	FAM175A
circRNA_03544	0	0	0.098138903	0	0	0	0	0	0	0	0	0	-	NC_000004.12	-	84243111	84293409	50299	50299	intergenic		
circRNA_03545	0	0	0.04361729	0	0	0	0	0	0	0	0	0	-	NC_000004.12	-	84266206	84293409	27204	27204	intergenic		
circRNA_03546	0	0	0	0.0412724	0	0	0	0	0	0	0	0	hsa_circ_0003680	NC_000004.12	-	84695970	84709347	13378	859	sense-overlappin	NM_014991.4	WDFY3
circRNA_03547	0	0	0	0.0206362	0	0	0	0	0	0	0	0	-	NC_000004.12	-	84718422	84757161	38740	2566	sense-overlappin	NM_014991.4	WDFY3
circRNA_03548	0	0.030704951	0	0.0206362	0	0	0	0	0	0	0	0	hsa_circ_0002283	NC_000004.12	-	84879406	84932363	52958	283	sense-overlappin	XM_005262858.3	WDFY3
circRNA_03549	0.030862537	0	0.032712968	0	0	0	0	0.020486295	0	0	0	0	hsa_circ_0002221	NC_000004.12	-	84896911	84932363	35453	194	sense-overlappin	NM_014991.4	WDFY3
circRNA_03550	0	0	0	0	0	0	0	0	0	0.030258724	0	0	-	NC_000004.12	-	86151991	86159467	7477	7477	sense-overlappin	XM_011532122.1	MAPK10
circRNA_03551	0	0	0	0	0	0.020712909	0	0	0	0	0	0	-	NC_000004.12	-	86354530	86384236	29707	29707	sense-overlappin	XM_011532117.1	MAPK10
circRNA_03552	0	0	0	0	0	0	0	0	0	0.040344966	0	0	-	NC_000004.12	-	86354530	86399806	45277	45277	sense-overlappin	XM_011532117.1	MAPK10
circRNA_03553	0	0	0	0.0515905	0	0	0	0.020486295	0	0	0	0	hsa_circ_0007263	NC_000004.12	+	86693587	86701801	8215	649	sense-overlappin	XM_011532165.1	PTPN13
circRNA_03554	0	0	0	0	0	0.031069364	0	0.051215739	0.020254528	0	0.02025523	0	hsa_circ_0007948	NC_000004.12	+	86764593	86767976	3384	487	sense-overlappin	XM_011532165.1	PTPN13
circRNA_03555	0	0	0	0	0.02006311	0	0	0.020486295	0	0	0	0	hsa_circ_0007324	NC_000004.12	+	86772778	86775652	2875	723	sense-overlappin	XM_011532165.1	PTPN13
circRNA_03556	0.102875123	0.153524756	0.13085187	0.0825448	0.03009467	0.134633911	0.070345399	0.102431477	0.050636319	0.131121138	0.030382845	0.051591	hsa_circ_0001423	NC_000004.12	+	87046166	87047594	1429	1021	sense-overlappin	XM_005263013.2	AFF1
circRNA_03557	0	0	0.032712968	0	0	0	0	0	0	0	0	0	-	NC_000004.12	+	87046166	87047682	1517	1109	sense-overlappin	XM_005263013.2	AFF1
circRNA_03558	0	0	0.032712968	0	0	0	0	0	0	0	0	0	-	NC_000004.12	+	87046166	87091829	45664	1190	sense-overlappin	XM_005263013.2	AFF1
circRNA_03559	0	0	0	0	0	0	0	0.020486295	0	0	0	0	hsa_circ_0070377	NC_000004.12	+	87046166	87094969	48804	1245	sense-overlappin	XM_005263013.2	AFF1
circRNA_03560	0	0	0	0	0	0	0	0.030729443	0	0	0	0	hsa_circ_0070382	NC_000004.12	+	87084120	87091829	7710	169	sense-overlappin	XM_005263013.2	AFF1
circRNA_03561	0	0	0	0.0206362	0	0	0	0	0	0	0	0	-	NC_000004.12	-	87163479	87195690	32212	2054	sense-overlappin	NM_001292003.1	KLHL8
circRNA_03562	0	0	0.04361729	0	0	0	0	0.020486295	0.020254528	0	0	0	-	NC_000004.12	-	87163558	87195690	32133	1975	sense-overlappin	NM_001292003.1	KLHL8
circRNA_03563	0	0	0.021808645	0	0	0	0	0	0	0	0	0	-	NC_000004.12	-	87170079	87170615	537	329	sense-overlappin	NM_001292003.1	KLHL8
circRNA_03564	0	0	0	0	0.03009467	0	0	0	0	0	0	0	hsa_circ_0070388	NC_000004.12	-	87170079	87176868	6790	441	sense-overlappin	NM_001292003.1	KLHL8
circRNA_03565	0	0	0.021808645	0.0206362	0	0	0	0	0	0	0	0	hsa_circ_0005384	NC_000004.12	-	87178477	87195690	17214	1247	sense-overlappin	NM_001292003.1	KLHL8
circRNA_03566	0	0	0	0	0	0	0	0.020486295	0	0.020172483	0	0	hsa_circ_0006866	NC_000004.12	-	87183203	87195690	12488	1103	sense-overlappin	NM_001292003.1	KLHL8
circRNA_03567	0.174887709	0.184229708	0.109043225	0.1238171	0.19059959	0.093208092	0.120592112	0.071702034	0.151908958	0.282414759	0.141786611	0.134136601	hsa_circ_0002538	NC_000004.12	-	87195324	87195690	367	367	exonic	NM_001292003.1	KLHL8
circRNA_03568	0.051437562	0	0	0	0	0	0	0	0	0	0	0	hsa_circ_0070396	NC_000004.12	+	87438277	87454455	16179	527	sense-overlappin	XM_011532032.1	NUDT9
circRNA_03569	0	0	0	0	0	0.020712909	0	0	0	0	0	0	hsa_circ_0004929	NC_000004.12	+	88038251	88052158	13908	873	sense-overlappin	XR_244632.2	
circRNA_03570	0	0	0	0.0309543	0	0	0	0	0	0	0	0	hsa_circ_0004050	NC_000004.12	+	88038251	88065879	27629	1515	sense-overlappin	NM_000297.3	PKD2
circRNA_03571	0	0	0	0.0206362	0	0	0	0	0	0	0	0	hsa_circ_0004577	NC_000004.12	+	88061906	88065879	3974	339	sense-overlappin	XM_011532028.1	PKD2
circRNA_03572	0	0	0.065425935	0	0	0.020712909	0	0	0	0	0	0	hsa_circ_0001425	NC_000004.12	-	88419302	88420428	1127	1127	antisense	XR_938752.1	
circRNA_03573	0	0	0	0	0	0	0	0	0	0.020172483	0	0	-	NC_000004.12	-	88448827	88453507	4681	4681	intergenic		
circRNA_03574	0	0	0	0.0206362	0	0	0	0	0	0	0	0	hsa_circ_0001426	NC_000004.12	+	88475841	88494331	18491	1052	sense-overlappin	NM_016323.3	HERC5
circRNA_03575	0	0	0	0.0206362	0	0	0	0	0	0	0	0	hsa_circ_0070438	NC_000004.12	-	88990973	89029649	38677	706	sense-overlappin	XM_011531518.1	FAM13A
circRNA_03576	0	0	0	0.0309543	0	0	0	0	0	0	0	0	hsa_circ_0070440	NC_000004.12	-	89020460	89029649	9190	400	sense-overlappin	NM_014883.3	FAM13A
circRNA_03577	0	0	0.098138903	0.0515905	0	0.041425819	0	0.020486295	0	0	0	0	-	NC_000004.12	+	90308244	90313047	4804	1550	sense-overlappin	XM_011531960.1	CCSER1
circRNA_03578	0	0	0.021808645	0	0	0	0	0	0	0	0	0	-	NC_000004.12	+	90723914	90727297	3384	172	sense-overlappin	XM_011531954.1	CCSER1
circRNA_03579	0	0	0	0	0	0	0	0	0	0.020172483	0	0	-	NC_000004.12	-	91339992	91341835	1844	1844	antisense	XM_011531936.1	CCSER1
circRNA_03580	0	0	0.021808645	0	0	0	0	0	0	0	0	0	-	NC_000004.12	+	94233954	94270818	36865	1204	sense-overlappin	NM_001128430.1	SMARCAD1
circRNA_03581	0.020575025	0	0	0	0	0	0	0	0	0	0	0	hsa_circ_0070463	NC_000004.12	+	94276339	94283303	6965	1107	sense-overlappin	NM_001128430.1	SMARCAD1
circRNA_03582	0.123450148	0.102349838	0.054521613	0.0825448	0.03009467	0.041425819	0.100493427	0.071702034	0.060763583	0.110948655	0.02025523	0.0412728	hsa_circ_0070467	NC_000004.12	+	94573351	94586444	13094	303	sense-overlappin	NM_001011516.2	PDLIM5
circRNA_03583	0.041150049	0	0	0	0.02006311	0	0.020098685	0	0.030381792	0	0	0	-	NC_000004.12	+	94573351	94618191	44841	887	sense-overlappin	XM_006714070.2	PDLIM5
circRNA_03584	0.020575025	0	0	0	0	0.020712909	0	0	0	0	0.02025523	0	hsa_circ_0005233	NC_000004.12	+	94573351	94640450	67100	1062	sense-overlappin	XM_006714070.2	PDLIM5
circRNA_03585	0	0	0.021808645	0	0	0	0	0	0	0	0	0	-	NC_000004.12	+	94875831	94899050	23220	23220	sense-overlappin	NM_001203.2	BMPR1B
circRNA_03586	0	0	0.021808645	0	0	0	0	0	0	0	0	0	hsa_circ_0070476	NC_000004.12	+	95104408	95131512	27105	1093	sense-overlappin	NM_001203.2	BMPR1B
circRNA_03587	0	0	0.021808645	0	0	0	0	0	0	0	0	0	-	NC_000004.12	+	95114720	95131512	16793	933	sense-overlappin	NM_001203.2	BMPR1B
circRNA_03588	0	0	0	0.0206362	0	0	0	0	0	0	0	0.0206364	-	NC_000004.12	-	98105953	98134459	28507	662	sense-overlappin	XM_011531887.1	STPG2
circRNA_03589	0	0	0	0	0	0	0	0	0	0.020172483	0	0	-	NC_000004.12	-	99880911	99892034	11124	1428	sense-overlappin	NM_001243736.1	LAMTOR3
circRNA_03590	0	0.020469968	0.032712968	0.0412724	0	0	0.030148028	0.040972591	0.091145375	0.040344966	0	0.0206364	hsa_circ_0002782	NC_000004.12	-	102304317	102315830	11514	621	sense-overlappin	NM_001135147.1	SLC39A8
circRNA_03591	0	0	0	0	0	0	0.020098685	0	0	0	0	0	-	NC_000004.12	-	102304317	102324998	20682	9789	sense-overlappin	NM_001135147.1	SLC39A8
circRNA_03592	0.082300098	0	0.065425935	0.0309543	0.05015779	0.031069364	0.020098685	0	0.020254528	0	0.030382845	0.0309546	hsa_circ_0008012	NC_000004.12	+	102525512	102537956	12445	262	sense-overlappin	NM_001165412.1	NFKB1

circRNA_03593	0.030862537	0	0	0	0.02006311	0	0	0	0.020254528	0.040344966	0.050638075	0	-	NC_000004.12	+	102580535	102582957	2423	197	sense-overlappin	NM_001165412.1	NFKB1
circRNA_03594	0	0	0	0	0	0.031069364	0	0	0	0	0	0	-	NC_000004.12	-	102664685	102679709	15025	6164	sense-overlappin	NM_005908.3	MANBA
circRNA_03595	0	0	0.032712968	0	0.03009467	0	0.020098685	0	0	0.050431207	0	0	hsa_circ_0001429	NC_000004.12	-	102689574	102690957	1384	365	sense-overlappin	XM_011531965.1	MANBA
circRNA_03596	0	0	0	0	0	0	0	0.040972591	0.030381792	0.030258724	0	0	hsa_circ_0001430	NC_000004.12	-	102689574	102726683	37110	783	sense-overlappin	NM_005908.3	MANBA
circRNA_03597	0.061725074	0.030704951	0.08723458	0.0515905	0.04012623	0.072495183	0.090444084	0.040972591	0.050636319	0.121034897	0.02025523	0	hsa_circ_0001432	NC_000004.12	-	102714438	102726683	12246	496	sense-overlappin	NM_005908.3	MANBA
circRNA_03598	0	0.020469968	0.08723458	0	0	0.020712909	0	0	0	0	0	0	-	NC_000004.12	-	102714438	102730736	16299	4549	sense-overlappin	NM_005908.3	MANBA
circRNA_03599	0	0	0.021808645	0	0	0.020712909	0	0	0	0	0	0	-	NC_000004.12	-	102714438	102736435	21998	10248	sense-overlappin	NM_005908.3	MANBA
circRNA_03600	0	0	0	0	0	0.020712909	0.020098685	0.020486295	0	0	0	0	hsa_circ_0006007	NC_000004.12	-	102722871	102726683	3813	372	sense-overlappin	NM_005908.3	MANBA
circRNA_03601	0	0.030704951	0	0.0515905	0	0	0	0	0	0	0	0	-	NC_000004.12	-	102726589	102730736	4148	4148	sense-overlappin	NM_005908.3	MANBA
circRNA_03602	0	0	0.065425935	0	0	0	0	0	0	0	0	0	-	NC_000004.12	+	103548743	103593432	44690	43198	antisense	NM_001059.2	TACR3
circRNA_03603	0	0	0.141756193	0	0	0	0	0	0	0.080689931	0.030382845	0	-	NC_000004.12	-	104518577	104519454	878	878	intergenic		
circRNA_03604	0.092587611	0.051174919	0.152660515	0.1547714	0.03009467	0.020712909	0.070345399	0.020486295	0.040509055	0.030258724	0.030382845	0.092863801	hsa_circ_0070562	NC_000004.12	+	105233897	105237351	3455	3455	exonic	NM_017628.4	TET2
circRNA_03605	0	0	0	0	0	0	0	0	0.020254528	0.030258724	0	0	-	NC_000004.12	-	105370837	105399164	28328	321	sense-overlappin	NM_006903.4	PPA2
circRNA_03606	0	0	0.021808645	0	0	0	0	0	0	0	0	0	-	NC_000004.12	-	105370837	105434161	63325	10287	sense-overlappin	NM_006903.4	PPA2
circRNA_03607	0	0	0	0	0.02006311	0	0	0	0	0	0	0	-	NC_000004.12	-	105396249	105424322	28074	341	sense-overlappin	NM_006903.4	PPA2
circRNA_03608	0	0	0	0	0	0	0	0.020486295	0	0	0	0	-	NC_000004.12	-	105396249	105438036	41788	428	sense-overlappin	NM_176869.2	PPA2
circRNA_03609	0	0	0	0	0	0	0	0	0.020254528	0	0	0	-	NC_000004.12	-	105396249	105453642	57394	647	sense-overlappin	NM_176869.2	PPA2
circRNA_03610	0	0	0.021808645	0	0	0	0	0	0	0	0	0.0412728	-	NC_000004.12	-	105424196	105449403	25208	388	sense-overlappin	NM_176869.2	PPA2
circRNA_03611	0	0	0	0	0	0	0	0	0	0.020172483	0	0	hsa_circ_0070565	NC_000004.12	-	105424196	105453642	29447	433	sense-overlappin	NM_176869.2	PPA2
circRNA_03612	0.082300098	0.020469968	0.109043225	0.0515905	0.03009467	0.082851637	0	0.020486295	0.070890847	0.040344966	0.030382845	0	hsa_circ_0001434	NC_000004.12	-	105424196	105456745	32550	498	sense-overlappin	NM_176869.2	PPA2
circRNA_03613	0	0.040939935	0.021808645	0.0619086	0.05015779	0	0	0.081945182	0.030381792	0.060517448	0.02025523	0.0206364	hsa_circ_0007477	NC_000004.12	-	105446383	105456745	10363	284	sense-overlappin	NM_006903.4	PPA2
circRNA_03614	0	0	0	0	0.02006311	0	0	0	0	0	0	0	-	NC_000004.12	+	105645188	105659365	14178	871	sense-overlappin	XR_938751.1	
circRNA_03615	0	0	0.021808645	0	0.02006311	0.031069364	0.070345399	0.051215739	0.030381792	0	0	0	hsa_circ_0007540	NC_000004.12	-	106171095	106212835	41741	461	sense-overlappin	NM_001163435.2	TBCK
circRNA_03616	0	0	0	0	0.02006311	0.020712909	0	0	0	0	0	0	hsa_circ_0070590	NC_000004.12	-	106193609	106212835	19227	285	sense-overlappin	NM_001163435.2	TBCK
circRNA_03617	0	0	0.032712968	0	0	0	0	0	0.020254528	0.020172483	0.02025523	0	hsa_circ_0003673	NC_000004.12	-	106295094	106308989	13896	441	sense-overlappin	XM_011532418.1	TBCK
circRNA_03618	0	0	0	0.0206362	0	0	0	0	0	0	0	0	-	NC_000004.12	-	107631631	107660072	28442	1067	sense-overlappin	XM_011532400.1	PAPSS1
circRNA_03619	0.020575025	0	0	0.0412724	0	0	0	0	0	0.020172483	0	0.0206364	-	NC_000004.12	-	107644802	107660072	15271	837	sense-overlappin	XM_011532400.1	PAPSS1
circRNA_03620	0	0	0	0.0206362	0	0	0	0	0	0	0	0	hsa_circ_0070610	NC_000004.12	-	107644802	107701285	56484	1446	sense-overlappin	XM_011532400.1	PAPSS1
circRNA_03621	0	0.102349838	0.119947548	0.1238171	0.03009467	0.072495183	0.020098685	0	0.060763583	0.030258724	0	0.0309546	hsa_circ_0005965	NC_000004.12	-	107682015	107694006	11992	494	sense-overlappin	XM_011532400.1	PAPSS1
circRNA_03622	0	0	0.098138903	0.1238171	0.08025246	0.041425819	0.030148028	0.030729443	0.060763583	0.040344966	0	0	hsa_circ_0006935	NC_000004.12	-	107682015	107701285	19271	609	sense-overlappin	XM_011532400.1	PAPSS1
circRNA_03623	0	0	0	0.0309543	0	0	0	0	0	0	0	0	-	NC_000004.12	+	107895310	107903386	8077	971	sense-overlappin	XM_011531701.1	SGMS2
circRNA_03624	0	0	0	0	0	0	0	0.040972591	0	0.030258724	0	0	hsa_circ_0004700	NC_000004.12	+	108655564	108657647	2084	292	sense-overlappin	NM_001267818.1	OSTC
circRNA_03625	0.020575025	0	0.076330258	0	0	0	0.020098685	0	0	0.030258724	0.050638075	0	hsa_circ_0003875	NC_000004.12	+	109462901	109463644	744	744	exonic	NM_001042734.2	SEC24B
circRNA_03626	0	0	0.021808645	0	0	0	0	0	0	0	0	0	hsa_circ_0070630	NC_000004.12	+	109462901	109473186	10286	927	sense-overlappin	NM_001042734.2	SEC24B
circRNA_03627	0.051437562	0	0	0	0.05015779	0.072495183	0	0	0	0.020172483	0.030382845	0	hsa_circ_0001436	NC_000004.12	+	109491327	109494856	3530	323	sense-overlappin	NM_001042734.2	SEC24B
circRNA_03628	0	0	0.032712968	0.0206362	0	0.031069364	0	0	0	0	0	0	hsa_circ_0070637	NC_000004.12	+	109491327	109516640	25314	961	sense-overlappin	NM_001042734.2	SEC24B
circRNA_03629	0	0	0	0	0.02006311	0	0	0	0	0	0	0	hsa_circ_0070648	NC_000004.12	+	109513747	109520484	6738	342	sense-overlappin	NM_001042734.2	SEC24B
circRNA_03630	0	0	0.04361729	0	0	0	0	0	0	0	0	0	hsa_circ_0007184	NC_000004.12	+	109530289	109531522	1234	314	sense-overlappin	NM_001042734.2	SEC24B
circRNA_03631	0	0.030704951	0	0.0206362	0	0	0.020098685	0	0	0	0	0	hsa_circ_0070659	NC_000004.12	+	109659011	109660365	1355	247	sense-overlappin	NM_017918.4	CCDC109B
circRNA_03632	0	0	0.076330258	0.0206362	0	0	0.020098685	0	0	0	0	0.0309546	hsa_circ_0070660	NC_000004.12	+	109659011	109664394	5384	352	sense-overlappin	NM_017918.4	CCDC109B
circRNA_03633	0	0	0.021808645	0	0	0	0	0	0	0	0	0	-	NC_000004.12	+	110488541	110520366	31826	1083	sense-overlappin	NM_001977.3	ENPEP
circRNA_03634	0	0	0	0	0	0	0	0	0	0	0.030382845	0	-	NC_000004.12	+	112426467	112441105	14639	3105	sense-overlappin	XM_005263247.3	ALPK1
circRNA_03635	0.061725074	0	0.054521613	0	0.02006311	0	0	0	0	0.020172483	0	0	hsa_circ_0070680	NC_000004.12	-	112562371	112585725	23355	781	sense-overlappin	NM_018392.4	ZGRF1
circRNA_03636	0	0	0.04361729	0.0309543	0	0	0	0	0.020254528	0	0	0	-	NC_000004.12	-	112562371	112589874	27504	1721	sense-overlappin	NM_018392.4	ZGRF1
circRNA_03637	0	0	0	0	0	0	0	0.020486295	0	0	0	0	-	NC_000004.12	-	113661713	113679496	17784	255	sense-overlappin	XM_005263261.3	CAMK2D
circRNA_03638	0	0.030704951	0	0.0412724	0	0	0	0	0	0	0	0	-	NC_000004.12	+	114619423	114623702	4280	927	sense-overlappin	XM_011532232.1	UGT8
circRNA_03639	0	0	0	0.0206362	0	0	0	0	0	0	0	0	-	NC_000004.12	+	118088039	118114960	26922	17222	sense-overlappin	XM_006714416.2	NDST3
circRNA_03640	0	0	0	0.0309543	0	0	0	0	0	0	0	0	-	NC_000004.12	+	118105018	118114960	9943	243	sense-overlappin	XM_006714416.2	NDST3
circRNA_03641	0	0	0	0.0515905	0	0	0	0	0	0	0	0	-	NC_000004.12	+	118105018	118143684	38667	558	sense-overlappin	XM_006714416.2	NDST3
circRNA_03642	0	0	0	0	0	0	0	0	0	0	0	0.0206364	-	NC_000004.12	-	118313201	118318556	5356	518	sense-overlappin	NM_003619.3	PRSS12
circRNA_03643	0	0	0.032712968	0	0	0	0	0	0	0.020172483	0	0	hsa_circ_0006472	NC_000004.12	-	118331716	118338314	6599	469	sense-overlappin	NM_003619.3	PRSS12
circRNA_03644	0	0	0	0	0	0	0	0	0	0	0	0.0309546	hsa_circ_0008493	NC_000004.12	+	118620414	118628226	7813	7813	intergenic		
circRNA_03645	0	0.020469968	0	0	0	0	0	0	0	0	0	0	hsa_circ_0070754	NC_000004.12	-	118797683	118817412	19730	793	sense-overlappin	NM_014822.2	SEC24D
circRNA_03646	0.020575025	0	0	0	0	0	0	0	0	0	0	0	-	NC_000004.12	+	118914428	118914556	129	129	intronic	NM_001286754.1	SYNPO2

circRNA_03647	0.030862537	0	0	0	0	0	0	0	0	0	0	0	-	NC_000004.12	+	119150872	119164394	13523	484	sense-overlappin	NM_016599.4	MYOZ2
circRNA_03648	0	0	0	0.0928628	0	0	0	0	0	0	0	0	hsa_circ_0070771	NC_000004.12	+	119239218	119273708	34491	2793	sense-overlappin	XM_005263077.2	USP53
circRNA_03649	0	0	0.04361729	0.0722267	0.02006311	0	0	0.020486295	0	0	0	0	hsa_circ_0070774	NC_000004.12	+	119245337	119273708	28372	2107	sense-overlappin	XM_011532038.1	USP53
circRNA_03650	0	0	0.021808645	0	0	0	0	0	0	0	0	0	-	NC_000004.12	+	119256246	119273708	17463	1879	sense-overlappin	XM_011532038.1	USP53
circRNA_03651	0	0	0	0.0206362	0	0	0	0	0	0	0	0	-	NC_000004.12	+	119259820	119269837	10018	866	sense-overlappin	XM_011532038.1	USP53
circRNA_03652	0	0	0	0.0309543	0	0	0	0	0	0	0	0	-	NC_000004.12	+	119268268	119272034	3767	1039	sense-overlappin	XM_011532038.1	USP53
circRNA_03653	0	0	0	0	0	0	0	0	0	0	0	0.0309546	-	NC_000004.12	-	119298274	119298385	112	112	exonic	NM_001001701.3	C4orf3
circRNA_03654	0	0	0	0	0	0	0	0.020486295	0	0	0	0	hsa_circ_0008144	NC_000004.12	-	119538960	119567144	28185	801	sense-overlappin	NM_033430.2	PDE5A
circRNA_03655	0	0.020469968	0	0.0206362	0	0.062138728	0	0	0.030381792	0	0.030382845	0	hsa_circ_0002474	NC_000004.12	-	119596523	119607297	10775	679	sense-overlappin	NM_033430.2	PDE5A
circRNA_03656	0	0.020469968	0	0	0	0	0	0	0	0	0	0	hsa_circ_0006647	NC_000004.12	-	120754553	120785091	30539	639	sense-overlappin	XR_938679.1	
circRNA_03657	0.051437562	0.040939935	0.065425935	0.0309543	0	0.031069364	0	0	0.040509055	0.080689931	0	0	hsa_circ_0005654	NC_000004.12	-	120754553	120811449	56897	995	sense-overlappin	XR_938679.1	
circRNA_03658	0	0.020469968	0	0	0	0	0	0	0	0	0	0	-	NC_000004.12	-	120754553	120812814	58262	2360	sense-overlappin	XR_938679.1	
circRNA_03659	0	0	0.021808645	0	0	0	0	0	0	0	0	0	-	NC_000004.12	-	120754553	120821345	66793	1560	sense-overlappin	XR_938679.1	
circRNA_03660	0	0	0.032712968	0	0	0	0	0	0	0	0	0	-	NC_000004.12	-	120798267	120821345	23079	921	sense-overlappin	XM_011531569.1	PRDM5
circRNA_03661	0	0	0.032712968	0	0	0	0	0	0	0	0	0	hsa_circ_0002314	NC_000004.12	-	120811370	120821345	9976	645	sense-overlappin	XM_011531569.1	PRDM5
circRNA_03662	0	0	0.021808645	0	0	0	0	0	0	0	0	0	-	NC_000004.12	-	120903261	120907557	4297	4297	sense-overlappin	XM_011531569.1	PRDM5
circRNA_03663	0	0	0	0	0	0.020712909	0	0	0	0	0	0	hsa_circ_0008244	NC_000004.12	+	121804622	121810099	5478	354	sense-overlappin	XM_011532035.1	EXOSC9
circRNA_03664	0	0	0	0	0	0	0	0	0	0	0	0.0309546	-	NC_000004.12	-	121833231	121854823	21593	1078	sense-overlappin	NM_176824.2	BBS7
circRNA_03665	0	0	0.021808645	0	0	0	0	0	0	0	0.02025523	0	-	NC_000004.12	+	122170615	122175914	5300	391	sense-overlappin	XR_938783.1	
circRNA_03666	0	0	0.04361729	0	0	0	0	0	0	0	0	0	-	NC_000004.12	+	122190000	122201126	11127	677	sense-overlappin	XR_938783.1	
circRNA_03667	0	0	0.021808645	0	0	0	0	0	0	0	0	0	-	NC_000004.12	+	122271018	122289192	18175	1895	sense-overlappin	XR_938783.1	
circRNA_03668	0	0	0.021808645	0	0	0	0	0	0	0	0	0	-	NC_000004.12	+	122927634	122947488	19855	1551	sense-overlappin	XM_005262783.3	SPATA5
circRNA_03669	0	0	0	0.0515905	0	0	0	0	0	0	0	0	-	NC_000004.12	+	122927634	122952270	24637	1623	sense-overlappin	XM_011531678.1	SPATA5
circRNA_03670	0	0.040939935	0.13085187	0.0515905	0	0.041425819	0	0	0	0.030258724	0	0.0206364	-	NC_000004.12	+	122927634	122979386	51753	1778	sense-overlappin	XM_011531678.1	SPATA5
circRNA_03671	0	0	0.032712968	0	0	0	0	0	0	0	0	0	-	NC_000004.12	+	122927637	122979386	51750	1775	sense-overlappin	XM_011531678.1	SPATA5
circRNA_03672	0	0	0	0.0206362	0	0	0	0	0	0	0	0	hsa_circ_0006112	NC_000004.12	+	123056387	123057288	902	134	sense-overlappin	XM_005262783.3	SPATA5
circRNA_03673	0	0	0.021808645	0	0	0	0	0	0	0	0	0	-	NC_000004.12	-	124251992	124326346	74355	74355	intergenic		
circRNA_03674	0	0	0.032712968	0	0	0	0	0	0	0	0	0.0206364	-	NC_000004.12	-	124500896	124556510	55615	55615	intergenic		
circRNA_03675	0	0	0	0	0	0	0	0	0	0	0	0.0206364	-	NC_000004.12	-	127094411	127106774	12364	12364	intergenic		
circRNA_03676	0	0	0	0	0	0	0	0	0	0.020172483	0	0	-	NC_000004.12	+	127643521	127656721	13201	622	sense-overlappin	XM_011531846.1	INTU
circRNA_03677	0	0	0.021808645	0	0	0.020712909	0	0	0.030381792	0	0	0	hsa_circ_0070905	NC_000004.12	+	127643521	127687867	44347	1303	sense-overlappin	XM_011531846.1	INTU
circRNA_03678	0.092587611	0.020469968	0.065425935	0.0309543	0	0.051782273	0	0.040972591	0.040509055	0.171466104	0	0.0309546	hsa_circ_0003390	NC_000004.12	+	127669036	127687867	18832	477	sense-overlappin	XM_011531846.1	INTU
circRNA_03679	0	0	0	0	0	0	0	0	0	0.020172483	0	0	-	NC_000004.12	-	127844809	127844921	113	113	intergenic		
circRNA_03680	0.030862537	0.030704951	0.08723458	0.1341352	0.06018934	0.113921001	0.070345399	0.061458886	0	0.040344966	0.030382845	0.051591	hsa_circ_0009133	NC_000004.12	-	127921524	127939997	18474	797	sense-overlappin	XM_005262893.1	MFSD8
circRNA_03681	0	0.020469968	0	0	0	0	0	0	0	0	0	0	hsa_circ_0006901	NC_000004.12	-	127930683	127933093	2411	244	sense-overlappin	XM_005262893.1	MFSD8
circRNA_03682	0	0	0	0	0	0	0	0	0	0	0.030382845	0	-	NC_000004.12	+	128017358	128028853	11496	715	sense-overlappin	XM_006714321.2	C4orf29
circRNA_03683	0.041150049	0.122819805	0.04361729	0.2063619	0.10031557	0.144990365	0.050246713	0.133160921	0.081018111	0.121034897	0.030382845	0.051591	hsa_circ_0001438	NC_000004.12	+	128074460	128077962	3503	294	sense-overlappin	NM_001278604.1	LARP1B
circRNA_03684	0.051437562	0.040939935	0.054521613	0.0412724	0.06018934	0.041425819	0.030148028	0.102431477	0.091145375	0.07060369	0	0.0309546	hsa_circ_0007619	NC_000004.12	+	128074460	128082305	7846	435	sense-overlappin	NM_001278604.1	LARP1B
circRNA_03685	0	0	0.021808645	0.0619086	0	0.031069364	0.040197371	0.030729443	0.030381792	0	0	0	hsa_circ_0070934	NC_000004.12	+	128074460	128091512	17053	745	sense-overlappin	NM_001278604.1	LARP1B
circRNA_03686	0	0	0	0	0.02006311	0	0	0	0	0	0	0	-	NC_000004.12	+	128074460	128093031	18572	2264	sense-overlappin	NM_001278604.1	LARP1B
circRNA_03687	0	0	0.032712968	0.0412724	0.02006311	0.041425819	0.020098685	0	0	0	0	0	hsa_circ_0070935	NC_000004.12	+	128074460	128098330	23871	890	sense-overlappin	NM_001278604.1	LARP1B
circRNA_03688	0	0	0	0	0.03009467	0	0	0	0	0	0	0	-	NC_000004.12	+	128074460	128114742	40283	1238	sense-overlappin	NM_178043.2	LARP1B
circRNA_03689	0	0	0	0.0309543	0	0	0	0	0	0	0	0	-	NC_000004.12	+	128074460	128122188	47729	1601	sense-overlappin	NM_178043.2	LARP1B
circRNA_03690	0	0	0	0	0	0	0	0	0	0.020172483	0	0	-	NC_000004.12	+	128077788	128082305	4518	316	sense-overlappin	NM_001278604.1	LARP1B
circRNA_03691	0	0	0	0	0.02006311	0	0	0	0	0	0	0	hsa_circ_0070939	NC_000004.12	+	128091001	128091512	512	310	sense-overlappin	NM_001278604.1	LARP1B
circRNA_03692	0	0	0	0.0206362	0	0	0	0	0	0	0	0	-	NC_000004.12	+	128091001	128114742	23742	803	sense-overlappin	NM_178043.2	LARP1B
circRNA_03693	0	0	0	0	0.03009467	0	0	0	0	0	0	0	-	NC_000004.12	+	128093504	128093805	302	302	intronic	NM_001278604.1	LARP1B
circRNA_03694	0	0	0	0	0	0.031069364	0	0	0	0	0	0	-	NC_000004.12	+	128114570	128178642	64073	992	sense-overlappin	XM_011532067.1	LARP1B
circRNA_03695	0	0	0	0	0.02006311	0	0	0	0	0	0	0	-	NC_000004.12	+	128121826	128122188	363	363	exonic	NM_178043.2	LARP1B
circRNA_03696	0	0	0	0.0722267	0.04012623	0.051782273	0.020098685	0	0.020254528	0.020172483	0	0	-	NC_000004.12	+	128121826	128162317	40492	487	sense-overlappin	NM_018078.3	LARP1B
circRNA_03697	0	0	0	0.0206362	0	0	0	0	0	0	0	0	-	NC_000004.12	+	128121826	128168845	47020	515	sense-overlappin	XM_011532067.1	LARP1B
circRNA_03698	0	0	0.032712968	0.0206362	0	0	0	0	0	0.020172483	0	0	hsa_circ_0070942	NC_000004.12	+	128121826	128179512	57687	926	sense-overlappin	XM_011532067.1	LARP1B
circRNA_03699	0	0	0.04361729	0	0	0	0	0	0	0	0	0	-	NC_000004.12	+	128162194	128179512	17319	563	sense-overlappin	XM_011532067.1	LARP1B
circRNA_03700	0	0	0	0	0	0	0.020098685	0	0	0	0	0	-	NC_000004.12	+	128460327	128470549	10223	10223	intergenic		

circRNA_03701	0	0	0.032712968	0	0	0	0	0	0	0	0	0	hsa_circ_0006563	NC_000004.12	+	128831733	128852268	20536	722	sense-overlappin	NM_024900.4	JADE1
circRNA_03702	0.020575025	0	0.032712968	0.0412724	0	0.020712909	0.020098685	0	0	0.030258724	0	0	hsa_circ_0070959	NC_000004.12	-	128936655	128959777	23123	1150	sense-overlappin	XR_938689.1	
circRNA_03703	0	0.040939935	0	0.0825448	0	0.020712909	0	0	0.060763583	0.030258724	0.02025523	0	hsa_circ_0070960	NC_000004.12	-	128936655	128970468	33814	1333	sense-overlappin	XR_938689.1	
circRNA_03704	0	0.040939935	0.109043225	0.0515905	0.02006311	0.041425819	0	0	0	0.020172483	0.02025523	0	-	NC_000004.12	-	128936655	129003876	67222	1729	sense-overlappin	XR_938689.1	
circRNA_03705	0	0	0.032712968	0	0	0.020712909	0.020098685	0	0	0	0	0	-	NC_000004.12	-	128946007	128970468	24462	943	sense-overlappin	XR_938689.1	
circRNA_03706	0	0	0	0	0	0	0	0	0	0.020172483	0	0	-	NC_000004.12	-	128946007	129003876	57870	1339	sense-overlappin	XR_938689.1	
circRNA_03707	0	0	0	0	0	0	0.020098685	0	0	0	0	0	-	NC_000004.12	-	128965227	128999794	34568	443	sense-overlappin	XM_011531592.1	SCLT1
circRNA_03708	0.236612783	0.32751948	1.035910638	0.4127238	0.37116763	0.683526008	0.291430938	0.184376659	0.384836027	0.423622139	0.202552301	0.196045802	hsa_circ_0001439	NC_000004.12	-	128992167	129003876	11710	396	sense-overlappin	XM_011531592.1	SCLT1
circRNA_03709	0	0	0	0	0	0	0	0	0	0.020172483	0	0	hsa_circ_0001440	NC_000004.12	-	128992167	129039096	46930	452	sense-overlappin	XM_011531592.1	SCLT1
circRNA_03710	0	0	0.04361729	0	0	0	0	0	0	0	0	0	hsa_circ_0070962	NC_000004.12	-	128992167	129082373	90207	652	sense-overlappin	XM_011531592.1	SCLT1
circRNA_03711	0	0	0.032712968	0	0	0.020712909	0	0	0.070890847	0	0	0	hsa_circ_0008426	NC_000004.12	-	128997874	129003876	6003	325	sense-overlappin	XM_011531592.1	SCLT1
circRNA_03712	0	0	0.141756193	0	0	0	0	0	0	0	0	0	-	NC_000004.12	-	129039041	129082373	43333	256	sense-overlappin	XM_011531592.1	SCLT1
circRNA_03713	0.051437562	0	0	0	0	0	0	0	0	0	0	0	-	NC_000004.12	-	133948459	133978893	30435	30435	intergenic		
circRNA_03714	0	0	0	0	0	0	0	0	0	0.020172483	0	0	-	NC_000004.12	-	139040750	139073567	32818	19741	sense-overlappin	NM_001276458.1	ELF2
circRNA_03715	0	0	0	0	0	0	0	0	0	0.030258724	0	0	-	NC_000004.12	-	139058669	139059607	939	939	exonic	NM_001276457.1	ELF2
circRNA_03716	0	0.020469968	0	0	0	0	0	0	0	0	0	0	-	NC_000004.12	-	139058669	139073567	14899	1822	sense-overlappin	NM_001276458.1	ELF2
circRNA_03717	0.030862537	0.040939935	0	0.0309543	0	0.020712909	0.040197371	0	0.040509055	0.050431207	0	0	hsa_circ_0007137	NC_000004.12	-	139060324	139073567	13244	883	sense-overlappin	NM_001276458.1	ELF2
circRNA_03718	0	0	0	0	0	0	0.020098685	0	0	0	0	0	-	NC_000004.12	-	139061865	139073567	11703	532	sense-overlappin	NM_001276458.1	ELF2
circRNA_03719	0	0	0	0	0.02006311	0.020712909	0	0.030729443	0.040509055	0	0	0	hsa_circ_0002594	NC_000004.12	-	139071866	139073567	1702	288	sense-overlappin	NM_001276457.1	ELF2
circRNA_03720	0.072012586	0.051174919	0.021808645	0.0722267	0.0702209	0.031069364	0	0	0.050636319	0	0	0.0206364	hsa_circ_0007710	NC_000004.12	-	139116649	139139497	22849	9004	sense-overlappin	XM_005262803.1	ELF2
circRNA_03721	0	0	0	0	0.02006311	0	0	0	0.020254528	0	0	0	-	NC_000004.12	-	139116649	139168042	51394	37549	sense-overlappin	XM_005262803.1	ELF2
circRNA_03722	0.082300098	0	0.098138903	0.0928628	0.06018934	0.041425819	0.060296056	0.020486295	0.030381792	0.080689931	0.030382845	0	hsa_circ_0001441	NC_000004.12	-	139125164	139139497	14334	489	sense-overlappin	XM_005262803.1	ELF2
circRNA_03723	0	0	0	0	0	0.031069364	0	0	0	0	0	0	hsa_circ_0008825	NC_000004.12	-	139125164	139168042	42879	29034	sense-overlappin	XM_005262803.1	ELF2
circRNA_03724	0.082300098	0.030704951	0.021808645	0.0515905	0.05015779	0	0.050246713	0	0.040509055	0.060517448	0	0.0309546	hsa_circ_0007466	NC_000004.12	-	139137630	139139497	1868	323	sense-overlappin	XM_005262803.1	ELF2
circRNA_03725	0	0	0.021808645	0	0	0	0	0	0.030381792	0	0	0	-	NC_000004.12	+	139393591	139404490	10900	10900	intergenic		
circRNA_03726	0.020575025	0	0	0	0	0	0	0	0	0	0	0	-	NC_000004.12	-	139517885	139533366	15482	750	sense-overlappin	XM_011532288.1	SETD7
circRNA_03727	0.041150049	0	0.065425935	0	0.03009467	0	0	0	0	0	0.030382845	0.0206364	-	NC_000004.12	+	139695197	139704196	9000	334	sense-overlappin	NM_002413.4	MGST2
circRNA_03728	0	0	0	0	0.02006311	0	0	0	0	0	0	0	hsa_circ_0070997	NC_000004.12	+	139703455	139704196	742	263	sense-overlappin	NM_001204368.1	MGST2
circRNA_03729	0	0.061409903	0	0.0515905	0	0.020712909	0	0	0.040509055	0	0	0	-	NC_000004.12	-	139889357	139890967	1611	1611	exonic	NM_018717.4	MAML3
circRNA_03730	0.030862537	0	0	0	0	0	0	0	0	0	0.030382845	0	hsa_circ_0071037	NC_000004.12	-	142402938	142462726	59789	619	sense-overlappin	XM_006714403.2	INPP4B
circRNA_03731	0.020575025	0	0	0.0825448	0	0.031069364	0	0	0	0	0	0	-	NC_000004.12	+	143415477	143440382	24906	1594	sense-overlappin	XM_005262881.3	GAB1
circRNA_03732	0.020575025	0	0.021808645	0.0515905	0	0	0	0	0	0	0	0	hsa_circ_0071040	NC_000004.12	+	143415477	143466225	50749	2025	sense-overlappin	XM_005262881.3	GAB1
circRNA_03733	0.020575025	0.020469968	0	0.0206362	0	0	0.020098685	0	0	0	0.02025523	0	-	NC_000004.12	+	143415477	143469277	53801	2272	sense-overlappin	XM_005262881.3	GAB1
circRNA_03734	0	0	0	0	0	0	0	0	0	0.030258724	0	0	-	NC_000004.12	+	143527868	143530526	2659	357	sense-overlappin	NM_003601.3	SMARCA5
circRNA_03735	1.111051329	1.095143263	1.003197671	1.1659447	1.00315575	1.398121381	0.773799386	1.014071627	0.962090066	1.956730831	0.627912132	0.660364805	hsa_circ_0001445	NC_000004.12	+	143543509	143543972	464	269	sense-overlappin	NM_003601.3	SMARCA5
circRNA_03736	0	0	0	0	0	0	0	0	0	0	0	0.0309546	hsa_circ_0071053	NC_000004.12	-	145064572	145081750	17179	212	sense-overlappin	XM_011531532.1	ANAPC10
circRNA_03737	0	0	0.021808645	0	0	0	0.030148028	0	0	0	0	0	-	NC_000004.12	-	145159503	145171720	12218	386	sense-overlappin	NM_001102653.1	OTUD4
circRNA_03738	0.041150049	0	0	0	0	0	0	0	0	0	0	0	hsa_circ_0007970	NC_000004.12	+	145514438	145515013	576	576	exonic	XM_006714217.2	SMAD1
circRNA_03739	0	0	0.021808645	0	0	0.072495183	0	0.040972591	0	0.030258724	0	0.0206364	hsa_circ_0001447	NC_000004.12	-	145823422	145849561	26140	402	sense-overlappin	XM_011531645.1	ZNF827
circRNA_03740	0	0	0.021808645	0.0309543	0	0	0	0	0.050636319	0.110948655	0	0	hsa_circ_0003187	NC_000004.12	-	145845956	145849561	3606	298	sense-overlappin	XM_011531645.1	ZNF827
circRNA_03741	0	0	0	0.0206362	0	0	0	0	0	0	0	0	hsa_circ_0071088	NC_000004.12	-	145870245	145903215	32971	1938	sense-overlappin	XM_011531635.1	ZNF827
circRNA_03742	0	0	0	0	0	0	0	0	0	0.020172483	0	0	hsa_circ_0002293	NC_000004.12	-	145885678	145903215	17538	1704	sense-overlappin	XM_011531635.1	ZNF827
circRNA_03743	0	0.020469968	0	0	0.02006311	0	0.020098685	0	0	0	0	0	-	NC_000004.12	-	146305926	146308975	3050	3050	sense-overlappin	NM_001029998.4	SLC10A7
circRNA_03744	0.020575025	0	0	0	0	0	0	0	0	0	0	0	-	NC_000004.12	-	146487934	146510049	22116	16128	sense-overlappin	NM_001029998.4	SLC10A7
circRNA_03745	0	0	0.021808645	0	0	0	0	0	0	0	0	0	-	NC_000004.12	+	147633765	147634558	794	562	sense-overlappin	NM_018241.2	TMEM184C
circRNA_03746	0	0	0	0	0	0	0	0	0	0.020172483	0	0	-	NC_000004.12	+	147633765	147634581	817	585	sense-overlappin	NM_018241.2	TMEM184C
circRNA_03747	0	0	0	0	0	0	0	0	0	0	0	0.0206364	hsa_circ_0071099	NC_000004.12	+	147822727	147822957	231	158	sense-overlappin	XM_005263217.2	ARHGAP10
circRNA_03748	0.020575025	0	0	0	0	0	0	0	0	0	0	0	-	NC_000004.12	+	147854676	147866816	12141	3195	sense-overlappin	XM_005263217.2	ARHGAP10
circRNA_03749	0.041150049	0	0	0.0515905	0	0.082851637	0	0.030729443	0	0.040344966	0.030382845	0.0206364	-	NC_000004.12	+	147854676	147881932	27257	3527	sense-overlappin	XM_005263217.2	ARHGAP10
circRNA_03750	0.298337857	0.153524756	0.13085187	0.2063619	0.05015779	0.15534682	0.040197371	0.112674625	0.121527166	0.191638587	0.091148535	0.051591	hsa_circ_0071106	NC_000004.12	+	147857553	147881932	24380	650	sense-overlappin	XM_005263217.2	ARHGAP10
circRNA_03751	0	0.020469968	0.032712968	0	0	0	0	0	0	0	0	0	-	NC_000004.12	+	147857553	147913139	55587	844	sense-overlappin	XM_005263217.2	ARHGAP10
circRNA_03752	0	0	0	0	0	0	0	0.020486295	0.020254528	0	0	0	hsa_circ_0008243	NC_000004.12	+	147864846	147881932	17087	548	sense-overlappin	XM_005263217.2	ARHGAP10
circRNA_03753	0.030862537	0	0	0	0	0	0	0	0	0	0	0	hsa_circ_0006638	NC_000004.12	+	147906638	147913139	6502	194	sense-overlappin	XM_005263217.2	ARHGAP10
circRNA_03754	0.020575025	0	0	0	0	0	0	0	0	0	0	0	hsa_circ_0071113	NC_000004.12	+	147906638	147946704	40067	317	sense-overlappin	XM_005263217.2	ARHGAP10

circRNA_03755	0.051437562	0	0	0	0	0	0	0	0	0	0.02025523	0	hsa_circ_0002177	NC_000004.12	+	147906638	147955374	48737	416	sense-overlappin	XM_005263215.2	ARHGAP10
circRNA_03756	0.020575025	0	0	0	0	0	0	0	0	0	0	0	hsa_circ_0071114	NC_000004.12	+	147906638	147966839	60202	682	sense-overlappin	XM_005263215.2	ARHGAP10
circRNA_03757	0.051437562	0.030704951	0.021808645	0	0	0.041425819	0.050246713	0.051215739	0.081018111	0.060517448	0	0.0309546	hsa_circ_0001449	NC_000004.12	+	147939825	147955374	15550	222	sense-overlappin	XM_005263215.2	ARHGAP10
circRNA_03758	0.041150049	0.020469968	0.021808645	0	0	0.020712909	0	0	0.020254528	0.040344966	0.030382845	0	hsa_circ_0007265	NC_000004.12	+	147939825	147966839	27015	488	sense-overlappin	XM_005263215.2	ARHGAP10
circRNA_03759	0.061725074	0	0.032712968	0.0206362	0.03009467	0.031069364	0	0	0.020254528	0	0.02025523	0	hsa_circ_0006473	NC_000004.12	+	147946617	147966839	20223	413	sense-overlappin	XM_005263215.2	ARHGAP10
circRNA_03760	0	0	0	0	0	0	0	0	0	0.020172483	0	0	hsa_circ_0071122	NC_000004.12	+	147955316	147966839	11524	325	sense-overlappin	XM_005263215.2	ARHGAP10
circRNA_03761	0	0	0	0	0	0	0	0	0	0.030258724	0	0	-	NC_000004.12	+	147965024	147966839	1816	266	sense-overlappin	XM_005263215.2	ARHGAP10
circRNA_03762	0.030862537	0	0	0	0	0	0	0	0	0	0	0	hsa_circ_0071123	NC_000004.12	+	148023263	148047051	23789	311	sense-overlappin	XM_005263215.2	ARHGAP10
circRNA_03763	0	0	0.13085187	0	0	0	0	0.030729443	0	0	0	0	hsa_circ_0071127	NC_000004.12	-	148435104	148436862	1759	1759	exonic	NM_000901.4	NR3C2
circRNA_03764	0.13373766	0.020469968	0.032712968	0.0206362	0	0	0	0	0	0	0	0	hsa_circ_0005480	NC_000004.12	+	150102478	150102812	335	335	exonic	NM_001040260.3	DCLK2
circRNA_03765	0.020575025	0	0	0	0	0	0	0	0	0	0	0	-	NC_000004.12	+	150203795	150221785	17991	280	sense-overlappin	NM_001040260.3	DCLK2
circRNA_03766	0.020575025	0	0	0	0	0	0	0	0	0	0	0	-	NC_000004.12	-	150302625	150350159	47535	823	sense-overlappin	NM_001199282.2	LRBA
circRNA_03767	0	0	0	0	0	0	0	0.030729443	0	0.030258724	0	0.051591	-	NC_000004.12	-	150415438	150491035	75598	864	sense-overlappin	NM_001199282.2	LRBA
circRNA_03768	0	0	0	0	0	0	0	0	0	0.020172483	0	0	-	NC_000004.12	-	150435589	150491035	55447	711	sense-overlappin	NM_001199282.2	LRBA
circRNA_03769	0.154312685	0.092114854	0	0.0928628	0	0.144990365	0	0	0.121527166	0.161379862	0	0.092863801	hsa_circ_0006867	NC_000004.12	-	150467673	150491035	23363	450	sense-overlappin	NM_001199282.2	LRBA
circRNA_03770	0	0	0.021808645	0	0	0	0	0	0	0	0	0	hsa_circ_0004780	NC_000004.12	-	150588048	150599131	11084	442	sense-overlappin	XM_005263372.2	LRBA
circRNA_03771	0	0	0.04361729	0.0412724	0.03009467	0	0	0	0.050636319	0.030258724	0	0.0206364	hsa_circ_0071174	NC_000004.12	-	150735258	150808398	73141	449	sense-overlappin	NM_001199282.2	LRBA
circRNA_03772	0.051437562	0	0.076330258	0.0515905	0	0	0	0.020486295	0.040509055	0.050431207	0.02025523	0.0206364	hsa_circ_0071185	NC_000004.12	-	150761783	150808398	46616	340	sense-overlappin	NM_001199282.2	LRBA
circRNA_03773	0	0	0	0	0	0	0	0	0	0.040344966	0	0	hsa_circ_0071186	NC_000004.12	-	150761783	150817257	55475	474	sense-overlappin	NM_001199282.2	LRBA
circRNA_03774	0	0	0.054521613	0	0	0.020712909	0.020098685	0.030729443	0	0.040344966	0	0	hsa_circ_0004636	NC_000004.12	-	150798081	150808398	10318	275	sense-overlappin	NM_001199282.2	LRBA
circRNA_03775	0	0	0.065425935	0.0722267	0.06018934	0.051782273	0	0.030729443	0.030381792	0	0	0	hsa_circ_0008618	NC_000004.12	-	150798081	150817257	19177	409	sense-overlappin	NM_001199282.2	LRBA
circRNA_03776	0	0	0	0	0	0	0	0	0	0	0.030382845	0	hsa_circ_0071189	NC_000004.12	-	150798081	150831976	33896	1011	sense-overlappin	NM_001199282.2	LRBA
circRNA_03777	0.041150049	0	0	0	0	0	0	0	0	0	0	0	hsa_circ_0071192	NC_000004.12	-	150798081	150852943	54863	2814	sense-overlappin	NM_001199282.2	LRBA
circRNA_03778	0	0	0	0	0	0	0.020098685	0	0	0	0	0	hsa_circ_0071200	NC_000004.12	-	150806271	150849575	43305	1514	sense-overlappin	NM_001199282.2	LRBA
circRNA_03779	0	0	0	0	0	0	0	0	0.020254528	0	0	0	hsa_circ_0071243	NC_000004.12	-	150896394	150916738	20345	1422	sense-overlappin	NM_001199282.2	LRBA
circRNA_03780	0.051437562	0	0	0.0206362	0.02006311	0	0	0.020486295	0.020254528	0	0	0	-	NC_000004.12	-	151132331	151135132	2802	315	sense-overlappin	NM_001128924.1	SH3D19
circRNA_03781	0	0	0	0	0	0	0	0	0	0.020172483	0	0	-	NC_000004.12	-	151147922	151149561	1640	327	sense-overlappin	NM_001128924.1	SH3D19
circRNA_03782	0	0	0.021808645	0	0	0	0	0	0	0	0	0	hsa_circ_0071260	NC_000004.12	-	151159240	151179397	20158	1571	sense-overlappin	XM_011531646.1	SH3D19
circRNA_03783	0.020575025	0	0	0	0	0.031069364	0	0.020486295	0.020254528	0.030258724	0	0	hsa_circ_0071261	NC_000004.12	-	151174670	151187463	12794	1432	sense-overlappin	XM_011531648.1	SH3D19
circRNA_03784	0	0	0.054521613	0	0	0	0	0	0	0	0	0	-	NC_000004.12	-	151352900	151353391	492	492	intergenic		
circRNA_03785	0	0.030704951	0	0.0206362	0	0	0.020098685	0	0	0	0	0	-	NC_000004.12	+	151415724	151482648	66925	203	sense-overlappin	XM_011532221.1	FAM160A1
circRNA_03786	0	0	0	0.0206362	0	0	0.040197371	0	0.020254528	0	0.030382845	0	-	NC_000004.12	+	151454701	151482648	27948	233	sense-overlappin	NM_001109977.1	FAM160A1
circRNA_03787	0	0	0.021808645	0	0	0	0	0	0	0	0	0	-	NC_000004.12	+	151566138	151578076	11939	854	sense-overlappin	XM_011532221.1	FAM160A1
circRNA_03788	0	0	0	0.0206362	0	0	0	0	0	0	0	0	hsa_circ_0071271	NC_000004.12	-	152332596	152337936	5341	259	sense-overlappin	NM_018315.4	FBXW7
circRNA_03789	0	0	0.032712968	0	0	0	0	0	0	0	0	0	-	NC_000004.12	-	152337802	152412529	74728	1158	sense-overlappin	XM_011532086.1	FBXW7
circRNA_03790	0	0	0	0	0	0	0.030148028	0.030729443	0	0.020172483	0	0	-	NC_000004.12	-	152382189	152412529	30341	798	sense-overlappin	XM_011532086.1	FBXW7
circRNA_03791	0.483513078	1.043968344	0.828728511	1.6199408	0.85268239	1.253131015	1.547598772	1.157475695	0.678526678	1.997075797	0.293700836	1.073092809	hsa_circ_0001451	NC_000004.12	-	152411303	152412529	1227	620	sense-overlappin	XM_011532083.1	FBXW7
circRNA_03792	0.020575025	0	0	0.0619086	0	0.103564547	0.030148028	0.040972591	0	0.040344966	0	0.113500201	-	NC_000004.12	-	152411303	152469988	58686	58079	sense-overlappin	XM_011532083.1	FBXW7
circRNA_03793	0	0	0	0	0	0	0	0	0	0.030258724	0	0	-	NC_000004.12	-	152411303	152482673	71371	70764	sense-overlappin	XM_011532083.1	FBXW7
circRNA_03794	0	0	0	0	0	0	0.020098685	0	0	0	0	0	-	NC_000004.12	-	152482370	152482673	304	304	intronic	NM_033632.3	FBXW7
circRNA_03795	0	0	0	0	0	0	0.020098685	0	0	0	0	0	hsa_circ_0071278	NC_000004.12	+	152829625	152872564	42940	420	sense-overlappin	NM_001025595.2	ARFIP1
circRNA_03796	0	0	0	0	0	0	0	0	0.020254528	0	0	0	-	NC_000004.12	+	152895215	152895295	81	81	intronic	NM_001025593.2	ARFIP1
circRNA_03797	0	0	0	0	0.03009467	0	0	0	0	0	0	0	hsa_circ_0005272	NC_000004.12	+	153270335	153276130	5796	423	sense-overlappin	XM_011531796.1	TRIM2
circRNA_03798	0	0	0	0	0	0	0	0	0	0	0.030382845	0	-	NC_000004.12	+	153270338	153276130	5793	420	sense-overlappin	XM_011531796.1	TRIM2
circRNA_03799	0	0	0.04361729	0.0309543	0	0	0	0	0	0	0	0	hsa_circ_0006811	NC_000004.12	+	153292982	153296036	3055	1057	sense-overlappin	XM_011531796.1	TRIM2
circRNA_03800	0	0	0.021808645	0	0	0	0	0	0	0.020172483	0	0	hsa_circ_0071291	NC_000004.12	+	153315485	153315999	515	272	sense-overlappin	XM_011531796.1	TRIM2
circRNA_03801	0	0	0	0	0	0	0.020098685	0	0	0	0	0	hsa_circ_0006225	NC_000004.12	+	153394262	153397333	3072	190	sense-overlappin	NM_032117.3	MND1
circRNA_03802	0	0	0	0	0.03009467	0	0	0	0	0	0	0	hsa_circ_0005686	NC_000004.12	+	153394262	153409015	14754	235	sense-overlappin	NM_032117.3	MND1
circRNA_03803	0	0	0	0	0	0	0	0	0	0.030258724	0	0	hsa_circ_0071298	NC_000004.12	+	153550073	153558368	8296	421	sense-overlappin	NM_001131007.1	KIAA0922
circRNA_03804	0	0	0	0	0.03009467	0	0	0	0	0	0	0	-	NC_000004.12	-	154269900	154315987	46088	1557	sense-overlappin	XM_011532049.1	DCHS2
circRNA_03805	0	0	0	0	0	0	0	0	0	0.020172483	0	0	-	NC_000004.12	-	154297851	154298708	858	858	exonic	XM_011532049.1	DCHS2
circRNA_03806	0	0	0	0	0.06018934	0	0	0	0	0	0	0	-	NC_000004.12	-	154297851	154315987	18137	1443	sense-overlappin	XM_011532049.1	DCHS2
circRNA_03807	0.041150049	0	0	0	0	0	0	0.020486295	0	0	0	0	-	NC_000004.12	+	155696756	155703993	7238	344	sense-overlappin	XM_005262956.1	GUCY1A3
circRNA_03808	0.020575025	0	0	0	0	0	0	0	0	0	0	0	-	NC_000004.12	+	155696756	155717302	20547	1743	sense-overlappin	XM_005262956.1	GUCY1A3
circRNA_03809	0.185175222	0.051174919	0.021808645	0.0206362	0	0.062138728	0	0.020486295	0.060763583	0.090776173	0.091148535	0	-	NC_000004.12	+	155696756	155722192	25437	1898	sense-overlappin	XM_005262956.1	GUCY1A3

circRNA_03810	0	0	0	0	0	0	0	0	0	0.020172483	0	0	-	NC_000004.12	+	155774968	155804747	29780	1698	sense-overlappin	NM_001291951.1	GUCY1B3
circRNA_03811	0	0	0	0	0	0	0.020098685	0	0	0	0	0	-	NC_000004.12	+	158237525	158244349	6825	391	sense-overlappin	XM_005263112.2	TMEM144
circRNA_03812	0	0	0.021808645	0	0	0.020712909	0	0	0	0	0	0	hsa_circ_0006432	NC_000004.12	+	158825916	158835476	9561	710	sense-overlappin	XM_005263158.1	FNIP2
circRNA_03813	0	0	0.032712968	0.0206362	0	0	0	0	0	0	0	0	-	NC_000004.12	+	158851321	158870469	19149	2222	sense-overlappin	NM_020840.1	FNIP2
circRNA_03814	0	0	0.021808645	0.0206362	0	0	0	0	0	0	0	0	-	NC_000004.12	+	158868102	158870469	2368	1484	sense-overlappin	NM_020840.1	FNIP2
circRNA_03815	0	0	0.021808645	0	0	0	0	0	0	0	0	0	-	NC_000004.12	+	159186642	159241368	54727	456	sense-overlappin	XM_005263358.2	RAPGEF2
circRNA_03816	0	0	0	0	0	0	0	0	0	0	0.030382845	0	hsa_circ_0007121	NC_000004.12	+	159238809	159241368	2560	244	sense-overlappin	XM_005263358.2	RAPGEF2
circRNA_03817	0	0	0	0	0	0	0	0	0	0	0	0.072227401	-	NC_000004.12	+	159238809	159243791	4983	262	sense-overlappin	XM_005263358.2	RAPGEF2
circRNA_03818	0	0	0	0.0206362	0	0	0	0	0	0	0	0	-	NC_000004.12	+	159304342	159332697	28356	1592	sense-overlappin	XM_005263358.2	RAPGEF2
circRNA_03819	0	0	0	0.0206362	0	0	0	0	0	0	0	0	hsa_circ_0071369	NC_000004.12	-	165085586	165103096	17511	650	sense-overlappin	NM_001100389.1	TMEM192
circRNA_03820	0	0	0	0	0	0.020712909	0	0	0	0	0	0	-	NC_000004.12	-	165100628	165103096	2469	412	sense-overlappin	NM_001100389.1	TMEM192
circRNA_03821	0	0	0.076330258	0	0	0	0	0	0	0	0	0	hsa_circ_0071384	NC_000004.12	+	165294359	165299656	5298	377	sense-overlappin	NM_007246.3	KLHL2
circRNA_03822	0	0.040939935	0	0	0	0	0	0	0	0	0	0	-	NC_000004.12	+	166039339	166078030	38692	1368	sense-overlappin	XM_011532213.1	TLL1
circRNA_03823	0	0	0.021808645	0	0	0	0	0	0	0	0	0	-	NC_000004.12	-	168220655	168225676	5022	506	sense-overlappin	NM_017631.5	DDX60
circRNA_03824	0	0	0.141756193	0	0.05015779	0	0	0	0	0	0	0	hsa_circ_0004303	NC_000004.12	-	168220655	168237795	17141	875	sense-overlappin	NM_017631.5	DDX60
circRNA_03825	0	0	0.04361729	0.0206362	0	0	0	0	0	0	0	0.0206364	hsa_circ_0005462	NC_000004.12	-	168236252	168237795	1544	369	sense-overlappin	NM_017631.5	DDX60
circRNA_03826	0	0	0.021808645	0	0	0	0	0	0	0	0	0	-	NC_000004.12	-	168251064	168252646	1583	181	sense-overlappin	NM_017631.5	DDX60
circRNA_03827	0	0	0.021808645	0	0	0	0	0	0	0	0	0	-	NC_000004.12	-	168302300	168311365	9066	829	sense-overlappin	NM_017631.5	DDX60
circRNA_03828	0	0	0.032712968	0	0.02006311	0	0	0	0	0.030258724	0	0	-	NC_000004.12	-	168400826	168458008	57183	2885	sense-overlappin	XM_005263345.3	DDX60L
circRNA_03829	0	0	0	0	0	0	0.020098685	0	0	0	0	0	-	NC_000004.12	-	168863880	168928677	64798	18420	antisense	XM_011531770.1	PALLD
circRNA_03830	0.051437562	0	0	0.0206362	0	0	0	0	0	0.020172483	0	0	hsa_circ_0006358	NC_000004.12	+	168890922	168903906	12985	658	sense-overlappin	XM_005262861.3	PALLD
circRNA_03831	0.030862537	0	0.021808645	0	0	0	0.020098685	0	0	0.020172483	0	0	hsa_circ_0071410	NC_000004.12	+	168890922	168916027	25106	886	sense-overlappin	XM_005262861.3	PALLD
circRNA_03832	0.020575025	0	0	0.0206362	0	0	0.020098685	0	0	0	0	0	-	NC_000004.12	-	169002071	169007756	5686	423	sense-overlappin	XR_938789.1	
circRNA_03833	0	0	0	0	0.04012623	0.020712909	0	0	0.040509055	0	0	0	hsa_circ_0071422	NC_000004.12	-	169155480	169156679	1200	372	sense-overlappin	NM_020870.3	SH3RF1
circRNA_03834	0.020575025	0	0	0	0	0	0	0	0	0	0	0	-	NC_000004.12	-	169463243	169508331	45089	838	sense-overlappin	NM_001199397.1	NEK1
circRNA_03835	0	0	0	0	0	0	0	0	0	0.020172483	0	0	hsa_circ_0071427	NC_000004.12	-	169463243	169537911	74669	1025	sense-overlappin	NM_001199397.1	NEK1
circRNA_03836	0	0	0	0.0309543	0	0.031069364	0	0	0	0	0	0	-	NC_000004.12	-	169463243	169556095	92853	1321	sense-overlappin	NM_001199397.1	NEK1
circRNA_03837	0	0	0	0.0412724	0	0.031069364	0	0	0	0	0	0	hsa_circ_0071430	NC_000004.12	-	169477124	169556095	78972	1168	sense-overlappin	NM_001199397.1	NEK1
circRNA_03838	0	0	0	0	0	0.020712909	0	0	0	0	0.030382845	0	hsa_circ_0001456	NC_000004.12	-	169507037	169508331	1295	258	sense-overlappin	NM_001199397.1	NEK1
circRNA_03839	0.030862537	0	0.065425935	0	0	0.062138728	0	0	0	0	0	0	-	NC_000004.12	-	169507037	169537911	30875	445	sense-overlappin	NM_001199397.1	NEK1
circRNA_03840	0	0	0	0.0412724	0.02006311	0	0	0	0	0.030258724	0	0	-	NC_000004.12	-	169507037	169556095	49059	741	sense-overlappin	NM_001199397.1	NEK1
circRNA_03841	0	0	0	0.0309543	0	0	0.020098685	0.020486295	0.020254528	0.030258724	0	0	hsa_circ_0003196	NC_000004.12	-	169537809	169556095	18287	399	sense-overlappin	NM_001199397.1	NEK1
circRNA_03842	0	0.020469968	0	0	0	0.020712909	0	0	0	0	0	0	-	NC_000004.12	-	169576928	169590809	13882	708	sense-overlappin	NM_001199397.1	NEK1
circRNA_03843	0	0	0	0	0	0	0	0	0.020254528	0	0	0	-	NC_000004.12	-	169576928	169599197	22270	806	sense-overlappin	NM_001199397.1	NEK1
circRNA_03844	0.020575025	0.020469968	0.021808645	0.0206362	0	0.031069364	0.030148028	0	0	0	0	0	hsa_circ_0071434	NC_000004.12	-	169580842	169590809	9968	556	sense-overlappin	NM_001199397.1	NEK1
circRNA_03845	0	0	0.021808645	0	0	0.062138728	0	0	0	0.030258724	0	0	-	NC_000004.12	-	169580842	169602678	21837	916	sense-overlappin	NM_001199397.1	NEK1
circRNA_03846	0	0	0	0.0309543	0	0.020712909	0	0	0	0	0	0	-	NC_000004.12	-	169585349	169599197	13849	593	sense-overlappin	NM_001199397.1	NEK1
circRNA_03847	0.030862537	0	0.163564838	0.0309543	0	0	0	0	0	0	0	0.0412728	hsa_circ_0001458	NC_000004.12	+	173247980	173248440	461	461	exonic	NM_017423.2	GALNT7
circRNA_03848	0	0	0.032712968	0	0	0	0	0	0	0	0	0	-	NC_000004.12	+	173247980	173304118	56139	1263	sense-overlappin	NM_017423.2	GALNT7
circRNA_03849	0	0	0.032712968	0.0412724	0	0	0	0	0	0	0	0	-	NC_000004.12	+	173292108	173304118	12011	802	sense-overlappin	NM_017423.2	GALNT7
circRNA_03850	0	0.020469968	0.032712968	0	0	0	0	0	0	0	0	0	-	NC_000004.12	+	173384651	173403950	19300	16523	antisense	XM_011531578.1	SCRG1
circRNA_03851	0	0	0.065425935	0	0.02006311	0.051782273	0	0	0	0	0	0	hsa_circ_0001459	NC_000004.12	+	177353308	177353728	421	421	exonic	NM_018248.2	NEIL3
circRNA_03852	0.020575025	0.030704951	0.163564838	0	0.08025246	0	0.030148028	0.040972591	0.030381792	0.050431207	0.04051046	0.0206364	hsa_circ_0001460	NC_000004.12	+	177353308	177360677	7370	596	sense-overlappin	NM_018248.2	NEIL3
circRNA_03853	0.020575025	0	0	0	0	0	0	0	0	0	0	0	hsa_circ_0002840	NC_000004.12	+	182323946	182324252	307	307	sense-overlappin	NM_001080477.2	TENM3
circRNA_03854	0	0	0	0	0	0.031069364	0	0	0	0	0	0	-	NC_000004.12	-	182877511	182915575	38065	14432	sense-overlappin	XM_005262778.2	DCTD
circRNA_03855	0	0	0.021808645	0	0	0.020712909	0	0	0	0.040344966	0	0	hsa_circ_0002212	NC_000004.12	-	182891403	182915575	24173	540	sense-overlappin	XM_005262778.2	DCTD
circRNA_03856	0	0	0.021808645	0	0	0	0	0	0	0	0	0	-	NC_000004.12	+	183683975	183685403	1429	555	sense-overlappin	XM_011532180.1	TRAPPC11
circRNA_03857	0	0	0	0	0	0	0	0.020486295	0	0	0	0	-	NC_000004.12	+	183683975	183686748	2774	686	sense-overlappin	XM_011532180.1	TRAPPC11
circRNA_03858	0.030862537	0	0	0	0	0	0	0	0	0	0	0	hsa_circ_0071510	NC_000004.12	+	183683975	183693147	9173	1030	sense-overlappin	XM_011532180.1	TRAPPC11
circRNA_03859	0	0	0	0.0206362	0	0	0	0	0	0	0	0	hsa_circ_0071515	NC_000004.12	+	183693589	183701808	8220	726	sense-overlappin	XM_011532180.1	TRAPPC11
circRNA_03860	0	0	0.021808645	0	0	0	0	0	0	0	0	0	-	NC_000004.12	+	183852880	183853843	964	964	intronic	XR_938767.1	
circRNA_03861	0	0	0	0.0206362	0	0	0	0	0.020254528	0	0	0	hsa_circ_0071519	NC_000004.12	+	184001325	184011423	10099	2419	sense-overlappin	XR_938767.1	
circRNA_03862	0	0	0	0.0206362	0	0	0	0	0	0	0	0	-	NC_000004.12	+	184001325	184011635	10311	2468	sense-overlappin	XR_938767.1	
circRNA_03863	0	0	0	0.0309543	0	0	0	0.030729443	0	0	0	0	hsa_circ_0071520	NC_000004.12	+	184009158	184011423	2266	2266	exonic	XR_938767.1	
circRNA_03864	0.041150049	0	0	0	0	0	0	0	0	0	0	0	-	NC_000004.12	+	184009158	184011635	2478	2315	sense-overlappin	XR_938767.1	

circRNA_03865	0	0	0.032712968	0	0	0	0	0	0	0	0	0	-	NC_000004.12	-	184398915	184419568	20654	607	sense-overlappin	XM_011531928.1	IRF2
circRNA_03866	0	0	0	0	0	0	0	0.030729443	0	0	0	0	-	NC_000004.12	-	184418167	184419568	1402	324	sense-overlappin	XM_011531928.1	IRF2
circRNA_03867	0	0	0	0	0	0	0	0.030729443	0.020254528	0.07060369	0	0.0309546	hsa_circ_0003788	NC_000004.12	+	184659340	184678394	19055	827	sense-overlappin	NM_001300767.1	PRIMPOL
circRNA_03868	0	0	0.032712968	0	0	0	0	0.020486295	0	0.030258724	0	0	hsa_circ_0004697	NC_000004.12	-	185175786	185176051	266	266	exonic	XM_011532152.1	CFAP97
circRNA_03869	0.030862537	0	0.228990773	0.0619086	0.02006311	0.041425819	0	0	0.040509055	0.020172483	0.030382845	0	hsa_circ_0004874	NC_000004.12	+	185247294	185267155	19862	662	sense-overlappin	XM_011532309.1	SNX25
circRNA_03870	0	0	0.032712968	0	0	0	0	0	0	0	0	0	hsa_circ_0003237	NC_000004.12	+	185247294	185288082	40789	832	sense-overlappin	XR_938776.1	
circRNA_03871	0	0	0.021808645	0	0	0	0	0	0	0	0	0	-	NC_000004.12	+	185247294	185346650	99357	1961	sense-overlappin	XR_938776.1	
circRNA_03872	0	0	0.021808645	0	0	0	0	0	0	0	0	0	hsa_circ_0071570	NC_000004.12	+	185339379	185342116	2738	273	sense-overlappin	XM_011532309.1	SNX25
circRNA_03873	0	0	0	0	0	0	0	0	0	0	0	0.0206364	-	NC_000004.12	-	185523362	185525171	1810	237	sense-overlappin	NM_001114107.4	PDLIM3
circRNA_03874	0	0	0	0	0	0.020712909	0	0	0	0	0	0	-	NC_000004.12	-	185611780	185630598	18819	2400	sense-overlappin	NM_001145674.1	SORBS2
circRNA_03875	0	0	0.04361729	0.0309543	0	0	0	0	0	0	0	0	-	NC_000004.12	+	186149173	186167262	18090	1682	sense-overlappin	XM_005262912.1	FAM149A
circRNA_03876	0	0	0	0	0	0	0	0	0.020254528	0	0	0	-	NC_000004.12	-	186617005	186621775	4771	4265	sense-overlappin	NM_005245.3	FAT1
circRNA_03877	0	0	0	0	0	0	0.030148028	0	0	0	0	0	hsa_circ_0071616	NC_000004.12	-	186663299	186709845	46547	3598	sense-overlappin	NM_005245.3	FAT1
circRNA_03878	0.195462734	0.214934659	1.035910638	0.3095428	0	0.372832368	0.160789483	0.153647216	0.070890847	0.231983552	0.02025523	0.113500201	hsa_circ_0001461	NC_000004.12	-	186706563	186709845	3283	3283	exonic	NM_005245.3	FAT1
circRNA_03879	0.030862537	0	0.021808645	0	0	0.020712909	0	0	0	0.030258724	0	0	-	NC_000005.10	+	228185	233645	5461	443	sense-overlappin	XM_005248331.2	SDHA
circRNA_03880	0	0	0.032712968	0	0	0	0	0	0	0	0	0	-	NC_000005.10	+	618990	633947	14958	609	sense-overlappin	NM_018140.3	CEP72
circRNA_03881	0	0	0.021808645	0	0	0	0	0	0	0	0	0	hsa_circ_0071660	NC_000005.10	+	633769	644425	10657	1154	sense-overlappin	NM_018140.3	CEP72
circRNA_03882	0	0	0.021808645	0	0	0	0	0	0	0	0	0	-	NC_000005.10	+	647805	655584	7780	445	sense-overlappin	XR_925630.1	
circRNA_03883	0	0	0.141756193	0	0	0	0	0	0	0	0	0	-	NC_000005.10	-	693278	770117	76840	19442	sense-overlappin	XM_011514172.1	ZDHHC11B
circRNA_03884	0	0	0.021808645	0	0	0	0	0	0	0	0	0	-	NC_000005.10	-	700645	733839	33195	10572	sense-overlappin	XM_011514179.1	ZDHHC11B
circRNA_03885	0	0	0.272608063	0	0	0	0	0	0	0	0	0	-	NC_000005.10	-	730434	733839	3406	123	sense-overlappin	XM_011514179.1	ZDHHC11B
circRNA_03886	0	0	0.021808645	0	0	0	0	0	0	0	0	0	-	NC_000005.10	-	730434	770117	39684	1626	sense-overlappin	XM_011514172.1	ZDHHC11B
circRNA_03887	0	0	0.021808645	0	0	0	0	0	0	0	0	0	-	NC_000005.10	-	751133	840650	89518	46876	sense-overlappin	XR_925654.1	
circRNA_03888	0	0	0.098138903	0	0	0	0	0	0	0	0	0	-	NC_000005.10	-	814761	825251	10491	927	sense-overlappin	XR_925654.1	
circRNA_03889	0.020575025	0	0	0	0	0	0	0	0	0	0	0	-	NC_000005.10	-	881107	886707	5601	325	sense-overlappin	NM_001009877.2	BRD9
circRNA_03890	0	0	0.021808645	0	0	0	0	0	0	0	0	0	-	NC_000005.10	-	1611669	1612072	404	404	intergenic		
circRNA_03891	0	0.051174919	0.04361729	0	0	0	0	0.020486295	0	0	0	0	hsa_circ_0001466	NC_000005.10	-	6604138	6605408	1271	356	sense-overlappin	NM_001193455.1	NSUN2
circRNA_03892	0	0	0	0	0	0.020712909	0	0	0	0	0	0	hsa_circ_0007380	NC_000005.10	-	6623214	6625669	2456	178	sense-overlappin	NM_001193455.1	NSUN2
circRNA_03893	0	0	0.109043225	0	0	0.031069364	0.030148028	0	0	0.050431207	0	0.0309546	hsa_circ_0008903	NC_000005.10	+	6737510	6746427	8918	743	sense-overlappin	XM_005248234.2	PAPD7
circRNA_03894	0	0	0	0	0.05015779	0	0	0	0	0	0	0	-	NC_000005.10	-	8783435	8783711	277	277	intergenic		
circRNA_03895	0	0	0.021808645	0	0	0	0	0	0	0	0	0	hsa_circ_0002753	NC_000005.10	-	10213491	10224061	10571	10571	intergenic		
circRNA_03896	0	0	0.032712968	0	0	0	0	0	0	0	0	0	hsa_circ_0003698	NC_000005.10	-	10213491	10227647	14157	14157	sense-overlappin	NM_001258388.1	FAM173B
circRNA_03897	0.072012586	0.092114854	0.141756193	0.0825448	0	0.072495183	0.020098685	0.040972591	0.040509055	0.090776173	0.06076569	0.0412728	hsa_circ_0001467	NC_000005.10	+	10415488	10417404	1917	317	sense-overlappin	NM_001270660.1	6-Mar
circRNA_03898	0	0	0	0	0	0	0	0.030729443	0	0	0	0	hsa_circ_0005645	NC_000005.10	+	10415488	10426522	11035	540	sense-overlappin	NM_001270660.1	6-Mar
circRNA_03899	0	0	0.021808645	0	0.03009467	0	0	0	0	0.020172483	0	0.0412728	-	NC_000005.10	-	13911386	13931244	19859	1587	sense-overlappin	NM_001369.2	DNAH5
circRNA_03900	0	0	0	0	0	0	0.030148028	0	0	0	0	0	hsa_circ_0005323	NC_000005.10	+	14270825	14287063	16239	383	sense-overlappin	XM_011514113.1	TRIO
circRNA_03901	0	0	0	0	0	0	0	0	0	0	0.02025523	0	hsa_circ_0005683	NC_000005.10	+	14290716	14336727	46012	1506	sense-overlappin	XM_011514113.1	TRIO
circRNA_03902	0	0	0.065425935	0	0.03009467	0	0.020098685	0	0	0	0	0	hsa_circ_0005260	NC_000005.10	+	14316513	14336727	20215	546	sense-overlappin	XM_011514113.1	TRIO
circRNA_03903	0	0	0.032712968	0	0.03009467	0	0	0	0	0	0	0	hsa_circ_0008646	NC_000005.10	+	14330778	14336727	5950	315	sense-overlappin	XM_011514113.1	TRIO
circRNA_03904	0.020575025	0	0	0	0	0	0	0	0	0	0	0	hsa_circ_0008697	NC_000005.10	+	14405848	14420021	14174	487	sense-overlappin	XM_011514113.1	TRIO
circRNA_03905	0.020575025	0	0.054521613	0	0	0.020712909	0.020098685	0	0	0	0	0	hsa_circ_0005145	NC_000005.10	+	14673642	14681607	7966	316	sense-overlappin	NM_138348.4	OTULIN
circRNA_03906	0	0	0.04361729	0.0206362	0	0.041425819	0	0	0.020254528	0.020172483	0.02025523	0	-	NC_000005.10	+	14673642	14693094	19453	953	sense-overlappin	NM_138348.4	OTULIN
circRNA_03907	0	0	0.021808645	0	0	0	0	0	0	0	0	0	-	NC_000005.10	+	14678681	14693094	14414	876	sense-overlappin	NM_138348.4	OTULIN
circRNA_03908	0	0	0.021808645	0	0	0	0	0	0	0	0	0	hsa_circ_0071973	NC_000005.10	-	14741827	14751239	9413	495	sense-overlappin	NM_054027.4	ANKH
circRNA_03909	0	0	0	0	0	0	0	0	0	0.020172483	0	0	-	NC_000005.10	+	15615983	15634464	18482	18482	sense-overlappin	XM_005248273.3	FBXL7
circRNA_03910	0	0	0.032712968	0	0	0	0	0	0	0	0	0	-	NC_000005.10	-	16477662	16483472	5811	542	sense-overlappin	XM_011514054.1	FAM134B
circRNA_03911	0	0	0	0	0.02006311	0.020712909	0	0	0	0.020172483	0	0	-	NC_000005.10	-	16684970	16685320	351	351	intronic	NM_012334.2	MYO10
circRNA_03912	0	0	0	0	0	0	0	0.061458886	0	0	0	0	-	NC_000005.10	-	16702543	16702976	434	98	sense-overlappin	NM_012334.2	MYO10
circRNA_03913	0	0	0	0	0	0	0.020098685	0	0	0	0	0	hsa_circ_0072013	NC_000005.10	-	16763481	16764396	916	315	sense-overlappin	NM_012334.2	MYO10
circRNA_03914	0	0	0	0	0.02006311	0	0	0.030729443	0	0	0	0	hsa_circ_0072014	NC_000005.10	-	16763481	16766198	2718	434	sense-overlappin	NM_012334.2	MYO10
circRNA_03915	0.123450148	0.040939935	0.119947548	0.0309543	0.34107295	0.041425819	0.090444084	0.061458886	0.091145375	0.030258724	0.030382845	0	-	NC_000005.10	-	16779545	16783469	3925	463	sense-overlappin	NM_012334.2	MYO10
circRNA_03916	0	0	0	0	0	0	0.020098685	0	0	0	0	0	-	NC_000005.10	-	16916055	16917042	988	988	intronic	NM_012334.2	MYO10
circRNA_03917	0	0	0.054521613	0.0619086	0.04012623	0.041425819	0.291430938	0.204862955	0.091145375	0.121034897	0.506380752	0.268273202	-	NC_000005.10	+	21491321	21497196	5876	5876	intergenic		
circRNA_03918	0	0	0.054521613	0.0412724	0.04012623	0	0.020098685	0.040972591	0.030381792	0.040344966	0	0	hsa_circ_0005524	NC_000005.10	-	31421272	31424471	3200	309	sense-overlappin	NM_001100412.1	DROSHA
circRNA_03919	0	0.040939935	0.065425935	0	0	0	0	0	0	0	0	0	hsa_circ_0004599	NC_000005.10	-	31448547	31451640	3094	308	sense-overlappin	NM_001100412.1	DROSHA

circRNA_03920	0	0	0.032712968	0	0	0	0	0	0	0	0	0	hsa_circ_0072056	NC_000005.10	-	31448547	31472232	23686	811	sense-overlappin	NM_001100412.1	DROSHA
circRNA_03921	0.030862537	0	0	0	0	0	0	0	0	0	0.02025523	0	-	NC_000005.10	+	31995576	32010482	14907	429	sense-overlappin	XM_005248269.3	PDZD2
circRNA_03922	0	0	0	0	0	0	0	0	0	0.030258724	0	0	-	NC_000005.10	-	32121495	32126636	5142	5142	sense-overlappin	NM_022130.3	GOLPH3
circRNA_03923	0.020575025	0.040939935	0	0	0.03009467	0	0	0	0	0	0	0	-	NC_000005.10	-	32121495	32143880	22386	5389	sense-overlappin	NM_022130.3	GOLPH3
circRNA_03924	0.113162635	0.08187987	0.250799418	0.1960438	0.24075738	0.144990365	0.090444084	0.163890364	0.070890847	0.131121138	0.06076569	0.165091201	hsa_circ_0001470	NC_000005.10	-	32135572	32143880	8309	247	sense-overlappin	NM_022130.3	GOLPH3
circRNA_03925	0	0	0	0	0.03009467	0	0	0.020486295	0	0	0	0	-	NC_000005.10	-	32143749	32170768	27020	27020	sense-overlappin	NM_022130.3	GOLPH3
circRNA_03926	0	0	0	0.0206362	0	0	0	0	0	0	0	0	hsa_circ_0072081	NC_000005.10	-	32268701	32276742	8042	502	sense-overlappin	XM_005248313.2	MTMR12
circRNA_03927	0	0.020469968	0	0	0	0	0	0	0	0	0	0	-	NC_000005.10	-	32273980	32276742	2763	204	sense-overlappin	XM_005248313.2	MTMR12
circRNA_03928	0	0	0	0	0	0	0	0	0	0	0	0.0206364	hsa_circ_0072088	NC_000005.10	-	32379115	32388674	9560	693	sense-overlappin	NM_016107.3	ZFR
circRNA_03929	0	0	0.032712968	0	0	0	0	0	0	0	0	0	-	NC_000005.10	-	32379115	32390437	11323	856	sense-overlappin	NM_016107.3	ZFR
circRNA_03930	0	0	0.032712968	0	0	0	0	0	0	0	0	0	-	NC_000005.10	-	32379115	32400203	21089	1319	sense-overlappin	NM_016107.3	ZFR
circRNA_03931	0	0	0	0	0	0.020712909	0	0	0	0	0	0	-	NC_000005.10	-	32390275	32397338	7064	429	sense-overlappin	NM_016107.3	ZFR
circRNA_03932	0	0	0	0.0309543	0	0.020712909	0	0	0	0	0	0	-	NC_000005.10	-	32395159	32400203	5045	463	sense-overlappin	NM_016107.3	ZFR
circRNA_03933	0	0	0	0	0	0	0	0	0	0	0	0.0206364	-	NC_000005.10	+	33466871	33470140	3270	2696	sense-overlappin	NM_001258437.1	TARS
circRNA_03934	0.020575025	0	0	0	0	0	0	0	0	0	0	0	-	NC_000005.10	-	33624231	33643470	19240	664	sense-overlappin	XM_011514145.1	ADAMTS12
circRNA_03935	0	0	0	0	0	0.041425819	0	0	0	0	0	0	-	NC_000005.10	-	34020587	34052110	31524	9722	sense-overlappin	NM_030945.3	C1QTNF3
circRNA_03936	0	0	0.13085187	0.0309543	0	0	0.040197371	0	0.050636319	0.07060369	0.04051046	0.082545601	-	NC_000005.10	-	34179013	34182867	3855	3855	intergenic		
circRNA_03937	0	0	0	0	0.04012623	0	0	0	0	0	0	0	hsa_circ_0006970	NC_000005.10	+	34918364	34923234	4871	504	sense-overlappin	NM_018321.3	BRIX1
circRNA_03938	0	0	0	0	0.02006311	0	0	0	0.030381792	0	0	0	-	NC_000005.10	+	34922217	34923234	1018	348	sense-overlappin	NM_018321.3	BRIX1
circRNA_03939	0	0	0.032712968	0	0	0	0	0	0	0	0	0	-	NC_000005.10	-	36207170	36227565	20396	722	sense-overlappin	NM_001287341.1	NADK2
circRNA_03940	0.030862537	0	0	0	0	0	0	0	0	0	0	0	hsa_circ_0002292	NC_000005.10	-	36211844	36227565	15722	560	sense-overlappin	NM_001287340.1	NADK2
circRNA_03941	0	0	0	0	0.03009467	0	0.020098685	0	0	0	0	0	hsa_circ_0072161	NC_000005.10	+	36953618	36962274	8657	689	sense-overlappin	NM_015384.4	NIPBL
circRNA_03942	0.082300098	0.061409903	0.21808645	0.1341352	0.11034713	0.093208092	0.120592112	0.081945182	0.081018111	0.080689931	0.070893305	0.0309546	hsa_circ_0001472	NC_000005.10	+	36953618	36976402	22785	1574	sense-overlappin	NM_015384.4	NIPBL
circRNA_03943	0	0	0	0.0309543	0	0	0	0	0	0.030258724	0	0	hsa_circ_0072162	NC_000005.10	+	36953618	36986301	32684	3200	sense-overlappin	NM_015384.4	NIPBL
circRNA_03944	0	0.061409903	0.250799418	0.1238171	0.19059959	0.062138728	0.080394741	0.051215739	0.070890847	0.14120738	0.091148535	0.0619092	-	NC_000005.10	+	36982165	36986301	4137	4137	sense-overlappin	NM_015384.4	NIPBL
circRNA_03945	0	0	0.054521613	0	0.02006311	0	0	0	0	0	0	0.0309546	-	NC_000005.10	+	36984676	36986301	1626	1626	exonic	NM_015384.4	NIPBL
circRNA_03946	0	0.040939935	0	0	0	0	0	0	0	0	0	0	-	NC_000005.10	+	36984676	36995804	11129	1809	sense-overlappin	NM_015384.4	NIPBL
circRNA_03947	0	0	0	0	0	0.020712909	0	0	0	0	0	0	-	NC_000005.10	+	37024585	37027412	2828	288	sense-overlappin	NM_015384.4	NIPBL
circRNA_03948	0	0	0	0	0	0	0	0	0.030381792	0	0	0	hsa_circ_0072202	NC_000005.10	-	37114960	37120340	5381	215	sense-overlappin	XR_427661.2	
circRNA_03949	0.020575025	0	0	0	0	0	0	0	0	0	0	0	-	NC_000005.10	-	37120216	37122488	2273	352	sense-overlappin	XR_427661.2	
circRNA_03950	0	0	0	0.0206362	0	0	0	0	0	0	0	0	-	NC_000005.10	-	37179361	37181005	1645	399	sense-overlappin	XR_427661.2	
circRNA_03951	0	0	0	0	0	0	0	0.020486295	0	0	0	0	-	NC_000005.10	-	37221324	37247745	26422	2793	sense-overlappin	XR_427661.2	
circRNA_03952	0	0	0.032712968	0.0412724	0	0	0	0	0	0	0	0	-	NC_000005.10	-	37238857	37239869	1013	261	sense-overlappin	XR_427661.2	
circRNA_03953	0	0	0	0	0	0	0	0.020486295	0	0.020172483	0	0	-	NC_000005.10	-	37238857	37247745	8889	985	sense-overlappin	XR_427661.2	
circRNA_03954	0	0	0.021808645	0	0	0	0	0	0	0	0	0	hsa_circ_0008755	NC_000005.10	-	37243013	37247745	4733	724	sense-overlappin	XR_427661.2	
circRNA_03955	0	0	0.021808645	0	0	0	0	0	0	0	0	0	hsa_circ_0003227	NC_000005.10	-	37301437	37303414	1978	399	sense-overlappin	NM_001278312.1	NUP155
circRNA_03956	0.030862537	0	0	0	0	0	0	0	0	0	0	0	hsa_circ_0072255	NC_000005.10	-	37325901	37328420	2520	278	sense-overlappin	NM_001278312.1	NUP155
circRNA_03957	0	0	0	0.0515905	0	0	0	0	0	0.020172483	0	0	-	NC_000005.10	-	38510464	38530666	20203	1010	sense-overlappin	XM_011514041.1	LIFR
circRNA_03958	0.041150049	0.102349838	0.032712968	0.3095428	0.08025246	0.144990365	0.060296056	0.112674625	0.040509055	0.100862414	0	0.134136601	hsa_circ_0072309	NC_000005.10	-	38523419	38530666	7248	580	sense-overlappin	XM_011514041.1	LIFR
circRNA_03959	0	0	0	0.0206362	0	0	0	0	0	0	0	0	-	NC_000005.10	-	38527155	38530666	3512	416	sense-overlappin	XM_011514041.1	LIFR
circRNA_03960	0	0	0.021808645	0	0	0	0	0	0	0	0	0	-	NC_000005.10	+	38869032	38885484	16453	852	sense-overlappin	NM_001168355.1	OSMR
circRNA_03961	0	0	0.04361729	0	0	0	0	0	0	0	0	0	-	NC_000005.10	+	38869032	38886190	17159	1004	sense-overlappin	NM_001168355.1	OSMR
circRNA_03962	0	0	0.021808645	0	0	0	0	0	0	0	0	0	hsa_circ_0072311	NC_000005.10	+	38903882	38924595	20714	1053	sense-overlappin	NM_003999.2	OSMR
circRNA_03963	0	0	0	0	0	0.051782273	0	0.020486295	0	0	0	0	hsa_circ_0072330	NC_000005.10	-	38967161	38978650	11490	465	sense-overlappin	XM_011514006.1	RICTOR
circRNA_03964	0	0.020469968	0	0	0	0	0	0	0	0	0	0	-	NC_000005.10	-	38970464	38970618	155	155	intronic	XM_011514007.1	RICTOR
circRNA_03965	0	0	0	0	0.02006311	0	0	0	0	0.020172483	0	0	hsa_circ_0072331	NC_000005.10	-	38971877	38978650	6774	219	sense-overlappin	XM_011514006.1	RICTOR
circRNA_03966	0.051437562	0	0.065425935	0.0412724	0	0	0	0	0.020254528	0	0	0.0206364	hsa_circ_0006157	NC_000005.10	-	38990949	39003622	12674	388	sense-overlappin	XM_011514006.1	RICTOR
circRNA_03967	0.041150049	0	0.032712968	0.0825448	0	0	0.020098685	0	0.020254528	0	0	0	hsa_circ_0006308	NC_000005.10	-	38990949	39021136	30188	486	sense-overlappin	NM_001285439.1	RICTOR
circRNA_03968	0	0	0.098138903	0	0	0.031069364	0.020098685	0	0	0.020172483	0	0	hsa_circ_0001475	NC_000005.10	-	39002535	39021136	18602	295	sense-overlappin	NM_001285439.1	RICTOR
circRNA_03969	0	0	0	0	0	0	0.020098685	0	0	0	0	0	-	NC_000005.10	-	39373390	39381616	8227	1013	sense-overlappin	NM_001244871.1	DAB2
circRNA_03970	0.041150049	0	0	0	0	0	0	0.030729443	0	0	0	0	hsa_circ_0002649	NC_000005.10	-	39382618	39388852	6235	771	sense-overlappin	NM_001343.3	DAB2
circRNA_03971	0	0	0.032712968	0	0	0	0	0	0	0.040344966	0	0	hsa_circ_0007795	NC_000005.10	-	40728345	40747019	18675	436	sense-overlappin	NM_012382.2	TTC33
circRNA_03972	0	0	0.021808645	0.0412724	0	0	0	0	0	0.030258724	0	0	hsa_circ_0003548	NC_000005.10	-	40764514	40771863	7350	1072	sense-overlappin	NM_006251.5	PRKAA1
circRNA_03973	0	0	0.021808645	0	0	0	0	0	0	0	0	0	hsa_circ_0072351	NC_000005.10	-	40764514	40775503	10990	1211	sense-overlappin	NM_206907.3	PRKAA1
circRNA_03974	0	0	0.04361729	0.0206362	0	0	0	0	0	0	0	0	hsa_circ_0001478	NC_000005.10	-	40764514	40777586	13073	1353	sense-overlappin	NM_206907.3	PRKAA1
circRNA_03975	0	0.051174919	0.032712968	0.1650895	0	0.062138728	0.020098685	0.102431477	0.050636319	0.100862414	0.050638075	0.0619092	hsa_circ_0005895	NC_000005.10	+	40852174	40854096	1923	1923	exonic	NM_032587.3	CARD6

circRNA_03976	0.051437562	0.030704951	0.021808645	0.0515905	0.03009467	0	0	0.040972591	0.020254528	0	0	0	hsa_circ_0004873	NC_000005.10	-	41794003	41807438	13436	516	sense-overlappin	NM_000436.3	OXCT1
circRNA_03977	0	0	0	0	0	0	0	0	0.020254528	0	0	0	hsa_circ_0072363	NC_000005.10	-	41840451	41862750	22300	654	sense-overlappin	XR_427658.2	
circRNA_03978	0	0.040939935	0	0	0	0	0	0	0.020254528	0	0	0	hsa_circ_0004608	NC_000005.10	+	41927013	41929917	2905	457	sense-overlappin	NM_001297437.1	FBXO4
circRNA_03979	0	0	0	0	0	0	0	0.020486295	0	0	0	0	-	NC_000005.10	+	42688890	42718974	30085	1331	sense-overlappin	NM_000163.4	GHR
circRNA_03980	0	0	0.021808645	0	0	0	0	0	0	0	0	0	hsa_circ_0007071	NC_000005.10	+	43122039	43139309	17271	386	sense-overlappin	XM_005248359.2	ZNF131
circRNA_03981	0	0	0.04361729	0	0	0	0	0	0	0	0	0	hsa_circ_0001479	NC_000005.10	+	43122039	43161931	39893	967	sense-overlappin	XM_005248365.2	ZNF131
circRNA_03982	0	0	0	0	0	0	0	0	0	0	0.02025523	0	-	NC_000005.10	+	43133373	43135093	1721	1721	intronic	XM_005248359.2	ZNF131
circRNA_03983	0	0	0	0	0	0	0	0	0	0	0.02025523	0	-	NC_000005.10	+	43136386	43139309	2924	2924	sense-overlappin	XM_005248359.2	ZNF131
circRNA_03984	0.030862537	0.020469968	0.119947548	0.0309543	0.09028402	0.031069364	0.030148028	0	0	0	0.070893305	0.0206364	hsa_circ_0072380	NC_000005.10	+	43161249	43161931	683	581	exonic	XM_005248365.2	ZNF131
circRNA_03985	0	0	0.054521613	0.0515905	0.04012623	0.031069364	0	0	0	0	0	0	hsa_circ_0008621	NC_000005.10	-	43292474	43297166	4693	899	sense-overlappin	NM_001098272.2	HMGCS1
circRNA_03986	0	0	0.04361729	0	0.03009467	0.020712909	0	0	0	0	0	0	hsa_circ_0072386	NC_000005.10	-	43292474	43298975	6502	1483	sense-overlappin	NM_001098272.2	HMGCS1
circRNA_03987	0	0	0.021808645	0	0.06018934	0	0	0	0	0	0	0	hsa_circ_0072387	NC_000005.10	-	43294056	43297166	3111	609	sense-overlappin	NM_001098272.2	HMGCS1
circRNA_03988	0.020575025	0.020469968	0.032712968	0.0309543	0.06018934	0.041425819	0.030148028	0.020486295	0	0.020172483	0	0	hsa_circ_0072391	NC_000005.10	-	43295752	43297166	1415	331	sense-overlappin	NM_001098272.2	HMGCS1
circRNA_03989	0	0	0.021808645	0	0	0	0	0	0	0	0	0	hsa_circ_0072396	NC_000005.10	-	43543004	43547913	4910	299	sense-overlappin	NM_006451.4	PAIP1
circRNA_03990	0	0	0.109043225	0.0515905	0.04012623	0.051782273	0	0.020486295	0.030381792	0.050431207	0	0.0206364	hsa_circ_0003832	NC_000005.10	+	43675511	43677806	2296	242	sense-overlappin	NM_012343.3	NNT
circRNA_03991	0.061725074	0	0.141756193	0.1238171	0.02006311	0.041425819	0	0	0.040509055	0.100862414	0.02025523	0.0412728	hsa_circ_0005585	NC_000005.10	+	43675511	43704400	28890	623	sense-overlappin	NM_012343.3	NNT
circRNA_03992	0	0	0.021808645	0	0	0	0	0	0	0	0	0	-	NC_000005.10	-	50319621	50411383	91763	80440	sense-overlappin	XM_011543146.1	EMB
circRNA_03993	0	0	0	0	0	0.020712909	0	0	0	0	0	0	-	NC_000005.10	-	50382571	50411383	28813	17490	sense-overlappin	XM_011543146.1	EMB
circRNA_03994	0	0.173994724	0.468885868	0.0928628	0.06018934	0.258911367	0.15074014	0.204862955	0.151908958	0.221897311	0.111403765	0.082545601	hsa_circ_0001481	NC_000005.10	-	50399107	50411383	12277	954	sense-overlappin	XM_011543146.1	EMB
circRNA_03995	0	0	0	0	0	0	0.030148028	0	0.020254528	0	0	0	-	NC_000005.10	-	50399107	50416090	16984	5661	sense-overlappin	XM_011543146.1	EMB
circRNA_03996	0	0	0	0	0	0.031069364	0	0.030729443	0	0	0	0	-	NC_000005.10	-	50399859	50411383	11525	770	sense-overlappin	XM_011543146.1	EMB
circRNA_03997	0	0	0.054521613	0	0	0.041425819	0.050246713	0	0	0	0.030382845	0	hsa_circ_0072428	NC_000005.10	-	50402286	50411383	9098	715	sense-overlappin	XM_011543146.1	EMB
circRNA_03998	0	0	0.04361729	0.0619086	0	0.041425819	0	0.030729443	0.020254528	0.080689931	0	0.0206364	hsa_circ_0005809	NC_000005.10	+	50750151	50763242	13092	469	sense-overlappin	XM_011543637.1	PARP8
circRNA_03999	0.020575025	0	0	0	0	0	0.020098685	0	0	0	0	0	hsa_circ_0072430	NC_000005.10	+	50750151	50778650	28500	621	sense-overlappin	XM_011543637.1	PARP8
circRNA_04000	0	0	0	0.0825448	0	0	0	0.020486295	0.030381792	0	0	0	hsa_circ_0072431	NC_000005.10	+	50750151	50797233	47083	1526	sense-overlappin	XM_011543637.1	PARP8
circRNA_04001	0.030862537	0	0.032712968	0.0206362	0	0.031069364	0.020098685	0	0	0.060517448	0	0.0309546	hsa_circ_0006787	NC_000005.10	+	50759643	50763242	3600	334	sense-overlappin	NM_001178055.1	PARP8
circRNA_04002	0	0	0.021808645	0	0	0	0	0	0	0	0	0	hsa_circ_0072433	NC_000005.10	+	50759643	50797233	37591	1391	sense-overlappin	NM_001178055.1	PARP8
circRNA_04003	0.030862537	0.040939935	0.032712968	0	0	0	0.030148028	0.020486295	0	0	0	0	hsa_circ_0072437	NC_000005.10	+	50778069	50797233	19165	1057	sense-overlappin	NM_001178055.1	PARP8
circRNA_04004	0	0	0	0	0	0	0	0	0.020254528	0	0	0	hsa_circ_0072440	NC_000005.10	+	50788523	50797233	8711	905	sense-overlappin	NM_001178055.1	PARP8
circRNA_04005	0.051437562	0	0	0.0206362	0	0.031069364	0.030148028	0	0.020254528	0.030258724	0	0.0619092	hsa_circ_0004840	NC_000005.10	+	52861447	52865817	4371	442	sense-overlappin	NM_181501.1	ITGA1
circRNA_04006	0	0.030704951	0	0	0	0	0	0	0	0	0	0	-	NC_000005.10	+	52861447	52882021	20575	591	sense-overlappin	NM_181501.1	ITGA1
circRNA_04007	0	0	0	0	0	0.020712909	0	0	0	0	0	0	-	NC_000005.10	+	52897286	52898383	1098	388	sense-overlappin	NM_181501.1	ITGA1
circRNA_04008	0	0	0	0	0.03009467	0	0	0	0	0	0	0	hsa_circ_0005593	NC_000005.10	+	53603452	53604895	1444	251	sense-overlappin	XM_011543414.1	NDUFS4
circRNA_04009	0	0	0	0.0309543	0	0	0	0	0.030381792	0	0	0.0206364	-	NC_000005.10	+	53646233	53658624	12392	247	sense-overlappin	NM_002495.2	NDUFS4
circRNA_04010	0	0	0	0.0309543	0	0	0	0	0	0	0	0	-	NC_000005.10	-	55269413	55281515	12103	1329	sense-overlappin	XM_006714653.1	DHX29
circRNA_04011	0	0	0	0	0	0.041425819	0	0.020486295	0	0	0	0	hsa_circ_0008935	NC_000005.10	-	55274614	55281515	6902	725	sense-overlappin	XM_006714653.1	DHX29
circRNA_04012	0	0	0	0	0	0.031069364	0	0	0	0	0	0	-	NC_000005.10	+	55327716	55345196	17481	769	sense-overlappin	NM_015360.4	SKIV2L2
circRNA_04013	0	0	0.021808645	0	0	0	0	0	0	0	0	0	-	NC_000005.10	+	55340010	55366875	26866	1295	sense-overlappin	NM_015360.4	SKIV2L2
circRNA_04014	0	0.020469968	0	0	0	0	0	0	0	0	0	0	-	NC_000005.10	+	55433595	55434064	470	470	antisense	NM_003711.3	PPAP2A
circRNA_04015	0	0	0	0	0	0	0	0	0	0.020172483	0	0	hsa_circ_0072492	NC_000005.10	-	55467869	55475450	7582	433	sense-overlappin	NM_003711.3	PPAP2A
circRNA_04016	0	0	0	0	0.03009467	0	0	0	0	0	0	0	-	NC_000005.10	-	55633754	55649314	15561	478	sense-overlappin	NM_001258286.1	SLC38A9
circRNA_04017	0	0.020469968	0	0.0515905	0	0	0	0	0	0.040344966	0.030382845	0	hsa_circ_0008731	NC_000005.10	-	55645789	55664863	19075	641	sense-overlappin	NM_001258286.1	SLC38A9
circRNA_04018	0	0	0	0	0.02006311	0	0	0	0	0	0	0	-	NC_000005.10	-	55649207	55656774	7568	363	sense-overlappin	NM_001258286.1	SLC38A9
circRNA_04019	0	0	0	0	0.02006311	0	0	0	0	0	0	0	-	NC_000005.10	-	55652529	55656774	4246	255	sense-overlappin	NM_001258286.1	SLC38A9
circRNA_04020	0	0	0	0	0.02006311	0	0	0	0	0	0	0	hsa_circ_0006456	NC_000005.10	-	55697846	55704434	6589	511	sense-overlappin	XM_011543174.1	SLC38A9
circRNA_04021	0	0.020469968	0	0	0	0	0	0	0	0	0	0	-	NC_000005.10	+	56856600	56859915	3316	352	sense-overlappin	NM_005921.1	MAP3K1
circRNA_04022	0	0	0	0	0	0	0.070345399	0.020486295	0	0	0	0	-	NC_000005.10	+	56856600	56865977	9378	819	sense-overlappin	NM_005921.1	MAP3K1
circRNA_04023	0	0	0	0.0412724	0.03009467	0	0.020098685	0	0	0	0	0.0206364	-	NC_000005.10	+	56856600	56888357	31758	3907	sense-overlappin	NM_005921.1	MAP3K1
circRNA_04024	0	0.051174919	0.04361729	0.0722267	0.04012623	0.093208092	0.190937511	0.09218833	0.020254528	0.090776173	0	0.092863801	hsa_circ_0001485	NC_000005.10	+	56864734	56865977	1244	467	sense-overlappin	NM_005921.1	MAP3K1
circRNA_04025	0	0	0.021808645	0.0309543	0	0.041425819	0.020098685	0.020486295	0	0.040344966	0	0.0206364	hsa_circ_0001486	NC_000005.10	+	57230846	57231321	476	348	sense-overlappin	XM_005248572.2	GPBP1
circRNA_04026	0	0.020469968	0.032712968	0.0412724	0.05015779	0.020712909	0.030148028	0.030729443	0.030381792	0	0	0.0309546	hsa_circ_0072546	NC_000005.10	+	57246300	57247215	916	326	sense-overlappin	XM_005248572.2	GPBP1
circRNA_04027	0	0.061409903	0	0.0412724	0.03009467	0.031069364	0.070345399	0.030729443	0	0.060517448	0	0.0309546	hsa_circ_0002775	NC_000005.10	+	57246300	57249576	3277	494	sense-overlappin	XM_005248572.2	GPBP1
circRNA_04028	0	0.040939935	0	0.0309543	0.0702209	0.020712909	0.020098685	0	0	0.040344966	0	0	hsa_circ_0001487	NC_000005.10	-	58988493	58993465	4973	631	sense-overlappin	NM_001197223.1	PDE4D
circRNA_04029	0	0	0	0	0	0	0	0	0.020254528	0.020172483	0	0.0206364	hsa_circ_0072568	NC_000005.10	-	59180595	59215968	35374	353	sense-overlappin	XM_011543479.1	PDE4D
circRNA_04030	0	0	0	0	0	0	0	0	0	0.020172483	0	0	-	NC_000005.10	-	59450981	59451199	219	219	intronic	XM_011543475.1	PDE4D
circRNA_04031	0	0	0	0	0	0	0.020098685	0	0	0	0	0	-	NC_000005.10	-	59468296	59532734	64439	64439	sense-overlappin	XM_011543478.1	PDE4D

circRNA_04032	0.123450148	0.061409903	0.119947548	0.0619086	0.05015779	0	0	0.030729443	0	0	0	0.051591	hsa_circ_0005399	NC_000005.10	+	61472681	61473037	357	357	exonic	NM_020928.1	ZSWIM6
circRNA_04033	0	0	0	0.0722267	0	0.041425819	0.020098685	0	0	0.040344966	0	0	hsa_circ_0006381	NC_000005.10	+	61472681	61494410	21730	657	sense-overlappin	NM_020928.1	ZSWIM6
circRNA_04034	0	0	0	0	0	0	0	0	0	0.020172483	0	0	-	NC_000005.10	+	62347130	62363899	16770	1403	sense-overlappin	NM_001098511.2	KIF2A
circRNA_04035	0.041150049	0	0	0	0	0	0	0	0	0	0	0	hsa_circ_0004230	NC_000005.10	+	62437274	62451933	14660	522	sense-overlappin	NM_016338.4	IPO11
circRNA_04036	0	0.030704951	0.04361729	0	0	0	0.030148028	0	0.030381792	0	0.02025523	0	hsa_circ_0007915	NC_000005.10	+	62449927	62451933	2007	277	sense-overlappin	NM_016338.4	IPO11
circRNA_04037	0	0	0.021808645	0	0	0	0	0	0	0	0	0	hsa_circ_0072629	NC_000005.10	+	62483101	62515501	32401	1068	sense-overlappin	NM_016338.4	IPO11
circRNA_04038	0	0	0	0	0	0.020712909	0	0	0	0	0	0	-	NC_000005.10	+	62517106	62517497	392	392	intronic	NM_016338.4	IPO11
circRNA_04039	0	0	0.032712968	0	0	0	0	0	0	0.040344966	0.030382845	0	hsa_circ_0001491	NC_000005.10	+	62550367	62561257	10891	332	sense-overlappin	NM_016338.4	IPO11
circRNA_04040	0	0	0.04361729	0.0206362	0	0	0	0	0	0	0	0	-	NC_000005.10	+	64200808	64217396	16589	1227	sense-overlappin	XM_006714589.2	RNF180
circRNA_04041	0.030862537	0	0.032712968	0.0412724	0	0	0.030148028	0	0.020254528	0.07060369	0	0	hsa_circ_0072654	NC_000005.10	+	64788951	64804386	15436	339	sense-overlappin	NM_001297645.1	CWC27
circRNA_04042	0.020575025	0	0	0	0	0	0	0	0.040509055	0.020172483	0	0	hsa_circ_0008667	NC_000005.10	-	65170617	65197151	26535	669	sense-overlappin	XM_011543124.1	ADAMTS6
circRNA_04043	0	0	0	0	0	0	0.020098685	0.020486295	0	0.030258724	0	0	hsa_circ_0072672	NC_000005.10	-	65224320	65226219	1900	339	sense-overlappin	XM_011543124.1	ADAMTS6
circRNA_04044	0	0	0	0	0	0	0	0	0	0	0.02025523	0	hsa_circ_0006489	NC_000005.10	-	65260600	65273447	12848	318	sense-overlappin	XM_011543124.1	ADAMTS6
circRNA_04045	0.051437562	0.092114854	0.152660515	0	0	0.031069364	0	0.051215739	0.081018111	0.131121138	0	0.0412728	hsa_circ_0072688	NC_000005.10	-	65451475	65473952	22478	1352	sense-overlappin	XM_011543114.1	ADAMTS6
circRNA_04046	0	0.051174919	0	0	0	0	0	0	0	0	0	0	-	NC_000005.10	-	65521475	65542848	21374	410	sense-overlappin	XR_948282.1	
circRNA_04047	0	0	0	0	0.03009467	0	0.030148028	0	0	0	0	0	-	NC_000005.10	-	65528452	65529199	748	309	sense-overlappin	XR_948282.1	
circRNA_04048	0.030862537	0	0	0	0	0	0	0	0	0	0	0	-	NC_000005.10	-	65528452	65542848	14397	356	sense-overlappin	XR_948282.1	
circRNA_04049	0	0	0	0	0.02006311	0	0	0	0	0	0	0	-	NC_000005.10	-	65528452	65551636	23185	429	sense-overlappin	XR_948282.1	
circRNA_04050	0	0	0.021808645	0	0	0	0	0	0.040509055	0.080689931	0	0	hsa_circ_0072697	NC_000005.10	+	65567513	65572286	4774	850	sense-overlappin	XM_006714583.2	PPWD1
circRNA_04051	0.123450148	0.122819805	0.272608063	0.1341352	0.1404418	0.082851637	0.200986853	0.133160921	0.172163486	0.171466104	0.04051046	0.113500201	hsa_circ_0001492	NC_000005.10	+	65988635	65994864	6230	364	sense-overlappin	XM_011543514.1	ERBB2IP
circRNA_04052	0.020575025	0.08187987	0.338033998	0.0722267	0.08025246	0	0.110542769	0.102431477	0.030381792	0.07060369	0	0.0412728	hsa_circ_0001493	NC_000005.10	+	65988635	66014725	26091	590	sense-overlappin	XM_011543514.1	ERBB2IP
circRNA_04053	0	0	0.119947548	0	0.03009467	0.020712909	0	0	0	0	0.02025523	0.0412728	hsa_circ_0001494	NC_000005.10	+	66012049	66014725	2677	226	sense-overlappin	XM_011543514.1	ERBB2IP
circRNA_04054	0	0	0.021808645	0	0	0	0	0	0	0	0	0	-	NC_000005.10	+	66012049	66023364	11316	365	sense-overlappin	XM_011543514.1	ERBB2IP
circRNA_04055	0	0	0.08723458	0	0	0	0	0	0	0	0	0	-	NC_000005.10	+	66012049	66054951	42903	3326	sense-overlappin	XM_011543514.1	ERBB2IP
circRNA_04056	0.020575025	0	0.21808645	0	0	0.031069364	0.030148028	0	0.030381792	0.020172483	0	0	-	NC_000005.10	+	66021322	66054951	33630	3100	sense-overlappin	XM_011543514.1	ERBB2IP
circRNA_04057	0	0	0	0.0412724	0	0	0	0	0	0	0	0	-	NC_000005.10	+	66038383	66050966	12584	881	sense-overlappin	XM_011543514.1	ERBB2IP
circRNA_04058	0	0	0	0	0	0	0.020098685	0	0	0	0	0	-	NC_000005.10	+	66046353	66054951	8599	2031	sense-overlappin	XM_011543514.1	ERBB2IP
circRNA_04059	0.051437562	0.061409903	0.08723458	0.0825448	0.09028402	0.051782273	0.070345399	0.112674625	0.040509055	0.121034897	0.030382845	0	hsa_circ_0072732	NC_000005.10	+	66053406	66054951	1546	1546	exonic	XM_011543514.1	ERBB2IP
circRNA_04060	0	0	0.04361729	0	0	0	0	0	0	0	0	0	hsa_circ_0006920	NC_000005.10	+	66202825	66208992	6168	6168	intergenic		
circRNA_04061	0	0	0.021808645	0	0	0	0	0	0	0	0.02025523	0.0206364	hsa_circ_0006411	NC_000005.10	+	68226290	68227009	720	720	exonic	NM_181523.2	PIK3R1
circRNA_04062	0	0	0	0	0	0	0	0	0	0.020172483	0	0	-	NC_000005.10	+	69117239	69129791	12553	1191	sense-overlappin	XM_005248569.2	SLC30A5
circRNA_04063	0	0	0.185373483	0	0.03009467	0.020712909	0.050246713	0.040972591	0	0	0	0	hsa_circ_0001495	NC_000005.10	+	69174877	69175537	661	378	sense-overlappin	NM_031966.3	CCNB1
circRNA_04064	0	0	0	0	0	0.031069364	0	0	0	0	0	0	hsa_circ_0007886	NC_000005.10	+	72335018	72343036	8019	360	sense-overlappin	NM_024754.4	PTCD2
circRNA_04065	0.030862537	0	0.032712968	0.0515905	0.02006311	0.051782273	0.040197371	0	0.040509055	0.080689931	0.02025523	0	hsa_circ_0002692	NC_000005.10	+	72861808	72865729	3922	241	sense-overlappin	NM_002270.3	TNPO1
circRNA_04066	0	0	0	0.0412724	0	0	0	0	0	0	0	0	hsa_circ_0072932	NC_000005.10	+	72887070	72888303	1234	379	sense-overlappin	NM_002270.3	TNPO1
circRNA_04067	0	0	0	0	0	0	0	0	0	0.020172483	0	0	hsa_circ_0006621	NC_000005.10	+	72887070	72889957	2888	551	sense-overlappin	NM_002270.3	TNPO1
circRNA_04068	0	0	0.08723458	0.0206362	0.04012623	0	0.060296056	0	0	0.030258724	0.04051046	0.0309546	hsa_circ_0072954	NC_000005.10	+	72989427	72990864	1438	370	sense-overlappin	NM_001146032.1	FCHO2
circRNA_04069	0	0	0.021808645	0	0.03009467	0	0	0	0	0	0	0	-	NC_000005.10	+	73051349	73054549	3201	271	sense-overlappin	NM_001146032.1	FCHO2
circRNA_04070	0	0	0.04361729	0	0	0	0	0	0	0	0	0	hsa_circ_0008047	NC_000005.10	+	73051349	73068779	17431	640	sense-overlappin	NM_001146032.1	FCHO2
circRNA_04071	0.051437562	0.020469968	0.032712968	0	0.04012623	0.041425819	0.020098685	0	0.030381792	0.020172483	0.050638075	0.051591	hsa_circ_0003571	NC_000005.10	+	73058433	73077493	19061	594	sense-overlappin	NM_001146032.1	FCHO2
circRNA_04072	0.298337857	0.286579545	0.283512385	0.3714514	0.34107295	0.31069364	0.381875022	0.256078694	0.374708763	0.393363415	0.374721756	0.206364002	hsa_circ_0002490	NC_000005.10	+	73074742	73077493	2752	268	sense-overlappin	NM_001146032.1	FCHO2
circRNA_04073	0	0	0	0	0	0.020712909	0	0.040972591	0	0	0	0	hsa_circ_0072975	NC_000005.10	+	73074742	73082825	8084	666	sense-overlappin	NM_001146032.1	FCHO2
circRNA_04074	0	0	0.032712968	0	0	0	0	0	0.030381792	0	0.050638075	0	hsa_circ_0072979	NC_000005.10	+	73078180	73082825	4646	398	sense-overlappin	NM_001146032.1	FCHO2
circRNA_04075	0	0	0	0	0	0	0	0	0	0.020172483	0	0	-	NC_000005.10	-	73554322	73561288	6967	516	sense-overlappin	NM_023039.4	ANKRA2
circRNA_04076	0	0	0	0.0309543	0	0	0	0	0	0	0	0	hsa_circ_0006874	NC_000005.10	+	73773855	73795391	21537	549	sense-overlappin	NM_001080479.2	ARHGEF28
circRNA_04077	0	0	0	0.0206362	0	0	0	0	0	0	0	0	-	NC_000005.10	-	73817841	73817976	136	136	antisense	NM_001080479.2	ARHGEF28
circRNA_04078	0	0	0	0.0412724	0	0	0	0	0	0	0	0	hsa_circ_0006969	NC_000005.10	+	73832338	73840760	8423	403	sense-overlappin	NM_001080479.2	ARHGEF28
circRNA_04079	0	0	0	0	0	0	0	0	0	0.020172483	0	0	hsa_circ_0003787	NC_000005.10	+	73832338	73852692	20355	766	sense-overlappin	NM_001080479.2	ARHGEF28
circRNA_04080	0	0	0	0.0412724	0	0	0	0	0	0	0	0	hsa_circ_0003035	NC_000005.10	+	73832338	73858219	25882	1023	sense-overlappin	NM_001080479.2	ARHGEF28
circRNA_04081	0	0.020469968	0	0.0928628	0.06018934	0.062138728	0.050246713	0.061458886	0	0.07060369	0	0.0412728	hsa_circ_0005777	NC_000005.10	+	73840480	73840760	281	281	exonic	NM_001080479.2	ARHGEF28
circRNA_04082	0.061725074	0	0.021808645	0	0.48151476	0.031069364	0.080394741	0.030729443	0.030381792	0	0	0	hsa_circ_0006859	NC_000005.10	-	74634684	74636498	1815	1815	exonic	NM_001256574.1	ENC1
circRNA_04083	0.020575025	0	0	0	0.02006311	0.031069364	0	0.030729443	0	0	0	0	hsa_circ_0007559	NC_000005.10	+	74715510	74716673	1164	268	sense-overlappin	NM_001292004.1	HEXB
circRNA_04084	0.020575025	0.061409903	0.032712968	0.0619086	0.04012623	0.072495183	0	0.030729443	0.030381792	0.090776173	0.02025523	0.0206364	-	NC_000005.10	-	74745678	74750667	4990	419	sense-overlappin	NM_001281302.1	GFM2
circRNA_04085	0	0	0.021808645	0	0	0	0	0	0	0	0	0	hsa_circ_0001497	NC_000005.10	-	74750579	74751493	915	215	sense-overlappin	NM_001281302.1	GFM2
circRNA_04086	0	0	0	0	0.03009467	0	0.030148028	0	0	0	0	0	hsa_circ_0007158	NC_000005.10	-	74813840	74841679	27840	673	sense-overlappin	XM_011543306.1	FAM169A

circRNA_04087	0.020575025	0	0	0	0	0	0.050246713	0	0	0.040344966	0	0.0206364	hsa_circ_0004405	NC_000005.10	-		74834426	74841679	7254	493	sense-overlappin	XM_011543306.1	FAM169A
circRNA_04088	0	0	0	0	0.03009467	0	0	0	0	0	0	0	-	NC_000005.10	-		75116566	75118297	1732	279	sense-overlappin	XM_011543302.1	ANKRD31
circRNA_04089	0	0	0	0	0.02006311	0	0	0	0	0	0	0	-	NC_000005.10	-		75379337	75411103	31767	1047	sense-overlappin	XM_006714513.1	COL4A3BP
circRNA_04090	0.020575025	0	0	0	0	0	0	0	0	0	0	0	-	NC_000005.10	-		75384642	75386034	1393	204	sense-overlappin	XM_006714513.1	COL4A3BP
circRNA_04091	0	0	0	0	0	0	0	0	0	0	0	0.0206364	-	NC_000005.10	-		75384642	75389687	5046	300	sense-overlappin	XM_006714513.1	COL4A3BP
circRNA_04092	0	0	0	0	0	0	0	0	0	0.020172483	0	0	hsa_circ_0007350	NC_000005.10	-		75400205	75411103	10899	273	sense-overlappin	XM_006714513.1	COL4A3BP
circRNA_04093	0	0	0.032712968	0	0	0	0	0	0	0	0	0	-	NC_000005.10	+		75543859	75552591	8733	3419	sense-overlappin	XM_011543466.1	POLK
circRNA_04094	0	0	0.021808645	0	0	0	0.030148028	0	0	0.030258724	0	0	hsa_circ_0073052	NC_000005.10	+		75547010	75552591	5582	268	sense-overlappin	XM_011543466.1	POLK
circRNA_04095	0	0	0	0	0	0	0	0.030729443	0	0	0	0	hsa_circ_0073057	NC_000005.10	-		75685207	75702810	17604	1100	sense-overlappin	NM_152408.2	POC5
circRNA_04096	0	0	0	0	0	0	0.040197371	0	0	0	0	0	-	NC_000005.10	+		76562396	76575769	13374	312	sense-overlappin	NM_006633.3	IQGAP2
circRNA_04097	0	0	0	0	0	0	0	0	0.030381792	0	0	0	-	NC_000005.10	+		76588906	76590586	1681	361	sense-overlappin	NM_006633.3	IQGAP2
circRNA_04098	0	0	0.021808645	0	0	0.020712909	0	0	0	0	0	0	-	NC_000005.10	+		76697987	76702590	4604	408	sense-overlappin	NM_006633.3	IQGAP2
circRNA_04099	0	0	0	0	0	0	0	0	0.040509055	0	0	0	hsa_circ_0073068	NC_000005.10	+		77034418	77036720	2303	471	sense-overlappin	NM_018046.4	AGGF1
circRNA_04100	0.020575025	0.061409903	0.054521613	0.0412724	0.02006311	0.082851637	0	0.020486295	0.030381792	0.040344966	0	0	hsa_circ_0008620	NC_000005.10	+		77046347	77048272	1926	443	sense-overlappin	NM_018046.4	AGGF1
circRNA_04101	0	0	0	0	0	0	0	0	0	0.030258724	0	0	-	NC_000005.10	+		77046347	77048987	2641	495	sense-overlappin	NM_018046.4	AGGF1
circRNA_04102	0	0	0	0	0	0	0	0	0.030381792	0	0	0	-	NC_000005.10	+		77046347	77055596	9250	846	sense-overlappin	NM_018046.4	AGGF1
circRNA_04103	0	0	0	0.0206362	0	0	0	0	0	0	0	0	-	NC_000005.10	+		77048936	77055596	6661	403	sense-overlappin	NM_018046.4	AGGF1
circRNA_04104	0	0	0	0.0206362	0	0	0	0	0	0	0	0	hsa_circ_0073076	NC_000005.10	+		77098655	77100391	1737	1737	intergenic		
circRNA_04105	0	0	0	0	0	0.031069364	0	0	0	0	0	0	-	NC_000005.10	+		77311994	77353406	41413	828	sense-overlappin	XM_005248623.3	PDE8B
circRNA_04106	0	0	0.021808645	0	0	0	0	0	0	0.020172483	0	0.0412728	hsa_circ_0008089	NC_000005.10	-		77449760	77463226	13467	481	sense-overlappin	XM_011543505.1	WDR41
circRNA_04107	0.13373766	0	0.228990773	0.0515905	0.05015779	0.082851637	0.030148028	0	0.030381792	0.060517448	0	0	hsa_circ_0008861	NC_000005.10	-		77449760	77464809	15050	530	sense-overlappin	XM_011543505.1	WDR41
circRNA_04108	0	0	0	0.0206362	0	0.051782273	0	0	0	0.030258724	0	0	hsa_circ_0073081	NC_000005.10	-		77449760	77489572	39813	646	sense-overlappin	XM_011543505.1	WDR41
circRNA_04109	0	0	0	0	0	0	0	0	0	0.030258724	0	0	hsa_circ_0001498	NC_000005.10	-		77463095	77464809	1715	181	sense-overlappin	XM_005248551.3	WDR41
circRNA_04110	0	0	0	0.0309543	0	0	0	0.030729443	0	0.020172483	0	0	-	NC_000005.10	-		78089393	78165672	76280	1410	sense-overlappin	NM_001271769.1	AP3B1
circRNA_04111	0	0	0.032712968	0	0	0.020712909	0.030148028	0	0.030381792	0.020172483	0	0	hsa_circ_0001501	NC_000005.10	-		78128030	78165672	37643	801	sense-overlappin	NM_001271769.1	AP3B1
circRNA_04112	0	0	0.021808645	0	0	0.020712909	0	0	0	0	0	0	-	NC_000005.10	-		78134923	78165672	30750	6703	sense-overlappin	NM_001271769.1	AP3B1
circRNA_04113	0.020575025	0	0.021808645	0	0	0	0	0	0	0	0	0	hsa_circ_0008938	NC_000005.10	-		78156258	78165672	9415	306	sense-overlappin	NM_001271769.1	AP3B1
circRNA_04114	0	0	0.054521613	0	0	0	0	0	0	0	0	0	hsa_circ_0073111	NC_000005.10	+		78388837	78421960	33124	575	sense-overlappin	NM_004866.5	SCAMP1
circRNA_04115	0	0	0	0.0206362	0	0	0	0	0	0	0	0	-	NC_000005.10	+		78388837	78459362	70526	795	sense-overlappin	NM_004866.5	SCAMP1
circRNA_04116	0	0.020469968	0.08723458	0	0.03009467	0	0	0	0.030381792	0.020172483	0	0	hsa_circ_0002186	NC_000005.10	+		78415520	78421960	6441	497	sense-overlappin	NM_001290229.1	SCAMP1
circRNA_04117	0	0	0	0	0	0	0	0	0	0	0.050638075	0	-	NC_000005.10	+		79077480	79083856	6377	977	sense-overlappin	NM_017614.4	BHMT2
circRNA_04118	0	0	0	0.0206362	0	0	0	0	0	0	0	0	-	NC_000005.10	-		79370612	79447145	76534	6168	sense-overlappin	NM_004272.4	HOMER1
circRNA_04119	0	0	0.021808645	0	0	0	0	0	0	0	0	0	hsa_circ_0073127	NC_000005.10	-		79396823	79402055	5233	349	sense-overlappin	NM_004272.4	HOMER1
circRNA_04120	0	0	0.032712968	0	0	0	0	0.020486295	0	0.050431207	0	0.0309546	hsa_circ_0073128	NC_000005.10	-		79396823	79457018	60196	871	sense-overlappin	NM_004272.4	HOMER1
circRNA_04121	0.041150049	0.020469968	0.065425935	0.0619086	0.06018934	0.051782273	0.070345399	0.051215739	0	0.110948655	0	0.051591	hsa_circ_0006916	NC_000005.10	-		79439010	79457018	18009	522	sense-overlappin	NM_001277078.1	HOMER1
circRNA_04122	0	0	0	0	0	0	0	0	0	0.040344966	0	0	-	NC_000005.10	+		79617530	79620083	2554	334	sense-overlappin	XM_011543220.1	PAPD4
circRNA_04123	0	0	0	0.0309543	0	0.051782273	0	0	0	0.040344966	0	0.0309546	hsa_circ_0001503	NC_000005.10	+		79618647	79620083	1437	1229	sense-overlappin	XM_011543231.1	PAPD4
circRNA_04124	0	0	0	0	0	0.020712909	0	0	0	0	0	0	-	NC_000005.10	+		79619612	79623355	3744	368	sense-overlappin	XM_011543231.1	PAPD4
circRNA_04125	0.020575025	0	0.076330258	0.0206362	0.05015779	0	0.080394741	0	0.020254528	0	0.06076569	0.0412728	hsa_circ_0002260	NC_000005.10	+		79640851	79669028	28178	818	sense-overlappin	XM_011543219.1	PAPD4
circRNA_04126	0	0.020469968	0	0	0	0	0.020098685	0	0	0	0	0	hsa_circ_0008517	NC_000005.10	+		79656958	79669028	12071	181	sense-overlappin	XM_011543219.1	PAPD4
circRNA_04127	0	0	0	0.0722267	0	0	0	0.020486295	0	0	0	0	hsa_circ_0005398	NC_000005.10	+		79668892	79682061	13170	309	sense-overlappin	XR_948240.1	
circRNA_04128	0	0	0	0	0.02006311	0	0	0	0	0	0	0	-	NC_000005.10	-		80146090	80175047	28958	781	sense-overlappin	NM_001174071.2	SERINC5
circRNA_04129	0	0	0	0.0309543	0	0	0	0	0	0	0	0.0206364	-	NC_000005.10	-		80174954	80178064	3111	356	sense-overlappin	NM_001174071.2	SERINC5
circRNA_04130	0	0	0	0	0	0.020712909	0	0	0	0	0	0	-	NC_000005.10	-		80202886	80205360	2475	2475	sense-overlappin	NM_001174071.2	SERINC5
circRNA_04131	0	0	0	0	0	0	0	0.020486295	0	0	0	0	-	NC_000005.10	+		80449591	80474830	25240	1358	sense-overlappin	XM_005248632.3	ZFYVE16
circRNA_04132	0.020575025	0.040939935	0.098138903	0.0515905	0.02006311	0.031069364	0	0	0	0	0	0	hsa_circ_0073177	NC_000005.10	+		80767933	80792844	24912	759	sense-overlappin	NM_002439.4	MSH3
circRNA_04133	0	0	0.04361729	0.0412724	0.02006311	0	0	0	0.050636319	0.020172483	0.030382845	0.0206364	hsa_circ_0001508	NC_000005.10	+		80775694	80792844	17151	402	sense-overlappin	NM_002439.4	MSH3
circRNA_04134	0	0	0	0	0	0	0	0	0.020254528	0	0.02025523	0	hsa_circ_0001509	NC_000005.10	-		81612562	81652326	39765	5118	sense-overlappin	NM_001256732.1	SSBP2
circRNA_04135	0	0.030704951	0	0	0	0	0	0.081945182	0.040509055	0.060517448	0.030382845	0	hsa_circ_0073222	NC_000005.10	-		81615473	81650339	34867	220	sense-overlappin	NM_001256732.1	SSBP2
circRNA_04136	0	0	0	0	0	0.031069364	0	0	0	0	0	0	-	NC_000005.10	-		81636557	81725049	88493	74845	sense-overlappin	NM_001256732.1	SSBP2
circRNA_04137	0	0	0	0.0412724	0	0	0	0	0	0	0	0	hsa_circ_0073230	NC_000005.10	+		82310157	82312851	2695	292	sense-overlappin	XM_011543719.1	ATP6AP1L
circRNA_04138	0	0	0.054521613	0	0	0	0	0	0	0	0	0	-	NC_000005.10	+		83195770	83204921	9152	430	sense-overlappin	XM_011543628.1	XRCC4
circRNA_04139	0	0	0.021808645	0	0	0	0	0	0	0	0	0	hsa_circ_0073236	NC_000005.10	+		83519349	83522309	2961	2681	antisense	XR_948513.1	
circRNA_04140	0	0	0.032712968	0	0	0	0	0	0.020254528	0	0	0	hsa_circ_0073237	NC_000005.10	+		83537007	83542268	5262	5262	exonic	NM_001164097.1	VCAN
circRNA_04141	0	0.030704951	0.021808645	0	0	0	0	0	0	0	0	0	hsa_circ_0073239	NC_000005.10	+		83537007	83555038	18032	5732	sense-overlappin	NM_001164097.1	VCAN
circRNA_04142	0.061725074	0	0.021808645	0.0309543	0	0	0	0.051215739	0.081018111	0.060517448	0	0.082545601	hsa_circ_0073244	NC_000005.10	-		84060300	84066606	6307	486	sense-overlappin	XM_011543089.1	EDIL3

circRNA_04143	0	0	0.239895095	0	0	0	0	0	0	0	0	0	-	NC_000005.10	-	85291399	85320889	29491	29491	intergenic		
circRNA_04144	0	0	0.239895095	0	0	0	0	0	0	0	0	0	-	NC_000005.10	-	85291399	85336266	44868	44868	intergenic		
circRNA_04145	0	0	0.076330258	0	0	0	0	0	0	0	0	0	-	NC_000005.10	-	85291399	85366680	75282	75282	intergenic		
circRNA_04146	0	0	0.021808645	0	0	0	0	0	0	0	0	0	-	NC_000005.10	-	85295174	85336266	41093	41093	intergenic		
circRNA_04147	0	0	0.021808645	0	0	0	0	0	0	0	0	0	-	NC_000005.10	-	85304086	85336266	32181	32181	intergenic		
circRNA_04148	0	0	0.021808645	0	0	0	0	0	0	0	0	0	-	NC_000005.10	-	85578621	85589009	10389	10389	intergenic		
circRNA_04149	0	0	0.926867413	0	0	0	0	0	0	0	0	0	-	NC_000005.10	-	85582431	85589009	6579	6579	intergenic		
circRNA_04150	0	0	0.032712968	0	0	0	0	0	0	0	0	0	-	NC_000005.10	-	85582431	85601266	18836	18836	intergenic		
circRNA_04151	0	0	0.04361729	0	0	0	0	0	0	0	0	0	-	NC_000005.10	-	85619937	85635252	15316	15316	intergenic		
circRNA_04152	0	0	0.065425935	0	0	0	0	0	0	0	0	0	-	NC_000005.10	-	85620098	85635252	15155	15155	intergenic		
circRNA_04153	0	0	0.032712968	0	0.04012623	0	0	0	0	0	0	0	-	NC_000005.10	+	85805307	85821466	16160	16160	intergenic		
circRNA_04154	0	0	0.021808645	0	0	0	0	0	0	0	0	0	-	NC_000005.10	+	87369878	87390929	21052	1515	sense-overlappin	NM_002890.2	RASA1
circRNA_04155	0.061725074	0	0.119947548	0.0515905	0	0.020712909	0.030148028	0	0	0.020172483	0	0	hsa_circ_0005476	NC_000005.10	-	88220563	88245792	25230	5406	sense-overlappin	NR_110266.1	
circRNA_04156	0	0	0	0	0	0	0	0.061458886	0	0.040344966	0.02025523	0	-	NC_000005.10	+	88282042	88292643	10602	10602	intergenic		
circRNA_04157	0	0	0	0.0515905	0	0	0	0	0	0	0	0	-	NC_000005.10	+	88905209	88965915	60707	60707	intergenic		
circRNA_04158	0	0	0	0.0206362	0	0	0	0	0	0	0	0	-	NC_000005.10	+	88943016	88965915	22900	22900	intergenic		
circRNA_04159	0	0	0.04361729	0	0	0	0	0	0	0	0	0	-	NC_000005.10	-	89832494	89909089	76596	76596	intergenic		
circRNA_04160	0	0	0.04361729	0	0	0	0	0	0	0	0	0	-	NC_000005.10	-	89908952	89943425	34474	34474	intergenic		
circRNA_04161	0	0	0	0	0	0	0	0.020486295	0	0	0	0	-	NC_000005.10	+	90488000	90497735	9736	267	sense-overlappin	XM_011543100.1	POLR3G
circRNA_04162	0	0	0	0.0206362	0	0	0	0	0	0	0	0	-	NC_000005.10	-	90516736	90525300	8565	3015	sense-overlappin	XM_005248421.3	LYSMD3
circRNA_04163	0	0	0	0	0	0	0	0	0	0.020172483	0	0	hsa_circ_0073360	NC_000005.10	-	93544795	93563463	18669	18669	intergenic		
circRNA_04164	0	0.040939935	0	0.0309543	0	0.031069364	0	0.020486295	0.030381792	0.060517448	0.02025523	0	hsa_circ_0007747	NC_000005.10	-	93553761	93563463	9703	9703	intergenic		
circRNA_04165	0.020575025	0	0	0	0	0	0	0	0	0	0	0	-	NC_000005.10	-	93718757	93718869	113	113	intronic	NM_001163417.1	FAM172A
circRNA_04166	0	0	0.032712968	0	0	0	0	0	0	0	0	0	-	NC_000005.10	-	94050731	94057203	6473	328	sense-overlappin	XM_011543672.1	FAM172A
circRNA_04167	0	0	0.021808645	0.0309543	0	0	0	0	0	0	0	0	-	NC_000005.10	-	94050731	94074769	24039	327	sense-overlappin	NM_032042.5	FAM172A
circRNA_04168	0	0	0.163564838	0	0.05015779	0.020712909	0.050246713	0	0	0	0.04051046	0	-	NC_000005.10	+	94643273	94654752	11480	724	sense-overlappin	XM_005272113.3	ANKRD32
circRNA_04169	0	0.051174919	0	0	0	0	0	0.020486295	0.020254528	0.060517448	0	0	hsa_circ_0005540	NC_000005.10	-	94868333	94912976	44644	1086	sense-overlappin	XM_005272083.3	MCTP1
circRNA_04170	0	0	0	0	0	0	0	0.051215739	0	0.050431207	0	0.0309546	hsa_circ_0006639	NC_000005.10	-	94868333	94924021	55689	1224	sense-overlappin	XM_005272083.3	MCTP1
circRNA_04171	0	0.08187987	0	0	0	0	0	0.020486295	0.060763583	0.050431207	0	0	hsa_circ_0001512	NC_000005.10	-	94888879	94912976	24098	583	sense-overlappin	XM_005272083.3	MCTP1
circRNA_04172	0	0	0	0	0	0	0	0.020486295	0.020254528	0	0	0	-	NC_000005.10	-	94888879	94924021	35143	721	sense-overlappin	XM_005272083.3	MCTP1
circRNA_04173	0	0	0	0.0309543	0	0	0	0.051215739	0	0.060517448	0	0	hsa_circ_0008417	NC_000005.10	-	94909251	94924021	14771	440	sense-overlappin	XM_005272083.3	MCTP1
circRNA_04174	0	0	0.021808645	0	0	0	0	0	0	0	0	0	hsa_circ_0073434	NC_000005.10	+	95736889	95763620	26732	933	sense-overlappin	NM_014899.3	RHOBTB3
circRNA_04175	0.041150049	0	0	0.0206362	0	0	0	0	0	0	0	0	hsa_circ_0073435	NC_000005.10	+	95736889	95783963	47075	1395	sense-overlappin	NM_014899.3	RHOBTB3
circRNA_04176	0	0.030704951	0	0	0	0	0	0	0	0	0	0	hsa_circ_0073440	NC_000005.10	+	95748333	95768166	19834	867	sense-overlappin	NM_014899.3	RHOBTB3
circRNA_04177	0.030862537	0	0	0	0	0	0	0	0	0	0	0	hsa_circ_0073441	NC_000005.10	+	95748333	95783963	35631	1208	sense-overlappin	NM_014899.3	RHOBTB3
circRNA_04178	0	0	0	0	0	0	0	0	0	0	0.02025523	0	-	NC_000005.10	+	95752239	95763620	11382	591	sense-overlappin	NM_014899.3	RHOBTB3
circRNA_04179	0.349775418	0.307049513	0.119947548	0.7532209	0.06018934	0.383188823	0.110542769	0.184376659	0.182290749	0.393363415	0.151914226	0.092863801	hsa_circ_0007444	NC_000005.10	+	95755396	95763620	8225	479	sense-overlappin	NM_014899.3	RHOBTB3
circRNA_04180	0	0	0	0	0	0	0	0	0.030381792	0	0	0.0309546	hsa_circ_0006725	NC_000005.10	+	95763508	95783963	20456	575	sense-overlappin	XM_011543279.1	RHOBTB3
circRNA_04181	0	0	0	0.0206362	0	0	0	0	0	0	0	0	-	NC_000005.10	-	95895628	95901080	5453	848	sense-overlappin	XM_011543280.1	ELL2
circRNA_04182	0	0	0	0.0206362	0	0	0	0	0	0	0	0	hsa_circ_0009176	NC_000005.10	-	95906523	95932713	26191	628	sense-overlappin	XM_006714575.1	ELL2
circRNA_04183	0	0	0	0	0.02006311	0	0	0	0	0	0	0	hsa_circ_0005153	NC_000005.10	+	96722639	96729725	7087	339	sense-overlappin	NM_001190442.1	CAST
circRNA_04184	0	0	0.032712968	0	0	0	0	0	0	0	0	0	hsa_circ_0007003	NC_000005.10	+	96722639	96730860	8222	420	sense-overlappin	NM_001190442.1	CAST
circRNA_04185	0	0	0	0	0	0	0	0.040972591	0	0	0	0	-	NC_000005.10	+	96757444	96767999	10556	558	sense-overlappin	NM_001190442.1	CAST
circRNA_04186	0	0	0	0.0309543	0	0	0	0	0	0	0	0	-	NC_000005.10	+	96912637	96913457	821	303	sense-overlappin	NM_001130140.1	ERAP2
circRNA_04187	0	0	0	0	0	0.031069364	0	0	0	0	0	0	hsa_circ_0001513	NC_000005.10	+	96979138	96986670	7533	1112	sense-overlappin	NM_005575.2	LNPEP
circRNA_04188	0	0	0	0.0206362	0	0.051782273	0	0	0	0	0	0	-	NC_000005.10	+	96985080	97028612	43533	2297	sense-overlappin	NM_005575.2	LNPEP
circRNA_04189	0	0	0	0	0	0.041425819	0	0.040972591	0	0	0	0	hsa_circ_0001516	NC_000005.10	+	97101759	97103094	1336	1336	antisense	NM_153234.4	LIX1
circRNA_04190	0	0	0	0	0	0	0	0	0.020254528	0	0	0	hsa_circ_0073486	NC_000005.10	-	98868495	98872555	4061	941	sense-overlappin	XM_005271866.3	CHD1
circRNA_04191	0	0.020469968	0	0	0	0.020712909	0	0	0	0	0	0	-	NC_000005.10	-	98873593	98876558	2966	334	sense-overlappin	NM_001270.2	CHD1
circRNA_04192	0	0	0.032712968	0.0309543	0	0.020712909	0	0.030729443	0.020254528	0	0.02025523	0	-	NC_000005.10	-	98879552	98881375	1824	370	sense-overlappin	NM_001270.2	CHD1
circRNA_04193	0.020575025	0	0	0	0	0	0	0	0	0	0	0	-	NC_000005.10	-	98898256	98899705	1450	506	sense-overlappin	NM_001270.2	CHD1
circRNA_04194	0	0	0	0	0	0	0	0	0.030381792	0	0	0	-	NC_000005.10	-	100856103	100895785	39683	684	sense-overlappin	NM_005668.5	ST8SIA4
circRNA_04195	0	0	0	0	0	0	0.020098685	0	0.020254528	0	0	0	-	NC_000005.10	-	100886343	100895785	9443	390	sense-overlappin	NM_005668.5	ST8SIA4
circRNA_04196	0.020575025	0.020469968	0.021808645	0.0825448	0.04012623	0.031069364	0	0.051215739	0.040509055	0.020172483	0	0.0206364	hsa_circ_0006499	NC_000005.10	-	103096541	103097781	1241	655	sense-overlappin	NM_017676.2	GIN1
circRNA_04197	0	0	0.04361729	0	0	0	0	0	0	0	0	0	-	NC_000005.10	+	104405311	104461152	55842	55842	intergenic		
circRNA_04198	0	0	0.04361729	0	0	0	0	0	0	0	0	0	-	NC_000005.10	-	105243293	105246366	3074	3074	intergenic		

circRNA_04199	0	0.020469968	0	0	0	0	0	0	0	0	0	0	-	NC_000005.10	-	108244106	108244185	80	80	intronic	NM_001163315.2	FBXL17
circRNA_04200	0	0	0.032712968	0.0206362	0	0.020712909	0.040197371	0	0.040509055	0.050431207	0	0	hsa_circ_0001517	NC_000005.10	-	108341927	108348530	6604	389	sense-overlappin	XM_011543580.1	FBXL17
circRNA_04201	0.051437562	0	0	0	0	0	0.070345399	0	0	0	0	0	hsa_circ_0001518	NC_000005.10	-	108348399	108367953	19555	513	sense-overlappin	NM_001163315.2	FBXL17
circRNA_04202	0	0	0	0	0	0.031069364	0	0	0	0	0	0	-	NC_000005.10	+	108768093	108798389	30297	412	sense-overlappin	XM_011543267.1	FER
circRNA_04203	0	0	0.021808645	0	0	0	0	0	0	0	0	0	hsa_circ_0006072	NC_000005.10	+	108798124	108798389	266	266	exonic	XM_011543267.1	FER
circRNA_04204	0	0	0	0	0	0	0	0	0	0.020172483	0	0	-	NC_000005.10	+	108867767	108883518	15752	565	sense-overlappin	XM_011543267.1	FER
circRNA_04205	0	0	0.021808645	0	0	0	0	0	0	0	0	0	hsa_circ_0073525	NC_000005.10	+	108867767	108959347	91581	1175	sense-overlappin	XM_011543267.1	FER
circRNA_04206	0	0	0	0	0	0.031069364	0	0	0	0	0	0	hsa_circ_0003032	NC_000005.10	+	108946130	108959347	13218	420	sense-overlappin	XM_011543267.1	FER
circRNA_04207	0	0	0	0	0	0.031069364	0	0	0	0	0	0	hsa_circ_0004691	NC_000005.10	+	109037422	109047198	9777	268	sense-overlappin	XM_011543267.1	FER
circRNA_04208	0	0	0	0	0	0	0	0.020486295	0	0	0	0	-	NC_000005.10	-	109355915	109383520	27606	1851	sense-overlappin	NM_014819.4	PJA2
circRNA_04209	0	0	0	0	0	0	0	0.020486295	0	0	0	0	-	NC_000005.10	-	109355915	109389901	33987	8232	sense-overlappin	NM_014819.4	PJA2
circRNA_04210	0.154312685	0.061409903	0.185373483	0.0722267	0.02006311	0.031069364	0.070345399	0.030729443	0.111399902	0.151293621	0.02025523	0.051591	hsa_circ_0008058	NC_000005.10	+	109713520	109716264	2745	400	sense-overlappin	NM_002372.3	MAN2A1
circRNA_04211	0.082300098	0.08187987	0.141756193	0.1031809	0.04012623	0.041425819	0.080394741	0	0.060763583	0.090776173	0.04051046	0.051591	hsa_circ_0001519	NC_000005.10	+	109713520	109729513	15994	572	sense-overlappin	NM_002372.3	MAN2A1
circRNA_04212	0	0.020469968	0	0.0412724	0	0	0.030148028	0	0.020254528	0.090776173	0	0	-	NC_000005.10	+	109713520	109755456	41937	700	sense-overlappin	NM_002372.3	MAN2A1
circRNA_04213	0	0	0	0	0	0	0	0	0	0.020172483	0	0	-	NC_000005.10	+	109713520	109774965	61446	1239	sense-overlappin	NM_002372.3	MAN2A1
circRNA_04214	0.030862537	0	0.032712968	0.0309543	0.02006311	0	0.020098685	0	0.030381792	0.020172483	0	0	hsa_circ_0004768	NC_000005.10	+	109781396	109789527	8132	569	sense-overlappin	NM_002372.3	MAN2A1
circRNA_04215	0	0	0	0	0.02006311	0	0	0	0	0	0	0	-	NC_000005.10	+	109781396	109830980	49585	1339	sense-overlappin	XM_011543395.1	MAN2A1
circRNA_04216	0.020575025	0	0	0	0	0	0.030148028	0	0	0	0	0	-	NC_000005.10	+	109817273	109823837	6565	623	sense-overlappin	NM_002372.3	MAN2A1
circRNA_04217	0	0	0	0.0206362	0	0	0	0	0	0	0	0	-	NC_000005.10	-	110605109	110627880	22772	775	sense-overlappin	XR_948287.1	
circRNA_04218	0	0	0	0.0412724	0	0	0	0.020486295	0	0	0	0	-	NC_000005.10	-	112234634	112240810	6177	292	sense-overlappin	XM_011543531.1	EPB41L4A
circRNA_04219	0	0	0	0	0	0	0	0	0	0.040344966	0	0	-	NC_000005.10	+	112891603	112893038	1436	1436	exonic	NM_001204199.1	SRP19
circRNA_04220	0	0	0	0	0.03009467	0	0	0	0	0.030258724	0	0	hsa_circ_0073608	NC_000005.10	+	112985835	113001674	15840	753	sense-overlappin	XM_005271914.3	DCP2
circRNA_04221	0	0	0.021808645	0	0.05015779	0.020712909	0	0	0	0.020172483	0	0	hsa_circ_0001520	NC_000005.10	+	112985835	113004077	18243	889	sense-overlappin	NM_001242377.1	DCP2
circRNA_04222	0	0	0	0	0	0	0	0.040972591	0	0.020172483	0	0	-	NC_000005.10	+	113532879	113535798	2920	427	sense-overlappin	XM_011543586.1	YTHDC2
circRNA_04223	0	0	0	0	0.02006311	0.051782273	0	0.040972591	0	0	0	0	-	NC_000005.10	+	113534305	113539181	4877	368	sense-overlappin	XM_011543586.1	YTHDC2
circRNA_04224	0.072012586	0	0	0	0	0	0	0	0	0	0	0	-	NC_000005.10	+	114404438	114404856	419	419	exonic	XM_011543387.1	KCNN2
circRNA_04225	0	0	0	0	0	0	0	0	0.020254528	0	0	0	-	NC_000005.10	-	115221824	115241606	19783	584	sense-overlappin	XM_011543491.1	PGGT1B
circRNA_04226	0	0	0.032712968	0	0	0	0	0	0	0	0	0	-	NC_000005.10	-	115539665	115542901	3237	3237	intronic	XM_011543515.1	FEM1C
circRNA_04227	0.020575025	0	0.021808645	0	0	0	0	0	0	0	0	0	-	NC_000005.10	+	116091079	116134178	43100	378	sense-overlappin	XM_011543458.1	COMMD10
circRNA_04228	0	0	0	0	0	0	0	0.020486295	0	0	0	0	-	NC_000005.10	+	116153149	116153289	141	141	intronic	XM_011543458.1	COMMD10
circRNA_04229	0	0	0	0.0206362	0	0	0	0	0	0	0	0	-	NC_000005.10	+	116291517	116303683	12167	470	sense-overlappin	XR_948271.1	
circRNA_04230	0	0	0	0	0	0.020712909	0	0	0	0	0	0	-	NC_000005.10	+	116317517	116318300	784	171	sense-overlappin	XR_948268.1	
circRNA_04231	0	0	0	0	0	0	0	0.020486295	0	0	0	0	-	NC_000005.10	+	116399482	116498555	99074	51240	antisense	XM_006714663.1	SEMA6A
circRNA_04232	0	0	0.04361729	0	0.02006311	0	0	0	0.030381792	0	0	0	hsa_circ_0004509	NC_000005.10	-	118928537	118944649	16113	379	sense-overlappin	XM_011543341.1	DTWD2
circRNA_04233	0	0	0	0	0	0	0	0	0.020254528	0	0	0	-	NC_000005.10	+	119101935	119116336	14402	530	sense-overlappin	XM_011543213.1	DMXL1
circRNA_04234	0	0	0	0	0	0	0.020098685	0	0	0	0	0	-	NC_000005.10	+	119189708	119197956	8249	610	sense-overlappin	XM_011543213.1	DMXL1
circRNA_04235	0	0.020469968	0	0	0	0	0	0.030729443	0	0.050431207	0.050638075	0	hsa_circ_0006500	NC_000005.10	+	119240419	119244576	4158	271	sense-overlappin	XM_011543213.1	DMXL1
circRNA_04236	0.020575025	0.020469968	0.119947548	0.0412724	0.04012623	0	0.050246713	0.040972591	0	0	0.030382845	0.0309546	-	NC_000005.10	+	122019260	122022407	3148	835	sense-overlappin	NM_152546.2	SRFBP1
circRNA_04237	0	0	0	0	0	0	0	0	0.030381792	0	0	0	-	NC_000005.10	+	122336788	122403865	67078	53400	sense-overlappin	XM_011543740.1	SNCAIP
circRNA_04238	0	0	0	0	0	0	0	0	0.020254528	0	0	0	-	NC_000005.10	+	122425352	122451601	26250	1752	sense-overlappin	XM_011543742.1	SNCAIP
circRNA_04239	0	0	0	0	0	0	0	0	0	0.030258724	0	0	-	NC_000005.10	+	122431969	122451601	19633	1572	sense-overlappin	XM_011543742.1	SNCAIP
circRNA_04240	0	0	0	0	0	0.031069364	0	0	0	0	0	0	-	NC_000005.10	+	122795266	122808355	13090	614	sense-overlappin	NM_001278199.1	SNX2
circRNA_04241	0	0.020469968	0	0.0206362	0	0	0	0	0	0	0	0	hsa_circ_0073692	NC_000005.10	+	122801869	122803613	1745	253	sense-overlappin	NM_001278199.1	SNX2
circRNA_04242	0	0	0	0	0	0.041425819	0	0	0	0	0	0	-	NC_000005.10	+	122931861	122946159	14299	5062	sense-overlappin	NM_014035.2	SNX24
circRNA_04243	0	0	0.032712968	0.0515905	0.02006311	0	0	0	0.020254528	0.030258724	0	0.0309546	-	NC_000005.10	+	123532569	123557564	24996	13384	sense-overlappin	XM_005271891.1	CSNK1G3
circRNA_04244	0.030862537	0	0	0	0	0	0	0	0	0	0	0	-	NC_000005.10	+	123545417	123545841	425	425	exonic	XM_005271891.1	CSNK1G3
circRNA_04245	0	0	0.032712968	0	0	0.020712909	0	0	0	0.020172483	0	0	-	NC_000005.10	+	123545417	123553147	7731	466	sense-overlappin	XM_005271891.1	CSNK1G3
circRNA_04246	0.390925468	0.532219156	0.806919866	0.5365409	0.44138853	0.704238918	0.613009903	0.399482762	0.435472346	0.806899312	0.394976987	0.299227802	hsa_circ_0001522	NC_000005.10	+	123545417	123557564	12148	536	sense-overlappin	XM_005271891.1	CSNK1G3
circRNA_04247	0	0.030704951	0.098138903	0.0412724	0.05015779	0	0.100493427	0.030729443	0.030381792	0.060517448	0.04051046	0.072227401	hsa_circ_0073706	NC_000005.10	+	123545417	123575963	30547	920	sense-overlappin	XM_005271891.1	CSNK1G3
circRNA_04248	0	0.020469968	0.021808645	0	0	0	0.020098685	0	0	0	0	0	hsa_circ_0005949	NC_000005.10	-	124639133	124644661	5529	827	sense-overlappin	XM_011543523.1	ZNF608
circRNA_04249	0	0	0.04361729	0.0619086	0	0	0	0	0	0	0	0	hsa_circ_0073716	NC_000005.10	-	124639133	124701269	62137	3626	sense-overlappin	XM_011543522.1	ZNF608
circRNA_04250	0	0	0	0	0	0	0.040197371	0	0	0	0	0	hsa_circ_0073717	NC_000005.10	-	124643511	124644661	1151	591	sense-overlappin	XM_011543523.1	ZNF608
circRNA_04251	0	0	0.032712968	0	0	0	0	0	0	0	0	0	hsa_circ_0073718	NC_000005.10	-	124643511	124701269	57759	3390	sense-overlappin	XM_011543522.1	ZNF608
circRNA_04252	0.205750246	0.102349838	0.152660515	0.0619086	0.02006311	0.041425819	0	0.020486295	0	0.121034897	0.12153138	0.0206364	hsa_circ_0001523	NC_000005.10	-	124701014	124701269	256	256	exonic	XM_011543522.1	ZNF608
circRNA_04253	0.020575025	0	0	0	0.02006311	0	0.030148028	0.030729443	0	0	0	0	-	NC_000005.10	+	126603570	126604183	614	614	exonic	NM_032177.3	PHAX

circRNA_04254	0	0	0	0	0	0	0	0	0	0.020172483	0	0	-	NC_000005.10	+	126793648	126794160	513	513	intronic	NM_005573.3	LMNB1
circRNA_04255	0	0	0.04361729	0	0	0	0.020098685	0	0	0	0	0	hsa_circ_0007726	NC_000005.10	+	126804776	126811898	7123	580	sense-overlappin	NM_005573.3	LMNB1
circRNA_04256	0	0	0.032712968	0	0	0	0	0	0	0	0	0	-	NC_000005.10	-	126914930	126918227	3298	449	sense-overlappin	XM_011543131.1	3-Mar
circRNA_04257	0.072012586	0	0.021808645	0.0412724	0	0	0	0	0.020254528	0.020172483	0	0.0206364	-	NC_000005.10	-	127956804	127966928	10125	10125	intergenic		
circRNA_04258	0	0.030704951	0	0.1031809	0.03009467	0.031069364	0.020098685	0	0.040509055	0	0.10127615	0	hsa_circ_0073762	NC_000005.10	+	128131067	128141981	10915	725	sense-overlappin	NM_001046.2	SLC12A2
circRNA_04259	0	0.040939935	0	0	0	0	0	0	0	0	0	0	hsa_circ_0073763	NC_000005.10	+	128131067	128152805	21739	1315	sense-overlappin	NM_001046.2	SLC12A2
circRNA_04260	0	0	0.04361729	0.0309543	0	0	0	0.020486295	0	0	0	0	-	NC_000005.10	+	128131067	128161800	30734	1568	sense-overlappin	NM_001046.2	SLC12A2
circRNA_04261	0	0.071644886	0.04361729	0.1547714	0.04012623	0.062138728	0.050246713	0.040972591	0	0.07060369	0.08102092	0	hsa_circ_0006034	NC_000005.10	+	128138597	128152805	14209	955	sense-overlappin	NM_001046.2	SLC12A2
circRNA_04262	0	0	0.021808645	0	0	0	0	0	0	0	0	0	hsa_circ_0073772	NC_000005.10	+	128141830	128161800	19971	995	sense-overlappin	NM_001046.2	SLC12A2
circRNA_04263	0	0	0	0.0309543	0	0	0	0	0	0	0	0	hsa_circ_0073775	NC_000005.10	+	128174541	128182941	8401	496	sense-overlappin	NM_001046.2	SLC12A2
circRNA_04264	0	0	0.185373483	0.0206362	0.02006311	0.020712909	0.020098685	0	0.020254528	0.020172483	0	0	hsa_circ_0007067	NC_000005.10	+	129104956	129107062	2107	441	sense-overlappin	NM_016048.2	ISOC1
circRNA_04265	0	0	0	0.0309543	0	0	0	0	0	0	0	0	-	NC_000005.10	-	129352072	129355537	3466	3466	intergenic		
circRNA_04266	0	0	0.054521613	0	0.02006311	0	0	0	0	0	0	0	-	NC_000005.10	-	130798539	130811692	13154	13154	intergenic		
circRNA_04267	0	0	0	0	0	0	0	0	0.030381792	0.020172483	0	0	-	NC_000005.10	+	131255095	131256192	1098	1098	intergenic		
circRNA_04268	0	0	0	0	0	0	0	0	0	0	0.02025523	0	-	NC_000005.10	+	131315976	131359547	43572	508	sense-overlappin	NM_020240.2	CDC42SE2
circRNA_04269	0	0	0	0.0206362	0	0.020712909	0.020098685	0	0	0	0	0	-	NC_000005.10	-	131504626	131548190	43565	903	sense-overlappin	NM_001164386.1	RAPGEF6
circRNA_04270	0.020575025	0	0	0	0	0	0	0	0	0	0	0	-	NC_000005.10	+	131660301	131661777	1477	1477	antisense	NM_001008738.2	FNIP1
circRNA_04271	0	0	0	0	0	0	0.030148028	0	0	0	0	0	hsa_circ_0073858	NC_000005.10	-	131670463	131677872	7410	1759	sense-overlappin	NM_001008738.2	FNIP1
circRNA_04272	0	0	0.021808645	0	0	0	0	0	0	0	0	0	hsa_circ_0073859	NC_000005.10	-	131670463	131679175	8713	1906	sense-overlappin	NM_001008738.2	FNIP1
circRNA_04273	0	0	0	0	0	0	0	0	0	0.030258724	0	0	hsa_circ_0008314	NC_000005.10	-	131677703	131699002	21300	403	sense-overlappin	NM_001008738.2	FNIP1
circRNA_04274	0	0	0	0.0309543	0	0.041425819	0	0	0	0.020172483	0	0.051591	hsa_circ_0001524	NC_000005.10	-	131677703	131709272	31570	813	sense-overlappin	NM_001008738.2	FNIP1
circRNA_04275	0	0.020469968	0	0	0	0	0	0	0	0	0	0	-	NC_000005.10	-	131698917	131719417	20501	848	sense-overlappin	NM_133372.2	FNIP1
circRNA_04276	0	0	0	0	0	0	0	0	0.040509055	0	0	0	hsa_circ_0001528	NC_000005.10	+	132616003	132618294	2292	353	sense-overlappin	NM_005732.3	RAD50
circRNA_04277	0	0	0	0	0	0	0.020098685	0	0	0	0	0	-	NC_000005.10	+	132876680	132876833	154	154	antisense	NM_014423.3	AFF4
circRNA_04278	0.051437562	0	0	0	0	0	0	0	0.030381792	0	0	0.0206364	hsa_circ_0007945	NC_000005.10	-	132883340	132885119	1780	265	sense-overlappin	NM_014423.3	AFF4
circRNA_04279	0	0	0.021808645	0	0	0	0	0	0	0	0	0	-	NC_000005.10	-	132889079	132897240	8162	1343	sense-overlappin	NM_014423.3	AFF4
circRNA_04280	0.102875123	0.030704951	0.054521613	0.1650895	0.09028402	0	0.040197371	0.071702034	0	0.07060369	0.04051046	0.0206364	hsa_circ_0001529	NC_000005.10	-	132892164	132893118	955	330	sense-overlappin	NM_014423.3	AFF4
circRNA_04281	0	0	0	0	0.03009467	0	0.020098685	0	0	0.030258724	0	0	hsa_circ_0073901	NC_000005.10	-	132892164	132897240	5077	1248	sense-overlappin	NM_014423.3	AFF4
circRNA_04282	0	0	0.021808645	0	0	0.020712909	0	0	0	0	0	0	hsa_circ_0073904	NC_000005.10	-	132927121	132934941	7821	927	sense-overlappin	NM_014423.3	AFF4
circRNA_04283	0	0	0.021808645	0.0309543	0	0.020712909	0	0	0	0.030258724	0	0	-	NC_000005.10	+	133064980	133070496	5517	322	sense-overlappin	NM_002154.3	HSPA4
circRNA_04284	0	0	0.032712968	0	0	0	0	0	0	0	0	0	hsa_circ_0002633	NC_000005.10	+	133064980	133076898	11919	801	sense-overlappin	NM_002154.3	HSPA4
circRNA_04285	0.020575025	0.061409903	0	0.0206362	0.02006311	0.041425819	0.030148028	0	0	0.020172483	0.04051046	0	hsa_circ_0001531	NC_000005.10	+	133091193	133092789	1597	272	sense-overlappin	NM_002154.3	HSPA4
circRNA_04286	0.041150049	0	0	0	0	0	0	0	0	0	0	0	-	NC_000005.10	+	133092700	133099652	6953	477	sense-overlappin	NM_002154.3	HSPA4
circRNA_04287	0	0	0	0	0	0	0	0	0	0	0.02025523	0.0309546	-	NC_000005.10	-	133959494	133960020	527	527	exonic	NM_020199.2	C5orf15
circRNA_04288	0	0	0.054521613	0	0	0	0	0	0	0	0	0	hsa_circ_0004145	NC_000005.10	-	134157707	134174022	16316	518	sense-overlappin	NM_170679.2	SKP1
circRNA_04289	0	0	0	0.0309543	0	0	0	0	0	0	0	0	hsa_circ_0002602	NC_000005.10	-	134199086	134206131	7046	755	sense-overlappin	NM_002715.2	PPP2CA
circRNA_04290	0	0	0	0	0	0	0	0	0	0.040344966	0	0	-	NC_000005.10	-	134319358	134360091	40734	663	sense-overlappin	XM_011543433.1	CDKL3
circRNA_04291	0	0	0	0	0	0	0	0	0	0.020172483	0	0	hsa_circ_0005608	NC_000005.10	+	134535858	134552209	16352	311	sense-overlappin	XM_011543293.1	JADE2
circRNA_04292	0	0	0	0	0	0	0	0	0	0	0.02025523	0	hsa_circ_0003319	NC_000005.10	+	134661119	134661586	468	468	exonic	NM_001252231.1	SEC24A
circRNA_04293	0	0	0	0.0309543	0	0.020712909	0	0	0	0	0	0	hsa_circ_0003180	NC_000005.10	+	134666823	134671886	5064	252	sense-overlappin	NM_001252231.1	SEC24A
circRNA_04294	0	0.020469968	0	0.0309543	0	0.020712909	0	0	0	0	0	0	hsa_circ_0004345	NC_000005.10	+	134674615	134676125	1511	437	sense-overlappin	NM_001252231.1	SEC24A
circRNA_04295	0	0	0	0	0.05015779	0	0	0	0	0	0	0	hsa_circ_0073970	NC_000005.10	+	134674615	134682482	7868	674	sense-overlappin	NM_001252231.1	SEC24A
circRNA_04296	0	0	0	0	0	0.020712909	0	0	0	0	0	0	hsa_circ_0073978	NC_000005.10	+	134682373	134688299	5927	342	sense-overlappin	NM_001252231.1	SEC24A
circRNA_04297	0	0	0	0	0	0	0.020098685	0	0	0	0	0	hsa_circ_0073992	NC_000005.10	+	134697126	134698057	932	280	sense-overlappin	XR_948228.1	
circRNA_04298	0	0	0.021808645	0	0	0	0	0	0	0	0	0	hsa_circ_0073993	NC_000005.10	+	134697126	134703932	6807	454	sense-overlappin	XR_948228.1	
circRNA_04299	0	0	0.065425935	0	0	0	0	0	0	0	0	0	hsa_circ_0073994	NC_000005.10	+	134697126	134705437	8312	565	sense-overlappin	XR_948228.1	
circRNA_04300	0.041150049	0.020469968	0.04361729	0.0309543	0.04012623	0.082851637	0.100493427	0.051215739	0.091145375	0.100862414	0.050638075	0	hsa_circ_0003154	NC_000005.10	+	134741063	134744052	2990	527	sense-overlappin	NM_001745.3	CAMLG
circRNA_04301	0	0	0.021808645	0	0	0	0	0	0	0	0	0	-	NC_000005.10	+	134777574	134788591	11018	930	sense-overlappin	NM_001300860.1	DDX46
circRNA_04302	0	0	0	0.0309543	0	0	0	0	0	0	0	0	-	NC_000005.10	+	134777574	134819004	41431	2481	sense-overlappin	NR_125341.1	
circRNA_04303	0.020575025	0	0	0	0	0	0	0	0	0	0	0	-	NC_000005.10	+	134964467	134975324	10858	9009	sense-overlappin	NM_178019.2	CATSPER3
circRNA_04304	0	0.020469968	0	0	0	0	0	0	0	0	0.030382845	0	hsa_circ_0005025	NC_000005.10	+	135052454	135075388	22935	22935	sense-overlappin	NM_001277348.1	C5orf66
circRNA_04305	0	0	0	0	0	0	0	0	0	0.020172483	0	0	-	NC_000005.10	+	135174042	135254012	79971	79971	sense-overlappin	NM_001277348.1	C5orf66
circRNA_04306	0.020575025	0	0	0	0	0	0	0	0	0	0	0	hsa_circ_0004854	NC_000005.10	-	135369406	135370142	737	305	sense-overlappin	NM_001040158.1	H2AFY
circRNA_04307	0.082300098	0	0.032712968	0.0825448	0	0.031069364	0.030148028	0	0	0	0.04051046	0.0309546	hsa_circ_0007930	NC_000005.10	+	136147832	136154163	6332	731	sense-overlappin	NM_001001419.2	SMAD5
circRNA_04308	0	0	0	0.0206362	0	0	0	0	0	0	0	0	hsa_circ_0003690	NC_000005.10	+	136147832	136163391	15560	1103	sense-overlappin	NM_001001419.2	SMAD5

circRNA_04309	0	0	0	0	0	0	0	0	0.020254528	0	0	0	hsa_circ_0074066	NC_000005.10	-	137692285	137709856	17572	392	sense-overlappin	NM_001257194.1	KLHL3
circRNA_04310	0.020575025	0.040939935	0	0.0206362	0	0	0	0	0	0.030258724	0	0	hsa_circ_0005222	NC_000005.10	-	137757867	137759020	1154	1154	intergenic		
circRNA_04311	0	0	0	0	0	0.031069364	0	0	0	0	0	0	-	NC_000005.10	-	137770003	137782226	12224	12224	intergenic		
circRNA_04312	0.020575025	0	0	0	0	0	0	0	0	0	0	0	-	NC_000005.10	-	137941944	137943216	1273	350	sense-overlappin	NM_001101800.1	FAM13B
circRNA_04313	0	0	0	0.0309543	0.03009467	0.020712909	0.040197371	0.020486295	0	0	0	0	hsa_circ_0001534	NC_000005.10	-	137942875	137943216	342	248	sense-overlappin	NM_001101800.1	FAM13B
circRNA_04314	0	0	0.021808645	0.0309543	0	0	0	0	0	0	0	0	-	NC_000005.10	-	137942875	137949184	6310	658	sense-overlappin	NM_016603.2	FAM13B
circRNA_04315	0	0	0	0.0619086	0	0	0	0	0	0	0	0.0309546	-	NC_000005.10	-	137945902	137954376	8475	833	sense-overlappin	NM_016603.2	FAM13B
circRNA_04316	0	0	0	0	0	0	0	0	0	0	0	0.0206364	-	NC_000005.10	-	137952628	137953465	838	212	sense-overlappin	NM_001101800.1	FAM13B
circRNA_04317	0.030862537	0	0	0	0.0702209	0	0	0	0.030381792	0	0	0	hsa_circ_0008177	NC_000005.10	-	137952628	137954376	1749	423	sense-overlappin	NM_001101800.1	FAM13B
circRNA_04318	0.792138448	0.235404627	0.665163673	0.5262228	0.56176722	0.507466279	0.371825679	0.378996467	0.303817916	0.595088243	0.293700836	0.278591402	hsa_circ_0001535	NC_000005.10	-	137985257	137988315	3059	331	sense-overlappin	NM_001101800.1	FAM13B
circRNA_04319	0	0	0	0	0	0	0	0	0.030381792	0	0	0	-	NC_000005.10	-	137985257	138019146	33890	1214	sense-overlappin	NM_001101800.1	FAM13B
circRNA_04320	0	0	0.032712968	0	0.02006311	0	0	0	0.020254528	0	0	0	hsa_circ_0004894	NC_000005.10	-	138006990	138011945	4956	478	sense-overlappin	NM_001101800.1	FAM13B
circRNA_04321	0	0	0	0	0	0	0	0	0	0	0	0.0619092	hsa_circ_0074082	NC_000005.10	-	138006990	138019146	12157	883	sense-overlappin	NM_001101800.1	FAM13B
circRNA_04322	0	0	0.021808645	0	0	0	0.020098685	0	0	0.030258724	0.030382845	0	hsa_circ_0007235	NC_000005.10	-	138006990	138021197	14208	1147	sense-overlappin	XM_011543449.1	FAM13B
circRNA_04323	0	0	0	0	0	0	0	0	0	0.030258724	0	0	-	NC_000005.10	+	138372674	138386621	13948	1188	sense-overlappin	NM_016604.3	KDM3B
circRNA_04324	0	0	0	0	0	0	0	0	0	0.040344966	0	0	hsa_circ_0074111	NC_000005.10	+	138377720	138381590	3871	306	sense-overlappin	NM_016604.3	KDM3B
circRNA_04325	0.020575025	0	0	0	0	0	0	0	0	0	0	0	-	NC_000005.10	+	138386022	138400012	13991	2419	sense-overlappin	NM_016604.3	KDM3B
circRNA_04326	0	0	0	0.0206362	0	0	0	0	0	0	0	0	-	NC_000005.10	+	138391013	138393372	2360	1451	sense-overlappin	NM_016604.3	KDM3B
circRNA_04327	0	0	0	0	0	0.020712909	0	0	0	0	0	0	hsa_circ_0074119	NC_000005.10	+	138398178	138400012	1835	368	sense-overlappin	NM_016604.3	KDM3B
circRNA_04328	0.020575025	0	0	0	0	0	0	0	0	0	0	0	hsa_circ_0006616	NC_000005.10	-	138517561	138529678	12118	440	sense-overlappin	NM_001291974.1	ETF1
circRNA_04329	0	0	0	0	0	0.041425819	0	0	0	0	0	0	hsa_circ_0008356	NC_000005.10	-	138561580	138567722	6143	647	sense-overlappin	NM_004134.6	HSPA9
circRNA_04330	0.041150049	0	0	0.0412724	0	0	0	0	0	0	0	0	hsa_circ_0003280	NC_000005.10	-	138568925	138571141	2217	307	sense-overlappin	NM_004134.6	HSPA9
circRNA_04331	0	0.030704951	0	0	0	0	0	0	0	0	0	0	-	NC_000005.10	-	138767325	138770668	3344	3344	antisense	NM_001290307.1	CTNNA1
circRNA_04332	0	0	0	0	0.02006311	0	0	0	0.020254528	0	0	0	hsa_circ_0074158	NC_000005.10	+	138781923	138783372	1450	303	sense-overlappin	NM_001290307.1	CTNNA1
circRNA_04333	0	0	0.032712968	0	0	0	0	0	0	0	0	0	hsa_circ_0074159	NC_000005.10	+	138781923	138827718	45796	1064	sense-overlappin	NM_001290307.1	CTNNA1
circRNA_04334	0	0	0	0	0	0	0	0.020486295	0	0	0	0	-	NC_000005.10	+	138781923	138827743	45821	1089	sense-overlappin	NM_001290307.1	CTNNA1
circRNA_04335	0	0	0.04361729	0.0309543	0.02006311	0	0	0	0.030381792	0.040344966	0	0	hsa_circ_0007440	NC_000005.10	+	138810038	138827718	17681	761	sense-overlappin	NM_001290307.1	CTNNA1
circRNA_04336	0	0	0.021808645	0	0	0	0	0	0	0	0	0	-	NC_000005.10	-	138947405	138951884	4480	331	sense-overlappin	NM_001037633.1	SIL1
circRNA_04337	0	0	0.021808645	0	0	0	0	0	0	0	0	0	-	NC_000005.10	-	139121035	139127853	6819	254	sense-overlappin	NM_001037633.1	SIL1
circRNA_04338	0	0	0.04361729	0	0	0	0.030148028	0.030729443	0	0.030258724	0	0	hsa_circ_0008922	NC_000005.10	+	139278327	139279129	803	161	sense-overlappin	NM_001282278.1	MATR3
circRNA_04339	0	0	0	0.0206362	0.02006311	0	0	0	0	0	0.02025523	0	hsa_circ_0074206	NC_000005.10	+	139317053	139319501	2449	473	sense-overlappin	NM_001194954.1	MATR3
circRNA_04340	0.082300098	0	0	0.0722267	0.04012623	0.031069364	0.060296056	0.081945182	0.040509055	0.090776173	0.02025523	0.0309546	hsa_circ_0001538	NC_000005.10	+	139363759	139364743	985	344	sense-overlappin	NM_016480.4	PAIP2
circRNA_04341	0	0	0	0	0.02006311	0	0	0	0	0	0	0	-	NC_000005.10	+	139440794	139441108	315	315	intergenic		
circRNA_04342	0	0	0	0	0	0	0	0	0	0	0	0.0206364	-	NC_000005.10	-	139442287	139443767	1481	1481	intergenic		
circRNA_04343	0.061725074	0	0.098138903	0.113499	0.0702209	0.124277456	0.040197371	0.051215739	0.020254528	0.121034897	0.030382845	0.0309546	hsa_circ_0005728	NC_000005.10	+	139600372	139614966	14595	280	sense-overlappin	NM_003339.2	UBE2D2
circRNA_04344	0	0	0	0	0	0	0.020098685	0	0.020254528	0	0	0	hsa_circ_0004503	NC_000005.10	+	139600372	139623461	23090	374	sense-overlappin	NM_003339.2	UBE2D2
circRNA_04345	0.020575025	0	0	0.0206362	0.04012623	0	0	0.020486295	0	0	0	0.0206364	hsa_circ_0006716	NC_000005.10	+	139614586	139614966	381	216	sense-overlappin	NM_003339.2	UBE2D2
circRNA_04346	0	0	0	0.0412724	0	0	0	0	0	0.020172483	0	0	hsa_circ_0074229	NC_000005.10	+	139614586	139623461	8876	310	sense-overlappin	NM_003339.2	UBE2D2
circRNA_04347	0.051437562	0.020469968	0	0	0	0	0	0	0	0	0.030382845	0	hsa_circ_0002423	NC_000005.10	+	140167365	140167802	438	438	intergenic		
circRNA_04348	0	0	0.021808645	0.0412724	0.0702209	0	0	0	0	0	0	0	-	NC_000005.10	+	140189064	140194652	5589	5589	sense-overlappin	NM_032412.3	CYSTM1
circRNA_04349	0	0.020469968	0	0.0619086	0.05015779	0	0	0	0.040509055	0.020172483	0	0	hsa_circ_0002449	NC_000005.10	+	140194446	140194652	207	207	exonic	NM_032412.3	CYSTM1
circRNA_04350	0	0	0.032712968	0.0722267	0.06018934	0.031069364	0	0	0.040509055	0	0	0.0309546	-	NC_000005.10	-	140210806	140211646	841	841	antisense	NM_032412.3	CYSTM1
circRNA_04351	0	0	0	0	0	0	0.020098685	0	0	0	0	0	-	NC_000005.10	+	140435398	140464776	29379	2149	sense-overlappin	NM_017978.2	ANKHD1
circRNA_04352	0	0	0.04361729	0	0	0.041425819	0	0	0.020254528	0.030258724	0	0	hsa_circ_0007862	NC_000005.10	+	140438461	140445975	7515	687	sense-overlappin	NM_001197030.1	ANKHD1
circRNA_04353	0.020575025	0	0	0	0	0	0	0	0	0	0	0	hsa_circ_0007402	NC_000005.10	+	140438461	140449305	10845	782	sense-overlappin	NM_001197030.1	ANKHD1
circRNA_04354	0	0.030704951	0.032712968	0.1031809	0.10031557	0.072495183	0.040197371	0.030729443	0.020254528	0.080689931	0	0.0309546	hsa_circ_0001540	NC_000005.10	+	140440119	140445975	5857	530	sense-overlappin	NM_001197030.1	ANKHD1
circRNA_04355	0.030862537	0.061409903	0.152660515	0.1341352	0.04012623	0.020712909	0.040197371	0	0	0	0.02025523	0	hsa_circ_0001541	NC_000005.10	+	140440119	140449305	9187	625	sense-overlappin	NM_001197030.1	ANKHD1
circRNA_04356	0	0	0.032712968	0	0	0	0	0	0	0	0	0	-	NC_000005.10	+	140440119	140459355	19237	1055	sense-overlappin	NM_001197030.1	ANKHD1
circRNA_04357	0	0.030704951	0	0	0	0	0	0	0	0	0	0	hsa_circ_0004025	NC_000005.10	+	140482580	140487060	4481	463	sense-overlappin	NM_017747.2	ANKHD1
circRNA_04358	0	0	0	0	0	0	0	0	0	0	0.02025523	0	hsa_circ_0074270	NC_000005.10	+	140504821	140505869	1049	404	sense-overlappin	NM_017747.2	ANKHD1
circRNA_04359	0	0.020469968	0	0	0	0	0	0	0	0	0	0	-	NC_000005.10	+	140504821	140539083	34263	4565	sense-overlappin	NM_017747.2	ANKHD1
circRNA_04360	0	0	0.032712968	0	0	0	0	0	0	0	0	0	-	NC_000005.10	+	140506835	140513479	6645	909	sense-overlappin	NM_017747.2	ANKHD1
circRNA_04361	0	0	0	0	0	0	0	0	0	0	0.02025523	0	hsa_circ_0007420	NC_000005.10	+	141409268	141410620	1353	1353	intergenic	NM_032100.1	PCDHGB6
circRNA_04362	0	0	0	0	0	0.031069364	0	0	0	0	0	0	hsa_circ_0074323	NC_000005.10	-	141580744	141583615	2872	422	sense-overlappin	XM_011537572.1	DIAPH1
circRNA_04363	0	0	0.032712968	0	0.03009467	0	0	0	0	0	0	0	-	NC_000005.10	-	141581915	141583615	1701	679	sense-overlappin	XM_011537572.1	DIAPH1

circRNA_04364	0	0.030704951	0.054521613	0.0206362	0.02006311	0.031069364	0	0.030729443	0.040509055	0.040344966	0	0	hsa_circ_0008982	NC_000005.10	-	141582312	141583615	1304	282	sense-overlappin	XM_011537572.1	DIAPH1
circRNA_04365	0	0	0	0	0.08025246	0.020712909	0	0	0	0	0	0	-	NC_000005.10	+	142131808	142144676	12869	605	sense-overlappin	NM_030571.3	NDFIP1
circRNA_04366	0	0	0	0	0	0.031069364	0	0	0	0.020172483	0	0	-	NC_000005.10	+	142135730	142140629	4900	280	sense-overlappin	NM_030571.3	NDFIP1
circRNA_04367	0.020575025	0	0	0	0	0	0	0	0	0	0.02025523	0	hsa_circ_0007558	NC_000005.10	+	142463970	142467831	3862	3862	intergenic		
circRNA_04368	0	0	0.032712968	0	0	0	0	0	0	0	0	0	-	NC_000005.10	+	142787460	142790458	2999	2999	intronic	XR_944312.1	
circRNA_04369	0	0	0	0	0	0	0.020098685	0	0	0	0	0	-	NC_000005.10	+	142787460	142792773	5314	5314	sense-overlappin	XR_944312.1	
circRNA_04370	0	0	0.076330258	0	0.02006311	0	0	0	0	0	0	0	-	NC_000005.10	+	142885298	142913293	27996	644	sense-overlappin	XR_944312.1	
circRNA_04371	0	0	0.109043225	0	0	0	0	0.030729443	0.050636319	0.020172483	0	0	hsa_circ_0074362	NC_000005.10	+	142885298	142932125	46828	723	sense-overlappin	XR_944312.1	
circRNA_04372	0.051437562	0.071644886	0.098138903	0.1031809	0.11034713	0.020712909	0.020098685	0.061458886	0.070890847	0.030258724	0.04051046	0.0206364	hsa_circ_0074368	NC_000005.10	+	143037196	143057747	20552	394	sense-overlappin	XR_944312.1	
circRNA_04373	0	0	0	0	0	0	0	0	0.020254528	0	0	0	-	NC_000005.10	+	143037196	143134105	96910	693	sense-overlappin	XR_944312.1	
circRNA_04374	0	0.061409903	0.283512385	0.0619086	0.1404418	0.082851637	0.100493427	0.112674625	0.101272639	0.030258724	0.06076569	0.103182001	hsa_circ_0074371	NC_000005.10	+	143054439	143057747	3309	253	sense-overlappin	XR_944312.1	
circRNA_04375	0	0	0.04361729	0	0	0.020712909	0.020098685	0	0	0	0	0	-	NC_000005.10	+	143054439	143121147	66709	413	sense-overlappin	XR_944312.1	
circRNA_04376	0.020575025	0	0	0	0	0	0	0	0	0	0	0	hsa_circ_0074373	NC_000005.10	+	143054439	143147381	92943	703	sense-overlappin	XR_944312.1	
circRNA_04377	0	0	0	0	0.04012623	0	0	0	0	0	0	0	-	NC_000005.10	-	143344835	143345297	463	463	intronic	NM_000176.2	NR3C1
circRNA_04378	0	0	0.04361729	0.0722267	0	0.031069364	0.020098685	0.030729443	0.030381792	0.110948655	0	0	hsa_circ_0001543	NC_000005.10	-	143399656	143400852	1197	1197	exonic	NM_000176.2	NR3C1
circRNA_04379	0	0	0.021808645	0.0206362	0	0	0	0	0	0	0	0	-	NC_000005.10	-	145438072	145473315	35244	35244	intronic	XR_944308.1	
circRNA_04380	0	0	0	0.1031809	0.04012623	0.051782273	0	0	0.020254528	0.07060369	0	0	-	NC_000005.10	-	145764931	145823134	58204	505	sense-overlappin	NM_182960.3	PRELID2
circRNA_04381	0	0.020469968	0.098138903	0.0825448	0.04012623	0	0	0	0	0	0.02025523	0	hsa_circ_0008132	NC_000005.10	-	145764931	145826200	61270	672	sense-overlappin	NM_138492.5	PRELID2
circRNA_04382	0	0	0.032712968	0.1031809	0	0	0	0	0	0.030258724	0	0	-	NC_000005.10	-	145796442	145823134	26693	435	sense-overlappin	NM_182960.3	PRELID2
circRNA_04383	0	0.092114854	0.163564838	0.2889066	0.02006311	0.103564547	0	0.081945182	0.040509055	0	0.06076569	0.0309546	hsa_circ_0008647	NC_000005.10	-	145796442	145826200	29759	602	sense-overlappin	NM_138492.5	PRELID2
circRNA_04384	0	0	0.021808645	0	0.02006311	0	0.030148028	0	0	0	0	0	hsa_circ_0074389	NC_000005.10	-	145817894	145823134	5241	329	sense-overlappin	NM_182960.3	PRELID2
circRNA_04385	0.020575025	0.071644886	0.338033998	0.113499	0	0.062138728	0	0.061458886	0	0.030258724	0	0	hsa_circ_0006528	NC_000005.10	-	145817894	145826200	8307	496	sense-overlappin	NM_138492.5	PRELID2
circRNA_04386	0	0	0	0	0	0	0	0.020486295	0	0	0	0	-	NC_000005.10	-	145817894	145831047	13154	5343	sense-overlappin	NM_138492.5	PRELID2
circRNA_04387	0	0	0	0	0	0	0.020098685	0	0	0	0	0	-	NC_000005.10	-	146149622	146153980	4359	438	sense-overlappin	NM_020117.9	LARS
circRNA_04388	0	0	0	0	0	0	0	0.030729443	0	0	0.030382845	0	-	NC_000005.10	-	146168128	146172774	4647	307	sense-overlappin	NM_020117.9	LARS
circRNA_04389	0.082300098	0.020469968	0.054521613	0.0515905	0.0702209	0.031069364	0.020098685	0.040972591	0	0.060517448	0	0.0619092	hsa_circ_0006087	NC_000005.10	+	146254943	146258593	3651	295	sense-overlappin	XR_944325.1	
circRNA_04390	0	0	0.054521613	0	0	0	0	0	0	0	0	0	-	NC_000005.10	-	148089354	148093742	4389	2846	antisense	XM_011537550.1	SPINK5
circRNA_04391	0	0	0.021808645	0	0	0	0	0	0	0	0	0	-	NC_000005.10	-	148089354	148100597	11244	902	antisense	XM_011537550.1	SPINK5
circRNA_04392	0	0	0	0	0	0	0	0	0.020254528	0	0	0	hsa_circ_0074471	NC_000005.10	+	148394714	148417204	22491	1681	sense-overlappin	NM_001271723.1	FBXO38
circRNA_04393	0	0	0	0	0	0.031069364	0	0	0	0	0	0	hsa_circ_0074472	NC_000005.10	+	148394714	148425701	30988	1981	sense-overlappin	NM_001271723.1	FBXO38
circRNA_04394	0	0.020469968	0	0	0	0	0	0	0	0	0	0	-	NC_000005.10	-	148548668	148550262	1595	327	sense-overlappin	NM_001040169.2	HTR4
circRNA_04395	0	0	0.021808645	0	0	0	0	0	0	0	0	0	-	NC_000005.10	+	149752813	149753221	409	409	intronic	NM_001172698.1	PPARGC1B
circRNA_04396	0	0.020469968	0	0	0	0	0	0	0	0	0	0	-	NC_000005.10	+	149835301	149845914	10614	1229	sense-overlappin	NM_001172698.1	PPARGC1B
circRNA_04397	0	0	0	0	0	0	0.020098685	0	0	0	0	0	-	NC_000005.10	+	150374954	150379408	4455	1380	sense-overlappin	NM_001008657.2	TCOF1
circRNA_04398	0	0	0.021808645	0	0	0	0	0	0	0	0	0	hsa_circ_0074532	NC_000005.10	-	150406259	150407324	1066	316	sense-overlappin	NM_001025158.2	CD74
circRNA_04399	0	0.020469968	0	0	0	0	0	0	0	0	0	0	-	NC_000005.10	-	150736578	150736755	178	178	intronic	NM_001135644.1	DCTN4
circRNA_04400	0	0	0.021808645	0	0	0	0	0	0	0	0	0	-	NC_000005.10	-	151060318	151063747	3430	299	sense-overlappin	NM_001258455.1	TNIP1
circRNA_04401	0	0	0	0	0	0.020712909	0	0.020486295	0	0.020172483	0	0	-	NC_000005.10	-	151198997	151205475	6479	271	sense-overlappin	NM_015621.2	CCDC69
circRNA_04402	0	0	0.032712968	0	0	0	0	0	0	0	0	0	hsa_circ_0003278	NC_000005.10	+	151786572	151791062	4491	400	sense-overlappin	NM_005754.2	G3BP1
circRNA_04403	0	0	0.021808645	0	0.02006311	0	0	0	0	0	0	0	-	NC_000005.10	+	151786603	151803996	17394	1324	sense-overlappin	NM_005754.2	G3BP1
circRNA_04404	0	0	0.032712968	0	0	0	0.020098685	0	0	0	0	0	-	NC_000005.10	+	151786603	151804446	17844	1774	sense-overlappin	NM_005754.2	G3BP1
circRNA_04405	0.082300098	0	0.04361729	0.0825448	0	0	0.030148028	0	0.020254528	0.020172483	0	0	hsa_circ_0003967	NC_000005.10	+	151790323	151791062	740	256	sense-overlappin	NM_005754.2	G3BP1
circRNA_04406	0	0.08187987	0.021808645	0.0515905	0	0	0	0	0.040509055	0	0	0	hsa_circ_0003152	NC_000005.10	-	153997803	154034967	37165	1343	sense-overlappin	XM_011537540.1	FAM114A2
circRNA_04407	0	0	0	0	0	0	0.020098685	0	0	0	0	0	-	NC_000005.10	-	154020663	154028283	7621	6154	sense-overlappin	XM_011537540.1	FAM114A2
circRNA_04408	0.041150049	0	0	0	0.02006311	0	0.030148028	0	0	0.020172483	0	0	-	NC_000005.10	-	154026399	154034967	8569	927	sense-overlappin	XM_011537540.1	FAM114A2
circRNA_04409	0.041150049	0.040939935	0.032712968	0.1238171	0.06018934	0.072495183	0.030148028	0.040972591	0.030381792	0.121034897	0.04051046	0.0309546	hsa_circ_0007773	NC_000005.10	-	154029489	154034967	5479	509	sense-overlappin	XM_011537540.1	FAM114A2
circRNA_04410	0	0	0	0.0206362	0	0	0	0	0	0	0	0	hsa_circ_0007691	NC_000005.10	-	154033791	154034946	1156	396	sense-overlappin	XM_011537540.1	FAM114A2
circRNA_04411	0.13373766	0.184229708	0.119947548	0.5571771	0.1404418	0.176059729	0.221085539	0.245835546	0.212672541	0.463967104	0.141786611	0.216682202	hsa_circ_0001546	NC_000005.10	-	154033791	154034967	1177	417	sense-overlappin	XM_011537540.1	FAM114A2
circRNA_04412	0	0	0.04361729	0	0	0	0	0	0	0	0	0	hsa_circ_0007069	NC_000005.10	-	154790325	154795319	4995	941	sense-overlappin	XM_005268407.3	LARP1
circRNA_04413	0	0	0.021808645	0	0	0	0	0	0	0	0	0	-	NC_000005.10	+	154790325	154811640	21316	2645	sense-overlappin	XM_005268407.3	LARP1
circRNA_04414	0	0	0	0	0	0	0	0	0.020254528	0.020172483	0	0	-	NC_000005.10	+	154871730	154872651	922	256	sense-overlappin	XM_011537708.1	CNOT8
circRNA_04415	0	0	0.021808645	0.0309543	0.03009467	0	0	0.030729443	0	0	0	0	-	NC_000005.10	-	154950821	154967237	16417	884	sense-overlappin	NM_014180.3	MRPL22
circRNA_04416	0	0	0	0	0	0	0	0	0	0.020172483	0	0	-	NC_000005.10	+	157241646	157244478	2833	464	sense-overlappin	NM_005546.3	ITK
circRNA_04417	0	0	0	0	0	0	0	0	0	0.020172483	0	0	-	NC_000005.10	-	157518823	157537991	19169	415	sense-overlappin	NM_033274.4	ADAM19
circRNA_04418	0	0	0	0	0	0	0	0	0	0	0.050638075	0	-	NC_000005.10	-	157841489	157841730	242	242	intronic	NM_001195555.1	CLINT1

circRNA_04419	0	0	0.054521613	0.0206362	0.08025246	0.031069364	0	0	0	0	0	0	hsa_circ_0074828	NC_000005.10	-	159194716	159203656	8941	332	sense-overlappin	NM_001199382.1	RNF145
circRNA_04420	0	0	0	0	0	0	0.020098685	0	0	0	0	0	-	NC_000005.10	+	160010500	160036803	26304	533	sense-overlappin	NM_001282500.1	TTC1
circRNA_04421	0	0	0	0	0	0	0	0	0	0.030258724	0	0	-	NC_000005.10	+	160010500	160051183	40684	774	sense-overlappin	NM_001282500.1	TTC1
circRNA_04422	0	0	0	0.0412724	0	0	0	0	0	0	0	0	-	NC_000005.10	+	160035140	160043169	8030	211	sense-overlappin	NM_001282500.1	TTC1
circRNA_04423	0	0.030704951	0.032712968	0	0.02006311	0	0	0	0	0	0	0	-	NC_000005.10	+	160035140	160051183	16044	415	sense-overlappin	NM_001282500.1	TTC1
circRNA_04424	0	0	0	0	0.02006311	0	0	0	0	0.020172483	0	0	hsa_circ_0074837	NC_000005.10	-	160093101	160094065	965	965	exonic	XM_011534424.1	PWWP2A
circRNA_04425	0	0	0	0	0.02006311	0	0	0	0	0	0	0	-	NC_000005.10	-	160412451	160417241	4791	785	sense-overlappin	XM_011534418.1	SLU7
circRNA_04426	0	0	0	0	0	0	0	0	0	0	0.050638075	0	-	NC_000005.10	-	160670463	160670667	205	205	exonic	XM_011534472.1	ATP10B
circRNA_04427	0	0.020469968	0	0.0309543	0	0	0.050246713	0	0	0	0	0	-	NC_000005.10	+	163439257	163444481	5225	1288	sense-overlappin	XR_941118.1	
circRNA_04428	0	0	0	0	0	0	0.020098685	0	0	0	0	0	-	NC_000005.10	+	163443674	163444481	808	397	exonic	XR_941118.1	
circRNA_04429	0	0	0.021808645	0	0	0	0	0	0	0	0	0	-	NC_000005.10	+	163473179	163478800	5622	735	sense-overlappin	NM_001142556.1	HMMR
circRNA_04430	0	0	0.021808645	0	0	0	0.020098685	0.020486295	0	0.040344966	0	0	hsa_circ_0009145	NC_000005.10	+	168488602	168492847	4246	324	sense-overlappin	NM_002887.3	RARS
circRNA_04431	0.185175222	0.184229708	0.905058768	0.5881314	0.52164099	0.31069364	0.482368448	0.286808137	0.283563388	0.453880863	0.131658996	0.175409401	hsa_circ_0001550	NC_000005.10	+	168488602	168494650	6049	534	sense-overlappin	NM_002887.3	RARS
circRNA_04432	0	0	0.021808645	0	0	0	0.020098685	0	0	0	0	0	hsa_circ_0074874	NC_000005.10	+	168488602	168495436	6835	656	sense-overlappin	NM_002887.3	RARS
circRNA_04433	0.020575025	0.040939935	0.098138903	0.0619086	0.13041025	0.031069364	0.040197371	0.020486295	0.070890847	0.030258724	0.04051046	0.0412728	hsa_circ_0001551	NC_000005.10	+	168488602	168497348	8747	777	sense-overlappin	NM_002887.3	RARS
circRNA_04434	0	0	0	0.0206362	0	0	0	0	0	0	0	0	-	NC_000005.10	+	168488602	168500720	12119	907	sense-overlappin	NM_002887.3	RARS
circRNA_04435	0	0	0.021808645	0.0412724	0	0	0	0	0	0	0	0.0206364	hsa_circ_0074875	NC_000005.10	+	168488602	168502105	13504	1012	sense-overlappin	NM_002887.3	RARS
circRNA_04436	0	0	0	0	0	0	0	0.020486295	0.020254528	0.020172483	0.04051046	0.0206364	hsa_circ_0007958	NC_000005.10	+	168500591	168502105	1515	235	sense-overlappin	NM_002887.3	RARS
circRNA_04437	0	0	0.054521613	0	0	0	0	0	0	0	0	0	hsa_circ_0074881	NC_000005.10	+	168500591	168510686	10096	630	sense-overlappin	NM_002887.3	RARS
circRNA_04438	0	0	0	0	0	0	0	0	0.020254528	0	0	0	hsa_circ_0074883	NC_000005.10	+	168500591	168518062	17472	1051	sense-overlappin	NM_002887.3	RARS
circRNA_04439	0	0.020469968	0.032712968	0	0	0	0	0	0	0.020172483	0.02025523	0	-	NC_000005.10	-	168559032	168566266	7235	681	sense-overlappin	NM_024594.3	PANK3
circRNA_04440	0	0	0.032712968	0	0	0	0	0	0	0.030258724	0	0	hsa_circ_0006386	NC_000005.10	-	168559032	168568998	9967	1034	sense-overlappin	NM_024594.3	PANK3
circRNA_04441	0	0	0	0	0	0	0	0	0	0	0	0.0206364	hsa_circ_0074944	NC_000005.10	+	169681744	169684350	2607	291	sense-overlappin	XM_005265830.2	DOCK2
circRNA_04442	0	0	0	0	0	0	0.020098685	0	0	0	0	0	hsa_circ_0074945	NC_000005.10	+	169681744	169689333	7590	373	sense-overlappin	XM_005265830.2	DOCK2
circRNA_04443	0	0	0	0	0	0	0	0	0	0.040344966	0	0	-	NC_000005.10	+	169759705	169764081	4377	2634	sense-overlappin	XM_005265830.2	DOCK2
circRNA_04444	0	0	0	0	0	0	0	0	0	0	0	0.0206364	-	NC_000005.10	+	169983068	169996164	13097	273	sense-overlappin	NM_004946.2	DOCK2
circRNA_04445	0	0	0	0.0309543	0	0	0	0	0	0	0	0	-	NC_000005.10	+	170878097	170892553	14457	405	sense-overlappin	NM_022897.4	RANBP17
circRNA_04446	0.041150049	0	0.21808645	0.0515905	0.10031557	0.041425819	0	0.030729443	0	0.020172483	0	0	hsa_circ_0001554	NC_000005.10	+	170878097	170896115	18019	471	sense-overlappin	NM_022897.4	RANBP17
circRNA_04447	0	0	0.021808645	0	0	0	0	0	0	0	0	0	-	NC_000005.10	+	170878097	170919613	41517	1256	sense-overlappin	NM_022897.4	RANBP17
circRNA_04448	0	0	0.04361729	0.0515905	0	0	0	0	0	0.050431207	0	0	hsa_circ_0004527	NC_000005.10	+	170878097	170924550	46454	1450	sense-overlappin	NM_022897.4	RANBP17
circRNA_04449	0	0	0.032712968	0	0	0	0	0	0	0	0	0	hsa_circ_0074978	NC_000005.10	+	170892387	170896115	3729	233	sense-overlappin	NM_022897.4	RANBP17
circRNA_04450	0	0	0.021808645	0	0	0	0	0	0	0	0	0	hsa_circ_0003696	NC_000005.10	+	170892387	170924550	32164	1212	sense-overlappin	NM_022897.4	RANBP17
circRNA_04451	0	0	0.04361729	0	0.02006311	0	0	0	0.030381792	0	0	0	hsa_circ_0002745	NC_000005.10	+	170909661	170924550	14890	979	sense-overlappin	NM_022897.4	RANBP17
circRNA_04452	0.030862537	0.020469968	0.13085187	0.0825448	0.06018934	0.031069364	0	0.071702034	0.081018111	0.080689931	0.02025523	0.0412728	hsa_circ_0003718	NC_000005.10	+	171183195	171205612	22418	338	sense-overlappin	NM_022897.4	RANBP17
circRNA_04453	0.051437562	0	0.054521613	0.0309543	0.02006311	0	0	0	0	0	0	0	hsa_circ_0002713	NC_000005.10	+	171183199	171205612	22414	334	sense-overlappin	NM_022897.4	RANBP17
circRNA_04454	0	0	0.054521613	0.0206362	0	0	0	0	0	0	0	0	hsa_circ_0004738	NC_000005.10	+	171240928	171242820	1893	354	sense-overlappin	NM_022897.4	RANBP17
circRNA_04455	0	0.030704951	0.076330258	0.0206362	0	0	0	0	0.020254528	0.030258724	0	0	hsa_circ_0002358	NC_000005.10	+	171391305	171392978	1674	386	sense-overlappin	XM_005265920.2	NPM1
circRNA_04456	0	0	0.021808645	0	0	0	0	0	0	0	0	0	hsa_circ_0075005	NC_000005.10	+	171391305	171400210	8906	444	sense-overlappin	XM_005265920.2	NPM1
circRNA_04457	0	0	0	0	0	0	0.030148028	0.020486295	0	0	0.02025523	0.0412728	hsa_circ_0004098	NC_000005.10	-	171910572	171914405	3834	289	sense-overlappin	XM_005265857.2	FBXW11
circRNA_04458	0	0.030704951	0	0	0	0	0	0	0	0	0	0	-	NC_000005.10	-	171994353	171997051	2699	2699	sense-overlappin	XM_005265856.1	FBXW11
circRNA_04459	0	0	0.021808645	0	0	0	0	0	0	0	0.02025523	0	-	NC_000005.10	-	172052929	172061268	8340	684	sense-overlappin	NM_005990.3	STK10
circRNA_04460	0	0	0	0	0	0	0	0	0	0.040344966	0.02025523	0	hsa_circ_0001555	NC_000005.10	-	172055588	172057473	1886	314	sense-overlappin	NM_005990.3	STK10
circRNA_04461	0	0	0	0.0206362	0	0	0	0	0	0	0	0	-	NC_000005.10	-	172211967	172234358	22392	498	sense-overlappin	XM_011534695.1	UBTD2
circRNA_04462	0	0	0	0	0	0	0	0	0.020254528	0	0	0	-	NC_000005.10	-	172234122	172234358	237	237	exonic	XM_011534695.1	UBTD2
circRNA_04463	0	0	0	0.0412724	0	0	0	0	0	0	0	0	hsa_circ_0004004	NC_000005.10	+	172932436	172935310	2875	224	sense-overlappin	NM_001031711.2	ERGIC1
circRNA_04464	0	0	0.021808645	0	0	0	0	0	0	0	0	0	hsa_circ_0008140	NC_000005.10	+	173080585	173091401	10817	1427	sense-overlappin	XM_005265821.3	CREBRF
circRNA_04465	0	0	0	0	0	0	0	0	0	0.030258724	0	0	hsa_circ_0075061	NC_000005.10	+	173080585	173123202	42618	2009	sense-overlappin	XM_005265821.3	CREBRF
circRNA_04466	0.041150049	0	0	0	0	0	0	0	0	0	0	0	-	NC_000005.10	+	173090315	173091401	1087	1087	exonic	XM_005265821.3	CREBRF
circRNA_04467	0.061725074	0.020469968	0.04361729	0	0	0	0	0.020486295	0	0.080689931	0	0	-	NC_000005.10	+	173108624	173112379	3756	459	sense-overlappin	XM_005265821.3	CREBRF
circRNA_04468	0	0	0.021808645	0.0515905	0.02006311	0	0	0	0	0	0	0	-	NC_000005.10	+	173108624	173123202	14579	582	sense-overlappin	XM_005265821.3	CREBRF
circRNA_04469	0	0	0	0	0	0.020712909	0	0	0	0	0	0	hsa_circ_0008042	NC_000005.10	+	173944967	173953272	8306	680	sense-overlappin	NM_030627.2	CPEB4
circRNA_04470	0	0	0	0.0309543	0	0	0	0	0	0	0	0.0206364	hsa_circ_0075092	NC_000005.10	+	176289654	176290955	1302	1302	exonic	XM_005265909.1	SIMC1
circRNA_04471	0	0	0	0	0.03009467	0	0	0	0	0	0	0	hsa_circ_0002171	NC_000005.10	+	176289654	176296320	6667	1605	sense-overlappin	XM_005265909.1	SIMC1
circRNA_04472	0	0	0	0	0.02006311	0	0	0	0	0	0	0	hsa_circ_0075097	NC_000005.10	+	176322273	176324757	2485	282	sense-overlappin	NM_198567.4	SIMC1
circRNA_04473	0	0	0	0	0	0	0	0	0.020254528	0	0	0	hsa_circ_0008823	NC_000005.10	-	176350613	176355749	5137	431	sense-overlappin	NM_001079684.2	KIAA1191

circRNA_04474	0	0	0	0	0	0	0	0	0	0.040344966	0	0	-	NC_000005.10	+	176573555	176576671	3117	1803	sense-overlappin	NM_001171976.1	CDHR2
circRNA_04475	0	0	0	0	0	0.020712909	0	0	0	0	0.04051046	0	hsa_circ_0004490	NC_000005.10	-	176943335	176951577	8243	258	sense-overlappin	NM_016290.4	UIMC1
circRNA_04476	0.092587611	0.092114854	0.30532103	0.2889066	0.16050492	0.176059729	0.080394741	0.081945182	0.26330886	0.272328518	0.04051046	0.092863801	hsa_circ_0001558	NC_000005.10	-	176943335	176958154	14820	397	sense-overlappin	NM_016290.4	UIMC1
circRNA_04477	0	0	0.04361729	0	0	0	0	0	0	0	0	0	-	NC_000005.10	-	176951474	176982623	31150	1154	sense-overlappin	XM_005265931.2	UIMC1
circRNA_04478	0	0	0	0	0.02006311	0	0	0	0	0	0	0	-	NC_000005.10	-	176975396	176980390	4995	4995	sense-overlappin	NM_016290.4	UIMC1
circRNA_04479	0	0	0	0	0.06018934	0.031069364	0	0	0.020254528	0.040344966	0	0.0206364	hsa_circ_0008631	NC_000005.10	-	176975396	176982623	7228	240	sense-overlappin	NM_016290.4	UIMC1
circRNA_04480	0	0	0	0	0	0	0.020098685	0	0	0	0	0	hsa_circ_0075143	NC_000005.10	+	177041126	177044533	3408	342	sense-overlappin	XM_005265862.2	ZNF346
circRNA_04481	0.020575025	0	0	0	0.04012623	0	0	0	0	0.020172483	0	0.0206364	hsa_circ_0001559	NC_000005.10	+	177041126	177050936	9811	576	sense-overlappin	XM_005265862.2	ZNF346
circRNA_04482	0	0.030704951	0	0.0206362	0	0	0	0	0	0.020172483	0	0	hsa_circ_0075157	NC_000005.10	+	177191884	177204292	12409	309	sense-overlappin	XM_005265959.1	NSD1
circRNA_04483	0	0	0	0.0206362	0	0.020712909	0.030148028	0.020486295	0	0	0	0	hsa_circ_0075158	NC_000005.10	+	177191884	177212195	20312	2869	sense-overlappin	XM_005265959.1	NSD1
circRNA_04484	0.051437562	0	0.076330258	0.0309543	0	0.020712909	0	0.020486295	0	0.020172483	0.02025523	0	hsa_circ_0075161	NC_000005.10	+	177209636	177212195	2560	2560	exonic	XM_005265959.1	NSD1
circRNA_04485	0.030862537	0	0.04361729	0.0206362	0.08025246	0.072495183	0.060296056	0	0.020254528	0.020172483	0.04051046	0.0412728	hsa_circ_0002972	NC_000005.10	-	177536358	177539147	2790	1020	sense-overlappin	XM_011534578.1	FAM193B
circRNA_04486	0.041150049	0	0	0	0	0	0	0	0	0	0	0	hsa_circ_0075236	NC_000005.10	-	177537873	177539147	1275	632	sense-overlappin	XM_011534578.1	FAM193B
circRNA_04487	0	0	0	0	0	0.020712909	0	0	0	0	0	0	hsa_circ_0004937	NC_000005.10	-	177619078	177627614	8537	8537	intergenic		
circRNA_04488	0	0	0	0.0206362	0.02006311	0.072495183	0.030148028	0	0.030381792	0	0	0	hsa_circ_0004138	NC_000005.10	-	177619078	177632738	13661	13661	intergenic		
circRNA_04489	0	0	0	0	0	0.020712909	0	0	0	0	0	0	hsa_circ_0007932	NC_000005.10	-	177626436	177632738	6303	6303	intergenic		
circRNA_04490	0	0	0	0	0	0	0.020098685	0	0	0	0	0	-	NC_000005.10	+	179562547	179567562	5016	220	sense-overlappin	NM_025158.4	RUFY1
circRNA_04491	0	0	0	0	0	0	0.020098685	0	0	0	0	0	-	NC_000005.10	+	179591625	179593645	2021	285	sense-overlappin	NM_025158.4	RUFY1
circRNA_04492	0	0	0	0.0206362	0	0	0	0	0	0	0	0	-	NC_000005.10	+	179593478	179594963	1486	266	sense-overlappin	NM_025158.4	RUFY1
circRNA_04493	0	0	0	0.0309543	0	0	0	0	0	0.020172483	0	0	hsa_circ_0075288	NC_000005.10	-	179623037	179623164	128	128	exonic	XM_011534541.1	HNRNPH1
circRNA_04494	0	0	0	0	0	0	0.030148028	0	0	0	0	0	hsa_circ_0075291	NC_000005.10	+	179705679	179706331	653	248	sense-overlappin	XM_011534665.1	CANX
circRNA_04495	0	0.030704951	0	0	0	0	0	0	0.020254528	0.020172483	0	0	hsa_circ_0002300	NC_000005.10	+	179705679	179707190	1512	307	sense-overlappin	XM_011534665.1	CANX
circRNA_04496	0	0	0.054521613	0	0.03009467	0	0	0	0	0	0	0	hsa_circ_0075292	NC_000005.10	+	179705679	179709059	3381	531	sense-overlappin	XM_011534665.1	CANX
circRNA_04497	0	0.040939935	0.032712968	0.0825448	0.04012623	0.041425819	0.030148028	0	0	0	0.070893305	0	hsa_circ_0001564	NC_000005.10	+	179705679	179710065	4387	724	sense-overlappin	XM_011534665.1	CANX
circRNA_04498	0	0	0	0	0.06018934	0	0	0	0	0	0	0	hsa_circ_0075298	NC_000005.10	+	179707132	179710065	2934	476	sense-overlappin	XM_011534665.1	CANX
circRNA_04499	0	0	0.021808645	0	0.02006311	0	0	0	0	0	0	0	hsa_circ_0005778	NC_000005.10	+	179708239	179709059	821	224	sense-overlappin	XM_011534665.1	CANX
circRNA_04500	0	0	0.032712968	0	0.04012623	0	0	0	0	0	0	0	-	NC_000005.10	+	179708239	179710065	1827	417	sense-overlappin	XM_011534665.1	CANX
circRNA_04501	0	0	0	0	0.03009467	0	0	0	0.020254528	0.040344966	0.050638075	0.0309546	hsa_circ_0001565	NC_000005.10	+	179709873	179716294	6422	383	sense-overlappin	XM_011534665.1	CANX
circRNA_04502	0.041150049	0	0.04361729	0.0206362	0.02006311	0	0	0.051215739	0.050636319	0	0	0.0309546	hsa_circ_0002051	NC_000005.10	+	179709873	179720560	10688	654	sense-overlappin	XM_011534665.1	CANX
circRNA_04503	0	0	0	0	0	0	0	0	0.030381792	0	0	0	hsa_circ_0075303	NC_000005.10	+	179719668	179719781	114	114	exonic	XM_011534665.1	CANX
circRNA_04504	0.051437562	0	0	0	0	0	0	0	0	0	0	0	-	NC_000005.10	+	179768850	179771243	2394	337	sense-overlappin	NM_014757.4	MAML1
circRNA_04505	0	0.030704951	0	0	0	0.031069364	0	0.020486295	0	0	0.030382845	0	-	NC_000005.10	+	179831570	179831878	309	309	intronic	NM_001142298.1	SQSTM1
circRNA_04506	0.030862537	0	0	0	0	0	0	0	0	0	0	0	hsa_circ_0075329	NC_000005.10	-	179888103	179888312	210	210	exonic	NM_015043.3	TBC1D9B
circRNA_04507	0	0.020469968	0	0	0	0	0	0	0	0	0	0	hsa_circ_0075338	NC_000005.10	-	179966806	179980200	13395	457	sense-overlappin	XM_011534593.1	RNF130
circRNA_04508	0	0.020469968	0.032712968	0.0206362	0.05015779	0	0	0	0.030381792	0.030258724	0.04051046	0.0412728	hsa_circ_0075341	NC_000005.10	-	180238332	180241155	2824	261	sense-overlappin	XM_005265940.3	MAPK9
circRNA_04509	0	0	0.021808645	0.0206362	0	0	0	0	0	0	0	0	hsa_circ_0075346	NC_000005.10	-	180248973	180280608	31636	904	sense-overlappin	XM_005265940.3	MAPK9
circRNA_04510	0.123450148	0.040939935	0.163564838	0.0515905	0.04012623	0.041425819	0.040197371	0.020486295	0.091145375	0.07060369	0.08102092	0.082545601	hsa_circ_0001566	NC_000005.10	-	180261684	180280608	18925	738	sense-overlappin	XM_005265940.3	MAPK9
circRNA_04511	0	0	0.021808645	0	0	0	0	0	0	0	0	0	-	NC_000005.10	-	180261684	180287067	25384	7197	sense-overlappin	XM_005265940.3	MAPK9
circRNA_04512	0	0	0.032712968	0	0.03009467	0	0	0	0	0	0.030382845	0	hsa_circ_0075357	NC_000005.10	+	180529275	180553471	24197	387	sense-overlappin	XM_011534607.1	CNOT6
circRNA_04513	0	0.020469968	0.054521613	0.0309543	0.0702209	0.031069364	0.020098685	0	0	0	0.02025523	0.0309546	hsa_circ_0008836	NC_000005.10	+	180549931	180553471	3541	273	sense-overlappin	XM_011534607.1	CNOT6
circRNA_04514	0	0.020469968	0.04361729	0	0	0	0	0	0	0	0	0	-	NC_000005.10	+	180908586	180911614	3029	624	sense-overlappin	NM_001040462.2	BTNL8
circRNA_04515	0.020575025	0	0	0	0	0	0	0	0	0.020172483	0	0	-	NC_000006.12	+	345854	348841	2988	320	sense-overlappin	NM_001286555.1	DUSP22
circRNA_04516	0	0	0	0	0.02006311	0	0	0	0	0	0	0	hsa_circ_0075434	NC_000006.12	-	576757	599225	22469	576	sense-overlappin	NM_018303.5	EXOC2
circRNA_04517	0	0	0	0	0	0	0	0	0	0	0	0.0412728	-	NC_000006.12	-	1887664	1887767	104	104	intronic	NM_001253846.1	GMDS
circRNA_04518	0	0	0	0	0	0.031069364	0	0	0	0	0	0.051591	-	NC_000006.12	-	1930103	1960966	30864	426	sense-overlappin	NM_001253846.1	GMDS
circRNA_04519	0	0	0	0	0	0	0	0	0	0	0	0.0206364	-	NC_000006.12	-	1959867	1960966	1100	298	sense-overlappin	NM_001253846.1	GMDS
circRNA_04520	0	0.020469968	0	0	0	0	0	0	0	0	0	0	hsa_circ_0075450	NC_000006.12	-	2115771	2117556	1786	198	sense-overlappin	NM_001253846.1	GMDS
circRNA_04521	0	0	0.054521613	0	0	0.051782273	0.050246713	0.030729443	0	0	0	0	hsa_circ_0075451	NC_000006.12	-	2115771	2124731	8961	243	sense-overlappin	NM_001253846.1	GMDS
circRNA_04522	0	0.071644886	0.021808645	0.0722267	0.04012623	0.062138728	0	0.030729443	0.030381792	0.030258724	0.030382845	0	-	NC_000006.12	+	2263601	2273417	9817	9817	intergenic		
circRNA_04523	0	0.061409903	0	0	0	0	0	0	0.040509055	0	0	0	hsa_circ_0008313	NC_000006.12	+	2397221	2399037	1817	1817	intergenic		
circRNA_04524	0.092587611	0.122819805	0.054521613	0.1650895	0.05015779	0.093208092	0.120592112	0.040972591	0.050636319	0.110948655	0	0.103182001	hsa_circ_0001571	NC_000006.12	+	3076764	3077935	1172	381	sense-overlappin	XM_005249458.1	RIPK1
circRNA_04525	0	0.020469968	0.021808645	0	0	0	0	0	0	0	0	0	hsa_circ_0003886	NC_000006.12	+	3076764	3081116	4353	519	sense-overlappin	XM_005249458.1	RIPK1
circRNA_04526	0	0	0	0.0206362	0.04012623	0	0	0	0	0	0	0	-	NC_000006.12	+	3085259	3104315	19057	318	sense-overlappin	XM_005249458.1	RIPK1
circRNA_04527	0	0	0	0	0	0	0	0.020486295	0	0	0	0	hsa_circ_0075499	NC_000006.12	+	3104225	3106051	1827	661	sense-overlappin	XM_005249458.1	RIPK1
circRNA_04528	0	0	0	0.0206362	0	0	0	0	0	0	0	0	hsa_circ_0075501	NC_000006.12	-	3283852	3298218	14367	645	sense-overlappin	XM_006715165.2	SLC22A23

circRNA_04529	0	0	0	0	0	0	0	0.030729443	0	0	0	0.0619092	hsa_circ_0075504	NC_000006.12	-	3410188	3415855	5668	259	sense-overlappin	NM_001286455.1	SLC22A23
circRNA_04530	0.792138448	0.450339286	0.948676058	0.5674952	0.48151476	0.341763004	0.482368448	0.286808137	0.303817916	0.302587242	0.192424686	0.227000402	hsa_circ_0008285	NC_000006.12	+	4891713	4892379	667	667	exonic	NR_026590.1	
circRNA_04531	0	0	0	0	0.02006311	0	0	0	0	0	0	0	hsa_circ_0005893	NC_000006.12	+	5396633	5431172	34540	8201	sense-overlappin	XM_005248811.1	FARS2
circRNA_04532	0.030862537	0	0.032712968	0.0619086	0.03009467	0.031069364	0	0.020486295	0.040509055	0.050431207	0.030382845	0.0412728	hsa_circ_0075533	NC_000006.12	+	5404542	5431172	26631	292	sense-overlappin	XM_005248811.1	FARS2
circRNA_04533	0.154312685	0.133054789	0.47979019	0.2373162	0.06018934	0.124277456	0.160789483	0.071702034	0.13165443	0.110948655	0.141786611	0.082545601	hsa_circ_0001573	NC_000006.12	+	7176655	7189322	12668	709	sense-overlappin	NM_001168344.1	RREB1
circRNA_04534	0.041150049	0.020469968	0.065425935	0.0412724	0.06018934	0	0.030148028	0	0.020254528	0.050431207	0.06076569	0	hsa_circ_0007429	NC_000006.12	+	7181124	7189322	8199	590	sense-overlappin	NM_001168344.1	RREB1
circRNA_04535	0	0	0.021808645	0	0	0	0	0	0	0	0	0	hsa_circ_0075561	NC_000006.12	+	7187434	7189322	1889	254	sense-overlappin	NM_001168344.1	RREB1
circRNA_04536	0	0	0	0	0	0	0.020098685	0	0	0	0	0	-	NC_000006.12	+	7187434	7211709	24276	536	sense-overlappin	NM_001168344.1	RREB1
circRNA_04537	0	0	0	0	0	0	0.020098685	0	0	0	0	0	-	NC_000006.12	+	7226467	7231907	5441	3101	sense-overlappin	NM_001168344.1	RREB1
circRNA_04538	0.030862537	0.030704951	0	0.0206362	0	0.020712909	0	0	0	0	0.030382845	0.0309546	hsa_circ_0001574	NC_000006.12	+	7602623	7606195	3573	376	sense-overlappin	NM_152551.3	SNRNP48
circRNA_04539	0	0	0	0	0	0	0	0	0	0.020172483	0	0	-	NC_000006.12	-	7884359	7888848	4490	357	sense-overlappin	NM_001145549.2	TXNDC5
circRNA_04540	0	0.030704951	0	0	0	0	0	0	0.020254528	0	0	0	-	NC_000006.12	-	7899576	7904723	5148	256	sense-overlappin	NM_001145549.2	TXNDC5
circRNA_04541	0	0	0	0	0	0.041425819	0	0	0	0	0	0	hsa_circ_0005917	NC_000006.12	+	10702369	10702639	271	196	sense-overlappin	XM_011514720.1	PAK1IP1
circRNA_04542	0	0	0	0.0309543	0.04012623	0.020712909	0.020098685	0	0	0	0	0	hsa_circ_0008053	NC_000006.12	+	10703405	10704844	1440	297	sense-overlappin	XM_011514720.1	PAK1IP1
circRNA_04543	0	0	0	0	0.02006311	0	0	0	0	0	0	0	-	NC_000006.12	-	11213281	11213727	447	447	exonic	NM_006403.3	NEDD9
circRNA_04544	0	0	0	0	0	0	0	0.020486295	0	0	0	0	hsa_circ_0075638	NC_000006.12	+	12119890	12125870	5981	5981	exonic	XM_011514546.1	HIVEP1
circRNA_04545	0.020575025	0	0	0	0.03009467	0	0	0	0	0	0	0	hsa_circ_0002268	NC_000006.12	+	13227816	13230193	2378	405	sense-overlappin	XM_011514390.1	PHACTR1
circRNA_04546	0.041150049	0.040939935	0.119947548	0.1031809	0.08025246	0.217485548	0	0.081945182	0	0.161379862	0.06076569	0.0412728	hsa_circ_0007218	NC_000006.12	+	13579451	13584225	4775	309	sense-overlappin	XM_005248968.3	SIRT5
circRNA_04547	0	0	0.054521613	0	0.06018934	0.031069364	0	0	0.020254528	0	0	0.0206364	hsa_circ_0075648	NC_000006.12	+	13579451	13600949	21499	1192	sense-overlappin	XM_011514444.1	SIRT5
circRNA_04548	0.072012586	0	0.13085187	0.1341352	0.22069426	0.093208092	0.040197371	0.09218833	0.060763583	0.07060369	0.10127615	0	hsa_circ_0001577	NC_000006.12	-	13632370	13644729	12360	1020	sense-overlappin	NM_005493.2	RANBP9
circRNA_04549	0	0	0.021808645	0	0	0.031069364	0	0	0	0.060517448	0	0	hsa_circ_0075650	NC_000006.12	-	13632370	13658832	26463	1264	sense-overlappin	NM_005493.2	RANBP9
circRNA_04550	0.051437562	0.020469968	0	0.0619086	0.0702209	0.020712909	0.030148028	0.030729443	0.020254528	0.060517448	0.04051046	0.072227401	hsa_circ_0001578	NC_000006.12	-	13639563	13644729	5167	598	sense-overlappin	NM_005493.2	RANBP9
circRNA_04551	0	0	0.04361729	0	0	0	0	0	0	0	0	0	hsa_circ_0008418	NC_000006.12	-	15615267	15637804	22538	327	sense-overlappin	XM_005249447.3	DTNBP1
circRNA_04552	0.154312685	0.204699675	0.26170374	0.330179	0.0702209	0.196772639	0.120592112	0.143404068	0.060763583	0.211811069	0.091148535	0.257955002	hsa_circ_0007132	NC_000006.12	-	16326394	16328470	2077	2077	exonic	NM_000332.3	ATXN1
circRNA_04553	0	0	0	0	0	0	0	0	0	0.060517448	0	0.051591	-	NC_000006.12	-	16391085	16392605	1521	1521	intronic	NM_000332.3	ATXN1
circRNA_04554	0	0	0.032712968	0	0	0	0.030148028	0	0	0	0	0	hsa_circ_0004032	NC_000006.12	-	16657776	16753347	95572	241	sense-overlappin	NM_000332.3	ATXN1
circRNA_04555	0	0	0	0	0.02006311	0	0	0	0	0	0	0	hsa_circ_0005000	NC_000006.12	-	16657776	16754091	96316	290	sense-overlappin	NM_000332.3	ATXN1
circRNA_04556	0	0	0.021808645	0	0.03009467	0	0	0	0	0	0	0	-	NC_000006.12	+	17540973	17551604	10632	524	sense-overlappin	XM_011514233.1	CAP2
circRNA_04557	0	0	0.021808645	0	0	0	0	0	0	0	0	0	-	NC_000006.12	-	17624561	17640064	15504	2454	sense-overlappin	NM_001278209.1	NUP153
circRNA_04558	0	0	0.054521613	0	0	0	0	0	0	0	0	0	hsa_circ_0007370	NC_000006.12	-	17646067	17649300	3234	325	sense-overlappin	NM_001278209.1	NUP153
circRNA_04559	0	0	0.04361729	0	0.03009467	0	0.020098685	0	0	0	0	0	hsa_circ_0003576	NC_000006.12	-	17661653	17675770	14118	1061	sense-overlappin	NM_001278209.1	NUP153
circRNA_04560	0	0	0.021808645	0	0	0	0	0	0	0.020172483	0	0	hsa_circ_0002492	NC_000006.12	-	17665239	17669028	3790	201	sense-overlappin	NM_001278209.1	NUP153
circRNA_04561	0	0	0.076330258	0	0.03009467	0.031069364	0	0	0.020254528	0	0	0	hsa_circ_0007268	NC_000006.12	-	17665239	17669546	4308	363	sense-overlappin	NM_001278209.1	NUP153
circRNA_04562	0	0	0.032712968	0	0	0	0	0	0	0	0	0	hsa_circ_0075734	NC_000006.12	-	17665239	17675770	10532	881	sense-overlappin	NM_001278209.1	NUP153
circRNA_04563	0	0	0	0	0	0	0.020098685	0	0.040509055	0	0	0	hsa_circ_0075737	NC_000006.12	-	17669293	17675033	5741	291	sense-overlappin	NM_001278209.1	NUP153
circRNA_04564	0	0	0	0	0	0	0	0	0	0.020172483	0	0	hsa_circ_0007867	NC_000006.12	-	17764155	17764946	792	792	exonic	NM_001105568.2	KIF13A
circRNA_04565	0	0	0.054521613	0	0	0	0	0	0	0	0.02025523	0	hsa_circ_0075748	NC_000006.12	-	17771908	17779099	7192	537	sense-overlappin	NM_001105568.2	KIF13A
circRNA_04566	0	0.040939935	0	0	0	0	0	0	0	0	0	0	-	NC_000006.12	-	17825768	17834071	8304	631	sense-overlappin	NM_001105568.2	KIF13A
circRNA_04567	0	0	0.021808645	0	0	0	0	0	0	0	0	0	-	NC_000006.12	+	18159883	18162924	3042	318	sense-overlappin	XM_005248929.2	KDM1B
circRNA_04568	0	0	0.098138903	0	0	0	0.030148028	0	0.040509055	0.020172483	0.030382845	0	hsa_circ_0001580	NC_000006.12	+	18159883	18166378	6496	430	sense-overlappin	XM_005248929.2	KDM1B
circRNA_04569	0	0	0.021808645	0	0	0	0	0	0	0	0	0	-	NC_000006.12	+	18171363	18205664	34302	1242	sense-overlappin	XM_005248925.1	KDM1B
circRNA_04570	0.030862537	0.061409903	0	0	0.02006311	0	0	0	0	0	0	0	hsa_circ_0075792	NC_000006.12	+	18212488	18213781	1294	243	sense-overlappin	NM_153042.3	KDM1B
circRNA_04571	0	0.020469968	0.076330258	0	0.0702209	0	0.030148028	0	0	0.030258724	0	0	-	NC_000006.12	-	18236452	18237516	1065	285	sense-overlappin	NM_001134709.1	DEK
circRNA_04572	0.185175222	0.092114854	0.501598835	0.2889066	0.4012623	0.186416184	0.160789483	0.163890364	0.172163486	0.201724828	0.151914226	0.113500201	hsa_circ_0075796	NC_000006.12	-	18236452	18258405	21954	998	sense-overlappin	XR_926307.1	
circRNA_04573	0	0.020469968	0	0	0	0	0	0	0	0	0	0	-	NC_000006.12	-	18237381	18249701	12321	283	sense-overlappin	XR_926307.1	
circRNA_04574	0	0	0.076330258	0	0	0	0	0	0	0.020172483	0	0	-	NC_000006.12	-	18249651	18258405	8755	617	sense-overlappin	NM_003472.3	DEK
circRNA_04575	0	0	0.032712968	0	0	0	0	0	0	0	0	0	-	NC_000006.12	-	18255731	18258405	2675	428	sense-overlappin	NM_003472.3	DEK
circRNA_04576	0.082300098	0.030704951	0.032712968	0.0206362	0.08025246	0	0.040197371	0	0	0.020172483	0.02025523	0	hsa_circ_0008846	NC_000006.12	-	18256361	18258405	2045	307	sense-overlappin	NM_003472.3	DEK
circRNA_04577	0	0	0.021808645	0	0	0	0	0	0	0	0	0	hsa_circ_0075799	NC_000006.12	-	20109598	20144315	34718	1038	sense-overlappin	XM_006714999.1	MBOAT1
circRNA_04578	0	0	0.021808645	0	0	0	0	0	0	0	0	0	-	NC_000006.12	-	20144220	20152769	8550	320	sense-overlappin	XM_006714999.1	MBOAT1
circRNA_04579	0	0	0.021808645	0	0	0	0	0	0	0	0	0	hsa_circ_0075806	NC_000006.12	+	20479846	20488248	8403	742	sense-overlappin	NM_001949.4	E2F3
circRNA_04580	0.030862537	0	0.032712968	0.0206362	0.02006311	0.031069364	0	0	0	0.050431207	0	0.0309546	hsa_circ_0001582	NC_000006.12	+	20546346	20548705	2360	291	sense-overlappin	XM_006715128.2	CDKAL1
circRNA_04581	0	0	0	0	0	0	0	0	0.020254528	0	0	0	-	NC_000006.12	+	20739519	20781265	41747	267	sense-overlappin	XM_006715128.2	CDKAL1
circRNA_04582	0	0	0	0	0.02006311	0	0	0	0	0	0	0	-	NC_000006.12	+	20771807	20781265	9459	9459	sense-overlappin	XM_006715128.2	CDKAL1
circRNA_04583	0	0	0	0.0206362	0	0	0	0.030729443	0	0	0	0	-	NC_000006.12	+	20771807	20846178	74372	9563	sense-overlappin	XM_006715128.2	CDKAL1
circRNA_04584	0	0.040939935	0	0	0	0	0	0	0	0	0	0	-	NC_000006.12	+	20781145	20786774	5630	5630	sense-overlappin	XM_006715128.2	CDKAL1
circRNA_04585	0	0	0	0	0.03009467	0	0	0	0	0	0	0	-	NC_000006.12	+	20781145	20805719	24575	24575	sense-overlappin	XM_006715128.2	CDKAL1
circRNA_04586	0	0	0	0.0309543	0	0	0	0	0	0	0	0	-	NC_000006.12	+	20781145	20820720	39576	39576	sense-overlappin	XM_006715128.2	CDKAL1

circRNA_04587	0.051437562	0	0.04361729	0	0.03009467	0	0	0	0	0.100862414	0.02025523	0.0206364	hsa_circ_0008086	NC_000006.12	+	20781145	20846178	65034	225	sense-overlappin	XM_006715128.2	CDKAL1
circRNA_04588	0	0	0.021808645	0.0412724	0.04012623	0.020712909	0	0	0	0	0	0	-	NC_000006.12	+	20781145	20874430	93286	28477	sense-overlappin	XM_006715128.2	CDKAL1
circRNA_04589	0	0.020469968	0	0	0	0	0	0	0	0.030258724	0	0	hsa_circ_0075814	NC_000006.12	+	21108401	21201274	92874	2114	sense-overlappin	XR_926265.1	
circRNA_04590	0	0	0	0.0309543	0	0	0	0	0	0	0	0	-	NC_000006.12	+	21198021	21201274	3254	2051	sense-overlappin	XR_926265.1	
circRNA_04591	0	0	0	0	0	0	0	0	0.020254528	0	0	0	hsa_circ_0075819	NC_000006.12	+	21783632	21802740	19109	19109	intergenic		
circRNA_04592	0	0	0	0	0	0	0.020098685	0	0	0	0	0	hsa_circ_0075828	NC_000006.12	+	22020339	22020542	204	204	intergenic		
circRNA_04593	0.041150049	0	0	0	0	0	0.020098685	0.020486295	0	0	0	0	hsa_circ_0075829	NC_000006.12	+	22020339	22056690	36352	36352	intergenic		
circRNA_04594	0.030862537	0	0	0	0	0	0	0	0	0	0	0	hsa_circ_0075832	NC_000006.12	+	22056546	22110920	54375	54375	intergenic		
circRNA_04595	0	0	0	0	0	0	0	0	0	0.050431207	0.02025523	0.0309546	hsa_circ_0075835	NC_000006.12	+	24416397	24418578	2182	388	sense-overlappin	NM_001286265.1	MRS2
circRNA_04596	0	0	0	0	0	0.041425819	0.020098685	0	0	0	0.04051046	0.0206364	hsa_circ_0075842	NC_000006.12	-	24475121	24479959	4839	288	sense-overlappin	NM_001503.3	GPLD1
circRNA_04597	0	0	0	0	0	0	0.040197371	0	0	0	0	0	-	NC_000006.12	+	24502523	24533687	31165	1268	sense-overlappin	NM_170740.1	ALDH5A1
circRNA_04598	0.020575025	0	0	0	0	0	0	0	0	0	0	0	-	NC_000006.12	+	24697883	24701767	3885	494	sense-overlappin	NM_001160094.1	ACOT13
circRNA_04599	0	0	0	0	0	0.020712909	0	0	0	0	0	0	-	NC_000006.12	-	24848025	24852618	4594	449	sense-overlappin	NM_014722.3	FAM65B
circRNA_04600	0	0.020469968	0	0	0	0	0	0	0	0	0	0	-	NC_000006.12	-	25255189	25264409	9221	9221	intergenic		
circRNA_04601	0	0	0.021808645	0	0	0	0	0	0	0	0	0	-	NC_000006.12	+	25465873	25488585	22713	451	sense-overlappin	XM_011514736.1	LRRC16A
circRNA_04602	0	0	0.021808645	0	0	0	0	0	0	0	0	0	-	NC_000006.12	+	25465873	25540078	74206	1714	sense-overlappin	XM_011514736.1	LRRC16A
circRNA_04603	0	0	0	0	0	0	0	0.020486295	0	0	0	0	-	NC_000006.12	+	25482257	25500235	17979	521	sense-overlappin	XM_011514736.1	LRRC16A
circRNA_04604	0	0	0.021808645	0	0	0	0	0	0	0	0	0	hsa_circ_0075865	NC_000006.12	+	25482257	25520337	38081	1094	sense-overlappin	XM_011514736.1	LRRC16A
circRNA_04605	0.041150049	0	0	0	0	0	0	0	0	0	0.02025523	0	hsa_circ_0075872	NC_000006.12	+	25515675	25520337	4663	336	sense-overlappin	XM_011514736.1	LRRC16A
circRNA_04606	0.030862537	0	0	0	0	0	0	0	0	0	0	0	hsa_circ_0075881	NC_000006.12	+	25604812	25606273	1462	277	sense-overlappin	NM_001173977.1	LRRC16A
circRNA_04607	0.020575025	0	0	0	0	0	0	0	0	0	0	0	-	NC_000006.12	+	25966335	25969420	3086	695	sense-overlappin	NM_006355.4	TRIM38
circRNA_04608	0	0	0	0	0	0	0	0	0	0	0	0.0206364	-	NC_000006.12	+	26372897	26445985	73089	71511	sense-overlappin	XM_011514268.1	BTN3A2
circRNA_04609	0	0	0	0	0	0	0	0	0.020254528	0	0	0	hsa_circ_0001590	NC_000006.12	+	26385015	26388294	3280	630	sense-overlappin	XM_006714953.2	BTN2A2
circRNA_04610	0	0	0.021808645	0	0	0	0	0	0	0	0	0.0206364	-	NC_000006.12	+	26422933	26458718	35786	35322	sense-overlappin	NM_007049.4	BTN2A1
circRNA_04611	0	0	0	0	0	0	0	0	0.030381792	0	0	0	-	NC_000006.12	+	26425808	26459828	34021	32795	sense-overlappin	NM_007049.4	BTN2A1
circRNA_04612	0	0	0.021808645	0	0	0	0	0	0	0	0	0	-	NC_000006.12	+	28080712	28088673	7962	1480	sense-overlappin	NM_003447.3	ZNF165
circRNA_04613	0	0	0	0	0	0	0	0	0	0	0	0.0206364	-	NC_000006.12	-	31271999	31356957	84959	83318	sense-overlappin	XM_011514556.1	HLA-B
circRNA_04614	0	0	0	0	0	0	0	1.577444753	0	0	0	0	-	NC_000006.12	-	32521905	32581838	59934	57617	intergenic	NM_002124.3	HLA-DRB1
circRNA_04615	0	0.030704951	0	0	0.02006311	0.041425819	0	0.020486295	0	0.020172483	0	0	hsa_circ_0006757	NC_000006.12	-	34606555	34606904	350	350	exonic	NM_022758.5	C6orf106
circRNA_04616	0	0.030704951	0.032712968	0.0309543	0	0.020712909	0.040197371	0.030729443	0.030381792	0.07060369	0	0	hsa_circ_0009095	NC_000006.12	-	34606555	34646798	40244	548	sense-overlappin	NM_024294.3	C6orf106
circRNA_04617	0	0	0.054521613	0	0	0	0	0	0.020254528	0	0	0	-	NC_000006.12	-	34646601	34654779	8179	353	sense-overlappin	NM_024294.3	C6orf106
circRNA_04618	0	0	0.054521613	0	0	0	0	0	0	0	0	0	-	NC_000006.12	+	34967239	35060253	93015	2050	sense-overlappin	XM_011514433.1	ANKS1A
circRNA_04619	0	0.030704951	0	0	0	0	0	0	0	0.030258724	0	0	-	NC_000006.12	+	35312214	35312772	559	472	sense-overlappin	NM_022047.3	DEF6
circRNA_04620	0	0.020469968	0.04361729	0.0309543	0	0	0.040197371	0	0	0.030258724	0	0.0309546	hsa_circ_0076125	NC_000006.12	+	35312302	35312772	471	384	sense-overlappin	NM_022047.3	DEF6
circRNA_04621	0	0	0.054521613	0.0309543	0	0.031069364	0	0	0	0.020172483	0	0	hsa_circ_0001599	NC_000006.12	-	35619096	35642843	23748	527	sense-overlappin	NM_001145776.1	FKBP5
circRNA_04622	0	0	0.04361729	0.0412724	0	0	0	0	0	0	0	0	hsa_circ_0001600	NC_000006.12	-	35619096	35646850	27755	4534	sense-overlappin	NM_001145776.1	FKBP5
circRNA_04623	0	0	0.04361729	0.0412724	0	0	0	0	0	0	0	0	-	NC_000006.12	-	35637014	35646850	9837	4276	sense-overlappin	NM_001145776.1	FKBP5
circRNA_04624	0	0	0	0	0	0	0.020098685	0	0	0	0	0	-	NC_000006.12	-	35672592	35679074	6483	6483	intronic	NM_001145776.1	FKBP5
circRNA_04625	0	0	0.021808645	0	0	0	0	0	0	0	0	0	-	NC_000006.12	+	35786998	35797459	10462	9716	sense-overlappin	NM_001010886.3	CLPSL1
circRNA_04626	0	0	0	0	0	0	0	0	0	0.020172483	0	0	-	NC_000006.12	+	35786998	35798941	11944	11198	sense-overlappin	NM_001010886.3	CLPSL1
circRNA_04627	0	0	0.021808645	0	0	0	0	0	0	0	0	0	hsa_circ_0076169	NC_000006.12	-	35857261	35870959	13699	869	sense-overlappin	NM_003137.4	SRPK1
circRNA_04628	0	0	0.032712968	0	0	0	0	0.040972591	0	0	0	0	hsa_circ_0002165	NC_000006.12	-	35857261	35872728	15468	1035	sense-overlappin	NM_003137.4	SRPK1
circRNA_04629	0	0	0.032712968	0	0	0	0	0	0	0	0	0	-	NC_000006.12	-	35869010	35891013	22004	1438	sense-overlappin	NM_003137.4	SRPK1
circRNA_04630	0	0	0.08723458	0	0.02006311	0.031069364	0	0	0	0	0	0	-	NC_000006.12	-	36158648	36158941	294	294	intergenic		
circRNA_04631	0.020575025	0	0	0	0.02006311	0	0	0	0	0	0	0	hsa_circ_0009139	NC_000006.12	+	36213880	36218010	4131	601	sense-overlappin	NM_015695.2	BRPF3
circRNA_04632	0	0	0	0	0	0	0	0	0	0.020172483	0	0	-	NC_000006.12	+	36465601	36475062	9462	578	sense-overlappin	XM_005248940.3	KCTD20
circRNA_04633	0	0	0	0	0	0	0	0	0.050636319	0	0	0	-	NC_000006.12	-	36496711	36507602	10892	598	sense-overlappin	XM_006714988.2	STK38
circRNA_04634	0	0	0	0	0	0	0	0	0	0.020172483	0	0	-	NC_000006.12	-	36499873	36507602	7730	283	sense-overlappin	XM_006714988.2	STK38
circRNA_04635	0	0	0	0	0	0	0	0	0.020254528	0	0	0	-	NC_000006.12	-	36515338	36521817	6480	363	sense-overlappin	XM_006714988.2	STK38
circRNA_04636	0	0	0	0	0	0	0	0	0.020254528	0	0	0	-	NC_000006.12	-	36515338	36540204	24867	671	sense-overlappin	XM_006714988.2	STK38
circRNA_04637	0	0.020469968	0	0	0.06018934	0	0	0	0.020254528	0.030258724	0	0	-	NC_000006.12	-	36524341	36540204	15864	308	sense-overlappin	XM_006714988.2	STK38
circRNA_04638	0.030862537	0	0	0.0206362	0	0	0.020098685	0	0	0.030258724	0	0.0206364	hsa_circ_0003131	NC_000006.12	-	36524341	36540207	15867	311	sense-overlappin	XM_006714988.2	STK38
circRNA_04639	0	0	0.021808645	0	0	0	0	0	0	0	0	0	hsa_circ_0009153	NC_000006.12	+	36601708	36603173	1466	1299	sense-overlappin	NM_003017.4	SRSF3
circRNA_04640	0	0	0	0	0	0	0	0	0.020254528	0	0	0	hsa_circ_0076196	NC_000006.12	-	36798165	36800070	1906	221	sense-overlappin	XM_005249247.1	CPNE5
circRNA_04641	0	0	0	0	0	0.020712909	0	0	0	0	0.02025523	0	-	NC_000006.12	+	37929959	37930576	618	618	sense-overlappin	XM_005249269.1	ZFAND3

circRNA_04642	0	0	0.032712968	0	0	0	0	0	0	0	0	0	-	NC_000006.12	+	38061593	38062525	933	933	sense-overlappin	XM_005249269.1	ZFAND3
circRNA_04643	0	0	0.04361729	0	0.08025246	0	0	0	0	0	0	0	hsa_circ_0076254	NC_000006.12	-	38577600	38598121	20522	1181	sense-overlappin	NM_001099272.1	BTBD9
circRNA_04644	0	0	0	0	0	0	0	0	0	0.030258724	0	0	-	NC_000006.12	-	39357277	39362518	5242	319	sense-overlappin	NM_001289024.1	KIF6
circRNA_04645	0	0	0	0	0.02006311	0	0	0	0	0	0	0	-	NC_000006.12	+	41090204	41094497	4294	549	sense-overlappin	NM_002505.4	NFYA
circRNA_04646	0	0	0	0	0.03009467	0	0	0	0	0	0	0.0619092	-	NC_000006.12	+	41736786	41736965	180	180	antisense	NM_002630.3	PGC
circRNA_04647	0	0	0	0.0515905	0	0	0	0	0	0	0	0	-	NC_000006.12	-	41741796	41742489	694	248	sense-overlappin	NM_001166424.1	PGC
circRNA_04648	0	0	0	0.0206362	0	0	0	0	0	0	0	0	-	NC_000006.12	+	41742384	41743352	969	188	antisense	NM_002630.3	PGC
circRNA_04649	0	0	0	0.0206362	0	0	0	0	0	0	0	0	-	NC_000006.12	+	41743353	41744772	1420	270	antisense	NM_002630.3	PGC
circRNA_04650	0	0	0	0	0	0	0	0.051215739	0	0	0	0	-	NC_000006.12	-	41840893	41891875	50983	30827	sense-overlappin	NM_018561.4	USP49
circRNA_04651	0	0	0	0	0	0	0	0.030729443	0	0	0.04051046	0	-	NC_000006.12	-	41871564	41891875	20312	156	sense-overlappin	NM_018561.4	USP49
circRNA_04652	0	0	0.032712968	0	0	0	0	0	0	0	0	0	hsa_circ_0006232	NC_000006.12	-	42268154	42269848	1695	1695	exonic	NM_001297573.1	TRERF1
circRNA_04653	0	0	0	0.0412724	0	0.020712909	0.020098685	0	0	0	0	0	hsa_circ_0076347	NC_000006.12	+	42573734	42594304	20571	453	sense-overlappin	XM_011514442.1	UBR2
circRNA_04654	0	0.030704951	0.054521613	0.0825448	0.06018934	0	0.070345399	0	0.040509055	0.030258724	0.050638075	0	hsa_circ_0001603	NC_000006.12	+	42592151	42594304	2154	193	sense-overlappin	XM_011514442.1	UBR2
circRNA_04655	0	0	0.04361729	0.0412724	0.04012623	0.031069364	0	0.040972591	0.040509055	0.020172483	0	0	hsa_circ_0003177	NC_000006.12	+	42603588	42606651	3064	333	sense-overlappin	XM_011514442.1	UBR2
circRNA_04656	0	0	0	0	0	0.020712909	0	0	0	0	0	0	-	NC_000006.12	+	42612171	42617507	5337	417	sense-overlappin	XM_011514442.1	UBR2
circRNA_04657	0	0.020469968	0	0	0	0	0	0	0	0	0	0	-	NC_000006.12	+	42612171	42632904	20734	681	sense-overlappin	XM_011514442.1	UBR2
circRNA_04658	0	0.030704951	0	0	0	0	0	0	0	0	0	0	-	NC_000006.12	+	42615071	42632904	17834	560	sense-overlappin	XM_011514442.1	UBR2
circRNA_04659	0	0	0	0	0	0	0	0	0.020254528	0	0.02025523	0	-	NC_000006.12	+	42644214	42650386	6173	468	sense-overlappin	XM_011514442.1	UBR2
circRNA_04660	0	0	0	0	0.02006311	0	0	0	0	0	0	0	-	NC_000006.12	+	42650284	42655723	5440	410	sense-overlappin	XM_011514442.1	UBR2
circRNA_04661	0	0.030704951	0	0.0309543	0.03009467	0	0.040197371	0.030729443	0	0	0	0.0412728	hsa_circ_0002906	NC_000006.12	+	42663258	42666245	2988	345	sense-overlappin	XM_005248965.3	UBR2
circRNA_04662	0	0	0	0	0	0	0.040197371	0	0	0.020172483	0.02025523	0	hsa_circ_0076405	NC_000006.12	+	42676056	42679832	3777	467	sense-overlappin	XM_005248965.3	UBR2
circRNA_04663	0	0	0	0	0.02006311	0	0	0	0	0	0	0	hsa_circ_0076408	NC_000006.12	+	42822018	42830172	8155	1844	sense-overlappin	XM_005248972.2	GLTSCR1L
circRNA_04664	0	0	0	0	0.02006311	0.051782273	0	0	0	0	0	0	hsa_circ_0076410	NC_000006.12	+	42822796	42830172	7377	1798	sense-overlappin	XM_005248972.2	GLTSCR1L
circRNA_04665	0	0	0	0	0	0	0.030148028	0.020486295	0.020254528	0	0	0	hsa_circ_0008801	NC_000006.12	+	42934475	42935670	1196	320	sense-overlappin	XM_005248815.2	CNPY3
circRNA_04666	0.020575025	0.040939935	0.032712968	0	0	0	0	0	0.020254528	0	0	0	hsa_circ_0005038	NC_000006.12	-	42974003	42975038	1036	248	sense-overlappin	NM_000287.3	PEX6
circRNA_04667	0.051437562	0.030704951	0.04361729	0	0.02006311	0.020712909	0.030148028	0.020486295	0	0.050431207	0.030382845	0.0412728	hsa_circ_0001608	NC_000006.12	-	43055545	43056445	901	440	sense-overlappin	NM_015950.4	MRPL2
circRNA_04668	0.041150049	0	0	0	0	0	0	0	0	0	0	0	-	NC_000006.12	+	43206007	43206510	504	419	sense-overlappin	XM_011514423.1	CUL9
circRNA_04669	0	0	0	0	0	0	0	0	0	0	0	0.0206364	hsa_circ_0076558	NC_000006.12	-	43533907	43551453	17547	871	sense-overlappin	NM_020750.2	XPO5
circRNA_04670	0	0	0.021808645	0	0	0	0	0	0	0	0	0	hsa_circ_0002037	NC_000006.12	-	43555836	43567354	11519	793	sense-overlappin	NM_020750.2	XPO5
circRNA_04671	0	0	0	0	0.04012623	0	0	0	0.030381792	0	0	0	-	NC_000006.12	-	43570502	43570994	493	321	sense-overlappin	NM_020750.2	XPO5
circRNA_04672	0	0	0	0	0	0	0	0	0.020254528	0	0	0	hsa_circ_0005451	NC_000006.12	+	43597696	43601091	3396	274	sense-overlappin	NM_001291969.1	POLH
circRNA_04673	0	0	0	0	0	0.020712909	0	0	0	0	0	0	hsa_circ_0003563	NC_000006.12	+	45431863	45438051	6189	262	sense-overlappin	XM_011514963.1	RUNX2
circRNA_04674	0	0	0	0	0	0.020712909	0	0	0	0	0	0	-	NC_000006.12	+	45431863	45443193	11331	4841	sense-overlappin	XM_011514967.1	RUNX2
circRNA_04675	0	0	0	0	0	0	0	0	0.040509055	0	0	0	-	NC_000006.12	-	46246748	46248896	2149	346	sense-overlappin	NM_005822.3	RCAN2
circRNA_04676	0	0	0	0	0	0.031069364	0	0	0	0	0	0	-	NC_000006.12	-	46456752	46456978	227	227	exonic	NM_001251974.1	RCAN2
circRNA_04677	0	0	0	0.0825448	0	0	0	0	0	0	0	0	-	NC_000006.12	-	46595987	46650244	54258	8774	sense-overlappin	NM_001278738.1	CYP39A1
circRNA_04678	0	0	0.032712968	0	0	0	0	0	0	0	0	0	-	NC_000006.12	+	46833074	46833538	465	465	exonic	NM_005588.2	MEP1A
circRNA_04679	0	0	0.04361729	0	0	0	0	0	0	0	0	0	hsa_circ_0005897	NC_000006.12	-	47253256	47286595	33340	1413	sense-overlappin	NM_014452.4	TNFRSF21
circRNA_04680	0	0	0.032712968	0	0	0	0	0	0	0	0	0	-	NC_000006.12	-	47267820	47286595	18776	1282	sense-overlappin	XM_011514495.1	TNFRSF21
circRNA_04681	0	0	0	0	0.03009467	0	0	0	0	0	0	0	-	NC_000006.12	+	47281687	47282114	428	428	antisense	NM_014452.4	TNFRSF21
circRNA_04682	0.452650542	0.214934659	1.515700829	0.1341352	0.39123074	0.124277456	0.281381595	0.102431477	0.121527166	0.171466104	0.12153138	0.154773001	hsa_circ_0001610	NC_000006.12	-	47283938	47286595	2658	1147	sense-overlappin	NM_014452.4	TNFRSF21
circRNA_04683	0.051437562	0.061409903	0.21808645	0.0206362	0.0702209	0.051782273	0.060296056	0.061458886	0	0.030258724	0.08102092	0.0206364	hsa_circ_0007936	NC_000006.12	+	47503280	47504758	1479	1479	sense-overlappin	XM_005248977.2	CD2AP
circRNA_04684	0	0	0	0	0.02006311	0	0.040197371	0	0	0	0	0	-	NC_000006.12	+	47503280	47515962	12683	12683	sense-overlappin	XM_005248977.2	CD2AP
circRNA_04685	0	0	0	0.0206362	0	0	0	0	0	0	0	0	-	NC_000006.12	+	47503280	47533755	30476	315	sense-overlappin	XM_005248977.2	CD2AP
circRNA_04686	0	0	0.065425935	0	0.05015779	0	0.050246713	0	0	0	0	0	hsa_circ_0076704	NC_000006.12	+	47503280	47554766	51487	537	sense-overlappin	XM_005248977.2	CD2AP
circRNA_04687	0	0.040939935	0.21808645	0	0	0	0	0	0	0	0	0	-	NC_000006.12	+	47526628	47554766	28139	7350	sense-overlappin	XM_005248977.2	CD2AP
circRNA_04688	0	0.030704951	0.316225353	0	0	0	0.020098685	0.020486295	0	0	0.050638075	0	-	NC_000006.12	+	47533602	47554766	21165	376	sense-overlappin	XM_005248977.2	CD2AP
circRNA_04689	0	0	0.04361729	0	0	0	0	0	0	0	0	0	-	NC_000006.12	+	47533602	47582065	48464	943	sense-overlappin	XM_005248977.2	CD2AP
circRNA_04690	0	0	0.076330258	0.0206362	0.05015779	0	0.040197371	0	0	0	0	0	-	NC_000006.12	+	47574064	47582065	8002	567	sense-overlappin	XM_005248977.2	CD2AP
circRNA_04691	0	0	0.021808645	0	0	0	0	0	0	0	0	0	-	NC_000006.12	+	47595861	47606277	10417	422	sense-overlappin	XM_005248977.2	CD2AP
circRNA_04692	0	0	0.065425935	0.0309543	0	0	0	0	0	0	0	0	-	NC_000006.12	+	47595861	47612536	16676	770	sense-overlappin	XM_005248976.1	CD2AP
circRNA_04693	0	0	0.04361729	0.0412724	0.04012623	0	0	0.020486295	0	0.040344966	0.02025523	0.0206364	-	NC_000006.12	+	47607927	47612536	4610	348	sense-overlappin	XM_005248976.1	CD2AP
circRNA_04694	0	0	0.021808645	0	0	0	0	0	0	0	0	0	-	NC_000006.12	+	49709145	49714438	5294	1245	antisense	XM_005249357.3	CRISP2
circRNA_04695	0.030862537	0	0	0	0	0.051782273	0	0	0	0	0	0	-	NC_000006.12	-	52010309	52035721	25413	2012	sense-overlappin	XM_011514683.1	PKHD1
circRNA_04696	0	0	0	0	0	0.031069364	0	0	0	0	0	0	hsa_circ_0076742	NC_000006.12	-	52267865	52269226	1362	245	sense-overlappin	NM_001270472.1	MCM3

circRNA_04697	0	0	0	0	0	0	0	0	0	0.020172483	0	0	-	NC_000006.12	-	52754943	52794266	39324	36484	sense-overlappin	XM_011514532.1	GSTA2
circRNA_04698	0	0	0	0	0	0	0	0.040972591	0	0	0	0	-	NC_000006.12	-	52792856	52848032	55177	44824	sense-overlappin	XM_005249034.2	GSTA1
circRNA_04699	0.020575025	0.040939935	0.08723458	0.1650895	0	0.113921001	0	0.020486295	0.040509055	0.07060369	0.04051046	0	hsa_circ_0003700	NC_000006.12	+	53071057	53076543	5487	304	sense-overlappin	XM_005248995.3	FBXO9
circRNA_04700	0	0	0.032712968	0.0206362	0	0	0.020098685	0	0	0	0	0	-	NC_000006.12	-	53498607	53522527	23921	1913	sense-overlappin	NM_001498.3	GCLC
circRNA_04701	0	0	0	0	0	0	0	0.020486295	0	0	0	0	-	NC_000006.12	-	56101472	56141983	40512	403	sense-overlappin	XR_241922.1	
circRNA_04702	0	0	0	0	0	0	0	0	0	0.020172483	0	0	-	NC_000006.12	-	56179578	56182656	3079	678	sense-overlappin	XM_011514926.1	COL21A1
circRNA_04703	0	0	0.065425935	0	0	0	0	0	0	0	0	0	hsa_circ_0006880	NC_000006.12	-	56699653	56735289	35637	422	sense-overlappin	XM_005249322.3	DST
circRNA_04704	0	0	0.04361729	0	0	0.041425819	0	0.020486295	0	0.020172483	0	0	-	NC_000006.12	+	57050774	57055797	5024	477	sense-overlappin	XM_011514767.1	KIAA1586
circRNA_04705	0	0	0	0	0	0	0	0	0	0	0.02025523	0	-	NC_000006.12	+	57052686	57055797	3112	396	sense-overlappin	XM_011514767.1	KIAA1586
circRNA_04706	0	0	0	0	0.02006311	0	0	0	0	0	0	0	hsa_circ_0076881	NC_000006.12	+	57124734	57134870	10137	516	sense-overlappin	XM_011514462.1	ZNF451
circRNA_04707	0	0	0.032712968	0.0412724	0	0.031069364	0	0	0	0	0	0	hsa_circ_0007587	NC_000006.12	+	57124734	57142095	17362	818	sense-overlappin	XM_011514462.1	ZNF451
circRNA_04708	0	0	0	0.0309543	0	0	0	0	0	0	0	0	hsa_circ_0076883	NC_000006.12	+	57141302	57148693	7392	1906	sense-overlappin	XM_011514462.1	ZNF451
circRNA_04709	0	0	0	0	0.02006311	0	0	0.020486295	0	0.020172483	0	0	hsa_circ_0002638	NC_000006.12	+	57152221	57161152	8932	542	sense-overlappin	XR_241885.1	
circRNA_04710	0	0	0.04361729	0	0	0	0	0	0	0	0	0	-	NC_000006.12	-	57190393	57210445	20053	847	sense-overlappin	NM_001278668.1	RAB23
circRNA_04711	0.257187808	0.030704951	0.185373483	0.0412724	0.08025246	0	0	0.030729443	0.081018111	0.121034897	0.10127615	0.0412728	hsa_circ_0004119	NC_000006.12	-	57193842	57210445	16604	639	sense-overlappin	NM_001278668.1	RAB23
circRNA_04712	0	0	0.054521613	0	0	0	0	0	0	0	0	0	hsa_circ_0008325	NC_000006.12	-	57207628	57210445	2818	306	sense-overlappin	NM_001278668.1	RAB23
circRNA_04713	0	0	0	0.0206362	0	0	0	0	0	0	0	0	-	NC_000006.12	+	57318437	57326045	7609	468	sense-overlappin	NM_001282488.1	PRIM2
circRNA_04714	0	0	0.021808645	0	0	0	0	0	0	0	0	0	hsa_circ_0076922	NC_000006.12	+	63646527	63685911	39385	2407	sense-overlappin	NM_001290259.1	PHF3
circRNA_04715	0.020575025	0.030704951	0.021808645	0	0.02006311	0	0.020098685	0.020486295	0	0.020172483	0.02025523	0	-	NC_000006.12	+	63680000	63685911	5912	1945	sense-overlappin	XM_005248702.2	PHF3
circRNA_04716	0	0	0.032712968	0	0	0	0	0	0	0	0	0	-	NC_000006.12	+	63684129	63692043	7915	2090	sense-overlappin	XM_005248702.2	PHF3
circRNA_04717	0	0	0	0.0412724	0	0	0	0	0	0	0.04051046	0	-	NC_000006.12	+	63694581	63700466	5886	603	sense-overlappin	XM_005248702.2	PHF3
circRNA_04718	0	0	0	0	0.02006311	0	0	0	0	0	0	0	-	NC_000006.12	-	63712756	63712930	175	175	antisense	XM_005248702.2	PHF3
circRNA_04719	0	0	0	0	0	0	0	0	0	0.020172483	0	0.0206364	hsa_circ_0076948	NC_000006.12	+	69014038	69076038	62001	551	sense-overlappin	XM_011536013.1	ADGRB3
circRNA_04720	0.020575025	0	0.021808645	0	0.03009467	0	0	0	0	0	0	0	-	NC_000006.12	-	69737942	69752356	14415	329	sense-overlappin	NM_018368.3	LMBRD1
circRNA_04721	0.102875123	0.020469968	0	0.0825448	0	0.031069364	0	0	0	0.050431207	0.02025523	0	-	NC_000006.12	-	69737942	69790472	52531	567	sense-overlappin	NM_018368.3	LMBRD1
circRNA_04722	0.020575025	0	0	0	0	0	0	0	0	0	0	0	-	NC_000006.12	+	70415291	70452571	37281	376	sense-overlappin	NM_001105531.2	FAM135A
circRNA_04723	0	0.040939935	0.076330258	0	0.02006311	0.051782273	0	0.051215739	0.030381792	0.050431207	0.04051046	0.051591	-	NC_000006.12	+	70426439	70452571	26133	290	sense-overlappin	NM_001105531.2	FAM135A
circRNA_04724	0	0	0	0	0	0.041425819	0	0	0	0	0	0	-	NC_000006.12	+	70426439	70502791	76353	1240	sense-overlappin	XM_005248744.3	FAM135A
circRNA_04725	0	0	0.04361729	0	0	0	0	0	0	0	0	0	-	NC_000006.12	+	70426570	70452571	26002	378	sense-overlappin	XM_011535994.1	FAM135A
circRNA_04726	0	0	0.032712968	0	0	0.020712909	0	0	0	0	0	0	hsa_circ_0076961	NC_000006.12	+	70475410	70502791	27382	950	sense-overlappin	NM_020819.4	FAM135A
circRNA_04727	0	0	0	0	0.03009467	0	0	0	0	0	0	0	-	NC_000006.12	+	70533160	70538401	5242	453	sense-overlappin	NM_001105531.2	FAM135A
circRNA_04728	0	0	0	0.0206362	0.03009467	0	0	0	0	0	0	0	-	NC_000006.12	+	70732378	70773425	41048	416	sense-overlappin	XM_011536048.1	SMAP1
circRNA_04729	0	0	0	0.0206362	0	0	0	0	0	0	0	0	-	NC_000006.12	+	72284047	72313672	29626	678	sense-overlappin	XM_011535605.1	RIMS1
circRNA_04730	0	0	0	0.0206362	0	0	0	0	0	0	0	0	hsa_circ_0076969	NC_000006.12	+	72284047	72333835	49789	914	sense-overlappin	XM_011535605.1	RIMS1
circRNA_04731	0.216037758	0.235404627	0.08723458	0.8873561	0.03009467	0.217485548	0	0.153647216	0.121527166	0.262242276	0.030382845	0.144454801	-	NC_000006.12	+	72295937	72333835	37899	546	sense-overlappin	XM_011535605.1	RIMS1
circRNA_04732	0	0.020469968	0	0	0	0.041425819	0	0	0	0	0	0	-	NC_000006.12	+	72647932	72648904	973	973	intronic	XM_011535944.1	KCNQ5
circRNA_04733	0	0	0	0	0	0.031069364	0.040197371	0	0	0	0	0	-	NC_000006.12	-	73425464	73445747	20284	675	sense-overlappin	XM_006715327.2	MB21D1
circRNA_04734	0	0	0	0	0	0.072495183	0.020098685	0	0	0	0	0	hsa_circ_0004092	NC_000006.12	-	73440209	73440445	237	237	exonic	XM_006715327.2	MB21D1
circRNA_04735	0	0	0	0	0	0	0	0	0.020254528	0	0	0.0309546	-	NC_000006.12	-	73440209	73445747	5539	457	sense-overlappin	XM_006715327.2	MB21D1
circRNA_04736	0.020575025	0	0.032712968	0.0309543	0	0.020712909	0	0	0.020254528	0.020172483	0	0	hsa_circ_0007874	NC_000006.12	+	73466209	73466606	398	318	sense-overlappin	XM_006715447.2	MTO1
circRNA_04737	0.041150049	0.030704951	0.032712968	0	0	0.020712909	0	0	0.030381792	0	0	0	hsa_circ_0004973	NC_000006.12	+	73480675	73482244	1570	456	sense-overlappin	NM_001123226.1	MTO1
circRNA_04738	0	0	0	0	0	0.031069364	0	0	0	0	0	0	hsa_circ_0002465	NC_000006.12	+	73723251	73736508	13258	386	sense-overlappin	XM_011535474.1	CD109
circRNA_04739	0	0	0	0	0.02006311	0	0	0	0	0	0	0	-	NC_000006.12	+	74369090	74372126	3037	3037	intergenic		
circRNA_04740	0	0	0	0	0	0	0	0	0	0	0	0.0309546	hsa_circ_0077068	NC_000006.12	-	75183050	75184144	1095	894	sense-overlappin	NM_004370.5	COL12A1
circRNA_04741	0	0	0	0.0206362	0	0	0	0	0	0	0	0	-	NC_000006.12	-	75312397	75315202	2806	2806	exonic	XM_005248713.2	FILIP1
circRNA_04742	0	0.040939935	0.04361729	0	0.05015779	0	0.030148028	0.020486295	0.020254528	0.020172483	0.030382845	0.051591	hsa_circ_0077078	NC_000006.12	+	75621532	75634811	13280	515	sense-overlappin	XM_006715457.2	SENP6
circRNA_04743	0.061725074	0	0.021808645	0.0309543	0	0	0	0	0	0.020172483	0	0	hsa_circ_0001612	NC_000006.12	+	75621532	75647801	26270	607	sense-overlappin	XM_006715457.2	SENP6
circRNA_04744	0	0	0	0	0.02006311	0	0	0	0	0	0	0	-	NC_000006.12	+	75622751	75634811	12061	421	sense-overlappin	XM_006715457.2	SENP6
circRNA_04745	0	0	0	0.0309543	0	0	0	0	0	0	0	0	hsa_circ_0077079	NC_000006.12	+	75633581	75634811	1231	251	sense-overlappin	NM_001304792.1	SENP6
circRNA_04746	0	0	0	0	0	0	0	0	0	0.030258724	0	0	hsa_circ_0077082	NC_000006.12	+	75634707	75640704	5998	126	sense-overlappin	NM_015571.3	SENP6
circRNA_04747	0	0.040939935	0.021808645	0.0928628	0	0	0	0	0	0.020172483	0	0.051591	hsa_circ_0077083	NC_000006.12	+	75634707	75663518	28812	641	sense-overlappin	NM_015571.3	SENP6
circRNA_04748	0	0	0.021808645	0.0309543	0.03009467	0	0	0	0.030381792	0.030258724	0	0	hsa_circ_0077084	NC_000006.12	+	75634707	75670720	36014	1039	sense-overlappin	NM_015571.3	SENP6
circRNA_04749	0	0	0.054521613	0.0412724	0	0.031069364	0	0.061458886	0	0.040344966	0	0	hsa_circ_0001613	NC_000006.12	+	75634707	75678927	44221	1722	sense-overlappin	NM_015571.3	SENP6
circRNA_04750	0	0	0	0	0.08025246	0	0	0	0	0	0	0	-	NC_000006.12	+	75637071	75663518	26448	4149	sense-overlappin	NM_015571.3	SENP6

circRNA_04751	0	0	0	0	0	0	0	0	0	0.020172483	0	0	hsa_circ_0007002	NC_000006.12	+	75647731	75659407	11677	217	sense-overlappin	NM_001304792.1	SENP6
circRNA_04752	0	0	0.032712968	0	0	0	0.030148028	0	0	0.040344966	0	0.0309546	hsa_circ_0077086	NC_000006.12	+	75647731	75670720	22990	913	sense-overlappin	NM_001304792.1	SENP6
circRNA_04753	0	0	0	0.0412724	0	0.031069364	0	0	0	0	0	0	hsa_circ_0077088	NC_000006.12	+	75647731	75678927	31197	1596	sense-overlappin	NM_001304792.1	SENP6
circRNA_04754	0	0	0	0	0	0	0.020098685	0	0	0	0	0	-	NC_000006.12	+	75647731	75697517	49787	1809	sense-overlappin	NM_001100409.2	SENP6
circRNA_04755	0	0	0	0	0	0	0.020098685	0	0	0	0	0	hsa_circ_0077089	NC_000006.12	+	75659262	75670720	11459	842	sense-overlappin	NM_001304792.1	SENP6
circRNA_04756	0	0	0.021808645	0.0619086	0	0	0	0	0	0.040344966	0.02025523	0	-	NC_000006.12	+	75659262	75678927	19666	1525	sense-overlappin	NM_001304792.1	SENP6
circRNA_04757	0	0	0	0.0206362	0	0	0.020098685	0	0	0	0	0	-	NC_000006.12	+	75675435	75678927	3493	683	sense-overlappin	NM_001304792.1	SENP6
circRNA_04758	0.13373766	0.08187987	0.065425935	0.0515905	0.0702209	0.15534682	0.040197371	0.040972591	0.081018111	0.080689931	0.091148535	0.072227401	hsa_circ_0001614	NC_000006.12	+	75702645	75703072	428	428	exonic	NM_001100409.2	SENP6
circRNA_04759	0	0	0	0	0	0	0.020098685	0	0	0	0	0	hsa_circ_0077095	NC_000006.12	+	75702645	75709630	6986	532	sense-overlappin	NM_001100409.2	SENP6
circRNA_04760	0	0	0	0.0928628	0.08025246	0	0.030148028	0	0.030381792	0.040344966	0.02025523	0.0412728	hsa_circ_0077096	NC_000006.12	+	75702645	75711416	8772	621	sense-overlappin	NM_001100409.2	SENP6
circRNA_04761	0	0.020469968	0	0	0	0	0	0	0	0	0	0	-	NC_000006.12	+	75822782	75832947	10166	380	sense-overlappin	XM_005248719.2	MYO6
circRNA_04762	0	0	0	0	0.02006311	0	0	0	0	0	0	0	hsa_circ_0077101	NC_000006.12	+	75840585	75841378	794	263	sense-overlappin	XM_005248719.2	MYO6
circRNA_04763	0.051437562	0.020469968	0	0.0206362	0.02006311	0.031069364	0	0.020486295	0.020254528	0.060517448	0	0	-	NC_000006.12	-	77271340	77273307	1968	1968	intergenic		
circRNA_04764	0	0	0	0	0	0	0	0	0.020254528	0	0	0	-	NC_000006.12	-	77271340	77316704	45365	45365	intergenic		
circRNA_04765	0.061725074	0.020469968	0.17446916	0.0206362	0.13041025	0	0.050246713	0	0.020254528	0.040344966	0	0.0206364	hsa_circ_0001615	NC_000006.12	-	79042843	79060818	17976	411	sense-overlappin	NM_017934.5	PHIP
circRNA_04766	0	0	0	0.0206362	0	0	0	0	0	0	0	0	-	NC_000006.12	-	79042843	79063910	21068	501	sense-overlappin	XM_011535918.1	PHIP
circRNA_04767	0	0	0.04361729	0	0.03009467	0	0	0	0	0.020172483	0	0	hsa_circ_0003810	NC_000006.12	-	79060478	79060818	341	250	sense-overlappin	NM_017934.5	PHIP
circRNA_04768	0	0	0	0	0.02006311	0	0	0	0.020254528	0	0	0	-	NC_000006.12	-	79489084	79493750	4667	511	sense-overlappin	XM_005248665.3	LCA5
circRNA_04769	0	0	0	0	0.02006311	0	0	0	0	0	0	0	-	NC_000006.12	-	79513212	79519085	5874	911	sense-overlappin	XM_005248665.3	LCA5
circRNA_04770	0	0	0	0.0206362	0	0	0	0	0	0	0	0	-	NC_000006.12	+	79654495	79673799	19305	19305	sense-overlappin	NM_031469.2	SH3BGRL2
circRNA_04771	0	0	0	0	0.02006311	0	0	0	0	0	0	0	-	NC_000006.12	+	80007945	80013366	5422	709	sense-overlappin	NM_001166691.1	TTK
circRNA_04772	0	0	0.032712968	0	0	0	0	0	0	0	0	0	-	NC_000006.12	-	82057939	82072245	14307	14307	intergenic		
circRNA_04773	0	0	0	0	0	0	0.020098685	0	0	0	0	0	hsa_circ_0003158	NC_000006.12	-	82957312	83038485	81174	552	sense-overlappin	NM_001304437.1	UBE3D
circRNA_04774	0.030862537	0.030704951	0.021808645	0	0	0	0	0	0	0	0	0.0412728	hsa_circ_0008236	NC_000006.12	-	82957312	83044659	87348	784	sense-overlappin	NM_001304437.1	UBE3D
circRNA_04775	0	0.051174919	0	0	0	0.031069364	0	0	0	0	0	0	hsa_circ_0004908	NC_000006.12	-	83018973	83044659	25687	645	sense-overlappin	NM_001304437.1	UBE3D
circRNA_04776	0	0	0	0.0206362	0	0	0	0	0	0	0	0	-	NC_000006.12	+	83100705	83145658	44954	5511	sense-overlappin	NM_001199942.1	DOPEY1
circRNA_04777	0	0	0	0	0	0	0	0	0	0	0.02025523	0	hsa_circ_0077203	NC_000006.12	+	83132176	83142046	9871	2925	sense-overlappin	XM_011535620.1	DOPEY1
circRNA_04778	0	0.030704951	0.076330258	0	0.10031557	0	0	0.020486295	0	0.020172483	0	0	hsa_circ_0003267	NC_000006.12	-	83398367	83407901	9535	284	sense-overlappin	NM_002395.5	ME1
circRNA_04779	0	0	0	0	0.02006311	0	0	0	0	0	0	0	-	NC_000006.12	+	83909009	83924592	15584	484	sense-overlappin	NM_016230.3	CYB5R4
circRNA_04780	0	0	0	0	0	0	0	0	0	0.030258724	0	0	-	NC_000006.12	+	83918005	83940601	22597	901	sense-overlappin	NM_016230.3	CYB5R4
circRNA_04781	0	0	0	0.0206362	0	0.031069364	0	0	0	0	0	0.0309546	-	NC_000006.12	-	84146687	84186623	39937	2761	sense-overlappin	NM_001286206.1	CEP162
circRNA_04782	0	0	0	0	0	0	0	0	0	0.020172483	0	0	-	NC_000006.12	-	84160812	84163270	2459	396	sense-overlappin	NM_001286206.1	CEP162
circRNA_04783	0	0	0	0	0	0.020712909	0.020098685	0	0	0	0.070893305	0	hsa_circ_0077223	NC_000006.12	-	84185187	84186623	1437	554	sense-overlappin	NM_001286206.1	CEP162
circRNA_04784	0.030862537	0.020469968	0	0.0206362	0.03009467	0.031069364	0.030148028	0.040972591	0	0.030258724	0	0	-	NC_000006.12	-	84185187	84204096	18910	1092	sense-overlappin	NM_001286206.1	CEP162
circRNA_04785	0	0	0.021808645	0	0	0	0	0	0	0	0	0	-	NC_000006.12	-	84185187	84215922	30736	1491	sense-overlappin	NM_001286206.1	CEP162
circRNA_04786	0.020575025	0	0	0	0	0	0	0	0	0	0	0	-	NC_000006.12	-	85514070	85558060	43991	2008	sense-overlappin	NM_001304479.1	SNX14
circRNA_04787	0	0	0	0.0722267	0.06018934	0	0	0	0	0.060517448	0	0.0206364	hsa_circ_0008093	NC_000006.12	-	85517756	85530275	12520	458	sense-overlappin	NM_001297614.2	SNX14
circRNA_04788	0	0.020469968	0	0	0	0	0	0	0	0	0	0	-	NC_000006.12	-	85528262	85542043	13782	606	sense-overlappin	NM_001304479.1	SNX14
circRNA_04789	0.051437562	0	0	0	0	0	0	0	0	0	0	0	-	NC_000006.12	-	85528262	85558060	29799	1446	sense-overlappin	NM_001304479.1	SNX14
circRNA_04790	0	0	0	0	0	0.020712909	0	0	0	0	0	0	hsa_circ_0077241	NC_000006.12	-	85530192	85542043	11852	505	sense-overlappin	NM_001304479.1	SNX14
circRNA_04791	0	0	0	0	0.02006311	0	0.020098685	0	0	0	0	0	-	NC_000006.12	-	85543182	85558060	14879	840	sense-overlappin	NM_001297614.2	SNX14
circRNA_04792	0	0.030704951	0	0	0	0	0	0	0	0	0	0	-	NC_000006.12	-	85557976	85565419	7444	173	sense-overlappin	NM_001297614.2	SNX14
circRNA_04793	0.041150049	0.051174919	0.032712968	0	0	0	0.020098685	0.051215739	0.040509055	0	0	0	hsa_circ_0001617	NC_000006.12	-	85557976	85574378	16403	494	sense-overlappin	NM_001297614.2	SNX14
circRNA_04794	0	0	0	0.0309543	0	0	0	0	0	0	0	0	hsa_circ_0004990	NC_000006.12	-	85572137	85574378	2242	277	sense-overlappin	NM_001297614.2	SNX14
circRNA_04795	0	0	0.021808645	0	0	0	0	0	0	0	0	0	-	NC_000006.12	-	85612832	85642890	30059	1740	sense-overlappin	NM_001159674.1	SYNCRIP
circRNA_04796	0	0.040939935	0.065425935	0.0206362	0	0.051782273	0.040197371	0.040972591	0	0.07060369	0	0	hsa_circ_0006131	NC_000006.12	-	85618818	85619417	600	272	sense-overlappin	NM_001159676.1	SYNCRIP
circRNA_04797	0.102875123	0.071644886	0.065425935	0.2269981	0.0702209	0.207129093	0.200986853	0.081945182	0.091145375	0.110948655	0.04051046	0.0412728	hsa_circ_0004383	NC_000006.12	+	87210451	87218731	8281	5822	sense-overlappin	NM_015021.1	ZNF292
circRNA_04798	0.349775418	0.092114854	0.065425935	0.1650895	0.17053648	0.082851637	0.070345399	0.122917773	0.13165443	0.221897311	0.12153138	0.113500201	hsa_circ_0004058	NC_000006.12	+	87215903	87218731	2829	370	sense-overlappin	NM_015021.1	ZNF292
circRNA_04799	0	0	0	0	0	0	0.020098685	0	0	0	0	0	-	NC_000006.12	+	87215903	87233527	17625	573	sense-overlappin	NM_015021.1	ZNF292
circRNA_04800	0.030862537	0	0	0	0	0	0	0	0	0	0	0	-	NC_000006.12	+	87240260	87240517	258	258	intronic	NM_015021.1	ZNF292
circRNA_04801	0	0	0.021808645	0	0	0	0	0	0	0	0	0	-	NC_000006.12	+	87356301	87375710	19410	10953	sense-overlappin	NM_001010868.2	C6orf163
circRNA_04802	0	0	0	0.0309543	0	0.020712909	0	0	0	0	0	0.0206364	-	NC_000006.12	-	87526440	87526780	341	341	intronic	XM_011535950.1	RARS2
circRNA_04803	0	0	0.021808645	0	0	0	0	0	0	0	0	0	hsa_circ_0077276	NC_000006.12	-	87555408	87569590	14183	359	sense-overlappin	XM_011535950.1	RARS2
circRNA_04804	0	0	0	0	0	0	0	0	0	0.020172483	0	0	-	NC_000006.12	+	87601784	87606021	4238	348	sense-overlappin	NM_012381.3	ORC3

circRNA_04805	0	0	0	0	0	0.020712909	0	0	0	0	0	0	hsa_circ_0077280	NC_000006.12	+	87601784	87636486	34703	1303	sense-overlappin	NM_012381.3	ORC3
circRNA_04806	0	0	0	0	0	0	0	0	0	0	0	0.0206364	-	NC_000006.12	+	87607673	87609229	1557	286	sense-overlappin	NM_001197259.1	ORC3
circRNA_04807	0	0	0	0	0.03009467	0	0.050246713	0	0	0	0	0	hsa_circ_0077292	NC_000006.12	-	88844357	88853764	9408	373	sense-overlappin	NM_001286426.1	RNGTT
circRNA_04808	0	0	0.032712968	0	0	0.031069364	0	0	0	0	0	0	-	NC_000006.12	-	88844357	88904955	60599	826	sense-overlappin	NM_001286426.1	RNGTT
circRNA_04809	0	0	0	0	0.02006311	0	0	0	0	0	0	0	hsa_circ_0003393	NC_000006.12	-	88890495	88941180	50686	832	sense-overlappin	NM_001286426.1	RNGTT
circRNA_04810	0	0	0.021808645	0.0309543	0	0	0	0	0	0.020172483	0	0	hsa_circ_0077296	NC_000006.12	-	88928985	88941180	12196	303	sense-overlappin	NM_001286426.1	RNGTT
circRNA_04811	0.072012586	0.040939935	0.04361729	0.0515905	0.06018934	0	0.030148028	0	0.060763583	0.07060369	0.050638075	0.0206364	-	NC_000006.12	+	89083753	89084643	891	891	exonic	NM_006813.2	PNRC1
circRNA_04812	0	0.020469968	0	0	0	0	0	0	0	0	0	0	-	NC_000006.12	-	89329783	89338543	8761	646	sense-overlappin	XM_011535888.1	UBE2J1
circRNA_04813	0	0.020469968	0	0	0	0	0	0	0	0	0	0	-	NC_000006.12	-	89342324	89343756	1433	206	sense-overlappin	XM_011535887.1	UBE2J1
circRNA_04814	0	0.040939935	0	0	0	0	0	0	0	0	0	0	-	NC_000006.12	-	89372437	89387590	15154	903	sense-overlappin	NM_021244.4	RRAGD
circRNA_04815	0.020575025	0	0	0	0	0	0	0	0	0	0	0	-	NC_000006.12	-	89377671	89387590	9920	754	sense-overlappin	NM_021244.4	RRAGD
circRNA_04816	0	0	0	0	0.02006311	0	0	0	0	0	0	0	-	NC_000006.12	-	89722955	89723619	665	297	sense-overlappin	XM_006715405.2	MDN1
circRNA_04817	0	0	0	0	0	0	0	0.020486295	0	0	0	0	-	NC_000006.12	-	89722955	89725396	2442	495	sense-overlappin	XM_006715405.2	MDN1
circRNA_04818	0	0	0	0	0	0	0.040197371	0	0	0	0.02025523	0.0206364	hsa_circ_0001619	NC_000006.12	-	89751431	89753622	2192	263	sense-overlappin	NM_014611.2	MDN1
circRNA_04819	0	0	0.04361729	0.0515905	0	0.051782273	0.040197371	0	0.020254528	0	0.02025523	0	hsa_circ_0003814	NC_000006.12	-	89751431	89762530	11100	1083	sense-overlappin	NM_014611.2	MDN1
circRNA_04820	0	0	0.021808645	0	0.03009467	0	0	0	0	0	0	0	hsa_circ_0001621	NC_000006.12	+	89846562	89857199	10638	444	sense-overlappin	NM_001137667.1	CASP8AP2
circRNA_04821	0	0	0	0	0.03009467	0	0	0	0	0	0	0	-	NC_000006.12	+	89854817	89857199	2383	292	sense-overlappin	NM_001137667.1	CASP8AP2
circRNA_04822	0	0	0	0.0412724	0	0	0	0	0	0.030258724	0	0	hsa_circ_0077385	NC_000006.12	+	89862126	89869082	6957	5376	sense-overlappin	NM_001137667.1	CASP8AP2
circRNA_04823	0.020575025	0	0.076330258	0.0619086	0	0	0	0	0	0.020172483	0.070893305	0	-	NC_000006.12	+	89862126	89871388	9263	5476	sense-overlappin	NM_001137667.1	CASP8AP2
circRNA_04824	0	0	0.021808645	0	0	0	0	0	0	0	0	0	hsa_circ_0077398	NC_000006.12	-	93356717	93358411	1695	492	sense-overlappin	NM_001288629.1	EPHA7
circRNA_04825	0	0	0	0.0206362	0	0	0	0	0	0	0	0	-	NC_000006.12	+	96047074	96114127	67054	67054	sense-overlappin	NM_006581.3	FUT9
circRNA_04826	0	0	0	0.0619086	0	0	0	0.020486295	0	0	0	0.0206364	-	NC_000006.12	+	96107740	96121121	13382	13382	sense-overlappin	NM_006581.3	FUT9
circRNA_04827	0	0	0	0	0	0	0	0.020486295	0	0	0	0	-	NC_000006.12	+	96584304	96584531	228	228	intronic	NM_001170807.1	FHL5
circRNA_04828	0	0	0	0.0412724	0	0	0	0.030729443	0	0	0	0	hsa_circ_0077409	NC_000006.12	-	97267872	97273062	5191	488	sense-overlappin	NM_198468.2	MMS22L
circRNA_04829	0	0	0	0	0	0	0	0	0	0	0	0.0412728	hsa_circ_0003364	NC_000006.12	-	98875415	98905670	30256	844	sense-overlappin	NM_001278716.1	FBXL4
circRNA_04830	0	0	0	0	0	0	0	0	0	0	0.02025523	0	-	NC_000006.12	-	98880553	98934879	54327	1697	sense-overlappin	NM_001278716.1	FBXL4
circRNA_04831	0	0.030704951	0	0	0	0	0	0	0	0	0.02025523	0	-	NC_000006.12	-	98899268	98917719	18452	805	sense-overlappin	NM_001278716.1	FBXL4
circRNA_04832	0	0	0.032712968	0.0515905	0	0	0	0	0	0	0	0	hsa_circ_0077417	NC_000006.12	-	98899268	98927822	28555	1507	sense-overlappin	NM_001278716.1	FBXL4
circRNA_04833	0	0	0.021808645	0	0	0	0.020098685	0	0	0	0	0	-	NC_000006.12	-	98899268	98934879	35612	1625	sense-overlappin	NM_001278716.1	FBXL4
circRNA_04834	0	0	0.021808645	0	0	0	0	0	0	0	0	0	hsa_circ_0003554	NC_000006.12	-	99375940	99383824	7885	746	sense-overlappin	XM_006715500.2	COQ3
circRNA_04835	0	0	0	0	0	0	0	0.040972591	0	0	0	0	-	NC_000006.12	-	99408081	99412739	4659	776	sense-overlappin	XM_005266917.3	PNISR
circRNA_04836	0.020575025	0	0	0	0.03009467	0.020712909	0.020098685	0	0.020254528	0.040344966	0	0.0206364	hsa_circ_0001630	NC_000006.12	-	99412551	99416428	3878	388	sense-overlappin	XM_005266915.3	PNISR
circRNA_04837	0	0	0	0.0206362	0	0	0	0	0	0	0	0	-	NC_000006.12	-	99435707	99510230	74524	2464	sense-overlappin	NM_001080481.1	USP45
circRNA_04838	0.020575025	0	0.032712968	0.0412724	0	0	0.030148028	0	0.040509055	0.020172483	0.04051046	0	hsa_circ_0077425	NC_000006.12	-	99439769	99465136	25368	1053	sense-overlappin	NM_001080481.1	USP45
circRNA_04839	0.020575025	0	0.032712968	0	0	0.031069364	0.050246713	0	0	0	0	0	hsa_circ_0004999	NC_000006.12	-	99464604	99468618	4015	375	sense-overlappin	NM_001080481.1	USP45
circRNA_04840	0	0	0	0	0	0.062138728	0	0	0.020254528	0	0.02025523	0	hsa_circ_0077426	NC_000006.12	-	99464604	99510230	45627	1318	sense-overlappin	NM_001080481.1	USP45
circRNA_04841	0	0	0	0	0	0	0	0	0	0.030258724	0	0	-	NC_000006.12	-	99558497	99562948	4452	314	sense-overlappin	NM_001013399.1	CCNC
circRNA_04842	0	0	0	0	0	0	0	0.020486295	0	0	0	0.0206364	hsa_circ_0077438	NC_000006.12	-	99561367	99562948	1582	262	sense-overlappin	NM_001013399.1	CCNC
circRNA_04843	0	0	0	0.0412724	0	0	0	0	0.030381792	0	0	0	hsa_circ_0008321	NC_000006.12	-	100715462	100725703	10242	414	sense-overlappin	NM_006828.3	ASCC3
circRNA_04844	0	0	0	0	0	0	0.020098685	0	0.030381792	0.060517448	0	0	hsa_circ_0001632	NC_000006.12	-	100766565	100805880	39316	936	sense-overlappin	NM_006828.3	ASCC3
circRNA_04845	0	0.020469968	0	0	0	0	0.020098685	0	0	0	0	0	hsa_circ_0077493	NC_000006.12	-	100766565	100848707	82143	1496	sense-overlappin	NM_006828.3	ASCC3
circRNA_04846	0.030862537	0	0.021808645	0	0.03009467	0.072495183	0.040197371	0	0.040509055	0.050431207	0	0	hsa_circ_0077495	NC_000006.12	-	100798713	100805880	7168	594	sense-overlappin	NM_006828.3	ASCC3
circRNA_04847	0	0	0	0	0	0	0	0	0.020254528	0	0	0	hsa_circ_0006993	NC_000006.12	-	104744160	104750472	6313	302	sense-overlappin	XR_942529.1	
circRNA_04848	0	0	0	0	0	0.020712909	0	0	0	0	0	0	hsa_circ_0004397	NC_000006.12	-	104744160	104785319	41160	1544	sense-overlappin	NR_104424.1	
circRNA_04849	0	0	0.021808645	0	0	0	0	0	0	0	0	0	hsa_circ_0077514	NC_000006.12	-	104811311	104833173	21863	309	sense-overlappin	NR_104424.1	
circRNA_04850	0	0	0	0	0	0.031069364	0	0	0	0	0	0	-	NC_000006.12	-	104824814	104843298	18485	302	sense-overlappin	NR_104424.1	
circRNA_04851	0.020575025	0	0	0	0.03009467	0	0	0	0.020254528	0	0	0	hsa_circ_0077519	NC_000006.12	-	104833042	104843298	10257	208	sense-overlappin	XR_942529.1	
circRNA_04852	0	0	0	0	0	0	0	0	0	0.020172483	0	0	hsa_circ_0077520	NC_000006.12	-	104833042	104849246	16205	313	sense-overlappin	XR_942529.1	
circRNA_04853	0	0	0	0	0	0	0.020098685	0	0	0	0	0	hsa_circ_0005932	NC_000006.12	-	104833042	104852371	19330	458	sense-overlappin	XR_942529.1	
circRNA_04854	0	0.051174919	0	0	0	0	0	0	0.020254528	0.020172483	0	0	hsa_circ_0006490	NC_000006.12	-	106248150	106293106	44957	337	sense-overlappin	NM_001286106.1	ATG5
circRNA_04855	0	0	0	0.0206362	0	0	0	0	0	0	0	0	-	NC_000006.12	-	106248150	106308491	60342	465	sense-overlappin	NM_001286106.1	ATG5
circRNA_04856	0	0	0	0	0	0	0	0	0	0.020172483	0	0	hsa_circ_0077535	NC_000006.12	-	106279661	106293106	13446	242	sense-overlappin	NR_104402.1	
circRNA_04857	0	0	0	0	0.02006311	0	0	0	0	0	0	0	hsa_circ_0077536	NC_000006.12	-	106279661	106308491	28831	370	sense-overlappin	NR_104402.1	
circRNA_04858	0	0	0	0	0.04012623	0	0	0	0	0	0	0	-	NC_000006.12	-	106755175	106755562	388	388	intergenic		
circRNA_04859	0	0.020469968	0	0	0	0	0	0	0	0	0	0	-	NC_000006.12	+	107039749	107044332	4584	555	sense-overlappin	NM_001142468.2	C6orf203

circRNA_04860	0	0	0	0	0	0	0.030148028	0	0	0	0.030382845	0	-	NC_000006.12	-	107098551	107099296	746	251	sense-overlappin	XM_011536007.1	BEND3
circRNA_04861	0	0	0	0	0.02006311	0	0	0	0	0	0	0	-	NC_000006.12	-	107193822	107197898	4077	4077	sense-overlappin	NM_020381.3	PDSS2
circRNA_04862	0.123450148	0.030704951	0	0.0206362	0.05015779	0.020712909	0	0	0.050636319	0.050431207	0.04051046	0	hsa_circ_0001633	NC_000006.12	+	107503657	107506427	2771	325	sense-overlappin	XM_005267042.3	SOBP
circRNA_04863	0	0	0	0	0	0	0	0	0	0.060517448	0	0	-	NC_000006.12	+	107503657	107533610	29954	630	sense-overlappin	XM_005267042.3	SOBP
circRNA_04864	0	0.040939935	0	0	0.05015779	0.041425819	0.020098685	0	0.030381792	0	0	0	hsa_circ_0077548	NC_000006.12	-	107881149	107883146	1998	261	sense-overlappin	NM_007214.4	SEC63
circRNA_04865	0	0	0.032712968	0	0.02006311	0	0	0	0	0	0	0	hsa_circ_0008925	NC_000006.12	-	107901370	107924932	23563	1133	sense-overlappin	NM_007214.4	SEC63
circRNA_04866	0.030862537	0	0	0	0.02006311	0	0.040197371	0.030729443	0	0.040344966	0	0.0309546	hsa_circ_0009144	NC_000006.12	-	107904629	107924932	20304	830	sense-overlappin	NM_007214.4	SEC63
circRNA_04867	0	0	0	0	0	0.031069364	0	0	0	0	0	0	-	NC_000006.12	-	107911346	107924932	13587	400	sense-overlappin	NM_007214.4	SEC63
circRNA_04868	0	0.020469968	0.04361729	0	0	0.031069364	0.020098685	0	0	0.030258724	0	0	hsa_circ_0005115	NC_000006.12	-	107912716	107924932	12217	349	sense-overlappin	NM_007214.4	SEC63
circRNA_04869	0	0	0	0	0	0.031069364	0	0	0	0	0	0	-	NC_000006.12	-	107920929	107921909	981	981	sense-overlappin	NM_007214.4	SEC63
circRNA_04870	0	0	0	0	0	0	0	0	0.020254528	0	0	0	-	NC_000006.12	-	107920929	107924932	4004	1096	sense-overlappin	NM_007214.4	SEC63
circRNA_04871	0	0.030704951	0	0	0.04012623	0.041425819	0.030148028	0	0	0.030258724	0	0	hsa_circ_0001635	NC_000006.12	-	107921797	107929514	7718	328	sense-overlappin	NM_007214.4	SEC63
circRNA_04872	0	0	0	0	0	0.051782273	0	0.020486295	0	0	0	0	hsa_circ_0077574	NC_000006.12	+	108928088	108936999	8912	246	sense-overlappin	XM_005267155.2	ARMC2
circRNA_04873	0	0	0.054521613	0	0	0	0	0	0	0	0	0	-	NC_000006.12	+	109140421	109146937	6517	5141	sense-overlappin	NM_001083535.1	CEP57L1
circRNA_04874	0	0	0.032712968	0	0	0	0	0	0	0.020172483	0	0	hsa_circ_0004131	NC_000006.12	+	109145219	109146937	1719	343	sense-overlappin	NM_001083535.1	CEP57L1
circRNA_04875	0	0.030704951	0	0.0515905	0	0	0	0	0.040509055	0	0	0	hsa_circ_0077605	NC_000006.12	-	109475399	109476930	1532	336	sense-overlappin	NM_014797.2	ZBTB24
circRNA_04876	0	0	0	0.0722267	0	0	0	0	0	0	0	0	hsa_circ_0001637	NC_000006.12	-	109610365	109619236	8872	588	sense-overlappin	XM_011535553.1	AK9
circRNA_04877	0	0	0	0	0.02006311	0	0	0	0	0	0	0	-	NC_000006.12	-	109656756	109675756	19001	770	sense-overlappin	XM_011535553.1	AK9
circRNA_04878	0	0	0	0	0	0	0	0.020486295	0	0	0	0	-	NC_000006.12	+	109715078	109716568	1491	223	sense-overlappin	NM_014845.5	FIG4
circRNA_04879	0	0.020469968	0.021808645	0	0	0	0	0	0	0.020172483	0	0	hsa_circ_0077607	NC_000006.12	+	109715078	109743772	28695	1071	sense-overlappin	NM_014845.5	FIG4
circRNA_04880	0	0	0	0	0	0.020712909	0	0	0	0.020172483	0	0	-	NC_000006.12	+	109727109	109743772	16664	848	sense-overlappin	NM_014845.5	FIG4
circRNA_04881	0	0	0	0	0	0	0	0	0	0.030258724	0	0	-	NC_000006.12	+	110210707	110213160	2454	312	sense-overlappin	NM_015891.2	CDC40
circRNA_04882	0	0.020469968	0	0	0	0	0	0	0	0	0	0	-	NC_000006.12	+	110217702	110219869	2168	352	sense-overlappin	NM_015891.2	CDC40
circRNA_04883	0.020575025	0	0	0	0	0	0	0	0	0	0	0	-	NC_000006.12	-	110298922	110322872	23951	468	sense-overlappin	NM_001123364.1	METTL24
circRNA_04884	0	0	0	0	0	0	0	0	0	0.020172483	0	0	-	NC_000006.12	-	110298922	110332565	33644	10161	sense-overlappin	NM_001123364.1	METTL24
circRNA_04885	0	0	0	0	0	0.020712909	0	0.030729443	0	0	0	0	-	NC_000006.12	-	110315342	110332565	17224	9932	sense-overlappin	NM_001123364.1	METTL24
circRNA_04886	0	0	0	0	0	0.020712909	0.020098685	0	0	0	0	0	hsa_circ_0002160	NC_000006.12	-	110667434	110746201	78768	328	sense-overlappin	NM_001300960.1	CDK19
circRNA_04887	0	0.040939935	0	0.0412724	0	0.020712909	0	0	0	0	0	0	-	NC_000006.12	-	110746126	110773937	27812	138	sense-overlappin	NM_001300963.1	CDK19
circRNA_04888	0.020575025	0	0	0.0515905	0	0	0	0	0	0	0.02025523	0	-	NC_000006.12	+	110879987	110890356	10370	7835	sense-overlappin	NM_001287215.1	AMD1
circRNA_04889	0.061725074	0.030704951	0.163564838	0.0619086	0.06018934	0	0.030148028	0.020486295	0.050636319	0.060517448	0.050638075	0	hsa_circ_0005954	NC_000006.12	+	110887505	110890356	2852	317	sense-overlappin	NM_001287215.1	AMD1
circRNA_04890	0	0	0	0.0206362	0	0	0	0	0	0	0	0	-	NC_000006.12	+	111172695	111177665	4971	599	sense-overlappin	XM_006715329.2	SLC16A10
circRNA_04891	0	0	0	0.0412724	0	0	0	0	0	0	0	0	-	NC_000006.12	+	111172695	111206735	34041	743	sense-overlappin	XM_005266818.2	SLC16A10
circRNA_04892	0	0	0.032712968	0.0309543	0.03009467	0.051782273	0.090444084	0.040972591	0.070890847	0.121034897	0.030382845	0.0206364	hsa_circ_0001638	NC_000006.12	+	111262257	111263946	1690	286	sense-overlappin	NM_153369.2	KIAA1919
circRNA_04893	0	0	0	0	0	0	0	0	0	0	0.02025523	0	-	NC_000006.12	+	111281249	111281580	332	332	intergenic		
circRNA_04894	0	0	0	0	0	0	0	0.020486295	0	0	0	0	-	NC_000006.12	+	111281275	111281530	256	256	intergenic		
circRNA_04895	0	0	0	0.0309543	0	0	0	0	0	0	0	0	-	NC_000006.12	-	111311069	111322678	11610	554	sense-overlappin	NM_001286431.1	REV3L
circRNA_04896	0	0	0	0	0	0.031069364	0	0	0	0	0	0	-	NC_000006.12	-	111343925	111351791	7867	354	sense-overlappin	NM_001286431.1	REV3L
circRNA_04897	0	0	0	0	0	0	0	0	0	0	0.030382845	0	hsa_circ_0003799	NC_000006.12	-	111343925	111357125	13201	544	sense-overlappin	XM_011536032.1	REV3L
circRNA_04898	0	0	0	0	0	0	0	0	0	0.020172483	0	0	hsa_circ_0077650	NC_000006.12	-	111363853	111368028	4176	1120	sense-overlappin	NM_001286431.1	REV3L
circRNA_04899	0	0	0	0	0	0.041425819	0	0	0	0	0	0	hsa_circ_0077655	NC_000006.12	-	111387765	111392972	5208	531	sense-overlappin	NM_001286431.1	REV3L
circRNA_04900	0	0	0	0	0	0	0	0	0	0.020172483	0	0	hsa_circ_0002519	NC_000006.12	-	111387765	111405630	17866	692	sense-overlappin	NM_001286431.1	REV3L
circRNA_04901	0	0	0.021808645	0	0	0	0	0	0	0	0	0	hsa_circ_0077656	NC_000006.12	-	111387765	111416472	28708	957	sense-overlappin	NM_001286431.1	REV3L
circRNA_04902	0	0	0.021808645	0	0	0	0	0.020486295	0	0	0	0	hsa_circ_0077657	NC_000006.12	-	111387765	111472140	84376	1192	sense-overlappin	NM_001286431.1	REV3L
circRNA_04903	0	0.030704951	0	0.0412724	0	0	0.020098685	0	0	0	0	0	hsa_circ_0077660	NC_000006.12	-	111405470	111472140	66671	661	sense-overlappin	NM_001286431.1	REV3L
circRNA_04904	0	0	0	0	0	0	0	0	0	0.050431207	0	0	-	NC_000006.12	-	111694375	111708020	13646	1085	sense-overlappin	XM_011535666.1	FYN
circRNA_04905	0	0	0	0.0206362	0	0	0	0	0	0	0	0	-	NC_000006.12	-	116003877	116076202	72326	967	sense-overlappin	XM_005266882.3	FRK
circRNA_04906	0	0	0.032712968	0.0206362	0.02006311	0.020712909	0	0	0	0.050431207	0	0	hsa_circ_0077705	NC_000006.12	-	116645714	116660846	15133	1130	sense-overlappin	XM_011535565.1	ZUFSP
circRNA_04907	0	0	0	0	0	0	0	0	0	0	0.030382845	0	-	NC_000006.12	+	116689320	116692156	2837	236	sense-overlappin	XM_011535805.1	KPNA5
circRNA_04908	0.020575025	0	0.065425935	0.0619086	0	0.031069364	0	0.020486295	0.020254528	0.121034897	0	0.051591	-	NC_000006.12	+	116689320	116705160	15841	652	sense-overlappin	XM_011535805.1	KPNA5
circRNA_04909	0	0	0.021808645	0	0	0	0	0	0	0	0	0	-	NC_000006.12	+	116692055	116702150	10096	429	sense-overlappin	XM_011535805.1	KPNA5
circRNA_04910	0	0	0	0	0	0	0	0	0	0.020172483	0	0	-	NC_000006.12	+	116692055	116705160	13106	518	sense-overlappin	XM_011535805.1	KPNA5
circRNA_04911	0	0.020469968	0	0.0206362	0.03009467	0	0.030148028	0	0.020254528	0	0	0	hsa_circ_0006146	NC_000006.12	+	116692055	116726622	34568	1115	sense-overlappin	XM_011535805.1	KPNA5
circRNA_04912	0.092587611	0	0.207182128	0.0515905	0.08025246	0.082851637	0.060296056	0.051215739	0.020254528	0.14120738	0.091148535	0.051591	hsa_circ_0001639	NC_000006.12	+	116698704	116705160	6457	316	sense-overlappin	XM_011535805.1	KPNA5
circRNA_04913	0	0	0.021808645	0	0	0	0	0	0	0	0	0	-	NC_000006.12	-	117130319	117181204	50886	50886	intergenic		
circRNA_04914	0	0	0	0	0	0	0.020098685	0	0	0	0	0	hsa_circ_0077717	NC_000006.12	+	117519816	117545597	25782	1290	sense-overlappin	XM_011535772.1	DCBLD1

circRNA_04915	0	0	0	0	0	0	0	0.020486295	0	0	0	0	-	NC_000006.12	+	117693042	117694180	1139	276	sense-overlappin	NM_138459.3	NUS1
circRNA_04916	0	0	0	0	0	0	0	0	0.020254528	0.030258724	0	0.0309546	-	NC_000006.12	-	118448404	118449870	1467	1467	intergenic		
circRNA_04917	0	0	0	0.0309543	0	0	0	0	0	0	0	0	-	NC_000006.12	-	118479871	118566316	86446	1682	sense-overlappin	XM_005266970.1	CEP85L
circRNA_04918	0.020575025	0.051174919	0.065425935	0.1754076	0.06018934	0.093208092	0.040197371	0.112674625	0.091145375	0.050431207	0	0.113500201	hsa_circ_0077736	NC_000006.12	-	118511298	118566316	55019	1025	sense-overlappin	XM_005266970.1	CEP85L
circRNA_04919	0	0	0	0	0	0	0	0.020486295	0	0	0	0	-	NC_000006.12	-	119180312	119210930	30619	6891	sense-overlappin	XM_011535833.1	MAN1A1
circRNA_04920	0	0	0	0	0	0	0.020098685	0	0	0	0	0	-	NC_000006.12	-	119290683	119306992	16310	294	sense-overlappin	XM_011535833.1	MAN1A1
circRNA_04921	0	0	0	0	0	0	0.030148028	0	0	0	0	0	-	NC_000006.12	+	119533881	119550395	16515	16515	intergenic		
circRNA_04922	0	0	0.032712968	0	0	0	0	0	0	0	0	0	-	NC_000006.12	-	121004170	121016795	12626	12626	intergenic		
circRNA_04923	0	0	0.054521613	0	0	0	0	0	0	0.020172483	0	0	hsa_circ_0077745	NC_000006.12	-	121170405	121242339	71935	932	sense-overlappin	NR_104452.1	
circRNA_04924	0	0	0	0	0	0	0	0	0	0.020172483	0	0	hsa_circ_0077747	NC_000006.12	-	121223236	121242339	19104	552	sense-overlappin	NR_104452.1	
circRNA_04925	0	0	0	0.0309543	0	0	0	0	0	0	0	0	-	NC_000006.12	-	124920188	124948098	27911	27911	intergenic		
circRNA_04926	0.051437562	0	0	0	0	0	0	0	0	0	0	0	hsa_circ_0008536	NC_000006.12	+	125045211	125058106	12896	454	sense-overlappin	XR_942305.1	
circRNA_04927	0	0	0	0	0.03009467	0	0	0	0	0	0	0	-	NC_000006.12	+	125915333	125956888	41556	28850	sense-overlappin	XM_006715340.2	NCOA7
circRNA_04928	0	0	0	0	0.09028402	0	0	0	0	0	0	0	-	NC_000006.12	+	125915333	125957178	41846	29140	sense-overlappin	XM_006715340.2	NCOA7
circRNA_04929	0	0	0.032712968	0	0	0	0	0	0	0	0	0	-	NC_000006.12	+	125993757	125999613	5857	607	sense-overlappin	NM_001031712.2	TRMT11
circRNA_04930	0	0	0.054521613	0	0	0	0	0	0.050636319	0.040344966	0	0	hsa_circ_0077789	NC_000006.12	+	125993757	126013101	19345	1067	sense-overlappin	NM_001031712.2	TRMT11
circRNA_04931	0	0	0.021808645	0	0	0	0	0	0	0	0	0	hsa_circ_0077795	NC_000006.12	+	126011253	126013101	1849	379	sense-overlappin	NM_001031712.2	TRMT11
circRNA_04932	0	0	0	0	0	0	0.020098685	0	0	0	0	0	-	NC_000006.12	+	126112866	126174911	62046	62046	intergenic		
circRNA_04933	0	0	0	0	0	0	0	0	0	0.040344966	0	0	hsa_circ_0003625	NC_000006.12	+	127148648	127155438	6791	537	sense-overlappin	NM_032784.4	RSPO3
circRNA_04934	0	0	0	0	0	0	0	0	0	0.020172483	0	0	-	NC_000006.12	+	129478693	129481439	2747	298	sense-overlappin	NM_000426.3	LAMA2
circRNA_04935	0.020575025	0	0	0	0	0	0	0	0	0	0	0	-	NC_000006.12	-	129502605	129528738	26134	13570	antisense	NM_000426.3	LAMA2
circRNA_04936	0	0	0	0.0825448	0	0	0	0	0.020254528	0	0	0	-	NC_000006.12	-	129502605	129558166	55562	42998	antisense	NM_000426.3	LAMA2
circRNA_04937	0	0	0	0.0206362	0	0	0.020098685	0	0.020254528	0	0	0	hsa_circ_0003804	NC_000006.12	-	129522477	129558166	35690	35690	intergenic		
circRNA_04938	0	0	0	0	0	0	0.040197371	0	0	0	0	0	hsa_circ_0006887	NC_000006.12	-	129611533	129618852	7320	336	sense-overlappin	XM_005267212.1	ARHGAP18
circRNA_04939	0	0	0	0	0	0.020712909	0	0	0.040509055	0.030258724	0	0.0206364	hsa_circ_0008833	NC_000006.12	-	130184103	130184623	521	271	sense-overlappin	XM_011535487.1	SAMD3
circRNA_04940	0.123450148	0.133054789	0.119947548	0.4952685	0.03009467	0.289980731	0.070345399	0.071702034	0.212672541	0.423622139	0.030382845	0.082545601	hsa_circ_0077837	NC_000006.12	-	130926605	130956499	29895	824	sense-overlappin	XR_942326.1	
circRNA_04941	0.102875123	0.071644886	0.065425935	0.1754076	0	0.144990365	0	0.163890364	0.050636319	0.110948655	0.050638075	0.072227401	hsa_circ_0001640	NC_000006.12	-	130955105	130956499	1395	719	sense-overlappin	XR_942326.1	
circRNA_04942	0	0.040939935	0.032712968	0.0515905	0	0	0.020098685	0	0	0.030258724	0.04051046	0	-	NC_000006.12	+	131145285	131169273	23989	570	sense-overlappin	NM_016377.3	AKAP7
circRNA_04943	0	0	0.021808645	0	0	0	0	0	0	0	0	0	-	NC_000006.12	+	131145285	131199573	54289	683	sense-overlappin	NM_016377.3	AKAP7
circRNA_04944	0	0	0	0	0	0	0	0	0	0.020172483	0	0	hsa_circ_0077843	NC_000006.12	-	131598287	131603204	4918	851	sense-overlappin	NM_001270521.1	MED23
circRNA_04945	0	0	0	0	0	0	0	0.020486295	0	0	0	0	-	NC_000006.12	+	131718672	131726200	7529	541	sense-overlappin	XR_245539.1	
circRNA_04946	0	0	0	0	0	0	0	0	0	0	0.02025523	0	-	NC_000006.12	-	132363282	132374777	11496	9725	sense-overlappin	NM_015529.3	MOXD1
circRNA_04947	0	0	0.021808645	0	0	0	0	0	0	0	0	0	-	NC_000006.12	+	133198782	133274813	76032	43066	sense-overlappin	XM_005266852.3	EYA4
circRNA_04948	0	0	0.021808645	0	0	0	0	0	0	0	0	0	-	NC_000006.12	+	133269869	133274813	4945	4945	sense-overlappin	XM_005266852.3	EYA4
circRNA_04949	0	0	0	0.0206362	0.04012623	0	0	0	0	0	0	0	hsa_circ_0077892	NC_000006.12	+	133980082	133984671	4590	525	sense-overlappin	NM_001253676.1	TBPL1
circRNA_04950	0	0	0.021808645	0.0515905	0	0	0	0	0	0	0	0	-	NC_000006.12	+	133980082	133987168	7087	733	sense-overlappin	NM_001253676.1	TBPL1
circRNA_04951	0	0	0	0	0	0	0	0	0	0.020172483	0	0	hsa_circ_0003504	NC_000006.12	-	134986066	134993875	7810	458	sense-overlappin	NM_001145158.1	HBS1L
circRNA_04952	0.030862537	0	0.021808645	0.0515905	0.06018934	0	0	0	0.030381792	0.07060369	0	0.0412728	hsa_circ_0001641	NC_000006.12	-	135039573	135042126	2554	321	sense-overlappin	NM_006620.3	HBS1L
circRNA_04953	0	0	0.021808645	0	0	0	0	0	0	0	0	0	-	NC_000006.12	-	135039573	135050647	11075	387	sense-overlappin	NM_006620.3	HBS1L
circRNA_04954	0	0	0.021808645	0	0	0	0	0	0	0	0	0	-	NC_000006.12	-	135290423	135323324	32902	1972	sense-overlappin	XR_942488.1	
circRNA_04955	0	0	0.08723458	0.0412724	0	0	0	0	0.050636319	0.020172483	0	0	hsa_circ_0005214	NC_000006.12	-	135300500	135323324	22825	1869	sense-overlappin	XR_942488.1	
circRNA_04956	0	0	0.054521613	0	0	0	0	0	0	0	0	0	hsa_circ_0077910	NC_000006.12	-	135300500	135394896	94397	2046	sense-overlappin	XR_942488.1	
circRNA_04957	0	0	0.021808645	0	0	0	0	0	0	0	0	0	-	NC_000006.12	-	135318519	135346796	28278	23733	sense-overlappin	NM_001134830.1	AHI1
circRNA_04958	0	0	0	0	0.02006311	0	0	0	0	0	0	0	hsa_circ_0077911	NC_000006.12	-	135318519	135394896	76378	438	sense-overlappin	NM_001134830.1	AHI1
circRNA_04959	0	0	0	0	0	0	0	0	0	0.020172483	0	0	-	NC_000006.12	-	135346675	135394896	48222	299	sense-overlappin	XR_942495.1	
circRNA_04960	0	0	0.021808645	0	0	0	0	0	0	0	0	0	hsa_circ_0077935	NC_000006.12	-	135438375	135457713	19339	1105	sense-overlappin	NM_001134830.1	AHI1
circRNA_04961	0	0	0.065425935	0	0	0	0	0	0	0	0	0	hsa_circ_0077936	NC_000006.12	-	135442582	135457713	15132	981	sense-overlappin	NM_001134830.1	AHI1
circRNA_04962	0	0	0	0	0.02006311	0.020712909	0	0	0	0	0	0	hsa_circ_0007341	NC_000006.12	-	135453341	135455926	2586	289	sense-overlappin	NM_001134830.1	AHI1
circRNA_04963	0	0	0.021808645	0	0	0	0	0	0	0	0	0	-	NC_000006.12	-	135463125	135492291	29167	985	sense-overlappin	NM_001134830.1	AHI1
circRNA_04964	0	0	0	0	0.02006311	0	0	0	0	0	0	0	-	NC_000006.12	-	135463125	135495898	32774	1241	sense-overlappin	XM_011535910.1	AHI1
circRNA_04965	0.164600197	0.071644886	0	0	0	0.031069364	0	0.020486295	0.020254528	0.040344966	0	0	hsa_circ_0002198	NC_000006.12	+	136151160	136155758	4599	329	sense-overlappin	XM_011535755.1	PDE7B
circRNA_04966	0	0	0.021808645	0	0	0	0	0	0	0	0	0	-	NC_000006.12	-	136360697	136361179	483	277	sense-overlappin	NM_001198615.1	MAP7
circRNA_04967	0	0	0.032712968	0	0.03009467	0	0	0	0	0	0	0	hsa_circ_0077957	NC_000006.12	-	136383671	136389517	5847	393	sense-overlappin	NM_001198615.1	MAP7
circRNA_04968	0	0.020469968	0.13085187	0.0309543	0	0.041425819	0	0.040972591	0.020254528	0.020172483	0	0.0206364	-	NC_000006.12	-	136388393	136389517	1125	282	sense-overlappin	NM_001198615.1	MAP7
circRNA_04969	0	0	0	0	0	0	0	0	0	0.020172483	0	0	-	NC_000006.12	-	136558800	136568069	9270	742	sense-overlappin	XM_011535837.1	MAP3K5

circRNA_04970	0	0	0	0.0206362	0	0	0	0	0	0	0	0	hsa_circ_0077967	NC_000006.12	-	136580301	136622981	42681	1504	sense-overlappin	XM_011535837.1	MAP3K5
circRNA_04971	0	0	0	0.0206362	0	0	0	0	0	0	0	0	-	NC_000006.12	-	136601022	136622981	21960	862	sense-overlappin	XM_011535837.1	MAP3K5
circRNA_04972	0	0	0	0.0309543	0	0	0	0	0	0	0	0	-	NC_000006.12	-	136601022	136635086	34065	12967	sense-overlappin	XM_011535837.1	MAP3K5
circRNA_04973	0	0	0.032712968	0	0	0	0	0	0	0	0	0	-	NC_000006.12	-	136601022	136698682	97661	2266	sense-overlappin	XM_011535838.1	MAP3K5
circRNA_04974	0	0	0.032712968	0	0	0	0	0	0	0	0	0	hsa_circ_0077975	NC_000006.12	-	136622848	136698682	75835	1538	sense-overlappin	XM_011535838.1	MAP3K5
circRNA_04975	0	0	0	0.0206362	0	0	0	0	0	0.020172483	0	0	-	NC_000006.12	-	136637325	136659378	22054	650	sense-overlappin	XM_011535838.1	MAP3K5
circRNA_04976	0	0	0.021808645	0	0	0.020712909	0	0	0	0	0	0	-	NC_000006.12	-	136637325	136698682	61358	1404	sense-overlappin	XM_011535838.1	MAP3K5
circRNA_04977	0	0	0	0	0	0	0.020098685	0	0	0	0	0	hsa_circ_0005378	NC_000006.12	-	136656307	136698682	42376	1068	sense-overlappin	XM_011535837.1	MAP3K5
circRNA_04978	0.030862537	0.020469968	0.04361729	0.0206362	0	0	0	0	0.050636319	0.020172483	0	0	hsa_circ_0009034	NC_000006.12	-	136669283	136698682	29400	754	sense-overlappin	XM_011535837.1	MAP3K5
circRNA_04979	0	0	0.021808645	0.0206362	0	0	0	0	0	0	0	0	hsa_circ_0006554	NC_000006.12	-	136694140	136697387	3248	447	sense-overlappin	XM_011535837.1	MAP3K5
circRNA_04980	0.020575025	0	0.098138903	0.1960438	0.05015779	0.176059729	0.030148028	0.020486295	0	0.050431207	0.06076569	0.0309546	hsa_circ_0006856	NC_000006.12	-	136694140	136698682	4543	641	sense-overlappin	XM_011535837.1	MAP3K5
circRNA_04981	0	0	0.098138903	0.0619086	0.04012623	0	0	0	0	0.040344966	0	0.0619092	hsa_circ_0007798	NC_000006.12	-	136694140	136720589	26450	805	sense-overlappin	XM_011535837.1	MAP3K5
circRNA_04982	0	0	0	0	0.02006311	0	0	0	0	0	0	0	-	NC_000006.12	+	138207042	138210041	3000	214	sense-overlappin	NM_020340.4	ARFGEF3
circRNA_04983	0	0.020469968	0.021808645	0.0619086	0	0	0.020098685	0	0	0	0	0	-	NC_000006.12	+	138333970	138335188	1219	1219	exonic	NM_020340.4	ARFGEF3
circRNA_04984	0	0	0	0	0.02006311	0	0	0	0	0	0	0	-	NC_000006.12	-	138441983	138447193	5211	325	sense-overlappin	XM_011535971.1	NHSL1
circRNA_04985	0	0	0.109043225	0	0	0	0.030148028	0	0.020254528	0	0	0	hsa_circ_0006835	NC_000006.12	-	138473306	138496371	23066	335	sense-overlappin	XM_011535974.1	NHSL1
circRNA_04986	0	0	0	0	0	0	0	0	0	0.020172483	0	0	hsa_circ_0078018	NC_000006.12	-	138907495	138914761	7267	602	sense-overlappin	NM_001128617.2	REPS1
circRNA_04987	0	0	0	0	0	0.020712909	0	0	0	0	0	0	-	NC_000006.12	-	138911276	138930098	18823	932	sense-overlappin	NM_001286611.1	REPS1
circRNA_04988	0.041150049	0.16375974	0.08723458	0.2992247	0.16050492	0.238198457	0.190937511	0.102431477	0.091145375	0.14120738	0.091148535	0.123818401	hsa_circ_0004368	NC_000006.12	-	138943513	138944622	1110	352	sense-overlappin	NM_001128617.2	REPS1
circRNA_04989	0	0	0	0.0206362	0	0	0	0	0	0	0	0	-	NC_000006.12	-	138975757	138978413	2657	2657	intronic	NM_001128617.2	REPS1
circRNA_04990	0	0	0	0	0	0	0	0	0	0.030258724	0	0	-	NC_000006.12	+	139429081	139436460	7380	7380	intergenic		
circRNA_04991	0	0	0	0	0	0	0	0	0	0.040344966	0	0	-	NC_000006.12	+	139429081	139451669	22589	22589	intergenic		
circRNA_04992	0	0	0.04361729	0	0	0	0	0	0	0	0	0	-	NC_000006.12	-	141772873	141798155	25283	25283	intergenic		
circRNA_04993	0	0	0.04361729	0	0	0	0	0	0.020254528	0	0	0	-	NC_000006.12	+	142166228	142170421	4194	299	sense-overlappin	NM_001286371.1	VTA1
circRNA_04994	0	0	0.021808645	0	0	0	0	0	0	0	0	0	-	NC_000006.12	+	142166228	142189534	23307	408	sense-overlappin	NM_001286371.1	VTA1
circRNA_04995	0	0	0	0	0	0	0	0	0	0.020172483	0	0	-	NC_000006.12	+	142405688	142438364	32677	1447	sense-overlappin	NM_001032394.2	ADGRG6
circRNA_04996	0	0	0	0.0309543	0	0	0	0	0	0	0	0	-	NC_000006.12	-	142764799	142837047	72249	6158	sense-overlappin	NM_006734.3	HIVEP2
circRNA_04997	0.020575025	0	0	0	0	0	0	0	0	0	0	0	-	NC_000006.12	-	142836935	142907058	70124	70124	sense-overlappin	NM_006734.3	HIVEP2
circRNA_04998	0.030862537	0	0.054521613	0.0412724	0	0	0	0	0	0	0	0	-	NC_000006.12	+	143470961	143474856	3896	487	sense-overlappin	NM_003630.2	PEX3
circRNA_04999	0	0	0	0.0206362	0	0	0	0	0	0	0	0	-	NC_000006.12	+	143485152	143505457	20306	12752	antisense	NM_032020.4	FUCA2
circRNA_05000	0	0	0.021808645	0	0	0	0	0	0	0	0	0	hsa_circ_0007769	NC_000006.12	+	143765261	143765798	538	538	exonic	NM_001100166.1	PHACTR2
circRNA_05001	0	0	0	0	0	0	0	0	0	0.040344966	0	0.0309546	-	NC_000006.12	+	143765261	143777383	12123	951	sense-overlappin	NM_001100166.1	PHACTR2
circRNA_05002	0	0	0	0	0	0.031069364	0	0	0	0	0	0	hsa_circ_0078060	NC_000006.12	+	143772258	143783280	11023	475	sense-overlappin	NM_001100166.1	PHACTR2
circRNA_05003	0	0	0	0	0	0	0	0	0	0	0.02025523	0	-	NC_000006.12	-	143960469	143985174	24706	508	sense-overlappin	NM_001289046.1	PLAGL1
circRNA_05004	0	0.020469968	0	0.0309543	0	0	0	0.030729443	0	0	0	0	hsa_circ_0009096	NC_000006.12	+	144451370	144459354	7985	635	sense-overlappin	XM_011536108.1	UTRN
circRNA_05005	0.030862537	0.020469968	0.08723458	0.0825448	0.02006311	0.031069364	0	0.051215739	0.040509055	0.020172483	0.04051046	0.0619092	hsa_circ_0004826	NC_000006.12	+	144487548	144493456	5909	771	sense-overlappin	XM_011536108.1	UTRN
circRNA_05006	0	0.020469968	0	0	0	0	0	0	0	0	0	0	hsa_circ_0004327	NC_000006.12	+	144487548	144511123	23576	1122	sense-overlappin	XM_011536108.1	UTRN
circRNA_05007	0	0	0	0	0	0	0	0	0	0.020172483	0	0	hsa_circ_0078098	NC_000006.12	+	144487548	144577288	89741	3657	sense-overlappin	XM_011536108.1	UTRN
circRNA_05008	0	0	0	0	0	0	0	0.020486295	0.030381792	0.050431207	0	0	-	NC_000006.12	+	144513909	144516948	3040	597	sense-overlappin	XM_011536108.1	UTRN
circRNA_05009	0	0	0	0	0	0.031069364	0	0	0	0	0	0	hsa_circ_0078110	NC_000006.12	+	144531052	144542870	11819	689	sense-overlappin	XM_011536108.1	UTRN
circRNA_05010	0	0	0	0	0	0	0	0	0.020254528	0	0	0	-	NC_000006.12	+	144531052	144557311	26260	1383	sense-overlappin	XM_011536108.1	UTRN
circRNA_05011	0.061725074	0.16375974	0	0.0619086	0.03009467	0.062138728	0.080394741	0.09218833	0.091145375	0.090776173	0.030382845	0.0619092	hsa_circ_0001647	NC_000006.12	+	144537582	144539443	1862	286	sense-overlappin	XM_011536108.1	UTRN
circRNA_05012	0.020575025	0	0	0.0825448	0	0.041425819	0	0.040972591	0.050636319	0.050431207	0	0	-	NC_000006.12	+	144537582	144542870	5289	362	sense-overlappin	XM_011536108.1	UTRN
circRNA_05013	0.020575025	0	0	0	0	0	0	0.020486295	0	0	0	0	-	NC_000006.12	+	144537582	144557311	19730	1056	sense-overlappin	XM_011536108.1	UTRN
circRNA_05014	0	0.030704951	0.054521613	0.0515905	0	0.041425819	0	0.040972591	0	0	0.02025523	0.051591	hsa_circ_0001648	NC_000006.12	+	144781922	144803147	21226	725	sense-overlappin	XM_011536101.1	UTRN
circRNA_05015	0	0	0.032712968	0	0	0	0	0	0	0.020172483	0	0	-	NC_000006.12	-	145804372	145806421	2050	2050	exonic	XM_011536177.1	FBXO30
circRNA_05016	0	0	0.076330258	0.0515905	0	0	0	0.020486295	0	0	0.030382845	0.0309546	-	NC_000006.12	-	145864388	145894977	30590	544	sense-overlappin	XM_011535721.1	SHPRH
circRNA_05017	0	0	0	0.0825448	0	0.031069364	0.020098685	0.030729443	0.020254528	0.040344966	0.131658996	0	hsa_circ_0001649	NC_000006.12	-	145888020	145894977	6958	440	sense-overlappin	XM_011535721.1	SHPRH
circRNA_05018	0	0	0	0.0309543	0	0	0	0	0	0	0	0	hsa_circ_0078143	NC_000006.12	-	145919348	145933178	13831	1162	sense-overlappin	XM_011535721.1	SHPRH
circRNA_05019	0	0	0	0	0	0	0	0	0	0	0.030382845	0	-	NC_000006.12	+	146635375	146656980	21606	538	sense-overlappin	XM_011536133.1	ADGB
circRNA_05020	0	0	0	0	0	0	0	0	0.030381792	0.020172483	0	0	hsa_circ_0078150	NC_000006.12	+	147205971	147239270	33300	281	sense-overlappin	NM_001127715.2	STXBP5
circRNA_05021	0	0	0	0	0.06018934	0	0	0	0	0	0	0	hsa_circ_0007828	NC_000006.12	+	147260615	147267167	6553	378	sense-overlappin	XM_011535453.1	STXBP5
circRNA_05022	0	0	0.021808645	0	0	0	0	0	0	0	0	0	hsa_circ_0007762	NC_000006.12	+	147260615	147278204	17590	502	sense-overlappin	XM_011535453.1	STXBP5
circRNA_05023	0	0.040939935	0	0	0	0	0	0	0	0	0	0	-	NC_000006.12	+	147669957	147733811	63855	63855	sense-overlappin	XM_011535819.1	SAMD5
circRNA_05024	0	0	0	0	0	0	0	0	0.020254528	0	0	0	hsa_circ_0078189	NC_000006.12	+	148440184	148487715	47532	444	sense-overlappin	XM_011535639.1	SASH1
circRNA_05025	0.030862537	0	0	0	0	0	0.020098685	0	0.020254528	0	0	0.082545601	-	NC_000006.12	+	148468545	148487715	19171	343	sense-overlappin	XM_011535639.1	SASH1

circRNA_05026	0	0	0	0	0	0	0	0	0	0.030258724	0	0	hsa_circ_0005753	NC_000006.12	+	149342685	149370099	27415	427	sense-overlappin	XM_006715403.2	TAB2
circRNA_05027	0.030862537	0	0	0.0206362	0.02006311	0	0.030148028	0	0	0	0	0.0309546	hsa_circ_0078200	NC_000006.12	+	149369909	149379518	9610	1692	sense-overlappin	NM_001292034.2	TAB2
circRNA_05028	0.030862537	0	0.032712968	0	0.02006311	0	0	0.020486295	0	0	0	0	hsa_circ_0078201	NC_000006.12	+	149378018	149379518	1501	1501	exonic	NM_001292035.2	TAB2
circRNA_05029	0	0.051174919	0.021808645	0	0	0	0	0	0	0	0	0.0206364	hsa_circ_0009074	NC_000006.12	-	149512155	149517450	5296	245	sense-overlappin	NM_139126.3	PPIL4
circRNA_05030	0.041150049	0	0.054521613	0.0309543	0	0	0	0	0	0	0	0	-	NC_000006.12	-	149512155	149541586	29432	1157	sense-overlappin	NM_139126.3	PPIL4
circRNA_05031	0	0	0	0	0	0.031069364	0	0.020486295	0	0	0	0	-	NC_000006.12	-	149517042	149517450	409	409	sense-overlappin	NM_139126.3	PPIL4
circRNA_05032	0	0	0.021808645	0	0	0	0	0	0	0	0	0	-	NC_000006.12	-	149517042	149541586	24545	1321	sense-overlappin	NM_139126.3	PPIL4
circRNA_05033	0	0	0	0	0.02006311	0	0	0.020486295	0	0	0	0	hsa_circ_0078208	NC_000006.12	-	149533458	149535738	2281	357	sense-overlappin	NM_139126.3	PPIL4
circRNA_05034	0	0	0	0	0	0	0	0.020486295	0	0	0	0	-	NC_000006.12	-	149533458	149541586	8129	608	sense-overlappin	NM_139126.3	PPIL4
circRNA_05035	0	0	0.021808645	0.0206362	0.02006311	0.051782273	0	0.030729443	0	0.030258724	0.04051046	0	-	NC_000006.12	+	149572285	149582603	10319	761	sense-overlappin	NM_138785.3	GINM1
circRNA_05036	0	0	0	0	0	0	0	0	0	0	0	0.0206364	hsa_circ_0001651	NC_000006.12	-	149603268	149604782	1515	228	sense-overlappin	XM_011535397.1	KATNA1
circRNA_05037	0	0	0.065425935	0	0	0	0	0	0	0	0	0	-	NC_000006.12	-	149623103	149638560	15458	514	sense-overlappin	XM_011535397.1	KATNA1
circRNA_05038	0.051437562	0.030704951	0	0	0.02006311	0	0	0	0.040509055	0	0	0	hsa_circ_0078218	NC_000006.12	-	149632759	149638560	5802	333	sense-overlappin	XM_011535397.1	KATNA1
circRNA_05039	0	0	0	0.0619086	0	0	0	0	0	0	0	0	-	NC_000006.12	-	149676260	149678745	2486	2298	sense-overlappin	XM_011536250.1	LATS1
circRNA_05040	0.030862537	0	0.021808645	0	0	0	0	0	0	0.030258724	0	0	hsa_circ_0078224	NC_000006.12	-	149676260	149680457	4198	873	sense-overlappin	XM_011536250.1	LATS1
circRNA_05041	0	0.030704951	0.065425935	0.0206362	0.0702209	0.020712909	0	0.030729443	0	0	0	0	hsa_circ_0078241	NC_000006.12	-	149738643	149742570	3928	317	sense-overlappin	XM_005266960.3	NUP43
circRNA_05042	0	0	0.032712968	0	0	0	0	0	0	0	0	0	-	NC_000006.12	+	149759328	149773169	13842	11971	sense-overlappin	NM_001252053.1	PCMT1
circRNA_05043	0	0	0.021808645	0	0	0	0.040197371	0	0.050636319	0.020172483	0	0	hsa_circ_0006936	NC_000006.12	+	149771162	149773169	2008	137	sense-overlappin	NM_001252053.1	PCMT1
circRNA_05044	0	0	0.065425935	0	0	0	0	0	0	0	0	0	hsa_circ_0007771	NC_000006.12	-	149826264	149853160	26897	735	sense-overlappin	XM_006715584.1	LRP11
circRNA_05045	0	0	0.076330258	0.0206362	0.03009467	0	0.020098685	0	0	0	0	0	hsa_circ_0003249	NC_000006.12	-	149836085	149853160	17076	639	sense-overlappin	XM_006715584.1	LRP11
circRNA_05046	0	0	0.021808645	0	0	0	0	0	0	0	0	0	hsa_circ_0078277	NC_000006.12	+	150905650	150926295	20646	476	sense-overlappin	NM_001242767.1	MTHFD1L
circRNA_05047	0	0	0.021808645	0	0	0	0	0	0	0	0	0	-	NC_000006.12	+	150905650	150949133	43484	946	sense-overlappin	NM_001242767.1	MTHFD1L
circRNA_05048	0	0	0.021808645	0	0	0	0	0	0	0	0	0	hsa_circ_0078293	NC_000006.12	+	150955995	150965037	9043	287	sense-overlappin	NM_001242767.1	MTHFD1L
circRNA_05049	0.226325271	0.102349838	0.032712968	0.0412724	0	0.062138728	0	0.051215739	0.050636319	0.171466104	0.08102092	0.0206364	hsa_circ_0078299	NC_000006.12	+	151348711	151353752	5042	5042	exonic	NM_005100.3	AKAP12
circRNA_05050	0	0	0	0	0	0	0	0	0	0	0.030382845	0	-	NC_000006.12	+	151348711	151356262	7552	5578	sense-overlappin	NM_005100.3	AKAP12
circRNA_05051	0	0	0	0	0.02006311	0	0	0	0	0	0	0	-	NC_000006.12	+	151407961	151413238	5278	5278	antisense	XR_942497.1	
circRNA_05052	0	0	0	0	0	0	0	0	0	0.020172483	0	0	-	NC_000006.12	+	151412673	151418603	5931	5931	antisense	XM_005267040.2	RMND1
circRNA_05053	0	0	0	0	0	0	0	0	0	0	0.030382845	0	hsa_circ_0078304	NC_000006.12	-	151422541	151433230	10690	389	sense-overlappin	XM_005267040.2	RMND1
circRNA_05054	0	0	0	0	0.02006311	0	0	0	0	0	0	0	-	NC_000006.12	-	151427482	151433230	5749	217	sense-overlappin	XM_005267040.2	RMND1
circRNA_05055	0	0	0	0	0	0	0.020098685	0	0	0	0	0	-	NC_000006.12	-	152239533	152256765	17233	1095	sense-overlappin	NM_033071.3	SYNE1
circRNA_05056	0.082300098	0.061409903	0.032712968	0.0206362	0.04012623	0.020712909	0	0.020486295	0	0.060517448	0.02025523	0	hsa_circ_0078328	NC_000006.12	-	152329730	152331890	2161	2161	exonic	NM_033071.3	SYNE1
circRNA_05057	0	0	0	0	0	0.020712909	0	0	0	0	0	0	hsa_circ_0078343	NC_000006.12	-	154410343	154411142	800	299	sense-overlappin	XM_011535485.1	CNKSR3
circRNA_05058	0	0	0.032712968	0	0	0	0	0	0	0	0	0	hsa_circ_0005497	NC_000006.12	-	154422506	154442290	19785	729	sense-overlappin	XM_011535485.1	CNKSR3
circRNA_05059	0	0	0	0.0206362	0	0	0	0	0	0	0	0	hsa_circ_0078348	NC_000006.12	-	154422506	154450258	27753	893	sense-overlappin	XM_011535485.1	CNKSR3
circRNA_05060	0	0	0.032712968	0	0	0	0	0	0	0	0	0	hsa_circ_0078352	NC_000006.12	-	154441292	154442290	999	291	sense-overlappin	XM_011535485.1	CNKSR3
circRNA_05061	0	0	0.021808645	0.0309543	0	0	0	0	0	0	0	0	hsa_circ_0007317	NC_000006.12	-	154441292	154450258	8967	455	sense-overlappin	XM_011535485.1	CNKSR3
circRNA_05062	0	0	0.054521613	0	0	0	0	0	0	0	0	0	hsa_circ_0078362	NC_000006.12	+	154773989	154778045	4057	129	sense-overlappin	NM_001286188.1	SCAF8
circRNA_05063	0	0	0	0	0.03009467	0	0	0	0	0	0	0	hsa_circ_0078364	NC_000006.12	+	154773989	154792976	18988	445	sense-overlappin	NM_001286188.1	SCAF8
circRNA_05064	0.061725074	0	0.228990773	0.0515905	0.11034713	0.041425819	0.090444084	0	0.070890847	0.090776173	0.02025523	0.072227401	hsa_circ_0001654	NC_000006.12	+	154773989	154795139	21151	576	sense-overlappin	NM_001286188.1	SCAF8
circRNA_05065	0	0	0.08723458	0.0309543	0.03009467	0	0	0.020486295	0	0.060517448	0	0	-	NC_000006.12	+	154773989	154833409	59421	3800	sense-overlappin	NM_001286188.1	SCAF8
circRNA_05066	0	0	0.032712968	0	0	0.020712909	0.030148028	0	0	0	0	0	-	NC_000006.12	+	154773989	154860998	87010	31389	sense-overlappin	NM_001286188.1	SCAF8
circRNA_05067	0	0	0.04361729	0	0.04012623	0	0.020098685	0.030729443	0	0	0.02025523	0	hsa_circ_0078373	NC_000006.12	+	154787861	154792976	5116	316	sense-overlappin	NM_001286188.1	SCAF8
circRNA_05068	0	0	0	0	0.04012623	0	0.030148028	0	0	0	0	0	hsa_circ_0007981	NC_000006.12	+	154787861	154795139	7279	447	sense-overlappin	NM_001286188.1	SCAF8
circRNA_05069	0	0	0	0	0	0.020712909	0	0	0	0	0	0	-	NC_000006.12	+	154787861	154833409	45549	3671	sense-overlappin	NM_001286188.1	SCAF8
circRNA_05070	0	0	0	0	0.02006311	0	0	0	0	0	0	0	-	NC_000006.12	+	154808070	154810208	2139	439	sense-overlappin	NM_001286188.1	SCAF8
circRNA_05071	0	0	0.021808645	0	0	0	0	0	0	0	0	0	hsa_circ_0078379	NC_000006.12	+	154808070	154824378	16309	1090	sense-overlappin	NM_001286188.1	SCAF8
circRNA_05072	0	0	0.032712968	0	0	0	0	0	0	0	0	0	hsa_circ_0005685	NC_000006.12	+	154860738	154860998	261	261	intergenic		
circRNA_05073	0	0	0	0	0	0	0	0	0	0.020172483	0	0	-	NC_000006.12	+	155016241	155022442	6202	6202	intergenic		
circRNA_05074	0	0	0.021808645	0	0	0	0	0	0	0	0	0	-	NC_000006.12	-	155231159	155253764	22606	11139	antisense	NM_012454.3	TIAM2
circRNA_05075	0	0	0.08723458	0	0	0	0.040197371	0	0.030381792	0	0	0	-	NC_000006.12	+	156829227	156835950	6724	6724	sense-overlappin	XM_005267069.3	ARID1B
circRNA_05076	0	0	0.032712968	0.0309543	0	0	0	0	0	0	0	0	hsa_circ_0008519	NC_000006.12	+	156829227	156901525	72299	384	sense-overlappin	NM_020732.3	ARID1B
circRNA_05077	0	0	0	0	0	0	0	0	0	0	0	0.0206364	hsa_circ_0078399	NC_000006.12	+	156901376	156935576	34201	261	sense-overlappin	XM_005267069.3	ARID1B
circRNA_05078	0.030862537	0.051174919	0.196277805	0.0309543	0.02006311	0	0.080394741	0	0.020254528	0.030258724	0	0.0309546	hsa_circ_0001658	NC_000006.12	+	157036835	157084905	48071	286	sense-overlappin	XM_011535984.1	ARID1B
circRNA_05079	0	0	0.04361729	0	0.03009467	0	0	0	0	0	0	0	hsa_circ_0004019	NC_000006.12	+	157036835	157110561	73727	376	sense-overlappin	XM_011535984.1	ARID1B
circRNA_05080	0	0	0.021808645	0	0	0	0	0	0	0	0	0	hsa_circ_0004381	NC_000006.12	+	157084662	157110561	25900	334	sense-overlappin	XM_005267069.3	ARID1B

circRNA_05081	0	0	0.032712968	0	0	0	0	0	0	0	0	0	hsa_circ_0006305	NC_000006.12	+	157110472	157133207	22736	270	sense-overlappin	XM_005267069.3	ARID1B
circRNA_05082	0	0.020469968	0	0	0	0	0	0	0	0	0	0	-	NC_000006.12	+	157131301	157131369	69	69	intronic	XM_005267069.3	ARID1B
circRNA_05083	0.020575025	0	0.08723458	0	0	0	0	0	0	0	0	0	hsa_circ_0002971	NC_000006.12	+	157867547	157875176	7630	288	sense-overlappin	XM_005267015.1	SNX9
circRNA_05084	0	0	0.04361729	0	0	0	0	0	0	0	0	0	hsa_circ_0078437	NC_000006.12	+	157867547	157910025	42479	937	sense-overlappin	XM_005267015.1	SNX9
circRNA_05085	0	0	0.032712968	0	0	0	0	0	0	0	0	0	hsa_circ_0003004	NC_000006.12	+	157896827	157910025	13199	649	sense-overlappin	XM_005267015.1	SNX9
circRNA_05086	0	0	0.021808645	0	0	0	0	0	0	0	0	0	-	NC_000006.12	+	158033455	158064991	31537	1040	sense-overlappin	XM_011536229.1	SYNJ2
circRNA_05087	0.051437562	0	0	0	0	0	0	0	0	0	0	0	hsa_circ_0008110	NC_000006.12	+	158081109	158081510	402	298	sense-overlappin	XM_011536227.1	SYNJ2
circRNA_05088	0	0	0.021808645	0	0	0	0	0	0	0	0	0	-	NC_000006.12	+	158256854	158256920	67	67	intronic	XM_011535946.1	TULP4
circRNA_05089	0	0	0	0	0	0	0	0	0	0	0.02025523	0	-	NC_000006.12	+	158260484	158260915	432	432	intronic	XM_011535946.1	TULP4
circRNA_05090	0	0	0.054521613	0	0	0	0	0	0	0	0	0	-	NC_000006.12	+	158262645	158314268	51624	51624	sense-overlappin	XM_011535946.1	TULP4
circRNA_05091	0	0	0.316225353	0.1960438	0.06018934	0.051782273	0	0	0	0	0.06076569	0	-	NC_000006.12	+	158267234	158314268	47035	47035	sense-overlappin	XM_011535946.1	TULP4
circRNA_05092	0.123450148	0.143289773	0.447077223	0.3714514	0.09028402	0.113921001	0.130641455	0.081945182	0.070890847	0.110948655	0.141786611	0.051591	-	NC_000006.12	+	158282263	158314268	32006	32006	sense-overlappin	XM_011535946.1	TULP4
circRNA_05093	0	0	0.054521613	0.0722267	0.02006311	0.020712909	0	0.020486295	0	0.030258724	0	0	-	NC_000006.12	+	158310366	158314268	3903	3903	sense-overlappin	XM_011535946.1	TULP4
circRNA_05094	0.164600197	0.16375974	0.512503158	0.5055866	0.09028402	0.207129093	0.140690797	0.081945182	0.121527166	0.221897311	0.08102092	0.185727601	-	NC_000006.12	+	158312051	158314268	2218	2218	exonic	XM_011535946.1	TULP4
circRNA_05095	0	0	0.021808645	0	0.02006311	0	0	0	0	0	0	0	hsa_circ_0001660	NC_000006.12	+	158429736	158431663	1928	1928	sense-overlappin	XM_011535946.1	TULP4
circRNA_05096	0	0	0.032712968	0	0	0	0	0	0	0	0	0	hsa_circ_0002073	NC_000006.12	+	158448996	158452268	3273	316	sense-overlappin	XM_011535946.1	TULP4
circRNA_05097	0	0.020469968	0	0	0	0.051782273	0.020098685	0	0	0	0	0	hsa_circ_0002163	NC_000006.12	+	158501678	158504178	2501	2501	exonic	XM_011535946.1	TULP4
circRNA_05098	0.020575025	0.040939935	0.08723458	0.1031809	0	0.031069364	0.020098685	0.030729443	0.040509055	0.040344966	0	0.0412728	hsa_circ_0004587	NC_000006.12	+	158573420	158589782	16363	508	sense-overlappin	XM_011536003.1	TMEM181
circRNA_05099	0	0	0	0.0515905	0	0	0	0	0	0	0	0	-	NC_000006.12	+	158573420	158608750	35331	912	sense-overlappin	XM_011536003.1	TMEM181
circRNA_05100	0	0	0.109043225	0	0	0.062138728	0.020098685	0.020486295	0	0	0	0	-	NC_000006.12	+	158573420	158625754	52335	1125	sense-overlappin	XM_011535999.1	TMEM181
circRNA_05101	0	0	0.04361729	0	0	0	0	0	0	0	0.02025523	0	hsa_circ_0078491	NC_000006.12	+	158580940	158585425	4486	269	sense-overlappin	XM_011536003.1	TMEM181
circRNA_05102	0.061725074	0.051174919	0.196277805	0.0722267	0.09028402	0	0	0.020486295	0.070890847	0.090776173	0.02025523	0.072227401	hsa_circ_0001661	NC_000006.12	+	158580940	158589782	8843	404	sense-overlappin	XM_011536003.1	TMEM181
circRNA_05103	0	0	0	0	0.02006311	0	0	0	0	0	0	0	hsa_circ_0003345	NC_000006.12	+	158583954	158585425	1472	213	sense-overlappin	XM_011536003.1	TMEM181
circRNA_05104	0.082300098	0.061409903	0.61064206	0.113499	0.06018934	0.072495183	0.060296056	0.051215739	0.121527166	0.080689931	0.04051046	0.0206364	hsa_circ_0001663	NC_000006.12	+	158583954	158589782	5829	348	sense-overlappin	XM_011536003.1	TMEM181
circRNA_05105	0	0	0.021808645	0	0	0	0	0	0	0	0	0	-	NC_000006.12	-	158637128	158641360	4233	224	sense-overlappin	NM_001291603.1	DYNLT1
circRNA_05106	0	0	0	0.0309543	0	0	0	0	0	0	0	0	-	NC_000006.12	+	158707230	158725637	18408	461	sense-overlappin	XM_005267214.3	SYTL3
circRNA_05107	0	0	0.054521613	0	0.03009467	0	0	0	0	0	0	0	hsa_circ_0078538	NC_000006.12	-	158776408	158789371	12964	783	sense-overlappin	NM_003379.4	EZR
circRNA_05108	0	0	0.054521613	0	0	0	0.020098685	0	0	0	0	0	hsa_circ_0078539	NC_000006.12	-	158783520	158789371	5852	686	sense-overlappin	NM_003379.4	EZR
circRNA_05109	0.061725074	0	0	0	0	0	0	0	0	0	0	0	-	NC_000006.12	-	159594103	159595204	1102	1102	intergenic		
circRNA_05110	0.061725074	0	0.032712968	0	0.13041025	0	0	0	0.040509055	0.030258724	0	0	hsa_circ_0004662	NC_000006.12	-	159682474	159688242	5769	462	sense-overlappin	NM_001024465.1	SOD2
circRNA_05111	0	0	0	0	0	0	0	0.040972591	0	0	0	0	hsa_circ_0005388	NC_000006.12	-	159784666	159788143	3478	606	sense-overlappin	NM_030752.2	TCP1
circRNA_05112	0	0	0	0	0	0	0.020098685	0	0	0	0	0	-	NC_000006.12	+	159991184	160010785	19602	364	sense-overlappin	NM_000876.2	IGF2R
circRNA_05113	0.041150049	0	0.032712968	0.0309543	0.02006311	0.031069364	0.020098685	0.040972591	0	0	0	0	-	NC_000006.12	+	160046498	160048543	2046	611	sense-overlappin	NM_000876.2	IGF2R
circRNA_05114	0	0	0.021808645	0	0.05015779	0	0	0	0	0	0	0	hsa_circ_0078607	NC_000006.12	+	160397979	160410846	12868	546	sense-overlappin	NM_021977.3	SLC22A3
circRNA_05115	0.020575025	0.020469968	0.054521613	0	0.02006311	0	0	0.020486295	0	0	0	0	-	NC_000006.12	+	161022010	161034449	12440	12440	sense-overlappin	NM_001291958.1	MAP3K4
circRNA_05116	0	0	0.228990773	0.0825448	0.03009467	0.062138728	0.050246713	0	0	0	0	0	hsa_circ_0078617	NC_000006.12	+	161034259	161049979	15721	1555	sense-overlappin	NM_001291958.1	MAP3K4
circRNA_05117	0	0	0.054521613	0.0309543	0	0	0	0.030729443	0	0	0	0	hsa_circ_0078619	NC_000006.12	+	161048616	161049979	1364	1364	exonic	NM_001291958.1	MAP3K4
circRNA_05118	0	0	0	0.0515905	0.05015779	0.051782273	0.020098685	0.051215739	0	0.050431207	0.02025523	0	hsa_circ_0005328	NC_000006.12	+	163455279	163478896	23618	260	sense-overlappin	NM_001301085.1	QKI
circRNA_05119	0	0	0	0.0309543	0	0.072495183	0	0	0	0	0	0	-	NC_000006.12	+	163455279	163535125	79847	404	sense-overlappin	NM_001301085.1	QKI
circRNA_05120	0	0	0	0	0	0	0	0	0.030381792	0	0	0	hsa_circ_0078654	NC_000006.12	-	166430453	166432490	2038	249	sense-overlappin	NM_021135.4	RPS6KA2
circRNA_05121	0	0	0	0	0	0.031069364	0	0	0	0	0	0	hsa_circ_0078663	NC_000006.12	-	166934091	166952548	18458	406	sense-overlappin	NM_003730.4	RNASET2
circRNA_05122	0	0	0.021808645	0	0	0	0	0	0	0	0	0	hsa_circ_0078665	NC_000006.12	-	166943019	166952548	9530	246	sense-overlappin	NM_003730.4	RNASET2
circRNA_05123	0	0.020469968	0.04361729	0.0206362	0	0	0	0	0	0	0	0	hsa_circ_0004556	NC_000006.12	+	167022409	167024894	2486	340	sense-overlappin	NM_001278690.1	FGFR1OP
circRNA_05124	0	0.020469968	0.109043225	0.0206362	0	0	0	0.020486295	0.030381792	0.060517448	0.050638075	0.0309546	hsa_circ_0004558	NC_000006.12	+	167022409	167033971	11563	546	sense-overlappin	NM_001278690.1	FGFR1OP
circRNA_05125	0	0	0	0	0	0	0	0	0	0	0	0.0412728	-	NC_000006.12	+	167022409	167040339	17931	838	sense-overlappin	NM_001278690.1	FGFR1OP
circRNA_05126	0	0	0.021808645	0	0	0	0	0.020486295	0	0	0.02025523	0	-	NC_000006.12	+	167022409	167044576	22168	5075	sense-overlappin	NM_001278690.1	FGFR1OP
circRNA_05127	0	0	0.04361729	0	0	0	0	0	0	0	0	0	-	NC_000006.12	+	167864551	167880517	15967	795	sense-overlappin	XM_006715493.2	MLLT4
circRNA_05128	0	0	0.141756193	0.0206362	0	0	0	0	0	0	0	0	hsa_circ_0078673	NC_000006.12	+	167864551	167891029	26479	1075	sense-overlappin	XM_006715493.2	MLLT4
circRNA_05129	0	0	0.021808645	0.0309543	0	0	0	0	0	0	0	0	-	NC_000006.12	+	167864551	167925091	60541	2997	sense-overlappin	XM_006715493.2	MLLT4
circRNA_05130	0	0	0.109043225	0	0	0	0	0	0	0	0	0	-	NC_000006.12	+	167870383	167880517	10135	599	sense-overlappin	XM_006715493.2	MLLT4
circRNA_05131	0	0.030704951	0.294416708	0.0412724	0.02006311	0.031069364	0	0	0.020254528	0.020172483	0.04051046	0	-	NC_000006.12	+	167870383	167889326	18944	711	sense-overlappin	XM_006715493.2	MLLT4
circRNA_05132	0	0	0.076330258	0	0.02006311	0	0.060296056	0	0	0	0	0	-	NC_000006.12	+	167870383	167898467	28085	1282	sense-overlappin	XM_006715493.2	MLLT4
circRNA_05133	0	0	0.163564838	0	0	0	0.030148028	0	0.030381792	0.020172483	0	0	-	NC_000006.12	+	167870383	167902386	32004	1352	sense-overlappin	XM_006715493.2	MLLT4
circRNA_05134	0	0	0.076330258	0	0	0	0	0	0	0	0	0	-	NC_000006.12	+	167870386	167880517	10132	596	sense-overlappin	XM_006715493.2	MLLT4
circRNA_05135	0	0	0.109043225	0	0	0	0	0	0	0	0	0	hsa_circ_0004342	NC_000006.12	+	167870386	167889326	18941	708	sense-overlappin	XM_006715493.2	MLLT4
circRNA_05136	0	0	0.021808645	0	0	0	0	0	0	0	0	0	-	NC_000006.12	+	167870386	167898467	28082	1279	sense-overlappin	XM_006715493.2	MLLT4
circRNA_05137	0	0	0.04361729	0	0	0	0	0	0	0	0	0	-	NC_000006.12	+	167870386	167902386	32001	1349	sense-overlappin	XM_006715493.2	MLLT4

circRNA_05138	0	0	0.054521613	0	0	0	0	0	0	0	0	0	-	NC_000006.12	+	167943129	167952187	9059	1734	sense-overlappin	XM_006715492.1	MLLT4
circRNA_05139	0.041150049	0	0.021808645	0	0	0	0	0	0	0	0.02025523	0	-	NC_000006.12	+	168509915	168549203	39289	553	sense-overlappin	NM_001166412.1	SMOC2
circRNA_05140	0.030862537	0	0	0	0	0	0	0	0	0	0	0	hsa_circ_0078696	NC_000006.12	+	168543625	168608239	64615	444	sense-overlappin	NM_001166412.1	SMOC2
circRNA_05141	0	0	0	0.0206362	0	0	0	0	0	0	0	0	-	NC_000006.12	-	169710236	169716088	5853	704	sense-overlappin	NM_018288.3	PHF10
circRNA_05142	0	0	0.021808645	0.0206362	0	0	0	0	0	0	0	0	hsa_circ_0078755	NC_000006.12	-	169710236	169721111	10876	1026	sense-overlappin	NM_018288.3	PHF10
circRNA_05143	0	0	0	0	0	0	0	0	0	0.030258724	0	0	hsa_circ_0078784	NC_000006.12	-	170537234	170549113	11880	427	sense-overlappin	NM_002793.3	PSMB1
circRNA_05144	0.041150049	0.071644886	0.021808645	0.0309543	0.06018934	0.072495183	0.030148028	0.040972591	0.030381792	0.020172483	0.030382845	0	hsa_circ_0006107	NC_000006.12	-	170543601	170549113	5513	320	sense-overlappin	NM_002793.3	PSMB1
circRNA_05145	0	0	0	0	0.03009467	0	0	0	0	0	0	0	hsa_circ_0078786	NC_000006.12	-	170546103	170549113	3011	190	sense-overlappin	NM_002793.3	PSMB1
circRNA_05146	0	0	0.021808645	0.0309543	0	0	0	0	0	0	0	0	hsa_circ_0079040	NC_000007.14	-	579256	607452	28197	451	sense-overlappin	XM_011515446.1	PRKAR1B
circRNA_05147	0	0	0.04361729	0	0	0	0	0	0	0	0	0	hsa_circ_0079048	NC_000007.14	-	677229	680726	3498	263	sense-overlappin	XM_011515446.1	PRKAR1B
circRNA_05148	0	0	0.13085187	0.0412724	0	0	0.060296056	0	0.020254528	0	0	0	hsa_circ_0008039	NC_000007.14	-	677229	711527	34299	462	sense-overlappin	NM_002735.2	PRKAR1B
circRNA_05149	0	0	0.021808645	0	0	0	0	0	0	0	0	0	-	NC_000007.14	-	722754	724864	2111	2111	intronic	NM_001164760.1	PRKAR1B
circRNA_05150	0	0	0.054521613	0	0.03009467	0	0.040197371	0	0	0	0	0	hsa_circ_0001667	NC_000007.14	+	770471	775162	4692	456	sense-overlappin	NM_017802.3	DNAAF5
circRNA_05151	0	0	0	0	0	0.041425819	0	0	0	0	0	0	hsa_circ_0004037	NC_000007.14	+	851384	853618	2235	605	sense-overlappin	NM_001130965.2	SUN1
circRNA_05152	0	0	0.021808645	0.0206362	0.04012623	0	0.020098685	0	0	0	0.04051046	0	hsa_circ_0079074	NC_000007.14	+	854920	857957	3038	261	sense-overlappin	NM_001171944.1	SUN1
circRNA_05153	0	0	0.032712968	0	0	0	0	0	0	0	0	0	hsa_circ_0079080	NC_000007.14	+	860128	866067	5940	456	sense-overlappin	NM_001171944.1	SUN1
circRNA_05154	0	0	0.021808645	0	0	0	0	0	0	0	0	0	-	NC_000007.14	-	904126	926644	22519	435	sense-overlappin	NM_001284310.1	ADAP1
circRNA_05155	0	0	0.021808645	0	0	0	0	0	0	0	0	0	-	NC_000007.14	-	919968	935505	15538	306	sense-overlappin	NM_001284309.1	ADAP1
circRNA_05156	0	0	0.021808645	0	0	0	0	0	0	0	0	0	-	NC_000007.14	-	1009950	1010300	351	351	sense-overlappin	XM_011515580.1	C7orf50
circRNA_05157	0	0	0.04361729	0	0	0	0	0	0	0	0	0	-	NC_000007.14	-	1936687	2002121	65435	448	sense-overlappin	NM_001013836.1	MAD1L1
circRNA_05158	0	0	0.021808645	0	0	0	0	0	0	0	0	0	-	NC_000007.14	-	2209947	2219456	9510	3780	sense-overlappin	NM_001013836.1	MAD1L1
circRNA_05159	0	0	0.076330258	0	0	0	0	0	0	0	0	0	hsa_circ_0079149	NC_000007.14	-	2213212	2219456	6245	515	sense-overlappin	NM_001013836.1	MAD1L1
circRNA_05160	0	0	0.021808645	0	0	0	0	0	0	0	0	0	hsa_circ_0005860	NC_000007.14	-	2213212	2222754	9543	834	sense-overlappin	NM_001304524.1	MAD1L1
circRNA_05161	0	0	0	0	0.02006311	0	0.020098685	0	0.020254528	0	0	0	hsa_circ_0004961	NC_000007.14	-	2229984	2230727	744	339	sense-overlappin	NM_001013836.1	MAD1L1
circRNA_05162	0	0	0.032712968	0	0	0	0.030148028	0	0	0	0.04051046	0	hsa_circ_0008809	NC_000007.14	+	2363632	2364529	898	287	sense-overlappin	XM_011515599.1	EIF3B
circRNA_05163	0	0	0.04361729	0	0	0	0	0	0	0	0	0	hsa_circ_0001671	NC_000007.14	+	2364372	2366448	2077	290	sense-overlappin	XM_011515599.1	EIF3B
circRNA_05164	0.030862537	0	0	0	0	0	0	0	0	0	0	0	-	NC_000007.14	+	2364372	2366591	2220	357	sense-overlappin	XM_011515599.1	EIF3B
circRNA_05165	0	0	0	0	0	0	0	0	0.030381792	0	0.02025523	0	-	NC_000007.14	+	2437804	2443746	5943	5943	intergenic		
circRNA_05166	0	0	0.08723458	0	0	0	0	0	0	0	0	0	-	NC_000007.14	+	3619080	3642105	23026	415	sense-overlappin	NM_152744.3	SDK1
circRNA_05167	0	0	0	0	0	0	0	0	0	0	0.02025523	0	-	NC_000007.14	+	4983671	4989177	5507	5507	intergenic		
circRNA_05168	0	0	0	0	0.02006311	0	0	0	0	0	0	0	hsa_circ_0006202	NC_000007.14	+	5048033	5057779	9747	282	sense-overlappin	NM_001204456.1	RBAK
circRNA_05169	0.020575025	0	0	0	0	0	0	0	0	0	0	0	hsa_circ_0002685	NC_000007.14	+	5216563	5217187	625	195	sense-overlappin	NM_001033518.1	WIPI2
circRNA_05170	0.061725074	0	0.021808645	0.0206362	0	0	0.040197371	0.030729443	0.030381792	0.060517448	0.030382845	0.0309546	hsa_circ_0079284	NC_000007.14	-	5641154	5652510	11357	321	sense-overlappin	NM_207111.3	RNF216
circRNA_05171	0	0	0.021808645	0	0	0	0	0	0	0	0	0	hsa_circ_0079292	NC_000007.14	-	5729432	5741644	12213	1017	sense-overlappin	NM_207111.3	RNF216
circRNA_05172	0	0.020469968	0	0	0	0	0	0	0	0	0	0	hsa_circ_0079294	NC_000007.14	-	5739276	5741644	2369	749	sense-overlappin	NM_207111.3	RNF216
circRNA_05173	0	0	0.021808645	0.0309543	0	0	0.030148028	0	0	0	0	0	hsa_circ_0007501	NC_000007.14	-	5739276	5741815	2540	920	sense-overlappin	NM_207111.3	RNF216
circRNA_05174	0	0	0.021808645	0	0.02006311	0.020712909	0	0	0	0	0	0	hsa_circ_0001673	NC_000007.14	-	6046071	6050045	3975	353	sense-overlappin	NM_001134335.1	EIF2AK1
circRNA_05175	0.020575025	0.020469968	0.021808645	0	0	0	0	0.020486295	0	0	0	0	hsa_circ_0007823	NC_000007.14	+	6111125	6115523	4399	451	sense-overlappin	XM_011515573.1	USP42
circRNA_05176	0	0	0	0	0	0	0	0	0.020254528	0	0	0	hsa_circ_0079340	NC_000007.14	+	6153756	6157055	3300	1742	sense-overlappin	XM_011515573.1	USP42
circRNA_05177	0.030862537	0	0	0	0	0	0	0	0	0	0	0	-	NC_000007.14	+	6379484	6386568	7085	7085	intronic	NM_006908.4	RAC1
circRNA_05178	0	0.030704951	0	0	0	0	0	0	0	0	0	0	-	NC_000007.14	-	6461097	6463175	2079	2079	exonic	NM_001100603.1	KDELR2
circRNA_05179	0	0	0	0	0	0	0	0	0	0.020172483	0	0	hsa_circ_0003948	NC_000007.14	-	6466071	6469754	3684	412	sense-overlappin	NM_006854.3	KDELR2
circRNA_05180	0	0	0	0	0	0	0	0	0	0	0	0.0206364	hsa_circ_0007281	NC_000007.14	-	6469596	6474284	4689	260	sense-overlappin	NM_001100603.1	KDELR2
circRNA_05181	0	0	0.021808645	0	0	0	0	0	0	0	0	0	hsa_circ_0079375	NC_000007.14	+	6578501	6585260	6760	994	sense-overlappin	XM_005249796.2	ZDHHC4
circRNA_05182	0	0	0	0	0	0	0.030148028	0	0	0	0	0	hsa_circ_0079380	NC_000007.14	+	6580555	6585260	4706	748	sense-overlappin	XM_005249796.2	ZDHHC4
circRNA_05183	0.030862537	0	0.032712968	0.0309543	0	0	0	0	0.030381792	0.040344966	0.02025523	0	hsa_circ_0079385	NC_000007.14	+	6585016	6585260	245	245	exonic	XM_005249796.2	ZDHHC4
circRNA_05184	0	0	0	0	0	0	0.070345399	0	0	0	0	0	hsa_circ_0007140	NC_000007.14	-	6697339	6705223	7885	288	sense-overlappin	NM_006956.2	ZNF12
circRNA_05185	0.082300098	0.030704951	0.065425935	0.0928628	0.15047336	0	0.030148028	0.051215739	0.020254528	0.07060369	0	0	hsa_circ_0001675	NC_000007.14	+	7234303	7243750	9448	1132	sense-overlappin	XM_005249812.1	C1GALT1
circRNA_05186	0	0	0.021808645	0.0515905	0	0	0	0	0	0	0	0	-	NC_000007.14	+	7238255	7243750	5496	895	sense-overlappin	XM_005249812.1	C1GALT1
circRNA_05187	0	0	0	0	0.04012623	0	0	0	0	0	0	0	-	NC_000007.14	-	7432473	7437462	4990	276	sense-overlappin	XR_926936.1	
circRNA_05188	0	0	0.032712968	0	0	0	0	0	0	0	0	0	hsa_circ_0079413	NC_000007.14	+	7572436	7574196	1761	1433	sense-overlappin	XM_005249780.2	MIOS
circRNA_05189	0	0	0	0.0309543	0	0	0	0	0	0	0	0	hsa_circ_0079416	NC_000007.14	+	7588498	7596461	7964	583	sense-overlappin	XM_005249780.2	MIOS
circRNA_05190	0	0.061409903	0	0.1031809	0.03009467	0.041425819	0.030148028	0.071702034	0	0.07060369	0.04051046	0.0619092	hsa_circ_0001676	NC_000007.14	+	7786788	7801743	14956	14956	sense-overlappin	NM_001302348.1	UMAD1
circRNA_05191	0	0.020469968	0	0	0	0.020712909	0	0	0	0	0	0	-	NC_000007.14	+	8003908	8008266	4359	4359	sense-overlappin	NM_138426.3	GLCCI1
circRNA_05192	0.020575025	0.040939935	0	0.0206362	0	0.082851637	0.040197371	0.020486295	0.030381792	0.110948655	0	0.0309546	hsa_circ_0001678	NC_000007.14	+	8003908	8060248	56341	509	sense-overlappin	NM_138426.3	GLCCI1
circRNA_05193	0	0.030704951	0	0	0	0.041425819	0	0	0.030381792	0.100862414	0	0.0412728	-	NC_000007.14	+	8003908	8071131	67224	720	sense-overlappin	NM_138426.3	GLCCI1

circRNA_05194	0	0.020469968	0	0.0206362	0	0.051782273	0.020098685	0.030729443	0	0.040344966	0	0.0619092	-	NC_000007.14	+	8022483	8071131	48649	568	sense-overlappin	NM_138426.3	GLCCI1
circRNA_05195	0	0	0	0.0309543	0	0.020712909	0	0	0	0.020172483	0	0	-	NC_000007.14	+	8055433	8071131	15699	481	sense-overlappin	NM_138426.3	GLCCI1
circRNA_05196	0	0	0	0	0	0	0.030148028	0.030729443	0	0	0	0	hsa_circ_0001679	NC_000007.14	+	8055436	8071131	15696	478	sense-overlappin	NM_138426.3	GLCCI1
circRNA_05197	0	0	0	0	0.02006311	0	0	0	0	0	0	0	-	NC_000007.14	-	8218305	8235940	17636	593	sense-overlappin	NM_022307.2	ICA1
circRNA_05198	0	0	0.08723458	0.0928628	0.04012623	0	0	0	0	0	0.050638075	0	hsa_circ_0079422	NC_000007.14	-	8218305	8236005	17701	658	sense-overlappin	NM_022307.2	ICA1
circRNA_05199	0	0	0.021808645	0	0	0	0.050246713	0	0	0	0.030382845	0	-	NC_000007.14	+	10982372	10990847	8476	933	sense-overlappin	XM_011515623.1	PHF14
circRNA_05200	0	0	0.021808645	0	0	0	0	0	0	0	0	0	-	NC_000007.14	+	11028681	11062085	33405	1337	sense-overlappin	XM_011515623.1	PHF14
circRNA_05201	0.082300098	0.061409903	0.054521613	0	0.05015779	0.041425819	0	0	0.060763583	0.050431207	0.030382845	0	hsa_circ_0079440	NC_000007.14	+	11061791	11111467	49677	321	sense-overlappin	XM_011515619.1	PHF14
circRNA_05202	0	0	0	0	0	0	0.020098685	0	0	0	0	0	hsa_circ_0079449	NC_000007.14	+	12224226	12229819	5594	301	sense-overlappin	NM_001134232.1	TMEM106B
circRNA_05203	0	0	0	0	0	0	0	0	0	0	0.04051046	0	-	NC_000007.14	+	12224226	12244885	20660	13454	sense-overlappin	NM_001134232.1	TMEM106B
circRNA_05204	0	0	0.032712968	0	0	0	0	0	0	0	0	0	-	NC_000007.14	+	13217545	13310323	92779	92779	intergenic		
circRNA_05205	0.030862537	0	0	0	0	0	0	0	0	0	0	0	-	NC_000007.14	-	14574212	14673027	98816	738	sense-overlappin	NM_004080.2	DGKB
circRNA_05206	0	0	0	0	0	0	0	0	0	0.020172483	0	0	-	NC_000007.14	-	16216066	16278226	62161	416	sense-overlappin	XM_011515498.1	ISPD
circRNA_05207	0.061725074	0.061409903	0.054521613	0.113499	0	0.031069364	0.040197371	0.030729443	0.081018111	0.161379862	0.04051046	0.051591	hsa_circ_0079480	NC_000007.14	-	16258390	16278226	19837	284	sense-overlappin	XM_011515498.1	ISPD
circRNA_05208	0.030862537	0	0.032712968	0	0	0.020712909	0	0	0.020254528	0	0	0	hsa_circ_0002228	NC_000007.14	-	16376092	16406337	30246	427	sense-overlappin	XM_011515498.1	ISPD
circRNA_05209	0	0	0	0	0	0.041425819	0	0	0	0	0	0	hsa_circ_0003637	NC_000007.14	-	16624983	16636455	11473	303	sense-overlappin	XM_011515458.1	ANKMY2
circRNA_05210	0	0	0	0	0.02006311	0	0.020098685	0	0	0	0	0	hsa_circ_0079492	NC_000007.14	+	16681301	16689906	8606	416	sense-overlappin	NM_001159767.1	BZW2
circRNA_05211	0	0	0	0	0	0	0.020098685	0	0	0	0	0	hsa_circ_0007102	NC_000007.14	+	16681301	16697061	15761	734	sense-overlappin	NM_001159767.1	BZW2
circRNA_05212	0	0	0.021808645	0	0	0	0	0	0	0	0	0	-	NC_000007.14	+	17305800	17340228	34429	6474	sense-overlappin	NM_001621.4	AHR
circRNA_05213	0	0	0.021808645	0	0	0	0.050246713	0	0	0	0	0	-	NC_000007.14	+	17309936	17335786	25851	1095	sense-overlappin	NM_001621.4	AHR
circRNA_05214	0	0	0.04361729	0	0	0	0.020098685	0	0	0	0	0	-	NC_000007.14	+	17309936	17340228	30293	2338	sense-overlappin	NM_001621.4	AHR
circRNA_05215	0	0.040939935	0	0	0	0	0	0	0	0	0	0	-	NC_000007.14	-	17814834	17850964	36131	1260	sense-overlappin	XM_011515232.1	SNX13
circRNA_05216	0	0.030704951	0.054521613	0.0206362	0.02006311	0	0.020098685	0	0	0.050431207	0.02025523	0	hsa_circ_0005535	NC_000007.14	-	17845595	17850964	5370	328	sense-overlappin	XM_011515232.1	SNX13
circRNA_05217	0	0	0	0	0	0	0	0	0.020254528	0	0	0	-	NC_000007.14	-	17850347	17875790	25444	625	sense-overlappin	XM_011515232.1	SNX13
circRNA_05218	0.030862537	0	0.065425935	0.0825448	0.04012623	0.020712909	0.040197371	0.030729443	0.030381792	0.040344966	0.04051046	0.0206364	hsa_circ_0005519	NC_000007.14	-	17868407	17875790	7384	397	sense-overlappin	XM_011515232.1	SNX13
circRNA_05219	0	0	0.076330258	0	0.06018934	0	0	0	0.030381792	0	0	0.0412728	hsa_circ_0004671	NC_000007.14	-	17868407	17897446	29040	892	sense-overlappin	XM_011515233.1	SNX13
circRNA_05220	0	0	0.04361729	0	0	0.020712909	0	0	0	0	0	0	-	NC_000007.14	-	17868407	17907340	38934	958	sense-overlappin	XM_011515231.1	SNX13
circRNA_05221	0	0	0.021808645	0	0.02006311	0.062138728	0.030148028	0	0.030381792	0	0.02025523	0	hsa_circ_0079524	NC_000007.14	-	17890363	17897446	7084	428	sense-overlappin	XM_011515232.1	SNX13
circRNA_05222	0	0	0	0	0	0.020712909	0	0	0	0.020172483	0	0	hsa_circ_0001680	NC_000007.14	+	18585281	18594029	8749	657	sense-overlappin	XM_011515631.1	HDAC9
circRNA_05223	0.082300098	0.030704951	0	0.0206362	0	0	0	0.020486295	0	0	0	0	hsa_circ_0007904	NC_000007.14	+	18666213	18666476	264	264	exonic	XM_011515631.1	HDAC9
circRNA_05224	0	0	0	0	0	0	0	0	0	0	0.02025523	0	-	NC_000007.14	-	20129120	20161870	32751	14393	sense-overlappin	NM_182762.3	MACC1
circRNA_05225	0.041150049	0	0.098138903	0	0	0	0	0	0	0	0.050638075	0	hsa_circ_0079534	NC_000007.14	-	20154193	20161870	7678	2354	sense-overlappin	NM_182762.3	MACC1
circRNA_05226	0	0	0.021808645	0	0	0	0	0	0	0	0	0	-	NC_000007.14	-	20154193	20170778	16586	2563	sense-overlappin	NM_182762.3	MACC1
circRNA_05227	0	0	0	0	0	0	0	0	0.020254528	0.020172483	0	0	hsa_circ_0079540	NC_000007.14	+	20367012	20380831	13820	588	sense-overlappin	XM_011515392.1	ITGB8
circRNA_05228	0	0	0	0	0	0	0	0	0	0.030258724	0	0	hsa_circ_0079546	NC_000007.14	+	21477079	21482123	5045	429	sense-overlappin	NM_003112.3	SP4
circRNA_05229	0	0	0.021808645	0	0	0	0	0	0	0	0	0	-	NC_000007.14	-	22219866	22318037	98172	765	sense-overlappin	NM_012294.3	RAPGEF5
circRNA_05230	0	0.071644886	0.250799418	0	0	0	0.080394741	0	0.030381792	0.030258724	0.02025523	0	hsa_circ_0079557	NC_000007.14	-	22266964	22318037	51074	565	sense-overlappin	NM_012294.3	RAPGEF5
circRNA_05231	0.072012586	0.102349838	0.861441478	0.1238171	0.05015779	0.020712909	0.321578966	0.051215739	0.141781694	0.090776173	0.10127615	0.072227401	hsa_circ_0001681	NC_000007.14	-	22291175	22318037	26863	516	sense-overlappin	NM_012294.3	RAPGEF5
circRNA_05232	0	0.020469968	0.119947548	0	0	0	0.050246713	0	0	0	0	0	-	NC_000007.14	-	22291175	22340886	49712	23365	sense-overlappin	NM_012294.3	RAPGEF5
circRNA_05233	0	0.051174919	0.796015543	0.0619086	0.03009467	0.020712909	0.281381595	0	0.060763583	0	0.091148535	0.0309546	-	NC_000007.14	-	22308339	22318037	9699	449	sense-overlappin	NM_012294.3	RAPGEF5
circRNA_05234	0.061725074	0.061409903	0.021808645	0.0722267	0.03009467	0.15534682	0.060296056	0.071702034	0.060763583	0.161379862	0.070893305	0.092863801	hsa_circ_0008951	NC_000007.14	-	22960256	22991139	30884	1019	sense-overlappin	XM_011515590.1	FAM126A
circRNA_05235	0	0	0	0	0	0	0	0	0	0.020172483	0	0	-	NC_000007.14	-	22964415	22991139	26725	771	sense-overlappin	XM_011515590.1	FAM126A
circRNA_05236	0	0	0.021808645	0.0206362	0	0.072495183	0	0	0.020254528	0.050431207	0	0	hsa_circ_0001971	NC_000007.14	-	22976210	22984045	7836	575	sense-overlappin	XM_011515590.1	FAM126A
circRNA_05237	0	0.030704951	0.021808645	0	0	0.051782273	0	0	0	0.040344966	0	0	hsa_circ_0005251	NC_000007.14	-	22976210	22991139	14930	654	sense-overlappin	XM_011515590.1	FAM126A
circRNA_05238	0	0	0	0	0	0	0	0	0	0	0.02025523	0	-	NC_000007.14	-	22977341	22991139	13799	442	sense-overlappin	XM_011515590.1	FAM126A
circRNA_05239	0	0	0	0	0	0	0	0	0	0.030258724	0	0	hsa_circ_0005096	NC_000007.14	+	23185070	23185298	229	229	exonic	NM_007342.2	NUPL2
circRNA_05240	0	0	0	0	0	0.020712909	0	0	0	0	0	0	-	NC_000007.14	+	23185070	23196766	11697	559	sense-overlappin	XR_926913.1	
circRNA_05241	0	0	0.032712968	0	0	0	0	0	0	0	0	0	-	NC_000007.14	-	23342064	23351586	9523	802	sense-overlappin	XM_011515093.1	IGF2BP3
circRNA_05242	0	0	0	0	0.03009467	0	0	0	0	0	0	0	-	NC_000007.14	+	23604086	23611553	7468	7468	sense-overlappin	NM_138771.3	CCDC126
circRNA_05243	0.226325271	0.102349838	0.34893832	0.21668	0.52164099	0.15534682	0.110542769	0.081945182	0.111399902	0.171466104	0.06076569	0.165091201	hsa_circ_0001684	NC_000007.14	+	23611171	23611553	383	383	exonic	NM_138771.3	CCDC126
circRNA_05244	0	0	0	0	0	0	0	0.020486295	0	0	0	0	-	NC_000007.14	+	23611171	23622227	11057	11057	sense-overlappin	NM_138771.3	CCDC126
circRNA_05245	0.030862537	0	0	0	0	0	0	0	0	0	0	0	hsa_circ_0004582	NC_000007.14	+	24620052	24650712	30661	822	sense-overlappin	NM_016447.3	MPP6
circRNA_05246	0.020575025	0.040939935	0.381651288	0	0.11034713	0.020712909	0	0	0	0.050431207	0	0	-	NC_000007.14	+	24620052	24668660	48609	1285	sense-overlappin	NM_016447.3	MPP6
circRNA_05247	0	0	0.021808645	0	0.04012623	0	0	0	0	0	0	0.0309546	hsa_circ_0079617	NC_000007.14	+	24620052	24680520	60469	1617	sense-overlappin	NM_016447.3	MPP6
circRNA_05248	0	0	0	0	0.04012623	0	0	0	0	0	0	0	-	NC_000007.14	+	24623663	24641868	18206	275	sense-overlappin	NM_001303037.1	MPP6

circRNA_05249	0.020575025	0.040939935	0	0.0206362	0	0	0	0.020486295	0	0	0	0	-	NC_000007.14	+	24623663	24650712	27050	656	sense-overlappin	NM_001303037.1	MPP6
circRNA_05250	0.061725074	0.051174919	0.21808645	0.1341352	0.1404418	0.020712909	0	0.051215739	0.030381792	0.090776173	0	0	-	NC_000007.14	+	24623663	24668660	44998	1119	sense-overlappin	NM_001303037.1	MPP6
circRNA_05251	0	0	0	0.0206362	0	0	0	0	0	0	0	0	-	NC_000007.14	+	24623663	24680520	56858	1451	sense-overlappin	NM_001303037.1	MPP6
circRNA_05252	0	0.030704951	0	0	0.02006311	0	0	0	0	0	0	0	hsa_circ_0079619	NC_000007.14	+	24623666	24641868	18203	272	sense-overlappin	NM_001303037.1	MPP6
circRNA_05253	0	0.071644886	0.076330258	0.0309543	0.10031557	0.062138728	0.020098685	0.040972591	0	0.020172483	0	0.0619092	hsa_circ_0001685	NC_000007.14	+	24623666	24650712	27047	653	sense-overlappin	NM_001303037.1	MPP6
circRNA_05254	0.020575025	0.020469968	0.370746965	0.1341352	0.22069426	0.051782273	0.050246713	0	0	0.100862414	0.111403765	0.0619092	hsa_circ_0001686	NC_000007.14	+	24623666	24668660	44995	1116	sense-overlappin	NM_001303037.1	MPP6
circRNA_05255	0	0.030704951	0.065425935	0.0206362	0.08025246	0.020712909	0	0	0	0	0	0	hsa_circ_0001687	NC_000007.14	+	24623666	24680520	56855	1448	sense-overlappin	NM_001303037.1	MPP6
circRNA_05256	0	0	0	0	0.02006311	0	0	0	0	0	0	0	-	NC_000007.14	+	24623666	24693848	70183	7859	sense-overlappin	XM_011515425.1	MPP6
circRNA_05257	0	0	0.021808645	0	0	0	0	0	0	0	0	0	hsa_circ_0079624	NC_000007.14	+	24641716	24650712	8997	534	sense-overlappin	NM_001303037.1	MPP6
circRNA_05258	0	0	0.021808645	0	0	0	0	0	0	0	0	0	hsa_circ_0079625	NC_000007.14	+	24641716	24668660	26945	997	sense-overlappin	NM_001303037.1	MPP6
circRNA_05259	0	0	0	0.0309543	0	0	0	0	0	0	0	0	hsa_circ_0079626	NC_000007.14	+	24641716	24680520	38805	1329	sense-overlappin	NM_001303037.1	MPP6
circRNA_05260	0	0	0.04361729	0	0	0	0	0	0	0	0	0	hsa_circ_0079627	NC_000007.14	+	24649612	24650712	1101	381	sense-overlappin	NM_001303037.1	MPP6
circRNA_05261	0	0	0.04361729	0	0	0	0	0	0	0	0	0	hsa_circ_0079628	NC_000007.14	+	24649612	24668660	19049	844	sense-overlappin	NM_001303037.1	MPP6
circRNA_05262	0	0	0.021808645	0	0	0	0	0	0	0	0	0	hsa_circ_0007078	NC_000007.14	-	24816610	24849176	32567	869	sense-overlappin	NM_145320.2	OSBPL3
circRNA_05263	0	0	0	0	0.02006311	0	0	0	0	0	0	0	-	NC_000007.14	-	24861613	24892621	31009	1523	sense-overlappin	XM_011515259.1	OSBPL3
circRNA_05264	0	0	0	0	0	0	0.020098685	0	0	0	0	0	-	NC_000007.14	-	24863200	24883167	19968	11872	sense-overlappin	NM_145321.2	OSBPL3
circRNA_05265	0	0	0	0.0206362	0	0	0	0	0.060763583	0	0	0	-	NC_000007.14	-	26192495	26197866	5372	1077	sense-overlappin	XM_005249729.1	HNRNPA2B1
circRNA_05266	0.030862537	0	0	0	0	0	0.020098685	0	0.040509055	0	0.02025523	0	hsa_circ_0001689	NC_000007.14	-	26193251	26193373	123	123	exonic	XM_005249729.1	HNRNPA2B1
circRNA_05267	0	0	0	0	0.02006311	0	0	0	0	0	0	0	-	NC_000007.14	-	26193287	26193598	312	111	sense-overlappin	XM_005249729.1	HNRNPA2B1
circRNA_05268	0	0	0.054521613	0.0206362	0.02006311	0	0	0	0	0.030258724	0	0	hsa_circ_0079651	NC_000007.14	-	26195847	26197732	1886	715	sense-overlappin	XM_005249729.1	HNRNPA2B1
circRNA_05269	0	0	0	0	0	0	0	0.020486295	0	0	0	0	-	NC_000007.14	-	26196401	26197732	1332	652	sense-overlappin	XM_005249729.1	HNRNPA2B1
circRNA_05270	0	0	0	0	0	0	0	0	0	0.020172483	0	0	-	NC_000007.14	+	26206368	26208555	2188	306	sense-overlappin	NM_007276.4	CBX3
circRNA_05271	0.020575025	0	0	0	0	0	0	0	0	0	0	0	hsa_circ_0079658	NC_000007.14	-	26684736	26690362	5627	191	sense-overlappin	NM_001303468.1	SKAP2
circRNA_05272	0.051437562	0	0	0	0	0.031069364	0	0.030729443	0	0	0	0	-	NC_000007.14	-	26844030	26854890	10861	240	sense-overlappin	NM_001303468.1	SKAP2
circRNA_05273	0	0	0.04361729	0.0206362	0.02006311	0.031069364	0.020098685	0	0	0.040344966	0.08102092	0	hsa_circ_0003958	NC_000007.14	-	27629371	27632445	3075	232	sense-overlappin	NM_152740.3	HIBADH
circRNA_05274	0.030862537	0.071644886	0.054521613	0.0206362	0	0.041425819	0	0	0.030381792	0.080689931	0.06076569	0.051591	hsa_circ_0006773	NC_000007.14	-	27629371	27649633	20263	393	sense-overlappin	NM_152740.3	HIBADH
circRNA_05275	0	0	0.04361729	0	0	0	0	0.020486295	0	0	0	0	-	NC_000007.14	+	27748518	27769834	21317	619	sense-overlappin	NM_001206901.1	TAX1BP1
circRNA_05276	0	0	0.021808645	0	0	0	0	0	0	0	0	0	-	NC_000007.14	+	27748518	27787603	39086	1045	sense-overlappin	NM_001206901.1	TAX1BP1
circRNA_05277	0.041150049	0	0.021808645	0.0309543	0.02006311	0	0	0.030729443	0	0.030258724	0	0	hsa_circ_0079668	NC_000007.14	+	27785163	27785489	327	240	sense-overlappin	NM_001206901.1	TAX1BP1
circRNA_05278	0.020575025	0.030704951	0.04361729	0.113499	0.06018934	0.020712909	0	0	0.050636319	0.080689931	0.050638075	0	-	NC_000007.14	+	27785163	27800090	14928	1152	sense-overlappin	NM_001206901.1	TAX1BP1
circRNA_05279	0	0	0.032712968	0	0	0	0	0	0	0	0	0	-	NC_000007.14	-	29926452	29936721	10270	347	sense-overlappin	NM_001145515.1	SCRN1
circRNA_05280	0	0	0	0	0	0	0	0	0	0	0	0.0309546	-	NC_000007.14	+	30355728	30366295	10568	447	sense-overlappin	NM_147128.3	ZNRF2
circRNA_05281	0.030862537	0	0	0	0	0	0	0	0	0	0	0	hsa_circ_0079701	NC_000007.14	-	30455137	30457042	1906	497	sense-overlappin	XM_011515080.1	NOD1
circRNA_05282	0.174887709	0.051174919	0.04361729	0.0309543	0.02006311	0	0.050246713	0.040972591	0.020254528	0.020172483	0	0	hsa_circ_0001693	NC_000007.14	-	30550636	30562128	11493	11493	intergenic		
circRNA_05283	0.041150049	0.102349838	0.04361729	0.113499	0.03009467	0.062138728	0.050246713	0.163890364	0.13165443	0.161379862	0.192424686	0.144454801	-	NC_000007.14	-	30550636	30574881	24246	24246	intergenic		
circRNA_05284	0	0	0.021808645	0.0619086	0	0	0	0	0.030381792	0	0	0	-	NC_000007.14	-	30573000	30574881	1882	1882	intergenic		
circRNA_05285	0	0	0.032712968	0.0309543	0.02006311	0	0.020098685	0.030729443	0	0.040344966	0	0	hsa_circ_0009000	NC_000007.14	+	30621393	30622462	1070	254	sense-overlappin	NM_002047.2	GARS
circRNA_05286	0	0	0	0	0	0	0.020098685	0	0	0	0	0	hsa_circ_0079733	NC_000007.14	+	30778432	30782212	3781	356	sense-overlappin	NM_032222.2	FAM188B
circRNA_05287	0	0	0	0	0	0	0.030148028	0	0	0	0	0	-	NC_000007.14	+	30778432	30785992	7561	600	sense-overlappin	NM_032222.2	FAM188B
circRNA_05288	0	0	0	0	0	0	0	0	0.020254528	0	0	0	-	NC_000007.14	-	31775664	31816154	40491	378	sense-overlappin	XM_011515412.1	PDE1C
circRNA_05289	0	0.020469968	0	0	0	0	0	0	0	0	0	0	-	NC_000007.14	-	31809031	31816154	7124	309	sense-overlappin	XM_011515412.1	PDE1C
circRNA_05290	0	0	0	0	0	0	0	0	0.040509055	0.020172483	0	0	-	NC_000007.14	-	31809031	31828373	19343	1706	sense-overlappin	XM_011515414.1	PDE1C
circRNA_05291	0.030862537	0	0	0	0	0	0	0	0	0.030258724	0	0	hsa_circ_0079753	NC_000007.14	-	31873292	31880860	7569	481	sense-overlappin	XM_011515412.1	PDE1C
circRNA_05292	0	0.020469968	0	0	0	0	0	0	0	0	0	0	hsa_circ_0005530	NC_000007.14	+	32543141	32552295	9155	436	sense-overlappin	NM_015060.2	AVL9
circRNA_05293	0	0	0.032712968	0	0	0	0	0	0	0	0	0	hsa_circ_0079762	NC_000007.14	+	32543141	32559464	16324	1122	sense-overlappin	NM_015060.2	AVL9
circRNA_05294	0	0	0	0.0309543	0	0.020712909	0	0	0.020254528	0.020172483	0	0	hsa_circ_0001694	NC_000007.14	+	32544694	32554596	9903	395	sense-overlappin	NM_015060.2	AVL9
circRNA_05295	0	0	0.021808645	0	0	0	0	0	0	0	0	0	hsa_circ_0079765	NC_000007.14	+	32544694	32559464	14771	1001	sense-overlappin	NM_015060.2	AVL9
circRNA_05296	0	0	0	0	0	0	0	0	0	0	0.02025523	0	hsa_circ_0079767	NC_000007.14	+	32548847	32552295	3449	229	sense-overlappin	NM_015060.2	AVL9
circRNA_05297	0.030862537	0	0	0	0	0	0	0	0	0	0	0	hsa_circ_0002399	NC_000007.14	+	32558559	32559464	906	606	sense-overlappin	NM_015060.2	AVL9
circRNA_05298	0.030862537	0	0	0	0	0	0	0	0	0	0	0	-	NC_000007.14	-	32620404	32639365	18962	18962	intergenic		
circRNA_05299	0	0	0	0.0412724	0	0	0	0	0	0	0	0	-	NC_000007.14	-	32620404	32679130	58727	58727	intergenic		
circRNA_05300	0	0	0.021808645	0	0.03009467	0	0	0	0	0	0	0	hsa_circ_0006010	NC_000007.14	-	32632543	32639365	6823	6823	intergenic		
circRNA_05301	0.020575025	0	0.08723458	0.0412724	0.04012623	0.031069364	0	0.040972591	0	0.060517448	0	0	-	NC_000007.14	-	32632543	32679130	46588	46588	intergenic		
circRNA_05302	0	0	0.021808645	0	0.02006311	0	0	0	0	0.020172483	0	0	-	NC_000007.14	-	32655987	32679130	23144	23144	intergenic		
circRNA_05303	0	0	0	0	0	0	0	0	0	0	0	0.0206364	-	NC_000007.14	+	32812757	32818611	5855	1528	sense-overlappin	XM_011515684.1	LOC102724668

circRNA_05304	0.041150049	0	0	0	0	0.072495183	0.020098685	0.020486295	0.030381792	0	0.050638075	0	hsa_circ_0005885	NC_000007.14	+	32812757	32820741	7985	249	sense-overlappin	XM_011515684.1	LOC102724668
circRNA_05305	0	0	0	0	0	0	0	0	0	0	0	0.0206364	-	NC_000007.14	+	32812757	32823221	10465	541	sense-overlappin	XM_011515684.1	LOC102724668
circRNA_05306	0	0	0	0	0	0	0	0	0	0.020172483	0	0.0309546	hsa_circ_0007400	NC_000007.14	+	32812757	32838666	25910	6200	sense-overlappin	XM_011515684.1	LOC102724668
circRNA_05307	0.041150049	0	0	0	0	0	0	0	0	0	0	0	-	NC_000007.14	+	32812757	32849121	36365	16655	sense-overlappin	XM_011515684.1	LOC102724668
circRNA_05308	0	0	0.021808645	0.0206362	0	0	0.030148028	0	0	0	0	0	-	NC_000007.14	+	32833008	32834919	1912	1912	sense-overlappin	XM_011515684.1	LOC102724668
circRNA_05309	0	0	0.021808645	0	0	0	0	0	0	0	0	0	-	NC_000007.14	+	32833008	32838666	5659	5659	sense-overlappin	XM_011515684.1	LOC102724668
circRNA_05310	0	0	0.021808645	0	0	0	0	0	0	0	0	0	-	NC_000007.14	-	33015670	33026915	11246	756	sense-overlappin	NM_001166118.2	NT5C3A
circRNA_05311	0.020575025	0	0	0.0515905	0	0	0	0	0	0	0	0	-	NC_000007.14	-	33015670	33035988	20319	897	sense-overlappin	NM_001166118.2	NT5C3A
circRNA_05312	0	0	0.021808645	0	0.02006311	0	0	0	0	0	0	0	hsa_circ_0079799	NC_000007.14	-	33017439	33035988	18550	696	sense-overlappin	NM_001166118.2	NT5C3A
circRNA_05313	0	0	0.065425935	0	0	0	0	0	0	0	0	0	hsa_circ_0004730	NC_000007.14	+	33146242	33155702	9461	339	sense-overlappin	XM_011515265.1	BBS9
circRNA_05314	0	0	0.08723458	0.0619086	0.04012623	0.041425819	0	0.051215739	0.050636319	0.060517448	0.030382845	0.0309546	hsa_circ_0003162	NC_000007.14	+	33146242	33177591	31350	453	sense-overlappin	XM_011515265.1	BBS9
circRNA_05315	0	0	0	0	0	0	0	0	0.030381792	0	0	0.0309546	-	NC_000007.14	+	33146242	33183405	37164	6267	sense-overlappin	XM_011515265.1	BBS9
circRNA_05316	0.020575025	0	0	0	0	0	0	0	0	0	0	0	hsa_circ_0079810	NC_000007.14	+	33344581	33367862	23282	514	sense-overlappin	XM_011515265.1	BBS9
circRNA_05317	0	0	0.032712968	0	0	0	0	0	0.030381792	0.020172483	0	0	hsa_circ_0079812	NC_000007.14	+	33349068	33388144	39077	786	sense-overlappin	XM_011515265.1	BBS9
circRNA_05318	0	0.020469968	0	0.0206362	0	0.020712909	0	0.020486295	0.050636319	0.040344966	0	0	hsa_circ_0079813	NC_000007.14	+	33351219	33388144	36926	683	sense-overlappin	XM_011515265.1	BBS9
circRNA_05319	0	0.020469968	0.021808645	0.0309543	0.02006311	0.062138728	0.030148028	0.051215739	0.060763583	0.080689931	0.02025523	0.0412728	hsa_circ_0008336	NC_000007.14	+	33357855	33388144	30290	563	sense-overlappin	XM_011515265.1	BBS9
circRNA_05320	0	0.051174919	0.04361729	0.0206362	0	0.031069364	0	0	0	0	0	0	hsa_circ_0007567	NC_000007.14	+	33367767	33388144	20378	422	sense-overlappin	XM_011515265.1	BBS9
circRNA_05321	0	0	0	0	0	0.051782273	0	0	0	0.040344966	0	0	-	NC_000007.14	+	33604865	33642918	38054	37832	sense-overlappin	XM_005249700.3	BBS9
circRNA_05322	0	0	0	0.0206362	0	0	0	0	0	0.050431207	0	0	-	NC_000007.14	+	33937289	33974784	37496	357	sense-overlappin	NM_133468.4	BMPER
circRNA_05323	0	0.020469968	0	0	0	0	0	0	0	0	0	0	hsa_circ_0004750	NC_000007.14	+	33966479	33974784	8306	257	sense-overlappin	NM_133468.4	BMPER
circRNA_05324	0	0	0	0	0	0	0	0	0.020254528	0	0	0	hsa_circ_0006083	NC_000007.14	+	34046306	34086092	39787	1169	sense-overlappin	NM_133468.4	BMPER
circRNA_05325	0	0	0.032712968	0	0	0	0	0	0	0	0	0	-	NC_000007.14	-	34952408	34973605	21198	2788	sense-overlappin	XM_011515245.1	DPY19L1
circRNA_05326	0	0	0.032712968	0	0.02006311	0	0	0.020486295	0	0	0	0	-	NC_000007.14	-	34966894	34973605	6712	270	sense-overlappin	XM_011515245.1	DPY19L1
circRNA_05327	0.051437562	0	0.065425935	0.0412724	0.03009467	0.020712909	0.341677651	0	0.030381792	0.030258724	0.050638075	0	-	NC_000007.14	-	35010468	35018596	8129	466	sense-overlappin	XM_011515245.1	DPY19L1
circRNA_05328	0.030862537	0	0.08723458	0.0309543	0	0.062138728	0	0	0.040509055	0.050431207	0	0	hsa_circ_0001696	NC_000007.14	-	35667434	35673278	5845	347	sense-overlappin	NM_022373.4	HERPUD2
circRNA_05329	0	0	0	0	0	0	0	0	0	0.020172483	0	0	-	NC_000007.14	-	35781209	35794022	12814	12814	intergenic		
circRNA_05330	0	0	0	0.0206362	0	0	0	0	0	0	0	0	-	NC_000007.14	+	35831492	35832900	1409	108	sense-overlappin	NM_001788.5	7-Sep
circRNA_05331	0	0	0	0.0206362	0	0	0	0	0	0	0	0	-	NC_000007.14	-	36335046	36336297	1252	277	sense-overlappin	NM_001199706.1	KIAA0895
circRNA_05332	0	0	0.032712968	0	0	0	0	0.020486295	0	0	0	0	hsa_circ_0004698	NC_000007.14	+	38207447	38219660	12214	707	sense-overlappin	NM_032016.3	STARD3NL
circRNA_05333	0	0	0	0	0.03009467	0.020712909	0	0	0	0	0	0	hsa_circ_0002808	NC_000007.14	-	38789801	38796864	7064	334	sense-overlappin	NM_014396.3	VPS41
circRNA_05334	0	0	0.13085187	0	0	0	0	0	0	0	0	0	hsa_circ_0079911	NC_000007.14	+	39204235	39207620	3386	321	sense-overlappin	XM_011515113.1	POU6F2
circRNA_05335	0.030862537	0.030704951	0.152660515	0	0	0	0.090444084	0.020486295	0.030381792	0	0.04051046	0.0206364	hsa_circ_0079929	NC_000007.14	+	39987599	39988258	660	660	exonic	XM_011515598.1	CDK13
circRNA_05336	0	0	0.054521613	0.0206362	0	0.041425819	0.020098685	0	0	0	0	0	-	NC_000007.14	+	39987599	39997664	10066	831	sense-overlappin	XM_011515598.1	CDK13
circRNA_05337	0.051437562	0.08187987	0.512503158	0.113499	0.03009467	0.062138728	0.160789483	0.020486295	0.070890847	0.040344966	0	0.0412728	hsa_circ_0001699	NC_000007.14	+	39987599	40002031	14433	1142	sense-overlappin	XM_011515598.1	CDK13
circRNA_05338	0	0	0.065425935	0	0	0	0	0	0	0	0	0	hsa_circ_0079931	NC_000007.14	+	39987599	40047877	60279	1389	sense-overlappin	XM_011515598.1	CDK13
circRNA_05339	0	0	0.032712968	0	0	0	0	0	0	0	0	0	hsa_circ_0079933	NC_000007.14	+	39987599	40063100	75502	1569	sense-overlappin	NM_003718.4	CDK13
circRNA_05340	0	0	0.021808645	0	0	0	0	0.020486295	0	0	0	0	hsa_circ_0003951	NC_000007.14	+	39997494	40002031	4538	482	sense-overlappin	XM_011515598.1	CDK13
circRNA_05341	0	0.020469968	0.032712968	0	0.04012623	0	0	0	0	0.030258724	0	0	hsa_circ_0005485	NC_000007.14	+	39997494	40047877	50384	729	sense-overlappin	XM_011515598.1	CDK13
circRNA_05342	0	0	0	0	0	0	0	0.030729443	0	0.060517448	0	0	-	NC_000007.14	+	39999361	40047877	48517	558	sense-overlappin	XM_011515598.1	CDK13
circRNA_05343	0	0	0	0	0	0.020712909	0	0.020486295	0	0	0	0	-	NC_000007.14	+	43595901	43596113	213	213	exonic	NM_004760.2	STK17A
circRNA_05344	0.041150049	0.030704951	0.054521613	0.0619086	0.09028402	0.041425819	0.100493427	0.061458886	0.020254528	0.080689931	0.06076569	0	hsa_circ_0001700	NC_000007.14	-	43639449	43640649	1201	310	sense-overlappin	XR_242093.2	
circRNA_05345	0	0	0	0.0206362	0	0	0	0	0	0	0	0.0412728	hsa_circ_0080000	NC_000007.14	+	44050225	44052941	2717	244	sense-overlappin	NM_001014436.2	DBNL
circRNA_05346	0	0	0.04361729	0	0	0	0	0	0	0	0	0	hsa_circ_0080035	NC_000007.14	-	44425900	44427703	1804	1804	sense-overlappin	NM_015332.3	NUDCD3
circRNA_05347	0.020575025	0	0.065425935	0	0	0	0.030148028	0	0	0.020172483	0	0	hsa_circ_0003340	NC_000007.14	+	44645327	44647759	2433	295	sense-overlappin	NM_001003941.2	OGDH
circRNA_05348	0	0	0.021808645	0	0.03009467	0.020712909	0	0	0	0.080689931	0	0	hsa_circ_0005004	NC_000007.14	+	44674411	44675268	858	238	sense-overlappin	NM_001003941.2	OGDH
circRNA_05349	0.020575025	0	0.04361729	0	0	0	0	0	0	0	0	0	hsa_circ_0001701	NC_000007.14	+	44674411	44676149	1739	418	sense-overlappin	NM_001003941.2	OGDH
circRNA_05350	0	0	0.032712968	0	0	0	0	0	0	0	0	0	hsa_circ_0080069	NC_000007.14	+	44674411	44681848	7438	547	sense-overlappin	NM_001165036.1	OGDH
circRNA_05351	0.020575025	0	0	0	0	0	0	0	0	0	0	0	hsa_circ_0008791	NC_000007.14	-	44840899	44843354	2456	192	sense-overlappin	NM_138635.3	H2AFV
circRNA_05352	0	0	0	0	0	0.020712909	0	0	0	0	0	0.0206364	hsa_circ_0007145	NC_000007.14	+	45063918	45064646	729	268	sense-overlappin	NR_030770.1	
circRNA_05353	0	0	0	0	0	0	0	0	0	0.020172483	0	0	-	NC_000007.14	-	45747215	45751646	4432	4432	intergenic		
circRNA_05354	0.072012586	0	0	0	0	0	0	0	0	0	0	0	hsa_circ_0006229	NC_000007.14	-	47344755	47346356	1602	369	sense-overlappin	XM_011515484.1	TNS3
circRNA_05355	0	0	0	0	0	0	0.030148028	0	0	0	0	0	hsa_circ_0008729	NC_000007.14	-	47435282	47442055	6774	399	sense-overlappin	XM_011515483.1	TNS3
circRNA_05356	0	0	0.021808645	0	0	0	0	0	0	0	0.030382845	0	-	NC_000007.14	+	48219354	48249351	29998	1718	sense-overlappin	XM_011515149.1	ABCA13
circRNA_05357	0	0	0	0	0	0	0	0.020486295	0	0	0	0	-	NC_000007.14	+	48410520	48412583	2064	389	sense-overlappin	XM_011515141.1	ABCA13
circRNA_05358	0.030862537	0	0	0	0.03009467	0.020712909	0	0	0	0	0	0	hsa_circ_0001707	NC_000007.14	+	48502126	48502552	427	427	intronic	XM_011515140.1	ABCA13
circRNA_05359	0	0.08187987	0	0.0515905	0	0.031069364	0.030148028	0.040972591	0.020254528	0.090776173	0	0.0309546	hsa_circ_0001708	NC_000007.14	+	50319048	50327757	8710	174	sense-overlappin	XM_011515078.1	IKZF1
circRNA_05360	0	0	0	0	0	0	0	0	0	0	0	0.0206364	-	NC_000007.14	+	50334358	50334492	135	135	intronic	XM_011515078.1	IKZF1

circRNA_05361	0	0	0	0	0	0.020712909	0	0	0	0	0	0	-	NC_000007.14	+	50376533	50382707	6175	429	sense-overlappin	XM_011515067.1	IKZF1
circRNA_05362	0	0	0	0	0	0	0	0	0	0	0.02025523	0	-	NC_000007.14	-	50495350	50504059	8710	230	sense-overlappin	XM_011515161.1	DDC
circRNA_05363	0	0	0	0	0	0.062138728	0	0	0	0	0	0	hsa_circ_0080209	NC_000007.14	-	50669722	50703908	34187	586	sense-overlappin	XM_011515321.1	GRB10
circRNA_05364	0	0	0	0	0	0	0.020098685	0	0	0	0	0	hsa_circ_0080210	NC_000007.14	-	50669722	50705323	35602	770	sense-overlappin	XM_011515321.1	GRB10
circRNA_05365	0	0	0.021808645	0	0	0	0	0	0	0	0	0	-	NC_000007.14	-	50669722	50756056	86335	1486	sense-overlappin	XM_011515321.1	GRB10
circRNA_05366	0	0	0.021808645	0	0	0	0	0	0	0	0	0	hsa_circ_0002033	NC_000007.14	-	50732272	50762014	29743	24039	sense-overlappin	XM_011515318.1	GRB10
circRNA_05367	0	0	0.021808645	0	0	0	0	0	0	0	0	0	hsa_circ_0002650	NC_000007.14	-	50779135	50780736	1602	405	sense-overlappin	XM_011515303.1	GRB10
circRNA_05368	0	0	0.021808645	0	0	0	0	0	0	0	0	0	hsa_circ_0080217	NC_000007.14	-	51190850	51193589	2740	440	sense-overlappin	XM_005271750.1	COBL
circRNA_05369	0	0	0	0	0	0	0.030148028	0	0	0	0	0	-	NC_000007.14	+	55911616	55923699	12084	762	sense-overlappin	NM_182633.2	ZNF713
circRNA_05370	0.041150049	0	0.04361729	0	0	0.062138728	0.050246713	0	0	0	0	0	hsa_circ_0001709	NC_000007.14	+	55982210	55983868	1659	212	sense-overlappin	NM_001483.2	GBAS
circRNA_05371	0	0.020469968	0	0.0515905	0	0	0	0	0.030381792	0	0	0.0412728	hsa_circ_0080250	NC_000007.14	+	55982210	55984878	2669	244	sense-overlappin	NM_001483.2	GBAS
circRNA_05372	0	0	0	0	0	0	0	0	0	0.020172483	0	0	hsa_circ_0080251	NC_000007.14	+	55982210	55994988	12779	339	sense-overlappin	NM_001483.2	GBAS
circRNA_05373	0	0	0	0	0	0	0	0	0	0.030258724	0	0	hsa_circ_0080285	NC_000007.14	-	56083278	56083715	438	230	sense-overlappin	NM_001258459.1	PHKG1
circRNA_05374	0.020575025	0	0	0	0	0	0	0	0	0	0	0	-	NC_000007.14	+	64068891	64077406	8516	354	sense-overlappin	NM_001159522.1	ZNF727
circRNA_05375	0	0	0	0	0	0	0	0	0.030381792	0	0	0	-	NC_000007.14	-	64075475	64076528	1054	1054	antisense	XR_927470.1	
circRNA_05376	0	0.020469968	0.065425935	0	0	0	0	0	0	0.030258724	0	0	hsa_circ_0008543	NC_000007.14	+	64336259	64336982	724	223	sense-overlappin	NM_001294255.1	ZNF736
circRNA_05377	0	0	0.163564838	0	0	0	0	0	0	0	0	0	-	NC_000007.14	-	64521064	64522500	1437	1437	exonic	XR_428175.2	
circRNA_05378	0	0	0	0	0.02006311	0	0	0	0	0	0	0	-	NC_000007.14	-	64521064	64543802	22739	1533	sense-overlappin	XR_428175.2	
circRNA_05379	0	0	0.065425935	0.0206362	0	0.020712909	0	0	0.020254528	0	0	0	-	NC_000007.14	-	64521064	64544432	23369	1660	sense-overlappin	XR_428175.2	
circRNA_05380	0	0	0.021808645	0	0	0	0	0	0	0	0	0	-	NC_000007.14	-	64535819	64544432	8614	8111	sense-overlappin	XR_428175.2	
circRNA_05381	0	0	0.021808645	0	0	0	0	0	0	0	0	0	-	NC_000007.14	-	64541730	64544432	2703	2200	sense-overlappin	XR_428175.2	
circRNA_05382	0.102875123	0.092114854	0.119947548	0.113499	0	0.093208092	0.040197371	0.030729443	0.081018111	0.14120738	0.08102092	0.0412728	hsa_circ_0002566	NC_000007.14	-	64543707	64544432	726	223	sense-overlappin	XR_428175.2	
circRNA_05383	0	0	0.021808645	0.0309543	0	0	0	0	0	0	0	0	-	NC_000007.14	+	64814918	64817335	2418	1887	sense-overlappin	NM_001160183.2	ZNF138
circRNA_05384	0	0	0	0	0	0.020712909	0	0	0	0	0	0.0309546	hsa_circ_0080302	NC_000007.14	+	65066253	65067050	798	798	intergenic		
circRNA_05385	0	0	0	0	0.04012623	0	0	0	0	0	0	0	hsa_circ_0001712	NC_000007.14	-	65964323	65967907	3585	313	sense-overlappin	XM_011516114.1	GUSB
circRNA_05386	0	0.040939935	0.065425935	0.0722267	0.08025246	0.062138728	0.020098685	0.051215739	0.111399902	0.090776173	0.06076569	0.0309546	hsa_circ_0004604	NC_000007.14	+	66127704	66134374	6671	353	sense-overlappin	NR_024548.1	
circRNA_05387	0	0	0	0.0412724	0	0	0	0	0	0	0	0	hsa_circ_0080340	NC_000007.14	+	66127704	66145500	17797	411	sense-overlappin	NR_024548.1	
circRNA_05388	0	0	0	0	0.03009467	0	0	0	0	0	0	0	hsa_circ_0080342	NC_000007.14	+	66130744	66134374	3631	194	sense-overlappin	NM_014478.4	CRCP
circRNA_05389	0.020575025	0	0.032712968	0	0.04012623	0.041425819	0	0	0	0.040344966	0	0	hsa_circ_0007672	NC_000007.14	+	66240325	66241270	946	946	exonic	NM_003596.3	TPST1
circRNA_05390	0.020575025	0	0.021808645	0.0412724	0	0	0	0	0	0.020172483	0	0	-	NC_000007.14	+	66240325	66248646	8322	8322	sense-overlappin	NM_003596.3	TPST1
circRNA_05391	0.041150049	0	0.08723458	0.0722267	0.03009467	0.051782273	0.040197371	0	0.050636319	0.151293621	0.02025523	0.0412728	hsa_circ_0006041	NC_000007.14	+	66240325	66286709	46385	1145	sense-overlappin	NM_003596.3	TPST1
circRNA_05392	0.030862537	0	0	0	0	0.020712909	0	0	0	0	0	0	hsa_circ_0006365	NC_000007.14	+	66286511	66286709	199	199	exonic	NM_003596.3	TPST1
circRNA_05393	0.020575025	0	0	0	0	0	0	0	0	0	0	0	-	NC_000007.14	+	66286511	66356871	70361	297	sense-overlappin	NM_003596.3	TPST1
circRNA_05394	0	0	0	0	0	0	0	0	0.020254528	0	0	0	-	NC_000007.14	-	66400923	66407364	6442	6442	intergenic		
circRNA_05395	0	0	0	0	0	0	0.030148028	0	0	0	0	0	-	NC_000007.14	-	66427949	66430017	2069	2069	intergenic		
circRNA_05396	0	0	0.021808645	0	0.05015779	0	0	0	0	0	0	0	-	NC_000007.14	-	66427949	66439304	11356	11356	intergenic		
circRNA_05397	0	0	0	0.0206362	0	0	0	0	0.020254528	0.030258724	0.02025523	0	-	NC_000007.14	-	66450071	66485153	35083	35083	intergenic		
circRNA_05398	0	0.030704951	0	0	0	0	0	0	0	0	0	0	-	NC_000007.14	-	66481541	66490107	8567	8567	intergenic		
circRNA_05399	0	0	0.021808645	0	0	0	0	0	0	0	0	0	-	NC_000007.14	-	66560579	66576932	16354	16354	intergenic		
circRNA_05400	0	0	0	0.0206362	0.05015779	0	0	0	0	0.020172483	0	0	-	NC_000007.14	-	66573385	66576929	3545	3545	intergenic		
circRNA_05401	0.082300098	0.102349838	0	0	0.04012623	0.041425819	0.040197371	0	0	0.020172483	0.02025523	0.0412728	hsa_circ_0007417	NC_000007.14	-	66573385	66576932	3548	3548	intergenic		
circRNA_05402	0	0	0	0	0	0	0	0	0.020254528	0	0	0	-	NC_000007.14	+	66743095	66743717	623	623	intronic	NM_001287060.1	RABGEF1
circRNA_05403	0	0	0	0.0206362	0	0	0	0	0	0	0	0	hsa_circ_0006032	NC_000007.14	+	66771883	66775393	3511	363	sense-overlappin	NM_001287060.1	RABGEF1
circRNA_05404	0	0	0.021808645	0	0.04012623	0.031069364	0.020098685	0	0	0.020172483	0	0.0206364	hsa_circ_0007992	NC_000007.14	+	66797374	66805396	8023	482	sense-overlappin	NM_001287060.1	RABGEF1
circRNA_05405	0	0	0	0	0.02006311	0	0	0	0	0	0	0	hsa_circ_0005592	NC_000007.14	+	66808886	66810847	1962	1962	exonic	NM_001287060.1	RABGEF1
circRNA_05406	0	0	0	0.0309543	0	0	0	0	0	0	0	0	-	NC_000007.14	+	66848489	66880864	32376	32376	intergenic		
circRNA_05407	0	0	0	0	0	0.031069364	0	0	0	0	0	0	-	NC_000007.14	+	66950952	66953369	2418	328	sense-overlappin	NM_017994.4	TMEM248
circRNA_05408	0.061725074	0.020469968	0	0.0412724	0	0	0.020098685	0.030729443	0	0	0.06076569	0	-	NC_000007.14	-	66991137	66994341	3205	496	sense-overlappin	NM_016038.2	SBDS
circRNA_05409	0	0	0	0	0	0.051782273	0.060296056	0.020486295	0.030381792	0.090776173	0.030382845	0	hsa_circ_0003203	NC_000007.14	-	66993217	66994341	1125	331	sense-overlappin	NM_016038.2	SBDS
circRNA_05410	0	0	0	0	0	0	0	0	0	0.020172483	0	0	-	NC_000007.14	+	67055835	67067403	11569	172	sense-overlappin	NM_018264.3	TYW1
circRNA_05411	0	0.020469968	0.021808645	0	0	0	0	0	0	0	0	0	hsa_circ_0003228	NC_000007.14	+	67098541	67117618	19078	314	sense-overlappin	NM_018264.3	TYW1
circRNA_05412	0	0	0	0.0206362	0	0	0	0	0	0	0	0	hsa_circ_0080414	NC_000007.14	+	69899286	69899498	213	213	exonic	XM_011516010.1	AUTS2
circRNA_05413	0.020575025	0	0	0	0	0	0	0	0	0.030258724	0	0	hsa_circ_0080420	NC_000007.14	+	70762870	70771644	8775	1109	sense-overlappin	XM_011516010.1	AUTS2
circRNA_05414	0	0	0	0	0	0	0	0	0	0.040344966	0	0	-	NC_000007.14	+	70762870	70777174	14305	1283	sense-overlappin	XM_011516010.1	AUTS2

circRNA_05415	0	0	0	0	0	0	0	0	0	0	0.02025523	0	-	NC_000007.14	+	71196932	71276721	79790	79790	intronic	XM_011516469.1	WBSCR17
circRNA_05416	0	0	0	0	0	0	0	0	0	0.030258724	0	0	hsa_circ_0080436	NC_000007.14	-	72616672	72628997	12326	279	sense-overlappin	XM_011516233.1	TYW1B
circRNA_05417	0	0	0.021808645	0	0	0	0	0	0	0	0	0	-	NC_000007.14	-	72616672	72639862	23191	11144	sense-overlappin	XM_011516233.1	TYW1B
circRNA_05418	0	0	0	0	0	0.020712909	0	0	0	0	0	0	hsa_circ_0003123	NC_000007.14	-	72728822	72744601	15780	228	sense-overlappin	XM_011516233.1	TYW1B
circRNA_05419	0	0	0	0	0	0	0	0.020486295	0	0.040344966	0	0.051591	-	NC_000007.14	-	72807066	72807356	291	291	exonic	NM_001145440.2	TYW1B
circRNA_05420	0.020575025	0	0	0	0	0	0	0	0.040509055	0.030258724	0.030382845	0	hsa_circ_0005878	NC_000007.14	+	72830692	72831798	1107	1107	intergenic		
circRNA_05421	0	0	0	0	0	0	0	0.040972591	0	0	0	0	-	NC_000007.14	+	72830692	72833928	3237	3237	intergenic		
circRNA_05422	0	0	0	0	0.02006311	0	0	0	0	0	0	0	hsa_circ_0006688	NC_000007.14	+	72830751	72831798	1048	1048	intergenic		
circRNA_05423	0	0	0	0	0.02006311	0	0	0	0	0	0	0	hsa_circ_0008708	NC_000007.14	+	72831602	72831798	197	197	intergenic		
circRNA_05424	0	0	0	0	0	0	0	0	0	0.030258724	0	0	hsa_circ_0080442	NC_000007.14	+	72831602	72833928	2327	2327	intergenic		
circRNA_05425	0	0	0.032712968	0	0	0	0	0	0	0	0	0	-	NC_000007.14	+	72890627	72901128	10502	10323	sense-overlappin	NM_001257190.2	POM121
circRNA_05426	0	0	0	0	0	0	0.020098685	0	0	0	0	0	-	NC_000007.14	+	72890627	72913828	23202	369	sense-overlappin	NM_001257190.2	POM121
circRNA_05427	0	0	0	0	0	0	0	0	0.020254528	0	0	0	-	NC_000007.14	-	73447264	73459718	12455	595	sense-overlappin	NM_032408.3	BAZ1B
circRNA_05428	0	0	0	0	0	0	0	0	0	0	0.02025523	0	-	NC_000007.14	-	73459536	73466401	6866	566	sense-overlappin	NM_032408.3	BAZ1B
circRNA_05429	0	0	0.076330258	0.0412724	0.04012623	0.031069364	0.020098685	0.020486295	0.030381792	0.030258724	0	0	hsa_circ_0004670	NC_000007.14	-	73459536	73470483	10948	839	sense-overlappin	NM_032408.3	BAZ1B
circRNA_05430	0	0	0.021808645	0.0206362	0.03009467	0	0	0	0	0	0	0	hsa_circ_0080483	NC_000007.14	-	73462922	73470483	7562	656	sense-overlappin	NM_032408.3	BAZ1B
circRNA_05431	0	0	0	0	0.03009467	0	0	0	0	0	0	0	hsa_circ_0006627	NC_000007.14	-	73465439	73470483	5045	478	sense-overlappin	NM_032408.3	BAZ1B
circRNA_05432	0	0	0.021808645	0	0.06018934	0	0.050246713	0	0	0.030258724	0	0	hsa_circ_0003866	NC_000007.14	-	73469517	73470483	967	273	sense-overlappin	NM_032408.3	BAZ1B
circRNA_05433	0	0	0	0	0.02006311	0	0	0	0	0	0	0	hsa_circ_0080496	NC_000007.14	-	73539882	73543644	3763	268	sense-overlappin	NR_036682.1	
circRNA_05434	0.020575025	0.030704951	0.119947548	0.0515905	0.05015779	0.072495183	0.020098685	0.040972591	0	0.030258724	0.030382845	0.0309546	hsa_circ_0005588	NC_000007.14	+	73686636	73687095	460	276	sense-overlappin	NM_001202560.2	WBSCR22
circRNA_05435	0	0	0.032712968	0	0	0	0	0	0	0	0	0	hsa_circ_0009012	NC_000007.14	-	73700378	73700931	554	309	sense-overlappin	XM_011516541.1	STX1A
circRNA_05436	0	0	0	0	0	0	0	0	0	0	0.02025523	0	-	NC_000007.14	+	74105875	74106243	369	273	sense-overlappin	NM_002314.3	LIMK1
circRNA_05437	0	0.040939935	0	0	0	0	0	0	0	0	0	0	hsa_circ_0080543	NC_000007.14	+	74219744	74220734	991	198	sense-overlappin	XM_011516558.1	LAT2
circRNA_05438	0	0	0	0	0	0	0	0	0	0.020172483	0	0	hsa_circ_0007842	NC_000007.14	-	74239938	74246763	6826	361	sense-overlappin	NM_001278791.1	RFC2
circRNA_05439	0	0.020469968	0	0	0	0	0	0	0	0	0	0	-	NC_000007.14	+	74295580	74297030	1451	1451	intronic	NM_003388.4	CLIP2
circRNA_05440	0.030862537	0	0.08723458	0	0.04012623	0	0	0	0	0	0.08102092	0	hsa_circ_0002755	NC_000007.14	+	74356410	74357477	1068	412	sense-overlappin	NM_003388.4	CLIP2
circRNA_05441	0	0	0	0	0.04012623	0	0	0	0	0	0	0	-	NC_000007.14	+	74539879	74545809	5931	204	sense-overlappin	NM_001199207.1	GTF2IRD1
circRNA_05442	0	0	0.021808645	0	0	0	0	0	0	0.040344966	0	0	-	NC_000007.14	+	74689124	74716950	27827	882	sense-overlappin	NM_001163636.2	GTF2I
circRNA_05443	0.041150049	0	0.021808645	0.0206362	0.02006311	0	0	0	0.050636319	0.040344966	0.030382845	0	hsa_circ_0006944	NC_000007.14	+	74705164	74716950	11787	291	sense-overlappin	NM_001163636.2	GTF2I
circRNA_05444	0	0.030704951	0.04361729	0.0825448	0.04012623	0.031069364	0	0.020486295	0.030381792	0	0	0.0206364	hsa_circ_0005513	NC_000007.14	+	74705164	74718941	13778	357	sense-overlappin	NM_032999.3	GTF2I
circRNA_05445	0	0	0	0	0	0	0	0	0	0	0	0.0206364	hsa_circ_0080668	NC_000007.14	-	75052711	75057616	4906	348	sense-overlappin	NM_148842.2	WBSCR16
circRNA_05446	0	0	0	0	0	0.020712909	0	0	0	0	0	0	-	NC_000007.14	-	75474704	75484300	9597	9418	sense-overlappin	NM_001099415.2	POM121C
circRNA_05447	0	0	0	0	0.02006311	0	0	0	0	0	0	0	hsa_circ_0080710	NC_000007.14	-	75548891	75554526	5636	443	sense-overlappin	XM_005250304.2	HIP1
circRNA_05448	0	0	0	0.0206362	0	0	0	0	0	0	0	0	hsa_circ_0080712	NC_000007.14	-	75553453	75554526	1074	332	sense-overlappin	XM_005250304.2	HIP1
circRNA_05449	0	0	0	0	0.03009467	0	0	0	0	0	0	0	-	NC_000007.14	-	76099440	76101414	1975	1975	intergenic		
circRNA_05450	0	0	0	0	0	0	0	0	0	0.020172483	0	0	-	NC_000007.14	+	77273694	77280653	6960	746	sense-overlappin	NM_020879.2	CCDC146
circRNA_05451	0.020575025	0	0.032712968	0	0	0	0	0	0	0.020172483	0	0	-	NC_000007.14	+	77274482	77280653	6172	650	sense-overlappin	NM_020879.2	CCDC146
circRNA_05452	0	0.071644886	0	0	0	0	0	0	0.020254528	0	0	0.072227401	-	NC_000007.14	+	77292952	77294938	1987	525	sense-overlappin	NM_020879.2	CCDC146
circRNA_05453	0	0	0	0	0	0	0	0	0.020254528	0	0	0	-	NC_000007.14	-	77360824	77375101	14278	286	sense-overlappin	XM_011516330.1	GSAP
circRNA_05454	0	0	0	0	0	0	0.020098685	0	0	0	0	0	-	NC_000007.14	-	77370443	77371516	1074	1074	intronic	XM_011516330.1	GSAP
circRNA_05455	0.030862537	0.061409903	0	0.0309543	0	0.031069364	0	0	0	0	0	0.0206364	hsa_circ_0080831	NC_000007.14	-	77376848	77404615	27768	555	sense-overlappin	NM_017439.3	GSAP
circRNA_05456	0	0	0	0.0206362	0.02006311	0	0	0	0	0	0	0	-	NC_000007.14	-	77396982	77404615	7634	181	sense-overlappin	NM_017439.3	GSAP
circRNA_05457	0	0	0	0	0	0.020712909	0	0	0	0	0	0.051591	-	NC_000007.14	-	77396982	77406105	9124	258	sense-overlappin	NM_017439.3	GSAP
circRNA_05458	0	0	0.04361729	0	0	0	0.020098685	0	0.020254528	0	0	0	-	NC_000007.14	+	77571078	77585581	14504	321	sense-overlappin	NM_002835.3	PTPN12
circRNA_05459	0	0	0.04361729	0	0	0	0.020098685	0	0	0	0.02025523	0	hsa_circ_0008901	NC_000007.14	+	77571078	77592256	21179	393	sense-overlappin	NM_002835.3	PTPN12
circRNA_05460	0	0	0	0	0	0	0	0	0	0.040344966	0	0	hsa_circ_0080835	NC_000007.14	+	77571078	77600806	29729	596	sense-overlappin	NM_002835.3	PTPN12
circRNA_05461	0	0	0.021808645	0	0	0	0	0	0	0.020172483	0	0	-	NC_000007.14	+	77571078	77611046	39969	840	sense-overlappin	NM_002835.3	PTPN12
circRNA_05462	0	0	0	0	0.02006311	0	0	0.030729443	0	0	0	0	hsa_circ_0008139	NC_000007.14	+	77581427	77583650	2224	173	sense-overlappin	NM_002835.3	PTPN12
circRNA_05463	0	0	0	0	0.03009467	0	0	0	0	0	0	0	-	NC_000007.14	+	77581427	77585581	4155	212	sense-overlappin	NM_002835.3	PTPN12
circRNA_05464	0.020575025	0	0.065425935	0	0.04012623	0	0	0.020486295	0	0	0	0	hsa_circ_0002458	NC_000007.14	+	77581427	77592256	10830	284	sense-overlappin	NM_002835.3	PTPN12
circRNA_05465	0	0	0.021808645	0	0.02006311	0	0	0	0	0	0	0	hsa_circ_0005197	NC_000007.14	+	77581427	77597901	16475	344	sense-overlappin	NM_002835.3	PTPN12
circRNA_05466	0	0	0	0	0.02006311	0	0	0	0	0	0	0	hsa_circ_0007411	NC_000007.14	+	77581427	77600806	19380	487	sense-overlappin	NM_002835.3	PTPN12
circRNA_05467	0.092587611	0.040939935	0.08723458	0.0619086	0.15047336	0.051782273	0.080394741	0.030729443	0.040509055	0.110948655	0.04051046	0.0206364	hsa_circ_0003764	NC_000007.14	+	77585543	77600806	15264	314	sense-overlappin	NM_002835.3	PTPN12
circRNA_05468	0	0	0	0	0.02006311	0	0	0	0	0	0	0	-	NC_000007.14	+	77585543	77611046	25504	558	sense-overlappin	NM_002835.3	PTPN12
circRNA_05469	0.030862537	0	0.021808645	0	0	0	0	0	0	0	0	0	hsa_circ_0080849	NC_000007.14	+	77607235	77627675	20441	1301	sense-overlappin	NM_002835.3	PTPN12
circRNA_05470	0	0	0	0	0.04012623	0	0	0	0	0	0	0	hsa_circ_0080850	NC_000007.14	+	77607235	77632425	25191	1379	sense-overlappin	NM_002835.3	PTPN12

circRNA_05471	0.020575025	0	0	0	0.02006311	0	0	0	0	0.020172483	0	0	hsa_circ_0080851	NC_000007.14	+	77607235	77635849	28615	1447	sense-overlappin	NM_002835.3	PTPN12
circRNA_05472	0.020575025	0	0	0	0	0	0.030148028	0	0	0	0	0	hsa_circ_0001720	NC_000007.14	+	77749424	77758078	8655	8655	sense-overlappin	NM_198467.2	RSBN1L
circRNA_05473	0	0	0.032712968	0.0309543	0.02006311	0.020712909	0.020098685	0	0	0	0	0	-	NC_000007.14	+	77749424	77765632	16209	779	sense-overlappin	NM_198467.2	RSBN1L
circRNA_05474	0	0	0	0	0	0	0	0.020486295	0.020254528	0	0	0	-	NC_000007.14	+	77765495	77773314	7820	449	sense-overlappin	NM_198467.2	RSBN1L
circRNA_05475	0	0.020469968	0	0	0	0	0	0	0	0	0	0	-	NC_000007.14	+	77765495	77778814	13320	843	sense-overlappin	NM_198467.2	RSBN1L
circRNA_05476	0	0.061409903	0.032712968	0.0412724	0.04012623	0.020712909	0	0.030729443	0	0	0.030382845	0	-	NC_000007.14	+	77778338	77778814	477	394	sense-overlappin	NM_198467.2	RSBN1L
circRNA_05477	0	0	0	0.0515905	0	0	0	0	0	0	0	0	hsa_circ_0004331	NC_000007.14	+	80199225	80212869	13645	571	sense-overlappin	NM_002069.5	GNAI1
circRNA_05478	0	0	0	0.0309543	0	0	0	0	0	0	0	0	hsa_circ_0002714	NC_000007.14	-	80789306	80805758	16453	816	sense-overlappin	XM_005250113.1	SEMA3C
circRNA_05479	0.051437562	0	0.065425935	0.1238171	0	0.082851637	0	0.020486295	0.030381792	0	0.02025523	0	hsa_circ_0004365	NC_000007.14	-	80789306	80810701	21396	907	sense-overlappin	XM_005250113.1	SEMA3C
circRNA_05480	0	0	0	0	0	0	0.020098685	0	0	0	0	0	hsa_circ_0080910	NC_000007.14	-	80818299	80828745	10447	344	sense-overlappin	XM_005250113.1	SEMA3C
circRNA_05481	0	0	0	0	0	0.020712909	0	0	0	0	0	0	-	NC_000007.14	-	81968887	81982627	13741	501	sense-overlappin	XM_006716121.2	CACNA2D1
circRNA_05482	0	0	0.021808645	0	0	0	0	0	0	0	0	0	-	NC_000007.14	-	82949476	82956935	7460	7095	sense-overlappin	NM_033026.5	PCLO
circRNA_05483	0	0	0.065425935	0	0	0	0	0	0	0	0	0	hsa_circ_0080941	NC_000007.14	-	83134250	83135656	1407	1407	exonic	NM_033026.5	PCLO
circRNA_05484	0.030862537	0	0	0	0	0	0	0	0	0	0	0	-	NC_000007.14	-	84129123	84194668	65546	415	sense-overlappin	XM_006715839.2	SEMA3A
circRNA_05485	0	0	0	0.0412724	0	0	0	0	0	0	0	0	-	NC_000007.14	-	85036889	85068284	31396	696	sense-overlappin	XM_011515961.1	SEMA3D
circRNA_05486	0	0	0.152660515	0	0	0	0	0	0	0	0	0	hsa_circ_0005515	NC_000007.14	+	87163495	87174669	11175	650	sense-overlappin	NM_001142327.1	DMTF1
circRNA_05487	0.041150049	0	0	0.0412724	0	0	0	0	0	0	0	0	hsa_circ_0080950	NC_000007.14	+	87163495	87185980	22486	1332	sense-overlappin	NM_001142327.1	DMTF1
circRNA_05488	0	0.020469968	0	0	0	0	0	0	0	0	0	0	hsa_circ_0080960	NC_000007.14	-	87439667	87440402	736	375	sense-overlappin	XM_011516315.1	ABCB4
circRNA_05489	0	0	0	0	0	0.051782273	0.050246713	0	0	0.030258724	0	0	hsa_circ_0004674	NC_000007.14	+	87964634	87978412	13779	210	sense-overlappin	XM_011516324.1	ADAM22
circRNA_05490	0	0	0	0	0	0	0	0	0	0	0.02025523	0	-	NC_000007.14	+	90286281	90304820	18540	728	sense-overlappin	NM_001039706.2	CFAP69
circRNA_05491	0	0	0	0	0	0	0	0	0	0.020172483	0	0	hsa_circ_0080973	NC_000007.14	+	90354458	90374354	19897	364	sense-overlappin	NM_033107.3	GTPBP10
circRNA_05492	0	0	0	0.0309543	0.03009467	0	0	0	0.030381792	0	0	0	hsa_circ_0007090	NC_000007.14	+	90372155	90378211	6057	313	sense-overlappin	NM_001042717.2	GTPBP10
circRNA_05493	0	0	0	0	0	0	0	0	0	0	0.02025523	0	-	NC_000007.14	+	90372155	90393458	21304	9004	sense-overlappin	NM_001042717.2	GTPBP10
circRNA_05494	0	0	0.021808645	0	0.02006311	0	0	0.030729443	0	0	0	0	hsa_circ_0080975	NC_000007.14	+	90374302	90378211	3910	239	sense-overlappin	NM_001042717.2	GTPBP10
circRNA_05495	0	0	0	0	0.03009467	0	0	0	0	0	0	0	-	NC_000007.14	+	90374302	90393458	19157	8930	sense-overlappin	NM_001042717.2	GTPBP10
circRNA_05496	0.030862537	0.030704951	0	0	0.04012623	0.051782273	0	0.040972591	0	0.040344966	0.030382845	0	hsa_circ_0001721	NC_000007.14	+	90726567	90726812	246	246	exonic	NM_001287135.1	CDK14
circRNA_05497	0	0	0	0	0	0	0.020098685	0	0	0	0	0	-	NC_000007.14	+	90726567	90735229	8663	8663	sense-overlappin	NM_001287135.1	CDK14
circRNA_05498	0	0	0	0	0	0.020712909	0	0.030729443	0	0.020172483	0	0	hsa_circ_0004015	NC_000007.14	+	90726567	90747775	21209	341	sense-overlappin	NM_001287135.1	CDK14
circRNA_05499	0	0	0	0	0	0.031069364	0	0	0.020254528	0	0.02025523	0	hsa_circ_0008645	NC_000007.14	+	90726567	90790652	64086	421	sense-overlappin	NM_001287135.1	CDK14
circRNA_05500	0	0	0	0	0	0.020712909	0	0.040972591	0	0	0	0	hsa_circ_0001722	NC_000007.14	+	90747681	90790652	42972	175	sense-overlappin	NM_001287135.1	CDK14
circRNA_05501	0	0	0	0.0309543	0	0	0	0	0	0	0	0	hsa_circ_0080986	NC_000007.14	+	90917601	90984241	66641	339	sense-overlappin	NM_001287135.1	CDK14
circRNA_05502	0	0	0	0	0.06018934	0	0	0	0	0	0	0	-	NC_000007.14	+	91493395	91496646	3252	3252	intergenic		
circRNA_05503	0	0	0	0	0.02006311	0	0	0	0	0	0	0	-	NC_000007.14	+	91941095	92017102	76008	3878	sense-overlappin	XM_011515720.1	AKAP9
circRNA_05504	0	0	0.021808645	0	0	0	0	0	0	0	0	0	-	NC_000007.14	+	91970473	91980333	9861	339	sense-overlappin	XM_011515720.1	AKAP9
circRNA_05505	0	0	0.04361729	0	0	0	0	0	0	0	0	0.0206364	-	NC_000007.14	+	91973711	91980958	7248	928	sense-overlappin	XM_011515711.1	AKAP9
circRNA_05506	0	0.040939935	0.04361729	0	0.02006311	0	0	0	0	0	0.02025523	0	-	NC_000007.14	+	91992885	92003235	10351	2913	sense-overlappin	XM_011515711.1	AKAP9
circRNA_05507	0	0	0	0	0.02006311	0	0	0	0	0	0	0	-	NC_000007.14	+	92077696	92080152	2457	1254	sense-overlappin	XM_011515711.1	AKAP9
circRNA_05508	0	0.030704951	0	0	0.02006311	0	0	0	0	0	0	0	-	NC_000007.14	+	92096689	92101056	4368	1368	sense-overlappin	XM_011515711.1	AKAP9
circRNA_05509	0	0.051174919	0	0.0206362	0.03009467	0	0	0	0.020254528	0	0	0	hsa_circ_0002085	NC_000007.14	-	92123120	92131872	8753	894	sense-overlappin	NM_000786.3	CYP51A1
circRNA_05510	0	0.030704951	0	0	0	0	0	0	0	0	0	0	hsa_circ_0081001	NC_000007.14	-	92126253	92127631	1379	302	sense-overlappin	NM_000786.3	CYP51A1
circRNA_05511	0	0	0	0.0412724	0	0	0	0	0	0	0	0	-	NC_000007.14	-	92201307	92214777	13471	579	sense-overlappin	NM_004912.3	KRIT1
circRNA_05512	0	0	0	0	0	0.020712909	0	0	0	0	0	0	-	NC_000007.14	-	92213195	92214777	1583	462	sense-overlappin	NM_004912.3	KRIT1
circRNA_05513	0	0.020469968	0	0.0515905	0.06018934	0.020712909	0	0	0.040509055	0.080689931	0	0.0206364	hsa_circ_0081006	NC_000007.14	-	92213195	92226682	13488	1036	sense-overlappin	NM_004912.3	KRIT1
circRNA_05514	0.020575025	0	0	0	0	0.020712909	0	0	0	0	0.02025523	0	-	NC_000007.14	-	92221902	92222978	1077	309	sense-overlappin	NM_004912.3	KRIT1
circRNA_05515	0	0	0	0	0	0	0	0.020486295	0	0	0	0	-	NC_000007.14	-	92225720	92242137	16418	1256	sense-overlappin	NM_004912.3	KRIT1
circRNA_05516	0	0	0.032712968	0	0.10031557	0.020712909	0.040197371	0	0.020254528	0.080689931	0.050638075	0.0309546	-	NC_000007.14	+	92294889	92307656	12768	576	sense-overlappin	XM_005250458.2	ANKIB1
circRNA_05517	0.030862537	0.020469968	0	0.0412724	0.03009467	0.041425819	0.040197371	0	0	0	0	0.0206364	hsa_circ_0001723	NC_000007.14	+	92294889	92319512	24624	759	sense-overlappin	XM_005250458.2	ANKIB1
circRNA_05518	0.082300098	0.08187987	0.34893832	0.1754076	0.21066271	0.082851637	0.080394741	0.133160921	0.070890847	0.161379862	0.04051046	0.092863801	-	NC_000007.14	+	92294889	92327900	33012	877	sense-overlappin	XM_005250458.2	ANKIB1
circRNA_05519	0	0.020469968	0.032712968	0	0	0	0	0	0	0	0	0	hsa_circ_0002497	NC_000007.14	+	92294889	92333720	38832	6697	sense-overlappin	XM_005250458.2	ANKIB1
circRNA_05520	0	0	0.076330258	0.0515905	0.03009467	0	0.030148028	0.020486295	0	0	0.02025523	0	hsa_circ_0002744	NC_000007.14	+	92294889	92352642	57754	1487	sense-overlappin	XM_005250458.2	ANKIB1
circRNA_05521	0	0	0.065425935	0	0	0	0.020098685	0	0.020254528	0	0	0	hsa_circ_0081011	NC_000007.14	+	92294889	92362273	67385	1576	sense-overlappin	XM_005250458.2	ANKIB1
circRNA_05522	0	0	0	0	0	0	0	0	0	0.020172483	0	0	hsa_circ_0081012	NC_000007.14	+	92294889	92371607	76719	1707	sense-overlappin	XM_005250458.2	ANKIB1
circRNA_05523	0.020575025	0	0	0	0	0	0	0	0	0.040344966	0	0	hsa_circ_0009112	NC_000007.14	+	92343024	92352642	9619	610	sense-overlappin	XM_005250458.2	ANKIB1
circRNA_05524	0	0	0	0	0	0	0	0.020486295	0	0	0	0	-	NC_000007.14	+	92350950	92362273	11324	401	sense-overlappin	XM_005250458.2	ANKIB1
circRNA_05525	0	0.030704951	0	0.0515905	0	0	0	0.030729443	0.020254528	0	0	0	-	NC_000007.14	+	92457552	92492286	34735	5060	sense-overlappin	XR_927498.1	

circRNA_05526	0	0	0	0	0	0	0	0	0	0	0.02025523	0	hsa_circ_0081024	NC_000007.14	-	92489293	92489911	619	329	sense-overlappin	NM_000466.2	PEX1
circRNA_05527	0	0	0.021808645	0	0.03009467	0	0	0	0	0.040344966	0	0	-	NC_000007.14	-	92517276	92518255	980	882	sense-overlappin	NM_000466.2	PEX1
circRNA_05528	0	0	0.054521613	0	0	0	0.030148028	0	0	0.030258724	0	0	hsa_circ_0081028	NC_000007.14	-	92517276	92519078	1803	966	sense-overlappin	NM_000466.2	PEX1
circRNA_05529	0	0	0	0.0309543	0	0	0	0	0	0	0	0	-	NC_000007.14	-	92517276	92522245	4970	1110	sense-overlappin	NM_000466.2	PEX1
circRNA_05530	0	0	0	0	0	0.031069364	0	0	0.020254528	0	0	0	-	NC_000007.14	+	93252653	93259632	6980	557	sense-overlappin	NM_024553.2	CCDC132
circRNA_05531	0	0	0.032712968	0	0	0	0	0	0	0	0	0	-	NC_000007.14	+	93252653	93311272	58620	1753	sense-overlappin	NM_001257998.1	CCDC132
circRNA_05532	0	0	0	0.0309543	0	0	0	0	0	0	0	0	-	NC_000007.14	+	93252653	93323732	71080	1875	sense-overlappin	NM_001257998.1	CCDC132
circRNA_05533	0	0	0.04361729	0	0	0	0	0	0	0	0	0	hsa_circ_0002620	NC_000007.14	+	93291703	93297243	5541	419	sense-overlappin	NM_001257998.1	CCDC132
circRNA_05534	0	0	0	0	0	0	0	0.030729443	0	0	0	0	hsa_circ_0003974	NC_000007.14	+	93291703	93311272	19570	913	sense-overlappin	NM_001257998.1	CCDC132
circRNA_05535	0	0	0.04361729	0	0	0	0	0	0	0.040344966	0	0	hsa_circ_0081171	NC_000007.14	-	94618758	94629841	11084	553	sense-overlappin	XM_005250675.3	SGCE
circRNA_05536	0.030862537	0	0	0	0	0	0	0	0	0	0	0	-	NC_000007.14	+	95111259	95120832	9574	254	sense-overlappin	XM_011516383.1	PPP1R9A
circRNA_05537	0	0	0	0	0.02006311	0	0	0	0	0	0	0	hsa_circ_0081181	NC_000007.14	-	96170045	96208977	38933	983	sense-overlappin	XM_006715831.2	SLC25A13
circRNA_05538	0	0	0	0	0.02006311	0	0	0	0	0	0	0	hsa_circ_0081183	NC_000007.14	-	96184277	96208977	24701	849	sense-overlappin	XM_006715831.2	SLC25A13
circRNA_05539	0	0	0	0	0	0	0.020098685	0	0.020254528	0	0	0	hsa_circ_0081188	NC_000007.14	-	96277196	96296951	19756	197	sense-overlappin	XM_006715831.2	SLC25A13
circRNA_05540	0	0.020469968	0	0	0	0	0	0	0	0	0	0	-	NC_000007.14	+	98137315	98159425	22111	554	sense-overlappin	XM_011515981.1	LMTK2
circRNA_05541	0.061725074	0.092114854	0.054521613	0.21668	0.04012623	0.103564547	0.020098685	0	0.040509055	0.100862414	0	0.051591	hsa_circ_0001725	NC_000007.14	+	98190728	98194572	3845	3109	sense-overlappin	XM_011515981.1	LMTK2
circRNA_05542	0	0	0	0	0	0	0.020098685	0	0	0	0	0	-	NC_000007.14	-	98390655	98390943	289	289	intronic	NM_018842.4	BAIAP2L1
circRNA_05543	0	0	0	0	0.10031557	0	0	0	0	0	0	0	-	NC_000007.14	+	98881090	98882024	935	211	sense-overlappin	NM_001244580.1	TRRAP
circRNA_05544	0	0	0	0	0.02006311	0	0	0	0	0	0	0	-	NC_000007.14	+	98881090	98893881	12792	511	sense-overlappin	NM_001244580.1	TRRAP
circRNA_05545	0	0	0	0	0.08025246	0	0	0	0	0	0	0	-	NC_000007.14	+	98881090	98897866	16777	694	sense-overlappin	NM_001244580.1	TRRAP
circRNA_05546	0.030862537	0	0	0	0	0	0	0	0	0	0	0	hsa_circ_0081234	NC_000007.14	+	98890335	98897866	7532	483	sense-overlappin	NM_001244580.1	TRRAP
circRNA_05547	0	0	0	0	0.02006311	0	0	0	0	0	0	0	hsa_circ_0081251	NC_000007.14	+	98903379	98906255	2877	218	sense-overlappin	NM_001244580.1	TRRAP
circRNA_05548	0	0	0	0	0.02006311	0	0	0	0	0	0	0	hsa_circ_0081258	NC_000007.14	+	98915723	98917679	1957	423	sense-overlappin	NM_001244580.1	TRRAP
circRNA_05549	0	0	0	0	0.03009467	0	0	0	0	0	0	0	-	NC_000007.14	+	98926927	98932208	5282	1660	sense-overlappin	NM_001244580.1	TRRAP
circRNA_05550	0	0	0	0	0	0	0	0	0	0.020172483	0	0	hsa_circ_0081288	NC_000007.14	+	98937156	98937820	665	293	sense-overlappin	NM_001244580.1	TRRAP
circRNA_05551	0	0	0	0	0	0.020712909	0	0	0	0	0	0	hsa_circ_0081301	NC_000007.14	+	98961261	98962427	1167	340	sense-overlappin	NM_001244580.1	TRRAP
circRNA_05552	0	0	0	0	0.04012623	0	0	0	0	0	0	0	hsa_circ_0081308	NC_000007.14	+	98964629	98971945	7317	1010	sense-overlappin	NM_001244580.1	TRRAP
circRNA_05553	0	0.040939935	0.021808645	0	0.06018934	0	0	0	0.020254528	0.020172483	0	0	hsa_circ_0081311	NC_000007.14	+	98965696	98971945	6250	863	sense-overlappin	NM_001244580.1	TRRAP
circRNA_05554	0	0	0	0	0.03009467	0	0	0	0	0	0	0	-	NC_000007.14	-	99045698	99047882	2185	303	sense-overlappin	XM_011516418.1	SMURF1
circRNA_05555	0	0	0	0	0.02006311	0	0	0	0	0	0	0	-	NC_000007.14	-	99081075	99082740	1666	1666	intronic	XM_011516418.1	SMURF1
circRNA_05556	0	0	0	0	0.02006311	0	0	0	0	0	0	0	hsa_circ_0081326	NC_000007.14	+	99333325	99348959	15635	527	sense-overlappin	NM_001190996.1	ARPC1A
circRNA_05557	0	0	0.021808645	0	0	0	0	0	0	0	0	0	hsa_circ_0008163	NC_000007.14	+	99386685	99388261	1577	328	sense-overlappin	NM_005720.3	ARPC1B
circRNA_05558	0.020575025	0	0	0	0	0	0.040197371	0	0	0	0	0	hsa_circ_0081343	NC_000007.14	+	99388039	99388261	223	223	exonic	NM_005720.3	ARPC1B
circRNA_05559	0	0	0	0	0.03009467	0	0	0	0	0	0	0	hsa_circ_0004299	NC_000007.14	+	99388039	99390012	1974	331	sense-overlappin	NM_005720.3	ARPC1B
circRNA_05560	0	0	0	0	0	0.041425819	0	0	0	0.020172483	0	0	hsa_circ_0007940	NC_000007.14	+	99390893	99391253	361	283	sense-overlappin	NM_005720.3	ARPC1B
circRNA_05561	0	0	0	0	0	0	0	0	0	0.020172483	0	0	-	NC_000007.14	-	99403398	99404953	1556	200	sense-overlappin	NM_014891.6	PDAP1
circRNA_05562	0	0	0.032712968	0	0	0	0	0	0	0	0	0	hsa_circ_0081360	NC_000007.14	+	99476403	99479787	3385	288	sense-overlappin	XM_011516067.1	ZNF789
circRNA_05563	0	0	0	0	0.03009467	0	0	0	0	0	0	0	-	NC_000007.14	-	99491077	99494631	3555	3555	sense-overlappin	NM_032164.2	ZNF394
circRNA_05564	0.082300098	0.173994724	0.250799418	0.21668	0.20063115	0.165703275	0.160789483	0.081945182	0.101272639	0.080689931	0.253190376	0.0206364	hsa_circ_0001726	NC_000007.14	-	99493040	99494631	1592	1592	sense-overlappin	NM_032164.2	ZNF394
circRNA_05565	0	0	0	0	0	0	0	0	0	0.050431207	0	0	hsa_circ_0004074	NC_000007.14	+	99597936	99610601	12666	1102	sense-overlappin	XM_011515696.1	GS1-259H13.2
circRNA_05566	0.360062931	0.603864042	0.948676058	0.7841752	2.11665863	0.445327551	1.105427694	0.419969057	0.638017623	0.494225829	0.394976987	0.340500603	hsa_circ_0001727	NC_000007.14	+	100023419	100024307	889	578	sense-overlappin	NM_001287054.1	ZKSCAN1
circRNA_05567	0	0	0	0	0	0.031069364	0	0	0	0	0	0	-	NC_000007.14	-	100060040	100060317	278	278	antisense	XM_005250566.2	ZSCAN21
circRNA_05568	0	0	0.04361729	0	0	0	0	0	0	0	0	0	hsa_circ_0081423	NC_000007.14	+	100102675	100102960	286	204	sense-overlappin	XM_005250689.3	AP4M1
circRNA_05569	0.030862537	0	0.021808645	0	0.03009467	0	0	0	0	0	0	0	hsa_circ_0004312	NC_000007.14	+	100103409	100103692	284	192	sense-overlappin	XM_005250689.3	AP4M1
circRNA_05570	0.154312685	0.092114854	0.08723458	0.0206362	0.28088361	0	0.100493427	0.09218833	0.060763583	0.050431207	0.070893305	0.0309546	hsa_circ_0005925	NC_000007.14	+	100355143	100355804	662	662	intergenic		
circRNA_05571	0	0	0	0	0	0	0	0	0	0.020172483	0	0	-	NC_000007.14	-	100415975	100416456	482	275	sense-overlappin	XM_011516366.1	ZCWPW1
circRNA_05572	0.061725074	0.102349838	0.185373483	0.0825448	0.09028402	0.051782273	0.090444084	0.09218833	0.070890847	0.07060369	0.08102092	0	hsa_circ_0001730	NC_000007.14	-	100812747	100813208	462	362	sense-overlappin	NM_004444.4	EPHB4
circRNA_05573	0	0	0	0	0	0	0	0	0	0.020172483	0	0	hsa_circ_0081523	NC_000007.14	-	100812747	100813716	970	427	sense-overlappin	NM_004444.4	EPHB4
circRNA_05574	0	0	0.021808645	0	0	0	0.020098685	0	0	0	0	0	hsa_circ_0081534	NC_000007.14	-	100819557	100820296	740	489	sense-overlappin	NM_004444.4	EPHB4
circRNA_05575	0	0	0	0	0.02006311	0	0	0	0	0	0	0	hsa_circ_0007639	NC_000007.14	+	100856868	100857176	309	309	exonic	XM_011516413.1	SLC12A9
circRNA_05576	0.020575025	0	0	0	0	0	0	0	0	0	0	0	-	NC_000007.14	+	101223477	101224141	665	455	sense-overlappin	NM_006349.2	ZNHIT1
circRNA_05577	0.041150049	0.020469968	0	0.0206362	0	0	0.030148028	0	0	0	0	0	hsa_circ_0005253	NC_000007.14	+	102158560	102178657	20098	343	sense-overlappin	NM_001202543.1	CUX1
circRNA_05578	0.144025172	0.071644886	0.076330258	0.1031809	0.09028402	0	0.080394741	0.061458886	0.040509055	0.030258724	0.06076569	0.051591	hsa_circ_0006501	NC_000007.14	+	102227367	102227669	303	303	exonic	NM_001202543.1	CUX1
circRNA_05579	0	0	0	0	0	0	0	0.020486295	0	0.030258724	0.02025523	0	hsa_circ_0007004	NC_000007.14	+	102399548	102407498	7951	252	sense-overlappin	NM_024653.3	PRKRIP1
circRNA_05580	0	0	0	0.0206362	0	0	0	0	0	0	0	0	-	NC_000007.14	+	102404598	102407498	2901	151	sense-overlappin	NM_024653.3	PRKRIP1
circRNA_05581	0.041150049	0	0	0	0.03009467	0	0.030148028	0	0	0	0	0	hsa_circ_0003327	NC_000007.14	+	102465817	102466270	454	352	sense-overlappin	NM_152892.1	LRWD1
circRNA_05582	0	0	0	0.0309543	0	0	0	0	0	0	0	0	-	NC_000007.14	-	102813277	102822203	8927	502	sense-overlappin	XR_927408.1	

circRNA_05583	0	0	0	0.0515905	0	0	0	0	0	0	0	0	-	NC_000007.14	-	102877467	102884312	6846	627	sense-overlappin	XR_927409.1	
circRNA_05584	0.020575025	0.030704951	0.076330258	0.0412724	0.06018934	0	0	0	0.040509055	0	0.050638075	0	hsa_circ_0007375	NC_000007.14	-	103025063	103029418	4356	495	sense-overlappin	XR_927409.1	
circRNA_05585	0	0	0	0	0.02006311	0	0	0	0	0	0	0	hsa_circ_0008540	NC_000007.14	+	103303842	103304490	649	279	sense-overlappin	NM_004279.2	PMPCB
circRNA_05586	0	0	0.021808645	0	0	0	0	0	0	0	0	0	hsa_circ_0081746	NC_000007.14	+	103308952	103310475	1524	305	sense-overlappin	NM_004279.2	PMPCB
circRNA_05587	0	0	0.021808645	0	0	0	0	0.020486295	0	0	0	0	hsa_circ_0081750	NC_000007.14	-	103319609	103322794	3186	523	sense-overlappin	NM_014377.1	DNAJC2
circRNA_05588	0	0	0.04361729	0.0619086	0.02006311	0	0.020098685	0.081945182	0	0.030258724	0.030382845	0.0206364	hsa_circ_0081751	NC_000007.14	-	103321932	103322794	863	364	sense-overlappin	NM_001129887.1	DNAJC2
circRNA_05589	0.020575025	0	0	0	0	0	0	0	0	0	0	0	hsa_circ_0002747	NC_000007.14	+	103353921	103355793	1873	220	sense-overlappin	XM_005250505.1	PSMC2
circRNA_05590	0	0	0	0	0	0	0.020098685	0	0	0	0	0	-	NC_000007.14	+	103361957	103362758	802	205	sense-overlappin	NM_002803.3	PSMC2
circRNA_05591	0	0	0	0	0	0	0	0	0	0.050431207	0	0	hsa_circ_0007612	NC_000007.14	-	104136781	104165282	28502	272	sense-overlappin	NM_002553.3	ORC5
circRNA_05592	0	0	0	0	0	0	0	0	0	0	0.02025523	0	hsa_circ_0081803	NC_000007.14	-	104188251	104195254	7004	243	sense-overlappin	NM_002553.3	ORC5
circRNA_05593	0	0.020469968	0	0	0	0.051782273	0	0	0	0	0	0	hsa_circ_0003000	NC_000007.14	-	104188251	104204234	15984	612	sense-overlappin	NM_002553.3	ORC5
circRNA_05594	0	0	0	0	0	0.020712909	0	0	0	0.020172483	0	0.0206364	-	NC_000007.14	+	105005318	105009209	3892	3892	intergenic		
circRNA_05595	0.13373766	0.040939935	0.021808645	0.0412724	0.03009467	0	0	0.040972591	0	0.030258724	0	0	hsa_circ_0007395	NC_000007.14	+	105038126	105041023	2898	400	sense-overlappin	XM_011516400.1	KMT2E
circRNA_05596	0	0	0	0.0309543	0	0	0	0	0	0	0	0	hsa_circ_0081808	NC_000007.14	+	105038126	105066807	28682	826	sense-overlappin	XM_011516400.1	KMT2E
circRNA_05597	0	0	0	0	0.03009467	0	0	0	0	0.030258724	0.030382845	0	hsa_circ_0081813	NC_000007.14	+	105062164	105066807	4644	426	sense-overlappin	NM_018682.3	KMT2E
circRNA_05598	0.041150049	0.020469968	0.076330258	0.0206362	0	0.124277456	0	0.051215739	0.060763583	0.090776173	0.02025523	0	-	NC_000007.14	+	105062164	105081797	19634	1287	sense-overlappin	NM_018682.3	KMT2E
circRNA_05599	0	0	0	0	0.02006311	0	0	0	0	0	0	0	-	NC_000007.14	+	105062164	105091314	29151	1651	sense-overlappin	NM_018682.3	KMT2E
circRNA_05600	0.061725074	0.030704951	0.04361729	0.0309543	0.02006311	0.041425819	0.040197371	0.030729443	0.020254528	0.131121138	0.02025523	0.0412728	hsa_circ_0001736	NC_000007.14	+	105073619	105077433	3815	633	sense-overlappin	NM_018682.3	KMT2E
circRNA_05601	0.020575025	0	0.032712968	0.113499	0.06018934	0.103564547	0	0	0.081018111	0.040344966	0.02025523	0.0309546	hsa_circ_0081819	NC_000007.14	+	105073619	105078963	5345	751	sense-overlappin	NM_018682.3	KMT2E
circRNA_05602	0	0.040939935	0	0	0	0.041425819	0	0	0.030381792	0	0	0	hsa_circ_0081820	NC_000007.14	+	105073619	105081797	8179	861	sense-overlappin	NM_018682.3	KMT2E
circRNA_05603	0	0.040939935	0	0	0	0	0	0	0	0	0	0	-	NC_000007.14	+	105078846	105091314	12469	592	sense-overlappin	NM_018682.3	KMT2E
circRNA_05604	0	0.020469968	0	0	0	0.031069364	0	0	0	0.020172483	0.02025523	0	hsa_circ_0081822	NC_000007.14	+	105090009	105091314	1306	364	sense-overlappin	NM_018682.3	KMT2E
circRNA_05605	0	0	0.021808645	0	0	0	0	0	0	0	0.02025523	0	-	NC_000007.14	+	105101425	105102194	770	474	sense-overlappin	NM_018682.3	KMT2E
circRNA_05606	0	0.020469968	0	0	0.02006311	0.041425819	0	0.020486295	0	0	0	0	hsa_circ_0081828	NC_000007.14	-	105142008	105143330	1323	730	sense-overlappin	XR_927524.1	
circRNA_05607	0.041150049	0	0.021808645	0	0	0	0	0	0	0	0	0	hsa_circ_0003656	NC_000007.14	-	105285009	105297533	12525	12525	sense-overlappin	XM_006716098.2	SRPK2
circRNA_05608	0	0	0	0.0206362	0	0	0	0	0	0	0	0	hsa_circ_0008628	NC_000007.14	-	105459168	105465414	6247	324	sense-overlappin	XM_005250463.2	PUS7
circRNA_05609	0	0	0.021808645	0	0	0	0	0	0	0	0	0	hsa_circ_0081840	NC_000007.14	-	105459168	105495253	36086	1119	sense-overlappin	XM_005250463.2	PUS7
circRNA_05610	0	0	0.032712968	0	0	0	0	0	0	0	0	0	hsa_circ_0081845	NC_000007.14	-	105462621	105495253	32633	1027	sense-overlappin	XM_005250463.2	PUS7
circRNA_05611	0	0	0	0	0	0	0.020098685	0	0	0	0	0	-	NC_000007.14	-	105472132	105502564	30433	670	sense-overlappin	XM_005250462.2	PUS7
circRNA_05612	0	0	0	0	0.02006311	0	0	0	0	0	0	0	hsa_circ_0007459	NC_000007.14	-	105481052	105502564	21513	608	sense-overlappin	XM_005250462.2	PUS7
circRNA_05613	0	0.020469968	0	0	0	0	0	0	0	0	0	0	-	NC_000007.14	-	105481052	105506056	25005	710	sense-overlappin	XM_005250462.2	PUS7
circRNA_05614	0	0	0	0.0412724	0.02006311	0	0	0	0	0	0	0	-	NC_000007.14	-	105481052	105506273	25222	795	sense-overlappin	XM_005250462.2	PUS7
circRNA_05615	0	0.020469968	0	0	0	0	0	0	0	0	0	0	hsa_circ_0081856	NC_000007.14	-	105481052	105508544	27493	1225	sense-overlappin	XM_005250462.2	PUS7
circRNA_05616	0	0	0.021808645	0	0	0	0	0	0	0	0	0	-	NC_000007.14	+	105974844	106004687	29844	1006	sense-overlappin	NM_152750.4	CDHR3
circRNA_05617	0	0	0	0	0	0	0.020098685	0	0	0.020172483	0	0	hsa_circ_0081872	NC_000007.14	-	106092949	106099282	6334	522	sense-overlappin	NM_182715.2	SYPL1
circRNA_05618	0	0	0.04361729	0	0.04012623	0	0	0	0	0.020172483	0	0	hsa_circ_0081873	NC_000007.14	-	106097690	106099282	1593	333	sense-overlappin	NM_182715.2	SYPL1
circRNA_05619	0	0	0	0	0	0	0	0	0	0.030258724	0	0	hsa_circ_0005937	NC_000007.14	-	111935540	111945798	10259	365	sense-overlappin	NM_014705.3	DOCK4
circRNA_05620	0	0	0	0	0	0	0	0	0	0.040344966	0	0	-	NC_000007.14	+	112455763	112457038	1276	315	sense-overlappin	NM_001007245.2	IFRD1
circRNA_05621	0	0	0.21808645	0	0	0	0	0	0	0	0	0	-	NC_000007.14	+	112617100	112635922	18823	18823	intergenic		
circRNA_05622	0	0	0.697876641	0	0	0	0	0	0	0	0	0	-	NC_000007.14	+	112617100	112640218	23119	23119	intergenic		
circRNA_05623	0	0	0.021808645	0	0	0	0	0	0	0	0	0	-	NC_000007.14	+	112617100	112707861	90762	90762	intergenic		
circRNA_05624	0	0	0.021808645	0	0	0	0	0	0	0	0	0	-	NC_000007.14	+	112620408	112640218	19811	19811	intergenic		
circRNA_05625	0	0	0.370746965	0	0	0	0	0	0	0	0	0	-	NC_000007.14	+	112620658	112640218	19561	19561	intergenic		
circRNA_05626	0	0	0.032712968	0	0	0	0	0	0	0	0	0	-	NC_000007.14	+	112620658	112699522	78865	78865	intergenic		
circRNA_05627	0	0	0.054521613	0	0	0	0	0	0	0	0	0	-	NC_000007.14	+	112632558	112635922	3365	3365	intergenic		
circRNA_05628	0	0	0.109043225	0	0	0	0	0	0	0	0	0	-	NC_000007.14	+	112632558	112640218	7661	7661	intergenic		
circRNA_05629	0	0	0.032712968	0	0	0	0	0	0	0	0	0	-	NC_000007.14	+	112632558	112707861	75304	75304	intergenic		
circRNA_05630	0.020575025	0	0	0	0	0	0	0	0	0	0	0	-	NC_000007.14	+	114498907	114538383	39477	35858	sense-overlappin	NR_033766.1	
circRNA_05631	0	0	0	0	0	0	0	0	0.020254528	0.030258724	0	0	-	NC_000007.14	-	116092137	116112038	19902	18837	sense-overlappin	XM_011515966.1	TFEC
circRNA_05632	0.041150049	0.061409903	0.021808645	0.0206362	0	0.331406549	0.190937511	0.143404068	0	0.322759725	0	0.0619092	-	NC_000007.14	-	116110708	116112038	1331	266	sense-overlappin	XM_011515966.1	TFEC
circRNA_05633	0	0	0	0	0	0	0	0	0	0	0	0.0206364	hsa_circ_0002699	NC_000007.14	+	116695750	116700284	4535	1264	sense-overlappin	XM_011516223.1	MET
circRNA_05634	0	0	0	0	0	0.020712909	0	0.020486295	0	0	0	0	hsa_circ_0082002	NC_000007.14	+	116699071	116700284	1214	1214	exonic	NM_000245.2	MET
circRNA_05635	0	0	0.032712968	0.0206362	0	0	0	0	0	0	0	0	hsa_circ_0001740	NC_000007.14	+	118185647	118188405	2759	176	sense-overlappin	NM_016200.4	LSM8
circRNA_05636	0.051437562	0	0.152660515	0.1547714	0	0.031069364	0	0	0.020254528	0	0	0	hsa_circ_0006919	NC_000007.14	-	122113118	122133741	20624	1293	sense-overlappin	NM_005763.3	AASS
circRNA_05637	0	0	0	0.0206362	0	0	0	0	0	0	0	0	-	NC_000007.14	+	122197950	122201396	3447	3447	intergenic		

circRNA_05638	0	0	0	0	0	0.051782273	0.020098685	0	0	0	0	0	hsa_circ_0003773	NC_000007.14	-	122379368	122393582	14215	656	sense-overlappin	XM_005250695.3	CADPS2
circRNA_05639	0	0	0	0.0206362	0	0	0	0	0	0	0	0	-	NC_000007.14	-	122615181	122629328	14148	437	sense-overlappin	NM_001009571.3	CADPS2
circRNA_05640	0.072012586	0	0.054521613	0.0309543	0.03009467	0.051782273	0.030148028	0.040972591	0	0	0.06076569	0	hsa_circ_0082096	NC_000007.14	-	127373342	127377329	3988	1837	sense-overlappin	XM_006715867.2	ZNF800
circRNA_05641	0	0.051174919	0	0.0206362	0	0.020712909	0	0.020486295	0.020254528	0.040344966	0	0	hsa_circ_0003655	NC_000007.14	+	127704839	127721400	16562	312	sense-overlappin	XM_011516051.1	SND1
circRNA_05642	0	0	0.04361729	0	0	0	0	0	0	0	0	0	hsa_circ_0082141	NC_000007.14	+	127807484	127904819	97336	375	sense-overlappin	XM_011516051.1	SND1
circRNA_05643	0	0	0.032712968	0	0	0	0	0	0	0	0	0	-	NC_000007.14	-	128310780	128315020	4241	509	sense-overlappin	XM_011516371.1	RBM28
circRNA_05644	0	0	0	0	0	0	0.020098685	0	0	0	0	0	-	NC_000007.14	-	128330819	128336042	5224	516	sense-overlappin	XM_011516371.1	RBM28
circRNA_05645	0	0	0	0	0	0	0	0	0	0	0	0.0206364	-	NC_000007.14	+	128572330	128574791	2462	2462	intergenic		
circRNA_05646	0	0	0.021808645	0	0	0	0	0	0	0	0	0	hsa_circ_0006771	NC_000007.14	+	128574720	128592017	17298	17298	intergenic		
circRNA_05647	0	0	0	0	0	0	0	0	0	0.020172483	0	0	-	NC_000007.14	+	128574720	128638897	64178	64178	intergenic		
circRNA_05648	0.020575025	0.020469968	0	0	0	0.020712909	0.030148028	0	0.040509055	0.060517448	0.04051046	0	-	NC_000007.14	+	128577953	128638897	60945	60945	intergenic		
circRNA_05649	0.102875123	0.030704951	0.021808645	0	0.04012623	0.020712909	0	0	0	0	0	0	hsa_circ_0005921	NC_000007.14	+	128641203	128648982	7780	7780	intergenic		
circRNA_05650	0	0	0	0	0	0	0.020098685	0	0	0	0	0.0309546	hsa_circ_0082179	NC_000007.14	+	128642217	128648982	6766	6766	intergenic		
circRNA_05651	0	0	0.021808645	0	0	0	0	0	0	0	0	0	hsa_circ_0082271	NC_000007.14	-	128997389	129001234	3846	462	sense-overlappin	NM_001191028.2	TNPO3
circRNA_05652	0	0	0	0	0	0	0	0	0	0.020172483	0	0	hsa_circ_0082273	NC_000007.14	-	129005016	129018157	13142	576	sense-overlappin	NM_001191028.2	TNPO3
circRNA_05653	0.082300098	0.16375974	0.39255561	0.3611333	0.17053648	0.113921001	0.130641455	0.061458886	0.081018111	0.181552345	0.10127615	0.175409401	hsa_circ_0001741	NC_000007.14	-	129014979	129018157	3179	432	sense-overlappin	NM_001191028.2	TNPO3
circRNA_05654	0.072012586	0	0.076330258	0.0309543	0	0.020712909	0.020098685	0.020486295	0.030381792	0	0	0	hsa_circ_0001742	NC_000007.14	+	129205203	129206587	1385	727	sense-overlappin	NM_005631.4	SMO
circRNA_05655	0	0	0	0.0412724	0	0	0	0	0	0	0	0	-	NC_000007.14	+	129368470	129379749	11280	194	sense-overlappin	XM_011515988.1	AHCYL2
circRNA_05656	0	0	0.021808645	0	0	0	0	0	0	0	0	0	-	NC_000007.14	-	129911291	129911747	457	457	intronic	NM_001202498.1	UBE2H
circRNA_05657	0	0	0	0	0	0	0.020098685	0	0	0	0	0	-	NC_000007.14	-	129921622	129922093	472	472	intronic	NM_001202498.1	UBE2H
circRNA_05658	0	0	0	0	0	0	0.020098685	0	0	0	0	0	-	NC_000007.14	-	130026158	130029029	2872	283	sense-overlappin	XM_011516290.1	ZC3HC1
circRNA_05659	0	0	0.021808645	0	0	0	0	0	0	0	0	0	hsa_circ_0007735	NC_000007.14	-	130026158	130049144	22987	630	sense-overlappin	NM_001282190.1	ZC3HC1
circRNA_05660	0	0	0.021808645	0	0	0	0	0	0	0	0	0	hsa_circ_0006889	NC_000007.14	-	130039464	130041473	2010	607	sense-overlappin	NM_001282190.1	ZC3HC1
circRNA_05661	0.041150049	0	0.021808645	0	0	0.062138728	0.020098685	0	0.050636319	0.030258724	0.02025523	0	hsa_circ_0004364	NC_000007.14	-	130039464	130049144	9681	347	sense-overlappin	NM_001282190.1	ZC3HC1
circRNA_05662	0	0	0	0	0	0	0	0	0.020254528	0	0	0	-	NC_000007.14	-	130040951	130049144	8194	263	sense-overlappin	NM_001282190.1	ZC3HC1
circRNA_05663	0.030862537	0	0	0	0	0	0.030148028	0	0	0	0	0	hsa_circ_0082333	NC_000007.14	+	130116445	130122202	5758	526	sense-overlappin	NM_014997.3	KLHDC10
circRNA_05664	0.030862537	0.030704951	0.032712968	0.0825448	0.04012623	0.051782273	0.020098685	0.071702034	0.070890847	0.100862414	0.02025523	0.082545601	hsa_circ_0002190	NC_000007.14	+	130120749	130122202	1454	304	sense-overlappin	NM_014997.3	KLHDC10
circRNA_05665	0	0	0	0	0	0	0.030148028	0	0	0	0	0	-	NC_000007.14	-	130652869	130667534	14666	286	sense-overlappin	NM_012133.5	COPG2
circRNA_05666	0	0	0.021808645	0	0	0	0	0	0	0	0	0	hsa_circ_0003551	NC_000007.14	+	131373710	131399433	25724	2319	sense-overlappin	NM_001145354.1	MKLN1
circRNA_05667	0	0	0	0.0206362	0	0	0	0	0	0	0	0	hsa_circ_0082398	NC_000007.14	+	131375424	131388972	13549	302	sense-overlappin	NM_001145354.1	MKLN1
circRNA_05668	0.030862537	0.030704951	0.370746965	0.1031809	0.06018934	0.082851637	0.140690797	0.020486295	0.030381792	0.060517448	0.04051046	0	hsa_circ_0001746	NC_000007.14	+	131375424	131399433	24010	605	sense-overlappin	NM_001145354.1	MKLN1
circRNA_05669	0	0.092114854	0.076330258	0.0722267	0.05015779	0	0.050246713	0.040972591	0.030381792	0.040344966	0.06076569	0	hsa_circ_0001747	NC_000007.14	+	131387120	131399433	12314	535	sense-overlappin	NM_001145354.1	MKLN1
circRNA_05670	0	0.061409903	0.021808645	0.113499	0.02006311	0.041425819	0	0	0	0.020172483	0.06076569	0	hsa_circ_0082415	NC_000007.14	+	131429033	131445903	16871	678	sense-overlappin	NM_001145354.1	MKLN1
circRNA_05671	0	0	0	0	0.03009467	0	0	0	0	0	0	0	hsa_circ_0082416	NC_000007.14	+	131429033	131478677	49645	1239	sense-overlappin	NM_001145354.1	MKLN1
circRNA_05672	0	0	0	0.0206362	0	0	0	0	0	0	0	0	hsa_circ_0001973	NC_000007.14	-	131508951	131511433	2483	1001	sense-overlappin	NM_001018111.2	PODXL
circRNA_05673	0.030862537	0	0	0	0	0	0	0	0	0	0	0	-	NC_000007.14	-	132507506	132508779	1274	1274	exonic	NM_020911.1	PLXNA4
circRNA_05674	0	0	0	0	0	0	0	0	0	0	0.02025523	0	-	NC_000007.14	-	133047011	133055157	8147	8147	intronic	NM_017812.2	CHCHD3
circRNA_05675	0	0.030704951	0	0	0	0	0	0	0	0	0	0	-	NC_000007.14	-	133732222	133743832	11611	11611	antisense	NM_021807.3	EXOC4
circRNA_05676	0	0	0	0	0	0.041425819	0	0	0	0	0	0	-	NC_000007.14	+	134137023	134163540	26518	642	sense-overlappin	XR_927362.1	
circRNA_05677	0	0	0	0	0.03009467	0	0	0	0	0	0	0	-	NC_000007.14	-	134294821	134302110	7290	664	sense-overlappin	XM_011516645.1	SLC35B4
circRNA_05678	0.030862537	0.030704951	0.021808645	0.0206362	0	0	0	0	0	0.050431207	0.030382845	0	hsa_circ_0003746	NC_000007.14	+	134947508	134950514	3007	403	sense-overlappin	XM_006716136.2	CALD1
circRNA_05679	0	0	0	0	0.02006311	0	0	0	0	0	0	0	-	NC_000007.14	+	134947695	134947769	75	75	exonic	XM_006716136.2	CALD1
circRNA_05680	0	0	0	0	0	0	0	0	0.020254528	0	0	0	-	NC_000007.14	+	134993493	135017159	23667	294	sense-overlappin	NM_178563.3	AGBL3
circRNA_05681	0	0.030704951	0	0	0	0	0	0	0	0	0	0	-	NC_000007.14	+	134993493	135081790	88298	1986	sense-overlappin	NM_178563.3	AGBL3
circRNA_05682	0	0	0	0	0	0	0	0	0	0.020172483	0	0	-	NC_000007.14	+	135044025	135081790	37766	610	sense-overlappin	NM_178563.3	AGBL3
circRNA_05683	0.041150049	0	0.04361729	0.0206362	0	0.031069364	0.030148028	0	0.081018111	0.020172483	0	0	hsa_circ_0001750	NC_000007.14	+	135577796	135593192	15397	1182	sense-overlappin	NM_015135.2	NUP205
circRNA_05684	0	0	0	0	0	0.020712909	0	0	0	0	0	0	-	NC_000007.14	+	135584832	135591600	6769	582	sense-overlappin	NM_015135.2	NUP205
circRNA_05685	0	0.020469968	0	0	0	0	0	0	0	0.060517448	0	0	-	NC_000007.14	+	136020701	136031033	10333	10333	intergenic		
circRNA_05686	0	0	0	0	0	0	0	0	0	0.020172483	0	0	-	NC_000007.14	+	136025657	136031033	5377	5377	intergenic		
circRNA_05687	0.051437562	0	0	0	0	0	0	0	0	0	0	0	hsa_circ_0003949	NC_000007.14	-	137251230	137254974	3745	452	sense-overlappin	XM_005250515.2	PTN
circRNA_05688	0	0	0	0	0	0	0	0	0	0	0	0.0309546	-	NC_000007.14	-	137901354	137908436	7083	460	sense-overlappin	XM_011516492.1	CREB3L2
circRNA_05689	0	0.030704951	0.021808645	0.0515905	0	0	0	0	0	0.030258724	0	0.0619092	hsa_circ_0005047	NC_000007.14	-	137901354	137916012	14659	724	sense-overlappin	XM_011516492.1	CREB3L2
circRNA_05690	0.041150049	0.051174919	0	0	0	0.020712909	0	0	0	0.060517448	0	0	hsa_circ_0001753	NC_000007.14	-	137912991	137916012	3022	264	sense-overlappin	XM_011516492.1	CREB3L2
circRNA_05691	0	0	0.054521613	0	0	0	0	0	0.020254528	0	0	0	-	NC_000007.14	+	138504290	138529230	24941	632	sense-overlappin	XM_011516650.1	TRIM24
circRNA_05692	0	0	0	0	0	0	0.020098685	0	0	0	0	0	hsa_circ_0082574	NC_000007.14	+	138504290	138538803	34514	779	sense-overlappin	XM_011516650.1	TRIM24
circRNA_05693	0	0	0	0.0206362	0	0.020712909	0	0	0	0	0	0	hsa_circ_0082580	NC_000007.14	+	138519189	138551180	31992	630	sense-overlappin	XM_011516650.1	TRIM24

circRNA_05694	0	0	0	0.0412724	0	0	0	0	0.040509055	0	0	0	hsa_circ_0082600	NC_000007.14	+	138576373	138579532	3160	571	sense-overlappin	NM_003852.3	TRIM24
circRNA_05695	0	0	0	0	0	0	0.020098685	0	0	0	0	0	hsa_circ_0082626	NC_000007.14	-	139073856	139089759	15904	1564	sense-overlappin	NM_020119.3	ZC3HAV1
circRNA_05696	0	0	0	0.0412724	0	0	0	0	0	0	0	0	-	NC_000007.14	+	139237005	139276147	39143	1556	sense-overlappin	XM_005250249.2	UBN2
circRNA_05697	0.020575025	0	0	0	0	0	0	0	0	0	0	0	-	NC_000007.14	+	139237005	139276151	39147	1560	sense-overlappin	XM_005250249.2	UBN2
circRNA_05698	0	0	0.032712968	0.0206362	0	0	0	0	0	0	0	0	-	NC_000007.14	+	139237005	139284574	47570	3201	sense-overlappin	XM_005250249.2	UBN2
circRNA_05699	0	0	0.021808645	0.0412724	0	0	0	0	0	0	0	0	-	NC_000007.14	+	139237005	139297964	60960	3704	sense-overlappin	XM_005250249.2	UBN2
circRNA_05700	0.030862537	0.030704951	0	0.0515905	0	0.031069364	0.040197371	0.020486295	0.020254528	0.030258724	0	0	hsa_circ_0005594	NC_000007.14	+	139266333	139272440	6108	320	sense-overlappin	XM_005250249.2	UBN2
circRNA_05701	0	0	0	0	0	0	0.020098685	0	0	0	0	0	-	NC_000007.14	+	139269394	139284574	15181	2203	sense-overlappin	XM_005250249.2	UBN2
circRNA_05702	0	0	0.021808645	0.0825448	0	0	0.040197371	0.030729443	0.040509055	0.050431207	0	0	-	NC_000007.14	+	139398599	139409654	11056	623	sense-overlappin	NM_001244585.1	LUC7L2
circRNA_05703	0.020575025	0	0	0	0	0	0	0.020486295	0	0	0	0	-	NC_000007.14	+	139398599	139417729	19131	845	sense-overlappin	NM_001244585.1	LUC7L2
circRNA_05704	0	0	0.032712968	0	0	0	0	0	0	0	0	0	-	NC_000007.14	+	139402137	139409654	7518	524	sense-overlappin	NM_001244585.1	LUC7L2
circRNA_05705	0	0	0	0	0	0	0	0	0	0	0.030382845	0	-	NC_000007.14	-	139402813	139404223	1411	1411	antisense	NM_001244585.1	LUC7L2
circRNA_05706	0.072012586	0.16375974	0.152660515	0.3611333	0.09028402	0.144990365	0.381875022	0.122917773	0.121527166	0.252156035	0.02025523	0.113500201	hsa_circ_0001756	NC_000007.14	-	139715932	139717015	1084	1084	exonic	XM_011516081.1	HIPK2
circRNA_05707	0	0	0	0	0	0	0.020098685	0	0	0	0	0	-	NC_000007.14	-	139715932	139741845	25914	1281	sense-overlappin	XM_006715935.2	HIPK2
circRNA_05708	0	0	0	0.0206362	0	0	0.050246713	0	0	0	0	0	-	NC_000007.14	-	139715932	139742038	26107	1357	sense-overlappin	XM_006715935.2	HIPK2
circRNA_05709	0	0	0.021808645	0	0	0	0	0	0	0	0	0	-	NC_000007.14	-	139740042	139771318	31277	31277	sense-overlappin	NM_001113239.2	HIPK2
circRNA_05710	0	0	0.032712968	0	0	0	0	0	0	0.030258724	0	0	-	NC_000007.14	-	140034235	140058034	23800	1095	sense-overlappin	NM_022750.3	PARP12
circRNA_05711	0	0.040939935	0	0.0206362	0	0	0	0	0.030381792	0	0	0	hsa_circ_0004684	NC_000007.14	-	140041644	140058034	16391	856	sense-overlappin	NM_022750.3	PARP12
circRNA_05712	0.061725074	0.020469968	0	0.0206362	0.04012623	0.031069364	0.050246713	0.040972591	0.020254528	0	0.02025523	0	hsa_circ_0082688	NC_000007.14	-	140046884	140058034	11151	660	sense-overlappin	NM_022750.3	PARP12
circRNA_05713	0	0	0	0	0	0	0.020098685	0	0	0	0	0	hsa_circ_0082689	NC_000007.14	-	140054662	140058034	3373	536	sense-overlappin	NM_022750.3	PARP12
circRNA_05714	0	0	0.021808645	0	0	0	0	0	0	0	0	0	-	NC_000007.14	-	140096555	140120529	23975	1323	sense-overlappin	XM_011516587.1	KDM7A
circRNA_05715	0	0.020469968	0	0	0	0	0	0	0	0	0	0	-	NC_000007.14	-	140111095	140120529	9435	377	sense-overlappin	XM_011516587.1	KDM7A
circRNA_05716	0	0	0.032712968	0	0	0	0	0	0	0	0	0	-	NC_000007.14	-	140113491	140133656	20166	1058	sense-overlappin	NM_030647.1	KDM7A
circRNA_05717	0	0	0	0.0206362	0	0	0	0	0	0	0	0	-	NC_000007.14	-	140119113	140120529	1417	195	sense-overlappin	XM_011516587.1	KDM7A
circRNA_05718	0	0	0	0	0	0	0.050246713	0	0	0.040344966	0	0	-	NC_000007.14	-	140127442	140129653	2212	303	sense-overlappin	NM_030647.1	KDM7A
circRNA_05719	0	0.020469968	0	0	0.03009467	0	0	0	0	0	0.04051046	0	hsa_circ_0001757	NC_000007.14	-	140334429	140337349	2921	1142	sense-overlappin	NM_001287498.1	SLC37A3
circRNA_05720	0.051437562	0	0	0	0	0	0	0	0.030381792	0.020172483	0.02025523	0	hsa_circ_0002494	NC_000007.14	-	140334634	140337349	2716	937	sense-overlappin	NM_001287498.1	SLC37A3
circRNA_05721	0	0	0	0	0	0.031069364	0	0	0	0	0	0	-	NC_000007.14	-	140334679	140337349	2671	892	sense-overlappin	NM_001287498.1	SLC37A3
circRNA_05722	0	0	0.08723458	0.0206362	0	0.031069364	0	0.020486295	0.030381792	0.07060369	0.02025523	0	hsa_circ_0004069	NC_000007.14	-	140355668	140358785	3118	243	sense-overlappin	NM_001287498.1	SLC37A3
circRNA_05723	0	0	0.032712968	0	0	0	0	0	0	0	0	0	hsa_circ_0082708	NC_000007.14	-	140355668	140369682	14015	420	sense-overlappin	NM_001287498.1	SLC37A3
circRNA_05724	0	0	0	0	0	0	0	0	0	0.020172483	0	0	-	NC_000007.14	-	140453799	140453930	132	132	exonic	NM_013446.3	MKRN1
circRNA_05725	0	0	0	0	0	0	0.020098685	0	0.020254528	0	0	0	-	NC_000007.14	-	140585589	140587780	2192	250	sense-overlappin	XM_011516053.1	DENND2A
circRNA_05726	0	0	0	0	0	0	0	0.051215739	0	0	0	0	hsa_circ_0002142	NC_000007.14	-	140601403	140602542	1140	1140	exonic	XM_011516053.1	DENND2A
circRNA_05727	0.123450148	0	0.032712968	0	0	0.020712909	0.040197371	0	0.030381792	0.040344966	0	0	hsa_circ_0007518	NC_000007.14	+	140702866	140704963	2098	249	sense-overlappin	NM_004546.2	NDUFB2
circRNA_05728	0	0	0	0	0	0	0	0	0	0.020172483	0	0	hsa_circ_0006013	NC_000007.14	-	140734617	140808995	74379	1980	sense-overlappin	XR_927521.1	
circRNA_05729	0	0	0	0	0	0	0.020098685	0	0	0	0	0	hsa_circ_0005106	NC_000007.14	-	140739812	140808995	69184	1826	sense-overlappin	XR_927521.1	
circRNA_05730	0	0	0.054521613	0	0	0	0	0	0	0	0	0	-	NC_000007.14	-	140776912	140787584	10673	554	sense-overlappin	XM_011516529.1	BRAF
circRNA_05731	0	0.030704951	0.021808645	0	0.02006311	0	0	0.020486295	0	0.030258724	0	0	hsa_circ_0006460	NC_000007.14	-	140776912	140794467	17556	714	sense-overlappin	XM_011516529.1	BRAF
circRNA_05732	0	0	0.065425935	0.0412724	0.03009467	0.020712909	0	0.020486295	0.030381792	0.040344966	0	0.0206364	hsa_circ_0007178	NC_000007.14	-	140776912	140808995	32084	1190	sense-overlappin	XM_011516529.1	BRAF
circRNA_05733	0.051437562	0.030704951	0.021808645	0	0	0	0	0	0	0	0	0	hsa_circ_0006961	NC_000007.14	-	140794308	140808995	14688	636	sense-overlappin	XM_011516529.1	BRAF
circRNA_05734	0	0	0	0	0	0	0	0	0	0.020172483	0	0	hsa_circ_0082779	NC_000007.14	+	141611195	141633980	22786	371	sense-overlappin	NM_018238.3	AGK
circRNA_05735	0	0	0	0	0	0	0	0.020486295	0	0.020172483	0	0	-	NC_000007.14	+	142463288	142482603	19316	19316	intergenic	TRBV5-4	
circRNA_05736	0	0	0.021808645	0	0	0	0	0	0	0	0	0	hsa_circ_0008612	NC_000007.14	+	143292300	143292698	399	250	sense-overlappin	NM_032982.3	CASP2
circRNA_05737	0.030862537	0	0	0	0	0	0	0	0	0	0	0	hsa_circ_0002339	NC_000007.14	-	143875989	143876622	634	634	exonic	NM_014719.2	TCAF1
circRNA_05738	0	0	0	0.0206362	0	0	0	0	0	0	0	0	-	NC_000007.14	-	144058520	144066472	7953	7953	intergenic		
circRNA_05739	0	0	0.021808645	0	0	0	0	0	0	0	0	0	-	NC_000007.14	+	145785062	145848416	63355	63355	intergenic		
circRNA_05740	0	0	0.08723458	0	0	0	0	0	0	0	0	0	-	NC_000007.14	+	145842491	145848416	5926	5926	intergenic		
circRNA_05741	0	0.020469968	0	0	0	0	0	0.030729443	0.020254528	0	0	0	hsa_circ_0006565	NC_000007.14	+	148766561	148767749	1189	294	sense-overlappin	XM_011516630.1	CUL1
circRNA_05742	0	0	0.021808645	0	0	0	0.030148028	0	0	0	0	0	hsa_circ_0008324	NC_000007.14	-	148832634	148847305	14672	507	sense-overlappin	XM_011515898.1	EZH2
circRNA_05743	0	0.030704951	0.08723458	0	0.03009467	0	0.020098685	0	0.020254528	0	0	0	hsa_circ_0006357	NC_000007.14	-	148846470	148847305	836	253	sense-overlappin	NM_001203249.1	EZH2
circRNA_05744	0	0.040939935	0.109043225	0.1031809	0.05015779	0.082851637	0.040197371	0.051215739	0	0.050431207	0.050638075	0.0619092	hsa_circ_0001766	NC_000007.14	-	149018992	149021147	2156	387	sense-overlappin	XM_006716185.1	PDIA4
circRNA_05745	0	0	0.021808645	0	0	0	0	0	0	0	0	0	hsa_circ_0082854	NC_000007.14	-	149074386	149080717	6332	280	sense-overlappin	NM_152411.3	ZNF786
circRNA_05746	0	0	0.054521613	0.0309543	0	0.020712909	0	0	0	0	0.02025523	0	hsa_circ_0004351	NC_000007.14	+	149153945	149154340	396	396	exonic	NM_020781.3	ZNF398
circRNA_05747	0	0	0	0	0	0	0	0	0.020254528	0	0	0	-	NC_000007.14	+	149235710	149236169	460	460	intergenic		
circRNA_05748	0	0	0	0.0722267	0	0	0.020098685	0	0	0	0	0	-	NC_000007.14	-	149492859	149558839	65981	62540	sense-overlappin	XM_005249957.1	ZNF746

circRNA_05749	0	0	0	0	0	0.031069364	0	0	0	0	0	0	-	NC_000007.14	-	149549193	149558839	9647	9647	intergenic		
circRNA_05750	0.061725074	0	0	0	0	0	0	0	0	0	0	0	-	NC_000007.14	-	149550868	149558839	7972	7972	intergenic		
circRNA_05751	0.030862537	0	0	0	0	0	0	0	0	0	0	0	-	NC_000007.14	-	150233035	150235253	2219	2219	sense-overlappin	XR_927516.1	
circRNA_05752	0.030862537	0	0	0.0309543	0	0	0	0	0	0	0	0	hsa_circ_0082888	NC_000007.14	-	150284753	150295347	10595	615	sense-overlappin	XR_927516.1	
circRNA_05753	0	0	0	0	0	0	0	0	0	0	0	0.0309546	hsa_circ_0082921	NC_000007.14	+	151116793	151123886	7094	890	sense-overlappin	NM_001281300.1	AGAP3
circRNA_05754	0	0.040939935	0	0	0	0.031069364	0.030148028	0	0.020254528	0	0	0	hsa_circ_0003027	NC_000007.14	+	151117095	151118632	1538	579	sense-overlappin	NM_001281300.1	AGAP3
circRNA_05755	0	0	0	0	0	0	0.020098685	0	0	0	0	0	-	NC_000007.14	+	151352812	151356222	3411	361	sense-overlappin	XM_011516303.1	NUB1
circRNA_05756	0	0	0.021808645	0.0309543	0	0	0	0	0	0	0	0	-	NC_000007.14	-	151470571	151498180	27610	502	sense-overlappin	XM_011516457.1	RHEB
circRNA_05757	0	0	0	0	0.03009467	0	0	0	0	0	0	0	hsa_circ_0002147	NC_000007.14	-	151477333	151498180	20848	315	sense-overlappin	XM_011516457.1	RHEB
circRNA_05758	0	0	0	0	0	0	0	0	0	0	0	0.0206364	-	NC_000007.14	-	151481549	151491014	9466	3328	sense-overlappin	XM_011516457.1	RHEB
circRNA_05759	0.030862537	0	0.054521613	0	0	0.020712909	0	0	0.030381792	0.020172483	0.02025523	0	hsa_circ_0004779	NC_000007.14	-	151484737	151498180	13444	232	sense-overlappin	XM_011516457.1	RHEB
circRNA_05760	0	0	0	0.0515905	0	0	0	0	0	0	0	0	-	NC_000007.14	-	151781152	151786541	5390	352	sense-overlappin	NM_001040633.1	PRKAG2
circRNA_05761	0.030862537	0	0	0	0	0	0	0	0	0	0	0	-	NC_000007.14	-	152138796	152149152	10357	1869	sense-overlappin	XM_011516455.1	KMT2C
circRNA_05762	0	0	0.021808645	0	0	0	0	0	0	0	0	0	-	NC_000007.14	-	152162117	152187476	25360	6667	sense-overlappin	XM_011516455.1	KMT2C
circRNA_05763	0	0	0	0	0.02006311	0	0.020098685	0	0	0	0	0	hsa_circ_0082993	NC_000007.14	-	152176191	152178010	1820	1820	exonic	XM_011516455.1	KMT2C
circRNA_05764	0	0	0	0.0206362	0	0	0.030148028	0	0	0	0	0	hsa_circ_0007123	NC_000007.14	-	152194009	152199459	5451	616	sense-overlappin	XM_011516455.1	KMT2C
circRNA_05765	0.051437562	0	0.119947548	0.0619086	0.02006311	0.020712909	0.020098685	0.081945182	0.020254528	0.030258724	0.02025523	0.0619092	hsa_circ_0002810	NC_000007.14	-	152220523	152238826	18304	1180	sense-overlappin	XM_011516454.1	KMT2C
circRNA_05766	0	0	0	0	0	0	0	0	0	0	0.02025523	0	-	NC_000007.14	-	152238707	152250966	12260	1031	sense-overlappin	XM_011516454.1	KMT2C
circRNA_05767	0	0	0	0	0	0	0	0	0.030381792	0	0	0	hsa_circ_0005823	NC_000007.14	-	152249876	152250966	1091	192	sense-overlappin	XM_011516454.1	KMT2C
circRNA_05768	0	0	0.04361729	0.0619086	0	0	0	0	0	0.040344966	0	0	hsa_circ_0006784	NC_000007.14	-	152263016	152315338	52323	910	sense-overlappin	XM_005250027.3	KMT2C
circRNA_05769	0	0	0.021808645	0	0	0	0	0	0	0	0	0	-	NC_000007.14	-	152263016	152358675	95660	1141	sense-overlappin	XM_005250027.3	KMT2C
circRNA_05770	0.092587611	0.112584821	0.26170374	0.1444533	0.10031557	0.134633911	0.060296056	0.051215739	0.070890847	0.121034897	0.06076569	0.0206364	hsa_circ_0001769	NC_000007.14	-	152309966	152315338	5373	460	sense-overlappin	XM_005250027.3	KMT2C
circRNA_05771	0	0	0.04361729	0	0	0	0	0	0	0	0	0	hsa_circ_0083055	NC_000007.14	-	152309966	152330739	20774	599	sense-overlappin	XM_005250027.3	KMT2C
circRNA_05772	0	0	0.098138903	0.0206362	0	0	0.030148028	0	0	0	0.02025523	0	hsa_circ_0007017	NC_000007.14	-	152309966	152358675	48710	691	sense-overlappin	XM_005250027.3	KMT2C
circRNA_05773	0	0	0.021808645	0	0	0	0	0	0	0	0	0	-	NC_000007.14	-	152315138	152358675	43538	432	sense-overlappin	XM_005250027.3	KMT2C
circRNA_05774	0	0	0.076330258	0	0	0	0.030148028	0	0	0	0	0	hsa_circ_0001770	NC_000007.14	-	152330601	152358675	28075	231	sense-overlappin	XM_005250027.3	KMT2C
circRNA_05775	0.030862537	0	0	0	0	0	0	0	0	0	0	0	-	NC_000007.14	+	153228447	153269967	41521	41521	intergenic		
circRNA_05776	0.082300098	0.051174919	0.065425935	0.0309543	0.02006311	0.072495183	0.020098685	0.061458886	0.030381792	0.060517448	0.030382845	0	hsa_circ_0001771	NC_000007.14	+	155665175	155680908	15734	524	sense-overlappin	XM_005249531.1	RBM33
circRNA_05777	0.174887709	0.32751948	2.289907727	0.3920876	0.61192501	0.227842003	0.361776336	0.174133512	0.182290749	0.524484553	0.344338911	0.123818401	hsa_circ_0001772	NC_000007.14	+	155672867	155680908	8042	445	sense-overlappin	XM_005249531.1	RBM33
circRNA_05778	0	0	0.021808645	0	0	0	0	0	0	0	0	0	-	NC_000007.14	+	155672867	155681386	8520	923	sense-overlappin	XM_005249531.1	RBM33
circRNA_05779	0	0	0.054521613	0	0	0	0	0	0	0	0	0	-	NC_000007.14	+	155672867	155718443	45577	1288	sense-overlappin	XM_005249531.1	RBM33
circRNA_05780	0	0	0	0	0	0	0	0	0	0.050431207	0	0	-	NC_000007.14	+	155700773	155707068	6296	381	sense-overlappin	XM_005249531.1	RBM33
circRNA_05781	0.020575025	0.020469968	0	0	0.05015779	0	0	0	0	0	0	0	hsa_circ_0083097	NC_000007.14	+	155700773	155718443	17671	723	sense-overlappin	XM_005249531.1	RBM33
circRNA_05782	0	0.040939935	0.141756193	0	0	0.062138728	0.020098685	0	0.020254528	0.020172483	0	0	hsa_circ_0002094	NC_000007.14	+	155706860	155718443	11584	551	sense-overlappin	XM_005249531.1	RBM33
circRNA_05783	0	0	0.021808645	0	0	0	0	0	0	0	0	0	hsa_circ_0083111	NC_000007.14	+	155763812	155774647	10836	485	sense-overlappin	XM_005249531.1	RBM33
circRNA_05784	0	0	0.04361729	0	0	0	0	0	0	0	0	0	-	NC_000007.14	-	156724112	156756465	32354	541	sense-overlappin	XR_927508.1	
circRNA_05785	0	0	0	0	0	0	0	0	0	0	0.02025523	0	-	NC_000007.14	-	156734177	156756465	22289	154	sense-overlappin	XR_927508.1	
circRNA_05786	0	0	0.04361729	0	0.04012623	0.041425819	0.020098685	0	0	0	0	0	hsa_circ_0003511	NC_000007.14	-	156756393	156826744	70352	578	sense-overlappin	XR_927508.1	
circRNA_05787	0	0	0	0	0	0	0	0	0.030381792	0	0	0	hsa_circ_0083122	NC_000007.14	-	156762134	156763795	1662	261	sense-overlappin	XR_927508.1	
circRNA_05788	0	0	0.021808645	0	0	0	0	0	0	0	0	0	hsa_circ_0083125	NC_000007.14	-	156796389	156826744	30356	244	sense-overlappin	XR_927508.1	
circRNA_05789	0.061725074	0.051174919	0.272608063	0.113499	0.13041025	0.062138728	0.110542769	0.020486295	0.070890847	0.060517448	0.02025523	0	hsa_circ_0005939	NC_000007.14	-	156826605	156836885	10281	253	sense-overlappin	XR_927508.1	
circRNA_05790	0	0	0.054521613	0.0619086	0.02006311	0.072495183	0	0.061458886	0.030381792	0.020172483	0.06076569	0.0412728	hsa_circ_0004210	NC_000007.14	+	156966270	156967092	823	265	sense-overlappin	NM_138400.1	NOM1
circRNA_05791	0	0	0.04361729	0	0	0	0.030148028	0	0	0	0	0.0206364	hsa_circ_0083142	NC_000007.14	+	157163810	157170450	6641	276	sense-overlappin	XR_927552.1	
circRNA_05792	0	0.020469968	0	0	0	0	0	0	0	0	0	0	-	NC_000007.14	+	157201721	157225539	23819	902	sense-overlappin	XM_005249564.3	UBE3C
circRNA_05793	0	0	0.021808645	0	0	0	0	0	0.020254528	0	0	0	hsa_circ_0001773	NC_000007.14	+	157201721	157231327	29607	1150	sense-overlappin	XM_005249564.3	UBE3C
circRNA_05794	0	0	0	0	0	0.020712909	0	0	0	0	0	0	-	NC_000007.14	+	157207398	157225539	18142	815	sense-overlappin	XM_005249564.3	UBE3C
circRNA_05795	0	0	0	0	0	0	0	0	0	0	0.030382845	0	hsa_circ_0003649	NC_000007.14	+	157220689	157248580	27892	780	sense-overlappin	XM_005249564.3	UBE3C
circRNA_05796	0.041150049	0	0	0	0	0	0	0	0	0	0	0	-	NC_000007.14	+	157223254	157248580	25327	692	sense-overlappin	XR_927552.1	
circRNA_05797	0.020575025	0	0.032712968	0.0206362	0.04012623	0	0	0.020486295	0	0	0	0	hsa_circ_0083176	NC_000007.14	+	157382246	157385611	3366	345	sense-overlappin	NM_005494.2	DNAJB6
circRNA_05798	0	0	0.032712968	0	0	0	0	0	0	0	0	0	-	NC_000007.14	-	157656357	157682937	26581	408	sense-overlappin	XM_011516445.1	PTPRN2
circRNA_05799	0	0	0	0.0309543	0	0	0	0	0	0	0	0	-	NC_000007.14	-	158133677	158138515	4839	646	sense-overlappin	XM_011516445.1	PTPRN2
circRNA_05800	0	0	0	0	0.03009467	0	0	0	0	0	0	0	hsa_circ_0083196	NC_000007.14	-	158735503	158739121	3619	337	sense-overlappin	NM_020728.2	ESYT2
circRNA_05801	0	0	0	0.0309543	0	0	0	0	0	0	0	0	-	NC_000007.14	-	158748194	158767774	19581	778	sense-overlappin	NM_020728.2	ESYT2
circRNA_05802	0.699550837	0.378694399	0.457981545	0.5571771	0.28088361	0.569605007	0.160789483	0.215106103	0.344326971	0.766554346	0.313956066	0.371455203	hsa_circ_0001776	NC_000007.14	-	158759486	158764853	5368	495	sense-overlappin	NM_020728.2	ESYT2
circRNA_05803	0	0	0	0	0	0.020712909	0	0	0	0	0	0	hsa_circ_0083210	NC_000007.14	-	158759486	158773396	13911	672	sense-overlappin	NM_020728.2	ESYT2
circRNA_05804	0	0	0.032712968	0	0	0	0.070345399	0	0	0	0.030382845	0	hsa_circ_0083213	NC_000007.14	-	158759486	158799072	39587	1089	sense-overlappin	NM_020728.2	ESYT2

circRNA_05805	0.020575025	0	0	0	0	0	0	0	0	0	0	0	-	NC_000007.14	-	158773341	158798076	24736	431	sense-overlappin	NM_020728.2	ESYT2
circRNA_05806	0.030862537	0	0.032712968	0	0.02006311	0	0	0	0	0.020172483	0.04051046	0.103182001	hsa_circ_0083220	NC_000007.14	-	158773341	158799072	25732	473	sense-overlappin	NM_020728.2	ESYT2
circRNA_05807	0.26747532	0.071644886	0.119947548	0.0825448	0.06018934	0.134633911	0.160789483	0.040972591	0.172163486	0.14120738	0.050638075	0.123818401	hsa_circ_0001777	NC_000007.14	-	158788004	158799072	11069	417	sense-overlappin	NM_020728.2	ESYT2
circRNA_05808	0	0	0	0	0.02006311	0	0	0	0	0	0	0	-	NC_000007.14	+	158869855	158871562	1708	475	sense-overlappin	NM_018051.4	WDR60
circRNA_05809	0.041150049	0.08187987	0.076330258	0.0412724	0.04012623	0.062138728	0.050246713	0.051215739	0.050636319	0.080689931	0.02025523	0.0206364	hsa_circ_0001778	NC_000007.14	+	158869855	158876691	6837	558	sense-overlappin	NM_018051.4	WDR60
circRNA_05810	0	0	0.021808645	0	0	0	0	0	0	0	0	0	-	NC_000007.14	+	158869855	158906091	36237	1445	sense-overlappin	NM_018051.4	WDR60
circRNA_05811	0	0	0	0.0206362	0	0	0	0	0	0	0	0	-	NC_000007.14	+	158869855	158918869	49015	1906	sense-overlappin	NM_018051.4	WDR60
circRNA_05812	0	0.030704951	0	0.0309543	0	0	0	0	0	0	0	0	hsa_circ_0083226	NC_000007.14	+	158879684	158891333	11650	486	sense-overlappin	NM_018051.4	WDR60
circRNA_05813	0.020575025	0.020469968	0.054521613	0	0	0	0	0	0	0	0	0	hsa_circ_0083228	NC_000007.14	+	158879684	158906091	26408	887	sense-overlappin	NM_018051.4	WDR60
circRNA_05814	0	0.020469968	0.076330258	0	0	0	0	0	0.020254528	0.020172483	0	0.0206364	hsa_circ_0083229	NC_000007.14	+	158879684	158918869	39186	1348	sense-overlappin	NM_018051.4	WDR60
circRNA_05815	0	0	0	0	0.04012623	0	0	0	0	0	0	0	-	NC_000007.14	+	158901739	158906091	4353	401	sense-overlappin	NM_018051.4	WDR60
circRNA_05816	0.020575025	0.030704951	0	0	0	0	0	0	0	0	0	0	-	NC_000007.14	-	159103757	159142545	38789	306	sense-overlappin	NM_001304522.1	VIPR2
circRNA_05817	0	0	0	0	0.02006311	0	0	0	0	0	0	0	-	NC_000008.11	+	431341	435707	4367	247	sense-overlappin	NM_183420.1	FBXO25
circRNA_05818	0.020575025	0	0	0	0	0.072495183	0	0	0	0	0	0.0206364	-	NC_000008.11	-	528895	531576	2682	2682	intronic	XM_011534725.1	TDRP
circRNA_05819	0.030862537	0.030704951	0.032712968	0.0619086	0.04012623	0	0	0	0.030381792	0.050431207	0.030382845	0	-	NC_000008.11	-	668598	674047	5450	1137	sense-overlappin	XM_011534732.1	ERICH1
circRNA_05820	0.020575025	0	0	0	0	0	0	0	0	0	0	0	-	NC_000008.11	+	2925060	2928287	3228	3228	intergenic		
circRNA_05821	0.020575025	0	0	0.0206362	0	0	0	0	0	0.020172483	0	0	-	NC_000008.11	+	6477594	6499929	22336	279	sense-overlappin	NM_024596.3	MCPH1
circRNA_05822	0	0	0	0	0.04012623	0.031069364	0	0	0.020254528	0	0	0	hsa_circ_0002682	NC_000008.11	+	6724870	6747828	22959	526	sense-overlappin	NM_018361.3	AGPAT5
circRNA_05823	0	0	0	0	0.02006311	0	0	0	0	0	0	0	-	NC_000008.11	+	8885812	8887062	1251	1251	antisense	XR_246634.2	
circRNA_05824	0	0	0	0.0412724	0	0	0	0	0	0	0	0.0309546	-	NC_000008.11	+	8922289	8922750	462	462	intergenic		
circRNA_05825	0	0	0	0	0	0	0	0	0	0	0	0.0206364	-	NC_000008.11	+	8991548	8994245	2698	2698	intergenic		
circRNA_05826	0	0	0	0	0.02006311	0	0	0	0	0	0	0	-	NC_000008.11	-	10497712	10498808	1097	1097	intergenic		
circRNA_05827	0	0	0	0	0	0	0	0	0.020254528	0	0	0	-	NC_000008.11	-	10820193	10834775	14583	452	sense-overlappin	NM_017884.5	PINX1
circRNA_05828	0.030862537	0	0	0	0	0	0	0	0	0	0	0	-	NC_000008.11	-	11080893	11082416	1524	1524	intronic	NM_173683.3	XKR6
circRNA_05829	0	0	0	0	0	0	0.030148028	0	0	0	0	0	-	NC_000008.11	-	12665278	12665976	699	699	intergenic		
circRNA_05830	0.061725074	0	0	0	0	0	0	0	0	0	0	0	hsa_circ_0006410	NC_000008.11	+	15650697	15673836	23140	490	sense-overlappin	NM_006765.3	TUSC3
circRNA_05831	0.061725074	0	0	0	0	0	0	0	0	0	0	0	-	NC_000008.11	+	17098458	17120401	21944	819	sense-overlappin	NM_181723.2	MICU3
circRNA_05832	0	0	0	0.0309543	0	0	0	0	0	0	0	0	hsa_circ_0008752	NC_000008.11	-	17234716	17237373	2658	307	sense-overlappin	XM_005273481.2	CNOT7
circRNA_05833	0	0.020469968	0	0.0412724	0	0	0	0	0	0.020172483	0	0	-	NC_000008.11	+	17265907	17286427	20521	1069	sense-overlappin	NM_152415.2	VPS37A
circRNA_05834	0	0	0.032712968	0	0	0	0	0.020486295	0	0	0	0	hsa_circ_0008555	NC_000008.11	+	17268258	17286427	18170	994	sense-overlappin	NM_001145152.1	VPS37A
circRNA_05835	0	0	0	0	0	0	0	0	0.020254528	0	0	0	-	NC_000008.11	-	17649846	17654666	4821	393	sense-overlappin	NM_020749.4	MTUS1
circRNA_05836	0	0	0	0	0	0	0	0	0	0.020172483	0	0	-	NC_000008.11	-	17653186	17656065	2880	479	sense-overlappin	NM_020749.4	MTUS1
circRNA_05837	0	0	0	0	0.02006311	0.041425819	0	0	0	0	0	0	-	NC_000008.11	-	17653186	17675252	22067	546	sense-overlappin	NM_020749.4	MTUS1
circRNA_05838	0	0	0	0.0206362	0.02006311	0.020712909	0	0	0	0	0	0	hsa_circ_0083441	NC_000008.11	-	17675186	17723833	48648	618	sense-overlappin	NM_001001924.2	MTUS1
circRNA_05839	0	0	0	0	0.05015779	0.031069364	0	0	0	0	0	0	-	NC_000008.11	-	17675186	17755961	80776	3059	sense-overlappin	NM_001001924.2	MTUS1
circRNA_05840	0	0	0	0	0.02006311	0	0	0	0	0	0	0	hsa_circ_0083442	NC_000008.11	-	17713214	17715901	2688	174	sense-overlappin	NM_001001931.2	MTUS1
circRNA_05841	0	0	0.04361729	0.113499	0.22069426	0.062138728	0.070345399	0	0.020254528	0.050431207	0	0.0206364	hsa_circ_0083443	NC_000008.11	-	17713214	17723833	10620	336	sense-overlappin	NM_001001924.2	MTUS1
circRNA_05842	0	0	0	0	0.03009467	0	0	0	0	0	0	0	-	NC_000008.11	-	17713214	17724162	10949	665	sense-overlappin	NM_001001924.2	MTUS1
circRNA_05843	0	0	0	0	0.03009467	0	0	0	0	0	0	0	-	NC_000008.11	-	17713214	17743799	30586	532	sense-overlappin	NM_001001924.2	MTUS1
circRNA_05844	0	0	0	0.0515905	0.19059959	0	0.020098685	0.020486295	0	0.040344966	0	0	-	NC_000008.11	-	17713214	17755961	42748	2777	sense-overlappin	NM_001001924.2	MTUS1
circRNA_05845	0	0	0	0.0309543	0.03009467	0	0	0	0	0	0	0	-	NC_000008.11	-	17723672	17755961	32290	2603	sense-overlappin	NM_001001924.2	MTUS1
circRNA_05846	0	0.040939935	0.098138903	0.4643142	0.49154632	0.144990365	0.080394741	0.143404068	0.050636319	0.090776173	0.02025523	0.092863801	hsa_circ_0083444	NC_000008.11	-	17743604	17755961	12358	2441	sense-overlappin	NM_001001924.2	MTUS1
circRNA_05847	0	0	0	0	0	0	0.040197371	0	0	0	0	0	-	NC_000008.11	-	17743604	17758204	14601	4684	sense-overlappin	NM_001001924.2	MTUS1
circRNA_05848	0.020575025	0.030704951	0.08723458	0.0412724	0.10031557	0	0.020098685	0	0	0.020172483	0	0	hsa_circ_0083445	NC_000008.11	-	17753717	17755961	2245	2245	exonic	NM_001001924.2	MTUS1
circRNA_05849	0	0	0	0	0	0.051782273	0	0	0	0	0	0	hsa_circ_0083455	NC_000008.11	+	17950615	17953186	2572	327	sense-overlappin	NM_006197.3	PCM1
circRNA_05850	0	0.08187987	0	0.0206362	0	0	0	0	0.040509055	0	0	0	hsa_circ_0002887	NC_000008.11	+	17989859	17993619	3761	417	sense-overlappin	NM_006197.3	PCM1
circRNA_05851	0.061725074	0.092114854	0.054521613	0.0928628	0.03009467	0.051782273	0.060296056	0.051215739	0.050636319	0.14120738	0.02025523	0.0619092	hsa_circ_0083465	NC_000008.11	+	18011237	18011827	591	291	sense-overlappin	NM_006197.3	PCM1
circRNA_05852	0	0	0	0	0	0	0	0	0	0	0.02025523	0	-	NC_000008.11	-	18046066	18046190	125	125	intergenic		
circRNA_05853	0	0	0.021808645	0.0206362	0	0.020712909	0	0	0	0	0	0	hsa_circ_0083470	NC_000008.11	-	18572528	18575285	2758	303	sense-overlappin	NM_206909.2	PSD3
circRNA_05854	0.061725074	0.122819805	0.109043225	0.1754076	0.05015779	0.082851637	0	0.030729443	0.202545277	0.231983552	0.030382845	0.175409401	hsa_circ_0002111	NC_000008.11	-	18765449	18804898	39450	538	sense-overlappin	NM_206909.2	PSD3
circRNA_05855	0	0	0	0.0412724	0	0	0	0	0	0	0	0	hsa_circ_0002189	NC_000008.11	-	18765449	18855382	89934	51022	sense-overlappin	XM_011544470.1	PSD3
circRNA_05856	0.113162635	0.153524756	0.272608063	0.7532209	0.15047336	0.238198457	0	0	0.39496329	0.494225829	0.04051046	0.144454801	hsa_circ_0004458	NC_000008.11	-	18799295	18804898	5604	448	sense-overlappin	NM_206909.2	PSD3
circRNA_05857	0	0.092114854	0.13085187	0.0619086	0.04012623	0.041425819	0	0.051215739	0.182290749	0.020172483	0.04051046	0.051591	-	NC_000008.11	-	18867674	18872733	5060	1504	sense-overlappin	XM_011544470.1	PSD3
circRNA_05858	0	0	0	0	0	0	0	0	0	0	0	0.0206364	-	NC_000008.11	-	18936034	18970598	34565	34565	sense-overlappin	XM_011544466.1	PSD3
circRNA_05859	0	0	0	0	0	0	0	0	0	0.020172483	0	0	-	NC_000008.11	-	19505201	19601865	96665	1675	sense-overlappin	XM_011544581.1	CSGALNACT1

circRNA_05860	0	0	0	0	0	0	0	0.020486295	0	0	0	0	-	NC_000008.11	-	19704761	19715024	10264	10264	intronic	XM_006716362.1	CSGALNACT1
circRNA_05861	0	0	0	0	0.02006311	0.020712909	0	0.030729443	0	0	0	0	hsa_circ_0001781	NC_000008.11	-	19714885	19732566	17682	17682	intronic	XM_006716362.1	CSGALNACT1
circRNA_05862	0	0	0	0	0.03009467	0	0	0	0	0	0	0	-	NC_000008.11	+	19824803	19826559	1757	304	sense-overlappin	XM_011544564.1	INTS10
circRNA_05863	0	0	0	0	0	0	0.020098685	0	0	0	0	0.0412728	hsa_circ_0083500	NC_000008.11	+	19826426	19837160	10735	636	sense-overlappin	XM_011544564.1	INTS10
circRNA_05864	0	0	0	0	0	0	0	0	0	0	0.030382845	0	hsa_circ_0083530	NC_000008.11	+	21974670	21976521	1852	271	sense-overlappin	NM_015024.4	XPO7
circRNA_05865	0	0	0.032712968	0	0	0	0	0	0	0	0	0	-	NC_000008.11	+	21974670	21980203	5534	465	sense-overlappin	NM_015024.4	XPO7
circRNA_05866	0.072012586	0.040939935	0.04361729	0	0	0	0	0.040972591	0.030381792	0.030258724	0	0	hsa_circ_0002075	NC_000008.11	+	22196680	22197420	741	342	sense-overlappin	XM_006716386.2	BMP1
circRNA_05867	0	0	0	0	0	0.031069364	0	0	0	0	0	0	-	NC_000008.11	+	22206854	22207516	663	342	sense-overlappin	NM_006129.4	BMP1
circRNA_05868	0	0.020469968	0	0	0	0	0	0	0	0	0	0	-	NC_000008.11	+	22461334	22475624	14291	13943	sense-overlappin	XR_949436.1	
circRNA_05869	0.051437562	0.071644886	0.141756193	0.0412724	0	0.041425819	0.040197371	0	0.030381792	0.030258724	0	0	hsa_circ_0083619	NC_000008.11	+	22474954	22475624	671	323	sense-overlappin	XR_949436.1	
circRNA_05870	0	0	0	0	0.02006311	0.020712909	0.020098685	0	0	0	0	0	hsa_circ_0006320	NC_000008.11	+	22474954	22498112	23159	435	sense-overlappin	XR_949436.1	
circRNA_05871	0	0	0	0.0515905	0	0	0.020098685	0	0	0	0	0	hsa_circ_0002081	NC_000008.11	+	22474954	22513432	38479	721	sense-overlappin	XR_949436.1	
circRNA_05872	0	0	0	0	0	0	0	0.030729443	0.020254528	0	0	0	-	NC_000008.11	+	22498001	22498489	489	489	sense-overlappin	XR_949436.1	
circRNA_05873	0	0	0	0	0.02006311	0	0	0	0	0	0	0	-	NC_000008.11	+	23021973	23022276	304	304	antisense	XR_949500.1	
circRNA_05874	0	0	0	0	0.02006311	0	0	0	0	0	0	0	-	NC_000008.11	-	23042041	23043243	1203	1203	sense-overlappin	XR_949500.1	
circRNA_05875	0	0.020469968	0	0	0	0	0	0	0	0	0	0	-	NC_000008.11	-	23434317	23439915	5599	740	sense-overlappin	NM_001128930.2	ENTPD4
circRNA_05876	0	0	0	0	0	0	0.020098685	0	0	0	0	0	-	NC_000008.11	+	23566108	23568378	2271	286	exonic	NM_016612.2	SLC25A37
circRNA_05877	0	0	0.021808645	0	0	0	0	0	0	0	0	0	hsa_circ_0006605	NC_000008.11	+	25366870	25369641	2772	401	sense-overlappin	NM_024940.6	DOCK5
circRNA_05878	0	0.030704951	0	0	0	0	0	0	0	0	0	0	hsa_circ_0083726	NC_000008.11	+	25374564	25380394	5831	301	sense-overlappin	NM_024940.6	DOCK5
circRNA_05879	0	0.020469968	0.032712968	0.0309543	0.04012623	0	0.030148028	0.051215739	0	0	0.02025523	0	hsa_circ_0007618	NC_000008.11	+	25407983	25408940	958	311	sense-overlappin	NM_024940.6	DOCK5
circRNA_05880	0	0	0.032712968	0	0	0	0	0	0	0	0	0	-	NC_000008.11	+	26338890	26354633	15744	335	sense-overlappin	XM_011544567.1	PPP2R2A
circRNA_05881	0	0	0	0	0	0	0.030148028	0	0	0.020172483	0	0	hsa_circ_0002131	NC_000008.11	+	26391243	26408376	17134	511	sense-overlappin	NM_004331.2	BNIP3L
circRNA_05882	0	0	0	0	0	0	0	0	0	0.030258724	0	0	-	NC_000008.11	-	26535955	26536587	633	633	antisense	NM_001197293.2	DPYSL2
circRNA_05883	0	0	0.054521613	0	0	0.020712909	0	0	0	0	0	0	hsa_circ_0005013	NC_000008.11	+	26581969	26583983	2015	274	sense-overlappin	NM_001197293.2	DPYSL2
circRNA_05884	0.061725074	0	0.065425935	0	0.06018934	0.020712909	0	0.040972591	0.030381792	0	0	0.0206364	hsa_circ_0083756	NC_000008.11	-	27294080	27294310	231	231	exonic	XM_011544453.1	TRIM35
circRNA_05885	0	0	0	0.0412724	0	0	0	0	0	0	0	0	hsa_circ_0083757	NC_000008.11	-	27294080	27298559	4480	327	sense-overlappin	XM_011544453.1	TRIM35
circRNA_05886	0	0	0.021808645	0.0206362	0	0	0	0	0	0	0	0	hsa_circ_0083766	NC_000008.11	+	27525362	27536855	11494	184	sense-overlappin	NM_001256482.1	EPHX2
circRNA_05887	0	0	0.021808645	0.0206362	0	0	0	0	0	0	0	0	hsa_circ_0008761	NC_000008.11	+	28129502	28137891	8390	483	sense-overlappin	NM_001284222.1	ELP3
circRNA_05888	0	0	0.054521613	0	0	0	0	0	0	0	0	0	hsa_circ_0001784	NC_000008.11	+	28129502	28162078	32577	950	sense-overlappin	NM_001284222.1	ELP3
circRNA_05889	0.041150049	0.08187987	0.13085187	0.0412724	0.15047336	0.041425819	0.070345399	0.030729443	0.060763583	0.050431207	0.030382845	0.0412728	hsa_circ_0001785	NC_000008.11	+	28155942	28162078	6137	467	sense-overlappin	NM_001284222.1	ELP3
circRNA_05890	0.030862537	0	0.04361729	0.0309543	0.03009467	0	0	0	0	0.020172483	0	0	hsa_circ_0003885	NC_000008.11	+	28713457	28718207	4751	2717	sense-overlappin	NM_001440.3	EXTL3
circRNA_05891	0	0	0	0	0.02006311	0	0	0	0	0	0	0	-	NC_000008.11	+	28734054	28734484	431	431	intronic	NM_001440.3	EXTL3
circRNA_05892	0	0	0	0	0.02006311	0	0	0	0	0	0	0	hsa_circ_0005411	NC_000008.11	+	28737519	28743214	5696	274	sense-overlappin	NM_001440.3	EXTL3
circRNA_05893	0	0	0.021808645	0	0	0	0	0	0	0	0	0	-	NC_000008.11	+	28970043	29018913	48871	828	sense-overlappin	XM_011544641.1	HMBOX1
circRNA_05894	0	0	0	0	0	0	0	0	0	0.020172483	0	0	hsa_circ_0083825	NC_000008.11	+	28980071	29018913	38843	351	sense-overlappin	XM_011544641.1	HMBOX1
circRNA_05895	0	0	0.04361729	0	0.03009467	0	0	0.020486295	0.040509055	0.020172483	0	0	hsa_circ_0083826	NC_000008.11	+	29009072	29018913	9842	265	sense-overlappin	XM_011544641.1	HMBOX1
circRNA_05896	0	0	0	0	0	0	0	0	0	0	0	0.0206364	-	NC_000008.11	+	29231044	29232120	1077	1077	antisense	XM_011544460.1	KIF13B
circRNA_05897	0	0	0.021808645	0	0	0	0	0	0	0	0	0	-	NC_000008.11	-	29245346	29250084	4739	4739	sense-overlappin	NM_015254.3	KIF13B
circRNA_05898	0	0	0	0	0	0	0	0	0	0	0	0.0206364	hsa_circ_0083834	NC_000008.11	-	30069642	30074055	4414	597	sense-overlappin	NM_016127.5	SARAF
circRNA_05899	0.030862537	0.061409903	0	0.0412724	0.06018934	0.051782273	0.140690797	0.061458886	0.030381792	0.151293621	0.04051046	0.0309546	hsa_circ_0007353	NC_000008.11	+	30101898	30104486	2589	263	sense-overlappin	NM_015344.2	LEPROTL1
circRNA_05900	0	0	0.021808645	0	0	0	0	0	0	0	0	0	hsa_circ_0006863	NC_000008.11	+	30175085	30180630	5546	386	sense-overlappin	NM_006571.3	DCTN6
circRNA_05901	0.030862537	0	0	0	0	0	0	0	0.020254528	0.030258724	0	0	hsa_circ_0001786	NC_000008.11	+	30177126	30180630	3505	280	sense-overlappin	NM_006571.3	DCTN6
circRNA_05902	0	0.020469968	0	0	0	0	0	0	0	0	0	0	-	NC_000008.11	+	30408302	30409403	1102	1102	intronic	XM_011544377.1	RBPMS
circRNA_05903	0.102875123	0.040939935	0.032712968	0	0	0.041425819	0	0	0.050636319	0.020172483	0	0	hsa_circ_0007409	NC_000008.11	+	30474779	30479377	4599	180	sense-overlappin	XM_011544373.1	RBPMS
circRNA_05904	0.226325271	0.112584821	0	0.0309543	0.04012623	0.041425819	0	0	0.070890847	0.191638587	0	0.0309546	hsa_circ_0006539	NC_000008.11	+	30474779	30504436	29658	331	sense-overlappin	XM_011544373.1	RBPMS
circRNA_05905	0	0	0	0.0619086	0	0	0	0	0	0	0	0	hsa_circ_0083854	NC_000008.11	-	30680904	30700135	19232	779	sense-overlappin	NM_000637.3	GSR
circRNA_05906	0	0	0	0	0.03009467	0	0	0	0	0	0	0	hsa_circ_0083861	NC_000008.11	-	30700081	30703240	3160	203	sense-overlappin	NM_000637.3	GSR
circRNA_05907	0	0	0	0	0	0	0	0	0	0.020172483	0	0	hsa_circ_0083863	NC_000008.11	-	30700081	30712088	12008	389	sense-overlappin	NM_000637.3	GSR
circRNA_05908	0.020575025	0	0.065425935	0	0	0	0	0	0	0	0	0	hsa_circ_0005060	NC_000008.11	-	30786211	30799755	13545	852	sense-overlappin	NM_001009552.1	PPP2CB
circRNA_05909	0	0	0	0.0309543	0	0	0	0	0	0	0	0	-	NC_000008.11	+	31080867	31096850	15984	1142	sense-overlappin	NM_000553.4	WRN
circRNA_05910	0	0	0	0	0	0	0	0.020486295	0	0	0	0	hsa_circ_0007279	NC_000008.11	+	32595828	32616885	21058	402	sense-overlappin	NM_001159999.1	NRG1
circRNA_05911	0	0	0	0	0	0.041425819	0	0	0	0	0	0	-	NC_000008.11	-	33372644	33389798	17155	997	sense-overlappin	XM_011544676.1	FUT10
circRNA_05912	0.092587611	0.051174919	0.185373483	0.113499	0.26082049	0.15534682	0.080394741	0.051215739	0.091145375	0.14120738	0.111403765	0.0619092	hsa_circ_0001788	NC_000008.11	+	37765526	37766355	830	220	sense-overlappin	NM_007198.3	PROSC
circRNA_05913	0.020575025	0	0	0	0	0	0	0	0	0	0	0	hsa_circ_0007884	NC_000008.11	+	37835174	37837939	2766	651	sense-overlappin	XM_005273471.3	ADGRA2

circRNA_05914	0	0	0	0	0	0	0	0	0.020254528	0	0	0	-	NC_000008.11	+	37860478	37862498	2021	2021	antisense	NM_001002814.2	RAB11FIP1
circRNA_05915	0	0	0.032712968	0	0	0	0	0	0	0	0	0	-	NC_000008.11	-	37862737	37877551	14815	3639	sense-overlappin	NM_001002814.2	RAB11FIP1
circRNA_05916	0.020575025	0.061409903	0.04361729	0.0619086	0.05015779	0.020712909	0.050246713	0.020486295	0.070890847	0	0.050638075	0	hsa_circ_0005630	NC_000008.11	-	37870420	37877551	7132	3262	sense-overlappin	NM_001002814.2	RAB11FIP1
circRNA_05917	0	0	0.032712968	0	0	0	0.030148028	0	0	0	0	0	hsa_circ_0083946	NC_000008.11	-	37874515	37877551	3037	1251	sense-overlappin	NM_001002814.2	RAB11FIP1
circRNA_05918	0.092587611	0.061409903	0.08723458	0.0515905	0.19059959	0.103564547	0.130641455	0.061458886	0.050636319	0.100862414	0.08102092	0.0619092	hsa_circ_0001789	NC_000008.11	-	37877109	37877551	443	443	exonic	NM_001002814.2	RAB11FIP1
circRNA_05919	0	0	0	0	0	0.020712909	0	0	0	0	0	0	-	NC_000008.11	+	38107021	38107610	590	590	sense-overlappin	XM_005273682.1	ASH2L
circRNA_05920	0	0	0	0	0.03009467	0	0	0	0	0	0	0	hsa_circ_0083952	NC_000008.11	+	38110379	38110833	455	184	sense-overlappin	XM_005273682.1	ASH2L
circRNA_05921	0	0	0.04361729	0.0619086	0.02006311	0.051782273	0.030148028	0	0.020254528	0.07060369	0.02025523	0	hsa_circ_0001790	NC_000008.11	+	38114192	38119363	5172	380	sense-overlappin	XM_005273682.1	ASH2L
circRNA_05922	0	0	0	0.0206362	0.03009467	0.020712909	0	0	0	0	0	0	hsa_circ_0006302	NC_000008.11	+	38114192	38121149	6958	598	sense-overlappin	XM_005273682.1	ASH2L
circRNA_05923	0	0	0	0	0.02006311	0	0	0	0	0	0	0	hsa_circ_0008385	NC_000008.11	+	38114192	38135766	21575	1152	sense-overlappin	XM_005273682.1	ASH2L
circRNA_05924	0	0	0	0	0	0	0	0	0.020254528	0	0	0	hsa_circ_0083964	NC_000008.11	+	38128291	38128951	661	362	sense-overlappin	XM_005273682.1	ASH2L
circRNA_05925	0	0	0	0.0206362	0	0.020712909	0	0.020486295	0.020254528	0	0	0	-	NC_000008.11	+	38133454	38135766	2313	192	sense-overlappin	XM_005273682.1	ASH2L
circRNA_05926	0	0	0.021808645	0	0	0	0	0	0	0	0	0	-	NC_000008.11	+	38233139	38261599	28461	3054	sense-overlappin	XM_005273455.2	DDHD2
circRNA_05927	0	0	0	0.0515905	0	0	0	0	0	0.050431207	0	0	-	NC_000008.11	+	38246233	38261599	15367	2141	sense-overlappin	XM_005273455.2	DDHD2
circRNA_05928	0	0	0	0	0.03009467	0	0	0	0	0	0	0	hsa_circ_0083974	NC_000008.11	+	38247713	38253718	6006	929	sense-overlappin	XM_005273455.2	DDHD2
circRNA_05929	0	0	0	0	0	0	0	0	0	0.020172483	0	0	-	NC_000008.11	+	38247713	38261599	13887	2073	sense-overlappin	XM_005273455.2	DDHD2
circRNA_05930	0	0	0.032712968	0	0	0	0	0	0	0	0	0	hsa_circ_0083992	NC_000008.11	-	38314647	38348215	33569	2286	sense-overlappin	XM_011544562.1	WHSC1L1
circRNA_05931	0	0.061409903	0.032712968	0	0.05015779	0.020712909	0	0	0	0.060517448	0.04051046	0	hsa_circ_0083995	NC_000008.11	-	38337305	38348215	10911	954	sense-overlappin	XM_011544562.1	WHSC1L1
circRNA_05932	0	0	0.076330258	0.0722267	0.03009467	0.062138728	0.040197371	0	0	0.07060369	0.02025523	0.0619092	hsa_circ_0083996	NC_000008.11	-	38347497	38348215	719	719	exonic	XM_011544562.1	WHSC1L1
circRNA_05933	0.020575025	0	0	0	0	0	0	0	0	0	0	0	hsa_circ_0005564	NC_000008.11	-	38424509	38426245	1737	315	sense-overlappin	NM_001174065.1	FGFR1
circRNA_05934	0.030862537	0.020469968	0	0	0	0	0	0	0	0.050431207	0	0	hsa_circ_0008016	NC_000008.11	-	38429682	38429948	267	267	exonic	NM_001174065.1	FGFR1
circRNA_05935	0	0	0	0	0	0.020712909	0	0	0	0	0	0	-	NC_000008.11	+	38742351	38788819	46469	43689	sense-overlappin	XM_011544632.1	TACC1
circRNA_05936	0	0	0.021808645	0	0	0	0	0	0	0	0	0	-	NC_000008.11	+	38785676	38788819	3144	364	sense-overlappin	XM_011544632.1	TACC1
circRNA_05937	0	0	0.021808645	0	0.03009467	0	0.020098685	0	0	0	0	0	-	NC_000008.11	+	38788704	38820635	31932	1230	sense-overlappin	NM_001146216.2	TACC1
circRNA_05938	0	0	0.065425935	0.0412724	0.02006311	0	0.040197371	0.051215739	0	0.020172483	0	0	hsa_circ_0003316	NC_000008.11	+	38819522	38820635	1114	1114	exonic	NM_001146216.2	TACC1
circRNA_05939	0	0	0.021808645	0	0	0	0	0	0	0	0	0	-	NC_000008.11	+	38825308	38836287	10980	448	sense-overlappin	NM_001146216.2	TACC1
circRNA_05940	0	0	0	0	0	0	0	0	0	0.020172483	0	0	hsa_circ_0008489	NC_000008.11	+	38836162	38838546	2385	203	sense-overlappin	NM_001146216.2	TACC1
circRNA_05941	0	0	0	0	0	0	0	0	0	0.030258724	0	0	hsa_circ_0084021	NC_000008.11	+	38943789	38946221	2433	147	sense-overlappin	NM_021623.1	PLEKHA2
circRNA_05942	0	0	0	0	0	0	0	0	0	0.030258724	0	0	hsa_circ_0001791	NC_000008.11	+	39021643	39023325	1683	242	sense-overlappin	XR_949497.1	
circRNA_05943	0	0	0.021808645	0	0	0	0	0	0	0	0	0	hsa_circ_0084049	NC_000008.11	+	39054481	39091346	36866	996	sense-overlappin	NM_003816.2	ADAM9
circRNA_05944	0.041150049	0	0.032712968	0	0	0.020712909	0	0.020486295	0	0	0	0	hsa_circ_0084051	NC_000008.11	+	39090047	39091346	1300	230	sense-overlappin	NM_003816.2	ADAM9
circRNA_05945	0	0	0.021808645	0	0.03009467	0	0	0	0	0	0	0	hsa_circ_0007461	NC_000008.11	+	41598292	41599304	1013	1013	exonic	NM_178819.3	AGPAT6
circRNA_05946	0	0	0	0	0	0	0	0	0	0	0.02025523	0	hsa_circ_0001792	NC_000008.11	-	41661430	41661941	512	201	sense-overlappin	NM_020478.4	ANK1
circRNA_05947	0	0	0	0	0	0	0	0	0	0	0.030382845	0	-	NC_000008.11	-	41962936	41963032	97	97	intronic	NM_001099412.1	KAT6A
circRNA_05948	0.061725074	0	0.021808645	0	0	0	0	0	0	0	0	0	-	NC_000008.11	-	41974704	41981954	7251	773	sense-overlappin	NM_001099412.1	KAT6A
circRNA_05949	0	0	0	0	0	0	0.020098685	0	0	0	0	0	hsa_circ_0084089	NC_000008.11	-	41974704	41987563	12860	882	sense-overlappin	NM_001099412.1	KAT6A
circRNA_05950	0.030862537	0.020469968	0.17446916	0	0	0	0.030148028	0	0	0.020172483	0	0	hsa_circ_0002754	NC_000008.11	-	42048378	42049707	1330	1057	sense-overlappin	NM_001099412.1	KAT6A
circRNA_05951	0	0	0	0.0309543	0	0	0	0	0	0	0	0	-	NC_000008.11	+	42314322	42316904	2583	433	sense-overlappin	XM_005273494.1	IKBKB
circRNA_05952	0.020575025	0	0.065425935	0.0206362	0.0702209	0.031069364	0.040197371	0.030729443	0	0.020172483	0.030382845	0.103182001	hsa_circ_0001793	NC_000008.11	+	42325970	42329214	3245	219	sense-overlappin	XM_005273490.1	IKBKB
circRNA_05953	0.030862537	0	0	0	0	0	0	0	0	0	0	0	hsa_circ_0001794	NC_000008.11	+	42344953	42349090	4138	142	sense-overlappin	NM_002690.2	POLB
circRNA_05954	0.020575025	0.030704951	0.021808645	0	0	0	0	0	0	0	0	0	hsa_circ_0084106	NC_000008.11	+	42344953	42352568	7616	251	sense-overlappin	NM_002690.2	POLB
circRNA_05955	0	0.030704951	0	0.0206362	0	0	0	0.040972591	0.030381792	0	0	0	-	NC_000008.11	+	42344953	42357392	12440	536	sense-overlappin	XM_005273538.2	POLB
circRNA_05956	0	0	0	0.0206362	0	0	0	0	0	0	0	0	-	NC_000008.11	+	42352519	42361365	8847	513	sense-overlappin	XR_428311.1	
circRNA_05957	0	0	0	0	0	0	0	0	0.020254528	0	0.02025523	0.0412728	-	NC_000008.11	+	42354425	42371662	17238	740	sense-overlappin	XM_005273538.2	POLB
circRNA_05958	0	0	0	0.0309543	0	0	0	0	0	0.040344966	0	0	-	NC_000008.11	+	42355516	42361365	5850	358	sense-overlappin	XR_428311.1	
circRNA_05959	0	0	0	0	0	0.020712909	0	0	0	0	0	0	hsa_circ_0084119	NC_000008.11	+	42398712	42403461	4750	585	sense-overlappin	XM_006716394.1	VDAC3
circRNA_05960	0	0	0	0	0	0	0	0.020486295	0.030381792	0	0.050638075	0	hsa_circ_0005927	NC_000008.11	+	42401788	42403461	1674	379	sense-overlappin	XM_006716394.1	VDAC3
circRNA_05961	0	0.040939935	0	0	0	0	0	0	0	0.040344966	0	0	hsa_circ_0009161	NC_000008.11	-	42459896	42472654	12759	1012	sense-overlappin	XM_006716391.2	SLC20A2
circRNA_05962	0.020575025	0.020469968	0.021808645	0.0206362	0.02006311	0.020712909	0	0.040972591	0	0.060517448	0.02025523	0	hsa_circ_0084143	NC_000008.11	+	42906173	42943445	37273	343	sense-overlappin	NM_032410.3	HOOK3
circRNA_05963	0	0	0	0	0	0.031069364	0	0	0.040509055	0	0	0	hsa_circ_0084144	NC_000008.11	+	42906173	42950455	44283	411	sense-overlappin	NM_032410.3	HOOK3
circRNA_05964	0	0	0	0	0	0.020712909	0	0	0	0	0	0	hsa_circ_0084145	NC_000008.11	+	42906173	42959314	53142	558	sense-overlappin	NM_032410.3	HOOK3
circRNA_05965	0	0	0	0	0	0	0.020098685	0	0	0	0	0	hsa_circ_0084147	NC_000008.11	+	42906173	42974194	68022	1264	sense-overlappin	NM_032410.3	HOOK3
circRNA_05966	0	0	0	0.0206362	0	0	0	0	0	0	0	0	hsa_circ_0084149	NC_000008.11	+	42906173	42986795	80623	1475	sense-overlappin	NM_032410.3	HOOK3
circRNA_05967	0	0	0.021808645	0	0	0	0	0	0	0.020172483	0	0	hsa_circ_0005376	NC_000008.11	+	42925557	42943445	17889	257	sense-overlappin	NM_032410.3	HOOK3

circRNA_05968	0.041150049	0.030704951	0	0	0	0.134633911	0.050246713	0	0	0.050431207	0.02025523	0	hsa_circ_0006376	NC_000008.11	+	42957094	42964474	7381	311	sense-overlappin	NM_032410.3	HOOK3
circRNA_05969	0	0	0	0	0	0	0	0	0.020254528	0	0	0	-	NC_000008.11	+	42957094	42986795	29702	1064	sense-overlappin	NM_032410.3	HOOK3
circRNA_05970	0	0	0.04361729	0	0	0	0	0	0	0	0	0	-	NC_000008.11	+	42964311	42997637	33327	1005	sense-overlappin	NM_032410.3	HOOK3
circRNA_05971	0	0	0	0	0	0.020712909	0	0	0	0	0	0	hsa_circ_0084164	NC_000008.11	+	42966473	42968214	1742	343	sense-overlappin	NM_032410.3	HOOK3
circRNA_05972	0	0	0	0	0	0	0.020098685	0	0	0	0	0	-	NC_000008.11	+	42966473	42974194	7722	542	sense-overlappin	NM_032410.3	HOOK3
circRNA_05973	0	0	0	0	0	0	0	0	0	0.050431207	0	0	-	NC_000008.11	+	43007847	43013400	5554	361	sense-overlappin	NM_032410.3	HOOK3
circRNA_05974	0	0	0	0	0	0	0	0	0	0	0.02025523	0	-	NC_000008.11	+	43020700	43021684	985	985	exonic	NM_032410.3	HOOK3
circRNA_05975	0.051437562	0	0.04361729	0	0.0702209	0	0.040197371	0	0.030381792	0.060517448	0.030382845	0.0619092	hsa_circ_0001796	NC_000008.11	+	43059092	43064215	5124	201	sense-overlappin	NM_002027.2	FNTA
circRNA_05976	0	0.020469968	0	0	0	0	0	0	0	0	0	0	hsa_circ_0084169	NC_000008.11	+	43059092	43069659	10568	306	sense-overlappin	NM_002027.2	FNTA
circRNA_05977	0	0	0.021808645	0	0	0	0	0	0	0	0	0	hsa_circ_0006586	NC_000008.11	+	43064101	43072307	8207	347	sense-overlappin	NM_002027.2	FNTA
circRNA_05978	0	0	0	0	0	0	0.020098685	0	0	0	0	0	hsa_circ_0008397	NC_000008.11	+	43064101	43077364	13264	496	sense-overlappin	NM_002027.2	FNTA
circRNA_05979	0	0.051174919	0	0.0412724	0	0.041425819	0	0.071702034	0	0.040344966	0	0.0206364	hsa_circ_0005847	NC_000008.11	+	43069555	43072307	2753	232	sense-overlappin	NM_002027.2	FNTA
circRNA_05980	0	0	0.032712968	0	0	0	0	0	0	0	0	0	-	NC_000008.11	+	43146948	43161507	14560	445	sense-overlappin	XM_005273412.2	HGSNAT
circRNA_05981	0.030862537	0	0	0	0.02006311	0	0	0	0	0	0	0	-	NC_000008.11	+	43158575	43173743	15169	617	sense-overlappin	XM_005273412.2	HGSNAT
circRNA_05982	0	0.030704951	0.861441478	0.0928628	0.12037869	0	0.020098685	0.040972591	0.040509055	0.080689931	0.050638075	0.051591	hsa_circ_0084188	NC_000008.11	+	47279862	47294030	14169	492	sense-overlappin	NM_001080394.3	SPIDR
circRNA_05983	0.072012586	0.051174919	0.359842643	0.1238171	0.12037869	0.051782273	0.040197371	0.051215739	0.060763583	0.14120738	0	0	hsa_circ_0002157	NC_000008.11	+	47284028	47294030	10003	336	sense-overlappin	NM_001080394.3	SPIDR
circRNA_05984	0	0	0	0.0206362	0	0	0	0	0	0	0	0	-	NC_000008.11	+	47284028	47330895	46868	37201	sense-overlappin	NM_001080394.3	SPIDR
circRNA_05985	0	0	0	0.0309543	0	0	0	0	0	0	0	0	hsa_circ_0084193	NC_000008.11	+	47291033	47294030	2998	269	sense-overlappin	NM_001080394.3	SPIDR
circRNA_05986	0	0	0.021808645	0.0206362	0	0	0.020098685	0	0	0.040344966	0	0	-	NC_000008.11	+	47394978	47407961	12984	396	sense-overlappin	XM_011517500.1	SPIDR
circRNA_05987	0.082300098	0.092114854	0.08723458	0.1857257	0.16050492	0.134633911	0.100493427	0.09218833	0.050636319	0.161379862	0.06076569	0.072227401	hsa_circ_0001798	NC_000008.11	+	47396376	47407961	11586	352	sense-overlappin	NM_001080394.3	SPIDR
circRNA_05988	0	0	0.032712968	0.0309543	0	0	0	0	0	0	0	0.051591	hsa_circ_0002003	NC_000008.11	+	47407861	47440542	32682	321	sense-overlappin	NM_001080394.3	SPIDR
circRNA_05989	0	0	0	0	0	0	0.020098685	0	0	0	0	0	-	NC_000008.11	+	47440323	47440542	220	220	exonic	NM_001080394.3	SPIDR
circRNA_05990	0	0	0.032712968	0	0	0	0	0	0	0	0	0	hsa_circ_0004511	NC_000008.11	+	47595811	47599196	3386	447	sense-overlappin	NM_001080394.3	SPIDR
circRNA_05991	0.041150049	0	0.04361729	0.0722267	0	0	0.020098685	0	0	0	0	0	hsa_circ_0001799	NC_000008.11	+	47700403	47702015	1613	292	sense-overlappin	NM_001080394.3	SPIDR
circRNA_05992	0	0	0	0	0	0.020712909	0	0	0	0	0	0	-	NC_000008.11	-	47777686	47783809	6124	935	sense-overlappin	XM_011517567.1	PRKDC
circRNA_05993	0	0	0.021808645	0	0	0	0	0	0	0	0	0	-	NC_000008.11	-	47783742	47789238	5497	505	sense-overlappin	XM_011517567.1	PRKDC
circRNA_05994	0	0	0.021808645	0	0	0	0	0	0	0	0	0	-	NC_000008.11	-	47783742	47794501	10760	717	sense-overlappin	XM_011517567.1	PRKDC
circRNA_05995	0	0	0	0	0	0	0	0	0.020254528	0	0	0	hsa_circ_0084304	NC_000008.11	-	47817450	47831926	14477	1405	sense-overlappin	XM_011517567.1	PRKDC
circRNA_05996	0	0	0	0	0.04012623	0	0	0	0	0	0	0	hsa_circ_0084443	NC_000008.11	-	47927194	47930012	2819	527	sense-overlappin	XM_011517567.1	PRKDC
circRNA_05997	0	0.030704951	0	0	0	0	0	0	0	0	0	0	-	NC_000008.11	-	47933020	47935058	2039	329	sense-overlappin	XM_011517567.1	PRKDC
circRNA_05998	0	0	0.032712968	0	0	0	0	0	0	0	0	0	hsa_circ_0084464	NC_000008.11	-	47953620	47957431	3812	567	sense-overlappin	XM_011517567.1	PRKDC
circRNA_05999	0	0	0	0	0	0	0	0.020486295	0	0	0	0	hsa_circ_0005346	NC_000008.11	+	47969798	47976866	7069	1506	sense-overlappin	NM_005914.3	MCM4
circRNA_06000	0.020575025	0	0.021808645	0	0	0.113921001	0	0	0	0	0	0	hsa_circ_0002537	NC_000008.11	+	48043033	48049978	6946	275	sense-overlappin	XM_011517583.1	UBE2V2
circRNA_06001	0	0	0	0	0	0	0	0	0	0.030258724	0	0	hsa_circ_0007598	NC_000008.11	+	48806783	48817083	10301	10301	intergenic		
circRNA_06002	0	0	0.021808645	0	0	0	0	0	0	0	0	0	-	NC_000008.11	-	51831444	51845763	14320	591	sense-overlappin	NM_001286783.1	PCMTD1
circRNA_06003	0	0.020469968	0.032712968	0	0.02006311	0	0.030148028	0	0	0.020172483	0	0	-	NC_000008.11	-	51831444	51861246	29803	16074	sense-overlappin	NM_001286783.1	PCMTD1
circRNA_06004	0.041150049	0.122819805	0.065425935	0.0309543	0.0702209	0.051782273	0.030148028	0.020486295	0	0.07060369	0	0	-	NC_000008.11	-	51845661	51861246	15586	505	sense-overlappin	NM_052937.3	PCMTD1
circRNA_06005	0	0	0	0	0.04012623	0.020712909	0.020098685	0	0	0	0	0	-	NC_000008.11	-	51857460	51861246	3787	3787	sense-overlappin	NM_052937.3	PCMTD1
circRNA_06006	0.730413374	0.491279221	0.468885868	0.4849504	0.96302952	1.046001922	0.391924364	0.665804603	0.232927069	0.837158036	0.486125522	0.484955404	hsa_circ_0001801	NC_000008.11	-	51860845	51861246	402	402	exonic	NM_052937.3	PCMTD1
circRNA_06007	0	0.040939935	0.054521613	0	0	0	0	0	0	0	0	0	hsa_circ_0001802	NC_000008.11	-	51860861	51861246	386	386	exonic	NM_052937.3	PCMTD1
circRNA_06008	0.020575025	0	0	0	0.04012623	0	0	0	0	0	0	0	hsa_circ_0084501	NC_000008.11	-	52683549	52686967	3419	755	sense-overlappin	XM_011517643.1	RB1CC1
circRNA_06009	0	0	0	0.0309543	0	0	0	0.030729443	0	0	0	0	-	NC_000008.11	-	53769618	53772167	2550	305	sense-overlappin	NM_213620.2	ATP6V1H
circRNA_06010	0	0	0.021808645	0	0	0	0	0	0	0	0	0	-	NC_000008.11	-	53769618	53801896	32279	596	sense-overlappin	NM_213620.2	ATP6V1H
circRNA_06011	0	0	0	0	0.02006311	0	0	0	0	0	0	0	-	NC_000008.11	-	53795647	53829533	33887	654	sense-overlappin	NM_213620.2	ATP6V1H
circRNA_06012	0	0	0	0	0.06018934	0	0	0	0	0	0	0	hsa_circ_0008328	NC_000008.11	-	53811164	53829533	18370	363	sense-overlappin	NM_213620.2	ATP6V1H
circRNA_06013	0	0	0	0	0	0	0	0	0.030381792	0	0	0	hsa_circ_0084538	NC_000008.11	+	55782748	55805036	22289	2042	sense-overlappin	NM_024831.6	TGS1
circRNA_06014	0	0	0	0	0	0	0.020098685	0	0	0.020172483	0	0	hsa_circ_0084546	NC_000008.11	+	55941855	55947723	5869	226	sense-overlappin	NM_001111097.2	LYN
circRNA_06015	0.020575025	0	0.021808645	0	0	0	0	0.020486295	0	0.020172483	0	0	hsa_circ_0001803	NC_000008.11	+	55941855	55953984	12130	732	sense-overlappin	NM_001111097.2	LYN
circRNA_06016	0	0	0	0	0	0	0	0.030729443	0.020254528	0	0	0.0309546	hsa_circ_0006022	NC_000008.11	+	55950459	55953984	3526	506	sense-overlappin	XR_928779.1	
circRNA_06017	0	0	0	0	0	0	0	0.020486295	0	0	0	0	hsa_circ_0003392	NC_000008.11	-	56050767	56050949	183	183	intergenic		
circRNA_06018	0	0	0.021808645	0	0	0	0	0	0	0	0	0	hsa_circ_0006683	NC_000008.11	-	58623220	58635546	12327	408	sense-overlappin	NM_001144772.1	NSMAF
circRNA_06019	0	0	0	0	0	0.031069364	0	0	0	0	0	0	-	NC_000008.11	-	58642984	58643096	113	113	sense-overlappin	NM_001144772.1	NSMAF
circRNA_06020	0.041150049	0	0	0.0206362	0	0	0	0	0	0	0	0	hsa_circ_0006786	NC_000008.11	-	58939302	58960008	20707	309	sense-overlappin	NM_014729.2	TOX
circRNA_06021	0	0	0	0	0.02006311	0	0	0	0	0	0	0.0206364	-	NC_000008.11	-	60208750	60226935	18186	395	sense-overlappin	XM_011517586.1	CA8
circRNA_06022	0	0	0	0	0.08025246	0	0	0.030729443	0.030381792	0.040344966	0.02025523	0	hsa_circ_0007581	NC_000008.11	+	60572046	60591969	19924	356	sense-overlappin	NM_001242644.1	RAB2A

circRNA_06023	0.051437562	0	0.04361729	0.0309543	0.03009467	0	0	0	0	0	0	0	hsa_circ_0003183	NC_000008.11	+	60584208	60591969	7762	288	sense-overlappin	NM_001242644.1	RAB2A
circRNA_06024	0.082300098	0.092114854	0.196277805	0.0928628	0.05015779	0	0.070345399	0.051215739	0.020254528	0.07060369	0	0.0309546	hsa_circ_0084582	NC_000008.11	+	60741259	60743097	1839	1839	exonic	XM_011517560.1	CHD7
circRNA_06025	0	0	0	0	0.02006311	0	0	0	0	0.020172483	0	0	-	NC_000008.11	+	60741259	60781430	40172	2270	sense-overlappin	XM_011517560.1	CHD7
circRNA_06026	0.030862537	0	0.065425935	0.0206362	0.04012623	0	0	0	0	0.030258724	0	0	-	NC_000008.11	+	60794986	60801593	6608	346	sense-overlappin	XM_011517560.1	CHD7
circRNA_06027	0	0	0.08723458	0	0	0	0	0	0	0	0	0	-	NC_000008.11	+	60794986	60808272	13287	402	sense-overlappin	XM_011517560.1	CHD7
circRNA_06028	0	0	0	0	0.04012623	0	0	0	0	0	0	0	-	NC_000008.11	-	61576772	61643433	66662	440	sense-overlappin	XM_005251248.1	ASPH
circRNA_06029	0	0	0	0	0	0.031069364	0	0	0	0	0	0	-	NC_000008.11	-	61576772	61644632	67861	530	sense-overlappin	XM_005251240.1	ASPH
circRNA_06030	0	0	0	0	2.5480156	0	0	0	0	0	0	0	-	NC_000008.11	-	61618978	61643433	24456	267	sense-overlappin	XM_005251248.1	ASPH
circRNA_06031	0.041150049	0	0.04361729	0.2682705	0.13041025	0.113921001	0.020098685	0.112674625	0.101272639	0.060517448	0	0	-	NC_000008.11	-	61618978	61644632	25655	357	sense-overlappin	XM_005251240.1	ASPH
circRNA_06032	0	0	0.021808645	0	0.04012623	0	0	0	0	0	0	0	-	NC_000008.11	-	61618978	61646878	27901	486	sense-overlappin	XM_005251240.1	ASPH
circRNA_06033	0.082300098	0.122819805	0.098138903	0.330179	0.30094672	0.124277456	0	0.061458886	0.121527166	0.262242276	0.04051046	0.0412728	hsa_circ_0084606	NC_000008.11	-	61618978	61653660	34683	654	sense-overlappin	XM_005251240.1	ASPH
circRNA_06034	0	0	0	0.0309543	0.02006311	0	0	0	0	0.020172483	0	0	-	NC_000008.11	-	61618978	61684188	65211	918	sense-overlappin	XM_005251240.1	ASPH
circRNA_06035	0	0	0	0	0.09028402	0	0	0	0	0	0	0	-	NC_000008.11	-	61633683	61643433	9751	225	sense-overlappin	XM_005251248.1	ASPH
circRNA_06036	0	0	0	0.0309543	0.03009467	0	0	0.020486295	0	0	0	0.0206364	hsa_circ_0009158	NC_000008.11	-	61633683	61653660	19978	612	sense-overlappin	XM_005251240.1	ASPH
circRNA_06037	0	0	0	0	0.02006311	0	0	0	0	0	0	0	-	NC_000008.11	-	61637947	61644632	6686	270	sense-overlappin	XM_005251240.1	ASPH
circRNA_06038	0	0	0	0	0.02006311	0	0	0	0	0	0	0	hsa_circ_0084609	NC_000008.11	-	61642888	61684188	41301	732	sense-overlappin	XM_005251240.1	ASPH
circRNA_06039	0	0.020469968	0	0	0	0.020712909	0	0	0	0	0.02025523	0	hsa_circ_0084620	NC_000008.11	+	63186147	63187745	1599	1599	exonic	NM_001277813.1	YTHDF3
circRNA_06040	0	0	0	0	0	0	0.020098685	0	0	0	0	0	hsa_circ_0084622	NC_000008.11	-	65622263	65627433	5171	310	sense-overlappin	NM_001286702.1	ARMC1
circRNA_06041	0	0	0	0	0.04012623	0	0	0	0	0	0	0	hsa_circ_0084635	NC_000008.11	-	65745407	65782843	37437	361	sense-overlappin	NM_002603.3	PDE7A
circRNA_06042	0	0	0	0	0	0.020712909	0	0	0	0	0	0	-	NC_000008.11	-	65819905	65820388	484	484	intronic	NM_001242318.2	PDE7A
circRNA_06043	0.020575025	0	0	0.0515905	0	0	0	0	0	0	0	0	hsa_circ_0002562	NC_000008.11	+	66471430	66473938	2509	2509	intergenic		
circRNA_06044	0	0	0	0.0412724	0	0	0	0	0	0.040344966	0	0	hsa_circ_0084648	NC_000008.11	+	66804375	66822459	18085	237	sense-overlappin	NM_001204173.1	C8orf44-SGK3
circRNA_06045	0	0	0	0	0	0	0	0	0	0	0.030382845	0	hsa_circ_0084650	NC_000008.11	+	66813853	66831311	17459	272	sense-overlappin	NM_001204173.1	C8orf44-SGK3
circRNA_06046	0	0	0	0	0	0.031069364	0	0	0	0	0	0	-	NC_000008.11	+	67076482	67095732	19251	848	sense-overlappin	XM_005251305.3	CSPP1
circRNA_06047	0	0.020469968	0	0	0.04012623	0	0	0	0	0	0	0	hsa_circ_0084663	NC_000008.11	+	67093543	67095732	2190	539	sense-overlappin	XM_005251305.3	CSPP1
circRNA_06048	0	0	0	0	0	0	0.020098685	0.040972591	0	0	0	0	hsa_circ_0084665	NC_000008.11	+	67095293	67095732	440	440	exonic	XM_005251305.3	CSPP1
circRNA_06049	0	0	0.021808645	0	0	0	0	0	0	0	0	0	-	NC_000008.11	+	67095293	67105975	10683	610	sense-overlappin	XM_005251305.3	CSPP1
circRNA_06050	0	0	0	0.0206362	0	0	0	0.020486295	0	0.020172483	0	0	-	NC_000008.11	+	67095293	67112065	16773	704	sense-overlappin	XM_005251305.3	CSPP1
circRNA_06051	0	0	0	0	0	0	0	0.020486295	0	0	0	0	hsa_circ_0084669	NC_000008.11	+	67103037	67118821	15785	774	sense-overlappin	XM_011517598.1	CSPP1
circRNA_06052	0	0	0	0	0	0	0	0	0	0	0.02025523	0	hsa_circ_0084670	NC_000008.11	+	67103037	67137603	34567	1052	sense-overlappin	XM_011517598.1	CSPP1
circRNA_06053	0	0.020469968	0	0.0206362	0.06018934	0.041425819	0	0.030729443	0.030381792	0	0	0	hsa_circ_0001806	NC_000008.11	+	67105905	67116122	10218	474	sense-overlappin	XM_011517598.1	CSPP1
circRNA_06054	0	0	0.04361729	0	0	0.020712909	0	0.020486295	0	0	0	0	hsa_circ_0084678	NC_000008.11	+	67118248	67137603	19356	479	sense-overlappin	XM_005251305.3	CSPP1
circRNA_06055	0.020575025	0.040939935	0.119947548	0.0722267	0	0	0	0	0	0.060517448	0	0	hsa_circ_0003388	NC_000008.11	+	67131951	67137603	5653	278	sense-overlappin	XM_005251305.3	CSPP1
circRNA_06056	0	0	0	0	0	0	0	0.030729443	0	0	0	0	-	NC_000008.11	+	67131951	67190759	58809	1633	sense-overlappin	XM_005251305.3	CSPP1
circRNA_06057	0	0	0	0.0412724	0	0	0	0	0	0	0	0	-	NC_000008.11	-	67240162	67288065	47904	2063	sense-overlappin	XM_005251136.2	ARFGEF1
circRNA_06058	0	0	0	0	0.03009467	0	0	0	0	0.060517448	0.02025523	0	hsa_circ_0001807	NC_000008.11	-	67251299	67259926	8628	727	sense-overlappin	XM_005251136.2	ARFGEF1
circRNA_06059	0.041150049	0	0	0.0412724	0.02006311	0	0	0	0.030381792	0	0	0	-	NC_000008.11	-	67266006	67277457	11452	1096	sense-overlappin	XM_005251136.2	ARFGEF1
circRNA_06060	0.102875123	0.030704951	0.04361729	0.0412724	0.06018934	0.041425819	0.030148028	0.020486295	0.020254528	0.030258724	0.02025523	0	-	NC_000008.11	-	67287955	67302466	14512	903	sense-overlappin	XM_005251136.2	ARFGEF1
circRNA_06061	0.020575025	0	0.065425935	0	0.02006311	0	0	0.040972591	0	0.030258724	0	0	hsa_circ_0001808	NC_000008.11	-	67299209	67302466	3258	335	sense-overlappin	XM_005251136.2	ARFGEF1
circRNA_06062	0	0	0	0	0	0	0	0	0.020254528	0	0	0	-	NC_000008.11	+	68022036	68038292	16257	503	sense-overlappin	XM_011517612.1	PREX2
circRNA_06063	0.061725074	0	0	0	0	0	0	0	0	0	0	0	-	NC_000008.11	+	68038159	68060779	22621	634	sense-overlappin	XM_011517612.1	PREX2
circRNA_06064	0	0	0	0	0	0	0	0	0	0.020172483	0	0	-	NC_000008.11	+	68044487	68060779	16293	500	sense-overlappin	XM_011517612.1	PREX2
circRNA_06065	0.030862537	0	0	0	0	0	0	0	0	0	0	0	hsa_circ_0084727	NC_000008.11	-	69738040	69761875	23836	516	sense-overlappin	XR_428341.2	
circRNA_06066	0.051437562	0	0	0	0	0	0	0.030729443	0	0	0	0	hsa_circ_0001809	NC_000008.11	-	69755424	69761875	6452	351	sense-overlappin	XM_005251313.2	SLCO5A1
circRNA_06067	0	0	0	0	0	0	0.020098685	0	0.020254528	0	0	0	-	NC_000008.11	-	70123884	70144848	20965	1688	sense-overlappin	XM_011517440.1	NCOA2
circRNA_06068	0.051437562	0.061409903	0.054521613	0.0412724	0.05015779	0.020712909	0.140690797	0.040972591	0.030381792	0.080689931	0.070893305	0	hsa_circ_0001810	NC_000008.11	-	70159505	70162854	3350	292	sense-overlappin	XM_011517440.1	NCOA2
circRNA_06069	0.164600197	0.051174919	0.163564838	0.113499	0.09028402	0.165703275	0.15074014	0.184376659	0.101272639	0.252156035	0.070893305	0.0309546	-	NC_000008.11	-	70213903	70216764	2862	278	sense-overlappin	XM_005251130.3	NCOA2
circRNA_06070	0	0	0	0	0	0	0.090444084	0	0	0	0	0	-	NC_000008.11	-	70583164	70583701	538	213	sense-overlappin	NM_014294.5	TRAM1
circRNA_06071	0.020575025	0.020469968	0.098138903	0	0	0	0	0	0	0	0	0	hsa_circ_0084746	NC_000008.11	-	70583164	70583793	630	305	sense-overlappin	NM_014294.5	TRAM1
circRNA_06072	0	0	0	0	0	0.041425819	0	0	0.040509055	0	0	0	hsa_circ_0084748	NC_000008.11	-	70583164	70598255	15092	864	sense-overlappin	NM_014294.5	TRAM1
circRNA_06073	0	0	0	0	0	0	0	0	0.020254528	0	0	0	hsa_circ_0084756	NC_000008.11	-	70594506	70598255	3750	383	sense-overlappin	NM_014294.5	TRAM1
circRNA_06074	0.072012586	0.051174919	0.054521613	0.0309543	0.05015779	0.051782273	0	0.040972591	0	0.060517448	0.02025523	0.0206364	-	NC_000008.11	-	70637815	70641050	3236	320	sense-overlappin	NM_016027.2	LACTB2
circRNA_06075	0	0	0.109043225	0.0206362	0	0	0	0	0	0	0	0	-	NC_000008.11	-	70637815	70644243	6429	499	sense-overlappin	NM_016027.2	LACTB2
circRNA_06076	0	0	0.021808645	0	0.03009467	0.020712909	0	0	0	0	0	0	-	NC_000008.11	+	70674818	70681330	6513	742	sense-overlappin	NM_001287259.1	XKR9

circRNA_06077	0.020575025	0	0.076330258	0	0.09028402	0	0	0	0	0	0	0	-	NC_000008.11	+	70674818	70707153	32336	971	sense-overlappin	NM_001287258.1	XKR9
circRNA_06078	0	0	0	0	0	0	0	0	0	0.020172483	0	0	hsa_circ_0007033	NC_000008.11	+	72052539	72062365	9827	1668	antisense	NM_007332.2	TRPA1
circRNA_06079	0.113162635	0	0	0.0309543	0.02006311	0	0.040197371	0.030729443	0.040509055	0.07060369	0.04051046	0	hsa_circ_0002503	NC_000008.11	+	72052539	72076627	24089	3089	antisense	XM_011517625.1	TRPA1
circRNA_06080	0	0	0	0.1547714	0	0	0	0	0	0	0	0	-	NC_000008.11	-	72205299	72211555	6257	6257	intergenic		
circRNA_06081	0	0	0	0.0206362	0	0	0	0	0	0	0	0	-	NC_000008.11	-	72211360	72238495	27136	27136	intergenic		
circRNA_06082	0	0	0	0	0	0	0	0	0	0.020172483	0	0	-	NC_000008.11	-	73595166	73603863	8698	270	sense-overlappin	NM_001164380.1	STAU2
circRNA_06083	0.13373766	0.08187987	0.076330258	0.1238171	0.11034713	0.082851637	0.110542769	0.102431477	0.121527166	0.110948655	0.111403765	0.092863801	hsa_circ_0001811	NC_000008.11	-	73673107	73688813	15707	296	sense-overlappin	NM_001164380.1	STAU2
circRNA_06084	0.030862537	0	0	0	0	0	0	0	0	0	0	0	hsa_circ_0006915	NC_000008.11	-	73673107	73709162	36056	427	sense-overlappin	NM_001164380.1	STAU2
circRNA_06085	0	0	0.04361729	0	0	0.031069364	0.040197371	0.040972591	0	0.050431207	0	0	hsa_circ_0084781	NC_000008.11	-	73673107	73739868	66762	606	sense-overlappin	NM_001164380.1	STAU2
circRNA_06086	0	0	0	0	0.02006311	0	0	0.020486295	0	0	0	0	hsa_circ_0002216	NC_000008.11	-	73688654	73738349	49696	357	sense-overlappin	NM_001164380.1	STAU2
circRNA_06087	0	0	0	0.0206362	0	0	0	0	0	0	0	0	hsa_circ_0084783	NC_000008.11	-	73688654	73739868	51215	470	sense-overlappin	NM_001164380.1	STAU2
circRNA_06088	0	0	0	0	0	0	0	0	0.020254528	0	0	0	-	NC_000008.11	-	73809040	73825249	16210	1693	sense-overlappin	NM_001271015.1	UBE2W
circRNA_06089	0	0	0	0	0	0	0.020098685	0	0	0	0	0	-	NC_000008.11	+	75481406	75490208	8803	230	sense-overlappin	XM_011517515.1	HNF4G
circRNA_06090	0	0	0	0	0	0	0.020098685	0	0	0	0	0	hsa_circ_0001812	NC_000008.11	+	78933743	78943071	9329	9329	intergenic		
circRNA_06091	0	0	0.032712968	0	0	0.031069364	0	0	0	0	0	0	-	NC_000008.11	-	79917978	79919148	1171	1171	sense-overlappin	NM_014018.2	MRPS28
circRNA_06092	0	0.040939935	0.032712968	0.0309543	0	0	0	0	0	0	0	0	-	NC_000008.11	-	80051527	80064593	13067	437	sense-overlappin	NR_105033.1	
circRNA_06093	0	0	0.021808645	0	0.02006311	0	0	0	0	0	0	0	-	NC_000008.11	+	80499494	80500382	889	889	exonic	NM_001277145.1	ZBTB10
circRNA_06094	0	0	0	0.0206362	0	0	0	0	0	0	0	0	hsa_circ_0005438	NC_000008.11	-	81025220	81030089	4870	4870	sense-overlappin	XM_011517561.1	PAG1
circRNA_06095	0	0	0.021808645	0	0	0	0	0	0	0	0	0	-	NC_000008.11	-	81713918	81718224	4307	425	sense-overlappin	NM_001170796.1	ZFAND1
circRNA_06096	0	0	0	0.0206362	0	0	0	0	0	0	0	0	-	NC_000008.11	+	85278388	85281639	3252	3252	sense-overlappin	NM_198584.2	CA13
circRNA_06097	0	0	0.021808645	0.0619086	0.02006311	0	0.030148028	0.030729443	0	0	0.030382845	0	-	NC_000008.11	+	85281230	85281639	410	410	exonic	NM_198584.2	CA13
circRNA_06098	0	0	0.021808645	0	0	0.031069364	0	0	0	0	0	0	-	NC_000008.11	+	86368939	86381629	12691	448	sense-overlappin	XM_005250761.2	WWP1
circRNA_06099	0	0	0	0	0	0	0	0	0.020254528	0	0	0	-	NC_000008.11	-	86377374	86383456	6083	5443	antisense	XM_005250760.2	WWP1
circRNA_06100	0	0	0	0	0	0	0	0.020486295	0	0	0	0	-	NC_000008.11	+	86425223	86425318	96	96	exonic	XM_005250760.2	WWP1
circRNA_06101	0.020575025	0	0	0	0	0	0	0.020486295	0	0	0	0	hsa_circ_0003175	NC_000008.11	-	86484872	86507112	22241	456	sense-overlappin	XM_011517090.1	RMDN1
circRNA_06102	0	0	0	0	0	0	0	0	0.020254528	0	0	0	hsa_circ_0084844	NC_000008.11	-	86486484	86507112	20629	366	sense-overlappin	XM_011517090.1	RMDN1
circRNA_06103	0	0	0.021808645	0	0	0.062138728	0	0	0	0	0	0	-	NC_000008.11	+	86528536	86532580	4045	469	sense-overlappin	NM_003909.4	CPNE3
circRNA_06104	0	0.030704951	0	0	0	0	0	0	0	0	0	0	-	NC_000008.11	+	86753519	86783980	30462	30462	intergenic		
circRNA_06105	0.030862537	0	0	0	0.03009467	0	0	0	0	0	0	0	hsa_circ_0002915	NC_000008.11	-	89587891	89596512	8622	8622	intergenic		
circRNA_06106	0	0	0	0.0206362	0	0	0	0	0	0	0	0	-	NC_000008.11	-	89647740	89725641	77902	77902	intergenic		
circRNA_06107	0	0	0	0	0	0	0.030148028	0	0	0	0	0	-	NC_000008.11	+	89660296	89660904	609	609	intergenic		
circRNA_06108	0.041150049	0.020469968	0.076330258	0	0	0	0	0.020486295	0	0	0.030382845	0	hsa_circ_0008587	NC_000008.11	-	89722015	89725641	3627	3627	intergenic		
circRNA_06109	0.020575025	0	0	0	0	0	0	0	0	0	0	0	-	NC_000008.11	+	89765341	89790412	25072	1292	sense-overlappin	XM_005251092.2	RIPK2
circRNA_06110	0.041150049	0	0	0	0	0	0	0	0	0	0	0	hsa_circ_0084867	NC_000008.11	-	89943253	89947892	4640	339	sense-overlappin	NM_002485.4	NBN
circRNA_06111	0	0	0	0	0	0	0	0	0	0.020172483	0	0	-	NC_000008.11	+	90042728	90044995	2268	220	sense-overlappin	NM_001359.1	DECR1
circRNA_06112	0.020575025	0	0	0	0	0	0	0	0	0	0	0	-	NC_000008.11	-	91008743	91024366	15624	3395	sense-overlappin	NM_018710.2	TMEM55A
circRNA_06113	0	0	0.032712968	0	0	0	0	0	0	0	0	0	-	NC_000008.11	+	91102659	91133305	30647	582	sense-overlappin	NM_001129890.1	LRRC69
circRNA_06114	0	0	0	0	0.02006311	0	0	0	0	0	0	0	-	NC_000008.11	-	91986124	92076137	90014	1446	sense-overlappin	NM_001198625.1	RUNX1T1
circRNA_06115	0	0	0	0	0	0	0	0	0.020254528	0.060517448	0	0	hsa_circ_0007443	NC_000008.11	-	92005116	92017363	12248	652	sense-overlappin	NM_175636.2	RUNX1T1
circRNA_06116	0	0	0	0.0206362	0	0	0	0	0	0	0	0	hsa_circ_0005643	NC_000008.11	-	92916929	92954564	37636	396	sense-overlappin	NM_001171796.1	TRIQK
circRNA_06117	0.030862537	0	0	0	0	0	0	0	0	0	0	0	-	NC_000008.11	+	93763842	93785378	21537	882	sense-overlappin	NM_001142301.1	TMEM67
circRNA_06118	0.020575025	0	0	0	0	0	0	0	0	0	0	0	hsa_circ_0084889	NC_000008.11	+	93786223	93791319	5097	287	sense-overlappin	NM_001142301.1	TMEM67
circRNA_06119	0	0	0	0	0.05015779	0	0	0	0	0	0	0	-	NC_000008.11	+	93786223	93822563	36341	1989	sense-overlappin	XR_928361.1	
circRNA_06120	0	0	0	0	0	0	0	0	0	0	0.02025523	0	-	NC_000008.11	-	94130626	94130992	367	231	sense-overlappin	XM_011516790.1	CDH17
circRNA_06121	0	0	0.021808645	0	0	0	0	0	0	0	0	0	hsa_circ_0084893	NC_000008.11	-	94378180	94411315	33136	2211	sense-overlappin	NM_001205263.1	RAD54B
circRNA_06122	0	0	0.032712968	0.0412724	0	0	0	0.020486295	0	0	0	0	hsa_circ_0084904	NC_000008.11	-	94506500	94512089	5590	1346	sense-overlappin	NM_015496.4	KIAA1429
circRNA_06123	0	0	0	0	0	0	0	0	0	0	0	0.0206364	-	NC_000008.11	-	94529094	94529168	75	75	exonic	NM_015496.4	KIAA1429
circRNA_06124	0	0	0	0	0	0	0	0	0.030381792	0	0	0	hsa_circ_0084920	NC_000008.11	-	94530963	94538346	7384	428	sense-overlappin	NM_015496.4	KIAA1429
circRNA_06125	0.020575025	0.030704951	0.076330258	0.0825448	0.03009467	0.020712909	0	0.040972591	0.040509055	0.020172483	0	0.0309546	hsa_circ_0006896	NC_000008.11	-	94537103	94538346	1244	136	sense-overlappin	NM_015496.4	KIAA1429
circRNA_06126	0	0	0.119947548	0.1547714	0.19059959	0	0	0.020486295	0	0	0.02025523	0.051591	hsa_circ_0084927	NC_000008.11	+	94664697	94665196	500	287	sense-overlappin	NM_001034915.2	ESRP1
circRNA_06127	0	0	0	0	0	0	0	0.020486295	0	0	0	0	-	NC_000008.11	+	94726331	94739790	13460	595	sense-overlappin	NM_181787.2	DPY19L4
circRNA_06128	0	0	0	0.0206362	0	0	0	0	0	0	0	0	hsa_circ_0001814	NC_000008.11	+	94827263	94842488	15226	955	sense-overlappin	XM_011517155.1	INTS8
circRNA_06129	0	0	0	0.0206362	0	0	0	0	0	0	0	0	-	NC_000008.11	+	94828975	94841591	12617	600	sense-overlappin	XM_011517155.1	INTS8
circRNA_06130	0.020575025	0	0	0	0	0.062138728	0	0	0	0	0	0	-	NC_000008.11	+	94851553	94866191	14639	788	sense-overlappin	XM_011517155.1	INTS8
circRNA_06131	0	0	0	0	0	0	0	0	0	0.020172483	0	0	-	NC_000008.11	+	94867140	94876489	9350	698	sense-overlappin	NR_073444.1	

circRNA_06132	0	0	0.021808645	0	0	0	0	0	0	0	0	0	hsa_circ_0007549	NC_000008.11	+	95031995	95052230	20236	795	sense-overlappin	XM_005250790.1	NDUFAF6
circRNA_06133	0	0	0.054521613	0.0515905	0	0	0	0	0	0	0	0	hsa_circ_0008303	NC_000008.11	+	96306444	96331095	24652	418	sense-overlappin	NM_001290225.1	PTDSS1
circRNA_06134	0	0	0.021808645	0	0	0	0	0	0	0	0	0	-	NC_000008.11	+	96680443	96682076	1634	1634	sense-overlappin	XM_011516793.1	CPQ
circRNA_06135	0.020575025	0	0	0	0	0.031069364	0	0.020486295	0	0	0	0	hsa_circ_0004762	NC_000008.11	+	96834973	96880005	45033	483	sense-overlappin	XR_428374.1	
circRNA_06136	0	0	0	0	0	0	0	0	0.020254528	0	0	0	hsa_circ_0084994	NC_000008.11	+	96879798	96966046	86249	320	sense-overlappin	XR_428374.1	
circRNA_06137	0	0	0	0	0	0	0	0	0	0.020172483	0	0.051591	hsa_circ_0002114	NC_000008.11	+	97661072	97691188	30117	667	sense-overlappin	XM_005251099.2	MTDH
circRNA_06138	0	0.040939935	0	0	0	0	0	0	0	0	0.02025523	0	-	NC_000008.11	+	97668195	97668649	455	455	intronic	XM_005251099.2	MTDH
circRNA_06139	0	0.020469968	0.021808645	0.0619086	0.06018934	0.020712909	0	0.020486295	0.030381792	0.030258724	0	0	hsa_circ_0002961	NC_000008.11	+	97686668	97691188	4521	565	sense-overlappin	XM_005251099.2	MTDH
circRNA_06140	0.041150049	0	0	0	0	0	0	0	0	0.020172483	0	0	hsa_circ_0003117	NC_000008.11	+	97713662	97719189	5528	249	sense-overlappin	XM_005251099.2	MTDH
circRNA_06141	0	0	0.032712968	0	0.03009467	0.031069364	0.020098685	0	0.030381792	0.020172483	0	0	hsa_circ_0002946	NC_000008.11	+	97713662	97723035	9374	406	sense-overlappin	XM_005251099.2	MTDH
circRNA_06142	0.030862537	0	0	0	0	0	0	0	0	0	0	0	hsa_circ_0007901	NC_000008.11	+	97815328	97825153	9826	392	sense-overlappin	NM_018407.4	LAPTM4B
circRNA_06143	0	0	0	0.0619086	0	0	0	0	0	0	0	0	-	NC_000008.11	-	98203108	98254087	50980	745	sense-overlappin	XM_005251069.3	NIPAL2
circRNA_06144	0	0	0.021808645	0	0	0	0.020098685	0	0	0	0	0	hsa_circ_0003593	NC_000008.11	-	98526742	98548161	21420	369	sense-overlappin	XM_011517247.1	STK3
circRNA_06145	0.041150049	0	0	0.0309543	0.03009467	0.020712909	0.020098685	0	0	0	0	0	hsa_circ_0002798	NC_000008.11	-	98526742	98596169	69428	633	sense-overlappin	XM_011517247.1	STK3
circRNA_06146	0.041150049	0	0	0	0	0	0.020098685	0	0	0	0	0	hsa_circ_0085043	NC_000008.11	-	98547969	98596169	48201	457	sense-overlappin	XM_011517247.1	STK3
circRNA_06147	0.020575025	0	0	0	0	0	0	0	0.020254528	0	0	0	-	NC_000008.11	-	98663930	98707311	43382	42870	sense-overlappin	XM_011517247.1	STK3
circRNA_06148	0.288050345	0.133054789	0.577929093	0.0619086	0.15047336	0.072495183	0.160789483	0.040972591	0.020254528	0.14120738	0.091148535	0.0206364	hsa_circ_0004592	NC_000008.11	-	98706467	98707311	845	333	sense-overlappin	XM_011517247.1	STK3
circRNA_06149	0	0	0	0	0	0	0.020098685	0	0	0	0	0	-	NC_000008.11	-	98706467	98722818	16352	496	sense-overlappin	XM_011517253.1	STK3
circRNA_06150	0.041150049	0	0	0	0	0	0.030148028	0	0	0.030258724	0	0	hsa_circ_0005934	NC_000008.11	-	98706467	98722969	16503	15991	sense-overlappin	XM_011517247.1	STK3
circRNA_06151	0	0	0	0	0.02006311	0	0	0	0	0	0	0	-	NC_000008.11	-	98706467	98728621	22155	21643	sense-overlappin	XM_011517247.1	STK3
circRNA_06152	0	0	0.032712968	0	0	0	0	0	0	0	0	0	-	NC_000008.11	-	98746710	98774819	28110	2891	sense-overlappin	XM_011517247.1	STK3
circRNA_06153	0	0	0.076330258	0	0	0	0	0	0	0	0	0	-	NC_000008.11	-	98749276	98774819	25544	325	sense-overlappin	XM_011517247.1	STK3
circRNA_06154	0	0	0.021808645	0.0206362	0	0	0	0	0	0	0	0	-	NC_000008.11	+	99134632	99193057	58426	1309	sense-overlappin	XM_005250801.3	VPS13B
circRNA_06155	0	0	0	0	0.02006311	0	0	0	0	0	0	0	-	NC_000008.11	+	99245606	99246795	1190	1190	intronic	XM_005250801.3	VPS13B
circRNA_06156	0	0	0.098138903	0	0.02006311	0	0.030148028	0	0	0.040344966	0.02025523	0	hsa_circ_0004366	NC_000008.11	+	99274198	99275254	1057	309	sense-overlappin	XM_005250801.3	VPS13B
circRNA_06157	0	0	0	0	0	0	0	0	0.020254528	0.020172483	0	0	hsa_circ_0005984	NC_000008.11	+	99431537	99481802	50266	788	sense-overlappin	XM_005250801.3	VPS13B
circRNA_06158	0	0	0	0.0412724	0	0	0	0	0	0	0	0	hsa_circ_0085086	NC_000008.11	+	99431537	99521010	89474	1805	sense-overlappin	XR_928304.1	
circRNA_06159	0.030862537	0	0.032712968	0	0	0.031069364	0	0	0.020254528	0.020172483	0	0	-	NC_000008.11	+	99502836	99511512	8677	733	sense-overlappin	XR_928304.1	
circRNA_06160	0.061725074	0	0.08723458	0.0206362	0.08025246	0	0.030148028	0	0.020254528	0	0	0	-	NC_000008.11	+	99575658	99585517	9860	634	sense-overlappin	XR_928301.1	
circRNA_06161	0	0	0	0.0206362	0	0	0	0	0	0	0	0	hsa_circ_0085097	NC_000008.11	+	99641811	99661491	19681	826	sense-overlappin	XM_005250801.3	VPS13B
circRNA_06162	0	0	0	0	0	0	0	0	0	0.020172483	0	0	-	NC_000008.11	-	100062591	100080355	17765	1397	sense-overlappin	XR_928317.1	
circRNA_06163	0	0	0.032712968	0	0.04012623	0	0	0	0	0	0	0	-	NC_000008.11	+	100220279	100233537	13259	580	sense-overlappin	XM_011517242.1	SPAG1
circRNA_06164	0	0	0	0	0	0	0	0	0	0	0.030382845	0	-	NC_000008.11	+	100220279	100240771	20493	1114	sense-overlappin	XM_011517242.1	SPAG1
circRNA_06165	0.020575025	0	0	0	0	0.051782273	0	0	0	0	0.02025523	0	-	NC_000008.11	-	100259107	100264785	5679	775	sense-overlappin	NM_015435.4	RNF19A
circRNA_06166	0	0	0.021808645	0.0412724	0.03009467	0.020712909	0	0	0	0	0	0	-	NC_000008.11	-	100259107	100275161	16055	1292	sense-overlappin	NM_015435.4	RNF19A
circRNA_06167	0	0	0	0.0825448	0	0.062138728	0	0	0	0.040344966	0	0	-	NC_000008.11	-	100259107	100288267	29161	2059	sense-overlappin	NM_015435.4	RNF19A
circRNA_06168	0.030862537	0	0	0	0	0	0	0	0	0	0	0	hsa_circ_0085124	NC_000008.11	-	100268785	100275161	6377	517	sense-overlappin	NM_015435.4	RNF19A
circRNA_06169	0	0	0	0	0.03009467	0	0	0	0	0	0	0	hsa_circ_0085125	NC_000008.11	-	100268785	100288267	19483	1284	sense-overlappin	NM_015435.4	RNF19A
circRNA_06170	0	0	0	0	0	0	0	0	0	0.020172483	0	0	hsa_circ_0085127	NC_000008.11	-	100274953	100288267	13315	976	sense-overlappin	NM_015435.4	RNF19A
circRNA_06171	0.030862537	0	0.021808645	0.0515905	0	0	0	0.020486295	0.020254528	0.040344966	0	0	hsa_circ_0085128	NC_000008.11	-	100287501	100288267	767	767	exonic	NM_015435.4	RNF19A
circRNA_06172	0	0	0	0.0309543	0	0	0	0.020486295	0	0	0.04051046	0	hsa_circ_0085129	NC_000008.11	-	100527845	100529860	2016	497	sense-overlappin	NM_001270379.1	ANKRD46
circRNA_06173	0	0	0	0	0.02006311	0	0	0	0	0	0	0	-	NC_000008.11	-	100548057	100548156	100	100	intronic	NM_001270379.1	ANKRD46
circRNA_06174	0	0.030704951	0	0	0	0.041425819	0.040197371	0	0	0	0	0	hsa_circ_0008496	NC_000008.11	-	100709133	100713181	4049	693	sense-overlappin	NM_002568.3	PABPC1
circRNA_06175	0.051437562	0	0.021808645	0.0309543	0.03009467	0.031069364	0.030148028	0.020486295	0	0	0.02025523	0	hsa_circ_0085173	NC_000008.11	+	101558419	101558812	394	394	exonic	XM_011517306.1	GRHL2
circRNA_06176	0	0	0	0	0	0.020712909	0	0	0	0	0	0	-	NC_000008.11	-	101946077	102025962	79886	79886	sense-overlappin	XM_011517333.1	NCALD
circRNA_06177	0	0	0	0	0	0	0	0.030729443	0	0	0	0	hsa_circ_0003794	NC_000008.11	-	102224046	102225018	973	229	sense-overlappin	NM_001172477.1	RRM2B
circRNA_06178	0	0	0	0	0	0.020712909	0	0	0	0	0	0	-	NC_000008.11	-	102271134	102272772	1639	356	sense-overlappin	NM_001282873.1	UBR5
circRNA_06179	0	0.020469968	0	0	0	0	0	0	0	0	0	0.0206364	-	NC_000008.11	-	102271134	102280467	9334	1158	sense-overlappin	NM_001282873.1	UBR5
circRNA_06180	0	0.020469968	0	0.0309543	0	0.020712909	0	0	0	0	0	0	hsa_circ_0001818	NC_000008.11	-	102287433	102288266	834	244	sense-overlappin	NM_001282873.1	UBR5
circRNA_06181	0	0	0	0	0	0	0.020098685	0	0	0	0	0	-	NC_000008.11	-	102299452	102300151	700	248	sense-overlappin	NM_001282873.1	UBR5
circRNA_06182	0	0	0	0.0206362	0	0	0	0	0	0	0	0	-	NC_000008.11	-	102307955	102308068	114	114	intronic	NM_001282873.1	UBR5
circRNA_06183	0	0	0.021808645	0	0	0	0	0	0	0	0	0	hsa_circ_0008217	NC_000008.11	-	102323311	102347092	23782	1398	sense-overlappin	NM_001282873.1	UBR5
circRNA_06184	0	0	0.032712968	0	0	0	0	0	0	0	0	0	-	NC_000008.11	-	102327742	102345545	17804	745	sense-overlappin	NM_001282873.1	UBR5
circRNA_06185	0.061725074	0.143289773	0.065425935	0.1650895	0.0702209	0.134633911	0.050246713	0.122917773	0.162036222	0.14120738	0.04051046	0.134136601	hsa_circ_0001819	NC_000008.11	-	102360071	102361626	1556	324	sense-overlappin	NM_001282873.1	UBR5
circRNA_06186	0	0.040939935	0	0	0	0	0	0	0	0	0	0	-	NC_000008.11	-	102401111	102401228	118	118	intronic	NM_001282873.1	UBR5
circRNA_06187	0	0	0	0	0	0	0	0	0.030381792	0	0	0	hsa_circ_0007374	NC_000008.11	-	102834189	102839823	5635	639	sense-overlappin	NM_001301668.1	AZIN1

circRNA_06188	0	0	0	0	0	0	0	0	0.020254528	0	0	0	hsa_circ_0003304	NC_000008.11	-	102838744	102843747	5004	544	sense-overlappin	NM_001301668.1	AZIN1
circRNA_06189	0	0	0	0.0206362	0	0	0	0	0	0	0	0	-	NC_000008.11	-	103246294	103246684	391	391	intergenic		
circRNA_06190	0	0	0	0	0.03009467	0	0.020098685	0	0	0.050431207	0.04051046	0	hsa_circ_0005499	NC_000008.11	-	103404776	103407784	3009	237	sense-overlappin	NM_030780.4	SLC25A32
circRNA_06191	0	0	0.021808645	0.0309543	0	0	0	0	0	0	0	0	hsa_circ_0005114	NC_000008.11	+	104068512	104148848	80337	768	sense-overlappin	XM_011517389.1	RIMS2
circRNA_06192	0.030862537	0.030704951	0.021808645	0.0619086	0.02006311	0.051782273	0.020098685	0.020486295	0	0.050431207	0.02025523	0	hsa_circ_0003380	NC_000008.11	+	105419144	105444381	25238	261	sense-overlappin	XM_011516946.1	ZFPM2
circRNA_06193	0	0	0.08723458	0	0.04012623	0	0.050246713	0	0	0	0	0	hsa_circ_0008320	NC_000008.11	+	106679210	106684359	5150	305	sense-overlappin	NM_001198533.1	OXR1
circRNA_06194	0	0.030704951	0.08723458	0	0.02006311	0	0	0	0	0	0	0	hsa_circ_0085311	NC_000008.11	+	106679210	106692877	13668	455	sense-overlappin	NM_001198533.1	OXR1
circRNA_06195	0	0	0	0	0	0	0	0	0	0.020172483	0	0	-	NC_000008.11	+	106702906	106707145	4240	949	sense-overlappin	NM_001198533.1	OXR1
circRNA_06196	0	0	0	0	0	0.031069364	0	0	0	0.020172483	0	0	-	NC_000008.11	-	107958080	107989244	31165	522	sense-overlappin	XM_011517020.1	RSPO2
circRNA_06197	0	0	0	0	0	0	0	0	0	0.020172483	0	0	-	NC_000008.11	-	108214607	108241913	27307	971	sense-overlappin	NM_001568.2	EIF3E
circRNA_06198	0	0	0.04361729	0	0	0	0.020098685	0	0	0.020172483	0	0	hsa_circ_0085323	NC_000008.11	-	108228267	108241913	13647	632	sense-overlappin	NM_001568.2	EIF3E
circRNA_06199	0	0	0	0.0515905	0.04012623	0.041425819	0	0.030729443	0.020254528	0.020172483	0.030382845	0	-	NC_000008.11	+	108449823	108450492	670	179	sense-overlappin	NM_014673.3	EMC2
circRNA_06200	0	0	0.021808645	0	0	0	0	0	0	0	0	0	-	NC_000008.11	+	108449823	108453147	3325	265	sense-overlappin	NM_014673.3	EMC2
circRNA_06201	0.030862537	0.153524756	0.109043225	0.1444533	0.16050492	0.093208092	0.070345399	0.030729443	0.030381792	0.151293621	0.08102092	0	-	NC_000008.11	+	108449823	108455930	6108	323	sense-overlappin	NM_014673.3	EMC2
circRNA_06202	0	0	0.021808645	0	0	0	0	0	0	0	0	0	-	NC_000008.11	-	111003414	111027433	24020	24020	intergenic		
circRNA_06203	0.020575025	0	0	0	0	0	0	0	0	0	0.04051046	0	hsa_circ_0085362	NC_000008.11	-	115587001	115623758	36758	2807	sense-overlappin	NM_001282903.2	TRPS1
circRNA_06204	0.020575025	0	0	0	0	0	0	0	0	0	0	0	hsa_circ_0085363	NC_000008.11	-	115603873	115623758	19886	2203	sense-overlappin	NM_001282903.2	TRPS1
circRNA_06205	0.061725074	0	0.032712968	0	0.02006311	0.020712909	0.030148028	0.030729443	0	0	0	0	hsa_circ_0005231	NC_000008.11	-	116655856	116658980	3125	418	sense-overlappin	NM_003756.2	EIF3H
circRNA_06206	0	0	0	0.0309543	0	0	0	0	0	0	0	0	-	NC_000008.11	-	116655856	116726172	70317	575	sense-overlappin	NM_003756.2	EIF3H
circRNA_06207	0	0	0	0	0	0.020712909	0	0	0	0	0	0	hsa_circ_0002312	NC_000008.11	-	116726016	116737287	11272	11272	sense-overlappin	NM_003756.2	EIF3H
circRNA_06208	0	0	0	0	0	0.020712909	0	0	0	0	0	0	hsa_circ_0008035	NC_000008.11	-	117818435	117837201	18767	670	sense-overlappin	NM_000127.2	EXT1
circRNA_06209	0	0	0.032712968	0	0	0	0	0	0	0.020172483	0	0	-	NC_000008.11	-	118375782	118379700	3919	216	sense-overlappin	XM_011517034.1	SAMD12
circRNA_06210	0	0	0	0.0206362	0	0	0	0	0.020254528	0	0	0	-	NC_000008.11	-	119783381	119791459	8079	835	sense-overlappin	XM_011517262.1	TAF2
circRNA_06211	0	0	0	0	0.05015779	0	0.020098685	0.051215739	0.030381792	0.060517448	0	0	-	NC_000008.11	-	119788338	119797846	9509	1001	sense-overlappin	XM_006716621.2	TAF2
circRNA_06212	0	0	0	0.0206362	0	0	0	0.051215739	0	0	0	0	-	NC_000008.11	-	119793366	119802025	8660	717	sense-overlappin	XM_006716621.2	TAF2
circRNA_06213	0	0	0	0	0	0	0.020098685	0	0	0	0	0	-	NC_000008.11	-	119801794	119831721	29928	699	sense-overlappin	XM_006716621.2	TAF2
circRNA_06214	0	0	0	0	0.02006311	0	0	0	0	0	0	0	hsa_circ_0002593	NC_000008.11	-	119817984	119819506	1523	1523	sense-overlappin	XM_006716621.2	TAF2
circRNA_06215	0	0	0	0.0309543	0	0	0	0	0.060763583	0	0	0	-	NC_000008.11	+	119928400	119929938	1539	303	sense-overlappin	NM_022783.3	DEPTOR
circRNA_06216	0.030862537	0	0	0	0	0	0	0	0	0	0	0	hsa_circ_0085412	NC_000008.11	+	120001525	120009133	7609	497	sense-overlappin	NM_001283012.1	DEPTOR
circRNA_06217	0	0	0	0	0.04012623	0	0	0	0	0	0	0	-	NC_000008.11	+	120006805	120009133	2329	176	sense-overlappin	NM_001283012.1	DEPTOR
circRNA_06218	0	0	0.021808645	0	0	0	0	0	0	0	0	0	-	NC_000008.11	+	120453847	120461255	7409	552	sense-overlappin	NM_022045.4	MTBP
circRNA_06219	0	0	0.04361729	0	0	0	0	0	0.040509055	0.040344966	0	0	hsa_circ_0005015	NC_000008.11	-	121628714	121629340	627	627	exonic	XM_011517000.1	HAS2
circRNA_06220	0	0	0.021808645	0	0	0	0	0	0	0	0	0	-	NC_000008.11	+	122807390	122863539	56150	56150	sense-overlappin	XM_005250837.3	ZHX2
circRNA_06221	0	0	0	0.0206362	0	0	0	0	0	0	0	0	-	NC_000008.11	-	123015393	123019305	3913	304	sense-overlappin	NM_001134671.2	DERL1
circRNA_06222	0	0	0	0	0.02006311	0	0	0	0	0	0	0	hsa_circ_0001980	NC_000008.11	-	123019195	123030716	11522	404	sense-overlappin	NM_001134671.2	DERL1
circRNA_06223	0.051437562	0	0.032712968	0	0.04012623	0.031069364	0	0	0	0.030258724	0	0	hsa_circ_0085434	NC_000008.11	-	123023713	123030716	7004	204	sense-overlappin	NM_001134671.2	DERL1
circRNA_06224	0.041150049	0	0.032712968	0	0	0.051782273	0	0	0	0.020172483	0	0.0206364	hsa_circ_0085438	NC_000008.11	+	123077111	123084340	7230	442	sense-overlappin	XR_928365.1	
circRNA_06225	0	0	0.021808645	0	0	0	0	0	0	0	0	0	-	NC_000008.11	+	123077111	123097441	20331	754	sense-overlappin	XR_928365.1	
circRNA_06226	0.020575025	0	0.08723458	0	0.04012623	0.031069364	0	0	0.050636319	0.050431207	0.04051046	0.0206364	hsa_circ_0085439	NC_000008.11	+	123077111	123105464	28354	1132	sense-overlappin	XR_928365.1	
circRNA_06227	0.061725074	0	0.04361729	0.0206362	0.02006311	0	0	0	0	0	0	0.0309546	hsa_circ_0085440	NC_000008.11	+	123077111	123109620	32510	1359	sense-overlappin	XR_928365.1	
circRNA_06228	0	0	0.04361729	0	0	0	0	0	0	0	0	0	hsa_circ_0085441	NC_000008.11	+	123077111	123120188	43078	1493	sense-overlappin	XR_928365.1	
circRNA_06229	0	0	0	0	0	0.031069364	0	0	0	0	0	0	-	NC_000008.11	+	123077111	123142456	65346	2758	sense-overlappin	NM_145647.3	TBC1D31
circRNA_06230	0	0	0.054521613	0	0	0	0	0	0	0	0	0	-	NC_000008.11	+	123077111	123144855	67745	2897	sense-overlappin	NM_145647.3	TBC1D31
circRNA_06231	0	0	0	0	0	0.020712909	0	0	0	0	0	0	hsa_circ_0001820	NC_000008.11	+	123109317	123120188	10872	361	sense-overlappin	XR_928365.1	
circRNA_06232	0	0.040939935	0	0	0	0	0	0	0	0	0	0	-	NC_000008.11	+	123140761	123142456	1696	336	sense-overlappin	NM_145647.3	TBC1D31
circRNA_06233	0	0	0.04361729	0.0412724	0	0	0.030148028	0	0	0.020172483	0	0	hsa_circ_0002663	NC_000008.11	-	123231300	123239144	7845	698	sense-overlappin	NM_032847.2	C8orf76
circRNA_06234	0	0	0	0	0.02006311	0	0	0	0	0	0.02025523	0	hsa_circ_0085458	NC_000008.11	-	123333878	123336532	2655	427	sense-overlappin	XM_011516996.1	ATAD2
circRNA_06235	0	0	0.021808645	0	0	0	0	0	0	0	0	0	hsa_circ_0085459	NC_000008.11	-	123333878	123337821	3944	624	sense-overlappin	XM_011516996.1	ATAD2
circRNA_06236	0	0	0.065425935	0.0206362	0	0	0.030148028	0	0	0.060517448	0.02025523	0	hsa_circ_0085465	NC_000008.11	-	123337625	123339446	1822	333	sense-overlappin	XM_011516996.1	ATAD2
circRNA_06237	0	0	0	0	0	0	0.020098685	0	0	0	0	0	-	NC_000008.11	-	123369821	123380677	10857	760	sense-overlappin	XM_011516994.1	ATAD2
circRNA_06238	0.030862537	0	0	0	0.02006311	0	0	0	0	0	0	0	hsa_circ_0085495	NC_000008.11	-	124320086	124327421	7336	272	sense-overlappin	XM_011516847.1	TMEM65
circRNA_06239	0	0	0	0	0.02006311	0	0	0	0	0	0	0	hsa_circ_0004759	NC_000008.11	-	124508474	124516030	7557	314	sense-overlappin	NM_001146160.1	TATDN1
circRNA_06240	0	0	0	0.0206362	0	0	0	0	0	0	0	0	-	NC_000008.11	-	124512348	124513024	677	677	intronic	NM_001146160.1	TATDN1
circRNA_06241	0	0	0	0.0206362	0	0	0	0	0.020254528	0	0	0	-	NC_000008.11	-	124585087	124591235	6149	252	sense-overlappin	NM_001282971.1	MTSS1
circRNA_06242	0	0	0	0	0	0	0	0	0	0	0.02025523	0	-	NC_000008.11	+	124975606	124978090	2485	1857	sense-overlappin	XM_011516828.1	ZNF572

circRNA_06243	0	0	0.021808645	0	0.04012623	0	0	0	0	0	0	0	-	NC_000008.11	+	125008971	125009343	373	286	sense-overlappin	NM_003129.3	SQLE
circRNA_06244	0	0	0.021808645	0	0	0	0	0	0	0.040344966	0	0	-	NC_000008.11	+	125147081	125195272	48192	17367	sense-overlappin	XM_011516975.1	NSMCE2
circRNA_06245	0	0	0	0	0.04012623	0	0	0	0	0	0	0	-	NC_000008.11	+	125182103	125195272	13170	13170	sense-overlappin	XM_011516975.1	NSMCE2
circRNA_06246	0	0	0	0	0	0	0.040197371	0	0	0	0	0	-	NC_000008.11	+	127806369	127807862	1494	1494	intergenic		
circRNA_06247	0	0	0	0	0	0	0	0	0	0	0	0.0206364	hsa_circ_0009143	NC_000008.11	+	127855155	127890998	35844	35844	intergenic		
circRNA_06248	0.061725074	0	0.250799418	0	0.03009467	0	0.050246713	0.030729443	0.040509055	0.020172483	0	0.0206364	hsa_circ_0001821	NC_000008.11	+	127890589	127890998	410	410	intergenic		
circRNA_06249	0	0	0.032712968	0	0	0	0	0	0	0	0	0	-	NC_000008.11	+	127989162	128010444	21283	21283	intergenic		
circRNA_06250	0	0	0	0	0	0	0.020098685	0	0	0	0	0	-	NC_000008.11	+	128060164	128061004	841	841	intergenic		
circRNA_06251	0	0	0.04361729	0	0	0	0	0	0	0	0	0	-	NC_000008.11	-	129871375	129904585	33211	597	sense-overlappin	NR_046359.1	
circRNA_06252	0	0	0	0	0	0	0	0	0	0.020172483	0	0	hsa_circ_0003244	NC_000008.11	-	130118161	130126089	7929	499	sense-overlappin	XM_006716565.2	ASAP1
circRNA_06253	0	0.020469968	0	0	0	0	0	0	0	0	0	0	hsa_circ_0085596	NC_000008.11	-	130134296	130169067	34772	480	sense-overlappin	XM_006716565.2	ASAP1
circRNA_06254	0.041150049	0	0.054521613	0.0515905	0.03009467	0.031069364	0.040197371	0.030729443	0.050636319	0.07060369	0	0.0619092	hsa_circ_0001824	NC_000008.11	-	130152736	130169067	16332	343	sense-overlappin	XM_006716565.2	ASAP1
circRNA_06255	0.174887709	0.071644886	0.152660515	0.1238171	0.10031557	0.186416184	0.070345399	0.163890364	0.202545277	0.36310469	0.10127615	0.0619092	hsa_circ_0008934	NC_000008.11	-	130152736	130180880	28145	559	sense-overlappin	XM_006716565.2	ASAP1
circRNA_06256	0	0	0.021808645	0	0	0	0.030148028	0	0	0	0	0	hsa_circ_0002330	NC_000008.11	-	130159864	130180880	21017	489	sense-overlappin	XM_006716565.2	ASAP1
circRNA_06257	0	0	0	0	0	0	0	0	0.020254528	0	0	0	hsa_circ_0006108	NC_000008.11	-	130179264	130187285	8022	266	sense-overlappin	XM_006716565.2	ASAP1
circRNA_06258	0	0.030704951	0.054521613	0	0.04012623	0.031069364	0.060296056	0	0.070890847	0.040344966	0	0.051591	hsa_circ_0085616	NC_000008.11	-	130358017	130361771	3755	229	sense-overlappin	XM_011517053.1	ASAP1
circRNA_06259	0.030862537	0	0.032712968	0	0.02006311	0	0.020098685	0	0	0.030258724	0	0	-	NC_000008.11	+	131940499	131946633	6135	425	sense-overlappin	XM_005250849.1	EFR3A
circRNA_06260	0	0	0.032712968	0	0	0	0	0	0	0	0	0	-	NC_000008.11	-	132610166	132615097	4932	226	sense-overlappin	XM_011516954.1	LRRC6
circRNA_06261	0	0	0	0.0206362	0	0	0	0	0	0	0	0	-	NC_000008.11	-	132610166	132661627	51462	1130	sense-overlappin	XM_011516953.1	LRRC6
circRNA_06262	0.051437562	0.030704951	0.065425935	0.0619086	0.06018934	0.041425819	0	0.030729443	0.081018111	0.040344966	0	0	-	NC_000008.11	-	132622611	132661627	39017	904	sense-overlappin	XM_011516953.1	LRRC6
circRNA_06263	0	0	0	0	0	0	0	0	0	0.020172483	0	0	-	NC_000008.11	-	132722040	132751997	29958	594	sense-overlappin	NM_144649.2	TMEM71
circRNA_06264	0	0	0	0	0	0	0	0	0	0	0	0.0206364	hsa_circ_0008205	NC_000008.11	-	132722040	132757294	35255	655	sense-overlappin	NM_144649.2	TMEM71
circRNA_06265	0	0	0	0	0	0	0	0	0	0.030258724	0	0	hsa_circ_0006050	NC_000008.11	-	132727798	132757294	29497	579	sense-overlappin	NM_144649.2	TMEM71
circRNA_06266	0	0	0	0.0206362	0	0.020712909	0	0	0	0.07060369	0	0	hsa_circ_0085644	NC_000008.11	+	132836540	132844318	7779	1002	sense-overlappin	NM_001277196.1	PHF20L1
circRNA_06267	0	0	0.021808645	0	0	0.020712909	0	0	0	0	0	0	hsa_circ_0007934	NC_000008.11	-	134509619	134521001	11383	377	sense-overlappin	XM_011517204.1	ZFAT
circRNA_06268	0.020575025	0	0.098138903	0.0206362	0	0	0.040197371	0	0.020254528	0	0	0	hsa_circ_0007209	NC_000008.11	-	134600436	134610655	10220	2027	sense-overlappin	XM_011517204.1	ZFAT
circRNA_06269	0	0	0	0	0	0.041425819	0	0	0	0	0	0	-	NC_000008.11	-	134808504	134813169	4666	4666	intergenic		
circRNA_06270	0.082300098	0.020469968	0.032712968	0.1444533	0	0.020712909	0	0	0.030381792	0.050431207	0.030382845	0.051591	-	NC_000008.11	-	134812610	134813169	560	560	intergenic		
circRNA_06271	0.041150049	0	0	0	0	0	0	0	0	0	0	0	-	NC_000008.11	+	137138847	137172005	33159	33159	intergenic		
circRNA_06272	0	0	0.054521613	0.0309543	0	0	0	0	0	0	0	0	hsa_circ_0085694	NC_000008.11	-	140275658	140291078	15421	510	sense-overlappin	XM_011517330.1	TRAPPC9
circRNA_06273	0	0	0.032712968	0.0206362	0	0	0	0	0	0	0	0	hsa_circ_0004023	NC_000008.11	-	140275658	140300614	24957	656	sense-overlappin	XM_011517330.1	TRAPPC9
circRNA_06274	0.020575025	0	0	0	0	0	0	0	0	0	0	0	hsa_circ_0085696	NC_000008.11	-	140283889	140287734	3846	260	sense-overlappin	XM_011517330.1	TRAPPC9
circRNA_06275	0	0	0.065425935	0.0309543	0.05015779	0	0	0	0	0	0	0.0206364	hsa_circ_0001826	NC_000008.11	-	140347757	140360193	12437	12437	sense-overlappin	XM_011517329.1	TRAPPC9
circRNA_06276	0	0	0.054521613	0	0	0	0	0	0	0	0	0	hsa_circ_0085703	NC_000008.11	-	140360050	140405698	45649	609	sense-overlappin	XM_011517329.1	TRAPPC9
circRNA_06277	0	0	0.032712968	0	0	0	0	0	0	0	0	0	hsa_circ_0004380	NC_000008.11	-	140397620	140405698	8079	248	sense-overlappin	XM_011517329.1	TRAPPC9
circRNA_06278	0	0	0.021808645	0	0	0	0	0	0	0	0	0	-	NC_000008.11	-	140397620	140409617	11998	4167	sense-overlappin	XM_011517329.1	TRAPPC9
circRNA_06279	0	0	0	0.0309543	0	0	0	0	0	0.030258724	0	0	hsa_circ_0002965	NC_000008.11	-	140572812	140585311	12500	314	sense-overlappin	XM_011516965.1	AGO2
circRNA_06280	0	0.020469968	0	0	0	0	0	0	0	0	0	0	-	NC_000008.11	-	140572812	140605914	33103	20917	sense-overlappin	XM_011516964.1	AGO2
circRNA_06281	0	0	0	0.0515905	0.04012623	0.041425819	0.050246713	0	0.030381792	0	0.050638075	0	hsa_circ_0007313	NC_000008.11	-	140585119	140605914	20796	20796	sense-overlappin	XM_011516964.1	AGO2
circRNA_06282	0	0	0.021808645	0	0	0	0	0	0	0	0.02025523	0	hsa_circ_0004686	NC_000008.11	-	140700891	140702707	1817	270	sense-overlappin	NM_001199649.1	PTK2
circRNA_06283	0.020575025	0	0.032712968	0	0.03009467	0	0	0	0.020254528	0	0.030382845	0	hsa_circ_0005273	NC_000008.11	-	140700891	140706205	5315	357	sense-overlappin	NM_001199649.1	PTK2
circRNA_06284	0	0	0	0.0309543	0	0	0	0	0	0	0	0	-	NC_000008.11	-	140700891	140764290	63400	1343	sense-overlappin	XM_005251003.1	PTK2
circRNA_06285	0	0.030704951	0.021808645	0	0.02006311	0	0	0	0	0	0.050638075	0	hsa_circ_0004238	NC_000008.11	-	140735251	140752316	17066	698	sense-overlappin	NM_001199649.1	PTK2
circRNA_06286	0	0.040939935	0.098138903	0	0	0.041425819	0	0	0	0.020172483	0	0	hsa_circ_0003171	NC_000008.11	-	140739018	140752316	13299	493	sense-overlappin	NM_001199649.1	PTK2
circRNA_06287	0	0	0	0	0	0	0	0	0.020254528	0	0	0	hsa_circ_0003066	NC_000008.11	-	140789474	140803650	14177	310	sense-overlappin	NM_001199649.1	PTK2
circRNA_06288	0	0	0	0	0	0	0.030148028	0	0	0.020172483	0	0	hsa_circ_0085756	NC_000008.11	-	140789474	140819020	29547	529	sense-overlappin	NM_001199649.1	PTK2
circRNA_06289	0	0	0	0	0	0	0	0	0	0.030258724	0	0	hsa_circ_0008305	NC_000008.11	-	140789474	140830526	41053	584	sense-overlappin	NM_001199649.1	PTK2
circRNA_06290	0	0	0	0	0.04012623	0	0	0	0	0.020172483	0	0	hsa_circ_0009102	NC_000008.11	-	140789474	140864399	74926	815	sense-overlappin	NM_001199649.1	PTK2
circRNA_06291	0	0	0	0	0	0	0	0	0	0.030258724	0	0	-	NC_000008.11	-	140789474	140879637	90164	982	sense-overlappin	NM_001199649.1	PTK2
circRNA_06292	0	0	0.021808645	0	0	0	0	0	0	0	0	0	hsa_circ_0003675	NC_000008.11	-	140800459	140864399	63941	731	sense-overlappin	NM_001199649.1	PTK2
circRNA_06293	0	0	0.021808645	0	0	0	0.040197371	0	0	0	0	0	hsa_circ_0007521	NC_000008.11	-	140800459	140890769	90311	1125	sense-overlappin	NM_001199649.1	PTK2
circRNA_06294	0	0	0	0	0	0	0	0	0	0	0.02025523	0	-	NC_000008.11	-	140818277	140830526	12250	274	sense-overlappin	NM_001199649.1	PTK2
circRNA_06295	0	0	0.13085187	0.0309543	0	0	0	0	0	0	0	0	hsa_circ_0008345	NC_000008.11	-	140818277	140864399	46123	505	sense-overlappin	NM_001199649.1	PTK2
circRNA_06296	0	0	0	0.0309543	0	0.020712909	0	0	0.020254528	0	0	0	hsa_circ_0085765	NC_000008.11	-	140818277	140879637	61361	672	sense-overlappin	NM_001199649.1	PTK2
circRNA_06297	0.072012586	0.020469968	0.359842643	0.1444533	0.17053648	0.072495183	0.070345399	0.051215739	0.040509055	0.100862414	0.08102092	0.0412728	hsa_circ_0005982	NC_000008.11	-	140818277	140890769	72493	899	sense-overlappin	NM_001199649.1	PTK2
circRNA_06298	0.061725074	0.051174919	0.08723458	0.0515905	0.04012623	0.020712909	0	0	0	0.050431207	0	0	hsa_circ_0002908	NC_000008.11	-	140830472	140864399	33928	286	sense-overlappin	NM_001199649.1	PTK2
circRNA_06299	0.030862537	0	0.021808645	0.0412724	0	0.031069364	0.030148028	0	0.030381792	0.030258724	0.02025523	0	hsa_circ_0007786	NC_000008.11	-	140830472	140890769	60298	680	sense-overlappin	NM_001199649.1	PTK2

circRNA_06300	0.030862537	0	0.065425935	0	0.05015779	0.020712909	0	0.030729443	0.020254528	0	0	0	hsa_circ_0002162	NC_000008.11	-	140846260	140864399	18140	231	sense-overlappin	NM_001199649.1	PTK2
circRNA_06301	0.051437562	0	0.032712968	0	0	0	0	0	0	0	0	0	hsa_circ_0085769	NC_000008.11	-	140846260	140879637	33378	398	sense-overlappin	NM_001199649.1	PTK2
circRNA_06302	0.164600197	0.040939935	0.239895095	0.1238171	0.0702209	0.103564547	0.060296056	0.040972591	0.070890847	0.171466104	0.08102092	0.051591	hsa_circ_0003221	NC_000008.11	-	140846260	140890769	44510	625	sense-overlappin	NM_001199649.1	PTK2
circRNA_06303	0	0	0	0.0309543	0	0	0	0	0	0	0	0	hsa_circ_0006299	NC_000008.11	-	140846599	140890769	44171	562	sense-overlappin	NM_001199649.1	PTK2
circRNA_06304	0.174887709	0.173994724	0.283512385	0.1960438	0.15047336	0.15534682	0.110542769	0.040972591	0.151908958	0.181552345	0.091148535	0.103182001	hsa_circ_0002483	NC_000008.11	-	140864312	140890769	26458	482	sense-overlappin	NM_001199649.1	PTK2
circRNA_06305	0	0	0.04361729	0	0	0	0	0	0	0	0	0	hsa_circ_0008778	NC_000008.11	-	140864312	140925749	61438	571	sense-overlappin	NM_001199649.1	PTK2
circRNA_06306	0.030862537	0.020469968	0.076330258	0.113499	0.04012623	0.020712909	0.090444084	0.040972591	0.091145375	0.040344966	0.070893305	0.0619092	hsa_circ_0006646	NC_000008.11	-	140879471	140890769	11299	394	sense-overlappin	NM_001199649.1	PTK2
circRNA_06307	0	0	0	0	0	0.031069364	0	0	0	0	0	0	hsa_circ_0085782	NC_000008.11	+	141151619	141155970	4352	341	sense-overlappin	NM_014957.2	DENND3
circRNA_06308	0	0	0	0	0	0	0.020098685	0	0	0	0	0	-	NC_000008.11	+	141160632	141163429	2798	253	sense-overlappin	NM_014957.2	DENND3
circRNA_06309	0.360062931	0.16375974	1.068623606	0.3817695	0.08025246	0.269267821	0.040197371	0.204862955	0.111399902	0.191638587	0.182297071	0.175409401	hsa_circ_0001829	NC_000008.11	-	141253989	141254629	641	641	exonic	NM_001286646.1	SLC45A4
circRNA_06310	0.030862537	0	0	0	0	0	0	0	0	0	0	0	-	NC_000008.11	-	142328903	142331831	2929	1448	sense-overlappin	XM_011516924.1	TSNARE1
circRNA_06311	0	0	0.032712968	0	0.02006311	0	0	0	0	0	0	0	hsa_circ_0004423	NC_000008.11	-	142330901	142345892	14992	805	sense-overlappin	NM_145003.4	TSNARE1
circRNA_06312	0	0	0.04361729	0	0	0	0	0	0	0	0	0	hsa_circ_0006060	NC_000008.11	-	142343966	142345892	1927	657	sense-overlappin	NM_145003.4	TSNARE1
circRNA_06313	0	0	0.054521613	0	0.02006311	0	0	0	0	0	0	0	hsa_circ_0001833	NC_000008.11	+	144190784	144200541	9758	579	sense-overlappin	NM_001099280.2	MROH1
circRNA_06314	0	0	0	0	0	0	0	0	0	0	0	0.0206364	-	NC_000008.11	+	144196198	144196451	254	254	intronic	NM_001099280.2	MROH1
circRNA_06315	0	0.020469968	0	0	0	0	0	0	0	0	0	0	-	NC_000008.11	-	144384202	144385673	1472	1472	antisense	NM_174922.4	ADCK5
circRNA_06316	0	0	0	0.0412724	0.02006311	0	0.020098685	0	0	0	0.04051046	0	-	NC_000008.11	-	144880365	144882836	2472	2472	exonic	NM_001109689.3	ZNF250
circRNA_06317	0	0	0	0	0	0.020712909	0	0	0	0	0	0	hsa_circ_0086188	NC_000009.12	+	271627	289581	17955	351	sense-overlappin	NM_203447.3	DOCK8
circRNA_06318	0.020575025	0	0	0	0	0	0	0	0	0	0	0	-	NC_000009.12	+	286461	340321	53861	1523	sense-overlappin	NM_203447.3	DOCK8
circRNA_06319	0.020575025	0	0.021808645	0	0	0.020712909	0	0	0.020254528	0.030258724	0	0	hsa_circ_0086190	NC_000009.12	+	325671	340321	14651	852	sense-overlappin	NM_203447.3	DOCK8
circRNA_06320	0	0	0	0	0.02006311	0	0	0	0	0	0	0	-	NC_000009.12	+	336582	340321	3740	394	sense-overlappin	NM_203447.3	DOCK8
circRNA_06321	0	0	0	0	0	0	0	0.020486295	0	0	0	0	hsa_circ_0086193	NC_000009.12	+	370230	372286	2057	312	sense-overlappin	NM_203447.3	DOCK8
circRNA_06322	0	0	0	0	0	0.020712909	0	0	0	0	0	0	-	NC_000009.12	+	376210	405073	28864	1281	sense-overlappin	NM_203447.3	DOCK8
circRNA_06323	0	0	0	0	0	0	0	0	0	0.020172483	0	0	hsa_circ_0086195	NC_000009.12	+	406930	418207	11278	450	sense-overlappin	NM_203447.3	DOCK8
circRNA_06324	0	0	0	0	0	0	0.020098685	0	0	0	0	0	-	NC_000009.12	+	420401	422135	1735	401	sense-overlappin	NM_203447.3	DOCK8
circRNA_06325	0.041150049	0	0.021808645	0.0928628	0	0.031069364	0.020098685	0.030729443	0.030381792	0.020172483	0	0	hsa_circ_0005062	NC_000009.12	+	710804	713464	2661	2661	exonic	NM_001256876.1	KANK1
circRNA_06326	0.020575025	0	0	0	0	0	0	0	0	0	0	0	hsa_circ_0007075	NC_000009.12	+	734748	740934	6187	451	sense-overlappin	NM_001256876.1	KANK1
circRNA_06327	0	0.030704951	0	0	0	0.020712909	0.020098685	0	0.020254528	0	0	0	hsa_circ_0006082	NC_000009.12	+	2191266	2192802	1537	242	sense-overlappin	NM_001289397.1	SMARCA2
circRNA_06328	0	0	0.021808645	0	0	0	0	0	0	0	0	0	-	NC_000009.12	-	2720344	2782846	62503	57736	antisense	XR_929203.1	
circRNA_06329	0	0	0	0	0	0	0	0	0	0.020172483	0	0	-	NC_000009.12	-	2782756	2824815	42060	22487	sense-overlappin	NM_014878.4	KIAA0020
circRNA_06330	0	0	0	0	0	0	0	0	0	0	0.030382845	0	-	NC_000009.12	-	2782756	2838517	55762	23532	sense-overlappin	NM_014878.4	KIAA0020
circRNA_06331	0	0	0	0	0	0	0.020098685	0	0	0	0	0	-	NC_000009.12	-	2828675	2838517	9843	966	sense-overlappin	NM_014878.4	KIAA0020
circRNA_06332	0	0	0.021808645	0	0	0	0	0	0	0	0	0	hsa_circ_0086241	NC_000009.12	-	3330259	3330517	259	259	exonic	XM_011518001.1	RFX3
circRNA_06333	0	0	0.021808645	0	0	0	0	0	0.020254528	0	0	0	hsa_circ_0086242	NC_000009.12	-	3330259	3395596	65338	576	sense-overlappin	XM_011518001.1	RFX3
circRNA_06334	0	0	0.04361729	0	0	0	0	0	0	0	0	0	-	NC_000009.12	-	3346667	3420870	74204	25591	sense-overlappin	XM_011518001.1	RFX3
circRNA_06335	0	0	0.021808645	0	0	0	0	0	0	0	0	0	-	NC_000009.12	-	3395472	3420870	25399	25399	sense-overlappin	XM_011518001.1	RFX3
circRNA_06336	0	0	0	0	0	0	0	0	0	0	0.02025523	0	-	NC_000009.12	-	3484974	3490345	5372	3986	sense-overlappin	XM_006716844.1	RFX3
circRNA_06337	0	0	0	0	0	0	0	0.020486295	0	0	0	0	-	NC_000009.12	+	3546778	3606738	59961	59961	intergenic		
circRNA_06338	0	0	0.04361729	0	0	0	0	0	0	0	0	0	-	NC_000009.12	+	3647338	3651867	4530	4530	intergenic		
circRNA_06339	0	0	0	0	0	0.020712909	0	0	0	0	0	0	-	NC_000009.12	-	3932360	3937189	4830	273	sense-overlappin	XM_011517766.1	GLIS3
circRNA_06340	0	0	0	0.0206362	0	0	0	0	0	0	0	0	hsa_circ_0006370	NC_000009.12	-	4117768	4118881	1114	1114	exonic	XM_011517766.1	GLIS3
circRNA_06341	0.123450148	0	0.032712968	0.0412724	0.05015779	0	0.030148028	0	0	0	0	0	hsa_circ_0002874	NC_000009.12	-	4286038	4286523	486	486	exonic	XM_005251387.3	GLIS3
circRNA_06342	0	0	0	0	0	0	0	0.020486295	0	0	0	0	hsa_circ_0086251	NC_000009.12	-	4617923	4629168	11246	878	sense-overlappin	XM_011517958.1	SPATA6L
circRNA_06343	0	0	0.032712968	0	0	0	0	0	0	0	0	0	-	NC_000009.12	-	4622408	4635399	12992	780	sense-overlappin	XM_011517958.1	SPATA6L
circRNA_06344	0	0	0	0	0.03009467	0	0	0	0	0	0	0	-	NC_000009.12	-	4629091	4646528	17438	245	sense-overlappin	XM_011517951.1	SPATA6L
circRNA_06345	0	0	0	0	0	0	0	0	0	0.020172483	0	0	-	NC_000009.12	+	4823531	4827033	3503	265	sense-overlappin	NM_005772.4	RCL1
circRNA_06346	0	0	0	0	0	0.051782273	0	0	0	0	0	0	-	NC_000009.12	+	4823531	4833228	9698	340	sense-overlappin	NM_005772.4	RCL1
circRNA_06347	0	0.061409903	0.032712968	0.0206362	0	0.031069364	0	0	0	0	0.04051046	0	hsa_circ_0006345	NC_000009.12	+	4823548	4827033	3486	248	sense-overlappin	NM_005772.4	RCL1
circRNA_06348	0.072012586	0	0.065425935	0	0	0	0.030148028	0.020486295	0.070890847	0.050431207	0	0	hsa_circ_0006134	NC_000009.12	+	4823548	4833228	9681	323	sense-overlappin	NM_005772.4	RCL1
circRNA_06349	0	0	0.032712968	0	0	0	0	0	0	0	0	0	-	NC_000009.12	+	4823548	4860901	37354	1612	sense-overlappin	NM_005772.4	RCL1
circRNA_06350	0	0	0	0	0.02006311	0	0	0	0	0	0	0	-	NC_000009.12	+	4833154	4860901	27748	1364	sense-overlappin	NM_001286699.1	RCL1
circRNA_06351	0.041150049	0.020469968	0.065425935	0.0309543	0.02006311	0.031069364	0	0	0	0.030258724	0.02025523	0.0206364	hsa_circ_0007592	NC_000009.12	+	4860125	4860901	777	777	exonic	NM_001286699.1	RCL1
circRNA_06352	0	0	0	0	0	0	0.030148028	0	0	0	0	0	-	NC_000009.12	+	5064883	5073785	8903	808	sense-overlappin	NM_004972.3	JAK2
circRNA_06353	0	0	0	0	0	0	0.030148028	0	0	0	0	0.0206364	-	NC_000009.12	+	5064883	5081861	16979	1515	sense-overlappin	NM_004972.3	JAK2
circRNA_06354	0	0	0.032712968	0	0	0	0	0	0	0	0	0	-	NC_000009.12	+	5066678	5090911	24234	1845	sense-overlappin	NM_004972.3	JAK2

circRNA_06355	0	0	0	0	0	0	0.020098685	0	0	0	0	0	hsa_circ_0086268	NC_000009.12	+	5069022	5090911	21890	1733	sense-overlappin	NM_004972.3	JAK2
circRNA_06356	0	0	0	0	0	0	0	0	0	0.020172483	0	0	-	NC_000009.12	+	5500162	5500813	652	652	intergenic		
circRNA_06357	0	0	0.032712968	0	0	0	0	0	0	0	0	0	hsa_circ_0003084	NC_000009.12	+	5732388	5738538	6151	181	sense-overlappin	XM_005251523.2	RIC1
circRNA_06358	0	0	0	0	0	0	0.020098685	0	0	0	0	0	hsa_circ_0005149	NC_000009.12	-	5787061	5787593	533	412	sense-overlappin	XR_428431.2	
circRNA_06359	0	0	0	0	0	0	0	0	0	0.020172483	0	0	-	NC_000009.12	-	5787061	5831028	43968	2509	sense-overlappin	XR_929338.1	
circRNA_06360	0	0	0	0.0412724	0	0	0	0	0	0	0	0	hsa_circ_0086291	NC_000009.12	-	5810011	5813035	3025	674	sense-overlappin	XR_428431.2	
circRNA_06361	0	0	0	0	0	0.020712909	0	0	0	0	0	0	-	NC_000009.12	-	5910615	5954095	43481	3538	sense-overlappin	XR_929194.1	
circRNA_06362	0	0	0.04361729	0	0	0	0	0	0	0	0	0	-	NC_000009.12	-	5920544	5923309	2766	2766	exonic	XR_929194.1	
circRNA_06363	0	0	0	0	0.02006311	0	0	0	0.020254528	0	0	0	-	NC_000009.12	-	5924658	5954095	29438	474	sense-overlappin	XR_929194.1	
circRNA_06364	0	0.020469968	0	0	0.04012623	0	0	0	0	0	0	0	-	NC_000009.12	-	5944871	5988545	43675	1789	sense-overlappin	XR_929194.1	
circRNA_06365	0	0	0	0.0515905	0.02006311	0.020712909	0	0.030729443	0.050636319	0.030258724	0	0	-	NC_000009.12	-	5954016	5988545	34530	1699	sense-overlappin	XR_929194.1	
circRNA_06366	0.020575025	0.020469968	0.08723458	0	0	0	0	0	0	0	0.02025523	0	-	NC_000009.12	-	5968019	5969436	1418	1418	exonic	XR_929194.1	
circRNA_06367	0.174887709	0.112584821	0.196277805	0.2992247	0.16050492	0.186416184	0.140690797	0.204862955	0.111399902	0.191638587	0.030382845	0.092863801	-	NC_000009.12	-	5968019	5988545	20527	1619	sense-overlappin	XR_929194.1	
circRNA_06368	0.020575025	0	0.13085187	0.0412724	0.04012623	0	0.110542769	0	0	0.110948655	0	0.051591	hsa_circ_0002359	NC_000009.12	+	6420912	6434173	13262	491	sense-overlappin	XR_929173.1	
circRNA_06369	0	0	0	0.0206362	0	0	0	0	0	0	0	0	-	NC_000009.12	+	6420912	6445540	24629	11858	sense-overlappin	XR_929173.1	
circRNA_06370	0	0	0	0	0	0	0.030148028	0	0	0	0	0	-	NC_000009.12	+	6460573	6477808	17236	516	sense-overlappin	XR_929173.1	
circRNA_06371	0	0.030704951	0	0	0	0	0	0	0.020254528	0.060517448	0	0	hsa_circ_0003124	NC_000009.12	+	6849507	6893232	43726	486	sense-overlappin	NM_001146696.1	KDM4C
circRNA_06372	0	0	0	0	0	0	0	0	0.030381792	0	0	0	hsa_circ_0086338	NC_000009.12	+	6980925	6990524	9600	1808	sense-overlappin	NR_130707.1	
circRNA_06373	0	0	0	0.0206362	0	0	0	0	0	0	0	0	-	NC_000009.12	-	13150511	13186386	35876	1266	sense-overlappin	XM_006716887.2	MPDZ
circRNA_06374	0	0	0	0.0206362	0	0	0	0	0	0.050431207	0	0	-	NC_000009.12	-	13216774	13250372	33599	1347	sense-overlappin	XM_006716887.2	MPDZ
circRNA_06375	0	0	0	0.0206362	0	0	0	0	0	0	0	0	-	NC_000009.12	-	14102422	14179780	77359	994	sense-overlappin	NM_001282787.1	NFIB
circRNA_06376	0	0	0	0	0.02006311	0	0	0	0	0	0	0	-	NC_000009.12	-	14120440	14155893	35454	629	sense-overlappin	NM_001282787.1	NFIB
circRNA_06377	0.030862537	0	0	0	0.02006311	0	0	0	0	0	0	0	-	NC_000009.12	-	14120440	14179780	59341	683	sense-overlappin	NM_001282787.1	NFIB
circRNA_06378	0	0	0	0	0	0	0.020098685	0	0	0	0	0	-	NC_000009.12	-	14120440	14180866	60427	1769	sense-overlappin	NM_001190737.1	NFIB
circRNA_06379	0	0	0	0	0.02006311	0	0	0	0	0	0	0	hsa_circ_0086375	NC_000009.12	-	14146689	14155893	9205	309	sense-overlappin	NM_001282787.1	NFIB
circRNA_06380	0.041150049	0.051174919	0	0.0928628	0.02006311	0	0	0.020486295	0.020254528	0.030258724	0	0.0412728	hsa_circ_0086376	NC_000009.12	-	14146689	14179780	33092	363	sense-overlappin	NM_001282787.1	NFIB
circRNA_06381	0	0	0	0.0309543	0.02006311	0	0	0	0	0	0	0	-	NC_000009.12	-	14150145	14179780	29636	244	sense-overlappin	NM_001282787.1	NFIB
circRNA_06382	0	0	0	0	0.03009467	0	0	0	0	0	0	0	-	NC_000009.12	-	14243315	14307520	64206	64206	sense-overlappin	NM_001190737.1	NFIB
circRNA_06383	0	0	0.119947548	0	0.03009467	0.031069364	0	0	0	0	0.030382845	0	-	NC_000009.12	-	14639896	14662326	22431	368	sense-overlappin	XM_011517851.1	ZDHHC21
circRNA_06384	0	0.061409903	0.021808645	0	0.02006311	0	0	0	0	0	0	0	hsa_circ_0002654	NC_000009.12	-	14639896	14674385	34490	666	sense-overlappin	XM_011517851.1	ZDHHC21
circRNA_06385	0.072012586	0.040939935	0.370746965	0.2269981	0.10031557	0.103564547	0.030148028	0.081945182	0.101272639	0.151293621	0.091148535	0.082545601	hsa_circ_0008952	NC_000009.12	-	14639896	14680162	40267	960	sense-overlappin	XM_011517851.1	ZDHHC21
circRNA_06386	0	0	0	0	0.02006311	0	0	0	0	0	0	0	-	NC_000009.12	-	14658749	14680162	21414	843	sense-overlappin	XM_011517851.1	ZDHHC21
circRNA_06387	0	0	0	0	0	0	0	0	0	0.040344966	0	0	-	NC_000009.12	-	14672830	14680162	7333	592	sense-overlappin	XM_011517851.1	ZDHHC21
circRNA_06388	0.020575025	0	0	0	0	0	0	0	0	0	0	0	-	NC_000009.12	-	15177697	15187035	9339	446	sense-overlappin	NM_001168342.1	TTC39B
circRNA_06389	0	0	0	0	0	0	0	0	0	0.060517448	0	0	-	NC_000009.12	-	15267914	15274712	6799	6799	sense-overlappin	NM_001168339.1	TTC39B
circRNA_06390	0.030862537	0	0	0	0	0	0	0	0	0	0	0	-	NC_000009.12	+	15457960	15460313	2354	703	sense-overlappin	XR_428427.2	
circRNA_06391	0.061725074	0.051174919	0	0.0309543	0	0.051782273	0	0.020486295	0.030381792	0.080689931	0.02025523	0	hsa_circ_0086414	NC_000009.12	-	16435555	16437524	1970	1970	exonic	XM_011517931.1	BNC2
circRNA_06392	0.318912882	0.08187987	0.098138903	0.1238171	0.05015779	0.207129093	0.080394741	0.133160921	0.172163486	0.211811069	0.141786611	0	hsa_circ_0008732	NC_000009.12	-	16727797	16738485	10689	327	sense-overlappin	XM_011517922.1	BNC2
circRNA_06393	0.030862537	0	0	0	0	0	0	0	0	0	0	0	-	NC_000009.12	-	16727797	16758273	30477	20115	sense-overlappin	XM_011517922.1	BNC2
circRNA_06394	0	0	0	0	0	0.041425819	0	0	0	0.020172483	0	0	-	NC_000009.12	+	17226203	17236588	10386	400	sense-overlappin	XM_006716793.2	CNTLN
circRNA_06395	0	0	0	0	0.03009467	0	0	0	0	0	0	0	hsa_circ_0086420	NC_000009.12	+	17298190	17342444	44255	903	sense-overlappin	XM_006716793.2	CNTLN
circRNA_06396	0	0	0	0	0.03009467	0	0	0	0	0.040344966	0	0	-	NC_000009.12	+	17309058	17342444	33387	740	sense-overlappin	XM_006716793.2	CNTLN
circRNA_06397	0	0	0	0	0	0	0	0	0	0	0.02025523	0	hsa_circ_0086422	NC_000009.12	+	17330632	17342444	11813	545	sense-overlappin	XM_006716793.2	CNTLN
circRNA_06398	0	0	0	0	0	0	0	0	0.020254528	0	0	0	hsa_circ_0086425	NC_000009.12	+	17388162	17395069	6908	628	sense-overlappin	XM_006716793.2	CNTLN
circRNA_06399	0	0	0	0	0	0.020712909	0	0	0	0	0	0	-	NC_000009.12	+	18082355	18082783	429	429	intronic	XM_011518064.1	ADAMTSL1
circRNA_06400	0	0	0.054521613	0.0412724	0.02006311	0	0	0	0	0.020172483	0	0	-	NC_000009.12	-	19070219	19096769	26551	1143	sense-overlappin	NM_001270890.1	HAUS6
circRNA_06401	0	0	0.021808645	0	0	0	0	0	0	0	0	0	hsa_circ_0086450	NC_000009.12	-	19086734	19089559	2826	263	sense-overlappin	NM_001270890.1	HAUS6
circRNA_06402	0.051437562	0.061409903	0.185373483	0.0412724	0.03009467	0.031069364	0	0.040972591	0.020254528	0.040344966	0	0	hsa_circ_0005684	NC_000009.12	+	19286769	19305527	18759	1182	sense-overlappin	NM_017925.6	DENND4C
circRNA_06403	0	0.030704951	0	0	0	0	0	0	0	0	0	0	-	NC_000009.12	+	19286769	19316839	30071	1502	sense-overlappin	NM_017925.6	DENND4C
circRNA_06404	0	0	0.04361729	0.0206362	0	0	0	0	0	0	0.04051046	0	-	NC_000009.12	+	19288596	19300331	11736	953	sense-overlappin	XM_006716806.2	DENND4C
circRNA_06405	0	0	0.021808645	0	0	0.031069364	0.020098685	0	0	0	0	0	hsa_circ_0086466	NC_000009.12	+	19316417	19316839	423	320	sense-overlappin	NM_017925.6	DENND4C
circRNA_06406	0.030862537	0	0	0	0.02006311	0	0	0	0	0	0	0	-	NC_000009.12	+	19350702	19352693	1992	492	sense-overlappin	NM_017925.6	DENND4C
circRNA_06407	0.020575025	0.030704951	0.032712968	0.0515905	0	0	0	0	0	0	0	0	hsa_circ_0006556	NC_000009.12	+	19356972	19358160	1189	379	sense-overlappin	NM_017925.6	DENND4C
circRNA_06408	0	0	0	0	0	0	0	0	0	0.020172483	0	0	hsa_circ_0005373	NC_000009.12	+	19356972	19360489	3518	625	sense-overlappin	NM_017925.6	DENND4C

circRNA_06409	0	0	0	0	0	0	0	0	0	0.020172483	0.050638075	0	-	NC_000009.12	+	19361846	19369987	8142	269	sense-overlappin	NM_017925.6	DENND4C
circRNA_06410	0	0.040939935	0	0	0.02006311	0.041425819	0.030148028	0	0	0	0	0.0309546	hsa_circ_0086482	NC_000009.12	+	19423862	19424841	980	257	sense-overlappin	XM_011517857.1	ACER2
circRNA_06411	0	0	0	0.0309543	0	0	0	0	0	0	0	0	hsa_circ_0086483	NC_000009.12	+	19423862	19435084	11223	395	sense-overlappin	XM_011517857.1	ACER2
circRNA_06412	0	0.020469968	0	0	0	0	0	0	0	0	0	0	hsa_circ_0086488	NC_000009.12	-	20353525	20365744	12220	450	sense-overlappin	NM_001286691.1	MLLT3
circRNA_06413	0	0	0	0	0	0	0.020098685	0	0	0	0	0	hsa_circ_0086490	NC_000009.12	-	20360742	20365744	5003	306	sense-overlappin	NM_001286691.1	MLLT3
circRNA_06414	0	0	0	0	0	0	0	0.040972591	0	0	0	0	hsa_circ_0086492	NC_000009.12	-	20413721	20414425	705	705	exonic	NM_001286691.1	MLLT3
circRNA_06415	0.041150049	0	0	0	0	0	0	0	0	0	0	0	-	NC_000009.12	+	20715322	20720534	5213	319	sense-overlappin	NM_017794.4	FOCAD
circRNA_06416	0	0	0.065425935	0	0	0	0.020098685	0	0	0.030258724	0	0	-	NC_000009.12	+	20715322	20740340	25019	424	sense-overlappin	NM_017794.4	FOCAD
circRNA_06417	0	0	0	0.0206362	0	0	0	0	0	0	0	0	-	NC_000009.12	+	20715322	20770238	54917	938	sense-overlappin	NM_017794.4	FOCAD
circRNA_06418	0	0	0	0	0	0	0	0	0	0	0	0.0309546	-	NC_000009.12	+	20715322	20789608	74287	1487	sense-overlappin	NM_017794.4	FOCAD
circRNA_06419	0	0	0.021808645	0	0	0	0	0	0	0	0	0	-	NC_000009.12	+	20758090	20765073	6984	307	sense-overlappin	NM_017794.4	FOCAD
circRNA_06420	0	0	0.021808645	0	0	0	0	0	0	0	0	0	-	NC_000009.12	+	20764869	20789608	24740	961	sense-overlappin	NM_017794.4	FOCAD
circRNA_06421	0	0	0.21808645	0.0825448	0.02006311	0	0	0	0.020254528	0	0.04051046	0.0412728	hsa_circ_0003295	NC_000009.12	+	20819796	20823115	3320	465	sense-overlappin	NM_017794.4	FOCAD
circRNA_06422	0	0.020469968	0.08723458	0	0	0	0	0	0	0	0.030382845	0	hsa_circ_0086536	NC_000009.12	+	20819796	20885230	65435	1170	sense-overlappin	NM_017794.4	FOCAD
circRNA_06423	0	0	0.021808645	0	0	0	0	0	0	0	0	0	hsa_circ_0005426	NC_000009.12	+	20881871	20916937	35067	535	sense-overlappin	NM_017794.4	FOCAD
circRNA_06424	0	0	0.021808645	0.0309543	0	0	0	0	0	0	0	0	-	NC_000009.12	+	20881871	20929596	47726	1000	sense-overlappin	NM_017794.4	FOCAD
circRNA_06425	0	0	0.032712968	0	0	0	0	0	0	0	0	0	hsa_circ_0002555	NC_000009.12	+	20881871	20933103	51233	1090	sense-overlappin	NM_017794.4	FOCAD
circRNA_06426	0	0	0.076330258	0	0	0	0	0	0	0	0.02025523	0	hsa_circ_0086564	NC_000009.12	+	20907150	20933103	25954	782	sense-overlappin	NM_017794.4	FOCAD
circRNA_06427	0	0	0.021808645	0	0	0	0	0	0	0.020172483	0	0	hsa_circ_0006641	NC_000009.12	+	20907150	20933132	25983	811	sense-overlappin	NM_017794.4	FOCAD
circRNA_06428	0.030862537	0	0.021808645	0.0515905	0	0	0	0.030729443	0	0.020172483	0	0	hsa_circ_0008678	NC_000009.12	+	20923660	20933103	9444	555	sense-overlappin	NM_017794.4	FOCAD
circRNA_06429	0	0	0.032712968	0	0	0	0	0	0	0	0	0	hsa_circ_0005646	NC_000009.12	+	20923660	20933132	9473	584	sense-overlappin	NM_017794.4	FOCAD
circRNA_06430	0	0	0.076330258	0.0309543	0	0	0.040197371	0	0	0	0	0	hsa_circ_0008599	NC_000009.12	-	21476899	21477292	394	394	intergenic		
circRNA_06431	0	0	0.065425935	0	0.02006311	0	0	0	0	0	0	0	hsa_circ_0004297	NC_000009.12	+	22046317	22097364	51048	51048	intergenic		
circRNA_06432	0	0	0.04361729	0	0.02006311	0	0	0	0	0	0	0	hsa_circ_0008574	NC_000009.12	+	22046751	22056387	9637	9637	intergenic		
circRNA_06433	0	0	0.076330258	0	0	0	0	0	0	0	0	0	hsa_circ_0005118	NC_000009.12	+	22046751	22064018	17268	17268	intergenic		
circRNA_06434	0.020575025	0	0.098138903	0	0	0	0.020098685	0	0	0	0	0	hsa_circ_0008796	NC_000009.12	+	22046751	22097364	50614	50614	intergenic		
circRNA_06435	0	0	0	0	0.02006311	0	0	0	0	0	0	0	-	NC_000009.12	-	26346247	26346891	645	645	intergenic		
circRNA_06436	0	0	0	0	0	0	0	0.030729443	0	0	0	0	hsa_circ_0002781	NC_000009.12	-	26861066	26884885	23820	202	sense-overlappin	XM_005251581.2	CAAP1
circRNA_06437	0	0	0.032712968	0	0	0	0	0	0	0	0	0	-	NC_000009.12	-	26861066	26887513	26448	488	sense-overlappin	XM_005251581.2	CAAP1
circRNA_06438	0	0	0.021808645	0	0	0	0	0	0	0	0	0	-	NC_000009.12	+	27008995	27060651	51657	1122	sense-overlappin	NM_001099223.1	IFT74
circRNA_06439	0	0	0.021808645	0	0.03009467	0	0	0	0	0	0	0	-	NC_000009.12	+	27009020	27018687	9668	387	sense-overlappin	NM_001099223.1	IFT74
circRNA_06440	0	0	0.021808645	0	0.02006311	0	0	0	0	0	0	0	-	NC_000009.12	+	27009020	27029104	20085	467	sense-overlappin	NM_001099223.1	IFT74
circRNA_06441	0	0.030704951	0.021808645	0.0206362	0	0	0	0.020486295	0	0.040344966	0.02025523	0	-	NC_000009.12	+	27009020	27048274	39255	746	sense-overlappin	NM_001099223.1	IFT74
circRNA_06442	0	0	0	0	0	0	0.020098685	0	0	0	0	0	-	NC_000009.12	-	27455133	27455748	616	616	exonic	NM_024761.4	MOB3B
circRNA_06443	0	0	0	0	0	0	0.020098685	0	0	0	0	0	-	NC_000009.12	-	27455133	27459872	4740	4740	sense-overlappin	NM_024761.4	MOB3B
circRNA_06444	0	0	0.04361729	0	0.02006311	0	0	0	0	0	0	0	-	NC_000009.12	-	31233747	31235419	1673	1673	intergenic		
circRNA_06445	0	0	0.021808645	0	0	0	0	0	0	0	0	0	-	NC_000009.12	+	31973015	31978603	5589	5589	intergenic		
circRNA_06446	0	0	0	0.0206362	0.03009467	0	0	0	0	0	0	0	hsa_circ_0004389	NC_000009.12	-	32480219	32481497	1279	294	sense-overlappin	NM_014314.3	DDX58
circRNA_06447	0	0	0	0.0412724	0	0	0	0	0	0	0	0	-	NC_000009.12	+	33026475	33026990	516	320	sense-overlappin	NM_001539.2	DNAJA1
circRNA_06448	0	0.020469968	0	0.0309543	0	0	0	0	0	0	0	0	hsa_circ_0003762	NC_000009.12	+	33036574	33037115	542	217	sense-overlappin	NM_001539.2	DNAJA1
circRNA_06449	0	0	0.021808645	0	0	0	0.030148028	0	0	0	0	0	-	NC_000009.12	-	33068824	33073806	4983	475	sense-overlappin	NM_018225.2	SMU1
circRNA_06450	0.041150049	0.030704951	0	0.0515905	0.06018934	0	0.050246713	0	0.030381792	0.020172483	0.030382845	0	hsa_circ_0003575	NC_000009.12	+	33271152	33278225	7074	294	sense-overlappin	NM_016410.5	CHMP5
circRNA_06451	0	0	0	0.0206362	0	0	0	0	0	0.020172483	0	0	hsa_circ_0086648	NC_000009.12	+	33318731	33319127	397	318	sense-overlappin	NM_147134.2	NFX1
circRNA_06452	0	0.071644886	0.021808645	0	0.02006311	0.072495183	0	0.030729443	0.020254528	0	0.050638075	0.0206364	hsa_circ_0005405	NC_000009.12	+	33351560	33352719	1160	305	sense-overlappin	NM_147133.2	NFX1
circRNA_06453	0	0	0.021808645	0	0	0	0	0.020486295	0.020254528	0	0	0	hsa_circ_0006728	NC_000009.12	+	33351560	33354892	3333	449	sense-overlappin	NM_147133.2	NFX1
circRNA_06454	0	0	0.021808645	0	0	0	0.020098685	0	0	0	0	0	hsa_circ_0003624	NC_000009.12	+	33886881	33900271	13391	185	sense-overlappin	XM_011517949.1	UBE2R2
circRNA_06455	0	0	0	0	0	0	0	0	0	0.050431207	0	0	hsa_circ_0086694	NC_000009.12	-	33932562	33933628	1067	206	sense-overlappin	NM_001282530.1	UBAP2
circRNA_06456	0	0	0.032712968	0.0206362	0	0	0	0	0	0	0	0	hsa_circ_0086708	NC_000009.12	-	33941649	33948587	6939	873	sense-overlappin	NM_001282529.1	UBAP2
circRNA_06457	0	0	0.04361729	0	0	0	0	0	0	0	0	0	hsa_circ_0086709	NC_000009.12	-	33941649	33953474	11826	1063	sense-overlappin	NM_001282529.1	UBAP2
circRNA_06458	0	0	0	0.0206362	0	0	0	0	0	0	0	0	hsa_circ_0086710	NC_000009.12	-	33941649	33956146	14498	1131	sense-overlappin	NM_018449.3	UBAP2
circRNA_06459	0	0	0	0.0515905	0	0	0.030148028	0	0	0	0	0	-	NC_000009.12	-	33944365	33948587	4223	489	sense-overlappin	NM_001282529.1	UBAP2
circRNA_06460	0	0	0.04361729	0.0515905	0.04012623	0.041425819	0	0	0	0	0.04051046	0	hsa_circ_0001846	NC_000009.12	-	33944365	33956146	11782	747	sense-overlappin	NM_018449.3	UBAP2
circRNA_06461	0	0	0	0	0.04012623	0	0	0	0	0	0	0	hsa_circ_0002976	NC_000009.12	-	33948374	33948587	214	214	exonic	NM_001282529.1	UBAP2
circRNA_06462	0	0.061409903	0.076330258	0	0.0702209	0.051782273	0	0.020486295	0.050636319	0.020172483	0.06076569	0	hsa_circ_0003496	NC_000009.12	-	33948374	33953474	5101	404	sense-overlappin	NM_001282529.1	UBAP2
circRNA_06463	0	0	0.065425935	0.0412724	0.06018934	0.031069364	0.020098685	0.020486295	0.081018111	0.020172483	0.070893305	0.0206364	hsa_circ_0007367	NC_000009.12	-	33948374	33956146	7773	472	sense-overlappin	NM_018449.3	UBAP2
circRNA_06464	0	0	0.032712968	0.0309543	0	0	0	0.030729443	0	0	0.02025523	0	hsa_circ_0004774	NC_000009.12	-	33948374	33963791	15418	591	sense-overlappin	NM_018449.3	UBAP2

circRNA_06465	0	0	0	0.0412724	0	0	0	0	0	0	0	0	hsa_circ_0086715	NC_000009.12	-	33948374	33996333	47960	1093	sense-overlappin	NM_018449.3	UBAP2
circRNA_06466	0	0	0	0	0	0.031069364	0	0	0	0	0.02025523	0	hsa_circ_0003945	NC_000009.12	-	33953285	33956146	2862	258	sense-overlappin	NM_018449.3	UBAP2
circRNA_06467	0.102875123	0.071644886	0.447077223	0.4127238	0.15047336	0.186416184	0.160789483	0.09218833	0.121527166	0.151293621	0.12153138	0.051591	hsa_circ_0001847	NC_000009.12	-	33953285	33963791	10507	377	sense-overlappin	NM_018449.3	UBAP2
circRNA_06468	0.030862537	0	0	0.0309543	0	0	0.030148028	0.020486295	0	0	0.02025523	0	hsa_circ_0003141	NC_000009.12	-	33953285	33973237	19953	536	sense-overlappin	NM_018449.3	UBAP2
circRNA_06469	0	0.040939935	0.076330258	0.0515905	0	0.113921001	0.030148028	0.030729443	0.030381792	0	0.08102092	0	hsa_circ_0086720	NC_000009.12	-	33953285	33996333	43049	879	sense-overlappin	NM_018449.3	UBAP2
circRNA_06470	0	0	0.04361729	0	0.04012623	0	0	0.030729443	0	0	0.030382845	0	hsa_circ_0005993	NC_000009.12	-	33956079	33963791	7713	187	sense-overlappin	NM_018449.3	UBAP2
circRNA_06471	0	0	0	0	0	0	0	0	0	0.050431207	0	0	hsa_circ_0001848	NC_000009.12	-	33956079	33973237	17159	346	sense-overlappin	NM_018449.3	UBAP2
circRNA_06472	0.020575025	0	0	0	0	0	0	0	0	0	0	0	-	NC_000009.12	-	33956079	33996333	40255	689	sense-overlappin	NM_018449.3	UBAP2
circRNA_06473	0.020575025	0.020469968	0	0.0515905	0	0	0	0.061458886	0.030381792	0	0	0	hsa_circ_0001849	NC_000009.12	-	33960826	33963791	2966	119	sense-overlappin	NM_001282529.1	UBAP2
circRNA_06474	0.061725074	0.092114854	0.13085187	0.113499	0.16050492	0.093208092	0.050246713	0.020486295	0.070890847	0.07060369	0.02025523	0.0412728	hsa_circ_0001850	NC_000009.12	-	33960826	33973237	12412	278	sense-overlappin	NM_018449.3	UBAP2
circRNA_06475	0.030862537	0	0.21808645	0.0515905	0.03009467	0	0.020098685	0.030729443	0	0.060517448	0.08102092	0.0412728	-	NC_000009.12	-	33960826	33989126	28301	510	sense-overlappin	NM_018449.3	UBAP2
circRNA_06476	0	0	0.021808645	0	0.09028402	0.020712909	0	0	0	0.030258724	0.050638075	0	hsa_circ_0086724	NC_000009.12	-	33960826	33996333	35508	621	sense-overlappin	NM_018449.3	UBAP2
circRNA_06477	0	0	0.021808645	0	0	0	0	0	0	0	0	0	hsa_circ_0086726	NC_000009.12	-	33960826	34017189	56364	839	sense-overlappin	NM_018449.3	UBAP2
circRNA_06478	0.051437562	0	0.054521613	0.0928628	0.03009467	0.082851637	0.030148028	0.081945182	0.060763583	0.050431207	0.06076569	0.082545601	hsa_circ_0001851	NC_000009.12	-	33971651	33973237	1587	159	sense-overlappin	NM_018449.3	UBAP2
circRNA_06479	0.051437562	0	0.021808645	0	0	0.031069364	0	0.020486295	0	0	0	0	hsa_circ_0086729	NC_000009.12	-	33971651	33989126	17476	391	sense-overlappin	NM_018449.3	UBAP2
circRNA_06480	0	0	0	0	0	0	0	0.040972591	0	0	0.02025523	0	hsa_circ_0086730	NC_000009.12	-	33971651	33996333	24683	502	sense-overlappin	NM_018449.3	UBAP2
circRNA_06481	0	0	0.021808645	0	0	0	0	0	0	0	0	0	hsa_circ_0086732	NC_000009.12	-	33971651	34017189	45539	720	sense-overlappin	NM_018449.3	UBAP2
circRNA_06482	0	0	0.065425935	0.0309543	0	0	0.020098685	0.051215739	0	0	0	0	hsa_circ_0001852	NC_000009.12	-	33986760	33998864	12105	421	sense-overlappin	NM_001282529.1	UBAP2
circRNA_06483	0	0	0.076330258	0.0515905	0.03009467	0.041425819	0.050246713	0.020486295	0	0.030258724	0	0.0206364	hsa_circ_0086735	NC_000009.12	-	33986760	34017189	30430	561	sense-overlappin	NM_018449.3	UBAP2
circRNA_06484	0	0	0	0	0.03009467	0	0	0	0	0	0	0	hsa_circ_0086736	NC_000009.12	-	33996223	33998864	2642	189	sense-overlappin	NM_001282529.1	UBAP2
circRNA_06485	0	0	0.109043225	0	0.02006311	0.020712909	0	0.030729443	0	0	0.030382845	0	hsa_circ_0003410	NC_000009.12	-	33996223	34017189	20967	329	sense-overlappin	NM_018449.3	UBAP2
circRNA_06486	0	0	0.04361729	0	0	0	0	0	0	0	0	0	-	NC_000009.12	-	34096716	34098517	1802	260	sense-overlappin	NM_015397.3	DCAF12
circRNA_06487	0	0	0	0	0	0	0.020098685	0	0	0	0	0	-	NC_000009.12	-	34192272	34192688	417	417	antisense	NM_001171201.1	UBAP1
circRNA_06488	0	0	0	0	0	0	0	0	0.020254528	0	0	0	-	NC_000009.12	+	34207324	34210442	3119	3119	intronic	NM_001171201.1	UBAP1
circRNA_06489	0	0	0.04361729	0	0	0	0	0.020486295	0	0	0	0	hsa_circ_0086742	NC_000009.12	+	34220908	34242108	21201	1090	sense-overlappin	NM_001171203.2	UBAP1
circRNA_06490	0	0	0.021808645	0	0	0	0	0	0	0	0	0	hsa_circ_0086743	NC_000009.12	+	34234216	34242108	7893	1049	sense-overlappin	NM_001171201.1	UBAP1
circRNA_06491	0	0	0.032712968	0	0	0	0	0	0	0	0	0	hsa_circ_0086748	NC_000009.12	-	34269257	34271930	2674	228	sense-overlappin	XM_005251450.2	KIF24
circRNA_06492	0	0	0.032712968	0	0	0	0	0	0	0	0	0	hsa_circ_0003153	NC_000009.12	-	34269257	34286704	17448	316	sense-overlappin	XM_005251450.2	KIF24
circRNA_06493	0	0	0.021808645	0	0	0	0	0	0	0	0	0	hsa_circ_0007356	NC_000009.12	-	34286617	34290389	3773	304	sense-overlappin	XM_005251450.2	KIF24
circRNA_06494	0	0.030704951	0	0	0	0	0	0	0	0	0	0	hsa_circ_0086754	NC_000009.12	+	34338713	34339166	454	278	sense-overlappin	NM_001161.4	NUDT2
circRNA_06495	0	0	0	0	0	0	0	0	0	0.020172483	0	0	-	NC_000009.12	+	35228015	35237826	9812	372	sense-overlappin	XM_011517684.1	UNC13B
circRNA_06496	0	0.030704951	0.228990773	0.1238171	0.05015779	0	0	0.020486295	0.040509055	0.040344966	0.030382845	0	-	NC_000009.12	+	35228015	35243364	15350	446	sense-overlappin	XM_011517684.1	UNC13B
circRNA_06497	0	0	0.021808645	0	0	0	0	0	0	0	0	0	-	NC_000009.12	+	35228015	35259050	31036	504	sense-overlappin	XM_011517684.1	UNC13B
circRNA_06498	0	0.030704951	0	0.0825448	0.03009467	0	0	0	0	0	0	0	-	NC_000009.12	+	35236469	35243364	6896	316	sense-overlappin	XM_011517684.1	UNC13B
circRNA_06499	0	0	0	0	0	0	0	0	0	0.040344966	0	0	-	NC_000009.12	+	35236469	35259050	22582	374	sense-overlappin	XM_011517684.1	UNC13B
circRNA_06500	0.020575025	0	0.04361729	0.1031809	0	0	0.020098685	0	0	0.050431207	0.04051046	0.0206364	hsa_circ_0008518	NC_000009.12	+	35295696	35313989	18294	641	sense-overlappin	XM_011517684.1	UNC13B
circRNA_06501	0	0.020469968	0	0	0	0	0	0	0	0	0	0	-	NC_000009.12	+	35295696	35366993	71298	688	sense-overlappin	XM_011517684.1	UNC13B
circRNA_06502	0.061725074	0.020469968	0.098138903	0.0722267	0.05015779	0.031069364	0	0	0.070890847	0.040344966	0.030382845	0.0412728	hsa_circ_0002702	NC_000009.12	+	35546430	35548535	2106	2106	exonic	NM_014806.3	RUSC2
circRNA_06503	0	0	0.04361729	0	0	0	0	0	0	0	0	0	-	NC_000009.12	-	35705750	35706111	362	252	sense-overlappin	NM_006289.3	TLN1
circRNA_06504	0	0	0	0.0206362	0	0	0	0	0	0	0	0	hsa_circ_0007812	NC_000009.12	+	36197551	36204179	6629	268	sense-overlappin	NM_001076677.2	CLTA
circRNA_06505	0	0	0.021808645	0	0	0	0	0	0	0	0	0	-	NC_000009.12	-	36344832	36390616	45785	1373	sense-overlappin	XM_011517713.1	RNF38
circRNA_06506	0	0	0.065425935	0	0	0	0	0	0	0	0	0	hsa_circ_0087015	NC_000009.12	-	36351115	36376127	25013	1101	sense-overlappin	XM_011517713.1	RNF38
circRNA_06507	0	0	0	0	0	0	0	0.020486295	0	0	0	0	hsa_circ_0008698	NC_000009.12	-	36352742	36353331	590	269	sense-overlappin	XM_011517713.1	RNF38
circRNA_06508	0	0	0.032712968	0	0	0	0	0	0	0	0	0	hsa_circ_0087023	NC_000009.12	-	36369719	36376127	6409	408	sense-overlappin	XM_011517713.1	RNF38
circRNA_06509	0	0	0	0	0	0	0	0	0	0	0.04051046	0	hsa_circ_0001856	NC_000009.12	-	36375934	36390616	14683	344	sense-overlappin	XM_011517713.1	RNF38
circRNA_06510	0	0	0	0	0.02006311	0	0	0	0	0	0	0	-	NC_000009.12	+	36581644	36651877	70234	1091	sense-overlappin	NM_001256685.1	MELK
circRNA_06511	0	0	0.021808645	0	0	0	0	0	0	0	0.04051046	0	hsa_circ_0007658	NC_000009.12	+	36597222	36643083	45862	639	sense-overlappin	NR_046337.1	
circRNA_06512	0	0	0.032712968	0	0	0	0	0	0	0	0	0	hsa_circ_0005517	NC_000009.12	+	36669310	36674937	5628	370	sense-overlappin	NM_001256685.1	MELK
circRNA_06513	0.041150049	0.071644886	0.228990773	0.0619086	0.05015779	0.113921001	0.080394741	0.09218833	0.091145375	0.121034897	0.091148535	0.0619092	hsa_circ_0001860	NC_000009.12	+	37126312	37126942	631	631	exonic	XM_005251612.2	ZCCHC7
circRNA_06514	0.061725074	0.092114854	0.065425935	0.0206362	0	0.020712909	0.050246713	0	0	0.080689931	0	0.0206364	-	NC_000009.12	+	37126312	37127263	952	952	sense-overlappin	XM_005251612.2	ZCCHC7
circRNA_06515	0	0.030704951	0.065425935	0.1547714	0.06018934	0.072495183	0.040197371	0.020486295	0.060763583	0.080689931	0.04051046	0.0412728	-	NC_000009.12	+	37126312	37147445	21134	21134	sense-overlappin	XM_005251612.2	ZCCHC7
circRNA_06516	0	0.030704951	0	0	0	0	0	0	0	0	0	0	-	NC_000009.12	+	37126312	37165935	39624	39624	sense-overlappin	XM_005251612.2	ZCCHC7
circRNA_06517	0.061725074	0.051174919	0.207182128	0.0825448	0.23072582	0.186416184	0.140690797	0.061458886	0.121527166	0.181552345	0.151914226	0.0412728	hsa_circ_0001861	NC_000009.12	+	37424845	37426654	1810	321	sense-overlappin	XR_929374.1	
circRNA_06518	0.041150049	0	0	0	0	0	0	0	0	0.060517448	0	0	-	NC_000009.12	+	37763629	37770739	7111	425	sense-overlappin	XR_929185.1	
circRNA_06519	0	0.020469968	0	0	0	0.041425819	0	0	0	0	0	0	hsa_circ_0087082	NC_000009.12	+	37768076	37770739	2664	300	sense-overlappin	XR_929185.1	

circRNA_06520	0	0	0	0	0	0	0	0	0	0	0.030382845	0	-	NC_000009.12	+	37768076	37778056	9981	853	sense-overlappin	XM_011517736.1	TRMT10B
circRNA_06521	0.020575025	0	0	0	0	0	0	0	0	0	0	0	-	NC_000009.12	+	68465093	68484048	18956	436	sense-overlappin	NM_021965.3	PGM5
circRNA_06522	0	0	0	0	0	0	0	0	0.020254528	0	0	0	-	NC_000009.12	+	69218257	69219701	1445	1445	sense-overlappin	NM_001170414.2	TJP2
circRNA_06523	0	0	0	0	0	0	0	0	0.020254528	0	0	0	hsa_circ_0004940	NC_000009.12	+	69236028	69237136	1109	399	sense-overlappin	NM_001170414.2	TJP2
circRNA_06524	0	0	0.032712968	0	0	0	0	0	0	0	0	0	hsa_circ_0002106	NC_000009.12	+	69236028	69238789	2762	575	sense-overlappin	NM_001170414.2	TJP2
circRNA_06525	0	0	0	0	0	0	0	0	0	0.020172483	0	0	hsa_circ_0005976	NC_000009.12	-	69516011	69517279	1269	1269	exonic	XM_006717093.2	APBA1
circRNA_06526	0	0	0.04361729	0	0	0	0	0	0	0	0	0	hsa_circ_0087159	NC_000009.12	-	69723326	69741858	18533	747	sense-overlappin	XM_011518639.1	PTAR1
circRNA_06527	0	0	0	0	0	0	0	0.020486295	0	0.040344966	0	0	-	NC_000009.12	+	70277310	70286271	8962	673	sense-overlappin	NM_015110.3	SMC5
circRNA_06528	0	0	0	0	0	0	0.040197371	0	0	0	0	0	-	NC_000009.12	+	70344144	70348038	3895	492	sense-overlappin	NM_015110.3	SMC5
circRNA_06529	0.041150049	0	0	0	0	0	0	0	0	0	0	0	hsa_circ_0003861	NC_000009.12	-	71694509	71698204	3696	319	sense-overlappin	NM_001135820.1	TMEM2
circRNA_06530	0	0	0	0	0	0	0	0	0	0	0	0.0309546	-	NC_000009.12	-	71709259	71725709	16451	936	sense-overlappin	NM_001135820.1	TMEM2
circRNA_06531	0	0	0.032712968	0	0	0	0	0	0	0	0	0	-	NC_000009.12	-	71716517	71725709	9193	386	sense-overlappin	NM_001135820.1	TMEM2
circRNA_06532	0.030862537	0	0	0	0	0	0	0	0	0	0	0	hsa_circ_0003290	NC_000009.12	-	71740063	71740232	170	170	exonic	NM_001135820.1	TMEM2
circRNA_06533	0	0	0	0	0	0	0.030148028	0	0	0	0	0	-	NC_000009.12	-	71749757	71750385	629	629	sense-overlappin	NM_001135820.1	TMEM2
circRNA_06534	0	0	0	0	0	0	0.020098685	0	0	0	0	0	hsa_circ_0087196	NC_000009.12	-	71750043	71750385	343	343	exonic	NM_001135820.1	TMEM2
circRNA_06535	0	0	0	0	0	0	0	0	0.020254528	0.020172483	0	0	hsa_circ_0003118	NC_000009.12	-	71862551	71875083	12533	915	sense-overlappin	NM_016014.2	ABHD17B
circRNA_06536	0	0	0.021808645	0	0	0	0	0	0	0	0	0	-	NC_000009.12	-	71907821	71908130	310	310	intronic	NM_016014.2	ABHD17B
circRNA_06537	0	0	0	0	0.03009467	0	0	0	0	0	0	0	-	NC_000009.12	+	71947006	71949618	2613	2613	sense-overlappin	XM_011518227.1	C9orf85
circRNA_06538	0	0.030704951	0	0	0	0	0	0	0	0	0	0	-	NC_000009.12	+	72195504	72219506	24003	520	sense-overlappin	NM_001242506.2	GDA
circRNA_06539	0	0	0.021808645	0	0	0	0	0	0	0	0	0	-	NC_000009.12	+	72202571	72213991	11421	480	sense-overlappin	XM_011519219.1	GDA
circRNA_06540	0	0	0.021808645	0	0	0	0	0	0	0	0	0	-	NC_000009.12	+	72208225	72213991	5767	308	sense-overlappin	XM_011519219.1	GDA
circRNA_06541	0	0	0	0	0	0	0	0.020486295	0	0	0	0	-	NC_000009.12	+	72210687	72219506	8820	222	sense-overlappin	NM_001242507.2	GDA
circRNA_06542	0	0	0.065425935	0.0206362	0.02006311	0.041425819	0.030148028	0	0	0.050431207	0	0	hsa_circ_0087220	NC_000009.12	-	74996447	75017448	21002	794	sense-overlappin	XM_005251702.1	C9orf41
circRNA_06543	0	0	0	0.0825448	0.06018934	0	0	0.081945182	0.020254528	0.07060369	0.06076569	0	hsa_circ_0004189	NC_000009.12	-	75016268	75017448	1181	360	sense-overlappin	XM_005251702.1	C9orf41
circRNA_06544	0	0.020469968	0	0	0	0	0.030148028	0	0	0.040344966	0.050638075	0	-	NC_000009.12	-	75077159	75080941	3783	2756	sense-overlappin	XM_006717162.1	NMRK1
circRNA_06545	0	0	0	0	0	0.031069364	0	0	0	0	0	0	-	NC_000009.12	+	75152132	75168266	16135	16135	intergenic		
circRNA_06546	0	0	0.021808645	0	0	0	0	0	0	0	0	0	hsa_circ_0087229	NC_000009.12	+	75986132	76027037	40906	335	sense-overlappin	XR_929806.1	
circRNA_06547	0	0	0.08723458	0	0.05015779	0.031069364	0	0	0	0	0	0	hsa_circ_0087232	NC_000009.12	+	76067955	76071898	3944	262	sense-overlappin	XR_929806.1	
circRNA_06548	0	0.061409903	0.17446916	0	0	0	0.020098685	0	0	0	0	0	hsa_circ_0087233	NC_000009.12	+	76067955	76096102	28148	475	sense-overlappin	XR_929806.1	
circRNA_06549	0	0.020469968	0.228990773	0	0	0	0	0.020486295	0	0	0	0	hsa_circ_0087234	NC_000009.12	+	76067955	76107351	39397	576	sense-overlappin	XR_929806.1	
circRNA_06550	0	0.040939935	0	0	0	0	0	0	0	0	0	0	-	NC_000009.12	+	76500916	76503756	2841	1664	sense-overlappin	NM_001097634.1	GCNT1
circRNA_06551	0	0	0	0.0309543	0	0.020712909	0	0	0	0.020172483	0	0.0309546	hsa_circ_0003654	NC_000009.12	+	76500916	76516776	15861	14684	sense-overlappin	NM_001097634.1	GCNT1
circRNA_06552	0	0.051174919	0	0.0309543	0	0.020712909	0	0	0	0	0	0.0412728	-	NC_000009.12	+	76500916	76527895	26980	25803	sense-overlappin	NM_001097634.1	GCNT1
circRNA_06553	0.051437562	0.030704951	0	0.0412724	0	0	0	0.040972591	0.040509055	0	0	0.0206364	-	NC_000009.12	+	76515528	76565815	50288	50288	intergenic		
circRNA_06554	0	0	0	0.0206362	0	0	0	0	0	0	0	0	-	NC_000009.12	+	76515532	76527895	12364	12364	intergenic		
circRNA_06555	0	0.020469968	0	0	0	0	0	0	0	0	0	0	-	NC_000009.12	-	76703337	76713721	10385	7520	sense-overlappin	NM_015225.2	PRUNE2
circRNA_06556	0	0	0.021808645	0	0	0	0	0	0	0	0	0	-	NC_000009.12	+	77199945	77213314	13370	596	sense-overlappin	XR_242580.3	
circRNA_06557	0.030862537	0	0.098138903	0	0.05015779	0.031069364	0.040197371	0.030729443	0	0.060517448	0.08102092	0	hsa_circ_0087243	NC_000009.12	+	77214329	77227485	13157	756	sense-overlappin	XR_242580.3	
circRNA_06558	0	0	0	0	0	0	0	0	0	0.030258724	0	0	hsa_circ_0087247	NC_000009.12	+	77238002	77238386	385	305	sense-overlappin	XR_242580.3	
circRNA_06559	0	0	0.021808645	0	0	0	0	0	0	0	0.04051046	0	hsa_circ_0087248	NC_000009.12	+	77238002	77252352	14351	693	sense-overlappin	XR_242580.3	
circRNA_06560	0	0	0	0	0	0	0	0	0	0	0.02025523	0	-	NC_000009.12	+	77275498	77293508	18011	995	sense-overlappin	XR_242580.3	
circRNA_06561	0	0	0.054521613	0.0309543	0	0	0	0.040972591	0.030381792	0	0.030382845	0.0206364	hsa_circ_0087255	NC_000009.12	+	77337255	77371149	33895	2999	sense-overlappin	XR_242580.3	
circRNA_06562	0.061725074	0.020469968	0.04361729	0.0722267	0.04012623	0	0	0.051215739	0	0.050431207	0	0	hsa_circ_0008075	NC_000009.12	+	77381976	77407607	25632	397	sense-overlappin	NM_001018037.1	VPS13A
circRNA_06563	0.020575025	0	0	0.0206362	0.06018934	0	0	0	0	0	0	0	hsa_circ_0006459	NC_000009.12	-	77794463	77797648	3186	259	sense-overlappin	NM_002072.4	GNAQ
circRNA_06564	0	0	0.021808645	0	0	0	0	0	0	0.040344966	0	0	-	NC_000009.12	-	77922161	77931856	9696	9696	sense-overlappin	NM_002072.4	GNAQ
circRNA_06565	0.030862537	0	0	0	0	0	0	0	0	0	0	0	-	NC_000009.12	+	78254836	78264316	9481	374	sense-overlappin	XM_005252263.3	CEP78
circRNA_06566	0	0	0	0	0	0.020712909	0.020098685	0	0	0	0	0	hsa_circ_0087302	NC_000009.12	+	79704783	79709699	4917	827	sense-overlappin	NM_001282748.1	TLE4
circRNA_06567	0	0	0	0.0206362	0	0	0	0	0	0	0	0	hsa_circ_0087319	NC_000009.12	-	81610220	81620557	10338	839	sense-overlappin	XM_006717261.2	TLE1
circRNA_06568	0	0	0	0.0206362	0	0	0	0	0	0	0	0	-	NC_000009.12	-	82990391	83025921	35531	1686	sense-overlappin	NM_152573.3	RASEF
circRNA_06569	0	0.030704951	0	0	0	0	0.020098685	0	0	0	0	0	hsa_circ_0008038	NC_000009.12	-	83659780	83669327	9548	2672	sense-overlappin	NM_013438.4	UBQLN1
circRNA_06570	0	0	0.141756193	0.0825448	0.06018934	0	0.090444084	0	0.020254528	0.050431207	0	0	-	NC_000009.12	-	83659780	83686155	26376	3597	sense-overlappin	NM_013438.4	UBQLN1
circRNA_06571	0.030862537	0.040939935	0	0	0	0	0	0	0	0.020172483	0.02025523	0.0412728	hsa_circ_0007698	NC_000009.12	-	83661777	83669327	7551	675	sense-overlappin	NM_013438.4	UBQLN1
circRNA_06572	0	0	0	0	0	0	0	0.030729443	0	0	0	0	hsa_circ_0008510	NC_000009.12	-	83665030	83677961	12932	578	sense-overlappin	NM_013438.4	UBQLN1
circRNA_06573	0	0	0	0	0	0	0	0	0	0.020172483	0	0	hsa_circ_0004725	NC_000009.12	-	83665030	83678599	13570	737	sense-overlappin	NM_013438.4	UBQLN1
circRNA_06574	0.123450148	0	0	0.0619086	0.0702209	0.082851637	0.060296056	0.051215739	0.081018111	0.050431207	0.02025523	0	hsa_circ_0001865	NC_000009.12	-	83677727	83678599	873	394	sense-overlappin	NM_013438.4	UBQLN1

circRNA_06575	0	0.040939935	0.04361729	0.0722267	0.0702209	0	0.040197371	0.030729443	0.020254528	0	0	0	hsa_circ_0003715	NC_000009.12	-	83677727	83686155	8429	925	sense-overlappin	NM_013438.4	UBQLN1
circRNA_06576	0	0	0	0	0	0.020712909	0	0	0	0	0	0	hsa_circ_0001866	NC_000009.12	-	83678441	83683066	4626	538	sense-overlappin	NM_013438.4	UBQLN1
circRNA_06577	0.13373766	0.122819805	0.119947548	0.1650895	0.15047336	0.103564547	0.130641455	0.071702034	0.091145375	0.171466104	0.08102092	0.0619092	hsa_circ_0087357	NC_000009.12	-	83678441	83686155	7715	690	sense-overlappin	NM_013438.4	UBQLN1
circRNA_06578	0	0.030704951	0.065425935	0.1341352	0.04012623	0.041425819	0.080394741	0.040972591	0.020254528	0.040344966	0	0	hsa_circ_0008207	NC_000009.12	-	83679775	83686155	6381	531	sense-overlappin	NM_013438.4	UBQLN1
circRNA_06579	0.041150049	0.040939935	0.04361729	0.0722267	0.03009467	0	0.080394741	0.030729443	0.030381792	0.040344966	0.050638075	0	hsa_circ_0005142	NC_000009.12	-	83682951	83686155	3205	268	sense-overlappin	NM_013438.4	UBQLN1
circRNA_06580	0.051437562	0	0	0.0515905	0	0	0	0	0	0	0	0	-	NC_000009.12	-	83739701	83768970	29270	512	sense-overlappin	XM_006717293.1	GKAP1
circRNA_06581	0	0	0	0	0	0.020712909	0	0	0	0	0	0	-	NC_000009.12	-	83753258	83788678	35421	480	sense-overlappin	XM_006717293.1	GKAP1
circRNA_06582	0	0	0	0	0	0	0.020098685	0	0	0	0	0	-	NC_000009.12	-	83753258	83799328	46071	691	sense-overlappin	XM_006717293.1	GKAP1
circRNA_06583	0.030862537	0	0.032712968	0.0928628	0	0	0	0	0	0	0	0	-	NC_000009.12	-	83780382	83806560	26179	628	sense-overlappin	XM_005252241.1	GKAP1
circRNA_06584	0	0	0	0	0	0.020712909	0	0	0	0	0	0	-	NC_000009.12	-	83799185	83806560	7376	403	sense-overlappin	XM_005252241.1	GKAP1
circRNA_06585	0	0	0	0	0	0	0.020098685	0	0	0	0	0	-	NC_000009.12	-	85575315	85579096	3782	338	sense-overlappin	XM_011518420.1	AGTPBP1
circRNA_06586	0	0	0	0	0.05015779	0	0	0	0.030381792	0.020172483	0	0	hsa_circ_0087386	NC_000009.12	-	85585463	85633374	47912	1863	sense-overlappin	XM_011518420.1	AGTPBP1
circRNA_06587	0	0	0	0	0	0	0	0	0	0.030258724	0	0	-	NC_000009.12	-	85586831	85655320	68490	2124	sense-overlappin	XM_005251848.2	AGTPBP1
circRNA_06588	0	0	0	0	0.03009467	0	0	0	0	0.040344966	0.030382845	0	hsa_circ_0007162	NC_000009.12	-	85596362	85633374	37013	1121	sense-overlappin	XM_011518420.1	AGTPBP1
circRNA_06589	0.061725074	0.040939935	0.17446916	0	0.10031557	0.062138728	0.070345399	0.133160921	0.101272639	0.090776173	0.070893305	0.0206364	hsa_circ_0001868	NC_000009.12	-	85618983	85633374	14392	1033	sense-overlappin	XM_011518420.1	AGTPBP1
circRNA_06590	0	0	0.032712968	0	0.02006311	0	0	0	0	0	0	0	-	NC_000009.12	-	85669485	85692813	23329	630	sense-overlappin	XM_005251848.2	AGTPBP1
circRNA_06591	0.020575025	0	0.119947548	0	0.09028402	0.041425819	0.130641455	0.030729443	0	0.040344966	0.02025523	0.0206364	hsa_circ_0087391	NC_000009.12	-	85669485	85712566	43082	695	sense-overlappin	XM_005251848.2	AGTPBP1
circRNA_06592	0	0.020469968	0.04361729	0	0.03009467	0	0	0	0	0	0	0.0206364	hsa_circ_0006687	NC_000009.12	-	85677436	85712566	35131	469	sense-overlappin	XM_005251848.2	AGTPBP1
circRNA_06593	0	0	0	0	0	0.020712909	0	0	0	0.020172483	0	0	hsa_circ_0007351	NC_000009.12	-	85692689	85712566	19878	190	sense-overlappin	XM_005251848.2	AGTPBP1
circRNA_06594	0	0	0	0	0	0	0	0	0	0	0	0.0206364	-	NC_000009.12	+	85881356	85883234	1879	1879	intergenic		
circRNA_06595	0	0	0	0	0.05015779	0.031069364	0	0.030729443	0	0	0	0.0412728	-	NC_000009.12	+	85959793	85996577	36785	783	sense-overlappin	NM_024635.3	NAA35
circRNA_06596	0	0	0.032712968	0	0	0	0	0	0	0	0	0	-	NC_000009.12	+	85962013	85978381	16369	529	sense-overlappin	NM_024635.3	NAA35
circRNA_06597	0	0	0	0	0	0	0.050246713	0	0	0	0	0.0619092	-	NC_000009.12	-	86033282	86040868	7587	662	sense-overlappin	NM_016548.3	GOLM1
circRNA_06598	0	0	0	0	0	0	0	0	0	0	0.02025523	0	-	NC_000009.12	-	86033282	86046572	13291	765	sense-overlappin	NM_016548.3	GOLM1
circRNA_06599	0	0.020469968	0	0	0	0	0	0	0	0	0	0	-	NC_000009.12	-	86033282	86052591	19310	820	sense-overlappin	NM_016548.3	GOLM1
circRNA_06600	0	0	0.032712968	0	0.02006311	0	0	0	0	0	0	0	-	NC_000009.12	-	86040739	86046572	5834	233	sense-overlappin	NM_016548.3	GOLM1
circRNA_06601	0	0	0.021808645	0	0.04012623	0	0	0	0	0	0	0	hsa_circ_0087400	NC_000009.12	-	86040739	86052591	11853	288	sense-overlappin	NM_016548.3	GOLM1
circRNA_06602	0	0.030704951	0	0	0.02006311	0	0	0	0.020254528	0.060517448	0	0	hsa_circ_0007037	NC_000009.12	-	86303086	86304947	1862	208	sense-overlappin	XM_006717283.1	ZCCHC6
circRNA_06603	0.041150049	0.102349838	0.207182128	0.1547714	0.10031557	0.113921001	0.060296056	0.09218833	0.162036222	0.181552345	0.08102092	0.092863801	hsa_circ_0001869	NC_000009.12	-	86305192	86310017	4826	508	sense-overlappin	XM_006717283.1	ZCCHC6
circRNA_06604	0	0	0	0	0	0.020712909	0	0	0	0.030258724	0	0	-	NC_000009.12	-	86343075	86346480	3406	566	sense-overlappin	XM_006717283.1	ZCCHC6
circRNA_06605	0	0	0	0	0	0	0	0.020486295	0	0	0	0	-	NC_000009.12	+	87637943	87658127	20185	1639	sense-overlappin	NM_001288731.1	DAPK1
circRNA_06606	0	0	0.021808645	0	0	0	0	0	0	0	0	0	hsa_circ_0087420	NC_000009.12	+	87668597	87686739	18143	490	sense-overlappin	NM_001288731.1	DAPK1
circRNA_06607	0	0	0.021808645	0.0309543	0	0	0.020098685	0	0.020254528	0.050431207	0	0	-	NC_000009.12	+	88394127	88395772	1646	1646	intronic	NM_006717.2	SPIN1
circRNA_06608	0.020575025	0	0	0	0	0	0	0	0	0	0	0	hsa_circ_0087432	NC_000009.12	+	88426382	88468605	42224	747	sense-overlappin	NM_006717.2	SPIN1
circRNA_06609	0	0	0	0	0	0	0.020098685	0	0	0	0	0	hsa_circ_0007738	NC_000009.12	-	89109048	89112626	3579	3579	sense-overlappin	NM_016848.5	SHC3
circRNA_06610	0	0	0	0.0309543	0	0.041425819	0	0	0	0.050431207	0	0.0309546	hsa_circ_0005432	NC_000009.12	+	89328660	89328886	227	227	exonic	NM_001282688.1	SECISBP2
circRNA_06611	0	0	0	0	0	0	0.020098685	0	0	0	0	0	-	NC_000009.12	-	91264917	91356155	91239	31497	sense-overlappin	NM_001698.2	AUH
circRNA_06612	0	0	0	0	0	0	0	0.020486295	0	0	0	0	hsa_circ_0003518	NC_000009.12	-	91296021	91356155	60135	393	sense-overlappin	NM_001698.2	AUH
circRNA_06613	0	0.030704951	0.207182128	0.0928628	0	0.020712909	0.040197371	0	0.020254528	0	0.02025523	0	hsa_circ_0006702	NC_000009.12	-	92256680	92269983	13304	932	sense-overlappin	NM_002161.5	IARS
circRNA_06614	0	0.020469968	0	0.0515905	0	0.041425819	0	0	0	0	0	0	hsa_circ_0087493	NC_000009.12	-	92256680	92285839	29160	1658	sense-overlappin	NM_002161.5	IARS
circRNA_06615	0	0	0	0	0	0	0	0	0	0.020172483	0	0.0206364	hsa_circ_0007416	NC_000009.12	-	92855811	92856318	508	220	sense-overlappin	XM_011519076.1	ZNF484
circRNA_06616	0	0	0	0	0	0	0	0	0.020254528	0	0	0	-	NC_000009.12	+	92975238	92976709	1472	670	sense-overlappin	NM_001083536.1	FGD3
circRNA_06617	0	0	0.054521613	0	0	0	0	0	0	0	0	0	-	NC_000009.12	+	93470329	93476338	6010	1142	sense-overlappin	NM_001286724.1	FAM120A
circRNA_06618	0	0	0.04361729	0	0	0	0	0	0	0	0	0	-	NC_000009.12	+	93470329	93498886	28558	1368	sense-overlappin	NM_001286724.1	FAM120A
circRNA_06619	0	0.143289773	0.228990773	0.1754076	0.0702209	0.165703275	0.200986853	0.081945182	0.060763583	0.181552345	0.06076569	0.0412728	hsa_circ_0008193	NC_000009.12	+	93471141	93476338	5198	330	sense-overlappin	NM_001286724.1	FAM120A
circRNA_06620	0	0	0.065425935	0	0	0	0	0	0	0.020172483	0	0	hsa_circ_0005218	NC_000009.12	+	93471141	93497599	26459	459	sense-overlappin	NM_001286724.1	FAM120A
circRNA_06621	0.102875123	0.112584821	1.232188443	0.1650895	0.13041025	0.072495183	0.281381595	0.102431477	0.070890847	0.161379862	0.131658996	0.134136601	hsa_circ_0001875	NC_000009.12	+	93471141	93498886	27746	556	sense-overlappin	NM_001286724.1	FAM120A
circRNA_06622	0	0	0	0	0	0	0.030148028	0	0	0	0.02025523	0	hsa_circ_0087531	NC_000009.12	+	93471141	93516269	45129	944	sense-overlappin	NM_001286724.1	FAM120A
circRNA_06623	0	0	0.032712968	0	0	0	0	0	0	0	0	0	hsa_circ_0003972	NC_000009.12	+	93476256	93498886	22631	309	sense-overlappin	NM_001286724.1	FAM120A
circRNA_06624	0	0	0	0	0	0	0	0	0	0	0.030382845	0	hsa_circ_0002407	NC_000009.12	+	93629970	93636525	6556	201	sense-overlappin	NM_005392.3	PHF2
circRNA_06625	0.051437562	0	0.021808645	0	0	0	0	0	0	0	0	0	hsa_circ_0008577	NC_000009.12	+	94064734	94095454	30721	598	sense-overlappin	NM_177995.2	PTPDC1
circRNA_06626	0.041150049	0.061409903	0	0	0	0	0	0	0	0	0	0	-	NC_000009.12	+	94338836	94438528	99693	99693	antisense	XM_011518795.1	NUTM2F
circRNA_06627	0	0	0.021808645	0	0.02006311	0	0.020098685	0	0	0	0	0	hsa_circ_0002191	NC_000009.12	+	94773002	94801002	28001	567	sense-overlappin	XM_011519131.1	C9orf3
circRNA_06628	0.030862537	0	0	0	0	0	0	0	0	0	0	0	-	NC_000009.12	+	94923986	94979427	55442	613	sense-overlappin	XM_011519122.1	C9orf3
circRNA_06629	0	0	0.021808645	0	0.02006311	0	0	0	0	0	0	0	hsa_circ_0002769	NC_000009.12	-	95101615	95111637	10023	615	sense-overlappin	NM_001243743.1	FANCC

circRNA_06630	0	0	0.021808645	0	0	0	0	0	0	0	0	0	hsa_circ_0007298	NC_000009.12	-	95240649	95249369	8721	423	sense-overlappin	NM_001243743.1	FANCC
circRNA_06631	0	0	0	0	0	0	0	0	0	0.030258724	0	0	hsa_circ_0087601	NC_000009.12	+	95880869	95881293	425	425	exonic	XR_929789.1	
circRNA_06632	0	0	0	0.0722267	0	0	0	0	0	0	0	0	hsa_circ_0087605	NC_000009.12	+	95897849	95928864	31016	1280	sense-overlappin	XR_929789.1	
circRNA_06633	0	0	0	0	0	0	0	0	0.030381792	0	0	0	-	NC_000009.12	+	95966562	95978215	11654	1712	sense-overlappin	XM_011518650.1	ERCC6L2
circRNA_06634	0	0.051174919	0.054521613	0	0	0.051782273	0.080394741	0.081945182	0.020254528	0.040344966	0.030382845	0.0206364	hsa_circ_0008720	NC_000009.12	+	95978061	96004701	26641	337	sense-overlappin	XM_011518643.1	ERCC6L2
circRNA_06635	0	0	0	0	0	0	0.040197371	0	0	0	0	0	hsa_circ_0004605	NC_000009.12	-	96320998	96324169	3172	506	sense-overlappin	XR_929705.1	
circRNA_06636	0	0	0.04361729	0	0	0	0	0	0.030381792	0.040344966	0	0	hsa_circ_0087616	NC_000009.12	-	96336717	96352109	15393	405	sense-overlappin	NR_104627.1	
circRNA_06637	0.020575025	0.030704951	0	0	0	0	0	0	0.050636319	0.030258724	0	0	hsa_circ_0006589	NC_000009.12	-	96360154	96368305	8152	189	sense-overlappin	XR_929705.1	
circRNA_06638	0	0	0.021808645	0	0	0	0.020098685	0	0	0	0	0	hsa_circ_0087626	NC_000009.12	-	96364464	96368305	3842	121	sense-overlappin	XR_929705.1	
circRNA_06639	0.061725074	0	0	0	0	0	0	0	0.020254528	0	0	0	hsa_circ_0009125	NC_000009.12	+	96458379	96458541	163	163	exonic	NM_014282.3	HABP4
circRNA_06640	0.061725074	0.051174919	0	0.0206362	0.04012623	0.072495183	0	0	0	0.050431207	0.04051046	0	hsa_circ_0087630	NC_000009.12	+	96458379	96465778	7400	394	sense-overlappin	NM_014282.3	HABP4
circRNA_06641	0.051437562	0.051174919	0.054521613	0	0.02006311	0.062138728	0.040197371	0	0.030381792	0.040344966	0	0.0206364	hsa_circ_0087631	NC_000009.12	+	96458379	96471094	12716	478	sense-overlappin	NM_014282.3	HABP4
circRNA_06642	0	0.020469968	0	0	0	0	0	0	0	0	0	0	-	NC_000009.12	+	96465337	96484633	19297	487	sense-overlappin	NM_014282.3	HABP4
circRNA_06643	0	0	0.021808645	0	0.0702209	0	0	0	0	0	0.02025523	0	hsa_circ_0087636	NC_000009.12	-	96509673	96565483	55811	1444	sense-overlappin	XR_242602.2	
circRNA_06644	0	0	0	0.0206362	0	0	0	0	0	0	0	0	hsa_circ_0087640	NC_000009.12	-	96515649	96565483	49835	1327	sense-overlappin	XM_005252288.2	CDC14B
circRNA_06645	0.041150049	0.051174919	0.032712968	0.0928628	0.08025246	0.020712909	0	0.040972591	0.030381792	0.100862414	0.02025523	0.0206364	hsa_circ_0087641	NC_000009.12	-	96522506	96565483	42978	1183	sense-overlappin	XR_929866.1	
circRNA_06646	0	0.020469968	0.021808645	0.0309543	0	0	0	0	0	0	0	0	hsa_circ_0005789	NC_000009.12	-	96551796	96565483	13688	337	sense-overlappin	XR_929866.1	
circRNA_06647	0	0	0.021808645	0	0	0	0	0	0	0	0	0	-	NC_000009.12	+	96719509	96721130	1622	1622	intergenic		
circRNA_06648	0	0	0.021808645	0	0	0	0	0	0	0	0	0	-	NC_000009.12	-	96816809	96827181	10373	3072	sense-overlappin	XM_011518321.1	ZNF782
circRNA_06649	0.020575025	0	0.098138903	0	0.0702209	0.031069364	0	0	0	0	0	0	-	NC_000009.12	-	96817365	96827181	9817	2516	sense-overlappin	XM_011518321.1	ZNF782
circRNA_06650	0	0	0.032712968	0	0.02006311	0	0	0	0	0	0	0	-	NC_000009.12	-	96817365	96845016	27652	2643	sense-overlappin	XM_005251744.2	ZNF782
circRNA_06651	0	0	0.04361729	0	0	0	0	0	0	0	0	0	-	NC_000009.12	-	96827080	96845016	17937	229	sense-overlappin	XM_005251744.2	ZNF782
circRNA_06652	0	0	0.032712968	0	0	0	0	0	0	0	0.02025523	0	hsa_circ_0005306	NC_000009.12	-	96949555	96972948	23394	287	sense-overlappin	XR_929724.1	
circRNA_06653	0	0	0.021808645	0	0	0	0	0	0	0	0	0	-	NC_000009.12	-	97170607	97200565	29959	29959	intergenic		
circRNA_06654	0	0	0	0.0206362	0	0	0	0	0	0.030258724	0	0	-	NC_000009.12	+	97330154	97330767	614	486	sense-overlappin	NM_020893.2	CCDC180
circRNA_06655	0	0	0.054521613	0	0	0	0	0	0	0	0	0	hsa_circ_0087659	NC_000009.12	+	97347314	97350555	3242	504	sense-overlappin	NM_020893.2	CCDC180
circRNA_06656	0	0	0	0.0412724	0	0	0	0	0	0	0	0	hsa_circ_0002796	NC_000009.12	+	97369922	97371706	1785	411	sense-overlappin	NM_020893.2	CCDC180
circRNA_06657	0	0	0	0	0	0.020712909	0	0	0	0	0	0	-	NC_000009.12	-	97976421	97977270	850	850	intergenic		
circRNA_06658	0	0	0.054521613	0	0.06018934	0	0.030148028	0	0	0.020172483	0	0	hsa_circ_0006609	NC_000009.12	+	97994631	97998678	4048	273	sense-overlappin	NM_006401.2	ANP32B
circRNA_06659	0	0	0.032712968	0	0	0	0	0	0	0	0	0	hsa_circ_0005739	NC_000009.12	+	97994631	98005153	10523	463	sense-overlappin	NM_006401.2	ANP32B
circRNA_06660	0	0.020469968	0	0	0	0	0	0	0	0.020172483	0.02025523	0	hsa_circ_0004036	NC_000009.12	+	98018289	98026451	8163	8163	intergenic		
circRNA_06661	0	0	0	0.0515905	0	0	0	0	0.020254528	0	0	0	-	NC_000009.12	+	98075570	98077017	1448	263	sense-overlappin	XM_011518787.1	NANS
circRNA_06662	0	0	0	0	0	0	0	0	0.040509055	0.020172483	0	0	hsa_circ_0002900	NC_000009.12	+	98075570	98078347	2778	418	sense-overlappin	XM_011518787.1	NANS
circRNA_06663	0	0	0	0	0.02006311	0	0	0	0	0	0	0	-	NC_000009.12	+	98076918	98078347	1430	255	sense-overlappin	NM_018946.3	NANS
circRNA_06664	0	0.020469968	0	0	0	0.031069364	0	0	0	0	0	0	-	NC_000009.12	+	99128855	99149301	20447	1411	sense-overlappin	XM_011518949.1	TGFBR1
circRNA_06665	0	0	0	0	0.02006311	0	0	0	0	0	0	0	hsa_circ_0002643	NC_000009.12	+	99844649	99847615	2967	379	sense-overlappin	NM_006981.3	NR4A3
circRNA_06666	0.041150049	0	0	0	0	0	0	0.030729443	0	0	0.02025523	0	hsa_circ_0004825	NC_000009.12	+	99915178	99928843	13666	251	sense-overlappin	NM_017919.2	STX17
circRNA_06667	0	0	0	0	0	0	0	0	0	0	0.02025523	0	-	NC_000009.12	+	99915178	99960155	44978	644	sense-overlappin	NM_017919.2	STX17
circRNA_06668	0.020575025	0.030704951	0.021808645	0	0.03009467	0	0	0	0	0.020172483	0	0.0309546	hsa_circ_0001876	NC_000009.12	+	99959917	99960155	239	167	sense-overlappin	NM_017919.2	STX17
circRNA_06669	0	0	0	0	0.05015779	0	0	0	0	0	0	0	-	NC_000009.12	-	100052417	100060172	7756	229	sense-overlappin	NM_015051.2	ERP44
circRNA_06670	0	0	0	0	0.02006311	0	0	0	0	0	0	0	-	NC_000009.12	+	100226062	100264928	38867	1298	sense-overlappin	XM_005251923.3	INVS
circRNA_06671	0	0	0.032712968	0.0206362	0	0	0	0	0	0	0	0	hsa_circ_0087765	NC_000009.12	-	100308500	100310379	1880	263	sense-overlappin	NM_001161584.1	TEX10
circRNA_06672	0	0	0	0	0.03009467	0	0	0	0	0	0	0	-	NC_000009.12	-	100310299	100349372	39074	2292	sense-overlappin	NM_001161584.1	TEX10
circRNA_06673	0	0	0.109043225	0	0.04012623	0	0	0	0	0	0	0	-	NC_000009.12	-	100320265	100349372	29108	2211	sense-overlappin	NM_001161584.1	TEX10
circRNA_06674	0	0	0	0	0.06018934	0	0	0	0.020254528	0.030258724	0	0	hsa_circ_0006388	NC_000009.12	+	100441906	100442356	451	451	exonic	NM_001198807.1	MSANTD3
circRNA_06675	0	0	0.032712968	0	0	0	0	0.030729443	0	0.020172483	0	0.0309546	hsa_circ_0004425	NC_000009.12	+	100498765	100516771	18007	364	sense-overlappin	NM_001198812.1	MSANTD3-TMEFF1
circRNA_06676	0	0	0.032712968	0	0	0	0	0	0	0.020172483	0	0	-	NC_000009.12	+	100498765	100550160	51396	579	sense-overlappin	NM_001198812.1	MSANTD3-TMEFF1
circRNA_06677	0	0	0	0	0	0	0	0	0	0.020172483	0	0	-	NC_000009.12	+	100498765	100561520	62756	703	sense-overlappin	NM_001198812.1	MSANTD3-TMEFF1
circRNA_06678	0	0	0	0.0206362	0	0	0	0	0	0	0	0	-	NC_000009.12	+	100561397	100586185	24789	9953	sense-overlappin	NM_001198812.1	MSANTD3-TMEFF1
circRNA_06679	0	0	0	0	0.02006311	0	0	0	0	0	0	0	hsa_circ_0087794	NC_000009.12	+	104095324	104111814	16491	1315	sense-overlappin	XM_011518149.1	SMC2
circRNA_06680	0	0.051174919	0	0.0412724	0.09028402	0.051782273	0	0	0	0	0	0	-	NC_000009.12	+	104750956	104759171	8216	607	sense-overlappin	NM_015469.1	NIPSNAP3A
circRNA_06681	0.030862537	0	0	0	0	0	0	0	0	0	0	0	-	NC_000009.12	-	104814118	104814475	358	163	sense-overlappin	XM_011518340.1	ABCA1
circRNA_06682	0	0	0.021808645	0	0	0	0	0	0	0.040344966	0	0	hsa_circ_0005443	NC_000009.12	-	104883039	104889195	6157	355	sense-overlappin	XM_011518340.1	ABCA1
circRNA_06683	0	0	0	0	0	0	0	0	0.020254528	0.050431207	0	0	-	NC_000009.12	+	105273497	105361330	87834	26587	sense-overlappin	XM_005251855.3	SLC44A1
circRNA_06684	0	0	0	0	0.03009467	0	0	0	0	0	0	0	hsa_circ_0087828	NC_000009.12	+	105362821	105374735	11915	732	sense-overlappin	XM_005251855.3	SLC44A1

circRNA_06685	0	0	0.021808645	0	0	0	0	0	0	0	0	0	-	NC_000009.12	+	105471904	105539351	67448	1161	sense-overlappin	XM_011519077.1	FSD1L
circRNA_06686	0	0	0	0	0.03009467	0	0	0	0	0	0	0	hsa_circ_0003939	NC_000009.12	+	105534493	105539351	4859	442	sense-overlappin	XM_011519078.1	FSD1L
circRNA_06687	0	0	0.04361729	0	0	0	0	0	0	0.020172483	0	0	hsa_circ_0005171	NC_000009.12	+	105705597	105722621	17025	570	sense-overlappin	XM_011518833.1	TMEM38B
circRNA_06688	0	0	0.065425935	0	0	0	0	0	0	0	0	0	hsa_circ_0087847	NC_000009.12	+	105721537	105722621	1085	273	sense-overlappin	NM_018112.2	TMEM38B
circRNA_06689	0	0	0	0	0	0	0	0	0	0	0.02025523	0	hsa_circ_0087855	NC_000009.12	+	107300141	107306647	6507	431	sense-overlappin	NM_002874.4	RAD23B
circRNA_06690	0.020575025	0	0.065425935	0.0515905	0	0	0.060296056	0	0.020254528	0.07060369	0.02025523	0	hsa_circ_0008812	NC_000009.12	+	107300141	107311737	11597	487	sense-overlappin	NM_002874.4	RAD23B
circRNA_06691	0	0	0	0.0206362	0	0	0.040197371	0	0	0	0	0	hsa_circ_0007271	NC_000009.12	+	107300141	107325004	24864	1050	sense-overlappin	NM_002874.4	RAD23B
circRNA_06692	0	0	0	0	0.03009467	0	0	0	0	0	0	0	hsa_circ_0001880	NC_000009.12	+	107321983	107325004	3022	435	sense-overlappin	NM_002874.4	RAD23B
circRNA_06693	0	0	0	0	0	0	0	0	0.030381792	0	0	0	-	NC_000009.12	-	109033307	109073438	40132	1145	sense-overlappin	NM_032012.3	TMEM245
circRNA_06694	0.102875123	0.051174919	0.04361729	0.1031809	0	0	0.080394741	0.040972591	0.060763583	0.100862414	0.070893305	0.0206364	hsa_circ_0087905	NC_000009.12	-	109050283	109050692	410	269	sense-overlappin	NM_032012.3	TMEM245
circRNA_06695	0.030862537	0.020469968	0	0.0412724	0	0.020712909	0	0	0.020254528	0.020172483	0	0	hsa_circ_0003965	NC_000009.12	-	109050283	109064567	14285	591	sense-overlappin	NM_032012.3	TMEM245
circRNA_06696	0	0.030704951	0.021808645	0	0.02006311	0.031069364	0	0	0	0.020172483	0	0.0309546	hsa_circ_0004010	NC_000009.12	-	109050283	109073438	23156	674	sense-overlappin	NM_032012.3	TMEM245
circRNA_06697	0	0	0	0.0309543	0	0	0	0	0	0	0	0	hsa_circ_0087912	NC_000009.12	-	109050283	109108570	58288	1544	sense-overlappin	NM_032012.3	TMEM245
circRNA_06698	0.072012586	0.030704951	0.021808645	0.0206362	0	0	0	0	0	0	0	0	hsa_circ_0004109	NC_000009.12	-	109057191	109064567	7377	322	sense-overlappin	NM_032012.3	TMEM245
circRNA_06699	0.020575025	0	0	0	0	0	0	0	0	0	0	0	-	NC_000009.12	-	109073356	109108570	35215	953	sense-overlappin	NM_032012.3	TMEM245
circRNA_06700	0	0	0	0	0	0	0.030148028	0	0	0	0	0.0206364	hsa_circ_0005821	NC_000009.12	-	109080839	109108570	27732	870	sense-overlappin	NM_032012.3	TMEM245
circRNA_06701	0.082300098	0	0.021808645	0.0412724	0	0	0	0	0	0.020172483	0	0	hsa_circ_0087938	NC_000009.12	-	109106508	109108570	2063	220	sense-overlappin	NM_032012.3	TMEM245
circRNA_06702	0	0	0	0.0206362	0	0	0	0	0	0	0	0	hsa_circ_0087943	NC_000009.12	-	109263050	109268433	5384	220	sense-overlappin	XM_011518790.1	EPB41L4B
circRNA_06703	0	0	0	0.0309543	0	0	0	0	0	0	0	0	hsa_circ_0087944	NC_000009.12	-	109263050	109279921	16872	325	sense-overlappin	XM_011518790.1	EPB41L4B
circRNA_06704	0	0.092114854	0.021808645	0	0	0.031069364	0	0	0.030381792	0	0	0	-	NC_000009.12	+	109942686	109943381	696	696	exonic	NM_001037293.2	PALM2
circRNA_06705	0	0	0.021808645	0	0	0	0	0	0	0	0	0	-	NC_000009.12	-	110200362	110201474	1113	1113	exonic	NM_001012993.2	C9orf152
circRNA_06706	0	0	0	0	0	0	0	0	0	0.020172483	0	0	-	NC_000009.12	-	110429143	110472323	43181	3208	sense-overlappin	NM_153366.3	SVEP1
circRNA_06707	0	0	0.021808645	0.0412724	0	0.031069364	0	0	0	0	0.02025523	0	hsa_circ_0003611	NC_000009.12	-	110941421	110973558	32138	974	sense-overlappin	XM_005251782.2	LPAR1
circRNA_06708	0	0	0	0	0	0	0	0	0	0.020172483	0	0	hsa_circ_0002287	NC_000009.12	-	110941421	111009201	67781	36617	sense-overlappin	XM_005251782.2	LPAR1
circRNA_06709	0.174887709	0.092114854	0.08723458	0.0722267	0.19059959	0.186416184	0.040197371	0.112674625	0.222799805	0.231983552	0.10127615	0.154773001	hsa_circ_0087960	NC_000009.12	-	110972073	110973558	1486	226	sense-overlappin	XM_005251782.2	LPAR1
circRNA_06710	0	0.020469968	0.054521613	0.0619086	0.04012623	0.031069364	0.020098685	0	0.020254528	0.050431207	0	0	hsa_circ_0087961	NC_000009.12	-	110972073	111011690	39618	38358	sense-overlappin	XM_005251782.2	LPAR1
circRNA_06711	0.195462734	0.040939935	0.109043225	0.1444533	0.39123074	0.227842003	0.130641455	0.102431477	0.040509055	0.191638587	0.10127615	0.0412728	hsa_circ_0001882	NC_000009.12	-	111386377	111391824	5448	435	sense-overlappin	NM_001080398.1	KIAA0368
circRNA_06712	0	0	0	0	0	0.020712909	0	0	0	0	0	0	-	NC_000009.12	-	111428042	111442424	14383	780	sense-overlappin	NM_001080398.1	KIAA0368
circRNA_06713	0	0	0	0	0	0	0	0	0	0	0.02025523	0	hsa_circ_0002526	NC_000009.12	-	111472897	111473959	1063	1063	sense-overlappin	XM_005251851.3	KIAA0368
circRNA_06714	0.082300098	0	0.04361729	0.0412724	0	0.031069364	0	0.020486295	0.040509055	0.020172483	0.070893305	0.0206364	-	NC_000009.12	+	111527268	111534353	7086	849	sense-overlappin	XM_011518299.1	ZNF483
circRNA_06715	0.061725074	0	0	0	0	0	0	0	0	0.040344966	0	0	hsa_circ_0001962	NC_000009.12	+	111533739	111534353	615	220	sense-overlappin	XM_011518301.1	ZNF483
circRNA_06716	0	0.030704951	0	0.113499	0	0.113921001	0	0	0	0.030258724	0	0.0412728	hsa_circ_0008043	NC_000009.12	-	111570091	111586165	16075	670	sense-overlappin	NM_001146109.1	PTGR1
circRNA_06717	0	0	0	0.0206362	0	0	0	0	0	0.020172483	0.030382845	0	hsa_circ_0088030	NC_000009.12	-	111574734	111586165	11432	551	sense-overlappin	NM_001146109.1	PTGR1
circRNA_06718	0	0	0	0	0	0	0	0	0	0.020172483	0	0	-	NC_000009.12	-	111578370	111589382	11013	987	sense-overlappin	XM_011518395.1	PTGR1
circRNA_06719	0	0.030704951	0	0	0	0	0.020098685	0	0	0	0.030382845	0	hsa_circ_0003731	NC_000009.12	-	111578796	111586165	7370	442	sense-overlappin	NM_001146109.1	PTGR1
circRNA_06720	0	0.020469968	0	0	0	0	0	0	0	0.020172483	0	0.0206364	-	NC_000009.12	-	111591220	111591479	260	260	intronic	NM_001146109.1	PTGR1
circRNA_06721	0.082300098	0	0	0	0	0	0	0	0.030381792	0.080689931	0.070893305	0.0412728	-	NC_000009.12	+	111786793	111787947	1155	1155	antisense	XM_006716974.2	C9orf84
circRNA_06722	0	0	0	0.0206362	0.02006311	0	0	0.020486295	0	0	0	0	hsa_circ_0006435	NC_000009.12	+	111914605	111915836	1232	220	sense-overlappin	XM_005252186.3	UGCG
circRNA_06723	0	0	0.021808645	0	0	0.031069364	0	0	0	0	0	0	hsa_circ_0088036	NC_000009.12	-	112078538	112080165	1628	279	sense-overlappin	NM_001282640.1	SUSD1
circRNA_06724	0	0	0	0.0206362	0	0	0	0	0	0	0	0	hsa_circ_0088041	NC_000009.12	-	112080074	112098662	18589	285	sense-overlappin	NM_001282640.1	SUSD1
circRNA_06725	0	0	0	0	0	0.031069364	0	0	0	0	0	0	hsa_circ_0088054	NC_000009.12	-	112111654	112157613	45960	1068	sense-overlappin	NM_001282640.1	SUSD1
circRNA_06726	0	0	0	0.0412724	0.06018934	0	0	0	0	0	0	0	hsa_circ_0088062	NC_000009.12	-	112149244	112157613	8370	270	sense-overlappin	NM_001282640.1	SUSD1
circRNA_06727	0	0	0	0	0.02006311	0	0	0	0	0	0	0	hsa_circ_0088075	NC_000009.12	-	112250929	112276013	25085	768	sense-overlappin	NM_001244898.1	PTBP3
circRNA_06728	0	0	0.239895095	0.0206362	0.02006311	0	0.080394741	0	0	0.020172483	0.04051046	0	hsa_circ_0003458	NC_000009.12	-	112250929	112297916	46988	844	sense-overlappin	NM_001244898.1	PTBP3
circRNA_06729	0	0	0.021808645	0	0	0	0	0	0	0	0	0	hsa_circ_0004553	NC_000009.12	-	112252678	112297916	45239	669	sense-overlappin	NM_001244898.1	PTBP3
circRNA_06730	0	0	0.08723458	0	0	0	0.060296056	0	0	0	0.02025523	0	hsa_circ_0008192	NC_000009.12	-	112262435	112297916	35482	558	sense-overlappin	NM_001244898.1	PTBP3
circRNA_06731	0	0	0.065425935	0	0.0702209	0	0.070345399	0	0.030381792	0	0	0	hsa_circ_0003500	NC_000009.12	-	112268049	112297916	29868	393	sense-overlappin	NM_001244898.1	PTBP3
circRNA_06732	0	0	0	0	0.02006311	0	0.020098685	0	0	0	0	0	hsa_circ_0088088	NC_000009.12	+	112403995	112409021	5027	378	sense-overlappin	XM_011519091.1	HSDL2
circRNA_06733	0.020575025	0	0.032712968	0	0.04012623	0	0	0	0	0.020172483	0	0	hsa_circ_0003597	NC_000009.12	+	112403995	112418958	14964	581	sense-overlappin	XM_011519091.1	HSDL2
circRNA_06734	0	0	0	0.0206362	0	0	0	0	0	0	0	0	-	NC_000009.12	+	112403995	112459577	55583	1127	sense-overlappin	NM_032303.4	HSDL2
circRNA_06735	0	0	0	0	0	0	0	0	0	0	0.030382845	0.0309546	hsa_circ_0088095	NC_000009.12	+	112438431	112441770	3340	267	sense-overlappin	NM_001195822.1	HSDL2
circRNA_06736	0	0	0	0.0206362	0	0	0	0	0	0	0	0	-	NC_000009.12	-	112484456	112486541	2086	2086	intergenic		
circRNA_06737	0.030862537	0	0.119947548	0.1031809	0	0.020712909	0	0.020486295	0	0.020172483	0.02025523	0	hsa_circ_0002925	NC_000009.12	+	112574057	112575251	1195	1195	exonic	XM_011518310.1	KIAA1958
circRNA_06738	0	0	0.021808645	0	0	0	0	0	0	0	0	0	-	NC_000009.12	+	112589522	112590312	791	791	intronic	XM_011518310.1	KIAA1958
circRNA_06739	0.030862537	0	0	0	0	0	0	0	0	0	0	0	hsa_circ_0007285	NC_000009.12	-	113198855	113206578	7724	463	sense-overlappin	XM_006717018.1	FKBP15

circRNA_06740	0	0	0	0	0.02006311	0	0	0	0	0	0	0	hsa_circ_0088137	NC_000009.12	+	113286232	113288264	2033	273	sense-overlappin	NM_001244926.1	PRPF4
circRNA_06741	0.020575025	0	0	0	0	0	0	0	0	0	0	0	-	NC_000009.12	-	113328881	113332050	3170	352	sense-overlappin	NM_001012361.2	WDR31
circRNA_06742	0	0	0.021808645	0	0	0	0	0	0	0	0	0	-	NC_000009.12	-	113328881	113336440	7560	505	sense-overlappin	NM_001012361.2	WDR31
circRNA_06743	0	0	0	0	0	0	0	0	0.020254528	0	0	0	hsa_circ_0088158	NC_000009.12	+	113969117	113988580	19464	304	sense-overlappin	NM_133374.2	ZNF618
circRNA_06744	0	0	0	0	0	0	0	0	0.020254528	0	0	0	-	NC_000009.12	-	114354636	114355285	650	650	intronic	XR_929844.1	
circRNA_06745	0	0	0.021808645	0	0	0	0	0	0	0	0	0	hsa_circ_0088190	NC_000009.12	+	114633768	114638726	4959	375	sense-overlappin	XM_011518351.1	C9orf91
circRNA_06746	0	0.040939935	0	0.0309543	0	0	0	0	0	0.040344966	0	0	hsa_circ_0002318	NC_000009.12	+	114636990	114638726	1737	264	sense-overlappin	XM_011518351.1	C9orf91
circRNA_06747	0	0.020469968	0	0	0	0	0	0	0	0	0	0	-	NC_000009.12	+	114966538	115049067	82530	57181	antisense	NM_002160.3	TNC
circRNA_06748	0	0	0	0	0	0	0.020098685	0	0	0	0	0	hsa_circ_0088265	NC_000009.12	-	116618324	116620443	2120	283	sense-overlappin	NM_198186.3	ASTN2
circRNA_06749	0	0	0.021808645	0	0	0	0	0	0	0	0	0	hsa_circ_0088281	NC_000009.12	-	120437295	120448126	10832	1162	sense-overlappin	NM_001011649.2	CDK5RAP2
circRNA_06750	0	0	0	0.0206362	0	0	0	0	0	0	0	0	-	NC_000009.12	-	120437295	120491477	54183	2644	sense-overlappin	NM_001011649.2	CDK5RAP2
circRNA_06751	0	0.030704951	0.021808645	0	0	0.051782273	0	0	0	0	0	0	hsa_circ_0088284	NC_000009.12	-	120467860	120491477	23618	795	sense-overlappin	NM_001011649.2	CDK5RAP2
circRNA_06752	0	0	0	0	0	0	0	0	0	0.020172483	0	0	-	NC_000009.12	-	120477350	120491477	14128	416	sense-overlappin	NM_001011649.2	CDK5RAP2
circRNA_06753	0	0.040939935	0.065425935	0	0	0	0	0	0	0.030258724	0	0	hsa_circ_0004883	NC_000009.12	-	120518427	120539164	20738	928	sense-overlappin	NM_001011649.2	CDK5RAP2
circRNA_06754	0	0	0	0	0	0	0.030148028	0	0	0	0	0	hsa_circ_0088300	NC_000009.12	-	120831331	120833456	2126	388	sense-overlappin	XM_011518866.1	PSMD5
circRNA_06755	0	0	0	0	0	0.020712909	0	0	0	0	0	0	hsa_circ_0001884	NC_000009.12	+	121152485	121160302	7818	1126	sense-overlappin	XM_011518167.1	CNTRL
circRNA_06756	0.041150049	0	0	0	0.04012623	0.031069364	0.030148028	0.030729443	0	0.030258724	0	0	hsa_circ_0001885	NC_000009.12	+	121161856	121162271	416	334	sense-overlappin	XM_011518167.1	CNTRL
circRNA_06757	0	0.020469968	0	0.0515905	0.03009467	0.103564547	0.080394741	0	0.020254528	0.050431207	0.04051046	0.0206364	hsa_circ_0088333	NC_000009.12	+	121161856	121166180	4325	566	sense-overlappin	XM_011518167.1	CNTRL
circRNA_06758	0	0.030704951	0	0	0	0	0	0	0	0	0	0	hsa_circ_0088353	NC_000009.12	-	121974009	121989770	15762	788	sense-overlappin	XM_005251729.3	TTLL11
circRNA_06759	0.041150049	0	0	0	0	0	0	0	0	0	0	0	-	NC_000009.12	-	122148112	122152408	4297	330	sense-overlappin	XM_005251998.2	NDUFA8
circRNA_06760	0	0	0.04361729	0.0619086	0.11034713	0	0.020098685	0.020486295	0.030381792	0.040344966	0	0	hsa_circ_0003250	NC_000009.12	+	122280443	122291840	11398	662	sense-overlappin	XR_428543.2	
circRNA_06761	0	0	0.032712968	0.0206362	0.03009467	0	0	0	0	0	0.030382845	0	hsa_circ_0008000	NC_000009.12	+	122285169	122286166	998	414	sense-overlappin	XR_428543.2	
circRNA_06762	0	0.020469968	0	0	0	0	0	0	0	0	0	0	hsa_circ_0088369	NC_000009.12	-	122853952	122855878	1927	663	sense-overlappin	NM_001100588.1	RC3H2
circRNA_06763	0	0	0	0	0	0	0	0	0	0.060517448	0	0	hsa_circ_0004688	NC_000009.12	-	122855184	122855878	695	361	sense-overlappin	NM_001100588.1	RC3H2
circRNA_06764	0	0	0	0	0	0	0.030148028	0	0	0	0	0	hsa_circ_0088378	NC_000009.12	-	122855184	122860131	4948	1181	sense-overlappin	NM_001100588.1	RC3H2
circRNA_06765	0	0	0	0	0	0.031069364	0	0	0	0	0	0	-	NC_000009.12	-	122865349	122883379	18031	1051	sense-overlappin	NM_001100588.1	RC3H2
circRNA_06766	0.020575025	0	0.04361729	0	0	0.041425819	0.030148028	0	0	0	0	0	hsa_circ_0088389	NC_000009.12	-	122877471	122883379	5909	742	sense-overlappin	NM_001100588.1	RC3H2
circRNA_06767	0.020575025	0.020469968	0	0	0	0.041425819	0	0	0	0	0	0	-	NC_000009.12	+	122957011	122998766	41756	1423	sense-overlappin	NM_012197.3	RABGAP1
circRNA_06768	0	0.030704951	0	0.0619086	0.02006311	0	0	0	0	0	0	0	-	NC_000009.12	+	122957011	123020459	63449	1843	sense-overlappin	NM_012197.3	RABGAP1
circRNA_06769	0	0	0.021808645	0	0	0	0	0	0	0	0	0	-	NC_000009.12	+	122984485	123020459	35975	1644	sense-overlappin	NM_012197.3	RABGAP1
circRNA_06770	0	0	0	0.0309543	0	0.031069364	0.020098685	0.030729443	0	0.040344966	0	0	hsa_circ_0002672	NC_000009.12	+	122989297	122990213	917	333	sense-overlappin	NM_012197.3	RABGAP1
circRNA_06771	0	0	0	0	0	0	0	0	0	0.020172483	0	0	-	NC_000009.12	+	122998597	123065461	66865	704	sense-overlappin	NM_012197.3	RABGAP1
circRNA_06772	0	0	0	0	0	0	0	0	0	0.040344966	0	0	-	NC_000009.12	+	123065348	123099549	34202	1095	sense-overlappin	NM_012197.3	RABGAP1
circRNA_06773	0	0	0	0.0206362	0	0	0	0	0	0	0	0	hsa_circ_0007661	NC_000009.12	+	123070350	123076762	6413	516	sense-overlappin	NM_012197.3	RABGAP1
circRNA_06774	0	0	0	0.0309543	0	0.041425819	0	0	0	0	0.02025523	0	hsa_circ_0088425	NC_000009.12	-	123169902	123173842	3941	311	sense-overlappin	XR_929817.1	
circRNA_06775	0	0	0	0	0.02006311	0	0.040197371	0	0	0	0	0	hsa_circ_0001888	NC_000009.12	-	123169902	123184298	14397	807	sense-overlappin	NM_001171137.1	STRBP
circRNA_06776	0	0	0	0	0.02006311	0	0	0	0	0	0	0	hsa_circ_0088427	NC_000009.12	-	123179007	123184298	5292	496	sense-overlappin	NM_001171137.1	STRBP
circRNA_06777	0	0.020469968	0	0	0	0	0	0	0	0	0	0.0206364	-	NC_000009.12	-	123452276	123454779	2504	113	sense-overlappin	NM_020946.1	DENND1A
circRNA_06778	0	0	0	0	0	0	0	0	0.030381792	0	0	0	-	NC_000009.12	-	123817950	123818214	265	265	intronic	NM_020946.1	DENND1A
circRNA_06779	0	0	0.032712968	0	0	0.031069364	0.030148028	0	0	0	0	0	hsa_circ_0088485	NC_000009.12	+	124301936	124313985	12050	323	sense-overlappin	NM_001166167.1	NEK6
circRNA_06780	0.020575025	0	0	0.0309543	0	0	0	0	0	0	0	0	hsa_circ_0008383	NC_000009.12	+	124301936	124327445	25510	651	sense-overlappin	NM_001166167.1	NEK6
circRNA_06781	0	0	0	0	0.04012623	0	0.020098685	0.020486295	0.020254528	0.020172483	0.030382845	0	hsa_circ_0002883	NC_000009.12	-	124908377	124912026	3650	222	sense-overlappin	NM_002077.3	GOLGA1
circRNA_06782	0	0	0	0	0	0	0	0	0	0.020172483	0	0	-	NC_000009.12	-	124923095	124938866	15772	716	sense-overlappin	NM_002077.3	GOLGA1
circRNA_06783	0	0	0	0.0309543	0	0	0	0	0	0	0	0	-	NC_000009.12	-	125003469	125028478	25010	637	sense-overlappin	NM_001144877.2	SCAI
circRNA_06784	0	0	0.021808645	0	0.03009467	0	0	0	0	0	0	0	hsa_circ_0009007	NC_000009.12	+	125213370	125220700	7331	315	sense-overlappin	NM_001174152.1	RABEPK
circRNA_06785	0	0	0	0	0	0	0.030148028	0	0	0	0	0	-	NC_000009.12	+	125229162	125230059	898	898	intronic	NM_001174152.1	RABEPK
circRNA_06786	0	0	0	0	0.02006311	0	0	0	0	0	0	0	hsa_circ_0088567	NC_000009.12	+	125295458	125299106	3649	2280	sense-overlappin	XM_011518503.1	GAPVD1
circRNA_06787	0	0	0	0	0	0.020712909	0	0	0	0	0	0	hsa_circ_0088572	NC_000009.12	+	125301983	125305149	3167	931	sense-overlappin	XM_011518501.1	GAPVD1
circRNA_06788	0	0	0	0	0	0	0	0	0	0	0.02025523	0	-	NC_000009.12	+	125324719	125324799	81	81	intronic	XM_011518501.1	GAPVD1
circRNA_06789	0	0	0	0	0	0.031069364	0	0	0	0	0	0	-	NC_000009.12	+	125326416	125330218	3803	315	sense-overlappin	XM_011518501.1	GAPVD1
circRNA_06790	0.061725074	0.092114854	0.04361729	0.1238171	0.11034713	0.207129093	0.120592112	0.102431477	0.040509055	0.07060369	0.131658996	0.072227401	hsa_circ_0003270	NC_000009.12	+	125337018	125337591	574	449	sense-overlappin	XM_011518501.1	GAPVD1
circRNA_06791	0	0	0	0	0	0	0	0	0.020254528	0	0	0	hsa_circ_0005249	NC_000009.12	+	125341177	125346941	5765	292	sense-overlappin	XM_011518501.1	GAPVD1
circRNA_06792	0	0.112584821	0.04361729	0.113499	0.06018934	0.020712909	0.030148028	0.030729443	0.040509055	0.020172483	0.030382845	0.0309546	hsa_circ_0001890	NC_000009.12	-	125657651	125672643	14993	567	sense-overlappin	NM_001006617.1	MAPKAP1
circRNA_06793	0	0	0	0.0206362	0	0	0	0	0	0	0	0	hsa_circ_0003991	NC_000009.12	+	125915686	125935607	19922	569	sense-overlappin	NM_006195.5	PBX3
circRNA_06794	0	0	0.021808645	0	0	0	0	0	0	0	0	0	hsa_circ_0088608	NC_000009.12	+	126340508	126386658	46151	328	sense-overlappin	NM_001011703.2	MVB12B

circRNA_06795	0	0.020469968	0.021808645	0	0	0	0	0	0	0.050431207	0.02025523	0	hsa_circ_0008240	NC_000009.12	+	126340508	126395697	55190	581	sense-overlappin	NM_001011703.2	MVB12B
circRNA_06796	0	0	0	0	0	0	0	0	0	0	0.02025523	0	hsa_circ_0001891	NC_000009.12	+	126340508	126421948	81441	811	sense-overlappin	XM_011519175.1	MVB12B
circRNA_06797	0	0	0	0	0.02006311	0	0	0	0	0	0	0	-	NC_000009.12	+	126386564	126395697	9134	348	sense-overlappin	NM_001011703.2	MVB12B
circRNA_06798	0	0	0	0.0309543	0	0	0	0	0	0	0	0	hsa_circ_0088621	NC_000009.12	+	126962225	126977745	15521	281	sense-overlappin	NM_001190730.1	RALGPS1
circRNA_06799	0	0	0.021808645	0	0.03009467	0	0	0.020486295	0	0	0	0	hsa_circ_0004403	NC_000009.12	+	127034431	127052939	18509	267	sense-overlappin	NM_001190730.1	RALGPS1
circRNA_06800	0	0	0.032712968	0	0	0	0.030148028	0.020486295	0	0	0	0	-	NC_000009.12	+	127034431	127069356	34926	394	sense-overlappin	NM_001190730.1	RALGPS1
circRNA_06801	0	0	0.021808645	0	0	0	0	0	0	0	0	0	hsa_circ_0088630	NC_000009.12	+	127318063	127320778	2716	129	sense-overlappin	XM_005252268.1	GARNL3
circRNA_06802	0	0	0	0	0.02006311	0	0	0	0	0	0	0	hsa_circ_0088633	NC_000009.12	+	127339645	127342334	2690	223	sense-overlappin	XM_005252268.1	GARNL3
circRNA_06803	0.030862537	0	0	0	0	0	0	0	0	0	0	0	hsa_circ_0088635	NC_000009.12	+	127344235	127345477	1243	180	sense-overlappin	XM_005252268.1	GARNL3
circRNA_06804	0.030862537	0	0.032712968	0.0515905	0	0.020712909	0.040197371	0	0	0.020172483	0	0	hsa_circ_0006984	NC_000009.12	+	127444029	127445249	1221	1221	exonic	NM_001286696.1	ZNF79
circRNA_06805	0	0	0	0	0	0	0	0	0.020254528	0.030258724	0	0	hsa_circ_0088708	NC_000009.12	-	127890637	127891043	407	407	exonic	NM_001287001.1	ST6GALNAC6
circRNA_06806	0	0	0.021808645	0	0	0	0	0	0	0	0	0	hsa_circ_0088737	NC_000009.12	-	128169052	128180799	11748	1524	sense-overlappin	NM_001131017.1	CIZ1
circRNA_06807	0	0.051174919	0	0	0.05015779	0	0	0	0	0	0	0	hsa_circ_0088744	NC_000009.12	-	128185547	128187934	2388	302	sense-overlappin	NM_001131016.1	CIZ1
circRNA_06808	0.082300098	0	0	0	0	0	0	0	0.020254528	0.020172483	0	0	hsa_circ_0088767	NC_000009.12	-	128267194	128268524	1331	354	sense-overlappin	NM_004486.4	GOLGA2
circRNA_06809	0	0	0	0	0.03009467	0	0	0	0	0	0	0	hsa_circ_0088777	NC_000009.12	-	128317152	128321730	4579	207	sense-overlappin	NM_015679.1	TRUB2
circRNA_06810	0.020575025	0	0	0	0	0	0	0	0	0	0	0.0206364	-	NC_000009.12	+	128434102	128434613	512	332	sense-overlappin	XM_005252035.1	CERCAM
circRNA_06811	0	0	0	0	0	0	0.020098685	0	0	0	0	0	-	NC_000009.12	+	128472913	128473741	829	262	sense-overlappin	NM_153436.1	ODF2
circRNA_06812	0	0.020469968	0.054521613	0	0.03009467	0	0	0.030729443	0	0.040344966	0	0	hsa_circ_0002675	NC_000009.12	+	128508876	128515639	6764	333	sense-overlappin	NM_001499.2	GLE1
circRNA_06813	0	0.020469968	0	0	0	0	0	0	0	0	0	0	-	NC_000009.12	-	128739931	128742745	2815	683	sense-overlappin	XM_005251646.2	ZER1
circRNA_06814	0	0	0	0	0.02006311	0	0	0	0	0	0	0	-	NC_000009.12	-	128752673	128755659	2987	1017	sense-overlappin	XM_005251646.2	ZER1
circRNA_06815	0	0	0	0	0.03009467	0	0	0	0	0	0	0	-	NC_000009.12	+	128956350	128986875	30526	2103	sense-overlappin	NM_015354.2	NUP188
circRNA_06816	0	0	0	0.0206362	0	0	0	0	0	0	0	0	-	NC_000009.12	+	129328293	129330059	1767	1767	intergenic		
circRNA_06817	0	0	0	0.0206362	0	0	0	0	0	0	0	0	-	NC_000009.12	-	129904046	129907756	3711	3711	intronic	XM_005251824.2	FNBP1
circRNA_06818	0	0	0	0	0	0	0	0.030729443	0	0	0	0	-	NC_000009.12	-	129957343	129959330	1988	962	sense-overlappin	XM_005251824.2	FNBP1
circRNA_06819	0.020575025	0	0	0	0	0	0	0	0	0	0	0	-	NC_000009.12	-	130022483	130025705	3223	3223	intronic	XM_005251824.2	FNBP1
circRNA_06820	0	0	0	0	0	0	0	0	0	0.020172483	0	0	hsa_circ_0002625	NC_000009.12	+	130079630	130086476	6847	235	sense-overlappin	NM_001287346.1	GPR107
circRNA_06821	0	0	0	0	0	0	0	0	0	0	0.030382845	0	-	NC_000009.12	+	130079630	130092381	12752	477	sense-overlappin	NM_001287346.1	GPR107
circRNA_06822	0	0	0	0	0	0	0	0	0.020254528	0	0	0	hsa_circ_0089166	NC_000009.12	+	131159383	131164144	4762	457	sense-overlappin	XM_011519057.1	NUP214
circRNA_06823	0.020575025	0	0	0.0928628	0.05015779	0.020712909	0.050246713	0.040972591	0.020254528	0	0.02025523	0	hsa_circ_0001895	NC_000009.12	+	131430090	131432794	2705	348	sense-overlappin	NM_013318.3	PRRC2B
circRNA_06824	0	0	0	0	0	0	0	0	0	0.020172483	0	0	-	NC_000009.12	+	131432617	131447804	15188	1005	sense-overlappin	NM_013318.3	PRRC2B
circRNA_06825	0.082300098	0.030704951	0	0	0.05015779	0.072495183	0.070345399	0.051215739	0.040509055	0.080689931	0.030382845	0.0309546	hsa_circ_0005871	NC_000009.12	+	131436620	131439061	2442	176	sense-overlappin	NM_013318.3	PRRC2B
circRNA_06826	0.041150049	0	0.032712968	0	0.03009467	0	0	0	0	0	0	0	hsa_circ_0007613	NC_000009.12	+	131455076	131459356	4281	284	sense-overlappin	NM_013318.3	PRRC2B
circRNA_06827	0.061725074	0.030704951	0.021808645	0	0	0.062138728	0	0	0	0.030258724	0.08102092	0	hsa_circ_0001897	NC_000009.12	+	131506114	131506453	340	158	sense-overlappin	XM_005272158.1	POMT1
circRNA_06828	0	0	0	0.0206362	0	0	0	0	0	0	0.030382845	0	hsa_circ_0089254	NC_000009.12	-	131643239	131650949	7711	433	sense-overlappin	XM_006717067.2	RAPGEF1
circRNA_06829	0	0	0	0	0	0	0.030148028	0	0	0	0	0	hsa_circ_0089282	NC_000009.12	-	132296887	132311856	14970	675	sense-overlappin	XM_011518405.1	SETX
circRNA_06830	0	0	0	0	0	0	0	0	0	0.040344966	0	0	-	NC_000009.12	-	132625664	132632091	6428	273	sense-overlappin	XM_011518923.1	DDX31
circRNA_06831	0	0	0	0	0	0	0.030148028	0	0	0	0	0	hsa_circ_0089309	NC_000009.12	-	132927201	132928952	1752	290	sense-overlappin	NM_000368.4	TSC1
circRNA_06832	0	0.020469968	0.021808645	0	0.03009467	0	0	0	0	0	0	0	hsa_circ_0089310	NC_000009.12	+	133042087	133043926	1840	419	sense-overlappin	NM_001122823.1	GTF3C5
circRNA_06833	0	0	0.032712968	0	0.02006311	0	0	0	0	0	0.02025523	0	hsa_circ_0089392	NC_000009.12	-	133806081	133812216	6136	387	sense-overlappin	NM_001134398.1	VAV2
circRNA_06834	0	0	0.04361729	0	0	0	0	0	0	0	0	0	hsa_circ_0004520	NC_000009.12	-	133925843	133939219	13377	13377	sense-overlappin	NM_001134398.1	VAV2
circRNA_06835	0.020575025	0	0	0	0	0.020712909	0	0	0.020254528	0	0	0	hsa_circ_0007482	NC_000009.12	+	134690912	134701333	10422	545	sense-overlappin	XR_929712.1	
circRNA_06836	0.020575025	0	0.032712968	0.0206362	0.08025246	0.051782273	0.030148028	0	0	0.020172483	0.02025523	0	hsa_circ_0089489	NC_000009.12	-	135850137	135866536	16400	493	sense-overlappin	XM_011518273.1	CAMSAP1
circRNA_06837	0	0	0.065425935	0	0	0.041425819	0.060296056	0.020486295	0	0.020172483	0	0	hsa_circ_0004338	NC_000009.12	-	135850137	135883078	32942	918	sense-overlappin	XM_005263396.2	CAMSAP1
circRNA_06838	0.020575025	0	0.032712968	0.0309543	0.04012623	0	0	0	0	0	0	0	hsa_circ_0006814	NC_000009.12	-	135866456	135883078	16623	506	sense-overlappin	NM_015447.3	CAMSAP1
circRNA_06839	0.349775418	0.214934659	0.894154446	0.3508152	0.27085205	0.258911367	0.291430938	0.153647216	0.31394518	0.252156035	0.344338911	0.330182403	hsa_circ_0001900	NC_000009.12	-	135881633	135883078	1446	425	sense-overlappin	NM_015447.3	CAMSAP1
circRNA_06840	0.020575025	0	0	0	0	0	0	0.020486295	0	0	0	0	-	NC_000009.12	-	135881633	135888273	6641	5620	sense-overlappin	NM_015447.3	CAMSAP1
circRNA_06841	0	0	0.021808645	0	0	0	0	0	0	0	0	0	-	NC_000009.12	-	135933057	135933515	459	459	exonic	NM_016172.2	UBAC1
circRNA_06842	0	0	0.032712968	0	0	0	0	0	0	0	0	0	-	NC_000009.12	-	136085232	136085479	248	248	intronic	NM_144653.4	NACC2
circRNA_06843	0.020575025	0	0	0	0	0	0	0	0	0	0	0	-	NC_000009.12	+	137622974	137624642	1669	1669	intronic	NM_001145527.1	EHMT1
circRNA_06844	0	0	0	0.0206362	0	0	0	0	0	0	0	0	hsa_circ_0009011	NC_000009.12	+	137716626	137717182	557	557	exonic	NM_001145527.1	EHMT1
circRNA_06845	0	0	0.021808645	0	0	0	0	0.020486295	0	0	0	0	hsa_circ_0002647	NC_000009.12	+	137716626	137752408	35783	1163	sense-overlappin	NM_001145527.1	EHMT1
circRNA_06846	0	0	0.021808645	0	0	0	0	0	0	0	0	0	hsa_circ_0004101	NC_000009.12	+	137728349	137744090	15742	528	sense-overlappin	NM_001145527.1	EHMT1
circRNA_06847	0	0	0.054521613	0	0	0	0	0	0	0.020172483	0.02025523	0	hsa_circ_0089715	NC_000009.12	+	137728349	137762820	34472	1005	sense-overlappin	NM_001145527.1	EHMT1
circRNA_06848	0.020575025	0	0	0	0	0	0	0	0	0	0	0	-	NC_000009.12	-	137728697	137728868	172	172	antisense	NM_001145527.1	EHMT1

circRNA_06849	0	0	0	0	0	0	0	0	0	0	0.02025523	0	-	NC_000009.12	+	137743392	137818138	74747	2696	sense-overlappin	NM_024757.4	EHMT1
circRNA_06850	0	0.030704951	0.054521613	0.0619086	0.05015779	0.020712909	0.020098685	0	0.040509055	0.040344966	0	0	hsa_circ_0001904	NC_000009.12	+	137752331	137758011	5681	331	sense-overlappin	NM_001145527.1	EHMT1
circRNA_06851	0	0	0	0.0206362	0	0	0	0	0	0	0	0	hsa_circ_0089722	NC_000009.12	+	137752331	137762820	10490	477	sense-overlappin	NM_001145527.1	EHMT1
circRNA_06852	0	0	0.021808645	0	0	0	0	0	0	0	0	0	-	NC_000009.12	+	137754171	137818138	63968	2292	sense-overlappin	NM_024757.4	EHMT1
circRNA_06853	0	0	0.065425935	0	0	0	0	0.020486295	0	0	0	0	hsa_circ_0001905	NC_000009.12	+	137790848	137800984	10137	330	sense-overlappin	NM_024757.4	EHMT1
circRNA_06854	0	0	0.032712968	0.0206362	0	0	0	0	0	0	0	0	hsa_circ_0009134	NC_000009.12	+	137790848	137818138	27291	1158	sense-overlappin	NM_024757.4	EHMT1
circRNA_06855	0.030862537	0	0.054521613	0	0.02006311	0	0	0	0.020254528	0	0.02025523	0.0309546	hsa_circ_0001906	NC_000009.12	+	137811461	137818138	6678	828	sense-overlappin	NM_024757.4	EHMT1
circRNA_06856	0.041150049	0	0.054521613	0	0	0	0.020098685	0.030729443	0.020254528	0	0	0	hsa_circ_0008225	NC_000010.11	+	179994	221356	41363	457	sense-overlappin	NM_006624.5	ZMYND11
circRNA_06857	0	0	0	0	0	0	0	0	0	0.020172483	0	0	-	NC_000010.11	+	179994	249088	69095	1705	sense-overlappin	NM_006624.5	ZMYND11
circRNA_06858	0	0	0	0	0	0	0	0	0.020254528	0	0	0	hsa_circ_0017361	NC_000010.11	+	209889	221356	11468	322	sense-overlappin	NM_006624.5	ZMYND11
circRNA_06859	0	0	0	0	0.02006311	0	0	0	0	0	0	0	-	NC_000010.11	-	245901	273499	27599	22753	antisense	NM_006624.5	ZMYND11
circRNA_06860	0.020575025	0	0	0	0	0	0	0	0.030381792	0	0	0	-	NC_000010.11	-	263204	263658	455	455	intergenic		
circRNA_06861	0	0	0	0	0.03009467	0	0	0	0	0	0	0	hsa_circ_0002042	NC_000010.11	-	366275	384146	17872	512	sense-overlappin	NM_014974.2	DIP2C
circRNA_06862	0	0	0	0	0.03009467	0	0	0	0	0	0	0	-	NC_000010.11	-	408926	423033	14108	755	sense-overlappin	NM_014974.2	DIP2C
circRNA_06863	0	0	0	0	0.02006311	0	0	0	0	0	0	0	-	NC_000010.11	-	440871	486530	45660	477	sense-overlappin	XM_011519428.1	DIP2C
circRNA_06864	0	0	0.032712968	0.0206362	0.04012623	0	0	0	0	0	0	0.0309546	hsa_circ_0008655	NC_000010.11	-	472439	486530	14092	351	sense-overlappin	XM_011519428.1	DIP2C
circRNA_06865	0	0	0.021808645	0	0	0	0	0	0	0	0	0	hsa_circ_0003023	NC_000010.11	-	484781	486530	1750	240	sense-overlappin	XM_011519428.1	DIP2C
circRNA_06866	0	0.020469968	0	0	0	0	0	0	0	0	0	0	-	NC_000010.11	-	829385	845055	15671	784	sense-overlappin	XM_011519436.1	LARP4B
circRNA_06867	0	0	0.021808645	0	0	0	0	0	0	0	0	0	-	NC_000010.11	-	836403	885760	49358	860	sense-overlappin	XM_005252435.2	LARP4B
circRNA_06868	0	0	0.04361729	0	0	0	0	0	0	0	0	0	hsa_circ_0000203	NC_000010.11	-	842932	885760	42829	756	sense-overlappin	XM_005252435.2	LARP4B
circRNA_06869	0.020575025	0	0.04361729	0.0412724	0.02006311	0	0	0	0	0	0	0	hsa_circ_0017412	NC_000010.11	-	863743	864270	528	289	sense-overlappin	NM_015155.2	LARP4B
circRNA_06870	0	0	0.065425935	0	0	0	0	0	0.020254528	0	0	0	hsa_circ_0005772	NC_000010.11	-	863743	885760	22018	540	sense-overlappin	XM_005252435.2	LARP4B
circRNA_06871	0	0	0	0	0.02006311	0	0	0	0	0	0	0	-	NC_000010.11	-	864123	885760	21638	399	sense-overlappin	XM_005252435.2	LARP4B
circRNA_06872	0	0	0	0	0.03009467	0	0	0	0	0	0	0	hsa_circ_0004995	NC_000010.11	-	884447	890829	6383	426	sense-overlappin	XM_011519434.1	LARP4B
circRNA_06873	0	0	0	0	0.02006311	0	0	0	0	0	0	0	hsa_circ_0017416	NC_000010.11	+	992489	999095	6607	606	sense-overlappin	NM_012341.2	GTPBP4
circRNA_06874	0	0	0	0	0.02006311	0	0	0	0	0	0	0	-	NC_000010.11	+	1072116	1093496	21381	689	sense-overlappin	NM_014023.3	WDR37
circRNA_06875	0	0.030704951	0.021808645	0	0	0	0	0	0	0.050431207	0	0	hsa_circ_0000205	NC_000010.11	+	1072116	1096246	24131	766	sense-overlappin	NM_014023.3	WDR37
circRNA_06876	0	0	0	0	0.04012623	0	0	0	0	0	0	0	hsa_circ_0017443	NC_000010.11	+	1077907	1096246	18340	588	sense-overlappin	NM_014023.3	WDR37
circRNA_06877	0	0	0	0	0	0.020712909	0	0	0	0	0	0	hsa_circ_0017446	NC_000010.11	+	1080011	1086357	6347	369	sense-overlappin	NM_014023.3	WDR37
circRNA_06878	0.041150049	0	0	0	0	0	0	0	0	0	0	0	hsa_circ_0017454	NC_000010.11	+	1084403	1086357	1955	208	sense-overlappin	NM_014023.3	WDR37
circRNA_06879	0.020575025	0	0.032712968	0.0619086	0.02006311	0	0	0.020486295	0	0	0	0.0309546	hsa_circ_0000206	NC_000010.11	+	1084403	1096246	11844	330	sense-overlappin	NM_014023.3	WDR37
circRNA_06880	0	0	0.076330258	0	0	0	0	0	0	0	0	0	hsa_circ_0007796	NC_000010.11	+	1084403	1105267	20865	707	sense-overlappin	NM_014023.3	WDR37
circRNA_06881	0	0	0	0	0.02006311	0	0	0	0	0	0	0	hsa_circ_0017469	NC_000010.11	+	1124218	1125024	807	250	sense-overlappin	NM_014023.3	WDR37
circRNA_06882	0	0	0	0	0.02006311	0	0	0	0	0	0	0	hsa_circ_0006608	NC_000010.11	+	3118782	3120044	1263	241	sense-overlappin	XM_005252465.3	PFKP
circRNA_06883	0	0	0.021808645	0.0412724	0.06018934	0	0	0	0	0	0	0.0309546	hsa_circ_0017510	NC_000010.11	-	3158914	3160330	1417	345	sense-overlappin	NM_001242307.1	PITRM1
circRNA_06884	0.030862537	0	0.021808645	0	0	0	0	0	0	0	0	0	hsa_circ_0017521	NC_000010.11	+	4830675	4842504	11830	798	sense-overlappin	XM_011519716.1	AKR1E2
circRNA_06885	0	0	0.021808645	0	0.08025246	0	0	0	0	0	0	0	hsa_circ_0017523	NC_000010.11	+	4830675	4847299	16625	950	sense-overlappin	XM_011519715.1	AKR1E2
circRNA_06886	0.061725074	0	0	0	0	0	0	0	0	0	0	0	-	NC_000010.11	+	4830675	4847660	16986	1054	sense-overlappin	XM_011519715.1	AKR1E2
circRNA_06887	0.144025172	0.020469968	0.076330258	0.113499	0.09028402	0.031069364	0	0	0	0.030258724	0	0	-	NC_000010.11	+	4830675	4873307	42633	27077	sense-overlappin	XR_930519.1	
circRNA_06888	0	0	0.054521613	0	0.05015779	0	0	0	0	0	0	0	-	NC_000010.11	+	4830675	4889923	59249	43693	sense-overlappin	XR_930519.1	
circRNA_06889	0.082300098	0	0.109043225	0.0722267	0.12037869	0.093208092	0	0	0.020254528	0.060517448	0	0	-	NC_000010.11	+	4830675	4908420	77746	62190	sense-overlappin	XR_930519.1	
circRNA_06890	0.123450148	0.030704951	0.065425935	0.0515905	0.25078894	0.031069364	0	0	0.040509055	0.050431207	0	0	-	NC_000010.11	+	4830675	4909606	78932	63376	sense-overlappin	XR_930519.1	
circRNA_06891	0	0	0	0	0.03009467	0	0	0	0	0	0	0	-	NC_000010.11	+	4873214	4908420	35207	35207	intergenic		
circRNA_06892	0	0	0	0	0	0	0	0	0	0	0.02025523	0	-	NC_000010.11	-	5115230	5123820	8591	8591	intergenic		
circRNA_06893	0	0	0	0	0	0	0	0	0	0.020172483	0	0	-	NC_000010.11	+	5699525	5712918	13394	589	sense-overlappin	XM_011519516.1	FAM208B
circRNA_06894	0.030862537	0.020469968	0.098138903	0.2476343	0.06018934	0.072495183	0.140690797	0.163890364	0.091145375	0.161379862	0.12153138	0.0412728	hsa_circ_0000209	NC_000010.11	+	5699525	5714207	14683	662	sense-overlappin	XM_011519516.1	FAM208B
circRNA_06895	0	0	0	0	0	0	0	0	0	0.030258724	0	0	hsa_circ_0002118	NC_000010.11	+	5709530	5712918	3389	376	sense-overlappin	XM_005252476.3	FAM208B
circRNA_06896	0	0.030704951	0	0.0412724	0	0	0.020098685	0	0	0	0	0	hsa_circ_0003755	NC_000010.11	+	5709530	5714207	4678	449	sense-overlappin	XM_005252476.3	FAM208B
circRNA_06897	0	0	0	0.0206362	0	0	0	0	0	0	0	0	-	NC_000010.11	+	5726885	5735546	8662	1096	sense-overlappin	XM_005252481.3	FAM208B
circRNA_06898	0	0	0	0.0206362	0	0	0	0	0	0	0	0	-	NC_000010.11	+	5730487	5742526	12040	2270	sense-overlappin	XM_005252481.3	FAM208B
circRNA_06899	0	0	0	0.0206362	0	0	0	0	0	0	0	0	-	NC_000010.11	-	5768213	5800705	32493	946	sense-overlappin	NM_001494.3	GDI2
circRNA_06900	0	0	0.076330258	0.0206362	0.06018934	0.020712909	0	0	0.020254528	0.040344966	0	0.0206364	hsa_circ_0017586	NC_000010.11	-	5773842	5800705	26864	774	sense-overlappin	NM_001494.3	GDI2
circRNA_06901	0.072012586	0.122819805	0.08723458	0.1341352	0.12037869	0.031069364	0.090444084	0	0.020254528	0.030258724	0.04051046	0	hsa_circ_0005379	NC_000010.11	-	5785142	5800705	15564	674	sense-overlappin	NM_001494.3	GDI2
circRNA_06902	0	0	0	0	0.04012623	0	0	0	0	0	0	0	hsa_circ_0003351	NC_000010.11	-	5785852	5800705	14854	542	sense-overlappin	NM_001494.3	GDI2
circRNA_06903	0.061725074	0.102349838	0.152660515	0.1857257	0.17053648	0.165703275	0.120592112	0.122917773	0.050636319	0.131121138	0.050638075	0.082545601	hsa_circ_0002665	NC_000010.11	-	5794885	5800705	5821	343	sense-overlappin	NM_001494.3	GDI2

circRNA_06904	0	0	0	0.0206362	0.04012623	0	0	0	0	0.020172483	0	0	hsa_circ_0007403	NC_000010.11	-	5959754	5960567	814	234	sense-overlappin	XM_011519462.1	IL15RA
circRNA_06905	0	0	0	0	0	0	0	0	0	0.020172483	0	0	-	NC_000010.11	-	5959754	5960755	1002	422	sense-overlappin	XM_011519462.1	IL15RA
circRNA_06906	0	0	0.021808645	0	0	0	0	0	0	0	0	0	hsa_circ_0017604	NC_000010.11	-	5959754	5966339	6586	528	sense-overlappin	XM_011519462.1	IL15RA
circRNA_06907	0.051437562	0.051174919	0	0	0	0	0	0.071702034	0	0	0	0	hsa_circ_0017628	NC_000010.11	-	7220411	7285954	65544	914	sense-overlappin	XM_006717490.2	SFMBT2
circRNA_06908	0	0	0	0	0	0	0	0	0	0.020172483	0	0	hsa_circ_0017632	NC_000010.11	-	7227855	7285954	58100	787	sense-overlappin	XM_006717490.2	SFMBT2
circRNA_06909	0	0	0	0	0	0.041425819	0	0	0	0	0	0	hsa_circ_0017635	NC_000010.11	-	7243558	7276989	33432	348	sense-overlappin	XM_006717490.2	SFMBT2
circRNA_06910	0.102875123	0.051174919	0.021808645	0.0619086	0	0.134633911	0.080394741	0.071702034	0.081018111	0.14120738	0.070893305	0.0412728	hsa_circ_0017636	NC_000010.11	-	7243558	7285954	42397	704	sense-overlappin	XM_006717490.2	SFMBT2
circRNA_06911	0.030862537	0	0	0	0	0	0	0.030729443	0	0	0	0	hsa_circ_0017639	NC_000010.11	-	7248548	7285954	37407	556	sense-overlappin	XM_006717490.2	SFMBT2
circRNA_06912	0.164600197	0.092114854	0.152660515	0.0722267	0.03009467	0.15534682	0.221085539	0.174133512	0.151908958	0.453880863	0.172169456	0.257955002	hsa_circ_0000211	NC_000010.11	-	7276892	7285954	9063	454	sense-overlappin	XM_006717490.2	SFMBT2
circRNA_06913	0.020575025	0	0	0	0	0	0	0	0	0	0	0	hsa_circ_0017643	NC_000010.11	-	7283904	7285954	2051	356	sense-overlappin	XM_006717490.2	SFMBT2
circRNA_06914	0	0	0.021808645	0	0	0.031069364	0.030148028	0	0	0	0	0	hsa_circ_0000212	NC_000010.11	-	7363878	7381949	18072	4258	sense-overlappin	NM_001018039.1	SFMBT2
circRNA_06915	0	0	0	0	0	0	0.030148028	0	0	0	0	0	hsa_circ_0017647	NC_000010.11	-	7367649	7370375	2727	336	sense-overlappin	NM_001018039.1	SFMBT2
circRNA_06916	0.020575025	0.051174919	0.032712968	0.0309543	0	0.051782273	0.050246713	0.020486295	0.050636319	0.030258724	0	0.0412728	hsa_circ_0017648	NC_000010.11	-	7367649	7381949	14301	487	sense-overlappin	NM_001018039.1	SFMBT2
circRNA_06917	0	0	0	0.0412724	0	0.031069364	0	0.020486295	0.020254528	0	0	0	hsa_circ_0017650	NC_000010.11	-	7615982	7655675	39694	849	sense-overlappin	NM_030569.6	ITIH5
circRNA_06918	0	0	0	0	0	0	0	0	0.020254528	0	0	0	-	NC_000010.11	-	7637228	7655675	18448	562	sense-overlappin	NM_030569.6	ITIH5
circRNA_06919	0	0	0	0.0412724	0	0	0	0	0	0.020172483	0	0	-	NC_000010.11	-	7759890	7766103	6214	321	sense-overlappin	NM_012311.3	KIN
circRNA_06920	0.144025172	0.225169643	0.447077223	0.4849504	0.31097828	0.279624276	0.110542769	0.215106103	0.141781694	0.302587242	0.172169456	0.0206364	hsa_circ_0007292	NC_000010.11	+	7797047	7802854	5808	799	sense-overlappin	NM_001001973.1	ATP5C1
circRNA_06921	0	0	0	0	0	0	0.020098685	0	0	0	0	0	hsa_circ_0017680	NC_000010.11	+	11270666	11288552	17887	358	sense-overlappin	XM_005252354.3	CELF2
circRNA_06922	0	0	0	0	0	0.031069364	0	0	0	0	0	0	hsa_circ_0017683	NC_000010.11	-	11481770	11485911	4142	414	sense-overlappin	NM_001080491.3	USP6NL
circRNA_06923	0	0	0	0.0206362	0.02006311	0	0	0	0	0	0	0	-	NC_000010.11	-	11481770	11527567	45798	1074	sense-overlappin	NM_001080491.3	USP6NL
circRNA_06924	0	0	0.021808645	0	0	0	0	0	0	0	0	0	-	NC_000010.11	-	11489102	11527567	38466	660	sense-overlappin	NM_001080491.3	USP6NL
circRNA_06925	0.020575025	0	0.04361729	0	0	0	0	0	0	0	0	0	hsa_circ_0017688	NC_000010.11	-	11509595	11527567	17973	272	sense-overlappin	NM_001080491.3	USP6NL
circRNA_06926	0	0	0	0	0	0	0	0	0	0.030258724	0	0	-	NC_000010.11	-	11509595	11597717	88123	443	sense-overlappin	XM_011519762.1	USP6NL
circRNA_06927	0	0	0	0	0	0	0.020098685	0	0	0	0	0	-	NC_000010.11	-	11518535	11597717	79183	362	sense-overlappin	XM_011519762.1	USP6NL
circRNA_06928	0	0	0	0.0206362	0.04012623	0	0	0	0	0	0	0	-	NC_000010.11	+	11851997	11852215	219	219	exonic	XM_011519438.1	PROSER2
circRNA_06929	0.020575025	0	0	0	0	0	0	0	0	0	0	0	hsa_circ_0017705	NC_000010.11	-	11936545	11952249	15705	696	sense-overlappin	XM_011519447.1	UPF2
circRNA_06930	0	0	0.021808645	0	0.02006311	0	0	0	0	0	0.02025523	0	hsa_circ_0017713	NC_000010.11	-	11979057	12014184	35128	808	sense-overlappin	XM_011519447.1	UPF2
circRNA_06931	0	0	0.04361729	0	0	0	0	0	0	0	0.030382845	0	hsa_circ_0007448	NC_000010.11	-	11997672	12004727	7056	538	sense-overlappin	XM_011519447.1	UPF2
circRNA_06932	0.041150049	0.030704951	0.098138903	0.0825448	0.05015779	0.072495183	0.070345399	0.030729443	0.070890847	0.060517448	0.050638075	0.0412728	hsa_circ_0000213	NC_000010.11	-	11997672	12014184	16513	699	sense-overlappin	XM_011519447.1	UPF2
circRNA_06933	0	0	0	0.0206362	0	0	0	0	0	0	0	0	hsa_circ_0017724	NC_000010.11	+	12081472	12084751	3280	368	sense-overlappin	NM_018706.6	DHTKD1
circRNA_06934	0	0	0	0.0206362	0	0	0	0	0	0	0	0	hsa_circ_0017725	NC_000010.11	+	12081472	12089255	7784	833	sense-overlappin	NM_018706.6	DHTKD1
circRNA_06935	0.020575025	0	0	0	0.0702209	0	0	0	0	0	0	0	hsa_circ_0003074	NC_000010.11	+	12081472	12091684	10213	1005	sense-overlappin	NM_018706.6	DHTKD1
circRNA_06936	0	0	0.04361729	0.0412724	0.02006311	0	0	0	0	0	0	0	hsa_circ_0004630	NC_000010.11	+	12081472	12094271	12800	1204	sense-overlappin	NM_018706.6	DHTKD1
circRNA_06937	0	0	0.032712968	0	0	0.020712909	0	0	0	0	0	0	hsa_circ_0017728	NC_000010.11	+	12081472	12101181	19710	1742	sense-overlappin	NM_018706.6	DHTKD1
circRNA_06938	0	0	0	0.0309543	0	0	0	0	0	0	0	0	hsa_circ_0017731	NC_000010.11	+	12081472	12113064	31593	2165	sense-overlappin	NM_018706.6	DHTKD1
circRNA_06939	0.020575025	0	0.163564838	0.1650895	0	0.020712909	0.040197371	0.020486295	0	0	0	0.051591	hsa_circ_0007813	NC_000010.11	+	12081472	12120267	38796	2504	sense-overlappin	NM_018706.6	DHTKD1
circRNA_06940	0	0	0	0	0.02006311	0	0	0	0	0	0	0	-	NC_000010.11	+	12112900	12120267	7368	504	sense-overlappin	NM_018706.6	DHTKD1
circRNA_06941	0	0	0	0	0	0	0	0	0	0.020172483	0	0	hsa_circ_0000214	NC_000010.11	+	12118749	12120267	1519	256	sense-overlappin	NM_018706.6	DHTKD1
circRNA_06942	0	0	0.04361729	0.0309543	0	0.031069364	0	0.020486295	0	0	0	0	-	NC_000010.11	+	12215740	12238485	22746	480	sense-overlappin	NM_006023.2	CDC123
circRNA_06943	0	0	0	0	0	0	0	0	0.020254528	0	0	0	-	NC_000010.11	+	12235048	12238485	3438	228	sense-overlappin	NM_006023.2	CDC123
circRNA_06944	0	0	0	0	0	0.031069364	0.020098685	0	0	0.040344966	0	0	hsa_circ_0000215	NC_000010.11	+	12400016	12403369	3354	3354	intronic	XM_006717482.2	CAMK1D
circRNA_06945	0	0	0	0	0	0	0.030148028	0	0	0	0	0	-	NC_000010.11	+	12765003	12766060	1058	1058	intronic	XM_006717482.2	CAMK1D
circRNA_06946	0	0	0	0	0	0	0	0	0	0.020172483	0	0	-	NC_000010.11	+	13108138	13110476	2339	601	sense-overlappin	NM_001008211.1	OPTN
circRNA_06947	0	0.020469968	0	0	0	0	0	0	0	0	0	0	hsa_circ_0017784	NC_000010.11	+	13110274	13119040	8767	595	sense-overlappin	XM_005252336.2	OPTN
circRNA_06948	0	0	0	0	0	0.041425819	0	0	0	0	0	0	-	NC_000010.11	+	13125946	13136897	10952	617	sense-overlappin	XM_005252336.2	OPTN
circRNA_06949	0	0.020469968	0	0	0	0	0	0	0	0	0	0	-	NC_000010.11	+	13127745	13133581	5837	370	sense-overlappin	XM_005252336.2	OPTN
circRNA_06950	0.061725074	0.071644886	0.065425935	0.0309543	0.05015779	0.051782273	0	0	0.030381792	0.060517448	0.10127615	0.0619092	hsa_circ_0007242	NC_000010.11	+	13127745	13136897	9153	523	sense-overlappin	XM_005252336.2	OPTN
circRNA_06951	0	0	0	0	0.03009467	0	0	0	0	0	0	0	hsa_circ_0002109	NC_000010.11	+	13172376	13172765	390	243	sense-overlappin	NM_018518.4	MCM10
circRNA_06952	0	0	0	0	0	0	0.020098685	0	0	0	0	0	-	NC_000010.11	-	13480899	13492876	11978	492	sense-overlappin	NM_152751.2	BEND7
circRNA_06953	0.030862537	0	0	0	0	0	0	0	0	0	0	0	-	NC_000010.11	-	13654416	13707113	52698	2327	sense-overlappin	XM_005252494.3	FRMD4A
circRNA_06954	0.030862537	0	0	0	0	0	0	0	0	0.020172483	0	0	hsa_circ_0004281	NC_000010.11	-	13659323	13663509	4187	463	sense-overlappin	NM_018027.3	FRMD4A
circRNA_06955	0	0	0	0	0	0	0	0	0	0.020172483	0	0	hsa_circ_0004373	NC_000010.11	-	13663453	13675044	11592	543	sense-overlappin	NM_018027.3	FRMD4A
circRNA_06956	0	0	0	0.0206362	0.02006311	0.020712909	0.020098685	0	0	0	0	0	-	NC_000010.11	-	14520977	14530515	9539	665	sense-overlappin	XM_011519731.1	FAM107B
circRNA_06957	0	0	0	0	0.02006311	0	0	0	0	0	0	0	-	NC_000010.11	-	14520977	14553387	32411	731	sense-overlappin	XM_011519738.1	FAM107B
circRNA_06958	0	0	0	0	0.02006311	0	0	0	0	0	0	0	-	NC_000010.11	-	14530332	14530515	184	184	exonic	XM_011519731.1	FAM107B

circRNA_06959	0.020575025	0	0	0	0.03009467	0	0	0	0	0	0	0	hsa_circ_0006780	NC_000010.11	+	14896846	14897517	672	672	exonic	NM_001193424.1	SUV39H2
circRNA_06960	0	0	0.021808645	0	0	0	0	0	0	0	0	0	hsa_circ_0004201	NC_000010.11	-	14919738	14935564	15827	794	sense-overlappin	NM_001033855.2	DCLRE1C
circRNA_06961	0	0	0.021808645	0	0	0	0	0	0	0	0	0	-	NC_000010.11	-	14926843	14935564	8722	610	sense-overlappin	NM_001033855.2	DCLRE1C
circRNA_06962	0	0	0	0	0.04012623	0	0	0	0	0	0	0	hsa_circ_0017829	NC_000010.11	-	14928016	14935564	7549	555	sense-overlappin	NM_001033855.2	DCLRE1C
circRNA_06963	0	0	0	0	0	0	0.020098685	0.030729443	0	0	0	0	-	NC_000010.11	-	15275855	15284105	8251	321	sense-overlappin	XM_011519378.1	FAM171A1
circRNA_06964	0	0	0	0	0.02006311	0	0	0	0	0	0	0	-	NC_000010.11	-	15308316	15308577	262	262	intronic	XM_011519378.1	FAM171A1
circRNA_06965	0	0	0	0	0	0	0	0	0.020254528	0	0	0	-	NC_000010.11	-	15531050	15586664	55615	691	sense-overlappin	NM_001291494.1	ITGA8
circRNA_06966	0	0.020469968	0	0	0	0	0	0	0	0	0	0	hsa_circ_0017836	NC_000010.11	-	15531050	15592304	61255	771	sense-overlappin	NM_001291494.1	ITGA8
circRNA_06967	0.061725074	0	0	0	0	0	0	0	0	0	0	0	-	NC_000010.11	-	15548455	15586664	38210	589	sense-overlappin	NM_001291494.1	ITGA8
circRNA_06968	0.020575025	0	0	0	0	0	0	0	0	0	0	0	-	NC_000010.11	-	15548455	15592304	43850	669	sense-overlappin	NM_001291494.1	ITGA8
circRNA_06969	0	0	0	0	0	0	0	0	0	0	0	0.0206364	-	NC_000010.11	-	15558074	15558202	129	129	exonic	NM_001291494.1	ITGA8
circRNA_06970	0	0	0	0	0	0.020712909	0	0	0	0	0	0	-	NC_000010.11	-	15575489	15592304	16816	267	sense-overlappin	NM_001291494.1	ITGA8
circRNA_06971	0.051437562	0	0.021808645	0	0	0	0	0	0	0	0	0	hsa_circ_0017848	NC_000010.11	-	15796100	15847943	51844	5544	sense-overlappin	XM_011519693.1	FAM188A
circRNA_06972	0	0	0	0.0309543	0	0	0	0	0	0	0	0	hsa_circ_0007671	NC_000010.11	-	15816835	15843272	26438	800	sense-overlappin	XM_011519693.1	FAM188A
circRNA_06973	0.216037758	0.071644886	0.196277805	0.2063619	0.12037869	0.103564547	0.060296056	0.122917773	0.202545277	0.201724828	0	0.165091201	hsa_circ_0000219	NC_000010.11	-	15816835	15847943	31109	5471	sense-overlappin	XM_011519693.1	FAM188A
circRNA_06974	0	0	0	0.0309543	0.03009467	0	0	0	0.030381792	0	0	0.0206364	hsa_circ_0008178	NC_000010.11	-	15821656	15843272	21617	719	sense-overlappin	XM_011519693.1	FAM188A
circRNA_06975	0	0.020469968	0.119947548	0	0	0.031069364	0	0	0	0.060517448	0	0.0309546	hsa_circ_0000220	NC_000010.11	-	15821656	15847943	26288	707	sense-overlappin	XM_006717508.1	FAM188A
circRNA_06976	0	0	0	0	0	0.020712909	0	0	0	0	0	0	hsa_circ_0005825	NC_000010.11	-	15833630	15843272	9643	648	sense-overlappin	XM_011519693.1	FAM188A
circRNA_06977	0.020575025	0.08187987	0.08723458	0.0928628	0.0702209	0.072495183	0.030148028	0.030729443	0.050636319	0.090776173	0.050638075	0.0412728	hsa_circ_0006665	NC_000010.11	-	15833630	15847943	14314	636	sense-overlappin	XM_006717508.1	FAM188A
circRNA_06978	0	0	0.054521613	0	0.04012623	0	0	0	0	0.030258724	0	0.0206364	hsa_circ_0017854	NC_000010.11	+	16484337	16486617	2281	746	sense-overlappin	NM_001001484.2	PTER
circRNA_06979	0.030862537	0	0	0.0309543	0	0	0	0.020486295	0	0	0	0	hsa_circ_0006577	NC_000010.11	-	16752539	16782084	29546	489	sense-overlappin	NM_012425.3	RSU1
circRNA_06980	0.041150049	0.020469968	0	0.0206362	0	0	0	0	0	0.020172483	0	0	hsa_circ_0006751	NC_000010.11	-	17603560	17604047	488	226	sense-overlappin	NM_014241.3	HACD1
circRNA_06981	0.082300098	0.071644886	0.04361729	0.0928628	0.05015779	0.124277456	0.040197371	0.040972591	0.070890847	0.151293621	0.030382845	0.0206364	hsa_circ_0008311	NC_000010.11	+	17704431	17705741	1311	297	sense-overlappin	NM_003473.3	STAM
circRNA_06982	0	0	0	0	0	0	0	0	0.030381792	0	0	0	-	NC_000010.11	+	17894210	17902122	7913	652	sense-overlappin	NM_002438.3	MRC1
circRNA_06983	0	0	0	0.0309543	0	0	0	0	0	0	0	0	-	NC_000010.11	+	18317822	18319096	1275	1275	intronic	NM_201593.2	CACNB2
circRNA_06984	0	0.020469968	0.04361729	0	0	0	0	0	0	0	0	0	-	NC_000010.11	+	18317904	18319096	1193	1193	intronic	NM_201593.2	CACNB2
circRNA_06985	0	0	0.04361729	0	0	0	0	0	0	0	0	0	-	NC_000010.11	+	18387365	18402043	14679	14679	sense-overlappin	NM_201593.2	CACNB2
circRNA_06986	0.020575025	0	0.04361729	0	0	0	0	0	0	0	0	0	-	NC_000010.11	+	18527588	18536196	8609	358	sense-overlappin	NM_201593.2	CACNB2
circRNA_06987	0	0	0	0	0	0	0	0.030729443	0	0.020172483	0	0	-	NC_000010.11	-	18585949	18616293	30345	611	sense-overlappin	NM_182543.3	NSUN6
circRNA_06988	0	0	0.032712968	0	0	0	0	0	0	0	0	0	-	NC_000010.11	-	18596208	18616293	20086	466	sense-overlappin	NM_182543.3	NSUN6
circRNA_06989	0	0	0	0	0	0	0	0	0	0	0.030382845	0	-	NC_000010.11	+	19123492	19136781	13290	717	sense-overlappin	XM_011519454.1	MALRD1
circRNA_06990	0	0	0	0	0	0	0.020098685	0	0	0	0	0	-	NC_000010.11	+	19866746	19866882	137	137	intronic	XM_011519750.1	PLXDC2
circRNA_06991	0	0	0	0	0	0	0	0	0	0.030258724	0	0	hsa_circ_0017924	NC_000010.11	+	20143295	20164567	21273	342	sense-overlappin	XM_011519750.1	PLXDC2
circRNA_06992	0	0	0	0	0	0	0	0.020486295	0	0	0	0	-	NC_000010.11	-	20840739	20840849	111	111	exonic	XM_005252343.3	NEBL
circRNA_06993	0	0	0.032712968	0	0	0	0	0	0	0	0	0	-	NC_000010.11	-	20880794	20899450	18657	472	sense-overlappin	XM_011519290.1	NEBL
circRNA_06994	0	0	0.021808645	0	0.04012623	0	0	0	0	0	0	0	-	NC_000010.11	-	20945338	20961779	16442	16442	sense-overlappin	NM_001173484.1	NEBL
circRNA_06995	0	0	0	0.0206362	0.05015779	0.020712909	0	0	0	0.020172483	0	0	-	NC_000010.11	-	20957731	20961779	4049	4049	sense-overlappin	NM_001173484.1	NEBL
circRNA_06996	0.020575025	0	0	0	0	0	0	0	0	0	0	0	-	NC_000010.11	+	21545053	21551027	5975	5975	intronic	XM_011519698.1	MLLT10
circRNA_06997	0	0	0	0	0	0.020712909	0	0	0	0	0	0	-	NC_000010.11	+	21670449	21682257	11809	904	sense-overlappin	XM_011519698.1	MLLT10
circRNA_06998	0.030862537	0	0	0	0	0	0	0	0	0	0	0	-	NC_000010.11	+	21673782	21713950	40169	497	sense-overlappin	XM_011519698.1	MLLT10
circRNA_06999	0	0	0	0	0	0	0	0	0	0.020172483	0	0	-	NC_000010.11	+	21681332	21682257	926	78	sense-overlappin	XM_011519698.1	MLLT10
circRNA_07000	0	0.030704951	0.054521613	0	0.02006311	0.041425819	0	0	0	0	0	0	hsa_circ_0007084	NC_000010.11	+	21713772	21727928	14157	364	sense-overlappin	XM_011519698.1	MLLT10
circRNA_07001	0.020575025	0	0.109043225	0	0.10031557	0.031069364	0.020098685	0	0.030381792	0	0.02025523	0	-	NC_000010.11	+	21713772	21735235	21464	1256	sense-overlappin	XM_011519698.1	MLLT10
circRNA_07002	0	0	0	0	0	0	0	0	0	0.020172483	0	0	-	NC_000010.11	+	21732899	21735235	2337	737	sense-overlappin	XM_011519698.1	MLLT10
circRNA_07003	0.030862537	0	0.032712968	0	0	0	0.020098685	0.040972591	0	0.030258724	0	0	hsa_circ_0002618	NC_000010.11	-	21882282	21920963	38682	607	sense-overlappin	NM_022365.3	DNAJC1
circRNA_07004	0.030862537	0.020469968	0.04361729	0	0	0	0.020098685	0.020486295	0.020254528	0	0	0.051591	hsa_circ_0004306	NC_000010.11	-	21904522	21920963	16442	449	sense-overlappin	NM_022365.3	DNAJC1
circRNA_07005	0.041150049	0.030704951	0.054521613	0	0.02006311	0	0	0	0	0	0	0	-	NC_000010.11	-	21904522	21929141	24620	598	sense-overlappin	NM_022365.3	DNAJC1
circRNA_07006	0.020575025	0	0.021808645	0	0	0	0	0.020486295	0	0.080689931	0	0	hsa_circ_0003583	NC_000010.11	-	22591629	22609717	18089	348	sense-overlappin	NM_005028.4	PIP4K2A
circRNA_07007	0	0.020469968	0	0	0	0	0	0	0	0	0	0	-	NC_000010.11	+	23005800	23012983	7184	5450	sense-overlappin	NM_001282746.1	ARMC3
circRNA_07008	0	0	0	0	0.05015779	0	0.020098685	0	0	0	0	0	-	NC_000010.11	+	24380869	24384423	3555	3555	sense-overlappin	NM_001098500.2	KIAA1217
circRNA_07009	0	0	0	0	0	0.041425819	0	0	0	0	0	0	hsa_circ_0006580	NC_000010.11	+	24524323	24528119	3797	626	sense-overlappin	NM_001098500.2	KIAA1217
circRNA_07010	0	0	0	0.0412724	0	0	0	0	0	0	0	0	-	NC_000010.11	+	24527936	24536893	8958	636	sense-overlappin	XM_011519567.1	KIAA1217
circRNA_07011	0	0	0.032712968	0	0	0	0	0	0	0	0	0	hsa_circ_0017995	NC_000010.11	-	24937184	24942436	5253	291	sense-overlappin	XM_011519587.1	PRTFDC1
circRNA_07012	0	0	0	0	0	0	0.020098685	0	0	0	0	0.0309546	hsa_circ_0006063	NC_000010.11	+	26500819	26503274	2456	371	sense-overlappin	XM_011519514.1	APBB1IP

circRNA_07013	0	0	0	0	0	0.041425819	0	0	0	0	0	0	-	NC_000010.11	+	26500819	26513660	12842	653	sense-overlappin	XM_011519514.1	APBB1IP
circRNA_07014	0	0	0	0	0	0	0	0.020486295	0	0	0	0	-	NC_000010.11	+	26511747	26513660	1914	282	sense-overlappin	XM_011519514.1	APBB1IP
circRNA_07015	0	0	0	0	0	0	0	0	0	0.030258724	0	0	-	NC_000010.11	+	26720135	26724127	3993	451	sense-overlappin	XR_930486.1	
circRNA_07016	0	0	0	0	0	0.020712909	0	0	0	0.020172483	0	0	-	NC_000010.11	-	26759062	26770345	11284	601	sense-overlappin	NM_001178116.1	ABI1
circRNA_07017	0	0	0	0	0	0	0	0.020486295	0	0	0	0	-	NC_000010.11	-	26762987	26763130	144	144	intronic	NM_001012750.2	ABI1
circRNA_07018	0	0	0.032712968	0	0	0	0	0	0	0	0	0	-	NC_000010.11	-	26765218	26770345	5128	343	sense-overlappin	NM_001012750.2	ABI1
circRNA_07019	0.041150049	0	0	0	0	0	0	0	0	0.020172483	0	0	-	NC_000010.11	-	26934413	26935627	1215	1215	intergenic		
circRNA_07020	0	0	0	0	0	0	0	0	0	0.020172483	0	0	hsa_circ_0018007	NC_000010.11	-	27012882	27014711	1830	447	sense-overlappin	XR_930483.1	
circRNA_07021	0	0	0	0	0	0	0.020098685	0	0	0	0	0	-	NC_000010.11	-	27012882	27017792	4911	738	sense-overlappin	XR_930483.1	
circRNA_07022	0.030862537	0	0	0	0	0	0	0	0	0	0.030382845	0	-	NC_000010.11	-	27022558	27033377	10820	561	sense-overlappin	XR_930483.1	
circRNA_07023	0	0	0.021808645	0	0.03009467	0.020712909	0	0	0	0	0.02025523	0	-	NC_000010.11	-	27022558	27048979	26422	2580	sense-overlappin	XR_930483.1	
circRNA_07024	0	0	0.032712968	0	0	0	0	0	0	0	0.050638075	0	hsa_circ_0018009	NC_000010.11	-	27037186	27048979	11794	1062	sense-overlappin	XR_930483.1	
circRNA_07025	0	0	0	0	0	0.031069364	0	0	0	0	0	0	-	NC_000010.11	-	27039965	27043567	3603	356	sense-overlappin	XR_930483.1	
circRNA_07026	0	0	0.021808645	0.0722267	0	0	0	0	0	0	0	0	-	NC_000010.11	-	27114521	27121448	6928	772	sense-overlappin	NM_001253866.1	YME1L1
circRNA_07027	0	0	0	0	0	0	0	0.030729443	0	0.030258724	0	0	hsa_circ_0006534	NC_000010.11	-	27117576	27121448	3873	484	sense-overlappin	NM_001253866.1	YME1L1
circRNA_07028	0	0	0	0	0	0.020712909	0	0	0	0	0	0	-	NC_000010.11	-	27117576	27136385	18810	1289	sense-overlappin	NM_001253866.1	YME1L1
circRNA_07029	0	0	0	0	0.02006311	0	0	0	0	0	0	0	-	NC_000010.11	-	27122841	27126786	3946	377	sense-overlappin	NM_001253866.1	YME1L1
circRNA_07030	0	0	0.021808645	0.0309543	0	0.020712909	0.020098685	0	0	0	0	0.0309546	hsa_circ_0005125	NC_000010.11	-	27131859	27136385	4527	428	sense-overlappin	NM_001253866.1	YME1L1
circRNA_07031	0	0	0.032712968	0	0	0	0	0	0	0	0	0	-	NC_000010.11	-	27138767	27145590	6824	3882	sense-overlappin	NM_014263.3	YME1L1
circRNA_07032	0	0.030704951	0.076330258	0.0825448	0.04012623	0.031069364	0	0	0.030381792	0.07060369	0.02025523	0	hsa_circ_0005633	NC_000010.11	-	27142387	27145590	3204	262	sense-overlappin	NM_014263.3	YME1L1
circRNA_07033	0.030862537	0	0	0	0.03009467	0	0	0	0	0	0	0	hsa_circ_0000225	NC_000010.11	+	27165064	27165539	476	258	sense-overlappin	NM_001172303.1	MASTL
circRNA_07034	0	0	0.04361729	0	0	0	0	0	0	0	0.02025523	0	hsa_circ_0018038	NC_000010.11	+	27169944	27173259	3316	1282	sense-overlappin	NM_001172303.1	MASTL
circRNA_07035	0	0	0	0	0	0	0.020098685	0	0.030381792	0	0	0	hsa_circ_0018039	NC_000010.11	+	27169944	27181581	11638	1510	sense-overlappin	XM_005252630.2	MASTL
circRNA_07036	0.051437562	0	0.065425935	0	0.02006311	0	0	0.020486295	0	0.030258724	0.02025523	0	hsa_circ_0000226	NC_000010.11	+	27532507	27533994	1488	259	sense-overlappin	NM_001256410.1	RAB18
circRNA_07037	0	0	0	0	0	0	0	0	0	0	0	0.0309546	-	NC_000010.11	-	27673960	27675554	1595	1490	sense-overlappin	XM_006717438.2	MKX
circRNA_07038	0.020575025	0	0	0	0	0	0	0	0	0	0	0	-	NC_000010.11	-	27935010	27936880	1871	398	sense-overlappin	NM_001290021.1	ARMC4
circRNA_07039	0	0	0.021808645	0	0	0	0	0	0	0	0	0	-	NC_000010.11	-	28119651	28131691	12041	637	sense-overlappin	NM_173496.3	MPP7
circRNA_07040	0	0	0.04361729	0	0.02006311	0	0.020098685	0	0	0	0	0	hsa_circ_0018054	NC_000010.11	+	28583399	28590832	7434	336	sense-overlappin	NM_100264.2	WAC
circRNA_07041	0.051437562	0	0.04361729	0.0412724	0.04012623	0.031069364	0.030148028	0	0.030381792	0.060517448	0.030382845	0	hsa_circ_0007503	NC_000010.11	+	28583399	28596041	12643	645	sense-overlappin	NM_100264.2	WAC
circRNA_07042	0	0	0.032712968	0	0	0	0	0	0	0	0	0	-	NC_000010.11	+	28589736	28596041	6306	538	sense-overlappin	NM_100264.2	WAC
circRNA_07043	0.13373766	0	0	0	0	0	0	0	0	0	0	0	hsa_circ_0018064	NC_000010.11	-	29512735	29531288	18554	1507	sense-overlappin	XM_011519630.1	SVIL
circRNA_07044	0.020575025	0	0.032712968	0	0	0	0	0	0	0	0	0	-	NC_000010.11	-	29512735	29532172	19438	1678	sense-overlappin	XM_011519630.1	SVIL
circRNA_07045	0	0	0	0	0	0	0	0	0	0	0	0.0206364	-	NC_000010.11	-	29554783	29555108	326	210	sense-overlappin	XM_011519630.1	SVIL
circRNA_07046	0.041150049	0	0	0	0	0	0	0	0	0	0	0	hsa_circ_0007338	NC_000010.11	-	29657969	29686651	28683	199	sense-overlappin	XM_011519634.1	SVIL
circRNA_07047	0	0	0.021808645	0	0	0	0	0	0	0	0	0	-	NC_000010.11	+	30450258	30450519	262	262	exonic	XM_011519309.1	MAP3K8
circRNA_07048	0	0	0	0	0	0	0	0	0	0	0	0.0206364	hsa_circ_0004737	NC_000010.11	+	31355144	31358333	3190	238	sense-overlappin	XM_011519641.1	ZEB1
circRNA_07049	0	0	0	0	0.02006311	0	0.020098685	0	0.040509055	0	0	0	hsa_circ_0000227	NC_000010.11	+	31355144	31387266	32123	699	sense-overlappin	XM_011519641.1	ZEB1
circRNA_07050	0	0	0	0	0	0	0	0	0	0.030258724	0	0	hsa_circ_0007045	NC_000010.11	+	31355144	31387798	32655	1231	sense-overlappin	XM_011519641.1	ZEB1
circRNA_07051	0	0	0	0	0	0	0	0	0.020254528	0.020172483	0.02025523	0	hsa_circ_0003519	NC_000010.11	+	31355147	31387266	32120	696	sense-overlappin	XM_011519641.1	ZEB1
circRNA_07052	0	0.020469968	0	0.0412724	0.03009467	0	0	0	0.040509055	0.030258724	0.02025523	0	hsa_circ_0000228	NC_000010.11	+	31373018	31387266	14249	461	sense-overlappin	XM_006717498.1	ZEB1
circRNA_07053	0.020575025	0	0	0.0412724	0	0	0	0	0	0.030258724	0.02025523	0.051591	hsa_circ_0003793	NC_000010.11	+	31373018	31387798	14781	378	sense-overlappin	XM_011519645.1	ZEB1
circRNA_07054	0.051437562	0.040939935	0.021808645	0.0515905	0.02006311	0.062138728	0.040197371	0.040972591	0.030381792	0.090776173	0.02025523	0	hsa_circ_0000230	NC_000010.11	+	31373018	31461237	88220	579	sense-overlappin	XM_011519645.1	ZEB1
circRNA_07055	0	0	0	0	0	0	0	0	0.020254528	0	0	0.0206364	-	NC_000010.11	+	31373122	31461237	88116	475	sense-overlappin	XM_011519645.1	ZEB1
circRNA_07056	0	0.020469968	0	0	0	0	0	0	0	0	0	0	hsa_circ_0018087	NC_000010.11	+	31387124	31461237	74114	423	sense-overlappin	XM_011519645.1	ZEB1
circRNA_07057	0	0	0	0	0	0	0	0	0	0	0.04051046	0	hsa_circ_0002140	NC_000010.11	+	31461037	31461237	201	201	exonic	XM_011519644.1	ZEB1
circRNA_07058	0	0	0	0	0	0	0	0	0	0	0.02025523	0	-	NC_000010.11	+	31807800	31808713	914	98	antisense	NM_001270699.1	ARHGAP12
circRNA_07059	0	0	0.054521613	0	0	0	0	0	0	0	0	0	-	NC_000010.11	-	31826304	31861658	35355	846	sense-overlappin	NM_018287.6	ARHGAP12
circRNA_07060	0	0	0.021808645	0	0	0	0	0	0	0	0	0	hsa_circ_0008251	NC_000010.11	-	31843461	31861658	18198	612	sense-overlappin	NM_001270695.1	ARHGAP12
circRNA_07061	0	0	0.021808645	0	0	0	0	0	0	0	0	0	hsa_circ_0018100	NC_000010.11	-	31852517	31861658	9142	486	sense-overlappin	NM_001270695.1	ARHGAP12
circRNA_07062	0.905301083	0.440104302	3.293105398	1.5477141	0.65205124	0.569605007	0.442171078	0.358510171	0.415217818	0.595088243	0.354466526	0.330182403	hsa_circ_0000231	NC_000010.11	-	31908172	31910563	2392	794	sense-overlappin	NM_001270695.1	ARHGAP12
circRNA_07063	0	0	0.032712968	0	0	0	0	0	0	0	0	0	hsa_circ_0002813	NC_000010.11	-	31908172	31922960	14789	13191	sense-overlappin	NM_001270695.1	ARHGAP12
circRNA_07064	0	0	0	0	0	0.031069364	0.020098685	0.030729443	0	0	0	0	hsa_circ_0007664	NC_000010.11	-	32017143	32019959	2817	557	sense-overlappin	NM_004521.2	KIF5B
circRNA_07065	0	0	0.021808645	0	0.03009467	0.020712909	0	0	0.030381792	0.050431207	0.06076569	0	hsa_circ_0006408	NC_000010.11	-	32019858	32021287	1430	274	sense-overlappin	NM_004521.2	KIF5B
circRNA_07066	0	0	0.021808645	0	0	0	0	0	0	0	0	0	-	NC_000010.11	-	32028428	32037607	9180	1227	sense-overlappin	NM_004521.2	KIF5B
circRNA_07067	0.041150049	0	0	0	0	0	0	0	0	0	0	0	-	NC_000010.11	-	32034690	32037607	2918	613	sense-overlappin	NM_004521.2	KIF5B
circRNA_07068	0	0	0	0	0.08025246	0	0	0	0	0	0	0	hsa_circ_0018131	NC_000010.11	-	32038163	32040457	2295	284	sense-overlappin	NM_004521.2	KIF5B

circRNA_07069	0	0	0	0	0	0	0	0	0.020254528	0	0	0	-	NC_000010.11	+	32451592	32456334	4743	507	sense-overlappin	NR_109827.1	
circRNA_07070	0.030862537	0	0	0	0	0.020712909	0	0	0	0	0	0	-	NC_000010.11	+	32451592	32463049	11458	561	sense-overlappin	NR_109827.1	
circRNA_07071	0.030862537	0.051174919	0.152660515	0.0722267	0	0.020712909	0.030148028	0.09218833	0.070890847	0.07060369	0	0.0206364	-	NC_000010.11	+	32451592	32474023	22432	847	sense-overlappin	NM_001026383.2	CCDC7
circRNA_07072	0	0	0.032712968	0	0	0	0	0	0	0.030258724	0	0	-	NC_000010.11	+	32451592	32481782	30191	8606	sense-overlappin	NM_001026383.2	CCDC7
circRNA_07073	0.020575025	0	0	0	0	0	0	0	0.020254528	0	0	0	-	NC_000010.11	+	32462683	32474023	11341	340	sense-overlappin	NM_001026383.2	CCDC7
circRNA_07074	0	0	0	0.0206362	0	0	0	0	0	0.040344966	0	0	hsa_circ_0002351	NC_000010.11	+	32471064	32474023	2960	286	sense-overlappin	NM_001026383.2	CCDC7
circRNA_07075	0	0	0	0	0	0	0	0	0	0	0.02025523	0	-	NC_000010.11	+	32472481	32544301	71821	457	sense-overlappin	NM_001026383.2	CCDC7
circRNA_07076	0.061725074	0.051174919	0.239895095	0.0722267	0.21066271	0.134633911	0.100493427	0.071702034	0.141781694	0.332845966	0.070893305	0.0619092	hsa_circ_0000233	NC_000010.11	+	32543300	32584304	41005	808	sense-overlappin	XM_006717504.1	CCDC7
circRNA_07077	0	0.020469968	0	0	0	0	0	0	0	0	0	0	-	NC_000010.11	+	32543300	32595194	51895	11698	sense-overlappin	XM_006717504.1	CCDC7
circRNA_07078	0	0	0	0	0.04012623	0	0	0.020486295	0.040509055	0	0	0	-	NC_000010.11	+	32543300	32623817	80518	40321	sense-overlappin	XM_006717504.1	CCDC7
circRNA_07079	0	0	0	0	0	0	0	0.020486295	0	0	0	0	-	NC_000010.11	+	32543300	32625144	81845	41648	sense-overlappin	XM_006717504.1	CCDC7
circRNA_07080	0	0	0.021808645	0	0	0	0	0	0	0	0	0	-	NC_000010.11	+	32558986	32584304	25319	7239	sense-overlappin	XM_006717504.1	CCDC7
circRNA_07081	0.051437562	0.061409903	0.152660515	0.0722267	0.10031557	0.041425819	0.050246713	0.040972591	0.070890847	0.151293621	0.030382845	0	hsa_circ_0008679	NC_000010.11	+	32565558	32584304	18747	667	sense-overlappin	XM_006717504.1	CCDC7
circRNA_07082	0	0	0	0	0	0	0	0	0	0.020172483	0	0	-	NC_000010.11	+	32565558	32623817	58260	40180	sense-overlappin	XM_006717504.1	CCDC7
circRNA_07083	0	0	0	0	0	0	0.020098685	0	0	0	0	0	-	NC_000010.11	-	34248667	34269899	21233	21233	sense-overlappin	XM_011519582.1	PARD3
circRNA_07084	0.020575025	0	0	0	0	0	0	0	0	0	0	0	-	NC_000010.11	-	34251602	34336243	84642	18914	sense-overlappin	XM_011519582.1	PARD3
circRNA_07085	0.154312685	0.040939935	0.196277805	0.1341352	0.06018934	0.082851637	0.040197371	0.061458886	0.091145375	0.080689931	0.030382845	0	hsa_circ_0018168	NC_000010.11	-	34269657	34284245	14589	354	sense-overlappin	XM_011519582.1	PARD3
circRNA_07086	0	0	0	0	0	0	0.020098685	0	0	0	0	0	-	NC_000010.11	-	34317107	34360259	43153	1367	sense-overlappin	XM_011519582.1	PARD3
circRNA_07087	0	0	0	0	0	0	0	0.020486295	0	0	0	0	-	NC_000010.11	-	34331117	34384254	53138	1952	sense-overlappin	XM_011519582.1	PARD3
circRNA_07088	0.020575025	0	0	0	0	0	0	0	0.030381792	0	0	0	hsa_circ_0005486	NC_000010.11	-	34359147	34384254	25108	1177	sense-overlappin	XM_011519582.1	PARD3
circRNA_07089	0.030862537	0.030704951	0	0	0	0	0	0	0	0	0	0	hsa_circ_0018180	NC_000010.11	-	34372498	34384254	11757	817	sense-overlappin	XM_011519582.1	PARD3
circRNA_07090	0	0	0	0	0.02006311	0	0	0	0	0	0	0	-	NC_000010.11	-	34459465	34460573	1109	1109	intronic	XM_011519574.1	PARD3
circRNA_07091	0.020575025	0	0	0	0	0	0	0	0	0	0	0	-	NC_000010.11	-	34470085	34517159	47075	360	sense-overlappin	XM_011519574.1	PARD3
circRNA_07092	0	0	0	0	0	0	0	0	0	0.020172483	0	0	-	NC_000010.11	-	35013699	35031619	17921	819	sense-overlappin	NM_001198778.1	CUL2
circRNA_07093	0	0	0	0	0	0.020712909	0	0	0	0	0	0	-	NC_000010.11	-	35029488	35033273	3786	537	sense-overlappin	NM_001198778.1	CUL2
circRNA_07094	0	0	0	0.0309543	0.02006311	0	0	0	0	0	0	0	hsa_circ_0018188	NC_000010.11	-	35032435	35044868	12434	664	sense-overlappin	NM_001198778.1	CUL2
circRNA_07095	0	0	0.021808645	0	0	0	0	0	0	0	0	0	-	NC_000010.11	-	35044566	35071339	26774	736	sense-overlappin	NM_001198778.1	CUL2
circRNA_07096	0.020575025	0	0.065425935	0.0206362	0.02006311	0.020712909	0.020098685	0	0.040509055	0.030258724	0.02025523	0	hsa_circ_0000234	NC_000010.11	-	35060874	35071339	10466	339	sense-overlappin	NM_001198778.1	CUL2
circRNA_07097	0	0	0	0	0.02006311	0	0	0	0	0	0	0	hsa_circ_0018195	NC_000010.11	+	35178889	35207051	28163	623	sense-overlappin	XM_011519326.1	CREM
circRNA_07098	0	0	0	0	0	0.020712909	0	0	0	0	0	0	-	NC_000010.11	+	35447185	35457675	10491	10491	sense-overlappin	XM_011519358.1	CCNY
circRNA_07099	0.030862537	0.020469968	0.032712968	0	0.03009467	0.020712909	0	0.030729443	0	0.020172483	0	0	hsa_circ_0000235	NC_000010.11	+	35516523	35530243	13721	315	sense-overlappin	XM_011519362.1	CCNY
circRNA_07100	0	0	0	0	0	0.020712909	0	0	0	0	0	0	hsa_circ_0018206	NC_000010.11	+	35516523	35553185	36663	482	sense-overlappin	XM_011519362.1	CCNY
circRNA_07101	0	0	0	0	0	0	0	0.020486295	0	0	0	0	-	NC_000010.11	+	35529973	35553185	23213	345	sense-overlappin	XM_011519362.1	CCNY
circRNA_07102	0	0	0	0	0	0	0	0	0	0	0.02025523	0	-	NC_000010.11	+	42787168	42790511	3344	269	sense-overlappin	NM_014753.3	BMS1
circRNA_07103	0	0	0	0	0.02006311	0	0	0	0	0	0	0	hsa_circ_0018242	NC_000010.11	+	42816599	42823784	7186	1127	sense-overlappin	NM_014753.3	BMS1
circRNA_07104	0.030862537	0	0.021808645	0	0.05015779	0.041425819	0.020098685	0	0.020254528	0.020172483	0	0.0206364	hsa_circ_0000236	NC_000010.11	+	43154897	43158931	4035	1131	sense-overlappin	XM_011539938.1	CSGALNACT2
circRNA_07105	0	0	0.021808645	0	0	0	0	0	0	0	0	0	-	NC_000010.11	+	43154897	43167098	12202	1625	sense-overlappin	XR_945783.1	
circRNA_07106	0.030862537	0.020469968	0.065425935	0.0515905	0.02006311	0	0.040197371	0	0.060763583	0.030258724	0	0	-	NC_000010.11	+	43154897	43176032	21136	1707	sense-overlappin	XR_945783.1	
circRNA_07107	0	0	0	0.0309543	0	0	0	0	0	0	0	0	-	NC_000010.11	-	45533110	45553204	20095	20095	sense-overlappin	XM_006717705.1	8-Mar
circRNA_07108	0	0	0.04361729	0.0309543	0	0	0	0	0	0	0	0	-	NC_000010.11	-	45625951	45627105	1155	1155	exonic	XM_011540368.1	ZFAND4
circRNA_07109	0	0	0	0.0206362	0	0	0	0	0	0.020172483	0	0	-	NC_000010.11	-	45625951	45639963	14013	1303	sense-overlappin	XM_011540368.1	ZFAND4
circRNA_07110	0	0	0.054521613	0	0	0	0	0	0	0	0	0	-	NC_000010.11	-	45639816	45653059	13244	714	sense-overlappin	NR_104256.1	
circRNA_07111	0	0	0.065425935	0.0309543	0	0	0.020098685	0	0	0.030258724	0	0	-	NC_000010.11	-	45639816	45663842	24027	936	sense-overlappin	NM_001282905.1	ZFAND4
circRNA_07112	0	0	0.04361729	0	0.02006311	0	0	0	0	0	0	0	hsa_circ_0018281	NC_000010.11	-	45663542	45663842	301	301	exonic	XM_011540366.1	ZFAND4
circRNA_07113	0	0	0	0	0	0.031069364	0.020098685	0	0	0.050431207	0	0	hsa_circ_0004915	NC_000010.11	-	45939361	45962994	23634	23634	intergenic		
circRNA_07114	0	0	0	0	0	0	0.030148028	0	0	0.030258724	0	0	-	NC_000010.11	-	45939361	45965855	26495	26495	intergenic		
circRNA_07115	0.020575025	0	0	0	0	0.020712909	0	0	0	0.020172483	0	0	-	NC_000010.11	-	46010223	46011206	984	984	exonic	NM_001145262.1	NCOA4
circRNA_07116	0	0	0	0	0	0	0	0.030729443	0	0	0	0	-	NC_000010.11	+	48325897	48404981	79085	370	sense-overlappin	XM_011539953.1	MAPK8
circRNA_07117	0	0	0.032712968	0	0	0	0	0	0	0	0	0	hsa_circ_0018315	NC_000010.11	+	48401612	48404981	3370	301	sense-overlappin	NM_001278547.1	MAPK8
circRNA_07118	0.051437562	0.040939935	0.021808645	0.0412724	0.04012623	0.072495183	0	0.030729443	0.050636319	0.080689931	0.050638075	0	hsa_circ_0002968	NC_000010.11	+	48401612	48410168	8557	499	sense-overlappin	NM_001278547.1	MAPK8
circRNA_07119	0	0	0	0	0.02006311	0	0	0	0	0	0	0	hsa_circ_0018341	NC_000010.11	-	49857306	49935142	77837	2136	sense-overlappin	XM_011540306.1	PARG
circRNA_07120	0.020575025	0.051174919	0	0	0	0	0.080394741	0	0.101272639	0.020172483	0	0.0309546	hsa_circ_0018343	NC_000010.11	-	49915917	49935142	19226	1520	sense-overlappin	XM_011540306.1	PARG
circRNA_07121	0	0	0	0	0	0	0	0	0	0	0.02025523	0	-	NC_000010.11	-	49932100	49934163	2064	1171	sense-overlappin	XM_011540306.1	PARG
circRNA_07122	0	0	0	0.0619086	0	0.020712909	0	0	0	0.020172483	0	0	-	NC_000010.11	-	49932100	49935142	3043	1238	sense-overlappin	XM_011540306.1	PARG
circRNA_07123	0	0	0	0.0206362	0	0.031069364	0	0	0	0.030258724	0	0	hsa_circ_0006820	NC_000010.11	+	49952126	49958480	6355	349	sense-overlappin	NM_001290117.1	TIMM23B

circRNA_07124	0	0.030704951	0	0	0	0.031069364	0.030148028	0.030729443	0	0.040344966	0.030382845	0	-	NC_000010.11	-		50433476	50466932	33457	266	sense-overlappin	NM_147156.3	SGMS1
circRNA_07125	0	0	0	0	0	0	0	0.020486295	0	0	0.02025523	0	hsa_circ_0018402	NC_000010.11	-		50460673	50466932	6260	185	sense-overlappin	NM_147156.3	SGMS1
circRNA_07126	0	0	0	0	0	0	0	0	0.020254528	0	0	0	hsa_circ_0001961	NC_000010.11	+		51148082	51157148	9067	9067	sense-overlappin	NM_001098512.2	PRKG1
circRNA_07127	0.030862537	0	0	0	0	0	0	0	0	0	0	0	hsa_circ_0003030	NC_000010.11	+		52280789	52290290	9502	559	sense-overlappin	NM_001098512.2	PRKG1
circRNA_07128	0	0	0	0	0	0	0	0	0.020254528	0	0	0	-	NC_000010.11	+		58394358	58395342	985	472	sense-overlappin	NM_001270782.1	TFAM
circRNA_07129	0	0	0.054521613	0	0	0	0	0	0	0	0.02025523	0	-	NC_000010.11	+		58588215	58620901	32687	28800	sense-overlappin	XM_011540187.1	BICC1
circRNA_07130	0	0.020469968	0	0	0	0	0	0	0	0	0	0	-	NC_000010.11	+		58789262	58793615	4354	579	sense-overlappin	XM_011540187.1	BICC1
circRNA_07131	0	0	0	0	0	0	0	0	0	0.020172483	0	0	-	NC_000010.11	-		60084602	60085253	652	326	sense-overlappin	NM_001149.3	ANK3
circRNA_07132	0	0	0	0	0	0	0	0	0.020254528	0	0	0	-	NC_000010.11	-		63183447	63184738	1292	254	sense-overlappin	XM_011539505.1	JMJD1C
circRNA_07133	0	0	0	0	0	0.031069364	0	0	0.020254528	0.020172483	0	0	hsa_circ_0002313	NC_000010.11	-		63380318	63404965	24648	24648	sense-overlappin	NM_032776.2	JMJD1C
circRNA_07134	0	0	0.021808645	0	0	0	0	0	0	0	0	0	hsa_circ_0005658	NC_000010.11	-		63453396	63455089	1694	1694	intronic	NM_032776.2	JMJD1C
circRNA_07135	0	0	0.021808645	0	0	0	0.020098685	0	0	0	0	0.0206364	hsa_circ_0007626	NC_000010.11	+		63610187	63619800	9614	294	sense-overlappin	NM_001001330.2	REEP3
circRNA_07136	0.030862537	0	0	0	0.09028402	0	0	0	0	0.020172483	0	0	-	NC_000010.11	+		67887417	67891554	4138	512	sense-overlappin	NM_012238.4	SIRT1
circRNA_07137	0	0	0	0	0.02006311	0	0	0	0	0	0	0	-	NC_000010.11	+		67894974	67895137	164	164	intronic	XM_006717737.2	SIRT1
circRNA_07138	0	0	0	0	0	0	0	0.020486295	0	0	0	0	-	NC_000010.11	+		67908046	67909442	1397	267	sense-overlappin	XM_006717737.2	SIRT1
circRNA_07139	0.030862537	0	0	0	0.03009467	0.041425819	0	0	0.030381792	0.020172483	0	0	hsa_circ_0018478	NC_000010.11	-		67954595	68014186	59592	1453	sense-overlappin	NM_001278185.1	HERC4
circRNA_07140	0	0	0	0.0206362	0	0	0	0	0	0	0	0	-	NC_000010.11	-		67954595	68038169	83575	1975	sense-overlappin	NM_001278185.1	HERC4
circRNA_07141	0	0	0	0	0	0.041425819	0	0.040972591	0	0	0	0	-	NC_000010.11	-		67966683	68014186	47504	1018	sense-overlappin	NM_001278185.1	HERC4
circRNA_07142	0	0	0	0	0.03009467	0	0.020098685	0	0.020254528	0	0	0	hsa_circ_0018484	NC_000010.11	-		67988663	68014186	25524	898	sense-overlappin	NM_001278185.1	HERC4
circRNA_07143	0	0	0	0.0309543	0	0	0	0	0	0	0	0	-	NC_000010.11	-		67988663	68044558	55896	1647	sense-overlappin	XM_011539595.1	HERC4
circRNA_07144	0	0	0	0	0	0	0	0	0	0	0.030382845	0	hsa_circ_0004472	NC_000010.11	-		68025546	68044558	19013	749	sense-overlappin	XM_011539595.1	HERC4
circRNA_07145	0.020575025	0	0.032712968	0.0309543	0	0	0.030148028	0.020486295	0.040509055	0	0	0.0412728	hsa_circ_0007113	NC_000010.11	-		68025546	68044563	19018	754	sense-overlappin	XM_011539595.1	HERC4
circRNA_07146	0	0	0.021808645	0	0	0.020712909	0	0	0	0	0	0	hsa_circ_0006031	NC_000010.11	-		68355353	68364113	8761	274	sense-overlappin	NR_103475.1	
circRNA_07147	0	0	0	0	0	0	0	0	0.020254528	0.030258724	0	0	-	NC_000010.11	-		68355353	68386128	30776	949	sense-overlappin	NR_103475.1	
circRNA_07148	0	0	0	0	0	0.020712909	0	0	0	0	0	0	-	NC_000010.11	-		68363590	68364113	524	225	sense-overlappin	NR_103475.1	
circRNA_07149	0	0.051174919	0	0.0309543	0	0.041425819	0.060296056	0	0.030381792	0	0	0.0309546	hsa_circ_0000239	NC_000010.11	-		68393138	68394451	1314	252	sense-overlappin	NR_103475.1	
circRNA_07150	0.020575025	0	0	0	0.02006311	0	0	0	0	0	0	0	-	NC_000010.11	-		68393138	68396881	3744	354	sense-overlappin	NR_103475.1	
circRNA_07151	0	0	0.04361729	0	0.02006311	0	0	0	0.020254528	0	0	0	-	NC_000010.11	-		68393138	68401737	8600	472	sense-overlappin	NR_103475.1	
circRNA_07152	0	0.020469968	0	0	0	0	0	0	0	0	0	0	-	NC_000010.11	-		68393138	68404844	11707	646	sense-overlappin	NR_103475.1	
circRNA_07153	0	0	0	0	0.02006311	0	0	0	0	0	0	0	hsa_circ_0005177	NC_000010.11	-		68394075	68394451	377	186	sense-overlappin	NR_103475.1	
circRNA_07154	0	0	0.032712968	0	0	0	0	0	0	0	0	0	-	NC_000010.11	-		68401620	68401737	118	118	exonic	NR_103475.1	
circRNA_07155	0	0	0.021808645	0	0	0	0	0	0	0	0	0	-	NC_000010.11	-		68442917	68470163	27247	1341	sense-overlappin	NM_001080449.2	DNA2
circRNA_07156	0	0	0.032712968	0.0206362	0.02006311	0	0.030148028	0	0	0	0	0	hsa_circ_0009172	NC_000010.11	-		68459104	68470163	11060	645	sense-overlappin	NM_001080449.2	DNA2
circRNA_07157	0.030862537	0.051174919	0.109043225	0.0619086	0.09028402	0	0.060296056	0.020486295	0	0.100862414	0.050638075	0	hsa_circ_0006151	NC_000010.11	-		68468123	68470163	2041	367	sense-overlappin	NM_001080449.2	DNA2
circRNA_07158	0	0	0.021808645	0.0619086	0	0	0	0	0	0	0	0	hsa_circ_0002920	NC_000010.11	-		68483420	68493570	10151	590	sense-overlappin	XM_011540212.1	SLC25A16
circRNA_07159	0	0	0.021808645	0.0928628	0	0.031069364	0.030148028	0	0.070890847	0.060517448	0	0	hsa_circ_0003459	NC_000010.11	-		68487144	68493570	6427	421	sense-overlappin	XM_011540212.1	SLC25A16
circRNA_07160	0	0	0	0.0206362	0	0	0	0	0	0	0	0	hsa_circ_0002624	NC_000010.11	-		68487144	68503695	16552	530	sense-overlappin	XM_005270183.3	SLC25A16
circRNA_07161	0	0	0.021808645	0.0722267	0	0	0	0	0	0.030258724	0	0	hsa_circ_0007778	NC_000010.11	-		68487144	68506718	19575	619	sense-overlappin	XM_011540212.1	SLC25A16
circRNA_07162	0	0	0	0	0	0	0	0	0	0.020172483	0	0	hsa_circ_0008564	NC_000010.11	-		68487144	68516843	29700	712	sense-overlappin	XM_011540212.1	SLC25A16
circRNA_07163	0.051437562	0	0	0	0	0	0	0	0	0	0	0	-	NC_000010.11	-		68522738	68523196	459	459	intronic	XM_011540212.1	SLC25A16
circRNA_07164	0.030862537	0.020469968	0.021808645	0	0	0.082851637	0.020098685	0.061458886	0.050636319	0.050431207	0.030382845	0.0309546	-	NC_000010.11	+		68644698	68647005	2308	2308	exonic	XM_011540206.1	TET1
circRNA_07165	0	0	0	0	0	0	0	0	0.030381792	0	0	0	-	NC_000010.11	+		68644698	68652594	7897	2493	sense-overlappin	XM_011540206.1	TET1
circRNA_07166	0	0	0	0	0	0	0	0	0	0.020172483	0	0	-	NC_000010.11	+		68644698	68667256	22559	2705	sense-overlappin	NM_030625.2	TET1
circRNA_07167	0	0.061409903	0.032712968	0.0619086	0.03009467	0	0	0	0.030381792	0	0.050638075	0	hsa_circ_0006272	NC_000010.11	+		68737845	68742569	4725	272	sense-overlappin	NM_018237.3	CCAR1
circRNA_07168	0	0	0	0.0206362	0	0	0	0	0	0	0	0	-	NC_000010.11	+		68740629	68749685	9057	827	sense-overlappin	NM_001282959.1	CCAR1
circRNA_07169	0	0.020469968	0	0.0206362	0	0	0	0	0	0	0	0	hsa_circ_0018553	NC_000010.11	+		68747161	68747566	406	308	sense-overlappin	NM_001282959.1	CCAR1
circRNA_07170	0.020575025	0	0	0.0206362	0	0	0	0	0	0	0	0	hsa_circ_0000240	NC_000010.11	+		68753852	68773099	19248	1532	sense-overlappin	NM_001282959.1	CCAR1
circRNA_07171	0	0	0	0.0412724	0.02006311	0	0	0	0	0.020172483	0	0.0206364	hsa_circ_0018557	NC_000010.11	+		68754714	68757377	2664	576	sense-overlappin	NM_001282959.1	CCAR1
circRNA_07172	0	0	0	0	0	0.031069364	0	0	0	0	0	0	-	NC_000010.11	+		68761007	68773099	12093	730	sense-overlappin	NM_001282959.1	CCAR1
circRNA_07173	0	0	0	0.0206362	0	0	0	0	0	0	0	0	-	NC_000010.11	+		68771206	68773099	1894	352	sense-overlappin	NM_001282959.1	CCAR1
circRNA_07174	0	0	0	0	0.02006311	0	0	0	0	0	0	0	hsa_circ_0002371	NC_000010.11	+		68786136	68786692	557	230	sense-overlappin	NM_001282959.1	CCAR1
circRNA_07175	0	0	0	0	0.02006311	0	0.020098685	0	0	0	0	0	-	NC_000010.11	+		68911068	68914204	3137	629	sense-overlappin	XM_011540144.1	DDX50
circRNA_07176	0	0	0	0	0	0	0.040197371	0	0	0	0	0	-	NC_000010.11	+		68913162	68914204	1043	450	sense-overlappin	XM_011540144.1	DDX50
circRNA_07177	0	0	0	0	0	0	0	0	0	0.020172483	0	0	hsa_circ_0007097	NC_000010.11	+		68936936	68943257	6322	340	sense-overlappin	XM_011540144.1	DDX50
circRNA_07178	0.092587611	0.071644886	0.13085187	0.0825448	0.15047336	0.051782273	0.130641455	0.081945182	0.060763583	0.121034897	0.10127615	0.0309546	hsa_circ_0008865	NC_000010.11	+		68959806	68960249	444	444	exonic	NM_004728.3	DDX21

circRNA_07179	0	0	0	0	0	0	0.050246713	0	0	0	0	0	-	NC_000010.11	+	68959806	68963469	3664	699	sense-overlappin	NM_004728.3	DDX21
circRNA_07180	0	0	0	0	0.02006311	0	0	0	0	0	0	0	-	NC_000010.11	+	68959806	68972052	12247	1461	sense-overlappin	NM_004728.3	DDX21
circRNA_07181	0	0	0	0	0.03009467	0	0	0	0	0	0	0	hsa_circ_0018600	NC_000010.11	+	69185987	69187756	1770	301	sense-overlappin	NM_001301683.1	SUPV3L1
circRNA_07182	0	0	0.032712968	0	0	0	0	0	0	0	0	0	hsa_circ_0018601	NC_000010.11	+	69185987	69189435	3449	470	sense-overlappin	NM_001301683.1	SUPV3L1
circRNA_07183	0	0	0.021808645	0	0	0	0	0	0	0	0	0	hsa_circ_0018620	NC_000010.11	+	69376934	69380095	3162	390	sense-overlappin	XM_005269736.1	HK1
circRNA_07184	0	0	0	0	0	0	0	0	0	0.020172483	0	0	-	NC_000010.11	+	69382487	69386418	3932	670	sense-overlappin	XM_005269736.1	HK1
circRNA_07185	0.030862537	0	0	0	0.02006311	0	0	0	0	0	0	0	hsa_circ_0018624	NC_000010.11	+	69392125	69398828	6704	574	sense-overlappin	XM_005269736.1	HK1
circRNA_07186	0.030862537	0	0.021808645	0.0515905	0.06018934	0	0	0.020486295	0.030381792	0.030258724	0.04051046	0	hsa_circ_0002758	NC_000010.11	+	69483691	69485215	1525	261	sense-overlappin	XM_006717738.2	TSPAN15
circRNA_07187	0	0	0	0	0.02006311	0	0	0	0	0	0	0	-	NC_000010.11	+	69483691	69495689	11999	357	sense-overlappin	XM_006717738.2	TSPAN15
circRNA_07188	0	0	0.032712968	0	0.02006311	0	0	0	0	0.030258724	0	0	-	NC_000010.11	+	69483691	69498396	14706	474	sense-overlappin	XM_006717738.2	TSPAN15
circRNA_07189	0	0	0	0	0.02006311	0	0	0	0	0	0	0	-	NC_000010.11	+	69498280	69511870	13591	5324	sense-overlappin	NM_012339.3	TSPAN15
circRNA_07190	0	0	0	0	0	0	0.020098685	0	0	0	0	0	-	NC_000010.11	-	70152263	70161069	8807	632	sense-overlappin	NM_001142648.1	SAR1A
circRNA_07191	0	0.030704951	0.032712968	0	0	0	0	0.020486295	0	0	0	0	hsa_circ_0005624	NC_000010.11	-	70152263	70161931	9669	826	sense-overlappin	NM_001142648.1	SAR1A
circRNA_07192	0	0	0	0	0	0	0	0	0	0.040344966	0.02025523	0	-	NC_000010.11	-	70204873	70209685	4813	327	sense-overlappin	NM_021129.3	PPA1
circRNA_07193	0.030862537	0	0	0	0	0	0	0	0	0	0	0	-	NC_000010.11	-	70209205	70218817	9613	602	sense-overlappin	NM_021129.3	PPA1
circRNA_07194	0	0	0	0	0	0.020712909	0	0	0	0	0	0	-	NC_000010.11	-	70213463	70218817	5355	388	sense-overlappin	NM_021129.3	PPA1
circRNA_07195	0	0	0	0	0	0	0	0	0	0	0.02025523	0	-	NC_000010.11	-	70339996	70340702	707	707	sense-overlappin	NM_207119.2	LRRC20
circRNA_07196	0	0	0	0	0	0	0	0	0	0.020172483	0	0	-	NC_000010.11	+	70427797	70428054	258	258	exonic	NM_004096.4	EIF4EBP2
circRNA_07197	0	0	0	0	0.02006311	0	0	0	0	0	0	0	hsa_circ_0004606	NC_000010.11	+	70702312	70708778	6467	348	sense-overlappin	XM_011539305.1	ADAMTS14
circRNA_07198	0.051437562	0.030704951	0.021808645	0	0	0	0.020098685	0	0.070890847	0	0	0	hsa_circ_0004977	NC_000010.11	+	70844473	70859499	15027	588	sense-overlappin	XM_011540316.1	SGPL1
circRNA_07199	0	0	0.021808645	0	0	0	0	0	0	0	0	0	-	NC_000010.11	+	70844473	70868433	23961	677	sense-overlappin	XM_011540316.1	SGPL1
circRNA_07200	0	0	0.021808645	0	0	0	0	0	0	0	0	0	hsa_circ_0008145	NC_000010.11	-	72196811	72213331	16521	606	sense-overlappin	NM_001198799.2	ASCC1
circRNA_07201	0	0	0	0	0	0	0.020098685	0	0	0	0	0	-	NC_000010.11	+	72223888	72230440	6553	244	sense-overlappin	NM_001242546.1	ANAPC16
circRNA_07202	0.102875123	0	0	0	0.03009467	0.031069364	0.040197371	0.020486295	0	0	0.04051046	0.0309546	hsa_circ_0008662	NC_000010.11	-	72340789	72341170	382	266	sense-overlappin	NM_001002762.2	DNAJB12
circRNA_07203	0	0	0	0	0	0	0	0	0	0	0.131658996	0	-	NC_000010.11	-	72475100	72566794	91695	952	sense-overlappin	XM_005269383.1	MICU1
circRNA_07204	0	0	0.065425935	0	0	0	0	0	0	0	0	0	-	NC_000010.11	-	72551179	72566794	15616	494	sense-overlappin	NM_001195518.1	MICU1
circRNA_07205	0	0	0.196277805	0	0	0	0	0	0.040509055	0.020172483	0.02025523	0	hsa_circ_0006628	NC_000010.11	-	72562895	72566794	3900	331	sense-overlappin	NM_001195518.1	MICU1
circRNA_07206	0.13373766	0.051174919	0.468885868	0.0309543	0.04012623	0.062138728	0.020098685	0.081945182	0.060763583	0.040344966	0.050638075	0.051591	hsa_circ_0000246	NC_000010.11	+	72708283	72715902	7620	6993	sense-overlappin	NR_073062.1	
circRNA_07207	0	0	0.021808645	0	0	0	0	0	0	0	0	0	-	NC_000010.11	+	72708283	72719445	11163	10536	sense-overlappin	NR_073062.1	
circRNA_07208	0	0	0.054521613	0	0	0	0.020098685	0	0	0	0	0	-	NC_000010.11	+	72708283	72741845	33563	32936	sense-overlappin	NR_073062.1	
circRNA_07209	0	0	0.021808645	0	0	0	0	0	0	0	0	0	-	NC_000010.11	+	72708283	72742273	33991	33364	sense-overlappin	NR_073062.1	
circRNA_07210	0.030862537	0.030704951	0.065425935	0	0.02006311	0.020712909	0	0.040972591	0.020254528	0.030258724	0.02025523	0	hsa_circ_0000247	NC_000010.11	+	72715111	72715902	792	165	sense-overlappin	NR_073062.1	
circRNA_07211	0	0	0.065425935	0	0	0	0	0	0	0	0	0	-	NC_000010.11	+	72715111	72719445	4335	3708	sense-overlappin	NR_073062.1	
circRNA_07212	0	0	0.021808645	0	0	0	0	0	0	0	0	0	-	NC_000010.11	+	72715111	72736571	21461	20834	sense-overlappin	NR_073062.1	
circRNA_07213	0	0	0	0	0	0.041425819	0	0	0	0	0	0	-	NC_000010.11	+	72741936	72799021	57086	57086	intronic	NM_001270679.1	MCU
circRNA_07214	0	0	0.032712968	0	0	0	0	0	0	0	0	0	-	NC_000010.11	+	72790176	72834428	44253	44253	sense-overlappin	NM_001270679.1	MCU
circRNA_07215	0	0	0	0	0.02006311	0	0	0	0	0	0	0	hsa_circ_0007093	NC_000010.11	-	73030271	73047101	16831	348	sense-overlappin	NM_000917.3	P4HA1
circRNA_07216	0	0	0	0	0	0	0	0	0	0.020172483	0	0	hsa_circ_0018774	NC_000010.11	-	73072029	73074915	2887	357	sense-overlappin	NM_000917.3	P4HA1
circRNA_07217	0	0	0.032712968	0	0	0	0	0	0	0	0	0	-	NC_000010.11	-	73134603	73148404	13802	1102	sense-overlappin	NM_001135752.1	ECD
circRNA_07218	0	0	0.032712968	0	0	0	0	0	0	0	0	0	hsa_circ_0007995	NC_000010.11	-	73146276	73163950	17675	1140	sense-overlappin	NM_001135752.1	ECD
circRNA_07219	0	0	0.021808645	0	0	0	0	0	0	0	0	0	hsa_circ_0018785	NC_000010.11	-	73154256	73156655	2400	460	sense-overlappin	NM_001135752.1	ECD
circRNA_07220	0	0	0.021808645	0	0	0	0	0	0	0	0	0	-	NC_000010.11	+	73174687	73193593	18907	493	sense-overlappin	XM_011539737.1	FAM149B1
circRNA_07221	0.092587611	0	0.316225353	0	0	0	0	0	0	0	0.050638075	0	hsa_circ_0006276	NC_000010.11	-	73396519	73400857	4339	436	sense-overlappin	NM_001156.3	ANXA7
circRNA_07222	0	0.020469968	0	0	0.02006311	0.031069364	0	0	0	0	0	0	hsa_circ_0018814	NC_000010.11	-	73454412	73479517	25106	1155	sense-overlappin	NM_001289968.1	PPP3CB
circRNA_07223	0	0	0	0.0206362	0	0	0	0	0	0.030258724	0	0	hsa_circ_0018815	NC_000010.11	-	73467553	73479517	11965	1077	sense-overlappin	NM_001289968.1	PPP3CB
circRNA_07224	0.041150049	0	0	0.0412724	0	0	0	0	0	0	0	0	-	NC_000010.11	-	73474919	73479517	4599	492	sense-overlappin	NM_001289968.1	PPP3CB
circRNA_07225	0	0	0.021808645	0	0	0	0	0	0	0	0	0	-	NC_000010.11	-	73516375	73539593	23219	3226	sense-overlappin	NM_152586.3	USP54
circRNA_07226	0	0	0.054521613	0	0	0	0	0	0	0	0	0	-	NC_000010.11	-	73529680	73530835	1156	745	sense-overlappin	NM_152586.3	USP54
circRNA_07227	0	0	0.021808645	0	0	0	0	0	0	0	0	0	-	NC_000010.11	-	73529680	73539593	9914	1235	sense-overlappin	NM_152586.3	USP54
circRNA_07228	0	0	0.098138903	0.0412724	0	0	0	0.020486295	0	0	0	0	hsa_circ_0004568	NC_000010.11	-	73536269	73539593	3325	319	sense-overlappin	NM_152586.3	USP54
circRNA_07229	0	0	0.17446916	0	0	0	0	0	0	0	0	0.0309546	hsa_circ_0007221	NC_000010.11	-	73571421	73576361	4941	943	sense-overlappin	XM_011539364.1	USP54
circRNA_07230	0	0	0.065425935	0	0	0	0.030148028	0	0	0	0	0	-	NC_000010.11	-	73575512	73576361	850	850	sense-overlappin	XM_011539364.1	USP54
circRNA_07231	0	0	0.021808645	0	0	0	0	0	0	0	0	0	-	NC_000010.11	-	73575512	73576621	1110	1110	sense-overlappin	XM_011539364.1	USP54
circRNA_07232	0	0	0	0.0515905	0	0	0	0	0	0	0	0	hsa_circ_0003397	NC_000010.11	-	73719160	73721504	2345	2345	intergenic		
circRNA_07233	0	0	0	0	0.04012623	0	0	0	0	0	0	0	hsa_circ_0004387	NC_000010.11	+	73746630	73747004	375	287	sense-overlappin	XM_011540381.1	SEC24C
circRNA_07234	0.020575025	0	0	0	0	0	0	0.040972591	0	0	0	0	-	NC_000010.11	-	73817015	73824084	7070	1074	sense-overlappin	XR_945823.1	

circRNA_07235	0.020575025	0.020469968	0	0	0	0.041425819	0	0	0	0.030258724	0	0	-	NC_000010.11	-	73817023	73824084	7062	1066	sense-overlappin	XR_945823.1	
circRNA_07236	0	0	0	0.0206362	0	0	0	0	0	0	0	0	-	NC_000010.11	+	73913290	73913758	469	312	sense-overlappin	XM_011539866.1	PLAU
circRNA_07237	0	0	0	0	0.02006311	0	0	0	0	0	0	0	hsa_circ_0018882	NC_000010.11	+	74070670	74083513	12844	786	sense-overlappin	XM_005270142.1	VCL
circRNA_07238	0	0	0	0	0	0	0.020098685	0	0	0	0	0	hsa_circ_0018889	NC_000010.11	+	74097204	74101097	3894	279	sense-overlappin	XM_005270142.1	VCL
circRNA_07239	0	0	0	0	0	0	0	0	0	0.030258724	0	0	-	NC_000010.11	+	74340621	74398579	57959	53802	sense-overlappin	NM_001202450.1	ADK
circRNA_07240	0	0	0	0	0	0	0	0	0	0	0.050638075	0	hsa_circ_0009121	NC_000010.11	+	74394141	74398579	4439	282	sense-overlappin	NM_001202450.1	ADK
circRNA_07241	0	0.040939935	0	0	0	0	0	0	0	0	0	0	hsa_circ_0018900	NC_000010.11	+	74589282	74600493	11212	151	sense-overlappin	NM_001202450.1	ADK
circRNA_07242	0.020575025	0	0	0.0309543	0	0	0	0	0	0	0	0	hsa_circ_0006148	NC_000010.11	+	75150514	75151106	593	593	exonic	XM_011539312.1	SAMD8
circRNA_07243	0.061725074	0	0	0	0	0	0	0	0	0	0	0	hsa_circ_0018904	NC_000010.11	+	75150514	75164740	14227	689	sense-overlappin	XM_011539312.1	SAMD8
circRNA_07244	0	0	0	0	0	0	0	0	0	0	0.04051046	0.0206364	hsa_circ_0007335	NC_000010.11	+	75219063	75219356	294	206	sense-overlappin	NM_001184823.1	VDAC2
circRNA_07245	0	0	0	0.0309543	0	0	0	0	0	0	0	0	-	NC_000010.11	+	76036008	76058783	22776	385	sense-overlappin	XM_011540258.1	C10orf11
circRNA_07246	0	0	0	0	0	0	0	0	0	0	0	0.0206364	-	NC_000010.11	-	76953801	76970067	16267	218	sense-overlappin	NM_001014797.2	KCNMA1
circRNA_07247	0.020575025	0	0	0	0	0	0	0	0	0	0	0	hsa_circ_0005967	NC_000010.11	-	77011967	77027891	15925	245	sense-overlappin	NM_001014797.2	KCNMA1
circRNA_07248	0	0	0	0	0.02006311	0	0	0	0	0	0	0	-	NC_000010.11	+	78037218	78037304	87	87	exonic	NM_001026.4	RPS24
circRNA_07249	0	0	0	0	0	0	0	0	0.030381792	0	0	0	hsa_circ_0018973	NC_000010.11	-	80053396	80053586	191	191	intergenic		
circRNA_07250	0	0	0	0	0	0	0	0	0.020254528	0	0	0	-	NC_000010.11	-	80161935	80166197	4263	436	sense-overlappin	XM_005269741.3	ANXA11
circRNA_07251	0.072012586	0	0	0	0	0	0	0	0	0	0	0	-	NC_000010.11	-	80170800	80176155	5356	497	sense-overlappin	NM_001278409.1	ANXA11
circRNA_07252	0	0	0	0	0	0	0	0	0	0.030258724	0	0	hsa_circ_0009033	NC_000010.11	+	80468547	80489314	20768	239	sense-overlappin	XM_011540219.1	TSPAN14
circRNA_07253	0.051437562	0.030704951	0.109043225	0.0206362	0.08025246	0.031069364	0.020098685	0.020486295	0.030381792	0	0.050638075	0	hsa_circ_0018992	NC_000010.11	+	84371014	84373815	2802	1653	sense-overlappin	XM_011539872.1	CCSER2
circRNA_07254	0	0	0.032712968	0	0	0	0	0	0	0	0	0	hsa_circ_0018993	NC_000010.11	+	84371014	84425893	54880	1907	sense-overlappin	XM_011539872.1	CCSER2
circRNA_07255	0	0.040939935	0	0	0	0	0	0	0	0	0	0	-	NC_000010.11	+	84380098	84477664	97567	38384	sense-overlappin	XM_005269905.2	CCSER2
circRNA_07256	0.092587611	0.051174919	0.152660515	0.0515905	0.0702209	0.186416184	0.080394741	0.09218833	0.101272639	0.191638587	0.06076569	0.0309546	hsa_circ_0006956	NC_000010.11	+	84417771	84425893	8123	254	sense-overlappin	XM_011539872.1	CCSER2
circRNA_07257	0.030862537	0.020469968	0.04361729	0.0206362	0.03009467	0	0	0	0	0.020172483	0.06076569	0	hsa_circ_0018996	NC_000010.11	+	84417771	84477664	59894	711	sense-overlappin	XM_005269905.2	CCSER2
circRNA_07258	0.020575025	0	0	0	0	0	0	0	0	0.020172483	0	0	-	NC_000010.11	+	84438282	84477664	39383	687	sense-overlappin	XM_005269905.2	CCSER2
circRNA_07259	0.102875123	0.020469968	0.098138903	0.0412724	0.05015779	0.062138728	0	0.020486295	0.070890847	0.050431207	0.030382845	0	hsa_circ_0018998	NC_000010.11	+	84438512	84477664	39153	457	sense-overlappin	XM_005269905.2	CCSER2
circRNA_07260	0	0	0	0.0309543	0.02006311	0	0.020098685	0	0	0.020172483	0	0	-	NC_000010.11	+	84463933	84477664	13732	261	sense-overlappin	XM_005269905.2	CCSER2
circRNA_07261	0	0	0	0	0	0	0	0	0	0.020172483	0	0	hsa_circ_0019005	NC_000010.11	-	86451967	86461287	9321	744	sense-overlappin	XM_011539547.1	WAPAL
circRNA_07262	0	0	0	0.0206362	0	0	0	0	0.020254528	0	0	0	hsa_circ_0019006	NC_000010.11	-	86451967	86473973	22007	1470	sense-overlappin	NM_015045.2	WAPAL
circRNA_07263	0	0	0	0.0309543	0	0	0	0	0	0	0	0	-	NC_000010.11	-	86467279	86473973	6695	726	sense-overlappin	NM_015045.2	WAPAL
circRNA_07264	0	0	0.032712968	0	0	0	0	0	0	0	0	0	hsa_circ_0002727	NC_000010.11	-	86470992	86473973	2982	498	sense-overlappin	NM_015045.2	WAPAL
circRNA_07265	0	0	0	0.0206362	0	0	0	0	0	0	0	0	-	NC_000010.11	-	87049401	87051870	2470	2470	sense-overlappin	NM_005271.3	GLUD1
circRNA_07266	0	0	0	0	0	0	0	0	0	0.020172483	0	0	-	NC_000010.11	-	87051583	87060241	8659	648	sense-overlappin	NM_005271.3	GLUD1
circRNA_07267	0	0.040939935	0	0	0.03009467	0.041425819	0.060296056	0	0	0	0	0	-	NC_000010.11	+	87151350	87152879	1530	1530	exonic	NM_019054.2	FAM35A
circRNA_07268	0	0	0	0	0.02006311	0	0	0	0	0	0	0	hsa_circ_0007229	NC_000010.11	-	87767673	87776427	8755	248	sense-overlappin	XM_011540302.1	ATAD1
circRNA_07269	0	0	0	0.2682705	0	0.072495183	0	0	0	0	0	0	-	NC_000010.11	+	88667287	88673734	6448	728	sense-overlappin	NM_001198828.1	LIPF
circRNA_07270	0	0	0	0.0515905	0	0	0	0	0	0	0	0	-	NC_000010.11	+	88667291	88673734	6444	724	sense-overlappin	NM_001198828.1	LIPF
circRNA_07271	0.041150049	0	0	0	0.02006311	0.020712909	0	0.020486295	0	0.030258724	0	0	hsa_circ_0008629	NC_000010.11	+	88893871	88905660	11790	530	sense-overlappin	XM_006717929.2	STAMBPL1
circRNA_07272	0	0	0	0	0	0	0	0	0.030381792	0	0	0	hsa_circ_0019061	NC_000010.11	+	88901656	88922436	20781	1307	sense-overlappin	XM_006717928.2	STAMBPL1
circRNA_07273	0	0	0.098138903	0.0309543	0.05015779	0.020712909	0.060296056	0.030729443	0	0.030258724	0.131658996	0.0619092	hsa_circ_0019079	NC_000010.11	+	89751346	89762835	11490	893	sense-overlappin	NM_001284259.1	KIF20B
circRNA_07274	0	0	0	0	0	0.031069364	0	0	0	0	0	0	hsa_circ_0019094	NC_000010.11	+	91240484	91271697	31214	611	sense-overlappin	NM_001257101.1	PCGF5
circRNA_07275	0.051437562	0	0.04361729	0	0.03009467	0	0	0	0.050636319	0	0	0	hsa_circ_0007168	NC_000010.11	+	91425281	91462184	36904	462	sense-overlappin	XM_011539337.1	HECTD2
circRNA_07276	0	0	0	0	0	0.051782273	0	0	0	0	0	0	hsa_circ_0019101	NC_000010.11	+	91460427	91462184	1758	332	sense-overlappin	XM_011539337.1	HECTD2
circRNA_07277	0	0	0	0	0	0	0	0.020486295	0	0	0	0	-	NC_000010.11	+	91817134	91836998	19865	1103	sense-overlappin	NM_025235.3	TNKS2
circRNA_07278	0	0.051174919	0.021808645	0.0309543	0	0	0	0	0	0	0	0	-	NC_000010.11	+	91819270	91842391	23122	1539	sense-overlappin	NM_025235.3	TNKS2
circRNA_07279	0	0	0	0.0309543	0	0	0	0	0.020254528	0	0	0	hsa_circ_0008332	NC_000010.11	+	91848383	91851336	2954	457	sense-overlappin	NM_025235.3	TNKS2
circRNA_07280	0	0	0	0	0	0	0	0.020486295	0	0	0	0	hsa_circ_0019120	NC_000010.11	+	91939952	91942568	2617	262	sense-overlappin	XM_011540329.1	BTAF1
circRNA_07281	0	0	0	0	0	0	0	0	0	0.020172483	0	0	-	NC_000010.11	+	91942422	91953873	11452	448	sense-overlappin	XM_011540329.1	BTAF1
circRNA_07282	0	0	0	0	0.02006311	0	0	0	0	0	0	0	hsa_circ_0019122	NC_000010.11	+	91951403	91953873	2471	301	sense-overlappin	XM_011540329.1	BTAF1
circRNA_07283	0.020575025	0	0	0	0	0.031069364	0	0	0	0.020172483	0	0	-	NC_000010.11	+	91951403	91966757	15355	1250	sense-overlappin	XM_011540329.1	BTAF1
circRNA_07284	0	0	0	0	0	0	0	0	0.020254528	0	0	0	hsa_circ_0005975	NC_000010.11	+	91992119	92008275	16157	1064	sense-overlappin	XR_246113.1	
circRNA_07285	0	0	0.021808645	0.0206362	0.12037869	0	0.040197371	0.030729443	0	0	0.02025523	0	hsa_circ_0008148	NC_000010.11	+	91994535	92008275	13741	719	sense-overlappin	XR_246113.1	
circRNA_07286	0	0.061409903	0	0	0	0	0	0	0	0	0	0	hsa_circ_0003584	NC_000010.11	+	91996369	91997751	1383	456	sense-overlappin	XR_246113.1	
circRNA_07287	0	0	0.032712968	0.0515905	0.03009467	0	0.030148028	0	0	0	0	0	hsa_circ_0007578	NC_000010.11	+	91996369	92008275	11907	609	sense-overlappin	XR_246113.1	
circRNA_07288	0	0	0	0	0.03009467	0	0	0	0	0	0	0	-	NC_000010.11	+	92008829	92009208	380	290	sense-overlappin	XM_011540326.1	BTAF1
circRNA_07289	0	0	0	0	0	0	0	0	0.020254528	0	0	0	hsa_circ_0004224	NC_000010.11	-	92081320	92091944	10625	297	sense-overlappin	XM_006717714.2	CPEB3

circRNA_07290	0	0	0	0	0	0	0	0	0	0.030258724	0	0	hsa_circ_0019146	NC_000010.11	-	92143029	92181019	37991	339	sense-overlappin	XM_006717714.2	CPEB3
circRNA_07291	0	0	0	0.0206362	0	0	0	0	0	0	0	0	hsa_circ_0019147	NC_000010.11	-	92143029	92192636	49608	499	sense-overlappin	XM_006717714.2	CPEB3
circRNA_07292	0	0	0	0	0	0	0	0	0	0.020172483	0	0	-	NC_000010.11	+	92313132	92319749	6618	6618	intronic	NM_017824.4	5-Mar
circRNA_07293	0	0	0	0	0	0	0	0	0	0.020172483	0	0	-	NC_000010.11	-	92504794	92510162	5369	646	sense-overlappin	NM_004969.3	IDE
circRNA_07294	0.061725074	0	0.032712968	0	0.10031557	0.041425819	0.060296056	0	0	0.110948655	0	0	hsa_circ_0000251	NC_000010.11	-	92531748	92537550	5803	563	sense-overlappin	NM_004969.3	IDE
circRNA_07295	0	0.020469968	0	0	0	0	0	0	0	0	0	0	-	NC_000010.11	+	92637184	92645642	8459	672	sense-overlappin	NM_004523.3	KIF11
circRNA_07296	0.020575025	0.020469968	0.152660515	0.0309543	0.06018934	0.093208092	0.020098685	0	0	0.060517448	0	0	hsa_circ_0019170	NC_000010.11	+	92893349	92899644	6296	357	sense-overlappin	NM_001013848.2	EXOC6
circRNA_07297	0	0	0.032712968	0	0.03009467	0	0	0	0	0.020172483	0.04051046	0	hsa_circ_0019171	NC_000010.11	+	92893349	92909631	16283	562	sense-overlappin	NM_001013848.2	EXOC6
circRNA_07298	0	0	0	0.0206362	0	0	0	0	0	0	0	0	-	NC_000010.11	+	92893349	92915913	22565	718	sense-overlappin	NM_001013848.2	EXOC6
circRNA_07299	0	0	0.032712968	0.0206362	0	0	0.020098685	0.030729443	0.030381792	0.030258724	0	0	hsa_circ_0019172	NC_000010.11	+	92893349	92920050	26702	787	sense-overlappin	NM_001013848.2	EXOC6
circRNA_07300	0.030862537	0	0	0	0	0	0	0	0	0	0	0	hsa_circ_0019201	NC_000010.11	-	93363961	93369776	5816	411	sense-overlappin	XM_011539633.1	MYOF
circRNA_07301	0	0	0.032712968	0	0	0.051782273	0	0.030729443	0	0	0.030382845	0.051591	hsa_circ_0005898	NC_000010.11	-	93381219	93389154	7936	420	sense-overlappin	XM_011539633.1	MYOF
circRNA_07302	0.030862537	0	0	0	0	0	0	0	0	0	0	0	-	NC_000010.11	-	93465811	93466713	903	903	intronic	XM_006717760.2	MYOF
circRNA_07303	0.051437562	0.040939935	0.021808645	0	0	0.041425819	0.020098685	0.020486295	0	0.060517448	0.030382845	0.0206364	-	NC_000010.11	-	93676653	93687449	10797	361	sense-overlappin	NM_145246.4	FRA10AC1
circRNA_07304	0	0	0	0	0	0	0	0	0	0	0.02025523	0	-	NC_000010.11	-	93681480	93687449	5970	322	sense-overlappin	NM_145246.4	FRA10AC1
circRNA_07305	0.030862537	0.020469968	0.065425935	0	0	0	0.020098685	0.020486295	0	0.020172483	0.030382845	0	hsa_circ_0019223	NC_000010.11	+	94030683	94032252	1570	1570	exonic	NM_001288989.1	PLCE1
circRNA_07306	0	0.020469968	0	0	0	0	0.020098685	0	0	0	0.050638075	0	hsa_circ_0019225	NC_000010.11	+	94245946	94246621	676	676	exonic	NM_001288989.1	PLCE1
circRNA_07307	0.041150049	0	0	0	0	0.031069364	0	0	0.020254528	0	0	0.072227401	hsa_circ_0019230	NC_000010.11	+	94258800	94259150	351	260	sense-overlappin	NM_001288989.1	PLCE1
circRNA_07308	0.030862537	0	0	0	0	0	0	0	0	0	0	0	hsa_circ_0019240	NC_000010.11	-	94350113	94352403	2291	270	sense-overlappin	XR_945799.1	
circRNA_07309	0	0	0	0	0.05015779	0.020712909	0	0	0.030381792	0	0	0	-	NC_000010.11	+	94441896	94443423	1528	1528	sense-overlappin	XR_945637.1	
circRNA_07310	0	0	0.04361729	0	0	0	0	0	0	0	0	0	-	NC_000010.11	+	94441896	94444539	2644	2644	sense-overlappin	XR_945637.1	
circRNA_07311	0.020575025	0	0	0	0	0	0	0	0	0	0	0	-	NC_000010.11	+	94441896	94451609	9714	4099	sense-overlappin	XM_006717733.1	TBC1D12
circRNA_07312	0	0	0.04361729	0.0309543	0.02006311	0	0.080394741	0	0.060763583	0	0.050638075	0.0206364	hsa_circ_0008874	NC_000010.11	+	94441896	94474783	32888	276	sense-overlappin	XM_006717733.1	TBC1D12
circRNA_07313	0	0	0.021808645	0	0	0	0	0	0	0	0	0	-	NC_000010.11	+	94441896	94522453	80558	1065	sense-overlappin	XM_006717733.1	TBC1D12
circRNA_07314	0	0	0	0	0	0	0	0.020486295	0	0.020172483	0	0	hsa_circ_0008528	NC_000010.11	+	94447635	94474783	27149	152	sense-overlappin	XM_006717733.1	TBC1D12
circRNA_07315	0	0	0.021808645	0	0	0	0	0	0	0	0	0	-	NC_000010.11	+	94474668	94511654	36987	666	sense-overlappin	XR_246080.2	
circRNA_07316	0	0	0	0	0.03009467	0	0	0	0	0	0	0	-	NC_000010.11	+	94493365	94507347	13983	389	sense-overlappin	XR_945636.1	
circRNA_07317	0.020575025	0	0.021808645	0	0.02006311	0	0	0	0	0	0.02025523	0	-	NC_000010.11	+	94510091	94522453	12363	400	sense-overlappin	XM_006717732.2	TBC1D12
circRNA_07318	0.051437562	0	0	0	0	0	0	0	0	0	0	0	hsa_circ_0000252	NC_000010.11	+	94588229	94594854	6626	922	sense-overlappin	NM_001289067.1	HELLS
circRNA_07319	0	0.133054789	0	0	0	0	0	0	0	0.07060369	0	0.072227401	-	NC_000010.11	+	94724346	94781997	57652	39179	sense-overlappin	NM_000769.2	CYP2C19
circRNA_07320	0	0	0	0	0	0	0	0	0.040509055	0.030258724	0	0	-	NC_000010.11	+	94733297	94775539	42243	29890	sense-overlappin	NM_000769.2	CYP2C19
circRNA_07321	0	0.061409903	0.032712968	0	0	0	0	0.051215739	0.040509055	0.020172483	0	0	-	NC_000010.11	+	94733297	94781997	48701	30228	sense-overlappin	NM_000769.2	CYP2C19
circRNA_07322	0	0.030704951	0	0	0	0	0	0	0	0	0	0	-	NC_000010.11	+	94775058	94781997	6940	651	sense-overlappin	NM_000769.2	CYP2C19
circRNA_07323	0	0	0	0.0206362	0	0	0.030148028	0.020486295	0.030381792	0	0	0.0206364	-	NC_000010.11	+	94775058	94850058	75001	1123	sense-overlappin	NM_000769.2	CYP2C19
circRNA_07324	0	0.040939935	0	0	0	0	0	0	0	0	0	0	-	NC_000010.11	+	94780499	94781997	1499	338	sense-overlappin	NM_000769.2	CYP2C19
circRNA_07325	0	0	0	0	0.02006311	0	0	0	0	0	0	0	-	NC_000010.11	+	94780499	94850058	69560	810	sense-overlappin	NM_000769.2	CYP2C19
circRNA_07326	0	0.030704951	0.032712968	0	0	0	0.040197371	0.030729443	0.040509055	0	0	0	-	NC_000010.11	+	94820496	94850058	29563	472	sense-overlappin	NM_000769.2	CYP2C19
circRNA_07327	0	0	0	0	0	0	0	0	0.030381792	0	0	0.0206364	-	NC_000010.11	+	94842837	94850058	7222	330	sense-overlappin	NM_000769.2	CYP2C19
circRNA_07328	0	0	0	0	0	0	0	0.020486295	0	0	0	0	-	NC_000010.11	+	94842837	94874809	31973	22407	sense-overlappin	NM_000769.2	CYP2C19
circRNA_07329	0	0	0	0	0	0	0	0.030729443	0	0	0	0	-	NC_000010.11	+	94938437	94949284	10848	1065	sense-overlappin	NM_000771.3	CYP2C9
circRNA_07330	0.092587611	0	0	0	0	0.082851637	0	0	0.020254528	0.030258724	0.02025523	0.0309546	-	NC_000010.11	-	95339133	95346452	7320	483	sense-overlappin	XM_011539169.1	SORBS1
circRNA_07331	0	0.020469968	0	0	0	0	0	0	0	0.030258724	0	0	-	NC_000010.11	-	95339133	95367699	28567	960	sense-overlappin	XM_011539169.1	SORBS1
circRNA_07332	0	0	0	0	0	0	0	0	0	0	0.02025523	0	-	NC_000010.11	-	95339133	95384285	45153	1505	sense-overlappin	XM_011539149.1	SORBS1
circRNA_07333	0	0	0	0.0206362	0	0.041425819	0	0	0	0.040344966	0.050638075	0	-	NC_000010.11	-	95351209	95384285	33077	1022	sense-overlappin	XM_011539149.1	SORBS1
circRNA_07334	0.030862537	0	0	0	0	0	0	0	0	0	0	0	-	NC_000010.11	-	95351216	95384285	33070	1015	sense-overlappin	XM_011539149.1	SORBS1
circRNA_07335	0.020575025	0	0	0	0	0	0	0	0	0	0	0	-	NC_000010.11	-	95367650	95410777	43128	1145	sense-overlappin	XM_011539149.1	SORBS1
circRNA_07336	0.174887709	0.051174919	0.032712968	0.0309543	0	0.041425819	0	0.040972591	0	0.050431207	0	0	-	NC_000010.11	-	95381685	95410777	29093	843	sense-overlappin	XM_011539149.1	SORBS1
circRNA_07337	0.020575025	0	0	0	0	0	0	0	0	0	0	0	-	NC_000010.11	-	95397220	95410777	13558	412	sense-overlappin	XM_011539149.1	SORBS1
circRNA_07338	0	0	0	0	0.02006311	0	0	0	0	0	0	0	-	NC_000010.11	+	95839691	95847706	8016	930	sense-overlappin	XM_011540371.1	ENTPD1
circRNA_07339	0.082300098	0	0	0	0.02006311	0.020712909	0.020098685	0	0.030381792	0.050431207	0	0	-	NC_000010.11	+	95922016	95927332	5317	300	sense-overlappin	NM_001130446.2	C10orf131
circRNA_07340	0	0	0	0	0	0	0	0	0	0.020172483	0	0	-	NC_000010.11	+	95955394	95974156	18763	18763	sense-overlappin	XM_011539789.1	CC2D2B
circRNA_07341	0.030862537	0	0.04361729	0	0.02006311	0	0	0	0.030381792	0.050431207	0	0	-	NC_000010.11	+	96155326	96160402	5077	4381	sense-overlappin	XM_011540413.1	ZNF518A
circRNA_07342	0	0	0	0	0	0	0	0	0	0	0	0.0206364	-	NC_000010.11	+	96155924	96160402	4479	4330	sense-overlappin	XM_011540411.1	ZNF518A
circRNA_07343	0	0	0	0.0206362	0	0	0	0	0	0	0	0	-	NC_000010.11	-	96160557	96160671	115	115	antisense	XM_011540411.1	ZNF518A

circRNA_07344	0	0	0	0	0	0	0	0	0	0	0	0.0206364	-	NC_000010.11	-	96200075	96209907	9833	419	sense-overlappin	NM_001258440.1	BLNK
circRNA_07345	0	0	0.13085187	0.0928628	0.0702209	0.020712909	0.040197371	0.153647216	0.040509055	0	0.06076569	0.0206364	hsa_circ_0005801	NC_000010.11	-	96544076	96565426	21351	887	sense-overlappin	XM_011539976.1	TM9SF3
circRNA_07346	0.020575025	0.040939935	0.228990773	0.0825448	0.16050492	0.031069364	0.050246713	0.020486295	0.070890847	0.07060369	0	0.0412728	-	NC_000010.11	-	96544076	96576829	32754	1083	sense-overlappin	XM_011539976.1	TM9SF3
circRNA_07347	0	0	0	0	0.03009467	0	0	0	0	0	0	0	hsa_circ_0019299	NC_000010.11	-	96552928	96576829	23902	690	sense-overlappin	XM_011539976.1	TM9SF3
circRNA_07348	0	0	0	0	0.05015779	0	0	0	0	0	0.02025523	0	hsa_circ_0006198	NC_000010.11	+	96858292	96907747	49456	49119	sense-overlappin	NM_001170765.1	LCOR
circRNA_07349	0	0	0.021808645	0	0	0	0	0	0	0	0	0	-	NC_000010.11	+	96884403	96907747	23345	23008	sense-overlappin	NM_001170765.1	LCOR
circRNA_07350	0	0	0	0.0206362	0	0	0	0	0	0	0	0	-	NC_000010.11	+	96893693	96894147	455	455	intronic	NM_001170765.1	LCOR
circRNA_07351	0	0	0	0.0206362	0	0	0	0	0	0.020172483	0	0	hsa_circ_0007707	NC_000010.11	+	96907265	96907747	483	146	sense-overlappin	NM_001170765.1	LCOR
circRNA_07352	0	0.020469968	0.021808645	0.0412724	0.02006311	0.041425819	0	0	0	0	0.02025523	0	hsa_circ_0008102	NC_000010.11	+	96944113	96952196	8084	515	sense-overlappin	NM_001170765.1	LCOR
circRNA_07353	0	0	0	0	0	0	0	0	0	0	0.04051046	0	-	NC_000010.11	+	96980793	96989839	9047	9047	sense-overlappin	NM_015652.2	C10orf12
circRNA_07354	0.288050345	0.16375974	0.04361729	0.1444533	0.04012623	0.134633911	0.040197371	0.040972591	0.121527166	0.121034897	0.08102092	0.134136601	hsa_circ_0005887	NC_000010.11	-	97436417	97437750	1334	271	sense-overlappin	NM_016046.3	EXOSC1
circRNA_07355	0	0	0	0.0619086	0	0.051782273	0	0	0.020254528	0.040344966	0	0	-	NC_000010.11	-	97436417	97438703	2287	305	sense-overlappin	NM_016046.3	EXOSC1
circRNA_07356	0	0	0.04361729	0.0206362	0	0.031069364	0	0	0	0	0	0	hsa_circ_0004896	NC_000010.11	-	97437191	97437750	560	136	sense-overlappin	NM_016046.3	EXOSC1
circRNA_07357	0	0	0.021808645	0	0	0	0	0	0	0.020172483	0	0	hsa_circ_0006520	NC_000010.11	-	97476683	97478389	1707	422	sense-overlappin	XM_006717944.2	MMS19
circRNA_07358	0	0	0	0	0.04012623	0	0	0.030729443	0	0.030258724	0.030382845	0	hsa_circ_0003168	NC_000010.11	+	97743094	97744915	1822	258	sense-overlappin	NM_001002262.3	ZFYVE27
circRNA_07359	0	0	0.021808645	0	0	0	0	0	0	0	0	0	hsa_circ_0004419	NC_000010.11	+	98156093	98163397	7305	253	sense-overlappin	NM_001256619.1	R3HCC1L
circRNA_07360	0.030862537	0	0.054521613	0	0	0	0	0	0	0	0	0	-	NC_000010.11	+	98156093	98209899	53807	9202	sense-overlappin	XM_011539642.1	R3HCC1L
circRNA_07361	0	0	0.021808645	0	0	0	0	0	0.040509055	0	0	0	-	NC_000010.11	+	98162883	98209899	47017	2412	sense-overlappin	XM_011539642.1	R3HCC1L
circRNA_07362	0	0	0	0	0	0	0.030148028	0	0	0	0	0	hsa_circ_0019408	NC_000010.11	-	98429573	98431291	1719	430	sense-overlappin	NM_000195.3	HPS1
circRNA_07363	0	0	0	0.0206362	0	0	0	0	0	0	0	0	-	NC_000010.11	-	99401760	99402026	267	267	intronic	XR_945681.1	
circRNA_07364	0	0	0	0	0	0	0	0	0	0	0.030382845	0	-	NC_000010.11	+	99782829	99784781	1953	223	sense-overlappin	NM_000392.4	ABCC2
circRNA_07365	0	0	0	0	0	0	0	0.020486295	0	0	0	0	-	NC_000010.11	-	100253421	100262063	8643	600	sense-overlappin	NM_001303404.1	CWF19L1
circRNA_07366	0	0	0	0	0	0.031069364	0	0	0.040509055	0.020172483	0	0	hsa_circ_0006718	NC_000010.11	-	100260218	100262063	1846	266	sense-overlappin	NM_001303404.1	CWF19L1
circRNA_07367	0	0	0.032712968	0.0206362	0	0	0	0	0	0	0	0	-	NC_000010.11	+	100915999	100926019	10021	1902	sense-overlappin	NM_001136123.1	FAM178A
circRNA_07368	0.030862537	0	0	0.0206362	0	0	0	0	0	0	0	0	hsa_circ_0019529	NC_000010.11	+	100915999	100931078	15080	2296	sense-overlappin	NM_001136123.1	FAM178A
circRNA_07369	0	0	0	0	0	0.031069364	0	0	0	0	0	0	hsa_circ_0019531	NC_000010.11	+	100915999	100938736	22738	2514	sense-overlappin	NM_001136123.1	FAM178A
circRNA_07370	0	0.030704951	0.065425935	0.1031809	0.06018934	0.072495183	0.120592112	0.030729443	0.070890847	0.040344966	0.070893305	0.0309546	hsa_circ_0006654	NC_000010.11	+	100923975	100926019	2045	1069	sense-overlappin	NM_001136123.1	FAM178A
circRNA_07371	0	0	0	0.0206362	0	0.020712909	0	0	0	0	0	0	hsa_circ_0019538	NC_000010.11	+	100923975	100931078	7104	1463	sense-overlappin	NM_001136123.1	FAM178A
circRNA_07372	0	0	0	0.0412724	0	0	0	0	0	0.040344966	0.04051046	0	-	NC_000010.11	+	100923978	100926019	2042	1066	sense-overlappin	NM_001136123.1	FAM178A
circRNA_07373	0	0	0.032712968	0.0515905	0	0	0	0	0	0	0	0	hsa_circ_0006409	NC_000010.11	+	100937402	100944128	6727	321	sense-overlappin	NM_001136123.1	FAM178A
circRNA_07374	0	0	0	0.0309543	0	0	0	0	0	0	0	0	hsa_circ_0019548	NC_000010.11	+	100979848	100981229	1382	707	sense-overlappin	XR_945792.1	
circRNA_07375	0	0	0	0	0	0.041425819	0	0	0	0	0	0	-	NC_000010.11	+	101430345	101479457	49113	276	sense-overlappin	NM_033637.3	BTRC
circRNA_07376	0	0	0.021808645	0.0206362	0	0	0.040197371	0	0	0	0	0	hsa_circ_0005741	NC_000010.11	-	101624745	101676436	51692	648	sense-overlappin	XM_005270053.2	FBXW4
circRNA_07377	0.257187808	0.143289773	0.228990773	0.1857257	0.13041025	0.176059729	0.110542769	0.051215739	0.162036222	0.14120738	0.131658996	0.134136601	hsa_circ_0008362	NC_000010.11	-	101667886	101676436	8551	510	sense-overlappin	NM_022039.3	FBXW4
circRNA_07378	0	0	0	0	0	0	0	0	0	0	0	0.0206364	hsa_circ_0019591	NC_000010.11	-	101672915	101673673	759	319	sense-overlappin	NM_022039.3	FBXW4
circRNA_07379	0.072012586	0.061409903	0.04361729	0.0309543	0.02006311	0.041425819	0.030148028	0.020486295	0.050636319	0.050431207	0.030382845	0	hsa_circ_0000255	NC_000010.11	-	101672915	101676436	3522	415	sense-overlappin	NM_022039.3	FBXW4
circRNA_07380	0	0.020469968	0	0.0619086	0	0	0	0	0	0.030258724	0	0	hsa_circ_0019606	NC_000010.11	-	101792839	101807901	15063	1695	sense-overlappin	NM_012215.3	MGEA5
circRNA_07381	0.092587611	0.030704951	0.08723458	0.0825448	0.08025246	0.093208092	0	0.030729443	0	0.050431207	0.04051046	0.0619092	hsa_circ_0019607	NC_000010.11	-	101792839	101810314	17476	1826	sense-overlappin	NM_012215.3	MGEA5
circRNA_07382	0	0	0	0.0206362	0	0	0	0	0	0	0	0	-	NC_000010.11	-	101792839	101813606	20768	1976	sense-overlappin	NM_012215.3	MGEA5
circRNA_07383	0	0	0	0	0	0	0	0.020486295	0	0	0	0	-	NC_000010.11	-	101797980	101800400	2421	948	sense-overlappin	NM_012215.3	MGEA5
circRNA_07384	0	0	0	0.0206362	0	0	0	0.020486295	0	0	0	0	hsa_circ_0019608	NC_000010.11	-	101797980	101807901	9922	1504	sense-overlappin	NM_012215.3	MGEA5
circRNA_07385	0.020575025	0	0	0	0	0.020712909	0.020098685	0	0.030381792	0	0	0	hsa_circ_0019609	NC_000010.11	-	101797980	101810314	12335	1635	sense-overlappin	NM_012215.3	MGEA5
circRNA_07386	0.030862537	0	0	0	0	0	0	0	0	0	0	0	hsa_circ_0019610	NC_000010.11	-	101797980	101813606	15627	1785	sense-overlappin	NM_012215.3	MGEA5
circRNA_07387	0	0	0	0	0	0	0	0	0.030381792	0	0	0	hsa_circ_0019611	NC_000010.11	-	101798842	101800400	1559	773	sense-overlappin	NM_012215.3	MGEA5
circRNA_07388	0	0	0	0	0	0	0.030148028	0	0	0	0	0	hsa_circ_0019612	NC_000010.11	-	101798842	101807901	9060	1329	sense-overlappin	NM_012215.3	MGEA5
circRNA_07389	0	0	0	0	0	0	0	0	0.020254528	0	0	0	-	NC_000010.11	-	101798842	101810314	11473	1460	sense-overlappin	NM_012215.3	MGEA5
circRNA_07390	0.020575025	0	0	0	0	0.051782273	0	0	0	0	0	0	hsa_circ_0019617	NC_000010.11	-	101806045	101807901	1857	271	sense-overlappin	NM_001142434.1	MGEA5
circRNA_07391	0	0	0.021808645	0	0	0	0	0	0	0	0	0	hsa_circ_0007564	NC_000010.11	-	101889412	101957732	68321	365	sense-overlappin	XM_011540156.1	C10orf76
circRNA_07392	0	0	0	0.0412724	0	0.020712909	0	0	0.040509055	0	0	0	hsa_circ_0004117	NC_000010.11	-	101939863	101957732	17870	286	sense-overlappin	XM_011540156.1	C10orf76
circRNA_07393	0	0	0	0	0	0	0	0.040972591	0	0.030258724	0	0	-	NC_000010.11	-	102023477	102025248	1772	255	sense-overlappin	XM_011540151.1	C10orf76
circRNA_07394	0	0	0.021808645	0	0	0	0	0	0	0	0	0	hsa_circ_0002523	NC_000010.11	-	102023477	102033339	9863	567	sense-overlappin	XM_011540151.1	C10orf76
circRNA_07395	0	0	0.021808645	0.0309543	0.0702209	0.093208092	0.040197371	0.020486295	0	0	0	0	hsa_circ_0000257	NC_000010.11	+	102157019	102158214	1196	487	sense-overlappin	NM_001284388.1	NOLC1
circRNA_07396	0	0	0	0	0	0	0	0	0	0.050431207	0	0	hsa_circ_0019701	NC_000010.11	+	102358039	102359435	1397	541	sense-overlappin	XR_945857.1	
circRNA_07397	0	0.030704951	0	0	0	0.020712909	0	0	0	0.050431207	0	0	-	NC_000010.11	+	102362475	102363798	1324	420	sense-overlappin	XR_945857.1	
circRNA_07398	0	0	0	0	0	0	0	0	0.030381792	0	0	0	-	NC_000010.11	-	102685816	102705489	19674	498	sense-overlappin	NM_004311.3	ARL3
circRNA_07399	0	0	0.021808645	0	0	0	0	0	0	0	0	0	hsa_circ_0019770	NC_000010.11	+	102809893	102810054	162	162	exonic	XM_011539912.1	WBP1L
circRNA_07400	0.020575025	0.051174919	0	0	0	0	0	0	0	0.030258724	0	0.0309546	hsa_circ_0019773	NC_000010.11	+	102872448	102874661	2214	358	sense-overlappin	NM_020682.3	AS3MT

circRNA_07401	0	0.020469968	0	0	0	0	0	0	0	0	0	0	-	NC_000010.11	+	102876954	102890678	13725	492	sense-overlappin	NM_020682.3	AS3MT
circRNA_07402	0.030862537	0	0.021808645	0	0.02006311	0.020712909	0	0	0	0	0.030382845	0	hsa_circ_0019779	NC_000010.11	-	103091564	103094455	2892	398	sense-overlappin	NM_001134373.2	NT5C2
circRNA_07403	0	0	0	0	0.04012623	0	0	0	0	0	0	0	-	NC_000010.11	-	103139406	103181328	41923	297	sense-overlappin	NM_012229.4	NT5C2
circRNA_07404	0	0	0	0	0.03009467	0	0	0	0	0	0	0	-	NC_000010.11	-	103174858	103181328	6471	223	sense-overlappin	NM_012229.4	NT5C2
circRNA_07405	0.041150049	0	0.04361729	0.0412724	0.05015779	0.041425819	0.070345399	0.030729443	0.060763583	0	0.08102092	0	hsa_circ_0000258	NC_000010.11	+	103438015	103438808	794	180	sense-overlappin	XM_005269647.2	PDCD11
circRNA_07406	0	0	0	0	0.02006311	0.031069364	0.030148028	0	0.020254528	0	0	0	hsa_circ_0003366	NC_000010.11	+	103439746	103440850	1105	532	sense-overlappin	XM_005269647.2	PDCD11
circRNA_07407	0.288050345	0.071644886	0.021808645	0.0619086	0.11034713	0.031069364	0.030148028	0	0.111399902	0.050431207	0.030382845	0.0412728	hsa_circ_0005620	NC_000010.11	-	103622470	103661114	38645	423	sense-overlappin	XM_006718080.2	SH3PXD2A
circRNA_07408	0	0	0	0.0206362	0	0	0	0	0	0	0	0	hsa_circ_0005321	NC_000010.11	+	103990675	103993133	2459	364	sense-overlappin	XM_011540401.1	SLK
circRNA_07409	0.020575025	0.040939935	0	0	0	0	0	0	0	0	0	0	-	NC_000010.11	+	104003094	104003177	84	84	exonic	NM_001304743.1	SLK
circRNA_07410	0.061725074	0.040939935	0.054521613	0.0825448	0.02006311	0	0.070345399	0.051215739	0.081018111	0.080689931	0.08102092	0	hsa_circ_0000259	NC_000010.11	+	104008177	104018908	10732	528	sense-overlappin	XM_011540401.1	SLK
circRNA_07411	0	0	0	0	0	0	0	0	0	0	0.02025523	0	hsa_circ_0009067	NC_000010.11	+	104259576	104263077	3502	322	sense-overlappin	NM_001191003.1	GSTO1
circRNA_07412	0	0	0.021808645	0	0	0	0.020098685	0	0	0	0	0	-	NC_000010.11	+	110100625	110112915	12291	417	sense-overlappin	XM_006717629.1	ADD3
circRNA_07413	0	0	0	0	0	0	0	0	0	0	0.02025523	0	-	NC_000010.11	+	110124017	110126503	2487	465	sense-overlappin	XM_011539288.1	ADD3
circRNA_07414	0	0.040939935	0.032712968	0.0206362	0	0.041425819	0	0.020486295	0	0.050431207	0.04051046	0	hsa_circ_0003357	NC_000010.11	+	110124017	110130486	6470	589	sense-overlappin	XM_011539288.1	ADD3
circRNA_07415	0	0	0	0.0309543	0	0.031069364	0	0	0.020254528	0	0.02025523	0	-	NC_000010.11	+	110124017	110132400	8384	685	sense-overlappin	XM_011539288.1	ADD3
circRNA_07416	0	0	0	0	0	0	0	0.020486295	0	0	0	0	-	NC_000010.11	+	110589605	110593223	3619	658	sense-overlappin	NM_005445.3	SMC3
circRNA_07417	0.072012586	0.051174919	0.08723458	0.0619086	0.1404418	0.124277456	0.070345399	0.051215739	0.070890847	0.121034897	0.04051046	0.0619092	hsa_circ_0000260	NC_000010.11	+	110596398	110598290	1893	305	sense-overlappin	NM_005445.3	SMC3
circRNA_07418	0	0	0	0	0	0.020712909	0	0	0	0	0	0	-	NC_000010.11	+	110881233	110883097	1865	398	sense-overlappin	NM_001199492.1	PDCD4
circRNA_07419	0	0.030704951	0.032712968	0.0412724	0	0.041425819	0	0.020486295	0.020254528	0.020172483	0.030382845	0	-	NC_000010.11	+	110881233	110887886	6654	734	sense-overlappin	NM_001199492.1	PDCD4
circRNA_07420	0	0	0	0.0825448	0	0.031069364	0	0.020486295	0	0.040344966	0	0	-	NC_000010.11	+	110881233	110890670	9438	947	sense-overlappin	NM_001199492.1	PDCD4
circRNA_07421	0	0	0	0	0	0	0	0	0.020254528	0	0	0	-	NC_000010.11	+	110881275	110890670	9396	905	sense-overlappin	NM_001199492.1	PDCD4
circRNA_07422	0	0.071644886	0	0.0309543	0	0.051782273	0	0.040972591	0	0.07060369	0.030382845	0.0619092	hsa_circ_0020024	NC_000010.11	+	110885253	110890670	5418	549	sense-overlappin	NM_001199492.1	PDCD4
circRNA_07423	0	0	0	0	0	0	0	0	0	0.040344966	0	0	-	NC_000010.11	+	110951608	110965061	13454	13454	sense-overlappin	NM_007373.3	SHOC2
circRNA_07424	0	0	0	0	0.02006311	0	0	0	0	0	0	0	-	NC_000010.11	+	110951608	110985765	34158	13592	sense-overlappin	NM_007373.3	SHOC2
circRNA_07425	0	0	0.054521613	0	0	0	0	0	0	0	0	0	hsa_circ_0000261	NC_000010.11	+	110964125	110965061	937	937	exonic	NM_007373.3	SHOC2
circRNA_07426	0.13373766	0.122819805	0.239895095	0.1960438	0.19059959	0.165703275	0.190937511	0.051215739	0.091145375	0.14120738	0.08102092	0.092863801	hsa_circ_0020028	NC_000010.11	+	110964125	110985765	21641	1075	sense-overlappin	NM_007373.3	SHOC2
circRNA_07427	0	0	0.021808645	0	0	0	0.030148028	0	0	0.040344966	0	0	hsa_circ_0002533	NC_000010.11	+	112527087	112538330	11244	184	sense-overlappin	XM_011539333.1	VTI1A
circRNA_07428	0.020575025	0	0	0	0	0	0	0	0	0	0	0	-	NC_000010.11	+	113767345	113774333	6989	555	sense-overlappin	XM_011540172.1	PLEKHS1
circRNA_07429	0.030862537	0.030704951	0.04361729	0.0206362	0	0.020712909	0.060296056	0	0	0.030258724	0	0	-	NC_000010.11	+	113876521	113884380	7860	708	sense-overlappin	XM_011539769.1	NHLRC2
circRNA_07430	0	0	0.021808645	0	0	0	0	0	0	0	0	0.0412728	hsa_circ_0006317	NC_000010.11	+	113898110	113908523	10414	1129	sense-overlappin	NM_198514.3	NHLRC2
circRNA_07431	0	0	0	0.0206362	0	0	0	0	0	0	0	0	hsa_circ_0020072	NC_000010.11	-	114487958	114491878	3921	147	sense-overlappin	NM_006720.3	ABLIM1
circRNA_07432	0	0	0	0	0	0	0	0	0	0.020172483	0	0	hsa_circ_0020078	NC_000010.11	+	114830852	114836246	5395	477	sense-overlappin	NM_020940.3	FAM160B1
circRNA_07433	0	0	0.032712968	0.0309543	0	0.020712909	0	0	0	0.020172483	0	0	hsa_circ_0020080	NC_000010.11	+	114830852	114848737	17886	1758	sense-overlappin	NM_020940.3	FAM160B1
circRNA_07434	0	0	0	0	0.06018934	0.031069364	0	0	0	0	0	0	hsa_circ_0006610	NC_000010.11	+	114843741	114848737	4997	987	sense-overlappin	NM_020940.3	FAM160B1
circRNA_07435	0	0	0.032712968	0.0412724	0.06018934	0.051782273	0	0	0	0.020172483	0	0	hsa_circ_0020091	NC_000010.11	+	114959726	114974385	14660	352	sense-overlappin	NM_139169.4	TRUB1
circRNA_07436	0.061725074	0.051174919	0	0.0515905	0.16050492	0.103564547	0	0.030729443	0.040509055	0.020172483	0.02025523	0	hsa_circ_0020092	NC_000010.11	+	114970368	114974385	4018	270	sense-overlappin	NM_139169.4	TRUB1
circRNA_07437	0	0	0.032712968	0	0	0	0	0.030729443	0.081018111	0.060517448	0	0	hsa_circ_0020093	NC_000010.11	+	115120185	115129535	9351	638	sense-overlappin	NR_074088.2	
circRNA_07438	0	0	0	0	0	0.062138728	0	0	0	0	0	0	hsa_circ_0020094	NC_000010.11	+	115120185	115165645	45461	901	sense-overlappin	NR_074088.2	
circRNA_07439	0	0	0	0	0	0	0	0	0.020254528	0.020172483	0	0	hsa_circ_0005239	NC_000010.11	-	116089741	116096764	7024	427	sense-overlappin	NM_145793.3	GFRA1
circRNA_07440	0	0	0.021808645	0	0	0	0	0	0	0	0	0	-	NC_000010.11	-	116728174	116732063	3890	3890	intronic	XM_011539580.1	HSPA12A
circRNA_07441	0	0	0.021808645	0	0	0	0	0	0	0	0	0	-	NC_000010.11	-	117318872	117341102	22231	226	sense-overlappin	NM_173791.4	PDZD8
circRNA_07442	0	0	0	0.0825448	0	0.020712909	0	0	0	0	0	0	-	NC_000010.11	-	118009082	118040565	31484	1162	sense-overlappin	XM_005269629.2	RAB11FIP2
circRNA_07443	0	0	0	0	0	0	0	0	0.030381792	0	0	0	-	NC_000010.11	-	118335114	118337408	2295	644	sense-overlappin	XM_006717939.2	FAM204A
circRNA_07444	0	0	0.032712968	0	0	0	0	0	0	0	0	0	-	NC_000010.11	-	118685983	118730410	44428	888	sense-overlappin	XR_945608.1	
circRNA_07445	0	0	0.04361729	0	0.03009467	0	0	0	0	0.020172483	0.02025523	0	hsa_circ_0020134	NC_000010.11	-	118729295	118730410	1116	230	sense-overlappin	XR_945608.1	
circRNA_07446	0	0	0	0	0.02006311	0	0	0	0	0	0.030382845	0	hsa_circ_0004792	NC_000010.11	-	119038238	119038439	202	202	exonic	NM_003750.2	EIF3A
circRNA_07447	0.041150049	0	0.021808645	0	0	0	0	0	0	0	0	0	-	NC_000010.11	-	119041994	119044142	2149	868	sense-overlappin	NM_003750.2	EIF3A
circRNA_07448	0	0	0	0	0.03009467	0.020712909	0	0.030729443	0.030381792	0	0.030382845	0	hsa_circ_0004350	NC_000010.11	-	119072890	119073937	1048	492	sense-overlappin	NM_003750.2	EIF3A
circRNA_07449	0	0	0	0	0.03009467	0	0	0	0	0	0	0	hsa_circ_0020165	NC_000010.11	-	119160915	119162414	1500	157	sense-overlappin	XM_011539282.1	SFXN4
circRNA_07450	0	0	0	0	0.05015779	0	0	0	0	0	0	0	-	NC_000010.11	-	119213434	119214056	623	623	antisense	NM_005308.2	GRK5
circRNA_07451	0	0	0	0	0	0	0.040197371	0	0	0	0	0	-	NC_000010.11	+	119326516	119396772	70257	287	sense-overlappin	NM_005308.2	GRK5
circRNA_07452	0	0.020469968	0.04361729	0	0	0.031069364	0	0.020486295	0	0.040344966	0	0	hsa_circ_0005516	NC_000010.11	-	119515509	119527424	11916	350	sense-overlappin	NM_002925.3	RGS10
circRNA_07453	0	0	0.032712968	0	0	0	0	0	0	0	0	0	hsa_circ_0004498	NC_000010.11	-	119575691	119580538	4848	1318	sense-overlappin	XR_945808.1	
circRNA_07454	0	0	0.04361729	0.0309543	0.04012623	0	0	0	0	0	0	0	hsa_circ_0020181	NC_000010.11	-	119578726	119588248	9523	592	sense-overlappin	XM_005270110.3	TIAL1
circRNA_07455	0	0	0	0	0	0	0	0	0.030381792	0.030258724	0	0	hsa_circ_0002298	NC_000010.11	+	119909041	119918511	9471	771	sense-overlappin	NM_007190.3	SEC23IP

circRNA_07456	0	0	0.021808645	0	0	0	0	0	0	0.020172483	0	0	hsa_circ_0020203	NC_000010.11	+	119926036	119933787	7752	902	sense-overlappin	NM_007190.3	SEC23IP
circRNA_07457	0	0	0	0	0.04012623	0	0	0	0	0	0	0	-	NC_000010.11	+	120181230	120208995	27766	27766	intergenic		
circRNA_07458	0	0	0	0	0.02006311	0	0	0	0	0	0	0	-	NC_000010.11	+	120187118	120208995	21878	21878	intergenic		
circRNA_07459	0	0	0	0	0.32100984	0	0	0	0	0	0	0	-	NC_000010.11	+	120204995	120208995	4001	4001	intergenic		
circRNA_07460	0	0	0	0	0.09028402	0	0	0	0	0	0	0	-	NC_000010.11	+	120204995	120294175	89181	89181	intergenic		
circRNA_07461	0	0	0	0	0.06018934	0	0	0	0	0	0	0	-	NC_000010.11	+	120288390	120294175	5786	5786	intergenic		
circRNA_07462	0	0.040939935	0.119947548	0.0515905	0.03009467	0.031069364	0.030148028	0	0.040509055	0.080689931	0.06076569	0	-	NC_000010.11	-	121538592	121565704	27113	639	sense-overlappin	NM_001144914.1	FGFR2
circRNA_07463	0	0	0	0	0	0	0.050246713	0	0	0	0	0	hsa_circ_0020238	NC_000010.11	-	121564502	121565704	1203	345	sense-overlappin	NM_001144914.1	FGFR2
circRNA_07464	0	0	0.04361729	0	0	0	0	0	0	0	0	0	hsa_circ_0020246	NC_000010.11	-	121841082	121924329	83248	1180	sense-overlappin	XM_005269458.1	ATE1
circRNA_07465	0	0	0.109043225	0	0.03009467	0	0	0	0	0	0	0.0309546	hsa_circ_0020249	NC_000010.11	-	121870006	121924329	54324	998	sense-overlappin	XM_005269458.1	ATE1
circRNA_07466	0	0	0.04361729	0	0	0	0	0	0	0	0	0	-	NC_000010.11	-	121898841	121926527	27687	3163	sense-overlappin	XM_005269458.1	ATE1
circRNA_07467	0	0	0	0	0	0.031069364	0	0	0	0	0	0	-	NC_000010.11	-	121902391	121911151	8761	476	sense-overlappin	NM_001001976.2	ATE1
circRNA_07468	0	0	0.021808645	0	0	0	0	0	0	0	0	0	hsa_circ_0002850	NC_000010.11	-	121902391	121924329	21939	707	sense-overlappin	NM_001001976.2	ATE1
circRNA_07469	0	0.030704951	0	0	0	0	0	0	0	0	0	0	-	NC_000010.11	+	121954142	121954459	318	318	intergenic		
circRNA_07470	0	0	0.032712968	0	0	0	0	0	0	0	0.030382845	0	hsa_circ_0020256	NC_000010.11	-	121959324	121961517	2194	323	sense-overlappin	NM_001167865.1	NSMCE4A
circRNA_07471	0	0	0	0	0	0	0	0	0	0	0.02025523	0	-	NC_000010.11	-	121959324	121963328	4005	414	sense-overlappin	NM_001167865.1	NSMCE4A
circRNA_07472	0	0.020469968	0	0.0619086	0	0	0	0	0	0	0	0	-	NC_000010.11	+	122082647	122088591	5945	5427	sense-overlappin	NM_001291876.1	TACC2
circRNA_07473	0	0	0.054521613	0	0	0	0	0	0	0	0	0	hsa_circ_0020264	NC_000010.11	+	122392303	122424959	32657	900	sense-overlappin	XM_005270021.3	PLEKHA1
circRNA_07474	0	0	0	0	0	0	0	0.020486295	0	0	0	0	-	NC_000010.11	+	122570890	122572361	1472	96	sense-overlappin	NM_004406.2	DMBT1
circRNA_07475	0	0	0	0	0	0	0	0	0	0	0.04051046	0	-	NC_000010.11	+	122577811	122631281	53471	5739	sense-overlappin	XM_006717660.2	DMBT1
circRNA_07476	0	0	0	0	0.03009467	0	0	0	0	0	0	0	-	NC_000010.11	+	122579578	122598012	18435	2277	sense-overlappin	NM_007329.2	DMBT1
circRNA_07477	0	0.020469968	0	0	0	0	0	0	0	0	0	0	-	NC_000010.11	+	122590665	122602120	11456	1560	sense-overlappin	NM_007329.2	DMBT1
circRNA_07478	0	0	0	0	0	0	0	0	0	0	0.050638075	0	hsa_circ_0020283	NC_000010.11	+	122591479	122618340	26862	3078	sense-overlappin	XM_006717660.2	DMBT1
circRNA_07479	0	0	0	0	0	0	0.030148028	0	0	0	0	0	-	NC_000010.11	+	122618017	122620291	2275	393	sense-overlappin	NM_004406.2	DMBT1
circRNA_07480	0	0	0	0	0	0	0	0	0	0	0.08102092	0	-	NC_000010.11	+	122619308	122621380	2073	393	sense-overlappin	NM_004406.2	DMBT1
circRNA_07481	0	0	0	0	0	0	0	0	0	0	0.02025523	0	-	NC_000010.11	+	122643122	122643394	273	273	exonic	NM_004406.2	DMBT1
circRNA_07482	0	0.020469968	0	0	0	0	0	0	0	0	0	0	hsa_circ_0020303	NC_000010.11	-	124012333	124046724	34392	2007	sense-overlappin	XR_945773.1	
circRNA_07483	0	0	0	0	0	0	0.020098685	0	0	0	0	0	hsa_circ_0020304	NC_000010.11	-	124021256	124046724	25469	1859	sense-overlappin	XR_945773.1	
circRNA_07484	0.113162635	0.020469968	0	0.0309543	0.0702209	0.062138728	0.020098685	0.020486295	0.030381792	0.040344966	0.02025523	0.0412728	hsa_circ_0000264	NC_000010.11	-	124038515	124046724	8210	1702	sense-overlappin	XR_945773.1	
circRNA_07485	0	0	0	0	0	0.031069364	0	0	0	0	0	0	-	NC_000010.11	-	124405436	124412200	6765	677	sense-overlappin	XM_011539833.1	OAT
circRNA_07486	0	0	0	0.0309543	0.09028402	0	0	0	0.030381792	0.020172483	0	0	hsa_circ_0008898	NC_000010.11	-	124408542	124412200	3659	549	sense-overlappin	XM_011539833.1	OAT
circRNA_07487	0	0.020469968	0	0	0	0	0	0	0	0	0	0	-	NC_000010.11	+	124462503	124462962	460	460	intronic	NM_001167880.1	LHPP
circRNA_07488	0	0	0	0	0	0	0	0	0	0	0.030382845	0	hsa_circ_0003766	NC_000010.11	+	124488422	124498128	9707	311	sense-overlappin	NM_001167880.1	LHPP
circRNA_07489	0.082300098	0.102349838	0.185373483	0.0206362	0.05015779	0.031069364	0.140690797	0.051215739	0.040509055	0.090776173	0	0.082545601	hsa_circ_0000267	NC_000010.11	-	124681607	124682379	773	773	exonic	NM_014661.3	FAM53B
circRNA_07490	0	0.051174919	0.021808645	0.0722267	0	0	0.020098685	0.020486295	0.020254528	0	0	0	hsa_circ_0003570	NC_000010.11	-	124681607	124696212	14606	828	sense-overlappin	NM_014661.3	FAM53B
circRNA_07491	0	0	0.054521613	0	0	0	0	0	0	0	0	0	hsa_circ_0002282	NC_000010.11	-	124681607	124706887	25281	1080	sense-overlappin	NM_014661.3	FAM53B
circRNA_07492	0	0	0.021808645	0	0	0	0	0.020486295	0.020254528	0.020172483	0	0	hsa_circ_0005734	NC_000010.11	-	124706636	124706887	252	252	exonic	NM_014661.3	FAM53B
circRNA_07493	0	0	0	0	0	0	0	0	0	0	0.02025523	0	-	NC_000010.11	-	124753392	124754142	751	751	intergenic		
circRNA_07494	0	0	0.021808645	0	0.03009467	0	0	0	0.030381792	0	0	0	hsa_circ_0008969	NC_000010.11	-	124774675	124790338	15664	293	sense-overlappin	NM_001304467.1	METTL10
circRNA_07495	0.041150049	0	0.163564838	0.0309543	0	0.062138728	0.020098685	0	0.020254528	0.020172483	0.030382845	0.0309546	hsa_circ_0006545	NC_000010.11	+	124940374	124943307	2934	1025	sense-overlappin	XM_005269925.3	ZRANB1
circRNA_07496	0.030862537	0.030704951	0.599737738	0.0825448	0.0702209	0.093208092	0.15074014	0.051215739	0	0.030258724	0.04051046	0.072227401	hsa_circ_0000268	NC_000010.11	+	124942457	124943307	851	851	exonic	XM_005269926.3	ZRANB1
circRNA_07497	0	0	0	0	0	0	0	0	0	0.020172483	0	0	hsa_circ_0004451	NC_000010.11	+	124942457	124962408	19952	19952	sense-overlappin	XM_005269926.3	ZRANB1
circRNA_07498	0	0	0.032712968	0	0	0	0	0	0	0	0	0	hsa_circ_0020336	NC_000010.11	+	124942457	124974398	31942	1464	sense-overlappin	XM_005269926.3	ZRANB1
circRNA_07499	0	0	0.021808645	0	0.02006311	0	0	0	0	0	0	0	hsa_circ_0020340	NC_000010.11	+	124973645	124974398	754	271	sense-overlappin	XM_005269926.3	ZRANB1
circRNA_07500	0	0	0.032712968	0	0.02006311	0	0	0	0.060763583	0	0	0	hsa_circ_0002133	NC_000010.11	-	125038997	125074843	35847	260	sense-overlappin	XM_011539351.1	CTBP2
circRNA_07501	0.030862537	0	0.04361729	0	0	0	0	0	0	0	0	0	hsa_circ_0003462	NC_000010.11	-	125038997	125088319	49323	389	sense-overlappin	XM_011539351.1	CTBP2
circRNA_07502	0.020575025	0	0.098138903	0.0515905	0.02006311	0	0.040197371	0.030729443	0.040509055	0	0.050638075	0	hsa_circ_0005418	NC_000010.11	-	125038997	125111093	72097	493	sense-overlappin	XM_011539351.1	CTBP2
circRNA_07503	0	0	0.04361729	0.0206362	0	0	0	0	0	0	0.02025523	0	hsa_circ_0002545	NC_000010.11	-	125038997	125122868	83872	12268	sense-overlappin	XM_011539351.1	CTBP2
circRNA_07504	0	0	0.04361729	0	0	0	0	0	0	0.020172483	0	0	-	NC_000010.11	-	125038997	125133612	94616	576	sense-overlappin	XM_011539350.1	CTBP2
circRNA_07505	0	0	0.04361729	0	0.02006311	0	0	0	0	0	0	0	hsa_circ_0009087	NC_000010.11	-	125088191	125111093	22903	233	sense-overlappin	XM_005269567.2	CTBP2
circRNA_07506	0	0	0	0	0	0	0	0	0	0	0.030382845	0	hsa_circ_0020354	NC_000010.11	-	125110990	125111093	104	104	exonic	NM_001290215.1	CTBP2
circRNA_07507	0	0	0.13085187	0	0	0	0.030148028	0	0	0	0	0	hsa_circ_0004875	NC_000010.11	-	125110990	125122868	11879	316	sense-overlappin	XM_011539350.1	CTBP2
circRNA_07508	0	0	0	0.0515905	0	0.031069364	0	0	0	0	0	0	-	NC_000010.11	-	125852293	125881071	28779	1598	sense-overlappin	NM_018180.2	DHX32
circRNA_07509	0	0	0.141756193	0.1341352	0.06018934	0	0.020098685	0	0	0.030258724	0.030382845	0	hsa_circ_0007142	NC_000010.11	+	126970702	126990603	19902	427	sense-overlappin	NM_001290223.1	DOCK1
circRNA_07510	0.092587611	0.112584821	0.316225353	0.1444533	0.11034713	0.062138728	0.050246713	0.051215739	0.070890847	0.060517448	0	0.0412728	hsa_circ_0002669	NC_000010.11	+	126970702	127000307	29606	939	sense-overlappin	NM_001290223.1	DOCK1
circRNA_07511	0	0	0.021808645	0	0	0	0	0	0	0	0	0	hsa_circ_0004102	NC_000010.11	+	126970702	127008804	38103	1012	sense-overlappin	NM_001290223.1	DOCK1
circRNA_07512	0	0	0.021808645	0	0	0	0	0	0	0	0	0	hsa_circ_0020385	NC_000010.11	+	126970702	127018835	48134	1281	sense-overlappin	NM_001290223.1	DOCK1

circRNA_07513	0	0	0.021808645	0	0	0	0	0	0	0	0	0	hsa_circ_0020388	NC_000010.11	+	126970702	127026424	55723	1578	sense-overlappin	NM_001290223.1	DOCK1
circRNA_07514	0	0	0.032712968	0	0	0	0	0	0	0	0	0	hsa_circ_0020390	NC_000010.11	+	126970702	127037816	67115	1964	sense-overlappin	NM_001290223.1	DOCK1
circRNA_07515	0	0	0.08723458	0	0	0	0	0	0	0	0	0	hsa_circ_0020392	NC_000010.11	+	126970702	127043164	72463	2155	sense-overlappin	NM_001290223.1	DOCK1
circRNA_07516	0	0	0.021808645	0	0	0	0	0	0	0	0	0	hsa_circ_0020393	NC_000010.11	+	126970702	127052815	82114	2290	sense-overlappin	NM_001290223.1	DOCK1
circRNA_07517	0	0.051174919	0.098138903	0.0206362	0	0.031069364	0	0	0	0	0	0	hsa_circ_0020394	NC_000010.11	+	126970702	127061776	91075	2399	sense-overlappin	NM_001290223.1	DOCK1
circRNA_07518	0	0	0.032712968	0	0	0	0	0	0	0	0	0	hsa_circ_0020427	NC_000010.11	+	126996748	126999435	2688	376	sense-overlappin	NM_001290223.1	DOCK1
circRNA_07519	0	0	0	0.0206362	0	0	0	0	0	0	0	0	hsa_circ_0003970	NC_000010.11	+	126996748	127000307	3560	512	sense-overlappin	NM_001290223.1	DOCK1
circRNA_07520	0	0	0	0	0	0	0	0	0	0	0	0.0309546	hsa_circ_0003499	NC_000010.11	+	127008732	127018835	10104	342	sense-overlappin	NM_001290223.1	DOCK1
circRNA_07521	0	0	0.032712968	0	0	0	0	0	0	0	0	0	hsa_circ_0002050	NC_000010.11	+	127031650	127037816	6167	386	sense-overlappin	NM_001290223.1	DOCK1
circRNA_07522	0	0	0	0	0	0	0	0	0	0.020172483	0	0	hsa_circ_0020457	NC_000010.11	+	127042625	127061776	19152	435	sense-overlappin	NM_001290223.1	DOCK1
circRNA_07523	0.030862537	0	0	0	0	0	0	0	0	0	0	0	hsa_circ_0020462	NC_000010.11	+	127061668	127110354	48687	287	sense-overlappin	NM_001290223.1	DOCK1
circRNA_07524	0.041150049	0	0.054521613	0	0	0	0	0	0	0	0	0	hsa_circ_0002446	NC_000010.11	+	127061668	127127764	66097	511	sense-overlappin	NM_001290223.1	DOCK1
circRNA_07525	0	0.020469968	0.021808645	0	0	0	0	0	0	0	0	0	-	NC_000010.11	+	129536241	129538293	2053	2053	sense-overlappin	NM_002412.3	MGMT
circRNA_07526	0	0	0	0	0	0.031069364	0	0	0	0	0	0	-	NC_000010.11	+	129536241	129538443	2203	2203	sense-overlappin	NM_002412.3	MGMT
circRNA_07527	0	0.020469968	0	0	0.0702209	0	0	0	0	0	0	0	hsa_circ_0020506	NC_000010.11	+	130166507	130171636	5130	346	sense-overlappin	NM_006541.4	GLRX3
circRNA_07528	0	0	0	0	0	0	0.020098685	0	0	0	0	0	-	NC_000010.11	+	131934458	131945459	11002	706	sense-overlappin	NR_033191.2	
circRNA_07529	0	0	0.054521613	0	0.03009467	0	0	0	0	0	0	0	-	NC_000010.11	+	131934458	131947791	13334	968	sense-overlappin	NR_033191.2	
circRNA_07530	0	0	0	0	0	0	0	0	0	0.040344966	0	0	hsa_circ_0020523	NC_000010.11	+	131943968	131945459	1492	343	sense-overlappin	NM_001291310.1	PPP2R2D
circRNA_07531	0	0	0	0	0.02006311	0.082851637	0	0	0.020254528	0	0	0	hsa_circ_0020545	NC_000010.11	+	132690392	132710456	20065	341	sense-overlappin	XR_945735.1	
circRNA_07532	0	0	0.032712968	0	0.02006311	0	0	0	0	0	0	0	hsa_circ_0000271	NC_000010.11	+	133395713	133402773	7061	640	sense-overlappin	NM_138384.2	MTG1
circRNA_07533	0	0	0.032712968	0	0	0	0	0	0	0	0	0	-	NC_000010.11	+	133493287	133498005	4719	4719	intergenic		
circRNA_07534	0	0	0	0.0206362	0	0	0	0	0	0	0	0	-	NC_000011.10	-	230452	233215	2764	334	sense-overlappin	NM_001017524.2	SIRT3
circRNA_07535	0	0	0	0	0.05015779	0	0	0	0	0	0	0	-	NC_000011.10	+	309018	314356	5339	5339	sense-overlappin	NM_006435.2	IFITM2
circRNA_07536	0	0	0.021808645	0	0	0	0	0	0	0	0	0	-	NC_000011.10	-	679688	681089	1402	256	sense-overlappin	NM_021008.3	DEAF1
circRNA_07537	0	0	0	0	0	0	0	0	0	0.020172483	0	0	hsa_circ_0003307	NC_000011.10	+	755879	760253	4375	364	sense-overlappin	NM_006755.1	TALDO1
circRNA_07538	0	0	0.04361729	0	0	0	0.020098685	0	0	0	0	0	hsa_circ_0008301	NC_000011.10	-	1286002	1295794	9793	577	sense-overlappin	NM_019009.3	TOLLIP
circRNA_07539	0.020575025	0	0	0	0	0	0	0	0	0	0	0	-	NC_000011.10	-	1470271	1480885	10615	598	sense-overlappin	NM_053005.5	MOB2
circRNA_07540	0	0	0	0	0.02006311	0	0	0	0	0	0	0	-	NC_000011.10	+	2453281	2455192	1912	1912	intronic	NM_000218.2	KCNQ1
circRNA_07541	0	0	0.021808645	0	0	0	0	0	0	0	0	0	-	NC_000011.10	+	2570628	2572986	2359	444	sense-overlappin	NM_000218.2	KCNQ1
circRNA_07542	0.020575025	0.030704951	0	0.113499	0.03009467	0	0.020098685	0.051215739	0.030381792	0	0.02025523	0	-	NC_000011.10	+	3395713	3397406	1694	1694	intergenic		
circRNA_07543	0	0	0.021808645	0	0	0	0	0	0	0	0	0	hsa_circ_0004146	NC_000011.10	-	3691347	3693375	2029	287	sense-overlappin	XM_011520135.1	NUP98
circRNA_07544	0	0	0.021808645	0	0	0	0	0	0	0.030258724	0	0	hsa_circ_0006430	NC_000011.10	-	3691347	3695606	4260	445	sense-overlappin	XM_011520135.1	NUP98
circRNA_07545	0.020575025	0	0	0	0	0	0	0	0	0	0	0	hsa_circ_0006548	NC_000011.10	-	3702463	3706627	4165	770	sense-overlappin	XM_011520135.1	NUP98
circRNA_07546	0	0	0	0	0.02006311	0	0	0	0	0	0	0	hsa_circ_0020875	NC_000011.10	-	3705200	3706627	1428	340	sense-overlappin	XM_011520135.1	NUP98
circRNA_07547	0	0	0	0	0	0	0.020098685	0	0	0	0	0	-	NC_000011.10	-	3719412	3753408	33997	1225	sense-overlappin	XM_011520135.1	NUP98
circRNA_07548	0	0	0	0	0.02006311	0	0	0	0	0	0	0	hsa_circ_0003944	NC_000011.10	-	3723157	3753408	30252	972	sense-overlappin	XM_011520135.1	NUP98
circRNA_07549	0	0	0.04361729	0	0.06018934	0.020712909	0	0	0	0.050431207	0	0	hsa_circ_0000274	NC_000011.10	-	3731391	3753408	22018	556	sense-overlappin	XM_011520135.1	NUP98
circRNA_07550	0	0	0	0	0.02006311	0	0	0	0	0	0.02025523	0	-	NC_000011.10	-	3756716	3757538	823	823	intronic	XM_011520135.1	NUP98
circRNA_07551	0	0	0	0	0.02006311	0	0	0	0	0	0	0	hsa_circ_0004251	NC_000011.10	-	3762902	3776021	13120	731	sense-overlappin	XM_011520135.1	NUP98
circRNA_07552	0.051437562	0	0.021808645	0	0.02006311	0.051782273	0	0.030729443	0	0.020172483	0	0	hsa_circ_0007401	NC_000011.10	-	3768581	3776021	7441	593	sense-overlappin	XM_011520135.1	NUP98
circRNA_07553	0	0	0	0	0.03009467	0	0	0	0	0	0	0	-	NC_000011.10	-	3771157	3774464	3308	1605	sense-overlappin	XM_011520135.1	NUP98
circRNA_07554	0	0	0	0.0309543	0	0	0.040197371	0	0	0	0	0	hsa_circ_0004960	NC_000011.10	-	3773632	3776021	2390	248	sense-overlappin	XM_011520135.1	NUP98
circRNA_07555	0	0	0	0	0	0.031069364	0	0	0	0	0	0	hsa_circ_0020936	NC_000011.10	+	4055526	4074679	19154	584	sense-overlappin	NM_001277961.1	STIM1
circRNA_07556	0	0	0	0	0	0	0	0	0	0.050431207	0	0	hsa_circ_0020982	NC_000011.10	+	5698315	5706616	8302	254	sense-overlappin	NM_001199573.1	TRIM22
circRNA_07557	0	0	0	0.0309543	0	0	0	0	0	0	0	0	-	NC_000011.10	+	6323585	6342391	18807	18807	intergenic		
circRNA_07558	0	0	0	0	0	0	0.020098685	0	0.030381792	0	0	0	-	NC_000011.10	+	6932094	6955782	23689	984	sense-overlappin	XM_011520358.1	ZNF215
circRNA_07559	0	0.092114854	0.098138903	0	0	0.041425819	0	0.061458886	0	0.151293621	0.02025523	0.0309546	hsa_circ_0008338	NC_000011.10	+	6941571	6955782	14212	405	sense-overlappin	XM_011520358.1	ZNF215
circRNA_07560	0	0	0	0	0	0	0.020098685	0	0	0	0	0	-	NC_000011.10	-	8137378	8140193	2816	397	sense-overlappin	XR_428851.2	
circRNA_07561	0	0	0.021808645	0	0	0	0	0	0	0	0	0	-	NC_000011.10	-	8286265	8294971	8707	8707	intergenic		
circRNA_07562	0.154312685	0.040939935	0.054521613	0.0619086	0.09028402	0.072495183	0	0.030729443	0.030381792	0.060517448	0.06076569	0.051591	-	NC_000011.10	-	8413495	8464822	51328	1005	sense-overlappin	NM_001289061.1	STK33
circRNA_07563	0	0	0.021808645	0	0	0.031069364	0	0	0	0	0	0	-	NC_000011.10	-	8413495	8474816	61322	1255	sense-overlappin	NM_001289061.1	STK33
circRNA_07564	0	0	0.04361729	0	0	0	0	0	0	0	0	0	-	NC_000011.10	-	8435494	8440753	5260	275	sense-overlappin	NM_001289061.1	STK33
circRNA_07565	0.061725074	0	0.109043225	0.0309543	0.04012623	0.020712909	0	0.020486295	0	0.020172483	0	0	hsa_circ_0021089	NC_000011.10	-	8435494	8464822	29329	807	sense-overlappin	NM_001289061.1	STK33
circRNA_07566	0	0.061409903	0.054521613	0	0	0	0	0	0	0	0	0	-	NC_000011.10	-	8452822	8464822	12001	532	sense-overlappin	NM_001289061.1	STK33
circRNA_07567	0.020575025	0	0	0	0	0	0	0	0	0	0	0	hsa_circ_0021102	NC_000011.10	-	8707085	8712735	5651	687	sense-overlappin	XR_930888.1	

circRNA_07568	0	0	0.032712968	0	0	0	0	0	0	0	0	0	hsa_circ_0005944	NC_000011.10	-	8710845	8712735	1891	365	sense-overlappin	XM_011520330.1	ST5
circRNA_07569	0	0	0.021808645	0	0	0	0	0	0	0	0	0	hsa_circ_0021110	NC_000011.10	-	8811247	8839350	28104	263	sense-overlappin	XM_011520312.1	ST5
circRNA_07570	0	0.020469968	0	0	0	0	0	0	0	0	0	0	-	NC_000011.10	+	8965904	8968041	2138	2138	intergenic		
circRNA_07571	0	0	0	0.0309543	0	0	0	0	0	0	0	0	-	NC_000011.10	-	9047943	9053215	5273	465	sense-overlappin	NM_020974.2	SCUBE2
circRNA_07572	0	0	0	0	0.02006311	0.041425819	0	0	0	0	0	0	hsa_circ_0021155	NC_000011.10	-	9286455	9295387	8933	474	sense-overlappin	XR_930870.1	
circRNA_07573	0	0	0.021808645	0	0	0	0	0	0	0	0	0.051591	hsa_circ_0021157	NC_000011.10	-	9287702	9295387	7686	335	sense-overlappin	XR_930870.1	
circRNA_07574	0.020575025	0	0	0	0	0	0	0	0	0	0	0	-	NC_000011.10	+	9414255	9420698	6444	427	sense-overlappin	NM_006391.2	IPO7
circRNA_07575	0	0	0.04361729	0.0206362	0.06018934	0	0.040197371	0	0	0.030258724	0	0	hsa_circ_0005092	NC_000011.10	+	9429674	9431003	1330	290	sense-overlappin	NM_006391.2	IPO7
circRNA_07576	0	0	0	0.0412724	0	0.051782273	0	0	0	0	0.02025523	0	hsa_circ_0004007	NC_000011.10	+	9440455	9442197	1743	324	sense-overlappin	NM_006391.2	IPO7
circRNA_07577	0	0	0.021808645	0	0	0.020712909	0	0	0	0.020172483	0	0	-	NC_000011.10	+	9713466	9732710	19245	840	sense-overlappin	NM_015055.3	SWAP70
circRNA_07578	0	0	0	0	0.03009467	0	0	0	0	0	0	0	-	NC_000011.10	+	9713466	9749183	35718	1411	sense-overlappin	NM_015055.3	SWAP70
circRNA_07579	0	0	0	0.0206362	0	0	0	0	0	0	0.02025523	0	-	NC_000011.10	-	9989497	10002689	13193	776	sense-overlappin	NM_030962.3	SBF2
circRNA_07580	0	0	0.021808645	0	0	0	0	0	0	0	0	0	-	NC_000011.10	-	9989497	10031170	41674	1116	sense-overlappin	NM_030962.3	SBF2
circRNA_07581	0	0	0.032712968	0	0	0	0	0.030729443	0	0	0	0	hsa_circ_0007055	NC_000011.10	-	9998266	10002689	4424	356	sense-overlappin	NM_030962.3	SBF2
circRNA_07582	0	0	0	0.0206362	0	0	0	0	0	0	0	0	hsa_circ_0021237	NC_000011.10	-	10028452	10042981	14530	478	sense-overlappin	NM_030962.3	SBF2
circRNA_07583	0	0	0	0	0	0	0	0	0.020254528	0	0	0	-	NC_000011.10	-	11327086	11341004	13919	420	sense-overlappin	XM_011520068.1	GALNT18
circRNA_07584	0	0	0	0	0	0	0	0.020486295	0	0	0	0	-	NC_000011.10	+	12319696	12327266	7571	303	sense-overlappin	NM_032867.2	MICALCL
circRNA_07585	0	0	0	0	0	0.031069364	0	0	0	0	0	0	-	NC_000011.10	+	12405866	12406765	900	900	intronic	XM_005253015.3	PARVA
circRNA_07586	0	0	0	0	0	0.020712909	0	0	0	0	0	0	-	NC_000011.10	+	12495061	12518517	23457	2039	sense-overlappin	XM_005253015.3	PARVA
circRNA_07587	0.041150049	0	0	0	0	0	0	0	0	0.030258724	0	0	-	NC_000011.10	+	12496458	12513360	16903	398	sense-overlappin	XM_005253015.3	PARVA
circRNA_07588	0.020575025	0	0.021808645	0.0825448	0.02006311	0	0.020098685	0	0	0	0	0	-	NC_000011.10	+	12764179	12764434	256	256	exonic	NM_021961.5	TEAD1
circRNA_07589	0.020575025	0	0	0	0	0	0	0	0	0	0	0	hsa_circ_0003015	NC_000011.10	+	12879708	12902113	22406	543	sense-overlappin	NM_021961.5	TEAD1
circRNA_07590	0	0	0	0.0412724	0	0	0	0	0	0	0	0	hsa_circ_0021310	NC_000011.10	+	12879708	12925052	45345	684	sense-overlappin	NM_021961.5	TEAD1
circRNA_07591	0.061725074	0	0.13085187	0.1031809	0.02006311	0.031069364	0.060296056	0.040972591	0.040509055	0.050431207	0.050638075	0.0309546	hsa_circ_0008967	NC_000011.10	-	13413530	13421838	8309	707	sense-overlappin	NM_001297742.1	BTBD10
circRNA_07592	0.041150049	0.020469968	0	0.0619086	0	0.072495183	0.040197371	0.020486295	0	0.050431207	0	0	hsa_circ_0003234	NC_000011.10	-	13413530	13445181	31652	865	sense-overlappin	NM_032320.6	BTBD10
circRNA_07593	0.030862537	0	0	0	0	0	0	0	0	0	0	0	-	NC_000011.10	+	14041521	14080021	38501	331	sense-overlappin	NM_006108.3	SPON1
circRNA_07594	0	0	0	0	0	0	0	0	0.020254528	0	0	0	-	NC_000011.10	-	14457784	14458687	904	256	sense-overlappin	NM_016451.4	COPB1
circRNA_07595	0	0	0.021808645	0	0	0	0	0	0	0	0	0	-	NC_000011.10	-	14457784	14498985	41202	2959	sense-overlappin	NM_016451.4	COPB1
circRNA_07596	0	0	0	0	0	0	0	0.020486295	0	0	0	0	hsa_circ_0021327	NC_000011.10	-	14469336	14477015	7680	607	sense-overlappin	NM_016451.4	COPB1
circRNA_07597	0	0	0.054521613	0	0	0	0	0.061458886	0.040509055	0.040344966	0.02025523	0	hsa_circ_0000277	NC_000011.10	+	14771937	14789242	17306	437	sense-overlappin	XM_011520183.1	PDE3B
circRNA_07598	0	0	0	0	0	0	0	0	0	0	0.02025523	0	hsa_circ_0021337	NC_000011.10	+	14771937	14819209	47273	829	sense-overlappin	XM_011520183.1	PDE3B
circRNA_07599	0	0	0.021808645	0	0	0	0	0	0	0	0	0	-	NC_000011.10	+	14843827	14853560	9734	9734	sense-overlappin	NM_000922.3	PDE3B
circRNA_07600	0	0	0	0	0	0	0	0	0.020254528	0	0	0	-	NC_000011.10	+	15177111	15190814	13704	396	sense-overlappin	NM_001278314.1	INSC
circRNA_07601	0	0	0	0	0	0.041425819	0	0	0	0	0	0	-	NC_000011.10	+	15344834	15345293	460	460	intergenic		
circRNA_07602	0	0.020469968	0	0	0	0	0	0	0	0	0	0	-	NC_000011.10	-	16111803	16186955	75153	363	sense-overlappin	NM_001145819.1	SOX6
circRNA_07603	0	0	0	0	0	0	0	0.020486295	0.020254528	0	0	0	-	NC_000011.10	-	16318446	16341252	22807	449	sense-overlappin	NM_001145819.1	SOX6
circRNA_07604	0.020575025	0	0	0	0	0	0	0	0	0	0	0	-	NC_000011.10	-	16800974	16803295	2322	402	sense-overlappin	XM_011519913.1	PLEKHA7
circRNA_07605	0	0	0.021808645	0	0	0	0	0	0	0	0	0	hsa_circ_0021369	NC_000011.10	-	16841547	16855914	14368	567	sense-overlappin	XM_011519913.1	PLEKHA7
circRNA_07606	0	0	0.021808645	0	0	0	0.020098685	0	0.030381792	0	0	0	-	NC_000011.10	+	17020473	17026982	6510	6510	intergenic		
circRNA_07607	0	0.040939935	0	0	0	0.041425819	0	0	0	0.030258724	0.04051046	0	hsa_circ_0021396	NC_000011.10	-	17131916	17145942	14027	671	sense-overlappin	XM_005252978.3	PIK3C2A
circRNA_07608	0	0	0	0	0.02006311	0	0	0	0	0	0	0	-	NC_000011.10	+	17295324	17296211	888	252	sense-overlappin	XM_011520127.1	NUCB2
circRNA_07609	0	0	0	0.0309543	0	0	0	0	0	0	0	0	-	NC_000011.10	+	17310825	17312120	1296	429	sense-overlappin	XM_011520127.1	NUCB2
circRNA_07610	0	0	0	0.0206362	0	0	0	0	0	0	0	0	-	NC_000011.10	+	17330127	17337534	7408	6804	sense-overlappin	XM_011520127.1	NUCB2
circRNA_07611	0	0	0	0	0.03009467	0	0	0	0	0	0	0	-	NC_000011.10	+	17336960	17337534	575	575	intronic	XM_011520127.1	NUCB2
circRNA_07612	0	0	0	0	0	0	0	0	0	0	0.030382845	0	-	NC_000011.10	-	17959470	18008076	48607	951	sense-overlappin	NM_012139.3	SERGEF
circRNA_07613	0	0	0	0	0	0	0.020098685	0.030729443	0	0	0	0	-	NC_000011.10	-	17959470	18010139	50670	3014	sense-overlappin	NM_012139.3	SERGEF
circRNA_07614	0	0.030704951	0.021808645	0	0	0	0	0	0	0	0	0	hsa_circ_0021412	NC_000011.10	-	17988597	18008076	19480	784	sense-overlappin	NM_012139.3	SERGEF
circRNA_07615	0	0	0.04361729	0	0.08025246	0	0	0	0.020254528	0	0	0	-	NC_000011.10	-	17988597	18010139	21543	2847	sense-overlappin	NM_012139.3	SERGEF
circRNA_07616	0	0	0	0	0	0	0	0	0	0.020172483	0	0	-	NC_000011.10	-	18000497	18010139	9643	2511	sense-overlappin	NM_012139.3	SERGEF
circRNA_07617	0	0	0	0	0	0	0	0	0.030381792	0	0	0	hsa_circ_0007484	NC_000011.10	-	18283795	18285459	1665	221	sense-overlappin	NM_007216.3	HPS5
circRNA_07618	0	0	0	0	0	0.020712909	0	0.020486295	0.040509055	0	0	0	hsa_circ_0021424	NC_000011.10	-	18283795	18286710	2916	341	sense-overlappin	NM_007216.3	HPS5
circRNA_07619	0.072012586	0.020469968	0.021808645	0.0928628	0.08025246	0.062138728	0.030148028	0.030729443	0.020254528	0.060517448	0	0	hsa_circ_0000279	NC_000011.10	-	18291442	18292019	578	578	exonic	NM_007216.3	HPS5
circRNA_07620	0.102875123	0.122819805	0.109043225	0.1960438	0.0702209	0.134633911	0.080394741	0.071702034	0.081018111	0.161379862	0.091148535	0.082545601	hsa_circ_0000280	NC_000011.10	-	18291442	18292976	1535	656	sense-overlappin	NM_007216.3	HPS5
circRNA_07621	0.030862537	0	0	0	0	0	0	0	0	0	0	0	-	NC_000011.10	+	18335754	18341607	5854	683	sense-overlappin	NM_001142307.1	GTF2H1

circRNA_07622	0	0	0	0	0.03009467	0	0	0	0	0	0	0	hsa_circ_0006469	NC_000011.10	+	18400837	18403811	2975	466	sense-overlappin	NM_001165415.1	LDHA
circRNA_07623	0	0	0.021808645	0	0	0	0	0	0	0	0	0	hsa_circ_0021460	NC_000011.10	-	18502486	18516164	13679	513	sense-overlappin	XM_005253108.2	TSG101
circRNA_07624	0	0	0	0	0	0	0.020098685	0	0	0	0	0	-	NC_000011.10	-	18502486	18519603	17118	598	sense-overlappin	XM_005253108.2	TSG101
circRNA_07625	0	0	0	0	0	0	0	0	0	0	0.02025523	0	hsa_circ_0021464	NC_000011.10	-	18514678	18516164	1487	230	sense-overlappin	XM_005253108.2	TSG101
circRNA_07626	0	0	0	0.0309543	0	0.041425819	0	0	0	0.040344966	0	0	hsa_circ_0002687	NC_000011.10	-	18514678	18519603	4926	315	sense-overlappin	XM_005253108.2	TSG101
circRNA_07627	0	0	0	0.0412724	0	0	0	0	0	0	0	0	hsa_circ_0021472	NC_000011.10	-	18570214	18578808	8595	315	sense-overlappin	NM_001261385.1	UEVLD
circRNA_07628	0.020575025	0	0	0	0	0	0	0	0	0	0	0	hsa_circ_0004819	NC_000011.10	+	20080064	20083179	3116	328	sense-overlappin	NM_001244963.1	NAV2
circRNA_07629	0.020575025	0	0	0	0	0	0	0	0	0	0	0	-	NC_000011.10	+	20095672	20097745	2074	265	sense-overlappin	XM_011520452.1	NAV2
circRNA_07630	0.030862537	0	0	0	0	0	0	0	0	0	0	0	hsa_circ_0021502	NC_000011.10	+	20402919	20426865	23947	288	sense-overlappin	NM_001145166.1	PRMT3
circRNA_07631	0	0	0	0	0	0	0	0.020486295	0.030381792	0	0	0	hsa_circ_0021506	NC_000011.10	+	22203804	22227586	23783	608	sense-overlappin	XM_005252822.3	ANO5
circRNA_07632	0.041150049	0.020469968	0.054521613	0.0619086	0	0.041425819	0	0	0	0.030258724	0.02025523	0	hsa_circ_0021507	NC_000011.10	+	22203804	22239684	35881	838	sense-overlappin	XM_005252822.3	ANO5
circRNA_07633	0	0	0.04361729	0	0	0	0	0	0	0	0	0	-	NC_000011.10	+	22203804	22263043	59240	1858	sense-overlappin	XM_005252822.3	ANO5
circRNA_07634	0	0	0.021808645	0	0	0	0	0	0	0	0	0	-	NC_000011.10	+	22221097	22239684	18588	698	sense-overlappin	XM_005252822.3	ANO5
circRNA_07635	0	0	0.04361729	0	0	0.082851637	0	0.020486295	0	0	0	0	-	NC_000011.10	+	22221097	22263043	41947	1718	sense-overlappin	XM_005252822.3	ANO5
circRNA_07636	0	0	0.032712968	0	0	0	0	0	0	0	0	0	-	NC_000011.10	+	22674850	22755953	81104	743	sense-overlappin	XM_011519972.1	GAS2
circRNA_07637	0	0	0.021808645	0	0	0	0	0	0	0	0	0	-	NC_000011.10	-	27047103	27066662	19560	18009	antisense	XM_005253160.2	BBOX1
circRNA_07638	0	0	0.021808645	0	0	0	0	0	0	0	0	0	hsa_circ_0021523	NC_000011.10	-	27371615	27392518	20904	1322	sense-overlappin	NM_018490.2	LGR4
circRNA_07639	0.020575025	0	0	0	0	0	0	0	0	0	0	0	-	NC_000011.10	+	28202935	28228931	25997	25997	sense-overlappin	XM_011519940.1	METTL15
circRNA_07640	0	0	0.054521613	0	0	0	0	0	0	0	0	0	-	NC_000011.10	+	28398788	28424364	25577	25577	sense-overlappin	XR_930849.1	
circRNA_07641	0.020575025	0	0.021808645	0	0.03009467	0	0	0	0	0	0	0	-	NC_000011.10	+	28407013	28424364	17352	17352	sense-overlappin	XR_930849.1	
circRNA_07642	0	0	0	0	0.04012623	0	0	0	0	0	0	0	-	NC_000011.10	+	28411898	28424364	12467	12467	sense-overlappin	XR_930849.1	
circRNA_07643	0	0	0	0	0	0	0	0.020486295	0	0	0	0	-	NC_000011.10	+	29383256	29384112	857	857	intergenic		
circRNA_07644	0	0	0.04361729	0	0	0	0	0	0	0	0	0	-	NC_000011.10	-	31241450	31307908	66459	22514	sense-overlappin	NM_181807.3	DCDC1
circRNA_07645	0	0	0	0.0309543	0	0	0	0	0	0	0	0.0309546	hsa_circ_0003712	NC_000011.10	-	31452718	31463305	10588	3892	sense-overlappin	NM_001304274.1	IMMP1L
circRNA_07646	0.020575025	0	0	0	0	0	0	0	0	0	0	0	hsa_circ_0007245	NC_000011.10	+	32097144	32098528	1385	373	sense-overlappin	NM_002901.2	RCN1
circRNA_07647	0	0.030704951	0.04361729	0.0206362	0.03009467	0	0	0	0.020254528	0.050431207	0	0	hsa_circ_0003119	NC_000011.10	+	32589547	32596047	6501	361	sense-overlappin	NM_006360.4	EIF3M
circRNA_07648	0.041150049	0.020469968	0.239895095	0.2476343	0.08025246	0.093208092	0.100493427	0.040972591	0.050636319	0.07060369	0.02025523	0.0206364	hsa_circ_0021570	NC_000011.10	+	32927157	32935435	8279	3968	sense-overlappin	NM_001076786.2	QSER1
circRNA_07649	0	0	0.021808645	0	0	0	0	0.030729443	0	0.030258724	0	0	hsa_circ_0000283	NC_000011.10	-	33105567	33106064	498	229	sense-overlappin	NM_001326.2	CSTF3
circRNA_07650	0.041150049	0	0	0	0	0	0	0	0	0	0	0	-	NC_000011.10	-	33105567	33108418	2852	360	sense-overlappin	NM_001326.2	CSTF3
circRNA_07651	0	0	0	0	0.03009467	0	0	0	0	0	0	0	-	NC_000011.10	-	33141667	33161369	19703	269	sense-overlappin	NM_001326.2	CSTF3
circRNA_07652	2.119227535	0.747153815	1.668361344	1.7643941	1.76555412	1.377408471	1.085329009	0.778479229	0.810181108	1.422160037	0.779826358	0.722274006	hsa_circ_0000284	NC_000011.10	+	33286413	33287511	1099	1099	exonic	NM_001278163.1	HIPK3
circRNA_07653	0.030862537	0	0	0	0	0	0	0	0	0.040344966	0	0	-	NC_000011.10	+	33286413	33306009	19597	19597	sense-overlappin	NM_001278163.1	HIPK3
circRNA_07654	0.030862537	0	0	0.0206362	0.0702209	0.020712909	0	0	0	0.020172483	0.02025523	0	hsa_circ_0008887	NC_000011.10	+	33286413	33328633	42221	1223	sense-overlappin	NM_001278163.1	HIPK3
circRNA_07655	0	0	0	0	0.02006311	0	0	0	0.020254528	0	0	0	hsa_circ_0021603	NC_000011.10	+	33337075	33341686	4612	676	sense-overlappin	NM_001278163.1	HIPK3
circRNA_07656	0.030862537	0	0	0	0	0	0	0	0	0	0	0	-	NC_000011.10	+	33337075	33348013	10939	1085	sense-overlappin	NM_001278163.1	HIPK3
circRNA_07657	0	0	0	0	0.02006311	0	0	0	0	0	0	0	hsa_circ_0021604	NC_000011.10	+	33338757	33341686	2930	556	sense-overlappin	NM_001278163.1	HIPK3
circRNA_07658	0.030862537	0.051174919	0.141756193	0.0928628	0.03009467	0.031069364	0.040197371	0.051215739	0.081018111	0.090776173	0	0.051591	hsa_circ_0005974	NC_000011.10	+	34071726	34076642	4917	472	sense-overlappin	XR_930869.1	
circRNA_07659	0.020575025	0	0	0	0	0	0	0	0	0	0	0	-	NC_000011.10	+	34079628	34086413	6786	543	sense-overlappin	XR_930869.1	
circRNA_07660	0	0	0.021808645	0	0	0	0	0	0	0	0	0	hsa_circ_0000286	NC_000011.10	+	34079628	34089456	9829	605	sense-overlappin	XR_930869.1	
circRNA_07661	0	0	0.065425935	0.0412724	0	0	0	0	0	0.020172483	0	0	hsa_circ_0021639	NC_000011.10	+	34082825	34089456	6632	467	sense-overlappin	XR_930869.1	
circRNA_07662	0	0.061409903	0	0	0	0	0	0	0	0	0	0	hsa_circ_0021644	NC_000011.10	+	34086064	34086413	350	265	sense-overlappin	XR_930869.1	
circRNA_07663	0	0	0.021808645	0	0	0	0	0.030729443	0	0	0	0	hsa_circ_0000287	NC_000011.10	+	34086064	34092056	5993	739	sense-overlappin	NM_203364.2	CAPRIN1
circRNA_07664	0	0	0	0	0	0.020712909	0	0	0	0	0	0	hsa_circ_0021652	NC_000011.10	+	34089395	34090678	1284	323	sense-overlappin	XR_930869.1	
circRNA_07665	0	0	0	0.0206362	0.0702209	0	0	0	0	0	0	0.0412728	hsa_circ_0002280	NC_000011.10	+	34089395	34092056	2662	474	sense-overlappin	NM_203364.2	CAPRIN1
circRNA_07666	0	0	0	0	0	0	0	0	0.020254528	0	0	0	hsa_circ_0000288	NC_000011.10	+	34090179	34090678	500	261	sense-overlappin	XR_930869.1	
circRNA_07667	0	0	0.04361729	0.0412724	0	0.020712909	0	0	0.020254528	0.030258724	0	0.0309546	hsa_circ_0004851	NC_000011.10	+	34090179	34092056	1878	412	sense-overlappin	NM_203364.2	CAPRIN1
circRNA_07668	0	0	0	0	0	0	0	0	0	0	0	0.0206364	-	NC_000011.10	+	34319946	34320951	1006	1006	antisense	NM_145804.2	ABTB2
circRNA_07669	0	0	0	0	0.02006311	0	0	0	0	0	0	0	hsa_circ_0021707	NC_000011.10	+	34931404	34970286	38883	804	sense-overlappin	XM_011520390.1	PDHX
circRNA_07670	0	0	0	0.0206362	0	0	0	0	0	0	0	0	-	NC_000011.10	+	34931404	34978182	46779	863	sense-overlappin	XM_011520390.1	PDHX
circRNA_07671	0	0	0.032712968	0	0.06018934	0	0	0	0	0	0	0.051591	hsa_circ_0021708	NC_000011.10	+	34931404	34984728	53325	1022	sense-overlappin	XM_011520390.1	PDHX
circRNA_07672	0	0	0	0.0206362	0	0	0	0	0	0	0	0	hsa_circ_0006254	NC_000011.10	+	34947506	34970286	22781	723	sense-overlappin	XM_011520390.1	PDHX
circRNA_07673	0	0	0	0	0	0	0.030148028	0	0	0	0	0	hsa_circ_0021709	NC_000011.10	+	34947506	34978182	30677	782	sense-overlappin	XM_011520390.1	PDHX
circRNA_07674	0.051437562	0.030704951	0.098138903	0.1238171	0.12037869	0.113921001	0.080394741	0.040972591	0.050636319	0.110948655	0.091148535	0	hsa_circ_0021712	NC_000011.10	+	34957384	34978182	20799	681	sense-overlappin	XM_011520390.1	PDHX
circRNA_07675	0	0	0	0.0412724	0	0	0	0	0	0	0.02025523	0	hsa_circ_0003768	NC_000011.10	+	34957384	34984728	27345	840	sense-overlappin	XM_011520390.1	PDHX
circRNA_07676	0	0	0.054521613	0	0.05015779	0	0	0	0	0	0.02025523	0	-	NC_000011.10	+	34957384	34992379	34996	905	sense-overlappin	XM_011520390.1	PDHX

circRNA_07677	0	0	0	0.0515905	0	0	0.020098685	0	0	0	0	0	hsa_circ_0000290	NC_000011.10	+	34970139	34984728	14590	366	sense-overlappin	XM_011520390.1	PDHX
circRNA_07678	0	0	0	0.0309543	0	0	0	0	0	0	0	0	hsa_circ_0004453	NC_000011.10	+	36081653	36098461	16809	261	sense-overlappin	NM_001304263.1	LDLRAD3
circRNA_07679	0.051437562	0.030704951	0.032712968	0	0	0.051782273	0	0.051215739	0.101272639	0.110948655	0.050638075	0.0412728	hsa_circ_0006988	NC_000011.10	+	36227085	36227430	346	346	exonic	NM_001304263.1	LDLRAD3
circRNA_07680	0	0	0	0	0.02006311	0.020712909	0.040197371	0	0	0.030258724	0	0	hsa_circ_0005044	NC_000011.10	+	36393846	36403378	9533	7521	sense-overlappin	NM_001160167.1	PRR5L
circRNA_07681	0	0	0	0	0	0	0	0	0.020254528	0	0	0	-	NC_000011.10	+	36393846	36419303	25458	7570	sense-overlappin	NM_001160167.1	PRR5L
circRNA_07682	0	0	0.021808645	0	0	0	0	0	0	0	0	0	hsa_circ_0021760	NC_000011.10	+	36610081	36648155	38075	521	sense-overlappin	XM_011519885.1	C11orf74
circRNA_07683	0	0	0	0	0	0.020712909	0	0	0	0	0	0	-	NC_000011.10	-	38870077	38885214	15138	15138	intergenic		
circRNA_07684	0.020575025	0	0	0	0	0	0	0	0	0	0	0	-	NC_000011.10	+	43327789	43327871	83	83	exonic	NM_001142930.1	API5
circRNA_07685	0	0	0	0	0	0	0	0.020486295	0	0	0	0	hsa_circ_0021773	NC_000011.10	+	43399888	43407577	7690	1006	sense-overlappin	XM_005253017.2	TTC17
circRNA_07686	0	0	0.032712968	0	0	0	0	0	0	0	0	0	-	NC_000011.10	+	43403998	43407577	3580	732	sense-overlappin	XM_005253017.2	TTC17
circRNA_07687	0	0	0.032712968	0	0	0	0	0	0	0	0.02025523	0	hsa_circ_0009018	NC_000011.10	+	44107683	44114301	6619	773	sense-overlappin	NM_001178083.1	EXT2
circRNA_07688	0.041150049	0	0	0.0619086	0.03009467	0	0.020098685	0.020486295	0	0.020172483	0	0.0412728	hsa_circ_0000295	NC_000011.10	+	45862060	45872233	10174	1132	sense-overlappin	NM_001127457.2	CRY2
circRNA_07689	0	0	0	0.0206362	0	0	0	0	0	0	0	0	-	NC_000011.10	-	46076754	46084302	7549	257	sense-overlappin	XM_011520165.1	PHF21A
circRNA_07690	0.13373766	0.102349838	0.370746965	0.2476343	0.12037869	0.196772639	0.140690797	0.030729443	0.101272639	0.262242276	0.070893305	0.0412728	hsa_circ_0000296	NC_000011.10	-	46076754	46092223	15470	389	sense-overlappin	XM_011520174.1	PHF21A
circRNA_07691	0	0	0	0.0825448	0.1404418	0	0	0	0.040509055	0	0	0	hsa_circ_0007719	NC_000011.10	+	46312325	46312919	595	278	sense-overlappin	NM_052854.3	CREB3L1
circRNA_07692	0	0	0	0.0206362	0	0	0	0	0	0	0	0	hsa_circ_0009155	NC_000011.10	-	46493608	46494204	597	182	sense-overlappin	NM_017749.3	AMBRA1
circRNA_07693	0	0	0.076330258	0	0.02006311	0	0	0	0	0	0	0	hsa_circ_0007001	NC_000011.10	-	46493608	46508370	14763	362	sense-overlappin	NM_017749.3	AMBRA1
circRNA_07694	0	0	0.065425935	0	0	0	0	0	0	0	0	0	hsa_circ_0007061	NC_000011.10	-	46493608	46512813	19206	449	sense-overlappin	NM_017749.3	AMBRA1
circRNA_07695	0	0	0.021808645	0.0206362	0	0	0	0	0	0	0	0	hsa_circ_0008415	NC_000011.10	-	46493608	46548500	54893	2650	sense-overlappin	NM_001267782.1	AMBRA1
circRNA_07696	0	0	0.065425935	0	0	0	0	0	0	0	0	0	-	NC_000011.10	-	46783274	46788773	5500	374	sense-overlappin	NM_001008938.3	CKAP5
circRNA_07697	0	0.051174919	0.021808645	0	0	0	0	0	0	0	0	0	-	NC_000011.10	-	46808031	46821268	13238	1015	sense-overlappin	NM_001008938.3	CKAP5
circRNA_07698	0	0	0	0	0	0.051782273	0	0	0	0	0	0	-	NC_000011.10	-	46889411	46890494	1084	518	sense-overlappin	NM_002334.3	LRP4
circRNA_07699	0	0	0	0	0	0	0	0	0	0	0	0.0206364	-	NC_000011.10	-	46894589	46895291	703	357	sense-overlappin	NM_002334.3	LRP4
circRNA_07700	0	0	0	0	0	0.020712909	0	0	0	0	0	0	hsa_circ_0000303	NC_000011.10	-	47358844	47375729	16886	448	sense-overlappin	NM_001080547.1	SPI1
circRNA_07701	0	0.030704951	0	0.0412724	0.05015779	0	0.040197371	0	0.020254528	0	0	0	hsa_circ_0004075	NC_000011.10	-	47752916	47754664	1749	324	sense-overlappin	NM_015308.2	FNBP4
circRNA_07702	0	0	0	0.0206362	0	0	0	0	0	0	0	0	hsa_circ_0022075	NC_000011.10	-	47797779	47801930	4152	514	sense-overlappin	XM_006718170.2	NUP160
circRNA_07703	0	0	0	0	0	0	0	0.030729443	0	0	0	0	-	NC_000011.10	-	47815479	47816029	551	255	sense-overlappin	XM_006718170.2	NUP160
circRNA_07704	0	0	0	0	0	0	0	0	0	0.030258724	0	0	-	NC_000011.10	-	47815479	47819458	3980	409	sense-overlappin	XM_006718170.2	NUP160
circRNA_07705	0	0	0	0	0.02006311	0	0	0	0	0	0	0	hsa_circ_0022103	NC_000011.10	+	48139486	48139776	291	291	exonic	NM_002843.3	PTPRJ
circRNA_07706	0	0	0	0	0	0	0.020098685	0	0	0	0	0	-	NC_000011.10	+	48153796	48156119	2324	300	sense-overlappin	NM_002843.3	PTPRJ
circRNA_07707	0.051437562	0.071644886	0.032712968	0.1444533	0.02006311	0.072495183	0.020098685	0	0.060763583	0.100862414	0	0.0309546	hsa_circ_0005204	NC_000011.10	-	57491224	57491862	639	322	sense-overlappin	XM_011545323.1	SLC43A1
circRNA_07708	0	0	0.021808645	0	0	0	0	0	0	0	0	0	hsa_circ_0022169	NC_000011.10	+	57692611	57696873	4263	462	sense-overlappin	NM_015457.2	ZDHHC5
circRNA_07709	0	0	0	0.0309543	0	0	0	0	0	0	0.02025523	0	hsa_circ_0004997	NC_000011.10	+	57698559	57700397	1839	1392	sense-overlappin	NM_015457.2	ZDHHC5
circRNA_07710	0	0	0	0	0	0	0	0	0	0	0.02025523	0	-	NC_000011.10	+	57796457	57802196	5740	1000	sense-overlappin	NM_001085458.1	CTNND1
circRNA_07711	0	0	0.021808645	0	0	0	0	0	0.020254528	0	0	0	hsa_circ_0002904	NC_000011.10	-	58549786	58551232	1447	424	sense-overlappin	NM_004811.2	LPXN
circRNA_07712	0	0	0	0	0	0	0	0	0	0	0.08102092	0	-	NC_000011.10	-	58956312	58999689	43378	43378	antisense	XM_011545355.1	GLYATL1
circRNA_07713	0	0	0.021808645	0	0	0	0	0.020486295	0	0	0	0	hsa_circ_0004988	NC_000011.10	-	59639048	59643035	3988	398	sense-overlappin	XM_005273835.3	PATL1
circRNA_07714	0	0.061409903	0.021808645	0	0	0.051782273	0.050246713	0	0.050636319	0	0	0	hsa_circ_0004162	NC_000011.10	-	59658866	59659469	604	299	sense-overlappin	XM_005273833.3	PATL1
circRNA_07715	0	0	0	0.0206362	0	0	0	0	0	0	0	0	-	NC_000011.10	+	59773211	59797396	24186	870	sense-overlappin	NM_004177.4	STX3
circRNA_07716	0	0	0	0.0515905	0	0	0	0	0	0.030258724	0	0	-	NC_000011.10	-	59835808	59844055	8248	952	sense-overlappin	XM_011544939.1	GIF
circRNA_07717	0	0	0.04361729	0	0	0	0	0	0	0	0	0	hsa_circ_0005701	NC_000011.10	+	60919686	60920988	1303	348	sense-overlappin	NM_024092.2	TMEM109
circRNA_07718	0.030862537	0	0	0	0	0	0	0	0	0	0	0	-	NC_000011.10	-	60919815	60919918	104	104	antisense	NM_024092.2	TMEM109
circRNA_07719	0	0	0.021808645	0.0206362	0	0	0	0	0	0	0	0	hsa_circ_0002489	NC_000011.10	-	61132420	61138835	6416	474	sense-overlappin	NM_017966.4	VPS37C
circRNA_07720	0	0	0.032712968	0	0	0	0	0	0	0	0	0	-	NC_000011.10	-	61313499	61316567	3069	844	sense-overlappin	NM_001923.4	DDB1
circRNA_07721	0.041150049	0	0.04361729	0.0206362	0.03009467	0	0	0	0	0.040344966	0	0	hsa_circ_0003900	NC_000011.10	-	61326779	61330074	3296	454	sense-overlappin	NM_001923.4	DDB1
circRNA_07722	0.020575025	0.071644886	0.065425935	0.1238171	0.05015779	0.082851637	0.090444084	0.081945182	0.081018111	0.121034897	0.030382845	0.051591	hsa_circ_0002058	NC_000011.10	+	61366045	61367998	1954	248	sense-overlappin	NM_016464.4	TMEM138
circRNA_07723	0	0	0	0	0	0.020712909	0	0	0	0	0	0	-	NC_000011.10	-	61410938	61416519	5582	670	sense-overlappin	XM_011545257.1	CPSF7
circRNA_07724	0	0	0.021808645	0	0	0	0	0	0	0	0	0	-	NC_000011.10	+	61436761	61438113	1353	1198	sense-overlappin	NM_017841.2	SDHAF2
circRNA_07725	0	0	0	0	0	0	0.020098685	0	0	0	0	0	-	NC_000011.10	+	61437625	61438080	456	301	sense-overlappin	NM_017841.2	SDHAF2
circRNA_07726	0.051437562	0.040939935	0.26170374	0.0825448	0.10031557	0.020712909	0.090444084	0.020486295	0.091145375	0.080689931	0.02025523	0	hsa_circ_0000311	NC_000011.10	+	61437625	61438113	489	334	sense-overlappin	NM_017841.2	SDHAF2
circRNA_07727	0	0	0.021808645	0	0.02006311	0	0	0	0	0	0	0	-	NC_000011.10	+	61437625	61438362	738	583	sense-overlappin	NM_017841.2	SDHAF2
circRNA_07728	0.154312685	0.358224432	0	0	0	0	0	0	0	0	0	0	-	NC_000011.10	-	62510526	62536567	26042	24918	sense-overlappin	NM_024060.3	AHNAK
circRNA_07729	0.030862537	0	0	0	0	0	0	0	0	0	0	0	-	NC_000011.10	-	62516866	62517009	144	144	exonic	XM_005274240.1	AHNAK
circRNA_07730	0.051437562	0.030704951	0.054521613	0.0309543	0	0.031069364	0.020098685	0	0.020254528	0.030258724	0	0	hsa_circ_0008194	NC_000011.10	-	62535945	62536567	623	253	sense-overlappin	NM_024060.3	AHNAK
circRNA_07731	0	0.020469968	0	0	0	0.041425819	0	0	0	0	0	0	-	NC_000011.10	-	62569397	62570171	775	775	intronic	NM_001404.4	EEF1G

circRNA_07732	0.020575025	0	0	0	0	0	0.020098685	0	0	0	0	0	hsa_circ_0022487	NC_000011.10	-	62632565	62639731	7167	1024	sense-overlappin	NM_198335.3	GANAB
circRNA_07733	0	0.030704951	0.065425935	0	0.02006311	0	0.020098685	0	0	0	0	0	hsa_circ_0008011	NC_000011.10	-	62638983	62639731	749	342	sense-overlappin	NM_198334.2	GANAB
circRNA_07734	0	0	0.032712968	0.0412724	0	0	0	0	0.020254528	0	0	0	hsa_circ_0007866	NC_000011.10	-	62723587	62724426	840	353	sense-overlappin	NM_001079559.2	HNRNPUL2
circRNA_07735	0	0	0	0.0206362	0	0	0	0	0	0	0	0	hsa_circ_0002340	NC_000011.10	+	62735382	62738490	3109	386	sense-overlappin	XM_005273931.1	TTC9C
circRNA_07736	0	0	0	0	0	0	0	0	0	0.020172483	0	0	hsa_circ_0022537	NC_000011.10	-	62827155	62827631	477	198	sense-overlappin	NM_001244666.1	STX5
circRNA_07737	0	0	0.032712968	0	0	0	0	0	0	0	0	0	hsa_circ_0022599	NC_000011.10	-	63651936	63652575	640	156	sense-overlappin	XM_006718493.1	ATL3
circRNA_07738	0	0	0	0	0	0	0	0.020486295	0	0	0	0	hsa_circ_0022601	NC_000011.10	-	63658761	63659252	492	359	sense-overlappin	XM_006718493.1	ATL3
circRNA_07739	0	0	0	0.0206362	0	0	0	0	0	0	0	0	-	NC_000011.10	-	63669078	63669316	239	239	intronic	XM_006718493.1	ATL3
circRNA_07740	0	0	0	0	0	0.041425819	0	0.020486295	0	0	0	0	hsa_circ_0022631	NC_000011.10	+	64193078	64197974	4897	1014	sense-overlappin	NM_001282652.1	STIP1
circRNA_07741	0	0	0	0	0	0	0	0	0	0	0	0.0206364	hsa_circ_0022723	NC_000011.10	-	64859924	64860336	413	413	exonic	NM_006795.3	EHD1
circRNA_07742	0	0.020469968	0	0	0	0	0	0	0	0	0	0	-	NC_000011.10	+	65100011	65103160	3150	3150	intronic	NM_013265.3	VPS51
circRNA_07743	0	0.020469968	0	0	0	0.041425819	0.030148028	0	0	0	0	0	hsa_circ_0003812	NC_000011.10	+	65430769	65437657	6889	6889	intergenic		
circRNA_07744	0	0	0	0	0	0.041425819	0	0	0	0.050431207	0	0	-	NC_000011.10	+	65499273	65499423	151	151	intergenic		
circRNA_07745	0	0	0.021808645	0	0	0	0	0.030729443	0.030381792	0	0	0	-	NC_000011.10	-	65499667	65499765	99	99	intergenic		
circRNA_07746	0	0	0	0	0	0	0	0	0	0	0	0.0206364	-	NC_000011.10	+	65499689	65500063	375	375	intergenic		
circRNA_07747	0.072012586	0	0	0	0	0	0	0	0	0	0.070893305	0	-	NC_000011.10	+	65499726	65499826	101	101	intergenic		
circRNA_07748	0	0	0	0	0	0	0	0	0	0.07060369	0	0	-	NC_000011.10	+	65499777	65499888	112	112	intergenic		
circRNA_07749	0	0	0	0	0.03009467	0	0	0	0	0	0	0	-	NC_000011.10	+	65499944	65500063	120	120	intergenic		
circRNA_07750	0.041150049	0	0	0	0	0	0.020098685	0	0.050636319	0	0	0	-	NC_000011.10	+	65500520	65500650	131	131	intergenic		
circRNA_07751	0	0	0	0	0	0	0	0	0	0.080689931	0	0	-	NC_000011.10	+	65500912	65501007	96	96	intergenic		
circRNA_07752	0	0	0	0	0	0	0	0	0	0	0	0.0206364	-	NC_000011.10	+	65501312	65501630	319	319	intergenic		
circRNA_07753	0	0	0	0	0.03009467	0	0	0	0	0	0	0	-	NC_000011.10	+	65502321	65504358	2038	2038	intergenic		
circRNA_07754	0	0	0	0.0206362	0	0	0	0	0	0	0	0	-	NC_000011.10	+	66063628	66068333	4706	388	sense-overlappin	XM_005273726.2	SF3B2
circRNA_07755	0	0	0.021808645	0	0	0	0	0	0	0	0	0.0206364	hsa_circ_0006857	NC_000011.10	+	66238804	66239277	474	179	sense-overlappin	NM_018026.3	PACS1
circRNA_07756	0	0	0	0	0	0	0	0	0.030381792	0	0	0	hsa_circ_0022983	NC_000011.10	-	66365936	66369532	3597	948	sense-overlappin	NM_001532.2	SLC29A2
circRNA_07757	0	0	0	0.0206362	0.03009467	0	0	0	0.040509055	0	0	0	hsa_circ_0022989	NC_000011.10	+	66486540	66488007	1468	307	sense-overlappin	NM_001256670.1	DPP3
circRNA_07758	0	0	0	0	0	0	0	0	0	0	0	0.0206364	hsa_circ_0004168	NC_000011.10	-	66668615	66669291	677	677	exonic	NM_031492.3	RBM4B
circRNA_07759	0	0	0.04361729	0	0	0	0	0	0	0	0	0	hsa_circ_0023029	NC_000011.10	+	66748379	66828364	79986	1491	sense-overlappin	NM_024650.3	C11orf80
circRNA_07760	0	0	0.021808645	0	0	0	0	0	0	0	0	0	-	NC_000011.10	+	66756353	66759096	2744	207	sense-overlappin	NM_024650.3	C11orf80
circRNA_07761	0	0	0.032712968	0	0	0	0	0	0	0	0.04051046	0	-	NC_000011.10	+	66759043	66762450	3408	3408	sense-overlappin	NM_001302084.1	C11orf80
circRNA_07762	0	0	0	0	0	0	0	0	0	0	0.030382845	0	-	NC_000011.10	+	66803995	66828364	24370	826	sense-overlappin	NM_001302084.1	C11orf80
circRNA_07763	0	0	0	0	0	0	0	0	0	0.030258724	0	0	hsa_circ_0023031	NC_000011.10	+	66816053	66822674	6622	380	sense-overlappin	NM_001302084.1	C11orf80
circRNA_07764	0	0	0	0	0	0	0.020098685	0	0	0	0	0	hsa_circ_0005793	NC_000011.10	+	66816053	66828364	12312	479	sense-overlappin	NM_001302084.1	C11orf80
circRNA_07765	0	0	0.021808645	0	0	0	0	0	0	0	0	0	-	NC_000011.10	-	67873206	67874022	817	817	intergenic		
circRNA_07766	0	0	0.065425935	0.0515905	0	0.031069364	0.020098685	0	0	0.030258724	0	0.051591	-	NC_000011.10	-	68166982	68190152	23171	1318	sense-overlappin	NM_001300907.1	SUV420H1
circRNA_07767	0	0.020469968	0	0	0	0	0	0	0	0	0	0	hsa_circ_0023176	NC_000011.10	-	68171543	68185928	14386	728	sense-overlappin	NM_001300907.1	SUV420H1
circRNA_07768	0	0	0.021808645	0	0	0	0	0	0	0	0	0	-	NC_000011.10	-	68175018	68185928	10911	451	sense-overlappin	NM_001300907.1	SUV420H1
circRNA_07769	0	0	0.021808645	0.0206362	0	0.031069364	0	0	0.020254528	0	0	0.0206364	-	NC_000011.10	-	68185781	68190152	4372	384	sense-overlappin	XM_011545091.1	SUV420H1
circRNA_07770	0	0	0.021808645	0	0	0	0	0	0	0	0	0	hsa_circ_0023181	NC_000011.10	+	68347847	68363943	16097	792	sense-overlappin	XR_949925.1	
circRNA_07771	0	0	0.13085187	0.0206362	0.02006311	0	0	0	0	0	0	0	hsa_circ_0023182	NC_000011.10	+	68347847	68365702	17856	924	sense-overlappin	XR_949925.1	
circRNA_07772	0.020575025	0.020469968	0	0	0	0	0	0	0	0	0	0	-	NC_000011.10	+	68438446	68438682	237	237	exonic	XR_949925.1	
circRNA_07773	0	0	0.021808645	0	0	0	0	0	0	0.030258724	0	0	hsa_circ_0004449	NC_000011.10	+	68505145	68537891	32747	707	sense-overlappin	XM_011545138.1	PPP6R3
circRNA_07774	0	0	0	0.0206362	0	0	0	0	0	0	0	0	-	NC_000011.10	+	68515028	68537891	22864	5053	sense-overlappin	XM_011545138.1	PPP6R3
circRNA_07775	0	0	0.021808645	0	0	0.041425819	0	0	0	0	0	0	hsa_circ_0023224	NC_000011.10	+	68519501	68537891	18391	580	sense-overlappin	XM_011545138.1	PPP6R3
circRNA_07776	0	0.020469968	0	0	0	0	0	0	0	0	0	0	-	NC_000011.10	-	68531452	68533586	2135	2135	antisense	NM_001164160.1	PPP6R3
circRNA_07777	0.061725074	0	0.032712968	0	0.04012623	0	0.040197371	0	0	0.07060369	0.030382845	0.0206364	hsa_circ_0007660	NC_000011.10	+	68551121	68564432	13312	423	sense-overlappin	NM_001164160.1	PPP6R3
circRNA_07778	0.020575025	0	0.032712968	0.0825448	0	0	0	0	0	0	0	0.0206364	hsa_circ_0002038	NC_000011.10	+	68567014	68576043	9030	570	sense-overlappin	NM_001164160.1	PPP6R3
circRNA_07779	0	0	0	0.0206362	0.03009467	0	0	0	0	0	0	0	-	NC_000011.10	+	68567014	68596218	29205	1063	sense-overlappin	NM_001164160.1	PPP6R3
circRNA_07780	0	0	0	0	0	0	0	0	0	0	0.02025523	0	hsa_circ_0023232	NC_000011.10	+	68567014	68603492	36479	1475	sense-overlappin	NM_001164160.1	PPP6R3
circRNA_07781	0	0.040939935	0	0	0	0	0	0	0	0	0	0	hsa_circ_0023233	NC_000011.10	+	68569748	68576043	6296	417	sense-overlappin	NM_001164160.1	PPP6R3
circRNA_07782	0	0	0.021808645	0	0	0	0	0	0	0	0	0	-	NC_000011.10	+	68583043	68596218	13176	493	sense-overlappin	NM_001164160.1	PPP6R3
circRNA_07783	0	0	0	0	0	0.041425819	0	0	0	0	0	0	-	NC_000011.10	+	68587927	68603492	15566	818	sense-overlappin	NM_001164160.1	PPP6R3
circRNA_07784	0	0	0	0	0	0	0	0.020486295	0.050636319	0	0	0.0206364	hsa_circ_0001968	NC_000011.10	+	68591576	68600494	8919	407	sense-overlappin	NM_001164160.1	PPP6R3
circRNA_07785	0	0	0.04361729	0	0	0	0	0	0	0.030258724	0	0	hsa_circ_0008391	NC_000011.10	+	68596097	68603492	7396	534	sense-overlappin	NM_001164160.1	PPP6R3
circRNA_07786	0	0	0	0	0	0	0	0	0	0.020172483	0	0	hsa_circ_0007464	NC_000011.10	+	68600341	68603492	3152	412	sense-overlappin	NM_001164160.1	PPP6R3

circRNA_07787	0	0	0.04361729	0	0	0	0	0	0	0	0	0.0412728	hsa_circ_0002417	NC_000011.10	+		68601863	68603492	1630	258	sense-overlappin	NM_001164160.1	PPP6R3
circRNA_07788	0.020575025	0	0	0.0309543	0	0.041425819	0	0	0.020254528	0.060517448	0	0	hsa_circ_0023249	NC_000011.10	-		68759569	68761687	2119	360	sense-overlappin	XM_005273763.1	CPT1A
circRNA_07789	0	0	0.04361729	0	0	0	0	0	0	0	0	0	hsa_circ_0023275	NC_000011.10	-		68799218	68812576	13359	552	sense-overlappin	XM_005273763.1	CPT1A
circRNA_07790	0	0	0.04361729	0	0	0	0	0	0	0	0	0	hsa_circ_0023276	NC_000011.10	-		68804000	68807638	3639	274	sense-overlappin	XM_005273763.1	CPT1A
circRNA_07791	0	0	0	0	0	0.020712909	0	0	0	0	0	0	hsa_circ_0005282	NC_000011.10	-		68804000	68812576	8577	414	sense-overlappin	XM_005273763.1	CPT1A
circRNA_07792	0	0	0	0	0	0.020712909	0	0	0	0	0	0	-	NC_000011.10	-		70132028	70149768	17741	111	antisense	NM_018043.5	ANO1
circRNA_07793	0.020575025	0	0.021808645	0.0206362	0.06018934	0.031069364	0.040197371	0	0.020254528	0.030258724	0.02025523	0	hsa_circ_0000337	NC_000011.10	+		70354301	70356254	1954	419	sense-overlappin	XM_011545307.1	PPFIA1
circRNA_07794	0	0	0.021808645	0	0	0	0	0	0	0	0	0	hsa_circ_0003429	NC_000011.10	+		70354301	70362488	8188	702	sense-overlappin	XM_011545307.1	PPFIA1
circRNA_07795	0	0.020469968	0	0.0309543	0	0	0	0	0	0	0	0	-	NC_000011.10	-		70659828	70661678	1851	208	sense-overlappin	NM_133266.4	SHANK2
circRNA_07796	0	0	0	0.0206362	0	0	0	0	0	0	0	0	hsa_circ_0023418	NC_000011.10	-		72062589	72069911	7323	5713	sense-overlappin	XM_011545059.1	NUMA1
circRNA_07797	0	0	0	0	0.02006311	0	0	0	0	0	0	0	-	NC_000011.10	-		72705805	72714321	8517	1300	sense-overlappin	NM_001135190.1	ARAP1
circRNA_07798	0	0.030704951	0.076330258	0.0309543	0.05015779	0	0	0	0.020254528	0.030258724	0	0	hsa_circ_0002383	NC_000011.10	-		72757767	72759381	1615	370	sense-overlappin	NM_006645.2	STARD10
circRNA_07799	0	0	0	0	0	0	0.020098685	0	0	0	0	0	hsa_circ_0007195	NC_000011.10	-		72887470	72889945	2476	222	sense-overlappin	XM_011545410.1	FCHSD2
circRNA_07800	0	0	0	0	0	0.020712909	0	0	0	0	0	0	hsa_circ_0005527	NC_000011.10	-		72887470	72902638	15169	318	sense-overlappin	XM_011545410.1	FCHSD2
circRNA_07801	0.020575025	0.061409903	0.054521613	0.1031809	0.04012623	0.15534682	0.030148028	0.051215739	0.030381792	0.201724828	0	0.051591	hsa_circ_0005918	NC_000011.10	-		72984088	72989097	5010	318	sense-overlappin	XM_011545410.1	FCHSD2
circRNA_07802	0	0	0	0	0	0	0	0.030729443	0	0	0	0	-	NC_000011.10	-		72984088	73015885	31798	540	sense-overlappin	XM_011545410.1	FCHSD2
circRNA_07803	0	0	0	0	0	0.020712909	0	0	0	0	0	0	hsa_circ_0023480	NC_000011.10	-		73000990	73015885	14896	222	sense-overlappin	XM_011545410.1	FCHSD2
circRNA_07804	0.051437562	0	0.076330258	0.0619086	0.13041025	0.041425819	0.120592112	0.071702034	0.030381792	0.121034897	0.070893305	0.0619092	hsa_circ_0000339	NC_000011.10	-		73707420	73718718	11299	312	sense-overlappin	NM_198896.1	RAB6A
circRNA_07805	0.030862537	0	0.054521613	0.0515905	0	0	0	0	0	0	0	0	hsa_circ_0004596	NC_000011.10	-		73707420	73718890	11471	312	sense-overlappin	NM_001243719.1	RAB6A
circRNA_07806	0.061725074	0.030704951	0.04361729	0.0412724	0.05015779	0.062138728	0.050246713	0.020486295	0.040509055	0.07060369	0.02025523	0	hsa_circ_0003908	NC_000011.10	-		73707420	73720899	13480	366	sense-overlappin	NM_001243719.1	RAB6A
circRNA_07807	0	0	0.021808645	0	0	0	0	0	0	0	0	0.0206364	hsa_circ_0023555	NC_000011.10	-		74118228	74123135	4908	303	sense-overlappin	NM_001286577.1	C2CD3
circRNA_07808	0	0	0	0	0	0.062138728	0	0	0	0	0	0	hsa_circ_0023558	NC_000011.10	-		74132844	74133557	714	262	sense-overlappin	NM_001286577.1	C2CD3
circRNA_07809	0	0	0	0.0619086	0	0	0	0	0	0	0	0	-	NC_000011.10	+		74203728	74204445	718	187	sense-overlappin	XR_428916.1	
circRNA_07810	0	0	0.021808645	0	0	0	0	0	0	0	0	0	-	NC_000011.10	+		74225205	74239256	14052	488	sense-overlappin	XR_428916.1	
circRNA_07811	0	0	0	0	0.02006311	0	0	0	0	0	0	0	hsa_circ_0023577	NC_000011.10	-		74285809	74289130	3322	393	sense-overlappin	XR_949888.1	
circRNA_07812	0.030862537	0.061409903	0.021808645	0.0515905	0.10031557	0.031069364	0.100493427	0.020486295	0.020254528	0.050431207	0.02025523	0	hsa_circ_0006705	NC_000011.10	+		74789626	74817714	28089	340	sense-overlappin	XM_011544889.1	RNF169
circRNA_07813	0.041150049	0	0	0	0	0	0	0	0	0	0	0	-	NC_000011.10	-		74837477	74840652	3176	3176	antisense	XM_011544889.1	RNF169
circRNA_07814	0.020575025	0	0	0	0	0	0	0	0	0	0	0	-	NC_000011.10	+		74850930	74874895	23966	12409	antisense	NM_182969.2	XRRA1
circRNA_07815	0	0.020469968	0	0.0206362	0	0	0	0	0	0	0	0	-	NC_000011.10	-		74859158	74863021	3864	167	sense-overlappin	NM_182969.2	XRRA1
circRNA_07816	0	0	0	0	0	0	0	0	0	0.030258724	0	0	-	NC_000011.10	+		74951718	74953287	1570	1570	intronic	NM_014752.2	SPCS2
circRNA_07817	0.030862537	0	0.021808645	0	0	0	0	0	0	0	0	0	hsa_circ_0023637	NC_000011.10	+		75879879	75912039	32161	323	sense-overlappin	XM_011545228.1	UVRAG
circRNA_07818	0.030862537	0	0	0	0	0	0.020098685	0.020486295	0	0	0	0	hsa_circ_0023644	NC_000011.10	+		76458183	76464080	5898	589	sense-overlappin	XR_949996.1	
circRNA_07819	0	0	0.032712968	0.0206362	0	0.020712909	0.030148028	0.020486295	0	0.020172483	0	0	hsa_circ_0007458	NC_000011.10	+		76463821	76472840	9020	537	sense-overlappin	XR_949996.1	
circRNA_07820	0	0.030704951	0.032712968	0	0.02006311	0	0	0	0	0	0	0	hsa_circ_0007583	NC_000011.10	+		76496215	76528466	32252	1086	sense-overlappin	XR_949996.1	
circRNA_07821	0	0	0.021808645	0.0309543	0	0.020712909	0.020098685	0	0	0	0	0	hsa_circ_0008189	NC_000011.10	+		76513386	76528466	15081	831	sense-overlappin	XR_949996.1	
circRNA_07822	0.030862537	0	0.021808645	0	0	0	0	0	0	0	0	0	hsa_circ_0003320	NC_000011.10	+		76958979	76998821	39843	422	sense-overlappin	XR_949983.1	
circRNA_07823	0	0	0	0	0.04012623	0	0	0	0	0	0	0	-	NC_000011.10	-		77227864	77258542	30679	818	sense-overlappin	NM_182833.1	GDPD4
circRNA_07824	0	0	0	0	0.05015779	0	0	0	0	0	0	0	-	NC_000011.10	-		77227864	77269024	41161	1002	sense-overlappin	NM_182833.1	GDPD4
circRNA_07825	0	0	0	0	0.02006311	0	0	0	0	0	0	0	-	NC_000011.10	-		77264778	77286831	22054	6080	sense-overlappin	NM_182833.1	GDPD4
circRNA_07826	0	0	0.021808645	0	0	0	0	0	0	0	0	0	hsa_circ_0023683	NC_000011.10	-		77358898	77379994	21097	407	sense-overlappin	XM_011545084.1	PAK1
circRNA_07827	0	0	0.021808645	0	0	0	0	0	0	0	0	0	hsa_circ_0023685	NC_000011.10	-		77374328	77392541	18214	498	sense-overlappin	XM_011545084.1	PAK1
circRNA_07828	0	0	0	0	0.05015779	0	0.020098685	0	0	0.020172483	0	0	hsa_circ_0008992	NC_000011.10	-		77379241	77379994	754	249	sense-overlappin	XM_011545084.1	PAK1
circRNA_07829	0.061725074	0.071644886	0.08723458	0.1857257	0.13041025	0.134633911	0.070345399	0.040972591	0.101272639	0.131121138	0.08102092	0.051591	hsa_circ_0023694	NC_000011.10	-		77619606	77625818	6213	474	sense-overlappin	NM_001293.2	CLNS1A
circRNA_07830	0.030862537	0.030704951	0.021808645	0.0515905	0.04012623	0.031069364	0	0.030729443	0	0	0.02025523	0.0412728	hsa_circ_0008342	NC_000011.10	-		77619606	77629899	10294	611	sense-overlappin	NM_001293.2	CLNS1A
circRNA_07831	0.041150049	0.020469968	0	0.0206362	0	0.082851637	0.030148028	0.040972591	0.081018111	0.151293621	0.030382845	0.0412728	hsa_circ_0000343	NC_000011.10	-		77624963	77625818	856	210	sense-overlappin	NM_001293.2	CLNS1A
circRNA_07832	0.020575025	0	0	0.0619086	0	0.051782273	0	0	0	0	0	0	hsa_circ_0004593	NC_000011.10	-		77624963	77629899	4937	347	sense-overlappin	NM_001293.2	CLNS1A
circRNA_07833	0	0	0	0	0	0	0.020098685	0	0	0	0	0	-	NC_000011.10	-		77672042	77678153	6112	686	sense-overlappin	XM_005274051.2	RSF1
circRNA_07834	0.020575025	0	0	0	0	0	0	0	0	0	0	0	hsa_circ_0023700	NC_000011.10	-		77672042	77693611	21570	1036	sense-overlappin	XM_005274051.2	RSF1
circRNA_07835	0	0.030704951	0	0	0	0	0	0	0	0.030258724	0	0	-	NC_000011.10	-		77675036	77678153	3118	497	sense-overlappin	XM_005274051.2	RSF1
circRNA_07836	0	0	0	0	0	0	0	0	0.020254528	0	0	0	hsa_circ_0023701	NC_000011.10	-		77675036	77693611	18576	847	sense-overlappin	XM_005274051.2	RSF1
circRNA_07837	0.113162635	0.051174919	0.021808645	0.0928628	0.03009467	0.093208092	0.110542769	0.030729443	0.070890847	0.100862414	0	0.0619092	hsa_circ_0000344	NC_000011.10	-		77683710	77693611	9902	350	sense-overlappin	XM_005274051.2	RSF1
circRNA_07838	0	0.020469968	0	0.0206362	0	0.020712909	0	0	0	0.030258724	0	0	hsa_circ_0002290	NC_000011.10	-		77685105	77693611	8507	240	sense-overlappin	XM_005274051.2	RSF1
circRNA_07839	0	0.030704951	0.021808645	0.0309543	0	0	0	0	0	0.020172483	0	0	-	NC_000011.10	-		77693507	77702495	8989	2078	sense-overlappin	XM_005274051.2	RSF1
circRNA_07840	0.051437562	0.061409903	0.065425935	0.0825448	0	0.093208092	0	0	0	0.060517448	0.02025523	0.0412728	hsa_circ_0000345	NC_000011.10	-		77698487	77702495	4009	1973	sense-overlappin	XM_005274051.2	RSF1
circRNA_07841	0.030862537	0	0	0	0	0	0	0	0	0	0	0	-	NC_000011.10	-		77722688	77725699	3012	3012	sense-overlappin	XM_005274051.2	RSF1

circRNA_07842	0	0	0.032712968	0	0	0	0	0	0	0	0	0	-	NC_000011.10	-	77740731	77764689	23959	391	sense-overlappin	XM_005274051.2	RSF1
circRNA_07843	0	0	0.021808645	0.0206362	0.02006311	0	0	0.020486295	0	0	0	0	hsa_circ_0023746	NC_000011.10	-	77955942	77961138	5197	447	sense-overlappin	XM_011545352.1	INTS4
circRNA_07844	0	0	0	0	0	0.031069364	0	0	0	0.020172483	0	0	hsa_circ_0023748	NC_000011.10	-	77955942	77981576	25635	672	sense-overlappin	XM_011545352.1	INTS4
circRNA_07845	0	0	0	0.0412724	0	0.020712909	0	0	0	0.020172483	0	0.051591	hsa_circ_0005935	NC_000011.10	-	78465876	78493195	27320	475	sense-overlappin	NM_001243251.1	NARS2
circRNA_07846	0.020575025	0	0.032712968	0	0.02006311	0	0	0	0	0.060517448	0	0.0206364	hsa_circ_0023812	NC_000011.10	-	78469247	78493195	23949	337	sense-overlappin	NM_001243251.1	NARS2
circRNA_07847	0	0	0.021808645	0	0	0	0	0	0	0	0	0	-	NC_000011.10	-	78489913	78493195	3283	3283	sense-overlappin	NM_001243251.1	NARS2
circRNA_07848	0	0	0	0.0206362	0	0	0	0	0	0	0	0	hsa_circ_0023814	NC_000011.10	-	78528842	78571444	42603	548	sense-overlappin	NM_001243251.1	NARS2
circRNA_07849	0	0	0	0	0	0	0.020098685	0	0	0	0	0	hsa_circ_0023843	NC_000011.10	-	82838387	82853278	14892	965	sense-overlappin	NM_005040.2	PRCP
circRNA_07850	0	0	0.021808645	0	0	0	0	0	0.030381792	0.020172483	0	0	-	NC_000011.10	-	82849049	82860117	11069	753	sense-overlappin	NM_005040.2	PRCP
circRNA_07851	0	0.08187987	0	0	0	0.051782273	0.050246713	0	0	0.090776173	0.02025523	0.0412728	hsa_circ_0000346	NC_000011.10	-	83111113	83117197	6085	6085	intergenic		
circRNA_07852	0	0	0	0	0	0	0	0	0	0	0	0.0206364	-	NC_000011.10	+	83163679	83169995	6317	3342	sense-overlappin	NM_015885.3	PCF11
circRNA_07853	0	0	0	0	0.04012623	0	0.030148028	0	0	0.030258724	0	0.051591	hsa_circ_0023858	NC_000011.10	+	83206058	83236509	30452	797	sense-overlappin	NM_001300975.1	ANKRD42
circRNA_07854	0	0	0	0	0	0	0	0	0	0.020172483	0	0	hsa_circ_0023859	NC_000011.10	+	83206058	83248087	42030	1245	sense-overlappin	NM_001300975.1	ANKRD42
circRNA_07855	0	0	0	0	0	0.031069364	0	0	0	0	0	0	-	NC_000011.10	+	83224855	83227872	3018	327	sense-overlappin	NM_001300975.1	ANKRD42
circRNA_07856	0	0	0	0	0.03009467	0.051782273	0	0	0	0.030258724	0	0	hsa_circ_0023861	NC_000011.10	+	83224855	83248087	23233	881	sense-overlappin	NM_001300975.1	ANKRD42
circRNA_07857	0	0.061409903	0	0.0515905	0	0.031069364	0.030148028	0.020486295	0	0.060517448	0	0.103182001	-	NC_000011.10	-	83273647	83278829	5183	374	sense-overlappin	NM_001286116.1	CCDC90B
circRNA_07858	0	0	0.04361729	0	0	0	0	0	0	0.040344966	0	0	-	NC_000011.10	-	85695128	85709500	14373	1155	sense-overlappin	NM_001289609.1	SYTL2
circRNA_07859	0	0	0.021808645	0	0	0	0	0	0	0	0	0	-	NC_000011.10	-	85733939	85758114	24176	1776	sense-overlappin	NM_032943.4	SYTL2
circRNA_07860	0.061725074	0	0.032712968	0.0412724	0.03009467	0.031069364	0.030148028	0.030729443	0.030381792	0.030258724	0	0.0206364	hsa_circ_0006629	NC_000011.10	-	85974708	85983973	9266	560	sense-overlappin	NM_001008660.2	PICALM
circRNA_07861	0.072012586	0	0.119947548	0.0515905	0.08025246	0.062138728	0.060296056	0.030729443	0.030381792	0	0	0.0619092	hsa_circ_0023891	NC_000011.10	-	85974708	86031611	56904	1838	sense-overlappin	XM_005274322.2	PICALM
circRNA_07862	0	0	0	0	0.02006311	0	0	0	0	0	0	0	-	NC_000011.10	-	85981129	85981988	860	248	sense-overlappin	NM_001008660.2	PICALM
circRNA_07863	0	0	0	0.0309543	0.05015779	0	0	0	0.030381792	0.030258724	0	0	hsa_circ_0023900	NC_000011.10	-	85981129	85982003	875	263	sense-overlappin	NM_001008660.2	PICALM
circRNA_07864	0	0	0.032712968	0	0	0.020712909	0	0	0	0	0	0	hsa_circ_0023903	NC_000011.10	-	85981129	86031611	50483	1628	sense-overlappin	NM_001206946.1	PICALM
circRNA_07865	0	0	0	0	0.02006311	0.020712909	0	0	0	0	0	0	hsa_circ_0008773	NC_000011.10	-	85981745	85983973	2229	271	sense-overlappin	NM_001008660.2	PICALM
circRNA_07866	0	0	0	0	0.02006311	0	0	0	0.040509055	0	0	0	hsa_circ_0002264	NC_000011.10	-	85981745	85996929	15185	504	sense-overlappin	NM_001206946.1	PICALM
circRNA_07867	0.041150049	0.020469968	0.021808645	0.0619086	0.03009467	0	0.070345399	0.040972591	0	0.030258724	0	0.082545601	hsa_circ_0023918	NC_000011.10	-	85990250	86031611	41362	1257	sense-overlappin	NM_001206946.1	PICALM
circRNA_07868	0	0	0.021808645	0	0	0	0	0.020486295	0	0	0	0	hsa_circ_0002513	NC_000011.10	-	85996826	86001158	4333	365	sense-overlappin	NM_001008660.2	PICALM
circRNA_07869	0.092587611	0.122819805	0.054521613	0.1341352	0.17053648	0.082851637	0.080394741	0.061458886	0.060763583	0.121034897	0.050638075	0.0619092	hsa_circ_0023919	NC_000011.10	-	85996826	86003451	6626	451	sense-overlappin	NM_001008660.2	PICALM
circRNA_07870	0.020575025	0	0	0	0	0	0	0	0	0	0	0	hsa_circ_0023920	NC_000011.10	-	85996826	86007583	10758	493	sense-overlappin	NM_001008660.2	PICALM
circRNA_07871	0.164600197	0.071644886	0.239895095	0.1754076	0.18056803	0.186416184	0.090444084	0.102431477	0.13165443	0.161379862	0.070893305	0.051591	hsa_circ_0023923	NC_000011.10	-	85996826	86031611	34786	1128	sense-overlappin	NM_001008660.2	PICALM
circRNA_07872	0	0	0.032712968	0	0.02006311	0	0	0	0	0.020172483	0	0	hsa_circ_0023928	NC_000011.10	-	86000643	86031611	30969	1024	sense-overlappin	NM_001008660.2	PICALM
circRNA_07873	0.061725074	0.173994724	0.196277805	0.0722267	0.08025246	0.041425819	0.060296056	0.030729443	0.101272639	0.050431207	0.02025523	0.0619092	hsa_circ_0023936	NC_000011.10	-	86007542	86031611	24070	677	sense-overlappin	NM_001008660.2	PICALM
circRNA_07874	0	0.030704951	0.119947548	0.0928628	0.10031557	0.051782273	0.060296056	0.071702034	0.030381792	0.030258724	0.070893305	0.0206364	hsa_circ_0000347	NC_000011.10	-	86011030	86031611	20582	635	sense-overlappin	NM_001008660.2	PICALM
circRNA_07875	0.041150049	0	0	0	0	0.020712909	0	0	0	0.030258724	0	0	hsa_circ_0023940	NC_000011.10	-	86012281	86031611	19331	528	sense-overlappin	NM_001008660.2	PICALM
circRNA_07876	0	0	0	0	0	0	0	0	0.020254528	0	0	0	hsa_circ_0023941	NC_000011.10	-	86014870	86031611	16742	416	sense-overlappin	NM_001008660.2	PICALM
circRNA_07877	0.123450148	0.061409903	0.141756193	0.0722267	0.1404418	0.051782273	0.120592112	0.020486295	0.070890847	0.060517448	0.091148535	0.113500201	hsa_circ_0023942	NC_000011.10	-	86022367	86031611	9245	322	sense-overlappin	NM_001008660.2	PICALM
circRNA_07878	0	0	0	0	0	0.020712909	0	0	0	0	0	0	hsa_circ_0002433	NC_000011.10	-	86026292	86031611	5320	219	sense-overlappin	NM_001008660.2	PICALM
circRNA_07879	0.041150049	0	0	0.0412724	0	0	0	0	0	0	0	0	hsa_circ_0007455	NC_000011.10	+	86250296	86256512	6217	438	sense-overlappin	XM_011545331.1	EED
circRNA_07880	0	0	0	0	0	0	0	0	0.020254528	0	0	0	hsa_circ_0023956	NC_000011.10	+	86306245	86306482	238	238	exonic	NM_016401.3	C11orf73
circRNA_07881	0	0	0	0	0.02006311	0	0	0	0	0	0	0	-	NC_000011.10	+	87067694	87075786	8093	4392	sense-overlappin	NM_001168724.1	TMEM135
circRNA_07882	0.030862537	0	0.032712968	0.0206362	0.08025246	0.051782273	0	0.030729443	0	0.030258724	0.030382845	0	hsa_circ_0008359	NC_000011.10	+	87067694	87091395	23702	255	sense-overlappin	NM_001168724.1	TMEM135
circRNA_07883	0	0	0	0.0206362	0	0	0	0	0	0	0	0	-	NC_000011.10	+	87067694	87157406	89713	321	sense-overlappin	NM_022918.3	TMEM135
circRNA_07884	0.041150049	0	0	0	0	0	0	0	0	0	0	0	hsa_circ_0023983	NC_000011.10	-	89373432	89402542	29111	506	sense-overlappin	NM_001291929.1	NOX4
circRNA_07885	0.041150049	0	0.021808645	0	0	0	0	0	0	0	0	0	hsa_circ_0023984	NC_000011.10	-	89400017	89402542	2526	445	sense-overlappin	NM_001291929.1	NOX4
circRNA_07886	0	0	0.021808645	0	0	0	0	0	0	0	0	0	-	NC_000011.10	+	93408231	93415114	6884	546	sense-overlappin	XR_947798.1	
circRNA_07887	0	0	0	0	0	0	0.020098685	0	0	0	0	0	-	NC_000011.10	+	93691683	93706897	15215	4413	sense-overlappin	NM_033395.1	CEP295
circRNA_07888	0	0.030704951	0	0	0.05015779	0	0	0	0	0	0	0	-	NC_000011.10	+	93696320	93700186	3867	3603	sense-overlappin	NM_033395.1	CEP295
circRNA_07889	0	0	0	0	0.02006311	0.031069364	0	0.020486295	0	0	0.030382845	0.0412728	-	NC_000011.10	+	93747297	93757465	10169	754	sense-overlappin	NM_001286067.1	C11orf54
circRNA_07890	0.061725074	0	0	0.0309543	0	0	0	0	0.030381792	0.020172483	0	0	-	NC_000011.10	+	93747297	93759858	12562	871	sense-overlappin	NM_001286067.1	C11orf54
circRNA_07891	0	0	0	0	0	0	0.020098685	0	0	0	0	0	-	NC_000011.10	+	93757316	93759858	2543	267	sense-overlappin	NM_001286067.1	C11orf54
circRNA_07892	0	0	0	0.0206362	0	0	0	0	0	0	0	0	-	NC_000011.10	+	93788001	93794035	6035	609	sense-overlappin	NM_004268.4	MED17
circRNA_07893	0	0	0.021808645	0	0	0.031069364	0.020098685	0	0	0	0	0	hsa_circ_0000349	NC_000011.10	+	93790574	93794035	3462	442	sense-overlappin	NM_004268.4	MED17
circRNA_07894	0	0	0	0	0	0	0.030148028	0.030729443	0	0	0	0	-	NC_000011.10	+	93790574	93801972	11399	1049	sense-overlappin	NM_004268.4	MED17
circRNA_07895	0	0	0	0	0	0	0	0	0	0.060517448	0	0	-	NC_000011.10	+	93809717	93812002	2286	310	sense-overlappin	NM_004268.4	MED17
circRNA_07896	0	0	0.04361729	0	0	0	0	0	0	0.030258724	0	0	hsa_circ_0024037	NC_000011.10	-	94445810	94447438	1629	304	sense-overlappin	XR_947828.1	

circRNA_07897	0	0	0	0	0.03009467	0	0	0	0	0	0	0	hsa_circ_0024043	NC_000011.10	-	94485924	94490965	5042	294	sense-overlappin	XR_947828.1	
circRNA_07898	0	0	0	0	0	0	0.020098685	0	0	0	0	0	-	NC_000011.10	-	94485928	94490965	5038	290	sense-overlappin	XR_947828.1	
circRNA_07899	0	0	0	0	0	0	0	0	0	0.030258724	0.050638075	0	-	NC_000011.10	+	94553196	94553775	580	580	intergenic		
circRNA_07900	0.020575025	0	0.021808645	0.0206362	0	0	0	0	0	0	0	0	hsa_circ_0004214	NC_000011.10	+	94799390	94800311	922	922	exonic	XM_006718772.2	AMOTL1
circRNA_07901	0	0	0	0	0	0	0	0.030729443	0	0	0	0	hsa_circ_0008648	NC_000011.10	+	94821530	94831541	10012	527	sense-overlappin	XM_006718772.2	AMOTL1
circRNA_07902	0	0	0	0	0	0	0	0	0	0.050431207	0	0	hsa_circ_0024053	NC_000011.10	+	94850114	94859715	9602	487	sense-overlappin	XM_005273798.3	AMOTL1
circRNA_07903	0	0.020469968	0	0	0	0	0	0	0.030381792	0	0	0	hsa_circ_0024067	NC_000011.10	+	95812932	95813589	658	302	sense-overlappin	NM_001243776.1	CEP57
circRNA_07904	0	0	0	0	0.02006311	0	0	0	0	0	0	0	-	NC_000011.10	+	95812932	95828027	15096	925	sense-overlappin	NM_001243776.1	CEP57
circRNA_07905	0	0.071644886	0	0.0619086	0.02006311	0	0.030148028	0.020486295	0.020254528	0.060517448	0	0.0309546	hsa_circ_0024085	NC_000011.10	-	96091892	96093517	1626	1626	exonic	XM_011543023.1	MAML2
circRNA_07906	0	0	0.032712968	0	0	0	0	0	0	0	0	0	-	NC_000011.10	+	100816715	100859625	42911	42911	sense-overlappin	NM_152432.2	ARHGAP42
circRNA_07907	0	0	0.021808645	0	0	0	0	0	0	0	0	0	-	NC_000011.10	+	100933156	100943868	10713	446	sense-overlappin	NM_152432.2	ARHGAP42
circRNA_07908	0	0	0.054521613	0	0	0	0	0	0	0	0	0	-	NC_000011.10	+	102075873	102081281	5409	240	sense-overlappin	NM_032930.2	C11orf70
circRNA_07909	0	0	0	0.0309543	0	0	0	0	0	0	0	0	hsa_circ_0024096	NC_000011.10	+	102162456	102206074	43619	418	sense-overlappin	XM_005271378.2	YAP1
circRNA_07910	0	0	0	0	0	0	0.030148028	0	0	0	0	0	hsa_circ_0006286	NC_000011.10	+	102205893	102209564	3672	242	sense-overlappin	XM_005271378.2	YAP1
circRNA_07911	0	0.040939935	0.04361729	0	0	0.031069364	0	0	0.030381792	0.050431207	0	0	hsa_circ_0004103	NC_000011.10	+	102362896	102368548	5653	371	sense-overlappin	NM_001166.4	BIRC2
circRNA_07912	0	0	0	0	0.03009467	0	0	0	0	0	0	0	-	NC_000011.10	+	103215721	103223086	7366	659	sense-overlappin	XM_006718903.2	DYNC2H1
circRNA_07913	0.020575025	0	0	0.0206362	0	0	0	0	0	0.040344966	0	0	-	NC_000011.10	+	103219915	103223086	3172	521	sense-overlappin	XM_006718903.2	DYNC2H1
circRNA_07914	0	0	0.021808645	0	0	0	0	0	0	0	0	0	-	NC_000011.10	+	103243693	103253448	9756	387	sense-overlappin	XM_006718903.2	DYNC2H1
circRNA_07915	0	0	0	0	0	0	0	0	0.020254528	0	0	0	hsa_circ_0006960	NC_000011.10	+	103280348	103287605	7258	400	sense-overlappin	XM_006718903.2	DYNC2H1
circRNA_07916	0	0	0	0	0	0	0	0	0	0.030258724	0	0	-	NC_000011.10	+	103280348	103358359	78012	1461	sense-overlappin	XM_006718903.2	DYNC2H1
circRNA_07917	0	0	0	0	0	0	0	0	0	0.040344966	0	0	hsa_circ_0024142	NC_000011.10	+	103303093	103312033	8941	554	sense-overlappin	XM_006718903.2	DYNC2H1
circRNA_07918	0.020575025	0	0	0.0206362	0	0	0	0	0	0	0	0	hsa_circ_0006864	NC_000011.10	+	103303093	103316620	13528	630	sense-overlappin	XM_006718903.2	DYNC2H1
circRNA_07919	0	0	0.065425935	0	0.03009467	0.051782273	0	0	0.040509055	0	0	0	hsa_circ_0024143	NC_000011.10	+	103303093	103323990	20898	944	sense-overlappin	XM_006718903.2	DYNC2H1
circRNA_07920	0	0	0.021808645	0	0	0	0	0	0	0	0	0	hsa_circ_0024144	NC_000011.10	+	103303093	103358359	55267	1061	sense-overlappin	XM_006718903.2	DYNC2H1
circRNA_07921	0	0	0	0	0	0	0	0	0	0	0.02025523	0	hsa_circ_0024152	NC_000011.10	-	106939460	106940178	719	719	exonic	NM_000855.2	GUCY1A2
circRNA_07922	0	0	0	0	0	0	0	0	0	0.030258724	0	0	-	NC_000011.10	-	106939460	106978740	39281	841	sense-overlappin	NM_000855.2	GUCY1A2
circRNA_07923	0	0	0	0	0	0	0.020098685	0	0	0	0	0	-	NC_000011.10	-	106939460	106986131	46672	903	sense-overlappin	NM_000855.2	GUCY1A2
circRNA_07924	0	0	0	0	0.03009467	0	0	0	0	0	0	0	hsa_circ_0008602	NC_000011.10	-	106978619	106986131	7513	184	sense-overlappin	NM_000855.2	GUCY1A2
circRNA_07925	0.030862537	0	0	0.0309543	0	0	0.020098685	0	0	0	0	0	-	NC_000011.10	-	107390074	107392895	2822	255	sense-overlappin	NM_152434.2	CWF19L2
circRNA_07926	0	0	0.054521613	0.0515905	0.02006311	0.020712909	0	0	0	0.030258724	0	0	hsa_circ_0006868	NC_000011.10	-	107390074	107418287	28214	439	sense-overlappin	NM_152434.2	CWF19L2
circRNA_07927	0	0	0.032712968	0	0	0	0	0	0	0	0	0	-	NC_000011.10	-	107428799	107443049	14251	1094	sense-overlappin	NM_152434.2	CWF19L2
circRNA_07928	0	0	0.032712968	0.1031809	0	0.031069364	0	0.020486295	0.020254528	0	0	0	hsa_circ_0024169	NC_000011.10	+	108046270	108054955	8686	646	sense-overlappin	NM_003478.3	CUL5
circRNA_07929	0	0	0	0	0	0	0	0	0.020254528	0	0	0	-	NC_000011.10	-	108069289	108069382	94	94	antisense	NM_003478.3	CUL5
circRNA_07930	0	0	0	0.0206362	0	0	0	0	0	0	0	0	-	NC_000011.10	+	108094391	108098529	4139	705	sense-overlappin	NM_003478.3	CUL5
circRNA_07931	0	0	0	0	0.02006311	0	0	0	0	0	0	0	hsa_circ_0007512	NC_000011.10	+	108131907	108134316	2410	262	sense-overlappin	NM_000019.3	ACAT1
circRNA_07932	0	0.030704951	0	0.0412724	0	0	0	0	0	0.020172483	0.030382845	0	-	NC_000011.10	+	108135142	108148858	13717	2418	sense-overlappin	NM_000019.3	ACAT1
circRNA_07933	0	0	0.021808645	0	0	0	0	0	0	0	0	0	-	NC_000011.10	-	108172199	108177090	4892	1879	sense-overlappin	XM_011542854.1	NPAT
circRNA_07934	0	0.040939935	0	0	0	0.031069364	0.020098685	0	0	0.020172483	0	0	hsa_circ_0006208	NC_000011.10	-	108176246	108177090	845	226	sense-overlappin	XM_011542854.1	NPAT
circRNA_07935	0	0	0	0	0	0.041425819	0	0	0.020254528	0	0	0	-	NC_000011.10	-	108185232	108190500	5269	616	sense-overlappin	XM_011542854.1	NPAT
circRNA_07936	0	0	0.021808645	0.0412724	0	0	0	0.030729443	0.020254528	0.030258724	0	0	hsa_circ_0024193	NC_000011.10	+	108227595	108229323	1729	361	sense-overlappin	XM_011542846.1	ATM
circRNA_07937	0	0	0	0.0309543	0	0.031069364	0	0.061458886	0.040509055	0.07060369	0	0	hsa_circ_0003641	NC_000011.10	+	108227595	108235834	8240	526	sense-overlappin	XM_011542846.1	ATM
circRNA_07938	0	0	0	0	0	0	0	0	0	0.050431207	0	0	-	NC_000011.10	+	108227595	108247127	19533	1095	sense-overlappin	XM_011542846.1	ATM
circRNA_07939	0	0	0	0	0	0	0	0	0	0.020172483	0	0	-	NC_000011.10	+	108235670	108245026	9357	570	sense-overlappin	XM_011542846.1	ATM
circRNA_07940	0	0	0	0	0	0	0.020098685	0	0	0	0	0	hsa_circ_0000356	NC_000011.10	+	108235670	108249102	13433	904	sense-overlappin	XM_011542846.1	ATM
circRNA_07941	0	0	0	0	0.02006311	0.020712909	0	0	0	0.050431207	0	0.0309546	hsa_circ_0007694	NC_000011.10	+	108267171	108267342	172	172	exonic	XM_011542846.1	ATM
circRNA_07942	0	0	0	0	0	0	0	0	0	0.020172483	0	0	hsa_circ_0024217	NC_000011.10	+	108267171	108268609	1439	372	sense-overlappin	XM_011542846.1	ATM
circRNA_07943	0	0	0	0	0	0.020712909	0	0.020486295	0.040509055	0.07060369	0	0.0412728	hsa_circ_0005583	NC_000011.10	+	108301648	108304852	3205	497	sense-overlappin	XM_011542843.1	ATM
circRNA_07944	0	0	0	0	0	0	0	0	0	0.030258724	0	0	hsa_circ_0024231	NC_000011.10	+	108315823	108335961	20139	2262	sense-overlappin	XM_011542843.1	ATM
circRNA_07945	0	0	0	0	0	0	0.030148028	0	0	0	0	0	hsa_circ_0024234	NC_000011.10	+	108329021	108332037	3017	699	sense-overlappin	XM_011542843.1	ATM
circRNA_07946	0	0	0	0	0	0.020712909	0	0	0	0	0	0.0412728	hsa_circ_0024251	NC_000011.10	+	108715879	108723462	7584	643	sense-overlappin	NM_004398.3	DDX10
circRNA_07947	0	0	0	0	0	0.020712909	0	0	0.020254528	0	0	0.0206364	hsa_circ_0003541	NC_000011.10	+	110136663	110159490	22828	1127	sense-overlappin	XM_011543056.1	ZC3H12C
circRNA_07948	0.041150049	0	0	0	0	0.031069364	0	0	0	0	0	0.0412728	-	NC_000011.10	-	110247703	110254109	6407	295	sense-overlappin	NM_001260493.1	RDX
circRNA_07949	0	0	0	0	0	0	0	0	0	0.040344966	0	0	hsa_circ_0002773	NC_000011.10	-	110263960	110264874	915	371	sense-overlappin	NM_001260493.1	RDX
circRNA_07950	0.020575025	0	0	0	0	0	0	0	0.020254528	0	0	0	-	NC_000011.10	+	110435834	110437138	1305	1305	sense-overlappin	NM_004109.4	FDX1
circRNA_07951	0	0	0	0.0206362	0	0	0	0	0	0	0	0	-	NC_000011.10	+	110435834	110457047	21214	255	sense-overlappin	NM_004109.4	FDX1

circRNA_07952	0	0	0.021808645	0	0	0	0	0	0	0.030258724	0	0	hsa_circ_0024271	NC_000011.10	-	111752159	111753577	1419	309	sense-overlappin	XR_947857.1	
circRNA_07953	0	0	0	0	0	0	0.020098685	0	0	0	0	0	hsa_circ_0024273	NC_000011.10	-	111754499	111755450	952	342	sense-overlappin	XR_947857.1	
circRNA_07954	0	0	0	0	0	0	0	0	0	0.020172483	0	0	-	NC_000011.10	-	111836165	111838378	2214	408	sense-overlappin	NM_001077691.1	ALG9
circRNA_07955	0	0	0	0	0	0.031069364	0	0	0	0	0	0.0206364	-	NC_000011.10	-	111836165	111857737	21573	1037	sense-overlappin	NM_001077691.1	ALG9
circRNA_07956	0	0	0	0	0	0	0	0	0	0	0	0.0206364	hsa_circ_0006244	NC_000011.10	-	111844601	111857737	13137	453	sense-overlappin	NM_001077691.1	ALG9
circRNA_07957	0	0	0	0	0	0.041425819	0	0	0	0	0	0	hsa_circ_0024281	NC_000011.10	-	111844601	111865251	20651	613	sense-overlappin	NM_001077691.1	ALG9
circRNA_07958	0	0	0	0	0	0	0	0	0	0	0	0.0309546	hsa_circ_0024295	NC_000011.10	+	112037273	112039397	2125	342	sense-overlappin	NM_001931.4	DLAT
circRNA_07959	0	0	0	0.0206362	0	0	0	0	0	0	0	0	-	NC_000011.10	+	112045138	112051349	6212	317	sense-overlappin	NM_001931.4	DLAT
circRNA_07960	0	0	0	0	0.02006311	0	0	0	0	0	0	0	hsa_circ_0024310	NC_000011.10	-	113741694	113744040	2347	311	sense-overlappin	NM_004724.3	ZW10
circRNA_07961	0	0	0	0	0	0	0	0	0.030381792	0	0	0	hsa_circ_0024323	NC_000011.10	-	113840598	113854335	13738	477	sense-overlappin	XM_005271633.3	USP28
circRNA_07962	0.020575025	0.020469968	0	0	0	0	0	0	0	0	0	0	hsa_circ_0005032	NC_000011.10	-	116757146	116758407	1262	406	sense-overlappin	NM_001159736.1	BUD13
circRNA_07963	0	0	0	0	0	0	0	0	0	0.020172483	0	0	hsa_circ_0006000	NC_000011.10	-	116762553	116765446	2894	799	sense-overlappin	NM_001159736.1	BUD13
circRNA_07964	0.020575025	0	0.076330258	0	0.0702209	0	0	0.020486295	0	0.050431207	0.02025523	0	hsa_circ_0007827	NC_000011.10	+	117152441	117163892	11452	418	sense-overlappin	NM_002572.3	PAFAH1B2
circRNA_07965	0.030862537	0	0	0.0412724	0	0	0	0	0	0.030258724	0.02025523	0	hsa_circ_0004962	NC_000011.10	+	117159934	117163892	3959	330	sense-overlappin	NM_002572.3	PAFAH1B2
circRNA_07966	0	0	0	0	0	0	0	0.020486295	0	0	0	0	hsa_circ_0024402	NC_000011.10	+	117238601	117239860	1260	571	sense-overlappin	NM_207343.3	RNF214
circRNA_07967	0	0	0	0	0	0	0.020098685	0	0	0	0	0	-	NC_000011.10	+	117238601	117246948	8348	852	sense-overlappin	NM_207343.3	RNF214
circRNA_07968	0	0.020469968	0	0	0	0	0	0	0	0	0	0	-	NC_000011.10	+	117244445	117246948	2504	281	sense-overlappin	NM_207343.3	RNF214
circRNA_07969	0	0	0	0	0.03009467	0	0	0	0	0	0	0	hsa_circ_0002743	NC_000011.10	+	117279908	117280259	352	186	sense-overlappin	NM_207343.3	RNF214
circRNA_07970	0	0	0	0	0	0	0	0	0	0	0	0.0412728	-	NC_000011.10	+	117993241	117995710	2470	443	sense-overlappin	NM_001558.3	IL10RA
circRNA_07971	0	0	0.032712968	0	0	0	0	0	0	0	0	0	-	NC_000011.10	+	118103101	118104820	1720	283	sense-overlappin	NM_001083947.1	TMPRSS4
circRNA_07972	0	0	0	0	0	0.020712909	0	0	0	0	0	0	-	NC_000011.10	-	118210487	118214886	4400	444	sense-overlappin	NM_001098526.1	AMICA1
circRNA_07973	0	0	0	0	0	0	0	0	0	0	0	0.0206364	-	NC_000011.10	+	118476805	118482495	5691	930	sense-overlappin	NM_001197104.1	KMT2A
circRNA_07974	0	0	0.021808645	0	0.03009467	0	0	0.020486295	0	0	0	0	-	NC_000011.10	+	118481715	118482495	781	452	sense-overlappin	NM_001197104.1	KMT2A
circRNA_07975	0	0.020469968	0	0	0.02006311	0.093208092	0	0.020486295	0	0	0.02025523	0	hsa_circ_0007372	NC_000011.10	-	118551786	118559864	8079	860	sense-overlappin	NM_020153.3	IFT46
circRNA_07976	0.051437562	0	0.021808645	0.0309543	0	0	0	0.030729443	0	0	0	0	hsa_circ_0008157	NC_000011.10	-	118554459	118559864	5406	671	sense-overlappin	NM_020153.3	IFT46
circRNA_07977	0.041150049	0	0	0	0	0	0	0	0	0	0	0	hsa_circ_0024470	NC_000011.10	-	118556906	118559864	2959	373	sense-overlappin	NM_020153.3	IFT46
circRNA_07978	0	0.040939935	0.08723458	0.0309543	0	0.031069364	0	0	0	0.050431207	0	0.0412728	hsa_circ_0006374	NC_000011.10	+	118581246	118584644	3399	815	sense-overlappin	XM_005271542.2	ARCN1
circRNA_07979	0	0	0.04361729	0.0206362	0.03009467	0	0.020098685	0	0	0.020172483	0	0	hsa_circ_0007383	NC_000011.10	+	118583179	118584644	1466	551	sense-overlappin	XM_005271542.2	ARCN1
circRNA_07980	0.041150049	0.030704951	0.032712968	0.0515905	0.02006311	0.041425819	0	0	0	0.030258724	0	0.0206364	hsa_circ_0005589	NC_000011.10	+	118583809	118584644	836	371	sense-overlappin	XM_005271542.2	ARCN1
circRNA_07981	0	0	0	0	0	0.020712909	0	0	0	0	0	0	hsa_circ_0024490	NC_000011.10	-	118754705	118757287	2583	466	sense-overlappin	XM_005271417.2	DDX6
circRNA_07982	0	0	0	0	0	0.020712909	0	0	0	0	0	0	hsa_circ_0002294	NC_000011.10	-	118754705	118758902	4198	595	sense-overlappin	XM_005271417.2	DDX6
circRNA_07983	0	0	0	0.0206362	0	0	0	0	0	0	0	0	hsa_circ_0008918	NC_000011.10	-	118765209	118768352	3144	277	sense-overlappin	XM_005271417.2	DDX6
circRNA_07984	0	0	0	0	0	0	0.030148028	0	0	0	0	0	-	NC_000011.10	-	118768223	118786518	18296	766	sense-overlappin	XM_005271417.2	DDX6
circRNA_07985	0	0	0	0.0206362	0	0.020712909	0	0	0	0	0	0	hsa_circ_0006588	NC_000011.10	-	118779632	118786518	6887	636	sense-overlappin	XM_005271417.2	DDX6
circRNA_07986	0.072012586	0.071644886	0.04361729	0.0619086	0.04012623	0.020712909	0.020098685	0.020486295	0	0	0.050638075	0	hsa_circ_0004293	NC_000011.10	-	118786052	118786518	467	467	exonic	XM_005271417.2	DDX6
circRNA_07987	0	0	0.054521613	0.0206362	0	0.020712909	0	0	0	0	0	0	hsa_circ_0006932	NC_000011.10	+	119131535	119133219	1685	728	sense-overlappin	NM_001243259.1	HINFP
circRNA_07988	0	0	0	0	0	0	0	0.020486295	0	0	0	0	hsa_circ_0007665	NC_000011.10	+	119232448	119232695	248	248	exonic	XM_011543057.1	CBL
circRNA_07989	0	0	0	0	0	0	0	0	0.020254528	0	0	0.0206364	-	NC_000011.10	+	119271735	119274953	3219	426	sense-overlappin	XM_011543057.1	CBL
circRNA_07990	0	0.061409903	0	0.0206362	0	0.020712909	0	0	0.030381792	0	0	0	hsa_circ_0000362	NC_000011.10	+	119273868	119274953	1086	279	sense-overlappin	XM_011543057.1	CBL
circRNA_07991	0.051437562	0.040939935	0.021808645	0.0206362	0.03009467	0	0	0.020486295	0.020254528	0	0	0.051591	-	NC_000011.10	+	119284969	119285566	598	510	sense-overlappin	NM_005188.3	CBL
circRNA_07992	0	0	0	0	0.02006311	0	0	0	0	0	0	0	-	NC_000011.10	+	119284969	119287946	2978	605	sense-overlappin	NM_005188.3	CBL
circRNA_07993	0	0	0.021808645	0	0	0	0	0	0	0	0	0	hsa_circ_0024596	NC_000011.10	-	119675159	119678765	3607	924	sense-overlappin	NM_203285.1	PVRL1
circRNA_07994	0	0	0	0.0309543	0.04012623	0	0.020098685	0	0	0.040344966	0	0	hsa_circ_0024604	NC_000011.10	+	120406118	120407823	1706	110	sense-overlappin	XM_011542720.1	ARHGEF12
circRNA_07995	0	0	0.032712968	0	0	0.041425819	0	0	0	0	0.02025523	0	hsa_circ_0024605	NC_000011.10	+	120406118	120409450	3333	167	sense-overlappin	XM_011542720.1	ARHGEF12
circRNA_07996	0.061725074	0	0.04361729	0	0.04012623	0	0.020098685	0	0.020254528	0.030258724	0	0	hsa_circ_0024609	NC_000011.10	+	120406118	120431911	25794	892	sense-overlappin	XM_011542720.1	ARHGEF12
circRNA_07997	0	0	0	0.0206362	0	0	0	0	0	0	0	0	hsa_circ_0024612	NC_000011.10	+	120406118	120460757	54640	2581	sense-overlappin	XM_011542720.1	ARHGEF12
circRNA_07998	0	0	0	0	0	0	0	0	0	0.020172483	0	0	hsa_circ_0024626	NC_000011.10	+	120440129	120445464	5336	346	sense-overlappin	XM_011542720.1	ARHGEF12
circRNA_07999	0	0.020469968	0	0	0	0	0	0	0	0	0	0	hsa_circ_0024632	NC_000011.10	+	120440129	120477526	37398	2533	sense-overlappin	XM_011542720.1	ARHGEF12
circRNA_08000	0	0	0	0.0309543	0.04012623	0	0	0	0.040509055	0	0	0	-	NC_000011.10	+	120465237	120467308	2072	241	sense-overlappin	XM_011542720.1	ARHGEF12
circRNA_08001	0	0	0	0	0	0.020712909	0.030148028	0	0	0	0	0	hsa_circ_0024648	NC_000011.10	+	120469288	120477526	8239	678	sense-overlappin	XM_011542720.1	ARHGEF12
circRNA_08002	0	0	0	0	0	0.031069364	0	0	0	0	0	0	-	NC_000011.10	+	120469378	120469531	154	154	sense-overlappin	XM_011542720.1	ARHGEF12
circRNA_08003	0.061725074	0	0.04361729	0.0412724	0.03009467	0.051782273	0	0.061458886	0.040509055	0.050431207	0.030382845	0.0309546	hsa_circ_0009021	NC_000011.10	+	120473050	120477526	4477	577	sense-overlappin	XM_011542720.1	ARHGEF12
circRNA_08004	0.102875123	0.030704951	0.065425935	0.1650895	0.06018934	0.113921001	0.040197371	0.040972591	0.060763583	0.131121138	0.070893305	0.0412728	hsa_circ_0002100	NC_000011.10	+	120474560	120477526	2967	499	sense-overlappin	XM_011542720.1	ARHGEF12
circRNA_08005	0	0.020469968	0	0	0	0.031069364	0	0	0	0.040344966	0	0.0206364	hsa_circ_0002955	NC_000011.10	+	120475340	120477526	2187	423	sense-overlappin	XM_011542720.1	ARHGEF12
circRNA_08006	0.030862537	0.071644886	0.054521613	0.0722267	0.04012623	0.051782273	0	0	0.020254528	0.030258724	0.050638075	0	hsa_circ_0002089	NC_000011.10	+	120476661	120477526	866	255	sense-overlappin	XM_011542720.1	ARHGEF12

circRNA_08007	0.041150049	0	0	0.0206362	0	0.020712909	0.050246713	0.030729443	0	0.07060369	0	0.051591	-	NC_000011.10	+	121036505	121060085	23581	10142	sense-overlappin	NM_001130047.1	TBCEL
circRNA_08008	0	0	0	0.0206362	0	0	0	0	0	0	0	0	hsa_circ_0000365	NC_000011.10	+	121045674	121053732	8059	472	sense-overlappin	NM_001130047.1	TBCEL
circRNA_08009	0.113162635	0.071644886	0.04361729	0.1238171	0.03009467	0.176059729	0.060296056	0.030729443	0.111399902	0.201724828	0.030382845	0.072227401	hsa_circ_0003302	NC_000011.10	+	121045674	121060085	14412	973	sense-overlappin	NM_001130047.1	TBCEL
circRNA_08010	0	0	0	0.0206362	0	0.020712909	0	0	0	0	0	0	hsa_circ_0004798	NC_000011.10	+	123594082	123596037	1956	285	sense-overlappin	XM_011542928.1	GRAMD1B
circRNA_08011	0	0	0	0.0206362	0	0	0.020098685	0	0	0	0	0	hsa_circ_0024737	NC_000011.10	+	124117497	124119089	1593	775	sense-overlappin	NM_198315.2	VWA5A
circRNA_08012	0.051437562	0	0	0.0309543	0	0	0	0	0.020254528	0.020172483	0.02025523	0	hsa_circ_0000367	NC_000011.10	-	124647365	124648175	811	244	sense-overlappin	NM_170601.4	SIAE
circRNA_08013	0	0	0	0	0	0	0	0	0	0	0.030382845	0	-	NC_000011.10	+	124986742	125005212	18471	1658	sense-overlappin	NM_025004.2	CCDC15
circRNA_08014	0.030862537	0	0	0	0	0	0	0	0	0	0	0	hsa_circ_0002019	NC_000011.10	+	126272969	126273454	486	230	sense-overlappin	NM_017547.3	FOXRED1
circRNA_08015	0	0	0	0	0	0.031069364	0	0	0	0	0	0	hsa_circ_0002083	NC_000011.10	-	128480191	128490576	10386	909	sense-overlappin	NM_005238.3	ETS1
circRNA_08016	0	0.020469968	0	0	0	0	0	0	0	0	0	0	hsa_circ_0000368	NC_000011.10	+	128758115	128768272	10158	538	sense-overlappin	NM_001271010.1	FLI1
circRNA_08017	0	0	0	0	0	0	0	0	0	0.040344966	0	0	hsa_circ_0000369	NC_000011.10	+	128758115	128772985	14871	742	sense-overlappin	NM_001271010.1	FLI1
circRNA_08018	0	0	0.054521613	0	0	0	0	0	0	0	0	0	hsa_circ_0024840	NC_000011.10	-	129062280	129066868	4589	432	sense-overlappin	XM_011543074.1	ARHGAP32
circRNA_08019	0	0.030704951	0	0	0	0	0	0	0	0	0	0	-	NC_000011.10	-	129062280	129093707	31428	519	sense-overlappin	XM_011543073.1	ARHGAP32
circRNA_08020	0	0	0.076330258	0	0	0	0	0	0	0	0.02025523	0	hsa_circ_0002984	NC_000011.10	-	129093621	129124894	31274	306	sense-overlappin	XM_011543073.1	ARHGAP32
circRNA_08021	0	0	0	0	0	0	0.030148028	0	0	0	0	0	-	NC_000011.10	-	129093621	129127305	33685	2717	sense-overlappin	XM_005271736.3	ARHGAP32
circRNA_08022	0	0	0.076330258	0	0	0	0.030148028	0	0	0	0	0	hsa_circ_0024843	NC_000011.10	-	129093621	129164427	70807	415	sense-overlappin	XM_005271736.3	ARHGAP32
circRNA_08023	0	0	0.119947548	0	0	0	0.020098685	0.020486295	0.040509055	0.030258724	0	0	hsa_circ_0000371	NC_000011.10	-	129123446	129124894	1449	219	sense-overlappin	XM_011543073.1	ARHGAP32
circRNA_08024	0	0	0.04361729	0	0	0	0	0	0	0	0	0	hsa_circ_0005027	NC_000011.10	-	129123446	129127305	3860	2630	sense-overlappin	XM_005271736.3	ARHGAP32
circRNA_08025	0.020575025	0	0.338033998	0.0309543	0	0.031069364	0.030148028	0	0	0	0	0	hsa_circ_0007843	NC_000011.10	-	129123446	129164427	40982	328	sense-overlappin	XM_005271736.3	ARHGAP32
circRNA_08026	0	0	0	0	0	0	0	0	0.020254528	0	0	0	-	NC_000011.10	+	129436751	129442919	6169	386	sense-overlappin	XM_011543044.1	BARX2
circRNA_08027	0	0.020469968	0.08723458	0	0	0	0	0	0	0	0	0	hsa_circ_0000372	NC_000011.10	+	130109429	130110661	1233	298	sense-overlappin	NM_001142276.1	APLP2
circRNA_08028	0	0	0	0	0	0	0.020098685	0	0	0	0	0	-	NC_000011.10	+	130109429	130122513	13085	817	sense-overlappin	NM_001142276.1	APLP2
circRNA_08029	0	0	0	0	0	0	0.020098685	0	0	0	0	0	-	NC_000011.10	+	130121746	130122350	605	111	sense-overlappin	NM_001142276.1	APLP2
circRNA_08030	0.40121298	0.204699675	1.144953863	0.3611333	0.21066271	0.414258187	0.321578966	0.133160921	0.222799805	0.302587242	0.172169456	0.206364002	hsa_circ_0002484	NC_000011.10	-	130260856	130261929	1074	1074	exonic	NM_001301098.1	ZBTB44
circRNA_08031	0	0	0	0	0	0	0	0	0	0	0	0.0309546	-	NC_000011.10	+	134283737	134345129	61393	27622	sense-overlappin	NM_001080407.2	GLB1L3
circRNA_08032	0	0	0.032712968	0	0	0	0	0	0	0.030258724	0	0	hsa_circ_0006599	NC_000012.12	-	350621	352324	1704	279	sense-overlappin	NM_001042603.2	KDM5A
circRNA_08033	0.041150049	0.020469968	0	0	0	0	0	0	0.050636319	0.020172483	0	0	hsa_circ_0002881	NC_000012.12	-	350621	354234	3614	438	sense-overlappin	NM_001042603.2	KDM5A
circRNA_08034	0	0	0	0	0	0	0	0.030729443	0	0.040344966	0	0	hsa_circ_0024946	NC_000012.12	-	350621	356537	5917	636	sense-overlappin	NM_001042603.2	KDM5A
circRNA_08035	0.030862537	0	0	0	0	0	0	0	0	0	0	0	-	NC_000012.12	-	350621	363097	12477	771	sense-overlappin	NM_001042603.2	KDM5A
circRNA_08036	0	0	0	0.0412724	0	0	0	0	0	0.020172483	0.02025523	0	-	NC_000012.12	-	350621	366104	15484	942	sense-overlappin	NM_001042603.2	KDM5A
circRNA_08037	0	0.061409903	0	0	0	0	0	0	0	0	0	0	-	NC_000012.12	+	409368	418636	9269	429	sense-overlappin	NM_032358.3	CCDC77
circRNA_08038	0	0	0	0.0206362	0	0	0	0	0	0	0	0	-	NC_000012.12	+	486485	487434	950	950	intronic	NM_173593.3	B4GALNT3
circRNA_08039	0	0	0	0.0206362	0	0	0	0	0	0.020172483	0	0	-	NC_000012.12	+	486485	487436	952	952	intronic	NM_173593.3	B4GALNT3
circRNA_08040	0	0	0	0	0	0	0	0	0	0	0	0.0206364	-	NC_000012.12	+	562623	562844	222	222	intergenic		
circRNA_08041	0	0	0	0	0	0	0	0	0	0	0.02025523	0	-	NC_000012.12	+	702949	711112	8164	8164	intergenic		
circRNA_08042	0	0	0	0	0	0	0	0.020486295	0	0	0.02025523	0	hsa_circ_0007892	NC_000012.12	+	813642	830160	16519	552	sense-overlappin	XM_006719003.1	WNK1
circRNA_08043	0.030862537	0	0	0.0206362	0	0	0	0	0	0.030258724	0	0	hsa_circ_0003251	NC_000012.12	+	894562	897681	3120	939	sense-overlappin	XM_006719003.1	WNK1
circRNA_08044	0	0	0	0.0206362	0	0	0	0	0	0	0	0	hsa_circ_0024989	NC_000012.12	-	925450	927263	1814	346	sense-overlappin	NR_123713.1	
circRNA_08045	0	0	0.021808645	0	0	0	0	0	0	0	0	0	hsa_circ_0005534	NC_000012.12	+	998324	1028572	30249	984	sense-overlappin	XM_011520943.1	ERC1
circRNA_08046	0	0	0.021808645	0	0	0	0	0	0	0	0	0	-	NC_000012.12	+	998324	1083580	85257	1401	sense-overlappin	XM_011520943.1	ERC1
circRNA_08047	0.030862537	0.102349838	0.141756193	0.0206362	0.03009467	0.020712909	0.020098685	0.030729443	0.040509055	0.050431207	0	0.0206364	hsa_circ_0000373	NC_000012.12	+	1027748	1028572	825	825	exonic	NM_001301248.1	ERC1
circRNA_08048	0	0	0.08723458	0	0	0	0	0	0	0	0	0	-	NC_000012.12	+	1103533	1141787	38255	1868	sense-overlappin	NM_001301248.1	ERC1
circRNA_08049	0	0	0.098138903	0	0	0	0	0	0	0	0	0	-	NC_000012.12	+	1103533	1190052	86520	2482	sense-overlappin	NM_001301248.1	ERC1
circRNA_08050	0	0	0.141756193	0	0	0	0	0	0	0	0	0	-	NC_000012.12	+	1104750	1141787	37038	651	sense-overlappin	NM_001301248.1	ERC1
circRNA_08051	0	0	0.032712968	0	0	0	0	0	0	0	0	0	-	NC_000012.12	+	1104750	1190052	85303	1265	sense-overlappin	NM_001301248.1	ERC1
circRNA_08052	0	0	0.04361729	0	0	0	0	0	0	0	0	0	-	NC_000012.12	+	1141620	1190052	48433	782	sense-overlappin	NM_001301248.1	ERC1
circRNA_08053	0.041150049	0.040939935	0.403459933	0.0515905	0	0.020712909	0.040197371	0	0	0	0	0.0206364	-	NC_000012.12	+	1180540	1190052	9513	614	sense-overlappin	NM_001301248.1	ERC1
circRNA_08054	0	0	0	0.0515905	0	0.020712909	0	0	0	0	0	0	hsa_circ_0024997	NC_000012.12	+	1289852	1371977	82126	306	sense-overlappin	NM_001301248.1	ERC1
circRNA_08055	0.020575025	0.030704951	0	0.0206362	0	0	0.020098685	0	0	0.020172483	0.02025523	0	hsa_circ_0006747	NC_000012.12	+	1371833	1444750	72918	498	sense-overlappin	NR_027946.2	
circRNA_08056	0	0	0	0	0.02006311	0	0	0	0	0	0	0	hsa_circ_0025006	NC_000012.12	+	1777854	1784073	6220	741	sense-overlappin	XM_005253789.1	ADIPOR2
circRNA_08057	0.030862537	0	0	0	0	0	0	0	0	0	0	0	-	NC_000012.12	+	2110180	2120430	10251	5472	sense-overlappin	XM_006719017.1	CACNA1C
circRNA_08058	0.030862537	0	0	0	0	0	0	0	0	0	0	0	hsa_circ_0025016	NC_000012.12	+	2115224	2120430	5207	428	sense-overlappin	XM_006719017.1	CACNA1C
circRNA_08059	0.020575025	0	0	0	0	0	0	0	0	0	0	0	hsa_circ_0003792	NC_000012.12	+	2486104	2512984	26881	633	sense-overlappin	XM_006719017.1	CACNA1C
circRNA_08060	0.020575025	0	0	0	0	0	0	0	0	0	0	0	-	NC_000012.12	+	2493190	2512984	19795	474	sense-overlappin	XM_006719017.1	CACNA1C

circRNA_08061	0	0.030704951	0	0	0	0	0	0	0	0	0	0	-	NC_000012.12	+	2582895	2612013	29119	1712	sense-overlappin	NM_001129827.1	CACNA1C
circRNA_08062	0	0	0	0	0	0	0	0	0	0.080689931	0	0.0412728	-	NC_000012.12	-	2681654	2682612	959	464	antisense	XM_011521021.1	CACNA1C
circRNA_08063	0	0	0	0	0	0	0	0	0	0	0.02025523	0.0206364	-	NC_000012.12	+	2686166	2742180	56015	51718	sense-overlappin	XM_006719017.1	CACNA1C
circRNA_08064	0.113162635	0.030704951	0.032712968	0.0619086	0.03009467	0.051782273	0.020098685	0	0.040509055	0.090776173	0	0.082545601	hsa_circ_0000374	NC_000012.12	+	2820086	2821792	1707	542	sense-overlappin	NM_018463.3	ITFG2
circRNA_08065	0	0	0	0	0	0	0	0	0	0	0	0.0206364	-	NC_000012.12	+	2917785	2918208	424	424	intronic	NM_001160408.1	TULP3
circRNA_08066	0	0	0	0	0	0	0	0	0.020254528	0.020172483	0	0	-	NC_000012.12	-	3821873	3863957	42085	652	sense-overlappin	XM_011520972.1	PARP11
circRNA_08067	0	0	0	0	0	0	0	0	0	0	0.04051046	0	-	NC_000012.12	-	4109922	4110234	313	313	intergenic		
circRNA_08068	0	0	0	0	0	0	0	0	0	0	0.02025523	0	hsa_circ_0008684	NC_000012.12	-	4517061	4518880	1820	434	sense-overlappin	NM_001304811.1	C12orf4
circRNA_08069	0	0	0.021808645	0	0	0	0	0	0	0	0	0	-	NC_000012.12	+	5160307	5171889	11583	11583	intergenic		
circRNA_08070	0	0	0	0	0	0	0	0	0.020254528	0	0	0	-	NC_000012.12	-	5732520	5750970	18451	502	intergenic	NM_001278596.1	ANO2
circRNA_08071	0.020575025	0	0	0	0	0	0	0	0	0	0	0	-	NC_000012.12	-	5799507	5832603	33097	422	intergenic	XR_931519.1	
circRNA_08072	0	0	0	0	0.04012623	0	0	0	0	0	0	0	-	NC_000012.12	-	5799507	5854141	54635	524	intergenic	NM_001278596.1	ANO2
circRNA_08073	0	0	0	0	0.03009467	0	0	0	0	0	0	0	-	NC_000012.12	-	5806052	5832603	26552	357	intergenic	XR_931519.1	
circRNA_08074	0	0	0	0	0	0	0	0	0.020254528	0	0	0	-	NC_000012.12	-	6110374	6123196	12823	532	sense-overlappin	NM_000552.3	VWF
circRNA_08075	0	0	0	0	0	0	0	0	0	0.020172483	0	0	hsa_circ_0025128	NC_000012.12	-	6333069	6334244	1176	512	sense-overlappin	NM_001065.3	TNFRSF1A
circRNA_08076	0.205750246	0.061409903	0.283512385	0.1754076	0.26082049	0.144990365	0.140690797	0.09218833	0.060763583	0.181552345	0.172169456	0.134136601	-	NC_000012.12	-	6510279	6510532	254	254	antisense	NM_014865.3	NCAPD2
circRNA_08077	0	0	0	0.1754076	0	0	0	0	0	0	0	0	-	NC_000012.12	-	6578846	6581349	2504	261	sense-overlappin	NM_001297553.1	CHD4
circRNA_08078	0	0	0	0	0	0.020712909	0	0	0	0	0	0	hsa_circ_0007845	NC_000012.12	-	6749848	6750766	919	343	sense-overlappin	NM_005439.2	MLF2
circRNA_08079	0	0	0	0	0	0	0	0	0	0	0.02025523	0	-	NC_000012.12	-	6946905	6946996	92	92	antisense	XM_011520988.1	PTPN6
circRNA_08080	0	0	0	0	0	0	0	0.020486295	0	0	0	0	-	NC_000012.12	+	7095426	7097996	2571	2571	antisense	NM_001297640.1	C1RL
circRNA_08081	0	0	0	0	0	0	0	0	0.020254528	0	0	0	-	NC_000012.12	-	7303278	7317157	13880	1695	antisense	NM_001080454.1	ACSM4
circRNA_08082	0.041150049	0	0	0	0	0	0	0	0	0	0	0	hsa_circ_0025359	NC_000012.12	+	8039819	8040165	347	347	exonic	XM_011520760.1	FOXJ2
circRNA_08083	0	0	0	0	0	0	0	0	0	0.020172483	0	0	-	NC_000012.12	+	8102908	8125677	22770	21170	sense-overlappin	NM_016184.3	CLEC4A
circRNA_08084	0	0	0	0	0	0	0	0	0	0	0.02025523	0	hsa_circ_0002373	NC_000012.12	-	8943405	8943910	506	241	sense-overlappin	NM_002355.3	M6PR
circRNA_08085	0	0	0	0	0	0	0	0	0	0	0	0.0206364	-	NC_000012.12	-	9072359	9072871	513	347	sense-overlappin	XM_006719056.2	A2M
circRNA_08086	0.082300098	0.051174919	0.021808645	0.0309543	0	0	0	0.030729443	0.020254528	0.020172483	0	0	-	NC_000012.12	-	9074560	9153343	78784	41250	sense-overlappin	XM_006719056.2	A2M
circRNA_08087	0	0	0	0	0	0	0	0	0.020254528	0	0	0	-	NC_000012.12	+	9606353	9622436	16084	16084	antisense	NM_002258.2	KLRB1
circRNA_08088	0	0	0	0	0	0.020712909	0	0	0	0.030258724	0	0	-	NC_000012.12	+	9680923	9694969	14047	692	sense-overlappin	NM_001004419.4	CLEC2D
circRNA_08089	0	0	0	0.0206362	0	0.041425819	0.030148028	0	0	0	0	0	-	NC_000012.12	-	10386903	10390027	3125	298	sense-overlappin	NM_007360.3	KLRK1
circRNA_08090	0	0.020469968	0	0	0	0	0.020098685	0.020486295	0.040509055	0	0	0.0309546	-	NC_000012.12	-	10386903	10393867	6965	328	sense-overlappin	NM_001199805.1	KLRC4-KLRK1
circRNA_08091	0.020575025	0.051174919	0.021808645	0.0206362	0	0.020712909	0	0.030729443	0.070890847	0	0	0.0206364	-	NC_000012.12	-	10420364	10435410	15047	11262	sense-overlappin	NM_002260.3	KLRC2
circRNA_08092	0	0	0.021808645	0	0	0	0	0	0	0	0	0	hsa_circ_0008221	NC_000012.12	-	11047020	11121180	74161	74161	sense-overlappin	NM_001291314.1	PRH1
circRNA_08093	0	0	0	0	0	0	0.020098685	0	0.020254528	0	0.050638075	0.051591	hsa_circ_0004123	NC_000012.12	+	11869424	11869969	546	546	exonic	NM_001987.4	ETV6
circRNA_08094	0	0	0	0.0309543	0	0	0	0	0	0	0	0	-	NC_000012.12	+	14396791	14396865	75	75	intronic	XM_011520759.1	ATF7IP
circRNA_08095	0.061725074	0	0.098138903	0.0206362	0.0702209	0.062138728	0.020098685	0	0.040509055	0.110948655	0.02025523	0.0309546	-	NC_000012.12	+	14423909	14425473	1565	1565	exonic	XM_011520759.1	ATF7IP
circRNA_08096	0	0	0.021808645	0	0.02006311	0	0.050246713	0	0	0	0.02025523	0	-	NC_000012.12	+	14423909	14466590	42682	2866	sense-overlappin	NM_001286515.1	ATF7IP
circRNA_08097	0	0	0.032712968	0	0	0	0	0	0	0	0	0	-	NC_000012.12	+	14434337	14438267	3931	371	sense-overlappin	XM_011520759.1	ATF7IP
circRNA_08098	0	0	0.021808645	0	0	0	0	0	0	0.020172483	0.02025523	0	hsa_circ_0025511	NC_000012.12	+	14446988	14457295	10308	229	sense-overlappin	NM_001286515.1	ATF7IP
circRNA_08099	0	0.040939935	0.065425935	0	0	0	0	0	0	0	0	0	hsa_circ_0025514	NC_000012.12	+	14456561	14466590	10030	867	sense-overlappin	NM_001286515.1	ATF7IP
circRNA_08100	0	0	0.021808645	0	0	0	0	0	0	0	0	0	-	NC_000012.12	-	14535659	14553412	17754	729	sense-overlappin	NM_024829.5	PLBD1
circRNA_08101	0	0	0	0	0	0	0	0	0	0	0.02025523	0	-	NC_000012.12	-	14540764	14553412	12649	443	sense-overlappin	NM_024829.5	PLBD1
circRNA_08102	0	0	0	0	0	0	0	0.020486295	0	0	0	0	-	NC_000012.12	-	14542970	14544283	1314	1314	intronic	NM_024829.5	PLBD1
circRNA_08103	0	0	0	0	0	0	0	0	0.020254528	0	0	0	hsa_circ_0025534	NC_000012.12	-	15665756	15681302	15547	677	sense-overlappin	NM_004447.5	EPS8
circRNA_08104	0	0	0.032712968	0	0.02006311	0	0	0	0	0.020172483	0	0	-	NC_000012.12	-	15681226	15713325	32100	217	sense-overlappin	XM_011520605.1	EPS8
circRNA_08105	0	0	0	0.1960438	0	0.020712909	0	0.040972591	0.040509055	0	0	0	-	NC_000012.12	+	18281023	18294015	12993	1277	sense-overlappin	XM_011520694.1	PIK3C2G
circRNA_08106	0	0	0	0.0412724	0	0	0	0	0	0	0	0	-	NC_000012.12	+	18281023	18314064	33042	1380	sense-overlappin	XM_011520694.1	PIK3C2G
circRNA_08107	0	0	0	0.1857257	0	0.062138728	0.050246713	0.030729443	0.050636319	0.020172483	0	0.0412728	-	NC_000012.12	+	18282004	18294015	12012	1112	sense-overlappin	XM_006719088.2	PIK3C2G
circRNA_08108	0	0	0	0.0206362	0	0	0	0	0	0	0	0	-	NC_000012.12	+	18290855	18325098	34244	511	sense-overlappin	XM_006719088.2	PIK3C2G
circRNA_08109	0	0	0	0.0309543	0	0	0	0	0	0	0	0	-	NC_000012.12	+	18338426	18371311	32886	608	sense-overlappin	XM_006719088.2	PIK3C2G
circRNA_08110	0	0	0	0.0309543	0	0	0	0	0	0	0	0	-	NC_000012.12	+	18562703	18567057	4355	421	sense-overlappin	XM_006719088.2	PIK3C2G
circRNA_08111	0.041150049	0	0	0	0	0	0	0	0	0	0	0	-	NC_000012.12	-	19253369	19253529	161	161	antisense	NM_001143821.2	PLEKHA5
circRNA_08112	0	0	0.021808645	0	0.05015779	0	0	0	0.030381792	0	0	0	hsa_circ_0006611	NC_000012.12	+	19253940	19261021	7082	383	sense-overlappin	NM_001143821.2	PLEKHA5
circRNA_08113	0	0	0	0	0	0	0	0	0.020254528	0	0	0	hsa_circ_0025544	NC_000012.12	+	19253940	19265850	11911	484	sense-overlappin	NM_001143821.2	PLEKHA5
circRNA_08114	0	0	0	0	0	0	0.020098685	0	0	0	0	0	hsa_circ_0025547	NC_000012.12	+	19253940	19287556	33617	1636	sense-overlappin	NM_001256470.1	PLEKHA5

circRNA_08115	0	0	0	0	0.02006311	0	0	0	0	0	0	0	-	NC_000012.12	+	19255045	19261021	5977	299	sense-overlappin	NM_001143821.2	PLEKHA5
circRNA_08116	0	0	0	0	0.02006311	0	0	0	0	0	0	0	hsa_circ_0025549	NC_000012.12	+	19269770	19283745	13976	1068	sense-overlappin	NM_001256470.1	PLEKHA5
circRNA_08117	0	0	0	0	0.05015779	0	0	0	0	0	0	0	-	NC_000012.12	+	19269816	19354002	84187	2381	sense-overlappin	NM_001256470.1	PLEKHA5
circRNA_08118	0	0	0	0	0.02006311	0	0	0	0.030381792	0	0	0	hsa_circ_0003844	NC_000012.12	+	19283280	19283745	466	466	exonic	NM_001143821.2	PLEKHA5
circRNA_08119	0	0	0.032712968	0	0	0	0	0	0	0	0	0	-	NC_000012.12	+	19320562	19354002	33441	984	sense-overlappin	NM_001256470.1	PLEKHA5
circRNA_08120	0	0	0	0	0	0	0	0	0	0	0.030382845	0	-	NC_000012.12	+	19343323	19348519	5197	469	sense-overlappin	NM_001256470.1	PLEKHA5
circRNA_08121	0	0	0	0	0.02006311	0	0	0	0	0	0	0	-	NC_000012.12	+	19345842	19348519	2678	357	sense-overlappin	NM_001256470.1	PLEKHA5
circRNA_08122	0	0	0	0	0	0	0	0	0	0.030258724	0	0	-	NC_000012.12	+	19345842	19354002	8161	476	sense-overlappin	NM_001256470.1	PLEKHA5
circRNA_08123	0	0	0	0	0	0	0	0	0	0.020172483	0	0	-	NC_000012.12	+	19348399	19366109	17711	856	sense-overlappin	NM_001143821.2	PLEKHA5
circRNA_08124	0.113162635	0.020469968	0.08723458	0.0412724	0.0702209	0.031069364	0.040197371	0.020486295	0	0.060517448	0	0	hsa_circ_0006420	NC_000012.12	+	19462510	19473355	10846	316	sense-overlappin	XM_006719031.2	AEBP2
circRNA_08125	0	0	0	0	0	0	0	0	0	0	0.02025523	0	-	NC_000012.12	+	19462510	19493986	31477	503	sense-overlappin	XM_006719031.2	AEBP2
circRNA_08126	0.041150049	0	0.032712968	0	0	0	0	0.020486295	0	0	0	0	hsa_circ_0006128	NC_000012.12	+	19462510	19500221	37712	628	sense-overlappin	XM_006719031.2	AEBP2
circRNA_08127	0	0	0.032712968	0	0	0	0	0	0	0	0	0	hsa_circ_0025554	NC_000012.12	+	19462510	19514784	52275	810	sense-overlappin	NM_001114176.1	AEBP2
circRNA_08128	0	0	0	0	0	0	0	0	0	0.020172483	0	0	hsa_circ_0005257	NC_000012.12	+	19473248	19500221	26974	420	sense-overlappin	XM_006719031.2	AEBP2
circRNA_08129	0	0	0	0	0.03009467	0	0	0	0	0	0	0	-	NC_000012.12	+	19923198	19944544	21347	21347	intergenic		
circRNA_08130	0	0	0	0	0	0	0	0	0	0	0.030382845	0	-	NC_000012.12	+	20008645	20011451	2807	2807	intergenic		
circRNA_08131	0	0	0	0	0.02006311	0	0	0	0	0	0	0	-	NC_000012.12	+	20099123	20127653	28531	28531	intergenic		
circRNA_08132	0.020575025	0	0	0	0	0	0	0	0	0	0	0	-	NC_000012.12	-	20490851	20490935	85	85	antisense	XM_006719086.2	PDE3A
circRNA_08133	0	0.030704951	0	0	0	0	0	0	0	0	0	0	-	NC_000012.12	+	20621296	20654205	32910	1760	sense-overlappin	XM_006719086.2	PDE3A
circRNA_08134	0.020575025	0	0	0	0	0	0	0	0	0	0	0	-	NC_000012.12	-	21805998	21818251	12254	843	sense-overlappin	NM_020297.3	ABCC9
circRNA_08135	0	0	0	0	0	0	0	0	0	0.030258724	0	0	hsa_circ_0025608	NC_000012.12	-	22469709	22506819	37111	1561	sense-overlappin	NM_001286175.1	C2CD5
circRNA_08136	0	0	0	0.0206362	0	0.031069364	0	0	0	0	0	0	-	NC_000012.12	-	22482557	22506819	24263	765	sense-overlappin	NM_001286175.1	C2CD5
circRNA_08137	0	0	0	0	0	0	0	0	0	0	0	0.0206364	hsa_circ_0025614	NC_000012.12	-	22493223	22506819	13597	290	sense-overlappin	NM_001286175.1	C2CD5
circRNA_08138	0	0.040939935	0.490694513	0	0.03009467	0.020712909	0.030148028	0	0.050636319	0.020172483	0	0	hsa_circ_0008664	NC_000012.12	+	22643763	22673660	29898	789	sense-overlappin	NM_018638.4	ETNK1
circRNA_08139	0	0	0.283512385	0	0	0	0	0	0	0	0	0	hsa_circ_0025619	NC_000012.12	+	22659014	22673660	14647	529	sense-overlappin	NM_018638.4	ETNK1
circRNA_08140	0	0	0	0	0	0	0	0.020486295	0	0	0	0	-	NC_000012.12	-	24213343	24249916	36574	36574	sense-overlappin	XM_011520839.1	SOX5
circRNA_08141	0	0	0	0	0	0.020712909	0	0	0	0	0	0	-	NC_000012.12	-	24451472	24460776	9305	9305	intronic	XM_011520839.1	SOX5
circRNA_08142	0	0	0	0	0	0	0	0	0	0.020172483	0	0	hsa_circ_0000381	NC_000012.12	+	25061592	25069431	7840	470	sense-overlappin	XM_011520667.1	LRMP
circRNA_08143	0	0	0.021808645	0	0	0	0	0	0	0	0	0	hsa_circ_0025633	NC_000012.12	+	25079244	25083493	4250	291	sense-overlappin	XM_005253372.2	LRMP
circRNA_08144	0	0	0	0	0	0	0.020098685	0	0	0	0	0	-	NC_000012.12	-	25225614	25245395	19782	461	sense-overlappin	NM_004985.4	KRAS
circRNA_08145	0	0	0	0	0.02006311	0	0	0	0	0	0	0	-	NC_000012.12	+	25995037	26003445	8409	8409	sense-overlappin	NM_007211.4	RASSF8
circRNA_08146	0.082300098	0	0	0	0.03009467	0	0	0	0	0	0	0.0412728	hsa_circ_0025638	NC_000012.12	+	26055236	26067713	12478	1246	sense-overlappin	NM_001164748.1	RASSF8
circRNA_08147	0	0	0	0.0206362	0	0.031069364	0	0	0	0	0	0	hsa_circ_0025640	NC_000012.12	+	26055279	26067713	12435	1203	sense-overlappin	NM_001164748.1	RASSF8
circRNA_08148	0.020575025	0.061409903	0	0	0.04012623	0	0.020098685	0	0	0.020172483	0	0	-	NC_000012.12	-	26550247	26602706	52460	1611	sense-overlappin	XM_011520646.1	ITPR2
circRNA_08149	0	0	0	0	0	0	0	0	0	0.020172483	0	0.0206364	-	NC_000012.12	-	26550247	26632059	81813	2333	sense-overlappin	XM_011520646.1	ITPR2
circRNA_08150	0	0	0	0	0	0.020712909	0	0	0	0	0	0	-	NC_000012.12	-	26578713	26602706	23994	1168	sense-overlappin	XM_011520646.1	ITPR2
circRNA_08151	0	0	0	0	0	0	0	0.030729443	0	0	0	0	-	NC_000012.12	-	26595465	26624356	28892	1316	sense-overlappin	XM_011520646.1	ITPR2
circRNA_08152	0	0	0	0	0	0.020712909	0	0	0	0	0	0	-	NC_000012.12	-	26595465	26655852	60388	1936	sense-overlappin	XM_011520646.1	ITPR2
circRNA_08153	0	0	0.032712968	0	0	0	0	0	0	0	0	0	-	NC_000012.12	-	26621123	26632059	10937	722	sense-overlappin	XM_011520646.1	ITPR2
circRNA_08154	0	0	0	0	0	0	0	0	0	0	0	0.0206364	-	NC_000012.12	-	26628033	26716242	88210	2539	sense-overlappin	XM_011520645.1	ITPR2
circRNA_08155	0	0	0	0	0.02006311	0	0	0	0	0	0	0	-	NC_000012.12	-	26790157	26818505	28349	28349	sense-overlappin	NM_002223.2	ITPR2
circRNA_08156	0	0	0	0.0309543	0	0	0	0	0	0.020172483	0	0	hsa_circ_0025705	NC_000012.12	-	26979784	26990627	10844	499	sense-overlappin	NM_016551.2	TM7SF3
circRNA_08157	0.030862537	0	0	0	0	0	0	0.030729443	0	0	0	0	-	NC_000012.12	-	26982773	26990627	7855	369	sense-overlappin	XM_011520692.1	TM7SF3
circRNA_08158	0	0	0	0	0	0	0	0	0	0.020172483	0	0	hsa_circ_0005824	NC_000012.12	-	26990450	27003390	12941	777	sense-overlappin	NM_016551.2	TM7SF3
circRNA_08159	0	0	0	0.0515905	0	0.020712909	0	0	0	0.080689931	0.04051046	0	hsa_circ_0007683	NC_000012.12	-	26996742	26999676	2935	272	sense-overlappin	NM_016551.2	TM7SF3
circRNA_08160	0	0	0.021808645	0	0	0	0	0	0	0	0	0	-	NC_000012.12	+	27030158	27032681	2524	2524	sense-overlappin	NM_001271811.1	MED21
circRNA_08161	0	0	0.032712968	0	0	0	0	0	0	0	0	0	-	NC_000012.12	+	27046158	27066226	20069	20069	intergenic		
circRNA_08162	0	0	0	0	0	0	0.020098685	0	0	0	0	0	hsa_circ_0009009	NC_000012.12	+	27368262	27370230	1969	253	sense-overlappin	NM_001248002.1	ARNTL2
circRNA_08163	0	0	0.054521613	0	0	0	0	0	0	0	0	0.0206364	hsa_circ_0001964	NC_000012.12	+	27368262	27380404	12143	453	sense-overlappin	NM_020183.4	ARNTL2
circRNA_08164	0	0	0.04361729	0	0	0	0	0	0	0	0	0	-	NC_000012.12	+	27380247	27426317	46071	7370	sense-overlappin	XM_006719112.2	ARNTL2
circRNA_08165	0	0	0	0	0	0	0	0	0	0.020172483	0	0	hsa_circ_0025725	NC_000012.12	+	27650010	27656730	6721	433	sense-overlappin	NM_001198915.1	PPFIBP1
circRNA_08166	0	0	0.021808645	0	0	0	0	0	0	0	0	0	-	NC_000012.12	+	27650010	27667320	17311	768	sense-overlappin	XM_006719159.2	PPFIBP1
circRNA_08167	0.030862537	0	0	0	0	0	0	0	0	0	0	0	-	NC_000012.12	+	27654722	27656730	2009	301	sense-overlappin	NM_001198915.1	PPFIBP1
circRNA_08168	0	0	0	0	0	0	0	0	0.030381792	0	0	0	-	NC_000012.12	+	27676428	27691928	15501	1455	sense-overlappin	NM_003622.3	PPFIBP1
circRNA_08169	0.051437562	0.08187987	0.152660515	0.0515905	0.13041025	0.103564547	0.030148028	0.040972591	0.040509055	0.151293621	0.04051046	0.0412728	hsa_circ_0000384	NC_000012.12	+	27714780	27724186	9407	410	sense-overlappin	NM_001190864.1	MRPS35

circRNA_08170	0	0	0	0	0.02006311	0	0	0	0	0	0	0	hsa_circ_0009017	NC_000012.12	+	27714780	27737608	22829	590	sense-overlappin	NM_021821.3	MRPS35
circRNA_08171	0	0	0	0	0.02006311	0	0	0	0	0	0	0	hsa_circ_0025735	NC_000012.12	+	27719808	27724186	4379	201	sense-overlappin	NM_001190864.1	MRPS35
circRNA_08172	0	0.16375974	0.283512385	0.1547714	0.15047336	0.217485548	0.070345399	0.09218833	0.13165443	0.242069794	0.08102092	0.123818401	hsa_circ_0000386	NC_000012.12	+	28225795	28259442	33648	332	sense-overlappin	XM_006719104.2	CCDC91
circRNA_08173	0	0	0.032712968	0	0	0.031069364	0	0	0	0	0	0	hsa_circ_0006742	NC_000012.12	+	28225795	28307749	81955	799	sense-overlappin	XM_006719104.2	CCDC91
circRNA_08174	0.020575025	0.030704951	0.032712968	0.0309543	0.03009467	0	0	0.061458886	0.030381792	0.050431207	0	0	hsa_circ_0000387	NC_000012.12	+	28255581	28259442	3862	205	sense-overlappin	XM_011520730.1	CCDC91
circRNA_08175	0.030862537	0.08187987	0.141756193	0.1341352	0.06018934	0.103564547	0	0.030729443	0.070890847	0.151293621	0	0.0309546	-	NC_000012.12	+	28255581	28307749	52169	672	sense-overlappin	XM_011520730.1	CCDC91
circRNA_08176	0.072012586	0.061409903	0.207182128	0.1341352	0.09028402	0.144990365	0.030148028	0.020486295	0.081018111	0.131121138	0.02025523	0.092863801	hsa_circ_0007723	NC_000012.12	+	28305649	28307749	2101	467	sense-overlappin	XM_011520729.1	CCDC91
circRNA_08177	0.020575025	0	0.04361729	0.0206362	0.06018934	0	0	0	0	0.020172483	0	0	hsa_circ_0008250	NC_000012.12	+	28305649	28391411	85763	653	sense-overlappin	XM_011520729.1	CCDC91
circRNA_08178	0	0	0	0	0.02006311	0	0	0	0	0	0	0	-	NC_000012.12	+	28391304	28452654	61351	447	sense-overlappin	XM_011520729.1	CCDC91
circRNA_08179	0	0	0	0	0	0.031069364	0	0	0	0	0	0	hsa_circ_0025750	NC_000012.12	+	29293300	29297200	3901	356	sense-overlappin	NM_001271783.1	FAR2
circRNA_08180	0	0	0	0.0206362	0	0	0	0	0	0	0	0	-	NC_000012.12	-	29338531	29341218	2688	2688	sense-overlappin	NM_016570.2	ERGIC2
circRNA_08181	0	0	0	0	0.05015779	0	0	0.040972591	0	0.100862414	0	0	-	NC_000012.12	-	29338531	29341816	3286	2771	sense-overlappin	NM_016570.2	ERGIC2
circRNA_08182	0	0	0	0	0.04012623	0.031069364	0.020098685	0	0	0.050431207	0	0	-	NC_000012.12	-	29339851	29341218	1368	1368	sense-overlappin	NM_016570.2	ERGIC2
circRNA_08183	0	0.020469968	0.021808645	0.0309543	0.03009467	0	0.050246713	0.030729443	0	0	0	0	-	NC_000012.12	-	29339851	29341816	1966	1451	sense-overlappin	NM_016570.2	ERGIC2
circRNA_08184	0	0	0.021808645	0	0	0	0	0	0	0	0	0	hsa_circ_0008318	NC_000012.12	-	29356382	29361685	5304	239	sense-overlappin	NM_016570.2	ERGIC2
circRNA_08185	0	0	0	0	0	0	0	0	0	0.040344966	0	0.0412728	hsa_circ_0025765	NC_000012.12	-	29659880	29660885	1006	186	sense-overlappin	XM_005253498.3	TMTC1
circRNA_08186	0.020575025	0	0	0.0515905	0	0	0.040197371	0	0	0	0	0	-	NC_000012.12	-	29659880	29661379	1500	252	sense-overlappin	XM_011520867.1	TMTC1
circRNA_08187	0	0	0	0	0	0	0	0	0	0	0.02025523	0	hsa_circ_0025767	NC_000012.12	-	29751666	29758777	7112	458	sense-overlappin	NM_001193451.1	TMTC1
circRNA_08188	0	0	0.021808645	0	0	0	0	0	0	0	0	0	-	NC_000012.12	-	30661141	30666251	5111	737	sense-overlappin	NM_001190995.1	IPO8
circRNA_08189	0	0	0	0.0206362	0	0	0	0	0	0	0	0	-	NC_000012.12	-	30663489	30676587	13099	955	sense-overlappin	NM_001190995.1	IPO8
circRNA_08190	0	0	0	0.0206362	0	0	0	0	0	0	0	0	-	NC_000012.12	-	30673990	30684457	10468	743	sense-overlappin	XM_011520547.1	IPO8
circRNA_08191	0	0	0	0.0309543	0	0.020712909	0	0	0	0	0	0	hsa_circ_0025783	NC_000012.12	-	30725966	30751133	25168	1485	sense-overlappin	NM_001002259.2	CAPRIN2
circRNA_08192	0	0	0	0.0206362	0	0	0	0	0	0	0	0	-	NC_000012.12	-	30728648	30751133	22486	1362	sense-overlappin	NM_001002259.2	CAPRIN2
circRNA_08193	0	0	0	0	0	0	0	0.020486295	0	0	0	0	hsa_circ_0004487	NC_000012.12	-	31392267	31392696	430	210	sense-overlappin	NM_144973.3	DENND5B
circRNA_08194	0	0	0	0	0	0	0	0.020486295	0	0	0	0	-	NC_000012.12	-	31402498	31409384	6887	268	sense-overlappin	NM_144973.3	DENND5B
circRNA_08195	0	0	0	0	0	0	0.020098685	0	0	0	0	0	-	NC_000012.12	-	31402498	31426424	23927	843	sense-overlappin	NM_144973.3	DENND5B
circRNA_08196	0	0	0	0	0	0	0	0	0	0.020172483	0	0	-	NC_000012.12	-	31413436	31426424	12989	575	sense-overlappin	NM_144973.3	DENND5B
circRNA_08197	0	0	0	0	0	0	0	0	0	0.020172483	0	0	hsa_circ_0025812	NC_000012.12	-	31423597	31426424	2828	364	sense-overlappin	NM_144973.3	DENND5B
circRNA_08198	0	0	0	0	0	0	0	0	0.020254528	0	0	0	hsa_circ_0025821	NC_000012.12	-	31433155	31442925	9771	245	sense-overlappin	NM_144973.3	DENND5B
circRNA_08199	0	0	0.021808645	0	0	0.072495183	0.020098685	0	0	0.020172483	0	0	hsa_circ_0008445	NC_000012.12	-	31442775	31447769	4995	383	sense-overlappin	NM_144973.3	DENND5B
circRNA_08200	0	0	0	0	0	0.031069364	0	0	0	0	0	0	-	NC_000012.12	-	31442775	31495919	53145	1885	sense-overlappin	NM_144973.3	DENND5B
circRNA_08201	0	0	0.021808645	0	0	0.031069364	0	0.030729443	0.030381792	0	0	0	hsa_circ_0025830	NC_000012.12	-	31451940	31495919	43980	1502	sense-overlappin	NM_144973.3	DENND5B
circRNA_08202	0	0	0	0	0.02006311	0	0	0	0	0	0	0	-	NC_000012.12	-	31451940	31499671	47732	1568	sense-overlappin	XM_011520589.1	DENND5B
circRNA_08203	0	0	0	0	0	0	0	0	0	0	0	0.0309546	-	NC_000012.12	-	31479589	31499671	20083	843	sense-overlappin	XM_011520589.1	DENND5B
circRNA_08204	0	0	0	0	0	0	0.020098685	0	0	0.020172483	0	0	hsa_circ_0007680	NC_000012.12	-	31495810	31499671	3862	176	sense-overlappin	XM_011520589.1	DENND5B
circRNA_08205	0	0	0.021808645	0	0	0	0	0	0	0	0	0	-	NC_000012.12	-	31701863	31719356	17494	487	sense-overlappin	NM_001278412.1	AMN1
circRNA_08206	0	0	0	0	0	0.051782273	0	0	0	0	0	0	hsa_circ_0000388	NC_000012.12	-	31796749	31802163	5415	5415	intergenic		
circRNA_08207	0	0	0	0	0	0	0	0	0	0.030258724	0	0	-	NC_000012.12	+	31992378	31996935	4558	4558	sense-overlappin	NM_018169.3	KIAA1551
circRNA_08208	0	0	0	0	0	0	0	0	0	0.040344966	0	0	-	NC_000012.12	+	32564137	32640453	76317	2585	sense-overlappin	NM_001304484.1	FGD4
circRNA_08209	0	0.030704951	0	0.0825448	0	0	0	0	0	0	0	0	hsa_circ_0025842	NC_000012.12	+	32576266	32611283	35018	1530	sense-overlappin	NM_001304483.1	FGD4
circRNA_08210	0	0	0	0	0	0	0.030148028	0	0	0	0	0	-	NC_000012.12	+	32581960	32602317	20358	1001	sense-overlappin	NM_001304483.1	FGD4
circRNA_08211	0	0	0	0	0	0	0	0	0	0.020172483	0	0	-	NC_000012.12	+	32581960	32611283	29324	1346	sense-overlappin	NM_001304483.1	FGD4
circRNA_08212	0.051437562	0	0	0.1238171	0.03009467	0.051782273	0.030148028	0	0.030381792	0.030258724	0.02025523	0.0309546	-	NC_000012.12	+	32598497	32601423	2927	336	sense-overlappin	NM_001304483.1	FGD4
circRNA_08213	0	0	0	0	0	0	0	0	0.030381792	0	0	0	-	NC_000012.12	+	32598497	32602317	3821	493	sense-overlappin	NM_001304483.1	FGD4
circRNA_08214	0.051437562	0.16375974	0.08723458	0.4849504	0.0702209	0.279624276	0.140690797	0.112674625	0.192418013	0.181552345	0.12153138	0.123818401	hsa_circ_0025843	NC_000012.12	+	32598497	32611283	12787	838	sense-overlappin	NM_001304483.1	FGD4
circRNA_08215	0	0	0.021808645	0.0515905	0	0	0	0	0.020254528	0	0	0	-	NC_000012.12	+	32598497	32625779	27283	1261	sense-overlappin	NM_001304483.1	FGD4
circRNA_08216	0	0	0	0.0206362	0	0	0	0	0	0	0	0	-	NC_000012.12	+	32598497	32633689	35193	1402	sense-overlappin	NM_001304483.1	FGD4
circRNA_08217	0	0	0	0	0	0	0	0.020486295	0	0	0	0	-	NC_000012.12	+	32599402	32599642	241	241	intronic	XM_005253304.3	FGD4
circRNA_08218	0.020575025	0	0.04361729	0.2373162	0.03009467	0.072495183	0.050246713	0.040972591	0.040509055	0.161379862	0.070893305	0.051591	hsa_circ_0000390	NC_000012.12	+	32607957	32611283	3327	345	sense-overlappin	XM_005253304.3	FGD4
circRNA_08219	0	0	0	0.0515905	0	0	0	0	0	0	0	0	-	NC_000012.12	+	32619698	32633689	13992	564	sense-overlappin	XM_005253304.3	FGD4
circRNA_08220	0	0	0	0	0	0	0	0	0	0	0.02025523	0	-	NC_000012.12	+	32624976	32625779	804	219	sense-overlappin	XM_005253304.3	FGD4
circRNA_08221	0	0	0	0	0	0	0	0	0	0.050431207	0	0	hsa_circ_0025850	NC_000012.12	+	32708153	32718763	10611	443	sense-overlappin	NM_001278463.1	DNM1L
circRNA_08222	0.020575025	0	0	0	0	0	0	0	0	0	0	0	hsa_circ_0025853	NC_000012.12	+	32713209	32722633	9425	623	sense-overlappin	NM_001278463.1	DNM1L
circRNA_08223	0	0	0.021808645	0	0	0	0	0	0	0	0	0	-	NC_000012.12	+	34381457	34387686	6230	6230	intergenic		
circRNA_08224	0	0	0	0	0	0	0	0	0	0.020172483	0	0	-	NC_000012.12	-	38760847	38776301	15455	315	sense-overlappin	NM_153634.2	CPNE8

circRNA_08225	0	0	0	0	0	0	0	0	0	0.020172483	0	0	hsa_circ_0025875	NC_000012.12	-	39579535	39586297	6763	231	sense-overlappin	XM_011538027.1	ABCD2
circRNA_08226	0	0	0	0.0619086	0	0	0	0	0	0.020172483	0	0	hsa_circ_0007918	NC_000012.12	-	40028301	40048210	19910	369	sense-overlappin	NM_052885.3	SLC2A13
circRNA_08227	0	0	0	0	0	0	0	0	0	0.030258724	0	0	hsa_circ_0004502	NC_000012.12	-	42210355	42210619	265	172	sense-overlappin	XR_944468.1	
circRNA_08228	0.072012586	0.061409903	0.065425935	0.0515905	0.04012623	0.020712909	0	0.051215739	0.101272639	0.110948655	0.030382845	0.0412728	hsa_circ_0000392	NC_000012.12	-	42210355	42210680	326	233	sense-overlappin	XR_944468.1	
circRNA_08229	0.020575025	0	0	0	0	0	0	0	0	0	0	0	-	NC_000012.12	-	42317340	42324104	6765	335	sense-overlappin	NM_033114.3	ZCRB1
circRNA_08230	0	0	0.054521613	0.0412724	0.03009467	0	0	0	0	0.020172483	0	0	hsa_circ_0004815	NC_000012.12	+	42351885	42398994	47110	837	sense-overlappin	NM_201439.1	PPHLN1
circRNA_08231	0	0	0.032712968	0.0206362	0	0	0	0.020486295	0	0.020172483	0	0	hsa_circ_0003961	NC_000012.12	+	42374863	42398994	24132	610	sense-overlappin	XM_005268965.2	PPHLN1
circRNA_08232	0	0	0	0	0.02006311	0	0	0	0	0	0	0	-	NC_000012.12	+	42374956	42398994	24039	517	sense-overlappin	XM_005268965.2	PPHLN1
circRNA_08233	0	0	0	0	0	0.062138728	0	0	0	0	0	0	hsa_circ_0025908	NC_000012.12	+	42384940	42398994	14055	398	sense-overlappin	XM_005268965.2	PPHLN1
circRNA_08234	0	0	0	0	0.05015779	0.020712909	0	0	0	0	0	0	hsa_circ_0003881	NC_000012.12	+	44143525	44211387	67863	198	sense-overlappin	XM_011538831.1	TMEM117
circRNA_08235	0	0	0.021808645	0	0	0	0	0	0	0	0	0	hsa_circ_0003433	NC_000012.12	+	45331295	45357424	26130	848	sense-overlappin	XM_005268707.2	ANO6
circRNA_08236	0	0	0	0.0309543	0	0	0	0	0	0	0.02025523	0	hsa_circ_0005192	NC_000012.12	+	45378053	45390498	12446	282	sense-overlappin	XM_005268707.2	ANO6
circRNA_08237	0	0.030704951	0.065425935	0	0	0	0	0	0	0.040344966	0	0	hsa_circ_0025951	NC_000012.12	+	45836589	45839496	2908	793	sense-overlappin	XR_944505.1	
circRNA_08238	0	0	0.021808645	0.0619086	0	0.031069364	0	0	0	0.030258724	0.02025523	0	hsa_circ_0025953	NC_000012.12	+	45836589	45860949	24361	4275	sense-overlappin	XR_944505.1	
circRNA_08239	0	0	0	0.0309543	0	0	0	0	0	0	0	0	-	NC_000012.12	+	45836589	45872962	36374	16288	sense-overlappin	XR_944505.1	
circRNA_08240	0	0	0	0	0	0	0	0	0	0	0.030382845	0	hsa_circ_0004985	NC_000012.12	+	45891780	45893721	1942	441	sense-overlappin	XR_944505.1	
circRNA_08241	0	0.040939935	0.04361729	0.1238171	0.03009467	0.031069364	0.020098685	0.061458886	0.060763583	0.080689931	0.02025523	0.072227401	hsa_circ_0025967	NC_000012.12	-	45926142	45928859	2718	2718	exonic	XM_011538987.1	SCAF11
circRNA_08242	0	0	0	0	0	0.020712909	0	0	0	0	0	0	hsa_circ_0025969	NC_000012.12	-	45931506	45951727	20222	622	sense-overlappin	XM_011538987.1	SCAF11
circRNA_08243	0	0	0	0	0.02006311	0	0	0	0	0	0	0	-	NC_000012.12	-	45945249	45953857	8609	274	sense-overlappin	XM_011538987.1	SCAF11
circRNA_08244	0	0	0.021808645	0	0	0	0	0	0	0	0	0	-	NC_000012.12	-	45945249	45964188	18940	484	sense-overlappin	XM_011538984.1	SCAF11
circRNA_08245	0.216037758	0.16375974	0.13085187	0.1650895	0.21066271	0.15534682	0.070345399	0.245835546	0.060763583	0.191638587	0.10127615	0.0619092	hsa_circ_0000396	NC_000012.12	-	46229153	46243314	14162	522	sense-overlappin	NM_001278389.1	SLC38A1
circRNA_08246	0	0.030704951	0	0	0.04012623	0.062138728	0	0	0.030381792	0.07060369	0	0.0206364	hsa_circ_0000397	NC_000012.12	-	46229153	46254936	25784	645	sense-overlappin	NM_001278387.1	SLC38A1
circRNA_08247	0.082300098	0.092114854	0.08723458	0	0.05015779	0.103564547	0.020098685	0.071702034	0	0.090776173	0.030382845	0.0309546	hsa_circ_0000398	NC_000012.12	-	46239679	46243314	3636	330	sense-overlappin	NM_001278389.1	SLC38A1
circRNA_08248	0	0	0	0	0.02006311	0	0.020098685	0	0	0.020172483	0	0.0309546	-	NC_000012.12	-	46239679	46254936	15258	453	sense-overlappin	NM_001278387.1	SLC38A1
circRNA_08249	0	0	0	0	0	0	0.020098685	0	0	0	0	0	-	NC_000012.12	+	46470153	46477340	7188	7188	intergenic		
circRNA_08250	0.020575025	0.020469968	0.065425935	0.0206362	0	0.020712909	0.030148028	0.030729443	0.020254528	0.030258724	0	0.0206364	hsa_circ_0004650	NC_000012.12	+	47104113	47108164	4052	251	sense-overlappin	XM_011538978.1	PCED1B
circRNA_08251	0	0	0	0	0	0.041425819	0	0	0	0	0	0	-	NC_000012.12	+	47212597	47216689	4093	261	sense-overlappin	XM_011538979.1	PCED1B
circRNA_08252	0	0	0	0	0	0	0	0	0	0	0	0.0206364	-	NC_000012.12	-	47226669	47228578	1910	1910	antisense	NM_001281429.1	PCED1B
circRNA_08253	0	0	0	0	0	0.020712909	0	0	0	0	0	0	hsa_circ_0007838	NC_000012.12	-	48071667	48074793	3127	443	sense-overlappin	NM_001267595.1	SENP1
circRNA_08254	0	0	0	0	0	0.020712909	0	0	0	0	0	0	hsa_circ_0006222	NC_000012.12	-	48083591	48098124	14534	679	sense-overlappin	NR_051991.1	
circRNA_08255	0	0	0	0	0	0	0	0	0	0	0	0.0206364	hsa_circ_0026028	NC_000012.12	-	48088801	48098124	9324	507	sense-overlappin	NR_051991.1	
circRNA_08256	0	0	0	0.0206362	0	0	0	0	0	0	0	0	hsa_circ_0026029	NC_000012.12	-	48096343	48101516	5174	264	sense-overlappin	NM_001267595.1	SENP1
circRNA_08257	0	0	0	0	0.02006311	0	0	0	0	0	0	0	-	NC_000012.12	-	48773469	48774518	1050	455	sense-overlappin	NM_015270.3	ADCY6
circRNA_08258	0	0	0.021808645	0	0	0	0	0	0	0	0	0	-	NC_000012.12	-	48939035	48941188	2154	551	sense-overlappin	XM_011538322.1	ARF3
circRNA_08259	0.020575025	0	0.021808645	0	0.0702209	0	0	0	0	0	0	0	hsa_circ_0003770	NC_000012.12	-	48939655	48941188	1534	477	sense-overlappin	XM_006719391.2	ARF3
circRNA_08260	0	0	0	0	0.05015779	0	0.020098685	0	0.040509055	0.020172483	0	0	hsa_circ_0004602	NC_000012.12	-	49097566	49098105	540	336	sense-overlappin	XM_011538563.1	LMBR1L
circRNA_08261	0	0	0	0	0.03009467	0	0	0.020486295	0	0	0	0	hsa_circ_0026150	NC_000012.12	+	49460770	49490731	29962	507	sense-overlappin	NM_023071.3	SPATS2
circRNA_08262	0	0	0	0	0	0	0.020098685	0	0	0	0	0	-	NC_000012.12	+	49460770	49500205	39436	1082	sense-overlappin	NM_023071.3	SPATS2
circRNA_08263	0	0	0	0	0	0.031069364	0	0	0	0	0	0	-	NC_000012.12	+	49465880	49466214	335	335	intronic	NM_023071.3	SPATS2
circRNA_08264	0	0.030704951	0	0.0206362	0.06018934	0.031069364	0	0	0.020254528	0	0	0	-	NC_000012.12	+	49486250	49490731	4482	3374	sense-overlappin	NM_023071.3	SPATS2
circRNA_08265	0	0.020469968	0	0	0	0	0	0	0	0	0	0	hsa_circ_0026153	NC_000012.12	+	49489465	49490731	1267	159	sense-overlappin	NM_023071.3	SPATS2
circRNA_08266	0	0	0	0	0	0.020712909	0.020098685	0	0	0	0	0	hsa_circ_0006238	NC_000012.12	+	49494741	49500205	5465	575	sense-overlappin	NM_023071.3	SPATS2
circRNA_08267	0	0	0	0	0	0.020712909	0	0	0.020254528	0	0	0	hsa_circ_0026154	NC_000012.12	+	49496833	49500205	3373	313	sense-overlappin	NM_023071.3	SPATS2
circRNA_08268	0	0	0.021808645	0	0	0	0	0	0	0	0	0	hsa_circ_0009035	NC_000012.12	-	49991998	49992655	658	375	sense-overlappin	XM_006719359.1	RACGAP1
circRNA_08269	0	0	0	0.0412724	0	0.072495183	0.060296056	0	0	0	0	0	hsa_circ_0006535	NC_000012.12	+	50094437	50096972	2536	259	sense-overlappin	XM_005269107.3	SMARCD1
circRNA_08270	0	0.040939935	0	0	0	0.041425819	0	0	0	0	0	0	-	NC_000012.12	+	50129527	50130217	691	691	antisense	NM_001281731.1	CERS5
circRNA_08271	0.051437562	0	0.04361729	0.0412724	0.09028402	0.041425819	0.040197371	0.040972591	0.030381792	0.040344966	0.030382845	0	hsa_circ_0003873	NC_000012.12	+	50427762	50429090	1329	304	sense-overlappin	NM_001170804.1	LARP4
circRNA_08272	0	0	0.08723458	0.0515905	0.06018934	0.020712909	0	0	0	0.020172483	0.02025523	0	hsa_circ_0003664	NC_000012.12	+	50427762	50430570	2809	380	sense-overlappin	NM_001170804.1	LARP4
circRNA_08273	0	0	0	0	0	0	0.020098685	0	0	0	0.04051046	0	-	NC_000012.12	+	50427762	50435624	7863	517	sense-overlappin	NM_001170804.1	LARP4
circRNA_08274	0	0	0.021808645	0	0	0	0	0	0	0	0.04051046	0	hsa_circ_0026227	NC_000012.12	+	50427762	50437838	10077	621	sense-overlappin	NM_001170804.1	LARP4
circRNA_08275	0.072012586	0.040939935	0.04361729	0.1754076	0.09028402	0.072495183	0	0.040972591	0.060763583	0.080689931	0	0	hsa_circ_0026228	NC_000012.12	+	50427762	50441643	13882	786	sense-overlappin	NM_001170804.1	LARP4
circRNA_08276	0	0	0	0.0412724	0	0	0	0	0	0	0	0	hsa_circ_0026229	NC_000012.12	+	50427762	50454417	26656	1103	sense-overlappin	NM_001170804.1	LARP4
circRNA_08277	0	0	0	0.0206362	0.04012623	0.020712909	0	0	0	0	0	0	hsa_circ_0026230	NC_000012.12	+	50427762	50461347	33586	1316	sense-overlappin	NM_001170804.1	LARP4
circRNA_08278	0	0	0	0	0	0	0	0.020486295	0	0	0	0	-	NC_000012.12	+	50427762	50467120	39359	1527	sense-overlappin	XM_005268613.1	LARP4
circRNA_08279	0	0	0	0.0619086	0	0	0	0	0	0	0	0	hsa_circ_0026233	NC_000012.12	+	50430495	50454417	23923	799	sense-overlappin	NM_001170804.1	LARP4
circRNA_08280	0	0	0	0.0206362	0.02006311	0	0	0	0	0	0	0	-	NC_000012.12	+	50430498	50454417	23920	796	sense-overlappin	NM_001170804.1	LARP4

circRNA_08281	0	0	0.032712968	0	0	0	0	0	0	0	0	0	hsa_circ_0026235	NC_000012.12	+	50435488	50441643	6156	406	sense-overlappin	NM_001170804.1	LARP4
circRNA_08282	0	0	0.021808645	0	0.09028402	0	0	0	0	0	0	0	-	NC_000012.12	+	50435488	50454417	18930	723	sense-overlappin	NM_001170804.1	LARP4
circRNA_08283	0	0.020469968	0	0	0	0	0	0	0.020254528	0	0	0	hsa_circ_0026238	NC_000012.12	+	50453460	50454417	958	317	sense-overlappin	NM_001170804.1	LARP4
circRNA_08284	0	0	0.021808645	0	0	0	0	0.020486295	0	0.030258724	0	0	hsa_circ_0003222	NC_000012.12	+	50454314	50461347	7034	317	sense-overlappin	NM_001170804.1	LARP4
circRNA_08285	0	0	0	0	0.02006311	0	0	0	0	0	0	0	-	NC_000012.12	+	50461135	50467120	5986	424	sense-overlappin	XM_005268613.1	LARP4
circRNA_08286	0.030862537	0	0	0	0	0	0	0	0	0	0	0	-	NC_000012.12	+	50462582	50474167	11586	502	sense-overlappin	XM_005268613.1	LARP4
circRNA_08287	0	0	0	0	0	0	0	0	0.020254528	0	0	0	-	NC_000012.12	+	50671186	50680763	9578	782	sense-overlappin	XM_005269044.1	DIP2B
circRNA_08288	0	0.040939935	0	0	0	0	0	0	0.020254528	0	0	0	-	NC_000012.12	+	50780140	50809589	29450	334	sense-overlappin	XM_011538387.1	ATF1
circRNA_08289	0	0	0	0	0	0	0.030148028	0	0	0	0	0.0412728	hsa_circ_0026285	NC_000012.12	+	50814010	50814439	430	343	sense-overlappin	XM_011538387.1	ATF1
circRNA_08290	0	0	0	0	0	0	0.020098685	0	0	0	0	0	-	NC_000012.12	-	50981639	51010766	29128	1910	sense-overlappin	XM_011538406.1	SLC11A2
circRNA_08291	0	0	0	0	0	0	0	0	0.020254528	0	0	0	hsa_circ_0026291	NC_000012.12	-	50996817	50999415	2599	295	sense-overlappin	XR_944555.1	
circRNA_08292	0.020575025	0.030704951	0	0	0.05015779	0	0	0.071702034	0	0.020172483	0	0	hsa_circ_0008802	NC_000012.12	-	51008476	51010766	2291	221	sense-overlappin	XR_944555.1	
circRNA_08293	0	0	0	0	0.02006311	0	0	0	0	0	0	0	hsa_circ_0003754	NC_000012.12	+	51053812	51056502	2691	491	sense-overlappin	NM_001243689.1	LETMD1
circRNA_08294	0	0	0	0.0309543	0	0.020712909	0.020098685	0	0	0.030258724	0.02025523	0	hsa_circ_0004313	NC_000012.12	+	51055835	51056502	668	442	sense-overlappin	NM_001243689.1	LETMD1
circRNA_08295	0	0	0	0	0	0	0	0	0	0.030258724	0	0	hsa_circ_0026300	NC_000012.12	-	51067673	51067969	297	297	exonic	XM_006719621.2	CSRNP2
circRNA_08296	0	0	0	0	0	0	0	0	0	0.020172483	0	0	hsa_circ_0026301	NC_000012.12	-	51067673	51074082	6410	557	sense-overlappin	XM_006719621.2	CSRNP2
circRNA_08297	0.061725074	0.102349838	0	0	0	0.020712909	0	0	0.030381792	0.050431207	0	0	hsa_circ_0004538	NC_000012.12	-	51073823	51074082	260	260	exonic	XM_006719621.2	CSRNP2
circRNA_08298	0	0	0	0	0	0	0	0.020486295	0	0	0	0	-	NC_000012.12	-	51103670	51118772	15103	938	sense-overlappin	NM_001173452.1	TFCP2
circRNA_08299	0	0.040939935	0	0	0	0	0	0	0	0	0	0	hsa_circ_0003729	NC_000012.12	+	51986818	51991993	5176	256	sense-overlappin	NM_004302.4	ACVR1B
circRNA_08300	0	0	0.032712968	0	0	0	0.080394741	0	0	0	0	0	-	NC_000012.12	-	52449469	52470630	21162	19629	sense-overlappin	NM_005555.3	KRT6B
circRNA_08301	0.102875123	0	0	0	0	0	0.050246713	0	0	0	0	0	-	NC_000012.12	-	52449469	52490068	40600	39067	sense-overlappin	NM_005555.3	KRT6B
circRNA_08302	0.082300098	0	0.032712968	0.0619086	0.03009467	0.020712909	0.020098685	0	0.020254528	0.080689931	0	0.0412728	hsa_circ_0007889	NC_000012.12	+	53027782	53028188	407	312	sense-overlappin	NM_001300821.1	EIF4B
circRNA_08303	0	0	0	0	0.05015779	0	0	0	0	0	0	0	hsa_circ_0026627	NC_000012.12	+	53381659	53409561	27903	1893	sense-overlappin	NM_001251825.1	SP1
circRNA_08304	0	0	0	0	0	0	0	0	0	0	0	0.0206364	-	NC_000012.12	-	53719739	53722183	2445	399	sense-overlappin	XM_011538603.1	CALCOCO1
circRNA_08305	0	0	0	0	0	0	0	0	0.020254528	0	0	0	hsa_circ_0004282	NC_000012.12	-	54241744	54257692	15949	629	sense-overlappin	NM_001127322.1	CBX5
circRNA_08306	0.329200394	0.133054789	0	0.0412724	0	0.103564547	0	0	0.070890847	0.040344966	0.091148535	0.082545601	hsa_circ_0026782	NC_000012.12	-	55700899	55701154	256	256	exonic	NM_001144996.1	ITGA7
circRNA_08307	0	0	0	0	0.03009467	0	0	0	0	0	0	0.0206364	hsa_circ_0002737	NC_000012.12	-	55789075	55803728	14654	465	sense-overlappin	NR_026723.1	
circRNA_08308	0	0	0	0	0	0	0	0.020486295	0	0	0	0	hsa_circ_0026810	NC_000012.12	+	56086531	56088868	2338	688	sense-overlappin	NM_001982.3	ERBB3
circRNA_08309	0	0	0	0	0.02006311	0	0	0	0	0	0	0.0206364	-	NC_000012.12	+	56099648	56100245	598	362	sense-overlappin	NM_001982.3	ERBB3
circRNA_08310	0	0	0	0.0309543	0.06018934	0	0.050246713	0	0.020254528	0	0	0	-	NC_000012.12	-	56169529	56170208	680	368	sense-overlappin	NM_001130420.1	SMARCC2
circRNA_08311	0	0	0	0	0	0	0	0	0	0	0	0.0412728	-	NC_000012.12	+	56298342	56298763	422	422	antisense	NM_004077.2	CS
circRNA_08312	0.061725074	0	0.04361729	0.0206362	0.03009467	0	0.020098685	0.030729443	0	0	0	0	hsa_circ_0008085	NC_000012.12	-	56348529	56349636	1108	515	sense-overlappin	NM_005419.3	STAT2
circRNA_08313	0	0	0	0	0	0	0	0	0	0	0	0.0206364	hsa_circ_0000408	NC_000012.12	-	56430881	56432524	1644	378	sense-overlappin	NM_003920.3	TIMELESS
circRNA_08314	0	0	0	0	0	0	0.030148028	0	0	0	0	0	hsa_circ_0004843	NC_000012.12	-	56603359	56605327	1969	786	sense-overlappin	NM_001300905.1	BAZ2A
circRNA_08315	0.030862537	0.020469968	0	0	0	0	0	0	0	0	0	0	hsa_circ_0027089	NC_000012.12	-	56666204	56670364	4161	153	sense-overlappin	NR_104219.1	
circRNA_08316	0	0	0	0	0	0	0.020098685	0	0	0	0	0	hsa_circ_0027090	NC_000012.12	-	56666204	56673065	6862	436	sense-overlappin	NR_104219.1	
circRNA_08317	0	0.051174919	0.04361729	0	0	0.020712909	0	0	0	0.040344966	0	0.0206364	hsa_circ_0027093	NC_000012.12	-	56670275	56673065	2791	373	sense-overlappin	NR_104219.1	
circRNA_08318	0	0	0	0	0.02006311	0	0	0	0	0	0	0	hsa_circ_0008137	NC_000012.12	-	56671749	56673065	1317	283	sense-overlappin	NR_104219.1	
circRNA_08319	0	0	0	0	0	0	0	0	0.020254528	0	0	0	-	NC_000012.12	-	57046552	57048343	1792	660	sense-overlappin	NM_005379.3	MYO1A
circRNA_08320	0	0	0	0	0.02006311	0	0	0	0	0	0	0	hsa_circ_0000411	NC_000012.12	-	57069234	57072912	3679	418	sense-overlappin	NM_001130963.1	TMEM194A
circRNA_08321	0	0	0	0	0.02006311	0	0	0	0	0	0	0	-	NC_000012.12	-	57280358	57303217	22860	1233	sense-overlappin	XM_011538030.1	R3HDM2
circRNA_08322	0	0	0	0	0.06018934	0	0	0	0	0	0	0	-	NC_000012.12	-	57288857	57296551	7695	432	sense-overlappin	XM_011538030.1	R3HDM2
circRNA_08323	0	0	0	0	0.02006311	0	0	0	0	0	0	0	hsa_circ_0027244	NC_000012.12	-	57289009	57296551	7543	378	sense-overlappin	XM_011538030.1	R3HDM2
circRNA_08324	0	0.020469968	0	0	0	0.020712909	0	0	0.020254528	0.020172483	0	0	-	NC_000012.12	-	57392125	57393185	1061	1061	intronic	XM_011538030.1	R3HDM2
circRNA_08325	0	0	0	0	0.03009467	0	0	0	0.020254528	0	0	0	-	NC_000012.12	-	57395749	57398198	2450	2450	sense-overlappin	XM_011538042.1	R3HDM2
circRNA_08326	0	0	0	0.0206362	0	0	0	0	0.040509055	0.050431207	0	0	hsa_circ_0005358	NC_000012.12	+	57467333	57468224	892	396	sense-overlappin	NM_001160045.1	GLI1
circRNA_08327	0	0	0	0	0.02006311	0	0	0	0	0	0	0	hsa_circ_0006104	NC_000012.12	-	57533953	57534452	500	306	sense-overlappin	NM_001261412.1	DCTN2
circRNA_08328	0	0	0	0	0.02006311	0	0	0	0	0	0	0	hsa_circ_0004034	NC_000012.12	-	57535056	57535845	790	258	sense-overlappin	NM_001261412.1	DCTN2
circRNA_08329	0	0	0	0.0206362	0	0	0	0	0	0	0	0	-	NC_000012.12	-	58885831	58914336	28506	1008	sense-overlappin	XM_011537893.1	LRIG3
circRNA_08330	0	0	0.021808645	0	0	0	0	0	0	0	0	0	hsa_circ_0027352	NC_000012.12	+	62314790	62325933	11144	335	sense-overlappin	NM_001252079.1	USP15
circRNA_08331	0	0	0	0	0.02006311	0	0	0	0	0	0	0	-	NC_000012.12	+	62470701	62499048	28348	532	sense-overlappin	NM_001278472.1	MON2
circRNA_08332	0.030862537	0	0	0	0.03009467	0	0	0	0	0	0	0.0206364	-	NC_000012.12	+	62484170	62501698	17529	759	sense-overlappin	XM_011538051.1	MON2
circRNA_08333	0.020575025	0	0	0	0.03009467	0	0	0	0	0	0	0	hsa_circ_0027364	NC_000012.12	+	62498919	62501698	2780	354	sense-overlappin	XM_011538052.1	MON2
circRNA_08334	0	0	0	0	0.03009467	0	0	0	0	0	0	0	hsa_circ_0027374	NC_000012.12	+	62565237	62588156	22920	958	sense-overlappin	NM_001278469.1	MON2
circRNA_08335	0	0	0	0.0206362	0	0	0	0	0	0	0	0	-	NC_000012.12	+	62580297	62588156	7860	415	sense-overlappin	XR_944510.1	

circRNA_08336	0	0.030704951	0	0	0	0	0	0	0	0	0	0	-	NC_000012.12	-		62879116	62912108	32993	32993	intronic	NM_020700.1	PPM1H
circRNA_08337	0	0	0	0	0	0	0	0	0	0	0.02025523	0	hsa_circ_0004909	NC_000012.12	-		63617304	63647365	30062	630	sense-overlappin	XM_011538215.1	DPY19L2
circRNA_08338	0	0	0	0	0.0702209	0	0	0	0	0	0	0	-	NC_000012.12	+		64409962	64420521	10560	917	sense-overlappin	XR_944484.1	
circRNA_08339	0	0	0	0	0	0	0	0	0	0.040344966	0	0	-	NC_000012.12	+		64648537	64649552	1016	1016	sense-overlappin	NM_178169.3	RASSF3
circRNA_08340	0	0	0	0	0.03009467	0	0	0	0	0	0	0	-	NC_000012.12	+		64684787	64698710	13924	4404	sense-overlappin	XM_011538195.1	RASSF3
circRNA_08341	0	0	0	0.0928628	0.10031557	0	0	0.020486295	0.020254528	0	0	0	hsa_circ_0027440	NC_000012.12	+		64864668	64866903	2236	253	sense-overlappin	NM_015279.1	TBC1D30
circRNA_08342	0	0	0	0	0.02006311	0	0	0	0	0	0	0	-	NC_000012.12	+		64866764	64875035	8272	442	sense-overlappin	XM_011538078.1	TBC1D30
circRNA_08343	0.051437562	0	0	0.0206362	0	0	0	0	0	0	0	0	hsa_circ_0027443	NC_000012.12	+		65308529	65369026	60498	343	sense-overlappin	NM_001031679.2	MSRB3
circRNA_08344	0	0	0	0	0	0.041425819	0	0	0	0	0	0	-	NC_000012.12	+		65326826	65369026	42201	216	sense-overlappin	NM_001031679.2	MSRB3
circRNA_08345	0	0	0.032712968	0	0.12037869	0.020712909	0	0	0	0	0	0	-	NC_000012.12	-		66145841	66153448	7608	367	sense-overlappin	NM_001282606.1	TMBIM4
circRNA_08346	0.030862537	0	0	0	0.08025246	0	0	0	0	0	0	0.0309546	hsa_circ_0005408	NC_000012.12	-		66145841	66161172	15332	1375	sense-overlappin	NM_001282606.1	TMBIM4
circRNA_08347	0	0	0	0	0.06018934	0	0.060296056	0	0	0	0	0	hsa_circ_0000414	NC_000012.12	+		66203711	66211597	7887	455	sense-overlappin	NM_007199.2	IRAK3
circRNA_08348	0	0	0	0	0.16050492	0.051782273	0.020098685	0.020486295	0	0	0	0.0206364	hsa_circ_0008381	NC_000012.12	+		66203711	66217235	13525	520	sense-overlappin	NM_007199.2	IRAK3
circRNA_08349	0	0	0.032712968	0.0722267	0.3310414	0.103564547	0.030148028	0.071702034	0.030381792	0.050431207	0	0	hsa_circ_0005505	NC_000012.12	+		66203711	66228370	24660	754	sense-overlappin	NM_007199.2	IRAK3
circRNA_08350	0.020575025	0	0	0.0309543	0.18056803	0.041425819	0	0	0	0	0	0	hsa_circ_0003753	NC_000012.12	+		66209456	66217235	7780	337	sense-overlappin	NM_001142523.1	IRAK3
circRNA_08351	0	0	0	0	0.03009467	0	0	0	0	0	0	0	-	NC_000012.12	+		66209456	66228370	18915	571	sense-overlappin	NM_001142523.1	IRAK3
circRNA_08352	0	0	0	0	0.02006311	0	0	0	0	0	0	0	-	NC_000012.12	+		66304731	66318792	14062	1968	sense-overlappin	NM_033647.3	HELB
circRNA_08353	0	0	0	0	0	0	0.020098685	0	0	0	0	0	hsa_circ_0027459	NC_000012.12	-		68302620	68302872	253	253	exonic	XM_005269026.1	MDM1
circRNA_08354	0	0	0	0	0	0	0	0	0	0	0.02025523	0	-	NC_000012.12	+		68632626	68632703	78	78	intronic	NM_001010942.2	RAP1B
circRNA_08355	0	0	0	0	0.05015779	0	0	0	0	0	0	0	hsa_circ_0027463	NC_000012.12	+		68650400	68652051	1652	126	sense-overlappin	NM_001010942.2	RAP1B
circRNA_08356	0	0	0	0	0.02006311	0	0	0	0	0	0	0	-	NC_000012.12	+		68690631	68700853	10223	493	sense-overlappin	NM_020401.2	NUP107
circRNA_08357	0	0	0	0	0.04012623	0	0	0	0	0	0	0	-	NC_000012.12	+		68710005	68715740	5736	282	sense-overlappin	NM_020401.2	NUP107
circRNA_08358	0	0	0.021808645	0.0928628	0	0	0	0	0	0	0	0	hsa_circ_0003599	NC_000012.12	+		68715627	68725796	10170	607	sense-overlappin	NM_020401.2	NUP107
circRNA_08359	0	0	0	0.0206362	0	0	0	0	0	0	0	0	hsa_circ_0027485	NC_000012.12	+		68726499	68727389	891	158	sense-overlappin	NM_020401.2	NUP107
circRNA_08360	0	0	0	0	0.02006311	0	0	0	0	0	0	0	-	NC_000012.12	+		68733452	68735344	1893	401	sense-overlappin	NM_020401.2	NUP107
circRNA_08361	0.020575025	0	0	0	0.16050492	0.041425819	0.060296056	0	0	0.020172483	0.02025523	0	hsa_circ_0027491	NC_000012.12	+		68816812	68824651	7840	349	sense-overlappin	NM_002392.5	MDM2
circRNA_08362	0	0	0	0	0.13041025	0	0	0	0	0	0.02025523	0	hsa_circ_0004448	NC_000012.12	+		68820325	68824651	4327	215	sense-overlappin	XM_006719400.2	MDM2
circRNA_08363	0	0	0	0	0.0702209	0	0	0	0	0.050431207	0	0	hsa_circ_0002770	NC_000012.12	+		68828771	68836749	7979	395	sense-overlappin	XM_006719400.2	MDM2
circRNA_08364	0	0	0	0	0.05015779	0	0	0	0	0	0	0	-	NC_000012.12	+		68828771	68839801	11031	923	sense-overlappin	XM_006719400.2	MDM2
circRNA_08365	0	0	0	0	0.02006311	0	0	0	0	0	0	0	-	NC_000012.12	+		68835829	68839801	3973	762	sense-overlappin	XM_006719400.2	MDM2
circRNA_08366	0	0	0	0	0.06018934	0	0	0	0	0	0	0	hsa_circ_0027502	NC_000012.12	+		69251129	69260197	9069	1520	sense-overlappin	NM_001300947.1	CPSF6
circRNA_08367	0.082300098	0.143289773	0.785111221	0.113499	1.21381845	0.062138728	0.060296056	0	0.030381792	0.050431207	0	0.051591	hsa_circ_0000417	NC_000012.12	+		69251129	69262562	11434	1710	sense-overlappin	NM_001300947.1	CPSF6
circRNA_08368	0	0	0.021808645	0	0	0	0	0	0	0	0	0	-	NC_000012.12	+		69251129	69269668	18540	1867	sense-overlappin	NM_001300947.1	CPSF6
circRNA_08369	0	0	0	0	0.03009467	0	0	0	0	0	0	0	hsa_circ_0027504	NC_000012.12	+		69258590	69262562	3973	965	sense-overlappin	NM_001300947.1	CPSF6
circRNA_08370	0	0	0	0	0.05015779	0	0	0	0	0	0	0	-	NC_000012.12	+		69258999	69262562	3564	556	sense-overlappin	NM_001300947.1	CPSF6
circRNA_08371	0	0	0	0.0412724	0	0	0	0	0	0	0	0	-	NC_000012.12	+		69365633	69370975	5343	343	sense-overlappin	XR_944742.1	
circRNA_08372	0.020575025	0	0	0	0	0	0	0	0	0	0	0	-	NC_000012.12	+		69459517	69462525	3009	3009	intergenic		
circRNA_08373	0.020575025	0	0.032712968	0	0.09028402	0	0	0	0	0	0	0	-	NC_000012.12	+		69530290	69532056	1767	714	sense-overlappin	NM_001042555.2	FRS2
circRNA_08374	0	0	0	0	0.06018934	0	0.020098685	0	0	0	0	0	-	NC_000012.12	+		69530865	69532056	1192	139	sense-overlappin	NM_001042555.2	FRS2
circRNA_08375	0.020575025	0.061409903	0.054521613	0.0722267	0.68214591	0.031069364	0.060296056	0.051215739	0.040509055	0.07060369	0.08102092	0.0309546	hsa_circ_0000418	NC_000012.12	+		69589485	69592159	2675	304	sense-overlappin	NM_006431.2	CCT2
circRNA_08376	0.061725074	0.020469968	0.021808645	0.0412724	0.38119918	0.041425819	0	0.051215739	0.020254528	0.030258724	0	0	hsa_circ_0002940	NC_000012.12	+		69589485	69593613	4129	536	sense-overlappin	NM_006431.2	CCT2
circRNA_08377	0	0	0	0	0.02006311	0	0	0	0	0	0	0	hsa_circ_0027528	NC_000012.12	+		69592059	69593613	1555	333	sense-overlappin	NM_006431.2	CCT2
circRNA_08378	0	0	0	0	0.05015779	0	0	0	0	0	0	0	-	NC_000012.12	+		69593022	69593586	565	159	sense-overlappin	NM_006431.2	CCT2
circRNA_08379	0	0	0	0	0.02006311	0	0	0	0	0	0	0	-	NC_000012.12	+		69750746	69756663	5918	5173	sense-overlappin	XR_944487.1	
circRNA_08380	0.030862537	0	0.065425935	0.0515905	0.4012623	0	0	0	0	0.020172483	0	0	-	NC_000012.12	+		69755384	69756663	1280	535	sense-overlappin	XR_944487.1	
circRNA_08381	0	0	0	0	0.03009467	0	0	0	0	0	0	0	-	NC_000012.12	+		69755384	69795344	39961	913	sense-overlappin	XR_944487.1	
circRNA_08382	0	0	0	0	0.02006311	0	0	0	0	0	0	0	-	NC_000012.12	+		69756405	69787142	30738	2682	sense-overlappin	XR_944487.1	
circRNA_08383	0	0	0	0	0.02006311	0	0	0	0	0	0	0	-	NC_000012.12	+		69784720	69795344	10625	378	sense-overlappin	XR_944487.1	
circRNA_08384	0	0	0.032712968	0	0.16050492	0	0	0	0	0	0	0	-	NC_000012.12	+		69784720	69801721	17002	787	sense-overlappin	XR_944491.1	
circRNA_08385	0.154312685	0.266109578	0.21808645	0.2682705	4.02265455	0.31069364	0.231134882	0.276564989	0.222799805	0.221897311	0.374721756	0.330182403	hsa_circ_0000419	NC_000012.12	+		69800209	69801721	1513	237	sense-overlappin	XR_944488.1	
circRNA_08386	0	0	0	0	0.04012623	0	0	0	0	0	0	0	-	NC_000012.12	+		69879028	69910930	31903	1465	sense-overlappin	XM_011539059.1	MYRFL
circRNA_08387	0	0	0	0	0.13041025	0	0	0	0	0	0	0	-	NC_000012.12	+		69890971	69893840	2870	273	sense-overlappin	XM_011539059.1	MYRFL
circRNA_08388	0	0	0	0	0.02006311	0	0	0	0	0	0	0	-	NC_000012.12	+		69952113	69955437	3325	226	sense-overlappin	XM_011539047.1	MYRFL
circRNA_08389	0.041150049	0.051174919	0.021808645	0.0206362	0.46145164	0.093208092	0.030148028	0.040972591	0.020254528	0.030258724	0	0	hsa_circ_0007127	NC_000012.12	+		70278132	70311017	32886	490	sense-overlappin	XM_006719429.2	CNOT2

circRNA_08390	0.051437562	0.030704951	0.032712968	0.0309543	0.04012623	0	0.020098685	0	0.020254528	0.030258724	0.02025523	0	hsa_circ_0027545	NC_000012.12	+	70278132	70319364	41233	557	sense-overlappin	XM_006719429.2	CNOT2
circRNA_08391	0	0	0	0	0.08025246	0	0	0	0	0	0	0	hsa_circ_0027547	NC_000012.12	+	70278132	70330469	52338	888	sense-overlappin	XM_006719429.2	CNOT2
circRNA_08392	0	0	0	0	0.04012623	0	0	0	0	0	0	0	hsa_circ_0027558	NC_000012.12	+	70329423	70346324	16902	1298	sense-overlappin	NM_014515.5	CNOT2
circRNA_08393	0	0	0	0	0.05015779	0	0	0	0	0	0	0	-	NC_000012.12	+	70507895	70522630	14736	14736	antisense	NM_001206971.1	PTPRB
circRNA_08394	0	0	0	0	0.02006311	0	0	0	0	0	0	0	-	NC_000012.12	+	70510212	70522630	12419	12419	antisense	NM_001206971.1	PTPRB
circRNA_08395	0	0	0	0	0.0702209	0	0	0	0	0	0	0	-	NC_000012.12	+	70628099	70628886	788	788	antisense	NM_001109754.2	PTPRB
circRNA_08396	0	0	0	0	0.04012623	0	0	0	0	0	0	0	-	NC_000012.12	-	70684127	70701323	17197	490	sense-overlappin	NM_130846.2	PTPRR
circRNA_08397	0	0	0	0	0.02006311	0	0	0	0	0	0	0	-	NC_000012.12	-	70684127	70754301	70175	870	sense-overlappin	NM_130846.2	PTPRR
circRNA_08398	0	0	0	0	0.03009467	0	0	0	0	0	0	0	hsa_circ_0008912	NC_000012.12	-	71626260	71629037	2778	491	sense-overlappin	NM_144982.4	ZFC3H1
circRNA_08399	0	0	0	0.0206362	0	0	0	0	0	0	0	0	-	NC_000012.12	-	71634155	71644318	10164	1231	sense-overlappin	NM_144982.4	ZFC3H1
circRNA_08400	0	0	0.021808645	0	0.04012623	0	0	0	0	0	0	0	-	NC_000012.12	-	71642436	71644318	1883	348	sense-overlappin	NM_144982.4	ZFC3H1
circRNA_08401	0	0	0	0	0	0	0	0	0	0.020172483	0.030382845	0	-	NC_000012.12	-	71642436	71660427	17992	4155	sense-overlappin	NM_144982.4	ZFC3H1
circRNA_08402	0	0	0.021808645	0	0	0	0	0	0	0	0	0	-	NC_000012.12	-	71658630	71660427	1798	1798	intronic	NM_144982.4	ZFC3H1
circRNA_08403	0	0.040939935	0	0	0.06018934	0.031069364	0.020098685	0	0	0	0	0.0206364	-	NC_000012.12	+	71769800	71774022	4223	233	sense-overlappin	XM_011538050.1	RAB21
circRNA_08404	0	0	0	0	0.04012623	0	0	0	0	0	0	0	-	NC_000012.12	+	71918451	71921454	3004	302	sense-overlappin	XM_006719566.2	TBC1D15
circRNA_08405	0	0	0	0.0206362	0	0	0	0	0	0	0	0	-	NC_000012.12	+	72562165	72575542	13378	533	sense-overlappin	XM_005268819.3	TRHDE
circRNA_08406	0	0	0.04361729	0	0	0	0	0	0	0	0	0	-	NC_000012.12	-	74285025	74306669	21645	21645	intergenic		
circRNA_08407	0	0	0	0.0206362	0	0	0	0	0	0	0	0	-	NC_000012.12	-	75692105	75694975	2871	2871	intergenic		
circRNA_08408	0	0	0	0	0	0	0	0	0	0.020172483	0	0	-	NC_000012.12	+	76845709	76849470	3762	431	sense-overlappin	NM_015336.2	ZDHHC17
circRNA_08409	0	0	0	0	0.02006311	0	0	0	0	0	0	0	-	NC_000012.12	+	76848233	76849470	1238	253	sense-overlappin	NM_015336.2	ZDHHC17
circRNA_08410	0	0	0	0	0	0.031069364	0	0	0	0	0	0	-	NC_000012.12	-	79574866	79596658	21793	18081	sense-overlappin	XM_006719435.2	PAWR
circRNA_08411	0	0	0	0	0	0.031069364	0	0	0	0	0	0	-	NC_000012.12	-	79613575	79621207	7633	167	sense-overlappin	XM_006719435.2	PAWR
circRNA_08412	0.020575025	0	0	0.0309543	0	0.041425819	0.030148028	0	0.020254528	0.050431207	0.02025523	0.0412728	-	NC_000012.12	-	79793863	79822190	28328	1974	sense-overlappin	XM_011538381.1	PPP1R12A
circRNA_08413	0.051437562	0	0	0	0	0	0	0	0	0	0	0	hsa_circ_0027668	NC_000012.12	-	79817394	79822190	4797	447	sense-overlappin	NM_001143886.1	PPP1R12A
circRNA_08414	0	0	0	0	0	0	0	0	0	0.050431207	0	0	-	NC_000012.12	-	79923967	79924538	572	572	intronic	NM_001244992.1	PPP1R12A
circRNA_08415	0	0	0	0	0	0	0	0	0	0.020172483	0	0	-	NC_000012.12	+	81199341	81233471	34131	469	sense-overlappin	XR_944720.1	
circRNA_08416	0	0	0	0.0206362	0	0	0	0	0	0	0	0	hsa_circ_0002797	NC_000012.12	-	81676791	81687585	10795	10795	sense-overlappin	NM_001220475.2	PPFIA2
circRNA_08417	0	0.08187987	0.054521613	0.0515905	0.03009467	0.072495183	0	0.030729443	0	0.020172483	0	0.0309546	-	NC_000012.12	+	82369387	82403130	33744	18436	sense-overlappin	XM_005269186.3	METTL25
circRNA_08418	0	0.020469968	0.021808645	0	0	0	0	0	0	0.020172483	0	0	-	NC_000012.12	+	82369387	82434724	65338	18616	sense-overlappin	XR_944768.1	
circRNA_08419	0	0	0	0	0	0	0	0	0	0	0.050638075	0	-	NC_000012.12	+	82369387	82438791	69405	22683	sense-overlappin	XR_944769.1	
circRNA_08420	0	0	0	0	0	0.020712909	0.040197371	0	0.030381792	0	0	0	-	NC_000012.12	+	82369387	82456820	87434	18729	sense-overlappin	XM_011538829.1	METTL25
circRNA_08421	0	0	0.021808645	0	0	0.020712909	0	0	0.040509055	0	0	0	-	NC_000012.12	+	82386803	82403130	16328	1020	sense-overlappin	XM_005269186.3	METTL25
circRNA_08422	0	0	0	0.0206362	0	0.031069364	0	0	0	0	0	0	-	NC_000012.12	+	82386803	82434724	47922	1200	sense-overlappin	XR_944768.1	
circRNA_08423	0	0	0	0	0	0	0	0.020486295	0	0	0	0	-	NC_000012.12	+	82386803	82456820	70018	1313	sense-overlappin	XM_011538829.1	METTL25
circRNA_08424	0.030862537	0.040939935	0.04361729	0.1238171	0.06018934	0.051782273	0.020098685	0	0.020254528	0.090776173	0.06076569	0.082545601	hsa_circ_0002886	NC_000012.12	+	82857010	82857580	571	571	exonic	NM_152588.1	TMTC2
circRNA_08425	0	0	0.021808645	0	0	0	0	0	0	0	0	0	-	NC_000012.12	+	85065262	85066898	1637	304	sense-overlappin	NM_001079910.1	LRRIQ1
circRNA_08426	0	0	0.021808645	0	0	0.051782273	0	0	0	0	0	0	-	NC_000012.12	-	88106675	88107098	424	334	sense-overlappin	NM_025114.3	CEP290
circRNA_08427	0	0	0.076330258	0.0412724	0	0.020712909	0	0	0.040509055	0.030258724	0.02025523	0	hsa_circ_0027689	NC_000012.12	+	88148288	88176319	28032	1460	sense-overlappin	NM_181783.3	TMTC3
circRNA_08428	0	0	0.065425935	0.0722267	0	0.020712909	0	0	0	0.020172483	0	0	hsa_circ_0002375	NC_000012.12	-	88505159	88545865	40707	844	sense-overlappin	NM_000899.4	KITLG
circRNA_08429	0.020575025	0	0	0.0206362	0	0	0	0	0	0.030258724	0	0	-	NC_000012.12	-	88515534	88545865	30332	589	sense-overlappin	NM_000899.4	KITLG
circRNA_08430	0	0	0	0	0	0	0	0	0	0.020172483	0	0	-	NC_000012.12	-	89459638	89467685	8048	326	sense-overlappin	NR_037659.1	
circRNA_08431	0	0	0	0.0309543	0	0	0	0	0	0.040344966	0	0	-	NC_000012.12	-	89459638	89472275	12638	684	sense-overlappin	NR_037659.1	
circRNA_08432	0	0.020469968	0.054521613	0.0515905	0	0	0.030148028	0	0	0.060517448	0	0.0206364	hsa_circ_0027702	NC_000012.12	-	89466770	89472275	5506	580	sense-overlappin	NM_001199777.1	POC1B
circRNA_08433	0	0	0.021808645	0	0	0	0	0	0	0.040344966	0	0	-	NC_000012.12	-	89466770	89497342	30573	932	sense-overlappin	NM_001199777.1	POC1B
circRNA_08434	0	0	0	0	0.02006311	0	0	0	0	0	0	0	hsa_circ_0027707	NC_000012.12	-	89609937	89635251	25315	2036	sense-overlappin	NM_001001323.1	ATP2B1
circRNA_08435	0	0	0	0	0	0	0	0	0	0.030258724	0.02025523	0	-	NC_000012.12	-	89619999	89635251	15253	1423	sense-overlappin	NM_001001323.1	ATP2B1
circRNA_08436	0	0.040939935	0	0	0	0	0	0	0	0	0	0	-	NC_000012.12	-	89627678	89635251	7574	561	sense-overlappin	NM_001001323.1	ATP2B1
circRNA_08437	0	0	0.021808645	0	0	0	0	0	0	0	0	0.0206364	hsa_circ_0007621	NC_000012.12	-	89655679	89656107	429	429	exonic	XM_005268919.1	ATP2B1
circRNA_08438	0	0	0.021808645	0	0	0	0	0	0	0	0	0	-	NC_000012.12	-	89662989	89677816	14828	14828	intronic	XM_005268919.1	ATP2B1
circRNA_08439	0	0	0	0	0	0	0.020098685	0	0	0	0	0	-	NC_000012.12	-	91158296	91164717	6422	327	sense-overlappin	NM_133503.2	DCN
circRNA_08440	0	0.040939935	0	0	0	0	0	0	0	0	0	0	-	NC_000012.12	-	92798892	92799086	195	195	exonic	XM_011538815.1	EEA1
circRNA_08441	0	0	0	0	0.02006311	0	0	0	0	0.020172483	0	0	-	NC_000012.12	-	92798892	92802734	3843	628	sense-overlappin	XM_011538815.1	EEA1
circRNA_08442	0	0	0	0	0	0	0	0	0	0.030258724	0	0	-	NC_000012.12	-	92798892	92809156	10265	768	sense-overlappin	XM_011538815.1	EEA1
circRNA_08443	0	0	0	0	0	0.031069364	0	0	0	0	0	0	-	NC_000012.12	-	92798892	92857485	58594	2722	sense-overlappin	XM_011538815.1	EEA1

circRNA_08444	0	0.020469968	0	0	0	0	0	0	0	0	0	0	-	NC_000012.12	-	92809017	92811434	2418	296	sense-overlappin	XM_011538815.1	EEA1
circRNA_08445	0	0	0	0.0206362	0	0	0	0	0	0	0	0	-	NC_000012.12	-	92826166	92853025	26860	1118	sense-overlappin	XM_011538815.1	EEA1
circRNA_08446	0	0	0	0	0	0	0.030148028	0.020486295	0	0	0	0	-	NC_000012.12	-	92832512	92853025	20514	848	sense-overlappin	XM_011538815.1	EEA1
circRNA_08447	0	0	0.032712968	0	0	0	0	0	0	0.020172483	0	0	-	NC_000012.12	-	92842465	92864987	22523	798	sense-overlappin	XM_011538815.1	EEA1
circRNA_08448	0	0	0	0.0206362	0	0	0	0	0	0	0	0	-	NC_000012.12	-	92851111	92853025	1915	392	sense-overlappin	XM_011538815.1	EEA1
circRNA_08449	0.082300098	0.102349838	0.054521613	0	0.0702209	0.072495183	0.160789483	0.081945182	0.040509055	0.131121138	0	0.103182001	-	NC_000012.12	+	94169153	94186473	17321	377	sense-overlappin	NM_005761.2	PLXNC1
circRNA_08450	0	0	0	0	0	0.020712909	0	0	0	0	0	0	-	NC_000012.12	+	94169153	94227235	58083	918	sense-overlappin	NM_005761.2	PLXNC1
circRNA_08451	0	0	0	0	0	0.020712909	0	0	0	0	0	0	-	NC_000012.12	+	94209590	94220163	10574	263	sense-overlappin	NM_005761.2	PLXNC1
circRNA_08452	0	0	0	0	0	0.031069364	0.020098685	0.030729443	0	0	0	0	-	NC_000012.12	+	94209590	94227235	17646	541	sense-overlappin	NM_005761.2	PLXNC1
circRNA_08453	0.041150049	0	0	0.0206362	0	0	0	0	0	0	0.02025523	0.0309546	-	NC_000012.12	+	94220016	94227235	7220	426	sense-overlappin	NM_005761.2	PLXNC1
circRNA_08454	0	0	0	0	0	0	0	0	0	0	0	0.051591	-	NC_000012.12	-	95028387	95057878	29492	987	sense-overlappin	NM_003297.3	NR2C1
circRNA_08455	0.020575025	0	0	0	0	0.020712909	0	0	0	0	0	0	-	NC_000012.12	-	95152811	95152993	183	99	sense-overlappin	NM_018351.3	FGD6
circRNA_08456	0.277762832	0.051174919	0.152660515	0.0309543	0.02006311	0.062138728	0.140690797	0.061458886	0.081018111	0.060517448	0.030382845	0.0206364	-	NC_000012.12	-	95208843	95211267	2425	2425	exonic	NM_018351.3	FGD6
circRNA_08457	0	0	0.021808645	0	0	0	0	0	0	0	0	0	-	NC_000012.12	+	95251940	95266632	14693	747	sense-overlappin	NR_038241.1	
circRNA_08458	0	0	0.021808645	0	0	0	0	0	0	0	0	0	hsa_circ_0027767	NC_000012.12	+	95251940	95274889	22950	1033	sense-overlappin	NR_038241.1	
circRNA_08459	0	0	0.021808645	0	0	0	0	0	0	0	0	0	-	NC_000012.12	+	95270039	95294372	24334	925	sense-overlappin	NM_017599.3	VEZT
circRNA_08460	0	0	0	0	0	0	0	0	0	0.020172483	0	0	hsa_circ_0008775	NC_000012.12	+	95294272	95296258	1987	309	sense-overlappin	NM_017599.3	VEZT
circRNA_08461	0	0	0	0	0	0.031069364	0.030148028	0	0	0	0	0	hsa_circ_0027775	NC_000012.12	+	95494056	95496098	2043	439	sense-overlappin	NM_006838.3	METAP2
circRNA_08462	0	0	0	0.0206362	0	0	0	0	0	0	0	0	hsa_circ_0027776	NC_000012.12	+	95494056	95504161	10106	536	sense-overlappin	NM_006838.3	METAP2
circRNA_08463	0	0	0	0	0	0	0.020098685	0	0.020254528	0	0	0	-	NC_000012.12	-	96294999	96300360	5362	454	sense-overlappin	NM_001170464.2	CDK17
circRNA_08464	0	0	0	0.0206362	0	0	0	0.020486295	0	0	0	0	hsa_circ_0007881	NC_000012.12	-	96298869	96300360	1492	172	sense-overlappin	NM_001170464.2	CDK17
circRNA_08465	0	0	0.109043225	0.0619086	0.08025246	0.062138728	0.030148028	0.030729443	0.040509055	0.050431207	0.04051046	0	hsa_circ_0002762	NC_000012.12	-	96323948	96334865	10918	312	sense-overlappin	NM_001170464.2	CDK17
circRNA_08466	0	0	0	0	0	0	0	0	0	0	0	0.0206364	-	NC_000012.12	+	96764151	96829088	64938	1131	sense-overlappin	XM_011539072.1	CFAP54
circRNA_08467	0	0	0	0	0.02006311	0	0	0	0	0	0	0	-	NC_000012.12	+	96909752	96920125	10374	497	sense-overlappin	NM_001135176.1	NEDD1
circRNA_08468	0.072012586	0.051174919	0.08723458	0.5365409	0	0.072495183	0	0.081945182	0.081018111	0.040344966	0	0.134136601	-	NC_000012.12	+	97492461	97561047	68587	68587	intergenic		
circRNA_08469	0.030862537	0	0.04361729	0	0.02006311	0	0	0	0	0	0	0	hsa_circ_0003517	NC_000012.12	+	98527886	98537572	9687	384	sense-overlappin	NM_001032283.2	TMPO
circRNA_08470	0	0	0	0	0	0.020712909	0	0	0	0	0	0	-	NC_000012.12	+	98538631	98542585	3955	3955	intronic	NM_001032283.2	TMPO
circRNA_08471	0	0	0	0	0.02006311	0	0	0	0	0	0	0	hsa_circ_0005781	NC_000012.12	+	98595433	98595661	229	229	sense-overlappin	NM_005888.3	SLC25A3
circRNA_08472	0	0	0	0	0	0	0	0	0	0.030258724	0	0	hsa_circ_0004901	NC_000012.12	+	98677425	98686873	9449	511	sense-overlappin	XM_011538254.1	APAF1
circRNA_08473	0	0	0	0	0	0.020712909	0	0	0	0	0	0	hsa_circ_0027836	NC_000012.12	+	98677425	98699569	22145	673	sense-overlappin	XR_429095.2	
circRNA_08474	0.020575025	0	0	0	0	0	0	0	0	0	0	0	hsa_circ_0007294	NC_000012.12	-	99772922	99782097	9176	459	sense-overlappin	XR_944629.1	
circRNA_08475	0	0	0	0	0	0	0	0	0	0.030258724	0	0	-	NC_000012.12	-	99772922	99806700	33779	756	sense-overlappin	XR_944629.1	
circRNA_08476	0	0	0	0	0	0	0	0	0	0.050431207	0	0	-	NC_000012.12	-	99772922	99812311	39390	913	sense-overlappin	XR_944629.1	
circRNA_08477	0	0	0	0	0	0	0	0	0.020254528	0	0	0	-	NC_000012.12	-	99772922	99825389	52468	994	sense-overlappin	XR_944629.1	
circRNA_08478	0	0	0	0	0	0.031069364	0	0	0	0	0	0	-	NC_000012.12	-	99806545	99825389	18845	394	sense-overlappin	XR_944629.1	
circRNA_08479	0	0	0.032712968	0	0	0	0	0	0	0	0	0	-	NC_000012.12	-	100072672	100103951	31280	1342	sense-overlappin	XM_005268738.1	UHRF1BP1L
circRNA_08480	0	0	0	0	0	0	0	0	0	0.050431207	0	0	hsa_circ_0027850	NC_000012.12	-	100095667	100103951	8285	633	sense-overlappin	XM_005268739.2	UHRF1BP1L
circRNA_08481	0	0	0.021808645	0	0	0	0	0	0	0	0	0	hsa_circ_0027851	NC_000012.12	-	100097365	100098541	1177	331	sense-overlappin	XM_005268739.2	UHRF1BP1L
circRNA_08482	0	0	0.021808645	0	0	0	0	0	0.020254528	0	0	0	hsa_circ_0027852	NC_000012.12	-	100097365	100103951	6587	462	sense-overlappin	XM_005268739.2	UHRF1BP1L
circRNA_08483	0	0	0	0	0	0	0	0.020486295	0	0	0	0	hsa_circ_0027857	NC_000012.12	+	100204940	100205744	805	187	sense-overlappin	NM_022496.4	ACTR6
circRNA_08484	0	0	0	0	0	0	0	0	0	0.040344966	0	0	-	NC_000012.12	+	100204940	100205757	818	200	sense-overlappin	NM_022496.4	ACTR6
circRNA_08485	0	0	0	0	0	0	0	0	0	0	0	0.0206364	-	NC_000012.12	+	100204940	100207786	2847	311	sense-overlappin	NM_022496.4	ACTR6
circRNA_08486	0.082300098	0.092114854	0.141756193	0.1960438	0.16050492	0.093208092	0.080394741	0.040972591	0.060763583	0.121034897	0.050638075	0.082545601	hsa_circ_0006258	NC_000012.12	+	100282943	100298175	15233	644	sense-overlappin	XM_011538559.1	SCYL2
circRNA_08487	0	0	0	0	0	0	0.030148028	0	0	0	0	0	-	NC_000012.12	-	100287408	100288582	1175	1175	antisense	XM_011538559.1	SCYL2
circRNA_08488	0	0	0	0	0	0	0	0.020486295	0	0	0	0	-	NC_000012.12	+	100298031	100317925	19895	1060	sense-overlappin	XM_011538559.1	SCYL2
circRNA_08489	0	0	0.021808645	0	0	0	0	0	0	0	0	0	-	NC_000012.12	+	100591736	100622382	30647	983	sense-overlappin	XM_005268804.2	GAS2L3
circRNA_08490	0	0	0	0	0	0	0	0	0	0.030258724	0	0	-	NC_000012.12	+	101356554	101366699	10146	873	sense-overlappin	NM_014503.2	UTP20
circRNA_08491	0	0	0	0	0	0	0	0	0	0.020172483	0	0	-	NC_000012.12	-	101398319	101398559	241	241	intronic	NM_001177.5	ARL1
circRNA_08492	0	0	0	0.0309543	0	0.020712909	0.020098685	0	0	0	0	0	hsa_circ_0006660	NC_000012.12	+	101714090	101716812	2723	568	sense-overlappin	XM_011538575.1	CHPT1
circRNA_08493	0.041150049	0	0	0	0	0.020712909	0	0	0	0	0	0	-	NC_000012.12	+	101723168	101730636	7469	2132	sense-overlappin	NM_020244.2	CHPT1
circRNA_08494	0.020575025	0.030704951	0	0.0309543	0	0	0	0	0	0	0	0	hsa_circ_0027910	NC_000012.12	-	101780152	101790057	9906	568	sense-overlappin	XM_011538731.1	GNPTAB
circRNA_08495	0	0	0	0.0206362	0	0	0	0	0	0	0	0	-	NC_000012.12	-	101780152	101796762	16611	654	sense-overlappin	XM_011538731.1	GNPTAB
circRNA_08496	0	0	0	0	0	0	0.020098685	0.040972591	0	0.020172483	0	0	hsa_circ_0004925	NC_000012.12	-	101786012	101790057	4046	368	sense-overlappin	XM_011538731.1	GNPTAB
circRNA_08497	0	0	0	0	0	0	0.020098685	0	0	0	0	0	hsa_circ_0004459	NC_000012.12	+	101897863	101901433	3571	211	sense-overlappin	NM_018370.2	DRAM1
circRNA_08498	0.020575025	0	0	0	0.02006311	0	0	0	0.020254528	0	0	0	hsa_circ_0000431	NC_000012.12	+	101908186	101914232	6047	237	sense-overlappin	NM_018370.2	DRAM1
circRNA_08499	0	0	0	0.0928628	0	0	0	0	0	0.040344966	0	0	hsa_circ_0002349	NC_000012.12	-	102039868	102046119	6252	282	sense-overlappin	NM_001301107.1	CCDC53
circRNA_08500	0	0	0	0	0	0	0	0	0	0.030258724	0	0	-	NC_000012.12	-	102039868	102050291	10424	4454	sense-overlappin	NM_001301107.1	CCDC53

circRNA_08501	0	0	0.021808645	0	0	0	0	0	0	0	0	0	-	NC_000012.12	+	102257215	102282298	25084	25084	intergenic		
circRNA_08502	0	0	0	0	0	0	0	0.051215739	0	0.060517448	0	0.0206364	-	NC_000012.12	-	103263326	103270308	6983	826	sense-overlappin	XR_944540.1	
circRNA_08503	0	0.030704951	0	0	0	0.020712909	0	0	0	0	0	0	-	NC_000012.12	-	103906138	103916083	9946	9946	intergenic		
circRNA_08504	0	0	0	0	0	0.020712909	0	0	0	0.020172483	0	0	-	NC_000012.12	+	103947311	103947397	87	87	exonic	NM_003299.2	HSP90B1
circRNA_08505	0	0	0	0	0	0.041425819	0	0	0	0.040344966	0	0	-	NC_000012.12	+	103947311	103947430	120	120	exonic	NM_003299.2	HSP90B1
circRNA_08506	0	0.040939935	0.021808645	0.0619086	0	0	0	0	0	0	0	0	hsa_circ_0008166	NC_000012.12	+	103982799	103985728	2930	612	sense-overlappin	NM_003211.4	TDG
circRNA_08507	0	0	0	0	0	0.020712909	0	0	0	0	0	0	-	NC_000012.12	+	104079445	104087014	7570	758	sense-overlappin	XR_944527.1	
circRNA_08508	0	0	0	0.1444533	0.02006311	0.072495183	0	0	0	0	0	0	hsa_circ_0027966	NC_000012.12	-	104844472	104928694	84223	1703	sense-overlappin	XM_011538811.1	SLC41A2
circRNA_08509	0	0	0	0.0412724	0	0	0	0	0	0	0	0	-	NC_000012.12	-	104844472	104931791	87320	1863	sense-overlappin	XM_006719630.2	SLC41A2
circRNA_08510	0	0	0.076330258	0	0	0	0	0	0	0	0	0	-	NC_000012.12	-	104861291	104895345	34055	592	sense-overlappin	XM_011538813.1	SLC41A2
circRNA_08511	0	0	0	0.2373162	0.05015779	0.062138728	0	0.020486295	0	0	0	0.0206364	hsa_circ_0027969	NC_000012.12	-	104861291	104928694	67404	1422	sense-overlappin	XM_011538811.1	SLC41A2
circRNA_08512	0	0	0.021808645	0.0825448	0	0	0.020098685	0	0	0	0	0	-	NC_000012.12	-	104861291	104931791	70501	1582	sense-overlappin	XM_006719630.2	SLC41A2
circRNA_08513	0	0	0.021808645	0.0928628	0	0	0	0	0	0	0	0	hsa_circ_0027971	NC_000012.12	-	104886293	104928694	42402	1194	sense-overlappin	XM_011538811.1	SLC41A2
circRNA_08514	0.020575025	0	0.032712968	0.0206362	0.02006311	0	0	0	0	0.030258724	0	0	-	NC_000012.12	-	104895274	104928694	33421	902	sense-overlappin	XM_011538811.1	SLC41A2
circRNA_08515	0	0	0	0.0206362	0	0	0	0	0	0	0	0	-	NC_000012.12	-	104895274	104931791	36518	1062	sense-overlappin	XM_006719630.2	SLC41A2
circRNA_08516	0	0	0.065425935	0.113499	0.02006311	0	0.040197371	0.061458886	0.050636319	0.040344966	0	0.0309546	hsa_circ_0003012	NC_000012.12	-	104909655	104928694	19040	830	sense-overlappin	XM_011538811.1	SLC41A2
circRNA_08517	0.051437562	0.030704951	0.17446916	0.1754076	0.11034713	0.051782273	0.020098685	0	0.040509055	0.07060369	0.070893305	0.0309546	hsa_circ_0006260	NC_000012.12	-	104927973	104928694	722	722	exonic	XM_011538811.1	SLC41A2
circRNA_08518	0	0	0.04361729	0.0722267	0.02006311	0.031069364	0	0.030729443	0	0	0	0	-	NC_000012.12	-	104927973	104931791	3819	882	sense-overlappin	XM_006719630.2	SLC41A2
circRNA_08519	0	0	0.239895095	0.0206362	0	0	0.020098685	0	0	0	0	0.0309546	hsa_circ_0009063	NC_000012.12	+	104991712	104996026	4315	1686	sense-overlappin	NM_152318.2	C12orf45
circRNA_08520	0	0	0.021808645	0	0	0	0	0	0	0	0	0	-	NC_000012.12	+	105149615	105160148	10534	546	sense-overlappin	NM_001293640.1	KIAA1033
circRNA_08521	0	0	0.021808645	0	0	0	0	0	0	0	0	0	-	NC_000012.12	+	105152343	105157322	4980	263	sense-overlappin	NM_001293640.1	KIAA1033
circRNA_08522	0	0	0.054521613	0.0206362	0	0	0	0.040972591	0	0	0	0.0309546	hsa_circ_0002154	NC_000012.12	+	106842589	106843947	1359	325	sense-overlappin	XM_011538527.1	RIC8B
circRNA_08523	0.93616362	0.255874594	0.17446916	0.2785885	0.32100984	0.383188823	0.331628308	0.276564989	0.475981401	0.534570794	0.212679916	0.247636802	hsa_circ_0000437	NC_000012.12	-	108652272	108654410	2139	274	sense-overlappin	XR_944514.1	
circRNA_08524	0	0.020469968	0	0	0	0	0	0	0	0.040344966	0	0	hsa_circ_0008092	NC_000012.12	-	108652272	108657423	5152	394	sense-overlappin	XR_944514.1	
circRNA_08525	0	0	0.032712968	0	0	0	0	0	0	0	0	0	hsa_circ_0007007	NC_000012.12	-	108658738	108662158	3421	312	sense-overlappin	XR_944514.1	
circRNA_08526	0	0	0.021808645	0	0	0	0	0	0	0	0	0	hsa_circ_0008081	NC_000012.12	-	108678272	108701323	23052	323	sense-overlappin	XR_944514.1	
circRNA_08527	0	0	0	0	0	0	0.030148028	0	0	0	0	0	-	NC_000012.12	-	108759938	108761063	1126	1126	intergenic		
circRNA_08528	0	0	0	0	0.02006311	0	0	0	0	0	0	0	hsa_circ_0028088	NC_000012.12	-	108817038	108823361	6324	291	sense-overlappin	NM_018984.3	SSH1
circRNA_08529	0	0	0	0	0.02006311	0	0.040197371	0	0	0	0	0	hsa_circ_0028090	NC_000012.12	-	108818249	108823361	5113	169	sense-overlappin	NM_018984.3	SSH1
circRNA_08530	0	0	0	0	0	0.020712909	0	0	0	0	0	0	hsa_circ_0028098	NC_000012.12	+	109102839	109103611	773	268	sense-overlappin	NM_080911.2	UNG
circRNA_08531	0	0.020469968	0	0	0	0	0	0	0	0	0	0	-	NC_000012.12	+	109171805	109180087	8283	893	sense-overlappin	XM_011538260.1	ACACB
circRNA_08532	0	0	0	0	0	0.020712909	0	0	0	0	0	0	-	NC_000012.12	+	109191613	109199552	7940	634	sense-overlappin	XM_011538260.1	ACACB
circRNA_08533	0	0.020469968	0	0	0	0.031069364	0	0	0	0	0	0	hsa_circ_0028123	NC_000012.12	+	109222507	109223904	1398	318	sense-overlappin	XM_011538260.1	ACACB
circRNA_08534	0	0	0	0	0	0	0	0	0	0	0.02025523	0	-	NC_000012.12	+	109222507	109227489	4983	437	sense-overlappin	XM_011538260.1	ACACB
circRNA_08535	0	0	0	0	0	0	0	0.020486295	0	0	0	0	-	NC_000012.12	+	109247606	109254334	6729	595	sense-overlappin	XM_011538260.1	ACACB
circRNA_08536	0.041150049	0.040939935	0	0.0309543	0	0	0	0.030729443	0	0	0	0	hsa_circ_0028129	NC_000012.12	+	109249984	109259108	9125	827	sense-overlappin	XM_011538260.1	ACACB
circRNA_08537	0	0	0	0	0	0.031069364	0	0.020486295	0	0	0	0	-	NC_000012.12	+	109252046	109254334	2289	376	sense-overlappin	XM_011538260.1	ACACB
circRNA_08538	0	0	0	0	0	0.041425819	0	0	0	0	0	0	-	NC_000012.12	+	109258268	109259108	841	233	sense-overlappin	XM_011538260.1	ACACB
circRNA_08539	0	0	0	0.0206362	0	0	0	0	0	0	0	0	hsa_circ_0028135	NC_000012.12	+	109488572	109507735	19164	1175	sense-overlappin	XR_429119.2	
circRNA_08540	0	0	0	0	0	0	0	0	0	0	0.02025523	0	-	NC_000012.12	-	109857526	109858741	1216	532	sense-overlappin	XM_011538434.1	GLTP
circRNA_08541	0	0	0	0.0206362	0.03009467	0	0	0	0	0.040344966	0	0	-	NC_000012.12	+	109907526	109908937	1412	354	sense-overlappin	XM_011538837.1	TCHP
circRNA_08542	0.041150049	0	0	0	0	0	0	0	0	0	0	0	-	NC_000012.12	+	110013125	110029635	16511	1008	sense-overlappin	XM_005253984.1	ANKRD13A
circRNA_08543	0	0	0	0	0	0	0	0	0.020254528	0	0	0	-	NC_000012.12	+	110016388	110025823	9436	529	sense-overlappin	XM_005253984.1	ANKRD13A
circRNA_08544	0	0.030704951	0	0	0	0	0	0	0	0	0	0	-	NC_000012.12	+	110018345	110030758	12414	951	sense-overlappin	XM_005253984.1	ANKRD13A
circRNA_08545	0	0	0	0	0	0	0	0	0	0	0.02025523	0	-	NC_000012.12	+	110024046	110033957	9912	778	sense-overlappin	XM_005253984.1	ANKRD13A
circRNA_08546	0	0	0	0	0.03009467	0	0	0	0	0	0	0	hsa_circ_0005591	NC_000012.12	+	110128950	110132636	3687	271	sense-overlappin	NM_031473.3	IFT81
circRNA_08547	0	0.030704951	0	0	0	0.031069364	0	0	0	0	0	0	-	NC_000012.12	+	110128950	110143545	14596	697	sense-overlappin	NM_031473.3	IFT81
circRNA_08548	0	0	0	0	0	0	0	0	0.030381792	0.030258724	0	0	-	NC_000012.12	+	110128950	110147048	18099	793	sense-overlappin	NM_031473.3	IFT81
circRNA_08549	0	0	0	0	0.02006311	0	0	0	0	0	0	0	-	NC_000012.12	+	110128950	110163065	34116	940	sense-overlappin	NM_031473.3	IFT81
circRNA_08550	0	0	0	0	0	0	0	0.051215739	0	0	0	0	hsa_circ_0005279	NC_000012.12	+	110326390	110328017	1628	551	sense-overlappin	NM_001681.3	ATP2A2
circRNA_08551	0	0	0.021808645	0	0	0	0	0	0	0	0	0	hsa_circ_0002628	NC_000012.12	+	110340659	110342448	1790	557	sense-overlappin	NM_001681.3	ATP2A2
circRNA_08552	0	0	0	0	0	0	0	0	0	0.030258724	0	0	hsa_circ_0005785	NC_000012.12	-	110381752	110396452	14701	1031	sense-overlappin	NM_016238.2	ANAPC7
circRNA_08553	0	0	0	0	0	0	0	0	0	0	0	0.0206364	-	NC_000012.12	-	110442570	110444057	1488	1488	intronic	NM_001278556.1	ARPC3
circRNA_08554	0	0	0	0.0206362	0	0	0	0	0	0.020172483	0	0	hsa_circ_0028202	NC_000012.12	-	110455586	110457634	2049	338	sense-overlappin	NM_001164373.1	GPN3
circRNA_08555	0	0	0.021808645	0	0	0	0	0	0.020254528	0	0	0	hsa_circ_0004516	NC_000012.12	+	110485078	110487943	2866	519	sense-overlappin	NM_013300.2	FAM216A
circRNA_08556	0	0	0	0	0	0	0.020098685	0	0	0	0	0	hsa_circ_0006741	NC_000012.12	+	111418119	111418877	759	759	exonic	XM_011537720.1	SH2B3

circRNA_08557	0	0	0.021808645	0	0.03009467	0	0	0	0	0.050431207	0.02025523	0	-	NC_000012.12	-	111479070	111486824	7755	6479	sense-overlappin	NM_002973.3	ATXN2
circRNA_08558	0.020575025	0	0	0	0	0	0	0.020486295	0	0.030258724	0	0	hsa_circ_0005981	NC_000012.12	-	111482787	111486824	4038	2762	sense-overlappin	NM_002973.3	ATXN2
circRNA_08559	0	0	0	0	0.04012623	0	0	0.030729443	0.020254528	0	0	0	hsa_circ_0005756	NC_000012.12	-	111485265	111486824	1560	284	sense-overlappin	NM_002973.3	ATXN2
circRNA_08560	0	0	0	0	0	0	0	0	0	0	0.02025523	0	-	NC_000012.12	-	111485265	111510582	25318	966	sense-overlappin	NM_002973.3	ATXN2
circRNA_08561	0	0	0	0	0	0.020712909	0	0	0	0	0	0	hsa_circ_0002273	NC_000012.12	-	111485271	111486824	1554	278	sense-overlappin	NM_002973.3	ATXN2
circRNA_08562	0	0	0	0	0	0	0	0.020486295	0	0	0	0	hsa_circ_0028255	NC_000012.12	-	111485271	111510582	25312	960	sense-overlappin	NM_002973.3	ATXN2
circRNA_08563	0.020575025	0.040939935	0	0	0.05015779	0.051782273	0.020098685	0.020486295	0.040509055	0.030258724	0	0	hsa_circ_0002601	NC_000012.12	-	111509549	111510582	1034	377	sense-overlappin	NM_002973.3	ATXN2
circRNA_08564	0.030862537	0	0	0.0206362	0	0	0	0	0	0	0	0	hsa_circ_0028263	NC_000012.12	-	111509549	111555919	46371	1684	sense-overlappin	NM_002973.3	ATXN2
circRNA_08565	0.041150049	0	0.032712968	0	0.02006311	0	0	0.020486295	0	0	0	0	hsa_circ_0000438	NC_000012.12	-	111513357	111520076	6720	770	sense-overlappin	NM_002973.3	ATXN2
circRNA_08566	0	0	0	0	0	0	0	0	0	0.030258724	0	0	hsa_circ_0028270	NC_000012.12	-	111552280	111552977	698	223	sense-overlappin	NM_002973.3	ATXN2
circRNA_08567	0.185175222	0.133054789	0.196277805	0.3920876	0.17053648	0.041425819	0.100493427	0.143404068	0.172163486	0.252156035	0.050638075	0.0619092	hsa_circ_0002457	NC_000012.12	-	111552280	111555919	3640	320	sense-overlappin	NM_002973.3	ATXN2
circRNA_08568	0.051437562	0.020469968	0.032712968	0	0.09028402	0	0.040197371	0.030729443	0	0	0	0.0206364	hsa_circ_0000439	NC_000012.12	-	111554158	111555919	1762	97	sense-overlappin	NM_002973.3	ATXN2
circRNA_08569	0	0	0	0.0206362	0	0	0	0	0	0	0	0	-	NC_000012.12	-	111649939	111660675	10737	519	sense-overlappin	XM_011538788.1	BRAP
circRNA_08570	0	0	0	0	0	0	0.020098685	0	0	0.020172483	0	0	hsa_circ_0007885	NC_000012.12	-	111658736	111659345	610	249	sense-overlappin	XM_011538788.1	BRAP
circRNA_08571	0	0	0	0	0	0.062138728	0	0	0	0	0	0	-	NC_000012.12	-	111660600	111683307	22708	890	sense-overlappin	XM_005253944.3	BRAP
circRNA_08572	0	0	0.021808645	0	0.05015779	0.020712909	0.030148028	0	0	0.030258724	0	0	hsa_circ_0006396	NC_000012.12	-	111679151	111683307	4157	551	sense-overlappin	XM_011538789.1	BRAP
circRNA_08573	0	0	0	0	0	0	0.020098685	0	0	0	0	0	hsa_circ_0028308	NC_000012.12	+	111792061	111798242	6182	453	sense-overlappin	NM_000690.3	ALDH2
circRNA_08574	0	0	0.032712968	0	0	0	0	0	0	0	0	0	hsa_circ_0004466	NC_000012.12	+	111865250	111871180	5931	543	sense-overlappin	NM_003668.3	MAPKAPK5
circRNA_08575	0	0	0	0	0.02006311	0	0	0	0	0	0	0	hsa_circ_0005985	NC_000012.12	+	111867572	111871180	3609	393	sense-overlappin	NM_003668.3	MAPKAPK5
circRNA_08576	0	0	0	0	0	0	0	0	0	0.020172483	0	0	hsa_circ_0028319	NC_000012.12	-	111932586	111943369	10784	597	sense-overlappin	XM_011538948.1	TMEM116
circRNA_08577	0	0	0	0	0	0.020712909	0	0	0	0	0	0	hsa_circ_0028322	NC_000012.12	-	111933886	111938210	4325	418	sense-overlappin	XM_011538948.1	TMEM116
circRNA_08578	0	0	0	0.0206362	0.02006311	0	0.030148028	0	0	0.030258724	0	0	hsa_circ_0004206	NC_000012.12	-	111933886	111943369	9484	523	sense-overlappin	XM_011538948.1	TMEM116
circRNA_08579	0	0	0	0	0	0	0	0	0	0	0	0.0206364	-	NC_000012.12	-	112033233	112093136	59904	2738	sense-overlappin	NM_024953.3	NAA25
circRNA_08580	0	0	0	0.0309543	0	0	0	0	0	0	0	0	hsa_circ_0028334	NC_000012.12	-	112071895	112078741	6847	559	sense-overlappin	NM_024953.3	NAA25
circRNA_08581	0	0	0.021808645	0	0	0.020712909	0	0	0	0.030258724	0	0	-	NC_000012.12	-	112075678	112078741	3064	299	sense-overlappin	NM_024953.3	NAA25
circRNA_08582	0	0.020469968	0	0.0206362	0	0	0	0	0	0	0	0	-	NC_000012.12	-	112075678	112093136	17459	718	sense-overlappin	NM_024953.3	NAA25
circRNA_08583	0	0.020469968	0	0	0	0	0	0	0	0	0	0	hsa_circ_0028350	NC_000012.12	+	112140819	112142295	1477	613	sense-overlappin	NM_001143906.1	TRAFD1
circRNA_08584	0	0	0	0	0	0	0	0	0	0.020172483	0	0	-	NC_000012.12	-	112178931	112179397	467	376	sense-overlappin	NM_001109662.3	HECTD4
circRNA_08585	0	0	0	0	0	0	0.020098685	0	0	0	0	0	-	NC_000012.12	-	112200638	112204623	3986	436	sense-overlappin	NM_001109662.3	HECTD4
circRNA_08586	0	0	0	0	0	0	0	0	0	0	0	0.0206364	hsa_circ_0028410	NC_000012.12	-	112269704	112273795	4092	520	sense-overlappin	NM_001109662.3	HECTD4
circRNA_08587	0	0	0	0.0206362	0	0	0	0	0	0	0	0	-	NC_000012.12	-	112306064	112313147	7084	550	sense-overlappin	NM_001109662.3	HECTD4
circRNA_08588	0.020575025	0	0	0.0309543	0	0	0.050246713	0	0	0.020172483	0	0	hsa_circ_0002584	NC_000012.12	-	112319225	112319742	518	518	exonic	NM_001109662.3	HECTD4
circRNA_08589	0	0	0	0	0.04012623	0	0	0	0	0	0	0	-	NC_000012.12	+	112446276	112456063	9788	742	sense-overlappin	NM_080601.1	PTPN11
circRNA_08590	0	0	0	0	0.03009467	0	0	0	0	0	0	0	hsa_circ_0008666	NC_000012.12	+	112477651	112478015	365	239	sense-overlappin	NM_080601.1	PTPN11
circRNA_08591	0	0	0	0	0.03009467	0	0	0.020486295	0	0	0	0	-	NC_000012.12	+	112916509	112917700	1192	384	sense-overlappin	NM_002534.2	OAS1
circRNA_08592	0	0.020469968	0	0	0	0	0	0	0	0	0	0	-	NC_000012.12	-	113103946	113104298	353	274	sense-overlappin	NM_001301202.1	RASAL1
circRNA_08593	0.030862537	0	0	0	0	0	0	0	0	0	0	0.0309546	hsa_circ_0002857	NC_000012.12	+	113267843	113269845	2003	334	sense-overlappin	XR_243017.2	
circRNA_08594	0	0.020469968	0	0	0	0	0	0	0	0	0	0	-	NC_000012.12	-	113399556	113399710	155	155	exonic	NM_006843.2	SDS
circRNA_08595	0	0.040939935	0.04361729	0.1031809	0	0.020712909	0.050246713	0.020486295	0.050636319	0.040344966	0	0	hsa_circ_0000441	NC_000012.12	-	116096669	116111512	14844	169	sense-overlappin	XM_011538082.1	MED13L
circRNA_08596	0	0.020469968	0	0	0	0	0	0	0	0	0	0	-	NC_000012.12	-	116096669	116118129	21461	6786	sense-overlappin	XM_011538082.1	MED13L
circRNA_08597	0	0	0	0	0	0.031069364	0	0	0	0	0	0	-	NC_000012.12	-	116096669	116119779	23111	8436	sense-overlappin	XM_011538082.1	MED13L
circRNA_08598	0.102875123	0.061409903	0.021808645	0.0309543	0.09028402	0.072495183	0.070345399	0.040972591	0.040509055	0.090776173	0.050638075	0.0206364	hsa_circ_0000442	NC_000012.12	-	116230433	116237705	7273	7273	sense-overlappin	XM_011538082.1	MED13L
circRNA_08599	0.216037758	0.194464692	0.21808645	0.4643142	0.29091517	0.352119459	0.190937511	0.276564989	0.13165443	0.353018449	0.08102092	0.103182001	hsa_circ_0000443	NC_000012.12	-	116230533	116237705	7173	7173	sense-overlappin	XM_011538082.1	MED13L
circRNA_08600	0	0.030704951	0.076330258	0.1960438	0	0	0.090444084	0.040972591	0.030381792	0.030258724	0.06076569	0.0412728	hsa_circ_0008151	NC_000012.12	-	116236821	116237705	885	885	sense-overlappin	XM_011538082.1	MED13L
circRNA_08601	0	0	0.021808645	0	0	0	0	0	0	0	0	0	-	NC_000012.12	-	116719763	116723223	3461	423	sense-overlappin	NM_024738.2	C12orf49
circRNA_08602	0	0	0	0	0	0	0	0	0	0	0	0.0206364	hsa_circ_0004632	NC_000012.12	+	116918531	116964854	46324	713	sense-overlappin	XM_011538172.1	FBXW8
circRNA_08603	0	0.020469968	0	0.0515905	0.02006311	0.020712909	0	0	0	0.040344966	0	0	hsa_circ_0028605	NC_000012.12	+	116928023	116949706	21684	359	sense-overlappin	XR_944518.1	
circRNA_08604	0	0.020469968	0.032712968	0.0206362	0	0	0	0	0	0	0	0	hsa_circ_0003677	NC_000012.12	+	116928023	116964854	36832	517	sense-overlappin	XR_944518.1	
circRNA_08605	0	0	0	0.0309543	0	0	0	0	0	0	0	0	hsa_circ_0028608	NC_000012.12	+	116945364	116964854	19491	412	sense-overlappin	XR_944518.1	
circRNA_08606	0.030862537	0	0	0	0.02006311	0	0	0	0	0	0	0	-	NC_000012.12	-	117038983	117046838	7856	446	sense-overlappin	XM_011538503.1	TESC
circRNA_08607	0	0	0.032712968	0	0	0	0	0	0	0	0	0	hsa_circ_0028630	NC_000012.12	+	118019072	118028030	8959	806	sense-overlappin	NM_001130112.2	RFC5
circRNA_08608	0	0	0	0	0.02006311	0	0.020098685	0	0	0	0.02025523	0	hsa_circ_0000446	NC_000012.12	-	118181371	118181607	237	237	exonic	XM_011538440.1	TAOK3
circRNA_08609	0	0	0	0	0	0	0	0	0.020254528	0	0	0	-	NC_000012.12	-	118243415	118244965	1551	174	sense-overlappin	XM_011538438.1	TAOK3
circRNA_08610	0	0	0	0	0	0	0.020098685	0	0	0	0	0	hsa_circ_0028671	NC_000012.12	-	118244894	118255655	10762	280	sense-overlappin	XM_011538438.1	TAOK3
circRNA_08611	0	0	0	0	0	0	0	0	0.020254528	0	0	0	-	NC_000012.12	-	118255448	118266759	11312	313	sense-overlappin	XM_005253898.1	TAOK3

circRNA_08612	0	0	0	0.0206362	0	0	0	0	0	0	0	0	hsa_circ_0028673	NC_000012.12	+	119673964	119676670	2707	343	sense-overlappin	XM_005253909.1	PRKAB1
circRNA_08613	0	0	0	0	0	0	0	0	0	0	0	0.0206364	-	NC_000012.12	-	119757371	119761055	3685	402	sense-overlappin	NM_001206999.1	CIT
circRNA_08614	0.236612783	0.051174919	0.163564838	0.1341352	0.19059959	0.082851637	0.050246713	0.071702034	0.081018111	0.100862414	0.02025523	0.0309546	hsa_circ_0000448	NC_000012.12	-	120154970	120155719	750	389	sense-overlappin	NM_006836.1	GCN1L1
circRNA_08615	0	0	0	0	0.02006311	0	0	0	0	0	0	0	-	NC_000012.12	-	120512133	120525209	13077	7652	sense-overlappin	XM_006719639.1	COQ5
circRNA_08616	0	0.040939935	0	0	0	0	0	0	0	0	0	0	-	NC_000012.12	+	120539368	120546601	7234	298	sense-overlappin	XM_005254013.1	RNF10
circRNA_08617	0.020575025	0	0.076330258	0.0412724	0.02006311	0.031069364	0.040197371	0	0.030381792	0.040344966	0.030382845	0	hsa_circ_0028899	NC_000012.12	+	120557282	120557682	401	322	sense-overlappin	XM_005254014.2	RNF10
circRNA_08618	0	0	0	0	0	0	0	0.020486295	0	0	0	0	hsa_circ_0005086	NC_000012.12	+	120565072	120566980	1909	376	sense-overlappin	XM_006719717.1	RNF10
circRNA_08619	0	0	0.032712968	0	0	0	0	0	0	0	0	0	hsa_circ_0005043	NC_000012.12	+	120565072	120571291	6220	477	sense-overlappin	XM_006719717.1	RNF10
circRNA_08620	0	0	0	0	0	0	0	0	0.030381792	0	0	0	-	NC_000012.12	+	120716628	120717042	415	285	sense-overlappin	NM_001080533.2	UNC119B
circRNA_08621	0.092587611	0.122819805	0.08723458	0.0619086	0.09028402	0.144990365	0.050246713	0.133160921	0.151908958	0.231983552	0.151914226	0.185727601	hsa_circ_0003472	NC_000012.12	-	120782655	120784593	1939	312	sense-overlappin	NM_139015.4	SPPL3
circRNA_08622	0	0.020469968	0.065425935	0.0309543	0.03009467	0	0.030148028	0	0	0	0	0.0412728	hsa_circ_0004479	NC_000012.12	-	120782655	120791557	8903	401	sense-overlappin	NM_139015.4	SPPL3
circRNA_08623	0	0	0	0	0	0	0	0	0	0.020172483	0	0	-	NC_000012.12	-	121023990	121027817	3828	390	sense-overlappin	NM_003733.3	OASL
circRNA_08624	0	0	0.021808645	0	0	0	0	0	0	0	0	0	-	NC_000012.12	-	121293259	121294014	756	756	intronic	XM_005253822.3	CAMKK2
circRNA_08625	0	0	0	0	0	0	0	0	0.020254528	0	0	0	hsa_circ_0028978	NC_000012.12	-	121335533	121347005	11473	663	sense-overlappin	NM_016237.4	ANAPC5
circRNA_08626	0	0.030704951	0	0.0515905	0	0	0.020098685	0	0.020254528	0	0	0.0412728	hsa_circ_0004513	NC_000012.12	+	121416159	121417911	1753	627	sense-overlappin	NM_025126.3	RNF34
circRNA_08627	0	0.020469968	0	0	0	0	0	0	0	0	0	0	hsa_circ_0006030	NC_000012.12	+	121743629	121752223	8595	392	sense-overlappin	XM_006719244.2	TMEM120B
circRNA_08628	0	0	0.021808645	0	0	0	0.020098685	0	0.030381792	0.020172483	0	0	hsa_circ_0000450	NC_000012.12	-	121780872	121781192	321	245	antisense	NM_019034.2	RHOF
circRNA_08629	0	0	0	0.0309543	0	0	0	0	0	0	0	0	hsa_circ_0003414	NC_000012.12	+	121942534	121949061	6528	271	sense-overlappin	NM_001178003.1	WDR66
circRNA_08630	0	0.020469968	0	0	0	0	0	0	0	0	0	0	hsa_circ_0029051	NC_000012.12	-	122216488	122218397	1910	340	sense-overlappin	NM_001278304.1	DIABLO
circRNA_08631	0	0	0	0	0	0	0	0.020486295	0	0	0	0	-	NC_000012.12	-	122288489	122316855	28367	281	sense-overlappin	XM_005253593.2	CLIP1
circRNA_08632	0	0	0	0	0	0.020712909	0	0	0	0	0	0	-	NC_000012.12	-	122327947	122333143	5197	539	sense-overlappin	XM_005253593.2	CLIP1
circRNA_08633	0	0	0	0	0	0	0.020098685	0	0	0	0	0	-	NC_000012.12	-	122332987	122334705	1719	299	sense-overlappin	XM_005253593.2	CLIP1
circRNA_08634	0.072012586	0.030704951	0.032712968	0.113499	0.03009467	0.062138728	0.080394741	0.030729443	0.050636319	0.060517448	0	0.0309546	hsa_circ_0029069	NC_000012.12	-	122340753	122341697	945	945	exonic	XM_005253593.2	CLIP1
circRNA_08635	0.020575025	0	0.04361729	0	0	0.041425819	0.030148028	0	0	0.020172483	0	0	hsa_circ_0005465	NC_000012.12	-	122377389	122380558	3170	763	sense-overlappin	XM_005253593.2	CLIP1
circRNA_08636	0.030862537	0	0	0	0	0.020712909	0	0	0	0.040344966	0	0	-	NC_000012.12	-	122511109	122515227	4119	328	sense-overlappin	XR_242959.1	
circRNA_08637	0	0	0	0	0	0.051782273	0	0	0	0	0	0	hsa_circ_0029111	NC_000012.12	+	122569681	122574380	4700	666	sense-overlappin	XM_011539030.1	KNTC1
circRNA_08638	0	0	0	0	0	0	0	0.040972591	0	0.030258724	0	0	hsa_circ_0004265	NC_000012.12	+	122577672	122580670	2999	261	sense-overlappin	XM_011539030.1	KNTC1
circRNA_08639	0.020575025	0	0	0	0	0	0	0	0.020254528	0	0	0	hsa_circ_0029168	NC_000012.12	-	123004394	123005548	1155	405	sense-overlappin	XM_011538590.1	PITPNM2
circRNA_08640	0.041150049	0	0.021808645	0	0	0	0	0	0	0	0	0	hsa_circ_0029170	NC_000012.12	-	123012613	123014042	1430	337	sense-overlappin	XM_011538590.1	PITPNM2
circRNA_08641	0	0	0	0	0	0	0	0	0	0.020172483	0	0	hsa_circ_0029184	NC_000012.12	-	123166655	123181210	14556	350	sense-overlappin	XM_006719192.2	MPHOSPH9
circRNA_08642	0	0	0.032712968	0	0.04012623	0.020712909	0	0	0	0	0	0	hsa_circ_0005310	NC_000012.12	-	123313620	123315660	2041	285	sense-overlappin	NM_001167856.1	SBNO1
circRNA_08643	0	0	0	0	0	0	0	0	0	0.020172483	0	0	-	NC_000012.12	-	123313620	123323831	10212	1247	sense-overlappin	NM_001167856.1	SBNO1
circRNA_08644	0	0	0.032712968	0	0	0	0	0	0	0	0	0	hsa_circ_0029221	NC_000012.12	-	123334053	123341088	7036	359	sense-overlappin	NM_001167856.1	SBNO1
circRNA_08645	0.092587611	0	0	0.0309543	0.04012623	0	0	0.030729443	0.070890847	0.050431207	0.04051046	0	hsa_circ_0007552	NC_000012.12	-	123498544	123499536	993	341	sense-overlappin	NM_178314.3	RILPL1
circRNA_08646	0.061725074	0.040939935	0.109043225	0.1341352	0.17053648	0.093208092	0.030148028	0.040972591	0.070890847	0.14120738	0.030382845	0.0412728	hsa_circ_0000458	NC_000012.12	+	123586747	123590449	3703	322	sense-overlappin	XM_005253544.2	TMED2
circRNA_08647	0	0	0	0	0	0	0	0.020486295	0	0	0	0	-	NC_000012.12	+	123639264	123652735	13472	473	sense-overlappin	NM_001271866.1	GTF2H3
circRNA_08648	0	0	0	0	0.06018934	0	0	0	0	0	0	0	hsa_circ_0003725	NC_000012.12	+	123645455	123652735	7281	393	sense-overlappin	NM_001271866.1	GTF2H3
circRNA_08649	0	0	0	0	0.02006311	0	0	0	0	0	0	0	-	NC_000012.12	+	123688051	123707073	19023	1220	sense-overlappin	XM_006719605.2	TCTN2
circRNA_08650	0	0.020469968	0.021808645	0	0	0	0	0	0	0	0	0	hsa_circ_0005082	NC_000012.12	-	124362126	124363799	1674	293	sense-overlappin	NM_001077261.3	NCOR2
circRNA_08651	0.041150049	0	0	0	0	0	0	0	0	0	0	0.0206364	-	NC_000012.12	-	124398119	124430787	32669	991	sense-overlappin	NM_001077261.3	NCOR2
circRNA_08652	0.061725074	0	0.032712968	0	0.03009467	0.062138728	0.020098685	0	0.040509055	0.080689931	0	0	hsa_circ_0029308	NC_000012.12	-	124419957	124430787	10831	597	sense-overlappin	NM_001077261.3	NCOR2
circRNA_08653	0	0	0	0.0206362	0	0.041425819	0	0.020486295	0.040509055	0.020172483	0.02025523	0.0309546	hsa_circ_0029309	NC_000012.12	-	124419957	124437996	18040	664	sense-overlappin	NM_001077261.3	NCOR2
circRNA_08654	0	0.020469968	0	0	0	0	0	0	0	0	0	0	-	NC_000012.12	-	124419957	124449867	29911	717	sense-overlappin	NM_001077261.3	NCOR2
circRNA_08655	0	0	0	0	0.03009467	0	0	0	0	0	0	0	hsa_circ_0007479	NC_000012.12	-	124437930	124457162	19233	177	sense-overlappin	NM_001077261.3	NCOR2
circRNA_08656	0	0	0.021808645	0	0	0	0	0	0	0	0	0	hsa_circ_0029340	NC_000012.12	-	124807761	124810289	2529	283	sense-overlappin	NM_001082959.1	SCARB1
circRNA_08657	0	0.020469968	0	0	0	0	0	0	0	0	0	0	-	NC_000012.12	-	126915354	126926291	10938	10938	intergenic		
circRNA_08658	0	0	0	0	0	0.031069364	0	0	0	0	0	0	-	NC_000012.12	-	127688597	127688766	170	170	intergenic		
circRNA_08659	0	0	0	0	0.02006311	0	0	0	0	0	0	0	hsa_circ_0029392	NC_000012.12	-	128799259	128815070	15812	1027	sense-overlappin	XM_011537895.1	SLC15A4
circRNA_08660	0	0.051174919	0.021808645	0.0309543	0	0	0	0	0	0.020172483	0.02025523	0.0412728	hsa_circ_0006689	NC_000012.12	-	128808788	128815070	6283	712	sense-overlappin	XM_011537895.1	SLC15A4
circRNA_08661	0.041150049	0	0.021808645	0	0	0	0.060296056	0.030729443	0.040509055	0.030258724	0.070893305	0.0206364	hsa_circ_0000462	NC_000012.12	-	128814775	128815070	296	296	exonic	XM_011537895.1	SLC15A4
circRNA_08662	0	0	0	0	0	0	0	0	0.030381792	0	0	0	hsa_circ_0005614	NC_000012.12	-	130806982	130813031	6050	258	sense-overlappin	XM_011538026.1	STX2
circRNA_08663	0	0.020469968	0	0	0	0	0	0	0	0.020172483	0	0	hsa_circ_0029434	NC_000012.12	+	131000383	131014340	13958	507	sense-overlappin	XM_011538210.1	ADGRD1
circRNA_08664	0	0	0	0	0.02006311	0	0	0	0	0	0	0	hsa_circ_0006903	NC_000012.12	-	132734385	132735512	1128	298	sense-overlappin	NM_015114.2	ANKLE2
circRNA_08665	0	0	0.04361729	0.0206362	0	0.020712909	0	0	0	0	0	0	hsa_circ_0008567	NC_000012.12	-	132747832	132755133	7302	1049	sense-overlappin	NM_015114.2	ANKLE2
circRNA_08666	0.020575025	0	0.032712968	0	0	0	0	0	0	0	0	0	hsa_circ_0029596	NC_000012.12	+	133191489	133192003	515	223	sense-overlappin	NM_001165887.1	ZNF268
circRNA_08667	0	0	0	0	0	0.041425819	0.070345399	0	0	0.040344966	0	0	-	NC_000013.11	-	19475573	19570288	94716	9267	sense-overlappin	NR_073485.1	
circRNA_08668	0	0	0	0	0.02006311	0	0	0	0	0	0	0	-	NC_000013.11	+	19642115	19650260	8146	1363	sense-overlappin	XM_005266436.3	MPHOSPH8

circRNA_08669	0	0	0	0	0	0.041425819	0	0	0	0	0	0	-	NC_000013.11	+	19658995	19661838	2844	356	sense-overlappin	XR_941609.1	
circRNA_08670	0.020575025	0	0	0	0.02006311	0	0	0	0	0	0	0	hsa_circ_0029614	NC_000013.11	-	19730239	19782791	52553	1192	sense-overlappin	XM_011535139.1	PSPC1
circRNA_08671	0	0	0	0	0	0.020712909	0	0	0	0	0	0	-	NC_000013.11	-	19741565	19751467	9903	282	sense-overlappin	XM_011535139.1	PSPC1
circRNA_08672	0	0	0	0	0	0	0.020098685	0	0	0	0	0	-	NC_000013.11	-	19741565	19759418	17854	378	sense-overlappin	XM_011535139.1	PSPC1
circRNA_08673	0.030862537	0	0	0	0	0	0	0	0	0	0	0	-	NC_000013.11	-	19824920	19825235	316	316	exonic	XM_006719894.2	ZMYM5
circRNA_08674	0	0.030704951	0.021808645	0.0206362	0	0	0.020098685	0	0	0	0	0	hsa_circ_0002673	NC_000013.11	-	19851355	19852190	836	596	sense-overlappin	XM_006719894.2	ZMYM5
circRNA_08675	0	0	0	0	0	0.020712909	0	0	0	0	0	0	-	NC_000013.11	+	19865471	19953138	87668	87668	intergenic		
circRNA_08676	0	0	0	0	0.02006311	0	0	0	0	0	0	0	hsa_circ_0029622	NC_000013.11	+	19970025	19993919	23895	904	sense-overlappin	XM_011535219.1	ZMYM2
circRNA_08677	0	0	0	0	0.03009467	0	0	0	0	0	0	0	hsa_circ_0029624	NC_000013.11	+	19993063	19993919	857	596	exonic	XM_011535216.1	ZMYM2
circRNA_08678	0	0.020469968	0.04361729	0	0.02006311	0	0.030148028	0.030729443	0	0	0	0	hsa_circ_0005966	NC_000013.11	+	20002850	20006586	3737	665	sense-overlappin	XM_011535219.1	ZMYM2
circRNA_08679	0.030862537	0	0.04361729	0.0309543	0.11034713	0.020712909	0	0	0	0.030258724	0	0	hsa_circ_0029633	NC_000013.11	+	20051433	20067390	15958	1161	sense-overlappin	XM_011535219.1	ZMYM2
circRNA_08680	0	0	0	0	0	0	0.020098685	0	0	0	0	0	-	NC_000013.11	+	20058575	20064545	5971	639	sense-overlappin	XM_011535219.1	ZMYM2
circRNA_08681	0.041150049	0.020469968	0.065425935	0	0.05015779	0	0	0	0	0.030258724	0	0	hsa_circ_0029636	NC_000013.11	+	20058575	20067390	8816	960	sense-overlappin	XM_011535219.1	ZMYM2
circRNA_08682	0.030862537	0	0	0	0.03009467	0	0	0	0	0	0	0	hsa_circ_0000465	NC_000013.11	+	20064451	20067390	2940	416	sense-overlappin	XM_011535219.1	ZMYM2
circRNA_08683	0	0	0	0	0.04012623	0	0	0	0	0	0	0	hsa_circ_0029638	NC_000013.11	+	20064451	20083032	18582	919	sense-overlappin	XR_941652.1	
circRNA_08684	0	0	0	0.0206362	0.03009467	0.072495183	0.030148028	0.020486295	0	0	0.030382845	0	-	NC_000013.11	+	20064451	20083776	19326	904	sense-overlappin	XM_011535219.1	ZMYM2
circRNA_08685	0	0	0	0	0	0	0	0	0	0	0	0.0206364	-	NC_000013.11	+	20066851	20083776	16926	809	sense-overlappin	XM_011535219.1	ZMYM2
circRNA_08686	0	0	0	0	0	0	0	0.020486295	0	0	0	0	-	NC_000013.11	+	20569953	20571088	1136	1136	intronic	XM_011535246.1	IFT88
circRNA_08687	0	0	0	0	0	0	0.020098685	0	0	0	0	0	-	NC_000013.11	+	20573021	20583019	9999	290	sense-overlappin	XM_011535242.1	IFT88
circRNA_08688	0	0	0.04361729	0	0	0	0	0	0	0	0	0	-	NC_000013.11	+	20574380	20615879	41500	1103	sense-overlappin	XM_005266549.2	IFT88
circRNA_08689	0	0	0.021808645	0	0	0	0	0	0	0	0	0	-	NC_000013.11	+	20596150	20615879	19730	699	sense-overlappin	XM_005266549.2	IFT88
circRNA_08690	0.030862537	0	0	0	0	0	0	0	0	0	0	0	-	NC_000013.11	+	20641290	20643605	2316	260	sense-overlappin	XM_011535245.1	IFT88
circRNA_08691	0.061725074	0	0	0.0412724	0.02006311	0	0	0	0.040509055	0.050431207	0.030382845	0.0206364	hsa_circ_0003285	NC_000013.11	-	20731841	20732121	281	281	exonic	XM_005266287.2	N6AMT2
circRNA_08692	0	0	0	0	0	0	0	0	0	0	0.02025523	0	hsa_circ_0003852	NC_000013.11	-	20731841	20737805	5965	364	sense-overlappin	XM_005266287.2	N6AMT2
circRNA_08693	0	0	0.021808645	0	0	0	0	0	0	0	0	0	hsa_circ_0008007	NC_000013.11	-	20799165	20800990	1826	505	sense-overlappin	NM_022459.4	XPO4
circRNA_08694	0	0	0.021808645	0	0	0	0	0	0	0	0	0	-	NC_000013.11	-	20800831	20809967	9137	804	sense-overlappin	NM_022459.4	XPO4
circRNA_08695	0	0	0.021808645	0	0	0	0	0.030729443	0	0	0	0	hsa_circ_0007547	NC_000013.11	-	21157922	21172681	14760	1016	sense-overlappin	XM_011534994.1	SKA3
circRNA_08696	0	0	0.032712968	0	0.02006311	0	0	0	0	0	0	0	hsa_circ_0029696	NC_000013.11	-	21161790	21172681	10892	726	sense-overlappin	XM_011534994.1	SKA3
circRNA_08697	0.030862537	0	0.239895095	0	0.1404418	0.031069364	0.050246713	0	0	0	0.04051046	0	hsa_circ_0000467	NC_000013.11	-	21167988	21168399	412	412	exonic	XM_005266288.2	SKA3
circRNA_08698	0	0	0	0	0.04012623	0	0	0	0	0	0	0	-	NC_000013.11	-	21167988	21172681	4694	640	sense-overlappin	XM_011534994.1	SKA3
circRNA_08699	0.041150049	0	0.032712968	0	0	0	0	0	0	0	0	0	hsa_circ_0029703	NC_000013.11	-	21381434	21391854	10421	730	sense-overlappin	XR_941546.1	
circRNA_08700	0	0	0	0.0206362	0.04012623	0	0	0	0	0.030258724	0	0	-	NC_000013.11	-	21381434	21425678	44245	1206	sense-overlappin	XR_941546.1	
circRNA_08701	0	0	0	0	0	0	0	0	0	0	0	0.0309546	-	NC_000013.11	-	21400373	21413772	13400	463	sense-overlappin	NR_104486.1	
circRNA_08702	0	0.020469968	0	0	0	0.031069364	0	0	0	0	0	0	-	NC_000013.11	-	21400373	21421164	20792	567	sense-overlappin	NR_104486.1	
circRNA_08703	0	0	0	0	0	0	0.020098685	0	0	0	0	0	-	NC_000013.11	-	21413652	21421164	7513	225	sense-overlappin	XM_006719795.1	ZDHHC20
circRNA_08704	0	0	0	0	0.02006311	0.031069364	0	0.020486295	0	0	0	0	hsa_circ_0006732	NC_000013.11	-	21413652	21425678	12027	252	sense-overlappin	XM_006719795.1	ZDHHC20
circRNA_08705	0	0	0	0.0206362	0	0	0	0	0	0	0	0	hsa_circ_0003408	NC_000013.11	-	23353785	23371165	17381	2014	sense-overlappin	NM_014363.5	SACS
circRNA_08706	0	0	0	0	0	0	0	0	0	0.040344966	0	0	hsa_circ_0002046	NC_000013.11	-	23365166	23371165	6000	286	sense-overlappin	NM_014363.5	SACS
circRNA_08707	0	0	0	0	0	0	0.020098685	0	0	0	0	0	-	NC_000013.11	-	23745853	23747850	1998	1998	intronic	XM_011535097.1	MIPEP
circRNA_08708	0	0	0.021808645	0	0	0	0	0	0	0	0	0	-	NC_000013.11	-	23836240	23886506	50267	1464	sense-overlappin	XM_011535097.1	MIPEP
circRNA_08709	0.020575025	0	0.34893832	0	0.02006311	0	0.020098685	0	0	0	0.050638075	0.0309546	hsa_circ_0004865	NC_000013.11	+	24222819	24224582	1764	1764	exonic	NM_001286792.1	SPATA13
circRNA_08710	0	0	0.021808645	0.0412724	0	0	0	0	0	0	0	0	hsa_circ_0003040	NC_000013.11	+	24249477	24251862	2386	511	sense-overlappin	NM_001286792.1	SPATA13
circRNA_08711	0	0	0	0.0206362	0	0	0	0	0	0	0	0	-	NC_000013.11	+	24294739	24297735	2997	503	sense-overlappin	NM_001286792.1	SPATA13
circRNA_08712	0	0.030704951	0	0	0.04012623	0.031069364	0	0.030729443	0	0.050431207	0	0	hsa_circ_0029762	NC_000013.11	-	24475472	24478276	2805	466	sense-overlappin	XM_011534932.1	PARP4
circRNA_08713	0	0	0	0.0206362	0	0	0	0	0	0	0	0	-	NC_000013.11	-	24477701	24478276	576	341	sense-overlappin	XM_011534932.1	PARP4
circRNA_08714	0	0	0	0	0	0	0.020098685	0.051215739	0	0.030258724	0	0	-	NC_000013.11	-	24477701	24493733	16033	1048	sense-overlappin	XM_011534932.1	PARP4
circRNA_08715	0	0	0	0.0206362	0	0	0	0	0	0	0	0	hsa_circ_0029767	NC_000013.11	-	24484653	24503777	19125	1449	sense-overlappin	XM_011534932.1	PARP4
circRNA_08716	0	0	0	0	0	0.020712909	0	0	0	0.020172483	0	0	hsa_circ_0029775	NC_000013.11	-	24903926	24913091	9166	2891	sense-overlappin	NM_018451.4	CENPJ
circRNA_08717	0.020575025	0	0	0	0.02006311	0	0.030148028	0	0	0	0	0	hsa_circ_0006541	NC_000013.11	+	26324772	26353880	29109	378	sense-overlappin	XM_005266216.3	CDK8
circRNA_08718	0.061725074	0	0.076330258	0	0	0	0	0	0	0.050431207	0	0	hsa_circ_0029803	NC_000013.11	+	26337567	26353880	16314	328	sense-overlappin	XM_005266215.1	CDK8
circRNA_08719	0	0	0.021808645	0	0	0	0	0	0	0	0	0	-	NC_000013.11	+	26349117	26401624	52508	1017	sense-overlappin	XM_005266215.1	CDK8
circRNA_08720	0.082300098	0.040939935	0.065425935	0.0619086	0.0702209	0.051782273	0.040197371	0.051215739	0.050636319	0	0.06076569	0	hsa_circ_0003489	NC_000013.11	+	26400453	26401624	1172	333	sense-overlappin	XM_005266215.1	CDK8
circRNA_08721	0.030862537	0	0	0	0.02006311	0	0	0	0	0	0	0	-	NC_000013.11	+	27427092	27430621	3530	287	sense-overlappin	NM_002097.2	GTF3A
circRNA_08722	0	0	0.021808645	0.0206362	0.10031557	0	0.020098685	0	0	0	0	0	hsa_circ_0029827	NC_000013.11	-	27581297	27581803	507	507	exonic	XM_011534995.1	LNX2
circRNA_08723	0.020575025	0.030704951	0.065425935	0.0412724	0.10031557	0.062138728	0.040197371	0	0.070890847	0.050431207	0	0.0309546	hsa_circ_0004372	NC_000013.11	+	28174272	28177935	3664	260	sense-overlappin	XM_011535034.1	PAN3
circRNA_08724	0	0	0.032712968	0.0412724	0.03009467	0	0	0.030729443	0	0	0	0	hsa_circ_0008760	NC_000013.11	+	28174272	28197346	23075	422	sense-overlappin	XM_011535034.1	PAN3

circRNA_08725	0	0.040939935	0.04361729	0.0412724	0	0	0.040197371	0.051215739	0.020254528	0.030258724	0.02025523	0.0206364	hsa_circ_0006597	NC_000013.11	+	28197185	28220378	23194	310	sense-overlappin	XM_011535034.1	PAN3
circRNA_08726	0.030862537	0	0.054521613	0.0206362	0.08025246	0.041425819	0.030148028	0.09218833	0.030381792	0	0.030382845	0	hsa_circ_0008902	NC_000013.11	+	28256292	28261458	5167	411	sense-overlappin	XM_011535034.1	PAN3
circRNA_08727	0	0	0.021808645	0	0	0	0	0	0	0	0	0	-	NC_000013.11	+	28256292	28272071	15780	1049	sense-overlappin	XM_011535034.1	PAN3
circRNA_08728	0	0	0.04361729	0	0	0	0	0	0	0	0	0	hsa_circ_0029852	NC_000013.11	+	28256292	28280541	24250	1319	sense-overlappin	XM_011535034.1	PAN3
circRNA_08729	0.051437562	0	0.08723458	0	0.02006311	0.031069364	0.030148028	0	0.020254528	0	0	0	hsa_circ_0029853	NC_000013.11	+	28256292	28281379	25088	1384	sense-overlappin	XM_011535034.1	PAN3
circRNA_08730	0	0	0	0.0206362	0	0	0	0	0	0	0	0	hsa_circ_0002552	NC_000013.11	+	28260447	28280541	20095	1071	sense-overlappin	XM_011535034.1	PAN3
circRNA_08731	0	0	0	0	0.02006311	0	0.020098685	0	0	0	0	0	hsa_circ_0000469	NC_000013.11	+	28266715	28270866	4152	547	sense-overlappin	XM_011535034.1	PAN3
circRNA_08732	0	0.030704951	0	0	0	0	0	0	0	0	0	0	-	NC_000013.11	-	28703943	28710843	6901	241	sense-overlappin	XM_005266361.1	SLC46A3
circRNA_08733	0	0	0	0	0.02006311	0	0	0	0	0	0	0	hsa_circ_0006290	NC_000013.11	-	29530538	29536202	5665	718	sense-overlappin	XM_005266507.2	SLC7A1
circRNA_08734	0	0	0	0	0.03009467	0	0	0	0	0	0	0	hsa_circ_0008598	NC_000013.11	-	29530538	29553860	23323	818	sense-overlappin	XM_005266507.2	SLC7A1
circRNA_08735	0	0	0	0.0309543	0	0.062138728	0	0.030729443	0	0.020172483	0	0	hsa_circ_0003587	NC_000013.11	-	30227412	30241086	13675	655	sense-overlappin	XM_006719881.2	KATNAL1
circRNA_08736	0.030862537	0.020469968	0.021808645	0	0	0	0	0	0	0	0	0	hsa_circ_0008068	NC_000013.11	-	30227412	30283791	56380	1161	sense-overlappin	XM_005266574.1	KATNAL1
circRNA_08737	0	0	0	0	0	0	0	0	0	0.020172483	0	0	hsa_circ_0002310	NC_000013.11	-	30240460	30283791	43332	740	sense-overlappin	XM_005266574.1	KATNAL1
circRNA_08738	0	0	0.032712968	0.0206362	0	0	0	0	0	0	0	0	hsa_circ_0008651	NC_000013.11	-	30280063	30283791	3729	337	sense-overlappin	XM_005266574.1	KATNAL1
circRNA_08739	0.082300098	0.071644886	0	0	0	0	0.020098685	0	0	0	0	0	hsa_circ_0005874	NC_000013.11	+	30630835	30631474	640	640	exonic	NM_005800.4	USPL1
circRNA_08740	0	0	0	0	0	0	0.020098685	0	0	0	0	0	hsa_circ_0029898	NC_000013.11	+	30637744	30647057	9314	370	sense-overlappin	NM_005800.4	USPL1
circRNA_08741	0	0	0.032712968	0	0.09028402	0.031069364	0	0	0	0	0.02025523	0	hsa_circ_0002635	NC_000013.11	+	31215051	31229294	14244	200	sense-overlappin	NM_194318.3	B3GALTL
circRNA_08742	0	0	0	0.0206362	0	0	0	0	0	0.030258724	0	0	hsa_circ_0029909	NC_000013.11	+	32078834	32117473	38640	394	sense-overlappin	NM_023037.2	FRY
circRNA_08743	0	0.020469968	0	0	0	0	0	0	0	0	0	0	hsa_circ_0029914	NC_000013.11	+	32124286	32149834	25549	1015	sense-overlappin	NM_023037.2	FRY
circRNA_08744	0	0.020469968	0	0	0	0	0	0	0	0	0	0	-	NC_000013.11	+	32151789	32151857	69	69	intronic	NM_023037.2	FRY
circRNA_08745	0	0	0	0	0.03009467	0	0	0	0	0	0	0	hsa_circ_0029930	NC_000013.11	+	32325076	32326613	1538	315	sense-overlappin	XM_011535203.1	BRCA2
circRNA_08746	0	0	0	0	0	0.031069364	0	0	0	0	0	0	hsa_circ_0029934	NC_000013.11	-	32438652	32444126	5475	1825	sense-overlappin	NM_001278432.1	N4BP2L2
circRNA_08747	0	0	0	0	0	0	0	0	0	0	0	0.0309546	-	NC_000013.11	-	32480590	32521449	40860	37491	sense-overlappin	NM_001278432.1	N4BP2L2
circRNA_08748	0.020575025	0	0	0	0.02006311	0	0	0	0	0	0	0	hsa_circ_0005723	NC_000013.11	-	32480590	32527532	46943	37655	sense-overlappin	NM_001278432.1	N4BP2L2
circRNA_08749	0.658400788	0.614099026	0.447077223	0.7222666	0.8727455	0.590317916	0.271332252	0.338023876	0.415217818	1.069141588	0.293700836	0.288909602	hsa_circ_0000471	NC_000013.11	-	32517857	32527532	9676	388	sense-overlappin	NM_001278432.1	N4BP2L2
circRNA_08750	0	0	0	0	0	0.020712909	0	0	0	0	0	0	-	NC_000013.11	-	32521373	32527532	6160	241	sense-overlappin	NM_001278432.1	N4BP2L2
circRNA_08751	0.051437562	0.071644886	0.021808645	0.0825448	0.06018934	0.051782273	0.040197371	0	0.040509055	0.090776173	0.030382845	0.0206364	-	NC_000013.11	-	32535769	32537027	1259	1259	exonic	NM_033111.4	N4BP2L2
circRNA_08752	0	0	0	0	0	0	0	0	0	0	0	0.0206364	-	NC_000013.11	-	32599346	32600661	1316	1316	antisense	NM_015032.3	PDS5B
circRNA_08753	0	0	0	0	0	0	0	0	0.040509055	0	0	0	hsa_circ_0029941	NC_000013.11	+	32648754	32678929	30176	1076	sense-overlappin	NM_015032.3	PDS5B
circRNA_08754	0	0	0	0.0206362	0	0	0	0	0	0	0	0	-	NC_000013.11	+	32673216	32678929	5714	352	sense-overlappin	NM_015032.3	PDS5B
circRNA_08755	0	0	0.04361729	0	0.02006311	0.051782273	0.020098685	0	0	0.050431207	0	0	hsa_circ_0004494	NC_000013.11	+	32732101	32735330	3230	283	sense-overlappin	NM_015032.3	PDS5B
circRNA_08756	0	0	0.032712968	0	0	0	0	0	0	0	0	0	hsa_circ_0029961	NC_000013.11	+	32732101	32746100	14000	613	sense-overlappin	NM_015032.3	PDS5B
circRNA_08757	0	0	0	0	0	0.020712909	0	0	0	0	0	0	-	NC_000013.11	+	32770121	32773324	3204	684	sense-overlappin	NM_015032.3	PDS5B
circRNA_08758	0	0	0	0	0	0	0	0	0.030381792	0	0	0	-	NC_000013.11	-	33270159	33329813	59655	59655	sense-overlappin	NM_001243476.2	STARD13
circRNA_08759	0	0	0	0	0	0	0	0.030729443	0	0	0	0	hsa_circ_0029976	NC_000013.11	+	35040933	35070852	29920	1277	sense-overlappin	XM_011535047.1	NBEA
circRNA_08760	0	0	0.098138903	0.0309543	0.10031557	0	0	0	0	0.020172483	0.02025523	0.0309546	hsa_circ_0000475	NC_000013.11	-	37040405	37051583	11179	663	sense-overlappin	XM_005266450.3	SUPT20H
circRNA_08761	0	0	0	0	0	0	0	0	0	0.030258724	0	0	-	NC_000013.11	-	38050769	38061453	10685	10685	intergenic		
circRNA_08762	0	0	0	0.0412724	0.03009467	0	0	0	0	0	0	0	-	NC_000013.11	-	38050769	38072976	22208	22208	intergenic		
circRNA_08763	0	0	0	0	0	0.020712909	0	0	0	0	0	0	-	NC_000013.11	-	40558386	40560860	2475	2001	sense-overlappin	NM_002015.3	FOXO1
circRNA_08764	0	0	0.109043225	0	0	0.072495183	0.020098685	0.030729443	0	0	0.02025523	0.0309546	hsa_circ_0030042	NC_000013.11	-	40559509	40560860	1352	1352	exonic	NM_002015.3	FOXO1
circRNA_08765	0	0	0.04361729	0	0	0	0	0	0	0	0	0	-	NC_000013.11	-	40559509	40562798	3290	3290	sense-overlappin	NM_002015.3	FOXO1
circRNA_08766	0	0	0	0	0.02006311	0	0	0.030729443	0	0.020172483	0	0	-	NC_000013.11	-	40559509	40645486	85978	85978	sense-overlappin	NM_002015.3	FOXO1
circRNA_08767	0	0	0	0	0	0	0	0	0.020254528	0	0	0	-	NC_000013.11	-	40749138	40767033	17896	806	sense-overlappin	NM_005830.3	MRPS31
circRNA_08768	0.020575025	0.020469968	0	0	0.02006311	0.020712909	0	0	0	0.050431207	0	0	hsa_circ_0030049	NC_000013.11	-	40826506	40836885	10380	10380	intergenic		
circRNA_08769	0	0	0	0	0.02006311	0.031069364	0	0	0	0	0	0	-	NC_000013.11	-	40826506	40855815	29310	29310	intergenic		
circRNA_08770	0	0	0.021808645	0	0	0	0	0	0	0.030258724	0	0	-	NC_000013.11	-	40836597	40855815	19219	19219	intergenic		
circRNA_08771	0.041150049	0	0.04361729	0.0206362	0.02006311	0	0	0	0	0	0	0	-	NC_000013.11	-	40908159	40914105	5947	5947	intergenic		
circRNA_08772	0	0	0.065425935	0.0412724	0	0	0	0	0	0	0	0	-	NC_000013.11	-	40933470	40941370	7901	1009	sense-overlappin	NM_001145353.1	ELF1
circRNA_08773	0	0	0.021808645	0	0	0	0	0	0	0	0	0	hsa_circ_0030050	NC_000013.11	-	40940921	40941370	450	450	exonic	NM_001145353.1	ELF1
circRNA_08774	0.020575025	0	0.04361729	0.0206362	0.02006311	0	0	0	0.020254528	0.07060369	0	0	hsa_circ_0030051	NC_000013.11	-	40940921	40943925	3005	727	sense-overlappin	NM_001145353.1	ELF1
circRNA_08775	0	0	0	0	0	0.020712909	0	0	0	0	0	0	-	NC_000013.11	-	40940921	40982282	41362	1603	sense-overlappin	XM_011534951.1	ELF1
circRNA_08776	0	0	0	0	0	0.031069364	0	0.030729443	0	0.020172483	0	0	-	NC_000013.11	-	40942952	40943925	974	277	sense-overlappin	NM_001145353.1	ELF1
circRNA_08777	0	0	0	0	0	0.020712909	0	0	0	0	0	0	-	NC_000013.11	-	41233890	41260915	27026	996	sense-overlappin	XM_006719898.1	MTRF1
circRNA_08778	0	0	0.021808645	0	0	0	0	0	0	0.020172483	0	0	-	NC_000013.11	-	41252645	41254620	1976	282	sense-overlappin	XM_006719898.1	MTRF1
circRNA_08779	0.041150049	0.020469968	0.054521613	0	0.10031557	0.020712909	0.040197371	0	0.030381792	0.030258724	0	0	hsa_circ_0008230	NC_000013.11	-	41252645	41260915	8271	705	sense-overlappin	XM_006719898.1	MTRF1
circRNA_08780	0	0	0	0	0	0	0	0.020486295	0	0	0	0	hsa_circ_0009152	NC_000013.11	+	41316846	41320824	3979	348	sense-overlappin	NM_001110798.1	NAA16

circRNA_08781	0	0	0	0	0	0	0	0	0	0	0	0.0206364	hsa_circ_0030065	NC_000013.11	+	41316846	41323190	6345	483	sense-overlappin	NM_001110798.1	NAA16
circRNA_08782	0	0	0	0	0	0	0.030148028	0	0	0	0	0	hsa_circ_0030067	NC_000013.11	+	41316846	41336756	19911	960	sense-overlappin	NM_001110798.1	NAA16
circRNA_08783	0	0	0	0	0	0	0	0	0	0.020172483	0	0	-	NC_000013.11	+	41318806	41320824	2019	263	sense-overlappin	NM_001110798.1	NAA16
circRNA_08784	0	0	0.032712968	0.0206362	0	0	0	0	0	0	0	0	hsa_circ_0030069	NC_000013.11	+	41318806	41336756	17951	875	sense-overlappin	NM_001110798.1	NAA16
circRNA_08785	0.020575025	0	0	0.0515905	0.02006311	0.041425819	0	0.040972591	0	0.050431207	0	0.0309546	-	NC_000013.11	+	41369090	41372830	3741	402	sense-overlappin	NM_024561.4	NAA16
circRNA_08786	0	0	0	0	0	0.020712909	0	0	0	0	0	0	-	NC_000013.11	-	41719591	41778056	58466	839	sense-overlappin	XM_006719791.2	VWA8
circRNA_08787	0	0	0.04361729	0	0	0.020712909	0	0	0	0	0	0	hsa_circ_0030101	NC_000013.11	-	41761128	41833531	72404	1001	sense-overlappin	XM_006719791.2	VWA8
circRNA_08788	0	0	0.08723458	0	0.02006311	0.031069364	0.020098685	0.040972591	0	0	0	0	hsa_circ_0004711	NC_000013.11	-	41811225	41819386	8162	363	sense-overlappin	XM_006719791.2	VWA8
circRNA_08789	0	0.020469968	0.098138903	0.0619086	0	0	0	0	0	0	0	0	hsa_circ_0006049	NC_000013.11	-	41811225	41833531	22307	638	sense-overlappin	XM_006719791.2	VWA8
circRNA_08790	0	0	0.021808645	0	0	0	0	0	0	0	0	0	-	NC_000013.11	-	41811225	41837250	26026	4357	sense-overlappin	XM_006719791.2	VWA8
circRNA_08791	0.041150049	0	0.098138903	0.1031809	0.06018934	0.051782273	0	0.030729443	0.030381792	0.040344966	0	0.0309546	hsa_circ_0000478	NC_000013.11	-	41865736	41868477	2742	345	sense-overlappin	XM_006719791.2	VWA8
circRNA_08792	0	0.020469968	0	0.0206362	0	0	0	0	0.020254528	0.040344966	0	0	hsa_circ_0030117	NC_000013.11	-	41865736	41887361	21626	774	sense-overlappin	XM_011535006.1	VWA8
circRNA_08793	0	0	0.021808645	0	0	0	0	0	0	0	0	0	-	NC_000013.11	+	42155291	42174144	18854	1068	sense-overlappin	NM_001204504.2	DGKH
circRNA_08794	0	0	0	0	0.02006311	0	0	0	0	0	0	0	hsa_circ_0030130	NC_000013.11	+	42159266	42160136	871	233	sense-overlappin	NM_001204504.2	DGKH
circRNA_08795	0	0	0.468885868	0.0309543	0.04012623	0	0	0	0	0	0	0	-	NC_000013.11	+	42285986	42308609	22624	5372	sense-overlappin	XM_011534904.1	AKAP11
circRNA_08796	0.030862537	0	0	0	0	0	0	0	0	0	0	0	-	NC_000013.11	-	42783183	42800041	16859	12556	antisense	NM_182508.2	FAM216B
circRNA_08797	0	0	0	0	0	0	0.020098685	0	0	0	0	0	-	NC_000013.11	-	42895009	42917624	22616	258	sense-overlappin	XM_011535314.1	EPSTI1
circRNA_08798	0	0	0	0	0	0	0.020098685	0	0	0	0	0	-	NC_000013.11	-	42900310	42970670	70361	660	sense-overlappin	XM_011535312.1	EPSTI1
circRNA_08799	0	0	0.054521613	0	0	0	0.090444084	0	0.030381792	0.030258724	0.08102092	0	-	NC_000013.11	-	42917541	42970670	53130	586	sense-overlappin	XM_011535312.1	EPSTI1
circRNA_08800	0	0	0	0	0.03009467	0	0.030148028	0	0	0	0	0	-	NC_000013.11	-	42926336	42964139	37804	326	sense-overlappin	XM_011535312.1	EPSTI1
circRNA_08801	0	0	0.04361729	0	0.02006311	0	0.030148028	0	0	0	0	0	-	NC_000013.11	-	42926336	42970670	44335	469	sense-overlappin	XM_011535312.1	EPSTI1
circRNA_08802	0.123450148	0.040939935	0.316225353	0.0722267	0.08025246	0.041425819	0.200986853	0.051215739	0.050636319	0.171466104	0.182297071	0	hsa_circ_0000479	NC_000013.11	-	42953948	42970670	16723	375	sense-overlappin	XM_011535312.1	EPSTI1
circRNA_08803	0.020575025	0	0.098138903	0	0	0	0	0	0	0	0	0	-	NC_000013.11	-	42963255	42970670	7416	301	sense-overlappin	XM_011535312.1	EPSTI1
circRNA_08804	0	0	0	0.0206362	0	0	0	0	0	0	0	0	-	NC_000013.11	-	43484009	43506226	22218	22218	sense-overlappin	XM_011535126.1	ENOX1
circRNA_08805	0	0	0	0.0309543	0	0.020712909	0	0.030729443	0	0	0.02025523	0	-	NC_000013.11	-	44510315	44510702	388	388	intronic	NM_001243799.1	TSC22D1
circRNA_08806	0.020575025	0	0	0	0	0.020712909	0	0	0	0	0	0	-	NC_000013.11	-	44538535	44539936	1402	524	sense-overlappin	XM_005266583.3	TSC22D1
circRNA_08807	0	0	0	0	0	0	0	0	0.030381792	0	0	0	-	NC_000013.11	+	45004305	45020428	16124	716	sense-overlappin	XM_005266443.1	GPALPP1
circRNA_08808	0	0	0	0.0309543	0	0	0	0	0	0	0	0	hsa_circ_0030154	NC_000013.11	+	45004305	45028003	23699	935	sense-overlappin	XM_005266443.1	GPALPP1
circRNA_08809	0.030862537	0	0.04361729	0.0206362	0	0	0.020098685	0.020486295	0	0	0.02025523	0	hsa_circ_0007259	NC_000013.11	+	45151687	45207505	55819	305	sense-overlappin	XM_011535052.1	GTF2F2
circRNA_08810	0	0	0.04361729	0	0	0	0	0	0	0	0	0	-	NC_000013.11	-	45336536	45337388	853	853	exonic	NM_001286272.1	TPT1
circRNA_08811	0	0.030704951	0	0	0	0.031069364	0	0.051215739	0.020254528	0.030258724	0	0	hsa_circ_0003401	NC_000013.11	+	45516143	45519094	2952	345	sense-overlappin	NM_031431.3	COG3
circRNA_08812	0	0	0	0.0412724	0	0	0.030148028	0	0	0.030258724	0.030382845	0	hsa_circ_0008937	NC_000013.11	+	45516143	45525051	8909	421	sense-overlappin	NM_031431.3	COG3
circRNA_08813	0	0	0.021808645	0	0	0	0	0	0	0	0	0	hsa_circ_0030188	NC_000013.11	-	45959483	45969971	10489	2267	sense-overlappin	XM_005266305.2	ZC3H13
circRNA_08814	0	0	0.021808645	0	0	0	0	0	0	0	0	0	-	NC_000013.11	-	45979813	46045513	65701	1918	sense-overlappin	XM_005266305.2	ZC3H13
circRNA_08815	0	0	0.076330258	0	0	0	0	0	0	0	0	0	-	NC_000013.11	-	45985297	46020557	35261	1381	sense-overlappin	XM_005266305.2	ZC3H13
circRNA_08816	0	0	0.098138903	0.0309543	0	0.051782273	0	0.020486295	0	0.030258724	0	0	-	NC_000013.11	-	46003139	46020557	17419	605	sense-overlappin	XM_005266305.2	ZC3H13
circRNA_08817	0	0	0.032712968	0	0	0	0	0	0	0	0	0	-	NC_000013.11	-	46003139	46045513	42375	950	sense-overlappin	XM_005266305.2	ZC3H13
circRNA_08818	0	0.030704951	0.032712968	0.0309543	0	0	0	0	0	0	0	0	-	NC_000013.11	-	46003139	46045516	42378	953	sense-overlappin	XM_005266305.2	ZC3H13
circRNA_08819	0	0	0.021808645	0	0	0	0	0	0	0	0	0	-	NC_000013.11	-	46020449	46045516	25068	457	sense-overlappin	XM_005266305.2	ZC3H13
circRNA_08820	0	0	0.021808645	0	0	0	0	0	0	0	0	0	hsa_circ_0030200	NC_000013.11	+	46650201	46669156	18956	272	sense-overlappin	XM_011535013.1	LRCH1
circRNA_08821	0	0	0.021808645	0	0	0	0	0	0	0	0	0	hsa_circ_0030207	NC_000013.11	+	46692536	46728984	36449	993	sense-overlappin	NM_001164211.1	LRCH1
circRNA_08822	0	0	0	0	0.02006311	0	0	0	0	0	0	0	-	NC_000013.11	+	46694893	46701207	6315	280	sense-overlappin	XM_011535013.1	LRCH1
circRNA_08823	0	0	0.032712968	0	0	0.041425819	0	0	0	0	0	0	hsa_circ_0030213	NC_000013.11	+	46711791	46733998	22208	558	sense-overlappin	NM_001164211.1	LRCH1
circRNA_08824	0.041150049	0	0	0	0	0	0	0	0	0	0	0	hsa_circ_0030214	NC_000013.11	+	46723221	46728984	5764	248	sense-overlappin	NM_001164211.1	LRCH1
circRNA_08825	0	0.051174919	0.185373483	0.0825448	0.05015779	0.031069364	0.040197371	0.051215739	0.040509055	0	0.06076569	0	hsa_circ_0002215	NC_000013.11	+	46723221	46733998	10778	326	sense-overlappin	NM_001164211.1	LRCH1
circRNA_08826	0.051437562	0	0.021808645	0	0.03009467	0	0.040197371	0.020486295	0.040509055	0.030258724	0	0	hsa_circ_0004936	NC_000013.11	-	48077153	48090418	13266	674	sense-overlappin	NM_001270629.1	MED4
circRNA_08827	0	0	0	0	0.02006311	0	0	0	0	0	0	0	hsa_circ_0030227	NC_000013.11	-	48079844	48083428	3585	277	sense-overlappin	NM_001270629.1	MED4
circRNA_08828	0	0	0	0	0	0	0	0	0	0	0	0.0206364	hsa_circ_0006620	NC_000013.11	+	48253808	48261321	7514	781	sense-overlappin	NM_021999.4	ITM2B
circRNA_08829	0.061725074	0	0	0.0206362	0.02006311	0.051782273	0	0	0	0	0	0	-	NC_000013.11	+	48256177	48258236	2060	318	sense-overlappin	NM_021999.4	ITM2B
circRNA_08830	0	0	0	0	0	0	0.020098685	0	0	0	0	0	-	NC_000013.11	+	49075289	49145935	70647	878	sense-overlappin	NM_001079673.1	FNDC3A
circRNA_08831	0	0	0	0.0309543	0	0	0	0	0	0	0	0	-	NC_000013.11	+	49114655	49145935	31281	802	sense-overlappin	NM_001079673.1	FNDC3A
circRNA_08832	0	0	0.032712968	0.0206362	0	0.051782273	0	0	0.040509055	0.040344966	0	0.0412728	hsa_circ_0000481	NC_000013.11	+	49138747	49145935	7189	217	sense-overlappin	NM_001079673.1	FNDC3A
circRNA_08833	0	0.030704951	0.021808645	0.0412724	0	0.124277456	0	0.030729443	0	0.090776173	0.030382845	0.0309546	hsa_circ_0004024	NC_000013.11	+	49167244	49175541	8298	553	sense-overlappin	NM_001079673.1	FNDC3A
circRNA_08834	0	0	0	0.0309543	0.03009467	0	0	0	0	0.040344966	0	0	hsa_circ_0005275	NC_000013.11	+	49168613	49175541	6929	493	sense-overlappin	NM_001079673.1	FNDC3A
circRNA_08835	0	0	0.021808645	0.0206362	0.05015779	0.051782273	0	0.030729443	0.020254528	0.100862414	0	0	hsa_circ_0006978	NC_000013.11	+	49168613	49178654	10042	580	sense-overlappin	NM_001079673.1	FNDC3A
circRNA_08836	0	0	0	0	0	0	0	0.020486295	0	0	0	0	hsa_circ_0007463	NC_000013.11	+	49187122	49188633	1512	188	sense-overlappin	NM_001079673.1	FNDC3A

circRNA_08837	0.051437562	0.040939935	0.04361729	0.0309543	0.06018934	0.041425819	0.020098685	0.061458886	0	0.020172483	0	0	hsa_circ_0005263	NC_000013.11	+	49196877	49198574	1698	761	sense-overlappin	NM_001079673.1	FNDC3A
circRNA_08838	0	0	0	0	0	0	0	0	0	0.020172483	0	0	-	NC_000013.11	+	49274291	49286282	11992	471	sense-overlappin	XM_011535253.1	CDADC1
circRNA_08839	0	0.020469968	0	0.0515905	0.05015779	0.051782273	0	0.020486295	0	0.050431207	0.04051046	0.0309546	hsa_circ_0030253	NC_000013.11	+	49451553	49451909	357	357	exonic	XM_011535270.1	SETDB2
circRNA_08840	0.020575025	0	0	0	0.03009467	0	0	0	0	0	0	0	-	NC_000013.11	+	49451553	49461162	9610	549	sense-overlappin	XM_011535270.1	SETDB2
circRNA_08841	0.020575025	0.061409903	0.065425935	0	0	0	0.020098685	0.030729443	0	0.040344966	0.050638075	0.0412728	hsa_circ_0030254	NC_000013.11	+	49451553	49467960	16408	682	sense-overlappin	XM_011535270.1	SETDB2
circRNA_08842	0	0	0	0.0515905	0	0	0	0	0	0	0	0	-	NC_000013.11	+	49451553	49490910	39358	2383	sense-overlappin	XM_011535270.1	SETDB2
circRNA_08843	0	0	0	0.0206362	0.02006311	0	0.020098685	0	0.020254528	0	0.02025523	0.0309546	hsa_circ_0000483	NC_000013.11	+	49480219	49483563	3345	613	sense-overlappin	XM_011535270.1	SETDB2
circRNA_08844	0	0	0.021808645	0	0	0	0	0	0	0	0	0	hsa_circ_0007232	NC_000013.11	-	49705621	49706372	752	340	sense-overlappin	NM_002267.3	KPNA3
circRNA_08845	0	0	0.08723458	0	0	0.041425819	0	0	0	0	0	0	hsa_circ_0006475	NC_000013.11	-	49997074	50044768	47695	33188	antisense	NM_001007278.2	TRIM13
circRNA_08846	0	0	0.04361729	0	0	0	0	0	0	0	0	0	-	NC_000013.11	-	49997074	50045232	48159	33652	antisense	NM_001007278.2	TRIM13
circRNA_08847	0	0	0	0	0	0	0.020098685	0	0	0.020172483	0	0	-	NC_000013.11	-	50044651	50075653	31003	31003	intergenic		
circRNA_08848	0	0	0	0.0206362	0	0	0	0	0	0.020172483	0	0	hsa_circ_0030281	NC_000013.11	-	50068097	50075653	7557	7557	intergenic		
circRNA_08849	0	0	0.021808645	0	0	0	0	0	0	0	0	0	-	NC_000013.11	+	50299751	50301148	1398	1398	intergenic		
circRNA_08850	0	0	0.021808645	0	0	0	0	0	0	0	0	0	-	NC_000013.11	+	50492651	50502880	10230	10230	intergenic		
circRNA_08851	0	0	0.04361729	0	0	0	0	0	0	0	0	0	-	NC_000013.11	+	50757763	50832130	74368	74368	antisense	XM_011534973.1	DLEU7
circRNA_08852	0	0	0	0	0	0.031069364	0	0	0	0.040344966	0	0	hsa_circ_0030286	NC_000013.11	+	50927407	50934999	7593	372	sense-overlappin	NM_024570.3	RNASEH2B
circRNA_08853	0.123450148	0.16375974	0.697876641	0.0722267	0.16050492	0.207129093	0.110542769	0.071702034	0.182290749	0.272328518	0.111403765	0.123818401	hsa_circ_0000489	NC_000013.11	+	50927407	50949505	22099	882	sense-overlappin	XM_011535233.1	RNASEH2B
circRNA_08854	0.020575025	0	0	0	0	0.031069364	0	0	0	0	0	0	-	NC_000013.11	+	50927407	50960171	32765	785	sense-overlappin	XM_005266524.2	RNASEH2B
circRNA_08855	0	0	0.021808645	0	0	0	0	0	0	0	0	0	-	NC_000013.11	+	52018913	52024937	6025	1163	sense-overlappin	NM_001004127.2	ALG11
circRNA_08856	0	0	0.032712968	0	0	0	0	0	0	0	0.04051046	0	-	NC_000013.11	-	52065484	52076143	10660	403	sense-overlappin	XM_011535067.1	NEK5
circRNA_08857	0	0	0	0	0	0	0	0	0	0.020172483	0	0	hsa_circ_0008776	NC_000013.11	-	52397232	52398194	963	963	exonic	NM_018676.3	THSD1
circRNA_08858	0.020575025	0	0.054521613	0	0	0	0	0	0	0	0	0	hsa_circ_0008581	NC_000013.11	-	52397232	52402681	5450	1102	sense-overlappin	NM_018676.3	THSD1
circRNA_08859	0.041150049	0.061409903	0.08723458	0.0412724	0.04012623	0.051782273	0.020098685	0	0	0.040344966	0	0.0206364	hsa_circ_0030342	NC_000013.11	-	52417992	52427219	9228	377	sense-overlappin	NM_001282169.1	VPS36
circRNA_08860	0	0.020469968	0	0	0	0	0	0	0	0	0	0	-	NC_000013.11	-	52423574	52429432	5859	2525	sense-overlappin	NM_001282169.1	VPS36
circRNA_08861	0.020575025	0	0	0	0	0	0	0	0	0	0	0	-	NC_000013.11	-	52426989	52434882	7894	288	sense-overlappin	NM_001282169.1	VPS36
circRNA_08862	0.041150049	0	0	0	0	0	0	0	0	0	0	0	-	NC_000013.11	-	52433662	52434882	1221	177	sense-overlappin	NM_001282169.1	VPS36
circRNA_08863	0	0	0	0	0	0.020712909	0	0	0	0	0	0	-	NC_000013.11	+	52657532	52659249	1718	360	sense-overlappin	XM_011534892.1	SUGT1
circRNA_08864	0	0	0	0	0	0.041425819	0	0	0	0	0	0	-	NC_000013.11	+	52658399	52665733	7335	428	sense-overlappin	NM_001130912.1	SUGT1
circRNA_08865	0	0	0.021808645	0	0	0	0	0	0.020254528	0	0	0	-	NC_000013.11	+	52658399	52680155	21757	809	sense-overlappin	NM_001130912.1	SUGT1
circRNA_08866	0	0	0	0	0.05015779	0	0	0	0	0	0	0	-	NC_000013.11	+	53049969	53057186	7218	7218	sense-overlappin	NM_006418.4	OLFM4
circRNA_08867	0	0	0.054521613	0	0	0	0	0	0	0	0	0	hsa_circ_0004878	NC_000013.11	-	59774189	59810923	36735	292	sense-overlappin	XM_011535262.1	DIAPH3
circRNA_08868	0	0	0.021808645	0	0.03009467	0	0	0	0	0	0	0	hsa_circ_0030369	NC_000013.11	-	59833107	59839448	6342	290	sense-overlappin	XM_011535262.1	DIAPH3
circRNA_08869	0	0	0.021808645	0	0	0	0	0	0	0	0	0	-	NC_000013.11	-	59833107	59916249	83143	857	sense-overlappin	XM_011535262.1	DIAPH3
circRNA_08870	0	0	0.021808645	0	0	0	0	0	0	0	0	0	-	NC_000013.11	-	59916155	59992583	76429	1251	sense-overlappin	XM_011535262.1	DIAPH3
circRNA_08871	0	0	0.021808645	0	0	0	0	0	0	0	0	0	hsa_circ_0030374	NC_000013.11	-	60093628	60112186	18559	282	sense-overlappin	XM_011535261.1	DIAPH3
circRNA_08872	0	0	0.021808645	0.0206362	0.03009467	0.031069364	0	0.051215739	0	0.020172483	0.030382845	0	hsa_circ_0004245	NC_000013.11	+	60439688	60460540	20853	308	sense-overlappin	NM_001146071.1	TDRD3
circRNA_08873	0.123450148	0.143289773	0.665163673	0.2682705	0.28088361	0.144990365	0.211036196	0.061458886	0.182290749	0.211811069	0.06076569	0.051591	hsa_circ_0003441	NC_000013.11	+	60439688	60467379	27692	450	sense-overlappin	NM_001146071.1	TDRD3
circRNA_08874	0	0	0.054521613	0	0	0	0	0	0	0	0	0.0206364	hsa_circ_0030377	NC_000013.11	+	60439688	60485948	46261	672	sense-overlappin	NM_001146071.1	TDRD3
circRNA_08875	0	0	0	0	0.02006311	0.041425819	0	0	0	0	0	0	-	NC_000013.11	+	60439688	60494575	54888	813	sense-overlappin	NM_001146071.1	TDRD3
circRNA_08876	0	0	0.021808645	0	0	0	0	0	0	0	0	0	-	NC_000013.11	+	60439688	60510755	71068	1096	sense-overlappin	NM_001146071.1	TDRD3
circRNA_08877	0	0.020469968	0	0	0	0	0	0	0	0	0	0	-	NC_000013.11	+	60483775	60510755	26981	646	sense-overlappin	NM_001146071.1	TDRD3
circRNA_08878	0	0	0	0	0.02006311	0	0	0	0	0	0	0	hsa_circ_0030385	NC_000013.11	+	60509763	60535233	25471	1260	sense-overlappin	NM_001146071.1	TDRD3
circRNA_08879	0	0	0.032712968	0	0	0	0	0	0	0	0	0	-	NC_000013.11	-	63990096	64003465	13370	13370	intergenic		
circRNA_08880	0	0	0.076330258	0	0	0	0	0	0	0	0	0	hsa_circ_0030388	NC_000013.11	-	63990096	64034537	44442	44442	intergenic		
circRNA_08881	0	0	0.196277805	0	0	0	0	0	0	0	0	0	hsa_circ_0030389	NC_000013.11	-	64002385	64034537	32153	32153	intergenic		
circRNA_08882	0	0	0.032712968	0	0	0	0	0	0	0	0	0	hsa_circ_0006762	NC_000013.11	-	72773684	72777493	3810	659	sense-overlappin	NM_001128226.2	DIS3
circRNA_08883	0	0	0	0	0	0	0	0.040972591	0	0	0	0	-	NC_000013.11	-	72778187	72778380	194	194	exonic	NM_001128226.2	DIS3
circRNA_08884	0	0	0	0	0.02006311	0	0	0	0	0	0	0	-	NC_000013.11	+	72792447	72835368	42922	971	sense-overlappin	NM_006346.2	PIBF1
circRNA_08885	0	0	0.021808645	0	0	0	0	0	0	0	0	0	-	NC_000013.11	+	72835243	72854155	18913	312	sense-overlappin	XM_011534885.1	PIBF1
circRNA_08886	0.020575025	0	0.109043225	0.0206362	0.02006311	0.051782273	0	0	0.030381792	0	0	0	-	NC_000013.11	+	72835243	72931267	96025	823	sense-overlappin	XM_011534885.1	PIBF1
circRNA_08887	0	0.020469968	0	0	0.02006311	0	0	0	0	0	0	0	-	NC_000013.11	+	72893784	72931267	37484	511	sense-overlappin	NM_006346.2	PIBF1
circRNA_08888	0	0	0	0	0.02006311	0	0	0	0	0	0	0	-	NC_000013.11	+	72908531	72939934	31404	7659	sense-overlappin	XM_011534883.1	PIBF1
circRNA_08889	0	0	0	0.0309543	0	0	0	0	0	0	0	0	hsa_circ_0030421	NC_000013.11	-	75302243	75306471	4229	318	sense-overlappin	NM_001286658.1	TBC1D4
circRNA_08890	0	0	0	0.0309543	0	0	0	0	0	0	0	0	hsa_circ_0002917	NC_000013.11	-	75326197	75349302	23106	758	sense-overlappin	NM_001286658.1	TBC1D4
circRNA_08891	0	0	0.054521613	0	0	0	0	0	0	0	0	0	hsa_circ_0030427	NC_000013.11	-	75326197	75362607	36411	1535	sense-overlappin	NM_001286658.1	TBC1D4

circRNA_08892	0	0	0.08723458	0	0	0	0	0	0	0	0	0	-	NC_000013.11	-	75356147	75362607	6461	777	sense-overlappin	NM_001286658.1	TBC1D4
circRNA_08893	0.030862537	0.071644886	0.08723458	0.0619086	0.05015779	0.082851637	0.060296056	0.040972591	0.040509055	0.100862414	0.030382845	0	hsa_circ_0000494	NC_000013.11	+	75560753	75569507	8755	420	sense-overlappin	XM_011535214.1	UCHL3
circRNA_08894	0	0	0	0	0	0	0	0	0.020254528	0	0	0	hsa_circ_0008259	NC_000013.11	+	75713182	75761038	47857	248	sense-overlappin	NM_005358.5	LMO7
circRNA_08895	0	0	0.021808645	0	0	0	0	0	0	0	0	0	-	NC_000013.11	+	75713186	75761038	47853	244	sense-overlappin	NM_005358.5	LMO7
circRNA_08896	0.030862537	0	0	0.0309543	0	0	0	0	0	0	0.04051046	0	hsa_circ_0008794	NC_000013.11	-	77015409	77021861	6453	644	sense-overlappin	XM_011535037.1	FBXL3
circRNA_08897	0	0	0	0	0	0	0	0	0	0.020172483	0	0	hsa_circ_0004850	NC_000013.11	-	77081427	77087633	6207	693	sense-overlappin	XM_011535005.1	MYCBP2
circRNA_08898	0	0	0.04361729	0	0	0	0.030148028	0	0	0.030258724	0	0	hsa_circ_0000495	NC_000013.11	-	77126318	77129230	2913	300	sense-overlappin	XM_011535005.1	MYCBP2
circRNA_08899	0	0	0	0	0	0	0	0	0	0	0	0.0206364	-	NC_000013.11	-	77157937	77171634	13698	1119	sense-overlappin	XM_011535005.1	MYCBP2
circRNA_08900	0	0	0	0.0206362	0	0	0	0	0	0	0	0	-	NC_000013.11	-	77176497	77180318	3822	531	sense-overlappin	XM_011535005.1	MYCBP2
circRNA_08901	0	0	0.032712968	0	0	0	0	0	0.020254528	0.020172483	0	0	hsa_circ_0030507	NC_000013.11	-	77205256	77212160	6905	786	sense-overlappin	XM_011535005.1	MYCBP2
circRNA_08902	0	0	0	0	0	0	0	0	0	0.020172483	0	0	hsa_circ_0030508	NC_000013.11	-	77205256	77217957	12702	904	sense-overlappin	XM_011535005.1	MYCBP2
circRNA_08903	0.061725074	0	0.207182128	0.0309543	0.04012623	0	0.040197371	0	0.020254528	0.040344966	0	0	hsa_circ_0030509	NC_000013.11	-	77205256	77243951	38696	1462	sense-overlappin	XM_011535004.1	MYCBP2
circRNA_08904	0	0	0	0.0206362	0	0	0	0	0	0	0	0	-	NC_000013.11	-	77205256	77288376	83121	3465	sense-overlappin	XM_011535004.1	MYCBP2
circRNA_08905	0	0	0.032712968	0	0	0	0	0	0	0	0	0	-	NC_000013.11	-	77211167	77243951	32785	1035	sense-overlappin	XM_011535004.1	MYCBP2
circRNA_08906	0.030862537	0.020469968	0.065425935	0	0.02006311	0	0	0	0	0.020172483	0	0	hsa_circ_0004217	NC_000013.11	-	77217840	77243951	26112	676	sense-overlappin	XM_011535004.1	MYCBP2
circRNA_08907	0	0	0.04361729	0	0	0	0	0	0	0	0	0	-	NC_000013.11	-	77224451	77243951	19501	558	sense-overlappin	XM_011535004.1	MYCBP2
circRNA_08908	0	0	0.032712968	0	0	0	0	0	0	0	0	0	-	NC_000013.11	-	77251151	77264002	12852	1024	sense-overlappin	XM_011535004.1	MYCBP2
circRNA_08909	0	0	0	0	0	0.020712909	0	0	0	0	0	0	-	NC_000013.11	+	77719517	77753358	33842	953	sense-overlappin	XM_011534929.1	SLAIN1
circRNA_08910	0	0	0	0.0515905	0	0	0	0	0	0	0	0	-	NC_000013.11	+	77719532	77746855	27324	782	sense-overlappin	XM_011534929.1	SLAIN1
circRNA_08911	0.041150049	0.051174919	0.109043225	0.0722267	0.11034713	0	0.020098685	0.030729443	0.020254528	0.050431207	0	0.0412728	hsa_circ_0000497	NC_000013.11	+	77719532	77753358	33827	938	sense-overlappin	XM_011534929.1	SLAIN1
circRNA_08912	0	0	0	0	0	0	0.030148028	0	0	0	0	0	-	NC_000013.11	+	77744283	77753358	9076	648	sense-overlappin	NM_001242868.1	SLAIN1
circRNA_08913	0	0.030704951	0	0	0	0	0	0	0	0	0	0	hsa_circ_0030533	NC_000013.11	-	79341123	79344794	3672	474	sense-overlappin	XM_005266497.1	RBM26
circRNA_08914	0	0	0	0	0	0.020712909	0	0	0	0	0	0	-	NC_000013.11	-	79341123	79378907	37785	2506	sense-overlappin	XM_005266497.1	RBM26
circRNA_08915	0	0	0	0	0	0	0	0.030729443	0	0	0	0	-	NC_000013.11	-	79344248	79378907	34660	2233	sense-overlappin	XM_005266497.1	RBM26
circRNA_08916	0	0	0	0	0	0.020712909	0	0	0	0	0	0	hsa_circ_0030536	NC_000013.11	-	79358274	79371930	13657	1407	sense-overlappin	XM_005266497.1	RBM26
circRNA_08917	0	0	0	0.0412724	0	0	0	0	0	0	0	0	hsa_circ_0004205	NC_000013.11	-	79370945	79371930	986	307	sense-overlappin	XM_005266497.1	RBM26
circRNA_08918	0	0	0.04361729	0	0	0	0	0	0	0	0	0	hsa_circ_0030548	NC_000013.11	+	79520810	79539775	18966	394	sense-overlappin	XR_245389.2	
circRNA_08919	0	0	0	0	0	0	0	0	0	0	0.030382845	0	-	NC_000013.11	+	79570563	79571217	655	655	intergenic		
circRNA_08920	0	0	0.021808645	0	0	0	0	0	0	0	0	0	-	NC_000013.11	+	81386480	81390453	3974	3974	intergenic		
circRNA_08921	0	0	0	0	0	0	0	0	0	0	0	0.0412728	-	NC_000013.11	-	95115922	95170628	54707	808	sense-overlappin	NM_005845.4	ABCC4
circRNA_08922	0	0	0	0	0.02006311	0	0	0	0	0	0	0.0309546	hsa_circ_0030582	NC_000013.11	-	95115922	95188542	72621	1272	sense-overlappin	NM_005845.4	ABCC4
circRNA_08923	0	0.020469968	0.032712968	0.0206362	0	0	0	0	0	0	0	0	hsa_circ_0007470	NC_000013.11	-	95161189	95166367	5179	631	sense-overlappin	NM_005845.4	ABCC4
circRNA_08924	0	0.051174919	0	0.0619086	0.02006311	0.031069364	0.020098685	0	0	0	0	0.0206364	hsa_circ_0006659	NC_000013.11	-	95161189	95170628	9440	728	sense-overlappin	NM_005845.4	ABCC4
circRNA_08925	0	0.020469968	0.021808645	0	0.02006311	0.041425819	0	0.051215739	0	0	0	0.0206364	hsa_circ_0030586	NC_000013.11	-	95161189	95188542	27354	1192	sense-overlappin	NM_005845.4	ABCC4
circRNA_08926	0	0	0.021808645	0	0	0	0	0	0	0	0	0	hsa_circ_0006318	NC_000013.11	-	95206532	95210781	4250	630	sense-overlappin	NM_001301829.1	ABCC4
circRNA_08927	0	0	0	0	0	0	0	0	0	0.020172483	0	0.0206364	-	NC_000013.11	-	95611445	95612177	733	190	sense-overlappin	NM_014934.4	DZIP1
circRNA_08928	0	0	0.021808645	0	0	0	0	0	0	0	0	0	-	NC_000013.11	+	95705548	95725252	19705	445	sense-overlappin	XM_011521105.1	DNAJC3
circRNA_08929	0	0	0.054521613	0	0	0	0	0	0	0	0	0	-	NC_000013.11	+	95709227	95725252	16026	311	sense-overlappin	XM_011521104.1	DNAJC3
circRNA_08930	0	0	0.08723458	0.1341352	0.12037869	0.093208092	0.080394741	0.061458886	0.020254528	0.060517448	0	0	hsa_circ_0002473	NC_000013.11	+	95723242	95725252	2011	200	sense-overlappin	XM_011521104.1	DNAJC3
circRNA_08931	0.061725074	0.122819805	0.26170374	0.4127238	0.09028402	0.15534682	0.060296056	0.061458886	0.050636319	0.14120738	0.10127615	0.144454801	-	NC_000013.11	+	95757644	95763953	6310	682	sense-overlappin	XM_011521104.1	DNAJC3
circRNA_08932	0	0.030704951	0.065425935	0	0.02006311	0.020712909	0	0.020486295	0.040509055	0.020172483	0	0	hsa_circ_0008274	NC_000013.11	-	95832927	95837202	4276	244	sense-overlappin	XM_011521097.1	UGGT2
circRNA_08933	0	0	0	0	0.05015779	0	0	0.020486295	0	0	0.02025523	0	-	NC_000013.11	-	95863629	95867423	3795	171	sense-overlappin	XM_011521097.1	UGGT2
circRNA_08934	0	0	0	0	0.03009467	0	0	0	0.020254528	0	0	0	-	NC_000013.11	-	95925680	95940091	14412	618	sense-overlappin	XM_011521097.1	UGGT2
circRNA_08935	0	0	0.021808645	0	0	0	0	0	0	0	0	0	-	NC_000013.11	-	95970112	95996135	26024	578	sense-overlappin	XM_011521097.1	UGGT2
circRNA_08936	0.041150049	0	0.04361729	0	0.16050492	0.062138728	0	0	0	0.020172483	0	0	-	NC_000013.11	-	95970112	95999307	29196	675	sense-overlappin	XM_011521097.1	UGGT2
circRNA_08937	0	0	0	0	0.02006311	0	0	0	0	0	0	0	-	NC_000013.11	-	95970112	96013481	43370	850	sense-overlappin	XM_011521097.1	UGGT2
circRNA_08938	0.030862537	0.092114854	0.250799418	0.0825448	0.3310414	0.072495183	0.040197371	0.040972591	0.030381792	0.080689931	0.08102092	0.123818401	hsa_circ_0030632	NC_000013.11	-	95983804	95999307	15504	432	sense-overlappin	XM_011521097.1	UGGT2
circRNA_08939	0	0	0.04361729	0.0412724	0.02006311	0	0	0	0.020254528	0	0	0	hsa_circ_0003836	NC_000013.11	-	95985239	95996135	10897	1368	sense-overlappin	XM_011521097.1	UGGT2
circRNA_08940	0	0	0.021808645	0	0	0	0	0	0	0	0	0.0206364	-	NC_000013.11	-	96013307	96031971	18665	502	sense-overlappin	XM_011521097.1	UGGT2
circRNA_08941	0	0	0	0	0	0	0	0	0	0	0	0.0412728	hsa_circ_0030638	NC_000013.11	+	97275632	97276409	778	778	exonic	XM_011521045.1	MBNL2
circRNA_08942	0	0	0	0	0.02006311	0	0	0	0	0	0	0	-	NC_000013.11	+	98153133	98213413	60281	60281	sense-overlappin	NM_001286839.1	FARP1
circRNA_08943	0	0	0	0	0	0.020712909	0	0	0	0	0	0	hsa_circ_0002557	NC_000013.11	+	98213220	98213413	194	194	exonic	NM_001286839.1	FARP1
circRNA_08944	0.020575025	0	0	0.0206362	0.03009467	0	0	0	0	0	0	0	hsa_circ_0030694	NC_000013.11	+	98409338	98424650	15313	491	sense-overlappin	NM_001286839.1	FARP1
circRNA_08945	0	0	0.021808645	0	0	0	0	0	0	0	0	0	hsa_circ_0030704	NC_000013.11	-	98457168	98466561	9394	662	sense-overlappin	NM_003576.4	STK24

circRNA_08946	0	0	0	0	0	0	0.020098685	0.020486295	0	0.020172483	0	0	hsa_circ_0030707	NC_000013.11	-	98474821	98482321	7501	324	sense-overlappin	NM_003576.4	STK24
circRNA_08947	0	0	0	0	0	0	0.030148028	0	0	0	0	0	hsa_circ_0005148	NC_000013.11	-	98474821	98519473	44653	555	sense-overlappin	NM_003576.4	STK24
circRNA_08948	0	0	0.021808645	0	0	0	0	0	0	0	0	0	hsa_circ_0003170	NC_000013.11	-	98504915	98519473	14559	14559	sense-overlappin	NM_003576.4	STK24
circRNA_08949	0	0	0	0	0	0	0	0	0	0.040344966	0	0	-	NC_000013.11	-	98519243	98519473	231	231	exonic	NM_003576.4	STK24
circRNA_08950	0	0	0	0	0	0.031069364	0	0	0	0	0	0	-	NC_000013.11	-	98797115	98800478	3364	431	sense-overlappin	NM_001130048.1	DOCK9
circRNA_08951	0.020575025	0	0	0	0	0	0	0	0	0	0	0	hsa_circ_0030711	NC_000013.11	-	98848592	98856031	7440	364	sense-overlappin	NM_001130048.1	DOCK9
circRNA_08952	0.041150049	0.061409903	0.13085187	0.0309543	0.08025246	0.103564547	0.020098685	0	0.070890847	0.040344966	0.02025523	0	hsa_circ_0030720	NC_000013.11	+	99238427	99244624	6198	358	sense-overlappin	NM_001144072.1	UBAC2
circRNA_08953	0	0	0	0	0	0.020712909	0	0	0	0	0	0	-	NC_000013.11	+	99539446	99543995	4550	434	sense-overlappin	NM_004800.2	TM9SF2
circRNA_08954	0	0	0	0	0	0	0	0	0	0	0	0.0309546	hsa_circ_0030741	NC_000013.11	+	99554304	99555647	1344	264	sense-overlappin	NM_004800.2	TM9SF2
circRNA_08955	0	0	0	0	0	0	0.020098685	0	0	0	0	0	-	NC_000013.11	+	100209332	100262831	53500	351	sense-overlappin	NM_000282.3	PCCA
circRNA_08956	0	0.020469968	0	0	0	0	0	0	0	0	0	0	-	NC_000013.11	+	100257595	100268783	11189	277	sense-overlappin	NM_000282.3	PCCA
circRNA_08957	0.082300098	0.08187987	0.065425935	0.1341352	0	0.082851637	0	0.051215739	0.050636319	0.090776173	0.02025523	0.0412728	hsa_circ_0000500	NC_000013.11	+	100257595	100273346	15752	428	sense-overlappin	NM_000282.3	PCCA
circRNA_08958	0	0.020469968	0	0.0412724	0	0	0	0	0	0	0	0	-	NC_000013.11	+	100257595	100309908	52314	792	sense-overlappin	NM_000282.3	PCCA
circRNA_08959	0	0	0.021808645	0.0515905	0	0.031069364	0	0	0	0	0	0	hsa_circ_0002664	NC_000013.11	+	100301460	100309908	8449	364	sense-overlappin	NM_000282.3	PCCA
circRNA_08960	0	0	0	0	0	0.031069364	0	0	0	0	0	0.0206364	hsa_circ_0030763	NC_000013.11	+	100301460	100340259	38800	578	sense-overlappin	NM_000282.3	PCCA
circRNA_08961	0.061725074	0	0.021808645	0.0928628	0	0.020712909	0	0.040972591	0	0.040344966	0	0	-	NC_000013.11	+	100301460	100368574	67115	681	sense-overlappin	NM_000282.3	PCCA
circRNA_08962	0	0	0	0.0206362	0	0	0	0	0	0	0	0	-	NC_000013.11	+	100305751	100368574	62824	1903	sense-overlappin	NM_000282.3	PCCA
circRNA_08963	0.061725074	0	0	0	0	0.031069364	0.020098685	0	0	0	0	0	hsa_circ_0030766	NC_000013.11	+	100307192	100340259	33068	359	sense-overlappin	NM_000282.3	PCCA
circRNA_08964	0	0	0	0.0619086	0	0	0	0.020486295	0	0	0	0	hsa_circ_0030767	NC_000013.11	+	100307192	100368574	61383	462	sense-overlappin	NM_000282.3	PCCA
circRNA_08965	0	0	0	0	0	0	0	0	0.020254528	0	0	0	hsa_circ_0008420	NC_000013.11	+	100330561	100340259	9699	214	sense-overlappin	NM_000282.3	PCCA
circRNA_08966	0	0	0	0.0309543	0	0	0	0	0	0	0	0	hsa_circ_0030771	NC_000013.11	+	100330561	100368574	38014	317	sense-overlappin	NM_000282.3	PCCA
circRNA_08967	0	0	0	0	0	0	0	0	0	0.050431207	0	0	hsa_circ_0007754	NC_000013.11	+	100368472	100449305	80834	256	sense-overlappin	NM_000282.3	PCCA
circRNA_08968	0	0	0	0	0	0	0	0	0	0	0.02025523	0	hsa_circ_0030777	NC_000013.11	+	100425633	100449305	23673	153	sense-overlappin	NM_000282.3	PCCA
circRNA_08969	0.020575025	0	0	0	0	0	0	0	0	0	0	0	-	NC_000013.11	-	100668579	100670569	1991	426	sense-overlappin	NM_032813.3	TMTC4
circRNA_08970	0	0	0	0.0206362	0	0	0	0	0	0	0	0	-	NC_000013.11	+	102618722	102647344	28623	2133	sense-overlappin	NM_003291.2	TPP2
circRNA_08971	0	0	0.032712968	0	0	0	0	0	0	0	0	0	-	NC_000013.11	+	102621293	102678288	56996	4851	sense-overlappin	XM_005254070.1	TPP2
circRNA_08972	0.051437562	0.040939935	0	0.0206362	0	0	0	0.020486295	0	0	0	0	hsa_circ_0008881	NC_000013.11	+	102622877	102627924	5048	396	sense-overlappin	NM_003291.2	TPP2
circRNA_08973	0	0	0.032712968	0	0	0.020712909	0	0	0	0	0	0	-	NC_000013.11	+	102807146	102834552	27407	1243	sense-overlappin	NM_017693.3	BIVM
circRNA_08974	0	0	0.021808645	0	0	0	0	0	0	0	0	0	hsa_circ_0006288	NC_000013.11	+	102816428	102822159	5732	423	sense-overlappin	NM_001159596.1	BIVM
circRNA_08975	0	0	0	0	0	0.020712909	0	0	0	0	0	0	hsa_circ_0000501	NC_000013.11	+	102852118	102875914	23797	3484	sense-overlappin	NM_001204425.1	BIVM-ERCC5
circRNA_08976	0	0	0.021808645	0	0	0	0.030148028	0	0	0	0	0	-	NC_000013.11	-	106492411	106512812	20402	1509	sense-overlappin	NM_004093.3	EFNB2
circRNA_08977	0	0	0	0	0	0	0	0.020486295	0	0	0	0	-	NC_000013.11	+	106505686	106505788	103	103	antisense	NM_004093.3	EFNB2
circRNA_08978	0	0.030704951	0	0	0	0	0	0	0	0	0	0	-	NC_000013.11	-	106512529	106512812	284	284	exonic	NM_004093.3	EFNB2
circRNA_08979	0	0	0	0	0.02006311	0	0	0	0	0	0	0	-	NC_000013.11	-	106557048	106559657	2610	368	sense-overlappin	XR_429269.2	
circRNA_08980	0.041150049	0.030704951	0	0	0	0	0	0	0	0	0	0	hsa_circ_0000502	NC_000013.11	+	108243532	108251316	7785	7785	intergenic		
circRNA_08981	0.030862537	0	0	0	0	0	0	0	0	0.040344966	0	0	hsa_circ_0030883	NC_000013.11	+	110622216	110624279	2064	197	sense-overlappin	NM_001242882.1	CARKD
circRNA_08982	0.030862537	0	0.021808645	0	0	0	0	0	0	0	0	0	hsa_circ_0030885	NC_000013.11	+	110622216	110627547	5332	395	sense-overlappin	NM_001242882.1	CARKD
circRNA_08983	0	0.020469968	0.021808645	0	0	0	0	0	0	0	0.030382845	0	hsa_circ_0030914	NC_000013.11	+	111205289	111233293	28005	519	sense-overlappin	XM_011521132.1	ARHGEF7
circRNA_08984	0	0	0.032712968	0	0.02006311	0	0	0	0	0	0	0	hsa_circ_0009141	NC_000013.11	+	111217679	111244294	26616	482	sense-overlappin	NM_001113511.1	ARHGEF7
circRNA_08985	0.082300098	0.030704951	0.185373483	0.0412724	0.05015779	0.051782273	0.040197371	0.071702034	0.091145375	0.030258724	0.04051046	0	hsa_circ_0000504	NC_000013.11	-	112516440	112527484	11045	751	sense-overlappin	XM_011537463.1	TUBGCP3
circRNA_08986	0	0.020469968	0	0	0	0	0	0	0	0	0	0	hsa_circ_0030982	NC_000013.11	-	113178174	113185560	7387	757	sense-overlappin	NM_001258213.1	PCID2
circRNA_08987	0.061725074	0	0	0	0	0	0	0	0	0	0	0	hsa_circ_0000506	NC_000013.11	-	113190872	113200516	9645	431	sense-overlappin	NM_001258213.1	PCID2
circRNA_08988	0	0	0.032712968	0	0	0	0	0	0	0	0	0	hsa_circ_0004408	NC_000013.11	+	113306485	113310867	4383	501	sense-overlappin	NM_005561.3	LAMP1
circRNA_08989	0	0	0.032712968	0	0	0	0	0	0	0	0	0	-	NC_000013.11	-	113344863	113355481	10619	381	sense-overlappin	NM_001286733.1	GRTP1
circRNA_08990	0	0	0.054521613	0	0	0	0	0	0	0	0	0	hsa_circ_0031017	NC_000013.11	+	113495502	113520733	25232	1423	sense-overlappin	NM_017905.4	TMCO3
circRNA_08991	0	0	0.021808645	0	0	0	0	0	0	0	0	0	-	NC_000013.11	+	113495502	113534240	38739	1619	sense-overlappin	NM_017905.4	TMCO3
circRNA_08992	0	0	0	0	0	0	0	0	0	0.020172483	0	0	hsa_circ_0008539	NC_000013.11	+	113510238	113520733	10496	305	sense-overlappin	NM_017905.4	TMCO3
circRNA_08993	0	0	0	0	0	0	0	0	0	0	0.02025523	0	-	NC_000013.11	-	114147992	114148962	971	971	intergenic		
circRNA_08994	0	0	0	0	0	0.031069364	0	0	0	0	0	0.0309546	hsa_circ_0031113	NC_000014.9	-	20395450	20395949	500	269	sense-overlappin	NM_007110.4	TEP1
circRNA_08995	0	0.020469968	0	0	0	0	0	0.030729443	0	0	0	0	hsa_circ_0005721	NC_000014.9	-	20403377	20406400	3024	699	sense-overlappin	NM_007110.4	TEP1
circRNA_08996	0.061725074	0.051174919	0.239895095	0.1031809	0.08025246	0.082851637	0.020098685	0.051215739	0.091145375	0.121034897	0.06076569	0	hsa_circ_0003643	NC_000014.9	-	21230319	21234229	3911	440	sense-overlappin	NM_001077442.1	HNRNPC
circRNA_08997	0	0	0.021808645	0	0	0	0	0	0	0	0	0	-	NC_000014.9	-	21357197	21358427	1231	359	sense-overlappin	NM_007192.3	SUPT16H
circRNA_08998	0	0	0	0.0309543	0	0	0	0	0	0	0	0	hsa_circ_0000522	NC_000014.9	-	21357197	21361213	4017	867	sense-overlappin	NM_007192.3	SUPT16H
circRNA_08999	0	0	0	0	0	0	0	0	0	0	0.050638075	0	hsa_circ_0031194	NC_000014.9	-	21415574	21429335	13762	1125	sense-overlappin	NM_001170629.1	CHD8
circRNA_09000	0	0	0	0	0	0	0	0	0	0.020172483	0	0	-	NC_000014.9	-	21432627	21432744	118	118	intronic	NM_020920.3	CHD8
circRNA_09001	0	0	0	0	0	0	0.020098685	0	0	0	0	0	-	NC_000014.9	-	21451489	21452150	662	662	intergenic		
circRNA_09002	0	0	0	0	0	0	0	0	0	0.020172483	0	0	hsa_circ_0005957	NC_000014.9	-	21500495	21501903	1409	581	intergenic	NM_019852.4	METTL3

circRNA_09003	0.123450148	0.153524756	0.228990773	0.1547714	0.06018934	0.144990365	0.110542769	0.081945182	0.13165443	0.221897311	0.070893305	0.092863801	hsa_circ_0000523	NC_000014.9	-	21503173	21503881	709	623	intergenic	NM_019852.4	METTL3
circRNA_09004	0	0	0.021808645	0	0	0	0	0	0	0.020172483	0	0	hsa_circ_0004137	NC_000014.9	-	22906195	22909595	3401	335	sense-overlappin	NM_001077351.1	RBM23
circRNA_09005	0.082300098	0.112584821	0.109043225	0.1857257	0.06018934	0.082851637	0.090444084	0.061458886	0.070890847	0.201724828	0.06076569	0.0619092	hsa_circ_0031241	NC_000014.9	-	22906195	22911403	5209	411	sense-overlappin	NM_001077351.1	RBM23
circRNA_09006	0.123450148	0.08187987	0.076330258	0.1031809	0.10031557	0.15534682	0.070345399	0.122917773	0.111399902	0.211811069	0.050638075	0.092863801	hsa_circ_0000524	NC_000014.9	-	22909483	22911403	1921	189	sense-overlappin	NM_001077351.1	RBM23
circRNA_09007	0	0.020469968	0.021808645	0	0.03009467	0	0	0	0	0	0	0	hsa_circ_0031250	NC_000014.9	-	22926133	22926814	682	327	sense-overlappin	NM_001039619.2	PRMT5
circRNA_09008	0.020575025	0.061409903	0.08723458	0.0206362	0.04012623	0.041425819	0	0.020486295	0.030381792	0	0.050638075	0.0206364	hsa_circ_0002928	NC_000014.9	-	22926133	22927660	1528	462	sense-overlappin	NM_001039619.2	PRMT5
circRNA_09009	0	0	0	0	0	0	0	0	0	0.020172483	0	0	-	NC_000014.9	-	22947171	22948033	863	366	sense-overlappin	NM_001166269.1	HAUS4
circRNA_09010	0	0	0	0	0	0	0	0	0	0.020172483	0	0	hsa_circ_0031258	NC_000014.9	-	22947171	22952683	5513	853	sense-overlappin	NM_001166269.1	HAUS4
circRNA_09011	0.020575025	0.020469968	0.021808645	0.0206362	0	0	0	0	0	0.040344966	0	0.051591	hsa_circ_0008758	NC_000014.9	-	23033368	23033674	307	307	exonic	XM_005267871.2	PSMB5
circRNA_09012	0.030862537	0	0	0	0	0	0	0	0	0	0	0	-	NC_000014.9	-	23081748	23090633	8886	321	sense-overlappin	XM_011536569.1	ACIN1
circRNA_09013	0.020575025	0	0	0	0	0	0	0	0	0	0	0	-	NC_000014.9	-	23526847	23527804	958	328	sense-overlappin	NM_033400.2	ZFHX2
circRNA_09014	0	0	0	0.0309543	0.02006311	0	0.020098685	0	0	0	0	0	-	NC_000014.9	+	24170834	24171379	546	546	intergenic		
circRNA_09015	0	0	0	0	0.02006311	0	0	0	0	0	0	0	-	NC_000014.9	-	24202383	24211829	9447	8132	sense-overlappin	NM_014169.3	CHMP4A
circRNA_09016	0	0	0	0	0	0	0	0.061458886	0.020254528	0	0	0	-	NC_000014.9	-	24209890	24211829	1940	625	sense-overlappin	NM_014169.3	CHMP4A
circRNA_09017	0	0	0	0.0309543	0.04012623	0.031069364	0.030148028	0	0	0.060517448	0	0.051591	hsa_circ_0031359	NC_000014.9	-	24211415	24211829	415	328	sense-overlappin	NM_014169.3	CHMP4A
circRNA_09018	0	0	0.021808645	0	0	0.031069364	0	0	0	0	0	0.0309546	hsa_circ_0007750	NC_000014.9	-	24266430	24268619	2190	655	sense-overlappin	XM_005267959.2	RABGGTA
circRNA_09019	0	0	0.032712968	0	0	0	0	0	0	0	0	0	-	NC_000014.9	-	24904768	24974850	70083	70083	sense-overlappin	XM_011536668.1	STXBP6
circRNA_09020	0	0	0.021808645	0	0	0	0	0	0	0	0	0	hsa_circ_0031408	NC_000014.9	-	24974665	24974850	186	186	exonic	XM_011536668.1	STXBP6
circRNA_09021	0	0	0.076330258	0	0	0	0	0	0	0	0	0	hsa_circ_0005040	NC_000014.9	-	24974665	24977243	2579	2579	sense-overlappin	XM_011536668.1	STXBP6
circRNA_09022	0	0	0.021808645	0	0	0	0	0	0	0	0	0	-	NC_000014.9	-	27256983	27259882	2900	2900	intergenic		
circRNA_09023	0	0	0.032712968	0	0	0	0	0	0	0	0	0	-	NC_000014.9	+	28405426	28411299	5874	5874	intergenic		
circRNA_09024	0	0	0	0	0	0	0	0	0	0.020172483	0	0	hsa_circ_0005185	NC_000014.9	-	29624152	29636494	12343	920	sense-overlappin	XM_011536965.1	PRKD1
circRNA_09025	0	0	0.032712968	0	0	0	0	0	0	0	0	0	-	NC_000014.9	-	30386012	30418812	32801	32801	intergenic		
circRNA_09026	0	0	0.021808645	0	0	0	0	0	0	0	0	0	-	NC_000014.9	-	30412171	30432992	20822	20822	intergenic		
circRNA_09027	0	0	0.021808645	0	0	0	0	0	0	0	0	0	-	NC_000014.9	+	30580864	30581116	253	253	sense-overlappin	XM_005267836.2	G2E3
circRNA_09028	0	0	0.032712968	0	0	0	0	0	0	0	0	0	-	NC_000014.9	+	30628209	30643405	15197	552	sense-overlappin	NM_016106.3	SCFD1
circRNA_09029	0.061725074	0	0	0	0	0	0	0	0	0	0	0	hsa_circ_0031419	NC_000014.9	+	30628209	30653588	25380	794	sense-overlappin	NM_016106.3	SCFD1
circRNA_09030	0	0	0	0	0	0	0	0	0.020254528	0	0	0	hsa_circ_0031420	NC_000014.9	+	30628209	30675065	46857	1181	sense-overlappin	NM_016106.3	SCFD1
circRNA_09031	0	0	0.021808645	0	0	0	0	0	0	0	0	0	-	NC_000014.9	+	30649528	30675065	25538	629	sense-overlappin	NM_001283031.1	SCFD1
circRNA_09032	0.061725074	0.030704951	0	0.0412724	0.06018934	0.031069364	0	0.030729443	0.030381792	0.040344966	0.02025523	0.0412728	hsa_circ_0009140	NC_000014.9	+	30670256	30675065	4810	387	sense-overlappin	NM_001283031.1	SCFD1
circRNA_09033	0.020575025	0.020469968	0.04361729	0.0619086	0.05015779	0.062138728	0.020098685	0	0.050636319	0.050431207	0	0	hsa_circ_0000528	NC_000014.9	+	30715924	30734858	18935	276	sense-overlappin	NM_001283031.1	SCFD1
circRNA_09034	0.030862537	0	0.032712968	0.0515905	0	0	0.020098685	0	0	0	0.02025523	0	hsa_circ_0031433	NC_000014.9	-	30902536	30911162	8627	539	sense-overlappin	XM_011536681.1	STRN3
circRNA_09035	0.020575025	0.030704951	0.098138903	0.0515905	0	0.041425819	0	0.020486295	0.030381792	0.121034897	0	0.0206364	hsa_circ_0031446	NC_000014.9	-	30935163	30956242	21080	706	sense-overlappin	XM_011536681.1	STRN3
circRNA_09036	0	0	0.021808645	0.0412724	0.03009467	0	0.020098685	0	0	0	0	0	hsa_circ_0031447	NC_000014.9	-	30947090	30956242	9153	434	sense-overlappin	XM_011536681.1	STRN3
circRNA_09037	0	0	0.032712968	0	0	0	0	0	0	0	0	0	-	NC_000014.9	-	30950863	30956242	5380	260	sense-overlappin	XM_011536681.1	STRN3
circRNA_09038	0	0	0	0	0	0.020712909	0	0	0.030381792	0	0	0	hsa_circ_0007656	NC_000014.9	-	30955620	30956242	623	178	sense-overlappin	XM_011536681.1	STRN3
circRNA_09039	0	0	0	0	0	0.041425819	0	0	0	0.040344966	0	0	hsa_circ_0004829	NC_000014.9	-	31112416	31114340	1925	660	sense-overlappin	NM_015382.3	HECTD1
circRNA_09040	0	0	0.032712968	0	0.02006311	0	0	0	0	0	0	0	hsa_circ_0031460	NC_000014.9	-	31113104	31133675	20572	2572	sense-overlappin	NM_015382.3	HECTD1
circRNA_09041	0	0	0	0.0206362	0	0	0	0	0	0	0	0	-	NC_000014.9	-	31113104	31172122	59019	4994	sense-overlappin	NM_015382.3	HECTD1
circRNA_09042	0	0.030704951	0.032712968	0.0206362	0	0	0	0	0	0	0	0	hsa_circ_0031465	NC_000014.9	-	31116236	31121505	5270	503	sense-overlappin	NM_015382.3	HECTD1
circRNA_09043	0	0.030704951	0	0	0	0	0	0	0	0	0	0	-	NC_000014.9	-	31122914	31133675	10762	1537	sense-overlappin	NM_015382.3	HECTD1
circRNA_09044	0.020575025	0.030704951	0.08723458	0.0412724	0.05015779	0	0.030148028	0.040972591	0.070890847	0.060517448	0.030382845	0	hsa_circ_0002301	NC_000014.9	-	31127785	31133675	5891	1403	sense-overlappin	NM_015382.3	HECTD1
circRNA_09045	0	0	0.021808645	0	0	0	0	0	0	0	0	0	-	NC_000014.9	-	31127785	31169468	41684	3649	sense-overlappin	NM_015382.3	HECTD1
circRNA_09046	0	0.030704951	0	0.0619086	0	0.031069364	0	0	0.040509055	0	0	0	hsa_circ_0031482	NC_000014.9	-	31127785	31172122	44338	3825	sense-overlappin	NM_015382.3	HECTD1
circRNA_09047	0	0.020469968	0	0.0825448	0	0.103564547	0.030148028	0.040972591	0	0.050431207	0.02025523	0	hsa_circ_0031485	NC_000014.9	-	31133238	31133675	438	344	sense-overlappin	NM_015382.3	HECTD1
circRNA_09048	0	0	0	0	0	0	0	0	0	0	0	0.0309546	hsa_circ_0031489	NC_000014.9	-	31133238	31172122	38885	2766	sense-overlappin	NM_015382.3	HECTD1
circRNA_09049	0	0	0	0	0	0.020712909	0	0	0	0	0	0	-	NC_000014.9	-	31141858	31172122	30265	1717	sense-overlappin	NM_015382.3	HECTD1
circRNA_09050	0	0	0	0	0	0.031069364	0.030148028	0	0	0	0	0	-	NC_000014.9	-	31144111	31172122	28012	1622	sense-overlappin	NM_015382.3	HECTD1
circRNA_09051	0	0	0	0	0	0	0	0	0	0.030258724	0	0	-	NC_000014.9	-	31157964	31158170	207	207	intronic	NM_015382.3	HECTD1
circRNA_09052	0	0	0	0	0.02006311	0	0.020098685	0	0	0	0	0	hsa_circ_0031554	NC_000014.9	-	31343896	31374968	31073	1520	sense-overlappin	NM_015473.3	HEATR5A
circRNA_09053	0	0	0	0	0	0.020712909	0	0	0	0	0	0	hsa_circ_0031557	NC_000014.9	-	31345087	31374968	29882	1350	sense-overlappin	NM_015473.3	HEATR5A
circRNA_09054	0	0	0	0	0	0	0	0	0	0.020172483	0	0	hsa_circ_0031564	NC_000014.9	-	31349789	31387375	37587	1775	sense-overlappin	NM_015473.3	HEATR5A
circRNA_09055	0	0	0	0.0412724	0	0	0	0	0	0	0	0	hsa_circ_0031569	NC_000014.9	-	31371810	31374968	3159	253	sense-overlappin	NM_015473.3	HEATR5A
circRNA_09056	0	0	0	0	0	0	0	0	0	0.020172483	0	0	hsa_circ_0031571	NC_000014.9	-	31380467	31387375	6909	775	sense-overlappin	NM_015473.3	HEATR5A
circRNA_09057	0.349775418	0.266109578	1.755595924	0.5159047	0.19059959	0.331406549	0.110542769	0.194619807	0.13165443	0.312673483	0.182297071	0.227000402	hsa_circ_0031583	NC_000014.9	+	32090502	32094386	3885	3885	exonic	XM_005267636.2	ARHGAP5

circRNA_09058	0	0	0.021808645	0	0	0	0	0	0	0	0	0	-	NC_000014.9	+	32090502	32094402	3901	3901	sense-overlappin	XM_005267636.2	ARHGAP5
circRNA_09059	0.030862537	0.051174919	0.381651288	0.1754076	0.11034713	0.103564547	0.020098685	0.051215739	0.091145375	0.121034897	0.030382845	0.072227401	hsa_circ_0031584	NC_000014.9	+	32090502	32117287	26786	4033	sense-overlappin	XM_005267636.2	ARHGAP5
circRNA_09060	0	0	0	0.0206362	0	0.041425819	0	0.020486295	0	0	0	0	hsa_circ_0005236	NC_000014.9	+	32117140	32152528	35389	464	sense-overlappin	XM_005267636.2	ARHGAP5
circRNA_09061	0	0.020469968	0	0	0	0	0	0	0	0	0	0	hsa_circ_0031586	NC_000014.9	+	32146263	32152528	6266	316	sense-overlappin	XM_005267636.2	ARHGAP5
circRNA_09062	0.041150049	0	0	0	0	0	0	0	0	0	0	0	-	NC_000014.9	+	32535554	32600792	65239	2406	sense-overlappin	NM_004274.4	AKAP6
circRNA_09063	0	0.020469968	0	0.0309543	0	0	0	0	0	0.050431207	0	0.0619092	-	NC_000014.9	+	33215182	33215587	406	296	sense-overlappin	XM_005267993.2	NPAS3
circRNA_09064	0	0	0	0	0.03009467	0	0	0	0	0	0	0	-	NC_000014.9	-	33925618	33927033	1416	379	sense-overlappin	NM_022073.3	EGLN3
circRNA_09065	0	0.020469968	0	0	0.02006311	0	0	0	0	0	0	0.0412728	-	NC_000014.9	-	33925618	33931215	5598	636	sense-overlappin	NM_022073.3	EGLN3
circRNA_09066	0.030862537	0	0	0	0	0	0	0	0	0	0	0	-	NC_000014.9	-	34533444	34536275	2832	278	sense-overlappin	NM_018453.3	EAPP
circRNA_09067	0.102875123	0.051174919	0.13085187	0.0309543	0.06018934	0.062138728	0	0	0.091145375	0.050431207	0	0	hsa_circ_0007379	NC_000014.9	-	34551714	34554912	3199	3199	intergenic		
circRNA_09068	0	0	0	0.0206362	0	0.041425819	0.040197371	0	0.040509055	0.020172483	0	0.0206364	-	NC_000014.9	-	34567686	34568013	328	246	sense-overlappin	XM_006720224.1	SNX6
circRNA_09069	0	0	0.163564838	0	0	0.041425819	0	0	0	0.020172483	0	0	hsa_circ_0031607	NC_000014.9	-	34575756	34609742	33987	867	sense-overlappin	NM_021249.3	SNX6
circRNA_09070	0	0	0.032712968	0	0	0	0	0	0.020254528	0	0	0	hsa_circ_0031608	NC_000014.9	-	34586230	34609742	23513	740	sense-overlappin	NM_021249.3	SNX6
circRNA_09071	0	0	0.021808645	0	0	0	0	0	0	0	0	0	-	NC_000014.9	-	34593045	34609742	16698	664	sense-overlappin	NM_021249.3	SNX6
circRNA_09072	0.020575025	0	0	0	0	0	0	0	0	0	0	0	-	NC_000014.9	-	34764707	34764933	227	227	exonic	XM_011536376.1	BAZ1A
circRNA_09073	0	0	0	0	0	0	0	0.020486295	0	0	0	0.0206364	hsa_circ_0006431	NC_000014.9	-	34783762	34792921	9160	634	sense-overlappin	XM_011536376.1	BAZ1A
circRNA_09074	0	0.030704951	0	0	0	0	0	0	0.030381792	0	0	0.0412728	hsa_circ_0031632	NC_000014.9	-	34792775	34862322	69548	1397	sense-overlappin	XM_011536375.1	BAZ1A
circRNA_09075	0	0	0.021808645	0	0.02006311	0.062138728	0.030148028	0	0.020254528	0	0	0	-	NC_000014.9	-	34800224	34802988	2765	402	sense-overlappin	XM_011536375.1	BAZ1A
circRNA_09076	0	0	0	0	0	0	0.020098685	0	0	0	0	0	-	NC_000014.9	-	34813869	34814731	863	863	intronic	XM_011536375.1	BAZ1A
circRNA_09077	0.030862537	0.030704951	0.08723458	0	0.02006311	0.113921001	0.050246713	0.051215739	0.060763583	0.020172483	0.06076569	0.0206364	hsa_circ_0006137	NC_000014.9	-	34862044	34862322	279	279	exonic	XM_011536375.1	BAZ1A
circRNA_09078	0	0	0.021808645	0	0	0	0.020098685	0.020486295	0	0	0	0	hsa_circ_0008568	NC_000014.9	-	34969170	34972064	2895	2895	intergenic		
circRNA_09079	0	0	0.04361729	0	0	0.020712909	0	0	0	0	0	0	hsa_circ_0031644	NC_000014.9	+	35007283	35008831	1549	230	sense-overlappin	XM_005268024.1	SRP54
circRNA_09080	0	0.040939935	0	0	0	0	0	0	0	0	0	0	-	NC_000014.9	+	35007283	35011659	4377	381	sense-overlappin	XM_005268024.1	SRP54
circRNA_09081	0	0	0	0	0	0	0	0	0	0	0.030382845	0	-	NC_000014.9	+	35013346	35019074	5729	520	sense-overlappin	XM_005268024.1	SRP54
circRNA_09082	0	0	0	0.0206362	0	0	0	0	0	0	0	0	hsa_circ_0006424	NC_000014.9	+	35050784	35053451	2668	2668	sense-overlappin	NM_001079519.1	FAM177A1
circRNA_09083	0	0	0	0	0	0	0	0	0.020254528	0	0.02025523	0	hsa_circ_0031657	NC_000014.9	-	35096558	35099384	2827	265	sense-overlappin	XM_011536885.1	PPP2R3C
circRNA_09084	0	0	0.021808645	0	0	0	0	0	0	0	0	0	-	NC_000014.9	-	35107304	35110629	3326	387	sense-overlappin	XM_011536885.1	PPP2R3C
circRNA_09085	0	0	0	0.0309543	0	0	0	0	0.020254528	0	0	0	-	NC_000014.9	+	35126735	35180777	54043	289	sense-overlappin	XM_011537411.1	KIAA0391
circRNA_09086	0	0	0.032712968	0	0	0.082851637	0	0	0	0	0	0	hsa_circ_0031677	NC_000014.9	-	35664642	35725153	60512	2113	sense-overlappin	NM_001283043.1	RALGAPA1
circRNA_09087	0	0	0.04361729	0	0	0	0	0	0	0	0	0	-	NC_000014.9	+	35831410	35834923	3514	299	sense-overlappin	XM_005268128.1	BRMS1L
circRNA_09088	0	0	0	0.0412724	0	0	0	0	0	0	0	0	-	NC_000014.9	-	36311573	36316812	5240	661	sense-overlappin	XM_005267755.2	MBIP
circRNA_09089	0	0.020469968	0	0.0412724	0	0	0	0.030729443	0	0	0	0	hsa_circ_0004455	NC_000014.9	+	37285318	37308519	23202	335	sense-overlappin	NM_001195296.1	MIPOL1
circRNA_09090	0	0	0.021808645	0	0	0	0	0	0	0.020172483	0	0	hsa_circ_0004204	NC_000014.9	+	37285318	37369624	84307	443	sense-overlappin	NM_001195296.1	MIPOL1
circRNA_09091	0	0	0	0.0206362	0	0	0	0	0	0	0	0	-	NC_000014.9	+	37680151	37701526	21376	632	sense-overlappin	XM_011537432.1	TTC6
circRNA_09092	0	0.020469968	0	0.0619086	0	0	0	0	0	0	0	0	-	NC_000014.9	+	37748939	37753235	4297	903	sense-overlappin	XM_011537432.1	TTC6
circRNA_09093	0	0	0	0.0206362	0	0	0	0	0	0	0	0	-	NC_000014.9	+	37748939	37796947	48009	1666	sense-overlappin	XR_943762.1	
circRNA_09094	0	0	0	0.0928628	0	0	0	0	0	0	0	0	-	NC_000014.9	+	37787468	37796947	9480	763	sense-overlappin	XR_943762.1	
circRNA_09095	0	0	0	0.0206362	0	0	0	0	0	0	0	0	-	NC_000014.9	+	37807320	37874563	67244	34324	sense-overlappin	XR_943762.1	
circRNA_09096	0	0	0	0.0206362	0	0	0	0	0	0	0	0	-	NC_000014.9	+	37874460	37968588	94129	94129	intergenic		
circRNA_09097	0	0	0	0	0	0	0	0.030729443	0	0	0	0	-	NC_000014.9	-	38711077	38749654	38578	38578	intergenic		
circRNA_09098	0	0	0	0	0.02006311	0	0	0	0	0	0	0	hsa_circ_0031709	NC_000014.9	-	39045163	39048729	3567	312	sense-overlappin	XM_005267262.1	SEC23A
circRNA_09099	0	0	0	0	0	0.031069364	0	0	0	0	0	0	hsa_circ_0003374	NC_000014.9	-	39048652	39055296	6645	232	sense-overlappin	NM_006364.2	SEC23A
circRNA_09100	0	0	0.032712968	0.0515905	0	0	0	0	0	0.020172483	0	0	hsa_circ_0031738	NC_000014.9	-	39151746	39159550	7805	364	sense-overlappin	NM_001079537.1	TRAPPC6B
circRNA_09101	0	0	0.032712968	0	0	0	0	0	0.020254528	0.040344966	0	0	-	NC_000014.9	-	39154211	39159550	5340	270	sense-overlappin	NM_001079537.1	TRAPPC6B
circRNA_09102	0	0	0	0.0309543	0	0	0	0	0	0.030258724	0	0	hsa_circ_0002395	NC_000014.9	-	39158285	39159550	1266	186	sense-overlappin	NM_001079537.1	TRAPPC6B
circRNA_09103	0.030862537	0.071644886	0.185373483	0.1031809	0.16050492	0.103564547	0.070345399	0.112674625	0.070890847	0.191638587	0.111403765	0.051591	-	NC_000014.9	+	39179091	39179462	372	295	sense-overlappin	NM_002687.3	PNN
circRNA_09104	0.051437562	0.061409903	0.207182128	0.0619086	0.13041025	0.031069364	0.070345399	0	0.050636319	0.100862414	0.030382845	0.0412728	hsa_circ_0000530	NC_000014.9	+	39276934	39279537	2604	243	sense-overlappin	NM_203354.2	CTAGE5
circRNA_09105	0	0	0.021808645	0	0.03009467	0	0	0	0	0	0	0	hsa_circ_0002835	NC_000014.9	+	39276934	39295029	18096	636	sense-overlappin	XM_011536778.1	CTAGE5
circRNA_09106	0	0.020469968	0.076330258	0.0619086	0.0702209	0	0	0	0.020254528	0	0.050638075	0	hsa_circ_0031741	NC_000014.9	+	39276934	39327022	50089	1795	sense-overlappin	XM_011536778.1	CTAGE5
circRNA_09107	0	0.030704951	0.316225353	0.0309543	0.09028402	0	0	0	0	0	0	0.0412728	hsa_circ_0031742	NC_000014.9	+	39276934	39348977	72044	2212	sense-overlappin	XM_011536778.1	CTAGE5
circRNA_09108	0	0	0	0	0	0	0	0	0	0	0	0.0309546	-	NC_000014.9	+	39291019	39308587	17569	914	sense-overlappin	XM_011536778.1	CTAGE5
circRNA_09109	0.041150049	0	0.04361729	0.0309543	0	0	0	0	0	0	0	0	hsa_circ_0031749	NC_000014.9	+	39313340	39327022	13683	638	sense-overlappin	NM_203354.2	CTAGE5
circRNA_09110	0	0	0	0	0	0.020712909	0	0	0	0	0	0	-	NC_000014.9	-	39334398	39334648	251	251	antisense	NM_203354.2	CTAGE5
circRNA_09111	0	0	0	0	0.06018934	0.031069364	0	0	0	0	0	0	-	NC_000014.9	+	39642713	39655808	13096	13096	intergenic		
circRNA_09112	0	0	0	0.0206362	0	0	0	0	0	0	0	0	-	NC_000014.9	+	39654845	39655808	964	964	intergenic		
circRNA_09113	0	0	0	0.0206362	0	0.020712909	0	0	0	0	0	0	-	NC_000014.9	+	44995746	45012075	16330	1192	sense-overlappin	NM_015091.2	FAM179B
circRNA_09114	0	0	0	0	0.02006311	0	0	0	0	0	0	0	-	NC_000014.9	+	44999363	45012075	12713	1035	sense-overlappin	NM_015091.2	FAM179B
circRNA_09115	0	0	0	0	0	0	0	0	0.030381792	0	0	0	hsa_circ_0031755	NC_000014.9	+	45006008	45025872	19865	748	sense-overlappin	XR_943403.1	

circRNA_09116	0	0.020469968	0	0	0	0	0	0	0	0	0	0	-	NC_000014.9	+	45008913	45027474	18562	664	sense-overlappin	XR_943403.1	
circRNA_09117	0	0	0	0	0	0	0	0	0	0.030258724	0	0	hsa_circ_0031757	NC_000014.9	+	45008913	45032376	23464	908	sense-overlappin	NM_015091.2	FAM179B
circRNA_09118	0	0	0.021808645	0	0	0	0	0	0	0	0	0	-	NC_000014.9	+	45008913	45046703	37791	1409	sense-overlappin	XM_011536572.1	FAM179B
circRNA_09119	0	0	0	0	0	0	0	0.020486295	0	0	0	0	hsa_circ_0031760	NC_000014.9	+	45011975	45054549	42575	1422	sense-overlappin	XM_005267451.1	FAM179B
circRNA_09120	0	0	0	0	0	0	0	0	0	0	0.02025523	0	-	NC_000014.9	+	45027299	45028329	1031	330	sense-overlappin	NM_015091.2	FAM179B
circRNA_09121	0.030862537	0.020469968	0.08723458	0.0309543	0.06018934	0.113921001	0.020098685	0.122917773	0.081018111	0.221897311	0.030382845	0.0619092	-	NC_000014.9	-	45118028	45130790	12763	502	sense-overlappin	NM_002013.3	FKBP3
circRNA_09122	0.020575025	0	0	0	0	0	0	0	0	0	0	0	-	NC_000014.9	+	45148837	45170746	21910	1401	sense-overlappin	NM_020937.2	FANCM
circRNA_09123	0	0	0	0	0	0.020712909	0	0	0	0	0	0	-	NC_000014.9	+	45175071	45176976	1906	1906	exonic	NM_020937.2	FANCM
circRNA_09124	0	0	0.021808645	0	0	0	0	0	0	0	0	0	-	NC_000014.9	-	45231141	45247377	16237	1685	sense-overlappin	XM_005267833.3	MIS18BP1
circRNA_09125	0	0	0	0.0515905	0.02006311	0	0	0.020486295	0	0	0	0.0309546	-	NC_000014.9	-	45246743	45247377	635	635	exonic	XM_005267833.3	MIS18BP1
circRNA_09126	0	0	0	0	0	0	0	0	0	0.050431207	0	0	-	NC_000014.9	+	49602370	49608121	5752	888	sense-overlappin	NR_037792.1	
circRNA_09127	0	0	0	0	0	0	0.020098685	0	0	0	0	0	hsa_circ_0008786	NC_000014.9	-	49653990	49655811	1822	424	sense-overlappin	NM_001197330.1	POLE2
circRNA_09128	0	0	0	0	0	0	0.030148028	0	0.020254528	0	0.02025523	0	hsa_circ_0008002	NC_000014.9	-	49663315	49674427	11113	510	sense-overlappin	NM_002692.3	POLE2
circRNA_09129	0	0	0	0	0	0.020712909	0	0	0	0	0	0	-	NC_000014.9	-	49664626	49665163	538	106	sense-overlappin	NM_001197330.1	POLE2
circRNA_09130	0	0	0	0	0	0	0.020098685	0	0.020254528	0	0	0	hsa_circ_0004904	NC_000014.9	-	49669524	49674427	4904	247	sense-overlappin	NM_002692.3	POLE2
circRNA_09131	0	0	0	0	0	0	0	0	0	0	0	0.0206364	-	NC_000014.9	+	49709159	49723952	14794	387	sense-overlappin	NM_172193.2	KLHDC1
circRNA_09132	0	0.040939935	0	0.0722267	0	0.020712909	0	0	0.020254528	0	0	0	-	NC_000014.9	+	49709159	49743805	34647	938	sense-overlappin	NM_172193.2	KLHDC1
circRNA_09133	0	0.020469968	0	0	0	0	0	0	0	0	0	0	hsa_circ_0007809	NC_000014.9	+	49723874	49725769	1896	163	sense-overlappin	NM_172193.2	KLHDC1
circRNA_09134	0	0	0	0.0206362	0	0	0	0.020486295	0	0.050431207	0	0	-	NC_000014.9	-	49795791	49800696	4906	524	sense-overlappin	XR_943556.1	
circRNA_09135	0	0	0	0	0	0	0	0	0	0	0	0.051591	-	NC_000014.9	-	49795791	49831361	35571	1737	sense-overlappin	XR_943556.1	
circRNA_09136	0	0	0	0	0	0	0.050246713	0	0	0	0	0	-	NC_000014.9	-	49799475	49806133	6659	721	sense-overlappin	XR_943556.1	
circRNA_09137	0	0	0	0.0206362	0.05015779	0	0.020098685	0.020486295	0	0	0	0	-	NC_000014.9	-	49799475	49831361	31887	1583	sense-overlappin	XR_943556.1	
circRNA_09138	0	0	0	0	0	0	0.020098685	0	0	0	0	0	-	NC_000014.9	-	49802453	49803294	842	238	sense-overlappin	XR_943556.1	
circRNA_09139	0	0	0	0.0206362	0	0	0	0	0	0	0	0	-	NC_000014.9	-	49816789	49818838	2050	2050	sense-overlappin	XR_943556.1	
circRNA_09140	0.030862537	0.051174919	0.054521613	0.1650895	0.04012623	0	0	0.030729443	0.060763583	0.060517448	0.10127615	0.0206364	-	NC_000014.9	-	49825867	49831361	5495	695	sense-overlappin	XR_943556.1	
circRNA_09141	0	0	0	0	0	0.031069364	0	0	0	0	0	0	-	NC_000014.9	-	49825867	49833496	7630	916	sense-overlappin	XR_943556.1	
circRNA_09142	0.020575025	0	0	0	0	0	0	0	0	0	0	0	-	NC_000014.9	-	49825867	49834449	8583	1003	sense-overlappin	XR_943556.1	
circRNA_09143	0	0.030704951	0	0	0	0	0	0	0	0	0	0	hsa_circ_0031814	NC_000014.9	-	49832051	49834449	2399	308	sense-overlappin	XR_943556.1	
circRNA_09144	0	0	0.04361729	0	0	0	0	0	0	0	0	0	-	NC_000014.9	-	49834363	49840866	6504	304	sense-overlappin	XR_943556.1	
circRNA_09145	0	0	0	0	0	0	0	0	0	0	0	0.072227401	-	NC_000014.9	+	49862555	49862850	296	296	intergenic		
circRNA_09146	0	0.020469968	0	0.0515905	0	0.062138728	0	0.030729443	0	0	0.02025523	0	-	NC_000014.9	-	50148285	50150230	1946	1946	sense-overlappin	XM_005268021.1	SOS2
circRNA_09147	0	0	0	0	0	0	0	0	0.020254528	0	0	0	-	NC_000014.9	-	50148285	50153173	4889	2050	sense-overlappin	XM_005268021.1	SOS2
circRNA_09148	0.041150049	0.020469968	0.021808645	0.0412724	0.06018934	0.031069364	0	0.112674625	0.030381792	0.050431207	0.030382845	0.0619092	hsa_circ_0007695	NC_000014.9	-	50150008	50150230	223	223	exonic	XM_005268021.1	SOS2
circRNA_09149	0	0	0	0.0412724	0.03009467	0.020712909	0	0	0	0	0	0	-	NC_000014.9	-	50150008	50153173	3166	327	sense-overlappin	XM_005268021.1	SOS2
circRNA_09150	0	0	0	0.0206362	0	0	0	0	0	0	0	0	hsa_circ_0031831	NC_000014.9	-	50180572	50204409	23838	882	sense-overlappin	XM_005268021.1	SOS2
circRNA_09151	0	0	0	0	0	0.020712909	0	0	0	0	0	0	hsa_circ_0031841	NC_000014.9	-	50265358	50284033	18676	656	sense-overlappin	NM_024884.2	L2HGDH
circRNA_09152	0	0	0.032712968	0	0	0	0	0	0	0	0	0	hsa_circ_0031842	NC_000014.9	-	50265358	50294246	28889	788	sense-overlappin	NM_024884.2	L2HGDH
circRNA_09153	0	0	0	0.0206362	0	0	0	0	0	0	0	0	-	NC_000014.9	-	50283871	50303017	19147	563	sense-overlappin	NM_024884.2	L2HGDH
circRNA_09154	0	0	0	0	0	0	0	0	0	0.030258724	0	0	-	NC_000014.9	-	50329833	50332432	2600	520	sense-overlappin	XM_005268160.3	CDKL1
circRNA_09155	0	0	0	0	0	0	0	0	0	0.030258724	0	0	-	NC_000014.9	-	50442732	50448832	6101	549	sense-overlappin	XM_011536380.1	MAP4K5
circRNA_09156	0	0	0	0	0	0.031069364	0	0	0	0	0	0	hsa_circ_0031857	NC_000014.9	-	50456516	50464133	7618	278	sense-overlappin	XM_011536379.1	MAP4K5
circRNA_09157	0	0.020469968	0	0	0	0	0	0	0	0	0	0	-	NC_000014.9	-	50466583	50476306	9724	359	sense-overlappin	XM_011536379.1	MAP4K5
circRNA_09158	0.020575025	0	0	0	0	0	0	0	0	0	0	0	-	NC_000014.9	-	50466583	50482416	15834	415	sense-overlappin	XM_011536379.1	MAP4K5
circRNA_09159	0	0	0	0	0	0	0	0	0	0	0	0.0309546	hsa_circ_0031863	NC_000014.9	-	50475077	50482416	7340	220	sense-overlappin	XM_011536379.1	MAP4K5
circRNA_09160	0.041150049	0	0	0.0412724	0	0.020712909	0	0	0	0.020172483	0	0	hsa_circ_0007101	NC_000014.9	-	50640750	50645014	4265	415	sense-overlappin	NM_021818.3	SAV1
circRNA_09161	0	0	0	0	0	0	0.020098685	0	0	0	0	0	hsa_circ_0031897	NC_000014.9	+	51243856	51247220	3365	291	sense-overlappin	NM_030755.4	TMX1
circRNA_09162	0.030862537	0	0	0	0	0	0	0	0	0	0	0	hsa_circ_0031933	NC_000014.9	-	52053579	52060356	6778	895	sense-overlappin	NM_007361.3	NID2
circRNA_09163	0	0.040939935	0.021808645	0	0	0	0	0	0	0	0	0	-	NC_000014.9	-	52470037	52491005	20969	862	sense-overlappin	NM_001160047.1	TXNDC16
circRNA_09164	0	0.030704951	0.021808645	0.0722267	0	0	0	0.020486295	0.040509055	0.050431207	0.030382845	0	hsa_circ_0031939	NC_000014.9	-	52511240	52544371	33132	922	sense-overlappin	NM_001160047.1	TXNDC16
circRNA_09165	0.020575025	0.051174919	0.032712968	0.0309543	0.02006311	0	0	0	0.050636319	0	0	0	hsa_circ_0031940	NC_000014.9	-	52536719	52544371	7653	573	sense-overlappin	NM_001160047.1	TXNDC16
circRNA_09166	0	0	0.021808645	0	0	0	0	0	0	0	0	0	-	NC_000014.9	-	52652239	52666495	14257	617	sense-overlappin	NM_014584.2	ERO1L
circRNA_09167	0	0	0.021808645	0	0	0	0	0	0	0	0	0	-	NC_000014.9	+	52744852	52759754	14903	447	sense-overlappin	NM_001130701.1	STYX
circRNA_09168	0	0	0	0	0	0.031069364	0	0	0	0.030258724	0	0	hsa_circ_0031959	NC_000014.9	+	52756551	52759754	3204	262	sense-overlappin	NM_001130701.1	STYX
circRNA_09169	0	0	0	0	0	0	0	0	0	0	0	0.0309546	hsa_circ_0031972	NC_000014.9	-	53058477	53061201	2725	226	sense-overlappin	NM_001160147.1	DDHD1
circRNA_09170	0	0	0	0	0.02006311	0	0	0	0	0	0.02025523	0	hsa_circ_0031977	NC_000014.9	-	53091785	53103856	12072	574	sense-overlappin	XM_011537188.1	DDHD1

circRNA_09171	0	0	0	0	0	0.020712909	0	0	0	0	0	0	hsa_circ_0006378	NC_000014.9	+	53156243	53189936	33694	33694	intergenic		
circRNA_09172	0.030862537	0	0.021808645	0.0309543	0	0.051782273	0	0	0.030381792	0.040344966	0.050638075	0	hsa_circ_0004846	NC_000014.9	+	54702062	54702580	519	519	exonic	NM_001161576.2	SAMD4A
circRNA_09173	0	0	0	0	0.04012623	0	0.020098685	0	0	0	0	0	hsa_circ_0000539	NC_000014.9	-	55181213	55183753	2541	342	sense-overlappin	NM_001146015.1	DLGAP5
circRNA_09174	0.020575025	0	0	0	0	0	0	0	0	0	0	0	-	NC_000014.9	-	55283660	55284295	636	636	antisense	NM_017943.3	FBXO34
circRNA_09175	0	0	0	0	0	0	0	0	0	0	0	0.0206364	-	NC_000014.9	+	55350381	55355222	4842	4842	sense-overlappin	NM_152231.1	FBXO34
circRNA_09176	0	0	0.109043225	0	0.11034713	0.031069364	0.040197371	0	0	0	0	0.0206364	hsa_circ_0032029	NC_000014.9	+	55612019	55619312	7294	993	sense-overlappin	NM_001079521.1	KTN1
circRNA_09177	0	0.040939935	0	0	0	0	0	0	0	0	0	0	hsa_circ_0004110	NC_000014.9	+	55648025	55648870	846	160	sense-overlappin	NM_001079521.1	KTN1
circRNA_09178	0	0	0.021808645	0	0	0	0	0	0	0.020172483	0	0	-	NC_000014.9	+	55653017	55658614	5598	267	sense-overlappin	NM_001079521.1	KTN1
circRNA_09179	0	0	0	0	0	0	0	0.030729443	0	0	0	0	-	NC_000014.9	+	56179614	56179748	135	135	intronic	XM_005267890.3	PELI2
circRNA_09180	0	0	0	0.0412724	0	0	0	0	0	0	0	0	hsa_circ_0000541	NC_000014.9	+	56279676	56290456	10781	489	sense-overlappin	XM_005267890.3	PELI2
circRNA_09181	0	0	0	0.0515905	0	0	0	0	0	0	0	0	-	NC_000014.9	+	56321486	56330487	9002	9002	intergenic		
circRNA_09182	0	0	0	0.0206362	0	0	0	0	0.020254528	0.020172483	0	0	hsa_circ_0032047	NC_000014.9	-	57229734	57247712	17979	1269	sense-overlappin	NM_006544.3	EXOC5
circRNA_09183	0	0	0	0	0	0	0	0	0.030381792	0	0	0	-	NC_000014.9	-	57268247	57268329	83	83	intronic	NM_006544.3	EXOC5
circRNA_09184	0	0	0	0	0	0	0	0	0	0	0.02025523	0	hsa_circ_0032051	NC_000014.9	+	57274244	57274889	646	646	exonic	NM_018229.3	AP5M1
circRNA_09185	0	0	0	0	0	0	0	0	0	0.020172483	0	0	hsa_circ_0032052	NC_000014.9	+	57274244	57280422	6179	874	sense-overlappin	NM_018229.3	AP5M1
circRNA_09186	0	0	0.04361729	0	0	0	0	0	0	0	0	0	hsa_circ_0032053	NC_000014.9	+	57274244	57283230	8987	1219	sense-overlappin	NM_018229.3	AP5M1
circRNA_09187	0	0	0	0	0.02006311	0	0	0	0	0	0	0	-	NC_000014.9	+	58252124	58257998	5875	295	sense-overlappin	NM_002788.3	PSMA3
circRNA_09188	0	0	0	0	0	0	0	0.020486295	0	0	0	0	-	NC_000014.9	+	58263705	58270998	7294	246	sense-overlappin	NM_002788.3	PSMA3
circRNA_09189	0	0	0.021808645	0	0	0	0	0	0	0	0	0	-	NC_000014.9	-	58291635	58293926	2292	2292	intergenic		
circRNA_09190	0	0	0	0	0	0	0	0.020486295	0	0	0	0	-	NC_000014.9	+	58306022	58330169	24148	723	sense-overlappin	XM_005267964.1	ARID4A
circRNA_09191	0	0.020469968	0	0.0309543	0.02006311	0.031069364	0.040197371	0.020486295	0	0.090776173	0	0	hsa_circ_0000542	NC_000014.9	+	58318542	58330169	11628	632	sense-overlappin	XM_005267964.1	ARID4A
circRNA_09192	0.020575025	0	0	0	0	0	0	0	0	0	0	0	hsa_circ_0009160	NC_000014.9	-	58410843	58424095	13253	249	sense-overlappin	NM_001304488.1	TIMM9
circRNA_09193	0	0	0	0	0	0	0.020098685	0	0	0	0	0	hsa_circ_0000544	NC_000014.9	+	60115719	60125823	10105	969	sense-overlappin	XR_943509.1	
circRNA_09194	0	0	0	0	0	0	0	0	0	0.020172483	0	0	-	NC_000014.9	-	60149353	60153178	3826	579	sense-overlappin	NM_016029.2	DHRS7
circRNA_09195	0	0	0	0	0	0	0	0.020486295	0	0.040344966	0	0.0206364	hsa_circ_0032109	NC_000014.9	+	60282684	60291454	8771	1139	sense-overlappin	NM_177952.2	PPM1A
circRNA_09196	0	0	0	0.0206362	0	0	0	0	0	0	0	0	-	NC_000014.9	-	60455001	60471739	16739	1180	sense-overlappin	XM_011536701.1	C14orf39
circRNA_09197	0	0	0	0	0	0.041425819	0	0	0	0	0	0	-	NC_000014.9	+	60791615	60879835	88221	5322	sense-overlappin	NM_002431.3	MNAT1
circRNA_09198	0	0	0	0.0206362	0	0	0	0	0	0	0	0	-	NC_000014.9	+	60793044	60818847	25804	3771	sense-overlappin	NM_002431.3	MNAT1
circRNA_09199	0	0.051174919	0.021808645	0	0.03009467	0.031069364	0	0	0	0.080689931	0	0	hsa_circ_0032116	NC_000014.9	+	60796217	60818847	22631	598	sense-overlappin	NM_002431.3	MNAT1
circRNA_09200	0.041150049	0	0	0	0	0	0	0	0	0.040344966	0	0	hsa_circ_0032117	NC_000014.9	+	60796217	60879835	83619	720	sense-overlappin	NM_002431.3	MNAT1
circRNA_09201	0	0	0	0.0309543	0	0	0	0	0	0	0	0	hsa_circ_0004244	NC_000014.9	+	60808325	60879835	71511	493	sense-overlappin	NM_002431.3	MNAT1
circRNA_09202	0.020575025	0	0	0	0	0	0	0	0	0	0	0	-	NC_000014.9	+	61391225	61457679	66455	915	sense-overlappin	NM_006255.4	PRKCH
circRNA_09203	0	0	0	0	0	0	0	0	0.020254528	0	0	0	-	NC_000014.9	+	61484258	61485656	1399	1399	sense-overlappin	NM_006255.4	PRKCH
circRNA_09204	0	0.020469968	0	0	0	0	0.020098685	0	0	0	0	0	hsa_circ_0006393	NC_000014.9	+	61720382	61721823	1442	422	sense-overlappin	NM_001530.3	HIF1A
circRNA_09205	0	0.040939935	0.109043225	0.0412724	0.03009467	0.020712909	0.040197371	0	0.020254528	0	0.02025523	0	hsa_circ_0007976	NC_000014.9	+	61721509	61721823	315	231	sense-overlappin	NM_001530.3	HIF1A
circRNA_09206	0	0	0	0	0	0	0	0	0	0	0	0.0309546	hsa_circ_0032140	NC_000014.9	+	61738087	61738373	287	287	exonic	NM_001530.3	HIF1A
circRNA_09207	0.020575025	0	0	0.0309543	0	0.041425819	0	0	0	0	0	0	hsa_circ_0003051	NC_000014.9	-	63598756	63599973	1218	1218	exonic	XM_011536383.1	WDR89
circRNA_09208	0	0	0	0	0	0	0.030148028	0	0	0	0	0	-	NC_000014.9	+	63982630	63986617	3988	477	sense-overlappin	XM_011536576.1	SYNE2
circRNA_09209	0	0	0.032712968	0	0	0	0	0	0	0	0	0	-	NC_000014.9	+	63998914	64003330	4417	1044	sense-overlappin	XM_011536576.1	SYNE2
circRNA_09210	0	0	0.076330258	0	0	0.020712909	0	0.030729443	0	0.020172483	0.02025523	0.051591	-	NC_000014.9	+	63998914	64016631	17718	1534	sense-overlappin	XM_011536576.1	SYNE2
circRNA_09211	0	0	0.021808645	0	0	0	0	0	0	0	0	0	-	NC_000014.9	+	63998914	64022028	23115	2171	sense-overlappin	XM_011536576.1	SYNE2
circRNA_09212	0.030862537	0.102349838	0.065425935	0.0412724	0.10031557	0.041425819	0.231134882	0.051215739	0.070890847	0.040344966	0.050638075	0.103182001	-	NC_000014.9	+	63998914	64022863	23950	2284	sense-overlappin	XM_011536576.1	SYNE2
circRNA_09213	0	0	0	0	0	0	0.040197371	0	0	0	0	0	-	NC_000014.9	+	64051557	64053657	2101	2101	exonic	XM_011536576.1	SYNE2
circRNA_09214	0	0	0.021808645	0	0	0	0	0	0	0	0	0	-	NC_000014.9	+	64055944	64093480	37537	2364	sense-overlappin	XM_011536576.1	SYNE2
circRNA_09215	0	0	0	0	0	0	0	0	0	0	0.02025523	0	-	NC_000014.9	+	64062751	64065650	2900	364	sense-overlappin	XM_011536576.1	SYNE2
circRNA_09216	0	0	0	0	0	0	0.030148028	0	0	0	0	0	-	NC_000014.9	+	64128452	64137983	9532	926	sense-overlappin	XM_011536576.1	SYNE2
circRNA_09217	0.020575025	0	0	0	0	0	0	0	0	0	0	0	-	NC_000014.9	+	64186424	64190237	3814	482	sense-overlappin	XM_011536576.1	SYNE2
circRNA_09218	0	0	0	0	0	0	0.040197371	0	0	0.020172483	0	0	-	NC_000014.9	+	64521487	64523402	1916	1916	exonic	XM_005267411.3	ZBTB1
circRNA_09219	0	0	0	0.0309543	0	0	0	0	0	0.040344966	0	0	-	NC_000014.9	-	65075774	65078036	2263	1115	sense-overlappin	NM_145113.2	MAX
circRNA_09220	0	0.020469968	0	0.1031809	0.03009467	0.051782273	0.030148028	0.081945182	0.070890847	0	0.02025523	0	-	NC_000014.9	+	65430216	65455718	25503	25503	sense-overlappin	NM_004480.4	FUT8
circRNA_09221	0	0	0	0.0206362	0	0	0	0	0	0.050431207	0	0.0619092	hsa_circ_0008468	NC_000014.9	+	65432037	65455718	23682	23682	sense-overlappin	NM_004480.4	FUT8
circRNA_09222	0	0	0.021808645	0	0	0	0	0.030729443	0	0	0	0.0206364	-	NC_000014.9	+	65455621	65458207	2587	2587	sense-overlappin	NM_004480.4	FUT8
circRNA_09223	0	0	0	0.0309543	0	0.020712909	0	0	0.020254528	0	0	0	-	NC_000014.9	+	65455621	65461151	5531	5531	sense-overlappin	NM_004480.4	FUT8
circRNA_09224	0.030862537	0	0	0	0	0.020712909	0	0	0	0	0	0	-	NC_000014.9	+	65455621	65464473	8853	8853	sense-overlappin	NM_004480.4	FUT8
circRNA_09225	0	0	0	0	0	0	0	0.020486295	0	0	0	0	-	NC_000014.9	+	65557609	65558054	446	446	intronic	NM_004480.4	FUT8

circRNA_09226	0.082300098	0.122819805	0.098138903	0.2476343	0.18056803	0.134633911	0.30148028	0.133160921	0.151908958	0.161379862	0.04051046	0.123818401	hsa_circ_0003028	NC_000014.9	+	65561337	65561766	430	430	exonic	NM_178155.2	FUT8
circRNA_09227	0	0	0.076330258	0.1341352	0.17053648	0.165703275	0.200986853	0.081945182	0.151908958	0	0	0.165091201	-	NC_000014.9	+	65561337	65629606	68270	824	sense-overlappin	NM_178155.2	FUT8
circRNA_09228	0.061725074	0	0.04361729	0.1238171	0.06018934	0.041425819	0.030148028	0.040972591	0.050636319	0.020172483	0	0.0412728	-	NC_000014.9	+	65615978	65629606	13629	394	sense-overlappin	NM_004480.4	FUT8
circRNA_09229	0	0	0	0.0206362	0	0	0	0	0	0	0	0	hsa_circ_0032248	NC_000014.9	+	65629492	65669480	39989	353	sense-overlappin	NM_004480.4	FUT8
circRNA_09230	0	0	0	0	0	0.062138728	0	0	0	0	0	0	hsa_circ_0032249	NC_000014.9	+	65721775	65733381	11607	575	sense-overlappin	NM_004480.4	FUT8
circRNA_09231	0	0	0.021808645	0	0	0	0	0	0	0	0	0	-	NC_000014.9	+	66922666	66924292	1627	372	sense-overlappin	NM_020806.4	GPHN
circRNA_09232	0	0	0.065425935	0	0	0.031069364	0	0	0	0	0	0	hsa_circ_0032253	NC_000014.9	+	66965191	67023675	58485	307	sense-overlappin	XM_011536340.1	GPHN
circRNA_09233	0	0	0	0.0412724	0.02006311	0.041425819	0	0.020486295	0.020254528	0	0	0	hsa_circ_0032254	NC_000014.9	+	66965191	67058786	93596	445	sense-overlappin	XM_011536340.1	GPHN
circRNA_09234	0	0	0	0	0	0	0	0.030729443	0.020254528	0	0	0	-	NC_000014.9	+	67128710	67129049	340	340	intronic	NM_001024218.1	GPHN
circRNA_09235	0.030862537	0.040939935	0	0.0722267	0.04012623	0.051782273	0.020098685	0.040972591	0	0.080689931	0	0	hsa_circ_0032261	NC_000014.9	+	67269701	67303599	33899	1045	sense-overlappin	NM_001256550.1	MPP5
circRNA_09236	0	0	0	0	0	0	0	0	0	0	0.02025523	0	-	NC_000014.9	+	67292511	67323812	31302	1484	sense-overlappin	NM_001256550.1	MPP5
circRNA_09237	0.030862537	0	0	0.0515905	0	0	0	0.030729443	0	0	0	0	hsa_circ_0005429	NC_000014.9	+	67301389	67303599	2211	465	sense-overlappin	NM_001256550.1	MPP5
circRNA_09238	0.092587611	0.020469968	0.021808645	0	0	0	0	0	0	0.030258724	0	0	hsa_circ_0006278	NC_000014.9	-	67685015	67690421	5407	400	sense-overlappin	NM_016026.3	RDH11
circRNA_09239	0	0.030704951	0.021808645	0.0206362	0	0	0	0	0	0	0	0	hsa_circ_0000545	NC_000014.9	-	67761366	67762819	1454	577	sense-overlappin	XM_006720093.2	ZFYVE26
circRNA_09240	0	0	0	0.0309543	0	0	0	0	0	0	0	0	hsa_circ_0032289	NC_000014.9	-	67762203	67769730	7528	885	sense-overlappin	XM_006720093.2	ZFYVE26
circRNA_09241	0	0.020469968	0	0	0	0	0	0	0	0	0	0	hsa_circ_0000546	NC_000014.9	-	67767704	67769730	2027	306	sense-overlappin	XM_006720093.2	ZFYVE26
circRNA_09242	0	0	0	0	0	0	0	0	0	0.020172483	0	0	hsa_circ_0032305	NC_000014.9	+	67823542	67835196	11655	317	sense-overlappin	XM_011537051.1	RAD51B
circRNA_09243	0.030862537	0	0	0	0	0	0	0	0	0	0	0	hsa_circ_0032321	NC_000014.9	-	68890139	68892283	2145	379	sense-overlappin	XM_011537267.1	ACTN1
circRNA_09244	0.020575025	0	0.04361729	0.0309543	0	0	0	0	0	0	0	0	hsa_circ_0007392	NC_000014.9	-	69116366	69122360	5995	451	sense-overlappin	NM_001284207.1	DCAF5
circRNA_09245	0	0	0	0	0	0	0.030148028	0	0	0	0	0	-	NC_000014.9	-	69122217	69149503	27287	217	sense-overlappin	XM_011537278.1	DCAF5
circRNA_09246	0	0	0	0.0206362	0	0	0.020098685	0	0	0	0	0	hsa_circ_0032353	NC_000014.9	+	69703395	69704742	1348	337	sense-overlappin	NM_014734.3	SUSD6
circRNA_09247	0	0	0	0	0	0	0	0	0	0	0	0.0206364	-	NC_000014.9	-	69768804	69770684	1881	381	antisense	XM_005267998.1	SRSF5
circRNA_09248	0	0	0	0	0	0.020712909	0	0	0	0	0	0	-	NC_000014.9	-	70584817	70597777	12961	736	sense-overlappin	NM_001284209.1	MED6
circRNA_09249	0	0	0	0	0	0	0	0	0	0.020172483	0	0	-	NC_000014.9	-	70800667	70801080	414	414	exonic	NM_001284230.1	MAP3K9
circRNA_09250	0	0	0	0	0	0	0	0	0.030381792	0	0	0	hsa_circ_0032384	NC_000014.9	+	70946915	70978648	31734	2158	sense-overlappin	NM_014982.2	PCNX
circRNA_09251	0	0	0	0	0	0	0	0	0	0	0.030382845	0	hsa_circ_0032396	NC_000014.9	+	70976942	70995925	18984	2025	sense-overlappin	NM_014982.2	PCNX
circRNA_09252	0	0	0.021808645	0	0	0	0	0	0	0	0	0	hsa_circ_0008859	NC_000014.9	+	71013000	71036157	23158	1074	sense-overlappin	NM_014982.2	PCNX
circRNA_09253	0	0	0	0	0	0.020712909	0	0	0	0.040344966	0	0	hsa_circ_0032436	NC_000014.9	+	71019009	71036157	17149	871	sense-overlappin	NM_014982.2	PCNX
circRNA_09254	0	0.030704951	0.04361729	0	0	0	0	0	0	0.040344966	0	0	hsa_circ_0007996	NC_000014.9	+	71026117	71036157	10041	684	sense-overlappin	NM_014982.2	PCNX
circRNA_09255	0	0	0	0	0	0	0	0	0	0	0	0.0309546	hsa_circ_0032446	NC_000014.9	+	71036065	71052012	15948	803	sense-overlappin	NM_014982.2	PCNX
circRNA_09256	0	0	0.021808645	0.0206362	0	0	0	0	0	0.030258724	0	0	hsa_circ_0001997	NC_000014.9	+	71045133	71057724	12592	985	sense-overlappin	NM_014982.2	PCNX
circRNA_09257	0	0	0.021808645	0	0	0	0	0	0	0	0	0	hsa_circ_0032448	NC_000014.9	+	71045133	71076419	31287	1470	sense-overlappin	NM_014982.2	PCNX
circRNA_09258	0	0	0	0	0	0	0	0	0.020254528	0	0	0	hsa_circ_0032457	NC_000014.9	+	71105235	71105440	206	206	exonic	NM_014982.2	PCNX
circRNA_09259	0	0	0	0.0309543	0	0	0	0	0	0	0	0	-	NC_000014.9	+	71351851	71418196	66346	62231	sense-overlappin	XM_011536630.1	SIPA1L1
circRNA_09260	0.030862537	0.030704951	0	0	0.02006311	0.041425819	0.040197371	0.040972591	0.030381792	0.030258724	0.050638075	0	hsa_circ_0002722	NC_000014.9	+	71400794	71418196	17403	13288	sense-overlappin	XM_011536630.1	SIPA1L1
circRNA_09261	0	0	0.021808645	0.0206362	0.02006311	0	0	0	0	0	0	0	-	NC_000014.9	+	71413948	71414680	733	733	sense-overlappin	XM_011536630.1	SIPA1L1
circRNA_09262	0	0.020469968	0	0	0	0	0	0.040972591	0	0.050431207	0	0.0412728	-	NC_000014.9	+	71413948	71418196	4249	134	sense-overlappin	XM_011536630.1	SIPA1L1
circRNA_09263	0	0	0	0	0	0.031069364	0	0	0	0	0	0	-	NC_000014.9	+	71413948	71512845	98898	237	sense-overlappin	XM_011536630.1	SIPA1L1
circRNA_09264	0.020575025	0	0	0	0	0	0	0	0	0	0	0	-	NC_000014.9	+	71512743	71589370	76628	2057	sense-overlappin	XM_011536634.1	SIPA1L1
circRNA_09265	0	0.020469968	0.032712968	0	0	0	0	0.020486295	0	0	0	0	hsa_circ_0001979	NC_000014.9	+	71587571	71589370	1800	1800	exonic	XM_011536635.1	SIPA1L1
circRNA_09266	0	0	0.021808645	0	0	0	0	0	0	0	0	0	hsa_circ_0032468	NC_000014.9	+	71650335	71672622	22288	1286	sense-overlappin	XM_011536635.1	SIPA1L1
circRNA_09267	0	0.071644886	0	0.0206362	0	0.031069364	0	0.030729443	0	0.030258724	0	0	-	NC_000014.9	+	73089049	73089455	407	407	intronic	XR_943501.1	
circRNA_09268	0.092587611	0.102349838	0.054521613	0.0206362	0.02006311	0.041425819	0.070345399	0.061458886	0.040509055	0.040344966	0.08102092	0.0619092	hsa_circ_0008521	NC_000014.9	+	73147795	73148094	300	210	sense-overlappin	NM_000021.3	PSEN1
circRNA_09269	0.041150049	0.040939935	0.076330258	0	0.05015779	0	0	0	0.020254528	0.030258724	0.030382845	0	hsa_circ_0003848	NC_000014.9	+	73147795	73148106	312	222	sense-overlappin	NM_000021.3	PSEN1
circRNA_09270	0	0	0	0.0309543	0	0	0	0	0	0	0	0	-	NC_000014.9	+	73147795	73165413	17619	17529	sense-overlappin	NM_000021.3	PSEN1
circRNA_09271	0.020575025	0	0.152660515	0.0206362	0.04012623	0.020712909	0	0	0.030381792	0.020172483	0.02025523	0.0309546	hsa_circ_0002564	NC_000014.9	+	73147795	73173707	25913	615	sense-overlappin	NM_000021.3	PSEN1
circRNA_09272	0	0	0.065425935	0.0309543	0	0	0	0.020486295	0.020254528	0.030258724	0	0	hsa_circ_0002289	NC_000014.9	+	73147795	73198129	50335	1003	sense-overlappin	NM_000021.3	PSEN1
circRNA_09273	0	0	0	0.0309543	0	0	0	0	0	0	0	0	hsa_circ_0008406	NC_000014.9	-	73282359	73297285	14927	862	sense-overlappin	XM_011537253.1	NUMB
circRNA_09274	0	0	0	0	0	0	0	0	0	0.020172483	0	0	-	NC_000014.9	+	73304330	73305565	1236	1236	antisense	XM_011537253.1	NUMB
circRNA_09275	0	0	0	0	0	0	0	0	0.030381792	0	0	0	-	NC_000014.9	+	73866415	73866812	398	398	intronic	NM_001146154.1	PTGR2
circRNA_09276	0	0	0	0	0	0	0	0	0.020254528	0	0	0	hsa_circ_0032526	NC_000014.9	+	73876998	73880176	3179	503	sense-overlappin	NM_001146154.1	PTGR2
circRNA_09277	0	0.020469968	0	0	0	0	0	0	0	0	0	0	-	NC_000014.9	-	73885212	73885856	645	645	antisense	NM_001146154.1	PTGR2
circRNA_09278	0	0	0	0.0206362	0	0	0	0	0	0	0	0	-	NC_000014.9	+	73892027	73905083	13057	1113	sense-overlappin	NM_001242924.1	ZNF410
circRNA_09279	0	0	0	0	0	0	0	0	0.020254528	0	0	0	hsa_circ_0032533	NC_000014.9	+	73953435	73955928	2494	318	sense-overlappin	NM_182480.2	COQ6
circRNA_09280	0	0	0.021808645	0	0	0	0	0	0.040509055	0	0	0	hsa_circ_0005182	NC_000014.9	+	74091232	74101238	10007	264	sense-overlappin	XM_011537320.1	LIN52

circRNA_09281	0	0	0	0	0	0.041425819	0	0	0	0	0	0	-	NC_000014.9	+	74119856	74120164	309	309	intronic	NM_001024674.2	LIN52
circRNA_09282	0	0	0	0.0206362	0	0.031069364	0	0	0	0	0	0	hsa_circ_0005030	NC_000014.9	-	74669649	74671483	1835	492	sense-overlappin	XM_011537415.1	AREL1
circRNA_09283	0	0	0	0	0	0	0	0	0	0.020172483	0	0	-	NC_000014.9	+	74669988	74670255	268	268	antisense	XM_011537415.1	AREL1
circRNA_09284	0	0	0	0	0	0	0	0	0.020254528	0	0	0	-	NC_000014.9	-	74672831	74674111	1281	342	sense-overlappin	XM_011537415.1	AREL1
circRNA_09285	0	0	0	0	0	0	0	0	0	0	0	0.0309546	-	NC_000014.9	+	74717264	74718568	1305	1305	intronic	XM_011536815.1	FCF1
circRNA_09286	0.030862537	0	0	0	0	0	0	0	0	0.020172483	0	0	-	NC_000014.9	-	74735984	74736325	342	342	antisense	NM_015962.4	FCF1
circRNA_09287	0.082300098	0	0.054521613	0.1238171	0	0	0.020098685	0.020486295	0	0.050431207	0.030382845	0	hsa_circ_0032649	NC_000014.9	-	75046376	75049718	3343	3343	exonic	NM_001040108.1	MLH3
circRNA_09288	0.030862537	0	0	0.0206362	0	0.020712909	0.100493427	0.051215739	0.060763583	0	0	0.0309546	hsa_circ_0000557	NC_000014.9	-	75109685	75110371	687	244	sense-overlappin	XM_005268208.1	NEK9
circRNA_09289	0	0.020469968	0	0	0	0	0	0	0	0	0	0	hsa_circ_0032689	NC_000014.9	+	75622079	75624752	2674	283	sense-overlappin	NM_017791.2	FLVCR2
circRNA_09290	0.020575025	0.030704951	0	0.0206362	0	0.020712909	0	0	0	0	0	0	hsa_circ_0008187	NC_000014.9	+	75732338	75735289	2952	239	sense-overlappin	NM_015072.4	TTLL5
circRNA_09291	0	0	0	0	0.02006311	0	0	0	0	0	0	0	hsa_circ_0007922	NC_000014.9	+	75732338	75737607	5270	2557	sense-overlappin	NM_015072.4	TTLL5
circRNA_09292	0	0	0.021808645	0	0	0	0	0	0	0	0	0	hsa_circ_0007946	NC_000014.9	+	75863667	75902224	38558	497	sense-overlappin	NM_015072.4	TTLL5
circRNA_09293	0	0	0	0.0515905	0	0	0	0	0	0	0	0	hsa_circ_0032731	NC_000014.9	+	75882685	75902224	19540	301	sense-overlappin	NM_015072.4	TTLL5
circRNA_09294	0	0	0	0	0	0	0.020098685	0	0	0	0	0	hsa_circ_0004176	NC_000014.9	+	76031955	76059373	27419	26767	sense-overlappin	NM_001102564.1	IFT43
circRNA_09295	0.020575025	0	0	0	0	0	0	0	0	0	0	0	-	NC_000014.9	+	76058642	76059373	732	80	sense-overlappin	NM_001102564.1	IFT43
circRNA_09296	0.030862537	0	0.098138903	0.0515905	0	0	0	0	0.030381792	0.040344966	0.02025523	0	hsa_circ_0005267	NC_000014.9	+	76166663	76180849	14187	616	sense-overlappin	XR_943490.1	
circRNA_09297	0.030862537	0	0.08723458	0	0	0.020712909	0	0	0.020254528	0.040344966	0	0	hsa_circ_0005252	NC_000014.9	+	76166663	76195972	29310	741	sense-overlappin	XR_943489.1	
circRNA_09298	0	0	0	0	0	0.041425819	0	0	0	0	0	0.0412728	hsa_circ_0003819	NC_000014.9	+	76171843	76180849	9007	551	sense-overlappin	XR_943490.1	
circRNA_09299	0	0	0.021808645	0	0	0	0	0	0	0	0	0	hsa_circ_0032745	NC_000014.9	+	76176623	76180849	4227	294	sense-overlappin	XR_943490.1	
circRNA_09300	0	0	0	0	0.02006311	0	0	0	0	0	0	0	hsa_circ_0032746	NC_000014.9	+	76176623	76195972	19350	419	sense-overlappin	XR_943489.1	
circRNA_09301	0.020575025	0	0	0	0	0	0	0	0	0	0	0.0206364	hsa_circ_0004433	NC_000014.9	-	77451187	77454102	2916	343	sense-overlappin	XM_011537066.1	VIPAS39
circRNA_09302	0	0	0	0	0	0	0	0	0	0.020172483	0	0	hsa_circ_0008404	NC_000014.9	+	77464598	77465667	1070	218	sense-overlappin	XM_011536361.1	AHSA1
circRNA_09303	0	0	0	0.0309543	0	0	0	0	0	0	0	0	hsa_circ_0004187	NC_000014.9	-	77557041	77576915	19875	474	sense-overlappin	NM_004863.3	SPTLC2
circRNA_09304	0	0	0	0	0	0	0.030148028	0	0	0	0	0	hsa_circ_0006261	NC_000014.9	-	80478254	80530886	52633	569	sense-overlappin	XM_011536491.1	CEP128
circRNA_09305	0.041150049	0.020469968	0.032712968	0.0412724	0	0.031069364	0.040197371	0	0	0.090776173	0	0	hsa_circ_0007332	NC_000014.9	-	80497469	80530886	33418	415	sense-overlappin	XM_011536491.1	CEP128
circRNA_09306	0	0	0.021808645	0	0	0	0	0	0	0	0	0	hsa_circ_0032815	NC_000014.9	-	80504912	80530886	25975	301	sense-overlappin	XM_011536491.1	CEP128
circRNA_09307	0	0	0.021808645	0	0	0.020712909	0	0	0	0	0	0	hsa_circ_0032821	NC_000014.9	-	80743075	80761613	18539	430	sense-overlappin	XM_011536491.1	CEP128
circRNA_09308	0	0.040939935	0.098138903	0	0	0.051782273	0.030148028	0.040972591	0.040509055	0.030258724	0	0.0412728	hsa_circ_0032822	NC_000014.9	-	80743075	80778046	34972	595	sense-overlappin	XM_011536491.1	CEP128
circRNA_09309	0	0	0	0	0	0	0	0	0	0.020172483	0	0	-	NC_000014.9	-	80743075	80838278	95204	1957	sense-overlappin	XM_011536491.1	CEP128
circRNA_09310	0	0	0.021808645	0	0	0	0	0	0	0.020172483	0	0	hsa_circ_0032825	NC_000014.9	-	80831143	80840768	9626	447	sense-overlappin	XM_011536491.1	CEP128
circRNA_09311	0	0	0	0	0.02006311	0	0	0	0	0	0	0	hsa_circ_0032826	NC_000014.9	-	80862757	80900029	37273	282	sense-overlappin	XM_011536491.1	CEP128
circRNA_09312	0	0.051174919	0.032712968	0	0	0	0	0	0	0	0	0	hsa_circ_0002499	NC_000014.9	-	80862757	80906081	43325	528	sense-overlappin	XM_011536491.1	CEP128
circRNA_09313	0	0	0.021808645	0.0206362	0	0.020712909	0	0	0.050636319	0.07060369	0	0	-	NC_000014.9	-	80895718	80906081	10364	411	sense-overlappin	XM_011536491.1	CEP128
circRNA_09314	0	0.020469968	0	0	0	0	0	0	0	0	0	0	-	NC_000014.9	-	80895718	80916562	20845	660	sense-overlappin	XM_011536491.1	CEP128
circRNA_09315	0	0	0	0	0	0.020712909	0	0	0.020254528	0	0	0	-	NC_000014.9	-	80939385	80942380	2996	2996	sense-overlappin	XM_011536492.1	CEP128
circRNA_09316	0	0	0	0	0	0.020712909	0	0	0	0	0	0	-	NC_000014.9	-	81518138	81518441	304	304	intronic	NM_005065.5	SEL1L
circRNA_09317	0	0	0	0	0.03009467	0	0	0	0	0	0	0	-	NC_000014.9	+	83646544	83666209	19666	19666	intergenic		
circRNA_09318	0	0	0.021808645	0.0412724	0	0.031069364	0.020098685	0	0	0.050431207	0.04051046	0	-	NC_000014.9	+	86028369	86041833	13465	13465	intergenic		
circRNA_09319	0	0	0	0	0	0	0	0	0	0.020172483	0	0	-	NC_000014.9	+	86031136	86070967	39832	39832	intergenic		
circRNA_09320	0	0	0.021808645	0	0	0	0	0	0	0	0	0	-	NC_000014.9	+	88415205	88437903	22699	1108	sense-overlappin	XM_005267855.1	SPATA7
circRNA_09321	0	0	0	0.0412724	0.03009467	0.020712909	0	0	0.030381792	0	0	0	-	NC_000014.9	+	88416711	88433212	16502	925	sense-overlappin	XM_011536953.1	SPATA7
circRNA_09322	0	0	0.119947548	0.0515905	0.03009467	0.020712909	0	0	0.060763583	0.060517448	0.030382845	0	-	NC_000014.9	+	88416711	88437903	21193	1046	sense-overlappin	XM_011536953.1	SPATA7
circRNA_09323	0	0	0	0.0206362	0	0	0	0	0	0	0	0	hsa_circ_0002559	NC_000014.9	-	88478920	88480352	1433	1433	exonic	XM_011536368.1	PTPN21
circRNA_09324	0	0	0	0	0	0	0	0	0	0	0	0.0309546	hsa_circ_0032851	NC_000014.9	-	88496413	88508020	11608	582	sense-overlappin	XM_011536369.1	PTPN21
circRNA_09325	0	0	0.021808645	0	0	0	0	0	0	0	0	0	-	NC_000014.9	+	88571084	88575940	4857	929	sense-overlappin	NM_001160103.1	ZC3H14
circRNA_09326	0.041150049	0.020469968	0	0.0412724	0.02006311	0.051782273	0.020098685	0.030729443	0.030381792	0.060517448	0	0	hsa_circ_0003164	NC_000014.9	+	88574693	88578140	3448	418	sense-overlappin	NM_001160103.1	ZC3H14
circRNA_09327	0	0	0	0	0	0	0	0	0	0	0	0.0412728	hsa_circ_0032869	NC_000014.9	-	88681890	88696952	15063	886	sense-overlappin	XM_006720070.2	EML5
circRNA_09328	0	0	0	0	0	0	0	0	0	0	0	0.0206364	hsa_circ_0032873	NC_000014.9	-	88712271	88715195	2925	470	sense-overlappin	XM_006720070.2	EML5
circRNA_09329	0	0	0	0	0	0	0	0	0	0.030258724	0	0	hsa_circ_0032876	NC_000014.9	-	88726541	88746283	19743	830	sense-overlappin	XM_006720070.2	EML5
circRNA_09330	0.041150049	0	0	0	0	0.020712909	0.060296056	0	0.020254528	0.030258724	0	0	hsa_circ_0000558	NC_000014.9	-	89161701	89162969	1269	1269	exonic	NM_005197.3	FOXN3
circRNA_09331	0	0	0	0.0515905	0	0	0	0.020486295	0	0	0	0	-	NC_000014.9	-	89161701	89180806	19106	1375	sense-overlappin	NM_005197.3	FOXN3
circRNA_09332	0	0	0.021808645	0	0	0	0	0	0	0	0	0	-	NC_000014.9	-	89280950	89350808	69859	202	sense-overlappin	NM_005197.3	FOXN3
circRNA_09333	0	0	0.054521613	0.0825448	0	0	0	0	0	0	0	0	hsa_circ_0005987	NC_000014.9	-	89411934	89412490	557	557	exonic	NM_005197.3	FOXN3
circRNA_09334	0	0	0.021808645	0	0	0	0	0	0	0	0	0	hsa_circ_0032891	NC_000014.9	-	89931541	89932627	1087	193	sense-overlappin	NM_001284269.1	EFCAB11
circRNA_09335	0	0	0	0	0	0	0	0	0.040509055	0	0	0	-	NC_000014.9	+	90404437	90404514	78	78	exonic	NM_006888.4	CALM1

circRNA_09336	0	0	0	0	0	0	0	0	0	0.030258724	0	0	-	NC_000014.9	-	90890487	90906299	15813	1030	sense-overlappin	XM_011537360.1	RPS6KA5
circRNA_09337	0	0	0	0	0	0	0	0	0	0.020172483	0	0	-	NC_000014.9	-	90920206	91001159	80954	703	sense-overlappin	NM_004755.2	RPS6KA5
circRNA_09338	0	0	0	0	0	0.062138728	0	0	0	0	0	0	hsa_circ_0032940	NC_000014.9	-	90943078	91001159	58082	515	sense-overlappin	NM_004755.2	RPS6KA5
circRNA_09339	0.020575025	0	0.04361729	0.0309543	0.02006311	0.041425819	0.020098685	0	0.020254528	0	0	0	hsa_circ_0032943	NC_000014.9	-	90978306	91001159	22854	291	sense-overlappin	NM_004755.2	RPS6KA5
circRNA_09340	0	0	0	0	0	0	0	0	0	0.020172483	0	0	-	NC_000014.9	-	90978306	91041893	63588	41025	sense-overlappin	NM_004755.2	RPS6KA5
circRNA_09341	0	0	0	0	0	0	0	0	0	0	0.050638075	0	-	NC_000014.9	-	91289105	91294318	5214	475	sense-overlappin	XM_011536796.1	CCDC88C
circRNA_09342	0	0	0	0	0.02006311	0	0	0	0	0	0	0	-	NC_000014.9	-	91297305	91300070	2766	331	sense-overlappin	XM_011536796.1	CCDC88C
circRNA_09343	0	0	0	0	0	0	0.050246713	0	0	0	0	0	-	NC_000014.9	-	91338005	91343657	5653	710	sense-overlappin	XM_011536796.1	CCDC88C
circRNA_09344	0	0	0	0	0.03009467	0	0	0	0	0	0	0	-	NC_000014.9	-	91476909	91490802	13894	851	sense-overlappin	NM_001284280.1	SMEK1
circRNA_09345	0.041150049	0.051174919	0.021808645	0.0515905	0.02006311	0.051782273	0	0.040972591	0.081018111	0.090776173	0.030382845	0.0412728	hsa_circ_0003045	NC_000014.9	-	91481576	91485730	4155	717	sense-overlappin	NM_001284280.1	SMEK1
circRNA_09346	0	0	0.021808645	0.0412724	0.05015779	0.093208092	0.030148028	0.040972591	0	0	0.02025523	0.051591	hsa_circ_0032965	NC_000014.9	-	91481576	91490802	9227	773	sense-overlappin	NM_001284280.1	SMEK1
circRNA_09347	0	0.020469968	0	0	0	0	0	0	0	0	0	0	-	NC_000014.9	-	91988284	92011795	23512	4074	sense-overlappin	XM_006720321.2	TRIP11
circRNA_09348	0	0	0.021808645	0	0	0	0	0	0	0	0	0	-	NC_000014.9	-	91999240	92011795	12556	3706	sense-overlappin	XM_006720321.2	TRIP11
circRNA_09349	0	0	0.021808645	0.0515905	0	0	0	0	0	0	0	0	-	NC_000014.9	-	92003419	92007852	4434	3243	sense-overlappin	XM_005268214.2	TRIP11
circRNA_09350	0	0	0	0.0206362	0	0	0	0	0	0	0	0	-	NC_000014.9	-	92003419	92011072	7654	3330	sense-overlappin	XM_006720321.2	TRIP11
circRNA_09351	0	0.030704951	0	0.0722267	0.02006311	0	0	0.020486295	0	0	0	0	-	NC_000014.9	-	92003419	92011795	8377	3371	sense-overlappin	XM_006720321.2	TRIP11
circRNA_09352	0	0	0.021808645	0	0.03009467	0	0.030148028	0	0	0	0	0	-	NC_000014.9	-	92007640	92011072	3433	300	sense-overlappin	XM_005268215.2	TRIP11
circRNA_09353	0	0	0	0.0309543	0.02006311	0	0	0	0	0.07060369	0	0	-	NC_000014.9	-	92014215	92033253	19039	1047	sense-overlappin	XM_005268215.2	TRIP11
circRNA_09354	0	0	0	0	0	0	0	0	0	0.030258724	0	0	-	NC_000014.9	-	92070935	92082466	11532	437	sense-overlappin	NR_028455.1	
circRNA_09355	0	0	0.032712968	0	0	0	0	0.030729443	0	0	0	0	-	NC_000014.9	+	92126122	92143294	17173	1233	sense-overlappin	NM_017437.2	CPSF2
circRNA_09356	0	0	0	0	0.02006311	0	0	0	0	0	0	0	-	NC_000014.9	+	92142164	92143294	1131	479	sense-overlappin	NM_017437.2	CPSF2
circRNA_09357	0.041150049	0	0	0	0	0	0	0	0.020254528	0	0	0	-	NC_000014.9	+	92555751	92577477	21727	323	sense-overlappin	NM_024832.3	RIN3
circRNA_09358	0	0	0	0.0206362	0	0	0	0.030729443	0	0	0	0	-	NC_000014.9	+	92809300	92816421	7122	719	sense-overlappin	XM_011537420.1	GOLGA5
circRNA_09359	0	0	0	0	0	0	0	0	0	0.020172483	0	0	-	NC_000014.9	+	92809300	92837449	28150	1343	sense-overlappin	NM_005113.3	GOLGA5
circRNA_09360	0	0	0	0.0206362	0	0	0	0	0	0	0	0	-	NC_000014.9	+	92809300	92840048	30749	2026	sense-overlappin	NM_005113.3	GOLGA5
circRNA_09361	0.020575025	0	0	0	0	0	0	0	0	0	0	0	-	NC_000014.9	+	94519341	94519840	500	500	intergenic		
circRNA_09362	0	0	0	0	0	0	0	0	0	0	0.04051046	0	hsa_circ_0000564	NC_000014.9	-	95140659	95141748	1090	201	sense-overlappin	XM_011536602.1	DICER1
circRNA_09363	0	0	0.021808645	0.0619086	0.04012623	0	0	0	0	0	0	0	hsa_circ_0002223	NC_000014.9	-	96341522	96347341	5820	762	sense-overlappin	NM_018036.5	ATG2B
circRNA_09364	0	0	0	0	0	0	0.030148028	0	0	0	0	0	hsa_circ_0005007	NC_000014.9	+	96520055	96521072	1018	241	sense-overlappin	NM_001252006.1	PAPOLA
circRNA_09365	0	0	0.04361729	0	0.02006311	0	0	0	0	0	0.030382845	0	hsa_circ_0002120	NC_000014.9	+	96520055	96525391	5337	323	sense-overlappin	NM_001252006.1	PAPOLA
circRNA_09366	0	0	0	0.0206362	0	0	0	0.020486295	0.030381792	0	0	0	hsa_circ_0002793	NC_000014.9	+	96520055	96528006	7952	487	sense-overlappin	NM_001252006.1	PAPOLA
circRNA_09367	0	0	0	0	0	0	0	0	0.020254528	0	0	0	-	NC_000014.9	+	96552480	96556413	3934	480	sense-overlappin	NM_001293627.1	PAPOLA
circRNA_09368	0.020575025	0	0.021808645	0.0206362	0	0.072495183	0.030148028	0	0	0	0	0	hsa_circ_0006717	NC_000014.9	+	96555847	96556413	567	340	sense-overlappin	NM_001293627.1	PAPOLA
circRNA_09369	0	0	0	0.0309543	0.03009467	0	0	0.020486295	0	0	0	0	-	NC_000014.9	+	96560649	96565214	4566	398	sense-overlappin	NM_001293627.1	PAPOLA
circRNA_09370	0.030862537	0.133054789	0.817824188	0.0515905	0.0702209	0.124277456	0.070345399	0.051215739	0.060763583	0.14120738	0.06076569	0.123818401	hsa_circ_0000566	NC_000014.9	+	96833467	96860735	27269	1073	sense-overlappin	NM_003384.2	VRK1
circRNA_09371	0	0	0.13085187	0	0	0	0	0	0	0	0	0	-	NC_000014.9	+	96846095	96860735	14641	852	sense-overlappin	NM_003384.2	VRK1
circRNA_09372	0	0	0.04361729	0	0	0	0	0	0	0	0	0	-	NC_000014.9	+	96852831	96860735	7905	694	sense-overlappin	NM_003384.2	VRK1
circRNA_09373	0	0	0.065425935	0	0	0	0.040197371	0.040972591	0.030381792	0.050431207	0	0	-	NC_000014.9	-	99257471	99257836	366	366	exonic	NM_001282237.1	BCL11B
circRNA_09374	0.030862537	0.020469968	0.141756193	0.0412724	0	0.041425819	0.040197371	0	0.070890847	0.020172483	0.030382845	0.0309546	hsa_circ_0033144	NC_000014.9	-	99257471	99257839	369	369	exonic	NM_001282237.1	BCL11B
circRNA_09375	0.26747532	0.204699675	1.166762508	0.4849504	0.27085205	0.269267821	0.271332252	0.174133512	0.243054333	0.302587242	0.374721756	0.185727601	hsa_circ_0000567	NC_000014.9	-	99458279	99465813	7535	683	sense-overlappin	XM_011537232.1	SETD3
circRNA_09376	0	0	0.021808645	0	0	0	0	0	0	0	0	0	-	NC_000014.9	-	99458279	99475334	17056	10204	sense-overlappin	XM_011537232.1	SETD3
circRNA_09377	0.082300098	0	0	0	0	0	0	0	0.020254528	0.020172483	0	0	-	NC_000014.9	+	99914179	99914697	519	258	sense-overlappin	XM_005267398.3	EML1
circRNA_09378	0	0	0	0	0.03009467	0	0	0	0	0	0	0	hsa_circ_0033161	NC_000014.9	+	99914179	99920877	6699	415	sense-overlappin	XM_005267398.3	EML1
circRNA_09379	0.020575025	0	0	0	0	0	0	0	0	0	0	0	-	NC_000014.9	+	100262304	100262466	163	163	exonic	NM_003403.4	YY1
circRNA_09380	0.030862537	0	0	0	0	0	0	0	0	0	0	0	hsa_circ_0033180	NC_000014.9	-	100337062	100343387	6326	428	sense-overlappin	XM_011537136.1	WARS
circRNA_09381	0	0	0	0	0	0	0	0	0.020254528	0	0	0	hsa_circ_0033184	NC_000014.9	-	100353687	100361921	8235	626	sense-overlappin	XM_011537136.1	WARS
circRNA_09382	0.030862537	0	0	0	0	0	0	0.020486295	0	0	0	0	-	NC_000014.9	+	100829709	100831094	1386	1386	intergenic		
circRNA_09383	0	0	0	0	0	0	0	0	0	0	0	0.0309546	-	NC_000014.9	+	100935772	100988790	53019	53019	intergenic		
circRNA_09384	0	0	0	0.0309543	0	0	0	0	0	0	0	0	hsa_circ_0003265	NC_000014.9	+	100937403	100949596	12194	12194	intergenic		
circRNA_09385	0	0	0	0	0	0	0	0	0	0.020172483	0	0	hsa_circ_0002571	NC_000014.9	+	100937403	100951494	14092	14092	intergenic		
circRNA_09386	0	0.030704951	0	0	0	0	0	0	0	0	0	0	hsa_circ_0004567	NC_000014.9	+	100972262	100977694	5433	5433	intergenic		
circRNA_09387	0	0	0	0	0	0	0.020098685	0	0	0	0	0	-	NC_000014.9	-	101874822	101911197	36376	9805	antisense	XM_005267827.1	PPP2R5C
circRNA_09388	0	0	0	0.0309543	0	0.051782273	0	0	0.030381792	0.050431207	0	0	hsa_circ_0003831	NC_000014.9	+	101901719	101906529	4811	299	sense-overlappin	XM_005267827.1	PPP2R5C
circRNA_09389	0	0	0	0	0	0	0.050246713	0	0	0	0	0	hsa_circ_0002060	NC_000014.9	+	101983019	101983609	591	500	sense-overlappin	NM_001376.4	DYNC1H1
circRNA_09390	0.041150049	0	0.021808645	0	0	0	0.020098685	0	0	0	0	0.0412728	hsa_circ_0008683	NC_000014.9	+	102019893	102022880	2988	294	sense-overlappin	NM_001376.4	DYNC1H1
circRNA_09391	0.26747532	0.092114854	0.163564838	0.1238171	0.12037869	0.134633911	0.180888168	0.09218833	0.081018111	0.242069794	0.06076569	0.082545601	hsa_circ_0002398	NC_000014.9	+	102040236	102040673	438	251	sense-overlappin	NM_001376.4	DYNC1H1

circRNA_09392	0	0	0	0	0	0	0	0.194619807	0	0	0	0	-	NC_000014.9	+	102084875	102085393	519	220	antisense	NM_005348.3	HSP90AA1
circRNA_09393	0.020575025	0.040939935	0	0	0.02006311	0.031069364	0.030148028	0	0	0	0.02025523	0	hsa_circ_0000569	NC_000014.9	+	102193463	102195120	1658	235	sense-overlappin	NR_038354.1	
circRNA_09394	0	0	0	0	0	0.031069364	0.030148028	0.020486295	0	0.020172483	0	0	hsa_circ_0003512	NC_000014.9	+	102194938	102197847	2910	276	sense-overlappin	XM_011537349.1	WDR20
circRNA_09395	0	0.030704951	0.141756193	0.0825448	0.03009467	0.041425819	0.050246713	0.040972591	0.020254528	0	0.050638075	0.051591	hsa_circ_0002553	NC_000014.9	+	102194938	102209862	14925	1277	sense-overlappin	XM_011537353.1	WDR20
circRNA_09396	0.020575025	0	0	0	0	0	0	0	0	0	0	0	-	NC_000014.9	+	102338816	102339223	408	268	sense-overlappin	NM_001267827.1	ZNF839
circRNA_09397	0	0	0	0	0	0	0	0	0.030381792	0	0	0	-	NC_000014.9	-	102343821	102355897	12077	5199	sense-overlappin	XM_011536830.1	CINP
circRNA_09398	0	0	0.032712968	0	0	0	0	0	0	0	0	0	-	NC_000014.9	+	102662323	102663938	1616	1616	sense-overlappin	XR_943406.1	
circRNA_09399	0	0	0	0	0	0	0	0	0	0.030258724	0	0	hsa_circ_0006016	NC_000014.9	+	102714423	102721377	6955	331	sense-overlappin	NM_015156.3	RCOR1
circRNA_09400	0	0	0.076330258	0	0	0	0.020098685	0	0	0	0	0	hsa_circ_0004166	NC_000014.9	+	102870185	102876525	6341	587	sense-overlappin	XR_943512.1	
circRNA_09401	0	0	0	0.0309543	0	0	0	0	0	0.030258724	0	0	hsa_circ_0006809	NC_000014.9	-	102968253	102970261	2009	462	sense-overlappin	NM_006035.3	CDC42BPB
circRNA_09402	0	0	0.032712968	0	0	0	0	0	0	0	0	0	hsa_circ_0005178	NC_000014.9	-	102999565	103012188	12624	421	sense-overlappin	NM_006035.3	CDC42BPB
circRNA_09403	0.051437562	0	0.054521613	0.0309543	0	0	0	0.020486295	0	0	0	0.0309546	hsa_circ_0000571	NC_000014.9	-	103003928	103012188	8261	272	sense-overlappin	NM_006035.3	CDC42BPB
circRNA_09404	0.020575025	0	0.021808645	0	0.03009467	0	0.030148028	0	0	0	0	0	hsa_circ_0033469	NC_000014.9	+	103336677	103339803	3127	917	sense-overlappin	NM_001969.4	EIF5
circRNA_09405	0	0.020469968	0.065425935	0.0309543	0.03009467	0	0.040197371	0	0	0.110948655	0	0.0206364	hsa_circ_0008282	NC_000014.9	+	103398951	103405267	6317	6317	sense-overlappin	NM_001128918.2	MARK3
circRNA_09406	0	0	0	0.0206362	0	0	0	0	0	0.020172483	0	0	hsa_circ_0008780	NC_000014.9	+	103398951	103428440	29490	6371	sense-overlappin	NM_001128918.2	MARK3
circRNA_09407	0	0	0	0	0	0	0	0	0.020254528	0	0.04051046	0	-	NC_000014.9	+	103441564	103442092	529	529	intronic	NM_001128918.2	MARK3
circRNA_09408	0.030862537	0	0	0	0	0	0	0	0.020254528	0	0	0	hsa_circ_0033475	NC_000014.9	+	103451918	103457212	5295	137	sense-overlappin	NM_001128918.2	MARK3
circRNA_09409	0	0	0.021808645	0	0	0	0	0	0	0	0	0	hsa_circ_0033480	NC_000014.9	+	103457142	103462461	5320	128	sense-overlappin	NM_001128918.2	MARK3
circRNA_09410	0	0.020469968	0	0	0	0	0	0	0	0	0	0	-	NC_000014.9	+	103467079	103492034	24956	847	sense-overlappin	NM_001128918.2	MARK3
circRNA_09411	0.030862537	0	0	0	0	0	0	0	0	0	0	0	-	NC_000014.9	+	103498502	103500200	1699	72	sense-overlappin	NM_001128918.2	MARK3
circRNA_09412	0	0	0.021808645	0	0	0	0	0	0	0	0	0	-	NC_000014.9	+	103571623	103574170	2548	262	sense-overlappin	NM_001302654.1	APOPT1
circRNA_09413	0	0	0	0	0	0	0.020098685	0	0.030381792	0	0	0	hsa_circ_0007737	NC_000014.9	+	103669511	103673431	3921	464	sense-overlappin	NM_005552.4	KLC1
circRNA_09414	0	0	0	0	0	0	0	0	0	0.020172483	0	0	-	NC_000014.9	+	103669511	103679545	10035	853	sense-overlappin	NM_005552.4	KLC1
circRNA_09415	0	0	0.021808645	0	0	0	0	0	0	0	0	0	-	NC_000014.9	+	103673014	103673431	418	274	sense-overlappin	NM_005552.4	KLC1
circRNA_09416	0.020575025	0	0	0	0	0	0	0	0	0	0	0	-	NC_000014.9	+	103675552	103679545	3994	389	sense-overlappin	NM_005552.4	KLC1
circRNA_09417	0	0	0	0	0	0.020712909	0	0	0	0	0	0	-	NC_000014.9	-	103746373	103754244	7872	694	sense-overlappin	NM_015316.2	PPP1R13B
circRNA_09418	0	0	0.021808645	0	0	0	0	0	0	0	0	0	-	NC_000014.9	-	103746373	103762805	16433	5850	sense-overlappin	NM_015316.2	PPP1R13B
circRNA_09419	0	0.030704951	0.032712968	0.0825448	0.06018934	0	0	0	0	0	0	0	hsa_circ_0000572	NC_000014.9	-	103753000	103754244	1245	372	sense-overlappin	NM_015316.2	PPP1R13B
circRNA_09420	0.082300098	0.051174919	0.4361729	0.2269981	0.12037869	0.041425819	0.060296056	0.040972591	0.070890847	0.050431207	0.04051046	0	hsa_circ_0005791	NC_000014.9	-	103778745	103797518	18774	345	sense-overlappin	NM_015316.2	PPP1R13B
circRNA_09421	0	0	0.032712968	0	0	0	0	0	0	0.020172483	0	0	-	NC_000014.9	-	103784795	103797518	12724	268	sense-overlappin	NM_015316.2	PPP1R13B
circRNA_09422	0	0.020469968	0	0	0	0	0	0	0	0	0	0	-	NC_000014.9	-	103913961	103915189	1229	148	sense-overlappin	NM_001127393.1	C14orf2
circRNA_09423	0	0	0.021808645	0	0	0	0	0	0	0	0	0	-	NC_000014.9	+	103963079	103975553	12475	689	sense-overlappin	XM_011536396.1	TDRD9
circRNA_09424	0	0	0.021808645	0	0	0	0	0	0	0	0	0	-	NC_000014.9	+	103966709	103970621	3913	204	sense-overlappin	XM_011536396.1	TDRD9
circRNA_09425	0	0	0.04361729	0	0	0	0	0	0	0	0	0	hsa_circ_0033530	NC_000014.9	+	104022157	104026939	4783	850	sense-overlappin	XM_011536396.1	TDRD9
circRNA_09426	0	0	0.021808645	0	0	0	0	0	0	0.020172483	0	0	hsa_circ_0006153	NC_000014.9	-	105226069	105226760	692	260	sense-overlappin	XM_005267563.2	BRF1
circRNA_09427	0	0.020469968	0	0	0	0	0	0	0	0	0	0	hsa_circ_0005309	NC_000014.9	-	105252507	105272894	20388	279	sense-overlappin	NM_001242788.1	BRF1
circRNA_09428	0	0	0.021808645	0	0	0	0	0	0	0	0	0	hsa_circ_0033614	NC_000014.9	+	105445418	105454310	8893	454	sense-overlappin	NM_001203258.1	MTA1
circRNA_09429	0.072012586	0	0	0	0	0	0	0	0	0	0	0	hsa_circ_0034088	NC_000015.10	-	22872825	22873729	905	387	sense-overlappin	NM_001033028.1	CYFIP1
circRNA_09430	0	0	0	0	0	0.031069364	0	0	0	0	0	0	hsa_circ_0034102	NC_000015.10	+	24962114	24968082	5969	247	intergenic	NM_022806.2	SNRPN
circRNA_09431	0.041150049	0	0	0	0	0	0	0	0	0.040344966	0	0	hsa_circ_0000585	NC_000015.10	+	25083396	25093957	10562	10562	intergenic		
circRNA_09432	0	0	0.021808645	0	0	0	0	0	0	0	0	0	-	NC_000015.10	-	25354353	25405502	51150	2334	intergenic	XM_011521995.1	UBE3A
circRNA_09433	0	0.020469968	0.13085187	0.0309543	0	0.031069364	0	0	0	0	0	0	-	NC_000015.10	-	25354353	25411971	57619	2827	intergenic	XM_005268267.3	UBE3A
circRNA_09434	0	0	0	0	0	0	0	0	0	0.030258724	0	0	-	NC_000015.10	-	25354353	25415842	61490	2716	intergenic	XM_011521995.1	UBE3A
circRNA_09435	0	0	0.021808645	0	0	0	0	0	0	0	0	0	-	NC_000015.10	-	25393746	25411971	18226	12250	intergenic	XM_005268267.3	UBE3A
circRNA_09436	0	0	0.021808645	0	0	0	0	0	0	0	0	0	hsa_circ_0006140	NC_000015.10	-	25393746	25415842	22097	12139	intergenic	XM_011521995.1	UBE3A
circRNA_09437	0.185175222	0.071644886	0.414364255	0.3095428	0.23072582	0.186416184	0.190937511	0.051215739	0.202545277	0.322759725	0.06076569	0.216682202	hsa_circ_0000586	NC_000015.10	-	25405461	25411971	6511	535	intergenic	XM_005268267.3	UBE3A
circRNA_09438	0	0	0.250799418	0.0825448	0.0702209	0.041425819	0.100493427	0.051215739	0.060763583	0.07060369	0.02025523	0	hsa_circ_0005147	NC_000015.10	-	25405461	25415842	10382	424	intergenic	XM_011521995.1	UBE3A
circRNA_09439	0.020575025	0	0.021808645	0	0	0	0	0	0.040509055	0	0	0	-	NC_000015.10	-	25405461	25426219	20759	10801	intergenic	XM_011521995.1	UBE3A
circRNA_09440	0	0	0.032712968	0	0	0	0	0	0	0	0	0	-	NC_000015.10	-	25408620	25415842	7223	382	intergenic	XM_011521995.1	UBE3A
circRNA_09441	0	0	0	0.0309543	0	0	0.020098685	0	0	0	0	0	-	NC_000015.10	-	32964107	32969477	5371	915	sense-overlappin	XM_011521512.1	FMN1
circRNA_09442	0	0	0.032712968	0	0	0	0	0	0	0	0.02025523	0	-	NC_000015.10	-	33153048	33155045	1998	1998	exonic	XM_011521504.1	FMN1
circRNA_09443	0	0	0	0	0.02006311	0	0	0	0	0	0	0	-	NC_000015.10	+	33722715	33750286	27572	1777	sense-overlappin	XM_011521880.1	RYR3
circRNA_09444	0	0	0	0	0	0	0.020098685	0	0	0	0	0	-	NC_000015.10	-	33896325	33896476	152	152	intronic	XM_005254563.2	AVEN
circRNA_09445	0	0	0	0	0	0	0	0	0	0.040344966	0.02025523	0	hsa_circ_0034346	NC_000015.10	-	34250298	34251057	760	316	sense-overlappin	NM_005135.2	SLC12A6
circRNA_09446	0	0	0	0.0206362	0	0	0	0	0	0	0	0	-	NC_000015.10	-	34860042	34867609	7568	375	sense-overlappin	NM_014691.2	AQR

circRNA_09447	0	0	0	0	0	0.020712909	0	0	0	0	0	0	-	NC_000015.10	-	34896897	34897705	809	217	sense-overlappin	NM_014691.2	AQR
circRNA_09448	0	0	0	0.0206362	0	0	0	0	0	0	0	0	-	NC_000015.10	-	34915038	34927138	12101	470	sense-overlappin	NM_014691.2	AQR
circRNA_09449	0	0	0	0	0	0	0	0	0	0.020172483	0	0	-	NC_000015.10	-	35096526	35118354	21829	21829	intergenic		
circRNA_09450	0	0	0.021808645	0	0.03009467	0	0	0	0	0	0	0	hsa_circ_0034398	NC_000015.10	+	36644278	36657905	13628	245	sense-overlappin	NM_001290233.1	C15orf41
circRNA_09451	0	0	0	0	0.03009467	0	0	0	0	0	0	0	hsa_circ_0008295	NC_000015.10	+	36691685	36709961	18277	1227	sense-overlappin	XR_931922.1	
circRNA_09452	0	0	0.032712968	0	0	0	0	0	0	0	0	0	-	NC_000015.10	+	37935257	37943398	8142	729	sense-overlappin	XM_005254168.2	TMCO5A
circRNA_09453	0	0	0.17446916	0	0	0	0	0	0	0	0	0	-	NC_000015.10	+	37935257	37947696	12440	770	sense-overlappin	XM_005254168.2	TMCO5A
circRNA_09454	0	0	0.021808645	0	0	0	0	0	0	0	0	0	-	NC_000015.10	+	37935257	37968677	33421	21751	sense-overlappin	XM_011521262.1	TMCO5A
circRNA_09455	0	0	0.021808645	0	0	0	0	0	0	0	0	0	-	NC_000015.10	+	37935257	37984835	49579	37909	sense-overlappin	XM_011521262.1	TMCO5A
circRNA_09456	0	0	0.076330258	0	0	0	0	0	0	0	0	0	-	NC_000015.10	+	37936314	37943398	7085	637	sense-overlappin	XM_005254168.2	TMCO5A
circRNA_09457	0	0	0.185373483	0	0	0	0	0	0	0	0	0	-	NC_000015.10	+	38020192	38021272	1081	1081	intergenic		
circRNA_09458	0.030862537	0	0	0	0	0	0	0	0	0	0	0	hsa_circ_0004229	NC_000015.10	+	38299373	38324809	25437	391	sense-overlappin	XM_005254202.2	SPRED1
circRNA_09459	0	0	0.04361729	0	0	0	0	0	0	0	0	0	hsa_circ_0003140	NC_000015.10	+	38299373	38339895	40523	550	sense-overlappin	XM_005254202.2	SPRED1
circRNA_09460	0	0	0.021808645	0	0	0.041425819	0	0.020486295	0	0.050431207	0	0	hsa_circ_0034414	NC_000015.10	-	38502312	38505920	3609	296	sense-overlappin	NM_005739.3	RASGRP1
circRNA_09461	0.061725074	0	0	0	0	0	0	0	0	0	0	0	-	NC_000015.10	+	39582471	39582680	210	210	exonic	NM_003246.2	THBS1
circRNA_09462	0	0	0.021808645	0	0	0	0	0	0	0	0	0	-	NC_000015.10	+	39990273	40008195	17923	1050	sense-overlappin	NM_001013703.3	EIF2AK4
circRNA_09463	0.020575025	0	0.021808645	0	0	0	0	0	0	0	0	0	hsa_circ_0034557	NC_000015.10	+	40009614	40019200	9587	597	sense-overlappin	NM_001013703.3	EIF2AK4
circRNA_09464	0	0	0.04361729	0	0	0	0	0	0	0	0	0	hsa_circ_0008471	NC_000015.10	+	40183714	40185642	1929	477	sense-overlappin	NM_001211.5	BUB1B
circRNA_09465	0	0	0.021808645	0	0	0	0	0	0	0	0	0	hsa_circ_0034565	NC_000015.10	+	40183714	40196774	13061	707	sense-overlappin	NM_001211.5	BUB1B
circRNA_09466	0	0	0.021808645	0	0	0	0	0	0	0	0	0	hsa_circ_0034568	NC_000015.10	+	40196545	40202694	6150	676	sense-overlappin	NM_001211.5	BUB1B
circRNA_09467	0	0	0.021808645	0	0	0	0	0	0	0	0	0	hsa_circ_0034576	NC_000015.10	+	40383228	40392023	8796	613	sense-overlappin	NM_001142761.1	KNSTRN
circRNA_09468	0	0	0.021808645	0	0	0	0	0	0	0	0	0	hsa_circ_0004818	NC_000015.10	+	40407636	40407990	355	142	sense-overlappin	NM_002225.3	IVD
circRNA_09469	0	0	0.021808645	0	0.03009467	0	0	0	0	0	0	0	hsa_circ_0034595	NC_000015.10	+	40571604	40571900	297	297	exonic	NM_001286407.1	RPUSD2
circRNA_09470	0	0	0.021808645	0	0	0	0	0	0	0	0	0	-	NC_000015.10	+	40602915	40611511	8597	379	sense-overlappin	NM_170589.4	CASC5
circRNA_09471	0	0	0.021808645	0	0	0	0	0	0	0	0	0	-	NC_000015.10	+	40608847	40619011	10165	318	sense-overlappin	NM_170589.4	CASC5
circRNA_09472	0	0	0	0	0	0	0	0	0.020254528	0	0	0	-	NC_000015.10	+	40628070	40628678	609	207	sense-overlappin	NM_144508.4	CASC5
circRNA_09473	0	0	0	0	0.04012623	0	0	0	0	0	0	0	-	NC_000015.10	+	40628070	40650819	22750	1072	sense-overlappin	NM_144508.4	CASC5
circRNA_09474	0	0	0.021808645	0	0	0	0	0	0	0	0	0	-	NC_000015.10	+	40641379	40650819	9441	4268	sense-overlappin	NM_144508.4	CASC5
circRNA_09475	0	0	0.021808645	0	0	0	0	0	0	0	0	0	hsa_circ_0034616	NC_000015.10	+	40709025	40728824	19800	301	sense-overlappin	XM_011521857.1	RAD51
circRNA_09476	0.020575025	0	0	0	0	0.041425819	0.020098685	0	0.040509055	0.050431207	0	0	hsa_circ_0004942	NC_000015.10	-	40744047	40745259	1213	386	sense-overlappin	NM_001304802.1	RMDN3
circRNA_09477	0	0	0	0.0309543	0	0	0	0	0	0	0	0	hsa_circ_0034651	NC_000015.10	-	40987091	40987974	884	262	sense-overlappin	XM_011521686.1	INO80
circRNA_09478	0	0	0	0.0206362	0	0	0	0	0	0	0	0	-	NC_000015.10	-	41047408	41050102	2695	461	sense-overlappin	XM_011521686.1	INO80
circRNA_09479	0.061725074	0	0	0	0	0	0	0	0	0	0	0	hsa_circ_0034669	NC_000015.10	-	41055247	41058781	3535	346	sense-overlappin	NM_017553.2	INO80
circRNA_09480	0.041150049	0	0	0	0	0	0.020098685	0.020486295	0	0	0.030382845	0	hsa_circ_0007489	NC_000015.10	-	41069570	41070547	978	177	sense-overlappin	NM_017553.2	INO80
circRNA_09481	0	0	0	0	0	0	0	0	0	0.020172483	0	0	-	NC_000015.10	-	41069570	41072058	2489	387	sense-overlappin	NM_017553.2	INO80
circRNA_09482	0	0	0	0	0	0	0	0	0.020254528	0	0	0	hsa_circ_0006525	NC_000015.10	-	41069570	41074565	4996	651	sense-overlappin	NM_017553.2	INO80
circRNA_09483	0.020575025	0	0	0.0206362	0	0	0	0	0	0	0	0.0309546	hsa_circ_0004279	NC_000015.10	-	41085369	41087682	2314	336	sense-overlappin	NM_017553.2	INO80
circRNA_09484	0	0	0.032712968	0	0	0	0	0	0	0	0	0	hsa_circ_0034693	NC_000015.10	+	41356039	41358258	2220	212	sense-overlappin	NM_001243142.1	NUSAP1
circRNA_09485	0	0	0.021808645	0	0.02006311	0	0	0	0	0	0	0	hsa_circ_0008926	NC_000015.10	+	41356039	41375828	19790	672	sense-overlappin	NM_001243142.1	NUSAP1
circRNA_09486	0	0	0.098138903	0	0.11034713	0.020712909	0	0	0	0	0	0	hsa_circ_0008346	NC_000015.10	+	41356039	41377304	21266	781	sense-overlappin	NM_001243142.1	NUSAP1
circRNA_09487	0	0	0	0	0	0.020712909	0	0	0	0	0	0	-	NC_000015.10	+	41475525	41477515	1991	454	sense-overlappin	NM_015138.4	RTF1
circRNA_09488	0.257187808	0.194464692	1.090432251	0.5055866	0.31097828	0.300337185	0.160789483	0.194619807	0.111399902	0.302587242	0.091148535	0.103182001	hsa_circ_0000591	NC_000015.10	+	41668828	41669958	1131	1131	exonic	XM_005254246.1	MGA
circRNA_09489	0	0.020469968	0.021808645	0.0206362	0	0	0	0	0	0	0	0	-	NC_000015.10	+	41668828	41699159	30332	2255	sense-overlappin	XM_005254246.1	MGA
circRNA_09490	0.051437562	0.122819805	0.545216125	0.1650895	0.26082049	0.082851637	0.050246713	0.051215739	0.030381792	0.14120738	0.02025523	0.0309546	hsa_circ_0000592	NC_000015.10	+	41696075	41699159	3085	1124	sense-overlappin	XM_006720445.2	MGA
circRNA_09491	0	0	0.065425935	0	0.04012623	0	0	0.040972591	0	0	0	0	hsa_circ_0000593	NC_000015.10	+	41696075	41713496	17422	2366	sense-overlappin	XM_006720445.2	MGA
circRNA_09492	0	0	0.021808645	0	0	0	0	0	0	0	0	0	-	NC_000015.10	+	41696075	41729349	33275	2779	sense-overlappin	XM_006720445.2	MGA
circRNA_09493	0	0	0	0.0206362	0	0	0	0	0	0	0	0	-	NC_000015.10	+	41707728	41713496	5769	1242	sense-overlappin	XM_006720445.2	MGA
circRNA_09494	0	0	0.021808645	0	0	0	0	0	0	0	0	0	-	NC_000015.10	+	41742546	41754567	12022	2557	sense-overlappin	XM_006720445.2	MGA
circRNA_09495	0	0	0	0	0	0.031069364	0	0	0	0	0	0	hsa_circ_0034754	NC_000015.10	+	41748637	41750615	1979	1796	sense-overlappin	XM_006720445.2	MGA
circRNA_09496	0	0	0	0	0	0.020712909	0	0	0	0	0	0	-	NC_000015.10	-	42184517	42199961	15445	678	sense-overlappin	XM_011521403.1	VPS39
circRNA_09497	0	0.040939935	0.119947548	0.0928628	0.06018934	0.103564547	0.030148028	0.051215739	0	0.14120738	0	0.051591	hsa_circ_0034803	NC_000015.10	-	42260958	42268032	7075	299	sense-overlappin	NM_001286487.1	TMEM87A
circRNA_09498	0	0	0	0	0	0	0	0	0	0	0	0.0206364	hsa_circ_0006343	NC_000015.10	-	42264090	42268032	3943	200	sense-overlappin	NM_001286487.1	TMEM87A
circRNA_09499	0	0	0.021808645	0	0	0	0	0	0	0	0	0	-	NC_000015.10	+	42321785	42330672	8888	684	sense-overlappin	NM_198141.2	GANC
circRNA_09500	0	0	0	0	0.02006311	0	0	0	0	0	0	0	hsa_circ_0034815	NC_000015.10	-	42435384	42439813	4430	1118	sense-overlappin	NM_001284306.1	ZNF106
circRNA_09501	0	0	0	0	0	0	0	0.020486295	0	0	0.04051046	0.0412728	hsa_circ_0034822	NC_000015.10	+	42511833	42515354	3522	280	sense-overlappin	XM_011522130.1	SNAP23
circRNA_09502	0	0	0.021808645	0.0206362	0	0	0	0	0	0.030258724	0	0	hsa_circ_0034825	NC_000015.10	+	42515237	42529819	14583	455	sense-overlappin	XM_011522129.1	SNAP23

circRNA_09503	0.020575025	0	0	0	0	0	0	0	0	0	0	0	-	NC_000015.10	+	42559339	42563857	4519	312	sense-overlappin	NM_001130447.1	HAUS2
circRNA_09504	0	0	0	0	0	0	0	0	0	0	0	0.0206364	-	NC_000015.10	+	42665253	42669337	4085	321	sense-overlappin	XR_931874.1	
circRNA_09505	0	0	0	0	0.02006311	0	0	0	0	0	0	0	hsa_circ_0034866	NC_000015.10	-	42794644	42830078	35435	689	sense-overlappin	XM_011521269.1	TTBK2
circRNA_09506	0.030862537	0	0.021808645	0.0309543	0	0	0	0	0	0.050431207	0	0	hsa_circ_0002466	NC_000015.10	-	42810614	42840433	29820	605	sense-overlappin	XM_011521269.1	TTBK2
circRNA_09507	0	0	0	0	0.02006311	0	0	0	0	0	0	0	hsa_circ_0034869	NC_000015.10	-	42810614	42872758	62145	753	sense-overlappin	NM_173500.3	TTBK2
circRNA_09508	0	0	0	0	0	0	0	0	0.020254528	0	0	0	-	NC_000015.10	-	42811688	42840433	28746	479	sense-overlappin	XM_011521269.1	TTBK2
circRNA_09509	0.072012586	0	0.152660515	0.0825448	0.05015779	0	0	0.071702034	0.040509055	0.090776173	0.02025523	0.0412728	hsa_circ_0008319	NC_000015.10	-	42827928	42840433	12506	320	sense-overlappin	XM_011521269.1	TTBK2
circRNA_09510	0	0	0.098138903	0	0	0	0	0	0	0	0	0	hsa_circ_0006297	NC_000015.10	-	42827928	42878684	50757	604	sense-overlappin	NM_173500.3	TTBK2
circRNA_09511	0	0	0	0	0	0	0	0.020486295	0	0	0	0	hsa_circ_0034878	NC_000015.10	-	42829938	42840433	10496	215	sense-overlappin	XM_011521269.1	TTBK2
circRNA_09512	0	0	0.04361729	0	0	0	0.020098685	0	0	0.07060369	0	0	hsa_circ_0034880	NC_000015.10	-	42872611	42878684	6074	284	sense-overlappin	NM_173500.3	TTBK2
circRNA_09513	0	0	0	0.0309543	0.02006311	0.020712909	0.020098685	0	0.020254528	0	0	0.0206364	hsa_circ_0008546	NC_000015.10	-	42950262	42966286	16025	651	sense-overlappin	NM_174916.2	UBR1
circRNA_09514	0	0	0	0	0	0	0	0	0	0.030258724	0	0	-	NC_000015.10	-	42958013	42960701	2689	135	sense-overlappin	NM_174916.2	UBR1
circRNA_09515	0	0	0	0	0	0	0.020098685	0	0	0	0	0	-	NC_000015.10	-	42976717	43025429	48713	1834	sense-overlappin	NM_174916.2	UBR1
circRNA_09516	0	0	0	0.0206362	0	0	0	0	0	0	0	0	-	NC_000015.10	-	42988819	43002704	13886	488	sense-overlappin	NM_174916.2	UBR1
circRNA_09517	0	0	0	0	0.02006311	0	0.020098685	0	0	0	0	0	hsa_circ_0034913	NC_000015.10	-	43002555	43025429	22875	1124	sense-overlappin	NM_174916.2	UBR1
circRNA_09518	0	0	0	0	0	0	0	0	0	0.040344966	0	0	-	NC_000015.10	-	43047161	43048491	1331	229	sense-overlappin	NM_174916.2	UBR1
circRNA_09519	0	0	0	0	0	0	0	0	0	0	0	0.0412728	-	NC_000015.10	+	43135512	43160794	25283	1004	sense-overlappin	XM_011522071.1	TMEM62
circRNA_09520	0	0	0	0.0515905	0.03009467	0	0	0	0	0.020172483	0	0	hsa_circ_0034947	NC_000015.10	+	43334945	43335826	882	357	sense-overlappin	NM_001012969.3	ADAL
circRNA_09521	0	0	0	0	0.02006311	0	0	0	0	0	0	0	-	NC_000015.10	+	43335696	43349045	13350	709	sense-overlappin	NM_001012969.3	ADAL
circRNA_09522	0	0	0	0	0	0	0	0	0	0	0.02025523	0	hsa_circ_0034967	NC_000015.10	+	43395107	43401850	6744	783	sense-overlappin	XR_243092.2	
circRNA_09523	0	0	0	0	0	0.031069364	0	0	0	0	0	0	hsa_circ_0034970	NC_000015.10	+	43398038	43401850	3813	518	sense-overlappin	XR_243092.2	
circRNA_09524	0.020575025	0.030704951	0	0	0	0	0	0	0.020254528	0	0	0	hsa_circ_0034972	NC_000015.10	+	43400044	43401850	1807	313	sense-overlappin	NM_001286414.1	TUBGCP4
circRNA_09525	0	0	0	0	0	0	0	0	0	0.030258724	0	0	-	NC_000015.10	-	43441526	43481022	39497	2727	sense-overlappin	XM_011521985.1	TP53BP1
circRNA_09526	0	0	0	0.0309543	0	0	0	0	0	0	0	0	hsa_circ_0006327	NC_000015.10	-	43455892	43457218	1327	1327	exonic	XM_011521985.1	TP53BP1
circRNA_09527	0	0	0	0.0206362	0	0	0	0	0	0	0	0	-	NC_000015.10	-	43455892	43470066	14175	1536	sense-overlappin	XM_011521985.1	TP53BP1
circRNA_09528	0	0	0	0	0	0	0	0	0	0.040344966	0	0	-	NC_000015.10	-	43647917	43744568	96652	95531	sense-overlappin	XM_011521277.1	STRC
circRNA_09529	0	0	0	0	0.03009467	0	0	0	0	0	0	0	hsa_circ_0002151	NC_000015.10	+	43753824	43756766	2943	197	sense-overlappin	NM_005313.4	PDIA3
circRNA_09530	0	0	0	0.0825448	0.02006311	0	0.020098685	0.020486295	0	0.040344966	0	0	hsa_circ_0002891	NC_000015.10	+	43756649	43763206	6558	356	sense-overlappin	NM_005313.4	PDIA3
circRNA_09531	0.020575025	0	0	0	0	0	0	0	0	0	0.04051046	0	hsa_circ_0003598	NC_000015.10	+	43761424	43763206	1783	238	sense-overlappin	NM_005313.4	PDIA3
circRNA_09532	0	0	0	0	0	0	0	0	0	0	0.02025523	0	-	NC_000015.10	-	43814501	43815074	574	318	sense-overlappin	NM_005926.2	MFAP1
circRNA_09533	0	0	0	0	0	0	0.020098685	0	0	0	0	0	hsa_circ_0004078	NC_000015.10	+	44328685	44332078	3394	194	sense-overlappin	XM_011521170.1	CASC4
circRNA_09534	0	0	0	0.0309543	0.03009467	0	0	0.020486295	0	0.040344966	0	0	hsa_circ_0035051	NC_000015.10	+	44328685	44338317	9633	420	sense-overlappin	XM_011521170.1	CASC4
circRNA_09535	0	0	0	0	0.05015779	0	0	0	0	0.030258724	0	0	hsa_circ_0035052	NC_000015.10	+	44328685	44380976	52292	690	sense-overlappin	NM_138423.3	CASC4
circRNA_09536	0.041150049	0.040939935	0	0.0309543	0.10031557	0.062138728	0	0.051215739	0.030381792	0.14120738	0	0.0412728	hsa_circ_0000596	NC_000015.10	+	44331988	44338317	6330	317	sense-overlappin	XM_011521170.1	CASC4
circRNA_09537	0	0	0	0	0	0	0	0.020486295	0	0	0	0	-	NC_000015.10	+	44331988	44339481	7494	1481	sense-overlappin	XM_011521170.1	CASC4
circRNA_09538	0	0	0	0	0.04012623	0	0	0	0	0	0	0	-	NC_000015.10	+	44331988	44380976	48989	587	sense-overlappin	NM_138423.3	CASC4
circRNA_09539	0	0	0	0	0	0	0.030148028	0	0	0	0	0	hsa_circ_0003829	NC_000015.10	+	44379690	44380976	1287	270	sense-overlappin	NM_138423.3	CASC4
circRNA_09540	0	0	0.021808645	0	0	0	0	0	0	0	0	0	-	NC_000015.10	+	44413336	44414615	1280	1280	exonic	NM_138423.3	CASC4
circRNA_09541	0.020575025	0	0.032712968	0	0	0	0	0	0	0	0	0	-	NC_000015.10	+	44484224	44486700	2477	286	sense-overlappin	XM_011521665.1	CTDSPL2
circRNA_09542	0	0	0	0	0	0	0	0	0.020254528	0	0	0	hsa_circ_0035060	NC_000015.10	+	44490784	44496458	5675	295	sense-overlappin	XM_011521665.1	CTDSPL2
circRNA_09543	0	0	0	0	0.02006311	0	0	0	0	0	0	0	-	NC_000015.10	+	44550876	44554667	3792	262	sense-overlappin	NM_001284335.1	EIF3J
circRNA_09544	0	0	0	0	0	0	0	0.030729443	0	0	0	0	-	NC_000015.10	+	44560249	44563225	2977	2282	sense-overlappin	NM_001284335.1	EIF3J
circRNA_09545	0	0	0	0	0	0	0	0.020486295	0	0	0	0	-	NC_000015.10	-	44565854	44570658	4805	656	sense-overlappin	XM_005254695.3	SPG11
circRNA_09546	0	0	0.021808645	0	0	0	0	0	0	0	0	0	hsa_circ_0035086	NC_000015.10	-	44592331	44596943	4613	742	sense-overlappin	XM_005254695.3	SPG11
circRNA_09547	0	0	0.021808645	0	0.02006311	0	0	0	0	0	0	0	hsa_circ_0007457	NC_000015.10	-	44606025	44610985	4961	375	sense-overlappin	XM_005254695.3	SPG11
circRNA_09548	0.030862537	0	0.021808645	0	0.11034713	0.062138728	0.030148028	0.030729443	0.020254528	0.040344966	0.030382845	0.0206364	hsa_circ_0003784	NC_000015.10	-	44615363	44620403	5041	418	sense-overlappin	XM_005254695.3	SPG11
circRNA_09549	0	0	0	0	0	0.020712909	0	0	0	0	0	0	-	NC_000015.10	-	44626331	44657263	30933	1544	sense-overlappin	XM_005254695.3	SPG11
circRNA_09550	0	0	0	0.0309543	0	0	0	0	0	0	0	0	hsa_circ_0035115	NC_000015.10	-	45175219	45178306	3088	349	sense-overlappin	NM_001301171.1	SHF
circRNA_09551	0	0	0	0	0	0	0	0	0	0	0	0.0206364	-	NC_000015.10	+	45476079	45476552	474	474	intergenic		
circRNA_09552	0	0	0.032712968	0	0	0	0	0	0	0	0	0	-	NC_000015.10	+	45682478	45689217	6740	431	sense-overlappin	NM_001271213.1	SQRDL
circRNA_09553	0	0	0.021808645	0	0.04012623	0	0	0	0	0	0	0	-	NC_000015.10	-	48717018	48748610	31593	779	sense-overlappin	XR_931770.1	
circRNA_09554	0	0	0	0	0	0.031069364	0	0	0	0	0	0	hsa_circ_0035184	NC_000015.10	-	49027365	49028682	1318	371	sense-overlappin	NM_001193489.1	SECISBP2L
circRNA_09555	0	0	0.239895095	0	0	0	0	0	0	0	0	0	hsa_circ_0007487	NC_000015.10	+	49225205	49239367	14163	383	sense-overlappin	XM_011521441.1	GALK2
circRNA_09556	0	0	0.196277805	0.0309543	0	0.020712909	0	0	0	0	0	0	-	NC_000015.10	+	49225205	49292537	67333	846	sense-overlappin	XM_011521441.1	GALK2
circRNA_09557	0	0	0.054521613	0	0	0	0	0	0	0	0	0	-	NC_000015.10	+	49225205	49308777	83573	17086	sense-overlappin	XM_011521441.1	GALK2

circRNA_09558	0	0	0.021808645	0	0	0	0	0	0	0	0	0	-	NC_000015.10	+	49235625	49239367	3743	464	sense-overlappin	NM_001001556.2	GALK2
circRNA_09559	0	0	0.032712968	0	0	0	0	0	0	0	0	0	-	NC_000015.10	+	49235625	49292537	56913	927	sense-overlappin	NM_001001556.2	GALK2
circRNA_09560	0.020575025	0.133054789	0.327129675	0.0309543	0.05015779	0.041425819	0.080394741	0.102431477	0.070890847	0.100862414	0.06076569	0.0412728	hsa_circ_0008488	NC_000015.10	+	49235851	49239367	3517	238	sense-overlappin	NM_001001556.2	GALK2
circRNA_09561	0	0	0.04361729	0.0412724	0.05015779	0	0	0.020486295	0	0	0	0	hsa_circ_0035188	NC_000015.10	+	49235851	49283718	47868	490	sense-overlappin	NM_001001556.2	GALK2
circRNA_09562	0.102875123	0.051174919	0.21808645	0.1444533	0	0.093208092	0.070345399	0.030729443	0.060763583	0	0.02025523	0.0309546	hsa_circ_0035189	NC_000015.10	+	49235851	49292537	56687	701	sense-overlappin	NM_001001556.2	GALK2
circRNA_09563	0	0	0.032712968	0	0	0	0	0	0	0	0	0	-	NC_000015.10	+	49235851	49308777	72927	871	sense-overlappin	XM_011521442.1	GALK2
circRNA_09564	0	0	0.098138903	0.0206362	0	0	0	0	0	0.020172483	0	0	hsa_circ_0005337	NC_000015.10	+	49235851	49328130	92280	1128	sense-overlappin	XR_931787.1	
circRNA_09565	0	0	0.021808645	0	0	0	0.020098685	0	0	0	0	0	-	NC_000015.10	+	49281987	49292537	10551	463	sense-overlappin	NM_001001556.2	GALK2
circRNA_09566	0	0.030704951	0	0	0	0	0	0	0.020254528	0.030258724	0	0	hsa_circ_0035194	NC_000015.10	+	49625113	49634794	9682	780	sense-overlappin	XM_011521813.1	DTWD1
circRNA_09567	0	0.040939935	0.032712968	0.0309543	0.06018934	0	0	0.040972591	0.030381792	0	0.030382845	0	hsa_circ_0002551	NC_000015.10	-	50300789	50301368	580	262	sense-overlappin	NM_005254.5	GABPB1
circRNA_09568	0	0	0	0	0	0.020712909	0	0	0	0.020172483	0	0	hsa_circ_0007983	NC_000015.10	-	50300789	50304133	3345	625	sense-overlappin	NM_005254.5	GABPB1
circRNA_09569	0	0	0.04361729	0	0.02006311	0.051782273	0	0.040972591	0	0	0	0	hsa_circ_0003501	NC_000015.10	-	50300789	50309798	9010	733	sense-overlappin	NM_005254.5	GABPB1
circRNA_09570	0	0	0	0	0.02006311	0	0	0	0	0	0	0	-	NC_000015.10	-	50300789	50314670	13882	5605	sense-overlappin	NM_005254.5	GABPB1
circRNA_09571	0	0	0	0	0.03009467	0	0	0	0	0	0	0	-	NC_000015.10	-	50300789	50346395	45607	757	sense-overlappin	XM_005254273.3	GABPB1
circRNA_09572	0	0	0.032712968	0	0	0	0	0	0	0	0	0	hsa_circ_0035211	NC_000015.10	+	50432338	50449485	17148	562	sense-overlappin	XM_006720761.2	USP8
circRNA_09573	0	0	0.021808645	0	0	0	0	0	0	0	0	0	-	NC_000015.10	+	50439009	50449485	10477	400	sense-overlappin	NM_001128610.2	USP8
circRNA_09574	0	0	0	0	0	0	0.020098685	0	0	0	0	0	-	NC_000015.10	+	50441349	50449485	8137	231	sense-overlappin	NM_001128610.2	USP8
circRNA_09575	0	0	0	0	0	0.020712909	0	0	0	0	0.02025523	0	hsa_circ_0035216	NC_000015.10	+	50465047	50471795	6749	308	sense-overlappin	NM_001128610.2	USP8
circRNA_09576	0	0	0.04361729	0	0	0	0	0	0	0	0	0	hsa_circ_0003879	NC_000015.10	+	50471633	50477499	5867	532	sense-overlappin	NM_001128610.2	USP8
circRNA_09577	0	0	0	0	0.02006311	0	0	0	0	0	0	0	-	NC_000015.10	-	50574274	50580908	6635	751	sense-overlappin	NM_001301212.1	TRPM7
circRNA_09578	0	0.040939935	0	0	0	0	0	0	0	0	0	0	-	NC_000015.10	-	50574274	50583159	8886	822	sense-overlappin	NM_001301212.1	TRPM7
circRNA_09579	0	0	0.032712968	0	0.02006311	0	0	0	0	0	0	0	hsa_circ_0008693	NC_000015.10	-	50574852	50575789	938	350	sense-overlappin	NM_001301212.1	TRPM7
circRNA_09580	0	0.071644886	0	0.0412724	0.05015779	0.031069364	0.040197371	0.020486295	0.040509055	0.060517448	0	0	hsa_circ_0035228	NC_000015.10	-	50583089	50592626	9538	1009	sense-overlappin	XM_005254486.2	TRPM7
circRNA_09581	0	0	0.032712968	0	0.04012623	0.020712909	0.060296056	0.030729443	0	0.080689931	0	0	-	NC_000015.10	-	50586392	50592626	6235	938	sense-overlappin	XM_005254486.2	TRPM7
circRNA_09582	0	0	0	0	0.02006311	0	0	0	0	0	0	0.0206364	hsa_circ_0008637	NC_000015.10	-	50631417	50648885	17469	1082	sense-overlappin	NM_001301212.1	TRPM7
circRNA_09583	0	0	0	0.0412724	0	0	0	0	0	0	0	0	hsa_circ_0035249	NC_000015.10	-	50631417	50663046	31630	1201	sense-overlappin	NM_001301212.1	TRPM7
circRNA_09584	0	0	0	0	0.02006311	0	0	0	0	0	0	0	-	NC_000015.10	-	50634382	50639548	5167	472	sense-overlappin	NM_001301212.1	TRPM7
circRNA_09585	0	0	0	0.0309543	0	0	0	0	0	0	0	0	hsa_circ_0007206	NC_000015.10	-	50637422	50639548	2127	297	sense-overlappin	NM_001301212.1	TRPM7
circRNA_09586	0	0	0	0	0.03009467	0.031069364	0	0	0	0	0	0	hsa_circ_0004951	NC_000015.10	-	50643340	50663046	19707	532	sense-overlappin	NM_001301212.1	TRPM7
circRNA_09587	0	0	0	0.0309543	0	0	0.020098685	0	0	0.030258724	0	0	hsa_circ_0007775	NC_000015.10	-	50648687	50663046	14360	318	sense-overlappin	NM_001301212.1	TRPM7
circRNA_09588	0	0	0	0	0	0	0	0	0	0	0.04051046	0	-	NC_000015.10	-	50722124	50749746	27623	1315	sense-overlappin	XM_005254722.2	SPPL2A
circRNA_09589	0	0	0	0	0.02006311	0	0	0	0	0	0	0	-	NC_000015.10	-	50732603	50749746	17144	948	sense-overlappin	NM_032802.3	SPPL2A
circRNA_09590	0.020575025	0	0	0	0.03009467	0.031069364	0.020098685	0	0	0.020172483	0	0.0309546	-	NC_000015.10	-	50747495	50748870	1376	407	sense-overlappin	NM_032802.3	SPPL2A
circRNA_09591	0	0	0.021808645	0	0	0	0	0	0.020254528	0	0	0	hsa_circ_0035269	NC_000015.10	+	50948020	50958794	10775	675	sense-overlappin	XM_005254264.2	AP4E1
circRNA_09592	0	0	0	0	0	0	0	0.020486295	0	0	0	0	hsa_circ_0035276	NC_000015.10	-	51451645	51453641	1997	145	sense-overlappin	NM_001174116.1	DMXL2
circRNA_09593	0	0	0	0	0	0	0	0	0	0	0.02025523	0	hsa_circ_0035277	NC_000015.10	-	51451645	51455228	3584	223	sense-overlappin	NM_001174116.1	DMXL2
circRNA_09594	0	0	0	0	0.03009467	0	0	0	0	0	0	0	hsa_circ_0035281	NC_000015.10	-	51498552	51500231	1680	1680	exonic	NM_001174116.1	DMXL2
circRNA_09595	0	0	0	0	0.04012623	0	0	0	0	0.030258724	0	0	-	NC_000015.10	-	51535663	51547408	11746	1869	sense-overlappin	NM_001174116.1	DMXL2
circRNA_09596	0.020575025	0	0	0	0	0	0	0	0	0	0	0	hsa_circ_0005566	NC_000015.10	+	51781044	51782828	1785	239	sense-overlappin	NM_014548.3	TMOD2
circRNA_09597	0	0.040939935	0.04361729	0.0206362	0.02006311	0.051782273	0	0.030729443	0.060763583	0.040344966	0.02025523	0.0206364	hsa_circ_0035292	NC_000015.10	+	51869217	51902036	32820	898	sense-overlappin	NM_014547.4	TMOD3
circRNA_09598	0	0	0.032712968	0	0	0	0	0	0	0	0	0	hsa_circ_0035294	NC_000015.10	+	51887589	51902036	14448	741	sense-overlappin	NM_014547.4	TMOD3
circRNA_09599	0	0	0	0	0	0	0	0.020486295	0	0	0	0	hsa_circ_0035296	NC_000015.10	+	51893815	51902036	8222	528	sense-overlappin	NM_014547.4	TMOD3
circRNA_09600	0.020575025	0	0	0	0	0	0	0	0	0	0	0	hsa_circ_0035298	NC_000015.10	+	51896419	51902036	5618	397	sense-overlappin	NM_014547.4	TMOD3
circRNA_09601	0	0	0	0	0.02006311	0	0	0	0	0	0	0	hsa_circ_0004773	NC_000015.10	+	51900155	51902036	1882	289	sense-overlappin	NM_014547.4	TMOD3
circRNA_09602	0	0	0.021808645	0	0	0	0	0	0	0	0	0	-	NC_000015.10	+	51921496	51934354	12859	12859	intergenic		
circRNA_09603	0	0	0.054521613	0.0309543	0	0	0	0	0.030381792	0.020172483	0	0	-	NC_000015.10	+	52045830	52050137	4308	1331	sense-overlappin	XM_005254536.3	MAPK6
circRNA_09604	0	0	0.04361729	0	0.03009467	0	0	0	0	0	0	0	-	NC_000015.10	-	52269753	52282892	13140	913	sense-overlappin	NM_018728.3	MYO5C
circRNA_09605	0	0	0	0.0206362	0	0	0	0	0	0	0	0	-	NC_000015.10	-	52387829	52389363	1535	210	sense-overlappin	XM_005254398.3	MYO5A
circRNA_09606	0	0	0	0	0	0	0	0	0	0.040344966	0	0	hsa_circ_0002207	NC_000015.10	-	52425830	52433285	7456	428	sense-overlappin	XM_005254398.3	MYO5A
circRNA_09607	0	0	0	0.0412724	0.02006311	0	0	0.020486295	0	0.020172483	0	0	-	NC_000015.10	-	52584745	52593736	8992	428	sense-overlappin	NM_001286495.1	FAM214A
circRNA_09608	0	0	0	0.0309543	0	0.020712909	0	0	0	0	0	0	hsa_circ_0035350	NC_000015.10	-	52605133	52611777	6645	2259	sense-overlappin	NM_001286495.1	FAM214A
circRNA_09609	0	0	0.054521613	0	0	0	0	0	0	0	0	0	-	NC_000015.10	-	53699750	53722908	23159	1612	sense-overlappin	XM_011521435.1	WDR72
circRNA_09610	0.051437562	0	0	0.0309543	0	0	0	0	0	0.020172483	0	0	-	NC_000015.10	+	54012648	54015886	3239	3239	exonic	XM_005254394.3	UNC13C
circRNA_09611	0	0.030704951	0.04361729	0	0	0	0.040197371	0.030729443	0	0	0	0.051591	hsa_circ_0003863	NC_000015.10	-	55223889	55234956	11068	489	sense-overlappin	XM_011521856.1	RAB27A
circRNA_09612	0	0	0	0	0	0.020712909	0	0	0	0	0	0	-	NC_000015.10	+	55327531	55334007	6477	377	sense-overlappin	XM_005254795.3	PIGB

circRNA_09613	0.051437562	0	0	0	0	0	0	0	0	0	0	0	hsa_circ_0035380	NC_000015.10	+	55327531	55341802	14272	706	sense-overlappin	XM_005254795.3	PIGB
circRNA_09614	0.030862537	0	0	0.0412724	0	0	0	0	0	0	0	0.0206364	-	NC_000015.10	-	55338520	55348725	10206	7999	antisense	XM_005254795.3	PIGB
circRNA_09615	0	0	0	0.0206362	0	0.041425819	0	0	0	0	0	0	-	NC_000015.10	-	55348332	55348725	394	394	antisense	XM_005254795.3	PIGB
circRNA_09616	0	0	0	0	0	0	0	0.020486295	0	0	0	0	-	NC_000015.10	-	55432497	55450367	17871	516	sense-overlappin	NM_130810.3	DYX1C1
circRNA_09617	0	0	0	0	0.02006311	0	0	0	0	0	0	0	-	NC_000015.10	-	55439472	55467161	27690	488	sense-overlappin	NM_001033560.1	DYX1C1
circRNA_09618	0	0	0.021808645	0	0	0	0	0	0	0	0	0	-	NC_000015.10	-	55833008	55834286	1279	265	sense-overlappin	NM_001284338.1	NEDD4
circRNA_09619	0	0	0	0	0.04012623	0	0	0	0	0.030258724	0	0	-	NC_000015.10	+	56373441	56412436	38996	844	sense-overlappin	XM_005254361.2	TEX9
circRNA_09620	0	0.040939935	0.021808645	0	0.02006311	0	0.020098685	0	0	0	0	0.0206364	-	NC_000015.10	+	56383952	56412436	28485	780	sense-overlappin	XM_005254361.2	TEX9
circRNA_09621	0	0.020469968	0.032712968	0.0825448	0.04012623	0.082851637	0.020098685	0.030729443	0.020254528	0.040344966	0	0.0309546	hsa_circ_0000603	NC_000015.10	+	56388472	56394834	6363	565	sense-overlappin	XM_005254361.2	TEX9
circRNA_09622	0	0	0	0	0	0	0	0	0	0.020172483	0	0	-	NC_000015.10	+	56388472	56412436	23965	700	sense-overlappin	XM_005254361.2	TEX9
circRNA_09623	0	0	0	0.0515905	0	0	0	0	0	0	0	0	-	NC_000015.10	+	56391243	56394834	3592	433	sense-overlappin	XM_005254361.2	TEX9
circRNA_09624	0.030862537	0	0	0.0619086	0	0	0	0.020486295	0	0	0.02025523	0.0206364	-	NC_000015.10	+	56394165	56394834	670	257	sense-overlappin	XM_005254361.2	TEX9
circRNA_09625	0	0	0.04361729	0	0	0	0	0	0	0	0	0	hsa_circ_0035410	NC_000015.10	-	56689041	56707306	18266	865	sense-overlappin	XM_011521702.1	ZNF280D
circRNA_09626	0	0	0	0	0	0	0	0.020486295	0	0	0	0	-	NC_000015.10	-	56700933	56707306	6374	466	sense-overlappin	XM_011521702.1	ZNF280D
circRNA_09627	0.030862537	0	0	0	0	0	0	0	0	0	0	0	-	NC_000015.10	-	56890014	56892300	2287	2287	intergenic		
circRNA_09628	0	0.020469968	0.032712968	0	0	0.020712909	0	0	0	0	0.02025523	0	hsa_circ_0004889	NC_000015.10	+	57091789	57095163	3375	3375	sense-overlappin	XM_005254613.3	TCF12
circRNA_09629	0	0	0.04361729	0.0309543	0	0	0	0.020486295	0.020254528	0.030258724	0	0	hsa_circ_0006380	NC_000015.10	+	57231152	57234107	2956	456	sense-overlappin	XM_005254613.3	TCF12
circRNA_09630	0.082300098	0.040939935	0	0.2373162	0	0.062138728	0	0	0	0.050431207	0.02025523	0	hsa_circ_0035431	NC_000015.10	+	57437985	57442478	4494	1818	sense-overlappin	XM_011522120.1	CGNL1
circRNA_09631	0	0	0	0.0206362	0	0	0	0	0	0	0	0	-	NC_000015.10	+	57437985	57453818	15834	2205	sense-overlappin	XM_011522120.1	CGNL1
circRNA_09632	0	0	0	0.0825448	0	0	0	0	0	0	0	0.0206364	hsa_circ_0035432	NC_000015.10	+	57437985	57461892	23908	2418	sense-overlappin	XM_011522120.1	CGNL1
circRNA_09633	0.030862537	0	0.04361729	0.1650895	0	0.020712909	0	0	0	0	0	0	hsa_circ_0035435	NC_000015.10	+	57451500	57461892	10393	600	sense-overlappin	XM_011522120.1	CGNL1
circRNA_09634	0	0	0.08723458	0	0	0	0	0	0	0	0	0	hsa_circ_0035443	NC_000015.10	-	57992705	58014281	21577	681	sense-overlappin	NM_001206897.1	ALDH1A2
circRNA_09635	0	0	0.119947548	0	0	0	0	0	0	0	0	0	hsa_circ_0035445	NC_000015.10	-	58010649	58014281	3633	376	sense-overlappin	NM_001206897.1	ALDH1A2
circRNA_09636	0	0	0	0	0.02006311	0	0	0	0	0	0	0	-	NC_000015.10	-	58336638	58420105	83468	83468	antisense	XM_011521551.1	LIPC
circRNA_09637	0	0	0	0	0.02006311	0	0	0	0	0	0	0	-	NC_000015.10	-	58610297	58682314	72018	1819	sense-overlappin	NM_001110.3	ADAM10
circRNA_09638	0	0	0	0	0.04012623	0	0.020098685	0	0	0	0	0	hsa_circ_0005678	NC_000015.10	-	58621471	58643978	22508	776	sense-overlappin	NM_001110.3	ADAM10
circRNA_09639	0	0	0.021808645	0	0	0	0	0	0	0	0	0	-	NC_000015.10	-	58627700	58682314	54615	1154	sense-overlappin	NM_001110.3	ADAM10
circRNA_09640	0	0	0	0	0.03009467	0	0	0	0	0	0	0	hsa_circ_0035455	NC_000015.10	-	58640777	58682314	41538	806	sense-overlappin	NM_001110.3	ADAM10
circRNA_09641	0	0	0	0	0.04012623	0	0	0	0	0	0	0	-	NC_000015.10	+	58771959	58847470	75512	1033	sense-overlappin	XM_011521687.1	FAM63B
circRNA_09642	0.030862537	0.030704951	0	0.0619086	0.08025246	0.051782273	0.020098685	0.040972591	0	0	0	0	hsa_circ_0000604	NC_000015.10	-	58886975	58887540	566	460	sense-overlappin	NM_001013843.1	SLTM
circRNA_09643	0	0	0	0	0	0	0	0.020486295	0	0	0	0	-	NC_000015.10	-	58893342	58893901	560	104	sense-overlappin	NM_001013843.1	SLTM
circRNA_09644	0	0	0	0	0	0.020712909	0	0	0	0	0	0	-	NC_000015.10	-	58897115	58916999	19885	923	sense-overlappin	NM_001013843.1	SLTM
circRNA_09645	0.030862537	0.020469968	0.054521613	0.0722267	0.06018934	0.093208092	0.070345399	0	0.060763583	0.060517448	0	0.0206364	hsa_circ_0003713	NC_000015.10	-	58912563	58913696	1134	246	sense-overlappin	NM_001013843.1	SLTM
circRNA_09646	0.370350443	0.358224432	0.719685286	0.4539961	0.43135697	0.352119459	0.231134882	0.22534925	0.354454235	0.494225829	0.202552301	0.175409401	hsa_circ_0000605	NC_000015.10	-	58912563	58916999	4437	311	sense-overlappin	NM_001013843.1	SLTM
circRNA_09647	0	0	0	0.0206362	0.05015779	0	0	0.020486295	0	0	0	0	-	NC_000015.10	-	58912563	58919972	7410	3284	sense-overlappin	NM_001013843.1	SLTM
circRNA_09648	0	0	0	0	0.02006311	0	0	0	0	0	0	0	-	NC_000015.10	-	58912563	58921584	9022	4896	sense-overlappin	NM_001013843.1	SLTM
circRNA_09649	0	0.030704951	0.021808645	0.0206362	0.02006311	0.020712909	0	0	0	0	0	0	hsa_circ_0001982	NC_000015.10	+	59030804	59031702	899	899	exonic	NM_001270528.1	RNF111
circRNA_09650	0	0	0	0	0	0	0	0	0	0.030258724	0	0	hsa_circ_0035472	NC_000015.10	+	59030804	59058550	27747	1385	sense-overlappin	NM_001270528.1	RNF111
circRNA_09651	0	0	0.021808645	0	0	0	0	0	0	0	0	0	hsa_circ_0035473	NC_000015.10	+	59030804	59067083	36280	1705	sense-overlappin	NM_001270528.1	RNF111
circRNA_09652	0	0	0	0.0206362	0	0	0	0	0	0.020172483	0	0	hsa_circ_0035479	NC_000015.10	+	59080936	59081284	349	349	exonic	NM_001270528.1	RNF111
circRNA_09653	0	0	0	0	0.03009467	0	0	0	0	0	0	0	-	NC_000015.10	-	59153590	59214720	61131	1973	sense-overlappin	NM_004998.3	MYO1E
circRNA_09654	0	0	0	0	0.02006311	0	0	0	0	0	0	0	-	NC_000015.10	-	59153590	59218087	64498	2170	sense-overlappin	NM_004998.3	MYO1E
circRNA_09655	0	0	0	0	0.02006311	0	0	0	0	0	0	0	-	NC_000015.10	-	59188118	59214720	26603	797	sense-overlappin	NM_004998.3	MYO1E
circRNA_09656	0	0	0	0	0	0	0	0	0.020254528	0	0	0	-	NC_000015.10	-	59195461	59214720	19260	698	sense-overlappin	NM_004998.3	MYO1E
circRNA_09657	0	0	0.032712968	0	0	0	0	0	0	0	0	0	hsa_circ_0004432	NC_000015.10	-	59195461	59218087	22627	895	sense-overlappin	NM_004998.3	MYO1E
circRNA_09658	0	0.030704951	0.021808645	0	0	0	0	0	0	0	0	0	hsa_circ_0005350	NC_000015.10	-	59202326	59214720	12395	591	sense-overlappin	NM_004998.3	MYO1E
circRNA_09659	0	0	0	0	0	0	0.020098685	0	0	0	0	0	hsa_circ_0035537	NC_000015.10	-	59671183	59672739	1557	235	sense-overlappin	XM_005254607.1	BNIP2
circRNA_09660	0.030862537	0	0.032712968	0.0515905	0	0.031069364	0	0.020486295	0.050636319	0.060517448	0	0	-	NC_000015.10	-	59671183	59680308	9126	657	sense-overlappin	XM_005254607.1	BNIP2
circRNA_09661	0.041150049	0	0	0	0	0	0	0	0	0	0	0	hsa_circ_0035554	NC_000015.10	-	60355919	60364523	8605	380	sense-overlappin	NM_001002857.1	ANXA2
circRNA_09662	0	0.020469968	0.032712968	0	0.10031557	0.124277456	0.030148028	0	0.060763583	0.020172483	0.04051046	0	hsa_circ_0000606	NC_000015.10	-	60442416	60445791	3376	3376	sense-overlappin	NM_001018089.2	ICE2
circRNA_09663	0	0	0	0	0.02006311	0	0	0	0	0	0	0	hsa_circ_0035572	NC_000015.10	-	60442416	60466713	24298	2017	sense-overlappin	NM_001018089.2	ICE2
circRNA_09664	0	0	0	0	0.03009467	0	0	0	0	0	0	0	hsa_circ_0035575	NC_000015.10	-	60453603	60466713	13111	717	sense-overlappin	NM_001018089.2	ICE2
circRNA_09665	0	0	0.032712968	0	0	0	0	0	0	0	0	0	-	NC_000015.10	-	61000892	61016035	15144	15144	sense-overlappin	XM_011521878.1	RORA
circRNA_09666	0	0	0	0	0	0	0	0	0	0.040344966	0	0	-	NC_000015.10	-	61913311	61927320	14010	2264	sense-overlappin	NM_017684.4	VPS13C
circRNA_09667	0	0	0	0	0	0	0	0	0	0.040344966	0	0	-	NC_000015.10	-	61927091	61931259	4169	648	sense-overlappin	NM_017684.4	VPS13C

circRNA_09668	0	0	0	0	0.02006311	0	0	0	0	0.020172483	0	0	hsa_circ_0004456	NC_000015.10	-	61929501	61931259	1759	418	sense-overlappin	NM_017684.4	VPS13C
circRNA_09669	0	0	0	0	0	0.041425819	0	0	0	0	0	0	-	NC_000015.10	-	61961589	61984999	23411	2330	sense-overlappin	NM_017684.4	VPS13C
circRNA_09670	0	0	0	0	0	0	0	0.020486295	0	0	0	0	-	NC_000015.10	-	61991000	61991802	803	225	sense-overlappin	NM_017684.4	VPS13C
circRNA_09671	0.020575025	0	0.032712968	0	0	0	0	0	0	0	0	0	-	NC_000015.10	-	61991000	62035052	44053	1391	sense-overlappin	NM_020821.2	VPS13C
circRNA_09672	0	0	0.021808645	0	0	0	0	0	0	0	0	0	-	NC_000015.10	-	62000564	62035052	34489	1166	sense-overlappin	NM_020821.2	VPS13C
circRNA_09673	0.164600197	0.092114854	0.119947548	0.1444533	0.11034713	0.238198457	0.040197371	0.081945182	0.091145375	0.161379862	0.111403765	0.051591	hsa_circ_0000607	NC_000015.10	-	62007308	62013992	6685	606	sense-overlappin	NM_017684.4	VPS13C
circRNA_09674	0	0	0	0.0309543	0	0	0	0	0	0	0	0	hsa_circ_0000609	NC_000015.10	-	62020479	62047814	27336	4143	sense-overlappin	NM_020821.2	VPS13C
circRNA_09675	0	0	0	0	0	0.031069364	0	0	0	0	0	0	-	NC_000015.10	-	62023411	62035052	11642	437	sense-overlappin	NM_020821.2	VPS13C
circRNA_09676	0	0.051174919	0.04361729	0	0.02006311	0.020712909	0	0.040972591	0.030381792	0.07060369	0.02025523	0.0206364	hsa_circ_0005761	NC_000015.10	-	62033441	62044255	10815	285	sense-overlappin	NM_017684.4	VPS13C
circRNA_09677	0	0	0	0	0	0	0	0	0.020254528	0	0	0	-	NC_000015.10	+	62589687	62657898	68212	1025	sense-overlappin	XM_005254710.3	TLN2
circRNA_09678	0.041150049	0.030704951	0	0	0	0	0	0	0	0.020172483	0	0	-	NC_000015.10	+	63056985	63062645	5661	532	sense-overlappin	NM_000366.5	TPM1
circRNA_09679	0	0	0	0	0.03009467	0	0	0	0	0	0	0	-	NC_000015.10	+	63126859	63129650	2792	690	sense-overlappin	NM_001288585.1	LACTB
circRNA_09680	0.020575025	0	0.021808645	0	0	0	0.030148028	0	0	0	0	0.0309546	-	NC_000015.10	+	63213989	63214386	398	398	intronic	NM_016530.2	RAB8B
circRNA_09681	0	0	0	0	0.02006311	0	0	0	0	0	0	0	hsa_circ_0035640	NC_000015.10	-	63338819	63346709	7891	768	sense-overlappin	NM_001218.4	CA12
circRNA_09682	0	0	0.032712968	0	0	0	0	0	0	0	0	0	hsa_circ_0008104	NC_000015.10	+	63529014	63537156	8143	462	sense-overlappin	XM_011522265.1	USP3
circRNA_09683	0	0	0.08723458	0.0206362	0.0702209	0	0.040197371	0.030729443	0.020254528	0	0	0	hsa_circ_0007349	NC_000015.10	+	63529014	63563008	33995	1077	sense-overlappin	XM_011522265.1	USP3
circRNA_09684	0	0	0.076330258	0.0206362	0.0702209	0.031069364	0	0	0	0	0	0.0206364	-	NC_000015.10	+	63529014	63574403	45390	1412	sense-overlappin	XM_011522265.1	USP3
circRNA_09685	0	0.051174919	0.032712968	0.0412724	0.0702209	0.051782273	0.020098685	0	0	0.030258724	0.04051046	0	hsa_circ_0000612	NC_000015.10	+	63532647	63537156	4510	309	sense-overlappin	NR_046342.1	
circRNA_09686	0	0	0	0	0	0	0	0	0	0.030258724	0	0	-	NC_000015.10	+	63532647	63538952	6306	2105	sense-overlappin	NR_046342.1	
circRNA_09687	0	0.030704951	0.032712968	0	0.03009467	0.020712909	0.060296056	0	0	0.020172483	0	0	hsa_circ_0035649	NC_000015.10	+	63532647	63559970	27324	810	sense-overlappin	XM_011522265.1	USP3
circRNA_09688	0.051437562	0.030704951	0.26170374	0.0825448	0.31097828	0.103564547	0.110542769	0.071702034	0	0.121034897	0.030382845	0.0412728	hsa_circ_0002138	NC_000015.10	+	63532647	63563008	30362	924	sense-overlappin	XM_011522265.1	USP3
circRNA_09689	0	0.020469968	0.032712968	0	0.02006311	0.020712909	0.040197371	0	0	0.030258724	0	0	hsa_circ_0035650	NC_000015.10	+	63532647	63574403	41757	1259	sense-overlappin	XM_011522265.1	USP3
circRNA_09690	0	0	0.021808645	0	0	0	0	0	0	0	0	0	hsa_circ_0000613	NC_000015.10	+	63553715	63559970	6256	363	sense-overlappin	NM_001256702.1	USP3
circRNA_09691	0	0.020469968	0.109043225	0.0722267	0.05015779	0.031069364	0.030148028	0.071702034	0.020254528	0.080689931	0	0	hsa_circ_0008153	NC_000015.10	+	63553715	63563008	9294	477	sense-overlappin	NM_001256702.1	USP3
circRNA_09692	0.020575025	0.020469968	0.04361729	0	0.03009467	0	0	0	0.020254528	0	0	0	hsa_circ_0035654	NC_000015.10	+	63553715	63574403	20689	812	sense-overlappin	NM_001256702.1	USP3
circRNA_09693	0	0	0	0	0	0	0	0	0	0.020172483	0	0	-	NC_000015.10	-	63622815	63624327	1513	413	sense-overlappin	XM_011522140.1	HERC1
circRNA_09694	0	0	0.021808645	0	0	0	0	0	0	0	0	0	-	NC_000015.10	-	63628677	63630635	1959	309	sense-overlappin	XM_011522140.1	HERC1
circRNA_09695	0.072012586	0.051174919	0.065425935	0.1857257	0.05015779	0.103564547	0.080394741	0.071702034	0.070890847	0.14120738	0.070893305	0.072227401	hsa_circ_0035796	NC_000015.10	-	63696124	63716473	20350	1170	sense-overlappin	XM_011522140.1	HERC1
circRNA_09696	0	0.040939935	0.032712968	0	0	0.031069364	0	0.040972591	0	0.050431207	0.030382845	0.072227401	hsa_circ_0035801	NC_000015.10	-	63706780	63716473	9694	685	sense-overlappin	XM_011522140.1	HERC1
circRNA_09697	0	0	0	0	0	0.020712909	0	0	0	0.020172483	0	0	hsa_circ_0035803	NC_000015.10	-	63712775	63716473	3699	633	sense-overlappin	XM_011522140.1	HERC1
circRNA_09698	0	0	0.032712968	0	0	0	0	0	0	0.040344966	0	0	hsa_circ_0007112	NC_000015.10	-	63774694	63775649	956	956	exonic	XM_011522140.1	HERC1
circRNA_09699	0	0.020469968	0	0	0	0	0	0	0	0	0	0	hsa_circ_0003867	NC_000015.10	+	64112573	64118854	6282	307	sense-overlappin	XM_005254611.1	SNX1
circRNA_09700	0	0	0	0.0309543	0	0.020712909	0.030148028	0	0	0	0	0	hsa_circ_0002178	NC_000015.10	+	64112573	64123546	10974	351	sense-overlappin	XM_005254611.1	SNX1
circRNA_09701	0	0	0	0.0206362	0	0	0	0	0	0	0	0	hsa_circ_0000614	NC_000015.10	+	64118117	64118854	738	195	sense-overlappin	XM_005254611.1	SNX1
circRNA_09702	0	0	0	0.0206362	0	0	0	0.030729443	0	0.040344966	0	0	hsa_circ_0035818	NC_000015.10	+	64118117	64123546	5430	239	sense-overlappin	XM_005254611.1	SNX1
circRNA_09703	0	0	0	0	0	0	0.020098685	0.020486295	0	0	0	0	hsa_circ_0035828	NC_000015.10	-	64204441	64204949	509	234	sense-overlappin	NM_022048.3	CSNK1G1
circRNA_09704	0	0	0	0	0	0	0.030148028	0	0	0	0	0.0206364	-	NC_000015.10	-	64204441	64216713	12273	707	sense-overlappin	NM_022048.3	CSNK1G1
circRNA_09705	0	0	0	0	0	0	0	0	0	0	0.02025523	0	-	NC_000015.10	+	64337164	64339407	2244	2244	antisense	NM_022048.3	CSNK1G1
circRNA_09706	0	0	0	0.0206362	0	0	0	0	0	0	0	0	-	NC_000015.10	+	64393946	64418728	24783	1257	sense-overlappin	NM_016213.4	TRIP4
circRNA_09707	0	0	0.032712968	0	0	0	0	0	0	0	0	0	-	NC_000015.10	+	64393946	64425631	31686	1474	sense-overlappin	NM_016213.4	TRIP4
circRNA_09708	0	0	0.021808645	0	0	0	0	0	0	0	0	0	-	NC_000015.10	+	64418541	64425631	7091	405	sense-overlappin	NM_016213.4	TRIP4
circRNA_09709	0.823000985	0.388929383	0.56702477	0.6294037	0.28088361	0.590317916	0.311529623	0.338023876	0.31394518	0.685864415	0.243062761	0.330182403	hsa_circ_0000615	NC_000015.10	+	64499293	64500166	874	874	exonic	XM_011521386.1	ZNF609
circRNA_09710	0.020575025	0	0	0	0	0	0	0	0	0	0	0	-	NC_000015.10	-	64750343	64751638	1296	117	sense-overlappin	NM_194272.1	RBPMS2
circRNA_09711	0	0	0.021808645	0	0	0	0	0	0	0	0	0	hsa_circ_0035855	NC_000015.10	-	64822266	64824354	2089	836	sense-overlappin	NM_001286496.1	PIF1
circRNA_09712	0.061725074	0.030704951	0.098138903	0.0412724	0.04012623	0.082851637	0.020098685	0.061458886	0.070890847	0.030258724	0.08102092	0.0412728	hsa_circ_0003526	NC_000015.10	-	64974602	64983593	8992	476	sense-overlappin	NM_001127889.4	SPG21
circRNA_09713	0	0	0	0	0	0	0	0.020486295	0	0	0	0	-	NC_000015.10	-	65023672	65027040	3369	333	sense-overlappin	NM_139242.3	MTFMT
circRNA_09714	0.072012586	0.153524756	0.098138903	0.0722267	0.12037869	0.031069364	0.040197371	0.020486295	0.040509055	0.030258724	0.070893305	0.0206364	hsa_circ_0004374	NC_000015.10	-	65178934	65180204	1271	279	sense-overlappin	NM_006660.3	CLPX
circRNA_09715	0	0	0	0	0	0	0	0	0	0	0	0.0206364	-	NC_000015.10	-	65459937	65462050	2114	2114	intronic	XM_005254505.1	DPP8
circRNA_09716	0	0	0.021808645	0	0.03009467	0	0.020098685	0	0	0	0	0	hsa_circ_0035897	NC_000015.10	-	65463761	65480399	16639	853	sense-overlappin	XM_005254505.1	DPP8
circRNA_09717	0	0	0.021808645	0	0	0	0	0	0	0	0	0	-	NC_000015.10	-	65474209	65515791	41583	1683	sense-overlappin	XM_011521734.1	DPP8
circRNA_09718	0	0	0	0	0.02006311	0	0.020098685	0	0	0	0	0	hsa_circ_0035901	NC_000015.10	-	65478880	65480399	1520	338	sense-overlappin	XM_005254505.1	DPP8
circRNA_09719	0	0	0	0.0309543	0	0.051782273	0	0	0	0	0	0	-	NC_000015.10	-	65485099	65512564	27466	1028	sense-overlappin	XM_011521734.1	DPP8
circRNA_09720	0	0	0	0	0.03009467	0	0.030148028	0	0	0	0	0	hsa_circ_0006487	NC_000015.10	-	65507243	65512564	5322	383	sense-overlappin	XM_011521730.1	DPP8
circRNA_09721	0	0	0	0	0	0	0	0	0	0	0	0.0206364	-	NC_000015.10	-	65584770	65605236	20467	1017	sense-overlappin	XM_006720709.2	VWA9
circRNA_09722	0	0	0	0	0	0	0	0	0	0	0.02025523	0	-	NC_000015.10	-	65641235	65654621	13387	4981	antisense	XM_006720764.2	SLC24A1
circRNA_09723	0.030862537	0.051174919	0	0	0	0	0	0	0.020254528	0	0	0	hsa_circ_0035923	NC_000015.10	-	65667449	65670188	2740	777	sense-overlappin	XM_005254122.3	DENND4A
circRNA_09724	0	0	0	0	0	0.020712909	0	0.051215739	0	0.050431207	0	0	hsa_circ_0035929	NC_000015.10	-	65669779	65670188	410	323	sense-overlappin	XM_005254122.3	DENND4A

circRNA_09725	0	0	0	0.0206362	0	0	0	0	0	0	0	0	hsa_circ_0035930	NC_000015.10	-	65669779	65761438	91660	4971	sense-overlappin	XM_011521155.1	DENND4A
circRNA_09726	0	0	0	0	0	0	0	0.020486295	0	0	0	0	-	NC_000015.10	-	65701133	65752439	51307	2119	sense-overlappin	XM_006720371.2	DENND4A
circRNA_09727	0	0	0	0.0206362	0	0.031069364	0	0	0.020254528	0.040344966	0	0	hsa_circ_0035938	NC_000015.10	-	65715478	65756472	40995	1975	sense-overlappin	XM_006720371.2	DENND4A
circRNA_09728	0	0	0	0	0	0.020712909	0	0	0.030381792	0	0	0	-	NC_000015.10	-	65715478	65761438	45961	2137	sense-overlappin	XM_011521155.1	DENND4A
circRNA_09729	0	0	0	0	0	0	0.040197371	0	0	0	0	0	hsa_circ_0035940	NC_000015.10	-	65722848	65741784	18937	1027	sense-overlappin	XM_006720371.2	DENND4A
circRNA_09730	0	0	0	0.0206362	0	0	0	0	0	0	0	0	hsa_circ_0004367	NC_000015.10	-	65722848	65756472	33625	1610	sense-overlappin	XM_006720371.2	DENND4A
circRNA_09731	0	0	0	0.0206362	0	0	0	0	0	0	0	0	hsa_circ_0035946	NC_000015.10	-	65729072	65756472	27401	1509	sense-overlappin	XM_006720371.2	DENND4A
circRNA_09732	0.030862537	0	0	0	0	0	0	0	0	0	0	0	hsa_circ_0035949	NC_000015.10	-	65737707	65738875	1169	409	sense-overlappin	XM_006720371.2	DENND4A
circRNA_09733	0.041150049	0	0.065425935	0.0515905	0	0	0	0	0	0	0.06076569	0	hsa_circ_0035957	NC_000015.10	-	65752379	65761438	9060	745	sense-overlappin	XM_011521155.1	DENND4A
circRNA_09734	0	0	0	0	0	0	0	0	0.020254528	0	0	0	-	NC_000015.10	-	65756140	65761438	5299	495	sense-overlappin	XM_011521155.1	DENND4A
circRNA_09735	0	0	0	0	0	0	0	0	0	0.020172483	0	0	hsa_circ_0035958	NC_000015.10	+	66306824	66308844	2021	265	sense-overlappin	XM_005254147.3	DIS3L
circRNA_09736	0	0	0.04361729	0	0	0	0	0.020486295	0	0	0	0	hsa_circ_0008798	NC_000015.10	+	66435027	66436892	1866	358	sense-overlappin	NM_002755.3	MAP2K1
circRNA_09737	0	0	0	0	0.04012623	0	0	0	0	0	0	0	hsa_circ_0008168	NC_000015.10	+	66535933	66546705	10773	461	sense-overlappin	NM_001287821.1	ZWILCH
circRNA_09738	0	0	0.021808645	0	0	0	0	0	0	0	0	0	hsa_circ_0005065	NC_000015.10	-	67226901	67236820	9920	512	sense-overlappin	XM_006720683.2	AAGAB
circRNA_09739	0	0	0.076330258	0	0	0	0	0.020486295	0	0	0	0	hsa_circ_0008636	NC_000015.10	-	67231230	67236820	5591	565	sense-overlappin	XM_005254664.2	AAGAB
circRNA_09740	0.102875123	0.071644886	0.316225353	0.113499	0.13041025	0.144990365	0.060296056	0.020486295	0.060763583	0.100862414	0.050638075	0.0309546	hsa_circ_0000620	NC_000015.10	-	67231814	67236820	5007	462	sense-overlappin	NM_001271886.1	AAGAB
circRNA_09741	0	0	0.032712968	0	0	0	0	0	0	0	0	0	hsa_circ_0005498	NC_000015.10	+	67580754	67600749	19996	293	sense-overlappin	XM_011521787.1	MAP2K5
circRNA_09742	0	0	0	0	0	0	0	0.020486295	0	0	0	0	-	NC_000015.10	+	67604825	67605108	284	284	intronic	XM_011521787.1	MAP2K5
circRNA_09743	0	0	0.021808645	0	0	0	0	0	0.020254528	0	0	0	-	NC_000015.10	+	67630888	67664645	33758	302	sense-overlappin	XM_011521787.1	MAP2K5
circRNA_09744	0.020575025	0	0	0	0	0	0	0	0	0	0	0	hsa_circ_0003440	NC_000015.10	+	67664597	67703408	38812	246	sense-overlappin	XM_011521785.1	MAP2K5
circRNA_09745	0.030862537	0	0	0	0	0	0	0	0	0	0	0	hsa_circ_0036036	NC_000015.10	+	67692479	67703408	10930	197	sense-overlappin	XM_011521785.1	MAP2K5
circRNA_09746	0	0	0	0	0	0	0	0	0.020254528	0	0	0	-	NC_000015.10	+	68117364	68118050	687	687	intronic	NM_016166.1	PIAS1
circRNA_09747	0	0	0.076330258	0.0206362	0	0.031069364	0	0	0.020254528	0	0	0	hsa_circ_0008378	NC_000015.10	+	68141946	68146700	4755	359	sense-overlappin	NM_016166.1	PIAS1
circRNA_09748	0	0	0.021808645	0	0	0	0	0.020486295	0	0	0	0	hsa_circ_0003916	NC_000015.10	+	68141946	68153695	11750	465	sense-overlappin	NM_016166.1	PIAS1
circRNA_09749	0	0.071644886	0	0.0412724	0.02006311	0	0	0	0.020254528	0	0	0	hsa_circ_0036044	NC_000015.10	+	68141946	68164804	22859	539	sense-overlappin	NM_016166.1	PIAS1
circRNA_09750	0	0	0	0	0	0	0	0	0	0	0	0.0206364	hsa_circ_0007088	NC_000015.10	+	68141946	68173892	31947	700	sense-overlappin	NM_016166.1	PIAS1
circRNA_09751	0	0	0	0	0.02006311	0	0	0	0	0	0	0	-	NC_000015.10	+	68145816	68173892	28077	567	sense-overlappin	NM_016166.1	PIAS1
circRNA_09752	0	0	0	0	0	0	0	0	0.030381792	0.020172483	0	0	-	NC_000015.10	+	68175637	68176654	1018	312	sense-overlappin	NM_016166.1	PIAS1
circRNA_09753	0	0	0.021808645	0	0	0	0	0	0	0.07060369	0	0	-	NC_000015.10	+	69210316	69261329	51014	933	sense-overlappin	XM_005254298.2	GLCE
circRNA_09754	0	0	0.021808645	0	0	0	0	0	0	0	0	0	hsa_circ_0004266	NC_000015.10	+	69421647	69422435	789	353	sense-overlappin	NM_004856.6	KIF23
circRNA_09755	0	0	0.032712968	0	0.02006311	0	0	0	0	0	0	0	hsa_circ_0003166	NC_000015.10	+	69436138	69436722	585	283	sense-overlappin	NM_001281301.1	KIF23
circRNA_09756	0	0	0.021808645	0	0	0	0	0	0	0	0	0	-	NC_000015.10	-	70682758	70695105	12348	610	sense-overlappin	NM_001008224.1	UACA
circRNA_09757	0	0	0.065425935	0	0	0	0	0	0	0	0	0	hsa_circ_0008722	NC_000015.10	-	70682758	70699660	16903	744	sense-overlappin	NM_001008224.1	UACA
circRNA_09758	0	0	0	0	0	0.031069364	0	0	0	0	0	0	-	NC_000015.10	-	70684900	70685035	136	136	intronic	NM_001008224.1	UACA
circRNA_09759	0	0	0.021808645	0	0	0	0	0	0	0	0	0	hsa_circ_0036103	NC_000015.10	-	70687540	70699660	12121	524	sense-overlappin	NM_001008224.1	UACA
circRNA_09760	0	0	0	0	0	0	0	0.020486295	0	0	0	0	-	NC_000015.10	+	70980101	70984257	4157	248	sense-overlappin	NM_001284357.1	LRRC49
circRNA_09761	0	0.030704951	0	0	0	0	0	0	0.030381792	0.020172483	0	0	hsa_circ_0005857	NC_000015.10	+	71242649	71256715	14067	551	sense-overlappin	XM_006720692.2	THSD4
circRNA_09762	0	0	0.054521613	0	0	0	0	0	0	0	0	0	hsa_circ_0036146	NC_000015.10	-	71848845	71888116	39272	1695	sense-overlappin	XM_011521623.1	MYO9A
circRNA_09763	0	0	0	0.0206362	0	0	0	0	0	0	0	0	-	NC_000015.10	-	71888004	71905006	17003	2786	sense-overlappin	XM_011521623.1	MYO9A
circRNA_09764	0.020575025	0	0	0	0	0	0	0	0	0	0	0	hsa_circ_0003454	NC_000015.10	-	71916370	71938927	22558	383	sense-overlappin	XM_011521614.1	MYO9A
circRNA_09765	0	0.020469968	0	0	0	0	0	0	0	0	0	0	-	NC_000015.10	-	71935341	71938927	3587	220	sense-overlappin	XM_011521614.1	MYO9A
circRNA_09766	0	0	0	0	0.02006311	0	0	0	0	0	0	0	hsa_circ_0036169	NC_000015.10	-	71991103	72010447	19345	567	sense-overlappin	XM_011521614.1	MYO9A
circRNA_09767	0	0	0.021808645	0	0	0	0	0	0	0	0	0	hsa_circ_0036172	NC_000015.10	-	71999851	72046634	46784	1541	sense-overlappin	XM_011521614.1	MYO9A
circRNA_09768	0.041150049	0.051174919	0.021808645	0.0309543	0	0.031069364	0	0	0	0.020172483	0	0	hsa_circ_0007266	NC_000015.10	-	72007826	72027793	19968	445	sense-overlappin	XM_011521614.1	MYO9A
circRNA_09769	0	0	0.021808645	0	0	0	0	0	0	0	0	0	hsa_circ_0036173	NC_000015.10	-	72007826	72032588	24763	540	sense-overlappin	XM_011521614.1	MYO9A
circRNA_09770	0	0	0.076330258	0	0	0.020712909	0	0	0	0	0	0	hsa_circ_0036174	NC_000015.10	-	72007826	72046634	38809	1451	sense-overlappin	XM_011521614.1	MYO9A
circRNA_09771	0	0.040939935	0	0	0.05015779	0	0	0.020486295	0	0	0	0	hsa_circ_0002506	NC_000015.10	-	72032494	72046634	14141	1006	sense-overlappin	XM_011521614.1	MYO9A
circRNA_09772	0.041150049	0	0.076330258	0	0	0	0	0	0	0.020172483	0	0	hsa_circ_0006509	NC_000015.10	-	72045724	72046634	911	911	exonic	XM_011521614.1	MYO9A
circRNA_09773	0	0	0.021808645	0	0	0	0	0	0	0	0	0	hsa_circ_0000627	NC_000015.10	+	72496791	72555907	59117	430	sense-overlappin	XM_011521439.1	ARIH1
circRNA_09774	0	0	0	0	0.02006311	0	0	0	0	0	0	0	hsa_circ_0006314	NC_000015.10	+	72518067	72544964	26898	213	sense-overlappin	NM_005744.3	ARIH1
circRNA_09775	0	0	0.065425935	0.0206362	0.02006311	0	0.040197371	0	0	0.020172483	0	0	hsa_circ_0000628	NC_000015.10	+	72518067	72555907	37841	362	sense-overlappin	NM_005744.3	ARIH1
circRNA_09776	0	0	0.032712968	0	0.02006311	0	0.020098685	0.020486295	0	0	0.030382845	0	hsa_circ_0000629	NC_000015.10	+	72518067	72561549	43483	429	sense-overlappin	NM_005744.3	ARIH1
circRNA_09777	0	0	0	0.0412724	0	0	0.020098685	0	0	0.040344966	0	0	-	NC_000015.10	+	72518067	72572165	54099	840	sense-overlappin	NM_005744.3	ARIH1
circRNA_09778	0	0	0.021808645	0	0	0	0	0	0	0	0	0	-	NC_000015.10	+	72544820	72555907	11088	294	sense-overlappin	XM_011521438.1	ARIH1
circRNA_09779	0	0	0.021808645	0	0	0	0	0	0	0	0	0	-	NC_000015.10	+	72561483	72570307	8825	420	sense-overlappin	XM_011521438.1	ARIH1

circRNA_09780	0	0	0.021808645	0	0	0	0	0	0	0	0	0	hsa_circ_0036234	NC_000015.10	+	72566563	72572165	5603	304	sense-overlappin	XM_011521438.1	ARIH1
circRNA_09781	0	0	0.065425935	0	0	0	0	0	0	0	0	0	hsa_circ_0036240	NC_000015.10	+	72695177	72716850	21674	439	sense-overlappin	NR_045565.1	
circRNA_09782	0	0	0	0	0	0	0.040197371	0	0.020254528	0	0	0.0206364	hsa_circ_0004033	NC_000015.10	-	72760407	72775097	14691	410	sense-overlappin	XM_006720713.2	ADPGK
circRNA_09783	0	0	0	0.0619086	0	0	0	0	0	0	0	0	hsa_circ_0005073	NC_000015.10	-	72760407	72780322	19916	5635	sense-overlappin	XM_006720713.2	ADPGK
circRNA_09784	0	0	0	0	0.03009467	0	0	0.030729443	0.020254528	0.030258724	0	0	hsa_circ_0000631	NC_000015.10	-	72771783	72775097	3315	289	sense-overlappin	XM_006720713.2	ADPGK
circRNA_09785	0	0	0	0.0206362	0	0	0	0	0	0	0	0	-	NC_000015.10	+	73116540	73126570	10031	748	sense-overlappin	XM_005254408.1	NEO1
circRNA_09786	0	0	0	0	0	0	0	0	0	0.030258724	0	0.0206364	-	NC_000015.10	+	73122525	73136027	13503	567	sense-overlappin	XM_005254408.1	NEO1
circRNA_09787	0.041150049	0	0	0	0	0	0	0	0	0	0	0	-	NC_000015.10	+	73176403	73178427	2025	276	sense-overlappin	XM_005254408.1	NEO1
circRNA_09788	0	0	0	0	0	0	0	0	0.020254528	0	0	0	hsa_circ_0007390	NC_000015.10	-	73561896	73573795	11900	493	sense-overlappin	NM_001161363.1	NPTN
circRNA_09789	0	0	0	0	0.04012623	0	0	0	0	0	0	0	hsa_circ_0036250	NC_000015.10	-	73570150	73573795	3646	408	sense-overlappin	NM_001161363.1	NPTN
circRNA_09790	0	0	0	0	0.02006311	0	0	0	0	0	0	0	-	NC_000015.10	+	74600501	74601411	911	911	exonic	XM_011521208.1	CLK3
circRNA_09791	0	0	0	0	0.03009467	0	0	0	0	0	0	0	hsa_circ_0004401	NC_000015.10	-	74655733	74656068	336	336	exonic	XM_011522092.1	EDC3
circRNA_09792	0	0	0.08723458	0.0309543	0	0.041425819	0	0	0	0	0	0.0412728	hsa_circ_0000633	NC_000015.10	-	74671455	74675142	3688	502	sense-overlappin	XM_011522091.1	EDC3
circRNA_09793	0	0	0.021808645	0	0.05015779	0	0.030148028	0	0	0.020172483	0.04051046	0	hsa_circ_0007840	NC_000015.10	-	74923648	74926887	3240	245	sense-overlappin	NM_004255.3	COX5A
circRNA_09794	0	0	0	0	0.08025246	0.031069364	0	0	0.030381792	0.030258724	0	0	hsa_circ_0006810	NC_000015.10	-	74923648	74929232	5585	362	sense-overlappin	NM_004255.3	COX5A
circRNA_09795	0	0	0	0.0206362	0.0702209	0	0.040197371	0	0.020254528	0.080689931	0	0	hsa_circ_0005725	NC_000015.10	-	74926766	74929232	2467	239	sense-overlappin	NM_004255.3	COX5A
circRNA_09796	0	0	0	0.0206362	0	0	0	0	0	0	0	0	hsa_circ_0007103	NC_000015.10	-	75394680	75396496	1817	423	sense-overlappin	NM_015477.2	SIN3A
circRNA_09797	0	0	0	0	0.02006311	0	0	0	0	0	0	0	hsa_circ_0036347	NC_000015.10	-	75400730	75413045	12316	1264	sense-overlappin	NM_015477.2	SIN3A
circRNA_09798	0	0	0	0	0	0	0	0	0	0	0.02025523	0	-	NC_000015.10	-	75400730	75414311	13582	1371	sense-overlappin	NM_015477.2	SIN3A
circRNA_09799	0.020575025	0	0	0	0	0	0	0	0	0	0	0	hsa_circ_0036351	NC_000015.10	-	75409836	75413045	3210	844	sense-overlappin	NM_015477.2	SIN3A
circRNA_09800	0	0.020469968	0.021808645	0.0206362	0	0	0	0	0	0	0	0	hsa_circ_0036353	NC_000015.10	-	75411492	75413045	1554	535	sense-overlappin	NM_015477.2	SIN3A
circRNA_09801	0	0	0.04361729	0	0	0	0	0	0	0	0	0	-	NC_000015.10	-	75490208	75527261	37054	999	sense-overlappin	NM_002833.3	PTPN9
circRNA_09802	0	0	0.021808645	0	0	0	0	0	0	0	0	0	-	NC_000015.10	-	75601138	75621056	19919	764	sense-overlappin	NM_001042581.1	SNUPN
circRNA_09803	0	0	0.021808645	0	0	0	0	0	0	0	0.02025523	0.0206364	-	NC_000015.10	+	75854386	75873568	19183	408	sense-overlappin	NM_173469.3	UBE2Q2
circRNA_09804	0	0	0.08723458	0.0412724	0.02006311	0	0	0.030729443	0	0.030258724	0.02025523	0.0309546	hsa_circ_0036372	NC_000015.10	+	75854386	75883424	29039	704	sense-overlappin	NM_173469.3	UBE2Q2
circRNA_09805	0.072012586	0.071644886	0.076330258	0.1031809	0.16050492	0.15534682	0.070345399	0.051215739	0.081018111	0.191638587	0.091148535	0	-	NC_000015.10	+	75859878	75873568	13691	306	sense-overlappin	NM_173469.3	UBE2Q2
circRNA_09806	0	0	0.021808645	0	0.02006311	0	0	0	0	0	0	0	hsa_circ_0036376	NC_000015.10	+	75868951	75873568	4618	201	sense-overlappin	NM_001284382.1	UBE2Q2
circRNA_09807	0	0	0	0	0	0	0	0.020486295	0	0	0	0	-	NC_000015.10	+	75873428	75883424	9997	437	sense-overlappin	NM_001284382.1	UBE2Q2
circRNA_09808	0	0	0	0	0.02006311	0.020712909	0.040197371	0.040972591	0	0	0	0	hsa_circ_0005151	NC_000015.10	+	75876187	75883424	7238	296	sense-overlappin	NM_001284382.1	UBE2Q2
circRNA_09809	0	0	0	0	0.02006311	0	0	0	0	0	0	0	-	NC_000015.10	+	75913203	75917394	4192	349	sense-overlappin	NM_012170.3	FBXO22
circRNA_09810	0	0	0	0	0	0	0	0	0	0	0	0.0309546	-	NC_000015.10	-	75943572	75971248	27677	9584	sense-overlappin	NM_138573.3	NRG4
circRNA_09811	0.041150049	0	0	0	0	0	0	0	0	0	0	0	hsa_circ_0036398	NC_000015.10	-	76274412	76286481	12070	365	sense-overlappin	XR_931766.1	
circRNA_09812	0	0	0	0.0206362	0	0	0	0	0	0	0	0	hsa_circ_0006842	NC_000015.10	-	76274412	76292700	18289	630	sense-overlappin	XR_931766.1	
circRNA_09813	0.051437562	0.08187987	0.065425935	0	0.04012623	0.020712909	0	0.020486295	0.070890847	0.050431207	0	0	hsa_circ_0000638	NC_000015.10	-	76274412	76295737	21326	777	sense-overlappin	XR_931766.1	
circRNA_09814	0	0	0.032712968	0.0309543	0	0	0	0.020486295	0	0.020172483	0	0	hsa_circ_0005036	NC_000015.10	-	76283757	76292700	8944	547	sense-overlappin	XR_931766.1	
circRNA_09815	0	0	0.076330258	0	0	0	0	0	0.030381792	0	0	0	hsa_circ_0002226	NC_000015.10	-	76283757	76295737	11981	694	sense-overlappin	XR_931766.1	
circRNA_09816	0	0	0.021808645	0	0	0	0	0	0	0	0	0.0206364	hsa_circ_0036405	NC_000015.10	-	76285637	76295737	10101	625	sense-overlappin	XR_931766.1	
circRNA_09817	0	0.040939935	0.054521613	0	0	0.020712909	0.040197371	0.020486295	0.020254528	0.030258724	0.030382845	0	hsa_circ_0000639	NC_000015.10	-	76287846	76292700	4855	265	sense-overlappin	XR_931766.1	
circRNA_09818	0	0.051174919	0.065425935	0.0206362	0	0	0.020098685	0	0.030381792	0	0.02025523	0	hsa_circ_0003620	NC_000015.10	-	76292431	76295737	3307	312	sense-overlappin	XR_931766.1	
circRNA_09819	0	0	0	0	0	0	0	0	0	0.050431207	0	0	-	NC_000015.10	-	76567270	76574284	7015	7015	sense-overlappin	NM_001145923.1	SCAPER
circRNA_09820	0	0	0.032712968	0	0	0	0	0	0	0	0	0	hsa_circ_0005143	NC_000015.10	-	76621764	76665789	44026	203	sense-overlappin	NM_001145923.1	SCAPER
circRNA_09821	0	0	0.021808645	0	0	0	0	0	0	0	0	0	-	NC_000015.10	-	76621764	76683061	61298	17475	sense-overlappin	NM_001145923.1	SCAPER
circRNA_09822	0	0	0	0	0	0	0	0.020486295	0	0	0	0	-	NC_000015.10	-	76661249	76665789	4541	4541	sense-overlappin	NM_001145923.1	SCAPER
circRNA_09823	0	0	0	0.0206362	0	0	0	0	0	0	0	0	-	NC_000015.10	-	76665653	76683061	17409	17409	sense-overlappin	NM_001145923.1	SCAPER
circRNA_09824	0	0	0.021808645	0	0	0	0	0	0	0.030258724	0	0	hsa_circ_0000640	NC_000015.10	-	76728595	76733384	4790	299	sense-overlappin	NM_001145923.1	SCAPER
circRNA_09825	0	0	0	0	0	0.020712909	0	0	0	0	0	0	-	NC_000015.10	-	76728595	76765638	37044	746	sense-overlappin	NM_001145923.1	SCAPER
circRNA_09826	0	0	0.021808645	0	0.02006311	0	0	0.020486295	0	0	0	0	-	NC_000015.10	-	76753808	76775117	21310	1094	sense-overlappin	NM_001145923.1	SCAPER
circRNA_09827	0	0	0.021808645	0	0	0	0	0	0	0	0	0	-	NC_000015.10	-	76771742	76795440	23699	796	sense-overlappin	NM_001145923.1	SCAPER
circRNA_09828	0	0	0	0	0	0	0	0	0	0	0.02025523	0	-	NC_000015.10	-	76857809	76862533	4725	189	sense-overlappin	NM_020843.2	SCAPER
circRNA_09829	0	0	0.021808645	0	0	0	0	0	0	0	0	0	hsa_circ_0036417	NC_000015.10	-	76857809	76883876	26068	254	sense-overlappin	NM_020843.2	SCAPER
circRNA_09830	0	0	0	0	0	0	0	0	0	0	0.02025523	0	-	NC_000015.10	-	77046776	77056255	9480	758	sense-overlappin	NM_005724.5	TSPAN3
circRNA_09831	0.020575025	0	0	0	0	0	0	0	0	0	0	0	-	NC_000015.10	-	77133005	77158696	25692	940	sense-overlappin	XM_011522040.1	PEAK1
circRNA_09832	0.020575025	0	0	0.0309543	0	0	0	0	0	0.020172483	0.02025523	0	hsa_circ_0036423	NC_000015.10	-	77178790	77182040	3251	3251	exonic	XM_011522040.1	PEAK1
circRNA_09833	0	0	0	0	0	0.051782273	0	0.030729443	0.020254528	0.030258724	0.02025523	0	-	NC_000015.10	-	77252367	77284030	31664	467	sense-overlappin	XM_011522040.1	PEAK1

circRNA_09834	0	0	0	0	0	0	0.020098685	0	0	0	0	0	hsa_circ_0007284	NC_000015.10	-	77252367	77286504	34138	670	sense-overlappin	XM_011522040.1	PEAK1
circRNA_09835	0	0	0	0	0	0	0	0	0.030381792	0	0	0	-	NC_000015.10	-	77283883	77286504	2622	351	sense-overlappin	XM_011522040.1	PEAK1
circRNA_09836	0	0	0	0	0.02006311	0	0	0	0	0	0	0	-	NC_000015.10	-	77361315	77371899	10585	10585	sense-overlappin	XM_011522040.1	PEAK1
circRNA_09837	0	0	0.04361729	0	0	0	0	0	0	0	0	0	-	NC_000015.10	-	77361315	77388802	27488	27488	sense-overlappin	XM_011522040.1	PEAK1
circRNA_09838	0	0	0.021808645	0	0	0.051782273	0	0.020486295	0	0	0	0	-	NC_000015.10	-	77364074	77365225	1152	1152	sense-overlappin	XM_011522040.1	PEAK1
circRNA_09839	0	0	0	0	0	0	0.030148028	0	0	0	0	0	-	NC_000015.10	-	77365163	77388802	23640	23640	sense-overlappin	XM_011522040.1	PEAK1
circRNA_09840	0.072012586	0	0.032712968	0.0619086	0.04012623	0.031069364	0	0.030729443	0	0.020172483	0	0	hsa_circ_0036429	NC_000015.10	+	77467095	77471814	4720	378	sense-overlappin	NM_001304504.1	HMG20A
circRNA_09841	0	0	0	0.0309543	0	0	0	0	0	0	0	0	hsa_circ_0036441	NC_000015.10	-	78044900	78054187	9288	323	sense-overlappin	NM_015079.5	TBC1D2B
circRNA_09842	0	0	0	0	0	0	0.020098685	0	0	0	0	0	hsa_circ_0008905	NC_000015.10	+	78160092	78162367	2276	437	sense-overlappin	XM_005254334.1	IDH3A
circRNA_09843	0	0	0.04361729	0	0	0	0	0	0	0	0	0	-	NC_000015.10	+	79411400	79413557	2158	2158	sense-overlappin	XR_931778.1	
circRNA_09844	0	0	0	0	0	0	0	0.020486295	0	0	0	0	-	NC_000015.10	+	79456098	79458445	2348	2348	exonic	XR_931778.1	
circRNA_09845	0	0	0	0.0309543	0.03009467	0	0	0	0	0	0	0	-	NC_000015.10	+	79456098	79469145	13048	3628	sense-overlappin	XR_931777.1	
circRNA_09846	0.020575025	0.061409903	0	0	0.11034713	0.020712909	0.040197371	0.040972591	0	0.050431207	0	0	hsa_circ_0000642	NC_000015.10	+	80098416	80122800	24385	676	sense-overlappin	XM_011521683.1	ZFAND6
circRNA_09847	0.174887709	0.225169643	0.359842643	0.1754076	0.52164099	0.227842003	0.100493427	0.112674625	0.101272639	0.252156035	0.050638075	0.072227401	hsa_circ_0000643	NC_000015.10	+	80120328	80122800	2473	381	sense-overlappin	NM_001242911.1	ZFAND6
circRNA_09848	0.030862537	0.051174919	0.021808645	0.0309543	0.02006311	0.124277456	0.030148028	0	0.050636319	0.060517448	0	0.051591	hsa_circ_0005558	NC_000015.10	-	80970462	80982182	11721	9249	sense-overlappin	NR_126327.1	
circRNA_09849	0	0	0	0	0	0	0	0	0.020254528	0.030258724	0	0	hsa_circ_0002255	NC_000015.10	-	80978766	80982182	3417	945	sense-overlappin	NR_126327.1	
circRNA_09850	0	0	0.032712968	0	0.04012623	0	0	0	0	0	0	0	hsa_circ_0000645	NC_000015.10	-	80978931	80982182	3252	780	sense-overlappin	NR_126327.1	
circRNA_09851	0	0	0.032712968	0	0.03009467	0	0	0	0	0	0	0	-	NC_000015.10	-	81925673	81926025	353	353	intergenic		
circRNA_09852	0	0	0.021808645	0	0	0	0	0	0	0	0	0	-	NC_000015.10	-	81925673	81967192	41520	41520	intergenic		
circRNA_09853	0	0.020469968	0	0	0	0	0	0	0	0	0	0	hsa_circ_0005745	NC_000015.10	-	82151465	82152423	959	959	exonic	NM_001040610.2	EFTUD1
circRNA_09854	0	0	0.021808645	0	0	0	0	0	0	0	0	0	hsa_circ_0036548	NC_000015.10	-	82151465	82163984	12520	1239	sense-overlappin	NM_001040610.2	EFTUD1
circRNA_09855	0	0	0	0.0928628	0	0	0	0	0	0	0	0	-	NC_000015.10	+	82813103	82819488	6386	661	sense-overlappin	XM_011521233.1	WHAMM
circRNA_09856	0	0	0	0	0	0	0	0	0	0.030258724	0	0	-	NC_000015.10	+	83885101	83899731	14631	740	sense-overlappin	NM_001301110.1	ADAMTSL3
circRNA_09857	0.041150049	0	0	0	0	0	0	0	0	0	0	0	-	NC_000015.10	+	83942596	83943082	487	373	sense-overlappin	NM_001301110.1	ADAMTSL3
circRNA_09858	0	0.020469968	0	0	0	0	0	0	0	0.020172483	0	0	-	NC_000015.10	-	84538532	84546277	7746	7746	intergenic		
circRNA_09859	0.082300098	0.051174919	0.04361729	0.0515905	0	0.020712909	0	0.030729443	0.020254528	0.121034897	0.02025523	0.0309546	-	NC_000015.10	-	84538532	84555070	16539	16539	intergenic		
circRNA_09860	0.030862537	0	0	0	0	0	0	0	0	0	0	0	hsa_circ_0006899	NC_000015.10	+	84637347	84641262	3916	3916	intergenic		
circRNA_09861	0	0.040939935	0.054521613	0	0.12037869	0.062138728	0.020098685	0.102431477	0.060763583	0.040344966	0.02025523	0.0206364	hsa_circ_0000647	NC_000015.10	-	84680713	84691644	10932	380	sense-overlappin	NM_001271918.1	SEC11A
circRNA_09862	0	0	0	0	0.04012623	0	0.020098685	0.020486295	0	0	0.02025523	0	hsa_circ_0036599	NC_000015.10	-	84687625	84691644	4020	260	sense-overlappin	NM_001271918.1	SEC11A
circRNA_09863	0	0	0	0.0206362	0	0	0	0	0	0	0	0	-	NC_000015.10	+	85017169	85019674	2506	2506	intronic	NM_001243137.1	PDE8A
circRNA_09864	0	0	0.065425935	0	0	0.020712909	0	0	0	0	0	0	hsa_circ_0036610	NC_000015.10	+	85064370	85067204	2835	248	sense-overlappin	NM_001243137.1	PDE8A
circRNA_09865	0	0	0.098138903	0.0206362	0	0	0	0	0	0	0	0	hsa_circ_0036615	NC_000015.10	+	85064370	85109130	44761	928	sense-overlappin	NM_001243137.1	PDE8A
circRNA_09866	0.020575025	0	0.032712968	0	0	0.062138728	0.030148028	0.030729443	0.020254528	0.020172483	0	0	hsa_circ_0036627	NC_000015.10	+	85113377	85126374	12998	1139	sense-overlappin	NM_001243137.1	PDE8A
circRNA_09867	0	0.040939935	0.098138903	0.0825448	0.08025246	0.113921001	0.030148028	0	0.081018111	0.050431207	0.030382845	0	hsa_circ_0003856	NC_000015.10	+	85113873	85115487	1615	214	sense-overlappin	NM_001243137.1	PDE8A
circRNA_09868	0.041150049	0	0	0	0	0	0	0	0	0	0	0	hsa_circ_0005889	NC_000015.10	+	85113873	85117839	3967	549	sense-overlappin	NM_001243137.1	PDE8A
circRNA_09869	0.061725074	0.071644886	0.065425935	0.0928628	0.0702209	0.062138728	0.030148028	0.051215739	0.030381792	0.040344966	0.06076569	0.051591	hsa_circ_0036629	NC_000015.10	+	85113873	85121014	7142	767	sense-overlappin	NM_001243137.1	PDE8A
circRNA_09870	0	0	0.04361729	0	0	0	0	0	0	0	0	0	hsa_circ_0008454	NC_000015.10	+	85521428	85543955	22528	629	sense-overlappin	NM_006738.5	AKAP13
circRNA_09871	0	0	0.109043225	0	0	0	0	0	0	0	0	0	-	NC_000015.10	+	85543772	85585823	42052	3683	sense-overlappin	NM_006738.5	AKAP13
circRNA_09872	0	0	0	0	0	0	0.020098685	0	0	0	0	0	-	NC_000015.10	+	85575131	85585823	10693	3499	sense-overlappin	NM_006738.5	AKAP13
circRNA_09873	0	0	0.032712968	0	0	0	0	0	0	0	0	0	-	NC_000015.10	+	85639374	85669830	30457	952	sense-overlappin	NM_006738.5	AKAP13
circRNA_09874	0	0	0	0.0309543	0	0	0	0	0	0	0	0	hsa_circ_0036646	NC_000015.10	+	85655417	85669830	14414	739	sense-overlappin	NM_006738.5	AKAP13
circRNA_09875	0	0	0	0	0.02006311	0	0	0	0	0	0	0	-	NC_000015.10	+	85662388	85710645	48258	866	sense-overlappin	NM_006738.5	AKAP13
circRNA_09876	0	0	0	0	0.02006311	0	0	0	0	0.040344966	0	0	hsa_circ_0036649	NC_000015.10	+	85664563	85669830	5268	302	sense-overlappin	NM_006738.5	AKAP13
circRNA_09877	0	0.051174919	0	0	0	0	0	0	0	0	0	0	-	NC_000015.10	+	85664563	85684873	20311	490	sense-overlappin	NM_006738.5	AKAP13
circRNA_09878	0	0	0	0.0309543	0	0	0	0	0	0.07060369	0	0	-	NC_000015.10	+	85664563	85693451	28889	665	sense-overlappin	NM_006738.5	AKAP13
circRNA_09879	0	0	0	0.0206362	0	0.020712909	0	0	0.020254528	0	0	0	-	NC_000015.10	+	85664563	85710645	46083	800	sense-overlappin	NM_006738.5	AKAP13
circRNA_09880	0	0	0	0.0206362	0	0	0	0	0	0	0	0	-	NC_000015.10	+	85684741	85710645	25905	443	sense-overlappin	NM_006738.5	AKAP13
circRNA_09881	0	0	0	0.0206362	0	0	0	0	0	0	0	0	-	NC_000015.10	+	85710579	85719326	8748	720	sense-overlappin	NM_006738.5	AKAP13
circRNA_09882	0	0	0	0.0206362	0	0	0	0	0	0	0	0	-	NC_000015.10	+	85715788	85741495	25708	2459	sense-overlappin	NM_006738.5	AKAP13
circRNA_09883	0	0	0	0	0	0	0	0	0	0.020172483	0	0	-	NC_000015.10	+	85740222	85741495	1274	501	sense-overlappin	NM_006738.5	AKAP13
circRNA_09884	0	0	0	0	0	0	0	0	0.020254528	0	0	0	-	NC_000015.10	+	86716977	86760623	43647	43647	intronic	XM_011521226.1	AGBL1
circRNA_09885	0.164600197	0.092114854	0.39255561	0.2476343	0.21066271	0.093208092	0.110542769	0.061458886	0.121527166	0.171466104	0.02025523	0.082545601	hsa_circ_0007099	NC_000015.10	+	89113725	89116521	2797	300	sense-overlappin	NM_007011.7	ABHD2
circRNA_09886	0	0	0.065425935	0	0	0	0.030148028	0	0	0	0	0	hsa_circ_0036683	NC_000015.10	+	89113725	89155534	41810	644	sense-overlappin	NM_007011.7	ABHD2
circRNA_09887	0	0	0.021808645	0	0	0	0	0	0	0	0	0	hsa_circ_0003679	NC_000015.10	+	89113732	89116521	2790	293	sense-overlappin	NM_007011.7	ABHD2
circRNA_09888	0	0	0	0	0	0	0.020098685	0	0	0	0	0	hsa_circ_0036749	NC_000015.10	-	89837615	89889140	51526	503	sense-overlappin	NR_023361.1	

[truncated: 511,199 more chars]
